# Genomic Portrait of a Sporadic Amyotrophic Lateral Sclerosis Case in a Large Spinocerebellar Ataxia Type 1 Family

**DOI:** 10.3390/jpm10040262

**Published:** 2020-12-02

**Authors:** Giovanna Morello, Giulia Gentile, Rossella Spataro, Antonio Gianmaria Spampinato, Maria Guarnaccia, Salvatore Salomone, Vincenzo La Bella, Francesca Luisa Conforti, Sebastiano Cavallaro

**Affiliations:** 1 Institute for Research and Biomedical Innovation (IRIB), Italian National Research Council (CNR), Via Paolo Gaifami, 18, 95125 Catania, Italy; giomor86@hotmail.it (G.M.); giulia.gentile@cnr.it (G.G.); antonio.spampinato@unict.it (A.G.S.); maria.guarnaccia@cnr.it (M.G.); 2 Department of Biomedical and Biotechnological Sciences, Section of Pharmacology, University of Catania, 95123 Catania, Italy; salomone@unict.it; 3 ALS Clinical Research Center and Neurochemistry Laboratory, BioNeC, University of Palermo, 90127 Palermo, Italy; rossella.spataro@unipa.it (R.S.); vincenzo.labella@unipa.it (V.L.B.); 4 Department of Mathematics and Computer Science, University of Catania, 95123 Catania, Italy; 5 Department of Pharmacy, Health and Nutritional Sciences, University of Calabria, Arcavacata di Rende, 87036 Rende, Italy

**Keywords:** spinocerebellar ataxia, amyotrophic lateral sclerosis, SCA1-MN, NGS, customized aCGH, multi-omics, pathway, network

## Abstract

Background: Repeat expansions in the spinocerebellar ataxia type 1 (SCA1) gene *ATXN1* increases the risk for amyotrophic lateral sclerosis (ALS), supporting a relationship between these disorders. We recently reported the co-existence, in a large SCA1 family, of a clinically definite ALS individual bearing an intermediate *ATXN1* expansion and SCA1 patients with a full expansion, some of which manifested signs of lower motor neuron involvement. Methods: In this study, we employed a systems biology approach that integrated multiple genomic analyses of the ALS patient and some SCA1 family members. Results: Our analysis identified common and distinctive candidate genes/variants and related biological processes that, in addition to or in combination with *ATXN1*, may contribute to motor neuron degeneration phenotype. Among these, we distinguished ALS-specific likely pathogenic variants in *TAF15* and *C9ORF72*, two ALS-linked genes involved in the regulation of RNA metabolism, similarly to *ATXN1*, suggesting a selective role for this pathway in ALS pathogenesis. Conclusions: Overall, our work supports the utility to apply personal genomic information for characterizing complex disease phenotypes.

## 1. Introduction

Amyotrophic lateral sclerosis (ALS) is a progressive neurodegenerative disorder characterized by severe muscle weakness with atrophy caused by the loss of motor neurons (MNs) in the motor cortex, brainstem, and spinal cord [1]. The genetic and molecular architecture of ALS is complex as the disease is associated with a multitude of causative genes and biological pathways. A limited number of genes, including Cu/Zn superoxide dismutase (*SOD1*), fused in sarcoma/translocated in liposarcoma (*FUS/TLS* or FUS), transactive response DNA binding protein 43 kDa (TDP-43), and chromosome 9 open reading frame 72 (*C9ORF72*), are responsible for a significant percentage of both familial (FALS) and sporadic ALS (SALS) cases [2,3,4]. In addition to multiple disease-associated genetic variants, there is evidence about putatively associated variants with a moderate or small effect size that may act as predisposing factors or modifiers of the disease phenotype [5,6,7]. Among the different genetic risk factors for ALS is ataxin-1 (*ATXN1*), a gene involved in transcriptional regulation that normally contains a segment of 22–23 CAG trinucleotide repeats, encoding for a polyglutamine (polyQ) tract. Intermediate-length (~29–33 CAG) repeats are consistently associated with increased risk for ALS, while high poly-Q repeat expansions (>34 CAG) cause spinocerebellar ataxia type 1 (SCA1), an adult-onset autosomal dominant neurodegenerative disease that is characterized by progressive cerebellar degeneration causing loss of motor coordination and balance [8,9]. 

**Figure 1:**
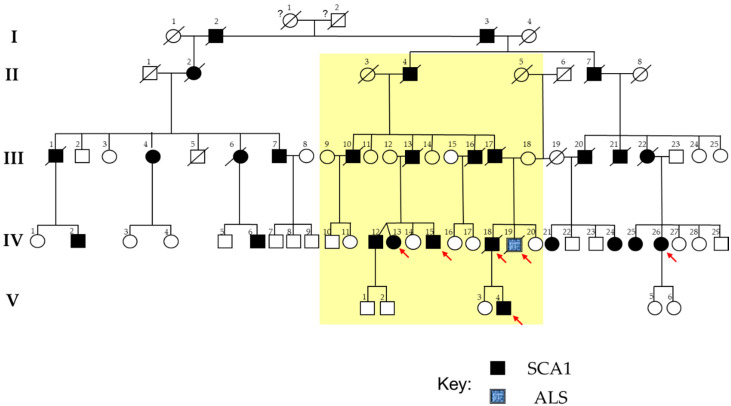
SCA1-ALS family pedigree. Square indicates male; circle female; slash deceased; black symbols indicate patients affected by SCA1; blue symbol indicates the patient affected by amyotrophic lateral sclerosis (ALS). The red arrows indicate the patients in whom genomic analyses were performed. The “central branch” of the genealogical tree, presenting patients showing ataxic-spastic phenotype with lower motor neuron (MN) signs or symptoms is highlighted in yellow.

In our previous work, we described a large SCA1 family, in which one non-SCA1 member, bearing an intermediate *ATXN1* poly-Q expansion, was instead affected by ALS (10) (Figure 1). The coexistence of ALS and SCA1 in the same family is very rare and supports a role for *ATXN1* in the pathogenesis of ALS [10]. Traditional genetic testing for the ALS patient did not identify mutations in the ALS-causing genes *SOD1*, *C9ORF72*, *FUS*, *TARDPB*, and *ANG*. The phenotypic variability of this family is further complicated by the presence of a “central branch” in the genealogical tree (termed as MN-branch), including SCA1 patients showing early signs and symptoms of lower MN involvement, reinforcing a putative pathogenic link between SCA1 and other degenerative MN diseases, including ALS (Figure 1).

In this study, we employed a systems biology approach that integrated multiple genomic data (sequence and copy number variations, CNV) from the ALS patient along with some SCA1 family members (with or without MN phenotype), to fully investigate the complex genetic factors and pathogenic mechanisms that may contribute to motor neuron dysfunctions. 

## 2. Materials and Methods

### 2.1. The SCA1 Family with a Member Affected by ALS

Figure 1 shows the large pedigree of the SCA1 family, spanning five generations [10]. The founder could not be identified. All patients belonging to the fourth-generation of this large family underwent an accurate clinical evaluation, which confirmed in all the presence of cerebellar ataxia [10]. Interestingly, patients belonging to a branch of the family, all descendants from the patient II-4, showed early signs and symptoms of lower MN involvement (this branch, termed as “MN-branch”, is highlighted in yellow in Figure 1). The ALS patient, bearing an *ATXN1* intermediate expansion, was in this branch too. None of the other fourth-generation patients, belonging to the other branches of this family, showed signs or symptoms of lower MN degeneration, even after years-long disease duration. After psychological and genetic counseling, we obtained blood from four of the five SCA1 individuals of the MN-branch (i.e., IV-13, IV-15, IV-18, and V-4), from the ALS patient (i.e., IV-19), and from a SCA1 patient (IV-26) without lower MN signs and symptoms. All of these subjects underwent a comprehensive clinical evaluation. 

The ALS member of this family was a 47-year-old worker affected by an upper-limb onset disease while having an *ATXN1* intermediate CAG expansion. His phenotype has been already fully described [10]. Shortly, he had a history of progressive atrophy and weakness of the right hand, which quickly spread to the contralateral limb. Genetic screening for the major ALS-related genes (*SOD1*, *C9ORF72*, *FUS*, *TARDPB*, and *ANG*) was negative. Analysis of *ATXN1* showed an intermediate CAG expansion in both alleles (33/33) with no CAT interruptions. He had a rapidly evolving disease (ΔFS > 3.33). Ten months after diagnosis, he died due to myocardial infarction. IV-18 was the ALS patient’s brother (Figure 1). When he was 30 years old, unsteadiness when walking, truncal titubation and slurred speech occurred. No symptoms or signs of lower MN degeneration were reported at the onset. Genetic testing revealed an expanded CAG repeat of 33/54 in *ATXN1*. As expected, cerebellar ataxia worsened over time. By the age of 45, a full-blown ALS phenotype occurred, with rapidly progressive distal, and then proximal, muscle atrophy, marked weakness, brisk reflexes in all four limbs, anarthria, dysphagia, and atrophy of the tongue with fasciculations. He died at the age of 46 years because of respiratory failure. 

The other three SCA1 members of the family, i.e., IV-13, IV-15, and V-4, showed, respectively, a clinical onset at age 37, 40, and 22 with an ataxic-spastic phenotype, and with early lower bulbar-related MN signs or symptoms (i.e., mixed ataxic and flaccid dysarthria, dysphagia and atrophy of the tongue with abundant fasciculations). Genetic testing revealed expanded *ATXN1* CAG repeats of 30/49 for IV-13, 30/49 for IV-15, and 29/57 for V-4, but not mutations in the four ALS-related genes. 

In the other SCA1 member of the family (i.e., IV-26), belonging to a branch not showing early lower MN signs or symptoms (Figure 1), the clinical onset was at the age of 25 with a prominent ataxic-spastic phenotype and slow progression. At the time of the clinical examination, she was 43-years old, wheelchair-bound, and with severe cerebellar ataxia. Genetic testing revealed expanded *ATXN1* CAG repeats of 30/45. 

All subjects described in this study signed informed consent. All samples were collected and all experiments were performed in accordance with the World Medical Association Declaration of Helsinki. This study was approved by the Ethics Committees of the University of Palermo (document 04/2019, 29 April 2019).

### 2.2. DNA Extraction 

Blood samples were obtained from all subjects. Genomic DNA was isolated from peripheral blood leukocytes, using the salting-out method, quantified by using the NanoDrop ND-1000 spectrophotometer and assessed for quality by microcapillary electrophoresis on a 2100 Bioanalyzer (Agilent Technologies, Palo Alto, CA, USA). 

### 2.3. Targeted Next-Generation Sequencing and Data Processing 

A custom-targeted NGS-based panel, encompassing 39 ALS-related genes and their 25 bp flanking regions, was used on an Ion Torrent™ Personal Genome Machine™ (PGM) sequencer (Thermo Fisher Scientific, Waltham, MA, USA), as previously described [11]. Briefly, genomic DNA (50 ng) from the ALS proband and 3 SCA1 patients (IV-15, IV-26, and V-4), two of which are SCA1-MN affected (IV-15 and V-4), was used for library preparation with the Ion AmpliSeq™ Library Kit 2.0. Libraries (Thermo Fisher Scientific) were then quantified by using the Qubit™ Fluorometer (Invitrogen, NY, USA) to determine the dilution factor resulting in a concentration of ~ 100 pM. The template preparation was performed with the Ion PGM™ Hi-Q™ View OT2 Kit on the ION OT2 instrument (Thermo Fisher Scientific), using an emulsion polymerase chain reaction (PCR) method. The enriched libraries were purified by using the Ion OneTouch™ ES (Thermo Fisher Scientific), according to the manufacturer’s protocol. The Ion Sphere Particles were loaded onto an Ion 316 chip and sequenced with the Ion PGM™ Hi-Q™ View Sequencing Kit (Thermo Fisher Scientific), using the ION PGM machine. Sequencing was performed by running 10 samples on an ION 316 chip. For more information about data related to the run, please refer to our previous work [11]. 

After sequencing, the raw data were processed by the Torrent Suite Software v5.10 (Thermo Fisher Scientific), using the standard pipeline parameters. Read alignment and variant identification were carried out with the Torrent Mapping Program (TMAP) v3.4.1 and Torrent Variant Caller (TVC) v5.0 software. The readings were mapped to the human reference sequence build GRCh37/Hg19 (Genome Reference Consortium Human Build 37, https://www.ncbi.nlm.nih.gov/assembly/GCF_000001405.13/), by limiting to the regions of target genes. The Coverage Analysis plugin was applied to all data and used to assess amplicon coverage for regions of interest. Initial variant calling from the Ion AmpliSeq™ sequencing data was generated using Torrent Suite and Ion Reporter Software (Thermo Fisher Scientific) with the plug-in “variant caller” program. To eliminate erroneous base calling, two filtering steps were used to generate the final variant calls. For basic filtering, raw variants were selected by using the following parameters: Phred quality score > 20, an average depth of total coverage > 20, each variant coverage > 5, and *p* < 0.0001. The second filter was employed by filtering out possible strand-specific errors (i.e., a mutation was detected only in one, but not both, strands of DNA).

### 2.4. Variant Prioritization and Assessment of Pathogenicity

Potential pathogenic variants were sequentially assessed by following the guidelines for the interpretation of sequence variants proposed by the ACMG [12]. In particular, we filtered only non-synonymous exonic variants that were either absent or had a minor allele frequency (MAF) ≤0.05 in ExAC Browser (http://exac.broadinstitute.org/). Moreover, the functional impact of the missense mutations was estimated by using PolyPhen-2 (http://genetics.bwh.harvard.edu/pph2/), SIFT (https://sift.bii.a-star.edu.sg/), Mutation Taster (http://www.mutationtaster.org/), and PhyloP (http://compgen.cshl.edu/phast/background.php) tools. Retained variants were revealed to be damaging by at least one of the prediction programs. 

### 2.5. NeuroArray aCGH Processing and Data Analysis 

High-resolution exon-centered analysis of CNVs was done by using an 8 × 60 K custom exon-centric *NeuroArray* platform v.1.0 (Agilent Technologies), tailored to detect single/multi-exon deletions and duplications in a large panel of genes associated with several neurological disorders, including ALS (*n* = 154) and SCA (*n* = 52) [13]. DNA labeling and hybridization on *NeuroArray* were performed according to the manufacturer’s protocol (Agilent Technologies). Briefly, DNA test from the ALS proband and some SCA1-MN affected family members (IV-18, IV-13, and IV15), together with a reference of the same sex (Euro Reference, Agilent Technologies), at the concentration of 500 ng, were double digested with RsaI and AluI for 2 h at 37 °C. After heat inactivation of the enzymes at 65 °C for 20 min, each digested sample was labeled by random priming by using the genomic DNA Enzymatic Labelling Kit (Agilent Technologies) for 2 h, using Cy5-dUTP for patient DNAs and Cy3-dUTP for reference DNAs. Labeled products were column purified by using the SureTag DNA Labeling Kit Purification Columns (Agilent Technologies). After probe denaturation and pre-annealing with Cot-1 DNA, hybridization was performed at 65 °C with rotation for 24 h. After two washing steps, arrays were scanned at 3 µm resolution, using an Agilent G4900DA SureScan Microarray Scanner System, and aCGH image data were processed by using Agilent’s Feature Extraction software to assess the array spot quality as well as check signal and background intensity statistics in the default setting. 

Feature-extracted raw data were normalized, analyzed, and visualized, using Agilent CytoGenomics v. 4.0.3.12 software (Agilent Technologies). Briefly, after filtering for saturated and non-uniform probes, data were normalized by GC correction with a window size of 2 kb and Diploid Peak Centralization. The Centralization Normalization Algorithm with a threshold of 6.0 and a bin size of 10 was also used for detecting aberrant regions or regions of constant CNVs. Aberrations were detected by the Aberration Detection Method II algorithm (ADM-2), with a sensitivity threshold of 6.0 and moving an average window of 2 Mb, which permits to identify all aberrant intervals in a given sample with consistently high or low log-ratios based on the statistical score. An aberration filter was applied for identifying copy number alterations; changes were considered as true positive events with a minimum log2 ratio test/control of ±0.25 and a minimum of 3 consecutive probes. A positive statistical score meant an amplification, while a negative score indicated a deletion.

Human reference sequence hg19 assembly was used to define the genomic coordinates of detected CNVs. To assess the effective relations between the detected CNVs and ALS pathogenesis, we compared identified aberrant regions with those previously associated with ALS via screening of publicly available databases and the published literature. Once identified, aberrations were manually assessed and classified into different categories (pathogenic, benign, likely benign, likely pathogenic, and uncertain clinical significance), according to the American College of Medical Genetics and Genomics (ACMG) guidelines for CNVs [12]. In addition, all CNVs that are absent both from the Database of Genomic Variants (DGV) or that are reported in very low frequency (<1%) were considered as rare.

### 2.6. CNV Validation

Ad hoc quantitative real-time polymerase chain reaction (qPCR) assays were performed to validate genomic imbalances detected by the *NeuroArray*. Briefly, we used DNA extracted from peripheral blood samples of 3 patients (IV-13, IV-18, and IV-19), assayed by *NeuroArray*, and additional 3 samples including a SCA1-MN patient (V-4) and two “pure” SCA1 family members (IV-2 and IV-26). Primers flanking the putative exonic imbalances were designed by using the PrimerBlast tool (http://www.ncbi.nlm.nih.gov/tools/primer-blast/). RT-qPCR was performed in triplicate, using the LightCycler 1.5 (Roche Diagnostics, Germany). Cycling conditions were 95 °C for 15 s, followed by 40 cycles of 95 °C (5 s), 60 °C (15 s) and one cycle of 95 °C (15 s), 60 °C (60 s), and 95 °C (15 s). The relative quantification was measured by using the ∆∆Ct method that requires a healthy control sample (diploid) as a calibrator in all amplifications. As calibrator control, we used the same DNA reference hybridized in the *NeuroArray* experiments. A control gene, checked as normal double-copies on *NeuroArray*, was used as a reference for normalization. Moreover, 2-ΔΔCt ≥ 1.4 or ≤0.6 was defined as copy number gain or loss, respectively, whereas 2-ΔΔCt values from 0.8 to 1.2 were considered as normal diploid. PCR products were visualized by agarose gel electrophoresis.

### 2.7. Functional Enrichment Analysis of the CNV-Associated Gene Sets

To analyze and visualize functional and biological shared or specific features with respect to ALS and/or MN phenotypes, genes located in the identified CNV regions, both in ALS patients and its SCA1-MN relatives, were used for ontology and pathway enrichment analyses. In particular, the CNV-associated gene sets for each patient were submitted to the bioinformatics resource ToppGene Suite (https://toppgene.cchmc.org/) and ToppCluster (https://toppcluster.cchmc.org/), which allow performing a gene list functional enrichment based on Gene Ontology, KEGG, Reactome, and Panther pathway [14,15]. The extent of statistical enrichment for each functional group was determined by applying a Fisher’s Exact Test then corrected by the Benjamini-Hochberg False Discovery Rate (FDR) procedure and the number of enriched genes > 2 and *p* < 0.05 were selected as cut-off criteria to consider statistically significant differences. 

### 2.8. Genomic Scale Profiling of ALS by Whole-Exome Sequencing

The ALS patient’s specific genomic profile was further investigated by performing a pathway enrichment analysis of whole-exome sequencing (WES) data, to verify also if a mutational signature extracted from WES data confirmed our previous pathway-based CNV characterization of the disease phenotype. Specifically, genomic DNA of the ALS patient was sequenced, using the Ion Chef and Ion S5 Next Generation Sequencing platform (Thermo Fisher Scientific), following the manufacturer’s recommended protocol. In brief, 100 ng of DNA was used as starting quantity for library preparation by using the Ion Ampliseq Exome RDY Kit 1 × 8 that permits to obtain a coverage >97% of the Consensus Coding Sequences (CCDS), >90% base on-target, and >90% coverage uniformity. After barcode ligation using an Ion Xpress Barcode Adapters kit (Thermo Fisher Scientific), library samples were purified by using Agencourt AMPure XP reagent (Beckman Coulter, Brea, CA, USA) and subsequently inspected for quality by using Bioanalyzer 2100 instrument and DNA High Sensitivity kit (Agilent Technologies). The final sequencing libraries were quantified, using a Qubit 4.0 Fluorometer (Thermo Fisher Scientific) and subsequently diluted to a concentration of 100 pM before being clonally amplified on the Ion Chef System, using the Ion 540 Chip kit-Chef before loading on an Ion 540 Chip with an additional sample for sequencing with the Ion S5 platform (Thermo Fisher Scientific). 

Data were processed by using Ion Torrent platform-specific pipeline software, Torrent Suite Software v5.10 (Thermo Fisher Scientific), using Germ Line—Low Stringency parameters to generate sequence reads, trim adapter sequences, filter, and remove poor signal-profile reads. The alignment was done against the human reference sequence build GRCh37/Hg19 (Genome Reference Consortium Human Build 37, https://www.ncbi.nlm.nih.gov/assembly/GCF_000001405.13/). Initial variant calling from the Ion AmpliSeq™ sequencing data was generated by Torrent Suite and Ion Reporter Software with a plug-in “variant caller” program. To eliminate erroneous base calling, two filtering steps were used to generate the final variant calling. For basic filtering, raw variants were selected by using the following parameters: Phred quality score > 20, an average depth of total coverage > 20, each variant coverage > 5, and *p* < 0.0001. The second filter was employed by filtering out possible strand-specific errors (i.e., a mutation was detected only in one, but not both, strands of DNA). 

Exome variants were further filtered for rare non-coding variants, nonsynonymous SNVs, frameshift INDELs, MNVs, and SNVs/INDELs affecting stop codons and splice sites. The KEGG pathway enrichment analysis of the genomic regions affected by the remaining variants was carried out, using the ToppFun tool (https://toppgene.cchmc.org/enrichment.jsp) to identify the most relevant functional pathways plausibly involved in driving the ALS phenotype. Pathway enrichment analysis was performed by using the human genome as background and applying a corrected Fisher’s exact test with a *p* < 0.05 as a threshold to consider statistically significant differences.

### 2.9. Visualization of the Protein–Protein Interaction Network

To further investigate the interaction and correlation between genes harboring rare non-coding and/or potentially pathogenic coding variants we constructed an extended protein–protein interaction (PPI) network of their encoding products by using the STRING database and visualized with the Cytoscape v.3.7.1 software. The extended network was constructed by using the candidate genes as seed molecules and setting a high level of confidence between molecular interactions (high confidence score of at least 0.8) and a maximum number of interactions to 100. To identify the “Hub” nodes, a network topology analysis was performed by using the Cytoscape plug-in NetworkAnalyzer based on topological parameters. The relative importance of the genes in each network, meaning their ability to hold together the communicating nodes in a biological network, was determined based on the node centrality measure setting the topological parameter “node degree” ≥10. Nodes with a high degree (hub genes) represented genes having important biological functions: the higher the value, the higher the relevance of the gene in connecting regulatory molecules. Likewise, values of edge betweenness were mapped with the edge size: high values of this parameter correspond to large edge size. After removing the nodes with a score of 0, the final PPI network was visualized based on node degree and edge betweenness parameters. Moreover, an additional PPI network was constructed to assess possible interactions between *ATXN1* and other SNP- and CNV-driven genes in the ALS proband. This *ATXN1*-centered network was constructed by setting a moderate level of confidence between molecular interactions (confidence score of at least 0.4) and a maximum number of interactions to 100. 

## 3. Results

### 3.1. Identification of Potentially Disease-Causing Sequence Variants in ALS Associated Genes

We utilized a 39-ALS gene panel NGS-based targeted sequencing [11] in the ALS patient and some SCA1 individuals (with or without lower MN signs and symptoms), to search for genetic variants that, in addition to or in combination with *ATXN1*, may influence ALS phenotype or susceptibility. Our analysis identified a total of 19 non-synonymous variants in the ALS patient (Supplementary Materials Table S1). After applying the filtering criteria (MAF < 5% and in silico pathogenicity prediction), 6 gene variants in *ALS2*, *CCNF*, *NEFH*, *NEK1*, *SETX*, *TAF15* genes were selected (Table 1). Among these, we distinguished three novel coding variants (previously not cataloged in dbSNP), including two missense SNVs in ALS2 and *NEFH* genes and a frameshift insertion in *TAF15* exon 15 that was selectively found in the ALS patient (absent in SCA1 individuals) (Table 1). Of note, the majority of variants (4/6) detected in the ALS proband were shared with SCA1 family members belonging to the MN-branch but not with the “pure” SCA1 patient (IV-26), suggesting these variants may contribute to induce motor neuron dysfunctions (Table 1). 

### 3.2. Identification of Copy Number Variants Related to ALS Phenotype

In addition to point mutations in ALS driver genes, we searched for numerical chromosomal aberrations in the ALS patient and SCA1 patients of the MN-branch. Using a customized exon-centric high-resolution aCGH platform “*NeuroArray v. 1.0*” [13,16], we identified 16 significant CNVs in the ALS patient, five of which encompass known ALS genes (*VPS54*, *SCN7A*, *CHMP2B*, *LPA*, *C9ORF72*) (Table 2). According to ACMG guidelines (12), 9 variants were classified as likely pathogenic, 4 as likely benign and 3 of uncertain clinical significance (Table 2). Most of the observed CNVs were rare deletions, including 3 novel deletions affecting parts of *C9orf72*, *SCN1A*, and *WRN* genes that did not overlap with any CNVs previously described in the DGV (Table 2). The large majority of these alterations were not detected in SCA1 patients, suggesting their specific relevance in ALS etiopathogenesis (Table 2 and Supplementary Materials Tables S2–S4). Four of the 15 deletions in the ALS patient were also detected in SCA1 family members belonging to MN-branch, suggesting these alterations may play a role in motor neuron dysfunction (Table 2 and Supplementary Materials Tables S2–S4). In particular, deletion of the *NSF* gene was identified and validated in the ALS patient as well as in all SCA1 patients of the MN-branch (IV-18, IV-13, IV-15, and V-4), while it was absent in patients with a “pure” SCA1 phenotype (IV-26 and IV-2).

### 3.3. Functional and Pathway Enrichment Analysis of CNV-Driven Genes Identified Disease-Specific Molecular Signatures 

To explore the overall contribution of CNV-driven dysregulated genes to motor neuron dysfunctions, we performed a functional enrichment analysis to characterize their aberrant functions in the ALS patient and SCA1 patients with MN signs. Gene Ontology and pathway enrichment analyses revealed both common and distinctive biological processes and signaling cascades significantly altered in ALS and SCA1-MN patients (Table 3 and Supplementary Materials Table S5). In particular, the regulation of synaptic transmission and membrane trafficking were overrepresented in all patients, whereas endocytosis, regulation of growth rate and cytoskeleton organization were specifically affected in the ALS patient (Table 3 and Supplementary Materials Table S5). 

### 3.4. WES-Based Mutation Profile Confirms the Functional Impact of Axon Guidance, Cell Adhesion and Immune Response in ALS

**Figure 2:**
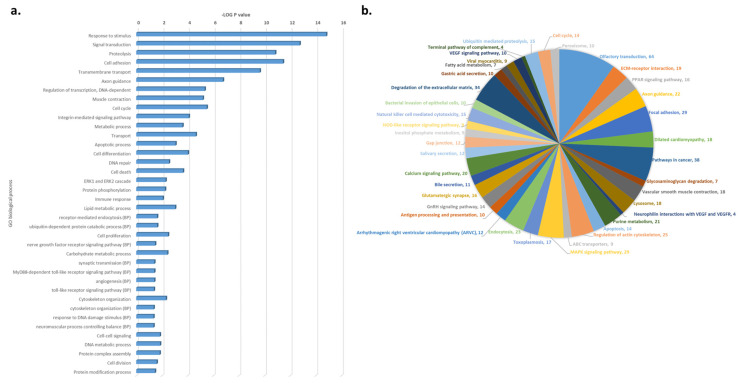
Functional enrichment analysis of the SNP mutant genes identified in the ALS patient by WES. The most representative (**a**) GO and (**b**) pathways terms that exhibit statistically significant differences are shown in the graphic (Fisher’s exact test, FDR; *p* < 0.05).

As both targeted panel sequencing and *NeuroArray* aCGH focuses on a limited set of known disease-associated genes, the ALS patient’s specific genomic profile was further investigated by performing WES analysis looking for novel disease-causing genes and mechanisms potentially associated with ALS pathogenesis (Supplementary Materials Table S6). Due to the complexity to identify likely damaged genes among the large number of genetic variants discovered by WES and considering that genes do not play independent roles but form biological function and pathway networks through their intricate interactions, we applied a systems biology approach to prioritize genes with variants and investigate their potential functional impact on ALS. Interestingly, this functional analysis confirmed our previous CNV-based molecular characterization of the ALS patient, highlighting cytoskeleton organization, transmembrane transport, axon guidance, and cell adhesion as the most significantly enriched GO terms and focal adhesion, extracellular matrix organization, and autophagy-lysosome as the most overrepresented pathways within the set of mutated genes in the ALS patient (Figure 2 and Supplementary Materials Table S7). 

**Figure 3:**
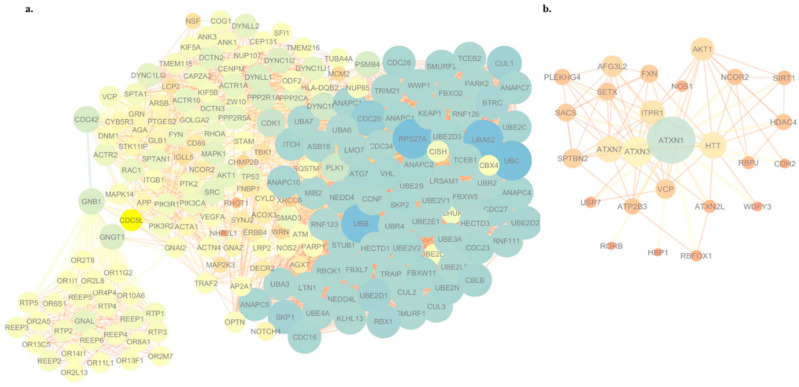
Integrative network analysis of the genetic variants in the ALS patient. (**a**) Protein–protein interaction (PPI) network of hub genes (degree ≥ 10) affected by rare missense mutations in the ALS patient. The node size was proportional to the degree and the edge width was proportional to the combined score based on the STRING database. Genes with higher degree values have a stronger capacity of modulating adjacent genes. (**b**) *ATXN-1*-gene-centered subnetwork. The subnetwork includes most of the *ATXN-1*-related genes involved in the Notch-specific miRNA-TF regulatory network. The node size was proportional to the degree, and the edge width was proportional to the combined score based on the STRING database.

Genes affected by rare and/or potentially pathogenic variants identified in the ALS patient were mapped to the PPI network to further investigate how these genes could jointly confer ALS susceptibility (Supplementary Materials Table S8 and Figure S1). In addition, to support the pathogenic role of genes already known to be associated with ALS (i.e., *CHMP2B*, *MAPT*, *DYNC1H1*, *ERBB4*, *GRN*, *OPTN*, *SQSTM1*, *TBK1*, *TUBA4A*, *VCP*, and *VEGFA*), our WES-based network analysis identified new potential causal genes, including RPS27A, UBA52, UBC, and *UBB* that were identified as the most significant bottleneck proteins connecting different complexes or pathways in the network (Figure 3a). Of note, 25 mutated genes in the ALS patient showed a direct interaction with *ATXN1* (Figure 3b). Between them, of note, we distinguished some SCA genes (*ATXN3*, *ATXN7*, and *ATXN2L*), as well as two genes already known to be associated with ALS (*SETX* and *VCP*) (Figure 3b).

## 4. Discussion

This study aimed to provide comprehensive genomic profiling of a clinically definite ALS individual, bearing an intermediate *ATXN1* poly-Q expansion and belonging to a large SCA1 family with a “central branch” of SCA1 patients showing early signs and symptoms of lower MN involvement [10]. In particular, we used a set of parallel high-throughput genomic approaches, including (i) an NGS-based targeted mutational analysis focused on a restricted number of ALS genes (exons and flanking regions) and characterized by high coverage, (ii) a high-resolution exon-targeted CNVs analysis of ALS-related genes expanded to those concerning other neurological disorders, and (iii) GO- and pathway-based analyses of genetic variants identified in the ALS patient by *NeuroArray* aCGH and WES.

With regard to targeted NGS panel analysis of the ALS patient, we identified six potentially deleterious exonic variants in genes traditionally associated with ALS (Table 1). Among these, we distinguished three novel heterozygous variants (c.238A > C in *ALS2*, c.2279A > G in *NEFH*, and c.1296_1297in *TAF15*) predicted to be pathogenic, using *in silico* tools (Table 1 and Supplementary Materials Table S1). In particular, the frameshift mutation in exon 15 of the TAF15 gene was detected exclusively in the ALS patient (absent in all SCA1 samples), suggesting a selective role for this variant in disease etiopathogenesis (Table 1). Similar to other ALS-linked RNA-binding proteins (including ATXN1) mutated *T*AF15 is more aggregation-prone in vitro, supporting a key role for RNA metabolism defects in ALS and suggesting that this class of proteins might contribute very broadly to the pathogenesis of the disease [15,16]. In addition to ALS-specific variants, two novel pathogenic missense mutations were found in *NEFH* and *ALS2* genes in the ALS patient and the SCA1-MN member (IV-15) but absents in the SCA1 patient without MN signs (IV-26), suggesting these variants or their combination may contribute to the MN phenotypic heterogeneity observed amongst family members (Table 1). In particular, previous studies reported an association between a lot of loss-of-function *NEFH* variants and motor neuronal injury, showing how the expression of mutated *NEFH* may interfere with neurofilament assembly via protein sequestration and cause neurotoxicity [17,18,19,20]. Several *ALS2* missense or in-frame deletion mutations have been demonstrated to be associated with different but relatively similar motor neuron disorders, including ALS [21]. *ALS2* is a gene responsible for producing the protein alsin, a guanine nucleotide exchange factor for the small GTPase protein Rab5, which is involved in neurite outgrowth and endosomal trafficking and whose loss-of-function leads to increased degradation, decreased signaling, and decreased turnover of membrane components that may underlie disease pathology [22,23].

Using the customized *NeuroArray* aCGH platform, we identified a list of chromosomal aberrations in genes previously implicated in a wide range of neurological disorders that could be responsible for motor neuron degeneration phenotypes observed in this family (Table 2). In particular, our analysis identified nine likely pathogenic deletions, some of which encompassed ALS genes, including a novel deletion in the *C9ORF72* gene that was detected exclusively in the ALS patient but absent in other SCA1 family members (Table 2). Apart from *C9ORF72* hexanucleotide (GGGGCC) repeat expansion, representing the most common genetic cause of both familial and sporadic ALS, decreased *C9ORF72* mRNA levels were found in patient-derived cells and tissue; the deletion of this gene locus leads to disruption in endosomal trafficking, synaptic vesicle function, regulation of the actin cytoskeleton, and formation of autophagosome, resulting in MN degeneration [19,20]. Moreover, previous results indicated that loss-of-function of C9ORF72 not only induces a deleterious effect on neuron survival by itself but synergizes with polyQ-Ataxin-2 toxicity to induce motor neuron dysfunction and neuronal cell death [19]. Considering that ataxin-1 has similarities with ataxin-2, our data prompted us to explore the potential contribution of a multiple-hit pathological mechanism in ALS involving *C9ORF72* haploinsufficiency and neuronal toxicity resulting from mutant *ATXN1*. 

In the context of a systems biology view, the assessment of the functional impact of CNVs detected in ALS and SCA1-MN patients revealed both specific and common disease-associated molecular signatures (Table 3). In particular, the regulation of synaptic transmission and vesicular trafficking to lysosomes were significantly enriched both in the ALS patient and SCA1-MN family members, suggesting that these mechanisms may be involved in motor neuron degenerative processes characterizing these individuals (Table 3). In this regard, the deletion of *NSF*, a gene encoding an enzyme that plays an important role in synaptic vesicle release, was found both in ALS and SCA1-MN patients (but absent in patients with “pure” SCA1 phenotype) (Table 2). Although further studies are needed to deepen the role of *NSF* in MN degenerative processes, decreased expression levels of *NSF* were previously found in the motor cortex of SALS patients, and its depletion is known to attenuate calcium-dependent delivery of adherens junction proteins to intercellular junctions, thereby producing defects of protein exocytosis and resulting in severe neuronal damage and eventually neuronal death [20,21,22]. Functional analysis also identified endosomal transport and regulation of cytoskeleton organization as signaling pathways selectively deregulated in the ALS patient, supporting previous reports indicating dysregulated autophagy and microtubule dynamic instability as biological processes implicated in familial and sporadic insults in ALS [23,24]. Of note, the *NeuroArray*-based genomic signature of the ALS proband was reproduced by functional enrichment analysis of WES data generated for this patient, further supporting the role of cytoskeletal defects in axons and aberrant transmembrane transport, as well as immune response and regulation of ubiquitin-mediated proteolysis in disease pathogenesis (Figure 2 and Supplementary Materials Table S7). The involvement of dysregulated immune system, proteasome activity, and altered cytoskeleton remodeling in driving the ALS phenotype also emerged in the PPI network analysis that highlights the central role of multiple ubiquitin coding genes (i.e., *UBA52*, *RPS27A*, *UBC*, and *UBB*) identified as the most interconnected nodes in the network (Figure 3a). Substantial contribution by these molecules to ALS pathogenesis was previously described [25], and their abnormal processing and assembling were demonstrated to confer cytotoxic effects, contributing to motor neuronal damage [26,27,28]. Interestingly, network analysis also revealed a strong interaction between some mutated genes in the ALS patient and *ATXN1* (Figure 3b). Among these, we distinguished SNV and indel variations in some SCA genes (*ATXN3*, *ATXN7*, and *ATXN2L*), as well as known ALS-linked genes (i.e., *SETX* and *VCP*), suggesting that genetic variations in these genes may play an additive role in potentiating the neuropathological effects mediated by *ATXN1* repeat expansion (Figure 3b and Supplementary Materials Table S6). 

Our integrative genomic analysis allowed us to comprehensively investigate genetic variations and molecular mechanisms occurring in ALS and SCA1-MN patients of a large SCA1 family, revealing specific and genomic signatures that may explain progressive degeneration of motor neurons observed in these patients. Overall, our study supports the utility of an individual genomics approach in identifying genetic modifiers for complex disorders characterized by different molecular mechanisms and extreme phenotypic heterogeneity.

## Figures and Tables

**Table 1:** Potential pathogenic variants identified in the ALS patient by targeted panel sequencing.

											Genotype		
Chr	Pos	Reference	Gene Symbol	Variant Class	Exonic	AA Change (Reference Gene)	ExAC_Freq	dbSNP	Polyphen/SIFT Prediction	IV-19 (ALS)	IV-15 (SCA1-MN)	V-4 (SCA1-MN)	V-26 (SCA1)
2	202626479	T	ALS2	nonsynonymous SNV	4	p.Ser80Arg (NM_00113574)	.	.	Damaging/Pathogenic	T/G	T/G	-	-
16	2498978	G	CCNF	nonsynonymous SNV	10	p.Arg406Gln (NM_001323538)	0.00	rs146438723	Damaging/Pathogenic	G/A	G/A	-	G/A
22	29885908	A	NEFH	nonsynonymous SNV	4	p.Asp760Gly (NM_021076)	.	.	Damaging/Pathogenic	A/G	A/G	-	-
4	170428901	C	NEK1	nonsynonymous SNV	22	p.Ala626Thr (NM_001199397.1)	0.05	rs33933790	Damaging/Pathogenic	C/T	-	C/T	-
9	135204010	T	SETX	nonsynonymous SNV	10	p.Lys992Arg (NM_001351527)	0.02	rs61742937	Damaging/Pathogenic	T/C	-	T/C	-
17	34171599	-	TAF15	frameshift insertion	15	p.Ser433fs (NM_139215)	.	.	Damaging/Pathogenic	C/CG	-	-	-

Chromosome coordinates are given according to hg19 assembly (UCSC genome browser https://genome.ucsc.edu/). *Chr: Chromosome; Pos: Position.*

**Table 2:** Copy number variations (CNVs) identified in the ALS proband by *NeuroArray* aCGH.

Chr	Start	Stop	Probes	Log2 Ratio (Test/Control)	*p*-Value	Gene	Common CNV (DGV Frequency)	qPCR Validation	Clinical Interpretation	Detected in SCA1-MN Family Members (Patient Code)
1	98,164,881.00	98,187,177.00	6	−0.63967	1.54 × 10^−10^	*DPYD*	Yes (0.005–0.04%)		Likely pathogenic	No
2	64,146,992	64,211,176.00	25	−0.3406	5.01 × 10^−13^	*VPS54*	Yes (0.003%)	X	Likely pathogenic	Yes (IV-18 *, IV-13, IV-15)
2	166,852,501	166,870,328.00	14	−0.5323	4.49 × 10^−14^	*SCN1A*	Not		Likely pathogenic	No
2	166,911,120	166,913,035.00	5	−0.67504	5.51 × 10^−11^	*SCN1A*	Yes (0.1%)		Likely pathogenic	No
2	167,328,904	167,334,011.00	6	−0.75482	4.24 × 10^−11^	*SCN7A*	Yes (0.005–1%)	X	Likely pathogenic	No
2	179,536,740	179,540,750.00	9	−0.57381	1.35 × 10^−10^	*TTN*	Yes (0.4%)		Likely pathogenic	No
3	87,299,007	89,814,870.00	10	−0.45231	1.51 × 10^−10^	*CHMP2B, EPHA3*	Yes (0.003–1%)	X	Uncertain clinical significance	No
3	93,772,085	113,652,487.00	20	−0.28537	4.68 × 10^−11^	*ARL13B*	Yes (0.006–0.03%)		Uncertain clinical significance	No
6	161,026,135	161,067,305.00	17	0.477799	2.80 × 10^−21^	*LPA*	Yes (>70%)		Likely benign	No
7	17,362,101	17,375,411.00	12	−0.64847	3.43 × 10^−22^	*AHR*	Yes (0.0034–0.04%)		Likely pathogenic	Yes (IV-13)
8	30,947,985	30,999,316.00	23	−0.30766	1.60 × 10^−11^	*WRN*	Not		Likely pathogenic	No
9	27,558,554	27,573,862.00	13	−0.39928	2.08 × 10^−11^	*C9orf72*	Not	X	Likely pathogenic	No
10	70,892,631	70,931,418.00	15	−0.36405	4.32 × 10^−10^	*VPS26A*	Yes (>10%)		Likely benign	No
17	44,301,037	44,771,900.00	16	−0.61264	4.50 × 10^−29^	*NSF*	Yes	X	Likely benign	Yes (IV-18, IV-13, IV-15)
21	38,791,571	38,865,493.00	15	−0.35809	8.53 × 10^−11^	*DYRK1A*	Yes (0.003–5%)		Uncertain clinical significance	Yes (IV-13)
X	108,902,635	108,906,573.00	6	−0.73236	5.03 × 10^−10^	*ACSL4*	Yes (2%)		Likely benign	No

Chromosome coordinates are given according to hg19 assembly (UCSC genome browser https://genome.ucsc.edu/). ALS genes inside CNVs are depicted in bold. Log2 ratio (test/ctrl) = duplications (red), deletions (blue). Database of Genomic Variants (DGV) frequency indicates the population frequency of respective CNV in the Database of Genomic Variants (DGV, http://dgv.tcag.ca/dgv/app/home). Clinical interpretation was manually assessed and classified into different categories, according to the American College of Medical Genetics and Genomics (ACMG) guidelines for CNVs. Of note, some of the alterations reported here were previously described to validate the *NeuroArray* v.1 platform as a genomic profiling assay for ALS [13]. * Sample IV-18 reported a duplication in the same genomic region.

**Table 3:** Functional enrichment analysis of CNV-associated gene sets in ALS and SCA1-MN patients.

**GO Biological Processes**	**ALS**	**SCA IV-18**	**SCA IV-13**	**SCA IV-15**
growth				
vacuolar transport				
circadian rhythm				
peptidyl-tyrosine modification, phosphorylation				
regulation of hydrolase activity				
cell morphogenesis				
regulation of endocytosis				
regulation of growth rate				
lysosomal transport				
membrane depolarization during action potential				
regulation of microtubule cytoskeleton organization				
neuronal action potential				
regulation of microtubule-based process				
endosomal transport				
Golgi vesicle transport				
autophagy				
regulation of microtubule cytoskeleton organization				
**Pathway Name**	**ALS**	**SCA IV-18**	**SCA IV-13**	**SCA IV-15**
Interaction between L1 and Ankyrins				
Phase 0 - rapid depolarization				
Retrograde transport at the Trans-Golgi-Network				
Muscle contraction				
L1CAM interactions				
Cardiac conduction				
Intra-Golgi and retrograde Golgi-to-ER traffic				
Axon guidance				
Membrane Trafficking				
Fatty acid, triacylglycerol, and ketone body metabolism				
Endocytosis				
Vesicle-mediated transport				

Green boxes represent processes significantly enriched in ALS and SCA1-MN patients; gray bars indicate no significant change.

## Supplementary Materials

The following are available online at https://www.mdpi.com/2075-4426/10/4/262/s1. Figure S1. PPI network including genes affected by rare non-coding and/or potentially pathogenic coding variants identified in the ALS patient. The node size was proportional to the degree and the edge width was proportional to the combined score based on the STRING database. Genes with higher degree values have a stronger capacity of modulating adjacent genes. Table S1. Results of customized targeted ALS NGS panel in the ALS patient. Table S2. CNVs identified in the SCA1-MN patient IV-18 by NeuroArray aCGH. Table S3. CNVs identified in the SCA1-MN patient IV-15 by NeuroArray aCGH. Table S4. CNVs identified in the SCA1-MN patient IV-13 by NeuroArray aCGH. Table S5. Functional enrichment analyses of the CNV-associated gene sets in ALS and SCA1-MN family members. Table S6. Results of whole-exome sequencing in the ALS proband. Table S7. Functional enrichment analyses of the CNV-associated gene sets in ALS and SCA1-MN family members. Table S8. Protein–protein interaction network properties.

## Abbreviations

ALS	amyotrophic lateral sclerosis
ATXN-1	ataxin-1
ATXN-2	ataxin-2
ATXN-3	Ataxin 3
ATXN7	Ataxin 7
ATXN2L	Ataxin 2 Like
SCA1	spinocerebellar ataxia type I
SCA2	spinocerebellar ataxia type II
aCGH	comparative genomic hybridization array
WES	whole-exome sequencing
NGS	next-generation sequencing
SOD1	Cu/Zn superoxide dismutase-1
FUS	fused in sarcoma/translocated in liposarcoma
TARDBP/TDP-43	transactive response DNA binding protein 43 kDa
C9ORF72	chromosome 9 open reading frame 72
ANG	Angiogenin
SNV	single-nucleotide variant
CNV	copy number variant
MN	motor neuron
CSF	cerebrospinal fluid
MRI	magnetic resonance imaging
EMG	Electromyography
MUPs	motor unit potentials
PSWs	positive sharp waves
DSF	disease-free survival
Cy5	cyanine 5
Cy3	cyanine 3
dUTP	deoxyuridine triphosphate
ADM-2	aberration detection method II algorithm
FDR	Flase Discovery Rate
CNVD	Copy Number Variation in Disease
ACMG	American College of Medical Genetics
DGV	Database of Genomic Variants
UTR	untranslated region
ncRNA	non coding RNA
LoF	loss-of-function
MAF	minor allele frequency
EVS	Exome Variant Server
HGMD	Human Gene Mutation Database
LOVD	Leiden Open Variation Database
OMIM	Online Mendelian Inheritance in Man
GO	Gene Ontology
BP	biological process
MF	molecular function
CC	Cellular Component
PPI	protein–protein interaction
SCN1A	sodium voltage-gated channel alpha subunit 1
WRN	Werner syndrome ATP-dependent helicase
LPA	Lipoprotein(A)
SCN7A	sodium voltage-gated channel alpha subunit 7
EPHA3	Ephrin type-A receptor 3
EPHA1	Ephrin type-A receptor 1
EPHA2	Ephrin type-A receptor 2
EPHA4	Ephrin type-A receptor 4
EPHA5	Ephrin type-A receptor 5
EPHA6	Ephrin type-A receptor 6
EPHB1	Ephrin type-B receptor 1
EPHX1	Epoxide Hydrolase 1
CHMP2B	Charged Multivesicular Body Protein 2B
MAPT	Microtubule Associated Protein Tau
VPS54	Vacuolar Protein Sorting-Associated Protein 54
TTN	Titin
AHR	Aryl Hydrocarbon Receptor
NSF	N-Ethylmaleimide Sensitive Factor, Vesicle Fusing ATPase
ARL13B	ADP Ribosylation Factor Like GTPase 13B
MNVs	Multiple nucleotide variants
INDEL	Insertion/Deletion
CAPN2	Calpain 2
DCC	DCC netrin 1 receptor
LRRK1	Leucine Rich Repeat Kinase 1
MMRN1	Multimerin 1
NOS2	Nitric Oxide Synthase 2
ARSB	Arylsulfatase B
TMEM216	Transmembrane Protein 216
LRP2	LDL Receptor Related Protein 2
NOTCH4	Notch Receptor 4
PCK1	Phosphoenolpyruvate Carboxykinase 1
TNK1	Tyrosine Kinase Non Receptor 1
CNTN4	Contactin 4
DIAPH3	Diaphanous Related Formin 3
CDH13	Cadherin 13
DYNC1H1	Dynein Cytoplasmic 1 Heavy Chain 1;
CPZ	Carboxypeptidase Z
NEFH	Neurofilament Heavy
TRPM7	Transient Receptor Potential Cation Channel Subfamily M Member 7
VPS26A	Vacuolar Protein Sorting-Associated Protein 26, Retromer Complex Component A
KIF1A	Kinesin Family Member 1A
CYP2D6	Cytochrome P450 Family 2 Subfamily D Member 6
GLE1	GLE1 RNA Export Mediator
SETX	Senataxin
TAF15	TATA-Box Binding Protein Associated Factor 15
ALS2	Alsin Rho Guanine Nucleotide Exchange Factor ALS2
CCNF	Cyclin F
PFN1	Profilin 1
CEP112	Centrosomal Protein 112
VAPB	Vesicle-Associated Membrane Protein Associated Protein B and C
AVS	ALS variant server
DPYD	Dihydropyrimidine Dehydrogenase
SNARE	soluble NSF attachment protein receptor
MATR3	Matrin 3
ERBB4	Erb-B2 Receptor Tyrosine Kinase 4
NEK1	NIMA Related Kinase 1
OPTN	Optineurin
SQSTM1	Sequestosome 1
TBK1	TANK Binding Kinase 1
TUBA4A	Tubulin Alpha 4a
VCP	Valosin Containing Protein
VEGFA	Vascular Endothelial Growth Factor A
RPS27A	Ribosomal Protein S27a
UBA52	Ubiquitin A-52 Residue Ribosomal Protein Fusion Product 1
UBC	Ubiquitin C
UBB	Ubiquitin B
ANO2	Anoctamin 2
CHCHD10	Coiled-Coil-Helix-Coiled-Coil-Helix Domain Containing 10
ELAVL4	Embryonic Lethal, Abnormal Vision, Drosophila (ELAV)-Like 4
GRIN1	Glutamate Ionotropic Receptor NMDA Type Subunit 1
NMDA	N-methyl-D-aspartate
ZNF280A	Zinc Finger Protein 280A
C22orf46	Chromosome 22 Open Reading Frame 46
APEH	Acylaminoacyl-Peptide Hydrolase
MST1	Macrophage Stimulating 1
DCHS2	Dachsous Cadherin-Related 2
FAT1	FAT Atypical Cadherin 1
BACE1	Beta-Secretase 1
