# Supplementary material for: Genomic Portrait of a Sporadic Amyotrophic Lateral Sclerosis Case in a Large Spinocerebellar Ataxia Type 1 Family
Source: J Pers Med. 2020 Dec 2;10(4):262. doi: 10.3390/jpm10040262 (PMC7712010; doi:10.3390/jpm10040262)
Supplement: Supplementary file 1 [file jpm-10-00262-s001.pdf]

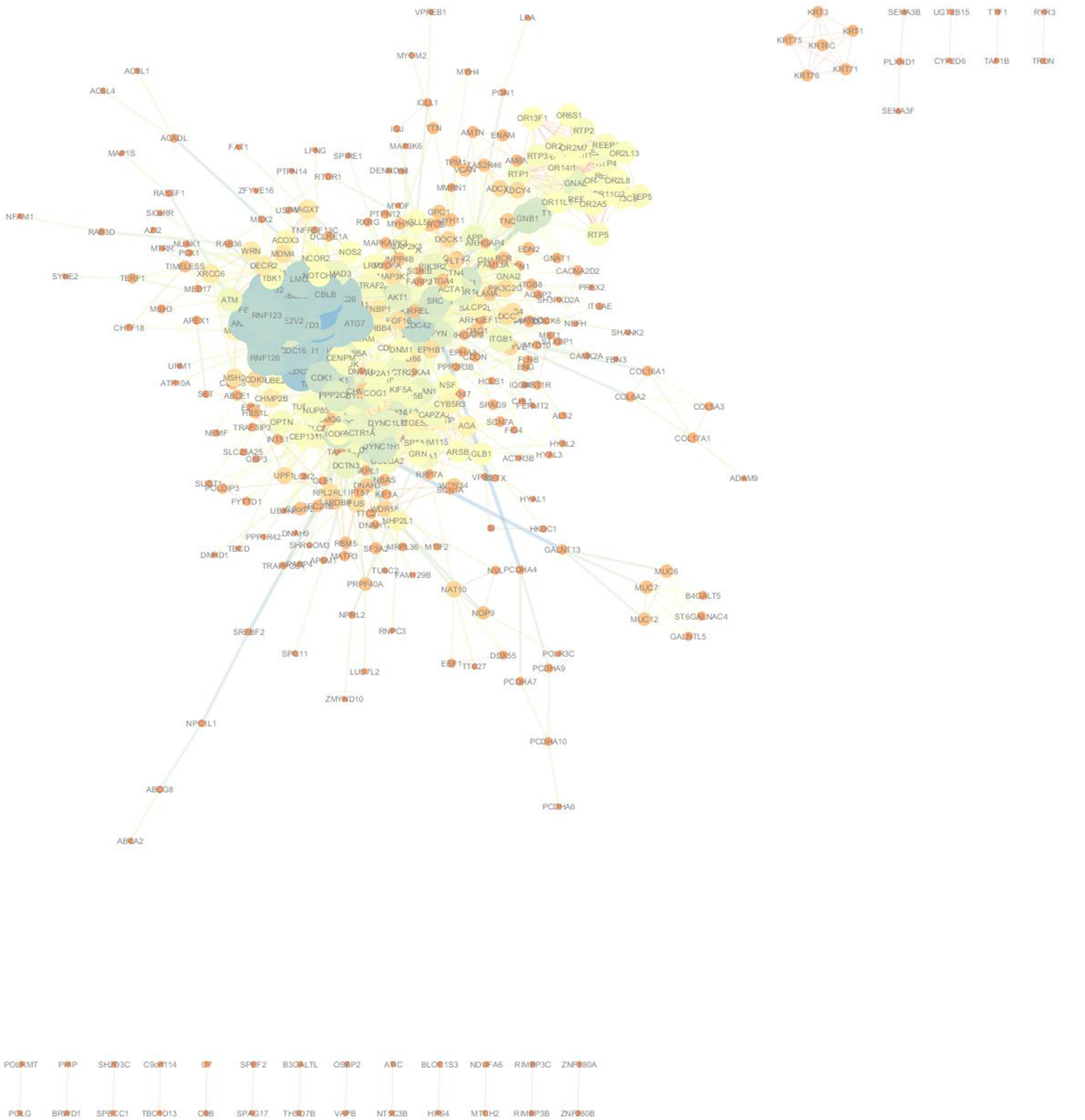
ATM
AKT1
SRC
FYN
ITGB1
CDK1
ATG7
RNF123
RNF126
CBLB
NOTCH
GNB1
RTP5
RTP2
OR6S1
OR13F1
OR2L13
OR2L8
OR2A5
ADAM9
COL17A1
MUC6
MUC7
MUC12
B4GALT5
ST6GALNAC4
GALNTL5
NAT10
NOP9
PCDHA4
PCDHA7
PCDHA9
PCDHA10
PCDHA6
NPC1L1
ABCG8
ABCA2
SREBF2
ZMYND10
SHANK2
KRT3
KRT75
KRT76
KRT71
KRT1
SEMA3B
PLXND1
SEMA3F
SYNE2
TIMELESS
XRCC6
MDM4
WRN
DOCK1
MYOM2
VPREB1

Supplementary Table 1. Results of customized targeted ALS NGS panel for the ALS patient

Chr	Start	End	Ref	Alt	Gene region	Gene symbol	Variant class	ExAC_Freq	dbSNP	SIFT_score	Polyphen2_score	Genotype	Quality	Read Depth
chr10	13166076	13166076	A	G	exonic	OPTN	nonsynonymous SNV	1,00	rs523747	.	.	hom	6072,78	641
chr12	5841733	5841733	A	C	exonic	ANO2	nonsynonymous SNV	0,10	rs1860961	0,449	0,03	het	3118,94	1465
chr14	20925154	20925154	T	G	exonic	APEX1	nonsynonymous SNV	0,43	rs1130409	1	0	het	3328,69	1444
chr16	2498978	2498978	G	A	exonic	CCNF	nonsynonymous SNV	0,00	rs146438723	0,053	0,977	het	1012,09	419
chr17	64023624	64023624	T	C	exonic	CEP112	nonsynonymous SNV	0,54	rs17704679	0,094	0,003	het	1799,19	946
chr17	34171599	34171599	-	G	exonic	TAF15	frameshift insertion					het	21,1871	51
chr2	202625615	202625615	C	T	exonic	ALS2	nonsynonymous SNV	0,91	rs3219156	0,191	0,006	hom	15661,7	1603
chr2	202626479	202626479	T	G	exonic	ALS2	nonsynonymous SNV	.	.	0,032	0,478	het	88,5808	2790
chr22	29885473	29885473	C	T	exonic	NEFH	nonsynonymous SNV	0,18	rs5763269	0,047	0,672	hom	10178,4	1085
chr22	29885908	29885908	A	G	exonic	NEFH	nonsynonymous SNV	.	.	0,002	0,094	het	32,8415	1156
chr4	170428901	170428901	C	T	exonic	NEK1	nonsynonymous SNV	0,05	rs33933790	0,083	0,146	het	858,64	358
chr6	110064928	110064928	A	T	exonic	FIG4	nonsynonymous SNV	0,07	rs2295837	1	0	het	1498,19	673
chr6	110107517	110107517	T	C	exonic	FIG4	nonsynonymous SNV	0,28	rs9885672	0,833	0	het	1253,96	651
chr7	94937446	94937446	T	C	exonic	PON1	nonsynonymous SNV	0,38	rs662	0,779	0	het	1495,79	689
chr9	135139901	135139901	T	C	exonic	SETX	nonsynonymous SNV	0,39	rs1056899	1	0	het	4995,29	2506
chr9	135202829	135202829	T	C	exonic	SETX	nonsynonymous SNV	0,73	rs543573	0,872	0	hom	5391,16	569
chr9	135203231	135203231	C	T	exonic	SETX	nonsynonymous SNV	0,73	rs1183768	0,133	0,796	hom	8217,42	898
chr9	135203409	135203409	A	C	exonic	SETX	nonsynonymous SNV	0,76	rs1185193	0,377	0,004	hom	1686,31	176
chr9	135204010	135204010	T	C	exonic	SETX	nonsynonymous SNV	0,02	rs61742937	0,039	0,102	het	3481,71	1491

Supplementary Table 2. CNVs identified in the SCA1-MN patient IV-18 by *NeuroArray* aCGH

Chr	Start	Stop	Cytoband	#Probes	Log2 ratio (Test/Ctrl)	pval	Gene Names
chr1	14,124,918	14,703,778	p36.21	5	1.448088	2.16E-10	PRDM2
chr1	41,475,871	41,478,153	p34.2	4	2.142642	2.20E-17	CTPS1
chr1	45,213,104	45,316,628	p34.1	34	0.61867	4.65E-11	EIF2B3
chr1	55,608,624	55,614,187	p32.3	11	0.978236	6.97E-10	USP24
chr1	57,528,551	57,537,314	p32.2	6	1.288615	7.43E-10	DAB1
chr1	115,251,231	115,258,361	p13.2	6	1.297375	5.66E-10	NRAS
chr1	163,038,607	165,172,712	q23.3	15	0.866144	5.27E-12	RGS4, LMX1A
chr1	197,065,106	197,091,313	q31.3	9	1.048951	9.03E-11	ASPM
chr2	27,590,586	27,591,343	p23.3	6	1.302483	5.05E-11	EIF2B4
chr2	36,774,108	37,362,697	p22.2	20	-0.692336	1.76E-10	CRIM1, EIF2AK2
chr2	44,173,246	44,174,449	p21	3	1.847663	4.42E-11	LRPPRC
chr2	48,925,810	49,216,197	p16.3	21	0.791682	9.64E-14	LHCGR
chr2	64,147,652	64,148,427	p14	3	1.799184	1.35E-10	VPS54
chr2	162,761,268	165,354,026	q24.2 - q24.3	42	0.643279	9.49E-13	SLC4A10, GRB14 (SLA)
chr2	179,474,676	179,478,649	q31.2	19	-0.728485	8.00E-14	TTN
chr2	179,671,916	202,137,517	q31.2 - q33.1	58	-0.326747	1.12E-11	TTN, HSPD1, MARS2 (SCA), CASP8
chr2	202,254,072	202,263,051	q33.1	9	1.190505	2.20E-11	TRAK2 (SLA)
chr2	222,290,299	222,307,610	q36.1	11	0.982995	1.96E-11	EPHA4 (SLA)
chr2	234,164,719	234,173,570	q37.1	7	1.456546	3.87E-15	ATG16L1
chr3	1,363,464	1,371,563	p26.3	7	1.17279	1.61E-10	CNTN6 (SLA)
chr3	4,715,908	4,776,981	p26.1	38	0.503688	1.57E-10	ITPR1 (SCA)
chr3	46,400,344	46,401,842	p21.31	3	2.048314	3.22E-13	CCR2
chr3	134,265,025	142,281,571	q22.2 - q23	147	0.342251	2.09E-17	CEP63, EPHB1, MRAS
chr3	178,928,066	178,936,110	q26.32	4	1.520756	3.57E-10	PIK3CA
chr4	15,556,669	15,569,439	p15.32	14	0.831871	1.42E-10	CC2D2A
chr4	56,744,114	57,356,837	q12	71	0.478281	1.55E-16	CEP135
chr4	99,399,826	99,403,350	q23	4	1.532546	2.63E-10	TSPAN5
chr4	112,312,260	114,095,683	q25	9	1.055257	2.08E-10	C4orf32, AP1AR, TIFA, ALPK1, NEUROG2, ZGRF1, LARP7, MIR367, MIR302D, MIR302A, MIR302C, MIR302B, ANK2, MIR1243, MIR8082
chr4	114,251,379	114,257,852	q26	8	1.065978	5.00E-10	ANK2
chr5	58,272,149	58,273,101	q11.2	4	1.816099	2.12E-13	PDE4D
chr5	74,014,636	74,016,371	q13.3	4	1.647444	1.21E-11	HEXB
chr5	74,652,139	74,655,854	q13.3	9	1.113502	6.43E-12	HMGCR
chr6	31,541,470	31,546,112	p21.33	10	1.054148	7.09E-12	LTA, TNF (SLA)
chr6	31,918,133	31,950,240	p21.33	14	0.892197	6.63E-12	CFB, C4A
chr6	112,015,870	112,025,232	q21	9	1.261277	7.03E-12	FYN
chr6	129,601,214	129,609,223	q22.33	3	2.104286	3.43E-12	LAMA2
chr6	152,734,473	152,737,603	q25.2	3	2.128005	1.90E-12	SYNE1 (SCA/SLA)
chr7	79,082,572	80,231,563	q21.11	3	2.06833	1.92E-13	MAGI2, CD36
chr7	87,173,559	92,122,449	q21.12 - q21.2	50	0.530343	1.55E-14	ABCB1
chr7	148,513,770	148,523,620	q36.1	9	1.078511	2.74E-11	EZH2
chr8	101,322,252	103,277,468	q22.2 - q22.3	16	-0.797031	5.21E-11	RNF19A (SLA)
chr8	120,631,456	126,096,269	q24.12 - q24.13	76	0.372195	2.42E-11	KIAA0196
chr9	2,644,822	6,251,257	p24.2 - p24.1	32	0.688947	1.39E-15	VLDLR, IL33
chr9	32,972,737	32,974,478	p21.1	3	1.895863	1.42E-11	APTX (SCA)
chr9	35,059,175	36,223,524	p13.3	40	0.579665	5.38E-14	VCP(SLA)
chr9	107,562,129	107,565,596	q31.1	6	1.416516	8.13E-11	ABCA1
chr9	128,002,429	130,422,368	q33.3 - q34.11	32	-0.402576	5.70E-10	HSPA5, LMX1B, STXBP1
chr10	6,525,419	13,174,214	p15.1 - p13	76	0.377882	1.22E-11	PRKCQ, USP6NL (SLA), OPTN (SLA)
chr10	69,672,624	70,360,755	q21.3	6	1.491475	3.50E-12	SIRT1,TET1
chr10	75,856,988	75,888,955	q22.2	26	0.772209	3.91E-12	VCL, AP3M1
chr10	79,743,724	79,744,014	q22.3	4	1.827961	1.66E-12	POLR3A
chr10	121,156,191	121,184,569	q26.11	5	1.324638	9.77E-10	GRK5
chr11	18,042,679	18,050,813	p15.1	10	0.949187	5.93E-10	TPH1
chr11	22,277,032	22,297,729	p14.3	15	0.935896	1.01E-13	ANO5
chr11	73,429,634	73,431,971	q13.4	4	1.893435	4.38E-13	RAB6A
chr11	95,647,385	102,662,127	q21 - q22.2	31	-0.437585	7.58E-11	MTMR2,MMP1
chr11	118,889,234	118,889,972	q23.3	3	1.872906	4.77E-10	TRAPPC4
chr12	40,872,902	40,884,200	q12	3	2.567741	9.72E-20	MUC19
chr12	57,957,270	88,452,738	q13.3 - q21.32	178	0.33966	2.05E-20	KIF5A, TMEM5, TBK1, IFNG, CEP290
chr12	110,771,860	110,777,530	q24.11	6	1.280322	1.04E-10	ATP2A2
chr12	111,086,828	111,158,740	q24.11	4	1.648149	1.19E-11	TCTN1, HVCN1, PPP1CC
chr13	60,413,451	60,490,355	q21.2	9	1.098949	1.18E-11	DIAPH3
chr14	20,923,430	20,923,829	q11.2	4	1.603993	3.98E-11	APEX1

chr14	51,202,244	51,224,083	q22.1	18	0.712369	4.56E-10	NIN
chr14	57,272,292	58,838,823	q22.3 - q23.1	46	0.514759	7.40E-13	OTX2
chr14	81,942,024	81,955,619	q31.1	12	1.289141	6.92E-20	SEL1L
chr14	92,563,016	97,321,686	q32.12 - q32.2	45	0.503338	3.87E-12	ATXN3, SERPINA1, SERPINA3
chr16	4,556,943	4,559,539	p13.3	5	1.624889	8.88E-14	HMOX2
chr16	7,102,031	7,382,963	p13.3	3	1.934426	5.58E-12	RBFOX1
chr16	50,745,620	50,750,591	q12.1	3	1.828845	6.85E-11	NOD2
chr16	81,397,375	81,991,597	q23.2 - q23.3	10	1.342749	3.72E-18	GAN
chr17	7,579,911	10,316,045	p13.1	170	0.299097	1.34E-15	TP53, PIK3R5, MYH13
chr17	29,258,933	29,280,360	q11.2	8	1.146308	2.50E-11	ADAP2
chr17	29,663,455	29,667,686	q11.2	13	0.935434	4.08E-12	NF1
chr17	44,301,037	44,714,881	q21.31	7	-1.279559	3.41E-12	KANSL1, NSF
chr17	61,563,975	61,564,394	q23.3	3	1.734921	5.69E-10	ACE
chr17	71,196,116	71,201,785	q25.1	11	0.99661	1.05E-11	COG1
chr18	20,516,844	20,529,682	q11.2	3	1.850036	4.18E-11	RBBP8
chr18	25,570,038	28,710,025	q12.1	24	0.646855	6.73E-11	CDH2, MIR302F, DSC3, DSC2, DSCAS, DSC1
chr18	67,671,113	67,672,489	q22.2	4	1.88366	1.12E-14	RTTN
chr20	2,635,038	2,637,811	p13	16	0.804268	3.52E-11	NOP56
chr21	22,804,521	22,839,062	q21.1	3	1.739517	5.14E-10	NCAM2
chr21	47,769,069	47,862,455	q22.3	68	0.472528	1.41E-15	PCNT
chr22	21,237,821	21,243,879	q11.21	3	2.12015	4.90E-14	SNAP29
chrX	13,762,578	13,778,335	p22.2	10	0.968702	2.69E-10	OFD1

Supplementary Table 3. CNVs identified in the SCA1-MN patient IV-15 by *NeuroArray* aCGH

Chr	Cytoband	Start	Stop	#Probes	Log2 ratio (Test/Ctrl)	pval	Gene Names
chr1	p36.22	10.408.695	10.420.998	5	0,893666	2,65E-14	KIF1B
chr1	p34.1	44.257.729	44.257.889	3	1,122156	2,49E-12	ST3GAL3
chr1	p32.3	55.545.118	55.545.426	3	0,989565	7,71E-10	USP24
chr1	p32.3	55.563.551	55.563.805	3	1,079846	1,68E-11	USP24
chr1	p32.3	55.609.725	55.611.721	5	0,927325	1,26E-12	USP24
chr1	p32.3	55.622.642	55.624.544	6	-0,588926	9,21E-10	USP24
chr1	p32.1	60.163.258	60.223.681	9	0,860767	1,70E-19	FGGY
chr1	p13.3	107.866.976	107.867.459	3	1,345618	3,00E-17	NTNG1
chr1	p12	120.298.103	120.306.915	13	0,649053	6,10E-19	HMGCS2
chr1	q21.3	154.562.255	154.562.379	3	1,109463	2,32E-13	ADAR
chr1	q23.3	163.038.486	165.398.181	81	0,344324	8,94E-32	RGS4,LMX1A, RXRG
chr1	q25.2	179.314.020	179.316.846	5	0,722773	5,89E-10	SOAT1
chr1	q32.2	207.758.222	207.780.654	12	-0,311482	4,37E-10	CR1
chr1	q41	223.957.574	223.962.678	12	0,565066	9,46E-14	CAPN2
chr1	q42.2	230.814.650	230.814.881	3	1,364047	5,91E-19	COG2
chr1	q43	236.969.356	236.972.106	6	0,85597	1,45E-15	MTR
chr1	q43	237.038.104	237.044.127	4	0,910529	4,45E-12	MTR
chr2	p22.2	36.623.723	36.771.719	42	0,391666	5,93E-22	CRIM1
chr2	p22.2	37.334.367	37.368.844	34	-0,339907	5,03E-14	EIF2AK2
chr2	p16.3	50.463.978	50.850.750	42	0,470815	8,76E-31	NRXN1
chr2	p14	64.139.743	64.140.379	3	0,959768	1,96E-10	VPS54
chr2	p14	64.140.484	64.211.189	50	-0,319625	9,86E-18	VPS54
chr2	q21.3	135.602.034	135.619.580	11	0,589522	9,07E-14	ACMSD
chr2	q22.3	145.158.681	145.187.597	14	0,475166	1,09E-11	ZEB2
chr2	q23.1	149.818.488	149.838.111	12	0,556012	2,05E-13	KIF5C
chr2	q23.3	152.830.215	152.830.476	3	0,996684	4,05E-11	CACNB4
chr2	q24.2	162.627.594	162.696.406	6	0,692336	8,63E-11	SLC4A10
chr2	q24.2	162.804.058	162.804.311	3	1,285422	3,69E-17	SLC4A10
chr2	q24.3	166.166.787	166.179.947	18	0,38856	2,73E-10	SCN2A
chr2	q24.3	166.903.209	166.903.544	3	1,130811	8,24E-14	SCN1A
chr2	q24.3	166.908.216	166.908.543	3	1,31996	1,03E-16	SCN1A
chr2	q24.3	167.060.582	167.083.334	8	-0,598635	9,22E-11	SCN9A
chr2	q24.3	167.144.880	167.149.930	5	1,089166	1,17E-17	SCN9A
chr2	q31.1	170.145.555	170.145.713	3	1,376674	4,04E-16	LRP2
chr2	q31.1	171.716.542	171.717.494	3	1,083174	4,22E-10	GAD1
chr2	q31.2	179.422.539	179.435.244	11	0,68023	9,02E-12	TTN
chr2	q31.2	179.447.689	179.447.951	3	1,115558	1,09E-10	TTN
chr2	q31.2	179.449.689	179.450.126	4	0,985455	1,07E-10	TTN
chr2	q31.2	179.466.844	179.467.181	3	1,242067	3,64E-13	TTN
chr2	q31.2	179.472.425	179.473.023	3	1,454167	5,70E-18	TTN
chr2	q31.2	179.481.716	179.481.871	3	1,127712	6,49E-11	TTN
chr2	q31.2	179.500.189	179.500.670	4	1,192055	1,30E-15	TTN
chr2	q31.2	179.518.558	179.530.601	20	0,755754	2,37E-25	TTN
chr2	q31.2	179.542.427	179.544.320	13	0,592621	6,83E-10	TTN
chr2	q31.2	179.588.396	179.592.414	16	0,631319	8,53E-14	TTN
chr2	q31.2	179.602.742	179.605.334	5	0,912392	4,43E-11	TTN
chr2	q31.2	179.659.057	179.671.941	22	0,410471	2,10E-13	TTN
chr2	q34	212.251.533	212.251.904	3	1,102381	3,66E-10	ERBB4
chr2	q35	215.813.359	215.815.705	7	0,784489	2,15E-10	ABCA12
chr2	q35	215.882.843	215.910.617	19	-0,365493	1,01E-09	ABCA12
chr2	q35	215.928.723	215.928.992	3	1,002473	3,15E-11	ABCA12
chr2	q36.1	222.290.299	222.321.569	32	0,627979	5,72E-41	EPHA4
chr2	q37.1	233.641.084	233.674.489	22	0,390484	2,68E-12	KCNJ13

chr3	p26.3	1.367.417	1.371.563	9	1,055758	1,49E-21	CNTN6
chr3	p26.3	1.414.461	1.415.419	5	1,030073	7,60E-13	CNTN6
chr3	p25.2	12.421.188	12.434.294	9	0,923251	1,12E-25	PPARG
chr3	p22.1	43.618.330	43.622.043	5	0,86345	1,80E-13	ANO10
chr3	p21.31	46.400.344	46.402.138	4	0,827945	2,29E-10	CCR2
chr3	q13.33	119.582.231	119.585.412	4	0,830659	2,01E-10	GSK3B
chr3	q22.2 - q22	134.270.791	138.119.480	98	0,250323	6,06E-21	EPHB1
chr3	q27.1	182.756.887	182.759.459	4	0,902022	5,54E-12	MCCC1
chr3	q27.2	184.646.199	184.646.374	3	1,413668	1,47E-20	VPS8
chr4	p15.31 - p1	20.751.367	23.891.711	42	0,530145	1,75E-38	PPARGC1A
chr4	p14	40.827.864	40.828.036	3	1,135549	6,53E-14	APBB2
chr4	q13.2	68.447.036	68.447.248	3	0,93362	5,77E-10	STAP1
chr4	q13.3	74.285.363	74.607.469	14	0,663878	4,19E-21	ALB, CXCL8
chr4	q22.3	96.137.149	96.140.527	6	0,980045	1,47E-13	UNC5C
chr4	q25	109.741.117	109.748.397	9	0,733568	7,99E-16	COL25A1
chr4	q25	109.841.686	109.841.804	3	1,006011	2,70E-11	COL25A1
chr4	q27	123.377.215	123.377.568	5	1,041958	8,62E-19	IL2
chr4	q32.3	166.385.567	166.408.704	13	0,520168	8,25E-13	CPE
chr4	q33	170.458.692	170.533.566	48	-0,30197	2,25E-15	NEK1
chr5	p12	42.549.653	42.711.480	25	0,416414	2,25E-15	GHR
chr5	q12.1	59.783.794	59.783.869	3	1,518787	1,94E-23	PDE4D
chr5	q12.1	59.965.756	61.658.380	44	-0,255721	1,40E-10	KIF2A
chr5	q13.3	74.650.500	74.655.854	21	0,453781	2,43E-15	HMGCR
chr5	q14.1	78.135.098	78.181.453	4	1,437911	3,40E-27	ARSB
chr5	q23.2	121.726.813	121.736.860	5	0,910903	8,63E-15	SNCAIP
chr5	q23.2	121.758.675	121.761.053	4	0,814435	4,37E-10	SNCAIP
chr5	q31.2 - q31	138.651.665	139.847.937	37	-0,310723	5,55E-13	MATR3
chr5	q32	147.817.920	147.820.133	9	0,759016	3,32E-10	FBXO38
chr5	q34	161.300.082	161.317.943	11	0,835569	3,50E-15	GABRA1
chr6	p25.1	6.152.036	6.182.446	12	0,539496	9,87E-13	F13A1
chr6	q15	88.226.523	88.228.479	7	0,63217	1,68E-10	RARS2
chr6	q16.2 - q21	99.847.160	110.037.670	41	-0,26846	4,87E-11	FIG4
chr6	q21	110.056.293	110.064.399	11	0,560556	1,42E-12	FIG4
chr6	q22.33	129.571.169	129.582.042	9	0,801232	2,76E-15	LAMA2
chr6	q22.33	129.612.698	129.637.185	22	-0,291519	8,03E-13	LAMA2
chr6	q22.33	129.766.878	129.774.317	6	0,98283	4,60E-16	LAMA2
chr6	q24.2 - q24	144.104.281	145.956.447	22	-0,322168	1,40E-14	EPM2A
chr6	q25.1 - q25	152.476.975	152.510.586	15	0,68572	1,92E-17	SYNE1
chr6	q25.2	152.680.570	152.686.014	7	0,713302	8,60E-10	SYNE1
chr6	q25.2	152.771.924	152.776.548	8	0,848306	1,10E-14	SYNE1
chr6	q25.2	152.783.795	152.784.007	3	1,05244	3,79E-10	SYNE1
chr7	p14.1	42.064.971	42.187.850	17	0,44925	1,54E-12	GLI3
chr7	q21.11	80.276.185	80.286.090	4	0,93529	9,42E-13	CD36
chr7	q21.12	87.145.767	87.146.033	3	1,274314	4,72E-17	ABCB1
chr7	q21.3	94.940.647	94.993.305	27	0,456222	2,80E-19	PON1, PON3
chr7	q22.1	103.237.033	103.243.880	4	0,837839	1,42E-10	RELN
chr7	q35 - q36.1	146.805.304	147.964.204	54	0,368161	1,11E-24	CNTNAP2
chr8	p21.3	22.210.528	22.213.116	7	0,786241	3,22E-10	PIWIL2
chr8	p21.2	27.361.265	27.362.726	4	0,977254	5,02E-10	EPHX2
chr8	p12	32.599.491	32.600.237	4	1,118452	3,65E-13	NRG1
chr8	q21.3	90.976.727	90.997.032	26	-0,337088	4,89E-11	NBN
chr8	q22.2	100.115.139	100.147.799	19	-0,493469	3,68E-16	VPS13B
chr8	q22.2	100.147.862	100.148.985	4	1,200881	7,50E-20	VPS13B
chr8	q22.2	100.789.121	100.821.812	11	0,611496	1,12E-14	VPS13B
chr8	q24.13	126.044.564	126.044.659	3	1,310243	6,37E-18	KIAA0196
chr9	p24.2	2.641.346	2.654.436	41	0,30024	2,27E-13	VLDLR

chr9	p13.3	35.057.322	35.062.078	23	0,337635	5,48E-10	VCP
chr9	q21.11	72.047.355	72.072.097	18	0,462864	7,12E-14	APBA1
chr9	q21.2	79.867.028	79.867.265	3	1,111834	2,07E-13	VPS13A
chr9	q21.2	79.874.891	79.895.235	15	-0,443334	5,11E-11	VPS13A
chr9	q31.1	107.554.266	107.620.810	102	0,273711	1,16E-25	ABCA1
chr9	q31.1	107.562.129	107.562.827	4	1,145454	2,63E-11	ABCA1
chr9	q34.11	131.337.057	131.337.451	3	1,032987	8,15E-12	SPTAN1
chr9	q34.11	131.344.717	131.344.956	4	0,881111	1,63E-11	SPTAN1
chr9	q34.13	135.139.370	135.221.685	61	-0,261254	7,76E-15	SETX
chr9	q34.13	135.786.332	135.786.570	3	1,392002	5,45E-20	TSC1
chr9	q34.13	135.797.329	135.798.705	3	1,506853	4,22E-23	TSC1
chr9	q34.13	135.810.326	135.810.501	3	0,970526	1,24E-10	TSC1
chr10	p15.1	6.549.372	6.553.187	6	0,856608	1,38E-15	PRKCQ
chr10	p14	10.191.129	11.569.652	40	-0,255614	5,83E-10	USP6NL
chr10	p14	11.574.221	11.574.301	3	1,171655	1,08E-14	USP6NL
chr10	p12.31	19.493.882	19.498.404	4	1,044622	6,35E-10	MALRD1
chr10	q21.2	61.802.537	61.815.739	4	0,844695	1,01E-10	ANK3
chr10	q21.2	61.868.549	62.039.435	74	0,288246	6,27E-21	ANK3
chr10	q21.3	67.726.469	69.366.829	68	0,257893	5,83E-16	CTNNA3
chr10	q21.3	69.672.219	69.677.273	5	0,909337	9,57E-15	SIRT1
chr10	q22.3	78.649.333	79.769.325	144	0,315735	2,07E-46	KCNMA1, POLR3A
chr10	q22.3	78.651.428	78.674.866	11	0,927444	2,40E-14	KCNMA1
chr10	q24.2	101.451.335	101.462.452	13	0,522481	6,58E-13	ENTPD7
chr10	q25.1	108.338.730	108.389.068	19	0,560044	7,92E-13	SORCS1
chr10	q25.3	114.903.601	114.912.237	14	0,576597	1,44E-10	TCF7L2
chr10	q26.2	127.753.392	127.786.935	12	0,678534	3,81E-13	ADAM12
chr10	q26.2	127.806.637	127.824.197	5	0,891653	6,96E-11	ADAM12
chr11	p15.4	9.875.144	9.880.001	8	0,5876	1,98E-10	SBF2
chr11	p15.4	10.019.877	10.293.941	25	-0,362794	4,27E-12	SBF2
chr11	p15.3 - p15	12.382.997	12.914.677	3	1,14549	7,88E-14	PARVA, TEAD1
chr11	p15.1	18.047.968	18.048.226	3	1,180566	1,37E-11	TPH1
chr11	p15.1	18.050.894	19.692.883	21	-0,409819	4,55E-23	TPH1
chr11	p13	35.313.870	35.323.226	5	0,949913	6,31E-16	SLC1A2
chr11	q12.1	57.944.568	59.622.110	20	0,477904	4,69E-16	CNTF, TCN1
chr11	q14.1	83.393.184	83.497.769	5	0,811908	4,10E-12	DLG2
chr11	q14.1	83.673.879	83.676.349	4	1,293031	1,35E-22	DLG2
chr11	q14.2	85.692.236	85.756.385	44	-0,25056	1,97E-10	PICALM
chr11	q21	95.569.256	95.571.253	4	0,891923	9,36E-12	MTMR2
chr11	q22.2	102.662.221	102.663.526	4	0,958716	7,68E-13	MMP1
chr12	p13.33	1.006.780	1.009.884	4	1,047321	1,54E-15	WNK1
chr12	p13.31	9.229.848	9.231.998	9	0,552705	2,17E-10	A2M
chr12	p12.1	21.990.912	21.997.905	12	0,632027	3,15E-10	ABCC9
chr12	p12.1	23.757.340	23.887.639	10	0,704057	1,35E-11	SOX5
chr12	p11.23	26.568.434	26.572.214	4	1,11505	2,27E-17	ITPR2
chr12	p11.23	26.592.113	26.648.259	30	-0,308876	1,10E-10	ITPR2
chr12	p11.23	26.809.414	26.810.859	4	0,865297	3,64E-11	ITPR2
chr12	q12	40.707.945	40.709.201	4	0,688781	3,60E-12	LRRK2
chr12	q21.33	91.501.889	91.502.565	3	0,936007	5,23E-10	LUM
chr12	q24.11	110.771.688	110.777.530	8	0,650486	2,15E-12	ATP2A2
chr12	q24.12	111.923.459	111.993.621	45	-0,275318	2,00E-12	ATXN2
chr12	q24.22	117.655.853	117.728.321	78	0,420835	9,94E-45	NOS1
chr13	q12.12	23.755.101	23.777.969	4	0,775915	7,29E-12	SGCG
chr13	q13.3	36.903.533	36.905.738	6	0,869143	5,43E-16	SPG20
chr13	q33.1	101.942.395	102.379.180	8	0,576877	4,09E-10	FGF14
chr14	q23.1	61.915.848	61.917.526	4	1,121493	1,50E-17	PRKCH
chr14	q24.3	73.822.253	73.876.874	9	-0,571939	5,27E-11	NUMB

chr14	q24.3	74.426.183	74.428.308	6	0,848958	2,43E-15	COQ6
chr14	q24.3	74.946.850	74.951.214	7	0,696152	2,04E-12	NPC2
chr14	q24.3	77.757.743	77.796.637	55	0,271624	1,66E-14	POMT2, GSTZ1
chr15	q12	26.806.083	26.861.259	18	0,651568	1,11E-25	GABRB3
chr15	q21.1	44.903.167	44.918.607	21	-0,420063	2,12E-13	SPG11
chr15	q21.1	49.061.054	49.076.416	15	-0,41372	8,15E-10	CEP152
chr15	q21.1	49.080.955	49.081.224	3	1,002759	9,23E-11	CEP152
chr15	q21.2	50.866.574	51.216.286	117	-0,345508	3,56E-45	TRPM7, AP4E1
chr15	q21.2	51.510.642	51.520.164	9	0,575489	4,04E-11	CYP19A1
chr15	q21.3	58.902.632	58.904.240	7	0,397546	6,13E-10	ADAM10
chr16	p12.2	23.403.744	23.409.560	8	0,785167	3,06E-17	COG7
chr16	q11.2 - q12	46.697.028	49.616.842	44	-0,267432	1,70E-11	VPS35
chr16	q22.1	70.516.049	70.530.353	13	0,459047	2,33E-10	COG4
chr17	p12 - p11.2	15.257.416	17.715.482	25	-0,335399	1,37E-10	UBB, SREBF1
chr17	p11.2	19.752.704	19.769.218	11	-0,599036	1,51E-13	ULK2
chr17	q11.2	30.219.712	30.221.916	7	0,591761	6,78E-14	UTP6
chr17	q12	34.207.327	34.416.675	5	0,748271	1,51E-10	CCL5, CCL3
chr17	q21.31	44.269.718	44.544.914	6	-0,65049	1,00E-09	KANSL1
chr17	q21.31	44.827.148	44.832.714	8	0,576508	4,19E-10	NSF
chr17	q22 - q23.2	56.469.890	58.372.232	56	-0,31351	5,71E-19	USP32
chr18	q12.1	25.414.110	25.907.443	3	0,972968	1,12E-10	CDH2
chr18	q21.2	52.924.644	52.927.290	3	1,167578	4,58E-10	TCF4
chr18	q21.31	56.002.754	56.024.395	18	0,477923	1,18E-14	NEDD4L
chr18	q21.33	59.781.936	59.814.450	19	-0,500296	3,36E-16	PIGN
chr18	q22.3	69.156.574	70.461.471	27	0,481562	2,53E-21	NETO1
chr20	p12.3	8.637.801	8.638.056	3	1,105537	2,81E-13	PLCB1
chr20	q13.31	56.136.582	56.138.760	12	0,476289	2,64E-10	PCK1
chr21	q21.1	22.707.857	22.710.787	4	1,041993	2,13E-15	NCAM2
chr21	q21.3	27.253.184	27.264.032	4	0,834233	1,69E-10	APP
chr22	q11.23	24.376.158	24.384.300	17	0,536646	3,97E-17	GSTT1
chr22	q12.1 - q12	26.117.138	29.670.389	30	-0,302039	2,38E-10	EWSR1
chr22	q12.3	32.215.198	32.230.062	10	0,572015	4,88E-12	DEPDC5
chr22	q12.3	32.297.679	32.302.125	6	0,957367	5,18E-19	DEPDC5
chrX	q12	66.905.876	66.941.894	10	0,849064	2,79E-24	AR
chrX	q21.1	76.829.691	76.972.627	74	-0,29073	8,66E-21	ATRX
chrX	q25	122.338.396	122.460.062	6	0,806328	5,97E-14	GRIA3

Supplementary Table 4. CNVs identified in the SCA1-MN patient IV-13 by NeuroArray aCGH

Chr	Start	Stop	Cytoband	#Probes	Log2 ratio (Test/Ctrl)	pval	Gene Names
chr1	41,475,871	41,478,153	p34.2	4	1.145444	2.52E-13	CTPS1
chr1	55,622,642	57,185,945	p32.3 - p32	57	-0.286809	4.31E-12	USP24, PRKAA2, C1orf168
chr1	107,691,190	107,867,345	p13.3	3	1.036483	1.55E-10	NTNG1
chr1	115,251,174	118,983,652	p13.2 - p12	16	0.501848	1.24E-10	NRAS, NGF
chr1	197,065,106	197,091,194	q31.3	8	0.762124	5.34E-12	ASPM
chr1	210,194,380	220,338,142	q32.2 - q41	51	-0.302199	5.06E-12	SYT14, TGFB2, RAB3GAP2
chr1	228,345,589	230,804,550	q42.13 - q4	16	-0.538107	5.73E-12	GJC2, COG2
chr2	36,774,108	44,145,293	p22.2 - p21	136	-0.260711	4.68E-22	CRIM1, EIF2AK2, CYP1B1, GEMIN6, SOS1, ABCG5, LRPPRC
chr2	64,161,055	64,211,176	p14	19	-0.570702	2.16E-15	VPS54
chr2	202,598,024	202,603,477	q33.1	3	1.072697	2.01E-10	ALS2
chr2	215,854,029	215,862,529	q35	6	0.722164	4.21E-10	ABCA12
chr2	234,164,719	234,173,570	q37.1	7	0.726776	2.47E-11	ATG16L1
chr3	46,400,344	46,401,842	p21.31	3	1.17811	6.24E-11	CCR2
chr3	178,928,066	178,928,315	q26.32	3	0.981386	4.52E-11	PIK3CA
chr4	8,575,302	15,534,912	p16.1 - p15	38	-0.359806	1.34E-12	CPZ, DRD5, CC2D2A
chr4	56,874,500	56,876,041	q12	3	1.487806	2.60E-14	CEP135
chr4	57,356,535	57,356,837	q12	3	1.30354	5.41E-13	SRP72
chr5	70,297,569	70,308,602	q13.2	5	1.016078	3.90E-13	NAIP
chr5	74,014,636	74,016,371	q13.3	4	1.016502	7.29E-11	HEXB
chr5	90,281,188	90,368,430	q14.3	4	1.000036	4.62E-13	ADGRV1
chr5	121,726,813	121,759,256	q23.2	6	0.697206	7.47E-11	SNCAIP
chr6	46,593,201	52,334,157	p12.3 - p12	53	-0.357582	1.10E-16	CYP39A1, PLA2G7, EFHC1
chr6	70,291,737	88,187,113	q13 - q15	58	-0.326518	2.21E-15	SLC17A5, NT5E, SLC35A1
chr6	129,601,214	129,609,223	q22.33	3	1.189709	4.10E-11	LAMA2
chr7	17,362,169	30,673,675	p21.1 - p14	94	-0.276554	1.38E-17	AHR, IL6, FAM126A, NPY, HNRNPA2B1, GARS
chr7	79,082,572	80,231,563	q21.11	3	1.079132	4.01E-11	MAGI2, CD36
chr8	90,949,230	90,955,613	q21.3	3	1.224934	2.51E-11	NBN
chr8	103,265,457	103,276,806	q22.3	12	-0.786355	4.30E-18	UBR5
chr9	79,234,236	79,244,169	q21.2	3	1.191424	3.85E-11	PRUNE2
chr10	112,363,966	114,711,039	q25.2	27	-0.428413	1.82E-12	SMC3, SHOC2, ADRA2A, TCF7L2
chr11	18,051,048	22,248,955	p15.1 - p14	23	-0.404755	4.70E-10	TPH1, SLC6A5, ANO5

chr11	73,429,634	73,431,971	q13.4	4	1.033085	3.65E-11	RAB6A
chr12	40,671,933	40,820,373	q12	77	-0.358128	2.01E-23	LRRK2, MUC19
chr12	40,872,902	40,884,200	q12	3	1.184417	4.97E-11	MUC19
chr12	40,937,600	40,963,628	q12	53	-0.261575	9.69E-10	MUC19
chr13	23,808,771	23,945,327	q12.12	26	-0.382282	4.00E-10	SGCG, SACS
chr13	102,527,580	102,568,879	q33.1	3	1.169215	8.58E-11	FGF14
chr14	58,772,789	58,785,518	q23.1	6	0.679213	1.47E-10	ARID4A
chr14	81,942,024	81,955,619	q31.1	12	0.617288	7.79E-12	SEL1L
chr14	92,547,363	92,560,197	q32.12	12	-0.598606	3.65E-11	ATXN3
chr15	58,853,063	58,891,915	q21.3	10	-0.690653	2.86E-12	LIPC, ADAM10
chr15	67,485,105	67,950,927	q22.33 - q2	22	-0.454228	9.23E-12	SMAD3, MAP2K5
chr15	78,764,067	78,775,789	q25.1	10	-0.609692	6.09E-10	IREB2
chr15	91,535,874	93,527,589	q26.1	50	-0.290729	4.54E-11	CHD2
chr16	81,397,375	81,423,887	q23.2	9	0.694104	2.60E-11	GAN
chr16	83,158,984	83,636,205	q23.3	14	-0.536419	1.24E-10	CDH13
chr17	30,267,441	30,327,458	q11.2	21	-0.439481	1.08E-10	SUZ12
chr17	44,833,747	45,216,140	q21.31 - q2	29	-0.38554	2.95E-11	NSF, GOSR2, CDC27
chr17	45,247,377	45,258,958	q21.32	4	1.064986	9.34E-12	CDC27
chr17	60,064,371	60,088,616	q23.2	8	-0.735341	2.73E-11	MED13
chr18	3,502,234	12,371,638	p11.31 - p1	83	-0.277312	8.08E-16	DLGAP1, NDUFV2, AFG3L2
chr18	20,516,844	20,529,682	q11.2	3	1.193868	3.52E-11	RBBP8
chr18	53,070,678	55,998,002	q21.2 - q21	54	-0.29206	6.57E-12	TCF4, NEDD4L
chr18	67,671,113	67,672,489	q22.2	4	1.055825	5.67E-11	RTTN
chr21	15,870,866	22,658,642	q11.2 - q21	73	-0.268836	2.59E-13	SAMSN1, TMPRSS15, NCAM2
chr21	36,259,743	40,192,279	q22.12 - q2	43	-0.37067	8.91E-15	RUNX1, DYRK1A, KCNJ6
chr22	24,376,158	24,384,300	q11.23	10	0.695766	1.99E-12	GSTT1

Supplementary Table 5. Functional enrichment analyses of the CNV-associated gene sets in ALS and SCA1-MN family members

GO biological process	ALS (n genes)	-LogP(FDR)_ALS	SCA IV-18 (n genes)	-LogP(FDR)_SCA IV-18	SCA IV-13 (n genes)	-LogP(FDR)_SCA IV-13	SCA IV-15 (n genes)	-LogP(FDR)_SCA IV-15
growth	6	3.712857495	15	3.55	18	3.76	37	10.00
vacuolar transport	3	3.701506184	4	2.33	5	2.76	6	2.73
circadian rhythm	3	3.105432655	6	3.14	5	1.90	10	4.37
peptidyl-tyrosine modification, phosphorylation	3	2.385413256	10	4.66	12	5.17	16	10.00
regulation of hydrolase activity	4	1.631396911	18	4.13	21	4.08	26	3.51
cell morphogenesis	4	1.918806224	15	3.56	22	5.92	40	10.00
regulation of endocytosis	2	1.814884574	6	3.08	5	0.00	14	10.00
regulation of growth rate	2	4.576499341	0	0.00	0	0.00	0	0.00
lysosomal transport	3	4.074514376	0	0.00	5	3.32	5	2.55
membrane depolarization during action potential	2	3.343652772	0	0.00	0	0.00	7	10.00
regulation of microtubule cytoskeleton organization	3	3.290290251	0	0.00	0	0.00	0	0.00
neuronal action potential	2	3.222013671	0	0.00	0	0.00	7	10.00
regulation of microtubule-based process	3	3.084191504	0	0.00	0	0.00	0	0.00
endosomal transport	3	3.078939214	0	0.00	0	0.00	0	4.29
Golgi vesicle transport	3	1.674052377	0	0.00	20	10.00	18	3.87
autophagy	3	2.116199709	0	0.00	11	3.58	14	3.66
regulation of microtubule cytoskeleton organization	3	3.290290251	0	0	0	0	0	0
cellular homeostasis	0	0	18	5.76	18	4.21	28	10.00
circulatory system development	0	0	19	5.17	18	3.17	26	4.07
regulation of cell adhesion	0	0	14	4.95	15	4.31	0	0.00
synaptic signaling	0	0	15	4.88	19	5.93	42	10.00
regulation of cytokine production	0	0	14	4.88	15	4.24	16	2.81
tube development	0	0	18	4.80	17	2.91	25	3.90
regulation of neurotransmitter levels	0	0	10	4.62	9	3.05	15	5.29
blood vessel development	0	0	14	4.53	0	0.00	15	2.12
negative regulation of multicellular organismal process	0	0	19	4.40	27	10.00	38	10.00
cytokine production	0	0	14	4.38	16	4.29	17	2.76
response to growth factor	0	0	13	4.38	0	0.00	18	3.89
neuron projection development	0	0	16	4.22	28	10.00	45	10.00
neuron development	0	0	17	4.14	32	10.00	51	10.00
regulation of cellular localization	0	0	15	4.06	23	10.00	32	10.00
lipid homeostasis	0	0	6	3.89	0	0.00	9	4.72
stem cell division	0	0	6	3.85	6	3.26	6	2.38
positive regulation of intracellular signal transduction	0	0	15	3.80	17	3.56	22	3.50
regulation of phosphatase activity	0	0	6	3.64	0	0.00	7	2.86
ion homeostasis	0	0	13	3.62	0	4.09	26	10.00
neurotransmitter biosynthetic process	0	0	5	3.58	4	2.21	5	2.33
positive regulation of response to external stimulus	0	0	11	3.52	14	4.29	13	2.12
chemotaxis	0	0	11	3.48	17	10.00	29	10.00
regulation of locomotion	0	0	15	3.46	15	2.35	24	3.88
positive regulation of cell death	0	0	12	3.34	14	3.37	22	5.32
leukocyte activation	0	0	16	3.30	27	10.00	23	2.65
behavior	0	0	11	3.27	14	3.97	39	10.00
extracellular structure organization	0	0	9	3.26	8	1.98	17	5.58
energy homeostasis	0	0	3	3.19	3	2.88	4	3.53
astrocyte cell migration	0	0	2	3.14	2	2.92	2	2.58
activation of protein kinase activity	0	0	8	3.13	0	0.00	9	1.89
Wnt signaling pathway	0	0	9	3.06	0	0.00	15	4.03
cell-cell junction organization	0	0	5	2.96	5	2.48	0	0.00
gliogenesis	0	0	7	2.94	16	10.00	17	10.00
axon development	0	0	9	2.86	20	10.00	31	10.00
response to toxic substance	0	0	9	2.85	11	3.19	24	10.00
regulation of neurogenesis	0	0	12	2.81	24	10.00	39	10.00
regulation of cell motility	0	0	13	2.77	15	2.64	22	3.50
NLRP3 inflammasome complex assembly	0	0	2	2.62	2	2.41	2	2.08
oxidation-reduction process	0	0	13	2.42	0	0.00	19	1.94
myelination	0	0	0	0.00	9	10.00	10	10.00
microtubule-based transport	0	0	0	0.00	15	10.00	14	3.56
cytoskeleton-dependent intracellular transport	0	0	0	0.00	15	10.00	14	3.53
neuron death	0	0	0	0.00	13	5.79	19	10.00
inflammatory response	0	0	0	0.00	14	3.58	16	2.72

Pathway	ALS (n genes)	-LogP(FDR)_ALS	SCAIV-18 (n genes)	-LogP(FDR)_SCA IV-18	SCAIV-13 (n genes)	-LogP(FDR)_SCA IV-13	SCAIV-15 (n genes)	-LogP(FDR)_SCA IV-15
Interaction between L1 and Ankyrins	2	3.238061438	0	0	0	0	7	10
Phase 0 - rapid depolarisation	2	2.847861988	0	0	0	0	7	5.756894864
Retrograde transport at the Trans-Golgi-Network	2	2.81188455	4	3.549747832	5	4.29925246	6	4.501697249
Muscle contraction	3	2.768150886	0	0	0	0	12	5.014852843
L1CAM interactions	2	2.186398695	0	0	0	0	9	5.290292512
Cardiac conduction	2	1.909870319	0	0	0	0	11	5.786655544
Intra-Golgi and retrograde Golgi-to-ER traffic	2	1.714692988	5	2.199612394	7	3.13977392	8	2.715164651
Axon guidance	3	1.572933582	0	0	10	1.78953366	20	4.713953877
Membrane Trafficking	3	1.4576996	8	1.339221403	11	1.902972508	17	2.751502687
Fatty acid, triacylglycerol, and ketone body metabolism	2	1.440235819	0	0	0	0	8	1.842758901
Endocytosis	2	1.418584584	0	0	0	0	0	0
Vesicle-mediated transport	3	1.377974439	9	1.566025496	12	2.08561641	19	3.22644193
Hypertrophic cardiomyopathy (HCM)	0	0	5	3.697080291	5	3.213115977	5	2.416117436
Tie2 Signaling	0	0	3	3.695146244	3	3.387231361	0	0
Pathways in cancer	0	0	10	3.634156092	0	0	12	2.390366419
Signaling by EGFR	0	0	3	3.554007351	2	1.932286973	0	0
AGE-RAGE signaling pathway	0	0	5	3.340738483	3	1.31180766	0	0
Signaling of FGFR3	0	0	3	3.259364613	2	1.745548625	0	0
Signaling by MET	0	0	4	2.981811752	3	1.711597877	0	0
Signaling events mediated by TCPTP	0	0	3	2.789743785	2	1.448780468	0	0
EGFR tyrosine kinase inhibitor resistance	0	0	4	2.763085095	3	1.55839167	0	0
Signaling by ERBB4	0	0	3	2.566280018	2	1.308532986	0	1.782158754
Ras signaling pathway	0	0	6	2.446456351	5	1.397027883	0	0
Signaling by ERBB2	0	0	3	2.355227742	3	2.066008908	3	1.586276633
Insulin resistance	0	0	4	2.286713776	5	2.717257062	6	2.648779781
Inflammatory bowel disease (IBD)	0	0	3	2.061763528	3	1.780339053	0	0
Nephrin interactions	0	0	2	2.051777847	3	3.121839935	0	0
PDGFR-alpha signaling pathway	0	0	2	2.051777847	2	1.852084832	0	0
Autophagy - animal	0	0	4	2.015779116	7	4.045542208	0	0
Downregulation of SMAD2/3:SMAD4 transcriptional activity	0	0	2	2.014093086	0	0	2	1.478506534
Fc epsilon RI signaling pathway	0	0	3	2.008105383	4	2.620142705	0	0
Adipocytokine signaling pathway	0	0	3	1.990810964	3	1.711597877	5	2.767618622
Asparagine N-linked glycosylation	0	0	6	1.976230205	7	2.0286482	9	2.012103396
Prolactin signaling pathway	0	0	3	1.973797892	3	1.695136556	0	0
Diseases of signal transduction	0	0	7	1.972378192	7	1.456252485	0	0
Downregulation of TGF-beta receptor signaling	0	0	2	1.879192692	0	0	2	1.350381709
Fluid shear stress and atherosclerosis	0	0	4	1.86296118	5	2.190889064	0	0
COPI-mediated anterograde transport	0	0	3	1.832107156	3	1.55839167	6	3.337382658
BARD1 signaling events	0	0	2	1.81961151	2	1.623420105	0	0
Post-translational protein modification	0	0	13	1.785809203	14	1.324187321	0	0
IL23-mediated signaling events	0	0	2	1.619178276	3	2.45795342	0	0
Estrogen signaling pathway	0	0	3	1.586034031	3	1.322639781	0	0
Class I PI3K signaling events	0	0	2	1.53607443	2	1.345814675	0	0
Fat digestion and absorption	0	0	2	1.53607443	2	1.345814675	0	0
T cell receptor signaling pathway	0	0	3	1.530504393	6	3.683944884	0	0
Chemokine signaling pathway	0	0	4	1.511700109	0	0	6	1.568438514
Transmembrane transport of small molecules	0	0	9	1.49389559	0	0	16	1.94936153
ABC transporters	0	0	2	1.461474304	0	0	4	2.625196314
Platelet activation, signaling and aggregation	0	0	5	1.436708185	11	4.681098616	11	3.073318005
Signaling by NOTCH	0	0	3	1.428518675	4	1.844562686	5	1.861016538
Metabolism of lipids and lipoproteins	0	0	10	1.416345594	0	0	22	3.277933415
Thyroid hormone signaling pathway	0	0	3	1.400028063	4	1.806546155	0	0
Interleukin-10 signaling	0	0	2	1.393908766	0	0	4	2.489076052
AMPK signaling pathway	0	0	3	1.3544806	4	1.745793053	8	3.860037234
IL2-mediated signaling events	0	0	2	1.347167867	3	2.042781471	0	0
PPARA activates gene expression	0	0	3	1.328243406	0	0	8	3.786137016
Signaling events mediated by focal adhesion kinase	0	0	2	1.31766402	3	1.997808736	0	0
Regulation of actin cytoskeleton	0	0	4	1.307125676	6	2.082642143	0	0
Reelin signalling pathway	0	0	0	0	3	5.264893266	2	2.817627177
Hemostasis	0	0	0	0	17	4.743406223	17	2.56307849
Reelin signaling pathway	0	0	0	0	4	4.048169145	3	2.255493464
VLDL interactions	0	0	0	0	3	3.063749566	2	1.478506534
Response to elevated platelet cytosolic Ca2+	0	0	0	0	6	3.022494397	7	2.776220451
Signaling by Interleukins	0	0	0	0	12	2.846087658	13	1.794777264
Cytokine Signaling in Immune system	0	0	0	0	15	2.832502339	16	1.527979617
Lipid digestion, mobilization, and transport	0	0	0	0	5	2.580327666	7	3.222373463
EPH-ephrin mediated repulsion of cells	0	0	0	0	3	2.138902897	3	1.655177564
Cytosolic DNA-sensing pathway	0	0	0	0	3	1.798293771	3	1.335702053
Glucocorticoid receptor regulatory network	0	0	0	0	3	1.516867285	4	1.709822355
Signaling by EGFR	0	0	0	0	7	1.488761782	9	1.382599589
Rheumatoid arthritis	0	0	0	0	3	1.414500827	4	1.578237116

Supplementary Table 6. Results of whole exome sequencing for the ALS proband.

Locus	Genotype	Ref	Observed Allele	Type	Genes	Location	Length	% Frequency	Exon	Variant Effect	Transcript	Coding	Amino Acid Change	PhyloP	SIFT	Grantham	PolyPhen	FATHMM	PFAM	dbSNP	DGV	EMAF	UCSC Com	ExAC EAAF	COSMIC	OMIM	Gene Ontc	DRA	DrugBank	ClinVar	Allele Cove	Allele Ratio	p-value	Phred QUA	Coverage	Ref+/Ref-/Var
chr1:100152443	C/C	T	C	SNV	PALMD	PALMD:intronic:NM_017734.4	1	100		intronic	NM_01773	c.367-43T	p.?	-0.15					Paralemmin	rs1338576		0.4273	YES	4.874E-1 (pos.100152443)		Palmdelphi	cytoplasm,dendritic spine,membrane,regulation of cell				T=0, C=86	T=0.0, C=1.0	0.00001	1406.83	86	T=0/0, C=53/3
chr1:100330237	G/G	A	G	SNV	AGL	AGL:intronic:NM_000642.2	1	100		intronic	NM_00064	c.664+92A	p.?	-0.8					Amylo-alpha-	rs538389			YES			Amylo-1,6-g	4-alpha-gl	Autoimmune Diseases;Carbohydrate Metabc			A=0, G=47	A=0.0, G=1.0	0.00001	785.908	47	A=0/0, G=19/2
chr1:220970028	G/G	A	G	SNV	MARC1	MARC1:exonic:NM_022746.3	1	100	3	missense	NM_02274	c.493A>G	p.Thr165Ala	3.9	1	58	0		MOSC N-term	rs2642438		0.2967	YES	7.948E-1 (pos.22097002	malignant_me	Molybdenu	catalytic a	Amphetamine-Related Disorders;Mental Dis			A=0, G=91	A=0.0, G=1.0	0.00001	1513.79	91	A=0/0, G=43/4
chr1:100340225	A/A	G	A	SNV	AGL	AGL:intronic:NM_000642.2	1	100		intronic	NM_00064	c.959-18G	p.?	-1.04					Amylo-alpha-	rs634880,rs8860383		0.297	YES	7.163E-1;0.0;0.0 (pos.100340225)		Amylo-1,6-g	4-alpha-gl	Autoimmune Diseases;Carboh		Benign	G=0, A=35	G=0.0, A=1.0	0.00001	548.283	35	G=0/0, A=25/1
chr1:100353675	A/A	G	A	SNV	AGL	AGL:intronic:NM_000642.2	1	100		intronic	NM_00064	c.2812+11	p.?	-0.76					Amylo-alpha-	rs555929,rs7793915		0.3153	YES	0.0 (pos.100353674),6.923E-1 (pos.1003		Amylo-1,6-g	4-alpha-gl	Autoimmune Diseases;Carboh		Benign	G=0, A=23	G=0.0, A=1.0	0.00001	316.747	23	G=0/0, A=15/8
chr1:100376448	G/A	G	A	SNV	AGL	AGL:intronic:NM_000642.2	1	56		intronic	NM_00064	c.3836+45	p.?	-0.8					Amylo-alpha-	rs2274570,rs386633		0.4942	YES	5.453E-1 (pos.100376448)		Amylo-1,6-g	4-alpha-gl	Autoimmune Diseases;Carboh		Benign	G=22, A=28	G=0.44, A=0.56	0.00001	341.079	50	G=13/9, A=9/1
chr1:100376456	T/A	T	A	SNV	AGL	AGL:intronic:NM_000642.2	1	56		intronic	NM_00064	c.3836+53	p.?	0.45					Amylo-alpha-	rs1541041,rs386633844			YES			Amylo-1,6-g	4-alpha-gl	Autoimmune Diseases;Carbohydrate Metabc			T=22, A=28	T=0.44, A=0.56	0.00001	341.078	50	T=13/9, A=9/1
chr2:217123958	C/C	T	C	SNV	MARCH4	MARCH4:utr_3:NM_020814.2	1	100	4	utr_3	NM_02081	c.1310A>G	p.?	-0.52					RING-variant	rs169616			YES			Membrane-	Golgi mem	Brain Diseases;Central Nervous System Dise			T=0, C=51	T=0.0, C=1.0	0.00001	754.299	51	T=0/0, C=22/2
chr1:101342497	C/C	T	C	SNV	EXTL2	EXTL2:intronic:NM_001439.3	1	100		intronic	NM_00143	c.434-77A	p.?	-1.58					Glycosyl tran	rs2295280			YES			Exostosin-li	IRE1-mediated unfolded protei		Uridine-5'-Diphosphate,I		T=0, C=23	T=0.0, C=1.0	0.00001	316.747	23	T=0/0, C=7/16
chr2:160604812	T/T	C	T	SNV	MARCH7	MARCH7:exonic:NM_022826.3	1	100	5	synonymous	NM_02282	c.1011C>T	p.(=)	-0.12				0.04(COSM37	RING-variant	rs1427329		0.3573	YES	3.43E-1 (pos.160604812)	acute_myeloic	Membrane-	negative r	Autoimmune Diseases;Female Urogenital Dis			C=0, T=62	C=0.0, T=1.0	0.00001	940.299	62	C=0/0, T=26/3
chr2:160605268	C/C	T	C	SNV	MARCH7	MARCH7:exonic:NM_022826.3	1	100	5	synonymous	NM_02282	c.1467T>C	p.(=)	-0.26					RING-variant	rs1365803		0.0897	YES	7.316E-1 (pos.160605268)		Membrane-	negative r	Autoimmune Diseases;Female Urogenital Dis			T=0, C=54	T=0.0, C=1.0	0.00001	798.148	54	T=0/0, C=13/4
chr5:132098242	G/G	A	G	SNV	SEPT8	SEPT8:exonic:NM_001098811.1	1	100	5	synonymous	NM_00109	c.630T>C	p.(=)	0.06					50S ribosome	rs30510		0.0086		9.98E-1 (pos.132098242)		Septin 8	GTP binding,cytoplasm,cytoskeleton,protein binding				A=0, G=47	A=0.0, G=1.0	0.00001	764.31	47	A=0/0, G=27/2
chr1:10342629	G/A	G	A	SNV	KIF1B	KIF1B:intronic:NM_015074.3	1	58.33		intronic	NM_01507	c.1296+38	p.?	-0.76					FHA domain,I	rs3748576		0.397	YES	5.402E-1 (pos.10342629)		Kinesin fam	ATP bindin	Adenocarcinoma;Autoimmune Diseases;Auto			G=10, A=14	G=0.4167, A=0.5833	0.00001	135.351	24	G=7/3, A=10/4
chr1:103427381	G/G	A	G	SNV	COL11A1	COL11A1:intronic:NM_001854.3	1	100		intronic	NM_00185	c.3168+41	p.?	-0.59					Collagen tripl	rs1012282		0.2629	YES	9.084E-2 (pos.103427381)		Collagen XI,	cartilage c	Abnormalities, Multiple;Adnex		Benign	A=0, G=23	A=0.0, G=1.0	0.00001	316.747	23	A=0/0, G=6/17
chr1:103444497	G/G	A	G	SNV	COL11A1	COL11A1:intronic:NM_001854.3	1	100		intronic	NM_00185	c.2656-28	p.?	-0.28					Collagen tripl	rs1903787		0.2637	YES	8.914E-2 (pos.103444497)		Collagen XI,	cartilage c	Abnormalities, Multiple;Adnex		Benign	A=0, G=26	A=0.0, G=1.0	0.00001	377	26	A=0/0, G=20/6
chr1:103449598	C/C	G	C	SNV	COL11A1	COL11A1:intronic:NM_001854.3	1	100		intronic	NM_00185	c.2556+96	p.?	-0.91					Collagen tripl	rs2615978			YES			Collagen XI,	cartilage c	Abnormalities, Multiple;Adnexal Diseases;Bc			G=0, C=29	G=0.0, C=1.0	0.00001	427.603	29	G=0/0, C=13/1
chr1:103455217	T/T	C	T	SNV	COL11A1	COL11A1:intronic:NM_001854.3	1	100		intronic	NM_00185	c.2341-90	p.?	-0.14					Collagen tripl	rs1463034			YES			Collagen XI,	cartilage c	Abnormalities, Multiple;Adnexal Diseases;Bc			C=0, T=63	C=0.0, T=1.0	0.00001	958.916	63	C=0/0, T=27/3
chr1:103467596	G/G	A	G	SNV	COL11A1	COL11A1:intronic:NM_001854.3	1	100		intronic	NM_00185	c.2098-71	p.?	1.5					Collagen tripl	rs12135531		0.1374	YES			Collagen XI,	cartilage c	Abnormalities, Multiple;Adnexal Diseases;Bc			A=0, G=20	A=0.0, G=1.0	0.00001	263.786	20	A=0/0, G=9/11
chr1:103467615	T/T	C	T	SNV	COL11A1	COL11A1:intronic:NM_001854.3	1	100		intronic	NM_00185	c.2098-90	p.?	-0.74					Collagen tripl	rs2929162			YES			Collagen XI,	cartilage c	Abnormalities, Multiple;Adnexal Diseases;Bc			C=0, T=20	C=0.0, T=1.0	0.00001	263.786	20	C=0/0, T=9/11
chr1:103471456	C/C	CCAT	C	INDEL	COL11A1	COL11A1:intronic:NM_001854.3	3	100		intronic	NM_00185	c.1792-9A	p.?	-0.46, -0.35, 2.88, -0.32					Collagen tripl	rs565657269,rs71752747,rs746			YES	0.0 (pos.103471455),5.658E-1 (pos.1034		Collagen XI,	cartilage c	Abnormalities, Multiple;Adnex		Benign/Likely	CCAT=0, C=	CCAT=0.0, C=1.0	0.00001	480.794	32	CCAT=0/0, C=1
chr1:103544191	T/T	C	T	SNV	COL11A1	COL11A1:intronic:NM_001854.3	1	100		intronic	NM_00185	c.488+23G	p.?	0.06					Collagen tripl	rs6668897		0.1553	YES	6.241E-3 (pos.103544191)		Collagen XI,	cartilage c	Abnormalities, Multiple;Adnex		Benign	C=0, T=112	C=0.0, T=1.0	0.00001	1776.37	112	C=0/0, T=71/4
chr1:103544434	G/G	A	G	SNV	COL11A1	COL11A1:intronic:NM_001854.3	1	100		intronic	NM_00185	c.275-7T>(	p.?	1.98					Collagen tripl	rs12136865		0.1507	YES	8.359E-3 (pos.103544434)		Collagen XI,	cartilage c	Abnormalities, Multiple;Adnex		Benign	A=0, G=25	A=0.0, G=1.0	0.00001	352.995	25	A=0/0, G=13/1
chr1:103548211	T/T	A	T	SNV	COL11A1	COL11A1:intronic:NM_001854.3	1	100		intronic	NM_00185	c.274+150	p.?	-0.52					Collagen tripl	rs1337184			YES			Collagen XI,	cartilage c	Abnormalities, Multiple;Adnexal Diseases;Bc			A=0, T=33	A=0.0, T=1.0	0.00001	504.586	33	A=0/0, T=12/2
chr1:104080169	C/C	G	C	SNV	RNPC3	RNPC3:intronic:NM_017619.3	1	100		intronic	NM_01761	c.767+63G	p.?	0.71					RNA recogniti	rs10736038			YES				RNA bindir	Bone Diseases;Bone Diseases, Development			G=0, C=46	G=0.0, C=1.0	0.00001	768.313	46	G=0/0, C=25/2
chr1:104086145	G/G	A	G	SNV	RNPC3	RNPC3:intronic:NM_017619.3	1	100		intronic	NM_01761	c.1045+76	p.?	-2.36					RNA recogniti	rs10785725							RNA bindir	Bone Diseases;Bone Diseases, Development			A=0, G=26	A=0.0, G=1.0	0.00001	371.399	26	A=0/0, G=7/19
chr1:104116312	G/G	A	G	SNV	AMY2B	AMY2B:intronic:NM_020978.4	1	100		intronic	NM_02097	c.514-18A	p.?	-1.01					Alpha amylas	rs12132040		0.1842	YES	1.392E-1 (pos.104116312)		Amylase, pa	alpha-amylase activity,alpha-a		1,4-dithio-alpha-D-gluco		A=0, G=58	A=0.0, G=1.0	0.00001	867.385	58	A=0/0, G=24/3
chr1:108679531	CT/CT	C	CT	INDEL	SLC25A24	SLC25A24:intronic:NM_013386.4	1	100		intronic	NM_01338	c.1250-72	p.?	-0.34					EF hand,EF-ha	rs1053504822							ADP transı	Autoimmune Diseases;Female Urogenital Dis			C=0, CT=47	C=0.0, CT=1.0	0.00001	420.876	47	C=0/0, CT=22/
chr1:108679532	T/T	C	T	SNV	SLC25A24	SLC25A24:intronic:NM_013386.4	1	100		intronic	NM_01338	c.1250-73	p.?	-0.33					EF hand,EF-ha	rs17513812			YES				ADP transı	Autoimmune Diseases;Female Urogenital Dis			C=0, T=49	C=0.0, T=1.0	0.00001	831.527	49	C=0/0, T=24/2
chr1:108724518	G/G	A	G	SNV	SLC25A24	SLC25A24:intronic:NM_013386.4	1	100		intronic	NM_01338	c.398+45T	p.?	0.02					EF hand,EF-ha	rs490680		0.3163	YES	6.737E-1 (pos.108724518)			ADP transı	Autoimmune Diseases;Female Urogenital Dis			A=0, G=29	A=0.0, G=1.0	0.00001	427.534	29	A=0/0, G=10/1
chr1:109255513	T/T	G	T	SNV	FNDC7	FNDC7:upstream:NM_001144937.1	1	100		intronic	NM_00114	c.-43G>T	p.?	-0.82						rs3006864		0.0141	YES	8.891E-1 (pos.109255513)			extracellular region				G=0, T=59	G=0.0, T=1.0	0.00001	885.368	59	G=0/0, T=35/2
chr1:109366370	T/T	C	T	SNV	AKNAD1	AKNAD1:intronic:NM_152763.4	1	100		intronic	NM_15276	c.1937-60	p.?	-2.69					AT-hook-cont	rs7522260			YES								C=0, T=103	C=0.0, T=1.0	0.00001	1613.76	103	C=0/0, T=53/5
chr12:9248233	C/C	T	C	SNV	A2M	A2M:exonic:NM_000014.5	1	100	16	missense	NM_00001	c.1915A>G	p.Asn639Asp	-1.81	0.95	23	0		A-macroglobı	rs226405		0		1.0 (pos.9248233)		Alpha-2-ma	GTPase ac	Acute Kidney Injury	Bacitracin,Becaplermin,		T=0, C=33	T=0.0, C=1.0	0.00001	504.598	33	T=0/0, C=17/1
chr1:109608874	T/T	TACTT	T	INDEL	TAF13	TAF13:intronic:NM_005645.3	4	100		intronic	NM_00564	c.107-20A	p.?	0.0, 0.36, -0.18, 1.71, 0.14					Transcriptior	rs201363933,rs3217403,rs7573			YES	1.057E-1;0.0 (pos.109608870)		TAF13 RNA	DNA binding,DNA binding transcription factor activity,DN				TACTT=0, T=	TACTT=0.0, T=1.0	0.00001	771.256	52	TACTT=0/0, T=
chr12:9246176	CTATGG/C	CTATGG	C	INDEL	A2M	A2M:splicesite_3:NM_000014.5	5	50	18	splicesite	NM_00001	c.2126-1C	p.?	1.72, 2.56, -0.56, -0.06, 0.03, -0.31				0.42(COSM50	A-macroglobı	rs1799759,rs762288		0.1555		0.0 (pos.9246177),3.826	adenocarcinoı	Alpha-2-ma	GTPase ac	Acute Kidney Injury	Bacitracin	Benign, risk fa	CTATGG=74	CTATGG=0.5, C=0.5	0.00001	831.217	148	CTATGG=18/5
chr12:9004512	A/A	G	A	SNV	A2ML1	A2ML1:exonic:NM_144670.5	1	100	19	synonymous	NM_14467	c.2367G>A	p.(=)	-3.57					A-macroglobı	rs1860927		0.1591	YES	9.999E-1 (pos.9004512)		Alpha-2-macroglobulin		Cardiovascular Abnormalities;		Benign	G=0, A=58	G=0.0, A=1.0	0.00001	866.629	58	G=0/0, A=18/4
chr12:9004892	A/A	C	A	SNV	A2ML1	A2ML1:exonic:NM_144670.5	1	100	20	missense	NM_14467	c.2550C>A	p.Asp850Glu	0.23	1	45	0		A-macroglobı	rs1860926		2.00E-04	YES	1.0 (pos.9004892)		Alpha-2-macroglobulin		Cardiovascular Abnormalities;Cardiovascula			C=0, A=57	C=0.0, A=1.0	0.00001	849.608	57	C=0/0, A=24/3
chr12:9016573	G/G	A	G	SNV	A2ML1	A2ML1:exonic:NM_144670.5	1	100	29	missense	NM_14467	c.3686A>G	p.His1229Arg	1.3	0.25	29	0	0.29(COSM50	A-macroglobı	rs10219561		5.00E-04		1.0 (pos.9016573)	osteosarcoma	Alpha-2-macroglobulin		Cardiovascular Abnormalities;Cardiovascula			A=0, G=33	A=0.0, G=1.0	0.00001	504.498	33	A=0/0, G=19/1
chr12:9020489	G/G	A	G	SNV	A2ML1	A2ML1:exonic:NM_144670.5	1	100	30	missense	NM_14467	c.3769A>G	p.Met1257Val	-0.08	0.12	21	0		A-macroglobı	rs7308811,rs765785		0.1607	YES	4.637E-4;0.0;0.0 (pos.9020488),9.998E-1		Alpha-2-macroglobulin		Cardiovascular Abnormalities;		Benign	A=0, G=32	A=0.0, G=1.0	0.00001	485.141	32	A=0/0, G=20/1
chr12:53703021	A/A	G	A	SNV	AAAS	AAAS:exonic:NM_015665.5	1	100	9	synonymous	NM_01566	c.855C>T	p.(=)	-0.36					WD domain,	rs1546808		0.0015	YES	9.999E-1 (pos.53703021)		Aladin	centroson	Adrenal Gland Diseases;Adren		Benign/Likely	G=0, A=51	G=0.0, A=1.0	0.00001	754.539	51	G=0/0, A=32/1
chr12:53700860	G/G	A	G	SNV	AAAS,C12orf10	AAAS:downstream:NM_015665.5, C12orf10:exonic:NM_021	1	100	7	missense	NM_02164	c.1058A>G	p.His353Arg, p.?	-0.44	0.64	29	0	0.09(COSM37	Uncharacteri	rs711316		5.00E-04	YES	9.999E-1 (pos.53700860)	squamous_cel	Aladin,Chro	centroson	Adrenal Gland Diseases;Adrenal Insufficiency			A=0, G=127	A=0.0, G=1.0	0.00001	2073.29	127	A=0/0, G=79/4
chr1:109839392	G/G	A	G	SNV	MYBPHL	MYBPHL:intronic:NM_001010985.2	1	100		intronic	NM_00101	c.730+13T	p.?	-1.34					Fibronectin t	rs604349		0.0949	YES	9.685E-1;0.0 (pos.109839392)			M band,Z disc,actin filament binding,actin filament orga				A=0, G=25	A=0.0, G=1.0	0.00001	353.019	25	A=0/0, G=10/1
chr12:53700848	T/T	C	T	SNV	AAAS,C12orf10	AAAS:downstream:NM_015665.5, C12orf10:exonic:NM_021	1	99.26	7	missense	NM_02164	c.1046C>T	p.Thr349Ile, p.?	4.74	1	89	0		Uncharacteri	rs1534282		0.001	YES	9.999E-1 (pos.53700848)		Aladin,Chro	centroson	Adrenal Gland Diseases;Adrenal Insufficiency			C=1, T=135	C=0.0074, T=0.9926	0.00001	2210.8	136	C=1/0, T=87/4
chr1:110018148	T/T	C	T	SNV	SYPL2	SYPL2:intronic:NM_001040709.1	1	100		intronic	NM_00104	c.130-55C	p.?	0.16					Membrane-a	rs530565							cellular calcium ion homeostasis,integral component of				C=0, T=20	C=0.0, T=1.0	0.00001	263.782	20	C=0/0, T=17/3
chr1:12776218	C/C	A	C	SNV	AADACL3	AADACL3:exonic:NM_001103170.2	1	100	1	stoploss	NM_00110	c.39A>C	p.Ter13Cys	-1.63					alpha/beta h	rs3000860			YES				catabolic process,hydrolase activity				A=0, C=29	A=0.0, C=1.0	0.00001	427.601	29	A=0/0, C=16/1
chr1:11015325	T/T	C	T	SNV	C1orf127	C1orf127:intronic:NM_001170754.1	1	100		intronic	NM_00117	c.779-82G	p.?	0.25					Domain of un	rs41274492			YES					Alopecia;Hair Diseases;Hypotrichosis;Skin Di			C=0, T=21	C=0.0, T=1.0	0.00001	281.235	21	C=0/0, T=7/14
chr1:12779560	C/C	T	C	SNV	AADACL3	AADACL3:exonic:NM_001103170.2	1	100	2	synonymous	NM_00110	c.252T>C	p.(=)	-4.13					alpha/beta h	rs3010876		0.1706	YES	7.77E-1 (pos.12779560)			catabolic process,hydrolase activity				T=0, C=58	T=0.0, C=1.0	0.00001	850.682	58	T=0/0, C=20/3
chr1:11017609	C/C	T	C	SNV	C1orf127	C1orf127:intronic:NM_001170754.1	1	100		intronic	NM_00117	c.693+64A	p.?	0.66					Domain of un	rs7511959,rs765006		0.2002	YES	0.0;0.0 (pos.11017605),2.523E-1 (pos.11017609)				Alopecia;Hair Diseases;Hypotrichosis;Skin Di			T=0, C=55	T=0.0, C=1.0	0.00001	813.784	55	T=0/0, C=23/3
chr1:11017617	C/C	G	C	SNV	C1orf127	C1orf127:intronic:NM_001170754.1	1	100		intronic	NM_00117	c.693+56C	p.?	1.12					Domain of un	rs34691090,rs765001		0.1992	YES	0.0;0.0 (pos.11017605),2.504E-1;0.0 (pos.11017617)				Alopecia;Hair Diseases;Hypotrichosis;Skin Di			G=0, C=55	G=0.0, C=1.0	0.00001	814.086	55	G=0/0, C=23/3
chr1:11017634	T/T	C	T	SNV	C1orf127	C1orf127:intronic:NM_001170754.1	1	100		intronic	NM_00117	c.693+39G	p.?	-0.54				0.08(COSM37	Domain of un	rs35950437		0.1998	YES	2.475E-1 (pos.11017634)	hepatocellular_carcinoma			Alopecia;Hair Diseases;Hypotrichosis;Skin Di			C=0, T=53	C=0.0, T=1.0	0.00001	784.043	53	C=0/0, T=23/3
chr1:110211835	C/C	T	C	SNV	GSTM2	GSTM2:intronic:NM_001142368.1	1	100		intronic	NM_00114	c.178-77T	p.?	-2.1						rs402505			YES			Glutathione	cellular de	Abnormalities, Mul	Glutathione,Glutathione		T=0, C=35	T=0.0, C=1.0	0.00001	543.903	35	T=0/0, C=12/2
chr1:11024353	A/G	A	G	SNV	C1orf127	C1orf127:intronic:NM_001170754.1	1	95.45		intronic	NM_00117	c.373-25T	p.?	0.37					Domain of un	rs6659231		0.2489	YES	4.701E-1 (pos.11024353)				Alopecia;Hair Diseases;Hypotrichosis;Skin Di			A=1, G=21	A=0.0455, G=0.9545	0.00001	271.509	22	A=0/1, G=5/16
chr1:11024381	G/G	A	G	SNV	C1orf127	C1orf127:intronic:NM_001170754.1	1	100		intronic	NM_00117	c.373-53T	p.?	0.01					Domain of un	rs6659326			YES					Alopecia;Hair Diseases;Hypotrichosis;Skin Di			A=0, G=26	A=0.0, G=1.0	0.00001	371.42	26	A=0/0, G=5/21
chr1:11024395	C/C	T	C	SNV	C1orf127	C1orf127:intronic:NM_001170754.1	1	100		intronic	NM_00117	c.373-67A	p.?	-0.05					Domain of un	rs11121663			YES					Alopecia;Hair Diseases;Hypotrichosis;Skin Di			T=0, C=26	T=0.0, C=1.0	0.00001	371.42	26	T=0/0, C=5/21
chr1:110295643	G/T	G	T	SNV	EPS8L3	EPS8L3:intronic:NM_139053.2	1	50		intronic	NM_13905	c.1203+96	p.?	1.77					Phosphotyro	rs1887546			YES			EPS8-like pr	Rac guany	Genital Diseases, Male;Genital Neoplasms, I			G=14, T=14	G=0.5, T=0.5	0.00001	131.924	28	G=8/6, T=9/5
chr1:12776344	A/T	A	T	SNV	AADACL3	AADACL3:exonic:NM_001103170.2	1	96.77	1	synonymous	NM_00110	c.165A>T	p.(=)	-2.33					alpha/beta h	rs3000859		0.1687	YES	6.424E-1 (pos.12776344)			catabolic process,hydrolase activity				A=1, T=30	A=0.0323, T=0.9677	0.00001	435.414	31	A=1/0, T=14/1
chr1:110467745	G/G	A	G	SNV	CSF1	CSF1:intronic:NM_000757.5	1	100		intronic	NM_00075	c.1623-24	p.?	-1.37					Macrophage	rs333972		0		1.0 (pos.110467745)		Colony-stim	CSF1-CSF1	Bone Diseases;Breast Diseases;Breast Neop			A=0, G=36	A=0.0, G=1.0	0.00001	563.744	36	A=0/0, G=14/2
chr2:69741854	G/G	T	G	SNV	AAK1	AAK1:exonic:NM_014911.3	1	100	13	missense	NM_01491	c.1525A>C	p.Lys509Gln	3.43	1	53	0	0.06(COSM45	Kinase-like,P	rs6715776			YES	1.0;0.0 (pos.69741854)	squamous_cel	Adaptor prc	AP-2 adaptor complex binding,ATP binding,Notch binding				T=0, G=34	T=0.0, G=1.0	0.00001	524.186	34	T=0/0, G=12/2
chr2:219128506	T/T	C	T	SNV	AAMP,GPBAR1	GPBAR1:utr_3:NM_170699.2, AAMP:downstream:NM_0010	1	100	2	utr_3	NM_17069	c.1059C>T	p.?, p.?	-0.6					7 transmemb	rs2292549			YES			Angio-assoc	G-protein	Crohn Disease;Digestive System Diseases;Ga			C=0, T=56	C=0.0, T=1.0	0.00001	832.098	56	C=0/0, T=32/2
chr17:41116234	A/A	G	A	SNV	AARSD1,PTGES3L-AARSD1	AARSD1:exonic:NM_001261434.1, PTGES3L-AARSD1:exonic:	1	100	7	synonymous	NM_00126	c.60C>T, c.	p.(=), p.(=)	0.67					Threonyl and	rs690941		8.00E-04	YES	9.995E-1 (pos.41116234)		Alanyl-tRNA	ATP binding,Ser-tRNA(Ala) hydrolase activity,alanine-tRN				G=0, A=21	G=0.0, A=1.0	0.00001	281.021	21	G=0/0, A=10/1
chr17:79095629	A/A	C	A	SNV	AATK	AATK:exonic:NM_001080395.2	1	100	11	missense	NM_00108	c.2107G>T	p.Gly703Cys	-2.38	0.14	159	0.002	0.02(COSM14	Protein kinas	rs386799844,rs7503		0.4816	YES	6.537E-1;0.0;0.0 (pos.79	adenocarcinoı	Apoptosis-a	ATP binding,biological_process,cellular_component,inte				C=0, A=25	C=0.0, A=1.0	0.00001	353.019	25	C=0/0, A=7/18
chr1:111861714	A/A	T	A	SNV	CHIA	CHIA:intronic:NM_201653.3	1	100		intronic	NM_20165	c.916-28T	p.?	0.32					Chitin bindir	rs2786161		0.3149	YES	4.422E-1 (pos.111861714)		Chitinase, a	apoptotic_	Abortion, Spontaneous;Female Urogenital Di			T=0, A=113	T=0.0, A=1.0	0.00001	1795.83	113	T=0/0, A=56/5
chr9:107586753	T/C	T	C	SNV	ABCA1	ABCA1:exonic:NM_005502.3	1	57.14	18	missense	NM_00550	c.2649A>G	p.Ile883Met	-1.12	0.12	10	0.002	0.08(COSM11	AAA domain,	rs2066714		0.1342	YES	7.204E-1 (pos.10758675	adenoma_neo	ATP-binding	ATP bindin	Arterial Occlusive D	ATP,Glybu	Benign	T=9, C=12	T=0.4286, C=0.5714	0.00001	109.102	21	T=3/6, C=5/7
chr17:67212423	A/A	G	A	SNV	ABCA10	ABCA10:exonic:NM_080282.3	1	100	8	missense	NM_08028	c.607C>T	p.Pro203Ser	0.18	0.81	74	0.006	0.03(COSM40	AAA domain,	rs9909216		0.3423	YES	2.993E-1;1.503E-3 (pos.6	acute_myeloic	ATP-binding	ATP bindin	Colonic Diseases;Colorectal Neoplasms;Dige			G=0, A=46	G=0.0, A=1.0	0.00001	768.313	46	G=0/0, A=29/1
chr1:112020238	C/C	A	C	SNV	C1orf162	C1orf162:intronic:NM_174896.3	1	100		intronic	NM_17489	c.203-35A	p.?	0.11						rs2211494				9.951E-1 (pos.112020238)			integral component of membrane				A=0, C=29	A=0.0, C=1.0	0.00001	427.28	29	A=0/0, C=17/1
chr2:215876166	T/T	A	T	SNV	ABCA12	ABCA12:exonic:NM_173076.2	1	100	17	missense	NM_17307	c.2329T>A	p.Ser777Thr	-1.41	1	58	0		AAA domain,	rs7560008		0		1.0 (pos.215876166)		ATP-binding	ATP bindin	Colonic Diseases;Colorectal N		Benign	A=0, T=29	A=0.0, T=1.0	0.00001	427.594	29	A=0/0, T=11/1
chr2:215820013	A/A	G	A	SNV	ABCA12,LOC101928103	ABCA12:exonic:NM_173076.2, LOC101928103:intronic_nc:N	1	100	43	synonymous	NR_11029	c.6306C>T	p.(=)	-0.51				0.07(COSM37	AAA domain,	rs10498027		0.4165	YES	2.32E-1 (pos.215820013)	adenocarcinoı	ATP-binding	ATP bindin	Colonic Diseases;Colorectal N		Benign	G=0, A=109	G=0.0, A=1.0	0.00001	1718.9	109	G=0/0, A=32/7
chr9:139904036	CG/CG	C	CG	INDEL	ABCA2	ABCA2:exonic:NM_001606.4	1	100	44	frameshiftInsertion	NM_00160	c.6690_66	p.Asp2231fs	1.79					AAA domain,	rs762693872				0.0 (pos.139904036)		ATP-binding	ATP binding,ATP-binding cassette (ABC) transporter comp				C=0, CG=97	C=0.0, CG=1.0	0.00001	1214.74	97	C=0/0, CG=58/
chr9:139906359	A/A	G	A	SNV	ABCA2	ABCA2:exonic:NM_001606.4	1	100	35	synonymous	NM_00160	c.5472C>T	p.(=)	3.13				0.92(COSM37	AAA domain,	rs2271862		0.3021	YES	8.083E-1 (pos.13990635	adenocarcinoı	ATP-binding	ATP binding,ATP-binding cassette (ABC) transporter comp				G=0, A=135	G=0.0, A=1.0	0.00001	2238.23	135	G=0/0, A=70/6
chr1:114339959	AATAAT/AATA	A	AATAAT	INDEL	RSBN1	RSBN1:intronic:NM_018364.4	5	100		intronic	NM_01836	c.1377+26	p.?	0.72												Round sper	nucleus				A=0, AATAA	A=0.0, AATAAT=1.0	0.00001	1745.89	121	A=0/0, AATAAT
chr9:139913239	G/G	T	G	SNV	ABCA2	ABCA2:exonic:NM_001606.4	1	100	13	missense	NM_00160	c.1751A>C	p.His584Pro	7.53	1	77	0		AAA domain,	rs908828		0		1.0 (pos.139913239)		ATP-binding	ATP binding,ATP-binding cassette (ABC) transporter comp				T=0, G=25	T=0.0, G=1.0	0.00001	353.018	25	T=0/0, G=20/5
chr1:94471075	G/G	A	G	SNV	ABCA4	ABCA4:exonic:NM_000350.2	1	100	44	synonymous	NM_00035	c.6069T>C	p.(=)	-1.4				0.02(COSM39	AAA domain,	rs1762114		0.0667	YES	8.885E-1 (pos.94471075)	acute_myeloic	ATP-binding	ATP bindin	Cleft Lip;Colonic Diseases;Colc		Benign	A=0, G=24	A=0.0, G=1.0	0.00001	334.783	24	A=0/0, G=5/19
chr1:115161140	T/T	C	T	SNV	DENND2C	DENND2C:intronic:NM_001256404.1	1	100		intronic	NM_00125	c.1228-37	p.?	-0.46					DENN (AEX-3)	rs12145066		0.2765	YES	1.25E-4 (pos.115161140)			Rab guanyl-nucleotide exchange factor activity,regulatic				C=0, T=55	C=0.0, T=1.0	0.00001	814.903	55	C=0/0, T=28/2
chr1:94578548	C/C	T	C	SNV	ABCA4	ABCA4:exonic:NM_000350.2	1	100	2	synonymous	NM_00035	c.141A>G	p.(=)	-3.09					AAA domain,	rs4847281		1.00E-04		1.0;0.0 (pos.94578548)		ATP-binding	ATP bindin	Cleft Lip;Colonic Diseases;Colc		Benign	T=0, C=25	T=0.0, C=1.0	0.00001	358.708	25	T=0/0, C=9/16
chr17:67260926	G/G	A	G	SNV	ABCA5	ABCA5:exonic:NM_172232.3	1	100	24	synonymous	NM_17223	c.3265T>C	p.(=)	4.73				0.95(COSM37	ABC transpor	rs12449649		0.4718	YES	3.81E-1 (pos.67260926)	acute_myeloic	ATP-binding	ATP bindin	Colonic Diseases;C	Tacrolimus		A=0, G=23	A=0.0, G=1.0	0.00001	316.738	23	A=0/0, G=14/9
chr1:115631924	T/T	C	T	SNV	TSPAN2	TSPAN2:intronic:NM_005725.5	1	100		intronic	NM_00572	c.69+61G>	p.?	-1.27					Tetraspanin f	rs4240540			YES			Tetraspanir	astrocyte development,axon development,brain develo				C=0, T=33	C=0.0, T=1.0	0.00001	509.092	33	C=0/0, T=9/24
chr1:11594400	C/C	T	C	SNV	DISP3	DISP3:intronic:NM_020780.1	1	100		intronic	NM_02078	c.3376-38	p.?	0.17					MMPL family	rs2235663		0.3727	YES	7.54E-1 (pos.11594400)		Patched do	cholesterol homeostasis,cholesterol metabolic process				T=0, C=22	T=0.0, C=1.0	0.00001	298.773	22	T=0/0, C=15/7
chr17:67101718	T/T	C	T	SNV	ABCA6	ABCA6:exonic:NM_080284.2	1	100	20	missense	NM_08028	c.2625G>A	p.Met875Ile	-2.11	0.86	10	0		ABC transpor	rs7212506		0.0591	YES	8.52E-1 (pos.67101718)		ATP-binding	ATP bindin	Colonic Diseases;Colorectal Neoplasms;Derr			C=0, T=106	C=0.0, T=1.0	0.00001	1662.78	106	C=0/0, T=61/4
chr17:67125840	T/T	C	T	SNV	ABCA6	ABCA6:exonic:NM_080284.2	1	100	7	missense	NM_08028	c.844G>A	p.Val282Ile	1.05	0.18	29	0	0.02(COSM40	ABC transpor	rs4968839		0.3025	YES	4.518E-1;0.0 (pos.67125	acute_myeloic	ATP-binding	ATP bindin	Colonic Diseases;Colorectal Neoplasms;Derr			C=0, T=181	C=0.0, T=1.0	0.00001	3004.1	181	C=0/0, T=82/9
chr19:1065018	T/T	G	T	SNV	ABCA7,ARHGAP45	ABCA7:exonic:NM_019112.3, ARHGAP45:upstream:NM_001	1	99.58	46	missense	NM_01911	c.6133G>T	p.Ala2045Ser, p.?	1.74	1	99	0.057	0.05(COSM37	AAA domain,	rs4147934		0.2258	YES	5.44E-1 (pos.1065018)	adenocarcinoı	ATP-binding	ATP bindin	Alzheimer Disease;Brain Diseases;Central Ne			G=1, T=236	G=0.0042, T=0.9958	0.00001	3935.67	237	G=1/0, T=109/
chr17:66985992	G/G	T	G	SNV	ABCA9	ABCA9:exonic:NM_080283.3	1	100	30	missense	NM_08028	c.3917A>C	p.Lys1306Thr	4.46	0.02	78	0.999	0.95(COSM44	AAA domain,	rs2302294		0.3576	YES	3.015E-1 (pos.66985992)	acute_myeloic	ATP-binding	ATP bindin	Colonic Diseases;Colorectal Neoplasms;Dige			T=0, G=34	T=0.0, G=1.0	0.00001	501.431	34	T=0/0, G=21/1
chr1:117142531	A/A	G	A	SNV	IGSF3	IGSF3:intronic:NM_001542.3	1	100		intronic	NM_00154	c.2089+32	p.?	-1.07					Immunoglobı	rs631461		0.4421	YES	5.303E-1 (pos.117142531)		Immunoglo	cell surface,cell surface receptor signaling pathway,inte				G=0, A=62	G=0.0, A=1.0	0.00001	843.389	62	G=0/0, A=34/2
chr17:67028260	C/C	T	C	SNV	ABCA9	ABCA9:exonic:NM_080283.3	1	100	10	synonymous	NM_08028	c.1434A>G	p.(=)	1.05				0.85(COSM14	AAA domain,	rs2302291		0.3112	YES	2.29E-1 (pos.67028260)	adenocarcinoı	ATP-binding	ATP bindin	Colonic Diseases;Colorectal Neoplasms;Dige			T=0, C=80	T=0.0, C=1.0	0.00001	1275.14	80	T=0/0, C=47/3
chr1:11854755	G/A	G	A	SNV	MTHFR	MTHFR:intronic:NM_005957.4	1	57.45		intronic	NM_00595	c.1166+31	p.?	-3.88					Methylenete	rs1994798		0.4124	YES	7.643E-1 (pos.11854755)		Methylenet	NADP bind	Abnormalities, Mul	Benazepril,Cyanocobala		G=20, A=27	G=0.4255, A=0.5745	0.00001	326.534	47	G=10/10, A=7/
chr1:118644338	C/A	C	A	SNV	SPAG17	SPAG17:intronic:NM_206996.3	1	52.27		intronic	NM_20699	c.634+25G	p.?	-1.25					Flagellar-ass	rs888062687						Sperm-asso	axonemal central apparatus,axonemal central apparatu				C=21, A=23	C=0.4773, A=0.5227	0.00001	259.557	44	C=14/7, A=10/
chr7:87082292	G/A	G	A	SNV	ABCB4	ABCB4:exonic:NM_018849.2	1	45.83	6	synonymous	NM_01884	c.504C>T	p.(=)	-0.52				0.08(COSM37	ABC transpor	rs1202283		0.4517	YES	3.729E-1 (pos.87082292)	adenocarcinoı	ATP-binding	ATP bindin	Adenocarcinoma;Bi	Colchicine	Benign	G=13, A=11	G=0.5417, A=0.4583	0.00001	93.5021	24	G=8/5, A=7/4
chr16:48227862	A/A	G	A	SNV	ABCC11	ABCC11:exonic:NM_032583.3	1	100	19	synonymous	NM_03258	c.2436C>T	p.(=)	1.41				0.28(COSM37	ABC transpor	rs11866251		0.1678	YES	2.663E-2 (pos.48227862)	adenocarcinoı	ATP-binding	ATP binding,ATP hydrolysis cou		Conjugated estrogens,C		G=0, A=25	G=0.0, A=1.0	0.00001	347.844	25	G=0/0, A=11/1
chr16:48122582	G/A	G	A	SNV	ABCC12	ABCC12:exonic:NM_033226.2	1	57.14	24	missense	NM_03322	c.3349C>T	p.Arg1117Cys	0.04	0.04	180	0.004		ABC transpor	rs7193955		0.2174	YES	8.961E-1 (pos.48122582)		ATP-binding	ATP binding,ATPase activity, coupled to transmembrane				G=27, A=36	G=0.4286, A=0.5714	0.00001	380.911	63	G=2/25, A=22/
chr13:95726541	G/G	A	G	SNV	ABCC4	ABCC4:exonic:NM_005845.4	1	100	23	synonymous	NM_00584	c.2844T>C	p.(=)	-0.2					ABC transpor	rs1189466		0.0409	YES	7.903E-1 (pos.95726541)		ATP-binding	15-hydrox	Arthritis;Arthritis, F	Adefovir Dipivoxil,Alpros		A=0, G=107	A=0.0, G=1.0	0.00001	1681.28	107	A=0/0, G=31/7
chr13:95727780	C/C	T	C	SNV	ABCC4	ABCC4:exonic:NM_005845.4	1	100	22	synonymous	NM_00584	c.2712A>G	p.(=)	-2.12					ABC transpor	rs1678339		0.041	YES	7.924E-1 (pos.95727780)		ATP-binding	15-hydrox	Arthritis;Arthritis, F	Adefovir Dipivoxil,Alpros		T=0, C=63	T=0.0, C=1.0	0.00001	958.917	63	T=0/0, C=42/2
chr3:183685534	G/G	A	G	SNV	ABCC5	ABCC5:exonic:NM_005688.3	1	100	12	synonymous	NM_00568	c.1782T>C	p.(=)	0.15					ABC transpor	rs939336		0.4353	YES	8.64E-1 (pos.183685534)		ATP-binding	ATP bindin	Arthritis;Arthritis, F	Adefovir Dipivoxil,Alpros		A=0, G=104	A=0.0, G=1.0	0.00001	1594.1	104	A=0/0, G=42/6
chr1:120293367	C/C	T	C	SNV	HMGCS2	HMGCS2:intronic:NM_005518.3	1	100		intronic	NM_00551	c.*1532+5	p.?	-0.32					Hydroxymeth	rs667246			YES			3-hydroxy-3	cholesterc	Cardiovascular Diseases;Colonic Diseases;Cc			T=0, C=53	T=0.0, C=1.0	0.00001	782.03	53	T=0/0, C=31/2
chr16:16271357	C/C	T	C	SNV	ABCC6	ABCC6:exonic:NM_001171.5	1	100	19	missense	NM_00117	c.2542A>G	p.Met848Val	1.47	0.14	21	0		ABC transpor	rs6416668,rs678673		5.00E-04	YES	3.12E-3 (pos.16271356),9.969E-1 (pos.16		ATP-binding	ATP bindin	Calcinosis;Calcium	Cisplatin,Dactinomycin,I		T=0, C=31	T=0.0, C=1.0	0.00001	465.819	31	T=0/0, C=18/1
chr1:12062205	G/G	A	G	SNV	MFN2	MFN2:intronic:NM_001127660.1	1	100		intronic	NM_00112	c.1160+45	p.?	-2.06					50S ribosome	rs2236057		0.3486	YES	6.703E-1 (pos.12062205)		Mitofusin 2	GTP bindir	Cardiomegaly;Cardiovascular Diseases;Char			A=0, G=75	A=0.0, G=1.0	0.00001	1187.57	75	A=0/0, G=35/4
chr6:30558477	GA/GA	G	GA	INDEL	ABCF1	ABCF1:exonic:NM_001025091.1	1	100	25	frameshiftInsertion	NM_00102	c.2538_25	p.Ter846fs	3.87					ABC transpor	rs4148252,rs725459		0.3225	YES	6.911E-1 (pos.30558477)		ATP-binding	ATP bindin	Autoimmune Diseases;Endocrine System Dis			G=0, GA=42	G=0.0, GA=1.0	0.00001	612.757	42	G=0/0, GA=10/
chr7:150921950	A/G	A	G	SNV	ABCF2	ABCF2:exonic:NM_005692.4	1	97.5	3	synonymous	NM_00569	c.279T>C	p.(=)	0.85					ABC transpor	rs6464133		0.3157	YES	8.88E-1 (pos.150921950)		ATP-binding	ATP binding,ATP-binding cassette (ABC) transporter comp				A=1, G=39	A=0.025, G=0.975	0.00001	613.862	40	A=0/1, G=23/1
chr1:12418700	C/C	T	C	SNV	VPS13D	VPS13D:intronic:NM_015378.3	1	100		intronic	NM_01537	c.10141+4	p.?	-1.56					N-terminal re	rs10864552		0.096	YES	9.998E-1 (pos.12418700)			extracellular exosome				T=0, C=80	T=0.0, C=1.0	0.00001	1288.96	80	T=0/0, C=22/5
chr7:150916228	G/A	G	A	SNV	ABCF2	ABCF2:exonic:NM_005692.4	1	57.58	8	synonymous	NM_00569	c.939C>T	p.(=)	0.3				0.31(COSM37	ABC transpor	rs6949587		0.4113	YES	7.267E-1 (pos.15091622	adenocarcinoı	ATP-binding	ATP binding,ATP-binding cassette (ABC) transporter comp				G=28, A=38	G=0.4242, A=0.5758	0.00001	408.204	66	G=16/12, A=16
chr2:44071743	A/G	A	G	SNV	ABCG8	ABCG8:exonic:NM_022437.2	1	57.14	2	missense	NM_02243	c.161A>G	p.Tyr54Cys	5.35	0	194	0.215	0.98(COSM37	ABC transpor	rs4148211		0.3852	YES	8.702E-1 (pos.44071743)	adenocarcinoı	ATP-binding	ATP bindin	Arterial Occlusive D	Ezetimibe	Benign	A=12, G=16	A=0.4286, G=0.5714	0.00001	161.769	28	A=1/11, G=3/1
chr2:27351948	G/G	T	G	SNV	ABHD1	ABHD1:exonic:NM_032604.3	1	100	3	missense	NM_03260	c.411T>G	p.Asp137Glu	0.09	1	45	0		Alpha/beta h	rs6715286		2.00E-04		1.0 (pos.27351948)		Abhydrolas	biological_process,carboxylic ester hydrolase activity,ce				T=0, G=26	T=0.0, G=1.0	0.00001	371.416	26	T=0/0, G=6/20
chr20:25288632	A/A	G	A	SNV	ABHD12	ABHD12:exonic:NM_015600.4	1	100	9	synonymous	NM_01560	c.837C>T	p.(=)	-2.17				0.04(COSM41	Alpha/beta h	rs6107027		0.4802	YES	1.024E-2 (pos.25288632)	neoplasm	Abhydrolas	AMPA glut	Ataxia;Cataract;Congenital, He		Benign/Likely	G=0, A=39	G=0.0, A=1.0	0.00001	623.979	39	G=0/0, A=22/1
chr14:51371150	C/T	C	T	SNV	ABHD12B,PYGL	PYGL:downstream:NM_002863.4, ABHD12B:utr_3:NM_0012	1	57.89	13	utr_3	NM_00120	c.1155C>T	p.?, p.?	-0.1					Alpha/beta h	rs11157781		0.1829	YES			Phosphoryl	5-phosphc	Adenocarcinoma;C	1-N-Acetyl-Beta-D-Gluco		C=16, T=22	C=0.4211, T=0.5789	0.00001	240.411	38	C=0/16, T=5/1
chr9:133761001	G/G	A	G	SNV	ABL1	ABL1:exonic:NM_005157.5	1	100	11	synonymous	NM_00515	c.3324A>G	p.(=)	-2.44				0.09(COSM50	F-actin bindir	rs1056171		0.4669	YES	5.973E-1;0.0 (pos.13376	diffuse_large_	Abelson mu	ATP bindin	Adenocarcinoma;B	1-4-(PYRIDIN-4-YLOXY)PH		A=0, G=20	A=0.0, G=1.0	0.00001	263.786	20	A=0/0, G=10/1
chr10:116307504	G/G	A	G	SNV	ABLIM1	ABLIM1:exonic:NM_002313.6	1	100	5	synonymous	NM_00231	c.705T>C	p.(=)	-0.19				0.11(COSM54	LIM domain,I	rs2247528		0.2715	YES	6.768E-1 (pos.11630750	acute_myeloid_leukaemia		actin bind	Mental Disorders;Substance-Related Disorde			A=0, G=100	A=0.0, G=1.0	0.00001	1702.26	100	A=0/0, G=53/4
chr17:970413	T/T	C	T	SNV	ABR	ABR:exonic:NM_021962.4	1	100	10	synonymous	NM_02196	c.1086G>A	p.(=)	1.28					C2 domain,PI	rs2262150		0.0963	YES	9.626E-1 (pos.970413)		Active BCR-	G-protein	Genital Diseases, Male;Genital Neoplasms, I			C=0, T=43	C=0.0, T=1.0	0.00001	705.844	43	C=0/0, T=15/2
chr12:109577735	T/T	C	T	SNV	ACACB	ACACB:exonic:NM_001093.3	1	100	1	synonymous	NM_00109	c.525C>T	p.(=)	-2.95				0.01(COSM41	Acetyl-CoA ca	rs2878960		0.3292	YES	2.506E-1 (pos.10957773	acute_myeloic	Acetyl-Coer	ATP bindin	Glucose Metabolisı	(2S)-2-(4-{3-CHLORO-5-(TF		C=0, T=29	C=0.0, T=1.0	0.00001	427.593	29	C=0/0, T=18/1
chr12:109577764	G/A	G	A	SNV	ACACB	ACACB:exonic:NM_001093.3	1	50	1	missense	NM_00109	c.554G>A	p.Arg185His	0.82	0.06	29	0.625		Acetyl-CoA ca	rs147024369		0		4.636E-4;0.0 (pos.109577764)		Acetyl-Coer	ATP bindin	Glucose Metabolisı	(2S)-2-(4-{3-CHLORO-5-(TF		G=15, A=15	G=0.5, A=0.5	0.00001	145.021	30	G=8/7, A=10/5
chr1:1289367	C/C	CTG	C	INDEL	MXRA8	MXRA8:intronic:NM_032348.3	2	100		intronic	NM_03234	c.1222+43	p.?	0.8, -0.16, 0.43					Immunoglobı	rs140777846,rs3506		0.0649	YES	0.0 (pos.1289366),0.0 (pos.1289369),0.0		Matrix remı	cell surface,cellular protein metabolic process,endopla				CTG=0, C=3	CTG=0.0, C=1.0	0.00001	583.661	37	CTG=0/0, C=19
chr12:121174899	T/C	T	C	SNV	ACADS	ACADS:exonic:NM_000017.3	1	59.09	3	synonymous	NM_00001	c.321T>C	p.(=)	-0.19				0.28(COSM37	Acyl-CoA deh	rs1064795254,rs391		0.4466	YES	5.701E-1 (pos.12117489	hepatocellular	Acyl-Coenzy	acyl-CoA d	Congenital, Heredit	Acetoacet	Benign	T=9, C=13	T=0.4091, C=0.5909	0.00001	125.258	22	T=3/6, C=12/1
chr1:1323078	G/G	A	G	SNV	CCNL2	CCNL2:intronic:NM_030937.5	1	100		intronic	NM_03093	c.1211+79	p.?	-0.04					Cyclin, C-tern	rs12729599			YES			Cyclin L2	cyclin-dependent protein kinase holoenzyme complex,cy				A=0, G=62	A=0.0, G=1.0	0.00001	940.298	62	A=0/0, G=22/4
chr1:1323143	C/C	CCT	C	INDEL	CCNL2	CCNL2:intronic:NM_030937.5	2	100		intronic	NM_03093	c.1211+14	p.?	-0.77, 1.91, -1.4					Cyclin, C-tern	rs113128675,rs3565		0.1834	YES	2.253E-2 (pos.1323143),3.914E-4 (pos.13		Cyclin L2	cyclin-dependent protein kinase holoenzyme complex,cy				CCT=0, C=5	CCT=0.0, C=1.0	0.00001	781.286	53	CCT=0/0, C=13
chr12:121176679	C/T	C	T	SNV	ACADS	ACADS:exonic:NM_000017.3	1	46.43	8	synonymous	NM_00001	c.990C>T	p.(=)	-1.49				0.02(COSM41	Acyl-CoA deh	rs3915,rs796051906		0.4462	YES	5.283E-1 (pos.12117667	neoplasm	Acyl-Coenzy	acyl-CoA d	Congenital, Heredit	Acetoacet	Benign	C=15, T=13	C=0.5357, T=0.4643	0.00001	117.914	28	C=4/11, T=9/4
chr15:89398329	CA/CA	C	CA	INDEL	ACAN	ACAN:exonic:NM_013227.3	1	100	12	frameshiftInsertion	NM_01322	c.2513_25	p.Glu839fs	0.6					Extracellular	rs776978647				0.0 (pos.89398329)		Aggrecan (chondroitin s		Arthritis;Arthritis, F	GM6001		C=0, CA=51	C=0.0, CA=1.0	0.00001	348.44	51	C=0/0, CA=22/
chr1:14149622	T/C	T	C	SNV	PRDM2	PRDM2:intronic:NM_012231.4	1	47.62		intronic	NM_01223	c.*5181-3	p.?	-4.05					C2H2-type zir	rs2697968		0.2383	YES	6.371E-1 (pos.14149622)		PR domain-	DNA bindi	Amphetamine-Related Disorders;Endocrine (			T=11, C=10	T=0.5238, C=0.4762	0.00001	82.2898	21	T=7/4, C=3/7
chr1:144618070	A/A	G	A	SNV	NBPF19,NBPF20,NBPF8,NBPF9	NBPF19:intronic:NM_001351365.1, NBPF8:intronic:NM_001	1	100		intronic	NM_00127	c.5306-20	p.?, p.?, p.?, p.?	-0.34					Repeat of unl	rs55646423			YES	4.938E-1 (pos.144618070)		Neuroblast	cytoplasm				G=0, A=78	G=0.0, A=1.0	0.00001	1249.35	78	G=0/0, A=44/3
chr1:144619483	C/C	A	C	SNV	NBPF19,NBPF20,NBPF8,NBPF9	NBPF19:intronic:NM_001351365.1, NBPF8:intronic:NM_001	1	100		intronic	NM_00127	c.5306-20	p.?, p.?, p.?, p.?	-2.69					Repeat of unl	rs4067648			YES			Neuroblast	cytoplasm				A=0, C=33	A=0.0, C=1.0	0.00001	504.557	33	A=0/0, C=19/1
chr15:89401615	T/G	T	G	SNV	ACAN	ACAN:exonic:NM_013227.3	1	50	12	synonymous	NM_01322	c.5799T>G	p.(=)	-2.48				0.03(COSM39	Extracellular	rs3825994		0.2538	YES	5.151E-1;0.0 (pos.89401	acute_myeloic	Aggrecan (chondroitin s		Arthritis;Arthritis, F	GM6001		T=46, G=46	T=0.5, G=0.5	0.00001	507.589	92	T=22/24, G=30
chr1:144930571	C/A	C	A	SNV	LOC100996724,NBPF19,NBPF20,NBPF9,PDE4DIP	LOC100996724:intronic_nc:NR_144516.1, NBPF19:intronic:	1	50		intronic	NM_00127	c.5357+10	p.?, p.?, p.?, p.?	1.27					Centrosomin	rs2762866			YES	5E-1 (pos.144930571)		Neuroblast	Golgi apparatus,cellular protein complex assembly,cent				C=22, A=22	C=0.5, A=0.5	0.00001	243.353	44	C=10/12, A=16

chr1:145016020	G/G	T	G	SNV	LOC100996724,NBPF19,NBPF20,NBPF9	LOC100996724:intronic nc:NR 144516.1, NBPF19:intronic:	1	100		intronic	NM 00127	c.5357+19	p.?, p.?, p.?	-0.08					Repeat of unl	rs657162	5.00E-04	YES	9.102E-1;0.0 (pos.145016020)		Neuroblast	cytoplasm				T=0, G=262	T=0.0, G=1.0	0.00001	4312.77	262	T=0/0, G=104/
chr1:145075854	G/C	G	C	SNV	NBPF19,NBPF20,NBPF9	NBPF19:intronic:NM 001351365.1, NBPF9:intronic:NM 001	1	57.14		intronic	NM 00127	c.5358-23	p.?, p.?, p.?	-0.32				0(COSM3723(	Repeat of unl	rs1663853		YES	5E-1 (pos.145075854)	carcinoma,nec	Neuroblast	cytoplasm				G=9, C=12	G=0.4286, C=0.5714	0.00001	108.039	21	G=7/2, C=9/3
chr1:145109468	G/G	A	G	SNV	NBPF19,NBPF20,NBPF9,SEC22B	NBPF19:intronic:NM 001351365.1, NBPF9:intronic:NM 001	1	100		intronic	NM 00127	c.5358-20	p.?, p.?, p.?, p.?	-0.05					Regulated-SN	rs2442652		YES			Neuroblast	COPII vesicle coating,ER to Golgi transport vesicle memb				A=0, G=407	A=0.0, G=1.0	0.00001	6750.96	407	A=0/0, G=261/
chr1:145311083	C/C	A	C	SNV	NBPF10,NBPF19,NBPF20,NBPF25P,NBPF9	NBPF19:intronic:NM 001351365.1, NBPF9:intronic:NM 001	1	100		intronic	NM 00127	c.5358-22	p.?, p.?, p.?, p.?	-0.97					Repeat of unl	rs4649871		YES	4.999E-1 (pos.145311083)		Neuroblast	cytoplasm				A=0, C=23	A=0.0, C=1.0	0.00001	316.747	23	A=0/0, C=8/15
chr1:145507826	T/T	A	T	SNV	LIX1L-AS1,NBPF10,NBPF19,NBPF20,RBM8A	NBPF19:intronic:NM 001351365.1, RBM8A:intronic:NM 00	1	100		intronic	NM 00127	c.10988+1	p.?, p.?, p.?, p.?	-1.61					RNA binding r	rs872786		YES			Neuroblast	RNA bindir	Blood Platelet Disorders;Cong		Benign	A=0, T=48	A=0.0, T=1.0	0.00001	809.696	48	A=0/0, T=22/2
chr1:145586780	C/C	T	C	SNV	NBPF10,NBPF19,NBPF20,NUDT17,PIAS3	PIAS3:downstream:NM 006099.3, NBPF19:intronic:NM 001	1	100		intronic	NM 00127	c.10988+2	p.?, p.?, p.?, p.?, p.?	0.21					NUDIX domai	rs10910829	0.3176	YES	4.102E-1 (pos.145586780)		Neuroblast	SUMO trar	Female Urogenital Diseases;Female Urogeni			T=0, C=69	T=0.0, C=1.0	0.00001	1070.5	69	T=0/0, C=39/3
chr1:145588536	T/T	C	T	SNV	NBPF10,NBPF19,NBPF20,NUDT17	NBPF19:intronic:NM 001351365.1, NBPF10:intronic:NM 0C	1	100		intronic	NM 00127	c.10988+2	p.?, p.?, p.?, p.?	0.76					NUDIX domai	rs9286836	0.3554	YES			Neuroblast	hydrolase activity,metal ion binding				C=0, T=35	C=0.0, T=1.0	0.00001	543.902	35	C=0/0, T=27/8
chr1:145601861	A/A	G	A	SNV	NBPF10,NBPF19,NBPF20,POLR3C	NBPF19:intronic:NM 001351365.1, POLR3C:intronic:NM 0C	1	100		intronic	NM 00127	c.10988+2	p.?, p.?, p.?, p.?	-0.1					RNA polymer	rs781822602,rs92868	3.00E-04		0.0 (pos.145601860),1.0 (pos.14560186		Neuroblast	DNA binding,DNA-directed 5'-3' RNA polymerase activity,				G=0, A=28	G=0.0, A=1.0	0.00001	407.513	28	G=0/0, A=10/1
chr1:1458073	A/G	A	G	SNV	ATAD3A	ATAD3A:intronic:NM 018188.4	1	52.38		intronic	NM 01818	c.895-51A	p.?	-3.69					ATPase famil	rs3118507	0.2144	YES	2.569E-3 (pos.1458073)		ATPase fam	ATP bindin	Genital Diseases, Male;Genital Neoplasms, I			A=20, G=22	A=0.4762, G=0.5238	0.00001	238.502	42	A=11/9, G=21/
chr16:67691668	G/G	A	G	SNV	ACD,CARMIL2	CARMIL2:downstream:NM 001013838.2, ACD:exonic:NM 0	1	100	11	missense	NM 00101	c.*247A>G	p.?, p.Val518Ala	-0.13	1	64	0	0.17(COSM44	Shelterin con	rs6979	0.489	YES	1.551E-1 (pos.67691668)	acute myeloic	Acd, mouse	DNA polyn	Congenital Abnormalities;Congenital, Heredi			A=0, G=37	A=0.0, G=1.0	0.00001	585.292	37	A=0/0, G=18/1
chr11:76637651	A/A	G	A	SNV	ACER3	ACER3:exonic:NM 018367.6	1	100	2	missense	NM 01836	c.154G>A	p.Val52Ile	1.02	1	29	0	0.09(COSM15	Ceramidase	rs4379869	0.264	YES	6.317E-1 (pos.76637651)	serous carcin	Alkaline cer	ceramide	Melanoma;Neoplasms;Neoplasms by Histolc			G=0, A=64	G=0.0, A=1.0	0.00001	977.633	64	G=0/0, A=25/3
chr1:148015876	T/T	C	T	SNV	NBPF19,NBPF26	NBPF19:intronic:NM 001351365.1, NBPF26:intronic:NM 0C	1	100		intronic	NM 00135	c.8252-40	p.?, p.?	0.8						rs61810765		YES			Neuroblastoma breakpoint family, member 19					C=0, T=29	C=0.0, T=1.0	0.00001	427.603	29	C=0/0, T=20/9
chr11:76637680	A/A	G	A	SNV	ACER3	ACER3:exonic:NM 018367.6	1	100	2	synonymous	NM 01836	c.183G>A	p.(=)	0					Ceramidase	rs4479014	0.267	YES	6.317E-1 (pos.76637680)		Alkaline cer	ceramide	Melanoma;Neoplasms;Neoplasms by Histolc			G=0, A=64	G=0.0, A=1.0	0.00001	977.63	64	G=0/0, A=25/3
chr7:100490797	G/T	G	T	SNV	ACHE	ACHE:exonic:NM 000665.4	1	57.58	2	missense	NM 00066	c.1057C>A	p.His353Asn	1.99	0.04	68	0.017	0.39(COSM37	Carboxyleste	rs1799805	0.0473	YES	2.369E-4 (pos.10049079	adenocarcino	Acetylcholi	DNA replic	Adenocarcinoma:A	(4ar,6s,8a	Benign	G=14, T=19	G=0.4242, T=0.5758	0.00001	204.329	33	G=10/4, T=9/1
chr1:150529322	TAA/TAA	T	TAA	INDEL	ADAMTSL4	ADAMTSL4:intronic:NM 019032.5	2	100		intronic	NM 01903	c.1749+53	p.?	0.24					ADAM-TS Spa	rs34379037		YES			ADAMTS-like	apoptotic	Congenital Abnormalities;Congenital, Heredi			T=0, TAA=2	T=0.0, TAA=1.0	0.00001	370.831	26	T=0/0, TAA=14
chr1:150531170	T/T	C	T	SNV	ADAMTSL4	ADAMTSL4:intronic:NM 019032.5	1	100		intronic	NM 01903	c.2559+45	p.?	-1.78				0.05(COSM41	ADAM-TS Spa	rs10749658	7.00E-04	YES	1.0 (pos.150531170)	neoplasm	ADAMTS-like	apoptotic	Congenital Abnormalities;Congenital, Heredi			C=0, T=22	C=0.0, T=1.0	0.00001	298.892	22	C=0/0, T=14/8
chr14:23549379	G/G	C	G	SNV	ACIN1	ACIN1:exonic:NM 014977.3	1	100	6	missense	NM 01497	c.1339G>C	p.Ala447Pro	2.78	1	27	0		RNSP1-SAP18	rs386775580,rs8793	0.0043		9.999E-1;1.156E-4;0.0 (pos.23549379)		Acinus	ASAP complex,ATPase activity,RNA binding,RNA splicing,a				C=0, G=24	C=0.0, G=1.0	0.00001	334.792	24	C=0/0, G=11/1
chr3:42907112	C/C	A	C	SNV	ACKR2	ACKR2:exonic:NM 001296.4	1	100	3	missense	NM 00129	c.1118A>C	p.Tyr373Ser	-0.13		144		0(COSM1423	7 transmemb	rs2228468	0.395	YES	6.769E-1;1.159E-4 (pos.4	acute myeloic	Atypical che	C-C chemo	Nutrition Disorders:Nutritional and Metaboli			A=0, C=29	A=0.0, C=1.0	0.00001	426.036	29	A=0/0, C=14/1
chr17:40024023	T/T	A	T	SNV	ACLY	ACLY:utr 3:NM 001096.2	1	100	29	utr 3	NM 00109	c.3346T>A	p.?	-0.97					ATP citrate ly	rs7217218	0.081	YES	9.999E-1 (pos.40024023)		ATP citrate	ATP bindin	Adenocarcinoma;Carcinoma;Carcinoma, Hep			A=0, T=77	A=0.0, T=1.0	0.00001	1229.4	77	A=0/0, T=48/2
chr1:55074922	G/A	G	A	SNV	ACOT11,FAM151A	FAM151A:utr 3:NM 176782.2, ACOT11:utr 3:NM 147161.3	1	50	16	utr 3	NM 14716	c.1922G>A	p.?, p.?	0.44					START domai	rs2289016	0.3342	YES	3.324E-1 (pos.55074922)		Acyl-CoA thi	acyl-CoA hydrolase activity,acyl-CoA metabolic process,I				G=10, A=10	G=0.5, A=0.5	0.00001	82.8855	20	G=6/4, A=5/5
chr14:74061968	C/C	T	C	SNV	ACOT4	ACOT4:exonic:NM 152331.3	1	100	3	synonymous	NM 15233	c.876T>C	p.(=)	-0.46					Acyl-CoA thio	rs2010070	0.2752	YES	9.475E-1 (pos.74061968)		Acyl-CoA thi	acyl-CoA hydrolase activity,acyl-CoA metabolic process,I				T=0, C=40	T=0.0, C=1.0	0.00001	644.222	40	T=0/0, C=13/2
chr3:58520859	G/G	A	G	SNV	ACOX2	ACOX2:utr 5:NM 003500.3	1	100	2	utr 5	NM 00350	c.-26T>C	p.?	-0.14					Acyl-CoA deh	rs4681862	0.0664	YES	1.0 (pos.58520859)		Acyl-Coenzy	3alpha,7alpha,12alpha-trihydroxy-5beta-cholestanoyl-(				A=0, G=57	A=0.0, G=1.0	0.00001	849.582	57	A=0/0, G=3/54
chr1:147119273	G/A	G	A	SNV	ACP6,NBPF19	NBPF19:intronic:NM 001351365.1, ACP6:exonic:NM 0163	1	45.83	10	synonymous	NM 00135	c.8810+45	p.?, p.(=)	0.64				0.08(COSM44	Histidine phc	rs1344	0.4315	YES	3.729E-1 (pos.14711927	acute myeloic	Acid phosph	acid phosphatase activity,cytoplasm,dephosphorylatior				G=13, A=11	G=0.5417, A=0.4583	0.00001	93.44	24	G=8/5, A=5/6
chr16:89167140	G/C	G	C	SNV	ACSF3	ACSF3:exonic:NM 001127214.3	1	96.67	2	synonymous	NM 00112	c.51G>C	p.(=)	0.76				0(COSM1380	AMP-binding	rs7201122	0.0885	YES	6.768E-1;1.191E-4 (pos.8	adenocarcino	Acyl-CoA sy	ATP bindin	Amino Acid Metabolism, Inbor		Benign	G=1, C=29	G=0.0333, C=0.9667	0.00001	415.52	30	G=0/1, C=14/1
chr1:151210789	T/T	C	T	SNV	PIP5K1A	PIP5K1A:intronic:NM 001135638.1	1	100		intronic	NM 00113	c.1229+48	p.?	0					Phosphatidyl	rs7532935	0.3069	YES	6.266E-1 (pos.151210789)		Phosphatid	1-phosphatidylinositol-3-phosphate 4-kinase activity,1-(				C=0, T=26	C=0.0, T=1.0	0.00001	371.42	26	C=0/0, T=11/1
chr1:151227371	G/G	A	G	SNV	PSMD4	PSMD4:intronic:NM 002810.2	1	100		intronic	NM 00281	c.26+87A>	p.?	-0.03					Ssl1-like,Ubic	rs111503421,rs3811405		YES			Proteasom	Fc-epsilon receptor signaling pathway,MAPK cascade,NI				A=0, G=29	A=0.0, G=1.0	0.00001	425.221	29	A=0/0, G=19/1
chr4:185701549	G/T	G	T	SNV	ACSL1	ACSL1:exonic:NM 001995.3	1	50	5	synonymous	NM 00199	c.414C>A	p.(=)	-0.07					AMP-binding	rs1803898	0.336	YES	6.438E-2 (pos.185701549)		Acyl-CoA sy	ATP bindin	Arthritis;Arthritis, E	ATP,Adenosine monophc		G=21, T=21	G=0.5, T=0.5	0.00001	228.871	42	G=17/4, T=14/
chr20:24987438	T/T	C	T	SNV	ACSS1	ACSS1:utr 3:NM 032501.3	1	100	14	utr 3	NM 03250	c.3030G>A	p.?	-2.89					AMP-binding	rs6083718		YES			Acyl-CoA sy	AMP binding,ATP binding,aceta		ATP,Adenosine monophc		C=0, T=31	C=0.0, T=1.0	0.00001	440.743	31	C=0/0, T=24/7
chr20:25011423	C/C	T	C	SNV	ACSS1	ACSS1:exonic:NM 032501.3	1	100	3	synonymous	NM 03250	c.603A>G	p.(=)	-5.74				0.02(COSM40	AMP-binding	rs6050259	0.3097	YES	9.752E-2 (pos.25011423)	acute myeloic	Acyl-CoA sy	AMP binding,ATP binding,aceta		ATP,Adenosine monophc		T=0, C=105	T=0.0, C=1.0	0.00001	1644.55	105	T=0/0, C=43/6
chr1:151373200	C/C	T	C	SNV	PSMB4	PSMB4:intronic:NM 002796.2	1	100		intronic	NM 00279	c.495-40T	p.?	-0.68					Proteasome	rs2495391	3.00E-04		1.0;0.0 (pos.151373200)		Proteasom	Fc-epsilon	HIV Infections:Imm	(3AR,6R,6AS)-6-((S)-((S)-C		T=0, C=22	T=0.0, C=1.0	0.00001	298.892	22	T=0/0, C=16/6
chr1:151384733	A/A	G	A	SNV	POGZ	POGZ:intronic:NM 015100.3	1	100		intronic	NM 01510	c.1779+39	p.?	0.07					DDE superfar	rs3748550	0.1733	YES	6.395E-1 (pos.151384733)		POGO trans	DNA binding,cell division,cytoplasm,cytosol,kinetochore				G=0, A=20	G=0.0, A=1.0	0.00001	263.786	20	G=0/0, A=9/11
chr1:151395782	C/C	T	C	SNV	POGZ	POGZ:intronic:NM 015100.3	1	100		intronic	NM 01510	c.1678+91	p.?	-1.8					DDE superfar	rs2274533		YES			POGO trans	DNA binding,cell division,cytoplasm,cytosol,kinetochore				T=0, C=30	T=0.0, C=1.0	0.00001	446.634	30	T=0/0, C=11/1
chr1:151402045	G/G	A	G	SNV	POGZ	POGZ:intronic:NM 015100.3	1	100		intronic	NM 01510	c.568+34T	p.?	-0.39					DDE superfar	rs6587577	0.1731	YES	6.361E-1 (pos.151402045)		POGO trans	DNA binding,cell division,cytoplasm,cytosol,kinetochore				A=0, G=35	A=0.0, G=1.0	0.00001	543.903	35	A=0/0, G=16/1
chr1:151551320	A/A	G	A	SNV	TUFT1	TUFT1:intronic:NM 020127.2	1	100		intronic	NM 02012	c.924+18G	p.?	-1.66						rs3748608	0.304	YES	4.524E-1 (pos.151551320)		Tuftelin 1	bone mineralization,cytoplasm,extracellular region,odo				G=0, A=20	G=0.0, A=1.0	0.00001	263.78	20	G=0/0, A=15/5
chr1:151743870	T/T	TA	T	INDEL	OAZ3	OAZ3:downstream:NM 016178.2	1	100		intronic	NM 01617	c.*175TA>	p.?	-0.86, -0.06						rs3833528,rs397725608		YES			Ornithine d	blood microparticle,cytoplasm		Ornithine		TA=0, T=33	TA=0.0, T=1.0	0.00001	503.014	33	TA=0/0, T=16/
chr1:151748425	A/A	C	A	SNV	TDRKH	TDRKH:intronic:NM 001083963.1	1	100		intronic	NM 00108	c.1218-65	p.?	-0.61					KH domain,Tu	rs3923394	0.3498	YES				DNA methylation involved in gamete generation,RNA bin				C=0, A=22	C=0.0, A=1.0	0.00001	298.889	22	C=0/0, A=13/9
chr1:151754112	T/C	T	C	SNV	TDRKH	TDRKH:intronic:NM 001083963.1	1	95.65		intronic	NM 00108	c.125-49A	p.?	-0.13					KH domain,Tu	rs35077383	0.4944	YES	2.317E-1 (pos.151754112)			DNA methylation involved in gamete generation,RNA bin				T=1, C=22	T=0.0435, C=0.9565	0.00001	282.77	23	T=0/1, C=9/13
chr20:24994275	G/A	G	A	SNV	ACSS1	ACSS1:exonic:NM 032501.3	1	52.38	10	synonymous	NM 03250	c.1476C>T	p.(=)	-0.53					AMP-binding	rs6115001	0.1019	YES	5.847E-4;0.0 (pos.24994275)		Acyl-CoA sy	AMP binding,ATP binding,aceta		ATP,Adenosine monophc		G=10, A=11	G=0.4762, A=0.5238	0.00001	95.3003	21	G=8/2, A=7/4
chr20:24994289	C/T	C	T	SNV	ACSS1	ACSS1:exonic:NM 032501.3	1	47.62	10	missense	NM 03250	c.1462G>A	p.Val488Met	-2.49	0.04	21	0.19	0.02(COSM41	AMP-binding	rs6050249	0.1816	YES	7.436E-2 (pos.24994289)	neoplasm	Acyl-CoA sy	AMP binding,ATP binding,aceta		ATP,Adenosine monophc		C=11, T=10	C=0.5238, T=0.4762	0.00001	82.289	21	C=7/4, T=8/2
chr17:79478019	A/A	G	A	SNV	ACTG1	ACTG1:exonic:NM 001614.3	1	100	5	synonymous	NM 00161	c.918C>T	p.(=)	3.09				0.82(COSM13	Actin	rs1139405	0.1838	YES	8.763E-1 (pos.79478019)	acute myeloic	Actin, gamn	ATP bindin	Acute Coronary Syndrome;Bon		Benign/Likely	G=0, A=62	G=0.0, A=1.0	0.00001	940.319	62	G=0/0, A=31/3
chr2:74128433	T/T	C	T	SNV	ACTG2	ACTG2:utr 5:NM 001615.3	1	100	2	utr 5	NM 00161	c.-6C>T	p.?	0.17					Actin	rs1050146	0.205	YES	5.583E-1 (pos.74128433)		Actin, gamn	ATP bindin	Abnormalities, Multiple;Colonic Diseases;Co			C=0, T=41	C=0.0, T=1.0	0.00001	664.697	41	C=0/0, T=27/1
chr9:111625913	G/G	A	G	SNV	ACTL7A	ACTL7A:utr 3:NM 006687.3	1	100	1	utr 3	NM 00668	c.1311A>G	p.?	-3.17					Actin,Actin-li	rs4978370	0.1556	YES	8.257E-1 (pos.111625913)		Actin-like 7	Golgi apparatus,cytoplasm,cytoskeleton,cytoskeleton o				A=0, G=40	A=0.0, G=1.0	0.00001	644.286	40	A=0/0, G=26/1
chr1:236883421	T/T	C	T	SNV	ACTN2	ACTN2:exonic:NM 001103.3	1	100	4	synonymous	NM 00110	c.378C>T	p.(=)	-0.34					CAMSAP CH d	rs1341863,rs201411	0.0047	YES	9.812E-1 (pos.236883421)		Actinin, alp	FATZ bindi	Aortic Stenosis, Subvalvular;Ac		Benign	C=0, T=27	C=0.0, T=1.0	0.00001	380.911	27	C=0/0, T=15/1
chr19:39196736	G/A	G	A	SNV	ACTN4	ACTN4:exonic:NM 004924.5	1	97.3	5	synonymous	NM 00492	c.537G>A	p.(=)	-2.99				0.09(COSM37	CAMSAP CH d	rs11553600	0.1885	YES	3.236E-3 (pos.39196736)	adenocarcino	Actinin, alp	RNA bindir	Female Urogenital Diseases:Fe		Benign	G=1, A=36	G=0.027, A=0.973	0.00001	551.897	37	G=0/1, A=23/1
chr2:65495887	A/A	G	A	SNV	ACTR2	ACTR2:utr 3:NM 001005386.2	1	100	10	utr 3	NM 00100	c.1219G>A	p.?	-0.54					Actin	rs268875	0.1272	YES	7.101E-1 (pos.65495887)		Actin-relate	ATP bindin	Autoimmune Disea	(2S)-2-(3-bromophenyl)-3		G=0, A=23	G=0.0, A=1.0	0.00001	316.748	23	G=0/0, A=10/1
chr20:37400490	G/A	G	A	SNV	ACTR5	ACTR5:utr 3:NM 024855.3	1	45.45	9	utr 3	NM 02485	c.1855G>A	p.?	-0.85					Actin	rs6128952	0.1671	YES	1.369E-1 (pos.37400490)			DNA recombination,Ino80 complex,UV-damage excision				G=18, A=15	G=0.5455, A=0.4545	0.00001	143.162	33	G=6/12, A=6/9
chr2:158626980	T/T	C	T	SNV	ACVR1	ACVR1:exonic:NM 001111067.2	1	100	7	synonymous	NM 00111	c.690G>A	p.(=)	0.7					Activin types	rs1146031	0.0113	YES	9.682E-1 (pos.158626980)		Activin A rec	ATP bindin	Acute Lung Injury;A	6-4-(2-pipe	Benign	C=0, T=30	C=0.0, T=1.0	0.00001	446.639	30	C=0/0, T=16/1
chr2:158636910	A/A	G	A	SNV	ACVR1	ACVR1:exonic:NM 001111067.2	1	100	4	synonymous	NM 00111	c.270C>T	p.(=)	-1.75					Activin types	rs2227861	0.2273	YES	9.279E-1 (pos.158636910)		Activin A rec	ATP bindin	Acute Lung Injury;A	6-4-(2-pipe	Benign	G=0, A=41	G=0.0, A=1.0	0.00001	662.083	41	G=0/0, A=20/2
chr14:75520065	C/C	T	C	SNV	ACYP1	ACYP1:utr 3:NM 001107.4	1	100	3	utr 3	NM 00110	c.382A>G	p.?	0.34					Acylphospha	rs7303		YES			Acylphosph	acylphosphatase activity,extracellular exosome,phosph				T=0, C=66	T=0.0, C=1.0	0.00001	1015.4	66	T=0/0, C=38/2
chr22:17662793	G/G	A	G	SNV	ADA2	ADA2:exonic:NM 001282225.1	1	100	9	synonymous	NM 00128	c.1359T>C	p.(=)	0.46				0.47(COSM37	Adenosine/A	rs7289170	0.3324	YES	4.892E-2 (pos.17662793)	adenocarcinoma,carcinom		adenosine catabolic process,adenosine d			Benign	A=0, G=35	A=0.0, G=1.0	0.00001	543.903	35	A=0/0, G=14/2
chr16:84229559	C/C	T	C	SNV	ADAD2,LOC654780	LOC654780:intronic nc:NR 147175.1, ADAD2:exonic:NM 0	1	100	7	synonymous	NM 00114	c.1191T>C	p.(=)	-3.8				0.01(COSM43	Adenosine-de	rs2303238	0.227	YES	7.131E-1 (pos.84229559)	adenocarcinoma,neoplasn		RNA binding,RNA processing,adenosine deaminase activ				T=0, C=20	T=0.0, C=1.0	0.00001	263.785	20	T=0/0, C=5/15
chr1:152193990	G/G	A	G	SNV	HRNR	HRNR:intronic:NM 001009931.2	1	100		intronic	NM 00100	c.139-24T	p.?	0.28					S-100/ICaBP t	rs11204938	0.1701	YES	3.881E-1;0.0 (pos.152193990)		Hornerin	azurophil granule lumen,calcium ion binding,cell envelo				A=0, G=39	A=0.0, G=1.0	0.00001	623.98	39	A=0/0, G=17/2
chr16:84230500	C/C	A	C	SNV	ADAD2,LOC654780	LOC654780:exonic nc:NR 147175.1, ADAD2:exonic:NM 00	1	100	10	synonymous	NM 00114	c.1672A>C	p.(=)	0.69				0.01(COSM43	Adenosine-de	rs2303243	0.2884	YES	2.154E-1;0.0 (pos.84230	hepatocellular carcinoma		RNA binding,RNA processing,adenosine deaminase activ				A=0, C=27	A=0.0, C=1.0	0.00001	389.932	27	A=0/0, C=11/1
chr15:43632549	T/C	T	C	SNV	ADAL	ADAL:exonic:NM 001159280.2	1	57.14	7	synonymous	NM 00115	c.342T>C	p.(=)	-0.39				0.43(COSM37	Adenosine/A	rs2278857	0.3168	YES	4.864E-1 (pos.43632549)	acute myeloid leukaemia		adenosine catabolic process,adenosine deaminase acti				T=9, C=12	T=0.4286, C=0.5714	0.00001	109.102	21	T=6/3, C=6/6
chr8:39496029	C/C	T	C	SNV	ADAM18	ADAM18:exonic:NM 014237.2	1	100	10	synonymous	NM 01423	c.873T>C	p.(=)	-0.54				0.01(COSM39	ADAM cysteir	rs12708194	0.3324	YES	2.627E-1 (pos.39496029)	acute myeloid leukaemia		cell differentiation,integral component of membrane,m				T=0, C=26	T=0.0, C=1.0	0.00001	371.419	26	T=0/0, C=11/1
chr5:156936364	C/C	T	C	SNV	ADAM19	ADAM19:exonic:NM 033274.4	1	100	9	missense	NM 03327	c.850A>G	p.Ser284Gly	0.03	0.09	56	0.897	0.12(COSM44	ADAM cysteir	rs1422795	0.3516	YES	1.386E-1 (pos.15693636	hepatocellula	A disintegri	Golgi apparatus,SH3 domain binding,extracellular matri				T=0, C=104	T=0.0, C=1.0	0.00001	1626.73	104	T=0/0, C=42/6
chr14:70924462	G/G	A	G	SNV	ADAM21	ADAM21:exonic:NM 003813.3	1	100	2	synonymous	NM 00381	c.246A>G	p.(=)	0.03				0(COSM3999	ADAM cysteir	rs3751523	0.1128	YES	5.398E-1 (pos.70924462)	acute myeloic	A disintegri	axon,binding of sperm to zona pellucida,integral compor				A=0, G=180	A=0.0, G=1.0	0.00001	2987.95	180	A=0/0, G=84/9
chr1:153283004	T/T	C	T	SNV	PGLYRP3	PGLYRP3:intronic:NM 052891.2	1	100		intronic	NM 05289	c.55+83G>	p.?	-1.87					N-acetylmura	rs821423		YES				N-acetylmuramoyl-L-alanine a		2-ACETAMIDO-2-DEOXY-B		C=0, T=22	C=0.0, T=1.0	0.00001	298.792	22	C=0/0, T=10/1
chr14:70924501	G/G	C	G	SNV	ADAM21	ADAM21:exonic:NM 003813.3	1	100	2	missense	NM 00381	c.285C>G	p.Asp95Glu	-1.97		45		0(COSM4590	ADAM cysteir	rs8010994		YES	6.112E-1;0.0 (pos.70924	squamous cel	A disintegri	axon,binding of sperm to zona pellucida,integral compor				C=0, G=182	C=0.0, G=1.0	0.00001	3024.74	182	C=0/0, G=83/9
chr8:24211331	A/A	G	A	SNV	ADAM28,LOC101929294	ADAM28:exonic:NM 014265.5, LOC101929294:intronic nc:	1	100	22	missense	NM 01426	c.2293G>A	p.Val765Met	0.1	1	21	0		ADAM cysteir	rs7814768	0.0041	YES	9.99E-1 (pos.24211331)		A disintegri	extracellular region,integral cc	(2R,3R)-N^1^-(1S)-2,2-DIN			G=0, A=118	G=0.0, A=1.0	0.00001	1893.99	118	G=0/0, A=42/7
chr8:24211993	A/A	ACTAT	A	INDEL	ADAM28,LOC101929294	ADAM28:utr 3:NM 014265.5, LOC101929294:intronic nc:N	4	100	23	utr 3	NM 01426	c.2405 24	p.?	0.68, 0.01, -0.67, 0.06, 0.06					ADAM cysteir	rs10584989,rs10610	0.005	YES			A disintegri	extracellular region,integral cc	(2R,3R)-N^1^-(1S)-2,2-DIN			ACTAT=0, A	ACTAT=0.0, A=1.0	0.00001	583.707	37	ACTAT=0/0, A=
chr8:24356818	C/C	A	C	SNV	ADAM7,LOC101929294,LOC101929315	LOC101929315:intronic nc:NR 125809.1, LOC101929294:i	1	100	17	missense	NR 12580	c.1912A>C	p.Asn638His	-1.27	0.35	68	0	0.02(COSM41	ADAM cysteir	rs13259668	0.3335	YES	1.826E-1 (pos.24356818)	carcinoma,nec	A disintegri	integral component of membrane,metal ion binding,met				A=0, C=33	A=0.0, C=1.0	0.00001	502.645	33	A=0/0, C=19/1
chr1:153604029	T/T	C	T	SNV	S100A1	S100A1:intronic:NM 006271.1	1	100		intronic	NM 00627	c.142-145	p.?	-0.17					EF hand,EF-ha	rs1046379,rs746372901		YES	1.161E-4 (pos.153604028),8.493E-1 (pos		S100 protei	ATPase bir	Brain Diseases:Car	Olopatadine		C=0, T=26	C=0.0, T=1.0	0.00001	370.189	26	C=0/0, T=19/7
chr1:153604112	A/A	G	A	SNV	S100A1	S100A1:intronic:NM 006271.1	1	100		intronic	NM 00627	c.142-62G	p.?	-1.77					EF hand,EF-ha	rs1046381		YES			S100 protei	ATPase bir	Brain Diseases:Car	Olopatadine		G=0, A=29	G=0.0, A=1.0	0.00001	427.602	29	G=0/0, A=20/9
chr1:153656352	G/G	T	G	SNV	NPR1	NPR1:intronic:NM 000906.3	1	100		intronic	NM 00090	c.1484+52	p.?	-2.38					Adenylate an	rs9662664	0.4511	YES	6.843E-1 (pos.153656352)		Natriuretic	ATP bindin	Cardiomegaly;Card	Amyl Nitrite,Erythrityl Te		T=0, G=62	T=0.0, G=1.0	0.00001	940.313	62	T=0/0, G=32/3
chr1:153656405	G/G	C	G	SNV	NPR1	NPR1:intronic:NM 000906.3	1	100		intronic	NM 00090	c.1484+10	p.?	0.03					Adenylate an	rs9661993		YES			Natriuretic	ATP bindin	Cardiomegaly;Card	Amyl Nitrite,Erythrityl Te		C=0, G=59	C=0.0, G=1.0	0.00001	888.299	59	C=0/0, G=29/3
chr1:153661374	C/C	CG	C	INDEL	MIR8083,NPR1	NPR1:intronic:NM 000906.3, MIR8083:downstream:NR 10	1	100		intronic	NM 00090	c.2418-55	p.?	-1.4, 0.1					Adenylate an	rs34402066,rs397815088,rs552		YES			Natriuretic	ATP bindin	Cardiomegaly;Card	Amyl Nitrite,Erythrityl Te		CG=0, C=26	CG=0.0, C=1.0	0.00001	360.892	26	CG=0/0, C=20/
chr1:153790501	C/T	C	T	SNV	GATAD2B	GATAD2B:intronic:NM 020699.3	1	59.09		intronic	NM 02069	c.729+15G	p.?	-0.82					Coiled-coil ar	rs4363451	0.2991	YES	2.741E-1 (pos.153790501)		GATA zinc fi	ATP-dependent chromatin remodeling,DNA binding trans				C=9, T=13	C=0.4091, T=0.5909	0.00001	122.567	22	C=7/2, T=5/8
chr8:24359068	A/A	G	A	SNV	ADAM7,LOC101929294,LOC101929315	LOC101929315:intronic nc:NR 125809.1, LOC101929294:i	1	100	20	synonymous	NR 12580	c.2187G>A	p.(=)	-1.16				0(COSM1456	ADAM cysteir	rs13277171	0.3323	YES	1.799E-1;1.164E-4 (pos.2	adenocarcino	A disintegri	integral component of membrane,metal ion binding,met				G=0, A=31	G=0.0, A=1.0	0.00001	465.819	31	G=0/0, A=11/2
chr1:154141908	G/G	A	G	SNV	TPM3	TPM3:intronic:NM 152263.3	1	100		intronic	NM 15226	c.776-49T	p.?	1.44					C-terminal le	rs4845364	0.4972	YES	8.769E-1 (pos.154141908)		Tropomyosi	actin cyto	Adenocarcinoma;Carcinoma;C		Benign	A=0, G=21	A=0.0, G=1.0	0.00001	281.235	21	A=0/0, G=10/1
chr1:154186918	C/C	G	C	SNV	C1orf43	C1orf43:intronic:NM 001098616.2	1	100		intronic	NM 00109	c.286+15C	p.?	-0.1					NICE-3 protei	rs12408458	0.3695	YES	4.194E-1 (pos.154186918)		Chromosom	integral component of membrane,oxidation-reduction p				G=0, C=47	G=0.0, C=1.0	0.00001	789.313	47	G=0/0, C=30/1
chr1:154228287	C/C	T	C	SNV	UBAP2L	UBAP2L:intronic:NM 014847.3	1	100		intronic	NM 01484	c.2157+56	p.?	-1.8					Ubiquitin-ass	rs1194603	0.4654	YES			Ubiquitin-a	Ada2/Gcn5/Ada3 transcription activator complex,PcG pr				T=0, C=40	T=0.0, C=1.0	0.00001	644.283	40	T=0/0, C=20/2
chr1:15427982	A/A	G	A	SNV	KAZN	KAZN:intronic:NM 201628.2	1	100		intronic	NM 20162	c.1548-57	p.?	-0.14					SAM domain	rs502918		YES				cornification,cornified envelope,cytoskeleton,cytosol,de				G=0, A=51	G=0.0, A=1.0	0.00001	754.575	51	G=0/0, A=26/2
chr1:154310048	G/G	C	G	SNV	ATP8B2	ATP8B2:intronic:NM 020452.3	1	100		intronic	NM 02045	c.1133+28	p.?	0.37				0.12(COSM37	Cation transp	rs1760795	0.4723	YES	6.073E-1 (pos.15431004	acute myeloic	ATPase, cla	ATP binding,Golgi apparatus,Golgi organization,endopla				C=0, G=128	C=0.0, G=1.0	0.00001	2094.85	128	C=0/0, G=59/6
chr10:135076596	C/C	T	C	SNV	ADAM8	ADAM8:utr 3:NM 001109.4	1	100	23	utr 3	NM 00110	c.2539A>G	p.?	-1.4					ADAM cysteir	rs2230576	0.1342	YES	9.368E-1 (pos.135076596)		A disintegri	alpha9-be	Digestive System Diseases;Drug-Induced Live			T=0, C=57	T=0.0, C=1.0	0.00001	823.033	57	T=0/0, C=31/2
chr1:154407419	A/C	A	C	SNV	IL6R	IL6R:intronic:NM 000565.3	1	50		intronic	NM 00056	c.641-47A	p.?	-0.16					Immunoglob	rs7518199	0.411	YES	4.037E-1;0.0 (pos.154407419)		Interleukin-	acute-pha	Adenocarcinoma;A	Tocilizumab		A=24, C=24	A=0.5, C=0.5	0.00001	273.105	48	A=19/5, C=17/
chr1:154459197	G/G	C	G	SNV	SHE	SHE:intronic:NM 001010846.2	1	100		intronic	NM 00101	c.1025-38	p.?	-1.59					SH2 domain	rs4845633	2.00E-04	YES	1.0 (pos.154459197)		SH2 domair	SH3/SH2 adaptor activity,positive regulation of signal tra				C=0, G=29	C=0.0, G=1.0	0.00001	427.603	29	C=0/0, G=16/1
chr10:135085426	G/G	A	G	SNV	ADAM8	ADAM8:exonic:NM 001109.4	1	100	11	synonymous	NM 00110	c.990T>C	p.(=)	0.58				0.02(COSM42	ADAM cysteir	rs1131719	0.1067	YES	9.541E-1 (pos.13508542	neoplasm	A disintegri	alpha9-be	Digestive System Diseases;Drug-Induced Live			A=0, G=50	A=0.0, G=1.0	0.00001	852.748	50	A=0/0, G=28/2
chr8:38869207	G/A	G	A	SNV	ADAM9	ADAM9:exonic:NM 003816.2	1	47.37	3	missense	NM 00381	c.226G>A	p.Glu76Lys	1.08	0.34	56	0		ADAM cysteir	rs61753672	0.0059		1.156E-4;0.0 (pos.38869207)		A disintegri	PMA-induc	Autoimmune Disea	INCB7839	Likely benign	G=39, A=36	G=0.52, A=0.48	0.00001	374.441	76	G=18/21, A=18
chr21:28216595	G/G	C	G	SNV	ADAMTS1	ADAMTS1:exonic:NM 006988.4	1	100	1	missense	NM 00698	c.679G>C	p.Ala227Pro	-0.07	0.25	27	0	0.02(COSM44	ADAM-TS Spa	rs428785,rs7661175	0.2314	YES	4.451E-1;0.0;0.0 (pos.28	neoplasm	A disintegri	basement	Abnormalities, Multiple;Adenocarcinoma;Au			C=0, G=21	C=0.0, G=1.0	0.00001	275.764	21	C=0/0, G=5/16
chr1:154580458	C/A	C	A	SNV	ADAR	ADAR:intronic:NM 001111.4	1	57.45		intronic	NM 00111	c.15+10G>	p.?	-0.12					Adenosine de	rs58655370	0.2106	YES	1.56E-1 (pos.154580458)		Adenosine	DNA bindi	Autoimmune Diseases;Autoim		Benign	C=20, A=27	C=0.4255, A=0.5745	0.00001	326.455	47	C=9/11, A=16/
chr21:28216692	T/T	G	T	SNV	ADAMTS1	ADAMTS1:exonic:NM 006988.4	1	100	1	synonymous	NM 00698	c.582C>A	p.(=)	0.41				0.06(COSM37	ADAM-TS Spa	rs445784	0.1964	YES	4.821E-1;0.0 (pos.28216	adenocarcino	A disintegri	basement	Abnormalities, Multiple;Adenocarcinoma;Au			G=0, T=29	G=0.0, T=1.0	0.00001	427.602	29	G=0/0, T=4/25
chr19:8669931	G/C	G	C	SNV	ADAMTS10	ADAMTS10:exonic:NM 030957.3	1	95.45	4	missense	NM 03095	c.401C>G	p.Thr134Ser	0.95	1	58	0	0.39(COSM37	ADAM-TS Spa	rs7255721	0.2976	YES	9.733E-1 (pos.8669931)	neoplasm,squ	A disintegri	biological	Abnormalities, Multiple;Bone I		Benign	G=1, C=21	G=0.0455, C=0.9545	0.00001	271.51	22	G=0/1, C=6/15
chr1:155106054	A/G	A	G	SNV	EFNA1	EFNA1:intronic:NM 004428.2	1	45.83		intronic	NM 00442	c.454+10A	p.?	-1.39				0.15(COSM39	Ephrin	rs4390169	0.4713	YES	1.997E-1 (pos.15510605	acute myeloic	eph-relatec	anchored	Breast Diseases;Breast Neoplasms;Neoplasr			A=26, G=22	A=0.5417, G=0.4583	0.00001	240.97	48	A=8/18, G=16/
chr9:136291063	T/T	C	T	SNV	ADAMTS13	ADAMTS13:exonic:NM 139025.4	1	100	5	synonymous	NM 13902	c.420C>T	p.(=)	-0.4				0.01(COSM14	Metallo-pept	rs3118667	0.4664	YES	8.449E-1 (pos.13629106	hepatocellula	A disintegri	calcium io	Autoimmune Diseases;Blood Coagulation Dis			C=0, T=63	C=0.0, T=1.0	0.00001	958.917	63	C=0/0, T=58/5
chr10:72513682	T/T	C	T	SNV	ADAMTS14	ADAMTS14:exonic:NM 139155.3	1	100	19	synonymous	NM 13915	c.2865C>T	p.(=)	1.67				0.7(COSM375	ADAM-TS Spa	rs2587475	0.434	YES	5.236E-1 (pos.72513682)	adenocarcino	A disintegri	collagen catabolic process,collagen fibril organization,e				C=0, T=47	C=0.0, T=1.0	0.00001	789.255	47	C=0/0, T=32/1
chr1:155294274	G/G	A	G	SNV	RUSC1,RUSC1-AS1	RUSC1-AS1:upstream:NR 145424.1, RUSC1:intronic:NM 00	1	100		intronic	NR 14542	c.1358-36	p.?	0.32				0.01(COSM41	RUN domain,	rs914616	7.00E-04	YES	1.0 (pos.155294274)	neoplasm	RUN domai	Golgi apparatus,SH3/SH2 adaptor activity,actin binding,a				A=0, G=40	A=0.0, G=1.0	0.00001	644.283	40	A=0/0, G=25/1
chr1:155294772	G/G	A	G	SNV	RUSC1,RUSC1-AS1	RUSC1-AS1:upstream:NR 145424.1, RUSC1:intronic:NM 00	1	100		intronic	NR 14542	c.1456+38	p.?	-0.98					RUN domain,	rs4971075	7.00E-04	YES	9.999E-1 (pos.155294772)		RUN domai	Golgi apparatus,SH3/SH2 adaptor activity,actin binding,a				A=0, G=25	A=0.0, G=1.0	0.00001	350.334	25	A=0/0, G=15/1
chr10:72517837	T/T	C	T	SNV	ADAMTS14	ADAMTS14:exonic:NM 139155.3	1	100	20	synonymous	NM 13915	c.3066C>T	p.(=)	-6.44					ADAM-TS Spa	rs6480463,rs775082	0.4115	YES	3.992E-1;0.0 (pos.72517837)		A disintegri	collagen catabolic process,collagen fibril organization,e				C=0, T=39	C=0.0, T=1.0	0.00001	623.98	39	C=0/0, T=20/1
chr16:77353973	G/T	G	T	SNV	ADAMTS18	ADAMTS18:exonic:NM 199355.3	1	50	16	missense	NM 19935	c.2305C>A	p.Leu769Ile	2.54	0.21	5	0	0.07(COSM37	ADAM-TS Spa	rs9930984	0.3948	YES	8.818E-1 (pos.77353973)	adenocarcino	A disintegri	eye development,integrin-mediated signaling pathway,i				G=15, T=15	G=0.5, T=0.5	0.00001	144.99	30	G=3/12, T=5/1
chr5:128863471	G/G	A	G	SNV	ADAMTS19	ADAMTS19:exonic:NM 133638.4	1	100	5	missense	NM 13363	c.1117A>G	p.Ser373Gly	4.07	1	56	0		ADAM-TS Spa	rs6595908	0.0056		1.0 (pos.128863471)		A disintegri	metalloendopeptidase activity,metallopeptidase activi				A=0, G=77	A=0.0, G=1.0	0.00001	1229.45	77	A=0/0, G=25/5
chr5:129072943	T/T	G	T	SNV	ADAMTS19	ADAMTS19:utr 3:NM 133638.4	1	100	23	utr 3	NM 13363	c.3674G>T	p.?	0.87					ADAM-TS Spa	rs1465686	0.0735	YES	9.981E-1 (pos.129072943)		A disintegri	metalloendopeptidase activity,metallopeptidase activi				G=0, T=63	G=0.0, T=1.0	0.00001	955.452	63	G=0/0, T=32/3
chr1:155903408	C/T	C	T	SNV	KIAA0907	KIAA0907:intronic:NM 014949.3	1	46.88		intronic	NM 01494	c.255+16G	p.?	0.09						rs4661151,rs774605	0.343	YES	2.31E-1;3.249E-4 (pos.155903408)			RNA binding,cytoplasm,mRNA splice site selection,nucle				C=17, T=15	C=0.5313, T=0.4688	0.00001	143.702	32	C=8/9, T=6/9
chr5:178634619	T/T	C	T	SNV	ADAMTS2	ADAMTS2:exonic:NM 014244.4	1	100	4	synonymous	NM 01424	c.786G>A	p.(=)	-5.17				0.03(COSM41	ADAM-TS Spa	rs423552	0.074	YES	9.985E-1 (pos.17863461	neoplasm	A disintegri	collagen c	Cardiovascular Diseases;Colla		Benign	C=0, T=20	C=0.0, T=1.0	0.00001	263.785	20	C=0/0, T=9/11
chr1:155924505	G/G	A	G	SNV	ARHGEF2	ARHGEF2:intronic:NM 001162383.1	1	100		intronic	NM 00116	c.1783+18	p.?	-0.77					RhoGEF doma	rs4661162		YES			RHO guanin	G-protein	Amyotrophic Lateral Sclerosis:Central Nervo			A=0, G=21	A=0.0, G=1.0	0.00001	281.233	21	A=0/0, G=13/8

chr5:178770981	A/G	A	G	SNV	ADAMTS2	ADAMTS2:exonic:NM_014244.4	1	50	2	synonymous	NM_01424	c.321T>C	p.(=)	-0.14				0.05(COSM37	ADAM-TS Spa	rs2271212	0.3524	YES	4.922E-1 (pos.17877098:	adenocarcino	A disintegri	collagen c	Cardiovascular Diseases;Colla		Benign	A=50, G=5C	A=0.5, G=0.5	0.00001	566.806	100	A=37/13, G=1!
chr5:178580549	G/A	G	A	SNV	ADAMTS2	ADAMTS2:exonic:NM_014244.4	1	47.62	9	synonymous	NM_01424	c.1458C>T	p.(=)	2.64				0.89(COSM37	ADAM-TS Spa	rs61757478	0.0047		0.0 (pos.178580549)	adenocarcino	A disintegri	collagen c	Cardiovascular Diseases;Colla		Conflicting in	G=11, A=1C	G=0.5238, A=0.4762	0.00001	82.2884	21	G=7/4, A=4/6
chr12:43860526	G/G	A	G	SNV	ADAMTS20	ADAMTS20:exonic:NM_025003.4	1	100	9	synonymous	NM_02500	c.1296T>C	p.(=)	0.16				0.81(COSM56	ADAM-TS Spa	rs11182088	0.3412	YES	7.561E-1 (pos.43860526)	diffuse_large_	A disintegri	extracellu	Melanoma:Neoplasms:Neoplasms by Histolo			A=0, G=74	A=0.0, G=1.0	0.00001	1159.76	74	A=0/0, G=41/3
chr1:15616183	T/C	T	C	SNV	FHAD1	FHAD1:intronic:NM_052929.1	1	96.77		intronic	NM_05292	c.568+21T	p.?	-2.58					FHA domain	rs487338		YES	3.168E-1 (pos.15616183)							T=1, C=30	T=0.0323, C=0.9677	0.00001	435.605	31	T=0/1, C=12/1
chr1:1562437	G/G	C	G	SNV	MIB2	MIB2:intronic:NM_080875.2	1	100		intronic	NM_08087	c.1586-17(	p.?	0.03					Ankyrin repea	rs28510084	0.2135	YES	3.846E-1;0.0;0.0 (pos.1562437)		Mindbomb,	Notch signaling pathway,actin binding,cytoplasm,cytosc				C=0, G=30	C=0.0, G=1.0	0.00001	446.578	30	C=0/0, G=20/1
chr21:28302355	G/G	A	G	SNV	ADAMTS5	ADAMTS5:exonic:NM_007038.4	1	100	7	missense	NM_00703	c.2075T>C	p.Leu692Pro	7.32	1	98	0	0.08(COSM44	ADAM-TS Spa	rs226794,rs7962203(	0.1081	YES	4.667E-1 (pos.28302355)	acute_myeloic	A disintegri	defense re	Abnormalities, Mul	(2R)-N~4~-hydroxy-2-(3-h		A=0, G=41	A=0.0, G=1.0	0.00001	664.693	41	A=0/0, G=22/1
chr15:79089007	C/C	T	C	SNV	ADAMTS7	ADAMTS7:exonic:NM_014272.4	1	100	4	synonymous	NM_01427	c.744A>G	p.(=)	1.91					ADAM-TS Spa	rs3743057	0.2459	YES	8.787E-1 (pos.79089007)		A disintegri	cell surfac	Adnexal Diseases:Arterial Occlusive Disease			T=0, C=27	T=0.0, C=1.0	0.00001	389.985	27	T=0/0, C=18/9
chr1:156503541	C/C	T	C	SNV	IQGAP3	IQGAP3:intronic:NM_178229.4	1	100		intronic	NM_17822	c.3976+24	p.?	0.07					Calponin hon	rs6699212	1.00E-04		1.0 (pos.156503541)			ERK1 and ERK2 cascade,G1/S transition of mitotic cell cy				T=0, C=29	T=0.0, C=1.0	0.00001	427.603	29	T=0/0, C=6/23
chr1:156504104	C/C	A	C	SNV	IQGAP3	IQGAP3:intronic:NM_178229.4	1	100		intronic	NM_17822	c.3741-17:	p.?	0.11					Calponin hon	rs7552082		YES				ERK1 and ERK2 cascade,G1/S transition of mitotic cell cy				A=0, C=34	A=0.0, C=1.0	0.00001	524.184	34	A=0/0, C=24/1
chr1:156508477	A/A	G	A	SNV	IQGAP3	IQGAP3:intronic:NM_178229.4	1	100		intronic	NM_17822	c.3281+12	p.?	0.27					Calponin hon	rs1177696		YES				ERK1 and ERK2 cascade,G1/S transition of mitotic cell cy				G=0, A=35	G=0.0, A=1.0	0.00001	543.903	35	G=0/0, A=16/1
chr1:156520173	A/A	T	A	SNV	IQGAP3	IQGAP3:intronic:NM_178229.4	1	100		intronic	NM_17822	c.1735-30,	p.?	0.19					Calponin hon	rs1778819	1.00E-04		1.0 (pos.156520173)			ERK1 and ERK2 cascade,G1/S transition of mitotic cell cy				T=0, A=26	T=0.0, A=1.0	0.00001	377.08	26	T=0/0, A=8/18
chr1:156524221	G/T	G	T	SNV	IQGAP3	IQGAP3:intronic:NM_178229.4	1	56		intronic	NM_17822	c.1291-37(	p.?	1.65					Calponin hon	rs6694580	0.3088	YES	5.128E-1 (pos.156524221)			ERK1 and ERK2 cascade,G1/S transition of mitotic cell cy				G=11, T=14	G=0.44, T=0.56	0.00001	134.35	25	G=2/9, T=6/8
chr11:130297957	C/C	T	C	SNV	ADAMTS8	ADAMTS8:exonic:NM_007037.5	1	100	1	synonymous	NM_00703	c.225A>G	p.(=)	-0.74				0.02(COSM37	ADAM-TS Spa	rs775882152,rs7942(	0.1428	YES	4.348E-3;0.0;0.0 (pos.13(	adenocarcino	A disintegri	extracellular matrix,heparin binding,integrin binding,lo				T=0, C=36	T=0.0, C=1.0	0.00001	563.482	36	T=0/0, C=7/29
chr1:156536123	C/C	T	C	SNV	IQGAP3	IQGAP3:intronic:NM_178229.4	1	100		intronic	NM_17822	c.282+59A	p.?	-0.2					Calponin hon	rs2066378		YES				ERK1 and ERK2 cascade,G1/S transition of mitotic cell cy				T=0, C=89	T=0.0, C=1.0	0.00001	1472.49	89	T=0/0, C=65/2
chr9:18504916	A/A	T	A	SNV	ADAMTSL1	ADAMTSL1:exonic:NM_001040272.5	1	100	2	synonymous	NM_00104	c.153T>A	p.(=)	0.13				0.29(COSM44	Immunoglob	rs2277160	0.4367	YES	4.642E-1 (pos.18504916)	hepatocellula	ADAMTS-lik	endoplasmic reticulum lumen,metalloendopeptidase a				T=0, A=28	T=0.0, A=1.0	0.00001	413.631	28	T=0/0, A=20/8
chr9:18776840	C/C	A	C	SNV	ADAMTSL1	ADAMTSL1:exonic:NM_001040272.5	1	100	19	synonymous	NM_00104	c.2613A>C	p.(=)	1.97				0.17(COSM37	Immunoglob	rs1549986	0.0845	YES	1.0 (pos.18776840)	adenocarcino	ADAMTS-lik	endoplasmic reticulum lumen,metalloendopeptidase a				A=0, C=50	A=0.0, C=1.0	0.00001	852.748	50	A=0/0, C=24/2
chr1:156536353	G/G	A	G	SNV	IQGAP3	IQGAP3:intronic:NM_178229.4	1	100		intronic	NM_17822	c.126-15T:	p.?	0.18					Calponin hon	rs947663	3.00E-04	YES	1.0 (pos.156536353)			ERK1 and ERK2 cascade,G1/S transition of mitotic cell cy				A=0, G=251	A=0.0, G=1.0	0.00001	4107.3	251	A=0/0, G=130/
chr1:156539154	C/C	T	C	SNV	IQGAP3	IQGAP3:intronic:NM_178229.4	1	100		intronic	NM_17822	c.125+14A	p.?	-1.55					Calponin hon	rs6427319	0.0026		9.996E-1 (pos.156539154)			ERK1 and ERK2 cascade,G1/S transition of mitotic cell cy				T=0, C=28	T=0.0, C=1.0	0.00001	408.718	28	T=0/0, C=22/6
chr9:18777368	C/C	T	C	SNV	ADAMTSL1	ADAMTSL1:exonic:NM_001040272.5	1	100	19	synonymous	NM_00104	c.3141T>C	p.(=)	-4.54				0.01(COSM45	Immunoglob	rs7033684	0.2849	YES	7.25E-1 (pos.18777368)	NS,adenocarci	ADAMTS-lik	endoplasmic reticulum lumen,metalloendopeptidase a				T=0, C=23	T=0.0, C=1.0	0.00001	316.685	23	T=0/0, C=13/1
chr9:136412170	T/T	C	T	SNV	ADAMTSL2	ADAMTSL2:exonic:NM_014694.3	1	100	9	synonymous	NM_01469	c.774C>T	p.(=)	-0.61					ADAM-TS Spa	rs2073874	0.0831	YES	5.216E-1 (pos.136412170)		ADAMTS-lik	extracellu	Bone Diseases;Bone Diseases,		Benign	C=0, T=20	C=0.0, T=1.0	0.00001	263.786	20	C=0/0, T=10/1
chr9:136412236	T/T	A	T	SNV	ADAMTSL2	ADAMTSL2:exonic:NM_014694.3	1	100	9	synonymous	NM_01469	c.840A>T	p.(=)	-2.2					ADAM-TS Spa	rs2073875	0.0828	YES	5.211E-1 (pos.136412236)		ADAMTS-lik	extracellu	Bone Diseases;Bone Diseases,		Benign	A=0, T=20	A=0.0, T=1.0	0.00001	263.786	20	A=0/0, T=10/1
chr1:156884365	C/C	T	C	SNV	PEAR1	PEAR1:intronic:NM_001080471.1	1	100		intronic	NM_00108	c.2963-74	p.?	-2.87					Human growt	rs749256		YES			Platelet en	integral component of membrane,negative regulation of				T=0, C=26	T=0.0, C=1.0	0.00001	371.418	26	T=0/0, C=15/1
chr9:136412255	C/C	A	C	SNV	ADAMTSL2	ADAMTSL2:exonic:NM_014694.3	1	100	9	synonymous	NM_01469	c.859A>C	p.(=)	3.05					ADAM-TS Spa	rs2073876	0.0828	YES	5.215E-1;0.0 (pos.136412255)		ADAMTS-lik	extracellu	Bone Diseases;Bone Diseases,		Benign	A=0, C=20	A=0.0, C=1.0	0.00001	263.786	20	A=0/0, C=10/1
chr1:157566019	A/A	G	A	SNV	FCRL4	FCRL4:intronic:NM_031282.2	1	100		intronic	NM_03128	c.52+99C>	p.?	0.11					Immunoglob	rs10489674		YES				adaptive immune response,extracellular exosome,inte				G=0, A=52	G=0.0, A=1.0	0.00001	765.058	52	G=0/0, A=39/1
chr1:157566211	G/G	A	G	SNV	FCRL4	FCRL4:intronic:NM_031282.2	1	100		intronic	NM_03128	c.32-73T>(	p.?	0.13					Immunoglob	rs12744410	0.3309	YES				adaptive immune response,extracellular exosome,inte				A=0, G=60	A=0.0, G=1.0	0.00001	886.906	60	A=0/0, G=34/2
chr1:150526406	C/T	C	T	SNV	ADAMTSL4	ADAMTSL4:exonic:NM_019032.5	1	50	6	synonymous	NM_01903	c.939C>T	p.(=)	0.53				0.15(COSM41	ADAM-TS Spa	rs6681639	0.2701	YES	3.056E-1;0.0;0.0 (pos.15(	carcinoma,ne	ADAMTS-lik	apoptotic	Congenital Abnormalities:Con		Benign	C=30, T=30	C=0.5, T=0.5	0.00001	277.133	60	C=16/14, T=16
chr1:157740186	G/G	GTCAG	G	INDEL	FCRL2	FCRL2:intronic:NM_030764.3	4	100		intronic	NM_03076	c.310+13C	p.?	-0.71, -2.21, -0.99, -0.66, -0.81					Immunoglob	rs10540465,rs38663!	0.4581	YES	0.0 (pos.157740185),0.0 (pos.15774019(		Fc receptor	SH3/SH2 adaptor activity,cell-cell signaling,integral com				GTCAG=0, (	GTCAG=0.0, G=1.0	0.00001	192.221	21	GTCAG=0/0, G
chr19:1510661	G/G	A	G	SNV	ADAMTSL5	ADAMTSL5:exonic:NM_213604.2	1	100	3	synonymous	NM_21360	c.168T>C	p.(=)	-6.98				0.01(COSM37	ADAM-TS Spa	rs265291	0.0687	YES	1.0 (pos.1510661)	adenocarcinoma,neoplasn		extracellular region,heparin binding,metalloendopeptic				A=0, G=29	A=0.0, G=1.0	0.00001	427.603	29	A=0/0, G=24/5
chr1:15789176	T/T	C	T	SNV	CELA2A	CELA2A:intronic:NM_033440.2	1	100		intronic	NM_03344	c.228-52C:	p.?	0.59					Trypsin,Tryps	rs3815786	0.2484	YES			Chymotryps	cornificati	Cardiovascular Dis	2-(2-HYDROXY-CYCLOPEN		C=0, T=100	C=0.0, T=1.0	0.00001	1689.31	100	C=0/0, T=57/4
chr1:154574820	C/C	T	C	SNV	ADAR	ADAR:exonic:NM_001111.4	1	100	2	missense	NM_00111	c.298A>G	p.Arg100Gly	-0.95	0.7	125	0		Adenosine de	rs1466731	1.00E-04		1.0 (pos.154574820)		Adenosine	DNA bindi	Autoimmune Diseases:Autoimmune Disease			T=0, C=36	T=0.0, C=1.0	0.00001	563.744	36	T=0/0, C=18/1
chr7:45753324	A/A	G	A	SNV	ADCY1	ADCY1:exonic:NM_021116.3	1	100	20	synonymous	NM_02111	c.3090G>A	p.(=)	1.51				0.56(COSM44	Adenylate an	rs2293106	0.1678	YES	1.071E-1 (pos.45753324)	acute_myeloic	Adenylate c	ATP bindin	Bronchial Neoplasr	ATP,Adenosine monophc		G=0, A=10C	G=0.0, A=1.0	0.00001	1562.79	100	G=0/0, A=36/6
chr2:25141534	A/AG	A	AG	INDEL	ADCY3	ADCY3:exonic:NM_004036.4	1	100	2	frameshiftInsertion	NM_00403	c.322_323	p.Leu108fs	2.51					Adenylate and Guanylate cyclase catalytic domain,Adenylyl cyclase N-terminal extracellular and tra						Adenylate c	ATP bindin	Digestive System Diseases:Gastroenteritis:G			A=0, AG=39	A=0.0, AG=1.0	0.00001	263.798	39	A=0/0, AG=26/
chr2:25141538	G/G	A	G	SNV	ADCY3	ADCY3:exonic:NM_004036.4	1	100	2	missense	NM_00403	c.319T>C	p.Ser107Pro	1.28	0.39	74	0	0.15(COSM41	Adenylate an	rs11676272	0.472	YES	4.337E-1 (pos.25141538)	acute_myeloic	Adenylate c	ATP bindin	Digestive System Diseases:Gastroenteritis:G			A=0, G=39	A=0.0, G=1.0	0.00001	620.537	39	A=0/0, G=26/1
chr1:158587784	G/G	T	G	SNV	SPTA1	SPTA1:intronic:NM_003126.2	1	100		intronic	NM_00312	c.6548+45	p.?	-0.15					Ca2+ insensit	rs857717	1.00E-04		1.0;0.0 (pos.158587784)		Spectrin, al	ER to Golgi	Anemia;Anemia, Hemolytic;Anemia, Hemolyt			T=0, G=35	T=0.0, G=1.0	0.00001	543.878	35	T=0/0, G=19/1
chr1:158607788	G/G	T	G	SNV	SPTA1	SPTA1:intronic:NM_003126.2	1	100		intronic	NM_00312	c.5189+35	p.?	-0.58					Ca2+ insensit	rs2518492	1.00E-04		1.0;0.0 (pos.158607788)		Spectrin, al	ER to Golgi	Anemia;Anemia, Hemolytic;Anemia, Hemolyt			T=0, G=30	T=0.0, G=1.0	0.00001	446.636	30	T=0/0, G=19/1
chr16:50326661	G/G	A	G	SNV	ADCY7,MIR6771	ADCY7:exonic:NM_001114.4. MIR6771:downstream:NR_10(	1	100	4	synonymous	NM_00111	c.612A>G	p.(=)	-0.48					Adenylate an	rs8051594	6.00E-04		1.0 (pos.50326661)		Adenylate c	ATP bindin	Arthritis:Arthritis, Juvenile:Autoimmune Dise			A=0, G=20	A=0.0, G=1.0	0.00001	268.322	20	A=0/0, G=5/15
chr1:158647631	A/A	T	A	SNV	SPTA1	SPTA1:intronic:NM_003126.2	1	100		intronic	NM_00312	c.813-7A>	p.?	2.46					Ca2+ insensit	rs325995	0.0029	YES	1.0 (pos.158647631)		Spectrin, al	ER to Golgi	Anemia;Anemia, Hemolytic;An		Benign/Likely	T=0, A=87	T=0.0, A=1.0	0.00001	1431.37	87	T=0/0, A=51/3
chr1:158647669	T/C	T	C	SNV	SPTA1	SPTA1:intronic:NM_003126.2	1	47.62		intronic	NM_00312	c.813-45A:	p.?	1.49					Ca2+ insensit	rs703122	0.4275	YES	5.485E-1 (pos.158647669)		Spectrin, al	ER to Golgi	Anemia;Anemia, Hemolytic;Anemia, Hemolyt			T=44, C=40	T=0.5238, C=0.4762	0.00001	419.846	84	T=32/12, C=17
chr8:131921956	A/G	A	G	SNV	ADCY8	ADCY8:exonic:NM_001115.2	1	50	6	synonymous	NM_00111	c.1638T>C	p.(=)	-1.75				0.05(COSM39	Adenylate an	rs12547243	0.3164	YES	6.891E-1 (pos.13192195(	acute_myeloic	Adenylyl cy	ATP bindin	Dyskinesias:Hyperkinesis:Mental Disorders:I			A=19, G=19	A=0.5, G=0.5	0.00001	199.994	38	A=12/7, G=7/1
chr1:158655162	A/A	C	A	SNV	SPTA1	SPTA1:intronic:NM_003126.2	1	100		intronic	NM_00312	c.25-25G>	p.?	-0.77					Ca2+ insensit	rs2564859	4.00E-04		1.0;0.0 (pos.158655162)		Spectrin, al	ER to Golgi	Anemia;Anemia, Hemolytic;Anemia, Hemolyt			C=0, A=26	C=0.0, A=1.0	0.00001	371.417	26	C=0/0, A=16/1
chr1:158656281	C/C	T	C	SNV	SPTA1	SPTA1:intronic:NM_003126.2	1	100		intronic	NM_00312	c.24+3A>G	p.?	0.74					Ca2+ insensit	rs2564858	4.00E-04		1.0 (pos.158656281)		Spectrin, al	ER to Golgi	Anemia;Anemia, Hemolytic;An		Benign/Likely	T=0, C=135	T=0.0, C=1.0	0.00001	2238.23	135	T=0/0, C=58/7
chr16:4016676	G/G	A	G	SNV	ADCY9	ADCY9:exonic:NM_001116.3	1	100	11	synonymous	NM_00111	c.3162T>C	p.(=)	-1.37				0.03(COSM41	Adenylate an	rs2230742	0.143	YES	8.45E-1;0.0 (pos.401667(	neoplasm	Adenylate c	ATP bindin	Brain Diseases;Central Nervous System Dise			A=0, G=32	A=0.0, G=1.0	0.00001	485.137	32	A=0/0, G=19/1
chr1:159002557	G/G	A	G	SNV	IFI16	IFI16:intronic:NM_001206567.1	1	100		intronic	NM_00120	c.1161+76	p.?	-0.1					HIN-200/IF12	rs1633256,rs763811747		YES			Interferon,	RNA bindir	Nutrition Disorders:Nutritional and Metaboli			A=0, G=69	A=0.0, G=1.0	0.00001	1072.78	69	A=0/0, G=31/3
chr1:159002577	T/T	TGGATTG	T	INDEL	IFI16	IFI16:intronic:NM_001206567.1	6	100		intronic	NM_00120	c.1161+96	p.?	0.15, 0.17, 0.0, 0.0, 0.0, 0.0, 0.0					HIN-200/IF12	rs147007436,rs200604720,rs20		YES			Interferon,	RNA bindir	Nutrition Disorders:Nutritional and Metaboli			TGGATTG=(	TGGATTG=0.0, T=1.0	0.00001	1012.99	66	TGGATTG=0/0,
chr16:4016377	T/C	T	C	SNV	ADCY9	ADCY9:exonic:NM_001116.3	1	50	11	missense	NM_00111	c.3461A>G	p.Asn1154Ser	2.62	0.08	46	0.001		Adenylate an	rs61731445	0.0616	YES	1.156E-4 (pos.4016377)		Adenylate c	ATP bindin	Brain Diseases;Central Nervous System Dise			T=13, C=13	T=0.5, C=0.5	0.00001	119.144	26	T=8/5, C=8/5
chr1:15909895	G/G	A	G	SNV	AGMAT	AGMAT:intronic:NM_024758.4	1	100		intronic	NM_02475	c.273-5T>(	p.?	0.72				0.11(COSM54	Arginase fam	rs6429758	0.4491	YES	6.399E-1 (pos.15909895)	adenocarcinoma		agmatinase activity,agmatine biosynthetic process,extr				A=0, G=26	A=0.0, G=1.0	0.00001	371.42	26	A=0/0, G=8/18
chr18:907710	G/G	A	G	SNV	ADCYAP1	ADCYAP1:exonic:NM_001117.4	1	100	2	missense	NM_00111	c.161A>G	p.Asp54Gly	1.32	0.08	94	0.002	0.76(COSM37	Peptide horn	rs2856966	0.2143	YES	8.291E-2 (pos.907710)	adenocarcino	Adenylate c	ATP metat	Carcinoma;Carcino	2,6-Diamino-Hexanoic A		A=0, G=29	A=0.0, G=1.0	0.00001	427.603	29	A=0/0, G=21/8
chr7:31146455	G/G	A	G	SNV	ADCYAP1R1	ADCYAP1R1:utr_3:NM_001199635.1	1	100	17	utr_3	NM_00119	c.1648A>G	p.?	-1.05					7 transmemb	rs2302476		YES			Adenylate c	ADP-ribos	Asthma:Bronchial Diseases:Hypersensitivity			A=0, G=34	A=0.0, G=1.0	0.00001	523.776	34	A=0/0, G=3/31
chr10:111886249	T/A	T	A	SNV	ADD3	ADD3:exonic:NM_016824.4	1	52.38	12	missense	NM_01682	c.1596T>A	p.Asp532Glu	0.84	0.29	45	0.391		Class II Aldol	rs143966199	0.0013		0.0 (pos.111886249)		Adducin-3,	actin bind	Brain Diseases:Brain Ischemia:Cardiovascula			T=10, A=11	T=0.4762, A=0.5238	0.00001	95.3003	21	T=4/6, A=5/6
chr10:134902314	G/G	A	G	SNV	ADGRA1	ADGRA1:utr_5:NM_001083909.2	1	100	2	utr_5	NM_00108	c.-100A>G	p.?	-0.33					7 transmemb	rs10745302		YES			Adhesion G	G-protein coupled receptor activity,G-protein coupled re				A=0, G=33	A=0.0, G=1.0	0.00001	504.574	33	A=0/0, G=22/1
chr1:159854392	A/A	C	A	SNV	CFAP45	CFAP45:intronic:NM_012337.2	1	100		intronic	NM_01233	c.768-37G	p.?	-0.57					Trichohyalin-	rs2501325	0.2341	YES	8.376E-1;0.0 (pos.159854392)			cilium,nucleoplasm,nucleus				C=0, A=132	C=0.0, A=1.0	0.00001	2164.44	132	C=0/0, A=49/8
chr8:143603418	C/C	G	C	SNV	ADGRB1	ADGRB1:exonic:NM_001702.2	1	100	20	synonymous	NM_00170	c.3117G>C	p.(=)	-3.47					7 transmemb	rs7460600	0.0236	YES	9.503E-1;1.165E-4 (pos.143603418)		Adhesion G	G-protein coupled receptor activity,G-protein coupled re				G=0, C=42	G=0.0, C=1.0	0.00001	676.327	42	G=0/0, C=5/37
chr1:159906156	G/G	A	G	SNV	IGSF9	IGSF9:intronic:NM_001135050.1	1	100		intronic	NM_00113	c.673+33T	p.?	-0.83					Fibronectin t	rs10908750	0.3537	YES	4.693E-1 (pos.159906156)		Immunoglo	axon,cell junction,cell-cell adhesion mediator activity,d				A=0, G=50	A=0.0, G=1.0	0.00001	852.697	50	A=0/0, G=41/9
chr1:160093222	C/T	C	T	SNV	ATP1A2	ATP1A2:intronic:NM_000702.3	1	53.13		intronic	NM_00070	c.381+16C	p.?	-0.54					Cation trans	rs2820581	9.00E-04	YES	1.0 (pos.160093222)		ATPase, Na	ATP bindin	Brain Diseases;Car	Rubidium	Benign	C=15, T=17	C=0.4688, T=0.5313	0.00001	173.56	32	C=9/6, T=10/7
chr12:131466562	C/C	T	C	SNV	ADGRD1	ADGRD1:exonic:NM_198827.4	1	100	5	synonymous	NM_19882	c.444T>C	p.(=)	0.39					7 transmemb	rs4759534	0.0047	YES	9.97E-1 (pos.131466562)		Adhesion G	G-protein coupled receptor activity,G-protein coupled re				T=0, C=76	T=0.0, C=1.0	0.00001	1209.6	76	T=0/0, C=50/2
chr1:160251792	G/G	A	G	SNV	PEX19	PEX19:intronic:NM_002857.3	1	100		intronic	NM_00285	c.594+37T	p.?	-1.59						rs2795067	0.4472	YES	4.852E-1 (pos.160251792)		Peroxisome	ATPase bir	Abnormalities, Multiple:Brain Diseases:Brair			A=0, G=22	A=0.0, G=1.0	0.00001	298.892	22	A=0/0, G=13/9
chr1:160263124	T/T	C	T	SNV	COPA	COPA:intronic:NM_001098398.1	1	100		intronic	NM_00109	c.2781+52	p.?	-0.96					Anaphase-pr	rs1317944	0.4499	YES			Coatomer s	COPI vesic	Arthritis:Autoimmune Diseases:Immune Syst			C=0, T=27	C=0.0, T=1.0	0.00001	389.988	27	C=0/0, T=12/1
chr1:160318940	G/G	A	G	SNV	NCSTN	NCSTN:intronic:NM_015331.2	1	100		intronic	NM_01533	c.314+28A	p.?	0.31					Nicastrin,Per	rs6669689	0.4957	YES	4.929E-1 (pos.160318940)		Nicastrin	Golgi appa	Alzheimer Disease:Brain Diseases:Central Ne			A=0, G=31	A=0.0, G=1.0	0.00001	465.819	31	A=0/0, G=13/1
chr1:16055732	T/T	C	T	SNV	PLEKHM2	PLEKHM2:intronic:NM_015164.3	1	100		intronic	NM_01516	c.2075+47	p.?	-0.15					PH domain,R	rs6429772	0.2305	YES	9.239E-2 (pos.16055732)		Pleckstrin h	Golgi organization,endosome membrane,kinesin bindin				C=0, T=40	C=0.0, T=1.0	0.00001	644.25	40	C=0/0, T=21/1
chr19:6919624	C/C	A	C	SNV	ADGRE1	ADGRE1:exonic:NM_001974.4	1	100	13	missense	NM_00197	c.1486A>C	p.Lys496Gln	-1.02	0.62	53	0	0(COSM5425:	7 transmemb	rs373533	0.2102	YES	7.859E-1 (pos.6919624)	acute_myeloic	Adhesion G	G-protein coupled receptor activity,G-protein coupled re				A=0, C=115	A=0.0, C=1.0	0.00001	1834.69	115	A=0/0, C=63/5
chr19:14862430	G/G	C	G	SNV	ADGRE2	ADGRE2:exonic:NM_013447.3	1	100	16	missense	NM_01344	c.1842G>C	p.Leu614Phe	1.82	1	22	0		7 transmemb	rs2524383	0.2976	YES	7.955E-1 (pos.14862430)		Adhesion G	G-protein coupled receptor activity,G-protein coupled re				C=0, G=60	C=0.0, G=1.0	0.00001	903.531	60	C=0/0, G=38/2
chr1:160722064	A/G	A	G	SNV	SLAMF7	SLAMF7:intronic:NM_021181.4	1	50		intronic	NM_02118	c.936+26A	p.?	0.01						rs527342	0.0255	YES	9.722E-1 (pos.160722064)		SLAM family	adaptive immune response,ce		Elotuzumab		A=12, G=12	A=0.5, G=0.5	0.00001	106.709	24	A=6/6, G=5/7
chr1:160786670	G/G	A	G	SNV	LY9	LY9:intronic:NM_002348.3	1	100		intronic	NM_00234	c.1342+17	p.?	-0.42						rs560681	0.3087	YES	3.591E-1 (pos.160786670)		T-lymphocy	T-helper 17 cell lineage commitment,cell adhesion,cell s				A=0, G=43	A=0.0, G=1.0	0.00001	705.844	43	A=0/0, G=30/1
chr1:160920813	G/G	A	G	SNV	ITLN2	ITLN2:intronic:NM_080878.2	1	100		intronic	NM_08087	c.441+20T	p.?	0.06						rs790059	0.0058		1.0 (pos.160920813)		Intelectin 2	carbohydrate binding,extracellular region,metal ion bin				A=0, G=20	A=0.0, G=1.0	0.00001	263.786	20	A=0/0, G=16/4
chr19:14752325	T/T	C	T	SNV	ADGRE3	ADGRE3:exonic:NM_032571.4	1	100	10	missense	NM_03257	c.1154G>A	p.Arg385Gln	0.73	1	43	0.01	0.06(COSM56	7 transmemb	rs45508602	0.1427	YES	8.711E-1 (pos.14752325)	diffuse_large_B_cell_lymph		G-protein coupled receptor activity,G-protein coupled re				C=0, T=58	C=0.0, T=1.0	0.00001	867.386	58	C=0/0, T=16/4
chr1:161136843	G/G	C	G	SNV	PPOX	PPOX:intronic:NM_000309.4	1	100		intronic	NM_00030	c.88-47C>(	p.?	-0.37					Flavin contai	rs2301287	0.3776	YES	4.288E-1;0.0 (pos.161136843)		Protoporph	flavin ader	Congenital, Hereditary, and Neonatal Diseas			C=0, G=33	C=0.0, G=1.0	0.00001	504.593	33	C=0/0, G=12/2
chr19:14769339	C/G	C	G	SNV	ADGRE3	ADGRE3:exonic:NM_032571.4	1	98.72	5	missense	NM_03257	c.379G>C	p.Glu127Gln	-0.76	0.61	29	0.006		7 transmemb	rs4606855	0.2763	YES	8.653E-1 (pos.14769339)			G-protein coupled receptor activity,G-protein coupled re				C=1, G=77	C=0.0128, G=0.9872	0.00001	1217.89	78	C=0/1, G=49/2
chr6:47646842	G/G	A	G	SNV	ADGRF2	ADGRF2:exonic:NM_153839.6	1	100	5	missense	NM_15383	c.239A>G	p.Gln80Arg	0.16	0.9	43	0		7 transmemb	rs6907125	0.3535	YES	2.978E-1 (pos.47646842)			G-protein coupled receptor activity,G-protein coupled re				A=0, G=36	A=0.0, G=1.0	0.00001	559.512	36	A=0/0, G=20/1
chr1:161822959	C/T	C	T	SNV	ATF6	ATF6:intronic:NM_007348.3	1	100		intronic	NM_00734	c.1434-55(	p.?	0.47					Basic region l	rs2340717		YES			Activating t	ATF6-medi	Asbestosis;Brain Di	Pseudoephedrine		C=0, T=45	C=0.0, T=1.0	0.00001	645.292	45	C=0/0, T=37/8
chr1:161823011	A/A	T	A	SNV	ATF6	ATF6:intronic:NM_007348.3	1	100		intronic	NM_00734	c.1434-3T:	p.?	0.52				0.93(COSM14	Basic region l	rs2257107	0.3356	YES	4.452E-1 (pos.16182301:	adenocarcino	Activating t	ATF6-medi	Asbestosis;Brain Di	Pseudoephedrine		T=0, A=73	T=0.0, A=1.0	0.00001	1150.51	73	T=0/0, A=31/4
chr6:47649572	GT/GT	G	GT	INDEL	ADGRF2	ADGRF2:exonic:NM_153839.6	1	100	7	frameshiftInsertion	NM_15383	c.1073_10	p.Lys359fs	-0.05					7 transmemb	rs71538333,rs759858870,rs879737514			1.161E-4 (pos.47649572)			G-protein coupled receptor activity,G-protein coupled re				G=0, GT=48	G=0.0, GT=1.0	0.00001	305.139	48	G=0/0, GT=29/
chr6:47649694	G/G	A	G	SNV	ADGRF2	ADGRF2:exonic:NM_153839.6	1	100	7	missense	NM_15383	c.1195A>G	p.Ile399Val	6.08	0.47	29	0.923	0.97(COSM41	7 transmemb	rs9381594	0.3526	YES	2.986E-1 (pos.47649694)	neoplasm		G-protein coupled receptor activity,G-protein coupled re				A=0, G=52	A=0.0, G=1.0	0.00001	767.013	52	A=0/0, G=28/2
chr1:162743418	T/T	G	T	SNV	DDR2	DDR2:intronic:NM_006182.2	1	100		intronic	NM_00618	c.1856+32	p.?	2.23					F5/8 type C d	rs1355287	0		1.0;0.0 (pos.162743418)		Discoidin d	ATP bindin	Adenocarcinoma;B	Regorafen	Benign	G=0, T=58	G=0.0, T=1.0	0.00001	867.365	58	G=0/0, T=25/3
chr1:163038853	A/A	G	A	SNV	RGS4	RGS4:intronic:NM_001102445.2	1	100		intronic	NM_00110	c.58+71G>	p.?	-0.12					Regulator of	rs10917671	0.4154	YES			Regulator o	G-protein	Autoimmune Diseases;Endometriosis;Femal			G=0, A=36	G=0.0, A=1.0	0.00001	563.735	36	G=0/0, A=18/1
chr1:163122528	C/C	G	C	SNV	RGS5	RGS5:intronic:NM_001254749.1	1	100		intronic	NM_00125	c.218-22C:	p.?	-0.06					Regulator of	rs1354939	0.3754	YES	6.32E-1 (pos.163122528)		Regulator o	GTPase ac	Cardiovascular Diseases;Hypertension;Vasc			G=0, C=36	G=0.0, C=1.0	0.00001	563.743	36	G=0/0, C=7/29
chr1:163315350	C/C	G	C	SNV	NUF2	NUF2:intronic:NM_031423.3	1	100		intronic	NM_03142	c.808-118(	p.?	-0.3					Nuf2 family	rs10753611		YES			NUF2, S. cer	Ndc80 complex,cell division,chromosome segregation,c				G=0, C=96	G=0.0, C=1.0	0.00001	1605.7	96	G=0/0, C=52/4
chr1:16359052	G/G	A	G	SNV	CLCNKA	CLCNKA:intronic:NM_004070.3	1	100		intronic	NM_00407	c.1929+31	p.?	0.77					CBS domain,\	rs10927893,rs71493!	0.1531	YES	9.998E-1;0.0 (pos.16359052)		Chloride ch	chloride cl	Adrenal Gland Dise	Niflumic Acid		A=0, G=32	A=0.0, G=1.0	0.00001	485.141	32	A=0/0, G=3/29
chr1:16370899	G/A	G	A	SNV	CLCNKB	CLCNKB:intronic:NM_000085.4	1	52.38		intronic	NM_00008	c.-6-82G>A	p.?	0.11						rs945394		YES			Chloride ch	catalytic a	Adrenal Gland Diseases;Adrenocortical Hype			G=9, A=11	G=0.45, A=0.55	0.00001	102.503	21	G=7/2, A=4/7
chr1:16372003	G/C	G	C	SNV	CLCNKB	CLCNKB:intronic:NM_000085.4	1	50		intronic	NM_00008	c.101-50G	p.?	0.68						rs6604910	0.3571	YES	6.876E-1 (pos.16372003)		Chloride ch	catalytic a	Adrenal Gland Diseases;Adrenocortical Hype			G=20, C=2C	G=0.5, C=0.5	0.00001	214.237	40	G=8/12, C=4/1
chr6:47654742	T/T	A	T	SNV	ADGRF2	ADGRF2:exonic:NM_153839.6	1	100	8	synonymous	NM_15383	c.1878A>T	p.(=)	0.97					7 transmemb	rs9296573	0.3537	YES	2.975E-1 (pos.47654742)			G-protein coupled receptor activity,G-protein coupled re				A=0, T=176	A=0.0, T=1.0	0.00001	2901.45	176	A=0/0, T=96/8
chr1:16375354	T/T	C	T	SNV	CLCNKB	CLCNKB:intronic:NM_000085.4	1	100		intronic	NM_00008	c.656-261(	p.?	-0.12						rs9442227		YES			Chloride ch	catalytic a	Adrenal Gland Diseases;Adrenocortical Hype			C=0, T=58	C=0.0, T=1.0	0.00001	867.354	58	C=0/0, T=40/1
chr6:46826508	A/A	G	A	SNV	ADGRF5	ADGRF5:exonic:NM_001098518.1	1	100	17	synonymous	NM_00109	c.3132C>T	p.(=)	1.88					7 transmemb	rs613870	0.0679	YES	9.902E-1 (pos.46826508)			G-protein coupled receptor activity,G-protein coupled re				G=0, A=23	G=0.0, A=1.0	0.00001	316.747	23	G=0/0, A=11/1
chr1:16382877	G/G	A	G	SNV	CLCNKB	CLCNKB:intronic:NM_000085.4	1	100		intronic	NM_00008	c.1930-40,	p.?	-2.53						rs746580799,rs7517	0.2093	YES	0.0 (pos.16382876),8.073E-1 (pos.16382		Chloride ch	catalytic a	Adrenal Gland Diseases;Adrenocortical Hype			A=0, G=21	A=0.0, G=1.0	0.00001	281.235	21	A=0/0, G=15/6
chr1:16382880	A/A	T	A	SNV	CLCNKB	CLCNKB:intronic:NM_000085.4	1	100		intronic	NM_00008	c.1930-37	p.?	-0.77						rs746580799,rs7512!	0.292	YES	0.0 (pos.16382876),8.443E-3 (pos.16382		Chloride ch	catalytic a	Adrenal Gland Diseases;Adrenocortical Hype			T=0, A=21	T=0.0, A=1.0	0.00001	281.219	21	T=0/0, A=15/6
chr1:16385979	TA/TA	T	TA	INDEL	FAM131C	FAM131C:intronic:NM_182623.2	1	100		intronic	NM_18262	c.562+10A	p.?	-1.58					Putative cell signalling							protein binding				T=0, TA=20	T=0.0, TA=1.0	0.00001	232.603	20	T=0/0, TA=12/
chr6:46827126	T/T	C	T	SNV	ADGRF5	ADGRF5:exonic:NM_001098518.1	1	100	17	synonymous	NM_00109	c.2514G>A	p.(=)	-1.09					7 transmemb	rs610856	0.068	YES	9.902E-1 (pos.46827126)			G-protein coupled receptor activity,G-protein coupled re				C=0, T=69	C=0.0, T=1.0	0.00001	1072.72	69	C=0/0, T=27/4
chr6:46834685	A/A	G	A	SNV	ADGRF5	ADGRF5:exonic:NM_001098518.1	1	100	13	missense	NM_00109	c.1811C>T	p.Thr604Met	-0.3	0.19	81	0.002		7 transmemb	rs586024	0.068	YES	9.902E-1 (pos.46834685)			G-protein coupled receptor activity,G-protein coupled re				G=0, A=113	G=0.0, A=1.0	0.00001	1795.82	113	G=0/0, A=74/3
chr1:164781110	AATATAAG/AA	A	AATATAAG	INDEL	PBX1	PBX1:intronic:NM_002585.3	7	100		intronic	NM_00258	c.838-117,	p.?	-1.02					Homeobox Kl	rs11280817,rs866647485		YES			Pre-B cell le	DNA bindi	Burkitt Lymphoma;Hemic and Lymphatic Dise			A=0, AATAT	A=0.0, AATATAAG=1.(	0.00001	373.583	26	A=0/0, AATATA
chr6:46856100	A/A	G	A	SNV	ADGRF5	ADGRF5:exonic:NM_001098518.1	1	100	4	synonymous	NM_00109	c.300C>T	p.(=)	-1.63					7 transmemb	rs678312	0.1244	YES	8.567E-1 (pos.46856100)			G-protein coupled receptor activity,G-protein coupled re				G=0, A=26	G=0.0, A=1.0	0.00001	311.819	26	G=0/0, A=11/1
chr16:57689883	C/C	T	C	SNV	ADGRG1	ADGRG1:exonic:NM_005682.6	1	100	8	synonymous	NM_00568	c.996T>C	p.(=)	-0.07				0.08(COSM43	7 transmemb	rs1376041	0.2941	YES	8.093E-1 (pos.57689883)	diffuse_large_	Adhesion G	G-protein coupled receptor activity,G-prot			Benign/Likely	T=0, C=24	T=0.0, C=1.0	0.00001	330.392	24	T=0/0, C=18/6
chr16:57697532	G/G	T	G	SNV	ADGRG1	ADGRG1:utr_3:NM_005682.6	1	100	15	utr_3	NM_00568	c.2120T>G	p.?	-0.36					7 transmemb	rs10852554	0.2888	YES	9.479E-1 (pos.57697532)		Adhesion G	G-protein coupled receptor activity,G-prot			Benign	T=0, G=77	T=0.0, G=1.0	0.00001	1229.42	77	T=0/0, G=46/3
chr1:16529117	C/C	T	C	SNV	ARHGEF19	ARHGEF19:intronic:NM_153213.4	1	100		intronic	NM_15321	c.1908-48,	p.?	-1.71					RhoGEF dom	rs221039	0.2573	YES	1.256E-1 (pos.16529117)		RHO guanin	G-protein coupled receptor signaling pathway,GTPase a				T=0, C=23	T=0.0, C=1.0	0.00001	316.733	23	T=0/0, C=6/17
chr1:1653028	T/T	C	T	SNV	CDK11A,CDK11B	CDK11A:intronic:NM_001313896.1. CDK11B:intronic:NM_0:	1	100		intronic	NM_03348	c.227+7G>	p.?, p.?	-1.14					Protein kinas	rs16825265,rs79626:	0.0702	YES	7.701E-1 (pos.1653028)		Cyclin-depe	ATP binding,apoptotic process,cyclin-dependent protein				C=0, T=34	C=0.0, T=1.0	0.00001	524.186	34	C=0/0, T=18/1
chrX:135426968	A/A	C	A	SNV	ADGRG4	ADGRG4:exonic:NM_153834.3	1	100	6	missense	NM_15383	c.1103C>A	p.Pro368His	0.3	0	77	0.875	0.03(COSM24	7 transmemb	rs5930931	0.4756	YES	2.666E-1 (pos.13542696:	adenocarcinoma,carcinom		G-protein coupled receptor activity,G-protein coupled re				C=0, A=36	C=0.0, A=1.0	0.00001	563.743	36	C=0/0, A=23/1
chr1:16532848	G/G	A	G	SNV	ARHGEF19	ARHGEF19:intronic:NM_153213.4	1	100		intronic	NM_15321	c.1138-13	p.?	0.1					RhoGEF dom	rs221051	0.2513	YES	1.443E-1;0.0 (pos.16532848)		RHO guanin	G-protein coupled receptor signaling pathway,GTPase a				A=0, G=139	A=0.0, G=1.0	0.00001	2321.01	139	A=0/0, G=64/7
chr1:16533817	C/C	T	C	SNV	ARHGEF19	ARHGEF19:intronic:NM_153213.4	1	100		intronic	NM_15321	c.875-112,	p.?	0.26					RhoGEF dom	rs170234		YES			RHO guanin	G-protein coupled receptor signaling pathway,GTPase a				T=0, C=92	T=0.0, C=1.0	0.00001	1534.54	92	T=0/0, C=44/4
chr1:16533830	A/A	AATTC	A	INDEL	ARHGEF19	ARHGEF19:intronic:NM_153213.4	4	100		intronic	NM_15321	c.875-125(	p.?	0.04, 0.64, 0.06, 1.58, 0.43					RhoGEF dom	rs149715516,rs199861124,rs38		YES			RHO guanin	G-protein coupled receptor signaling pathway,GTPase a				AATTC=0, A	AATTC=0.0, A=1.0	0.00001	1419.13	86	AATTC=0/0, A=
chrX:135429503	A/A	C	A	SNV	ADGRG4	ADGRG4:exonic:NM_153834.3	1	100	6	missense	NM_15383	c.3638C>A	p.Thr1213Asn	-0.31	0	65	0.001	0.01(COSM40	7 transmemb	rs4829830	0.4786	YES	3.254E-1 (pos.13542950:	acute_myeloid_leukaemia		G-protein coupled receptor activity,G-protein coupled re				C=0, A=86	C=0.0, A=1.0	0.00001	1410.91	86	C=0/0, A=33/5
chrX:135430044	C/C	T	C	SNV	ADGRG4	ADGRG4:exonic:NM_153834.3	1	100	6	synonymous	NM_15383	c.4179T>C	p.(=)	0.15				0.03(COSM37	7 transmemb	rs877761	0.4802	YES	3.45E-1 (pos.135430044)	acute_myeloid_leukaemia		G-protein coupled receptor activity,G-protein coupled re				T=0, C=30	T=0.0, C=1.0	0.00001	446.639	30	T=0/0, C=13/1
chr1:165376043	C/C	T	C	SNV	RXRG	RXRG:intronic:NM_006917.4	1	100		intronic	NM_00691	c.1244+6A	p.?	-0.77					Ligand-bindir	rs283696	0.2197	YES	9.255E-1 (pos.165376043)		Retinoid X r	9-cis retin	Bipolar Disorder;Bi	Acitretin,Adapalene,Alit		T=0, C=39	T=0.0, C=1.0	0.00001	623.975	39	T=0/0, C=18/2
chr1:165377389	C/C	T	C	SNV	RXRG	RXRG:intronic:NM_006917.4	1	100		intronic	NM_00691	c.1138+75	p.?	-1.11					Ligand-bindir	rs283692					Retinoid X r	9-cis retin	Bipolar Disorder;Bi	Acitretin,Adapalene,Alit		T=0, C=27	T=0.0, C=1.0	0.00001	389.986	27	T=0/0, C=16/1
chr1:165859630	G/G	C	G	SNV	UCK2	UCK2:intronic:NM_012474.4	1	100		intronic	NM_01247	c.259+30C	p.?	-0.01					Dephospho-C	rs477033,rs753780993,rs777413066			0.0 (pos.165859627),0.0 (pos.16585962!		Uridine/cyt	ATP bindin	Endocrine Gland Ne	Citric Acid,Cytidine,Cytid		C=0, G=54	C=0.0, G=1.0	0.00001	798.148	54	C=0/0, G=22/3
chr1:1664019	C/C	T	C	SNV	SLC35E2	SLC35E2:intronic:NM_182838.2	1	100		intronic	NM_18283	c.733-57A:	p.?	-0.98					Triose-phosp	rs2294488	0.0733	YES				integral component of membrane				T=0, C=20	T=0.0, C=1.0	0.00001	263.784	20	T=0/0, C=5/15
chr1:166831404	A/C	A	C	SNV	TADA1	TADA1:intronic:NM_053053.3	1	56		intronic	NM_05305	c.540+36T	p.?	0.53					Transcription	rs2075947	0.3777	YES	2.939E-1;0.0 (pos.166831404)			SAGA complex,STAGA complex,cytosol,focal adhesion,his				A=11, C=14	A=0.44, C=0.56	0.00001	134.349	25	A=3/8, C=6/8
chrX:135496398	A/A	G	A	SNV	ADGRG4	ADGRG4:exonic:NM_153834.3	1	100	25	synonymous	NM_15383	c.9117G>A	p.(=)	0.03				0(COSM6351(	7 transmemb	rs5930942	0.3936	YES	9.068E-1 (pos.13549639:	carcinoma		G-protein coupled receptor activity,G-protein coupled re				G=0, A=52	G=0.0, A=1.0	0.00001	767.015	52	G=0/0, A=31/2
chr6:142758631	G/G	A	G	SNV	ADGRG6	ADGRG6:exonic:NM_198569.2	1	100	23	missense	NM_19856	c.3380A>G	p.Gln1127Arg	3.59	1	43	0		7 transmemb	rs1262686	0.0023		1.0;0.0 (pos.142758631)		Adhesion G	G-protein coupled receptor activity,G-protein coupled re				A=0, G=78	A=0.0, G=1.0	0.00001	1249.35	78	A=0/0, G=27/5
chr4:62598689	T/T	C	T	SNV	ADGRL3	ADGRL3:exonic:NM_015236.5	1	100	7	synonymous	NM_01523	c.612C>T	p.(=)	-0.36				0.15(COSM11	7 transmemb	rs10434219	0.3956	YES	9.387E-1 (pos.62598689)	adenocarcino	Adhesion G	G-protein coupled receptor activity,G-protein coupled re				C=0, T=39	C=0.0, T=1.0	0.00001	623.979	39	C=0/0, T=25/1
chr1:169494196	T/T	C	T	SNV	F5	F5:intronic:NM_000130.4	1	100		intronic	NM_00013	c.5717-50(	p.?	0.01					Coagulation	rs2301515	0.3179	YES	8.694E-1 (pos.169494196)		Coagulation	COPII vesi	Abortion, Habitual;	Anti-inhibitor coagulant		C=0, T=26	C=0.0, T=1.0	0.00001	371.42	26	C=0/0, T=16/1
chr1:169498834	C/C	T	C	SNV	F5	F5:intronic:NM_000130.4	1	100		intronic	NM_00013	c.5419+12	p.?	0.34					Coagulation	rs386636637,rs6009.	0.0744	YES	9.999E-1 (pos.169498834)		Coagulation	COPII vesi	Abortion, Habitual;	Anti-inhibi	Benign/Likely	T=0, C=116	T=0.0, C=1.0	0.00001	1852.92	116	T=0/0, C=16/1
chr4:62845388	G/C	G	C	SNV	ADGRL3	ADGRL3:exonic:NM_015236.5	1	53.33	17	synonymous	NM_01523	c.2709G>C	p.(=)	-2.48					7 transmemb	rs1397547	0.0538	YES	8.128E-1;4.64E-4 (pos.62845388)		Adhesion G	G-protein coupled receptor activity,G-protein coupled re				G=35, C=4C	G=0.4667, C=0.5333	0.00001	433.78	75	G=16/19, C=2:
chr1:79392756	A/A	C	A	SNV	ADGRL4	ADGRL4:exonic:NM_022159.3	1	100	8	missense	NM_02215	c.898G>T	p.Val300Leu	1.54	1	32	0	0.2(COSM375	7 transmemb	rs12754818	0.3604	YES	6.999E-1 (pos.79392756)	adenocarcino	Adhesion G	G-protein coupled receptor activity,G-protein coupled re				C=0, A=121	C=0.0, A=1.0	0.00001	1939.85	121	C=0/0, A=43/7
chr1:169513436	A/A	C	A	SNV	F5	F5:intronic:NM_000130.4	1	100		intronic	NM_00013	c.1975+98	p.?	0.09					Coagulation	rs9287092		YES			Coagulation	COPII vesi	Abortion, Habitual;	Anti-inhibitor coagulant		C=0, A=42	C=0.0, A=1.0	0.00001	685.22	42	C=0/0, A=16/2
chr5:89979589	A/A	G	A	SNV	ADGRV1	ADGRV1:exonic:NM_032119.3	1	100	28	missense	NM_03211	c.5851G>A	p.Val1951Ile	0.57	0.55	29	0	0.05(COSM37	7 transmemb	rs4916684	0.3552	YES	8.022E-1 (pos.89979589)	basal_cell_car	Adhesion G	G-protein coupled receptor activity,G-prot			Benign/Likely	G=0, A=103	G=0.0, A=1.0	0.00001	1609.48	103	G=0/0, A=50/5
chr1:169665632	C/C	T	C	SNV	SELL	SELL:intronic:NM_000655.4	1	100		intronic	NM_00065	c.1139+44	p.?	-1.3					Human growt	rs2223286	0.3066	YES	1.351E-1 (pos.169665632)		Selectin L (l	carbohydr	Autoimmune Diseases:Cardiovascular Disea			T=0, C=24	T=0.0, C=1.0	0.00001	334.792	24	T=0/0, C=12/1
chr1:1696659	G/G	A	G	SNV	NADK	NADK:intronic:NM_001198994.1	1	100		intronic	NM_00119	c.179+8T>(	p.?	0.73					ATP-NAD kina	rs5021859	0.0545	YES	8.054E-1 (pos.1696659)		NAD kinase	ATP binding,ATP metabolic process,NAD metabolic proce				A=0, G=30	A=0.0, G=1.0	0.00001	446.612	30	A=0/0, G=21/9
chr1:169679472	G/GCT	G	GCT	INDEL	SELL	SELL:intronic:NM_000655.4	2	50		intronic	NM_00065	c.124+105	p.?	-0.21					Human growt	rs397815888,rs4987281		YES			Selectin L (l	carbohydr	Autoimmune Diseases;Cardiovascular Disea			G=57, GCT=	G=0.5, GCT=0.5	0.00001	589.199	114	G=10/47, GCT=
chr1:169831993	G/G	A	G	SNV	SCYL3	SCYL3:intronic:NM_181093.3	1	100		intronic	NM_18109	c.956-55T:	p.?	-0.14					Protein kinas	rs7525259		YES			SCY1-like 3,	ATP binding,Golgi apparatus,cell migration,cytoplasm,ki				A=0, G=40	A=0.0, G=1.0	0.00001	644.286	40	A=0/0, G=12/2
chr1:169930049	T/T	C	T	SNV	KIFAP3	KIFAP3:intronic:NM_014970.3	1	100		intronic	NM_01497	c.2173+13	p.?	0.18					Kinesin-asso	rs386636674,rs386636675,rs54		YES			Kinesin-ass	Golgi appa	Amyotrophic Lateral Sclerosis;Central Nervo			C=0, T=62	C=0.0, T=1.0	0.00001	940.316	62	C=0/0, T=35/2
chr1:169930051	C/C	T	C	SNV	KIFAP3	KIFAP3:intronic:NM_014970.3	1	100		intronic	NM_01497	c.2173+13	p.?	-0.05					Kinesin-asso	rs386636674,rs386636675,rs61		YES			Kinesin-ass	Golgi appa	Amyotrophic Lateral Sclerosis;Central Nervo			T=0, C=62	T=0.0, C=1.0	0.00001	940.32	62	T=0/0, C=35/2
chr1:169952385	T/T	G	T	SNV	KIFAP3	KIFAP3:intronic:NM_014970.3	1	100		intronic	NM_01497	c.1506+26	p.?	-0.02					Kinesin-asso	rs2057249	0.0966	YES	9.984E-1 (pos.169952385)		Kinesin-ass	Golgi appa	Amyotrophic Lateral Sclerosis;Central Nervo			G=0, T=22	G=0.0, T=1.0	0.00001	298.457	22	G=0/0, T=9/13
chr5:90151589	G/A	G	A	SNV	ADGRV1	ADGRV1:exonic:NM_032119.3	1	50	82	missense	NM_03211	c.17626G>	p.Val5876Ile	2.9	0.06	29	0.576	0.76(COSM14	7 transmemb	rs2247870	0.4528	YES	5.499E-1 (pos.90151589)	adenocarcino	Adhesion G	G-protein coupled receptor activity,G-prot			Benign/Likely	G=22, A=22	G=0.5, A=0.5	0.00001	242.66	44	G=8/14, A=7/1
chr1:171077198	A/A	G	A	SNV	FMO3	FMO3:intronic:NM_006894.5	1	100		intronic	NM_00689	c.485-22G	p.?	-0.07					Flavin-bindin	rs1920149	0.4679	YES	4.037E-1 (pos.171077198)		Flavin-cont	N,N-dimet	Adenoma:Adenoma	Almotripta	Benign	G=0, A=94	G=0.0, A=1.0	0.00001	1576.17	94	G=0/0, A=58/3
chr1:171162438	C/T	C	T	SNV	FMO2	FMO2:intronic:NM_001460.4	1	46.88		intronic	NM_00146	c.133-36C:	p.?	-1.91					Flavin-bindin	rs28969551	0.0479	YES	4.733E-4 (pos.171162438)		Flavin-cont	N,N-dimet	Endometriosis:Female Urogenital Diseases:F			C=17, T=15	C=0.5313, T=0.4688	0.00001	143.712	32	C=8/9, T=4/11
chr4:100235053	G/G	A	G	SNV	ADH1B	ADH1B:exonic:NM_000668.5	1	100	6	synonymous	NM_00066	c.753T>C	p.(=)	-0.02					Alcohol dehy	rs1789882	0.1675	YES	9.148E-1 (pos.100235053)		Alcohol deh	alcohol de	Alcohol-Related Dis	2'-Monophosphoadenos		A=0, G=73	A=0.0, G=1.0	0.00001	1145.79	73	A=0/0, G=29/4
chr4:100268188	TC/TC	T	TC	INDEL	ADH1C	ADH1C:exonic:NM_000669.4	1	100	3	stoploss	NM_00066	c.233_234	p.Ter78Trp	-1.26					Alcohol dehydrogenase GroES-like domain,Zinc-binding dehydrogenase						Alcohol deh	alcohol de	Alcohol-Related Dis	(R)-N-(1-Methyl-Hexyl)-Fo		T=0, TC=77	T=0.0, TC=1.0	0.00001	824.148	77	T=0/0, TC=40/3
chr4:100266371	A/G	A	G	SNV	ADH1C	ADH1C:exonic:NM_000669.4	1	58.33	4	synonymous	NM_00066	c.312T>C	p.(=)	-0.04					Alcohol dehy	rs1789915	0.2933	YES	1.957E-2 (pos.100266371)		Alcohol deh	alcohol de	Alcohol-Related Dis	(R)-N-(1-Methyl-Hexyl)-Fo		A=20, G=28	A=0.4167, G=0.5833	0.00001	344.614	48	A=9/11, G=17/
chr8:67380528	C/C	T	C	SNV	ADHFE1	ADHFE1:exonic:NM_144650.2	1	100	14	missense	NM_14465	c.1345T>C	p.Cys449Arg	1.92	1	180	0	0.14(COSM40	Iron-containi	rs1060242	0.4737	YES	4.136E-1 (pos.67380528)	carcinoma,duc	Alcohol deh	2-oxoglutarate metabolic proc		Sodium oxybate		T=0, C=52	T=0.0, C=1.0	0.00001	767.453	52	T=0/0, C=27/2
chr2:3502709	G/G	A	G	SNV	ADI1	ADI1:utr_3:NM_018269.3	1	100	4	utr_3	NM_01826	c.565T>C	p.?	-0.78					ARD/ARD' far	rs10208494	0.1512	YES	1.941E-1;0.0 (pos.3502709)		Acireductor	L-methion	Genital Diseases, N	Isopropyl Alcohol		A=0, G=21	A=0.0, G=1.0	0.00001	281.235	21	A=0/0, G=4/17
chr2:3504687	G/G	A	G	SNV	ADI1	ADI1:exonic:NM_018269.3	1	100	3	synonymous	NM_01826	c.318T>C	p.(=)	-0.46				0.08(COSM14	ARD/ARD' far	rs9950	0.3555	YES	4.316E-1 (pos.3504687)	acute_myeloic	Acireductor	L-methion	Genital Diseases, N	Isopropyl Alcohol		A=0, G=149	A=0.0, G=1.0	0.00001	2530.58	149	A=0/0, G=66/8
chr1:112042632	G/G	A	G	SNV	ADORA3,TMIGD3	TMIGD3:intronic:NM_020683.6. ADORA3:exonic:NM_00067	1	100	2	synonymous	NM_02068	c.350+299	p.?, p.(=)	1.7					7 transmemb	rs2229155	0.1853	YES	7.683E-1 (pos.112042632)		Adenosine	G-protein	Autoimmune Disea	Adenosine,Aminophyllin		A=0, G=28	A=0.0, G=1.0	0.00001	408.699	28	A=0/0, G=22/6
chr1:173556699	T/T	G	T	SNV	SLC9C2	SLC9C2:intronic:NM_178527.3	1	100		intronic	NM_17852	c.523+105	p.?	-1.44					Cyclic nucleo	rs301698		YES				hydrogen ion transmembrane transport,integral compo				G=0, T=53	G=0.0, T=1.0	0.00001	768.761	53	G=0/0, T=28/2

chr15:73067234	C/C	T	C	SNV	ADPGK	ADPGK:exonic:NM_031284.4	1	100	2	synonymous	NM_03128	c.438A>G	p.(=)	-3.81					ADP-specific	rs11634630	4.00E-04	YES	9.999E-1;0.0;0.0 (pos.73067234)		ADP-depen	ADP-specific glucokinase activity,canonical glycolysis,ca	T=0, C=86	T=0.0, C=1.0	0.00001	1410.92	86	T=0/0, C=58/2
chr1:17402116	G/T	G	T	SNV	PADI2	PADI2:intronic:NM_007365.2	1	45.83		intronic	NM_00736	c.1455+58	p.?	-1.22					Protein-argin	rs3818035		YES			Peptidylarg	azurophil granule lumen,calciu L-Citrulline	G=13, T=11	G=0.5417, T=0.4583	0.00001	93.1297	24	G=11/2, T=7/4
chr1:17418883	G/G	A	G	SNV	PADI2	PADI2:intronic:NM_007365.2	1	100		intronic	NM_00736	c.655+20T	p.?	-0.4					Protein-argin	rs2647184,rs775198	0.0538	YES	0.0;0.0 (pos.17418878),9.984E-1 (pos.17		Peptidylarg	azurophil granule lumen,calciu L-Citrulline	A=0, G=22	A=0.0, G=1.0	0.00001	298.878	22	A=0/0, G=18/4
chr1:175097331	G/A	G	A	SNV	TNN	TNN:intronic:NM_022093.1	1	50		intronic	NM_02209	c.3178+31	p.?	-2.05					Fibrinogen be	rs10912891	0.3785	YES	3.149E-1 (pos.175097331)		Tenascin N	axonogenesis,cell growth,cell migration,cell surface,cel	G=16, A=16	G=0.5, A=0.5	0.00001	158.382	32	G=6/10, A=4/1
chr1:36553673	G/G	A	G	SNV	ADPRHL2,TEKT2	ADPRHL2:upstream:NM_017825.2, TEKT2:exonic:NM_0144	1	100	10	synonymous	NM_01446	c.1179A>G	p.(=), p.?	-1.7					Tektin family	rs399393	0.0816	YES	9.992E-1 (pos.36553673)		ADP-ribosyl	cellular response to superoxide,cilium assembly,cilium	A=0, G=20	A=0.0, G=1.0	0.00001	263.78	20	A=0/0, G=8/12
chr20:4202041	A/A	T	A	SNV	ADRA1D	ADRA1D:utr_3:NM_000678.3	1	100	2	utr_3	NM_00067	c.1848A>T	p.?	-0.68					7 transmemb	rs2236554		YES			Adrenergic.	DNA metabolic process,G-prot Alfuzosin,Amitriptyline,A	T=0, A=48	T=0.0, A=1.0	0.00001	810.158	48	T=0/0, A=15/3
chr1:175336292	A/A	C	A	SNV	TNR	TNR:intronic:NM_003285.2	1	100		intronic	NM_00328	c.2053+52	p.?	0.3					EGF-like dom	rs1872440	0.2427	YES			Tenascin R	associativ Abortion, Spontaneous;Female Urogenital Di	C=0, A=63	C=0.0, A=1.0	0.00001	958.917	63	C=0/0, A=38/2
chr1:175336305	C/C	G	C	SNV	TNR	TNR:intronic:NM_003285.2	1	100		intronic	NM_00328	c.2053+39	p.?	-1.39					EGF-like dom	rs1872439	0.2434	YES	9.08E-2 (pos.175336305)		Tenascin R	associativ Abortion, Spontaneous;Female Urogenital Di	G=0, C=63	G=0.0, C=1.0	0.00001	958.805	63	G=0/0, C=38/2
chr1:17550225	T/C	T	C	SNV	PADI1	PADI1:intronic:NM_013358.2	1	95.45		intronic	NM_01335	c.346+37T	p.?	-0.83					Protein-argin	rs3003481	2.00E-04		1.0 (pos.17550225)		Peptidylarg	calcium ion binding,chromatin L-Citrulline	T=3, C=63	T=0.0455, C=0.9545	0.00001	922.715	66	T=2/1, C=19/4
chr1:176012807	A/A	G	A	SNV	RFWD2	RFWD2:intronic:NM_022457.6	1	100		intronic	NM_02245	c.1530+39	p.?	0.23					RING-type zin	rs4233165	0.0099	YES	1.0 (pos.176012807)		Ring finger-	Cul4A-RIN Autistic Disorder;Child Development Disorde	G=0, A=22	G=0.0, A=1.0	0.00001	298.889	22	G=0/0, A=19/3
chr1:176153658	T/C	T	C	SNV	RFWD2	RFWD2:intronic:NM_022457.6	1	97.5		intronic	NM_02245	c.467+111	p.?	-0.04					RING-type zin	rs791744		YES			Ring finger-	Cul4A-RIN Autistic Disorder;Child Development Disorde	T=1, C=39	T=0.025, C=0.975	0.00001	611.635	40	T=1/0, C=29/1
chr1:176857347	G/G	A	G	SNV	ASTN1	ASTN1:intronic:NM_004319.2	1	100		intronic	NM_00431	c.2941-7T	p.?	0.57				0.01(COSM54	Coagulation	rs2072935	0.1664	YES	7.902E-1 (pos.17685734	adenocarcino	Astrotactin 1		A=0, G=37	A=0.0, G=1.0	0.00001	583.707	37	A=0/0, G=18/1
chr1:17723476	CCA/CCA	C	CCA	INDEL	PADI6	PADI6:intronic:NM_207421.4	2	100		intronic	NM_20742	c.1619-91	p.?	-0.33					Protein-argin	rs61476146		YES			Peptidylarg	calcium io Carcinoma;Carcino L-Citrulline	C=0, CCA=3	C=0.0, CCA=1.0	0.00001	485.141	32	C=0/0, CCA=18
chr1:17725358	G/G	C	G	SNV	PADI6	PADI6:intronic:NM_207421.4	1	100		intronic	NM_20742	c.1851+15	p.?	0.27					Protein-argin	rs2244936	0.3501	YES	5.278E-1 (pos.17725358)		Peptidylarg	calcium io Carcinoma;Carcino L-Citrulline	C=0, G=22	C=0.0, G=1.0	0.00001	298.892	22	C=0/0, G=15/7
chr2:96780716	G/G	T	G	SNV	ADRA2B	ADRA2B:exonic:NM_000682.6	1	100	2	synonymous	NM_00068	c.1173A>C	p.(=)	-1.9				0.09(COSM56	7 transmemb	rs2229169	0.3174	YES	5.587E-1;0.0 (pos.96780	carcinoma,duc	Adrenergic.	G-protein Brain Diseases:Car Amoxapine,Amphetamir	T=0, G=31	T=0.0, G=1.0	0.00001	465.818	31	T=0/0, G=20/1
chr1:178780648	A/AG	A	AG	INDEL	RALGPS2	RALGPS2:intronic:NM_152663.4	1	100		intronic	NM_15266	c.387+109	p.?	0.54					PH domain,RasGEF domain							cytoplasm,guanyl-nucleotide exchange factor activity,in	A=0, AG=38	A=0.0, AG=1.0	0.00001	217.41	38	A=0/0, AG=22/
chr1:178854162	T/T	C	T	SNV	RALGPS2	RALGPS2:intronic:NM_152663.4	1	100		intronic	NM_15266	c.905-49C	p.?	0.04					PH domain,R	rs6425503	0.4215	YES	5.292E-1 (pos.178854162)			cytoplasm,guanyl-nucleotide exchange factor activity,in	C=0, T=22	C=0.0, T=1.0	0.00001	298.891	22	C=0/0, T=19/3
chr20:60881330	A/A	G	A	SNV	ADRM1	ADRM1:exonic:NM_175573.2	1	100	4	synonymous	NM_17557	c.408G>A	p.(=)	-1.58					Proteasome	rs2427273	0.072	YES	9.816E-1 (pos.60881330)			cytoplasm,cytosol,endopeptidase activator activity,inte	G=0, A=20	G=0.0, A=1.0	0.00001	263.778	20	G=0/0, A=11/9
chr1:179503698	G/A	G	A	SNV	AXDND1	AXDND1:intronic:NM_144696.5	1	50		intronic	NM_14469	c.2799-16	p.?	-0.12					Axonemal dy	rs6425571		YES					G=10, A=10	G=0.5, A=0.5	0.00001	82.886	20	G=1/9, A=7/3
chr14:105196365	C/C	T	C	SNV	ADSSL1	ADSSL1:exonic:NM_199165.2	1	100	1	synonymous	NM_19916	c.136T>C	p.(=)	0.45				0(COSM3753	Adenylosucci	rs33958252	0.4558	YES	6.665E-1 (pos.10519636	adenocarcinoma,hepatoce		'de novo' AMP biosynthetic pro 6-O-Phosphoryl Inosine	T=0, C=22	T=0.0, C=1.0	0.00001	298.89	22	T=0/0, C=13/9
chr14:105213343	G/G	A	G	SNV	ADSSL1	ADSSL1:utr_3:NM_199165.2	1	100	13	utr_3	NM_19916	c.1506A>G	p.?	-2.15					Adenylosucci	rs6644	0.4583	YES	6.336E-1 (pos.105213343)			'de novo' AMP biosynthetic pro 6-O-Phosphoryl Inosine	A=0, G=35	A=0.0, G=1.0	0.00001	543.882	35	A=0/0, G=15/2
chr7:44148553	G/G	A	G	SNV	AEBP1	AEBP1:exonic:NM_001129.4	1	100	7	synonymous	NM_00112	c.996A>G	p.(=)	-0.66				0.02(COSM41	Carboxypepti	rs2595701	0.3179	YES	8.591E-1 (pos.44148553)	hepatocellular_carcinoma		DNA binding transcription factor activity,RNA polymeras	A=0, G=26	A=0.0, G=1.0	0.00001	371.364	26	A=0/0, G=15/1
chr1:180022760	T/T	A	T	SNV	CEP350	CEP350:intronic:NM_014810.4	1	100		intronic	NM_01481	c.4990-12	p.?	0.79					CAP-Gly dom	rs2040083		YES				cell projection,centriole,centrosome,membrane,microt	A=0, T=95	A=0.0, T=1.0	0.00001	1597.02	95	A=0/0, T=61/3
chr1:180067988	G/A	G	A	SNV	CEP350	CEP350:intronic:NM_014810.4	1	50		intronic	NM_01481	c.9067-10	p.?	-0.8					CAP-Gly dom	rs12121845	0.139	YES	7.277E-2 (pos.180067988)			cell projection,centriole,centrosome,membrane,microt	G=10, A=10	G=0.5, A=0.5	0.00001	83.006	20	G=5/5, A=5/5
chr15:89169858	G/G	A	G	SNV	AEN	AEN:exonic:NM_022767.3	1	100	2	missense	NM_02276	c.418A>G	p.Asn140Asp	2.02	1	23	0	0.16(COSM48	Exonuclease	rs8027765	0.0669	YES	7.933E-1 (pos.89169858)	adenocarcino	Interferon-s	exonuclease activity,intrinsic apoptotic signaling pathw	A=0, G=70	A=0.0, G=1.0	0.00001	1092.06	70	A=0/0, G=44/2
chr1:180257692	C/C	A	C	SNV	ACBD6	ACBD6:intronic:NM_032360.3	1	100		intronic	NM_03236	c.695-40T	p.?	-3.34					Acyl CoA bind	rs2254465	0.0612	YES	9.846E-1;0.0 (pos.180257692)		Acyl-CoA-bi	acyl-CoA n Intellectual Disability;Mental Disorders;Nerv	A=0, C=24	A=0.0, C=1.0	0.00001	334.791	24	A=0/0, C=5/19
chr1:180803922	T/C	T	C	SNV	XPR1	XPR1:intronic:NM_004736.3	1	56		intronic	NM_00473	c.1135-88	p.?	0.45					EXS family,SP	rs2271669		YES			Xenotropic	G-protein Basal Ganglia Diseases;Brain Diseases;Calci	T=22, C=28	T=0.44, C=0.56	0.00001	341.073	50	T=14/8, C=21/
chr1:180897521	T/T	C	T	SNV	KIAA1614	KIAA1614:intronic:NM_020950.1	1	100		intronic	NM_02095	c.1062-45	p.?	1.8					Domain of un	rs7544551	0.0013	YES	1.0 (pos.180897521)				C=0, T=40	C=0.0, T=1.0	0.00001	644.247	40	C=0/0, T=21/1
chr15:89172558	C/C	G	C	SNV	AEN	AEN:exonic:NM_022767.3	1	100	3	synonymous	NM_02276	c.642G>C	p.(=)	-0.05				0.02(COSM41	Exonuclease	rs3743475	0.0771	YES	7.928E-1;0.0 (pos.89172	neoplasm	Interferon-s	exonuclease activity,intrinsic apoptotic signaling pathw	G=0, C=88	G=0.0, C=1.0	0.00001	1448.76	88	G=0/0, C=65/2
chr15:89173657	T/T	A	T	SNV	AEN	AEN:utr_3:NM_022767.3	1	100	4	utr_3	NM_02276	c.1110A>T	p.?	-3.33					Exonuclease	rs3826038		YES	7.398E-1;0.0 (pos.89173657)		Interferon-s	exonuclease activity,intrinsic apoptotic signaling pathw	A=0, T=32	A=0.0, T=1.0	0.00001	485.13	32	A=0/0, T=10/2
chr1:182501933	A/A	C	A	SNV	RGSL1	RGSL1:intronic:NM_001137669.1	1	100		intronic	NM_00113	c.2494+7C	p.?	0.17				0.03(COSM44	Regulator of	rs266534	0.3253	YES	5.077E-1 (pos.18250193	acute_myeloid	Regulator o	GTPase activator activity,cytoplasm,integral component	C=0, A=56	C=0.0, A=1.0	0.00001	832.096	56	C=0/0, A=27/2
chr1:182509269	A/A	G	A	SNV	RGSL1	RGSL1:intronic:NM_001137669.1	1	100		intronic	NM_00113	c.2495-11	p.?	-0.89					Regulator of	rs266531		YES			Regulator o	GTPase activator activity,cytoplasm,integral component	G=0, A=21	G=0.0, A=1.0	0.00001	281.231	21	G=0/0, A=15/6
chr1:182509292	G/G	A	G	SNV	RGSL1	RGSL1:intronic:NM_001137669.1	1	100		intronic	NM_00113	c.2495-90	p.?	0.55					Regulator of	rs3856115		YES			Regulator o	GTPase activator activity,cytoplasm,integral component	A=0, G=21	A=0.0, G=1.0	0.00001	281.235	21	A=0/0, G=15/6
chr1:182509617	A/A	C	A	SNV	RGSL1	RGSL1:intronic:NM_001137669.1	1	100		intronic	NM_00113	c.2669+61	p.?	-0.06					Regulator of	rs386637388,rs3911280		YES			Regulator o	GTPase activator activity,cytoplasm,integral component	C=0, A=49	C=0.0, A=1.0	0.00001	830.281	49	C=0/0, A=26/2
chr10:116060387	C/C	T	C	SNV	AFAP1L2	AFAP1L2:exonic:NM_001001936.2	1	100	14	synonymous	NM_00100	c.1605A>G	p.(=)	-0.45				0.3(COSM414	PH domain	rs2286396	0.1647	YES	7.271E-1 (pos.11606038	neoplasm	Actin filame	SH2 domain binding,SH3 domain binding,cytoplasm,cyto	T=0, C=84	T=0.0, C=1.0	0.00001	1370.17	84	T=0/0, C=41/4
chr2:100343557	T/T	C	T	SNV	AFF3	AFF3:exonic:NM_001025108.1	1	100	10	missense	NM_00102	c.1148G>A	p.Ser383Asn	3.28	1	46	0	0.02(COSM45	AF-4 proto-or	rs4851223			1.0 (pos.100343557)	squamous_cell_carcinoma		cytosol,do Arthritis;Arthritis, Rheumatoid;Autoimmune	C=0, T=50	C=0.0, T=1.0	0.00001	852.748	50	C=0/0, T=33/1
chr1:183099701	G/A	G	A	SNV	LAMC1	LAMC1:intronic:NM_002293.3	1	45.45		intronic	NM_00229	c.3486+17	p.?	-0.96					Laminin B (Do	rs2296300	0.4306	YES	6.322E-1 (pos.183099701)		Laminin, ga	basement Abnormalities, Multiple;Aortic Diseases;Bon	G=24, A=20	G=0.5455, A=0.4545	0.00001	211.641	44	G=10/14, A=1
chr1:183775638	T/T	A	T	SNV	RGL1	RGL1:intronic:NM_015149.4	1	100		intronic	NM_01514	c.243+19A	p.?	0.98					Ras associati	rs11579867	0.3121	YES	6.945E-2 (pos.183775638)		Ral guanine	Ral guanyl-nucleotide exchange factor activity,cellular	A=0, T=21	A=0.0, T=1.0	0.00001	281.235	21	A=0/0, T=4/17
chr18:12329536	G/G	C	G	SNV	AFG3L2	AFG3L2:utr_3:NM_006796.2	1	100	17	utr_3	NM_00679	c.2422G>C	p.?	0.01					ATPase famil	rs1129115	0.218	YES	3.845E-1 (pos.12329536)		ATPase fam	ATP bindin Ataxia;Brain Diseas ATP Benign	C=0, G=64	C=0.0, G=1.0	0.00001	977.613	64	C=0/0, G=29/3
chr1:1850428	G/G	A	G	SNV	TMEM52	TMEM52:intronic:NM_178545.3	1	100		intronic	NM_17854	c.129-55T	p.?	0.11					Transmembr	rs2295363		YES				integral component of membrane	A=0, G=39	A=0.0, G=1.0	0.00001	623.977	39	A=0/0, G=15/2
chr17:76183043	A/G	A	G	SNV	AFMID,TK1	AFMID:upstream:NM_001145526.2, TK1:exonic:NM_00325	1	50	1	synonymous	NM_00325	c.33T>C, c.	p.(=), p.?	-0.04					Thymidine ki	rs1071664	0.3551	YES	6.711E-1 (pos.76183043)		Thymidine	ATP bindin Colonic Diseases;C Dithioerythritol,Thymidi	A=21, G=21	A=0.5, G=0.5	0.00001	228.892	42	A=13/8, G=11/
chr1:186039006	T/T	C	T	SNV	HMCN1	HMCN1:intronic:NM_031935.2	1	100		intronic	NM_03193	c.7993+98	p.?	1.22					Calcium-bind	rs761905		YES			Hemicentin	basement Eye Diseases;Macular Degeneration;Retinal	C=0, T=20	C=0.0, T=1.0	0.00001	257.855	20	C=0/0, T=7/13
chr1:186086578	A/A	G	A	SNV	HMCN1	HMCN1:intronic:NM_031935.2	1	100		intronic	NM_03193	c.11684-1	p.?	-0.58					Calcium-bind	rs10157742	0.4985	YES	6.389E-1;0.0 (pos.186086578)		Hemicentin	basement Eye Diseases;Macular Degene Likely benign	G=0, A=25	G=0.0, A=1.0	0.00001	353.016	25	G=0/0, A=12/1
chr4:178359960	C/C	G	C	SNV	AGA	AGA:exonic:NM_000027.3	1	100	4	missense	NM_00002	c.446C>G	p.Thr149Ser	-0.15	0.51	58	0		Asparaginase	rs2228119	0.001	YES	9.999E-1 (pos.178359960)		Aspartylglu	N4-(beta-N Aspartylglucosaminuria;Conge Benign	G=0, C=45	G=0.0, C=1.0	0.00001	747.364	45	G=0/0, C=23/2
chr10:88768657	T/T	C	T	SNV	AGAP11	AGAP11:exonic:NM_133447.1	1	100	12	synonymous	NM_13344	c.648C>T	p.(=)	0.2					Ankyrin repe	rs1745901	0.3188	YES	9.101E-1 (pos.88768657)			GTPase activator activity,metal ion binding,positive reg	C=0, T=36	C=0.0, T=1.0	0.00001	563.744	36	C=0/0, T=23/1
chr1:186316656	C/C	G	C	SNV	TPR	TPR:intronic:NM_003292.2	1	100		intronic	NM_00329	c.2777-66	p.?	-0.49					TPR/MLP1/M	rs3753564		YES			Tumor pote	MAPK imp Carcinoma;Endocri Flavin adenine dinucleot	G=0, C=35	G=0.0, C=1.0	0.00001	543.903	35	G=0/0, C=16/1
chr1:186329869	G/G	C	G	SNV	TPR	TPR:intronic:NM_003292.2	1	100		intronic	NM_00329	c.1099+28	p.?	-0.04					TPR/MLP1/M	rs2026187	0.3919	YES	6.607E-1 (pos.186329869)		Tumor pote	MAPK imp Carcinoma;Endocri Flavin adenine dinucleot	C=0, G=24	C=0.0, G=1.0	0.00001	334.791	24	C=0/0, G=7/17
chr1:186330643	C/C	T	C	SNV	TPR	TPR:intronic:NM_003292.2	1	100		intronic	NM_00329	c.958+111	p.?	-0.07					TPR/MLP1/M	rs10494586		YES			Tumor pote	MAPK imp Carcinoma;Endocri Flavin adenine dinucleot	T=0, C=40	T=0.0, C=1.0	0.00001	644.286	40	T=0/0, C=27/1
chr1:186357523	C/C	T	C	SNV	C1orf27	C1orf27:intronic:NM_017847.5	1	100		intronic	NM_01784	c.331-51T	p.?	-0.74					Olfactory rec	rs1290390,rs956768557		YES				integral component of membrane	T=0, C=22	T=0.0, C=1.0	0.00001	298.892	22	T=0/0, C=16/6
chr7:150815705	G/A	G	A	SNV	AGAP3	AGAP3:exonic:NM_031946.6	1	46.51	7	missense	NM_03194	c.955G>A	p.Val319Met	7.44	0	21	0.999		Ankyrin repe	rs61741392	1.00E-04		0.0;0.0 (pos.150815705)		ARF GTPase	GTP binding,GTPase activator activity,GTPase activity,cy	G=23, A=20	G=0.5349, A=0.4651	0.00001	212.325	43	G=6/17, A=9/1
chr1:186645927	T/T	C	T	SNV	PTGS2	PTGS2:intronic:NM_000963.3	1	100		intronic	NM_00096	c.723+38G	p.?	-1.75					Animal haem	rs2066826	0.1392	YES	3.804E-2 (pos.186645927)		Prostaglanc	NAD biosy Adenocarcinoma;A 1-Phenylsulfonamide-3-	C=0, T=23	C=0.0, T=1.0	0.00001	316.747	23	C=0/0, T=11/1
chr15:87217613	G/G	A	G	SNV	AGBL1	AGBL1:exonic:NM_152336.3	1	100	23	missense	NM_15233	c.3167A>G	p.Gln1056Arg	0	0.94	43	0		Zinc carboxyp	rs8028043	0.0917	YES	9.115E-1;0.0;0.0 (pos.87217613)		ATP/GTP-binding protein-like 1		A=0, G=73	A=0.0, G=1.0	0.00001	1150.5	73	A=0/0, G=26/4
chr1:18807339	T/C	T	C	SNV	KLHDC7A	KLHDC7A:upstream:NM_152375.2	1	47.37		intronic	NM_15237	c.-137T>C	p.?	0.56						rs2992756		YES				Cul3-RING ubiquitin ligase complex,integral component	T=30, C=27	T=0.5263, C=0.4737	0.00001	256	57	T=9/21, C=15/
chr11:47701528	A/A	C	A	SNV	AGBL2	AGBL2:exonic:NM_024783.3	1	100	13	missense	NM_02478	c.2013G>T	p.Met671Ile	0.47	0.1	10	0	0.53(COSM37	Zinc carboxy	rs12286721	0.4539	YES	6.171E-1 (pos.47701528)	adenocarcino	ATP/GTP-bir	centriole,ciliary basal body,cytosol,metallocarboxypept	C=0, A=24	C=0.0, A=1.0	0.00001	334.792	24	C=0/0, A=5/19
chr7:134701856	C/C	G	C	SNV	AGBL3	AGBL3:exonic:NM_178563.3	1	100	5	missense	NM_17856	c.364G>C	p.Glu122Gln	3.58	0.17	29	0.232		Zinc carboxy	rs4236655	3.00E-04	YES	9.905E-1 (pos.134701856)		ATP/GTP-bir	cytosol,metallocarboxypeptidase activity,protein side c	G=0, C=144	G=0.0, C=1.0	0.00001	2433.71	144	G=0/0, C=64/8
chr2:27293150	T/T	G	T	SNV	AGBL5,OST4	AGBL5:utr_3:NM_021831.5, OST4:downstream:NM_001134	1	100	15	utr_3	NM_02183	c.2680G>T	p.?, p.?	1.23					Zinc carboxy	rs1053609	0.3798	YES	7.626E-1 (pos.27293150)		AATP/GTP-b	cytoplasm,cytosol,defense response to virus,integral co	G=0, T=54	G=0.0, T=1.0	0.00001	798.148	54	G=0/0, T=16/3
chr2:228416712	G/G	A	G	SNV	AGFG1	AGFG1:exonic:NM_001135187.1	1	100	12	synonymous	NM_00113	c.1488A>G	p.(=)	-0.21					Putative GTP	rs13426457	0.2783	YES	7.023E-1 (pos.228416712)		ADP-ribosyl	DNA binding,GTPase activator activity,RNA binding,acros	A=0, G=48	A=0.0, G=1.0	0.00001	810.422	48	A=0/0, G=36/1
chr5:76359024	C/A	C	A	SNV	AGGF1	AGGF1:exonic:NM_018046.4	1	50	14	missense	NM_01804	c.2092C>A	p.Pro698Thr	-0.21	1	38	0.22		FHA domain,	rs34400049	0.2906	YES	2.65E-1 (pos.76359024)		Angiogenic	angiogene Angiomatosis;Cardiovascular Diseases;Klipp	C=21, A=21	C=0.5, A=0.5	0.00001	228.879	42	C=13/8, A=4/1
chr1:100336361	T/T	C	T	SNV	AGL	AGL:exonic:NM_000642.2	1	100	7	synonymous	NM_00064	c.894C>T	p.(=)	1.36					Amylo-alpha-	rs2230306	0.3043	YES	6.811E-1 (pos.100336361)		Amylo-1,6-g	4-alpha-gl Autoimmune Diseases;Carboh Benign	C=0, T=97	C=0.0, T=1.0	0.00001	1637.63	97	C=0/0, T=32/6
chr1:19202770	C/C	T	C	SNV	ALDH4A1	ALDH4A1:intronic:NM_003748.3	1	100		intronic	NM_00374	c.1338+39	p.?	-0.2					Aldehyde deh	rs7550822	0.0205	YES	1.0 (pos.19202770)		Aldehyde de	1-pyrroline Amino Acid Metabo NADH	T=0, C=39	T=0.0, C=1.0	0.00001	623.924	39	T=0/0, C=31/8
chr1:15909850	G/G	C	G	SNV	AGMAT	AGMAT:exonic:NM_024758.4	1	100	2	missense	NM_02475	c.313G>C	p.Gly105Arg	0.6	1	125	0	0.15(COSM37	Arginase fam	rs6429757	0.4471	YES	6.322E-1 (pos.15909850)	adenocarcinoma		agmatinase activity,agmatine biosynthetic process,extr	C=0, G=27	C=0.0, G=1.0	0.00001	389.983	27	C=0/0, G=9/18
chr1:36316571	C/C	A	C	SNV	AGO4	AGO4:exonic:NM_017629.3	1	100	17	synonymous	NM_01762	c.2394A>C	p.(=)	-2					Argonaute lir	rs4652895	0.1407	YES	1.941E-1;0.0 (pos.36316571)		Eukaryotic t	P-body,RISC complex,RISC-loading complex,RNA seconda	A=0, C=22	A=0.0, C=1.0	0.00001	298.892	22	A=0/0, C=16/6
chr9:139566823	G/G	A	G	SNV	AGPAT2,EGFL7	EGFL7:utr_3:NM_201446.2, AGPAT2:downstream:NM_0064	1	100	9	utr_3	NM_20144	c.907A>G,	p.?, p.?	-1.63					Calcium-bind	rs1051828	0.099	YES			1-acylglycer	1-acylglyc Congenital, Hereditary, and Neonatal Diseas	A=0, G=23	A=0.0, G=1.0	0.00001	316.632	23	A=0/0, G=10/1
chr6:161557508	A/A	AAGG	A	INDEL	AGPAT4	AGPAT4:utr_3:NM_020133.2	3	100	9	utr_3	NM_02013	c.1198_12	p.?	0.66, 0.48, 0.76, 0.69					Acyltransfera	rs1005814125,rs145549477		YES			1-acylglycer	1-acylglycerol-3-phosphate O-acyltransferase activity,CI	AAGG=0, A=	AAGG=0.0, A=1.0	0.00001	334.496	24	AAGG=0/0, A=
chr1:19243589	G/G	A	G	SNV	IFFO2	IFFO2:intronic:NM_001136265.1	1	100		intronic	NM_00113	c.964-53T	p.?	3.6					Intermediate	rs3748586		YES				intermedi Digestive System Diseases;Eosinophilia;Eosi	A=0, G=31	A=0.0, G=1.0	0.00001	465.79	31	A=0/0, G=5/26
chr7:16834597	A/G	A	G	SNV	AGR2	AGR2:exonic:NM_006408.3	1	45.45	7	synonymous	NM_00640	c.441T>C	p.(=)	0.24				0.32(COSM37	Thioredoxin-l	rs6842	0.4969	YES	2.938E-1 (pos.16834597)	adenocarcino	Anterior gra	cell differe Breast Diseases;Breast Neoplasms;Genital D	A=36, G=30	A=0.5455, G=0.4545	0.00001	285.783	66	A=17/19, G=1
chr1:230841687	C/C	T	C	SNV	AGT	AGT:exonic:NM_000029.3	1	100	3	synonymous	NM_00002	c.1116A>G	p.(=)	-0.75					Serpin (serine	rs7080	0.0813	YES	1.0 (pos.230841687)		Angiotensin	ERK1 and E Acid-Base Imbalanc Sparsenta Benign	T=0, C=21	T=0.0, C=1.0	0.00001	281.234	21	T=0/0, C=13/8
chr5:35033605	A/A	G	A	SNV	AGXT2	AGXT2:exonic:NM_031900.3	1	100	6	missense	NM_03190	c.635C>T	p.Thr212Ile	-0.97	0.14	89	0	0.05(COSM36	Aminotransfe	rs180749	0.0013	YES	7.873E-1 (pos.35033605)	acute_myeloid	Alanine-gly	(R)-3-amin Congenital, Heredit Glycine,L-Alanine,Pyrido	G=0, A=69	G=0.0, A=1.0	0.00001	1072.78	69	G=0/0, A=37/3
chr5:35039486	T/T	C	T	SNV	AGXT2	AGXT2:exonic:NM_031900.3	1	100	3	missense	NM_03190	c.305G>A	p.Ser102Asn	0.48	0.56	46	0		Aminotransfe	rs37370	0.0938	YES	5.279E-1 (pos.35039486)		Alanine-gly	(R)-3-amin Congenital, Heredit Glycine,L-Alanine,Pyrido	C=0, T=38	C=0.0, T=1.0	0.00001	603.765	38	C=0/0, T=21/1
chr1:19487656	C/C	T	C	SNV	UBR4	UBR4:intronic:NM_020765.2	1	100		intronic	NM_02076	c.5176-15	p.?	-3.02					E3 ubiquitin-	rs3748759	0.43	YES	8.054E-1 (pos.19487656)		Ubiquitin p	calmodulin binding,centrosom HspE7,PCL-016	T=0, C=24	T=0.0, C=1.0	0.00001	334.792	24	T=0/0, C=5/19
chr1:19499835	C/C	G	C	SNV	UBR4	UBR4:intronic:NM_020765.2	1	100		intronic	NM_02076	c.3166-35	p.?	-0.32					E3 ubiquitin-	rs951908	0.3993	YES	8.111E-1 (pos.19499835)		Ubiquitin p	calmodulin binding,centrosom HspE7,PCL-016	G=0, C=45	G=0.0, C=1.0	0.00001	747.359	45	G=0/0, C=23/2
chr1:19510394	G/G	A	G	SNV	UBR4	UBR4:intronic:NM_020765.2	1	100		intronic	NM_02076	c.2099-4T	p.?	0.26					E3 ubiquitin-	rs3762396	0.3979	YES	8.129E-1 (pos.19510394)		Ubiquitin p	calmodulin binding,centrosom HspE7,PCL-016	A=0, G=45	A=0.0, G=1.0	0.00001	747.391	45	A=0/0, G=24/2
chr1:19549864	C/C	T	C	SNV	EMC1,LOC101927895	LOC101927895:intronic_nc:NR_135114.1, EMC1:intronic:NI	1	100		intronic	NR_13511	c.2376+26	p.?	-1.89					PQQ-like dom	rs2073105	0.2443	YES	6.644E-1 (pos.19549864)		Endoplasmi	ER membrane protein complex,integral component of m	T=0, C=85	T=0.0, C=1.0	0.00001	1379.1	85	T=0/0, C=63/2
chr1:27875824	T/T	C	T	SNV	AHDC1	AHDC1:exonic:NM_001029882.3	1	100	6	missense	NM_00102	c.2803G>A	p.Ala935Thr	-0.69		58			Domain of un	rs4908364	0.0066		1.0 (pos.27875824)		AT-hook DN	DNA binding	C=0, T=27	C=0.0, T=1.0	0.00001	389.983	27	C=0/0, T=14/1
chr1:196642072	C/T	C	T	SNV	CFH	CFH:intronic:NM_000186.3	1	50		intronic	NM_00018	c.59-36C>	p.?	-0.2					Sushi repeat	rs551397	0.2187	YES	4.369E-1 (pos.196642072)		Complemer	blood mici Anemia;Anemia, Hemolytic;Atypical Hemolyt	C=16, T=16	C=0.5, T=0.5	0.00001	158.383	32	C=8/8, T=5/11
chr11:62292882	T/T	G	T	SNV	AHNAK	AHNAK:exonic:NM_001620.2	1	100	5	missense	NM_00162	c.9007C>A	p.Gln3003Lys	0.2	0.86	53	0	0.04(COSM4591032),0.04(C		rs566144		YES	1.0 (pos.62292882)	squamous_cel	AHNAK nucl	RNA bindir Acneiform Eruptions;Adenocarcinoma;Carcin	G=0, T=34	G=0.0, T=1.0	0.00001	516.982	34	G=0/0, T=17/1
chr1:196705926	A/A	C	A	SNV	CFH	CFH:intronic:NM_000186.3	1	100		intronic	NM_00018	c.2414-28	p.?	-0.37					Sushi repeat	rs375046		YES	9.545E-1 (pos.196705926)		Complemer	blood mici Anemia;Anemia, Hemolytic;Atypical Hemolyt	C=0, A=25	C=0.0, A=1.0	0.00001	353.018	25	C=0/0, A=18/7
chr14:105410183	T/C	T	C	SNV	AHNAK2	AHNAK2:exonic:NM_138420.3	1	57.14	7	missense	NM_13842	c.11605A>	p.Met3869Val	-3.23	0.83	21	0	0(COSM4147874)		rs10438246	0.4551	YES	4.359E-1 (pos.10541018	neoplasm		T-tubule,Z disc,costamere,cytoplasm,cytoplasmic vesicl	T=24, C=32	T=0.4286, C=0.5714	0.00001	331.797	56	T=9/15, C=15/
chr14:105408955	A/G	A	G	SNV	AHNAK2	AHNAK2:exonic:NM_138420.3	1	50	7	missense	NM_13842	c.12833T>	p.Val4278Ala	-1.02	1	64	0	0(COSM3765732)		rs2819422	0.4574	YES	4.357E-1 (pos.10540895	hepatocellular_carcinoma		T-tubule,Z disc,costamere,cytoplasm,cytoplasmic vesicl	A=17, G=17	A=0.5, G=0.5	0.00001	172.008	34	A=14/3, G=13/
chr14:105416220	T/A	T	A	SNV	AHNAK2	AHNAK2:exonic:NM_138420.3	1	50	7	missense	NM_13842	c.5568A>T	p.Glu1856Asp	-14.02	1	45	0	0(COSM3765739),0(COSM4		rs2819435	0.1758	YES	6.048E-1;2.061E-2;2.329	ductal_carcinoma,hepatoc		T-tubule,Z disc,costamere,cytoplasm,cytoplasmic vesicl	T=11, A=11	T=0.5, A=0.5	0.00001	94.6067	22	T=1/10, A=9/2
chr5:311478	C/C	T	C	SNV	AHRR,PDCD6	PDCD6:exonic:NM_013232.3, AHRR:intronic:NM_00124241	1	100	5	synonymous	NM_01323	c.438T>C,	p.(=), p.?	0.07				0.14(COSM14	EF hand,EF-ha	rs2244029	0.2923	YES	2.552E-1 (pos.311478)	adenocarcino	Arylhydroca	COPII vesicle coat,COPII vesicle Calcium Citrate,Calcium	T=0, C=62	T=0.0, C=1.0	0.00001	940.317	62	T=0/0, C=23/3
chr3:186337713	C/C	T	C	SNV	AHSG	AHSG:exonic:NM_001622.2	1	100	6	missense	NM_00162	c.743T>C	p.Met248Thr	-0.51	1	81	0	0.02(COSM40	Cystatin dom	rs4917	0.348	YES	7.248E-1 (pos.18633771	acute_myeloid	Alpha-2HS-	Golgi appa Calcinosis;Calcium Metabolism risk factor	T=0, C=181	T=0.0, C=1.0	0.00001	3008.45	181	T=0/0, C=88/9
chr1:196920299	T/A	T	A	SNV	CFHR2	CFHR2:intronic:NM_005666.3	1	50		intronic	NM_00566	c.430+141	p.?	-1.28					Sushi repeat	rs3790414		YES			Complemer	extracellular region,negative regulation of protein bindi	T=12, A=12	T=0.5, A=0.5	0.00001	106.691	24	T=2/10, A=5/7
chr1:197026672	G/G	A	G	SNV	F13B	F13B:intronic:NM_001994.2	1	100		intronic	NM_00199	c.806-77T	p.?	-0.1					Sushi repeat	rs1759009		YES			Coagulation	blood coa Blood Coagulation Disorders;Blood Coagulati	A=0, G=85	A=0.0, G=1.0	0.00001	1360.85	85	A=0/0, G=44/4
chr1:197030317	A/G	A	G	SNV	F13B	F13B:intronic:NM_001994.2	1	47.37		intronic	NM_00199	c.452-112	p.?	-0.27					Sushi repeat	rs1615413		YES			Coagulation	blood coa Blood Coagulation Disorders;Blood Coagulati	A=20, G=18	A=0.5263, G=0.4737	0.00001	184.539	38	A=9/11, G=13/
chr12:8765447	C/C	T	C	SNV	AICDA	AICDA:utr_5:NM_020661.3	1	100	1	utr_5	NM_02066	c.-84A>G	p.?	-2.06						rs1345004	3.00E-04				Activation-i	B cell diffe Autoimmune Diseases;Blast C Benign	T=0, C=20	T=0.0, C=1.0	0.00001	263.786	20	T=0/0, C=4/16
chr1:197059878	A/A	G	A	SNV	ASPM	ASPM:intronic:NM_018136.4	1	100		intronic	NM_01813	c.9636+10	p.?	-1.52					Abnormal spi	rs4915315		YES			Abnormal s	calmoduli Adenocarcinoma;Adnexal Diseases;Autoimm	G=0, A=35	G=0.0, A=1.0	0.00001	543.901	35	G=0/0, A=26/9
chr1:197059892	G/G	A	G	SNV	ASPM	ASPM:intronic:NM_018136.4	1	100		intronic	NM_01813	c.9636+88	p.?	-0.13					Abnormal spi	rs10754214		YES			Abnormal s	calmoduli Adenocarcinoma;Adnexal Diseases;Autoimm	A=0, G=35	A=0.0, G=1.0	0.00001	522.956	35	A=0/0, G=26/9
chr1:197059928	G/G	A	G	SNV	ASPM	ASPM:intronic:NM_018136.4	1	100		intronic	NM_01813	c.9636+52	p.?	0.02					Abnormal spi	rs4915316	0.0934	YES			Abnormal s	calmoduli Adenocarcinoma;Adnexal Diseases;Autoimm	A=0, G=36	A=0.0, G=1.0	0.00001	563.744	36	A=0/0, G=26/1
chr22:21331043	T/T	A	T	SNV	AIFM3	AIFM3:exonic:NM_144704.2	1	100	12	synonymous	NM_14470	c.1134A>T	p.(=)	-0.1				0.1(COSM4135184),0.1(COS		rs178269		YES	1.0 (pos.21331043)	neoplasm,squ	Apoptosis-i	2 iron, 2 sulfur cluster binding,cytosol,endoplasmic retic	A=0, T=32	A=0.0, T=1.0	0.00001	485.132	32	A=0/0, T=22/1
chr1:197109042	T/T	C	T	SNV	ASPM	ASPM:intronic:NM_018136.4	1	100		intronic	NM_01813	c.1922-41	p.?	-0.73					Abnormal spi	rs1332663	0.0921	YES	9.996E-1 (pos.197109042)		Abnormal s	calmoduli Adenocarcinoma;Adnexal Dise Likely benign	C=0, T=72	C=0.0, T=1.0	0.00001	1130.95	72	C=0/0, T=33/3
chr7:6063283	C/T	C	T	SNV	AIMP2,EIF2AK1	AIMP2:exonic:NM_006303.3, EIF2AK1:utr_3:NM_014413.3	1	50	4	synonymous	NM_00630	c.924C>T,	p.(=), p.?	2.29				0.94(COSM41	AIMP2 lysyl-t	rs4560	0.4301	YES	3.815E-1 (pos.6063283)	neoplasm,oste	Aminoacyl t	ATP binding,Type II pneumocyte differentiation,acute inf	C=27, T=27	C=0.5, T=0.5	0.00001	262.867	54	C=13/14, T=22
chr1:197141243	C/C	A	C	SNV	ZBTB41	ZBTB41:intronic:NM_194314.2	1	100		intronic	NM_19431	c.2074+47	p.?	0.71					BTB/POZ dom	rs6428389,rs764655	0.1	YES	9.794E-1;0.0;0.0 (pos.197141243)			DNA binding,metal ion binding,nucleus,regulation of tra	A=0, C=23	A=0.0, C=1.0	0.00001	316.746	23	A=0/0, C=14/9
chr17:6331803	C/C	T	C	SNV	AIPL1	AIPL1:exonic:NM_014336.4	1	100	3	synonymous	NM_01433	c.300A>G	p.(=)	-0.34				0.07(COSM37	FKBP-type pe	rs8075035	0.3408	YES	5.4E-1 (pos.6331803)	adenocarcino	Arylhydroca	cytoplasm Congenital, Hereditary, and Ne Benign/Likely	T=0, C=34	T=0.0, C=1.0	0.00001	524.182	34	T=0/0, C=21/1
chr1:197482167	G/G	C	G	SNV	DENND1B	DENND1B:intronic:NM_001195215.1	1	100		intronic	NM_00119	c.1516-84	p.?	-1.37					DENN (AEX-3)	rs2013617		YES			DENN/MADI	Rab guany Bile Duct Diseases;Biliary Tract Diseases;Chc	C=0, G=28	C=0.0, G=1.0	0.00001	408.362	28	C=0/0, G=9/19
chr1:197576307	AT/AT	A	AT	INDEL	DENND1B	DENND1B:intronic:NM_001195215.1	1	100		intronic	NM_00119	c.820-3T>A	p.?	2.48					DENN (AEX-3)	rs11411505	8.00E-04		9.994E-1 (pos.197576307)		DENN/MADI	Rab guany Bile Duct Diseases;Biliary Tract Diseases;Chc	A=0, AT=25	A=0.0, AT=1.0	0.00001	352.017	25	A=0/0, AT=10/
chr1:197742171	T/T	C	T	SNV	DENND1B	DENND1B:intronic:NM_001195215.1	1	100		intronic	NM_00119	c.18-109G	p.?	-1.36					DENN (AEX-3)	rs2488396		YES	8.753E-1 (pos.197742171)		DENN/MADI	Rab guany Bile Duct Diseases;Biliary Tract Diseases;Chc	C=0, T=80	C=0.0, T=1.0	0.00001	1289.37	80	C=0/0, T=41/3
chr9:130630233	T/T	G	T	SNV	AK1	AK1:utr_3:NM_000476.2	1	100	7	utr_3	NM_00047	c.639C>A	p.?	-0.01					AAA domain,	rs4226	0.2024	YES			Adenylate k	ADP biosy Anemia;Anemia, He Bis(Adenosine)-5'-Penta	G=0, T=27	G=0.0, T=1.0	0.00001	389.988	27	G=0/0, T=11/1
chr1:197890422	G/G	C	G	SNV	LHX9	LHX9:intronic:NM_020204.2	1	100		intronic	NM_02020	c.378-12C	p.?	1.15					Homeobox d	rs12122995,rs76863	0.0287	YES	0.0 (pos.197890421),9.995E-1 (pos.1978		LIM homeo	DNA binding,cell proliferation,female gonad developme	C=0, G=96	C=0.0, G=1.0	0.00001	1617.82	96	C=0/0, G=77/1
chr1:200182654	T/C	T	C	SNV			1	50		intronic				-1.85						rs386638408,rs5313	0.0885	YES	0.0 (pos.200182644),9.618E-1;0.0;0.0 (pos.200182654)				T=14, C=14	T=0.5, C=0.5	0.00001	131.94	28	T=13/1, C=12/
chr1:200182664	C/T	C	T	SNV			1	50		intronic				-1.41						rs386638408,rs5313	0.3997	YES	0.0 (pos.200182644),9.124E-1 (pos.200182664)				C=14, T=14	C=0.5, T=0.5	0.00001	131.941	28	C=13/1, T=12/
chr1:200182834	A/G	A	G	SNV			1	46.88		intronic				0.12						rs12028402	0.3613	YES	8.829E-1 (pos.200182834)				A=17, G=15	A=0.5313, G=0.4688	0.00001	143.729	32	A=10/7, G=9/6
chr1:2005740	C/C	T	C	SNV	PRKCZ	PRKCZ:intronic:NM_002744.5	1	100		intronic	NM_00274	c.334+147	p.?	-1.04					PB1 domain,	rs2459986		YES	1.0 (pos.2005740)		Protein kina	14-3-3 pro Genital Diseases, M Tamoxifen	T=0, C=33	T=0.0, C=1.0	0.00001	504.596	33	T=0/0, C=20/1
chr1:200574959	C/C	T	C	SNV	KIF14	KIF14:intronic:NM_014875.2	1	100		intronic	NM_01487	c.1638+33	p.?	1.77					FHA domain,	rs2808238	0.3367	YES	1.591E-1 (pos.200574959)		Kinesin fam	ATP bindin Autoimmune Diseases;Female Urogenital Di	T=0, C=66	T=0.0, C=1.0	0.00001	1015.4	66	T=0/0, C=33/3
chr1:200578093	A/A	G	A	SNV	KIF14	KIF14:intronic:NM_014875.2	1	100		intronic	NM_01487	c.1456-37	p.?	-0.35					FHA domain,	rs2794409	0.0379	YES	8.412E-1 (pos.200578093)		Kinesin fam	ATP bindin Autoimmune Diseases;Female Urogenital Di	G=0, A=27	G=0.0, A=1.0	0.00001	389.977	27	G=0/0, A=11/1
chr9:130630639	G/G	A	G	SNV	AK1	AK1:exonic:NM_000476.2	1	100	6	synonymous	NM_00047	c.477T>C	p.(=)	-4.95					AAA domain,	rs763200874,rs9139	1.00E-04		0.0 (pos.130630638),1.0 (pos.13063063		Adenylate k	ADP biosy Anemia;Anemia, He Bis(Adeno Benign	A=0, G=23	A=0.0, G=1.0	0.00001	316.728	23	A=0/0, G=11/1
chr1:200943794	A/A	AGGAA	A	INDEL	KIF21B	KIF21B:intronic:NM_001252100.1	4	100		intronic	NM_00125	c.4814+48	p.?	0.0, 0.0, 0.0, 0.0, -0.75					Kinesin moto	rs201185144,rs201905676,rs53		YES	(pos.200943795),1.0;0.0 (pos.20094379		Kinesin fam	ATP bindin Ankylosis;Arthritis;Bone Diseases;Colitis;Col	AGGAA=0,	AGGAA=0.0, A=1.0	0.00001	693.868	42	AGGAA=0/0, A
chr1:201044748	G/G	A	G	SNV	CACNA1S	CACNA1S:intronic:NM_000069.2	1	100		intronic	NM_00006	c.1828-5T	p.?	-1.19					Ion transport	rs1998721	0.223	YES	9.57E-1 (pos.201044748)		Calcium cha	I band,L-ty ACMG Recommend Amlodipin Benign	A=0, G=107	A=0.0, G=1.0	0.00001	1681.19	107	A=0/0, G=41/6
chr1:77763542	A/A	G	A	SNV	AK5	AK5:exonic:NM_174858.2	1	100	5	synonymous	NM_17485	c.609G>A	p.(=)	1.52					AAA domain,	rs1167206	1.00E-04		1.0 (pos.77763542)		Adenylate k	ADP biosy Amphetamine-Related Disorde Benign	G=0, A=54	G=0.0, A=1.0	0.00001	787.244	54	G=0/0, A=18/3
chr1:201113118	G/G	A	G	SNV	TMEM9	TMEM9:intronic:NM_016456.4	1	100		intronic	NM_01645	c.268-39T	p.?	-0.7					TMEM9	rs3738266,rs752766	0.0469	YES	0.0 (pos.201113117),8.59E-1 (pos.20111		Transmemt	biological_process,integral component of membrane,la	A=0, G=21	A=0.0, G=1.0	0.00001	281.235	21	A=0/0, G=9/12
chr1:201116018	G/G	C	G	SNV	TMEM9	TMEM9:intronic:NM_016456.4	1	100		intronic	NM_01645	c.159-42G	p.?	-0.89					TMEM9	rs3752979	0.0468	YES	8.612E-1 (pos.201116018)		Transmemt	biological_process,integral component of membrane,la	C=0, G=37	C=0.0, G=1.0	0.00001	583.477	37	C=0/0, G=13/2
chr1:201122947	G/G	T	G	SNV	TMEM9	TMEM9:intronic:NM_016456.4	1	100		intronic	NM_01645	c.66+39A>	p.?	-0.23				0(COSM4143	TMEM9	rs1925361	0.048	YES	8.703E-1 (pos.20112294	neoplasm	Transmemt	biological_process,integral component of membrane,la	T=0, G=40	T=0.0, G=1.0	0.00001	644.165	40	T=0/0, G=26/1
chr1:201170824	G/G	C	G	SNV	IGFN1	IGFN1:intronic:NM_001164586.1	1	100		intronic	NM_00116	c.634-23C	p.?	-0.82					Fibronectin t	rs10158976	0.0676	YES	9.984E-1 (pos.201170824)		Immunoglo	Z disc,biological_process,nucleus,protein binding	C=0, G=32	C=0.0, G=1.0	0.00001	485.141	32	C=0/0, G=9/23
chr1:201171007	T/G	T	G	SNV	IGFN1	IGFN1:intronic:NM_001164586.1	1	52.38		intronic	NM_00116	c.747+47T	p.?	-0.24					Fibronectin t	rs10800762	0.2577	YES	9.446E-1;0.0 (pos.201171007)		Immunoglo	Z disc,biological_process,nucleus,protein binding	T=20, G=22	T=0.4762, G=0.5238	0.00001	245.162	42	T=3/17, G=6/1
chr14:96922752	G/G	C	G	SNV	AK7	AK7:exonic:NM_152327.4	1	100	11	missense	NM_15232	c.1167C>G	p.Asn389Lys	-0.06	1	94	0	0.01(COSM56	Adenylate kir	rs2369679	0.1684	YES	9.618E-1 (pos.96922752)	ductal_carcinc	Adenylate k	ATP binding,adenylate kinase activity,cell Benign	C=0, G=95	C=0.0, G=1.0	0.00001	1597	95	C=0/0, G=57/3
chr17:55182811	CT/C	CT	C	INDEL	AKAP1	AKAP1:utr_5:NM_003488.3	1	46.51	2	utr_5	NM_00348	c.-15CT>C	p.?	1.75, 0.13					KH domain,Tu	rs3833091,rs397747	0.4135	YES	8.136E-1 (pos.55182811)		A-kinase an	RNA bindir Nutrition Disorders;Nutritional and Metaboli	CT=23, C=2	CT=0.5349, C=0.465	0.00001	192.257	43	CT=14/9, C=12
chr1:201187828	C/C	T	C	SNV	IGFN1	IGFN1:intronic:NM_001164586.1	1	100		intronic	NM_00116	c.9898+42	p.?	-2.1					Fibronectin t	rs3738268	0.1245	YES	9.979E-1 (pos.201187828)		Immunoglo	Z disc,biological_process,nucleus,protein binding	T=0, C=47	T=0.0, C=1.0	0.00001	786.243	47	T=0/0, C=24/2
chr1:201286678	C/T	C	T	SNV	PKP1	PKP1:intronic:NM_000299.3	1	57.89		intronic	NM_00029	c.847-22C	p.?	-0.66					Armadillo/be	rs1794867	0.2114	YES	2.301E-1 (pos.201286678)		Plakophilin	cell adhes Abnormalities, Multiple;Adenocarcinoma;An	C=16, T=22	C=0.4211, T=0.5789	0.00001	249.057	38	C=8/8, T=9/13
chr1:201288898	C/C	A	C	SNV	PKP1	PKP1:intronic:NM_000299.3	1	100		intronic	NM_00029	c.1233-46	p.?	-0.01					Armadillo/be	rs1722759	0.001		1.0 (pos.201288898)		Plakophilin	cell adhes Abnormalities, Multiple;Adenocarcinoma;An	A=0, C=38	A=0.0, C=1.0	0.00001	603.775	38	A=0/0, C=19/1
chr6:151670897	C/C	T	C	SNV	AKAP12	AKAP12:exonic:NM_005100.3	1	100	4	synonymous	NM_00510	c.1371T>C	p.(=)	-3.11				0.03(COSM44	RII binding d	rs900654	0.215	YES	6.135E-1 (pos.15167089	acute_myeloid_leukaemia		G-protein Breast Diseases;Breast Neoplasms;Neoplasr	T=0, C=33	T=0.0, C=1.0	0.00001	504.596	33	T=0/0, C=20/1
chr1:201966273	A/A	G	A	SNV	RNPEP	RNPEP:intronic:NM_020216.3	1	100		intronic	NM_02021	c.855-174	p.?	-5.27					Leukotriene	rs4950757		YES			Arginyl ami	aminopep Mammary Neoplasms, Animal;Mammary Nec	G=0, A=129	G=0.0, A=1.0	0.00001	2115.17	129	G=0/0, A=107/
chr6:151674326	A/C	A	C	SNV	AKAP12	AKAP12:exonic:NM_005100.3	1	58.33	4	missense	NM_00510	c.4800A>C	p.Glu1600Asp	-0.73	0.37	45	0.003	0.15(COSM41	RII binding d	rs3823310	0.3309	YES	4.532E-1 (pos.15167432	basal_cell_carcinoma,hep		G-protein Breast Diseases;Breast Neoplasms;Neoplasr	A=30, C=42	A=0.4167, C=0.5833	0.00001	460.914	72	A=17/13, C=24
chr15:86122779	C/C	T	C	SNV	AKAP13	AKAP13:exonic:NM_006738.5	1	100	7	missense	NM_00673	c.1480T>C	p.Trp494Arg	-0.09	0.12	101	0	0.01(COSM39	PH domain,R	rs2061822	0.3251	YES	5.788E-1;1.158E-4 (pos.8	acute_myeloid	A-kinase an	G-protein Adenocarcinoma;Carcinoma;Carcinoma, Ren	T=0, C=34	T=0.0, C=1.0	0.00001	524.185	34	T=0/0, C=18/1
chr1:201981354	CC/CC	TG	CC	MNV	ELF3	ELF3:intronic:NM_004433.4	2	100		intronic	NM_00443	c.385+48T	p.?	-2.94, -0.22					AT hook moti	rs386638521,rs5566	0.1935	YES	0.0 (pos.201981353),3.486E-1;0.0 (pos.2		E74-like fac	DNA bindi Biliary Tract Diseases;Biliary Tract Neoplasm	TG=0, CC=3	TG=0.0, CC=1.0	0.00001	563.206	36	TG=0/0, CC=12
chr15:86122916	C/C	T	C	SNV	AKAP13	AKAP13:exonic:NM_006738.5	1	100	7	synonymous	NM_00673	c.1617T>C	p.(=)	0.04				0.01(COSM63	PH domain,R	rs2061823	0.3561	YES	5.786E-1 (pos.86122916)	carcinoma	A-kinase an	G-protein Adenocarcinoma;Carcinoma;Carcinoma, Ren	T=0, C=26	T=0.0, C=1.0	0.00001	371.42	26	T=0/0, C=7/19
chr15:86123833	C/C	T	C	SNV	AKAP13	AKAP13:exonic:NM_006738.5	1	100	7	missense	NM_00673	c.2534T>C	p.Val845Ala	-1.25	1	64	0	0.01(COSM63	PH domain,R	rs2061824	0.3503	YES	5.791E-1;0.0 (pos.86123833)	carcinoma	A-kinase an	G-protein Adenocarcinoma;Carcinoma;Carcinoma, Ren	T=0, C=76	T=0.0, C=1.0	0.00001	768.313	76	T=0/0, C=33/43
chr1:202163364	G/G	A	G	SNV	LGR6	LGR6:intronic:NM_001017403.1	1	100		intronic	NM_00101	c.212+35C	p.?	-0.12					Leucine Rich	rs2077598	0.1651	YES	6.814E-1 (pos.202163364)		Leucine-rich	G-protein Breast Diseases;Breast Neoplasms;Colonic C	G=0, A=66	G=0.0, A=1.0	0.00001	1015.4	66	G=0/0, A=33/3
chr1:202386075	C/C	T	C	SNV	PPP1R12B	PPP1R12B:intronic:NM_002481.3	1	100		intronic	NM_00248	c.422+30T	p.?	-1.51					Ankyrin repe	rs2843413	0.0056	YES	1.0 (pos.202386075)		Protein phos	A band,G2 Cardiovascular Diseases;Celiac Disease;Cor	T=0, C=20	T=0.0, C=1.0	0.00001	263.786	20	T=0/0, C=7/13
chr15:86123988	A/A	G	A	SNV	AKAP13	AKAP13:exonic:NM_006738.5	1	100	7	missense	NM_00673	c.2689G>A	p.Val897Met	0.66	0	21	0.033	0.15(COSM63	PH domain,R	rs4075254	0.3557	YES	5.777E-1 (pos.86123988)	carcinoma	A-kinase an	G-protein Adenocarcinoma;Carcinoma;Carcinoma, Ren	G=0, A=37	G=0.0, A=1.0	0.00001	583.706	37	G=0/0, A=21/1

chr15:86124419	T/T	C	T	SNV	AKAP13	AKAP13:exonic:NM 006738.5	1	100	7	synonymous	NM 00673	c.3120C>T	p.(=)	-2.29				0.05(COSM39	PH domain,R	rs4843073	0.3559	YES	5.784E-1 (pos.86124419)	acute_myeloic	A-kinase an	G-protein	Adenocarcinoma;Carcinoma;Carcinoma, Ren		C=0, T=29	C=0.0, T=1.0	0.00001	427.603	29	C=0/0, T=10/1
chr1:202743892	C/C	T	C	SNV	KDM5B	KDM5B:intronic:NM 006618.4	1	100		intronic	NM 00661	c.283-29A	p.?	0.73					ARID/BRIGHT	rs17497253	0.2843	YES	1.604E-1;0.0 (pos.202743892)		Lysine-spec	DNA binding,DNA binding transcription factor activity,br			T=0, C=76	T=0.0, C=1.0	0.00001	1209.6	76	T=0/0, C=49/2
chr1:203023122	A/C	A	C	SNV	PPFIA4	PPFIA4:intronic:NM 001304331.1	1	50		intronic	NM 00130	c.1829+33	p.?	-0.64					SAM domain	rs2291691	0.2248	YES	1.521E-1 (pos.203023122)		Protein-tyrc	cell surface,cytosol,glutamate secretion,intracellular,ne			A=16, C=16	A=0.5, C=0.5	0.00001	158.116	32	A=7/9, C=7/9
chr1:203465396	C/C	T	C	SNV	OPTC	OPTC:intronic:NM 014359.3	1	100		intronic	NM 01435	c.231+32T	p.?	-0.22					Leucine rich r	rs2242199	0.3847	YES	4.888E-1 (pos.203465396)			extracellular matrix disassembly,extracellular matrix st			T=0, C=64	T=0.0, C=1.0	0.00001	971.815	64	T=0/0, C=33/3
chr15:86124483	G/G	C	G	SNV	AKAP13	AKAP13:exonic:NM 006738.5	1	100	7	missense	NM 00673	c.3184C>G	p.Pro1062Ala	-0.51	0.01	27	0.356	0.06(COSM39	PH domain,R	rs4843074	0.3565	YES	5.787E-1 (pos.86124483)	acute_myeloic	A-kinase an	G-protein	Adenocarcinoma;Carcinoma;Carcinoma, Ren		C=0, G=35	C=0.0, G=1.0	0.00001	520.013	35	C=0/0, G=10/2
chr1:203740458	C/A	C	A	SNV	LAX1	LAX1:intronic:NM 017773.3	1	52.38		intronic	NM 01777	c.200-37C	p.?	0.91					Lymphocyte a	rs3820196	0.0664	YES	6.008E-1 (pos.203740458)			B cell activation,Golgi apparatus,SH2 domain binding,ad			C=10, A=11	C=0.4762, A=0.5238	0.00001	95.3098	21	C=9/1, A=9/2
chr15:86124946	C/C	T	C	SNV	AKAP13	AKAP13:exonic:NM 006738.5	1	100	7	missense	NM 00673	c.3647T>C	p.Met1216Thr	-0.69	0.45	81	0	0.02(COSM63	PH domain,R	rs7162168	0.3557	YES	5.775E-1 (pos.86124946)	carcinoma	A-kinase an	G-protein	Adenocarcinoma;Carcinoma;Carcinoma, Ren		T=0, C=23	T=0.0, C=1.0	0.00001	316.747	23	T=0/0, C=10/1
chr1:204094902	C/C	T	C	SNV	SOX13	SOX13:intronic:NM 005686.2	1	100		intronic	NM 00568	c.1593-84	p.?	-3.18					HMG (high mo	rs2692010		YES			SRY-box 13	DNA binding transcription factor activity,RNA polymeras			T=0, C=33	T=0.0, C=1.0	0.00001	497.046	33	T=0/0, C=15/1
chr15:86124968	A/A	G	A	SNV	AKAP13	AKAP13:exonic:NM 006738.5	1	100	7	synonymous	NM 00673	c.3669G>A	p.(=)	0.77				0.15(COSM41	PH domain,R	rs11633087	0.3579	YES	5.749E-1 (pos.86124968)	carcinoma,ne	A-kinase an	G-protein	Adenocarcinoma;Carcinoma;Carcinoma, Ren		G=0, A=23	G=0.0, A=1.0	0.00001	316.747	23	G=0/0, A=10/1
chr15:86125031	G/A	G	A	SNV	AKAP13	AKAP13:exonic:NM 006738.5	1	95.65	7	synonymous	NM 00673	c.3732G>A	p.(=)	0.02				0(COSM4128	PH domain,R	rs7178065	0.3558	YES	5.73E-1 (pos.86125031)	acute_myeloic	A-kinase an	G-protein	Adenocarcinoma;Carcinoma;Carcinoma, Ren		G=1, A=22	G=0.0435, A=0.9565	0.00001	289.386	23	G=1/0, A=8/14
chr15:86123170	G/T	G	T	SNV	AKAP13	AKAP13:exonic:NM 006738.5	1	52.38	7	missense	NM 00673	c.1871G>T	p.Gly624Val	0.12	0.19	109	0.001	0.11(COSM37	PH domain,R	rs745191	0.284	YES	2.492E-2 (pos.86123170)	adenocarcinoı	A-kinase an	G-protein	Adenocarcinoma;Carcinoma;Carcinoma, Ren		G=10, T=11	G=0.4762, T=0.5238	0.00001	95.3003	21	G=6/4, T=6/5
chrX:1719897	G/G	C	G	SNV	AKAP17A	AKAP17A:exonic:NM 005088.2	1	100	5	missense	NM 00508	c.1498C>G	p.Pro500Ala	-0.1	1	27	0.376			rs28661622	0.4477	YES	6.023E-1;0.0 (pos.1719897)		A kinase (PF	B cell activation,RNA binding,RNA splicing,cytosol,mRNA			C=0, G=39	C=0.0, G=1.0	0.00001	623.974	39	C=0/0, G=19/2
chr1:204434277	C/C	T	C	SNV	PIK3C2B	PIK3C2B:intronic:NM 002646.3	1	100		intronic	NM 00264	c.1034+70	p.?	0.92					C2 domain,PI	rs2942149		YES			Phosphatid	1-phospha	Neoplasm Metastasis;Neoplasms;Neoplastic		T=0, C=22	T=0.0, C=1.0	0.00001	298.886	22	T=0/0, C=16/6
chr1:204515859	CT/CT	C	CT	INDEL	MDM4	MDM4:intronic:NM 002393.4	1	100		intronic	NM 00239	c.823-66C	p.?	-0.65					SWIB/MDM2 domain,Zinc finger, C3HC4 type (RING finger),Zn-finger in Ran binding protein and others						Mouse doul	DNA dama	Astrocytoma;Breast Diseases;Breast Neopla		C=0, CT=37	C=0.0, CT=1.0	0.00001	451.438	37	C=0/0, CT=18/
chr1:204515863	T/T	C	T	SNV	MDM4	MDM4:intronic:NM 002393.4	1	100		intronic	NM 00239	c.823-62C	p.?	-1.37					SWIB/MDM2	rs2290855		YES			Mouse doul	DNA dama	Astrocytoma;Breast Diseases;Breast Neopla		C=0, T=41	C=0.0, T=1.0	0.00001	664.572	41	C=0/0, T=21/2
chr1:204516025	G/G	A	G	SNV	MDM4	MDM4:intronic:NM 002393.4	1	100		intronic	NM 00239	c.903+20A	p.?	0.32					SWIB/MDM2	rs2290854	0.3122	YES	6.842E-1 (pos.204516025)		Mouse doul	DNA dama	Astrocytoma;Breast Diseases;Breast Neopla		A=0, G=43	A=0.0, G=1.0	0.00001	705.844	43	A=0/0, G=20/2
chr9:112811015	TCCCCCGGAG	TCCCCCGG	T	INDEL	AKAP2,PALM2-AKAP2	PALM2-AKAP2:intronic:NM 007203.4, AKAP2:exonic:NM 00	18	46.58	1	nonframeshiftDeletion	NM 00720	c.582+326	p.?, p.Pro13_Gly18del	-0.51, -1.29, 0.91, -1.25, -0.24, -0.69, 0.04, 0.71,				0.01(COSM62	A-kinase ancl	rs754326938,rs759764997,rs764654508,rs			0.0 (pos.112811013),0.0	adenocarcinoı	A-kinase anchor protein 2			Benign	TCCCCCGG	TCCCCCGGAGTCTCCT	0.00001	346.039	73	TCCCCCGGAGT
chr12:4737318	G/G	A	G	SNV	AKAP3	AKAP3:exonic:NM 006422.3	1	100	5	synonymous	NM 00642	c.750T>C	p.(=)	0.17					A-kinase ancl	rs7960207	0.0337	YES	7.211E-1 (pos.4737318)			acrosomal vesicle,acrosome reaction,cytoplasm,moven			A=0, G=21	A=0.0, G=1.0	0.00001	281.234	21	A=0/0, G=6/15
chr12:4737715	T/T	C	T	SNV	AKAP3	AKAP3:exonic:NM 006422.3	1	100	5	missense	NM 00642	c.353G>A	p.Gly118Glu	-0.3	0.84	98	0		A-kinase ancl	rs2072355	0.1912	YES	6.507E-1 (pos.4737715)			acrosomal vesicle,acrosome reaction,cytoplasm,moven			C=0, T=40	C=0.0, T=1.0	0.00001	644.286	40	C=0/0, T=22/1
chr1:204943285	T/T	A	T	SNV	NFASC	NFASC:intronic:NM 001005388.2	1	100		intronic	NM 00100	c.1280-22	p.?	-0.21					Bravo-like int	rs2595945	0.0058	YES	9.944E-1 (pos.204943285)		Neurofascir	Schwann cell microvillus,axon guidance,axon initial segr			A=0, T=31	A=0.0, T=1.0	0.00001	460.666	31	A=0/0, T=15/1
chr14:64935720	T/T	C	T	SNV	AKAP5	AKAP5:exonic:NM 004857.3	1	100	2	missense	NM 00485	c.608C>T	p.Thr203Ile	-0.28		89			WSK motif	rs1256149	0		9.464E-1 (pos.64935720)		A-kinase an	SH3 doma	Cardiomegaly;Cardiovascular Diseases;Hear		C=0, T=56	C=0.0, T=1.0	0.00001	832.057	56	C=0/0, T=35/2
chr14:33015014	A/A	G	A	SNV	AKAP6	AKAP6:exonic:NM 004274.4	1	100	4	synonymous	NM 00427	c.1155G>A	p.(=)	-0.52						rs7150894	0.3424	YES	6.144E-1;0.0 (pos.33015014)			T-tubule,action potential,adenylate cyclase binding,cAN			G=0, A=72	G=0.0, A=1.0	0.00001	1130.94	72	G=0/0, A=29/4
chr1:204951209	C/T	C	T	SNV	NFASC	NFASC:intronic:NM 001005388.2	1	56		intronic	NM 00100	c.2470+61	p.?	-1.19					Bravo-like int	rs2278796		YES			Neurofascir	Schwann cell microvillus,axon guidance,axon initial segr			C=11, T=14	C=0.44, T=0.56	0.00001	134.349	25	C=3/8, T=10/4
chr1:204960445	A/G	A	G	SNV	NFASC	NFASC:intronic:NM 001005388.2	1	45.83		intronic	NM 00100	c.2782+11	p.?	2.07					Bravo-like int	rs72753447	0.0359	YES	1.336E-4 (pos.204960445)		Neurofascir	Schwann cell microvillus,axon guidance,axon initial segr			A=13, G=11	A=0.5417, G=0.4583	0.00001	93.4358	24	A=7/6, G=2/9
chr19:15508362	G/C	G	C	SNV	AKAP8L	AKAP8L:exonic:NM 014371.3	1	97.5	11	missense	NM 01437	c.1374C>G	p.His458Gln	0.34	1	24	0	0.42(COSM45	A-kinase ancl	rs2058322		YES	1.0 (pos.15508362)	squamous_cel	A-kinase an	DEAD/H-box RNA helicase binding,DNA binding,PML body			G=1, C=39	G=0.025, C=0.975	0.00001	611.656	40	G=1/0, C=23/1
chr1:205240913	G/G	A	G	SNV	TMCC2	TMCC2:intronic:NM 014858.3	1	100		intronic	NM 01485	c.1819-28	p.?	-0.66					Predicted tra	rs386638735,rs9659	0.0938	YES	1.0 (pos.205240913)			amyloid precursor protein metabolic process,endoplasr			A=0, G=22	A=0.0, G=1.0	0.00001	298.892	22	A=0/0, G=19/3
chr7:91714911	T/T	C	T	SNV	AKAP9	AKAP9:exonic:NM 005751.4	1	100	36	missense	NM 00575	c.8935C>T	p.Pro2979Ser	0.63	1	74	0		Pericentrin-A	rs1063242	0.0038		1.0 (pos.91714911)		A-kinase an	DNA bindi	Arrhythmias, Cardiac;Arsenic F	Benign/Likely	C=0, T=62	C=0.0, T=1.0	0.00001	943.86	62	C=0/0, T=27/3
chr1:205766159	C/C	CA	C	INDEL	SLC41A1	SLC41A1:intronic:NM 173854.5	1	100		intronic	NM 17385	c.993-28T	p.?	-0.3, 0.99					Divalent cati	rs374392479,rs3979	0.11	YES	7.978E-1 (pos.205766159)		Solute carri	basolater	Basal Ganglia Diseases;Brain Diseases;Centr		CA=0, C=38	CA=0.0, C=1.0	0.00001	543.413	38	CA=0/0, C=20/
chr7:91691601	C/T	C	T	SNV	AKAP9	AKAP9:exonic:NM 005751.4	1	52.38	24	synonymous	NM 00575	c.5778C>T	p.(=)	0.25				0.81(COSM44	Pericentrin-A	rs10236397	0.4031	YES	1.746E-1 (pos.91691601)	acute_myeloic	A-kinase an	DNA bindi	Arrhythmias, Cardiac;Arsenic F	Benign/Likely	C=10, T=11	C=0.4762, T=0.5238	0.00001	95.3002	21	C=6/4, T=2/9
chr1:109395105	T/T	C	T	SNV	AKNAD1	AKNAD1:exonic:NM 152763.4	1	100	2	missense	NM 15276	c.182G>A	p.Ser61Asn	-0.41	1	46	0		AT-hook-cont	rs1277207	0.1837	YES	9.383E-1 (pos.109395105)						C=0, T=97	C=0.0, T=1.0	0.00001	1606.13	97	C=0/0, T=70/2
chr1:205890679	C/C	T	C	SNV	SLC26A9	SLC26A9:intronic:NM 134325.2	1	100		intronic	NM 13432	c.2055+15	p.?	-1.07					STAS domain,	rs12759719,rs34323	0.1024	YES	9.235E-1 (pos.205890679)		Solute carri	ATPase bir	Digestive System Diseases;Ga	Benign	T=0, C=56	T=0.0, C=1.0	0.00001	828.196	56	T=0/0, C=32/2
chr1:205896475	T/T	C	T	SNV	SLC26A9	SLC26A9:intronic:NM 134325.2	1	100		intronic	NM 13432	c.1216-59	p.?	0.84					STAS domain,	rs7366204		YES			Solute carri	ATPase bir	Digestive System Diseases;Gastrointestinal		C=0, T=34	C=0.0, T=1.0	0.00001	524.186	34	C=0/0, T=13/2
chr7:134215403	T/C	T	C	SNV	AKR1B10	AKR1B10:exonic:NM 020299.4	1	50	2	synonymous	NM 02029	c.75T>C	p.(=)	-2.21				0.06(COSM37	Aldo/keto rec	rs1722883	0.4598	YES	4.435E-1 (pos.13421540	acute_myeloic	Aldo-keto re	aldo-keto	Adenocarcinoma;C	2'-Monophosphoadenos	T=17, C=17	T=0.5, C=0.5	0.00001	172.008	34	T=9/8, C=7/10
chr1:206567100	C/T	C	T	SNV	SRGAP2,SRGAP2D	SRGAP2D:intronic_nc:NR 120535.1, SRGAP2:intronic:NM 0	1	46.51		intronic	NM 01532	c.571+70C	p.?	-0.02					Fes/CIP4, anc	rs2480377		YES			SLIT-ROBO R	GTPase activator activity,Rac GTPase binding,actin filam			C=23, T=20	C=0.5349, T=0.4651	0.00001	212.257	43	C=14/9, T=10/
chr10:5139685	A/A	G	A	SNV	AKR1C3	AKR1C3:exonic:NM 001253908.1	1	100	3	synonymous	NM 00125	c.312G>A	p.(=)	-0.45					Aldo/keto rec	rs12387,rs78255158	0.1702	YES	0.0 (pos.5139684),8.697E-1;0.0 (pos.513		Aldo-keto re	15-hydrox	Adnexal Diseases;C	2'-Monophosphoadenos	G=0, A=194	G=0.0, A=1.0	0.00001	3274.72	194	G=0/0, A=120/
chr10:5255025	G/G	A	G	SNV	AKR1C4	AKR1C4:exonic:NM 001818.3	1	100	7	missense	NM 00181	c.749A>G	p.Gln250Arg	0.24	1	43	0	0(COSM4590	Aldo/keto rec	rs4880718			1.0 (pos.5255025)	malignant_me	Aldo-keto re	aldo-keto	46, XY Disorders of	NADH	A=0, G=44	A=0.0, G=1.0	0.00001	726.56	44	A=0/0, G=22/2
chr19:49967981	G/G	T	G	SNV	ALDH16A1	ALDH16A1:exonic:NM 153329.3	1	100	12	synonymous	NM 15332	c.1530T>G	p.(=)	-1.66					Aldehyde del	rs11558188	0.3505	YES	2.428E-3;0.0 (pos.49967981)		Aldehyde d	aldehyde	Arthritis;Congenital, Hereditary, and Neonat		T=0, G=39	T=0.0, G=1.0	0.00001	623.98	39	T=0/0, G=24/1
chr9:38396065	T/T	G	T	SNV	ALDH1B1	ALDH1B1:exonic:NM 000692.4	1	100	2	missense	NM 00069	c.320G>T	p.Arg107Leu	2.23		102		0.79(COSM14	Aldehyde del	rs2073478	0.3963	YES	3.123E-1;0.0 (pos.38396	adenocarcinoı	Aldehyde d	NAD bindi	Colonic Diseases;C	NADH Benign	G=0, T=40	G=0.0, T=1.0	0.00001	644.286	40	G=0/0, T=13/2
chr1:207135546	G/A	G	A	SNV	FCAMR	FCAMR:intronic:NM 001170631.1	1	45.83		intronic	NM 00117	c.652+12C	p.?	-5.07					Immunoglobu	rs1572808	0.2746	YES	5.496E-2 (pos.207135546)		Fc fragment of IgA and IgM, receptor for				G=26, A=22	G=0.5417, A=0.4583	0.00001	240.97	48	G=21/5, A=13/
chr1:207235954	A/A	G	A	SNV	PFKFB2	PFKFB2:intronic:NM 006212.2	1	100		intronic	NM 00621	c.212-11G	p.?	-0.72					6-phosphofru	rs2075863	0.4272	YES	1.677E-1 (pos.207235954)		Fructose-2,	6-phosphofructo-2-kinase activity,ATP binding,carbohyd			G=0, A=37	G=0.0, A=1.0	0.00001	583.326	37	G=0/0, A=19/1
chr1:207510596	A/A	G	A	SNV	CD55	CD55:intronic:NM 001114752.2	1	100		intronic	NM 00111	c.980-78G	p.?	-2.02					Sushi repeat	rs10746463		YES			CD55 antige	CD4-positi	Amphetamine-Rela	Chloramphenicol	G=0, A=24	G=0.0, A=1.0	0.00001	334.792	24	G=0/0, A=12/1
chr1:2075570	T/C	T	C	SNV	PRKCZ	PRKCZ:intronic:NM 002744.5	1	50		intronic	NM 00274	c.421-79T	p.?	-0.51					PB1 domain,I	rs377283		YES			Protein kina	14-3-3 pro	Genital Diseases, N	Tamoxifen	T=20, C=20	T=0.5, C=0.5	0.00001	214.332	40	T=4/16, C=16/
chr9:38396502	A/A	G	A	SNV	ALDH1B1	ALDH1B1:exonic:NM 000692.4	1	100	2	missense	NM 00069	c.757G>A	p.Val253Met	2.73		21			Aldehyde del	rs4878199	0.0462	YES	9.999E-1 (pos.38396502)		Aldehyde d	NAD bindi	Colonic Diseases;C	NADH Benign	G=0, A=58	G=0.0, A=1.0	0.00001	867.38	58	G=0/0, A=48/1
chr1:208211887	C/C	T	C	SNV	PLXNA2	PLXNA2:intronic:NM 025179.3	1	100		intronic	NM 02517	c.4661-68	p.?	0.86					IPT/TIG doma	rs905100		YES			Plexin A2 (C	branchion	Mental Disorders;Schizophrenia;Schizophren		T=0, C=33	T=0.0, C=1.0	0.00001	504.596	33	T=0/0, C=6/27
chr1:208213125	G/G	A	G	SNV	PLXNA2	PLXNA2:intronic:NM 025179.3	1	100		intronic	NM 02517	c.4354-13	p.?	0.07					IPT/TIG doma	rs1751580	2.00E-04	YES	1.0 (pos.208213125)		Plexin A2 (C	branchion	Mental Disorders;Schizophrenia;Schizophren		A=0, G=26	A=0.0, G=1.0	0.00001	371.42	26	A=0/0, G=15/1
chr1:208255877	C/C	T	C	SNV	PLXNA2	PLXNA2:intronic:NM 025179.3	1	100		intronic	NM 02517	c.2299-24	p.?	-0.41					IPT/TIG doma	rs6540453	0.3015	YES	6.871E-1 (pos.208255877)		Plexin A2 (C	branchion	Mental Disorders;Schizophrenia;Schizophren		T=0, C=38	T=0.0, C=1.0	0.00001	603.785	38	T=0/0, C=18/2
chr11:67789293	GC/GC	G	GC	INDEL	ALDH3B1	ALDH3B1:exonic:NM 001030010.2	1	100	6	unknown	NM 001030010.2			5.62					Aldehyde del	rs397695802,rs5286	1.00E-04	YES	9.899E-1;1.006E-2 (pos.67789293)		Aldehyde d	3-chloroallyl aldehyde dehydrc		NADH	G=0, GC=54	G=0.0, GC=1.0	0.00001	789.342	54	G=0/0, GC=27/
chr11:67432804	C/C	T	C	SNV	ALDH3B2	ALDH3B2:exonic:NM 001031615.1	1	100	7	missense	NM 00103	c.658A>G	p.Ser220Gly	0.94	1	56	0		Aldehyde del	rs2447571	0		9.907E-1 (pos.67432804)		Aldehyde d	3-chloroallyl aldehyde dehydrc		NADH	T=0, C=27	T=0.0, C=1.0	0.00001	389.988	27	T=0/0, C=17/1
chr1:209786078	A/A	G	A	SNV	CAMK1G	CAMK1G:intronic:NM 020439.2	1	100		intronic	NM 02043	c.1341-52	p.?	-3.36					Kinase-like,P	rs2272880	0.3758	YES			Calcium/ca	ATP binding,Golgi membrane,c	5-(E)-(5-CHLORO-2-OXO-1		G=0, A=102	G=0.0, A=1.0	0.00001	1593.32	102	G=0/0, A=52/5
chr1:20978832	C/C	T	C	SNV	DDOST,PINK1,PINK1-AS	PINK1:downstream:NM 032409.2, PINK1-AS:upstream:NR	1	100		intronic	NM 03240	c.*1648T>	p.?, p.?	-0.33					Oligosacchar	rs6893	0.1187	YES	8.308E-1;0.0 (pos.20978832)		Dolichyl-dic	ATP bindin	Basal Ganglia Diseases;Brain I	Benign	T=0, C=23	T=0.0, C=1.0	0.00001	316.639	23	T=0/0, C=11/1
chr11:67432854	C/C	T	C	SNV	ALDH3B2	ALDH3B2:exonic:NM 001031615.1	1	100	7	missense	NM 00103	c.608A>G	p.His203Arg	4.56	1	29	0		Aldehyde del	rs6591270	4.00E-04	YES	9.999E-1 (pos.67432854)		Aldehyde d	3-chloroallyl aldehyde dehydrc		NADH	T=0, C=21	T=0.0, C=1.0	0.00001	281.21	21	T=0/0, C=11/1
chr11:67434416	A/A	C	A	SNV	ALDH3B2	ALDH3B2:utr 5:NM 001031615.1	1	100	3	utr 5	NM 00103	c.-10G>T	p.?	0.42					Aldehyde del	rs4646823	0.1015	YES	8.878E-1 (pos.67434416)		Aldehyde d	3-chloroallyl aldehyde dehydrc		NADH	C=0, A=44	C=0.0, A=1.0	0.00001	729.745	44	C=0/0, A=9/35
chr1:209804095	A/A	G	A	SNV	LAMB3	LAMB3:intronic:NM 000228.2	1	100		intronic	NM 00022	c.823-15C	p.?	-2.23					Laminin EGF	rs4844863	0.1515	YES	8.278E-1 (pos.209804095)		Laminin, be	brown fat	Amelogenesis Imperfecta;Con	Benign	G=0, A=53	G=0.0, A=1.0	0.00001	782.03	53	G=0/0, A=20/3
chr1:20992606	G/T	G	T	SNV	KIF17	KIF17:intronic:NM 020816.3	1	57.14		intronic	NM 02081	c.2911+10	p.?	0					Kinesin moto	rs630484		YES			Kinesin fam	ATP bindin	Digestive System Diseases;Eosinophilia;Eosi		G=12, T=16	G=0.4286, T=0.5714	0.00001	161.768	28	G=9/3, T=9/7
chr1:209950677	GC/GC	G	GC	INDEL	TRAF3IP3	TRAF3IP3:intronic:NM 025228.3	1	100		intronic	NM 02522	c.1054-20	p.?	-0.03											TRAF3-inter	integral component of membrane,protein binding			G=0, GC=32	G=0.0, GC=1.0	0.00001	222.52	32	G=0/0, GC=22/
chr1:209950681	C/C	T	C	SNV	TRAF3IP3	TRAF3IP3:intronic:NM 025228.3	1	100		intronic	NM 02522	c.1054-16	p.?	-0.14						rs649275	0.3649	YES	6.438E-1 (pos.209950681)		TRAF3-inter	integral component of membrane,protein binding			T=0, C=32	T=0.0, C=1.0	0.00001	484.13	32	T=0/0, C=22/1
chr1:19202917	C/C	T	C	SNV	ALDH4A1	ALDH4A1:exonic:NM 003748.3	1	100	12	synonymous	NM 00374	c.1230A>G	p.(=)	-5.52				0.02(COSM13	Aldehyde del	rs7550938	0.3062	YES	7.275E-1 (pos.19202917)	adenocarcinoı	Aldehyde d	1-pyrrolin	Amino Acid Metabo	NADH Benign	T=0, C=32	T=0.0, C=1.0	0.00001	485.123	32	T=0/0, C=7/25
chr1:210003402	C/C	T	C	SNV	DIEXF	DIEXF:intronic:NM 014388.6	1	100		intronic	NM 01438	c.108-51T	p.?	0.28					Utp25, U3 sm	rs599649	0.2384	YES	3.957E-1 (pos.210003402)			RNA binding,U3 snoRNA binding,maturation of SSU-rRNA			T=0, C=20	T=0.0, C=1.0	0.00001	263.786	20	T=0/0, C=10/1
chr1:19202926	C/C	T	C	SNV	ALDH4A1	ALDH4A1:exonic:NM 003748.3	1	100	12	synonymous	NM 00374	c.1221A>G	p.(=)	0.24				0.67(COSM13	Aldehyde del	rs2230706	0.3081	YES	7.31E-1 (pos.19202926)	clear_cell_ren	Aldehyde d	1-pyrrolin	Amino Acid Metabo	NADH Benign	T=0, C=31	T=0.0, C=1.0	0.00001	465.819	31	T=0/0, C=6/25
chr1:210015568	G/G	GCT	G	INDEL	DIEXF	DIEXF:intronic:NM 014388.6	2	100		intronic	NM 01438	c.1486-42	p.?	-0.1, 0.0, 0.0					Utp25, U3 sm	rs10570454,rs20032	0.2173	YES	8.001E-1 (pos.210015568)			RNA binding,U3 snoRNA binding,maturation of SSU-rRNA			GCT=0, G=1	GCT=0.0, G=1.0	0.00001	3231.48	193	GCT=0/0, G=1
chr1:19203997	G/G	C	G	SNV	ALDH4A1	ALDH4A1:exonic:NM 003748.3	1	100	10	synonymous	NM 00374	c.1050G>C	p.(=)	1.51				0.18(COSM14	Aldehyde del	rs2230705	0.2925	YES	5.004E-1 (pos.19203997)	adenocarcinoı	Aldehyde d	1-pyrrolin	Amino Acid Metabo	NADH Benign	C=0, G=66	C=0.0, G=1.0	0.00001	1015.4	66	C=0/0, G=34/3
chr13:52603775	C/T	C	T	SNV	ALG11,UTP14C	ALG11:utr 3:NM 001004127.2, UTP14C:exonic:NM 021645	1	59.09	2	synonymous	NM 00100	c.2528C>T	p.?, p.(=)	0.11				0.03(COSM37	ALG11 mann	rs3742291	0.2948	YES	5.911E-1 (pos.52603775)	adenocarcinoı	Alg11, S. cei	GDP-Man:Man3GlcNAc2-PP-Dol alpha-1,2-		Benign	C=18, T=26	C=0.4091, T=0.5909	0.00001	312.069	44	C=8/10, T=9/1
chr11:77811990	C/C	T	C	SNV	ALG8	ALG8:utr 3:NM 024079.4	1	100	13	utr 3	NM 02407	c.1601A>G	p.?	0.23					ALG6, ALG8 g	rs1263505	0.1485	YES	7.542E-1 (pos.77811990)		Alg8, S. cere	alpha-1,3-	Carbohydrate Metabolism, Inb	Benign	T=0, C=92	T=0.0, C=1.0	0.00001	1524.47	92	T=0/0, C=56/3
chr1:210578141	A/G	A	G	SNV	HHAT	HHAT:intronic:NM 001122834.3	1	51.92		intronic	NM 00112	c.684+118	p.?	1.15					MBOAT, mem	rs2294850		YES			Hedgehog a	GTP bindir	46, XY Disorders of Sex Development;Bone Di		A=25, G=27	A=0.4808, G=0.5192	0.00001	270.41	52	A=14/11, G=1
chr1:210761462	C/C	G	C	SNV	HHAT	HHAT:intronic:NM 001122834.3	1	100		intronic	NM 00112	c.1245+19	p.?	-3.06					MBOAT, mem	rs4297293	0.0013	YES	1.0;0.0 (pos.210761462)		Hedgehog a	GTP bindir	46, XY Disorders of Sex Development;Bone Di		G=0, C=21	G=0.0, C=1.0	0.00001	281.235	21	G=0/0, C=5/16
chr2:29416572	C/C	T	C	SNV	ALK	ALK:exonic:NM 004304.4	1	100	29	missense	NM 00430	c.4381A>G	p.Ile1461Val	-0.73	0.84	29	0		Glycine rich p	rs1670283	3.00E-04		1.0 (pos.29416572)		Anaplastic l	ATP bindin	Adenocarcinoma;A	ATP,Alecti Benign	T=0, C=21	T=0.0, C=1.0	0.00001	285.522	21	T=0/0, C=13/8
chr2:30143499	C/C	G	C	SNV	ALK	ALK:exonic:NM 004304.4	1	100	1	synonymous	NM 00430	c.27C>G	p.(=)	0.32				0.07(COSM37	Glycine rich p	rs4358080	0.0773	YES	1.0 (pos.30143499)	adenocarcinoı	Anaplastic l	ATP bindin	Adenocarcinoma;A	ATP,Alecti Benign	G=0, C=31	G=0.0, C=1.0	0.00001	465.778	31	G=0/0, C=14/1
chr1:211452678	A/A	G	A	SNV	RCOR3	RCOR3:intronic:NM 001136223.2	1	100		intronic	NM 00113	c.720+20G	p.?	0.72					ELM2 domain	rs3767377	0.3928	YES	6.288E-1 (pos.211452678)			DNA binding,DNA binding transcription factor activity,ne			G=0, A=49	G=0.0, A=1.0	0.00001	831.13	49	G=0/0, A=24/2
chr1:211452728	A/A	G	A	SNV	RCOR3	RCOR3:intronic:NM 001136223.2	1	100		intronic	NM 00113	c.720+70G	p.?	0					ELM2 domain	rs3767378		YES				DNA binding,DNA binding transcription factor activity,ne			G=0, A=49	G=0.0, A=1.0	0.00001	831.633	49	G=0/0, A=24/2
chr14:78174473	C/C	A	C	SNV	ALKBH1,SLIRP	ALKBH1:upstream:NM 006020.2, SLIRP:exonic:NM 031210	1	100	1	synonymous	NM 00602	c.-126T>G,	p.?, p.(=)	0.07				0.04(COSM44	RNA recognit	rs11159286	0.0363	YES	8.221E-1;0.0 (pos.78174	acute_myeloic	AlkB, E. coli,	1-ethyladenine demethylase activity,DNA dealkylation i			A=0, C=31	A=0.0, C=1.0	0.00001	465.725	31	A=0/0, C=9/22
chr11:43911365	C/C	T	C	SNV	ALKBH3	ALKBH3:exonic:NM 139178.3	1	100	6	synonymous	NM 13917	c.357T>C	p.(=)	-2.07					2OG-Fe(II) ox	rs2434478	0		1.0 (pos.43911365)		AlkB, E. coli,	DNA dealkylation involved in D		Vitamin C	T=0, C=116	T=0.0, C=1.0	0.00001	1854.51	116	T=0/0, C=81/3
chr1:212519292	T/G	T	G	SNV	PPP2R5A	PPP2R5A:intronic:NM 006243.3	1	57.14		intronic	NM 00624	c.704+17T	p.?	1.17					Protein phosphatase 2A regulatory B subunit (B56 family)						Protein pho	M band,Z disc,chromosome, centromeric region,cytopla			T=21, G=28	T=0.4286, G=0.5714	0.00001	342.296	49	T=8/13, G=15/
chr1:212616037	G/A	G	A	SNV	NENF	NENF:intronic:NM 013349.4	1	52.27		intronic	NM 01334	c.238+70G	p.?	0.66					Cytochrome l	rs377685630	3.00E-04				Neuron-der	endomembrane system,extracellular space,growth fact			G=21, A=23	G=0.4773, A=0.5227	0.00001	260.142	44	G=6/15, A=12/
chr2:73679866	T/C	T	C	SNV	ALMS1	ALMS1:exonic:NM 015120.4	1	51.85	8	missense	NM 01512	c.6209T>C	p.Ile2070Thr	-0.19		89	0.034		ALMS motif	rs10496192	0.1673	YES	5.393E-3 (pos.73679866)		Alstrom syn	G2/M tran	Abnormalities, Multiple;Alstro	Benign	T=13, C=14	T=0.4815, C=0.5185	0.00001	132.665	27	T=8/5, C=4/10
chr1:213032568	A/A	T	A	SNV	FLVCR1	FLVCR1:intronic:NM 014053.3	1	100		intronic	NM 01405	c.738+36T	p.?	-0.05					Major Facilit	rs12023052	0.4994	YES	5.295E-1 (pos.213032568)		Feline leuke	blood vess	Ataxia;Congenital, Hereditary, and Neonatal		T=0, A=24	T=0.0, A=1.0	0.00001	334.163	24	T=0/0, A=6/18
chr17:6899559	C/G	C	G	SNV	ALOX12,ALOX12-AS1	ALOX12:exonic:NM 000697.2, ALOX12-AS1:intronic_nc:NR	1	96.77	1	synonymous	NR 04008	c.123C>G	p.(=)	-1.45				0(COSM3749	Lipoxygenase	rs312467		YES	9.821E-1;0.0 (pos.68995	adenocarcinoı	Arachidona	aging,arac	Arthritis;Arthritis, Juvenile;Autoimmune Dise		C=1, G=30	C=0.0323, G=0.9677	0.00001	426.944	31	C=1/0, G=22/8
chr1:213139748	T/G	T	G	SNV	VASH2	VASH2:intronic:NM 024749.4	1	100		intronic	NM 02474	c.365+515	p.?	-0.07					Vasohibin	rs3738805,rs935491	0.4214	YES			Vasohibin 2	cytoplasm,extracellular region,labyrinthine layer blood			T=0, G=50	T=0.0, G=1.0	0.00001	821.339	50	T=0/0, G=25/2
chr1:213341353	AT/A	AT	A	INDEL	RPS6KC1	RPS6KC1:intronic:NM 012424.5	1	58.33		intronic	NM 01242	c.951+37A	p.?	-0.15, -0.12					MIT (microtul	rs900180687					Ribosomal	ATP binding,early endosome,membrane,phosphatidylin			AT=10, A=1	AT=0.4167, A=0.583	0.00001	79.0301	24	AT=7/3, A=5/9
chr1:213405414	G/G	GGCAACTG	G	INDEL	RPS6KC1	RPS6KC1:intronic:NM 012424.5	12	100		intronic	NM 01242	c.1093-52	p.?	7.79, 6.08, 3.48, 4.56, 1.73, 6.18, 1.18, 4.06, 3.63, 6.0, 7.79, 5.					MIT (microtul	rs11271079,rs39805	0.3118	YES	0.0 (pos.213405422),9.921E-4 (pos.2134		Ribosomal	ATP binding,early endosome,membrane,phosphatidylin			GGCAACTG	GGCAACTGAGGAT=0.	0.00001	887.69	58	GGCAACTGAG
chr1:214491536	C/C	G	C	SNV	SMYD2	SMYD2:intronic:NM 020197.2	1	100		intronic	NM 02019	c.409+54G	p.?	-0.11					MYND finger,	rs55760458	0.3341	YES			SET and MYI	RNA polymerase II core binding,cytoplasm,cytosol,heart			G=0, C=34	G=0.0, C=1.0	0.00001	524.186	34	G=0/0, C=16/1
chr4:113352628	A/G	A	G	SNV	ALPK1	ALPK1:exonic:NM 001102406.1	1	57.14	11	missense	NM 00110	c.1925A>G	p.His642Arg	0.25	0.51	29	0		Alpha-kinase	rs13148353	0.3429	YES	7.159E-1 (pos.113352628)		Alpha-kinas	ATP bindin	Nutrition Disorders;Nutritional and Metaboli		A=42, G=56	A=0.4286, G=0.5714	0.00001	673.819	98	A=22/20, G=2
chr1:214637881	AG/AG	A	AG	INDEL	PTPN14	PTPN14:intronic:NM 005401.4	1	100		intronic	NM 00540	c.174+92T	p.?	-0.44					Dual specificity phosphatase, catalytic domain,FERM C-terminal PH-like domain,FERM N-terminal do						Protein tyrc	cytoplasm	Ear Diseases;Hearing Disorders;Hearing Loss		A=0, AG=28	A=0.0, AG=1.0	0.00001	348.249	28	A=0/0, AG=18/
chr4:113359703	A/G	A	G	SNV	ALPK1	ALPK1:exonic:NM 001102406.1	1	45.45	13	synonymous	NM 00110	c.3252A>G	p.(=)	-0.44				0.05(COSM41	Alpha-kinase	rs231247	0.3442	YES	7.173E-1 (pos.11335970	neoplasm	Alpha-kinas	ATP bindin	Nutrition Disorders;Nutritional and Metaboli		A=12, G=10	A=0.5455, G=0.4545	0.00001	81.7625	22	A=7/5, G=5/5
chr18:56247180	C/C	T	C	SNV	ALPK2	ALPK2:exonic:NM 052947.3	1	100	4	synonymous	NM 05294	c.828A>G	p.(=)	-0.36				0.01(COSM37	Alpha-kinase	rs9958735	0.4988	YES	6.077E-1;0.0 (pos.56247	adenocarcinoma,neoplasn		ATP binding,cytoplasm,protein phosphorylation,protein			T=0, C=175	T=0.0, C=1.0	0.00001	2881.01	175	T=0/0, C=95/8
chr1:216370130	G/G	T	G	SNV	USH2A	USH2A:intronic:NM 206933.2	1	100		intronic	NM 20693	c.4082-66	p.?	-0.17					Concanavalir	rs386851		YES			Usherin	USH2 com	Abnormalities, Multiple;Blindness;Congenita		T=0, G=50	T=0.0, G=1.0	0.00001	852.261	50	T=0/0, G=20/3
chr18:56247567	G/G	A	G	SNV	ALPK2	ALPK2:exonic:NM 052947.3	1	100	4	synonymous	NM 05294	c.441T>C	p.(=)	-0.34				0.03(COSM37	Alpha-kinase	rs9956630	0.498	YES	6.072E-1 (pos.56247567)	adenocarcinoma,neoplasn		ATP binding,cytoplasm,protein phosphorylation,protein			A=0, G=29	A=0.0, G=1.0	0.00001	427.586	29	A=0/0, G=16/1
chr18:56204250	A/C	A	C	SNV	ALPK2	ALPK2:exonic:NM 052947.3	1	52.27	5	missense	NM 05294	c.3169T>G	p.Leu1057Val	-0.23	0.01	32	0.675	0.06(COSM49	Alpha-kinase	rs3809976	0.2316	YES	7.845E-1 (pos.56204250)	ductal_carcinoma		ATP binding,cytoplasm,protein phosphorylation,protein			A=21, C=23	A=0.4773, C=0.5227	0.00001	260.156	44	A=10/11, C=14
chr1:21799489	C/C	T	C	SNV	NBPF3	NBPF3:intronic:NM 032264.4	1	100		intronic	NM 03226	c.734+81T	p.?	-3.69					Repeat of unl	rs1971327		YES			Neuroblast	cytoplasm,positive regulation of protein targeting to mit			T=0, C=23	T=0.0, C=1.0	0.00001	316.747	23	T=0/0, C=7/16
chr1:21801346	A/A	G	A	SNV	NBPF3	NBPF3:intronic:NM 032264.4	1	100		intronic	NM 03226	c.941-47G	p.?	0.22					Repeat of unl	rs7531217	0.2213	YES	8.079E-1 (pos.21801346)		Neuroblast	cytoplasm,positive regulation of protein targeting to mit			G=0, A=21	G=0.0, A=1.0	0.00001	281.215	21	G=0/0, A=6/15
chr1:21805730	G/G	A	G	SNV	NBPF3	NBPF3:intronic:NM 032264.4	1	100		intronic	NM 03226	c.1157-11	p.?	-0.5					Repeat of unl	rs2316504		YES			Neuroblast	cytoplasm,positive regulation of protein targeting to mit			A=0, G=81	A=0.0, G=1.0	0.00001	1309.45	81	A=0/0, G=51/3
chr18:56203891	G/T	G	T	SNV	ALPK2	ALPK2:exonic:NM 052947.3	1	50	5	synonymous	NM 05294	c.3528C>A	p.(=)	-0.32				0.01(COSM48	Alpha-kinase	rs3809978	0.2319	YES	7.827E-1 (pos.56203891)	squamous_cell_carcinoma		ATP binding,cytoplasm,protein phosphorylation,protein			G=21, T=21	G=0.5, T=0.5	0.00001	228.879	42	G=16/5, T=8/1
chr1:21928344	G/G	C	G	SNV	RAP1GAP	RAP1GAP:intronic:NM 001145658.2	1	100		intronic	NM 00114	c.1731-54	p.?	0.01					GoLoco motif	rs2275366		YES				GTPase ac	Digestive System Diseases;Digestive System		C=0, G=47	C=0.0, G=1.0	0.00001	776.955	47	C=0/0, G=19/2
chr1:219383867	C/C	T	C	SNV	LYPLAL1	LYPLAL1:intronic:NM 138794.4	1	100		intronic	NM 13879	c.362-7T>C	p.?	1.1					Dienelactone	rs940571	3.00E-04	YES	1.0 (pos.219383867)		Lysophospl	biological_process,carboxylic ester hydrolase activity,cy			T=0, C=79	T=0.0, C=1.0	0.00001	1239.52	79	T=0/0, C=32/4
chr18:56203898	T/G	T	G	SNV	ALPK2	ALPK2:exonic:NM 052947.3	1	50	5	missense	NM 05294	c.3521A>C	p.His1174Pro	-0.33	0.25	77	0.937		Alpha-kinase	rs3809977	0.2324	YES	7.826E-1;0.0 (pos.56203898)			ATP binding,cytoplasm,protein phosphorylation,protein			T=21, G=21	T=0.5, G=0.5	0.00001	228.879	42	T=16/5, G=8/1
chr1:21889635	C/C	T	C	SNV	ALPL	ALPL:exonic:NM 000478.5	1	100	5	synonymous	NM 00047	c.330T>C	p.(=)	-0.92				0.01(COSM37	Alkaline phos	rs1780316,rs200166	0.0507	YES	9.878E-1 (pos.21889635)	Ewings_sarcor	Alkaline ph	C-termina	Alveolar Bone Loss;	Amifostine Benign	T=0, C=36	T=0.0, C=1.0	0.00001	563.743	36	T=0/0, C=21/1
chr1:22013771	A/A	G	A	SNV	USP48	USP48:intronic:NM 032236.7	1	100		intronic	NM 03223	c.3010-39	p.?	-0.92					Ubiquitin car	rs10493008	0.4398	YES	6.832E-1 (pos.22013771)		Ubiquitin-s	cytosol,mi	Amphetamine-Related Disorders;Mental Dis		G=0, A=34	G=0.0, A=1.0	0.00001	523.724	34	G=0/0, A=14/2
chr1:220205943	A/A	G	A	SNV	EPRS,RNU5F-1	RNU5F-1:intronic_nc:NR 002753.5, EPRS:intronic:NM 0044	1	100		intronic	NR 00275	c.389-75C	p.?	0.14					Anticodon bir	rs2789816		YES			Glutamyl-p	ATP binding,GAIT complex,GTP	'5'-O-(N-(L-Alanyl)-Sulfam		G=0, A=43	G=0.0, A=1.0	0.00001	704.114	43	G=0/0, A=21/2
chr1:22032472	AT/AT	A	AT	INDEL	USP48	USP48:intronic:NM 032236.7	1	100		intronic	NM 03223	c.2274-14	p.?	0.55					Ubiquitin car	rs11426922,rs397764126					Ubiquitin-s	cytosol,mi	Amphetamine-Related Disorders;Mental Dis		A=0, AT=60	A=0.0, AT=1.0	0.00001	677.576	60	A=0/0, AT=5/5
chr1:220789236	T/T	C	T	SNV	MARK1	MARK1:intronic:NM 018650.4	1	100		intronic	NM 01865	c.496-45C	p.?	-3.98					Kinase assoc	rs2786599	7.00E-04	YES	1.0 (pos.220789236)		MAP/micro	ATP bindin	Autistic Disorder;Child Development Disorde		C=0, T=40	C=0.0, T=1.0	0.00001	644.254	40	C=0/0, T=16/2
chr2:233245141	C/C	T	C	SNV	ALPP	ALPP:exonic:NM 001632.4	1	100	7	synonymous	NM 00163	c.804T>C	p.(=)	-4.44					Alkaline phos	rs2260309	0.4952	YES	7.911E-1;0.0 (pos.233245141)		Alkaline ph	alkaline phosphatase activity,	METHYL-PHOSPHONIC AC		T=0, C=39	T=0.0, C=1.0	0.00001	613.135	39	T=0/0, C=14/2
chr1:221055463	A/A	G	A	SNV	HLX	HLX:intronic:NM 021958.3	1	100		intronic	NM 02195	c.773-43G	p.?	-3.8					Homeobox do	rs2247213	0.3523	YES	6.702E-2 (pos.221055463)			cell differentiation,embryonic digestive tract morphoge			G=0, A=43	G=0.0, A=1.0	0.00001	705.839	43	G=0/0, A=23/2
chr2:202625615	T/T	C	T	SNV	ALS2	ALS2:exonic:NM 020919.3	1	100	4	missense	NM 02091	c.1102G>A	p.Val368Met	-2.36	0.13	21	0		MORN repeat	rs3219156	0.1016	YES	9.999E-1 (pos.202625615)		Alsin	Rab GTPas	Amyotrophic Lateral Sclerosis;	Benign	C=0, T=78	C=0.0, T=1.0	0.00001	1249.34	78	C=0/0, T=40/3
chr11:44297054	C/C	T	C	SNV	ALX4	ALX4:exonic:NM 021926.3	1	100	2	synonymous	NM 02192	c.621A>G	p.(=)	-6.83				0.01(COSM41	Homeobox do	rs10769028	2.00E-04		9.999E-1;0.0 (pos.44297	neoplasm	Aristaless-l	DNA bindi	Bone Diseases;Bone Diseases,	Benign	T=0, C=58	T=0.0, C=1.0	0.00001	870.82	58	T=0/0, C=34/2
chr12:96346594	C/C	T	C	SNV	AMDHD1	AMDHD1:exonic:NM 152435.2	1	100	2	synonymous	NM 15243	c.237T>C	p.(=)	-0.06					Amidohydrol	rs1982138	0.2411	YES	4.401E-1 (pos.96346594)			cytosol,histidine catabolic process,histidine catabolic p			T=0, C=59	T=0.0, C=1.0	0.00001	885.286	59	T=0/0, C=19/4
chr19:2249477	T/T	G	T	SNV	AMH,SF3A2	SF3A2:downstream:NM 007165.4, AMH:exonic:NM 00047	1	100	1	missense	NM 00716	c.*932G>T	p.?, p.Ser49Ile	2.14	0.04	142	0.838	0.42(COSM41	Anti-Mulleria	rs10407022	0.169	YES	6.45E-1 (pos.2249477)	neoplasm	Anti-Muller	BMP signa	46, XY Disorders of Sex Development;Adnexa		G=0, T=36	G=0.0, T=1.0	0.00001	563.744	36	G=0/0, T=19/1
chr14:103388999	C/C	T	C	SNV	AMN	AMN:utr 5:NM 030943.3	1	100	1	utr 5	NM 03094	c.-27T>C	p.?	-3.59					Amnionless	rs2295828	0.4825	YES	2.283E-1;0.0;0.0 (pos.103388999)		ATP-binding	Golgi to pl	Acute Kidney Injury	Cyanocobalamin,Hydrox	T=0, C=88	T=0.0, C=1.0	0.00001	1451.91	88	T=0/0, C=48/4
chr3:134077470	G/G	C	G	SNV	AMOTL2	AMOTL2:exonic:NM 016201.3	1	100	9	missense	NM 01620	c.2196G>C	p.Glu732Asp	-2.81	1	45	0	0.02(COSM44	Angiomotin C	rs1353776	0.343	YES	9.218E-1;0.0 (pos.13407	hepatocellula	Angiomotin	Wnt signaling pathway,apical plasma membrane,bicellu			C=0, G=44	C=0.0, G=1.0	0.00001	726.443	44	C=0/0, G=22/2
chr1:110170896	C/C	T	C	SNV	AMPD2	AMPD2:exonic:NM 001257360.1	1	100	11	synonymous	NM 00125	c.1434T>C	p.(=)	-0.77				0.12(COSM41	Adenosine/A	rs863978	0.3526	YES	9.82E-1 (pos.110170896)	ductal_carcin	Adenosine	AMP deaminase activity,IMP biosynthetic process,IMP s			T=0, C=45	T=0.0, C=1.0	0.00001	747.392	45	T=0/0, C=29/1
chr4:71388551	A/G	A	G	SNV	AMTN	AMTN:exonic:NM 212557.3	1	45.52	3	missense	NM 21255	c.134A>G	p.Asn45Ser	-0.25	0.09	46	0.102	0.38(COSM37	Amelotin	rs7660807	0.0821	YES	1.96E-1;0.0 (pos.713885	acute_myeloic	Amelotin	basal lamina,biomineral tissue development,cell adhes			A=73, G=61	A=0.5448, G=0.4552	0.00001	630.549	134	A=38/35, G=2
chr17:66246416	G/G	A	G	SNV	AMZ2	AMZ2:exonic:NM 001033571.1	1	100	3	missense	NM 00103	c.88A>G	p.Asn30Asp	0.39	1	23	0	0.01(COSM40	Peptidase fai	rs3213690	0.3264	YES	7.766E-1 (pos.66246416)	acute_myeloic	Archaelysin	cellular_component,metallopeptidase activity,peptidas			A=0, G=100	A=0.0, G=1.0	0.00001	1702.26	100	A=0/0, G=58/4
chr8:6378763	T/T	C	T	SNV	ANGPT2,MCPH1	ANGPT2:exonic:NM 001147.2, MCPH1:intronic:NM 024596	1	100	4	synonymous	NM 02459	c.2214+21	p.?, p.(=)	0.03					BRCA1 C Term	rs3020221	0.3807	YES	2.312E-1 (pos.6378763)		Angiopoieti	Tie signali	Brain Diseases;Cardiovascular	Likely benign	C=0, T=20	C=0.0, T=1.0	0.00001	263.786	20	C=0/0, T=14/6
chr1:22174426	G/G	T	G	SNV	HSPG2	HSPG2:intronic:NM 005529.6	1	100		intronic	NM 00552	c.7873+25	p.?	0					EGF-like dom	rs2290501	0.3108	YES	1.72E-1 (pos.22174426)		Heparan su	Golgi lume	Bone Diseases;Bon	Palifermin	T=0, G=55	T=0.0, G=1.0	0.00001	814.874	55	T=0/0, G=20/3
chr1:22199081	T/T	A	T	SNV	HSPG2	HSPG2:intronic:NM 005529.6	1	100		intronic	NM 00552	c.4029+32	p.?	-0.48					EGF-like dom	rs2501266	7.00E-04		1.0 (pos.22199081)		Heparan su	Golgi lume	Bone Diseases;Bon	Palifermin	A=0, T=45	A=0.0, T=1.0	0.00001	747.351	45	A=0/0, T=32/1
chr1:22208030	C/T	C	T	SNV	HSPG2	HSPG2:intronic:NM 005529.6	1	95.45		intronic	NM 00552	c.1655-35	p.?	-0.49					EGF-like dom	rs921847	0.0163	YES	1.0 (pos.22208030)		Heparan su	Golgi lume	Bone Diseases;Bon	Palifermin Benign	C=1, T=21	C=0.0455, T=0.9545	0.00001	271.505	22	C=1/0, T=6/15
chr1:22211222	C/C	T	C	SNV	HSPG2	HSPG2:intronic:NM 005529.6	1	100		intronic	NM 00552	c.1507+38	p.?	-0.99					EGF-like dom	rs4654994	0.0652	YES	9.639E-1 (pos.22211222)		Heparan su	Golgi lume	Bone Diseases;Bon	Palifermin	T=0, C=37	T=0.0, C=1.0	0.00001	583.384	37	T=0/0, C=21/1
chr8:6389889	A/A	C	A	SNV	ANGPT2,MCPH1	ANGPT2:exonic:NM 001147.2, MCPH1:intronic:NM 024596	1	100	2	synonymous	NM 02459	c.2214+32	p.?, p.(=)	-1.8				0.02(COSM41	BRCA1 C Term	rs6559167	0.4506	YES	4.607E-1;4.022E-1;0.0 (pc	adenocarcinoı	Angiopoieti	Tie signali	Brain Diseases;Cardiovascular	Likely benign	C=0, A=31	C=0.0, A=1.0	0.00001	465.662	31	C=0/0, A=17/1
chr8:41585438	A/A	G	A	SNV	ANK1	ANK1:exonic:NM 001142446.1	1	100	4	synonymous	NM 00114	c.414C>T	p.(=)	-0.65				0.12(COSM37	Ankyrin repe	rs2304871	0.2795	YES	1.949E-1 (pos.41585438)	adenocarcinoı	Ankyrin-1, e	ATPase bir	Anemia;Anemia, Hemolytic;An	Benign/Likely	G=0, A=78	G=0.0, A=1.0	0.00001	1249.34	78	G=0/0, A=40/3
chr10:61868716	A/A	C	A	SNV	ANK3	ANK3:exonic:NM 020987.4	1	100	27	synonymous	NM 02098	c.3045G>T	p.(=)	-2.37					Ankyrin repe	rs3750800	0.3264	YES	6.08E-2;0.0 (pos.61868716)		Ankyrin-3, n	ER to Golgi	Amyotrophic Lateral Sclerosis;	Likely benign	C=0, A=58	C=0.0, A=1.0	0.00001	867.385	58	C=0/0, A=34/2
chr1:222751034	T/C	T	C	SNV	TAF1A	TAF1A:intronic:NM 001201536.1	1	97.3		intronic	NM 00120	c.406-49A	p.?	-1.04					TAF RNA Polyi	rs2936033	0.2955	YES	5.73E-1 (pos.222751034)		TATA box-bi	DNA binding,RNA polymerase I transcription factor comp			T=1, C=36	T=0.027, C=0.973	0.00001	551.74	37	T=0/1, C=22/1
chr1:222825717	C/C	T	C	SNV	MIA3	MIA3:intronic:NM 198551.3	1	100		intronic	NM 19855	c.4086+43	p.?	-4.37					Variant SH3 c	rs10495198	0.2826	YES	5.975E-1 (pos.222825717)		Melanoma	ER to Golgi	Arterial Occlusive Diseases;Arteriosclerosis;		T=0, C=142	T=0.0, C=1.0	0.00001	2383.55	142	T=0/0, C=49/9
chr1:222826481	A/A	G	A	SNV	MIA3	MIA3:intronic:NM 198551.3	1	100		intronic	NM 19855	c.4209+9G	p.?	-0.33				0(COSM1467	Variant SH3 c	rs2291832	0.2827	YES	5.79E-1;0.0 (pos.222826	adenocarcinoı	Melanoma	ER to Golgi	Arterial Occlusive Diseases;Arteriosclerosis;		G=0, A=118	G=0.0, A=1.0	0.00001	1893.97	118	G=0/0, A=52/6
chr2:190595258	T/T	G	T	SNV	ANKAR	ANKAR:exonic:NM 144708.3	1	100	16	missense	NM 14470	c.3231G>T	p.Leu1077Phe	-1.3	1	22	0		Ankyrin repe	rs1225090,rs760199	3.00E-04		2.32E-4 (pos.190595256),9.998E-1 (pos.190595258)			cytoplasm,integral component of membrane,nucleus			G=0, T=43	G=0.0, T=1.0	0.00001	705.67	43	G=0/0, T=23/2
chr1:22304712	G/G	A	G	SNV	CELA3B	CELA3B:intronic:NM 007352.3	1	100		intronic	NM 00735	c.44-150A	p.?	0.45					Trypsin	rs10917088		YES				cholesterc	Digestive System Diseases;Exocrine Pancrea		A=0, G=31	A=0.0, G=1.0	0.00001	465.817	31	A=0/0, G=16/1
chr17:54450038	A/A	C	A	SNV	ANKFN1	ANKFN1:exonic:NM 153228.2	1	100	6	synonymous	NM 15322	c.642C>A	p.(=)	0.53					Ankyrin repe	rs957724	0.3291	YES	9.689E-1 (pos.54450038)				Marijuana Abuse;Mental Disorders;Substanc		C=0, A=22	C=0.0, A=1.0	0.00001	298.892	22	C=0/0, A=5/17
chr1:22310933	G/G	C	G	SNV	CELA3B	CELA3B:intronic:NM 007352.3	1	100		intronic	NM 00735	c.642+109	p.?	-0.23					Trypsin	rs12130164		YES				cholesterc	Digestive System Diseases;Exocrine Pancrea		C=0, G=23	C=0.0, G=1.0	0.00001	316.664	23	C=0/0, G=6/17

chr17:54450134	A/A	G	A	SNV	ANKFN1	ANKFN1:exonic:NM 153228.2	1	100	6	synonymous	NM 15322	c.738G>A	p.(=)	0.14					Ankyrin repe	rs8069322	0.2723	YES	9.691E-1 (pos.54450134)				Marijuana Abuse:Mental Disorders;Substanc			G=0, A=36	G=0.0, A=1.0	0.00001	563.744	36	G=0/0, A=4/32
chr1:22336371	A/A	C	A	SNV	CELA3A	CELA3A:intronic:NM 005747.4	1	100		intronic	NM 00574	c.795+21C	p.?	-4.01						rs3806297	0.2959	YES	3.045E-1;0.0 (pos.22336371)			cholesterol metabolic process.digestion.extracellular s				C=0, A=34	C=0.0, A=1.0	0.00001	524.187	34	C=0/0, A=17/1
chr1:2234903	T/T	C	T	SNV	SKI	SKI:intronic:NM 003036.3	1	100		intronic	NM 00303	c.1211+64	p.?	-1.4					SKI/SNO/DAC	rs2256178		YES			Avian sarco	BMP signa	Abnormalities, Multiple:Aneurysm:Aortic Ane			C=0, T=42	C=0.0, T=1.0	0.00001	685.187	42	C=0/0, T=9/33
chr1:223717379	C/C	A	C	SNV	CAPN8	CAPN8:intronic:NM 001143962.1	1	100		intronic	NM 00114	c.1703+50	p.?	-0.43					Calpain famil	rs61823524	0.0019		9.443E-1 (pos.223717379)			Golgi apparatus,calcium ion binding,calcium-dependent				A=0, C=28	A=0.0, C=1.0	0.00001	408.699	28	A=0/0, C=22/6
chr1:223853099	C/A	C	A	SNV	CAPN8	CAPN8:intronic:NM 001143962.1	1	57.14		intronic	NM 00114	c.237+13G	p.?	-0.38					Calpain famil	rs66700641	0.0937	YES	0.0;0.0 (pos.223853099)			Golgi apparatus,calcium ion binding,calcium-dependent				C=9, A=12	C=0.4286, A=0.5714	0.00001	109.102	21	C=6/3, A=4/8
chr17:54534634	A/A	G	A	SNV	ANKFN1	ANKFN1:exonic:NM 153228.2	1	100	12	missense	NM 15322	c.1333G>A	p.Val445Ile	4.13	0.01	29	0.659		Ankyrin repe	rs10852985	0.2757	YES	9.734E-1 (pos.54534634)				Marijuana Abuse:Mental Disorders;Substanc			G=0, A=61	G=0.0, A=1.0	0.00001	921.854	61	G=0/0, A=42/1
chr17:4088291	T/T	C	T	SNV	ANKFY1	ANKFY1:exonic:NM 001257999.2	1	100	12	synonymous	NM 00125	c.1647G>A	p.(=)	-4.24					Ankyrin repe	rs2020118	0.0751	YES	9.398E-1 (pos.4088291)		Ankyrin rep	Golgi to lys	Female Urogenital Diseases:Female Urogeni			C=0, T=75	C=0.0, T=1.0	0.00001	1189.47	75	C=0/0, T=30/4
chr1:224463189	A/G	A	G	SNV	NVL	NVL:intronic:NM 002533.3	1	50		intronic	NM 00253	c.1963-29	p.?	0.26					AAA domain (	rs199926089	0.0022		0.0 (pos.224463189)		Nuclear val	ATP binding,RNA binding,membrane,nucleolus,nucleopl				A=15, G=15	A=0.5, G=0.5	0.00001	145.459	30	A=0/15, G=8/7
chr11:113270015	C/C	G	C	SNV	ANKK1	ANKK1:exonic:NM 178510.1	1	100	8	missense	NM 17851	c.1324G>C	p.Gly442Arg	-0.71	0.7	125	0	0(COSM4284	Ankyrin repe	rs4938016	0.3046	YES	5.025E-1;0.0;1.537E-4 (p	neoplasm	Ankyrin rep	ATP bindin	Communication Disorders;Language Disorde			G=0, C=29	G=0.0, C=1.0	0.00001	427.546	29	G=0/0, C=25/4
chr1:224517907	T/T	A	T	SNV	NVL	NVL:upstream:NM 002533.3	1	100			NM 00253	c.-78T>A	p.?	0.71						rs4146965		YES			Nuclear val	ATP binding,RNA binding,membrane,nucleolus,nucleopl				A=0, T=68	A=0.0, T=1.0	0.00001	1053.56	68	A=0/0, T=36/3
chr1:224621368	C/C	G	C	SNV	WDR26	WDR26:intronic:NM 025160.6	1	100		intronic	NM 02516	c.422+18C	p.?	-0.54					WD domain, (	rs2405033	0	YES	1.0 (pos.224621368)		WD repeat-	cytoplasm,cytosol,nucleoplasm,protein binding				G=0, C=24	G=0.0, C=1.0	0.00001	333.441	24	G=0/0, C=17/7
chr1:225492845	T/T	C	T	SNV	DNAH14	DNAH14:intronic:NM 001373.1	1	100		intronic	NM 00137	c.8726+54	p.?	-1.4					ATP-binding c	rs12034925		YES			Dynein, axo	ATP bindin	Congenital, Hereditary, and Neonatal Diseas			C=0, T=36	C=0.0, T=1.0	0.00001	563.739	36	C=0/0, T=17/1
chr1:225510641	C/C	T	C	SNV	DNAH14	DNAH14:intronic:NM 001373.1	1	100		intronic	NM 00137	c.9216+11	p.?	-0.26					ATP-binding c	rs1532771		YES			Dynein, axo	ATP bindin	Congenital, Hereditary, and Neonatal Diseas			T=0, C=30	T=0.0, C=1.0	0.00001	446.639	30	T=0/0, C=9/21
chr1:225511905	T/C	T	C	SNV	DNAH14	DNAH14:intronic:NM 001373.1	1	53.13		intronic	NM 00137	c.9217-19	p.?	-1.08					ATP-binding c	rs10799300	0.2131	YES	1.447E-1 (pos.225511905)		Dynein, axo	ATP bindin	Congenital, Hereditary, and Neonatal Diseas			T=15, C=17	T=0.4688, C=0.5313	0.00001	173.559	32	T=9/6, C=7/10
chr19:17393504	C/C	G	C	SNV	ANKLE1	ANKLE1:exonic:NM 152363.5	1	100	3	synonymous	NM 15236	c.417G>C	p.(=)	0.18				0(COSM3749	Ankyrin repe	rs1864113	0.1584	YES	3.882E-1 (pos.17393504)	adenocarcinoma,neoplasn		DNA repai	Adnexal Diseases;Breast Diseases;Breast Ne			G=0, C=34	G=0.0, C=1.0	0.00001	528.567	34	G=0/0, C=21/1
chr19:17393015	C/T	C	T	SNV	ANKLE1	ANKLE1:exonic:NM 152363.5	1	96.77	2	missense	NM 15236	c.374C>T	p.Ala125Val	0.6	0.85	64	0.155	0.09(COSM37	Ankyrin repe	rs1864116	0.128	YES	3.776E-1 (pos.17393015)	adenocarcinoma,neoplasn		DNA repai	Adnexal Diseases;Breast Diseases;Breast Ne			C=2, T=60	C=0.0323, T=0.9677	0.00001	877.531	62	C=2/0, T=14/4
chr1:225611845	C/T	C	T	SNV	LBR	LBR:intronic:NM 002296.3	1	50		intronic	NM 00229	c.-13-54G>	p.?	-0.47					Ergosterol bi	rs1340867		YES			Lamin B rec	DNA bindi	Bone Diseases:Bone Diseases, Development			C=11, T=11	C=0.5, T=0.5	0.00001	92.9105	22	C=3/8, T=6/5
chr1:225742588	A/G	A	G	SNV	ENAH	ENAH:intronic:NM 001008493.2	1	47.62		intronic	NM 00100	c.349+20T	p.?	1.18					VASP tetrame	rs17561228	0.0508	YES	1.564E-2 (pos.225742588)		Enabled, Dr	SH3 doma	Autoimmune Diseases:Female Urogenital Di			A=33, G=30	A=0.5238, G=0.4762	0.00001	289.4	63	A=14/19, G=16
chr16:89350178	A/A	G	A	SNV	ANKRD11	ANKRD11:exonic:NM 001256183.1	1	100	9	synonymous	NM 00125	c.2772C>T	p.(=)	-3.21					Ankyrin repe	rs2279349	0.1501	YES	6.153E-1 (pos.89350178)		Ankyrin rep	bone deve	Abnormalities, Multiple;Bone Diseases;Bone			G=0, A=25	G=0.0, A=1.0	0.00001	353.018	25	G=0/0, A=3/22
chr1:226044766	A/A	G	A	SNV	TMEM63A	TMEM63A:intronic:NM 014698.2	1	100		intronic	NM 01469	c.1378-49	p.?	-1.68					Calcium-depe	rs9919303	0.2887	YES	5.628E-1 (pos.226044766)			extracellu	Digestive System Diseases;Digestive System			G=0, A=23	G=0.0, A=1.0	0.00001	316.747	23	G=0/0, A=6/17
chr1:226550924	C/C	G	C	SNV	PARP1	PARP1:intronic:NM 001618.3	1	100		intronic	NM 00161	c.2787-63	p.?	-0.65					BRCA1 C Term	rs747657	0.3375	YES			Poly(ADP-ril	ATP genera	Alopecia;Anemia;A	2-(3'-Methoxyphenyl) Ber		G=0, C=25	G=0.0, C=1.0	0.00001	353.018	25	G=0/0, C=7/18
chr1:226578098	G/GC	G	GC	INDEL	PARP1	PARP1:intronic:NM 001618.3	1	100		intronic	NM 00161	c.617+13C	p.?	-0.11					BRCA1 C Terminus (BRCT) domain.PADR1 (NUC008) domain.Poly(ADP-ribose) polymerase and DNA-Lig						Poly(ADP-ril	ATP genera	Alopecia;Anemia;A	2-(3'-Methoxyphenyl) Ber		G=0, GC=42	G=0.0, GC=1.0	0.00001	223.31	42	G=0/0, GC=28/
chr1:226578099	C/C	T	C	SNV	PARP1	PARP1:intronic:NM 001618.3	1	100		intronic	NM 00161	c.617+12A	p.?	-5.63					BRCA1 C Term	rs1805403	0.2331	YES	9.365E-1 (pos.226578099)		Poly(ADP-ril	ATP genera	Alopecia;Anemia;A	2-(3'-Methoxyphenyl) Ber		T=0, C=49	T=0.0, C=1.0	0.00001	829.408	49	T=0/0, C=28/2
chr18:9254785	T/T	C	T	SNV	ANKRD12	ANKRD12:exonic:NM 015208.4	1	100	9	missense	NM 01520	c.1520C>T	p.Thr507Ile	7.29	0.02	89	0.437		Ankyrin repe	rs17498752	0.0749	YES	2.642E-2 (pos.9254785)		Ankyrin rep	cytosol,nucleoplasm				C=0, T=99	C=0.0, T=1.0	0.00001	1685.3	99	C=0/0, T=31/6
chr1:227071377	C/C	T	C	SNV	PSEN2	PSEN2:intronic:NM 000447.2	1	100		intronic	NM 00044	c.142-29T	p.?	-3.99					Presenilin	rs1295644	0.225	YES	5.277E-1;0.0 (pos.227071377)		Presenilin 2	Golgi appa	Alzheimer Disease:Asphyxia Neonatorum:Bra			T=0, C=20	T=0.0, C=1.0	0.00001	263.786	20	T=0/0, C=8/12
chr1:227073410	C/C	G	C	SNV	PSEN2	PSEN2:intronic:NM 000447.2	1	100		intronic	NM 00044	c.498+30G	p.?	-1.4					Presenilin	rs2236910,rs753615	0.22	YES	0.0 (pos.227073409),5.616E-1:0.0:0.0 (p		Presenilin 2	Golgi appa	Alzheimer Disease:Asphyxia Neonatorum:Bra			G=0, C=33	G=0.0, C=1.0	0.00001	501.062	33	G=0/0, C=19/1
chr18:9275542	T/T	C	T	SNV	ANKRD12	ANKRD12:exonic:NM 015208.4	1	100	11	synonymous	NM 01520	c.5784C>T	p.(=)	0.19					Ankyrin repe	rs59633812	0.076	YES	2.635E-2 (pos.9275542)		Ankyrin rep	cytosol,nucleoplasm				C=0, T=79	C=0.0, T=1.0	0.00001	1269.33	79	C=0/0, T=54/2
chr18:9275563	G/G	T	G	SNV	ANKRD12	ANKRD12:exonic:NM 015208.4	1	100	11	synonymous	NM 01520	c.5805T>G	p.(=)	0.85				0.88(COSM56	Ankyrin repe	rs17499116	0.0759	YES	2.635E-2 (pos.9275563)	ductal_carcinc	Ankyrin rep	cytosol,nucleoplasm				T=0, G=80	T=0.0, G=1.0	0.00001	1289.37	80	T=0/0, G=54/2
chr18:9258531	T/T	C	T	SNV	ANKRD12	ANKRD12:exonic:NM 015208.4	1	99.26	9	synonymous	NM 01520	c.5266C>T	p.(=)	1.58					Ankyrin repe	rs3744823	0.076	YES	2.634E-2 (pos.9258531)		Ankyrin rep	cytosol,nucleoplasm				C=1, T=135	C=0.0074, T=0.9926	0.00001	2220.23	136	C=1/0, T=107/
chr11:67068859	C/C	T	C	SNV	ANKRD13D	ANKRD13D:exonic:NM 207354.2	1	100	12	synonymous	NM 20735	c.1338T>C	p.(=)	-0.59					Ankyrin repe	rs2298815	0.0336	YES	8.843E-1 (pos.67068859)		Ankyrin rep	cytoplasm,late endosome,plasma membrane				T=0, C=58	T=0.0, C=1.0	0.00001	867.197	58	T=0/0, C=21/3
chr10:5920121	C/C	T	C	SNV	ANKRD16	ANKRD16:exonic:NM 019046.2	1	100	7	missense	NM 01904	c.1058A>G	p.Gln353Arg	-0.03	0.34	43	0.001		Ankyrin repe	rs1052420	0.1241	YES	8.55E-1 (pos.5920121)							T=0, C=20	T=0.0, C=1.0	0.00001	263.786	20	T=0/0, C=10/1
chr1:22832743	T/T	A	T	SNV	ZBTB40	ZBTB40:intronic:NM 014870.3	1	100		intronic	NM 01487	c.1360+9A	p.?	0.89				0.02(COSM37	BTB/POZ dom	rs209732	0.3836	YES	8.964E-1 (pos.22832743)	hepatocellula	Zinc finger-	DNA bindi	Intellectual Disability:Mental Disorders:Nerv			A=0, T=85	A=0.0, T=1.0	0.00001	1355.71	85	A=0/0, T=27/5
chr10:5925971	A/A	C	A	SNV	ANKRD16	ANKRD16:exonic:NM 019046.2	1	100	4	synonymous	NM 01904	c.648G>T	p.(=)	-1.88					Ankyrin repe	rs631947	0.1021	YES	9.572E-1 (pos.5925971)							C=0, A=24	C=0.0, A=1.0	0.00001	334.789	24	C=0/0, A=13/1
chr4:73991006	C/C	T	C	SNV	ANKRD17	ANKRD17:exonic:NM 032217.4	1	100	17	synonymous	NM 03221	c.3258A>G	p.(=)	-0.82					Ankyrin repe	rs6822576	1.00E-04		1.0 (pos.73991006)		Ankyrin rep	RNA binding,blood vessel maturation,chromatin,chroma				T=0, C=27	T=0.0, C=1.0	0.00001	389.988	27	T=0/0, C=12/1
chr1:228404668	C/C	T	C	SNV	OBSCN	OBSCN:intronic:NM 001271223.2	1	100		intronic	NM 00127	c.2378-46	p.?	-4.77					Fibronectin t	rs3795777	0.3954	YES	3.91E-1 (pos.228404668)							T=0, C=27	T=0.0, C=1.0	0.00001	389.987	27	T=0/0, C=11/1
chr9:38588602	T/C	T	C	SNV	ANKRD18A	ANKRD18A:exonic:NM 147195.3	1	57.45	11	missense	NM 14719	c.2063A>G	p.Tyr688Cys	-0.52	0.08	194	0.991	0.01(COSM45	Ankyrin repe	rs2799163		YES	4.855E-1 (pos.38588602)	acute_myeloid_leukaemia,adenocarci			Lung Diseases:Lung Neoplasms:Neoplasms:N			T=20, C=27	T=0.4255, C=0.5745	0.00001	326.525	47	T=18/2, C=25/
chr9:33548690	G/A	G	A	SNV	ANKRD18B	ANKRD18B:exonic:NM 001353432.1	1	51.85	9	missense	NM 00135	c.1715G>A	p.Arg572His	1.52	1	29	0.001	0(COSM4163	Ankyrin repe	rs41313776		YES	3.537E-2 (pos.33548690)	neoplasm,squamous_cell_carcinoma						G=13, A=14	G=0.4815, A=0.5185	0.00001	133.048	27	G=3/10, A=9/5
chr9:33548689	C/CA	C	CA	INDEL	ANKRD18B	ANKRD18B:exonic:NM 001353432.1	1	50	9	frameshiftInsertion	NM 00135	c.1714_17	p.Arg572fs	-0.04					Ankyrin repe	rs760567222			0.0 (pos.33548689)							C=10, CA=1	C=0.5, CA=0.5	0.00001	63.9364	20	C=0/10, CA=7/
chr10:90580144	T/T	C	T	SNV	ANKRD22,LIPM	ANKRD22:utr 3:NM 144590.2, LIPM:exonic:NM 00112821	1	100	9	synonymous	NM 00112	c.1158C>T	p.(=), p.?	-0.86				0.03(COSM41	Ankyrin repe	rs10788615	0.3149	YES	4.444E-1 (pos.90580144)	acute_myeloic	Lipase fami	cornification,extracellular region,lipid catabolic process				C=0, T=31	C=0.0, T=1.0	0.00001	464.245	31	C=0/0, T=14/1
chr1:228464842	G/G	C	G	SNV	OBSCN	OBSCN:intronic:NM 001271223.2	1	100		intronic	NM 00127	c.7726-19	p.?	-3.01					Fibronectin t	rs936885	0.3047	YES	7.475E-1 (pos.228464842)							C=0, G=31	C=0.0, G=1.0	0.00001	465.53	31	C=0/0, G=18/1
chr1:228466750	T/C	T	C	SNV	OBSCN	OBSCN:intronic:NM 001271223.2	1	97.06		intronic	NM 00127	c.8413+94	p.?	-2.78					Fibronectin t	rs3795782		YES								T=1, C=33	T=0.0294, C=0.9706	0.00001	493.065	34	T=0/1, C=28/5
chr19:33106621	T/T	C	T	SNV	ANKRD27	ANKRD27:exonic:NM 032139.2	1	100	22	synonymous	NM 03213	c.2118G>A	p.(=)	-4.18				0.02(COSM37	Ankyrin repe	rs200055396,rs4058	0.318	YES	3.244E-1 (pos.33106621)	adenocarcinoma		GTPase ac	Depressive Disorder;Depressive Disorder, M			C=0, T=50	C=0.0, T=1.0	0.00001	852.748	50	C=0/0, T=29/2
chr19:33117666	C/C	T	C	SNV	ANKRD27	ANKRD27:exonic:NM 032139.2	1	100	16	synonymous	NM 03213	c.1488A>G	p.(=)	-0.3				0.17(COSM37	Ankyrin repe	rs6510271	0.3617	YES	6.931E-1 (pos.33117666)	adenocarcinoma		GTPase ac	Depressive Disorder:Depressive Disorder, M			T=0, C=80	T=0.0, C=1.0	0.00001	1288.34	80	T=0/0, C=27/5
chr1:22849003	C/C	A	C	SNV	ZBTB40	ZBTB40:intronic:NM 014870.3	1	100		intronic	NM 01487	c.3298+47	p.?	-6.29					BTB/POZ dom	rs998978	0.3691	YES	4.561E-1 (pos.22849003)		Zinc finger-	DNA bindi	Intellectual Disability:Mental Disorders:Nerv			A=0, C=29	A=0.0, C=1.0	0.00001	427.599	29	A=0/0, C=16/1
chr1:228494357	T/T	C	T	SNV	OBSCN	OBSCN:intronic:NM 001271223.2	1	100		intronic	NM 00127	c.14797+1	p.?	-0.48					Fibronectin t	rs3795795	0.1716	YES	1.608E-1 (pos.228494357)							C=0, T=23	C=0.0, T=1.0	0.00001	316.746	23	C=0/0, T=9/14
chr1:228494382	C/C	T	C	SNV	OBSCN	OBSCN:intronic:NM 001271223.2	1	100		intronic	NM 00127	c.14797+4	p.?	-0.59					Fibronectin t	rs1150911	0.378	YES	2.161E-1;5.149E-1 (pos.228494382)							T=0, C=23	T=0.0, C=1.0	0.00001	316.698	23	T=0/0, C=9/14
chr18:14850375	G/A	G	A	SNV	ANKRD30B	ANKRD30B:exonic:NM 001145029.1	1	46.51	35	synonymous	NM 00114	c.3201G>A	p.(=)	-0.43				0.03(COSM37	Ankyrin repe	rs3126404		YES	4.497E-1 (pos.14850375)	acute_myeloic	Ankyrin rep	biological_process,cellular_component,molecular_func				G=23, A=20	G=0.5349, A=0.4651	0.00001	212.326	43	G=10/13, A=1
chr2:97849219	C/C	T	C	SNV	ANKRD36	ANKRD36:exonic:NM 001164315.1	1	100	27	missense	NM 00116	c.1861T>C	p.Trp621Arg	1.81	0.03	101	0.797	0.03(COSM14	Ankyrin repe	rs12996352		YES	4.289E-1 (pos.97849219)	acute_myeloid_leukaemia,adenocarcinoma,neoplasm,squamous_cell_carcinoma						T=0, C=53	T=0.0, C=1.0	0.00001	782.03	53	T=0/0, C=26/2
chr2:97864333	AT/AT	CC	AT	MNV	ANKRD36	ANKRD36:exonic:NM 001164315.1	2	100	44	missense	NM 00116	c.2693_26	p.Ala898Asp	-0.65, -0.1	1	126	0.055	0(COSM4591	Ankyrin repe	rs56835800,rs61218502,rs7132		YES	3.006E-1 (pos.97864333)	acute_myeloid_leukaemia,neoplasm,squamous_cell_carcinoma						CC=0, AT=8	CC=0.0, AT=1.0	0.00001	1431.39	87	CC=0/0, AT=39
chr2:97877292	A/A	C	A	SNV	ANKRD36	ANKRD36:exonic:NM 001164315.1	1	100	57	missense	NM 00116	c.3377C>A	p.Pro1126Gln	0.19	0.79	76	0	0(COSM3991	Ankyrin repe	rs10171441		YES	5.189E-1;1.225E-4 (pos.9	acute_myeloid_leukaemia,papillary_renal_cell_carcinoma,squamous_cell_carcinor						C=0, A=244	C=0.0, A=1.0	0.00001	4124.13	244	C=0/0, A=32/2
chr4:125592065	C/A	C	A	SNV	ANKRD50	ANKRD50:exonic:NM 020337.2	1	59.09	4	synonymous	NM 02033	c.2367G>T	p.(=)	0.03					Ankyrin repe	rs10018651,rs77644	0.2093	YES	2.681E-1;1.157E-4;0.0 (pos.125592065)			endosome,protein binding,protein transport,retrograde				C=9, A=13	C=0.4091, A=0.5909	0.00001	122.539	22	C=4/5, A=8/5
chr4:125591687	A/G	A	G	SNV	ANKRD50	ANKRD50:exonic:NM 020337.2	1	47.37	4	synonymous	NM 02033	c.2745T>C	p.(=)	0.21				0.77(COSM41	Ankyrin repe	rs3733471	0.2097	YES	2.685E-1 (pos.12559168	neoplasm		endosome,protein binding,protein transport,retrograde				A=40, G=36	A=0.5263, G=0.4737	0.00001	364.044	76	A=14/26, G=1
chr12:56647911	C/C	G	C	SNV	ANKRD52	ANKRD52:exonic:NM 173595.3	1	100	8	synonymous	NM 17359	c.846C>G	p.(=)	2.84					Ankyrin repe	rs808919	0.0664	YES	9.724E-1;0.0 (pos.56647911)			protein binding				G=0, C=37	G=0.0, C=1.0	0.00001	583.686	37	G=0/0, C=19/1
chr2:71211989	G/G	A	G	SNV	ANKRD53	ANKRD53:exonic:NM 001115116.1	1	100	6	synonymous	NM 00111	c.1152A>G	p.(=)	-0.32				0.01(COSM56	Ankyrin repe	rs3796099	0.384	YES	4.733E-1 (pos.71211989)	diffuse_large_	Ankyrin rep	cell division,cytoplasm,mitotic metaphase plate congre				A=0, G=32	A=0.0, G=1.0	0.00001	485.117	32	A=0/0, G=6/26
chr1:228613052	C/C	T	C	SNV	HIST3H3	HIST3H3:upstream:NM 003493.2	1	100		intronic	NM 00349	c.-26A>G	p.?	-2.71						rs1188474	0.1051	YES	6.702E-1 (pos.228613052)		Histone 3, H	double-str	Intellectual Disability;Lung Diseases;Lung Di			T=0, C=26	T=0.0, C=1.0	0.00001	371.415	26	T=0/0, C=10/1
chr1:229568002	AG/A	AG	A	INDEL	ACTA1	ACTA1:intronic:NM 001100.3	1	100		intronic	NM 00110	c.616+15C	p.?	-0.69, -2.18					Actin						Actin, alpha	ADP bindir	Bone Diseases;Carc	4-Methyl-Histidine,Aplyr		AG=0, A=27	AG=0.0, A=1.0	0.00001	268.265	27	AG=0/0, A=9/1
chr6:90333599	G/G	A	G	SNV	ANKRD6	ANKRD6:exonic:NM 001242809.1	1	100	12	synonymous	NM 00124	c.1041A>G	p.(=)	-0.91				0.34(COSM41	Ankyrin repe	rs17292811	0.1631	YES	9.974E-1 (pos.90333599)	neoplasm	Ankyrin rep	cytoplasm,intracellular membrane-bounded organelle,r				A=0, G=23	A=0.0, G=1.0	0.00001	316.748	23	A=0/0, G=10/1
chr15:40573716	G/G	A	G	SNV	ANKRD63	ANKRD63:exonic:NM 001190479.2	1	100	1	missense	NM 00119	c.1072T>C	p.Ser358Pro	1.89	1	74	0		Ankyrin repe	rs4924446			1.0 (pos.40573716)							A=0, G=77	A=0.0, G=1.0	0.00001	1229.33	77	A=0/0, G=14/6
chr6:35027927	C/C	T	C	SNV	ANKS1A	ANKS1A:exonic:NM 015245.2	1	100	13	missense	NM 01524	c.2081T>C	p.Leu694Ser	3.78	1	145	0		Ankyrin repe	rs820085	1.00E-04		1.0 (pos.35027927)			cytosol,ep	Arterial Occlusive Diseases:Arteriosclerosis:			T=0, C=32	T=0.0, C=1.0	0.00001	485.14	32	T=0/0, C=23/9
chr6:35056443	G/G	A	G	SNV	ANKS1A	ANKS1A:utr 3:NM 015245.2	1	100	24	utr 3	NM 01524	c.3462A>G	p.?	-0.82					Ankyrin repe	rs2005	0.0893	YES				cytosol,ep	Arterial Occlusive Diseases;Arteriosclerosis;			A=0, G=93	A=0.0, G=1.0	0.00001	1555.33	93	A=0/0, G=88/5
chr1:230825942	A/G	A	G	SNV	COG2	COG2:intronic:NM 007357.2	1	57.14		intronic	NM 00735	c.1934+43	p.?	0.39					COG (conserv	rs3000064	0.4328	YES	1.146E-1 (pos.230825942)		Component	ER to Golgi vesicle-mediated transport,Golgi membrane,				A=9, G=12	A=0.4286, G=0.5714	0.00001	109.102	21	A=7/2, G=8/4
chr11:69934085	A/A	G	A	SNV	ANO1	ANO1:exonic:NM 018043.5	1	100	2	synonymous	NM 01804	c.336G>A	p.(=)	-1.13				0.07(COSM50	Calcium-activ	rs10898112	0.2468	YES	4.032E-2;0.0 (pos.69934(	Wilms_tumou	Anoctamin	apical plasma membrane,calc		Crofelemer		G=0, A=67	G=0.0, A=1.0	0.00001	956.88	67	G=0/0, A=37/3
chr1:231057310	T/T	C	T	SNV	TTC13	TTC13:intronic:NM 024525.4	1	100		intronic	NM 02452	c.1815-60	p.?	0.35					Tetratricope	rs2275334		YES								C=0, T=76	C=0.0, T=1.0	0.00001	1209.6	76	C=0/0, T=43/3
chr11:70007311	G/G	A	G	SNV	ANO1	ANO1:exonic:NM 018043.5	1	100	17	synonymous	NM 01804	c.1623A>G	p.(=)	-0.01				0.32(COSM41	Calcium-activ	rs2276066	0.3398	YES	7.704E-1 (pos.70007311)	acute_myeloic	Anoctamin	apical plasma membrane,calc		Crofelemer		A=0, G=80	A=0.0, G=1.0	0.00001	1289.07	80	A=0/0, G=47/3
chr11:70007392	G/G	A	G	SNV	ANO1	ANO1:exonic:NM 018043.5	1	100	17	synonymous	NM 01804	c.1704A>G	p.(=)	-8.35				0.01(COSM44	Calcium-activ	rs2276067	0.3352	YES	7.699E-1 (pos.70007392)	acute_myeloic	Anoctamin	apical plasma membrane,calc		Crofelemer		A=0, G=84	A=0.0, G=1.0	0.00001	1370.17	84	A=0/0, G=41/4
chr1:232568293	G/G	A	G	SNV	SIPA1L2	SIPA1L2:intronic:NM 020808.4	1	100		intronic	NM 02080	c.4032-76	p.?	0.3					C-terminal dc	rs1547741		YES			SIPA1-like p	GTPase ac	Basal Ganglia Diseases;Brain Diseases;Centr			A=0, G=45	A=0.0, G=1.0	0.00001	747.392	45	A=0/0, G=20/2
chr1:232576998	T/A	T	A	SNV	SIPA1L2	SIPA1L2:intronic:NM 020808.4	1	45.45		intronic	NM 02080	c.3642+39	p.?	0.15					C-terminal dc	rs12036020,rs75173	0.033	YES	0.0 (pos.232576997),5.624E-1 (pos.2325		SIPA1-like p	GTPase ac	Basal Ganglia Diseases:Brain Diseases:Centr			T=12, A=10	T=0.5455, A=0.4545	0.00001	81.7621	22	T=12/0, A=6/4
chr11:26587133	C/C	T	C	SNV	ANO3,MUC15	MUC15:exonic:NM 001135091.1, ANO3:intronic:NM 03141	1	100	3	synonymous	NM 03141	c.1447+58	p.?, p.(=)	-0.41				0(COSM4416	Calcium-activ	rs293980,rs7475840	0.2597	YES	4.571E-1;0.0 (pos.26587	acute_myeloic	Anoctamin	Golgi lume	Brain Diseases:Central Nervous System Dise			T=0, C=84	T=0.0, C=1.0	0.00001	1370.17	84	T=0/0, C=23/6
chr11:22242729	C/C	T	C	SNV	ANO5	ANO5:exonic:NM 213599.2	1	100	5	synonymous	NM 21359	c.267T>C	p.(=)	-1.04				0.02(COSM39	Calcium-activ	rs4312063	0.2004	YES	5.576E-1 (pos.22242729)	acute_myeloic	Anoctamin	chloride tr	Bone Diseases;Bone Diseases,		Benign	T=0, C=47	T=0.0, C=1.0	0.00001	789.324	47	T=0/0, C=17/3
chr2:242163359	A/A	G	A	SNV	ANO7	ANO7:exonic:NM 001001891.3	1	100	24	missense	NM 00100	c.2734G>A	p.Glu912Lys	1	0.1	56	0	0.05(COSM41	Calcium-activ	rs7590653	0.2206	YES	5.78E-4 (pos.242163359)	neoplasm		calcium activated galactosylceramide scrambling,calciu				G=0, A=44	G=0.0, A=1.0	0.00001	726.558	44	G=0/0, A=32/1
chr1:23399932	T/T	C	T	SNV	KDM1A	KDM1A:intronic:NM 001009999.2	1	100		intronic	NM 00100	c.1734+36	p.?	-0.53					FAD binding c	rs967605	0.1709	YES	8.099E-1;0.0 (pos.23399932)		Lysine-spec	DNA repai	Bronchial Neoplasms:Carcinoma, Bronchoge			C=0, T=31	C=0.0, T=1.0	0.00001	465.814	31	C=0/0, T=16/1
chr19:17438642	G/G	A	G	SNV	ANO8	ANO8:exonic:NM 020959.2	1	100	14	synonymous	NM 02095	c.2274T>C	p.(=)	-0.96					Calcium-activ	rs8102944	0.2919	YES	2.611E-1 (pos.17438642)			cellular pr	Arthritis:Arthritis, Rheumatoid:Autoimmune			A=0, G=61	A=0.0, G=1.0	0.00001	918.908	61	A=0/0, G=22/3
chr1:234454405	C/C	A	C	SNV	SLC35F3	SLC35F3:intronic:NM 173508.3	1	100		intronic	NM 17350	c.955-92A	p.?	-0.66					EamA-like tra	rs657010						integral component of membrane,thiamine transport				A=0, C=21	A=0.0, C=1.0	0.00001	280.985	21	A=0/0, C=12/9
chr1:234454412	G/A	G	A	SNV	SLC35F3	SLC35F3:intronic:NM 173508.3	1	47.62		intronic	NM 17350	c.955-85G	p.?	-1.07					EamA-like tra	rs116179211						integral component of membrane,thiamine transport				G=11, A=10	G=0.5238, A=0.4762	0.00001	82.29	21	G=7/4, A=5/5
chr1:235394541	C/C	A	C	SNV	ARID4B	ARID4B:intronic:NM 016374.5	1	100		intronic	NM 01637	c.666-37T	p.?	0.02					ARID/BRIGHT	rs7518225,rs758507	0.4185	YES	2.696E-1;5.862E-4 (pos.235394541)		AT-rich inte	DNA bindi	Adenocarcinoma;Carcinoma;Carcinoma, Ade			A=0, C=47	A=0.0, C=1.0	0.00001	671.621	47	A=0/0, C=22/2
chr1:235582924	T/G	T	G	SNV	TBCE	TBCE:intronic:NM 001079515.2	1	51.85		intronic	NM 00107	c.460+48T	p.?	-0.07					CAP-Gly doma	rs13374091	0.0295	YES	4.877E-4 (pos.235582924)		Tubulin-spe	adult loco	Abnormalities, Multiple:Bone Diseases:Bone			T=26, G=28	T=0.4815, G=0.5185	0.00001	277.148	54	T=13/13, G=11
chr11:433387	G/G	A	G	SNV	ANO9	ANO9:exonic:NM 001012302.2	1	100	4	missense	NM 00101	c.277T>C	p.Phe93Leu	3.61	1	22	0	0(COSM4145	Calcium-activ	rs7395065,rs766579871			4.634E-4 (pos.433376),9.	malignant_melanoma.neo		calcium activated galactosylceramide scrambling,calciu				A=0, G=43	A=0.0, G=1.0	0.00001	705.844	43	A=0/0, G=26/1
chr1:235647631	T/T	G	T	SNV	B3GALNT2	B3GALNT2:intronic:NM 152490.4	1	100		intronic	NM 15249	c.555+7C>	p.?	-1.93				0(COSM5904	Galactosyltra	rs291387	0.4985	YES	8.609E-1 (pos.23564763	basal_cell_car	Beta-1,3-N-	Golgi mem	Cobblestone Lissencephaly;Congenital Abno			G=0, T=42	G=0.0, T=1.0	0.00001	685.22	42	G=0/0, T=20/2
chr4:165118652	T/T	C	T	SNV	ANP32C,MARCH1	MARCH1:intronic:NM 001166373.1, ANP32C:exonic:NM 01	1	100	1	missense	NM 00116	c.-112-858	p.?, p.Arg71Lys	3.47	0	26	0.57	0.1(COSM499	Leucine Rich	rs2288675	0.4941	YES	1.8E-1 (pos.165118652)	carcinoma,duc	Acidic leuci	MHC protein binding,antigen processing and presentatic				C=0, T=32	C=0.0, T=1.0	0.00001	485.141	32	C=0/0, T=4/28
chr1:235860340	T/T	C	T	SNV	LYST,MIR5096	MIR5096:intronic nc:NR 039973.1, LYST:intronic:NM 0000	1	100		intronic	NR 03997	c.10564+4	p.?	-1.5					Beige/BEACH	rs7541057	0.4907	YES	5.353E-2 (pos.235860340)		Lysosomal t	cytoplasm	Chediak-Higashi Syndrome:He		Benign	C=0, T=58	C=0.0, T=1.0	0.00001	867.074	58	C=0/0, T=30/2
chr1:235909485	C/C	T	C	SNV	LYST	LYST:intronic:NM 000081.3	1	100		intronic	NM 00008	c.7972+15	p.?	-0.03					Beige/BEACH	rs10926565		YES			Lysosomal t	cytoplasm	Chediak-Higashi Syndrome;Hematologic Dise			T=0, C=20	T=0.0, C=1.0	0.00001	263.784	20	T=0/0, C=13/7
chr4:165118796	A/A	G	A	SNV	ANP32C,MARCH1	MARCH1:intronic:NM 001166373.1, ANP32C:exonic:NM 01	1	100	1	missense	NM 00116	c.-112-859	p.?, p.Ala23Val	4.21	1	64	0.007		Leucine Rich	rs2288674	0.4936	YES	1.775E-1;0.0 (pos.165118796)		Acidic leuci	MHC protein binding,antigen processing and presentatic				G=0, A=57	G=0.0, A=1.0	0.00001	849.607	57	G=0/0, A=23/3
chr15:90349558	T/T	C	T	SNV	ANPEP	ANPEP:exonic:NM 001150.2	1	100	2	missense	NM 00115	c.257G>A	p.Arg86Gln	-0.4	0.39	43	0	0.02(COSM49	ERAP1-like C-	rs25653	0.4749	YES	4.66E-1 (pos.90349558)	rhabdomyosar	Alanyl (men	aminopep	Female Urogenital I	Ezetimibe,Icatibant		C=0, T=25	C=0.0, T=1.0	0.00001	352.765	25	C=0/0, T=8/17
chr4:80905990	G/G	C	G	SNV	ANTXR2	ANTXR2:exonic:NM 001145794.1	1	100	13	missense	NM 00114	c.1069G>C	p.Ala357Pro	2.24	1	27	0	0.01(COSM37	Anthrax rece	rs12647691,rs38667	0.2926	YES	1.143E-2;0.0;0.0 (pos.80	adenocarcino	Anthrax tox	endoplasr	Ankylosis;Arthritis;Bone Disea		Benign	C=0, G=76	C=0.0, G=1.0	0.00001	1209.6	76	C=0/0, G=18/5
chr4:169086441	G/G	A	G	SNV	ANXA10	ANXA10:exonic:NM 007193.4	1	100	6	synonymous	NM 00719	c.444A>G	p.(=)	-1.26				0.03(COSM14	Annexin	rs4405979	0.4164	YES	2.296E-1 (pos.16908644	adenocarcino	Annexin A1(	calcium ion binding,calcium-dependent phospholipid bi				A=0, G=20	A=0.0, G=1.0	0.00001	263.786	20	A=0/0, G=7/13
chr8:124693444	T/T	C	T	SNV	ANXA13	ANXA13:utr 3:NM 001003954.2	1	100	12	utr 3	NM 00100	c.1110G>A	p.?	-0.6					Annexin	rs3739283	0.2793	YES	4.138E-1 (pos.124693444)			apical plasma membrane,basolateral plasma membran				C=0, T=28	C=0.0, T=1.0	0.00001	413.152	28	C=0/0, T=13/1
chr1:236706352	C/C	A	C	SNV	LGALS8	LGALS8:intronic:NM 006499.4	1	100		intronic	NM 00649	c.675+12A	p.?	0.22					Galactoside-	rs2758174	0.3187	YES	4.912E-1 (pos.236706352)		Lectin, gala	carbohydrate binding,cellular response to virus,cytopla				A=0, C=20	A=0.0, C=1.0	0.00001	263.786	20	A=0/0, C=6/14
chr1:236706780	T/T	C	T	SNV	LGALS8	LGALS8:intronic:NM 006499.4	1	100		intronic	NM 00649	c.676-80C	p.?	-0.62					Galactoside-	rs2243527		YES	6.947E-1 (pos.236706780)		Lectin, gala	carbohydrate binding,cellular response to virus,cytopla				C=0, T=70	C=0.0, T=1.0	0.00001	1090.58	70	C=0/0, T=45/2
chr1:236707007	T/T	C	T	SNV	LGALS8	LGALS8:intronic:NM 006499.4	1	100		intronic	NM 00649	c.764+59C	p.?	-1.18					Galactoside-	rs2799410		YES			Lectin, gala	carbohydrate binding,cellular response to virus,cytopla				C=0, T=66	C=0.0, T=1.0	0.00001	1015.3	66	C=0/0, T=30/3
chr1:236708224	C/C	A	C	SNV	LGALS8	LGALS8:intronic:NM 006499.4	1	100		intronic	NM 00649	c.930+9A>	p.?	-0.55				0.19(COSM39	Galactoside-	rs2244063,rs751465	0.3177	YES	0.0 (pos.236708223),4.9	acute_myeloic	Lectin, gala	carbohydrate binding,cellular response to virus,cytopla				A=0, C=115	A=0.0, C=1.0	0.00001	1830.96	115	A=0/0, C=48/6
chr8:124749610	T/T	C	T	SNV	ANXA13	ANXA13:utr 5:NM 001003954.2	1	100	1	utr 5	NM 00100	c.-36G>A	p.?	-0.48					Annexin	rs6470179	0.0772	YES	9.987E-1 (pos.124749610)			apical plasma membrane,basolateral plasma membran				C=0, T=20	C=0.0, T=1.0	0.00001	263.782	20	C=0/0, T=12/8
chr22:29727866	T/T	C	T	SNV	AP1B1	AP1B1:exonic:NM 001127.3	1	100	18	synonymous	NM 00112	c.2349G>A	p.(=)	-5.1				0.01(COSM37	Adaptin C-ter	rs174765	0.4456	YES	6.915E-1;0.0 (pos.29727	adenocarcino	Adaptor-rel	Golgi apparatus,Golgi membrane,antigen processing an				C=0, T=24	C=0.0, T=1.0	0.00001	334.789	24	C=0/0, T=14/1
chr1:236719259	A/A	G	A	SNV	HEATR1	HEATR1:intronic:NM 018072.5	1	100		intronic	NM 01807	c.5515-20	p.?	1.88					BP28CT (NUC	rs1885534	0.3772	YES	3.99E-1 (pos.236719259)			90S preribosome,RNA binding,fibrillar center,maturatio				G=0, A=85	G=0.0, A=1.0	0.00001	1388.95	85	G=0/0, A=57/2
chr22:29727886	C/C	T	C	SNV	AP1B1	AP1B1:exonic:NM 001127.3	1	100	18	missense	NM 00112	c.2329A>G	p.Thr777Ala	2.48	1	58	0	0.09(COSM41	Adaptin C-ter	rs2857465	1.00E-04		9.998E-1 (pos.29727886)	neoplasm	Adaptor-rel	Golgi apparatus,Golgi membrane,antigen processing an				T=0, C=23	T=0.0, C=1.0	0.00001	314.079	23	T=0/0, C=12/1
chr22:29755888	T/C	T	C	SNV	AP1B1	AP1B1:exonic:NM 001127.3	1	46.88	4	synonymous	NM 00112	c.204A>G	p.(=)	1.95				0.3(COSM375	Adaptin C-ter	rs2072051	0.4533	YES	7.146E-1 (pos.29755888)	adenocarcino	Adaptor-rel	Golgi apparatus,Golgi membrane,antigen processing an				T=34, C=30	T=0.5313, C=0.4688	0.00001	288.133	64	T=9/25, C=17/
chr19:2138654	T/G	T	G	SNV	AP3D1	AP3D1:exonic:NM 001261826.1	1	58.33	2	synonymous	NM 00126	c.156A>C	p.(=)	0.77				0.36(COSM41	AP-3 complex	rs25672	0.4269	YES	2.057E-1 (pos.2138654)	ductal_carcinc	Adaptor-rel	AP-3 adap	Albinism:Amino Acid Metabolism, Inborn Errc			T=10, G=14	T=0.4167, G=0.5833	0.00001	135.337	24	T=4/6, G=4/10
chr1:236746275	C/C	G	C	SNV	HEATR1	HEATR1:intronic:NM 018072.5	1	100		intronic	NM 01807	c.2425+38	p.?	-1.3					BP28CT (NUC	rs2794769,rs563091	0.3109	YES	0.0 (pos.236746264),6.298E-1 (pos.236746275)			90S preribosome,RNA binding,fibrillar center,maturatio				G=0, C=22	G=0.0, C=1.0	0.00001	298.892	22	G=0/0, C=12/1
chr1:114424601	C/T	C	T	SNV	AP4B1-AS1,BCL2L15	AP4B1-AS1:intronic nc:NR 037864.1, BCL2L15:exonic:NM (	1	98.72	3	missense	NR 03786	c.268G>A	p.Asp90Asn	-1.53	1	23	0.001			rs1217381	1.00E-04		1.0 (pos.114424601)			apoptotic process,cytosol,nucleus,protein binding,regul				C=1, T=77	C=0.0128, T=0.9872	0.00001	1213.92	78	C=0/1, T=33/4
chr1:236749817	T/T	C	T	SNV	HEATR1	HEATR1:intronic:NM 018072.5	1	100		intronic	NM 01807	c.1716-65	p.?	-0.31					BP28CT (NUC	rs2799420		YES				90S preribosome,RNA binding,fibrillar center,maturatio				C=0, T=39	C=0.0, T=1.0	0.00001	616.245	39	C=0/0, T=16/2
chr1:236759060	C/C	T	C	SNV	HEATR1	HEATR1:intronic:NM 018072.5	1	100		intronic	NM 01807	c.956+73A	p.?	0.76					BP28CT (NUC	rs2794750		YES				90S preribosome,RNA binding,fibrillar center,maturatio				T=0, C=42	T=0.0, C=1.0	0.00001	685.139	42	T=0/0, C=13/2
chr14:57755564	A/A	G	A	SNV	AP5M1	AP5M1:exonic:NM 018229.3	1	100	8	missense	NM 01822	c.1435G>A	p.Ala479Thr	7.05	0	58	0.773	0.99(COSM37	Adaptor com	rs35759976	0.071	YES	1.156E-4 (pos.57755564)	adenocarcino	Adaptor-rel	AP-type m	Adenocarcinoma;Carcinoma;Carcinoma, Ren			G=0, A=46	G=0.0, A=1.0	0.00001	752.181	46	G=0/0, A=16/3
chr19:3752874	G/G	A	G	SNV	APBA3,MIR1268A	MIR1268A:intronic nc:NR 031672.1, APBA3:exonic:NM 004	1	100	7	missense	NR 03167	c.1126T>C	p.Cys376Arg	0.93	0.07	180	0	0.07(COSM32	PDZ domain (	rs8102086	0.4483	YES	6.541E-1;0.0 (pos.37528	adenocarcino	Amyloid bet	amyloid-beta binding,chemical synaptic transmission,e				A=0, G=25	A=0.0, G=1.0	0.00001	356.859	25	A=0/0, G=18/7
chr1:236883564	A/A	G	A	SNV	ACTN2	ACTN2:intronic:NM 001103.3	1	100		intronic	NM 00110	c.448+73G	p.?	-0.49					CAMSAP CH d	rs1341862		YES			Actinin, alp	FATZ bindi	Aortic Stenosis, Subvalvular:Aortic Valve Ster			G=0, A=125	G=0.0, A=1.0	0.00001	2034.05	125	G=0/0, A=34/9
chr1:236902594	G/G	C	G	SNV	ACTN2	ACTN2:intronic:NM 001103.3	1	100		intronic	NM 00110	c.877-8C>	p.?	1.53					CAMSAP CH d	rs2288601	0.2237	YES	7.994E-1;0.0 (pos.236902594)		Actinin, alp	FATZ bindi	Aortic Stenosis, Subvalvular:Ac		Benign/Likely	C=0, G=39	C=0.0, G=1.0	0.00001	623.98	39	C=0/0, G=19/2
chr1:236902865	A/C	A	C	SNV	ACTN2	ACTN2:intronic:NM 001103.3	1	57.58		intronic	NM 00110	c.1107+33	p.?	0.5					CAMSAP CH d	rs2288602	0.343	YES	5.204E-1 (pos.236902865)		Actinin, alp	FATZ bindi	Aortic Stenosis, Subvalvular;Aortic Valve Ster			A=14, C=19	A=0.4242, C=0.5758	0.00001	204.394	33	A=11/3, C=9/1
chr1:236902955	C/C	T	C	SNV	ACTN2	ACTN2:intronic:NM 001103.3	1	100		intronic	NM 00110	c.1107+12	p.?	-0.37					CAMSAP CH d	rs7544174		YES			Actinin, alp	FATZ bindi	Aortic Stenosis, Subvalvular;Aortic Valve Ster			T=0, C=32	T=0.0, C=1.0	0.00001	485.126	32	T=0/0, C=18/1
chr1:23710805	G/G	A	G	SNV	TCEA3	TCEA3:intronic:NM 003196.2	1	100		intronic	NM 00319	c.1038+34	p.?	-0.1					TFIIS helical b	rs605468	0.0825	YES	9.177E-1 (pos.23710805)		Transcriptic	DNA binding,nucleic acid binding,nucleus,regulation of t				A=0, G=80	A=0.0, G=1.0	0.00001	1289.34	80	A=0/0, G=21/5
chr1:23735241	T/T	G	T	SNV	TCEA3	TCEA3:intronic:NM 003196.2	1	100		intronic	NM 00319	c.381-22C	p.?	1.64					TFIIS helical b	rs1198423	0.1283	YES	8.998E-1 (pos.23735241)		Transcriptic	DNA binding,nucleic acid binding,nucleus,regulation of t				G=0, T=25	G=0.0, T=1.0	0.00001	353.019	25	G=0/0, T=10/1
chr1:23765799	GC/G	GC	G	INDEL	ASAP3	ASAP3:intronic:NM 017707.3	1	50		intronic	NM 01770	c.945-76G	p.?	1.11, -0.72					Ankyrin repeat,Ankyrin repeats (3 copies),Ankyrin repeats (many copies),BAR domain of APPL family,						ARF GTPase	GTPase activator activity,cell migration,cytosol,focal ad				GC=11, G=1	GC=0.5, G=0.5	0.00001	77.2508	22	GC=1/10, G=8/
chr1:237670140	A/G	A	G	SNV	RYR2	RYR2:intronic:NM 001035.2	1	50		intronic	NM 00103	c.2718+26	p.?	0.33					EF-hand dom	rs2618702	0.3422	YES	7.145E-1 (pos.237670140)		Ryanodine r	BMP signa	ACMG Recommend	Calcium C	Benign	A=11, G=11	A=0.5, G=0.5	0.00001	94.6067	22	A=6/5, G=4/7
chr3:49721532	A/A	G	A	SNV	APEH,MST1	MST1:exonic:NM 020998.3, APEH:downstream:NM 00164C	1	100	18	missense	NM 00164	c.*757G>A	p.?, p.Arg703Cys	1.07	0.01	180	0.727	0.65(COSM14	Kringle doma	rs3197999	0.2936	YES	5.194E-2 (pos.49721532)	adenocarcino	Macrophag	RNA bindir	Bile Duct Diseases;Biliary Tract Diseases;Chc			G=0, A=20	G=0.0, A=1.0	0.00001	263.786	20	G=0/0, A=9/11
chr1:237765470	A/T	A	T	SNV	RYR2	RYR2:intronic:NM 001035.2	1	56		intronic	NM 00103	c.4683+59	p.?	0.45					EF-hand dom	rs791541	0.4598	YES			Ryanodine r	BMP signa	ACMG Recommend	Calcium Citrate,Calcium		A=11, T=14	A=0.44, T=0.56	0.00001	128.801	25	A=3/8, T=7/7
chr11:34937813	A/A	G	A	SNV	APIP,PDHX	PDHX:utr 5:NM 003477.2, APIP:exonic:NM 015957.3	1	100	1	missense	NM 01595	c.19C>T, c.	p.Arg7Trp, p.?	0.29	0.02	101	0.74	0.07(COSM41	2-oxoacid del	rs2956114	0.3546	YES	7.498E-1 (pos.34937813)	neoplasm	APAF1-inter	L-methion	Acid-Base Imbalance:Acidosis:		Benign	G=0, A=31	G=0.0, A=1.0	0.00001	465.814	31	G=0/0, A=6/25
chr1:237893674	T/T	C	T	SNV	RYR2	RYR2:intronic:NM 001035.2	1	100		intronic	NM 00103	c.10935+1	p.?	-0.02					EF-hand dom	rs2797445	2.00E-04		1.0 (pos.237893674)		Ryanodine r	BMP signa	ACMG Recommend	Calcium C	Benign	C=0, T=55	C=0.0, T=1.0	0.00001	814.898	55	C=0/0, T=22/3
chr1:237951451	G/G	A	G	SNV	RYR2	RYR2:intronic:NM 001035.2	1	100		intronic	NM 00103	c.13476+1	p.?	-0.18					EF-hand dom	rs2256242	0.4557	YES	6.762E-1 (pos.237951451)		Ryanodine r	BMP signa	ACMG Recommend	Calcium C	Benign	A=0, G=39	A=0.0, G=1.0	0.00001	623.98	39	A=0/0, G=11/2
chr1:237957146	A/A	G	A	SNV	RYR2	RYR2:intronic:NM 001035.2	1	100		intronic	NM 00103	c.13783-2	p.?	-1.33					EF-hand dom	rs790902	0.3421	YES	9.371E-1 (pos.237957146)		Ryanodine r	BMP signa	ACMG Recommend	Calcium C	Benign	G=0, A=167	G=0.0, A=1.0	0.00001	2719.1	167	G=0/0, A=69/9
chr1:237965094	T/T	G	T	SNV	RYR2	RYR2:intronic:NM 001035.2	1	100		intronic	NM 00103	c.14091-6	p.?	-0.62					EF-hand dom	rs2794820		YES			Ryanodine r	BMP signa	ACMG Recommend	Calcium Citrate,Calcium		G=0, T=23	G=0.0, T=1.0	0.00001	316.653	23	G=0/0, T=13/1
chr1:237965131	A/A	G	A	SNV	RYR2	RYR2:intronic:NM 001035.2	1	100		intronic	NM 00103	c.14091-2	p.?	0.32					EF-hand dom	rs761038693,rs9428	0.4032	YES	0.0 (pos.237965129),6.195E-1 (pos.2379		Ryanodine r	BMP signa	ACMG Recommend	Calcium C	Benign	G=0, A=23	G=0.0, A=1.0	0.00001	316.612	23	G=0/0, A=13/1
chr20:24973363	A/G	A	G	SNV	APMAP	APMAP:utr 5:NM 020531.2	1	46.88	1	utr 5	NM 02053	c.-39T>C	p.?	-2.9					SMP-30/Gluc	rs11087492	0.0801	YES	5E-3 (pos.24973363)		Adipocyte p	arylesterase activity,biological_process,biosynthetic pr				A=17, G=15	A=0.5313, G=0.4688	0.00001	143.727	32	A=10/7, G=10/
chr11:116662579	T/T	C	T	SNV	APOA5	APOA5:utr 5:NM 001166598.1	1	100	2	utr 5	NM 00116	c.-3G>A	p.?	-0.03					Apolipoprote	rs651821	0.0687	YES	7.203E-1 (pos.116662579)		Apolipoprot	acylglycer	Arterial Occlusive Diseases;Arteriosclerosis;			C=0, T=34	C=0.0, T=1.0	0.00001	528.536	34	C=0/0, T=11/2
chr1:240421383	A/A	G	A	SNV	FMN2	FMN2:intronic:NM 020066.4	1	100		intronic	NM 02006	c.4153+51	p.?	0.48					Formin Homc	rs10926181	0.3713	YES	8.356E-1 (pos.240421383)		Formin 2	actin bind	Adenocarcinoma;Alzheimer Disease;Brain Di			G=0, A=44	G=0.0, A=1.0	0.00001	726.559	44	G=0/0, A=14/3
chr1:240975213	T/C	T	C	SNV	RGS7	RGS7:intronic:NM 002924.6	1	53.49		intronic	NM 00292	c.1082+5A	p.?	0.45				0.04(COSM14	Domain foun	rs2275742	0.4234	YES	4.555E-1 (pos.24097521	acute_myeloic	Regulator o	G-protein	Diabetes Mellitus:Diabetes Mellitus, Type 2:			T=13, C=23	T=0.3611, C=0.6389	0.00001	363.282	43	T=4/9, C=16/7
chr2:21225281	T/T	C	T	SNV	APOB	APOB:exonic:NM 000384.2	1	100	29	missense	NM 00038	c.13013G>	p.Ser4338Asn	0.64	1	46	0	0.02(COSM40	Apolipoprote	rs1042034	0.2176	YES	2.804E-1 (pos.21225281)	acute_myeloic	Apolipoprot	artery mo	ACMG Recommendations...:At		Benign/Likely	C=0, T=33	C=0.0, T=1.0	0.00001	504.598	33	C=0/0, T=17/1
chr1:241667244	T/T	G	T	SNV	FH	FH:intronic:NM 000143.3	1	100		intronic	NM 00014	c.1108+98	p.?	-0.63					Fumarase C C	rs4659607		YES			Fumarate h	cytoplasm	Congenital, Hereditary, and Neonatal Diseas			G=0, T=30	G=0.0, T=1.0	0.00001	446.639	30	G=0/0, T=20/1
chr1:241847018	T/T	C	T	SNV	WDR64	WDR64:intronic:NM 144625.4	1	100		intronic	NM 14462	c.809+15C	p.?	-0.95					WD domain, (	rs10802979		YES	5.786E-1;0.0 (pos.241847018)							C=0, T=33	C=0.0, T=1.0	0.00001	504.593	33	C=0/0, T=5/28
chr1:241933876	G/G	A	G	SNV	WDR64	WDR64:intronic:NM 144625.4	1	100		intronic	NM 14462	c.2154-47	p.?	-0.8					WD domain, (	rs11809239		YES	5.997E-1 (pos.241933876)							A=0, G=51	A=0.0, G=1.0	0.00001	754.584	51	A=0/0, G=16/3
chr2:21235475	C/C	T	C	SNV	APOB	APOB:exonic:NM 000384.2	1	100	26	missense	NM 00038	c.4265A>G	p.Tyr1422Cys	0.57	0.18	194	0		Apolipoprote	rs568413	2.00E-04		1.0 (pos.21235475)		Apolipoprot	artery mo	ACMG Recommendations...:Ataxia:Avitaminc			T=0, C=165	T=0.0, C=1.0	0.00001	2679.21	165	T=0/0, C=81/8

chr2:21232803	T/C	T	C	SNV	APOB	APOB:exonic:NM_000384.2	1	95.83	26	missense	NM_00038	c.6937A>G	p.Ile2313Val	-0.2	1	29	0.002		Apolipoprote	rs386643884,rs5845	6.00E-04		9.994E-1;1.156E-4 (pos.21232803)		Apolipoprot	artery mo	ACMG Recommendations...;Ataxia;Avitaminc		T=1, C=23	T=0.0417, C=0.9583	0.00001	306.709	24	T=1/0, C=11/1
chr12:7805236	G/G	C	G	SNV	APOBEC1	APOBEC1:exonic:NM_001644.4	1	100	3	missense	NM_00164	c.240G>C	p.Met80Ile	-0.22	0.68	10	0	0(COSM3736	APOBEC-like (	rs2302515	0.1095	YES	5.671E-1 (pos.7805236)	malignant_me	Apolipoprot	AU-rich ele	Nutritional and Metabolic Diseases		C=0, G=83	C=0.0, G=1.0	0.00001	1349.87	83	C=0/0, G=42/4
chr22:39382079	A/A	C	A	SNV	APOBEC3A_B,APOBEC3B	APOBEC3A_B:intronic:NM_001193289.1, APOBEC3B:exonic:I	1	100	3	missense	NM_00119	c.586-635	p.?, p.Thr146Lys	-0.84	1	78	0		APOBEC-like (	rs5995649	0.0439	YES	9.702E-1 (pos.39382079)		Apolipoprot	DNA cytos	Adenocarcinoma;Autoimmune Diseases;Brea		C=0, A=34	C=0.0, A=1.0	0.00001	524.186	34	C=0/0, A=18/1
chr22:39477123	C/C	T	C	SNV	APOBEC3G	APOBEC3G:exonic:NM_021822.3	1	100	3	synonymous	NM_02182	c.357T>C	p.(=)	-2.41				0(COSM149298),0(COSM37		rs5757465	0.4348	YES	2.304E-1 (pos.39477123)	acute_myeloic	Apolipoprot	DNA cytosine deamination,P-body,RNA binding,apolipop			T=0, C=38	T=0.0, C=1.0	0.00001	603.783	38	T=0/0, C=19/1
chr16:28508716	C/C	T	C	SNV	APOBR	APOBR:exonic:NM_018690.3	1	100	2	missense	NM_01869	c.2354T>C	p.Val785Ala	-1.06	1	64	0		Nuclear pore	rs40832	6.00E-04		9.999E-1 (pos.28508716)		Apolipoprot	cholesterol metabolic process,chylomicron,lipid transp			T=0, C=77	T=0.0, C=1.0	0.00001	1229.41	77	T=0/0, C=31/4
chr1:2422614	A/A	G	A	SNV	PLCH2	PLCH2:intronic:NM_014638.3	1	100		intronic	NM_01463	c.1516-17	p.?	-1.83					C2 domain.EF	rs2477699	0.2752	YES	9.139E-1 (pos.2422614)		Phospholip	biological_	Mental Disorders;Schizophrenia;Schizophren		G=0, A=22	G=0.0, A=1.0	0.00001	298.891	22	G=0/0, A=11/1
chr1:242511563	A/A	ACAAAGA	A	INDEL	PLD5	PLD5:intronic:NM_152666.2	6	100		intronic	NM_15266	c.190-19T	p.?	1.44, -0.69, 0.37, 0.57, 0.36, 1.14, 0.5					PLD-like dom	rs112794932,rs60071576,rs756833029,rs7			0.0 (pos.242511560),0.0 (pos.242511563),0.0 (pos.2			catalytic a	Autistic Disorder;Child Development Disorde		ACAAAGA=(	ACAAAGA=0.0, A=1.0	0.00001	297.945	22	ACAAAGA=0/0,
chr11:116701535	C/C	T	C	SNV	APOC3	APOC3:exonic:NM_000040.1	1	100	3	synonymous	NM_00004	c.102T>C	p.(=)	-0.48				0.01(COSM41	Apolipoprote	rs4520	0.2597	YES	3.968E-1 (pos.11670153	neoplasm	Apolipoprot	G-protein	Arterial Occlusive Diseases;Arteriosclerosis;		T=0, C=33	T=0.0, C=1.0	0.00001	504.567	33	T=0/0, C=12/2
chr11:116703640	G/C	G	C	SNV	APOC3	APOC3:utr_3:NM_000040.1	1	95.45	4	utr_3	NM_00004	c.340G>C	p.?	-2.17					Apolipoprote	rs5128	0.0942	YES	6.939E-1 (pos.116703640)		Apolipoprot	G-protein	Arterial Occlusive Diseases;Arteriosclerosis;		G=1, C=21	G=0.0455, C=0.9545	0.00001	271.51	22	G=1/0, C=11/1
chr1:24394424	A/A	G	A	SNV	MYOM3	MYOM3:intronic:NM_152372.3	1	100		intronic	NM_15237	c.3355+36	p.?	0.65					Fibronectin t	rs7542151	0.1382	YES	1.943E-1 (pos.24394424)		Myomesin 3	M band,Z disc,actin filament binding,actin filament orga			G=0, A=34	G=0.0, A=1.0	0.00001	524.186	34	G=0/0, A=29/5
chr17:64208229	A/A	T	A	SNV	APOH	APOH:utr_3:NM_000042.2	1	100	8	utr_3	NM_00004	c.1060A>T	p.?	-1.15					Beta-2-glycop	rs190137879	0.0069		0.0;0.0 (pos.64208229)		Apolipoprot	blood coa	Cardiovascular Dise	LJP 1082	T=0, A=41	T=0.0, A=1.0	0.00001	667.298	41	T=0/0, A=24/1
chr22:36661536	A/A	C	A	SNV	APOL1	APOL1:exonic:NM_001136540.1	1	100	6	synonymous	NM_00113	c.654C>A	p.(=)	-1.86					Apolipoprote	rs136174	0.2024	YES	8.35E-1;0.0 (pos.36661536)		Apolipoprot	blood mic	Mental Disorders;Schizophrenia;Schizophren		C=0, A=28	C=0.0, A=1.0	0.00001	408.718	28	C=0/0, A=14/1
chr1:246712033	G/G	A	G	SNV	TFB2M	TFB2M:intronic:NM_022366.2	1	100		intronic	NM_02236	c.796-124	p.?	-0.14					Ribosomal RI	rs1890160		YES			Transcriptic	RNA binding,cell junction,mitochondrial matrix,mitocho			A=0, G=74	A=0.0, G=1.0	0.00001	1170.13	74	A=0/0, G=39/3
chr1:246714631	TG/AA	TG	AA	MNV	TFB2M	TFB2M:intronic:NM_022366.2	2	57.14		intronic	NM_02236	c.706-27C	p.?	-0.52, 0.74					Ribosomal RI	rs35073704,rs75477023,rs7627		YES	0.0 (pos.246714630),3.218E-1;0.0 (pos.2		Transcriptic	RNA binding,cell junction,mitochondrial matrix,mitocho			TG=18, AA=	TG=0.4286, AA=0.57	0.00001	279.086	42	TG=7/11, AA=1
chr1:246714636	T/A	T	A	SNV	TFB2M	TFB2M:intronic:NM_022366.2	1	57.14		intronic	NM_02236	c.706-32A	p.?	-0.19					Ribosomal RI	rs34850511,rs76273	0.4663	YES	0.0 (pos.246714630),3.223E-1 (pos.2467		Transcriptic	RNA binding,cell junction,mitochondrial matrix,mitocho			T=18, A=24	T=0.4286, A=0.5714	0.00001	279.086	42	T=7/11, A=13/
chr1:246903654	C/C	G	C	SNV	SCCPDH	SCCPDH:intronic:NM_016002.2	1	100		intronic	NM_01600	c.514+51G	p.?	-2.17					Saccharopine	rs3007322	0.3977	YES	2.433E-1;0.0;0.0 (pos.246903654)			extracellular region,lipid droplet,membrane,midbody,m			G=0, C=25	G=0.0, C=1.0	0.00001	352.99	25	G=0/0, C=8/17
chr1:246929483	G/G	A	G	SNV	SCCPDH	SCCPDH:intronic:NM_016002.2	1	100		intronic	NM_01600	c.1184+42	p.?	-2.73					Saccharopine	rs12144408	0.4323	YES	2.444E-1 (pos.246929483)			extracellular region,lipid droplet,membrane,midbody,m			A=0, G=140	A=0.0, G=1.0	0.00001	2340.88	140	A=0/0, G=48/9
chr22:36661566	A/A	G	A	SNV	APOL1	APOL1:exonic:NM_001136540.1	1	100	6	missense	NM_00113	c.684G>A	p.Met228Ile	0.25	0.28	10	0.001	0.03(COSM41	Apolipoprote	rs136175	0.2022	YES	8.35E-1 (pos.36661566)	neoplasm	Apolipoprot	blood mic	Mental Disorders;Schizophrenia;Schizophren		G=0, A=109	G=0.0, A=1.0	0.00001	1718.93	109	G=0/0, A=48/6
chr22:36661646	A/A	G	A	SNV	APOL1	APOL1:exonic:NM_001136540.1	1	100	6	missense	NM_00113	c.764G>A	p.Arg255Lys	-4.57	1	26	0		Apolipoprote	rs136176	0.2022	YES	8.339E-1 (pos.36661646)		Apolipoprot	blood mic	Mental Disorders;Schizophrenia;Schizophren		G=0, A=84	G=0.0, A=1.0	0.00001	1369.93	84	G=0/0, A=35/4
chr22:36661842	A/A	G	A	SNV	APOL1	APOL1:exonic:NM_001136540.1	1	100	6	synonymous	NM_00113	c.960G>A	p.(=)	-0.47					Apolipoprote	rs136177	0.2027	YES	8.346E-1 (pos.36661842)		Apolipoprot	blood mic	Mental Disorders;Schizophrenia;Schizophren		G=0, A=70	G=0.0, A=1.0	0.00001	1092.09	70	G=0/0, A=41/2
chr22:36623731	C/C	T	C	SNV	APOL2	APOL2:exonic:NM_145637.2	1	100	6	missense	NM_14563	c.733A>G	p.Ile245Val	-0.17	1	29	0	0.02(COSM45	Apolipoprote	rs132760,rs776634268			0.0 (pos.36623730),1.0 (	squamous_cel	Apolipoprot	acute-pha	Cocaine-Related Disorders;Marijuana Abuse		T=0, C=40	T=0.0, C=1.0	0.00001	644.286	40	T=0/0, C=11/2
chr22:36537500	T/T	A	T	SNV	APOL3	APOL3:exonic:NM_145640.2	1	100	3	synonymous	NM_14564	c.957T>A	p.(=)	-0.19				0.02(COSM14	Apolipoprote	rs132618	0.4855	YES	3.275E-1 (pos.36537500)	adenocarcino	Apolipoprot	cytoplasm,extracellular region,inflammatory response,I			A=0, T=58	A=0.0, T=1.0	0.00001	867.385	58	A=0/0, T=29/2
chr22:36556823	T/T	G	T	SNV	APOL3	APOL3:exonic:NM_145640.2	1	100	1	missense	NM_14564	c.117C>A	p.Ser39Arg	-0.71	0	110	0.976	0.07(COSM50	Apolipoprote	rs132653	0.1736	YES	9.76E-1 (pos.36556823)	ductal_carcin	Apolipoprot	cytoplasm,extracellular region,inflammatory response,I			G=0, T=24	G=0.0, T=1.0	0.00001	334.786	24	G=0/0, T=14/1
chr22:36587704	C/C	T	C	SNV	APOL4	APOL4:exonic:NM_145660.2	1	100	6	missense	NM_14566	c.473A>G	p.Met158Val	-2.02		21		0(COSM1492	Apolipoprote	rs132700	0.2786	YES	1.106E-1 (pos.36587704)	adenocarcino	Apolipoprot	cellular_c	Mental Disorders;Schizophrenia;Schizophren		T=0, C=81	T=0.0, C=1.0	0.00001	1309.46	81	T=0/0, C=28/5
chrX:23858452	A/A	G	A	SNV	APOO	APOO:utr_3:NM_024122.4	1	100	8	utr_3	NM_02412	c.604C>T	p.?	0.34					Apolipoprote	rs768247035,rs8680	0.2939	YES	0.0 (pos.23858448),6.251E-1 (pos.23858		Apolipoprot	Golgi membrane,MICOS complex,cristae formation,cyto			G=0, A=40	G=0.0, A=1.0	0.00001	644.28	40	G=0/0, A=21/1
chr1:24779855	T/C	T	C	SNV	NIPAL3	NIPAL3:intronic:NM_020448.4	1	97.5		intronic	NM_02044	c.541-43T	p.?	-0.84					Magnesium t	rs2272934	0.2633	YES	4.806E-1 (pos.24779855)			integral cc	Genital Diseases, Male;Genital Neoplasms, I		T=1, C=39	T=0.025, C=0.975	0.00001	602.464	40	T=0/1, C=16/2
chr1:247835352	T/T	C	T	SNV	OR13G1	OR13G1:downstream:NM_001005487.1	1	100		intronic	NM_00100	c.*68G>A	p.?	-0.22						rs1151641		YES			Olfactory re	G-protein coupled receptor activity,G-protein coupled re			C=0, T=31	C=0.0, T=1.0	0.00001	465.819	31	C=0/0, T=18/1
chr12:105568122	A/A	G	A	SNV	APPL2	APPL2:exonic:NM_001251904.1	1	100	21	synonymous	NM_00125	c.1983C>T	p.(=)	0.74					BAR domain (	rs935241	0.1706	YES	9.994E-1 (pos.105568122)		Adaptor prc	NuRD com	Autoimmune Diseases;Female Urogenital Dis		G=0, A=58	G=0.0, A=1.0	0.00001	867.355	58	G=0/0, A=32/2
chr12:105568176	A/A	G	A	SNV	APPL2	APPL2:exonic:NM_001251904.1	1	100	21	synonymous	NM_00125	c.1929C>T	p.(=)	-0.02				0.1(COSM381	BAR domain (	rs935240	0.1706	YES	9.994E-1 (pos.10556817(	adenocarcino	Adaptor prc	NuRD com	Autoimmune Diseases;Female Urogenital Dis		G=0, A=51	G=0.0, A=1.0	0.00001	752.266	51	G=0/0, A=26/2
chr9:33447424	G/G	C	G	SNV	AQP3	AQP3:exonic:NM_004925.4	1	100	1	synonymous	NM_00492	c.105G>C	p.(=)	-0.08					Major intrins	rs591810	0.2844	YES	7.486E-1;0.0 (pos.33447424)		Aquaporin-3	basolatera	Adnexal Diseases;Carcinoma;Carcinoma, Sq		C=0, G=23	C=0.0, G=1.0	0.00001	316.747	23	C=0/0, G=14/9
chr15:58476281	G/G	A	G	SNV	AQP9	AQP9:exonic:NM_020980.4	1	100	6	missense	NM_02098	c.835A>G	p.Thr279Ala	-0.06	1	58	0		Major intrins	rs1867380	0.0428	YES	8.418E-1 (pos.58476281)		Aquaporin 9	amine tra	Arsenic Poisoning;Arthritis;Arthritis, Juvenile		A=0, G=162	A=0.0, G=1.0	0.00001	2619.57	162	A=0/0, G=101/
chr4:36081878	T/T	C	T	SNV	ARAP2	ARAP2:exonic:NM_015230.3	1	100	31	missense	NM_01523	c.4568G>A	p.Arg1523Gln	1.99	1	43	0		PH domain,P	rs4833069	0.0105		1.0 (pos.36081878)		ArfGAP with	GTPase ac	Amyotrophic Lateral Sclerosis;Central Nervo		C=0, T=22	C=0.0, T=1.0	0.00001	298.892	22	C=0/0, T=13/9
chr5:141059868	C/C	T	C	SNV	ARAP3	ARAP3:exonic:NM_022481.5	1	100	2	synonymous	NM_02248	c.186A>G	p.(=)	0.24				0.05(COSM37	PH domain,P	rs11167756	0.4201	YES	4.675E-1 (pos.14105986	adenocarcino	ArfGAP with	GTPase ac	HIV Infections;Hemic and Lymphatic Disease		T=0, C=29	T=0.0, C=1.0	0.00001	427.591	29	T=0/0, C=9/20
chr12:49334762	C/C	G	C	SNV	ARF3	ARF3:exonic:NM_001659.2	1	100	2	synonymous	NM_00165	c.117C>G	p.(=)	-0.89					50S ribosome	rs12122	0.0265	YES	9.999E-1 (pos.49334762)		ADP-ribosyl	GTP binding,GTPase activity,Golgi membrane,extracellu			G=0, C=28	G=0.0, C=1.0	0.00001	408.715	28	G=0/0, C=20/8
chr17:36646386	G/G	A	G	SNV	ARHGAP23	ARHGAP23:exonic:NM_001199417.1	1	100	18	synonymous	NM_00119	c.3030A>G	p.(=)	-1.81				0.04(COSM41	PDZ domain.I	rs9891156	0.0277	YES	1.0 (pos.36646386)	neoplasm	RHO GTPase	GTPase ac	Adnexal Diseases;Cysts;Endocrine System Di		A=0, G=21	A=0.0, G=1.0	0.00001	281.235	21	A=0/0, G=14/7
chr5:142421415	G/G	T	G	SNV	ARHGAP26	ARHGAP26:exonic:NM_015071.5	1	100	14	synonymous	NM_01507	c.1245T>G	p.(=)	0.59				0.13(COSM56	BAR domain (	rs2270068,rs587778	0.0015	YES	5.729E-1 (pos.14242141	ductal_carcin	RHO GTPase	GTPase ac	Bone Marrow Diseases;Hematologic Disease		T=0, G=40	T=0.0, G=1.0	0.00001	644.285	40	T=0/0, G=18/2
chr5:142593652	T/T	C	T	SNV	ARHGAP26	ARHGAP26:exonic:NM_015071.5	1	100	22	synonymous	NM_01507	c.2355C>T	p.(=)	-0.72					BAR domain (	rs258819	0.005		9.998E-1 (pos.142593652)		RHO GTPase	GTPase ac	Bone Marrow Diseases;Hematologic Disease		C=0, T=44	C=0.0, T=1.0	0.00001	718.568	44	C=0/0, T=17/2
chr1:248005212	TC/TC	T	TC	INDEL	OR11L1	OR11L1:upstream:NM_001001959.1	1	100		intronic	NM_00100	c.-14A>GA	p.?	0.09												G-protein coupled receptor activity,G-protein coupled re			T=0, TC=35	T=0.0, TC=1.0	0.00001	265.043	35	T=0/0, TC=11/
chr18:6889973	G/G	A	G	SNV	ARHGAP28	ARHGAP28:exonic:NM_001010000.2	1	100	12	synonymous	NM_00101	c.1146A>G	p.(=)	0.06					RhoGAP dom	rs4239328	0.428	YES	6.385E-1 (pos.6889973)		RHO GTPase-activating		Endometriosis;Female Urogenital Diseases;F		A=0, G=43	A=0.0, G=1.0	0.00001	705.665	43	A=0/0, G=16/2
chr1:248058755	G/G	A	G	SNV	OR2W3	OR2W3:upstream:NM_001001957.2	1	100		intronic	NM_00100	c.-134A>G	p.?	0.11						rs1337876		YES			Olfactory re	G-protein coupled receptor activity,G-protein coupled re			A=0, G=25	A=0.0, G=1.0	0.00001	353.012	25	A=0/0, G=7/18
chr1:248058797	G/G	T	G	SNV	OR2W3	OR2W3:upstream:NM_001001957.2	1	100		intronic	NM_00100	c.-92T>G	p.?	-1.23						rs1856248		YES			Olfactory re	G-protein coupled receptor activity,G-protein coupled re			T=0, G=25	T=0.0, G=1.0	0.00001	353.019	25	T=0/0, G=7/18
chr1:248058845	C/C	A	C	SNV	OR2W3	OR2W3:upstream:NM_001001957.2	1	100		intronic	NM_00100	c.-44A>C	p.?	0.24						rs4500353,rs780386	0.019	YES	0.0;0.0 (pos.248058842),9.47E-1;0.0;1.4		Olfactory re	G-protein coupled receptor activity,G-protein coupled re			A=0, C=26	A=0.0, C=1.0	0.00001	371.419	26	A=0/0, C=7/19
chr1:248058879	T/T	A	T	SNV	OR2W3	OR2W3:upstream:NM_001001957.2	1	100		intronic	NM_00100	c.-10A>T	p.?	0.21						rs6587387	0.0197	YES	9.442E-1 (pos.248058879)		Olfactory re	G-protein coupled receptor activity,G-protein coupled re			A=0, T=25	A=0.0, T=1.0	0.00001	353.019	25	A=0/0, T=6/19
chr18:6912143	C/C	A	C	SNV	ARHGAP28	ARHGAP28:exonic:NM_001010000.2	1	100	17	missense	NM_00101	c.1703A>C	p.Gln568Pro	1.17	0.66	76	0	0.01(COSM56	RhoGAP dom	rs1056408	0.1405	YES	6.784E-1 (pos.6912143)	ductal_carcin	RHO GTPase-activating		Endometriosis;Female Urogenital Diseases;F		A=0, C=36	A=0.0, C=1.0	0.00001	561.401	36	A=0/0, C=12/2
chr1:94639446	AT/AT	A	AT	INDEL	ARHGAP29	ARHGAP29:exonic:NM_004815.3	1	100	23	nonsense	NM_00481	c.3764_37	p.Glu1256Ter	0.28					Phorbol este	rs755542411			0.0 (pos.94639446)		RHO GTPase	GTPase ac	Hemic and Lymphatic Diseases;Immune Syst		A=0, AT=22	A=0.0, AT=1.0	0.00001	163.318	22	A=0/0, AT=9/1
chr1:248085340	TA/TA	T	TA	INDEL	OR2T8	OR2T8:downstream:NM_001005522.1	1	100		intronic	NM_00100	c.*82T>TA	p.?	0.1												G-protein coupled receptor activity,G-protein coupled re			T=0, TA=65	T=0.0, TA=1.0	0.00001	852.547	65	T=0/0, TA=23/
chr20:37278612	A/A	T	A	SNV	ARHGAP40	ARHGAP40:utr_3:NM_001164431.1	1	100	15	utr_3	NM_00116	c.2146T>A	p.?	-1					RhoGAP dom	rs220522		YES							T=0, A=24	T=0.0, A=1.0	0.00001	334.79	24	T=0/0, A=5/19
chr19:1077985	G/G	A	G	SNV	ARHGAP45	ARHGAP45:exonic:NM_001258328.2	1	100	11	missense	NM_00125	c.1363A>G	p.Ser455Gly	1.2	0.26	56	0.001		RhoGAP dom	rs7251797	0.358	YES	3.842E-1 (pos.1077985)			GTPase activator activity,azurophil granule lumen,cytos			A=0, G=38	A=0.0, G=1.0	0.00001	601.921	38	A=0/0, G=7/31
chr19:1081617	G/G	A	G	SNV	ARHGAP45	ARHGAP45:exonic:NM_001258328.2	1	100	18	synonymous	NM_00125	c.2307A>G	p.(=)	1.4				0.11(COSM37	RhoGAP dom	rs10404947	0.2026	YES	7.063E-1 (pos.1081617)	adenocarcinoma,neoplasn		GTPase activator activity,azurophil granule lumen,cytos			A=0, G=30	A=0.0, G=1.0	0.00001	443.473	30	A=0/0, G=18/1
chr12:15103605	G/G	A	G	SNV	ARHGDIB	ARHGDIB:exonic:NM_001175.6	1	100	2	synonymous	NM_00117	c.42T>C	p.(=)	-1.47					RHO protein (	rs2430711	0	YES	1.0 (pos.15103605)		Rho GDP dis	GTPase ac	Acute Coronary Syndrome;Cardiovascular Dis		A=0, G=25	A=0.0, G=1.0	0.00001	352.898	25	A=0/0, G=12/1
chr1:3397188	C/C	T	C	SNV	ARHGEF16	ARHGEF16:utr_3:NM_014448.3	1	100	15	utr_3	NM_01444	c.2167T>C	p.?	-2.87					PH domain,R	rs2487677,rs755077	1.00E-04		0.0 (pos.3397184),1.0 (pos.3397188)			G-protein coupled receptor signaling pathway,PDZ doma			T=0, C=21	T=0.0, C=1.0	0.00001	281.235	21	T=0/0, C=8/13
chr1:16525056	T/T	G	T	SNV	ARHGEF19	ARHGEF19:utr_3:NM_153213.4	1	100	16	utr_3	NM_15321	c.2435C>A	p.?	-1.56					RhoGEF dom	rs451260	0.2564	YES	1.234E-1 (pos.16525056)		RHO guanin	G-protein coupled receptor signaling pathway,GTPase a			G=0, T=23	G=0.0, T=1.0	0.00001	316.746	23	G=0/0, T=10/1
chr1:16532803	A/A	G	A	SNV	ARHGEF19	ARHGEF19:exonic:NM_153213.4	1	100	7	synonymous	NM_15321	c.1170C>T	p.(=)	-1.89					RhoGEF dom	rs221050	0.2545	YES	1.535E-1 (pos.16532803)		RHO guanin	G-protein coupled receptor signaling pathway,GTPase a			G=0, A=137	G=0.0, A=1.0	0.00001	2279.45	137	G=0/0, A=62/7
chr1:16534255	G/G	C	G	SNV	ARHGEF19	ARHGEF19:exonic:NM_153213.4	1	100	4	missense	NM_15321	c.712G>C	p.Glu238Gln	3.81	0.01	29	0.041	0.8(COSM374	RhoGEF dom	rs221057	0.2606	YES	1.274E-1;0.0 (pos.16534	ductal_carcin	RHO guanin	G-protein coupled receptor signaling pathway,GTPase a			C=0, G=41	C=0.0, G=1.0	0.00001	669.658	41	C=0/0, G=25/1
chr1:16534646	G/G	C	G	SNV	ARHGEF19	ARHGEF19:exonic:NM_153213.4	1	100	3	missense	NM_15321	c.487G>C	p.Gly163Arg	0.67	0.01	125	0.014	0.19(COSM13	RhoGEF dom	rs221058	0.2597	YES	1.269E-1;0.0 (pos.16534	neoplasm,squ	RHO guanin	G-protein coupled receptor signaling pathway,GTPase a			C=0, G=24	C=0.0, G=1.0	0.00001	334.698	24	C=0/0, G=12/1
chr5:73218954	C/T	C	T	SNV	ARHGEF28	ARHGEF28:exonic:NM_001080479.2	1	50	36	missense	NM_00108	c.4991C>T	p.Pro1664Leu	-1	1	98	0		Phorbol este	rs423333		YES	1.364E-1 (pos.73218954)			RNA binding,Rho guanyl-nucleotide excha		Benign	C=8, T=16	C=0.3333, T=0.6667	0.00001	287.671	32	C=1/7, T=13/3
chr2:39187190	C/C	T	C	SNV	ARHGEF33,LOC375196	ARHGEF33:exonic:NM_001145451.2, LOC375196:exonic_nc	1	100	14	missense	NM_00114	c.1744T>C	p.Ser582Pro	0.12	1	74	0	0.07(COSM37	RhoGEF dom	rs6755527	0.0661	YES	8.623E-1 (pos.39187190)	adenocarcinoma		Rho guanyl-nucleotide exchange factor activity,regulatic			T=0, C=129	T=0.0, C=1.0	0.00001	2090.98	129	T=0/0, C=91/3
chr2:39187519	G/G	A	G	SNV	ARHGEF33,LOC375196	ARHGEF33:exonic:NM_001145451.2, LOC375196:upstream	1	100	14	synonymous	NM_00114	c.2073A>G	p.(=)	2.52				0.05(COSM37	RhoGEF dom	rs958412		YES	8.028E-1 (pos.39187519)	adenocarcinoma		Rho guanyl-nucleotide exchange factor activity,regulatic			A=0, G=41	A=0.0, G=1.0	0.00001	664.696	41	A=0/0, G=26/1
chr9:35660990	G/G	A	G	SNV	ARHGEF39,CCDC107	ARHGEF39:utr_3:NM_032818.2, CCDC107:exonic:NM_17492	1	100	5	missense	NM_17492	c.658A>G,	p.Ile220Val, p.?	-0.11	1	29	0		RhoGEF dom	rs1339374,rs747714	0.2012	YES	0.0 (pos.35660987),9.864E-1 (pos.35660990)			Rho guanyl-nucleotide exchange factor activity,integral (			A=0, G=27	A=0.0, G=1.0	0.00001	389.979	27	A=0/0, G=15/1
chr14:21551069	C/C	T	C	SNV	ARHGEF40	ARHGEF40:exonic:NM_018071.4	1	100	16	missense	NM_01807	c.3566T>C	p.Leu1189Ser	0.72	0.56	145	0		RhoGEF dom	rs1958396	0.094	YES	5.902E-1 (pos.21551069)			Rho guanyl-nucleotide exchange factor activity,cytoplas			T=0, C=21	T=0.0, C=1.0	0.00001	281.232	21	T=0/0, C=7/14
chr19:965043	C/C	T	C	SNV	ARID3A	ARID3A:exonic:NM_005224.2	1	100	6	synonymous	NM_00522	c.1161T>C	p.(=)	-0.18					ARID/BRIGHT	rs12608658	0.0229	YES	9.999E-1 (pos.965043)			DNA binding,DNA damage response, signal transduction			T=0, C=40	T=0.0, C=1.0	0.00001	644.286	40	T=0/0, C=23/1
chr14:58831142	G/G	A	G	SNV	ARID4A	ARID4A:exonic:NM_002892.3	1	100	20	missense	NM_00289	c.2335A>G	p.Thr779Ala	-0.11	0.4	58	0	0.01(COSM50	ARID/BRIGHT	rs1051858,rs386777	0.3304	YES	5.209E-1 (pos.58831142)	ductal_carcin	AT-rich inte	DNA binding,DNA binding transcription factor activity,cy			A=0, G=23	A=0.0, G=1.0	0.00001	321.141	23	A=0/0, G=18/5
chr13:50205025	C/C	T	C	SNV	ARL11	ARL11:exonic:NM_138450.5	1	100	2	missense	NM_13845	c.442T>C	p.Cys148Arg	-1.35		180		0.01(COSM50	50S ribosome	rs3803185	0.486	YES	1.82E-1 (pos.50205025)	carcinoma	ADP-ribosyl	GTP bindir	Adnexal Diseases;Endocrine Gland Neoplasm		T=0, C=38	T=0.0, C=1.0	0.00001	603.784	38	T=0/0, C=25/1
chr5:53606295	T/C	T	C	SNV	ARL15	ARL15:exonic:NM_019087.2	1	95.83	1	synonymous	NM_01908	c.15A>G	p.(=)	0.16				0.18(COSM41	ADP-ribosyla	rs35941	0.156	YES	7.206E-1 (pos.53606295)	carcinoma,neoplasm		GTP bindir	Alcohol-Related Disorders;Alcoholism;Diabe		T=1, C=23	T=0.0417, C=0.9583	0.00001	307.284	24	T=0/1, C=14/9
chr11:64789194	C/C	T	C	SNV	ARL2,ARL2-SNX15	ARL2:exonic:NM_001667.3, ARL2-SNX15:intronic_nc:NR_03	1	100	5	missense	NM_00166	c.422T>C	p.Val141Ala	2.19	1	64	0	0.05(COSM11	50S ribosome	rs664226	0.3724	YES	1.631E-1 (pos.64789194)	primitive_neu	ADP=ribosy	GTP bindir	Melanoma;Neoplasms;Neoplasms by Histolc		T=0, C=33	T=0.0, C=1.0	0.00001	504.591	33	T=0/0, C=11/2
chr12:123463812	C/T	C	T	SNV	ARL6IP4,OGFOD2	OGFOD2:exonic:NM_024623.2, ARL6IP4:upstream:NM_018	1	57.14	8	synonymous	NM_02462	c.792C>T,	p.(=), p.?	-3.24						rs34010146	0.0018	YES	3.499E-4 (pos.123463812)		ADP-ribosyl	L-ascorbic acid binding,RNA bir		Vitamin C	C=9, T=12	C=0.4286, T=0.5714	0.00001	109.101	21	C=7/2, T=10/2
chr6:35705892	C/C	T	C	SNV	ARMC12	ARMC12:exonic:NM_145028.4	1	100	2	synonymous	NM_14502	c.333T>C	p.(=)	0.48				0.02(COSM41	Armadillo-lik	rs2817041,rs386700	0.195	YES	6.05E-1 (pos.35705892)	neoplasm		nucleus			T=0, C=41	T=0.0, C=1.0	0.00001	664.699	41	T=0/0, C=27/1
chr1:248789454	C/C	G	C	SNV	OR2T11	OR2T11:downstream:NM_001001964.1	1	100		intronic	NM_00100	c.*25C>G	p.?	-0.26						rs1892441	0.0107	YES	1.0;0.0 (pos.248789454)			G-protein coupled receptor activity,G-protein coupled re			G=0, C=29	G=0.0, C=1.0	0.00001	427.603	29	G=0/0, C=17/1
chr10:23297301	T/T	C	T	SNV	ARMC3	ARMC3:exonic:NM_173081.4	1	100	15	synonymous	NM_17308	c.1926C>T	p.(=)	1.25					Armadillo/be	rs11817610	0.3871	YES	5.586E-1;0.0 (pos.23297301)		Armadillo r	cytoplasm,extracellular exosome,nucleus			C=0, T=23	C=0.0, T=1.0	0.00001	316.747	23	C=0/0, T=11/1
chrX:100749585	T/T	C	T	SNV	ARMCX4	ARMCX4:exonic:NM_001256155.2	1	100	2	synonymous	NM_00125	c.6009C>T	p.(=)	0.01				0.03(COSM63	Armadillo-lik	rs3174476		YES	4.233E-1 (pos.10074958	carcinoma					C=0, T=41	C=0.0, T=1.0	0.00001	664.7	41	C=0/0, T=22/1
chr1:248813135	A/A	G	A	SNV	OR2T27	OR2T27:downstream:NM_001001824.1	1	100		intronic	NM_00100	c.*97C>T	p.?	0.02						rs4916153		YES				G-protein coupled receptor activity,G-protein coupled re			G=0, A=30	G=0.0, A=1.0	0.00001	446.623	30	G=0/0, A=26/4
chr3:9870857	A/A	G	A	SNV	ARPC4-TTLL3,TTLL3	TTLL3:exonic:NM_001025930.3, ARPC4-TTLL3:exonic:NM_0(	1	100	10	synonymous	NM_00119	c.1515G>A	p.(=), p.(=)	0.3				0.24(COSM37	Tubulin-tyros	rs2290303	0.4951	YES	1.388E-1 (pos.9870857)	adenocarcinoma,ductal_ca		ATP binding,Arp2/3 complex-mediated actin nucleation,			G=0, A=49	G=0.0, A=1.0	0.00001	835.827	49	G=0/0, A=27/2
chr3:35725250	T/T	C	T	SNV	ARPP21	ARPP21:exonic:NM_001267619.1	1	100	4	synonymous	NM_00126	c.204C>T	p.(=)	-0.5				0.02(COSM40	R3H domain	rs969818	0.3519	YES	6.54E-1 (pos.35725250)	acute_myeloid_leukaemia		biological_process,calmodulin binding,cellular response			C=0, T=40	C=0.0, T=1.0	0.00001	623.158	40	C=0/0, T=18/2
chr15:98504100	C/C	G	C	SNV	ARRDC4	ARRDC4:exonic:NM_183376.2	1	100	1	synonymous	NM_18337	c.9G>C	p.(=)	-1.45				0.19(COSM37	Arrestin (or S	rs4965046	0.1903	YES	8.831E-1 (pos.98504100)	adenocarcinoma,neoplasn		early endosome,endosome,extracellular vesicle,plasma			G=0, C=26	G=0.0, C=1.0	0.00001	371.42	26	G=0/0, C=4/22
chr5:78181477	T/T	C	T	SNV	ARSB	ARSB:exonic:NM_000046.4	1	100	5	missense	NM_00004	c.1072G>A	p.Val358Met	3.99	0	21	0.997	0.93(COSM44	Sulfatase	rs1065757	0.4324	YES	3.804E-1 (pos.78181477)	acute_myeloic	Arylsulfatas	Golgi appa	Carbohydrate Metabolism, Inb	Benign	C=0, T=25	C=0.0, T=1.0	0.00001	353.017	25	C=0/0, T=8/17
chr1:2528133	C/C	T	C	SNV	MMEL1	MMEL1:intronic:NM_033467.3	1	100		intronic	NM_03346	c.1273-5A	p.?	-3.59				0(COSM5428	Peptidase fa	rs2843401	0.32	YES	5.136E-1 (pos.2528133)	acute_myeloid_leukaemia		extracellu	Arthritis;Arthritis, Rheumatoid;Autoimmune		T=0, C=29	T=0.0, C=1.0	0.00001	427.533	29	T=0/0, C=26/3
chr1:25648349	T/C	T	C	SNV	RHD	RHD:intronic:NM_016124.4	1	57.89		intronic	NM_01612	c.1154-31	p.?	-0.22					Ammonium T	rs28669938	0.2489	YES	2.535E-1 (pos.25648349)		Rhesus syst	ammoniur	Anemia;Anemia, He	Human Rho(D) immune g	T=16, C=22	T=0.4211, C=0.5789	0.00001	249.054	38	T=7/9, C=9/13
chrX:2835985	GAAAA/AAAAC	GAAAA	AAAAC	MNV	ARSD	ARSD:exonic:NM_001669.3	5	50	5	missense	NM_00166	c.719_723	p.Phe240Cys	-0.29, 3.19	0.01	205	0.045	0.06(COSM40	C-terminal re	rs143238998,rs748243474,rs759462760,rs			0.0 (pos.2835984),0.0 (pc	adenocarcino	Arylsulfatas	arylsulfatase activity,catalytic activity,endoplasmic reti			GAAAA=51,	GAAAA=0.4904, AAA	0.00001	562.921	106	GAAAA=42/9,
chr1:25889672	A/A	G	A	SNV	LDLRAP1	LDLRAP1:intronic:NM_015627.2	1	100		intronic	NM_01562	c.616+28G	p.?	0.21					Phosphotyro	rs6688931	0.4885	YES	4.495E-1 (pos.25889672)		Low density	AP-1 adap	Dyslipidemias;Hypercholesterolemia;Hyperl		G=0, A=24	G=0.0, A=1.0	0.00001	334.722	24	G=0/0, A=19/5
chrX:2836047	C/T	C	T	SNV	ARSD	ARSD:exonic:NM_001669.3	1	50	5	missense	NM_00166	c.661G>A	p.Gly221Ser	-0.25	0.06	56	0.994	0.03(COSM33	C-terminal re	rs67359049			5.168E-3 (pos.2836047)	acute_myeloic	Arylsulfatas	arylsulfatase activity,catalytic activity,endoplasmic reti			C=34, T=34	C=0.5, T=0.5	0.00001	342.383	68	C=28/6, T=17/
chr1:26098107	A/A	G	A	SNV	MAN1C1	MAN1C1:intronic:NM_020379.3	1	100		intronic	NM_02037	c.1142-41	p.?	-5.81					Glycosyl hydr	rs2072747,rs762860	0.3553	YES	7.615E-1;0.0 (pos.26098107)		Mannosida	Golgi apparatus mannose trimming,Golgi membrane,N-g			G=0, A=65	G=0.0, A=1.0	0.00001	996.464	65	G=0/0, A=19/4
chr1:26098333	A/A	G	A	SNV	MAN1C1	MAN1C1:intronic:NM_020379.3	1	100		intronic	NM_02037	c.1257+70	p.?	-1.34					Glycosyl hydr	rs807264		YES			Mannosida	Golgi apparatus mannose trimming,Golgi membrane,N-g			G=0, A=20	G=0.0, A=1.0	0.00001	263.786	20	G=0/0, A=10/1
chr1:26110143	A/A	G	A	SNV	MAN1C1	MAN1C1:intronic:NM_020379.3	1	100		intronic	NM_02037	c.1767-11	p.?	-0.73					Glycosyl hydr	rs3767877	0.3684	YES	7.377E-1 (pos.26110143)		Mannosida	Golgi apparatus mannose trimming,Golgi membrane,N-g			G=0, A=22	G=0.0, A=1.0	0.00001	298.892	22	G=0/0, A=10/1
chr1:26138136	A/A	C	A	SNV	SELENON	SELENON:intronic:NM_206926.1	1	100		intronic	NM_20692	c.991-46C	p.?	0.91						rs760598	0.2279	YES	9.645E-1 (pos.26138136)		Selenoprot	calcium ion binding,calcium ion homeosta		Benign	C=0, A=26	C=0.0, A=1.0	0.00001	371.42	26	C=0/0, A=19/7
chr1:26138451	G/G	A	G	SNV	SELENON	SELENON:intronic:NM_206926.1	1	100		intronic	NM_20692	c.1179+81	p.?	-0.06						rs11247744,rs202103730		YES			Selenoprot	calcium ion binding,calcium ion homeostasis,endoplasn			A=0, G=23	A=0.0, G=1.0	0.00001	316.746	23	A=0/0, G=7/16
chr1:26158337	T/T	C	T	SNV	MTFR1L	MTFR1L:intronic:NM_001099625.1	1	100		intronic	NM_00109	c.774-75C	p.?	0.04					Mitochondria	rs1961580		YES				aerobic respiration,mitochondrial fission,mitochondrio			C=0, T=36	C=0.0, T=1.0	0.00001	563.738	36	C=0/0, T=17/1
chrX:2836060	G/A	G	A	SNV	ARSD	ARSD:exonic:NM_001669.3	1	50	5	synonymous	NM_00166	c.648C>T	p.(=)	-1.44				0.01(COSM33	C-terminal re	rs113318393		YES	8.573E-3 (pos.2836060)	adenocarcino	Arylsulfatas	arylsulfatase activity,catalytic activity,endoplasmic reti			G=36, A=36	G=0.5, A=0.5	0.00001	368.32	72	G=27/9, A=17/
chr1:26511700	G/G	A	G	SNV	CNKSR1	CNKSR1:intronic:NM_006314.2	1	100		intronic	NM_00631	c.1308+23	p.?	-0.95					Connector er	rs2783680	0.3981	YES	9.68E-1 (pos.26511700)		Connector e	Ras protei	Intellectual Disability;Mental Disorders;Nerv		A=0, G=82	A=0.0, G=1.0	0.00001	1326.32	82	A=0/0, G=33/4
chr17:66364749	C/C	T	C	SNV	ARSG	ARSG:exonic:NM_014960.4	1	100	7	synonymous	NM_01496	c.765T>C	p.(=)	-0.21				0.04(COSM44	C-terminal re	rs1558877	0.4793	YES	2.867E-1 (pos.66364749)	acute_myeloic	Arylsulfatas	arylsulfata	Central Nervous System Diseases;Dystonic D		T=0, C=73	T=0.0, C=1.0	0.00001	1150.46	73	T=0/0, C=28/4
chr1:26609307	T/T	G	T	SNV	UBXN11	UBXN11:intronic:NM_183008.2	1	100		intronic	NM_18300	c.1152-27	p.?	-0.9					SEP domain.U	rs6598952	0.0625	YES	4.453E-1;0.0 (pos.26609307)		UBX domair	cytoplasm,cytoskeleton,proteasome-mediated ubiquiti			G=0, T=55	G=0.0, T=1.0	0.00001	814.907	55	G=0/0, T=32/2
chr17:66364804	C/C	T	C	SNV	ARSG	ARSG:exonic:NM_014960.4	1	100	7	missense	NM_01496	c.820T>C	p.Trp274Arg	0.49	0.56	101	0	0.02(COSM44	C-terminal re	rs1558878	0.4972	YES	2.435E-1 (pos.66364804)	acute_myeloic	Arylsulfatas	arylsulfata	Central Nervous System Diseases;Dystonic D		T=0, C=70	T=0.0, C=1.0	0.00001	1092.09	70	T=0/0, C=25/4
chr4:77033725	A/A	C	A	SNV	ART3	ART3:utr_3:NM_001130016.2	1	100	12	utr_3	NM_00113	c.1223C>A	p.?	0.15					NAD:arginine	rs14773		YES			ADP-ribosyl	C-terminal protein lipidation,NAD(P)+-protein-arginine A			C=0, A=82	C=0.0, A=1.0	0.00001	1329.65	82	C=0/0, A=43/3
chr1:231114945	T/C	T	C	SNV	ARV1,TTC13	ARV1:exonic:NM_022786.2, TTC13:upstream:NM_024525.4	1	46.79	1	missense	NM_02452	c.-369A>G,	p.?, p.Tyr32His	6.26	0	83	0.999		Arv1-like fam	rs34745784	6.00E-04		0.0 (pos.231114945)		ARV1, S. cer	Golgi appa	Blindness;Eye Diseases;Nervo	Likely benign	T=58, C=51	T=0.5321, C=0.4679	0.00001	504.646	109	T=18/40, C=24
chr8:17918934	G/G	A	G	SNV	ASAH1	ASAH1:exonic:NM_004315.5	1	100	10	missense	NM_00431	c.785T>C	p.Val262Ala	2.68	1	64	0		Linear amide	rs10103355	0.0091	YES	8.697E-1 (pos.17918934)		N-acylsphin	catalytic a	Brain Diseases;Brain Diseases	Benign	A=0, G=86	A=0.0, G=1.0	0.00001	1410.92	86	A=0/0, G=51/3
chr8:17927327	T/C	T	C	SNV	ASAH1	ASAH1:exonic:NM_004315.5	1	97.5	4	missense	NM_00431	c.325A>G	p.Ile109Val	-1.94	0.65	29	0	0.01(COSM37	Linear amide	rs1049874	0.4919	YES	3.699E-1 (pos.17927327)	acute_myeloic	N-acylsphin	catalytic a	Brain Diseases;Brain Diseases	Benign	T=1, C=39	T=0.025, C=0.975	0.00001	608.066	40	T=1/0, C=11/2
chr10:5682641	T/T	C	T	SNV	ASB13	ASB13:utr_3:NM_024701.3	1	100	6	utr_3	NM_02470	c.862G>A	p.?	-0.47					Ankyrin repe	rs749619086,rs7896	3.00E-04		0.0 (pos.5682639),1.0 (pos.5682641)		Ankyrin rep	cytosol,intracellular signal transduction,post-translatio			C=0, T=24	C=0.0, T=1.0	0.00001	334.792	24	C=0/0, T=20/4
chr7:123256427	T/T	C	T	SNV	ASB15,LOC102724555	LOC102724555:intronic_nc:NR_111922.1, ASB15:exonic:NM	1	100	4	missense	NM_08092	c.170C>T	p.Pro57Leu	1.2	0.66	98	0	0.08(COSM40	Ankyrin repe	rs6962756	0.232	YES	7.738E-1 (pos.12325642	acute_myeloid_leukaemia		cytoplasm	Adenocarcinoma;Carcinoma;Carcinoma, Ren		C=0, T=107	C=0.0, T=1.0	0.00001	1680.99	107	C=0/0, T=45/6
chr2:237149941	T/T	C	T	SNV	ASB18	ASB18:exonic:NM_212556.3	1	100	2	missense	NM_21255	c.310G>A	p.Ala104Thr	2.68	0.03	58	0.76	0.89(COSM37	Ankyrin repe	rs6756597	0.4807	YES	5.641E-1 (pos.23714994	acute_myeloid_leukaemia		cytoplasm,cytosol,integral component of membrane,int			C=0, T=23	C=0.0, T=1.0	0.00001	314.955	23	C=0/0, T=10/1
chr14:94417586	A/A	G	A	SNV	ASB2	ASB2:exonic:NM_001202429.1	1	100	6	synonymous	NM_00120	c.639C>T	p.(=)	0.03				0.19(COSM37	Ankyrin repe	rs4483793	0.352	YES	7.951E-1 (pos.94417586)	acute_myeloic	Ankyrin rep	Cul5-RING ubiquitin ligase complex,cytosol,intracellular			G=0, A=21	G=0.0, A=1.0	0.00001	281.223	21	G=0/0, A=9/12
chr12:48543584	G/C	G	C	SNV	ASB8	ASB8:exonic:NM_024095.4	1	57.89	4	synonymous	NM_02409	c.432C>G	p.(=)	0.72					Ankyrin repe	rs3936179	0.0844	YES	1.156E-4 (pos.48543584)		Ankyrin rep	cytosol,intracellular signal transduction,post-translatio			G=24, C=33	G=0.4211, C=0.5789	0.00001	344.915	57	G=14/10, C=24
chr1:27686063	C/C	G	C	SNV	MAP3K6	MAP3K6:intronic:NM_004672.4	1	100		intronic	NM_00467	c.2320-50	p.?	-0.35					Domain of un	rs7521552	0.004		1.0;0.0 (pos.27686063)		Mitogen-ac	ATP bindin	Digestive System Diseases;Digestive System		G=0, C=29	G=0.0, C=1.0	0.00001	427.56	29	G=0/0, C=8/21
chr6:101296389	A/A	G	A	SNV	ASCC3	ASCC3:exonic:NM_006828.3	1	100	4	missense	NM_00682	c.436C>T	p.Leu146Phe	2.42	0.09	22	0.001	0.97(COSM15	DEAD/DEAH b	rs9390698	0.4558	YES	2.457E-1 (pos.10129638	adenocarcino	Activating s	ATP bindin	Autoimmune Diseases;Female Urogenital Dis		G=0, A=22	G=0.0, A=1.0	0.00001	298.892	22	G=0/0, A=6/16
chr6:101166095	G/A	G	A	SNV	ASCC3	ASCC3:exonic:NM_006828.3	1	50	12	synonymous	NM_00682	c.1935C>T	p.(=)	0.93				0.61(COSM15	DEAD/DEAH b	rs41288423	0.4969	YES	3.019E-1 (pos.10116609	adenocarcino	Activating s	ATP bindin	Autoimmune Diseases;Female Urogenital Dis		G=14, A=14	G=0.5, A=0.5	0.00001	131.939	28	G=8/6, A=11/3
chr1:155408635	C/C	T	C	SNV	ASH1L	ASH1L:exonic:NM_018489.2	1	100	5	missense	NM_01848	c.5311A>G	p.Thr1771Ala	2.26		58	0		BAH domain,	rs4971053	5.00E-04	YES	1.0 (pos.155408635)		ash1 (abser	DNA binding,DNA packaging,Golgi apparatus,bicellular ti			T=0, C=47	T=0.0, C=1.0	0.00001	789.315	47	T=0/0, C=23/2
chr8:37985897	A/G	A	G	SNV	ASH2L	ASH2L:exonic:NM_004674.4	1	98.36	11	synonymous	NM_00467	c.1254A>G	p.(=)	-0.69					SPRY domain	rs2843740	0.016	YES	7.488E-1 (pos.37985897)		ASH2, Dros	DNA binding,MLL1 complex,MLL3/4 complex,Set1C/COM			A=1, G=60	A=0.0164, G=0.9836	0.00001	889.398	61	A=0/1, G=32/2
chr2:220404381	G/G	A	G	SNV	ASIC4,CHPF	ASIC4:downstream:NM_182847.2, CHPF:exonic:NM_02453	1	100	4	synonymous	NM_18284	c.*1613A>	p.?, p.(=)	0.47				0.26(COSM41	Chondroitin N	rs1976616	3.00E-04		1.0 (pos.220404381)	neoplasm	Cation char	Golgi cisterna membrane,Golgi membrane,N-acetylgala			A=0, G=29	A=0.0, G=1.0	0.00001	427.603	29	A=0/0, G=9/20
chrX:1554649	C/C	T	C	SNV	ASMTL	ASMTL:exonic:NM_004192.3	1	100	4	synonymous	NM_00419	c.276A>G	p.(=)	-1.05				0.04(COSM40	Dimerisation	rs3183025,rs751576	0.3304	YES	0.0 (pos.1554638),3.888	carcinoma		O-methylt	Leukemia;Leukemia, Myeloid;Leukemia, Mye		T=0, C=50	T=0.0, C=1.0	0.00001	852.736	50	T=0/0, C=26/2
chr1:29345009	CTT/CTT	C	CTT	INDEL	EPB41	EPB41:intronic:NM_001166005.1	2	100		intronic	NM_00116	c.1124+55	p.?	-0.74					4.1 protein C	rs55946958,rs5773229		YES			Erythrocyte	1-phospha	Anemia;Anemia, Hemolytic;Anemia, Hemolyt		C=0, CTT=9	C=0.0, CTT=1.0	0.00001	1528.92	92	C=0/0, CTT=31
chr1:32155523	C/A	C	A	SNV	COL16A1	COL16A1:intronic:NM_001856.3	1	57.14		intronic	NM_00185	c.1510-71	p.?	-0.09					Collagen trip	rs1044058942					Collagen XV	cell adhesion,cell adhesion mediated by integrin,cellula			C=9, A=12	C=0.4286, A=0.5714	0.00001	109.102	21	C=0/9, A=7/5
chr1:32156869	T/T	A	T	SNV	COL16A1	COL16A1:intronic:NM_001856.3	1	100		intronic	NM_00185	c.1399-42	p.?	0.01					Collagen trip	rs10798884	0		1.0 (pos.32156869)		Collagen XV	cell adhesion,cell adhesion mediated by integrin,cellula			A=0, T=36	A=0.0, T=1.0	0.00001	563.743	36	A=0/0, T=9/27
chr1:197070901	G/G	A	G	SNV	ASPM	ASPM:exonic:NM_018136.4	1	100	18	missense	NM_01813	c.7480T>C	p.Tyr2494His	3	0.97	83	0		Abnormal spi	rs964201	0.0059		1.0 (pos.197070901)		Abnormal s	calmoduli	Adenocarcinoma;Adnexal Dise	Benign	A=0, G=20	A=0.0, G=1.0	0.00001	263.631	20	A=0/0, G=8/12
chr1:197112533	A/A	G	A	SNV	ASPM	ASPM:exonic:NM_018136.4	1	100	3	synonymous	NM_01813	c.849C>T	p.(=)	-0.08					Abnormal spi	rs6677082,rs779857	0.0932	YES	9.834E-1;0.0 (pos.197112533)		Abnormal s	calmoduli	Adenocarcinoma;Adnexal Dise	Benign/Likely	G=0, A=24	G=0.0, A=1.0	0.00001	334.792	24	G=0/0, A=6/18
chr9:95219597	A/A	G	A	SNV	ASPN,CENPP	CENPP:intronic:NM_001012267.2, ASPN:exonic:NM_01768	1	100	9	synonymous	NM_00101	c.564+774	p.?, p.(=)	-1.07					CENP-A-nucle	rs4744132	0.0172	YES	1.0 (pos.95219597)		Asporin,Cer	CENP-A co	Arthritis;Bone Diseases;Collagen Diseases;C		G=0, A=40	G=0.0, A=1.0	0.00001	644.283	40	G=0/0, A=22/1
chr1:33138605	C/C	T	C	SNV	RBBP4	RBBP4:intronic:NM_005610.2	1	100		intronic	NM_00561	c.1212+10	p.?	1.79					Histone-bind	rs1320511		YES				ATP-dependent chromatin remodeling,CAF-1 complex,CE			T=0, C=253	T=0.0, C=1.0	0.00001	4138.88	253	T=0/0, C=123/
chr2:70188676	C/C	T	C	SNV	ASPRV1,PCBP1-AS1	PCBP1-AS1:downstream:NR_033872.1, ASPRV1:exonic:NM_	1	100	1	missense	NM_15279	c.145A>G	p.Thr49Ala	-0.87		58		0.01(COSM63	Aspartyl prot	rs3796097	0.4791	YES	6.955E-1 (pos.70188676)	carcinoma	Aspartic pe	aspartic-type endopeptidase activity,integral componer			T=0, C=30	T=0.0, C=1.0	0.00001	446.639	30	T=0/0, C=27/3
chr1:33276424	A/A	G	A	SNV	YARS	YARS:intronic:NM_003680.3	1	100		intronic	NM_00368	c.205-57C	p.?	-0.31					Putative tRN	rs4951787		YES			Tyrosyl-tRN	ATP bindin	Charcot-Marie-Toot	4-(2,2,2-TRIFLUOROETHYL	G=0, A=31	G=0.0, A=1.0	0.00001	465.818	31	G=0/0, A=16/1
chr1:33330432	G/G	A	G	SNV	FNDC5	FNDC5:intronic:NM_001171941.2	1	100		intronic	NM_00117	c.275-34T	p.?	0.96					Domain of un	rs1284368	8.00E-04		1.0 (pos.33330432)		Fibronectin	biological_process,endoplasmic reticulum,extracellular			A=0, G=44	A=0.0, G=1.0	0.00001	726.236	44	A=0/0, G=29/1
chr1:33560338	G/G	A	G	SNV	AZIN2	AZIN2:intronic:NM_052998.3	1	100		intronic	NM_05299	c.753+24A	p.?	-2.71					Pyridoxal-de	rs804428	0.0552	YES	9.999E-1 (pos.33560338)		Antizyme in	agmatine biosynthetic process		L-Arginine,Pyridoxal Pho	A=0, G=47	A=0.0, G=1.0	0.00001	789.259	47	A=0/0, G=29/1
chr9:119495697	C/C	T	C	SNV	ASTN2	ASTN2:exonic:NM_014010.4	1	100	13	synonymous	NM_01401	c.2349A>G	p.(=)	-1.64					Coagulation I	rs7863560	0		1.0 (pos.119495697)		Astrotactin	calcium io	Amphetamine-Related Disorders;Attention D		T=0, C=26	T=0.0, C=1.0	0.00001	371.42	26	T=0/0, C=14/1
chr9:120053776	C/C	T	C	SNV	ASTN2	ASTN2:exonic:NM_014010.4	1	100	2	synonymous	NM_01401	c.459A>G	p.(=)	-4.49					Coagulation I	rs7848630	0.0069	YES	9.909E-1 (pos.120053776)		Astrotactin	calcium io	Amphetamine-Related Disorders;Attention D		T=0, C=100	T=0.0, C=1.0	0.00001	1701.76	100	T=0/0, C=35/6
chr2:24108652	C/C	T	C	SNV	ATAD2B	ATAD2B:exonic:NM_017552.3	1	100	5	missense	NM_01755	c.620A>G	p.Gln207Arg	3.64	1	43	0		ATPase famil	rs7579201	0		1.0 (pos.24108652)		ATPase fam	ATP bindin	Nutrition Disorders;Nutritional and Metaboli		T=0, C=23	T=0.0, C=1.0	0.00001	316.747	23	T=0/0, C=13/1
chr1:1469546	G/A	G	A	SNV	ATAD3A,TMEM240	ATAD3A:utr_3:NM_018188.4, TMEM240:downstream:NM_0	1	95.45	16	utr_3	NM_01818	c.1999G>A	p.?, p.?	-1.65					ATPase famil	rs3128344		YES			ATPase fam	ATP bindin	Brain Diseases:Central Nervous System Dise		G=1, A=21	G=0.0455, A=0.9545	0.00001	271.53	22	G=0/1, A=12/9

chr10:123673339	C/C	T	C	SNV	ATE1	ATE1:exonic:NM_001001976.2	1	100	4	synonymous	NM_00100	c.303A>G	p.(=)	0.37					Arginine-tRN	rs10749435	0.0717	YES	9.313E-1 (pos.123673339)			arginyltransferase activity,cytoplasm,nucleus,proteaso				T=0, C=91	T=0.0, C=1.0	0.00001	1509.14	91	T=0/0, C=60/3
chr1:34038413	C/C	A	C	SNV	CSMD2	CSMD2:intronic:NM_052896.4	1	100		intronic	NM_05289	c.7583-12	p.?	-0.34					CUB domain,:	rs2794602		YES			Cub and Sus	integral cc	Attention Deficit Disorder with Hyperactivity			A=0, C=53	A=0.0, C=1.0	0.00001	781.813	53	A=0/0, C=6/47
chr1:161928327	G/G	A	G	SNV	ATF6	ATF6:exonic:NM_007348.3	1	100	16	synonymous	NM_00734	c.1896A>G	p.(=)	-4.2				0.04(COSM13	Basic region l	rs9482	0.2549	YES	5.405E-1 (pos.16192832	acute_myeloic	Activating t	ATF6-medi	Asbestosis:Brain Di	Pseudoephedrine		A=0, G=24	A=0.0, G=1.0	0.00001	334.792	24	A=0/0, G=21/3
chr11:64680819	A/A	G	A	SNV	ATG2A	ATG2A:exonic:NM_015104.2	1	100	5	synonymous	NM_01510	c.645C>T	p.(=)	-0.24				0.43(COSM37	Autophagy-re	rs618006	0.2737	YES	5.213E-1 (pos.64680819)	adenocarcinoi	Autophagy	autophagosome assembly,autophagy,autophagy of mitc				G=0, A=42	G=0.0, A=1.0	0.00001	685.091	42	G=0/0, A=22/2
chr11:64677293	G/C	G	C	SNV	ATG2A	ATG2A:exonic:NM_015104.2	1	98.23	14	missense	NM_01510	c.1967C>G	p.Pro656Arg	0.97	0.66	103	0	0.06(COSM41	Autophagy-re	rs656195	0		1.0;0.0 (pos.64677293)	neoplasm	Autophagy	autophagosome assembly,autophagy,autophagy of mitc				G=2, C=111	G=0.0177, C=0.9823	0.00001	1681.69	113	G=2/0, C=58/5
chr1:3566706	G/A	G	A	SNV	WRAP73	WRAP73:upstream:NM_017818.3	1	100		intronic	NM_01781	c.-143C>T	p.?	0.15						rs2251098		YES				cell projection organization,centriole,centrosome,ciliar				G=0, A=46	G=0.0, A=1.0	0.00001	738.401	46	G=0/0, A=7/39
chr1:36205166	G/G	A	G	SNV	CLSPN	CLSPN:intronic:NM_022111.3	1	100		intronic	NM_02211	c.3144-36	p.?	0.87						rs577483	0.1336	YES	2.057E-1 (pos.36205166)			DNA binding,DNA damage checkpoint,DNA repair,DNA re				A=0, G=31	A=0.0, G=1.0	0.00001	465.81	31	A=0/0, G=23/8
chr14:96797724	A/A	G	A	SNV	ATG2B	ATG2B:exonic:NM_018036.6	1	100	11	synonymous	NM_01803	c.1719C>T	p.(=)	0.09					Autophagy-re	rs1822372	0.0103	YES	5.581E-1 (pos.96797724)		Autophagy	autophagc	Bone Marrow Diseases:Hematologic Disease			G=0, A=39	G=0.0, A=1.0	0.00001	623.951	39	G=0/0, A=17/2
chr2:242610773	A/A	T	A	SNV	ATG4B	ATG4B:exonic:NM_013325.4	1	100	12	missense	NM_01332	c.1061T>A	p.Leu354Gln	1.37	0.75	113	0		Peptidase fa	rs7601000	0.2132	YES	8.446E-1 (pos.242610773)		Autophagy	C-terminal protein lipidation,autophagosome assembly				T=0, A=25	T=0.0, A=1.0	0.00001	353.019	25	T=0/0, A=14/1
chr1:36565584	G/G	C	G	SNV	COL8A2	COL8A2:intronic:NM_005202.3	1	100		intronic	NM_00520	c.193+67G	p.?	-0.07					C1q domain,(	rs274753		YES			Collagen VII	angiogene	Congenital, Hereditary, and Neonatal Diseas			C=0, G=28	C=0.0, G=1.0	0.00001	408.717	28	C=0/0, G=15/1
chr1:36565617	A/A	G	A	SNV	COL8A2	COL8A2:intronic:NM_005202.3	1	100		intronic	NM_00520	c.193+34C	p.?	-0.78					C1q domain,(	rs274754	0.215	YES	2.092E-1 (pos.36565617)		Collagen VII	angiogene	Congenital, Hereditary, and Neonatal Diseas			G=0, A=36	G=0.0, A=1.0	0.00001	563.723	36	G=0/0, A=15/2
chr1:36773819	G/G	C	G	SNV	SH3D21	SH3D21:intronic:NM_001162530.1	1	100		intronic	NM_00116	c.639+9C>	p.?	0.16					SH3 domain,\	rs11263871	9.00E-04	YES	9.77E-1 (pos.36773819)			extracellular exosome,nucleoplasm,nucleus,plasma me				C=0, G=35	C=0.0, G=1.0	0.00001	543.438	35	C=0/0, G=22/1
chr1:3684107	A/A	T	A	SNV	CCDC27	CCDC27:intronic:NM_152492.2	1	100		intronic	NM_15249	c.1743+98	p.?	-1.02						rs1181872		YES								T=0, A=20	T=0.0, A=1.0	0.00001	263.786	20	T=0/0, A=7/13
chr3:11400019	C/C	T	C	SNV	ATG7	ATG7:exonic:NM_006395.2	1	100	13	missense	NM_00639	c.1412T>C	p.Val471Ala	7.53	0.01	64	0.794		ThiF family,U	rs36117895	0.0376	YES	2.312E-4 (pos.11400019)		Autophagy	Atg12 acti	Anemia;Hematologic Diseases;Hemic and Ly			T=0, C=73	T=0.0, C=1.0	0.00001	1150.51	73	T=0/0, C=38/3
chr1:36923494	C/C	G	C	SNV	MRPS15	MRPS15:intronic:NM_031280.3	1	100		intronic	NM_03128	c.444+30C	p.?	1.31					Ribosomal pr	rs12023818	0.3357	YES	9.685E-2;8.1E-4 (pos.36923494)		Mitochondr	RNA binding,mitochondrial inner membrane,mitochondr				G=0, C=181	G=0.0, C=1.0	0.00001	3004.06	181	G=0/0, C=103/
chr14:51057727	A/A	G	A	SNV	ATL1	ATL1:exonic:NM_001127713.1	1	100	4	synonymous	NM_00112	c.351G>A	p.(=)	2.32					Guanylate-bi	rs1060197	0.2712	YES	8.206E-1 (pos.51057727)		Atlastin GTI	GTP bindir	Congenital Abnormalities:Con		Benign/Likely	G=0, A=209	G=0.0, A=1.0	0.00001	3400.43	209	G=0/0, A=109/
chr11:108183167	G/G	A	G	SNV	ATM	ATM:exonic:NM_000051.3	1	100	40	missense	NM_00005	c.5948A>G	p.Asn1983Ser	-1.06		46	0	0.01(COSM45	Anaphase-pro	rs659243		YES	1.0 (pos.108183167)	squamous_cel	Ataxia-telai	1-phospha	Adenocarcinoma:A	Caffeine	Benign	A=0, G=99	A=0.0, G=1.0	0.00001	1681.12	99	A=0/0, G=48/5
chr1:3732103	C/C	T	C	SNV	CEP104	CEP104:intronic:NM_014704.3	1	100		intronic	NM_01470	c.2663-22	p.?	-2.56						rs4648409	0.4453	YES	5.891E-1 (pos.3732103)		Centrosom	centriole,cilium,glutamate binding,glycine binding,prote				T=0, C=37	T=0.0, C=1.0	0.00001	583.701	37	T=0/0, C=16/2
chr1:37335458	A/G	A	G	SNV	GRIK3	GRIK3:intronic:NM_000831.3	1	96.77		intronic	NM_00083	c.733-56T	p.?	-0.62					Bacterial ext	rs577094	0.0852	YES			Glutamate	G-protein	Bronchial Neoplasr	Glutamic Acid		A=1, G=30	A=0.0323, G=0.9677	0.00001	435.398	31	A=1/0, G=9/21
chr1:37335485	G/G	A	G	SNV	GRIK3	GRIK3:intronic:NM_000831.3	1	100		intronic	NM_00083	c.733-83T	p.?	-0.65					Bacterial ext	rs509284		YES			Glutamate	G-protein	Bronchial Neoplasr	Glutamic Acid		A=0, G=31	A=0.0, G=1.0	0.00001	464.967	31	A=0/0, G=10/2
chr1:37335494	G/T	G	T	SNV	GRIK3	GRIK3:intronic:NM_000831.3	1	96.77		intronic	NM_00083	c.733-92C	p.?	1.78					Bacterial ext	rs577813		YES			Glutamate	G-protein	Bronchial Neoplasr	Glutamic Acid		G=1, T=30	G=0.0323, T=0.9677	0.00001	435.345	31	G=0/1, T=10/2
chr1:3746589	G/G	C	G	SNV	CEP104	CEP104:intronic:NM_014704.3	1	100		intronic	NM_01470	c.1837-28	p.?	0.23						rs2275826	0.0463	YES	4.859E-1 (pos.3746589)		Centrosom	centriole,cilium,glutamate binding,glycine binding,prote				C=0, G=20	C=0.0, G=1.0	0.00001	263.777	20	C=0/0, G=10/1
chr1:3747603	C/C	T	C	SNV	CEP104	CEP104:intronic:NM_014704.3	1	100		intronic	NM_01470	c.1836+7A	p.?	-1.47				0.02(COSM5766584)		rs6413779,rs763310	0.0462	YES	0.0 (pos.3747598),0.0 (p	adenocarcinoi	Centrosom	centriole,cilium,glutamate binding,glycine binding,prote				T=0, C=53	T=0.0, C=1.0	0.00001	783.583	53	T=0/0, C=31/2
chr1:3764216	C/T	C	T	SNV	CEP104	CEP104:intronic:NM_014704.3	1	58.33		intronic	NM_01470	c.288-39G	p.?	-0.65						rs10492942	0.2473	YES	4.633E-4 (pos.3764216)		Centrosom	centriole,cilium,glutamate binding,glycine binding,prote				C=10, T=14	C=0.4167, T=0.5833	0.00001	135.338	24	C=4/6, T=3/11
chr1:3774138	T/C	T	C	SNV	CEP104,DFFB	CEP104:upstream:NM_014704.3, DFFB:utr_5:NM_004402.3	1	53.13		intronic	NM_01470	c.-5167A>(	p.?, p.?	-0.21					CIDE-N domai	rs12723005	0.2539	YES	2.301E-1 (pos.3774138)		Centrosom	DNA binding,DNA catabolic process,apoptotic DNA fragm				T=15, C=17	T=0.4688, C=0.5313	0.00001	173.497	32	T=9/6, C=2/15
chr15:25925094	C/G	C	G	SNV	ATP10A	ATP10A:exonic:NM_024490.3	1	51.85	21	missense	NM_02449	c.3894G>C	p.Arg1298Ser	-1.4	1	110	0	0.01(COSM39	Cation trans	rs3816800	0.4756	YES	3.742E-1 (pos.25925094)	acute_myeloic	ATPase, cla	ATP bindin	Autistic Disorder:Child Development Disorde			C=9, G=14	C=0.3913, G=0.6087	0.00001	172.026	27	C=3/6, G=11/3
chr1:38301250	G/G	A	G	SNV	MTF1	MTF1:intronic:NM_005955.2	1	100		intronic	NM_00595	c.853+93T	p.?	-0.6					C2H2-type zir	rs3912368		YES			Metal-regul	DNA bindii	Arthritis;Arthritis, F	Copper		A=0, G=47	A=0.0, G=1.0	0.00001	786.649	47	A=0/0, G=24/2
chr5:160097632	A/A	G	A	SNV	ATP10B	ATP10B:exonic:NM_025153.2	1	100	7	synonymous	NM_02515	c.513C>T	p.(=)	-0.47				0.05(COSM37	Cation trans	rs958911	0.1336	YES	8.826E-1 (pos.16009763	adenocarcinoma		ATP binding,cytoplasmic vesicle membrane,endoplasmi				G=0, A=43	G=0.0, A=1.0	0.00001	705.843	43	G=0/0, A=20/2
chr1:38464639	G/G	C	G	SNV	FHL3	FHL3:intronic:NM_004468.4	1	100		intronic	NM_00446	c.331+7G>	p.?	0.05					LIM domain	rs4570384	0		1.0 (pos.38464639)		Four-and-a-	Z disc,actin binding,actin cytoskeleton organization,foca				C=0, G=27	C=0.0, G=1.0	0.00001	389.988	27	C=0/0, G=24/3
chr1:39305403	T/T	C	T	SNV	RRAGC	RRAGC:intronic:NM_022157.3	1	100		intronic	NM_02215	c.1049-27	p.?	0.25					ADP-ribosyla	rs17499125	0.2088	YES	1.256E-1 (pos.39305403)		RAS-related	EGO complex,GDP binding,GTP binding,GTPase activity,G				C=0, T=25	C=0.0, T=1.0	0.00001	352.857	25	C=0/0, T=13/1
chr1:39325063	C/C	T	C	SNV	RRAGC	RRAGC:intronic:NM_022157.3	1	100		intronic	NM_02215	c.237+19A	p.?	-0.38					ADP-ribosyla	rs1925678		YES	4.076E-1 (pos.39325063)		RAS-related	EGO complex,GDP binding,GTP binding,GTPase activity,G				T=0, C=42	T=0.0, C=1.0	0.00001	683.615	42	T=0/0, C=18/2
chr1:39766041	A/G	A	G	SNV	MACF1	MACF1:intronic:NM_012090.5	1	45.45		intronic	NM_01209	c.2649+7A	p.?	1.13					Calponin hon	rs41270803	0.2208	YES	1.956E-1 (pos.39766041)		Microtubul	ATPase activity,Golgi apparatus,Golgi to plasma membr				A=18, G=15	A=0.5455, G=0.4545	0.00001	145.253	33	A=5/13, G=1/1
chr13:113479820	G/G	A	G	SNV	ATP11A	ATP11A:exonic:NM_032189.3	1	100	11	missense	NM_03218	c.949A>G	p.Met317Val	3.16	1	21	0	0.24(COSM14	Cation trans	rs368865	0.2764	YES	8.455E-1;1.156E-4 (pos.1	acute_myeloic	ATPase, cla	ATP bindin	Idiopathic Interstitial Pneumonias;Idiopathi			A=0, G=20	A=0.0, G=1.0	0.00001	263.785	20	A=0/0, G=15/5
chr1:40036847	G/C	G	C	SNV	LOC101929516,PABPC4	PABPC4:intronic:NM_001135653.1, LOC101929516:intronic	1	50		intronic	NM_00113	c.503+59C	p.?	2.02					Poly-adenyla	rs2293476		YES			Polyadenyla	RNA bindir	Autoimmune Diseases:Female Urogenital Di			G=14, C=14	G=0.5, C=0.5	0.00001	127.758	28	G=8/6, C=9/5
chr1:40149515	G/G	A	G	SNV	HPCAL4	HPCAL4:intronic:NM_016257.3	1	100		intronic	NM_01625	c.378+94T	p.?	-0.69					EF hand,EF-ha	rs113549324,rs784633		YES				calcium channel regulator activity,calcium ion binding,c				A=0, G=22	A=0.0, G=1.0	0.00001	298.884	22	A=0/0, G=15/7
chr1:40149550	AC/AC	A	AC	INDEL	HPCAL4	HPCAL4:intronic:NM_016257.3	1	100		intronic	NM_01625	c.378+59T	p.?	-1.84					EF hand,EF-hand domain,EF-hand domain pair							calcium channel regulator activity,calcium ion binding,c				A=0, AC=27	A=0.0, AC=1.0	0.00001	342.986	27	A=0/0, AC=15/
chr1:116932921	G/A	G	A	SNV	ATP1A1	ATP1A1:exonic:NM_000701.7	1	52.38	9	synonymous	NM_00070	c.1110G>A	p.(=)	-1.1					Cation trans	rs11540956	0.0266	YES	1.165E-4 (pos.116932921)		ATPase, Na	ADP bindir	Adenoma:Adrenal (	Acetyldigitoxin,Almitrine		G=10, A=11	G=0.4762, A=0.5238	0.00001	88.7811	21	G=6/4, A=4/7
chr19:42489516	C/C	A	C	SNV	ATP1A3	ATP1A3:exonic:NM_152296.4	1	100	7	synonymous	NM_15229	c.666T>G	p.(=)	-4.11				0.02(COSM41	Cation trans	rs2217342	0.0985	YES	8.74E-1;0.0 (pos.424895	neoplasm	ATPase, Na	ATP bindin	Arthritis;Ataxia;Bas	Rubidium	Benign/Likely	A=0, C=51	A=0.0, C=1.0	0.00001	754.582	51	A=0/0, C=23/2
chr1:40536074	CT/CT	C	CT	INDEL	CAP1	CAP1:intronic:NM_001105530.1	1	100		intronic	NM_00110	c.1118-28	p.?	0.1					Adenylate cy	rs138880025,rs5819	0	YES	1.0 (pos.40536074)		TNF recepto	actin bind	Bone Diseases;Bone Diseases, Metabolic;Mu			C=0, CT=21	C=0.0, CT=1.0	0.00001	281.122	21	C=0/0, CT=11/
chr1:40556922	C/C	T	C	SNV	PPT1	PPT1:intronic:NM_000310.3	1	100		intronic	NM_00031	c.433+79A	p.?	-0.38					Palmitoyl prc	rs3122439					Palmitoyl-p	Golgi appa	Congenital, Heredit	1-Hexadecylsulfonyl Fluc		T=0, C=23	T=0.0, C=1.0	0.00001	320.984	23	T=0/0, C=12/1
chr1:41101572	G/G	A	G	SNV	RIMS3	RIMS3:intronic:NM_014747.2	1	100		intronic	NM_01474	c.359+16T	p.?	0.63					C2 domain	rs2253835	0.2029	YES	5.562E-1 (pos.41101572)		Protein regi	calcium ion regulated exocytosis,calcium ion-regulated				A=0, G=47	A=0.0, G=1.0	0.00001	789.122	47	A=0/0, G=20/2
chr1:41107360	C/C	T	C	SNV	RIMS3	RIMS3:intronic:NM_014747.2	1	100		intronic	NM_01474	c.217+21A	p.?	-2.61					C2 domain	rs491619	0.2156	YES	5.427E-1 (pos.41107360)		Protein regi	calcium ion regulated exocytosis,calcium ion-regulated				T=0, C=53	T=0.0, C=1.0	0.00001	784.972	53	T=0/0, C=26/2
chr1:160124875	A/A	G	A	SNV	ATP1A4	ATP1A4:exonic:NM_144699.3	1	100	3	missense	NM_14469	c.248G>A	p.Gly83Asp	6.09	1	94	0	0.07(COSM54	Cation trans	rs6427504	0.2847	YES	6.695E-1;0.0 (pos.16012	acute_myeloic	ATPase, Na	ATP binding,ATP hydrolysis cou		Rubidium chloride Rb-82		G=0, A=61	G=0.0, A=1.0	0.00001	921.854	61	G=0/0, A=26/3
chr1:41540853	T/T	A	T	SNV	SCMH1	SCMH1:intronic:NM_001172219.1	1	100		intronic	NM_00117	c.978+38T	p.?	-1.14					RNA binding F	rs2011981	0.2163	YES	9.607E-1 (pos.41540853)		Sex comb o	DNA binding,DNA binding trans		Beta-Mercaptoethanol		A=0, T=36	A=0.0, T=1.0	0.00001	563.743	36	A=0/0, T=33/3
chr1:41540861	C/C	T	C	SNV	SCMH1	SCMH1:intronic:NM_001172219.1	1	100		intronic	NM_00117	c.978+30A	p.?	0.3					RNA binding F	rs2011982	0.2167	YES	9.614E-1 (pos.41540861)		Sex comb o	DNA binding,DNA binding trans		Beta-Mercaptoethanol		T=0, C=38	T=0.0, C=1.0	0.00001	603.617	38	T=0/0, C=33/5
chr1:41949595	A/A	G	A	SNV	EDN2	EDN2:intronic:NM_001956.4	1	100		intronic	NM_00195	c.221+123	p.?	-1.03					Endothelin fa	rs2759256					Endothelin-	G-protein	Arrhythmias, Cardiac;Bradycardia;Cardiovas			G=0, A=30	G=0.0, A=1.0	0.00001	446.399	30	G=0/0, A=21/9
chr12:58335626	A/T	A	T	SNV	ATP23	ATP23:exonic:NM_033276.3	1	50	1	missense	NM_03327	c.142A>T	p.Ser48Cys	0.66	0.15	112	0.369	0.07(COSM43	Peptidase M	rs3751325	0.3179	YES	6.731E-1;0.0 (pos.58335	angioimmunoblastic_T_cel		DNA-dependent protein kinase activity,DNA-dependent				A=25, T=25	A=0.5, T=0.5	0.00001	288.96	50	A=4/21, T=3/2
chr16:28911946	G/A	G	A	SNV	ATP2A1	ATP2A1:exonic:NM_173201.3	1	55.77	15	synonymous	NM_17320	c.1809G>A	p.(=)	-0.75					Cation trans	rs151309999	0.0072		0.0 (pos.28911946)		ATPase, Ca	ATP bindin	Cardiovascular Dis	(6AR,11AS	Benign/Likely	G=23, A=29	G=0.4423, A=0.5577	0.00001	299.78	52	G=21/2, A=26/
chr3:10413715	A/A	G	A	SNV	ATP2B2	ATP2B2:exonic:NM_001001331.2	1	100	12	synonymous	NM_00100	c.1437C>T	p.(=)	0.83				0.81(COSM14	Cation trans	rs2289274	0.3088	YES	3.059E-1 (pos.10413715)	adenocarcinoi	ATPase, Ca	ATP bindin	Ear Diseases;Hearing Disorders;Hearing Loss			G=0, A=24	G=0.0, A=1.0	0.00001	334.792	24	G=0/0, A=19/5
chr1:203652444	G/G	A	G	SNV	ATP2B4	ATP2B4:exonic:NM_001684.4	1	100	2	synonymous	NM_00168	c.111A>G	p.(=)	-3.59					Cation trans	rs1419114	0.1023	YES	9.995E-1 (pos.203652444)		ATPase, Ca	ATP binding,ATP hydrolysis coupled cation transmembra				A=0, G=46	A=0.0, G=1.0	0.00001	766.702	46	A=0/0, G=23/2
chr16:84444349	C/C	A	C	SNV	ATP2C2	ATP2C2:exonic:NM_014861.3	1	100	6	missense	NM_01486	c.493A>C	p.Met165Leu	1.11	1	15	0		Cation trans	rs247818	0		1.0 (pos.84444349)		ATPase, Ca	ATP bindin	Attention Deficit Disorder with Hyperactivity			A=0, C=20	A=0.0, C=1.0	0.00001	259.948	20	A=0/0, C=8/12
chr16:84485573	A/A	C	A	SNV	ATP2C2	ATP2C2:exonic:NM_014861.3	1	100	18	synonymous	NM_01486	c.1707C>A	p.(=)	1.59					Cation trans	rs247885	0		1.0 (pos.84485573)		ATPase, Ca	ATP bindin	Attention Deficit Disorder with Hyperactivity			C=0, A=86	C=0.0, A=1.0	0.00001	1410.91	86	C=0/0, A=28/5
chr1:42656819	A/A	T	A	SNV	FOXJ3	FOXJ3:intronic:NM_014947.4	1	100		intronic	NM_01494	c.1351+15	p.?	0.27					Forkhead dor	rs16829212		YES			Forkhead b	DNA binding,transcription factor activity,RNA polymeras				T=0, A=28	T=0.0, A=1.0	0.00001	408.717	28	T=0/0, A=14/1
chr10:7849688	T/C	T	C	SNV	ATP5C1	ATP5C1:utr_3:NM_001001973.2	1	57.14	10	utr_3	NM_00100	c.994T>C	p.?	1.39					ATP synthase	rs4655	0.362	YES			ATP synthas	ATP biosynthetic process,ATP		1-ACETYL-2-CARBOXYPIPE		T=9, C=12	T=0.4286, C=0.5714	0.00001	109.102	21	T=6/3, C=5/7
chr1:43300663	C/C	T	C	SNV	ERMAP	ERMAP:intronic:NM_018538.3	1	100		intronic	NM_01853	c.434-46T	p.?	-0.15					Immunoglobu	rs4660672	0.283	YES	3.756E-1 (pos.43300663)		Erythroblas	cytoplasm,integral component of membrane,plasma me				T=0, C=28	T=0.0, C=1.0	0.00001	406.557	28	T=0/0, C=18/1
chr12:124210782	C/C	T	C	SNV	ATP6V0A2	ATP6V0A2:exonic:NM_012463.3	1	100	5	synonymous	NM_01246	c.471T>C	p.(=)	-1.99				0.03(COSM37	V-type ATPas	rs1399961	0.3793	YES	9.453E-1 (pos.12421078	adenocarcinoi	ATPase, H+	ATP hydrol	Congenital, Hereditary, and Ne		Benign	T=0, C=98	T=0.0, C=1.0	0.00001	1651.37	98	T=0/0, C=35/6
chr7:138418910	G/A	G	A	SNV	ATP6V0A4	ATP6V0A4:exonic:NM_130841.2	1	50	15	synonymous	NM_13084	c.1662C>T	p.(=)	-0.15					V-type ATPas	rs1026435	0.2785	YES	8.087E-1 (pos.138418910)		ATPase, H+	ATP hydrol	Acid-Base Imbalance;Acidosis;		Benign	G=30, A=31	G=0.4918, A=0.5082	0.00001	311.577	62	G=10/20, A=8/
chr2:71187104	G/A	G	A	SNV	ATP6V1B1	ATP6V1B1:exonic:NM_001692.3	1	52.38	6	missense	NM_00169	c.481G>A	p.Glu161Lys	9.65	0.01	56	0.001		ATP synthase	rs114234874	0.0219	YES	1.157E-4 (pos.71187104)			ATP bindin	Acid-Base Imbalance;Acidosis;		Benign/Likely	G=10, A=11	G=0.4762, A=0.5238	0.00001	95.3003	21	G=6/4, A=7/4
chr6:31506624	C/C	T	C	SNV	ATP6V1G2-DDX39B,DDX39B	DDX39B:exonic:NM_080598.5, ATP6V1G2-DDX39B:exonic_r	1	100	4	synonymous	NR_03785	c.348A>G	p.(=)	-0.13				0.39(COSM12	DEAD/DEAH b	rs1129640	0.1809	YES	9.252E-1 (pos.31506624)	neuroblastom	DEAD box p	ATP binding,ATP-dependent RN		Isopropyl Alcohol		T=0, C=27	T=0.0, C=1.0	0.00001	389.988	27	T=0/0, C=13/1
chr1:43903589	G/T	G	T	SNV	SZT2	SZT2:intronic:NM_015284.3	1	50		intronic	NM_01528	c.6337+16	p.?	-0.74						rs2782648	0.414	YES	7.166E-2;0.0 (pos.43903589)		Seizure thre	GATOR1 complex,GATOR2 complex,KICSTO			Benign	G=16, T=16	G=0.5, T=0.5	0.00001	158.379	32	G=6/10, T=12/
chr1:44070691	A/G	A	G	SNV	PTPRF	PTPRF:intronic:NM_002840.4	1	47.62		intronic	NM_00284	c.3135+20	p.?	-0.58					Fibronectin t	rs17371903	0.3723	YES	2.351E-1 (pos.44070691)		Protein tyrc	cell adhes	Digestive System Diseases;Digestive System			A=11, G=10	A=0.5238, G=0.4762	0.00001	82.29	21	A=7/4, G=5/5
chr6:31497706	T/T	C	T	SNV	ATP6V1G2-DDX39B,DDX39B,MCCD1	DDX39B:downstream:NM_080598.5, MCCD1:exonic:NM_00	1	100	2	synonymous	NM_00101	c.354C>T,	p.(=), p.?	-3.39					Mitochondria	rs2075582	0.2423	YES	7.523E-1 (pos.31497706)		DEAD box p	ATP binding,ATP-dependent RN		Isopropyl Alcohol		C=0, T=46	C=0.0, T=1.0	0.00001	768.311	46	C=0/0, T=19/2
chr1:44086080	C/C	T	C	SNV	PTPRF	PTPRF:intronic:NM_002840.4	1	100		intronic	NM_00284	c.5239-45	p.?	-0.73					Fibronectin t	rs568639	0.251	YES	7.863E-1;0.0 (pos.44086080)		Protein tyrc	cell adhes	Digestive System Diseases;Digestive System			T=0, C=22	T=0.0, C=1.0	0.00001	298.892	22	T=0/0, C=17/5
chr1:44386615	G/G	A	G	SNV	ST3GAL3	ST3GAL3:intronic:NM_174964.3	1	100		intronic	NM_17496	c.1083+15	p.?	-1.19					Glycosyltrans	rs3120803	0.0761	YES	8.484E-1 (pos.44386615)		ST3 beta-ga	Golgi ciste	Amyotrophic Lateral Sclerosis;		Benign	A=0, G=44	A=0.0, G=1.0	0.00001	726.558	44	A=0/0, G=11/3
chr1:44395786	T/T	C	T	SNV	ST3GAL3	ST3GAL3:intronic:NM_174964.3	1	100		intronic	NM_17496	c.1084-18	p.?	1.21					Glycosyltrans	rs2108202	0.2215	YES	6.337E-1 (pos.44395786)		ST3 beta-ga	Golgi ciste	Amyotrophic Lateral Sclerosis;		Benign	C=0, T=28	C=0.0, T=1.0	0.00001	398.601	28	C=0/0, T=12/1
chr1:44426948	A/A	G	A	SNV	IPO13	IPO13:intronic:NM_014652.3	1	100		intronic	NM_01465	c.2344+14	p.?	-0.18					Exportin 1-lik	rs2906596	0.0655	YES	8.45E-1 (pos.44426948)		Importin 13	Ran GTPase binding,cytoplasm,nuclear localization sequ				G=0, A=38	G=0.0, A=1.0	0.00001	608.192	38	G=0/0, A=16/2
chr6:31497744	A/A	G	A	SNV	ATP6V1G2-DDX39B,DDX39B,MCCD1	DDX39B:downstream:NM_080598.5, MCCD1:utr_3:NM_001	1	100	2	utr_3	NM_00101	c.392G>A,	p.?, p.?	-2.03					Mitochondria	rs3093979	0.1528	YES	9E-1 (pos.31497744)		DEAD box p	ATP binding,ATP-dependent RN		Isopropyl Alcohol		G=0, A=40	G=0.0, A=1.0	0.00001	648.647	40	G=0/0, A=14/2
chr1:45276427	A/A	G	A	SNV	BTBD19	BTBD19:intronic:NM_001136537.1	1	100		intronic	NM_00113	c.354+148	p.?	-0.38					BTB And C-ter	rs3795718		YES								G=0, A=38	G=0.0, A=1.0	0.00001	603.682	38	G=0/0, A=20/1
chr13:52515354	A/G	A	G	SNV	ATP7B	ATP7B:exonic:NM_000053.3	1	55.77	16	missense	NM_00005	c.3419T>C	p.Val1140Ala	0.11	0.88	64	0	0(COSM3765	E1-E2 ATPase	rs1801249	0.4088	YES	3.938E-1 (pos.52515354)	ductal_carcinc	ATPase, Cu	ATP bindin	ACMG Recommend	Carboplat	Benign/Likely	A=23, G=29	A=0.4423, G=0.5577	0.00001	296.101	52	A=11/12, G=1
chr13:26273385	C/C	G	C	SNV	ATP8A2	ATP8A2:exonic:NM_016529.5	1	100	25	synonymous	NM_01652	c.2286G>C	p.(=)	0.57					Cation trans	rs6491088	2.00E-04		1.0 (pos.26273385)		ATPase, cla	ATP bindin	Ataxia;Brain Diseases;Central		Likely benign	G=0, C=28	G=0.0, C=1.0	0.00001	408.718	28	G=0/0, C=20/8
chr1:45294156	C/C	T	C	SNV	PTCH2	PTCH2:intronic:NM_003738.4	1	100		intronic	NM_00373	c.1590+22	p.?	-1.92					Patched fami	rs2273940	0.3426	YES	4.835E-1 (pos.45294156)		Patched, Dr	cell fate de	Abnormalities, Multiple;Basal Cell Nevus Syn			T=0, C=31	T=0.0, C=1.0	0.00001	469.966	31	T=0/0, C=13/1
chr1:45474157	A/A	G	A	SNV	HECTD3	HECTD3:intronic:NM_024602.5	1	100		intronic	NM_02460	c.1238+51	p.?	-0.24					Anaphase-pro	rs2298006	0.3037	YES	6.056E-1 (pos.45474157)			cytoplasm,perinuclear region of cytoplasm,proteasome				G=0, A=33	G=0.0, A=1.0	0.00001	504.562	33	G=0/0, A=12/2
chr1:45475566	G/G	A	G	SNV	HECTD3	HECTD3:intronic:NM_024602.5	1	100		intronic	NM_02460	c.759+92T	p.?	-0.2					Anaphase-pro	rs6429552		YES				cytoplasm,perinuclear region of cytoplasm,proteasome				A=0, G=23	A=0.0, G=1.0	0.00001	316.747	23	A=0/0, G=16/7
chr1:45476069	A/A	C	A	SNV	HECTD3	HECTD3:intronic:NM_024602.5	1	100		intronic	NM_02460	c.531-104	p.?	-0.03					Anaphase-pro	rs7533668						cytoplasm,perinuclear region of cytoplasm,proteasome				C=0, A=20	C=0.0, A=1.0	0.00001	263.746	20	C=0/0, A=12/8
chr18:55364852	G/G	A	G	SNV	ATP8B1	ATP8B1:exonic:NM_005603.5	1	100	8	synonymous	NM_00560	c.696T>C	p.(=)	0.06					Cation trans	rs319438	1.00E-04		9.998E-1;0.0 (pos.55364852)		ATPase, cla	ATP bindin	Bile Duct Diseases;Biliary Trac		Benign	A=0, G=38	A=0.0, G=1.0	0.00001	603.784	38	A=0/0, G=19/1
chr1:46080947	C/C	A	C	SNV	NASP	NASP:intronic:NM_002482.3	1	100		intronic	NM_00248	c.1856-30	p.?	-0.97					SHNi-TPR,Tet	rs1972410	0.292	YES	6.677E-1;0.0 (pos.46080947)		Nuclear aut	DNA replication,DNA replication-dependent nucleosome				A=0, C=65	A=0.0, C=1.0	0.00001	996.464	65	A=0/0, C=24/4
chr1:46083706	G/G	C	G	SNV	NASP	NASP:intronic:NM_002482.3	1	100		intronic	NM_00248	c.2287-27	p.?	0.43					SHNi-TPR,Tet	rs41310432	0.2261	YES	1.817E-2;0.0 (pos.46083706)		Nuclear aut	DNA replication,DNA replication-dependent nucleosome				C=0, G=56	C=0.0, G=1.0	0.00001	832.093	56	C=0/0, G=26/3
chr18:55335411	CG/CG	C	CG	INDEL	ATP8B1,LOC100505549	LOC100505549:utr_3:NM_001242804.1, ATP8B1:intronic:N	1	100	3	utr_3	NM_00124	c.313_314	p.?, p.?	-1.07					Cation trans	rs138781802,rs386803631			0.0 (pos.55335411)		ATPase, cla	ATP bindin	Bile Duct Diseases;Biliary Tract Diseases;Chc			C=0, CG=45	C=0.0, CG=1.0	0.00001	508.103	45	C=0/0, CG=27/
chr1:46089225	G/G	C	G	SNV	CCDC17	CCDC17:intronic:NM_001114938.2	1	100		intronic	NM_00111	c.270+4G>	p.?	0.16						rs3014240		YES	6.9E-1 (pos.46089225)			protein binding				C=0, G=27	C=0.0, G=1.0	0.00001	389.975	27	C=0/0, G=16/1
chr1:154317153	T/T	C	T	SNV	ATP8B2	ATP8B2:exonic:NM_020452.3	1	100	21	synonymous	NM_02045	c.2277C>T	p.(=)	-3.74				0.04(COSM37	Cation trans	rs1194587	0.4726	YES	6.057E-1 (pos.15431715	acute_myeloic	ATPase, cla	ATP binding,Golgi apparatus,Golgi organization,endopla				C=0, T=37	C=0.0, T=1.0	0.00001	583.704	37	C=0/0, T=9/28
chr20:50287790	G/G	A	G	SNV	ATP9A	ATP9A:exonic:NM_006045.2	1	100	12	synonymous	NM_00604	c.1044T>C	p.(=)	-0.75					Cation trans	rs2255342	0.1956	YES	8.035E-1 (pos.50287790)			ATP binding,early endosome,early endosome membrane				A=0, G=39	A=0.0, G=1.0	0.00001	623.973	39	A=0/0, G=17/2
chr3:142222284	A/G	A	G	SNV	ATR	ATR:exonic:NM_001184.3	1	47.62	30	synonymous	NM_00118	c.5208T>C	p.(=)	1.33				0.8(COSM400	FAT domain,F	rs2227931	0.4189	YES	3.579E-1 (pos.14222228	acute_myeloic	Anthrax tox	ATP bindin	Bone Diseases;Bone Diseases,		Benign/Likely	A=11, G=10	A=0.5238, G=0.4762	0.00001	82.2879	21	A=3/8, G=6/4
chr3:142277536	A/G	A	G	SNV	ATR	ATR:exonic:NM_001184.3	1	45.83	8	synonymous	NM_00118	c.1815T>C	p.(=)	0.6				0.14(COSM40	FAT domain,F	rs2227929	0.414	YES	3.554E-1 (pos.14227753	NS,acute_mye	Anthrax tox	ATP bindin	Bone Diseases;Bone Diseases,		Benign	A=26, G=22	A=0.5417, G=0.4583	0.00001	240.969	48	A=8/18, G=9/1
chr2:27435250	A/G	A	G	SNV	ATRAID,SLC5A6	SLC5A6:upstream:NM_021095.2, ATRAID:exonic:NM_00117	1	95.65	1	missense	NM_02109	c.-4732T>(	p.?, p.Asp5Gly	0.03	1	94	0	0(COSM4133	Human growt	rs1275533	0.0151	YES	9.974E-1 (pos.27435250)	neoplasm	Solute carri	biotin metabolic process,bioti		Biotin,Gabapentin Enaca		A=1, G=22	A=0.0435, G=0.9565	0.00001	288.969	23	A=1/0, G=11/1
chr1:46818416	AT/CC	AT	CC	MNV	NSUN4	NSUN4:intronic:NM_199044.3	2	50		intronic	NM_19904	c.593-124	p.?	0.1, -3.31					16S rRNA met	rs17413694,rs386630894,rs771		YES			NOP2/SUN	mature ribosome assembly,methyltransferase activity,r				AT=31, CC=	AT=0.5, CC=0.5	0.00001	305.391	62	AT=11/20, CC=
chr1:46827214	C/C	A	C	SNV	NSUN4	NSUN4:intronic:NM_199044.3	1	100		intronic	NM_19904	c.879-28A	p.?	-0.36					16S rRNA met	rs324414	0.0203	YES	1.0 (pos.46827214)		NOP2/SUN	mature ribosome assembly,methyltransferase activity,r				A=0, C=39	A=0.0, C=1.0	0.00001	623.972	39	A=0/0, C=20/1
chr20:3564672	T/T	C	T	SNV	ATRN	ATRN:exonic:NM_139321.2	1	100	17	synonymous	NM_13932	c.2892C>T	p.(=)	-1.04				0.18(COSM56	CUB domain,	rs235540	0.2629	YES	9.988E-1 (pos.3564672)	ductal_carcinc	Attractin (m	carbohydrate binding,cerebellum development,cytopla				C=0, T=71	C=0.0, T=1.0	0.00001	1111.48	71	C=0/0, T=35/3
chr1:47138444	T/T	C	T	SNV	TEX38	TEX38:intronic:NM_001145474.3	1	100		intronic	NM_00114	c.38-101C	p.?	0.13					Testis highly	rs616249						integral component of membrane				C=0, T=25	C=0.0, T=1.0	0.00001	353.019	25	C=0/0, T=16/9
chr1:47582292	T/G	T	G	SNV	CYP4Z1	CYP4Z1:intronic:NM_178134.2	1	46.58		intronic	NM_17813	c.1267-30	p.?	-0.67					Cytochrome	rs10890465	0.336	YES	9.67E-1 (pos.47582292)			aromatase activity,endoplasmic reticulum membrane,h				T=78, G=68	T=0.5342, G=0.4658	0.00001	725.987	146	T=41/37, G=37
chr10:117075175	A/A	G	A	SNV	ATRNL1	ATRNL1:exonic:NM_207303.4	1	100	18	missense	NM_20730	c.2966G>A	p.Ser989Asn	-0.62	0.58	46	0		CUB domain,	rs1953758	0.0315	YES	9.97E-1 (pos.117075175)		Attractin-lik	G-protein coupled receptor signaling pathway,carbohyd				G=0, A=151	G=0.0, A=1.0	0.00001	2418.29	151	G=0/0, A=83/6
chr6:16327615	G/G	A	G	SNV	ATXN1	ATXN1:exonic:NM_001128164.1	1	100	7	synonymous	NM_00112	c.927T>C	p.(=)	-1.56				0.03(COSM37	Ataxin-1 and	rs179990	0.3349	YES	8.624E-1;0.0 (pos.16327	adenocarcinoi	Ataxin-1	DNA bindii	Ataxia;Brain Diseases;Central		Likely benign	A=0, G=31	A=0.0, G=1.0	0.00001	465.819	31	A=0/0, G=22/9
chr3:63968083	A/G	A	G	SNV	ATXN7	ATXN7:exonic:NM_001177387.1	1	50	6	missense	NM_00117	c.974A>G	p.Asn325Ser	0.65	0.67	46	0	0.35(COSM59	SCA7, zinc-bir	rs61736574	0.0108	YES	1.445E-4 (pos.63968083)	basal_cell_car	Ataxin 7	chromatin	Arsenic Poisoning;Ataxia;Brain Diseases;Cen			A=25, G=25	A=0.5, G=0.5	0.00001	288.837	50	A=16/9, G=13/
chr1:49332969	G/G	A	G	SNV	AGBL4	AGBL4:intronic:NM_032785.3	1	100		intronic	NM_03278	c.595-67T	p.?	1.97					Zinc carboxy	rs61785986	0.3636	YES			ATP/GTP-bir	C-termina	Mental Disorders:Substance-Related Disorde			A=0, G=32	A=0.0, G=1.0	0.00001	485.141	32	A=0/0, G=16/1
chr17:42268274	T/T	C	T	SNV	ATXN7L3,TMUB2	ATXN7L3:downstream:NM_020218.1, TMUB2:utr_3:NM_00	1	100	4	utr_3	NM_00107	c.1008C>T	p.?, p.?	-0.05					Ubiquitin fan	rs757430	0.1353	YES	9.949E-1 (pos.42268274)			SAGA complex,histone deubiquitination,integral compo				C=0, T=35	C=0.0, T=1.0	0.00001	543.434	35	C=0/0, T=17/1
chr1:52489322	A/A	G	A	SNV	TXNDC12	TXNDC12:intronic:NM_015913.3	1	100		intronic	NM_01591	c.356-76C	p.?	0.89					Thioredoxin,	rs2783178,rs386631154,rs8798		YES			Thioredoxir	cell differentiation,cell redox h		Glutathione		G=0, A=20	G=0.0, A=1.0	0.00001	263.786	20	G=0/0, A=9/11
chr1:52820226	A/A	G	A	SNV	CC2D1B	CC2D1B:intronic:NM_032449.2	1	100		intronic	NM_03244	c.2448+54	p.?	-0.42					C2 domain	rs2820334	0					RNA polymerase II core promoter proximal region seque				G=0, A=53	G=0.0, A=1.0	0.00001	782.027	53	G=0/0, A=24/2
chr1:53072741	A/G	A	G	SNV	GPX7	GPX7:intronic:NM_015696.4	1	47.37		intronic	NM_01569	c.400+124	p.?	0.72					AhpC/TSA fan	rs1970950		YES			Glutathione	catalase activity,cellular oxida		Glutathione		A=50, G=45	A=0.5263, G=0.4737	0.00001	484.222	95	A=2/48, G=1/4
chr20:54961463	C/C	T	C	SNV	AURKA	AURKA:exonic:NM_003600.3	1	100	3	missense	NM_00360	c.169A>G	p.Ile57Val	-0.46	1	29	0		Protein kinas	rs1047972	0.1624	YES	8.776E-1 (pos.54961463)		Aurora kina	ATP bindin	Autoimmune Disea	1-(3-chlorophenyl)-3-(5-2		T=0, C=34	T=0.0, C=1.0	0.00001	524.186	34	T=0/0, C=7/27
chr17:8108331	G/G	A	G	SNV	AURKB	AURKB:exonic:NM_004217.3	1	100	9	missense	NM_00421	c.893T>C	p.Met298Thr	1.25	0.34	81	0	0.12(COSM41	Protein kinas	rs1059476	0.1171	YES	5.485E-1 (pos.8108331)	acute_myeloid_leukaemia		ATP bindin	Autoimmune Disea	AT9283,HESPERIDIN,N~6		A=0, G=21	A=0.0, G=1.0	0.00001	281.235	21	A=0/0, G=10/1
chr7:32535202	G/G	C	G	SNV	AVL9	AVL9:utr_5:NM_015060.2	1	100	1	utr_5	NM_01506	c.-120C>G	p.?	0.01					Docking dom	rs56981934		YES			AVL9, S. cer	cell migra	Arthritis;Arthritis, Juvenile;Autoimmune Dise			C=0, G=67	C=0.0, G=1.0	0.00001	1032.11	67	C=0/0, G=36/3
chr7:32613020	G/G	A	G	SNV	AVL9	AVL9:exonic:NM_015060.2	1	100	12	synonymous	NM_01506	c.1560A>G	p.(=)	-2.08				0.07(COSM15	Docking dom	rs1993050	0.3383	YES	6.524E-1 (pos.32613020)	adenocarcinoi	AVL9, S. cer	cell migra	Arthritis;Arthritis, Juvenile;Autoimmune Dise			A=0, G=22	A=0.0, G=1.0	0.00001	298.891	22	A=0/0, G=11/1
chr1:54344282	A/A	C	A	SNV	YIPF1	YIPF1:intronic:NM_018982.4	1	100		intronic	NM_01898	c.276+33G	p.?	-0.16					Yip1 domain	rs2272928	0.4464	YES	8.626E-1 (pos.54344282)		YIP1 domai	Golgi apparatus,Golgi medial cisterna,Golgi trans cister				C=0, A=259	C=0.0, A=1.0	0.00001	4256.79	259	C=0/0, A=110/
chr1:179347832	T/C	T	C	SNV	AXDND1	AXDND1:exonic:NM_144696.5	1	52.38	5	synonymous	NM_14469	c.435T>C	p.(=)	-0.05				0.19(COSM37	Axonemal dy	rs10798674	0.4641	YES	7.243E-1 (pos.17934783	adenocarcinoma,neoplasm						T=20, C=22	T=0.4762, C=0.5238	0.00001	245.185	42	T=13/7, C=10/
chr1:54607157	A/A	G	A	SNV	CDCP2	CDCP2:intronic:NM_201546.4	1	100		intronic	NM_20154	c.428-51C	p.?	-0.19					CUB domain	rs671539	0.147	YES	9.802E-1;0.0 (pos.54607157)		Cub domair	extracellular region,integral component of membrane				G=0, A=30	G=0.0, A=1.0	0.00001	446.626	30	G=0/0, A=3/27
chr1:54618435	T/T	A	T	SNV	CDCP2	CDCP2:intronic:NM_201546.4	1	100		intronic	NM_20154	c.79+82T>	p.?	-0.17					CUB domain	rs7528345		YES			Cub domair	extracellular region,integral component of membrane				A=0, T=44	A=0.0, T=1.0	0.00001	730.965	44	A=0/0, T=29/1
chr1:179520506	A/A	G	A	SNV	AXDND1,NPHS2	NPHS2:exonic:NM_014625.3, AXDND1:intronic:NM_144696	1	100	8	synonymous	NM_14469	c.3032-31	p.?, p.(=)	-0.56				0.03(COSM14	Axonemal dy	rs1410592	0.3738	YES	4.806E-1;0.0 (pos.17952	adenocarcinoi	Podocin	actin cyto	Albuminuria;Female Urogenita		Benign/Likely	G=0, A=33	G=0.0, A=1.0	0.00001	504.547	33	G=0/0, A=13/2
chr1:235652513	C/C	T	C	SNV	B3GALNT2	B3GALNT2:exonic:NM_152490.4	1	100	3	synonymous	NM_15249	c.321A>G	p.(=)	0.05				0.54(COSM41	Galactosyltra	rs291396	0.4985	YES	8.609E-1 (pos.23565251	neoplasm	Beta-1,3-N-	Golgi mem	Cobblestone Lissencephaly;Cc		Benign	T=0, C=86	T=0.0, C=1.0	0.00001	1410.92	86	T=0/0, C=52/3
chr21:41032740	C/C	T	C	SNV	B3GALT5	B3GALT5:exonic:NM_033170.2	1	100	3	missense	NM_03317	c.254T>C	p.Met85Thr	-3.51		81		0.05(COSM40	Galactosyltra	rs3746887	0.1899	YES	6.571E-1 (pos.41032740)	acute_myeloic	UDP-galact	Golgi appa	Abnormalities, Multiple;Chromosome Disord			T=0, C=43	T=0.0, C=1.0	0.00001	705.842	43	T=0/0, C=20/2
chr21:41032804	A/A	G	A	SNV	B3GALT5	B3GALT5:exonic:NM_033170.2	1	100	3	synonymous	NM_03317	c.318G>A	p.(=)	-0.06				0.09(COSM14	Galactosyltra	rs734413	0.191	YES	6.55E-1 (pos.41032804)	acute_myeloic	UDP-galact	Golgi appa	Abnormalities, Multiple;Chromosome Disord			G=0, A=58	G=0.0, A=1.0	0.00001	867.219	58	G=0/0, A=22/3
chr13:31821992	C/C	T	C	SNV	B3GLCT	B3GLCT:exonic:NM_194318.3	1	100	6	synonymous	NM_19431	c.348T>C	p.(=)	-0.55					Fringe-like	rs4943266	0.0179	YES	1.0 (pos.31821992)		Beta 3-gluc	endoplasmic reticulum membrane,fucose			Benign	T=0, C=30	T=0.0, C=1.0	0.00001	451.746	30	T=0/0, C=16/1
chr13:31891746	A/A	G	A	SNV	B3GLCT	B3GLCT:exonic:NM_194318.3	1	100	13	missense	NM_19431	c.1108G>A	p.Glu370Lys	5.98	0.02	56	0.964		Fringe-like	rs1041073	0.222	YES	7.885E-1 (pos.31891746)		Beta 3-gluc	endoplasmic reticulum membrane,fucose			Benign	G=0, A=57	G=0.0, A=1.0	0.00001	852.941	57	G=0/0, A=43/1
chr19:17922795	A/A	G	A	SNV	B3GNT3	B3GNT3:exonic:NM_014256.3	1	100	3	missense	NM_01425	c.983G>A	p.Arg328His	-0.54	0.13	29	0.448	0.12(COSM41	Galactosyltra	rs36686	0.245	YES	4.866E-1 (pos.17922795)	neoplasm	Transmemb	Golgi membrane,N-acetyllactosaminide beta-1,3-N-acet				G=0, A=23	G=0.0, A=1.0	0.00001	316.744	23	G=0/0, A=16/7
chr12:122691738	A/A	C	A	SNV	B3GNT4,DIABLO	DIABLO:downstream:NM_019887.5, B3GNT4:exonic:NM_03	1	100	3	synonymous	NM_03076	c.940C>A,	p.(=), p.?	0.93					Fringe-like,G	rs4758675,rs753666	1.00E-04		0.0 (pos.122691735),9.999E-1;1.156E-4 (		Beta-1,3-N-	CD40 rece	Basal Ganglia Diseases;Brain Diseases;Brain			C=0, A=47	C=0.0, A=1.0	0.00001	789.328	47	C=0/0, A=26/2
chr1:55557864	T/T	C	T	SNV	USP24	USP24:intronic:NM_015306.2	1	100		intronic	NM_01530	c.6451-65	p.?	-2.14					Ubiquitin car	rs3736992		YES			Ubiquitin-s	nucleoplasm,protein deubiquitination,thiol-dependent				C=0, T=57	C=0.0, T=1.0	0.00001	849.61	57	C=0/0, T=4/53
chr1:55558661	G/G	A	G	SNV	USP24	USP24:intronic:NM_015306.2	1	100		intronic	NM_01530	c.6355-72	p.?	0.39					Ubiquitin car	rs585203		YES			Ubiquitin-s	nucleoplasm,protein deubiquitination,thiol-dependent				A=0, G=37	A=0.0, G=1.0	0.00001	583.704	37	A=0/0, G=19/1
chr1:55567410	G/G	A	G	SNV	USP24	USP24:intronic:NM_015306.2	1	100		intronic	NM_01530	c.5026-34	p.?	-0.13					Ubiquitin car	rs615652	0.1482	YES	9.967E-1 (pos.55567410)		Ubiquitin-s	nucleoplasm,protein deubiquitination,thiol-dependent				A=0, G=23	A=0.0, G=1.0	0.00001	316.741	23	A=0/0, G=9/14
chr1:55614037	A/A	ATA	A	INDEL	USP24	USP24:intronic:NM_015306.2	2	100		intronic	NM_01530	c.1968+99	p.?	0.16, 0.34, -0.5					Ubiquitin car	rs199779813,rs34030121,rs573		YES			Ubiquitin-s	nucleoplasm,protein deubiquitination,thiol-dependent				ATA=0, A=4	ATA=0.0, A=1.0	0.00001	730.942	44	ATA=0/0, A=22
chr1:57185818	G/G	A	G	SNV	FYB2	FYB2:intronic:NM_001004303.4	1	100		intronic	NM_00100	c.2131+28	p.?	-0.84					Helically-ext	rs857141	0.3065	YES	8.374E-1 (pos.57185818)			T cell receptor signaling pathway,cell adhesion mediate				A=0, G=130	A=0.0, G=1.0	0.00001	2135.57	130	A=0/0, G=63/6
chr1:57395251	C/C	T	C	SNV	C8B	C8B:intronic:NM_000066.3	1	100		intronic	NM_00006	c.1622-20	p.?	-1.6					Low-density	rs605648	0.1448	YES	9.388E-1;0.0 (pos.57395251)		Complemer	compleme	Arthritis;Arthritis, Juvenile;Autoimmune Dise			T=0, C=31	T=0.0, C=1.0	0.00001	465.819	31	T=0/0, C=8/23

chr1:57417591	CA/CA	C	CA	INDEL	C8B	C8B:intronic:NM 000066.3	1	100		intronic	NM 00006	c.666+130	p.?	0.49					Low-density lipoprotein receptor domain class A,MAC/Perforin domain,Thrombospondin type 1 dom						Complemer	compleme	Arthritis;Arthritis, Juvenile;Autoimmune Dise	C=0, CA=60	C=0.0, CA=1.0	0.00001	531.629	60	C=0/0, CA=53/
chr17:81006387	A/A	G	A	SNV	B3GNTL1	B3GNTL1:exonic:NM 001009905.2	1	100	3	synonymous	NM 00100	c.237C>T	p.(=)	-1.6				0.02(COSM41	Glycosyl tran	rs1143006	0.2756	YES	4.013E-1 (pos.81006387)	acute_myeloic	Beat-1.3-N-	transferase activity, transferring glycosyl groups		G=0, A=93	G=0.0, A=1.0	0.00001	1555.33	93	G=0/0, A=50/4
chr17:80899281	A/A	G	A	SNV	B3GNTL1,TBCD	TBCD:exonic:NM 005993.4, B3GNTL1:downstream:NM 001	1	100	38	synonymous	NM 00599	c.3486G>	p.(=), p.?	-3.22				0.01(COSM53	Tubulin foldir	rs9390	0.4828	YES	5.681E-1 (pos.80899281)	Wilms_tumou	Beat-1.3-N-	GTPase activator activity,adherens junction,adherens ju		G=0, A=40	G=0.0, A=1.0	0.00001	644.285	40	G=0/0, A=16/2
chr1:59160885	G/G	A	G	SNV	MYSM1	MYSM1:intronic:NM 001085487.2	1	100		intronic	NM 00108	c.69-6T>C	p.?	2.06				0.72(COSM50	JAB1/Mov34/	rs2064374	0.42	YES	3.574E-1 (pos.59160885)	carcinoma,ost	MYB-like, SV	DNA bindi	Cardiovascular Diseases;Diabetes Complicat	A=0, G=25	A=0.0, G=1.0	0.00001	353.017	25	A=0/0, G=8/17
chr12:58019472	G/G	T	G	SNV	B4GALNT1,SLC26A10	B4GALNT1:downstream:NM 001478.4, SLC26A10:exonic:NI	1	100	14	missense	NM 13348	c.1636T>G	p.Leu546Val, p.?	0.27	1	32	0	0.06(COSM41	STAS domain,	rs774895	9.00E-04	YES	1.0 (pos.58019472)	neoplasm	Beta-1.4-N-	(N-acetyln	Congenital Abnormalities;Congenital, Heredi	T=0, G=31	T=0.0, G=1.0	0.00001	465.783	31	T=0/0, G=17/1
chr11:380315	C/T	C	T	SNV	B4GALNT4	B4GALNT4:exonic:NM 178537.4	1	50	18	synonymous	NM 17853	c.2739C>T	p.(=)	-0.02					Chondroitin N	rs117291242	0.0319	YES	3.509E-4 (pos.380315)			Golgi cisterna membrane,N-acetyl-beta-glucosaminyl-gl		C=22, T=22	C=0.5, T=0.5	0.00001	242.813	44	C=14/8, T=5/1
chr3:118931389	T/T	C	T	SNV	B4GALT4	B4GALT4:utr 3:NM 003778.3	1	100	8	utr 3	NM 00377	c.1042G>	p.?	0.04					N-terminal do	rs6784208	0.0422	YES	9.997E-1 (pos.118931389)		Beta-1,4-ga	Golgi appa	Genital Diseases, N N-Acetylglucosamine	C=0, T=43	C=0.0, T=1.0	0.00001	705.799	43	C=0/0, T=22/2
chr1:6211257	A/A	C	A	SNV	CHD5	CHD5:intronic:NM 015557.2	1	100		intronic	NM 01555	c.871-42G	p.?	-0.31					CHDCT2 (NUC	rs6700735	0.0622	YES	0.0;0.0 (pos.6211257)		Chromodon	ATP bindin	Colonic Diseases;Colorectal Neoplasms;Dige	C=0, A=84	C=0.0, A=1.0	0.00001	1338.55	84	C=0/0, A=38/4
chr5:177031348	C/C	T	C	SNV	B4GALT7	B4GALT7:exonic:NM 007255.2	1	100	2	synonymous	NM 00725	c.219T>C	p.(=)	-0.22				0.06(COSM37	N-terminal do	rs11537644	0.3782	YES	7.514E-1 (pos.17703134	acute_myeloic	Xylosylprot	Golgi appa	Cardiovascular Diseases:Colla Benign	T=0, C=30	T=0.0, C=1.0	0.00001	446.636	30	T=0/0, C=19/1
chr9:104133628	T/T	C	T	SNV	BAAT	BAAT:exonic:NM 001127610.1	1	100	2	missense	NM 00112	c.59G>A	p.Arg20Gln	-2.05	0.34	43	0.048	0.01(COSM39	Acyl-CoA thio	rs1572983	0.3042	YES	6.056E-1 (pos.10413362	acute_myeloic	Bile acid co	N-acyltran	Congenital, Heredit Glycine,Ol Benign	T=0, C=28	C=0.0, T=1.0	0.00001	408.718	28	C=0/0, T=11/1
chr1:6257856	A/A	G	A	SNV	RPL22	RPL22:intronic:NM 000983.3	1	100		intronic	NM 00098	c.13-40C>	p.?	-0.16					Ribosomal L2	rs2294713	0.4257	YES	6.765E-1 (pos.6257856)		Ribosomal	RNA binding,SRP-dependent cotranslational protein targ		G=0, A=29	G=0.0, A=1.0	0.00001	427.602	29	G=0/0, A=8/21
chr11:117160347	G/G	A	G	SNV	BACE1	BACE1:exonic:NM 012104.4	1	100	9	missense	NM 01210	c.1441T>C	p.Cys481Arg	7.63	1	180	0		Eukaryotic as	rs539765,rs7519434	7.00E-04		0.0;0.0 (pos.117160338),1.0 (pos.11716		Beta-site ar	Golgi appa	Alzheimer Disease; (2S)-1-(2,5-dimethylphen	A=0, G=54	A=0.0, G=1.0	0.00001	798.146	54	A=0/0, G=23/3
chr17:79409434	G/G	A	G	SNV	BAHCC1	BAHCC1:exonic:NM 001291324.1	1	100	4	synonymous	NM 00129	c.894A>G	p.(=)	-1.97					BAH domain	rs12939225	0.4253	YES	2.234E-1 (pos.79409434)		BAH domair	chromatin,chromatin binding,chromatin silencing,chron		A=0, G=26	A=0.0, G=1.0	0.00001	375.812	26	A=0/0, G=8/18
chr17:79414307	G/G	A	G	SNV	BAHCC1	BAHCC1:exonic:NM 001291324.1	1	100	11	missense	NM 00129	c.3337A>G	p.Thr1113Ala	0.23		58		0.01(COSM43	BAH domain	rs7213444	0.2096	YES	5.309E-1 (pos.79414307)	acute_myeloic	BAH domair	chromatin,chromatin binding,chromatin silencing,chron		A=0, G=30	A=0.0, G=1.0	0.00001	446.247	30	A=0/0, G=11/1
chr1:6272813	C/T	C	T	SNV	RNF207	RNF207:intronic:NM 207396.2	1	50		intronic	NM 20739	c.1534+20	p.?	-0.1					Apolipoprote	rs2294711	0.0397	YES	3.827E-2;0.0 (pos.6272813)		RING finger	Hsp70 pro	Neoplasm Invasiveness;Neoplasms;Neoplas	C=16, T=16	C=0.5, T=0.5	0.00001	158.383	32	C=4/12, T=6/1
chr17:79424516	G/G	A	G	SNV	BAHCC1	BAHCC1:exonic:NM 001291324.1	1	100	17	missense	NM 00129	c.4867A>G	p.Thr1623Ala	-1.16		58		0(COSM4130	BAH domain	rs12601317	0.2003	YES	4.881E-1 (pos.79424516)	neoplasm	BAH domair	chromatin,chromatin binding,chromatin silencing,chron		A=0, G=21	A=0.0, G=1.0	0.00001	281.226	21	A=0/0, G=15/6
chr17:79414634	G/A	G	A	SNV	BAHCC1	BAHCC1:exonic:NM 001291324.1	1	57.14	11	missense	NM 00129	c.3664G>	p.Gly1222Arg	-1.15		125		0.07(COSM41	BAH domain	rs61745233	0.087	YES	9.454E-3 (pos.79414634)	neoplasm	BAH domair	chromatin,chromatin binding,chromatin silencing,chron		G=18, A=24	G=0.4286, A=0.5714	0.00001	278.995	42	G=11/7, A=13/
chr17:79427311	G/A	G	A	SNV	BAHCC1	BAHCC1:exonic:NM 001291324.1	1	57.14	24	missense	NM 00129	c.5842G>	p.Gly1948Ser	1.02		56			BAH domain	rs368436933	0		0.0 (pos.79427311)		BAH domair	chromatin,chromatin binding,chromatin silencing,chron		G=9, A=12	G=0.4286, A=0.5714	0.00001	109.102	21	G=4/5, A=3/9
chr1:62729015	A/A	G	A	SNV	KANK4	KANK4:intronic:NM 181712.4	1	100		intronic	NM 18171	c.2320-32	p.?	0.35					Ankyrin repea	rs2262106	0.2655	YES	8.5E-1 (pos.62729015)		KN motif-ar	cytosol,microtubule cytoskeleton		G=0, A=50	G=0.0, A=1.0	0.00001	852.748	50	G=0/0, A=18/3
chr7:97933601	T/T	C	T	SNV	BAIAP2L1	BAIAP2L1:exonic:NM 018842.4	1	100	12	synonymous	NM 01884	c.1329G>	p.(=)	2.13				0.59(COSM40	IRSp53/MIM I	rs1045916	0.2571	YES	7.034E-1 (pos.97933601)	carcinoma,ne	BAI1-associ	actin bind	Arthritis;Arthritis, Rheumatoid;Autoimmune	C=0, T=62	C=0.0, T=1.0	0.00001	939.178	62	C=0/0, T=31/3
chr1:62740930	G/G	A	G	SNV	KANK4	KANK4:intronic:NM 181712.4	1	100		intronic	NM 18171	c.17-171T	p.?	1.16					Ankyrin repea	rs2666508		YES			KN motif-ar	cytosol,microtubule cytoskeleton		A=0, G=22	A=0.0, G=1.0	0.00001	288.363	22	A=0/0, G=9/13
chr1:62939634	T/T	C	T	SNV	DOCK7	DOCK7:intronic:NM 001271999.1	1	100		intronic	NM 00127	c.6179+19	p.?	-0.26					C2 domain in	rs1168007	3.00E-04	YES	1.0 (pos.62939634)		Dedicator o	COP9 signalosome,Rac GTPase binding,activation of GTP		C=0, T=28	C=0.0, T=1.0	0.00001	408.713	28	C=0/0, T=25/3
chr1:62993620	T/C	T	C	SNV	DOCK7	DOCK7:intronic:NM 001271999.1	1	50		intronic	NM 00127	c.3936+20	p.?	2.92					C2 domain in	rs12403207		YES			Dedicator o	COP9 signalosome,Rac GTPase binding,activation of GTP		T=22, C=23	T=0.4889, C=0.5111	0.00001	255.789	46	T=16/6, C=19/
chr22:38482354	G/G	GCGGGAG	G	INDEL	BAIAP2L2	BAIAP2L2:exonic:NM 025045.5	42	100	12	nonframeshiftDeletion	NM 02504	c.1320 13	p.Ile441 Arg454del	-1.36, 0.64, 1.46, 0.27, 2.1, 0.2, -1.09, 0.78, 2.03, 0.61, 2.51, 0.(					IRSp53/MIM I	rs113792005,rs369923129,rs37		YES	0.0 (pos.38482353),0.0 (	hairy_cell_leu	Bai1-associ	actin crosslink formation,actin cytoskeleton,actin filame		GCGGGAG(	GCGGGAGCGGGACT(	0.00001	726.599	49	GCGGGAGCGG
chr1:63307303	G/G	A	G	SNV	ATG4C	ATG4C:intronic:NM 032852.3	1	100		intronic	NM 03285	c.1209+85	p.?	-1.26					Peptidase fai	rs3737892		YES			Autophagy-	C-terminal protein lipidation,autophagosome assembly		A=0, G=23	A=0.0, G=1.0	0.00001	316.726	23	A=0/0, G=13/1
chr1:64707243	C/C	G	C	SNV	UBE2U	UBE2U:intronic:NM 152489.2	1	100		intronic	NM 15248	c.596-92G	p.?	-0.18					Ubiquitin-cor	rs825189		YES				ATP binding,DNA repair,HULC complex,cytoplasm,histon		G=0, C=21	G=0.0, C=1.0	0.00001	281.231	21	G=0/0, C=16/5
chr1:65068638	C/C	T	C	SNV	CACHD1	CACHD1:intronic:NM 020925.2	1	100		intronic	NM 02092	c.364+43T	p.?	0.14					VWA N-termi	rs6665437	4.00E-04		1.0 (pos.65068638)			calcium ion transport,integral component of membrane		T=0, C=51	T=0.0, C=1.0	0.00001	754.584	51	T=0/0, C=26/2
chr1:65113351	C/C	T	C	SNV	CACHD1	CACHD1:intronic:NM 020925.2	1	100		intronic	NM 02092	c.1004-13	p.?	-0.79					VWA N-termi	rs7534210						calcium ion transport,integral component of membrane		T=0, C=20	T=0.0, C=1.0	0.00001	263.786	20	T=0/0, C=7/13
chr1:66036501	T/T	G	T	SNV	LEPR	LEPR:intronic:NM 002303.5	1	100		intronic	NM 00230	c.370+16G	p.?	-0.13					Ig-like C2-typ	rs2186248	0		1.0 (pos.66036501)		Leptin rece	T cell diffe	Albuminuria;Brain I Leptin,Me Benign	G=0, T=124	G=0.0, T=1.0	0.00001	2013.88	124	G=0/0, T=59/6
chr1:6631319	A/A	G	A	SNV	TAS1R1	TAS1R1:intronic:NM 138697.3	1	100		intronic	NM 13869	c.498+44G	p.?	1.25					7 transmemb	rs11122096	0.0126	YES	9.423E-1 (pos.6631319)		Taste recep	G-protein coupled receptor activity,G-protein coupled re		G=0, A=62	G=0.0, A=1.0	0.00001	939.801	62	G=0/0, A=37/2
chr16:1394507	C/C	A	C	SNV	BAIAP3	BAIAP3:exonic:NM 003933.4	1	100	18	missense	NM 00393	c.1745A>C	p.Asp582Ala	0.77	0.84	126	0	0(COSM4128	C2 domain,M	rs1132356	0.1016	YES	9.994E-1 (pos.1394507)	neoplasm	BAI1-associ	G-protein coupled receptor signaling pathway,endoplas		A=0, C=56	A=0.0, C=1.0	0.00001	832.099	56	A=0/0, C=36/2
chr4:102965043	C/C	T	C	SNV	BANK1	BANK1:exonic:NM 017935.4	1	100	11	missense	NM 01793	c.1948T>C	p.Cys650Arg	0.49	0.97	180	0		Dof, BCAP, an	rs3113676	0.0131		1.0 (pos.102965043)		B-cell scaffc	B cell activ	Autoimmune Diseases;Connective Tissue Dis	T=0, C=32	T=0.0, C=1.0	0.00001	484.207	32	T=0/0, C=17/1
chr1:66829262	G/G	A	G	SNV	PDE4B	PDE4B:intronic:NM 002600.3	1	100		intronic	NM 00260	c.1284+8A	p.?	0.38				0.2(COSM374	3'5'-cyclic nuc	rs783039	0.2705	YES	9.477E-1 (pos.66829262)	hepatocellula	Phosphodie	3',5'-cyclic	Alcohol-Related Dis (R)-Rolipram,(S)-Rolipran	A=0, G=37	A=0.0, G=1.0	0.00001	583.706	37	A=0/0, G=18/1
chr16:88052161	A/A	C	A	SNV	BANP	BANP:exonic:NM 001173539.1	1	100	7	synonymous	NM 00117	c.783C>A	p.(=)	-0.02				0.45(COSM37	BEN domain	rs7499814	0.187	YES	5.154E-1 (pos.88052161)	adenocarcino	BTG3-assoc	DNA binding,cell cycle,covalent chromatin modification,		C=0, A=31	C=0.0, A=1.0	0.00001	465.813	31	C=0/0, A=7/24
chr2:160294842	A/G	A	G	SNV	BAZ2B	BAZ2B:exonic:NM 013450.3	1	46.88	8	missense	NM 01345	c.1265T>C	p.Leu422Ser	1.94	0.26	145	0	0.07(COSM41	Bromodomai	rs3213790	0.1247	YES	1.142E-1 (pos.16029484	neoplasm	Bromodom	DNA bindi	Cardiovascular Diseases;Death, Sudden, Car	A=34, G=30	A=0.5313, G=0.4688	0.00001	288.14	64	A=20/14, G=1
chr16:56548501	T/T	C	T	SNV	BBS2	BBS2:exonic:NM 031885.3	1	100	2	missense	NM 03188	c.209G>A	p.Ser70Asn	5.01	1	46	0		Ciliary BBSom	rs4784677	0.0077		9.999E-1 (pos.56548501)		Bardet-Bied	BBSome,G	Abnormalities, Multiple;Barde Benign	C=0, T=32	C=0.0, T=1.0	0.00001	485.14	32	C=0/0, T=22/1
chr1:67396222	G/G	T	G	SNV	MIER1	MIER1:intronic:NM 001077700.2	1	100		intronic	NM 00107	c.168+429	p.?	0.58					ELM2 domair	rs3008861		YES				DNA binding,cytoplasm,histone deacetylase activity,his		T=0, G=35	T=0.0, G=1.0	0.00001	543.903	35	T=0/0, G=16/1
chr1:67560897	G/G	A	G	SNV	C1orf141	C1orf141:intronic:NM 001276351.1	1	100		intronic	NM 00127	c.603+21T	p.?	0.97					Domain of un	rs10889653	0.1432	YES	9.719E-1 (pos.67560897)				Crohn Disease;Digestive System Diseases;Ga	A=0, G=38	A=0.0, G=1.0	0.00001	603.786	38	A=0/0, G=17/2
chr15:73023937	C/C	T	C	SNV	BBS4	BBS4:exonic:NM 033028.4	1	100	12	synonymous	NM 03302	c.906T>C	p.(=)	0.33					TPR repeat,T	rs12914333	1.00E-04	YES	1.0 (pos.73023937)		BBS4 gene	BBSome,R	Abnormalities, Multiple;Barde Benign	T=0, C=87	T=0.0, C=1.0	0.00001	1430.99	87	T=0/0, C=32/5
chr15:73027478	C/C	T	C	SNV	BBS4	BBS4:exonic:NM 033028.4	1	100	13	missense	NM 03302	c.1061T>C	p.Ile354Thr	-0.24	0.31	89	0	0.04(COSM39	TPR repeat,T	rs2277598	0.3596	YES	2.454E-1 (pos.73027478)	acute_myeloic	BBS4 gene	BBSome,R	Abnormalities, Multiple;Barde Benign/Likely	T=0, C=34	T=0.0, C=1.0	0.00001	524.186	34	T=0/0, C=20/1
chr17:58786625	G/G	A	G	SNV	BCAS3	BCAS3:exonic:NM 001099432.2	1	100	5	missense	NM 00109	c.260A>G	p.Asn87Ser	2.65	0.65	46	0.007		Breast carcin	rs2643103	0.0069	YES	9.122E-1 (pos.58786625)			Golgi orga	Alzheimer Disease;Brain Diseases;Central Ne	A=0, G=125	A=0.0, G=1.0	0.00001	2034.04	125	A=0/0, G=75/5
chr17:58824567	C/C	T	C	SNV	BCAS3	BCAS3:exonic:NM 001099432.2	1	100	6	synonymous	NM 00109	c.330T>C	p.(=)	-0.32					Breast carcin	rs7501675	5.00E-04		1.0 (pos.58824567)			Golgi orga	Alzheimer Disease;Brain Diseases;Central Ne	T=0, C=129	T=0.0, C=1.0	0.00001	2114.99	129	T=0/0, C=53/7
chr1:67890526	A/G	A	G	SNV	SERBP1	SERBP1:intronic:NM 001018067.1	1	50		intronic	NM 00101	c.695+45T	p.?	0.2					Hyaluronan /	rs6702742	0.4294	YES	3.285E-1 (pos.67890526)		Serpine1 m	RNA bindir	Autoimmune Diseases;Digestive System Dise	A=43, G=43	A=0.5, G=0.5	0.00001	464.372	86	A=22/21, G=1
chr1:68151980	G/G	T	G	SNV	GADD45A	GADD45A:intronic:NM 001924.3	1	100		intronic	NM 00192	c.147-53T	p.?	0.68					Ribosomal pr	rs2759219		YES				DNA dama	Acneiform Eruptions;Adnexal Diseases;Arthri	T=0, G=67	T=0.0, G=1.0	0.00001	1028.05	67	T=0/0, G=38/2
chr1:68173264	T/T	C	T	SNV	GNG12	GNG12:intronic:NM 018841.5	1	100		intronic	NM 01884	c.93+13G>	p.?	0.11					GGL domain	rs2295942,rs748743	0.4616	YES	4.377E-1;0.0 (pos.68173264)		Guanine nu	G-protein	Autoimmune Diseases;Female Urogenital Di	C=0, T=33	C=0.0, T=1.0	0.00001	504.596	33	C=0/0, T=16/1
chr1:71419499	T/T	C	T	SNV	PTGER3	PTGER3:intronic:NM 198718.1	1	100		intronic	NM 19871	c.1078-27	p.?	0.06					7 transmemb	rs571705	0.1657	YES	9.384E-1 (pos.71419499)		Prostaglanc	G-protein	Adnexal Diseases;C Bimatoprost,Dinoprosto	C=0, T=25	C=0.0, T=1.0	0.00001	353.007	25	C=0/0, T=12/1
chr22:18165995	T/G	T	G	SNV	BCL2L13	BCL2L13:exonic:NM 015367.3	1	47.37	3	missense	NM 01536	c.137T>G	p.Ile46Arg	-2.23	0.03	97	0.09	0.02(COSM49	Apoptosis re	rs2587070	0.2022	YES	6.215E-3;0.0 (pos.18165	Hurthle_cell_carcinoma,ac		activation of cysteine-type endopeptidase activity invol		T=30, G=27	T=0.5263, G=0.4737	0.00001	256	57	T=19/11, G=1
chr1:72242039	A/A	G	A	SNV	NEGR1	NEGR1:intronic:NM 173808.2	1	100		intronic	NM 17380	c.410-59C	p.?	0.84					Immunoglob	rs6424448		YES			Neuronal gr	C-termina	Nutrition Disorders;Nutritional and Metaboli	G=0, A=25	G=0.0, A=1.0	0.00001	353.019	25	G=0/0, A=9/16
chr1:74664149	T/C	T	C	SNV	FPGT,FPGT-TNNI3K,LRRIQ3	LRRIQ3:upstream:NM 001105659.1, FPGT:intronic:NM 001	1	50		intronic	NM 00110	c.-14781A	p.?, p.?, p.?	-1.05					Ankyrin repea	rs4422957		YES	7.752E-1 (pos.74664149)		Fucose-1-pl	ATP bindin	Adenocarcinoma;Carcinoma;Carcinoma, Ren	T=12, C=12	T=0.5, C=0.5	0.00001	106.709	24	T=5/7, C=5/7
chr1:74819077	G/G	T	G	SNV	FPGT-TNNI3K,TNNI3K	FPGT-TNNI3K:intronic:NM 001112808.2, TNNI3K:intronic:NI	1	100		intronic	NM 00111	c.1369+33	p.?, p.?	-0.07					Ankyrin repea	rs6424586	0.0577	YES	9.981E-1 (pos.74819077)		TNNI3-inter	ATP bindin	Nutrition Disorders;Nutritional and Metaboli	T=0, G=23	T=0.0, G=1.0	0.00001	316.747	23	T=0/0, G=8/15
chr12:12240199	T/T	C	T	SNV	BCL2L14	BCL2L14:exonic:NM 138723.1	1	100	3	synonymous	NM 13872	c.486C>T	p.(=)	-0.91						rs879732	0.4235	YES	4.587E-1 (pos.12240199)			apoptotic process,cytosol,endomembrane system,intra		C=0, T=91	C=0.0, T=1.0	0.00001	1513.81	91	C=0/0, T=35/5
chr1:75180401	C/T	C	T	SNV	CRYZ	CRYZ:intronic:NM 001889.3	1	45.83		intronic	NM 00188	c.429-87G	p.?	-0.03					Alcohol dehy	rs74095154		YES			Crystallin, z	NADH binding,NADPH binding,N 2'-Monophosphoadenos		C=13, T=11	C=0.5417, T=0.4583	0.00001	93.44	24	C=7/6, T=8/3
chr1:75229488	C/G	C	G	SNV	TYW3	TYW3:intronic:NM 138467.2	1	52.38		intronic	NM 13846	c.561-90C	p.?	0.27					Methyltransf	rs566939330					tRNA-wybut	methylation,methyltransferase activity,tRNA processin		C=10, G=11	C=0.4762, G=0.5238	0.00001	95.3	21	C=8/2, G=5/6
chr1:75603055	G/G	A	G	SNV	LHX8	LHX8:intronic:NM 001256114.1	1	100		intronic	NM 00125	c.237+109	p.?	-0.79					Homeobox KI	rs1526503					LIM homeol	female germ cell nucleus,female gonad development,fo		A=0, G=33	A=0.0, G=1.0	0.00001	504.556	33	A=0/0, G=17/1
chr1:75603101	A/G	A	G	SNV	LHX8	LHX8:intronic:NM 001256114.1	1	57.58		intronic	NM 00125	c.237+155	p.?	-1.88					Homeobox KI	rs6695423		YES			LIM homeol	female germ cell nucleus,female gonad development,fo		A=14, G=19	A=0.4242, G=0.5758	0.00001	204.483	33	A=10/4, G=7/1
chr1:75609049	A/C	A	C	SNV	LHX8	LHX8:intronic:NM 001256114.1	1	45.83		intronic	NM 00125	c.580+26A	p.?	1.22					Homeobox KI	rs12041465	0.1936	YES	7.942E-1;1.722E-3 (pos.75609049)		LIM homeol	female germ cell nucleus,female gonad development,fo		A=13, C=11	A=0.5417, C=0.4583	0.00001	93.4399	24	A=5/8, C=8/3
chr14:23777374	G/G	A	G	SNV	BCL2L2,BCL2L2-PABPN1	BCL2L2-PABPN1:exonic:NM 001199864.1, BCL2L2:exonic:N	1	100	3	missense	NM 00119	c.398A>G,	p.Gln133Arg, p.Gln133A	2.44	1.0, 0.28	43.0, 43.0	0.0, 0.0	0.44(COSM41	Apolipoprote	rs200485369,rs9103	0.003		1.0 (pos.23777374)	neoplasm	BCL2-like 2	BH domain binding,Bcl-2 family	Navitoclax	A=0, G=68	A=0.0, G=1.0	0.00001	1053.56	68	A=0/0, G=34/3
chr1:75707652	C/T	C	T	SNV	SLC44A5	SLC44A5:intronic:NM 152697.4	1	50		intronic	NM 15269	c.532+34G	p.?	0.6					Plasma-mem	rs386632487,rs7556	0.2667	YES	0.0;0.0 (pos.75707651),6.767E-2 (pos.75707652)			choline transmembrane transporter activity,choline tra		C=21, T=21	C=0.5, T=0.5	0.00001	231.539	42	C=8/13, T=7/1
chr1:76194306	G/G	A	G	SNV	ACADM	ACADM:intronic:NM 001127328.2	1	100		intronic	NM 00112	c.130+133	p.?	-0.01					Acyl-CoA deh	rs1146572		YES			Acyl-Coenzy	acyl-CoA d	Congenital, Heredit 3-Thiaoctanoyl-Coenzym	A=0, G=46	A=0.0, G=1.0	0.00001	768.312	46	A=0/0, G=24/2
chr1:76363505	A/C	A	C	SNV	MSH4	MSH4:intronic:NM 002440.3	1	46.88		intronic	NM 00244	c.2356-87	p.?	-0.13					MutS domain	rs2029680		YES			mutS, E. col	ATP binding,DNA binding,DNA-dependent ATPase activity		A=17, C=15	A=0.5313, C=0.4688	0.00001	143.734	32	A=9/8, C=12/3
chr1:77620035	C/C	T	C	SNV	PIGK	PIGK:intronic:NM 005482.2	1	100		intronic	NM 00548	c.986+99A	p.?	0.02					Peptidase C1	rs839832		YES			Phosphatid	GPI anchor binding,GPI-anchor transamidase activity,GP		T=0, C=22	T=0.0, C=1.0	0.00001	298.89	22	T=0/0, C=6/16
chr3:187447032	A/A	G	A	SNV	BCL6,LOC100131635	LOC100131635:intronic nc:NR 034062.1, BCL6:exonic:NM	1	100	5	synonymous	NR 03406	c.1161C>T	p.(=)	0.74				0.77(COSM50	BTB/POZ dom	rs1056932	0.3456	YES	8.06E-1 (pos.187447032)	ductal_carcin	B-cell CLL/ly	B cell diffe	Hemic and Lymphatic Diseases;Immune Syst	G=0, A=152	G=0.0, A=1.0	0.00001	2432.9	152	G=0/0, A=76/7
chr16:81279120	T/C	T	C	SNV	BCO1	BCO1:exonic:NM 017429.2	1	50	2	synonymous	NM 01742	c.105T>C	p.(=)	-2.12				0.02(COSM14	Retinal pigm	rs28370522	0.3207	YES	3.63E-1 (pos.81279120)	adenocarcino	Beta-carote	beta-carot	Avitaminosis;Deficiency Diseases;Malnutriti	T=27, C=31	T=0.4655, C=0.5345	0.00001	361.697	62	T=10/17, C=1
chr1:78045369	G/A	G	A	SNV	ZZZ3	ZZZ3:intronic:NM 015534.5	1	59.09		intronic	NM 01553	c.1981-56	p.?	0.32					Myb-like DNA	rs1565852		YES			Ada2/Gcn5	Nutrition Disorders;Nutritional and Metaboli		G=9, A=13	G=0.4091, A=0.5909	0.00001	121.951	22	G=4/5, A=1/12
chr1:78383467	A/A	G	A	SNV	NEXN	NEXN:intronic:NM 144573.3	1	100		intronic	NM 14457	c.219+25G	p.?	0.07					Immunoglob	rs1749913	0.1392	YES	7.99E-1 (pos.78383467)		Nexilin, rat	Z disc,acti	Aortic Stenosis, Subvalvular;Aortic Valve Ster	G=0, A=30	G=0.0, A=1.0	0.00001	446.639	30	G=0/0, A=17/1
chr1:78481640	G/A	G	A	SNV	DNAJB4	DNAJB4:intronic:NM 007034.4	1	97.56		intronic	NM 00703	c.781-58G	p.?	0.22					DnaJ C termi	rs1039863		YES			DNAJ/HSP4	ATPase activator activity,chaperone binding,cytosol,ext		G=1, A=40	G=0.0244, A=0.9756	0.00001	631.814	41	G=1/0, A=16/2
chr1:78591740	TAAC/T	TAAC	T	INDEL	GIPC2	GIPC2:intronic:NM 017655.5	3	47.37		intronic	NM 01765	c.796+94T	p.?	0.62, 1.46, 1.06, 0.27					PDZ domain (	rs139844601,rs201382691,rs36		YES				biological_process,cytoplasm,extracellular exosome,id		TAAC=20, T	TAAC=0.5263, T=0.47	0.00001	184.537	38	TAAC=4/16, T=
chr11:112065434	C/C	T	C	SNV	BCO2	BCO2:exonic:NM 031938.5	1	100	5	missense	NM 03193	c.692T>C	p.Leu231Pro	2.59	1	98	0		Retinal pigm	rs10891338	0.017	YES	9.995E-1 (pos.112065434)		Beta-carotene dioxygenase 2			T=0, C=26	T=0.0, C=1.0	0.00001	369.081	26	T=0/0, C=8/18
chr1:79128320	G/G	A	G	SNV	IFI44	IFI44:intronic:NM 006417.4	1	100		intronic	NM 00641	c.1114-69	p.?	-0.41					TLD	rs11577395		YES			Interferon-i	cytoplasm	Abortion, Spontaneous;Female Urogenital Di	A=0, G=43	A=0.0, G=1.0	0.00001	705.842	43	A=0/0, G=24/1
chr1:79128570	GT/GT	G	GT	INDEL	IFI44	IFI44:intronic:NM 006417.4	1	100		intronic	NM 00641	c.1288+7G	p.?	0.42					TLD	rs368139253	0.4299	YES	3.753E-1 (pos.79128570)		Interferon-i	cytoplasm	Abortion, Spontaneous;Female Urogenital Di	G=0, GT=25	G=0.0, GT=1.0	0.00001	352.643	25	G=0/0, GT=14/
chr1:79383458	T/T	A	T	SNV	ADGRL4	ADGRL4:intronic:NM 022159.3	1	100		intronic	NM 02215	c.1682+57	p.?	-0.03					7 transmemb	rs12569007		YES			Adhesion G	G-protein coupled receptor activity,G-protein coupled re		A=0, T=26	A=0.0, T=1.0	0.00001	371.42	26	A=0/0, T=14/1
chr11:27679662	C/C	T	C	SNV	BDNF,BDNF-AS	BDNF-AS:intronic nc:NR 002832.2, BDNF:exonic:NM 00114	1	100	3	synonymous	NR 00283	c.696A>G	p.(=)	-2.33					Nerve growth	rs2353512	0		1.0 (pos.27679662)		Brain-deriv	axon guida	46, XY Disorders of CX717 Benign	T=0, C=23	T=0.0, C=1.0	0.00001	316.747	23	T=0/0, C=13/1
chr1:79401927	G/G	A	G	SNV	ADGRL4	ADGRL4:intronic:NM 022159.3	1	100		intronic	NM 02215	c.877+53T	p.?	-0.05					7 transmemb	rs61770681		YES			Adhesion G	G-protein coupled receptor activity,G-protein coupled re		A=0, G=30	A=0.0, G=1.0	0.00001	446.637	30	A=0/0, G=12/1
chr1:82416220	A/A	G	A	SNV	ADGRL2	ADGRL2:intronic:NM 012302.4	1	100		intronic	NM 01230	c.1492+54	p.?	-0.24					7 transmemb	rs2148630		YES			Adhesion G	G-protein coupled receptor activity,G-protein coupled re		G=0, A=36	G=0.0, A=1.0	0.00001	563.742	36	G=0/0, A=12/2
chr1:82417552	G/G	T	G	SNV	ADGRL2	ADGRL2:intronic:NM 012302.4	1	100		intronic	NM 01230	c.1597-89	p.?	2.58					7 transmemb	rs7413275		YES			Adhesion G	G-protein coupled receptor activity,G-protein coupled re		T=0, G=41	T=0.0, G=1.0	0.00001	664.7	41	T=0/0, G=18/2
chr5:70751818	G/G	T	G	SNV	BDP1	BDP1:exonic:NM 018429.2	1	100	1	missense	NM 01842	c.114T>G	p.Asp38Glu	-0.47		45	0	0.01(COSM44	Myb DNA-bin	rs3748043	0.1678	YES	8.875E-1 (pos.70751818)	hepatocellula	B double pr	DNA binding,RNA polymerase III transcriptional preinitia		T=0, G=70	T=0.0, G=1.0	0.00001	1092.06	70	T=0/0, G=40/3
chr5:70806958	A/A	G	A	SNV	BDP1	BDP1:exonic:NM 018429.2	1	100	17	missense	NM 01842	c.4039G>	p.Val1347Met	-1.81		21	0.001	0.01(COSM44	Myb DNA-bin	rs6886336	0.1686	YES	8.811E-1 (pos.70806958)	hepatocellula	B double pr	DNA binding,RNA polymerase III transcriptional preinitia		G=0, A=49	G=0.0, A=1.0	0.00001	831.632	49	G=0/0, A=26/2
chr5:70858194	C/T	C	T	SNV	BDP1	BDP1:exonic:NM 018429.2	1	58.33	38	synonymous	NM 01842	c.7590C>T	p.(=)	0.2				0.12(COSM37	Myb DNA-bin	rs277941	0.3443	YES	5.012E-1 (pos.70858194)	acute_myeloic	B double pr	DNA binding,RNA polymerase III transcriptional preinitia		C=10, T=14	C=0.4167, T=0.5833	0.00001	135.339	24	C=4/6, T=8/6
chr16:66514858	C/A	C	A	SNV	BEAN1	BEAN1:utr 3:NM 001178020.2	1	52.27	5	utr 3	NM 00117	c.810C>A	p.?	0.19						rs185675010			0.0 (pos.66514858)			integral cc	Ataxia;Brain Diseases;Central Nervous Syste	C=21, A=23	C=0.4773, A=0.5227	0.00001	260.032	44	C=5/16, A=10/
chr1:84640691	G/G	T	G	SNV	PRKACB	PRKACB:intronic:NM 182948.3	1	100		intronic	NM 18294	c.188-416	p.?	0.94					Kinase-like,P	rs582201		YES	9.346E-1 (pos.84640691)		Protein kina	ATP bindin	Adenocarcinoma;B Phosphonothreonine	T=0, G=27	T=0.0, G=1.0	0.00001	389.988	27	T=0/0, G=15/1
chr1:84640834	G/G	T	G	SNV	PRKACB	PRKACB:intronic:NM 182948.3	1	100		intronic	NM 18294	c.188-402	p.?	1.77					Kinase-like,P	rs12132002		YES			Protein kina	ATP bindin	Adenocarcinoma;B Phosphonothreonine	T=0, G=23	T=0.0, G=1.0	0.00001	316.747	23	T=0/0, G=11/1
chr1:85131982	T/G	T	G	SNV	SSX2IP	SSX2IP:intronic:NM 001166293.1	1	58.33		intronic	NM 00116	c.427-67A	p.?	0.65					Afadin- and a	rs3096445		YES				cell adhes	Autoimmune Diseases;Female Urogenital Di	T=10, G=14	T=0.4167, G=0.5833	0.00001	135.339	24	T=3/7, G=7/7
chr1:85592126	T/T	G	T	SNV	WDR63	WDR63:intronic:NM 145172.4	1	100		intronic	NM 14517	c.2113-68	p.?	-0.65						rs817474		YES				ATP-dependent microtubule motor activity, plus-end-dir		G=0, T=23	G=0.0, T=1.0	0.00001	316.747	23	G=0/0, T=15/8
chrX:102632458	G/A	G	A	SNV	BEX3	BEX3:exonic:NM 014380.2	1	96.67	1	synonymous	NM 01438	c.39G>A	p.(=)	0.72					Brain expressed X-linked like family						Nerve grow	activation of cysteine-type endopeptidase activity invol		G=1, A=29	G=0.0333, A=0.9667	0.00001	416.517	30	G=1/0, A=13/1
chr20:17474690	G/G	T	G	SNV	BFSP1	BFSP1:utr 3:NM 001195.4	1	100	8	utr 3	NM 00119	c.2027A>C	p.?	0.01					Intermediate	rs6105762	0.4789	YES	4.603E-1 (pos.17474690)		Beaded fila	biological	Cataract;Eye Diseases;Lens Diseases	T=0, G=42	T=0.0, G=1.0	0.00001	685.163	42	T=0/0, G=20/2
chr20:17474968	C/C	T	C	SNV	BFSP1	BFSP1:exonic:NM 001195.4	1	100	8	synonymous	NM 00119	c.1749A>G	p.(=)	-5.59					Intermediate	rs6080718	0.2901	YES	6.5E-1;0.0 (pos.17474968)		Beaded fila	biological	Cataract;Eye Diseases;Lens Di Benign	T=0, C=44	T=0.0, C=1.0	0.00001	726.569	44	T=0/0, C=31/1
chr20:17475217	T/T	C	T	SNV	BFSP1	BFSP1:exonic:NM 001195.4	1	100	8	synonymous	NM 00119	c.1500G>	p.(=)	-0.44					Intermediate	rs6136118	0.2358	YES	3.954E-1 (pos.17475217)		Beaded fila	biological	Cataract;Eye Diseases;Lens Di Benign	C=0, T=53	C=0.0, T=1.0	0.00001	782.024	53	C=0/0, T=18/3
chr10:60588553	C/C	T	C	SNV	BICC1	BICC1:exonic:NM 001080512.2	1	100	21	missense	NM 00108	c.2827T>C	p.Ser943Pro	3.59	1	74	0	0.27(COSM41	KH domain,S	rs4948550	0.2876	YES	7.456E-1 (pos.60588553)	acute_myeloic	Bicaudal C	RNA bindir	Cardiovascular Abnormalities;Cardiovascula	T=0, C=26	T=0.0, C=1.0	0.00001	370.953	26	T=0/0, C=12/1
chr12:51681903	C/C	T	C	SNV	BIN2	BIN2:exonic:NM 016293.3	1	100	11	missense	NM 01629	c.1681A>G	p.Asn561Asp	0.26	1	23	0		BAR domain	rs7954976	0.1036	YES	9.37E-1 (pos.51681903)		Bridging int	cell chemotaxis,cell cortex,cell junction,cell projection,		T=0, C=53	T=0.0, C=1.0	0.00001	782.013	53	T=0/0, C=37/1
chr1:86173296	AT/AT	A	AT	INDEL	ZNHIT6	ZNHIT6:intronic:NM 017953.3	1	100		intronic	NM 01795	c.656+16T	p.?	-0.02					HIT zinc finger							ATPase binding,TFIID-class transcription factor binding,b		A=0, AT=77	A=0.0, AT=1.0	0.00001	1104.25	77	A=0/0, AT=36/
chr1:86210546	A/A	G	A	SNV	COL24A1	COL24A1:intronic:NM 152890.6	1	100		intronic	NM 15289	c.4504-29	p.?	1.73					Collagen trip	rs7547023	0.0338	YES	9.396E-1 (pos.86210546)		Collagen, ty	collagen trimer,endoplasmic reticulum lumen,extracell		G=0, A=24	G=0.0, A=1.0	0.00001	334.792	24	G=0/0, A=9/15
chr1:86249911	A/G	A	G	SNV	COL24A1	COL24A1:intronic:NM 152890.6	1	97.06		intronic	NM 15289	c.4167+31	p.?	-0.28					Collagen trip	rs313725	0.0365	YES	9.508E-1 (pos.86249911)		Collagen, ty	collagen trimer,endoplasmic reticulum lumen,extracell		A=1, G=33	A=0.0294, G=0.9706	0.00001	492.97	34	A=0/1, G=21/1
chr2:32713706	T/T	A	T	SNV	BIRC6	BIRC6:exonic:NM 016252.3	1	100	42	missense	NM 01625	c.8020A>T	p.Thr2674Ser	1.24	0.5	58	0	0.39(COSM14	Baculoviral I	rs2366894	0.3647	YES	3.503E-1 (pos.32713706)	adenocarcino	Baculoviral	Flemming body,Golgi apparatus,apoptotic process,cell c		A=0, T=118	A=0.0, T=1.0	0.00001	1893.93	118	A=0/0, T=53/6
chr19:53793042	A/A	G	A	SNV	BIRC8	BIRC8:exonic:NM 033341.4	1	100	1	missense	NM 03334	c.586C>T	p.His196Tyr	-0.48		83		0(COSM4000	Inhibitor of A	rs8109165	0.447	YES	5.03E-1 (pos.53793042)	acute_myeloid_leukaemia		apoptotic process,cysteine-type endopeptidase inhibitc		G=0, A=26	G=0.0, A=1.0	0.00001	371.419	26	G=0/0, A=18/8
chr1:86818813	C/C	T	C	SNV	ODF2L	ODF2L:intronic:NM 001007022.2	1	100		intronic	NM 00100	c.1768-14	p.?	-0.34						rs272497		YES				centrosome		T=0, C=124	T=0.0, C=1.0	0.00001	2019.17	124	T=0/0, C=70/5
chr19:53793574	A/G	A	G	SNV	BIRC8	BIRC8:exonic:NM 033341.4	1	97.3	1	synonymous	NM 03334	c.54T>C	p.(=)	1.51					Inhibitor of A	rs2865248	0.4403	YES	5.067E-1 (pos.53793574)			apoptotic process,cysteine-type endopeptidase inhibitc		A=1, G=36	A=0.027, G=0.973	0.00001	551.74	37	A=1/0, G=16/2
chr13:103504517	C/C	T	C	SNV	BIVM-ERCC5,ERCC5	ERCC5:exonic:NM 000123.3, BIVM-ERCC5:exonic:NM 0012(	1	100	2	synonymous	NM 00120	c.1500T>C	p.(=), p.(=)	0.1				0.73(COSM37	XPG I-region,	rs1047768	0.4134	YES	2.66E-1 (pos.103504517)	adenocarcino	Excision-re	DNA replic	Abnormalities, Multiple;Agene Benign/Likely	T=0, C=40	T=0.0, C=1.0	0.00001	644.286	40	T=0/0, C=17/2
chr13:103513944	G/G	A	G	SNV	BIVM-ERCC5,ERCC5	ERCC5:exonic:NM 000123.3, BIVM-ERCC5:exonic:NM 0012(	1	100	7	missense	NM 00120	c.2122A>G	p.Met708Val, p.Met254	2.93	0.02, 0.0	21.0, 21.0	0.858, 0.858	0.94(COSM49	XPG I-region,	rs1047769	0.0359	YES	4.625E-4 (pos.10351394	haemangiobla	Excision-re	DNA replic	Abnormalities, Multiple;Agene Benign/Likely	A=0, G=62	A=0.0, G=1.0	0.00001	940.306	62	A=0/0, G=44/1
chr1:86939390	A/A	G	A	SNV	CLCA1	CLCA1:intronic:NM 001285.3	1	100		intronic	NM 00128	c.304-22G	p.?	0.92					Calcium-acti	rs2180762	0.0337	YES	9.874E-1 (pos.86939390)		Chloride ch	calcium ion transport,cellular response to hypoxia,chlor		G=0, A=20	G=0.0, A=1.0	0.00001	263.786	20	G=0/0, A=9/11
chr1:86959014	C/C	T	C	SNV	CLCA1	CLCA1:intronic:NM 001285.3	1	100		intronic	NM 00128	c.1465-53	p.?	-1.9					Calcium-acti	rs2753346		YES			Chloride ch	calcium ion transport,cellular response to hypoxia,chlor		T=0, C=49	T=0.0, C=1.0	0.00001	831.629	49	T=0/0, C=34/1
chr13:103527930	C/C	G	C	SNV	BIVM-ERCC5,ERCC5	ERCC5:exonic:NM 000123.3, BIVM-ERCC5:exonic:NM 0012(	1	100	15	missense	NM 00120	c.4600G>C	p.Gly1534Arg, p.Gly108(	-0.37	0.17, 0.16	125.0, 125	0.001, 0.0		XPG I-region,	rs587778291,rs9514	0		1.0;0.0 (pos.103527930)		Excision-re	DNA replic	Abnormalities, Multiple;Agene not provided	G=0, C=29	G=0.0, C=1.0	0.00001	427.601	29	G=0/0, C=22/7
chr1:86964131	C/C	CT	C	INDEL	CLCA1	CLCA1:intronic:NM 001285.3	1	100		intronic	NM 00128	c.2114-12	p.?	-0.05, 0.07					Calcium-acti	rs11302985,rs200238737,rs201		YES			Chloride ch	calcium ion transport,cellular response to hypoxia,chlor		CT=0, C=31	CT=0.0, C=1.0	0.00001	465.391	31	CT=0/0, C=14/
chr1:87549858	G/G	A	G	SNV	HS2ST1	HS2ST1:intronic:NM 012262.3	1	100		intronic	NM 01226	c.364-19A	p.?	0.27					Sulfotransfer	rs2798675	0.0684	YES	9.958E-1 (pos.87549858)		Heparan su	Golgi membrane,glycosaminoglycan biosynthetic proces		A=0, G=46	A=0.0, G=1.0	0.00001	768.306	46	A=0/0, G=10/3
chr1:87563668	A/A	G	A	SNV	HS2ST1	HS2ST1:intronic:NM 012262.3	1	100		intronic	NM 01226	c.686+50G	p.?	-2.83					Sulfotransfer	rs1199677	0.0352	YES	9.961E-1 (pos.87563668)		Heparan su	Golgi membrane,glycosaminoglycan biosynthetic proces		G=0, A=65	G=0.0, A=1.0	0.00001	996.464	65	G=0/0, A=34/3
chr7:43810764	A/A	G	A	SNV	BLVRA	BLVRA:exonic:NM 000712.3	1	100	2	missense	NM 00071	c.7G>A	p.Ala3Thr	-0.55	0.45	58	0	0.03(COSM50	Biliverdin rec	rs699512	0.235	YES	6.939E-1 (pos.43810764)	adenocarcino	Biliverdin re	biliverdin	Bile Duct Diseases; NADH	G=0, A=21	G=0.0, A=1.0	0.00001	281.235	21	G=0/0, A=5/16
chr4:81967188	T/T	C	T	SNV	BMP3	BMP3:exonic:NM 001201.3	1	100	2	missense	NM 00120	c.613C>T	p.Leu205Phe	-0.01	0.18	22	0.015		TGF-beta pro	rs6831040	0		1.0 (pos.81967188)		Bone morpl	BMP receptor binding,BMP signaling pathway,SMAD prot		C=0, T=126	C=0.0, T=1.0	0.00001	2053.62	126	C=0/0, T=55/7
chr14:54417522	A/G	A	G	SNV	BMP4	BMP4:exonic:NM 130851.3	1	50	4	missense	NM 13085	c.455T>C	p.Val152Ala	5.07	0.07	64	0.002	0.94(COSM37	TGF-beta pro	rs17563	0.4517	YES	2.717E-1 (pos.54417522)	adenocarcino	Bone morpl	BMP recep	ACTH-Secreting Pituitary Adenc Benign/Likely	A=13, G=13	A=0.5, G=0.5	0.00001	119.158	26	A=3/10, G=5/8
chr1:89427252	A/A	G	A	SNV	KYAT3	KYAT3:intronic:NM 001008661.2	1	100		intronic	NM 00100	c.541-63C	p.?	0.24					Aminotransfe	rs10922530		YES				2-oxoglutarate metabolic process,L-kynurenine metabo		G=0, A=27	G=0.0, A=1.0	0.00001	389.987	27	G=0/0, A=7/20
chr1:89475229	TG/TG	T	TG	INDEL	GBP3	GBP3:intronic:NM 018284.2	1	100		intronic	NM 01828	c.1363-52	p.?	0.17					Guanylate-binding protein, C-terminal domain,Guanylate-binding protein, N-terminal domain						Guanylate-l	GTP binding,GTPase activity,Golgi membrane,defense re		T=0, TG=60	T=0.0, TG=1.0	0.00001	598.447	60	T=0/0, TG=25/
chr7:34192762	C/C	G	C	SNV	BMPER	BMPER:exonic:NM 133468.4	1	100	16	synonymous	NM 13346	c.1935G>C	p.(=)	-0.24				0.05(COSM41	C8 domain,Tr	rs6968741	0.0623	YES	9.997E-1;0.0 (pos.34192	neoplasm	BMP bindin	blood vess	Alzheimer Disease;Bone Disea Benign	G=0, C=46	G=0.0, C=1.0	0.00001	767.05	46	G=0/0, C=24/2
chr1:151019180	G/G	A	G	SNV	BNIPL	BNIPL:utr 3:NM 138278.3	1	100	10	utr 3	NM 13827	c.1091A>G	p.?	-0.57					Bcl2-/adenov	rs6587553	0.1915	YES	9.244E-1 (pos.151019180)		BCL2/adenc	apoptotic process,cytosol,identical protein binding,neg		A=0, G=39	A=0.0, G=1.0	0.00001	601.536	39	A=0/0, G=17/2
chr1:900285	CA/TG	CA	TG	MNV	KLHL17	KLHL17:intronic:NM 198317.2	2	53.33		intronic	NM 19831	c.1701-58	p.?	-1.34, -0.32					BTB And C-ter	rs202081469,rs4970434,rs4970		YES				Cul3-RING ubiquitin ligase complex,POZ domain binding,		CA=21, TG=	CA=0.4667, TG=0.53	0.00001	276.154	45	CA=14/7, TG=1
chr3:112998268	G/A	G	A	SNV	BOC	BOC:exonic:NM 033254.3	1	50	12	synonymous	NM 03325	c.1986G>	p.(=)	-1.42				0.11(COSM37	Fibronectin t	rs2650100	0.1743	YES	1.447E-1 (pos.11299826	acute_myeloid_leukaemia		axon guidance,axonal growth cone,cell adhesion,integra		G=11, A=11	G=0.5, A=0.5	0.00001	94.6061	22	G=10/1, A=8/3
chr12:12618715	C/C	T	C	SNV	BORCS5	BORCS5:utr 3:NM 058169.5	1	100	4	utr 3	NM 05816	c.596T>C	p.?	-2					Tumour supp	rs967648	6.00E-04	YES	1.0 (pos.12618715)		BLOC1-relat	BORC complex,cytoplasmic side of lysosomal membrane		T=0, C=29	T=0.0, C=1.0	0.00001	420.188	29	T=0/0, C=12/1
chr20:36952342	C/T	C	T	SNV	BPI	BPI:exonic:NM 001725.2	1	50	8	missense	NM 00172	c.839C>T	p.Ala280Val	0.52	0.01	64	0.005	0.01(COSM44	LBP / BPI / CE	rs5741804	0.0451	YES	2.447E-1 (pos.36952342)	acute_myeloic	Bactericida	antimicrobial humoral respon	Di-Stearoyl-3-Sn-Phosph	C=23, T=23	C=0.5, T=0.5	0.00001	258.607	46	C=9/14, T=18/
chr1:91728202	C/C	T	C	SNV	HFM1	HFM1:intronic:NM 001017975.4	1	100		intronic	NM 00101	c.3975-53	p.?	-0.69					DEAD/DEAH b	rs555432					Hfm1, ATP-c	ATP binding,ATP-dependent RNA helicase activity,RNA se		T=0, C=43	T=0.0, C=1.0	0.00001	705.842	43	T=0/0, C=16/2
chr1:91739208	T/T	C	T	SNV	HFM1	HFM1:intronic:NM 001017975.4	1	100		intronic	NM 00101	c.3772+61	p.?	0.89					DEAD/DEAH b	rs281964					Hfm1, ATP-c	ATP binding,ATP-dependent RNA helicase activity,RNA se		C=0, T=27	C=0.0, T=1.0	0.00001	389.988	27	C=0/0, T=15/1
chr1:91781304	T/C	T	C	SNV	HFM1	HFM1:intronic:NM 001017975.4	1	96.67		intronic	NM 00101	c.3140+68	p.?	-0.04					DEAD/DEAH b	rs113665292,rs281991					Hfm1, ATP-c	ATP binding,ATP-dependent RNA helicase activity,RNA se		T=1, C=29	T=0.0333, C=0.9667	0.00001	410.682	30	T=1/0, C=19/1
chr1:91846374	A/A	C	A	SNV	HFM1	HFM1:intronic:NM 001017975.4	1	100		intronic	NM 00101	c.873+95G	p.?	-0.73					DEAD/DEAH b	rs282033					Hfm1, ATP-c	ATP binding,ATP-dependent RNA helicase activity,RNA se		C=0, A=54	C=0.0, A=1.0	0.00001	798.094	54	C=0/0, A=28/2

chr20:31876681	A/G	A	G	SNV	BPIFB1	BPIFB1:exonic:NM 033197.2	1	45.83	3	missense	NM 03319	c.250A>G	p.Ile84Val	0.79	0.17	29	0.016	0.05(COSM44	LBP / BPI / CE	rs1078761	0.2881	YES	2.306E-1;0.0 (pos.31876(	adenocarcinoma,neoplasn	antimicrobial humoral response,extracellular exosome,	A=13, G=11	A=0.5417, G=0.4583	0.00001	93.4231	24	A=5/8, G=2/9
chr20:31622083	G/A	G	A	SNV	BPIFB6	BPIFB6:exonic:NM 174897.2	1	52.38	3	missense	NM 17489	c.289G>A	p.Val97Ile	2.18	1	29	0.001	0.03(COSM37	LBP / BPI / CE	rs2070317	0.4897	YES	8.102E-1 (pos.31622083)	adenocarcinoı	BPI fold-con extracellular region,lipid binding	G=20, A=22	G=0.4762, A=0.5238	0.00001	245.205	42	G=13/7, A=7/1
chr7:140449150	C/C	T	C	SNV	BRAF	BRAF:exonic:NM 004333.4	1	100	16	synonymous	NM 00433	c.1929A>G	p.(=)	1.5				0.54(COSM41	Kinase-like.P	rs9648696	0.1463	YES	1.812E-1 (pos.14044915(	Wilms_tumou	Murine sarc ATP bindin Abnormalities, Mul (1E)-5-(1-o Benign	T=0, C=136	T=0.0, C=1.0	0.00001	2258.68	136	T=0/0, C=71/6
chr1:92447004	G/G	A	G	SNV	BRDT	BRDT:intronic:NM 001242806.2	1	100		intronic	NM 00124	c.1876+66	p.?	-1.04					Bromodomai	rs10875104		YES			Bromodom RNA splicing,cell differentiation,chromatin remodeling,c	A=0, G=32	A=0.0, G=1.0	0.00001	485.14	32	A=0/0, G=11/2
chr13:32890572	A/A	G	A	SNV	BRCA2	BRCA2:utr 5:NM 000059.3	1	100	2	utr 5	NM 00005	c.-26G>A	p.?	0.5					BRCA2 repea	rs1064795074,rs179	0.2633	YES	3.805E-1 (pos.32890572)		BRCA2 gene BRCA2-MA ACMG Recommendations...;Ad Benign	G=0, A=20	G=0.0, A=1.0	0.00001	263.786	20	G=0/0, A=11/9
chr13:32915005	C/C	G	C	SNV	BRCA2	BRCA2:exonic:NM 000059.3	1	100	11	synonymous	NM 00005	c.6513G>C	p.(=)	-0.88				0.02(COSM41	BRCA2 repea	rs1064794089,rs206	6.00E-04		9.999E-1;0.0 (pos.32915(	neoplasm	BRCA2 gene BRCA2-MA ACMG Recommendations...;Ad Benign	G=0, C=38	G=0.0, C=1.0	0.00001	603.751	38	G=0/0, C=23/1
chr1:92755943	A/A	G	A	SNV	GLMN	GLMN:intronic:NM 053274.2	1	100		intronic	NM 05327	c.286-80C	p.?	1.25					Uncharacteri	rs3103178		YES			Cul2-RING Glomus Tumor;Neoplasms;Neoplasms by His	G=0, A=125	G=0.0, A=1.0	0.00001	2033.96	125	G=0/0, A=77/4
chr1:92765848	T/T	A	T	SNV	RPAP2	RPAP2:intronic:NM 024813.2	1	100		intronic	NM 02481	c.119+52A	p.?	2.27					Rtr1/RPAP2 f	rs1542685	0.4448	YES	9.711E-1 (pos.92765848)		RNA polyme CTD phosphatase activity,DNA-directed RNA polymerase	A=0, T=30	A=0.0, T=1.0	0.00001	446.639	30	A=0/0, T=13/1
chr13:32929231	CG/CG	C	CG	INDEL	BRCA2	BRCA2:exonic:NM 000059.3	1	100	14	frameshiftInsertion	NM 00005	c.7241_72	p.His2415fs	3.19					BRCA2 repea	rs80358951				endometrioid	BRCA2 gene BRCA2-MA ACMG Recommendations...;Adnexal Disease	C=0, CG=22	C=0.0, CG=1.0	0.00001	250.196	22	C=0/0, CG=9/1
chr5:891839	C/G	C	G	SNV	BRD9	BRD9:exonic:NM 023924.4	1	59.18	2	missense	NM 02392	c.183G>C	p.Glu61Asp	0.33	0.28	45	0.997		Bromodomai	rs199607133	3.00E-04		0.0 (pos.891839)		biological Hemic and Lymphatic Diseases;Immune Syst	C=20, G=29	C=0.4082, G=0.5918	0.00001	360.518	49	C=16/4, G=22/
chr1:93576302	G/G	T	G	SNV	MTF2	MTF2:intronic:NM 007358.3	1	100		intronic	NM 00735	c.286+119	p.?	-0.39					PHD-finger.P	rs494745					Metal-regul ESC/E(Z) complex,RNA polymerase II regulatory region se	T=0, G=45	T=0.0, G=1.0	0.00001	746.536	45	T=0/0, G=29/1
chr1:93621571	C/A	C	A	SNV	TMED5	TMED5:intronic:NM 016040.4	1	58.33		intronic	NM 01604	c.471+286	p.?	-2.3					emp24/gp25	rs142621868			0.0 (pos.93621571)		Transmemb Golgi appa Nervous System Diseases:Neuromuscular Di	C=15, A=21	C=0.4167, A=0.5833	0.00001	234.325	36	C=13/2, A=10/
chr5:870619	A/G	A	G	SNV	BRD9	BRD9:exonic:NM 023924.4	1	57.14	14	synonymous	NM 02392	c.1494T>C	p.(=)	1.26					Bromodomai	rs1051630	0.0563	YES	1.503E-3 (pos.870619)		biological Hemic and Lymphatic Diseases;Immune Syst	A=9, G=12	A=0.4286, G=0.5714	0.00001	109.539	21	A=4/5, G=9/3
chr1:92428495	A/A	C	A	SNV	BRDT	BRDT:exonic:NM 001242806.2	1	100	2	missense	NM 00124	c.184C>A	p.Gln62Lys	1.07	0.49	53	0	0.01(COSM44	Bromodomai	rs10783071	0.0586	YES	6.033E-1 (pos.92428495)	acute_myeloic	Bromodom RNA splicing,cell differentiation,chromatin remodeling,c	C=0, A=30	C=0.0, A=1.0	0.00001	446.639	30	C=0/0, A=16/1
chr1:92457843	T/T	C	T	SNV	BRDT	BRDT:exonic:NM 001242806.2	1	100	14	missense	NM 00124	c.2099C>T	p.Pro700Leu	-0.91	0.67	98	0		Bromodomai	rs10747493	0.1445	YES	6.111E-1 (pos.92457843)		Bromodom RNA splicing,cell differentiation,chromatin remodeling,c	C=0, T=25	C=0.0, T=1.0	0.00001	353.019	25	C=0/0, T=7/18
chr14:105716377	C/T	C	T	SNV	BRF1,BTBD6	BRF1:intronic:NM 001519.3, BTBD6:exonic:NM 033271.2	1	50	5	synonymous	NM 00151	c.544+246	p.?, p.(=)	-0.1					BTB And C-ter	rs45452103	0.0253	YES	1.169E-4 (pos.105716377)		BRF1, S. cer DNA-templated transcriptional preinitiation complex as	C=13, T=13	C=0.5, T=0.5	0.00001	117.747	26	C=1/12, T=5/8
chr11:66105194	A/A	G	A	SNV	BRMS1	BRMS1:exonic:NM 001024957.1	1	100	10	missense	NM 00102	c.818C>T	p.Ala273Val	-0.95	0.33	64	0	0.02(COSM37	Sds3-like	rs1052566	0.2984	YES	3.732E-1 (pos.66105194)	adenocarcinoı	Breast cancer metastasis suppressor 1	G=0, A=20	G=0.0, A=1.0	0.00001	263.786	20	G=0/0, A=11/9
chr11:66107674	G/G	A	G	SNV	BRMS1	BRMS1:exonic:NM 001024957.1	1	100	7	synonymous	NM 00102	c.546T>C	p.(=)	0.07					Sds3-like	rs1784035	0.0012		1.0 (pos.66107674)		Breast cancer metastasis suppressor 1	A=0, G=37	A=0.0, G=1.0	0.00001	583.693	37	A=0/0, G=26/1
chr1:94980671	G/G	C	G	SNV	ABCD3	ABCD3:intronic:NM 002858.3	1	100		intronic	NM 00285	c.1846-31(	p.?	-0.84					ABC transpor	rs3118109	0		1.0 (pos.94980671)		ATP-binding ATP bindin Abnormalities, Multiple;Brain Diseases;Brair	C=0, G=79	C=0.0, G=1.0	0.00001	1269.26	79	C=0/0, G=42/3
chr1:95305440	G/G	A	G	SNV	SLC44A3	SLC44A3:intronic:NM 001114106.2	1	100		intronic	NM 00111	c.671-64A	p.?	-0.92					Plasma-mem	rs859120		YES			choline transmembrane transı Choline	A=0, G=30	A=0.0, G=1.0	0.00001	446.639	30	A=0/0, G=20/1
chr21:40571246	G/G	A	G	SNV	BRWD1	BRWD1:exonic:NM 018963.4	1	100	40	missense	NM 01896	c.5096T>C	p.Leu1699Pro	-1.37	0.54	98	0	0.01(COSM63	Bromodomai	rs1041439	0.0655	YES	6.113E-1 (pos.40571246)	carcinoma	chromatin Abnormalities, Multiple;Cardiovascular Dise	A=0, G=21	A=0.0, G=1.0	0.00001	281.235	21	A=0/0, G=9/12
chr1:9658465	G/G	T	G	SNV	TMEM201	TMEM201:intronic:NM 001130924.2	1	100		intronic	NM 00113	c.430-42T	p.?	-1.36					Ima1 N-termi	rs9430181	0.1797	YES	7.235E-1 (pos.9658465)		actin filament binding,centrosome localization,cortical	T=0, G=28	T=0.0, G=1.0	0.00001	408.718	28	T=0/0, G=7/21
chr21:40574305	G/G	A	G	SNV	BRWD1	BRWD1:exonic:NM 018963.4	1	100	38	missense	NM 01896	c.4531T>C	p.Ser1511Pro	1.87	0	74	0.984	0.92(COSM54	Bromodomai	rs2183573	0.4094	YES	5.782E-1 (pos.40574305)	acute_myeloid_leukaemia	chromatin Abnormalities, Multiple;Cardiovascular Dise	A=0, G=46	A=0.0, G=1.0	0.00001	765.975	46	A=0/0, G=20/2
chr1:987200	T/T	C	T	SNV	AGRN	AGRN:intronic:NM 198576.3	1	100		intronic	NM 19857	c.5651+5C	p.?	-0.94					Agrin NtA dor	rs9803031	0.09	YES	9.968E-1 (pos.987200)		Agrin G-protein Congenital, Hereditary, and Ne Benign	C=0, T=26	C=0.0, T=1.0	0.00001	371.205	26	C=0/0, T=15/1
chr21:40670460	G/C	G	C	SNV	BRWD1	BRWD1:exonic:NM 018963.4	1	97.06	5	missense	NM 01896	c.247C>G	p.Gln83Glu	3.5	0.52	29	0.003	0.92(COSM40	Bromodomai	rs2056844	0.4592	YES	4.402E-1;0.0 (pos.40670(	acute_myeloid_leukaemia	chromatin Abnormalities, Multiple;Cardiovascular Dise	G=1, C=33	G=0.0294, C=0.9706	0.00001	492.932	34	G=1/0, C=18/1
chr3:49690199	A/A	G	A	SNV	BSN	BSN:exonic:NM 003458.3	1	100	5	synonymous	NM 00345	c.3210G>A	p.(=)	-9.43					Piccolo Zn-fin	rs4855885	0.1823	YES	9.953E-1 (pos.49690199)		Bassoon, m axon,cell i Cardiovascular Diseases;Colitis;Colitis, Ulce	G=0, A=25	G=0.0, A=1.0	0.00001	352.734	25	G=0/0, A=13/1
chr10:101372410	AC/AC	A	AC	INDEL	SLC25A28	SLC25A28:intronic:NM 031212.3	1	100		intronic	NM 03121	c.521-54T	p.?	-0.07					Mitochondrial carrier protein						Solute carri integral component of membrane,iron ion homeostasis,	A=0, AC=40	A=0.0, AC=1.0	0.00001	509.073	40	A=0/0, AC=10/
chr10:101980459	G/G	A	G	SNV	CHUK	CHUK:intronic:NM 001278.4	1	100		intronic	NM 00127	c.386-26T	p.?	-0.13					I-kappa-kinas	rs986266	3.00E-04		9.999E-1 (pos.101980459)		Conserved ATP bindin Autoimmune Disea Acetylcysteine,Aminosal	A=0, G=65	A=0.0, G=1.0	0.00001	1006.77	65	A=0/0, G=31/3
chr3:49690496	T/T	C	T	SNV	BSN	BSN:exonic:NM 003458.3	1	100	5	synonymous	NM 00345	c.3507C>T	p.(=)	-1.42					Piccolo Zn-fin	rs11709525	0.2972	YES	4.859E-2 (pos.49690496)		Bassoon, m axon,cell i Cardiovascular Diseases;Colitis;Colitis, Ulce	C=0, T=31	C=0.0, T=1.0	0.00001	460.908	31	C=0/0, T=26/5
chr1:55474262	A/A	G	A	SNV	BSND	BSND:exonic:NM 057176.2	1	100	4	synonymous	NM 05717	c.924G>A	p.(=)	-4.39				0(COSM3751	Bartter syndr	rs33938617	0.2056	YES	2.315E-4 (pos.55474262)	adenocarcinoı	Barttin basolater Adrenal Gland Diseases:Adren Benign/Likely	G=0, A=71	G=0.0, A=1.0	0.00001	1111.48	71	G=0/0, A=37/3
chr10:124089036	G/A	G	A	SNV	BTBD16	BTBD16:exonic:NM 144587.3	1	52.38	11	missense	NM 14458	c.953G>A	p.Arg318Gln	1.55	1	43	0	0.01(COSM4418949)		rs2421013	0.4891	YES	8.593E-1 (pos.12408903(	acute_myeloid_leukaemia	nucleus,ubiquitin ligase complex	G=20, A=22	G=0.4762, A=0.5238	0.00001	245.209	42	G=10/10, A=1
chr10:124089075	G/A	G	A	SNV	BTBD16	BTBD16:exonic:NM 144587.3	1	52.38	11	missense	NM 14458	c.992G>A	p.Gly331Asp	0.49	0.28	94	0.994	0.23(COSM915571)		rs986178	0.1271	YES	1.619E-1 (pos.12408907!	endometrioid_carcinoma	nucleus,ubiquitin ligase complex	G=20, A=22	G=0.4762, A=0.5238	0.00001	245.164	42	G=10/10, A=1
chr10:104157727	T/T	C	T	SNV	NFKB2	NFKB2:intronic:NM 001077494.3	1	100		intronic	NM 00107	c.662-11C	p.?	0.17					Ankyrin repe	rs4919632	0.0244	YES	9.993E-1 (pos.104157727)		Nuclear fac Bcl3/NF-ka Brain Diseases;Cen Acetylsali Benign	C=0, T=114	C=0.0, T=1.0	0.00001	1815.31	114	C=0/0, T=63/5
chr19:1997363	G/G	A	G	SNV	BTBD2	BTBD2:exonic:NM 017797.3	1	100	2	synonymous	NM 01779	c.507T>C	p.(=)	-2.08				0.1(COSM375	BTB And C-ter	rs1610045	0.3226	YES	4.594E-1;0.0 (pos.19973(	acute_myeloid_leukaemia	P-body,SCF ubiquitin ligase complex,cytosol,neurogenes	A=0, G=20	A=0.0, G=1.0	0.00001	263.773	20	A=0/0, G=7/13
chr10:104229057	CA/CA	C	CA	INDEL	MFSD13A	MFSD13A:intronic:NM 024789.3	1	100		intronic	NM 02478	c.168+103	p.?	0.3					MFS/sugar tra	rs35597970,rs397758277		YES			integral component of membrane	C=0, CA=30	C=0.0, CA=1.0	0.00001	440.438	30	C=0/0, CA=10/
chr3:112190137	T/T	G	T	SNV	BTLA	BTLA:exonic:NM 181780.3	1	100	3	missense	NM 18178	c.469C>A	p.Arg157Ser	-1.43	1	110	0		Immunoglob	rs2931761	0		9.949E-1 (pos.112190137)		B- and T-lym T cell costimulation,adaptive immune response,integral	G=0, T=26	G=0.0, T=1.0	0.00001	364.463	26	G=0/0, T=7/19
chr10:104232716	A/A	G	A	SNV	MFSD13A	MFSD13A:intronic:NM 024789.3	1	100		intronic	NM 02478	c.925+9G>	p.?	1.62				0.04(COSM37	MFS/sugar tra	rs3740415	0.4466	YES	3.123E-1 (pos.10423271(	rhabdomyosarcoma	integral component of membrane	G=0, A=23	G=0.0, A=1.0	0.00001	316.735	23	G=0/0, A=10/1
chr10:104241683	T/T	G	T	SNV	ACTR1A	ACTR1A:intronic:NM 005736.3	1	100		intronic	NM 00573	c.926-28C	p.?	0.47					Actin	rs2296580	0.2944	YES	1.443E-1 (pos.104241683)		Actin-relate ATP binding,COPI-coated vesicle,ER to Golgi vesicle-medi	G=0, T=28	G=0.0, T=1.0	0.00001	408.089	28	G=0/0, T=17/1
chr10:104250278	G/G	A	G	SNV	ACTR1A	ACTR1A:intronic:NM 005736.3	1	100		intronic	NM 00573	c.113+28T	p.?	0.1					Actin	rs17709610	0.2947	YES	1.446E-1 (pos.104250278)		Actin-relate ATP binding,COPI-coated vesicle,ER to Golgi vesicle-medi	A=0, G=38	A=0.0, G=1.0	0.00001	580.843	38	A=0/0, G=21/1
chr10:104572276	G/G	A	G	SNV	WBP1L	WBP1L:intronic:NM 001083913.1	1	100		intronic	NM 00108	c.356-76A	p.?	0.42					WW domain-	rs284861,rs386747117		YES			integral component of membrane,protein binding	A=0, G=24	A=0.0, G=1.0	0.00001	334.785	24	A=0/0, G=15/9
chr6:26501897	C/C	T	C	SNV	BTN1A1	BTN1A1:exonic:NM 001732.2	1	100	2	synonymous	NM 00173	c.159T>C	p.(=)	0.02				0.03(COSM14	CD80-like C2-	rs1321479	0.4662	YES	7.398E-1 (pos.26501897)	adenocarcinoı	Butyrophili extracellular space,integral component of membrane,in	T=0, C=117	T=0.0, C=1.0	0.00001	1874.22	117	T=0/0, C=57/6
chr10:105201565	G/A	G	A	SNV	PDCD11	PDCD11:intronic:NM 014976.1	1	45.45		intronic	NM 01497	c.4558-18(	p.?	-4.32					PPR repeat,S	rs2281861	0.3707	YES	3.424E-1 (pos.105201565)		Programme RNA binding,cytosol,mRNA processing,maturation of 5.8	G=12, A=10	G=0.5455, A=0.4545	0.00001	81.7445	22	G=7/5, A=8/2
chr10:105201577	C/T	C	T	SNV	PDCD11	PDCD11:intronic:NM 014976.1	1	45.45		intronic	NM 01497	c.4558-6C	p.?	0.34					PPR repeat,S	rs2281860	0.3845	YES	3.426E-1 (pos.105201577)		Programme RNA binding,cytosol,mRNA processing,maturation of 5.8	C=12, T=10	C=0.5455, T=0.4545	0.00001	81.7564	22	C=7/5, T=8/2
chr6:26505362	A/A	G	A	SNV	BTN1A1	BTN1A1:exonic:NM 001732.2	1	100	3	missense	NM 00173	c.637G>A	p.Ala213Thr	-1.6	0.27	58	0.005	0.01(COSM63	CD80-like C2-	rs3736781	0.4702	YES	7.479E-1 (pos.26505362)	neoplasm	Butyrophili extracellular space,integral component of membrane,in	G=0, A=73	G=0.0, A=1.0	0.00001	1150.51	73	G=0/0, A=25/4
chr6:26393021	G/A	G	A	SNV	BTN2A2	BTN2A2:exonic:NM 006995.4	1	50	8	synonymous	NM 00699	c.1398G>A	p.(=)	-4.11					Immunoglob	rs1614887	0.4584	YES	3.402E-1 (pos.26393021)		Butyrophili extracellular exosome,integral component of membran	G=13, A=13	G=0.5, A=0.5	0.00001	119.163	26	G=7/6, A=8/5
chr6:26413744	C/A	C	A	SNV	BTN3A1	BTN3A1:exonic:NM 007048.5	1	56.36	10	missense	NM 00704	c.1366C>A	p.Pro456Thr	-1.35	0.28	38	0.904	0.05(COSM37	Immunoglob	rs4712990	0.1219	YES	2.623E-2;0.0 (pos.26413	adenocarcinoı	Butyrophili T cell receptor signaling pathway,activated T cell prolife	C=24, A=31	C=0.4364, A=0.5636	0.00001	320.165	55	C=17/7, A=14/
chr10:124457906	C/T	C	T	SNV	C10orf120	C10orf120:exonic:NM 001010912.3	1	52.38	3	synonymous	NM 00101	c.351G>A	p.(=)	-0.6				0(COSM3751583)		rs2901343	0.413	YES	5.082E-1 (pos.12445790(	adenocarcinoma	nucleus,ubiquitin ligase complex	C=20, T=22	C=0.4762, T=0.5238	0.00001	245.209	42	C=6/14, T=3/1
chr10:105428462	A/A	AGA	A	INDEL	SH3PXD2A	SH3PXD2A:intronic:NM 014631.2	2	100		intronic	NM 01463	c.428-52T(	p.?	1.42, 0.46, -0.42					PX domain.Sl	rs3068778,rs34311211,rs5787496					cell junction,cell projection,cytosol,extracellular matrix	AGA=0, A=2	AGA=0.0, A=1.0	0.00001	408.311	28	AGA=0/0, A=8/
chr10:50534599	A/A	G	A	SNV	C10orf71	C10orf71:exonic:NM 001135196.1	1	100	3	missense	NM 00113	c.4009G>A	p.Gly1337Ser	4.34		56		0.48(COSM37	Domain of un	rs10857472	0.4488	YES	2.432E-1 (pos.50534599)	adenocarcinoma,carcinoma,ductal_carcinoma		G=0, A=133	G=0.0, A=1.0	0.00001	2196.98	133	G=0/0, A=89/4
chr10:50532683	T/C	T	C	SNV	C10orf71	C10orf71:exonic:NM 001135196.1	1	95.38	3	missense	NM 00113	c.2093T>C	p.Phe698Ser	2.4		155			Domain of un	rs7921186	7.00E-04	YES	1.0 (pos.50532683)			T=3, C=62	T=0.0462, C=0.9538	0.00001	896.12	65	T=2/1, C=23/3
chr10:105768271	A/A	C	A	SNV	SLK	SLK:intronic:NM 014720.3	1	100		intronic	NM 01472	c.2784+15	p.?	0.75					Polo kinase k	rs1965595		YES			STE20-like ATP bindin Mental Disorders;S 4-((4-(5-CYCLOPROPYL-1H	C=0, A=24	C=0.0, A=1.0	0.00001	334.782	24	C=0/0, A=15/9
chr10:105796399	C/C	G	C	SNV	COL17A1	COL17A1:intronic:NM 000494.3	1	100		intronic	NM 00049	c.3278-9C	p.?	0.68					Collagen trip	rs2274098	0.167	YES	8.563E-1 (pos.105796399)		Collagen XV basement Cardiomegaly;Cardiovascular Benign	G=0, C=39	G=0.0, C=1.0	0.00001	623.98	39	G=0/0, C=10/2
chr10:105796738	C/C	A	C	SNV	COL17A1	COL17A1:intronic:NM 000494.3	1	100		intronic	NM 00049	c.3277+65	p.?	-0.01					Collagen trip	rs805714	0	YES			Collagen XV basement Cardiomegaly;Cardiovascular Diseases;Cong	A=0, C=45	A=0.0, C=1.0	0.00001	747.375	45	A=0/0, C=25/2
chr10:128209959	T/T	G	T	SNV	C10orf90	C10orf90:utr 5:NM 001004298.3	1	100	1	utr 5	NM 00100	c.-70C>A	p.?	-0.88					ALMS motif	rs4962342	0.1484	YES			Chromosom actin cytoskeleton,alpha-actinin binding,centrosome,cy	G=0, T=102	G=0.0, T=1.0	0.00001	1594.29	102	G=0/0, T=81/2
chr10:105814826	A/A	C	A	SNV	COL17A1	COL17A1:intronic:NM 000494.3	1	100		intronic	NM 00049	c.1718-61(	p.?	-0.11					Collagen trip	rs805691	0.3001	YES			Collagen XV basement Cardiomegaly;Cardiovascular Diseases;Cong	C=0, A=43	C=0.0, A=1.0	0.00001	705.794	43	C=0/0, A=17/2
chr11:8942999	A/A	G	A	SNV	C11orf16	C11orf16:exonic:NM 020643.2	1	100	6	missense	NM 02064	c.1268C>T	p.Ala423Val	-1.28	0.52	64	0		Domain of un	rs2568023	0.0019	YES	9.998E-1 (pos.8942999)			G=0, A=25	G=0.0, A=1.0	0.00001	353.018	25	G=0/0, A=11/1
chr10:105821957	T/C	T	C	SNV	COL17A1	COL17A1:intronic:NM 000494.3	1	50		intronic	NM 00049	c.910+59A	p.?	-0.51					Collagen trip	rs150881647	0.0031				Collagen XV basement Cardiomegaly;Cardiovascular Diseases;Cong	T=15, C=15	T=0.5, C=0.5	0.00001	145.105	30	T=14/1, C=11/
chr11:8942942	C/T	C	T	SNV	C11orf16	C11orf16:exonic:NM 020643.2	1	45.83	6	missense	NM 02064	c.1325G>A	p.Arg442Gln	-0.18	1	43	0	0(COSM1471	Domain of un	rs2653601	0.3549	YES	2.275E-1 (pos.8942942)	acute_myeloid_leukaemia,adenocarcinoma,neoplasm		C=13, T=11	C=0.5417, T=0.4583	0.00001	93.4399	24	C=5/8, T=6/5
chr11:4592706	T/TAC	T	TAC	INDEL	C11orf40	C11orf40:exonic:NM 144663.1	2	46.43	4	frameshiftInsertion	NM 14466	c.600_601	p.Met201fs	-0.23						rs141600462,rs8031(	0.4644	YES	3.699E-1;0.0 (pos.4592706)			T=45, TAC=	T=0.5357, TAC=0.464	0.00001	404.497	84	T=24/21, TAC=
chr10:106214135	CA/C	CA	C	INDEL	CFAP58	CFAP58:intronic:NM 001008723.1	1	50		intronic	NM 00100	c.2511-45(	p.?	-0.36, 0.76											cilium,extracellular space,protein binding	CA=10, C=1	CA=0.5, C=0.5	0.00001	52.4171	20	CA=5/5, C=3/7
chr10:106970848	A/A	AG	A	INDEL	SORCS3	SORCS3:intronic:NM 014978.2	1	100		intronic	NM 01497	c.2262-47	p.?	-0.84, -0.36					PKD domain,	rs3214966,rs762981	0.3493	YES	2.481E-1 (pos.106970848)		SORCS rece integral component of membrane,learning,membrane,n	AG=0, A=22	AG=0.0, A=1.0	0.00001	304.308	22	AG=0/0, A=15/
chr10:108466412	T/T	C	T	SNV	SORCS1	SORCS1:intronic:NM 001013031.2	1	100		intronic	NM 00101	c.1144-20(	p.?	-1.48					PKD domain.	rs2756231	0.002	YES	8.478E-1 (pos.108466412)		SORCS rece integral component of membrane,membrane,neuropep	C=0, T=33	C=0.0, T=1.0	0.00001	504.564	33	C=0/0, T=12/2
chr10:111625108	C/C	T	C	SNV	XPNPEP1	XPNPEP1:intronic:NM 020383.3	1	100		intronic	NM 02038	c.1873-38	p.?	-0.08					C-terminal re	rs483072,rs7580222	5.00E-04		0.0 (pos.111625105),1.0 (pos.11162510		X-prolyl am aminopeptidase activity,bradykinin catabolic process,c	T=0, C=142	T=0.0, C=1.0	0.00001	2383.55	142	T=0/0, C=89/5
chr12:88440676	C/C	G	C	SNV	C12orf29	C12orf29:exonic:NM 001009894.2	1	100	6	missense	NM 00100	c.712G>C	p.Val238Leu	-1.17	1	32	0	0.04(COSM3753520)		rs9262	0.4112	YES	7.551E-1;0.0 (pos.88440(	adenocarcinoma,ductal_ca	hematopoietic progenitor cell differentiation	G=0, C=39	G=0.0, C=1.0	0.00001	623.98	39	G=0/0, C=22/1
chr10:112338377	T/C	T	C	SNV	SMC3	SMC3:intronic:NM 005445.3	1	45.45		intronic	NM 00544	c.351-9T>C	p.?	4.13				0.94(COSM50	AAA ATPase d	rs78663177	0.0438	YES	4.583E-4 (pos.11233837	haemangiobla	Structural n ATP bindin Abnormalities, Multiple;Chron Benign	T=24, C=20	T=0.5455, C=0.4545	0.00001	211.709	44	T=17/7, C=10/
chr12:121454257	A/A	T	A	SNV	C12orf43	C12orf43:exonic:NM 022895.2	1	100	1	synonymous	NM 02289	c.21A>T	p.(=)	-0.15						rs2258227	0.1827	YES	8.755E-1;0.0;0.0 (pos.121454257)		protein binding	T=0, A=29	T=0.0, A=1.0	0.00001	427.584	29	T=0/0, A=6/23
chr10:114181683	G/G	A	G	SNV	ACSL5	ACSL5:intronic:NM 016234.3	1	100		intronic	NM 01623	c.1556-22	p.?	0.02					AMP-binding	rs2255740	0.0876	YES	9.91E-1 (pos.114181683)		Acyl-CoA sy ATP bindin Arthritis;Arthritis, Experimental;Joint Diseas	A=0, G=28	A=0.0, G=1.0	0.00001	408.704	28	A=0/0, G=8/20
chr12:48888594	C/T	C	T	SNV	C12orf54	C12orf54:exonic:NM 152319.3	1	45.45	8	missense	NM 15231	c.256C>T	p.Pro86Ser	0.03	0.55	74	0.06	0.14(COSM37	Domain of un	rs11458	0.4612	YES	1.449E-1 (pos.48888594)	adenocarcinoma,carcinoma		C=18, T=15	C=0.5455, T=0.4545	0.00001	142.791	33	C=4/14, T=10/
chr12:64669090	A/A	C	A	SNV	C12orf56	C12orf56:exonic:NM 001170633.1	1	100	10	synonymous	NM 00117	c.1497G>T	p.(=)	0.99				0.02(COSM54	Protein of un	rs1002392	0.1936	YES	5.277E-1 (pos.64669090)	adenocarcinoma		C=0, A=25	C=0.0, A=1.0	0.00001	353.017	25	C=0/0, A=11/1
chr12:64587633	A/A	C	A	SNV	C12orf66	C12orf66:exonic:NM 152440.4	1	100	3	missense	NM 15244	c.1327G>T	p.Ala443Ser	6.22	1	99	0		Eukaryotic pr	rs699638	0		1.0 (pos.64587633)		Chromosom KICSTOR cc Adnexal Diseases;Cysts;Endocrine System Di	C=0, A=27	C=0.0, A=1.0	0.00001	389.988	27	C=0/0, A=14/1
chr10:115401264	G/G	A	G	SNV	NRAP	NRAP:intronic:NM 001261463.1	1	100		intronic	NM 00126	c.1216-33	p.?	1.8					LIM domain,	rs3121465	0.3851	YES	7.176E-1 (pos.115401264)		Nebulin-rel actin binding,biological_process,fascia adherens,metal	A=0, G=164	A=0.0, G=1.0	0.00001	2663.08	164	A=0/0, G=83/8
chr12:64609562	T/T	C	T	SNV	C12orf66	C12orf66:exonic:NM 152440.4	1	100	2	missense	NM 15244	c.417G>A	p.Met139Ile	-0.09	0.83	10	0		Eukaryotic pr	rs2335390	0		1.0 (pos.64609562)		Chromosom KICSTOR cc Adnexal Diseases;Cysts;Endocrine System Di	C=0, T=72	C=0.0, T=1.0	0.00001	1130.95	72	C=0/0, T=33/3
chr14:60903757	A/A	G	A	SNV	C14orf39	C14orf39:exonic:NM 174978.2	1	100	18	missense	NM 17497	c.1570C>T	p.Leu524Phe	4.27	1	22	0	0.02(COSM39	Six6 opposite	rs1254319	0.2948	YES	6.808E-1 (pos.60903757)	acute_myeloic	Chromosom cellular_c Eye Diseases;Refractive Errors	G=0, A=32	G=0.0, A=1.0	0.00001	484.484	32	G=0/0, A=12/2
chr14:60923783	C/T	C	T	SNV	C14orf39	C14orf39:exonic:NM 174978.2	1	57.14	15	missense	NM 17497	c.1210G>A	p.Val404Ile	-0.24	0.98	29	0	0(COSM6385	Six6 opposite	rs11625921	0.0171	YES	8.71E-1;0.0 (pos.609237	carcinoma	Chromosom cellular_c Eye Diseases;Refractive Errors	C=9, T=12	C=0.4286, T=0.5714	0.00001	109.098	21	C=6/3, T=9/3
chr14:23456315	C/C	T	C	SNV	C14orf93	C14orf93:utr 3:NM 001130708.2	1	100	7	utr 3	NM 00113	c.1726A>G	p.?	0.84					Domain of un	rs1043675		YES			RNA binding,extracellular region,protein binding	T=0, C=55	T=0.0, C=1.0	0.00001	814.877	55	T=0/0, C=15/4
chr10:115515120	G/A	G	A	SNV	PLEKHS1		1	52.27		intronic				-0.16						rs3981351	0.2351	YES			Nutrition Disorders;Nutritional and Metaboli	G=42, A=46	G=0.4773, A=0.5227	0.00001	511.555	88	G=15/27, A=8/
chr15:93015517	G/GT	G	GT	INDEL	C15orf32,LOC104613533	C15orf32:exonic:NM 153040.2, LOC104613533:upstream:N	1	50	1	frameshiftInsertion	NR 12650	c.139_140	p.Ala47fs	-0.37											Alcohol-Related Disorders;Alcoholism;Menta	G=26, GT=2	G=0.5, GT=0.5	0.00001	256.385	52	G=12/14, GT=
chr15:75499602	A/A	C	A	SNV	C15orf39	C15orf39:exonic:NM 015492.4	1	100	2	synonymous	NM 01549	c.1213C>A	p.(=)	0.63				0.32(COSM4128402),0.32(C		rs12591506	0.0088	YES	7.991E-1 (pos.75499602)	neoplasm,squamous_cell	cytosol	C=0, A=30	C=0.0, A=1.0	0.00001	453.409	30	C=0/0, A=21/9
chr15:75499619	C/C	T	C	SNV	C15orf39	C15orf39:exonic:NM 015492.4	1	100	2	synonymous	NM 01549	c.1230T>C	p.(=)	-1.55				0.01(COSM4128404),0.01(C		rs11072531	0.002	YES	8.074E-1 (pos.75499619)	ductal_carcinoma,neoplas	cytosol	T=0, C=31	T=0.0, C=1.0	0.00001	471.676	31	T=0/0, C=21/1
chr15:75503147	G/A	G	A	SNV	C15orf39	C15orf39:exonic:NM 015492.4	1	95.83	3	missense	NM 01549	c.2834G>A	p.Gly945Asp	0.2	1	94	0.017			rs3743211	0.001	YES	8.073E-1 (pos.75503147)		cytosol	G=1, A=23	G=0.0417, A=0.9583	0.00001	306.631	24	G=0/1, A=14/9
chr10:116068176	G/G	A	G	SNV	AFAP1L2	AFAP1L2:intronic:NM 001001936.2	1	100		intronic	NM 00100	c.967+16T	p.?	-3.24					PH domain	rs581327	0.0767	YES	9.82E-1 (pos.116068176)		Actin filame SH2 domain binding,SH3 domain binding,cytoplasm,cyto	A=0, G=44	A=0.0, G=1.0	0.00001	723.771	44	A=0/0, G=13/3
chr10:116075228	G/G	A	G	SNV	AFAP1L2	AFAP1L2:intronic:NM 001001936.2	1	100		intronic	NM 00100	c.612+91T	p.?	-0.3					PH domain	rs694883		YES			Actin filame SH2 domain binding,SH3 domain binding,cytoplasm,cyto	A=0, G=39	A=0.0, G=1.0	0.00001	623.98	39	A=0/0, G=21/1
chr15:74032783	G/G	A	G	SNV	C15orf59	C15orf59:exonic:NM 001039614.2	1	100	2	synonymous	NM 00103	c.357T>C	p.(=)	-0.06				0.43(COSM43	Domain of un	rs28594180	0.4042	YES	5.909E-1 (pos.74032783)	ductal_carcinc	Chromosome 15 open reading frame 59	A=0, G=49	A=0.0, G=1.0	0.00001	828.318	49	A=0/0, G=9/40
chr10:116920090	C/C	A	C	SNV	ATRNL1	ATRNL1:intronic:NM 207303.4	1	100		intronic	NM 20730	c.1004+11	p.?	-0.41					CUB domain,	rs2065719,rs35990212		YES			Attractin-lik G-protein coupled receptor signaling pathway,carbohyd	A=0, C=26	A=0.0, C=1.0	0.00001	371.419	26	A=0/0, C=9/17
chr16:19680546	T/C	T	C	SNV	C16orf62	C16orf62:exonic:NM 020314.5	1	53.13	27	synonymous	NM 02031	c.2553T>C	p.(=)	-1.18				0.11(COSM3754749),0.11(C		rs957676	0.3315	YES	3.237E-1 (pos.19680546)	adenocarcinoma,osteosar	Golgi to plasma membrane transport,early endosome,fi	T=15, C=17	T=0.4688, C=0.5313	0.00001	173.543	32	T=9/6, C=6/11
chr10:117309098	A/A	G	A	SNV	ATRNL1	ATRNL1:intronic:NM 207303.4	1	100		intronic	NM 20730	c.3795+52	p.?	-0.25					CUB domain,	rs1336108	0.2707	YES	3.253E-1 (pos.117309098)		Attractin-lik G-protein coupled receptor signaling pathway,carbohyd	G=0, A=94	G=0.0, A=1.0	0.00001	1576.15	94	G=0/0, A=52/4
chr16:4796393	G/G	A	G	SNV	C16orf71	C16orf71:exonic:NM 139170.2	1	100	7	missense	NM 13917	c.1061A>G	p.Gln354Arg	-1.22	0.84	43	0		Domain of un	rs737700	0.3394	YES	6.73E-1 (pos.4796393)			A=0, G=147	A=0.0, G=1.0	0.00001	2463.78	147	A=0/0, G=135/
chr17:32904586	T/T	C	T	SNV	C17orf102	C17orf102:exonic:NM 207454.2	1	100	2	missense	NM 20745	c.464G>A	p.Arg155Lys	-0.06	1	26	0	0(COSM5438078)		rs887230	0.1488	YES	7.667E-1 (pos.32904586)	squamous_cell_carcinoma		C=0, T=20	C=0.0, T=1.0	0.00001	263.779	20	C=0/0, T=8/12
chr10:11789511	G/G	C	G	SNV	ECHDC3	ECHDC3:intronic:NM 024693.4	1	100		intronic	NM 02469	c.292+42C	p.?	0.17					Enoyl-CoA hy	rs4750092	0.0017	YES	9.999E-1 (pos.11789511)		catalytic activity,metabolic process,mitochondrion	C=0, G=96	C=0.0, G=1.0	0.00001	1622.02	96	C=0/0, G=51/4
chr10:11797619	G/G	C	G	SNV	ECHDC3	ECHDC3:intronic:NM 024693.4	1	100		intronic	NM 02469	c.591+12C	p.?	0.23					Enoyl-CoA hy	rs902892	0.0014	YES	9.999E-1;0.0;0.0 (pos.11797619)		catalytic activity,metabolic process,mitochondrion	C=0, G=24	C=0.0, G=1.0	0.00001	272.938	24	C=0/0, G=20/4
chr10:11797758	A/A	G	A	SNV	ECHDC3	ECHDC3:intronic:NM 024693.4	1	100		intronic	NM 02469	c.591+151	p.?	-0.38					Enoyl-CoA hy	rs3750694		YES			catalytic activity,metabolic process,mitochondrion	G=0, A=25	G=0.0, A=1.0	0.00001	353.017	25	G=0/0, A=21/4
chr10:118357490	T/T	C	T	SNV	PNLIPRP1	PNLIPRP1:intronic:NM 006229.3	1	100		intronic	NM 00622	c.694+31C	p.?	-1.32					Lipase,PLAT/	rs2915764	8.00E-04	YES	9.998E-1;0.0 (pos.118357490)		Pancreatic calcium ion binding,extracellular region,extracellular sp	C=0, T=81	C=0.0, T=1.0	0.00001	1309.1	81	C=0/0, T=35/4
chr10:118452044	T/T	G	T	SNV	HSPA12A	HSPA12A:intronic:NM 025015.2	1	100		intronic	NM 02501	c.547-66C	p.?	1.14						rs2240711		YES			Heat-shock ATP bindin Colonic Diseases;Colorectal Neoplasms;Dige	G=0, T=66	G=0.0, T=1.0	0.00001	987.125	66	G=0/0, T=22/4
chr17:56621359	T/C	T	C	SNV	C17orf47,SEPT4-AS1	C17orf47:exonic:NM 001038704.2, SEPT4-AS1:intronic_nc:N	1	53.33	1	synonymous	NR 11081	c.189A>G	p.(=)	0.38					Domain of un	rs6503867	0.2153	YES	9.995E-1 (pos.56621359)			T=28, C=32	T=0.4667, C=0.5333	0.00001	323.552	60	T=7/21, C=14/
chr10:118633548	G/G	A	G	SNV	ENO4	ENO4:intronic:NM 001242699.1	1	100		intronic	NM 00124	c.1216-39	p.?	-1.74					Enolase, C-te	rs1637574			1.0 (pos.118633548)		Genital Diseases, Male;Infertility;Infertility, I	A=0, G=22	A=0.0, G=1.0	0.00001	298.892	22	A=0/0, G=16/6
chr10:118646129	C/C	G	C	SNV	SHTN1	SHTN1:intronic:NM 001127211.2	1	100		intronic	NM 00112	c.1674-52(	p.?	-0.42						rs2531685	0.2594	YES	4.772E-1 (pos.118646129)		Cdc42 protein signal transduction,Ras protein signal tra	G=0, C=29	G=0.0, C=1.0	0.00001	427.603	29	G=0/0, C=11/1
chr10:119014023	T/T	C	T	SNV	SLC18A2	SLC18A2:intronic:NM 003054.4	1	100		intronic	NM 00305	c.700+15C	p.?	-1.66					Major Facilit	rs2072362	0.0998	YES	7.034E-1 (pos.119014023)		Solute carri aminergic Basal Ganglia Dise 3,4-Methylenedioxymetl	C=0, T=20	C=0.0, T=1.0	0.00001	263.783	20	C=0/0, T=3/17
chr10:119014931	T/T	C	T	SNV	SLC18A2	SLC18A2:intronic:NM 003054.4	1	100		intronic	NM 00305	c.790+54C	p.?	-0.85					Major Facilit	rs363420	0.1678	YES			Solute carri aminergic Basal Ganglia Dise 3,4-Methylenedioxymetl	C=0, T=20	C=0.0, T=1.0	0.00001	263.782	20	C=0/0, T=13/7
chr10:119014948	A/A	C	A	SNV	SLC18A2	SLC18A2:intronic:NM 003054.4	1	100		intronic	NM 00305	c.791-42C	p.?	0.71					Major Facilit	rs363343	0.1743	YES	4.029E-1 (pos.119014948)		Solute carri aminergic Basal Ganglia Dise 3,4-Methylenedioxymetl	C=0, A=22	C=0.0, A=1.0	0.00001	298.892	22	C=0/0, A=15/7
chr10:119026401	T/C	T	C	SNV	SLC18A2	SLC18A2:intronic:NM 003054.4	1	96.67		intronic	NM 00305	c.1070+79	p.?	-1.24					Major Facilit	rs363266		YES			Solute carri aminergic Basal Ganglia Dise 3,4-Methylenedioxymetl	T=1, C=29	T=0.0333, C=0.9667	0.00001	416.556	30	T=1/0, C=15/1
chr17:56620167	C/T	C	T	SNV	C17orf47,SEPT4-AS1	C17orf47:exonic:NM 001038704.2, SEPT4-AS1:intronic_nc:N	1	46.43	1	missense	NR 11081	c.1381G>A	p.Gly461Ser	-0.17	0.59	56	0.077	0.01(COSM37	Domain of un	rs17822735	0.1992	YES	1.712E-1;2.312E-4 (pos.5	acute_myeloid_leukaemia,adenocarcinoma,basal_cell_carcinoma		C=45, T=39	C=0.5357, T=0.4643	0.00001	404.489	84	C=27/18, T=20
chr17:42225547	C/C	A	C	SNV	C17orf53	C17orf53:exonic:NM 024032.4	1	100	3	missense	NM 02403	c.376A>C	p.Thr126Pro	0.1	1	38	0	0.01(COSM44	Domain of un	rs227584	0.31	YES	7.852E-1 (pos.42225547)	acute_myeloid_leukaemia		A=0, C=49	A=0.0, C=1.0	0.00001	831.361	49	A=0/0, C=19/3
chr17:72588326	C/C	A	C	SNV	C17orf77,CD300LD	C17orf77:exonic:NM 152460.2, CD300LD:exonic:NM 0011	1	100	3	missense	NM 00111	c.16T>G, c.	p.Ser6Ala, p.Arg47Ser	-0.79	1	99.0, 110.	0	0(COSM1130	Immunoglob	rs493430	0.3337	YES	5.23E-1 (pos.72588326)	acute_myeloic	CD300 anti extracellular region,immune system process,integral co	A=0, C=27	A=0.0, C=1.0	0.00001	389.988	27	A=0/0, C=12/1
chr10:120816754	C/C	A	C	SNV	EIF3A	EIF3A:intronic:NM 003750.2	1	100		intronic	NM 00375	c.1978-20	p.?	-0.31					PCI domain	rs4752244		YES			Eukaryotic IRES-depe Genital Diseases, Male;Genital Neoplasms,	A=0, C=22	A=0.0, C=1.0	0.00001	298.892	22	A=0/0, C=15/7
chr17:71232804	A/A	C	A	SNV	C17orf80	C17orf80:exonic:NM 001100621.2	1	100	3	missense	NM 00110	c.1183C>A	p.His395Asn	-1.28	1	68	0			rs745732821,rs9043	2.00E-04		9.999E-1;1.156E-4;0.0 (pos.71232804)		biological_process,extracellular exosome,integral comp	C=0, A=50	C=0.0, A=1.0	0.00001	852.707	50	C=0/0, A=29/2
chr17:71232881	G/C	G	C	SNV	C17orf80	C17orf80:exonic:NM 001100621.2	1	50	3	missense	NM 00110	c.1260G>C	p.Gln420His	-0.01	0.36	24	0.504	0.13(COSM3755798),0.13(C		rs745142	0.456	YES	6.26E-1 (pos.71232881)	adenocarcinoma,neoplasn	biological_process,extracellular exosome,integral comp	G=26, C=26	G=0.5, C=0.5	0.00001	256.249	52	G=12/14, C=1
chr10:120917269	T/T	A	T	SNV	SFXN4	SFXN4:intronic:NM 213649.1	1	100		intronic	NM 21364	c.472-24T	p.?	-0.88					Tricarboxylat	rs2420494,rs762507	9.00E-04		0.0 (pos.120917266),1.0 (pos.12091726		Sideroflexir integral component of membrane,ion transmembrane t	A=0, T=39	A=0.0, T=1.0	0.00001	623.129	39	A=0/0, T=11/2
chr10:121201626	C/C	T	C	SNV	GRK5	GRK5:intronic:NM 005308.2	1	100		intronic	NM 00530	c.1057+26	p.?	-0.49					Protein kinas	rs2275044	0.1359	YES	8.365E-1 (pos.121201626)		G protein-c ATP bindin Diabetes Mellitus;Diabetes Mellitus, Type 2;	T=0, C=44	T=0.0, C=1.0	0.00001	726.567	44	T=0/0, C=19/2
chr10:12126490	A/T	A	T	SNV	DHTKD1	DHTKD1:intronic:NM 018706.6	1	47.62		intronic	NM 01870	c.311-49A	p.?	-0.23					2-oxoglutara	rs11257527	0.4107	YES	4.617E-1;0.0 (pos.12126490)		Dehydroger cellular nitrogen compound metabolic process,cytosol,g	A=11, T=10	A=0.5238, T=0.4762	0.00001	82.2892	21	A=3/8, T=3/7
chr10:121551581	A/C	A	C	SNV	INPP5F	INPP5F:intronic:NM 014937.3	1	58.33		intronic	NM 01493	c.612+33A	p.?	0.01				0(COSM1470	Inositol phos	rs2289306	0.4442	YES	2.444E-1 (pos.12155158	acute_myeloic	Inositol pol adult loco Basal Ganglia Diseases;Brain Diseases;Centr	A=25, C=35	A=0.4167, C=0.5833	0.00001	370.434	60	A=9/16, C=23/
chr17:71232687	T/C	T	C	SNV	C17orf80	C17orf80:exonic:NM 001100621.2	1	47.37	3	missense	NM 00110	c.1066T>C	p.Phe356Leu	-0.22	0.72	22	0	0.07(COSM3755794),0.07(C		rs745143	0.46	YES	6.256E-1 (pos.71232687)	adenocarcinoma,neoplasn	biological_process,extracellular exosome,integral comp	T=30, C=27	T=0.5263, C=0.4737	0.00001	256.712	57	T=11/19, C=13
chr10:122659490	C/T	C	T	SNV	WDR11	WDR11:intronic:NM 018117.11	1	56		intronic	NM 01811	c.2516-51(	p.?	-0.36						rs2289336	0.3259	YES	4.216E-1 (pos.122659490)		WD repeat- cytoplasm 46, XY Disorders of Sex Development;Congen	C=11, T=14	C=0.44, T=0.56	0.00001	134.206	25	C=7/4, T=9/5
chr10:122664967	T/T	C	T	SNV	WDR11	WDR11:intronic:NM 018117.11	1	100		intronic	NM 01811	c.3291+39	p.?	-0.04						rs1866516	0.4181	YES	5.226E-1 (pos.122664967)		WD repeat- cytoplasm 46, XY Disorders of Sex Development;Congen	C=0, T=35	C=0.0, T=1.0	0.00001	543.869	35	C=0/0, T=11/2
chr18:72021717	C/C	T	C	SNV	C18orf63	C18orf63:exonic:NM 001174123.1	1	100	13	missense	NM 00117	c.2027T>C	p.Leu676Pro	-0.06	1	98	0		Domain of un	rs12606060			1.0 (pos.72021717)			T=0, C=37	T=0.0, C=1.0	0.00001	583.682	37	T=0/0, C=25/1
chr10:123724767	G/G	T	G	SNV	NSMCE4A	NSMCE4A:intronic:NM 017615.2	1	100		intronic	NM 01761	c.753+34A	p.?	-0.3					Binding doma	rs4752626	0.0013	YES	9.999E-1 (pos.123724767)		Non-SMC el DNA recombination,DNA repair,Smc5-Smc6 complex,chr	T=0, G=52	T=0.0, G=1.0	0.00001	766.983	52	T=0/0, G=40/1
chr10:123733511	C/G	C	G	SNV	NSMCE4A	NSMCE4A:intronic:NM 017615.2	1	47.62		intronic	NM 01761	c.370+8G>	p.?	-1.1					Binding doma	rs10887034	0.1616	YES	2.562E-1 (pos.123733511)		Non-SMC el DNA recombination,DNA repair,Smc5-Smc6 complex,chr	C=11, G=10	C=0.5238, G=0.4762	0.00001	82.2867	21	C=3/8, G=5/5
chr18:72021145	T/A	T	A	SNV	C18orf63	C18orf63:exonic:NM 001174123.1	1	45.45	12	missense	NM 00117	c.1643T>A	p.Phe548Tyr	0.17	0.03	22	0.731	0.11(COSM40	Domain of un	rs8093952		YES	6.566E-2 (pos.72021145)	acute_myeloid_leukaemia,diffuse_large_B_cell_lymphoma		T=12, A=10	T=0.5455, A=0.4545	0.00001	81.7625	22	T=6/6, A=2/8
chr18:21100240	T/T	C	T	SNV	C18orf8	C18orf8:exonic:NM 013326.4	1	100	10	synonymous	NM 01332	c.924C>T	p.(=)	-1.65					Colon cancer	rs1367083	0.4771	YES	9.074E-1 (pos.21100240)		lysosomal membrane	C=0, T=22	C=0.0, T=1.0	0.00001	298.89	22	C=0/0, T=12/1
chr19:1277275	C/C	G	C	SNV	C19orf24	C19orf24:exonic:NM 017914.3	1	100	2	synonymous	NM 01791	c.375G>C	p.(=)	0.07				0.07(COSM43	Protein of un	rs757292	0.2134	YES	9.662E-1 (pos.1277275)	clear_cell_renal_cell_carci	cytoplasm,extracellular region,integral component of m	G=0, C=32	G=0.0, C=1.0	0.00001	484.983	32	G=0/0, C=14/1
chr19:51159571	G/G	T	G	SNV	C19orf81	C19orf81:exonic:NM 001195076.1	1	100	3	missense	NM 00119	c.229T>G	p.Ser77Ala	1.14	1	99	0	0.01(COSM45	Domain of un	rs4801844		YES	6.85E-1 (pos.51159571)	squamous_cell_carcinoma		T=0, G=39	T=0.0, G=1.0	0.00001	627.763	39	T=0/0, G=10/2
chr10:124216397	T/C	T	C	SNV	ARMS2	ARMS2:intronic:NM 001099667.1	1	57.14		intronic	NM 00109	c.298-26T	p.?	2.23						rs36213074	0.1298	YES	9.677E-3 (pos.124216397)		mitochonc Eye Diseases;Macular Degeneration;Retinal	T=9, C=12	T=0.4286, C=0.5714	0.00001	109.102	21	T=2/7, C=5/7
chr10:124271589	G/A	G	A	SNV	HTRA1	HTRA1:intronic:NM 002775.4	1	95.45		intronic	NM 00277	c.1274+8G	p.?	-1.14					Insulin-like g	rs2672586	0		1.0 (pos.124271589)		HTRA serine chorionic Alopecia;Bone Diseases;Brain Likely benign	G=1, A=21	G=0.0455, A=0.9545	0.00001	271.509	22	G=1/0, A=7/14
chr10:124325409	G/G	T	G	SNV	DMBT1	DMBT1:intronic:NM 007329.2	1	100		intronic	NM 00732	c.62-74T>C	p.?	-0.7					CUB domain,	rs3019515	0.3231	YES			Deleted in calcium-d Colonic Diseases;Colonic Neoplasms;Colorec	T=0, G=43	T=0.0, G=1.0	0.00001	708.402	43	T=0/0, G=28/1
chr10:124325569	G/G	A	G	SNV	DMBT1	DMBT1:intronic:NM 007329.2	1	100		intronic	NM 00732	c.91+57A>	p.?	0.07					CUB domain,	rs3019516	0.3231	YES			Deleted in calcium-d Colonic Diseases;Colonic Neoplasms;Colorec	A=0, G=48	A=0.0, G=1.0	0.00001	810.428	48	A=0/0, G=27/2
chr10:124329672	CT/CT	TC	CT	MNV	DMBT1	DMBT1:intronic:NM 007329.2	2	100		intronic	NM 00732	c.92-6TC>C	p.?	0.1, 0.07					CUB domain,	rs34118835,rs37402	0.2544	YES	7.871E-1 (pos.124329673),7.871E-1;0.0;(		Deleted in calcium-d Colonic Diseases;Colonic Neoplasms;Colorec	TC=0, CT=2	TC=0.0, CT=1.0	0.00001	261.827	20	TC=0/0, CT=8/
chr2:43903354	C/C	T	C	SNV	C1GALT1C1L,PLEKHH2	PLEKHH2:intronic:NM 172069.3, C1GALT1C1L:exonic:NM 0	1	100	1	synonymous	NM 17206	c.124-264	p.?, p.(=)	-0.26					FERM central	rs4952675	0.3024	YES	1.885E-1 (pos.43903354)		Pleckstrin h actin binding,cortical actin cytoskeleton,cytoplasm,cyto	T=0, C=48	T=0.0, C=1.0	0.00001	810.435	48	T=0/0, C=33/1
chr1:172437592	A/A	G	A	SNV	C1orf105	C1orf105:exonic:NM 139240.3	1	100	7	missense	NM 13924	c.410G>A	p.Ser137Asn	-0.32	0	46	0.04	0.05(COSM37	Domain of un	rs1129942	0.2151	YES	9.992E-1 (pos.17243759	adenocarcinoma	protein binding	G=0, A=66	G=0.0, A=1.0	0.00001	1003.34	66	G=0/0, A=33/3

chr10:124594153	G/G	A	G	SNV	CUZD1,FAM24B-CUZD1	FAM24B-CUZD1:intronic_nc:NR_037915.1, CUZD1:intronic:N	1	100		intronic	NM_02203	c.1382+69	p.?	4.12					CUB domain.:	rs11248321		YES			Cub and zor	cell cycle,cell division,cell proliferation,integral compon				A=0, G=12	A=0.0, G=1.0	0.00001	1932.36	120	A=0/0, G=65/5
chr1:11015165	G/G	A	G	SNV	C1orf127	C1orf127:exonic:NM_001170754.1	1	100	9	missense	NM_00117	c.857T>C	p.Ile286Thr	-1.25	0.31	89	0.001	0.02(COSM37	Domain of un	rs75130475,rs76075	0.1517	YES	0.0 (pos.11015163),2.48	adenocarcinoma,hepatocellular_carci			Alopecia:Hair Diseases:Hypotrichosis:Skin Di			A=0, G=128	A=0.0, G=1.0	0.00001	2094.83	128	A=0/0, G=59/6
chr1:67560956	T/T	C	T	SNV	C1orf141	C1orf141:exonic:NM_001276351.1	1	100	7	missense	NM_00127	c.565G>A	p.Val189Ile	0.83	1	29	0		Domain of un	rs11208997	0.143	YES	9.72E-1 (pos.67560956)				Crohn Disease:Digestive System Diseases:Ga			C=0, T=168	C=0.0, T=1.0	0.00001	2733.33	168	C=0/0, T=74/9
chr10:125426627	T/T	G	T	SNV	GPR26	GPR26:intronic:NM_153442.3	1	100		intronic	NM_15344	c.668+36G	p.?	-0.84					7 transmemb	rs3808964	0.3438	YES	3.778E-1 (pos.125426627)		G protein-c	G-protein coupled receptor activity,adenylate cyclase-a				G=0, T=39	G=0.0, T=1.0	0.00001	601.927	39	G=0/0, T=23/1
chr1:67561090	T/C	T	C	SNV	C1orf141	C1orf141:exonic:NM_001276351.1	1	57.58	7	missense	NM_00127	c.431A>G	p.Gln144Arg	-1	0.42	43	0.624	0.02(COSM37	Domain of un	rs72933970	0.0468	YES	8.523E-2 (pos.67561090)	adenocarcinoma			Crohn Disease:Digestive System Diseases:Ga			T=56, C=76	T=0.4242, C=0.5758	0.00001	882.232	132	T=33/23, C=24
chr1:3807593	C/C	G	C	SNV	C1orf174	C1orf174:exonic:NM_207356.2	1	100	3	missense	NM_20735	c.158C>G	p.Thr53Arg	0.1	1	71	0		UPF0688 fam	rs4274008	0.0078		9.999E-1 (pos.3807593)			nucleus,protein binding				G=0, C=42	G=0.0, C=1.0	0.00001	685.216	42	G=0/0, C=19/2
chr10:125526689	A/A	G	A	SNV	CPXM2	CPXM2:intronic:NM_198148.2	1	100		intronic	NM_19814	c.1300-21	p.?	0.31					Carboxypepti	rs1896392	0.2156	YES	5.729E-1 (pos.125526689)		Carboxypep	extracellular matrix,extracellular space,metallocarboxy				G=0, A=34	G=0.0, A=1.0	0.00001	524.186	34	G=0/0, A=19/1
chr10:125622250	CA/C	CA	C	INDEL	CPXM2	CPXM2:intronic:NM_198148.2	1	50		intronic	NM_19814	c.404-11T	p.?	-0.61, 2.54					Carboxypepti	rs11299200,rs20158	0.2211	YES	7.882E-1 (pos.125622250)		Carboxypep	extracellular matrix,extracellular space,metallocarboxy				CA=53, C=5	CA=0.5, C=0.5	0.00001	498.562	106	CA=28/25, C=2
chr10:125622263	A/T	A	T	SNV	CPXM2	CPXM2:intronic:NM_198148.2	1	50		intronic	NM_19814	c.404-24T	p.?	-0.13					Carboxypepti	rs28469012	0.1205	YES	3.221E-1 (pos.125622263)		Carboxypep	extracellular matrix,extracellular space,metallocarboxy				A=53, T=53	A=0.5, T=0.5	0.00001	540.551	106	A=28/25, T=21
chr10:126093991	C/C	T	C	SNV	OAT	OAT:intronic:NM_000274.3	1	100		intronic	NM_00027	c.648+14A	p.?	-1.87					Aminotransfe	rs9422807	0.1791	YES	9.545E-1 (pos.126093991)		Ornithine a	L-proline b	Choroid Diseases:C	Canaline,(	Benign	T=0, C=83	T=0.0, C=1.0	0.00001	1349.88	83	T=0/0, C=27/5
chr1:186363119	G/G	C	G	SNV	C1orf27	C1orf27:exonic:NM_017847.5	1	100	9	missense	NM_01784	c.752C>G	p.Ser251Cys	0.05	0.14	112	0	0.19(COSM42	Olfactory rec	rs12084264	0.3178	YES	1.078E-1 (pos.18636311	carcinoma		integral component of membrane				C=0, G=47	C=0.0, G=1.0	0.00001	789.326	47	C=0/0, G=26/2
chr10:126660781	T/T	A	T	SNV	ZRANB1	ZRANB1:intronic:NM_017580.2	1	100		intronic	NM_01758	c.1156+94	p.?	0.38					OTU-like cyst	rs3802700		YES			Zinc finger-	K63-linked polyubiquitin modification-dependent protei				A=0, T=40	A=0.0, T=1.0	0.00001	649.195	40	A=0/0, T=13/2
chr10:126681688	C/C	T	C	SNV	CTBP2	CTBP2:intronic:NM_022802.2	1	100		intronic	NM_02280	c.2659+83	p.?	-1.71					D-isomer spe	rs7097802		YES			C-terminal-	NAD bindi	Genital Diseases, Male;Genital Neoplasms, I			T=0, C=53	T=0.0, C=1.0	0.00001	782.027	53	T=0/0, C=41/1
chr22:37578579	T/T	C	T	SNV	C1QTNF6	C1QTNF6:exonic:NM_031910.3	1	100	3	synonymous	NM_03191	c.486G>A	p.(=)	-0.03				0.4(COSM149	C1q domain.(	rs229519	0.2643	YES	5.028E-1 (pos.37578579)	acute_myeloic	C1q- and tu	collagen t	Autoimmune Diseases:Diabetes Mellitus:Dia			C=0, T=55	C=0.0, T=1.0	0.00001	814.896	55	C=0/0, T=27/2
chr13:24465743	G/G	A	G	SNV	C1QTNF9B,PCOTH	PCOTH:intronic:NM_001135816.2, C1QTNF9B:exonic:NM_0(	1	100	3	synonymous	NM_00113	c.33-9A>G,	p.?, p.(=)	-1.65					C1q domain.(	rs3864971	2.00E-04		9.999E-1;1.159E-4 (pos.24465743)		C1QTNF9B a	collagen trimer,extracellular exosome,protein binding				A=0, G=53	A=0.0, G=1.0	0.00001	782.018	53	A=0/0, G=19/3
chr10:127426835	T/T	G	T	SNV	EDRF1	EDRF1:intronic:NM_001202438.1	1	100		intronic	NM_00120	c.1831-29	p.?	-0.58						rs3943225	0.1169	YES	9.144E-1 (pos.127426835)			cellular_component,nucleus,positive regulation of trans				G=0, T=20	G=0.0, T=1.0	0.00001	263.786	20	G=0/0, T=10/1
chr10:127515069	T/T	C	T	SNV	BCCIP	BCCIP:intronic:NM_016567.3	1	100		intronic	NM_01656	c.166-91C	p.?	-0.59					p21-C-termin	rs10159992		YES			BRCA2- and	DNA repair,RNA binding,centriole,centrosome,cytosol,e				C=0, T=71	C=0.0, T=1.0	0.00001	1109.41	71	C=0/0, T=40/3
chr10:127548587	G/G	C	G	SNV	DHX32	DHX32:intronic:NM_018180.2	1	100		intronic	NM_01818	c.477-43G	p.?	-0.7					Helicase ass	rs1539315,rs565702	0.0575	YES	0.0 (pos.127548584),0.0;0.0;0.0 (pos.12		DEAH (Asp-(	ATP binding,ATP-dependent RNA helicase activity,RNA bi				C=0, G=36	C=0.0, G=1.0	0.00001	563.743	36	C=0/0, G=13/2
chr10:128209797	T/T	C	T	SNV	C10orf90	C10orf90:intronic:NM_001004298.3	1	100		intronic	NM_00100	c.22+71G>	p.?	-1.03					ALMS motif	rs2099553	0.1483	YES			Chromosom	actin cytoskeleton,alpha-actinin binding,centrosome,cy				C=0, T=102	C=0.0, T=1.0	0.00001	1593.29	102	C=0/0, T=82/2
chr20:18810705	C/T	C	T	SNV	C20orf78	C20orf78:utr_5:NM_001242671.2	1	45.45	1	utr_5	NM_00124	c.-10G>A	p.?	0.97						rs6136530		YES	5.537E-1 (pos.18810705)							C=12, T=10	C=0.5455, T=0.4545	0.00001	81.7626	22	C=5/7, T=1/9
chr21:45553596	C/C	T	C	SNV	C21orf33	C21orf33:exonic:NM_004649.7	1	100	1	missense	NM_00464	c.17T>C	p.Val6Ala	-1.25	1	64	0	0(COSM444566)		rs763258982,rs9687	0.1938	YES	0.0;0.0 (pos.45553595),7	neoplasm,squ	ES1, zebrafi	mitochon	Colitis;Colitis, Ulcerative;Colonic Diseases;C			T=0, C=20	T=0.0, C=1.0	0.00001	263.73	20	T=0/0, C=14/6
chr10:128840836	T/TG	T	TG	INDEL	DOCK1	DOCK1:intronic:NM_001380.4	1	100		intronic	NM_00138	c.1948-53	p.?	-0.18					C2 domain in Dock180 and Zizimin proteins,DOCK N-terminus,Dock homology region 2,SH3 domain,V						Dedicator o	Fc-gamma	Mental Disorders:Substance-Related Disorde			T=0, TG=23	T=0.0, TG=1.0	0.00001	215.745	23	T=0/0, TG=10/
chr10:128840842	G/G	A	G	SNV	DOCK1	DOCK1:intronic:NM_001380.4	1	100		intronic	NM_00138	c.1948-47	p.?	-1.02					C2 domain in	rs880272	0.0897	YES	9.15E-1 (pos.128840842)		Dedicator o	Fc-gamma	Mental Disorders:Substance-Related Disorde			A=0, G=23	A=0.0, G=1.0	0.00001	312.804	23	A=0/0, G=10/1
chr10:129202729	T/T	G	T	SNV	DOCK1	DOCK1:intronic:NM_001380.4	1	100		intronic	NM_00138	c.4059+36	p.?	-0.25					C2 domain in	rs2296630	0.2289	YES	2.865E-1 (pos.129202729)		Dedicator o	Fc-gamma	Mental Disorders:Substance-Related Disorde			G=0, T=26	G=0.0, T=1.0	0.00001	371.42	26	G=0/0, T=12/1
chr10:129242555	A/A	G	A	SNV	DOCK1	DOCK1:intronic:NM_001380.4	1	100		intronic	NM_00138	c.5350+12	p.?	-1.55					C2 domain in	rs2275053	0.4426	YES	4.462E-1 (pos.129242555)		Dedicator o	Fc-gamma	Mental Disorders;Substance-Related Disorde			G=0, A=73	G=0.0, A=1.0	0.00001	1150.44	73	G=0/0, A=45/2
chr10:129536642	A/A	G	A	SNV	FOXI2	FOXI2:intronic:NM_207426.2	1	100		intronic	NM_20742	c.512-142	p.?	-1.28					Forkhead dor	rs7085541		YES			Forkhead b	RNA polymerase II transcription factor activity, sequenc				G=0, A=29	G=0.0, A=1.0	0.00001	427.603	29	G=0/0, A=24/5
chr10:129536648	T/T	G	T	SNV	FOXI2	FOXI2:intronic:NM_207426.2	1	100		intronic	NM_20742	c.512-136	p.?	-0.22					Forkhead dor	rs7085544		YES			Forkhead b	RNA polymerase II transcription factor activity, sequenc				G=0, T=29	G=0.0, T=1.0	0.00001	427.603	29	G=0/0, T=24/5
chr10:129846010	A/A	G	A	SNV	PTPRE	PTPRE:intronic:NM_006504.5	1	100		intronic	NM_00650	c.210-18G	p.?	-5.66					Protein-tyros	rs7911904	0.2165	YES	8.269E-1 (pos.129846010)		Protein tyrc	cytoplasm	Autoimmune Disea	Alendronic acid		G=0, A=53	G=0.0, A=1.0	0.00001	782.03	53	G=0/0, A=41/1
chr10:129854369	C/C	T	C	SNV	PTPRE	PTPRE:intronic:NM_006504.5	1	100		intronic	NM_00650	c.421-18T	p.?	-1.36					Protein-tyros	rs4424589	0.2159	YES	8.307E-1 (pos.129854369)		Protein tyrc	cytoplasm	Autoimmune Disea	Alendronic acid		T=0, C=55	T=0.0, C=1.0	0.00001	814.907	55	T=0/0, C=24/3
chr10:129854550	A/A	G	A	SNV	PTPRE	PTPRE:intronic:NM_006504.5	1	100		intronic	NM_00650	c.511+73G	p.?	0.01					Protein-tyros	rs11016041		YES			Protein tyrc	cytoplasm	Autoimmune Disea	Alendronic acid		G=0, A=49	G=0.0, A=1.0	0.00001	831.542	49	G=0/0, A=16/3
chr10:129859992	C/C	T	C	SNV	AS-PTPRE,PTPRE	PTPRE:intronic:NM_006504.5, AS-PTPRE:intronic_nc:NR_136	1	100		intronic	NM_00650	c.625+13T	p.?	0.07					Protein-tyros	rs4462251	0.4751	YES	6.024E-1 (pos.129859992)		Protein tyrc	cytoplasm	Autoimmune Disea	Alendronic acid		T=0, C=26	T=0.0, C=1.0	0.00001	371.42	26	T=0/0, C=15/1
chr10:129907741	A/A	G	A	SNV	MKI67	MKI67:intronic:NM_002417.4	1	100		intronic	NM_00241	c.2417-54	p.?	0.04					FHA domain.I	rs2065718		YES			Proliferatio	ATP bindin	Adenocarcinoma:Adnexal Diseases:Autoimm			G=0, A=200	G=0.0, A=1.0	0.00001	3401.19	200	G=0/0, A=114/
chr21:34166190	T/T	A	T	SNV	C21orf62,C21orf62-AS1	C21orf62:exonic:NM_001162496.2, C21orf62-AS1:intronic	1	100	2	missense	NR_02462	c.543T>A	p.Phe181Leu	0.74		22		0.1(COSM459	Domain of un	rs11390393,rs74617086		YES	1.0 (pos.34166190)	squamous_cell_carcinoma		biological_process,cellular_component,molecular_func				A=0, T=41	A=0.0, T=1.0	0.00001	645.92	41	A=0/0, T=27/1
chr22:42089623	C/C	T	C	SNV	C22orf46	C22orf46:exonic:NM_001142964.1	1	100	2	missense	NM_00114	c.373T>C	p.Trp125Arg	-0.02	0.01	101	0.002	0.03(COSM37	Domain of un	rs739134	0.197	YES	9.18E-1 (pos.42089623)	adenocarcinoma,ductal_ca		extracellular region				T=0, C=50	T=0.0, C=1.0	0.00001	852.738	50	T=0/0, C=24/2
chr10:13214812	G/G	A	G	SNV	MCM10	MCM10:intronic:NM_182751.2	1	100		intronic	NM_18275	c.595+47A	p.?	-2.88					Mcm10 replic	rs6602637,rs772473	0.1893	YES	0.0 (pos.13214810),5.696E-1 (pos.13214		Minichrom	DNA replication,DNA replication initiation,DNA replicati				A=0, G=94	A=0.0, G=1.0	0.00001	1580.4	94	A=0/0, G=61/3
chr10:13237015	A/A	G	A	SNV	MCM10	MCM10:intronic:NM_182751.2	1	100		intronic	NM_18275	c.1749-26	p.?	-0.56					Mcm10 replic	rs10796036	0.1237	YES	7.618E-1 (pos.13237015)		Minichrom	DNA replication,DNA replication initiation,DNA replicati				G=0, A=22	G=0.0, A=1.0	0.00001	298.856	22	G=0/0, A=15/7
chr10:13275514	G/G	A	G	SNV	UCMA	UCMA:intronic:NM_145314.2	1	100		intronic	NM_14531	c.220+24T	p.?	0					Unique cartil	rs4750327,rs771362	2.00E-04		0.0 (pos.13275513),1.0;0.0 (pos.13275514)			aggresome,extracellular space,negative regulation of os				A=0, G=24	A=0.0, G=1.0	0.00001	334.245	24	A=0/0, G=13/1
chr21:43327117	T/T	C	T	SNV	C2CD2	C2CD2:exonic:NM_015500.1	1	100	10	synonymous	NM_01550	c.1302G>A	p.(=)	-1.72					C2 domain	rs2277795,rs754767	0.1486	YES	2.67E-1;0.0;0.0 (pos.43327117)		C2 calcium-	cytosol,int	Abnormalities, Multiple:Chromosome Disord			C=0, T=63	C=0.0, T=1.0	0.00001	958.917	63	C=0/0, T=59/4
chr10:13275676	T/C	T	C	SNV	UCMA	UCMA:intronic:NM_145314.2	1	57.58		intronic	NM_14531	c.125-43A	p.?	0.19					Unique cartil	rs3829926	0.3079	YES	5.257E-1 (pos.13275676)			aggresome,extracellular space,negative regulation of os				T=14, C=19	T=0.4242, C=0.5758	0.00001	203.769	33	T=7/7, C=9/10
chr10:133966434	C/T	C	T	SNV	JAKMIP3	JAKMIP3:intronic:NM_001105521.3	1	57.45		intronic	NM_00110	c.2002-28	p.?	-0.13					JAKMIP CC3 d	rs11146215	0.0211	YES	5.462E-1 (pos.133966434)		Janus kinas	Golgi apparatus,kinase binding,microtubule binding				C=20, T=27	C=0.4255, T=0.5745	0.00001	326.535	47	C=9/11, T=8/1
chr21:43327793	C/T	C	T	SNV	C2CD2	C2CD2:exonic:NM_015500.1	1	57.45	9	synonymous	NM_01550	c.1119G>A	p.(=)	-2.35				0.05(COSM62	C2 domain	rs3746904	0.3505	YES	4.042E-1 (pos.43327793)	neoplasm	C2 calcium-	cytosol,int	Abnormalities, Multiple:Chromosome Disord			C=20, T=27	C=0.4255, T=0.5745	0.00001	320.794	47	C=7/13, T=7/2
chr10:134038647	G/G	A	G	SNV	STK32C	STK32C:intronic:NM_173575.3	1	100		intronic	NM_17357	c.876+90T	p.?	0.48					Kinase-like.P	rs902628		YES				ATP binding,intracellular,intracellular signal transductic				A=0, G=64	A=0.0, G=1.0	0.00001	976.937	64	A=0/0, G=31/3
chr10:134038715	G/G	A	G	SNV	STK32C	STK32C:intronic:NM_173575.3	1	100		intronic	NM_17357	c.876+22T	p.?	-1.29					Kinase-like.P	rs764448871,rs9026	0.1635	YES	9.551E-1;1.256E-3 (pos.134038715)			ATP binding,intracellular,intracellular signal transductic				A=0, G=75	A=0.0, G=1.0	0.00001	1192.49	75	A=0/0, G=26/4
chr12:22602796	G/G	A	G	SNV	C2CD5	C2CD5:exonic:NM_014802.2	1	100	25	synonymous	NM_01480	c.2901T>C	p.(=)	0.03				0.24(COSM63	C2 domain	rs2063573	0.0973	YES	1.366E-1 (pos.22602796)	carcinoma		calcium ion binding,calcium-dependent phospholipid bi				A=0, G=34	A=0.0, G=1.0	0.00001	523.973	34	A=0/0, G=16/1
chr10:134648048	T/T	C	T	SNV	CFAP46	CFAP46:intronic:NM_001200049.2	1	100		intronic	NM_00120	c.6866+11	p.?	0.15					Peptidase fa	rs3793688		YES				axoneme,axoneme assembly,cilium movement involvec				C=0, T=48	C=0.0, T=1.0	0.00001	810.433	48	C=0/0, T=27/2
chr10:134713176	A/A	G	A	SNV	CFAP46	CFAP46:intronic:NM_001200049.2	1	100		intronic	NM_00120	c.2925-6C	p.?	1.48					Peptidase fa	rs67123080		YES	5.992E-2 (pos.134713176)			axoneme,axoneme assembly,cilium movement involvec				G=0, A=26	G=0.0, A=1.0	0.00001	371.274	26	G=0/0, A=12/1
chr10:134750604	A/A	C	A	SNV	CFAP46	CFAP46:intronic:NM_001200049.2	1	100		intronic	NM_00120	c.661-45G	p.?	-1.36					Peptidase fa	rs10870211	0.0229	YES	1.0 (pos.134750604)			axoneme,axoneme assembly,cilium movement involvec				C=0, A=34	C=0.0, A=1.0	0.00001	524.186	34	C=0/0, A=14/2
chr2:202410300	T/T	A	T	SNV	C2CD6	C2CD6:exonic:NM_001168221.1	1	100	11	missense	NM_00116	c.1128T>A	p.His376Gln	-1.67	1	24	0		Amyotrophic	rs10804117	0.4072	YES	7.403E-1;0.0 (pos.202410300)			protein binding				A=0, T=24	A=0.0, T=1.0	0.00001	334.792	24	A=0/0, T=11/1
chr2:27801493	C/C	T	C	SNV	C2orf16	C2orf16:exonic:NM_032266.3	1	100	1	missense	NM_03226	c.2054T>C	p.Val685Ala	0.41		64		0.02(COSM4001640),0.02(C		rs1919127	0.2696	YES	4.866E-1 (pos.27801493)	acute_myeloid_leukaemia		extracellular exosome,nucleus				T=0, C=32	T=0.0, C=1.0	0.00001	485.138	32	T=0/0, C=17/1
chr2:27801759	G/G	A	G	SNV	C2orf16	C2orf16:exonic:NM_032266.3	1	100	1	missense	NM_03226	c.2320A>G	p.Ile774Val	2.26		29		0.13(COSM3758182),0.13(C		rs1919128,rs868020	0.2707	YES	4.867E-1 (pos.27801759)	acute_myeloid_leukaemia		extracellular exosome,nucleus				A=0, G=25	A=0.0, G=1.0	0.00001	353.019	25	A=0/0, G=7/18
chr10:135053123	G/G	A	G	SNV	VENTX	VENTX:intronic:NM_014468.3	1	100		intronic	NM_01446	c.242-57A	p.?	-0.58					Homeobox do	rs880340		YES			VENT home	multicellular organism development,nucleus,positive re				A=0, G=26	A=0.0, G=1.0	0.00001	371.417	26	A=0/0, G=19/7
chr2:29287708	C/T	C	T	SNV	C2orf71	C2orf71:utr_3:NM_001029883.2	1	57.14	2	utr_3	NM_00102	c.3894G>A	p.?	-1.29					Retinal prote	rs10182566	0.2664	YES	4.811E-1 (pos.29287708)		Chromosom	cilium,pho	Congenital, Hereditary, and Ne		Likely benign	C=9, T=12	C=0.4286, T=0.5714	0.00001	109.101	21	C=4/5, T=5/7
chr10:135076758	C/C	T	C	SNV	ADAM8	ADAM8:intronic:NM_001109.4	1	100		intronic	NM_00110	c.2398-21	p.?	-0.33					ADAM cystei	rs3008318	0.1097	YES	9.529E-1 (pos.135076758)		A disintegri	alpha9-be	Digestive System Diseases:Drug-Induced Live			T=0, C=73	T=0.0, C=1.0	0.00001	1150.49	73	T=0/0, C=23/5
chr10:135081684	C/C	T	C	SNV	ADAM8	ADAM8:intronic:NM_001109.4	1	100		intronic	NM_00110	c.2064-62	p.?	-5.17					ADAM cystei	rs2995309		YES			A disintegri	alpha9-be	Digestive System Diseases;Drug-Induced Live			T=0, C=30	T=0.0, C=1.0	0.00001	440.862	30	T=0/0, C=17/1
chr10:135081754	C/C	G	C	SNV	ADAM8	ADAM8:intronic:NM_001109.4	1	100		intronic	NM_00110	c.2064-13	p.?	-1.06					ADAM cystei	rs11101672		YES			A disintegri	alpha9-be	Digestive System Diseases;Drug-Induced Live			G=0, C=32	G=0.0, C=1.0	0.00001	485.049	32	G=0/0, C=14/1
chr10:135082874	A/A	G	A	SNV	ADAM8	ADAM8:intronic:NM_001109.4	1	100		intronic	NM_00110	c.1948+75	p.?	-2.05					ADAM cystei	rs2995311		YES			A disintegri	alpha9-be	Digestive System Diseases;Drug-Induced Live			G=0, A=20	G=0.0, A=1.0	0.00001	263.784	20	G=0/0, A=10/1
chr10:135083812	A/A	C	A	SNV	ADAM8	ADAM8:intronic:NM_001109.4	1	100		intronic	NM_00110	c.1785+52	p.?	0.07					ADAM cystei	rs885023	0.1844	YES			A disintegri	alpha9-be	Digestive System Diseases;Drug-Induced Live			C=0, A=22	C=0.0, A=1.0	0.00001	297.653	22	C=0/0, A=16/6
chr10:135084322	G/G	A	G	SNV	ADAM8	ADAM8:intronic:NM_001109.4	1	100		intronic	NM_00110	c.1565-13	p.?	-0.15					ADAM cystei	rs3008321	0.1085	YES	9.54E-1 (pos.135084322)		A disintegri	alpha9-be	Digestive System Diseases;Drug-Induced Live			A=0, G=28	A=0.0, G=1.0	0.00001	408.61	28	A=0/0, G=11/1
chr2:29295676	G/A	G	A	SNV	C2orf71	C2orf71:exonic:NM_001029883.2	1	56.36	1	synonymous	NM_00102	c.1452C>T	p.(=)	-0.78					Retinal prote	rs13385188	0.2714	YES	4.39E-1 (pos.29295676)		Chromosom	cilium,pho	Congenital, Hereditary, and Ne		Likely benign	G=48, A=62	G=0.4364, A=0.5636	0.00001	676.019	110	G=16/32, A=1
chr10:135086380	T/T	C	T	SNV	ADAM8	ADAM8:intronic:NM_001109.4	1	100		intronic	NM_00110	c.637-10G	p.?	-0.92				0.02(COSM37	ADAM cystei	rs3008324	0.109	YES	0.0 (pos.135086370),9.5	hepatocellula	A disintegri	alpha9-be	Digestive System Diseases;Drug-Induced Live			C=0, T=32	C=0.0, T=1.0	0.00001	485.135	32	C=0/0, T=13/1
chr19:6690703	G/A	G	A	SNV	C3	C3:exonic:NM_000064.3	1	58.33	27	synonymous	NM_00006	c.3426C>T	p.(=)	-0.08					A-macroglob	rs749973019			0.0 (pos.6690703)		Complemer	C5L2 anap	Anemia:Anemia, He	Cyclo-Tetrametavanadat		G=15, A=21	G=0.4167, A=0.5833	0.00001	234.328	36	G=6/9, A=8/13
chr3:14724413	G/A	G	A	SNV	C3orf20	C3orf20:exonic:NM_032137.4	1	96.77	3	missense	NM_03213	c.193G>A	p.Asp65Asn	-0.3	0	23	0.385	0.01(COSM37	FAM194 prot	rs9821143	0.3477	YES	2.382E-1 (pos.14724413)	adenocarcinoma,ductal_ca		cytoplasm,integral component of membrane				G=1, A=30	G=0.0323, A=0.9677	0.00001	435.383	31	G=1/0, A=13/1
chr10:135202594	C/C	T	C	SNV	PAOX	PAOX:intronic:NM_152911.3	1	100		intronic	NM_15291	c.1234+22	p.?	-1.59					Flavin contai	rs10776679	0.0581	YES	9.747E-1 (pos.135202594)		Polyamine	N(1),N(12)	Brain Diseases:Cardiovascular Diseases:Cen			T=0, C=91	T=0.0, C=1.0	0.00001	1513.55	91	T=0/0, C=50/4
chr10:135204799	T/T	C	T	SNV	PAOX	PAOX:intronic:NM_152911.3	1	100		intronic	NM_15291	c.1393-17	p.?	-0.27					Flavin contai	rs6537600,rs765944	0.1259	YES	1.161E-4 (pos.135204797),9.696E-1 (pos		Polyamine	N(1),N(12)	Brain Diseases;Cardiovascular Diseases;Cen			C=0, T=54	C=0.0, T=1.0	0.00001	798.069	54	C=0/0, T=30/2
chr3:155481609	A/A	G	A	SNV	C3orf33	C3orf33:exonic:NM_173657.2	1	100	6	synonymous	NM_17365	c.453C>T	p.(=)	-0.37						rs7653384	0.3727	YES	3.681E-1 (pos.155481609)			extracellular space,integral component of membrane,n				G=0, A=126	G=0.0, A=1.0	0.00001	2054.14	126	G=0/0, A=93/3
chr3:88199298	C/C	T	C	SNV	C3orf38	C3orf38:exonic:NM_173824.3	1	100	1	synonymous	NM_17382	c.96T>C	p.(=)	-0.47					Domain of un	rs6551278	0.1466	YES	8.474E-1 (pos.88199298)			apoptotic process,molecular_function,nucleus,positive				T=0, C=35	T=0.0, C=1.0	0.00001	548.282	35	T=0/0, C=29/6
chr10:135209105	G/G	A	G	SNV	MTG1	MTG1:intronic:NM_138384.2	1	100		intronic	NM_13838	c.113-112	p.?	0.29					50S ribosome	rs4838742		YES				GTP binding,GTPase activity,mitochondrial inner membr				A=0, G=43	A=0.0, G=1.0	0.00001	705.843	43	A=0/0, G=21/2
chr4:81529518	C/C	T	C	SNV	C4orf22	C4orf22:exonic:NM_001206997.1	1	100	4	missense	NM_00120	c.365T>C	p.Ile122Thr	-0.01	0.16	89	0		Domain of un	rs2867782		YES	1.0 (pos.81529518)							T=0, C=32	T=0.0, C=1.0	0.00001	485.141	32	T=0/0, C=12/2
chr4:130030652	G/G	A	G	SNV	C4orf33	C4orf33:exonic:NM_001099783.1	1	100	5	missense	NM_00109	c.319A>G	p.Met107Val	4.88	1	21	0	0.13(COSM1131254)		rs337277	0.1836	YES	7.635E-1 (pos.13003065	acute_myeloid_leukaemia		protein binding				A=0, G=73	A=0.0, G=1.0	0.00001	1150.5	73	A=0/0, G=28/4
chr10:135352153	G/G	A	G	SNV	CYP2E1	CYP2E1:intronic:NM_000773.3	1	100		intronic	NM_00077	c.1298-13	p.?	-0.33					Cytochrome I	rs2249694		YES			Cytochrome	Golgi mem	Adenocarcinoma;A	Acetaminophen,Ademet		A=0, G=20	A=0.0, G=1.0	0.00001	263.06	20	A=0/0, G=9/11
chr10:135352168	C/C	T	C	SNV	CYP2E1	CYP2E1:intronic:NM_000773.3	1	100		intronic	NM_00077	c.1298-11	p.?	0.2					Cytochrome I	rs2249695		YES			Cytochrome	Golgi mem	Adenocarcinoma:A	Acetaminophen,Ademet		T=0, C=21	T=0.0, C=1.0	0.00001	281.222	21	T=0/0, C=9/12
chr5:37227819	G/A	G	A	SNV	C5orf42	C5orf42:exonic:NM_023073.3	1	50	10	missense	NM_02307	c.1222C>T	p.Arg408Trp	1.26	0	101	0.036		Joubert synd	rs189796608			0.0 (pos.37227819)		Chromosom	cerebellu	Abnormalities, Multiple:Bone Diseases:Bone			G=49, A=49	G=0.5, A=0.5	0.00001	551.833	98	G=24/25, A=2
chr5:41158863	A/A	G	A	SNV	C6	C6:exonic:NM_000065.3	1	100	13	synonymous	NM_00006	c.1881C>T	p.(=)	0.11				0.08(COSM45	Low-density	rs6866352			9.937E-1 (pos.41158863)	squamous_cel	Complemer	compleme	Autoimmune Diseases;Autoimmune Disease			G=0, A=190	G=0.0, A=1.0	0.00001	3190.95	190	G=0/0, A=112/
chr10:13696570	G/G	A	G	SNV	FRMD4A	FRMD4A:intronic:NM_018027.4	1	100		intronic	NM_01802	c.2954-58	p.?	0.82					Domain of un	rs10906442		YES			FERM doma	adherens	Alzheimer Disease;Brain Diseases;Central N			A=0, G=92	A=0.0, G=1.0	0.00001	1537.17	92	A=0/0, G=38/5
chr10:13717076	G/G	C	G	SNV	FRMD4A	FRMD4A:intronic:NM_018027.4	1	100		intronic	NM_01802	c.1118-32	p.?	1.8					Domain of un	rs3814663	0.4627	YES	3.252E-2 (pos.13717076)		FERM doma	adherens	Alzheimer Disease;Brain Diseases;Central N			C=0, G=32	C=0.0, G=1.0	0.00001	482.65	32	C=0/0, G=15/1
chr10:15713507	G/G	A	G	SNV	ITGA8	ITGA8:intronic:NM_003638.2	1	100		intronic	NM_00363	c.847+95T	p.?	0.04					FG-GAP repe	rs7100228		YES			Integrin, al	apical par	Adnexal Diseases:Congenital Abnormalities:			A=0, G=79	A=0.0, G=1.0	0.00001	1254.31	79	A=0/0, G=54/2
chr10:15713541	C/C	T	C	SNV	ITGA8	ITGA8:intronic:NM_003638.2	1	100		intronic	NM_00363	c.847+61A	p.?	-0.64					FG-GAP repe	rs6602051	0.3187	YES			Integrin, al	apical par	Adnexal Diseases:Congenital Abnormalities:			T=0, C=81	T=0.0, C=1.0	0.00001	1309.47	81	T=0/0, C=54/2
chr10:15713667	G/G	C	G	SNV	ITGA8	ITGA8:intronic:NM_003638.2	1	100		intronic	NM_00363	c.803-21G	p.?	-1.2					FG-GAP repe	rs7100510	0.3164	YES	7.07E-1;0.0 (pos.15713667)		Integrin, al	apical par	Adnexal Diseases;Congenital Abnormalities;			C=0, G=61	C=0.0, G=1.0	0.00001	921.852	61	C=0/0, G=16/4
chr10:15729923	T/T	C	T	SNV	ITGA8	ITGA8:intronic:NM_003638.2	1	100		intronic	NM_00363	c.444+14G	p.?	-0.37					FG-GAP repe	rs1954181	0.1381	YES	7.726E-1 (pos.15729923)		Integrin, al	apical par	Adnexal Diseases;Congenital Abnormalities;			C=0, T=63	C=0.0, T=1.0	0.00001	958.917	63	C=0/0, T=34/2
chr6:42074106	G/G	A	G	SNV	C6orf132	C6orf132:exonic:NM_001164446.2	1	100	4	missense	NM_00116	c.1544T>C	p.Leu515Pro	0.18	0.48	98	0	0(COSM4161186),0(COSM4		rs9394866		YES	2.993E-1 (pos.42074106)	neoplasm,squamous_cell_carcinoma						A=0, G=39	A=0.0, G=1.0	0.00001	614.21	39	A=0/0, G=11/2
chr6:42072692	G/T	G	T	SNV	C6orf132	C6orf132:exonic:NM_001164446.2	1	50	4	synonymous	NM_00116	c.2958C>A	p.(=)	0.21						rs74901308		YES	1.263E-1 (pos.42072692)							G=15, T=15	G=0.5, T=0.5	0.00001	145.021	30	G=11/4, T=5/1
chr10:16948177	A/G	A	G	SNV	CUBN	CUBN:intronic:NM_001081.3	1	97.5		intronic	NM_00108	c.7912+25	p.?	0.12					CUB domain.	rs780807	0.1903	YES	8.181E-1 (pos.16948177)		Cubilin (intr	Golgi appa	Amphetamine-Rela	Cyanocobalamin,Hydrox		A=2, G=78	A=0.025, G=0.975	0.00001	1196.3	80	A=0/2, G=33/4
chr7:47835027	A/G	A	G	SNV	C7orf69,PKD1L1	PKD1L1:intronic:NM_138295.4, C7orf69:exonic:NM_025031	1	97.06	1	missense	NM_13829	c.8355+56	p.?, p.Lys32Glu	-1.49	1	56	0		PKD domain,	rs9719534		YES	6.493E-1 (pos.47835027)		Polycystin 1	calcium channel activity,calcium channel complex,calci				A=1, G=33	A=0.0294, G=0.9706	0.00001	492.984	34	A=1/0, G=22/1
chr1:57422484	T/T	C	T	SNV	C8B	C8B:exonic:NM_000066.3	1	100	3	missense	NM_00006	c.349G>A	p.Gly117Arg	0.53	0.97	125	0		Low-density	rs1013579	0.0328	YES	9.999E-1 (pos.57422484)		Complemer	compleme	Arthritis:Arthritis, Juvenile:Autoimmune Dise			C=0, T=102	C=0.0, T=1.0	0.00001	1593.37	102	C=0/0, T=37/6
chr10:17110595	A/A	G	A	SNV	CUBN	CUBN:intronic:NM_001081.3	1	100		intronic	NM_00108	c.2791+9C	p.?	0.6					CUB domain.	rs10795440,rs37579	0.1984	YES	0.0 (pos.17110587),9.342E-1 (pos.17110		Cubilin (intr	Golgi appa	Amphetamine-Rela	Cyanocob	Benign	G=0, A=29	G=0.0, A=1.0	0.00001	427.603	29	G=0/0, A=5/24
chr9:139840560	A/A	C	A	SNV	C8G	C8G:exonic:NM_000606.2	1	100	4	missense	NM_00060	c.370C>A	p.His124Asn	0.41	0.28	68	0.001		Lipocalin / cy	rs17613,rs93134249	0.1404	YES	2.036E-2 (pos.139840560)		Complemer	blood microparticle,compleme		Citric Acid,Lauric Acid		C=0, A=56	C=0.0, A=1.0	0.00001	831.693	56	C=0/0, A=19/3
chr10:17146652	A/A	C	A	SNV	CUBN	CUBN:intronic:NM_001081.3	1	100		intronic	NM_00108	c.1231-48	p.?	-0.38					CUB domain.	rs7068549,rs754572	0.2695	YES	2.495E-1;0.0;0.0 (pos.17146652)		Cubilin (intr	Golgi appa	Amphetamine-Rela	Cyanocobalamin,Hydrox		C=0, A=116	C=0.0, A=1.0	0.00001	1861.48	116	C=0/0, A=8/10
chr10:17171265	C/C	G	C	SNV	CUBN	CUBN:intronic:NM_001081.3	1	100		intronic	NM_00108	c.123-16C	p.?	-0.02					CUB domain.	rs2281648	0.0043	YES	7.364E-1 (pos.17171265)		Cubilin (intr	Golgi appa	Amphetamine-Rela	Cyanocobalamin,Hydrox		G=0, C=51	G=0.0, C=1.0	0.00001	754.582	51	G=0/0, C=37/1
chr9:139840580	T/T	C	T	SNV	C8G	C8G:exonic:NM_000606.2	1	100	4	synonymous	NM_00060	c.390C>T	p.(=)	-2.65					Lipocalin / cy	rs20574	0.1413	YES	2.108E-2 (pos.139840580)		Complemer	blood microparticle,compleme		Citric Acid,Lauric Acid		C=0, T=45	C=0.0, T=1.0	0.00001	747.393	45	C=0/0, T=6/39
chr8:40011079	A/A	G	A	SNV	C8orf4	C8orf4:exonic:NM_020130.4	1	100	1	missense	NM_02013	c.28G>A	p.Val10Ile	-1.83		29			Thyroid canc	rs6474226	2.00E-04		1.0 (pos.40011079)		Transcriptic	Notch binding,apoptotic process,cellular response to he				G=0, A=20	G=0.0, A=1.0	0.00001	263.786	20	G=0/0, A=11/9
chr8:67592152	T/C	T	C	SNV	C8orf44,C8orf44-SGK3	C8orf44-SGK3:utr_5:NM_001204173.1, C8orf44:exonic:NM	1	50	3	missense	NM_01960	c.443T>C,	p.Phe148Ser, p.?	1.33	0	155	0.713	0.04(COSM3982563)		rs1057463	0.2949	YES	2.234E-1 (pos.67592152)	acute_myeloid_leukaemia		ATP binding,calcium channel regulator activity,chloride				T=10, C=10	T=0.5, C=0.5	0.00001	82.8856	20	T=3/7, C=6/4
chr10:17735209	CT/C	CT	C	INDEL	STAM	STAM:intronic:NM_003473.3	1	100		intronic	NM_00347	c.445-12C	p.?	-0.2, 0.93					SH3 domain.I	rs782029189			0.0 (pos.17735210)		Signal trans	ESCRT-0 complex,SH3/SH2 adaptor activity,cytosol,early				CT=0, C=43	CT=0.0, C=1.0	0.00001	373.674	43	CT=0/0, C=22/
chr10:18122716	G/G	A	G	SNV	MRC1	MRC1:intronic:NM_002438.3	1	100		intronic	NM_00243	c.803-690	p.?	0.63				0.12(COSM41	Fibronectin t	rs111490411	0.2307	YES	3.702E-1 (pos.18122716)	lymphoid_neo	Mannose re	cell surfac	Colonic Diseases:Colorectal Neoplasms:Derr			A=0, G=23	A=0.0, G=1.0	0.00001	316.399	23	A=0/0, G=11/1
chr8:13424583	T/T	C	T	SNV	C8orf48	C8orf48:exonic:NM_001007090.2	1	100	1	missense	NM_00100	c.83C>T	p.Ser28Phe	-0.07		155			Domain of un	rs13273355	0.1813	YES	9.629E-1 (pos.13424583)			protein binding				C=0, T=101	C=0.0, T=1.0	0.00001	1579.75	101	C=0/0, T=54/4
chr5:39364554	G/A	G	A	SNV	C9	C9:exonic:NM_001737.4	1	57.58	1	missense	NM_00173	c.13C>T	p.Arg5Trp	-2.55	0.13	101	0		Low-density	rs700233	0.4143	YES	5.804E-2 (pos.39364554)		Complemer	blood mici	Adenocarcinoma;Carcinoma;C		Benign	G=14, A=19	G=0.4242, A=0.5758	0.00001	204.394	33	G=8/6, A=6/13
chr10:18439926	C/C	T	C	SNV	CACNB2	CACNB2:intronic:NM_201596.2	1	100		intronic	NM_20159	c.213+22T	p.?	-0.02					Guanylate ki	rs79819217	0.1213	YES	2E-2 (pos.18439926)		Calcium cha	L-type volt	Arrhythmias, Cardi	Amlodipine,Calcium Citr		T=0, C=111	T=0.0, C=1.0	0.00001	1760.73	111	T=0/0, C=56/5
chr10:20290956	T/A	T	A	SNV	PLXDC2	PLXDC2:intronic:NM_032812.8	1	97.92		intronic	NM_03281	c.324+41T	p.?	-0.96					Plexin repeat	rs1409344	0.4736	YES	6.153E-1 (pos.20290956)		Plexin dom	extracellu	Cardiovascular Diseases:Diabetes Complicat			T=1, A=47	T=0.0208, A=0.9792	0.00001	776.186	48	T=0/1, A=23/2
chr9:139739643	C/C	T	C	SNV	C9orf172	C9orf172:exonic:NM_001080482.3	1	100	3	synonymous	NM_00108	c.777T>C	p.(=)	-2.29				0.02(COSM3750164)		rs10870132		YES	8.866E-1;0.0 (pos.13973	adenocarcinoma,neoplasm,squamous_cell_carcinoma						T=0, C=35	T=0.0, C=1.0	0.00001	539.578	35	T=0/0, C=11/2
chr10:21141469	C/C	T	C	SNV	NEBL	NEBL:intronic:NM_006393.2	1	100		intronic	NM_00639	c.1008+5A	p.?	-0.49				0(COSM5438	Nebulin repe	rs386741736,rs7030	6.00E-04	YES	9.169E-1;0.0 (pos.21141	squamous_cel	Nebulette	I band,Z di	Cardiovascular Diseases;Embc		Benign	T=0, C=45	T=0.0, C=1.0	0.00001	747.373	45	T=0/0, C=22/2
chr9:114454580	G/G	A	G	SNV	C9orf84	C9orf84:exonic:NM_173521.4	1	100	25	missense	NM_17352	c.3485T>C	p.Ile1162Thr	0.43	1	89	0			rs1475110,rs759208	0.0038		0.0 (pos.114454578),1.0 (pos.114454580)							A=0, G=39	A=0.0, G=1.0	0.00001	623.795	39	A=0/0, G=22/1
chr9:114462322	G/G	A	G	SNV	C9orf84	C9orf84:exonic:NM_173521.4	1	100	22	missense	NM_17352	c.2903T>C	p.Leu968Pro	0.33	0.22	98	0			rs6477845	0.2143	YES	9.261E-1 (pos.114462322)							A=0, G=59	A=0.0, G=1.0	0.00001	885.368	59	A=0/0, G=29/3
chr10:23297336	T/T	A	T	SNV	ARMC3	ARMC3:intronic:NM_173081.4	1	100		intronic	NM_17308	c.1928+33	p.?	-0.12					Armadillo/be	rs11819616	0.3833	YES	5.36E-1 (pos.23297336)		Armadillo r	cytoplasm,extracellular exosome,nucleus				A=0, T=22	A=0.0, T=1.0	0.00001	297.241	22	A=0/0, T=10/1
chr10:24790491	GC/TT	GC	TT	MNV	KIAA1217	KIAA1217:intronic:NM_019590.4	2	58.33		intronic	NM_01959	c.2001+17	p.?	-0.09, -1.85					Actin interac	rs2366916,rs386741	0.2558	YES	1.458E-1 (pos.24790491),1.458E-1 (pos.2		KIAA1217 g	cytoplasm,embryonic skeletal system development,mol				GC=10, TT=	GC=0.4167, TT=0.58	0.00001	137.407	24	GC=0/10, TT=4
chr9:114464487	C/C	T	C	SNV	C9orf84	C9orf84:exonic:NM_173521.4	1	100	21	missense	NM_17352	c.2795A>G	p.Tyr932Cys	-1.04	1	194	0	0.04(COSM5618300),0.04(C		rs1407390	0.2121	YES	9.262E-1 (pos.11446448	carcinoma,ductal_carcinoma						T=0, C=31	T=0.0, C=1.0	0.00001	465.816	31	T=0/0, C=17/1
chr9:114490308	C/C	T	C	SNV	C9orf84	C9orf84:exonic:NM_173521.4	1	100	11	missense	NM_17352	c.1247A>G	p.His416Arg	-0.27	0.61	29	0	0.01(COSM5765422),0.01(C		rs7470491	0.223	YES	9.256E-1 (pos.11449030	adenocarcinoma						T=0, C=41	T=0.0, C=1.0	0.00001	664.7	41	T=0/0, C=9/32
chr9:114454544	T/C	T	C	SNV	C9orf84	C9orf84:exonic:NM_173521.4	1	97.56	25	missense	NM_17352	c.3521A>G	p.Glu1174Gly	2.03	0	98	0.893	0.66(COSM3763536),0.66(C		rs7869279	0.4458	YES	7.807E-1 (pos.11445454	adenocarcinoma,diffuse_large_B_cell_lymphoma						T=1, C=40	T=0.0244, C=0.9756	0.00001	631.376	41	T=1/0, C=23/1
chr10:26357625	C/C	T	C	SNV	MYO3A	MYO3A:intronic:NM_017433.4	1	100		intronic	NM_01743	c.1054-72	p.?	-0.58					IQ calmoduli	rs3817421		YES			Myosin IIIA	ADP bindi	Ear Diseases:Hearing Disorders:Hearing Loss			T=0, C=23	T=0.0, C=1.0	0.00001	316.735	23	T=0/0, C=16/7
chr17:49713300	A/A	G	A	SNV	CA10	CA10:exonic:NM_001082533.1	1	100	8	synonymous	NM_00108	c.705C>T	p.(=)	-0.94				0.03(COSM14	Eukaryotic-ty	rs11870209	0.4843	YES	3.408E-1 (pos.49713300)	adenocarcino	Carbonic ar	brain development,cellular_cc		Zonisamide		G=0, A=30	G=0.0, A=1.0	0.00001	446.317	30	G=0/0, A=18/1
chr10:26357820	T/T	C	T	SNV	MYO3A	MYO3A:intronic:NM_017433.4	1	100		intronic	NM_01743	c.1170+7C	p.?	-0.49					IQ calmoduli	rs3817419	0.4505	YES	2.782E-1 (pos.26357820)		Myosin IIIA	ADP bindi	Ear Diseases;Hearing Disorder	Benign/Likely		C=0, T=31	C=0.0, T=1.0	0.00001	465.819	31	C=0/0, T=13/1
chr10:26359180	G/G	T	G	SNV	MYO3A	MYO3A:intronic:NM_017433.4	1	100		intronic	NM_01743	c.1275+36	p.?	-0.31					IQ calmoduli	rs3758441	0.4507	YES	2.804E-1;0.0 (pos.26359180)		Myosin IIIA	ADP bindi	Ear Diseases;Hearing Disorders;Hearing Loss			T=0, G=32	T=0.0, G=1.0	0.00001	485.137	32	T=0/0, G=23/9
chr10:26359200	TA/TA	T	TA	INDEL	MYO3A	MYO3A:intronic:NM_017433.4	1	100		intronic	NM_01743	c.1276-47	p.?	2.83					IQ calmoduli	rs3834403,rs397779	0.4507	YES	2.789E-1 (pos.26359200)		Myosin IIIA	ADP bindi	Ear Diseases:Hearing Disorders:Hearing Loss			T=0, TA=29	T=0.0, TA=1.0	0.00001	382.859	29	T=0/0, TA=21/
chr8:86351997	A/A	G	A	SNV	CA3	CA3:exonic:NM_005181.3	1	100	2	missense	NM_00518	c.91G>A	p.Val31Ile	4.5	1	29	0	0.06(COSM39	Eukaryotic-ty	rs20571	0.3926	YES	4.28E-1 (pos.86351997)	acute_myeloic	Carbonic ar	bicarbona	Digestive System Di	Acetazolamide,Ellagic Ac		G=0, A=76	G=0.0, A=1.0	0.00001	1209.6	76	G=0/0, A=37/3
chr10:26457637	C/C	T	C	SNV	MYO3A	MYO3A:intronic:NM_017433.4	1	100		intronic	NM_01743	c.3112-4T	p.?	0.31					IQ calmoduli	rs16926628	0.1174	YES	2.008E-3 (pos.26457637)		Myosin IIIA	ADP bindi	Ear Diseases;Hearing Disorder	Benign/Likely		T=0, C=25	T=0.0, C=1.0	0.00001	352.31	25	T=0/0, C=6/19
chr10:26463600	A/A	G	A	SNV	MYO3A	MYO3A:intronic:NM_017433.4	1	100		intronic	NM_01743	c.4293+11	p.?	-0.62					IQ calmoduli	rs11812370		YES			Myosin IIIA	ADP bindi	Ear Diseases;Hearing Disorders;Hearing Loss			G=0, A=21	G=0.0, A=1.0	0.00001	281.062	21	G=0/0, A=6/15
chr10:26506752	G/T	G	T	SNV	GAD2	GAD2:intronic:NM_001134366.1	1	47.62		intronic	NM_00113	c.137-19G	p.?	-0.2					Pyridoxal-de	rs112021218	6.00E-04	YES	0.0 (pos.26506752)		Glutamate	Golgi mem	Autoimmune Disea	Glutamic Acid,Pyridoxal		G=55, T=50	G=0.5238, T=0.4762	0.00001	499.643	105	G=29/26, T=2
chr10:27009334	G/G	A	G	SNV	PDSS1	PDSS1:intronic:NM_014317.4	1	100		intronic	NM_01431	c.609+46A	p.?	-0.07					Polyprenyl sy	rs1780196,rs750746	0.1413	YES	9.232E-1;0.0 (pos.27009334)		Prenyl diph	isoprenoid biosynthetic process,metal ion binding,mito				A=0, G=20	A=0.0, G=1.0	0.00001	263.784	20	A=0/0, G=5/15
chr10:27065976	G/A	G	A	SNV	ABI1	ABI1:intronic:NM_005470.3	1	50		intronic	NM_00547	c.462+18C	p.?	-0.02					Abl-interacto	rs7096584	0.1867	YES	9.909E-2 (pos.27065976)		ABL-interac	Fc-gamma	Autoimmune Diseases:Female Urogenital Di			G=12, A=12	G=0.5, A=0.5	0.00001	106.695	24	G=6/6, A=5/7
chr10:27326999	C/C	T	C	SNV	ANKRD26	ANKRD26:intronic:NM_014915.2	1	100		intronic	NM_01491	c.2376-16	p.?	-0.98					Ankyrin repe	rs7096505	0.1867	YES	9.907E-1 (pos.27326999)		Ankyrin rep	centrosom	Blood Platelet Disorders;Chror		Benign	T=0, C=42	T=0.0, C=1.0	0.00001	685.209	42	T=0/0, C=23/1
chr10:27431564	A/A	G	A	SNV	YME1L1	YME1L1:intronic:NM_014263.3	1	100		intronic	NM_01426	c.332-150	p.?	-0.81					ATPase famil	rs10829195		YES			Mitochondr	ATP binding,ATP-dependent peptidase activity,cell prolif				G=0, A=78	G=0.0, A=1.0	0.00001	1249.26	78	G=0/0, A=41/3
chr10:27520829	T/C	T	C	SNV	ACBD5	ACBD5:intronic:NM_145698.4	1	59.09		intronic	NM_14569	c.303-80A	p.?	-0.66					Acyl CoA bind	rs74126945		YES			Acyl-CoA-bi	aggrephagy,autophagy of peroxisome,fatty acid cataboli				T=18, C=26	T=0.4091, C=0.5909	0.00001	312.069	44	T=11/7, C=4/2
chr10:27529565	C/C	T	C	SNV	ACBD5	ACBD5:intronic:NM_145698.4	1	100		intronic	NM_14569	c.15+38A>	p.?	0.04					Acyl CoA bind	rs2489372,rs386742227		YES	9.924E-1 (pos.27529565)		Acyl-CoA-bi	aggrephagy,autophagy of peroxisome,fatty acid cataboli				T=0, C=52	T=0.0, C=1.0	0.00001	766.776	52	T=0/0, C=32/2
chrX:15800751	A/A	G	A	SNV	CA5B	CA5B:exonic:NM_007220.3	1	100	8	synonymous	NM_00722	c.918G>A	p.(=)	-1.24				0.01(COSM15	Eukaryotic-ty	rs1808	0.3383	YES	4.055E-1 (pos.15800751)	acute_myeloic	Carbonic ar	bicarbonate transport,carbona		Ellagic Acid,Zonisamide		G=0, A=40	G=0.0, A=1.0	0.00001	644.279	40	G=0/0, A=22/1
chr8:61178574	C/C	T	C	SNV	CA8	CA8:exonic:NM_004056.5	1	100	3	synonymous	NM_00405	c.327A>G	p.(=)	0.9				0.6(COSM376	Eukaryotic-ty	rs7464181	0.4097	YES	2.567E-1 (pos.61178574)	acute_myeloic	Carbonic ar	carbonate	Ataxia;Brain Diseas	Zonisamid	Likely benign	T=0, C=43	T=0.0, C=1.0	0.00001	705.844	43	T=0/0, C=16/2
chr10:28233120	C/C	A	C	SNV	ARMC4	ARMC4:intronic:NM_018076.4	1	100		intronic	NM_01807	c.1743+31	p.?	-1.22					Armadillo/be	rs3824590	0.4658	YES	6E-1;0.0 (pos.28233120)		Armadillo r	axoneme,	Bronchial Diseases;Bronchiectasis;Cardiova			A=0, C=40	A=0.0, C=1.0	0.00001	634.847	40	A=0/0, C=19/2
chr10:292927	A/A	C	A	SNV	ZMYND11	ZMYND11:intronic:NM_001202468.1	1	100		intronic	NM_00120	c.1158+14	p.?	-0.93					Bromodomai	rs2303990	0.0272	YES	9.398E-1;0.0 (pos.292927)		Zinc finger M	RNA polym	Autistic Disorder:Child Development Disorde			C=0, A=29	C=0.0, A=1.0	0.00001	427.603	29	C=0/0, A=7/22
chr10:29580743	A/A	G	A	SNV	LYZL1	LYZL1:intronic:NM_032517.4	1	100		intronic	NM_03251	c.114-29G	p.?	-0.67					C-type lysozy	rs2148902,rs796961693		YES	7.71E-1 (pos.29580743)			cell wall macromolecule catabolic process,defense resp				G=0, A=91	G=0.0, A=1.0	0.00001	1513.8	91	G=0/0, A=37/5

chr10:29581440	T/T	C	T	SNV	LYZL1	LYZL1:intronic:NM_032517.4	1	100		intronic	NM_03251	c.278-8C>	p.?	-0.37				0.04(COSM54	C-type lysozy	rs3818550	0.4233	YES	5.372E-1 (pos.29581440)	acute_myeloid_leukaemia		cell wall macromolecule catabolic process,defense resp				C=0, T=25	C=0.0, T=1.0	0.00001	353.019	25	C=0/0, T=18/7
chr10:29783813	A/A	G	A	SNV	SVIL	SVIL:intronic:NM_021738.2	1	100		intronic	NM_02173	c.3841+30	p.?	0.31					Gelsolin repe	rs6481600	0.4465	YES	3.664E-1 (pos.29783813)		Supervillin	actin cyto:	Leukemia:Leukemia, Myeloid:Leukemia, Mye			G=0, A=63	G=0.0, A=1.0	0.00001	958.917	63	G=0/0, A=40/2
chr19:48543862	A/A	G	A	SNV	CABP5	CABP5:exonic:NM_019855.4	1	100	3	synonymous	NM_01985	c.238C>T	p.(=)	0.3				0.54(COSM41	EF hand,EF-ha	rs8105198	0.1844	YES	8.674E-1:0.0 (pos.48543	neoplasm	Calcium-bir	calcium ion binding,cytosol,protein binding,signal transc				G=0, A=39	G=0.0, A=1.0	0.00001	623.98	39	G=0/0, A=27/1
chr4:71201388	T/T	C	T	SNV	CABS1	CABS1:exonic:NM_033122.3	1	100	1	missense	NM_03312	c.632C>T	p.Thr211Ile	-0.28		89			Calcium-bind	rs1351419	0.0492	YES	1.0 (pos.71201388)			calcium ion binding,mitochondrial inner membrane,mot				C=0, T=25	C=0.0, T=1.0	0.00001	353.018	25	C=0/0, T=11/1
chr18:21736933	G/G	T	G	SNV	CABYR	CABYR:exonic:NM_012189.3	1	100	4	missense	NM_01218	c.1468T>G	p.Ser490Ala	1.33	1	99	0		Regulatory su	rs1049683	0.1652	YES	8.324E-1 (pos.21736933)		Calcium-bir	SH3 doma	Colonic Diseases;Colorectal Neoplasms;Dige			T=0, G=31	T=0.0, G=1.0	0.00001	465.783	31	T=0/0, G=14/1
chr10:3147508	T/T	C	T	SNV	PFKP	PFKP:intronic:NM_002627.4	1	100		intronic	NM_00262	c.666-77C	p.?	-0.72					Phosphofruct	rs4881082		YES			Phosphofru	6-phosphofructokinase activity,ATP binding,cadherin bir				C=0, T=37	C=0.0, T=1.0	0.00001	574.979	37	C=0/0, T=11/2
chr10:3154534	G/G	A	G	SNV	PFKP	PFKP:intronic:NM_002627.4	1	100		intronic	NM_00262	c.1154+56	p.?	-0.4					Phosphofruct	rs2306304		YES			Phosphofru	6-phosphofructokinase activity,ATP binding,cadherin bir				A=0, G=41	A=0.0, G=1.0	0.00001	664.7	41	A=0/0, G=19/2
chr10:3155540	G/G	A	G	SNV	PFKP	PFKP:intronic:NM_002627.4	1	100		intronic	NM_00262	c.1225-24	p.?	-1.41					Phosphofruct	rs4881085	0.1067	YES	9.868E-1;0.0 (pos.3155540)		Phosphofru	6-phosphofructokinase activity,ATP binding,cadherin bir				A=0, G=26	A=0.0, G=1.0	0.00001	371.42	26	A=0/0, G=11/1
chr9:136333786	G/G	GC	G	INDEL	CACFD1	CACFD1:exonic:NM_001242369.1	1	100	6	frameshiftDeletion	NM_00124	c.659delC	p.Pro220fs	-2.48, -4.64					Uncharacteri	rs867108595						biological_process,cellular_component,integral compor				GC=0, G=2(	GC=0.0, G=1.0	0.00001	212.283	20	GC=0/0, G=7/1
chr9:141008876	T/T	C	T	SNV	CACNA1B	CACNA1B:exonic:NM_000718.3	1	100	40	synonymous	NM_00071	c.5583C>T	p.(=)	-1.14				0.08(COSM44	Ion transport	rs2229948	0.245	YES	3.746E-1 (pos.14100887(	acute_myeloid	Calcium cha	ATP bindin	Nervous System Dis	Agmatine,Amlodipine,G		C=0, T=43	C=0.0, T=1.0	0.00001	705.843	43	C=0/0, T=23/2
chr10:3187924	C/C	T	C	SNV	PITRM1,PITRM1-AS1	PITRM1:intronic:NM_001242307.1, PITRM1-AS1:exonic_nc:N	1	100		intronic	NM_00124	c.2340-16	p.?	-3.08					Insulinase (P	rs3758631	0.2777	YES	7.292E-1 (pos.3187924)			catalytic activity,enzyme activator activity,metal ion bin				T=0, C=23	T=0.0, C=1.0	0.00001	316.747	23	T=0/0, C=17/6
chr9:141016262	G/G	T	G	SNV	CACNA1B	CACNA1B:exonic:NM_000718.3	1	100	46	synonymous	NM_00071	c.6831T>G	p.(=)	-0.67				0.01(COSM37	Ion transport	rs2278973	0.0906	YES	7.974E-1:1.165E-4 (pos.1	adenocarcino	Calcium cha	ATP bindin	Nervous System Dis	Agmatine,Amlodipine,G		T=0, G=20	T=0.0, G=1.0	0.00001	263.782	20	T=0/0, G=4/16
chr10:3199555	T/T	A	T	SNV	PITRM1	PITRM1:intronic:NM_001242307.1	1	100		intronic	NM_00124	c.1347+72	p.?	1.35					Insulinase (P	rs7086470		YES				catalytic activity,enzyme activator activity,metal ion bin				A=0, T=49	A=0.0, T=1.0	0.00001	831.625	49	A=0/0, T=35/1
chr10:3200155	G/C	G	C	SNV	PITRM1	PITRM1:intronic:NM_001242307.1	1	56		intronic	NM_00124	c.1250+77	p.?	-0.49					Insulinase (P	rs9423705		YES				catalytic activity,enzyme activator activity,metal ion bin				G=11, C=14	G=0.44, C=0.56	0.00001	134.35	25	G=5/6, C=8/6
chr12:2791130	T/T	C	T	SNV	CACNA1C,CACNA1C-AS1	CACNA1C-AS1:intronic_nc:NR_045725.1, CACNA1C:exonic:N	1	100	45	missense	NM_19946	c.5603C>T	p.Pro1868Leu	-1.32	0.58	98	0.002	0(COSM1582	Ion transport	rs10848683,rs71441	0.1787	YES	5.925E-1 (pos.2791130)	acute_myeloid	Calcium cha	L-type volt	Arrhythmias, Cardi	Amlodipin	Benign	C=0, T=25	C=0.0, T=1.0	0.00001	353.019	25	C=0/0, T=11/1
chr10:3205851	G/G	C	G	SNV	PITRM1	PITRM1:intronic:NM_001242307.1	1	100		intronic	NM_00124	c.791+66G	p.?	-1.18					Insulinase (P	rs6602034		YES				catalytic activity,enzyme activator activity,metal ion bin				C=0, G=20	C=0.0, G=1.0	0.00001	263.786	20	C=0/0, G=6/14
chr12:2791132	G/G	A	G	SNV	CACNA1C,CACNA1C-AS1	CACNA1C-AS1:intronic_nc:NR_045725.1, CACNA1C:exonic:N	1	100	45	missense	NM_19946	c.5605A>G	p.Met1869Val	-0.38	0.62	21	0	0(COSM3998	Ion transport	rs10774053,rs71441	0.169	YES	6.216E-1:0.0 (pos.27911	acute_myeloid	Calcium cha	L-type volt	Arrhythmias, Cardi	Amlodipin	Benign	A=0, G=25	A=0.0, G=1.0	0.00001	353.019	25	A=0/0, G=11/1
chr10:32307536	A/A	T	A	SNV	KIF5B	KIF5B:intronic:NM_004521.2	1	100		intronic	NM_00452	c.2307-46	p.?	-0.3					Kinesin moto	rs200346000,rs397948232,rs55938482,rs6			1.0;0.0 (pos.32307536)		Kinesin 1	ATP bindin	Bronchial Neoplasms;Carcinoma, Bronchoge			T=0, A=27	T=0.0, A=1.0	0.00001	389.988	27	T=0/0, A=14/1
chr10:32311969	G/G	A	G	SNV	KIF5B	KIF5B:intronic:NM_004521.2	1	100		intronic	NM_00452	c.1726-5T	p.?	0.39					Kinesin moto	rs176924	3.00E-04		1.0;0.0 (pos.32311969)		Kinesin 1	ATP bindin	Bronchial Neoplasms;Carcinoma, Bronchoge			A=0, G=48	A=0.0, G=1.0	0.00001	810.422	48	A=0/0, G=45/3
chr10:32751901	A/A	G	A	SNV	CCDC7	CCDC7:intronic:NM_001026383.2	1	100		intronic	NM_00102	c.478-44G	p.?	-1.03					Spermatoger	rs2505380	0.0015	YES	1.0;0.0 (pos.32751901)							G=0, A=109	G=0.0, A=1.0	0.00001	1722.53	109	G=0/0, A=47/6
chr10:32807182	T/T	C	T	SNV	CCDC7	CCDC7:intronic:NM_001026383.2	1	100		intronic	NM_00102	c.904-162	p.?	0.52					Spermatoger	rs2947052		YES								C=0, T=21	C=0.0, T=1.0	0.00001	281.235	21	C=0/0, T=9/12
chr10:33015782	A/A	G	A	SNV	CCDC7		1	100		intronic				1.3						rs928701	1.00E-04		1.0 (pos.33015782)							G=0, A=110	G=0.0, A=1.0	0.00001	1731.69	110	G=0/0, A=16/9
chr10:33018351	C/C	G	C	SNV	CCDC7		1	100		intronic				-0.09						rs1414548	0.0014	YES	1.0 (pos.33018351)							G=0, C=20	G=0.0, C=1.0	0.00001	263.786	20	G=0/0, C=4/16
chr10:33123824	C/C	G	C	SNV	CCDC7		1	100		intronic				-1.45						rs4448627	2.00E-04		1.0;0.0 (pos.33123824)							G=0, C=41	G=0.0, C=1.0	0.00001	664.7	41	G=0/0, C=18/2
chr12:2791204	AG/AG	A	AG	INDEL	CACNA1C,CACNA1C-AS1	CACNA1C-AS1:intronic_nc:NR_045725.1, CACNA1C:exonic:N	1	100	45	frameshiftInsertion	NM_19946	c.5677_56	p.Lys1893fs	0.28					Ion transport protein,Polycystin cation channel,Voltage gated calcium channel IQ domain,Voltage-d						Calcium cha	L-type volt	Arrhythmias, Cardi	Amlodipine,Calcium,Cal		A=0, AG=24	A=0.0, AG=1.0	0.00001	241.278	24	A=0/0, AG=10/
chrX:49071964	G/G	A	G	SNV	CACNA1F	CACNA1F:exonic:NM_001256789.2	1	100	28	synonymous	NM_00125	c.3276T>C	p.(=)	-1.6					Ion transport	rs2075866	0.293	YES	6.695E-1 (pos.49071964)		Calcium cha	T cell hom	Albinism;Albinism,	Calcium Citrate,Calcium		A=0, G=67	A=0.0, G=1.0	0.00001	1034.27	67	A=0/0, G=36/3
chr10:34666864	T/T	C	T	SNV	PARD3	PARD3:intronic:NM_019619.3	1	100		intronic	NM_01961	c.1539+31	p.?	-1.79					N-terminal of	rs10740890	0.4723	YES	4.339E-1 (pos.34666864)		Partitioning	adherens junction,asymmetric cell division,axonal grow				C=0, T=34	C=0.0, T=1.0	0.00001	524.161	34	C=0/0, T=19/1
chr16:1261282	C/C	T	C	SNV	CACNA1H	CACNA1H:exonic:NM_021098.2	1	100	22	synonymous	NM_02109	c.4338T>C	p.(=)	-0.14				0.16(COSM37	Ion transport	rs4984637	0.1833	YES	9.994E-1 (pos.1261282)	hepatocellula	Calcium cha	aldosteroi	Adrenal Gland Dise	Amiodarone,Bepridil,Cal		T=0, C=94	T=0.0, C=1.0	0.00001	1576.16	94	T=0/0, C=62/3
chr10:35322024	A/A	G	A	SNV	CUL2	CUL2:intronic:NM_001198779.1	1	100		intronic	NM_00119	c.1149+70	p.?	0.22					Cullin family,	rs12261654		YES			Cullin 2	Cul2-RING	Digestive System Diseases;Gastroenteritis;G			G=0, A=34	G=0.0, A=1.0	0.00001	524.17	34	G=0/0, A=23/1
chr10:35328088	G/G	A	G	SNV	CUL2	CUL2:intronic:NM_001198779.1	1	100		intronic	NM_00119	c.754-78T	p.?	0.38					Cullin family,	rs11010077		YES			Cullin 2	Cul2-RING	Digestive System Diseases;Gastroenteritis;G			A=0, G=30	A=0.0, G=1.0	0.00001	446.638	30	A=0/0, G=14/1
chr16:1270349	C/C	T	C	SNV	CACNA1H	CACNA1H:exonic:NM_021098.2	1	100	35	synonymous	NM_02109	c.6417T>C	p.(=)	-0.63				0.01(COSM37	Ion transport	rs4247094,rs759086	0.1668	YES	0.0 (pos.1270341),9.992	adenocarcino	Calcium cha	aldosteroi	Adrenal Gland Dise	Amiodaro	Benign	T=0, C=34	T=0.0, C=1.0	0.00001	524.179	34	T=0/0, C=16/1
chr10:38306079	C/C	T	C	SNV	ZNF33A	ZNF33A:intronic:NM_001278177.1	1	100		intronic	NM_00127	c.217+136	p.?	-1.04					C2H2-type zir	rs2738207		YES			Zinc finger c	DNA binding,DNA binding transcription factor activity,int				T=0, C=45	T=0.0, C=1.0	0.00001	745.413	45	T=0/0, C=34/1
chr10:395266	C/C	T	C	SNV	DIP2C	DIP2C:intronic:NM_014974.2	1	100		intronic	NM_01497	c.3109+5A	p.?	-1.04					AMP-binding	rs4880611	5.00E-04		1.0 (pos.395266)		Disco-interi	biological	Bronchial Neoplasms;Carcinoma, Bronchoge			T=0, C=53	T=0.0, C=1.0	0.00001	770.421	53	T=0/0, C=22/3
chr16:1272038	A/A	G	A	SNV	CACNA1H,TPSG1	TPSG1:exonic:NM_012467.3, CACNA1H:downstream:NM_02	1	100	6	missense	NM_02109	c.*1044G>	p.?, p.Thr239Ile	3.12	1	89	0	0(COSM4346	Trypsin	rs11248860,rs38678	0.2468	YES	9.994E-1 (pos.1272038)	basal_cell_car	Calcium cha	aldosteroi	Adrenal Gland Dise	Amiodarone,Bepridil,Cal		G=0, A=24	G=0.0, A=1.0	0.00001	331.444	24	G=0/0, A=8/16
chr1:201047075	G/G	A	G	SNV	CACNA1S	CACNA1S:exonic:NM_000069.2	1	100	11	synonymous	NM_00006	c.1551T>C	p.(=)	-1.51				0.02(COSM41	Ion transport	rs4915477	0.3395	YES	2.245E-1 (pos.20104707	neoplasm	Calcium cha	I band,L-ty	ACMG Recommend	Amlodipin	Benign	A=0, G=29	A=0.0, G=1.0	0.00001	427.584	29	A=0/0, G=6/23
chr3:54354510	C/C	T	C	SNV	CACNA2D3	CACNA2D3:exonic:NM_018398.2	1	100	3	synonymous	NM_01839	c.246T>C	p.(=)	-3.77				0.01(COSM44	Neuronal vol	rs9879885	0.2621	YES	6.563E-1 (pos.54354510)	adenocarcino	Calcium cha	calcium channel activity,calciu		Amlodipine,Nilvadipine,		T=0, C=52	T=0.0, C=1.0	0.00001	766.974	52	T=0/0, C=32/2
chr12:1995403	C/C	T	C	SNV	CACNA2D4	CACNA2D4:exonic:NM_172364.4	1	100	8	missense	NM_17236	c.979A>G	p.Ile327Val	3.14	1	29	0	0.23(COSM41	Neuronal vol	rs10735005	0.1137	YES	9.994E-1:0.0 (pos.19954	neoplasm	Calcium cha	calcium io	Congenital, Hereditary, and Ne		Benign	T=0, C=38	T=0.0, C=1.0	0.00001	603.782	38	T=0/0, C=31/7
chr10:444997	T/T	C	T	SNV	DIP2C	DIP2C:intronic:NM_014974.2	1	100		intronic	NM_01497	c.1260+52	p.?	-1.1					AMP-binding	rs6560836	0.4628	YES			Disco-interi	biological	Bronchial Neoplasms;Carcinoma, Bronchoge			C=0, T=51	C=0.0, T=1.0	0.00001	754.584	51	C=0/0, T=27/2
chr11:115085401	A/G	A	G	SNV	CADM1	CADM1:exonic:NM_014333.3	1	45.83	7	synonymous	NM_01433	c.921T>C	p.(=)	2.24				0.64(COSM36	CD80-like C2-	rs139604912	0.0038		0.0 (pos.115085401)	adenocarcino	Cell adhesi	PDZ doma	Autistic Disorder;Breast Diseases;Breast Nec			A=13, G=11	A=0.5417, G=0.4583	0.00001	93.4392	24	A=6/7, G=8/3
chr10:45939136	C/C	T	C	SNV	ALOX5,LOC102724323	ALOX5:intronic:NM_000698.4, LOC102724323:downstream	1	100		intronic	NM_00069	c.1574-40	p.?	-0.04					Lipoxygenase	rs7900977	0.2552	YES	7.991E-1 (pos.45939136)		Arachidona	arachidon	Adenoma;Arterial C	Aminosalicylic Acid,Balsa		T=0, C=26	T=0.0, C=1.0	0.00001	371.42	26	T=0/0, C=3/23
chr7:122303321	C/T	C	T	SNV	CADPS2	CADPS2:exonic:NM_001009571.3	1	97.01	3	synonymous	NM_00100	c.756G>A	p.(=)	-0.01				0.51(COSM40	Domain of Ur	rs2251761	0.2414	YES	7.424E-1 (pos.12230332	acute_myeloid	Calcium-de	cell juncti	Autistic Disorder;Cl	Calcium Citrate,Calcium		C=2, T=65	C=0.0299, T=0.9701	0.00001	973.933	67	C=0/2, T=36/2
chr11:14991539	C/C	T	C	SNV	CALCA	CALCA:exonic:NM_001033952.2	1	100	3	missense	NM_00103	c.169A>G	p.Asn57Asp	2	0.87	23	0		Calcitonin / C	rs5239	1.00E-04		1.0 (pos.14991539)		Calcitonin/	G-protein	Abnormalities, Mul	Olcegepant,Telcagepant		T=0, C=34	T=0.0, C=1.0	0.00001	524.117	34	T=0/0, C=15/1
chr10:48414570	G/G	A	G	SNV	GDF2	GDF2:intronic:NM_016204.3	1	100		intronic	NM_01620	c.347-49T	p.?	0.05					TGF-beta pro	rs7923671	0.068	YES	8.253E-1 (pos.48414570)		Growth diff	BMP signa	Adenocarcinoma;Carcinoma, Hep			A=0, G=33	A=0.0, G=1.0	0.00001	504.586	33	A=0/0, G=15/1
chr10:4889525	C/C	T	C	SNV	AKR1E2	AKR1E2:intronic:NM_001040177.2	1	100		intronic	NM_00104	c.920+103	p.?	-1					Aldo/keto rec	rs2961597,rs775619299		YES			Aldo-keto re	1,5-anhydro-D-fructose reductase activity,Golgi apparat				T=0, C=72	T=0.0, C=1.0	0.00001	1130.93	72	T=0/0, C=6/66
chr10:49658248	A/A	G	A	SNV	ARHGAP22	ARHGAP22:intronic:NM_001256025.2	1	100		intronic	NM_00125	c.1886+56	p.?	-0.04					PH domain,R	rs1867585		YES			Rho GTPase	GTPase ac	Autoimmune Diseases;Cardiovascular Disea			G=0, A=33	G=0.0, A=1.0	0.00001	504.598	33	G=0/0, A=19/1
chr10:49667713	A/A	G	A	SNV	ARHGAP22	ARHGAP22:intronic:NM_001256025.2	1	100		intronic	NM_00125	c.677+14C	p.?	-1.84					PH domain,R	rs1867575	0.007	YES	9.999E-1 (pos.49667713)		Rho GTPase	GTPase ac	Autoimmune Diseases;Cardiovascular Disea			G=0, A=73	G=0.0, A=1.0	0.00001	1150.1	73	G=0/0, A=51/2
chr10:49860258	A/G	A	G	SNV	ARHGAP22	ARHGAP22:intronic:NM_001256025.2	1	50		intronic	NM_00125	c.52+21T>	p.?	2.04					PH domain,R	rs2377614		YES	6.418E-1 (pos.49860258)		Rho GTPase	GTPase ac	Autoimmune Diseases;Cardiovascular Disea			A=12, G=12	A=0.5, G=0.5	0.00001	106.71	24	A=6/6, G=8/4
chr10:49983890	T/T	G	T	SNV	WDFY4	WDFY4:intronic:NM_020945.1	1	100		intronic	NM_02094	c.2863+39	p.?	0.18					Beige/BEACH	rs1871604	0.0581	YES	9.952E-1;1.603E-3 (pos.49983890)		WD repeat-	integral cc	Arthritis;Arthritis, Rheumatoid;Autoimmune			G=0, T=31	G=0.0, T=1.0	0.00001	465.812	31	G=0/0, T=20/1
chr10:105215016	G/G	A	G	SNV	CALHM1	CALHM1:utr_3:NM_001001412.3	1	100	2	utr_3	NM_00100	c.1044T>C	p.?	-1.69					Calcium hom	rs2986016	0.2544	YES	8.954E-1 (pos.105215016)		Calcium ho	ATP transc	Female Urogenital Diseases;Female Urogeni			A=0, G=28	A=0.0, G=1.0	0.00001	408.71	28	A=0/0, G=19/9
chr10:50227870	T/T	C	T	SNV	VSTM4	VSTM4:intronic:NM_001031746.4	1	100		intronic	NM_00103	c.838-50G	p.?	-1.57					Immunoglob	rs1913524	0.0601	YES	9.857E-1 (pos.50227870)			extracellu	Brain Diseases;Central Nervous System Dise			C=0, T=28	C=0.0, T=1.0	0.00001	408.718	28	C=0/0, T=9/19
chr10:105218252	G/G	A	G	SNV	CALHM1	CALHM1:exonic:NM_001001412.3	1	100	1	missense	NM_00100	c.257T>C	p.Leu86Pro	0.12	0.23	98	0	0.01(COSM41	Calcium hom	rs2986017,rs386747	0.2701	YES	0.0 (pos.105218244),8.8	adenocarcino	Calcium ho	ATP transc	Female Urogenital Diseases;Female Urogeni			A=0, G=23	A=0.0, G=1.0	0.00001	316.741	23	A=0/0, G=6/17
chr19:16593318	C/T	C	T	SNV	CALR3	CALR3:exonic:NM_145046.4	1	52.38	7	synonymous	NM_14504	c.861G>A	p.(=)	-7.15					Calreticulin f	rs118131979	0.0167	YES	0.0;0.0 (pos.16593318)		Calreticulin	calcium io	Arrhythmogenic Rig	Calcium C	Benign	C=20, T=22	C=0.4762, T=0.5238	0.00001	245.209	42	C=11/9, T=8/1
chr10:50701156	A/A	G	A	SNV	ERCC6	ERCC6:intronic:NM_000124.3	1	100		intronic	NM_00012	c.1821+7C	p.?	-0.59					Helicase con	rs4253132	0.1181	YES	9.992E-1;0.0 (pos.50701156)		Excision rep	ATP bindin	Abnormalities, Multiple;Agene		Benign	G=0, A=91	G=0.0, A=1.0	0.00001	1513.6	91	G=0/0, A=53/3
chr10:12867598	C/G	C	G	SNV	CAMK1D	CAMK1D:exonic:NM_153498.3	1	53.13	10	synonymous	NM_15349	c.948C>G	p.(=)	0.35					Kinase-like,P	rs1757051	0.2459	YES	8.426E-1 (pos.12867598)		Calcium/ca	ATP bindin	Nutrition Disorders	N-(5-METHYL-1H-PYRAZOL		C=15, G=17	C=0.4688, G=0.5313	0.00001	173.556	32	C=11/4, G=6/1
chr5:149602608	G/G	A	G	SNV	CAMK2A	CAMK2A:exonic:NM_015981.3	1	100	18	synonymous	NM_01598	c.1410T>C	p.(=)	-0.64				0.05(COSM41	Calcium/caln	rs2241694	0.0817	YES	8.329E-1 (pos.14960260	hepatocellula	Calcium/calmodulin-de		Basal Ganglia Disea	(2Z,3E)-2,3'-BIINDOLE-2',3		A=0, G=20	A=0.0, G=1.0	0.00001	263.786	20	A=0/0, G=7/13
chr3:183976108	C/C	T	C	SNV	CAMK2N2,EEF1AKMT4,EEF1AKMT4-ECE2	EEF1AKMT4-ECE2:intronic:NM_014693.3, EEF1AKMT4:exonic	1	100	3	synonymous	NM_03233	c.513T>C,(	p.(=), p.?, p.?	1.03				0.07(COSM40	Methyltransf	rs902415	0.2295	YES	3.017E-1 (pos.18397610	acute_myeloid	Calcium/ca	Golgi membrane,brain development,calcium-dependen				T=0, C=58	T=0.0, C=1.0	0.00001	867.369	58	T=0/0, C=32/2
chr5:110819753	C/C	G	C	SNV	CAMK4	CAMK4:exonic:NM_001744.5	1	100	11	synonymous	NM_00174	c.1011G>C	p.(=)	-0.05				0.16(COSM37	Protein kinas	rs25925	0.2113	YES	5.534E-1 (pos.11081975	adenocarcino	Ca(2+)-calm	ATP bindin	Cocaine-Related Di	3-{(3S)-3,4-dihydroxybut		G=0, C=27	G=0.0, C=1.0	0.00001	388.71	27	G=0/0, C=16/1
chr19:39228244	C/C	T	C	SNV	CAPN12	CAPN12:exonic:NM_144691.4	1	100	9	missense	NM_14469	c.1000A>G	p.Thr334Ala	0.57	0.01	58	0.953	0.45(COSM43	Calpain famil	rs73038948	0.1828	YES	3.68E-3 (pos.39228244)	neoplasm	Calpain 12	calcium ion binding,calcium-dependent cysteine-type er				T=0, C=38	T=0.0, C=1.0	0.00001	603.665	38	T=0/0, C=15/2
chr16:601632	C/C	T	C	SNV	CAPN15	CAPN15:exonic:NM_005632.2	1	100	9	synonymous	NM_00563	c.2313T>C	p.(=)	-1.42				0.03(COSM37	Calpain famil	rs3213574	0.4792	YES	7.857E-1 (pos.601632)	acute_myeloid_leukaemia		DNA binding transcription factor activity,calcium-depen				T=0, C=63	T=0.0, C=1.0	0.00001	962.65	63	T=0/0, C=32/3
chr10:50863565	T/T	C	T	SNV	CHAT	CHAT:intronic:NM_020549.4	1	100		intronic	NM_02054	c.1839+71	p.?	-4.04					Choline/Carn	rs7076926	0.0896	YES			Choline ace	choline O-	Alzheimer Disease;	Choline,Nicotine		C=0, T=20	C=0.0, T=1.0	0.00001	263.786	20	C=0/0, T=7/13
chr10:50947918	G/G	A	G	SNV	OGDHL	OGDHL:intronic:NM_018245.2	1	100		intronic	NM_01824	c.2141-33	p.?	-1.12					2-oxoglutara	rs1258259	0.0317	YES	9.778E-1 (pos.50947918)		Oxoglutarat	cytosol,glycolytic process,metal ion binding,mitochondr				A=0, G=43	A=0.0, G=1.0	0.00001	705.844	43	A=0/0, G=20/2
chr16:602313	A/A	C	A	SNV	CAPN15	CAPN15:exonic:NM_005632.2	1	100	11	synonymous	NM_00563	c.2520C>A	p.(=)	-6.11				0.01(COSM43	Calpain famil	rs9930550	0.3637	YES	8.497E-1:0.0 (pos.60231	NS,adenocarcinoma		DNA binding transcription factor activity,calcium-depen				C=0, A=22	C=0.0, A=1.0	0.00001	298.878	22	C=0/0, A=7/15
chr10:5139815	A/A	C	A	SNV	AKR1C3	AKR1C3:intronic:NM_001253908.1	1	100		intronic	NM_00125	c.369+73C	p.?	-0.08					Aldo/keto rec	rs2245191		YES	2.08E-1;0.0 (pos.5139815)		Aldo-keto re	15-hydrox	Adnexal Diseases;C	2'-Monophosphoadenos		C=0, A=166	C=0.0, A=1.0	0.00001	2702.96	166	C=0/0, A=93/7
chr10:51549545	T/T	G	T	SNV	MSMB,TIMM23B	TIMM23B:intronic_nc:NR_110767.1, MSMB:upstream:NM_0	1	100		intronic	NR_11076	c.-40G>T	p.?	-1.1					Tim17/Tim22	rs4935176	2.00E-04	YES	1.0 (pos.51549545)		Microsemir	biological	Genital Diseases, Male;Genital Neoplasms, I			G=0, T=32	G=0.0, T=1.0	0.00001	485.027	32	G=0/0, T=13/1
chr10:5242346	A/A	G	A	SNV	AKR1C4	AKR1C4:intronic:NM_001818.3	1	100		intronic	NM_00181	c.252+35G	p.?	-4.27					Aldo/keto rec	rs7099651	0		1.0 (pos.5242346)		Aldo-keto re	aldo-keto	46, XY Disorders of	NADH		G=0, A=33	G=0.0, A=1.0	0.00001	504.597	33	G=0/0, A=18/1
chr1:223900408	G/G	C	G	SNV	CAPN2	CAPN2:exonic:NM_001748.4	1	100	1	missense	NM_00174	c.66C>G	p.Asp22Glu	-0.18	1	45	0	0.01(COSM41	Calpain famil	rs25655	2.00E-04		1.0 (pos.223900408)	neoplasm	Calpain, lar	Golgi appa	Leukemia:Leukemia, Myeloid:Leukemia, Mye			C=0, G=23	C=0.0, G=1.0	0.00001	316.744	23	C=0/0, G=8/15
chr10:5255179	C/A	C	A	SNV	AKR1C4	AKR1C4:intronic:NM_001818.3	1	52.38		intronic	NM_00181	c.846+57C	p.?	-0.98					Aldo/keto rec	rs55673448		YES			Aldo-keto re	aldo-keto	46, XY Disorders of	NADH		C=20, A=22	C=0.4762, A=0.5238	0.00001	245.209	42	C=7/13, A=8/1
chr10:52576068	A/A	G	A	SNV	A1CF	A1CF:intronic:NM_138932.2	1	100		intronic	NM_13893	c.868-29C	p.?	-1.07					RNA recognit	rs4245008	0.1492	YES	9.797E-1 (pos.52576068)			RNA binding,apolipoprotein B mRNA editing enzyme com				G=0, A=28	G=0.0, A=1.0	0.00001	408.71	28	G=0/0, A=11/1
chr10:53814229	T/T	G	T	SNV	PRKG1	PRKG1:intronic:NM_006258.3	1	100		intronic	NM_00625	c.763-15G	p.?	-1.47					Cyclic nucleo	rs1904010,rs777750	0.3826	YES	2.6E-1;0.0 (pos.53814229)		Protein kina	ATP bindin	Amphetamine-Related Disorde		Benign	G=0, T=48	G=0.0, T=1.0	0.00001	810.435	48	G=0/0, T=21/2
chr10:53814383	T/T	C	T	SNV	PRKG1	PRKG1:intronic:NM_006258.3	1	100		intronic	NM_00625	c.840+62C	p.?	-0.41					Cyclic nucleo	rs12778630	0.4434	YES			Protein kina	ATP bindin	Amphetamine-Related Disorders;Asthma;Bra			C=0, T=47	C=0.0, T=1.0	0.00001	793.664	47	C=0/0, T=19/2
chr1:223954080	A/C	A	C	SNV	CAPN2	CAPN2:exonic:NM_001748.4	1	57.14	16	missense	NM_00174	c.1702A>C	p.Lys568Gln	7.16	0.04	53	0.007	0.99(COSM14	Calpain famil	rs17599	0.2447	YES	2.448E-1 (pos.22395408	acute_myeloid	Calpain, lar	Golgi appa	Leukemia:Leukemia, Myeloid:Leukemia, Mye			A=21, C=28	A=0.4286, C=0.5714	0.00001	342.29	49	A=14/7, C=13/
chr17:78175608	C/T	C	T	SNV	CARD14	CARD14:exonic:NM_024110.4	1	56	14	synonymous	NM_02411	c.1917C>T	p.(=)	-2.95					Caspase recr	rs79407194	3.00E-04		0.0 (pos.78175608)		Caspase rec	CARD dom	Pityriasis;Pityriasis Rubra Pilar		Benign	C=11, T=14	C=0.44, T=0.56	0.00001	133.925	25	C=6/5, T=8/6
chr5:40841741	T/T	C	T	SNV	CARD6	CARD6:exonic:NM_032587.3	1	100	1	missense	NM_03258	c.257C>T	p.Ser86Leu	0.48	0.06	145	0.989		Caspase recr	rs10512747	0.1205	YES	2.66E-3 (pos.40841741)		Caspase rec	apoptotic process,protein binding,regulation of apoptot				C=0, T=51	C=0.0, T=1.0	0.00001	754.58	51	C=0/0, T=20/3
chr10:54528298	C/C	G	C	SNV	MBL2	MBL2:intronic:NM_000242.2	1	100		intronic	NM_00024	c.374-28C	p.?	0.07					Collagen trip	rs930508	0.1813	YES	7.595E-1 (pos.54528298)		Mannose-b	acute-pha	Autoimmune Disea	Alpha-Methyl-N-Acetyl-D		G=0, C=29	G=0.0, C=1.0	0.00001	427.603	29	G=0/0, C=7/22
chr19:48722180	T/T	C	T	SNV	CARD8	CARD8:exonic:NM_001184900.2	1	100	12	synonymous	NM_00118	c.1251G>A	p.(=)	-0.49					Caspase recr	rs10500299	0.2824	YES	2.072E-1 (pos.48722180)		Caspase rec	CARD dom	Arthritis;Congenital, Hereditary, and Neonat			C=0, T=29	C=0.0, T=1.0	0.00001	427.345	29	C=0/0, T=26/3
chr10:5498248	T/T	G	T	SNV	NET1	NET1:intronic:NM_001047160.2	1	100		intronic	NM_00104	c.1384+12	p.?	-0.04					PH domain,P	rs4242790	0.3594	YES	6.771E-1 (pos.5498248)		Solute carri	G-protein coupled receptor signaling pathway,GTP-Rho b				G=0, T=37	G=0.0, T=1.0	0.00001	583.706	37	G=0/0, T=27/1
chr10:55617177	C/C	T	C	SNV	PCDH15	PCDH15:intronic:NM_001142763.1	1	100		intronic	NM_00114	c.3733-15	p.?	-1.57					Cadherin dor	rs7083307		YES			Protocadherin 15		Abnormalities, Multiple;Blindness;Cochlear I			T=0, C=40	T=0.0, C=1.0	0.00001	644.286	40	T=0/0, C=8/32
chr10:56089263	A/A	G	A	SNV	PCDH15	PCDH15:intronic:NM_001142763.1	1	100		intronic	NM_00114	c.720+93C	p.?	-1.09					Cadherin dor	rs857395		YES			Protocadherin 15		Abnormalities, Multiple;Blindness;Cochlear I			G=0, A=61	G=0.0, A=1.0	0.00001	921.797	61	G=0/0, A=31/3
chr19:48737764	G/G	A	G	SNV	CARD8	CARD8:exonic:NM_001184900.2	1	100	6	synonymous	NM_00118	c.246T>C	p.(=)	-2.32				0(COSM3990	Caspase recr	rs2288876	0.3278	YES	2.314E-1 (pos.48737764)	adenocarcino	Caspase rec	CARD dom	Arthritis;Congenital, Hereditary, and Neonat			A=0, G=31	A=0.0, G=1.0	0.00001	465.819	31	A=0/0, G=17/1
chr10:5766337	A/A	G	A	SNV	FAM208B	FAM208B:intronic:NM_017782.4	1	100		intronic	NM_01778	c.248-56G	p.?	-0.97					Protein of un	rs10737018		YES				protein binding				G=0, A=45	G=0.0, A=1.0	0.00001	747.379	45	G=0/0, A=28/1
chr6:25420344	G/G	C	G	SNV	CARMIL1	CARMIL1:exonic:NM_017640.5	1	100	3	synonymous	NM_01764	c.141C>G	p.(=)	2.41					CARMIL C-ter	rs913455	0.0657	YES	1.0 (pos.25420344)			actin filament network formation,actin filament organiza				C=0, G=81	C=0.0, G=1.0	0.00001	1309.48	81	C=0/0, G=49/3
chr2:202050677	A/G	A	G	SNV	CASP10	CASP10:exonic:NM_032977.3	1	51.92	2	synonymous	NM_03297	c.177A>G	p.(=)	-1.12				0.02(COSM37	Caspase dom	rs3900115	0.479	YES	2.213E-1 (pos.20205067	neoplasm,prir	Caspase 10	CD95 deat	Autoimmune Diseases;Autoim		Benign	A=25, G=27	A=0.4808, G=0.5192	0.00001	270.39	52	A=10/15, G=1
chr11:104761206	T/T	C	T	SNV	CASP12	CASP12:exonic:NM_001191016.2	1	100	5	missense	NM_00119	c.712G>A	p.Gly238Ser	-0.18	0.81	56	0.012	0.01(COSM45	Caspase dom	rs647039			1.0 (pos.104761206)	squamous_cel	Caspase 12	AIM2 infla	Autoimmune Diseases;Brain Diseases;Brain			C=0, T=20	C=0.0, T=1.0	0.00001	263.786	20	C=0/0, T=11/9
chr11:104877927	C/C	T	C	SNV	CASP5	CASP5:exonic:NM_001136112.1	1	100	3	missense	NM_00113	c.355A>G	p.Thr119Ala	0.17	1	58	0	0(COSM3998	Caspase dom	rs507879	0.4406	YES	2.178E-1 (pos.10487792	acute_myeloid	Caspase 5,	AIM2 inflammasome complex,IPAF inflammasome comp				T=0, C=44	T=0.0, C=1.0	0.00001	726.563	44	T=0/0, C=34/1
chr10:5958072	G/G	A	G	SNV	FBXO18	FBXO18:intronic:NM_032807.4	1	100		intronic	NM_03280	c.1719-12	p.?	-2.6					AAA domain,I	rs668498		YES			F-box only p	3'-5' DNA helicase activity,ATP binding,ATP-dependent DI				A=0, G=83	A=0.0, G=1.0	0.00001	1349.88	83	A=0/0, G=48/3
chr10:5966190	A/A	G	A	SNV	FBXO18	FBXO18:intronic:NM_032807.4	1	100		intronic	NM_03280	c.2552-84	p.?	0.24					AAA domain,I	rs2274029,rs386740405		YES			F-box only p	3'-5' DNA helicase activity,ATP binding,ATP-dependent DI				G=0, A=66	G=0.0, A=1.0	0.00001	1015.39	66	G=0/0, A=18/4
chr10:5966192	G/G	A	G	SNV	FBXO18	FBXO18:intronic:NM_032807.4	1	100		intronic	NM_03280	c.2552-82	p.?	-0.15					AAA domain,I	rs111226338,rs2274030,rs3867		YES			F-box only p	3'-5' DNA helicase activity,ATP binding,ATP-dependent DI				A=0, G=66	A=0.0, G=1.0	0.00001	1009.99	66	A=0/0, G=18/4
chr10:5978411	C/C	T	C	SNV	FBXO18	FBXO18:intronic:NM_032807.4	1	100		intronic	NM_03280	c.2983-8T	p.?	-0.04					AAA domain,I	rs646406	0.1338	YES	8.582E-1 (pos.5978411)		F-box only p	3'-5' DNA helicase activity,ATP binding,ATP-dependent DI				T=0, C=57	T=0.0, C=1.0	0.00001	849.566	57	T=0/0, C=38/1
chr10:60559047	C/C	G	C	SNV	BICC1	BICC1:intronic:NM_001080512.2	1	100		intronic	NM_00108	c.1725+35	p.?	-2.81					KH domain,S	rs2893789	0.2308	YES	8.774E-1 (pos.60559047)		Bicaudal C	RNA bindir	Cardiovascular Abnormalities;Cardiovascula			G=0, C=40	G=0.0, C=1.0	0.00001	644.285	40	G=0/0, C=23/1
chr10:60563062	C/C	G	C	SNV	BICC1	BICC1:intronic:NM_001080512.2	1	100		intronic	NM_00108	c.2181+60	p.?	-0.37					KH domain,S	rs4245600		YES			Bicaudal C	RNA bindir	Cardiovascular Abnormalities;Cardiovascula			G=0, C=60	G=0.0, C=1.0	0.00001	861.14	60	G=0/0, C=40/2
chr10:60573753	T/T	G	T	SNV	BICC1	BICC1:intronic:NM_001080512.2	1	100		intronic	NM_00108	c.2533+7G	p.?	0.09				0.07(COSM36	KH domain,S	rs9416746	0.278	YES	7.589E-1 (pos.60573753)	acute_myeloid	Bicaudal C	RNA bindir	Cardiovascular Abnormalities;Cardiovascula			G=0, T=128	G=0.0, T=1.0	0.00001	2084.69	128	G=0/0, T=57/7
chr6:90577706	TGACATCTTTG	TGACATCT	T	INDEL	CASP8AP2	CASP8AP2:exonic:NM_001137668.1	15	50	8	nonframeshiftDeletion	NM_00113	c.4698_47	p.Thr1567_Arg1571del	0.64, 2.58, 0.56, 0.28, -0.25, 1.5, 1.36, 0.35, 1.07, 0.67, -0.11, 0.63, 1.75, -1.3						rs200213073,rs2011	0.0838	YES	0.0 (pos.90577710),3.45	astrocytoma_(	Caspase 8-a	DNA binding,Fas signaling pathway,PML body,SUMO poly				TGACATCTT	TGACATCTTTGCCCAG	0.00001	145.218	30	TGACATCTTTG
chr10:6062051	A/A	T	A	SNV	IL2RA	IL2RA:intronic:NM_000417.2	1	100		intronic	NM_00041	c.584-147	p.?	0.04					Sushi repeat	rs9804284		YES			Interleukin-	MAPK casc	Alopecia;Alopecia /	Aldesleukin,Basiliximab,		T=0, A=65	T=0.0, A=1.0	0.00001	996.46	65	T=0/0, A=29/3
chr10:61014056	A/A	C	A	SNV	FAM13C	FAM13C:intronic:NM_198215.3	1	100		intronic	NM_19821	c.1332+52	p.?	0.91						rs1903252	0.1556	YES	9.077E-1 (pos.61014056)			protein binding				C=0, A=77	C=0.0, A=1.0	0.00001	1229.11	77	C=0/0, A=49/2
chr10:61022054	A/A	G	A	SNV	FAM13C	FAM13C:intronic:NM_198215.3	1	100		intronic	NM_19821	c.1236+14	p.?	-0.05						rs1459990		YES				protein binding				G=0, A=39	G=0.0, A=1.0	0.00001	623.978	39	G=0/0, A=24/1
chr10:61444100	A/G	A	G	SNV	SLC16A9	SLC16A9:intronic:NM_194298.2	1	57.58		intronic	NM_19429	c.-35-15T>	p.?	1.71					Major Facilit	rs74152113	0.0455	YES	1.331E-1 (pos.61444100)		Solute carri	integral component of plasma membrane,monocarboxy				A=14, G=19	A=0.4242, G=0.5758	0.00001	204.403	33	A=1/13, G=5/1
chr6:90577725	TGGA/T	TGGA	T	INDEL	CASP8AP2	CASP8AP2:exonic:NM_001137668.1	3	50	8	nonframeshiftDeletion	NM_00113	c.4717_47	p.Gly1573del	-0.08, 1.71, -0.36, 1.24						rs199515704,rs1996	0.0383	YES	0.0 (pos.90577725),3.466E-2 (pos.90577		Caspase 8-a	DNA binding,Fas signaling pathway,PML body,SUMO poly				TGGA=15, T	TGGA=0.5, T=0.5	0.00001	145.021	30	TGGA=6/9, T=1
chr3:122003832	C/C	G	C	SNV	CASR	CASR:exonic:NM_001178065.1	1	100	7	missense	NM_00117	c.3061G>C	p.Glu1021Gln	1.17	1	29	0		7 transmemb	rs1801726,rs386665	0.0405	YES	9.835E-1 (pos.122003832)		Calcium-ser	G-protein	Brain Diseases;Calc	Calcium C	Benign	G=0, C=23	G=0.0, C=1.0	0.00001	314.019	23	G=0/0, C=15/8
chr10:61802561	T/T	G	T	SNV	ANK3	ANK3:intronic:NM_020987.4	1	100		intronic	NM_02098	c.13066-4	p.?	0.69					Ankyrin repe	rs2393607,rs548779	0.2293	YES	0.0 (pos.61802557),8.173E-1:0.0:0.0 (po		Ankyrin-3, n	ER to Golgi	Amyotrophic Lateral Sclerosis;Bipolar Disord			G=0, T=35	G=0.0, T=1.0	0.00001	543.015	35	G=0/0, T=5/30
chr10:61802577	TC/TC	T	TC	INDEL	ANK3	ANK3:intronic:NM_020987.4	1	100		intronic	NM_02098	c.13066-6	p.?	2.31					Ankyrin repe	rs397956283,rs5785	0.0038				Ankyrin-3, n	ER to Golgi	Amyotrophic Lateral Sclerosis;Bipolar Disord			T=0, TC=34	T=0.0, TC=1.0	0.00001	502.311	34	T=0/0, TC=4/3
chr3:122004098	T/T	A	T	SNV	CASR	CASR:utr_3:NM_001178065.1	1	100	7	utr_3	NM_00117	c.3327A>T	p.?	-0.63					7 transmemb	rs4677948		YES			Calcium-ser	G-protein	Brain Diseases;Calc	Calcium C	Benign	A=0, T=33	A=0.0, T=1.0	0.00001	504.573	33	A=0/0, T=14/1
chr10:61926728	G/G	A	G	SNV	ANK3	ANK3:intronic:NM_020987.4	1	100		intronic	NM_02098	c.2479-74	p.?	2.93					Ankyrin repe	rs11814723		YES			Ankyrin-3, n	ER to Golgi	Amyotrophic Lateral Sclerosis;Bipolar Disord			A=0, G=75	A=0.0, G=1.0	0.00001	1189.82	75	A=0/0, G=43/3
chr10:62540063	A/A	T	A	SNV	CDK1	CDK1:intronic:NM_001786.4	1	100		intronic	NM_00178	c.37+103T	p.?	-0.39					Protein kinas	rs2448344		YES			Cyclin-depe	ATP bindin	Autoimmune Disea	AT7519,Alsterpaullone,A		T=0, A=31	T=0.0, A=1.0	0.00001	465.819	31	T=0/0, A=9/22
chr10:62551889	GT/GT	AA	GT	MNV	CDK1	CDK1:intronic:NM_001786.4	2	100		intronic	NM_00178	c.654-17A	p.?	0.77, -0.42					Protein kinas	rs2456777,rs245677	0.2545	YES	8.513E-1 (pos.62551890),8.514E-1 (pos.6		Cyclin-depe	ATP bindin	Autoimmune Disea	AT7519,Alsterpaullone,A		AA=0, GT=2	AA=0.0, GT=1.0	0.00001	337.075	24	AA=0/0, GT=1
chr10:6258015	C/C	T	C	SNV	PFKFB3	PFKFB3:intronic:NM_004566.3	1	100		intronic	NM_00456	c.300-73T	p.?	-1.72					6-phosphofru	rs111691148,rs2148293		YES			6-phosphof	6-phosphc	Arsenic Poisoning;Cardiovascular Diseases;H			T=0, C=22	T=0.0, C=1.0	0.00001	298.824	22	T=0/0, C=12/1
chr10:6258052	A/A	G	A	SNV	PFKFB3	PFKFB3:intronic:NM_004566.3	1	100		intronic	NM_00456	c.300-36G	p.?	-2.88					6-phosphofru	rs2148294,rs769357	0.0483	YES	0.0 (pos.6258040),7.893E-1 (pos.625805		6-phosphof	6-phosphc	Arsenic Poisoning;Cardiovascular Diseases;H			G=0, A=29	G=0.0, A=1.0	0.00001	427.466	29	G=0/0, A=12/1
chr10:6258796	C/C	T	C	SNV	PFKFB3	PFKFB3:intronic:NM_004566.3	1	100		intronic	NM_00456	c.441+53T	p.?	0.14					6-phosphofru	rs2182412,rs768663	9.00E-04	YES	2.315E-4 (pos.6258785)		6-phosphof	6-phosphc	Arsenic Poisoning;Cardiovascular Diseases;H			T=0, C=21	T=0.0, C=1.0	0.00001	281.232	21	T=0/0, C=9/12
chr10:63699895	A/C	A	C	SNV	ARID5B	ARID5B:intronic:NM_032199.2	1	52.27		intronic	NM_03219	c.277-47A	p.?	-0.56					ARID/BRIGHT	rs7073837	0.4022	YES	6.196E-1 (pos.63699895)		AT-rich inte	DNA bindi	Adenocarcinoma;Arthritis;Arthritis, Rheuma			A=21, C=23	A=0.4773, C=0.5227	0.00001	260.06	44	A=10/11, C=1
chr11:65788072	T/T	C	T	SNV	CATSPER1	CATSPER1:exonic:NM_053054.3	1	100	7	missense	NM_05305	c.1954G>A	p.Val652Ile	1.35	0.77	29	0.147		Ion transport	rs3814747	6.00E-04	YES	8.919E-1 (pos.65788072)		Cation char	CatSper cc	Asthenozoospermia	Calcium C	Benign	C=0, T=37	C=0.0, T=1.0	0.00001	583.706	37	C=0/0, T=18/1
chr10:64415413	A/A	T	A	SNV	ZNF365	ZNF365:intronic:NM_199451.2	1	100		intronic	NM_19945	c.1130-74	p.?	-0.12						rs7094595	0.3723	YES	5.989E-1 (pos.64415413)		Zinc finger p	centroson	Autism Spectrum Disorder;Breast Diseases;B			T=0, A=24	T=0.0, A=1.0	0.00001	334.788	24	T=0/0, A=8/16
chr14:92088016	A/A	G	A	SNV	CATSPERB	CATSPERB:exonic:NM_024764.3	1	100	19	synonymous	NM_02476	c.2196C>T	p.(=)	-0.35					Cation chann	rs1296082	0.2524	YES	9.743E-1 (pos.92088016)		Cation char	CatSper complex,cilium,multicellular organism developi				G=0, A=37	G=0.0, A=1.0	0.00001	583.702	37	G=0/0, A=21/1
chr10:64953311	A/A	G	A	SNV	JMJD1C	JMJD1C:intronic:NM_032776.2	1	100		intronic	NM_03277	c.5735-79	p.?	-2.7					JmjC domain	rs4747098,rs765980997		YES			Jumonji dor	blood coa	Adenocarcinoma;Arthritis;Arthritis, Juvenile			G=0, A=47	G=0.0, A=1.0	0.00001	789.321	47	G=0/0, A=19/2
chr11:6340525	T/T	A	T	SNV	CAVIN3	CAVIN3:exonic:NM_145040.2	1	100	2	synonymous	NM_14504	c.654T>A	p.(=)	-0.14				0.01(COSM11	PTRF/SDPR fa	rs12570,rs78084990	0.3133	YES	7.626E-1:8.268E-4:0.0 (pc	adenocarcinoma,carcinom		caveola,circadian regulation of gene expression,cortical				A=0, T=64	A=0.0, T=1.0	0.00001	977.633	64	A=0/0, T=16/4
chr10:65225244	CG/CG	GA	CG	MNV	JMJD1C,JMJD1C-AS1	JMJD1C-AS1:exonic_nc:NR_027182.1, JMJD1C:intronic:NM_(	2	100		intronic	NM_03277	c.168+11T	p.?	-0.24, 1.45					JmjC domain	rs1061259,rs107617	0.437	YES	1.0 (pos.65225245),6.982E-1 (pos.65225		Jumonji dor	blood coa	Adenocarcinoma;Arthritis;Arth		Benign	GA=0, CG=4	GA=0.0, CG=1.0	0.00001	789.324	47	GA=0/0, CG=3
chr21:37507745	T/T	C	T	SNV	CBR3,CBR3-AS1	CBR3-AS1:intronic_nc:NR_038892.1, CBR3:exonic:NM_0012	1	100	1	synonymous	NR_03889	c.255C>T	p.(=)	-0.02				0.19(COSM62	Enoyl-(Acyl ca	rs881711	0.1197	YES	8.556E-1:0.0 (pos.37507	neoplasm	Carbonyl re	3-keto ste	Abnormalities, Mul	Deutetrabenazine,Doxo		C=0, T=63	C=0.0, T=1.0	0.00001	958.917	63	C=0/0, T=27/3
chr10:69648569	T/A	T	A	SNV	SIRT1	SIRT1:intronic:NM_012238.4	1	57.14		intronic	NM_01223	c.548-71T	p.?	1.3					Sir2 family	rs2236318		YES			Sirtuin, S, c	DNA repai	Acute Kidney Injury	SRT501		T=9, A=12	T=0.4286, A=0.5714	0.00001	109.06	21	T=6/3, A=6/6
chr21:44480616	G/A	G	A	SNV	CBS,CBSL	CBS:exonic:NM_001178008.2, CBSL:exonic:NM_001354007	1	50	13	synonymous	NM_00117	c.1080C>T	p.(=), p.(=)	-2.62				0.05(COSM37	CBS domain,F	rs1801181	0.3592	YES	6.207E-1 (pos.44480616)	adenocarcino	Cystathioni	DNA prote	Amino Acid Disorde	Ademetio	Benign	G=15, A=15	G=0.5, A=0.5	0.00001	144.63	30	G=11/4, A=11/
chr9:154795	C/C	T	C	SNV	CBWD1	CBWD1:exonic:NM_018491.3	1	100	8	synonymous	NM_01849	c.576A>G	p.(=)	1.11				0.01(COSM44	CobW/HypB/	rs2785333	0.2437		7.08E-1 (pos.154795)	basal_cell_car	Cobalamin	ATP binding				T=0, C=26	T=0.0, C=1.0	0.00001	371.413	26	T=0/0, C=14/1
chr10:70652197	T/T	C	T	SNV	STOX1	STOX1:intronic:NM_001130161.2	1	100		intronic	NM_00113	c.2823-14	p.?	-0.47					Winged helix	rs10740310		YES			Storkhead b	RNA polym	Female Urogenital Diseases and Pregnancy C			C=0, T=29	C=0.0, T=1.0	0.00001	427.119	29	C=0/0, T=10/1
chr10:70696908	T/T	G	T	SNV	DDX50	DDX50:intronic:NM_024045.1	1	100		intronic	NM_02404	c.1755+57	p.?	-0.71					DEAD/DEAH b	rs1539166		YES			DEAD/H box	ATP binding,ATP-dependent RNA helicase activity,RNA bi				G=0, T=41	G=0.0, T=1.0	0.00001	664.7	41	G=0/0, T=28/1

chr10:71010026	C/C	T	C	SNV	HKDC1	HKDC1:intronic:NM 025130.3	1	100		intronic	NM 02513	c.1571-20	p.?	-1.21					Hexokinase	rs4745978	1.00E-04		9.966E-1 (pos.71010026)		Hexokinase	ATP binding,carbohydrate phosphorylation,cellular gluc				T=0, C=33	T=0.0, C=1.0	0.00001	504.598	33	T=0/0, C=11/2
chr10:71392495	A/A	T	A	SNV	FAM241B	FAM241B:intronic:NM 145306.2	1	100		intronic	NM 14530	c.97-51T>A	p.?	-0.11					Domain of un	rs2616089	0.3388	YES	8.897E-1 (pos.71392495)			integral component of membrane,protein binding				T=0, A=35	T=0.0, A=1.0	0.00001	543.903	35	T=0/0, A=24/1
chr10:71392501	C/C	G	C	SNV	FAM241B	FAM241B:intronic:NM 145306.2	1	100		intronic	NM 14530	c.97-45G>	p.?	-0.39					Domain of un	rs2616088	0.339	YES	8.897E-1;0.0 (pos.71392501)			integral component of membrane,protein binding				G=0, C=35	G=0.0, C=1.0	0.00001	543.903	35	G=0/0, C=24/1
chr10:70502288	A/A	G	A	SNV	CCAR1	CCAR1:exonic:NM 018237.3	1	100	6	synonymous	NM 01823	c.480G>A	p.(=)	-3.04				0.04(COSM41	DBC1,S1-like,	rs1300253	0.1403	YES	7.39E-1 (pos.70502288)	neoplasm	Cell divisior	RNA binding,apoptotic process,cell cycle,core promoter				G=0, A=48	G=0.0, A=1.0	0.00001	810.435	48	G=0/0, A=30/1
chr8:22471824	A/A	G	A	SNV	CCAR2	CCAR2:exonic:NM 021174.5	1	100	9	synonymous	NM 02117	c.924G>A	p.(=)	-0.3					DBC1,S1-like	rs3736147	0.3235	YES	2.558E-1;1.165E-4 (pos.22471824)		Cell divisior	DBIRD complex,RNA binding,RNA polymerase II core bind				G=0, A=25	G=0.0, A=1.0	0.00001	352.174	25	G=0/0, A=21/4
chr19:56160399	C/C	T	C	SNV	CCDC106	CCDC106:utr 5:NM 013301.2	1	100	2	utr 5	NM 01330	c.-14T>C	p.?	-0.1					Coiled-coil dc	rs7246799	0.025	YES	9.951E-1 (pos.56160399)		Coiled-coil (	cytosol,nucleus,protein binding				T=0, C=235	T=0.0, C=1.0	0.00001	3946.68	235	T=0/0, C=175/
chr10:71868763	G/A	G	A	SNV	H2AFY2	H2AFY2:intronic:NM 018649.2	1	52.38		intronic	NM 01864	c.779-26G	p.?	-0.62					C-terminus o'	rs2271698,rs773388:	0.2952	YES	0.0 (pos.71868762),3.768E-1 (pos.71868		H2A histone	Barr body,DNA binding,ESC/E(Z) complex,RNA polymerase				G=10, A=11	G=0.4762, A=0.5238	0.00001	95.2997	21	G=6/4, A=10/1
chr10:71969062	A/A	G	A	SNV	PPA1	PPA1:intronic:NM 021129.3	1	100		intronic	NM 02112	c.640-16C:	p.?	0.55					Inorganic pyr	rs9415006	0.1353	YES	9.991E-1 (pos.71969062)			cytoplasm,cytosol,diphosphate metabolic process,extra				G=0, A=49	G=0.0, A=1.0	0.00001	831.633	49	G=0/0, A=20/2
chr10:7230524	G/T	G	T	SNV	SFMBT2	SFMBT2:intronic:NM 001018039.1	1	50		intronic	NM 00101	c.1808+62	p.?	0.17					SAM domain	rs2462712		YES			SCM-like pr	aggresome,histone binding,intracellular membrane-bou				G=14, T=14	G=0.5, T=0.5	0.00001	131.933	28	G=12/2, T=12/
chr19:56162776	T/T	C	T	SNV	CCDC106	CCDC106:exonic:NM 013301.2	1	100	5	synonymous	NM 01330	c.441C>T	p.(=)	-0.06				0.01(COSM13	Coiled-coil dc	rs2287791,rs751609:	0.0807	YES	2.496E-4 (pos.56162762)	adenocarcinor	Coiled-coil (	cytosol,nucleus,protein binding				C=0, T=27	C=0.0, T=1.0	0.00001	389.945	27	C=0/0, T=11/1
chr19:48800569	G/A	G	A	SNV	CCDC114	CCDC114:exonic:NM 144577.3	1	46.88	14	synonymous	NM 14457	c.1677C>T	p.(=)	-2.3						rs147072026	0.0072		1.161E-4 (pos.48800569)		Coiled-coil (	axoneme,	Bronchial Diseases:Bronchiect		Benign/Likely	G=34, A=30	G=0.5313, A=0.4688	0.00001	288.102	64	G=18/16, A=1
chr10:73104056	G/G	A	G	SNV	SLC29A3	SLC29A3:intronic:NM 018344.5	1	100		intronic	NM 01834	c.383+8A>	p.?	-0.11					Nucleoside tr	rs2243540	0.1049	YES	6.91E-1 (pos.73104056)		Solute carri	Golgi appa	Autoimmune Diseases:Bone D		Benign/Likely	A=0, G=23	A=0.0, G=1.0	0.00001	306.357	23	A=0/0, G=14/9
chr7:31611758	T/T	G	T	SNV	CCDC129	CCDC129:exonic:NM 001257967.2	1	100	7	synonymous	NM 00125	c.381G>T	p.(=)	1.45					Ki-ras-induce	rs67930674	0.4184	YES	3.371E-1 (pos.31611758)			receptor binding				G=0, T=66	G=0.0, T=1.0	0.00001	1015.4	66	G=0/0, T=16/5
chr7:31682453	T/T	C	T	SNV	CCDC129	CCDC129:exonic:NM 001257967.2	1	100	12	missense	NM 00125	c.1499C>T	p.Ala500Val	1.28	0.96	64	0.156	0.02(COSM55	Ki-ras-induce	rs4141001	0.2237	YES	8.368E-1 (pos.31682453)	adenocarcinoma,malignan		receptor binding				C=0, T=81	C=0.0, T=1.0	0.00001	1309.41	81	C=0/0, T=50/3
chr7:31683410	A/A	G	A	SNV	CCDC129	CCDC129:exonic:NM 001257967.2	1	100	12	missense	NM 00125	c.2456G>A	p.Cys819Tyr	1.76	0.04	194	0.016	0.67(COSM37	Ki-ras-induce	rs10247620	0.2233	YES	8.378E-1 (pos.31683410)	adenocarcinoma		receptor binding				G=0, A=22	G=0.0, A=1.0	0.00001	298.891	22	G=0/0, A=7/15
chr2:109429146	A/A	T	A	SNV	CCDC138	CCDC138:exonic:NM 144978.2	1	100	8	synonymous	NM 14497	c.915T>A	p.(=)	0.15				0.61(COSM4001127)		rs975597	0.2403	YES	9.037E-1 (pos.10942914(	acute_myeloid_leukaemia,neoplasm						T=0, A=21	T=0.0, A=1.0	0.00001	281.232	21	T=0/0, A=4/17
chr10:73570334	G/A	G	A	SNV	CDH23	CDH23:intronic:NM 022124.5	1	56		intronic	NM 02212	c.9077+8G	p.?	0.58					Cadherin dor	rs11818398	0.1799	YES	2.337E-1;0.0 (pos.73570334)		Cadherin-2:	calcium io	Abnormalities, Multiple:Blindr		Benign/Likely	G=22, A=28	G=0.44, A=0.56	0.00001	341.078	50	G=12/10, A=1
chr10:73578882	T/C	T	C	SNV	PSAP	PSAP:intronic:NM 002778.3	1	50		intronic	NM 00277	c.1351-14	p.?	-0.56					Saposin A-typ	rs4747203	0.273	YES	6.241E-1 (pos.73578882)		Mitochondr	G-protein	Asthma:Autoimmu	Di-Stearoy	Benign/Likely	T=23, C=23	T=0.5, C=0.5	0.00001	260.758	46	T=9/14, C=14/
chr10:73823019	A/G	A	G	SNV	SPOCK2	SPOCK2:intronic:NM 001244950.1	1	53.49		intronic	NM 00124	c.992-99T:	p.?	-0.95					Kazal-type se	rs1668172		YES			SPARC/oste	calcium ion binding,cellular response to leukemia inhibi				A=40, G=46	A=0.4651, G=0.5349	0.00001	514.082	86	A=16/24, G=1
chr3:123633612	A/A	G	A	SNV	CCDC14	CCDC14:utr 3:NM 022757.4	1	100	12	utr 3	NM 02275	c.2753C>T	p.?	-0.99					Coiled-coil dc	rs11551801	0.2644	YES	1.588E-1 (pos.123633612)		Coiled-coil (	centriolar satellite,centrosome,protein binding,protein				G=0, A=31	G=0.0, A=1.0	0.00001	465.817	31	G=0/0, A=14/1
chr10:75863750	T/T	C	T	SNV	VCL	VCL:intronic:NM 014000.2	1	100		intronic	NM 01400	c.2131+64	p.?	0.5					Vinculin fami	rs2270552		YES			Vinculin	actin bind	Acute Coronary Syndrome:Aortic Stenosis, Su			C=0, T=53	C=0.0, T=1.0	0.00001	782.027	53	C=0/0, T=18/3
chr10:75873892	T/T	C	T	SNV	VCL	VCL:intronic:NM 014000.2	1	100		intronic	NM 01400	c.2950-50(	p.?	-1.32					Vinculin fami	rs10824072	0.4047	YES	2.629E-1 (pos.75873892)		Vinculin	actin bind	Acute Coronary Syndrome:Aortic Stenosis, Su			C=0, T=95	C=0.0, T=1.0	0.00001	1597.04	95	C=0/0, T=51/4
chr10:75874192	C/C	T	C	SNV	VCL	VCL:intronic:NM 014000.2	1	100		intronic	NM 01400	c.3153+47	p.?	-3.15					Vinculin fami	rs138018528,rs2270550,rs6091		YES	0.0 (pos.75874174),0.0 (pos.75874176),(		Vinculin	actin bind	Acute Coronary Syndrome:Aortic Stenosis, Su			T=0, C=21	T=0.0, C=1.0	0.00001	265.749	21	T=0/0, C=13/8
chr10:7622047	G/G	A	G	SNV	ITIH5	ITIH5:intronic:NM 030569.6	1	100		intronic	NM 03056	c.1109-20	p.?	-0.25					Inter-alpha-t	rs12256183	0.1045	YES	9.995E-1;0.0 (pos.7622047)		Inter-alpha	extracellular region,hyaluronan metabolic process,nega				A=0, G=23	A=0.0, G=1.0	0.00001	316.463	23	A=0/0, G=7/16
chr10:76429870	C/C	T	C	SNV	ADK	ADK:intronic:NM 006721.3	1	100		intronic	NM 00672	c.878-71T:	p.?	-0.89					pfkB family ca	rs10740443		YES			Adenosine I	AMP salva	Brain Diseases:Car	5-4-(DIMETHYLAMINO)PH		T=0, C=54	T=0.0, C=1.0	0.00001	798.141	54	T=0/0, C=24/3
chr10:7659273	TAACTT/T	TAACTT	T	INDEL	ITIH5	ITIH5:intronic:NM 030569.6	5	52.38		intronic	NM 03056	c.653-28A	p.?	-0.09, 0.47, 0.33, -1.57, 0.7, 0.74					Inter-alpha-t	rs150273439,rs559412977,rs58		YES	0.0 (pos.7659268),2.727E-1 (pos.765927		Inter-alpha	extracellular region,hyaluronan metabolic process,nega				TAACTT=20	TAACTT=0.4762, T=0.	0.00001	240.345	42	TAACTT=11/9,
chr3:123634046	A/A	G	A	SNV	CCDC14	CCDC14:exonic:NM 022757.4	1	100	12	synonymous	NM 02275	c.2319C>T	p.(=)	0.69				0(COSM3759(	Coiled-coil dc	rs2700373	0.2514	YES	9.139E-1 (pos.12363404(	adenocarcinor	Coiled-coil (	centriolar satellite,centrosome,protein binding,protein				G=0, A=52	G=0.0, A=1.0	0.00001	768.599	52	G=0/0, A=20/3
chr2:223161889	G/G	A	G	SNV	CCDC140,PAX3	PAX3:exonic:NM 181459.3, CCDC140:upstream:NM 15303	1	100	2	synonymous	NM 18145	c.129T>C, (	p.(=), p.?	-2.34					'Paired box' d	rs12623857	0.1526	YES	1.0 (pos.223161889)		Paired box I	DNA bindi	Abnormalities, Multiple;Carcir		Benign	A=0, G=25	A=0.0, G=1.0	0.00001	352.984	25	A=0/0, G=16/9
chr10:7769604	G/G	A	G	SNV	ITIH2	ITIH2:intronic:NM 002216.2	1	99.26		intronic	NM 00221	c.1154-62	p.?	-0.15					Inter-alpha-t	rs2277226		YES			Inter-alpha	blood microparticle,cellular protein metabolic process,(				A=1, G=135	A=0.0074, G=0.9926	0.00001	2219.49	136	A=1/0, G=98/3
chr10:7773976	G/G	A	G	SNV	ITIH2	ITIH2:intronic:NM 002216.2	1	100		intronic	NM 00221	c.1647+17	p.?	-0.05					Inter-alpha-t	rs386740613,rs7098:	0.2284	YES	0.0:0.0 (pos.7773960),1.158E-4 (pos.777		Inter-alpha	blood microparticle,cellular protein metabolic process,(				A=0, G=30	A=0.0, G=1.0	0.00001	446.639	30	A=0/0, G=20/1
chr17:20799119	G/G	A	G	SNV	CCDC144NL,CCDC144NL-AS1	CCDC144NL-AS1:intronic nc:NR 104185.1, CCDC144NL:exo	1	100	1	missense	NM 00100	c.215T>C	p.Leu72Pro	-1.28	0.67	98	0	0(COSM3755335)		rs2279263		YES	4.784E-1 (pos.20799119)	adenocarcinoma,carcinoma,neoplasm,squamous_cell_carcinoma						A=0, G=24	A=0.0, G=1.0	0.00001	334.792	24	A=0/0, G=3/21
chr10:7866268	G/G	C	G	SNV	TAF3	TAF3:intronic:NM 031923.3	1	100		intronic	NM 03192	c.167-13C:	p.?	0.11					Bromodomai	rs199762284,rs3867(	0.1866,5.(	YES	2.945E-2 (pos.7866266),9.719E-1 (pos.78		TAF3 RNA pc	maintenance of protein location in nucleus,metal ion bi				C=0, G=62	C=0.0, G=1.0	0.00001	940.319	62	C=0/0, G=38/2
chr10:7866576	A/T	A	T	SNV	TAF3	TAF3:intronic:NM 031923.3	1	50		intronic	NM 03192	c.409+53A	p.?	0.12					Bromodomai	rs1244472		YES			TAF3 RNA pc	maintenance of protein location in nucleus,metal ion bi				A=18, T=18	A=0.5, T=0.5	0.00001	187.207	36	A=5/13, T=12/
chr10:79565655	A/A	G	A	SNV	DLG5	DLG5:intronic:NM 004747.3	1	100		intronic	NM 00474	c.4968-36(	p.?	0.57					Guanylate kir	rs2289311	0.3462	YES	1.89E-1 (pos.79565655)		Discs large,	apical pro	Digestive System Diseases:Gastroenteritis;G			G=0, A=37	G=0.0, A=1.0	0.00001	583.706	37	G=0/0, A=18/1
chr10:79566407	C/C	T	C	SNV	DLG5	DLG5:intronic:NM 004747.3	1	100		intronic	NM 00474	c.4967+10	p.?	-0.29					Guanylate kir	rs10762765		YES			Discs large,	apical pro	Digestive System Diseases:Gastroenteritis;G			T=0, C=29	T=0.0, C=1.0	0.00001	424.573	29	T=0/0, C=11/1
chr17:20798992	C/T	C	T	SNV	CCDC144NL,CCDC144NL-AS1	CCDC144NL-AS1:intronic nc:NR 104185.1, CCDC144NL:exo	1	53.33	1	synonymous	NM 00100	c.342G>A	p.(=)	-0.85				0.01(COSM3755334)		rs762029355,rs8073(	0.1415	YES	0.0:0.0:0.0 (pos.2079899	acute_myeloid_leukaemia,adenocarcinoma,hepatocellular_carcinoma						C=14, T=16	C=0.4667, T=0.5333	0.00001	159.925	30	C=4/10, T=5/1
chr10:79590685	T/T	C	T	SNV	DLG5	DLG5:intronic:NM 004747.3	1	100		intronic	NM 00474	c.1749-54(	p.?	-1.29					Guanylate kir	rs10824579,rs539418253		YES			Discs large,	apical pro	Digestive System Diseases:Gastroenteritis;G			C=0, T=97	C=0.0, T=1.0	0.00001	1647.29	97	C=0/0, T=21/7
chr7:76916819	A/A	G	A	SNV	CCDC146	CCDC146:exonic:NM 020879.2	1	100	17	synonymous	NM 02087	c.2340G>A	p.(=)	0.33				0.77(COSM5619418)		rs3763402	0.1858	YES	1.124E-1 (pos.76916819)	ductal_carcinoma		centriole,protein binding				G=0, A=24	G=0.0, A=1.0	0.00001	334.782	24	G=0/0, A=15/9
chr10:79628875	A/G	A	G	SNV	DLG5	DLG5:intronic:NM 004747.3	1	95.45		intronic	NM 00474	c.373+12T	p.?	-0.25					Guanylate kir	rs1248655	0.3307	YES	7.476E-1 (pos.79628875)		Discs large,	apical pro	Digestive System Diseases:Gastroenteritis;G			A=1, G=21	A=0.0455, G=0.9545	0.00001	271.191	22	A=1/0, G=18/3
chr16:1484473	G/G	A	G	SNV	CCDC154	CCDC154:exonic:NM 001143980.1	1	100	17	missense	NM 00114	c.1940T>C	p.Leu647Pro	-1.71	0.33	98	0	0(COSM4128'	Coiled-coil dc	rs7192331	0.0695	YES	9.984E-1 (pos.1484473)	neoplasm		early endosome				A=0, G=86	A=0.0, G=1.0	0.00001	1410.93	86	A=0/0, G=33/5
chr10:81319354	T/T	C	T	SNV	SFTPA2	SFTPA2:intronic:NM 001098668.3	1	100		intronic	NM 00109	c.-22-92G>	p.?	-2.04					Lectin C-type	rs4268452		YES			Surfactant,	carbohydr	Idiopathic Interstitial Pneumonias:Idiopathi			C=0, T=27	C=0.0, T=1.0	0.00001	389.522	27	C=0/0, T=15/1
chr10:82095968	G/G	T	G	SNV	DYDC1	DYDC1:intronic:NM 001269053.1	1	100		intronic	NM 00126	c.505-27A	p.?	-0.2					Dpy-30 motif	rs1340383	0.3485	YES	9.779E-1 (pos.82095968)		DPY30 dom	Set1C/COMPASS complex,chromatin silencing at telomei				T=0, G=27	T=0.0, G=1.0	0.00001	389.988	27	T=0/0, G=11/1
chr10:86004736	A/G	A	G	SNV	RGR	RGR:upstream:NM 002921.3	1	98.41		intronic	NM 00292	c.-111A>G	p.?	1.93						rs2279228		YES			Ral guanine	G-protein	Congenital, Hereditary, and Neonatal Diseas			A=1, G=62	A=0.0159, G=0.9841	0.00001	925.366	63	A=1/0, G=24/3
chr19:49899076	A/A	G	A	SNV	CCDC155	CCDC155:exonic:NM 144688.4	1	100	5	missense	NM 14468	c.386G>A	p.Arg129Gln	-0.13	1	43	0	0.04(COSM50	Coiled-coil re	rs10405154	0.4517	YES	5.847E-1 (pos.49899076)	ductal_carcinoma		LINC complex,actin filament organization,chromosome I				G=0, A=35	G=0.0, A=1.0	0.00001	543.902	35	G=0/0, A=13/2
chr19:49901318	C/C	T	C	SNV	CCDC155	CCDC155:exonic:NM 144688.4	1	100	7	synonymous	NM 14468	c.547T>C	p.(=)	0.3					Coiled-coil re	rs10421748	0.4517	YES	5.161E-1 (pos.49901318)			LINC complex,actin filament organization,chromosome I				T=0, C=21	T=0.0, C=1.0	0.00001	281.234	21	T=0/0, C=5/16
chr10:88220866	T/T	C	T	SNV	WAPL	WAPL:intronic:NM 015045.3	1	100		intronic	NM 01504	c.2482+67	p.?	-0.84					Wings apart-l	rs7922658		YES			Wings apar	cell division,chromatin,chromosome,chromosome, cent				C=0, T=72	C=0.0, T=1.0	0.00001	1130.95	72	C=0/0, T=47/2
chr22:30771554	G/G	T	G	SNV	CCDC157,KIAA1656	KIAA1656:exonic nc:NR 046312.1, CCDC157:exonic:NM 00	1	100	10	missense	NM 00101	c.1759T>G	p.Ser587Ala	4.14	1	99	0			rs2015035	0.0487	YES	1.0 (pos.30771554)							T=0, G=23	T=0.0, G=1.0	0.00001	316.747	23	T=0/0, G=16/7
chr13:103388220	GC/GT	AT	GT	SNV	CCDC168	CCDC168:exonic:NM 001146197.2	2	97.8	4	missense	NM 00114	c.14827T>	p.Ser4943Pro, p.Ser494	-0.76, 0.44		74.0, 74.0	0.002, 0.002		Coiled-coil dc	rs9300758	6.00E-04		1.0 (pos.103388220)							AT=0, GC=2	AT=0.0, GC=0.022, G	0.00001	1502.69	91	AT=0/0, GC=2/
chr13:103385015	G/G	A	G	SNV	CCDC168	CCDC168:exonic:NM 001146197.2	1	100	4	missense	NM 00114	c.18032T>	p.Leu6011Pro	-0.05		98	0		Coiled-coil dc	rs6491707	0		1.0 (pos.103385015)							A=0, G=29	A=0.0, G=1.0	0.00001	427.603	29	A=0/0, G=19/1
chr13:103385534	T/T	C	T	SNV	CCDC168	CCDC168:exonic:NM 001146197.2	1	100	4	missense	NM 00114	c.17513G>	p.Arg5838Gln	-1.31		43	0	0(COSM3753!	Coiled-coil dc	rs17592438	0.1587	YES	2.244E-2 (pos.10338553(	adenocarcinoma						C=0, T=63	C=0.0, T=1.0	0.00001	958.066	63	C=0/0, T=35/2
chr10:88917974	G/G	A	G	SNV	FAM35A	FAM35A:intronic:NM 019054.2	1	100		intronic	NM 01905	c.1633+62	p.?	1.09					Protein famil	rs4933430		YES								A=0, G=20	A=0.0, G=1.0	0.00001	263.158	20	A=0/0, G=14/6
chr10:89419873	C/G	C	G	SNV	PAPSS2	PAPSS2:intronic:NM 004670.3	1	50		intronic	NM 00467	c.27+108C	p.?	-3					ATP-sulfuryla	rs2255682		YES			3'-phospho:	3'-phosph	Bone Diseases:Bone Diseases, Development			C=11, G=11	C=0.5, G=0.5	0.00001	94.5937	22	C=4/7, G=1/10
chr10:89419910	A/G	A	G	SNV	PAPSS2	PAPSS2:intronic:NM 004670.3	1	95.65		intronic	NM 00467	c.27+145A	p.?	-0.06					ATP-sulfuryla	rs2255683		YES			3'-phospho:	3'-phosph	Bone Diseases:Bone Diseases, Development			A=1, G=22	A=0.0435, G=0.9565	0.00001	288.807	23	A=1/0, G=5/17
chr10:90122512	T/T	C	T	SNV	RNLS	RNLS:intronic:NM 001031709.2	1	100		intronic	NM 00103	c.527-30G	p.?	0.27					Flavin contai	rs3740279	0.1792	YES	3.534E-1 (pos.90122512)		Renalase	NAD biosy	Female Urogenital Diseases:Female Urogeni			C=0, T=24	C=0.0, T=1.0	0.00001	334.357	24	C=0/0, T=9/15
chr13:103386116	G/G	C	G	SNV	CCDC168	CCDC168:exonic:NM 001146197.2	1	100	4	missense	NM 00114	c.16931G>	p.Arg5644Thr	0.13		71	0.05		Coiled-coil dc	rs7982465	3.00E-04	YES	1.0 (pos.103386116)							C=0, G=45	C=0.0, G=1.0	0.00001	747.384	45	C=0/0, G=18/2
chr13:103394003	A/A	G	A	SNV	CCDC168	CCDC168:exonic:NM 001146197.2	1	100	4	missense	NM 00114	c.9044C>T	p.Thr3015Ile	0.03		89	0.046		Coiled-coil dc	rs1449707	0.2003	YES	6.278E-1 (pos.103394003)							G=0, A=75	G=0.0, A=1.0	0.00001	1162.57	75	G=0/0, A=56/1
chr13:103394613	G/G	T	G	SNV	CCDC168	CCDC168:exonic:NM 001146197.2	1	100	4	missense	NM 00114	c.8434A>C	p.Thr2812Pro	0.11		38	0		Coiled-coil dc	rs9585986	0.2	YES	6.318E-1 (pos.103394613)							T=0, G=49	T=0.0, G=1.0	0.00001	829.565	49	T=0/0, G=25/2
chr13:103394716	C/C	T	C	SNV	CCDC168	CCDC168:exonic:NM 001146197.2	1	100	4	synonymous	NM 00114	c.8331A>G	p.(=)	0.05					Coiled-coil dc	rs9585987	0.2009	YES	6.343E-1 (pos.103394716)							T=0, C=50	T=0.0, C=1.0	0.00001	852.748	50	T=0/0, C=24/2
chr13:103396716	G/G	C	G	SNV	CCDC168	CCDC168:exonic:NM 001146197.2	1	100	4	missense	NM 00114	c.6331G>C	p.Glu2111Gln	-0.31		29	0.043		Coiled-coil dc	rs6491708	0.2	YES	6.306E-1 (pos.103396716)							C=0, G=51	C=0.0, G=1.0	0.00001	754.584	51	C=0/0, G=19/3
chr13:103397583	G/G	C	G	SNV	CCDC168	CCDC168:exonic:NM 001146197.2	1	100	4	missense	NM 00114	c.5464G>C	p.Ala1822Pro	-0.46		27	0.184		Coiled-coil dc	rs9582626	0.2005	YES	6.306E-1 (pos.103397583)							C=0, G=21	C=0.0, G=1.0	0.00001	281.234	21	C=0/0, G=9/12
chr13:103400083	A/A	G	A	SNV	CCDC168	CCDC168:exonic:NM 001146197.2	1	100	4	synonymous	NM 00114	c.2964C>T	p.(=)	-0.27					Coiled-coil dc	rs1026158	0.3422	YES	5.193E-1 (pos.103400083)							G=0, A=34	G=0.0, A=1.0	0.00001	529.622	34	G=0/0, A=13/2
chr10:91528370	C/T	C	T	SNV	KIF20B	KIF20B:intronic:NM 016195.3	1	45.45		intronic	NM 01619	c.4972-12!	p.?	-0.48					Kinesin moto	rs7912458		YES				ATP binding,ATP-dependent microtubule motor activity,				C=12, T=10	C=0.5455, T=0.4545	0.00001	81.7625	22	C=12/0, T=7/3
chr10:91528384	C/T	C	T	SNV	KIF20B	KIF20B:intronic:NM 016195.3	1	45.45		intronic	NM 01619	c.4972-11:	p.?	-0.04					Kinesin moto	rs7912464		YES				ATP binding,ATP-dependent microtubule motor activity,				C=12, T=10	C=0.5455, T=0.4545	0.00001	81.6797	22	C=12/0, T=7/3
chr10:93716899	C/C	G	C	SNV	BTAF1	BTAF1:intronic:NM 003972.2	1	100		intronic	NM 00397	c.832-83G	p.?	-3.64					Domain of un	rs7894570		YES			BTAF1 RNA (	ATP binding,DNA binding,DNA binding transcription facto				G=0, C=49	G=0.0, C=1.0	0.00001	831.615	49	G=0/0, C=38/1
chr13:103402522	T/T	C	T	SNV	CCDC168	CCDC168:exonic:NM 001146197.2	1	100	4	synonymous	NM 00114	c.525G>A	p.(=)	0.38					Coiled-coil dc	rs9300760	0.2005	YES	6.286E-1 (pos.103402522)							C=0, T=22	C=0.0, T=1.0	0.00001	298.89	22	C=0/0, T=12/1
chr13:103385798	T/C	T	C	SNV	CCDC168	CCDC168:exonic:NM 001146197.2	1	97.92	4	missense	NM 00114	c.17249A>	p.Asn5750Ser	-0.67		46	0		Coiled-coil dc	rs7983175	3.00E-04	YES	1.0 (pos.103385798)							T=1, C=47	T=0.0208, C=0.9792	0.00001	776.183	48	T=0/1, C=25/2
chr10:94816807	GA/GA	G	GA	INDEL	EXOC6	EXOC6:intronic:NM 019053.5	1	100		intronic	NM 01905	c.2282+14	p.?	-0.07					Exocyst comp	rs60582805	0.104	YES	9.898E-1 (pos.94816807)		Exocyst con	Flemming body,cytosol,exocyst,growth cone,perinuclea				G=0, GA=3(	G=0.0, GA=1.0	0.00001	364.675	30	G=0/0, GA=26/
chr10:94821374	G/G	A	G	SNV	CYP26C1	CYP26C1:intronic:NM 183374.2	1	100		intronic	NM 18337	c.204+150	p.?	-0.26					Cytochrome I	rs2422138		YES			Cytochrome	anterior/posterior pattern specification,central nervous				A=0, G=33	A=0.0, G=1.0	0.00001	504.595	33	A=0/0, G=11/2
chr10:95097537	C/C	T	C	SNV	MYOF	MYOF:intronic:NM 013451.3	1	100		intronic	NM 01345	c.4437+35	p.?	-1.45					C2 domain,Fe	rs1614065	0.2575	YES	9.728E-1;0.0 (pos.95097537)		Myoferlin	blood circulation,caveola,cellular response to heat,cyto				T=0, C=23	T=0.0, C=1.0	0.00001	316.746	23	T=0/0, C=17/6
chr10:95162496	T/T	A	T	SNV	MYOF	MYOF:intronic:NM 013451.3	1	100		intronic	NM 01345	c.874+121	p.?	-1.82					C2 domain,Fe	rs701875		YES			Myoferlin	blood circulation,caveola,cellular response to heat,cyto				A=0, T=30	A=0.0, T=1.0	0.00001	370.95	30	A=0/0, T=20/1
chr10:95216622	AG/AG	A	AG	INDEL	MYOF	MYOF:intronic:NM 013451.3	1	100		intronic	NM 01345	c.144+17T	p.?	1.6					C2 domain,FerA (NUC095) domain,FerB (NUC096) domain,FerI (NUC094) domain,Ferlin C-terminus						Myoferlin	blood circulation,caveola,cellular response to heat,cyto				A=0, AG=21	A=0.0, AG=1.0	0.00001	237.275	21	A=0/0, AG=7/1
chr10:95266683	A/G	A	G	SNV	CEP55	CEP55:intronic:NM 001127182.1	1	45.45		intronic	NM 00112	c.460-62A	p.?	-0.06					TSG101 and A	rs947599	0.4337	YES			Centrosom:	Flemming	Adnexal Diseases:Autoimmune Diseases:Cys			A=24, G=20	A=0.5455, G=0.4545	0.00001	211.733	44	A=9/15, G=8/1
chr13:36801415	T/T	C	T	SNV	CCDC169,CCDC169-SOHLH2	CCDC169:exonic:NM 001198908.1, CCDC169-SOHLH2:intro	1	100	8	missense	NM 00119	c.279+70G	p.?, p.Gly217Arg	0.3	0	125	0.999		Domain of un	rs9546785	0.4755	YES	3.698E-1 (pos.36801415)			cell differentiation,core promoter sequence-specific DN				C=0, T=139	C=0.0, T=1.0	0.00001	2313.84	139	C=0/0, T=60/7
chr10:95360109	C/G	C	G	SNV	RBP4	RBP4:intronic:NM 006744.3	1	55.77		intronic	NM 00674	c.355+41G	p.?	-0.65					Lipocalin / cy	rs57477607	0.0826	YES	1.393E-3 (pos.95360109)		Retinol-bin	cardiac m	Abortion, Spontane	2-((4-2-(trifluoromethyl)p		C=23, G=29	C=0.4423, G=0.5577	0.00001	299.667	52	C=15/8, G=12/
chr1:46086077	T/T	C	T	SNV	CCDC17	CCDC17:exonic:NM 001114938.2	1	100	13	missense	NM 00111	c.1739G>A	p.Ser580Asn	-0.3	0.72	46	0.001	0.02(COSM3746981),0.02(C		rs3014246	0.2927	YES	6.771E-1 (pos.46086077)	carcinoma,hepatocellular		protein binding				C=0, T=49	C=0.0, T=1.0	0.00001	831.632	49	C=0/0, T=22/2
chr10:95405665	C/C	CTA	C	INDEL	PDE6C	PDE6C:intronic:NM 006204.3	2	100		intronic	NM 00620	c.1848-52(	p.?	0.01, 0.37, -0.25					3'5'-cyclic nu	rs201850249,rs3085:	0.4773	YES			Phosphodie	3',5'-cyclic	Amphetamine-Rela	Caffeine,Trapidil		CTA=0, C=7	CTA=0.0, C=1.0	0.00001	1170.13	74	CTA=0/0, C=41
chr10:95444931	G/G	T	G	SNV	FRA10AC1	FRA10AC1:intronic:NM 145246.4	1	100		intronic	NM 14524	c.625+72A	p.?	0.97					Folate-sensit	rs767701		YES			Chromosom	nucleus,protein binding				T=0, G=40	T=0.0, G=1.0	0.00001	644.271	40	T=0/0, G=17/2
chr6:151894505	T/C	T	C	SNV	CCDC170	CCDC170:exonic:NM 025059.3	1	45.83	6	missense	NM 02505	c.971T>C	p.Phe324Ser	-0.17	1	155	0	0.01(COSM4407030)		rs953767	0.1175	YES	9.957E-1;0.0 (pos.15189(	hepatocellular_carcinoma		protein bi	Breast Diseases;Breast Neoplasms;Neoplasr			T=39, C=33	T=0.5417, C=0.4583	0.00001	323.029	72	T=18/21, C=20
chr10:95459698	C/C	G	C	SNV	FRA10AC1	FRA10AC1:intronic:NM 145246.4	1	100		intronic	NM 14524	c.77+89C>	p.?	-0.51					Folate-sensit	rs2275441		YES			Chromosom	nucleus,protein binding				G=0, C=54	G=0.0, C=1.0	0.00001	798.121	54	G=0/0, C=31/2
chr18:30804756	T/T	C	T	SNV	CCDC178	CCDC178:exonic:NM 001105528.1	1	100	16	missense	NM 00110	c.1801G>A	p.Asp601Asn	0.1	1	23	0.001			rs113589354,rs3868(	0		1.0 (pos.30804756)							C=0, T=48	C=0.0, T=1.0	0.00001	810.416	48	C=0/0, T=27/2
chr18:30846895	T/T	A	T	SNV	CCDC178	CCDC178:exonic:NM 001105528.1	1	100	13	missense	NM 00110	c.1394T>A	p.Val465Glu	-0.84	1	121	0			rs747171150,rs9965(	0.0718	YES	0.0 (pos.30846894),9.999E-1 (pos.30846895)							A=0, T=37	A=0.0, T=1.0	0.00001	583.688	37	A=0/0, T=22/1
chr1:93672723	A/C	A	C	SNV	CCDC18	CCDC18:exonic:NM 206886.4	1	96.77	9	missense	NM 20688	c.977A>C	p.Asn326Thr	1.75	0.05	65	0.196			rs2815413	0.1779	YES	9.593E-1 (pos.93672723)							A=1, C=30	A=0.0323, C=0.9677	0.00001	431.028	31	A=0/1, C=14/1
chr1:169391154	A/G	A	G	SNV	CCDC181	CCDC181:exonic:NM 021179.2	1	45.45	3	missense	NM 02117	c.515T>C	p.Phe172Ser	0.12	0.41	155	0.001			rs3820059	0.3545	YES	9.284E-1 (pos.169391154)			cytoplasm,manchette,microtubule,microtubule binding				A=12, G=1(	A=0.5455, G=0.4545	0.00001	81.7625	22	A=11/1, G=8/2
chr10:96305729	T/C	T	C	SNV	HELLS	HELLS:intronic:NM 018063.4	1	50		intronic	NM 01806	c.31+20T>	p.?	-0.44					Helicase con:	rs10882474	0.4183	YES	8.086E-1 (pos.96305729)		Helicase, ly	ATP bindin	Adnexal Diseases:Cysts:Endocrine System Di			T=14, C=14	T=0.5, C=0.5	0.00001	131.94	28	T=11/3, C=6/8
chr10:96466824	T/T	C	T	SNV	CYP2C18	CYP2C18:intronic:NM 000772.2	1	100		intronic	NM 00077	c.819+107	p.?	-1.15						rs7904929		YES			Cytochrome	arachidon	Cardiovascular Dis	4-METHYL-N-METHYL-N-(2		C=0, T=35	C=0.0, T=1.0	0.00001	543.101	35	C=0/0, T=18/1
chr9:139700605	C/C	T	C	SNV	CCDC183,CCDC183-AS1	CCDC183-AS1:intronic nc:NR 024580.1, CCDC183:exonic:N	1	100	10	missense	NM 00103	c.1024T>C	p.Trp342Arg	-0.68	0.83	101	0	0.01(COSM4163579)		rs2811795,rs770286(	0.1979	YES	0.0 (pos.139700590),8.8:	neoplasm	Coiled-coil (	axoneme,cilium movement,outer dynein arm assembly				T=0, C=34	T=0.0, C=1.0	0.00001	524.173	34	T=0/0, C=16/1
chr10:97023552	A/A	G	A	SNV	PDLIM1	PDLIM1:intronic:NM 020992.3	1	100		intronic	NM 02099	c.533+69C	p.?	-1.73					Domain of un	rs4917682		YES			PDZ and LIN	cadherin binding involved in cell-cell adhesion,cell-cell a				G=0, A=73	G=0.0, A=1.0	0.00001	1150.5	73	G=0/0, A=27/4
chr10:97023895	T/T	C	T	SNV	PDLIM1	PDLIM1:intronic:NM 020992.3	1	100		intronic	NM 02099	c.334-75G	p.?	-2.42					Domain of un	rs2296962		YES			PDZ and LIN	cadherin binding involved in cell-cell adhesion,cell-cell a				C=0, T=55	C=0.0, T=1.0	0.00001	814.907	55	C=0/0, T=20/3
chr9:139701108	C/C	A	C	SNV	CCDC183,CCDC183-AS1	CCDC183-AS1:intronic nc:NR 024580.1, CCDC183:exonic:N	1	100	11	missense	NM 00103	c.1262A>C	p.Asn421Thr	0.82	0.92	65	0			rs2254143	0.2037	YES	8.721E-1 (pos.139701108)		Coiled-coil (	axoneme,cilium movement,outer dynein arm assembly				A=0, C=21	A=0.0, C=1.0	0.00001	281.234	21	A=0/0, C=13/8
chr10:97370843	T/C	T	C	SNV	ALDH18A1	ALDH18A1:intronic:NM 002860.3	1	50		intronic	NM 00286	c.2110+17	p.?	-1.22					Aldehyde del	rs2296689		YES			Aldehyde de	ATP bindin	Cataract:Congenita	Glutamic Acid		T=13, C=13	T=0.5, C=0.5	0.00001	119.158	26	T=9/4, C=7/6
chr10:115922774	G/A	G	A	SNV	CCDC186	CCDC186:exonic:NM 018017.3	1	57.45	2	missense	NM 01801	c.254C>T	p.Thr85Ile	-0.82	0.35	89	0	0.01(COSM3751543)		rs1061159	0.2353	YES	6.993E-2;0.0 (pos.11592:	adenocarcinoma,rhabdomyosarcoma						G=40, A=54	G=0.4255, A=0.5745	0.00001	643.119	94	G=30/10, A=2
chr10:97772253	T/T	C	T	SNV	CC2D2B,ENTPD1-AS1	ENTPD1-AS1:intronic nc:NR 038444.1, CC2D2B:intronic:NM	1	100		intronic	NR 03844	c.3229-36(	p.?	0.1						rs3748224,rs774130	0.407	YES	8.254E-1 (pos.97772253)							C=0, T=55	C=0.0, T=1.0	0.00001	814.771	55	C=0/0, T=28/2
chr10:97772496	T/A	T	A	SNV	CC2D2B,ENTPD1-AS1	ENTPD1-AS1:intronic nc:NR 038444.1, CC2D2B:intronic:NM	1	98.23		intronic	NR 03844	c.3426+10	p.?	-1.76						rs10748652	0.408	YES	8.26E-1 (pos.97772496)							T=2, A=111	T=0.0177, A=0.9823	0.00001	1725.78	113	T=1/1, A=51/6
chr10:97779281	A/A	G	A	SNV	CC2D2B,ENTPD1-AS1	ENTPD1-AS1:intronic nc:NR 038444.1, CC2D2B:intronic:NM	1	100		intronic	NR 03844	c.3766-17:	p.?	0.17						rs4918984		YES								G=0, A=53	G=0.0, A=1.0	0.00001	775.657	53	G=0/0, A=18/3
chr16:30772040	G/G	A	G	SNV	CCDC189,PHKG2,RNF40	CCDC189:intronic:NM 001014979.2, PHKG2:utr 3:NM 001	1	100	11	utr 3	NM 00117	c.1684A>G	p.?, p.?, p.?	-1.15					Flagellar C1a	rs4889505	4.00E-04	YES	1.0 (pos.30772040)		Phosphoryl	ATP bindin	Carbohydrate Metabolism, Inborn Errors:Con			A=0, G=23	A=0.0, G=1.0	0.00001	316.523	23	A=0/0, G=17/6
chr10:98408714	G/G	A	G	SNV	PIK3AP1	PIK3AP1:intronic:NM 152309.2	1	100		intronic	NM 15230	c.989-102	p.?	0.46					Dof, BCAP, an	rs912483		YES			Phosphoinc	cytosol,identical protein binding,membrane,phosphatic				A=0, G=124	A=0.0, G=1.0	0.00001	2013.68	124	A=0/0, G=62/6
chr10:98989454	G/G	A	G	SNV	ARHGAP19,ARHGAP19-SLIT1	ARHGAP19-SLIT1:intronic nc:NR 037909.1, ARHGAP19:intrc	1	100		intronic	NR 03790	c.1395+67	p.?	-0.66					RhoGAP dom:	rs2297987		YES			RHO GTPase	GTPase activator activity,cytosol,nucleus,positive regul:				A=0, G=21	A=0.0, G=1.0	0.00001	281.235	21	A=0/0, G=17/4
chr10:99003613	C/C	A	C	SNV	ARHGAP19,ARHGAP19-SLIT1	ARHGAP19-SLIT1:intronic nc:NR 037909.1, ARHGAP19:intrc	1	100		intronic	NR 03790	c.1185+11	p.?	-2.86					RhoGAP dom:	rs793528		YES			RHO GTPase	GTPase activator activity,cytosol,nucleus,positive regul:				A=0, C=21	A=0.0, C=1.0	0.00001	281.235	21	A=0/0, C=6/15
chr10:99025611	A/A	T	A	SNV	ARHGAP19,ARHGAP19-SLIT1	ARHGAP19-SLIT1:intronic nc:NR 037909.1, ARHGAP19:intrc	1	100		intronic	NR 03790	c.322+6A>	p.?	-0.01				0.03(COSM59	RhoGAP dom:	rs10882884	0.4087	YES	7.344E-1 (pos.99025611)	basal_cell_car	RHO GTPase	GTPase activator activity,cytosol,nucleus,positive regul:				T=0, A=42	T=0.0, A=1.0	0.00001	685.22	42	T=0/0, A=25/1
chr10:99145616	G/A	G	A	SNV	RRP12	RRP12:intronic:NM 015179.3	1	45.45		intronic	NM 01517	c.1116+36	p.?	-1.05					NUC173 dom	rs75905387	0.1022	YES	1.376E-3 (pos.99145616)		Ribosomal I	RNA binding,integral component of membrane,nuclear n				G=18, A=15	G=0.5455, A=0.4545	0.00001	143.162	33	G=12/6, A=8/7
chr3:113737568	G/A	G	A	SNV	CCDC191	CCDC191:exonic:NM 020817.1	1	50	8	missense	NM 02081	c.1120C>T	p.Arg374Trp	2.29	0	101	1			rs17603649	0.0421	YES	3.467E-4 (pos.113737568)							G=35, A=35	G=0.5, A=0.5	0.00001	355.317	70	G=14/21, A=1
chr14:57938260	C/C	T	C	SNV	CCDC198	CCDC198:exonic:NM 018168.3	1	100	6	missense	NM 01816	c.704A>G	p.Tyr235Cys	-0.13	0.12	194	0.043		Domain of un	rs1152530	0.0012	YES	9.999E-1 (pos.57938260)			protein binding				T=0, C=31	T=0.0, C=1.0	0.00001	465.819	31	T=0/0, C=16/1
chr10:99517355	G/G	A	G	SNV	ZFYVE27	ZFYVE27:intronic:NM 144588.6	1	100		intronic	NM 14458	c.1090-44	p.?	-0.77					FYVE zinc fing	rs3750612	0.2126	YES	4.993E-1 (pos.99517355)		Zinc finger F	axon,denc	Congenital Abnormalities:Congenital, Heredi			A=0, G=45	A=0.0, G=1.0	0.00001	747.392	45	A=0/0, G=30/1
chr10:99682981	C/C	T	C	SNV	CRTAC1	CRTAC1:intronic:NM 018058.6	1	100		intronic	NM 01805	c.558+40A	p.?	-0.88					ASPIC and Un	rs894375	0.2066	YES	9.268E-1 (pos.99682981)			axonal fasciculation,calcium ion binding,extracellular e:				T=0, C=25	T=0.0, C=1.0	0.00001	353.003	25	T=0/0, C=19/6
chr8:27593650	C/C	T	C	SNV	CCDC25	CCDC25:utr 3:NM 018246.2	1	100	9	utr 3	NM 01824	c.710A>G	p.?	1.26					Domain of un	rs10081527	0.0987	YES				extracellular exosome,protein binding				T=0, C=81	T=0.0, C=1.0	0.00001	1319.05	81	T=0/0, C=48/3
chr11:100211032	G/G	T	G	SNV	CNTN5	CNTN5:intronic:NM 001243270.1	1	100		intronic	NM 00124	c.2731-16:	p.?	0.39					Fibronectin t	rs12289431		YES			Contactin 5	C-terminal protein lipidation,anchored component of m				T=0, G=42	T=0.0, G=1.0	0.00001	683.191	42	T=0/0, G=21/2
chr11:100812751	C/C	T	C	SNV	ARHGAP42	ARHGAP42:intronic:NM 152432.3	1	100		intronic	NM 15243	c.933+136	p.?	0.15					BAR domain (	rs693760		YES			Rho GTPase	GTPase ac	Cardiovascular Diseases:Hypertension:Vasc			T=0, C=25	T=0.0, C=1.0	0.00001	352.936	25	T=0/0, C=14/1
chr11:100814409	A/A	G	A	SNV	ARHGAP42	ARHGAP42:intronic:NM 152432.3	1	100		intronic	NM 15243	c.934-81G	p.?	-1.38					BAR domain (	rs619066		YES			Rho GTPase	GTPase ac	Cardiovascular Diseases:Hypertension:Vasc			G=0, A=20	G=0.0, A=1.0	0.00001	263.786	20	G=0/0, A=5/15
chr11:100814609	T/T	G	T	SNV	ARHGAP42	ARHGAP42:intronic:NM 152432.3	1	100		intronic	NM 15243	c.1043+10	p.?	-0.5					BAR domain (	rs618155	0.1417	YES	9.968E-1 (pos.100814609)		Rho GTPase	GTPase ac	Cardiovascular Diseases;Hypertension;Vasc			G=0, T=36	G=0.0, T=1.0	0.00001	563.744	36	G=0/0, T=26/1
chr3:49273996	T/T	C	T	SNV	CCDC36	CCDC36:exonic:NM 001135197.1	1	100	3	synonymous	NM 00113	c.72C>T	p.(=)	0.54					Interactor of	rs12631989,rs74745	0.3844	YES	1.269E-4 (pos.49273984),9.548E-1 (pos.49273996)			DNA recombination,condensed nuclear chromosome,m				C=0, T=29	C=0.0, T=1.0	0.00001	427.038	29	C=0/0, T=13/1
chr11:101762478	G/A	G	A	SNV	ANGPTL5	ANGPTL5:intronic:NM 178127.4	1	50		intronic	NM 17812	c.848-149(	p.?	-0.43					Fibrinogen be	rs11225049		YES			Angiopoieti	extracellular region,protein binding				G=26, A=26	G=0.5, A=0.5	0.00001	256.25	52	G=11/15, A=7/
chr12:96284650	A/G	A	G	SNV	CCDC38	CCDC38:exonic:NM 182496.2	1	47.62	9	synonymous	NM 18249	c.831T>C	p.(=)	-2.65				0(COSM6350!	Domain of un	rs6538681	0.2981	YES	7.387E-1 (pos.96284650)	carcinoma		centrosome,cytoplasm				A=33, G=30	A=0.5238, G=0.4762	0.00001	289.406	63	A=18/15, G=20

chr3:180334623	T/T	C	T	SNV	CCDC39	CCDC39:exonic:NM 181426.1	1	100	17	synonymous	NM 18142	c.2397G>A	p.(=)	0.4						rs7612917	0.1954	YES	1.659E-3 (pos.180334623)		Coiled-coil	axonemal	Ciliary Motility Disorders;Otorl		Benign/Likely	C=0, T=44	C=0.0, T=1.0	0.00001	726.569	44	C=0/0, T=24/2
chr3:180365956	A/A	G	A	SNV	CCDC39	CCDC39:exonic:NM 181426.1	1	100	10	synonymous	NM 18142	c.1359C>T	p.(=)	0.22						rs6769457	0.2199	YES	4.241E-3;4.104E-4 (pos.180365956)		Coiled-coil	axonemal	Ciliary Motility Disorders;Otorl		Benign	G=0, A=20	G=0.0, A=1.0	0.00001	263.786	20	G=0/0, A=12/8
chr11:102398434	C/T	C	T	SNV	MMP7	MMP7:intronic:NM 002423.4	1	46.51		intronic	NM 00242	c.336-31G	p.?	0					Matrixin.Puta	rs17884405		YES			Matrix meta	aging.cell	Abortion. Spontane	(1R)-N.6-DIHYDROXY-7-M		C=23. T=20	C=0.5349, T=0.4651	0.00001	212.325	43	C=10/13, T=14
chr11:102398436	C/CT	C	CT	INDEL	MMP7	MMP7:intronic:NM 002423.4	1	46.51		intronic	NM 00242	c.336-33G	p.?	-1.06					Matrixin.Puta	rs17884615.rs39809	0.2204	YES	2.497E-1 (pos.102398436)		Matrix meta	aging.cell	Abortion. Spontane	(1R)-N.6-DIHYDROXY-7-M		C=23. CT=2	C=0.5349. CT=0.4651	0.00001	178.559	43	C=10/13, CT=1
chr11:102449650	A/A	T	A	SNV	MMP20	MMP20:intronic:NM 004771.3	1	100		intronic	NM 00477	c.1351+12	p.?	-0.28					Hemopexin,M	rs7934921		YES			Matrix meta	amelogen	Amelogenesis Impe	Marimastat,N-ISOBUTYL-		T=0, A=340	T=0.0, A=1.0	0.00001	5735.88	340	T=0/0, A=181/
chr17:78055758	G/G	T	G	SNV	CCDC40	CCDC40:exonic:NM 017950.3	1	100	12	synonymous	NM 01795	c.1890T>G	p.(=)	-2.83				0.04(COSM37	BRE1 E3 ubiq	rs35578653,rs38679	0.4904	YES	2.769E-1 (pos.78055758)	adenocarcino	Coiled-coil	axonemal	Bronchial Diseases;Bronchiect		Benign	T=0, G=35	T=0.0, G=1.0	0.00001	543.854	35	T=0/0, G=6/29
chr10:61552692	T/T	G	T	SNV	CCDC6	CCDC6:exonic:NM 005436.4	1	100	9	missense	NM 00543	c.1408C>A	p.Pro470Thr	8.85	0.02	38	0.998	1(COSM1469	Uncharacteri	rs1053266	0.4966	YES	6.035E-1;0.0 (pos.61552	acute_myeloid	Coiled-coil	SH3 doma	Carcinoma:Endocrine Gland Neoplasms:Endc			G=0, T=43	G=0.0, T=1.0	0.00001	687.008	43	G=0/0, T=19/2
chr10:61552774	T/T	C	T	SNV	CCDC6	CCDC6:exonic:NM 005436.4	1	100	9	synonymous	NM 00543	c.1326G>A	p.(=)	-3.15				0.06(COSM13	Uncharacteri	rs1053265	0.2308	YES	6.541E-1 (pos.61552774)	acute_myeloid	Coiled-coil	SH3 doma	Carcinoma:Endocrine Gland Neoplasms:Endc			C=0, T=45	C=0.0, T=1.0	0.00001	747.36	45	C=0/0, T=18/2
chr11:102647310	G/G	T	G	SNV	MMP10	MMP10:intronic:NM 002425.2	1	100		intronic	NM 00242	c.787+33A	p.?	-0.47					Hemopexin,M	rs470154	0.07	YES	8.926E-1 (pos.102647310)		Matrix meta	collagen c	Arthritis;Arthritis, F	Marimastat,N-ISOBUTYL-		T=0, G=82	T=0.0, G=1.0	0.00001	1329.64	82	T=0/0, G=25/5
chr11:102647536	C/C	T	C	SNV	MMP10	MMP10:intronic:NM 002425.2	1	100		intronic	NM 00242	c.623-29A	p.?	-0.66					Hemopexin,M	rs2276108	0.1455	YES	8.815E-3 (pos.102647536)		Matrix meta	collagen c	Arthritis;Arthritis, F	Marimastat,N-ISOBUTYL-		T=0, C=25	T=0.0, C=1.0	0.00001	353.018	25	T=0/0, C=12/1
chr11:102647552	T/T	C	T	SNV	MMP10	MMP10:intronic:NM 002425.2	1	100		intronic	NM 00242	c.623-45G	p.?	-1.41					Hemopexin,M	rs3819099	0.1452	YES	8.845E-3 (pos.102647552)		Matrix meta	collagen c	Arthritis;Arthritis, F	Marimastat,N-ISOBUTYL-		C=0, T=24	C=0.0, T=1.0	0.00001	334.771	24	C=0/0, T=11/1
chr3:56597954	T/T	C	T	SNV	CCDC66	CCDC66:exonic:NM 001141947.2	1	100	4	synonymous	NM 00114	c.345C>T	p.(=)	-1.4				0.03(COSM40	Coiled-coil do	rs6769055	0.1534	YES	5.516E-1 (pos.56597954)	acute_myeloid_leukaemia						C=0, T=26	C=0.0, T=1.0	0.00001	371.42	26	C=0/0, T=10/1
chr11:102711338	T/C	T	C	SNV	MMP3	MMP3:intronic:NM 002422.4	1	53.49		intronic	NM 00242	c.626-14A	p.?	-0.43					Hemopexin,M	rs591058	0.4992	YES	6.689E-1 (pos.102711338)		Matrix meta	cellular re	Arterial Occlusive D	(1n)-4-N-B	Benign	T=20. C=23	T=0.4651, C=0.5349	0.00001	261.019	43	T=10/10, C=11
chr3:56627048	G/G	A	G	SNV	CCDC66	CCDC66:exonic:NM 001141947.2	1	100	8	synonymous	NM 00114	c.987A>G	p.(=)	0.57				0.03(COSM40	Coiled-coil do	rs1388256,rs765062	0.1029	YES	0.0 (pos.56627047),6.60	acute_myeloid_leukaemia,adenocarcinoma						A=0, G=41	A=0.0, G=1.0	0.00001	664.7	41	A=0/0, G=19/2
chr3:56628031	A/A	G	A	SNV	CCDC66	CCDC66:exonic:NM 001141947.2	1	100	10	missense	NM 00114	c.1379G>A	p.Arg460Gln	4.88	0	43	1	0.93(COSM50	Coiled-coil do	rs7637449	0.4627	YES	3.826E-2 (pos.56628031)	ductal_carcinoma						G=0, A=26	G=0.0, A=1.0	0.00001	371.418	26	G=0/0, A=11/1
chr11:103182774	C/A	C	A	SNV	DYNC2H1	DYNC2H1:intronic:NM 001080463.1	1	53.49		intronic	NM 00108	c.11670+1	p.?	-1.35					AAA domain (	rs79978663	0.1459	YES	2.001E-1;0.0 (pos.103182774)		Dynein, cyto	ATP bindin	Abnormalities, Multiple:Bone I		Benign	C=20, A=23	C=0.4651, A=0.5349	0.00001	261.021	43	C=4/16, A=15/
chr11:103192039	T/C	T	C	SNV	DYNC2H1	DYNC2H1:intronic:NM 001080463.1	1	57.14		intronic	NM 00108	c.11955+7	p.?	-0.59					AAA domain (	rs313407		YES			Dynein, cyto	ATP bindin	Abnormalities, Multiple:Bone Diseases:Bone			T=12, C=16	T=0.4286, C=0.5714	0.00001	161.767	28	T=9/3, C=2/14
chr11:104758035	A/A	G	A	SNV	CASP12	CASP12:intronic:NM 001191016.2	1	100		intronic	NM 00119	c.822-7C>T	p.?	2.23					Caspase dom	rs484438		YES	1.0 (pos.104758035)		Caspase 12	AIM2 infla	Autoimmune Diseases:Brain Diseases:Brain			G=0, A=55	G=0.0, A=1.0	0.00001	814.907	55	G=0/0, A=16/3
chr3:49200627	G/G	A	G	SNV	CCDC71	CCDC71:exonic:NM 022903.3	1	100	2	missense	NM 02290	c.1015T>C	p.Trp339Arg	-0.5		101		0.01(COSM41	Coiled-coil do	rs4955418,rs773541	0.384	YES	0.0 (pos.49200613),9.57	acute_lymphoblastic_leukaemia,neoplasm						A=0, G=25	A=0.0, G=1.0	0.00001	357.398	25	A=0/0, G=13/1
chr11:104877803	A/G	A	G	SNV	CASP5	CASP5:intronic:NM 001136112.1	1	46.88		intronic	NM 00113	c.472+7T>	p.?	-1.51				0(COSM3998	Caspase dom	rs2282657	0.3355	YES	2.496E-1 (pos.10487780	acute_myeloid	Caspase 5,	AIM2 inflammasome complex,IPAF inflammasome comp				A=32, G=30	A=0.5161, G=0.4839	0.00001	317.421	64	A=14/18, G=3/
chr3:49200692	A/A	T	A	SNV	CCDC71	CCDC71:exonic:NM 022903.3	1	100	2	missense	NM 02290	c.950A>T	p.Gln317Leu	-2.68		113		0.04(COSM41	Coiled-coil do	rs4955419.rs768066	0.3849	YES	0.0 (pos.49200676).9.54	acute_lymphoblastic_leukaemia,neoplasm						T=0, A=20	T=0.0, A=1.0	0.00001	263.776	20	T=0/0, A=6/14
chr11:104971210	T/T	C	T	SNV	CARD17	CARD17:intronic:NM 001007232.1	1	100		intronic	NM 00100	c.274+30G	p.?	1.08					Caspase recr	rs1792771	0.35	YES	4.078E-1 (pos.104971210)			caspase binding,cellular response to lipopolysaccharide				C=0, T=35	C=0.0, T=1.0	0.00001	543.903	35	C=0/0, T=22/1
chr11:104972190	T/T	C	T	SNV	CARD17	CARD17:upstream:NM 001007232.1	1	100		intronic	NM 00100	c.-49G>A	p.?	-0.99						rs1792773	0.349	YES	4.065E-1 (pos.104972190)			caspase binding,cellular response to lipopolysaccharide				C=0, T=112	C=0.0, T=1.0	0.00001	1776.42	112	C=0/0, T=64/4
chr11:10526060	T/T	C	T	SNV	AMPD3	AMPD3:intronic:NM 000480.2	1	100		intronic	NM 00048	c.2044-36	p.?	-0.58					Adenosine/A	rs7943804	0.1061	YES	8.677E-1 (pos.10526060)		Adenosine	ADP metal	Congenital, Hereditary, and Neonatal Diseas			C=0, T=21	C=0.0, T=1.0	0.00001	281.235	21	C=0/0, T=11/1
chr11:105844923	C/A	C	A	SNV	GRIA4	GRIA4:intronic:NM 000829.3	1	50		intronic	NM 00082	c.2410-11	p.?	-0.13					Bacterial ext	rs509512		YES			Glutamate	AMPA glut	Eye Diseases:Ment	CX717.Ethanol.Glutamic		C=11, A=11	C=0.5, A=0.5	0.00001	94.6068	22	C=8/3, A=9/2
chr11:10631247	C/T	C	T	SNV	MRVI1	MRVI1:intronic:NM 001098579.2	1	46.88		intronic	NM 00109	c.1547+28	p.?	0.45					MRVI1 protei	rs3741042.rs908697	0.4984	YES	7.045E-1 (pos.10631247)		Murine retr	endoplasr	Brain Diseases:Central Nervous System Disea			C=17, T=15	C=0.5313, T=0.4688	0.00001	148.221	32	C=8/9, T=8/7
chr11:10653426	G/G	A	G	SNV	MRVI1	MRVI1:intronic:NM 001098579.2	1	100		intronic	NM 00109	c.373+112	p.?	-0.58					MRVI1 protei	rs10840450		YES			Murine retr	endoplasr	Brain Diseases:Central Nervous System Disea			A=0, G=37	A=0.0, G=1.0	0.00001	577.768	37	A=0/0, G=22/1
chr2:56411816	CT/CT	C	CT	INDEL	CCDC85A,LOC100129434	LOC100129434:intronic_nc:NR 125368.1. CCDC85A:exonic:	1	100	1	frameshiftInsertion	NR 12536	c.57_58ins	p.Pro20fs	0.1					CCDC85 famil	rs928493595										C=0, CT=75	C=0.0, CT=1.0	0.00001	825.787	75	C=0/0, CT=58/
chr11:10715124	C/C	T	C	SNV	MRVI1		1	100		intronic				1.6						rs10770136		YES	9.03E-1 (pos.10715124)		Murine retrovirus inte		Brain Diseases:Central Nervous System Disea			T=0, C=53	T=0.0, C=1.0	0.00001	782.03	53	T=0/0, C=30/2
chr11:107326340	C/A	C	A	SNV	CWF19L2	CWF19L2:intronic:NM 152434.2	1	59.09		intronic	NM 15243	c.216+52G	p.?	0.1					Protein simil	rs618078		YES				protein binding				C=9, A=13	C=0.4091, A=0.5909	0.00001	122.568	22	C=3/6, A=7/6
chr19:4251069	C/C	T	C	SNV	CCDC94	CCDC94:exonic:NM 018074.5	1	100	3	synonymous	NM 01807	c.171T>C	p.(=)	-0.63				0.12(COSM14	Family of unk	rs1045750	0.2858	YES	4.668E-1 (pos.4251069)	acute_myeloid_leukaemia		protein binding				T=0, C=20	T=0.0, C=1.0	0.00001	263.785	20	T=0/0, C=9/11
chr11:107904646	C/T	C	T	SNV	CUL5	CUL5:intronic:NM 003478.4	1	47.62		intronic	NM 00347	c.134+9C>	p.?	0.13					Cullin family,	rs10890800	0.3443	YES	9.015E-1 (pos.107904646)		Cullin-5 (va	Cul4-RING	Breast Diseases;Breast Neoplasms;Neoplasr			C=22, T=20	C=0.5238, T=0.4762	0.00001	212.953	42	C=12/10, T=8/
chr11:107925729	G/A	G	A	SNV	CUL5	CUL5:intronic:NM 003478.4	1	51.85		intronic	NM 00347	c.780+47G	p.?	-0.21					Cullin family,	rs7125285	0.1212	YES	3.366E-2 (pos.107925729)		Cullin-5 (va	Cul4-RING	Breast Diseases;Breast Neoplasms;Neoplasr			G=13, A=14	G=0.4815, A=0.5185	0.00001	132.493	27	G=9/4, A=8/6
chr11:107948858	T/A	T	A	SNV	CUL5	CUL5:intronic:NM 003478.4	1	50		intronic	NM 00347	c.1114-45	p.?	0.48					Cullin family,	rs10890807	0.4051	YES	4.51E-1 (pos.107948858)		Cullin-5 (va	Cul4-RING	Breast Diseases;Breast Neoplasms;Neoplasr			T=16, A=16	T=0.5, A=0.5	0.00001	158.383	32	T=12/4, A=11/
chr11:108061379	T/T	C	T	SNV	NPAT	NPAT:intronic:NM 002519.2	1	100		intronic	NM 00251	c.291-152	p.?	-0.11					NPAT C termi	rs659584					Nuclear pro	Cajal body,Gemini of coiled bodies,cytoplasm,negative r				C=0, T=21	C=0.0, T=1.0	0.00001	281.231	21	C=0/0, T=4/17
chr6:31111180	C/C	T	C	SNV	CCHCR1	CCHCR1:exonic:NM 001105563.1	1	100	16	synonymous	NM 00110	c.2070A>G	p.(=)	0.19				0.06(COSM37	Alpha helical	rs130073	0.2905	YES	7.042E-1 (pos.31111180)	adenocarcino	Coiled-coil	cell differe	Amphetamine-Related Disorders;Colitis;Coli			T=0, C=52	T=0.0, C=1.0	0.00001	770.542	52	T=0/0, C=33/1
chr11:108216401	TA/T	TA	T	INDEL	ATM	ATM:intronic:NM 000051.3	1	100		intronic	NM 00005	c.8419-69	p.?	-0.44, 2.96					Anaphase-promoting complex subunit 5.FAT domain.FATC domain.Phosphatidylinositol 3- and 4-kina						Ataxia-telai	1-phospha	Adenocarcinoma:A	Caffeine		TA=0, T=25	TA=0.0, T=1.0	0.00001	206.841	25	TA=0/0, T=10/
chr11:10822202	A/A	G	A	SNV	EIF4G2,SNORD97	SNORD97:downstream:NR 004403.1. EIF4G2:intronic:NM (	1	100		intronic	NM 00141	c.1645-8C	p.?	0.02				0.4(COSM441	MA3 domain,	rs4910190	0.4336	YES	5.686E-1 (pos.10822202)	acute_myeloid	Eukaryotic t	RNA binding,axon,cadherin binding,cell cycle arrest,cell				G=0, A=35	G=0.0, A=1.0	0.00001	543.904	35	G=0/0, A=20/1
chr6:31113030	G/G	A	G	SNV	CCHCR1	CCHCR1:exonic:NM 001105563.1	1	100	13	synonymous	NM 00110	c.1681T>C	p.(=)	2.02				0.01(COSM41	Alpha helical	rs3132539,rs758053	0.2006	YES	0.0 (pos.31113029),8.14	neoplasm	Coiled-coil	cell differe	Amphetamine-Related Disorders;Colitis;Coli			A=0, G=77	A=0.0, G=1.0	0.00001	1229.44	77	A=0/0, G=48/2
chr11:10824540	A/A	G	A	SNV	EIF4G2	EIF4G2:intronic:NM 001418.3	1	100		intronic	NM 00141	c.996+37C	p.?	-1.08					MA3 domain,	rs10743150,rs77024	0.0817	YES	0.0 (pos.10824537),9.927E-1;0.0 (pos.10		Eukaryotic t	RNA binding,axon,cadherin binding,cell cycle arrest,cell				G=0, A=27	G=0.0, A=1.0	0.00001	389.988	27	G=0/0, A=11/1
chr11:10824731	T/T	C	T	SNV	EIF4G2	EIF4G2:intronic:NM 001418.3	1	100		intronic	NM 00141	c.897+27G	p.?	-0.51					MA3 domain,	rs2270620	0.4014	YES	5.717E-1;0.0 (pos.10824731)		Eukaryotic t	RNA binding,axon,cadherin binding,cell cycle arrest,cell				C=0, T=23	C=0.0, T=1.0	0.00001	316.747	23	C=0/0, T=12/1
chr11:10827418	C/C	T	C	SNV	EIF4G2	EIF4G2:intronic:NM 001418.3	1	100		intronic	NM 00141	c.248+36A	p.?	-1.25					MA3 domain,	rs11042964.rs76913	0.4342	YES	1.156E-4 (pos.10827407).5.699E-1 (pos.1		Eukaryotic t	RNA binding,axon,cadherin binding,cell cycle arrest,cell				T=0, C=41	T=0.0, C=1.0	0.00001	660.741	41	T=0/0, C=11/3
chr6:31118565	G/G	C	G	SNV	CCHCR1	CCHCR1:exonic:NM 001105563.1	1	100	6	synonymous	NM 00110	c.930G>C	p.(=)	-0.63					Alpha helical	rs130078	0.2518	YES	7.02E-1 (pos.31118565)		Coiled-coil	cell differe	Amphetamine-Related Disorders;Colitis;Coli			C=0, G=33	C=0.0, G=1.0	0.00001	504.597	33	C=0/0, G=19/1
chr9:36169494	A/A	G	A	SNV	CCIN	CCIN:utr 5:NM 005893.2	1	100	1	utr 5	NM 00589	c.-6G>A	p.?	0.27					BTB And C-ter	rs3739610,rs767219	0.2992	YES	0.0;0.0 (pos.36169487),3.988E-1 (pos.36		Calicin	Cul3-RING ubiquitin ligase complex,actin binding,actin c				G=0, A=28	G=0.0, A=1.0	0.00001	408.704	28	G=0/0, A=12/1
chr9:36169598	C/C	T	C	SNV	CCIN	CCIN:exonic:NM 005893.2	1	100	1	synonymous	NM 00589	c.99T>C	p.(=)	-1.05				0.05(COSM63	BTB And C-ter	rs3739609	0.447	YES	4.697E-1 (pos.36169598)	carcinoma	Calicin	Cul3-RING ubiquitin ligase complex,actin binding,actin c				T=0, C=29	T=0.0, C=1.0	0.00001	427.603	29	T=0/0, C=8/21
chr17:34313674	G/A	G	A	SNV	CCL14,CCL15-CCL14	CCL14:exonic:NM 032962.4. CCL15-CCL14:exonic_nc:NR 02	1	45.83	1	synonymous	NM 03296	c.12C>T	p.(=)	0.69					Small cytokin	rs113937434	0.0293	YES	2.752E-2 (pos.34313674)		Chemokine	CCR chemokine receptor binding,G-protein coupled rece				G=39, A=33	G=0.5417, A=0.4583	0.00001	323.033	72	G=20/19, A=1
chr11:1103991	G/C	G	C	SNV	MUC2	MUC2:intronic:NM 002457.4	1	56		intronic	NM 00245	c.8235-53	p.?	-1.48					C8 domain.M	rs41437546		YES			Mucin 2, intestinal/tra		Adenocarcinoma:A	Pranlukast		G=11, C=14	G=0.44, C=0.56	0.00001	134.313	25	G=7/4, C=11/3
chr11:110453250	C/G	C	G	SNV	ARHGAP20	ARHGAP20:intronic:NM 020809.3	1	57.58		intronic	NM 02080	c.1606-91	p.?	-0.89					Ras associati	rs4142632		YES				GTPase ac	Alzheimer Disease;Brain Diseases;Central Ne			C=14, G=19	C=0.4242, G=0.5758	0.00001	193.573	33	C=5/9, G=7/12
chr11:111228050	T/T	C	T	SNV	POU2AF1	POU2AF1:intronic:NM 006235.2	1	100		intronic	NM 00623	c.456+120	p.?	-0.8					POU domain,	rs1815950		YES			POU domai	RNA polym	Bile Duct Diseases;Biliary Tract Diseases;Chc			C=0, T=28	C=0.0, T=1.0	0.00001	408.718	28	C=0/0, T=18/1
chr17:34328461	A/G	A	G	SNV	CCL15,CCL15-CCL14	CCL15-CCL14:exonic_nc:NR 027921.3. CCL15:exonic:NM 0	1	51.85	1	missense	NR 02792	c.71T>C	p.Ile24Thr	-3.3	1	89	0		Small cytokin	rs854625	0.0398	YES	6.657E-1;0.0 (pos.34328461)		Chemokine	CCR chemokine receptor binding,G-protein coupled rece				A=13, G=14	A=0.4815, G=0.5185	0.00001	132.607	27	A=9/4, G=9/5
chr11:111889635	G/G	C	G	SNV	DIXDC1	DIXDC1:intronic:NM 033425.4	1	100		intronic	NM 03342	c.1338-45	p.?	-0.67					DIX domain	rs650461	0.3596	YES	5.839E-1 (pos.111889635)		DIX domain	Wnt signaling pathway,actin binding,canonical Wnt sign				C=0, G=34	C=0.0, G=1.0	0.00001	524.183	34	C=0/0, G=18/1
chr11:111889654	T/T	C	T	SNV	DIXDC1	DIXDC1:intronic:NM 033425.4	1	100		intronic	NM 03342	c.1338-26	p.?	-2.52					DIX domain	rs476581	0.2694	YES	8.198E-1 (pos.111889654)		DIX domain	Wnt signaling pathway,actin binding,canonical Wnt sign				C=0, T=34	C=0.0, T=1.0	0.00001	524.185	34	C=0/0, T=18/1
chr11:111909819	A/A	C	A	SNV	DLAT	DLAT:intronic:NM 001931.4	1	100		intronic	NM 00193	c.976-149	p.?	0.97					2-oxoacid de	rs6589260		YES			Dihydrolipo	acetyl-CoA	Acid-Base Imbalanc	Dihydrolipoic Acid.NADH		C=0, A=35	C=0.0, A=1.0	0.00001	543.903	35	C=0/0, A=30/5
chr11:111916001	A/A	AG	A	INDEL	DLAT	DLAT:intronic:NM 001931.4	1	100		intronic	NM 00193	c.1290+47	p.?	0.29, 0.52					2-oxoacid de	rs11335731.rs373885044.rs782		YES	0.0 (pos.111916002).1.0 (pos.11191600		Dihydrolipo	acetyl-CoA	Acid-Base Imbalanc	Dihydrolipoic Acid.NADH		AG=0. A=59	AG=0.0, A=1.0	0.00001	808.404	59	AG=0/0, A=27/
chr11:111933088	G/G	A	G	SNV	DLAT	DLAT:intronic:NM 001931.4	1	100		intronic	NM 00193	c.1815-42	p.?	-0.33					2-oxoacid de	rs10891315	0.3529	YES	5.843E-1 (pos.111933088)		Dihydrolipo	acetyl-CoA	Acid-Base Imbalanc	Dihydrolipoic Acid.NADH		A=0, G=45	A=0.0, G=1.0	0.00001	751.808	45	A=0/0, G=15/3
chr11:112064431	C/C	T	C	SNV	BCO2	BCO2:intronic:NM 031938.5	1	100		intronic	NM 03193	c.517+11T	p.?	0.19					Retinal pigm	rs7105056	0.0172	YES	9.994E-1 (pos.112064431)		Beta-carotene dioxygenase 2					T=0, C=60	T=0.0, C=1.0	0.00001	903.519	60	T=0/0, C=35/2
chr17:34398495	C/T	C	T	SNV	CCL18	CCL18:utr 3:NM 002988.3	1	50	3	utr 3	NM 00298	c.364C>T	p.?	-0.52					Small cytokin	rs14304		YES			Chemokine	CCR chem	Carcinogenesis;Cell Transformation, Neoplas			C=17, T=17	C=0.5, T=0.5	0.00001	172.019	34	C=4/13, T=1/1
chr4:122741807	C/T	C	T	SNV	CCNA2	CCNA2:exonic:NM 001237.4	1	46.43	4	synonymous	NM 00123	c.684G>A	p.(=)	1.24				0.75(COSM56	Cyclin, C-tern	rs769243	0.0227	YES	2.832E-2 (pos.12274180	diffuse_large_B_cell_lymph		G2/M tran	Intellectual Disabil	(2R)-2-((9-(1-methylethyl		C=13, T=13	C=0.5, T=0.5	0.00001	146.553	28	C=10/3, T=4/9
chr11:11348794	C/T	C	T	SNV	GALNT18	GALNT18:intronic:NM 198516.2	1	57.45		intronic	NM 19851	c.1417-66	p.?	-0.3					Glycosyl tran	rs75793419		YES			UPD-N-acet	Golgi membrane,carbohydrate binding,cellular compon				C=60, T=81	C=0.4255, T=0.5745	0.00001	956.102	141	C=14/46, T=28
chr11:113618470	A/A	T	A	SNV	ZW10	ZW10:intronic:NM 004724.3	1	100		intronic	NM 00472	c.1090-35	p.?	-0.26					Centromere/	rs2459977	0.0431	YES	9.574E-1 (pos.113618470)		Zeste-white	Dsl1/NZR	Neoplasms:Neoplasms by Histologic Type:Ne			T=0, A=29	T=0.0, A=1.0	0.00001	402.887	29	T=0/0, A=17/1
chr11:113618968	C/C	G	C	SNV	ZW10	ZW10:intronic:NM 004724.3	1	100		intronic	NM 00472	c.1089+11	p.?	1.29					Centromere/	rs2513584	0	YES	1.0;0.0 (pos.113618968)		Zeste-white	Dsl1/NZR	Neoplasms:Neoplasms by Histologic Type:Ne			G=0, C=51	G=0.0, C=1.0	0.00001	754.582	51	G=0/0, C=46/5
chr12:4388084	C/G	C	G	SNV	CCND2	CCND2:exonic:NM 001759.3	1	47.37	3	synonymous	NM 00175	c.570C>G	p.(=)	-0.1				0.59(COSM50	Cyclin, C-tern	rs3217805	0.3928	YES	8.397E-2;0.0;0.0 (pos.43	haemangiobla	Cyclin D2	adult loco	Adnexal Diseases;Brain Disea		Benign	C=20, G=18	C=0.5263, G=0.4737	0.00001	184.235	38	C=3/17, G=6/1
chr2:135711516	T/T	C	T	SNV	CCNT2	CCNT2:exonic:NM 058241.2	1	100	9	synonymous	NM 05824	c.1491C>T	p.(=)	0.11					Cyclin, N-term	rs3814355	0.47	YES	8.755E-1 (pos.135711516)		Cyclin T2	7SK snRNA binding,RNA polymerase binding,cell cycle,ce				C=0, T=116	C=0.0, T=1.0	0.00001	1858.41	116	C=0/0, T=72/4
chr16:19547747	G/G	C	G	SNV	CCP110	CCP110:exonic:NM 001199022.2	1	100	4	missense	NM 00119	c.756C>G	p.Ile252Met	-1.05	0.34	10	0.008	0.02(COSM14	Calcium-depe	rs226891	0.0828	YES	7.864E-1 (pos.19547747)	adenocarcino	Centrosom	G2/M transition of mitotic cell cycle,centr			Benign	C=0, G=65	C=0.0, G=1.0	0.00001	996.463	65	C=0/0, G=18/4
chr4:91229430	T/G	T	G	SNV	CCSER1	CCSER1:utr 5:NM 001145065.1	1	57.89	2	utr 5	NM 00114	c.-6T>G	p.?	2.27						rs28592243	0.0066	YES	4.061E-2 (pos.91229430)				Cocaine-Related Disorders:Mental Disorders			T=16, G=22	T=0.4211, G=0.5789	0.00001	249.054	38	T=11/5, G=16/
chr5:10256161	T/C	T	C	SNV	CCT5	CCT5:exonic:NM 012073.4	1	51.85	4	synonymous	NM 01207	c.426T>C	p.(=)	0.02				0.44(COSM55	TCP-1/cpn60	rs1042392	0.1376	YES	8.777E-1;0.0 (pos.10256	carcinoma	chaperonin	'de novo' p	Autoimmune Diseases;Breast		Benign	T=13, C=14	T=0.4815, C=0.5185	0.00001	132.665	27	T=5/8, C=9/5
chr6:74493432	C/C	A	C	SNV	CD109	CD109:exonic:NM 133493.4	1	100	19	missense	NM 13349	c.2108A>C	p.Tyr703Ser	0.73	1	144	0	0.12(COSM50	A-macroglob	rs10455097	0.489	YES	5.183E-1 (pos.74493432)	ductal_carcin	CD109 anti	C-termina	Breast Diseases;Breast Neoplasms;Colonic D			A=0, C=44	A=0.0, C=1.0	0.00001	726.561	44	A=0/0, C=18/2
chr11:116732512	T/T	G	T	SNV	SIK3	SIK3:intronic:NM 025164.4	1	100		intronic	NM 02516	c.2171+45	p.?	-0.7					Kinase-like.P	rs2075292	0.1169	YES	4.957E-1 (pos.116732512)		Salt-inducib	ATP binding.cytoplasm.magnesium ion binding.protein b				G=0, T=22	G=0.0, T=1.0	0.00001	298.826	22	G=0/0, T=8/14
chr11:117059552	A/A	G	A	SNV	SIDT2	SIDT2:intronic:NM 001040455.1	1	100		intronic	NM 00104	c.1278+10	p.?	-3.54				0.04(COSM44	dsRNA-gated	rs474339	0.2421	YES	3.588E-1;0.0 (pos.11705	acute_myeloid	SID1 transm	AP-1 adaptor complex binding.AP-2 adaptor complex bin				G=0, A=48	G=0.0, A=1.0	0.00001	810.421	48	G=0/0, A=21/2
chr6:74497009	G/G	A	G	SNV	CD109	CD109:exonic:NM 133493.4	1	100	21	missense	NM 13349	c.2390A>G	p.Asn797Ser	1.82	0.65	46	0.004	0.92(COSM14	A-macroglob	rs2351528	0.3555	YES	3.949E-1 (pos.74497009)	acute_myeloid	CD109 anti	C-termina	Breast Diseases;Breast Neoplasms;Colonic D			A=0, G=20	A=0.0, G=1.0	0.00001	263.333	20	A=0/0, G=12/8
chr11:117332352	G/G	A	G	SNV	DSCAML1	DSCAML1:intronic:NM 020693.3	1	100		intronic	NM 02069	c.3446-40	p.?	0.16					Fibronectin t	rs588048	5.00E-04		9.669E-1 (pos.117332352)		Down syndr	axonogenesis,brain development,cell adhesion,cell fate				A=0, G=42	A=0.0, G=1.0	0.00001	685.218	42	A=0/0, G=9/33
chr11:117342462	C/C	T	C	SNV	DSCAML1	DSCAML1:intronic:NM 020693.3	1	100		intronic	NM 02069	c.3133+12	p.?	0.08					Fibronectin t	rs540659		YES			Down syndr	axonogenesis,brain development,cell adhesion,cell fate				T=0, C=24	T=0.0, C=1.0	0.00001	322.643	24	T=0/0, C=18/6
chr11:117774146	C/G	C	G	SNV	TMPRSS13	TMPRSS13:intronic:NM 001077263.2	1	57.14		intronic	NM 00107	c.1677+22	p.?	-0.04					Domain of un	rs2186627		YES	1.833E-1 (pos.117774146)		Transmembrane protein, serine 13					C=21, G=28	C=0.4286, G=0.5714	0.00001	342.312	49	C=4/17, G=20/
chr12:7649653	C/C	T	C	SNV	CD163	CD163:exonic:NM 004244.5	1	100	5	synonymous	NM 00424	c.855A>G	p.(=)	-1.41				0.18(COSM56	Scavenger re	rs4883264	0.2387	YES	3.816E-1 (pos.7649653)	diffuse_large	CD163 anti	acute-pha	Abortion. Spontane	WF10		T=0, C=28	T=0.0, C=1.0	0.00001	408.718	28	T=0/0, C=24/4
chr11:117965595	T/T	C	T	SNV	TMPRSS4	TMPRSS4:intronic:NM 019894.3	1	100		intronic	NM 01989	c.43+25C>	p.?	-0.21					Domain of un	rs2276123	0.3444	YES	3.239E-1 (pos.117965595)		Transmemb	integral component of membrane,membrane,protein bi				C=0, T=43	C=0.0, T=1.0	0.00001	705.84	43	C=0/0, T=22/2
chr11:117965646	A/A	G	A	SNV	TMPRSS4	TMPRSS4:intronic:NM 019894.3	1	100		intronic	NM 01989	c.43+76G>	p.?	-0.15					Domain of un	rs2276125	0.3385	YES			Transmemb	integral component of membrane,membrane,protein bi				G=0, A=42	G=0.0, A=1.0	0.00001	685.174	42	G=0/0, A=21/2
chr11:118067426	A/G	A	G	SNV	JAML	JAML:intronic:NM 001098526.1	1	57.14		intronic	NM 00109	c.1092+24	p.?	-0.29					Immunoglob	rs648947	0.411	YES	5.743E-1 (pos.118067426)		Adhesion m	bicellular tight junction,cell adhesion molecule binding,				A=15, G=20	A=0.4286, G=0.5714	0.00001	218.716	35	A=10/5, G=13/
chr11:118083664	G/G	A	G	SNV	JAML	JAML:intronic:NM 001098526.1	1	100		intronic	NM 00109	c.44-388T	p.?	-0.62					Immunoglob	rs1715429	0.2416	YES	6.96E-1 (pos.118083664)		Adhesion m	bicellular tight junction.cell adhesion molecule binding.				A=0, G=22	A=0.0, G=1.0	0.00001	298.697	22	A=0/0, G=12/1
chr11:118100699	CAT/C	CAT	C	INDEL	MPZL3	MPZL3:intronic:NM 198275.2	2	59.09		intronic	NM 19827	c.682-64A	p.?	-0.81, -0.35, -1.64					Immunoglob	rs113464612.rs2010	0.2193	YES			Myelin prot	cell adhesion.extracellular matrix organization.hair cycl				CAT=9. C=1	CAT=0.4091. C=0.59	0.00001	126.644	22	CAT=6/3, C=6/
chr12:7551050	C/C	A	C	SNV	CD163L1	CD163L1:exonic:NM 174941.5	1	100	7	synonymous	NM 17494	c.1539T>G	p.(=)	-1.75					Scavenger re	rs6488269	1.00E-04		1.0;0.0 (pos.7551050)			extracellu	Melanoma;Neoplasms;Neoplasms by Histolo			A=0, C=50	A=0.0, C=1.0	0.00001	852.748	50	A=0/0, C=9/41
chr11:118134785	T/G	T	G	SNV	MPZL2	MPZL2:intronic:NM 005797.3	1	45.83		intronic	NM 00579	c.58+26A>	p.?	0.13					Immunoglob	rs1624780	0.3888	YES	4.682E-1 (pos.118134785)		Myelin prot	anatomical structure morphogenesis,cytoskeleton,hom				T=13, G=11	T=0.5417, G=0.4583	0.00001	93.3764	24	T=8/5, G=5/6
chr11:118210106	C/C	G	C	SNV	CD3D	CD3D:intronic:NM 000732.4	1	100		intronic	NM 00073	c.450+60C	p.?	-0.79					Immunorece	rs2276423	0.2612	YES			CD3D antig	T cell costi	Bile Duct Diseases:	Blinatumomab.Muromo		G=0, C=99	G=0.0, C=1.0	0.00001	1680.95	99	G=0/0, C=58/4
chr12:7551022	A/T	A	T	SNV	CD163L1	CD163L1:exonic:NM 174941.5	1	98.85	7	missense	NM 17494	c.1567T>A	p.Leu523Met	-5.32	0.2	15	0.42		Scavenger re	rs6488268	1.00E-04		1.0 (pos.7551022)			extracellu	Melanoma:Neoplasms:Neoplasms by Histolo			A=1, T=86	A=0.0115, T=0.9885	0.00001	1395	87	A=0/1, T=34/5
chr11:118920453	C/C	T	C	SNV	HYOU1	HYOU1:intronic:NM 001130991.2	1	100		intronic	NM 00113	c.1726+36	p.?	-4.63					Hsp70 protei	rs673768	0.1421	YES	9.535E-1 (pos.118920453)		Hypoxia-up	ATP bindin	Ataxia;Brain Diseases;Central Nervous Syste			T=0, C=31	T=0.0, C=1.0	0.00001	465.816	31	T=0/0, C=15/1
chr6:109689431	A/G	A	G	SNV	CD164	CD164:utr 3:NM 006016.5	1	58.33	6	utr 3	NM 00601	c.1217T>C	p.?	2.87					Multi-glycosy	rs974034		YES			CD164 anti	cell adhes	Abortion, Spontaneous;Female Urogenital Di			A=90, G=12	A=0.4167, G=0.5833	0.00001	1458.95	216	A=57/33, G=7
chr11:118949799	G/A	G	A	SNV	VPS11	VPS11:intronic:NM 021729.5	1	47.62		intronic	NM 02172	c.2266-40	p.?	-0.3					Region in Cla	rs1168568	0.4072	YES	1.804E-1 (pos.118949799)		Vacuolar pr	AP-3 adaptor complex,CORVET complex,HOPS complex,a				G=22, A=20	G=0.5238, A=0.4762	0.00001	212.952	42	G=17/5, A=4/1
chr5:66480004	C/C	T	C	SNV	CD180	CD180:exonic:NM 005582.2	1	100	3	missense	NM 00558	c.667A>G	p.Ile223Val	-0.78	0.66	29	0		Leucine Rich	rs1697144	1.00E-04		9.74E-1 (pos.66480004)		CD180 anti	B cell proliferation involved in immune response,cellula				T=0, C=35	T=0.0, C=1.0	0.00001	543.892	35	T=0/0, C=15/2
chr11:119181674	C/C	G	C	SNV	MCAM	MCAM:intronic:NM 006500.2	1	100		intronic	NM 00650	c.1646-61	p.?	-0.52					CD80-like C2-	rs2510146		YES	6.327E-1 (pos.119181674)		Melanoma	anatomica	Autoimmune Diseases:Autoimmune Disease			G=0, C=129	G=0.0, C=1.0	0.00001	2092.04	129	G=0/0, C=83/4
chr11:119215165	T/T	C	T	SNV	C1QTNF5,MFRP	MFRP:intronic:NM 031433.3, C1QTNF5:intronic:NM 01564	1	100		intronic	NM 01564	c.-1737-64	p.?, p.?	0.73					CUB domain,I	rs10790289	0.4931	YES			C1q- and tu	apical pla	Congenital Abnormalities;Congenital, Heredi			C=0, T=163	C=0.0, T=1.0	0.00001	2639.29	163	C=0/0, T=59/1
chr3:112642568	G/G	C	G	SNV	CD200R1	CD200R1:exonic:NM 138806.3	1	100	8	missense	NM 13880	c.1003G>C	p.Glu335Gln	0.07	1	29	0	0(COSM3759	CD80-like C2-	rs9865242	0.4672	YES	6.792E-1 (pos.11264256	adenocarcino	CD200 rece	external side of plasma membrane,extracellular region,				C=0, G=34	C=0.0, G=1.0	0.00001	524.186	34	C=0/0, G=22/1
chr11:119252151	C/T	C	T	SNV	USP2,USP2-AS1	USP2-AS1:upstream:NR 034160.1. USP2:intronic:NM 0042	1	57.58		intronic	NM 00420	c.-41+32G	p.?	0.53					Ubiquitin car	rs565296		YES	9.4E-1 (pos.119252151)			cell cortex	Adenocarcinoma:Carcinoma:Carcinoma, Hep			C=14, T=19	C=0.4242, T=0.5758	0.00001	204.392	33	C=8/6, T=10/9
chr3:112648222	C/C	T	C	SNV	CD200R1	CD200R1:exonic:NM 138806.3	1	100	4	missense	NM 13880	c.335A>G	p.Lys112Arg	-1.32	1	26	0	0(COSM3759	CD80-like C2-	rs2171509	0.4679	YES	6.792E-1 (pos.11264822	adenocarcino	CD200 rece	external side of plasma membrane,extracellular region,				T=0, C=24	T=0.0, C=1.0	0.00001	334.792	24	T=0/0, C=17/7
chr11:119510644	C/C	A	C	SNV	NECTIN1		1	100		intronic				0.07						rs7940667	0.0693	YES	9.487E-1 (pos.119510644)		Nectin 1					A=0, C=42	A=0.0, C=1.0	0.00001	685.16	42	A=0/0, C=24/1
chr3:112546473	A/A	G	A	SNV	CD200R1L	CD200R1L:exonic:NM 001008784.2	1	100	3	synonymous	NM 00100	c.171C>T	p.(=)	-0.18				0.02(COSM37	CD80-like C2-	rs1997281	0.3992	YES	3.716E-1 (pos.11254647	adenocarcinoma		external side of plasma membrane.integral component				G=0, A=47	G=0.0, A=1.0	0.00001	789.324	47	G=0/0, A=31/1
chr2:71062648	G/A	G	A	SNV	CD207	CD207:exonic:NM 015717.4	1	59.09	2	missense	NM 01571	c.164C>T	p.Ala55Val	2.13	0.45	64	0.267	0.43(COSM14	Lectin C-type	rs10489990	0.3489	YES	2.802E-1 (pos.71062648)	acute_myeloid	CD207 anti	antigen processing and presentation of exogenous pepti				G=9, A=13	G=0.4091, A=0.5909	0.00001	124.344	22	G=4/5, A=7/6
chr18:67531642	C/C	T	C	SNV	CD226	CD226:exonic:NM 006566.3	1	100	7	missense	NM 00656	c.919A>G	p.Ser307Gly	-0.19	0.39	56	0.001	0.02(COSM11	Immunoglob	rs763361	0.4794	YES	6.367E-1;1.157E-4 (pos.6	adenoma,basa	DNAX acces	cell adhes	Abortion, Spontaneous;Arthritis;Arthritis, Rh			T=0, C=120	T=0.0, C=1.0	0.00001	1932.33	120	T=0/0, C=81/3
chr11:120319064	C/C	A	C	SNV	ARHGEF12	ARHGEF12:intronic:NM 015313.2	1	100		intronic	NM 01531	c.1737+7A	p.?	-0.49				0.02(COSM44	PDZ domain,I	rs2305008	0.3939	YES	3.226E-1 (pos.12031906	acute_myeloid	Rho guanin	G-protein	Leukemia;Leukemia, Myeloid;Leukemia, Mye			A=0, C=41	A=0.0, C=1.0	0.00001	664.7	41	A=0/0, C=30/1
chr12:6554628	G/A	G	A	SNV	CD27,CD27-AS1	CD27:exonic:NM 001242.4. CD27-AS1:intronic_nc:NR 0153	1	50	2	missense	NR 01538	c.175G>A	p.Ala59Thr	0.84	0.31	58	0.473	0.08(COSM37	TNFR/NGFR c	rs25680	0.2299	YES	5.916E-2 (pos.6554628)	malignant_me	CD27 antig	cell surfac	Nervous System Diseases:Neuromuscular Di			G=18, A=18	G=0.5, A=0.5	0.00001	185.885	36	G=16/2, A=15/
chr12:6560473	G/G	A	G	SNV	CD27,CD27-AS1,TAPBPL	CD27:exonic:NM 001242.4, TAPBPL:upstream:NM 018009.	1	100	6	missense	NR 01538	c.698A>G,	p.His233Arg, p.?	0.36	0.84	29	0	0.04(COSM41	TNFR/NGFR c	rs2532502	0.0107		1.0 (pos.6560473)	neoplasm	CD27 antig	Golgi mem	Nervous System Diseases;Neu		Benign	A=0, G=36	A=0.0, G=1.0	0.00001	563.744	36	A=0/0, G=14/2
chr12:6560573	T/T	G	T	SNV	CD27,CD27-AS1,TAPBPL	CD27:utr 3:NM 001242.4. TAPBPL:upstream:NM 018009.4	1	100	1	utr 3	NR 01538	c.798G>T.	p.?, p.?	0.05					TNFR/NGFR c	rs1059501	0.4366	YES	4.366E-1;0.0 (pos.6560573)		CD27 antig	Golgi mem	Nervous System Diseases:Neu		Benign	G=0, T=29	G=0.0, T=1.0	0.00001	427.603	29	G=0/0, T=4/25
chr6:47445927	A/A	T	A	SNV	CD2AP	CD2AP:utr 5:NM 012120.2	1	100	1	utr 5	NM 01212	c.-54T>A	p.?	-0.75					SH3 domain,	rs9349406	0.0032	YES			CD2-associ	SH3 doma	Alzheimer Disease;Brain Disea		Benign	T=0, A=22	T=0.0, A=1.0	0.00001	298.891	22	T=0/0, A=13/9
chr11:121414456	A/C	A	C	SNV	SORL1	SORL1:intronic:NM 003105.5	1	50		intronic	NM 00310	c.1864+21	p.?	0.82					Fibronectin t	rs2276345	0.0194	YES	2.059E-1 (pos.121414456)		Sortilin-rela	ADP-ribos	Alzheimer Disease;Brain Diseases;Central Ne			A=31, C=31	A=0.5, C=0.5	0.00001	305.358	62	A=19/12, C=18
chr11:121437571	G/G	A	G	SNV	SORL1	SORL1:intronic:NM 003105.5	1	100		intronic	NM 00310	c.3050-78	p.?	-0.41					Fibronectin t	rs1784920		YES			Sortilin-rela	ADP-ribos	Alzheimer Disease;Brain Diseases;Central Ne			A=0, G=21	A=0.0, G=1.0	0.00001	281.215	21	A=0/0, G=5/16
chr6:47563692	T/T	C	T	SNV	CD2AP	CD2AP:exonic:NM 012120.2	1	100	12	synonymous	NM 01212	c.1204C>T	p.(=)	-0.29					SH3 domain,	rs2039503	1.00E-04		1.0 (pos.47563692)		CD2-associ	SH3 doma	Alzheimer Disease;Brain Disea		Benign	C=0, T=43	C=0.0, T=1.0	0.00001	705.839	43	C=0/0, T=22/2
chr17:72700943	G/G	A	G	SNV	CD300LF	CD300LF:exonic:NM 139018.4	1	100	2	missense	NM 13901	c.56T>C	p.Val19Ala	0.03	1	64	0		Immunoglob	rs35489971	0.1863	YES	7.437E-1 (pos.72700943)		CD300 anti	TRIF-dependent toll-like receptor signaling pathway,cera				A=0, G=44	A=0.0, G=1.0	0.00001	726.545	44	A=0/0, G=26/1
chr2:160639970	C/C	T	C	SNV	CD302,LY75-CD302	CD302:exonic:NM 014880.4, LY75-CD302:exonic:NM 0011	1	100	35	synonymous	NM 01488	c.78A>G, c	p.(=), p.(=)	-0.39				0.31(COSM37	Fibronectin t	rs2114626	0.3516	YES	3.433E-1 (pos.16063997	acute_myeloid	CD302 anti	carbohydrate binding,cell cortex,endocytosis,extracellu				T=0, C=107	T=0.0, C=1.0	0.00001	1681.3	107	T=0/0, C=54/5
chr11:12246204	A/A	G	A	SNV	MICAL2	MICAL2:intronic:NM 014632.3	1	100		intronic	NM 01463	c.1541-16	p.?	-1.06					CAMSAP CH d	rs78986346	0.1325	YES	5.331E-1 (pos.12246204)		Microtubul	FAD binding,NADPH:sulfur oxidoreductase activity,actin				G=0, A=40	G=0.0, A=1.0	0.00001	644.28	40	G=0/0, A=27/1
chr1:160654786	A/A	G	A	SNV	CD48	CD48:exonic:NM 001256030.1	1	100	2	synonymous	NM 00125	c.276C>T	p.(=)	-4.24				0.01(COSM37	Immunoglob	rs1980606	0.2227	YES	1.438E-1 (pos.16065478	adenocarcino	CD48 antigen (B-cell membrane protein)					G=0, A=34	G=0.0, A=1.0	0.00001	524.183	34	G=0/0, A=14/2
chr1:26646726	G/G	A	G	SNV	CD52	CD52:exonic:NM 001803.2	1	100	2	missense	NM 00180	c.119A>G	p.Asn40Ser	0.16	1	46	0.009	0.01(COSM11	CAMPATH-1 a	rs1071849	0.2345	YES	4.292E-1 (pos.26646726)	carcinoma,ne	CD52 molec	C-terminal protein lipidation,a		Alemtuzumab		A=0, G=40	A=0.0, G=1.0	0.00001	644.286	40	A=0/0, G=29/1
chr1:26646730	G/G	A	G	SNV	CD52	CD52:exonic:NM 001803.2	1	100	2	missense	NM 00180	c.123A>G	p.Ile41Met	0.09	0.24	10	0	0(COSM1180	CAMPATH-1 a	rs17645	0.2345	YES	4.293E-1 (pos.26646730)	acute_myeloid	CD52 molec	C-terminal protein lipidation,a		Alemtuzumab		A=0, G=40	A=0.0, G=1.0	0.00001	644.285	40	A=0/0, G=29/1
chr1:111441829	T/T	C	T	SNV	CD53	CD53:utr 3:NM 001040033.1	1	100	9	utr 3	NM 00104	c.672C>T	p.?	-0.45					Tetraspanin f	rs1803259	0.2365	YES	4.83E-1;0.0 (pos.111441829)		CD53 antig	cell surfac	Autoimmune Diseases;Female Urogenital Di			C=0, T=97	C=0.0, T=1.0	0.00001	1638.04	97	C=0/0, T=51/4
chr12:9906067	C/C	T	C	SNV	CD69	CD69:utr 3:NM 001781.2	1	100	5	utr 3	NM 00178	c.610A>G	p.?	-0.91					Lectin C-type	rs1051065.rs765367	0.2695	YES	0.0 (pos.9906065).0.0 (pos.9906066).9.5		CD69 antig	calcium io	Abortion. Spontaneous:Arthritis:Arthritis. Ex			T=0, C=71	T=0.0, C=1.0	0.00001	1099.03	71	T=0/0, C=40/3
chr17:62006497	G/G	A	G	SNV	CD79B	CD79B:utr 3:NM 001039933.2	1	100	6	utr 3	NM 00103	c.782T>C	p.?	-0.03					Immunoglob	rs1051684		YES			CD79B anti	B cell diffe	Digestive System Diseases:Hemic and Lymph			A=0, G=110	A=0.0, G=1.0	0.00001	1737.98	110	A=0/0, G=93/1
chr11:12379792	C/C	G	C	SNV	MICALCL	MICALCL:intronic:NM 032867.2	1	100		intronic	NM 03286	c.1904-50	p.?	0.23					Protein of un	rs10741583	0.1038	YES	9.361E-1;0.0 (pos.12379792)		MICAL C ter	biological_process,cell differentiation,cytoplasm,mitog				G=0, C=37	G=0.0, C=1.0	0.00001	583.706	37	G=0/0, C=22/1
chr17:62007498	G/G	A	G	SNV	CD79B	CD79B:exonic:NM 001039933.2	1	100	3	synonymous	NM 00103	c.369T>C	p.(=)	0.73				0(COSM1483	Immunoglob	rs2070776	0.3474	YES	4.596E-1 (pos.62007498)	adenocarcino	CD79B anti	B cell diffe	Digestive System Diseases:He		Benign	A=0, G=40	A=0.0, G=1.0	0.00001	644.285	40	A=0/0, G=16/2
chr3:121825197	A/A	G	A	SNV	CD86	CD86:exonic:NM 175862.4	1	100	4	missense	NM 17586	c.553G>A	p.Val185Ile	-1.16	0.44	29	0.009	0.01(COSM41	Immunoglob	rs2681417	0.0695	YES	9.983E-1 (pos.12182519	neoplasm	CD86 antig	B cell acti	Autoimmune Disea	Abatacept,Antithymocyt		G=0, A=22	G=0.0, A=1.0	0.00001	298.892	22	G=0/0, A=12/1
chr20:23065209	A/A	G	A	SNV	CD93	CD93:exonic:NM 012072.3	1	100	1	missense	NM 01207	c.1621C>T	p.Pro541Ser	2.36	0.06	74	0.192	0.25(COSM37	Calcium-bind	rs3746731	0.4334	YES	6.466E-1 (pos.23065209)	acute_myeloid	Complemer	calcium ion binding,carbohydrate binding,cell surface,ce				G=0, A=62	G=0.0, A=1.0	0.00001	940.32	62	G=0/0, A=42/2
chr3:111356083	C/C	G	C	SNV	CD96	CD96:exonic:NM 005816.4	1	100	11	synonymous	NM 00581	c.1362G>C	p.(=)	0.04						rs1533270	0		1.0 (pos.111356083)		CD96 antig	adherens	Bone Diseases:Bone Diseases,		Benign	G=0, C=39	G=0.0, C=1.0	0.00001	621.178	39	G=0/0, C=18/2
chr1:43824865	C/T	C	T	SNV	CDC20	CDC20:utr 5:NM 001255.2	1	50	2	utr 5	NM 00125	c.-22C>T	p.?	0.37					Anaphase-pr	rs1051097	0.0329	YES	2.271E-2 (pos.43824865)		Cell division	anaphase	Autoimmune Diseases;Female Urogenital Di			C=21, T=21	C=0.5, T=0.5	0.00001	228.883	42	C=14/7, T=17/
chr5:54410120	C/C	A	C	SNV	CDC20B	CDC20B:exonic:NM 001170402.1	1	100	12	missense	NM 00117	c.1486T>G	p.Ser496Ala	0.69	1	99	0		WD domain,	rs3104230	0.0174	YES	9.999E-1 (pos.54410120)			anaphase-promoting complex binding,positive regulatio				A=0, C=46	A=0.0, C=1.0	0.00001	772.682	46	A=0/0, C=15/3
chr5:137665323	G/A	G	A	SNV	CDC25C	CDC25C:exonic:NM 001790.4	1	52.38	3	missense	NM 00179	c.208C>T	p.Arg70Cys	-0.84	0.37	180	0.003	0.11(COSM37	M-phase indu	rs3734166	0.2777	YES	6.048E-1 (pos.13766532	acute_myeloid	Cell division	DNA damage response, signal transduction by p53 class				G=10, A=11	G=0.4762, A=0.5238	0.00001	95.3001	21	G=6/4, A=5/6
chr1:227182575	G/G	A	G	SNV	CDC42BPA	CDC42BPA:exonic:NM 003607.3	1	100	35	synonymous	NM 00360	c.4977T>C	p.(=)	-4.61					CNH domain,	rs1045247	0.1897	YES	8.427E-1 (pos.227182575)		CDC42-bind	ATP binding,actin cytoskeleton organization,actin cytosk				A=0, G=49	A=0.0, G=1.0	0.00001	831.501	49	A=0/0, G=32/1

chr1:151028234	T/T	G	T	SNV	CDC42SE1	CDC42SE1:utr_5:NM_020239.3	1	100	2	utr_5	NM_02023	c.-28C>A	p.?	-0.16				
chr3:45134822	C/C	T	C	SNV	CDCP1	CDCP1:exonic:NM_022842.4	1	100	6	missense	NM_02284	c.1574A>G	p.Gln525Arg	-0.24	0.88	43	0	
chr1:54607051	G/G	A	G	SNV	CDCP2	CDCP2:exonic:NM_201546.4	1	100	4	synonymous	NM_20154	c.483T>C	p.(=)	3.96				
chr16:65016087	C/C	A	C	SNV	CDH11	CDH11:exonic:NM_001797.3	1	100	8	missense	NM_00179	c.1117T>G	p.Ser373Ala	-0.18	0.55	99	0	
chr11:124856951	T/T	C	T	SNV	CCDC15	CCDC15:intronic:NM_025004.2	1	100		intronic	NM_02500	c.901-72C	p.?	0				
chr16:83065664	A/A	G	A	SNV	CDH13	CDH13:exonic:NM_001220488.1	1	100	4	synonymous	NM_00122	c.348G>A	p.(=)	-0.2				0.16(COSM37
chr11:125298871	G/G	A	G	SNV	PKNOX2	PKNOX2:intronic:NM_022062.2	1	100		intronic	NM_02206	c.937-37A	p.?	-1.71				
chr11:12534808	G/G	A	G	SNV	PARVA	PARVA:intronic:NM_018222.4	1	100		intronic	NM_01822	c.857-38A	p.?	-0.56				
chr8:95143186	G/G	C	G	SNV	CDH17	CDH17:exonic:NM_004063.3	1	100	16	missense	NM_00406	c.2202G>C	p.Glu734Asp	0.91	0.93	45	0	0(COSM3763
chr11:125647712	C/C	T	C	SNV	PATE2	PATE2:intronic:NM_212555.2	1	100		intronic	NM_21255	c.205+57A	p.?	-2.16				
chr11:1257867	C/C	T	C	SNV	MUC5B	MUC5B:intronic:NM_002458.2	1	100		intronic	NM_00245	c.3057+75	p.?	-0.7				
chr11:1257886	T/T	C	T	SNV	MUC5B	MUC5B:intronic:NM_002458.2	1	100		intronic	NM_00245	c.3057+94	p.?	-5				
chr8:95188850	T/C	T	C	SNV	CDH17	CDH17:exonic:NM_004063.3	1	95.45	5	missense	NM_00406	c.343A>G	p.Lys115Glu	0.54	1	56	0	
chr8:95161102	G/A	G	A	SNV	CDH17	CDH17:exonic:NM_004063.3	1	57.58	14	synonymous	NM_00406	c.1797C>T	p.(=)	-1.36				0.02(COSM39
chr11:125864973	A/A	AAC	A	INDEL	CDON	CDON:intronic:NM_001243597.1	2	100		intronic	NM_00124	c.2363-26	p.?	0.97, 0.72, -1.09				
chr11:126139100	C/C	T	C	SNV	FOXRED1,SRPRA	SRPRA:upstream:NM_003139.3, FOXRED1:utr_5:NM_01754	1	100		intronic	NM_00313	c.-401A>G	p.?, p.?	-0.71				
chr18:64176365	T/T	G	T	SNV	CDH19	CDH19:exonic:NM_021153.3	1	100	11	synonymous	NM_02115	c.1695C>A	p.(=)	-0.36				0.28(COSM41
chr18:25543387	A/A	G	A	SNV	CDH2	CDH2:exonic:NM_001792.4	1	100	15	synonymous	NM_00179	c.2448C>T	p.(=)	-2.5				0.1(COSM148
chr18:25565082	G/G	A	G	SNV	CDH2	CDH2:exonic:NM_001792.4	1	100	13	synonymous	NM_00179	c.2091T>C	p.(=)	-2.97				
chr11:1275592	AA/AA	GC	AA	MNV	MUC5B	MUC5B:intronic:NM_002458.2	2	100		intronic	NM_00245	c.15477+1	p.?	-2.02, -1.12				
chr11:1279486	G/G	A	G	SNV	MUC5B	MUC5B:intronic:NM_002458.2	1	100		intronic	NM_00245	c.16555+5	p.?	1.3				
chr11:1279527	T/T	A	T	SNV	MUC5B	MUC5B:intronic:NM_002458.2	1	100		intronic	NM_00245	c.16556-3	p.?	-0.34				
chr10:73520632	C/C	A	C	SNV	CDH23,VSIR	CDH23:intronic:NM_022124.5, VSIR:exonic:NM_022153.1	1	100	3	missense	NM_02212	c.4846-16	p.?, p.Asp187Glu	-0.7	1	45	0	0.09(COSM37
chr11:1281787	A/A	G	A	SNV	MUC5B	MUC5B:intronic:NM_002458.2	1	100		intronic	NM_00245	c.16967-6	p.?	-0.51				
chr16:68721533	G/C	G	C	SNV	CDH3	CDH3:exonic:NM_001793.5	1	50	12	missense	NM_00179	c.1689G>C	p.Gln563His	-0.05	0.26	24	0.029	0.11(COSM37
chr20:60485626	AT/AT	A	AT	INDEL	CDH4	CDH4:exonic:NM_001794.4	1	100	9	frameshiftInsertion	NM_00179	c.1337_13	p.Val448fs	8.7				
chr20:60512028	G/C	G	C	SNV	CDH4	CDH4:utr_3:NM_001794.4	1	45.83	16	utr_3	NM_00179	c.2778G>C	p.?	-0.81				
chr11:128782214	A/A	G	A	SNV	KCNJ5	KCNJ5:intronic:NM_000890.3	1	100		intronic	NM_00089	c.937+109	p.?	-1.41				
chr11:129743012	A/G	A	G	SNV	NFRKB	NFRKB:intronic:NM_006165.3	1	50		intronic	NM_00616	c.2626-21	p.?	-0.27				
chr11:130064223	G/G	A	G	SNV	ST14	ST14:intronic:NM_021978.3	1	100		intronic	NM_02197	c.1015+40	p.?	-0.56				
chr11:130064705	G/G	A	G	SNV	ST14	ST14:intronic:NM_021978.3	1	100		intronic	NM_02197	c.1113+73	p.?	-0.57				
chr20:60503350	A/G	A	G	SNV	CDH4	CDH4:exonic:NM_001794.4	1	45.45	12	missense	NM_00179	c.1874A>G	p.Lys625Arg	0.4	0.84	26	0	0.21(COSM37
chr11:13399791	G/G	A	G	SNV	ARNTL	ARNTL:intronic:NM_001178.5	1	100		intronic	NM_00117	c.1412-93	p.?	0.01				
chr16:66420885	T/T	C	T	SNV	CDH5	CDH5:exonic:NM_001795.4	1	100	3	synonymous	NM_00179	c.384C>T	p.(=)	0.14				0.11(COSM50
chr5:31302288	T/T	C	T	SNV	CDH6	CDH6:exonic:NM_004932.3	1	100	6	synonymous	NM_00493	c.882C>T	p.(=)	-0.49				
chr11:13716530	A/G	A	G	SNV	FAR1	FAR1:intronic:NM_032228.5	1	57.63		intronic	NM_03222	c.189+29A	p.?	0.03				
chr11:14501296	C/T	C	T	SNV	COPB1	COPB1:intronic:NM_001144062.1	1	50		intronic	NM_00114	c.1213-36	p.?	-0.39				
chr5:31317494	T/T	C	T	SNV	CDH6	CDH6:exonic:NM_004932.3	1	100	10	synonymous	NM_00493	c.1525C>T	p.(=)	1.23				
chr11:15198772	T/T	G	T	SNV	INSC	INSC:intronic:NM_001031853.4	1	100		intronic	NM_00103	c.596+63G	p.?	1.54				
chr5:31317529	G/G	A	G	SNV	CDH6	CDH6:exonic:NM_004932.3	1	100	10	synonymous	NM_00493	c.1560A>G	p.(=)	0.03				
chr11:16823059	C/C	T	C	SNV	PLEKHA7	PLEKHA7:intronic:NM_175058.4	1	100		intronic	NM_17505	c.2307+15	p.?	-0.1				
chr5:31317952	C/C	T	C	SNV	CDH6	CDH6:exonic:NM_004932.3	1	100	11	synonymous	NM_00493	c.1803T>C	p.(=)	-0.09				
chr5:176005477	T/T	C	T	SNV	CDHR2	CDHR2:exonic:NM_017675.4	1	100	16	synonymous	NM_01767	c.1686C>T	p.(=)	-3.32				0.24(COSM37
chr11:17415190	C/G	C	G	SNV	ABCC8	ABCC8:intronic:NM_000352.4	1	59.09		intronic	NM_00035	c.4608+54	p.?	-0.06				
chr11:17436936	G/G	A	G	SNV	ABCC8	ABCC8:intronic:NM_000352.4	1	100		intronic	NM_00035	c.2256-50	p.?	-0.25				
chr5:176011889	A/A	G	A	SNV	CDHR2	CDHR2:exonic:NM_017675.4	1	100	19	synonymous	NM_01767	c.2607G>A	p.(=)	-0.6				0.04(COSM56
chr11:17742681	C/C	G	C	SNV	MYOD1	MYOD1:intronic:NM_002478.4	1	100		intronic	NM_00247	c.710-121	p.?	-1.32				
chr11:1776024	T/T	TC	T	INDEL	CTSD	CTSD:intronic:NM_001909.4	1	100		intronic	NM_00190	c.827+112	p.?	0.05, 0.0				
chr7:105658451	G/A	G	A	SNV	CDHR3	CDHR3:exonic:NM_152750.4	1	47.62	12	missense	NM_15275	c.1586G>A	p.Cys529Tyr	3.35	1	194	0	
chr1:1650845	G/A	G	A	SNV	CDK11A,CDK11B	CDK11A:exonic:NM_001313896.1, CDK11B:exonic:NM_0334	1	96.77	4	missense	NM_03348	c.277C>T,	p.Arg93Trp, p.Arg93Trp	6.45	0.0, 0.0	101.0, 101	0.999, 1.0	0.97(COSM41
chr12:56360876	G/A	G	A	SNV	CDK2	CDK2:exonic:NM_001798.4	1	47.37	1	synonymous	NM_00179	c.84G>A	p.(=)	1.11				0.45(COSM50
chr4:184367558	T/T	TCTG	T	INDEL	CDKN2AIP	CDKN2AIP:exonic:NM_017632.3	3	100	3	nonframeshiftDeletion	NM_01763	c.723_725	p.Ala242del	-0.26, 4.25, 0.05, 0.83				
chr19:10679240	G/G	C	G	SNV	CDKN2D	CDKN2D:exonic:NM_001800.3	1	100	1	synonymous	NM_00180	c.90G>C	p.(=)	0.93				0.27(COSM37
chr19:10676423	A/A	G	A	SNV	CDKN2D,KRI1	CDKN2D:downstream:NM_001800.3, KRI1:exonic:NM_0230	1	100	2	synonymous	NM_02300	c.156C>T,	p.(=), p.?	0.76				
chr11:18267027	T/T	C	T	SNV	SAA2,SAA2-SAA4	SAA2:intronic:NM_001127380.2, SAA2-SAA4:intronic:NM_00	1	100		intronic	NM_00119	c.230+430	p.?, p.?	-1.21				
chr11:18267353	G/G	A	G	SNV	SAA2,SAA2-SAA4	SAA2:intronic:NM_001127380.2, SAA2-SAA4:intronic:NM_00	1	100		intronic	NM_00119	c.230+104	p.?, p.?	-2.76				
chr11:18267356	G/G	A	G	SNV	SAA2,SAA2-SAA4	SAA2:intronic:NM_001127380.2, SAA2-SAA4:intronic:NM_00	1	100		intronic	NM_00119	c.230+101	p.?, p.?	-3.27				
chr11:18267373	T/T	C	T	SNV	SAA2,SAA2-SAA4	SAA2:intronic:NM_001127380.2, SAA2-SAA4:intronic:NM_00	1	100		intronic	NM_00119	c.230+84G	p.?, p.?	-3.39				
chr11:18290902	CC/CC	TT	CC	MNV	SAA1	SAA1:intronic:NM_199161.4	2	100		intronic	NM_19916	c.228+24T	p.?	-0.08, -0.67				
chr11:18290906	A/A	G	A	SNV	SAA1	SAA1:intronic:NM_199161.4	1	100		intronic	NM_19916	c.228+28G	p.?	-0.07				
chr11:18303404	T/T	C	T	SNV	HPS5	HPS5:intronic:NM_181507.1	1	100		intronic	NM_18150	c.3329+93	p.?	-1.72				
chr19:10676681	C/C	T	C	SNV	CDKN2D,KRI1	CDKN2D:downstream:NM_001800.3, KRI1:exonic:NM_0230	1	100	1	missense	NM_02300	c.13A>G, c	p.Thr5Ala, p.?	0.89	1	58	0	0(COSM3749
chr11:18456181	G/G	A	G	SNV	LDHC	LDHC:intronic:NM_002301.4	1	100		intronic	NM_00230	c.419-106	p.?	-1.31				
chr11:1855854	C/C	G	C	SNV	SYT8	SYT8:intronic:NM_138567.4	1	100		intronic	NM_13856	c.30+23G>	p.?	-0.64				
chr11:125830970	T/T	A	T	SNV	CDON	CDON:exonic:NM_001243597.1	1	100	20	missense	NM_00124	c.3731T>A	p.Ile1244Asn	0.44	1	149	0	
chr11:125831701	G/A	G	A	SNV	CDON	CDON:exonic:NM_001243597.1	1	53.13	19	synonymous	NM_00124	c.3549C>T	p.(=)	-0.08				
chr11:18735321	C/C	A	C	SNV	IGSF22	IGSF22:intronic:NM_173588.3	1	100		intronic	NM_17358	c.2095+78	p.?	-0.01				
chr11:18735396	T/T	G	T	SNV	IGSF22	IGSF22:intronic:NM_173588.3	1	100		intronic	NM_17358	c.2095+3C	p.?	0.23				0.14(COSM54
chr11:18735753	A/A	G	A	SNV	IGSF22	IGSF22:intronic:NM_173588.3	1	100		intronic	NM_17358	c.1799-58	p.?	-1.66				
chr11:1902635	T/T	G	T	SNV	LSP1	LSP1:intronic:NM_001242932.1	1	100		intronic	NM_00124	c.576-27G	p.?	-1.35				
chr6:31084964	C/C	T	C	SNV	CDSN,PSORS1C1	CDSN:exonic:NM_001264.4, PSORS1C1:intronic:NM_014068	1	100	2	missense	NM_01406	c.-228+22	p.?, p.Asn143Ser	-1.16	1	46	0	0(COSM4006
chr11:19256250	T/T	C	T	SNV	E2F8	E2F8:intronic:NM_001256371.1	1	100		intronic	NM_00125	c.766+41G	p.?	-0.5				
chr11:19853992	A/T	A	T	SNV	NAV2	NAV2:intronic:NM_001244963.1	1	59.09		intronic	NM_00124	c.268-38A	p.?	0.09				
chr11:20072779	T/T	C	T	SNV	NAV2	NAV2:intronic:NM_001244963.1	1	100		intronic	NM_00124	c.4506-56	p.?	-0.67				
chr6:31085200	G/G	A	G	SNV	CDSN,PSORS1C1	CDSN:exonic:NM_001264.4, PSORS1C1:intronic:NM_014068	1	100	2	synonymous	NM_01406	c.-228+25	p.?, p.(=)	0.1				
chr6:31084943	CTTC/C	CTTC	C	INDEL	CDSN,PSORS1C1	CDSN:exonic:NM_001264.4, PSORS1C1:intronic:NM_014068	3	50	2	nonframeshiftDeletion	NM_01406	c.-228+22	p.?, p.Ser150del	0.09, -0.06, -0.68, 2.33				
chr11:20101924	G/G	T	G	SNV	NAV2	NAV2:intronic:NM_001244963.1	1	100		intronic	NM_00124	c.5497+16	p.?	-0.47				
chr16:88872145	C/C	T	C	SNV	CDT1	CDT1:exonic:NM_030928.3	1	100	5	missense	NM_03092	c.700T>C	p.Cys234Arg	1.02	1	180	0.001	
chr19:45016990	C/C	CT	C	INDEL	CEACAM20	CEACAM20:splicesite_3:NM_001102600.2	1	100	9	splicesite	NM_001102600.2			0.27, -0.77				
chr2:37438993	A/A	G	A	SNV	CEBPZ,CEBPZOS	CEBPZOS:intronic_nc:NR_136316.1, CEBPZ:exonic:NM_0057	1	100	12	synonymous	NR_13631	c.2793C>T	p.(=)	1.01				
chr11:20127046	A/A	C	A	SNV	NAV2	NAV2:intronic:NM_001244963.1	1	100		intronic	NM_00124	c.6822-31	p.?	-0.37				
chr11:20403825	T/T	A	T	SNV	HTATIP2	HTATIP2:intronic:NM_001098520.1	1	100		intronic	NM_00109	c.605+40A	p.?	-0.21				
chr2:37458711	A/A	G	A	SNV	CEBPZ,NDUFAF7	NDUFAF7:upstream:NM_144736.4, CEBPZ:utr_5:NM_00576	1	100	1	utr_5	NM_00576	c.-1C>T, c.-	p.?, p.?	-1.93				
chr1:15789297	C/C	A	C	SNV	CELA2A	CELA2A:exonic:NM_033440.2	1	100	4	synonymous	NM_03344	c.297A>C	p.(=)	-3.51				0.01(COSM30
chr11:20448531	A/A	G	A	SNV	PRMT3	PRMT3:intronic:NM_005788.3	1	100		intronic	NM_00578	c.993+120	p.?	-0.9				
chr11:20483748	C/C	T	C	SNV	PRMT3	PRMT3:intronic:NM_005788.3	1	100		intronic	NM_00578	c.1260+35	p.?	-0.8				
chr11:20515555	A/A	G	A	SNV	PRMT3	PRMT3:intronic:NM_005788.3	1	100		intronic	NM_00578	c.1398+40	p.?	-0.29				
chr1:15812432	G/G	A	G	SNV	CELA2B	CELA2B:exonic:NM_015849.2	1	100	6	missense	NM_01584	c.530A>G	p.Gln177Arg	-2.05	0.15	43	0	
chr11:205757	AACTCTG/A	AACTCTG	A	INDEL	BET1L	BET1L:intronic:NM_001098787.1	6	47.37		intronic	NM_00109	c.112-90C	p.?	-0.36, -0.14, -0.59, -0.69, 0.35, -0.0, 0.79				
chr1:22310824	C/C	T	C	SNV	CELA3B	CELA3B:exonic:NM_007352.3	1	100	6	synonymous	NM_00735	c.642T>C	p.(=)	-3.36				0.01(COSM57
chr11:20639479	A/A	G	A	SNV	SLC6A5	SLC6A5:intronic:NM_004211.4	1	100		intronic	NM_00421	c.1260+49	p.?	2.46				
chr11:2182360	C/C	T	C	SNV	INS,INS-IGF2	INS-IGF2:intronic:NM_001042376.2, INS:intronic:NM_0002C	1	100		intronic	NM_00104	c.-17+38A	p.?, p.?	-1.02				
chr11:2189184	CA/CA	C	CA	INDEL	TH	TH:intronic:NM_199292.2	1	100		intronic	NM_19929	c.670-21G	p.?	-1.99				
chr11:2192798	C/T	C	T	SNV	TH	TH:intronic:NM_199292.2	1	50		intronic	NM_19929	c.102+117	p.?	-2.81				
chr1:22313161	C/C	T	C	SNV	CELA3B	CELA3B:exonic:NM_007352.3	1	100	7	synonymous	NM_00735	c.780T>C	p.(=)	-1.89				
chr11:22759168	A/G	A	G	SNV	GAS2	GAS2:intronic:NM_005256.3	1	56		intronic	NM_00525	c.410-83A	p.?	-0.17				
chr22:46929692	G/G	A	G	SNV	CELSR1	CELSR1:exonic:NM_014246.1	1	100	1	missense	NM_01424	c.3376T>C	p.Cys1126Arg	2.06	1	180	0	0.44(COSM37
chr11:2436733	G/A	G	A	SNV	TRPM5	TRPM5:intronic:NM_014555.3	1	50		intronic	NM_01455	c.1129-32	p.?	0.13				
chr22:46931077	C/C	G	C	SNV	CELSR1	CELSR1:exonic:NM_014246.1	1	100	1	missense	NM_01424	c.1991C>G	p.Ser664Trp	0.62	0.16	177	0.999	0.2(COSM375
chr1:109794252	C/C	T	C	SNV	CELSR2	CELSR2:exonic:NM_001408.2	1	100	1	synonymous	NM_00140	c.1551T>C	p.(=)	-0.14				
chr1:109795026	C/C	T	C	SNV	CELSR2	CELSR2:exonic:NM_001408.2	1	100	1	synonymous	NM_00140	c.2325T>C	p.(=)	-4.01				
chr1:109795608	C/C	T	C	SNV	CELSR2	CELSR2:exonic:NM_001408.2	1	100	1	synonymous	NM_00140	c.2907T>C	p.(=)	0.44				
chr4:68338199	C/C	G	C	SNV	CENPC	CENPC:utr_3:NM_001812.2	1	100	19	utr_3	NM_00181	c.2956C>G	p.?	-0.45				
chr4:104062223	T/T	C	T	SNV	CENPE	CENPE:exonic:NM_001813.2	1	100	36	synonymous	NM_00181	c.5502G>A	p.(=)	-0.52				0.03(COSM41
chr4:104082349	T/T	C	T	SNV	CENPE	CENPE:exonic:NM_001813.2	1	100	20	synonymous	NM_00181	c.2025G>A	p.(=)	-1.43				0.03(COSM44
chrX:100395663	T/T	G	T	SNV	CENPI	CENPI:exonic:NM_006733.3	1	100	15	synonymous	NM_00673	c.1479G>T	p.(=)	2.19				0.02(COSM14
chr11:244414	T/T	C	T	SNV	PSMD13	PSMD13:intronic:NM_175932.2	1	100		intronic	NM_17593	c.272-12C	p.?	0.68				
chr11:244552	G/G	A	G	SNV	PSMD13	PSMD13:intronic:NM_175932.2	1	100		intronic	NM_17593	c.315+83A	p.?	-0.61				
chr11:24759855	A/A	G	A	SNV	LUZP2	LUZP2:intronic:NM_001009909.3	1	100		intronic	NM_00100	c.333+7G>	p.?	0.63				0.65(COSM5926223)
chr13:25466955	C/C	T	C	SNV	CENPJ	CENPJ:exonic:NM_018451.4	1	100	10	synonymous	NM_01845	c.3042A>G	p.(=)	0.18				0.58(COSM41
chr5:64814175	T/G	T	G	SNV	CENPK	CENPK:utr_3:NM_001267038.1	1	50	11	utr_3	NM_00126	c.937A>C	p.?	0.34				
chr11:26645894	T/T	A	T	SNV	ANO3	ANO3:intronic:NM_031418.3	1	100		intronic	NM_03141	c.1837-11	p.?	-0.16				
chr16:81050965	T/T	A	T	SNV	CENPN	CENPN:exonic:NM_001100625.2	1	100	4	missense	NM_00110	c.252A>T	p.Glu84Asp	1.45	1	45	0	
chr16:81061827	A/A	G	A	SNV	CENPN	CENPN:exonic:NM_001100625.2	1	100	10	missense	NM_00110	c.862G>A	p.Glu288Lys	0.12	0.66	56	0.001	0.34(COSM39
chr9:95279985	C/A	C	A	SNV	CENPP,ECM2	ECM2:exonic:NM_001393.3, CENPP:intronic:NM_00101226	1	52.38	3	synonymous	NM_00101	c.565-936	p.?, p.(=)	-3.47				0.01(COSM63
chr6:126661502	T/T	G	T	SNV	CENPW	CENPW:exonic:NM_001012507.3	1	100	1	missense	NM_00101	c.83G>T	p.Arg28Leu	-0.2	0.01	102	0.001	0.01(COSM62
chr17:64023624	T/C	T	C	SNV	CEP112	CEP112:exonic:NM_001199165.3	1	50	16	missense	NM_00119	c.1651A>G	p.Lys551Glu	3.24	0.04	56	0.003	
chr5:122682348	G/G	A	G	SNV	CEP120	CEP120:exonic:NM_153223.3	1	100	21	synonymous	NM_15322	c.2826T>C	p.(=)	0.34				0.74(COSM14
chr11:101833644	T/T	C	T	SNV	CEP126	CEP126:exonic:NM_020802.3	1	100	6	synonymous	NM_02080	c.1878C>T	p.(=)	-0.72				

chr11:28104605	T/T	C	T	SNV	KIF18A	KIF18A:intronic:NM_031217.3	1	100		intronic	NM_03121	c.1150-90	p.?	-0.72					Kinesin moto	rs7937778		YES			Kinesin fam	ATP bindin Autoimmune Diseases;Female Urogenital Di	C=0, T=24	C=0.0, T=1.0	0.00001	334.788	24	C=0/0, T=6/18
chr11:101857758	A/A	G	A	SNV	CEP126	CEP126:exonic:NM_020802.3	1	100	9	missense	NM_02080	c.3230G>A	p.Ser1077Asn	3.11	1	46	0		Susceptibility	rs6590942	0.0269	YES	9.995E-1 (pos.101857758)			centrosome,ciliary base,cilium assembly,cytoplasm,cyt	G=0, A=53	G=0.0, A=1.0	0.00001	777.994	53	G=0/0, A=22/3
chr14:81297635	A/G	A	G	SNV	CEP128	CEP128:exonic:NM_152446.3	1	50	12	missense	NM_15244	c.1061T>C	p.Val354Ala	0.39	0.32	64	0.07			rs116610923	0.0116		4.627E-4 (pos.81297635)			centriolar subdistal appendage,centriole,protein localiz	A=20, G=20	A=0.5, G=0.5	0.00001	214.326	40	A=2/18, G=8/1
chr17:79171967	G/G	A	G	SNV	CEP131	CEP131:exonic:NM_014984.3	1	100	12	missense	NM_01498	c.1418T>C	p.Val473Ala	0.61	0.52	64	0	0.02(COSM4130802),0.02(C		rs2659016	8.00E-04	YES	9.997E-1 (pos.79171967)	neoplasm		G2/M transition of mitotic cell cycle,acrosomal vesicle,c	A=0, G=23	A=0.0, G=1.0	0.00001	316.702	23	A=0/0, G=8/15
chr11:3026692	G/G	T	G	SNV	CARS	CARS:intronic:NM_001014437.2	1	100		intronic	NM_00101	c.2150-29	p.?	-1.25					tRNA synthet	rs4758458	0.2148	YES	8.645E-1;1.156E-4 (pos.3026692)		Cysteinyl-tR	ATP binding,cysteine-tRNA liga: L-Cysteine	T=0, G=118	T=0.0, G=1.0	0.00001	1897.74	118	T=0/0, G=73/4
chr11:31653956	A/A	C	A	SNV	ELP4	ELP4:intronic:NM_019040.4	1	100		intronic	NM_01904	c.927+4C>	p.?	0.43					PAXNEB prote	rs4922872	4.00E-04		1.0 (pos.31653956)		Elongation	Elongator Aniridia;Congenital Abnormalities;Congenita	C=0, A=27	C=0.0, A=1.0	0.00001	389.988	27	C=0/0, A=8/19
chr11:33054754	C/G	C	G	SNV	DEPDC7	DEPDC7:intronic:NM_001077242.1	1	57.14		intronic	NM_00107	c.1343-54	p.?	-0.81					Domain foun	rs2273539		YES			DEP domair	GTPase activator activity,biological_process,cellular_co	C=21, G=28	C=0.4286, G=0.5714	0.00001	342.312	49	C=11/10, G=17
chr11:33773161	C/C	G	C	SNV	FBXO3	FBXO3:intronic:NM_012175.3	1	100		intronic	NM_01217	c.725-8C>	p.?	-0.25					ApaG domair	rs7479064	9.00E-04	YES	9.999E-1;0.0 (pos.33773161)		F-box only p	cytosol,nucleoplasm,protein binding,protein ubiquitinat	G=0, C=37	G=0.0, C=1.0	0.00001	577.047	37	G=0/0, C=14/2
chr11:3382069	G/G	C	G	SNV	ZNF195	ZNF195:intronic:NM_001130520.2	1	100		intronic	NM_00113	c.374-51G	p.?	0.13					C2H2-type zir	rs6578335	0.0101	YES			Zinc finger p	DNA binding,intracellular,metal ion binding,nucleic acid	C=0, G=29	C=0.0, G=1.0	0.00001	439.051	29	C=0/0, G=18/1
chr11:33886547	T/T	C	T	SNV	LMO2	LMO2:intronic:NM_005574.3	1	100		intronic	NM_00557	c.249-184	p.?	-2.06					LIM domain	rs3824848		YES			LIM domain	E-box bind Hemic and Lymphatic Diseases;Immune Syst	C=0, T=96	C=0.0, T=1.0	0.00001	1618.01	96	C=0/0, T=29/6
chr11:34158411	G/G	A	G	SNV	NAT10	NAT10:intronic:NM_024662.2	1	100		intronic	NM_02466	c.2162+89	p.?	0.54					Domain of un	rs2982615					N-acetyltra	90S preribosome,ATP binding,DNA polymerase binding,N	A=0, G=25	A=0.0, G=1.0	0.00001	353.018	25	A=0/0, G=16/9
chr11:34158431	C/C	T	C	SNV	NAT10	NAT10:intronic:NM_024662.2	1	100		intronic	NM_02466	c.2163-94	p.?	-1.65					Domain of un	rs2957521					N-acetyltra	90S preribosome,ATP binding,DNA polymerase binding,N	T=0, C=25	T=0.0, C=1.0	0.00001	353.018	25	T=0/0, C=16/9
chr11:34189563	TG/TG	T	TG	INDEL	ABTB2	ABTB2:intronic:NM_145804.2	1	100		intronic	NM_14580	c.1564-24	p.?	0.64					Ankyrin repeat,Ankyrin repeats (3 copies),Ankyrin repeats (many copies),BTB/POZ domain							SCF ubiquitin ligase complex,cellular response to toxic s	T=0, TG=12	T=0.0, TG=1.0	0.00001	1821.9	129	T=0/0, TG=72/
chr11:34533002	A/C	A	C	SNV	ELF5	ELF5:intronic:NM_001422.3	1	50		intronic	NM_00142	c.-4+2222	p.?	-3.1					Ets-domain,S	rs28395819		YES			E74-like fac	RNA polymerase II regulatory region sequence-specific D	A=44, C=44	A=0.5, C=0.5	0.00001	478.649	88	A=18/26, C=15
chr11:34678435	G/G	C	G	SNV	EHF	EHF:intronic:NM_001206616.1	1	100		intronic	NM_00120	c.611-20C	p.?	0.04					Ets-domain,S	rs386752371,rs4355	8.00E-04	YES	1.0 (pos.34678435)		ETS homolo	DNA bindi Genital Diseases, Male;Genital Neoplasms, M	C=0, G=48	C=0.0, G=1.0	0.00001	810.241	48	C=0/0, G=16/3
chr11:34904399	T/T	C	T	SNV	APIP	APIP:intronic:NM_015957.3	1	100		intronic	NM_01595	c.630-36G	p.?	-0.62					Class II Aldol	rs2288667	0.309	YES	7.489E-1;0.0 (pos.34904399)		APAF1-inter	L-methionine salvage from methylthioadenosine,apopto	C=0, T=42	C=0.0, T=1.0	0.00001	685.22	42	C=0/0, T=22/2
chr11:34916710	G/G	A	G	SNV	APIP	APIP:intronic:NM_015957.3	1	100		intronic	NM_01595	c.58-53T>	p.?	-0.31					Class II Aldol	rs1326941		YES			APAF1-inter	L-methionine salvage from methylthioadenosine,apopto	A=0, G=39	A=0.0, G=1.0	0.00001	623.977	39	A=0/0, G=18/2
chr11:34937697	C/C	T	C	SNV	APIP,PDHX	PDHX:utr_5:NM_003477.2, APIP:intronic:NM_015957.3	1	100		intronic	NM_01595	c.57+78A>	p.?, p.?	-1.44					2-oxoacid de	rs2016814,rs386752391		YES			APAF1-inter	L-methion Acid-Base Imbalance;Acidosis; Benign	T=0, C=34	T=0.0, C=1.0	0.00001	524.186	34	T=0/0, C=12/2
chr17:79173184	C/C	T	C	SNV	CEP131	CEP131:exonic:NM_014984.3	1	100	10	missense	NM_01498	c.1189A>G	p.Thr397Ala	-2.25	0.67	58	0	0(COSM3756015),0(COSM3		rs2659015	0.0272	YES	9.612E-1 (pos.79173184)	adenocarcinoma,neoplasm		G2/M transition of mitotic cell cycle,acrosomal vesicle,c	T=0, C=36	T=0.0, C=1.0	0.00001	563.713	36	T=0/0, C=27/9
chr11:35323200	G/G	A	G	SNV	SLC1A2	SLC1A2:intronic:NM_004171.3	1	100		intronic	NM_00417	c.731-8T>	p.?	0.5				0.02(COSM57	Sodium:dicar	rs2273689,rs776609	0.35	YES	0.0 (pos.35323199),7.35	adenocarcino	Solute carri	D-asparta Amyotrophic Latera Glutamic Acid	A=0, G=60	A=0.0, G=1.0	0.00001	903.516	60	A=0/0, G=23/3
chr11:36311014	G/T	G	T	SNV	COMMD9	COMMD9:upstream:NM_014186.3	1	95.45		intronic	NM_01418	c.-52C>A	p.?	-0.93						rs7939948	0.4727	YES	5.419E-1;0.0 (pos.36311014)			Golgi apparatus,cholesterol homeostasis,cytosol,extrac	G=1, T=21	G=0.0455, T=0.9545	0.00001	270.17	22	G=0/1, T=13/8
chr11:36422532	G/G	T	G	SNV	PRR5L	PRR5L:intronic:NM_024841.4	1	100		intronic	NM_02484	c.-124-15T	p.?	0.58					HbrB-like	rs59943655		YES			Proline-rich	TORC2 complex,TORC2 signaling,cellular response to oxi	T=0, G=21	T=0.0, G=1.0	0.00001	281.235	21	T=0/0, G=13/8
chr11:36424967	A/G	A	G	SNV	PRR5L	PRR5L:intronic:NM_024841.4	1	50		intronic	NM_02484	c.245+39A	p.?	0.39					HbrB-like	rs12279091	0.0285	YES	3.522E-2 (pos.36424967)		Proline-rich	TORC2 complex,TORC2 signaling,cellular response to oxi	A=24, G=24	A=0.5, G=0.5	0.00001	273.817	48	A=5/19, G=6/1
chr14:105349388	C/C	A	C	SNV	CEP170B	CEP170B:exonic:NM_001112726.2	1	100	8	synonymous	NM_00111	c.594A>C	p.(=)	-2.73				0.02(COSM37	CEP170 C-ter	rs2028414	0.3426	YES	3.008E-1 (pos.10534938	adenocarcinoma,hepatoce		cytoplasm,microtubule	A=0, C=44	A=0.0, C=1.0	0.00001	700.773	44	A=0/0, C=25/1
chr11:396701	T/T	C	T	SNV	PKP3	PKP3:intronic:NM_007183.3	1	100		intronic	NM_00718	c.312+14C	p.?	-1.93					Armadillo/be	rs11246147	0.4502	YES	4.328E-1 (pos.396701)		Plakophilin	RNA bindir Genital Diseases, Male;Genital Neoplasms, M	C=0, T=46	C=0.0, T=1.0	0.00001	768.313	46	C=0/0, T=25/2
chr11:403939	G/G	A	G	SNV	PKP3	PKP3:intronic:NM_007183.3	1	100		intronic	NM_00718	c.2078-4A	p.?	-0.62					Armadillo/be	rs7479832	0.2383	YES	9.998E-1 (pos.403939)		Plakophilin	RNA bindir Genital Diseases, Male;Genital Neoplasms, M	A=0, G=32	A=0.0, G=1.0	0.00001	485.129	32	A=0/0, G=14/1
chr14:105354293	G/G	A	G	SNV	CEP170B	CEP170B:exonic:NM_001112726.2	1	100	12	synonymous	NM_00111	c.3717A>G	p.(=)	-1.16				0.02(COSM11	CEP170 C-ter	rs2582548	0.2146	YES	2.978E-1 (pos.10535429	adenocarcinoma,neoplasm		cytoplasm,microtubule	A=0, G=26	A=0.0, G=1.0	0.00001	371.087	26	A=0/0, G=6/20
chr11:4133317	A/A	C	A	SNV	RRM1	RRM1:intronic:NM_001033.4	1	100		intronic	NM_00103	c.650+25C	p.?	-0.09					ATP cone don	rs56336381	0.0692	YES	7.467E-1;0.0 (pos.4133317)		Ribonucleo	ATP bindin Adenocarcinoma;A Cladribine,Clofarabine,F	C=0, A=57	C=0.0, A=1.0	0.00001	848.608	57	C=0/0, A=32/2
chr18:13095609	C/C	T	C	SNV	CEP192	CEP192:exonic:NM_032142.3	1	100	35	missense	NM_03214	c.6362T>C	p.Leu2121Pro	1.6	0.3	98	0.003			rs474337	0.3802	YES	7.961E-1 (pos.13095609)		Centrosom	G2/M transition of mitotic cell cycle,centriole,centroson	T=0, C=160	T=0.0, C=1.0	0.00001	2579.56	160	T=0/0, C=86/7
chr18:13116432	T/T	G	T	SNV	CEP192	CEP192:exonic:NM_032142.3	1	100	43	missense	NM_03214	c.7346G>T	p.Arg2449Leu	0.75	1	102	0.001	0.04(COSM5001134),0.04(C		rs1786263	0.3841	YES	7.95E-1;1.156E-4 (pos.13	ductal_carcinc	Centrosom	G2/M transition of mitotic cell cycle,centriole,centroson	G=0, T=78	G=0.0, T=1.0	0.00001	1249.35	78	G=0/0, T=30/4
chr12:88505078	C/C	T	C	SNV	CEP290	CEP290:exonic:NM_025114.3	1	100	22	synonymous	NM_02511	c.2268A>G	p.(=)	0.25					Coiled-coil re	rs2468255	0.0774	YES	9.758E-1;0.0 (pos.88505078)		Centrosom	G2/M tran Abnormalities, Multiple;Barde Likely benign	T=0, C=53	T=0.0, C=1.0	0.00001	781.97	53	T=0/0, C=26/2
chr11:43775734	A/G	A	G	SNV	HSD17B12	HSD17B12:intronic:NM_016142.2	1	46.43		intronic	NM_01614	c.283+63A	p.?	-0.11					Enoyl-(Acyl ca	rs4643069	0.314	YES				3-oxo-arac Neoplasms;Neoplasms by Histologic Type;Ne	A=30, G=26	A=0.5357, G=0.4643	0.00001	244.121	56	A=14/16, G=10
chr11:93402096	A/A	C	A	SNV	CEP295	CEP295:exonic:NM_033395.1	1	100	4	synonymous	NM_03339	c.432C>A	p.(=)	0.17				0.59(COSM45	ALMS motif	rs1944101		YES	9.614E-1 (pos.93402096)	squamous_cel	Centrosom	centriole,centrosome,cytoplasm,cytoskeleton,positive	C=0, A=30	C=0.0, A=1.0	0.00001	446.634	30	C=0/0, A=6/24
chr11:44089161	A/A	G	A	SNV	ACCS	ACCS:intronic:NM_001127219.1	1	100		intronic	NM_00112	c.1-17G>A	p.?	-0.01					Aminotransfe	rs178514,rs3867530	0.2079	YES	8.641E-1 (pos.44089161)		1-aminocyc	1-aminocyclopropane-1-carbo: Vinylglycine	G=0, A=30	G=0.0, A=1.0	0.00001	445.982	30	G=0/0, A=18/1
chr11:4409511	A/G	A	G	SNV	TRIM21	TRIM21:intronic:NM_003141.3	1	56.36		intronic	NM_00314	c.735+19T	p.?	-1.25					B-box zinc fin	rs12805578	0.1937	YES	8.634E-1 (pos.4409511)		Tripartite m	DNA binding,P-body,RNA binding,SCF ubiquitin ligase con	A=24, G=31	A=0.4364, G=0.5636	0.00001	309.113	55	A=17/7, G=18/
chr11:44104885	G/G	T	G	SNV	ACCS	ACCS:intronic:NM_001127219.1	1	100		intronic	NM_00112	c.1254+24	p.?	-0.77					Aminotransfe	rs3107274	0.2836	YES	3.543E-1;1.165E-4 (pos.44104885)		1-aminocyc	1-aminocyclopropane-1-carbo: Vinylglycine	T=0, G=44	T=0.0, G=1.0	0.00001	726.568	44	T=0/0, G=21/2
chr10:95279506	T/T	A	T	SNV	CEP55	CEP55:exonic:NM_001127182.1	1	100	8	missense	NM_00112	c.1133A>T	p.His378Leu	6.08	0	99	0.483	0.98(COSM50	TSG101 and A	rs2293277	0.2449	YES	5.675E-1 (pos.95279506)	adenocarcino	Centrosom	Flemming Adnexal Diseases;Autoimmune Diseases;Cys	A=0, T=96	A=0.0, T=1.0	0.00001	1618.01	96	A=0/0, T=39/5
chr6:118973953	G/G	A	G	SNV	CEP85L	CEP85L:utr_5:NM_001178035.1	1	100	2	utr_5	NM_00117	c.-9T>C	p.?	-0.23						rs9489486		YES	3.416E-1 (pos.118973953)			centrosome,cytoplasm	A=0, G=52	A=0.0, G=1.0	0.00001	767.015	52	A=0/0, G=30/2
chr19:33390796	A/A	G	A	SNV	CEP89	CEP89:exonic:NM_032816.4	1	100	16	synonymous	NM_03281	c.1842C>T	p.(=)	-0.89						rs10418340	0.1407	YES	8.92E-1 (pos.33390796)		Centrosom	centriole,centrosome,chemical synaptic transmission,c	G=0, A=125	G=0.0, A=1.0	0.00001	2034.03	125	G=0/0, A=64/6
chr11:45877688	G/G	C	G	SNV	CRY2	CRY2:intronic:NM_021117.3	1	100		intronic	NM_02111	c.387+47C	p.?	-0.88					DNA photolya	rs2292912	0.197	YES	3.667E-1;0.0 (pos.45877688)		Cryptochro	DNA (6-4) Bone Marrow Diseases;Hematologic Disease	C=0, G=21	C=0.0, G=1.0	0.00001	281.148	21	C=0/0, G=16/5
chr19:33444707	C/T	C	T	SNV	CEP89	CEP89:exonic:NM_032816.4	1	50	4	synonymous	NM_03281	c.306G>A	p.(=)	-0.06				0(COSM4416657),0(COSM4		rs10411735,rs78131	0.3276	YES	4.219E-1;1.163E-4 (pos.3	acute_myeloic	Centrosom	centriole,centrosome,chemical synaptic transmission,c	C=11, T=11	C=0.5, T=0.5	0.00001	95.5709	22	C=1/10, T=6/5
chr3:101443461	C/C	T	C	SNV	CEP97	CEP97:utr_5:NM_024548.3	1	100	1	utr_5	NM_02454	c.-60T>C	p.?	-0.04						rs994573	0				Centrosom	calmodulin binding,centrosome,ciliary basal body-plasn	T=0, C=65	T=0.0, C=1.0	0.00001	996.457	65	T=0/0, C=45/2
chr11:46822686	A/A	C	A	SNV	CKAP5	CKAP5:intronic:NM_001008938.3	1	100		intronic	NM_00100	c.1083+64	p.?	0.36					CLASP N term	rs10734549		YES			Cytoskeletc	G2/M transition of mitotic cell cycle,RNA transport,cadh	C=0, A=21	C=0.0, A=1.0	0.00001	281.233	21	C=0/0, A=11/1
chr11:46897995	T/T	G	T	SNV	LRP4	LRP4:intronic:NM_002334.3	1	100		intronic	NM_00233	c.3536+22	p.?	0.61					Calcium-bind	rs964551	0.2284	YES	3.794E-1;0.0 (pos.46897995)		Low density	BMP signa Bone Diseases;Bone Diseases, Development	G=0, T=27	G=0.0, T=1.0	0.00001	383.405	27	G=0/0, T=10/1
chr11:46911704	T/T	C	T	SNV	LRP4	LRP4:intronic:NM_002334.3	1	100		intronic	NM_00233	c.1916-33	p.?	-0.23					Calcium-bind	rs2306036	0.1478	YES	3.751E-1 (pos.46911704)		Low density	BMP signa Bone Diseases;Bone Diseases, Development	C=0, T=22	C=0.0, T=1.0	0.00001	298.779	22	C=0/0, T=19/3
chr22:47083010	C/C	T	C	SNV	CERK	CERK:utr_3:NM_022766.5	1	100	13	utr_3	NM_02276	c.1635A>G	p.?	0.11					Diacylglycerc	rs2542014,rs386822	0.4237	YES	3.534E-1 (pos.47083010)		Ceramide k	ATP binding,NAD+ kinase activity,ceramide kinase activit	T=0, C=24	T=0.0, C=1.0	0.00001	334.79	24	T=0/0, C=13/1
chr22:47083012	A/A	T	A	SNV	CERK	CERK:utr_3:NM_022766.5	1	100	13	utr_3	NM_02276	c.1633A>T	p.?	0.28					Diacylglycerc	rs2748349,rs386822	0.4238	YES	3.53E-1 (pos.47083012)		Ceramide k	ATP binding,NAD+ kinase activity,ceramide kinase activit	T=0, A=24	T=0.0, A=1.0	0.00001	334.791	24	T=0/0, A=13/1
chr11:47204175	T/T	A	T	SNV	PACSIN3	PACSIN3:intronic:NM_016223.4	1	100		intronic	NM_01622	c.54+50T>	p.?	-0.63					Fes/CIP4, anc	rs2279438	0.2353	YES	3.437E-1;0.0 (pos.47204175)		Protein kin	calcium channel inhibitor activity,cytoplasm,cytoskelet	A=0, T=43	A=0.0, T=1.0	0.00001	705.84	43	A=0/0, T=15/2
chr11:47259668	C/C	T	C	SNV	DDB2	DDB2:intronic:NM_000107.2	1	100		intronic	NM_00010	c.1189-21	p.?	0.19					WD domain,	rs326222	0.2915	YES	2.511E-1 (pos.47259668)		Damage-sp	Cul4-RING Congenital Abnormalities;Congenital, Heredi	T=0, C=21	T=0.0, C=1.0	0.00001	281.235	21	T=0/0, C=7/14
chr2:182403851	A/A	G	A	SNV	CERKL,ITGA4	CERKL:exonic:NM_201548.4, ITGA4:downstream:NM_00088	1	100	12	synonymous	NM_00088	c.*3597G>	p.?, p.(=)	0.74					Diacylglycerc	rs10180793	2.00E-04		1.0 (pos.182403851)		Ceramide k	B cell diffe Autistic Disorder;Au ATL1102,C Benign	G=0, A=20	G=0.0, A=1.0	0.00001	263.782	20	G=0/0, A=13/7
chr1:150938616	A/A	G	A	SNV	CERS2	CERS2:utr_3:NM_022075.4	1	100	11	utr_3	NM_02207	c.1151C>T	p.?	0.06					Homeobox do	rs267739	0		1.0 (pos.150938616)		Ceramide s	DNA binding,ceramide biosynthetic process,endoplasmi	G=0, A=31	G=0.0, A=1.0	0.00001	465.816	31	G=0/0, A=16/1
chr11:47361084	C/C	T	C	SNV	MYBPC3	MYBPC3:intronic:NM_000256.3	1	100		intronic	NM_00025	c.2067+11	p.?	-2.72					Fibronectin t	rs2856653		YES			Myosin-bin	A band,ATI ACMG Recommendations...;Aortic Stenosis, S	T=0, C=35	T=0.0, C=1.0	0.00001	543.903	35	T=0/0, C=16/1
chr11:47364762	G/G	A	G	SNV	MYBPC3	MYBPC3:intronic:NM_000256.3	1	100		intronic	NM_00025	c.1224-63	p.?	-1.75					Fibronectin t	rs896818	0.0018	YES	1.0;0.0 (pos.47364762)		Myosin-bin	A band,ATI ACMG Recommendations...;Ao Benign	A=0, G=25	A=0.0, G=1.0	0.00001	345.903	25	A=0/0, G=11/1
chr11:47371484	A/A	AG	A	INDEL	MYBPC3	MYBPC3:intronic:NM_000256.3	1	100		intronic	NM_00025	c.506-11C	p.?	-0.28, 1.43					Fibronectin t	rs11570050,rs18184	0.2752	YES	0.0 (pos.47371485),6.438E-1 (pos.47371		Myosin-bin	A band,ATI ACMG Recommendations...;Ao Benign/Likely	AG=0, A=20	AG=0.0, A=1.0	0.00001	257.733	20	AG=0/0, A=11/
chr11:47397353	A/A	G	A	SNV	SPI1	SPI1:intronic:NM_001080547.1	1	100		intronic	NM_00108	c.46-70C>	p.?	0.59					Ets-domain	rs56030824		YES			Oncogene S	DNA bindi Leukemia;Leukemia, Myeloid;Leukemia, Mye	G=0, A=34	G=0.0, A=1.0	0.00001	492.279	34	G=0/0, A=21/1
chr16:55862717	T/C	T	C	SNV	CES1	CES1:exonic:NM_001025195.1	1	57.14	2	synonymous	NM_00102	c.222A>G	p.(=)	-1.63				0.04(COSM41	Carboxyleste	rs76828834		YES	1.799E-1 (pos.55862717)	acute_myeloic	Carboxylest	carboxylic Cocaine-Related Di (1R)-1,2,2-trimethylprop	T=9, C=12	T=0.4286, C=0.5714	0.00001	109.091	21	T=5/4, C=8/4
chr16:55862720	T/C	T	C	SNV	CES1	CES1:exonic:NM_001025195.1	1	57.14	2	synonymous	NM_00102	c.219A>G	p.(=)	-2.21				0.02(COSM13	Carboxyleste	rs74019278		YES	1.801E-1 (pos.55862720)	astrocytoma_(	Carboxylest	carboxylic Cocaine-Related Di (1R)-1,2,2-trimethylprop	T=9, C=12	T=0.4286, C=0.5714	0.00001	109.102	21	T=5/4, C=8/4
chr16:55862762	C/G	C	G	SNV	CES1	CES1:exonic:NM_001025195.1	1	50	2	synonymous	NM_00102	c.177G>C	p.(=)	-3.69				0.01(COSM13	Carboxyleste	rs3826194		YES	1.524E-1;0.0;0.0 (pos.55	NS,adenocarci	Carboxylest	carboxylic Cocaine-Related Di (1R)-1,2,2-trimethylprop	C=12, G=12	C=0.5, G=0.5	0.00001	106.71	24	C=5/7, G=8/4
chr11:47587577	T/T	TC	T	INDEL	PTPMT1	PTPMT1:intronic:NM_175732.2	1	100		intronic	NM_17573	c.255+39T	p.?	-0.33, 0.0					Dual specifi	rs398016003,rs574046003,rs58		YES	0.0 (pos.47587565),1.0 (pos.47587577)			cardiolipin biosynthetic process,mitochondrial inner me	TC=0, T=40	TC=0.0, T=1.0	0.00001	471.913	40	TC=0/0, T=21/
chr16:55862883	C/A	C	A	SNV	CES1	CES1:exonic:NM_001025195.1	1	50	2	missense	NM_00102	c.56G>T	p.Gly19Val	1.88	0	109	0.972	0.9(COSM112	Carboxyleste	rs3826190		YES	1.751E-1 (pos.55862883)	Merkel_cell_c	Carboxylest	carboxylic Cocaine-Related Di (1R)-1,2,2-trimethylprop	C=11, A=11	C=0.5, A=0.5	0.00001	94.6067	22	C=1/10, A=7/4
chr16:55862824	C/T	C	T	SNV	CES1	CES1:exonic:NM_001025195.1	1	45.83	2	missense	NM_00102	c.115G>A	p.Val39Ile	0.56	0.42	29	0.053	0.06(COSM44	Carboxyleste	rs3826192		YES	1.187E-1;1.315E-4 (pos.5	adenocarcino	Carboxylest	carboxylic Cocaine-Related Di (1R)-1,2,2-trimethylprop	C=13, T=11	C=0.5417, T=0.4583	0.00001	93.4394	24	C=3/10, T=7/4
chr11:47752775	G/G	A	G	SNV	FNBP4	FNBP4:intronic:NM_015308.4	1	100		intronic	NM_01530	c.2008+15	p.?	-0.33					WW domain	rs17788930		YES			Formin-bin	nuclear speck,protein binding	A=0, G=51	A=0.0, G=1.0	0.00001	754.584	51	A=0/0, G=27/2
chr16:55862836	G/A	G	A	SNV	CES1	CES1:exonic:NM_001025195.1	1	45.83	2	synonymous	NM_00102	c.103C>T	p.(=)	1.47				0.82(COSM13	Carboxyleste	rs3826191		YES	1.232E-1 (pos.55862836)	astrocytoma_(	Carboxylest	carboxylic Cocaine-Related Di (1R)-1,2,2-trimethylprop	G=13, A=11	G=0.5417, A=0.4583	0.00001	93.439	24	G=3/10, A=7/4
chr11:47837638	A/A	G	A	SNV	NUP160	NUP160:intronic:NM_015231.2	1	100		intronic	NM_01523	c.1432-57	p.?	-0.41					Nucleoporin	rs7130715	0.0013	YES			Nucleopori	cytosol,host cell,intracellular transport of virus,kinetoch	G=0, A=40	G=0.0, A=1.0	0.00001	624.932	40	G=0/0, A=16/2
chr16:55883618	C/C	T	C	SNV	CES5A	CES5A:exonic:NM_001190158.1	1	100	12	synonymous	NM_00119	c.1428A>G	p.(=)	-0.57				0.11(COSM37	Carboxyleste	rs2160276,rs762392	0.2732	YES	7.788E-1;0.0;0.0 (pos.55	adenocarcinoma		carboxylic ester hydrolase activity,extracellular region,l	T=0, C=55	T=0.0, C=1.0	0.00001	814.894	55	T=0/0, C=31/2
chr11:48171793	C/C	T	C	SNV	PTPRJ	PTPRJ:intronic:NM_002843.3	1	100		intronic	NM_00284	c.3138+58	p.?	-1.25					Fibronectin t	rs10734565	9.00E-04	YES			Protein tyrc	T cell rece Colonic Diseases;Colorectal Neoplasms;Dige	T=0, C=24	T=0.0, C=1.0	0.00001	341.702	24	T=0/0, C=8/16
chr16:57016092	A/A	G	A	SNV	CETP	CETP:exonic:NM_000078.2	1	100	14	missense	NM_00007	c.1264G>A	p.Val422Ile	-2.55	1	29	0.001	0(COSM3754	LBP / BPI / CE	rs5882	0.3186	YES	5.481E-1 (pos.57016092)	adenocarcino	Cholesteryl	cholester Arterial Occlusive Diseases;Ar Benign, assoc	G=0, A=45	G=0.0, A=1.0	0.00001	747.391	45	G=0/0, A=14/3
chr11:48266582	C/C	A	C	SNV	OR4X2	OR4X2:upstream:NM_001004727.1	1	100		intronic	NM_00100	c.-74A>C	p.?	-1.96						rs7120549		YES				G-protein coupled receptor activity,G-protein coupled re	A=0, C=34	A=0.0, C=1.0	0.00001	524.144	34	A=0/0, C=22/1
chr3:126137558	T/T	C	T	SNV	CFAP100	CFAP100:exonic:NM_182628.2	1	100	7	synonymous	NM_18262	c.591C>T	p.(=)	-2.99					Domain of un	rs386665601,rs4679	0.2124	YES	1.784E-1 (pos.126137558)			axonemal outer doublet,cilium movement,cytoplasm,dy	C=0, T=86	C=0.0, T=1.0	0.00001	1410.88	86	C=0/0, T=34/5
chr2:120388412	A/A	G	A	SNV	CFAP221	CFAP221:exonic:NM_001271049.1	1	100	19	missense	NM_00127	c.1909G>A	p.Val637Ile	-0.95	0.6	29	0		Flagellar-ass	rs2272058	2.00E-04		9.902E-1 (pos.120388412)			axoneme, Attention Deficit Disorder with Hyperactivity	G=0, A=33	G=0.0, A=1.0	0.00001	504.502	33	G=0/0, A=18/1
chr11:48373632	A/A	T	A	SNV	OR4C45	OR4C45:intronic:NM_001005513.1	1	100		intronic	NM_00100	c.298+70A	p.?	0.1					7 transmemb	rs4882248		YES					T=0, A=25	T=0.0, A=1.0	0.00001	353.018	25	T=0/0, A=10/1
chr3:113022870	C/C	T	C	SNV	CFAP44	CFAP44:exonic:NM_001164496.1	1	100	32	missense	NM_00116	c.4970A>G	p.His1657Arg	-0.1	0.54	29	0		WD domain,	rs4682484	0.0163	YES	1.0 (pos.113022870)		Cilia- and fl	cell projection,cilium-dependent cell motility	T=0, C=29	T=0.0, C=1.0	0.00001	427.603	29	T=0/0, C=16/1
chr17:9515777	A/A	G	A	SNV	CFAP52	CFAP52:exonic:NM_145054.4	1	100	8	missense	NM_14505	c.1006G>A	p.Glu336Lys	0.84	0.49	56	0	0.1(COSM148	WD domain,	rs6503235	0.2442	YES	5.709E-1 (pos.9515777)	adenocarcinoma		cell projection,cytoplasm,protein binding	G=0, A=443	G=0.0, A=1.0	0.00001	7500.61	443	G=0/0, A=196/
chr1:43675467	A/A	G	A	SNV	CFAP57	CFAP57:exonic:NM_001195831.2	1	100	11	synonymous	NM_00119	c.1809G>A	p.(=)	-2.36				0.04(COSM54	WD domain,	rs2453412	0.1242	YES	7.23E-1 (pos.43675467)	squamous_cell_carcinoma		Bipolar Disorder;Bipolar and Related Disorde	G=0, A=45	G=0.0, A=1.0	0.00001	747.367	45	G=0/0, A=21/2
chr11:48997592	T/T	C	T	SNV			1	100		intronic				-2.71						rs1813231			1.0 (pos.48997592)				C=0, T=20	C=0.0, T=1.0	0.00001	263.786	20	C=0/0, T=8/12
chr11:49000423	G/G	C	G	SNV			1	100		intronic				1.62						rs1965370		YES	6.283E-1 (pos.49000423)				C=0, G=101	C=0.0, G=1.0	0.00001	1567.06	101	C=0/0, G=59/4
chr11:49000518	CT/CT	C	CT	INDEL			1	100		intronic				-0.33						rs760082474			0.0 (pos.49000518)				C=0, CT=95	C=0.0, CT=1.0	0.00001	1108.45	95	C=0/0, CT=52/
chr11:49000522	T/T	C	T	SNV			1	100		intronic				-0.59				0(COSM4591499)		rs1963629		YES	6.265E-1 (pos.49000522)	squamous_cell_carcinoma			C=0, T=95	C=0.0, T=1.0	0.00001	1595.28	95	C=0/0, T=52/4
chr11:49000754	A/A	G	A	SNV			1	100		intronic				-0.61						rs1027750		YES					G=0, A=68	G=0.0, A=1.0	0.00001	1053.46	68	G=0/0, A=45/2
chr11:49053601	G/G	A	G	SNV	TRIM49B	TRIM49B:intronic:NM_001206626.1	1	100		intronic	NM_00120	c.411+39A	p.?	-1.3					B-box zinc fin	rs2696923		YES	6.317E-1 (pos.49053601)			intracellular,zinc ion binding	A=0, G=37	A=0.0, G=1.0	0.00001	583.688	37	A=0/0, G=18/1
chr2:219903258	G/G	T	G	SNV	CFAP65	CFAP65:exonic:NM_194302.3	1	100	4	missense	NM_19430	c.196A>C	p.Met66Leu	-0.44	1	15	0	0.01(COSM149068),0.01(CC		rs6736922	0.2349	YES	4.35E-1 (pos.219903258)	adenocarcino	Cilia- and fl	RNA binding,integral component of membrane,motile cil	T=0, G=44	T=0.0, G=1.0	0.00001	726.573	44	T=0/0, G=20/2
chr2:219903723	T/T	C	T	SNV	CFAP65	CFAP65:exonic:NM_194302.3	1	100	3	synonymous	NM_19430	c.48G>A	p.(=)	-1.01				0(COSM149070)		rs4674354	0.2348	YES	4.364E-1 (pos.21990372	acute_myeloic	Cilia- and fl	RNA binding,integral component of membrane,motile cil	C=0, T=70	C=0.0, T=1.0	0.00001	1092.09	70	C=0/0, T=32/3
chr7:89938588	G/G	C	G	SNV	CFAP69	CFAP69:exonic:NM_001039706.2	1	100	22	missense	NM_00103	c.2562C>G	p.Ser854Arg	0.39	1	110	0	0.18(COSM5749947),0.18(C		rs7803620	0.157	YES	9.989E-1;0.0 (pos.89938	germ_cell_tumour			C=0, G=29	C=0.0, G=1.0	0.00001	427.582	29	C=0/0, G=11/1
chr11:49196332	CG/TC	CG	TC	MNV	FOLH1	FOLH1:intronic:NM_004476.2	2	56		intronic	NM_00447	c.1105+11	p.?	-0.41, -2.26					PA domain,Pe	rs1042462986,rs34943739,rs49		YES			Folate hydr	Ac-Asp-Glu Genital Diseases, M (2S)-2-[HYDROXY(4-IODOE	CG=11, TC=	CG=0.44, TC=0.56	0.00001	129.703	25	CG=6/5, TC=13
chr7:89938680	T/T	C	T	SNV	CFAP69	CFAP69:exonic:NM_001039706.2	1	100	22	missense	NM_00103	c.2654C>T	p.Thr885Met	4.33	0	81	1	0.96(COSM3762891),0.96(C		rs1134956	0.4281	YES	2.094E-1 (pos.89938680)	acute_myeloid_leukaemia,adenocarcinoma,carcinoma,neoplasm			C=0, T=29	C=0.0, T=1.0	0.00001	427.603	29	C=0/0, T=11/1
chr1:196659237	T/T	C	T	SNV	CFH	CFH:exonic:NM_000186.3	1	100	9	missense	NM_00018	c.1204C>T	p.His402Tyr	-6.72	1	83	0	0(COSM4249	Sushi repeat	rs1061170	0.3823	YES	9.506E-1 (pos.19665923	adenocarcino	Complemer	blood mici Anemia;Anemia, Hemolytic;At Benign	C=0, T=34	C=0.0, T=1.0	0.00001	524.183	34	C=0/0, T=18/1
chr1:196801042	T/T	G	T	SNV	CFHR1	CFHR1:exonic:NM_002113.2	1	100	6	synonymous	NM_00211	c.906G>T	p.(=)	-1.08				0(COSM3750729)		rs4230	0.4063	YES	5.462E-1 (pos.19680104	acute_myeloic	Complemer	blood mici Anemia;Anemia, Hemolytic;Atypical Hemolyt	G=0, T=36	G=0.0, T=1.0	0.00001	563.744	36	G=0/0, T=19/1
chr1:196801078	T/T	A	T	SNV	CFHR1	CFHR1:exonic:NM_002113.2	1	100	6	synonymous	NM_00211	c.942A>T	p.(=)	1.05				0.01(COSM3750730)		rs414628	0.405	YES	5.255E-1 (pos.19680107	acute_myeloic	Complemer	blood mici Anemia;Anemia, Hemolytic;Atypical Hemolyt	A=0, T=22	A=0.0, T=1.0	0.00001	298.889	22	A=0/0, T=11/1
chr1:196801190	A/A	C	A	SNV	CFHR1	CFHR1:utr_3:NM_002113.2	1	100	6	utr_3	NM_00211	c.1054C>A	p.?	-1						rs390679		YES			Complemer	blood mici Anemia;Anemia, Hemolytic;Atypical Hemolyt	C=0, A=22	C=0.0, A=1.0	0.00001	298.889	22	C=0/0, A=11/1
chr1:196801226	C/T	C	T	SNV	CFHR1	CFHR1:utr_3:NM_002113.2	1	47.62	6	utr_3	NM_00211	c.1090C>T	p.?	0.41											Complemer	blood mici Anemia;Anemia, Hemolytic;Atypical Hemolyt	C=11, T=10	C=0.5238, T=0.4762	0.00001	82.2896	21	C=5/6, T=5/5
chr11:51412453	T/T	C	T	SNV	OR4A5	OR4A5:upstream:NM_001005272.3	1	100		intronic	NM_00100	c.-58G>A	p.?	-0.05						rs545033		YES				G-protein coupled receptor activity,G-protein coupled re	C=0, T=95	C=0.0, T=1.0	0.00001	1597.06	95	C=0/0, T=27/6
chr1:196874356	T/T	G	T	SNV	CFHR4	CFHR4:exonic:NM_001201550.2	1	100	3	missense	NM_00120	c.375G>T	p.Glu125Asp	0.04	0.96	45	0			rs10801578			1.0 (pos.196874356)		Complemer	extracellular region,lipid transport,lipid transporter act	G=0, T=76	G=0.0, T=1.0	0.00001	1209.6	76	G=0/0, T=26/5
chr11:5344423	A/A	T	A	SNV	OR51B2	OR51B2:downstream:NM_033180.4	1	100		intronic	NM_03318	c.*166A>T	p.?	-1.29						rs7951549		YES				G-protein coupled receptor activity,G-protein coupled re	T=0, A=31	T=0.0, A=1.0	0.00001	465.792	31	T=0/0, A=18/1
chr11:5344499	T/T	C	T	SNV	OR51B2	OR51B2:downstream:NM_033180.4	1	100		intronic	NM_03318	c.*90G>A	p.?	-1.01						rs7934005		YES				G-protein coupled receptor activity,G-protein coupled re	C=0, T=38	C=0.0, T=1.0	0.00001	603.785	38	C=0/0, T=19/1
chr4:110678925	C/C	T	C	SNV	CFI	CFI:exonic:NM_000204.4	1	100	7	missense	NM_00020	c.898A>G	p.Thr300Ala	-0.25	0.94	58	0		Low-density l	rs11098044	9.00E-04	YES	1.0 (pos.110678925)		Complemer	compleme Afibrinogenemia;Anemia;Anemia, Hemolytic	T=0, C=66	T=0.0, C=1.0	0.00001	1014.59	66	T=0/0, C=26/4
chr7:117307108	G/A	G	A	SNV	CFTR	CFTR:exonic:NM_000492.3	1	57.14	27	synonymous	NM_00049	c.4389G>A	p.(=)	0.01				0.11(COSM37	ABC transpor	rs1800136	0.2164	YES	1.902E-2 (pos.11730710	adenocarcino	Cystic fibro	ATP bindin Azoospermia;Bronc Bumetani Benign/Likely	G=24, A=32	G=0.4286, A=0.5714	0.00001	331.838	56	G=13/11, A=17
chr15:57731335	C/C	A	C	SNV	CGNL1	CGNL1:exonic:NM_032866.4	1	100	2	missense	NM_03286	c.1138A>C	p.Thr380Pro	3.81	1	38	0		Myosin tail	rs1280395	0		1.0 (pos.57731335)		Cingulin-lik	actin filam Bipolar Disorder;Bipolar and Related Disorde	A=0, C=126	A=0.0, C=1.0	0.00001	2051.53	126	A=0/0, C=55/7
chr19:4442999	G/G	T	G	SNV	CHAF1A	CHAF1A:exonic:NM_005483.2	1	100	15	missense	NM_00548	c.2848T>G	p.Ser950Ala	-1.35	1	99	0		CAF1 comple	rs243383	0.019	YES	9.68E-1;0.0 (pos.4442999)		Chromatin	CAF-1 com Mouth Diseases;Stomatognathic Diseases	T=0, G=42	T=0.0, G=1.0	0.00001	685.22	42	T=0/0, G=20/2
chr10:50863147	C/C	T	C	SNV	CHAT	CHAT:exonic:NM_020549.4	1	100	12	synonymous	NM_02054	c.1641T>C	p.(=)	-0.16				0.01(COSM41	Choline/Carn	rs8178992	0.0883	YES	7.947E-1;0.0 (pos.50863	neoplasm	Choline ace	choline O- Alzheimer Disease; Choline,Ni Benign	T=0, C=25	T=0.0, C=1.0	0.00001	353.019	25	T=0/0, C=13/1
chr10:50856652	G/A	G	A	SNV	CHAT	CHAT:exonic:NM_020549.4	1	98.41	9	missense	NM_02054	c.1381G>A	p.Val461Met	5.32	1	21	0.048		Choline/Carn	rs4838544	7.00E-04	YES	1.0 (pos.50856652)		Choline ace	choline O- Alzheimer Disease; Choline,Ni Benign	G=2, A=124	G=0.0159, A=0.9841	0.00001	1982.69	126	G=0/2, A=68/5
chr15:93510603	G/G	A	G	SNV	CHD2	CHD2:exonic:NM_001271.3	1	100	17	synonymous	NM_00127	c.2049A>G	p.(=)	0.4					Chromo (CHR	rs4777755	0.158	YES	9.973E-1 (pos.93510603)		Chromodon	ATP bindin Brain Diseases;Central Nervou Benign	A=0, G=34	A=0.0, G=1.0	0.00001	524.186	34	A=0/0, G=22/1
chr15:93536197	C/T	C	T	SNV	CHD2	CHD2:exonic:NM_001271.3	1	50	28	synonymous	NM_00127	c.3564C>T	p.(=)	0.05				0.65(COSM37	Chromo (CHR	rs2272457	0.274	YES	1.395E-1 (pos.93536197)	acute_myeloic	Chromodon	ATP bindin Brain Diseases;Central Nervou Benign	C=12, T=12	C=0.5, T=0.5	0.00001	106.71	24	C=4/8, T=5/7
chr3:53856653	T/T	C	T	SNV	CHDH	CHDH:exonic:NM_018397.4	1	100	4	synonymous	NM_01839	c.720G>A	p.(=)	-3.22				0.01(COSM41	GMC oxidore	rs2241808	0.4516	YES	5.655E-1 (pos.53856653)	neoplasm		choline catabolic process,chol Choline	C=0, T=29	C=0.0, T=1.0	0.00001	427.603	29	C=0/0, T=13/1
chr19:16633950	C/C	T	C	SNV	CHERP	CHERP:exonic:NM_006387.5	1	100	11	synonymous	NM_00638	c.1893A>G	p.(=)	-1.58				0.05(COSM17	G-patch dom	rs1043481	0.0833	YES	7.297E-1 (pos.16633950)	carcinoma,transitional_ce		RNA binding,RNA processing,cellular calcium ion homeo	T=0, C=23	T=0.0, C=1.0	0.00001	316.747	23	T=0/0, C=16/7
chr20:5903517	G/G	A	G	SNV	CHGB	CHGB:exonic:NM_001819.2	1	100	4	missense	NM_00181	c.727A>G	p.Thr243Ala	-0.36	0.53	58	0		Granin (chror	rs236151	0.2572	YES	9.99E-1;0.0 (pos.5903517)		Chromogra	cellular protein metabolic process,endoplasmic reticulu	A=0, G=31	A=0.0, G=1.0	0.00001	472.031	31	A=0/0, G=13/1
chr1:111783996	G/G	A	G	SNV	CHI3L2	CHI3L2:exonic:NM_004000.2	1	100	9	synonymous	NM_00400	c.966A>G	p.(=)	1.47					Glycosyl hydr	rs2494007	0		1.0 (pos.111783996)		Chitinase 3	carbohydrate binding,carbohydrate metabolic process,(	A=0, G=43	A=0.0, G=1.0	0.00001	705.844	43	A=0/0, G=22/2
chr1:111861841	G/G	A	G	SNV	CHIA	CHIA:exonic:NM_201653.3	1	100	10	missense	NM_20165	c.1015A>G	p.Ile339Val	-0.48	0.72	29	0	0.01(COSM11	Chitin bindin	rs2275253	0.3034	YES	4.476E-1 (pos.11186184	adenoma	Chitinase, a	apoptotic Abortion, Spontaneous;Female Urogenital Di	A=0, G=29	A=0.0, G=1.0	0.00001	427.603	29	A=0/0, G=10/1
chr1:111861822	G/A	G	A	SNV	CHIA	CHIA:exonic:NM_201653.3	1	51.85	10	synonymous	NM_20165	c.996G>A	p.(=)	0.93					Chitin bindin	rs17027410	0.1178	YES	2.313E-4 (pos.111861822)		Chitinase, a	apoptotic Abortion, Spontaneous;Female Urogenital Di	G=13, A=14	G=0.4815, A=0.5185	0.00001	132.666	27	G=4/9, A=6/8
chr1:111854937	A/AT	A	AT	INDEL	CHIA	CHIA:exonic:NM_201653.3	1	50	4	frameshiftInsertion	NM_20165	c.181_182	p.Arg61fs	8.36					Chitin binding Peritrophin-A domain,Glycosyl hydrolases family 18						Chitinase, a	apoptotic Abortion, Spontaneous;Female Urogenital Di	A=14, AT=14	A=0.5, AT=0.5	0.00001	74.1739	28	A=5/9, AT=11/
chr11:903070	C/C	G	C	SNV	CHID1	CHID1:exonic:NM_023947.3	1	100	3	synonymous	NM_02394	c.153C>G	p.(=)	0.97					Glycosyl hydr	rs28380110	1.00E-04		1.0 (pos.903070)		Chitinase d	carbohydrate metabolic process,chitin binding,chitin ca	G=0, C=27	G=0.0, C=1.0	0.00001	389.987	27	G=0/0, C=8/19
chr3:439963	G/G	A	G	SNV	CHL1	CHL1:exonic:NM_006614.3	1	99.58	25	missense	NM_00661	c.3148A>G	p.Ile1050Val	1	0.41	29	0.001		Bravo-like int	rs6442827	3.00E-04	YES	1.0 (pos.439963)		CHL1, mous	adult loco Intellectual Disability;Mental Disorders;Nerv	A=1, G=235	A=0.0042, G=0.9958	0.00001	3915.3	236	A=1/0, G=104/
chr11:552547	C/C	T	C	SNV	LRRC56	LRRC56:intronic:NM_198075.3	1	100		intronic	NM_19807	c.1182-22	p.?	-1.06						rs3740652	0.0639	YES	9.679E-1;0.0 (pos.552547)				T=0, C=30	T=0.0, C=1.0	0.00001	446.615	30	T=0/0, C=16/1
chr3:87295049	C/C	T	C	SNV	CHMP2B	CHMP2B:exonic:NM_014043.3	1	100	3	synonymous	NM_01404	c.312T>C	p.(=)	-0.63					Involucrin re	rs11540913	0.0552	YES	8.974E-1 (pos.87295049)		CHMP famil	ESCRT III cc Amyotrophic Lateral Sclerosis; Benign	T=0, C=20	T=0.0, C=1.0	0.00001	263.786	20	T=0/0, C=14/6
chr14:24679877	T/T	C	T	SNV	CHMP4A	CHMP4A:exonic:NM_014169.3	1	100	4	missense	NM_01416	c.586G>A	p.Gly196Arg	5.53	0	125	0.969	0.99(COSM39	Endomembra	rs2295322	0.4834	YES	2.355E-1 (pos.24679877)	acute_myeloic	CHMP famil	ATPase binding,ESCRT III complex,cell separation after cy	C=0, T=23	C=0.0, T=1.0	0.00001	316.747	23	C=0/0, T=13/1
chr11:55406788	CA/C	CA	C	INDEL	OR4P4	OR4P4:downstream:NM_001004124.2	1	46.51		intronic	NM_00100	c.*16CA>C	p.?	0.74, 0.06												G-protein coupled receptor activity,G-protein coupled re	CA=23, C=2	CA=0.5349, C=0.465	0.00001	211.33	43	CA=9/14, C=7/
chr8:23104195	T/T	G	T	SNV	CHMP7	CHMP7:utr_5:NM_152272.4	1	100	2	utr_5	NM_15227	c.-14G>T	p.?	3.31					FLYWCH-type	rs2294123	0.345	YES	3.633E-1 (pos.23104195)		CHMP famil	ESCRT III complex,ESCRT III complex disassembly,cell sep	G=0, T=42	G=0.0, T=1.0	0.00001	685.22	42	G=0/0, T=26/1
chr2:220406722	C/C	T	C	SNV	CHPF	CHPF:exonic:NM_024536.5	1	100	2	synonymous	NM_02453	c.504A>G	p.(=)	0.69				0.22(COSM44	Chondroitin	rs3731912	0.2784	YES	7.839E-1 (pos.22040672	adenocarcino	Chondroitir	Golgi cisterna membrane,Golgi membrane,N-acetylgala	T=0, C=79	T=0.0, C=1.0	0.00001	1269.33	79	T=0/0, C=29/5
chr12:102117589	T/T	C	T	SNV	CHPT1	CHPT1:exonic:NM_020244.2	1	100	7	synonymous	NM_02024	c.1029C>T	p.(=)	1.46					CDP-alcohol	rs11082	0.4765	YES	8.979E-1 (pos.102117589)		Choline phc	CDP-cholir Lung Diseases;Respiration Disorders;Respira	C=0, T=26	C=0.0, T=1.0	0.00001	371.418	26	C=0/0, T=11/1
chr11:556457	C/C	T	C	SNV	LMNTD2	LMNTD2:intronic:NM_173573.2	1	100		intronic	NM_17357	c.1073+35	p.?	-0.06					Lamin Tail Dc	rs28407128,rs75243	0.0662	YES	9.461E-1;0.0 (pos.556457)			positive regulation of mRNA splicing, via spliceosome	T=0, C=132	T=0.0, C=1.0	0.00001	2165.49	132	T=0/0, C=46/8
chr11:74419357	T/T	C	T	SNV	CHRDL2	CHRDL2:exonic:NM_015424.5	1	100	5	synonymous	NM_01542	c.516G>A	p.(=)	0.87				0.78(COSM11	von Willebra	rs1944937	0.2534	YES	8.214E-1 (pos.74419357)	adenocarcino	Chordin-like	cartilage development,cell differentiation,cytoplasm,ex	C=0, T=21	C=0.0, T=1.0	0.00001	280.493	21	C=0/0, T=13/8
chr11:55656483	T/A	T	A	SNV	TRIM51	TRIM51:intronic:NM_032681.3	1	50		intronic	NM_03268	c.761+6T>	p.?	0.19					RING-type zin	rs7483200	0.1647	YES	8.895E-1 (pos.55656483)			intracellular,zinc ion binding	T=30, A=30	T=0.5, A=0.5	0.00001	293.739	60	T=16/14, A=17
chr11:74419378	T/T	C	T	SNV	CHRDL2	CHRDL2:exonic:NM_015424.5	1	100	5	synonymous	NM_01542	c.495G>A	p.(=)	-5.77				0.01(COSM37	von Willebra	rs7948433	0.3763	YES	5.573E-1 (pos.74419378)	adenocarcino	Chordin-like	cartilage development,cell differentiation,cytoplasm,ex	C=0, T=24	C=0.0, T=1.0	0.00001	324.886	24	C=0/0, T=13/1
chr11:74414440	G/A	G	A	SNV	CHRDL2	CHRDL2:exonic:NM_015424.5	1	56.36	8	missense	NM_01542	c.856C>T	p.Arg286Cys	1.47	0.02	180	0.939		von Willebra	rs146734849	1.00E-04		1.164E-4 (pos.74414440)		Chordin-like	cartilage development,cell differentiation,cytoplasm,ex	G=24, A=31	G=0.4364, A=0.5636	0.00001	319.004	55	G=16/8, A=18/
chr15:78909452	C/C	T	C	SNV	CHRNA3	CHRNA3:exonic:NM_000743.4	1	100	4	synonymous	NM_00074	c.291A>G	p.(=)	0.85					Neurotransm	rs3743075	0.3682	YES	5.661E-1;0.0 (pos.78909452)		Cholinergic	acetylchol Adenocarcinoma;Al Bupropion,Cytisine,Dext	T=0, C=64	T=0.0, C=1.0	0.00001	977.632	64	T=0/0, C=26/3
chr20:61981536	G/G	A	G	SNV	CHRNA4	CHRNA4:exonic:NM_000744.6	1	100	5	synonymous	NM_00074	c.1227T>C	p.(=)	-0.53				0(COSM4134	Neurotransm	rs1064793841,rs222	0.0513	YES	9.997E-1 (pos.61981536)	neoplasm	Cholinergic	B cell activ Attention Deficit Di Amobarbi Benign/Likely	A=0, G=31	A=0.0, G=1.0	0.00001	465.805	31	A=0/0, G=12/1
chr11:55860780	A/A	G	A	SNV	OR8I2	OR8I2:upstream:NM_001003750.1	1	100		intronic	NM_00100	c.-4G>A	p.?	0.1						rs1842700	7.00E-04		1.0 (pos.55860780)			G-protein coupled receptor activity,G-protein coupled re	G=0, A=52	G=0.0, A=1.0	0.00001	767.014	52	G=0/0, A=35/1
chr20:61981554	A/A	C	A	SNV	CHRNA4	CHRNA4:exonic:NM_000744.6	1	100	5	synonymous	NM_00074	c.1209G>T	p.(=)	-2.57				0.02(COSM41	Neurotransm	rs1064795858,rs200	0.1194	YES	0.0;0.0 (pos.61981553),8	neoplasm	Cholinergic	B cell activ Attention Deficit Di Amobarbi Benign/Likely	C=0, A=31	C=0.0, A=1.0	0.00001	465.817	31	C=0/0, A=12/1
chr11:55944048	C/C	G	C	SNV	OR5J2	OR5J2:upstream:NM_001005492.1	1	100		intronic	NM_00100	c.-46G>C	p.?	-0.59						rs10896103	0.3409	YES	8.529E-1 (pos.55944048)			G-protein coupled receptor activity,G-protein coupled re	G=0, C=175	G=0.0, C=1.0	0.00001	2881.03	175	G=0/0, C=90/8
chr4:40356041	C/T	C	T	SNV	CHRNA9	CHRNA9:exonic:NM_017581.3	1	55.77	5	missense	NM_01758	c.944C>T	p.Ala315Val	5.77	0.33	64	0.84	0.96(COSM37	Neurotransm	rs55633891	0.131	YES	1.286E-1;0.0 (pos.40356	acute_myeloic	Cholinergic	acetylcholine-gated cation-sel ATG003,Ethanol,Galanta	C=23, T=29	C=0.4423, T=0.5577	0.00001	299.666	52	C=2/21, T=7/2
chr11:45672261	G/G	A	G	SNV	CHST1	CHST1:exonic:NM_003654.5	1	100	4	synonymous	NM_00365	c.213T>C	p.(=)	-0.59				0.03(COSM41	Sulfotransfer	rs3802762	0.1477	YES	6.804E-1 (pos.45672261)	neoplasm	Carbohydra	Golgi membrane,N-acetylglucosamine 6-O-sulfotransfer	A=0, G=47	A=0.0, G=1.0	0.00001	789.326	47	A=0/0, G=13/3
chr2:101010082	C/C	G	C	SNV	CHST10	CHST10:exonic:NM_004854.4	1	100	7	synonymous	NM_00485	c.696C>G	p.(=)	0.8				0.9(COSM561	Sulfotransfer	rs3748930	0.4953	YES	7.811E-1;0.0 (pos.10101	ductal_carcinc	Carbohydra	Golgi apparatus,Golgi membrane,HNK-1 sulfotransferas	G=0, C=45	G=0.0, C=1.0	0.00001	747.25	45	G=0/0, C=17/2
chr16:71571282	A/G	A	G	SNV	CHST4	CHST4:exonic:NM_001166395.1	1	46.43	2	synonymous	NM_00116	c.702A>G	p.(=)	-0.22					Sulfotransfer	rs117753658	0.0167	YES	0.0 (pos.71571282)			Golgi membrane,N-acetylglucosamine 6-O-sulfotransfer	A=11, G=13	A=0.4583, G=0.5417	0.00001	140.967	28	A=5/6, G=9/4
chr16:75563746	T/G	T	G	SNV	CHST5	CHST5:exonic:NM_024533.4	1	53.33	3	synonymous	NM_02453	c.537A>C	p.(=)	0.68				0.12(COSM37	Sulfotransfer	rs8048818	0.1963	YES	1.719E-1 (pos.75563746)	acute_myeloic	Carbohydra	Golgi appa Nervous System Diseases;Neuromuscular Dis	T=14, G=16	T=0.4667, G=0.5333	0.00001	159.925	30	T=4/10, G=10/
chr5:129240972	C/C	G	C	SNV	CHSY3	CHSY3:exonic:NM_175856.4	1	100	1	synonymous	NM_17585	c.450G>C	p.(=)	-2.22				0.02(COSM37	Chondroitin	rs33917		YES	6E-1 (pos.129240972)	adenocarcino	Chondroitir	Golgi cisterna membrane,Golgi membrane,N-acetylgala	G=0, C=30	G=0.0, C=1.0	0.00001	444.982	30	G=0/0, C=11/1

chr5:129521126	G/G	A	G	SNV	CHSY3	CHSY3:exonic:NM_175856.4	1	100	3	missense	NM_17585	c.2291A>G	p.Asp764Gly	2.7	0.06	94	0.001	0.84(COSM40	Chondroitin N	rs2015018	0.251	YES	6.49E-1 (pos.129521126)	acute_myeloic	Chondroitir	Golgi cisterna membrane,Golgi membrane,N-acetylgala			A=0, G=26	A=0.0, G=1.0	0.00001	371.42	26	A=0/0, G=10/1
chr15:90774447	T/T	TAGAG	T	INDEL	CIB1	CIB1:splicesite_3:NM_001277764.1	4	100	5	splicesite	NM_00127	c.467-2CT(	p.?	7.67, 1.04, 0.59, -0.64, -0.17					EF hand,EF-ha	rs1010477466,rs199557034,rs2		YES	0.0 (pos.90774448),0.0 (pos.90774449),0.0 (pos.907		Golgi apparatus,Ras GTPase bi	Calcium Citrate,Calcium			TAGAG=0,1	TAGAG=0.0, T=1.0	0.00001	1953.69	121	TAGAG=0/0, T=
chr19:16275655	T/T	C	T	SNV	CIB3	CIB3:exonic:NM_054113.3	1	100	5	missense	NM_05411	c.416G>A	p.Gly139Glu	6.03	1	98	0	0.19(COSM49	EF hand,EF-ha	rs6512087	0.2091	YES	9.582E-1:0.0:0.0 (pos.162	ductal_carcinoma		calcium ion binding,magnesium ion binding,protein bind			C=0, T=47	C=0.0, T=1.0	0.00001	774.253	47	C=0/0, T=15/3
chr16:11002904	A/A	G	A	SNV	CIITA	CIITA:exonic:NM_000246.3	1	100	12	synonymous	NM_00024	c.2676G>A	p.(=)	-1.76					Leucine Rich	rs2228238	0.3009	YES	8.514E-1 (pos.11002904)		Class II maj	ATP bindin	Addison Disease;Adrenal Glan	Benign	G=0, A=34	G=0.0, A=1.0	0.00001	524.149	34	G=0/0, A=20/1
chr16:11002927	G/G	A	G	SNV	CIITA	CIITA:exonic:NM_000246.3	1	100	12	missense	NM_00024	c.2699A>G	p.Gln900Arg	0.88	0.54	43	0		Leucine Rich	rs7197779,rs745976	6.00E-04	YES	0.0;0.0 (pos.11002919),1.0 (pos.110029		Class II maj	ATP bindin	Addison Disease;Adrenal Glan	Benign	A=0, G=35	A=0.0, G=1.0	0.00001	543.903	35	A=0/0, G=20/1
chr15:65489688	C/C	T	C	SNV	CILP	CILP:exonic:NM_003613.3	1	100	9	missense	NM_00361	c.2936A>G	p.Gln979Arg	-0.9	1	43	0		Immunoglobu	rs2679117	2.00E-04		9.994E-1 (pos.65489688)		Cartilage in	alkaline pl	Bone Diseases;Intervertebral Disc Degenera		T=0, C=27	T=0.0, C=1.0	0.00001	389.951	27	T=0/0, C=12/1
chr15:65490901	C/C	T	C	SNV	CILP	CILP:exonic:NM_003613.3	1	100	9	missense	NM_00361	c.1723A>G	p.Lys575Glu	0.31	1	56	0		Immunoglobu	rs2679118	1.00E-04		1.0 (pos.65490901)		Cartilage in	alkaline pl	Bone Diseases;Intervertebral Disc Degenera		T=0, C=21	T=0.0, C=1.0	0.00001	281.235	21	T=0/0, C=14/7
chr15:65494212	G/G	A	G	SNV	CILP	CILP:exonic:NM_003613.3	1	100	8	missense	NM_00361	c.1184T>C	p.Ile395Thr	4.09	0.13	89	0	0.98(COSM37	Immunoglobu	rs2073711	0.3637	YES	1.521E-1 (pos.65494212)	adenocarcinoı	Cartilage in	alkaline pl	Bone Diseases;Intervertebral I	risk factor	A=0, G=29	A=0.0, G=1.0	0.00001	427.595	29	A=0/0, G=7/22
chr13:53035783	C/C	T	C	SNV	CKAP2	CKAP2:exonic:NM_001098525.2	1	100	4	synonymous	NM_00109	c.825T>C	p.(=)	-0.99					Cytoskeleton	rs7337054,rs7723659	0		0.0 (pos.53035782),1.0 (pos.53035783)		Cytoskeletc	apoptotic process,centrosome,cytoplasmic microtubule			T=0, C=92	T=0.0, C=1.0	0.00001	1534.53	92	T=0/0, C=42/5
chr13:53035925	G/G	A	G	SNV	CKAP2	CKAP2:exonic:NM_001098525.2	1	100	4	missense	NM_00109	c.967A>G	p.Ile323Val	-0.29	1	29	0		Cytoskeleton	rs7335867	0		1.0 (pos.53035925)		Cytoskeletc	apoptotic process,centrosome,cytoplasmic microtubule			A=0, G=96	A=0.0, G=1.0	0.00001	1617.54	96	A=0/0, G=40/5
chr11:5653417	GA/GA	AT	GA	MNV	TRIM34,TRIM6-TRIM34	TRIM6-TRIM34:intronic:NM_001003819.3, TRIM34:intronic:	2	100		intronic	NM_00100	c.986-68A	p.?, p.?	-0.81, 0.5					B-box zinc fin	rs4287358,rs4601818,rs714721		YES			Tripartite m	cytoplasm,cytosol,defense response to virus,interferon-			AT=0, GA=8	AT=0.0, GA=1.0	0.00001	1458.44	88	AT=0/0, GA=60
chr15:43891512	C/T	C	T	SNV	CKMT1B,STRC	STRC:downstream:NM_153700.2, CKMT1B:utr_3:NM_02099	1	52.38	10	utr_3	NM_02099	c.1295C>T	p.?, p.?	0.38					ATP:guanido	rs2439	0.0993	YES	2.952E-1;0.0 (pos.43891512)		Creatine kir	ATP bindin	Ear Diseases;Genital Diseases, Male;Hearing		C=20, T=22	C=0.4762, T=0.5238	0.00001	245.209	42	C=6/14, T=7/1
chr11:56950264	G/G	A	G	SNV	LRRC55	LRRC55:intronic:NM_001005210.2	1	100		intronic	NM_00100	c.790+107	p.?	-1.22					Leucine Rich	rs609763		YES			Leucine-ricl	axonogenesis,extracellular space,integral component o			A=0, G=21	A=0.0, G=1.0	0.00001	281.224	21	A=0/0, G=12/9
chr14:105461067	T/T	C	T	SNV	CLBA1	CLBA1:exonic:NM_174891.3	1	100	5	synonymous	NM_17489	c.949C>T	p.(=)	-1.01				0.02(COSM63	Clathrin-bind	rs894039	0.1911	YES	5.415E-1 (pos.10546106)	carcinoma					C=0, T=20	C=0.0, T=1.0	0.00001	263.786	20	C=0/0, T=14/6
chr11:57097924	G/A	G	A	SNV	SSRP1	SSRP1:intronic:NM_003146.2	1	50		intronic	NM_00314	c.1297-21	p.?	-1.2					HMG (high mo	rs10896605	0.1628	YES	1.575E-2 (pos.57097924)		Structure-sı	DNA binding,DNA repair,DNA replication,RNA binding,chı			G=11, A=11	G=0.5, A=0.5	0.00001	94.6067	22	G=8/3, A=5/6
chr11:57148053	G/T	G	T	SNV	PRG3	PRG3:intronic:NM_006093.3	1	96.67		intronic	NM_00609	c.61+68C>	p.?	-3.58					Lectin C-type	rs571651	0.0022	YES			Proteoglyca	basophil activation,carbohydrate binding,extracellular			G=1, T=29	G=0.0333, T=0.9667	0.00001	405.753	30	G=1/0, T=14/1
chr19:40224986	A/G	A	G	SNV	CLC	CLC:exonic:NM_001828.5	1	57.89	3	synonymous	NM_00182	c.240T>C	p.(=)	-2.52				0(COSM1129	Galactoside-l	rs384138	0.3333	YES	3.253E-1 (pos.40224986)	acute_myeloic	Cardiotropl	T cell apoptotic process,carbol	N-Ethylmaleimide,Para-I		A=24, G=33	A=0.4211, G=0.5789	0.00001	345.084	57	A=7/17, G=10/
chr1:86939130	T/T	C	T	SNV	CLCA1	CLCA1:exonic:NM_001285.3	1	100	2	missense	NM_00128	c.193C>T	p.Leu65Phe	2.69	1	22	0		Calcium-acti	rs2145412	0.0376	YES	9.873E-1;0.0 (pos.86939130)		Chloride ch	calcium ion transport,cellular response to hypoxia,chlor			C=0, T=81	C=0.0, T=1.0	0.00001	1307.09	81	C=0/0, T=53/2
chr11:57265400	T/T	C	T	SNV	SLC43A1	SLC43A1:intronic:NM_001198810.1	1	100		intronic	NM_00119	c.466-82G	p.?	-1.81					Major Facilit	rs2581922		YES			Solute carri	L-alpha-amino acid transmembrane transport,L-amino a			C=0, T=36	C=0.0, T=1.0	0.00001	563.744	36	C=0/0, T=10/2
chr11:57414387	G/G	A	G	SNV	YPEL4	YPEL4:intronic:NM_145008.2	1	100		intronic	NM_14500	c.141+53T	p.?	-0.16					Yippee zinc-b	rs7947357		YES			Yippee-like	metal ion binding,nucleolus			A=0, G=22	A=0.0, G=1.0	0.00001	298.892	22	A=0/0, G=9/13
chr1:86959173	C/C	T	C	SNV	CLCA1	CLCA1:exonic:NM_001285.3	1	100	10	missense	NM_00128	c.1571T>C	p.Met524Thr	-0.15	0.86	81	0	0(COSM3747	Calcium-acti	rs2791494	0.1821	YES	9.213E-1 (pos.86959173)	adenocarcinoı	Chloride ch	calcium ion transport,cellular response to hypoxia,chlor			T=0, C=35	T=0.0, C=1.0	0.00001	543.902	35	T=0/0, C=10/2
chr7:143017807	T/T	G	T	SNV	CLCN1	CLCN1:exonic:NM_000083.2	1	100	3	missense	NM_00008	c.352G>T	p.Gly118Trp	5.97	1	184	0		Voltage gatec	rs10282312	0.025	YES	1.0 (pos.143017807)		Chloride ch	chloride cl	Congenital, Hereditary, and Neonatal Diseas		G=0, T=44	G=0.0, T=1.0	0.00001	726.569	44	G=0/0, T=28/1
chr1:11884555	G/G	A	G	SNV	CLCN6	CLCN6:exonic:NM_001286.3	1	100	8	missense	NM_00128	c.593A>G	p.Glu198Gly	7.67	1	98	0		CBS domain.\	rs198400	0.001		1.0 (pos.11884555)		Chloride ch	ATP binding,antiporter activity,cell volume homeostasis			A=0, G=20	A=0.0, G=1.0	0.00001	263.786	20	A=0/0, G=13/7
chr1:11896141	A/G	A	G	SNV	CLCN6	CLCN6:exonic:NM_001286.3	1	45.83	18	synonymous	NM_00128	c.1911A>G	p.(=)	-6.91					CBS domain.\	rs41275498	0.0273	YES	0.0 (pos.11896141)		Chloride ch	ATP binding,antiporter activity,cell volume homeostasis			A=13, G=11	A=0.5417, G=0.4583	0.00001	93.4402	24	A=9/4, G=4/7
chr1:16373124	G/G	A	G	SNV	CLCNKB	CLCNKB:exonic:NM_000085.4	1	100	4	synonymous	NM_00008	c.324A>G	p.(=)	-4.72						rs5257	0.1883	YES	9.898E-1 (pos.16373124)		Chloride ch	catalytic a	Adrenal Gland Diseases;Adren	Benign	A=0, G=49	A=0.0, G=1.0	0.00001	831.633	49	A=0/0, G=15/3
chr1:16380196	C/C	T	C	SNV	CLCNKB	CLCNKB:exonic:NM_000085.4	1	100	16	missense	NM_00008	c.1685T>C	p.Met562Thr	-0.92	0.48	81	0			rs5253	0.0156	YES	8.081E-1 (pos.16380196)		Chloride ch	catalytic a	Adrenal Gland Diseases;Adren	Benign	T=0, C=31	T=0.0, C=1.0	0.00001	465.819	31	T=0/0, C=17/1
chr16:3065924	G/A	G	A	SNV	CLDN6	CLDN6:exonic:NM_021195.4	1	50	2	synonymous	NM_02119	c.99C>T	p.(=)	-1.95				0.06(COSM14	PMP-22/EMP	rs2269911	0.1903	YES	3.78E-1 (pos.3065924)	adenocarcinoı	Claudin 6	apicolateral plasma membrane,bicellular tight junction			G=30, A=30	G=0.5, A=0.5	0.00001	293.739	60	G=12/18, A=1
chr12:10165469	G/G	A	G	SNV	CLEC12B	CLEC12B:exonic:NM_001129998.2	1	100	2	synonymous	NM_00112	c.177A>G	p.(=)	-1.22					Lectin C-type	rs476474,rs7758888	0.0845	YES	0.0 (pos.10165466),9.73E-1 (pos.101654		C-type lecti	carbohydrate binding,external side of plasma membran			A=0, G=23	A=0.0, G=1.0	0.00001	316.742	23	A=0/0, G=14/9
chr11:58381686	G/G	A	G	SNV	ZFP91,ZFP91-CNTF	ZFP91:intronic:NM_053023.4, ZFP91-CNTF:intronic_nc:NR_0	1	100		intronic	NM_05302	c.988-16A	p.?	-1.15					C2H2-type zir	rs1938596	0.4256	YES	3.365E-1 (pos.58381686)			activation	Hemic and Lymphatic Diseases;Immune Syst		A=0, G=27	A=0.0, G=1.0	0.00001	389.631	27	A=0/0, G=10/1
chr12:10167277	C/C	G	C	SNV	CLEC12B,LOC10272402	CLEC12B:exonic:NM_001129998.2, LOC102724020:downstr	1	100	3	missense	NM_00112	c.346G>C	p.Val116Leu	1.11	1	32	0		Lectin C-type	rs637790	0.0851	YES	9.728E-1;0.0 (pos.10167277)		C-type lecti	carbohydrate binding,external side of plasma membran			G=0, C=121	G=0.0, C=1.0	0.00001	1953.42	121	G=0/0, C=61/6
chr11:59210480	T/T	C	T	SNV	OR5A1	OR5A1:upstream:NM_001004728.1	1	100		intronic	NM_00100	c.-162C>T	p.?	-2.96						rs7948562		YES				G-protein coupled receptor activity,G-protein coupled re			C=0, T=24	C=0.0, T=1.0	0.00001	334.783	24	C=0/0, T=5/19
chr11:59210550	A/A	G	A	SNV	OR5A1	OR5A1:upstream:NM_001004728.1	1	100		intronic	NM_00100	c.-92G>A	p.?	-1.89						rs7947825		YES				G-protein coupled receptor activity,G-protein coupled re			G=0, A=23	G=0.0, A=1.0	0.00001	316.726	23	G=0/0, A=4/19
chr19:14721019	C/T	C	T	SNV	CLEC17A	CLEC17A:utr_3:NM_001204118.1	1	57.89	14	utr_3	NM_00120	c.1148C>T	p.?	-0.59					Lectin C-type	rs6511944		YES	8.284E-1 (pos.14721019)		C-type lecti	cell surface,fucose binding,integral component of memb			C=16, T=22	C=0.4211, T=0.5789	0.00001	249.053	38	C=11/5, T=13/
chr16:74455302	G/G	A	G	SNV	CLEC18B	CLEC18B:utr_5:NM_001011880.2	1	100	1	utr_5	NM_00101	c.-134T>C	p.?	-1.09					Cysteine-rich	rs3851723		YES			C-type lecti	Golgi apparatus,carbohydrate binding,endosome,extrac			A=0, G=163	A=0.0, G=1.0	0.00001	2639.49	163	A=0/0, G=62/1
chr12:10149851	T/T	C	T	SNV	CLEC1B	CLEC1B:exonic:NM_016509.3	1	100	3	missense	NM_01650	c.191G>A	p.Gly64Asp	-0.74	0.33	94	0		Lectin C-type	rs583903	4.00E-04		1.0 (pos.10149851)			carbohydrate binding,cell surface receptor signaling pat			C=0, T=29	C=0.0, T=1.0	0.00001	427.604	29	C=0/0, T=10/1
chr12:9833628	T/T	C	T	SNV	CLEC2D	CLEC2D:exonic:NM_001004419.4	1	100	2	synonymous	NM_00100	c.171C>T	p.(=)	-0.58				0(COSM1365	Lectin C-type	rs3764021	0.4741	YES	3.822E-1 (pos.9833628)	adenocarcinoı	C-type lecti	carbohydr	Autoimmune Diseases;Diabetes Mellitus;Dia		C=0, T=152	C=0.0, T=1.0	0.00001	2432.53	152	C=0/0, T=79/7
chr12:8667897	G/G	A	G	SNV	CLEC4D	CLEC4D:exonic:NM_080387.4	1	100	2	missense	NM_08038	c.94A>G	p.Ser32Gly	-0.18	0.49	56	0.003	0.02(COSM41	Lectin C-type	rs4304840	0.231	YES	1.426E-1 (pos.8667897)	neoplasm	C-type lecti	Fc-gamma	Lung Diseases;Respiratory Tra	Benign	A=0, G=22	A=0.0, G=1.0	0.00001	298.889	22	A=0/0, G=8/14
chr12:10206925	A/G	A	G	SNV	CLEC9A	CLEC9A:exonic:NM_207345.3	1	50	5	synonymous	NM_20734	c.147A>G	p.(=)	0.56				0.03(COSM37	Lectin C-type	rs7315231	0.3129	YES	2.216E-1 (pos.10206925)	acute_myeloic	C-type lecti	carbohydrate binding,cell surface,integral component o			A=26, G=26	A=0.5, G=0.5	0.00001	254.878	52	A=16/10, G=9/
chr12:9885708	TAAGTT/TAAG	T	TAAGTT	INDEL	CLECL1	CLECL1:exonic:NM_001253750.1	5	100	1	frameshiftInsertion	NM_00125	c.152_153	p.Ser52fs	0.48											C-type lecti	B cell adhe	Autoimmune Diseases;Autoimmune Disease		T=0, TAAGT	T=0.0, TAAGTT=1.0	0.00001	298.892	22	T=0/0, TAAGTT
chr2:55407754	T/T	C	T	SNV	CLHC1	CLHC1:exonic:NM_152385.3	1	100	11	missense	NM_15238	c.1276G>A	p.Val426Ile	1.57	1	29	0		Clathrin-H-lir	rs6716066	0.0231	YES	9.769E-1 (pos.55407754)			protein bi	Gait Disorders, Neurologic;Nervous System D		C=0, T=89	C=0.0, T=1.0	0.00001	1472.43	89	C=0/0, T=61/2
chr11:60215066	C/A	C	A	SNV	MS4A5	MS4A5:intronic:NM_023945.2	1	50		intronic	NM_02394	c.493-56C	p.?	-0.65					CD20-like fan	rs12787520		YES			Membrane-	integral component of membrane			C=14, A=14	C=0.5, A=0.5	0.00001	131.941	28	C=6/8, A=10/4
chr7:73814749	C/C	G	C	SNV	CLIP2	CLIP2:exonic:NM_003388.4	1	100	15	missense	NM_00338	c.2930G>C	p.Arg977Pro	5.45	1	103	0		CAP-Gly doma	rs2522943	0.0249	YES	1.0;0.0 (pos.73814749)		CAP-GLY dor	cytoplasm	Aortic Stenosis, Supravalvular;Aortic Valve St		G=0, C=240	G=0.0, C=1.0	0.00001	3994.54	240	G=0/0, C=116/
chr11:122955402	C/T	C	T	SNV	CLMP	CLMP:exonic:NM_024769.3	1	45.83	3	missense	NM_02476	c.206G>A	p.Arg69His	0.56	0.12	29	0.001	0.26(COSM37	CD47 immun	rs2276348	0.0186	YES	1.706E-1 (pos.12295540)	adenocarcinoı	Coxsackievi	bicellular	Digestive System Diseases;Gastrointestinal		C=13, T=11	C=0.5417, T=0.4583	0.00001	93.44	24	C=7/6, T=3/8
chr11:60296834	G/G	A	G	SNV	MS4A13	MS4A13:intronic:NM_001012417.2	1	100		intronic	NM_00101	c.307-4A>	p.?	0.62						rs12802514	0.0507	YES	1.447E-1 (pos.60296834)			integral component of membrane			A=0, G=27	A=0.0, G=1.0	0.00001	389.988	27	A=0/0, G=15/1
chr11:60561613	C/T	C	T	SNV	MS4A10	MS4A10:intronic:NM_206893.3	1	47.62		intronic	NM_20689	c.492+37C	p.?	-1.84					CD20-like fan	rs751026	0.4957	YES	8.7E-1 (pos.60561613)		Membrane-	integral component of membrane			C=11, T=10	C=0.5238, T=0.4762	0.00001	82.2938	21	C=8/3, T=3/7
chr11:60567275	A/A	G	A	SNV	MS4A10	MS4A10:intronic:NM_206893.3	1	100		intronic	NM_20689	c.723-26G	p.?	-0.42					CD20-like fan	rs579967	1.00E-04		1.0 (pos.60567275)		Membrane-	integral component of membrane			G=0, A=67	G=0.0, A=1.0	0.00001	1034.43	67	G=0/0, A=45/2
chr4:17517099	AG/G	A	G	SNV	CLRN2	CLRN2:exonic:NM_001079827.2	1	72.73	1	synonymous	NM_00107	c.211_212	p.Val71fs, p.(=)	1.19						rs1551091	1.00E-04		1.0 (pos.17517099)			integral component of membrane			A=0, AG=9,	A=0.0, AG=0.2727, G	0.00001	470.469	33	A=0/0, AG=9/0
chr3:140285115	C/C	T	C	SNV	CLSTN2	CLSTN2:utr_3:NM_022131.2	1	100	17	utr_3	NM_02213	c.2888T>C	p.?	-0.87					Cadherin dor	rs4683510	0.0931	YES	1.733E-1 (pos.140285115)		Calsyntenir	Golgi mem	Autoimmune Diseases;Autoimmune Disease		T=0, C=22	T=0.0, C=1.0	0.00001	297.19	22	T=0/0, C=9/13
chr11:60667907	A/G	A	G	SNV	PRPF19	PRPF19:intronic:NM_014502.4	1	50		intronic	NM_01450	c.828+147	p.?	-0.28					Prp19/Pso4-l	rs7928208		YES			Precursor m	DNA replic	Digestive System Diseases;Digestive System		A=15, G=15	A=0.5, G=0.5	0.00001	144.073	30	A=5/10, G=7/8
chr11:60890563	T/T	C	T	SNV	CD5	CD5:intronic:NM_014207.3	1	100		intronic	NM_01420	c.1225+59	p.?	0.01					Scavenger re	rs4245224		YES			CD5 antiger	T cell costi	Arthritis;Arthritis, Rheumatoid;Autoimmune		C=0, T=29	C=0.0, T=1.0	0.00001	427.603	29	C=0/0, T=19/1
chr3:140178381	T/C	T	C	SNV	CLSTN2	CLSTN2:exonic:NM_022131.2	1	50	7	missense	NM_02213	c.992T>C	p.Ile331Thr	2.72	0.59	89	0.01	0.89(COSM14	Cadherin dor	rs17348572	0.0524	YES	2.563E-2 (pos.14017838)	adenocarcinoı	Calsyntenir	Golgi mem	Autoimmune Diseases;Autoimmune Disease		T=29, C=29	T=0.5, C=0.5	0.00001	282.576	58	T=12/17, C=18
chr8:27462481	G/G	A	G	SNV	CLU	CLU:exonic:NM_001831.3	1	100	5	synonymous	NM_00183	c.789T>C	p.(=)	-0.72				0(COSM3763	Clusterin	rs7982	0.397	YES	7.831E-1 (pos.27462481)	adenocarcinoı	Clusterin (c	ATPase ac	Acute Kidney Injury;Alzheimer Disease;Amyo		A=0, G=49	A=0.0, G=1.0	0.00001	831.251	49	A=0/0, G=32/1
chr17:2595964	G/A	G	A	SNV	CLUH,MIR6776	CLUH:exonic:NM_015229.3, MIR6776:downstream:NR_106	1	50	21	synonymous	NM_01522	c.3222C>T	p.(=)	1.52				0.77(COSM43	Clustered mi	rs2302199	0.3679	YES	5.521E-1 (pos.2595964)	adenocarcinoı	Clustered m	cytoplasm,intracellular distribution of mitochondria,mR			G=40, A=40	G=0.5, A=0.5	0.00001	424.167	80	G=12/28, A=1
chr14:24975435	T/T	G	T	SNV	CMA1	CMA1:exonic:NM_001836.4	1	100	4	synonymous	NM_00183	c.399C>A	p.(=)	-0.81					Trypsin	rs5249	0.0012	YES	1.0 (pos.24975435)		Chymase-1,	angiotens	Cardiomegaly;Card	(1S)-1-(5-CHLORO-1-BENZ	G=0, T=34	G=0.0, T=1.0	0.00001	524.186	34	G=0/0, T=12/2
chr1:47842431	A/A	G	A	SNV	CMPK1	CMPK1:utr_3:NM_016308.2	1	100	6	utr_3	NM_01630	c.701G>A	p.?	0.26					AAA domain,i	rs3125648	0.0017	YES	1.0 (pos.47842431)		Cytidine mc	'de novo' p	Autoimmune Disea	Cytidine-5'-Monophosph	G=0, A=57	G=0.0, A=1.0	0.00001	849.604	57	G=0/0, A=32/2
chr14:23848311	T/T	C	T	SNV	CMTM5	CMTM5:exonic:NM_138460.2	1	100	3	synonymous	NM_13846	c.351C>T	p.(=)	-1.04				0.05(COSM14	Membrane-a	rs723840	0.4705	YES	2.55E-1 (pos.23848311)	adenocarcinoı	CKLF-like m	chemotaxis,cytokine activity,extracellular space,integra			C=0, T=36	C=0.0, T=1.0	0.00001	563.744	36	C=0/0, T=20/1
chr11:61205434	A/A	G	A	SNV	SDHAF2	SDHAF2:intronic:NM_017841.2	1	100		intronic	NM_01784	c.261-42G	p.?	0.22					Flavinator of	rs879647	0.1522	YES	9.616E-1 (pos.61205434)		Succinate d	cytosol,mi	ACMG Recommendations...;Ba	Benign	G=0, A=117	G=0.0, A=1.0	0.00001	1874.15	117	G=0/0, A=69/4
chr11:61253973	G/G	A	G	SNV	PPP1R32	PPP1R32:intronic:NM_145017.2	1	100		intronic	NM_14501	c.748-23A	p.?	0.03					Protein phos	rs2957858	0.1606	YES	9.621E-1 (pos.61253973)			phosphatase binding,protein binding			A=0, G=35	A=0.0, G=1.0	0.00001	540.631	35	A=0/0, G=14/2
chr11:612967	A/G	A	G	SNV	IRF7,PHRF1	IRF7:intronic:NM_001572.3, PHRF1:downstream:NM_0209(	1	57.14		intronic	NM_02090	c.*1190A>	p.?, p.?	-1.65					Interferon re	rs11246213	0.2926	YES	2.771E-2 (pos.612967)		Interferon r	DNA bindi	Autoimmune Diseases;Connective Tissue Dis		A=9, G=12	A=0.4286, G=0.5714	0.00001	96.1681	21	A=3/6, G=5/7
chr11:61491097	G/G	A	G	SNV	DAGLA	DAGLA:intronic:NM_006133.2	1	100		intronic	NM_00613	c.548+53A	p.?	1.45					Lipase (class	rs199604095,rs566009		YES			Diacylglyce	G-protein coupled glutamate receptor signaling pathway			A=0, G=59	A=0.0, G=1.0	0.00001	885.368	59	A=0/0, G=31/2
chr11:61548559	G/G	A	G	SNV	MYRF	MYRF:intronic:NM_001127392.2	1	100		intronic	NM_00112	c.2572+42	p.?	0.18					Chaperone of	rs509360	0.3265	YES	5.54E-1 (pos.61548559)			DNA bindi	Colonic Diseases;Colorectal Neoplasms;Dige		A=0, G=22	A=0.0, G=1.0	0.00001	298.892	22	A=0/0, G=6/16
chr11:61658629	C/C	T	C	SNV	FADS3	FADS3:intronic:NM_021727.4	1	100		intronic	NM_02172	c.213+12A	p.?	-1.26					Cytochrome I	rs693672	0.1718	YES	6.497E-1 (pos.61658629)		Fatty acid d	endoplasmic reticulum membrane,integral component			T=0, C=22	T=0.0, C=1.0	0.00001	298.891	22	T=0/0, C=7/15
chr11:61670037	C/T	C	T	SNV	RAB3IL1	RAB3IL1:intronic:NM_013401.3	1	46.43		intronic	NM_01340	c.900-24G	p.?	-0.14					GDP/GTP excl	rs174471	0.412	YES	3.839E-2 (pos.61670037)			Rab guanyl-nucleotide exchange factor activity,cytosol,c			C=15, T=13	C=0.5357, T=0.4643	0.00001	117.929	28	C=7/8, T=7/6
chr11:61731534	T/T	G	T	SNV	BEST1,FTH1	FTH1:downstream:NM_002032.2, BEST1:downstream:NM_(	1	100		intronic	NM_00113	c.*913G>T	p.?, p.?	-0.35						rs195155,rs7497989	1.00E-04	YES	9.999E-1;1.157E-4 (pos.61731534)		Bestrophin	autolysosı	Choroid Diseases;C	Ferric pyrophosphate,Ga	G=0, T=34	G=0.0, T=1.0	0.00001	524.186	34	G=0/0, T=19/1
chr11:61906299	G/G	A	G	SNV	INCENP	INCENP:intronic:NM_001040694.1	1	100		intronic	NM_00104	c.1173+57	p.?	-1.74					Chromosome	rs1614060	0.0867	YES			Inner centr	central ele	Melanoma;Neopla	N~6~-cyclohexyl-N~2~-(4	A=0, G=29	A=0.0, G=1.0	0.00001	427.52	29	A=0/0, G=12/1
chr16:71318577	T/C	T	C	SNV	CMTR2	CMTR2:exonic:NM_001099642.1	1	51.85	3	missense	NM_00109	c.1247A>G	p.Asn416Ser	0.15		46		0.88(COSM43	FtsJ-like meth	rs3803704	0.2513	YES	2.456E-1 (pos.71318577)	acute_myeloic	CAP methyl	7-methylg	Adenocarcinoma;Adnexal Diseases;Carcinon		T=39, C=42	T=0.4815, C=0.5185	0.00001	453.408	81	T=20/19, C=24
chr3:128890350	A/A	G	A	SNV	CNBP	CNBP:exonic:NM_001127192.1	1	100	3	synonymous	NM_00112	c.156C>T	p.(=)	3.18					Zinc knuckle	rs4303883	0.0305	YES	8.834E-1;0.0 (pos.128890350)			DNA bindi	Congenital, Hereditary, and Neonatal Diseas		G=0, A=87	G=0.0, A=1.0	0.00001	1431.39	87	G=0/0, A=44/4
chr18:72228124	G/G	A	G	SNV	CNDP1	CNDP1:exonic:NM_032649.5	1	100	4	missense	NM_03264	c.337A>G	p.Ile113Val	0.01	1	29	0		Peptidase dir	rs4263028	1.00E-04		1.0 (pos.72228124)		Carnosine c	carboxype	Amino Acid Metabolism, Inborn Errors;Brain I		A=0, G=34	A=0.0, G=1.0	0.00001	524.137	34	A=0/0, G=17/1
chr11:62380732	C/C	T	C	SNV	EML3,ROM1	ROM1:utr_5:NM_000327.3, EML3:upstream:NM_153265.2	1	100		intronic	NM_15326	c.-803A>G,	p.?, p.?	0.15					Tetraspanin f	rs4387351	0.0021	YES	9.998E-1 (pos.62380732)		Rod outer s	camera-ty	Congenital, Hereditary, and Neonatal Diseas		T=0, C=55	T=0.0, C=1.0	0.00001	814.883	55	T=0/0, C=37/1
chr2:99012444	C/G	C	G	SNV	CNGA3	CNGA3:exonic:NM_001298.2	1	50	8	missense	NM_00129	c.811C>G	p.Pro271Ala	7.32	0	27	0.576	0.99(COSM37	C-terminal le	rs149802213	6.00E-04		0.0;0.0 (pos.99012444)	acute_leukaer	Cyclic nucle	cGMP binc	Color Vision Defects;Eye Diseases;Nervous Sy		C=28, G=28	C=0.5, G=0.5	0.00001	272.138	56	C=8/20, G=17/
chr16:57937856	C/C	G	C	SNV	CNGB1	CNGB1:exonic:NM_001297.4	1	100	27	synonymous	NM_00129	c.2664C>G	p.(=)	-7.95					Cyclic nucleo	rs413562	0.1453	YES	9.175E-1;8.19E-2;0.0 (pos.57937856)		Cyclic nucle	Golgi mem	Congenital, Hereditary, and Ne	Benign	G=0, C=25	G=0.0, C=1.0	0.00001	352.669	25	G=0/0, C=11/1
chr11:62394881	A/G	A	G	SNV	GANAB	GANAB:intronic:NM_198335.3	1	46.88		intronic	NM_19833	c.2247-56	p.?	-0.24					Galactose mu	rs7949030	0.3906	YES			Neutral alp	Golgi apparatus,RNA binding,c		Miglitol	A=17, G=15	A=0.5313, G=0.4688	0.00001	143.733	32	A=11/6, G=4/1
chr11:62397177	G/G	C	G	SNV	GANAB	GANAB:intronic:NM_198335.3	1	100		intronic	NM_19833	c.1804-21	p.?	0.18					Galactose mu	rs948683	0.0019	YES	1.0 (pos.62397177)		Neutral alp	Golgi apparatus,RNA binding,c		Miglitol	C=0, G=34	C=0.0, G=1.0	0.00001	504.138	34	C=0/0, G=16/1
chr11:62430659	A/A	T	A	SNV	C11orf98,LBHD1	C11orf98:intronic:NM_001286086.1, LBHD1:intronic:NM_0	1	100		intronic	NM_00128	c.263-28A	p.?, p.?	0.65					Cardiac trans	rs597259	0.3025	YES	9.217E-1;0.0 (pos.62430659)						T=0, A=23	T=0.0, A=1.0	0.00001	316.738	23	T=0/0, A=8/15
chr7:135095270	G/G	A	G	SNV	CNOT4	CNOT4:exonic:NM_001190850.1	1	100	7	synonymous	NM_00119	c.816T>C	p.(=)	0.91					RING/Ubox lil	rs2696877	0.001		1.0 (pos.135095270)		CCR4-NOT ti	CCR4-NOT complex,DNA damage response, signal transd			A=0, G=27	A=0.0, G=1.0	0.00001	389.988	27	A=0/0, G=7/20
chr11:6245555	C/T	C	T	SNV	FAM160A2	FAM160A2:intronic:NM_032127.3	1	50		intronic	NM_03212	c.138+54G	p.?	-1.14					Retinoic acid	rs325608		YES				FHF complex,cytosol,early endosome to late endosome			C=30, T=30	C=0.5, T=0.5	0.00001	296.425	60	C=5/25, T=16/
chr11:62459994	A/G	A	G	SNV	BSCL2,HNRNPUL2-BSCL2	BSCL2:intronic:NM_001122955.3, HNRNPUL2-BSCL2:introni	1	96.67		intronic	NR_03794	c.766-49T	p.?	0.18					Putative adip	rs2850597	0.2415	YES	7.709E-1 (pos.62459994)		Seipin	fat cell dif	Asthenozoospermia;Central N	Benign	A=2, G=58	A=0.0333, G=0.9667	0.00001	841.438	60	A=2/0, G=24/3
chr11:62460066	C/C	T	C	SNV	BSCL2,HNRNPUL2-BSCL2	BSCL2:intronic:NM_001122955.3, HNRNPUL2-BSCL2:introni	1	100		intronic	NR_03794	c.765+69A	p.?	-0.59					Putative adip	rs2850596		YES			Seipin	fat cell dif	Asthenozoospermia;Central Nervous System		T=0, C=86	T=0.0, C=1.0	0.00001	1410.64	86	T=0/0, C=50/3
chr11:62488916	T/TG	T	TG	INDEL	HNRNPUL2,HNRNPUL2-BSCL2	HNRNPUL2:intronic:NM_001079559.2, HNRNPUL2-BSCL2:in	1	100		intronic	NR_03794	c.1483-21	p.?	-0.67					AAA domain,!	rs200449447,rs3442	0.2426	YES	5.674E-1;0.0 (pos.62488916)			RNA binding,biological_process,membrane,nucleoplasm			T=0, TG=24	T=0.0, TG=1.0	0.00001	306.604	24	T=0/0, TG=9/1
chr2:220037666	G/G	A	G	SNV	CNPPD1	CNPPD1:exonic:NM_015680.5	1	100	8	missense	NM_01568	c.875T>C	p.Leu292Pro	0.94	0.15	98	0	0.02(COSM39	Cyclin	rs1127102	0.3992	YES	2.723E-1 (pos.22003766)	acute_myeloid_leukaemia		integral component of membrane,protein kinase bindin			A=0, G=65	A=0.0, G=1.0	0.00001	996.464	65	A=0/0, G=27/3
chr1:24201643	G/A	G	A	SNV	CNR2	CNR2:exonic:NM_001841.2	1	57.14	2	synonymous	NM_00184	c.465C>T	p.(=)	-0.1					7 transmemb	rs2501431	0.4262	YES	5.412E-1 (pos.24201643)		Cannabinoi	G-protein	Arterial Occlusive D	Dronabinol,Lasofoxifene	G=9, A=12	G=0.4286, A=0.5714	0.00001	109.102	21	G=5/4, A=4/8
chr11:62847562	T/T	G	T	SNV	SLC22A24	SLC22A24:intronic:NM_001136506.2	1	100		intronic	NM_00113	c.1599-90	p.?	-3.81					Major Facilit	rs7926818		YES			Solute carrier family 22		Acute Kidney Injury;Female Urogenital Disea		G=0, T=47	G=0.0, T=1.0	0.00001	789.328	47	G=0/0, T=13/3
chr11:62848367	C/C	A	C	SNV	SLC22A24	SLC22A24:intronic:NM_001136506.2	1	100		intronic	NM_00113	c.1598+25	p.?	-0.16					Major Facilit	rs10897341	0.0016	YES	9.332E-1;0.0 (pos.62848367)		Solute carrier family 22		Acute Kidney Injury;Female Urogenital Disea		A=0, C=25	A=0.0, C=1.0	0.00001	353.012	25	A=0/0, C=10/1
chr1:24201262	A/G	A	G	SNV	CNR2	CNR2:exonic:NM_001841.2	1	52.38	2	synonymous	NM_00184	c.846T>C	p.(=)	0.24				0.23(COSM14	7 transmemb	rs2502993	0.4263	YES	5.389E-1 (pos.24201262)	adenocarcinoı	Cannabinoi	G-protein	Arterial Occlusive D	Dronabinol,Lasofoxifene	A=10, G=11	A=0.4762, G=0.5238	0.00001	95.2977	21	A=7/3, G=9/2
chr1:24201448	C/T	C	T	SNV	CNR2	CNR2:exonic:NM_001841.2	1	50	2	synonymous	NM_00184	c.660G>A	p.(=)	-1.07				0.04(COSM14	7 transmemb	rs3003336	0.4256	YES	5.402E-1 (pos.24201448)	adenocarcinoı	Cannabinoi	G-protein	Arterial Occlusive D	Dronabinol,Lasofoxifene	C=11, T=11	C=0.5, T=0.5	0.00001	94.6047	22	C=6/5, T=4/7
chr1:24201109	C/T	C	T	SNV	CNR2	CNR2:exonic:NM_001841.2	1	45.45	2	synonymous	NM_00184	c.999G>A	p.(=)	-2.59				0.06(COSM14	7 transmemb	rs2229580	0.4262	YES	5.391E-1 (pos.24201109)	adenocarcinoı	Cannabinoi	G-protein	Arterial Occlusive D	Dronabinol,Lasofoxifene	C=60, T=50	C=0.5455, T=0.4545	0.00001	478.69	110	C=30/30, T=25
chr11:62848995	C/C	G	C	SNV	SLC22A24	SLC22A24:intronic:NM_001136506.2	1	100		intronic	NM_00113	c.1394+35	p.?	-0.15					Major Facilit	rs6591733		YES	9.357E-1 (pos.62848995)		Solute carrier family 22		Acute Kidney Injury;Female Urogenital Disea		G=0, C=26	G=0.0, C=1.0	0.00001	371.419	26	G=0/0, C=15/1
chr2:68546449	G/A	G	A	SNV	CNRIP1	CNRIP1:exonic:NM_015463.2	1	50	1	synonymous	NM_01546	c.84C>T	p.(=)	2.67				0.79(COSM37	CB1 cannabir	rs13025842	0.3101	YES	1.126E-1 (pos.68546449)	hepatocellular_carcinoma		myelin sheath			G=14, A=14	G=0.5, A=0.5	0.00001	131.941	28	G=6/8, A=5/9
chr11:62984697	C/C	T	C	SNV	SLC22A25	SLC22A25:intronic:NM_199352.4	1	100		intronic	NM_19935	c.830+89A	p.?	-0.2					Major Facilit	rs7949840		YES				inorganic anion exchanger activity,inorganic anion trans			T=0, C=48	T=0.0, C=1.0	0.00001	810.426	48	T=0/0, C=27/2
chr9:17394996	C/C	T	C	SNV	CNTLN	CNTLN:exonic:NM_017738.3	1	100	15	synonymous	NM_01773	c.2544T>C	p.(=)	0.61						rs3739489	0.0851	YES	8.155E-1 (pos.17394996)		Centlein	centriole,centriole-centriole cohesion,centrosome,cyto			T=0, C=79	T=0.0, C=1.0	0.00001	1269.33	79	T=0/0, C=37/4
chr11:62996584	A/A	G	A	SNV	SLC22A25	SLC22A25:intronic:NM_199352.4	1	100		intronic	NM_19935	c.402+139	p.?	-0.46					Major Facilit	rs2510590		YES				inorganic anion exchanger activity,inorganic anion trans			G=0, A=36	G=0.0, A=1.0	0.00001	563.739	36	G=0/0, A=13/2
chr9:17409366	A/A	G	A	SNV	CNTLN	CNTLN:exonic:NM_017738.3	1	100	16	synonymous	NM_01773	c.2691G>A	p.(=)	0.9				0.01(COSM3982792)		rs2780211	0.0885	YES	8.148E-1 (pos.17409366)	acute_myeloic	Centlein	centriole,centriole-centriole cohesion,centrosome,cyto			G=0, A=26	G=0.0, A=1.0	0.00001	371.42	26	G=0/0, A=11/1
chr3:74413676	T/C	T	C	SNV	CNTN3	CNTN3:exonic:NM_020872.2	1	56	9	synonymous	NM_02087	c.1155A>G	p.(=)	-0.09				0.09(COSM40	Fibronectin t	rs6549590	0.4547	YES	4.72E-1 (pos.74413676)	acute_myeloic	Contactin 3	C-termina	Autistic Disorder;Child Development Disorde		T=11, C=14	T=0.44, C=0.56	0.00001	134.286	25	T=9/2, C=7/7
chr11:6341748	T/T	C	T	SNV	CAVIN3	CAVIN3:upstream:NM_145040.2	1	100		intronic	NM_14504	c.-42G>A	p.?	0.5						rs1994519	0.4602	YES	6.451E-1 (pos.6341748)			caveola,circadian regulation of gene expression,cortical			C=0, T=58	C=0.0, T=1.0	0.00001	867.383	58	C=0/0, T=22/3
chr11:63668557	T/T	C	T	SNV	MARK2	MARK2:intronic:NM_001039469.2	1	100		intronic	NM_00103	c.1101+16	p.?	-1.17					Kinase assoc	rs224174	1.00E-04		1.0 (pos.63668557)		MAP/microt	ATP bindin	Adenocarcinoma;Carcinoma;Carcinoma, Ade		C=0, T=46	C=0.0, T=1.0	0.00001	768.313	46	C=0/0, T=28/1
chr3:3081750	T/T	A	T	SNV	CNTN4,CNTN4-AS1	CNTN4:exonic:NM_175607.2, CNTN4-AS1:exonic_nc:NR_046	1	100	20	synonymous	NM_17560	c.2193A>T	p.(=)	0.4				0.62(COSM37	Fibronectin t	rs6802588	0.3976	YES	5.663E-1 (pos.3081750)	acute_myeloic	Contactin 4	C-termina	Autistic Disorder;Biliary Tract Diseases;Biliar		A=0, T=42	A=0.0, T=1.0	0.00001	684.948	42	A=0/0, T=16/2
chr11:63720086	G/G	T	G	SNV	NAA40	NAA40:intronic:NM_024771.3	1	100		intronic	NM_02477	c.410+49T	p.?	-1.23					Acetyltransfe	rs4980513	3.00E-04		1.0 (pos.63720086)			H2A histone acetyltransferase activity,H4 histone acety			T=0, G=44	T=0.0, G=1.0	0.00001	726.536	44	T=0/0, G=30/1
chr3:3081825	C/C	T	C	SNV	CNTN4,CNTN4-AS1	CNTN4:exonic:NM_175607.2, CNTN4-AS1:exonic_nc:NR_046	1	100	20	synonymous	NM_17560	c.2268T>C	p.(=)	-3.02					Fibronectin t	rs339285	0.0858	YES	8.894E-1 (pos.3081825)		Contactin 4	C-termina	Autistic Disorder;Biliary Tract Diseases;Biliar		T=0, C=38	T=0.0, C=1.0	0.00001	603.777	38	T=0/0, C=11/2
chr2:125192068	C/C	T	C	SNV	CNTNAP5	CNTNAP5:exonic:NM_130773.3	1	100	5	synonymous	NM_13077	c.537T>C	p.(=)	-1.33				0.05(COSM40	F5/8 type C d	rs1425745	0.0329	YES	8.914E-1 (pos.12519206)	acute_myeloic	Contactin-a	cell adhesion,integral component of membrane			T=0, C=38	T=0.0, C=1.0	0.00001	603.786	38	T=0/0, C=35/3
chr17:71196809	G/G	A	G	SNV	COG1	COG1:exonic:NM_018714.2	1	100	6	missense	NM_01871	c.1175A>G	p.Asn392Ser	1.24	1	46	0	0.43(COSM37	Vps51/Vps67	rs1026128	0.4603	YES	6.143E-1 (pos.71196809)	adenocarcinoı	Component	ER to Golgi	Carbohydrate Metabolism, Inb	Benign	A=0, G=31	A=0.0, G=1.0	0.00001	465.819	31	A=0/0, G=13/1
chr17:71197439	T/T	C	T	SNV	COG1	COG1:exonic:NM_018714.2	1	100	7	synonymous	NM_01871	c.1473C>T	p.(=)	-1.8				0.03(COSM37	Vps51/Vps67	rs1551036	0.1931	YES	1.696E-1 (pos.71197439)	acute_myeloic	Component	ER to Golgi	Carbohydrate Metabolism, Inb	Benign/Likely	C=0, T=26	C=0.0, T=1.0	0.00001	377.038	26	C=0/0, T=18/8
chr11:64052306	G/G	T	G	SNV	BAD,GPR137	BAD:upstream:NM_032989.2, GPR137:intronic:NM_001170	1	100		intronic	NM_03298	c.-466A>C,	p.?, p.?	0.79						rs652611		YES			BCL1 antag	14-3-3 pro	Brain Diseases;Brai	Navitoclax	T=0, G=44	T=0.0, G=1.0	0.00001	726.569	44	T=0/0, G=18/2
chr17:71197748	A/A	G	A	SNV	COG1	COG1:exonic:NM_018714.2	1	100	7	synonymous	NM_01871	c.1782G>A	p.(=)	-0.71				0.02(COSM37	Vps51/Vps67	rs1037256	0.4603	YES	6.133E-1;0.0;0.0 (pos.71	adenocarcinoı	Component	ER to Golgi	Carbohydrate Metabolism, Inb	Benign	G=0, A=76	G=0.0, A=1.0	0.00001	1209.59	76	G=0/0, A=32/4
chr11:64088435	G/G	A	G	SNV	PRDX5	PRDX5:intronic:NM_012094.4	1	100		intronic	NM_01209	c.478-44A	p.?	0.5					AhpC/TSA fam	rs566049	7.00E-04	YES	1.0 (pos.64088435)		Peroxiredo	NADPH oxi	Alopecia;Alopecia A	Auranofin,Benzoic Acid,E	A=0, G=39	A=0.0, G=1.0	0.00001	623.975	39	A=0/0, G=9/30
chr13:46108853	CA/CG	TG	CG	SNV	COG3	COG3:exonic:NM_031431.3	2	75	23	missense	NM_03143	c.2474T>C	p.Leu825Ser, p.Leu825S	1.23, -3.7	1.0, 1.0	145.0, 14	0.0, 0.0		Sec34-like fa	rs2985989,rs301490	0	YES	1.0 (pos.46108853),8.798E-1 (pos.46108		Component	ER to Golgi vesicle-mediated transport,Golgi apparatus,(			TG=0, CA=8	TG=0.0, CA=0.25, CG	0.00001	485.135	32	TG=0/0, CA=4/
chr11:64127876	CG/CG	C	CG	INDEL	RPS6KA4	RPS6KA4:intronic:NM_003942.2	1	100		intronic	NM_00394	c.346+23C	p.?	0.75					Protein kinas	rs35455832,rs745439541,rs764308310			0.0 (pos.64127876),0.0;0.0 (pos.641278		Ribosomal	ATP bindin	Bile Duct Diseases;	Riboflavin Monophospha	C=0, CG=46	C=0.0, CG=1.0	0.00001	572.131	46	C=0/0, CG=38/
chr11:64127902	G/A	G	A	SNV	RPS6KA4	RPS6KA4:intronic:NM_003942.2	1	47.37		intronic	NM_00394	c.347-47G	p.?	0.01					Protein kinas	rs2289573	0.1296	YES	1.552E-1 (pos.64127902)		Ribosomal	ATP bindin	Bile Duct Diseases;	Riboflavin Monophospha	G=30, A=27	G=0.5263, A=0.4737	0.00001	255.997	57	G=22/8, A=16/
chr11:64337410	C/A	C	A	SNV	SLC22A11	SLC22A11:intronic:NM_018484.3	1	50		intronic	NM_01848	c.1589+80	p.?	0.2					Major Facilitator Superfamily,Sugar (and other) transporter						Solute carri	apical plasma membrane,exte		Aminohippuric acid,Aspa	C=18, A=19	C=0.4865, A=0.5135	0.00001	208.321	38	C=15/3, A=15/
chr16:70548297	A/A	G	A	SNV	COG4	COG4:exonic:NM_015386.2	1	100	4	missense	NM_01538	c.485C>T	p.Thr162Ile	6.98	1	89	0		COG4 transpc	rs3931036	0.0605	YES	9.998E-1 (pos.70548297)		Component	ER to Golgi	Neoplasms;Precancerous Con	Benign/Likely	G=0, A=22	G=0.0, A=1.0	0.00001	298.891	22	G=0/0, A=13/9
chr11:64507315	C/C	T	C	SNV	RASGRP2	RASGRP2:intronic:NM_001098670.1	1	100		intronic	NM_00109	c.523-34A	p.?	-1.47					EF hand,EF-ha	rs550930	0.0014	YES	1.0 (pos.64507315)		Ras guanyl	Ras protein signal transduction,calcium ion binding,cell			T=0, C=65	T=0.0, C=1.0	0.00001	982.907	65	T=0/0, C=37/2
chr11:64525464	T/T	C	T	SNV	PYGM	PYGM:intronic:NM_005609.3	1	100		intronic	NM_00560	c.529-82G	p.?	-4.17					Carbohydrate	rs490980		YES			Phosphoryl	SHG alpha	Carbohydrate Meta	(2S)-N-(3S)-1-(2-AMINO-2-	C=0, T=45	C=0.0, T=1.0	0.00001	747.37	45	C=0/0, T=10/3
chr7:107002806	G/G	A	G	SNV	COG5	COG5:exonic:NM_006348.3	1	100	9	missense	NM_00634	c.988T>C	p.Phe330Leu	6.03	1	22	0	0.14(COSM15	Golgi transpc	rs2269970	0.4719	YES	6.564E-1 (pos.10700280)	adenocarcinoı	Component	ER to Golgi	Arthritis;Joint Diseases;Muscu	Benign	A=0, G=95	A=0.0, G=1.0	0.00001	1596.89	95	A=0/0, G=50/4
chr11:64594490	G/G	A	G	SNV	CDC42BPG	CDC42BPG:intronic:NM_017525.2	1	100		intronic	NM_01752	c.4389+32	p.?	-0.42					CNH domain,	rs646709	0		9.995E-1 (pos.64594490)		CDC42-bind	ATP binding,actin cytoskeleton reorganization,cell leadi			A=0, G=24	A=0.0, G=1.0	0.00001	334.792	24	A=0/0, G=15/9
chr11:64596916	T/T	C	T	SNV	CDC42BPG	CDC42BPG:intronic:NM_017525.2	1	100		intronic	NM_01752	c.3967+27	p.?	0.48					CNH domain,	rs10897529,rs74554	0.342	YES	0.0 (pos.64596906),4.958E-2 (pos.64596		CDC42-bind	ATP binding,actin cytoskeleton reorganization,cell leadi			C=0, T=22	C=0.0, T=1.0	0.00001	298.892	22	C=0/0, T=6/16
chr11:64598944	G/G	A	G	SNV	CDC42BPG	CDC42BPG:intronic:NM_017525.2	1	100		intronic	NM_01752	c.3304+33	p.?	-1.27					CNH domain,	rs684071	1.00E-04		1.0 (pos.64598944)		CDC42-bind	ATP binding,actin cytoskeleton reorganization,cell leadi			A=0, G=54	A=0.0, G=1.0	0.00001	798.146	54	A=0/0, G=40/1
chr1:103354138	G/G	A	G	SNV	COL11A1	COL11A1:exonic:NM_001854.3	1	100	62	missense	NM_00185	c.4603T>C	p.Ser1535Pro	6.35	1	74	0	0.09(COSM54	Collagen tripl	rs1676486	0.1887	YES	7.304E-1 (pos.10335413)	squamous_cel	Collagen XI,	cartilage c	Abnormalities, Multiple;Adnex	Benign, risk f	A=0, G=35	A=0.0, G=1.0	0.00001	543.903	35	A=0/0, G=22/1
chr1:103379918	A/A	G	A	SNV	COL11A1	COL11A1:exonic:NM_001854.3	1	100	52	missense	NM_00185	c.3968C>T	p.Pro1323Leu	9.37	0.09	98	0.044	1(COSM3747	Collagen tripl	rs3753841	0.3884	YES	6.892E-1 (pos.10337991)	hepatocellula	Collagen XI,	cartilage c	Abnormalities, Multiple;Adnex	Benign	G=0, A=78	G=0.0, A=1.0	0.00001	1248.85	78	G=0/0, A=32/4
chr6:33139328	T/T	C	T	SNV	COL11A2	COL11A2:exonic:NM_080680.2	1	100	43	synonymous	NM_08068	c.3174G>A	p.(=)	-3.76				0.04(COSM41	Collagen tripl	rs1799910	0.4524	YES	3.312E-1 (pos.33139328)	neoplasm	Collagen XI,	cartilage c	Abnormalities, Multiple;Arthri	Benign	C=0, T=52	C=0.0, T=1.0	0.00001	766.583	52	C=0/0, T=28/2

chr11:64702200	T/T	C	T	SNV	GPHA2,PPP2R5B	PPP2R5B:downstream:NM_006244.3, GPHA2:utr_3:NM_13(	1	100		intronic	NM_00624	c.*884C>T	p.?, p.?	-0.71						rs673995	0.3018	YES	5.511E-1 (pos.64702200)		Glycoprotei	G-protein	Melanoma:Neoplasms:Neoplasms by Histolc			C=0, T=55	C=0.0, T=1.0	0.00001	814.907	55	C=0/0, T=34/2
chr6:75841722	T/T	A	T	SNV	COL12A1	COL12A1:exonic:NM_004370.5	1	100	35	synonymous	NM_00437	c.5871T>A	p.(=)	-2.7					Collagen tripl	rs594012	0.1154	YES	8.741E-1 (pos.75841722)		Collagen, tv	cell adhes	Congenital, Hereditary, and Ne		Benign	A=0, T=39	A=0.0, T=1.0	0.00001	623.975	39	A=0/0, T=22/1
chr10:71657272	G/G	A	G	SNV	COL13A1	COL13A1:exonic:NM_001130103.1	1	100	13	synonymous	NM_00113	c.699A>G	p.(=)	1.75					Collagen tripl	rs1227746	0		1.0 (pos.71657272)		Collagen XII	cell differe	Abortion, Spontaneous;Basal Ganglia Diseas			A=0, G=54	A=0.0, G=1.0	0.00001	798.148	54	A=0/0, G=22/3
chr11:64977644	T/T	C	T	SNV	CAPN1	CAPN1:intronic:NM_001198868.1	1	100		intronic	NM_00119	c.1942+77	p.?	-0.16					Calpain famil	rs625750		YES			Calpain, lar	calcium io	Cranial Nerve Dise:	(2S)-4-METHYL-2-(3-PHEN		C=0, T=36	C=0.0, T=1.0	0.00001	563.637	36	C=0/0, T=20/1
chr10:105796817	AG/AG	A	AG	INDEL	COL17A1	COL17A1:exonic:NM_000494.3	1	100	47	frameshiftInsertion	NM_00049	c.3262_32	p.Leu1088fs	5.38					Collagen triple helix repeat (20 copies)						Collagen XV	basement	Cardiomegaly;Cardiovascular Diseases;Cong			A=0, AG=55	A=0.0, AG=1.0	0.00001	359.685	55	A=0/0, AG=25/
chr10:105819956	A/A	G	A	SNV	COL17A1	COL17A1:exonic:NM_000494.3	1	100	14	synonymous	NM_00049	c.1062C>T	p.(=)	3.45					Collagen tripl	rs805701	0.326	YES	6.937E-1 (pos.105819956)		Collagen XV	basement	Cardiomegaly;Cardiovascular I		Benign	G=0, A=82	G=0.0, A=1.0	0.00001	1329.59	82	G=0/0, A=17/6
chr10:105824333	A/A	G	A	SNV	COL17A1	COL17A1:exonic:NM_000494.3	1	100	10	missense	NM_00049	c.629C>T	p.Thr210Met	6.83	0.02	81	1		Collagen tripl	rs805708	0.297	YES	6.933E-1 (pos.105824333)		Collagen XV	basement	Cardiomegaly;Cardiovascular I		Benign	G=0, A=22	G=0.0, A=1.0	0.00001	298.89	22	G=0/0, A=9/13
chr21:46876083	A/G	A	G	SNV	COL18A1	COL18A1:exonic:NM_030582.3	1	47.37	1	synonymous	NM_03058	c.639A>G	p.(=)	-2.23				0(COSM4004	Collagen tripl	rs2236451	0.325	YES	3.322E-1 (pos.46876083)	carcinoma	Collagen XV	angiogene	Calcinosis;Calcium Metabolisr		Benign	A=20, G=18	A=0.5263, G=0.4737	0.00001	183.803	38	A=10/10, G=1
chr6:70866609	T/T	C	T	SNV	COL19A1	COL19A1:exonic:NM_001858.5	1	100	34	synonymous	NM_00185	c.2286C>T	p.(=)	-2.49				0.02(COSM37	Collagen tripl	rs2229799	0.47	YES	3.229E-1 (pos.70866609)	adenocarcinor	Collagen XI	cell adhesion,cell differentiation,cell-cell adhesion,colla				C=0, T=22	C=0.0, T=1.0	0.00001	298.892	22	C=0/0, T=10/1
chr17:48268223	G/G	A	G	SNV	COL1A1	COL1A1:exonic:NM_000088.3	1	100	33	synonymous	NM_00008	c.2298T>C	p.(=)	1.51				0.53(COSM41	Collagen tripl	rs2734272	1.00E-04		9.995E-1 (pos.48268223)	neoplasm	Collagen I, a	Golgi appa	Abortion, Spontane	Collagena	Benign/Likely	A=0, G=20	A=0.0, G=1.0	0.00001	263.785	20	A=0/0, G=4/16
chr7:94041937	C/C	A	C	SNV	COL1A2	COL1A2:exonic:NM_000089.3	1	100	25	synonymous	NM_00008	c.1446A>C	p.(=)	-0.01				0.64(COSM14	Collagen tripl	rs412777	0.3707	YES	3.269E-1;0.0 (pos.94041!	adenocarcinor	Collagen I, a	Rho protei	Abortion, Spontane	Collagena	Benign	A=0, C=29	A=0.0, C=1.0	0.00001	426.116	29	A=0/0, C=18/1
chr7:94043239	G/G	C	G	SNV	COL1A2	COL1A2:exonic:NM_000089.3	1	100	28	missense	NM_00008	c.1645C>G	p.Pro549Ala	1.25	1	27	0	0.19(COSM37	Collagen tripl	rs42524	0.2512	YES	9.151E-1;0.0;0.0 (pos.94(	adenocarcinor	Collagen I, a	Rho protei	Abortion, Spontane	Collagena	Benign	C=0, G=43	C=0.0, G=1.0	0.00001	705.844	43	C=0/0, G=21/2
chr5:177675217	T/T	C	T	SNV	COL23A1	COL23A1:exonic:NM_173465.3	1	100	20	synonymous	NM_17346	c.1188G>A	p.(=)	-1.72				0.01(COSM14	Collagen tripl	rs2913851,rs747701!	0.4972	YES	0.0 (pos.177675208),0.0	NS,acute_mye	Collagen, ty	collagen tı	Neoplasms;Precancerous Conditions			C=0, T=20	C=0.0, T=1.0	0.00001	263.782	20	C=0/0, T=8/12
chr11:65767153	G/G	A	G	SNV	EIF1AD	EIF1AD:intronic:NM_001242485.1	1	100		intronic	NM_00124	c.197-7T>C	p.?	0.35					Translation ir	rs507672	0.1816	YES	8.363E-1 (pos.65767153)			nucleus,pı	Melanoma:Neoplasms:Neoplasms by Histolc			A=0, G=28	A=0.0, G=1.0	0.00001	408.718	28	A=0/0, G=17/1
chr1:86590915	A/A	G	A	SNV	COL24A1	COL24A1:exonic:NM_152890.6	1	100	3	synonymous	NM_15289	c.1104C>T	p.(=)	0.17					Collagen tripl	rs2174767	7.00E-04	YES	9.693E-1 (pos.86590915)		Collagen, tv	collagen trimer,endoplasmic reticulum lumen,extracell				G=0, A=64	G=0.0, A=1.0	0.00001	977.635	64	G=0/0, A=33/3
chr11:6579539	G/G	A	G	SNV	DNHD1	DNHD1:intronic:NM_144666.2	1	100		intronic	NM_14466	c.9002+12	p.?	0.23					Dynein heavy	rs10839583	0.4233	YES	2.37E-1 (pos.6579539)			ATP bindin	Adenocarcinoma:Carcinoma:Carcinoma, Ren			A=0, G=39	A=0.0, G=1.0	0.00001	623.979	39	A=0/0, G=17/2
chr7:101200835	G/G	A	G	SNV	COL26A1	COL26A1:utr_3:NM_133457.4	1	100	14	utr_3	NM_13345	c.1344A>G	p.?	0.19					Collagen tripl	rs7809926	0.0327	YES	9.996E-1 (pos.101200835)			Golgi apparatus,collagen catabolic process,collagen trir				A=0, G=21	A=0.0, G=1.0	0.00001	281.231	21	A=0/0, G=5/16
chr11:66006323	G/C	G	C	SNV	PACS1	PACS1:intronic:NM_018026.3	1	57.14		intronic	NM_01802	c.2293+6G	p.?	-0.44					PACS-1 cytoso	rs476551	0.167	YES	3.53E-1 (pos.66006323)		Phosphofur	COPI-coat	Mental Disorders;Nutrition Dis		Likely benign	G=12, C=16	G=0.4286, C=0.5714	0.00001	161.676	28	G=2/10, C=9/7
chr11:66010633	C/C	T	C	SNV	PACS1	PACS1:intronic:NM_018026.3	1	100		intronic	NM_01802	c.2777-3T	p.?	-0.34					PACS-1 cytoso	rs524268	2.00E-04		1.0 (pos.66010633)		Phosphofur	COPI-coat	Mental Disorders;Nutrition Dis		Likely benign	T=0, C=37	T=0.0, C=1.0	0.00001	583.32	37	T=0/0, C=18/1
chr9:117002517	T/T	C	T	SNV	COL27A1	COL27A1:exonic:NM_032888.3	1	100	20	synonymous	NM_03288	c.2745C>T	p.(=)	-0.54				0.13(COSM44	Collagen tripl	rs2014134	0.3787	YES	2.135E-1 (pos.11700251;	acute_myeloi	Collagen, tv	endoplasr	Basal Ganglia Diseases;Brain Diseases;Centr			C=0, T=20	C=0.0, T=1.0	0.00001	263.786	20	C=0/0, T=16/4
chr9:117073006	T/T	C	T	SNV	COL27A1	COL27A1:utr_3:NM_032888.3	1	100	61	utr_3	NM_03288	c.5614C>T	p.?	-0.44						rs1249756	0.4203	YES	6.034E-1 (pos.117073006)		Collagen, tv	endoplasr	Basal Ganglia Diseases;Brain Diseases;Centr			C=0, T=37	C=0.0, T=1.0	0.00001	583.707	37	C=0/0, T=16/2
chr2:189875421	G/G	T	G	SNV	COL3A1	COL3A1:exonic:NM_000090.3	1	100	50	missense	NM_00009	c.4059T>G	p.His1353Gln	1.66	1	24	0		Collagen tripl	rs1516446	0		1.0 (pos.189875421)		Collagen III,	SMAD binc	ACMG Recommend:	Collagena	Benign	T=0, G=29	T=0.0, G=1.0	0.00001	427.603	29	T=0/0, G=19/1
chr11:66107527	A/A	C	A	SNV	BRMS1	BRMS1:intronic:NM_001024957.1	1	100		intronic	NM_00102	c.628+65G	p.?	-0.11					Sds3-like	rs1785633		YES			Breast cancer metastasis suppressor 1					C=0, A=30	C=0.0, A=1.0	0.00001	438.694	30	C=0/0, A=26/4
chr13:110827580	C/T	C	T	SNV	COL4A1	COL4A1:exonic:NM_001845.5	1	57.89	37	synonymous	NM_00184	c.3183G>A	p.(=)	-0.74				0.03(COSM50	C-terminal ta	rs874204	0.3626	YES	2.877E-1;0.0 (pos.11082;	osteosarcoma	Collagen IV,	basement	Brain Diseases;CADASIL;Cardic		Benign	C=16, T=22	C=0.4211, T=0.5789	0.00001	250.913	38	C=6/10, T=10/
chr11:66133311	C/C	T	C	SNV	SLC29A2	SLC29A2:intronic:NM_001532.2	1	100		intronic	NM_00153	c.1059+96	p.?	-0.18					Nucleoside tı	rs4930358		YES			Solute carri	basolateral plasma membrane		Didanosine,Ethanol,Gen		T=0, C=28	T=0.0, C=1.0	0.00001	403.273	28	T=0/0, C=25/3
chr11:66133336	G/G	A	G	SNV	SLC29A2	SLC29A2:intronic:NM_001532.2	1	100		intronic	NM_00153	c.1059+71	p.?	-0.18					Nucleoside tı	rs2279861		YES			Solute carri	basolateral plasma membrane		Didanosine,Ethanol,Gen		A=0, G=28	A=0.0, G=1.0	0.00001	408.656	28	A=0/0, G=25/3
chr13:111155773	C/C	T	C	SNV	COL4A2,COL4A2-AS1	COL4A2-AS1:intronic_nc:NR_046583.1, COL4A2:exonic:NM_(	1	100	43	synonymous	NM_00184	c.4083T>C	p.(=)	-0.42					C-terminal ta	rs438758	7.00E-04		9.755E-1;3.385E-4 (pos.111155773)		Collagen IV,	angiogene	Bronchial Neoplasms;Carcinoma, Bronchoge			T=0, C=27	T=0.0, C=1.0	0.00001	389.972	27	T=0/0, C=19/8
chr13:111158874	G/G	A	G	SNV	COL4A2,COL4A2-AS1	COL4A2-AS1:intronic_nc:NR_046583.1, COL4A2:exonic:NM_(	1	100	46	synonymous	NM_00184	c.4515A>G	p.(=)	-9.57					C-terminal ta	rs445348	0.0944	YES	5.86E-1 (pos.111158874)		Collagen IV,	angiogene	Bronchial Neoplasms;Carcinor		Benign	A=0, G=22	A=0.0, G=1.0	0.00001	291.2	22	A=0/0, G=5/17
chr9:137619195	T/T	C	T	SNV	COL5A1	COL5A1:exonic:NM_001278074.1	1	100	5	synonymous	NM_00127	c.738C>T	p.(=)	-6.9				0.01(COSM37	Collagen tripl	rs3124299	0.4527	YES	3.976E-1;0.0 (pos.13761!	acute_myeloi	Collagen V,	alpha-1 pol	Abortion, Spontaneous;Cardio		Benign	C=0, T=28	C=0.0, T=1.0	0.00001	408.718	28	C=0/0, T=15/1
chr11:66481264	T/T	C	T	SNV	SPTBN2	SPTBN2:intronic:NM_006946.2	1	100		intronic	NM_00694	c.657-47G	p.?	-0.61					CAMSAP CH d	rs615536	0.0553	YES	8.95E-1 (pos.66481264)		Spectrin, be	ER to Golgi	Ataxia;Brain Diseases;Central Nervous Syste			C=0, T=55	C=0.0, T=1.0	0.00001	814.907	55	C=0/0, T=14/4
chr2:189904203	G/G	A	G	SNV	COL5A2	COL5A2:exonic:NM_000393.4	1	100	51	synonymous	NM_00039	c.3720T>C	p.(=)	-0.59				0.09(COSM40	Collagen tripl	rs10208525	0.1034	YES	1.048E-1 (pos.18990420;	acute_myeloi	Collagen V,	SMAD binc	Abortion, Spontaneous;Cardio		Benign/Likely	A=0, G=46	A=0.0, G=1.0	0.00001	765.308	46	A=0/0, G=30/1
chr2:189904233	G/G	T	G	SNV	COL5A2	COL5A2:exonic:NM_000393.4	1	100	51	synonymous	NM_00039	c.3690A>C	p.(=)	-1.99				0.17(COSM44	Collagen tripl	rs10197596	0.1026	YES	1.23E-1 (pos.189904233)	acute_myeloi	Collagen V,	SMAD binc	Abortion, Spontaneous;Cardio		Benign/Likely	T=0, G=46	T=0.0, G=1.0	0.00001	768.313	46	T=0/0, G=30/1
chr2:189932831	C/C	T	C	SNV	COL5A2	COL5A2:exonic:NM_000393.4	1	100	21	synonymous	NM_00039	c.1311A>G	p.(=)	-7.08					Collagen tripl	rs2229495	0.0178	YES	9.658E-1 (pos.189932831)		Collagen V,	SMAD binc	Abortion, Spontaneous;Cardiovascular Disea			T=0, C=35	T=0.0, C=1.0	0.00001	543.903	35	T=0/0, C=18/1
chr19:10079093	T/T	C	T	SNV	COL5A3	COL5A3:exonic:NM_015719.3	1	100	59	missense	NM_01571	c.4282G>A	p.Val1428Met	-0.27	0.06	21	0.015	0.08(COSM40	Collagen tripl	rs3815746	0.2229	YES	2.424E-1;0.0 (pos.10079(	acute_myeloi	Collagen, tv	cell-matrix adhesion,collagen binding,collagen catabolic				C=0, T=36	C=0.0, T=1.0	0.00001	563.743	36	C=0/0, T=28/8
chr11:67071584	G/G	C	G	SNV	SSH3	SSH3:intronic:NM_017857.3	1	100		intronic	NM_01785	c.67-5C>G	p.?	-3.44					DEK C termin	rs4244822	0.0332	YES	8.926E-1;0.0 (pos.67071584)		Slingshot, D	actin binding,cytoplasm,cytoskeleton,nucleus,peptidyl-				C=0, G=49	C=0.0, G=1.0	0.00001	829.763	49	C=0/0, G=21/2
chr11:67196544	G/G	C	G	SNV	RPS6KB2	RPS6KB2:intronic:NM_003952.2	1	100		intronic	NM_00395	c.120-47C	p.?	-1.64					Protein kinas	rs917570	0.4108	YES	1.404E-1;0.0 (pos.67196544)			ATP bindin	Breast Diseases;Breast Neoplasms;Neoplasr			C=0, G=38	C=0.0, G=1.0	0.00001	603.772	38	C=0/0, G=5/33
chr11:67257037	T/T	C	T	SNV	AIP,MIR6752	AIP:intronic:NM_003977.3, MIR6752:upstream:NR_106810	1	100		intronic	NM_00397	c.468+111	p.?	-1.76					FKBP-type pe	rs4084113		YES	2.844E-1;1.272E-3 (pos.67257037)		Aryl hydroc	GAF doma	ACTH-Secreting Pituitary Adenoma;Acromega			C=0, T=26	C=0.0, T=1.0	0.00001	371.419	26	C=0/0, T=4/22
chr19:10085054	A/A	G	A	SNV	COL5A3	COL5A3:exonic:NM_015719.3	1	100	46	missense	NM_01571	c.3373C>T	p.Pro1125Ser	6.79	0.03	74	0.993	0.98(COSM40	Collagen tripl	rs3815745	0.2266	YES	2.355E-1 (pos.10085054)	acute_myeloi	Collagen, ty	cell-matrix adhesion,collagen binding,collagen catabolic				G=0, A=20	G=0.0, A=1.0	0.00001	263.781	20	G=0/0, A=5/15
chr19:10088271	G/G	C	G	SNV	COL5A3	COL5A3:exonic:NM_015719.3	1	100	42	missense	NM_01571	c.3125G>C	p.Arg1042Pro	4.93	1	103	0.018		Collagen tripl	rs2161468	0.404	YES	3.362E-1;0.0 (pos.10088271)		Collagen, ty	cell-matrix adhesion,collagen binding,collagen catabolic				C=0, G=25	C=0.0, G=1.0	0.00001	352.576	25	C=0/0, G=17/8
chr19:10112346	C/C	T	C	SNV	COL5A3	COL5A3:exonic:NM_015719.3	1	100	8	missense	NM_01571	c.964A>G	p.Arg322Gly	0.04	0.5	125	0	0(COSM4131	Collagen tripl	rs2287803	0.025	YES	8.169E-1 (pos.10112346)	acute_myeloi	Collagen, ty	cell-matrix adhesion,collagen binding,collagen catabolic				T=0, C=117	T=0.0, C=1.0	0.00001	1863.94	117	T=0/0, C=58/5
chr11:67434010	T/T	C	T	SNV	ALDH3B2	ALDH3B2:intronic:NM_001031615.1	1	100		intronic	NM_00103	c.151+35G	p.?	-0.77					Aldehyde del	rs1871032	0.4973	YES	6.257E-1 (pos.67434010)		Aldehyde de	3-chloroallyl aldehyde dehydrc		NADH		C=0, T=22	C=0.0, T=1.0	0.00001	298.892	22	C=0/0, T=14/8
chr21:47545768	A/A	G	A	SNV	COL6A2	COL6A2:exonic:NM_001849.3	1	100	26	missense	NM_00184	c.2039G>A	p.Arg680His	4.76	0.03	29	1	0.86(COSM37	Collagen tripl	rs1042917,rs106479!	0.4988	YES	4.363E-1 (pos.47545768)	adenocarcinor	Collagen VI,	cell adhes	Arthritis;Congenital, Hereditar		Benign	G=0, A=70	G=0.0, A=1.0	0.00001	1092.01	70	G=0/0, A=32/3
chr11:674454	T/T	G	T	SNV	DEAF1	DEAF1:intronic:NM_021008.3	1	100		intronic	NM_02100	c.1503+82	p.?	-0.69					MYND finger,!	rs11246258		YES			Deaf1, Dros	DNA bindir	Intellectual Disability;Mental Disorders;Nerv			G=0, T=60	G=0.0, T=1.0	0.00001	903.529	60	G=0/0, T=41/1
chr11:674458	A/A	G	A	SNV	DEAF1	DEAF1:intronic:NM_021008.3	1	100		intronic	NM_02100	c.1503+78	p.?	-0.44					MYND finger,!	rs6597997		YES			Deaf1, Dros	DNA bindir	Intellectual Disability;Mental Disorders;Nerv			G=0, A=64	G=0.0, A=1.0	0.00001	977.623	64	G=0/0, A=41/2
chr11:674499	A/A	G	A	SNV	DEAF1	DEAF1:intronic:NM_021008.3	1	100		intronic	NM_02100	c.1503+37	p.?	-0.12					MYND finger,!	rs6597996	0.452	YES	7.175E-1 (pos.674499)		Deaf1, Dros	DNA bindir	Intellectual Disability;Mental Disorders;Nerv			G=0, A=74	G=0.0, A=1.0	0.00001	1174.96	74	G=0/0, A=40/3
chr21:47545826	T/T	C	T	SNV	COL6A2	COL6A2:exonic:NM_001849.3	1	100	26	synonymous	NM_00184	c.2097C>T	p.(=)	-1.45				0.08(COSM37	Collagen tripl	rs13046639	0.4995	YES	4.324E-1 (pos.47545826)	adenocarcinor	Collagen VI,	cell adhes	Arthritis;Congenital, Hereditar		Benign	C=0, T=70	C=0.0, T=1.0	0.00001	1091.41	70	C=0/0, T=32/3
chr11:67804156	T/T	C	T	SNV	NDUFS8	NDUFS8:downstream:NM_002496.3	1	100		intronic	NM_00249	c.*96C>T	p.?	0.08						rs3133269		YES			NADH dehy	4 iron, 4 st	Arthritis;Brain Dise	NADH		C=0, T=46	C=0.0, T=1.0	0.00001	768.31	46	C=0/0, T=31/1
chr2:238243285	G/A	G	A	SNV	COL6A3	COL6A3:exonic:NM_004369.3	1	50	41	synonymous	NM_00436	c.9213C>T	p.(=)	-3.21					Collagen tripl	rs2270671	0.1052	YES	3.557E-2 (pos.238243285)		Collagen VI,	cell adhes	Abortion, Spontaneous;Conger		Benign/Likely	G=37, A=37	G=0.5, A=0.5	0.00001	381.538	74	G=32/5, A=26/
chr11:6789207	G/C	G	C	SNV	OR2AG2	OR2AG2:downstream:NM_001004490.1	1	50		intronic	NM_00100	c.*31C>G	p.?	-0.29						rs72914876	0.0623	YES	0.0 (pos.6789207)			G-protein coupled receptor activity,G-protein coupled re				G=16, C=16	G=0.5, C=0.5	0.00001	158.378	32	G=9/7, C=10/6
chr3:130124491	A/A	G	A	SNV	COL6A5	COL6A5:exonic:NM_001278298.1	1	100	14	synonymous	NM_00127	c.4341G>A	p.(=)	-0.63				0.01(COSM14	Collagen tripl	rs10934940	0.2448	YES	1.903E-1 (pos.13012449;	adenocarcinor	Collagen, tv	cell adhesion,collagen catabolic process,collagen trime				G=0, A=67	G=0.0, A=1.0	0.00001	1034.43	67	G=0/0, A=41/2
chr3:130124989	T/T	C	T	SNV	COL6A5	COL6A5:exonic:NM_001278298.1	1	100	16	synonymous	NM_00127	c.4395C>T	p.(=)	-7.22				0(COSM6352	Collagen tripl	rs4688761	0.2901	YES	1.865E-1 (pos.13012498!	carcinoma	Collagen, tv	cell adhesion,collagen catabolic process,collagen trime				C=0, T=164	C=0.0, T=1.0	0.00001	2659.32	164	C=0/0, T=95/6
chr3:130125116	C/C	G	C	SNV	COL6A5	COL6A5:exonic:NM_001278298.1	1	100	17	missense	NM_00127	c.4430G>C	p.Cys1477Ser	-0.62	0.75	112	0	0.01(COSM41	Collagen tripl	rs1497312		YES	2.016E-1 (pos.13012511(	carcinoma,ne	Collagen, tv	cell adhesion,collagen catabolic process,collagen trime				G=0, C=78	G=0.0, C=1.0	0.00001	1249.35	78	G=0/0, C=27/5
chr3:130162395	G/G	A	G	SNV	COL6A5	COL6A5:exonic:NM_001278298.1	1	100	36	missense	NM_00127	c.6563A>G	p.Gln2188Arg	-0.93	0.27	43	0		Collagen tripl	rs9883988	0.0519	YES	8.459E-1 (pos.130162395)		Collagen, ty	cell adhesion,collagen catabolic process,collagen trime				A=0, G=43	A=0.0, G=1.0	0.00001	622.727	43	A=0/0, G=20/2
chr11:68131167	T/T	A	T	SNV	LRP5	LRP5:intronic:NM_002335.3	1	100		intronic	NM_00233	c.687-48A	p.?	-1.41					Coagulation I	rs10791978			1.0 (pos.68131167)		Low density	Wnt signa	Bone Diseases;Bone Diseases, Development			A=0, T=28	A=0.0, T=1.0	0.00001	413.102	28	A=0/0, T=20/8
chr3:130174334	A/A	G	A	SNV	COL6A5	COL6A5:exonic:NM_001278298.1	1	100	37	missense	NM_00127	c.6614G>A	p.Gly2205Asp	1.08	0.56	94	0		Collagen tripl	rs819085	0.0541	YES	9.46E-1 (pos.130174334)		Collagen, tv	cell adhesion,collagen catabolic process,collagen trime				G=0, A=107	G=0.0, A=1.0	0.00001	1681.3	107	G=0/0, A=69/3
chr11:68177708	A/A	G	A	SNV	LRP5	LRP5:intronic:NM_002335.3	1	100		intronic	NM_00233	c.2318+10	p.?	-3.67					Coagulation I	rs576118		YES			Low density	Wnt signa	Bone Diseases;Bone Diseases, Development			G=0, A=189	G=0.0, A=1.0	0.00001	3170.07	189	G=0/0, A=83/1
chr11:68331715	G/G	C	G	SNV	PPP6R3	PPP6R3:intronic:NM_001164160.1	1	100		intronic	NM_00116	c.846-56C	p.?	-1.55					SIT4 phospha	rs10501398		YES				COPII vesicle coating,Golgi membrane,cytoplasm,cytoso				C=0, G=211	C=0.0, G=1.0	0.00001	3432.82	211	C=0/0, G=61/1
chr3:130187817	C/C	T	C	SNV	COL6A5	COL6A5:exonic:NM_001278298.1	1	100	38	synonymous	NM_00127	c.6969T>C	p.(=)	-0.54				0.02(COSM41	Collagen tripl	rs322113	0.0915	YES	8.958E-1 (pos.13018781;	neoplasm	Collagen, ty	cell adhesion,collagen catabolic process,collagen trime				T=0, C=24	T=0.0, C=1.0	0.00001	334.78	24	T=0/0, C=7/17
chr11:68548260	A/A	C	A	SNV	CPT1A	CPT1A:intronic:NM_001876.3	1	100		intronic	NM_00187	c.1353-47	p.?	-0.84					Carnitine O-p	rs3019576,rs750602!	0.0546	YES	9.105E-1;0.0 (pos.68548260)		Carnitine pa	carnitine (	Breast Diseases;Br	Glyburide,L-Carnitine,Pe		C=0, A=50	C=0.0, A=1.0	0.00001	852.748	50	C=0/0, A=21/2
chr6:70984436	G/G	A	G	SNV	COL9A1	COL9A1:exonic:NM_001851.4	1	100	11	missense	NM_00185	c.1015T>C	p.Ser339Pro	2.38	1	74	0.001	0.02(COSM11	Collagen tripl	rs592121	0.3776	YES	3.442E-1 (pos.70984436)	acute_myeloi	Collagen IX,	animal org	Bone Diseases;Bone Diseases,		Benign	A=0, G=64	A=0.0, G=1.0	0.00001	977.277	64	A=0/0, G=31/3
chr11:6866815	T/T	C	T	SNV	OR10A5	OR10A5:upstream:NM_178168.1	1	100		intronic	NM_17816	c.-99C>T	p.?	-0.29						rs2741801,rs990924309		YES				G-protein coupled receptor activity,G-protein coupled re				C=0, T=34	C=0.0, T=1.0	0.00001	524.042	34	C=0/0, T=16/1
chr11:6866834	C/C	T	C	SNV	OR10A5	OR10A5:upstream:NM_178168.1	1	100		intronic	NM_17816	c.-80T>C	p.?	-0.03						rs2595439		YES				G-protein coupled receptor activity,G-protein coupled re				T=0, C=33	T=0.0, C=1.0	0.00001	504.598	33	T=0/0, C=16/1
chr18:334758	C/C	T	C	SNV	COLEC12	COLEC12:exonic:NM_130386.2	1	100	6	synonymous	NM_13038	c.1800A>G	p.(=)	-1.22				0.03(COSM41	Collagen tripl	rs2305026	0.1686	YES	9.92E-1 (pos.334758)	neoplasm	Collectin 12	carbohydr	Mental Disorders;Substance-Related Disorde			T=0, C=48	T=0.0, C=1.0	0.00001	810.432	48	T=0/0, C=34/1
chr11:68825037	C/C	A	C	SNV	TPCN2	TPCN2:intronic:NM_139075.3	1	100		intronic	NM_13907	c.430-9A>C	p.?	-0.32				0.01(COSM63	Ion transport	rs3750973,rs764882(	0.009	YES	0.0 (pos.68825035),5.40;	neoplasm	Two-pore se	NAADP-sensitive calcium-release channel activity,calciu				A=0, C=26	A=0.0, C=1.0	0.00001	371.391	26	A=0/0, C=13/1
chr11:68831293	G/G	C	G	SNV	TPCN2	TPCN2:intronic:NM_139075.3	1	100		intronic	NM_13907	c.654-70C	p.?	-0.8					Ion transport	rs11228472		YES			Two-pore se	NAADP-sensitive calcium-release channel activity,calciu				C=0, G=25	C=0.0, G=1.0	0.00001	353.019	25	C=0/0, G=8/17
chr11:68834968	C/C	T	C	SNV	TPCN2	TPCN2:intronic:NM_139075.3	1	100		intronic	NM_13907	c.727-3T>C	p.?	-2.52					Ion transport	rs6591368	0.0016	YES	8.629E-1 (pos.68834968)		Two-pore se	NAADP-sensitive calcium-release channel activity,calciu				T=0, C=46	T=0.0, C=1.0	0.00001	768.312	46	T=0/0, C=22/2
chr5:115469773	C/T	C	T	SNV	COMMD10	COMMD10:exonic:NM_016144.3	1	50	5	synonymous	NM_01614	c.408C>T	p.(=)	-0.47				0.04(COSM14	COMM domai	rs1129496,rs754675	0.3574	YES	4.017E-1;0.0 (pos.11546!	adenocarcinor	COMM dom	cytoplasm,nucleus,protein binding,regulation of transcr				C=17, T=17	C=0.5, T=0.5	0.00001	172.008	34	C=12/5, T=16/
chr20:31291161	T/T	C	T	SNV	COMMD7	COMMD7:utr_3:NM_053041.2	1	100	9	utr_3	NM_05304	c.626G>A	p.?	-1.87					COMM domai	rs9421	0.2363	YES	4.646E-1 (pos.31291161)		COMM dom	NF-kappaB binding,cytoplasmic vesicle,extracellular exc				C=0, T=38	C=0.0, T=1.0	0.00001	603.785	38	C=0/0, T=18/2
chr17:30183857	C/C	T	C	SNV	COPRS	COPRS:exonic:NM_018405.3	1	100	2	missense	NM_01840	c.127A>G	p.Ser43Gly	-3.03	0.78	56	0		Cooperator o	rs8068049	0.1241	YES	8.757E-1 (pos.30183857)			cytosol,histone H4-R3 methylation,histone binding,mus				T=0, C=35	T=0.0, C=1.0	0.00001	543.902	35	T=0/0, C=21/1
chr4:84191031	G/G	A	G	SNV	COQ2	COQ2:exonic:NM_015697.7	1	100	5	synonymous	NM_01569	c.894T>C	p.(=)	1.09					UbiA prenyltr	rs6535454	0.2975	YES	8.45E-1 (pos.84191031)		CoQ2, S. cer	4-hydroxyl	Ataxia;Autonomic Nervous Sys		Benign/Likely	A=0, G=48	A=0.0, G=1.0	0.00001	810.381	48	A=0/0, G=26/2
chr9:131085373	G/C	G	C	SNV	COQ4,TRUB2	COQ4:exonic:NM_016035.4, TRUB2:upstream:NM_015679.:	1	96.77	2	missense	NM_01567	c.-686C>G,	p.?, p.Gly50Ala	0.04	1	60	0		Coenzyme Q (	rs3003601	4.00E-04		1.0;0.0 (pos.131085373)		Coenzyme (	RNA binding,cytosol,mRNA processing,mitochondrial inn				G=1, C=30	G=0.0323, C=0.9677	0.00001	435.594	31	G=0/1, C=14/1
chr14:74428445	A/A	G	A	SNV	COQ6	COQ6:exonic:NM_182476.2	1	100	11	missense	NM_18247	c.1216G>A	p.Val406Met	2.06	0.74	21	0.003	0.39(COSM37	FAD binding d	rs8500	0.4722	YES	1.883E-1 (pos.74428445)	acute_myeloi	Coq6, S. cer	FAD bindir	Congenital, Hereditary, and Ne		Benign/Likely	G=0, A=33	G=0.0, A=1.0	0.00001	504.598	33	G=0/0, A=22/1
chr11:69954412	G/G	A	G	SNV	ANO1	ANO1:intronic:NM_018043.5	1	100		intronic	NM_01804	c.748-47A	p.?	-0.18					Calcium-activ	rs2509182	8.00E-04	YES	1.0 (pos.69954412)		Anoctamin	apical plasma membrane,calci		Crofelemer		A=0, G=42	A=0.0, G=1.0	0.00001	664.914	42	A=0/0, G=19/2
chr11:69962642	G/G	A	G	SNV	ANO1	ANO1:intronic:NM_018043.5	1	100		intronic	NM_01804	c.897+37A	p.?	-2.05					Calcium-activ	rs2515245	0.1233	YES	8.695E-1 (pos.69962642)		Anoctamin	apical plasma membrane,calci		Crofelemer		A=0, G=28	A=0.0, G=1.0	0.00001	408.709	28	A=0/0, G=16/1
chr11:69970637	G/G	A	G	SNV	ANO1	ANO1:intronic:NM_018043.5	1	100		intronic	NM_01804	c.962+117	p.?	0.02					Calcium-activ	rs2515258		YES			Anoctamin	apical plasma membrane,calci		Crofelemer		A=0, G=72	A=0.0, G=1.0	0.00001	1130.92	72	A=0/0, G=37/3
chr16:19079037	A/A	G	A	SNV	COQ7,LOC102723385	COQ7:exonic:NM_016138.4, LOC102723385:upstream:NR_:	1	100	1	synonymous	NR_11937!	c.51G>A	p.(=)	-0.71				0.03(COSM37	Ubiquinone b	rs17357834	0.1777	YES	1.516E-1;0.0 (pos.19079(	adenocarcinor	COQ7, S. cer	chromatin binding,extrinsic component of mitochondria				G=0, A=38	G=0.0, A=1.0	0.00001	529.816	38	G=0/0, A=16/2
chr1:227149203	A/A	G	A	SNV	COQ8A	COQ8A:exonic:NM_020247.4	1	100	2	synonymous	NM_02024	c.117G>A	p.(=)	-3.43				0.01(COSM37	ABC1 family	rs11549708	0.239	YES	4.641E-4 (pos.22714920;	adenocarcinoma		ADP binding,ATP binding,integral compone			Benign/Likely	G=0, A=33	G=0.0, A=1.0	0.00001	504.398	33	G=0/0, A=12/2
chr11:70007484	C/G	C	G	SNV	ANO1	ANO1:intronic:NM_018043.5	1	58.33		intronic	NM_01804	c.1780+16	p.?	-0.4					Calcium-activ	rs2276068	0.4847	YES	7.008E-1 (pos.70007484)		Anoctamin	apical plasma membrane,calci		Crofelemer		C=10, G=14	C=0.4167, G=0.5833	0.00001	135.339	24	C=6/4, G=7/7
chr11:70208350	A/A	G	A	SNV	PPFIA1	PPFIA1:intronic:NM_003626.3	1	100		intronic	NM_00362	c.2665-44	p.?	-3.13					SAM domain	rs10751231	0.0013		1.0 (pos.70208350)		Protein-tyrc	axon,cell-matrix adhesion,cytoplasm,cytosol,dendrite,f				G=0, A=50	G=0.0, A=1.0	0.00001	852.734	50	G=0/0, A=27/2
chr1:227174210	C/C	T	C	SNV	COQ8A	COQ8A:exonic:NM_020247.4	1	100	15	synonymous	NM_02024	c.1716T>C	p.(=)	-5.97				0.07(COSM39	ABC1 family	rs3738725	0.4947	YES	2.27E-1 (pos.227174210)	acute_myeloid_leukaemia		ADP binding,ATP binding,integral compone			Benign/Likely	T=0, C=39	T=0.0, C=1.0	0.00001	623.98	39	T=0/0, C=30/9
chr11:70349061	G/G	A	G	SNV	SHANK2	SHANK2:intronic:NM_012309.4	1	100		intronic	NM_01230	c.1871-25	p.?	0.22					Ankyrin repe	rs7110158,rs782628;	2.00E-04		0.0;0.0 (pos.70349046),1.0 (pos.703490(		SH3 and mu	GKAP/Hon	Autistic Disorder;Child Development Disorde			A=0, G=41	A=0.0, G=1.0	0.00001	664.7	41	A=0/0, G=17/2
chr4:47667064	C/C	T	C	SNV	CORIN	CORIN:exonic:NM_006587.3	1	100	11	missense	NM_00658	c.1574A>G	p.His525Arg	1.5	1	29	0		Fz domain,Lo	rs11934749	0.1509	YES	9.764E-1 (pos.47667064)		Corin, serin	actin cyto	Cardiovascular Diseases;Female Urogenital I			T=0, C=145	T=0.0, C=1.0	0.00001	2446.33	145	T=0/0, C=61/8
chr4:47682174	G/G	A	G	SNV	CORIN	CORIN:exonic:NM_006587.3	1	100	8	synonymous	NM_00658	c.1116T>C	p.(=)	-1.46					Fz domain,Lo	rs10517195	0.4066	YES	1.19E-1 (pos.47682174)		Corin, serin	actin cyto	Cardiovascular Diseases;Female Urogenital I			A=0, G=37	A=0.0, G=1.0	0.00001	583.707	37	A=0/0, G=23/1
chr11:7070723	G/T	G	T	SNV	NLRP14	NLRP14:intronic:NM_176822.3	1	50		intronic	NM_17682	c.2124-17!	p.?	1.08					Leucine Rich	rs10839700		YES				ATP binding,cell differentiation,multicellular organism d				G=31, T=31	G=0.5, T=0.5	0.00001	305.324	62	G=15/16, T=13
chr16:30198151	G/G	A	G	SNV	CORO1A	CORO1A:exonic:NM_007074.3	1	100	4	synonymous	NM_00707	c.336A>G	p.(=)	-5.51				0.06(COSM37	Domain of un	rs1132812	0.4466	YES	9.299E-1 (pos.30198151)	neoplasm,squ	Coronin 1A	RNA bindir	Mammary Neoplasms, Animal;		Benign	A=0, G=27	A=0.0, G=1.0	0.00001	389.988	27	A=0/0, G=17/1
chr11:71149856	T/T	C	T	SNV	DHCR7	DHCR7:intronic:NM_001360.2	1	100		intronic	NM_00136	c.831+69G	p.?	-0.98					Ergosterol bi	rs1792265		YES			Delta-7-deh	7-dehydro	Abnormalities, Mul	NADH		C=0, T=58	C=0.0, T=1.0	0.00001	867.38	58	C=0/0, T=27/3
chr14:70793004	G/G	A	G	SNV	COX16,SYNJ2BP-COX16	SYNJ2BP-COX16:utr_3:NM_001202547.2, COX16:utr_3:NM_(	1	100	4	utr_3	NM_01646	c.367T>C, (	p.?, p.?	0.56					Cytochrome (	rs4749	0.2506	YES	9.3E-1 (pos.70793004)			biological_process,cellular_component,integral compor				A=0, G=49	A=0.0, G=1.0	0.00001	831.568	49	A=0/0, G=31/1
chr11:71714526	C/C	G	C	SNV	IL18BP,NUMA1	NUMA1:utr_3:NM_006185.3, IL18BP:downstream:NM_001(	1	100		intronic	NM_00103	c.*1619G>	p.?, p.?	0.8						rs386754843,rs9493;	0.056	YES	8.39E-1;0.0 (pos.71714526)		Interleukin	Golgi mem	Dermatitis;Dermatitis, Allergic Contact;Derm			G=0, C=45	G=0.0, C=1.0	0.00001	747.391	45	G=0/0, C=42/3
chr11:71714528	G/G	T	G	SNV	IL18BP,NUMA1	NUMA1:utr_3:NM_006185.3, IL18BP:downstream:NM_001(	1	100		intronic	NM_00103	c.*1621T>	p.?, p.?	-0.18						rs386754843,rs9493;	0.0655	YES	8.388E-1 (pos.71714528)		Interleukin	Golgi mem	Dermatitis;Dermatitis, Allergic Contact;Derm			T=0, G=45	T=0.0, G=1.0	0.00001	746.908	45	T=0/0, G=42/3
chr11:71727422	C/C	A	C	SNV	LOC100128494,NUMA1	NUMA1:intronic:NM_006185.3, LOC100128494:exonic_nc:N	1	100		intronic	NM_00618	c.1242+32	p.?	-1.02						rs1057992	0.0218	YES	8.445E-1 (pos.71727422)		Nuclear mit	Golgi mem	Dermatitis;Dermatitis, Allergic Contact;Derm			A=0, C=23	A=0.0, C=1.0	0.00001	316.747	23	A=0/0, C=9/14
chr11:71733519	C/T	C	T	SNV	NUMA1	NUMA1:intronic:NM_006185.3	1	50		intronic	NM_00618	c.292-54G	p.?	-0.88						rs10128658	0.0534	YES			Nuclear mit	Golgi mem	Dermatitis;Dermatitis, Allergic Contact;Derm			C=24, T=24	C=0.5, T=0.5	0.00001	262.016	48	C=15/9, T=11/
chr11:72295906	G/G	A	G	SNV	PDE2A	PDE2A:intronic:NM_002599.4	1	100		intronic	NM_00259	c.1359+10	p.?	-0.87					3'5'-cyclic nu	rs452228	5.00E-04		1.0 (pos.72295906)		Phosphodie	3',5'-cyclic	Arthritis;Arthritis, F	3-isobutyl-1-methyl-7H-x		A=0, G=27	A=0.0, G=1.0	0.00001	389.988	27	A=0/0, G=5/22
chr16:85838653	C/C	T	C	SNV	COX4I1	COX4I1:exonic:NM_001861.4	1	100	3	synonymous	NM_00186	c.184T>C	p.(=)	1.14					Cytochrome (	rs2599091	2.00E-04		1.0;0.0 (pos.85838653)		Cytochrome	cytochrome-c oxidase activity,		Cholic Acid,N-Formylmet		T=0, C=24	T=0.0, C=1.0	0.00001	334.79	24	T=0/0, C=9/15
chr7:129916515	C/C	T	C	SNV	CPA2	CPA2:exonic:NM_001869.2	1	100	7	synonymous	NM_00186	c.633T>C	p.(=)	0.33				0(COSM6217	Carboxypepti	rs17590	0.3885	YES	7.804E-1 (pos.12991651!	adenocarcinor	Carboxypep	carboxypeptidase activity,extracellular region,extracell				T=0, C=69	T=0.0, C=1.0	0.00001	1072.78	69	T=0/0, C=35/3
chr7:129962360	G/G	A	G	SNV	CPA4	CPA4:exonic:NM_016352.3	1	100	11	synonymous	NM_01635	c.1110A>G	p.(=)	-3.37					Carboxypepti	rs1569133	2.00E-04		1.0 (pos.129962360)		Carboxypep	cellular_component,extracellular space,histone acetyla				A=0, G=47	A=0.0, G=1.0	0.00001	793.676	47	A=0/0, G=29/1
chr13:46629944	G/G	A	G	SNV	CPB2,CPB2-AS1	CPB2:exonic:NM_001872.4, CPB2-AS1:intronic_nc:NR_0462:	1	100	10	missense	NR_04622(	c.1040T>C	p.Ile347Thr	-0.82	0.82	89	0	0(COSM5959	Carboxypepti	rs1926447	0.2969	YES	8.175E-1 (pos.46629944)	carcinoma	Carboxypep	blood coa	Nutrition Disorders	AZD-9684,Prothrombin		A=0, G=67	A=0.0, G=1.0	0.00001	1034.43	67	A=0/0, G=25/4
chr11:74407676	T/T	C	T	SNV	CHRDL2	CHRDL2:intronic:NM_015424.5	1	100		intronic	NM_01542	c.1269-46	p.?	-0.49					von Willebra	rs3819242	0.2517	YES	4.628E-1 (pos.74407676)		Chordin-like	cartilage development,cell differentiation,cytoplasm,ex				C=0, T=25	C=0.0, T=1.0	0.00001	353.017	25	C=0/0, T=10/1
chr11:74408384	T/T	C	T	SNV	CHRDL2	CHRDL2:intronic:NM_015424.5	1	100		intronic	NM_01542	c.1176-42	p.?	-1.12					von Willebra	rs10751240	0.1382	YES	7.847E-1 (pos.74408384)		Chordin-like	cartilage development,cell differentiation,cytoplasm,ex				C=0, T=43	C=0.0, T=1.0	0.00001	670.125	43	C=0/0, T=33/1
chr13:46641481	C/C	T	C	SNV	CPB2,CPB2-AS1	CPB2:exonic:NM_001872.4, CPB2-AS1:intronic_nc:NR_0462:	1	100	7	synonymous	NR_04622(	c.663A>G	p.(=)	-1.8				0.03(COSM39	Carboxypepti	rs7337140	0.2648	YES	8.338E-1 (pos.46641481)	acute_myeloi	Carboxypep	blood coa	Nutrition Disorders	AZD-9684,Prothrombin		T=0, C=21	T=0.0, C=1.0	0.00001	281.235	21	T=0/0, C=17/4
chr15:83215251	C/C	T	C	SNV	CPEB1	CPEB1:exonic:NM_030594.4	1	100	11	synonymous	NM_03059	c.1515A>G	p.(=)	-0.18				0.31(COSM37	Cytoplasmic	rs1267657	0.3749	YES	4.777E-1 (pos.83215251)	adenocarcinor	Cytoplasmi	P-body,cel	Hemic and Lymphatic Diseases;Immune Syst			T=0, C=79	T=0.0, C=1.0	0.00001	1269.33	79	T=0/0, C=24/5
chr15:83224696	T/T	C	T	SNV	CPEB1	CPEB1:exonic:NM_030594.4	1	100	5	synonymous	NM_03059	c.783G>A	p.(=)	0.12				0.32(COSM45	Cytoplasmic	rs1145171		YES	1.0 (pos.83224696)	squamous_cel	Cytoplasmi	P-body,cel	Hemic and Lymphatic Diseases;Immune Syst			C=0, T=62	C=0.0, T=1.0	0.00001	940.318	62	C=0/0, T=50/1
chr11:74676794	C/C	G	C	SNV	SPCS2	SPCS2:intronic:NM_014752.2	1	100		intronic	NM_01475	c.199-14G	p.?	-0.33					Microsomal s	rs514534	0.2018	YES	7.51E-1 (pos.74676794)			endoplasmic reticulum membrane,integral component (				G=0, C=62	G=0.0, C=1.0	0.00001	928.709	62	G=0/0, C=35/2
chr11:75167704	C/C	T	C	SNV	GDPD5	GDPD5:intronic:NM_030792.7	1	100		intronic	NM_03079	c.375+98A	p.?	-6.58					Glycerophos	rs1793410		YES			Glycerophо	axon,cerebral cortex neuron differentiation,glycerophos				T=0, C=31	T=0.0, C=1.0	0.00001	465.817	31	T=0/0, C=20/1
chr10:93841227	G/G	A	G	SNV	CPEB3	CPEB3:exonic:NM_014912.4	1	100	9	synonymous	NM_01491	c.1719T>C	p.(=)	0.25				0.57(COSM45	Cytoplasmic	rs2676811			1.0 (pos.93841227)	squamous_cel	Cytoplasmi	3'-UTR-mediated mRNA destabilization,CCR4-NOT comple				A=0, G=25	A=0.0, G=1.0	0.00001	353.018	25	A=0/0, G=12/1
chr7:120764477	G/C	G	C	SNV	CPED1	CPED1:exonic:NM_024913.4	1	50	8	synonymous	NM_02491	c.1011G>C	p.(=)	-0.93				0.06(COSM56	Cadherin-like	rs1524498	0.444	YES	8.392E-1;0.0;0.0 (pos.12(	diffuse_large_B_cell_lymph		endoplasr	Brain Diseases;Central Nervous System Dise:			G=34, C=34	G=0.5, C=0.5	0.00001	342.021	68	G=10/24, C=16
chr6:36733132	A/A	G	A	SNV	CPNE5	CPNE5:exonic:NM_020939.1	1	100	11	synonymous	NM_02093	c.759C>T	p.(=)	-0.25				0.41(COSM37	C2 domain,Ca	rs763048	0.3471	YES	6.301E-1 (pos.36733132)	adenocarcinor	Copine V	extracellular exosome,molecular_function,neuron proje				G=0, A=24	G=0.0, A=1.0	0.00001	334.792	24	G=0/0, A=15/9
chr11:75430950	G/G	A	G	SNV	MOGAT2	MOGAT2:intronic:NM_025098.3	1	100		intronic	NM_02509	c.92-87A>C	p.?	-0.53					Diacylglycerc	rs650861		YES			Monoacylgl	2-acylglycerol O-acyltransferase activity,acetyltransfera				A=0, G=26	A=0.0, G=1.0	0.00001	370.516	26	A=0/0, G=18/8
chr11:7614492	G/G	A	G	SNV	PPFIBP2	PPFIBP2:intronic:NM_003621.3	1	100		intronic	NM_00362	c.372+37A	p.?	0.25					SAM domain	rs7115173	0.3561	YES	4.59E-1 (pos.7614492)			cytosol,ex	Genital Diseases, Male;Genital Neoplasms, I			A=0, G=41	A=0.0, G=1.0	0.00001	664.69	41	A=0/0, G=22/1
chr11:7614530	CA/CA	C	CA	INDEL	PPFIBP2	PPFIBP2:intronic:NM_003621.3	1	100		intronic	NM_00362	c.372+75C	p.?	-2.63					SAM domain (Sterile alpha motif)							cytosol,ex	Genital Diseases, Male;Genital Neoplasms, I			C=0, CA=29	C=0.0, CA=1.0	0.00001	334.867	29	C=0/0, CA=10/
chr11:76183924	A/G	A	G	SNV	EMSY	EMSY:intronic:NM_020193.4	1	50		intronic	NM_02019	c.1108+40	p.?	-3.69					ENT domain	rs4245443	0.411	YES	7.213E-1 (pos.76183924)		EMSY gene	DNA repair,covalent chromatin modification,nucleoplas				A=15, G=15	A=0.5, G=0.5	0.00001	145.019	30	A=6/9, G=7/8
chr16:89662990	T/C	T	C	SNV	CPNE7	CPNE7:exonic:NM_153636.2	1	47.62	15	synonymous	NM_15363	c.1638T>C	p.(=)	-1.15				0(COSM1380	C2 domain,Ca	rs694285,rs7621856;	0.1857	YES	8.127E-1;0.0 (pos.89662!	adenocarcinor	Copine VII	cellular response to calcium ion,cytoplasm,extracellula				T=11, C=10	T=0.5238, C=0.4762	0.00001	82.2887	21	T=6/5, C=7/3
chr3:98304467	C/C	T	C	SNV	CPOX	CPOX:exonic:NM_000097.5	1	100	5	synonymous	NM_00009	c.990A>G	p.(=)	0.91					Coproporphy	rs1729995	0.3217	YES	7.608E-1 (pos.98304467)		Coproporph	coproporp	Congenital, Hereditary, and Ne		Benign	T=0, C=51	T=0.0, C=1.0	0.00001	754.584	51	T=0/0, C=25/2
chr16:12758966	C/C	T	C	SNV	CPPED1	CPPED1:exonic:NM_018340.2	1	100	4	missense	NM_01834	c.722A>G	p.Lys241Arg	0.27	0.59	26	0.006	0.74(COSM44	Calcineurin-li	rs1713480	0.0561	YES	5.519E-1 (pos.12758966)	acute_myeloi	Calcineurin	azurophil granule lumen,cytosol,extracellular exosome,				T=0, C=77	T=0.0, C=1.0	0.00001	1229.44	77	T=0/0, C=28/4
chr2:211481257	G/G	C	G	SNV	CPS1	CPS1:exonic:NM_001875.4	1	100	21	synonymous	NM_00187	c.2679C>G	p.(=)	-0.23				0.07(COSM50	ATP-grasp do	rs2287599	0.4384	YES	4.966E-1;0.0 (pos.21148;	osteosarcoma	Carbamoyl-	'de novo' p	Amino Acid Metabo	Carglumic	Benign/Likely	C=0, G=26	C=0.0, G=1.0	0.00001	371.3	26	C=0/0, G=13/1
chr14:92588002	G/G	T	G	SNV	CPSF2,NDUFB1	CPSF2:upstream:NM_017437.2, NDUFB1:exonic:NM_00454!	1	100	1	synonymous	NM_00454	c.120A>C,	p.(=), p.?	-1.3				0(COSM1563	MNLL subuni	rs3818263	0.3391	YES	8.334E-1 (pos.92588002)	adenocarcinor	NADH-ubiqu	NADH dehydrogenase (ubiquin		NADH		T=0, G=31	T=0.0, G=1.0	0.00001	465.81	31	T=0/0, G=20/1
chr11:76637730	G/G	C	G	SNV	ACER3	ACER3:intronic:NM_018367.6	1	100		intronic	NM_01836	c.214+19C	p.?	-0.13					Ceramidase	rs4604914	0.2671	YES	6.32E-1 (pos.76637730)		Alkaline cer	ceramide	Melanoma:Neoplasms:Neoplasms by Histolc			C=0, G=64	C=0.0, G=1.0	0.00001	977.63	64	C=0/0, G=25/3
chr11:76687399	C/C	A	C	SNV	ACER3	ACER3:intronic:NM_018367.6	1	100		intronic	NM_01836	c.320+14A	p.?	1.11					Ceramidase	rs1517909	0.2364	YES	6.852E-1 (pos.76687399)		Alkaline cer	ceramide	Melanoma:Neoplasms:Neoplasms by Histolc			A=0, C=44	A=0.0, C=1.0	0.00001	726.569	44	A=0/0, C=26/1
chr11:76687448	C/C	A	C	SNV	ACER3	ACER3:intronic:NM_018367.6	1	100		intronic	NM_01836	c.320+63A	p.?	1.16					Ceramidase	rs1517908	0.2415	YES			Alkaline cer	ceramide	Melanoma:Neoplasms:Neoplasms by Histolc			A=0, C=45	A=0.0, C=1.0	0.00001	747.393	45	A=0/0, C=26/1
chr11:76687516	C/C	T	C	SNV	ACER3	ACER3:intronic:NM_018367.6	1	100		intronic	NM_01836	c.320+131	p.?	0.76					Ceramidase	rs1517907		YES			Alkaline cer	ceramide	Melanoma:Neoplasms:Neoplasms by Histolc			T=0, C=45	T=0.0, C=1.0	0.00001	747.384	45	T=0/0, C=26/1
chr11:76701657	G/A	G	A	SNV	ACER3	ACER3:intronic:NM_018367.6	1	52.38		intronic	NM_01836	c.438+39G	p.?	-0.2					Ceramidase	rs3740766	0.4505	YES	2.656E-1 (pos.76701657)		Alkaline cer	ceramide	Melanoma:Neoplasms:Neoplasms by Histolc			G=10, A=11	G=0.4762, A=0.5238	0.00001	95.2994	21	G=6/4, A=5/6

chr11:61179285	A/A	G	A	SNV	CPSF7	CPSF7:exonic:NM_024811.3	1	100	8	synonymous	NM_02481	c.1338C>T	p.(=)	-0.79					RNA recogniti	rs3019198	0.1506	YES	9.622E-1 (pos.61179285)			RNA binding,mRNA 3'-end processing,mRNA cleavage fac				G=0, A=42	G=0.0, A=1.0	0.00001	685.208	42	G=0/0, A=21/2
chr11:76796159	T/T	C	T	SNV	CAPN5	CAPN5:intronic:NM_004055.4	1	100		intronic	NM_00405	c.165+62C	p.?	-0.08					Calpain famil	rs2276029		YES			Calpain 5	calcium-d	Digestive System Diseases:Eosinophilia:Eosi			C=0, T=66	C=0.0, T=1.0	0.00001	1015.4	66	C=0/0, T=26/4
chr11:68549340	G/G	A	G	SNV	CPT1A	CPT1A:exonic:NM_001876.3	1	100	11	synonymous	NM_00187	c.1251T>C	p.(=)	0.25					Carnitine O-p	rs2228502,rs755361	0.0547	YES	0.0 (pos.68549331),9.112E-1 (pos.68549		Carnitine pa	carnitine (	Breast Diseases:Br	Glyburide,	Benign	A=0, G=71	A=0.0, G=1.0	0.00001	1111.44	71	A=0/0, G=34/3
chr1:1262966	C/T	C	T	SNV	CPTP	CPTP:exonic:NM_001029885.1	1	97.5	3	synonymous	NM_00102	c.468C>T	p.(=)	0.16				0.67(COSM37	Glycolipid tra	rs307349	0.0687	YES	8.336E-1 (pos.1262966)	adenocarcinor	Glycolipid t	Golgi apparatus,ceramide 1-phosphate binding,ceramid				C=1, T=39	C=0.025, T=0.975	0.00001	611.603	40	C=0/1, T=16/2
chr11:7687669	C/C	A	C	SNV	CYB5R2	CYB5R2:intronic:NM_016229.4	1	100		intronic	NM_01622	c.658+13T	p.?	-1.05					Ferric reduct	rs12796110	0.2295	YES	7.894E-1 (pos.7687669)		Cytochrome	bicarbonate transport,cytochrome-b5 reductase activit				A=0, C=33	A=0.0, C=1.0	0.00001	504.595	33	A=0/0, C=19/1
chr7:29152371	G/A	G	A	SNV	CPVL	CPVL:exonic:NM_019029.3	1	53.33	3	synonymous	NM_01902	c.237C>T	p.(=)	-1.4				0.02(COSM37	Serine carbo	rs323182	0.1176	YES	3.205E-2;0.0 (pos.29152	adenocarcinor	Carboxypep	extracellu	Mental Disorders;Psychotic Disorders;Schizo			G=14, A=16	G=0.4667, A=0.5333	0.00001	159.925	30	G=7/7, A=7/9
chr11:76885779	A/A	G	A	SNV	MYO7A	MYO7A:intronic:NM_000260.3	1	100		intronic	NM_00026	c.1936-23	p.?	-0.24					FERM central	rs2276283	0.457	YES	5.468E-1 (pos.76885779)		Myosin VIIA	ADP bindir	Abnormalities, Multiple:Blindr		Benign	G=0, A=338	G=0.0, A=1.0	0.00001	5696.88	338	G=0/0, A=162/
chr11:76894235	C/C	G	C	SNV	MYO7A	MYO7A:intronic:NM_000260.3	1	100		intronic	NM_00026	c.3375+33	p.?	-1.21					FERM central	rs948972	0.4259	YES	4.533E-1;0.0 (pos.76894235)		Myosin VIIA	ADP bindir	Abnormalities, Multiple:Blindr		Benign	G=0, C=23	G=0.0, C=1.0	0.00001	302.424	23	G=0/0, C=11/1
chr11:76895770	G/G	GGGAGGC	G	INDEL	MYO7A	MYO7A:intronic:NM_000260.3	22	100		intronic	NM_00026	c.3503+10	p.?	-2.39, 0.22, 0.56, -0.08, 0.41, 1.34, 0.33, -2.75, 0.5, 0.28, 0.58, (					FERM central	rs111033223,rs1119	0.3973	YES	0.0 (pos.76895773),0.0 (pos.76895777),(		Myosin VIIA	ADP bindir	Abnormalities, Multiple:Blindr		Benign	GGGAGGC(	GGGAGGCGGGGACA	0.00001	642.863	40	GGGAGGCGG(
chr11:7689633	C/C	A	C	SNV	CYB5R2	CYB5R2:intronic:NM_016229.4	1	100		intronic	NM_01622	c.472+76T	p.?	-0.23					Ferric reduct	rs10743030					Cytochrome	bicarbonate transport,cytochrome-b5 reductase activit				A=0, C=43	A=0.0, C=1.0	0.00001	681.494	43	A=0/0, C=29/1
chr11:76901273	T/T	C	T	SNV	MYO7A	MYO7A:intronic:NM_000260.3	1	100		intronic	NM_00026	c.3750+89	p.?	0.77					FERM central	rs2276286		YES			Myosin VIIA	ADP bindir	Abnormalities, Multiple:Blindness:Congenita			C=0, T=24	C=0.0, T=1.0	0.00001	334.792	24	C=0/0, T=16/8
chr11:76905604	T/T	G	T	SNV	MYO7A	MYO7A:intronic:NM_000260.3	1	100		intronic	NM_00026	c.4323+35	p.?	-1.44					FERM central	rs1109977	0.4632	YES	4.984E-1 (pos.76905604)		Myosin VIIA	ADP bindir	Abnormalities, Multiple:Blindr		Benign	G=0, T=54	G=0.0, T=1.0	0.00001	798.148	54	G=0/0, T=22/3
chr7:29152416	G/A	G	A	SNV	CPVL	CPVL:exonic:NM_019029.3	1	53.33	3	synonymous	NM_01902	c.192C>T	p.(=)	0.09				0.08(COSM37	Serine carbo	rs2011337	0.1183	YES	3.221E-2 (pos.29152416)	adenocarcinor	Carboxypep	extracellu	Mental Disorders:Psychotic Disorders;Schizo			G=14, A=16	G=0.4667, A=0.5333	0.00001	159.942	30	G=7/7, A=7/9
chr11:76916967	G/G	A	G	SNV	MYO7A	MYO7A:intronic:NM_000260.3	1	100		intronic	NM_00026	c.5637-17	p.?	-1.77					FERM central	rs4945160		YES			Myosin VIIA	ADP bindir	Abnormalities, Multiple;Blindness;Congenita			A=0, G=21	A=0.0, G=1.0	0.00001	281.232	21	A=0/0, G=11/1
chr10:125506302	C/C	T	C	SNV	CPXM2	CPXM2:exonic:NM_198148.2	1	100	14	missense	NM_19814	c.2249A>G	p.Gln750Arg	2.75	1	43	0		Carboxypepti	rs7088479,rs777252	0.0015		0.0 (pos.125506301),1.0 (pos.12550630		Carboxypep	extracellular matrix,extracellular space,metallocarboxy				T=0, C=34	T=0.0, C=1.0	0.00001	524.186	34	T=0/0, C=14/2
chr11:76925075	T/T	G	T	SNV	MYO7A	MYO7A:intronic:NM_000260.3	1	100		intronic	NM_00026	c.6558+51	p.?	-0.4					FERM central	rs745519631,rs9489	0.0288	YES	0.0 (pos.76925066)		Myosin VIIA	ADP bindir	Abnormalities, Multiple:Blindness:Congenita			G=0, T=20	G=0.0, T=1.0	0.00001	263.778	20	G=0/0, T=8/12
chr10:125521590	A/A	G	A	SNV	CPXM2	CPXM2:exonic:NM_198148.2	1	100	11	synonymous	NM_19814	c.1575C>T	p.(=)	3.92				0.97(COSM41	Carboxypepti	rs10794567	0.2471	YES	5.534E-1 (pos.12552159	neoplasm	Carboxypep	extracellular matrix,extracellular space,metallocarboxy				G=0, A=21	G=0.0, A=1.0	0.00001	273.904	21	G=0/0, A=11/1
chr11:77775466	C/G	C	G	SNV	NDUFC2-KCTD14,THRSP	THRSP:intronic:NM_003251.3, NDUFC2-KCTD14:intronic:NM	1	52.38		intronic	NM_00120	c.310+857	p.?, p.?	0.74					NADH-ubiqui	rs545869		YES			Thyroid hor	NADH deh	Mammary Neoplasms, Animal;Mammary Nec			C=10, G=11	C=0.4762, G=0.5238	0.00001	95.2978	21	C=8/2, G=5/6
chr4:8602884	C/C	T	C	SNV	CPZ	CPZ:exonic:NM_001014447.2	1	100	3	synonymous	NM_00101	c.156T>C	p.(=)	-3.15				0.04(COSM41	Carboxypepti	rs4301095,rs756131	1.00E-04		0.0 (pos.8602870),1.0 (pc	neoplasm	Carboxypep	Wnt signaling pathway,extracellular exosome,extracell				T=0, C=23	T=0.0, C=1.0	0.00001	316.746	23	T=0/0, C=6/17
chr11:77850517	A/A	C	A	SNV	ALG8,KCTD21-AS1	ALG8:intronic:NM_024079.4, KCTD21-AS1:upstream:NR_10	1	100		intronic	NM_02407	c.95+23G>	p.?	1.21					ALG6, ALG8 g	rs10793289	0.4556	YES	3.989E-1 (pos.77850517)		Alg8, S. cere	alpha-1,3-	Carbohydrate Metabolism, Inb		Benign	C=0, A=24	C=0.0, A=1.0	0.00001	334.792	24	C=0/0, A=15/9
chr4:8602935	G/G	A	G	SNV	CPZ	CPZ:exonic:NM_001014447.2	1	100	3	synonymous	NM_00101	c.207A>G	p.(=)	-1.28				0.04(COSM41	Carboxypepti	rs4431213	1.00E-04		1.0;0.0 (pos.8602935)	neoplasm	Carboxypep	Wnt signaling pathway,extracellular exosome,extracell				A=0, G=27	A=0.0, G=1.0	0.00001	389.988	27	A=0/0, G=8/19
chr11:78189558	C/C	T	C	SNV	NARS2	NARS2:intronic:NM_024678.5	1	100		intronic	NM_02467	c.922-37A	p.?	-0.2					OB-fold nucle	rs4944202	0.1841	YES	5.773E-1 (pos.78189558)		Asparaginyl	ATP bindin	Brain Diseases:Brai	L-Asparagine		T=0, C=95	T=0.0, C=1.0	0.00001	1601.41	95	T=0/0, C=54/4
chr4:8621386	G/G	A	G	SNV	CPZ	CPZ:utr_3:NM_001014447.2	1	100	11	utr_3	NM_00101	c.2001A>G	p.?	-1.15					Carboxypepti	rs1050464	0.4309	YES	5.13E-1 (pos.8621386)		Carboxypep	Wnt signaling pathway,extracellular exosome,extracell				A=0, G=55	A=0.0, G=1.0	0.00001	814.906	55	A=0/0, G=26/2
chr4:8621480	C/C	G	C	SNV	CPZ	CPZ:utr_3:NM_001014447.2	1	100	11	utr_3	NM_00101	c.2095G>C	p.?	0.16					Carboxypepti	rs6816032					Carboxypep	Wnt signaling pathway,extracellular exosome,extracell				G=0, C=45	G=0.0, C=1.0	0.00001	747.392	45	G=0/0, C=16/2
chr1:197390368	G/G	A	G	SNV	CRB1	CRB1:exonic:NM_201253.2	1	100	6	synonymous	NM_20125	c.1410A>G	p.(=)	-3.4					Calcium-bind	rs3902057	0.0014	YES	9.995E-1 (pos.197390368)		Crumbs, Dro	apical pla:	Asthma;Bronchial Diseases;Ca		Benign	A=0, G=26	A=0.0, G=1.0	0.00001	371.42	26	A=0/0, G=13/1
chr9:126125318	A/A	C	A	SNV	CRB2	CRB2:exonic:NM_173689.6	1	100	2	missense	NM_17368	c.269C>A	p.Thr90Asn	-1.48	1	65	0	0(COSM3763	Calcium-bind	rs2808415	0		1.0;0.0 (pos.126125318)	adenocarcinor	Crumbs, Dr	apical pla:	Brain Diseases;Central Nervous System Dise			C=0, A=65	C=0.0, A=1.0	0.00001	996.446	65	C=0/0, A=32/3
chr9:126133547	C/C	T	C	SNV	CRB2	CRB2:exonic:NM_173689.6	1	100	8	missense	NM_17368	c.2126T>C	p.Val709Ala	-2.34	1	64	0.001		Calcium-bind	rs2488602	0		1.0 (pos.126133547)		Crumbs, Dr	apical pla:	Brain Diseases:Central Nervous System Dise			T=0, C=36	T=0.0, C=1.0	0.00001	563.71	36	T=0/0, C=19/1
chr1:152488063	T/T	C	T	SNV	CRCT1	CRCT1:exonic:NM_019060.2	1	100	2	synonymous	NM_01906	c.204C>T	p.(=)	-0.45				0.26(COSM37	Cysteine-rich	rs1053588	0.3273	YES	7.383E-1;0.0 (pos.15248	adenocarcinor	Cysteine-ric	protein binding				C=0, T=32	C=0.0, T=1.0	0.00001	485.14	32	C=0/0, T=19/1
chr11:802125	C/C	T	C	SNV	PIDD1	PIDD1:intronic:NM_145886.3	1	100		intronic	NM_14588	c.1177-35	p.?	-2.34					Death domai	rs28498421	0.0321	YES	9.983E-1 (pos.802125)		p53-induce	DNA dama	Adenocarcinoma:Carcinoma:Carcinoma, Ren			T=0, C=41	T=0.0, C=1.0	0.00001	664.698	41	T=0/0, C=17/2
chr11:8115762	C/G	C	G	SNV	TUB	TUB:intronic:NM_003320.4	1	96.67		intronic	NM_00332	c.562+26C	p.?	0.62					Tub family,Tu	rs7396690	0.0569	YES	7.785E-1 (pos.8115762)		Tubby, mou	cilium,cyt	Congenital, Heredit	L-Alpha-Glycerophospho		C=2, G=58	C=0.0333, G=0.9667	0.00001	821.5	60	C=1/1, G=18/4
chr11:8117228	G/G	A	G	SNV	TUB	TUB:intronic:NM_003320.4	1	100		intronic	NM_00332	c.730+16A	p.?	0.25					Tub family,Tu	rs11041739	0		1.0 (pos.8117228)		Tubby, mou	cilium,cyt	Congenital, Heredit	L-Alpha-Glycerophospho		A=0, G=21	A=0.0, G=1.0	0.00001	280.463	21	A=0/0, G=4/17
chr11:8122234	A/A	G	A	SNV	TUB	TUB:intronic:NM_003320.4	1	100		intronic	NM_00332	c.1380+86	p.?	-1.24					Tub family,Tu	rs145905715,rs7394561		YES			Tubby, mou	cilium,cyt	Congenital, Heredit	L-Alpha-Glycerophospho		G=0, A=54	G=0.0, A=1.0	0.00001	798.147	54	G=0/0, A=26/2
chr11:82976874	G/C	G	C	SNV	CCDC90B	CCDC90B:intronic:NM_021825.4	1	45.45		intronic	NM_02182	c.709+33C	p.?	-0.14					Protein of uni	rs99146	0.273	YES	2.57E-1;1.198E-4 (pos.82976874)			integral component of membrane,mitochondrial membr				G=24, C=20	G=0.5455, C=0.4545	0.00001	211.72	44	G=14/10, C=4/
chr11:83810113	G/G	T	G	SNV	DLG2	DLG2:intronic:NM_001142699.1	1	100		intronic	NM_00114	c.625-23A	p.?	1.38					Guanylate kir	rs3750917	0.0746	YES	7.813E-1 (pos.83810113)		Discs large,	GDP meta	Basal Ganglia Diseases:Brain Diseases:Centr			T=0, G=30	T=0.0, G=1.0	0.00001	446.639	30	T=0/0, G=11/1
chr7:137600690	T/T	C	T	SNV	CREB3L2	CREB3L2:exonic:NM_194071.3	1	100	3	missense	NM_19407	c.388G>A	p.Val130Ile	0.42	0.46	29	0		Basic region l	rs273957	0.3867	YES	9.243E-1 (pos.137600690)		cAMP respo	DNA binding transcription factor activity,ER to Golgi vesi				C=0, T=40	C=0.0, T=1.0	0.00001	644.278	40	C=0/0, T=19/2
chr3:9974809	A/A	G	A	SNV	CRELD1,IL17RC	CRELD1:upstream:NM_001031717.3, IL17RC:exonic:NM_15	1	100	19	synonymous	NM_15346	c.1908G>A	p.(=), p.?	-1.14					Interleukin-1	rs76999397	0.0486	YES	6.833E-2 (pos.9974809)		Cysteine-ric	calcium io	Abnormalities, Multiple:Candidiasis, Chronic			G=0, A=125	G=0.0, A=1.0	0.00001	2033.69	125	G=0/0, A=78/4
chr11:864626	G/G	A	G	SNV	TSPAN4	TSPAN4:intronic:NM_001025238.1	1	100		intronic	NM_00102	c.330+115	p.?	-3.04					Tetraspanin f	rs28377158,rs796783336					Tetraspanin	antigen binding,cell surface receptor signaling pathway,				A=0, G=23	A=0.0, G=1.0	0.00001	321.141	23	A=0/0, G=16/7
chr11:87032215	C/C	T	C	SNV	TMEM135	TMEM135:intronic:NM_022918.3	1	100		intronic	NM_02291	c.1245-28	p.?	-1.2					N-terminal cy	rs7112344			1.0 (pos.87032215)		Transmemb	integral cc	Autoimmune Diseases;Female Urogenital Di:			T=0, C=41	T=0.0, C=1.0	0.00001	664.601	41	T=0/0, C=29/1
chr3:9975247	G/G	C	G	SNV	CRELD1,IL17RC	CRELD1:upstream:NM_001031717.3, IL17RC:exonic:NM_15	1	100	19	synonymous	NM_15346	c.2346C>G	p.(=), p.?	0					Interleukin-1	rs183956,rs7586906	0.0109	YES	0.0:0.0 (pos.9975244),9.972E-1:0.0 (pos.		Cysteine-ric	calcium io	Abnormalities, Multiple:Candidiasis, Chronic			C=0, G=22	C=0.0, G=1.0	0.00001	298.656	22	C=0/0, G=13/9
chr10:35426755	G/G	A	G	SNV	CREM	CREM:utr_5:NM_181571.2	1	100	2	utr_5	NM_18157	c.-9A>G	p.?	2.52					Basic region l	rs17499247,rs98497	0.3424	YES	2.846E-1 (pos.35426755)		cAMP-respo	DNA bindi	Acneiform Eruptions:Arthritis:Arthritis, Juven			A=0, G=21	A=0.0, G=1.0	0.00001	274.497	21	A=0/0, G=13/8
chr11:89223616	G/G	C	G	SNV	NOX4	NOX4:intronic:NM_016931.4	1	100		intronic	NM_01693	c.153+10G	p.?	-2.19					FAD-binding (	rs490934	0.0559	YES	7.797E-1 (pos.89223616)		NADPH oxid	NAD(P)H o	Cardiovascular Diseases:Colonic Diseases:C			C=0, G=155	C=0.0, G=1.0	0.00001	2484.96	155	C=0/0, G=92/6
chr11:8934101	G/G	A	G	SNV	AKIP1	AKIP1:intronic:NM_020642.3	1	100		intronic	NM_02064	c.303+21A	p.?	1.43						rs2742518,rs759742	0.0022	YES	0.0;0.0 (pos.8934100),9.991E-1;8.089E-4		Protein kin	nucleoplasm,protein binding,regulation of NIK/NF-kappa				A=0, G=31	A=0.0, G=1.0	0.00001	470.193	31	A=0/0, G=7/24
chr6:49815997	C/T	C	T	SNV	CRISP1	CRISP1:exonic:NM_001131.2	1	57.14	4	missense	NM_00113	c.211G>A	p.Ala71Thr	3.04	0.05	58	0.996		Crisp,Cystein	rs150372078,rs7502	8.00E-04		0.0 (pos.49815995),0.0;7.705E-3;0.0 (po:		Cysteine-ric	binding of sperm to zona pellucida,calcium channel regu				C=45, T=60	C=0.4286, T=0.5714	0.00001	654.696	105	C=28/17, T=24
chr17:29113016	A/G	A	G	SNV	CRLF3	CRLF3:exonic:NM_015986.3	1	57.89	7	synonymous	NM_01598	c.993T>C	p.(=)	2.98				0.06(COSM44	Apolipoprote	rs34756112	0.1156	YES	1.33E-1:0.0 (pos.291130	acute_myeloic	Cytokine re	G1/S trans	Autoimmune Diseases:Female Urogenital Di:			A=24, G=33	A=0.4211, G=0.5789	0.00001	344.741	57	A=8/16, G=14/
chr11:89451071	C/A	C	A	SNV	TRIM77	TRIM77:downstream:NM_001146162.1	1	46.51		intronic	NM_00114	c.*31C>A	p.?	-1.12						rs3881254		YES	3.117E-1 (pos.89451071)			intracellular,zinc ion binding				C=23, A=20	C=0.5349, A=0.4651	0.00001	212.324	43	C=5/18, A=11/
chr11:89936093	C/C	T	C	SNV	CHORDC1	CHORDC1:intronic:NM_012124.2	1	100		intronic	NM_01212	c.790-50A	p.?	-0.28					CHORD,CS do	rs3758757	0.4342	YES	6.166E-1 (pos.89936093)			ADP binding,ATP binding,Hsp90 protein binding,cellular				T=0, C=164	T=0.0, C=1.0	0.00001	2659.32	164	T=0/0, C=72/9
chr11:89938615	A/A	AAG	A	INDEL	CHORDC1	CHORDC1:intronic:NM_012124.2	2	100		intronic	NM_01212	c.669+13C	p.?	1.13, 2.1, 0.1					CHORD,CS do	rs36109540,rs39804	0.4366	YES	6.141E-1 (pos.89938615)			ADP binding,ATP binding,Hsp90 protein binding,cellular				AAG=0, A=4	AAG=0.0, A=1.0	0.00001	810.435	48	AAG=0/0, A=2
chr15:91083353	G/A	G	A	SNV	CRTC3	CRTC3:exonic:NM_022769.4	1	57.14	2	missense	NM_02276	c.215G>A	p.Ser72Asn	1.23	0.23	46	0.955	0.47(COSM39	Transducer o	rs8033595	0.4523	YES	1.495E-1 (pos.91083353)	acute_myeloic	CREB-regula	cAMP resp	Digestive System Diseases;Gastroenteritis;G			G=18, A=24	G=0.4286, A=0.5714	0.00001	279.085	42	G=13/5, A=12/
chr12:107395106	G/G	A	G	SNV	CRY1	CRY1:exonic:NM_004075.4	1	100	5	synonymous	NM_00407	c.636T>C	p.(=)	-3.18					DNA photolya	rs8192440	0.3759	YES	9.742E-1;0.0 (pos.107395106)		Cryptochroi	DNA (6-4) (	Bone Marrow Diseases:Hematologic Disease			A=0, G=63	A=0.0, G=1.0	0.00001	958.917	63	A=0/0, G=48/1
chr11:9072139	AT/AT	A	AT	INDEL	SCUBE2	SCUBE2:intronic:NM_020974.2	1	100		intronic	NM_02097	c.1639+14	p.?	0.23					CUB domain,Calcium-binding EGF domain,Coagulation Factor Xa inhibitory site,Complement Clr-like						Signal pepti	calcium ion binding,cell differentiation,cell surface,extr				A=0, AT=78	A=0.0, AT=1.0	0.00001	1009.2	78	A=0/0, AT=64/
chr11:9072140	C/T	C	T	SNV	SCUBE2	SCUBE2:intronic:NM_020974.2	1	98.72		intronic	NM_02097	c.1639+13	p.?	-0.04					CUB domain,	rs3751052	0.4166	YES	6.027E-1 (pos.9072140)		Signal pepti	calcium ion binding,cell differentiation,cell surface,extr				C=1, T=77	C=0.0128, T=0.9872	0.00001	1115.7	78	C=1/0, T=63/1
chr11:9075020	T/T	G	T	SNV	SCUBE2	SCUBE2:intronic:NM_020974.2	1	100		intronic	NM_02097	c.1330+16	p.?	0.58					CUB domain,	rs3763904		YES			Signal pepti	calcium ion binding,cell differentiation,cell surface,extr				G=0, T=30	G=0.0, T=1.0	0.00001	446.639	30	G=0/0, T=27/3
chr11:9167399	C/C	T	C	SNV	DENND5A	DENND5A:intronic:NM_015213.3	1	100		intronic	NM_01521	c.2858-37	p.?	-0.16					DENN (AEX-3)	rs10732517	1.00E-04		1.0 (pos.9167399)		DENN doma	Golgi membrane,Rab guanyl-nucleotide exchange factor				T=0, C=50	T=0.0, C=1.0	0.00001	852.729	50	T=0/0, C=17/3
chr21:44589215	T/T	C	T	SNV	CRYAA,LOC102724652	LOC102724652:exonic:NM_001314050.2, CRYAA:exonic:NM	1	100	1	synonymous	NM_00039	c.6C>T, c.6	p.(=), p.(=)	0.77				0.42(COSM41	Alpha crystal	rs872331	0.3994	YES	1.993E-1 (pos.44589215)	neoplasm	Crystallin, a	cytoplasm	Autoimmune Diseases:Catarai		Benign	C=0, T=28	C=0.0, T=1.0	0.00001	408.713	28	C=0/0, T=16/1
chr1:26670752	T/T	C	T	SNV	CRYBG2	CRYBG2:exonic:NM_001039775.3	1	100	2	synonymous	NM_00103	c.2397G>A	p.(=)	-5.71					Beta/Gamma	rs11247923		YES	1.528E-1 (pos.26670752)			carbohydrate binding				C=0, T=27	C=0.0, T=1.0	0.00001	389.988	27	C=0/0, T=22/5
chr1:26673076	A/A	G	A	SNV	CRYBG2	CRYBG2:exonic:NM_001039775.3	1	100	2	missense	NM_00103	c.73C>T	p.Arg25Trp	0.7	0	101	0.995		Beta/Gamma	rs11247924	0.1941	YES	2.421E-1 (pos.26673076)			carbohydrate binding				G=0, A=44	G=0.0, A=1.0	0.00001	702.442	44	G=0/0, A=29/1
chr11:92564767	G/G	T	G	SNV	FAT3	FAT3:intronic:NM_001008781.2	1	100		intronic	NM_00100	c.9482-21	p.?	-0.58					Cadherin dor	rs6483189	0.1543	YES	6.457E-1 (pos.92564767)		FAT tumor s	calcium io	Carcinoma:Carcinoma, Squamous Cell:Diges			T=0, G=36	T=0.0, G=1.0	0.00001	563.431	36	T=0/0, G=30/6
chr11:92599851	A/A	G	A	SNV	FAT3	FAT3:intronic:NM_001008781.2	1	100		intronic	NM_00100	c.11659-5	p.?	-0.23					Cadherin dor	rs3847553		YES			FAT tumor s	calcium io	Carcinoma:Carcinoma, Squamous Cell:Diges			G=0, A=29	G=0.0, A=1.0	0.00001	427.603	29	G=0/0, A=18/1
chr11:92615715	T/T	C	T	SNV	FAT3	FAT3:intronic:NM_001008781.2	1	100		intronic	NM_00100	c.12282-1	p.?	-0.74					Cadherin dor	rs12224637		YES			FAT tumor s	calcium io	Carcinoma:Carcinoma, Squamous Cell:Diges			C=0, T=46	C=0.0, T=1.0	0.00001	768.283	46	C=0/0, T=20/2
chr11:9316937	A/A	G	A	SNV	TMEM41B	TMEM41B:intronic:NM_015012.3	1	100		intronic	NM_01501	c.240-3C>T	p.?	1.16					SNARE associ	rs4601780	0.0012	YES	1.0 (pos.9316937)			integral component of membrane,nervous system devel				G=0, A=103	G=0.0, A=1.0	0.00001	1603.66	103	G=0/0, A=45/5
chr1:26673108	T/T	C	T	SNV	CRYBG2	CRYBG2:exonic:NM_001039775.3	1	100	2	missense	NM_00103	c.41G>A	p.Arg14Gln	0.09	0	43	0.853	0.22(COSM41	Beta/Gamma	rs11247925	0.1902	YES	2.383E-1 (pos.26673108)	neoplasm		carbohydrate binding				C=0, T=28	C=0.0, T=1.0	0.00001	408.416	28	C=0/0, T=12/1
chr11:93417437	T/T	C	T	SNV	CEP295	CEP295:intronic:NM_033395.1	1	100		intronic	NM_03339	c.1114+14	p.?	-0.72					ALMS motif	rs4753098		YES			Centrosom	centriole,centrosome,cytoplasm,cytoskeleton,positive				C=0, T=34	C=0.0, T=1.0	0.00001	524.186	34	C=0/0, T=19/1
chr11:93463332	G/G	A	G	SNV	CEP295,SNORA25,SNORA32	CEP295:intronic:NM_033395.1, SNORA25:downstream:NR_(	1	100		intronic	NM_03339	c.7767+18	p.?	-1					ALMS motif	rs7101779	0.0126	YES	9.984E-1 (pos.93463332)		Centrosom	centriole,centrosome,cytoplasm,cytoskeleton,positive				A=0, G=34	A=0.0, G=1.0	0.00001	524.185	34	A=0/0, G=11/2
chr2:209007559	G/G	T	G	SNV	CRYGB,LOC100507443	CRYGB:exonic:NM_005210.3, LOC100507443:intronic_nc:NI	1	100	3	missense	NR_03843	c.331A>C	p.Ile111Leu	0.83	0.13	5	0	0.01(COSM37	Beta/Gamma	rs796287	0.2635	YES	5.404E-1 (pos.20900755	acute_myeloic	Crystallin, g	cellular_c	Cataract;Eye Diseases;Lens Diseases			T=0, G=27	T=0.0, G=1.0	0.00001	389.979	27	T=0/0, G=18/9
chr2:208986637	C/C	T	C	SNV	CRYGD,LOC100507443	CRYGD:exonic:NM_006891.3, LOC100507443:intronic_nc:N	1	100	3	synonymous	NR_03843	c.285A>G	p.(=)	-0.4					Beta/Gamma	rs2305430	0.0848	YES	5.756E-1;0.0 (pos.208986637)		Crystallin, g	cellular re	Cataract;Corneal Diseases;Eye		Benign	T=0, C=33	T=0.0, C=1.0	0.00001	504.598	33	T=0/0, C=18/1
chr11:93521405	T/T	C	T	SNV	MED17	MED17:intronic:NM_004268.4	1	100		intronic	NM_00426	c.417+72C	p.?	-1.62					Subunit 17 of	rs676586		YES			Mediator cc	RNA polym	Endometriosis;Female Urogenital Diseases;F			C=0, T=24	C=0.0, T=1.0	0.00001	332.259	24	C=0/0, T=11/1
chr11:93527099	GT/G	GT	G	INDEL	MED17	MED17:intronic:NM_004268.4	1	50		intronic	NM_00426	c.775-18G	p.?	0.02, 0.23					Subunit 17 of	rs755576982			0.0 (pos.93527093)		Mediator cc	RNA polym	Endometriosis;Female Urogenital Diseases;F			GT=17, G=1	GT=0.5, G=0.5	0.00001	71.3717	34	GT=12/5, G=1
chr11:93527219	T/C	T	C	SNV	MED17	MED17:intronic:NM_004268.4	1	97.3		intronic	NM_00426	c.859+18T	p.?	-1.12					Subunit 17 of	rs657177	8.00E-04	YES	1.0;0.0 (pos.93527219)		Mediator cc	RNA polym	Endometriosis;Female Urogenital Diseases;F			T=1, C=36	T=0.027, C=0.973	0.00001	551.82	37	T=1/0, C=20/1
chr11:93583790	G/G	T	G	SNV	VSTM5	VSTM5:upstream:NM_001144871.1	1	100		intronic	NM_00114	c.-122A>C	p.?	0.14						rs10831106,rs536485018		YES				axon,dendrite,integral component of membrane,multic				T=0, G=24	T=0.0, G=1.0	0.00001	334.524	24	T=0/0, G=6/18
chrX:1422868	G/A	G	A	SNV	CSF2RA	CSF2RA:exonic:NM_001161530.1	1	47.37	10	synonymous	NM_00116	c.1101G>A	p.(=)	1.25				0.04(COSM40	Interleukin-6	rs28460440	0.4301	YES	3.569E-1 (pos.1422868)	acute_myeloic	Colony-stim	MAPK casc	Congenital, Heredit	KB002,Sar	Benign	G=20, A=18	G=0.5263, A=0.4737	0.00001	184.537	38	G=17/3, A=6/1
chr22:37333794	G/G	A	G	SNV	CSF2RB	CSF2RB:exonic:NM_000395.2	1	100	14	synonymous	NM_00039	c.1944A>G	p.(=)	-0.19				0(COSM3766	Interferon-al	rs131840	0.1858	YES	9.514E-1 (pos.37333794)	hepatocellula	Colony-stim	MAPK casc	Lung Diseases;Lung	NPI 32101	Benign	A=0, G=38	A=0.0, G=1.0	0.00001	603.766	38	A=0/0, G=14/2
chr11:94197260	G/G	A	G	SNV	MRE11	MRE11:intronic:NM_005591.3	1	100		intronic	NM_00559	c.1225+19	p.?	-1.92					Calcineurin-li	rs641936	0.3399	YES	4.152E-1 (pos.94197260)		Meiotic rec	3'-5' exonuclease activity,ATP-dependent			Benign	A=0, G=27	A=0.0, G=1.0	0.00001	389.986	27	A=0/0, G=14/1
chr11:94203868	A/T	A	T	SNV	MRE11	MRE11:intronic:NM_005591.3	1	95.45		intronic	NM_00559	c.846-60T	p.?	-3.06					Calcineurin-li	rs610611		YES			Meiotic rec	3'-5' exonuclease activity,ATP-dependent DNA helicase a				A=1, T=21	A=0.0455, T=0.9545	0.00001	271.51	22	A=1/0, T=10/1
chr11:94225920	T/T	C	T	SNV	MRE11	MRE11:intronic:NM_005591.3	1	100		intronic	NM_00559	c.20+28G>	p.?	0.25					Calcineurin-li	rs497763	0.429	YES	4.99E-1;0.0 (pos.94225920)		Meiotic rec	3'-5' exonuclease activity,ATP-dependent			Benign	C=0, T=24	C=0.0, T=1.0	0.00001	334.792	24	C=0/0, T=20/4
chr22:37328901	C/G	C	G	SNV	CSF2RB	CSF2RB:exonic:NM_000395.2	1	51.85	9	missense	NM_00039	c.1107C>G	p.Asp369Glu	-3.21	1	45	0		Interferon-al	rs139685237	0		0.0 (pos.37328901)		Colony-stim	MAPK casc	Lung Diseases;Lung	NPI 32101,Sargramostim		C=13, G=14	C=0.4815, G=0.5185	0.00001	132.664	27	C=11/2, G=6/8
chr22:37329999	C/T	C	T	SNV	CSF2RB	CSF2RB:exonic:NM_000395.2	1	47.62	10	synonymous	NM_00039	c.1278C>T	p.(=)	-1.36				0.08(COSM37	Interferon-al	rs1801117,rs766552	0.2277,1.(	YES	6.199E-2;0.0 (pos.37329	adenocarcinor	Colony-stim	MAPK casc	Lung Diseases;Lung	NPI 32101	Benign	C=22, T=20	C=0.5238, T=0.4762	0.00001	212.935	42	C=8/14, T=8/1
chr1:36937059	G/G	A	G	SNV	CSF3R	CSF3R:exonic:NM_156039.3	1	100	10	synonymous	NM_15603	c.1260T>C	p.(=)	-0.84				0(COSM1748	Fibronectin t	rs3917981	0.4465	YES	4.048E-1 (pos.36937059)	acute_myeloic	Colony-stim	amelogen	Agranulocytosis;Bo	Favld,Filgr	Benign	A=0, G=32	A=0.0, G=1.0	0.00001	485.141	32	A=0/0, G=23/9
chr11:95825037	T/T	G	T	SNV	MAML2	MAML2:intronic:NM_032427.3	1	100		intronic	NM_03242	c.2139+19	p.?	0.04					MamL-1 dom	rs77959876	0.0253	YES	4.926E-4 (pos.95825037)		Mastermin	Notch sign	Arthritis:Arthritis, Juvenile:Autoimmune Dise			G=0, T=39	G=0.0, T=1.0	0.00001	623.819	39	G=0/0, T=16/2
chr1:36937065	G/G	A	G	SNV	CSF3R	CSF3R:exonic:NM_156039.3	1	100	10	synonymous	NM_15603	c.1254T>C	p.(=)	-2.3				0.01(COSM41	Fibronectin t	rs3917980	0.3487	YES	1.025E-1 (pos.36937065)	neoplasm	Colony-stim	amelogen	Agranulocytosis;Bo	Favld,Filgr	Benign	A=0, G=32	A=0.0, G=1.0	0.00001	484.879	32	A=0/0, G=23/9
chr11:96106661	G/G	C	G	SNV	CCDC82	CCDC82:intronic:NM_024725.3	1	100		intronic	NM_02472	c.992-30G	p.?	-0.64					Domain of un	rs11021560	0.3933	YES	2.83E-1;0.0 (pos.96106661)				Basal Ganglia Diseases;Brain Diseases;Centr			C=0, G=21	C=0.0, G=1.0	0.00001	281.235	21	C=0/0, G=15/6
chr11:96123734	TG/TG	T	TG	INDEL	CCDC82,JRKL	JRKL:utr_5:NM_001261833.1, CCDC82:upstream:NM_02472	1	100		intronic	NM_02472	c.-5823A>(	p.?, p.?	-0.11					CENP-B N-terr	rs3842515,rs397763454		YES			Jerky, mous	DNA bindi	Basal Ganglia Diseases:Brain Diseases:Centr			T=0, TG=22	T=0.0, TG=1.0	0.00001	289.691	22	T=0/0, TG=11/
chr11:99941322	T/T	C	T	SNV	CNTN5	CNTN5:intronic:NM_001243270.1	1	100		intronic	NM_00124	c.1299+30	p.?	-1.58					Fibronectin t	rs2059014	0.2625	YES	9.964E-1 (pos.99941322)		Contactin 5	C-terminal protein lipidation,anchored component of m				C=0, T=21	C=0.0, T=1.0	0.00001	279.822	21	C=0/0, T=6/15
chr12:100219207	G/G	A	G	SNV	ANKS1B	ANKS1B:intronic:NM_152788.4	1	100		intronic	NM_15278	c.135-40T	p.?	1.02					Ankyrin repe	rs7138513	0.3855	YES	8.166E-2 (pos.100219207)		Ankyrin rep	Cajal body	Basal Ganglia Diseases;Brain Diseases;Brain			A=0, G=60	A=0.0, G=1.0	0.00001	903.531	60	A=0/0, G=18/4
chr8:19362768	T/T	C	T	SNV	CSGALNACT1	CSGALNACT1:exonic:NM_018371.4	1	100	4	missense	NM_01837	c.578G>A	p.Ser193Asn	-0.84	1	46	0	0.06(COSM41	Chondroitin N	rs7017776	0.1927	YES	8.532E-1 (pos.19362768)	neoplasm	Chondroitin	Golgi cisterna membrane,Golgi membrane,UDP-N-acetv				C=0, T=29	C=0.0, T=1.0	0.00001	427.603	29	C=0/0, T=16/1
chr12:1005162	T/C	T	C	SNV	WNK1	WNK1:intronic:NM_001184985.1	1	50		intronic	NM_00118	c.6364-75	p.?	0.32					Oxidative-str	rs35956200		YES			WNK lysine	ATP bindin	Cardiovascular Diseases:Charcot-Marie-Toot			T=10, C=14	T=0.4167, C=0.5833	0.00001	156.491	28	T=5/5, C=4/10
chr10:43678796	T/T	C	T	SNV	CSGALNACT2	CSGALNACT2:exonic:NM_018590.4	1	100	8	missense	NM_01859	c.1435C>T	p.Pro479Ser	2.88	0.2	74	0.128	0.88(COSM37	Chondroitin N	rs2435381	0.2666	YES	2.321E-1 (pos.43678796)	acute_myeloic	Chondroitin	Golgi cisterna membrane,Golgi membrane,acetylgalact				C=0, T=76	C=0.0, T=1.0	0.00001	1208.65	76	C=0/0, T=26/5
chr17:61987570	T/T	G	T	SNV	CSHL1	CSHL1:exonic:NM_022579.2	1	100	4	missense	NM_02257	c.423C>A	p.Asp141Glu	1.43	0.01	45	0.389	0.81(COSM11	Somatotropi	rs2727307	0.4142	YES	3.96E-1 (pos.61987570)	acute_myeloic	Chorionic s	biological_process,cellular_component,extracellular re				G=0, T=22	G=0.0, T=1.0	0.00001	298.892	22	G=0/0, T=13/9
chr17:61987576	A/A	G	A	SNV	CSHL1	CSHL1:exonic:NM_022579.2	1	100	4	synonymous	NM_02257	c.417C>T	p.(=)	-0.64				0.01(COSM11	Somatotropi	rs2246207	0.4152	YES	3.963E-1:1.159E-4 (pos.6	acute_myeloic	Chorionic s	biological_process,cellular_component,extracellular re				G=0, A=22	G=0.0, A=1.0	0.00001	298.882	22	G=0/0, A=13/9
chr12:101728166	A/G	A	G	SNV	UTP20	UTP20:intronic:NM_014503.2	1	59.09		intronic	NM_01450	c.3562-37	p.?	-1.62					Down-regula	rs2002895,rs776346	0.268	YES	0.0 (pos.101728156),9.163E-1 (pos.1017		UTP20, S. ce	90S prerib	Alcohol-Related Disorders:Alcoholism:Menta			A=9, G=13	A=0.4091, G=0.5909	0.00001	122.568	22	A=3/6, G=12/1
chr12:101734277	T/C	T	C	SNV	UTP20	UTP20:intronic:NM_014503.2	1	51.85		intronic	NM_01450	c.4014-24	p.?	-0.23					Down-regula	rs7963896	0.2685	YES	9.148E-1 (pos.101734277)		UTP20, S. ce	90S prerib	Alcohol-Related Disorders;Alcoholism;Menta			T=13, C=14	T=0.4815, C=0.5185	0.00001	132.665	27	T=9/4, C=4/10
chr8:2807783	A/A	G	A	SNV	CSMD1	CSMD1:exonic:NM_033225.5	1	100	67	synonymous	NM_03322	c.10284C>	p.(=)	0.88				0.35(COSM37	CUB domain,	rs35043129	0.1244	YES	1.398E-1 (pos.2807783)	acute_myeloic	Cub and Sus	glucose hc	Alzheimer Disease;Amphetamine-Related Di			G=0, A=21	G=0.0, A=1.0	0.00001	280.99	21	G=0/0, A=3/18
chr12:102046595	C/C	T	C	SNV	MYBPC1	MYBPC1:intronic:NM_001254718.1	1	100		intronic	NM_00125	c.1558+33	p.?	-0.1					Fibronectin t	rs1997204	0.052	YES	9.95E-1 (pos.102046595)		Myosin-bin	M band,Z (	Arthrogryposis;Congenital Abnormalities;Cor			T=0, C=24	T=0.0, C=1.0	0.00001	334.69	24	T=0/0, C=18/6
chr8:3253768	T/T	C	T	SNV	CSMD1	CSMD1:exonic:NM_033225.5	1	100	17	synonymous	NM_03322	c.2541G>A	p.(=)	-3.1				0.02(COSM14	CUB domain,	rs3802303	0.4062	YES	1.837E-1:0.0 (pos.32537	adenocarcinor	Cub and Sus	glucose hc	Alzheimer Disease:Amphetamine-Related Di			C=0, T=32	C=0.0, T=1.0	0.00001	485.141	32	C=0/0, T=25/7
chr12:102078094	C/C	G	C	SNV	MYBPC1	MYBPC1:intronic:NM_001254718.1	1	100		intronic	NM_00125	c.3472-12	p.?	0.41					Fibronectin t	rs2303629	0.34	YES			Myosin-bin	M band,Z (	Arthrogryposis:Congenital Abnormalities:Cor			G=0, C=22	G=0.0, C=1.0	0.00001	298.892	22	G=0/0, C=15/7
chr8:3265590	T/T	C	T	SNV	CSMD1	CSMD1:exonic:NM_033225.5	1	100	14	synonymous	NM_03322	c.1902G>A	p.(=)	0.21				0.72(COSM37	CUB domain,	rs10088378	0.3324	YES	3.647E-1 (pos.3265590)	acute_myeloic	Cub and Sus	glucose hc	Alzheimer Disease;Amphetamine-Related Di			C=0, T=40	C=0.0, T=1.0	0.00001	644.264	40	C=0/0, T=13/2
chr1:34038214	C/C	T	C	SNV	CSMD2	CSMD2:exonic:NM_052896.4	1	100	51	missense	NM_05289	c.7660A>G	p.Met2554Val	0.28	0.59	21	0	0.03(COSM37	CUB domain,	rs2641962	0.4681	YES	3.764E-1 (pos.34038214)	acute_myeloic	Cub and Sus	integral cc	Attention Deficit Disorder with Hyperactivity			T=0, C=64	T=0.0, C=1.0	0.00001	977.613	64	T=0/0, C=25/3
chr12:102295218	C/C	T	C	SNV	DRAM1	DRAM1:intronic:NM_018370.2	1	100		intronic	NM_01837	c.342+7T>	p.?	-0.58					Frag1/DRAM/	rs201692573,rs3867	0.386	YES	9.206E-1 (pos.102295218)		Damage-re	apoptotic process,autophagy,cytoplasm,integral compo				T=0, C=25	T=0.0, C=1.0	0.00001	353.015	25	T=0/0, C=13/1
chr12:102433811	C/C	T	C	SNV	WASHC3	WASHC3:intronic:NM_016053.3	1	100		intronic	NM_01605	c.325-55A	p.?	0.22					Subunit CCDC	rs4237982		YES				WASH complex,biological_process,early endosome,prot				T=0, C=20	T=0.0, C=1.0	0.00001	268.184	20	T=0/0, C=8/12
chr1:34329897	C/C	T	C	SNV	CSMD2,HMGB4	CSMD2:intronic:NM_052896.4, HMGB4:exonic:NM_145205.	1	100	2	synonymous	NM_05289	c.801-173	p.?, p.(=)	-0.32					CUB domain,	rs911218	0.0455	YES	1.0;0.0 (pos.34329897)		Cub and Sus	DNA bindi	Attention Deficit Disorder with Hyperactivity			T=0, C=103	T=0.0, C=1.0	0.00001	1611.39	103	T=0/0, C=45/5
chr12:103271350	A/A	G	A	SNV	PAH	PAH:intronic:NM_000277.1	1	100		intronic	NM_00027	c.353-22C	p.?	-0.23					ACT domain,E	rs2037639	0.2309	YES	7.108E-1 (pos.103271350)		Phenylalan	L-phenylal	Amino Acid Disorde	Beta(2-Thi	Benign	G=0, A=54	G=0.0, A=1.0	0.00001	798.148	54	G=0/0, A=10/4
chr12:104149251	T/T	C	T	SNV	STAB2	STAB2:intronic:NM_017564.9	1	100		intronic	NM_01756	c.6880+6C	p.?	-2.17					EGF domain,E	rs7296626	0.0616	YES	9.999E-1 (pos.104149251)			angiogenesis,calcium ion bindi		Hyaluronic acid		C=0, T=88	C=0.0, T=1.0	0.00001	1451.91	88	C=0/0, T=49/3
chr12:104174192	C/T	C	T	SNV	NT5DC3	NT5DC3:intronic:NM_001031701.2	1	57.58		intronic	NM_00103	c.1330-50	p.?	0.06					5' nucleotida	rs1866292	0.2643	YES	4.11E-1 (pos.104174192)		5' nucleotid	5'-nucleotidase activity,dephosphorylation,metal ion bi				C=14, T=19	C=0.4242, T=0.5758	0.00001	204.394	33	C=7/7, T=8/11
chr12:10461883	G/G	A	G	SNV	KLRD1	KLRD1:intronic:NM_002262.4	1	100		intronic	NM_00226	c.8-104A>	p.?	-0.27					Lectin C-type	rs10734825		YES			Killer cell le	MHC class I protein complex binding,MHC class Ib protei				A=0, G=50	A=0.0, G=1.0	0.00001	852.705	50	A=0/0, G=32/1
chr1:34330067	C/C	A	C	SNV	CSMD2,HMGB4	CSMD2:intronic:NM_052896.4, HMGB4:exonic:NM_145205.	1	100	2	missense	NM_05289	c.801-174	p.?, p.Glu92Ala	3.89		107		0.02(COSM44	CUB domain,	rs10379	0.2401	YES	8.863E-1 (pos.34330067)	acute_myeloic	Cub and Sus	DNA bindi	Attention Deficit Disorder with Hyperactivity			A=0, C=53	A=0.0, C=1.0	0.00001	782.024	53	A=0/0, C=22/3
chr8:113241088	G/G	T	G	SNV	CSMD3	CSMD3:exonic:NM_198123.1	1	100	70	missense	NM_19812	c.10861A>	p.Asn3621His	4.19	0.47	68	0.883	0.94(COSM11	CUB domain,	rs1592624	0.4415	YES	7.6E-1 (pos.113241088)	adenocarcinor	Cub and Sus	integral cc	Adenocarcinoma:Autistic Disorder:Carcinom			T=0, G=122	T=0.0, G=1.0	0.00001	1973.7	122	T=0/0, G=55/6
chr12:105446719	T/T	G	T	SNV	ALDH1L2	ALDH1L2:intronic:NM_001034173.3	1	100		intronic	NM_00103	c.1288-10	p.?	1.27				0.03(COSM14	Aldehyde del	rs56118650	0.4843	YES	3.834E-1 (pos.10544671	adenocarcinor	Aldehyde de	10-formyltetrahydrofolate catabolic process,aldehyde d				G=0, T=87	G=0.0, T=1.0	0.00001	1431.38	87	G=0/0, T=41/4
chr12:105509160	T/T	G	T	SNV	WASHC4	WASHC4:intronic:NM_015275.2	1	100		intronic	NM_01527	c.367+153	p.?	-0.5					WASH compl	rs1196801		YES			WASH comp	BLOC-1 complex,WASH complex,early endosome,endosc				G=0, T=41	G=0.0, T=1.0	0.00001	664.7	41	G=0/0, T=19/2
chr12:105514480	G/G	A	G	SNV	WASHC4	WASHC4:intronic:NM_015275.2	1	100		intronic	NM_01527	c.561+105	p.?	-1.63					WASH compl	rs1196837		YES			WASH comp	BLOC-1 complex,WASH complex,early endosome,endosc				A=0, G=114	A=0.0, G=1.0	0.00001	1812.57	114	A=0/0, G=67/4
chr12:105534025	A/A	G	A	SNV	WASHC4	WASHC4:intronic:NM_015275.2	1	100		intronic	NM_01527	c.1453-47	p.?	-0.35					WASH compl	rs1196808,rs386765	0.1708	YES	9.993E-1 (pos.105534025)		WASH comp	BLOC-1 complex,WASH complex,early endosome,endosc				G=0, A=59	G=0.0, A=1.0	0.00001	885.361	59	G=0/0, A=28/3
chr15:75982428	C/T	C	T	SNV	CSPG4	CSPG4:exonic:NM_001897.4	1	46.43	3	synonymous	NM_00189	c.978G>A	p.(=)	-1.48				0.11(COSM43	Cadherin-like	rs7182906		YES	1.972E-1 (pos.75982428)	astrocytoma (	Chondroitin	Golgi lume	Brain Diseases:Cardiovascular Diseases:Cen			C=14, T=13	C=0.5185, T=0.4815	0.00001	125.566	28	C=10/4, T=7/6
chr3:39185077	A/A	G	A	SNV	CSRNP1	CSRNP1:exonic:NM_033027.3	1	100	5	synonymous	NM_03302	c.1239C>T	p.(=)	-1.93				0.1(COSM635	Cysteine/seri	rs1274957	0.3495	YES	6E-1 (pos.39185077)	carcinoma		DNA bindi	Cardiomegaly;Cardiomyopathies;Cardiomyo			G=0, A=32	G=0.0, A=1.0	0.00001	485.141	32	G=0/0, A=24/8
chr3:39185248	G/G	A	G	SNV	CSRNP1	CSRNP1:exonic:NM_033027.3	1	100	5	synonymous	NM_03302	c.1068T>C	p.(=)	0.02				0.03(COSM41	Cysteine/seri	rs784519	0.1263	YES	8.968E-1 (pos.39185248)	neoplasm		DNA bindi	Cardiomegaly;Cardiomyopathies;Cardiomyo			A=0, G=57	A=0.0, G=1.0	0.00001	849.51	57	A=0/0, G=30/2
chr3:39186728	T/T	C	T	SNV	CSRNP1	CSRNP1:exonic:NM_033027.3	1	100	3	synonymous	NM_03302	c.225G>A	p.(=)	1.83					Cysteine/seri	rs784517	0.4351	YES	5.872E-1 (pos.39186728)			DNA bindi	Cardiomegaly;Cardiomyopathies;Cardiomyo			C=0, T=38	C=0.0, T=1.0	0.00001	603.786	38	C=0/0, T=15/2
chr20:23618395	C/C	T	C	SNV	CST3	CST3:exonic:NM_000099.3	1	100	1	synonymous	NM_00009	c.105A>G	p.(=)	-1.44				0.02(COSM37	Cystatin dom	rs6138024	0.0017	YES	1.0 (pos.23618395)	adenocarcinor	Cystatin C	Sertoli cel	Acute Kidney Injury;Alzheimer Disease;Amylc			T=0, C=21	T=0.0, C=1.0	0.00001	281.235	21	T=0/0, C=15/6
chr20:23472390	C/CG	CA	CG	SNV	CST8	CST8:exonic:NM_005492.3	1	97.67	2	synonymous	NM_00549	c.87delA, (	p.Val31fs, p.(=)	-0.48, -0.28					Cystatin dom	rs114856939,rs2983	2.00E-04		0.0;0.0 (pos.23472390),1.0 (pos.23472391)			cell surfac	Genital Diseases, Male:Infertility:Infertility, I			CA=0, C=1,	CA=0.0, C=0.0233, C(	0.00001	707.04	43	CA=0/0, C=0/1
chr12:10571106	C/C	G	C	SNV	KLRC3	KLRC3:intronic:NM_007333.2	1	100		intronic	NM_00733	c.332-9C>(	p.?	-0.4					Lectin C-type	rs2682493,rs746095526			1.157E-4:0.0 (pos.10571105),9.999E-1 (p		Killer cell le	carbohydrate binding,cellular defense response,integra				G=0, C=37	G=0.0, C=1.0	0.00001	583.706	37	G=0/0, C=17/2
chr12:10571158	T/T	C	T	SNV	KLRC3	KLRC3:intronic:NM_007333.2	1	100		intronic	NM_00733	c.332-61G	p.?	0.42					Lectin C-type	rs10743890,rs796095553,rs796		YES			Killer cell le	carbohydrate binding,cellular defense response,integra				C=0, T=25	C=0.0, T=1.0	0.00001	353.016	25	C=0/0, T=4/21
chr12:10571177	G/G	A	G	SNV	KLRC3	KLRC3:intronic:NM_007333.2	1	100		intronic	NM_00733	c.332-80T	p.?	-0.2					Lectin C-type	rs4394921		YES			Killer cell le	carbohydrate binding,cellular defense response,integra				A=0, G=24	A=0.0, G=1.0	0.00001	332.176	24	A=0/0, G=3/21
chr3:122056432	T/T	C	T	SNV	CSTA	CSTA:exonic:NM_005213.3	1	100	2	synonymous	NM_00521	c.105C>T	p.(=)	-1.08					Cystatin dom	rs17589	0.433	YES	6.899E-2;0.0 (pos.122056432)		Cystatin A (	cell-cell ac	Astrocytoma:Carcinoma:Congenital, Heredit			C=0, T=84	C=0.0, T=1.0	0.00001	1370.17	84	C=0/0, T=59/2
chr12:105742457	G/A	G	A	SNV	C12orf75	C12orf75:intronic:NM_001145199.1	1	52.38		intronic	NM_00114	c.71+53G>	p.?	-0.72					OCC1 family	rs75924647	0.0374	YES								G=10, A=11	G=0.4762, A=0.5238	0.00001	95.3003	21	G=5/5, A=3/8

chr12:10588647	G/G	A	G	SNV	KLRC2	KLRC2:upstream:NM_002260.3	1	100		intronic	NM_00226	c.-62T>C	p.?	-0.07						rs28730284					Killer cell le	MHC class	Brain Diseases;Central Nervous System Dise	A=0, G=21	A=0.0, G=1.0	0.00001	279.959	21	A=0/0, G=12/9
chr20:23424613	T/C	T	C	SNV	CSTL1	CSTL1:exonic:NM_138283.1	1	50	3	missense	NM_13828	c.262T>C	p.Trp88Arg	-0.27	1	101	0	0.01(COSM37	Cystatin dom	rs3746736	0.4959	YES	7.279E-1;0.0 (pos.23424(	adenocarcinoma		cysteine-type endopeptidase inhibitor activity,extracell		T=13, C=13	T=0.5, C=0.5	0.00001	113.922	26	T=5/8, C=4/9
chr18:19995753	C/C	T	C	SNV	CTAGE1	CTAGE1:exonic:NM_172241.2	1	100	1	synonymous	NM_17224	c.2022A>G	p.(=)	-0.14				0.02(COSM6248072),0.02(C		rs9946145	0.0911	YES	8.275E-1 (pos.19995753)	neoplasm		biological_process,cellular_component,integral compor		T=0, C=25	T=0.0, C=1.0	0.00001	353.019	25	T=0/0, C=17/8
chr10:126714966	G/G	A	G	SNV	CTBP2	CTBP2:exonic:NM_022802.2	1	100	1	missense	NM_02280	c.1363T>C	p.Tyr455His	4.9	0	83	0.001	0.48(COSM37	D-isomer spe	rs3012075	0.4972	YES	6.376E-1 (pos.12671496(	adenocarcinor	C-terminal-	NAD bindi	Genital Diseases, Male;Genital Neoplasms, M	A=0, G=24	A=0.0, G=1.0	0.00001	334.788	24	A=0/0, G=19/5
chr10:126716346	T/T	C	T	SNV	CTBP2	CTBP2:utr_5:NM_022802.2	1	100	1	utr_5	NM_02280	c.-18G>A	p.?	-1.06					D-isomer spe	rs2938006	0.4937	YES	6.632E-1 (pos.126716346)		C-terminal-	NAD bindi	Genital Diseases, Male;Genital Neoplasms, M	C=0, T=30	C=0.0, T=1.0	0.00001	446.634	30	C=0/0, T=15/1
chr12:10763425	A/A	ATGTAGAC	A	INDEL	MAGOHB	MAGOHB:intronic:NM_018048.4	8	100		intronic	NM_01804	c.95-146T(	p.?	-0.06, 0.54, 1.36, 0.24, 0.65, -0.33, 0.88, -0.02, 1.57					Mago nashi p	rs200742244,rs375482462,rs38		YES				RNA bindir	Adnexal Diseases;Cysts;Endocrine System Di	ATGTAGAC/	ATGTAGACA=0.0, A=1	0.00001	730.012	50	ATGTAGACA=0
chr12:108136172	G/G	A	G	SNV	LOC101929162,PRDM4	PRDM4:intronic:NM_012406.3, LOC101929162:intronic_nc:	1	100		intronic	NM_01240	c.1482-47	p.?	0.44					C2H2-type zir	rs2302734	0.4085	YES	1.201E-1 (pos.108136172)		PR domain-	RNA polymerase II core promoter proximal region seque		A=0, G=34	A=0.0, G=1.0	0.00001	524.186	34	A=0/0, G=19/1
chr12:108140032	C/C	T	C	SNV	LOC101929162,PRDM4	PRDM4:intronic:NM_012406.3, LOC101929162:intronic_nc:	1	100		intronic	NM_01240	c.1276+20	p.?	-0.38					C2H2-type zir	rs3803120	0.408	YES	1.209E-1 (pos.108140032)		PR domain-	RNA polymerase II core promoter proximal region seque		T=0, C=53	T=0.0, C=1.0	0.00001	782.03	53	T=0/0, C=23/3
chr17:8132763	C/C	T	C	SNV	CTC1	CTC1:exonic:NM_025099.5	1	100	19	missense	NM_02509	c.3013A>G	p.Ile1005Val	-0.77	1	29	0	0(COSM4130	CST, telomere	rs3826543,rs761130	0.1859	YES	0.0 (pos.8132762),3.844	neoplasm	Conserved t	CST compl	Angiodysplasia;Ataxia;Bone Di Benign/Likely	T=0, C=64	T=0.0, C=1.0	0.00001	977.634	64	T=0/0, C=26/3
chr12:108919390	C/C	T	C	SNV	SART3	SART3:intronic:NM_014706.3	1	100		intronic	NM_01470	c.2371-4A	p.?	-1.54				0.07(COSM63	LSM-interacti	rs7963063	0.4131	YES	9.054E-2 (pos.10891939(	carcinoma	Squamous c	Cajal body	Congenital, Hereditary, and Neonatal Diseas	T=0, C=35	T=0.0, C=1.0	0.00001	543.903	35	T=0/0, C=16/1
chr12:108960906	T/T	G	T	SNV	ISCU	ISCU:intronic:NM_213595.3	1	100		intronic	NM_21359	c.340-60G	p.?	-1.52					NifU-like N te	rs10861945	0.1675	YES			Iron-sulfur c	2 iron, 2 su	Acid-Base Imbalance;Acidosis;Acidosis, Lacti	G=0, T=28	G=0.0, T=1.0	0.00001	408.718	28	G=0/0, T=20/8
chr12:109338835	T/T	C	T	SNV	SVOP	SVOP:intronic:NM_018711.4	1	100		intronic	NM_01871	c.483+44G	p.?	0.79					Major Facilit	rs36131538	0.3891	YES	5.422E-1 (pos.109338835)		SV2-related	cell juncti	Nervous System Diseases;Neuromuscular Di	C=0, T=20	C=0.0, T=1.0	0.00001	263.786	20	C=0/0, T=16/4
chr17:8135061	C/C	T	C	SNV	CTC1	CTC1:exonic:NM_025099.5	1	100	14	missense	NM_02509	c.2458A>G	p.Ile820Val	0.38	1	29	0		CST, telomere	rs3027238	0.0357	YES	9.92E-1 (pos.8135061)		Conserved t	CST compl	Angiodysplasia;Ataxia;Bone Di Benign/Likely	T=0, C=36	T=0.0, C=1.0	0.00001	563.741	36	T=0/0, C=18/1
chr17:7154534	A/A	G	A	SNV	CTDNEP1,ELP5	CTDNEP1:exonic:NM_001143775.1, ELP5:upstream:NM_20	1	100	1	synonymous	NM_00114	c.82C>T, c.	p.(=), p.?	4.28				0.21(COSM43	NLI interactir	rs414206	0.3794	YES	6.522E-1 (pos.7154534)	ductal_carcinc	C-terminal c	Elongator holoenzyme complex,Nem1-Spo7 phosphatase		G=0, A=29	G=0.0, A=1.0	0.00001	427.604	29	G=0/0, A=6/23
chr18:77513721	C/C	T	C	SNV	CTDP1	CTDP1:exonic:NM_004715.4	1	100	13	synonymous	NM_00471	c.2817T>C	p.(=)	0.04				0.56(COSM41	FCP1, C-termi	rs626169	0.0066	YES	9.998E-1 (pos.77513721)	neoplasm	C-terminal c	CTD phosp	Cataract;Congenital Abnormal Benign/Likely	T=0, C=29	T=0.0, C=1.0	0.00001	427.586	29	T=0/0, C=21/8
chr12:109520876	A/A	G	A	SNV	USP30	USP30:intronic:NM_032663.4	1	100		intronic	NM_03266	c.1168+9G	p.?	-0.71					Ubiquitin car	rs59162254	0.0826	YES	1.471E-1 (pos.109520876)		Ubiquitin-sp	autophagy of mitochondrion,cysteine-type endopeptida		G=0, A=33	G=0.0, A=1.0	0.00001	504.598	33	G=0/0, A=16/1
chr8:104394744	C/C	T	C	SNV	CTHRC1	CTHRC1:exonic:NM_138455.3	1	100	4	synonymous	NM_13845	c.648T>C	p.(=)	-0.93				0.05(COSM3762968),0.05(C		rs3098233	0.2131	YES	6.683E-1 (pos.10439474(	acute_myeloic	Collagen tri	Wnt signaling pathway, planar cell polarity pathway,Wn		T=0, C=42	T=0.0, C=1.0	0.00001	689.969	42	T=0/0, C=31/1
chr18:46385948	G/G	A	G	SNV	CTIF	CTIF:utr_3:NM_001142397.1	1	100	13	utr_3	NM_00114	c.1821A>G	p.?	-0.18				0.08(COSM40	MIF4G domai	rs3752082	0.4371	YES	2.528E-1 (pos.46385948)	acute_myeloic	KIAA0427 ge	RNA binding,cytosol,nuclear-transcribed mRNA catabolic		A=0, G=22	A=0.0, G=1.0	0.00001	293.485	22	A=0/0, G=14/8
chr11:57571232	T/T	C	T	SNV	CTNND1,TMX2-CTNND1	CTNND1:exonic:NM_001085458.1, TMX2-CTNND1:exonic_n	1	100	8	synonymous	NR_03764(	c.1560C>T	p.(=)	0.07					Armadillo/be	rs11229137	1.00E-04		1.0 (pos.57571232)		Catenin (ca	Wnt signaling pathway,adherens junction organization,t		C=0, T=34	C=0.0, T=1.0	0.00001	514.035	34	C=0/0, T=10/2
chr12:109637188	C/C	G	C	SNV	ACACB	ACACB:intronic:NM_001093.3	1	100		intronic	NM_00109	c.2628-19	p.?	-3.11					Acetyl-CoA ca	rs7299171	0.4235	YES	3.216E-1;1.173E-4 (pos.109637188)		Acetyl-Coer	ATP bindin	Glucose Metabolisi (2S)-2-(4-(3-CHLORO-5-(TF	G=0, C=29	G=0.0, C=1.0	0.00001	426.674	29	G=0/0, C=18/1
chr12:109685545	G/G	A	G	SNV	ACACB	ACACB:intronic:NM_001093.3	1	100		intronic	NM_00109	c.5669+37	p.?	-1.21					Acetyl-CoA ca	rs2284689	0.1826	YES	7.634E-1 (pos.109685545)		Acetyl-Coer	ATP bindin	Glucose Metabolisi (2S)-2-(4-(3-CHLORO-5-(TF	A=0, G=20	A=0.0, G=1.0	0.00001	263.773	20	A=0/0, G=4/16
chr5:10973727	G/A	G	A	SNV	CTNND2	CTNND2:exonic:NM_001332.3	1	50	22	synonymous	NM_00133	c.3516C>T	p.(=)	0.26				0.71(COSM24	Armadillo/be	rs1566622	0.087	YES	2.098E-1 (pos.10973727)	acute_myeloic	Catenin, de	Wnt signa	Abnormalities, Multiple;Amyotrophic Lateral	G=14, A=14	G=0.5, A=0.5	0.00001	127.768	28	G=14/0, A=9/5
chr17:3564068	G/A	G	A	SNV	CTNS	CTNS:utr_3:NM_001031681.2	1	58.33	13	utr_3	NM_00103	c.1243G>A	p.?	0.3					PQ loop repe	rs760273	0.2036	YES	2.479E-1 (pos.3564068)		Cystinosin	ATP metab	Congenital, Heredit Cystine Likely benign	G=10, A=14	G=0.4167, A=0.5833	0.00001	135.339	24	G=3/7, A=2/12
chr12:111317904	C/C	T	C	SNV	CCDC63	CCDC63:intronic:NM_152591.2	1	100		intronic	NM_15259	c.671+13T	p.?	-0.97						rs35762292	0.2084	YES	1.734E-3 (pos.111317904)			axoneme,	Melanoma;Neoplasms;Neoplasms by Histolo	T=0, C=25	T=0.0, C=1.0	0.00001	357.906	25	T=0/0, C=12/1
chr12:11215029	T/T	C	T	SNV	PRH1,PRH1-PRR4,PRH1-TAS2R14,TAS2R46	PRH1-PRR4:intronic_nc:NR_037918.2, TAS2R46:upstream:N	1	100		intronic	NR_03791	c.-293-152	p.?, p.?, p.?	-2.21						rs2708379					Proline-rich	G-protein coupled receptor activity,G-protein coupled re		C=0, T=26	C=0.0, T=1.0	0.00001	371.417	26	C=0/0, T=10/1
chr12:11215033	T/T	G	T	SNV	PRH1,PRH1-PRR4,PRH1-TAS2R14,TAS2R46	PRH1-PRR4:intronic_nc:NR_037918.2, TAS2R46:upstream:N	1	100		intronic	NR_03791	c.-293-152	p.?, p.?, p.?	-2.08						rs61912070					Proline-rich	G-protein coupled receptor activity,G-protein coupled re		G=0, T=26	G=0.0, T=1.0	0.00001	371.419	26	G=0/0, T=10/1
chr12:11215038	A/A	G	A	SNV	PRH1,PRH1-PRR4,PRH1-TAS2R14,TAS2R46	PRH1-PRR4:intronic_nc:NR_037918.2, TAS2R46:upstream:N	1	100		intronic	NR_03791	c.-293-152	p.?, p.?, p.?	-1.95						rs2708378					Proline-rich	G-protein coupled receptor activity,G-protein coupled re		G=0, A=26	G=0.0, A=1.0	0.00001	371.419	26	G=0/0, A=10/1
chr12:112610714	A/A	G	A	SNV	HECTD4	HECTD4:intronic:NM_001109662.3	1	100		intronic	NM_00110	c.11193-4	p.?	-0.81					HECT-domain	rs11066188	0.4402	YES	8.155E-4;0.0 (pos.112610714)				Arterial Occlusive Diseases;Arteriosclerosis;	G=0, A=74	G=0.0, A=1.0	0.00001	1170.13	74	G=0/0, A=41/3
chr17:3564073	A/G	A	G	SNV	CTNS	CTNS:utr_3:NM_001031681.2	1	58.33	13	utr_3	NM_00103	c.1248A>G	p.?	0.34					PQ loop repe	rs3826496	0.2035	YES	2.482E-1 (pos.3564073)		Cystinosin	ATP metab	Congenital, Heredit Cystine Likely benign	A=10, G=14	A=0.4167, G=0.5833	0.00001	135.339	24	A=3/7, G=2/12
chr17:3559823	G/A	G	A	SNV	CTNS	CTNS:exonic:NM_001031681.2	1	50	8	synonymous	NM_00103	c.504G>A	p.(=)	-0.77				0.09(COSM54	PQ loop repe	rs1800528	0.1815	YES	2.129E-1 (pos.3559823)	acute_myeloic	Cystinosin	ATP metab	Congenital, Heredit Cystine Benign/Likely	G=14, A=14	G=0.5, A=0.5	0.00001	131.691	28	G=6/8, A=6/8
chr11:10786142	T/T	C	T	SNV	CTR9	CTR9:exonic:NM_014633.4	1	100	12	synonymous	NM_01463	c.1461C>T	p.(=)	0.22				0.48(COSM41	Tetratricopep	rs3736326	0.4109	YES	8.313E-1 (pos.10786142)	neoplasm		Cdc73/Paf1 complex,JAK-STAT cascade,RNA polymerase		C=0, T=39	C=0.0, T=1.0	0.00001	623.98	39	C=0/0, T=17/2
chr1:206327510	T/C	T	C	SNV	CTSE	CTSE:exonic:NM_001910.3	1	50	6	synonymous	NM_00191	c.699T>C	p.(=)	-2.11				0.06(COSM37	A1 Propeptid	rs61742539	0.1823	YES	7.511E-3 (pos.20632751(	adenocarcinor	Cathepsin E	antigen processing and presentation of exogenous pepti		T=10, C=10	T=0.5, C=0.5	0.00001	77.4894	20	T=7/3, C=3/7
chr15:79217690	C/C	G	C	SNV	CTSH	CTSH:exonic:NM_004390.4	1	100	10	synonymous	NM_00439	c.792C>G	p.(=)	-2.28					Cathepsin pr	rs2044226	0		1.0;0.0 (pos.79217690)		Cathepsin H	ERK1 and E	Autoimmune Disea CARBOBENZYLOXY-(L)-LEU	G=0, C=43	G=0.0, C=1.0	0.00001	690.654	43	G=0/0, C=22/2
chr1:150727539	G/A	G	A	SNV	CTSS	CTSS:exonic:NM_004079.4	1	50	4	missense	NM_00407	c.337C>T	p.Arg113Trp	0.43	0.07	101	0.904	0.19(COSM14	Cathepsin pr	rs2230061	0.3549	YES	3.312E-1 (pos.15072753(	adenocarcinor	Cathepsin S	adaptive i	Autoimmune Disea (1R)-2-(CYANOMETHYLIAN	G=10, A=10	G=0.5, A=0.5	0.00001	82.8804	20	G=7/3, A=5/5
chr12:113836715	T/T	C	T	SNV	SDS	SDS:intronic:NM_006843.2	1	100		intronic	NM_00684	c.194-64G	p.?	-0.32					Pyridoxal-ph	rs73194896		YES			SDS gene,	L-serine ammonia-lyase activit L-Serine,Pyridoxal Phosp		C=0, T=47	C=0.0, T=1.0	0.00001	789.289	47	C=0/0, T=25/2
chr12:113836721	T/T	C	T	SNV	SDS	SDS:intronic:NM_006843.2	1	100		intronic	NM_00684	c.194-70G	p.?	-0.34					Pyridoxal-ph	rs73194898		YES			SDS gene,	L-serine ammonia-lyase activit L-Serine,Pyridoxal Phosp		C=0, T=47	C=0.0, T=1.0	0.00001	787.139	47	C=0/0, T=25/2
chr11:65649774	G/G	A	G	SNV	CTSW	CTSW:exonic:NM_001335.3	1	100	4	missense	NM_00133	c.415A>G	p.Ser139Gly	-0.76	1	56	0		Cathepsin pr	rs604630	8.00E-04	YES	9.998E-1;0.0 (pos.65649774)		Cathepsin W	cysteine-type endopeptidase activity,cysteine-type pep		A=0, G=24	A=0.0, G=1.0	0.00001	334.792	24	A=0/0, G=14/1
chr16:88782676	G/G	A	G	SNV	CTU2,MIR4722,PIEZO1	CTU2:downstream:NM_001012759.2, MIR4722:downstrear	1	100	49	synonymous	NM_00101	c.*1017A>	p.?, p.(=)	-0.27				0.03(COSM41	Piezo, Piezo n	rs2290902		YES	8.912E-1 (pos.88782676)	neoplasm,squ	PIEZO1 ion c	cation cha	Anemia;Anemia, Hemolytic;An Benign	A=0, G=22	A=0.0, G=1.0	0.00001	298.892	22	A=0/0, G=19/3
chr12:117477104	T/T	C	T	SNV	TESC	TESC:intronic:NM_017899.3	1	100		intronic	NM_01789	c.568-89G	p.?	0.61					EF hand,EF-ha	rs2270794		YES			Tescalcin, n	calcium ion binding,cellular response to retinoic acid,cy		C=0, T=22	C=0.0, T=1.0	0.00001	298.891	22	C=0/0, T=3/19
chr10:16882518	C/C	T	C	SNV	CUBN	CUBN:exonic:NM_001081.3	1	100	62	synonymous	NM_00108	c.9843A>G	p.(=)	-0.06				0.49(COSM14	CUB domain,	rs703064	0.2476	YES	5.401E-1 (pos.16882518)	adenocarcinor	Cubilin (intr	Golgi app	Amphetamine-Rela Cyanocob Benign	T=0, C=27	T=0.0, C=1.0	0.00001	389.988	27	T=0/0, C=12/1
chr10:16979661	C/C	T	C	SNV	CUBN	CUBN:exonic:NM_001081.3	1	100	39	synonymous	NM_00108	c.5856A>G	p.(=)	-0.52					CUB domain,	rs1801234	0.4212	YES	5.016E-1 (pos.16979661)		Cubilin (intr	Golgi app	Amphetamine-Rela Cyanocob Benign	T=0, C=36	T=0.0, C=1.0	0.00001	563.58	36	T=0/0, C=15/2
chr12:118676940	G/G	A	G	SNV	TAOK3	TAOK3:intronic:NM_016281.3	1	100		intronic	NM_01628	c.340+92T	p.?	0.19					Protein kinas	rs16948197		YES			TAO kinase	ATP binding,DNA repair,MAP kinase kinase kinase activit		A=0, G=24	A=0.0, G=1.0	0.00001	334.784	24	A=0/0, G=4/20
chr10:17088006	T/C	T	C	SNV	CUBN	CUBN:exonic:NM_001081.3	1	46.43	24	synonymous	NM_00108	c.3417A>G	p.(=)	-2.3				0.06(COSM37	CUB domain,	rs1801228	0.0392	YES	1.942E-2;0.0 (pos.17088(	adenocarcinor	Cubilin (intr	Golgi app	Amphetamine-Rela Cyanocob Likely benign	T=15, C=13	T=0.5357, C=0.4643	0.00001	117.908	28	T=11/4, C=3/1
chr12:118817877	A/A	G	A	SNV	SUDS3	SUDS3:intronic:NM_022491.2	1	100		intronic	NM_02249	c.143-90G	p.?	0.11					Sds3-like	rs10850967		YES				Sin3 complex,Sin3-type complex,apoptotic process,cyto		G=0, A=56	G=0.0, A=1.0	0.00001	832.1	56	G=0/0, A=23/3
chr12:119568670	A/A	C	A	SNV	SRRM4	SRRM4:intronic:NM_194286.3	1	100		intronic	NM_19428	c.771+31C	p.?	-0.1					Serine/argini	rs7298583	0.432	YES	5.806E-1 (pos.119568670)		Serine/argi	RNA splicing,cell differentiation,mRNA binding,mRNA pro		C=0, A=36	C=0.0, A=1.0	0.00001	563.744	36	C=0/0, A=21/1
chr12:119568691	G/G	A	G	SNV	SRRM4	SRRM4:intronic:NM_194286.3	1	100		intronic	NM_19428	c.771+52A	p.?	0.44					Serine/argini	rs4298970	0.3322	YES			Serine/argi	RNA splicing,cell differentiation,mRNA binding,mRNA pro		A=0, G=36	A=0.0, G=1.0	0.00001	563.546	36	A=0/0, G=21/1
chr10:17146590	A/G	A	G	SNV	CUBN	CUBN:exonic:NM_001081.3	1	46.43	12	synonymous	NM_00108	c.1245T>C	p.(=)	-0.07				0.58(COSM14	CUB domain,	rs12571671	0.0257	YES	2.241E-1 (pos.17146590)	adenocarcinor	Cubilin (intr	Golgi app	Amphetamine-Rela Cyanocob Likely benign	A=90, G=78	A=0.5357, G=0.4643	0.00001	795.634	168	A=25/65, G=3
chr12:120588852	G/G	A	G	SNV	GCN1	GCN1:intronic:NM_006836.1	1	100		intronic	NM_00683	c.4309+97	p.?	-0.36						rs4503602		YES				RNA binding,cadherin binding,cellular response to amin		A=0, G=24	A=0.0, G=1.0	0.00001	334.791	24	A=0/0, G=16/8
chr6:43014298	CC/CC	TT	CC	MNV	CUL7	CUL7:exonic:NM_001168370.1	2	100	11	missense	NM_00116	c.2690_26	p.Gln897Arg	0.45, 1.45	0.79	43	0		Anaphase-pr	rs61748654,rs87941	0.0322	YES	9.997E-1 (pos.43014298),9.997E-1 (pos.4		Cullin 7	3M compl	Adenocarcinoma;Autistic Diso Benign	TT=0, CC=3	TT=0.0, CC=1.0	0.00001	563.744	36	TT=0/0, CC=17
chr12:120606163	C/C	T	C	SNV	GCN1	GCN1:intronic:NM_006836.1	1	100		intronic	NM_00683	c.1520-59	p.?	-0.28						rs6490286		YES				RNA binding,cadherin binding,cellular response to amin		T=0, C=60	T=0.0, C=1.0	0.00001	903.531	60	T=0/0, C=26/3
chr12:120613484	C/T	C	T	SNV	GCN1	GCN1:intronic:NM_006836.1	1	52.38		intronic	NM_00683	c.1042+65	p.?	0.98						rs61945823		YES				RNA binding,cadherin binding,cellular response to amin		C=10, T=11	C=0.4762, T=0.5238	0.00001	94.4154	21	C=7/3, T=8/3
chr6:43154091	G/G	C	G	SNV	CUL9	CUL9:exonic:NM_015089.3	1	100	4	synonymous	NM_01508	c.1149C>G	p.(=)	0.93					Anaphase-pr	rs941849	8.00E-04		1.0 (pos.43154091)		Cullin 9	ATP binding,cullin-RING ubiquitin ligase complex,cytosol		C=0, G=38	C=0.0, G=1.0	0.00001	603.786	38	C=0/0, G=22/1
chr12:120800823	A/G	A	G	SNV	MSI1	MSI1:intronic:NM_002442.3	1	46.88		intronic	NM_00244	c.402+23T	p.?	-0.5					RNA recognit	rs757123	0.1498	YES	9.997E-1 (pos.120800823)		Musashi, D	RNA bindir	Brain Diseases;Brain Ischemia;Cardiovascula	A=17, G=15	A=0.5313, G=0.4688	0.00001	143.694	32	A=10/7, G=14/
chr12:121097622	A/A	G	A	SNV	CABP1	CABP1:intronic:NM_001033677.1	1	100		intronic	NM_00103	c.655-59G	p.?	-0.14					EF hand,EF-ha	rs529584	0.1358	YES				Golgi membrane,calcium ion binding,calcium-dependen		G=0, A=40	G=0.0, A=1.0	0.00001	644.285	40	G=0/0, A=23/1
chr11:94699507	AT/AT	A	AT	INDEL	CWC15	CWC15:exonic:NM_016403.3	1	100	7	unknown	NM_016403.3			0.95					Cwf15/Cwc15	rs200166995,rs3480	0.0033		1.0 (pos.94699507)			RNA binding,catalytic step 2 spliceosome,mRNA splicing		A=0, AT=42	A=0.0, AT=1.0	0.00001	625.516	42	A=0/0, AT=24/
chr4:49063872	A/A	C	A	SNV	CWH43	CWH43:exonic:NM_025087.2	1	100	16	missense	NM_02508	c.2065C>A	p.His689Asn	3.68	0.09	68	0.961	0.86(COSM4159099)		rs1051447	0.2866	YES	3.925E-1 (pos.49063872)	acute_myeloid_leukaemia		GPI anchor biosynthetic process,integral component of r		C=0, A=76	C=0.0, A=1.0	0.00001	1209.58	76	C=0/0, A=42/3
chr4:76955914	C/C	T	C	SNV	CXCL11	CXCL11:utr_3:NM_005409.4	1	100	4	utr_3	NM_00540	c.317A>G	p.?	1.61					Small cytokin	rs6532111	0.464	YES	9.456E-1 (pos.76955914)		Chemokine	CXCR3 che	Cystitis;Cystitis, Interstitial;Female Urogenit	T=0, C=31	T=0.0, C=1.0	0.00001	465.819	31	T=0/0, C=6/25
chr12:121438844	C/C	T	C	SNV	HNF1A	HNF1A:intronic:NM_000545.6	1	100		intronic	NM_00054	c.1769-24	p.?	0.51					Hepatocyte r	rs735396	0.369	YES	4.983E-1 (pos.121438844)		HNF1 home	DNA bindi	Adenocarcinoma;A Norleucine	T=0, C=48	T=0.0, C=1.0	0.00001	810.432	48	T=0/0, C=26/2
chr4:74863997	C/C	T	C	SNV	CXCL5	CXCL5:exonic:NM_002994.4	1	100	2	synonymous	NM_00299	c.168A>G	p.(=)	-5.44					Small cytokin	rs425535	0.1121	YES	9.671E-1 (pos.74863997)		Chemokine	CXCR chen	Hypersensitivity;Immune System Diseases;Li	T=0, C=42	T=0.0, C=1.0	0.00001	684.837	42	T=0/0, C=21/2
chr12:121615283	C/C	T	C	SNV	P2RX7	P2RX7:intronic:NM_002562.5	1	100		intronic	NM_00256	c.1188+34	p.?	0.8					ATP P2X rece	rs1653598	0.3978	YES	1.112E-1 (pos.121615283)		Purinergic r	ATP bindin	Autoimmune Diseases;Autoimmune Disease	T=0, C=88	T=0.0, C=1.0	0.00001	1451.9	88	T=0/0, C=55/3
chr12:121659684	T/T	C	T	SNV	P2RX4	P2RX4:intronic:NM_001256796.1	1	100		intronic	NM_00125	c.331-32C	p.?	0.26					ATP P2X rece	rs7298368	0.1701	YES	2.944E-1 (pos.121659684)		Purinergic r	ATP bindin	Brain Diseases;Cen Eslicarbazepine acetate	C=0, T=29	C=0.0, T=1.0	0.00001	426.492	29	C=0/0, T=17/1
chr12:121670064	T/T	C	T	SNV	P2RX4	P2RX4:intronic:NM_001256796.1	1	100		intronic	NM_00125	c.933-15(	p.?	-0.24					ATP P2X rece	rs2071271		YES			Purinergic r	ATP bindin	Brain Diseases;Cen Eslicarbazepine acetate	C=0, T=43	C=0.0, T=1.0	0.00001	705.783	43	C=0/0, T=29/1
chr12:121690548	C/C	T	C	SNV	CAMKK2	CAMKK2:intronic:NM_001270485.1	1	100		intronic	NM_00127	c.1108-31	p.?	-0.41					Kinase-like,P	rs2686344	0.2371	YES	6.831E-1 (pos.121690548)		Calcium/ca	ATP binding,CAMKK-AMPK signaling cascade,MAPK casca		T=0, C=44	T=0.0, C=1.0	0.00001	726.568	44	T=0/0, C=34/1
chr12:121690555	C/C	T	C	SNV	CAMKK2	CAMKK2:intronic:NM_001270485.1	1	100		intronic	NM_00127	c.1108-38	p.?	-0.27					Kinase-like,P	rs2686345	0.2372	YES	6.831E-1 (pos.121690555)		Calcium/ca	ATP binding,CAMKK-AMPK signaling cascade,MAPK casca		T=0, C=44	T=0.0, C=1.0	0.00001	726.569	44	T=0/0, C=34/1
chr11:7686596	A/A	G	A	SNV	CYB5R2	CYB5R2:utr_3:NM_016229.4	1	100	9	utr_3	NM_01622	c.840C>T	p.?	-0.04					Ferric reduct	rs13172	0.2452	YES	7.462E-1 (pos.7686596)		Cytochrome	bicarbonate transport,cytochrome-b5 reductase activit		G=0, A=39	G=0.0, A=1.0	0.00001	623.941	39	G=0/0, A=32/7
chr11:7686674	C/C	T	C	SNV	CYB5R2	CYB5R2:exonic:NM_016229.4	1	100	9	synonymous	NM_01622	c.762A>G	p.(=)	5.79				0.13(COSM41	Ferric reduct	rs13854	0.2322	YES	7.626E-1 (pos.7686674)	neoplasm	Cytochrome	bicarbonate transport,cytochrome-b5 reductase activit		T=0, C=45	T=0.0, C=1.0	0.00001	747.26	45	T=0/0, C=31/1
chr11:7687715	C/C	T	C	SNV	CYB5R2	CYB5R2:exonic:NM_016229.4	1	100	8	missense	NM_01622	c.625A>G	p.Asn209Asp	1.34	0.09	23	0		Ferric reduct	rs12801394	0.2294	YES	7.9E-1;0.0 (pos.7687715)		Cytochrome	bicarbonate transport,cytochrome-b5 reductase activit		T=0, C=33	T=0.0, C=1.0	0.00001	504.597	33	T=0/0, C=19/1
chr12:122361929	C/C	CT	C	INDEL	WDR66	WDR66:intronic:NM_144668.5	1	100		intronic	NM_14466	c.747+33C	p.?	0.01, 0.0					WD domain,	rs34885077,rs39782	0.1236	YES	8.457E-1 (pos.122361929)			axoneme,cilium movement,cytoplasm,motile cilium,rac		CT=0, C=51	CT=0.0, C=1.0	0.00001	708.812	51	CT=0/0, C=21/
chr12:122391963	G/G	A	G	SNV	WDR66	WDR66:intronic:NM_144668.5	1	100		intronic	NM_14466	c.1321-63	p.?	-0.45					WD domain,	rs1154513		YES				axoneme,cilium movement,cytoplasm,motile cilium,rac		A=0, G=20	A=0.0, G=1.0	0.00001	263.78	20	A=0/0, G=13/7
chr16:88709712	G/G	A	G	SNV	CYBA	CYBA:utr_3:NM_000101.3	1	100	6	utr_3	NM_0001C	c.637T>C	p.?	-0.57					Cytochrome b	rs7195830	0.2801	YES	6.97E-1 (pos.88709712)		Cytochrome	Golgi app	Arterial Occlusive C Dextromethorphan	A=0, G=64	A=0.0, G=1.0	0.00001	977.355	64	A=0/0, G=20/4
chr12:12247360	A/G	A	G	SNV	BCL2L14	BCL2L14:intronic:NM_138723.1	1	97.06		intronic	NM_13872	c.679-23	p.?	-0.7						rs1641728		YES				apoptotic process,cytosol,endomembrane system,intra		A=1, G=33	A=0.0294, G=0.9706	0.00001	493.098	34	A=0/1, G=24/9
chr12:1224917	G/G	A	G	SNV	ERC1	ERC1:intronic:NM_178040.3	1	100		intronic	NM_17804	c.1402-11	p.?	0.93					FIP domain,R	rs7973282		YES				Golgi mem	Carcinoma;Endocrine Gland Neoplasms;Endc	A=0, G=34	A=0.0, G=1.0	0.00001	524.169	34	A=0/0, G=12/2
chr12:1224972	C/C	A	C	SNV	ERC1	ERC1:intronic:NM_178040.3	1	100		intronic	NM_17804	c.1402-60	p.?	-0.53					FIP domain,R	rs7973392		YES				Golgi mem	Carcinoma;Endocrine Gland Neoplasms;Endc	A=0, C=38	A=0.0, C=1.0	0.00001	603.501	38	A=0/0, C=11/2
chr12:1224980	A/A	G	A	SNV	ERC1	ERC1:intronic:NM_178040.3	1	100		intronic	NM_17804	c.1402-52	p.?	0.22					FIP domain,R	rs61912159	0.2374	YES				Golgi mem	Carcinoma;Endocrine Gland Neoplasms;Endc	G=0, A=39	G=0.0, A=1.0	0.00001	623.979	39	G=0/0, A=11/2
chr12:122684966	C/C	G	C	SNV	LRRC43	LRRC43:intronic:NM_001098519.1	1	100		intronic	NM_00109	c.1491+89	p.?	-0.53					Leucine Rich	rs11060679								G=0, C=47	G=0.0, C=1.0	0.00001	789.33	47	G=0/0, C=20/2
chr2:172411273	A/A	G	A	SNV	CYBRD1	CYBRD1:exonic:NM_024843.3	1	100	4	missense	NM_02484	c.797G>A	p.Ser266Asn	-1.72	0.73	46	0	0.14(COSM62	Eukaryotic cy	rs10455	0.3407	YES	3.709E-1 (pos.17241127	neoplasm		brush border membrane,cellular iron ion homeostasis,e		G=0, A=21	G=0.0, A=1.0	0.00001	281.234	21	G=0/0, A=10/1
chr12:122966373	C/C	T	C	SNV	ZCCHC8	ZCCHC8:intronic:NM_017612.4	1	100		intronic	NM_01761	c.875+119	p.?	-0.39					PSP,Zinc knuc	rs4758692		YES			Zinc finger C	RNA bindi	Intellectual Disability;Mental Disorders;Nerv	T=0, C=25	T=0.0, C=1.0	0.00001	352.848	25	T=0/0, C=13/1
chr12:122983460	G/G	A	G	SNV	ZCCHC8	ZCCHC8:intronic:NM_017612.4	1	100		intronic	NM_01761	c.202-46T	p.?	-0.87					PSP,Zinc knuc	rs7314638	0.2526	YES	8.422E-1;2.52E-4 (pos.122983460)		Zinc finger C	RNA bindi	Intellectual Disability;Mental Disorders;Nerv	A=0, G=51	A=0.0, G=1.0	0.00001	754.584	51	A=0/0, G=29/2
chr12:122991478	G/G	C	G	SNV	RSRC2	RSRC2:intronic:NM_023012.5	1	100		intronic	NM_02301	c.1036-8G	p.?	0.23					Small acidic p	rs9988963	0.2653	YES	8.408E-1;0.0 (pos.122991478)			RNA binding,protein binding		C=0, G=29	C=0.0, G=1.0	0.00001	427.603	29	C=0/0, G=13/1
chr12:122993011	T/T	C	T	SNV	RSRC2	RSRC2:intronic:NM_023012.5	1	100		intronic	NM_02301	c.806-17G	p.?	-6.68					Small acidic p	rs10773230	0.459	YES	4.437E-1 (pos.122993011)			RNA binding,protein binding		C=0, T=48	C=0.0, T=1.0	0.00001	810.203	48	C=0/0, T=4/44
chr12:123003317	T/C	T	C	SNV	RSRC2	RSRC2:intronic:NM_023012.5	1	53.33		intronic	NM_02301	c.398+69A	p.?	-0.06					Small acidic p	rs11058562		YES				RNA binding,protein binding		T=14, C=16	T=0.4667, C=0.5333	0.00001	159.925	30	T=7/7, C=7/9
chr12:12303637	T/T	C	T	SNV	LRP6	LRP6:intronic:NM_002336.2	1	100		intronic	NM_00233	c.2994+13	p.?	-0.39					Coagulation f	rs7979485		YES			Low densit	Golgi app	Arterial Occlusive Diseases;Arteriosclerosis;	C=0, T=49	C=0.0, T=1.0	0.00001	831.63	49	C=0/0, T=23/2
chr12:123089746	A/ATATACT	A	ATATACT	INDEL	KNTC1	KNTC1:intronic:NM_014708.5	6	51.92		intronic	NM_0147C	c.5387-10	p.?	0.76					Rough deal p	rs146383155,rs376139602		YES			Kinetochor	RZZ complex,actin cytoskeleton,cell division,condensed		A=75, ATAT	A=0.4808, ATATACT=	0.00001	50	156	
chr12:123110654	C/C	T	C	SNV	KNTC1	KNTC1:intronic:NM_014708.5	1	100		intronic	NM_0147C	c.6607-98	p.?	0.56					Rough deal p	rs3803005		YES			Kinetochor	RZZ complex,actin cytoskeleton,cell division,condensed		T=0, C=76	T=0.0, C=1.0	0.00001	1209.58	76	T=0/0, C=50/2
chr5:156721863	TC/TC	T	TC,TCC	INDEL	CYFIP2	CYFIP2:exonic:NM_001037333.2	1	100	4	frameshiftInsertion	NM_00103	c.282_283	p.Pro94fs	-1.06					Cytoplasmic	rs200051515,rs3977	0.001		1.0 (pos.156721863)	ductal_carcinc	Cytoplasmi	Fc-gamma receptor signaling pathway involved in phago		T=0, TC=34	T=0.0, TC=1.0, TCC=0	0.00001	372.466	34	T=0/0, TC=23/
chr12:123281967	A/A	G	A	SNV	CCDC62	CCDC62:intronic:NM_201435.4	1	100		intronic	NM_20143	c.861+25G	p.?	-2.18						rs10744394	0.0457	YES	9.872E-1 (pos.123281967)		Coiled-coil c	cellular re	Basal Ganglia Diseases;Brain Diseases;Centr	G=0, A=63	G=0.0, A=1.0	0.00001	958.915	63	G=0/0, A=21/4
chr8:145690308	T/T	C	T	SNV	CYHR1	CYHR1:utr_5:NM_138496.1	1	100	1	utr_5	NM_13849	c.-24G>A	p.?	-0.38						rs2721166	0.0041	YES	7.976E-1;0.0 (pos.145690308)		Cysteine-a	integral component of membrane,nucleoplasm,perinucl		C=0, T=52	C=0.0, T=1.0	0.00001	764.645	52	C=0/0, T=13/3
chr12:123339344	G/G	A	G	SNV	HIP1R	HIP1R:intronic:NM_003959.2	1	100		intronic	NM_00395	c.719-108	p.?	-2.61					ANTH domair	rs2292136		YES			Huntingtin-	SH3 domain binding,actin binding,actin filament binding		A=0, G=56	A=0.0, G=1.0	0.00001	832.099	56	A=0/0, G=34/2
chr12:123339712	G/G	A	G	SNV	HIP1R	HIP1R:intronic:NM_003959.2	1	100		intronic	NM_00395	c.852+37A	p.?	-0.71					ANTH domair	rs2292138,rs774146	0.1066	YES	0.0 (pos.123339693),7.532E-1 (pos.1233		Huntingtin-	SH3 domain binding,actin binding,actin filament binding		A=0, G=24	A=0.0, G=1.0	0.00001	334.745	24	A=0/0, G=9/15
chr12:123345347	A/A	G	A	SNV	HIP1R	HIP1R:intronic:NM_003959.2	1	100		intronic	NM_00395	c.2766+16	p.?	-0.39					ANTH domair	rs7306948	0.0488	YES	9.892E-1 (pos.123345347)		Huntingtin-	SH3 domain binding,actin binding,actin filament binding		G=0, A=49	G=0.0, A=1.0	0.00001	831.606	49	G=0/0, A=34/1
chr12:123345360	C/C	T	C	SNV	HIP1R	HIP1R:intronic:NM_003959.2	1	100		intronic	NM_00395	c.2766+29	p.?	-1.73					ANTH domair	rs7298176	0.0479	YES	9.894E-1 (pos.123345360)		Huntingtin-	SH3 domain binding,actin binding,actin filament binding		T=0, C=49	T=0.0, C=1.0	0.00001	831.255	49	T=0/0, C=33/1
chr8:143995761	T/T	C	T	SNV	CYP11B2	CYP11B2:exonic:NM_000498.3	1	100	5	synonymous	NM_00049	c.873G>A	p.(=)	-5.22					Cytochrome I	rs4536	0.0207	YES	4.798E-1 (pos.143995761)		Cytochrome	C21-steroi	Adrenal Gland Dise Aldosterone Benign	C=0, T=68	C=0.0, T=1.0	0.00001	1053.56	68	C=0/0, T=35/3
chr12:123352179	T/T	C	T	SNV	VPS37B	VPS37B:intronic:NM_024667.2	1	100		intronic	NM_02466	c.367-25G	p.?	-2.31					Modifier of r	rs3825143	0.1092	YES	7.539E-1 (pos.123352179)		Vacuolar pr	ESCRT I complex,calcium-dependent protein binding,cyto		C=0, T=32	C=0.0, T=1.0	0.00001	484.84	32	C=0/0, T=4/28
chr15:75047426	T/T	C	T	SNV	CYP1A2	CYP1A2:exonic:NM_000761.4	1	100	7	synonymous	NM_00076	c.1548C>T	p.(=)	0.58					Cytochrome I	rs2470890	0.346	YES	1.628E-1 (pos.75047426)		Cytochrome	alkaloid m	Carcinoma;Carcino 2-PHENYL-4H-BENZOCHI	C=0, T=65	C=0.0, T=1.0	0.00001	992.365	65	C=0/0, T=25/4
chr12:123477311	C/C	T	C	SNV	PITPNM2	PITPNM2:intronic:NM_020845.2	1	100		intronic	NM_02084	c.2234-95	p.?	-1.95					DDHD domai	rs937564		YES				calcium ion binding,cytosol,endomembrane system,inte		T=0, C=27	T=0.0, C=1.0	0.00001	394.498	27	T=0/0, C=14/1
chr12:123665695	A/G	A	G	SNV	MPHOSPH9	MPHOSPH9:intronic:NM_022782.3	1	52.27		intronic	NM_02278	c.2289+15	p.?	-0.19						rs1727306	0.2071	YES	9.991E-1 (pos.123665695)		M-phase ph	Golgi app	Autoimmune Diseases;Autoimmune Disease	A=21, G=23	A=0.4773, G=0.5227	0.00001	260.154	44	A=11/10, G=1
chr12:123812207	T/T	C	T	SNV	SBNO1	SBNO1:intronic:NM_001167856.2	1	100		intronic	NM_00116	c.1538+47	p.?	-0.17					C-terminal dc	rs7959415	0.0384	YES	7.463E-1 (pos.123812207)		Strawberry	biological	Mental Disorders;Schizophrenia;Schizophren	C=0, T=64	C=0.0, T=1.0	0.00001	982.934	64	C=0/0, T=24/4
chr12:123812611	G/G	A	G	SNV	SBNO1	SBNO1:intronic:NM_001167856.2	1	100		intronic	NM_00116	c.1297-37	p.?	1.01					C-terminal dc	rs10846517	0.0384	YES	7.463E-1 (pos.123812611)		Strawberry	biological	Mental Disorders;Schizophrenia;Schizophren	A=0, G=49	A=0.0, G=1.0	0.00001	831.632	49	A=0/0, G=20/2
chr12:123813493	A/A	C	A	SNV	SBNO1	SBNO1:intronic:NM_001167856.2	1	100		intronic	NM_00116	c.1135-51	p.?	0.34					C-terminal dc	rs7301163	0.0377	YES	7.421E-1 (pos.123813493)		Strawberry	biological	Mental Disorders;Schizophrenia;Schizophren	C=0, A=68	C=0.0, A=1.0	0.00001	1043.21	68	C=0/0, A=26/4
chr2:204154552	T/T	C	T	SNV	CYP20A1	CYP20A1:exonic:NM_177538.2	1	100	10	missense	NM_17753	c.1036C>T	p.Leu346Phe	4.72	0.02	22	0.005	0.91(COSM44	Cytochrome I	rs1048013	0.4376	YES	2.977E-1 (pos.20415455)	acute_myeloic_leukaemia		heme binding,integral component of membrane,iron ion		C=0, T=71	C=0.0, T=1.0	0.00001	1115.27	71	C=0/0, T=23/4
chr12:124090453	G/G	A	G	SNV	DDX55	DDX55:intronic:NM_020936.2	1	100		intronic	NM_02093	c.109-25A	p.?	-2.75					DEAD/DEAH b	rs786436	1.00E-04		9.999E-1 (pos.124090453)			ATP binding,ATP-dependent RNA helicase activity,RNA bi		A=0, G=67	A=0.0, G=1.0	0.00001	1032.85	67	A=0/0, G=31/3
chr20:52786219	G/A	G	A	SNV	CYP24A1	CYP24A1:exonic:NM_000782.4	1	57.63	4	synonymous	NM_00078	c.552C>T	p.(=)	-1.72				0.02(COSM4C	Cytochrome I	rs2296241	0.461	YES	4.212E-1 (pos.52786219)	acute_myeloic	Cytochrome	1-alpha,2!	Abortion, Spontane CTA018,Ca Benign	G=25, A=34	G=0.4237, A=0.5763	0.00001	356.499	59	G=19/6, A=15/
chr12:124220055	C/C	T	C	SNV	ATP6V0A2	ATP6V0A2:intronic:NM_012463.3	1	100		intronic	NM_01246	c.732-23T	p.?	-0.59					V-type ATPas	rs2271660,rs748705	0.3931	YES	0.0;0.0 (pos.124220054),9.439E-1;0.0 (p		ATPase, H+	ATP hydrol	Congenital, Hereditary, and Ne Benign	T=0, C=52	T=0.0, C=1.0	0.00001	767.014	52	T=0/0, C=41/1
chr20:52781091	C/T	C	T	SNV	CYP24A1	CYP24A1:exonic:NM_000782.4	1	50	6	synonymous	NM_00078	c.744G>A	p.(=)	-4.36					Cytochrome I	rs6068816	0.1038	YES	3.67E-1 (pos.52781091)		Cytochrome	1-alpha,2!	Abortion, Spontane CTA018,Ca Benign	C=17, T=17	C=0.5, T=0.5	0.00001	172.009	34	C=6/11, T=6/1
chr12:124267638	G/G	A	G	SNV	DNAH10	DNAH10:intronic:NM_207437.3	1	100		intronic	NM_20743	c.659-16A	p.?	-1.19					AAA domain (	rs11057354	0.0983	YES	9.997E-1 (pos.124267638)		Dynein, axo	ATP binding,ATP-dependent microtubule n Benign		A=0, G=109	A=0.0, G=1.0	0.00001	1718.9	109	A=0/0, G=45/6
chr2:219679475	G/T	G	T	SNV	CYP27A1	CYP27A1:exonic:NM_000784.3	1	53.13	8	missense	NM_00078	c.1471G>T	p.Ala491Ser	-0.23	1	99	0.085	0.15(COSM3C	Cytochrome I	rs72551323	0.0019		0.0;0.0 (pos.219679475)	squamous_cel	Cytochrome	bile acid b	Arterial Occlusive C Chenodeo Conflicting in	G=15, T=17	G=0.4688, T=0.5313	0.00001	173.404	32	G=9/6, T=7/10
chr12:124268458	G/G	A	G	SNV	DNAH10	DNAH10:intronic:NM_207437.3	1	100		intronic	NM_20743	c.817-36A	p.?	-0.7					AAA domain (	rs7303002	0.3952	YES	9.99E-1 (pos.124268458)		Dynein, axo	ATP binding,ATP-dependent microtubule motor activity,		A=0, G=51	A=0.0, G=1.0	0.00001	754.584	51	A=0/0, G=20/3
chr19:41350664	T/T	A	T	SNV	CYP2A6	CYP2A6:exonic:NM_000762.5	1	100	8	missense	NM_00076	c.1175T>A	p.Phe392Tyr	1.7	0.46	22	0.012	0.32(COSM45	Cytochrome I	rs1809810		YES	9.876E-1 (pos.41350664)	squamous_cel	Cytochrome	arachidon	Adenocarcinoma;B (5-(PYRIDIN-3-YL)FURAN-2	A=0, T=30	A=0.0, T=1.0	0.00001	446.635	30	A=0/0, T=15/1
chr12:124285804	T/G	T	G	SNV	DNAH10	DNAH10:intronic:NM_207437.3	1	59.09		intronic	NM_20743	c.2109-24	p.?	1.33					AAA domain (	rs111937173	0.0041	YES	2.429E-3 (pos.124285804)		Dynein, axo	ATP binding,ATP-dependent microtubule motor activity,		T=9, G=13	T=0.4091, G=0.5909	0.00001	122.568	22	T=5/4, G=3/10
chr12:124330440	G/A	G	A	SNV	DNAH10	DNAH10:intronic:NM_207437.3	1	50		intronic	NM_20743	c.5276+24	p.?	-0.5					AAA domain (	rs4930728	0.2528	YES	3.478E-4 (pos.124330440)		Dynein, axo	ATP binding,ATP-dependent microtubule motor activity,		G=11, A=11	G=0.5, A=0.5	0.00001	94.7167	22	G=5/6, A=6/5
chr12:124408244	A/G	A	G	SNV	DNAH10	DNAH10:intronic:NM_207437.3	1	47.62		intronic	NM_20743	c.11153-6	p.?	-2.28					AAA domain (	rs75587926		YES			Dynein, axo	ATP binding,ATP-dependent microtubule motor activity,		A=11, G=10	A=0.5238, G=0.4762	0.00001	82.2806	21	A=8/3, G=7/3
chr12:124408255	A/G	A	G	SNV	DNAH10	DNAH10:intronic:NM_207437.3	1	45.45		intronic	NM_20743	c.11153-5	p.?	-0.16					AAA domain (	rs76233642		YES			Dynein, axo	ATP binding,ATP-dependent microtubule motor activity,		A=12, G=10	A=0.5455, G=0.4545	0.00001	81.7626	22	A=8/4, G=7/3
chr12:124412881	C/T	C	T	SNV	DNAH10	DNAH10:intronic:NM_207437.3	1	57.14		intronic	NM_20743	c.11752-5	p.?	-1.97					AAA domain (	rs78688187		YES			Dynein, axo	ATP binding,ATP-dependent microtubule motor activity,		C=15, T=20	C=0.4286, T=0.5714	0.00001	218.729	35	C=9/6, T=14/6
chr10:96741053	A/C	A	C	SNV	CYP2C9	CYP2C9:exonic:NM_000771.3	1	57.14	7	missense	NM_00077	c.1075A>C	p.Ile359Leu	0.03	0.01	5	0.002		Cytochrome I	rs1057910	0.0659	YES	3.379E-2 (pos.96741053)		Cytochrome	arachidon	Acute Kidney Injury 4-Hydroxy drug respons	A=12, C=16	A=0.4286, C=0.5714	0.00001	161.77	28	A=10/2, C=12/
chr12:124838772	G/G	C	G	SNV	NCOR2	NCOR2:intronic:NM_006312.5	1	100		intronic	NM_00631	c.3590-30	p.?	-0.16					G-protein pat	rs1244082,rs386767	2.00E-04		0.0;0.0 (pos.124838771),1.0 (pos.12483		Nuclear rec	DNA bindi	Cocaine-Related Disorders;Mental Disorders	C=0, G=33	C=0.0, G=1.0	0.00001	502.561	33	C=0/0, G=12/2
chr12:124838774	A/A	T	A	SNV	NCOR2	NCOR2:intronic:NM_006312.5	1	100		intronic	NM_00631	c.3590-32	p.?	-0.75					G-protein pat	rs3817108,rs386767	0.2728	YES	5.26E-1 (pos.124838774)		Nuclear rec	DNA bindi	Cocaine-Related Disorders;Mental Disorders	T=0, A=32	T=0.0, A=1.0	0.00001	490.551	32	T=0/0, A=12/2
chr22:42523943	G/G	A	G	SNV	CYP2D6,LOC101929829	CYP2D6:exonic:NM_000106.5, LOC101929829:exonic_nc:NI	1	100	6	missense	NM_0001C	c.886T>C	p.Cys296Arg	-1.2	1	180	0	0(COSM1130	Cytochrome I	rs16947	0.3427	YES	8.515E-1 (pos.42523943)	adenocarcinor	Cytochrome	alkaloid ca	Acute Kidney Injury 1-(2-Phenylethyl)-4-phen	A=0, G=33	A=0.0, G=1.0	0.00001	504.598	33	A=0/0, G=15/1
chr12:124885249	T/T	C	T	SNV	NCOR2	NCOR2:intronic:NM_006312.5	1	100		intronic	NM_00631	c.1641-30	p.?	-2.24					G-protein pat	rs2293514	0.3311	YES	7.38E-1 (pos.124885249)		Nuclear rec	DNA bindi	Cocaine-Related Disorders;Mental Disorders	C=0, T=20	C=0.0, T=1.0	0.00001	263.691	20	C=0/0, T=11/9
chr12:124885290	C/C	T	C	SNV	NCOR2	NCOR2:intronic:NM_006312.5	1	100		intronic	NM_00631	c.1641-71	p.?	-0.18					G-protein pat	rs2293513,rs386767259		YES			Nuclear rec	DNA bindi	Cocaine-Related Disorders;Mental Disorders	T=0, C=26	T=0.0, C=1.0	0.00001	371.42	26	T=0/0, C=11/1

chr10:135351362	C/C	T	C	SNV	CYP2E1	CYP2E1:exonic:NM_000773.3	1	100	8	synonymous	NM_00077	c.1263T>C	p.(=)	0.1					Cytochrome I	rs2515641	0.1173	YES	8.274E-1 (pos.135351362)		Cytochrome	Golgi mem	Adenocarcinoma;A	Acetaminc	Benign	T=0, C=46	T=0.0, C=1.0	0.00001	768.313	46	T=0/0, C=19/2
chr12:125299830	C/T	C	T	SNV	SCARB1	SCARB1:intronic:NM_005505.4	1	95.65		intronic	NM_00550	c.285-170	p.?	-2.23					CD36 family	rs4765615		YES			Scavenger r	1-phospha	Adenocarcinoma;C	Phosphatidyl serine		C=1, T=22	C=0.0435, T=0.9565	0.00001	288.995	23	C=1/0, T=3/19
chr10:135352509	A/A	T	A	SNV	CYP2E1	CYP2E1:utr_3:NM_000773.3	1	100	9	utr_3	NM_00077	c.1523T>A	p.?	0.34					Cytochrome I	rs2480257	0.2072	YES	5.689E-1;0.0 (pos.135352509)		Cytochrome	Golgi mem	Adenocarcinoma;A	Acetaminophen,Ademet		T=0, A=67	T=0.0, A=1.0	0.00001	1034.43	67	T=0/0, A=18/4
chr10:135352514	G/G	A	G	SNV	CYP2E1	CYP2E1:utr_3:NM_000773.3	1	100	9	utr_3	NM_00077	c.1528A>G	p.?	0.17					Cytochrome I	rs2480256	0.2071	YES	5.719E-1 (pos.135352514)		Cytochrome	Golgi mem	Adenocarcinoma;A	Acetaminophen,Ademet		A=0, G=67	A=0.0, G=1.0	0.00001	1034.39	67	A=0/0, G=18/4
chr19:16040292	A/G	A	G	SNV	CYP4F11	CYP4F11:exonic:NM_001128932.1	1	53.13	3	synonymous	NM_00112	c.318T>C	p.(=)	-0.53				0(COSM4131	Cytochrome I	rs3765070	0.4027	YES	4.156E-1 (pos.16040292)	acute_myeloic	Cytochrome	blood coagulation,endoplasmic reticulum membrane,fa				A=15, G=17	A=0.4688, G=0.5313	0.00001	173.559	32	A=9/6, G=7/10
chr12:125437160	A/A	G	A	SNV	DHX37	DHX37:intronic:NM_032656.3	1	100		intronic	NM_03265	c.2696-44	p.?	-0.17					DEAD/DEAH b	rs74403469	0.0384	YES	5.168E-2 (pos.125437160)		DEAH box p	ATP binding,ATP-dependent RNA helicase activity,RNA bi				G=0, A=55	G=0.0, A=1.0	0.00001	814.563	55	G=0/0, A=21/3
chr12:125438414	G/G	A	G	SNV	DHX37	DHX37:intronic:NM_032656.3	1	100		intronic	NM_03265	c.2695+12	p.?	-1.04					DEAD/DEAH b	rs12367953	0.4588	YES	8.41E-1 (pos.125438414)		DEAH box p	ATP binding,ATP-dependent RNA helicase activity,RNA bi				A=0, G=39	A=0.0, G=1.0	0.00001	623.943	39	A=0/0, G=26/1
chr19:16034714	A/G	A	G	SNV	CYP4F11	CYP4F11:exonic:NM_001128932.1	1	51.85	7	missense	NM_00112	c.826T>C	p.Cys276Arg	-0.38	1	180	0	0(COSM4131	Cytochrome I	rs8104361	0.1962	YES	9.92E-1 (pos.16034714)	neoplasm	Cytochrome	blood coagulation,endoplasmic reticulum membrane,fa				A=13, G=14	A=0.4815, G=0.5185	0.00001	132.17	27	A=7/6, G=4/10
chr19:15784386	T/T	C	T	SNV	CYP4F12	CYP4F12:exonic:NM_023944.3	1	100	2	missense	NM_02394	c.47C>T	p.Thr16Met	0.42	0.01	81	0.941	0.03(COSM41	Cytochrome I	rs16995378	0.0697	YES	1.0 (pos.15784386)	neoplasm	Cytochrome	alkane 1-n	Nervous System Dis	Fingolimod		C=0, T=45	C=0.0, T=1.0	0.00001	747.385	45	C=0/0, T=20/2
chr12:125438549	G/G	A	G	SNV	DHX37	DHX37:intronic:NM_032656.3	1	100		intronic	NM_03265	c.2579-7T	p.?	-0.19					DEAD/DEAH b	rs4606565	0.4208	YES	8.449E-1 (pos.125438549)		DEAH box p	ATP binding,ATP-dependent RNA helicase activity,RNA bi				A=0, G=35	A=0.0, G=1.0	0.00001	534.442	35	A=0/0, G=19/1
chr12:125451767	A/A	G	A	SNV	DHX37	DHX37:intronic:NM_032656.3	1	100		intronic	NM_03265	c.1409-3C	p.?	4.59					DEAD/DEAH b	rs60252902	0.1042	YES	1.333E-1 (pos.125451767)		DEAH box p	ATP binding,ATP-dependent RNA helicase activity,RNA bi				G=0, A=27	G=0.0, A=1.0	0.00001	389.986	27	G=0/0, A=5/22
chr12:125453542	T/T	G	T	SNV	DHX37	DHX37:intronic:NM_032656.3	1	100		intronic	NM_03265	c.1192-28	p.?	-0.48					DEAD/DEAH b	rs11614657	0.129	YES	2.006E-1 (pos.125453542)		DEAH box p	ATP binding,ATP-dependent RNA helicase activity,RNA bi				G=0, T=20	G=0.0, T=1.0	0.00001	263.784	20	G=0/0, T=16/4
chr19:15807305	T/C	T	C	SNV	CYP4F12	CYP4F12:exonic:NM_023944.3	1	51.85	12	synonymous	NM_02394	c.1380T>C	p.(=)	-0.17				0.02(COSM37	Cytochrome I	rs688755	0.2462	YES	2.081E-2 (pos.15807305)	adenocarcinoi	Cytochrome	alkane 1-n	Nervous System Dis	Fingolimod		T=13, C=14	T=0.4815, C=0.5185	0.00001	125.583	27	T=9/4, C=7/7
chr12:126003799	T/C	T	C	SNV	TMEM132B	TMEM132B:intronic:NM_052907.3	1	45.45		intronic	NM_05290	c.1092-18	p.?	-0.8					Mature oligo	rs2254785,rs386767400,rs8796		YES				biological	Melanoma;Neoplasms;Neoplasms by Histolc			T=30, C=25	T=0.5455, C=0.4545	0.00001	232.566	55	T=22/8, C=14/
chr19:15793235	T/C	T	C	SNV	CYP4F12	CYP4F12:exonic:NM_023944.3	1	45.83	6	missense	NM_02394	c.562T>C	p.Cys188Arg	0.3	1	180	0	0(COSM3756	Cytochrome I	rs2285888	0.4659	YES	7.201E-1 (pos.15793235)	adenocarcinoi	Cytochrome	alkane 1-n	Nervous System Dis	Fingolimod		T=13, C=11	T=0.5417, C=0.4583	0.00001	93.4398	24	T=6/7, C=5/6
chr19:15763721	A/A	G	A	SNV	CYP4F3	CYP4F3:exonic:NM_001199209.1	1	100	9	synonymous	NM_00119	c.1074G>A	p.(=)	0.53				0.21(COSM14	Cytochrome I	rs1805042	0.3542	YES	6.456E-1 (pos.15763721)	adenocarcinoi	Cytochrome	20-aldehyde-leukotriene B4 20-monooxygenase activity				G=0, A=26	G=0.0, A=1.0	0.00001	371.42	26	G=0/0, A=9/17
chr19:15730498	GC/GC	G	GC	INDEL	CYP4F8	CYP4F8:exonic:NM_007253.3	1	100	5	unknown	NM_007253.3			2.81				0.83(COSM42	Cytochrome I	rs55848814	4.00E-04	YES	1.0 (pos.15730498)	small_cell_car	Cytochrome	alkane 1-monooxygenase activity,aromatase activity,en				G=0, GC=57	G=0.0, GC=1.0	0.00001	842.895	57	G=0/0, GC=16/
chr4:187122319	T/G	T	G	SNV	CYP4V2	CYP4V2:exonic:NM_207352.3	1	52.38	7	synonymous	NM_20735	c.810T>G	p.(=)	-0.5				0.14(COSM14	Cytochrome I	rs207482233,rs3736	0.3492	YES	2.313E-3;0.0;0.0 (pos.187	acute_myeloic	Cytochrome	endoplasr	Congenital, Hereditary, and Ne		Benign	T=10, G=11	T=0.4762, G=0.5238	0.00001	95.3002	21	T=4/6, G=4/7
chr12:129299675	C/G	C	G	SNV	SLC15A4	SLC15A4:intronic:NM_145648.3	1	56.36		intronic	NM_14564	c.547-60G	p.?	-0.79					Major Facilit	rs2306683		YES			Solute carri	integral cc	Autoimmune Diseases;Connective Tissue Dis			C=24, G=31	C=0.4364, G=0.5636	0.00001	319.019	55	C=9/15, G=13/
chr7:91743150	A/G	A	G	SNV	CYP51A1	CYP51A1:exonic:NM_000786.3	1	45.45	10	synonymous	NM_00078	c.1359T>C	p.(=)	3.13				0.89(COSM44	Cytochrome I	rs7797834	0.4015	YES	1.758E-1 (pos.91743150)	acute_myeloic	Cytochrome	cholesterol biosynthetic proce		1-(2S)-2-(4-CHLOROBENZY		A=18, G=15	A=0.5455, G=0.4545	0.00001	143.145	33	A=10/8, G=12/
chr12:130847389	G/G	A	G	SNV	PIWIL1	PIWIL1:intronic:NM_004764.4	1	100		intronic	NM_00476	c.2041+8A	p.?	-1.57				0.01(COSM44	Argonaute lir	rs2242363	0.3172	YES	3.648E-1 (pos.13084738	acute_myeloic	Piwi, Droso	P granule,	Mental Disorders:Substance-Related Disorde			A=0, G=76	A=0.0, G=1.0	0.00001	1209.58	76	A=0/0, G=31/4
chr3:42917047	G/G	A	G	SNV	CYP8B1	CYP8B1:exonic:NM_004391.2	1	100	1	missense	NM_00439	c.262T>C	p.Ser88Pro	0.81		74			Cytochrome I	rs9865715	6.00E-04		1.0;0.0 (pos.42917047)		Cytochrome	7alpha-hy	Bile Duct Diseases;Biliary Tract Diseases;Chc			A=0, G=23	A=0.0, G=1.0	0.00001	316.748	23	A=0/0, G=20/3
chr22:37699377	T/C	T	C	SNV	CYTH4	CYTH4:exonic:NM_013385.4	1	50	8	synonymous	NM_01338	c.630T>C	p.(=)	-2.13				0.05(COSM37	PH domain,Se	rs2239822	0.3792	YES	7.015E-1 (pos.37699377)	carcinoma,he	Cytohesin 4	ARF guanyl-nucleotide exchange factor activity,Golgi me				T=15, C=15	T=0.5, C=0.5	0.00001	144.939	30	T=11/4, C=11/
chr4:5021021	A/A	G	A	SNV	CYTL1	CYTL1:exonic:NM_018659.2	1	100	1	missense	NM_01865	c.152C>T	p.Ser51Leu	-0.7	0.03	145	0.067	0.01(COSM17	Cytokine-like	rs35755546	0.022	YES	0.0 (pos.5021021)	carcinoma,tra	Cytokine-lik	cartilage homeostasis,chondrocyte differentiation,chon				G=0, A=27	G=0.0, A=1.0	0.00001	389.969	27	G=0/0, A=24/3
chr12:131471608	C/C	T	C	SNV	ADGRD1	ADGRD1:intronic:NM_198827.4	1	100		intronic	NM_19882	c.491-32T	p.?	-0.08					7 transmemb	rs1547577	0.0047	YES	9.966E-1 (pos.131471608)		Adhesion G	G-protein coupled receptor activity,G-protein coupled re				T=0, C=34	T=0.0, C=1.0	0.00001	524.186	34	T=0/0, C=17/1
chr14:23044002	T/T	G	T	SNV	DAD1	DAD1:utr_3:NM_001344.3	1	100	2	utr_3	NM_00134	c.343C>A	p.?	-0.05					DAD family	rs386775535,rs7621	0.2938	YES	7.831E-1;0.0 (pos.23044002)		Defender ai	apoptotic process,blastocyst development,dolichyl-dipl				G=0, T=61	G=0.0, T=1.0	0.00001	921.822	61	G=0/0, T=26/3
chr3:49548008	G/G	C	G	SNV	DAG1	DAG1:exonic:NM_001177639.2	1	100	2	missense	NM_00117	c.41C>G	p.Ser14Trp	2.07	0.8	177	0		Dystroglycan	rs2131107	5.00E-04		1.0 (pos.49548008)		Dystrophin-	Golgi lume	Cobblestone Lissencephaly;Cc		Benign	C=0, G=71	C=0.0, G=1.0	0.00001	1106.17	71	C=0/0, G=51/2
chr3:49570200	T/T	C	T	SNV	DAG1	DAG1:exonic:NM_001177639.2	1	100	3	synonymous	NM_00117	c.2256C>T	p.(=)	1.75					Dystroglycan	rs1801143	0.3138	YES	5.919E-2 (pos.49570200)		Dystrophin-	Golgi lume	Cobblestone Lissencephaly;Cc		Benign/Likely	C=0, T=63	C=0.0, T=1.0	0.00001	958.599	63	C=0/0, T=23/4
chr3:49053219	G/G	A	G	SNV	DALRD3,WDR6	DALRD3:intronic:NM_001009996.2, WDR6:utr_3:NM_01803	1	100	6	utr_3	NM_01803	c.3954A>G	p.?, p.?	-5.62					DALR anticod	rs7100	0.2421	YES	9.999E-1 (pos.49053219)		WD repeat-	ATP binding,COP9 signalosome,RNA binding,arginine-tRN				A=0, G=26	A=0.0, G=1.0	0.00001	371.42	26	A=0/0, G=13/1
chr9:90265015	T/T	C	T	SNV	DAPK1	DAPK1:exonic:NM_004938.3	1	100	16	synonymous	NM_00493	c.1608C>T	p.(=)	0.66				0.83(COSM16	Ankyrin repe	rs3818584	0.3793	YES	6.017E-1 (pos.90265015)	carcinoma	Death-asso	ATP bindin	Adenocarcinoma;A	5,6-Dihydro-BenzoHCinn		C=0, T=30	C=0.0, T=1.0	0.00001	446.639	30	C=0/0, T=6/24
chr12:132414769	T/T	G	T	SNV	PUS1	PUS1:intronic:NM_025215.5	1	100		intronic	NM_02521	c.303+89G	p.?	-0.43					tRNA pseudo	rs10902480		YES			Pseudourin	RNA bindir	Brain Diseases:Brain Diseases, Metabolic:Br			G=0, T=58	G=0.0, T=1.0	0.00001	866.818	58	G=0/0, T=17/4
chr12:132635521	C/C	T	C	SNV	NOC4L	NOC4L:intronic:NM_024078.2	1	100		intronic	NM_02407	c.902-5T>C	p.?	-0.03				0.09(COSM37	CBF/Mak21 fa	rs7397057,rs762464	0.2935	YES	0.0;0.0 (pos.132635520),	carcinoma,he	Nucleolar c	Noc4p-Nop14p complex,RNA binding,integral componen				T=0, C=21	T=0.0, C=1.0	0.00001	281.235	21	T=0/0, C=6/15
chr12:133196773	T/G	T	G	SNV	P2RX2	P2RX2:intronic:NM_170683.3	1	50		intronic	NM_17068	c.555-80T	p.?	-1.15					ATP P2X rece	rs7964872		YES			Purinergic r	ATP bindin	Brain Diseases;Central Nervous System Dise			T=27, G=27	T=0.5, G=0.5	0.00001	262.28	54	T=18/9, G=20/
chr12:133210985	C/T	C	T	SNV	POLE	POLE:intronic:NM_006231.3	1	50		intronic	NM_00623	c.5812-21	p.?	-0.26					DNA polymer	rs5745005	0.4152	YES	8.129E-1 (pos.133210985)			3'-5' exonu	Colonic Diseases;C	Cladribine		C=19, T=19	C=0.5, T=0.5	0.00001	199.911	38	C=13/6, T=10/
chr2:159660870	C/T	C	T	SNV	DAPL1	DAPL1:exonic:NM_001017920.2	1	50	2	synonymous	NM_00101	c.135C>T	p.(=)	-0.28					Death-associ	rs17810398	0.1343	YES	7.817E-4;0.0 (pos.159660870)			G-protein	Nutrition Disorders:Nutritional and Metaboli			C=61, T=61	C=0.5, T=0.5	0.00001	626.471	122	C=30/31, T=23
chr12:133349893	G/G	A	G	SNV	GOLGA3	GOLGA3:intronic:NM_005895.3	1	100		intronic	NM_00589	c.4308-13	p.?	0.43						rs3741484,rs759668	0.0684	YES	5.317E-1;0.0 (pos.133349893)		Golgi autoa	Golgi apparatus,Golgi cisterna membrane,Golgi membra				A=0, G=50	A=0.0, G=1.0	0.00001	852.711	50	A=0/0, G=31/1
chr12:51636259	T/T	C	T	SNV	DAZAP2	DAZAP2:exonic:NM_001136266.1	1	100	4	synonymous	NM_00113	c.447C>T	p.(=)	0.74					DAZ associat	rs1049467	0.2317	YES	8.91E-1 (pos.51636259)			WW domain binding,cytoplasm,identical protein bindin				C=0, T=32	C=0.0, T=1.0	0.00001	485.141	32	C=0/0, T=11/2
chr12:133625276	A/T	A	T	SNV	ZNF84	ZNF84:intronic:NM_001127372.2	1	95.35		intronic	NM_00112	c.143-63A	p.?	-0.23					C2H2-type zir	rs7314140		YES				DNA binding,face morphogenesis,hemopoiesis,intracell				A=2, T=41	A=0.0465, T=0.9535	0.00001	643.453	43	A=0/2, T=21/2
chr17:42786170	G/A	G	A	SNV	DBF4B	DBF4B:utr_5:NM_145663.2	1	97.06	1	utr_5	NM_14566	c.-19G>A	p.?	0.12					DBF zinc finge	rs115803922	0.0343	YES	0.0 (pos.42786170)		DBF4, S. cer	activation of protein kinase activity,aggresome,cell cycle				G=1, A=33	G=0.0294, A=0.9706	0.00001	492.96	34	G=0/1, A=31/2
chr12:13366599	C/T	C	T	SNV	EMP1	EMP1:intronic:NM_001423.2	1	54.46		intronic	NM_00142	c.176-16C	p.?	-0.83					PMP-22/EMP	rs2277395	0.2043	YES	1.444E-1 (pos.13366599)		Epithelial m	bleb asser	Abortion, Spontaneous:Female Urogenital Di			C=143, T=1	C=0.4554, T=0.5446	0.00001	1924.94	314	C=80/63, T=81
chr12:133795960	G/G	A	G	SNV	ANHX	ANHX:intronic:NM_001191054.1	1	100		intronic	NM_00119	c.969-7T>C	p.?	-0.13					Homeobox KI	rs36140725		YES	7.819E-1 (pos.133795960)			nucleus,regulation of transcription, DNA-templated,seq				A=0, G=21	A=0.0, G=1.0	0.00001	281.235	21	A=0/0, G=13/8
chr12:13720272	G/G	A	G	SNV	GRIN2B	GRIN2B:intronic:NM_000834.4	1	100		intronic	NM_00083	c.2360-75	p.?	0.19					Bacterial ext	rs4764010		YES			Glutamate	MAPK casc	Brain Diseases;Car	Acamprosate,Acetylcyst		A=0, G=25	A=0.0, G=1.0	0.00001	352.877	25	A=0/0, G=6/19
chr9:136505114	G/G	A	G	SNV	DBH	DBH:exonic:NM_000787.3	1	100	2	synonymous	NM_00078	c.486A>G	p.(=)	7.4				0.11(COSM14	Copper type I	rs1108580	0.4664	YES	1.283E-1 (pos.13650511	adenocarcinoi	Dopamine-l	L-ascorbic	Autonomic Nervous	Capsaicin,	Benign	A=0, G=47	A=0.0, G=1.0	0.00001	785.808	47	A=0/0, G=22/2
chr5:176894756	T/T	C	T	SNV	DBN1	DBN1:exonic:NM_004395.3	1	100	4	synonymous	NM_00439	c.318G>A	p.(=)	-2				0.06(COSM17	Cofilin/tropo	rs2545794	0.4748	YES	2.514E-1 (pos.17689475	neoplasm,trar	Drebrin-1	actin bind	Brain Diseases;Central Nervous System Dise			C=0, T=25	C=0.0, T=1.0	0.00001	353.019	25	C=0/0, T=9/16
chr3:137881265	A/A	T	A	SNV	DBR1	DBR1:exonic:NM_016216.3	1	100	8	synonymous	NM_01621	c.1101A>T	p.(=)	-0.54					Calcineurin-l	rs3732839	0.31	YES	5.929E-1 (pos.137881265)		Debranchin	RNA bindir	Amyotrophic Lateral Sclerosis;Central Nervo			T=0, A=71	T=0.0, A=1.0	0.00001	1111.48	71	T=0/0, A=37/3
chr12:45410075	G/G	A	G	SNV	DBX2	DBX2:exonic:NM_001004329.2	1	100	4	synonymous	NM_00100	c.1014T>C	p.(=)	-1.95					Homeobox d	rs1224442	0.4074	YES	8.142E-1 (pos.45410075)			nucleus,regulation of transcription, DNA-templated,seq				A=0, G=21	A=0.0, G=1.0	0.00001	280.94	21	A=0/0, G=9/12
chr12:15273932	T/T	C	T	SNV	RERG	RERG:intronic:NM_032918.2	1	100		intronic	NM_03291	c.118+65G	p.?	-0.71					50S ribosome	rs2247574	0.3369	YES			Ras-like anc	GDP binding,GTP binding,GTPase activity,cytosol,estroge				C=0, T=71	C=0.0, T=1.0	0.00001	1111.48	71	C=0/0, T=39/3
chr12:15273957	G/G	A	G	SNV	RERG	RERG:intronic:NM_032918.2	1	100		intronic	NM_03291	c.118+40T	p.?	0.38					50S ribosome	rs2247568	0.3399	YES	5.756E-1 (pos.15273957)		Ras-like anc	GDP binding,GTP binding,GTPase activity,cytosol,estroge				A=0, G=70	A=0.0, G=1.0	0.00001	1092.09	70	A=0/0, G=37/3
chr12:15652220	G/G	A	G	SNV	PTPRO	PTPRO:intronic:NM_030667.2	1	100		intronic	NM_03066	c.509-156	p.?	0.04					Fibronectin t	rs2193165		YES			Protein tyrc	Wnt-prote	Digestive System Diseases;Digestive System			A=0, G=25	A=0.0, G=1.0	0.00001	352.982	25	A=0/0, G=9/16
chr12:15652330	C/C	T	C	SNV	PTPRO	PTPRO:intronic:NM_030667.2	1	100		intronic	NM_03066	c.509-46T	p.?	-0.07					Fibronectin t	rs4764197	0.033	YES	8.658E-1 (pos.15652330)		Protein tyrc	Wnt-prote	Digestive System Diseases;Digestive System			T=0, C=33	T=0.0, C=1.0	0.00001	504.598	33	T=0/0, C=8/25
chr12:15702007	C/T	C	T	SNV	PTPRO	PTPRO:intronic:NM_030667.2	1	45.45		intronic	NM_03066	c.2305-21	p.?	-0.14					Fibronectin t	rs7132826	0.2931	YES	2.91E-1 (pos.15702007)		Protein tyrc	Wnt-prote	Digestive System Diseases;Digestive System			C=12, T=10	C=0.5455, T=0.4545	0.00001	81.7626	22	C=2/10, T=1/9
chr12:15748020	T/T	C	T	SNV	PTPRO	PTPRO:intronic:NM_030667.2	1	100		intronic	NM_03066	c.*3667+2	p.?	0.29					Fibronectin t	rs1558787	0.0043	YES	9.615E-1;0.0 (pos.15748020)		Protein tyrc	Wnt-prote	Digestive System Diseases;Digestive System			C=0, T=77	C=0.0, T=1.0	0.00001	1229.07	77	C=0/0, T=31/4
chr12:45417666	C/C	T	C	SNV	DBX2	DBX2:exonic:NM_001004329.2	1	100	3	missense	NM_00100	c.511A>G	p.Met171Val	-0.14	1	21	0		Homeobox d	rs2731038	0.4119	YES	8.329E-1;0.0;0.0 (pos.45417666)			nucleus,regulation of transcription, DNA-templated,seq				T=0, C=32	T=0.0, C=1.0	0.00001	485.141	32	T=0/0, C=18/1
chrX:125299188	G/G	T	G	SNV	DCAF12L2	DCAF12L2:exonic:NM_001013628.2	1	100	1	synonymous	NM_00101	c.720A>C	p.(=)	-1.4				0(COSM6352	WD domain,	rs12014937	0.2966	YES	5.023E-1 (pos.12529918	carcinoma						T=0, G=34	T=0.0, G=1.0	0.00001	523.402	34	T=0/0, G=16/1
chrX:125299467	T/T	G	T	SNV	DCAF12L2	DCAF12L2:exonic:NM_001013628.2	1	100	1	missense	NM_00101	c.441C>A	p.His147Gln	0.74		24		0.25(COSM37	WD domain,	rs10126452	0.2974	YES	5.014E-1 (pos.12529946	adenocarcinoma,carcinoma						G=0, T=60	G=0.0, T=1.0	0.00001	902.708	60	G=0/0, T=15/4
chr14:73404680	A/C	A	C	SNV	DCAF4	DCAF4:utr_5:NM_001163509.1	1	52.38	2	utr_5	NM_00116	c.-41A>C	p.?	1.83					WD domain,	rs3815500	0.2022	YES	4.446E-1 (pos.73404680)		DDB1- and (	Cul4-RING E3 ubiquitin ligase complex,RNA binding,RNA s				A=10, C=11	A=0.4762, C=0.5238	0.00001	95.299	21	A=7/3, C=8/3
chr12:18641326	C/C	T	C	SNV	PIK3C2G	PIK3C2G:intronic:NM_004570.5	1	100		intronic	NM_00457	c.2382-57	p.?	-0.05					C2 domain,PI	rs7137852					Phosphatid	1-phosphatidylinositol-3-kinase activity,1-phosphatidyli				T=0, C=106	T=0.0, C=1.0	0.00001	1662.7	106	T=0/0, C=33/7
chr12:18848131	C/C	T	C	SNV	PLCZ1	PLCZ1:intronic:NM_033123.3	1	100		intronic	NM_03312	c.1292-11	p.?	-0.74					C2 domain,PI	rs1487070		YES			Phospholip	calcium ion binding,calcium ion transport,cytosol,egg ac				T=0, C=70	T=0.0, C=1.0	0.00001	1092.08	70	T=0/0, C=29/4
chr12:1889823	A/A	G	A	SNV	ADIPOR2	ADIPOR2:intronic:NM_024551.2	1	100		intronic	NM_02455	c.650+20G	p.?	-1.34					Haemolysin-l	rs767870	0.1526	YES	9.09E-1 (pos.1889823)		Adiponectir	G-protein	Female Urogenital Diseases;Female Urogeni			G=0, A=24	G=0.0, A=1.0	0.00001	334.792	24	G=0/0, A=10/1
chr12:1950011	AC/AC	A	AC	INDEL	CACNA2D4	CACNA2D4:intronic:NM_172364.4	1	100		intronic	NM_17236	c.2471-26	p.?	2.76					Neuronal vol	rs58188909	0.1238	YES	9.999E-1 (pos.1950011)		Calcium cha	calcium io	Congenital, Hereditary, and Neonatal Diseas			A=0, AC=39	A=0.0, AC=1.0	0.00001	625.417	39	A=0/0, AC=26/
chr12:1955664	T/T	G	T	SNV	CACNA2D4	CACNA2D4:intronic:NM_172364.4	1	100		intronic	NM_17236	c.2342+96	p.?	-3.09					Neuronal vol	rs7137553		YES			Calcium cha	calcium io	Congenital, Hereditary, and Neonatal Diseas			G=0, T=148	G=0.0, T=1.0	0.00001	2442.4	148	G=0/0, T=100/
chr12:1955667	GA/GA	G	GA	INDEL	CACNA2D4	CACNA2D4:intronic:NM_172364.4	1	100		intronic	NM_17236	c.2342+93	p.?	0.41					Neuronal voltage-dependent calcium channel alpha 2acd,VWA N-terminal,von Willebrand factor typ						Calcium cha	calcium io	Congenital, Hereditary, and Neonatal Diseas			G=0, GA=15	G=0.0, GA=1.0	0.00001	1971.02	156	G=0/0, GA=10
chr12:1955890	C/C	T	C	SNV	CACNA2D4	CACNA2D4:intronic:NM_172364.4	1	100		intronic	NM_17236	c.2247-35	p.?	-1.33					Neuronal vol	rs7137876	0.1916	YES	9.988E-1 (pos.1955890)		Calcium cha	calcium io	Congenital, Hereditary, and Neonatal Diseas			T=0, C=39	T=0.0, C=1.0	0.00001	613.294	39	T=0/0, C=20/1
chr12:1969262	C/C	T	C	SNV	CACNA2D4	CACNA2D4:intronic:NM_172364.4	1	100		intronic	NM_17236	c.1940+49	p.?	-0.54					Neuronal vol	rs10848582	0.4724	YES	3.45E-1 (pos.1969262)		Calcium cha	calcium io	Congenital, Hereditary, and Neonatal Diseas			T=0, C=22	T=0.0, C=1.0	0.00001	298.892	22	T=0/0, C=14/8
chr14:73404752	G/C	G	C	SNV	DCAF4	DCAF4:exonic:NM_001163509.1	1	50	2	missense	NM_00116	c.32G>C	p.Gly11Ala	1.14	1	60	0.004	0.08(COSM36	WD domain,	rs2302588	0.1047	YES	1.963E-1;0.0 (pos.73404	acute_myeloic	DDB1- and (	Cul4-RING E3 ubiquitin ligase complex,RNA binding,RNA s				G=11, C=11	G=0.5, C=0.5	0.00001	94.6067	22	G=8/3, C=7/4
chr12:20724988	A/A	G	A	SNV	PDE3A	PDE3A:intronic:NM_000921.4	1	100		intronic	NM_00092	c.1011+15	p.?	0.44					3'5'-cyclic nu	rs7966459		YES			Phosphodie	3',5'-cyclic	Brachydactyly;Card	Aminophylline,Amrinone		G=0, A=35	G=0.0, A=1.0	0.00001	540.937	35	G=0/0, A=26/9
chr4:41984118	T/T	C	T	SNV	DCAF4L1	DCAF4L1:exonic:NM_001029955.3	1	100	1	synonymous	NM_00102	c.309C>T	p.(=)	0.87				0.24(COSM4159076)		rs2660320	0.2234	YES	9.685E-1 (pos.41984118)	ductal_carcinoma,neoplasm						C=0, T=33	C=0.0, T=1.0	0.00001	504.594	33	C=0/0, T=13/2
chr12:20803323	C/C	A	C	SNV	PDE3A	PDE3A:intronic:NM_000921.4	1	100		intronic	NM_00092	c.2770-56	p.?	-1.09					3'5'-cyclic nu	rs9804769		YES			Phosphodie	3',5'-cyclic	Brachydactyly;Card	Aminophylline,Amrinone		A=0, C=32	A=0.0, C=1.0	0.00001	489.523	32	A=0/0, C=11/2
chr12:20868247	C/T	C	T	SNV	SLCO1C1	SLCO1C1:intronic:NM_001145946.1	1	47.62		intronic	NM_00114	c.676+28C	p.?	0.05					Kazal-type se	rs34288910	0.1317	YES	8.218E-3 (pos.20868247)		Solute carri	bile acid and bile salt transpor		Conjugated estrogens,D		C=11, T=10	C=0.5238, T=0.4762	0.00001	82.29	21	C=6/5, T=8/2
chr12:20968828	T/T	C	T	SNV	SLCO1B3	SLCO1B3:intronic:NM_019844.3	1	100		intronic	NM_01984	c.84+72C>	p.?	0.81					Kazal-type se	rs10734710		YES			Solute carri	basolater	Colonic Diseases;C	Ambrisentan,Atorvastat		C=0, T=47	C=0.0, T=1.0	0.00001	789.327	47	C=0/0, T=21/2
chr12:21011235	C/C	T	C	SNV	SLCO1B3	SLCO1B3:intronic:NM_019844.3	1	100		intronic	NM_01984	c.227-138	p.?	-1.49					Kazal-type se	rs4149114		YES			Solute carri	basolater	Colonic Diseases;C	Ambrisentan,Atorvastat		T=0, C=24	T=0.0, C=1.0	0.00001	334.777	24	T=0/0, C=9/15
chr12:21011296	G/G	A	G	SNV	SLCO1B3	SLCO1B3:intronic:NM_019844.3	1	100		intronic	NM_01984	c.227-77A	p.?	1.86					Kazal-type se	rs4149115		YES			Solute carri	basolater	Colonic Diseases;C	Ambrisentan,Atorvastat		A=0, G=24	A=0.0, G=1.0	0.00001	334.703	24	A=0/0, G=9/15
chr12:21011310	C/C	T	C	SNV	SLCO1B3	SLCO1B3:intronic:NM_019844.3	1	100		intronic	NM_01984	c.227-63T	p.?	0.33					Kazal-type se	rs4149116		YES			Solute carri	basolater	Colonic Diseases;C	Ambrisentan,Atorvastat		T=0, C=24	T=0.0, C=1.0	0.00001	334.772	24	T=0/0, C=9/15
chr18:50432602	G/G	C	G	SNV	DCC	DCC:exonic:NM_005215.3	1	100	3	missense	NM_00521	c.601C>G	p.Arg201Gly	-0.02	0.05	125	0.798	0.73(COSM50	Fibronectin t	rs2229080	0.3671	YES	5.527E-1 (pos.50432602)	adenocarcinoi	Deleted in (	anterior/p	Biliary Tract Diseases;Biliary Tract Neoplasm			C=0, G=27	C=0.0, G=1.0	0.00001	389.945	27	C=0/0, G=7/20
chr12:21030582	A/A	G	A	SNV	SLCO1B3	SLCO1B3:intronic:NM_019844.3	1	100		intronic	NM_01984	c.971-124	p.?	0.25					Kazal-type se	rs386761023,rs4149135		YES			Solute carri	basolater	Colonic Diseases;C	Ambrisentan,Atorvastat		G=0, A=20	G=0.0, A=1.0	0.00001	263.786	20	G=0/0, A=11/9
chr12:21036270	G/G	T	G	SNV	SLCO1B3	SLCO1B3:intronic:NM_019844.3	1	100		intronic	NM_01984	c.1498-82	p.?	0.07					Kazal-type se	rs2053097		YES			Solute carri	basolater	Colonic Diseases;C	Ambrisentan,Atorvastat		T=0, G=21	T=0.0, G=1.0	0.00001	281.214	21	T=0/0, G=9/12
chr6:24174955	C/C	G	C	SNV	DCDC2	DCDC2:utr_3:NM_016356.4	1	100	10	utr_3	NM_01635	c.1434C>G	p.?	0.11					Doublecortin	rs9358755	0.1834	YES	9.728E-1 (pos.24174955)		Doublecort	axoneme,	Communication Disorders:Deafness;Dyslexia			G=0, C=20	G=0.0, C=1.0	0.00001	263.786	20	G=0/0, C=12/8
chr12:21609127	C/C	T	C	SNV	PYROXD1	PYROXD1:intronic:NM_024854.4	1	100		intronic	NM_02485	c.750+98T	p.?	-1.23					Pyridine nucl	rs4367993		YES			Pyridine nu	cellular response to oxidative stress,nucleus,oxidation-				T=0, C=37	T=0.0, C=1.0	0.00001	583.707	37	T=0/0, C=15/2
chr12:21629987	C/C	T	C	SNV	RECQL	RECQL:intronic:NM_032941.2	1	100		intronic	NM_03294	c.868-61A	p.?	-0.48					DEAD/DEAH b	rs917854	0.0013	YES			RecQ helica	ATP bindin	Autoimmune Diseases;Breast Diseases;Brea			T=0, C=51	T=0.0, C=1.0	0.00001	754.584	51	T=0/0, C=14/3
chr12:21665207	A/G	A	G	SNV	GOLT1B	GOLT1B:intronic:NM_016072.4	1	45.45		intronic	NM_01607	c.297-22A	p.?	-0.31					Got1/Sft2-like	rs61926342	0.1276	YES	1.044E-2 (pos.21665207)		Golgi trans	Golgi membrane,endoplasmic reticulum,integral compo				A=36, G=30	A=0.5455, G=0.4545	0.00001	285.787	66	A=14/22, G=1
chr12:21695584	A/G	A	G	SNV	GYS2	GYS2:intronic:NM_021957.3	1	58.33		intronic	NM_02195	c.1550-59	p.?	0.68					Glycogen syn	rs3741841		YES			Glycogen sy	cell cortex	Carbohydrate Metabolism, Inborn Errors;Con			A=20, G=28	A=0.4167, G=0.5833	0.00001	343.108	48	A=10/10, G=1
chr12:22068849	T/T	G	T	SNV	ABCC9	ABCC9:intronic:NM_020297.3	1	100		intronic	NM_02029	c.574-5C>	p.?	1.66					ABC transpor	rs3759236	0.4075	YES	7.864E-1 (pos.22068849)		ATP-binding	ATP bindin	Acromegaly;Arrhytl	ATP,Glybu	Benign/Likely	G=0, T=36	G=0.0, T=1.0	0.00001	563.739	36	G=0/0, T=24/1
chr12:22078838	G/G	T	G	SNV	ABCC9	ABCC9:intronic:NM_020297.3	1	100		intronic	NM_02029	c.406+38A	p.?	-0.36					ABC transpor	rs2277405	0.3926	YES	7.898E-1 (pos.22078838)		ATP-binding	ATP bindin	Acromegaly;Arrhytl	ATP,Glybu	Benign	T=0, G=25	T=0.0, G=1.0	0.00001	352.953	25	T=0/0, G=15/1
chr4:155156207	A/A	G	A	SNV	DCHS2	DCHS2:exonic:NM_017639.3	1	100	25	synonymous	NM_01763	c.8232C>T	p.(=)	-0.44					Cadherin dor	rs7655799	0.077	YES	7.861E-1;0.0 (pos.155156207)		Dachsous, I	biological	Alzheimer Disease;Brain Diseases;Central Ne			G=0, A=24	G=0.0, A=1.0	0.00001	334.792	24	G=0/0, A=12/1
chr4:155256205	A/A	G	A	SNV	DCHS2	DCHS2:exonic:NM_017639.3	1	100	8	missense	NM_01763	c.1031C>T	p.Ser344Leu	0.69	0.39	145	0.002		Cadherin dor	rs6858712	6.00E-04		1.0 (pos.155256205)		Dachsous, I	biological	Alzheimer Disease;Brain Diseases;Central Ne			G=0, A=89	G=0.0, A=1.0	0.00001	1471.04	89	G=0/0, A=25/6
chr12:25242776	T/T	C	T	SNV	LRMP	LRMP:intronic:NM_001204126.1	1	100		intronic	NM_00120	c.465+52C	p.?	-0.24					MRVI1 protei	rs10743538,rs75588	0.2319	YES	0.0 (pos.25242773),8.837E-1 (pos.25242		Lymphoid-r	azurophil granule membrane,chromosome,endoplasmic				C=0, T=36	C=0.0, T=1.0	0.00001	563.439	36	C=0/0, T=9/27
chr3:36779707	A/A	G	A	SNV	DCLK3	DCLK3:exonic:NM_033403.1	1	100	2	synonymous	NM_03340	c.444C>T	p.(=)	-1.19				0.04(COSM14	Protein kinas	rs3796187	0.4316	YES	1.592E-1 (pos.36779707)	NS,acute_mye	Doublecort	ATP binding,cytoplasm,intracellular,intracellular signal				G=0, A=41	G=0.0, A=1.0	0.00001	664.569	41	G=0/0, A=28/1
chr12:26592288	A/A	G	A	SNV	ITPR2	ITPR2:intronic:NM_002223.3	1	100		intronic	NM_00222	c.6451-36	p.?	0.74					Inositol 1,4,5	rs2270960	0.4107	YES	3.625E-1 (pos.26592288)		Inositol 1,4	calcium io	Bone Diseases;Bon	Caffeine		G=0, A=94	G=0.0, A=1.0	0.00001	1568.71	94	G=0/0, A=47/4
chr12:27059224	A/A	G	A	SNV	INTS13	INTS13:intronic:NM_018164.2	1	100		intronic	NM_01816	c.2081+11	p.?	0.29					Cell cycle anc	rs1488052	0.2288	YES	9.997E-1 (pos.27059224)			cell division,centrosome localization,cytoplasm,flagella				G=0, A=41	G=0.0, A=1.0	0.00001	664.65	41	G=0/0, A=19/2
chr12:27059422	C/C	T	C	SNV	INTS13	INTS13:intronic:NM_018164.2	1	100		intronic	NM_01816	c.1946-52	p.?	-0.04					Cell cycle anc	rs1001545219,rs148	0.3695	YES	0.0;0.0 (pos.27059421),9.548E-1 (pos.27059422)			cell division,centrosome localization,cytoplasm,flagella				T=0, C=25	T=0.0, C=1.0	0.00001	341.957	25	T=0/0, C=15/1
chr10:115609915	G/G	C	G	SNV	DCLRE1A	DCLRE1A:exonic:NM_001271816.1	1	100	3	missense	NM_00127	c.949G>C	p.Asp317His	0.91	0.01	81	0.993		DNA repair m	rs3750898	0.2322	YES	8.435E-1 (pos.115609915)		DNA cross-l	5'-3' exodeoxyribonuclease activity,cell cycle,cell divisio				C=0, G=61	C=0.0, G=1.0	0.00001	921.779	61	C=0/0, G=30/3
chr5:112328377	G/G	A	G	SNV	DCP2	DCP2:exonic:NM_152624.5	1	100	4	synonymous	NM_15262	c.342A>G	p.(=)	0.7				0.09(COSM44	Dcp2, box A d	rs3733969	0.2424	YES	7.893E-1 (pos.11232837	hepatocellula	Decapping (	5'-3' exoribonuclease activity,P-body,RISC complex,RNA r				A=0, G=28	A=0.0, G=1.0	0.00001	408.718	28	A=0/0, G=4/24
chr12:27175640	G/G	A	G	SNV	MED21	MED21:intronic:NM_004264.4	1	100		intronic	NM_00426	c.42+86A>	p.?	-0.05					Subunit 21 of	rs1872191		YES	9.998E-1 (pos.27175640)		Mediator cc	DNA-directed 5'-3' RNA polymerase activity,RNA polymer				A=0, G=48	A=0.0, G=1.0	0.00001	796.624	48	A=0/0, G=23/2
chr12:27234505	T/T	A	T	SNV	C12orf71	C12orf71:intronic:NM_001080406.1	1	100		intronic	NM_00108	c.517-105	p.?	-0.28					Domain of un	rs34457591		YES								A=0, T=45	A=0.0, T=1.0	0.00001	747.392	45	A=0/0, T=13/3
chr12:274572	C/C	G	C	SNV	IQSEC3	IQSEC3:intronic:NM_001170738.1	1	100		intronic	NM_00117	c.2710-28	p.?	-1.47					PH domain,Se	rs11061700	0.0016	YES	1.0;0.0 (pos.274572)		IQ motif- an	ARF guanyl-nucleotide exchange factor activity,actin cytc				G=0, C=38	G=0.0, C=1.0	0.00001	603.783	38	G=0/0, C=15/2
chr12:27461984	C/C	T	C	SNV	STK38L	STK38L:intronic:NM_015000.3	1	100		intronic	NM_01500	c.310-63T	p.?	-1.74					Protein kinas	rs6487599		YES			Serine/thre	ATP binding,actin binding,actin cytoskeleton,cytoplasm,				T=0, C=24	T=0.0, C=1.0	0.00001	334.792	24	T=0/0, C=11/1
chr5:112349070	C/C	T	C	SNV	DCP2	DCP2:exonic:NM_152624.5	1	100	11	synonymous	NM_15262	c.1152T>C	p.(=)	-4.64				0.02(COSM41	Dcp2, box A d	rs9326869	0.2437	YES	7.975E-1 (pos.11234907	hepatocellula	Decapping (	5'-3' exoribonuclease activity,P-body,RISC complex,RNA r				T=0, C=36	T=0.0, C=1.0	0.00001	563.744	36	T=0/0, C=19/1
chr16:20873767	G/G	T	G	SNV	DCUN1D3	DCUN1D3:exonic:NM_173475.3	1	100	2	synonymous	NM_17347	c.94A>C	p.(=)	1.4				0.7(COSM399	Cullin binding	rs7187522	0.2815	YES	3.758E-1;0.0 (pos.20873	acute_myeloic	DCN1 doma	cullin family protein binding,molecular_function,negativ				T=0, G=29	T=0.0, G=1.0	0.00001	427.603	29	T=0/0, G=13/1
chr1:85787114	T/T	A	T	SNV	DDAH1	DDAH1:utr_3:NM_012137.3	1	100	6	utr_3	NM_01213	c.879T>A	p.?	-0.04					Amidinotrans	rs233116			1.0;0.0 (pos.85787114)		Dimethylar	amino aci	Digestive System Di	L-Citrulline		A=0, T=36	A=0.0, T=1.0	0.00001	563.742	36	A=0/0, T=26/1
chr1:85787115	AC/AC	A	AC	INDEL	DDAH1	DDAH1:utr_3:NM_012137.3	1	100	6	utr_3	NM_01213	c.877_878	p.?	0.49					Amidinotrans	rs763169081			0.0 (pos.85787115)		Dimethylar	amino aci	Digestive System Di	L-Citrulline		A=0, AC=34	A=0.0, AC=1.0	0.00001	479.943	34	A=0/0, AC=25/
chr1:85816134	C/C	T	C	SNV	DDAH1	DDAH1:exonic:NM_012137.3	1	100	4	synonymous	NM_01213	c.561A>G	p.(=)	-0.27				0.57(COSM37	Amidinotrans	rs2230820	0.1998	YES	1.423E-1 (pos.85816134)	acute_myeloic	Dimethylar	amino aci	Digestive System Di	L-Citrulline		T=0, C=30	T=0.0, C=1.0	0.00001	446.582	30	T=0/0, C=19/1
chr14:53529669	T/T	A	T	SNV	DDHD1	DDHD1:exonic:NM_001160148.1	1	100	7	synonymous	NM_00116	c.1758T>A	p.(=)	-3.74					DDHD domai	rs4898778	0.1613	YES	9.77E-1;2.776E-3 (pos.53529669)		DDHD doma	cytoplasm	Nervous System Diseases;Neu		Benign	A=0, T=38	A=0.0, T=1.0	0.00001	603.786	38	A=0/0, T=12/2
chr14:53619463	A/A	G	A	SNV	DDHD1,LOC101927620	LOC101927620:upstream:NR_110062.1, DDHD1:exonic:NM	1	100	1	synonymous	NM_00116	c.354C>T	p.(=)	0.14				0.42(COSM37	DDHD domai	rs2358188		YES	9.754E-1 (pos.53619463)	adenocarcinoi	DDHD doma	cytoplasm	Nervous System Diseases;Neu		Benign	G=0, A=44	G=0.0, A=1.0	0.00001	726.537	44	G=0/0, A=15/2
chr14:53619544	A/A	G	A	SNV	DDHD1,LOC101927620	LOC101927620:upstream:NR_110062.1, DDHD1:exonic:NM	1	100	1	synonymous	NM_00116	c.273C>T	p.(=)	2.88				0.76(COSM41	DDHD domai	rs2358189	0.1622	YES	9.739E-1;0.0 (pos.536195	neoplasm	DDHD doma	cytoplasm	Nervous System Diseases;Neu		Benign	G=0, A=42	G=0.0, A=1.0	0.00001	678.271	42	G=0/0, A=11/3
chr6:30864829	C/C	T	C	SNV	DDR1	DDR1:exonic:NM_001954.4	1	100	15	synonymous	NM_00195	c.1797T>C	p.(=)	0.59				0.04(COSM11	F5/8 type C d	rs1049623	0.3822	YES	6.532E-1 (pos.30864829)	adenocarcinoma,adenoma		ATP bindin	Adenocarcinoma;C	Imatinib		T=0, C=84	T=0.0, C=1.0	0.00001	1370.17	84	T=0/0, C=42/4
chr6:30867106	T/T	C	T	SNV	DDR1	DDR1:utr_3:NM_001954.4	1	100	19	utr_3	NM_00195	c.2664C>T	p.?	-0.4					F5/8 type C d	rs1049628	0.1847	YES	5.768E-1 (pos.30867106)			ATP bindin	Adenocarcinoma;C	Imatinib		C=0, T=58	C=0.0, T=1.0	0.00001	867.369	58	C=0/0, T=28/3
chr12:31132479	C/C	A	C	SNV	TSPAN11	TSPAN11:intronic:NM_001080509.2	1	100		intronic	NM_00108	c.352-22A	p.?	0.34					Tetraspanin f	rs35096	0.0429	YES	8.976E-1 (pos.31132479)			cell surface receptor signaling pathway,integral compon				A=0, C=51	A=0.0, C=1.0	0.00001	753.967	51	A=0/0, C=29/2
chr12:31244846	C/G	C	G	SNV	DDX11	DDX11:intronic:NM_001257144.1	1	50		intronic	NM_00125	c.1242+41	p.?	0.18					DEAD_2,Helic	rs1808348	0.4377	YES	8.164E-1 (pos.31244846)		DEAD/H box	4 iron, 4 sulfur cluster binding,ATP binding,ATP-depender				C=39, G=39	C=0.5, G=0.5	0.00001	412.504	78	C=32/7, G=34/
chr12:31250804	G/A	G	A	SNV	DDX11	DDX11:intronic:NM_001257144.1	1	47.62		intronic	NM_00125	c.1763-15	p.?	-3.39					DEAD_2,Helic	rs12366526	0.115	YES	6.94E-2;0.0 (pos.31250804)		DEAD/H box	4 iron, 4 sulfur cluster binding,ATP binding,ATP-depender				G=11, A=10	G=0.5238, A=0.4762	0.00001	81.6893	21	G=4/7, A=7/3
chr12:3149483	G/G	A	G	SNV	TEAD4	TEAD4:intronic:NM_201443.2	1	100		intronic	NM_20144	c.805-48A	p.?	-2.16					TEA/ATTS dor	rs7294804	0.2722	YES	9.36E-1;0.0 (pos.3149483)		TEA domain	DNA binding,DNA binding transcription factor activity,RN				A=0, G=33	A=0.0, G=1.0	0.00001	504.598	33	A=0/0, G=15/1
chr12:31551350	T/T	C	T	SNV	DENND5B	DENND5B:intronic:NM_144973.3	1	100		intronic	NM_14497	c.3069-54	p.?	0					DENN (AEX-3)	rs1798769		YES			DENN doma	Rab guanyl-nucleotide exchange factor activity,calcium (				C=0, T=36	C=0.0, T=1.0	0.00001	563.744	36	C=0/0, T=14/2
chr12:31632449	C/C	T	C	SNV	DENND5B	DENND5B:intronic:NM_144973.3	1	100		intronic	NM_14497	c.904+74A	p.?	-1.04					DENN (AEX-3)	rs3741878		YES			DENN doma	Rab guanyl-nucleotide exchange factor activity,calcium (				T=0, C=20	T=0.0, C=1.0	0.00001	263.782	20	T=0/0, C=6/14
chr12:31850220	T/T	C	T	SNV	AMN1	AMN1:intronic:NM_001113402.1	1	100		intronic	NM_00111	c.591+75G	p.?	-0.19					Leucine Rich	rs2388926		YES								C=0, T=34	C=0.0, T=1.0	0.00001	524.186	34	C=0/0, T=24/1
chr12:49230035	T/G	T	G	SNV	DDX23	DDX23:exonic:NM_004818.2	1	58.33	11	synonymous	NM_00481	c.1251A>C	p.(=)	3.78				0.1(COSM375	DEAD/DEAH b	rs1057908	0.4886	YES	6.003E-1;0.0 (pos.49230	Wilms_tumou	Dead box p	ATP binding,ATP-dependent RNA helicase activity,ATP-de				T=20, G=28	T=0.4167, G=0.5833	0.00001	343.411	48	T=10/10, G=11
chr16:68057096	C/C	T	C	SNV	DDX28,DUS2	DDX28:exonic:NM_018380.3, DUS2:utr_5:NM_001271762.1	1	100	1	missense	NM_01838	c.10A>G, c	p.Thr4Ala, p.?	-1.87		58		0(COSM3754	DEAD/DEAH b	rs237831	0.074	YES	9.996E-1;2.366E-4;1.183	adenocarcinoi	DEAD/H box	ATP binding,ATP-dependent RNA helicase activity,RNA bi				T=0, C=38	T=0.0, C=1.0	0.00001	603.772	38	T=0/0, C=18/2
chr17:35984410	C/C	T	C	SNV	DDX52	DDX52:exonic:NM_007010.4	1	100	9	missense	NM_00701	c.1207A>G	p.Met403Val	3.27	0.38	21	0.004	0.95(COSM54	DEAD/DEAH b	rs7216445	0.1976	YES	5.662E-1 (pos.35984410)	adenocarcinoi	DEAD box p	ATP bindin	Nervous System Diseases:Neuromuscular Dis			T=0, C=80	T=0.0, C=1.0	0.00001	1289.37	80	T=0/0, C=44/3
chr12:32778581	T/C	T	C	SNV	FGD4	FGD4:intronic:NM_139241.3	1	51.92		intronic	NM_13924	c.1636-7T	p.?	0.03					FYVE zinc fing	rs11052113	0.1083	YES	9.96E-3 (pos.32778581)		FYVE, RhoGI	G-protein	Charcot-Marie-Tooth Disease:(		Benign/Likely	T=25, C=27	T=0.4808, C=0.5192	0.00001	261.273	52	T=14/11, C=13
chr12:32854602	A/A	G	A	SNV	DNM1L	DNM1L:intronic:NM_012062.4	1	100		intronic	NM_01206	c.250+106	p.?	0.21					Dynamin GTP	rs9737752					Dynamin 1-	BH2 doma	Acid-Base Imbalance;Acidosis;Acidosis, Lacti			G=0, A=45	G=0.0, A=1.0	0.00001	747.392	45	G=0/0, A=26/1
chrX:23019317	T/T	G	T	SNV	DDX53,PTCHD1-AS	PTCHD1-AS:intronic_nc:NR_073010.2, DDX53:exonic:NM_18	1	100	1	missense	NR_07301	c.1143G>T	p.Met381Ile	8.51		10		1(COSM4002	DEAD/DEAH b	rs5925720	0.1173	YES	2.576E-1 (pos.23019317)	acute_myeloid_leukaemia		ATP binding,ATP-dependent RNA helicase activity,RNA bi				G=0, T=26	G=0.0, T=1.0	0.00001	371.418	26	G=0/0, T=7/19
chr12:32948934	T/C	T	C	SNV	PKP2	PKP2:intronic:NM_004572.3	1	50		intronic	NM_00457	c.2489+10	p.?	-0.65					Armadillo/be	rs12228193		YES			Plakophilin	adherens	ACMG Recommendations...:Arrhythmogenic I			T=17, C=17	T=0.5, C=0.5	0.00001	172.009	34	T=8/9, C=9/8
chr12:347068	A/A	T	A	SNV	SLC6A13	SLC6A13:intronic:NM_016615.4	1	100		intronic	NM_01661	c.563+24A	p.?	-0.41					Sodium:neur	rs2289957	0.466	YES	1.135E-1 (pos.347068)		Solute carri	extracellu	Female Urogenital I	Guvacine		T=0, A=23	T=0.0, A=1.0	0.00001	316.747	23	T=0/0, A=16/7
chr12:3736734	T/T	C	T	SNV	CRACR2A	CRACR2A:intronic:NM_001144958.1	1	100		intronic	NM_00114	c.1818-18	p.?	-3.06					50S ribosome	rs10848893	0.2354	YES	3.669E-1 (pos.3736734)		EF-hand cal	activation of store-operated calcium channel activity,ad				C=0, T=39	C=0.0, T=1.0	0.00001	628.35	39	C=0/0, T=19/2
chr12:3736745	C/C	T	C	SNV	CRACR2A	CRACR2A:intronic:NM_001144958.1	1	100		intronic	NM_00114	c.1818-29	p.?	-2.41					50S ribosome	rs10848894	0.3661	YES	5.909E-1 (pos.3736745)		EF-hand cal	activation of store-operated calcium channel activity,ad				T=0, C=40	T=0.0, C=1.0	0.00001	644.286	40	T=0/0, C=19/2
chr12:40037727	G/G	A	G	SNV	C12orf40	C12orf40:intronic:NM_001031748.3	1	100		intronic	NM_00103	c.202+8A>	p.?	-0.27					Domain of un	rs10735878	0.1333	YES	6.11E-1 (pos.40037727)			protein binding				A=0, G=33	A=0.0, G=1.0	0.00001	504.584	33	A=0/0, G=19/1
chr12:40077028	G/G	A	G	SNV	C12orf40	C12orf40:intronic:NM_001031748.3	1	100		intronic	NM_00103	c.1216+86	p.?	-1.69					Domain of un	rs10047563		YES				protein binding				A=0, G=164	A=0.0, G=1.0	0.00001	2663.08	164	A=0/0, G=78/8
chrX:23019346	T/T	G	T	SNV	DDX53,PTCHD1-AS	PTCHD1-AS:intronic_nc:NR_073010.2, DDX53:exonic:NM_18	1	100	1	missense	NR_07301	c.1172G>T	p.Arg391Met	0.91		91		0.03(COSM40	DEAD/DEAH b	rs5926203	0.1216	YES	2.577E-1 (pos.23019346)	acute_myeloid_leukaemia		ATP binding,ATP-dependent RNA helicase activity,RNA bi				G=0, T=26	G=0.0, T=1.0	0.00001	371.42	26	G=0/0, T=7/19
chr12:40657537	G/G	A	G	SNV	LRRK2	LRRK2:intronic:NM_198578.3	1	100		intronic	NM_19857	c.1544-54	p.?	2.65					ADP-ribosyla	rs10784461		YES			Leucine-ricl	ATP bindin	Adenocarcinoma;Basal Ganglia Diseases;Bra			A=0, G=57	A=0.0, G=1.0	0.00001	853.037	57	A=0/0, G=36/2
chr12:40681057	T/T	C	T	SNV	LRRK2	LRRK2:intronic:NM_198578.3	1	100		intronic	NM_19857	c.2501-96	p.?	-0.13					ADP-ribosyla	rs4473003		YES			Leucine-ricl	ATP bindin	Adenocarcinoma;Basal Ganglia Diseases;Bra			C=0, T=62	C=0.0, T=1.0	0.00001	940.315	62	C=0/0, T=25/3
chr12:40681142	CT/CT	C	CT	INDEL	LRRK2	LRRK2:intronic:NM_198578.3	1	100		intronic	NM_19857	c.2501-11	p.?	-0.07					ADP-ribosyla	rs201211726,rs3622	0.4627	YES	3.43E-1;0.0 (pos.40681142)		Leucine-ricl	ATP bindin	Adenocarcinoma;Basal Ganglia		Benign	C=0, CT=64	C=0.0, CT=1.0	0.00001	670.618	64	C=0/0, CT=25/

chr12:40702846	T/T	A	T	SNV	LRRK2	LRRK2:intronic:NM 198578.3	1	100		intronic	NM 19857	c.4190-62	p.?	-3.49					ADP-ribosyla	rs7305344,rs928530607		YES			Leucine-ricl	ATP bindin	Adenocarcinoma;Basal Ganglia Diseases;Bra			A=0, T=24	A=0.0, T=1.0	0.00001	249.654	24	A=0/0, T=11/1
chr12:40713759	C/C	T	C	SNV	LRRK2	LRRK2:intronic:NM 198578.3	1	100		intronic	NM 19857	c.4828-31	p.?	0.01					ADP-ribosyla	rs1896252	0.4595	YES	4.756E-1 (pos.40713759)		Leucine-ricl	ATP bindin	Adenocarcinoma;Basal Ganglia Diseases;Bra			T=0, C=48	T=0.0, C=1.0	0.00001	810.185	48	T=0/0, C=23/2
chr1:200618238	C/C	T	C	SNV	DDX59	DDX59:exonic:NM 001031725.5	1	100	6	synonymous	NM 00103	c.1431A>G	p.(=)	-3.29				0.03(COSM37	DEAD/DEAH b	rs6665604	0.0372	YES	1.0 (pos.200618238)	hepatocellula	DEAD box p	ATP bindin	Abnormalities. Multiple:Bone Diseases:Bone			T=0, C=58	T=0.0, C=1.0	0.00001	866.978	58	T=0/0, C=31/2
chr4:169315760	A/A	C	A	SNV	DDX60L	DDX60L:exonic:NM 001012967.2	1	100	28	missense	NM 00101	c.3666G>T	p.Glu1222Asp	-0.33	0.86	45	0.001	0.01(COSM41	DEAD/DEAH b	rs3749499	0.0079	YES	9.108E-1 (pos.16931576(	neoplasm	DEAD box p	ATP binding.RNA binding.helicase activity.nucleic acid bi				C=0, A=31	C=0.0, A=1.0	0.00001	465.817	31	C=0/0, A=14/1
chr4:169341435	G/G	A	G	SNV	DDX60L	DDX60L:exonic:NM 001012967.2	1	100	18	missense	NM 00101	c.2491T>C	p.Cys831Arg	1.18	0.92	180	0		DEAD/DEAH b	rs2130145,rs796305(	2.00E-04		1.0 (pos.169341435)		DEAD box p	ATP binding.RNA binding.helicase activity.nucleic acid bi				A=0, G=68	A=0.0, G=1.0	0.00001	1053.56	68	A=0/0, G=46/2
chr12:40753303	G/G	A	G	SNV	LRRK2	LRRK2:intronic:NM 198578.3	1	100		intronic	NM 19857	c.7028+57	p.?	-1.46					ADP-ribosyla	rs3789330		YES			Leucine-ricl	ATP bindin	Adenocarcinoma;Basal Ganglia Diseases;Bra			A=0, G=36	A=0.0, G=1.0	0.00001	563.744	36	A=0/0, G=11/2
chr4:169382991	C/A	C	A	SNV	DDX60L	DDX60L:exonic:NM 001012967.2	1	53.33	5	synonymous	NM 00101	c.465G>T	p.(=)	-1.12				0(COSM3336	DEAD/DEAH b	rs17612630,rs75737	0.1786	YES	0.0 (pos.169382990),1.2	adenocarcino	DEAD box p	ATP binding.RNA binding.helicase activity.nucleic acid bi				C=28, A=32	C=0.4667, A=0.5333	0.00001	323.555	60	C=11/17, A=25
chr12:41323480	C/T	C	T	SNV	CNTN1	CNTN1:intronic:NM 175038.2	1	50.99		intronic	NM 17503	c.464-118	p.?	-0.58					Fibronectin t	rs17622354		YES			Contactin 1	Notch sign	Basal Ganglia Diseases:Brain Diseases:Centr			C=74, T=77	C=0.4901, T=0.5099	0.00001	813.396	151	C=40/34, T=44
chr12:41946453	C/C	T	C	SNV	PDZRN4	PDZRN4:intronic:NM 001164595.1	1	100		intronic	NM 00116	c.1204-5T	p.?	1.94					PDZ domain,I	rs285585	0.0034		1.0 (pos.41946453)		PDZ domair	metal ion binding				T=0, C=72	T=0.0, C=1.0	0.00001	1130.95	72	T=0/0, C=40/3
chr12:43819298	A/A	G	A	SNV	ADAMTS20	ADAMTS20:intronic:NM 025003.4	1	100		intronic	NM 02500	c.4284+19	p.?	-0.17					ADAM-TS Spa	rs10785430	0.467	YES	8.587E-1 (pos.43819298)		A disintegri	extracellu	Melanoma;Neoplasms;Neoplasms by Histolo			G=0, A=34	G=0.0, A=1.0	0.00001	524.187	34	G=0/0, A=21/1
chr12:4383158	T/C	T	C	SNV	CCND2,CCN D2-AS1	CCND2-AS1:intronic nc:NR 125790.1, CCND2:utr 5:NM 00	1	95.45		intronic	NR 12579	c.-49T>C	p.?	-0.8					Cyclin, C-tern	rs3217786	5.00E-04		1.0 (pos.4383158)		Cyclin D2	adult loco	Adnexal Diseases:Brain Diseases:Cardiomeg			T=1, C=21	T=0.0455, C=0.9545	0.00001	245.686	22	T=1/0, C=13/8
chr6:35285720	C/C	A	C	SNV	DEF6	DEF6:exonic:NM 022047.3	1	100	6	missense	NM 02204	c.860A>C	p.Asn287Thr	1.68	0.32	65	0.017		PH domain	rs2395617	0.1142	YES	9.949E-1;0.0 (pos.35285720)		Def6, mous	cytoskeleton.cytosol.filopodium.membrane.nucleoplas				A=0, C=23	A=0.0, C=1.0	0.00001	316.717	23	A=0/0, C=9/14
chr8:6793540	T/T	C	T	SNV	DEFA4	DEFA4:utr 3:NM 001925.2	1	100	3	utr 3	NM 00192	c.296G>A	p.?	-1.78					Defensin pro	rs736227	0.321	YES	6.151E-1 (pos.6793540)		Defensin, a	Golgi lumen,antibacterial humoral response,antifungal				C=0, T=71	C=0.0, T=1.0	0.00001	1111.39	71	C=0/0, T=35/3
chr12:43945603	C/C	A	C	SNV	ADAMTS20	ADAMTS20:intronic:NM 025003.4	1	100		intronic	NM 02500	c.91+31T>	p.?	-0.88					ADAM-TS Spa	rs275602	0.014	YES	9.983E-1 (pos.43945603)		A disintegri	extracellu	Melanoma;Neoplasms;Neoplasms by Histolo			A=0, C=100	A=0.0, C=1.0	0.00001	1702.08	100	A=0/0, C=34/6
chr12:44139805	A/T	A	T	SNV	PUS7L	PUS7L:intronic:NM 001098614.2	1	53.13		intronic	NM 00109	c.1263+44	p.?	0.16					tRNA pseudo	rs11182246	0.1131	YES	1.903E-1 (pos.44139805)			RNA binding.protein binding.pseudouridine synthase act				A=30, T=34	A=0.4688, T=0.5313	0.00001	347.787	64	A=17/13, T=12
chr12:44338153	A/A	T	A	SNV	TMEM117	TMEM117:intronic:NM 032256.2	1	100		intronic	NM 03225	c.410+8T>	p.?	-0.37					TMEM117 pro	rs6582491	1.00E-04		1.0 (pos.44338153)			endoplasmic reticulum.integral component of membran				T=0, A=30	T=0.0, A=1.0	0.00001	446.639	30	T=0/0, A=12/1
chr8:6793581	A/G	A	G	SNV	DEFA4	DEFA4:exonic:NM 001925.2	1	45.45	3	synonymous	NM 00192	c.255T>C	p.(=)	-0.6				0.01(COSM39	Defensin pro	rs2738100	0.3799	YES	4.603E-1 (pos.6793581)	acute myeloic	Defensin, a	Golgi lumen.antibacterial humoral response.antifungal				A=36, G=30	A=0.5455, G=0.4545	0.00001	285.782	66	A=23/13, G=5/
chr8:6735399	T/T	C	T	SNV	DEFB1	DEFB1:utr 5:NM 005218.3	1	100	1	utr 5	NM 00521	c.-20G>A	p.?	-1.03					Beta defensi	rs11362	0.4393	YES	3.772E-1 (pos.6735399)		Defensin, b	CCR6 chen	Dermatitis;Dermatitis, Contact;Diabetes Me			C=0, T=27	C=0.0, T=1.0	0.00001	389.988	27	C=0/0, T=12/1
chr12:45444238	A/A	T	A	SNV	DBX2	DBX2:intronic:NM 001004329.2	1	100		intronic	NM 00100	c.403+70A	p.?	-0.59					Homeobox d	rs11182810		YES				nucleus,regulation of transcription, DNA-templated,seq				T=0, A=72	T=0.0, A=1.0	0.00001	1130.95	72	T=0/0, A=31/4
chr8:6735423	G/G	C	G	SNV	DEFB1	DEFB1:utr 5:NM 005218.3	1	100	1	utr 5	NM 00521	c.-44G>C	p.?	-1.15					Beta defensi	rs1800972	0.2051	YES	8.811E-1 (pos.6735423)		Defensin, b	CCR6 chen	Dermatitis:Dermatitis, Contact:Diabetes Me			C=0, G=27	C=0.0, G=1.0	0.00001	389.989	27	C=0/0, G=12/1
chr20:76962	C/C	T	C	SNV	DEFB125	DEFB125:exonic:NM 153325.3	1	100	2	synonymous	NM 15332	c.375T>C	p.(=)	-1.84					Beta defensi	rs6111385	0.1857	YES	6.921E-1 (pos.76962)			cell surface.defense response to bacterium.extracellula				T=0, C=93	T=0.0, C=1.0	0.00001	1554.66	93	T=0/0, C=52/4
chr12:45664213	A/A	G	A	SNV	ANO6	ANO6:intronic:NM 001204803.1	1	100		intronic	NM 00120	c.71-32G>	p.?	-0.77					Calcium-acti	rs10748415			1.0 (pos.45664213)		Anoctamin	activation	Ankylosis;Arthritis;Blood Coagulation Disord			G=0, A=23	G=0.0, A=1.0	0.00001	316.747	23	G=0/0, A=7/16
chr12:45695735	A/A	C	A	SNV	ANO6	ANO6:intronic:NM 001204803.1	1	100		intronic	NM 00120	c.134-62C	p.?	-0.76					Calcium-acti	rs4140756		YES			Anoctamin	activation	Ankylosis;Arthritis;Blood Coagulation Disord			C=0, A=23	C=0.0, A=1.0	0.00001	316.748	23	C=0/0, A=16/7
chr12:46215163	A/A	G	A	SNV	ARID2	ARID2:intronic:NM 152641.3	1	100		intronic	NM 15264	c.638-40G	p.?	-0.73					ARID/BRIGHT	rs2059404	0.4751	YES	4.397E-1 (pos.46215163)		AT-rich inte	DNA bindi	Adenocarcinoma:Carcinoma:Carcinoma, Her			G=0, A=80	G=0.0, A=1.0	0.00001	1294.65	80	G=0/0, A=43/3
chr12:464254	C/C	T	C	SNV	KDM5A	KDM5A:intronic:NM 001042603.2	1	100		intronic	NM 00104	c.870+70A	p.?	-0.04					ARID/BRIGHT	rs3759371		YES			Lysine(K)-sp	DNA bindi	Intellectual Disability:Mental Disorders:Nen			T=0, C=111	T=0.0, C=1.0	0.00001	1736.75	111	T=0/0, C=70/4
chr12:46755112	T/T	A	T	SNV	SLC38A2	SLC38A2:intronic:NM 018976.4	1	100		intronic	NM 01897	c.1423-12	p.?	-3.87					Transmembr	rs7961994		YES			Solute carri	alanine tr	Autoimmune Diseases:Female Urogenital Di			A=0, T=27	A=0.0, T=1.0	0.00001	389.988	27	A=0/0, T=14/1
chr12:46758494	G/G	A	G	SNV	SLC38A2	SLC38A2:intronic:NM 018976.4	1	100		intronic	NM 01897	c.647-9T>	p.?	-0.4					Transmembr	rs2307061,rs770634	0.3865	YES	8.156E-1;0.0;0.0 (pos.46758494)		Solute carri	alanine tr	Autoimmune Diseases;Female Urogenital Di			A=0, G=78	A=0.0, G=1.0	0.00001	1249.35	78	A=0/0, G=43/3
chr12:46759017	A/A	T	A	SNV	SLC38A2	SLC38A2:intronic:NM 018976.4	1	100		intronic	NM 01897	c.564-45A	p.?	0.9					Transmembr	rs7968173	0.3793	YES	8.196E-1 (pos.46759017)		Solute carri	alanine tr	Autoimmune Diseases;Female Urogenital Di			T=0, A=49	T=0.0, A=1.0	0.00001	831.633	49	T=0/0, A=21/2
chr12:46761127	A/A	G	A	SNV	SLC38A2	SLC38A2:intronic:NM 018976.4	1	100		intronic	NM 01897	c.315-4C>	p.?	-0.01					Transmembr	rs10880959	0.3795	YES	8.159E-1 (pos.46761127)		Solute carri	alanine tr	Autoimmune Diseases:Female Urogenital Di			G=0, A=48	G=0.0, A=1.0	0.00001	810.435	48	G=0/0, A=14/3
chr20:238436	T/T	TGGTCTT	T	INDEL	DEFB132	DEFB132:exonic:NM 207469.2	6	100	1	nonframeshiftDeletion	NM 20746	c.20 25de	p.Val7_Leu8del	1.67, 0.92, -0.14, 0.49, 0.14, 1.68, 0.51					Beta defensi	rs371825938,rs66489228,rs746		YES	6.749E-1 (pos.238435)			cell surface.defense response to Gram-negative bacteri				TGGTCTT=0	TGGTCTT=0.0, T=1.0	0.00001	485.141	32	TGGTCTT=0/0,
chr12:47170621	A/A	C	A	SNV	SLC38A4	SLC38A4:intronic:NM 001143824.1	1	100		intronic	NM 00114	c.1174+66	p.?	-0.27					Transmembr	rs12316767		YES			Solute carri	amino acid transmembrane transport.amino acid transr				C=0, A=23	C=0.0, A=1.0	0.00001	316.747	23	C=0/0, A=7/16
chr12:47173830	A/A	G	A	SNV	SLC38A4	SLC38A4:intronic:NM 001143824.1	1	100		intronic	NM 00114	c.494-17C	p.?	1.12					Transmembr	rs2287471	0.2118	YES	1.338E-1 (pos.47173830)		Solute carri	amino acid transmembrane transport.amino acid transr				G=0, A=33	G=0.0, A=1.0	0.00001	504.593	33	G=0/0, A=20/1
chr7:140244560	T/T	C	T	SNV	DENND2A	DENND2A:exonic:NM 015689.4	1	100	14	missense	NM 01568	c.2185G>A	p.Glu729Lys	4.77	0.02	56	0.068	0.96(COSM37	DENN (AEX-3)	rs2293177	0.3049	YES	1.725E-1 (pos.14024456(	adenocarcinoma,hepatoce	Rab guanyl-nucleotide exchange factor activity,actin cyt					C=0, T=42	C=0.0, T=1.0	0.00001	685.206	42	C=0/0, T=24/1
chr1:115168600	T/T	A	T	SNV	DENND2C	DENND2C:exonic:NM 001256404.1	1	100	4	missense	NM 00125	c.6T>A	p.Asp2Glu	0.65	0.33	45	0.084	0.25(COSM37	DENN (AEX-3)	rs7541738	0.276	YES	1.165E-4;0.0 (pos.11516	adenocarcinoma,malignan	Rab guanyl-nucleotide exchange factor activity,regulatic					A=0, T=33	A=0.0, T=1.0	0.00001	504.569	33	A=0/0, T=23/1
chr12:48084556	T/T	C	T	SNV	RPAP3	RPAP3:intronic:NM 024604.2	1	100		intronic	NM 02460	c.546-134	p.?	-0.06					Potential Mo	rs1233054		YES			RNA polyme	R2TP complex,protein binding				C=0, T=51	C=0.0, T=1.0	0.00001	754.584	51	C=0/0, T=23/2
chr12:48110608	G/G	A	G	SNV	ENDOU	ENDOU:intronic:NM 001172439.1	1	100		intronic	NM 00117	c.551+65T	p.?	-0.19					Endoribonucl	rs2524318		YES			Endonuclea	RNA binding.RNA phosphodiester bond hydrolysis, endor				A=0, G=224	A=0.0, G=1.0	0.00001	3585.36	224	A=0/0, G=115/
chr12:48132451	C/C	T	C	SNV	RAPGEF3	RAPGEF3:intronic:NM 001098531.2	1	100		intronic	NM 00109	c.2526+22	p.?	-2.9					Cyclic nucleo	rs2072116,rs774212	0.1701	YES	0.0 (pos.48132450),9.837E-1 (pos.48132451)				Acute Kidney Injury;Female Urogenital Disea			T=0, C=87	T=0.0, C=1.0	0.00001	1431.39	87	T=0/0, C=39/4
chr17:54921494	C/C	A	C	SNV	DGKE	DGKE:exonic:NM 003647.2	1	100	3	synonymous	NM 00364	c.579A>C	p.(=)	-0.41				0(COSM6353	Diacylglycerc	rs3760158	0.2642	YES	9.235E-1;2.317E-4 (pos.5	carcinoma	Diacylglyce	ATP bindin	Anemia:Anemia, Hemolytic:At		Benign	A=0, C=29	A=0.0, C=1.0	0.00001	427.526	29	A=0/0, C=16/1
chr12:48537624	G/G	A	G	SNV	PFKM	PFKM:intronic:NM 001166686.1	1	100		intronic	NM 00116	c.2093+27	p.?	0.11					Phosphofruct	rs4075913	0.17	YES	4.123E-1 (pos.48537624)		Phosphofru	6-phosphc	Carbohydrate Metabolism, Inb		Benign	A=0, G=21	A=0.0, G=1.0	0.00001	281.235	21	A=0/0, G=9/12
chr12:48537629	G/C	G	C	SNV	PFKM	PFKM:intronic:NM 001166686.1	1	47.62		intronic	NM 00116	c.2093+32	p.?	0.7					Phosphofruct	rs4075914	0.0844	YES	1.159E-4;0.0 (pos.48537629)		Phosphofru	6-phosphc	Carbohydrate Metabolism, Inb		Benign	G=11, C=10	G=0.5238, C=0.4762	0.00001	82.2901	21	G=6/5, C=3/7
chr3:185990096	T/T	C	T	SNV	DGKG	DGKG:exonic:NM 001346.2	1	100	11	missense	NM 00134	c.947G>A	p.Arg316Lys	0.84	1	26	0		Diacylglycerc	rs2193587	0.2122	YES	7.292E-1;0.0 (pos.185990096)		Diacylglyce	ATP binding,NAD+ kinase activi		Phosphatidyl serine		C=0, T=31	C=0.0, T=1.0	0.00001	465.82	31	C=0/0, T=16/1
chr1:12640650	C/T	C	T	SNV	DHRS3	DHRS3:exonic:NM 004753.6	1	52.27	2	synonymous	NM 00475	c.240G>A	p.(=)	-4.96				0.06(COSM37	Enoyl-(Acyl ca	rs11540058	0.2071	YES	1.041E-3 (pos.12640650)	adenocarcino	Short-chain	NADP-retinol dehydrogenase a		Vitamin A		C=21, T=23	C=0.4773, T=0.5227	0.00001	260.079	44	C=11/10, T=13
chr2:169952233	T/C	T	C	SNV	DHRS9	DHRS9:exonic:NM 001142271.1	1	50	5	synonymous	NM 00114	c.916T>C	p.(=)	0.29				0.53(COSM37	Enoyl-(Acyl ca	rs1059261	0.2009	YES	3.182E-1 (pos.16995223	acute myeloic	Short-chain	9-cis-retinoic acid biosynthetic process.alcohol dehydro				T=13, C=13	T=0.5, C=0.5	0.00001	119.163	26	T=5/8, C=9/4
chrX:2161129	G/G	C	G	SNV	DHRSX	DHRSX:exonic:NM 145177.2	1	100	6	missense	NM 14517	c.739G>C	p.Val247Leu	4.48	1	32	0.003	0.01(COSM44	Enoyl-(Acyl ca	rs1127915	0.2668	YES	2.345E-1;0.0 (pos.21611	acute myeloid leukaemia		extracellular region.oxidation-reduction process.oxidor				C=0, G=124	C=0.0, G=1.0	0.00001	2017.48	124	C=0/0, G=74/5
chr12:125434580	T/T	C	T	SNV	DHX37	DHX37:exonic:NM 032656.3	1	100	25	missense	NM 03265	c.3242G>A	p.Arg1081Gln	-0.03	0.33	43	0.024	0.07(COSM37	DEAD/DEAH b	rs4447263	0.4126	YES	8.449E-1 (pos.12543458(	adenocarcino	DEAH box p	ATP binding.ATP-dependent RNA helicase activity.RNA bi				C=0, T=24	C=0.0, T=1.0	0.00001	334.792	24	C=0/0, T=14/1
chr12:48741219	T/T	A	T	SNV	ZNF641	ZNF641:intronic:NM 001172681.1	1	100		intronic	NM 00117	c.185-93T	p.?	-0.06					KRAB box,Zin	rs901962		YES			Zinc finger p	DNA binding,RNA polymerase II transcription factor activ				A=0, T=58	A=0.0, T=1.0	0.00001	867.385	58	A=0/0, T=35/2
chr12:125437019	C/C	T	C	SNV	DHX37	DHX37:exonic:NM 032656.3	1	100	21	synonymous	NM 03265	c.2793A>G	p.(=)	-1.53				0.2(COSM372	DEAD/DEAH b	rs4429156	0.4126	YES	8.44E-1 (pos.125437019)	neoplasm,squ	DEAH box p	ATP binding,ATP-dependent RNA helicase activity,RNA bi				T=0, C=62	T=0.0, C=1.0	0.00001	940.271	62	T=0/0, C=30/3
chr12:125438516	C/C	T	C	SNV	DHX37	DHX37:exonic:NM 032656.3	1	100	20	missense	NM 03265	c.2605A>G	p.Ser869Gly	5.54	1	56	0	0.15(COSM20	DEAD/DEAH b	rs4516060	0.4144	YES	8.399E-1 (pos.12543851(	adenocarcino	DEAH box p	ATP binding,ATP-dependent RNA helicase activity,RNA bi				T=0, C=35	T=0.0, C=1.0	0.00001	543.899	35	T=0/0, C=19/1
chr12:125438523	T/T	C	T	SNV	DHX37	DHX37:exonic:NM 032656.3	1	100	20	synonymous	NM 03265	c.2598G>A	p.(=)	0.02					DEAD/DEAH b	rs4258464	0.4172	YES	8.399E-1 (pos.125438523)		DEAH box p	ATP binding,ATP-dependent RNA helicase activity,RNA bi				C=0, T=35	C=0.0, T=1.0	0.00001	543.872	35	C=0/0, T=19/1
chr12:49212806	C/T	C	T	SNV	CACNB3	CACNB3:intronic:NM 001206917.1	1	46.43		intronic	NM 00120	c.7-4321C	p.?	-0.03					Guanylate ki	rs12369114	0.1767	YES	2.475E-3 (pos.49212806)		Calcium cha	L-type volt	Adnexal Diseases:E	Calcium Citrate.Calcium		C=15, T=13	C=0.5357, T=0.4643	0.00001	117.914	28	C=6/9, T=11/2
chr2:39050141	C/C	A	C	SNV	DHX57	DHX57:exonic:NM 198963.2	1	100	17	synonymous	NM 19896	c.3285T>G	p.(=)	0.12					DEAD/DEAH b	rs3770681	0.3505	YES	8.34E-1 (pos.39050141)			ATP binding,ATP-dependent RNA helicase activity,RNA bi				A=0, C=45	A=0.0, C=1.0	0.00001	747.377	45	A=0/0, C=19/2
chr2:39082344	C/C	T	C	SNV	DHX57	DHX57:exonic:NM 198963.2	1	100	8	missense	NM 19896	c.1760A>G	p.Asn587Ser	0.62	1	46	0		DEAD/DEAH b	rs7598922	0.3678	YES	8.34E-1 (pos.39082344)			ATP binding,ATP-dependent RNA helicase activity,RNA bi				T=0, C=57	T=0.0, C=1.0	0.00001	849.609	57	T=0/0, C=26/3
chr12:49580752	AC/A	AC	A	INDEL	TUBA1A	TUBA1A:intronic:NM 006009.3	1	57.14		intronic	NM 00600	c.4-136GT	p.?	0.05, -0.44					Tubulin C-ter	rs34741313,rs76409127,rs7762		YES			Tubulin, alp	G2/M tran	Cardiovascular Dis	2-MERCAPTO-N-1,2,3,10-		AC=18, A=2	AC=0.4286, A=0.571	0.00001	279.086	42	AC=11/7, A=8/
chr12:50291735	C/C	G	C	SNV	FAIM2	FAIM2:intronic:NM 012306.3	1	100		intronic	NM 01230	c.315+35C	p.?	-1.1					Inhibitor of a	rs297937	0.466	YES	7.852E-1 (pos.50291735)		FAS apopto	Golgi apparatus.apoptotic process.cell junction.cerebel				G=0, C=51	G=0.0, C=1.0	0.00001	754.426	51	G=0/0, C=41/1
chr12:50744094	A/A	G	A	SNV	FAM186A	FAM186A:intronic:NM 001145475.2	1	100		intronic	NM 00114	c.6503+18	p.?	-0.03						rs7296277	0	YES	1.0 (pos.50744094)							G=0, A=50	G=0.0, A=1.0	0.00001	852.725	50	G=0/0, A=33/1
chr1:182850727	C/C	T	C	SNV	DHX9	DHX9:exonic:NM 001357.4	1	100	24	synonymous	NM 00135	c.2859T>C	p.(=)	-0.05					DEAD/DEAH b	rs6677840	0		1.0 (pos.182850727)		DEAH (Asp-(	ATP binding,ATP-dependent 3'-5' DNA helicase activity,A				T=0, C=61	T=0.0, C=1.0	0.00001	921.096	61	T=0/0, C=8/53
chr20:61512606	C/C	T	C	SNV	DIDO1	DIDO1:exonic:NM 001193369.1	1	100	16	missense	NM 00119	c.4702A>G	p.Thr1568Ala	-0.37	0.64	58	0	0.03(COSM37	PHD-finger,SF	rs910148	0.0136		1.0 (pos.61512606)	adenocarcinoma.neoplasn	RNA binding.apoptotic signaling pathway.cytoplasm.me					T=0, C=42	T=0.0, C=1.0	0.00001	685.207	42	T=0/0, C=16/2
chr1:210006603	T/T	C	T	SNV	DIEXF	DIEXF:exonic:NM 014388.6	1	100	4	synonymous	NM 01438	c.462C>T	p.(=)	-5.17				0.01(COSM14	Utp25, U3 sn	rs656566	0.2381	YES	3.937E-1 (pos.21000660	acute myeloid leukaemia	RNA binding.U3 snoRNA binding.maturation of SSU-rRNA					C=0, T=35	C=0.0, T=1.0	0.00001	543.903	35	C=0/0, T=11/2
chr12:50855195	A/G	A	G	SNV	LARP4	LARP4:intronic:NM 052879.4	1	95.65		intronic	NM 05287	c.1334+65	p.?	-0.43					La domain	rs4351895		YES				RNA bindir	Adnexal Diseases:Cysts:Endocrine System Di			A=1, G=22	A=0.0435, G=0.9565	0.00001	290.32	23	A=0/1, G=5/17
chr12:51089572	A/A	G	A	SNV	DIP2B	DIP2B:intronic:NM 173602.2	1	100		intronic	NM 17360	c.1814-59	p.?	-0.92					AMP-binding	rs2684890		YES			Disco-inter	biological	Chromosome Disorders;Congenital Abnorma			G=0, A=37	G=0.0, A=1.0	0.00001	583.705	37	G=0/0, A=23/1
chr12:51089613	TC/TC	T	TC	INDEL	DIP2B	DIP2B:intronic:NM 173602.2	1	100		intronic	NM 17360	c.1814-18	p.?	0.65					AMP-binding enzyme,DMAP1-binding Domain						Disco-inter	biological	Chromosome Disorders;Congenital Abnorma			T=0, TC=34	T=0.0, TC=1.0	0.00001	447.615	34	T=0/0, TC=19/
chr12:51089616	T/C	T	C	SNV	DIP2B	DIP2B:intronic:NM 173602.2	1	100		intronic	NM 17360	c.1814-15	p.?	0.17					AMP-binding	rs2684891	0.0245	YES	6.807E-1 (pos.51089616)		Disco-inter	biological	Chromosome Disorders;Congenital Abnorma			T=0, C=40	T=0.0, C=1.0	0.00001	546.897	40	T=0/0, C=23/1
chr12:51135275	C/C	T	C	SNV	DIP2B	DIP2B:exonic:NM 173602.2	1	100	37	synonymous	NM 17360	c.4431T>C	p.(=)	-1.85					AMP-binding	rs1866422	5.00E-04	YES	1.0 (pos.51135275)		Disco-inter	biological	Chromosome Disorders;Congenital Abnorma			T=0, C=71	T=0.0, C=1.0	0.00001	1111.39	71	T=0/0, C=35/3
chr10:445061	T/T	C	T	SNV	DIP2C	DIP2C:exonic:NM 014974.2	1	100	10	synonymous	NM 01497	c.1248G>A	p.(=)	-0.21				0.24(COSM37	AMP-binding	rs6560837	0.0033	YES	9.822E-1 (pos.445061)	adenocarcino	Disco-inter	biological	Bronchial Neoplasms:Carcinoma, Bronchoge			C=0, T=56	C=0.0, T=1.0	0.00001	831.56	56	C=0/0, T=27/2
chr12:51399050	T/G	T	G	SNV	SLC11A2	SLC11A2:intronic:NM 001174125.1	1	50		intronic	NM 00117	c.396+44A	p.?	-0.11					Natural resis	rs224589	0.2606	YES	8.672E-1;0.0 (pos.51399050)		Solute carri	activation	Anemia;Anemia, Hy	Copper,Mangafodipir		T=18, G=18	T=0.5, G=0.5	0.00001	184.883	36	T=13/5, G=13/
chr12:51458558	A/A	G	A	SNV	CSRNP2	CSRNP2:intronic:NM 030809.2	1	100		intronic	NM 03080	c.709-106	p.?	0.33					Cysteine/seri	rs2029147		YES				DNA binding transcription factor activity,apoptotic proce				G=0, A=34	G=0.0, A=1.0	0.00001	524.186	34	G=0/0, A=21/1
chr12:51461425	G/G	C	G	SNV	CSRNP2	CSRNP2:intronic:NM 030809.2	1	100		intronic	NM 03080	c.708+31G	p.?	-0.15					Cysteine/seri	rs4768956		YES	1.0 (pos.51461425)			DNA binding transcription factor activity,apoptotic proce				C=0, G=41	C=0.0, G=1.0	0.00001	664.699	41	C=0/0, G=18/2
chr12:51504799	T/C	T	C	SNV	TFCP2	TFCP2:intronic:NM 005653.4	1	96.67		intronic	NM 00565	c.458-33A	p.?	-0.34					CP2 transcric	rs7978782	8.00E-04	YES	1.0 (pos.51504799)		Transcriptic	DNA binding.DNA binding transcription factor activity.RN				T=1, C=29	T=0.0333, C=0.9667	0.00001	416.641	30	T=0/1, C=8/21
chr10:459940	A/A	G	A	SNV	DIP2C	DIP2C:exonic:NM 014974.2	1	100	8	synonymous	NM 01497	c.970C>T	p.(=)	1.88				0.73(COSM37	AMP-binding	rs4881274	0.459	YES	6.9E-1 (pos.459940)	adenocarcino	Disco-inter	biological	Bronchial Neoplasms:Carcinoma, Bronchoge			G=0, A=26	G=0.0, A=1.0	0.00001	371.419	26	G=0/0, A=16/1
chr12:51681846	T/T	C	T	SNV	BIN2	BIN2:intronic:NM 016293.3	1	100		intronic	NM 01629	c.1692+46	p.?	-0.34					BAR domain	rs4611251	0.1038	YES	9.365E-1 (pos.51681846)		Bridging int	cell chemotaxis,cell cortex,cell junction,cell projection,(				C=0, T=51	C=0.0, T=1.0	0.00001	754.565	51	C=0/0, T=37/1
chr19:49867913	A/A	G	A	SNV	DKKL1	DKKL1:exonic:NM 014419.3	1	100	2	missense	NM 01441	c.85G>A	p.Ala29Thr	-1.72	1	58	0	0(COSM5001917)		rs919364	0.3172	YES	1.737E-1 (pos.49867913)	ductal carcinc	Dickkopf-lik	acrosoma	Autoimmune Diseases;Autoimmune Disease			G=0, A=55	G=0.0, A=1.0	0.00001	814.905	55	G=0/0, A=22/3
chr12:51890721	G/A	G	A	SNV	SLC4A8	SLC4A8:intronic:NM 001039960.2	1	47.62		intronic	NM 00103	c.2944-50	p.?	0.34					Band 3 cytop	rs10783448	0.478	YES	3.674E-1 (pos.51890721)		Solute carri	anion tran	Alzheimer Disease:	Sodium bicarbonate		G=11, A=10	G=0.5238, A=0.4762	0.00001	82.2521	21	G=10/1, A=6/4
chr12:52163789	A/A	G	A	SNV	SCN8A	SCN8A:intronic:NM 014191.3	1	100		intronic	NM 01419	c.3490+20	p.?	-0.49					Cytoplasmic	rs303808	0.1568	YES	8.958E-1 (pos.52163789)		Sodium cha	ATP bindin	Ataxia:Brain Diseas	Brivaracet	Benign	G=0, A=23	G=0.0, A=1.0	0.00001	316.746	23	G=0/0, A=9/14
chr8:13357587	A/A	G	A	SNV	DLC1	DLC1:utr 5:NM 182643.2	1	100	2	utr 5	NM 18264	c.-7C>T	p.?	0.06					RhoGAP dom	rs1383942	0.0684	YES	6.009E-1 (pos.13357587)		Deleted in l	GTPase ac	Adnexal Diseases;Digestive System Diseases			G=0, A=62	G=0.0, A=1.0	0.00001	939.224	62	G=0/0, A=29/3
chr17:7099811	A/A	G	A	SNV	DLG4	DLG4:exonic:NM 001128827.2	1	100	10	synonymous	NM 00112	c.1158C>T	p.(=)	-1.71				0.21(COSM37	Guanylate ki	rs17203281	0.3056	YES	3.041E-1 (pos.7099811)	acute myeloic	Discs large,	AMPA glut	Adnexal Diseases;A	Guanidine,Guanosine-5'		G=0, A=81	G=0.0, A=1.0	0.00001	1309.48	81	G=0/0, A=46/3
chr12:52635443	C/C	T	C	SNV	KRT7	KRT7:intronic:NM 005556.3	1	100		intronic	NM 00555	c.858+23T	p.?	-0.28					Intermediate	rs2243588	0.1963	YES	8.972E-1 (pos.52635443)		Keratin 7	cornificati	Adenocarcinoma;A	HspE7,Primaquine		T=0, C=26	T=0.0, C=1.0	0.00001	371.419	26	T=0/0, C=6/20
chr12:52709032	T/C	T	C	SNV	KRT83	KRT83:intronic:NM 002282.3	1	46.43		intronic	NM 00228	c.1294+64	p.?	-0.75					Intermediate	rs3741721	0.3338	YES			Keratin 83	aging.corn	Abnormalities, Multiple:Congenital Abnorma			T=15, C=13	T=0.5357, C=0.4643	0.00001	117.927	28	T=6/9, C=5/8
chr12:52789509	T/C	T	C	SNV	KRT82	KRT82:intronic:NM 033033.3	1	50		intronic	NM 03303	c.1321+34	p.?	0.49					Intermediate	rs1732264	0.2441	YES	7.626E-1 (pos.52789509)		Keratin 82	biological process,cornification.cytosol.keratin filamen				T=14, C=14	T=0.5, C=0.5	0.00001	131.941	28	T=8/6, C=3/11
chr12:52789597	A/G	A	G	SNV	KRT82	KRT82:intronic:NM 033033.3	1	53.33		intronic	NM 03303	c.1290-23	p.?	-2.66					Intermediate	rs1732265	0.244	YES	7.581E-1 (pos.52789597)		Keratin 82	biological process,cornification,cytosol,keratin filamen				A=11, G=16	A=0.4074, G=0.5926	0.00001	175.554	30	A=7/4, G=3/13
chr12:52789605	T/A	T	A	SNV	KRT82	KRT82:intronic:NM 033033.3	1	50		intronic	NM 03303	c.1290-31	p.?	-3.33					Intermediate	rs1732266	0.2436	YES	7.576E-1 (pos.52789605)		Keratin 82	biological process,cornification,cytosol,keratin filamen				T=16, A=16	T=0.5, A=0.5	0.00001	158.383	32	T=8/8, A=3/13
chr10:79566632	A/A	G	A	SNV	DLG5	DLG5:exonic:NM 004747.3	1	100	26	synonymous	NM 00474	c.4851C>T	p.(=)	-1.27				0.04(COSM17	Guanylate ki	rs1058198	0.3463	YES	1.881E-1 (pos.79566632)	acute myeloic	Discs large,	apical pro	Digestive System Diseases;Gastroenteritis;G			G=0, A=28	G=0.0, A=1.0	0.00001	408.683	28	G=0/0, A=17/1
chr10:79616605	C/C	T	C	SNV	DLG5	DLG5:exonic:NM 004747.3	1	100	3	missense	NM 00474	c.419A>G	p.Gln140Arg	1.77	1	43	0		Guanylate ki	rs1248696	0.1037	YES	9.994E-1 (pos.79616605)		Discs large,	apical pro	Digestive System Diseases:Gastroenteritis:G			T=0, C=64	T=0.0, C=1.0	0.00001	977.635	64	T=0/0, C=39/2
chr12:52824302	A/A	C	A	SNV	KRT75	KRT75:intronic:NM 004693.2	1	100		intronic	NM 00469	c.1035+23	p.?	0.03					Intermediate	rs423949	0.4021	YES	6.953E-1 (pos.52824302)		Keratin 75	cornificati	Alopecia:Hair Diseases:Hypotrichosis:Loose			C=0, A=32	C=0.0, A=1.0	0.00001	485.139	32	C=0/0, A=21/1
chr8:1514009	A/A	C	A	SNV	DLGAP2,DL GAP2-AS1	DLGAP2-AS1:intronic nc:NR 103863.1, DLGAP2:exonic:NM	1	100	6	missense	NM 00134	c.1391C>A	p.Pro464Gln	3.41	0.15	76	0.943	0.88(COSM41	Guanylate-ki	rs2301963	0.4474	YES	4.829E-1 (pos.1514009)	neoplasm	Discs large	associated	Autistic Disorder;Child Development Disorde			C=0, A=43	C=0.0, A=1.0	0.00001	704.917	43	C=0/0, A=36/7
chr14:101198417	G/G	A	G	SNV	DLK1	DLK1:exonic:NM 003836.6	1	100	4	missense	NM 00383	c.301A>G	p.Arg101Gly	0.59	0.94	125	0		EGF-like dom	rs6575799	0		1.0 (pos.101198417)		Delta, Dros	calcium io	Bone Marrow Diseases:Hematologic Disease			A=0, G=27	A=0.0, G=1.0	0.00001	389.988	27	A=0/0, G=24/3
chr12:52863091	G/T	G	T	SNV	KRT6C	KRT6C:intronic:NM 173086.4	1	51.92		intronic	NM 17308	c.1460-10	p.?	-0.08					Intermediate	rs35481773	0.2179	YES	1.516E-1 (pos.52863091)		Keratin 6C	cornification.cytosol.extracellular exosome.intermedia				G=25, T=27	G=0.4808, T=0.5192	0.00001	270.397	52	G=10/15, T=11
chr12:52863160	G/A	G	A	SNV	KRT6C	KRT6C:intronic:NM 173086.4	1	47.62		intronic	NM 17308	c.1459+35	p.?	-1.42					Intermediate	rs34838670	0.218	YES	1.517E-1 (pos.52863160)		Keratin 6C	cornification,cytosol,extracellular exosome,intermedia				G=11, A=10	G=0.5238, A=0.4762	0.00001	82.2899	21	G=6/5, A=5/5
chr6:170591923	T/G	T	G	SNV	DLL1	DLL1:utr 3:NM 005618.3	1	50	11	utr 3	NM 00561	c.2211A>C	p.?	1.03					Calcium-bind	rs1033583	0.3447	YES	2.344E-1 (pos.170591923)			Notch bind	Abnormalities, Multiple;Agenesis of Corpus C			T=17, G=17	T=0.5, G=0.5	0.00001	172.009	34	T=7/10, G=13/
chr19:39994711	C/C	T	C	SNV	DLL3	DLL3:exonic:NM 016941.3	1	100	5	missense	NM 01694	c.653T>C	p.Leu218Pro	1.88	0.56	98	0	0.05(COSM37	EGF-like dom	rs1110627	0.4764	YES	5.734E-1 (pos.39994711)	acute myeloic	Delta, Dros	Notch bind	Abnormalities, Multiple;Astro		Benign	T=0, C=86	T=0.0, C=1.0	0.00001	1410.62	86	T=0/0, C=45/4
chr17:48070878	C/T	C	T	SNV	DLX3	DLX3:exonic:NM 005220.2	1	57.14	2	synonymous	NM 00522	c.402G>A	p.(=)	0.34				0.59(COSM37	Homeobox d	rs2303466	0.1735	YES	5.102E-1 (pos.48070878)	adenocarcino	Distal-less l	DNA bindi	Amelogenesis Imperfecta;Con		Benign	C=9, T=12	C=0.4286, T=0.5714	0.00001	109.102	21	C=4/5, T=4/8
chr17:48046775	G/A	G	A	SNV	DLX4	DLX4:utr 5:NM 138281.2	1	45.45	1	utr 5	NM 13828	c.-58G>A	p.?	2.99					Homeobox d	rs58769681		YES			Distal-less l	DNA bindi	Neoplasm Invasiveness:Neoplasms:Neoplas			G=12, A=10	G=0.5455, A=0.4545	0.00001	81.7631	22	G=7/5, A=3/7
chr10:124402677	G/G	A	G	SNV	DMBT1	DMBT1:exonic:NM 007329.2	1	100	53	synonymous	NM 00732	c.7005A>G	p.(=)	-7.54					CUB domain,	rs1052715	0.4182	YES	6.887E-1 (pos.124402677)		Deleted in r	calcium-d	Colonic Diseases;Colonic Neoplasms;Colorec			A=0, G=41	A=0.0, G=1.0	0.00001	664.697	41	A=0/0, G=9/32
chr5:78340286	G/G	A	G	SNV	DMGDH	DMGDH:exonic:NM 013391.3	1	100	6	missense	NM 01339	c.835T>C	p.Ser279Pro	7.83	1	74	0	0.1(COSM416	Aminomethyl	rs532964	0.4737	YES	7.781E-1 (pos.78340286)	ductal carcinc	Dimethylgly	RNA bindir	Amino Acid Metabolism, Inborn Errors;Conge			A=0, G=80	A=0.0, G=1.0	0.00001	1288.93	80	A=0/0, G=38/4
chr12:52942998	T/T	C	T	SNV	KRT71	KRT71:intronic:NM 033448.2	1	100		intronic	NM 03344	c.717+79G	p.?	-1.58					Intermediate	rs635206					Keratin 71	cornificati	Hair Diseases:Mammary Neoplasms, Animal			C=0, T=23	C=0.0, T=1.0	0.00001	316.747	23	C=0/0, T=12/1
chr19:36004106	G/G	A	G	SNV	DMKN	DMKN:exonic:NM 033317.4	1	100	1	missense	NM 03331	c.272T>C	p.Val91Ala	-0.08	0.6	64	0	0.02(COSM3756663),0.02(C		rs4806163	0.2283	YES	8.698E-1;0.0 (pos.36004	adenocarcino	Dermokine	cornified envelope assembly.extracellular exosome.ext				A=0, G=37	A=0.0, G=1.0	0.00001	583.706	37	A=0/0, G=25/1
chr9:991114	G/G	A	G	SNV	DMRT3	DMRT3:utr 3:NM 021240.3	1	100	2	utr 3	NM 02124	c.1528A>G	p.?	1.5					DM DNA bind	rs279877		YES			Doublesex-	DNA binding transcription factor activity,adult walking b				A=0, G=23	A=0.0, G=1.0	0.00001	316.11	23	A=0/0, G=15/8
chr12:53071338	A/G	A	G	SNV	KRT1	KRT1:intronic:NM 006121.3	1	95.65		intronic	NM 00612	c.964-74T	p.?	0.09					Intermediate	rs626480					Keratin 1	blood mic	Carcinoma;Carcinoma, Squamous Cell;Conge			A=1, G=22	A=0.0435, G=0.9565	0.00001	289.407	23	A=0/1, G=12/1
chr7:86800351	G/G	A	G	SNV	DMTF1	DMTF1:exonic:NM 001142327.1	1	100	5	synonymous	NM 00114	c.273A>G	p.(=)	0.43				0.63(COSM41	Myb-like DNA	rs3747807	0.1364	YES	6.293E-1 (pos.86800351)	carcinoma,ne	Cyclin D-bin	DNA binding,DNA binding transcription factor activity,RN				A=0, G=30	A=0.0, G=1.0	0.00001	446.635	30	A=0/0, G=8/22
chr19:55671337	T/T	C	T	SNV	DNAAF3	DNAAF3:exonic:NM 001256714.1	1	100	10	missense	NM 00125	c.1294G>A	p.Asp432Asn	0.18	0.83	23	0.007	0.03(COSM45	Domain of un	rs890872		YES	1.0 (pos.55671337)	squamous cel	Dynein, axo	axonemal	Bronchial Diseases:Bronchiect		Likely benign	C=0, T=36	C=0.0, T=1.0	0.00001	563.625	36	C=0/0, T=19/1
chr12:124265687	C/C	T	C	SNV	DNAH10	DNAH10:exonic:NM 207437.3	1	100	6	missense	NM 20743	c.499T>C	p.Ser167Pro	-2.2	0.26	74	0		AAA domain (	rs11057353	0.37	YES	9.473E-1 (pos.124265687)		Dynein, axo	ATP binding,ATP-dependent microtubule n			Benign	T=0, C=49	T=0.0, C=1.0	0.00001	831.615	49	T=0/0, C=26/2
chr12:124267703	C/C	T	C	SNV	DNAH10	DNAH10:exonic:NM 207437.3	1	100	7	synonymous	NM 20743	c.708T>C	p.(=)	-3.86					AAA domain (	rs11057355	0.3953	YES	9.992E-1 (pos.124267703)		Dynein, axo	ATP binding,ATP-dependent microtubule n			Benign	T=0, C=112	T=0.0, C=1.0	0.00001	1776.42	112	T=0/0, C=42/7
chr12:53167313	C/C	T	C	SNV	KRT76	KRT76:intronic:NM 015848.4	1	100		intronic	NM 01584	c.876+53A	p.?	-0.52					Intermediate	rs7399317	0.0097	YES			Keratin 76,	cornification,cytoskeleton organization,cytosol,extracel				T=0, C=24	T=0.0, C=1.0	0.00001	324.605	24	T=0/0, C=8/16
chr12:124274474	G/G	A	G	SNV	DNAH10	DNAH10:exonic:NM 207437.3	1	100	11	missense	NM 20743	c.1438A>G	p.Ile480Val	3.35	1	29	0		AAA domain (	rs10846559	0.3303	YES	9.994E-1 (pos.124274474)		Dynein, axo	ATP binding,ATP-dependent microtubule n			Benign	A=0, G=58	A=0.0, G=1.0	0.00001	867.019	58	A=0/0, G=22/3
chr12:53216184	A/G	A	G	SNV	KRT79	KRT79:intronic:NM 175834.2	1	95.45		intronic	NM 17583	c.1368-21	p.?	-0.33					Intermediate	rs4415849	1.00E-04		1.0 (pos.53216184)		Keratin 79	cornification.cytosol.enzyme binding.extracellular exos				A=1, G=21	A=0.0455, G=0.9545	0.00001	271.679	22	A=0/1, G=13/8
chr12:53416251	T/T	C	T	SNV	EIF4B	EIF4B:intronic:NM 001417.6	1	100		intronic	NM 00141	c.533-26C	p.?	-4.58					RNA recogniti	rs10506315,rs77553	0.0736	YES	0.0 (pos.53416250),8.348E-1 (pos.53416		Eukaryotic t	RNA binding,RNA strand annealing activity,RNA strand-e				C=0, T=45	C=0.0, T=1.0	0.00001	746.931	45	C=0/0, T=18/2
chr12:53421507	A/A	G	A	SNV	EIF4B	EIF4B:intronic:NM 001417.6	1	100		intronic	NM 00141	c.668-59G	p.?	-1.83					RNA recogniti	rs4919697		YES			Eukaryotic t	RNA binding,RNA strand annealing activity,RNA strand-e				G=0, A=39	G=0.0, A=1.0	0.00001	623.941	39	G=0/0, A=27/1
chr7:21628237	T/T	C	T	SNV	DNAH11	DNAH11:exonic:NM 001277115.1	1	100	11	synonymous	NM 00127	c.1956C>T	p.(=)	0					AAA domain (	rs6963535	0.0131		1.0 (pos.21628237)		Dynein, axo	ATP bindin	Blood Protein Disorders;Bronc		Benign	C=0, T=97	C=0.0, T=1.0	0.00001	1643.07	97	C=0/0, T=55/4
chr7:21640361	C/C	T	C	SNV	DNAH11	DNAH11:exonic:NM 001277115.1	1	100	16	missense	NM 00127	c.3068T>C	p.Val1023Ala	2.37	0.69	64	0.174		AAA domain (	rs10269582	0.4344	YES	1.496E-2 (pos.21640361)		Dynein, axo	ATP bindin	Blood Protein Disorders;Bronc		Benign/Likely	T=0, C=52	T=0.0, C=1.0	0.00001	767.001	52	T=0/0, C=24/2
chr12:53509812	G/A	G	A	SNV	SOAT2	SOAT2:intronic:NM 003578.3	1	50		intronic	NM 00357	c.709-69G	p.?	0.15					MBOAT, mem	rs61929189		YES			Sterol O-acy	O-acyltran	Female Urogenital	Hesperetin		G=30, A=30	G=0.5, A=0.5	0.00001	291.517	60	G=23/7, A=20/
chr7:21640405	G/G	A	G	SNV	DNAH11	DNAH11:exonic:NM 001277115.1	1	100	16	missense	NM 00127	c.3112A>G	p.Thr1038Ala	0.94	0.51	58	0	0.02(COSM40	AAA domain (	rs10224537	0.1622	YES	8.035E-1 (pos.21640405)	acute myeloic	Dynein, axo	ATP bindin	Blood Protein Disorders;Bronc		Benign	A=0, G=53	A=0.0, G=1.0	0.00001	781.918	53	A=0/0, G=25/2
chr7:21775443	A/A	G	A	SNV	DNAH11	DNAH11:exonic:NM 001277115.1	1	100	46	synonymous	NM 00127	c.7626G>A	p.(=)	-0.3				0.03(COSM56	AAA domain (	rs2072221	0.138	YES	5.236E-1;0.0 (pos.21775	ductal carcinc	Dynein, axo	ATP bindin	Blood Protein Disorders;Bronc		Benign	G=0, A=23	G=0.0, A=1.0	0.00001	316.747	23	G=0/0, A=18/5
chr7:21901566	C/C	T	C	SNV	DNAH11	DNAH11:exonic:NM 001277115.1	1	100	69	synonymous	NM 00127	c.11298T>	p.(=)	-2.46				0.03(COSM40	AAA domain (	rs4722067	0.1755	YES	5.326E-1 (pos.21901566)	acute myeloic	Dynein, axo	ATP bindin	Blood Protein Disorders;Bronc		Benign	T=0, C=94	T=0.0, C=1.0	0.00001	1576.17	94	T=0/0, C=46/4
chr7:21924014	G/G	A	G	SNV	DNAH11	DNAH11:exonic:NM 001277115.1	1	100	76	missense	NM 00127	c.12493A>	p.Met4165Val	3.7	0.11	21	0.1	0.92(COSM56	AAA domain (	rs6461613	0.1522	YES	6.806E-1;0.0 (pos.21924(	ductal carcinc	Dynein, axo	ATP bindin	Blood Protein Disorders;Bronc		Benign	A=0, G=87	A=0.0, G=1.0	0.00001	1431.38	87	A=0/0, G=36/5
chr7:21939032	C/A	C	A	SNV	DNAH11	DNAH11:exonic:NM 001277115.1	1	47.62	80	synonymous	NM 00127	c.13128C>	p.(=)	-1.53				0.2(COSM597	AAA domain (	rs56333627	0.3228	YES	1.683E-1;0.0 (pos.21939(	acute myeloic	Dynein, axo	ATP bindin	Blood Protein Disorders:Bronc		Benign/Likely	C=11, A=10	C=0.5238, A=0.4762	0.00001	82.2897	21	C=4/7, A=4/6
chr1:225534219	C/C	T	C	SNV	DNAH14	DNAH14:exonic:NM 001373.1	1	100	69	missense	NM 00137	c.10880T>	p.Val3627Ala	0.07	0.89	64	0		ATP-binding c	rs7527925	0.4423	YES	5.032E-1 (pos.225534219)		Dynein, axo	ATP bindin	Congenital, Hereditary, and Ne		Benign	T=0, C=59	T=0.0, C=1.0	0.00001	885.366	59	T=0/0, C=34/2
chr12:53702371	C/C	T	C	SNV	AAAS	AAAS:intronic:NM 015665.5	1	100		intronic	NM 01566	c.1088-59	p.?	-2.92					WD domain,	rs4759287		YES			Aladin	centrosom	Adrenal Gland Diseases;Adrenal Insufficiency			T=0, C=30	T=0.0, C=1.0	0.00001	446.338	30	T=0/0, C=16/1
chr17:76525565	G/G	A	G	SNV	DNAH17	DNAH17:exonic:NM 173628.3	1	100	22	synonymous	NM 17362	c.3496T>C	p.(=)	-0.01				0.01(COSM44	AAA domain (	rs4273108	0.3753	YES	6.044E-1 (pos.76525565)	acute myeloic	Dynein, axonemal, heavy chain 17				Benign	A=0, G=37	A=0.0, G=1.0	0.00001	583.694	37	A=0/0, G=15/2
chr17:76557862	C/C	T	C	SNV	DNAH17	DNAH17:exonic:NM 173628.3	1	100	12	synonymous	NM 17362	c.1770A>G	p.(=)	0.51				0.09(COSM41	AAA domain (	rs894966	0.0405	YES	9.063E-1;0.0 (pos.76557	neoplasm	Dynein, axonemal, heavy chain 17				Benign	T=0, C=76	T=0.0, C=1.0	0.00001	1209.6	76	T=0/0, C=46/3
chr17:76491127	CC/CC	TG	CC	MNV	DNAH17,DN AH17-AS1	DNAH17:exonic:NM 173628.3, DNAH17-AS1:intronic nc:NR	2	100	39	missense	NM 17362	c.5955 59	p.Met1986Val	5.72, 0.79	1	21	0	0.03(COSM41	AAA domain (	rs34426129,rs60694	0.186	YES	9.753E-1 (pos.76491128)	neoplasm	Dynein, axonemal, heavy chain 17					TG=0, CC=1	TG=0.0, CC=1.0	0.00001	2860.64	174	TG=0/0, CC=66
chr17:7681412	G/G	C	G	SNV	DNAH2	DNAH2:exonic:NM 020877.3	1	100	34	synonymous	NM 02087	c.5265C>G	p.(=)	0.2					AAA domain (	rs7359662	0.0642	YES	8.037E-1 (pos.7681412)		Dynein, axo	ATP bindin	Autoimmune Diseases;Diabet		Benign	C=0, G=62	C=0.0, G=1.0	0.00001	939.969	62	C=0/0, G=27/3
chr12:53817916	A/A	T	A	SNV	AMHR2	AMHR2:intronic:NM 020547.3	1	100		intronic	NM 02054	c.49+149T	p.?	-0.06					Activin types	rs2272002		YES			Anti-Muller	ATP bindin	46, XY Disorders of:	ATP		T=0, A=52	T=0.0, A=1.0	0.00001	766.654	52	T=0/0, A=15/3
chr12:53819467	T/T	C	T	SNV	AMHR2	AMHR2:intronic:NM 020547.3	1	100		intronic	NM 02054	c.622-6C>	p.?	0.43					Activin types	rs2071558	0.1815	YES	1.786E-1 (pos.53819467)		Anti-Muller	ATP bindin	46, XY Disorders of:	ATP		C=0, T=36	C=0.0, T=1.0	0.00001	563.738	36	C=0/0, T=18/1
chr12:53824143	G/G	A	G	SNV	AMHR2	AMHR2:intronic:NM 020547.3	1	100		intronic	NM 02054	c.1425+77	p.?	-0.01					Activin types	rs11170555		YES			Anti-Muller	ATP bindin	46, XY Disorders of:	ATP		A=0, G=44	A=0.0, G=1.0	0.00001	726.569	44	A=0/0, G=23/2
chr5:13944512	C/C	A	C	SNV	DNAH5	DNAH5:exonic:NM 001369.2	1	100	1	missense	NM 00136	c.36T>G	p.His12Gln	1.07	1	24	0		AAA domain (	rs339445	0.058	YES	8.855E-1 (pos.13944512)		Dynein, axo	ATP bindin	Asthma;Bronchial Diseases;Br		Benign	A=0, C=71	A=0.0, C=1.0	0.00001	1111.39	71	A=0/0, C=46/2
chr12:53877562	T/T	C	T	SNV	MAP3K12	MAP3K12:intronic:NM 001193511.1	1	100		intronic	NM 00119	c.1359-55	p.?	-0.5					Protein kinas	rs784565		YES			Mitogen-ac	ATP binding,JNK cascade,MAP kinase kinase kinase activ				C=0, T=48	C=0.0, T=1.0	0.00001	810.433	48	C=0/0, T=31/1
chr12:53878012	G/G	A	G	SNV	MAP3K12	MAP3K12:intronic:NM 001193511.1	1	100		intronic	NM 00119	c.1248+29	p.?	-0.14					Protein kinas	rs7313459	0.0359	YES	0.0 (pos.53878012)		Mitogen-ac	ATP binding,JNK cascade,MAP kinase kinase kinase activ				A=0, G=66	A=0.0, G=1.0	0.00001	1015.4	66	A=0/0, G=33/3
chr12:53880122	C/C	T	C	SNV	MAP3K12	MAP3K12:intronic:NM 001193511.1	1	100		intronic	NM 00119	c.630-91A	p.?	0.14					Protein kinas	rs3816806		YES			Mitogen-ac	ATP binding.JNK cascade.MAP kinase kinase kinase activ				T=0, C=31	T=0.0, C=1.0	0.00001	465.814	31	T=0/0, C=19/1
chr12:53901105	A/A	G	A	SNV	NPFF,TARBP 2	NPFF:intronic:NM 003717.3, TARBP2:downstream:NM 134	1	100		intronic	NM 13432	c.*1173G>	p.?, p.?	0.64					Neuropeptid	rs8192593	0.0347				TAR RNA-bir	G-protein coupled receptor binding,G-protein coupled re				G=0, A=23	G=0.0, A=1.0	0.00001	316.747	23	G=0/0, A=20/3
chr12:540994	G/G	A	G	SNV	CCDC77	CCDC77:intronic:NM 032358.3	1	100		intronic	NM 03235	c.584-38A	p.?	0.09						rs7310504	0.268	YES	8.254E-1 (pos.540994)			centrosom	Nutrition Disorders;Nutritional and Metaboli			A=0, G=30	A=0.0, G=1.0	0.00001	446.639	30	A=0/0, G=15/1
chr5:13829799	G/A	G	A	SNV	DNAH5	DNAH5:exonic:NM 001369.2	1	53.33	38	synonymous	NM 00136	c.6264C>T	p.(=)	-0.82				0.07(COSM37	AAA domain (	rs1348689	0.3645	YES	6.157E-1;0.0 (pos.13829	adenocarcino	Dynein, axo	ATP bindin	Asthma:Bronchial Diseases:Br		Benign	G=14, A=16	G=0.4667, A=0.5333	0.00001	159.925	30	G=2/12, A=4/1
chr12:547325	G/G	A	G	SNV	CCDC77	CCDC77:intronic:NM 032358.3	1	100		intronic	NM 03235	c.822-176	p.?	0.94						rs758539		YES				centrosom	Nutrition Disorders;Nutritional and Metaboli			A=0, G=22	A=0.0, G=1.0	0.00001	298.892	22	A=0/0, G=10/1

chr2:84756049	A/A	G	A	SNV	DNAH6	DNAH6:exonic:NM_001370.1	1	100	4	missense	NM_00137	c.421G>A	p.Val141Met	-1.33	0.24	21	0.001		AAA domain (	rs4832089	0.0541	YES	9.984E-1 (pos.84756049)		Dynein, axo	ATP bindin	Congenital Abnormalities;Con		Benign	G=0, A=84	G=0.0, A=1.0	0.00001	1370.15	84	G=0/0, A=30/5
chr12:54805753	T/T	G	T	SNV	ITGA5,LOC102724050	LOC102724050:intronic_nc:NR_120486.1, ITGA5:intronic:NI	1	100		intronic	NR_12048	c.219-5C>/	p.?	1.93				0.28(COSM54	FG-GAP repe	rs1922254		YES	9.514E-1 (pos.54805753)	acute_myeloic	Integrin, alı	Golgi appa	Carcinoma;Carcinoma, Squamous Cell;Cardic			G=0, T=39	G=0.0, T=1.0	0.00001	583.257	39	G=0/0, T=13/2
chr12:54805765	T/T	C	T	SNV	ITGA5,LOC102724050	LOC102724050:intronic_nc:NR_120486.1, ITGA5:intronic:NI	1	100		intronic	NR_12048	c.219-17G	p.?	-0.09					FG-GAP repe	rs1249181	0.0829	YES	9.513E-1 (pos.54805765)		Integrin, alı	Golgi appa	Carcinoma;Carcinoma, Squamous Cell;Cardic			C=0, T=39	C=0.0, T=1.0	0.00001	623.972	39	C=0/0, T=13/2
chr12:54852940	A/G	A	G	SNV	GTSF1,LOC102724050	LOC102724050:intronic_nc:NR_120486.1, GTSF1:intronic:N	1	50		intronic	NR_12048	c.488-31T	p.?	1.87					U11-48K-like	rs374376021	3.00E-04		0.0 (pos.54852940)		Gametocyt	cell differentiation,cytoplasm,metal ion binding,sperma				A=16, G=16	A=0.5, G=0.5	0.00001	158.383	32	A=6/10, G=7/9
chr2:84771480	A/A	G	A	SNV	DNAH6	DNAH6:exonic:NM_001370.1	1	100	5	synonymous	NM_00137	c.786G>A	p.(=)	-3.63					AAA domain (	rs1037863	0.0544	YES	9.984E-1 (pos.84771480)		Dynein, axo	ATP bindin	Congenital Abnormalities;Con		Benign	G=0, A=29	G=0.0, A=1.0	0.00001	427.603	29	G=0/0, A=9/20
chr2:84800605	A/A	C	A	SNV	DNAH6	DNAH6:exonic:NM_001370.1	1	100	12	synonymous	NM_00137	c.1818C>A	p.(=)	0.38					AAA domain (	rs11891970	0.0555	YES	9.979E-1 (pos.84800605)		Dynein, axo	ATP bindin	Congenital Abnormalities;Con		Benign	C=0, A=64	C=0.0, A=1.0	0.00001	977.633	64	C=0/0, A=39/2
chr2:84897517	G/G	A	G	SNV	DNAH6	DNAH6:exonic:NM_001370.1	1	100	39	synonymous	NM_00137	c.6372A>G	p.(=)	-0.05					AAA domain (	rs1881095	0.0488	YES	1.0 (pos.84897517)		Dynein, axo	ATP bindin	Congenital Abnormalities;Con		Benign	A=0, G=31	A=0.0, G=1.0	0.00001	448.626	31	A=0/0, G=18/1
chr12:56091617	A/G	A	G	SNV	ITGA7	ITGA7:intronic:NM_001144996.1	1	50		intronic	NM_00114	c.1294-11	p.?	0.01					FG-GAP repe	rs2293412	0.487	YES	9.483E-1 (pos.56091617)		Integrin, alı	cell adhes	Colonic Diseases;Colonic Neoc		Benign	A=10, G=10	A=0.5, G=0.5	0.00001	82.8864	20	A=5/5, G=3/7
chr2:84921433	A/G	A	G	SNV	DNAH6	DNAH6:exonic:NM_001370.1	1	50	46	synonymous	NM_00137	c.7353A>G	p.(=)	-0.66					AAA domain (	rs1192295	0.0544	YES	1.603E-3 (pos.84921433)		Dynein, axo	ATP bindin	Congenital Abnormalities;Con		Benign	A=15, G=15	A=0.5, G=0.5	0.00001	145.019	30	A=7/8, G=11/4
chr2:196673533	G/G	A	G	SNV	DNAH7	DNAH7:exonic:NM_018897.2	1	100	53	missense	NM_01889	c.9956T>C	p.Leu3319Pro	7.03	1	98	0		ATP-binding c	rs13411834	4.00E-04	YES	1.0 (pos.196673533)		Dynein, axo	ATP binding,ATP-dependent microtubule n			Benign	A=0, G=57	A=0.0, G=1.0	0.00001	849.571	57	A=0/0, G=39/1
chr12:56417403	T/C	T	C	SNV	IKZF4,LOC105369781	IKZF4:intronic:NM_022465.3, LOC105369781:intronic_nc:N	1	53.13		intronic	NR_13502	c.88-52T>C	p.?	0.01					C2H2-type zir	rs2658479	0.0839	YES			Ikaros famil	DNA bindi	Alopecia;Alopecia Areata;Asthma;Autoimmu			T=15, C=17	T=0.4688, C=0.5313	0.00001	173.56	32	T=12/3, C=9/8
chr17:11523082	G/G	A	G	SNV	DNAH9	DNAH9:exonic:NM_001372.3	1	100	6	missense	NM_00137	c.1334A>G	p.Gln445Arg	3.77	1	43	0		AAA domain (	rs9892256	1.00E-04		1.0 (pos.11523082)		Dynein, axo	ATP binding,ATP-dependent microtubule motor activity,				A=0, G=51	A=0.0, G=1.0	0.00001	754.542	51	A=0/0, G=31/2
chr12:56650742	T/T	C	T	SNV	ANKRD52	ANKRD52:intronic:NM_173595.3	1	100		intronic	NM_17359	c.261+57G	p.?	0.01					Ankyrin repe	rs1274491	0	YES				protein binding				C=0, T=43	C=0.0, T=1.0	0.00001	705.844	43	C=0/0, T=20/2
chr17:11583089	C/C	T	C	SNV	DNAH9	DNAH9:exonic:NM_001372.3	1	100	18	synonymous	NM_00137	c.3369T>C	p.(=)	0.25				0.4(COSM400	AAA domain (	rs8071438	0.012	YES	9.149E-1 (pos.11583089)	acute_myeloic	Dynein, axo	ATP binding,ATP-dependent microtubule n			Benign	T=0, C=142	T=0.0, C=1.0	0.00001	2387.74	142	T=0/0, C=70/7
chr12:56726518	A/A	G	A	SNV	PAN2	PAN2:intronic:NM_001127460.2	1	100		intronic	NM_00112	c.282+79C	p.?	0.6					Exonuclease,	rs2643626		YES			NACHT dom	3'-5'-exoribonuclease activity,P-body,PAN complex,RNA				G=0, A=28	G=0.0, A=1.0	0.00001	408.717	28	G=0/0, A=23/5
chr12:56818538	G/A	G	A	SNV	TIMELESS	TIMELESS:intronic:NM_003920.4	1	50		intronic	NM_00392	c.1868+8C	p.?	0.9					Timeless pro	rs150742725	0.0031		2.317E-4 (pos.56818538)		Timeless, D	DNA replic	Autoimmune Diseases;Female Urogenital Dis			G=29, A=29	G=0.5, A=0.5	0.00001	274.179	58	G=14/15, A=1
chr17:11648332	T/T	C	T	SNV	DNAH9	DNAH9:exonic:NM_001372.3	1	100	31	synonymous	NM_00137	c.6330C>T	p.(=)	0.61				0.7(COSM376	AAA domain (	rs3744578	0.2008	YES	9.103E-1 (pos.11648332)	acute_myeloic	Dynein, axo	ATP binding,ATP-dependent microtubule n			Benign	C=0, T=76	C=0.0, T=1.0	0.00001	1214.11	76	C=0/0, T=33/4
chr17:11833365	A/A	G	A	SNV	DNAH9	DNAH9:exonic:NM_001372.3	1	100	63	synonymous	NM_00137	c.12060G>	p.(=)	-4.89				0.02(COSM37	AAA domain (	rs1859887	0.332	YES	1.964E-1 (pos.11833365)	adenocarcino	Dynein, axo	ATP binding,ATP-dependent microtubule n			Benign	G=0, A=28	G=0.0, A=1.0	0.00001	399.044	28	G=0/0, A=13/1
chr17:11835331	A/A	G	A	SNV	DNAH9	DNAH9:exonic:NM_001372.3	1	100	64	missense	NM_00137	c.12106G>	p.Asp4036Asn	5.39	0.11	23	0.19	0.98(COSM37	AAA domain (	rs17612861	0.3344	YES	1.968E-1 (pos.11835331)	adenocarcino	Dynein, axo	ATP binding,ATP-dependent microtubule n			Benign	G=0, A=22	G=0.0, A=1.0	0.00001	298.396	22	G=0/0, A=8/14
chr17:72308319	A/A	G	A	SNV	DNAI2	DNAI2:exonic:NM_023036.5	1	100	12	missense	NM_02303	c.1672G>A	p.Ala558Thr	-2.58	0.52	58	0.002	0.03(COSM4130570)		rs1979370	0.0897	YES	8.515E-1 (pos.72308319)	Wilms_tumou	Dynein, axo	ATP-deper	Bronchial Diseases;Bronchiect		Benign	G=0, A=44	G=0.0, A=1.0	0.00001	726.561	44	G=0/0, A=31/1
chr15:78556589	G/G	C	G	SNV	DNAJA4	DNAJA4:utr_5:NM_018602.3	1	100	1	utr_5	NM_01860	c.-67C>G	p.?	0.69					DnaJ C termi	rs12898295,rs200375873		YES				ATP binding,chaperone binding,cytosol,heat shock prote				C=0, G=37	C=0.0, G=1.0	0.00001	583.621	37	C=0/0, G=33/4
chr1:6704635	G/G	A	G	SNV	DNAJC11	DNAJC11:exonic:NM_018198.3	1	100	10	synonymous	NM_01819	c.1080T>C	p.(=)	-1.26				0.12(COSM37	DnaJ domain	rs200458	0.0883	YES	5.977E-1 (pos.6704635)	hepatocellula	DNAJ/HSP4(	MICOS complex,cristae formation,extracellular exosome				A=0, G=35	A=0.0, G=1.0	0.00001	543.598	35	A=0/0, G=14/2
chr5:138775173	C/C	T	C	SNV	DNAJC18	DNAJC18:utr_5:NM_152686.3	1	100	1	utr_5	NM_15268	c.-39A>G	p.?	-1.48					DnaJ domain	rs3923059	0.2777	YES	3.627E-1 (pos.138775173)			integral component of membrane				T=0, C=24	T=0.0, C=1.0	0.00001	334.791	24	T=0/0, C=9/15
chr21:34860756	ATTAAA/A	ATTAAA	A,ATTTAAA	INDEL	DNAJC28	DNAJC28:exonic:NM_001040192.2	5	48.15	2	frameshiftDeletion	NM_00104	c.940_944	p.Leu316fs	1.26, 7.09, 7.09, 0.72, 5.05, 8.26					DnaJ domain	rs139967340,rs7494	1.00E-04		0.0;0.0 (pos.34860756)			Golgi orga	Abnormalities, Multiple;Chromosome Disord			ATTAAA=14	ATTAAA=0.5185, A=0	0.00001	117.885	27	ATTAAA=8/6, A
chrX:153629155	G/G	A	G	SNV	DNASE1L1,RPL10,SNORA70	SNORA70:downstream:NR_000011.1, RPL10:exonic:NM_00(	1	100	7	missense	NM_00601	c.605A>G,	p.Asn202Ser, p.?	1.28	0.39	46	0	0.81(COSM45	Ribosomal pr	rs12012747,rs4909		YES	9.291E-1 (pos.15362915!	ductal_carcinc	Deoxyribon	DNA bindi	Autistic Disorder;Child Develo		Benign/Likely	A=0, G=121	A=0.0, G=1.0	0.00001	1953.68	121	A=0/0, G=65/5
chr12:57137828	A/A	T	A	SNV	PRIM1	PRIM1:intronic:NM_000946.2	1	100		intronic	NM_00094	c.638+21A	p.?	0.11					DNA primase	rs1026565	0.378	YES	7.613E-1;0.0 (pos.57137828)		Primase, pc	DNA primase activity,DNA replication initiation,DNA rep				T=0, A=56	T=0.0, A=1.0	0.00001	832.1	56	T=0/0, A=28/2
chr12:57592557	T/T	C	T	SNV	LRP1	LRP1:intronic:NM_002332.2	1	100		intronic	NM_00233	c.9676+10	p.?	0.3					Calcium-bind	rs1800168		YES			Low density	RNA bindir	Alzheimer Disease;	Antihemophilic factor, h		C=0, T=104	C=0.0, T=1.0	0.00001	1629.13	104	C=0/0, T=43/6
chr5:140052271	G/G	A	G	SNV	DND1	DND1:exonic:NM_194249.2	1	100	3	synonymous	NM_19424	c.363T>C	p.(=)	1.12				0.28(COSM37	RNA recogniti	rs2563333,rs759113911		YES	5.333E-1;0.0 (pos.14005;	adenocarcino	Dead end, z	AU-rich element binding,cytoplasm,germ cell developme				A=0, G=21	A=0.0, G=1.0	0.00001	281.235	21	A=0/0, G=18/3
chr12:57869447	T/C	T	C	SNV	ARHGAP9	ARHGAP9:intronic:NM_032496.3	1	50		intronic	NM_03249	c.1312-49	p.?	0.2					PH domain,R	rs2277318	0.3135	YES	3.818E-1 (pos.57869447)		Rho GTPase	GTPase ac	Cardiovascular Diseases;Coronary Disease;C			T=14, C=14	T=0.5, C=0.5	0.00001	131.941	28	T=1/13, C=4/1
chr12:57869476	G/A	G	A	SNV	ARHGAP9	ARHGAP9:intronic:NM_032496.3	1	50		intronic	NM_03249	c.1312-78	p.?	-1.74					PH domain,R	rs2277317		YES			Rho GTPase	GTPase ac	Cardiovascular Diseases;Coronary Disease;C			G=14, A=14	G=0.5, A=0.5	0.00001	131.941	28	G=1/13, A=4/1
chr12:57869500	C/C	A	C	SNV	ARHGAP9	ARHGAP9:intronic:NM_032496.3	1	100		intronic	NM_03249	c.1312-10	p.?	-0.4					PH domain,R	rs524522					Rho GTPase	GTPase ac	Cardiovascular Diseases;Coronary Disease;C			A=0, C=29	A=0.0, C=1.0	0.00001	427.603	29	A=0/0, C=5/24
chr12:57870121	C/C	A	C	SNV	ARHGAP9	ARHGAP9:intronic:NM_032496.3	1	100		intronic	NM_03249	c.1116+26	p.?	0.04					PH domain,R	rs486699	0.0526	YES	8.426E-1;0.0 (pos.57870121)		Rho GTPase	GTPase ac	Cardiovascular Diseases;Coronary Disease;C			A=0, C=27	A=0.0, C=1.0	0.00001	389.768	27	A=0/0, C=3/24
chr12:57940749	T/T	C	T	SNV	DCTN2	DCTN2:intronic:NM_006400.4	1	100		intronic	NM_00640	c.36+62G>	p.?	-0.49					Dynamitin	rs775325	0.0019	YES			Dynactin 2	ER to Golgi vesicle-mediated transport,G2/M transition (				C=0, T=69	C=0.0, T=1.0	0.00001	1072.78	69	C=0/0, T=48/2
chr12:57975570	G/G	A	G	SNV	KIF5A	KIF5A:intronic:NM_004984.2	1	100		intronic	NM_00498	c.2910-83	p.?	-1.24					Kinesin moto	rs775247					Kinesin fam	ATP bindin	Arthritis;Arthritis, Rheumatoid;Autoimmune			A=0, G=31	A=0.0, G=1.0	0.00001	465.724	31	A=0/0, G=15/1
chr11:6549779	C/C	A	C	SNV	DNHD1	DNHD1:exonic:NM_144666.2	1	100	11	missense	NM_14466	c.1865A>C	p.Asp622Ala	-1.22	0.62	126	0		Dynein heavy	rs4633449	0.3281	YES	8.762E-1;0.0 (pos.6549779)			ATP bindin	Adenocarcinoma;Carcinoma;Carcinoma, Ren			A=0, C=56	A=0.0, C=1.0	0.00001	832.1	56	A=0/0, C=46/1
chr12:58135659	C/C	T	C	SNV	AGAP2		1	100		intronic				-1.23						rs238530	2.00E-04		1.0;0.0 (pos.58135659)		ARF-GAP with	GTP-bind	Autoimmune Diseases;Autoimmune Disease			T=0, C=48	T=0.0, C=1.0	0.00001	810.153	48	T=0/0, C=41/7
chr12:58166403	T/G	T	G	SNV	EEF1AKMT3,METTL1	METTL1:upstream:NM_005371.5, EEF1AKMT3:utr_5:NM_01!	1	50		intronic	NM_00537	c.-537A>C,	p.?, p.?	0.89					Lysine methy	rs2291617		YES			Methyltran	centrosom	Autoimmune Diseases;Autoimmune Disease			T=14, G=14	T=0.5, G=0.5	0.00001	131.941	28	T=11/3, G=10/
chr11:6555365	G/G	A	G	SNV	DNHD1	DNHD1:exonic:NM_144666.2	1	100	14	missense	NM_14466	c.2960A>G	p.Asp987Gly	0.28	0.5	94	0	0.02(COSM63	Dynein heavy	rs7103810	0.1301	YES	9.211E-1 (pos.6555365)	carcinoma		ATP bindin	Adenocarcinoma;Carcinoma;Carcinoma, Ren			A=0, G=37	A=0.0, G=1.0	0.00001	583.706	37	A=0/0, G=17/2
chr12:6085513	G/G	A	G	SNV	VWF	VWF:intronic:NM_000552.4	1	100		intronic	NM_00055	c.7288-87	p.?	0.09					C8 domain,Tr	rs216855		YES			Coagulatior	Weibel-Pa	Arrhythmias, Cardi	Antihemophilic factor, h		A=0, G=30	A=0.0, G=1.0	0.00001	446.63	30	A=0/0, G=11/1
chr12:6090892	G/A	G	A	SNV	VWF	VWF:intronic:NM_000552.4	1	45.45		intronic	NM_00055	c.7287+60	p.?	-0.84					C8 domain,Tr	rs2286937		YES			Coagulatior	Weibel-Pa	Arrhythmias, Cardi	Antihemophilic factor, h		G=12, A=10	G=0.5455, A=0.4545	0.00001	81.7622	22	G=7/5, A=4/6
chr11:6588228	A/A	G	A	SNV	DNHD1	DNHD1:exonic:NM_144666.2	1	100	36	missense	NM_14466	c.11489G>	p.Arg3830His	0.3	0.07	29	0.029	0.06(COSM37	Dynein heavy	rs10769699	0.3847	YES	2.452E-1 (pos.6588228)	acute_myeloid	leukaemia	ATP bindin	Adenocarcinoma;Carcinoma;Carcinoma, Ren			G=0, A=102	G=0.0, A=1.0	0.00001	1593.06	102	G=0/0, A=68/3
chr12:6138702	G/A	G	A	SNV	VWF	VWF:intronic:NM_000552.4	1	50		intronic	NM_00055	c.2821-48	p.?	-2.13					C8 domain,Tr	rs552884819,rs6083	0.0794	YES	0.0 (pos.6138698),6.573E-2 (pos.613870		Coagulatior	Weibel-Pa	Arrhythmias, Cardi	Antihemophilic factor, h		G=31, A=31	G=0.5, A=0.5	0.00001	306.848	62	G=18/13, A=1
chr11:6524072	A/C	A	C	SNV	DNHD1	DNHD1:exonic:NM_144666.2	1	50	4	missense	NM_14466	c.836A>C	p.Gln279Pro	0.12	0.01	76	0.838		Dynein heavy	rs11605196	0.1904	YES	1.445E-2 (pos.6524072)			ATP bindin	Adenocarcinoma;Carcinoma;Carcinoma, Ren			A=17, C=17	A=0.5, C=0.5	0.00001	171.561	34	A=10/7, C=6/1
chr9:130984755	T/T	C	T	SNV	DNM1	DNM1:exonic:NM_004408.3	1	100	8	synonymous	NM_00440	c.1008C>T	p.(=)	0.58				0.32(COSM41	Dynamin GTP	rs3003609	0.4579	YES	6.009E-1 (pos.13098475!	neoplasm	Dynamin-1	D2 dopam	Brain Diseases;Central Nervou		Benign	C=0, T=94	C=0.0, T=1.0	0.00001	1563.56	94	C=0/0, T=34/6
chr12:62861111	C/C	T	C	SNV	MON2	MON2:intronic:NM_015026.2	1	100		intronic	NM_01502	c.111+13T	p.?	0.08					C-terminal re	rs6581450	0.3574	YES	6.539E-1 (pos.62861111)		Mon2, S. ce	Golgi to endosome transport,cytosol,extracellular exosc				T=0, C=49	T=0.0, C=1.0	0.00001	831.632	49	T=0/0, C=20/2
chr12:62888729	T/T	C	T	SNV	MON2	MON2:intronic:NM_015026.2	1	100		intronic	NM_01502	c.304-67C	p.?	0.51					C-terminal re	rs7135893		YES			Mon2, S. ce	Golgi to endosome transport,cytosol,extracellular exosc				C=0, T=24	C=0.0, T=1.0	0.00001	333.607	24	C=0/0, T=11/1
chr12:32866157	T/A	T	A	SNV	DNM1L	DNM1L:exonic:NM_012062.4	1	57.14	6	missense	NM_01206	c.471T>A	p.Asp157Glu	1.27	0	45	0.996		Dynamin GTP	rs776124108			0.0 (pos.32866157)		Dynamin 1-	BH2 doma	Acid-Base Imbalance;Acidosis;Acidosis, Lacti			T=12, A=16	T=0.4286, A=0.5714	0.00001	161.77	28	T=5/7, A=6/10
chr12:6422272	C/C	T	C	SNV	PLEKHG6	PLEKHG6:intronic:NM_018173.3	1	100		intronic	NM_01817	c.139-523	p.?	-0.54					RhoGEF doma	rs10849440		YES	1.0 (pos.6422272)		Pleckstrin h	GTPase activator activity,Rho guanyl-nucleotide exchan				T=0, C=25	T=0.0, C=1.0	0.00001	352.965	25	T=0/0, C=14/1
chr12:6428116	G/G	T	G	SNV	PLEKHG6	PLEKHG6:intronic:NM_018173.3	1	100		intronic	NM_01817	c.1409-43	p.?	-0.81					RhoGEF doma	rs4764497	0.3887	YES	3.05E-1 (pos.6428116)		Pleckstrin h	GTPase activator activity,Rho guanyl-nucleotide exchan				T=0, G=23	T=0.0, G=1.0	0.00001	316.747	23	T=0/0, G=13/1
chr12:6439255	G/G	A	G	SNV	TNFRSF1A	TNFRSF1A:intronic:NM_001065.3	1	100		intronic	NM_00106	c.769-23T	p.?	0.18					Death domai	rs10849443		YES	1.0 (pos.6439255)		Tumor necr	Golgi mem	Arthritis;Autoimmu	6-3-(4-Morpholinyl)Propy		A=0, G=55	A=0.0, G=1.0	0.00001	814.905	55	A=0/0, G=33/2
chr12:6442346	G/G	A	G	SNV	TNFRSF1A	TNFRSF1A:intronic:NM_001065.3	1	100		intronic	NM_00106	c.473-33T	p.?	-0.51					Death domai	rs1800692	0.4023	YES	5.164E-1 (pos.6442346)		Tumor necr	Golgi mem	Arthritis;Autoimmu	6-3-(4-Morpholinyl)Propy		A=0, G=56	A=0.0, G=1.0	0.00001	831.967	56	A=0/0, G=29/2
chr1:172356437	G/G	A	G	SNV	DNM3	DNM3:exonic:NM_015569.4	1	100	19	synonymous	NM_01556	c.2223A>G	p.(=)	-4.88					Dynamin GTP	rs6688654	0.0144		1.0 (pos.172356437)		Dynamin 3	GTP binding,GTPase activity,Golgi apparatus,axon,dendı				A=0, G=31	A=0.0, G=1.0	0.00001	465.819	31	A=0/0, G=9/22
chr2:220250147	G/G	A	G	SNV	DNPEP	DNPEP:exonic:NM_012100.3	1	100	8	missense	NM_01210	c.773T>C	p.Val258Ala	3	0.94	64	0		Aminopeptid	rs907679	0		1.0 (pos.220250147)		Aspartyl am	aminopeptidase activity,blooc		Glutamic Acid		A=0, G=70	A=0.0, G=1.0	0.00001	1092.09	70	A=0/0, G=48/2
chr8:25267622	G/G	A	G	SNV	DOCK5	DOCK5:exonic:NM_024940.7	1	100	51	synonymous	NM_02494	c.5412A>G	p.(=)	-0.33				0.48(COSM37	C2 domain in	rs2709618	0.3723	YES	6.299E-1 (pos.25267622)	acute_myeloic	Dedicator c	cytoplasm,cytosol,guanyl-nucleotide exchange factor ac				A=0, G=23	A=0.0, G=1.0	0.00001	321.214	23	A=0/0, G=13/1
chr12:64668891	A/A	G	A	SNV	C12orf56	C12orf56:intronic:NM_001170633.1	1	100		intronic	NM_00117	c.1510-13	p.?	-0.24					Protein of unl	rs2010893		YES								G=0, A=31	G=0.0, A=1.0	0.00001	465.311	31	G=0/0, A=17/1
chr12:64668948	A/A	G	A	SNV	C12orf56	C12orf56:intronic:NM_001170633.1	1	100		intronic	NM_00117	c.1509+13	p.?	0.17					Protein of unl	rs2010889		YES								G=0, A=23	G=0.0, A=1.0	0.00001	316.738	23	G=0/0, A=11/1
chr12:64669018	A/A	AT	A	INDEL	C12orf56	C12orf56:intronic:NM_001170633.1	1	100		intronic	NM_00117	c.1509+60	p.?	-0.02, 0.12					Protein of unl	rs112622378,rs5798	0.2021	YES								AT=0, A=24	AT=0.0, A=1.0	0.00001	334.792	24	AT=0/0, A=11/
chr8:25234858	A/G	A	G	SNV	DOCK5	DOCK5:exonic:NM_024940.7	1	97.01	38	missense	NM_02494	c.3854A>G	p.Lys1285Arg	4.79	1	26	0		C2 domain in	rs2659585	0		1.0 (pos.25234858)		Dedicator c	cytoplasm,cytosol,guanyl-nucleotide exchange factor ac				A=2, G=65	A=0.0299, G=0.9701	0.00001	968.056	67	A=0/2, G=31/3
chr12:64818341	G/G	A	G	SNV	XPOT	XPOT:intronic:NM_007235.4	1	100		intronic	NM_00723	c.1183-38	p.?	1.56					Exportin 1-lik	rs61931551	0.2626	YES	2.289E-1;0.0 (pos.64818341)			Ran GTPase binding,cytoplasm,cytosol,intracellular prot				A=0, G=103	A=0.0, G=1.0	0.00001	1609.52	103	A=0/0, G=37/6
chr12:65134536	G/G	C	G	SNV	GNS	GNS:intronic:NM_002076.3	1	100		intronic	NM_00207	c.793-68G	p.?	-0.79					Sulfatase	rs1690277		YES			N-acetylglu	N-acetylgl	Autoimmune Diseases;Carbohydrate Metabc			C=0, G=44	C=0.0, G=1.0	0.00001	726.569	44	C=0/0, G=23/2
chr12:65460368	G/G	A	G	SNV	WIF1	WIF1:intronic:NM_007191.4	1	100		intronic	NM_00719	c.730+53T	p.?	-0.14					Human growt	rs3782498	0.3482	YES			WNT inhibit	Wnt signa	Colonic Diseases;Colorectal Neoplasms;Dige			A=0, G=27	A=0.0, G=1.0	0.00001	389.976	27	A=0/0, G=16/1
chr19:11358799	A/A	G	A	SNV	DOCK6	DOCK6:exonic:NM_020812.3	1	100	7	missense	NM_02081	c.749C>T	p.Pro250Leu	7.2	0	98	0.982	0.96(COSM37	C2 domain in	rs12978266	0.299	YES	5.162E-1 (pos.11358799)	acute_myeloic	Dedicator c	blood coa	Abnormalities, Multiple;Conge		Benign	G=0, A=42	G=0.0, A=1.0	0.00001	685.208	42	G=0/0, A=27/1
chr9:429719	C/C	T	C	SNV	DOCK8	DOCK8:exonic:NM_203447.3	1	100	36	synonymous	NM_20344	c.4491T>C	p.(=)	0.26					C2 domain in	rs7854035	0.0044		9.999E-1 (pos.429719)		Dedicator c	blood coa	Developmental Disabilities;He		Benign	T=0, C=20	T=0.0, C=1.0	0.00001	263.728	20	T=0/0, C=11/9
chr18:67365668	G/G	A	G	SNV	DOK6	DOK6:exonic:NM_152721.5	1	100	5	synonymous	NM_15272	c.438A>G	p.(=)	-0.84					PTB domain (	rs4426448	0.4658	YES	7.528E-1 (pos.67365668)			axon guida	Bone Diseases;Bone Diseases, Metabolic;Mu			A=0, G=28	A=0.0, G=1.0	0.00001	408.718	28	A=0/0, G=16/1
chr21:37617724	C/A	C	A	SNV	DOPEY2	DOPEY2:exonic:NM_005128.3	1	57.14	19	missense	NM_00512	c.3446C>A	p.Pro1149His	4.23	0	77	0.001	0.96(COSM44	Dopey, N-ter	rs3746866	0.1309	YES	2.454E-1 (pos.37617724)	ductal_carcinc	Dopey fami	Golgi mem	Abnormalities, Multiple;Chromosome Disord			C=9, A=12	C=0.4286, A=0.5714	0.00001	109.102	21	C=7/2, A=9/3
chr14:73360819	TTGTC/TTGTC	T	TTGTC	INDEL	DPF3	DPF3:utr_5:NM_012074.4	4	100	1	utr_5	NM_01207	c.-23A>GA	p.?	7.35						rs1038020539,rs580	0.0189	YES	1.0;0.0 (pos.73360819)		D4, zinc anc	biological	Nervous System Diseases;Neuromuscular Di			T=0, TTGTC	T=0.0, TTGTC=1.0	0.00001	316.747	23	T=0/0, TTGTC=
chr12:6567787	C/C	T	C	SNV	TAPBPL	TAPBPL:intronic:NM_018009.4	1	100		intronic	NM_01800	c.905-24T	p.?	0.93					Immunoglobu	rs2243750	0.3047	YES	3.436E-1 (pos.6567787)			Golgi membrane,endoplasmic reticulum membrane,imr				T=0, C=33	T=0.0, C=1.0	0.00001	504.598	33	T=0/0, C=29/4
chr12:66517828	A/A	T	A	SNV	LLPH	LLPH:intronic:NM_032338.3	1	100		intronic	NM_03233	c.212-30A	p.?	0.74					Learning-ass	rs2464304		YES	1.0 (pos.66517828)		LLP, Aplysia	RNA binding,basal RNA polymerase II transcription mach				T=0, A=27	T=0.0, A=1.0	0.00001	389.988	27	T=0/0, A=18/9
chr17:1943888	C/C	T	C	SNV	DPH1	DPH1:exonic:NM_001383.4	1	100	9	synonymous	NM_00138	c.1011T>C	p.(=)	-4.8					Putative diph	rs2236375	0.2227	YES	9.342E-1 (pos.1943888)		DPH1, S. cei	cell junction,cell proliferation,cytosol,nucleoplasm,pep				T=0, C=25	T=0.0, C=1.0	0.00001	353.019	25	T=0/0, C=3/22
chr12:66717692	T/G	T	G	SNV	HELB	HELB:intronic:NM_033647.4	1	47.62		intronic	NM_03364	c.2298-71	p.?	0.56					AAA domain,I	rs7969887		YES			Helicase, D	ATP binding,ATP-dependent 5'-3' DNA helicase activity,AT				T=11, G=10	T=0.5238, G=0.4762	0.00001	82.29	21	T=6/5, G=5/5
chr12:66717716	C/T	C	T	SNV	HELB	HELB:intronic:NM_033647.4	1	47.62		intronic	NM_03364	c.2298-47	p.?	-0.01					AAA domain,I	rs7957877	0.2562	YES	4.551E-1 (pos.66717716)		Helicase, D	ATP binding,ATP-dependent 5'-3' DNA helicase activity,AT				C=11, T=10	C=0.5238, T=0.4762	0.00001	82.29	21	C=6/5, T=5/5
chr12:6762026	C/C	T	C	SNV	ING4	ING4:intronic:NM_001127582.1	1	100		intronic	NM_00112	c.391+76A	p.?	-0.98					Inhibitor of gı	rs4764506		YES			Inhibitor of	DNA damage response, signal transduction by p53 class				T=0, C=49	T=0.0, C=1.0	0.00001	820.895	49	T=0/0, C=29/2
chr15:35664298	G/G	C	G	SNV	DPH6,MIR3942	MIR3942:downstream:NR_037507.1, DPH6:utr_3:NM_0806	1	100	9	utr_3	NM_08065	c.857G>C	p.?	-0.06					Diphthamide	rs3759846		YES	3.12E-1;0.0 (pos.35664298)			ATP binding,cytosol,diphthine-ammonia ligase activity,n				C=0, G=77	C=0.0, G=1.0	0.00001	1236.1	77	C=0/0, G=29/4
chr12:68719216	A/A	G	A	SNV	MDM1	MDM1:intronic:NM_017440.4	1	100		intronic	NM_01744	c.633+5C>	p.?	0.02				0.01(COSM44	Nuclear prot	rs2870812	0.2863	YES	4.221E-1 (pos.68719216)	acute_myeloic	Mouse doul	centriole,centrosome,cytosol,microtubule,microtubule				G=0, A=20	G=0.0, A=1.0	0.00001	263.786	20	G=0/0, A=12/8
chr12:69115003	TTAAA/T	TTAAA	T	INDEL	NUP107	NUP107:intronic:NM_020401.3	4	57.14		intronic	NM_02040	c.1311+46	p.?	0.1, -1.3, -0.47, -0.2, 1.74					Nuclear pore	rs747075812,rs765260259,rs780350662			0.0 (pos.69114999),0.0 (pos.69115007),:		Nucleopori	centrosome,condensed chromosome kinetochore,cytos				TTAAA=15,	TTAAA=0.4286, T=0.5	0.00001	217.689	35	TTAAA=7/8, T=
chr12:69250481	C/C	T	C	SNV	CPM	CPM:intronic:NM_001005502.2	1	100		intronic	NM_00100	c.1090-22	p.?	0.26					Carboxypept	rs3741599	0.2058	YES	1.077E-2 (pos.69250481)		Carboxypep	C-termina	Endometriosis;Female Urogenital Diseases;F			T=0, C=95	T=0.0, C=1.0	0.00001	1597.06	95	T=0/0, C=26/6
chr12:69279736	G/G	A	G	SNV	CPM	CPM:intronic:NM_001005502.2	1	100		intronic	NM_00100	c.161-67T	p.?	0.01					Carboxypept	rs2701085		YES			Carboxypep	C-termina	Endometriosis;Female Urogenital Diseases;F			A=0, G=49	A=0.0, G=1.0	0.00001	831.628	49	A=0/0, G=7/42
chr9:130698029	C/C	G	C	SNV	DPM2	DPM2:exonic:NM_003863.3	1	100	4	missense	NM_00386	c.227C>G	p.Thr76Ser	0.14	1	58	0	0.16(COSM39	Dolichol pho	rs7997	0.1319	YES	4.533E-1 (pos.130698029)	acute_myeloic	Dolichyl-ph	GPI anchor biosynthetic process,dolichol			Benign	G=0, C=31	G=0.0, C=1.0	0.00001	465.818	31	G=0/0, C=15/1
chr9:130698043	G/G	A	G	SNV	DPM2	DPM2:exonic:NM_003863.3	1	100	4	synonymous	NM_00386	c.213T>C	p.(=)	-0.52				0.02(COSM55	Dolichol pho	rs6781	0.1305	YES	6.674E-1;1.16E-4 (pos.13	carcinoma,nec	Dolichyl-ph	GPI anchor biosynthetic process,dolichol			Benign	A=0, G=31	A=0.0, G=1.0	0.00001	465.784	31	A=0/0, G=15/1
chr12:69753847	C/C	T	C	SNV	YEATS4	YEATS4:intronic:NM_006530.3	1	100		intronic	NM_00653	c.51+44T>	p.?	-0.14					YEATS family	rs554591	0.4893	YES	4.4E-1 (pos.69753847)		YEATS doma	DNA bindi	Cardiovascular Diseases;Embolism and Thro			T=0, C=26	T=0.0, C=1.0	0.00001	371.42	26	T=0/0, C=9/17
chr12:69764849	AT/AT	A	AT	INDEL	YEATS4	YEATS4:intronic:NM_006530.3	1	100		intronic	NM_00653	c.514+94A	p.?	4.43					YEATS family	rs80056268		YES			YEATS doma	DNA bindi	Cardiovascular Diseases;Embolism and Thro			A=0, AT=97	A=0.0, AT=1.0	0.00001	908.109	97	A=0/0, AT=53/
chr12:70070942	G/G	A	G	SNV	BEST3	BEST3:intronic:NM_032735.2	1	100		intronic	NM_03273	c.714+18T	p.?	-1.68					Bestrophin, F	rs710718	0.0012	YES	9.999E-1 (pos.70070942)		Bestrophin	biological process,chloride channel activity,chloride ch				A=0, G=78	A=0.0, G=1.0	0.00001	1249.35	78	A=0/0, G=35/4
chr12:7055860	T/C	T	C	SNV	C12orf57,PTPN6	C12orf57:downstream:NM_138425.3, PTPN6:utr_5:NM_08(	1	47.62		intronic	NM_13842	c.*775T>C,	p.?, p.?	0.25					Protein-tyros	rs2301262	0.2655	YES	7.737E-1 (pos.7055860)		Chromosom	B cell rece	Agenesis of Corpus Callosum;Brain Diseases:			T=11, C=10	T=0.5238, C=0.4762	0.00001	84.378	21	T=5/6, C=2/8
chr12:7061466	G/G	A	G	SNV	PTPN6	PTPN6:intronic:NM_080548.4	1	100		intronic	NM_08054	c.332+126	p.?	-1.87					Protein-tyros	rs4963514		YES			Protein tyrc	B cell rece	Calcinosis;Calcium Metabolism Disorders;Ca			A=0, G=21	A=0.0, G=1.0	0.00001	281.235	21	A=0/0, G=9/12
chr12:7065576	A/A	G	A	SNV	PTPN6	PTPN6:intronic:NM_080548.4	1	100		intronic	NM_08054	c.931-6G>	p.?	-3.73					Protein-tyros	rs2110071	0.0595	YES	9.969E-1 (pos.7065576)		Protein tyrc	B cell rece	Calcinosis;Calcium Metabolism Disorders;Ca			G=0, A=20	G=0.0, A=1.0	0.00001	263.786	20	G=0/0, A=11/9
chr12:7069620	C/C	T	C	SNV	PTPN6	PTPN6:intronic:NM_080548.4	1	100		intronic	NM_08054	c.1679+22	p.?	-2.33					Protein-tyros	rs759052,rs7820638	0.1206	YES	0.0 (pos.7069617),8.619E-1 (pos.706962		Protein tyrc	B cell rece	Calcinosis;Calcium Metabolism Disorders;Ca			T=0, C=23	T=0.0, C=1.0	0.00001	316.743	23	T=0/0, C=6/17
chr12:7069654	A/A	G	A	SNV	PTPN6	PTPN6:intronic:NM_080548.4	1	100		intronic	NM_08054	c.1679+56	p.?	-2.1					Protein-tyros	rs759053	0.1204	YES			Protein tyrc	B cell rece	Calcinosis;Calcium Metabolism Disorders;Ca			G=0, A=23	G=0.0, A=1.0	0.00001	316.747	23	G=0/0, A=6/17
chr12:71960584	T/C	T	C	SNV	LGR5	LGR5:intronic:NM_003667.3	1	53.33		intronic	NM_00366	c.999-37T	p.?	-1.12					Leucine Rich	rs1726469	0.0156	YES	9.958E-1 (pos.71960584)		G protein-c	G-protein	Colonic Diseases;Colonic Neoplasms;Colorec			T=28, C=32	T=0.4667, C=0.5333	0.00001	323.557	60	T=15/13, C=16
chr2:162929979	G/G	A	G	SNV	DPP4	DPP4:exonic:NM_001935.3	1	100	2	synonymous	NM_00193	c.24T>C	p.(=)	0.13				0.04(COSM37	Dienelactone	rs17574	0.3464	YES	3.595E-2 (pos.16292997!	adenocarcino	Dipeptidylp	T cell activ	Arthritis;Arthritis, F	(1S)-2-(2S,5R)-2-(AMINOM		A=0, G=20	A=0.0, G=1.0	0.00001	263.769	20	A=0/0, G=11/9
chr19:32930069	G/A	G	A	SNV	DPY19L3	DPY19L3:exonic:NM_001172774.1	1	57.14	7	synonymous	NM_00117	c.648G>A	p.(=)	-0.17				0.14(COSM40	Q-cell neurot	rs6510249	0.3784	YES	6.307E-1 (pos.32930069)	acute_myeloic	DPY19-like	integral component of membrane,mannosyltransferase				G=12, A=16	G=0.4286, A=0.5714	0.00001	163.127	28	G=8/4, A=10/6
chr12:72287159	C/C	T	C	SNV	TBC1D15	TBC1D15:intronic:NM_022771.4	1	100		intronic	NM_02277	c.657+55T	p.?	-0.85					Domain of un	rs10467177		YES			TBC1 domai	GTPase activator activity,Rab GTPase binding,activation				T=0, C=26	T=0.0, C=1.0	0.00001	371.418	26	T=0/0, C=11/1
chr12:72307722	C/T	C	T	SNV	TBC1D15	TBC1D15:intronic:NM_022771.4	1	50		intronic	NM_02277	c.1452+16	p.?	0.01					Domain of un	rs3759172	0.0626	YES	1.769E-1 (pos.72307722)		TBC1 domai	GTPase activator activity,Rab GTPase binding,activation				C=19, T=19	C=0.5, T=0.5	0.00001	199.992	38	C=11/8, T=13/
chr12:72388374	T/T	G	T	SNV	TPH2	TPH2:intronic:NM_173353.3	1	100		intronic	NM_17335	c.1068+29	p.?	-0.11					Biopterin-de	rs1007023	0.1565	YES	9.569E-1 (pos.72388374)		Tryptophan	aromatic a	Attention Deficit Di	L-Tryptophan,Telotristat		G=0, T=47	G=0.0, T=1.0	0.00001	789.327	47	G=0/0, T=20/2
chr12:72955901	C/T	C	T	SNV	TRHDE	TRHDE:intronic:NM_013381.2	1	57.14		intronic	NM_01338	c.1654-44	p.?	1.29					ERAP1-like C-	rs2272507	0.1191	YES	5.454E-1 (pos.72955901)		Thyrotropin	aminopeptidase activity,cell-cell signaling,cytoplasm,e				C=9, T=12	C=0.4286, T=0.5714	0.00001	109.102	21	C=3/6, T=6/6
chr12:7360229	C/G	C	G	SNV	PEX5	PEX5:intronic:NM_001131023.1	1	59.09		intronic	NM_00113	c.1012-26	p.?	-4.09					Tetratricope	rs3816424	0.2843	YES	1.448E-1 (pos.7360229)		Peroxisome	Golgi appa	Abnormalities, Multiple;Bone Diseases;Bone			C=18, G=26	C=0.4091, G=0.5909	0.00001	312.069	44	C=7/11, G=11/
chr1:98348885	A/A	G	A	SNV	DPYD	DPYD:exonic:NM_000110.3	1	100	2	missense	NM_00011	c.85C>T	p.Arg29Cys	2.24	0.11	180	0	0.52(COSM37	4Fe-4S diclus	rs1801265	0.2249	YES	9.258E-1 (pos.98348885)	malignant_me	Dihydropyri	4 iron, 4 su	Adenocarcinoma;A	(5s)-5-Iodc	not provided	G=0, A=34	G=0.0, A=1.0	0.00001	524.186	34	G=0/0, A=12/2
chr8:26481771	C/C	T	C	SNV	DPYSL2	DPYSL2:exonic:NM_001197293.2	1	100	4	synonymous	NM_00119	c.741T>C	p.(=)	-4.39					Amidohydrol	rs327222	0.0101	YES	9.058E-1 (pos.26481771)		Dihydropyri	axon guida	Alzheimer Disease;Arthritis;Brain Diseases;C			T=0, C=36	T=0.0, C=1.0	0.00001	563.739	36	T=0/0, C=27/9
chr12:75687185	A/A	G	A	SNV	CAPS2	CAPS2:intronic:NM_032606.3	1	100		intronic	NM_03260	c.1102-38	p.?	-0.53					EF-hand dom	rs7967562	0.3926	YES	7.033E-1 (pos.75687185)		Calcyphosir	calcium ion binding,calcium ion homeostasis,calcium:sc				G=0, A=33	G=0.0, A=1.0	0.00001	504.597	33	G=0/0, A=7/26
chr10:134013990	A/A	G	A	SNV	DPYSL4	DPYSL4:exonic:NM_006426.2	1	100	9	synonymous	NM_00642	c.942G>A	p.(=)	-9.13				0(COSM4144	Amidohydrol	rs2247705	0.1727	YES	9.396E-1 (pos.13401399(	neoplasm	Dihydropyri	cytosol,filamin binding,hydrolase activity, acting on carb				G=0, A=25	G=0.0, A=1.0	0.00001	353.019	25	G=0/0, A=10/1
chr2:26647277	G/G	T	G	SNV	DRC1	DRC1:exonic:NM_145038.4	1	100	4	synonymous	NM_14503	c.495T>G	p.(=)	-2.4				0.01(COSM44	Sperm tail,Sp	rs7423300	0.1095	YES	3.044E-1;0.0;0.0 (pos.26(	acute_myeloic	Dynein regu	axonemal	Bronchial Diseases;Bronchiect		Benign	T=0, G=26	T=0.0, G=1.0	0.00001	371.401	26	T=0/0, G=12/1
chr12:76453861	A/A	G	A	SNV	NAP1L1	NAP1L1:intronic:NM_004537.6	1	100		intronic	NM_00453	c.348+57C	p.?	-0.07					Nucleosome	rs7956821		YES			Nucleosom	DNA replic	Nutrition Disorders;Nutritional and Metaboli			G=0, A=27	G=0.0, A=1.0	0.00001	389.988	27	G=0/0, A=15/1
chr12:76453873	T/T	C	T	SNV	NAP1L1	NAP1L1:intronic:NM_004537.6	1	100		intronic	NM_00453	c.348+45G	p.?	0.01					Nucleosome	rs10880030	0.3975	YES	9.58E-1 (pos.76453873)		Nucleosom	DNA replic	Nutrition Disorders;Nutritional and Metaboli			C=0, T=27	C=0.0, T=1.0	0.00001	389.986	27	C=0/0, T=15/1
chr2:26676322	G/G	A	G	SNV	DRC1	DRC1:exonic:NM_145038.4	1	100	14	synonymous	NM_14503	c.1824A>G	p.(=)	-0.89					Sperm tail,Sp	rs1402962	1.00E-04		1.0 (pos.26676322)		Dynein regu	axonemal	Bronchial Diseases;Bronchiect		Benign	A=0, G=36	A=0.0, G=1.0	0.00001	563.728	36	A=0/0, G=17/1
chr2:26667615	T/C	T	C	SNV	DRC1	DRC1:exonic:NM_145038.4	1	97.56	10	missense	NM_14503	c.1195T>C	p.Trp399Arg	1.54	1	101	0		Sperm tail,Sp	rs939820	0		1.0 (pos.26667615)		Dynein regu	axonemal	Bronchial Diseases;Bronchiect		Benign	T=1, C=40	T=0.0244, C=0.9756	0.00001	634.555	41	T=0/1, C=20/2
chr17:17896205	C/T	C	T	SNV	DRC3	DRC3:exonic:NM_031294.3	1	50	6	missense	NM_03129	c.571C>T	p.Arg191Trp	3.33	0	101	1		Leucine rich r	rs4584886	0.3264	YES	8.804E-1 (pos.17896205)			axoneme,cytoplasm				C=21, T=21	C=0.5, T=0.5	0.00001	228.869	42	C=14/7, T=12/
chr3:113858350	T/T	C	T	SNV	DRD3	DRD3:exonic:NM_000796.5	1	100	5	synonymous	NM_00079	c.720G>A	p.(=)	-0.15					7 transmemb	rs2251177	2.00E-04	YES	9.995E-1;0.0 (pos.113858350)		Dopamine r	D1 dopam	Amphetamine-Rela	ACP-104,A	Likely benign	C=0, T=26	C=0.0, T=1.0	0.00001	371.407	26	C=0/0, T=17/9
chr12:78511755	C/C	T	C	SNV	NAV3	NAV3:intronic:NM_014903.5	1	100		intronic	NM_01490	c.2770-52	p.?	-1.37					ATPase famil	rs300442	0.1399	YES			Neuron nav	ATP bindin	Adenocarcinoma;Carcinoma;Carcinoma, Ren			T=0, C=41	T=0.0, C=1.0	0.00001	664.695	41	T=0/0, C=17/2
chr12:80175764	A/A	G	A	SNV	PPP1R12A	PPP1R12A:intronic:NM_002480.2	1	100		intronic	NM_00248	c.2908-12	p.?	0.26					Ankyrin repe	rs2111687		YES			Protein pho	14-3-3 protein binding,A band,G2/M transition of mitotic				G=0, A=24	G=0.0, A=1.0	0.00001	334.792	24	G=0/0, A=4/20
chr12:80216060	A/A	C	A	SNV	PPP1R12A	PPP1R12A:intronic:NM_002480.2	1	100		intronic	NM_00248	c.793-90G	p.?	0.29					Ankyrin repe	rs3849223		YES			Protein pho	14-3-3 protein binding,A band,G2/M transition of mitotic				C=0, A=159	C=0.0, A=1.0	0.00001	2553.09	159	C=0/0, A=94/6
chr12:80648399	T/T	C	T	SNV	OTOGL	OTOGL:intronic:NM_173591.3	1	100		intronic	NM_17359	c.1414+49	p.?	-0.3					Alpha-L-arab	rs7295623	0.0073		1.0 (pos.80648399)		Otogelin-like protein					C=0, T=20	C=0.0, T=1.0	0.00001	263.784	20	C=0/0, T=10/1
chr4:9784631	T/T	C	T	SNV	DRD5	DRD5:exonic:NM_000798.4	1	100	1	synonymous	NM_00079	c.978C>T	p.(=)	-1.81				0.04(COSM37	7 transmemb	rs6283		YES	6.133E-1 (pos.9784631)	acute_myeloic	Dopamine r	G-protein	Attention Deficit Di	Apomorphine,Aripiprazo		C=0, T=42	C=0.0, T=1.0	0.00001	677.013	42	C=0/0, T=21/2
chr18:28720147	T/T	C	T	SNV	DSC1,DSCAS	DSCAS:intronic_nc:NR_110785.1, DSC1:exonic:NM_024421.	1	100	10	missense	NR_11078	c.1378G>A	p.Val460Ile	3.86	0.1	29	0.999	0.91(COSM14	Cadherin cyt	rs17800159	0.1288	YES	4.674E-1 (pos.28720147)	adenocarcino	Desmocolli	calcium io	Melanoma;Neoplasms;Neoplasms by Histolc			C=0, T=45	C=0.0, T=1.0	0.00001	747.393	45	C=0/0, T=25/2
chr18:28935375	T/T	C	T	SNV	DSG1,DSG1-AS1	DSG1:utr_3:NM_001942.3, DSG1-AS1:intronic_nc:NR_11078	1	100	15	utr_3	NM_00194	c.3216C>T	p.?	0.35					Cadherin cyt	rs8099566		YES			Desmogleir	apical pla	Congenital, Hereditary, and Neonatal Diseas			C=0, T=28	C=0.0, T=1.0	0.00001	408.717	28	C=0/0, T=17/1
chr18:28968371	A/A	G	A	SNV	DSG1-AS1,DSG4	DSG4:exonic:NM_177986.4, DSG1-AS1:intronic_nc:NR_1107	1	100	4	synonymous	NR_11078	c.258G>A	p.(=)	-0.65					Cadherin cyt	rs16959856	0.2374	YES	9.02E-1 (pos.28968371)		Desmogleir	BMP signa	Abnormalities, Multiple;Conge		Benign	G=0, A=68	G=0.0, A=1.0	0.00001	1053.51	68	G=0/0, A=41/2
chr18:28968959	T/T	C	T	SNV	DSG1-AS1,DSG4	DSG4:exonic:NM_177986.4, DSG1-AS1:intronic_nc:NR_1107	1	100	5	synonymous	NR_11078	c.495C>T	p.(=)	-0.09					Cadherin cyt	rs9956865	0.237	YES	9.558E-1 (pos.28968959)		Desmogleir	BMP signa	Abnormalities, Multiple;Conge		Benign	C=0, T=172	C=0.0, T=1.0	0.00001	2818.98	172	C=0/0, T=75/9
chr18:28986333	C/C	A	C	SNV	DSG1-AS1,DSG4	DSG4:exonic:NM_177986.4, DSG1-AS1:intronic_nc:NR_1107	1	100	12	missense	NR_11078	c.1930A>C	p.Ile644Leu	1.6	1	5	0		Cadherin cyt	rs4799570,rs750850(	0.1017	YES	0.0 (pos.28986332),9.998E-1 (pos.28986		Desmogleir	BMP signa	Abnormalities, Multiple;Conge		Benign	A=0, C=78	A=0.0, C=1.0	0.00001	1218.11	78	A=0/0, C=37/4
chr6:7585967	C/C	G	C	SNV	DSP	DSP:exonic:NM_004415.3	1	100	24	synonymous	NM_00441	c.8472G>C	p.(=)	-0.05				0.11(COSM62	Plectin repea	rs2744380,rs397516	0.2827	YES	0.0 (pos.7585964),0.0:0.(	neoplasm	Desmoplak	RNA bindir	ACMG Recommendations...;Ar		Benign	G=0, C=114	G=0.0, C=1.0	0.00001	1755.88	114	G=0/0, C=60/5
chr4:88534235	G/G	A	G	SNV	DSPP	DSPP:exonic:NM_014208.3	1	100	4	synonymous	NM_01420	c.897A>G	p.(=)	0.35				0.05(COSM3760802)		rs2736982,rs386677(	0.3932	YES	6.909E-1 (pos.88534235)	acute_myeloic	Dentin sialc	biominera	Carcinoma;Carcinoma, Squam		Benign	A=0, G=24	A=0.0, G=1.0	0.00001	334.783	24	A=0/0, G=10/1
chr4:88537737	A/A	G	A	SNV	DSPP	DSPP:utr_3:NM_014208.3	1	100	5	utr_3	NM_01420	c.3923G>A	p.?	-0.05						rs2615489	0.125	YES	8.923E-1 (pos.88537737)		Dentin sialc	biominera	Carcinoma;Carcinoma, Squam		Benign	G=0, A=63	G=0.0, A=1.0	0.00001	958.917	63	G=0/0, A=24/3
chr12:80890325	TG/TG	T	TG	INDEL	PTPRO	PTPRO:intronic:NM_001145026.1	1	100		intronic	NM_00114	c.1756+15	p.?	-0.05					Fibronectin t	rs113908903,rs5692	0.0706	YES	4.699E-1 (pos.80890325)		Protein-tyrosine phosphatase, receptor-type, Q					T=0, TG=26	T=0.0, TG=1.0	0.00001	369.217	26	T=0/0, TG=12/
chr6:56417282	T/T	C	T	SNV	DST	DST:exonic:NM_001144769.2	1	100	55	missense	NM_00114	c.9951G>A	p.Met3317Ile	-0.39	0.15	10	0		Calponin hon	rs4715630	0.2577	YES	7.978E-1 (pos.56417282)		Dystonin (b	H zone,Z disc,actin binding,actin cytoskeleton,axon,axor				C=0, T=22	C=0.0, T=1.0	0.00001	298.892	22	C=0/0, T=15/7

chr4:36345377	G/A	G	A	SNV	DTHD1	DTHD1:exonic:NM_001170700.2	1	50	9	synonymous	NM_00117	c.2277G>A	p.(=)	0.48					Death domai	rs2270880	0.1653	YES	1.17E-1 (pos.36345377)		Death domi	signal transduction				G=13, A=13	G=0.5, A=0.5	0.00001	119.163	26	G=11/2, A=7/6
chr12:81751813	C/T	C	T	SNV	PPFIA2	PPFIA2:intronic:NM_003625.4	1	56.36		intronic	NM_00362	c.1773+48	p.?	-0.26					SAM domain	rs11835074	0.2202	YES	3.941E-1 (pos.81751813)			cell surface,cell-matrix adhesion,cytoplasm,cytosol,ext				C=24, T=31	C=0.4364, T=0.5636	0.00001	319.023	55	C=11/13, T=19
chr1:212274413	C/C	A	C	SNV	DTL	DTL:exonic:NM_016448.3	1	100	14	missense	NM_01644	c.2081A>C	p.Lys694Thr	-0.57	1	78	0		WD domain,	rs6540718	0.013	YES	1.0 (pos.212274413)		Denticleles	Cul4-RING	Adenocarcinoma;Autoimmune Diseases:Car			A=0, C=39	A=0.0, C=1.0	0.00001	623.98	39	A=0/0, C=23/1
chr2:242615462	C/C	T	C	SNV	DTYMK	DTYMK:utr_3:NM_012145.3	1	100	5	utr_3	NM_01214	c.719A>G	p.?	-2.58					Thymidylate	rs5860		YES			Deoxythym	ATP binding,cell cycle,cell proli		2',3'-Dideoxythymidine-5		T=0, C=28	T=0.0, C=1.0	0.00001	408.718	28	T=0/0, C=21/7
chr12:82832418	A/A	G	A	SNV	METTL25	METTL25:intronic:NM_032230.2	1	100		intronic	NM_03223	c.1405-79	p.?	0.8					Methyltransf	rs2717448						methylation,methyltransferase activity				G=0, A=24	G=0.0, A=1.0	0.00001	334.792	24	G=0/0, A=20/4
chr12:82850427	G/G	A	G	SNV	METTL25	METTL25:intronic:NM_032230.2	1	100		intronic	NM_03223	c.1479-79	p.?	0.3					Methyltransf	rs2731290		YES				methylation,methyltransferase activity				A=0, G=42	A=0.0, G=1.0	0.00001	685.22	42	A=0/0, G=15/2
chr15:45400358	G/G	C	G	SNV	DUOX2	DUOX2:exonic:NM_014080.4	1	100	13	synonymous	NM_01408	c.1461G>C	p.(=)	-0.23				0.63(COSM41	Animal haem	rs269860	0.0286	YES	9.791E-1;1.156E-4 (pos.4	neoplasm	Dual oxidas	NAD(P)H o	Bone Diseases:Bone Diseases,		Benign	C=0, G=30	C=0.0, G=1.0	0.00001	446.639	30	C=0/0, G=6/24
chr12:863516	CA/CA	C	CA	INDEL	WNK1	WNK1:intronic:NM_001184985.1	1	100		intronic	NM_00118	c.759+26C	p.?	-1.28					Oxidative-stress-responsive kinase 1 C-terminal domain,Protein kinase domain,Protein tyrosine kin						WNK lysine	ATP bindin	Cardiovascular Diseases:Charcot-Marie-Toot			C=0, CA=51	C=0.0, CA=1.0	0.00001	519.815	51	C=0/0, CA=36/
chr5:172196711	T/T	G	T	SNV	DUSP1	DUSP1:exonic:NM_004417.3	1	100	3	synonymous	NM_00441	c.600C>A	p.(=)	5.7					Dual specific	rs2431663	0.0444	YES	9.999E-1 (pos.172196711)		Dual specifi	MAP kinas	Arthritis;Arthritis, Juvenile;Autoimmune Dise			G=0, T=46	G=0.0, T=1.0	0.00001	768.3	46	G=0/0, T=30/1
chr10:76854564	T/T	C	T	SNV	DUSP13	DUSP13:exonic:NM_001007273.1	1	100	6	missense	NM_00100	c.746G>A	p.Cys249Tyr	-0.12	1	194	0.052		Dual specific	rs3088142	0.4259	YES	9.114E-1 (pos.76854564)		Dual-specificity phosph		Nervous System Diseases;Neuromuscular Di			C=0, T=20	C=0.0, T=1.0	0.00001	263.786	20	C=0/0, T=15/5
chr10:76861680	C/C	T	C	SNV	DUSP13	DUSP13:exonic:NM_001007273.1	1	100	3	missense	NM_00100	c.223A>G	p.Ser75Gly	-0.41	1	56	0		Dual specific	rs6480771	0.4294	YES	9.108E-1 (pos.76861680)		Dual-specificity phosph		Nervous System Diseases;Neuromuscular Di			T=0, C=22	T=0.0, C=1.0	0.00001	298.889	22	T=0/0, C=13/9
chr17:41856188	CG/C	CG	C	INDEL	DUSP3	DUSP3:exonic:NM_004090.3	1	95.45	1	frameshiftDeletion	NM_00409	c.104delC	p.Pro35fs	0.0, 8.45					Dual specificity phosphatase, catalytic domain						Dual specifi	MAP kinas	Bronchial Neoplasms;Carcinoma, Bronchoge			CG=1, C=21	CG=0.0455, C=0.954	0.00001	216.246	22	CG=0/1, C=3/1
chr12:89743223	A/A	G	A	SNV	DUSP6	DUSP6:exonic:NM_001946.3	1	100	3	synonymous	NM_00194	c.954C>T	p.(=)	0.42					Dual specific	rs704075	0		1.0 (pos.89743223)		Dual-specif	MAP kinas	46, XY Disorders of Sex Development;Astrocy			G=0, A=60	G=0.0, A=1.0	0.00001	903.529	60	G=0/0, A=33/2
chr19:57665750	C/C	T	C	SNV	DUXA	DUXA:utr_3:NM_001012729.1	1	100	6	utr_3	NM_00101	c.645A>G	p.?	0.09					Homeobox KI	rs8108591	0.4674	YES	2.74E-1 (pos.57665750)		Double hon	DNA bindi	Connective Tissue Diseases;Contracture;Dup			T=0, C=30	T=0.0, C=1.0	0.00001	446.637	30	T=0/0, C=18/1
chr1:1277533	C/C	T	C	SNV	DVL1	DVL1:exonic:NM_004421.2	1	100	4	synonymous	NM_00442	c.366A>G	p.(=)	-5.37				0.12(COSM37	DIX domain,D	rs307362	0.0013		9.977E-1;0.0 (pos.12775	adenocarcinoi	Dishevellec	Rac GTPase binding,Wnt signaling pathway,Wnt signalin				T=0, C=89	T=0.0, C=1.0	0.00001	1472.44	89	T=0/0, C=57/3
chr14:102514227	C/C	T	C	SNV	DYNC1H1	DYNC1H1:exonic:NM_001376.4	1	100	73	synonymous	NM_00137	c.13080T>	p.(=)	-2.12				0.07(COSM14	AAA domain (	rs13749	0.1659	YES	2.899E-1 (pos.10251422	acute_myeloi	Dynein, cyt	ATP bindin	Central Nervous System Disea:		Benign/Likely	T=0, C=27	T=0.0, C=1.0	0.00001	389.988	27	T=0/0, C=7/20
chr20:18393312	G/G	C	G	SNV	DZANK1	DZANK1:exonic:NM_001099407.1	1	100	13	synonymous	NM_00109	c.1410G>C	p.(=)	0.83				0.14(COSM39	Chitobiase/b	rs6075337	0.2132	YES	2.886E-1 (pos.18393312)	carcinoma		metal ion binding				C=0, G=28	C=0.0, G=1.0	0.00001	408.716	28	C=0/0, G=17/1
chr1:23847464	C/A	C	A	SNV	E2F2,LOC101928163	E2F2:exonic:NM_004091.3, LOC101928163:upstream:NR_1	1	50	4	missense	NM_00409	c.678G>T	p.Gln226His	0.03	0.26	24	0.985	0.78(COSM37	E2F transcrip	rs2075995	0.4972	YES	6.199E-1 (pos.23847464)	acute_myeloi	E2F transcri	DNA binding,DNA binding transcription factor activity,RN				C=16, A=16	C=0.5, A=0.5	0.00001	158.383	32	C=10/6, A=9/7
chr16:2282256	A/A	G	A	SNV	E4F1	E4F1:exonic:NM_004424.4	1	100	4	missense	NM_00442	c.500G>A	p.Arg167His	-0.66	0.36	29	0.245	0.03(COSM41	C2H2-type zir	rs26839	0.0097		1.0;0.0 (pos.2282256)	neoplasm	E4F transcri	DNA binding,DNA binding transcription factor activity,DN				G=0, A=28	G=0.0, A=1.0	0.00001	408.718	28	G=0/0, A=22/6
chr12:91358169	C/C	T	C	SNV	EPYC	EPYC:intronic:NM_004950.4	1	100		intronic	NM_00495	c.799-66A	p.?	0.43					Leucine Rich	rs1920741		YES			Epiphycan	axonogenesis,extracellular space,female pregnancy,gly				T=0, C=20	T=0.0, C=1.0	0.00001	263.499	20	T=0/0, C=13/7
chr3:15476011	G/G	C	G	SNV	EAF1	EAF1:exonic:NM_033083.6	1	100	4	synonymous	NM_03308	c.492C>G	p.(=)	0.54				0.82(COSM44	RNA polymer	rs1563415	0.2637	YES	3.005E-1;0.0;0.0 (pos.15	NS,acute_mye	ELL-associa	Cajal body,ELL-EAF complex,intercellular bridge,intracel				C=0, G=36	C=0.0, G=1.0	0.00001	563.573	36	C=0/0, G=22/1
chr16:23536684	C/C	T	C	SNV	EARS2	EARS2:exonic:NM_001083614.1	1	100	8	missense	NM_00108	c.1369A>G	p.Ser457Gly	-0.62	0.61	56	0		tRNA synthet	rs6497671	0.1414	YES	7.088E-1 (pos.23536684)		Glutamyl-tF	ATP binding,cytoplasm,glutam		Glutamic /	Benign	T=0, C=32	T=0.0, C=1.0	0.00001	485.141	32	T=0/0, C=14/1
chr16:23546561	C/C	G	C	SNV	EARS2	EARS2:exonic:NM_001083614.1	1	100	4	synonymous	NM_00108	c.606C>G	p.(=)	-1.12				0.04(COSM41	tRNA synthet	rs2073951	0.1401	YES	7.103E-1 (pos.23546561)	neoplasm	Glutamyl-tF	ATP binding,cytoplasm,glutam		Glutamic /	Benign	G=0, C=26	G=0.0, C=1.0	0.00001	371.411	26	G=0/0, C=18/8
chr12:9313169	CAG/C	CAG	C	INDEL	PZP	PZP:intronic:NM_002864.2	2	51.85		intronic	NM_00286	c.2873-83	p.?	-0.39, -0.17, -1.6					A-macroglob	rs10540786,rs199590841,rs398055299					Pregnancy zone protei		Digestive System Diseases;Drug-Induced Live			CAG=26, C=	CAG=0.4815, C=0.51	0.00001	277.174	54	CAG=10/16, C=
chr16:23563501	T/T	C	T	SNV	EARS2	EARS2:exonic:NM_001083614.1	1	100	2	synonymous	NM_00108	c.264G>A	p.(=)	-4.9				0.02(COSM50	tRNA synthet	rs7187920	0.1889	YES	6.881E-1 (pos.23563501)	osteosarcoma	Glutamyl-tF	ATP binding,cytoplasm,glutam		Glutamic /	Benign	C=0, T=70	C=0.0, T=1.0	0.00001	1092.06	70	C=0/0, T=30/4
chr12:93206878	A/A	G	A	SNV	EEA1	EEA1:intronic:NM_003566.3	1	100		intronic	NM_00356	c.1930-9C	p.?	0.83					FYVE zinc fing	rs3925797	0.0709	YES	9.589E-1 (pos.93206878)		Early endos	1-phosphatidylinositol binding		Inositol 1,3-Bisphosphat		G=0, A=25	G=0.0, A=1.0	0.00001	353.018	25	G=0/0, A=7/18
chr12:9344878	G/G	A	G	SNV	PZP	PZP:intronic:NM_002864.2	1	100		intronic	NM_00286	c.1483-26	p.?	-3.48					A-macroglob	rs2075394	0.2803	YES	4.255E-1 (pos.9344878)		Pregnancy zone protei		Digestive System Diseases;Drug-Induced Live			A=0, G=88	A=0.0, G=1.0	0.00001	1444.42	88	A=0/0, G=44/4
chr10:22498749	T/T	A	T	SNV	EBLN1	EBLN1:exonic:NM_001199938.1	1	100	1	missense	NM_00119	c.164T>A	p.Val55Asp	-0.42	1	152	0		Borna diseas	rs2478476			1.0 (pos.22498749)		Endogenous	Borna-like N element-containing protein 1				A=0, T=35	A=0.0, T=1.0	0.00001	543.897	35	A=0/0, T=24/1
chr3:73111809	A/G	A	G	SNV	EBLN2,PPP4R2	PPP4R2:intronic:NM_174907.3, EBLN2:exonic:NM_018029.4	1	57.14	1	missense	NM_17490	c.420-101	p.?, p.Ile193Val	-2.66		29		0(COSM4158	Borna diseas	rs2231926	0.486	YES	4.351E-1;0.0 (pos.73111	ductal_carcino	Endogenou	RNA splicing,cellular protein modification process,centr				A=12, G=16	A=0.4286, G=0.5714	0.00001	161.77	28	A=2/10, G=6/1
chr12:936468	G/G	C	G	SNV	WNK1	WNK1:intronic:NM_001184985.1	1	100		intronic	NM_00118	c.1153+40	p.?	0.01					Oxidative-str	rs2158502	0.2508	YES	8.661E-1 (pos.936468)		WNK lysine	ATP bindin	Cardiovascular Diseases:Charcot-Marie-Toot			C=0, G=104	C=0.0, G=1.0	0.00001	1631.88	104	C=0/0, G=38/6
chr1:43636484	C/G	C	G	SNV	EBNA1BP2,MIR6733	EBNA1BP2:exonic:NM_001159936.1, MIR6733:downstream	1	58.33	5	synonymous	NM_00115	c.555G>C	p.(=)	-0.08				0.3(COSM502	Eukaryotic rR	rs1049276	0.2577	YES	4.622E-4;1.156E-4 (pos.4	osteosarcoma	EBNA1-bind	RNA binding,nuclear periphery,nucleolus,nucleus,prerib				C=10, G=14	C=0.4167, G=0.5833	0.00001	135.336	24	C=8/2, G=12/2
chr12:94649103	C/C	T	C	SNV	PLXNC1	PLXNC1:intronic:NM_005761.2	1	100		intronic	NM_00576	c.3087+31	p.?	3.19					IPT/TIG doma	rs865569	0.1579	YES	6.202E-1 (pos.94649103)			cell adhes	Endometriosis;Female Urogenital Diseases:F			T=0, C=48	T=0.0, C=1.0	0.00001	810.415	48	T=0/0, C=5/43
chr12:95676053	TG/TG	T	TG	INDEL	VEZT	VEZT:intronic:NM_017599.3	1	100		intronic	NM_01759	c.997-36T	p.?	0.29					Myosin-bindi	rs201794726,rs32150	0.4795	YES	4.883E-1 (pos.95676053)			acrosomal vesicle,adherens junction,cell-cell adhesion,				T=0, TG=40	T=0.0, TG=1.0	0.00001	643.802	40	T=0/0, TG=20/
chr12:95897997	C/C	T	C	SNV	METAP2	METAP2:intronic:NM_006838.3	1	100		intronic	NM_00683	c.964+60T	p.?	0.04					Metallopepti	rs2769469		YES			Methionine	N-termina	Autoimmune Disea	(2s,3r)-3-Amino-2-Hydrox		T=0, C=22	T=0.0, C=1.0	0.00001	298.892	22	T=0/0, C=9/13
chrX:48385586	T/T	C	T	SNV	EBP	EBP:exonic:NM_006579.2	1	100	4	synonymous	NM_00657	c.382C>T	p.(=)	0.12					Emopamil bir	rs142881014	7.00E-04		0.0 (pos.48385586)		Emopamil-b	C-8 sterol	Bone Diseases;Bon	Tamoxifen	Uncertain sig	C=0, T=34	C=0.0, T=1.0	0.00001	524.185	34	C=0/0, T=28/6
chr2:233349588	A/A	G	A	SNV	ECEL1	ECEL1:exonic:NM_004826.3	1	100	5	missense	NM_00482	c.982C>T	p.His328Tyr	5.86	0.27	83	0	0.16(COSM41	Peptidase far	rs1529874	0.0145	YES	1.0 (pos.233349588)	neoplasm	Endothelin-	integral component of plasma membrane			Benign	G=0, A=40	G=0.0, A=1.0	0.00001	644.274	40	G=0/0, A=23/1
chr12:96387621	G/G	A	G	SNV	HAL	HAL:intronic:NM_002108.3	1	100		intronic	NM_00210	c.485-5T>C	p.?	1.01				0.67(COSM59	Aromatic ami	rs2302629	0.4093	YES	5.021E-1 (pos.96387621)	basal_cell_car	Histidine ar	ammonia-	Amino Acid Metabo	L-Histidine	Benign	A=0, G=49	A=0.0, G=1.0	0.00001	831.604	49	A=0/0, G=27/2
chr12:96415888	G/A	G	A	SNV	LTA4H	LTA4H:intronic:NM_000895.2	1	58.33		intronic	NM_00089	c.585+37C	p.?	0.83					Leukotriene A	rs11108379	0.0499	YES	8.242E-2 (pos.96415888)		Leukotriene	RNA bindir	Arterial Occlusive D	(2S)-2-amino-5-oxo-5-(4-		G=10, A=14	G=0.4167, A=0.5833	0.00001	135.325	24	G=7/3, A=7/7
chr12:96691355	T/TA	T	TA	INDEL	CDK17	CDK17:intronic:NM_001170464.2	1	50		intronic	NM_00117	c.810+50A	p.?	0.99					Haspin like ki	rs397802261,rs5800	0.3479	YES	5.778E-1;0.0 (pos.96691355)		Cyclin-depe	ATP binding,cyclin-dependent protein serine/threonine				T=10, TA=10	T=0.5, TA=0.5	0.00001	72.7857	20	T=3/7, TA=5/5
chr10:11789382	A/A	G	A	SNV	ECHDC3	ECHDC3:exonic:NM_024693.4	1	100	2	missense	NM_02469	c.205G>A	p.Ala69Thr	6.39	0.08	58	0.989		Enoyl-CoA hy	rs4750090	0.0021	YES	9.999E-1 (pos.11789382)			catalytic activity,metabolic process,mitochondrion				G=0, A=65	G=0.0, A=1.0	0.00001	996.302	65	G=0/0, A=58/7
chr10:11789405	A/A	G	A	SNV	ECHDC3	ECHDC3:exonic:NM_024693.4	1	100	2	synonymous	NM_02469	c.228G>A	p.(=)	0.31					Enoyl-CoA hy	rs4750091	0.0012	YES	9.999E-1 (pos.11789405)			catalytic activity,metabolic process,mitochondrion				G=0, A=77	G=0.0, A=1.0	0.00001	1229.26	77	G=0/0, A=58/1
chr10:135184126	A/A	G	A	SNV	ECHS1,MIR3944	MIR3944:downstream:NR_037509.1, ECHS1:exonic:NM_004	1	100	2	missense	NM_00409	c.224C>T	p.Thr75Ile	1.01	0.14	89	0.04		Enoyl-CoA hy	rs1049951	0.0598	YES	9.744E-1 (pos.135184126)		Enoyl-CoA h	enoyl-CoA	Brain Diseases:Brai	4-(N,N-Din	Benign	G=0, A=28	G=0.0, A=1.0	0.00001	408.715	28	G=0/0, A=14/1
chr12:9885526	T/T	C	T	SNV	CLECL1	CLECL1:intronic:NM_001253750.1	1	100		intronic	NM_00125	c.316+19G	p.?	0.64						rs12304510	0.4898	YES	3.913E-1 (pos.9885526)		C-type lecti	B cell adhe	Autoimmune Diseases;Autoimmune Disease			C=0, T=25	C=0.0, T=1.0	0.00001	353.017	25	C=0/0, T=6/19
chr2:109513321	A/A	C	A	SNV	EDAR	EDAR:utr_3:NM_022336.3	1	100	12	utr_3	NM_02233	c.1389G>T	p.?	-0.35						rs7607563	0.0989	YES	9.977E-1 (pos.109513321)		Ectodysplas	apical par	Abnormalities, Multiple;Conge		Benign	C=0, A=42	C=0.0, A=1.0	0.00001	685.22	42	C=0/0, A=24/1
chr2:109513654	A/A	G	A	SNV	EDAR	EDAR:exonic:NM_022336.3	1	100	12	synonymous	NM_02233	c.1056C>T	p.(=)	1.65						rs12623957	0.1695	YES	9.604E-1 (pos.109513654)		Ectodysplas	apical par	Abnormalities, Multiple;Conge		Conflicting in	G=0, A=61	G=0.0, A=1.0	0.00001	921.845	61	G=0/0, A=32/2
chr1:236645609	C/T	C	T	SNV	EDARADD	EDARADD:exonic:NM_145861.2	1	45.83	6	missense	NM_14586	c.308C>T	p.Ser103Phe	2.57	0	155	0.924		Death domai	rs114632254	0.0274	YES	1.156E-4 (pos.236645609)		EDAR-assoc	cell differe	Abnormalities, Multiple;Conge		Benign	C=13, T=11	C=0.5417, T=0.4583	0.00001	93.4277	24	C=6/7, T=6/5
chr13:101288992	C/T	C	T	SNV	TMTC4	TMTC4:intronic:NM_032813.4	1	50		intronic	NM_03281	c.1000-4G	p.?	1.14					Domain of un	rs3803256	0.4284	YES	6.076E-1 (pos.101288992)			integral component of membrane				C=17, T=17	C=0.5, T=0.5	0.00001	172.007	34	C=11/6, T=9/8
chr13:101289998	C/A	C	A	SNV	TMTC4	TMTC4:intronic:NM_032813.4	1	50		intronic	NM_03281	c.835-42G	p.?	2.09					Domain of un	rs7985718	0.3744	YES	9.862E-1 (pos.101289998)			integral component of membrane				C=14, A=14	C=0.5, A=0.5	0.00001	131.941	28	C=8/6, A=6/8
chr3:5241309	A/A	C	A	SNV	EDEM1	EDEM1:exonic:NM_014674.2	1	100	3	synonymous	NM_01467	c.615C>A	p.(=)	-1.96					Glycosyl hydr	rs410509	0.0244	YES	9.394E-1 (pos.5241309)			Golgi membrane,IRE1-mediated unfolded protein respor				C=0, A=60	C=0.0, A=1.0	0.00001	907.072	60	C=0/0, A=41/1
chr13:103271206	A/A	G	A	SNV	TPP2	TPP2:intronic:NM_003291.3	1	100		intronic	NM_00329	c.620+10G	p.?	-0.43				0.01(COSM39	Subtilase fan	rs4576935	0.4812	YES	4.429E-1 (pos.10327120	acute_myeloi	Tripeptidyl	aminopeptidase activity,cytoplasm,cytosol,endopeptid				G=0, A=96	G=0.0, A=1.0	0.00001	1618.01	96	G=0/0, A=47/4
chr13:78477674	G/G	A	G	SNV	EDNRB,EDNRB-AS1	EDNRB:exonic:NM_001201397.1, EDNRB-AS1:intronic_nc:NR	1	100	3	synonymous	NR_10385	c.822T>C	p.(=)	-1.82					7 transmemb	rs5348	2.00E-04		9.999E-1 (pos.78477674)		Endothelin	G-protein	Abnormalities, Mul	Ambrisent	Benign	A=0, G=34	A=0.0, G=1.0	0.00001	530.494	34	A=0/0, G=12/2
chr13:103289338	T/T	C	T	SNV	TPP2	TPP2:intronic:NM_003291.3	1	100		intronic	NM_00329	c.1679-94	p.?	-0.29					Subtilase fan	rs2274463		YES			Tripeptidyl	aminopeptidase activity,cytoplasm,cytosol,endopeptid				C=0, T=29	C=0.0, T=1.0	0.00001	427.603	29	C=0/0, T=21/8
chr13:103290535	C/C	G	C	SNV	TPP2	TPP2:intronic:NM_003291.3	1	100		intronic	NM_00329	c.1837-54	p.?	-0.2					Subtilase fan	rs613926		YES			Tripeptidyl	aminopeptidase activity,cytoplasm,cytosol,endopeptid				G=0, C=71	G=0.0, C=1.0	0.00001	1109.02	71	G=0/0, C=40/3
chr13:103290574	A/A	G	A	SNV	TPP2	TPP2:intronic:NM_003291.3	1	100		intronic	NM_00329	c.1837-15	p.?	-1.9					Subtilase fan	rs614290	0.4821	YES	4.429E-1 (pos.103290574)		Tripeptidyl	aminopeptidase activity,cytoplasm,cytosol,endopeptid				G=0, A=83	G=0.0, A=1.0	0.00001	1349.56	83	G=0/0, A=40/4
chr8:144671685	C/C	G	C	SNV	EEF1D	EEF1D:exonic:NM_001130053.3	1	100	3	missense	NM_00113	c.567C>G	p.Asp189Glu	-0.16	1	45	0	0.04(COSM37	EF-1 guanine	rs6985081	5.00E-04	YES	1.0 (pos.144671685)	adenocarcinoi	Eukaryotic t	DNA binding,activating transcription factor binding,cadh				G=0, C=27	G=0.0, C=1.0	0.00001	384.848	27	G=0/0, C=14/1
chr19:3977486	G/G	A	G	SNV	EEF2,MIR1268A	MIR1268A:intronic_nc:NR_031672.1, EEF2:exonic:NM_0019	1	100	13	synonymous	NR_03167	c.2190T>C	p.(=)	-2.58				0.44(COSM37	50S ribosome	rs36527	0.2014	YES	9.975E-1 (pos.3977486)	adenocarcinoi	Eukaryotic t	5S rRNA bi	Adenocarcinoma;A	Adenosine	Likely benign	A=0, G=33	A=0.0, G=1.0	0.00001	504.523	33	A=0/0, G=23/1
chr19:3979408	A/G	A	G	SNV	EEF2,MIR1268A	MIR1268A:intronic_nc:NR_031672.1, EEF2:exonic:NM_0019	1	97.3	11	synonymous	NR_03167	c.1632T>C	p.(=)	1.62				0.58(COSM41	50S ribosome	rs36526	0.2016	YES	9.98E-1 (pos.3979408)	neoplasm	Eukaryotic t	5S rRNA bi	Adenocarcinoma;A	Adenosine	Likely benign	A=1, G=36	A=0.027, G=0.973	0.00001	551.433	37	A=0/1, G=4/32
chr16:5139217	C/G	C	G	SNV	EEF2KMT	EEF2KMT:exonic:NM_201400.3	1	95.83	7	synonymous	NM_20140	c.783G>C	p.(=)	-0.81				0.05(COSM37	Family of unk	rs9745857	0.1042	YES	9.249E-1 (pos.5139217)	adenocarcinoi	Eukaryotic e	cytoplasm,cytosol,peptidyl-lysine trimethylation,protei				C=1, G=23	C=0.0417, G=0.9583	0.00001	306.64	24	C=0/1, G=10/1
chr16:5140561	C/T	C	T	SNV	EEF2KMT	EEF2KMT:exonic:NM_201400.3	1	47.37	5	synonymous	NM_20140	c.348G>A	p.(=)	-3.48				0.02(COSM43	Family of unk	rs9673770	0.126	YES	1.451E-1;0.0 (pos.51405	acute_myeloi	Eukaryotic e	cytoplasm,cytosol,peptidyl-lysine trimethylation,protei				C=28, T=27	C=0.5091, T=0.4909	0.00001	283.011	57	C=12/16, T=7/
chr7:36338841	C/C	T	C	SNV	EEPD1	EEPD1:utr_3:NM_030636.2	1	100	8	utr_3	NM_03063	c.1736T>C	p.?	-1.13					Helix-hairpin	rs196614	0.4964	YES	4.458E-1;0.0 (pos.36338841)		Endonuclea	DNA binding,DNA repair				T=0, C=31	T=0.0, C=1.0	0.00001	465.819	31	T=0/0, C=21/1
chr17:45438889	T/T	TGAGT	T	INDEL	EFCAB13	EFCAB13:splicesite_5:NM_152347.4	4	100	10	utr_5	NM_15234	c.805+2TG	p.?	2.88, 0.21, 3.35, 3.54, 1.58						rs10538163,rs201433080,rs559		YES	0.0 (pos.45438892),2.445E-1 (pos.45438886)							TGAGT=0, T	TGAGT=0.0, T=1.0	0.00001	689.659	42	TGAGT=0/0, T=
chr17:45438886	C/C	CAGTG	C	INDEL	EFCAB13	EFCAB13:splicesite_5:NM_152347.4	4	95.45	10	splicesite	NM_152347.4			0.44, 0.36, 3.54, 2.88, 0.21						rs10538163,rs20143	0.4586	YES	0.0 (pos.45438887),2.44	astrocytoma_Grade_IV						CAGTG=2, (	CAGTG=0.0455, C=0.	0.00001	50	44	
chr22:44112845	T/T	C	T	SNV	EFCAB6	EFCAB6:exonic:NM_022785.3	1	100	9	synonymous	NM_02278	c.765G>A	p.(=)	-2.4					EF-hand dom	rs5764234	0.2365	YES	8.798E-1 (pos.44112845)			calcium ion binding,nucleoplasm,regulation of transcrip				C=0, T=25	C=0.0, T=1.0	0.00001	352.973	25	C=0/0, T=17/8
chr3:128755953	A/A	G	A	SNV	EFCC1	EFCC1:exonic:NM_024768.2	1	100	6	missense	NM_02476	c.1583G>A	p.Arg528Gln	3.65	0.03	43	0.975	0.88(COSM37	Coiled-coil do	rs3732430	0.4171	YES	2.44E-1 (pos.128755953)	adenocarcinoma		calcium ion binding				G=0, A=30	G=0.0, A=1.0	0.00001	446.638	30	G=0/0, A=16/1
chr11:65636053	C/C	T	C	SNV	EFEMP2	EFEMP2:exonic:NM_016938.4	1	100	8	missense	NM_01693	c.775A>G	p.Ile259Val	0.54	1	29	0		Calcium-bind	rs601314	0.0015	YES	1.0 (pos.65636053)		EGF-contair	basement	Congenital, Hereditary, and Ne		Benign	T=0, C=32	T=0.0, C=1.0	0.00001	485.141	32	T=0/0, C=5/27
chr8:132982824	G/G	A	G	SNV	EFR3A	EFR3A:exonic:NM_015137.5	1	100	10	missense	NM_01513	c.1093A>G	p.Asn365Asp	0.55	0.2	23	0.015	0.22(COSM4006844)		rs1051221	0.4878	YES	3.306E-1 (pos.13298282	carcinoma	EFR3, S. cer	cytosol,ex	Autoimmune Diseases;Female Urogenital Di			A=0, G=29	A=0.0, G=1.0	0.00001	427.598	29	A=0/0, G=14/1
chr4:110914427	T/T	A	T	SNV	EGF	EGF:exonic:NM_001963.5	1	100	19	missense	NM_00196	c.2759A>T	p.Glu920Val	-1.92	1	121	0		Calcium-bind	rs4698803	0.2099	YES	9.993E-1 (pos.110914427)		Epidermal g	DNA replic	Adenocarcinoma;A	Alpha-Ami	Benign	A=0, T=52	A=0.0, T=1.0	0.00001	767.844	52	A=0/0, T=34/1
chr13:103402772	C/C	T	C	SNV	CCDC168	CCDC168:intronic:NM_001146197.2	1	100		intronic	NM_00114	c.309-34A	p.?	-0.58					Coiled-coil do	rs9284197		YES	6.786E-1 (pos.103402772)							T=0, C=24	T=0.0, C=1.0	0.00001	334.774	24	T=0/0, C=10/1
chr13:103445609	GT/GT	G	GT	INDEL	KDELC1	KDELC1:intronic:NM_024089.2	1	100		intronic	NM_02408	c.672+82C	p.?	0.76					Filamin/ABP2	rs11441956,rs397830647					KDEL motif-	endoplasmic reticulum lumen,glucosyltransferase activ				G=0, GT=66	G=0.0, GT=1.0	0.00001	950.683	66	G=0/0, GT=48/
chr7:55214348	T/T	C	T	SNV	EGFR	EGFR:exonic:NM_005228.4	1	100	4	synonymous	NM_00522	c.474C>T	p.(=)	1.22				0.83(COSM14	Furin-like cys	rs2072454	0.4748	YES	3.742E-1 (pos.55214348)	acute_myeloi	Epidermal g	AP-2 adap	Acneiform Eruption	Afatinib,Al	Benign/Likely	C=0, T=40	C=0.0, T=1.0	0.00001	644.285	40	C=0/0, T=9/31
chr11:65347636	C/C	G	C	SNV	EHBP1L1	EHBP1L1:exonic:NM_001099409.2	1	100	5	missense	NM_00109	c.397G>C	p.Val133Leu	0.37	1	32	0		CAMSAP CH d	rs1194100	0		1.0 (pos.65347636)			biological_process,endosome,membrane,molecular_fu				G=0, C=63	G=0.0, C=1.0	0.00001	958.862	63	G=0/0, C=48/1
chr13:103514091	C/C	T	C	SNV	BIVM-ERCC5,ERCC5	ERCC5:intronic:NM_000123.3, BIVM-ERCC5:intronic:NM_001	1	100		intronic	NM_00120	c.2242+27	p.?, p.?	-0.47					XPG I-region,	rs7982448	0		1.0 (pos.103514091)		Excision-rep	DNA replic	Abnormalities, Multiple;Agenesis of Corpus C			T=0, C=34	T=0.0, C=1.0	0.00001	524.177	34	T=0/0, C=24/1
chr11:64621856	G/G	A	G	SNV	EHD1	EHD1:exonic:NM_006795.3	1	100	5	synonymous	NM_00679	c.1554T>C	p.(=)	-1.49					50S ribosome	rs3897618	1.00E-04		1.0 (pos.64621856)		EH domain-	ATP binding,GTP binding,Rab GTPase binding,blood coag				A=0, G=26	A=0.0, G=1.0	0.00001	371.207	26	A=0/0, G=13/1
chr13:107165255	G/G	C	G	SNV	EFNB2	EFNB2:intronic:NM_004093.3	1	100		intronic	NM_00409	c.123-95G	p.?	-0.09					Ephrin	rs9520090		YES			eph-relatec	T cell costi	Cardiovascular Diseases;Digestive System Di			C=0, G=25	C=0.0, G=1.0	0.00001	353.006	25	C=0/0, G=14/1
chr13:109446080	ATTG/A	ATTG	A	INDEL	MYO16	MYO16:intronic:NM_015011.2	3	57.89		intronic	NM_01501	c.675+92A	p.?	-1.22, -0.11, -1.13, -0.2					Ankyrin repe	rs148553956,rs57874094		YES			Myosin XVI	ATP bindin	Alzheimer Disease;Brain Diseases;Central Ne			ATTG=16, A	ATTG=0.4211, A=0.57	0.00001	249.181	38	ATTG=10/6, A=
chr13:109459020	G/A	G	A	SNV	MYO16	MYO16:intronic:NM_015011.2	1	50		intronic	NM_01501	c.676-7G>	p.?	-4.63					Ankyrin repe	rs9301323	0.2853	YES	7.784E-1 (pos.109459020)		Myosin XVI	ATP bindin	Alzheimer Disease;Brain Diseases;Central Ne			G=15, A=15	G=0.5, A=0.5	0.00001	145.021	30	G=4/11, A=10/
chr19:48244401	G/A	G	A	SNV	EHD2	EHD2:exonic:NM_014601.3	1	58.33	6	synonymous	NM_01460	c.1344G>A	p.(=)	3				0.89(COSM37	50S ribosome	rs35910701	0.1957	YES	6.267E-3 (pos.48244401)	adenocarcinoi	EH domain-	ATP binding,GTP binding,blood coagulation,calcium ion b				G=10, A=14	G=0.4167, A=0.5833	0.00001	130.484	24	G=3/7, A=13/1
chr19:48244194	A/G	A	G	SNV	EHD2	EHD2:exonic:NM_014601.3	1	50	6	synonymous	NM_01460	c.1137A>G	p.(=)	3.2				0.09(COSM44	50S ribosome	rs7255434	0.3017	YES	5.396E-1 (pos.48244194)	acute_myeloi	EH domain-	ATP binding,GTP binding,blood coagulation,calcium ion b				A=16, G=16	A=0.5, G=0.5	0.00001	158.379	32	A=2/14, G=10/
chr13:109817127	A/A	G	A	SNV	MYO16,MYO16-AS1	MYO16-AS1:intronic_nc:NR_047700.1, MYO16:intronic:NM_	1	100		intronic	NM_01501	c.5099-12	p.?	-0.81					Ankyrin repe	rs496081		YES			Myosin XVI	ATP bindin	Alzheimer Disease;Brain Diseases;Central Ne			G=0, A=48	G=0.0, A=1.0	0.00001	810.435	48	G=0/0, A=27/2
chr13:109858890	AT/AT	A	AT	INDEL	MYO16	MYO16:intronic:NM_015011.2	1	100		intronic	NM_01501	c.5350-67	p.?	-1.69					Ankyrin repe	rs11430851		YES			Myosin XVI	ATP bindin	Alzheimer Disease;Brain Diseases;Central Ne			A=0, AT=35	A=0.0, AT=1.0	0.00001	507.951	35	A=0/0, AT=19/
chr19:40023308	G/G	A	G	SNV	EID2B	EID2B:exonic:NM_152361.2	1	100	1	synonymous	NM_15236	c.135T>C	p.(=)	0.09				0(COSM439481)		rs1123301	0.2685	YES	8.074E-1 (pos.40023308)	NS,adenocarcinoma		cell differentiation,identical protein binding,muscle org				A=0, G=82	A=0.0, G=1.0	0.00001	1329.65	82	A=0/0, G=33/4
chr13:110813523	T/T	C	T	SNV	COL4A1	COL4A1:intronic:NM_001845.5	1	100		intronic	NM_00184	c.4640+16	p.?	-0.43					C-terminal ta	rs2275842,rs747382	0.1615	YES	3.042E-1;0.0 (pos.110813523)		Collagen IV,	basement	Brain Diseases;CADASIL;Cardic		Benign	C=0, T=35	C=0.0, T=1.0	0.00001	543.902	35	C=0/0, T=14/2
chr13:110813532	A/A	G	A	SNV	COL4A1	COL4A1:intronic:NM_001845.5	1	100		intronic	NM_00184	c.4640+7C	p.?	-3.98				0.03(COSM14	C-terminal ta	rs2275843	0.1614	YES	3.049E-1 (pos.11081353	acute_myeloi	Collagen IV,	basement	Brain Diseases;CADASIL;Cardic		Benign	G=0, A=37	G=0.0, A=1.0	0.00001	582.526	37	G=0/0, A=14/2
chr15:40226495	C/C	T	C	SNV	EIF2AK4	EIF2AK4:exonic:NM_001013703.3	1	100	1	synonymous	NM_00101	c.99T>C	p.(=)	0.29				0.05(COSM37	Anticodon bir	rs566792	0.1355	YES	1.0 (pos.40226495)	adenocarcinoi	Eukaryotic t	ATP bindin	Amino Acid Metabolism, Inbor		Benign	T=0, C=24	T=0.0, C=1.0	0.00001	334.792	24	T=0/0, C=13/1
chr13:110845314	A/A	G	A	SNV	COL4A1	COL4A1:intronic:NM_001845.5	1	100		intronic	NM_00184	c.1382-54	p.?	-0.18					C-terminal ta	rs685884,rs77246740	0.2879	YES	0.0;0.0 (pos.110845305)		Collagen IV,	basement	Brain Diseases;CADASIL;Cardiovascular Dise			G=0, A=87	G=0.0, A=1.0	0.00001	1431.35	87	G=0/0, A=49/3
chr13:110859690	G/G	A	G	SNV	COL4A1	COL4A1:intronic:NM_001845.5	1	100		intronic	NM_00184	c.780+60T	p.?	-1.05					C-terminal ta	rs598819		YES			Collagen IV,	basement	Brain Diseases;CADASIL;Cardiovascular Dise			A=0, G=36	A=0.0, G=1.0	0.00001	563.744	36	A=0/0, G=20/1
chr13:111155711	T/T	C	T	SNV	COL4A2,COL4A2-AS1	COL4A2-AS1:intronic_nc:NR_046583.1, COL4A2:intronic:NM	1	100		intronic	NM_00184	c.4040-19	p.?	-0.84					C-terminal ta	rs4773198	0.361	YES	2.316E-1;0.0 (pos.111155711)		Collagen IV,	angiogene	Bronchial Neoplasms;Carcinoma, Bronchoge			C=0, T=27	C=0.0, T=1.0	0.00001	389.987	27	C=0/0, T=20/7
chr10:120828969	G/G	A	G	SNV	EIF3A	EIF3A:exonic:NM_003750.2	1	100	6	synonymous	NM_00375	c.939T>C	p.(=)	0.23					PCI domain	rs7908387	0.0265	YES	8.894E-1;0.0 (pos.120828969)		Eukaryotic t	IRES-depe	Genital Diseases, Male;Genital Neoplasms, I			A=0, G=39	A=0.0, G=1.0	0.00001	623.98	39	A=0/0, G=20/1
chr11:8014479	T/T	C	T	SNV	EIF3F	EIF3F:exonic:NM_003754.2	1	100	4	synonymous	NM_00375	c.561C>T	p.(=)	-0.51				0.11(COSM41	JAB1/Mov34/	rs4758267	0.2732	YES	8.357E-1 (pos.8014479)	neoplasm	Eukaryotic t	IRES-dependent viral translational initiation,cytosol,euk				C=0, T=51	C=0.0, T=1.0	0.00001	754.583	51	C=0/0, T=39/1
chr13:111164198	C/C	T	C	SNV	COL4A2	COL4A2:intronic:NM_001846.3	1	100		intronic	NM_00184	c.4882-83	p.?	-0.94					C-terminal ta	rs2479426		YES			Collagen IV,	angiogene	Bronchial Neoplasms;Carcinoma, Bronchoge			T=0, C=38	T=0.0, C=1.0	0.00001	605.867	38	T=0/0, C=26/1
chr19:10226256	A/G	A	G	SNV	EIF3G,P2RY11,PPAN-P2RY11	PPAN-P2RY11:downstream:NM_001040664.2, P2RY11:dow	1	51.92	10	synonymous	NM_00104	c.*842A>G	p.?, p.?, p.(=)	-0.19				0.59(COSM37	Eukaryotic tr	rs7710	0.4549	YES	3.361E-1 (pos.10226256)	adenocarcinoi	Eukaryotic t	ATP-activa	Disorders of Excessive Somnolence;Dyssomn			A=25, G=27	A=0.4808, G=0.5192	0.00001	253.173	52	A=16/9, G=16/
chr17:7477938	C/C	G	C	SNV	EIF4A1,SENP3-EIF4A1,SNORA48	EIF4A1:exonic:NM_001204510.1, SENP3-EIF4A1:exonic_nc:N	1	100	3	synonymous	NR_03792	c.147G>C	p.(=)	-0.12					DEAD/DEAH b	rs1057086	0.0323	YES	7.878E-1 (pos.7477938)		Eukaryotic t	ATP bindin	HIV Infections;Immune System Diseases;Imm			G=0, C=23	G=0.0, C=1.0	0.00001	316.747	23	G=0/0, C=9/14
chr13:111955465	C/C	T	C	SNV	ARHGEF7		1	100		intronic				-3.55						rs1555751,rs386774	0.0651	YES	9.171E-1 (pos.111955465)		Rho guanine nucleotide exchange factor 7 (PAK-interacting exchange					T=0, C=68	T=0.0, C=1.0	0.00001	1053.47	68	T=0/0, C=22/4
chr13:111980478	G/G	A	G	SNV	TEX29	TEX29:intronic:NM_152324.2	1	100		intronic	NM_15232	c.59-52A>	p.?	-0.6					Testis-expres	rs2479955	0.1878	YES				integral component of membrane				A=0, G=26	A=0.0, G=1.0	0.00001	371.42	26	A=0/0, G=20/6
chr12:53433089	A/A	C	A	SNV	EIF4B	EIF4B:exonic:NM_001417.6	1	100	13	synonymous	NM_00141	c.1644C>A	p.(=)	1.76					RNA recogniti	rs1548204	2.00E-04		1.0 (pos.53433089)		Eukaryotic t	RNA binding,RNA strand annealing activity,RNA strand-e				C=0, A=27	C=0.0, A=1.0	0.00001	389.988	27	C=0/0, A=10/1
chr13:113170683	G/A	G	A	SNV	TUBGCP3	TUBGCP3:intronic:NM_006322.5	1	59.09		intronic	NM_00632	c.2086+71	p.?	-0.17					Spc97 / Spc98	rs2182268		YES				centriole,centrosome,centrosome duplication,cytoplas				G=9, A=13	G=0.4091, A=0.5909	0.00001	118.482	22	G=6/3, A=7/6
chr12:53433486	T/T	C	T	SNV	EIF4B	EIF4B:exonic:NM_001417.6	1	100	14	synonymous	NM_00141	c.1755C>T	p.(=)	-0.88					RNA recogniti	rs8916	0.0727	YES	8.37E-1 (pos.53433486)		Eukaryotic t	RNA binding,RNA strand annealing activity,RNA strand-e				C=0, T=60	C=0.0, T=1.0	0.00001	903.531	60	C=0/0, T=29/3
chr13:113740706	G/G	A	G	SNV	MCF2L	MCF2L:intronic:NM_001112732.2	1	100		intronic	NM_00111	c.2373+14	p.?	-0.44					Divergent CR	rs1745949		YES			MCF2-like p	G-protein	Arthritis;Joint Diseases;Musculoskeletal Dis			A=0, G=25	A=0.0, G=1.0	0.00001	352.984	25	A=0/0, G=6/19
chr13:113792754	C/C	T	C	SNV	F10	F10:intronic:NM_000504.3	1	100		intronic	NM_00050	c.232-17T	p.?	-1.37					Coagulation I	rs2251102	0.1547	YES	8.893E-1 (pos.113792754)		Coagulatior	ER to Golgi	Blood Coagulation I	(2R)-2-(5-C	Benign	T=0, C=34	T=0.0, C=1.0	0.00001	524.186	34	T=0/0, C=29/5
chr13:113889474	T/T	C	T	SNV	CUL4A	CUL4A:intronic:NM_001008895.2	1	100		intronic	NM_00100	c.848+15C	p.?	-0.3					Cullin family,	rs2287250	0.2335	YES	4.361E-1 (pos.113889474)		Cullin 4A	Cul4-RING	Dermatitis;Drug Eruptions;Drug Hypersensiti			C=0, T=31	C=0.0, T=1.0	0.00001	465.819	31	C=0/0, T=13/1
chr13:113891075	A/A	G	A	SNV	CUL4A	CUL4A:intronic:NM_001008895.2	1	100		intronic	NM_00100	c.849-62G	p.?	-0.6					Cullin family,	rs2287253	0.2278	YES			Cullin 4A	Cul4-RING	Dermatitis;Drug Eruptions;Drug Hypersensiti			G=0, A=72	G=0.0, A=1.0	0.00001	1130.95	72	G=0/0, A=36/3
chr13:113915194	G/G	A	G	SNV	CUL4A	CUL4A:intronic:NM_001008895.2	1	100		intronic	NM_00100	c.2184+12	p.?	1.02					Cullin family,	rs61967893		YES			Cullin 4A	Cul4-RING	Dermatitis;Drug Eruptions;Drug Hypersensiti			A=0, G=35	A=0.0, G=1.0	0.00001	543.899	35	A=0/0, G=20/1
chr13:114098655	C/T	C	T	SNV	ADPRHL1	ADPRHL1:intronic:NM_138430.4	1	45.45		intronic	NM_13843	c.379+85G	p.?	-3.16					ADP-ribosylgl	rs73579439		YES			ADP-ribosyl	ADP-ribosylarginine hydrolase activity,GTPase activator				C=12, T=10	C=0.5455, T=0.4545	0.00001	81.7504	22	C=6/6, T=5/5
chr13:114507836	G/G	A	G	SNV	TMEM255B	TMEM255B:intronic:NM_182614.3	1	100		intronic	NM_18261	c.670-22A	p.?	-0.43					FAM70 protei	rs7323732	0.3438	YES	8.548E-1 (pos.114507836)			integral component of membrane				A=0, G=28	A=0.0, G=1.0	0.00001	413.104	28	A=0/0, G=13/1
chr13:114787031	G/G	T	G	SNV	RASA3	RASA3:intronic:NM_007368.3	1	100		intronic	NM_00736	c.681-47A	p.?	-1.78					BTK motif,C2	rs2274713	0.3672	YES	6.753E-1 (pos.114787031)		Ras p21 pro	GTPase activator activity,MAPK cascade,calcium-releas				T=0, G=21	T=0.0, G=1.0	0.00001	281.235	21	T=0/0, G=3/18
chr13:115010330	G/T	G	T	SNV	CDC16,MIR548AR	MIR548AR:downstream:NR_049839.1, CDC16:intronic:NM_	1	50		intronic	NM_00107	c.768-35G	p.?	-1.2					Anaphase-pr	rs2039092,rs75367	0.1472	YES	1.051E-1;0.0 (pos.115010330)		Cell divisior	anaphase-promoting complex,anaphase-promoting com				G=21, T=21	G=0.5, T=0.5	0.00001	227.968	42	G=12/9, T=15/
chr13:115027462	A/A	T	A	SNV	CDC16	CDC16:intronic:NM_001078645.2	1	100		intronic	NM_00107	c.1376+39	p.?	0.2					Anaphase-pr	rs7985356	0.2285	YES	1.538E-1 (pos.115027462)		Cell divisior	anaphase-promoting complex,anaphase-promoting com				T=0, A=29	T=0.0, A=1.0	0.00001	431.986	29	T=0/0, A=4/25
chr13:115037613	A/A	G	A	SNV	CDC16	CDC16:intronic:NM_001078645.2	1	100		intronic	NM_00107	c.1604-46	p.?	0.1					Anaphase-pr	rs201681245,rs2274	0.2869	YES	4.946E-1;2.577E-4 (pos.115037613)		Cell divisior	anaphase-promoting complex,anaphase-promoting com				G=0, A=31	G=0.0, A=1.0	0.00001	465.821	31	G=0/0, A=15/1
chr13:19756026	G/G	C	G	SNV	LOC101928697,TUBA3C	TUBA3C:upstream:NM_006001.2, LOC101928697:upstream	1	100		intronic	NM_00600	c.-139G>C	p.?	0.02						rs783595		YES				GTP binding,GTPase activity,cil		CYT997,Epothilone D,Pat		C=0, G=35	C=0.0, G=1.0	0.00001	539.387	35	C=0/0, G=19/1
chr13:20041323	T/T	G	T	SNV	TPTE2	TPTE2:intronic:NM_199254.2	1	100		intronic	NM_19925	c.512+42C	p.?	-4.15					C2 domain of	rs2497251	0.044	YES	9.998E-1 (pos.20041323)		Transmemb	Golgi membrane,endoplasmic reticulum membrane,inte				G=0, T=41	G=0.0, T=1.0	0.00001	664.697	41	G=0/0, T=12/2
chr4:99850126	G/G	A	G	SNV	EIF4E	EIF4E:utr_5:NM_001130678.2	1	100	1	utr_5	NM_00113	c.-3T>C	p.?	1.03					Eukaryotic ini	rs11723037	0.4334	YES	6.791E-1 (pos.99850126)		Eukaryotic t	G1/S trans	Cardiovascular Dis	2,2,5,5-TETRAMETHYL-3-(		A=0, G=36	A=0.0, G=1.0	0.00001	541.304	36	A=0/0, G=10/2
chr3:184039666	G/G	A	G	SNV	EIF4G1	EIF4G1:exonic:NM_001194947.1	1	100	10	missense	NM_00119	c.1315A>G	p.Met439Val	0.72	1	21	0		MA3 domain,	rs2178403	0.2421	YES	6.582E-1 (pos.184039666)		Eukaryotic translation initiation factor 4 gamma, 1					A=0, G=25	A=0.0, G=1.0	0.00001	353.016	25	A=0/0, G=3/22
chr13:21732348	T/C	T	C	SNV	SKA3	SKA3:intronic:NM_145061.5	1	47.62		intronic	NM_14506	c.916-84A	p.?	-0.51						rs4111077		YES				cell division,chromosome segregation,condensed chron				T=33, C=30	T=0.5238, C=0.4762	0.00001	289.404	63	T=21/12, C=15
chr13:21746453	C/C	T	C	SNV	SKA3	SKA3:intronic:NM_145061.5	1	100		intronic	NM_14506	c.331+25A	p.?	-0.22						rs2026600	0.0553	YES	9.988E-1 (pos.21746453)			cell division,chromosome segregation,condensed chron				T=0, C=31	T=0.0, C=1.0	0.00001	465.818	31	T=0/0, C=17/1
chr13:22096843	C/C	T	C	SNV	MICU2	MICU2:intronic:NM_152726.2	1	100		intronic	NM_15272	c.467-54A	p.?	-2.75					EF-hand dom	rs1200028	3.00E-04	YES			Mitochondr	calcium channel complex,calcium ion binding,mitochon				T=0, C=26	T=0.0, C=1.0	0.00001	371.419	26	T=0/0, C=12/1

chr11:10822350	T/T	C	T	SNV	EIF4G2,SNORD97	SNORD97:downstream:NR_004403.1, EIF4G2:exonic:NM_00	1	100	16	synonymous	NM_00141	c.1572G>	p.(=)	0.93					MA3 domain,	rs7951708	0		1.0 (pos.10822350)		Eukaryotic t	RNA binding,axon,cadherin binding,cell cycle arrest,cell			C=0, T=79	C=0.0, T=1.0	0.00001	1273.3	79	C=0/0, T=55/2
chr13:22255167	C/T	C	T	SNV	FGF9	FGF9:intronic:NM_002010.2	1	46.51		intronic	NM_00201	c.278-14C	p.?	0.08					Fibroblast gr	rs3818460	0.3383	YES	4.009E-1 (pos.22255167)		Fibroblast g	1-phospha	Adenocarcinoma;Bone Diseas	Benign	C=23, T=20	C=0.5349, T=0.4651	0.00001	212.33	43	C=14/9, T=7/1
chr13:22275127	T/T	A	T	SNV	FGF9	FGF9:intronic:NM_002010.2	1	100		intronic	NM_00201	c.382-202	p.?	0					Fibroblast gr	rs647236		YES			Fibroblast g	1-phospha	Adenocarcinoma;Bone Diseases;Bone Diseas		A=0, T=40	A=0.0, T=1.0	0.00001	642.279	40	A=0/0, T=36/4
chr13:22275193	C/C	T	C	SNV	FGF9	FGF9:intronic:NM_002010.2	1	100		intronic	NM_00201	c.382-136	p.?	-1.63					Fibroblast gr	rs518544		YES			Fibroblast g	1-phospha	Adenocarcinoma;Bone Diseases;Bone Diseas		T=0, C=43	T=0.0, C=1.0	0.00001	705.842	43	T=0/0, C=36/7
chr13:22275210	C/C	T	C	SNV	FGF9	FGF9:intronic:NM_002010.2	1	100		intronic	NM_00201	c.382-119	p.?	-0.21					Fibroblast gr	rs519234		YES			Fibroblast g	1-phospha	Adenocarcinoma;Bone Diseases;Bone Diseas		T=0, C=43	T=0.0, C=1.0	0.00001	705.844	43	T=0/0, C=36/7
chr1:21167404	G/G	A	G	SNV	EIF4G3	EIF4G3:exonic:NM_001198801.1	1	100	28	synonymous	NM_00119	c.3946T>C	p.(=)	2.01					MA3 domain,	rs2230573	6.00E-04	YES	1.0 (pos.21167404)		Eukaryotic t	RNA binding,RNA cap binding,cytosol,eukaryotic translat			A=0, G=20	A=0.0, G=1.0	0.00001	263.786	20	A=0/0, G=11/9
chr17:12898295	T/C	T	C	SNV	ELAC2	ELAC2:exonic:NM_001165962.1	1	51.85	19	synonymous	NM_00116	c.1773A>G	p.(=)	-3.97				0.07(COSM37	Beta-lactama	rs17552022	0.1273	YES	2.312E-4 (pos.12898295)	hepatocellula	elaC, E. coli	3'-tRNA pro	Genital Diseases, Male;Genita	Benign	T=13, C=14	T=0.4815, C=0.5185	0.00001	132.666	27	T=3/10, C=8/6
chr1:50666513	GT/GT	G	GT	INDEL	ELAVL4	ELAVL4:exonic:NM_021952.4	1	100	7	frameshiftInsertion	NM_02195	c.807_808	p.Pro270fs	4.28					RNA recognition motif. (a.k.a. RRM, RBD, or RNP domain)						Embryonic l	AU-rich ele	Nervous System Diseases		G=0, GT=48	G=0.0, GT=1.0	0.00001	713.547	48	G=0/0, GT=33/
chr13:41515286	A/A	T	A	SNV	ELF1	ELF1:exonic:NM_172373.3	1	100	8	missense	NM_17237	c.1027A>T	p.Thr343Ser	0.63	0.64	58	0.008	0.88(COSM13	Ets-domain,T	rs1056820	0.3216	YES	1.679E-1;1.156E-3 (pos.4	adenocarcino	E74-like fac	DNA bindi	Autoimmune Diseases;Connective Tissue Dis		T=0, A=73	T=0.0, A=1.0	0.00001	1150.49	73	T=0/0, A=29/4
chr13:41517985	G/G	A	G	SNV	ELF1	ELF1:exonic:NM_172373.3	1	100	6	synonymous	NM_17237	c.606T>C	p.(=)	2.27					Ets-domain,T	rs3764056	0.3212	YES	1.688E-1 (pos.41517985)		E74-like fac	DNA bindi	Autoimmune Diseases;Connective Tissue Dis		A=0, G=41	A=0.0, G=1.0	0.00001	664.7	41	A=0/0, G=11/3
chr1:201981218	G/G	C	G	SNV	ELF3	ELF3:exonic:NM_004433.4	1	100	3	synonymous	NM_00443	c.297C>G	p.(=)	-0.46				0.05(COSM14	AT hook moti	rs11543979	0.2185	YES	3.509E-1;0.0 (pos.20198	adenocarcino	E74-like fac	DNA bindi	Biliary Tract Diseases:Biliary Tract Neoplasm		C=0, G=32	C=0.0, G=1.0	0.00001	485.141	32	C=0/0, G=20/1
chr1:201970562	C/C	T	C	SNV	ELF3-AS1,RNPEP	ELF3-AS1:intronic_nc:NR_146472.1, RNPEP:exonic:NM_0202	1	100	7	synonymous	NM_02021	c.1263T>C	p.(=)	-1.15					Leukotriene	rs4332408	3.00E-04		1.0 (pos.201970562)		Arginyl ami	aminopep	Mammary Neoplasms, Animal;Mammary Ne		T=0, C=24	T=0.0, C=1.0	0.00001	334.792	24	T=0/0, C=12/1
chr13:24865029	G/A	G	A	SNV	SPATA13	SPATA13:intronic:NM_001166271.2	1	50		intronic	NM_00116	c.3080+7G	p.?	2.01					PH domain,R	rs41287032	0.1042	YES	2.6E-4 (pos.24865029)		Spermatoge	Rac guanyl-nucleotide exchange factor activity,Rho guan			G=10, A=10	G=0.5, A=0.5	0.00001	82.8871	20	G=5/5, A=5/5
chr18:44560429	A/A	C	A	SNV	ELOA2,KATNAL2	KATNAL2:intronic:NM_031303.3, ELOA2:exonic:NM_016427	1	100	1	missense	NM_03130	c.-93-1882	p.?, p.Ala403Ser	-0.52		99			ATPase famil	rs892586	0.4717	YES	6.537E-1;0.0 (pos.44560429)		Katanin p6	ATP binding,ATPase activity,cytoplasm,elongin complex,			C=0, A=40	C=0.0, A=1.0	0.00001	644.215	40	C=0/0, A=23/1
chr18:33718326	C/C	A	C	SNV	ELP2	ELP2:exonic:NM_018255.3	1	100	4	missense	NM_01825	c.382A>C	p.Thr128Pro	0.65	0.11	38	0	0.08(COSM50	WD domain,	rs1785934	0.3713	YES	1.893E-1 (pos.33718326)	ductal_carcinc	Elongator a	Elongator	Intellectual Disability;Mental Disorders;Nerv		A=0, C=91	A=0.0, C=1.0	0.00001	1512.32	91	A=0/0, C=34/5
chr18:33750046	T/T	C	T	SNV	ELP2	ELP2:exonic:NM_018255.3	1	100	20	synonymous	NM_01825	c.2097C>T	p.(=)	-2.8				0.03(COSM13	WD domain,	rs8299	0.3844	YES	1.889E-1 (pos.33750046)	adenocarcino	Elongator a	Elongator	Intellectual Disability;Mental Disorders;Nerv		C=0, T=20	C=0.0, T=1.0	0.00001	263.785	20	C=0/0, T=12/8
chr8:27950670	C/T	C	T	SNV	ELP3	ELP3:utr_5:NM_018091.5	1	57.14	1	utr_5	NM_01809	c.-62C>T	p.?	-0.08					Acetyltransfe	rs2305451	0.3143	YES			Elongation	Elongator holoenzyme complex,H3 histone acetyltransfe			C=57, T=76	C=0.4286, T=0.5714	0.00001	880.848	133	C=24/33, T=38
chr19:48519241	C/G	C	G	SNV	ELSPBP1	ELSPBP1:exonic:NM_022142.4	1	47.37	4	missense	NM_02214	c.300C>G	p.Cys100Trp	-0.22	0	215	1	0.1(COSM139	Fibronectin t	rs3745751	0.0626	YES	7.502E-2 (pos.48519241)	adenocarcino	Epididymal	cell surface,extracellular regio	N,N,N-Trimethyl-2-(phos		C=20, G=18	C=0.5263, G=0.4737	0.00001	184.537	38	C=9/11, G=10/
chr17:48452776	C/C	A	C	SNV	EME1	EME1:exonic:NM_001166131.1	1	100	2	missense	NM_00116	c.207A>C	p.Glu69Asp	-1.56	1	45	0		ERCC4 domai	rs3760413	0.1058	YES	2.296E-1 (pos.48452776)		Essential m	DNA binding,DNA repair,Holliday junction resolvase com			A=0, C=28	A=0.0, C=1.0	0.00001	408.718	28	A=0/0, C=12/1
chr13:25363776	T/T	C	T	SNV	RNF17	RNF17:intronic:NM_031277.2	1	100		intronic	NM_03127	c.861-60C	p.?	0.07					Tudor domair	rs6490979	0	YES			RING finger	cytoplasm,metal ion binding,multicellular organism dev			C=0, T=66	C=0.0, T=1.0	0.00001	1011.19	66	C=0/0, T=42/2
chr20:39990700	T/C	T	C	SNV	EMILIN3	EMILIN3:exonic:NM_052846.1	1	57.14	4	synonymous	NM_05284	c.1509A>G	p.(=)	0.04				0.03(COSM37	EMI domain	rs41278104	0.1735	YES	3.826E-3 (pos.39990700)	adenocarcinoma,ductal_ca		cytoplasm,extracellular matrix,proteinaceous extracell			T=9, C=12	T=0.4286, C=0.5714	0.00001	109.102	21	T=2/7, C=4/8
chr13:25373432	T/T	C	T	SNV	RNF17	RNF17:intronic:NM_031277.2	1	100		intronic	NM_03127	c.1400-10	p.?	0.2					Tudor domair	rs2166431		YES			RING finger	cytoplasm,metal ion binding,multicellular organism dev			C=0, T=34	C=0.0, T=1.0	0.00001	524.186	34	C=0/0, T=17/1
chr13:25399752	G/G	A	G	SNV	RNF17	RNF17:intronic:NM_031277.2	1	100		intronic	NM_03127	c.2092-5A	p.?	-0.06					Tudor domair	rs2121253	0.1358	YES	8.536E-1 (pos.25399752)		RING finger	cytoplasm,metal ion binding,multicellular organism dev			A=0, G=98	A=0.0, G=1.0	0.00001	1660.04	98	A=0/0, G=50/4
chr13:25404743	C/C	T	C	SNV	RNF17	RNF17:intronic:NM_031277.2	1	100		intronic	NM_03127	c.2361+6T	p.?	-0.19					Tudor domair	rs4769382,rs772251	9.00E-04	YES	0.0;0.0 (pos.25404740),9.474E-1 (pos.25		RING finger	cytoplasm,metal ion binding,multicellular organism dev			T=0, C=51	T=0.0, C=1.0	0.00001	754.584	51	T=0/0, C=12/3
chr13:25448120	A/A	G	A	SNV	RNF17	RNF17:intronic:NM_031277.2	1	100		intronic	NM_03127	c.4448-13	p.?	-1.62					Tudor domair	rs8001764		YES			RING finger	cytoplasm,metal ion binding,multicellular organism dev			G=0, A=87	G=0.0, A=1.0	0.00001	1431.39	87	G=0/0, A=58/2
chr13:25458650	A/A	G	A	SNV	CENPJ	CENPJ:intronic:NM_018451.4	1	100		intronic	NM_01845	c.3478-49	p.?	-0.27					T-complex pr	rs1530876	0.1683	YES	8.458E-1 (pos.25458650)		Centromeri	DNA dama	Bone Diseases;Bone Diseases,	Likely benign	G=0, A=41	G=0.0, A=1.0	0.00001	664.686	41	G=0/0, A=21/2
chr13:25466774	C/C	T	C	SNV	CENPJ	CENPJ:intronic:NM_018451.4	1	100		intronic	NM_01845	c.3216+7A	p.?	-0.27					T-complex pr	rs9318917	0.1692	YES	8.46E-1 (pos.25466774)		Centromeri	DNA dama	Bone Diseases;Bone Diseases,	Benign	T=0, C=24	T=0.0, C=1.0	0.00001	334.179	24	T=0/0, C=17/7
chr11:62381106	C/C	G	C	SNV	EML3,ROM1	ROM1:exonic:NM_000327.3, EML3:upstream:NM_153265.2	1	100	1	missense	NM_15326	c.-1177C>	p.?, p.Gly118Ala	0.1	1	60	0	0.02(COSM37	Tetraspanin f	rs1799959,rs763179	0.0021	YES	1.187E-4 (pos.62381089)	adenocarcino	Rod outer s	camera-ty	Congenital, Hereditary, and Ne	Benign	G=0, C=61	G=0.0, C=1.0	0.00001	915.182	61	G=0/0, C=11/5
chr13:25848404	C/C	A	C	SNV	MTMR6	MTMR6:intronic:NM_004685.3	1	100		intronic	NM_00468	c.25-79T>	p.?	-1.56					Myotubularir	rs4387458		YES			Myotubular	calcium-activated potassium channel activity,cytoplasm			A=0, C=23	A=0.0, C=1.0	0.00001	316.747	23	A=0/0, C=8/15
chr13:25905416	C/C	T	C	SNV	NUP58	NUP58:intronic:NM_014089.3	1	100		intronic	NM_01408	c.1234-79	p.?	-0.92					Nucleoporin	rs9553582		YES			Nucleopori	host cell,intracellular transport of virus,mRNA export fro			T=0, C=66	T=0.0, C=1.0	0.00001	1015.4	66	T=0/0, C=35/3
chr13:26133009	C/T	C	T	SNV	ATP8A2	ATP8A2:intronic:NM_016529.5	1	50		intronic	NM_01652	c.1264-10	p.?	-1.47					Cation transı	rs12862551		YES			ATPase, cla	ATP bindin	Ataxia;Brain Diseases;Central Nervous Syste		C=17, T=17	C=0.5, T=0.5	0.00001	172.009	34	C=11/6, T=10/
chr13:26155953	C/C	G	C	SNV	ATP8A2	ATP8A2:intronic:NM_016529.5	1	100		intronic	NM_01652	c.2008-4G	p.?	0.16				0(COSM5764	Cation transı	rs7335339	0.3001	YES	8.845E-3 (pos.26155953)	adenocarcino	ATPase, cla	ATP bindin	Ataxia;Brain Diseases;Central Nervous Syste		G=0, C=24	G=0.0, C=1.0	0.00001	334.792	24	G=0/0, C=19/5
chr2:55186394	C/C	A	C	SNV	EML6	EML6:exonic:NM_001039753.2	1	100	33	synonymous	NM_00103	c.4849A>C	p.(=)	1.26				0.63(COSM40	Anaphase-pr	rs17346629	0.2847	YES	6.896E-1 (pos.55186394)	acute_myeloid_leukaemia		cytoplasm	Nutrition Disorders:Nutritional and Metaboli		A=0, C=174	A=0.0, C=1.0	0.00001	2860.61	174	A=0/0, C=60/1
chr13:27829310	A/A	G	A	SNV	RPL21,RPL21P28,SNORA27,SNORD102	RPL21P28:intronic_nc:NR_026911.1, RPL21:intronic:NM_00	1	100		intronic	NM_00098	c.130-69G	p.?	-1.14					Ribosomal pr	rs3094293	5.00E-04		1.0 (pos.27829310)		Ribosomal	RNA bindir	Hair Diseases:Hypotrichosis;Skin Diseases:Sl		G=0, A=33	G=0.0, A=1.0	0.00001	508.994	33	G=0/0, A=17/1
chr5:73930751	T/T	C	T	SNV	ENC1	ENC1:exonic:NM_003633.3	1	100	2	synonymous	NM_00363	c.1560G>	p.(=)	1.09				0.77(COSM63	BTB And C-ter	rs300239	0.3456	YES	6.544E-1 (pos.73930751)	carcinoma	Ectodermal	Cul3-RING ubiquitin ligase complex,actin binding,cytopla			C=0, T=44	C=0.0, T=1.0	0.00001	692.658	44	C=0/0, T=19/2
chr13:28537537	G/G	T	G	SNV	CDX2	CDX2:intronic:NM_001265.4	1	100		intronic	NM_00126	c.688-31A	p.?	0.3					Caudal like p	rs2504212	0.1805	YES	8.163E-1 (pos.28537537)		Caudal type	DNA binding transcription factor activity,RNA polymeras			T=0, G=70	T=0.0, G=1.0	0.00001	1092.02	70	T=0/0, G=11/5
chr13:28592546	T/C	T	C	SNV	FLT3	FLT3:intronic:NM_004119.2	1	52.38		intronic	NM_00411	c.2541+58	p.?	0.14					Immunoglob	rs17086226		YES	2.482E-1 (pos.28592546)		fms-related	ATP bindin	Cardiovascular Dis	ABT-869,Amuvatinib,Mid	T=10, C=11	T=0.4762, C=0.5238	0.00001	95.3003	21	T=8/2, C=9/2
chr13:28609846	A/T	A	T	SNV	FLT3	FLT3:intronic:NM_004119.2	1	56		intronic	NM_00411	c.1419-36	p.?	-0.28					Immunoglob	rs2491228	0.2179	YES	7.436E-1 (pos.28609846)		fms-related	ATP bindin	Cardiovascular Dis	ABT-869,Amuvatinib,Mid	A=11, T=14	A=0.44, T=0.56	0.00001	134.346	25	A=7/4, T=6/8
chr13:28609991	T/C	T	C	SNV	FLT3	FLT3:intronic:NM_004119.2	1	57.14		intronic	NM_00411	c.1418+81	p.?	0.1					Immunoglob	rs2491229		YES			fms-related	ATP bindin	Cardiovascular Dis	ABT-869,Amuvatinib,Mid	T=9, C=12	T=0.4286, C=0.5714	0.00001	109.063	21	T=2/7, C=6/6
chr13:28622356	A/A	G	A	SNV	FLT3	FLT3:intronic:NM_004119.2	1	100		intronic	NM_00411	c.1205+56	p.?	0.54					Immunoglob	rs2153000		YES			fms-related	ATP bindin	Cardiovascular Dis	ABT-869,Amuvatinib,Mid	G=0, A=32	G=0.0, A=1.0	0.00001	461.192	32	G=0/0, A=15/1
chr13:28886113	G/G	A	G	SNV	FLT1	FLT1:intronic:NM_002019.4	1	100		intronic	NM_00201	c.3492+17	p.?	-0.37					Immunoglob	rs7328692	2.00E-04		1.0;0.0 (pos.28886113)		fms-related	ATP bindin	Adenocarcinoma;B	ABT-869,Axitinib,IMC-1C	A=0, G=24	A=0.0, G=1.0	0.00001	334.792	24	A=0/0, G=17/7
chr13:29275102	C/C	T	C	SNV	SLC46A3	SLC46A3:intronic:NM_001135919.1	1	100		intronic	NM_00113	c.1383-9A	p.?	-0.31					Major Facilit	rs947027	0		1.0 (pos.29275102)		Solute carri	biological_process,extracellular exosome,integral comp			T=0, C=22	T=0.0, C=1.0	0.00001	290.645	22	T=0/0, C=19/3
chr13:29291886	G/G	A	G	SNV	SLC46A3	SLC46A3:intronic:NM_001135919.1	1	100		intronic	NM_00113	c.189+61T	p.?	-1.21					Major Facilit	rs1300235					Solute carri	biological_process,extracellular exosome,integral comp			A=0, G=20	A=0.0, G=1.0	0.00001	263.784	20	A=0/0, G=11/9
chr13:29674840	C/C	T	C	SNV	MTUS2	MTUS2:intronic:NM_001033602.2	1	100		intronic	NM_00103	c.2477-70	p.?	-0.7					RNA polymer	rs1536720		YES				centrosome,cytoplasmic microtubule,intercellular brid			T=0, C=64	T=0.0, C=1.0	0.00001	977.604	64	T=0/0, C=34/3
chr13:29674855	G/G	A	G	SNV	MTUS2	MTUS2:intronic:NM_001033602.2	1	100		intronic	NM_00103	c.2477-55	p.?	-0.38					RNA polymer	rs1536719		YES				centrosome,cytoplasmic microtubule,intercellular brid			A=0, G=66	A=0.0, G=1.0	0.00001	1015.38	66	A=0/0, G=34/3
chr13:30090404	C/C	T	C	SNV	SLC7A1	SLC7A1:intronic:NM_003045.4	1	100		intronic	NM_00304	c.1678-21	p.?	-2.79					Amino acid p	rs2989592	0.0151	YES	9.469E-1 (pos.30090404)		Solute carri	amino aci	Genital Diseases, N	L-Arginine,L-Lysine,Ornit	T=0, C=151	T=0.0, C=1.0	0.00001	2416.89	151	T=0/0, C=17/1
chr5:73932315	C/C	T	C	SNV	ENC1	ENC1:utr_5:NM_003633.3	1	100	2	utr_5	NM_00363	c.-5A>G	p.?	0.2					BTB And C-ter	rs9176	0.3452	YES	7.086E-1 (pos.73932315)		Ectodermal	Cul3-RING ubiquitin ligase complex,actin binding,cytopla			T=0, C=82	T=0.0, C=1.0	0.00001	1329.65	82	T=0/0, C=35/4
chr13:30805627	G/G	A	G	SNV	KATNAL1	KATNAL1:intronic:NM_001014380.2	1	100		intronic	NM_00101	c.727-18T	p.?	-0.65					AAA domain (	rs202087	0.1141	YES	9.588E-1;0.0;0.0 (pos.30805627)		Katanin p6(	ATP binding,ATPase activity,cytoplasm,identical protein			A=0, G=36	A=0.0, G=1.0	0.00001	563.716	36	A=0/0, G=27/9
chr11:94862577	G/T	G	T	SNV	ENDOD1	ENDOD1:exonic:NM_015036.2	1	50	2	missense	NM_01503	c.1337G>T	p.Gly446Val	6.27	0.19	109	0.995	0.94(COSM37	DNA/RNA nor	rs3740861	0.2767	YES	1.258E-1;0.0 (pos.94862	adenocarcinoma		cytosol,endonuclease activity,extracellular exosome,ex			G=14, T=14	G=0.5, T=0.5	0.00001	131.94	28	G=5/9, T=5/9
chr13:31531195	C/C	T	C	SNV	TEX26	TEX26:intronic:NM_152325.2	1	100		intronic	NM_15232	c.469+29T	p.?	0.29						rs6560988	0		1.0 (pos.31531195)						T=0, C=20	T=0.0, C=1.0	0.00001	260.977	20	T=0/0, C=6/14
chr10:118620459	C/C	A	C	SNV	ENO4	ENO4:exonic:NM_001242699.1	1	100	5	synonymous	NM_00124	c.789A>C	p.(=)	-1.09				0.05(COSM37	Enolase, C-te	rs11197835		YES	9.917E-2 (pos.11862045	adenocarcinoma,carcinoma			Genital Diseases, Male:Infertility:Infertility, I		A=0, C=72	A=0.0, C=1.0	0.00001	1130.94	72	A=0/0, C=40/3
chr13:43788158	A/A	G	A	SNV	ENOX1	ENOX1:exonic:NM_001127615.2	1	100	17	synonymous	NM_00112	c.1900C>T	p.(=)	2.85				0.05(COSM37	RNA recogniti	rs703207	0.1323	YES	7.003E-1 (pos.43788158)	hepatocellula	ECTO-NOX d	extracellu	Depressive Disorder:Depressive Disorder, M		G=0, A=29	G=0.0, A=1.0	0.00001	427.602	29	G=0/0, A=7/22
chr8:120575184	A/A	G	A	SNV	ENPP2	ENPP2:exonic:NM_006209.4	1	100	25	synonymous	NM_00620	c.2490C>T	p.(=)	3.15					DNA/RNA nor	rs1058913	0.2321	YES	4.123E-2 (pos.120575184)		Ectonucleo	G-protein	Digestive System Diseases:Endocrine Gland I		G=0, A=59	G=0.0, A=1.0	0.00001	883.753	59	G=0/0, A=15/4
chr6:131995313	A/G	A	G	SNV	ENPP3	ENPP3:exonic:NM_005021.4	1	58.33	8	synonymous	NM_00502	c.654A>G	p.(=)	-0.42				0.15(COSM37	DNA/RNA nor	rs9493048	0.1094	YES	3.38E-1 (pos.131995313)	adenocarcino	Ectonucleo	NADH pyrophosphatase activity,catalytic activity,extrac			A=10, G=14	A=0.4167, G=0.5833	0.00001	135.339	24	A=6/4, G=12/2
chr13:32335840	A/T	A	T	SNV	RXFP2	RXFP2:intronic:NM_130806.4	1	51.85		intronic	NM_13080	c.242-21A	p.?	-0.6					7 transmemb	rs9532478	0.1783	YES	2.469E-1 (pos.32335840)			G-protein	Congenital Abnormalities;Congenital, Heredi		A=13, T=14	A=0.4815, T=0.5185	0.00001	132.662	27	A=7/6, T=3/11
chr13:32348859	C/C	T	C	SNV	RXFP2	RXFP2:intronic:NM_130806.4	1	100		intronic	NM_13080	c.569+31T	p.?	-0.22					7 transmemb	rs9549049	0.2337	YES	2.842E-1 (pos.32348859)			G-protein	Congenital Abnormalities;Congenital, Heredi		T=0, C=35	T=0.0, C=1.0	0.00001	543.877	35	T=0/0, C=16/1
chr13:32355693	GC/GC	G	GC	INDEL	RXFP2	RXFP2:intronic:NM_130806.4	1	100		intronic	NM_13080	c.786-115	p.?	0					7 transmemb	rs5802617		YES				G-protein	Congenital Abnormalities;Congenital, Heredi		G=0, GC=30	G=0.0, GC=1.0	0.00001	452.291	30	G=0/0, GC=15/
chr17:77705045	C/C	T	C	SNV	ENPP7	ENPP7:exonic:NM_178543.4	1	100	1	synonymous	NM_17854	c.144T>C	p.(=)	-0.45					Type I phosph	rs8074728	0.3828	YES	5.059E-1 (pos.77705045)		Ectonucleo	Golgi apparatus,glycosphingolipid metabolic process,int			T=0, C=22	T=0.0, C=1.0	0.00001	298.892	22	T=0/0, C=12/1
chr14:74433552	T/T	A	T	SNV	ENTPD5	ENTPD5:utr_3:NM_001249.3	1	100	16	utr_3	NM_00124	c.1366T>A	p.?	0.75					GDA1/CD39 (	rs13743		YES			Ectonucleo	'de novo' posttranslational protein folding,ATP metaboli			A=0, T=30	A=0.0, T=1.0	0.00001	446.623	30	A=0/0, T=15/1
chr13:32900933	A/A	T	A	SNV	BRCA2	BRCA2:intronic:NM_000059.3	1	100		intronic	NM_00005	c.631+183	p.?	-0.05					BRCA2 repeat	rs3752451		YES			BRCA2 gene	BRCA2-MA	ACMG Recommendations...;Ad	Benign	T=0, A=27	T=0.0, A=1.0	0.00001	389.967	27	T=0/0, A=11/1
chr14:74441684	A/A	G	A	SNV	ENTPD5	ENTPD5:exonic:NM_001249.3	1	100	11	synonymous	NM_00124	c.727C>T	p.(=)	1.52				0.3(COSM375	GDA1/CD39 (	rs2074932	0.3767	YES	2.466E-1 (pos.74441684)	acute_myeloic	Ectonucleo	'de novo' posttranslational protein folding,ATP metaboli			G=0, A=20	G=0.0, A=1.0	0.00001	263.733	20	G=0/0, A=9/11
chr18:5410574	G/G	A	G	SNV	EPB41L3	EPB41L3:exonic:NM_012307.3	1	100	14	synonymous	NM_01230	c.2112T>C	p.(=)	-0.61				0.09(COSM45	4.1 protein C	rs3817466		YES	9.181E-1 (pos.5410574)	astrocytoma_(	Erythrocyte	actin bind	Breast Diseases;Breast Neoplasms;Neoplasr		A=0, G=22	A=0.0, G=1.0	0.00001	298.892	22	A=0/0, G=14/8
chr13:32936646	T/C	T	C	SNV	BRCA2	BRCA2:intronic:NM_000059.3	1	97.06		intronic	NM_00005	c.7806-14	p.?	-0.98					BRCA2 repeat	rs755777054,rs9534	0.4963	YES	2.328E-4 (pos.32936645),5.544E-1 (pos.3		BRCA2 gene	BRCA2-MA	ACMG Recommendations...;Ad	Benign	T=1, C=33	T=0.0294, C=0.9706	0.00001	492.868	34	T=1/0, C=18/1
chr13:32953388	C/C	T	C	SNV	BRCA2	BRCA2:intronic:NM_000059.3	1	100		intronic	NM_00005	c.8755-66	p.?	-1.25					BRCA2 repeat	rs4942486		YES			BRCA2 gene	BRCA2-MA	ACMG Recommendations...;Ad	Benign	T=0, C=33	T=0.0, C=1.0	0.00001	509.934	33	T=0/0, C=18/1
chr5:111504479	T/T	C	T	SNV	EPB41L4A	EPB41L4A:exonic:NM_022140.4	1	100	22	missense	NM_02214	c.1889G>A	p.Arg630His	3.28	0.03	29	1		FERM C-termi	rs7703522	0.3593	YES	9.987E-1 (pos.111504479)		Erythrocyte	actomyosin structure organization,biological_process,c			C=0, T=33	C=0.0, T=1.0	0.00001	504.598	33	C=0/0, T=14/1
chr5:111519740	C/C	T	C	SNV	EPB41L4A	EPB41L4A:exonic:NM_022140.4	1	100	18	missense	NM_02214	c.1595A>G	p.Asn532Ser	2.19	0.86	46	0		FERM C-termi	rs1560058	0.3049	YES	9.908E-1 (pos.111519740)		Erythrocyte	actomyosin structure organization,biological_process,c			T=0, C=173	T=0.0, C=1.0	0.00001	2840.14	173	T=0/0, C=96/7
chr13:33635939	A/A	T	A	SNV	KL	KL:intronic:NM_004795.3	1	100		intronic	NM_00479	c.2701+22	p.?	-1.79					Glycosyl hydr	rs650439	0.132	YES	6.009E-1 (pos.33635939)		Klotho	1-phospha	Arterial Occlusive Diseases;Arteriosclerosis:		T=0, A=119	T=0.0, A=1.0	0.00001	1913.83	119	T=0/0, A=57/6
chr13:35618990	TG/TG	T	TG	INDEL	NBEA	NBEA:intronic:NM_015678.4	1	100		intronic	NM_01567	c.527-94T	p.?	0.59					Beige/BEACH domain,Concanavalin A-like lectin/glucanases superfamily,Domain of Unknown Functic						Neurobeacl	cytosol,en	Autistic Disorder;Child Development Disorde		T=0, TG=93	T=0.0, TG=1.0	0.00001	1187.24	93	T=0/0, TG=55/
chr13:35738430	G/G	A	G	SNV	NBEA	NBEA:intronic:NM_015678.4	1	100		intronic	NM_01567	c.4080-63	p.?	1.66					Beige/BEACH	rs9543506		YES			Neurobeacl	cytosol,en	Autistic Disorder;Child Development Disorde		A=0, G=125	A=0.0, G=1.0	0.00001	2034.04	125	A=0/0, G=53/7
chr13:36801256	C/C	T	C	SNV	CCDC169,CCDC169-SOHLH2	CCDC169:utr_3:NM_001198908.1, CCDC169-SOHLH2:intron	1	100		intronic	NM_00119	c.279+229	p.?, p.?	-0.64					Domain of un	rs9546784		YES				cell differentiation,core promoter sequence-specific DN			T=0, C=87	T=0.0, C=1.0	0.00001	1431.39	87	T=0/0, C=57/3
chr5:111530284	G/G	A	G	SNV	EPB41L4A	EPB41L4A:exonic:NM_022140.4	1	100	17	missense	NM_02214	c.1483T>C	p.Tyr495His	5.12	1	83	0		FERM C-termi	rs7719346	0.287	YES	9.99E-1 (pos.111530284)		Erythrocyte	actomyosin structure organization,biological_process,c			A=0, G=33	A=0.0, G=1.0	0.00001	504.595	33	A=0/0, G=13/2
chr13:36801579	G/G	A	G	SNV	CCDC169,CCDC169-SOHLH2	CCDC169:intronic:NM_001198908.1, CCDC169-SOHLH2:intr	1	100		intronic	NM_00119	c.246-61T	p.?, p.?	0.35					Domain of un	rs9546786		YES				cell differentiation,core promoter sequence-specific DN			A=0, G=39	A=0.0, G=1.0	0.00001	623.98	39	A=0/0, G=16/2
chr13:36801587	A/A	T	A	SNV	CCDC169,CCDC169-SOHLH2	CCDC169:intronic:NM_001198908.1, CCDC169-SOHLH2:intr	1	100		intronic	NM_00119	c.246-69A	p.?, p.?	0.31					Domain of un	rs9546787		YES				cell differentiation,core promoter sequence-specific DN			T=0, A=37	T=0.0, A=1.0	0.00001	583.706	37	T=0/0, A=16/2
chr13:36822711	T/T	G	T	SNV	CCDC169,CCDC169-SOHLH2	CCDC169:intronic:NM_001198908.1, CCDC169-SOHLH2:intr	1	100		intronic	NM_00119	c.245+32C	p.?, p.?	-0.02					Domain of un	rs9315402		YES	3.682E-1;0.0 (pos.36822711)			cell differentiation,core promoter sequence-specific DN			G=0, T=38	G=0.0, T=1.0	0.00001	603.786	38	G=0/0, T=22/1
chr13:36822849	T/T	C	T	SNV	CCDC169,CCDC169-SOHLH2	CCDC169:intronic:NM_001198908.1, CCDC169-SOHLH2:intr	1	100		intronic	NM_00119	c.169-30G	p.?, p.?	0.53					Domain of un	rs9315403	0.4727	YES	3.754E-1 (pos.36822849)			cell differentiation,core promoter sequence-specific DN			C=0, T=37	C=0.0, T=1.0	0.00001	583.707	37	C=0/0, T=21/1
chr13:36822884	T/T	C	T	SNV	CCDC169,CCDC169-SOHLH2	CCDC169:intronic:NM_001198908.1, CCDC169-SOHLH2:intr	1	100		intronic	NM_00119	c.169-65G	p.?, p.?	-5.15					Domain of un	rs9315404		YES				cell differentiation,core promoter sequence-specific DN			C=0, T=35	C=0.0, T=1.0	0.00001	543.875	35	C=0/0, T=20/1
chr13:36822908	T/T	C	T	SNV	CCDC169,CCDC169-SOHLH2	CCDC169:intronic:NM_001198908.1, CCDC169-SOHLH2:intr	1	100		intronic	NM_00119	c.169-89G	p.?, p.?	-0.68					Domain of un	rs9315405		YES				cell differentiation,core promoter sequence-specific DN			C=0, T=35	C=0.0, T=1.0	0.00001	543.257	35	C=0/0, T=20/1
chr13:36827833	T/T	C	T	SNV	CCDC169,CCDC169-SOHLH2	CCDC169:intronic:NM_001198908.1, CCDC169-SOHLH2:intr	1	100		intronic	NM_00119	c.168+107	p.?, p.?	0.56					Domain of un	rs9546886		YES				cell differentiation,core promoter sequence-specific DN			C=0, T=27	C=0.0, T=1.0	0.00001	379.343	27	C=0/0, T=11/1
chr13:36827896	T/T	C	T	SNV	CCDC169,CCDC169-SOHLH2	CCDC169:intronic:NM_001198908.1, CCDC169-SOHLH2:intr	1	100		intronic	NM_00119	c.168+44G	p.?, p.?	-3.81					Domain of un	rs9546887		YES	3.793E-1 (pos.36827896)			cell differentiation,core promoter sequence-specific DN			C=0, T=27	C=0.0, T=1.0	0.00001	389.988	27	C=0/0, T=11/1
chr13:37524255	C/C	A	C	SNV	ALG5	ALG5:intronic:NM_013338.4	1	100		intronic	NM_01333	c.860-61T	p.?	0.05					Glycosyl tran	rs3818380		YES			Alg5, S. cere	determination of left/right symmetry,dolichyl-phosphat			A=0, C=24	A=0.0, C=1.0	0.00001	334.792	24	A=0/0, C=5/19
chr13:37581051	G/G	T	G	SNV	EXOSC8	EXOSC8:intronic:NM_181503.2	1	100		intronic	NM_18150	c.391-61T	p.?	0.21					3' exoribonuc	rs9576154		YES			Exosome co	AU-rich ele	Autonomic Nervous System Diseases:Basal G		T=0, G=20	T=0.0, G=1.0	0.00001	263.786	20	T=0/0, G=14/6
chr13:37584838	A/A	G	A	SNV	SUPT20H	SUPT20H:intronic:NM_001014286.2	1	100		intronic	NM_00101	c.2099-46	p.?	3.08					Spt20 family	rs7317750	0.1264	YES	9.997E-1 (pos.37584838)			SAGA complex,SAGA-type complex,autophagy,fibrillar ce			G=0, A=24	G=0.0, A=1.0	0.00001	334.557	24	G=0/0, A=8/16
chr13:38153351	T/C	T	C	SNV	POSTN	POSTN:intronic:NM_006475.2	1	50		intronic	NM_00647	c.1791+15	p.?	-0.01					Fasciclin dom	rs7323378	0.4621	YES	1.437E-1 (pos.38153351)		Periostin	bone rege	Asthma;Asthma, Aspirin-Induced;Bronchial D		T=16, C=16	T=0.5, C=0.5	0.00001	158.383	32	T=8/8, C=12/4
chr13:38237872	A/G	A	G	SNV	TRPC4	TRPC4:intronic:NM_003306.1	1	95.65		intronic	NM_00330	c.1375-6T	p.?	0.43					Ion transport	rs2985171	5.00E-04		1.0 (pos.38237872)		Transient re	basolatera	Nervous System Diseases;Neuromuscular Dis		A=1, G=22	A=0.0435, G=0.9565	0.00001	288.994	23	A=1/0, G=7/15
chr13:38924083	T/T	A	T	SNV	UFM1	UFM1:intronic:NM_016617.3	1	100		intronic	NM_01661	c.2+25A>T	p.?	-0.14					Ubiquitin folc	rs2231330	0.0286	YES	9.884E-1 (pos.38924083)		Ubiquitin-fo	cytoplasm,endoplasmic reticulum,extracellular exosom			A=0, T=21	A=0.0, T=1.0	0.00001	281.231	21	A=0/0, T=14/7
chr5:111545670	G/G	C	G	SNV	EPB41L4A	EPB41L4A:exonic:NM_022140.4	1	100	13	missense	NM_02214	c.1097G>C	p.Ser366Thr	0.19	0.33	58	0.006		FERM C-termi	rs756743798,rs8907	0.3013	YES	0.0 (pos.111545655),9.907E-1 (pos.1115		Erythrocyte	actomyosin structure organization,biological_process,c			C=0, G=28	C=0.0, G=1.0	0.00001	408.718	28	C=0/0, G=5/23
chr9:111936732	A/A	G	A	SNV	EPB41L4B	EPB41L4B:utr_3:NM_019114.4	1	100	26	utr_3	NM_01911	c.2805C>T	p.?	0.67					FERM C-termi	rs10733569		YES	7.699E-1 (pos.111936732)		Erythrocyte	actomyosin structure organization,apical part of cell,bic			G=0, A=33	G=0.0, A=1.0	0.00001	503.873	33	G=0/0, A=16/1
chr13:40233404	C/C	T	C	SNV	COG6	COG6:intronic:NM_020751.2	1	100		intronic	NM_02075	c.154-97T	p.?	-1.4					Conserved ol	rs7330515		YES			Component	ER to Golgi	Hypohidrosis;Skin Diseases;Skin and Connect		T=0, C=22	T=0.0, C=1.0	0.00001	298.891	22	T=0/0, C=13/9
chr13:40239139	A/A	G	A	SNV	COG6,MIR4305	COG6:intronic:NM_020751.2, MIR4305:upstream:NR_0361	1	100		intronic	NM_02075	c.370-94G	p.?	0.39					Conserved ol	rs7991151		YES			Component	ER to Golgi	Hypohidrosis;Skin Diseases;Skin and Connect		G=0, A=31	G=0.0, A=1.0	0.00001	465.816	31	G=0/0, A=18/1
chr13:40263979	C/C	T	C	SNV	COG6	COG6:intronic:NM_020751.2	1	100		intronic	NM_02075	c.1074+18	p.?	0.25					Conserved ol	rs9548882	0.2781	YES	2.993E-1 (pos.40263979)		Component	ER to Golgi	Hypohidrosis;Skin Diseases;Sk	Benign	T=0, C=42	T=0.0, C=1.0	0.00001	683.998	42	T=0/0, C=18/2
chr2:120885310	A/A	G	A	SNV	EPB41L5	EPB41L5:exonic:NM_020909.3	1	100	17	missense	NM_02090	c.1384G>A	p.Ala462Thr	0.6	1	58	0	0.03(COSM37	FERM C-termi	rs1034489	0.2783	YES	7.101E-1 (pos.12088531	adenocarcino	Erythrocyte	actomyosin structure organization,apical constriction,a			G=0, A=26	G=0.0, A=1.0	0.00001	371.42	26	G=0/0, A=12/1
chr7:143097100	G/G	A	G	SNV	EPHA1	EPHA1:exonic:NM_005232.4	1	100	4	missense	NM_00523	c.479T>C	p.Val160Ala	0.99	0.39	64	0	0.01(COSM41	Ephrin recept	rs4725617	0.0773	YES	9.914E-1 (pos.14309710	neoplasm	eph tyrosin	ATP bindin	Alzheimer Disease;Brain Diseases;Central Ne		A=0, G=23	A=0.0, G=1.0	0.00001	316.685	23	A=0/0, G=12/1
chr1:16464671	GA/GA	G	GA	INDEL	EPHA2	EPHA2:exonic:NM_004431.4	1	100	5	frameshiftInsertion	NM_00443	c.988_989	p.Ser330fs	7.96					Ephrin recept	rs757313469			0.0 (pos.16464671)		Ephrin rece	ATP bindin	Abnormalities, Mul	Dasatinib,Phosphoamin	G=0, GA=30	G=0.0, GA=1.0	0.00001	386.714	30	G=0/0, GA=11/
chr13:41791440	A/A	T	A	SNV	LOC101929140,MTRF1	MTRF1:intronic:NM_004294.2, LOC101929140:intronic_nc:	1	100		intronic	NR_12042	c.1225-76	p.?	-0.34					PCRF domain	rs2274903		YES			Translation	mitochondrion,regulation of translational termination,t			T=0, A=113	T=0.0, A=1.0	0.00001	1626.76	113	T=0/0, A=32/8
chr13:41835089	G/G	T	G	SNV	MTRF1	MTRF1:intronic:NM_004294.2	1	100		intronic	NM_00429	c.-7-38A>C	p.?	0.84					PCRF domain	rs1332940	0.2892	YES	5.779E-1 (pos.41835089)		Translation	mitochondrion,regulation of translational termination,t			T=0, G=93	T=0.0, G=1.0	0.00001	1555.33	93	T=0/0, G=29/6
chr13:42390740	G/C	G	C	SNV	VWA8	VWA8:intronic:NM_015058.1	1	51.85		intronic	NM_01505	c.1947+94	p.?	-0.03					AAA domain (	rs78527162		YES			von Willebr	ATP bindin	Autistic Disorder;Child Development Disorde		G=13, C=14	G=0.4815, C=0.5185	0.00001	132.665	27	G=3/10, C=8/6
chr13:42461189	A/A	C	A	SNV	VWA8	VWA8:intronic:NM_015058.1	1	100		intronic	NM_01505	c.816+144	p.?	0.06					AAA domain (	rs7328609		YES			von Willebr	ATP bindin	Autistic Disorder;Child Development Disorde		C=0, A=48	C=0.0, A=1.0	0.00001	810.435	48	C=0/0, A=35/1
chr13:42481712	T/T	C	T	SNV	VWA8	VWA8:intronic:NM_015058.1	1	100		intronic	NM_01505	c.483+10G	p.?	-0.74				0.01(COSM37	AAA domain (	rs2003064	0.1386	YES	9.994E-1 (pos.42481712)	hepatocellula	von Willebr	ATP bindin	Autistic Disorder;Child Development Disorde		C=0, T=38	C=0.0, T=1.0	0.00001	603.785	38	C=0/0, T=14/2
chr13:42780367	A/T	A	T	SNV	DGKH	DGKH:intronic:NM_178009.4	1	58.33		intronic	NM_17800	c.2601+85	p.?	-0.34					Diacylglycerc	rs17063019		YES			Diacylglyce	ATP bindin	Bipolar Disorder;Bipolar and Related Disorde		A=10, T=14	A=0.4167, T=0.5833	0.00001	123.993	24	A=1/9, T=4/10
chr13:42780398	T/C	T	C	SNV	DGKH	DGKH:intronic:NM_178009.4	1	50		intronic	NM_17800	c.2601+11	p.?	-1.51					Diacylglycerc	rs9594704		YES			Diacylglyce	ATP bindin	Bipolar Disorder;Bipolar and Related Disorde		T=12, C=12	T=0.5, C=0.5	0.00001	105.397	24	T=2/10, C=2/1
chr13:43155443	A/A	G	A	SNV	TNFSF11	TNFSF11:intronic:NM_003701.3	1	100		intronic	NM_00370	c.387+14G	p.?	1.29					TNF(Tumour I	rs2277439	0.1678	YES	6.968E-1 (pos.43155443)		Tumor necr	ERK1 and E	Alveolar Bone Loss;	Denosuma Benign	G=0, A=59	G=0.0, A=1.0	0.00001	885.107	59	G=0/0, A=23/3
chr6:94067981	C/T	C	T	SNV	EPHA7	EPHA7:exonic:NM_004440.3	1	47.62	4	synonymous	NM_00444	c.981G>A	p.(=)	-2.42				0.02(COSM37	Ephrin recept	rs164544	0.4633	YES	8.3E-1 (pos.94067981)	adenocarcino	Ephrin rece	ATP bindin	Bronchial Neoplasr	5-(2-methyl-5-{3-(trifluor	C=11, T=10	C=0.5238, T=0.4762	0.00001	82.29	21	C=3/8, T=7/3
chr1:23219493	C/C	T	C	SNV	EPHB2	EPHB2:exonic:NM_004442.7	1	100	7	synonymous	NM_00444	c.1545T>C	p.(=)	-0.02					Ephrin recept	rs309472	0.0077		1.0;0.0 (pos.23219493)		eph tyrosin	ATP bindin	Ependymoma;Geni	Phosphoaminophosphor	T=0, C=20	T=0.0, C=1.0	0.00001	263.785	20	T=0/0, C=7/13
chr13:45010283	G/G	T	G	SNV	TSC22D1	TSC22D1:intronic:NM_183422.3	1	100		intronic	NM_18342	c.2913-52	p.?	0.2					TSC-22/dip/b	rs1555781	0.0013	YES	9.557E-1;0.0;0.0 (pos.45010283)		TSC22 doma	DNA bindi	Carcinogenesis:Cell Transformation, Neoplas		T=0, G=123	T=0.0, G=1.0	0.00001	1996.64	123	T=0/0, G=44/7
chr1:226026406	A/G	A	G	SNV	EPHX1	EPHX1:exonic:NM_001136018.3	1	50	4	missense	NM_00113	c.416A>G	p.His139Arg	0.82	1	29	0	0.04(COSM37	Epoxide hydr	rs2234922	0.205	YES	1.284E-1;0.0 (pos.22602	acute_myeloic	Epoxide hyc	aromatic (	Adenocarcinoma;Amphetamin	drug respons	A=18, G=18	A=0.5, G=0.5	0.00001	185.885	36	A=10/8, G=10/
chr17:19232078	C/C	T	C	SNV	EPN2	EPN2:exonic:NM_014964.4	1	100	8	missense	NM_01496	c.1202T>C	p.Val401Ala	2.21	0.73	64	0		ANTH domair	rs6587220	0.0117	YES	8.419E-1 (pos.19232078)		Epsin 2	cadherin binding,clathrin coat of endocytic vesicle,cytos			T=0, C=32	T=0.0, C=1.0	0.00001	485.139	32	T=0/0, C=20/1
chr13:46619316	C/C	T	C	SNV	ZC3H13	ZC3H13:intronic:NM_015070.5	1	100		intronic	NM_01507	c.118-117	p.?	0.84					Zinc finger C-	rs3736944		YES			Zinc finger (	RNA binding,metal ion binding,nuclear speck,nucleoplas			T=0, C=51	T=0.0, C=1.0	0.00001	749.031	51	T=0/0, C=24/2

chr17:48613837	G/G	A	G	SNV	EPN3,LOC105371824	EPN3:utr_5:NM_017957.2, LOC105371824:downstream:NR	1	100	2	utr_5	NM_01795	c.-81A>G	p.?	-3.83				0(COSM4590	ANTH domair	rs3785915		YES	9.194E-1 (pos.48613837)	adenocarcinoi	Epsin 3	EH domain binding,clathrin-coated pit,clathrin-coated v				A=0, G=46	A=0.0, G=1.0	0.00001	768.262	46	A=0/0, G=21/2
chr13:46629995	A/A	C	A	SNV	CPB2,CPB2-AS1	CPB2:intronic:NM_001872.4, CPB2-AS1:intronic_nc:NR_046	1	100		intronic	NR_04622	c.1000-11	p.?	-2.44					Carboxypept	rs1926446	0.2991	YES	8.17E-1 (pos.46629995)		Carboxypep	blood coa	Nutrition Disorders	AZD-9684,Prothrombin		C=0, A=62	C=0.0, A=1.0	0.00001	946.31	62	C=0/0, A=22/4
chr13:46632226	C/C	A	C	SNV	CPB2,CPB2-AS1	CPB2:intronic:NM_001872.4, CPB2-AS1:intronic_nc:NR_046	1	100		intronic	NR_04622	c.999+88T	p.?	2.9					Carboxypept	rs2296641		YES			Carboxypep	blood coa	Nutrition Disorders	AZD-9684,Prothrombin		A=0, C=31	A=0.0, C=1.0	0.00001	465.796	31	A=0/0, C=18/1
chr8:144941419	C/T	C	T	SNV	EPPK1	EPPK1:exonic:NM_031308.3	1	50	2	synonymous	NM_03130	c.6003G>A	p.(=)	-4.86				0.05(COSM45	Plectin repea	rs12681478	0.4003	YES	1.402E-1;0.0 (pos.14494	adenocarcinoi	Epiplakin 1	RNA bindir	Acneiform Eruptions;Chloracne;Skin Disease			C=25, T=25	C=0.5, T=0.5	0.00001	288.835	50	C=6/19, T=6/1
chr13:47224514	G/A	G	A	SNV	LRCH1	LRCH1:intronic:NM_001164211.1	1	53.33		intronic	NM_00116	c.452+34G	p.?	-2.45					Calponin hon	rs41284183	0.114	YES	6.544E-2 (pos.47224514)		Leucine-ricl	cellular response to chemokine,cytoplasm,negative regi				G=49, A=56	G=0.4667, A=0.5333	0.00001	589.827	105	G=27/22, A=3
chr13:47261970	C/C	T	C	SNV	LRCH1	LRCH1:intronic:NM_001164211.1	1	100		intronic	NM_00116	c.823-17T	p.?	1.25					Calponin hon	rs842375	0.2564	YES	8.521E-1 (pos.47261970)		Leucine-ricl	cellular response to chemokine,cytoplasm,negative regi				T=0, C=24	T=0.0, C=1.0	0.00001	334.792	24	T=0/0, C=9/15
chr1:110295772	C/C	T	C	SNV	EPS8L3	EPS8L3:exonic:NM_139053.2	1	100	13	synonymous	NM_13905	c.1170A>G	p.(=)	-2.06				0.01(COSM37	Phosphotyro	rs1887547	0.401	YES	8.389E-1 (pos.11029577	acute_myeloic	EPS8-like pr	Rac guany	Genital Diseases, Male;Genital Neoplasms, I			T=0, C=29	T=0.0, C=1.0	0.00001	427.603	29	T=0/0, C=13/1
chr13:43462422	T/T	A	T	SNV	EPSTI1	EPSTI1:exonic:NM_001002264.3	1	100	13	missense	NM_00100	c.1197T>A	p.Asn399Lys	1.48	0	94	0.992	0.81(COSM3753699)		rs1044856,rs756592	0.3412	YES	1.474E-1;0.0 (pos.43462	adenocarcinoi	Epithelial stromal interaction 1					A=0, T=55	A=0.0, T=1.0	0.00001	814.903	55	A=0/0, T=32/2
chr13:48563008	A/A	G	A	SNV	SUCLA2	SUCLA2:intronic:NM_003850.2	1	100		intronic	NM_00385	c.371+9C>	p.?	0.89					ATP-grasp do	rs6561424	0.182	YES	7.925E-1 (pos.48563008)		Succinate-C	ATP bindin	Nutrition Disorders	Succinic a	Benign	G=0, A=26	G=0.0, A=1.0	0.00001	371.418	26	G=0/0, A=20/6
chr13:48563246	G/G	A	G	SNV	SUCLA2	SUCLA2:intronic:NM_003850.2	1	100		intronic	NM_00385	c.272-130	p.?	-0.16					ATP-grasp do	rs6561425		YES			Succinate-C	ATP bindin	Nutrition Disorders	Succinic acid		A=0, G=20	A=0.0, G=1.0	0.00001	263.764	20	A=0/0, G=14/6
chr13:48575292	T/T	C	T	SNV	SUCLA2	SUCLA2:intronic:NM_003850.2	1	100		intronic	NM_00385	c.90+24G>	p.?	-0.64					ATP-grasp do	rs9567972	0.1788	YES	8.286E-1 (pos.48575292)		Succinate-C	ATP bindin	Nutrition Disorders	Succinic acid		C=0, T=42	C=0.0, T=1.0	0.00001	685.219	42	C=0/0, T=39/3
chr13:48919358	G/G	T	G	SNV	RB1	RB1:intronic:NM_000321.2	1	100		intronic	NM_00032	c.500+23T	p.?	-0.7					Domain of un	rs198617	0.02	YES	9.973E-1 (pos.48919358)		Retinoblast	DNA bindi	ACMG Recommend	Insulin Hu	Benign	T=0, G=25	T=0.0, G=1.0	0.00001	348.557	25	T=0/0, G=17/8
chr13:48921884	G/G	A	G	SNV	RB1	RB1:intronic:NM_000321.2	1	100		intronic	NM_00032	c.501-77A	p.?	-0.72					Domain of un	rs198616	0.0191	YES			Retinoblast	DNA bindi	ACMG Recommend	Insulin Human,Insulin Pc		A=0, G=68	A=0.0, G=1.0	0.00001	1053.56	68	A=0/0, G=44/2
chr13:48947469	T/T	G	T	SNV	RB1	RB1:intronic:NM_000321.2	1	100		intronic	NM_00032	c.1128-72	p.?	0.53					Domain of un	rs185587		YES			Retinoblast	DNA bindi	ACMG Recommend	Insulin Human,Insulin Pc		G=0, T=43	G=0.0, T=1.0	0.00001	705.814	43	G=0/0, T=30/1
chr13:49033747	A/A	G	A	SNV	RB1	RB1:intronic:NM_000321.2	1	100		intronic	NM_00032	c.1961-77	p.?	0.29					Domain of un	rs198580,rs894277694		YES			Retinoblast	DNA bindi	ACMG Recommend	Insulin Human,Insulin Pc		G=0, A=27	G=0.0, A=1.0	0.00001	389.975	27	G=0/0, A=17/1
chr13:49051012	T/T	C	T	SNV	RB1	RB1:intronic:NM_000321.2	1	100		intronic	NM_00032	c.2663+33	p.?	0.48					Domain of un	rs3020646	0.0209	YES	9.975E-1 (pos.49051012)		Retinoblast	DNA bindi	ACMG Recommend	Insulin Hu	Benign	C=0, T=27	C=0.0, T=1.0	0.00001	389.894	27	C=0/0, T=14/1
chr13:50115669	T/T	C	T	SNV	RCBTB1	RCBTB1:intronic:NM_018191.3	1	100		intronic	NM_01819	c.1324+14	p.?	-0.01					BTB/POZ dom	rs1536195		YES			RCC1 doma	cell cycle,	Alcohol-Related Disorders;Alcoholism;Menta			C=0, T=27	C=0.0, T=1.0	0.00001	381.997	27	C=0/0, T=14/1
chr17:56271094	C/C	G	C	SNV	EPX	EPX:exonic:NM_000502.5	1	100	4	missense	NM_00050	c.366G>C	p.Gln122His	2.52	0.03	24	1	0.75(COSM49	Animal haem	rs11652709,rs34702	0.3176	YES	1.07E-1 (pos.56271094)	adenocarcinoi	Eosinophil	cellular ox	Hematologic Disea	Melatonin		G=0, C=23	G=0.0, C=1.0	0.00001	316.743	23	G=0/0, C=19/4
chr17:56272390	G/G	A	G	SNV	EPX	EPX:exonic:NM_000502.5	1	100	6	synonymous	NM_00050	c.660A>G	p.(=)	-0.21				0.01(COSM63	Animal haem	rs2240815	0.4413	YES	2.401E-1 (pos.56272390)	carcinoma	Eosinophil	cellular ox	Hematologic Disea	Melatonin		A=0, G=30	A=0.0, G=1.0	0.00001	446.639	30	A=0/0, G=22/8
chr17:56270442	A/G	A	G	SNV	EPX	EPX:exonic:NM_000502.5	1	59.18	2	missense	NM_00050	c.120A>G	p.Ile40Met	-0.65	0.32	10	0.006	0.02(COSM44	Animal haem	rs11079339	0.1409	YES	1.138E-1 (pos.56270442)	acute_myeloic	Eosinophil	cellular ox	Hematologic Disea	Melatonin		A=20, G=29	A=0.4082, G=0.5918	0.00001	360.526	49	A=9/11, G=14/
chr13:50244041	C/C	T	C	SNV	EBPL	EBPL:intronic:NM_032565.4	1	100		intronic	NM_03256	c.172-59A	p.?	-0.84					Emopamil bir	rs9535292	0.2728	YES			Emopamil-E	cholestenol delta-isomerase activity,endoplasmic retic				T=0, C=27	T=0.0, C=1.0	0.00001	383.861	27	T=0/0, C=10/1
chr13:50296026	C/C	T	C	SNV	KPNA3	KPNA3:intronic:NM_002267.3	1	100		intronic	NM_00226	c.726+65A	p.?	-0.4					Armadillo/be	rs9535310		YES			Karyopheri	NLS-bearing protein import into nucleus,cytosol,host cel				T=0, C=115	T=0.0, C=1.0	0.00001	1834.74	115	T=0/0, C=73/4
chr17:27187636	T/T	G	T	SNV	ERAL1,MIR144,MIR451A,MIR451B	MIR144:downstream:NR_029685.1, MIR451B:upstream:NR	1	100	10	utr_3	NM_00570	c.1379G>T	p.?	-0.15					50S ribosome	rs9279		YES	6.783E-1;1.168E-4 (pos.27187636)		ERA G-prote	GTP binding,RNA binding,cytosol,mitochondrial inner me				G=0, T=25	G=0.0, T=1.0	0.00001	353.019	25	G=0/0, T=3/22
chr13:51953840	T/T	C	T	SNV	INTS6	INTS6:intronic:NM_012141.2	1	100		intronic	NM_01214	c.1276-13	p.?	-0.15					INTS6/SAGE1,	rs3818360		YES			Integrator c	actin cytoskeleton,integrator complex,nucleoplasm,nuc				C=0, T=80	C=0.0, T=1.0	0.00001	1289.34	80	C=0/0, T=40/4
chr13:52293532	C/C	T	C	SNV	WDFY2	WDFY2:intronic:NM_052950.3	1	100		intronic	NM_05295	c.485+48T	p.?	-1.17					FYVE zinc fing	rs912097	0.002	YES	1.0 (pos.52293532)		WD repeat-	early endosome,metal ion binding,positive regulation of				T=0, C=21	T=0.0, C=1.0	0.00001	280.986	21	T=0/0, C=8/13
chr5:96118852	C/C	G	C	SNV	ERAP1	ERAP1:exonic:NM_016442.4	1	100	15	missense	NM_01644	c.2188C>G	p.Gln730Glu	0.98	0.96	29	0.038	0.3(COSM441	ERAP1-like C-	rs27044	0.2914	YES	4.948E-1 (pos.96118852)	acute_myeloic	Endoplasmi	adaptive i	Ankylosis;Arthritis;Behcet Syndrome;Bone Di			G=0, C=61	G=0.0, C=1.0	0.00001	903.157	61	G=0/0, C=36/2
chr13:52373814	C/C	G	C	SNV	DHRS12	DHRS12:intronic:NM_001270424.1	1	100		intronic	NM_00127	c.57-11C>	p.?	-0.18					Enoyl-(Acyl ca	rs9535761	0.1284	YES	6.488E-1 (pos.52373814)		Short-chain	oxidation-reduction process,oxidoreductase activity				G=0, C=22	G=0.0, C=1.0	0.00001	298.891	22	G=0/0, C=12/1
chr5:96129512	G/G	A	G	SNV	ERAP1	ERAP1:exonic:NM_016442.4	1	100	6	synonymous	NM_01644	c.1068T>C	p.(=)	-0.14				0.16(COSM44	ERAP1-like C-	rs27434	0.2203	YES	4.808E-1;0.0 (pos.96129	acute_myeloic	Endoplasmi	adaptive i	Ankylosis;Arthritis;Behcet Syndrome;Bone Di			A=0, G=38	A=0.0, G=1.0	0.00001	603.786	38	A=0/0, G=19/1
chr5:96139250	G/G	C	G	SNV	ERAP1	ERAP1:exonic:NM_016442.4	1	100	2	missense	NM_01644	c.380G>C	p.Arg127Pro	0.18	0.47	103	0	0(COSM4160	ERAP1-like C-	rs26653,rs74800676	0.2914	YES	4.533E-1;0.0 (pos.96139	acute_myeloic	Endoplasmi	adaptive i	Ankylosis;Arthritis;Behcet Syndrome;Bone Di			C=0, G=42	C=0.0, G=1.0	0.00001	683.807	42	C=0/0, G=29/1
chr3:56330368	C/T	C	T	SNV	ERC2	ERC2:exonic:NM_015576.2	1	56	3	synonymous	NM_01557	c.753G>A	p.(=)	-2.49				0.03(COSM41	RIM-binding	rs56227347	0.0176	YES	3.478E-4 (pos.56330368)	neoplasm	ELKS/RAB6-i	cell junction,cytoplasm,cytoskeleton,growth cone,presy				C=11, T=14	C=0.44, T=0.56	0.00001	134.35	25	C=5/6, T=8/6
chr19:45855524	G/A	G	A	SNV	ERCC2,KLC3	KLC3:downstream:NM_177417.2, ERCC2:exonic:NM_00040	1	50	22	synonymous	NM_17741	c.*909G>A	p.?, p.(=)	-0.97				0.11(COSM37	DEAD_2,Helic	rs1052555	0.3331	YES	6.486E-2 (pos.45855524)	adenocarcinoi	Excision rec	4 iron, 4 su	Abnormalities, Multiple;Agene		Benign	G=71, A=71	G=0.5, A=0.5	0.00001	786.665	142	G=33/38, A=3
chr13:53240884	C/C	G	C	SNV	SUGT1	SUGT1:intronic:NM_001130912.2	1	100		intronic	NM_00113	c.616-63G	p.?	-1.15					CS domain,SC	rs9316580					Suppressor	cytosol,kinetochore,mitotic cell cycle,nucleus,positive r				G=0, C=40	G=0.0, C=1.0	0.00001	644.281	40	G=0/0, C=17/2
chr13:60385061	A/C	A	C	SNV	DIAPH3	DIAPH3:intronic:NM_001042517.1	1	50		intronic	NM_00104	c.3028-4T	p.?	2.98				0.94(COSM42	DRF Autoregu	rs201365358,rs33982149			(pos.60385061)	basal_cell_car	Diaphanous	Rho GTPas	Auditory Diseases, Central;Brain Diseases;Ce			A=10, C=10	A=0.5, C=0.5	0.00001	85.1284	20	A=3/7, C=8/2
chr10:50740600	C/T	C	T	SNV	ERCC6	ERCC6:exonic:NM_000124.3	1	47.62	2	synonymous	NM_00012	c.411G>A	p.(=)	0.35				0.87(COSM50	Helicase con	rs4253013	0.0642	YES	3.894E-1 (pos.50740600)	acute_myeloic	Excision rec	ATP bindin	Abnormalities, Multiple;Agene		Benign/Likely	C=11, T=10	C=0.5238, T=0.4762	0.00001	82.2899	21	C=2/9, T=1/9
chr13:61988107	G/G	T	G	SNV	PCDH20	PCDH20:intronic:NM_022843.3	1	100		intronic	NM_02284	c.133-8A>	p.?	-0.74					Cadherin dor	rs4281600	2.00E-04		1.0;0.0 (pos.61988107)		Protocadhe	RNA binding,calcium ion binding,homophilic cell adhesic				T=0, G=30	T=0.0, G=1.0	0.00001	440.375	30	T=0/0, G=20/1
chr10:50723684	A/A	G	A	SNV	ERCC6,PGBD3	PGBD3:exonic:NM_170753.3, ERCC6:intronic:NM_000124.3	1	100	2	synonymous	NM_00012	c.1397+83	p.?, p.(=)	-0.35					Helicase con	rs4253073	0.0957	YES	9.992E-1 (pos.50723684)		Excision rec	ATP bindin	Abnormalities, Multiple;Agenesis of Corpus C			G=0, A=69	G=0.0, A=1.0	0.00001	1072.13	69	G=0/0, A=31/3
chr13:70681305	G/A	G	A	SNV	ATXN8OS,KLHL1	KLHL1:intronic:NM_020866.2, ATXN8OS:upstream:NR_0027	1	45.45		intronic	NM_02086	c.497+30C	p.?	1.22					BTB And C-ter	rs9564649	0.229	YES	1.674E-1;0.0 (pos.70681305)		Ataxin 8 op	Cul3-RING ubiquitin ligase complex,actin binding,actin c				G=12, A=10	G=0.5455, A=0.4545	0.00001	81.7613	22	G=8/4, A=8/2
chr10:50724016	T/T	C	T	SNV	ERCC6,PGBD3	PGBD3:exonic:NM_170753.3, ERCC6:intronic:NM_000124.3	1	100	2	missense	NM_00012	c.1397+80	p.?, p.Arg382Lys	-0.54		26			Helicase con	rs4253072	0		1.0 (pos.50724016)		Excision rec	ATP bindin	Abnormalities, Multiple;Agenesis of Corpus C			C=0, T=49	C=0.0, T=1.0	0.00001	818.038	49	C=0/0, T=21/2
chr13:76398089	C/C	A	C	SNV	LMO7	LMO7:intronic:NM_005358.5	1	100		intronic	NM_00535	c.2103+80	p.?	-0.29					Calponin hon	rs7982790		YES			LIM domain	actinin bir	Autoimmune Diseases;Diabetes Mellitus;Dia			A=0, C=69	A=0.0, C=1.0	0.00001	1072.78	69	A=0/0, C=39/3
chr13:76417101	C/T	C	T	SNV	LMO7	LMO7:intronic:NM_005358.5	1	46.43		intronic	NM_00535	c.3251+49	p.?	0.06					Calponin hon	rs920701	0.3574	YES	3.778E-1 (pos.76417101)		LIM domain	actinin bir	Autoimmune Diseases;Diabetes Mellitus;Dia			C=15, T=13	C=0.5357, T=0.4643	0.00001	117.927	28	C=5/10, T=3/1
chr5:60241142	G/A	G	A	SNV	ERCC8,NDUFAF2	NDUFAF2:exonic:NM_174889.4, ERCC8:upstream:NM_0000	1	96.67	1	synonymous	NM_00008	c.-307C>T	p.?, p.(=)	0.8				0.7(COSM376	NADH ubiqui	rs158921		YES	9.284E-1 (pos.60241142)	adenocarcinoi	Excision rec	Cul4-RING	Abnormalities, Multiple;Bone I		Benign/Likely	G=1, A=29	G=0.0333, A=0.9667	0.00001	398.235	30	G=0/1, A=12/1
chr12:29521193	A/G	A	G	SNV	ERGIC2	ERGIC2:exonic:NM_016570.2	1	50	4	synonymous	NM_01657	c.243T>C	p.(=)	0.39				0.8(COSM441	Endoplasmic	rs12831730	0.1505	YES	2.033E-1 (pos.29521193)	acute_myeloic	Endoplasmi	Golgi appa	Autoimmune Diseases;Female Urogenital Di			A=23, G=23	A=0.5, G=0.5	0.00001	258.573	46	A=9/14, G=15/
chr8:8860620	C/C	T	C	SNV	ERI1	ERI1:exonic:NM_153332.3	1	100	1	missense	NM_15333	c.47T>C	p.Leu16Pro	0.03	1	98	0	0(COSM3750	Exonuclease,	rs2288672	0.0761	YES	7.222E-1 (pos.8860620)	NS,adenocarcinoma,neopl		3'-5' exonuclease activity,3'-5'-exoribonuclease activity,				T=0, C=24	T=0.0, C=1.0	0.00001	334.792	24	T=0/0, C=12/1
chr13:78191824	T/T	G	T	SNV	SCEL	SCEL:intronic:NM_144777.2	1	100		intronic	NM_14477	c.1511+31	p.?	0.71						rs4142585	1.00E-04		1.0 (pos.78191824)		Sciellin	cornified envelope,cytoplasm,embryo development,epi				G=0, T=49	G=0.0, T=1.0	0.00001	831.472	49	G=0/0, T=29/2
chr16:20810067	C/C	T	C	SNV	ERI2	ERI2:exonic:NM_001142725.1	1	100	9	missense	NM_00114	c.1055A>G	p.Tyr352Cys	-0.08	0.24	194	0	0.02(COSM37	Exonuclease,	rs3213646	0.428	YES	1.516E-1 (pos.20810067)	basal_cell_carcinoma,carc		DNA metabolic process,exonuclease activity,nucleic aci				T=0, C=111	T=0.0, C=1.0	0.00001	1757.15	111	T=0/0, C=44/6
chr13:78477897	C/C	T	C	SNV	EDNRB,EDNRB-AS1	EDNRB:intronic:NM_001201397.1, EDNRB-AS1:intronic_nc:N	1	100		intronic	NR_10385	c.754-155	p.?	-2.87					7 transmemt	rs3027108		YES			Endothelin	G-protein	Abnormalities, Mul	Ambrisentan,Bosentan,I		T=0, C=66	T=0.0, C=1.0	0.00001	1017.59	66	T=0/0, C=40/2
chr1:75037845	G/G	A	G	SNV	ERICH3	ERICH3:exonic:NM_001002912.4	1	100	14	synonymous	NM_00100	c.3549T>C	p.(=)	-1.65				0.03(COSM37	Domain of un	rs12723334	0.3694	YES	5.808E-1;0.0 (pos.75037	adenocarcinoma,neoplasm						A=0, G=63	A=0.0, G=1.0	0.00001	958.914	63	A=0/0, G=33/3
chr17:62141455	G/G	A	G	SNV	ERN1	ERN1:exonic:NM_001433.3	1	100	10	synonymous	NM_00143	c.978T>C	p.(=)	-1.25				0.13(COSM41	Protein kinas	rs187826	8.00E-04	YES	9.999E-1 (pos.62141455)	neoplasm	Endoplasmi	ADP bindir	Brain Diseases;Cen	N~2~-1H-benzimidazol-5-		A=0, G=28	A=0.0, G=1.0	0.00001	408.704	28	A=0/0, G=22/6
chr17:62132160	C/T	C	T	SNV	ERN1	ERN1:exonic:NM_001433.3	1	57.14	14	synonymous	NM_00143	c.1701G>A	p.(=)	-0.02					Protein kinas	rs55653398	0.0229	YES	4.786E-4 (pos.62132160)		Endoplasmi	ADP bindir	Brain Diseases;Cen	N~2~-1H-benzimidazol-5-		C=12, T=16	C=0.4286, T=0.5714	0.00001	161.718	28	C=5/7, T=2/14
chr13:95673939	G/G	A	G	SNV	ABCC4	ABCC4:intronic:NM_005845.4	1	100		intronic	NM_00584	c.3871-3T	p.?	0.28				0.02(COSM54	ABC transpor	rs766564344,rs9524	0.0036	YES	2.267E-2 (pos.95673938)	squamous_cel	ATP-binding	15-hydrox	Arthritis;Arthritis, F	Adefovir Dipivoxil,Alpros		A=0, G=24	A=0.0, G=1.0	0.00001	334.789	24	A=0/0, G=14/1
chr1:236413230	A/A	T	A	SNV	ERO1B	ERO1B:exonic:NM_019891.3	1	100	5	missense	NM_01989	c.386A>T	p.Asp129Val	4.28	0.01	152	0.1	0.94(COSM37	Endoplasmic	rs2477599	0.404	YES	1.208E-1 (pos.23641323	adenocarcinoma		4-hydroxyproline metabolic pr	1-Ethyl-Pyrrolidine-2,5-D			T=0, A=431	T=0.0, A=1.0	0.00001	7255.15	431	T=0/0, A=201/
chr7:64438403	A/A	G	A	SNV	ERV3-1-ZNF117,ZNF117	ERV3-1-ZNF117:utr_3:NM_001348050.1, ZNF117:utr_3:NM_	1	100	4	utr_3	NM_01585	c.1546C>T	p.?, p.?	-0.86					C2H2-type zir	rs4718175		YES			Zinc finger	DNA binding,DNA binding transcription factor activity,nu				G=0, A=138	G=0.0, A=1.0	0.00001	2292.69	138	G=0/0, A=81/5
chr13:95735604	T/T	G	T	SNV	ABCC4	ABCC4:intronic:NM_005845.4	1	100		intronic	NM_00584	c.2536-60	p.?	0.34					ABC transpor	rs1189437		YES			ATP-binding	15-hydrox	Arthritis;Arthritis, F	Adefovir Dipivoxil,Alpros		G=0, T=26	G=0.0, T=1.0	0.00001	375.815	26	G=0/0, T=9/17
chr13:95899354	G/G	A	G	SNV	ABCC4	ABCC4:intronic:NM_005845.4	1	100		intronic	NM_00584	c.186-5T>	p.?	0.26				0.07(COSM39	ABC transpor	rs4148437	0.3652	YES	1.797E-1 (pos.95899354)	acute_myeloic	ATP-binding	15-hydrox	Arthritis;Arthritis, F	Adefovir Dipivoxil,Alpros		A=0, G=26	A=0.0, G=1.0	0.00001	371.406	26	A=0/0, G=6/20
chr13:96599271	C/T	C	T	SNV	UGGT2	UGGT2:intronic:NM_020121.3	1	50		intronic	NM_02012	c.1677+20	p.?	1.25					Glycosyl tran	rs779957268,rs7986	0.4137	YES	0.0 (pos.96599268),3.123E-1 (pos.96599		UDP-glucos	'de novo' posttranslational protein folding,ER-associatec				C=28, T=28	C=0.5, T=0.5	0.00001	272.14	56	C=12/16, T=14
chr7:64438666	CA/CA	C	CA	INDEL	ERV3-1-ZNF117,ZNF117	ERV3-1-ZNF117:exonic:NM_001348050.1, ZNF117:exonic:NI	1	100	4	frameshiftInsertion, frameshiftIns	NM_01585	c.1282_12	p.Arg428fs, p.Arg428fs	-3.58					C2H2-type zir	rs7457502	0		0.0 (pos.64438666)		Zinc finger	DNA binding,DNA binding transcription factor activity,nu				C=0, CA=56	C=0.0, CA=1.0	0.00001	603.984	56	C=0/0, CA=22/
chr13:99537202	A/A	C	A	SNV	DOCK9	DOCK9:intronic:NM_015296.2	1	100		intronic	NM_01529	c.2385+23	p.?	0.06					C2 domain in	rs2274641	0.2165	YES	5.247E-1 (pos.99537202)			biological_process,blood coagulation,cadherin binding,				C=0, A=83	C=0.0, A=1.0	0.00001	1335.24	83	C=0/0, A=35/4
chr13:99537217	T/T	G	T	SNV	DOCK9	DOCK9:intronic:NM_015296.2	1	100		intronic	NM_01529	c.2385+8C	p.?	0.08					C2 domain in	rs2274642	0.2165	YES	5.207E-1 (pos.99537217)			biological_process,blood coagulation,cadherin binding,				G=0, T=85	G=0.0, T=1.0	0.00001	1385.64	85	G=0/0, T=35/5
chr20:13695464	C/C	T	C	SNV	ESF1	ESF1:utr_3:NM_001276380.1	1	100	14	utr_3	NM_00127	c.2613A>G	p.?	-1					NUC153 dom	rs6079145		YES				RNA binding,extracellular space,nucleolus,nucleoplasm				T=0, C=20	T=0.0, C=1.0	0.00001	263.775	20	T=0/0, C=8/12
chr14:100599164	A/A	G	A	SNV	EVL	EVL:intronic:NM_016337.2	1	100		intronic	NM_01633	c.900+48G	p.?	-0.83					VASP tetrame	rs941897	0.4172	YES	2.359E-1 (pos.100599164)		ENAH/VASP-	SH3 doma	Breast Diseases;Breast Neoplasms;Colonic D			G=0, A=24	G=0.0, A=1.0	0.00001	332.555	24	G=0/0, A=6/18
chr20:13695676	CT/CT	C	CT	INDEL	ESF1	ESF1:exonic:NM_001276380.1	1	100	14	frameshiftInsertion	NM_00127	c.2400_24	p.Glu801fs	0.72					NUC153 domain							RNA binding,extracellular space,nucleolus,nucleoplasm				C=0, CT=47	C=0.0, CT=1.0	0.00001	429.774	47	C=0/0, CT=34/
chr14:100803364	G/G	A	G	SNV	WARS	WARS:intronic:NM_173701.1	1	100		intronic	NM_17370	c.1254+35	p.?	-0.29					WHEP-TRS do	rs2234530	0.2165	YES	8.621E-1 (pos.100803364)		Tryptophan	ATP binding,aminoacyl-tRNA lig	L-Tryptophan,L-Tryptoph			A=0, G=40	A=0.0, G=1.0	0.00001	644.281	40	A=0/0, G=20/2
chr14:100813077	C/C	T	C	SNV	WARS	WARS:intronic:NM_173701.1	1	100		intronic	NM_17370	c.826+6A>	p.?	0.31					WHEP-TRS do	rs941923	0.2506	YES	7.852E-1 (pos.100813077)		Tryptophan	ATP binding,aminoacyl-tRNA lig	L-Tryptophan,L-Tryptoph			T=0, C=31	T=0.0, C=1.0	0.00001	465.819	31	T=0/0, C=27/4
chr20:13695680	T/T	C	T	SNV	ESF1	ESF1:exonic:NM_001276380.1	1	100	14	synonymous	NM_00127	c.2397G>A	p.(=)	0.2				0.43(COSM37	NUC153 dom	rs6110019	0.3705	YES	2.348E-1 (pos.13695680)	acute_myeloid_leukaemia		RNA binding,extracellular space,nucleolus,nucleoplasm				C=0, T=47	C=0.0, T=1.0	0.00001	788.193	47	C=0/0, T=34/1
chr20:13747441	G/G	A	G	SNV	ESF1	ESF1:exonic:NM_001276380.1	1	100	8	missense	NM_00127	c.1649T>C	p.Ile550Thr	0.02	0.53	89	0.094	0.04(COSM37	NUC153 dom	rs3180370	0.3308	YES	8.917E-1 (pos.13747441)	adenocarcinoma		RNA binding,extracellular space,nucleolus,nucleoplasm				A=0, G=21	A=0.0, G=1.0	0.00001	281.235	21	A=0/0, G=4/17
chr14:102349540	T/T	C	T	SNV	PPP2R5C	PPP2R5C:intronic:NM_001161726.1	1	100		intronic	NM_00116	c.571-54C	p.?	0.27					Protein phos	rs1741125,rs386780	0.1811	YES			Protein pho	DNA dama	Hemic and Lympha	(2S,3S,4E,6E,8S,9S)-3-am		C=0, T=54	C=0.0, T=1.0	0.00001	798.148	54	C=0/0, T=31/2
chr14:102349542	T/T	G	T	SNV	PPP2R5C	PPP2R5C:intronic:NM_001161726.1	1	100		intronic	NM_00116	c.571-52G	p.?	1.27					Protein phos	rs1741126,rs386780	0.1781	YES			Protein pho	DNA dama	Hemic and Lympha	(2S,3S,4E,6E,8S,9S)-3-am		G=0, T=54	G=0.0, T=1.0	0.00001	798.145	54	G=0/0, T=31/2
chr14:102349907	G/G	A	G	SNV	PPP2R5C	PPP2R5C:intronic:NM_001161726.1	1	100		intronic	NM_00116	c.794+8A>	p.?	-0.06				0(COSM1478	Protein phos	rs2720207	0.1867	YES	2.745E-1 (pos.10234990	acute_myeloic	Protein pho	DNA dama	Hemic and Lympha	(2S,3S,4E,6E,8S,9S)-3-am		A=0, G=36	A=0.0, G=1.0	0.00001	563.733	36	A=0/0, G=10/2
chr14:102466253	T/T	C	T	SNV	DYNC1H1	DYNC1H1:intronic:NM_001376.4	1	100		intronic	NM_00137	c.3805-73	p.?	-0.37					AAA domain (	rs8015117		YES			Dynein, cyt	ATP bindin	Central Nervous System Diseases;Charcot-M			C=0, T=25	C=0.0, T=1.0	0.00001	353.019	25	C=0/0, T=12/1
chr14:102500244	G/T	G	T	SNV	DYNC1H1	DYNC1H1:intronic:NM_001376.4	1	57.89		intronic	NM_00137	c.10414-6	p.?	-1.46					AAA domain (	rs17512713		YES			Dynein, cyt	ATP bindin	Central Nervous System Diseases;Charcot-M			G=16, T=22	G=0.4211, T=0.5789	0.00001	249.1	38	G=9/7, T=6/16
chr12:53686703	A/A	G	A	SNV	ESPL1	ESPL1:exonic:NM_012291.4	1	100	30	missense	NM_01229	c.6110G>A	p.Arg2037His	2.28	1	29	0.004		Peptidase fa	rs1056685	5.00E-04	YES	9.999E-1 (pos.53686703)		Extra spindl	apoptotic	Adenocarcinoma;Carcinoma;Carcinoma, Ade			G=0, A=23	G=0.0, A=1.0	0.00001	316.732	23	G=0/0, A=9/14
chr14:102515015	A/A	G	A	SNV	DYNC1H1	DYNC1H1:intronic:NM_001376.4	1	100		intronic	NM_00137	c.13372+9	p.?	0.88				0(COSM1478	AAA domain (	rs1004903	0.187	YES	2.531E-1 (pos.10251501	acute_myeloic	Dynein, cyt	ATP bindin	Central Nervous System Disea		Benign/Likely	G=0, A=53	G=0.0, A=1.0	0.00001	782.03	53	G=0/0, A=33/2
chr14:102516324	G/A	G	A	SNV	DYNC1H1	DYNC1H1:intronic:NM_001376.4	1	46.43		intronic	NM_00137	c.13685-8	p.?	0.3					AAA domain (	rs373150429,rs879817539					Dynein, cyt	ATP bindin	Central Nervous System Diseases;Charcot-M			G=15, A=13	G=0.5357, A=0.4643	0.00001	117.927	28	G=6/9, A=6/7
chr12:53682326	G/A	G	A	SNV	ESPL1	ESPL1:exonic:NM_012291.4	1	58.33	20	synonymous	NM_01229	c.4551G>A	p.(=)	1.54				0.15(COSM17	Peptidase fa	rs1110720	0.3645	YES	8.044E-1 (pos.53682326)	carcinoma,nei	Extra spindl	apoptotic	Adenocarcinoma;Carcinoma;Carcinoma, Ade			G=10, A=14	G=0.4167, A=0.5833	0.00001	136.342	24	G=5/5, A=3/11
chr12:53662624	C/A	C	A	SNV	ESPL1	ESPL1:exonic:NM_012291.4	1	50	2	missense	NM_01229	c.74C>A	p.Ala25Asp	3.45	1	126	0		Peptidase fa	rs6580942	0.3215	YES	9.988E-1 (pos.53662624)		Extra spindl	apoptotic	Adenocarcinoma;Carcinoma;Carcinoma, Ade			C=14, A=14	C=0.5, A=0.5	0.00001	131.939	28	C=6/8, A=5/9
chr12:53670545	C/A	C	A	SNV	ESPL1	ESPL1:exonic:NM_012291.4	1	50	8	missense	NM_01229	c.1842C>A	p.Ser614Arg	-0.16	0.08	110	0.998	0.8(COSM414	Peptidase fa	rs1318648	0.3612	YES	8.059E-1 (pos.53670545)	neoplasm	Extra spindl	apoptotic	Adenocarcinoma;Carcinoma;Carcinoma, Ade			C=12, A=12	C=0.5, A=0.5	0.00001	106.703	24	C=11/1, A=9/3
chr14:103923414	G/G	A	G	SNV	MARK3	MARK3:intronic:NM_001128918.2	1	100		intronic	NM_00112	c.413-65A	p.?	-0.05					Kinase assoc	rs2273700	0.3616	YES			MAP/micro	ATP binding,MAPK cascade,cytosol,establishment of cell				A=0, G=40	A=0.0, G=1.0	0.00001	644.285	40	A=0/0, G=19/2
chr14:103923475	A/G	A	G	SNV	MARK3	MARK3:intronic:NM_001128918.2	1	97.5		intronic	NM_00112	c.413-4A>	p.?	0.02				0.37(COSM44	Kinase assoc	rs2273699	0.362	YES	1.269E-1 (pos.10392347	acute_myeloic	MAP/micro	ATP binding,MAPK cascade,cytosol,establishment of cell				A=1, G=39	A=0.025, G=0.975	0.00001	611.631	40	A=1/0, G=18/2
chr2:239016539	G/G	C	G	SNV	ESPNL	ESPNL:exonic:NM_194312.3	1	100	4	synonymous	NM_19431	c.780C>G	p.(=)	0.08				0.33(COSM41	Ankyrin repe	rs935415	0.3015	YES	7.095E-1 (pos.23901653	neoplasm		actin filament binding,actin filament bundle assembly,c				C=0, G=34	C=0.0, G=1.0	0.00001	524.022	34	C=0/0, G=14/2
chr14:104027557	T/T	C	T	SNV	BAG5	BAG5:intronic:NM_001015049.2	1	100		intronic	NM_00101	c.96-28G>	p.?	-0.24					BAG domain	rs11625397	0.3459	YES	4.452E-1 (pos.104027557)			Golgi organization,adenyl-nucleotide exchange factor ac				C=0, T=58	C=0.0, T=1.0	0.00001	867.384	58	C=0/0, T=29/2
chr14:104139570	T/T	G	T	SNV	KLC1	KLC1:intronic:NM_001130107.1	1	100		intronic	NM_00113	c.1161+46	p.?	-0.19					PPR repeat,T	rs11160757	0.0225	YES	9.839E-1 (pos.104139570)		Kinesin, ligl	antigen processing and presentation of exogenous pepti				G=0, T=26	G=0.0, T=1.0	0.00001	371.419	26	G=0/0, T=9/17
chr14:104167075	T/T	C	T	SNV	KLC1,XRCC3	XRCC3:intronic:NM_001100118.1, KLC1:intronic:NM_00113	1	100		intronic	NM_00113	c.1854+11	p.?, p.?	0.35					AAA domain,I	rs861537	0.3017	YES	4.1E-1 (pos.104167075)		Kinesin, ligl	ATP bindin	Adenoma;Breast Diseases;Breast Neoplasm			C=0, T=30	C=0.0, T=1.0	0.00001	446.533	30	C=0/0, T=16/1
chr14:76957887	C/C	T	C	SNV	ESRRB	ESRRB:exonic:NM_004452.3	1	100	8	synonymous	NM_00445	c.885T>C	p.(=)	-1.86				0.04(COSM41	Ligand-bindir	rs2361293	1.00E-04		9.998E-1 (pos.76957887)	neoplasm	Estrogen-re	DNA bindi	Ear Diseases;Hearing Disorder		Benign	T=0, C=55	T=0.0, C=1.0	0.00001	814.901	55	T=0/0, C=22/3
chr14:104481016	A/A	G	A	SNV	TDRD9	TDRD9:intronic:NM_153046.2	1	100		intronic	NM_15304	c.2107-46	p.?	-0.49					DEAD/DEAH b	rs7150795	0.0022	YES	9.874E-1 (pos.104481016)			ATP binding,ATP-dependent RNA helicase activity,ATPase				G=0, A=43	G=0.0, A=1.0	0.00001	710.594	43	G=0/0, A=28/1
chr14:104563874	T/T	C	T	SNV	ASPG	ASPG:intronic:NM_001080464.2	1	100		intronic	NM_00108	c.430-17C	p.?	-2.47					Ankyrin repe	rs7156718	0.348	YES	5.653E-1 (pos.104563874)			1-alkyl-2-a	Anaphylaxis;Anemia;Bile Duct Diseases;Bilia			C=0, T=27	C=0.0, T=1.0	0.00001	389.988	27	C=0/0, T=21/6
chr14:104570116	G/G	C	G	SNV	ASPG	ASPG:intronic:NM_001080464.2	1	100		intronic	NM_00108	c.753+104	p.?	-1.77					Ankyrin repe	rs34878411		YES				1-alkyl-2-a	Anaphylaxis;Anemia;Bile Duct Diseases;Bilia			C=0, G=29	C=0.0, G=1.0	0.00001	432.521	29	C=0/0, G=3/26
chr14:104571826	G/G	A	G	SNV	ASPG	ASPG:intronic:NM_001080464.2	1	100		intronic	NM_00108	c.1173+39	p.?	-1.09					Ankyrin repe	rs1744300	0.0016	YES	9.973E-1;2.664E-3 (pos.104571826)			1-alkyl-2-a	Anaphylaxis;Anemia;Bile Duct Diseases;Bilia			A=0, G=24	A=0.0, G=1.0	0.00001	334.321	24	A=0/0, G=15/9
chr2:67630980	A/A	G	A	SNV	ETAA1	ETAA1:exonic:NM_019002.3	1	100	5	missense	NM_01900	c.1166G>A	p.Ser389Asn	2.21	1	46	0	0.03(COSM14	Ewing's tumo	rs3770657	0.2846	YES	5.651E-1 (pos.67630980)	adenocarcinoi	Ewing tumo	DNA repai	Digestive System Diseases;Digestive System			G=0, A=22	G=0.0, A=1.0	0.00001	298.879	22	G=0/0, A=5/17
chr2:67632125	T/T	C	T	SNV	ETAA1	ETAA1:exonic:NM_019002.3	1	100	5	missense	NM_01900	c.2311C>T	p.Pro771Ser	-0.23	0.7	74	0	0.05(COSM14	Ewing's tumo	rs3770655	0.2836	YES	5.65E-1;0.0 (pos.676321	adenocarcinoi	Ewing tumo	DNA repai	Digestive System Diseases;Digestive System			C=0, T=118	C=0.0, T=1.0	0.00001	1893.79	118	C=0/0, T=66/5
chr4:159601676	T/T	C	T	SNV	ETFDH	ETFDH:exonic:NM_004453.3	1	100	2	missense	NM_00445	c.92C>T	p.Thr31Ile	1.12	0.16	89	0	0.84(COSM41	Electron tran	rs11559290	0.14	YES	9.75E-1 (pos.159601676)	neoplasm	Electron tra	4 iron, 4 su	Amino Acid Metabo	2-Hexylox	Benign	C=0, T=62	C=0.0, T=1.0	0.00001	940.32	62	C=0/0, T=37/2
chr21:40191638	A/G	A	G	SNV	ETS2	ETS2:exonic:NM_001256295.1	1	52.27	9	synonymous	NM_00125	c.1443A>G	p.(=)	-0.88					Ets-domain,S	rs461155	0.1521	YES	4.59E-1 (pos.40191638)		Oncogene ETS-2		Abnormalities, Multiple;Breast Diseases;Bre			A=21, G=23	A=0.4773, G=0.5227	0.00001	260.151	44	A=11/10, G=1
chr14:105063253	T/T	C	T	SNV	TMEM179	TMEM179:intronic:NM_001286389.1	1	100		intronic	NM_00128	c.443+74G	p.?	0.19						rs11629201		YES				integral component of membrane				C=0, T=22	C=0.0, T=1.0	0.00001	298.855	22	C=0/0, T=18/4
chr1:92979354	G/G	A	G	SNV	EVI5	EVI5:exonic:NM_005665.5	1	100	18	synonymous	NM_00566	c.2292T>C	p.(=)	-0.07				0.01(COSM41	Rab-GTPase-	rs6603979	0.2058	YES	9.633E-1 (pos.92979354)	neoplasm	Ecotropic vi	GTPase ac	Autoimmune Diseases;Autoim		Benign	A=0, G=40	A=0.0, G=1.0	0.00001	643.832	40	A=0/0, G=33/7
chr1:93089823	T/C	T	C	SNV	EVI5	EVI5:exonic:NM_005665.5	1	95.45	14	synonymous	NM_00566	c.1689A>G	p.(=)	1.11					Rab-GTPase-	rs7514716	0.1024	YES	9.635E-1 (pos.93089823)		Ecotropic vi	GTPase ac	Autoimmune Diseases;Autoim		Benign	T=1, C=21	T=0.0455, C=0.9545	0.00001	271.495	22	T=1/0, C=18/3
chr14:105170222	G/A	G	A	SNV	INF2	INF2:intronic:NM_022489.3	1	59.09		intronic	NM_02248	c.668-31G	p.?	-3.35					Diaphanous	rs7145851	0.2189	YES	6.618E-2 (pos.105170222)		Inverted for	Rho GTPas	Female Urogenital Diseases;Female Urogeni			G=9, A=13	G=0.4091, A=0.5909	0.00001	121.892	22	G=5/4, A=10/3
chr19:7913974	C/C	T	C	SNV	EVI5L	EVI5L:exonic:NM_001159944.2	1	100	3	synonymous	NM_00115	c.495T>C	p.(=)	-0.45				0.06(COSM41	Rab-GTPase-	rs555609	0.2366	YES	6.033E-1 (pos.7913974)	neoplasm		GTPase activator activity,Rab GTPase binding,activation				T=0, C=23	T=0.0, C=1.0	0.00001	316.745	23	T=0/0, C=6/17
chr19:7928462	C/C	T	C	SNV	EVI5L	EVI5L:exonic:NM_001159944.2	1	100	19	synonymous	NM_00115	c.2292T>C	p.(=)	1.49				0.02(COSM41	Rab-GTPase-	rs10410459			9.997E-1 (pos.7928462)	neoplasm,squamous_cell		GTPase activator activity,Rab GTPase binding,activation				T=0, C=39	T=0.0, C=1.0	0.00001	623.98	39	T=0/0, C=32/7
chr17:18284262	A/A	G	A	SNV	EVPLL	EVPLL:exonic:NM_001145127.1	1	100	2	missense	NM_00114	c.11G>A	p.Ser4Asn	0.42	1	46	0	0(COSM3766024)		rs386795970,rs5701	0.3931	YES	3.923E-1 (pos.18284262)	hepatocellular_carcinoma		cornified envelope,epidermis development,intermediat				G=0, A=24	G=0.0, A=1.0	0.00001	334.682	24	G=0/0, A=15/9
chr17:18291544	G/G	A	G	SNV	EVPLL	EVPLL:exonic:NM_001145127.1	1	100	10	synonymous	NM_00114	c.888A>G	p.(=)	-2.29				0(COSM3766025)		rs586322		YES	1.873E-1 (pos.18291544)	acute_myeloid_leukaemia		cornified envelope,epidermis development,intermediat				A=0, G=21	A=0.0, G=1.0	0.00001	281.235	21	A=0/0, G=11/1
chr14:105242966	T/C	T	C	SNV	AKT1	AKT1:intronic:NM_001014431.1	1	50		intronic	NM_00101	c.287+30A	p.?	-3.98					Kinase-like,P	rs2494735	0.3587	YES	6.49E-1 (pos.105242966)		Murine thy	14-3-3 pro	Abnormalities, Mul	5-(5-chloro-7H-pyrrolo2,3		T=22, C=22	T=0.5, C=0.5	0.00001	243.562	44	T=6/16, C=10/
chr15:41476101	C/C	T	C	SNV	EXD1	EXD1:utr_3:NM_152596.3	1	100	10	utr_3	NM_15259	c.1573A>G	p.?	-3.27					3'-5' exonucle	rs521088	0		1.0 (pos.41476101)			3'-5' exonuclease activity,P granule,PET complex,RNA bir				T=0, C=45	T=0.0, C=1.0	0.00001	747.387	45	T=0/0, C=28/1
chr15:41476465	A/A	G	A	SNV	EXD1	EXD1:exonic:NM_152596.3	1	100	10	synonymous	NM_15259	c.1209C>T	p.(=)	-0.03					3'-5' exonucle	rs690733	0.4294	YES	2.99E-1 (pos.41476465)			3'-5' exonuclease activity,P granule,PET complex,RNA bir				G=0, A=24	G=0.0, A=1.0	0.00001	334.792	24	G=0/0, A=15/9
chr14:105359827	C/C	T	C	SNV	CEP170B	CEP170B:intronic:NM_001112726.2	1	100		intronic	NM_00111	c.4039-33	p.?	-1.26					CEP170 C-ter	rs2247712	0.2071	YES	2.718E-1;0.0 (pos.105359827)			cytoplasm,microtubule				T=0, C=32	T=0.0, C=1.0	0.00001	484.67	32	T=0/0, C=21/1
chr9:140243844	T/T	C	T	SNV	EXD3	EXD3:exonic:NM_017820.4	1	100	15	missense	NM_01782	c.1634G>A	p.Cys545Tyr	0.77	0.02	194	0.092	0.06(COSM11	3'-5' exonucle	rs28545754	0.3638	YES	8.36E-1 (pos.140243844)	adenocarcinoma		3'-5' exonuclease activity,cytoplasm,metal ion binding,n				C=0, T=65	C=0.0, T=1.0	0.00001	996.411	65	C=0/0, T=43/2
chr9:140247143	G/G	T	G	SNV	EXD3	EXD3:exonic:NM_017820.4	1	100	11	missense	NM_01782	c.966A>C	p.Glu322Asp	1.12	0.23	45	0	0(COSM1489	3'-5' exonucle	rs7020732	0.1072	YES	9.989E-1 (pos.14024714	adenocarcinoma		3'-5' exonuclease activity,cytoplasm,metal ion binding,n				T=0, G=65	T=0.0, G=1.0	0.00001	996.352	65	T=0/0, G=56/9
chr1:242030151	G/G	A	G	SNV	EXO1	EXO1:exonic:NM_130398.3	1	100	11	missense	NM_13039	c.1061A>G	p.His354Arg	0.38	1	29	0	0.21(COSM50	XPG I-region,	rs735943	0.4603	YES	7.996E-1 (pos.24203015	diffuse_large	Exonucleas	5'-3' exode	Breast Diseases;Breast Neoplasms;Colonic D			A=0, G=63	A=0.0, G=1.0	0.00001	958.554	63	A=0/0, G=28/3
chr6:656143	C/A	C	A	SNV	EXOC2,HUS1B	HUS1B:exonic:NM_148959.3, EXOC2:intronic:NM_018303.5	1	47.37	1	missense	NM_01830	c.-42-1828	p.?, p.Asp268Tyr	2.28		160			Exocyst comp	rs1211554	0.0977	YES	8.378E-1 (pos.656143)		Exocyst con	Flemming	Psoriasis;Skin Diseases;Skin Diseases, Papul			C=50, A=45	C=0.5263, A=0.4737	0.00001	489.605	95	C=31/19, A=13
chr10:94695617	T/T	C	T	SNV	EXOC6	EXOC6:exonic:NM_019053.5	1	100	12	missense	NM_01905	c.1187C>T	p.Thr396Ile	6.29	1	89	0	0.05(COSM37	Exocyst comp	rs1326331	0.311	YES	9.972E-1 (pos.94695617)	adenocarcinoi	Exocyst con	Flemming body,cytosol,exocyst,growth cone,perinuclea				C=0, T=64	C=0.0, T=1.0	0.00001	977.237	64	C=0/0, T=37/2
chr14:105618670	T/C	T	C	SNV	JAG2	JAG2:intronic:NM_002226.4	1	50		intronic	NM_00222	c.789-42A	p.?	-0.07					Calcium-bind	rs2238287,rs780009	0.3353	YES	5.499E-1;0.0 (pos.105618670)		Jagged 2	Notch binc	Adenocarcinoma;Astrocytoma;Bone Disease			T=13, C=13	T=0.5, C=0.5	0.00001	119.162	26	T=10/3, C=7/6
chr14:105686570	C/C	T	C	SNV	BRF1	BRF1:intronic:NM_001519.3	1	100		intronic	NM_00151	c.1316-10	p.?	1.24					Brf1-like TBP-	rs8005486		YES			BRF1, S. cer	DNA-templated transcriptional preinitiation complex as				T=0, C=20	T=0.0, C=1.0	0.00001	263.677	20	T=0/0, C=15/5
chr9:37782111	T/T	C	T	SNV	EXOSC3	EXOSC3:exonic:NM_016042.3	1	100	3	synonymous	NM_01604	c.498G>A	p.(=)	-0.69				0.12(COSM37	KH domain	rs7158	0.4972	YES	5.307E-1 (pos.37782111)	adenocarcinoi	Exosome co	3'-5'-exoril	Autonomic Nervous System Dis		Benign/Likely	C=0, T=102	C=0.0, T=1.0	0.00001	1594.11	102	C=0/0, T=64/3
chr14:105924786	A/A	G	A	SNV	MTA1	MTA1:intronic:NM_004689.3	1	100		intronic	NM_00468	c.653+77G	p.?	0.4					BAH domain,	rs4074001		YES			Metastasis	DNA bindi	Endometriosis;Female Urogenital Diseases;F			G=0, A=68	G=0.0, A=1.0	0.00001	1038.26	68	G=0/0, A=24/4
chr9:37780558	C/A	C	A	SNV	EXOSC3	EXOSC3:utr_3:NM_016042.3	1	53.33	4	utr_3	NM_01604	c.946G>T	p.?	1.96					KH domain	rs1059059		YES			Exosome co	3'-5'-exoril	Autonomic Nervous System Dis		Benign	C=21, A=24	C=0.4667, A=0.5333	0.00001	276.182	45	C=6/15, A=11/
chr11:108380335	T/C	T	C	SNV	EXPH5	EXPH5:exonic:NM_015065.2	1	98.84	6	missense	NM_01506	c.5899A>G	p.Asn1967Asp	3.81	1	23	0			rs1943382	0		1.0 (pos.108380335)		Exophilin 5					T=1, C=85	T=0.0116, C=0.9884	0.00001	1374.75	86	T=0/1, C=34/5
chr1:26357656	C/A	C	A	SNV	EXTL1	EXTL1:exonic:NM_004455.2	1	46.43	5	missense	NM_00445	c.1135C>A	p.His379Asn	1.29	0	68	0.029		Exostosin fan	rs2736831	0.3217	YES	7.315E-1 (pos.26357656)		Exostosin-li	IRE1-mediated unfolded protein response,endoplasmic				C=15, A=13	C=0.5357, A=0.4643	0.00001	117.927	28	C=3/12, A=6/7
chr6:66005888	T/T	C	T	SNV	EYS	EYS:exonic:NM_001142800.1	1	100	12	missense	NM_00114	c.1891G>A	p.Gly631Ser	2.14	0.53	56	0		EGF-like dom	rs9342464	0.4167	YES	8.866E-1 (pos.66005888)		Eyes shut, D	calcium io	Congenital, Hereditary, and Ne		Benign	C=0, T=156	C=0.0, T=1.0	0.00001	2503.77	156	C=0/0, T=75/8
chr6:66005970	A/A	G	A	SNV	EYS	EYS:exonic:NM_001142800.1	1	100	12	synonymous	NM_00114	c.1809C>T	p.(=)	1.33					EGF-like dom	rs9345601	0.4167	YES	8.875E-1 (pos.66005970)		Eyes shut, D	calcium io	Congenital, Hereditary, and Ne		Benign	G=0, A=159	G=0.0, A=1.0	0.00001	2557.81	159	G=0/0, A=76/8
chr17:40854534	GT/GT	G	GT	INDEL	EZH1	EZH1:utr_3:NM_001991.4	1	100	21	utr_3	NM_00199	c.2259_22	p.?	-0.45					SET domain,V	rs3841620,rs397791	0.4819	YES	5.036E-1;0.0 (pos.40854534)		Enhancer of	ESC/E(Z) complex,anatomical structure morphogenesis,c				G=0, GT=82	G=0.0, GT=1.0	0.00001	1277.13	82	G=0/0, GT=53/
chr5:176831826	G/G	C	G	SNV	F12	F12:exonic:NM_000505.3	1	100	7	missense	NM_00050	c.619G>C	p.Ala207Pro	0.25	0.59	27	0	0.01(COSM44	EGF-like dom	rs17876030	0.0144	YES	8.922E-1;0.0 (pos.17683	neoplasm	Coagulatior	Factor XII	Angioedema;Angio	Conestat	Benign	C=0, G=27	C=0.0, G=1.0	0.00001	389.989	27	C=0/0, G=18/9

chr14:20767618	A/A	G	A	SNV	TTC5	TTC5:intronic:NM_138376.2	1	100		intronic	NM_13837	c.397-11C	p.?	3.16					Tetratricope	rs2318864	0.266	YES	7.962E-1 (pos.20767618)			DNA binding,DNA repair,chromatin binding,cytoplasm,nu	G=0, A=23	G=0.0, A=1.0	0.00001	316.667	23	G=0/0, A=4/19
chr5:176836532	G/G	A	G	SNV	F12	F12:utr_5:NM_000505.3	1	100	1	utr_5	NM_00050	c.-4T>C	p.?	1.75					EGF-like dom	rs1801020	0.2374	YES	2.52E-1 (pos.176836532)		Coagulatior	Factor XII ; Angioedema:Angio(Conestat ; Benign	A=0, G=81	A=0.0, G=1.0	0.00001	1309.48	81	A=0/0, G=38/4
chr14:20824859	T/C	T	C	SNV	PARP2	PARP2:intronic:NM_005484.3	1	45.83		intronic	NM_00548	c.1368+11	p.?	-1.13					Poly(ADP-ribo	rs878156	0.083	YES	1.666E-1 (pos.20824859)		Poly(ADP-ril	DNA bindi Lipid Metabolism D Niraparib,Olaparib,Ruca	T=13, C=11	T=0.5417, C=0.4583	0.00001	93.44	24	T=7/6, C=8/3
chr14:20843889	G/A	G	A	SNV	TEP1	TEP1:intronic:NM_007110.4	1	57.89		intronic	NM_00711	c.6363+25	p.?	-0.18					Domain of un	rs1760908	0.2165	YES	2.656E-1;0.0 (pos.20843889)		Telomerase	ATP bindin Lung Diseases:Lung Neoplasms:Neoplasms:N	G=16, A=22	G=0.4211, A=0.5789	0.00001	249.15	38	G=8/8, A=11/1
chr1:197031021	T/T	C	T	SNV	F13B	F13B:exonic:NM_001994.2	1	100	3	missense	NM_00199	c.344G>A	p.Arg115His	-0.46	0.42	29	0		Sushi repeat	rs6003	0.0929	YES	9.837E-1 (pos.197031021)		Coagulatior	blood coa; Blood Coagulation Disorders;B Benign, risk fa	C=0, T=33	C=0.0, T=1.0	0.00001	504.069	33	C=0/0, T=21/1
chr5:76129053	C/C	T	C	SNV	F2RL1	F2RL1:exonic:NM_005242.5	1	100	2	synonymous	NM_00524	c.621T>C	p.(=)	0.28				0.58(COSM63	7 transmemb	rs631465	0.0464	YES	5.168E-1 (pos.76129053)	carcinoma	Coagulatior	G-protein Cardiovascular Diseases;Digestive System Di	T=0, C=52	T=0.0, C=1.0	0.00001	750.27	52	T=0/0, C=20/3
chr1:169511734	C/C	T	C	SNV	F5	F5:exonic:NM_000130.4	1	100	13	missense	NM_00013	c.2594A>G	p.His865Arg	-0.17	0.31	29	0.001	0.06(COSM37	Coagulation f	rs4525	0.274	YES	2.184E-1 (pos.16951173	acute_myeloic	Coagulatior	COPII vesi Abortion, Habitual; Anti-inhibi Benign/Likely	T=0, C=51	T=0.0, C=1.0	0.00001	754.584	51	T=0/0, C=22/2
chr1:169511755	C/C	T	C	SNV	F5	F5:exonic:NM_000130.4	1	100	13	missense	NM_00013	c.2573A>G	p.Lys858Arg	-0.45	0.48	26	0	0(COSM3750	Coagulation f	rs4524	0.2738	YES	2.189E-1 (pos.16951175	acute_myeloic	Coagulatior	COPII vesi Abortion, Habitual; Anti-inhibi Benign/Likely	T=0, C=51	T=0.0, C=1.0	0.00001	754.584	51	T=0/0, C=22/2
chr1:169519049	C/C	T	C	SNV	F5	F5:exonic:NM_000130.4	1	100	10	missense	NM_00013	c.1601A>G	p.Gln534Arg	2.02	1	43	0		Coagulation f	rs6025	0.0301	YES	1.0 (pos.169519049)		Coagulatior	COPII vesi Abortion, Habitual; Anti-inhibi drug respons	T=0, C=90	T=0.0, C=1.0	0.00001	1493.11	90	T=0/0, C=49/4
chr16:74750405	A/A	G	A	SNV	FA2H	FA2H:exonic:NM_024306.4	1	100	6	synonymous	NM_0243C	c.879C>T	p.(=)	-2.62					Cytochrome b	rs2301865	0.1945	YES	6.609E-1 (pos.74750405)		Fatty acid 2	central ne Congenital Abnormalities;Con; Benign	G=0, A=21	G=0.0, A=1.0	0.00001	296.847	21	G=0/0, A=9/12
chr1:46871986	C/C	T	C	SNV	FAAH	FAAH:exonic:NM_001441.2	1	100	7	synonymous	NM_00144	c.897T>C	p.(=)	-0.03				0.57(COSM37	Amidase	rs324419	0.163	YES	9.979E-1 (pos.46871986)	hepatocellula	Fatty acid 2	acylglycer Basal Ganglia Disea 1-DODECANOL,4-(3-(5-(tri	T=0, C=48	T=0.0, C=1.0	0.00001	810.335	48	T=0/0, C=28/2
chr17:79508400	C/C	T	C	SNV	FAAP100	FAAP100:exonic:NM_025161.5	1	100	8	missense	NM_02516	c.2449A>G	p.Thr817Ala	0.67	1	58	0	0.02(COSM41	Fanconi aner	rs14422	0.0933	YES	9.998E-1;0.0 (pos.79508	neoplasm	Chromosom	DNA binding,DNA repair,Fanconi anaemia nuclear compl	T=0, C=33	T=0.0, C=1.0	0.00001	504.596	33	T=0/0, C=22/1
chr14:21488046	C/C	G	C	SNV	NDRG2	NDRG2:intronic:NM_201539.1	1	100		intronic	NM_20153	c.612+33C	p.?	-0.98					Ndr family	rs1243448	0.1379	YES	9.978E-1 (pos.21488046)		N-myc dowr	Golgi appa Brain Diseases:Brain Ischemia:Cardiovascula	G=0, C=29	G=0.0, C=1.0	0.00001	427.565	29	G=0/0, C=24/5
chr19:33467620	C/C	T	C	SNV	FAAP24	FAAP24:utr_3:NM_152266.4	1	100	5	utr_3	NM_15226	c.680T>C	p.?	-0.79					Helix-hairpin	rs7247715	0.1466	YES	9.406E-1 (pos.33467620)		Chromosom	DNA binding,DNA repair,Fanconi anaemia nuclear compl	T=0, C=38	T=0.0, C=1.0	0.00001	603.786	38	T=0/0, C=24/1
chr16:1877698	C/G	C	G	SNV	FAHD1,HAGH	FAHD1:exonic:NM_001018104.2, HAGH:upstream:NM_005	1	57.58	1	synonymous	NM_00532	c.-910G>C	p.?, p.(=)	0.7				0.59(COSM13	Fumarylacet	rs3743852	0.1363	YES	1.106E-1 (pos.1877698)	acute_myeloic	Fumarylace	acetylpyruvate hydrolase activ Glutathione,S-(N-Hydrox	C=14, G=19	C=0.4242, G=0.5758	0.00001	204.392	33	C=8/6, G=7/12
chr14:21556963	AT/AT	A	AT	INDEL	ARHGEF40	ARHGEF40:intronic:NM_018071.4	1	100		intronic	NM_01807	c.4519-26	p.?	0.37					RhoGEF doma	rs201303935,rs3518	0.2289	YES	5.589E-1 (pos.21556963)			Rho guanyl-nucleotide exchange factor activity,cytoplas	A=0, AT=31	A=0.0, AT=1.0	0.00001	456.582	31	A=0/0, AT=17/
chr14:21624197	C/C	T	C	SNV	OR5AU1	OR5AU1:upstream:NM_001004731.1	1	100		intronic	NM_00100	c.-13A>G	p.?	0.09						rs10135366	0.2451	YES	4.092E-1 (pos.21624197)			G-protein coupled receptor activity,G-protein coupled re	T=0, C=39	T=0.0, C=1.0	0.00001	623.98	39	T=0/0, C=19/2
chr9:130707081	G/G	A	G	SNV	FAM102A	FAM102A:exonic:NM_001035254.2	1	100	9	synonymous	NM_00103	c.1014T>C	p.(=)	4.08				0.18(COSM56	N-terminal C2	rs2274426	0.1312	YES	4.578E-1 (pos.13070708	ductal_carcinc	Family with	protein binding	A=0, G=28	A=0.0, G=1.0	0.00001	408.715	28	A=0/0, G=12/1
chr14:21821821	C/C	A	C	SNV	SUPT16H	SUPT16H:intronic:NM_007192.3	1	100		intronic	NM_00719	c.2920+41	p.?	0.65					FACT comple	rs3736824,rs770546	0.4301	YES	0.0 (pos.21821817),4.789E-1;0.0 (pos.21		Suppressor	DNA repair,DNA replication,DNA replication-independer	A=0, C=33	A=0.0, C=1.0	0.00001	504.592	33	A=0/0, C=10/2
chr14:21828520	A/A	T	A	SNV	SUPT16H	SUPT16H:intronic:NM_007192.3	1	100		intronic	NM_00719	c.2175+54	p.?	-0.64					FACT comple	rs927412		YES			Suppressor	DNA repair,DNA replication,DNA replication-independer	T=0, A=23	T=0.0, A=1.0	0.00001	316.747	23	T=0/0, A=14/9
chr17:71205854	G/G	A	G	SNV	FAM104A	FAM104A:exonic:NM_001098832.1	1	100	4	synonymous	NM_00109	c.438T>C	p.(=)	2.5				0.11(COSM37	Family 104	rs8387	0.3574	YES	6.336E-1 (pos.71205854)	adenocarcinoma,ductal_ca		protein binding	A=0, G=54	A=0.0, G=1.0	0.00001	798.148	54	A=0/0, G=19/3
chr14:21831112	A/A	G	A	SNV	SUPT16H	SUPT16H:intronic:NM_007192.3	1	100		intronic	NM_00719	c.1512-6C	p.?	-0.22					FACT comple	rs10146717	0.0922	YES	8.793E-1 (pos.21831112)		Suppressor	DNA repair,DNA replication,DNA replication-independer	G=0, A=23	G=0.0, A=1.0	0.00001	315.872	23	G=0/0, A=13/1
chr14:21833147	A/A	G	A	SNV	SUPT16H	SUPT16H:intronic:NM_007192.3	1	100		intronic	NM_00719	c.1121-49	p.?	-0.82					FACT comple	rs2295866	0.0924	YES	8.794E-1 (pos.21833147)		Suppressor	DNA repair,DNA replication,DNA replication-independer	G=0, A=26	G=0.0, A=1.0	0.00001	370.488	26	G=0/0, A=11/1
chr14:21836400	C/C	T	C	SNV	SUPT16H	SUPT16H:intronic:NM_007192.3	1	100		intronic	NM_00719	c.955+28A	p.?	-0.03					FACT comple	rs8008670	0.064	YES	8.796E-1;0.0 (pos.21836400)		Suppressor	DNA repair,DNA replication,DNA replication-independer	T=0, C=40	T=0.0, C=1.0	0.00001	644.128	40	T=0/0, C=32/8
chr14:21861211	A/A	C	A	SNV	CHD8,SNORD9	SNORD9:upstream:NR_003029.2, CHD8:intronic:NM_00117	1	100		intronic	NM_00117	c.6468+54	p.?	0.14					BRK domain,(	rs7152833		YES			Chromodon	ATP bindin Autistic Disorder;Child Development Disorde	C=0, A=143	C=0.0, A=1.0	0.00001	2408.86	143	C=0/0, A=64/7
chr14:21864124	A/A	C	A	SNV	CHD8	CHD8:intronic:NM_001170629.1	1	100		intronic	NM_00117	c.5052-73	p.?	-0.31					BRK domain,(	rs10134108		YES			Chromodon	ATP bindin Autistic Disorder;Child Development Disorde	C=0, A=33	C=0.0, A=1.0	0.00001	504.491	33	C=0/0, A=16/1
chr14:21874068	C/C	G	C	SNV	CHD8	CHD8:intronic:NM_001170629.1	1	100		intronic	NM_00117	c.2908-45	p.?	0.74					BRK domain,(	rs7155123	0.0913	YES	8.807E-1 (pos.21874068)		Chromodon	ATP bindin Autistic Disorder;Child Development Disorde	G=0, C=27	G=0.0, C=1.0	0.00001	389.988	27	G=0/0, C=13/1
chr14:21930596	A/A	G	A	SNV	RAB2B	RAB2B:intronic:NM_032846.3	1	100		intronic	NM_03284	c.475-19C	p.?	0.43					50S ribosome	rs10143000	0.0616	YES	8.634E-1 (pos.21930596)		Ras-associa	GTP binding,GTPase activity,Golgi membrane,endoplasn	G=0, A=39	G=0.0, A=1.0	0.00001	623.979	39	G=0/0, A=13/2
chr14:21971729	C/G	C	G	SNV	METTL3	METTL3:intronic:NM_019852.4	1	45.83		intronic	NM_01985	c.319-9G>	p.?	0.25					MT-A70	rs2242526	0.4334	YES	3.011E-1;0.0 (pos.21971729)		Methyltran	RNA N6-methyladenosine methyltransferase complex,RI	C=13, G=11	C=0.5417, G=0.4583	0.00001	93.4399	24	C=3/10, G=6/5
chr20:825678	C/C	T	C	SNV	FAM110A	FAM110A:exonic:NM_001042353.2	1	100	2	synonymous	NM_00104	c.231T>C	p.(=)	0.29				0.07(COSM44	Centrosome-	rs538094	0.1309	YES	8.893E-1 (pos.825678)	neoplasm	Family with	cytoplasm,microtubule organizing center,protein bindin	T=0, C=28	T=0.0, C=1.0	0.00001	408.49	28	T=0/0, C=14/1
chr2:45895	G/G	A	G	SNV	FAM110C	FAM110C:exonic:NM_001077710.2	1	100	1	missense	NM_00107	c.491T>C	p.Ile164Thr	-0.4	0.6	89	0	0(COSM3749	Centrosome-	rs4241318	0.0032		1.0 (pos.45895)	adenocarcinoi	Family with	alpha-tubulin binding,cell cortex,microtubule,microtubu	A=0, G=64	A=0.0, G=1.0	0.00001	916.64	64	A=0/0, G=24/4
chr4:38937372	C/C	T	C	SNV	FAM114A1	FAM114A1:exonic:NM_138389.3	1	100	13	synonymous	NM_13838	c.1497T>C	p.(=)	-0.87				0.09(COSM40	Protein of unl	rs2271031	0.4043	YES	6.698E-1 (pos.38937372)	acute_myeloid	leukaemia	Golgi apparatus,cytosol,nucleoplasm,nucleus,protein b	T=0, C=62	T=0.0, C=1.0	0.00001	939.471	62	T=0/0, C=36/2
chr2:225244235	C/C	A	C	SNV	FAM124B	FAM124B:utr_3:NM_001122779.1	1	100	2	utr_3	NM_00112	c.1423T>G	p.?	-0.65					FAM124 fami	rs16865905		YES				mitochondrion,nucleoplasm,protein binding	A=0, C=33	A=0.0, C=1.0	0.00001	504.59	33	A=0/0, C=11/2
chr14:23239880	A/A	G	A	SNV	OXA1L	OXA1L:intronic:NM_005015.3	1	100		intronic	NM_00501	c.1014+46	p.?	-0.17					60Kd inner m	rs1957502	0.1536	YES	8.773E-1 (pos.23239880)		Oxidase (cy	aerobic respiration,integral component of membrane,in	G=0, A=25	G=0.0, A=1.0	0.00001	353.019	25	G=0/0, A=11/1
chr19:17648350	C/C	T	C	SNV	FAM129C	FAM129C:splicesite_5:NM_173544.4	1	100	6	splicesite	NM_17354	c.685+1T>	p.?	1.56						rs8107859	0.0282	YES	7.496E-1 (pos.17648350)				T=0, C=36	T=0.0, C=1.0	0.00001	563.744	36	T=0/0, C=8/28
chr14:23443962	C/C	T	C	SNV	AJUBA	AJUBA:intronic:NM_032876.5	1	100		intronic	NM_03287	c.1422+86	p.?	0.67					LIM domain	rs941721		YES			G2/M tran	Carcinoma;Carcinoma, Squamous Cell;Diges	T=0, C=57	T=0.0, C=1.0	0.00001	848.713	57	T=0/0, C=52/5
chr19:17651370	A/G	A	G	SNV	FAM129C	FAM129C:exonic:NM_173544.4	1	46.88	10	synonymous	NM_17354	c.1242A>G	p.(=)	-4.65				0(COSM1480754),0(COSM3		rs11878663	0.1137	YES	5.753E-1 (pos.17651370)	adenocarcinoma			A=17, G=15	A=0.5313, G=0.4688	0.00001	143.697	32	A=8/9, G=9/6
chr14:23459156	A/A	G	A	SNV	C14orf93	C14orf93:intronic:NM_001130708.2	1	100		intronic	NM_00113	c.981-102	p.?	-0.02					Domain of un	rs11624528		YES				RNA binding,extracellular region,protein binding	G=0, A=127	G=0.0, A=1.0	0.00001	2076.31	127	G=0/0, A=58/6
chr1:16386416	A/A	G	A	SNV	FAM131C	FAM131C:exonic:NM_182623.2	1	100	5	synonymous	NM_18262	c.399C>T	p.(=)	-1.52					Putative cell	rs9442230	0.2432	YES	8.056E-1 (pos.16386416)			protein binding	G=0, A=101	G=0.0, A=1.0	0.00001	1579.74	101	G=0/0, A=44/5
chr8:139165068	C/T	C	T	SNV	FAM135B	FAM135B:exonic:NM_015912.3	1	50	13	synonymous	NM_01591	c.1650G>A	p.(=)	-1.19				0.01(COSM41	PGAP1-like pr	rs3763590	0.3433	YES	5.277E-1 (pos.13916506	carcinoma,neoplasm		carboxylic ester hydrolase activity,cellular lipid metabo	C=28, T=28	C=0.5, T=0.5	0.00001	272.138	56	C=9/19, T=16/
chr4:187077206	G/G	A	G	SNV	FAM149A	FAM149A:exonic:NM_001006655.3	1	100	7	missense	NM_00100	c.436A>G	p.Lys146Glu	2.57	0.09	56	0.061	0.24(COSM37	Protein of unl	rs4862653,rs777381	0.1214	YES	0.0;0.0 (pos.187077205),	adenocarcinoma			A=0, G=20	A=0.0, G=1.0	0.00001	263.786	20	A=0/0, G=8/12
chr14:23816998	C/C	T	C	SNV	SLC22A17	SLC22A17:intronic:NM_020372.3	1	100		intronic	NM_02037	c.945-58A	p.?	-0.06					Major Faciliti	rs12896494		YES			Solute carri	cellular iron ion homeostasis,integral component of mer	T=0, C=60	T=0.0, C=1.0	0.00001	903.53	60	T=0/0, C=54/6
chr4:187078785	G/G	A	G	SNV	FAM149A	FAM149A:exonic:NM_001006655.3	1	100	8	missense	NM_00100	c.641A>G	p.His214Arg	0.29	1	29	0.001	0(COSM3760	Protein of unl	rs2276924	0.1191	YES	8.134E-1 (pos.18707878	adenocarcinoma			A=0, G=27	A=0.0, G=1.0	0.00001	389.984	27	A=0/0, G=19/8
chr4:187074833	A/G	A	G	SNV	FAM149A	FAM149A:exonic:NM_001006655.3	1	97.3	5	missense	NM_00100	c.121A>G	p.Lys41Glu	3.39	1	56	0	0.01(COSM37	Protein of unl	rs4862650	0.123	YES	8.125E-1 (pos.18707483	adenocarcinoma			A=1, G=36	A=0.027, G=0.973	0.00001	551.739	37	A=1/0, G=25/1
chr14:23872666	C/C	T	C	SNV	MYH6	MYH6:intronic:NM_002471.3	1	100		intronic	NM_00247	c.800-11A	p.?	-0.16					Myosin N-ter	rs434273	0.232	YES	6.85E-1 (pos.23872666)		Myosin, hea	ATP bindin Aortic Stenosis, Subvalvular;Ac Benign/Likely	T=0, C=52	T=0.0, C=1.0	0.00001	767.011	52	T=0/0, C=22/3
chr14:24031694	A/A	AC	A	INDEL	AP1G2,LOC102724814	LOC102724814:intronic_nc:NR_110555.1, AP1G2:intronic:N	1	100		intronic	NM_00391	c.1500+19	p.?	0.17, -0.37					Adaptin C-ter	rs398099231,rs5878	0.1921	YES	1.139E-1 (pos.24031694)			AP-1 adap Cardiovascular Diseases:Death, Sudden, Car	AC=0, A=48	AC=0.0, A=1.0	0.00001	737.503	48	AC=0/0, A=23/
chr14:24033070	T/T	C	T	SNV	AP1G2,LOC102724814	LOC102724814:intronic_nc:NR_110555.1, AP1G2:intronic:N	1	100		intronic	NM_00391	c.1092-5G	p.?	-1.11				0.06(COSM37	Adaptin C-ter	rs2281680	0.1905	YES	1.131E-1 (pos.24033070)	carcinoma,hepatocellular		AP-1 adap Cardiovascular Diseases:Death, Sudden, Car	C=0, T=66	C=0.0, T=1.0	0.00001	1015.36	66	C=0/0, T=28/3
chr14:24525634	A/A	C	A	SNV	CARMIL3	CARMIL3:intronic:NM_138360.3	1	100		intronic	NM_13836	c.865+32C	p.?	-2.58					CARMIL C-ter	rs4982851	0.1251	YES	9.985E-1 (pos.24525634)			cytoplasm,plasma membrane	C=0, A=25	C=0.0, A=1.0	0.00001	356.41	25	C=0/0, A=10/1
chr14:24526478	A/G	A	G	SNV	CARMIL3	CARMIL3:intronic:NM_138360.3	1	52.38		intronic	NM_13836	c.1140+25	p.?	-0.07					CARMIL C-ter	rs12879859	0.2785	YES	4.379E-1 (pos.24526478)			cytoplasm,plasma membrane	A=10, G=11	A=0.4762, G=0.5238	0.00001	95.3003	21	A=6/4, G=9/2
chr5:79815658	T/C	T	C	SNV	FAM151B	FAM151B:exonic:NM_205548.2	1	53.13	4	missense	NM_20554	c.464T>C	p.Ile155Thr	1.17	1	89	0	0.01(COSM54	Uncharacteri	rs369998	0.2124	YES	6.087E-1 (pos.79815658)	diffuse_large_B_cell_lymph		lipid metabolic process,phosphoric diester hydrolase ac	T=15, C=17	T=0.4688, C=0.5313	0.00001	173.559	32	T=10/5, C=9/8
chr14:24647957	C/T	C	T	SNV	REC8	REC8:intronic:NM_001048205.1	1	57.58		intronic	NM_00104	c.1061-26	p.?	-0.4					Conserved re	rs1885710	0.486	YES	9.077E-1 (pos.24647957)		Rec8, S. por	chromatin Genital Diseases, Male;Genital Neoplasms, I	C=28, T=38	C=0.4242, T=0.5758	0.00001	408.256	66	C=10/18, T=29
chr5:63986351	G/C	G	C	SNV	FAM159B	FAM159B:utr_5:NM_001164442.1	1	47.62	1	utr_5	NM_00116	c.-100G>C	p.?	0.23					Wnt and FGF	rs10471637		YES				integral component of membrane	G=11, C=10	G=0.5238, C=0.4762	0.00001	82.4872	21	G=9/2, C=9/1
chr11:6239344	A/A	G	A	SNV	FAM160A2	FAM160A2:exonic:NM_032127.3	1	100	9	missense	NM_03212	c.1514C>T	p.Thr505Met	1.77	0.12	81	0.051	0.28(COSM37	Retinoic acid	rs3750944	0.4442	YES	5.013E-1;0.0 (pos.62393	squamous_cell_carcinoma		FHF complex,cytosol,early endosome to late endosome	G=0, A=110	G=0.0, A=1.0	0.00001	1737.97	110	G=0/0, A=51/5
chr11:6243804	A/G	A	G	SNV	FAM160A2	FAM160A2:exonic:NM_032127.3	1	53.13	6	synonymous	NM_03212	c.1059T>C	p.(=)	2.51					Retinoic acid	rs3750945	0.1924	YES	9.651E-1 (pos.6243804)			FHF complex,cytosol,early endosome to late endosome	A=15, G=17	A=0.4688, G=0.5313	0.00001	173.559	32	A=12/3, G=10/
chr14:24771067	C/T	C	T	SNV	NOP9	NOP9:intronic:NM_174913.2	1	52.38		intronic	NM_17491	c.809-104	p.?	-0.15												RNA bindir Breast Diseases:Breast Neoplasms:Neoplasr	C=10, T=11	C=0.4762, T=0.5238	0.00001	95.297	21	C=7/3, T=8/3
chr14:24771080	T/C	T	C	SNV	NOP9	NOP9:intronic:NM_174913.2	1	52.38		intronic	NM_17491	c.809-91T	p.?	-0.54						rs4374085		YES				RNA bindir Breast Diseases:Breast Neoplasms:Neoplasr	T=10, C=11	T=0.4762, C=0.5238	0.00001	95.2991	21	T=7/3, C=8/3
chr14:24771137	A/A	G	A	SNV	NOP9	NOP9:intronic:NM_174913.2	1	100		intronic	NM_17491	c.809-34G	p.?	-0.51						rs4247000	0.0352	YES	8.565E-1;0.0 (pos.24771137)			RNA bindir Breast Diseases;Breast Neoplasms;Neoplasr	G=0, A=21	G=0.0, A=1.0	0.00001	281.235	21	G=0/0, A=13/8
chr2:62066927	G/G	A	G	SNV	FAM161A	FAM161A:exonic:NM_001201543.1	1	100	3	synonymous	NM_00120	c.1212T>C	p.(=)	-0.2					Uncharacteri	rs4672457	0.0281	YES	9.961E-1 (pos.62066927)		Family with	centroson Congenital, Hereditary, and Ne Benign	A=0, G=20	A=0.0, G=1.0	0.00001	263.783	20	A=0/0, G=8/12
chr14:24793600	A/A	G	A	SNV	ADCY4	ADCY4:intronic:NM_001198592.1	1	100		intronic	NM_00119	c.1824-3C	p.?	0.77					Adenylate an	rs1884366	0.0012	YES	1.0 (pos.24793600)		Adenylate c	ATP binding,G-protein coupled receptor signaling pathw	G=0, A=32	G=0.0, A=1.0	0.00001	485.136	32	G=0/0, A=15/1
chr14:24795115	T/T	G	T	SNV	ADCY4	ADCY4:intronic:NM_001198592.1	1	100		intronic	NM_00119	c.1656-22	p.?	2.22					Adenylate an	rs11622527	0.422	YES	1.983E-1 (pos.24795115)		Adenylate c	ATP binding,G-protein coupled receptor signaling pathw	G=0, T=24	G=0.0, T=1.0	0.00001	334.718	24	G=0/0, T=13/1
chr14:24802263	G/G	A	G	SNV	ADCY4	ADCY4:intronic:NM_001198592.1	1	100		intronic	NM_00119	c.160-69T	p.?	-0.36					Adenylate an	rs8003158					Adenylate c	ATP binding,G-protein coupled receptor signaling pathw	A=0, G=53	A=0.0, G=1.0	0.00001	782.03	53	A=0/0, G=31/2
chr14:24806229	A/A	G	A	SNV	RIPK3	RIPK3:intronic:NM_006871.3	1	100		intronic	NM_00687	c.1275+63	p.?	-0.18					Protein kinas	rs724165		YES			Receptor-in	ATP bindin Calcinosis;Calcium Metabolism Disorders;Ca	G=0, A=81	G=0.0, A=1.0	0.00001	1309.45	81	G=0/0, A=16/6
chr14:24806800	C/C	G	C	SNV	RIPK3	RIPK3:intronic:NM_006871.3	1	100		intronic	NM_00687	c.900+101	p.?	1.35					Protein kinas	rs1950501	0.4254	YES	4.411E-1;5.933E-3;0.0 (pos.24806800)		Receptor-in	ATP bindin Calcinosis;Calcium Metabolism Disorders;Ca	G=0, C=36	G=0.0, C=1.0	0.00001	563.62	36	G=0/0, C=11/2
chr2:62081012	C/C	A	C	SNV	FAM161A	FAM161A:exonic:NM_001201543.1	1	100	1	synonymous	NM_00120	c.165T>G	p.(=)	0.53					Uncharacteri	rs4270331	0.0276	YES	9.965E-1 (pos.62081012)		Family with	centroson Congenital, Hereditary, and Ne Benign	A=0, C=45	A=0.0, C=1.0	0.00001	747.329	45	A=0/0, C=11/3
chr14:74404756	C/C	T	C	SNV	FAM161B	FAM161B:exonic:NM_152445.2	1	100	6	missense	NM_15244	c.1649A>G	p.Lys550Arg	1.11	0.42	26	0.327	0.81(COSM17	Uncharacteri	rs28927675	0.4714	YES	4.848E-1 (pos.74404756)	adenocarcinoma,rhabdomyosarcoma			T=0, C=33	T=0.0, C=1.0	0.00001	504.598	33	T=0/0, C=21/1
chr14:24843699	T/T	C	T	SNV	NFATC4	NFATC4:intronic:NM_001136022.2	1	100		intronic	NM_00113	c.2062+27	p.?	-0.75					IPT/TIG doma	rs12890614	0.395	YES	1.436E-1;0.0 (pos.24843699)		Nuclear fac	DNA binding,DNA binding transcription factor activity,RN	C=0, T=37	C=0.0, T=1.0	0.00001	583.706	37	C=0/0, T=13/2
chr14:24900209	C/G	C	G	SNV	KHNYN	KHNYN:intronic:NM_015299.2	1	50		intronic	NM_01529	c.201+72C	p.?	0					Zc3h12a-like	rs1956905,rs550907537		YES				RNA binding,biological_process,cellular_component,mo	C=10, G=10	C=0.5, G=0.5	0.00001	82.8532	20	C=5/5, G=5/5
chr9:140139184	G/G	A	G	SNV	FAM166A	FAM166A:exonic:NM_001001710.2	1	100	5	synonymous	NM_00100	c.657T>C	p.(=)	-0.15					Protein of unl	rs4073074	0.03	YES	1.467E-1 (pos.140139184)			nucleus	A=0, G=40	A=0.0, G=1.0	0.00001	644.232	40	A=0/0, G=24/1
chr9:140140111	G/A	G	A	SNV	FAM166A	FAM166A:exonic:NM_001001710.2	1	50	2	missense	NM_00100	c.251C>T	p.Pro84Leu	5.23	0	98	1	0.88(COSM13	Protein of unl	rs74872755	0.0242	YES	4.635E-4 (pos.14014011	acute_myeloid_leukaemia		nucleus	G=17, A=17	G=0.5, A=0.5	0.00001	172.009	34	G=6/11, A=9/8
chr9:35562549	A/A	G	A	SNV	FAM166B,RUSC2	FAM166B:exonic:NM_001164310.2, RUSC2:downstream:NM	1	100	5	synonymous	NM_01480	c.*1170G>	p.?, p.(=)	-1.42				0.03(COSM54	Protein of unl	rs755774	0.468	YES	3.566E-1 (pos.35562549)	squamous_cel	RUN and SH	Rab GTPase binding,biological_process,cytoplasmic vesi	G=0, A=79	G=0.0, A=1.0	0.00001	1269.33	79	G=0/0, A=46/3
chr14:24910846	T/T	C	T	SNV	KHNYN,SDR39U1	KHNYN:downstream:NM_015299.2, SDR39U1:intronic:NM_(	1	100		intronic	NM_01529	c.*4355C>	p.?, p.?	-0.43					Domain of un	rs11628143,rs77597	0.088	YES	8.183E-1;0.0 (pos.24910846)		Short-chain	RNA binding,biological_process,cellular_component,mo	C=0, T=35	C=0.0, T=1.0	0.00001	543.871	35	C=0/0, T=19/1
chr14:24910876	A/A	G	A	SNV	KHNYN,SDR39U1	KHNYN:downstream:NM_015299.2, SDR39U1:intronic:NM_(	1	100		intronic	NM_01529	c.*4385G>	p.?, p.?	-0.1					Domain of un	rs11158703	0.0877	YES	8.195E-1 (pos.24910876)		Short-chain	RNA binding,biological_process,cellular_component,mo	G=0, A=39	G=0.0, A=1.0	0.00001	623.979	39	G=0/0, A=19/2
chr8:11302061	G/G	A	G	SNV	FAM167A	FAM167A:utr_5:NM_053279.2	1	100	2	utr_5	NM_05327	c.-141T>C	p.?	-0.68					FAM167	rs7830445		YES			Family with	endoplasmic reticulum,protein binding	A=0, G=47	A=0.0, G=1.0	0.00001	789.316	47	A=0/0, G=19/2
chr14:24911764	A/A	G	A	SNV	LOC101927045,SDR39U1	SDR39U1:intronic:NM_020195.2, LOC101927045:upstream	1	100		intronic	NM_02019	c.17-106C	p.?	-1.64					Domain of un	rs1956903		YES	8.448E-1 (pos.24911764)		Short-chain	nucleus,oxidation-reduction process,oxidoreductase ac	G=0, A=20	G=0.0, A=1.0	0.00001	263.785	20	G=0/0, A=10/1
chr5:118970770	T/T	C	T	SNV	FAM170A	FAM170A:utr_3:NM_182761.3	1	100	4	utr_3	NM_18276	c.1031C>T	p.?	0.2				0.18(COSM4419066)		rs2434600,rs779144	0.4215	YES	0.0 (pos.118970769),8.1	acute_myeloid_leukaemia		DNA binding,metal ion binding,nucleus,positive regulati	C=0, T=102	C=0.0, T=1.0	0.00001	1588.91	102	C=0/0, T=40/6
chr14:24975509	C/C	T	C	SNV	CMA1	CMA1:intronic:NM_001836.4	1	100		intronic	NM_00183	c.346-21A	p.?	0.17					Trypsin	rs1885108	0.2116	YES	6.474E-1;0.0 (pos.24975509)		Chymase-1,	angiotens Cardiomegaly;Card (1S)-1-(5-CHLORO-1-BENZ	T=0, C=42	T=0.0, C=1.0	0.00001	682.304	42	T=0/0, C=15/2
chr14:25044089	C/T	C	T	SNV	CTSG	CTSG:intronic:NM_001911.2	1	45.45		intronic	NM_00191	c.204-73G	p.?	0.55					Trypsin,Tryps	rs2070697		YES			Cathepsin (	angiotensin maturation,antiba 2-3-((Methyl1-(2-Naphth	C=12, T=10	C=0.5455, T=0.4545	0.00001	81.7606	22	C=7/5, T=8/2
chr5:10227548	A/A	C	A	SNV	FAM173B	FAM173B:utr_3:NM_199133.3	1	100	5	utr_3	NM_19913	c.707G>T	p.?	-0.87						rs2136197	0.0344	YES	8.81E-1 (pos.10227548)			integral component of membrane	C=0, A=48	C=0.0, A=1.0	0.00001	797.775	48	C=0/0, A=31/1
chr14:30098349	C/C	T	C	SNV	PRKD1	PRKD1:intronic:NM_002742.2	1	100		intronic	NM_00274	c.1673-50	p.?	-0.56					Kinase-like,P	rs1191601	0.4094	YES	6.894E-1;0.0 (pos.30098349)		Protein kina	ATP bindin Adenocarcinoma;Carcinoma:Colonic Disease	T=0, C=23	T=0.0, C=1.0	0.00001	316.746	23	T=0/0, C=14/9
chr14:35515606	T/T	TGG	T	INDEL	FAM177A1	FAM177A1:utr_5:NM_173607.4	2	100	1	utr_5	NM_17360	c.-64TGG>	p.?	-0.42, 0.0, 0.0						rs4007475							TGG=0, T=2	TGG=0.0, T=1.0	0.00001	370.95	26	TGG=0/0, T=16
chr1:222923351	G/G	A	G	SNV	FAM177B	FAM177B:exonic:NM_207468.2	1	100	6	missense	NM_20746	c.428A>G	p.Gln143Arg	-2.11	1	43	0	0(COSM1467	FAM177 fami	rs6683071	0.1898	YES	7.926E-1 (pos.22292335	adenocarcinoma			A=0, G=24	A=0.0, G=1.0	0.00001	334.787	24	A=0/0, G=10/1
chr2:97637905	C/C	T	C	SNV	FAM178B	FAM178B:exonic:NM_001122646.2	1	100	3	missense	NM_00112	c.297A>G	p.Ile99Met	0.57	1	10	0	0.01(COSM45	Family of unk	rs6715329		YES	4.299E-1 (pos.97637905)	carcinoma,squamous_cell_carcinoma			T=0, C=39	T=0.0, C=1.0	0.00001	623.959	39	T=0/0, C=29/1
chr4:17634213	G/G	A	G	SNV	FAM184B	FAM184B:exonic:NM_015688.1	1	100	18	missense	NM_01568	c.3125T>C	p.Val1042Ala	-0.09	1	64	0	0.04(COSM45	Family with s	rs6825562,rs945086910			1.0 (pos.17634213)	squamous_cell_carcinoma		Mental Disorders:Substance-Related Disorde	A=0, G=93	A=0.0, G=1.0	0.00001	1547.53	93	A=0/0, G=63/3
chr4:17660082	T/T	C	T	SNV	FAM184B	FAM184B:exonic:NM_015688.1	1	100	10	missense	NM_01568	c.1928G>A	p.Arg643His	-0.42	0.02	29	0.001		Family with s	rs1860596	0.3916	YES	8.657E-1 (pos.17660082)			Mental Disorders:Substance-Related Disorde	C=0, T=46	C=0.0, T=1.0	0.00001	768.306	46	C=0/0, T=16/3
chr14:32563640	G/G	A	G	SNV	ARHGAP5	ARHGAP5:intronic:NM_001030055.1	1	100		intronic	NM_00103	c.3717+48	p.?	-0.86					FF domain,Ra	rs771566041,rs8004	0.0385	YES	0.0 (pos.32563637),9.999E-1 (pos.32563		RHO GTPase	GTP binding,GTPase activator activity,GTPase activity,Rh	A=0, G=31	A=0.0, G=1.0	0.00001	465.819	31	A=0/0, G=17/1
chr4:17694943	G/G	A	G	SNV	FAM184B	FAM184B:exonic:NM_015688.1	1	100	6	synonymous	NM_01568	c.1470T>C	p.(=)	-0.38				0.09(COSM45	Family with s	rs3733579		YES	8.939E-1 (pos.17694943)	squamous_cell_carcinoma		Mental Disorders;Substance-Related Disorde	A=0, G=20	A=0.0, G=1.0	0.00001	263.739	20	A=0/0, G=8/12
chr14:33046471	T/G	T	G	SNV	AKAP6	AKAP6:intronic:NM_004274.4	1	52.27		intronic	NM_00427	c.2469+23	p.?	0.24						rs768787	0.3429	YES	8.651E-1 (pos.33046471)			T-tubule,action potential,adenylate cyclase binding,cAN	T=21, G=23	T=0.4773, G=0.5227	0.00001	260.15	44	T=15/6, G=19/
chr14:33165182	T/T	C	T	SNV	AKAP6	AKAP6:intronic:NM_004274.4	1	100		intronic	NM_00427	c.2880-14	p.?	1.29						rs3742929	0.4801	YES	8.549E-1 (pos.33165182)			T-tubule,action potential,adenylate cyclase binding,cAN	C=0, T=52	C=0.0, T=1.0	0.00001	766.996	52	C=0/0, T=34/1
chr14:35477785	A/A	G	A	SNV	SRP54	SRP54:intronic:NM_003136.3	1	100		intronic	NM_00313	c.361-48G	p.?	-0.55					CobW/HypB/	rs1712351	0.1224	YES	7.896E-1 (pos.35477785)		Signal recog	7S RNA binding,GDP binding,GTP binding,GTPase activity	G=0, A=25	G=0.0, A=1.0	0.00001	353.016	25	G=0/0, A=8/17
chr12:50744119	A/A	G	A	SNV	FAM186A	FAM186A:exonic:NM_001145475.2	1	100	4	missense	NM_00114	c.6496C>T	p.His2166Tyr	-0.05	0.11	83	0	0.15(COSM4147145)		rs7296291	0.3504	YES	7.588E-1 (pos.50744119)	neoplasm			G=0, A=51	G=0.0, A=1.0	0.00001	754.584	51	G=0/0, A=33/1
chr14:35576458	C/C	A	C	SNV	LOC101927178,PPP2R3C	PPP2R3C:intronic:NM_017917.3, LOC101927178:intronic_n	1	100		intronic	NR_11041	c.573+52T	p.?	0.7					EF hand	rs10132769	0.4837	YES				B cell homeostasis,Golgi apparatus,T cell homeostasis,a	A=0, C=24	A=0.0, C=1.0	0.00001	334.792	24	A=0/0, C=15/9
chr12:50744171	C/C	T	C	SNV	FAM186A	FAM186A:exonic:NM_001145475.2	1	100	4	synonymous	NM_00114	c.6444A>G	p.(=)	-0.32				0.02(COSM147504)		rs7312252	0.3504	YES	7.597E-1 (pos.50744171)	adenocarcinoma,neoplasm			T=0, C=129	T=0.0, C=1.0	0.00001	2114.32	129	T=0/0, C=70/5
chr12:50749221	C/C	G	C	SNV	FAM186A	FAM186A:exonic:NM_001145475.2	1	100	4	missense	NM_00114	c.1394C>G	p.Ala465Gly	-1.24		60	0			rs4435082	0	YES	1.0 (pos.50749221)				G=0, C=92	G=0.0, C=1.0	0.00001	1533.56	92	G=0/0, C=45/4
chr14:36781037	T/T	C	T	SNV	MBIP	MBIP:intronic:NM_016586.2	1	100		intronic	NM_01658	c.638-106	p.?	-0.34						rs7154340		YES			MAP3K12-b	Ada2/Gcn5/Ada3 transcription activator complex,cytosc	C=0, T=117	C=0.0, T=1.0	0.00001	1874.21	117	C=0/0, T=53/6
chr14:38016099	C/T	C	T	SNV	MIPOL1	MIPOL1:intronic:NM_138731.6	1	95.83		intronic	NM_13873	c.1263-11	p.?	1.53						rs991786	0.1164	YES	4.707E-4 (pos.38016099)		MIPOL1 gen	identical ; Abnormalities, Multiple;Congenital Abnorma	C=1, T=23	C=0.0417, T=0.9583	0.00001	306.624	24	C=1/0, T=15/8
chr19:35719257	A/A	G	A	SNV	FAM187B	FAM187B:exonic:NM_152481.1	1	100	1	synonymous	NM_15248	c.327C>T	p.(=)	0.47				0.54(COSM1392803)		rs11673347	0.4029	YES	3.27E-2 (pos.35719257)	adenocarcinoma		integral component of membrane	G=0, A=25	G=0.0, A=1.0	0.00001	353.016	25	G=0/0, A=10/1
chr14:39514274	T/G	T	G	SNV	SEC23A	SEC23A:intronic:NM_006364.3	1	50		intronic	NM_00636	c.1899+93	p.?	0.9					Gelsolin repe	rs77051476		YES			Sec23, S. ce	COPII vesi Arthritis;Autoimmune Diseases;Bone Diseas	T=12, G=13	T=0.48, G=0.52	0.00001	130.755	26	T=11/1, G=10/
chr14:39748480	A/A	G	A	SNV	MIA2	MIA2:intronic:NM_001329214.2	1	100		intronic	NM_00132	c.2020-61	p.?	1.41						rs7158558	0.0639	YES				ER to Golgi vesicle-mediated transport,cargo loading int	G=0, A=29	G=0.0, A=1.0	0.00001	431.978	29	G=0/0, A=19/1
chr14:39762347	C/C	T	C	SNV	MIA2	MIA2:intronic:NM_001329214.2	1	100		intronic	NM_00132	c.2209-12	p.?	0.71						rs2025029		YES				ER to Golgi vesicle-mediated transport,cargo loading int	T=0, C=82	T=0.0, C=1.0	0.00001	1329.63	82	T=0/0, C=56/2
chr14:39784042	G/G	A	G	SNV	MIA2	MIA2:intronic:NM_001329214.2	1	100		intronic	NM_00132	c.3180+39	p.?	-0.79						rs2415540,rs57139462,rs74851		YES	0.0 (pos.39784028),0.0 (pos.39784034),0.0 (pos.3978			ER to Golgi vesicle-mediated transport,cargo loading int	A=0, G=22	A=0.0, G=1.0	0.00001	297.961	22	A=0/0, G=5/17
chr15:29415698	G/G	A	G	SNV	FAM189A1	FAM189A1:exonic:NM_015307.1	1	100	11	synonymous	NM_01530	c.1464T>C	p.(=)	2.57				0.07(COSM41	CD20-like fan	rs3751563		YES	9.151E-1 (pos.29415698)	neoplasm,squamous_cell		integral component of membrane	A=0, G=20	A=0.0, G=1.0	0.00001	263.606	20	A=0/0, G=10/1
chr15:29418520	T/T	C	T	SNV	FAM189A1	FAM189A1:exonic:NM_015307.1	1	100	9	missense	NM_01530	c.1178G>A	p.Arg393His	0.11	0.03	29	0.005	0.01(COSM37	CD20-like fan	rs2256273	0.2184	YES	5.348E-1 (pos.29418520)	carcinoma,hepatocellular		integral component of membrane	C=0, T=49	C=0.0, T=1.0	0.00001	831.575	49	C=0/0, T=29/2
chr14:50241258	C/C	A	C	SNV	KLHDC2	KLHDC2:intronic:NM_014315.2	1	100		intronic	NM_01431	c.234-21A	p.?	0.67					Galactose ox	rs4499141	7.00E-04	YES	1.0;0.0 (pos.50241258)		KELCH doma	nuclear body,nuclear membrane,nucleus	A=0, C=91	A=0.0, C=1.0	0.00001	1513.72	91	A=0/0, C=47/4
chr15:29418573	GC/GC	AT	GC	MNV	FAM189A1	FAM189A1:exonic:NM_015307.1	2	100	9	missense	NM_01530	c.1124_11	p.His375Arg	-0.34, -2.5	1	29	0	0(COSM3754	CD20-like fan	rs2256277,rs2672680,rs342321		YES	5.304E-1 (pos.29418573)	adenocarcinoma,neoplasm		integral component of membrane	AT=0, GC=4	AT=0.0, GC=1.0	0.00001	710.218	43	AT=0/0, GC=23
chr14:50307238	C/T	C	T	SNV	NEMF	NEMF:intronic:NM_004713.5	1	50		intronic	NM_00471	c.506+198	p.?	-0.36					Domain of un	rs116145698					Nuclear exp	nuclear export,nucleus	C=44, T=44	C=0.5, T=0.5	0.00001	478.658	88	C=22/22, T=20
chr1:155220114	A/G	A	G	SNV	FAM189B	FAM189B:exonic:NM_006589.2	1	47.62	9	missense	NM_00658	c.1463T>C	p.Leu488Pro	2.6	0	98	0.995	0.76(COSM53	CD20-like fan	rs142630415	0.0028	YES	0.0 (pos.155220114)	Wilms_tumour		WW domain binding,biological_process,integral compor	A=11, G=10	A=0.5238, G=0.4762	0.00001	82.2771	21	A=3/8, G=5/5
chr14:51058234	T/T	G	T	SNV	ATL1	ATL1:intronic:NM_001127713.1	1	100		intronic	NM_00112	c.418-19G	p.?	0.93					Guanylate-bi	rs2934684	0.2722	YES	8.205E-1 (pos.51058234)		Atlastin GTI	GTP bindir Congenital Abnormalities;Congenital, Heredi	G=0, T=47	G=0.0, T=1.0	0.00001	789.328	47	G=0/0, T=22/2
chr14:51192659	G/A	G	A	SNV	NIN	NIN:intronic:NM_020921.3	1	58.33		intronic	NM_02092	c.6192+12	p.?	-1.3						rs1983764	0.355	YES	3.161E-1 (pos.51192659)		Ninein	GTP bindir Autoimmune Diseases;Connective Tissue Dis	G=10, A=14	G=0.4167, A=0.5833	0.00001	135.341	24	G=7/3, A=5/9
chr5:176963455	G/A	G	A	SNV	FAM193B	FAM193B:exonic:NM_001190946.2	1	45.45	4	missense	NM_00119	c.980C>T	p.Ser327Leu	6.89	0.05	145	0.308		FAM193 family	C-terminal					Family with	sequence similarity 193, member B	G=30, A=25	G=0.5455, A=0.4545	0.00001	229.499	55	G=17/13, A=2/
chr3:43074430	G/G	A	G	SNV	FAM198A	FAM198A:exonic:NM_001129908.2	1	100	2	synonymous	NM_00112	c.675A>G	p.(=)	0.08					FAM198 prot	rs2948619	0.4469	YES	7.664E-1 (pos.43074430)			extracellular region,intracellular membrane-bounded o	A=0, G=51	A=0.0, G=1.0	0.00001	754.584	51	A=0/0, G=25/2
chr14:51228682	A/A	G	A	SNV	NIN	NIN:intronic:NM_020921.3	1	100		intronic	NM_02092	c.1775-53	p.?	-0.32						rs6572694		YES			Ninein	GTP bindir Autoimmune Diseases;Connective Tissue Dis	G=0, A=21	G=0.0, A=1.0	0.00001	281.23	21	G=0/0, A=8/13
chr14:51353493	C/C	T	C	SNV	ABHD12B	ABHD12B:intronic:NM_001206673.1	1	100		intronic	NM_00120	c.700+91T	p.?	0.01					Alpha/beta h	rs7158183		YES				hydrolase activity	T=0, C=330	T=0.0, C=1.0	0.00001	5577.86	330	T=0/0, C=155/
chr14:51355517	A/A	C	A	SNV	ABHD12B	ABHD12B:intronic:NM_001206673.1	1	100		intronic	NM_00120	c.701-25C	p.?	-0.67					Alpha/beta h	rs10144606,rs77633	0.3078	YES	4.34E-1 (pos.51355517),8.323E-4;1.189E-4;0.0 (pos.5			hydrolase activity	C=0, A=97	C=0.0, A=1.0	0.00001	1643.16	97	C=0/0, A=52/4
chr9:34726524	A/A	G	A	SNV	FAM205A	FAM205A:exonic:NM_001141917.1	1	100	4	missense	NM_00114	c.713C>T	p.Thr238Met	-0.13	1	81	0	0(COSM4406	Domain of un	rs1810820,rs90836	0.1744	YES	7.258E-2 (pos.34726524)	hepatocellular_carcinoma		integral component of membrane,nucleus	G=0, A=21	G=0.0, A=1.0	0.00001	281.235	21	G=0/0, A=6/15
chr14:51492135	CCACACAGGT(	C	CCACACAGGTGTTTT	INDEL	TRIM9	TRIM9:intronic:NM_015163.5	14	100		intronic	NM_01516	c.823-57G	p.?	0.21					B-box zinc fin	rs11268748,rs202209972,rs583		YES			Tripartite m	SNARE bin Mental Disorders:Psychotic Disorders:Schizo	C=0, CCAC	C=0.0, CCACACAGGT(	0.00001	297.943	22	C=0/0, CCACAC
chr14:52194407	G/A	G	A	SNV	FRMD6	FRMD6:intronic:NM_001267046.1	1	51.85		intronic	NM_00126	c.1585-56	p.?	1.87					FERM C-termi	rs4433726		YES			FERM doma	actomyosin structure organization,apical constriction,a	G=13, A=14	G=0.4815, A=0.5185	0.00001	132.562	27	G=9/4, A=4/10
chr9:34729452	G/G	A	G	SNV	FAM205A	FAM205A:utr_5:NM_001141917.1	1	100	1	utr_5	NM_00114	c.-28T>C	p.?	-0.13					Domain of un	rs10115191	0.3422	YES	7.201E-1 (pos.34729452)			integral component of membrane,nucleus	A=0, G=40	A=0.0, G=1.0	0.00001	644.281	40	A=0/0, G=22/1
chr14:52496091	C/C	A	C	SNV	NID2	NID2:intronic:NM_007361.3	1	100		intronic	NM_00736	c.2401+17	p.?	-0.42					Calcium-bind	rs1497087		YES			Nidogen 2	basement membrane,basement membrane organizatio	A=0, C=28	A=0.0, C=1.0	0.00001	408.13	28	A=0/0, C=14/1
chr10:5781969	T/T	A	T	SNV	FAM208B	FAM208B:exonic:NM_017782.4	1	100	13	synonymous	NM_01778	c.1836A>T	p.(=)	-1.07					Protein of unl	rs2797486	0.1678	YES	8.117E-1 (pos.5781969)			protein binding	A=0, T=50	A=0.0, T=1.0	0.00001	845.942	50	A=0/0, T=30/2
chr20:55108617	A/A	C	A	SNV	FAM209B	FAM209B:exonic:NM_001013646.3	1	100	1	missense	NM_00101	c.220C>A	p.Gln74Lys	2.04	1	53	0		FAM209 fami	rs3209183	0.3903	YES	5.532E-1 (pos.55108617)			integral component of membrane,nucleus	C=0, A=64	C=0.0, A=1.0	0.00001	977.635	64	C=0/0, A=41/2
chr14:52535387	C/C	A	C	SNV	NID2	NID2:intronic:NM_007361.3	1	100		intronic	NM_00736	c.228+98T	p.?	-1.97					Calcium-bind	rs984051		YES			Nidogen 2	basement membrane,basement membrane organizatio	A=0, C=65	A=0.0, C=1.0	0.00001	983.355	65	A=0/0, C=51/1
chr17:66533655	G/G	A	G	SNV	FAM20A	FAM20A:exonic:NM_017565.3	1	100	11	missense	NM_01756	c.1589T>C	p.Leu530Ser	0.36	1	145	0		Golgi casein l	rs2907373	0.3379	YES	9.048E-1 (pos.66533655)		Family with	Golgi appa Amelogenesis Imperfecta;Arth Benign	A=0, G=50	A=0.0, G=1.0	0.00001	852.746	50	A=0/0, G=32/1
chr7:299850	G/T	G	T	SNV	FAM20C	FAM20C:exonic:NM_020223.3	1	50	10	synonymous	NM_02022	c.1659G>T	p.(=)	0.06					Golgi casein l	rs36170987	0.4437	YES	4.787E-1 (pos.299850)		Family with	ATP bindin Abnormalities, Multiple;Bone I Benign	G=20, T=20	G=0.5, T=0.5	0.00001	214.332	40	G=8/12, T=14/
chr7:299881	A/G	A	G	SNV	FAM20C	FAM20C:exonic:NM_020223.3	1	50	10	missense	NM_02022	c.1690A>G	p.Asn564Asp	3.87	0.93	23	0	0.05(COSM62	Golgi casein l	rs36139924	0.4428	YES	4.743E-1;0.0 (pos.29988	neoplasm	Family with	ATP bindin Abnormalities, Multiple;Bone I Benign	A=20, G=20	A=0.5, G=0.5	0.00001	214.097	40	A=8/12, G=14/

chr14:53133036	C/C	T	C	SNV	ERO1A	ERO1A:intronic:NM_014584.2	1	100		intronic	NM_01458	c.629+57A	p.?	0.77					Endoplasmic	rs12885338		YES				4-hydroxyproline metabolic process,animal organ senes		T=0, C=30	T=0.0, C=1.0	0.00001	446.558	30	T=0/0, C=14/1
chr1:112269872	A/A	G	A	SNV	FAM212B	FAM212B:exonic:NM_019099.4	1	100	2	synonymous	NM_01909	c.612C>T	p.(=)	0.42					FAM212 fami	rs197434	0.1813	YES	8.253E-1;0.0 (pos.112269872)			negative regulation of protein serine/threonine kinase a		G=0, A=51	G=0.0, A=1.0	0.00001	754.583	51	G=0/0, A=26/2
chr14:53326454	A/A	C	A	SNV	FERMT2	FERMT2:intronic:NM_001134999.1	1	100		intronic	NM_00113	c.1749-22	p.?	-1.6					FERM central	rs3818453	0.095	YES	7.456E-1 (pos.53326454)		Fermitin far	I band,Wn	Alzheimer Disease:Brain Diseases:Central Ne	C=0, A=23	C=0.0, A=1.0	0.00001	316.747	23	C=0/0, A=8/15
chr14:53342148	C/T	C	T	SNV	FERMT2	FERMT2:intronic:NM_001134999.1	1	51.85		intronic	NM_00113	c.964-73G	p.?	0.15					FERM central	rs113177107					Fermitin far	I band,Wn	Alzheimer Disease:Brain Diseases:Central Ne	C=26, T=28	C=0.4815, T=0.5185	0.00001	277.174	54	C=14/12, T=18
chr15:52901638	C/C	T	C	SNV	FAM214A	FAM214A:exonic:NM_019600.3	1	100	6	synonymous	NM_01960	c.1473A>G	p.(=)	-2.33				0.08(COSM14	Chromosome	rs3751614	0.2688	YES	1.067E-1 (pos.52901638)	acute_myeloid_leukaemia		protein binding		T=0, C=51	T=0.0, C=1.0	0.00001	754.53	51	T=0/0, C=19/3
chr14:53522324	TA/TA	T	TA	INDEL	DDHD1	DDHD1:intronic:NM_001160148.1	1	100		intronic	NM_00116	c.2245+54	p.?	0.75					DDHD domai	rs112807709,rs397690043,rs58		YES			DDHD doma	cytoplasm	Nervous System Diseases;Neurologic Manife	T=0, TA=57	T=0.0, TA=1.0	0.00001	806.083	57	T=0/0, TA=8/4
chr14:53525392	T/T	C	T	SNV	DDHD1	DDHD1:intronic:NM_001160148.1	1	100		intronic	NM_00116	c.1843-48	p.?	1.95					DDHD domai	rs915436	0		1.0 (pos.53525392)		DDHD doma	cytoplasm	Nervous System Diseases;Neurologic Manife	C=0, T=40	C=0.0, T=1.0	0.00001	644.285	40	C=0/0, T=8/32
chr6:4069166	T/T	C	T	SNV	FAM217A	FAM217A:exonic:NM_173563.2	1	100	7	missense	NM_17356	c.1291G>A	p.Val431Ile	0.21	0	29	0.319	0.11(COSM40	FAM217 fami	rs595413,rs77169710	0.262	YES	0.0 (pos.4069165),6.245	acute_myeloid_leukaemia				C=0, T=45	C=0.0, T=1.0	0.00001	747.381	45	C=0/0, T=26/1
chr2:24387178	GC/GC	G	GC	INDEL	FAM228B	FAM228B:exonic:NM_001145710.1	1	100	9	unknown	NM_001145710.1			1.3						rs113322110,rs3978	0.0278	YES	9.359E-1 (pos.24387178)					G=0, GC=30	G=0.0, GC=1.0	0.00001	425.579	30	G=0/0, GC=13/
chr12:13221606	C/C	T	C	SNV	FAM234B	FAM234B:exonic:NM_020853.1	1	100	9	synonymous	NM_02085	c.1329T>C	p.(=)	1.47						rs1386005	7.00E-04		1.0 (pos.13221606)			integral component of membrane		T=0, C=23	T=0.0, C=1.0	0.00001	307.672	23	T=0/0, C=10/1
chr3:46658737	G/G	A	G	SNV	FAM240A	FAM240A:exonic:NM_001195442.1	1	98.84	2	missense	NM_00119	c.98A>G	p.His33Arg	1.47	0.47	29	0			rs9832679		YES	1.0 (pos.46658737)					A=2, G=170	A=0.0116, G=0.9884	0.00001	2734.29	172	A=2/0, G=147/
chr14:55530120	T/C	T	C	SNV	MAPK1IP1L	MAPK1IP1L:intronic:NM_144578.3	1	50		intronic	NM_14457	c.726+77T	p.?	-0.01					MAPK-interac	rs3742564		YES				protein binding		T=24, C=24	T=0.5, C=0.5	0.00001	273.703	48	T=13/11, C=18
chr14:55902542	A/A	G	A	SNV	TBPL2	TBPL2:intronic:NM_199047.2	1	100		intronic	NM_19904	c.696+23C	p.?	0.22					Transcriptior	rs7149317	0.3321	YES	7.196E-1 (pos.55902542)			DNA binding,DNA binding transcription factor activity,DN		G=0, A=44	G=0.0, A=1.0	0.00001	713.076	44	G=0/0, A=24/2
chr14:56126456	A/A	C	A	SNV	KTN1	KTN1:intronic:NM_001079521.1	1	100		intronic	NM_00107	c.2999+35	p.?	0.27						rs7160678	1.00E-04		1.0 (pos.56126456)		Kinectin	RNA binding,cadherin binding,cellular protein metabolic		C=0, A=67	C=0.0, A=1.0	0.00001	1034.34	67	C=0/0, A=21/4
chr14:56142480	G/G	A	G	SNV	KTN1	KTN1:intronic:NM_001079521.1	1	100		intronic	NM_00107	c.3772-73	p.?	1.52						rs3736877		YES			Kinectin	RNA binding,cadherin binding,cellular protein metabolic		A=0, G=20	A=0.0, G=1.0	0.00001	263.737	20	A=0/0, G=8/12
chr10:71392557	T/T	C	T	SNV	FAM241B	FAM241B:exonic:NM_145306.2	1	100	4	synonymous	NM_14530	c.108C>T	p.(=)	0				0.51(COSM37	Domain of un	rs12020	0.3338	YES	8.933E-1 (pos.71392557)	adenocarcinoma,neoplasn		integral component of membrane,protein binding		C=0, T=34	C=0.0, T=1.0	0.00001	524.186	34	C=0/0, T=23/1
chr14:57092037	TG/TG	T	TG	INDEL	TMEM260	TMEM260:intronic:NM_017799.3	1	100		intronic	NM_01779	c.1399-63	p.?	0.32					Dolichyl-phos	rs750735531					Transmemt	integral component of membrane		T=0, TG=45	T=0.0, TG=1.0	0.00001	652.743	45	T=0/0, TG=16/
chr14:57101682	G/G	A	G	SNV	TMEM260	TMEM260:intronic:NM_017799.3	1	100		intronic	NM_01779	c.1778+12	p.?	-0.26					Dolichyl-phos	rs913742	0.2285	YES	7.647E-1 (pos.57101682)		Transmemt	integral component of membrane		A=0, G=65	A=0.0, G=1.0	0.00001	996.464	65	A=0/0, G=37/2
chr10:71392692	C/C	T	C	SNV	FAM241B	FAM241B:exonic:NM_145306.2	1	100	4	synonymous	NM_14530	c.243T>C	p.(=)	-3.99				0.01(COSM37	Domain of un	rs1052152	0.3331	YES	8.943E-1 (pos.71392692)	adenocarcinoma,neoplasn		integral component of membrane,protein binding		T=0, C=78	T=0.0, C=1.0	0.00001	1249.36	78	T=0/0, C=33/4
chr10:124610027	A/A	G	A	SNV	FAM24B,FAM24B-CUZD1	FAM24B:exonic:NM_001204364.1, FAM24B-CUZD1:intronic	1	100	3	missense	NR_03791	c.5C>T	p.Pro2Leu	1.24	1	98	0	0(COSM3751	CUB domain,	rs1891110	0.4451	YES	5.103E-1 (pos.12461002	adenocarcinoma,carcinom		extracellular region		G=0, A=38	G=0.0, A=1.0	0.00001	603.764	38	G=0/0, A=20/1
chr10:120896027	A/A	G	A	SNV	FAM45A,FAM45BP	FAM45A:exonic:NM_207009.3, FAM45BP:exonic_nc:NR_027	1	100	9	synonymous	NM_20700	c.942G>A	p.(=)	-1.73				0.01(COSM45	Stabilization	rs11198799		YES	9.59E-1 (pos.120896027)	squamous_cell_carcinoma				G=0, A=39	G=0.0, A=1.0	0.00001	623.98	39	G=0/0, A=24/1
chr14:57957664	A/A	T	A	SNV	CCDC198	CCDC198:intronic:NM_018168.3	1	100		intronic	NM_01816	c.306+43A	p.?	1.82					Domain of un	rs1203338	0.0013	YES	9.999E-1 (pos.57957664)			protein binding		T=0, A=93	T=0.0, A=1.0	0.00001	1555.33	93	T=0/0, A=38/5
chr6:82461520	A/G	A	G	SNV	FAM46A	FAM46A:exonic:NM_017633.2	1	50	2	synonymous	NM_01763	c.339T>C	p.(=)	0.71				0.1(COSM376	Nucleotidyltr	rs3186631	0.0733	YES	1.078E-1 (pos.82461520)	adenocarcinoma,neoplasn		RNA binding,nucleotidyltransferase activity,protein binc		A=21, G=21	A=0.5, G=0.5	0.00001	228.844	42	A=16/5, G=11/
chr14:58869510	C/C	T	C	SNV	TOMM20L	TOMM20L:intronic:NM_207377.2	1	100		intronic	NM_20737	c.262+31T	p.?	-0.46					MAS20 protei	rs7153357	1.00E-04		9.998E-1 (pos.58869510)			P-P-bond-hydrolysis-driven protein transmembrane tran		T=0, C=70	T=0.0, C=1.0	0.00001	1092.09	70	T=0/0, C=26/4
chr14:58923395	A/A	G	A	SNV	KIAA0586	KIAA0586:intronic:NM_001244189.1	1	100		intronic	NM_00124	c.1413-25	p.?	3.17					Hedgehog sig	rs1743721	7.00E-04	YES	9.875E-1 (pos.58923395)		KIAA0586 g	centriole,c	Congenital Abnormalities;Congenital, Heredi	G=0, A=50	G=0.0, A=1.0	0.00001	852.648	50	G=0/0, A=35/1
chr14:58927984	A/A	G	A	SNV	KIAA0586	KIAA0586:intronic:NM_001244189.1	1	100		intronic	NM_00124	c.2218+10	p.?	-0.14					Hedgehog sig	rs1743719		YES			KIAA0586 g	centriole,c	Congenital Abnormalities;Congenital, Heredi	G=0, A=84	G=0.0, A=1.0	0.00001	1369.27	84	G=0/0, A=47/3
chr14:58934371	A/A	G	A	SNV	KIAA0586	KIAA0586:intronic:NM_001244189.1	1	100		intronic	NM_00124	c.2414-82	p.?	0.36					Hedgehog sig	rs1632390		YES			KIAA0586 g	centriole,c	Congenital Abnormalities;Congenital, Heredi	G=0, A=65	G=0.0, A=1.0	0.00001	996.443	65	G=0/0, A=24/4
chr14:59946094	G/C	G	C	SNV	L3HYPDH	L3HYPDH:intronic:NM_144581.1	1	57.14		intronic	NM_14458	c.509-25C	p.?	-1.44					Proline racer	rs1752435,rs771295	0.0545	YES	0.0:0.0 (pos.59946091),1.972E-2 (pos.59		L-3-hydroxy	hydro-lyase activity,metabolic_L-Proline		G=15, C=20	G=0.4286, C=0.5714	0.00001	218.716	35	G=9/6, C=7/13
chr14:60074264	G/A	G	A	SNV	RTN1	RTN1:intronic:NM_021136.2	1	45.45		intronic	NM_02113	c.1766-54	p.?	-0.4					Reticulon	rs8009556		YES			Reticulon 1	endoplasr	Cardiovascular Diseases:Heart Diseases:My	G=42, A=35	G=0.5455, A=0.4545	0.00001	348.038	77	G=26/16, A=2
chr4:1656801	C/C	A	C	SNV	FAM53A	FAM53A:exonic:NM_001174070.2	1	100	4	synonymous	NM_00117	c.786T>G	p.(=)	-3.9					Family of FAN	rs62287700,rs75768	0.3399	YES	7.194E-2;0.0 (pos.1656801)		Family with	nucleus		A=0, C=49	A=0.0, C=1.0	0.00001	831.633	49	A=0/0, C=27/2
chr14:60921673	C/C	T	C	SNV	C14orf39	C14orf39:intronic:NM_174978.2	1	100		intronic	NM_17497	c.1503+46	p.?	-1.31					Six6 opposite	rs1262175	0		9.968E-1 (pos.60921673)		Chromosom	cellular_c	Eye Diseases;Refractive Errors	T=0, C=51	T=0.0, C=1.0	0.00001	754.471	51	T=0/0, C=10/4
chr4:1657281	A/A	G	A	SNV	FAM53A	FAM53A:exonic:NM_001174070.2	1	100	4	synonymous	NM_00117	c.306C>T	p.(=)	-0.47				0.03(COSM54	Family of FAN	rs79735962	0.3114	YES	9.416E-2 (pos.1657281)	squamous_cel	Family with	nucleus		G=0, A=61	G=0.0, A=1.0	0.00001	921.854	61	G=0/0, A=28/3
chr14:61497147	T/T	A	T	SNV	SLC38A6	SLC38A6:intronic:NM_001172702.1	1	100		intronic	NM_00117	c.404-16A	p.?	-0.14					Transmembr	rs1465146,rs757536	1.00E-04		0.0 (pos.61497145),1.0 (pos.61497147)		Solute carri	amino acid transmembrane transport,amino acid transr		A=0, T=26	A=0.0, T=1.0	0.00001	371.42	26	A=0/0, T=15/1
chr14:61497285	C/C	T	C	SNV	SLC38A6	SLC38A6:intronic:NM_001172702.1	1	100		intronic	NM_00117	c.482+44T	p.?	-0.5					Transmembr	rs1465145	1.00E-04		1.0 (pos.61497285)		Solute carri	amino acid transmembrane transport,amino acid transr		T=0, C=25	T=0.0, C=1.0	0.00001	353.016	25	T=0/0, C=14/1
chr5:137682714	G/G	T	G	SNV	FAM53C	FAM53C:utr_3:NM_001350195.1	1	100	5	utr_3	NM_00135	c.1215T>G	p.?	2.24					Family of FAN	rs2269953		YES			Family with	sequence similarity 53, member C		T=0, G=135	T=0.0, G=1.0	0.00001	2228.09	135	T=0/0, G=53/8
chr5:156589399	T/T	G	T	SNV	FAM71B	FAM71B:utr_3:NM_130899.2	1	100	2	utr_3	NM_13089	c.1877C>A	p.?	0.04					Protein of unl	rs2278385		YES				nucleus		G=0, T=20	G=0.0, T=1.0	0.00001	263.786	20	G=0/0, T=12/8
chr14:62208005	C/C	T	C	SNV	HIF1A,LOC105370526	LOC105370526:intronic_nc:NR_144368.1, HIF1A:intronic:NI	1	100		intronic	NM_00153	c.2093+99	p.?	-0.74					HIF-1 alpha C	rs4902080		YES			Hypoxia-inc	B-1 B cell h	Adenocarcinoma:A 2-Methoxyestradiol,Car	T=0, C=29	T=0.0, C=1.0	0.00001	427.576	29	T=0/0, C=5/24
chr19:55870289	T/T	C	T	SNV	FAM71E2	FAM71E2:exonic:NM_001145402.1	1	100	9	synonymous	NM_00114	c.1947G>A	p.(=)	0.12				0.03(COSM41	Protein of unl	rs12974907	0.1348	YES	9.019E-1 (pos.55870289)	neoplasm		nucleus		C=0, T=40	C=0.0, T=1.0	0.00001	644.054	40	C=0/0, T=21/1
chr14:64457079	A/A	G	A	SNV	SYNE2	SYNE2:intronic:NM_182914.2	1	100		intronic	NM_18291	c.2314-50	p.?	0.44					CAMSAP CH d	rs1255907	0.4461	YES	5.332E-1 (pos.64457079)		Spectrin rep	LINC comp	Adenocarcinoma;Arrhythmias, Cardiac;Atrial	G=0, A=41	G=0.0, A=1.0	0.00001	655.06	41	G=0/0, A=15/2
chr14:64457638	G/G	T	G	SNV	SYNE2	SYNE2:intronic:NM_182914.2	1	100		intronic	NM_18291	c.2473-22	p.?	1.25					CAMSAP CH d	rs1255908	0.2989	YES	3.645E-1 (pos.64457638)		Spectrin rep	LINC comp	Adenocarcinoma;Arrhythmias, Cardiac;Atrial	T=0, G=26	T=0.0, G=1.0	0.00001	371.361	26	T=0/0, G=16/1
chr14:64462007	C/C	A	C	SNV	SYNE2	SYNE2:intronic:NM_182914.2	1	100		intronic	NM_18291	c.2940+87	p.?	-0.26					CAMSAP CH d	rs1255912		YES			Spectrin rep	LINC comp	Adenocarcinoma;Arrhythmias, Cardiac;Atrial	A=0, C=35	A=0.0, C=1.0	0.00001	543.87	35	A=0/0, C=15/2
chr14:64462071	T/T	G	T	SNV	SYNE2	SYNE2:intronic:NM_182914.2	1	100		intronic	NM_18291	c.2940+15	p.?	0.04					CAMSAP CH d	rs1255913		YES			Spectrin rep	LINC comp	Adenocarcinoma;Arrhythmias, Cardiac;Atrial	G=0, T=30	G=0.0, T=1.0	0.00001	446.638	30	G=0/0, T=10/2
chr19:55870351	C/C	T	C	SNV	FAM71E2	FAM71E2:exonic:NM_001145402.1	1	100	9	missense	NM_00114	c.1885A>G	p.Lys629Glu	0	1	56	0	0.02(COSM41	Protein of unl	rs12976922	0.1348	YES	9.013E-1 (pos.55870351)	neoplasm		nucleus		T=0, C=37	T=0.0, C=1.0	0.00001	583.706	37	T=0/0, C=18/1
chr19:55870600	G/G	C	G	SNV	FAM71E2	FAM71E2:exonic:NM_001145402.1	1	100	9	missense	NM_00114	c.1636G>C	p.Glu546Gln	2.51	0.96	29	0		Protein of unl	rs12460416	0.0075	YES	9.156E-1 (pos.55870600)			nucleus		C=0, G=26	C=0.0, G=1.0	0.00001	371.404	26	C=0/0, G=14/1
chr19:55872397	G/G	A	G	SNV	FAM71E2	FAM71E2:exonic:NM_001145402.1	1	100	6	synonymous	NM_00114	c.729T>C	p.(=)	-1.42				0.01(COSM41	Protein of unl	rs2124918	0.006	YES	9.078E-1 (pos.55872397)	neoplasm		nucleus		A=0, G=47	A=0.0, G=1.0	0.00001	789.328	47	A=0/0, G=17/3
chr7:128315791	G/G	C	G	SNV	FAM71F2	FAM71F2:exonic:NM_001012454.5	1	100	2	synonymous	NM_00101	c.243C>G	p.(=)	0.66				0.06(COSM57	Protein of unl	rs61738927	0.2981	YES	5.436E-1 (pos.12831579	adenocarcinoma,carcinom		nucleus		C=0, G=25	C=0.0, G=1.0	0.00001	353.018	25	C=0/0, G=16/9
chr14:64537295	G/G	T	G	SNV	SYNE2	SYNE2:intronic:NM_182914.2	1	100		intronic	NM_18291	c.10432-6	p.?	-0.08					CAMSAP CH d	rs1255965		YES			Spectrin rep	LINC comp	Adenocarcinoma;Arrhythmias, Cardiac;Atrial	T=0, G=43	T=0.0, G=1.0	0.00001	710.796	43	T=0/0, G=25/1
chr14:64537657	A/A	G	A	SNV	SYNE2	SYNE2:intronic:NM_182914.2	1	100		intronic	NM_18291	c.10697+2	p.?	1.2					CAMSAP CH d	rs1255964	0.0668	YES	8.492E-1;0.0 (pos.64537657)		Spectrin rep	LINC comp	Adenocarcinoma;Arrhythmias, Cardiac;Atrial	G=0, A=99	G=0.0, A=1.0	0.00001	1681.13	99	G=0/0, A=22/7
chr7:128317658	T/T	C	T	SNV	FAM71F2	FAM71F2:exonic:NM_001012454.5	1	100	3	missense	NM_00101	c.406C>T	p.Arg136Trp	0.27	0.01	101	0.999	0.07(COSM40	Protein of unl	rs6467210	0.2958	YES	5.6E-1 (pos.128317658)	acute_myeloid_leukaemia		nucleus		C=0, T=87	C=0.0, T=1.0	0.00001	1431.39	87	C=0/0, T=45/4
chr9:134136677	G/G	A	G	SNV	FAM78A	FAM78A:exonic:NM_033387.3	1	100	2	synonymous	NM_03338	c.384T>C	p.(=)	-0.42				0.07(COSM5426344),0.07(C		rs352951	0.0027	YES	9.621E-1 (pos.13413667	acute_myeloid_leukaemia				A=0, G=40	A=0.0, G=1.0	0.00001	644.286	40	A=0/0, G=10/3
chr14:65234585	G/A	G	A	SNV	SPTB	SPTB:intronic:NM_001024858.2	1	50		intronic	NM_00102	c.6023-8C	p.?	0.11					CAMSAP CH d	rs56181906	0.1064	YES	7.729E-3 (pos.65234585)		Spectrin, be	ER to Golgi	Anemia:Anemia, Hemolytic:An Benign/Likely	G=25, A=25	G=0.5, A=0.5	0.00001	288.698	50	G=18/7, A=16/
chr5:94749723	A/A	G	A	SNV	FAM81B	FAM81B:exonic:NM_152548.2	1	100	4	synonymous	NM_15254	c.366G>A	p.(=)	0.1						rs7726891	0.2892	YES	1.255E-1 (pos.94749723)			nucleus,protein binding		G=0, A=28	G=0.0, A=1.0	0.00001	408.718	28	G=0/0, A=13/1
chr5:94772540	C/T	C	T	SNV	FAM81B	FAM81B:exonic:NM_152548.2	1	97.01	7	missense	NM_15254	c.823C>T	p.Pro275Ser	1.33	1	74	0	0.01(COSM4591005)		rs6878669			1.0;0.0 (pos.94772540)	squamous_cell_carcinoma		nucleus,protein binding		C=2, T=65	C=0.0299, T=0.9701	0.00001	959.956	67	C=0/2, T=34/3
chr14:66028505	C/C	T	C	SNV	FUT8	FUT8:intronic:NM_178156.2	1	100		intronic	NM_17815	c.203+21T	p.?	0.19					Variant SH3 c	rs3825639	0.3189	YES	8.979E-1 (pos.66028505)		Fucosyltran	GDP-L-fucose metabolic process,Golgi apparatus,Golgi c		T=0, C=130	T=0.0, C=1.0	0.00001	2135.6	130	T=0/0, C=60/7
chr14:68038697	G/G	C	G	SNV	PLEKHH1	PLEKHH1:intronic:NM_020715.2	1	100		intronic	NM_02071	c.1585+78	p.?	-0.01					FERM central	rs2236234		YES				cytoskeleton		C=0, G=35	C=0.0, G=1.0	0.00001	543.903	35	C=0/0, G=17/1
chr8:124206324	A/A	G	A	SNV	FAM83A	FAM83A:exonic:NM_032899.6	1	100	4	missense	NM_03289	c.709G>A	p.Ala237Thr	1.32	1	58	0.048	0.16(COSM37	Protein of unl	rs7813708	0.2083	YES	2.385E-1;0.0 (pos.12420	acute_myeloid_leukaemia		cell proliferation,cytoplasm,epidermal growth factor rec		G=0, A=36	G=0.0, A=1.0	0.00001	563.742	36	G=0/0, A=18/1
chr20:37555116	C/C	G	C	SNV	FAM83D	FAM83D:exonic:NM_030919.2	1	100	1	missense	NM_03091	c.31G>C	p.Val11Leu	0.28	1	32	0	0.18(COSM44	PLD-like dom	rs3752290	0.2973	YES	4.101E-1 (pos.37555116)	adenocarcinoma,neoplasn		cell division,cell migration,cell proliferation,cytoplasm,		G=0, C=44	G=0.0, C=1.0	0.00001	726.551	44	G=0/0, C=30/1
chr14:68232767	C/C	T	C	SNV	ZFYVE26	ZFYVE26:intronic:NM_015346.3	1	100		intronic	NM_01534	c.6011+17	p.?	-1.91					FYVE zinc fing	rs8006137		YES			Zinc finger F	centrosom	Amyotrophic Lateral Sclerosis;Central Nervo	T=0, C=32	T=0.0, C=1.0	0.00001	485.136	32	T=0/0, C=12/2
chr14:68232797	C/G	C	G	SNV	ZFYVE26	ZFYVE26:intronic:NM_015346.3	1	50		intronic	NM_01534	c.6011+14	p.?	-0.41					FYVE zinc fing	rs17782871		YES			Zinc finger F	centrosom	Amyotrophic Lateral Sclerosis;Central Nervo	C=17, G=17	C=0.5, G=0.5	0.00001	172.008	34	C=5/12, G=7/1
chr14:69695792	A/A	G	A	SNV	EXD2	EXD2:intronic:NM_001193361.1	1	100		intronic	NM_00119	c.590+3G>	p.?	0.28					3'-5' exonucle	rs3742899	0.3884	YES	9.347E-1 (pos.69695792)		Exonucleas	3'-5' exonuclease activity,DNA double-strand break proc		G=0, A=48	G=0.0, A=1.0	0.00001	810.392	48	G=0/0, A=21/2
chr14:69992873	G/G	A	G	SNV	PLEKHD1	PLEKHD1:intronic:NM_001161498.1	1	100		intronic	NM_00116	c.923+34A	p.?	-0.1					PH domain	rs8004574	0.0446	YES	9.932E-1 (pos.69992873)					A=0, G=34	A=0.0, G=1.0	0.00001	527.968	34	A=0/0, G=14/2
chr14:69994473	G/G	C	G	SNV	PLEKHD1	PLEKHD1:intronic:NM_001161498.1	1	100		intronic	NM_00116	c.1202-27	p.?	-0.5					PH domain	rs1885400			1.0;0.0 (pos.69994473)					C=0, G=30	C=0.0, G=1.0	0.00001	446.639	30	C=0/0, G=14/1
chr20:37570725	T/T	C	T	SNV	FAM83D	FAM83D:exonic:NM_030919.2	1	100	2	synonymous	NM_03091	c.607C>T	p.(=)	-0.82				0.17(COSM41	PLD-like dom	rs3752293	0.3216	YES	4.333E-1 (pos.37570725)	acute_myeloid_leukaemia		cell division,cell migration,cell proliferation,cytoplasm,		C=0, T=48	C=0.0, T=1.0	0.00001	810.281	48	C=0/0, T=34/1
chr19:49116555	G/G	A	G	SNV	FAM83E	FAM83E:exonic:NM_017708.3	1	100	1	synonymous	NM_01770	c.75T>C	p.(=)	1.5				0.02(COSM43	Protein of unl	rs368654	0.2759	YES	9.977E-1 (pos.49116555)	adenocarcinoma,neoplasn		protein kinase binding		A=0, G=32	A=0.0, G=1.0	0.00001	485.072	32	A=0/0, G=14/1
chr12:8374770	A/A	G	A	SNV	FAM90A1	FAM90A1:exonic:NM_018088.3	1	100	7	missense	NM_01808	c.1043C>T	p.Thr348Ile	-0.68	0.81	89	0		Zinc knuckle	rs9668474	2.00E-04		1.0 (pos.8374770)		Family with	protein binding		G=0, A=37	G=0.0, A=1.0	0.00001	583.706	37	G=0/0, A=15/2
chr14:72944943	G/G	T	G	SNV	RGS6	RGS6:intronic:NM_001204424.1	1	100		intronic	NM_00120	c.793-33T	p.?	1.74					Domain foun	rs2239277	0.3288	YES	6.809E-1 (pos.72944943)		Regulator o	G-protein	Mental Disorders:Schizophrenia:Schizophren	T=0, G=22	T=0.0, G=1.0	0.00001	298.567	22	T=0/0, G=10/1
chr15:31229420	T/C	T	C	SNV	FAN1	FAN1:exonic:NM_014967.4	1	45.45	14	synonymous	NM_01496	c.3015T>C	p.(=)	-0.82					VRR-NUC dor	rs2955795	0.4403	YES	8.515E-1 (pos.31229420)		FANCD2/FA	5'-3' exonu	Female Urogenital Diseases;Fe Benign	T=12, C=10	T=0.5455, C=0.4545	0.00001	81.7558	22	T=2/10, C=7/3
chr14:73198506	C/C	T	C	SNV	DPF3	DPF3:intronic:NM_012074.4	1	100		intronic	NM_01207	c.429+9A>	p.?	-0.42					N-terminal d	rs6574094	0.045	YES	9.997E-1 (pos.73198506)		D4, zinc anc	biological	Nervous System Diseases;Neuromuscular Di	T=0, C=27	T=0.0, C=1.0	0.00001	389.988	27	T=0/0, C=12/1
chr15:89838236	A/A	G	A	SNV	FANCI	FANCI:exonic:NM_001113378.1	1	100	24	synonymous	NM_00111	c.2547G>A	p.(=)	1.47					FANCI helical	rs7183618	0.0535	YES	1.0 (pos.89838236)		FANCI gene	DNA bindi	Anemia:Anemia, Aplastic:Aner Benign	G=0, A=46	G=0.0, A=1.0	0.00001	768.129	46	G=0/0, A=30/1
chr15:89836228	G/C	G	C	SNV	FANCI	FANCI:exonic:NM_001113378.1	1	47.62	22	missense	NM_00111	c.2225G>C	p.Cys742Ser	4.93	0.74	112	0.007	0.96(COSM11	FANCI helical	rs2283432	0.3895	YES	3.258E-1:0.0:0.0 (pos.89	acute_myeloic	FANCI gene	DNA bindi	Anemia:Anemia, Aplastic:Aner Benign	G=44, C=40	G=0.5238, C=0.4762	0.00001	418.342	84	G=33/11, C=2
chr2:242312572	G/A	G	A	SNV	FARP2	FARP2:exonic:NM_014808.3	1	51.85	2	missense	NM_01480	c.50G>A	p.Arg17His	7.65	0	29	1	0.92(COSM37	FERM C-termi	rs61739702	0.0252	YES	2.314E-4 (pos.24231257	adenocarcinoi	FERM, ARHG	Rac guany	Autistic Disorder;Child Development Disorde	G=13, A=14	G=0.4815, A=0.5185	0.00001	132.665	27	G=5/8, A=10/4
chr14:73406668	C/T	C	T	SNV	DCAF4	DCAF4:intronic:NM_001163509.1	1	45.45		intronic	NM_00116	c.159+58C	p.?	-0.34					WD domain,	rs887936		YES			DDB1- and (	Cul4-RING E3 ubiquitin ligase complex,RNA binding,RNA		C=12, T=10	C=0.5455, T=0.4545	0.00001	81.7611	22	C=1/11, T=4/6
chr5:7866967	C/C	G	C	SNV	FASTKD3	FASTKD3:exonic:NM_024091.3	1	100	2	synonymous	NM_02409	c.1230C>G	p.(=)	-0.12					FAST kinase-l	rs162030	0.1755	YES	7.023E-1;1.156E-4 (pos.7866967)		Fast kinase	RNA binding,cellular respiration,mitochondrion,nucleus		G=0, C=30	G=0.0, C=1.0	0.00001	446.638	30	G=0/0, C=25/5
chr14:73733410	G/G	A	G	SNV	PAPLN	PAPLN:intronic:NM_173462.3	1	100		intronic	NM_17346	c.3311-21	p.?	0.07					ADAM-TS Spa	rs909216	0.1728	YES	7.073E-1 (pos.73733410)		Papilin, pro	extracellular region,metalloendopeptidase activity,neg		A=0, G=92	A=0.0, G=1.0	0.00001	1534.54	92	A=0/0, G=40/5
chr4:187519206	G/G	C	G	SNV	FAT1	FAT1:exonic:NM_005245.3	1	100	23	missense	NM_00524	c.12177G>	p.Lys4059Asn	0.3	1	94	0		Cadherin dor	rs1280097	0.0064		1.0 (pos.187519206)		FAT tumor s	actin filan	Adenocarcinoma;Astrocytoma;Carcinoma;Ca	C=0, G=60	C=0.0, G=1.0	0.00001	900.128	60	C=0/0, G=26/3
chr4:187534363	A/A	G	A	SNV	FAT1	FAT1:exonic:NM_005245.3	1	100	13	synonymous	NM_00524	c.9363C>T	p.(=)	-0.7					Cadherin dor	rs2249916	0.4204	YES	6.866E-1 (pos.187534363)		FAT tumor s	actin filan	Adenocarcinoma;Astrocytoma;Carcinoma;Ca	G=0, A=27	G=0.0, A=1.0	0.00001	389.983	27	G=0/0, A=15/1
chr4:187534375	G/G	A	G	SNV	FAT1	FAT1:exonic:NM_005245.3	1	100	13	synonymous	NM_00524	c.9351T>C	p.(=)	-0.12				0.11(COSM40	Cadherin dor	rs2249917	0.4481	YES	2.862E-1 (pos.18753437	acute_myeloic	FAT tumor s	actin filan	Adenocarcinoma;Astrocytoma;Carcinoma;Ca	A=0, G=27	A=0.0, G=1.0	0.00001	389.988	27	A=0/0, G=15/1
chr4:187557893	C/C	T	C	SNV	FAT1	FAT1:exonic:NM_005245.3	1	100	5	missense	NM_00524	c.3818A>G	p.His1273Arg	5.94	1	29	0		Cadherin dor	rs328418	0.4478	YES	6.868E-1 (pos.187557893)		FAT tumor s	actin filan	Adenocarcinoma;Astrocytoma;Carcinoma;Ca	T=0, C=100	T=0.0, C=1.0	0.00001	1702.2	100	T=0/0, C=41/5
chr14:74428286	G/G	A	G	SNV	COQ6	COQ6:intronic:NM_182476.2	1	100		intronic	NM_18247	c.1210+13	p.?	-0.03					FAD binding c	rs7141392	0.1301	YES	9.398E-1;0.0 (pos.74428286)		Coq6, S. cer	FAD bindir	Congenital, Hereditary, and Ne Benign	A=0, G=21	A=0.0, G=1.0	0.00001	281.235	21	A=0/0, G=11/1
chr4:187628398	G/G	C	G	SNV	FAT1	FAT1:exonic:NM_005245.3	1	100	2	missense	NM_00524	c.2584G>C	p.Val862Leu	1.61	1	32	0	0.14(COSM40	Cadherin dor	rs1877731	0.2043	YES	4.98E-1:0.0 (pos.187628	acute_myeloic	FAT tumor s	actin filan	Adenocarcinoma;Astrocytoma;Carcinoma;Ca	C=0, G=27	C=0.0, G=1.0	0.00001	389.988	27	C=0/0, G=11/1
chr4:187629140	C/C	G	C	SNV	FAT1	FAT1:exonic:NM_005245.3	1	100	2	missense	NM_00524	c.1842C>G	p.Phe614Leu	1.45	1	22	0	0.48(COSM40	Cadherin dor	rs367863	0.2047	YES	4.986E-1 (pos.18762914	acute_myeloic	FAT tumor s	actin filan	Adenocarcinoma;Astrocytoma;Carcinoma;Ca	G=0, C=79	G=0.0, C=1.0	0.00001	1269.12	79	G=0/0, C=46/3
chr4:187630590	G/A	G	A	SNV	FAT1	FAT1:exonic:NM_005245.3	1	58.33	2	missense	NM_00524	c.392C>T	p.Ala131Val	6.18	0.03	64	0.904	0.94(COSM40	Cadherin dor	rs3733415	0.1388	YES	2.676E-1 (pos.18763059	acute_myeloic	FAT tumor s	actin filan	Adenocarcinoma;Astrocytoma;Carcinoma;Ca	G=15, A=21	G=0.4167, A=0.5833	0.00001	234.328	36	G=7/8, A=8/13
chr14:74564468	G/T	G	T	SNV	LIN52	LIN52:intronic:NM_001024674.2	1	50		intronic	NM_00102	c.145-29G	p.?	-0.94					Retinal tissu	rs2098032	0.1877	YES	2.464E-1 (pos.74564468)			DRM complex,cell cycle,nucleoplasm,regulation of cell c		G=13, T=13	G=0.5, T=0.5	0.00001	119.163	26	G=5/8, T=9/4
chr14:74665543	A/A	G	A	SNV	LIN52	LIN52:intronic:NM_001024674.2	1	100		intronic	NM_00102	c.296-82G	p.?	-0.35					Retinal tissu	rs10145171		YES				DRM complex,cell cycle,nucleoplasm,regulation of cell c		G=0, A=24	G=0.0, A=1.0	0.00001	334.792	24	G=0/0, A=18/6
chr14:74976986	G/G	A	G	SNV	LTBP2	LTBP2:intronic:NM_000428.2	1	100		intronic	NM_00042	c.3029-70	p.?	-0.83					Calcium-bind	rs7160756		YES			Latent tran	calcium io	Abnormalities, Multiple;Bone Diseases;Bone	A=0, G=48	A=0.0, G=1.0	0.00001	810.435	48	A=0/0, G=42/6
chr14:75200697	A/A	G	A	SNV	FCF1	FCF1:intronic:NM_015962.4	1	100		intronic	NM_01596	c.454-82G	p.?	-0.76					Fcf1	rs3825708		YES				RNA binding,endonucleolytic cleavage in 5'-ETS of tricistr		G=0, A=80	G=0.0, A=1.0	0.00001	1289.37	80	G=0/0, A=54/2
chr5:150886725	A/A	G	A	SNV	FAT2	FAT2:exonic:NM_001447.2	1	100	22	synonymous	NM_00144	c.12507C>	p.(=)	-0.9					Cadherin dor	rs7723266	0.1929	YES	9.772E-1 (pos.150886725)		Fat tumor s	calcium io	Carcinoma;Carcinoma, Squamous Cell;Diges	G=0, A=61	G=0.0, A=1.0	0.00001	921.843	61	G=0/0, A=36/2
chr5:150891733	G/G	A	G	SNV	FAT2	FAT2:exonic:NM_001447.2	1	100	20	synonymous	NM_00144	c.11898T>	p.(=)	-3.16					Cadherin dor	rs2304028	0.2815	YES	9.716E-1 (pos.150891733)		Fat tumor s	calcium io	Carcinoma;Carcinoma, Squamous Cell;Diges	A=0, G=36	A=0.0, G=1.0	0.00001	563.731	36	A=0/0, G=21/1
chr5:150925144	G/G	A	G	SNV	FAT2	FAT2:exonic:NM_001447.2	1	100	9	synonymous	NM_00144	c.5544T>C	p.(=)	0.12					Cadherin dor	rs3797875,rs386693	0.1835	YES	7.342E-1 (pos.150925144)		Fat tumor s	calcium io	Carcinoma;Carcinoma, Squamous Cell;Diges	A=0, G=21	A=0.0, G=1.0	0.00001	281.235	21	A=0/0, G=7/14
chr5:150942919	G/G	A	G	SNV	FAT2	FAT2:exonic:NM_001447.2	1	100	2	missense	NM_00144	c.3541T>C	p.Tyr1181His	2.75	0.17	83	0		Cadherin dor	rs6872614	0.0053		1.0 (pos.150942919)		Fat tumor s	calcium io	Carcinoma;Carcinoma, Squamous Cell;Diges	A=0, G=31	A=0.0, G=1.0	0.00001	465.819	31	A=0/0, G=16/1
chr14:75505008	C/C	G	C	SNV	MLH3	MLH3:intronic:NM_001040108.1	1	100		intronic	NM_00104	c.3643+35	p.?	1.18					DNA mismatc	rs3742780	0.486	YES	8.28E-1 (pos.75505008)		Mismatch r	ATP bindin	Colonic Diseases;Colorectal Neoplasms;Colo	G=0, C=59	G=0.0, C=1.0	0.00001	885.362	59	G=0/0, C=26/3
chr14:75505016	G/G	A	G	SNV	MLH3	MLH3:intronic:NM_001040108.1	1	100		intronic	NM_00104	c.3643+27	p.?	-1.66					DNA mismatc	rs175075	0		1.0 (pos.75505016)		Mismatch r	ATP bindin	Colonic Diseases;Colorectal Neoplasms;Colo	A=0, G=60	A=0.0, G=1.0	0.00001	903.531	60	A=0/0, G=26/3
chr5:150945483	T/T	C	T	SNV	FAT2	FAT2:exonic:NM_001447.2	1	100	1	missense	NM_00144	c.3010G>A	p.Gly1004Ser	0.36	0.25	56	0.001	0.1(COSM376	Cadherin dor	rs3734055	0.4772	YES	3.452E-1 (pos.15094548	adenocarcinoi	Fat tumor s	calcium io	Carcinoma;Carcinoma, Squamous Cell;Diges	C=0, T=38	C=0.0, T=1.0	0.00001	603.786	38	C=0/0, T=23/1
chr5:150946132	A/A	G	A	SNV	FAT2	FAT2:exonic:NM_001447.2	1	100	1	synonymous	NM_00144	c.2361C>T	p.(=)	1.41				0.74(COSM40	Cadherin dor	rs3734059	0.4771	YES	3.457E-1 (pos.15094613	acute_myeloic	Fat tumor s	calcium io	Carcinoma;Carcinoma, Squamous Cell;Diges	G=0, A=64	G=0.0, A=1.0	0.00001	977.633	64	G=0/0, A=45/1
chr14:75601737	G/G	A	G	SNV	TMED10	TMED10:intronic:NM_006827.5	1	100		intronic	NM_00682	c.539-28T	p.?	0.14					emp24/gp25	rs12435391	0.4851	YES	8.287E-1;4.634E-4;0.0 (pos.75601737)		Transmemt	COPI coating of Golgi vesicle,COPI-coated vesicle,COPI-cc		A=0, G=41	A=0.0, G=1.0	0.00001	664.65	41	A=0/0, G=12/2
chr5:150946436	G/G	A	G	SNV	FAT2	FAT2:exonic:NM_001447.2	1	100	1	missense	NM_00144	c.2057T>C	p.Phe686Ser	4.41	1	155	0	0.04(COSM37	Cadherin dor	rs9324700	0.4764	YES	3.46E-1 (pos.150946436)	acute_myeloic	Fat tumor s	calcium io	Carcinoma;Carcinoma, Squamous Cell;Diges	A=0, G=54	A=0.0, G=1.0	0.00001	798.148	54	A=0/0, G=18/3
chr5:150946964	TA/TA	T	TA	INDEL	FAT2	FAT2:exonic:NM_001447.2	1	100	1	frameshiftInsertion	NM_00144	c.1528_15	p.Tyr510fs	1.75					Cadherin domain,EGF-like domain,Human growth factor-like EGF,Laminin G domain						Fat tumor s	calcium io	Carcinoma;Carcinoma, Squamous Cell;Diges	T=0, TA=31	T=0.0, TA=1.0	0.00001	419.485	31	T=0/0, TA=16/
chr5:150901261	T/T	C	T	SNV	FAT2,MIR6499	FAT2:exonic:NM_001447.2, MIR6499:downstream:NR_1067	1	100	18	missense	NM_00144	c.10893G>	p.Met3631Ile	0.36	0.69	10	0.002		Cadherin dor	rs6650971	0.2841	YES	9.722E-1 (pos.150901261)		Fat tumor s	calcium io	Carcinoma;Carcinoma, Squamous Cell;Diges	C=0, T=22	C=0.0, T=1.0	0.00001	298.892	22	C=0/0, T=12/1
chr5:150901300	A/A	G	A	SNV	FAT2,MIR6499	FAT2:exonic:NM_001447.2, MIR6499:downstream:NR_1067	1	100	18	synonymous	NM_00144	c.10854C>	p.(=)	-0.69					Cadherin dor	rs3734046	0.2841	YES	9.718E-1 (pos.150901300)		Fat tumor s	calcium io	Carcinoma;Carcinoma, Squamous Cell;Diges	G=0, A=21	G=0.0, A=1.0	0.00001	281.235	21	G=0/0, A=11/1
chr5:150901111	A/G	A	G	SNV	FAT2,MIR6499	FAT2:exonic:NM_001447.2, MIR6499:downstream:NR_1067	1	96.67	18	synonymous	NM_00144	c.11043T>	p.(=)	-1.79					Cadherin dor	rs3822699	0.2824	YES	9.718E-1 (pos.150901111)		Fat tumor s	calcium io	Carcinoma;Carcinoma, Squamous Cell;Diges	A=1, G=29	A=0.0333, G=0.9667	0.00001	416.515	30	A=0/1, G=18/1
chr14:76072028	C/C	T	C	SNV	FLVCR2	FLVCR2:intronic:NM_017791.2	1	100		intronic	NM_01779	c.670-163	p.?	0.06					Major Faciliti	rs4903337		YES			Feline leuke	heme binc	Congenital Abnormalities;Congenital, Heredi	T=0, C=120	T=0.0, C=1.0	0.00001	1933.62	120	T=0/0, C=82/3
chr11:92088177	T/T	C	T	SNV	FAT3	FAT3:exonic:NM_001008781.2	1	100	1	synonymous	NM_00100	c.2899C>T	p.(=)	-0.49				0.04(COSM13	Cadherin dor	rs7479732	0.2154	YES	1.656E-1 (pos.92088177)	adenocarcinoi	FAT tumor s	calcium io	Carcinoma;Carcinoma, Squamous Cell;Diges	C=0, T=108	C=0.0, T=1.0	0.00001	1700.02	108	C=0/0, T=46/6
chr11:92532677	C/C	T	C	SNV	FAT3	FAT3:exonic:NM_001008781.2	1	100	9	synonymous	NM_00100	c.6498T>C	p.(=)	-1.78				0.06(COSM37	Cadherin dor	rs12362722	0.1585	YES	7.477E-1 (pos.92532677)	squamous_cel	FAT tumor s	calcium io	Carcinoma;Carcinoma, Squamous Cell;Diges	T=0, C=23	T=0.0, C=1.0	0.00001	316.716	23	T=0/0, C=13/1
chr11:92534442	G/G	A	G	SNV	FAT3	FAT3:exonic:NM_001008781.2	1	100	9	missense	NM_00100	c.8263A>G	p.Ile2755Val	2.02	1	29	0		Cadherin dor	rs3847531	0.1451	YES	9.416E-1 (pos.92534442)		FAT tumor s	calcium io	Carcinoma;Carcinoma, Squamous Cell;Diges	A=0, G=67	A=0.0, G=1.0	0.00001	1034.43	67	A=0/0, G=41/2
chr4:126329789	C/C	T	C	SNV	FAT4	FAT4:exonic:NM_024582.4	1	100	4	synonymous	NM_02458	c.5760T>C	p.(=)	-0.77				0.15(COSM11	Cadherin dor	rs958415	9.00E-04		9.999E-1 (pos.12632978	adenocarcinoi	FAT tumor s	Notch sign	Abnormalities, Multiple;Adenc Benign	T=0, C=284	T=0.0, C=1.0	0.00001	4769.6	284	T=0/0, C=111/
chr4:126369872	C/C	G	C	SNV	FAT4	FAT4:exonic:NM_024582.4	1	100	9	synonymous	NM_02458	c.7701G>C	p.(=)	0.13					Cadherin dor	rs988863	0		1.0;0.0 (pos.126369872)		FAT tumor s	Notch sign	Abnormalities, Multiple;Adenc Benign	G=0, C=52	G=0.0, C=1.0	0.00001	767.014	52	G=0/0, C=33/1
chr4:126373789	A/A	G	A	SNV	FAT4	FAT4:exonic:NM_024582.4	1	100	9	missense	NM_02458	c.11618G>	p.Ser3873Asn	7.21	1	46	0		Cadherin dor	rs12650153	0		1.0 (pos.126373789)		FAT tumor s	Notch sign	Abnormalities, Multiple;Adenc Benign	G=0, A=38	G=0.0, A=1.0	0.00001	603.771	38	G=0/0, A=14/2
chr14:77934758	A/G	A	G	SNV	AHSA1	AHSA1:intronic:NM_012111.2	1	50		intronic	NM_01211	c.793-42A	p.?	-2.28					Activator of H	rs4903584	0.4778	YES	9.288E-1 (pos.77934758)		Activator of	ATPase activator activity,cadherin binding,chaperone bi		A=30, G=30	A=0.5, G=0.5	0.00001	293.739	60	A=15/15, G=1
chr6:99771540	C/C	T	C	SNV	FAXC	FAXC:exonic:NM_032511.3	1	100	4	synonymous	NM_03251	c.603A>G	p.(=)	-1.75					Glutathione S	rs221527	0.1564	YES	9.994E-1 (pos.99771540)			integral component of membrane		T=0, C=44	T=0.0, C=1.0	0.00001	726.564	44	T=0/0, C=21/2
chr22:45923827	G/G	A	G	SNV	FBLN1	FBLN1:exonic:NM_006486.2	1	100	4	missense	NM_00648	c.422A>G	p.Gln141Arg	5.51	1	43	0		Calcium-bind	rs136730	0.0016		1.0 (pos.45923827)		Fibulin 1	basement	Congenital Abnormalities;Congenital, Heredi	A=0, G=34	A=0.0, G=1.0	0.00001	524.176	34	A=0/0, G=23/1
chr14:78325675	C/T	C	T	SNV	ADCK1	ADCK1:intronic:NM_020421.3	1	53.13		intronic	NM_02042	c.423+53C	p.?	-0.23					ABC1 family	rs28498552		YES				ATP binding,extracellular region,protein phosphorylatio		C=15, T=17	C=0.4688, T=0.5313	0.00001	173.972	32	C=14/1, T=5/1
chr14:81297385	A/A	G	A	SNV	CEP128	CEP128:intronic:NM_152446.3	1	100		intronic	NM_15244	c.1209+10	p.?	-0.17						rs7156572		YES	7.864E-1 (pos.81297385)			centriolar subdistal appendage,centriole,protein localiz		G=0, A=31	G=0.0, A=1.0	0.00001	465.814	31	G=0/0, A=13/1
chr15:48807637	T/T	C	T	SNV	FBN1	FBN1:exonic:NM_000138.4	1	100	12	missense	NM_00013	c.1415G>A	p.Cys472Tyr	5.96	1	194	0	0.12(COSM45	Calcium-bind	rs113936729,rs4775765		YES	1.0 (pos.48807637)	squamous_cel	Fibrillin-1	basement	ACMG Recommendations...:Ab Benign	C=0, T=24	C=0.0, T=1.0	0.00001	334.784	24	C=0/0, T=9/15
chr14:81574959	G/G	A	G	SNV	TSHR	TSHR:intronic:NM_000369.2	1	100		intronic	NM_00036	c.692+163	p.?	0.45					7 transmemb	rs3783942	0.3434	YES	3.397E-1 (pos.81574959)		Thyroid-stir	B cell diffe	Adenoma;Adrenal (Thyrotropin Alfa	A=0, G=104	A=0.0, G=1.0	0.00001	1626.74	104	A=0/0, G=51/5
chr14:81575005	A/A	C	A	SNV	TSHR	TSHR:intronic:NM_000369.2	1	100		intronic	NM_00036	c.692+209	p.?	-0.99					7 transmemb	rs3783941	0.3434	YES	3.403E-1;0.0 (pos.81575005)		Thyroid-stir	B cell diffe	Adenoma;Adrenal (Thyrotropi not provided	C=0, A=100	C=0.0, A=1.0	0.00001	1702.26	100	C=0/0, A=45/5
chr19:8145921	T/T	C	T	SNV	FBN3	FBN3:exonic:NM_032447.4	1	100	59	synonymous	NM_03244	c.7419G>A	p.(=)	-1.62					Calcium-bind	rs2303168	0.4613	YES	3.722E-1;0.0 (pos.8145921)			anatomical structure morphog	Calcium Citrate,Calcium	C=0, T=29	C=0.0, T=1.0	0.00001	427.603	29	C=0/0, T=17/1
chr19:8152725	G/G	A	G	SNV	FBN3	FBN3:exonic:NM_032447.4	1	100	53	synonymous	NM_03244	c.6603T>C	p.(=)	0.25				0.03(COSM44	Calcium-bind	rs12460643	0.2657	YES	5.064E-1 (pos.8152725)	acute_myeloid_leukaemia		anatomical structure morphog	Calcium Citrate,Calcium	A=0, G=49	A=0.0, G=1.0	0.00001	831.602	49	A=0/0, G=39/1
chr19:8176569	A/A	G	A	SNV	FBN3	FBN3:exonic:NM_032447.4	1	100	32	synonymous	NM_03244	c.4047C>T	p.(=)	1.98					Calcium-bind	rs35306870	0.2416	YES	4.433E-1 (pos.8176569)			anatomical structure morphog	Calcium Citrate,Calcium	G=0, A=24	G=0.0, A=1.0	0.00001	334.726	24	G=0/0, A=5/19
chr14:88406404	C/C	T	C	SNV	GALC	GALC:intronic:NM_000153.3	1	100		intronic	NM_00015	c.1835-79	p.?	-1.29					Glycosyl hydr	rs417276		YES			Galactosylc	extracellu	Brain Diseases:Brain Diseases, Metabolic:Br	T=0, C=46	T=0.0, C=1.0	0.00001	768.21	46	T=0/0, C=22/2
chr19:8136991	T/C	T	C	SNV	FBN3	FBN3:exonic:NM_032447.4	1	55.38	63	missense	NM_03244	c.8029A>G	p.Lys2677Glu	7.8	0	56	0.991		Calcium-bind	rs148428757	0		0.0 (pos.8136991)			anatomical structure morphog	Calcium Citrate,Calcium	T=29, C=36	T=0.4462, C=0.5538	0.00001	377.619	65	T=14/15, C=18
chr14:88407917	G/A	G	A	SNV	GALC	GALC:intronic:NM_000153.3	1	57.58		intronic	NM_00015	c.1671-15	p.?	-1.99					Glycosyl hydr	rs12432149,rs78051	0.4822	YES	0.0 (pos.88407915),7.554E-1 (pos.88407		Galactosylc	extracellu	Brain Diseases:Brain Diseases, Metabolic:Br	G=14, A=19	G=0.4242, A=0.5758	0.00001	204.394	33	G=6/8, A=6/13
chr14:88459795	C/T	C	T	SNV	GALC	GALC:upstream:NM_000153.3	1	56						-0.42				0.03(COSM4597249)		rs73312836		YES	7.971E-2 (pos.88459795)		Galactosylc		Brain Diseases:Brain Diseases, Metabolic:Br	C=11, T=14	C=0.44, T=0.56	0.00001	134.35	25	C=6/5, T=3/11
chr19:8175770	T/A	T	A	SNV	FBN3	FBN3:exonic:NM_032447.4	1	52.27	34	missense	NM_03244	c.4292A>T	p.Asn1431Ile	2.89	0	149	0.026		Calcium-bind	rs17160194	3.00E-04		0.0 (pos.8175770)			anatomical structure morphog	Calcium Citrate,Calcium	T=21, A=23	T=0.4773, A=0.5227	0.00001	260.185	44	T=6/15, A=17/
chr7:5540769	C/C	A	C	SNV	FBXL18	FBXL18:exonic:NM_024963.5	1	100	1	synonymous	NM_02496	c.1113T>G	p.(=)	-1.93				0.01(COSM37	Protein of unl	rs6953442	0.0958	YES	9.222E-1 (pos.5540769)	adenocarcinoma,neoplasm		F-box and leucine-rich repeat protein 18		A=0, C=46	A=0.0, C=1.0	0.00001	744.039	46	A=0/0, C=25/2
chr14:88792816	G/G	T	G	SNV	KCNK10	[illegible]	1	100		intronic	[illegible]	[illegible]	[illegible]	0.21						rs3742690,rs746789	0.0263	YES	0.0:0.0 (pos.88792809),7.431E-1 (pos.88		Potassium channel, su		Nervous System Diseases	T=0, G=52	T=0.0, G=1.0	0.00001	767.015	52	T=0/0, G=29/2
chr9:37537555	GA/GA	G	GA	INDEL	FBXO10	FBXO10:exonic:NM_012166.2	1	100	3	frameshiftInsertion	NM_01216	c.970_971	p.Ser324fs	2.62					F-box domain,F-box-like,Periplasmic copper-binding protein (NosD),Right handed beta helix region						F-box only	apoptotic process,cytoplasm,cytosol,post-translational		G=0, GA=41	G=0.0, GA=1.0	0.00001	403.747	41	G=0/0, GA=20/
chr14:88895669	T/T	G	T	SNV	SPATA7	SPATA7:intronic:NM_018418.4	1	100		intronic	NM_01841	c.913-24G	p.?	-0.23					Spermatogen	rs888669		YES	1.0 (pos.88895669)		Spermatog	axoneme,	Genetic Diseases, Inborn	G=0, T=81	G=0.0, T=1.0	0.00001	1309.46	81	G=0/0, T=[illegible]
chr14:89192854	A/T	A	T	SNV	EML5	EML5:intronic:NM_183387.2	1	57.14		intronic	NM_18338	c.1187+31	p.?	0.03					Anaphase-pr	rs186666841	0.0103	YES	2.234E-4 (pos.89192854)			biological_process,catalytic activity,cytoplasm,extracel		A=33, T=44	A=0.4286, T=0.5714	0.00001	490.74	77	A=20/13, T=22

chr14:90456188	T/T	G	T	SNV	TDP1	TDP1:intronic:NM 001008744.1	1	100		intronic	NM 00100	c.1366+79	p.?	-0.34					Tyrosyl-DNA	rs2401863,rs386779856		YES			Tyrosyl-DN/	3'-tyrosyl-l Brain Diseases;Central Nervous System Dise	G=0, T=24	G=0.0, T=1.0	0.00001	334.768	24	G=0/0, T=15/9
chr14:90723059	T/C	T	C	SNV	PSMC1	PSMC1:intronic:NM 002802.2	1	52.38		intronic	NM 00280	c.3+115T>	p.?	0.37					AAA domain (	rs2277514		YES			Proteasom	ATP binding,ATPase activity,Fc-epsilon receptor signalin	T=10, C=11	T=0.4762, C=0.5238	0.00001	95.3003	21	T=1/9, C=3/8
chr14:90729984	C/T	C	T	SNV	PSMC1	PSMC1:intronic:NM 002802.2	1	50		intronic	NM 00280	c.280-22C	p.?	-0.71					AAA domain (	rs751611393,rs8015	0.4052	YES	0.0 (pos.90729983),4.756E-1 (pos.90729		Proteasom	ATP binding,ATPase activity,Fc-epsilon receptor signalin	C=26, T=26	C=0.5, T=0.5	0.00001	232.175	52	C=15/11, T=25
chr14:91948643	A/A	G	A	SNV	PPP4R3A	PPP4R3A:intronic:NM 001284280.1	1	100		intronic	NM 00128	c.298-106	p.?	-0.65					Component c	rs6575195		YES			Protein pho	cytoplasm,microtubule organizing center,nucleoplasm	G=0, A=26	G=0.0, A=1.0	0.00001	371.416	26	G=0/0, A=14/1
chr1:11710561	G/G	T	G	SNV	FBXO2	FBXO2:exonic:NM 012168.5	1	100	2	missense	NM 01216	c.353A>C	p.Lys118Thr	2.09	0.01	78	0.998	0.3(COSM375	F-box associa	rs9614	0.1923	YES	5.018E-1 (pos.11710561)	adenocarcinoi	F-box only p	SCF ubiquitin ligase complex,SCF-dependent proteasom	T=0, G=21	T=0.0, G=1.0	0.00001	281.081	21	T=0/0, G=3/18
chr14:92185731	G/A	G	A	SNV	CATSPERB	CATSPERB:intronic:NM 024764.3	1	95.65		intronic	NM 02476	c.370+31C	p.?	-0.14					Cation chann	rs17783516	0.321	YES	4.637E-1 (pos.92185731)		Cation char	CatSper complex,cilium,multicellular organism develop	G=1, A=22	G=0.0435, A=0.9565	0.00001	288.971	23	G=1/0, A=11/1
chr14:92265160	A/A	G	A	SNV	TC2N	TC2N:intronic:NM 152332.5	1	100		intronic	NM 15233	c.637+173	p.?	-0.1					C2 domain	rs10873413		YES				calcium ion binding,calcium ion-regulated exocytosis of	G=0, A=35	G=0.0, A=1.0	0.00001	543.899	35	G=0/0, A=27/8
chr17:6690164	A/G	A	G	SNV	FBXO39	FBXO39:exonic:NM 153230.2	1	51.85	3	missense	NM 15323	c.1089A>G	p.Ile363Met	-0.13	0.09	10	0.001	0.13(COSM37	F-box domair	rs7213731	0.1984	YES	8.091E-4 (pos.6690164)	adenocarcinoi	F-box prote	SCF ubiquitin ligase complex,SCF-dependent proteasom	A=13, G=14	A=0.4815, G=0.5185	0.00001	132.662	27	A=11/2, G=9/5
chr17:18653070	G/A	G	A	SNV	FBXW10	FBXW10:exonic:NM 001267585.1	1	95.83	3	missense	NM 00126	c.706G>A	p.Glu236Lys	0.49	0	56	0.111	0.1(COSM400	WD domain,	rs9895749	0.402	YES	2.639E-1 (pos.18653070)	acute_myeloid	F-box and W	cytosol,post-translational protein modification,protein	G=1, A=23	G=0.0417, A=0.9583	0.00001	298.177	24	G=1/0, A=7/16
chr14:92547425	C/G	C	G	SNV	ATXN3	ATXN3:intronic:NM 004993.5	1	47.62		intronic	NM 00499	c.776-20G	p.?	-1.13					Josephin,Ubi	rs761553	0.2429	YES	4.476E-1 (pos.92547425)		Ataxin-3 (jo	ATPase bir Ataxia;Brain Diseases;Central Nervous Syste	C=11, G=10	C=0.5238, G=0.4762	0.00001	82.2897	21	C=1/10, G=6/4
chr9:139835740	A/T	A	T	SNV	FBXW5	FBXW5:exonic:NM 018998.3	1	45.45	8	missense	NM 01899	c.1420T>A	p.Phe474Ile	8.38	0	21	0.726		F-box domair	rs143554180,rs7769	9.00E-04		0.0 (pos.139835740),0.0:0.0 (pos.13983		F-box and W	Cul4-RING E3 ubiquitin ligase complex,SCF ubiquitin ligas	A=36, T=30	A=0.5455, T=0.4545	0.00001	285.786	66	A=13/23, T=21
chr12:117465857	C/C	T	C	SNV	FBXW8	FBXW8:exonic:NM 153348.2	1	100	11	synonymous	NM 15334	c.1677T>C	p.(=)	-2.33					F-box domair	rs4529958	0.1583	YES	9.957E-1 (pos.117465857)		F-box and W	3M compl Female Urogenital Diseases;Female Urogeni	T=0, C=26	T=0.0, C=1.0	0.00001	371.42	26	T=0/0, C=13/1
chr14:92588219	A/A	G	A	SNV	CPSF2,NDUFB1	CPSF2:upstream:NM 017437.2, NDUFB1:upstream:NM 004	1	100		intronic	NM 00454	c.-98C>T, c	p.?, p.?	0.74						rs7148709		YES			NADH-ubiqu	NADH dehydrogenase (ubiquin NADH	G=0, A=33	G=0.0, A=1.0	0.00001	504.598	33	G=0/0, A=7/26
chr19:40408821	C/T	C	T	SNV	FCGBP	FCGBP:exonic:NM 003890.2	1	50	8	missense	NM 00389	c.4018G>A	p.Val1340Met	-0.88	0.03	21	0.824	0.06(COSM11	C8 domain,Ig	rs11083543	0.3461	YES	2.467E-1 (pos.40408821)	adenocarcinoi	Fc fragment	extracellu Digestive System Diseases;Digestive System	C=16, T=16	C=0.5, T=0.5	0.00001	158.383	32	C=6/10, T=8/8
chr1:161561151	A/A	G	A	SNV	FCGR2C	FCGR2C:exonic nc:NR 047648.1	1	100	4	exonic nc	NR 047648.1			-0.66						rs74341264	0.0629	YES	4.215E-1 (pos.161561151)		Fc fragment	of IgG, low Autoimmune Disea Abciximab,Adalimumab,	G=0, A=47	G=0.0, A=1.0	0.00001	789.311	47	G=0/0, A=25/2
chr19:17892591	G/G	A	G	SNV	FCHO1	FCHO1:exonic:NM 001161357.1	1	100	23	synonymous	NM 00116	c.1899A>G	p.(=)	-10.14				0(COSM4131	Fes/CIP4, anc	rs2042298	0.0555	YES	8.12E-1 (pos.17892591)	neoplasm	FCH domair	AP-2 adaptor complex binding,clathrin coat assembly,cl	A=0, G=47	A=0.0, G=1.0	0.00001	789.288	47	A=0/0, G=33/1
chr14:93170901	C/C	A	C	SNV	LGMN	LGMN:intronic:NM 005606.6	1	100		intronic	NM 00560	c.1259+84	p.?	-0.38					Peptidase C1	rs2402189		YES			Legumain ((	antigen processing and presentation of exogenous pepti	A=0, C=29	A=0.0, C=1.0	0.00001	431.406	29	A=0/0, C=14/1
chr1:157768000	G/C	G	C	SNV	FCRL1	FCRL1:exonic:NM 052938.4	1	52.27	8	synonymous	NM 05293	c.1065C>G	p.(=)	-0.78				0.06(COSM48	Immunoglob	rs3811024	0.244	YES	1.689E-1 (pos.15776800(	squamous_cel	Fc receptor	integral component of membrane,plasma membrane	G=21, C=23	G=0.4773, C=0.5227	0.00001	260.157	44	G=6/15, C=11/
chr1:157670290	G/G	C	G	SNV	FCRL3	FCRL3:utr 5:NM 052939.3	1	100	2	utr 5	NM 05293	c.-11G>C	p.?	-0.45					Immunoglob	rs945635	0.4384	YES	4.024E-1;0.0 (pos.157670290)			integral cc Autoimmune Diseases;Endocrine System Dis	C=0, G=35	C=0.0, G=1.0	0.00001	542.96	35	C=0/0, G=19/1
chr14:94419888	G/G	A	G	SNV	ASB2	ASB2:intronic:NM 001202429.1	1	100		intronic	NM 00120	c.479-35T	p.?	-0.82					Ankyrin repe	rs4243701	0.1097	YES	9.994E-1 (pos.94419888)		Ankyrin rep	Cul5-RING ubiquitin ligase complex,cytosol,intracellular	A=0, G=41	A=0.0, G=1.0	0.00001	664.669	41	A=0/0, G=10/3
chr14:94568776	C/T	C	T	SNV	IFI27L1	IFI27L1:intronic:NM 145249.2	1	45.83		intronic	NM 14524	c.224-47C	p.?	-1.69					Interferon-in	rs2295217	0.2305	YES	5.069E-1 (pos.94568776)			integral component of membrane	C=13, T=11	C=0.5417, T=0.4583	0.00001	93.4409	24	C=13/0, T=7/4
chr14:94700138	G/G	C	G	SNV	PPP4R4	PPP4R4:intronic:NM 058237.1	1	100		intronic	NM 05823	c.623+42C	p.?	-0.32						rs4905172	0.0371	YES	1.0 (pos.94700138)		Protein pho	cytoplasm,cytosol,negative regulation of phosphoprotei	C=0, G=97	C=0.0, G=1.0	0.00001	1638.99	97	C=0/0, G=59/3
chr1:159785477	G/A	G	A	SNV	FCRL6	FCRL6:utr 3:NM 001004310.2	1	50	10	utr 3	NM 00100	c.1331G>A	p.?	-0.52					Immunoglob	rs61823163	0.1373	YES	1.555E-2;0.0 (pos.159785477)			MHC class II protein binding,external side of plasma mer	G=13, A=13	G=0.5, A=0.5	0.00001	119.499	26	G=8/5, A=2/11
chr1:159785518	G/C	G	C	SNV	FCRL6	FCRL6:utr 3:NM 001004310.2	1	50	10	utr 3	NM 00100	c.1372G>C	p.?	-0.3					Immunoglob	rs61823164		YES				MHC class II protein binding,external side of plasma mer	G=13, C=13	G=0.5, C=0.5	0.00001	120.956	26	G=8/5, C=2/11
chr14:94930984	G/T	G	T	SNV	SERPINA9	SERPINA9:intronic:NM 175739.3	1	57.14		intronic	NM 17573	c.1104+60	p.?	-1.88					Serpin (serin	rs13379439		YES			Serpin pept	cytoplasm,extracellular space,membrane,negative regu	G=24, T=32	G=0.4286, T=0.5714	0.00001	331.729	56	G=9/15, T=7/2
chr1:161683136	A/A	G	A	SNV	FCRLA	FCRLA:exonic:NM 001184866.1	1	100	6	missense	NM 00118	c.1115G>A	p.Arg372Gln	-0.77	0.34	43	0.021	0.03(COSM14	Immunoglob	rs11746	0.4819	YES	2.6E-1 (pos.161683136)	adenocarcinoma,basal_ce		cell differentiation,cytoplasm	G=0, A=40	G=0.0, A=1.0	0.00001	632.423	40	G=0/0, A=11/2
chr8:11683653	C/C	T	C	SNV	FDFT1	FDFT1:exonic:NM 004462.4	1	100	5	synonymous	NM 00446	c.631T>C	p.(=)	3.65					Squalene/ph	rs904011	2.00E-04		1.0 (pos.11683653)		Farnesyl-di	biosynthe Adenocarcinoma;C TAK-475	T=0, C=37	T=0.0, C=1.0	0.00001	583.707	37	T=0/0, C=22/1
chr8:11689119	G/C	G	C	SNV	FDFT1	FDFT1:exonic:NM 004462.4	1	50	7	synonymous	NM 00446	c.972G>C	p.(=)	-0.27				0.42(COSM37	Squalene/ph	rs9205	0.2094	YES	7.41E-1 (pos.11689119)	adenocarcinoi	Farnesyl-di	biosynthe Adenocarcinoma;C TAK-475	G=35, C=35	G=0.5, C=0.5	0.00001	355.233	70	G=28/7, C=14/
chr8:125072426	C/C	T	C	SNV	FER1L6,FER1L6-AS2	FER1L6-AS2:exonic nc:NR 103547.1, FER1L6:exonic:NM 00	1	100	23	synonymous	NM 00103	c.2880T>C	p.(=)	-1.19					C2 domain,Fe	rs4483140	0.1387	YES	7.012E-1 (pos.125072426)			integral component of membrane	T=0, C=35	T=0.0, C=1.0	0.00001	543.903	35	T=0/0, C=18/1
chr8:125076589	G/G	T	G	SNV	FER1L6,FER1L6-AS2	FER1L6-AS2:intronic nc:NR 103547.1, FER1L6:exonic:NM 0	1	100	26	missense	NM 00103	c.3330T>G	p.Asp1110Glu	-0.31	1	45	0	0.02(COSM54	C2 domain,Fe	rs7012186	0.1372	YES	6.825E-1 (pos.12507658(	squamous_cell_carcinoma		integral component of membrane	T=0, G=49	T=0.0, G=1.0	0.00001	831.515	49	T=0/0, G=37/1
chr14:96813703	T/T	A	T	SNV	ATG2B	ATG2B:intronic:NM 018036.6	1	100		intronic	NM 01803	c.163-25T	p.?	-1.01					Autophagy-re	rs4905483	0.01	YES	5.597E-1;0.0 (pos.96813703)		Autophagy	autophag Bone Marrow Diseases;Hematologic Disease	A=0, T=20	A=0.0, T=1.0	0.00001	263.785	20	A=0/0, T=3/17
chr14:53325090	T/T	C	T	SNV	FERMT2	FERMT2:utr 3:NM 001134999.1	1	100	16	utr 3	NM 00113	c.2069G>A	p.?	0.97					FERM central	rs6572868,rs768341	0.0926	YES	0.0 (pos.53325084),7.951E-1:0.0 (pos.53		Fermitin far	I band,Wn Alzheimer Disease;Brain Diseases;Central Ne	C=0, T=21	C=0.0, T=1.0	0.00001	281.232	21	C=0/0, T=13/8
chr14:99876505	G/G	A	G	SNV	SETD3	SETD3:intronic:NM 032233.2	1	100		intronic	NM 03223	c.849+278	p.?	-0.32					Rubisco LSM	rs1047351	0.4335	YES	5.581E-1 (pos.99876505)		SET domain	RNA polymerase II activating transcription factor bindin	A=0, G=50	A=0.0, G=1.0	0.00001	852.746	50	A=0/0, G=22/2
chr14:99929801	A/A	G	A	SNV	SETD3	SETD3:intronic:NM 032233.2	1	100		intronic	NM 03223	c.196+22C	p.?	0.56					Rubisco LSM	rs2273386	0.4137	YES	5.988E-1 (pos.99929801)		SET domain	RNA polymerase II activating transcription factor bindin	G=0, A=23	G=0.0, A=1.0	0.00001	316.747	23	G=0/0, A=19/4
chr15:100214958	T/T	A	T	SNV	MEF2A	MEF2A:intronic:NM 005587.3	1	100		intronic	NM 00558	c.616+141	p.?	0.21					Holliday junc	rs2033547		YES			MADS box t	DNA bindi Arterial Occlusive Diseases;Arteriosclerosis;	A=0, T=73	A=0.0, T=1.0	0.00001	1113.76	73	A=0/0, T=23/5
chr14:53345378	A/A	G	A	SNV	FERMT2	FERMT2:exonic:NM 001134999.1	1	100	7	synonymous	NM 00113	c.885C>T	p.(=)	1.39					FERM central	rs2357947	0.0936	YES	7.448E-1 (pos.53345378)		Fermitin far	I band,Wn Alzheimer Disease;Brain Diseases;Central Ne	G=0, A=29	G=0.0, A=1.0	0.00001	427.603	29	G=0/0, A=17/1
chr15:91430436	C/C	T	C	SNV	FES	FES:exonic:NM 002005.3	1	100	5	synonymous	NM 00200	c.504T>C	p.(=)	-0.16					Protein kinas	rs2071383	0.0319	YES	9.181E-1 (pos.91430436)		Oncogene F	ATP bindin Cardiovascular Diseases;Hypertension;Ment	T=0, C=20	T=0.0, C=1.0	0.00001	263.786	20	T=0/0, C=7/13
chr9:95738781	A/A	C	A	SNV	FGD3	FGD3:exonic:NM 033086.2	1	100	3	synonymous	NM 03308	c.243C>A	p.(=)	-1.44				0(COSM4007	FYVE zinc fing	rs3802381	0.3994	YES	5.539E-1 (pos.95738781)	carcinoma,her	FYVE, RhoGE	G-protein coupled receptor signaling pathway,Golgi app	C=0, A=36	C=0.0, A=1.0	0.00001	563.711	36	C=0/0, A=24/1
chr9:95780465	G/G	A	G	SNV	FGD3	FGD3:exonic:NM 033086.2	1	100	11	synonymous	NM 03308	c.1323A>G	p.(=)	-1.38					FYVE zinc fing	rs7048975	0.0035		1.0 (pos.95780465)		FYVE, RhoGE	G-protein coupled receptor signaling pathway,Golgi app	A=0, G=27	A=0.0, G=1.0	0.00001	388.402	27	A=0/0, G=12/1
chr15:100516084	AT/AT	A	AT	INDEL	ADAMTS17	ADAMTS17:intronic:NM 139057.3	1	100		intronic	NM 13905	c.3127+16	p.?	1.94					ADAM-TS Spa	rs11463271,rs201623305,rs397		YES			A disintegri	metalloen Bone Diseases;Bone Diseases, Development	A=0, AT=31	A=0.0, AT=1.0	0.00001	435.601	31	A=0/0, AT=24/
chr15:101110356	T/C	T	C	SNV	LINS1	LINS1:intronic:NM 001040616.2	1	45.83		intronic	NM 00104	c.1395-34	p.?	-0.06					Lines C-termi	rs2411838	0.4634	YES	2.074E-1 (pos.101110356)		Lines, Dros	cognition	T=26, C=22	T=0.5417, C=0.4583	0.00001	240.98	48	T=12/14, C=9/
chr9:95782667	T/T	C	T	SNV	FGD3	FGD3:exonic:NM 033086.2	1	100	13	synonymous	NM 03308	c.1455C>T	p.(=)	-0.2				0.21(COSM37	FYVE zinc fing	rs11790517	0.2782	YES	2.822E-1 (pos.95782667)	adenocarcinoi	FYVE, RhoGE	G-protein coupled receptor signaling pathway,Golgi app	C=0, T=26	C=0.0, T=1.0	0.00001	371.412	26	C=0/0, T=15/1
chr15:101115448	A/A	C	A	SNV	LINS1	LINS1:intronic:NM 001040616.2	1	100		intronic	NM 00104	c.490-115	p.?	0.17					Lines C-termi	rs4965678		YES			Lines, Dros	cognition	C=0, A=37	C=0.0, A=1.0	0.00001	583.707	37	C=0/0, A=8/29
chr15:101566118	T/T	C	T	SNV	LRRK1	LRRK1:intronic:NM 024652.5	1	100		intronic	NM 02465	c.2233-52	p.?	-0.05					ADP-ribosyla	rs4427776	0.2416	YES			Leucine-ricl	ATP binding,GTP binding,bone resorption,cytoplasm,cyt	C=0, T=35	C=0.0, T=1.0	0.00001	543.769	35	C=0/0, T=18/1
chr19:49259529	G/G	A	G	SNV	FGF21,FUT1	FUT1:upstream:NM 000148.3, FGF21:exonic:NM 019113.3	1	100	2	synonymous	NM 00014	c.-4991T>C	p.?, p.(=)	-0.08					Fibroblast gr	rs838133	0.4399	YES	9.935E-1 (pos.49259529)		Fibroblast g	Golgi appa Autoimmune Diseases;Diabetes Mellitus;Dia	A=0, G=34	A=0.0, G=1.0	0.00001	523.943	34	A=0/0, G=20/1
chr15:101595629	G/G	A	G	SNV	LRRK1	LRRK1:intronic:NM 024652.5	1	100		intronic	NM 02465	c.4332+20	p.?	-0.63					ADP-ribosyla	rs2924844		YES			Leucine-ricl	ATP binding,GTP binding,bone resorption,cytoplasm,cyt	A=0, G=66	A=0.0, G=1.0	0.00001	1014	66	A=0/0, G=29/3
chr12:4553332	G/G	A	G	SNV	FGF6	FGF6:exonic:NM 020996.2	1	100	2	synonymous	NM 02099	c.417T>C	p.(=)	-0.09				0.36(COSM54	Fibroblast gr	rs2241280	0.3991	YES	4.197E-1 (pos.4553332)	acute_myeloid	Fibroblast g	1-phospha Amyotrophic Lateral Sclerosis;Central Nervo	A=0, G=137	A=0.0, G=1.0	0.00001	2279.04	137	A=0/0, G=97/4
chr15:101970308	A/A	G	A	SNV	PCSK6	PCSK6:intronic:NM 002570.4	1	100		intronic	NM 00257	c.656-40C	p.?	-0.31					Growth facto	rs2643354,rs796552	0.0863	YES	6.341E-1 (pos.101970308)		Proprotein	Golgi lumen,cell surface,cornification,determination of l	G=0, A=21	G=0.0, A=1.0	0.00001	281.206	21	G=0/0, A=10/1
chr12:4553383	G/G	A	G	SNV	FGF6	FGF6:exonic:NM 020996.2	1	100	2	synonymous	NM 02099	c.366T>C	p.(=)	-2.31				0.05(COSM37	Fibroblast gr	rs2241279	0.3992	YES	4.183E-1 (pos.4553383)	adenocarcinoi	Fibroblast g	1-phospha Amyotrophic Lateral Sclerosis;Central Nervo	A=0, G=135	A=0.0, G=1.0	0.00001	2238.23	135	A=0/0, G=93/4
chr10:103530255	CGGGC/CGGG	CGGGGC	CGGG,CGGGC	INDEL	FGF8	FGF8:exonic:NM 033163.3	1	100	6	frameshiftDeletion	NM 03316	c.565delC	p.Arg189fs	3.52, 2.35, -0.12, 9.42, 9.42, 0.06				0.92(COSM54	Fibroblast gr	rs377240055,rs7487	1.00E-04		0.0 (pos.103530257)	carcinoma	Fibroblast g	1-phospha 22q11 Deletion Syndrome;46, XY Disorders o	CGGGGC=0	CGGGGC=0.0, CGGG	0.00001	270.144	26	CGGGGC=0/0,
chr13:22275583	A/A	G	A	SNV	FGF9	FGF9:utr 3:NM 002010.2	1	100	3	utr 3	NM 00201	c.636G>A	p.?	0.02					Fibroblast gr	rs9509842	3.00E-04		9.999E-1 (pos.22275583)		Fibroblast g	1-phospha Adenocarcinoma;Bone Disease Benign	G=0, A=191	G=0.0, A=1.0	0.00001	3211.64	191	G=0/0, A=77/1
chr4:15937731	C/C	T	C	SNV	FGFBP1	FGFBP1:exonic:NM 005130.4	1	100	3	synonymous	NM 00513	c.525A>G	p.(=)	-0.36					FGF binding p	rs2072313	0.0358	YES	8.277E-1 (pos.15937731)		Fibroblast g	cell surface,cell-cell signaling,extracellular region,extra	T=0, C=21	T=0.0, C=1.0	0.00001	259.495	21	T=0/0, C=12/9
chr4:15964485	A/A	G	A	SNV	FGFBP2	FGFBP2:exonic:NM 031950.3	1	100	1	missense	NM 03195	c.268C>T	p.Pro90Ser	0.7		74		0.29(COSM44	FGF binding p	rs758329	0.462	YES	4.943E-1;0.0 (pos.15964	acute_myeloid_leukaemia		extracellular space,growth factor binding	G=0, A=21	G=0.0, A=1.0	0.00001	281.231	21	G=0/0, A=15/6
chr1:60228310	TTC/TTC	T	TTC	INDEL	FGGY	FGGY:utr 3:NM 001113411.1	2	100	17	utr 3	NM 00111	c.1782 17	p.?	-0.35					FGGY family c	rs10626387,rs53612	0.1632	YES			FGGY carbo	D-ribulokinase activity,carbohydrate metabolic process	T=0, TTC=6	T=0.0, TTC=1.0	0.00001	925.584	61	T=0/0, TTC=21,
chr1:15616139	A/A	G	A	SNV	FHAD1	FHAD1:exonic:NM 052929.1	1	100	4	missense	NM 05292	c.545G>A	p.Arg182His	-0.85	0.49	29	0	0.01(COSM37	FHA domain	rs486557		YES	3.795E-2 (pos.15616139)	adenocarcinoma,carcinoma,neoplasm,squamous_cell_carcinoma			G=0, A=31	G=0.0, A=1.0	0.00001	465.819	31	G=0/0, A=12/1
chr1:15723843	A/A	G	A	SNV	FHAD1	FHAD1:exonic:NM 052929.1	1	100	31	synonymous	NM 05292	c.4173G>A	p.(=)	-0.1				0.04(COSM39	FHA domain	rs2294490	0.462	YES	4.633E-1 (pos.15723843)	acute_myeloid_leukaemia			G=0, A=21	G=0.0, A=1.0	0.00001	275.324	21	G=0/0, A=16/5
chr15:22929906	G/G	A	G	SNV	CYFIP1	CYFIP1:intronic:NM 014608.5	1	100		intronic	NM 01460	c.569+11A	p.?	-2.31					Cytoplasmic	rs2305093,rs386781	0.2662	YES	8.734E-1 (pos.22929906)		Cytoplasmi	Fc-gamma receptor signaling pathway involved in phago	A=0, G=25	A=0.0, G=1.0	0.00001	353.019	25	A=0/0, G=17/8
chr15:22940866	T/T	C	T	SNV	CYFIP1	CYFIP1:intronic:NM 014608.5	1	100		intronic	NM 01460	c.1110+21	p.?	0.54					Cytoplasmic	rs45464395	0.2829	YES	5.247E-1 (pos.22940866)		Cytoplasmi	Fc-gamma receptor signaling pathway involved in phago	C=0, T=66	C=0.0, T=1.0	0.00001	1015.39	66	C=0/0, T=28/3
chr15:22945314	T/T	C	T	SNV	CYFIP1	CYFIP1:intronic:NM 014608.5	1	100		intronic	NM 01460	c.1233+15	p.?	0.13					Cytoplasmic	rs8029108		YES			Cytoplasmi	Fc-gamma receptor signaling pathway involved in phago	C=0, T=67	C=0.0, T=1.0	0.00001	1033.39	67	C=0/0, T=25/4
chr4:153897770	G/A	G	A	SNV	FHDC1	FHDC1:exonic:NM 033393.2	1	95.45	11	synonymous	NM 03339	c.3327G>A	p.(=)	-1.74				0.03(COSM41	Formin Homc	rs3811832	0.3047	YES	5.824E-1 (pos.15389777(	acute_myeloid_leukaemia,neoplasm			G=1, A=21	G=0.0455, A=0.9545	0.00001	271.51	22	G=0/1, A=10/1
chr15:25925195	T/C	T	C	SNV	ATP10A	ATP10A:intronic:NM 024490.3	1	51.85		intronic	NM 02449	c.3866+73	p.?	-2.2					Cation transp	rs3736186		YES			ATPase, cla	ATP bindin Autistic Disorder;Child Development Disorde	T=13, C=14	T=0.4815, C=0.5185	0.00001	132.665	27	T=7/6, C=11/3
chr15:25937004	T/T	C	T	SNV	ATP10A	ATP10A:intronic:NM 024490.3	1	100		intronic	NM 02449	c.3089-66	p.?	-1.46					Cation transp	rs2066705		YES			ATPase, cla	ATP bindin Autistic Disorder;Child Development Disorde	C=0, T=53	C=0.0, T=1.0	0.00001	782.029	53	C=0/0, T=22/3
chr15:25967085	C/C	T	C	SNV	ATP10A	ATP10A:intronic:NM 024490.3	1	100		intronic	NM 02449	c.1111-29	p.?	-2.02					Cation transp	rs1564828	1.00E-04		9.999E-1;0.0 (pos.25967085)		ATPase, cla	ATP bindin Autistic Disorder;Child Development Disorde	T=0, C=37	T=0.0, C=1.0	0.00001	583.705	37	T=0/0, C=6/31
chr15:26866265	GA/GA	G	GA	INDEL	GABRB3	GABRB3:intronic:NM 000814.5	1	100		intronic	NM 00081	c.461+196	p.?	1.14					Neurotransm	rs112294385		YES			Gamma-am	GABA-A re Abnormalities, Mul Acamprosate,Adinazolar	G=0, GA=1	G=0.0, GA=1.0	0.00001	2083.19	129	G=0/0, GA=79/
chr9:133779448	A/G	A	G	SNV	FIBCD1	FIBCD1:utr 3:NM 032843.4	1	57.58	7	utr 3	NM 03284	c.1389T>C	p.?	-1.73					Fibrinogen be	rs11792889,rs76942	0.2373	YES	0.0 (pos.133779435),2.17E-1 (pos.13377		Fibrinogene	chitin binding,integral component of membrane,membr	A=14, G=19	A=0.4242, G=0.5758	0.00001	204.389	33	A=4/10, G=14/
chr15:28211758	G/G	A	G	SNV	OCA2	OCA2:intronic:NM 000275.2	1	100		intronic	NM 00027	c.1636+78	p.?	-0.78					Citrate trans	rs4778218		YES			Pink-eye dil	L-tyrosine Albinism;Albinism, Oculocutaneous;Amino A	A=0, G=63	A=0.0, G=1.0	0.00001	958.901	63	A=0/0, G=40/2
chr2:71012604	G/A	G	A	SNV	FIGLA	FIGLA:exonic:NM 001004311.3	1	57.14	3	synonymous	NM 00100	c.552C>T	p.(=)	-1.24				0.07(COSM37	Helix-loop-he	rs7566541	0.4372	YES	9.106E-1 (pos.71012604)	adenocarcinoi	Factor in ge	RNA polym Adnexal Diseases;Endocrine Sy Benign	G=21, A=28	G=0.4286, A=0.5714	0.00001	330.833	49	G=8/13, A=11/
chr15:28228644	A/A	G	A	SNV	OCA2	OCA2:intronic:NM 000275.2	1	100		intronic	NM 00027	c.1365-15	p.?	0.04					Citrate trans	rs12910433	0.2999	YES	1.383E-1 (pos.28228644)		Pink-eye dil	L-tyrosine Albinism;Albinism, Oculocutan Benign	G=0, A=21	G=0.0, A=1.0	0.00001	281.228	21	G=0/0, A=4/17
chr15:28231793	C/C	T	C	SNV	OCA2	OCA2:intronic:NM 000275.2	1	100		intronic	NM 00027	c.1183-4A	p.?	-0.27					Citrate trans	rs10852218,rs74639	0.181	YES	0.0 (pos.28231779),9.991E-1 (pos.28231		Pink-eye dil	L-tyrosine Albinism;Albinism, Oculocutan Benign	T=0, C=27	T=0.0, C=1.0	0.00001	389.957	27	T=0/0, C=15/1
chr3:58112440	G/G	A	G	SNV	FLNB	FLNB:exonic:NM 001164317.1	1	100	24	synonymous	NM 00116	c.4173A>G	p.(=)	-0.26				0.35(COSM41	Calponin hon	rs2362903	0.3583	YES	9.99E-1 (pos.58112440)	ductal_carcinc	Filamin B	RNA bindir Abnormalities, Multiple;Bone I Benign/Likely	A=0, G=33	A=0.0, G=1.0	0.00001	504.594	33	A=0/0, G=11/2
chr15:28378122	G/G	T	G	SNV	HERC2	HERC2:intronic:NM 004667.5	1	100		intronic	NM 00466	c.12231-1	p.?	-1.62					Anaphase-pr	rs4778141		YES			HECT domai	SUMO binc Asthma;Asthma, Occupational;Bronchial Dis	T=0, G=29	T=0.0, G=1.0	0.00001	427.477	29	T=0/0, G=17/1
chr3:58118555	A/A	G	A	SNV	FLNB	FLNB:exonic:NM 001164317.1	1	100	27	missense	NM 00116	c.4504G>A	p.Val1502Met	2.95	0.01	21	0.588	0.93(COSM41	Calponin hon	rs12632456	0.2452	YES	9.736E-1 (pos.58118555)	ductal_carcinc	Filamin B	RNA bindir Abnormalities, Multiple;Bone I Benign/Likely	G=0, A=76	G=0.0, A=1.0	0.00001	1209.59	76	G=0/0, A=38/3
chr15:28419497	T/T	TA	T	INDEL	HERC2	HERC2:intronic:NM 004667.5	1	100		intronic	NM 00466	c.10057+4	p.?	-0.84, 0.11					Anaphase-pr	rs397783208,rs5848	5.00E-04		1.0 (pos.28419497)		HECT domai	SUMO binc Asthma;Asthma, Occupational;Bronchial Dis	TA=0, T=26	TA=0.0, T=1.0	0.00001	361.608	26	TA=0/0, T=13/
chr3:58154327	T/T	C	T	SNV	FLNB,FLNB-AS1	FLNB-AS1:intronic nc:NR 135534.1, FLNB:exonic:NM 0011	1	100	45	synonymous	NM 00116	c.7452C>T	p.(=)	-1.1				0.11(COSM50	Calponin hon	rs8640	0.252	YES	9.155E-1 (pos.58154327)	haemangiobla	Filamin B	RNA bindir Abnormalities, Multiple;Bone I Benign/Likely	C=0, T=55	C=0.0, T=1.0	0.00001	814.907	55	C=0/0, T=27/2
chr11:63883985	C/C	T	C	SNV	FLRT1,MACROD1	FLRT1:exonic:NM 013280.4, MACROD1:intronic:NM 01406	1	100	2	synonymous	NM 01406	c.517+347	p.?, p.(=)	-2					Leucine Rich	rs614397	0.14	YES	4.216E-1 (pos.63883985)		Fibronectin	cell adhesion,cell-cell junction,cellular response to DNA	T=0, C=24	T=0.0, C=1.0	0.00001	334.792	24	T=0/0, C=11/1
chr11:63884027	T/T	C	T	SNV	FLRT1,MACROD1	FLRT1:exonic:NM 013280.4, MACROD1:intronic:NM 01406	1	100	2	synonymous	NM 01406	c.517+346	p.?, p.(=)	-6.77				0.01(COSM37	Leucine Rich	rs614035	0.3166	YES	3.265E-1 (pos.63884027)	carcinoma,he	Fibronectin	cell adhesion,cell-cell junction,cellular response to DNA	C=0, T=38	C=0.0, T=1.0	0.00001	603.786	38	C=0/0, T=16/2
chr15:29428556	G/G	C	G	SNV	FAM189A1	FAM189A1:intronic:NM 015307.1	1	100		intronic	NM 01530	c.927+13G	p.?	-0.33					CD20-like fan	rs8023368		YES	3.229E-1;0.0 (pos.29428556)			integral component of membrane	C=0, G=26	C=0.0, G=1.0	0.00001	363.054	26	C=0/0, G=20/6
chr11:63885704	T/T	C	T	SNV	FLRT1,MACROD1	FLRT1:exonic:NM 013280.4, MACROD1:intronic:NM 01406	1	100	2	synonymous	NM 01406	c.517+330	p.?, p.(=)	-1.06				0.17(COSM37	Leucine Rich	rs677447	0.3172	YES	3.275E-1 (pos.63885704)	carcinoma,he	Fibronectin	cell adhesion,cell-cell junction,cellular response to DNA	C=0, T=24	C=0.0, T=1.0	0.00001	334.787	24	C=0/0, T=20/4
chr15:31203093	C/T	C	T	SNV	FAN1	FAN1:intronic:NM 014967.4	1	50		intronic	NM 01496	c.1577+75	p.?	-0.79					VRR-NUC dor	rs3794594	0.4177	YES	7.887E-1 (pos.31203093)		FANCD2/FA	5'-3' exonu Female Urogenital Diseases;Female Urogeni	C=17, T=17	C=0.5, T=0.5	0.00001	172.008	34	C=4/13, T=4/1
chr15:31218164	T/C	T	C	SNV	FAN1	FAN1:intronic:NM 014967.4	1	50		intronic	NM 01496	c.2488+22	p.?	-0.12					VRR-NUC dor	rs2959037,rs769105	0.41	YES	9.809E-1;0.0 (pos.31218164)		FANCD2/FA	5'-3' exonu Female Urogenital Diseases;Female Urogeni	T=11, C=11	T=0.5, C=0.5	0.00001	94.6067	22	T=6/5, C=3/8
chr14:86088056	C/C	T	C	SNV	FLRT2	FLRT2:exonic:NM 013231.5	1	100	2	synonymous	NM 01323	c.198T>C	p.(=)	2.4				0.66(COSM45	Fibronectin t	rs10141266			9.72E-1 (pos.86088056)	squamous_cel	Fibronectin	axon guidance,basement membrane organization,biolo	T=0, C=57	T=0.0, C=1.0	0.00001	849.613	57	T=0/0, C=29/2
chr15:31245880	T/T	C	T	SNV	MTMR10	MTMR10:intronic:NM 017762.2	1	100		intronic	NM 01776	c.1067-46	p.?	-0.2					Myotubularir	rs1404467,rs748105	0.2149	YES	1.718E-3 (pos.31245878),8.469E-1 (pos.31245880)			cytosol	C=0, T=23	C=0.0, T=1.0	0.00001	316.747	23	C=0/0, T=10/1
chr15:31341461	A/A	G	A	SNV	TRPM1	TRPM1:intronic:NM 001252020.1	1	100		intronic	NM 00125	c.1623+11	p.?	-1.54					Ion transport	rs10851487		YES			Transient re	G-protein Congenital, Hereditary, and Neonatal Diseas	G=0, A=73	G=0.0, A=1.0	0.00001	1149.87	73	G=0/0, A=44/2
chr15:31341516	T/T	C	T	SNV	TRPM1	TRPM1:intronic:NM 001252020.1	1	100		intronic	NM 00125	c.1623+62	p.?	-0.36					Ion transport	rs2288241		YES			Transient re	G-protein Congenital, Hereditary, and Neonatal Diseas	C=0, T=73	C=0.0, T=1.0	0.00001	1150.5	73	C=0/0, T=44/2
chr15:31359457	G/G	T	G	SNV	TRPM1	TRPM1:intronic:NM 001252020.1	1	100		intronic	NM 00125	c.545-67A	p.?	-0.48					Ion transport	rs35345394,rs3784599		YES			Transient re	G-protein Congenital, Hereditary, and Neonatal Diseas	T=0, G=28	T=0.0, G=1.0	0.00001	408.718	28	T=0/0, G=13/1
chr1:213068595	T/T	C	T	SNV	FLVCR1	FLVCR1:exonic:NM 014053.3	1	100	10	missense	NM 01405	c.1631C>T	p.Thr544Met	-0.36	0.02	81	0.863	0.02(COSM15	Major Facilit	rs3207090,rs886044	0.4751	YES	4.787E-1 (pos.21306859	carcinoma	Feline leuk	blood ves Ataxia;Congenital, Hereditary, Benign/Likely	C=0, T=89	C=0.0, T=1.0	0.00001	1472.36	89	C=0/0, T=46/4
chr15:31369223	T/T	C	T	SNV	TRPM1	TRPM1:intronic:NM 001252020.1	1	100		intronic	NM 00125	c.55-36G	p.?	-1.17					Ion transport	rs4779817		YES	9.817E-1 (pos.31369223)		Transient re	G-protein Congenital, Hereditary, and Neonatal Diseas	C=0, T=73	C=0.0, T=1.0	0.00001	1142.97	73	C=0/0, T=29/4
chr15:33842222	G/G	C	G	SNV	RYR3	RYR3:intronic:NM 001036.4	1	100		intronic	NM 00103	c.816-139	p.?	0.11					EF hand,EF-h	rs1865496		YES			Ryanodine	calcium ion binding,calcium io Calcium Citrate,Calcium	C=0, G=29	C=0.0, G=1.0	0.00001	427.498	29	C=0/0, G=19/1
chr15:33876543	T/T	C	T	SNV	RYR3	RYR3:intronic:NM 001036.4	1	100		intronic	NM 00103	c.1574-53	p.?	-5.09					EF hand,EF-h	rs624954	0.2388	YES			Ryanodine	calcium ion binding,calcium io Calcium Citrate,Calcium	C=0, T=82	C=0.0, T=1.0	0.00001	1329.65	82	C=0/0, T=64/1
chr16:2946433	C/T	C	T	SNV	FLYWCH2	FLYWCH2:utr 5:NM 001142500.1	1	45.45	3	utr 5	NM 00114	c.-18C>T	p.?	0.27					FLYWCH-type	rs2285824	0.0769	YES	2.324E-1 (pos.2946433)			RNA binding,biological_process,cellular_component	C=12, T=10	C=0.5455, T=0.4545	0.00001	81.7497	22	C=6/6, T=8/2
chr1:171168584	TG/TG	CA	TG	MNV	FMO2	FMO2:exonic:NM 001460.4	2	100	5	missense	NM 00146	c.584 585	p.Ser195Leu	5.98, -4.4		145		0.99(COSM14	Flavin-bindin	rs2020861,rs202086	0.3201	YES	5.607E-1:0.0 (pos.17116	acute_myeloid	Flavin-cont	N,N-dimet Endometriosis;Female Urogenital Diseases;F	CA=0, TG=6	CA=0.0, TG=1.0	0.00001	1072.78	69	CA=0/0, TG=23
chr15:34016419	T/C	T	C	SNV	RYR3	RYR3:intronic:NM 001036.4	1	46.43		intronic	NM 00103	c.6912+42	p.?	0.42					EF hand,EF-h	rs16957866	0.0239	YES	1.106E-1 (pos.34016419)		Ryanodine	calcium ion binding,calcium io Calcium Citrate,Calcium	T=15, C=13	T=0.5357, C=0.4643	0.00001	117.921	28	T=3/12, C=1/1
chr15:34064043	C/C	T	C	SNV	RYR3	RYR3:intronic:NM 001036.4	1	100		intronic	NM 00103	c.8817-78	p.?	-0.51					EF hand,EF-h	rs1429754					Ryanodine	calcium ion binding,calcium io Calcium Citrate,Calcium	T=0, C=30	T=0.0, C=1.0	0.00001	446.631	30	T=0/0, C=17/1
chr15:34537435	T/T	C	T	SNV	SLC12A6	SLC12A6:intronic:NM 133647.1	1	100		intronic	NM 13364	c.1943+51	p.?	-1.93					Amino acid p	rs4371122	6.00E-04	YES	1.0 (pos.34537435)		Solute carri	angiogene Agenesis of Corpus Potassium Chloride	C=0, T=66	C=0.0, T=1.0	0.00001	1015.4	66	C=0/0, T=25/4
chr2:216235089	T/T	C	T	SNV	FN1	FN1:exonic:NM 212482.2	1	100	41	missense	NM 21248	c.6781G>A	p.Val2261Ile	3.8	0.9	29	0.002		Fibronectin t	rs1250209,rs773415	0		0.0 (pos.216235088),1.0 (pos.21623508		Fibronectin	acute-pha Abortion, Spontane Ocriplasmin	C=0, T=52	C=0.0, T=1.0	0.00001	767.015	52	C=0/0, T=19/3
chr15:35196492	T/T	C	T	SNV	AQR	AQR:intronic:NM 014691.2	1	100		intronic	NM 01469	c.2001+45	p.?	-0.12					AAA domain,I	rs12898639	0.2695	YES	6.3E-1 (pos.35196492)		Aquarius, n	RNA binding,catalytic step 2 spliceosome,mRNA splicing	C=0, T=59	C=0.0, T=1.0	0.00001	884.762	59	C=0/0, T=44/1
chr2:216249587	T/T	C	T	SNV	FN1	FN1:exonic:NM 212482.2	1	100	29	synonymous	NM 21248	c.4725G>A	p.(=)	0.84					Fibronectin t	rs13652	0.1273	YES	9.986E-1 (pos.216249587)		Fibronectin	acute-pha Abortion, Spontane Ocriplasmin	C=0, T=29	C=0.0, T=1.0	0.00001	427.603	29	C=0/0, T=15/1
chr15:35674184	G/G	T	G	SNV	DPH6	DPH6:intronic:NM 080650.3	1	100		intronic	NM 08065	c.568-67A	p.?	0.15					Diphthamide	rs80019930		YES				ATP binding,cytosol,diphthine-ammonia ligase activity,n	T=0, G=51	T=0.0, G=1.0	0.00001	754.584	51	T=0/0, G=28/2
chr17:80708601	G/G	C	G	SNV	FN3K	FN3K:exonic:NM 022158.3	1	100	6	synonymous	NM 02215	c.900C>G	p.(=)	-0.93				0.09(COSM41	Fructosamine	rs1056534	0.3937	YES	6.327E-1 (pos.80708601)	adenocarcinoi	Fructosami	cellular_component,cytosol,epithelial cell differentiatic	C=0, G=22	C=0.0, G=1.0	0.00001	298.892	22	C=0/0, G=16/6
chr15:40282625	C/C	T	C	SNV	EIF2AK4	EIF2AK4:intronic:NM 001013703.3	1	100		intronic	NM 00101	c.2631+47	p.?	-0.15					Anticodon bir	rs4144472	0.0015	YES	9.999E-1;0.0 (pos.40282625)		Eukaryotic t	ATP bindin Amino Acid Metabolism, Inborn Errors;Cardio	T=0, C=60	T=0.0, C=1.0	0.00001	903.529	60	T=0/0, C=15/4
chr15:40300214	C/C	G	C	SNV	EIF2AK4	EIF2AK4:intronic:NM 001013703.3	1	100		intronic	NM 00101	c.3408-14	p.?	1.55					Anticodon bir	rs2279580	0.371	YES	3.035E-1 (pos.40300214)		Eukaryotic t	ATP bindin Amino Acid Metabolism, Inbor Benign	G=0, C=52	G=0.0, C=1.0	0.00001	767.011	52	G=0/0, C=29/2
chr15:40322552	T/T	G	T	SNV	EIF2AK4	EIF2AK4:intronic:NM 001013703.3	1	100		intronic	NM 00101	c.4562-8G	p.?	0.3				0.01(COSM63	Anticodon bir	rs2250402,rs746660	0.0773	YES	0.0 (pos.40322550),0.0;0	neoplasm	Eukaryotic t	ATP bindin Amino Acid Metabolism, Inbor Benign	G=0, T=21	G=0.0, T=1.0	0.00001	281.136	21	G=0/0, T=12/9
chr15:40565055	A/A	G	A	SNV	BUB1B-PAK6,PAK6	PAK6:intronic:NM 001276717.1, BUB1B-PAK6:intronic:NM	1	100		intronic	NM 00112	c.1357-12	p.?, p.?	-1					Kinase-like,P	rs2253603	0.257	YES	2.928E-1 (pos.40565055)		p21-activat	ATP bindin Genital Diseases, Male;Genital Neoplasms, I	G=0, A=24	G=0.0, A=1.0	0.00001	334.772	24	G=0/0, A=9/15
chr6:159652931	C/C	G	C	SNV	FNDC1	FNDC1:exonic:NM 032532.2	1	100	11	missense	NM 03253	c.1387G>C	p.Glu463Gln	-0.11	1	29	0	0(COSM4508	Fibronectin t	rs420137	0.104	YES	6.39E-1 (pos.159652931)	acute_myeloid	Fibronectin	extracellu Cardiovascular Diseases;Coronary Disease;H	G=0, C=39	G=0.0, C=1.0	0.00001	623.98	39	G=0/0, C=27/1
chr15:40583458	G/G	A	G	SNV	PLCB2	PLCB2:intronic:NM 004573.2	1	100		intronic	NM 00457	c.2870+8T	p.?	-1.25					C2 domain,PI	rs936209	0.0067	YES	9.257E-1 (pos.40583458)		Phospholip	G-protein coupled receptor signaling pathway,Wnt signa	A=0, G=22	A=0.0, G=1.0	0.00001	298.892	22	A=0/0, G=11/1
chr15:40595414	C/C	G	C	SNV	PLCB2	PLCB2:intronic:NM 004573.2	1	100		intronic	NM 00457	c.231+75C	p.?	-0.28					C2 domain,PI	rs1869902		YES			Phospholip	G-protein coupled receptor signaling pathway,Wnt signa	G=0, C=42	G=0.0, C=1.0	0.00001	685.221	42	G=0/0, C=15/2
chr15:40595565	C/T	C	T	SNV	PLCB2	PLCB2:intronic:NM 004573.2	1	50		intronic	NM 00457	c.163-8G>	p.?	-4.01				0.01(COSM44	C2 domain,PI	rs2305645	0.3302	YES	1.355E-1 (pos.40595565)	acute_myeloid	Phospholip	G-protein coupled receptor signaling pathway,Wnt signa	C=29, T=30	C=0.4915, T=0.5085	0.00001	304.345	60	C=12/17, T=2/
chr15:40703609	G/G	A	G	SNV	IVD	IVD:intronic:NM 002225.3	1	100		intronic	NM 00222	c.559+57A	p.?	0.1					Acyl-CoA deh	rs4924466		YES			Isovaleryl C	acyl-CoA d Amino Acid Metabo Coenzyme Benign	A=0, G=40	A=0.0, G=1.0	0.00001	644.286	40	A=0/0, G=24/1
chr6:159654487	T/T	G	T	SNV	FNDC1	FNDC1:exonic:NM 032532.2	1	100	11	synonymous	NM 03253	c.2943G>T	p.(=)	-1.28				0(COSM4407	Fibronectin t	rs404435	0.103	YES	6.488E-1 (pos.15965448	hepatocellula	Fibronectin	extracellu Cardiovascular Diseases;Coronary Disease;H	G=0, T=42	G=0.0, T=1.0	0.00001	685.18	42	G=0/0, T=22/2
chr6:159655084	G/G	C	G	SNV	FNDC1	FNDC1:exonic:NM 032532.2	1	100	11	missense	NM 03253	c.3540C>G	p.Asp1180Glu	-0.07	1	45	0	0.02(COSM41	Fibronectin t	rs420054,rs7764438	0.104	YES	6.143E-1;0.0 (pos.15965	neoplasm	Fibronectin	extracellu Cardiovascular Diseases;Coronary Disease;H	C=0, G=29	C=0.0, G=1.0	0.00001	427.602	29	C=0/0, G=13/1
chr6:159655326	C/C	T	C	SNV	FNDC1	FNDC1:exonic:NM 032532.2	1	100	11	missense	NM 03253	c.3782T>C	p.Leu1261Pro	-1.11	0.19	98	0	0.01(COSM37	Fibronectin t	rs3003174	0.1048	YES	6.61E-1 (pos.159655326)	acute_myeloid	Fibronectin	extracellu Cardiovascular Diseases;Coronary Disease;H	T=0, C=69	T=0.0, C=1.0	0.00001	1071.8	69	T=0/0, C=45/2
chr15:41146581	C/C	T	C	SNV	SPINT1	SPINT1:intronic:NM 181642.2	1	100		intronic	NM 18164	c.989-14T	p.?	-0.57					Kunitz/Bovin	rs3736287	0.2219	YES	6.964E-1 (pos.41146581)			branching involved in labyrinthine layer morphogenesis,	T=0, C=31	T=0.0, C=1.0	0.00001	461.085	31	T=0/0, C=23/8
chr15:41165739	C/C	A	C	SNV	RHOV	RHOV:intronic:NM 133639.3	1	100		intronic	NM 13363	c.268-40T	p.?	-0.56					Ras family,Ra	rs1814692	0.238	YES	8.739E-1 (pos.41165739)			GTP binding,GTPase activity,cytosol,endosome membra	A=0, C=25	A=0.0, C=1.0	0.00001	353.019	25	A=0/0, C=16/9
chr15:41276158	A/A	G	A	SNV	INO80	INO80:intronic:NM 017553.2	1	100		intronic	NM 01755	c.4078-39	p.?	0.38					DNA-binding	rs11855248	0.4549	YES	2.822E-1 (pos.41276158)		INO80 com	ATP binding,ATPase activity,DNA binding,DNA duplex unv	G=0, A=26	G=0.0, A=1.0	0.00001	376.993	26	G=0/0, A=12/1
chr15:41346092	C/C	T	C	SNV	INO80	INO80:intronic:NM 017553.2	1	100		intronic	NM 01755	c.2274+35	p.?	-0.97					DNA-binding	rs2899010	0.013	YES	9.996E-1;0.0 (pos.41346092)		INO80 com	ATP binding,ATPase activity,DNA binding,DNA duplex unv	T=0, C=38	T=0.0, C=1.0	0.00001	603.784	38	T=0/0, C=18/2
chr6:159655383	G/G	A	G	SNV	FNDC1	FNDC1:exonic:NM 032532.2	1	100	11	missense	NM 03253	c.3839A>G	p.Gln1280Arg	-0.28	1	43	0	0(COSM4003	Fibronectin t	rs2501176	0.1041	YES	6.537E-1 (pos.15965538	acute_myeloid	Fibronectin	extracellu Cardiovascular Diseases;Coronary Disease;H	A=0, G=62	A=0.0, G=1.0	0.00001	940.282	62	A=0/0, G=36/2
chr6:159655402	G/G	A	G	SNV	FNDC1	FNDC1:exonic:NM 032532.2	1	100	11	synonymous	NM 03253	c.3858A>G	p.(=)	-1.19				0.01(COSM40	Fibronectin t	rs2932988	0.1041	YES	6.502E-1 (pos.15965540	acute_myeloid	Fibronectin	extracellu Cardiovascular Diseases;Coronary Disease;H	A=0, G=56	A=0.0, G=1.0	0.00001	832.1	56	A=0/0, G=30/2
chr15:41483636	T/T	C	T	SNV	EXD1	EXD1:intronic:NM 152596.3	1	100		intronic	NM 15259	c.690+4G>	p.?	2.01					3'-5' exonucle	rs200607195,rs2412	0		1.0 (pos.41483636)			3'-5' exonuclease activity,P granule,PET complex,RNA bir	C=0, T=24	C=0.0, T=1.0	0.00001	334.792	24	C=0/0, T=15/9
chr15:41605653	C/C	G	C	SNV	OIP5	OIP5:intronic:NM 007280.1	1	100		intronic	NM 00728	c.513-101	p.?	0.48					Yippee zinc-b	rs8032846		YES				CENP-A coi Autoimmune Diseases;Female Urogenital Dis	G=0, C=62	G=0.0, C=1.0	0.00001	940.289	62	G=0/0, C=22/4
chr17:33454415	A/A	C	A	SNV	FNDC8	FNDC8:exonic:NM 017559.3	1	100	2	synonymous	NM 01755	c.564C>A	p.(=)	-5.37					Fibronectin t	rs2306508,rs755729	0.4241	YES	0.0 (pos.33454411),7.02E-1;0.0;0.0 (pos.33454415)			nucleus	C=0, A=39	C=0.0, A=1.0	0.00001	623.98	39	C=0/0, A=12/2
chr5:131008194	C/C	T	C	SNV	FNIP1	FNIP1:exonic:NM 133372.2	1	100	14	missense	NM 13337	c.1943A>G	p.Gln648Arg	-0.12	1	43	0	0.17(COSM3683670)		rs26008	0.062	YES	7.947E-1 (pos.13100819	acute_myeloid	Folliculin-in	ATPase inhibitor activity,TOR signaling,cellular response	T=0, C=71	T=0.0, C=1.0	0.00001	1111.48	71	T=0/0, C=56/1
chr14:75745551	A/A	T	A	SNV	FOS	FOS:utr 5:NM 005252.3	1	100	1	utr 5	NM 00525	c.-135T>A	p.?	0.41					Basic region	rs2239615		YES			FBJ murine	DNA bindi Adenocarcinoma;A Nadroparin,Pseudoephe	T=0, A=116	T=0.0, A=1.0	0.00001	1861.16	116	T=0/0, A=111/
chr15:41796901	T/T	G	T	SNV	LTK	LTK:intronic:NM 002344.5	1	100		intronic	NM 00234	c.2121-63	p.?	0.52					Glycine rich	rs170296		YES			Leukocyte t	ATP bindin Adnexal Diseases;Cysts;Endocrine System Di	G=0, T=26	G=0.0, T=1.0	0.00001	371.416	26	G=0/0, T=14/1
chr15:41805115	G/A	G	A	SNV	LTK	LTK:intronic:NM 002344.5	1	95.83		intronic	NM 00234	c.188-39C	p.?	1.54					Glycine rich	rs2253936	0.4506	YES	3.105E-1 (pos.41805115)		Leukocyte t	ATP bindin Adnexal Diseases;Cysts;Endocrine System Di	G=1, A=23	G=0.0417, A=0.9583	0.00001	306.64	24	G=1/0, A=19/4
chr14:75745624	CT/CT	C	CT	INDEL	FOS	FOS:utr 5:NM 005252.3	1	100	1	utr 5	NM 00525	c.-62C>CT	p.?	0.72					Basic region leucine zipper,bZIP Maf transcription factor,bZIP transcription factor						FBJ murine	DNA bindi Adenocarcinoma;A Nadroparin,Pseudoephe	C=0, CT=11	C=0.0, CT=1.0	0.00001	747.408	119	C=0/0, CT=66/
chr15:41816190	G/G	T	G	SNV	RPAP1	RPAP1:intronic:NM 015540.3	1	100		intronic	NM 01554	c.2235-20	p.?	0.43					RPAP1-like, C	rs2297378	0.4843	YES	3.4E-1 (pos.41816190)		RNA polyme	DNA binding,DNA-directed 5'-3' RNA polymerase activity,	T=0, G=43	T=0.0, G=1.0	0.00001	705.843	43	T=0/0, G=19/2
chr15:41817367	G/G	A	G	SNV	RPAP1	RPAP1:intronic:NM 015540.3	1	100		intronic	NM 01554	c.1918-21	p.?	0.02					RPAP1-like, C	rs2274858	0.4845	YES	3.569E-1 (pos.41817367)		RNA polyme	DNA binding,DNA-directed 5'-3' RNA polymerase activity,	A=0, G=25	A=0.0, G=1.0	0.00001	353.019	25	A=0/0, G=3/22

chr14:75745626	T/T	C	T	SNV	FOS	FOS:utr_5:NM_005252.3	1	100	1	utr_5	NM_00525	c.-60C>T	p.?	0.15					Basic region l	rs7101		YES			FBJ murine	DNA bindi	Adenocarcinoma;A	Nadroparin,Pseudoephe			C=0, T=119	C=0.0, T=1.0	0.00001	1805.17	119	C=0/0, T=66/5
chr15:41856543	G/G	A	G	SNV	TYRO3	TYRO3:intronic:NM_006293.3	1	100		intronic	NM_00629	c.667+75A	p.?	-1.42					Fibronectin t	rs11070339		YES			TYRO3 prote	ATP bindin	Cardiovascular Diseases:Embolism and Thro				A=0, G=22	A=0.0, G=1.0	0.00001	298.892	22	A=0/0, G=6/16
chr14:75746690	T/T	C	T	SNV	FOS	FOS:exonic:NM_005252.3	1	100	2	synonymous	NM_00525	c.252C>T	p.(=)	-1.75				0.19(COSM41	Basic region l	rs1046117	0.2723	YES	5.617E-1 (pos.75746690)	neoplasm	FBJ murine	DNA bindi	Adenocarcinoma;A	Nadroparin,Pseudoephe			C=0, T=21	C=0.0, T=1.0	0.00001	280.64	21	C=0/0, T=15/6
chr14:38060646	T/T	C	T	SNV	FOXA1	FOXA1:exonic:NM_004496.3	1	100	2	missense	NM_00449	c.1343G>A	p.Ser448Asn	2.07	0.32	46	0.001	0.88(COSM50	Forkhead N-t	rs33984772	0.0938	YES	6.943E-4 (pos.38060646)	haemangiobla	Forkhead b	DNA bindi	Breast Diseases;Breast Neoplasms;Digestive				C=0, T=28	C=0.0, T=1.0	0.00001	408.718	28	C=0/0, T=12/1
chr20:22562674	C/C	T	C	SNV	FOXA2	FOXA2:exonic:NM_021784.4	1	100	2	synonymous	NM_02178	c.1206A>G	p.(=)	0.15					Forkhead N-t	rs1212275,rs777271	0.1365	YES	8.126E-4 (pos.22562672),9.979E-1 (pos.2		Forkhead b	DNA bindi	Lung Diseases;Respiratory Tract Diseases				T=0, C=22	T=0.0, C=1.0	0.00001	298.892	22	T=0/0, C=9/13
chr9:116800	G/G	C	G	SNV	FOXD4	FOXD4:exonic:NM_207305.4	1	100	1	stoploss	NM_20730	c.1320G>C	p.Ter440Tyr	1.31				0.19(COSM30	Forkhead dor	rs79220013	0.2427	YES	2.938E-1 (pos.116800)	acute_myeloic	Forkhead b	DNA binding transcription factor activity,DNA binding, be					C=0, G=38	C=0.0, G=1.0	0.00001	603.77	38	C=0/0, G=20/1
chr15:42115426	G/G	A	G	SNV	MAPKBP1	MAPKBP1:intronic:NM_001128608.1	1	100		intronic	NM_00112	c.3616+6A	p.?	0.83					WD domain,	rs1197688	0.1522	YES	3.23E-1 (pos.42115426)		Mitogen-ac	RNA bindir	Nervous System Diseases:Neuromuscular Di				A=0, G=23	A=0.0, G=1.0	0.00001	316.746	23	A=0/0, G=12/1
chr15:42129158	G/A	G	A	SNV	JMJD7,JMJD7-PLA2G4B	JMJD7-PLA2G4B:intronic:NM_005090.3, JMJD7:intronic:NM	1	52.38		intronic	NM_00111	c.862+20G	p.?, p.?	-0.85					C2 domain,C	rs890505	0.3396	YES	1.712E-1 (pos.42129158)			IRE1-mediated unfolded protein response,arachidonic a					G=10, A=11	G=0.4762, A=0.5238	0.00001	95.3463	21	G=6/4, A=5/6
chr15:42129176	T/C	T	C	SNV	JMJD7,JMJD7-PLA2G4B	JMJD7-PLA2G4B:intronic:NM_005090.3, JMJD7:intronic:NM	1	50		intronic	NM_00111	c.862+38T	p.?, p.?	-0.87					C2 domain,C	rs890504	0.4714	YES	2.994E-1 (pos.42129176)			IRE1-mediated unfolded protein response,arachidonic a					T=11, C=11	T=0.5, C=0.5	0.00001	94.6046	22	T=6/5, C=5/6
chr15:42134301	C/C	A	C	SNV	JMJD7-PLA2G4B,PLA2G4B	JMJD7-PLA2G4B:intronic:NM_005090.3, PLA2G4B:intronic:N	1	100		intronic	NM_00509	c.1315-90A	p.?, p.?	-0.57					C2 domain,C	rs1648835		YES			Phospholip	IRE1-mediated unfolded protein response,arachidonic a					A=0, C=31	A=0.0, C=1.0	0.00001	464.999	31	A=0/0, C=13/1
chr9:116832	C/C	T	C	SNV	FOXD4	FOXD4:exonic:NM_207305.4	1	100	1	missense	NM_20730	c.1288A>G	p.Ser430Gly	-2.1		56		0.01(COSM37	Forkhead dor	rs4742632	0.2587	YES	3.176E-1 (pos.116832)	acute_myeloic	Forkhead b	DNA binding transcription factor activity,DNA binding, be					T=0, C=34	T=0.0, C=1.0	0.00001	524.186	34	T=0/0, C=16/1
chr15:42137576	T/T	C	T	SNV	JMJD7-PLA2G4B,PLA2G4B	JMJD7-PLA2G4B:intronic:NM_005090.3, PLA2G4B:intronic:N	1	100		intronic	NM_00509	c.2050+58	p.?, p.?	-0.37					C2 domain,C	rs1648828		YES			Phospholip	IRE1-mediated unfolded protein response,arachidonic a					C=0, T=135	C=0.0, T=1.0	0.00001	2238.23	135	C=0/0, T=68/6
chr15:42137581	C/C	A	C	SNV	JMJD7-PLA2G4B,PLA2G4B	JMJD7-PLA2G4B:intronic:NM_005090.3, PLA2G4B:intronic:N	1	100		intronic	NM_00509	c.2050+63	p.?, p.?	-0.13					C2 domain,C	rs1648827		YES			Phospholip	IRE1-mediated unfolded protein response,arachidonic a					A=0, C=135	A=0.0, C=1.0	0.00001	2236.48	135	A=0/0, C=68/6
chr15:42139038	A/A	G	A	SNV	JMJD7-PLA2G4B,PLA2G4B	JMJD7-PLA2G4B:intronic:NM_005090.3, PLA2G4B:intronic:N	1	100		intronic	NM_00509	c.2640+5G	p.?, p.?	1.73					C2 domain,C	rs890508	0.1885	YES	8.679E-1;0.0 (pos.42139038)		Phospholip	IRE1-mediated unfolded protein response,arachidonic a					G=0, A=20	G=0.0, A=1.0	0.00001	263.786	20	G=0/0, A=5/15
chr7:4802029	G/G	T	G	SNV	FOXK1	FOXK1:exonic:NM_001037165.1	1	100	9	synonymous	NM_00103	c.2136T>G	p.(=)	0.72				0.09(COSM37	FHA domain,I	rs6976790	0.0257	YES	1.0 (pos.4802029)	adenocarcino	Forkhead b	RNA polymerase II regulatory region sequence-specific D					T=0, G=26	T=0.0, G=1.0	0.00001	366.781	26	T=0/0, G=20/6
chr15:42147206	C/C	T	C	SNV	SPTBN5	SPTBN5:intronic:NM_016642.3	1	100		intronic	NM_01664	c.9424-32A	p.?	-0.38					Calponin hon	rs1648826	0.112	YES	9.96E-1;0.0 (pos.42147206)		Spectrin, be	ER to Golgi vesicle-mediated transport,Golgi organizatio					T=0, C=25	T=0.0, C=1.0	0.00001	353.015	25	T=0/0, C=6/19
chr12:2968458	T/C	T	C	SNV	FOXM1	FOXM1:exonic:NM_021953.3	1	95.83	9	synonymous	NM_02195	c.1638A>G	p.(=)	-0.03					Forkhead dor	rs2215292	0.0187		1.0 (pos.2968458)		Forkhead b	DNA bindi	Adenocarcinoma;Autoimmune Diseases;Bre				T=1, C=23	T=0.0417, C=0.9583	0.00001	306.484	24	T=1/0, C=12/1
chr15:42171877	G/G	A	G	SNV	SPTBN5	SPTBN5:intronic:NM_016642.3	1	100		intronic	NM_01664	c.2942+55	p.?	-1.83					Calponin hon	rs1197685		YES			Spectrin, be	ER to Golgi vesicle-mediated transport,Golgi organizatio					A=0, G=39	A=0.0, G=1.0	0.00001	623.976	39	A=0/0, G=12/2
chr15:42285301	G/G	A	G	SNV	PLA2G4E,PLA2G4E-AS1	PLA2G4E-AS1:intronic_nc:NR_120334.1, PLA2G4E:intronic:N	1	100		intronic	NR_12033	c.1248-14	p.?	1.09					C2 domain,Ly	rs937993		YES				cytosol,lysophospholipase activity,lysosomal membran					A=0, G=48	A=0.0, G=1.0	0.00001	810.414	48	A=0/0, G=27/2
chr2:48602252	C/T	C	T	SNV	FOXN2	FOXN2:exonic:NM_002158.3	1	50	7	synonymous	NM_00215	c.966C>T	p.(=)	0.57				0.64(COSM44	Forkhead dor	rs17855177	0.3227	YES	4.4E-1 (pos.48602252)	acute_myeloid_leukaemia		DNA binding transcription factor activity,cell differentiat					C=13, T=13	C=0.5, T=0.5	0.00001	119.168	26	C=4/9, T=11/2
chr15:42306014	C/G	C	G	SNV	PLA2G4E	PLA2G4E:intronic:NM_001206670.1	1	57.89		intronic	NM_00120	c.184-59G	p.?	-0.27					C2 domain,Ly	rs892055810						cytosol,lysophospholipase activity,lysosomal membran					C=16, G=22	C=0.4211, G=0.5789	0.00001	184.81	38	C=5/11, G=14/
chr22:36900271	C/C	T	C	SNV	FOXRED2	FOXRED2:exonic:NM_001102371.1	1	100	4	missense	NM_00110	c.923A>G	p.Asn308Ser	-0.32	0.2	46	0	0.09(COSM41	NAD(P)-bindi	rs2277841	0.1979	YES	2.481E-1 (pos.36900271)	acute_myeloic	FAD-depenc	endoplasmic reticulum lumen,flavin adenine dinucleoti					T=0, C=37	T=0.0, C=1.0	0.00001	583.706	37	T=0/0, C=24/1
chr15:42377744	G/G	C	G	SNV	PLA2G4D	PLA2G4D:intronic:NM_178034.3	1	100		intronic	NM_17803	c.388-15G	p.?	0.4					C2 domain,Ly	rs605253	0.2195	YES	6.279E-1 (pos.42377744)		Phospholip	calcium-dependent phospholipase A2 activity,cytosol,gl					C=0, G=43	C=0.0, G=1.0	0.00001	704.284	43	C=0/0, G=14/2
chr15:42437711	C/C	T	C	SNV	PLA2G4F	PLA2G4F:intronic:NM_213600.3	1	100		intronic	NM_21360	c.1780+62	p.?	0.29					C2 domain,Ly	rs621560		YES				arachidonic acid secretion,calcium-dependent phospho					T=0, C=35	T=0.0, C=1.0	0.00001	543.9	35	T=0/0, C=16/1
chr22:36900806	G/G	A	G	SNV	FOXRED2	FOXRED2:exonic:NM_001102371.1	1	100	3	missense	NM_00110	c.535T>C	p.Phe179Leu	0.73	1	22	0	0.07(COSM44	NAD(P)-bindi	rs760718	0.2993	YES	2.476E-1 (pos.36900806)	acute_myeloic	FAD-depenc	endoplasmic reticulum lumen,flavin adenine dinucleoti					A=0, G=21	A=0.0, G=1.0	0.00001	281.227	21	A=0/0, G=15/6
chr19:52250216	G/G	A	G	SNV	FPR1	FPR1:exonic:NM_002029.3	1	100	2	missense	NM_00202	c.32T>C	p.Ile11Thr	-2.07		89			7 transmemb	rs5030878	0.2373	YES	9.626E-1 (pos.52250216)		Formyl pept	G-protein coupled receptor sig			Nedocrom	Benign	A=0, G=35	A=0.0, G=1.0	0.00001	543.902	35	A=0/0, G=29/6
chr15:42523341	TC/TC	T	TC	INDEL	TMEM87A	TMEM87A:intronic:NM_015497.4	1	100		intronic	NM_01549	c.1133+47	p.?	2.52					Lung seven tr	rs369314626,rs397725100,rs56119841			1.0 (pos.42523341)			Golgi cisterna membrane,cytosol,integral component of					T=0, TC=22	T=0.0, TC=1.0	0.00001	258.21	22	T=0/0, TC=3/1
chr19:52327784	T/T	C	T	SNV	FPR3	FPR3:exonic:NM_002030.4	1	100	2	synonymous	NM_00203	c.783C>T	p.(=)	-0.3					7 transmemb	rs3752125	0.3067	YES	2.558E-1 (pos.52327784)		Formyl pept	G-protein	Autoimmune Diseases:Female Urogenital Dis				C=0, T=38	C=0.0, T=1.0	0.00001	603.785	38	C=0/0, T=23/1
chr15:42568630	A/A	G	A	SNV	GANC	GANC:intronic:NM_198141.2	1	100		intronic	NM_19814	c.92+22G>	p.?	-0.61					Galactose mu	rs8023678	0.001	YES	9.473E-1 (pos.42568630)		Neutral alp	alpha-1,4-glucosidase activity,			Miglitol		G=0, A=44	G=0.0, A=1.0	0.00001	726.569	44	G=0/0, A=16/2
chr15:42584909	T/T	C	T	SNV	GANC	GANC:intronic:NM_198141.2	1	100		intronic	NM_19814	c.330-24C	p.?	0.48					Galactose mu	rs4923939	7.00E-04	YES	9.474E-1 (pos.42584909)		Neutral alp	alpha-1,4-glucosidase activity,			Miglitol		C=0, T=37	C=0.0, T=1.0	0.00001	583.706	37	C=0/0, T=20/1
chr15:42603083	T/T	C	T	SNV	GANC	GANC:intronic:NM_198141.2	1	100		intronic	NM_19814	c.1057+39	p.?	-1.6					Galactose mu	rs2412694	0.0012	YES	9.474E-1 (pos.42603083)		Neutral alp	alpha-1,4-glucosidase activity,			Miglitol		C=0, T=41	C=0.0, T=1.0	0.00001	664.411	41	C=0/0, T=27/1
chr15:42619508	T/T	C	T	SNV	GANC	GANC:intronic:NM_198141.2	1	100		intronic	NM_19814	c.1421-53	p.?	0.39					Galactose mu	rs1659215	0.0758	YES			Neutral alp	alpha-1,4-glucosidase activity,			Miglitol		C=0, T=53	C=0.0, T=1.0	0.00001	782.029	53	C=0/0, T=36/1
chr15:42820451	T/T	C	T	SNV	SNAP23	SNAP23:intronic:NM_003825.3	1	100		intronic	NM_00382	c.267-9C>T	p.?	-0.07					SNAP-25 fami	rs9302112	0.2554	YES	7.816E-1 (pos.42820451)		Synaptosom	SNAP rece	Cardiovascular Diseases:Heart Diseases:Mv				C=0, T=37	C=0.0, T=1.0	0.00001	583.597	37	C=0/0, T=28/9
chr15:43244699	G/G	A	G	SNV	UBR1	UBR1:intronic:NM_174916.2	1	100		intronic	NM_17491	c.4836-53	p.?	-1					ATP-depende	rs2277532,rs764510888		YES	0.0 (pos.43244696)		Ubiquitin-p	cellular re	Abnormalities, Multiple;Anus, Imperforate;C				A=0, G=24	A=0.0, G=1.0	0.00001	334.791	24	A=0/0, G=11/1
chr15:43352041	A/A	G	A	SNV	UBR1	UBR1:intronic:NM_174916.2	1	100		intronic	NM_17491	c.862-18C	p.?	1.13					ATP-depende	rs4924704	0.1181	YES	7.922E-1 (pos.43352041)		Ubiquitin-p	cellular re	Abnormalities, Multiple;Anus,		Benign		G=0, A=22	G=0.0, A=1.0	0.00001	298.891	22	G=0/0, A=12/1
chr10:95458141	G/G	A	G	SNV	FRA10AC1	FRA10AC1:exonic:NM_145246.4	1	100	3	synonymous	NM_14524	c.90T>C	p.(=)	0.84					Folate-sensit	rs11187597	0.4837	YES	4.022E-1 (pos.95458141)		Chromosom	nucleus,protein binding					A=0, G=32	A=0.0, G=1.0	0.00001	485.141	32	A=0/0, G=18/1
chr10:95459817	T/T	C	T	SNV	FRA10AC1	FRA10AC1:exonic:NM_145246.4	1	100	2	missense	NM_14524	c.47G>A	p.Arg16His	0.54	0.99	29	0	0.01(COSM63	Folate-sensit	rs726817	0.2579	YES	7.216E-1 (pos.95459817)	carcinoma	Chromosom	nucleus,protein binding					C=0, T=34	C=0.0, T=1.0	0.00001	524.166	34	C=0/0, T=10/2
chr4:79455714	G/G	C	G	SNV	FRAS1	FRAS1:exonic:NM_025074.6	1	100	71	synonymous	NM_02507	c.11037C>	p.(=)	0.49				0.83(COSM40	Cadherin-like	rs4975070	0.4359	YES	7.841E-1 (pos.79455714)	carcinoma,diff	FRAS1 gene	basement	Abnormalities, Multiple;Conge			Benign	C=0, G=38	C=0.0, G=1.0	0.00001	603.775	38	C=0/0, G=13/2
chr4:79455733	T/T	C	T	SNV	FRAS1	FRAS1:exonic:NM_025074.6	1	100	71	synonymous	NM_02507	c.11056C>	p.(=)	1.13				0.55(COSM40	Cadherin-like	rs4975139	0.4359	YES	7.833E-1 (pos.79455733)	carcinoma,ne	FRAS1 gene	basement	Abnormalities, Multiple;Conge			Benign	C=0, T=39	C=0.0, T=1.0	0.00001	623.945	39	C=0/0, T=13/2
chr15:44068160	G/G	C	G	SNV	ELL3	ELL3:intronic:NM_025165.2	1	100		intronic	NM_02516	c.282-39G	p.?	-0.06					Occludin hom	rs3844075	0.0867	YES	9.983E-1 (pos.44068160)		Elongation f	DNA-templated transcription, elongation,enhancer bind					C=0, G=37	C=0.0, G=1.0	0.00001	583.707	37	C=0/0, G=17/2
chr13:39263023	T/T	C	T	SNV	FREM2	FREM2:exonic:NM_207361.5	1	100	1	synonymous	NM_20736	c.1542C>T	p.(=)	-6.35				0.03(COSM37	Cadherin-like	rs12874397	0.2606	YES	1.201E-3 (pos.39263023)	adenocarcino	FRAS1-relat	basement	Abnormalities, Multiple:Conge			Benign	C=0, T=24	C=0.0, T=1.0	0.00001	334.703	24	C=0/0, T=10/1
chr13:39262057	G/A	G	A	SNV	FREM2	FREM2:exonic:NM_207361.5	1	97.3	1	synonymous	NM_20736	c.576G>A	p.(=)	0.21				0.59(COSM41	Cadherin-like	rs1868464	0		1.0 (pos.39262057)	neoplasm	FRAS1-relat	basement	Abnormalities, Multiple:Conge			Benign	G=1, A=36	G=0.027, A=0.973	0.00001	540.031	37	G=1/0, A=18/1
chr4:144617842	T/T	G	T	SNV	FREM3	FREM3:exonic:NM_001168235.1	1	100	1	synonymous	NM_00116	c.3987C>A	p.(=)	0.04				0.14(COSM44	Cadherin-like	rs1545437	0.3325	YES	5.294E-2 (pos.14461784	acute_myeloic	FRAS1-relat	basement	Depressive Disorder;Depressive Disorder, M				G=0, T=29	G=0.0, T=1.0	0.00001	427.602	29	G=0/0, T=17/1
chr15:45439910	G/G	A	G	SNV	DUOX1	DUOX1:intronic:NM_175940.2	1	100		intronic	NM_17594	c.2548+54	p.?	-1.79					Animal haem	rs938129		YES			Dual oxidas	NAD(P)H oxidase activity,NADP binding,apical plasma m					A=0, G=30	A=0.0, G=1.0	0.00001	446.61	30	A=0/0, G=11/1
chr15:45558083	A/A	G	A	SNV	LOC101928414,SLC28A2	LOC101928414:intronic_nc:NR_120335.1, SLC28A2:intronic	1	100		intronic	NR_12033	c.862-196	p.?	-4.01					Na+ depende	rs11854365		YES			Solute carri	integral component of plasma			Mercaptopurine		G=0, A=43	G=0.0, A=1.0	0.00001	705.837	43	G=0/0, A=24/1
chr15:45968535	C/C	A	C	SNV	SQOR	SQOR:intronic:NM_001271213.1	1	100		intronic	NM_00127	c.864+27A	p.?	-0.56					Pyridine nucl	rs12914266	0.1806	YES	7.7E-1 (pos.45968535)		Sulfide quin	hydrogen sulfide metabolic process,mitochondrial inner					A=0, C=59	A=0.0, C=1.0	0.00001	885.367	59	A=0/0, C=23/3
chr15:48058929	A/A	G	A	SNV	SEMA6D	SEMA6D:intronic:NM_153618.1	1	100		intronic	NM_15361	c.1708+55	p.?	0.87					Plexin repeat	rs603569	0.2711	YES			Semaphorir	Golgi appa	Mental Disorders;Substance-Related Disorde				G=0, A=29	G=0.0, A=1.0	0.00001	427.6	29	G=0/0, A=16/1
chr4:190876195	C/T	C	T	SNV	FRG1	FRG1:exonic:NM_004477.2	1	57.58	5	synonymous	NM_00447	c.321C>T	p.(=)	0.52					FRG1-like dor	rs7684300		YES	4.955E-1;0.0 (pos.190876195)		Facioscapu	Cajal body	Congenital, Hereditary, and Neonatal Diseas				C=14, T=19	C=0.4242, T=0.5758	0.00001	204.394	33	C=5/9, T=2/17
chr15:48460786	C/C	T	C	SNV	MYEF2	MYEF2:intronic:NM_016132.4	1	100		intronic	NM_01613	c.370+42A	p.?	-1.94					RNA recognit	rs11070626	9.00E-04	YES	9.236E-1 (pos.48460786)			RNA binding,RNA polymerase II transcription factor activ					T=0, C=21	T=0.0, C=1.0	0.00001	281.235	21	T=0/0, C=13/8
chr3:75713564	C/C	T	C	SNV	FRG2C,FRG2EP	FRG2EP:exonic_nc:NR_037925.1, FRG2C:exonic:NM_001124	1	100	1	missense	NM_00112	c.34T>C	p.Cys12Arg	0.74	0.02	180	0.189	0.02(COSM17	Facioscapulo	rs13073018		YES		transitional_cell_carcinom		nucleus					T=0, C=61	T=0.0, C=1.0	0.00001	921.854	61	T=0/0, C=36/2
chr3:75715124	C/T	C	T	SNV	FRG2C,FRG2EP	FRG2EP:exonic_nc:NR_037925.1, FRG2C:exonic:NM_001124	1	50	4	missense	NM_00112	c.781C>T	p.Pro261Ser	-0.1	0.02	74	0.996	0(COSM5425	Facioscapulo	rs72503535		YES		acute_myeloid_leukaemia		nucleus					C=50, T=50	C=0.5, T=0.5	0.00001	564.854	100	C=25/25, T=22
chr15:48736684	T/T	A	T	SNV	FBN1	FBN1:intronic:NM_000138.4	1	100		intronic	NM_00013	c.6037+54	p.?	-0.1					Calcium-bind	rs2303502		YES			Fibrillin-1	basement	ACMG Recommendations...:Abnormalities, M				A=0, T=25	A=0.0, T=1.0	0.00001	353.013	25	A=0/0, T=9/16
chr6:168459845	C/C	G	C	SNV	FRMD1	FRMD1:exonic:NM_024919.4	1	100	10	missense	NM_02491	c.1366C>G	p.Gln456Glu	-0.64	1	29	0	0.04(COSM57	FERM N-term	rs1548349	0.1012	YES	8.44E-1;0.0 (pos.168459	carcinoma		cytoskeleton					G=0, C=28	G=0.0, C=1.0	0.00001	408.689	28	G=0/0, C=18/1
chr15:49083577	T/T	C	T	SNV	CEP152	CEP152:intronic:NM_001194998.1	1	100		intronic	NM_00119	c.833-4G>	p.?	-0.09				0.03(COSM6352425),0.03(C		rs58156069	0.4601	YES	3.523E-1 (pos.49083577)	carcinoma	Centrosom	G2/M tran	Bone Diseases;Bone Diseases,			Benign	C=0, T=33	C=0.0, T=1.0	0.00001	504.598	33	C=0/0, T=17/1
chr15:49149828	T/T	C	T	SNV	SHC4	SHC4:intronic:NM_203349.3	1	100		intronic	NM_20334	c.1070+61	p.?	-0.92					Phosphotyro	rs4774526		YES			SHC transfo	apoptotic process,cell junction,intracellular,intracellula					C=0, T=39	C=0.0, T=1.0	0.00001	623.96	39	C=0/0, T=4/35
chr15:49301293	C/C	A	C	SNV	SECISBP2L	SECISBP2L:intronic:NM_001193489.1	1	100		intronic	NM_00119	c.2027+12	p.?	0.49					Ribosomal pr	rs7167402		YES			Selenocyste	RNA binding,intracellular ribonucleoprotein complex,mi					A=0, C=86	A=0.0, C=1.0	0.00001	1410.89	86	A=0/0, C=34/5
chr9:85863091	G/G	A	G	SNV	FRMD3	FRMD3:exonic:NM_174938.5	1	100	14	synonymous	NM_17493	c.1536T>C	p.(=)	3.07				0.77(COSM37	FERM C-termi	rs10114696	0.4134	YES	2.213E-1 (pos.85863091)	acute_myeloic	FERM doma	actomyosi	Acneiform Eruptions;Chloracne;Skin Disease				A=0, G=29	A=0.0, G=1.0	0.00001	427.603	29	A=0/0, G=19/1
chr15:49659983	G/G	A	G	SNV	FAM227B	FAM227B:intronic:NM_152647.2	1	100		intronic	NM_15264	c.1111-17	p.?	-3.06					Protein of unl	rs8038399		YES									A=0, G=33	A=0.0, G=1.0	0.00001	504.598	33	A=0/0, G=20/1
chr15:49867122	A/G	A	G	SNV	FAM227B	FAM227B:intronic:NM_152647.2	1	97.92		intronic	NM_15264	c.645+86T	p.?	-0.64					Protein of unl	rs12901416		YES									A=1, G=47	A=0.0208, G=0.9792	0.00001	776.226	48	A=1/0, G=30/1
chr15:50288858	G/G	T	G	SNV	ATP8B4	ATP8B4:intronic:NM_024837.3	1	100		intronic	NM_02483	c.589+16A	p.?	0.58					Cation transp	rs71469670	0.0806	YES	1.532E-3 (pos.50288858)			ATP binding,Golgi apparatus,Golgi organization,integral					T=0, G=60	T=0.0, G=1.0	0.00001	903.531	60	T=0/0, G=34/2
chr3:69230801	A/A	G	A	SNV	FRMD4B	FRMD4B:exonic:NM_015123.2	1	100	21	synonymous	NM_01512	c.2100C>T	p.(=)	0.59					Domain of un	rs9836305	0.0071	YES	6.001E-1 (pos.69230801)		FERM doma	adherens	Celiac Disease:Digestive System Diseases:Ga				G=0, A=311	G=0.0, A=1.0	0.00001	5140.36	311	G=0/0, A=159/
chr15:50731390	C/T	C	T	SNV	USP8	USP8:intronic:NM_005154.4	1	57.14		intronic	NM_00515	c.104+16C	p.?	0.54					Rhodanese-li	rs11632708	0.1819	YES	2.615E-3 (pos.50731390)		Ubiquitin-s	SH3 doma	ACTH-Secreting Pituitary Adenoma;Adenoma				C=15, T=20	C=0.4286, T=0.5714	0.00001	218.705	35	C=5/10, T=7/1
chr11:30255185	T/T	C	T	SNV	FSHB	FSHB:exonic:NM_001018080.1	1	100	3	synonymous	NM_00101	c.228C>T	p.(=)	-1.41				0.01(COSM39	Cystine-knot	rs6169	0.4358	YES	6.746E-1 (pos.30255185)	acute_myeloic	Follicle-stin	G-protein	Alcohol-Related Disorders;Alc			Benign/Likely	C=0, T=25	C=0.0, T=1.0	0.00001	353.019	25	C=0/0, T=19/6
chr2:49191041	T/T	C	T	SNV	FSHR	FSHR:exonic:NM_000145.3	1	98.99	10	missense	NM_00014	c.919G>A	p.Ala307Thr	-0.11	0.28	58	0	0.04(COSM39	7 transmemb	rs6165	0.4585	YES	6.596E-1;0.0 (pos.49191(	adenocarcino	Follicle stin	G-protein	46, XX Disorders of		Choriogon	Benign, drug	C=1, T=98	C=0.0101, T=0.9899	0.00001	1643.59	99	C=1/0, T=49/4
chr15:50878509	T/C	T	C	SNV	TRPM7	TRPM7:intronic:NM_017672.5	1	45.83		intronic	NM_01767	c.4486+80	p.?	-1.2					Alpha-kinase	rs1395297,rs910057799		YES			Transient re	ATP bindin	Adenoma;Amyotro	1,4-Dithiothreitol,Magne			T=65, C=55	T=0.5417, C=0.4583	0.00001	551.15	120	T=28/37, C=37
chr15:50885976	G/T	G	T	SNV	TRPM7	TRPM7:intronic:NM_017672.5	1	57.14		intronic	NM_01767	c.3476-30	p.?	-0.4					Alpha-kinase	rs567109	0.4369	YES	5.528E-1;0.0 (pos.50885976)		Transient re	ATP bindin	Adenoma;Amyotro	1,4-Dithiothreitol,Magne			G=15, T=20	G=0.4286, T=0.5714	0.00001	218.716	35	G=5/10, T=10/
chr15:50888618	AT/AT	A	AT	INDEL	TRPM7	TRPM7:intronic:NM_017672.5	1	100		intronic	NM_01767	c.3164-40	p.?	0.84					Alpha-kinase family,Ion transport protein,Tetramerisation domain of TRPM						Transient re	ATP bindin	Adenoma;Amyotro	1,4-Dithiothreitol,Magne			A=0, AT=42	A=0.0, AT=1.0	0.00001	540.306	42	A=0/0, AT=19/
chr15:50905780	A/A	G	A	SNV	TRPM7	TRPM7:intronic:NM_017672.5	1	100		intronic	NM_01767	c.1770+12	p.?	-0.58					Alpha-kinase	rs511736		YES			Transient re	ATP bindin	Adenoma;Amyotro	1,4-Dithiothreitol,Magne			G=0, A=97	G=0.0, A=1.0	0.00001	1638.92	97	G=0/0, A=60/3
chr15:52516942	A/A	C	A	SNV	MYO5C	MYO5C:intronic:NM_018728.3	1	100		intronic	NM_01872	c.3446+15	p.?	-0.26					DIL domain,I	rs11070886		YES			Myosin Vc	ATP bindin	Mental Disorders;Substance-Related Disorde				C=0, A=71	C=0.0, A=1.0	0.00001	1111.38	71	C=0/0, A=45/2
chr2:186697876	G/G	A	G	SNV	FSIP2	FSIP2:exonic:NM_173651.3	1	100	23	missense	NM_17365	c.20647A>	p.Thr6883Ala	1.08	1	58	0		Fibrous shea	rs1468999	0.0021	YES	9.387E-1 (pos.186697876)		Fibrous she	mitochondrion					A=0, G=20	A=0.0, G=1.0	0.00001	263.786	20	A=0/0, G=12/8
chr2:186672527	T/A	T	A	SNV	FSIP2	FSIP2:exonic:NM_173651.3	1	56	17	missense	NM_17365	c.18494T>	p.Leu6165Gln	0	0.1	113	0.274	0(COSM3757	Fibrous shea	rs72900086	0.2398	YES	2.25E-2 (pos.186672527)	adenocarcino	Fibrous she	mitochondrion					T=11, A=14	T=0.44, A=0.56	0.00001	131.102	25	T=9/2, A=8/6
chr2:186668935	A/G	A	G	SNV	FSIP2	FSIP2:exonic:NM_173651.3	1	50	17	missense	NM_17365	c.14902A>	p.Met4968Val	0.03	1	21	0.004		Fibrous shea	rs13003744		YES	3.763E-1 (pos.186668935)		Fibrous she	mitochondrion					A=15, G=15	A=0.5, G=0.5	0.00001	145.02	30	A=5/10, G=6/9
chr15:52553007	G/G	C	G	SNV	MYO5C	MYO5C:intronic:NM_018728.3	1	100		intronic	NM_01872	c.1313+52	p.?	-1.03					DIL domain,I	rs4497629	3.00E-04	YES			Myosin Vc	ATP bindin	Mental Disorders;Substance-Related Disorde				C=0, G=67	C=0.0, G=1.0	0.00001	1034.43	67	C=0/0, G=41/2
chr2:186658438	C/T	C	T	SNV	FSIP2,FSIP2-AS1	FSIP2-AS1:intronic_nc:NR_144453.1, FSIP2:exonic:NM_1736	1	51.85	16	missense	NM_17365	c.6575C>T	p.Thr2192Met	-1.69	0.24	81	0.16		Fibrous shea	rs10490391		YES	5.361E-1 (pos.186658438)		Fibrous she	mitochondrion					C=39, T=42	C=0.4815, T=0.5185	0.00001	453.453	81	C=23/16, T=22
chr2:186664473	T/C	T	C	SNV	FSIP2,FSIP2-AS1	FSIP2-AS1:intronic_nc:NR_144453.1, FSIP2:exonic:NM_1736	1	51.85	17	synonymous	NM_17365	c.10440T>	p.(=)	1.25					Fibrous shea	rs11686997		YES	5.579E-1 (pos.186664473)		Fibrous she	mitochondrion					T=26, C=28	T=0.4815, C=0.5185	0.00001	277.152	54	T=13/13, C=16
chr2:186656840	T/C	T	C	SNV	FSIP2,FSIP2-AS1	FSIP2-AS1:intronic_nc:NR_144453.1, FSIP2:exonic:NM_1736	1	50	16	synonymous	NM_17365	c.4977T>C	p.(=)	-0.01					Fibrous shea	rs17815052		YES	5.376E-2 (pos.186656840)		Fibrous she	mitochondrion					T=26, C=26	T=0.5, C=0.5	0.00001	254.431	52	T=12/14, C=12
chr15:52681573	G/G	A	G	SNV	MYO5A	MYO5A:intronic:NM_000259.3	1	100		intronic	NM_00025	c.1543-13	p.?	-1.77					DIL domain,I	rs1724623,rs374120080,rs6758		YES	1.784E-2 (pos.52681572),9.827E-1 (pos.5		Myosin Va	ATP bindin	Abnormalities, Multiple:Albini			Benign	A=0, G=46	A=0.0, G=1.0	0.00001	750.065	46	A=0/0, G=8/38
chr2:186656956	T/C	T	C	SNV	FSIP2,FSIP2-AS1	FSIP2-AS1:intronic_nc:NR_144453.1, FSIP2:exonic:NM_1736	1	50	16	missense	NM_17365	c.5093T>C	p.Met1698Thr	0.3	1	81	0		Fibrous shea	rs17229201		YES	5.309E-1 (pos.186656956)		Fibrous she	mitochondrion					T=15, C=15	T=0.5, C=0.5	0.00001	145.02	30	T=3/12, C=1/1
chr19:680378	C/T	C	T	SNV	FSTL3	FSTL3:exonic:NM_005860.2	1	57.45	3	synonymous	NM_00586	c.394C>T	p.(=)	1.03				0.44(COSM41	Follistatin/O	rs7246311		YES	2.5E-1 (pos.680378)	neoplasm	Follistatin-l	Golgi apparatus,activin binding,adrenal gland developm					C=20, T=27	C=0.4255, T=0.5745	0.00001	325.978	47	C=11/9, T=11/
chr19:5866585	T/T	C	T	SNV	FUT5	FUT5:utr_3:NM_002034.2	1	100	2	utr_3	NM_00203	c.1152G>A	p.?	-0.7					Fucosyltrans	rs778982	0.2213	YES	8.905E-1 (pos.5866585)		Fucosyltran	3-galactosyl-N-acetylglucosaminide 4-alpha-L-fucosyltra					C=0, T=45	C=0.0, T=1.0	0.00001	747.393	45	C=0/0, T=30/1
chr15:54008795	C/C	T	C	SNV	WDR72	WDR72:intronic:NM_182758.3	1	100		intronic	NM_18275	c.339+9A>	p.?	-1.64					WD domain,	rs690337	0.2268	YES	7.364E-1 (pos.54008795)		WD repeat-	cytoplasm	Amelogenesis Imperfecta;Con			Benign	T=0, C=21	T=0.0, C=1.0	0.00001	281.235	21	T=0/0, C=6/15
chr19:5866654	G/G	A	G	SNV	FUT5	FUT5:exonic:NM_002034.2	1	100	2	synonymous	NM_00203	c.1083T>C	p.(=)	0.64				0(COSM4132	Fucosyltrans	rs778983	0.2228	YES	8.9E-1 (pos.5866654)	adenocarcino	Fucosyltran	3-galactosyl-N-acetylglucosaminide 4-alpha-L-fucosyltra					A=0, G=42	A=0.0, G=1.0	0.00001	685.22	42	A=0/0, G=27/1
chr15:54008889	G/G	GA	G	INDEL	WDR72	WDR72:intronic:NM_182758.3	1	100		intronic	NM_18275	c.261-7TC	p.?	1.66, 0.0					WD domain,	rs34828731	0.4908	YES	1.917E-1 (pos.54008889)		WD repeat-	cytoplasm	Amelogenesis Imperfecta;Con			Benign/Likely	GA=0, G=22	GA=0.0, G=1.0	0.00001	297.917	22	GA=0/0, G=6/1
chr15:54624313	A/A	T	A	SNV	UNC13C	UNC13C:intronic:NM_001080534.2	1	100		intronic	NM_00108	c.4494+4T	p.?	-0.07				0.7(COSM399	C2 domain,D	rs11856987	0.2992	YES	1.127E-1 (pos.54624313)	acute_myeloic	Unc13, C. el	calyx of He	Nervous System Diseases;Neuromuscular Di				T=0, A=46	T=0.0, A=1.0	0.00001	772.927	46	T=0/0, A=20/2
chr15:54685463	A/A	G	A	SNV	UNC13C	UNC13C:intronic:NM_001080534.2	1	100		intronic	NM_00108	c.4847+84	p.?	0.06					C2 domain,D	rs489526		YES			Unc13, C. el	calyx of He	Nervous System Diseases;Neuromuscular Di				G=0, A=20	G=0.0, A=1.0	0.00001	263.779	20	G=0/0, A=4/16
chr15:55669392	T/C	T	C	SNV	CCPG1,DNAAF4-CCPG1	DNAAF4-CCPG1:intronic_nc:NR_037923.1, CCPG1:intronic:N	1	45.45		intronic	NR_03792	c.253-44A	p.?	-0.28						rs6493781	0.0895	YES	8.008E-3 (pos.55669392)		Cell cycle p	cell cycle,integral component of membrane,molecular_f					T=12, C=10	T=0.5455, C=0.4545	0.00001	81.7622	22	T=6/6, C=2/8
chr19:5866736	C/C	T	C	SNV	FUT5	FUT5:exonic:NM_002034.2	1	100	2	missense	NM_00203	c.1001A>G	p.His334Arg	-0.27		29		0.01(COSM41	Fucosyltrans	rs778984		YES	8.676E-1 (pos.5866736)	neoplasm,squ	Fucosyltran	3-galactosyl-N-acetylglucosaminide 4-alpha-L-fucosyltra					T=0, C=21	T=0.0, C=1.0	0.00001	281.228	21	T=0/0, C=17/4
chr19:5867748	T/T	G	T	SNV	FUT5	FUT5:utr_5:NM_002034.2	1	100	2	utr_5	NM_00203	c.-12C>A	p.?	0.24					Fucosyltrans	rs778971	0.4442	YES	7.8E-1 (pos.5867748)		Fucosyltran	3-galactosyl-N-acetylglucosaminide 4-alpha-L-fucosyltra					G=0, T=34	G=0.0, T=1.0	0.00001	524.186	34	G=0/0, T=11/2
chr15:56719797	T/T	G	T	SNV	TEX9	TEX9:intronic:NM_198524.2	1	100		intronic	NM_19852	c.964-6G>T	p.?	0.13						rs795785	0.1066	YES	9.216E-1 (pos.56719797)			protein binding					G=0, T=62	G=0.0, T=1.0	0.00001	939.429	62	G=0/0, T=25/3
chr15:56736208	C/C	T	C	SNV	MNS1,TEX9	TEX9:intronic:NM_198524.2, MNS1:intronic:NM_018365.2	1	100		intronic	NM_19852	c.*1206-1	p.?, p.?	-0.56					Trichohyalin-	rs795793		YES			Meiosis-spe	axoneme,cilium organization,identical protein binding,i					T=0, C=26	T=0.0, C=1.0	0.00001	371.403	26	T=0/0, C=11/1
chr15:56946296	A/A	C	A	SNV	ZNF280D	ZNF280D:intronic:NM_017661.3	1	100		intronic	NM_01766	c.2213+10	p.?	-0.24					Domain of un	rs8028457		YES				DNA binding,metal ion binding,nucleic acid binding,nucl					C=0, A=33	C=0.0, A=1.0	0.00001	510.896	33	C=0/0, A=7/26
chr15:57524857	T/C	T	C	SNV	TCF12	TCF12:intronic:NM_207037.1	1	52.38		intronic	NM_20703	c.826-53T	p.?	0.25					Helix-loop-he	rs2290938		YES			Transcriptic	DNA bindi	Bone Diseases;Bone Diseases, Development				T=20, C=22	T=0.4762, C=0.5238	0.00001	245.194	42	T=14/6, C=14/
chr15:57555523	A/A	C	A	SNV	TCF12	TCF12:intronic:NM_207037.1	1	100		intronic	NM_20703	c.1745+51	p.?	0.81					Helix-loop-he	rs7182038	0.2494	YES	8.265E-1;1.191E-4 (pos.57555523)		Transcriptic	DNA bindi	Bone Diseases;Bone Diseases, Development				C=0, A=92	C=0.0, A=1.0	0.00001	1527.56	92	C=0/0, A=38/5
chr14:66028446	G/G	A	G	SNV	FUT8	FUT8:exonic:NM_178156.2	1	100	2	synonymous	NM_17815	c.165A>G	p.(=)	0.77					Variant SH3 c	rs2229677	0.3184	YES	8.972E-1 (pos.66028446)		Fucosyltran	GDP-L-fucose metabolic process,Golgi apr			Benign		A=0, G=132	A=0.0, G=1.0	0.00001	2176.53	132	A=0/0, G=63/6
chr15:57836823	A/A	G	A	SNV	CGNL1	CGNL1:intronic:NM_032866.4	1	100		intronic	NM_03286	c.3500+28	p.?	-0.4					Myosin tail	rs8029295	0		1.0 (pos.57836823)		Cingulin-lik	actin filam	Bipolar Disorder;Bipolar and Related Disorde				G=0, A=77	G=0.0, A=1.0	0.00001	1229.44	77	G=0/0, A=30/4
chr15:58471626	G/A	G	A	SNV	AQP9	AQP9:intronic:NM_020980.4	1	57.45		intronic	NM_02098	c.713+82G	p.?	-0.32					Major intrins	rs17821117		YES			Aquaporin 9	amine trai	Arsenic Poisoning;Arthritis;Arthritis, Juvenile				G=20, A=27	G=0.4255, A=0.5745	0.00001	326.381	47	G=8/12, A=16/
chr9:71650752	G/G	A	G	SNV	FXN	FXN:exonic:NM_000144.4	1	100	1	synonymous	NM_00014	c.54A>G	p.(=)	-0.47					Frataxin-like	rs2481598,rs763799314			1.0;0.0 (pos.71650752)		Frataxin	2 iron, 2 su	Brain Diseases;Central Nervou			Benign/Likely	A=0, G=91	A=0.0, G=1.0	0.00001	1501.22	91	A=0/0, G=54/3
chr19:35660508	A/A	G	A	SNV	FXYD5	FXYD5:exonic:NM_014164.5	1	100	9	missense	NM_01416	c.527G>A	p.Arg176His	-1.8	0.49	29	0.001		ATP1G1/PLM	rs12110	0.2221	YES	7.805E-1 (pos.35660508)		FXYD domai	actin binding,cadherin binding,integral component of m					G=0, A=20	G=0.0, A=1.0	0.00001	263.772	20	G=0/0, A=15/5
chr15:58837910	C/C	T	C	SNV	LIPC	LIPC:intronic:NM_000236.2	1	100		intronic	NM_00023	c.575-31T	p.?	0.46					Lipase,PLAT/	rs2414592	8.00E-04	YES	1.0 (pos.58837910)		Lipase C, he	apolipopro	Cardiovascular Diseases;Congenital, Heredit				T=0, C=32	T=0.0, C=1.0	0.00001	484.984	32	T=0/0, C=18/1
chr3:45996761	A/A	G	A	SNV	FYCO1	FYCO1:exonic:NM_024513.3	1	100	14	synonymous	NM_02451	c.3924C>T	p.(=)	-0.92					FYVE zinc fing	rs1463680	0.2807	YES	9.579E-1 (pos.45996761)		FYVE and co	'de novo' p	Cataract;Eye Diseases;Lens Di			Benign	G=0, A=63	G=0.0, A=1.0	0.00001	958.913	63	G=0/0, A=50/1
chr3:46010077	T/T	C	T	SNV	FYCO1	FYCO1:exonic:NM_024513.3	1	100	8	missense	NM_02451	c.749G>A	p.Arg250Gln	2.19	0.18	43	0		FYVE zinc fing	rs4683158	0.2253	YES	9.997E-1;0.0 (pos.46010077)		FYVE and co	'de novo' p	Cataract;Eye Diseases;Lens Di			Benign	C=0, T=20	C=0.0, T=1.0	0.00001	263.786	20	C=0/0, T=13/7
chr15:59381769	A/T	A	T	SNV	RNF111	RNF111:intronic:NM_001270528.1	1	45.83		intronic	NM_00127	c.2578-97	p.?	0.14					E3 ubiquitin-l	rs2289895		YES			Ring finger p	PML body,	Congenital, Hereditary, and Neonatal Diseas				A=13, T=11	A=0.5417, T=0.4583	0.00001	93.4385	24	A=11/2, T=7/4
chr15:59515446	G/G	C	G	SNV	MYO1E	MYO1E:intronic:NM_004998.3	1	100		intronic	NM_00499	c.778-56G	p.?	-1.7					IQ calmodulin	rs2306780		YES			Myosin IE	ATP binding,ATPase activity, co			Imidazole		C=0, G=72	C=0.0, G=1.0	0.00001	1134.62	72	C=0/0, G=32/4
chr15:59524054	AC/AC	A	AC	INDEL	MYO1E	MYO1E:intronic:NM_004998.3	1	100		intronic	NM_00499	c.421-64T	p.?	0.91					IQ calmodulin-binding motif,Myosin head (motor domain),SH3 domain,Unconventional myosin tail, a						Myosin IE	ATP binding,ATPase activity, co			Imidazole		A=0, AC=65	A=0.0, AC=1.0	0.00001	741.343	65	A=0/0, AC=26/
chr15:59548475	T/T	C	T	SNV	MYO1E	MYO1E:intronic:NM_004998.3	1	100		intronic	NM_00499	c.332+8G>	p.?	0.3					IQ calmodulin	rs4508371	2.00E-04		1.0 (pos.59548475)		Myosin IE	ATP binding,ATPase activity, co			Imidazole		C=0, T=33	C=0.0, T=1.0	0.00001	504.145	33	C=0/0, T=24/9
chr15:59931429	C/C	G	C	SNV	GTF2A2	GTF2A2:intronic:NM_004492.2	1	100		intronic	NM_00449	c.305-73C	p.?	-0.09					Transcription	rs2306354	0.3634	YES			General tra	RNA polymerase II repressing transcription factor bindin					G=0, C=59	G=0.0, C=1.0	0.00001	885.341	59	G=0/0, C=20/3
chr15:59964798	G/G	C	G	SNV	BNIP2	BNIP2:intronic:NM_004330.3	1	100		intronic	NM_00433	c.938+38G	p.?	-1.04					Bcl2-/adenov	rs4774342	1.00E-04		1.0 (pos.59964798)		BCL2/adenc	GTPase activator activity,apoptotic process,blastocyst c					C=0, G=28	C=0.0, G=1.0	0.00001	408.708	28	C=0/0, G=24/4
chr15:60678298	A/A	T	A	SNV	ANXA2	ANXA2:intronic:NM_001002857.1	1	100		intronic	NM_00100	c.-10-13A>	p.?	-3.48					Annexin	rs11855679,rs74702	0.1774	YES	0.0 (pos.60678297),9.998E-1 (pos.60678		Annexin A2	PCSK9-Anx	Bone Diseases;Bon		Tenecteplase		T=0, A=33	T=0.0, A=1.0	0.00001	504.147	33	T=0/0, A=14/1
chr15:60678328	T/T	C	T	SNV	ANXA2	ANXA2:intronic:NM_001002857.1	1	100		intronic	NM_00100	c.-10-43G>	p.?	-0.5					Annexin	rs11858864	0.1776	YES	9.995E-1 (pos.60678328)		Annexin A2	PCSK9-Anx	Bone Diseases;Bon		Tenecteplase		C=0, T=31	C=0.0, T=1.0	0.00001	465.808	31	C=0/0, T=11/2
chr15:60678350	C/C	T	C	SNV	ANXA2	ANXA2:intronic:NM_001002857.1	1	100		intronic	NM_00100	c.-10-65A>	p.?	1.34					Annexin	rs7182242	0.1766	YES			Annexin A2	PCSK9-Anx	Bone Diseases;Bon		Tenecteplase		T=0, C=31	T=0.0, C=1.0	0.00001	465.8	31	T=0/0, C=10/2
chr15:60690089	G/G	A	G	SNV	ANXA2	ANXA2:intronic:NM_001002857.1	1	100		intronic	NM_00100	c.-92+53T	p.?	-2.62				0(COSM1129	Annexin	rs12904657	0.2674	YES	9.026E-1 (pos.60690089)	adenocarcino	Annexin A2	PCSK9-Anx	Bone Diseases;Bon		Tenecteplase		A=0, G=50	A=0.0, G=1.0	0.00001	852.738	50	A=0/0, G=32/1
chr3:46021218	T/T	G	T	SNV	FYCO1	FYCO1:exonic:NM_024513.3	1	100	4	synonymous	NM_02451	c.267C>A	p.(=)	-0.37					FYVE zinc fing	rs4682801	0.2285	YES	9.997E-1 (pos.46021218)		FYVE and co	'de novo' p	Cataract;Eye Diseases;Lens Di			Benign	G=0, T=25	G=0.0, T=1.0	0.00001	353.019	25	G=0/0, T=15/1

chr15:62259772	C/T	C	T	SNV	VPS13C	VPS13C:intronic:NM 020821.2	1	50		intronic	NM 02082	c.2912-12	p.?	-1.5					Autophagy-re	rs3784633		YES			Vacuolar pr	Golgi to endosome transport,cytoplasm,cytosol,extrace				C=15, T=15	C=0.5, T=0.5	0.00001	145.021	30	C=5/10, T=11/
chr15:62967395	T/T	C	T	SNV	TLN2	TLN2:intronic:NM 015059.2	1	100		intronic	NM 01505	c.853-21C	p.?	-1.18					FERM N-term	rs1878784	0.0034	YES	9.554E-1 (pos.62967395)		Talin 2	actin bind	Nervous System Diseases:Neuromuscular Di			C=0, T=28	C=0.0, T=1.0	0.00001	408.712	28	C=0/0, T=14/1
chr15:62967554	C/C	T	C	SNV	TLN2	TLN2:intronic:NM 015059.2	1	100		intronic	NM 01505	c.957+34T	p.?	-1.6					FERM N-term	rs1878783	0.0015	YES	9.628E-1 (pos.62967554)		Talin 2	actin bind	Nervous System Diseases:Neuromuscular Di			T=0, C=25	T=0.0, C=1.0	0.00001	353.015	25	T=0/0, C=10/1
chr15:62979016	C/C	A	C	SNV	TLN2	TLN2:intronic:NM 015059.2	1	100		intronic	NM 01505	c.1113+21	p.?	-2.78					FERM N-term	rs12917611	0.0522	YES	9.571E-1;0.0;0.0 (pos.62979016)		Talin 2	actin bind	Nervous System Diseases:Neuromuscular Di			A=0, C=40	A=0.0, C=1.0	0.00001	644.281	40	A=0/0, C=15/2
chr15:63014548	G/G	A	G	SNV	TLN2	TLN2:intronic:NM 015059.2	1	100		intronic	NM 01505	c.2992-4A	p.?	0.26				0.08(COSM50	FERM N-term	rs10775181	0.0409	YES	9.596E-1 (pos.63014548)	osteosarcoma	Talin 2	actin bind	Nervous System Diseases:Neuromuscular Di			A=0, G=20	A=0.0, G=1.0	0.00001	263.786	20	A=0/0, G=11/9
chr15:63335248	C/A	C	A	SNV	TPM1	TPM1:intronic:NM 001018020.1	1	57.14		intronic	NM 00101	c.114+106	p.?	0.15					Tropomyosin,Tropomyosin like						Tropomyosi	ACMG Recommendations...;Aortic Stenosis, S				C=15, A=20	C=0.4286, A=0.5714	0.00001	218.22	35	C=0/15, A=14/
chr3:46008087	G/A	G	A	SNV	FYCO1	FYCO1:exonic:NM 024513.3	1	58.33	8	synonymous	NM 02451	c.2739C>T	p.(=)	-0.43				0.05(COSM41	FYVE zinc fing	rs13079869	0.1101	YES	3.012E-3 (pos.46008087)	neoplasm	FYVE and co	'de novo' p	Cataract:Eye Diseases:Lens Di		Benign	G=10, A=14	G=0.4167, A=0.5833	0.00001	135.329	24	G=4/6, A=1/13
chr15:63848805	G/G	C	G	SNV	USP3	USP3:intronic:NM 006537.3	1	100		intronic	NM 00653	c.369-61C	p.?	-0.23					Ubiquitin car	rs3751043		YES			Ubiquitin-s	DNA repair,Flemming body,chromatin binding,core prom				C=0, G=31	C=0.0, G=1.0	0.00001	465.819	31	C=0/0, G=19/1
chr3:197495334	A/A	G	A	SNV	FYTTD1	FYTTD1:exonic:NM 032288.6	1	100	3	missense	NM 03228	c.260G>A	p.Arg87His	2.15	0.05	29	0.073	0.85(COSM37	Forty-two-thr	rs3205525	0.1566	YES	9.44E-1 (pos.197495334)	adenocarcinom	Forty-two-tl	RNA binding,intracellular,mRNA binding,mRNA export fro				G=0, A=47	G=0.0, A=1.0	0.00001	789.328	47	G=0/0, A=27/2
chr8:28384712	G/G	A	G	SNV	FZD3	FZD3:exonic:NM 145866.1	1	100	4	synonymous	NM 14586	c.435A>G	p.(=)	0.75				0.66(COSM37	Frizzled/Smo	rs2241802	0.4323	YES	5.878E-1 (pos.28384712)	adenocarcinom	Frizzled, Dr	G-protein coupled receptor activity,G-protein coupled re				A=0, G=49	A=0.0, G=1.0	0.00001	831.63	49	A=0/0, G=30/1
chr15:63984772	C/C	T	C	SNV	HERC1	HERC1:intronic:NM 003922.3	1	100		intronic	NM 00392	c.5675-7A	p.?	0.41				0.2(COSM147	HECT-domain	rs11630720	0.4543	YES	6.964E-2 (pos.63984772)	acute_myeloic	HECT domai	ARF guanyl-nucleotide exchange factor activity,Golgi apc				T=0, C=29	T=0.0, C=1.0	0.00001	427.53	29	T=0/0, C=12/1
chr2:208633413	C/C	T	C	SNV	FZD5	FZD5:exonic:NM 003468.3	1	100	2	synonymous	NM 00346	c.51A>G	p.(=)	0.03				0.03(COSM37	Frizzled/Smo	rs4675711	0		1.0 (pos.208633413)	adenocarcinom	Polarity ger	G-protein coupled receptor activity,G-protein coupled re				T=0, C=58	T=0.0, C=1.0	0.00001	865.219	58	T=0/0, C=31/2
chr15:64050300	A/A	G	A	SNV	HERC1	HERC1:intronic:NM 003922.3	1	100		intronic	NM 00392	c.1221+74	p.?	-0.02					HECT-domain	rs12912999		YES			HECT domai	ARF guanyl-nucleotide exchange factor activity,Golgi apc				G=0, A=21	G=0.0, A=1.0	0.00001	281.095	21	G=0/0, A=9/12
chr8:104337096	G/G	A	G	SNV	FZD6	FZD6:exonic:NM 001164615.1	1	100	4	synonymous	NM 00116	c.762A>G	p.(=)	0.44				0.55(COSM37	Frizzled/Smo	rs3808554	0.4662	YES	6.256E-1 (pos.10433709	adenocarcinom	Frizzled, Dr	G-protein	Congenital, Hereditary, and Neonatal Diseas			A=0, G=75	A=0.0, G=1.0	0.00001	1175.01	75	A=0/0, G=46/2
chr15:64410484	T/T	G	T	SNV	SNX1	SNX1:intronic:NM 001242933.1	1	100		intronic	NM 00124	c.399+41G	p.?	-0.02					PX domain,Sc	rs2290117	0.2102	YES	8.223E-1 (pos.64410484)		Sorting nexi	Golgi apparatus,cadherin binding,cytoplasm,cytosol,ear				G=0, T=81	G=0.0, T=1.0	0.00001	1299.89	81	G=0/0, T=36/4
chr15:64428657	A/A	G	A	SNV	SNX1	SNX1:intronic:NM 001242933.1	1	100		intronic	NM 00124	c.1446+48	p.?	-0.12					PX domain,Sc	rs2290122	0.2108	YES	8.268E-1 (pos.64428657)		Sorting nexi	Golgi apparatus,cadherin binding,cytoplasm,cytosol,ear				G=0, A=43	G=0.0, A=1.0	0.00001	705.84	43	G=0/0, A=26/1
chr15:64706259	A/A	G	A	SNV	TRIP4	TRIP4:intronic:NM 016213.4	1	100		intronic	NM 01621	c.1044-25	p.?	-0.07					ASCH domain	rs2046356,rs771474	0.1176	YES	0.0 (pos.64706244),9.55E-1 (pos.647062		Thyroid hor	activating	Alzheimer Disease:Brain Diseases:Central Ne			G=0, A=25	G=0.0, A=1.0	0.00001	353.018	25	G=0/0, A=18/7
chr8:104337367	C/C	A	C	SNV	FZD6	FZD6:exonic:NM 001164615.1	1	100	4	missense	NM 00116	c.1033A>C	p.Met345Leu	3.77	0.32	15	0.008	0.91(COSM37	Frizzled/Smo	rs3808553	0.4663	YES	6.262E-1 (pos.10433736	acute_myeloic	Frizzled, Dr	G-protein	Congenital, Hereditary, and Neonatal Diseas			A=0, C=30	A=0.0, C=1.0	0.00001	446.639	30	A=0/0, C=19/1
chr8:104343686	A/A	G	A	SNV	FZD6	FZD6:exonic:NM 001164615.1	1	100	7	synonymous	NM 00116	c.2070G>A	p.(=)	-4.8				0.01(COSM39	Frizzled/Smo	rs1053917	0.4642	YES	6.114E-1 (pos.10434368	acute_myeloic	Frizzled, Dr	G-protein	Congenital, Hereditary, and Neonatal Diseas			G=0, A=23	G=0.0, A=1.0	0.00001	316.747	23	G=0/0, A=7/16
chr15:65449169	T/T	C	T	SNV	CLPX	CLPX:intronic:NM 006660.4	1	100		intronic	NM 00666	c.1146+13	p.?	1.26					AAA domain (	rs2946670			1.0 (pos.65449169)		Caseinolyti	ATP binding,ATP metabolic process,ATP-dependent pept				C=0, T=57	C=0.0, T=1.0	0.00001	839.825	57	C=0/0, T=32/2
chr17:78078709	C/C	T	C	SNV	GAA	GAA:exonic:NM 001079804.2	1	100	2	synonymous	NM 00107	c.324T>C	p.(=)	1.99				0.18(COSM37	Galactose mu	rs1800300	0.251	YES	6.37E-1 (pos.78078709)	adenocarcinom	Glucosidase	alpha-1,4-	Aortic Stenosis, Sub	AT2220.Ac	Benign	T=0, C=24	T=0.0, C=1.0	0.00001	334.792	24	T=0/0, C=4/20
chr17:78091405	A/A	G	A	SNV	GAA	GAA:exonic:NM 001079804.2	1	100	17	missense	NM 00107	c.2338G>A	p.Val780Ile	-0.39	0.54	29	0.001	0.03(COSM37	Galactose mu	rs1126690	0.2501	YES	6.212E-1;0.0 (pos.780914	adenocarcinom	Glucosidase	alpha-1,4-	Aortic Stenosis, Sub	AT2220.Ac	Benign	G=0, A=22	G=0.0, A=1.0	0.00001	298.892	22	G=0/0, A=9/13
chr5:161119071	T/T	G	T	SNV	GABRA6	GABRA6:exonic:NM 000811.2	1	100	8	synonymous	NM 00081	c.951G>T	p.(=)	-0.13					Neurotransm	rs12522663	5.00E-04	YES	1.0 (pos.161119071)		Gamma-am	GABA-A rec	Mental Disorders;S	Acamprosate,Adipiplon,		G=0, T=22	G=0.0, T=1.0	0.00001	298.889	22	G=0/0, T=10/1
chr15:65495820	G/G	A	G	SNV	CILP	CILP:intronic:NM 003613.3	1	100		intronic	NM 00361	c.920-12T	p.?	0.35					Immunoglob	rs2072048	0.3578	YES	2.306E-1 (pos.65495820)		Cartilage in	alkaline pl	Bone Diseases;Intervertebral Disc Degenera			A=0, G=22	A=0.0, G=1.0	0.00001	298.892	22	A=0/0, G=8/14
chr15:65495827	G/G	A	G	SNV	CILP	CILP:intronic:NM 003613.3	1	100		intronic	NM 00361	c.920-19T	p.?	1.63					Immunoglob	rs2073707	0.3623	YES	2.255E-1 (pos.65495827)		Cartilage in	alkaline pl	Bone Diseases;Intervertebral Disc Degenera			A=0, G=22	A=0.0, G=1.0	0.00001	298.89	22	A=0/0, G=8/14
chr6:89907873	A/A	G	A	SNV	GABRR1	GABRR1:exonic:NM 002042.4	1	100	5	synonymous	NM 00204	c.438C>T	p.(=)	-0.03				0.53(COSM37	Neurotransm	rs422751	0.1695	YES	9.537E-1 (pos.89907873)	adenocarcinom	Gamma-am	GABA-A receptor activity,GABA		Adinazolam,Bromazepar		G=0, A=59	G=0.0, A=1.0	0.00001	885.359	59	G=0/0, A=27/3
chr15:65684422	T/TC	T	TC	INDEL	IGDCC4	IGDCC4:intronic:NM 020962.2	1	50		intronic	NM 02096	c.2122+50	p.?	-1.04					Fibronectin t	rs3216754	0.2084	YES	2.495E-1 (pos.65684422)		Immunoglo	integral component of membrane,plasma membrane				T=17, TC=1	T=0.5, TC=0.5	0.00001	154.072	34	T=6/11, TC=3/
chr6:89981413	C/C	T	C	SNV	GABRR2	GABRR2:exonic:NM 002043.4	1	100	3	synonymous	NM 00204	c.249A>G	p.(=)	-0.01				0.28(COSM37	Neurotransm	rs282117	0.3999	YES	6.911E-1 (pos.89981413)	adenocarcinom	Gamma-am	GABA-A rec	Digestive System Di	Adinazolam,Bromazepar		T=0, C=64	T=0.0, C=1.0	0.00001	977.631	64	T=0/0, C=22/4
chr4:843695	G/G	A	G	SNV	GAK	GAK:exonic:NM 005255.3	1	100	27	synonymous	NM 00525	c.3819T>C	p.(=)	-4.75				0.03(COSM37	C2 domain of	rs8093	0.3155	YES	8.864E-1 (pos.843695)	adenocarcinom	Cyclin G ass	ATP bindin	Basal Ganglia Diseases;Brain Diseases;Centr			A=0, G=55	A=0.0, G=1.0	0.00001	814.886	55	A=0/0, G=27/2
chr4:860192	G/G	A	G	SNV	GAK	GAK:exonic:NM 005255.3	1	100	22	synonymous	NM 00525	c.3003T>C	p.(=)	-1.08				0.04(COSM41	C2 domain of	rs1064207	0.3674	YES	6.583E-1 (pos.860192)	neoplasm	Cyclin G ass	ATP bindin	Basal Ganglia Diseases;Brain Diseases;Centr			A=0, G=33	A=0.0, G=1.0	0.00001	504.597	33	A=0/0, G=8/25
chr11:68458470	C/C	T	C	SNV	GAL	GAL:utr 3:NM 015973.4	1	100	6	utr 3	NM 01597	c.387T>C	p.?	-2.61					Galanin,Gala	rs1042577	0.3498	YES	7.777E-1 (pos.68458470)		Galanin	G-protein	Arrhythmias, Cardiac:Cardiovascular Disease			T=0, C=101	T=0.0, C=1.0	0.00001	1579.73	101	T=0/0, C=55/4
chr15:67463013	AG/A	AG	A	INDEL	SMAD3	SMAD3:intronic:NM 005902.3	1	100		intronic	NM 00590	c.658+71A	p.?	1.15, 0.13					MH1 domain,MH2 domain						Mothers ag	DEAD/H-bo	ACMG Recommendations...:Abnormalities, M			AG=0, A=32	AG=0.0, A=1.0	0.00001	262.099	32	AG=0/0, A=5/2
chr7:99757612	A/A	G	A	SNV	GAL3ST4	GAL3ST4:exonic:NM 024637.4	1	100	4	missense	NM 02463	c.1400C>T	p.Ala467Val	1.5	0.04	64	0.008	0.83(COSM37	Galactose-3-	rs3823646	0.4317	YES	6.351E-1 (pos.99757612)	adenocarcinom	Galactose-3	3'-phosphoadenosine 5'-phosphosulfate binding,Golgi ap				G=0, A=25	G=0.0, A=1.0	0.00001	353.019	25	G=0/0, A=10/1
chr15:67782440	A/A	AGGAAAT	A	INDEL	IQCH,IQCH-AS1	IQCH-AS1:intronic_nc:NR 040051.1, IQCH:intronic:NM 001(	6	100		intronic	NM 00103	c.2861+38	p.?	1.07, -1.03, -0.78, 0.29, 2.41, 0.92, -0.99					IQ calmoduli	rs10574862,rs11230	0.1307	YES	0.0 (pos.67782446),7.836E-1 (pos.67782		IQ motif-cor	biological	Melanoma;Neoplasms;Neoplasms by Histolc			AGGAAAT=(	AGGAAAT=0.0, A=1.0	0.00001	262.239	20	AGGAAAT=0/0
chr15:67985931	C/C	T	C	SNV	MAP2K5	MAP2K5:intronic:NM 145160.2	1	100		intronic	NM 14516	c.972+25T	p.?	2.59					PB1 domain,I	rs8034680	0		1.0 (pos.67985931)		Mitogen-ac	ATP bindin	Amphetamine-Related Disorders:Dyssomnia			T=0, C=65	T=0.0, C=1.0	0.00001	996.462	65	T=0/0, C=39/2
chr15:68121483	C/C	A	C	SNV	SKOR1	SKOR1:intronic:NM 001258024.1	1	100		intronic	NM 00125	c.2026-34	p.?	-0.06					SKI/SNO/DAC	rs7183168,rs754105	0.4249	YES	0.0;0.0 (pos.68121482),9.57E-1 (pos.68121483)			SMAD binding,dendrite,negative regulation of BMP signa				A=0, C=28	A=0.0, C=1.0	0.00001	405.153	28	A=0/0, C=21/7
chr15:68510770	A/G	A	G	SNV	CLN6	CLN6:intronic:NM 017882.2	1	59.09		intronic	NM 01788	c.198+104	p.?	-0.7					Ceroid-lipofu	rs8025947		YES			CLN6 gene	cellular m	Brain Diseases;Central Nervous System Dise			A=54, G=78	A=0.4091, G=0.5909	0.00001	913.451	132	A=41/13, G=1
chr15:68618961	C/C	T	C	SNV	ITGA11	ITGA11:intronic:NM 001004439.1	1	100		intronic	NM 00100	c.2211+31	p.?	-2.04					FG-GAP repea	rs2271722	0.2153	YES	8.36E-1 (pos.68618961)		Integrin, alp	cell adhes	Attention Deficit Disorder with Hyperactivity			T=0, C=20	T=0.0, C=1.0	0.00001	263.773	20	T=0/0, C=5/15
chr7:99758136	G/G	T	G	SNV	GAL3ST4	GAL3ST4:exonic:NM 024637.4	1	100	4	synonymous	NM 02463	c.876A>C	p.(=)	-0.01				0.08(COSM37	Galactose-3-	rs3800951	0.428	YES	6.364E-1 (pos.99758136)	acute_myeloic	Galactose-3	3'-phosphoadenosine 5'-phosphosulfate binding,Golgi ap				T=0, G=75	T=0.0, G=1.0	0.00001	1189.83	75	T=0/0, G=26/4
chr14:88407875	A/A	T	A	SNV	GALC	GALC:exonic:NM 000153.3	1	100	15	synonymous	NM 00015	c.1698A>T	p.(=)	-0.03					Glycosyl hydr	rs386779713,rs42146	6.00E-04	YES	9.374E-1;0.0 (pos.88407875)		Galactosylc	extracellu	Brain Diseases:Brain Diseases		Benign	T=0, A=33	T=0.0, A=1.0	0.00001	504.598	33	T=0/0, A=12/2
chr15:69003871	T/T	G	T	SNV	CORO2B	CORO2B:intronic:NM 001190456.1	1	100		intronic	NM 00119	c.469-10G	p.?	-0.06					Domain of un	rs3736272	0.283	YES	8.716E-1 (pos.69003871)		Coronin 2B	actin binding,actin cytoskeleton,actin cytoskeleton orga				G=0, T=36	G=0.0, T=1.0	0.00001	563.744	36	G=0/0, T=7/29
chr16:88908306	G/A	G	A	SNV	GALNS	GALNS:exonic:NM 000512.4	1	50	3	synonymous	NM 00051	c.318C>T	p.(=)	-1.24				0.13(COSM63	C-terminal re	rs34278797	0.0307	YES	0.0 (pos.88908306)	astrocytoma (	Galactosam	N-acetylga	Carbohydrate Meta	Elosulfase	Conflicting in	G=13, A=13	G=0.5, A=0.5	0.00001	119.163	26	G=0/13, A=8/5
chr7:151818777	C/C	T	C	SNV	GALNT11	GALNT11:utr 3:NM 022087.3	1	100	12	utr 3	NM 02208	c.1842T>C	p.?	-0.26					Glycosyl tran	rs967093	0.0098		9.999E-1;0.0 (pos.151818777)		UDP-N-acet	Golgi membrane,Notch binding,Notch receptor processi				T=0, C=30	T=0.0, C=1.0	0.00001	446.289	30	T=0/0, C=11/1
chr15:69732483	A/A	G	A	SNV	KIF23	KIF23:intronic:NM 138555.3	1	100		intronic	NM 13855	c.1887+67	p.?	-0.35					Arf6-interact	rs4776448		YES			Kinesin fam	ATP bindin	Anemia:Anemia, Dyserythropoietic, Congenit			G=0, A=51	G=0.0, A=1.0	0.00001	748.359	51	G=0/0, A=29/2
chr15:71146170	T/A	T	A	SNV	LARP6	LARP6:intronic:NM 018357.3	1	50		intronic	NM 01835	c.200+58A	p.?	-0.47					La domain,SL	rs531031621					La ribonucl	RNA processing,cytoplasm,intracellular ribonucleoprote				T=10, A=10	T=0.5, A=0.5	0.00001	82.8786	20	T=4/6, A=7/3
chr15:71175296	G/G	A	G	SNV	THAP10	THAP10:intronic:NM 020147.3	1	100		intronic	NM 02014	c.430-49T	p.?	-0.12					THAP domain	rs1528828	0		1.0 (pos.71175296)		THAP doma	DNA binding,metal ion binding,protein binding				A=0, G=55	A=0.0, G=1.0	0.00001	811.961	55	A=0/0, G=31/2
chr3:16261420	C/T	C	T	SNV	GALNT15	GALNT15:exonic:NM 054110.4	1	50	8	missense	NM 05411	c.1528C>T	p.His510Tyr	1.69	0.57	83	0.01	0.5(COSM175	Glycosyl tran	rs2271077	0.059	YES	2.058E-1 (pos.16261420)	acute_myeloic	UDP-N-acet	Golgi membrane,O-glycan processing,carbohydrate bind				C=14, T=14	C=0.5, T=0.5	0.00001	131.115	28	C=7/7, T=7/7
chr12:4881766	C/T	C	T	SNV	GALNT8	GALNT8:utr 3:NM 017417.1	1	57.14	11	utr 3	NM 01741	c.1917C>T	p.?	-0.19					Glycosyl tran	rs2286581	0.4151	YES	3.633E-1 (pos.4881766)		UDP-N-acet	Golgi membrane,O-glycan processing,carbohydrate bind				C=18, T=24	C=0.4286, T=0.5714	0.00001	278.84	42	C=12/6, T=4/2
chr7:151680072	C/C	T	C	SNV	GALNTL5	GALNTL5:exonic:NM 145292.3	1	100	4	missense	NM 14529	c.370T>C	p.Cys124Arg	3.52	0.01	180	1		Glycosyl tran	rs6960270	0.1442	YES	8.539E-1 (pos.151680072)		UDP-N-acet	integral component of membrane,late endosome memb				T=0, C=102	T=0.0, C=1.0	0.00001	1592.97	102	T=0/0, C=61/4
chr15:72454729	G/G	C	G	SNV	GRAMD2A	GRAMD2A:intronic:NM 001012642.2	1	100		intronic	NM 00101	c.957-11G	p.?	-0.02					GRAM domai	rs897731	0		1.0 (pos.72454729)			integral component of membrane				C=0, G=95	C=0.0, G=1.0	0.00001	1597.06	95	C=0/0, G=36/5
chr15:72462165	C/C	A	C	SNV	GRAMD2A	GRAMD2A:intronic:NM 001012642.2	1	100		intronic	NM 00101	c.134+23T	p.?	-0.35					GRAM domai	rs11072348	8.00E-04	YES	1.0 (pos.72462165)			integral component of membrane				A=0, C=41	A=0.0, C=1.0	0.00001	663.727	41	A=0/0, C=16/2
chr15:72543656	C/C	T	C	SNV	PARP6	PARP6:intronic:NM 020214.3	1	100		intronic	NM 02021	c.1260-60	p.?	-0.34					Poly(ADP-ribo	rs2959904	0	YES				NAD+ ADP-ribosyltransferase activity				T=0, C=29	T=0.0, C=1.0	0.00001	424.156	29	T=0/0, C=8/21
chr15:72545876	C/C	T	C	SNV	PARP6	PARP6:intronic:NM 020214.3	1	100		intronic	NM 02021	c.1192-31	p.?	-1.46					Poly(ADP-ribo	rs1426166	0		9.999E-1 (pos.72545876)			NAD+ ADP-ribosyltransferase activity				T=0, C=27	T=0.0, C=1.0	0.00001	389.959	27	T=0/0, C=8/19
chr18:74962645	C/C	T	C	SNV	GALR1	GALR1:exonic:NM 001480.3	1	100	1	synonymous	NM 00148	c.141T>C	p.(=)	-0.3				0.12(COSM13	7 transmemb	rs5374	0.355	YES	3.66E-1 (pos.74962645)	adenocarcinom	Galanin rec	G-protein coupled receptor signaling pathway,adenylate				T=0, C=89	T=0.0, C=1.0	0.00001	1472.49	89	T=0/0, C=53/3
chr15:73002035	A/A	G	A	SNV	BBS4	BBS4:intronic:NM 033028.4	1	100		intronic	NM 03302	c.77-6G>A	p.?	-2.12					TPR repeat,T	rs8033604	3.00E-04	YES	1.0 (pos.73002035)		BBS4 gene	BBSome,R	Abnormalities, Multiple;Barde		Benign	G=0, A=23	G=0.0, A=1.0	0.00001	316.747	23	G=0/0, A=16/7
chr18:74980809	A/A	G	A	SNV	GALR1	GALR1:exonic:NM 001480.3	1	100	3	missense	NM 00148	c.1001G>A	p.Ser334Asn	0.05	0.56	46	0		7 transmemb	rs5376	0.0012	YES	1.0 (pos.74980809)		Galanin rec	G-protein coupled receptor signaling pathway,adenylate				G=0, A=39	G=0.0, A=1.0	0.00001	621.952	39	G=0/0, A=21/1
chr15:42568547	G/G	C	G	SNV	GANC	GANC:exonic:NM 198141.2	1	100	2	missense	NM 19814	c.31C>G	p.Leu11Val	1.63		32	0	0.03(COSM54	Galactose mu	rs8043515	8.00E-04	YES	9.474E-1;0.0 (pos.42568	adenocarcinom	Neutral alp	alpha-1,4-glucosidase activity,		Miglitol		C=0, G=26	C=0.0, G=1.0	0.00001	361.019	26	C=0/0, G=16/1
chr9:128074807	T/C	T	C	SNV	GAPVD1	GAPVD1:exonic:NM 015635.3	1	95.45	7	synonymous	NM 01563	c.1518T>C	p.(=)	-0.05					GTPase-activ	rs432757	0.0569	YES	1.0 (pos.128074807)		GTPase-act	GTPase activating protein binding,GTPase activator acti				T=1, C=21	T=0.0455, C=0.9545	0.00001	271.509	22	T=1/0, C=11/1
chr15:73551250	G/G	A	G	SNV	NEO1	NEO1:intronic:NM 002499.3	1	100		intronic	NM 00249	c.2203+33	p.?	-0.41					Fibronectin t	rs2251205	0.4808	YES	3.116E-1 (pos.73551250)		Neogenin, c	Golgi apparatus,axon guidance,cell adhesion,co-recepto				A=0, G=28	A=0.0, G=1.0	0.00001	408.702	28	A=0/0, G=18/1
chr2:26410214	C/T	C	T	SNV	GAREM2	GAREM2:exonic:NM 001168241.1	1	58.33	6	synonymous	NM 00116	c.1713C>T	p.(=)	-4.86					Cell-cycle sus	rs1019972	0.0493	YES	0.0;0.0 (pos.26410214)			extracellular exosome				C=15, T=21	C=0.4167, T=0.5833	0.00001	229.596	36	C=8/7, T=20/1
chr21:34897113	T/T	C	T	SNV	GART	GART:exonic:NM 000819.4	1	100	11	missense	NM 00081	c.1261G>A	p.Val421Ile	5.79	1	29	0		AIR synthase	rs8788	0.2394	YES	9.923E-1 (pos.34897113)		Phosphorib	'de novo' II	Abnormalities, Mul	10-CF3C(OH)2-DDACTHF,(		C=0, T=21	C=0.0, T=1.0	0.00001	280.532	21	C=0/0, T=11/1
chr16:90102835	A/G	A	G	SNV	GAS8	GAS8:exonic:NM 001481.2	1	55.77	6	synonymous	NM 00148	c.597A>G	p.(=)	2.26					Growth-arres	rs868045	0.4107	YES	7.63E-3 (pos.90102835)		Growth arre	Golgi apparatus,Rab GTPase binding,axon			Benign	A=23, G=29	A=0.4423, G=0.5577	0.00001	294.59	52	A=8/15, G=8/2
chr15:74033033	G/G	A	G	SNV	C15orf59	C15orf59:intronic:NM 001039614.2	1	100		intronic	NM 00103	c.157-50T	p.?	-0.78					Domain of un	rs11858458	0.1032	YES	8.978E-1 (pos.74033033)		Chromosome 15 open reading frame 59					A=0, G=146	A=0.0, G=1.0	0.00001	2467.38	146	A=0/0, G=84/6
chr19:19606634	A/A	G	A	SNV	GATAD2A	GATAD2A:exonic:NM 017660.3	1	100	6	synonymous	NM 01766	c.696G>A	p.(=)	-1.37				0.15(COSM37	Coiled-coil ar	rs1047361	0.3524	YES	2.948E-1 (pos.19606634)	adenocarcinom	GATA zinc fi	DNA binding transcription factor activity,DNA methylatic				G=0, A=54	G=0.0, A=1.0	0.00001	799.421	54	G=0/0, A=37/1
chr15:74477216	G/A	G	A	SNV	STRA6	STRA6:intronic:NM 001199042.1	1	97.06		intronic	NM 00119	c.1283+10	p.?	0.29					Retinol bindi	rs351238		YES			Stimulated	adrenal gl	Anophthalmos;Cardiovascular Abnormalities			G=1, A=33	G=0.0294, A=0.9706	0.00001	493.406	34	G=1/0, A=13/2
chr15:74477239	G/A	G	A	SNV	STRA6	STRA6:intronic:NM 001199042.1	1	100		intronic	NM 00119	c.1283+82	p.?	1.28					Retinol bindi	rs351237		YES			Stimulated	adrenal gl	Anophthalmos;Cardiovascular Abnormalities			G=0, A=28	G=0.0, A=1.0	0.00001	408.666	28	G=0/0, A=8/20
chr15:74659609	T/T	G	T	SNV	CYP11A1	CYP11A1:intronic:NM 000781.2	1	100		intronic	NM 00078	c.269+49C	p.?	-0.2					Cytochrome I	rs28681535	0.1583	YES	4.246E-1 (pos.74659609)		Cytochrome	C21-steroi	46, XY Disorders of	Aminoglutethimide,Chol		G=0, T=27	G=0.0, T=1.0	0.00001	394.375	27	G=0/0, T=19/8
chr7:99821685	T/T	C	T	SNV	GATS	GATS:exonic:NM 178831.6	1	100	3	synonymous	NM 17883	c.231G>A	p.(=)	-2.38				0.06(COSM17	ACT domain	rs863450	0.2585	YES	3.8E-1 (pos.99821685)	adenocarcinoma						C=0, T=44	C=0.0, T=1.0	0.00001	726.555	44	C=0/0, T=27/1
chr7:99818639	ACCCCC/ACCC	ACCCCC	ACCCC,ACCCCG	INDEL	GATS,PVRIG	PVRIG:exonic:NM 024070.3, GATS:intronic:NM 178831.6	1	73.33	6	frameshiftDeletion	NM 17883	c.*540+25	p.?, p.His251fs	0.03, -0.33, -1.23, -1.26, 0.46, 0.15					ACT domain	rs184351940,rs7688	0		0.0 (pos.99818640),0.0;0.0 (pos.99818		Poliovirus r	integral component of membrane,negative regulation of				ACCCCC=6,	ACCCCC=0.2, ACCCC=	0.00001	169.17	30	ACCCCC=2/4, A
chr7:99817859	G/G	A	G	SNV	GATS,PVRIG	PVRIG:exonic:NM 024070.3, GATS:intronic:NM 178831.6	1	100	3	missense	NM 17883	c.*540+33	p.?, p.Asn81Asp	0.36	1	23	0	0.01(COSM11	ACT domain	rs2906645	0.2619	YES	4.591E-1 (pos.99817859)	adenocarcinom	Poliovirus r	integral component of membrane,negative regulation of				A=0, G=22	A=0.0, G=1.0	0.00001	298.876	22	A=0/0, G=14/8
chr4:22820504	T/T	A	T	SNV	GBA3	GBA3:exonic:NM 020973.4	1	100	5	stoploss	NM 02097	c.1368A>T	p.Ter456Tyr	0.36					Glycosyl hydr	rs358231	0.1508	YES	7.789E-1 (pos.22820504)		Glucosidase	beta-galac	Mental Disorders:Substance-Related Disorde			A=0, T=33	A=0.0, T=1.0	0.00001	504.598	33	A=0/0, T=12/2
chr10:104136469	C/C	T	C	SNV	GBF1	GBF1:exonic:NM 004193.2	1	100	32	synonymous	NM 00419	c.4197T>C	p.(=)	-1.06					Guanine nucl	rs2180707	0		1.0 (pos.104136469)		Golgi-specif	ARF guanyl-nucleotide exchange factor activity,COPI coat				T=0, C=48	T=0.0, C=1.0	0.00001	810.435	48	T=0/0, C=16/3
chr15:75653378	C/C	T	C	SNV	MAN2C1	MAN2C1:intronic:NM 001256494.1	1	100		intronic	NM 00125	c.1460+9A	p.?	-0.06					Alpha manno	rs7174408	3.00E-04		1.0 (pos.75653378)		Mannosida	alpha-mannosidase activity,carbohydrate binding,cytos				T=0, C=23	T=0.0, C=1.0	0.00001	316.736	23	T=0/0, C=16/7
chr1:89849742	T/T	A	T	SNV	GBP6	GBP6:exonic:NM 198460.2	1	100	10	missense	NM 19846	c.1559A>T	p.Asp520Val	3.71	0.02	152	0.011		Guanylate-bi	rs959460	0.1087	YES	7.505E-1 (pos.89849742)		Guanylate-l	GTP binding,GTPase activity,cellular response to interfe				A=0, T=43	A=0.0, T=1.0	0.00001	705.844	43	A=0/0, T=14/2
chr15:76673716	T/C	T	C	SNV	SCAPER	SCAPER:intronic:NM 020843.3	1	46.88		intronic	NM 02084	c.3705+3A	p.?	7.38					S phase cycli	rs3765115	0.3125	YES	5.96E-1 (pos.76673716)		S-phase cyc	cytosol,en	Intellectual Disability;Mental Disorders:Nerv			T=17, C=15	T=0.5313, C=0.4688	0.00001	143.732	32	T=9/8, C=4/11
chr15:76957958	G/G	A	G	SNV	SCAPER	SCAPER:intronic:NM 020843.3	1	100		intronic	NM 02084	c.2645+36	p.?	-0.67					S phase cycli	rs280021	5.00E-04	YES	9.998E-1 (pos.76957958)		S-phase cyc	cytosol,en	Intellectual Disability;Mental Disorders:Nerv			A=0, G=35	A=0.0, G=1.0	0.00001	543.903	35	A=0/0, G=12/2
chr15:77087785	T/C	T	C	SNV	SCAPER	SCAPER:intronic:NM 020843.3	1	53.33		intronic	NM 02084	c.612-4A>	p.?	-1.35					S phase cycli	rs11852395	0.2846	YES	5.455E-1 (pos.77087785)		S-phase cyc	cytosol,en	Intellectual Disability;Mental Disorders:Nerv			T=14, C=16	T=0.4667, C=0.5333	0.00001	159.925	30	T=3/11, C=8/8
chr15:78474517	C/C	T	C	SNV	ACSBG1	ACSBG1:intronic:NM 015162.4	1	100		intronic	NM 01516	c.895-30A	p.?	-1.7					AMP-binding	rs6495277	0.1784	YES	8.921E-1 (pos.78474517)		Acyl-CoA syn	ATP binding,catalytic activity,c		2-(6-HYDROXY-1,3-BENZO		T=0, C=31	T=0.0, C=1.0	0.00001	465.745	31	T=0/0, C=18/1
chr1:89850843	G/A	G	A	SNV	GBP6	GBP6:exonic:NM 198460.2	1	50.99	11	missense	NM 19846	c.1717G>A	p.Ala573Thr	-0.17	0.32	58	0.003		Guanylate-bi	rs76159539	1.00E-04		0.0 (pos.89850843)		Guanylate-l	GTP binding,GTPase activity,cellular response to interfe				G=74, A=77	G=0.4901, A=0.5099	0.00001	817.244	151	G=20/54, A=2
chr15:78587910	G/G	A	G	SNV	WDR61	WDR61:intronic:NM 025234.2	1	100		intronic	NM 02523	c.12+107T	p.?	0.29					Anaphase-pro	rs1040262		YES	9.995E-1 (pos.78587910)			Cdc73/Paf1 complex,Ski complex,Wnt signaling pathway				A=0, G=25	A=0.0, G=1.0	0.00001	353.019	25	A=0/0, G=17/8
chr17:79770740	C/T	C	T	SNV	GCGR	GCGR:exonic:NM 000160.4	1	53.13	12	synonymous	NM 00016	c.1095C>T	p.(=)	-1.09				0.05(COSM49	7 transmemb	rs5384	0.1741	YES	1.608E-1 (pos.79770740)	carcinoma,du	Glucagon re	G-protein	Diabetes Mellitus:I	Glucagon recombinant		C=15, T=17	C=0.4688, T=0.5313	0.00001	172.885	32	C=7/8, T=4/13
chr15:78786410	C/C	A	C	SNV	IREB2	IREB2:intronic:NM 004136.3	1	100		intronic	NM 00413	c.2472+12	p.?	-0.19					Aconitase C-t	rs4887060	2.00E-04		1.0 (pos.78786410)		Iron-respon	4 iron, 4 su	Lung Diseases;Lung Diseases, Obstructive;Ne			A=0, C=60	A=0.0, C=1.0	0.00001	903.531	60	A=0/0, C=21/3
chr12:120589160	G/G	A	G	SNV	GCN1	GCN1:exonic:NM 006836.1	1	100	34	synonymous	NM 00683	c.4098T>C	p.(=)	-5.28						rs2286046	0.217	YES	9.845E-1 (pos.120589160)			RNA binding,cadherin binding,cellular response to amin				A=0, G=50	A=0.0, G=1.0	0.00001	852.745	50	A=0/0, G=15/3
chr15:78909480	A/A	G	A	SNV	CHRNA3	CHRNA3:intronic:NM 000743.4	1	100		intronic	NM 00074	c.268-5C>	p.?	-0.59				0.05(COSM54	Neurotransm	rs3743074	0.37	YES	5.703E-1 (pos.78909480)	adenocarcinom	Cholinergic	acetylchol	Adenocarcinoma:A	Bupropion,Cytisine,Dext		G=0, A=69	G=0.0, A=1.0	0.00001	1072.78	69	G=0/0, A=29/4
chr15:78911261	C/C	T	C	SNV	CHRNA3	CHRNA3:intronic:NM 000743.4	1	100		intronic	NM 00074	c.83-4A>G	p.?	-0.95					Neurotransm	rs2869547,rs750158	3.00E-04		1.0;0.0 (pos.78911261)		Cholinergic	acetylchol	Adenocarcinoma:A	Bupropion,Cytisine,Dext		T=0, C=26	T=0.0, C=1.0	0.00001	371.362	26	T=0/0, C=8/18
chr15:79066932	A/G	A	G	SNV	ADAMTS7	ADAMTS7:intronic:NM 014272.4	1	50		intronic	NM 01427	c.1876+34	p.?	-0.05					ADAM-TS Spa	rs12900168		YES	4.762E-1 (pos.79066932)		A disintegri	cell surfac	Adnexal Diseases;Arterial Occlusive Disease			A=13, G=13	A=0.5, G=0.5	0.00001	119.163	26	A=11/2, G=11/
chr6:10587038	G/G	C	G	SNV	GCNT2	GCNT2:exonic:NM 145655.3	1	100	1	missense	NM 14565	c.816C>G	p.Asp272Glu	-0.62	1	45	0		Core-2/I-Bran	rs539351	0.0013		9.065E-1 (pos.10587038)		Glucosamin	Golgi mem	Cataract;Eye Diseases;Lens Diseases			C=0, G=31	C=0.0, G=1.0	0.00001	465.819	31	C=0/0, G=22/9
chr15:79185877	A/G	A	G	SNV	MORF4L1	MORF4L1:intronic:NM 206839.2	1	56.36		intronic	NM 20683	c.658-4A>	p.?	-0.66					MRG,RNA bin	rs1836556	0.1271	YES	2.733E-1 (pos.79185877)		Mortality fa	NuA4 histo	Adenocarcinoma;Carcinoma;Carcinoma, Ade			A=24, G=31	A=0.4364, G=0.5636	0.00001	319.021	55	A=10/14, G=1
chr8:75262798	C/C	G	C	SNV	GDAP1	GDAP1:exonic:NM 018972.2	1	100	1	synonymous	NM 01897	c.102G>C	p.(=)	0.13				0.01(COSM41	Glutathione	rs7828201			1.0 (pos.75262798)	neoplasm,squ	Ganglioside	cellular re	Charcot-Marie-Tooth Disease:Congenital Abr			G=0, C=38	G=0.0, C=1.0	0.00001	603.03	38	G=0/0, C=7/31
chr19:18497024	C/C	G	C	SNV	GDF15,MIR3189	GDF15:exonic:NM 004864.3, MIR3189:upstream:NR 0361	1	100	1	missense	NM 00486	c.25G>C	p.Val9Leu	-0.41	1	32	0	0.06(COSM13	Transforming	rs1059519	0.3407	YES	6.6E-1 (pos.18497024)	adenocarcinom	Growth/diff	BMP signa	Adenocarcinoma;Anemia;Anemia, Sideroblas			G=0, C=20	G=0.0, C=1.0	0.00001	263.698	20	G=0/0, C=10/1
chr20:34022387	C/C	A	C	SNV	GDF5,GDF5OS	GDF5OS:exonic:NM 001350519.1, GDF5:exonic:NM 00055	1	100	2	synonymous	NM 00135	c.798A>C	p.(=), p.Ser276Ala	1.59	1	99	0	0.13(COSM14	TGF-beta pro	rs224331	0.346	YES	2.941E-1;1.285E-4 (pos.3	adenocarcinom	Growth/diff	BMP bindi	Arthritis:Arthritis, F	Isopropyl	Benign	A=0, C=31	A=0.0, C=1.0	0.00001	464.198	31	A=0/0, C=16/1
chr2:20871190	C/C	A	C	SNV	GDF7	GDF7:utr 3:NM 182828.3	1	100	2	utr 3	NM 18282	c.1358A>C	p.?	-0.45					TGF-beta pro	rs4666457	0		1.0 (pos.20871190)		Growth/diff	BMP signaling pathway,SMAD protein signal transductio				A=0, C=32	A=0.0, C=1.0	0.00001	476.521	32	A=0/0, C=12/2
chr15:79606408	T/T	C	T	SNV	TMED3	TMED3:intronic:NM 007364.3	1	100		intronic	NM 00736	c.417+61C	p.?	-0.73					emp24/gp25	rs3784544		YES				COPI vesicle coat,ER to Golgi vesicle-mediated transport				C=0, T=28	C=0.0, T=1.0	0.00001	408.717	28	C=0/0, T=18/1
chr2:20871293	G/G	A	G	SNV	GDF7	GDF7:utr 3:NM 182828.3	1	100	2	utr 3	NM 18282	c.1461A>G	p.?	0.3						rs1564696					Growth/diff	BMP signaling pathway,SMAD protein signal transductio				A=0, G=36	A=0.0, G=1.0	0.00001	557.74	36	A=0/0, G=12/2
chr15:80452268	A/A	G	A	SNV	FAH	FAH:intronic:NM 000137.2	1	100		intronic	NM 00013	c.314+49G	p.?	-0.62					Fumarylacet	rs1370273	0.404	YES	7.836E-1;0.0 (pos.80452268)		Fumarylace	L-phenylal	Amino Acid Disorde	4-Hydroxy	Benign	G=0, A=21	G=0.0, A=1.0	0.00001	281.234	21	G=0/0, A=7/14
chr15:80452886	G/G	T	G	SNV	FAH	FAH:intronic:NM 000137.2	1	100		intronic	NM 00013	c.364+85T	p.?	-0.54					Fumarylacet	rs733679		YES	5.254E-1 (pos.80452886)		Fumarylace	L-phenylal	Amino Acid Disorde	4-Hydroxy-Methyl-Phosp		T=0, G=24	T=0.0, G=1.0	0.00001	334.792	24	T=0/0, G=10/1
chr10:5808086	A/A	G	A	SNV	GDI2	GDI2:exonic:NM 001494.3	1	100	11	synonymous	NM 00149	c.1221C>T	p.(=)	-0.12				0.81(COSM37	GDP dissocia	rs1129614	0.2083	YES	1.564E-1 (pos.5808086)	acute_myeloic	GDP dissoci	GTPase ac	Carcinoma:Carcinoma, Squamous Cell:Diges			G=0, A=30	G=0.0, A=1.0	0.00001	446.639	30	G=0/0, A=19/1
chr15:90784250	C/C	T	C	SNV	GDPGP1	GDPGP1:exonic:NM 001013657.2	1	100	4	missense	NM 00101	c.110T>C	p.Met37Thr	0.12		81		0.11(COSM3999684)		rs7171194,rs752791	0.3517	YES	1.156E-4 (pos.90784234)	acute_myeloid_leukaemia		GDP-D-glucose phosphorylase activity,cytoplasm,glucos				T=0, C=33	T=0.0, C=1.0	0.00001	504.595	33	T=0/0, C=9/24
chr17:648498	T/T	C	T	SNV	GEMIN4	GEMIN4:exonic:NM 015721.2	1	100	2	missense	NM 01572	c.2785G>A	p.Asp929Asn	1.2	1	23	0	0.1(COSM4130507),0.1(COS		rs2740349	0.1742	YES	8.881E-1 (pos.648498)	acute_myeloid_leukaemia		Gemini of coiled bodies,SMN complex,SMN-Sm protein c				C=0, T=23	C=0.0, T=1.0	0.00001	316.747	23	C=0/0, T=6/17
chr17:649935	C/C	G	C	SNV	GEMIN4	GEMIN4:exonic:NM 015721.2	1	100	2	missense	NM 01572	c.1348C>G	p.Gln450Glu	2.12	1	29	0			rs2740348	0.1741	YES	8.873E-1 (pos.649935)			Gemini of coiled bodies,SMN complex,SMN-Sm protein c				G=0, C=88	G=0.0, C=1.0	0.00001	1451.91	88	G=0/0, C=26/6
chrX:14027179	C/C	A	C	SNV	GEMIN8	GEMIN8:exonic:NM 001042480.1	1	100	4	synonymous	NM 00104	c.582T>G	p.(=)	-2.99					Gemini of Cajal bodies-associated protein 8						GEM-associ	Gemini of coiled bodies,SMN complex,SMN-Sm protein c				A=0, C=46	A=0.0, C=1.0	0.00001	768.313	46	A=0/0, C=26/2
chr15:83240293	C/C	A	C	SNV	CPEB1	CPEB1:intronic:NM 030594.4	1	100		intronic	NM 03059	c.191-11T	p.?	-0.16					Cytoplasmic	rs783544	0.2389	YES	7.948E-1 (pos.83240293)		Cytoplasmi	P-body,cel	Hemic and Lymphatic Diseases:Immune Syst			A=0, C=73	A=0.0, C=1.0	0.00001	1150.51	73	A=0/0, C=36/3
chr15:84473531	A/A	G	A	SNV	ADAMTSL3	ADAMTSL3:intronic:NM 207517.2	1	100		intronic	NM 20751	c.363+84G	p.?	-1.09					Immunoglob	rs7179507		YES			ADAMTS-lik	intracellul	Mental Disorders;Schizophrenia;Schizophren			G=0, A=30	G=0.0, A=1.0	0.00001	446.639	30	G=0/0, A=15/1
chr2:17962518	C/T	C	T	SNV	GEN1	GEN1:exonic:NM 182625.4	1	58.33	14	missense	NM 18262	c.2039C>T	p.Thr680Ile	0.18	1	89	0		XPG I-region,	rs300169	0.3755	YES	9.843E-1 (pos.17962518)		GEN1, Dros	DNA binding,DNA repair,centrosome,crossover junction				C=10, T=14	C=0.4167, T=0.5833	0.00001	135.338	24	C=1/9, T=4/10
chr15:85407564	A/C	A	C	SNV	ALPK3	ALPK3:intronic:NM 020778.4	1	52.27		intronic	NM 02077	c.5106-10	p.?	-0.18					Alpha-kinase	rs2289138		YES			Alpha kinas	ATP binding,heart development,nucleus,protein phosph				A=21, C=23	A=0.4773, C=0.5227	0.00001	260.157	44	A=13/8, C=7/1
chr3:158366900	A/A	G	A	SNV	GFM1	GFM1:exonic:NM 024996.5	1	100	5	missense	NM 02499	c.643G>A	p.Val215Ile	3.32	0.74	29	0.002	0.25(COSM40	Elongation Fa	rs2303909	0.4612	YES	3.25E-1 (pos.158366900)	NS,acute_mye	Mitochondr	GTP bindir	Brain Diseases;Brain Diseases		Benign	G=0, A=45	G=0.0, A=1.0	0.00001	747.386	45	G=0/0, A=25/2
chr5:74021847	ACTCAAA/A	ACTCAAA	A	INDEL	GFM2	GFM2:exonic:NM 032380.4	6	47.62	18	nonframeshiftDeletion	NM 03238	c.1825_18	p.Phe609_Glu610del	4.71, -0.93, 3.23, 0.42, -0.22, 3.79, 2.21					Elongation Fa	rs141759476,rs5868753,rs7633		YES	1.899E-1 (pos.74021846)			GTP binding,GTPase activity,mitochondrial matrix,mitoc				ACTCAAA=2	ACTCAAA=0.5238, A=	0.00001	210.722	42	ACTCAAA=10/
chr5:179729519	G/G	A	G	SNV	GFPT2	GFPT2:exonic:NM 005110.3	1	100	18	synonymous	NM 00511	c.1908T>C	p.(=)	-0.36					Glutamine ar	rs1035411	0.3804	YES	9.157E-1 (pos.179729519)		Glutamine:	UDP-N-acetylglucosamine bios		L-Glutamine		A=0, G=30	A=0.0, G=1.0	0.00001	446.533	30	A=0/0, G=10/2
chr16:23489711	G/G	C	G	SNV	GGA2	GGA2:exonic:NM 015044.4	1	100	13	missense	NM 01504	c.1270G>C	p.Ala424Pro	0.67	1	27	0	0.02(COSM59	Adaptin C-ter	rs1135045	0.1936	YES	6.863E-1 (pos.23489711)	basal_cell_carcinoma		ADP-ribosylation factor binding,Golgi apparatus,cellular				C=0, G=27	C=0.0, G=1.0	0.00001	389.637	27	C=0/0, G=12/1
chr2:85781318	G/G	T	G	SNV	GGCX	GGCX:exonic:NM 000821.6	1	100	7	synonymous	NM 00082	c.837A>C	p.(=)	0.34					Vitamin K-de	rs1254896	0.0153	YES	1.0 (pos.85781318)		Gamma-glu	blood coag	Blood Coagulation I	Anisindione,Coagulation		T=0, G=22	T=0.0, G=1.0	0.00001	298.617	22	T=0/0, G=18/4
chr17:4463023	A/A	G	A	SNV	GGT6	GGT6:exonic:NM 001122890.2	1	100	2	missense	NM 00112	c.173C>T	p.Ala58Val	2.36	0.03	64	0.437		Gamma-gluta	rs11657054	0.229	YES	9.147E-1 (pos.4463023)		Gamma-glu	biological process,extracellular exosome,glutathione b				G=0, A=34	G=0.0, A=1.0	0.00001	524.185	34	G=0/0, A=17/1
chr17:61996204	C/C	T	C	SNV	GH1	GH1:utr 5:NM 000515.4	1	100	1	utr 5	NM 00051	c.-68A>G	p.?	-1.61						rs6171	0.4271	YES			Growth hor	JAK-STAT c	Acromegaly:Adenoma:Azotemi		Likely benign	T=0, C=88	T=0.0, C=1.0	0.00001	1451.89	88	T=0/0, C=57/3
chr5:42700044	G/G	A	G	SNV	GHR	GHR:exonic:NM 000163.4	1	100	6	synonymous	NM 00016	c.558A>G	p.(=)	0.32					Erythropoieti	rs6179	0.2709	YES	8.565E-1 (pos.42700044)		Growth hor	2-oxogluta	Autistic Disorder;B	Pegvisoma	Benign	A=0, G=34	A=0.0, G=1.0	0.00001	523.566	34	A=0/0, G=13/2
chr7:100285974	C/C	A	C	SNV	GIGYF1	GIGYF1:utr 5:NM 022574.5	1	100	1	utr 5	NM 02257	c.-113T>G	p.?	-0.68					GYF domain	rs221797		YES			GRB10-inte	biological process,insulin-like growth factor receptor si				A=0, C=85	A=0.0, C=1.0	0.00001	1368.28	85	A=0/0, C=41/4
chr7:150434700	A/G	A	G	SNV	GIMAP1-GIMAP5,GIMAP5	GIMAP1-GIMAP5:exonic:NM 001199577.1, GIMAP5:utr 5:N	1	46.88	4	synonymous	NM 00119	c.510A>G,	p.(=), p.?	-1.02					50S ribosome	rs11977474		YES	0.0 (pos.150434700)		GTPase, IM	GTP bindir	Immune System Diseases			A=170, G=1	A=0.5313, G=0.4688	0.00001	1558.28	320	A=66/104, G=
chr15:86253768	A/A	T	A	SNV	AKAP13	AKAP13:intronic:NM 006738.5	1	100		intronic	NM 00673	c.5545-42	p.?	-0.95					PH domain,R	rs8037947	0.1575	YES	6.369E-1 (pos.86253768)		A-kinase an	G-protein	Adenocarcinoma;Carcinoma;Carcinoma, Ren			T=0, A=43	T=0.0, A=1.0	0.00001	705.844	43	T=0/0, A=19/2
chr15:86253911	G/G	T	G	SNV	AKAP13	AKAP13:intronic:NM 006738.5	1	100		intronic	NM 00673	c.5611+35	p.?	-0.26					PH domain,R	rs8038249	0.2199	YES	6.333E-1 (pos.86253911)		A-kinase an	G-protein	Adenocarcinoma;Carcinoma;Carcinoma, Ren			T=0, G=47	T=0.0, G=1.0	0.00001	789.327	47	T=0/0, G=19/2
chr7:150324976	T/C	T	C	SNV	GIMAP6	GIMAP6:exonic:NM 001244072.1	1	53.13	3	missense	NM 00124	c.920A>G	p.Gln307Arg	-0.18	0.15	43	0.013		50S ribosome	rs11977216	5.00E-04	YES	0.0 (pos.150324976)		GTPase, IM	GTP binding,cytosol				T=15, C=17	T=0.4688, C=0.5313	0.00001	173.092	32	T=10/5, C=11/
chr15:87217488	C/C	T	C	SNV	AGBL1	AGBL1:intronic:NM 152336.3	1	100		intronic	NM 15233	c.3058-16	p.?	0.72					Zinc carboxy	rs11853730	0.4156	YES	3.296E-1 (pos.87217488)		ATP/GTP-binding protein-like 1					T=0, C=30	T=0.0, C=1.0	0.00001	446.598	30	T=0/0, C=27/3
chr7:150217248	G/G	A	G	SNV	GIMAP7	GIMAP7:exonic:NM 153236.3	1	100	2	synonymous	NM 15323	c.186A>G	p.(=)	-2.63				0.02(COSM37	50S ribosome	rs3735081	0.3748	YES	7.175E-1 (pos.15021724	acute_myeloic	GTPase IMA	GTP binding,GTP metabolic process,GTPase activity,Golg				A=0, G=21	A=0.0, G=1.0	0.00001	281.235	21	A=0/0, G=6/15
chr19:46181392	G/C	G	C	SNV	GIPR	GIPR:exonic:NM 000164.3	1	45.83	12	missense	NM 00016	c.1060G>C	p.Glu354Gln	3.27	0.06	29	1	0.79(COSM41	7 transmemb	rs1800437	0.2035	YES	2.236E-1 (pos.46181392)	carcinoma,ne	Gastric inhi	G-protein	Bronchial Neoplasms;Carcinoma, Bronchoge			G=13, C=11	G=0.5417, C=0.4583	0.00001	93.2642	24	G=3/10, C=2/9
chr3:33110383	G/A	G	A	SNV	GLB1	GLB1:exonic:NM 000404.3	1	50	3	missense	NM 00040	c.325C>T	p.Arg109Trp	1.29	0	101	0.999		Beta-galacto	rs35289681	0.0208	YES	0.0 (pos.33110383)		Galactosida	Golgi appa	Bone Diseases;Bone Diseases,		Benign/Likely	G=33, A=33	G=0.5, A=0.5	0.00001	328.386	66	G=10/23, A=8/
chr15:89172352	T/T	C	T	SNV	AEN	AEN:intronic:NM 022767.3	1	100		intronic	NM 02276	c.541-105	p.?	-0.55					Exonuclease	rs3826040		YES			Interferon-s	exonuclease activity,intrinsic apoptotic signaling pathw				C=0, T=35	C=0.0, T=1.0	0.00001	543.903	35	C=0/0, T=30/5
chr11:134182375	A/A	G	A	SNV	GLB1L3	GLB1L3:exonic:NM 001080407.2	1	100	14	missense	NM 00108	c.1420G>A	p.Val474Met	-2.06	0.09	21	0.002	0(COSM4145	Beta-galacto	rs2509062	4.00E-04		9.962E-1 (pos.13418237	neoplasm		beta-galactosidase activity,carbohydrate metabolic pro				G=0, A=27	G=0.0, A=1.0	0.00001	395.52	27	G=0/0, A=12/1
chr11:134158745	A/G	A	G	SNV	GLB1L3	GLB1L3:exonic:NM 001080407.2	1	46.88	7	synonymous	NM 00108	c.690A>G	p.(=)	-1.57				0.02(COSM11	Beta-galacto	rs3824995	0.184	YES	3.138E-1;1.173E-4 (pos.1	adenocarcinoma,squamou		beta-galactosidase activity,carbohydrate metabolic pro				A=17, G=15	A=0.5313, G=0.4688	0.00001	143.733	32	A=7/10, G=5/1
chr7:8125964	T/T	C	T	SNV	GLCCI1	GLCCI1:exonic:NM 138426.3	1	100	8	synonymous	NM 13842	c.1440C>T	p.(=)	-0.14				0.31(COSM15	Protein Famil	rs929509	0.3097	YES	7.464E-1 (pos.8125964)	adenocarcinom	Glucocorticoid-induced transcript 1					C=0, T=62	C=0.0, T=1.0	0.00001	940.32	62	C=0/0, T=33/2
chr7:8126194	G/G	A	G	SNV	GLCCI1	GLCCI1:utr 3:NM 138426.3	1	100	8	utr 3	NM 13842	c.1670A>G	p.?	-0.17					Protein Famil	rs12847	0.4606	YES	5.834E-1 (pos.8126194)		Glucocorticoid-induced transcript 1					A=0, G=29	A=0.0, G=1.0	0.00001	427.597	29	A=0/0, G=4/25
chr9:6533010	G/G	C	G	SNV	GLDC	GLDC:utr 3:NM 000170.2	1	100	25	utr 3	NM 00017	c.3070G>C	p.?	0.33					Beta-elimina	rs2228098	0.3823	YES	9.503E-1 (pos.6533010)		Glycine deh	cellular re	Amino Acid Metabo	Glycine,Py	Benign	C=0, G=221	C=0.0, G=1.0	0.00001	3647.21	221	C=0/0, G=94/1

chr7:42088222	C/C	T	C	SNV	GLI3	GLI3:exonic:NM_000168.5	1	100	5	missense	NM_00016	c.547A>G	p.Thr183Ala	3.14	1	58	0	0.57(COSM37	Zinc finger, C	rs846266	0.4371	YES	5.606E-1 (pos.42088222)	adenocarcinoi	GLI-Kruppel	DNA bindii	Abnormalities, Multiple;Acroc		Benign	T=0, C=51	T=0.0, C=1.0	0.00001	754.578	51	T=0/0, C=33/1
chr7:42003792	A/T	A	T	SNV	GLI3	GLI3:utr_3:NM_000168.5	1	52.27	15	utr_3	NM_00016	c.4879T>A	p.?	2.2					Zinc finger, C	rs78794712		YES			GLI-Kruppel	DNA bindii	Abnormalities, Multiple;Acroc		Likely benign	A=21, T=23	A=0.4773, T=0.5227	0.00001	254.638	44	A=13/8, T=12/
chr1:156263940	T/C	T	C	SNV	GLMP	GLMP:exonic:NM_144580.2	1	50	4	missense	NM_14458	c.667A>G	p.Ile223Val	-0.07	0.89	29	0	0.01(COSM37	Lysosomal tr	rs10908495	0.2933	YES	2.486E-1 (pos.15626394(	adenocarcinoma,neoplasn		DNA binding transcription factor activity,cytosol,integra				T=11, C=11	T=0.5, C=0.5	0.00001	96.6901	22	T=3/8, C=3/8
chr15:89864317	C/CTACC	C	CTACC	INDEL	POLG	POLG:intronic:NM_001126131.1	4	53.33		intronic	NM_00112	c.2734+39	p.?	-0.45					DNA polymerase family A						Polymerase	3'-5' exonu	Articulation Disorders;Ataxia;Autoimmune D			C=14, CTAC	C=0.4667, CTACC=0.5	0.00001	159.925	30	C=11/3, CTACC
chr17:685640	G/T	G	T	SNV	GLOD4,MRM3	GLOD4:upstream:NM_016080.3, MRM3:exonic:NM_018146	1	58.33	1	missense	NM_01608	c.-145C>A,	p.?, p.Ala8Ser	-0.78	0.48	99	0	0.01(COSM37	SpoU rRNA M	rs2273454	0.1997	YES	4.429E-1 (pos.685640)	adenocarcinoma		RNA binding,RNA methylation,RNA methyltransferase ac				G=25, T=35	G=0.4167, T=0.5833	0.00001	370.432	60	G=7/18, T=5/3
chr17:685752	G/A	G	A	SNV	GLOD4,MRM3	GLOD4:upstream:NM_016080.3, MRM3:exonic:NM_018146	1	46.58	1	missense	NM_01608	c.-257C>T,	p.?, p.Gly45Glu	1.42	0.14	98	0.261	0.07(COSM63	SpoU rRNA M	rs2249542	0.2622	YES	1.021E-1 (pos.685752)	carcinoma		RNA binding,RNA methylation,RNA methyltransferase ac				G=39, A=34	G=0.5342, A=0.4658	0.00001	336.844	73	G=8/31, A=11/
chr15:90144851	C/C	T	C	SNV	TICRR	TICRR:intronic:NM_152259.3	1	100		intronic	NM_15225	c.2327+52	p.?	-0.93					Treslin N-terr	rs4932233	0.0899	YES	7.04E-1 (pos.90144851)			DNA repai	Nervous System Diseases;Neuromuscular Di			T=0, C=26	T=0.0, C=1.0	0.00001	371.42	26	T=0/0, C=11/1
chr15:90144894	C/C	T	C	SNV	TICRR	TICRR:intronic:NM_152259.3	1	100		intronic	NM_15225	c.2328-74	p.?	-1.04					Treslin N-terr	rs4932234		YES				DNA repai	Nervous System Diseases;Neuromuscular Di			T=0, C=32	T=0.0, C=1.0	0.00001	485.141	32	T=0/0, C=17/1
chr15:90174955	T/T	C	T	SNV	KIF7	KIF7:intronic:NM_198525.2	1	100		intronic	NM_19852	c.2896-14	p.?	-1.02					Kinesin moto	rs9672296	0.4131	YES	3.172E-1 (pos.90174955)		Kinesin fam	ATP bindin	Abnormalities, Multiple;Acroc		Benign	C=0, T=37	C=0.0, T=1.0	0.00001	583.678	37	C=0/0, T=25/1
chr6:39040654	A/C	A	C	SNV	GLP1R	GLP1R:exonic:NM_002062.4	1	57.14	6	synonymous	NM_00206	c.526A>C	p.(=)	2.37				0.42(COSM15	7 transmemb	rs2235868	0.4771	YES	4.43E-1;0.0 (pos.3904065	acute_myeloic	Glucagon-li	G-protein	Cardiovascular Dise	Albiglutide,Dulaglutide,I		A=9, C=12	A=0.4286, C=0.5714	0.00001	109.039	21	A=6/3, C=9/3
chr6:39041502	A/C	A	C	SNV	GLP1R	GLP1R:exonic:NM_002062.4	1	51.85	7	missense	NM_00206	c.780A>C	p.Leu260Phe	0.91	1	22	0		7 transmemb	rs1042044	0.4412	YES	5.565E-1 (pos.39041502)		Glucagon-li	G-protein	Cardiovascular Dise	Albiglutide,Dulaglutide,I		A=13, C=14	A=0.4815, C=0.5185	0.00001	132.663	27	A=6/7, C=5/9
chr4:175598334	C/C	T	C	SNV	GLRA3	GLRA3:exonic:NM_006529.3	1	100	7	synonymous	NM_00652	c.822A>G	p.(=)	-0.66					Neurotransm	rs6812439	3.00E-04		1.0 (pos.175598334)	malignant_me	Glycine rec	cell junction,chemical synaptic		Glycine,Ivermectin,Linda		T=0, C=39	T=0.0, C=1.0	0.00001	623.976	39	T=0/0, C=15/2
chr10:131959150	C/T	C	T	SNV	GLRX3	GLRX3:exonic:NM_006541.4	1	54.46	4	missense	NM_00654	c.367C>T	p.Pro123Ser	0.67	0.26	74	0	0.24(COSM36	Glutaredoxin	rs2274217	0.2609	YES	1.066E-1 (pos.13195915(	acute_myeloic	Glutaredox	2Fe-2S clu	Autoimmune Diseases;Cardiomegaly;Cardio			C=46, T=55	C=0.4554, T=0.5446	0.00001	592.065	101	C=24/22, T=25
chr15:90270567	T/C	T	C	SNV	WDR93	WDR93:intronic:NM_020212.1	1	97.92		intronic	NM_02021	c.1052+8T	p.?	0.01				0(COSM5765111)		rs3743117	0.4457	YES	2.092E-1 (pos.90270567)	adenocarcinoma		electron transport chain,oxidoreductase activity, acting				T=1, C=47	T=0.0208, C=0.9792	0.00001	776.185	48	T=0/1, C=18/2
chr9:138518010	G/G	A	G	SNV	GLT6D1	GLT6D1:exonic:NM_182974.2	1	100	4	synonymous	NM_18297	c.162T>C	p.(=)	-6.66				0(COSM4163	Glycosyltrans	rs1333240	5.00E-04		9.984E-1 (pos.13851801(	neoplasm	Glycosyltra	Golgi appa	Mouth Diseases;Pe	Beta-D-Glucose,Lactose,		A=0, G=81	A=0.0, G=1.0	0.00001	1309.48	81	A=0/0, G=32/4
chr15:90764359	T/G	T	G	SNV	SEMA4B	SEMA4B:intronic:NM_198925.2	1	57.58		intronic	NM_19892	c.595+34T	p.?	-0.49					Plexin repeat	rs3826001,rs552532	0.3111	YES	8.334E-1 (pos.90764359)		Semaphorir	cell differentiation,extracellular space,integral compon				T=28, G=38	T=0.4242, G=0.5758	0.00001	411.843	66	T=2/26, G=1/3
chr15:90768156	G/G	A	G	SNV	SEMA4B	SEMA4B:intronic:NM_198925.2	1	100		intronic	NM_19892	c.1195-44	p.?	-1.05					Plexin repeat	rs1040083,rs202077	0.1691	YES	9.972E-1;2.32E-4 (pos.90768156)		Semaphorir	cell differentiation,extracellular space,integral compon				A=0, G=81	A=0.0, G=1.0	0.00001	1314.06	81	A=0/0, G=20/6
chr6:49494241	G/G	A	G	SNV	GLYATL3	GLYATL3:exonic:NM_001010904.1	1	100	6	missense	NM_00101	c.481A>G	p.Asn161Asp	3.04	0.33	23	0	0.14(COSM37	Aralkyl acyl-C	rs9367359	0.3558	YES	5.161E-1 (pos.49494241)	adenocarcinoi	Glycine N-a	glycine N-acyltransferase activity,mitochondrion				A=0, G=72	A=0.0, G=1.0	0.00001	1130.94	72	A=0/0, G=38/3
chr15:90773782	T/T	C	T	SNV	CIB1,SEMA4B	SEMA4B:downstream:NM_198925.2, CIB1:intronic:NM_001	1	100		intronic	NM_19892	c.*1907C>	p.?, p.?	-1.14					EF hand,EF-ha	rs2073706	0.433	YES	8.42E-1 (pos.90773782)		Semaphorir	Golgi apparatus,Ras GTPase bi		Calcium Citrate,Calcium		C=0, T=47	C=0.0, T=1.0	0.00001	789.328	47	C=0/0, T=27/2
chr16:4861709	C/C	G	C	SNV	GLYR1	GLYR1:exonic:NM_032569.3	1	100	14	missense	NM_03256	c.1377C>G	p.His459Gln	0.1	0.79	24	0	0.64(COSM45	AT hook moti	rs2085329		YES	1.0 (pos.4861709)	squamous_cell_carcinoma		DNA binding,Golgi apparatus,NAD binding,cytosol,histor				G=0, C=28	G=0.0, C=1.0	0.00001	407.566	28	G=0/0, C=13/1
chr5:150639409	G/G	A	G	SNV	GM2A	GM2A:exonic:NM_000405.4	1	100	2	missense	NM_00040	c.175A>G	p.Ile59Val	-1	0.34	29	0	0(COSM5426	ML domain	rs153477	0.3735	YES	6.402E-1 (pos.15063940	acute_myeloic	GM2 ganglic	apical cort	Brain Diseases;Brai	Choline al	Benign	A=0, G=43	A=0.0, G=1.0	0.00001	705.795	43	A=0/0, G=19/2
chr15:90809633	A/A	G	A	SNV	NGRN	NGRN:intronic:NM_001033088.1	1	100		intronic	NM_00103	c.275+3G>	p.?	-0.46				0.02(COSM63	Neugrin	rs755416585,rs9635	0.0463	YES	0.0 (pos.90809623),8.70	carcinoma	Neurite out	RNA binding,extracellular region,neuron differentiation,				G=0, A=61	G=0.0, A=1.0	0.00001	921.805	61	G=0/0, A=25/3
chr5:150639439	G/G	A	G	SNV	GM2A	GM2A:exonic:NM_000405.4	1	100	2	missense	NM_00040	c.205A>G	p.Met69Val	0.53	0.37	21	0		ML domain	rs153478	0.3428	YES	9.988E-1 (pos.150639439)		GM2 ganglic	apical cort	Brain Diseases;Brai	Choline al	Benign	A=0, G=43	A=0.0, G=1.0	0.00001	705.811	43	A=0/0, G=19/2
chr15:91341599	T/G	T	G	SNV	BLM	BLM:intronic:NM_000057.3	1	53.13		intronic	NM_00005	c.3358+32	p.?	-0.01					BDHCT (NUC0	rs17273842	0.1823	YES	1.735E-1 (pos.91341599)		DNA helicas	8-hydroxy-	Abnormalities, Multiple;Bloom		Benign	T=15, G=17	T=0.4688, G=0.5313	0.00001	173.549	32	T=4/11, G=9/8
chr15:91358271	GGAA/GGAA	G	GGAA	INDEL	BLM	BLM:intronic:NM_000057.3	3	100		intronic	NM_00005	c.4077-61	p.?	-0.35					BDHCT (NUC0	rs10685387,rs202095393		YES			DNA helicas	8-hydroxy-	Abnormalities, Multiple;Bloom		Likely benign	G=0, GGAA	G=0.0, GGAA=1.0	0.00001	281.235	21	G=0/0, GGAA=
chr3:179137273	A/G	A	G	SNV	GNB4	GNB4:exonic:NM_021629.3	1	50	4	synonymous	NM_02162	c.117T>C	p.(=)	-2.75				0.02(COSM44	WD domain,	rs1362650	0.4693	YES	4.446E-1 (pos.17913727	acute_myeloic	Guanine nu	cellular re	Colonic Diseases;Colorectal N		Benign	A=18, G=18	A=0.5, G=0.5	0.00001	185.884	36	A=10/8, G=8/1
chr15:91433265	C/G	C	G	SNV	FES	FES:intronic:NM_002005.3	1	57.14		intronic	NM_00200	c.1050-57	p.?	0.56					Protein kinas	rs4932376		YES			Oncogene F	ATP bindin	Cardiovascular Diseases;Hypertension;Ment			C=9, G=12	C=0.4286, G=0.5714	0.00001	112.47	21	C=3/6, G=4/8
chr15:91482935	G/C	G	C	SNV	UNC45A	UNC45A:intronic:NM_018671.4	1	45.45		intronic	NM_01867	c.427-26G	p.?	-0.89					Myosin-bindi	rs12440774	0.4236	YES	9.963E-1;0.0 (pos.91482935)		UNC45, C. e	Golgi apparatus,Hsp90 protein binding,cadherin binding				G=12, C=10	G=0.5455, C=0.4545	0.00001	75.4731	22	G=8/4, C=6/4
chr7:93540153	A/A	G	A	SNV	GNGT1	GNGT1:exonic:NM_021955.4	1	100	3	missense	NM_02195	c.148G>A	p.Glu50Lys	6.79	0.02	56	0.799		GGL domain	rs17243826	0.0514	YES	1.161E-4;0.0 (pos.93540153)		Guanine nu	G-protein coupled receptor signaling pathway,GTPase a				G=0, A=28	G=0.0, A=1.0	0.00001	408.712	28	G=0/0, A=21/7
chr6:30521137	A/A	G	A	SNV	GNL1	GNL1:exonic:NM_005275.3	1	100	6	synonymous	NM_00527	c.798C>T	p.(=)	-0.1				0.3(COSM416	50S ribosome	rs2074505	0.3391	YES	6.649E-1 (pos.30521137)	neoplasm	Guanine nu	GTP binding,GTPase activity,T cell mediated immunity,ce				G=0, A=45	G=0.0, A=1.0	0.00001	747.392	45	G=0/0, A=12/3
chr5:141391532	A/A	G	A	SNV	GNPDA1	GNPDA1:exonic:NM_005471.4	1	100	2	synonymous	NM_00547	c.69C>T	p.(=)	0.61				0.79(COSM14	Glucosamine	rs164080	0.4566	YES	6.197E-1 (pos.14139153	adenocarcinoi	Glucosamir	N-acetylglucosamine metaboli		2-Deoxy-2-Amino Glucitc		G=0, A=77	G=0.0, A=1.0	0.00001	1229.44	77	G=0/0, A=58/1
chr12:102158763	C/C	T	C	SNV	GNPTAB	GNPTAB:exonic:NM_024312.4	1	100	13	synonymous	NM_02431	c.1932A>G	p.(=)	-3.4				0.02(COSM11	DMAP1-bindi	rs10778148,rs38676	0.4777	YES	9.014E-1 (pos.10215876	adenocarcinoi	N-acetylglu	Golgi appa	Abnormalities, Multiple;Bone I		Benign	T=0, C=82	T=0.0, C=1.0	0.00001	1323.89	82	T=0/0, C=51/3
chr12:133349620	G/G	C	G	SNV	GOLGA3	GOLGA3:utr_3:NM_005895.3	1	100	24	utr_3	NM_00589	c.4568G>C	p.?	0.04						rs3741483,rs925896556		YES			Golgi autoa	Golgi apparatus,Golgi cisterna membrane,Golgi membra				C=0, G=61	C=0.0, G=1.0	0.00001	921.854	61	C=0/0, G=31/3
chr15:93552330	T/T	C	T	SNV	CHD2	CHD2:intronic:NM_001271.3	1	100		intronic	NM_00127	c.4414-45	p.?	-0.13					Chromo (CHR	rs12915582	0.1623	YES	9.971E-1 (pos.93552330)		Chromodon	ATP bindin	Brain Diseases;Central Nervous System Dise			C=0, T=32	C=0.0, T=1.0	0.00001	485.141	32	C=0/0, T=9/23
chr17:45000547	C/C	G	C	SNV	GOSR2	GOSR2:utr_5:NM_054022.3	1	100	1	utr_5	NM_05402	c.-12G>C	p.?	-0.79					Snare region	rs183199,rs3867974	6.00E-04	YES	1.0;0.0 (pos.45000547)		Golgi snap r	COPII vesicle coating,ER to Golgi transport			Benign	G=0, C=36	G=0.0, C=1.0	0.00001	563.544	36	G=0/0, C=31/5
chr15:99456553	G/A	G	A	SNV	IGF1R	IGF1R:intronic:NM_000875.4	1	97.3		intronic	NM_00087	c.1828+42	p.?	-2.31					Furin-like cys	rs951715	0.3506	YES	4.998E-1 (pos.99456553)		Insulin-like	ATP bindin	Abnormalities, Mul	(4Z)-6-bromo-4-{(4-(pyrro		G=1, A=36	G=0.027, A=0.973	0.00001	551.74	37	G=0/1, A=21/1
chr16:10788129	G/T	G	T	SNV	TEKT5	TEKT5:intronic:NM_144674.1	1	45.45		intronic	NM_14467	c.564+38C	p.?	-3.04					Tektin family	rs2251985	0.4792	YES	6.195E-1 (pos.10788129)			cilium ass	Mental Disorders;Schizophrenia;Schizophren			G=12, T=10	G=0.5455, T=0.4545	0.00001	81.7627	22	G=6/6, T=8/2
chr16:10855341	C/T	C	T	SNV	NUBP1	NUBP1:intronic:NM_002484.3	1	46.43		intronic	NM_00248	c.717+10C	p.?	-1.14					Anion-transp	rs2233539	0.2485	YES	4.455E-1 (pos.10855341)		Nucleotide-	4 iron, 4 sulfur cluster binding,ATP binding,cell growth,ce				C=15, T=13	C=0.5357, T=0.4643	0.00001	117.919	28	C=10/5, T=5/8
chr16:10992715	T/G	T	G	SNV	CIITA	CIITA:intronic:NM_000246.3	1	56		intronic	NM_00024	c.359-67T	p.?	-0.97					Leucine Rich	rs6498121		YES			Class II maj	ATP bindin	Addison Disease;Adrenal Gland Diseases;Adi			T=11, G=14	T=0.44, G=0.56	0.00001	133.955	25	T=7/4, G=8/6
chr16:58750604	A/A	G	A	SNV	GOT2	GOT2:exonic:NM_002080.3	1	100	7	synonymous	NM_00208	c.816C>T	p.(=)	-0.26				0.15(COSM11	Aminotransfe	rs1058192	0.3519	YES	6.438E-1 (pos.58750604)	adenocarcinoi	Glutamic-o:	2-oxoglutarate metabolic proc		4'-Deoxy-4'-Acetylyamin(		G=0, A=25	G=0.0, A=1.0	0.00001	353.015	25	G=0/0, A=9/16
chr16:58757668	C/C	A	C	SNV	GOT2	GOT2:exonic:NM_002080.3	1	100	2	synonymous	NM_00208	c.228T>G	p.(=)	0.86				0.57(COSM39	Aminotransfe	rs14221	0.351	YES	6.348E-1 (pos.58757668)	acute_myeloic	Glutamic-o:	2-oxoglutarate metabolic proc		4'-Deoxy-4'-Acetylyamin		A=0, C=104	A=0.0, C=1.0	0.00001	1626.74	104	A=0/0, C=42/6
chr16:113493	C/C	G	C	SNV	RHBDF1	RHBDF1:intronic:NM_022450.3	1	100		intronic	NM_02245	c.462+92C	p.?	-0.19					Rhomboid fai	rs743724		YES			Rhomboid 5	Golgi membrane,cell migration,cell proliferation,endopl				G=0, C=27	G=0.0, C=1.0	0.00001	389.988	27	G=0/0, C=14/1
chr16:58757683	G/G	A	G	SNV	GOT2	GOT2:exonic:NM_002080.3	1	100	2	synonymous	NM_00208	c.213T>C	p.(=)	-0.63				0.09(COSM41	Aminotransfe	rs257636	0.3512	YES	6.056E-1 (pos.58757683)	neoplasm	Glutamic-o:	2-oxoglutarate metabolic proc		4'-Deoxy-4'-Acetylyamin		A=0, G=104	A=0.0, G=1.0	0.00001	1626.75	104	A=0/0, G=42/6
chr16:20335325	G/A	G	A	SNV	GP2	GP2:exonic:NM_001502.3	1	50	3	synonymous	NM_00150	c.348C>T	p.(=)	-1.3					Zona pellucic	rs12930599	0.2198	YES	9.486E-3 (pos.20335325)		Glycoprotei	C-terminal protein lipidation,anchored component of m				G=16, A=16	G=0.5, A=0.5	0.00001	158.383	32	G=11/5, A=10/
chr16:11444425	C/T	C	T	SNV	RMI2	RMI2:intronic:NM_152308.2	1	45.83		intronic	NM_15230	c.296-74C	p.?	0.61					RecQ-mediat	rs28627609		YES			BLM-associ	DNA binding,DNA replication,DNA synthesis involved in D				C=13, T=11	C=0.5417, T=0.4583	0.00001	93.4399	24	C=4/9, T=2/9
chr16:11444430	G/G	T	G	SNV	RMI2	RMI2:intronic:NM_152308.2	1	100		intronic	NM_15230	c.296-69T	p.?	-0.23					RecQ-mediat	rs2032930		YES			BLM-associ	DNA binding,DNA replication,DNA synthesis involved in D				T=0, G=24	T=0.0, G=1.0	0.00001	334.791	24	T=0/0, G=6/18
chr16:11444469	T/T	C	T	SNV	RMI2	RMI2:intronic:NM_152308.2	1	100		intronic	NM_15230	c.296-30C	p.?	-0.08					RecQ-mediat	rs2032931	0.1873	YES	9.463E-1 (pos.11444469)		BLM-associ	DNA binding,DNA replication,DNA synthesis involved in D				C=0, T=24	C=0.0, T=1.0	0.00001	334.508	24	C=0/0, T=4/20
chr19:55525497	G/G	A	G	SNV	GP6	GP6:exonic:NM_001083899.2	1	100	8	missense	NM_00108	c.1816T>C	p.Phe606Leu	-1.85	1	22	0		Immunoglobu	rs1671150	0.1297	YES	8.361E-1 (pos.55525497)		Glycoprotei	blood coagulation,cell surface,collagen bi			Benign	A=0, G=57	A=0.0, G=1.0	0.00001	831.166	57	A=0/0, G=31/2
chr19:55530035	T/T	C	T	SNV	GP6	GP6:exonic:NM_001083899.2	1	100	6	missense	NM_00108	c.709G>A	p.Glu237Lys	-0.15	0.16	56	0.01		Immunoglobu	rs1654416,rs747661	0.1671	YES	0.0;0.0 (pos.55530032),1.159E-4 (pos.55		Glycoprotei	blood coagulation,cell surface,collagen bi			Benign	C=0, T=45	C=0.0, T=1.0	0.00001	747.325	45	C=0/0, T=22/2
chr16:11828018	G/G	A	G	SNV	TXNDC11	TXNDC11:intronic:NM_015914.6	1	100		intronic	NM_01591	c.472-83T	p.?	-0.19					Thioredoxin	rs4780398		YES			Thioredoxir	cell,cell redox homeostasis,endoplasmic reticulum,end				A=0, G=26	A=0.0, G=1.0	0.00001	371.024	26	A=0/0, G=9/17
chr5:56526783	A/A	G	A	SNV	GPBP1	GPBP1:exonic:NM_001127236.2	1	100	3	synonymous	NM_00112	c.195G>A	p.(=)	0.13					Vascular prot	rs1130203	0.3201	YES	9.983E-1 (pos.56526783)		GC-rich pro	DNA binding,DNA binding transcription factor activity,cyt				G=0, A=56	G=0.0, A=1.0	0.00001	831.938	56	G=0/0, A=32/2
chr16:12371915	C/C	T	C	SNV	SNX29	SNX29:intronic:NM_032167.4	1	100		intronic	NM_03216	c.1782+22	p.?	-3.38					PX domain,R	rs12448443	0.3881	YES	4.983E-1 (pos.12371915)			extracellu	Mental Disorders;Schizophrenia;Schizophren			T=0, C=20	T=0.0, C=1.0	0.00001	263.785	20	T=0/0, C=6/14
chr13:92345579	C/T	C	T	SNV	GPC5	GPC5:exonic:NM_004466.5	1	45.45	3	missense	NM_00446	c.464C>T	p.Ala155Val	2.73	0.27	64	0.013	0.87(COSM37	Glypican	rs553717	0.1128	YES	2.914E-1 (pos.92345579)	acute_myeloic	Glypican 5	Golgi lume	Albuminuria;Autoimmune Diseases;Autoimm			C=42, T=35	C=0.5455, T=0.4545	0.00001	347.799	77	C=21/21, T=15
chr6:24437445	A/A	G	A	SNV	GPLD1	GPLD1:exonic:NM_001503.3	1	100	21	missense	NM_00150	c.2093C>T	p.Thr698Ile	0.32	0	89	0.022		FG-GAP repea	rs1772256	0.0214	YES	9.993E-1 (pos.24437445)		Phospholip	C-terminal protein lipidation,GPI anchor release,cell mi				G=0, A=26	G=0.0, A=1.0	0.00001	371.42	26	G=0/0, A=12/1
chr1:27210721	C/C	T	C	SNV	GPN2	GPN2:exonic:NM_018066.3	1	100	4	missense	NM_01806	c.790A>G	p.Arg264Gly	5.79	1	125	0		Conserved hy	rs3170660	0.3735	YES	9.985E-1 (pos.27210721)			GTP binding,GTPase activity,RNA polymerase II complex				T=0, C=66	T=0.0, C=1.0	0.00001	1022.08	66	T=0/0, C=36/3
chr16:1272472	T/T	C	T	SNV	CACNA1H,TPSG1	TPSG1:intronic:NM_012467.3, CACNA1H:downstream:NM_0	1	100		intronic	NM_02109	c.*1478C>	p.?, p.?	-0.07					Trypsin	rs7198064		YES			Calcium cha	aldosteroi	Adrenal Gland Dise	Amiodarone,Bepridil,Cal		C=0, T=54	C=0.0, T=1.0	0.00001	798.148	54	C=0/0, T=49/5
chr19:6737481	C/C	T	C	SNV	GPR108,MIR6791	MIR6791:upstream:NR_106849.1, GPR108:exonic:NM_001	1	100	1	missense	NM_00108	c.107A>G	p.Gln36Arg	0.19	0.8	43	0	0.28(COSM37	Lung seven tr	rs340138	0.1128	YES	9.788E-1 (pos.6737481)	adenocarcinoma,neoplasn		integral component of membrane				T=0, C=24	T=0.0, C=1.0	0.00001	334.764	24	T=0/0, C=9/15
chr19:6737509	A/A	G	A	SNV	GPR108,MIR6791	MIR6791:upstream:NR_106849.1, GPR108:exonic:NM_001	1	100	1	synonymous	NM_00108	c.79C>T	p.(=)	-0.81				0.11(COSM37	Lung seven tr	rs339401	0.1137	YES	9.793E-1 (pos.6737509)	adenocarcinoma,neoplasn		integral component of membrane				G=0, A=22	G=0.0, A=1.0	0.00001	298.891	22	G=0/0, A=5/17
chr1:6313938	T/T	C	T	SNV	GPR153	GPR153:exonic:NM_207370.3	1	100	3	missense	NM_20737	c.626G>A	p.Arg209His	0.37	0.31	29	0.012	0.51(COSM38	7 transmemb	rs12735670		YES	5.941E-1 (pos.6313938)	carcinoma,sou	G protein-c	G-protein	Mental Disorders;Neurodevelopmental Diso			C=0, T=22	C=0.0, T=1.0	0.00001	298.892	22	C=0/0, T=10/1
chr16:1370303	C/C	T	C	SNV	UBE2I	UBE2I:intronic:NM_194260.2	1	100		intronic	NM_19426	c.333+19T	p.?	-0.98					Ubiquitin-cor	rs909916	0.1157	YES	9.992E-1 (pos.1370303)		Ubiquitin-c	ATP bindin	Mammary Neoplasms, Animal;Mammary Nec			T=0, C=52	T=0.0, C=1.0	0.00001	767.001	52	T=0/0, C=30/2
chr16:1370309	G/G	C	G	SNV	UBE2I	UBE2I:intronic:NM_194260.2	1	100		intronic	NM_19426	c.333+25C	p.?	-0.57					Ubiquitin-cor	rs909917	0.1155	YES	9.997E-1;0.0;0.0 (pos.1370309)		Ubiquitin-c	ATP bindin	Mammary Neoplasms, Animal;Mammary Nec			C=0, G=54	C=0.0, G=1.0	0.00001	795.186	54	C=0/0, G=30/2
chr3:119886548	C/C	G	C	SNV	GPR156	GPR156:exonic:NM_153002.2	1	100	9	synonymous	NM_15300	c.1776C>G	p.(=)	-0.46					7 transmemb	rs9858566	0.0829	YES	9.171E-1 (pos.119886548)		G protein-c	G-protein coupled GABA receptor activity,G-protein couc				G=0, C=146	G=0.0, C=1.0	0.00001	2457.91	146	G=0/0, C=71/7
chr16:1388888	T/T	C	T	SNV	BAIAP3	BAIAP3:intronic:NM_003933.4	1	100		intronic	NM_00393	c.237-15C	p.?	-1.41					C2 domain,M	rs7197416	0.1197	YES	9.998E-1 (pos.1388888)		BAI1-associ	G-protein coupled receptor signaling pathway,endoplas				C=0, T=27	C=0.0, T=1.0	0.00001	389.742	27	C=0/0, T=17/1
chr3:119886776	A/A	T	A	SNV	GPR156	GPR156:exonic:NM_153002.2	1	100	9	missense	NM_15300	c.1548A>T	p.Glu516Asp	-0.61	0.86	45	0	0.01(COSM41	7 transmemb	rs902790	0.0834	YES	9.168E-1 (pos.11988677	neoplasm	G protein-c	G-protein coupled GABA receptor activity,G-protein couc				T=0, A=42	T=0.0, A=1.0	0.00001	685.22	42	T=0/0, A=24/1
chr16:1394551	G/G	A	G	SNV	BAIAP3	BAIAP3:intronic:NM_003933.4	1	100		intronic	NM_00393	c.1764+25	p.?	-2.32					C2 domain,M	rs11248872	0.1009	YES	9.949E-1;4.393E-3 (pos.1394551)		BAI1-associ	G-protein coupled receptor signaling pathway,endoplas				A=0, G=61	A=0.0, G=1.0	0.00001	921.703	61	A=0/0, G=36/2
chr16:14026007	A/A	G	A	SNV	ERCC4	ERCC4:intronic:NM_005236.2	1	100		intronic	NM_00523	c.974-7G>	p.?	-4.2					ERCC4 domai	rs254942	0.021	YES	7.878E-1;0.0 (pos.14026007)		Excision-rej	3' overhan	Abnormalities, Multiple;Anem		Benign	G=0, A=45	G=0.0, A=1.0	0.00001	747.393	45	G=0/0, A=16/2
chr1:9165669	A/A	G	A	SNV	GPR157	GPR157:exonic:NM_024980.4	1	100	3	missense	NM_02498	c.668C>T	p.Ala223Val	6.94	0	64	0.992		7 transmemb	rs12075362	0.0441	YES	0.0 (pos.9165669)			G-protein coupled receptor activity,G-protein coupled re				G=0, A=28	G=0.0, A=1.0	0.00001	408.716	28	G=0/0, A=17/1
chr16:1447319	A/A	G	A	SNV	UNKL	UNKL:intronic:NM_001193388.3	1	100		intronic	NM_00119	c.735-23C	p.?	-1.21					Zinc finger C-	rs79486249		YES	2E-2;0.0 (pos.1447319)		Unkempt fa	cytosol,metal ion binding,nucleus,protein binding,prote				G=0, A=21	G=0.0, A=1.0	0.00001	281.235	21	G=0/0, A=8/13
chr16:1479250	T/T	C	T	SNV			1	100		intronic				-0.79				0(COSM148004),0.01(COSM		rs11646599	0.3741	YES	2.751E-1 (pos.1479250)	acute_myeloid_leukaemia,adenocarcinoma,neoplasm						C=0, T=34	C=0.0, T=1.0	0.00001	524.184	34	C=0/0, T=14/2
chr16:1484338	G/G	A	G	SNV	CCDC154	CCDC154:downstream:NM_001143980.1	1	100		intronic	NM_00114	c.*71T>C	p.?	-3.52						rs7192100		YES				early endosome				A=0, G=67	A=0.0, G=1.0	0.00001	1017.79	67	A=0/0, G=33/3
chr10:25701341	G/G	C	G	SNV	GPR158	GPR158:exonic:NM_020752.2	1	100	4	missense	NM_02075	c.1274C>G	p.Ala425Gly	3.21	0.33	60	0.004	0.83(COSM56	7 transmemb	rs2480345	0.2716	YES	1.475E-1 (pos.25701341)	ductal_carcinc	G protein-c	G-protein coupled receptor activity,G-protein coupled re				C=0, G=32	C=0.0, G=1.0	0.00001	477.32	32	C=0/0, G=8/24
chr16:15092116	C/T	C	T	SNV	PDXDC1	PDXDC1:intronic:NM_015027.3	1	45.83		intronic	NM_01502	c.96-81C>	p.?	-0.77					Pyridoxal-dei	rs6498535					Pyridoxal-d	Golgi appa	Adenocarcinoma:A	Pyridoxal Phosphate		C=13, T=11	C=0.5417, T=0.4583	0.00001	93.44	24	C=2/11, T=6/5
chr16:1510567	G/G	T	G	SNV	CLCN7	CLCN7:intronic:NM_001287.5	1	100		intronic	NM_00128	c.485-39A	p.?	-1.81					CBS domain,\	rs2072950	0.316	YES	3.655E-1 (pos.1510567)		Chloride ch	ATP bindin	Bone Diseases;Bone Diseases, Development			T=0, G=43	T=0.0, G=1.0	0.00001	705.841	43	T=0/0, G=17/2
chr16:15135071	A/A	G	A	SNV	NTAN1	NTAN1:intronic:NM_173474.3	1	100		intronic	NM_17347	c.488-93C	p.?	-0.47					Protein N-ter	rs7498279					N-terminal	adult locomotory behavior,cytoplasm,memory,nucleus,				G=0, A=42	G=0.0, A=1.0	0.00001	685.192	42	G=0/0, A=21/2
chr13:99948178	A/A	C	A	SNV	GPR183,UBAC2	GPR183:exonic:NM_004951.4, UBAC2:intronic:NM_001144	1	100	2	synonymous	NM_00114	c.390-181	p.?, p.(=)	0.55					7 transmemb	rs2230342	0.3734	YES	1.827E-1 (pos.99948178)		G protein-c	B cell activ	Autoimmune Diseases;Behcet Syndrome;Car			C=0, A=21	C=0.0, A=1.0	0.00001	281.235	21	C=0/0, A=9/12
chr16:15705605	G/G	A	G	SNV	MARF1,MIR6506	MARF1:intronic:NM_001184998.1, MIR6506:upstream:NR_	1	100		intronic	NM_00118	c.3475-14	p.?	1.19					Limkain b1,N	rs7185421	0.3476	YES	5.236E-1 (pos.15705605)		Meiosis reg	Golgi apparatus,RNA binding,double-strand break repair				A=0, G=67	A=0.0, G=1.0	0.00001	1034.41	67	A=0/0, G=30/3
chr16:15841708	C/CG	C	CG	INDEL	MYH11	MYH11:intronic:NM_001040114.1	1	57.14		intronic	NM_00104	c.2271+23	p.?	-0.16					IQ calmodulin-binding motif,Myosin N-terminal SH3-like domain,Myosin head (motor domain),Myosi						Myosin, hea	ATP bindin	ACMG Recommend	Tetrafluoroaluminate Ioi		C=18, CG=2	C=0.4286, CG=0.571	0.00001	168.924	42	C=11/7, CG=2
chr16:15854571	A/G	A	G	SNV	MYH11	MYH11:intronic:NM_001040114.1	1	59.09		intronic	NM_00104	c.1151-56	p.?	0.28					IQ calmodulin-binding motif,Myosin N-terminal SH3-like domain,Myosin head (motor domain),Myosi						Myosin, hea	ATP bindin	ACMG Recommend	Tetrafluoroaluminate Ioi		A=9, G=13	A=0.4091, G=0.5909	0.00001	122.566	22	A=8/1, G=5/8
chr16:16184232	G/G	C	G	SNV	ABCC1	ABCC1:intronic:NM_004996.3	1	100		intronic	NM_00499	c.2461-30	p.?	0.5					ABC transpor	rs2074087	0.156	YES	8.283E-1 (pos.16184232)		ATP-binding	ATP bindin	Breast Diseases:Br	Abiraterone,Aminohippu		C=0, G=21	C=0.0, G=1.0	0.00001	281.234	21	C=0/0, G=17/4
chr16:16263361	A/A	G	A	SNV	ABCC6	ABCC6:intronic:NM_001171.5	1	100		intronic	NM_00117	c.2995+14	p.?	-0.45					ABC transpor	rs7194043		YES			ATP-binding	ATP bindin	Calcinosis;Calcium	Cisplatin,Dactinomycin,I		G=0, A=59	G=0.0, A=1.0	0.00001	885.326	59	G=0/0, A=33/2
chr12:12814818	T/T	C	T	SNV	GPR19	GPR19:exonic:NM_006143.2	1	100	4	missense	NM_00614	c.565G>A	p.Val189Ile	-0.37		29			7 transmemb	rs4763862	0.0672	YES	9.901E-1 (pos.12814818)		G protein-c	G-protein	Autoimmune Diseases;Connective Tissue Dis			C=0, T=65	C=0.0, T=1.0	0.00001	996.451	65	C=0/0, T=18/4
chr16:16286614	C/C	G	C	SNV	ABCC6	ABCC6:intronic:NM_001171.5	1	100		intronic	NM_00117	c.1431+73	p.?	-0.1					ABC transpor	rs6498618		YES			ATP-binding	ATP bindin	Calcinosis;Calcium	Cisplatin,Dactinomycin,I		G=0, C=40	G=0.0, C=1.0	0.00001	644.286	40	G=0/0, C=26/1
chr16:1810116	C/A	C	A	SNV	MAPK8IP3	MAPK8IP3:intronic:NM_015133.4	1	57.14		intronic	NM_01513	c.1301+10	p.?	-1.71					JNK-interacti	rs57876690		YES			Mitogen-ac	Golgi mem	Adnexal Diseases;Cysts;Endocrine System Di			C=9, A=12	C=0.4286, A=0.5714	0.00001	109.102	21	C=2/7, A=6/6
chr16:1818680	G/C	G	C	SNV	MAPK8IP3	MAPK8IP3:intronic:NM_015133.4	1	52.38		intronic	NM_01513	c.3890-24	p.?	-0.17					JNK-interacti	rs2072989,rs928457	0.1897	YES	6.162E-1 (pos.1818680)		Mitogen-ac	Golgi mem	Adnexal Diseases;Cysts;Endocrine System Di			G=10, C=11	G=0.4762, C=0.5238	0.00001	94.1426	21	G=4/6, C=6/5
chr8:142367335	C/C	T	C	SNV	GPR20	GPR20:exonic:NM_005293.2	1	100	2	missense	NM_00529	c.689A>G	p.His230Arg	0.94	0.97	29	0	0.57(COSM37	7 transmemb	rs10875472	0.1942	YES	6.031E-1 (pos.14236733	adenocarcinoi	G protein-c	G-protein coupled receptor activity,G-protein coupled re				T=0, C=46	T=0.0, C=1.0	0.00001	768.302	46	T=0/0, C=27/1
chr16:1837900	C/C	T	C	SNV	NUBP2	NUBP2:intronic:NM_012225.3	1	100		intronic	NM_01222	c.490-42T	p.?	-0.58					AAA domain,	rs344358	1.00E-04	YES	1.0 (pos.1837900)		Nucleotide-	4 iron, 4 sulfur cluster binding,ATP binding,cell projectio				T=0, C=29	T=0.0, C=1.0	0.00001	427.6	29	T=0/0, C=10/1
chr8:142367400	A/A	G	A	SNV	GPR20	GPR20:exonic:NM_005293.2	1	100	2	synonymous	NM_00529	c.624C>T	p.(=)	-4.95				0.06(COSM45	7 transmemb	rs11167054,rs76682	0.1913	YES	0.0 (pos.142367399),5.8	adenocarcinoi	G protein-c	G-protein coupled receptor activity,G-protein coupled re				G=0, A=64	G=0.0, A=1.0	0.00001	977.635	64	G=0/0, A=36/2
chr16:1877913	C/G	C	G	SNV	FAHD1,HAGH	FAHD1:intronic:NM_001018104.2, HAGH:upstream:NM_00	1	50		intronic	NM_00532	c.-1125G>	p.?, p.?	-0.05					Fumarylacet	rs13166	0.136	YES	1.099E-1;0.0 (pos.1877913)		Fumarylace	acetylpyruvate hydrolase activ		Glutathione,S-(N-Hydrox		C=32, G=32	C=0.5, G=0.5	0.00001	317.417	64	C=16/16, G=1
chr8:142367559	A/A	G	A	SNV	GPR20	GPR20:exonic:NM_005293.2	1	100	2	synonymous	NM_00529	c.465C>T	p.(=)	-1.21					7 transmemb	rs11785629	0.3111	YES	5.991E-1 (pos.142367559)		G protein-c	G-protein coupled receptor activity,G-protein coupled re				G=0, A=28	G=0.0, A=1.0	0.00001	408.717	28	G=0/0, A=9/19
chr10:125426565	C/C	G	C	SNV	GPR26	GPR26:exonic:NM_153442.3	1	100	1	synonymous	NM_15344	c.642G>C	p.(=)	-1.72					7 transmemb	rs4980313	1.00E-04		1.0;0.0 (pos.125426565)		G protein-c	G-protein coupled receptor activity,adenylate cyclase-a				G=0, C=39	G=0.0, C=1.0	0.00001	623.975	39	G=0/0, C=26/1
chr16:18861879	T/T	TCATA	T	INDEL	SMG1	SMG1:intronic:NM_015092.4	4	100		intronic	NM_01509	c.5053-90	p.?	0.26, -0.26, 1.03, -0.57, -0.79					FATC domain,	rs35992129,rs756668955,rs796		YES			SMG1, C. el	ATP binding,DNA repair,RNA binding,cytoplasm,cytosol,r				TCATA=0, T	TCATA=0.0, T=1.0	0.00001	1053.4	67	TCATA=0/0, T=
chr16:18870270	C/C	A	C	SNV	SMG1	SMG1:intronic:NM_015092.4	1	100		intronic	NM_01509	c.4113+74	p.?	-0.46					FATC domain	rs3752679		YES			SMG1, C. el	ATP binding,DNA repair,RNA binding,cytoplasm,cytosol,r				A=0, C=27	A=0.0, C=1.0	0.00001	314.218	27	A=0/0, C=17/1
chr7:124387374	G/G	A	G	SNV	GPR37	GPR37:exonic:NM_005302.4	1	100	2	synonymous	NM_00530	c.1047T>C	p.(=)	-0.82					7 transmemb	rs724356	0.2402	YES	9.992E-1 (pos.124387374)		G protein-c	G-protein coupled peptide receptor activity,G-protein cc				A=0, G=31	A=0.0, G=1.0	0.00001	465.733	31	A=0/0, G=16/1
chr16:19079095	G/G	T	G	SNV	COQ7,LOC102723385	COQ7:intronic:NM_016138.4, LOC102723385:upstream:NR	1	100		intronic	NR_11937	c.73+36T>	p.?	0.22					Ubiquinone b	rs4531717	0.0426	YES	9.662E-1 (pos.19079095)		COQ7, S. ce	chromatin binding,extrinsic component of mitochondria				T=0, G=45	T=0.0, G=1.0	0.00001	747.393	45	T=0/0, G=15/3
chr16:19083221	G/G	A	G	SNV	COQ7	COQ7:intronic:NM_016138.4	1	100		intronic	NM_01613	c.74-29A>	p.?	-0.1					Ubiquinone b	rs4782202	0.0364	YES	9.658E-1 (pos.19083221)		COQ7, S. ce	chromatin binding,extrinsic component of mitochondria				A=0, G=46	A=0.0, G=1.0	0.00001	768.275	46	A=0/0, G=20/2
chr1:202092360	A/A	G	A	SNV	GPR37L1	GPR37L1:exonic:NM_004767.3	1	100	1	missense	NM_00476	c.269G>A	p.Gly90Asp	-0.4	1	94	0	0.02(COSM39	7 transmemb	rs3795595	0.1065	YES	8.242E-1 (pos.20209236	acute_myeloic	G protein-c	G-protein coupled peptide receptor activity,adenylate c				G=0, A=25	G=0.0, A=1.0	0.00001	352.932	25	G=0/0, A=14/1
chr16:19460842	G/G	A	G	SNV	TMC5	TMC5:intronic:NM_001261841.1	1	100		intronic	NM_00126	c.959-22A	p.?	-0.12					TMC domain	rs6497373	0.1982	YES	8.642E-1 (pos.19460842)		Transmemb	extracellular exosome,integral component of membran				A=0, G=27	A=0.0, G=1.0	0.00001	389.956	27	A=0/0, G=13/1
chr16:19471665	C/C	T	C	SNV	TMC5	TMC5:intronic:NM_001261841.1	1	100		intronic	NM_00126	c.1148+9T	p.?	-0.69					TMC domain	rs6497375	0.2083	YES	8.646E-1 (pos.19471665)		Transmemb	extracellular exosome,integral component of membran				T=0, C=30	T=0.0, C=1.0	0.00001	446.639	30	T=0/0, C=7/23
chr1:202092580	C/C	T	C	SNV	GPR37L1	GPR37L1:exonic:NM_004767.3	1	100	1	synonymous	NM_00476	c.489T>C	p.(=)	-2.81					7 transmemb	rs7516762	0.1062	YES	8.298E-1 (pos.202092580)		G protein-c	G-protein coupled peptide receptor activity,adenylate c				T=0, C=35	T=0.0, C=1.0	0.00001	543.903	35	T=0/0, C=15/2
chr16:19580699	A/G	A	G	SNV	C16orf62	C16orf62:intronic:NM_020314.5	1	58.33		intronic	NM_02031	c.385-47A	p.?	0.02						rs121235	0.356	YES	3.94E-1 (pos.19580699)			Golgi to plasma membrane transport,early endosome,fi				A=10, G=14	A=0.4167, G=0.5833	0.00001	135.339	24	A=6/4, G=10/4
chr16:19580711	G/C	G	C	SNV	C16orf62	C16orf62:intronic:NM_020314.5	1	58.33		intronic	NM_02031	c.385-35G	p.?	0.31						rs8062210	0.0612	YES	2.55E-2;0.0 (pos.19580711)			Golgi to plasma membrane transport,early endosome,fi				G=10, C=14	G=0.4167, C=0.5833	0.00001	135.339	24	G=6/4, C=10/4
chr16:19590334	G/A	G	A	SNV	C16orf62	C16orf62:intronic:NM_020314.5	1	59.09		intronic	NM_02031	c.701-40G	p.?	-0.13						rs11861774	0.0177	YES	3.98E-2 (pos.19590334)			Golgi to plasma membrane transport,early endosome,fi				G=9, A=13	G=0.4091, A=0.5909	0.00001	122.568	22	G=9/0, A=10/3
chr2:133174764	C/T	C	T	SNV	GPR39	GPR39:exonic:NM_001508.2	1	47.37	1	missense	NM_00150	c.149C>T	p.Ala50Val	4.96	1	64	0	0.22(COSM37	7 transmemb	rs2241764	0.3267	YES	2.269E-1 (pos.13317476	acute_myeloic	G protein-c	G-protein	Cardiovascular Diseases;Hypertension;Vasc			C=20, T=18	C=0.5263, T=0.4737	0.00001	184.536	38	C=4/16, T=7/1
chr14:88477413	T/T	C	T	SNV	GPR65	GPR65:exonic:NM_003608.3	1	100	2	synonymous	NM_00360	c.222C>T	p.(=)	-0.37					7 transmemb	rs6574978	0.329	YES	9.795E-1 (pos.88477413)		G protein-c	G-protein	Autoimmune Diseases;Crohn Disease;Digest			C=0, T=39	C=0.0, T=1.0	0.00001	621.329	39	C=0/0, T=28/1
chr14:88477882	A/C	A	C	SNV	GPR65	GPR65:exonic:NM_003608.3	1	53.49	2	missense	NM_00360	c.691A>C	p.Ile231Leu	2.46		5		0.6(COSM399	7 transmemb	rs3742704	0.0879	YES	1.625E-1 (pos.88477882)	acute_myeloic	G protein-c	G-protein	Autoimmune Diseases;Crohn Disease;Digest			A=20, C=23	A=0.4651, C=0.5349	0.00001	260.164	43	A=6/14, C=15/
chr16:20352756	G/G	A	G	SNV	UMOD	UMOD:intronic:NM_001008389.2	1	100		intronic	NM_00100	c.1332-98	p.?	-3.62					Calcium-bind	rs9646256		YES			Uromodulir	Golgi lume	Arthritis;Cardiovascular Diseases;Congenita			A=0, G=26	A=0.0, G=1.0	0.00001	371.42	26	A=0/0, G=14/1
chr4:8582742	C/T	C	T	SNV	GPR78	GPR78:exonic:NM_080819.4	1	50	1	synonymous	NM_08081	c.33C>T	p.(=)	-0.84				0.15(COSM37	7 transmemb	rs17844776	0.335	YES	2.248E-2 (pos.8582742)	adenocarcinoi	G protein-c	G-protein coupled receptor activity,G-protein coupled re				C=17, T=17	C=0.5, T=0.5	0.00001	171.712	34	C=9/8, T=7/10
chr16:20410669	A/G	A	G	SNV	PDILT	PDILT:intronic:NM_174924.1	1	53.49		intronic	NM_17492	c.-6-40T>C	p.?	-1.28					Thioredoxin,'	rs764499875,rs9928	0.2866	YES	1.568E-1;0.0 (pos.20410669)			cell migration,cell redox homeostasis,endoplasmic retic				A=20, G=23	A=0.4651, G=0.5349	0.00001	261.077	43	A=7/13, G=15/
chr16:20497775	C/C	G	C	SNV	ACSM2A	ACSM2A:intronic:NM_001308954.1	1	100		intronic	NM_00130	c.1630-12	p.?	-0.25					AMP-binding	rs4783532		YES			Acyl-CoA sy	ATP binding,acyl-CoA ligase activity,butyrate-CoA ligase				G=0, C=81	G=0.0, C=1.0	0.00001	1308.54	81	G=0/0, C=39/4
chr16:20792188	C/C	T	C	SNV	ACSM3	ACSM3:intronic:NM_005622.3	1	100		intronic	NM_00562	c.782+9T>	p.?	-0.35				0.01(COSM39	AMP-binding	rs5715	0.2707	YES	4.784E-1 (pos.20792188)	acute_myeloic	Acyl-CoA sy	ATP bindin	Cardiovascular Diseases;Genital Diseases, N			T=0, C=46	T=0.0, C=1.0	0.00001	768.28	46	T=0/0, C=28/1
chr4:8582856	T/G	T	G	SNV	GPR78	GPR78:exonic:NM_080819.4	1	46.88	1	synonymous	NM_08081	c.147T>G	p.(=)	-0.67				0.12(COSM37	7 transmemb	rs17844777	0.3448	YES	1.757E-2 (pos.8582856)	adenocarcinoi	G protein-c	G-protein coupled receptor activity,G-protein coupled re				T=34, G=30	T=0.5313, G=0.4688	0.00001	288.123	64	T=15/19, G=6/
chr7:112724417	G/G	A	G	SNV	GPR85	GPR85:exonic:NM_001146267.1	1	100	3	synonymous	NM_00114	c.360T>C	p.(=)	6.75					7 transmemb	rs2256044	0.1114	YES	9.782E-1 (pos.112724417)		G protein-c	G-protein coupled receptor activity,G-protein coupled re				A=0, G=30	A=0.0, G=1.0	0.00001	446.603	30	A=0/0, G=20/1
chr7:112724525	G/G	A	G	SNV	GPR85	GPR85:exonic:NM_001146267.1	1	100	3	synonymous	NM_00114	c.252T>C	p.(=)	4.63					7 transmemb	rs1608890	0.1116	YES	9.779E-1 (pos.112724525)		G protein-c	G-protein coupled receptor activity,G-protein coupled re				A=0, G=32	A=0.0, G=1.0	0.00001	485.133	32	A=0/0, G=17/1
chr16:21030897	C/C	T	C	SNV	DNAH3	DNAH3:intronic:NM_017539.2	1	100		intronic	NM_01753	c.6022+49	p.?	-0.76					AAA domain (	rs3095922			9.994E-1;0.0 (pos.21030897)		Dynein, axo	ATP binding,ATP-dependent microtubule motor activity,				T=0, C=29	T=0.0, C=1.0	0.00001	427.604	29	T=0/0, C=12/1
chr16:21136477	A/G	A	G	SNV	DNAH3	DNAH3:intronic:NM_017539.2	1	57.14		intronic	NM_01753	c.1404+19	p.?	-1.67					AAA domain (	rs7191215	0.036	YES	9.999E-1 (pos.21136477)		Dynein, axo	ATP binding,ATP-dependent microtubule motor activity,				A=9, G=12	A=0.4286, G=0.5714	0.00001	109.102	21	A=7/2, G=10/2
chr3:151012497	C/C	T	C	SNV	GPR87,MED12L	GPR87:exonic:NM_023915.3, MED12L:intronic:NM_053002	1	100	3	synonymous	NM_05300	c.2146-55	p.?, p.(=)	-2.04				0.06(COSM50	7 transmemb	rs2242353	0.2217	YES	7.099E-1 (pos.15101249	carcinoma,dif	G protein-c	G-protein coupled purinergic nucleotide receptor activit				T=0, C=35	T=0.0, C=1.0	0.00001	537.041	35	T=0/0, C=19/1
chr3:151011969	G/A	G	A	SNV	GPR87,MED12L	GPR87:exonic:NM_023915.3, MED12L:intronic:NM_053002	1	50	3	synonymous	NM_05300	c.2146-55	p.?, p.(=)	-0.77				0.02(COSM37	7 transmemb	rs1048408	0.4698	YES	4.728E-1 (pos.15101196	adenocarcinoi	G protein-c	G-protein coupled purinergic nucleotide receptor activit				G=28, A=28	G=0.5, A=0.5	0.00001	272.138	56	G=11/17, A=1
chr16:223735	G/G	A	G	SNV	HBA2	HBA2:downstream:NM_000517.4	1	100		intronic	NM_00051	c.*136A>G	p.?	0.37						rs2685121		YES			Hemoglobir	bicarbona	Acneiform Eruptions;Anemia;A		Benign	A=0, G=21	A=0.0, G=1.0	0.00001	281.235	21	A=0/0, G=9/12
chr17:72443287	G/G	C	G	SNV	GPRC5C	GPRC5C:utr_3:NM_018653.3	1	100	4	utr_3	NM_01865	c.1482C>G	p.?	0.34					7 transmemb	rs6980		YES			G protein-c	G-protein coupled receptor activity,G-protein coupled re				C=0, G=35	C=0.0, G=1.0	0.00001	543.745	35	C=0/0, G=20/1
chr6:117121894	A/A	G	A	SNV	GPRC6A	GPRC6A:exonic:NM_148963.3	1	100	4	synonymous	NM_14896	c.1401C>T	p.(=)	-0.39					7 transmemb	rs6917467	8.00E-04		1.0 (pos.117121894)			G-protein coupled receptor activity,G-protein coupled re				G=0, A=40	G=0.0, A=1.0	0.00001	644.284	40	G=0/0, A=24/1
chr16:23205657	G/C	G	C	SNV	SCNN1G	SCNN1G:intronic:NM_001039.3	1	47.62		intronic	NM_00103	c.913+62G	p.?	0.28					Amiloride-se	rs4247210		YES			Sodium cha	WW doma	Bronchial Diseases	Amiloride,Triamterene		G=11, C=10	G=0.5238, C=0.4762	0.00001	82.2901	21	G=2/9, C=1/9
chr5:176025938	C/C	T	C	SNV	GPRIN1	GPRIN1:exonic:NM_052899.2	1	100	2	missense	NM_05289	c.898A>G	p.Met300Val	-2.16		21			G protein-reg	rs6556276	0		9.999E-1 (pos.176025938)		G protein-re	growth cone,neuron projection development,phosphop				T=0, C=77	T=0.0, C=1.0	0.00001	1233.48	77	T=0/0, C=37/4
chr10:47000188	G/G	A	G	SNV	GPRIN2	GPRIN2:exonic:NM_014696.4	1	100	3	synonymous	NM_01469	c.1308A>G	p.(=)	-7.59				0.01(COSM37	G protein-reg	rs3127824	0.0014	YES	1.0 (pos.47000188)	adenocarcinoi	G protein-re	protein binding				A=0, G=103	A=0.0, G=1.0	0.00001	1609.34	103	A=0/0, G=48/5

chr9:139252495	A/A	G	A	SNV	GPSM1	GPSM1:exonic:NM_001145638.2	1	100	14	synonymous	NM_00114	c.1851G>	p.(=)	-1.52				0.09(COSM37	GoLoco motif	rs3812547	0.333	YES	9.506E-1 (pos.13925249	adenocarcinoma,squamou		GDP-disso	Cocaine-Related Disorders;Diabetes Mellitu			G=0, A=27	G=0.0, A=1.0	0.00001	389.884	27	G=0/0, A=8/19
chr3:49395757	A/A	G	A	SNV	GPX1,RHOA	GPX1:utr_5:NM_000581.3, RHOA:downstream:NM_001664.	1	100	1	utr_5	NM_00058	c.-46C>T, c	p.?, p.?	-1.78					Glutathione	rs1800668,rs775819324		YES	0.0 (pos.49395755),8.85E-2 (pos.493957		Glutathione	G-protein	Autistic Disorder:Au	Glutathione,Guanosine-		G=0, A=31	G=0.0, A=1.0	0.00001	465.67	31	G=0/0, A=11/2
chr16:24135112	C/C	T	C	SNV	PRKCB	PRKCB:intronic:NM_002738.6	1	100		intronic	NM_00273	c.919-44T	p.?	0.17					C2 domain,PI	rs1013316	0.4041	YES	8.047E-1 (pos.24135112)			ATP bindin	Autistic Disorder:C	Ellagic Acid,Phosphorylc		T=0, C=45	T=0.0, C=1.0	0.00001	744.957	45	T=0/0, C=36/9
chr16:24886893	T/T	G	T	SNV	SLC5A11	SLC5A11:intronic:NM_052944.4	1	100		intronic	NM_05294	c.373-55G	p.?	0.12					Sodium:solut	rs34534629,rs386789920		YES			Solute carri	apoptotic	Autoimmune Diseases;Connective Tissue Dis			G=0, T=48	G=0.0, T=1.0	0.00001	808.657	48	G=0/0, T=24/2
chr19:35510102	C/C	G	C	SNV	GRAMD1A	GRAMD1A:exonic:NM_020895.4	1	100	12	synonymous	NM_02089	c.1221G>C	p.(=)	0.18				0.14(COSM40	Domain of un	rs2290646	0.3706	YES	2.463E-1 (pos.35510102)	acute_myeloid leukaemia		integral component of membrane,protein binding				G=0, C=40	G=0.0, C=1.0	0.00001	644.286	40	G=0/0, C=19/2
chr16:2510603	T/T	C	T	SNV	TEDC2	TEDC2:intronic:NM_025108.2	1	100		intronic	NM_02510	c.126-21C	p.?	-0.25					Domain of un	rs4786284	0.3365	YES	7.829E-1 (pos.2510603)			protein binding				C=0, T=27	C=0.0, T=1.0	0.00001	389.753	27	C=0/0, T=16/1
chr5:125696140	A/A	G	A	SNV	GRAMD2B	GRAMD2B:exonic:NM_001146319.2	1	100	1	synonymous	NM_00114	c.117G>A	p.(=)	0.11					GRAM domai	rs11241887	0.0409	YES	1.0 (pos.125696140)			cytoplasmic microtubule,identical protein binding,integ				G=0, A=33	G=0.0, A=1.0	0.00001	504.598	33	G=0/0, A=14/1
chr2:165477836	G/A	G	A	SNV	GRB14	GRB14:utr_5:NM_004490.2	1	50	1	utr_5	NM_00449	c.-17C>T	p.?	0.2					BPS (Betweer	rs10200142	0.0818	YES	4.387E-2;0.0 (pos.165477836)		Growth fact	SH3/SH2 a	Diabetes Mellitus;Diabetes Mellitus, Type 2;			G=12, A=13	G=0.48, A=0.52	0.00001	125.267	26	G=7/5, A=5/8
chr16:27777831	G/G	A	G	SNV	KIAA0556	KIAA0556:intronic:NM_015202.3	1	100		intronic	NM_01520	c.3975+36	p.?	0.94					Domain of un	rs8043555	0.142	YES	8.689E-1 (pos.27777831)		Katanin-int	cell projection,cytoplasm,cytoskeleton,extracellular spa				A=0, G=25	A=0.0, G=1.0	0.00001	353.019	25	A=0/0, G=3/22
chr2:11738091	C/C	T	C	SNV	GREB1	GREB1:exonic:NM_014668.3	1	100	14	synonymous	NM_01466	c.2139T>C	p.(=)	-1.01					Gene regulat	rs1435547	0.0049	YES	9.757E-1 (pos.11738091)		GREB1 prot	extracellu	Endometriosis;Female Urogenital Diseases:F			T=0, C=141	T=0.0, C=1.0	0.00001	2362.6	141	T=0/0, C=69/7
chr16:2825594	A/G	A	G	SNV	ELOB	ELOB:intronic:NM_207013.2	1	53.13		intronic	NM_20701	c.139-37T	p.?	-0.65					Ubiquitin fam	rs3094774	0.1591	YES	4.34E-1 (pos.2825594)		Transcriptic	Cul2-RING ubiquitin ligase complex,Cul5-RING ubiquitin l				A=15, G=17	A=0.4688, G=0.5313	0.00001	175.887	32	A=12/3, G=3/1
chr18:19079853	G/G	A	G	SNV	GREB1L	GREB1L:exonic:NM_001142966.2	1	100	22	synonymous	NM_00114	c.3555A>G	p.(=)	3.83				0.06(COSM41	Gene regulat	rs4800747	3.00E-04		1.0 (pos.19079853)	neoplasm	GREB1-like	integral component of membrane				A=0, G=25	A=0.0, G=1.0	0.00001	353.019	25	A=0/0, G=11/1
chr16:28604686	A/G	A	G	SNV	SULT1A2	SULT1A2:intronic:NM_001054.3	1	47.37		intronic	NM_00105	c.500-19T	p.?	-0.08					Nuclear pore	rs3743963	0.4645	YES	2.75E-1 (pos.28604686)		Sulfotransfe	3'-phosphoadenosine 5'-phosphosulfate metabolic proc				A=20, G=18	A=0.5263, G=0.4737	0.00001	190.618	38	A=11/9, G=8/1
chr8:102504974	G/G	C	G	SNV	GRHL2	GRHL2:utr_5:NM_024915.3	1	100	1	utr_5	NM_02491	c.-24C>G	p.?	5.57					CP2 transcrip	rs515622	0.001	YES	9.019E-1 (pos.102504974)		Grainyhead	DNA bindi	Ear Diseases;Hearing Disorders;Hearing Loss			C=0, G=81	C=0.0, G=1.0	0.00001	1280.93	81	C=0/0, G=14/6
chr16:28631585	G/G	T	G	SNV	SULT1A1		1	100		intronic				-2.06					Nuclear pore	rs1968752		YES			Sulfotransferase family		Cardiovascular Dise	Acetaminophen,Adenosi		T=0, G=20	T=0.0, G=1.0	0.00001	263.786	20	T=0/0, G=8/12
chr4:158238830	C/C	T	C	SNV	GRIA2	GRIA2:exonic:NM_000826.3	1	100	5	synonymous	NM_00082	c.687T>C	p.(=)	1.61				0.91(COSM11	Bacterial ext	rs4302506	0.3532	YES	8.16E-1 (pos.158238830)	adenocarcino	Glutamate	AMPA glut	Brain Diseases:Cen	(3R)-3-cyclopentyl-6-met		T=0, C=85	T=0.0, C=1.0	0.00001	1394.81	85	T=0/0, C=36/4
chr16:28847246	T/C	T	C	SNV	ATXN2L	ATXN2L:intronic:NM_148414.2	1	58.33		intronic	NM_14841	c.2896-8T	p.?	-0.82				0.03(COSM62	Ataxin 2 SM d	rs12928404	0.3876	YES	4.469E-1 (pos.28847246)	neoplasm	Ataxin 2-like	RNA bindir	Digestive System Diseases:Gastroenteritis;G			T=10, C=14	T=0.4167, C=0.5833	0.00001	134.438	24	T=7/3, C=7/7
chr9:140051375	TA/TA	T	TA	INDEL	GRIN1	GRIN1:exonic:NM_001185090.1	1	100	7	frameshiftInsertion	NM_00118	c.917_918	p.Val308fs	7.41					Bacterial extracellular solute-binding proteins, family 3,Calmodulin-binding domain C0 of NMDA rec						G protein-re	MAPK casc	Cocaine-Related Di	Acamprosate,Acetylcyst		T=0, TA=41	T=0.0, TA=1.0	0.00001	554.094	41	T=0/0, TA=23/
chr17:72851133	G/G	A	G	SNV	GRIN2C	GRIN2C:exonic:NM_000835.4	1	100	2	synonymous	NM_00083	c.99T>C	p.(=)	-0.55				0.02(COSM14	Bacterial ext	rs689730	0.0956	YES	7.401E-1 (pos.72851133)	adenocarcino	Glutamate	MAPK cascade,NMDA glutamat		Acamprosate,Atomoxeti		A=0, G=203	A=0.0, G=1.0	0.00001	3292.37	203	A=0/0, G=53/1
chr16:28913787	T/C	T	C	SNV	ATP2A1	ATP2A1:intronic:NM_173201.3	1	53.33		intronic	NM_17320	c.2524+80	p.?	-2.4					Cation transı	rs7189927		YES			ATPase, Ca+	ATP bindin	Cardiovascular Dise	(6AR,11AS,11BR)-10-ACE1		T=14, C=16	T=0.4667, C=0.5333	0.00001	159.916	30	T=2/12, C=8/8
chr16:28962755	G/A	G	A	SNV	NFATC2IP	NFATC2IP:intronic:NM_032815.3	1	57.14		intronic	NM_03281	c.387+36G	p.?	0.41					Nuclear pore	rs12449272	0.2107	YES	3.654E-1 (pos.28962755)		NFATC2-inte	cytokine production,cytoplasm,nucleus,positive regulat				G=33, A=44	G=0.4286, A=0.5714	0.00001	491.665	77	G=18/15, A=29
chr9:104499575	C/C	T	C	SNV	GRIN3A	GRIN3A:exonic:NM_133445.2	1	100	1	synonymous	NM_13344	c.687A>G	p.(=)	0.05				0.15(COSM63	Bacterial ext	rs1337677	0.0522	YES	5.462E-1 (pos.10449957	carcinoma	Glutamate	NMDA glut	Erectile Dysfunctio	Acamprosate,Acetylcyst		T=0, C=40	T=0.0, C=1.0	0.00001	644.213	40	T=0/0, C=30/1
chr16:29790767	G/G	A	G	SNV	ZG16	ZG16:intronic:NM_152338.3	1	100		intronic	NM_15233	c.56-59A>	p.?	-0.76					Jacalin-like le	rs235635		YES			Zymogen gr	Golgi lumen,carbohydrate binding,cytoplasmic vesicle lu				A=0, G=20	A=0.0, G=1.0	0.00001	263.786	20	A=0/0, G=6/14
chr19:1004740	C/C	T	C	SNV	GRIN3B	GRIN3B:exonic:NM_138690.2	1	100	3	missense	NM_13869	c.1240T>C	p.Trp414Arg	0.58	0.54	101	0	0(COSM3756	Bacterial ext	rs2240157	0.2881	YES	4.967E-1 (pos.1004740)	acute_myeloid leukaemia		NMDA selective glutamate rec		Acamprosate,Atomoxeti		T=0, C=50	T=0.0, C=1.0	0.00001	844.318	50	T=0/0, C=26/2
chr19:1004710	C/T	C	T	SNV	GRIN3B	GRIN3B:exonic:NM_138690.2	1	51.92	3	missense	NM_13869	c.1210C>T	p.Arg404Trp	-1.68	0.11	101	0.033	0.05(COSM37	Bacterial ext	rs4807399	0.4798	YES	8.617E-2 (pos.1004710)	adenocarcinoma,rhabdom		NMDA selective glutamate rec		Acamprosate,Atomoxeti		C=25, T=27	C=0.4808, T=0.5192	0.00001	270.395	52	C=16/9, T=14/
chr16:30119525	A/A	C	A	SNV	GDPD3	GDPD3:intronic:NM_024307.2	1	100		intronic	NM_02430	c.819+9G>	p.?	-0.04				0(COSM5021	Glycerophosı	rs8061772	0.1351	YES	9.838E-1 (pos.30119525)	osteosarcoma	Glycerophos	endoplasr	Congenital, Hereditary, and Neonatal Diseas			C=0, A=33	C=0.0, A=1.0	0.00001	504.591	33	C=0/0, A=14/1
chr16:3016885	G/G	A	G	SNV	KREMEN2	KREMEN2:intronic:NM_172229.2	1	100		intronic	NM_17222	c.641-26A	p.?	0.14					CUB domain,	rs58462745	0.0016		1.0 (pos.3016885)		Kringle dom	Wnt signaling pathway,cell communication,early endos				A=0, G=21	A=0.0, G=1.0	0.00001	281.235	21	A=0/0, G=11/1
chr11:67050291	TA/TA	T	TA	INDEL	GRK2	GRK2:splicesite_5:NM_001619.3	1	100	14	splicesite	NM_00161	c.1227+2T	p.?	7.15					PH domain,Protein kinase domain,Protein tyrosine kinase,Regulator of G protein signaling domain							ATP binding,Edg-2 lysophosphatidic acid receptor bindin				T=0, TA=32	T=0.0, TA=1.0	0.00001	401.582	32	T=0/0, TA=15/
chr11:88242024	G/G	T	G	SNV	GRM5,GRM5-AS1	GRM5-AS1:exonic_nc:NR_049724.1, GRM5:exonic:NM_0011	1	100	9	synonymous	NR_04972	c.3375A>C	p.(=)	0.13				0.04(COSM41	7 transmemb	rs2941563	0.0696	YES	9.994E-1 (pos.88242024)	neoplasm	Glutamate	G-protein	Attention Deficit Di	ADX10059,Acamprosate		T=0, G=28	T=0.0, G=1.0	0.00001	408.718	28	T=0/0, G=3/25
chr16:30500761	A/A	G	A	SNV	ITGAL	ITGAL:intronic:NM_002209.2	1	100		intronic	NM_00220	c.1213+54	p.?	-1.35					FG-GAP repe	rs3087438		YES			Integrin, al	ICAM-3 rec	Behcet Syndrome;C	(S)-2-((S)-3-ISOBUTYL-2,5-		G=0, A=60	G=0.0, A=1.0	0.00001	892.292	60	G=0/0, A=28/3
chr16:30505513	G/G	A	G	SNV	ITGAL	ITGAL:intronic:NM_002209.2	1	100		intronic	NM_00220	c.1214-20	p.?	-0.9					FG-GAP repe	rs386790266,rs4350	0.3377	YES	9.893E-1 (pos.30505513)		Integrin, al	ICAM-3 rec	Behcet Syndrome;C	(S)-2-((S)-3-ISOBUTYL-2,5-		A=0, G=53	A=0.0, G=1.0	0.00001	782.029	53	A=0/0, G=23/3
chr5:178415982	G/G	A	G	SNV	GRM6	GRM6:exonic:NM_000843.3	1	100	6	synonymous	NM_00084	c.1308T>C	p.(=)	-3.37					7 transmemb	rs4701014,rs797044	2.00E-04		1.0 (pos.178415982)		Glutamate	G-protein	Congenital, Hereditary, and Ne		Benign	A=0, G=24	A=0.0, G=1.0	0.00001	334.792	24	A=0/0, G=16/8
chr16:3071420	A/A	T	A	SNV	TNFRSF12A	TNFRSF12A:intronic:NM_016639.2	1	100		intronic	NM_01663	c.199+100	p.?	-0.2					Tumour necr	rs2232798		YES			Tumor necr	angiogene	Acute Kidney Injury;Cardiovascular Diseases			T=0, A=36	T=0.0, A=1.0	0.00001	564.588	36	T=0/0, A=18/1
chr5:178416288	G/A	G	A	SNV	GRM6	GRM6:exonic:NM_000843.3	1	96.77	5	synonymous	NM_00084	c.1131C>T	p.(=)	-2.55				0.03(COSM41	7 transmemb	rs2071246	0.2681	YES	6.989E-1 (pos.17841628	carcinoma,ne	Glutamate	G-protein	Congenital, Hereditary, and Ne		Benign	G=1, A=30	G=0.0323, A=0.9677	0.00001	435.398	31	G=0/1, A=8/22
chr5:178413947	T/C	T	C	SNV	GRM6	GRM6:exonic:NM_000843.3	1	50	7	synonymous	NM_00084	c.1392A>G	p.(=)	-0.83				0.14(COSM41	7 transmemb	rs11746675	0.3626	YES	5.848E-1 (pos.17841394	carcinoma,ne	Glutamate	G-protein	Congenital, Hereditary, and Ne		Benign	T=10, C=10	T=0.5, C=0.5	0.00001	82.8861	20	T=9/1, C=6/4
chr16:30977799	A/A	G	A	SNV	SETD1A	SETD1A:intronic:NM_014712.2	1	100		intronic	NM_01471	c.2505+92	p.?	0.06					COMPASS (Co	rs11150601		YES			SET domain	RNA binding,Set1C/COMPASS complex,beta-catenin bind				G=0, A=54	G=0.0, A=1.0	0.00001	800.525	54	G=0/0, A=22/3
chr16:30980902	C/C	T	C	SNV	SETD1A	SETD1A:intronic:NM_014712.2	1	100		intronic	NM_01471	c.2929-21	p.?	0.05					COMPASS (Co	rs781236353,rs8979	0.3566	YES	0.0 (pos.30980894),9.146E-1 (pos.30980		SET domain	RNA binding,Set1C/COMPASS complex,beta-catenin bind				T=0, C=29	T=0.0, C=1.0	0.00001	427.603	29	T=0/0, C=18/1
chr16:31049155	A/G	A	G	SNV	STX4	STX4:intronic:NM_001272096.1	1	46.88		intronic	NM_00127	c.373-92A	p.?	-0.9					SNARE domai	rs55979739		YES			Syntaxin 4	SNAP receptor activity,SNARE binding,SNARE complex,SN				A=51, G=45	A=0.5313, G=0.4688	0.00001	487.598	96	A=42/9, G=36/
chr3:7494417	T/T	A	T	SNV	GRM7	GRM7:exonic:NM_181874.2	1	100	6	missense	NM_18187	c.1298A>T	p.Tyr433Phe	2.34	0.26	22	0.001	0.82(COSM37	7 transmemb	rs2229902	0.4352	YES	8.242E-2 (pos.7494417)	adenocarcino	Glutamate	G-protein	Attention Deficit Di	Glutamic Acid		A=0, T=30	A=0.0, T=1.0	0.00001	446.639	30	A=0/0, T=16/1
chr4:7069804	A/A	C	A	SNV	GRPEL1	GRPEL1:utr_5:NM_025196.3	1	100	1	utr_5	NM_02519	c.-45G>T	p.?	0.84						rs3822270	0.2073	YES	7.323E-1;0.0;0.0 (pos.7069804)		GrpE, E. coli	adenyl-nu	HIV Infections;Immune System Diseases;Imm			C=0, A=38	C=0.0, A=1.0	0.00001	603.786	38	C=0/0, A=29/9
chr16:31141993	C/T	C	T	SNV	KAT8,PRSS8	KAT8:intronic:NM_182958.2, PRSS8:downstream:NM_0027	1	57.14		intronic	NM_18295	c.1157+66	p.?, p.?	-3.19					MOZ/SAS fam	rs1549293		YES			K(lysine) ac	MLL1 com	Congenital Abnorm	1-4-(hydroxymethyl)pher		C=18, T=24	C=0.4286, T=0.5714	0.00001	279.083	42	C=6/12, T=8/1
chr4:7069901	C/C	T	C	SNV	GRPEL1	GRPEL1:utr_5:NM_025196.3	1	100	1	utr_5	NM_02519	c.-142A>G	p.?	-0.53						rs3822269		YES			GrpE, E. coli	adenyl-nu	HIV Infections;Immune System Diseases;Imm			T=0, C=23	T=0.0, C=1.0	0.00001	316.747	23	T=0/0, C=15/8
chr19:48949286	T/T	G	T	SNV	GRWD1	GRWD1:exonic:NM_031485.3	1	100	1	synonymous	NM_03148	c.24G>T	p.(=)	-0.24				0.03(COSM62	Histone-bind	rs1643487	0.0865	YES	6.532E-1;0.0 (pos.48949	neoplasm	Glutamate-	DNA replication,DNA replication origin binding,RNA bind				G=0, T=21	G=0.0, T=1.0	0.00001	281.228	21	G=0/0, T=14/7
chr16:31273129	C/C	T	C	SNV	ITGAM	ITGAM:intronic:NM_001145808.1	1	100		intronic	NM_00114	c.134+11T	p.?	-0.66					FG-GAP repe	rs3764327	0.2827	YES	7.308E-1 (pos.31273129)		Integrin, al	amyloid-b	Autoimmune Diseases;Connective Tissue Dis			T=0, C=55	T=0.0, C=1.0	0.00001	814.906	55	T=0/0, C=32/2
chr16:31371193	G/G	C	G	SNV	ITGAX	ITGAX:intronic:NM_000887.4	1	100		intronic	NM_00088	c.561+42C	p.?	0.44					FG-GAP repe	rs4264407	0.0709	YES	9.942E-1 (pos.31371193)		Integrin, al	animal org	Autoimmune Diseases;Connective Tissue Dis			C=0, G=28	C=0.0, G=1.0	0.00001	408.713	28	C=0/0, G=12/1
chr16:31427678	T/T	C	T	SNV	ITGAD	ITGAD:intronic:NM_005353.2	1	100		intronic	NM_00535	c.2357+71	p.?	-0.64					FG-GAP repe	rs8056122		YES			Integrin, al	cell surface,extracellular matrix organization,heterotyp				C=0, T=36	C=0.0, T=1.0	0.00001	563.736	36	C=0/0, T=10/2
chr16:31476458	T/T	C	T	SNV	ARMC5	ARMC5:intronic:NM_001105247.1	1	100		intronic	NM_00110	c.1864+25	p.?	-1.17				0.02(COSM1599969)		rs11150624	0.4252	YES	4.547E-1 (pos.31476458)	acute_myeloic	Armadillo re	cytoplasm,cytosol,focal adhesion,nucleoplasm				C=0, T=33	C=0.0, T=1.0	0.00001	504.598	33	C=0/0, T=16/1
chr16:31519048	G/G	A	G	SNV	C16orf58	C16orf58:intronic:NM_022744.3	1	100		intronic	NM_02274	c.415+37T	p.?	-0.46					Vitamin B6 pl	rs11861164	0.098	YES	7.985E-1 (pos.31519048)			integral component of membrane,membrane				A=0, G=23	A=0.0, G=1.0	0.00001	316.747	23	A=0/0, G=7/16
chr19:48954420	C/CA	C	CA	INDEL	GRWD1	GRWD1:exonic:NM_031485.3	1	59.09	6	frameshiftInsertion	NM_03148	c.955_956	p.Arg319fs	0.53					Histone-bind	rs145223989	0		0.0 (pos.48954420)		Glutamate-	DNA replication,DNA replication origin binding,RNA bind				C=18, CA=2	C=0.4091, CA=0.590	0.00001	218.971	44	C=8/10, CA=16
chr19:48954421	G/A	G	A	SNV	GRWD1	GRWD1:exonic:NM_031485.3	1	52.27	6	missense	NM_03148	c.956G>A	p.Arg319Gln	0.69	0.65	43	0		Histone-bind	rs2302951	0.137	YES	1.683E-1 (pos.48954421)		Glutamate-	DNA replication,DNA replication origin binding,RNA bind				G=21, A=23	G=0.4773, A=0.5227	0.00001	260.216	44	G=9/12, A=15/
chr17:38121993	A/A	G	A	SNV	GSDMA	GSDMA:exonic:NM_178171.4	1	100	2	missense	NM_17817	c.53G>A	p.Arg18Gln	0.89	0.23	43	0.016	0.16(COSM37	Gasdermin fa	rs3894194	0.456	YES	5.768E-1 (pos.38121993)	adenocarcino	Gasdermin	apoptotic	Asthma;Bronchial Diseases;Hypersensitivity			G=0, A=59	G=0.0, A=1.0	0.00001	884.918	59	G=0/0, A=19/4
chr16:3726160	G/G	A	G	SNV	TRAP1	TRAP1:intronic:NM_016292.2	1	100		intronic	NM_01629	c.705-14T	p.?	0.72					Histidine kina	rs8050400	0.1065	YES	1.0 (pos.3726160)		Transformir	ATP bindin	Arthritis;Digestive System Dise		Benign	A=0, G=40	A=0.0, G=1.0	0.00001	643.779	40	A=0/0, G=15/2
chr10:106058901	G/T	G	T	SNV	GSTO2	GSTO2:exonic:NM_183239.1	1	52.38	7	synonymous	NM_18323	c.591G>T	p.(=)	0				0.29(COSM39	Glutathione	rs3758572	0.019	YES	1.169E-1 (pos.10605890	acute_myeloic	Glutathione	L-ascorbic	Female Urogenital	Glutathione		G=10, T=11	G=0.4762, T=0.5238	0.00001	95.2915	21	G=8/2, T=3/8
chr13:28367061	G/G	A	G	SNV	GSX1	GSX1:exonic:NM_145657.2	1	100	1	synonymous	NM_14565	c.234A>G	p.(=)	-0.09				0.03(COSM37	Homeobox d	rs1231058	0.1732	YES	1.0 (pos.28367061)	adenocarcino	GS homeob	RNA polymerase II core promoter proximal region seque				A=0, G=20	A=0.0, G=1.0	0.00001	259.882	20	A=0/0, G=17/3
chr16:4027423	T/T	C	T	SNV	ADCY9	ADCY9:intronic:NM_001116.3	1	100		intronic	NM_00111	c.2828+60	p.?	-1.38					Adenylate an	rs2072346		YES			Adenylate c	ATP bindin	Brain Diseases;Central Nervous System Dise			C=0, T=62	C=0.0, T=1.0	0.00001	915.02	62	C=0/0, T=37/2
chr16:4039141	A/G	A	G	SNV	ADCY9	ADCY9:intronic:NM_001116.3	1	57.58		intronic	NM_00111	c.2208-44	p.?	-1					Adenylate an	rs2072341	0.0631	YES	8.403E-1 (pos.4039141)		Adenylate c	ATP bindin	Brain Diseases;Central Nervous System Dise			A=14, G=19	A=0.4242, G=0.5758	0.00001	204.394	33	A=2/12, G=8/1
chr16:4057637	G/G	A	G	SNV	ADCY9	ADCY9:intronic:NM_001116.3	1	100		intronic	NM_00111	c.1694-78	p.?	0.13					Adenylate an	rs73490521		YES			Adenylate c	ATP bindin	Brain Diseases;Central Nervous System Dise			A=0, G=43	A=0.0, G=1.0	0.00001	705.844	43	A=0/0, G=25/1
chr16:418429	A/G	A	G	SNV	MRPL28	MRPL28:intronic:NM_006428.4	1	53.33		intronic	NM_00642	c.577-15T	p.?	0.38					Ribosomal L2	rs11642334	0.2791	YES	1.772E-1 (pos.418429)		Mitochondr	RNA binding,cytosol,mitochondrial inner membrane,mit				A=14, G=16	A=0.4667, G=0.5333	0.00001	159.912	30	A=8/6, G=5/11
chr16:418462	G/G	C	G	SNV	MRPL28	MRPL28:intronic:NM_006428.4	1	100		intronic	NM_00642	c.576+39G	p.?	0.13					Ribosomal L2	rs11640313,rs76560	0.4608	YES	0.0;0.0 (pos.418447),3.362E-1;0.0 (pos.4		Mitochondr	RNA binding,cytosol,mitochondrial inner membrane,mit				C=0, G=32	C=0.0, G=1.0	0.00001	485.033	32	C=0/0, G=11/2
chr4:54966830	A/A	G	A	SNV	GSX2	GSX2:exonic:NM_133267.2	1	100	1	missense	NM_13326	c.319G>A	p.Gly107Ser	0.55	1	56	0	0.37(COSM41	Homeobox d	rs13144341	1.00E-04		1.0 (pos.54966830)	neoplasm	GS homeob	forebrain	Amyotrophic Lateral Sclerosis;Central Nervo			G=0, A=28	G=0.0, A=1.0	0.00001	408.698	28	G=0/0, A=3/25
chr16:426476	C/C	T	C	SNV	TMEM8A	TMEM8A:intronic:NM_021259.2	1	100		intronic	NM_02125	c.905-21A	p.?	1.06					Protein of un	rs2071916	0.4333	YES	3.136E-1 (pos.426476)			biological_process,extracellular exosome,integral comp				T=0, C=79	T=0.0, C=1.0	0.00001	1249.41	79	T=0/0, C=41/3
chr4:54966667	C/T	C	T	SNV	GSX2	GSX2:exonic:NM_133267.2	1	58.33	1	synonymous	NM_13326	c.156C>T	p.(=)	-0.27				0.64(COSM37	Homeobox d	rs3194383	0.3843	YES	8.365E-1 (pos.54966667)	adenocarcino	GS homeob	forebrain	Amyotrophic Lateral Sclerosis;Central Nervo			C=10, T=14	C=0.4167, T=0.5833	0.00001	135.339	24	C=9/1, T=5/9
chr16:427657	A/A	G	A	SNV	TMEM8A	TMEM8A:intronic:NM_021259.2	1	100		intronic	NM_02125	c.299+14C	p.?	-2.73					Protein of un	rs11641693	0.4243	YES	4.02E-1 (pos.427657)			biological_process,extracellular exosome,integral comp				G=0, A=75	G=0.0, A=1.0	0.00001	1193.13	75	G=0/0, A=29/4
chr2:48906625	C/C	T	C	SNV	GTF2A1L,STON1-GTF2A1L	GTF2A1L:utr_3:NM_006872.4, STON1-GTF2A1L:utr_3:NM_00	1	100	8	utr_3	NM_00119	c.3452T>C	p.?, p.?	0.64					Adaptor com	rs1965508	0.03	YES	9.999E-1 (pos.48906625)		General tra	DNA bindi	Adnexal Diseases;Cysts;Endocrine System Di			T=0, C=80	T=0.0, C=1.0	0.00001	1272.99	80	T=0/0, C=36/4
chr16:4409439	C/A	C	A	SNV	CORO7,CORO7-PAM16	CORO7-PAM16:intronic:NM_001201479.1, CORO7:intronic:N	1	45.83		intronic	NM_00120	c.2250+42	p.?, p.?	-0.13					Domain of un	rs144121826,rs7773	0.0099		0.0;0.0;1.167E-4 (pos.4409433),0.0;8.16		Coronin 7	Golgi apparatus,Golgi membrane,Golgi to endosome tra				C=13, A=11	C=0.5417, A=0.4583	0.00001	93.6526	24	C=4/9, A=2/9
chr16:456291	G/GC	G	GC	INDEL	DECR2	DECR2:intronic:NM_020664.3	1	100		intronic	NM_02066	c.150-55G	p.?	0.18					Enoyl-(Acyl ca	rs11391098					2,4-dienoyl	2,4-dienoyl-CoA reductase (NADPH) activity,fatty acid be				G=0, GC=3	G=0.0, GC=1.0	0.00001	376.605	34	G=0/0, GC=4/3
chr1:89322982	A/A	G	A	SNV	GTF2B	GTF2B:exonic:NM_001514.5	1	100	6	synonymous	NM_00151	c.724C>T	p.(=)	-0.24					Cyclin, N-terr	rs2794318	1.00E-04		9.999E-1 (pos.89322982)		General tra	DNA-templated transcriptional preinitiation complex as				G=0, A=39	G=0.0, A=1.0	0.00001	623.979	39	G=0/0, A=20/1
chr7:73969541	G/G	A	G	SNV	GTF2IRD1	GTF2IRD1:exonic:NM_001199207.1	1	100	18	missense	NM_00119	c.2050A>G	p.Met684Val	-0.98	0.87	21	0	0.02(COSM39	GTF2I-like rep	rs2301895	0.2629	YES	5.157E-1 (pos.73969541)	acute_myeloic	GTF21 repe	DNA bindi	Aortic Stenosis, Supravalvular;Aortic Valve St			A=0, G=43	A=0.0, G=1.0	0.00001	705.83	43	A=0/0, G=37/6
chr16:4715012	C/T	C	T	SNV	MGRN1	MGRN1:intronic:NM_015246.3	1	53.33		intronic	NM_01524	c.629-91C	p.?	-1.64					Prokaryotic R	rs3813746		YES			Mahogunin	cytoplasm,cytosol,early endosome,endosome to lysoso				C=91, T=10	C=0.4667, T=0.5333	0.00001	1189.04	195	C=67/24, T=70
chr16:47486817	C/T	C	T	SNV	ITFG1	ITFG1:intronic:NM_030790.4	1	56.36		intronic	NM_03079	c.428-116	p.?	-0.21					Repeat doma	rs2111227		YES			Integrin-alp	extracellu	Autoimmune Diseases;Female Urogenital Di			C=72, T=93	C=0.4364, T=0.5636	0.00001	1044.6	165	C=22/50, T=28
chr16:47549597	G/A	G	A	SNV	PHKB	PHKB:intronic:NM_000293.2	1	50		intronic	NM_00029	c.594+85G	p.?	-0.42					Glycosyl hydr	rs78297758		YES			Phosphoryl	calmoduli	Carbohydrate Metabolism, Inborn Errors;Con			G=15, A=15	G=0.5, A=0.5	0.00001	144.064	30	G=10/5, A=7/8
chr7:89983808	G/G	T	G	SNV	GTPBP10	GTPBP10:exonic:NM_033107.3	1	100	3	missense	NM_03310	c.264T>G	p.Cys88Trp	1.54	0.15	215	0.959		50S ribosome	rs42663	0.2055	YES	8.944E-1 (pos.89983808)		GTP-binding	GTP binding,RNA binding,chromosome,mitochondrion,nu				T=0, G=21	T=0.0, G=1.0	0.00001	281.233	21	T=0/0, G=6/15
chr16:4796871	G/G	A	G	SNV	C16orf71	C16orf71:intronic:NM_139170.2	1	100		intronic	NM_13917	c.1182-57	p.?	-0.27					Domain of un	rs2341988		YES								A=0, G=28	A=0.0, G=1.0	0.00001	408.715	28	A=0/0, G=21/7
chr1:42630364	A/A	G	A	SNV	GUCA2A	GUCA2A:exonic:NM_033553.2	1	100	1	missense	NM_03355	c.20C>T	p.Ser7Phe	0.77	0.14	155	0.845	0.03(COSM41	Guanylin pre	rs2071499	0.4035	YES	4.305E-1 (pos.42630364)	carcinoma,ne	Guanylate c	digestion,extracellular exosome,extracellular region,gu				G=0, A=36	G=0.0, A=1.0	0.00001	563.66	36	G=0/0, A=10/2
chr4:156632390	AG/AG	A	AG	INDEL	GUCY1A3	GUCY1A3:exonic:NM_001130682.2	1	100	6	frameshiftInsertion	NM_00113	c.1073_10	p.Ser360fs	2.44					Adenylate an	rs113215543					Guanylate c	GTP bindir	Adenocarcinoma;Arterial Occlusive Diseases			A=0, AG=35	A=0.0, AG=1.0	0.00001	483.456	35	A=0/0, AG=6/2
chr12:14794062	A/A	G	A	SNV	GUCY2C	GUCY2C:exonic:NM_004963.3	1	100	18	synonymous	NM_00496	c.2022C>T	p.(=)	0.56					Adenylate an	rs10772800	0.0138	YES	9.899E-1 (pos.14794062)		Guanylate c	ATP bindin	Colonic Diseases:C	Linaclotid	Benign	G=0, A=40	G=0.0, A=1.0	0.00001	634.794	40	G=0/0, A=26/1
chr12:14829893	C/C	A	C	SNV	GUCY2C	GUCY2C:exonic:NM_004963.3	1	100	7	missense	NM_00496	c.843T>G	p.Phe281Leu	0.12	0.17	22	0.011		Adenylate an	rs1420635	0		1.0 (pos.14829893)		Guanylate c	ATP bindin	Colonic Diseases:C	Linaclotide		A=0, C=26	A=0.0, C=1.0	0.00001	371.416	26	A=0/0, C=18/8
chr16:4933301	T/T	A	T	SNV	PPL,UBN1	UBN1:downstream:NM_001079514.2, PPL:utr_3:NM_0027C	1	100		intronic	NM_00107	c.*3168A>	p.?, p.?	-0.22						rs2734732		YES			Periplakin,U	DNA bindi	Congenital, Hereditary, and Neonatal Diseas			A=0, T=48	A=0.0, T=1.0	0.00001	810.435	48	A=0/0, T=31/1
chrX:2779570	G/G	A	G	SNV	GYG2	GYG2:exonic:NM_003918.2	1	100	9	missense	NM_00391	c.938A>G	p.His313Arg	-1.46	0.7	29	0		Glycosyl tran	rs2306735	0.2794	YES	8.128E-1 (pos.2779570)		Glycogenin	UDP-alpha-D-glucose:glucosyl-glycogenin			Benign	A=0, G=49	A=0.0, G=1.0	0.00001	835.806	49	A=0/0, G=26/2
chr4:144920596	A/A	G	A	SNV	GYPB	GYPB:exonic:NM_001304382.1	1	100	4	missense	NM_00130	c.65C>T	p.Thr22Met	-5.84	0.11	81	0.002	0(COSM3760415),0(COSM3		rs7683365	0.3184	YES	4.717E-2 (pos.14492059	adenocarcino	Glycophorin	integral component of membrane,integral component o				G=0, A=45	G=0.0, A=1.0	0.00001	747.391	45	G=0/0, A=25/2
chr14:25078914	C/C	A	C	SNV	GZMH	GZMH:utr_5:NM_033423.4	1	100	1	utr_5	NM_03342	c.-95T>G	p.?	-0.89					Trypsin,Tryps	rs1957526		YES			Granzyme H	apoptotic	Nervous System Diseases;Neuromuscular Di			A=0, C=45	A=0.0, C=1.0	0.00001	729.696	45	A=0/0, C=10/3
chr12:48723784	C/T	C	T	SNV	H1FNT	H1FNT:exonic:NM_181788.1	1	45.83	1	missense	NM_18178	c.710C>T	p.Ser237Phe	0.4		155		0.18(COSM147490)		rs2291483	0.2082	YES	1.26E-1 (pos.48723784)	adenocarcinoma		ATP binding,DNA binding,chromosome condensation,mu				C=13, T=11	C=0.5417, T=0.4583	0.00001	93.439	24	C=6/7, T=3/8
chr16:50059500	C/T	C	T	SNV	CNEP1R1	CNEP1R1:intronic:NM_153261.5	1	53.33		intronic	NM_15326	c.26-62C>	p.?	-0.47					Transmembr	rs7194313	0.1047	YES			C-terminal	Nem1-Spo7 phosphatase complex,cytoplasm,cytosol,int				C=14, T=16	C=0.4667, T=0.5333	0.00001	159.916	30	C=12/2, T=5/1
chr16:5009433	G/A	G	A	SNV	SEC14L5	SEC14L5:intronic:NM_014692.1	1	95.83		intronic	NM_01469	c.63+46G>	p.?	0.1					CRAL/TRIO do	rs7200245	0.4928	YES	7.862E-1 (pos.5009433)			integral component of membrane,intracellular,transpor				G=1, A=23	G=0.0417, A=0.9583	0.00001	306.798	24	G=0/1, A=13/1
chr16:50134008	T/T	TA	T	INDEL	HEATR3	HEATR3:intronic:NM_182922.3	1	100		intronic	NM_18292	c.1600-13	p.?	0.61, -1.0					HEAT-like rep	rs11355227,rs750381874,rs769		YES			HEAT repea	intracellul	Digestive System Diseases;Digestive System			TA=0, T=28	TA=0.0, T=1.0	0.00001	399.474	28	TA=0/0, T=19/
chr12:48723718	AG/A	AG	A	INDEL	H1FNT	H1FNT:exonic:NM_181788.1	1	45.45	1	frameshiftDeletion	NM_18178	c.648delG	p.Arg217fs	1.53, 0.09												ATP binding,DNA binding,chromosome condensation,mu				AG=12, A=1	AG=0.5455, A=0.454	0.00001	51.9954	22	AG=5/7, A=1/9
chr16:50334840	AG/A	AG	A	INDEL	ADCY7	ADCY7:intronic:NM_001114.4	1	47.62		intronic	NM_00111	c.1235+56	p.?	-2.75, -0.02					Adenylate an	rs3833062,rs397708017,rs9536		YES			Adenylate c	ATP bindin	Arthritis;Arthritis, Juvenile;Autoimmune Dise			AG=11, A=1	AG=0.5238, A=0.476	0.00001	80.1299	21	AG=7/4, A=7/3
chr16:50388657	A/A	G	A	SNV	BRD7	BRD7:intronic:NM_001173984.2	1	100		intronic	NM_00117	c.388+47C	p.?	1					Bromodomai	rs4407056	0		1.0 (pos.50388657)			Wnt signaling pathway,cell cycle,cytoplasm,cytosol,hist				G=0, A=87	G=0.0, A=1.0	0.00001	1430.2	87	G=0/0, A=75/1
chrX:103267865	T/T	C	T	SNV	H2BFWT	H2BFWT:exonic:NM_001002916.4	1	100	1	missense	NM_00100	c.368G>A	p.Arg123His	0.78	0.12	29	0.016	0.32(COSM49	Core histone	rs553509	0.3346	YES	5.975E-1 (pos.10326786	adenocarcino	H2B histone	DNA binding,nuclear membrane,nucleosome,nucleosom				C=0, T=26	C=0.0, T=1.0	0.00001	370.816	26	C=0/0, T=16/1
chr10:115312811	C/C	T	C	SNV	HABP2	HABP2:utr_5:NM_004132.4	1	100	1	utr_5	NM_00413	c.-70T>C	p.?	-1.95					EGF-like dom	rs2240879,rs797005084,rs8799		YES			Hyaluronan	calcium io	Abortion, Spontane	Hyaluroni	Likely benign	T=0, C=22	T=0.0, C=1.0	0.00001	270.555	22	T=0/0, C=13/9
chr16:50759547	T/T	A	T	SNV	NOD2	NOD2:intronic:NM_022162.2	1	100		intronic	NM_02216	c.2966+64	p.?	-2.42					Caspase recr	rs1077861		YES			Nucleotide-	ATP bindin	Arthritis;Arthritis, Psoriatic;Autoimmune Dis			A=0, T=32	A=0.0, T=1.0	0.00001	485.08	32	A=0/0, T=18/1
chr16:5078841	C/C	T	C	SNV	NAGPA	NAGPA:intronic:NM_016256.3	1	100		intronic	NM_01625	c.920+40A	p.?	0.03					Phosphodies	rs1995278	0		9.998E-1;0.0 (pos.5078841)		N-acetylglu	Golgi cisterna membrane,N-ac		N-Acetylgl	Benign	T=0, C=58	T=0.0, C=1.0	0.00001	863.573	58	T=0/0, C=27/3
chr16:50827600	TG/TG	T	TG	INDEL	CYLD	CYLD:intronic:NM_001042355.1	1	100		intronic	NM_00104	c.2460+25	p.?	-0.41					CAP-Gly dom	rs761991818			0.0 (pos.50827600)		CYLD gene	CD4-positi	Adenocarcinoma;Bronchial Neoplasms;Carci			T=0, TG=72	T=0.0, TG=1.0	0.00001	933.931	72	T=0/0, TG=33/
chr10:115341846	G/A	G	A	SNV	HABP2	HABP2:exonic:NM_004132.4	1	45.45	9	synonymous	NM_00413	c.1050G>	p.(=)	-2.83					EGF-like dom	rs2245058	0.4203	YES	3.057E-1;0.0 (pos.115341846)		Hyaluronan	calcium io	Abortion, Spontane	Hyaluroni	Likely benign	G=18, A=15	G=0.5455, A=0.4545	0.00001	143.088	33	G=7/11, A=4/1
chr10:17659131	A/A	C	A	SNV	HACD1	HACD1:exonic:NM_014241.3	1	100	1	missense	NM_01424	c.208G>T	p.Val70Phe	0.51	0.73	50	0	0.02(COSM37	Protein tyros	rs11254692		YES	1.0 (pos.17659131)	adenocarcino	3-hydroxya	3-hydroxy-	Adnexal Diseases;Cysts;Endocrine System Di			C=0, A=24	C=0.0, A=1.0	0.00001	327.101	24	C=0/0, A=13/1
chr10:17659149	T/T	C	T	SNV	HACD1	HACD1:exonic:NM_014241.3	1	100	1	missense	NM_01424	c.190G>A	p.Glu64Lys	1.14	1	56	0	0.03(COSM37	Protein tyros	rs7895850	0.0482	YES	8.467E-1;1.533E-1 (pos.1	adenocarcino	3-hydroxya	3-hydroxy-	Adnexal Diseases;Cysts;Endocrine System Di			C=0, T=22	C=0.0, T=1.0	0.00001	298.888	22	C=0/0, T=11/1
chr4:108931039	T/C	T	C	SNV	HADH	HADH:exonic:NM_001184705.2	1	97.92	2	missense	NM_00118	c.257T>C	p.Leu86Pro	4.8	1	98	0		3-hydroxyacy	rs4956145	0.0763	YES	9.733E-1 (pos.108931039)			3-hydroxy	3-hydroxyacyl-CoA	3-Hydroxy	Benign	T=1, C=47	T=0.0208, C=0.9792	0.00001	775.002	48	T=1/0, C=10/3
chr16:53672355	C/T	C	T	SNV	RPGRIP1L	RPGRIP1L:intronic:NM_015272.4	1	97.5		intronic	NM_01527	c.2959-32	p.?	-0.14					C2 domain,Fi	rs7203525	0.3006	YES	6.031E-1 (pos.53672355)		RPGRIP1-lik	axonemal	Abnormalities, Multiple;Ataxia		Benign/Likely	C=1, T=39	C=0.025, T=0.975	0.00001	615.958	40	C=0/1, T=17/2
chr16:53878247	G/G	A	G	SNV	FTO	FTO:intronic:NM_001080432.2	1	100		intronic	NM_00108	c.895+37A	p.?	-3.19					FTO C-termin	rs62033438	0.3791	YES	4.921E-1 (pos.53878247)		Fat mass-a	DNA dealk	Abnormalities, Multiple;Bone Diseases;Bone			A=0, G=47	A=0.0, G=1.0	0.00001	789.328	47	A=0/0, G=27/2
chr16:54318102	T/T	C	T	SNV	IRX3	IRX3:intronic:NM_024336.2	1	100		intronic	NM_02433	c.1451+56	p.?	-0.2					Homeobox KI	rs8050831	6.00E-04	YES			Iroquois ho	axon,cyto	Lung Diseases;Respiratory Tract Diseases			C=0, T=20	C=0.0, T=1.0	0.00001	261.433	20	C=0/0, T=11/9
chr17:39890876	T/C	T	C	SNV	HAP1	HAP1:exonic:NM_177977.2	1	50	1	missense	NM_17797	c.11A>G	p.Lys4Arg	0.54	0	26	0.004	0.04(COSM37	HAP1 N-termi	rs4796604	0.4481	YES	7.282E-1 (pos.39890876)	adenocarcino	Huntingtin-	actin cytoskeleton,anterograde axonal transport,autopl				T=16, C=16	T=0.5, C=0.5	0.00001	158.371	32	T=3/13, C=6/1
chr17:3628212	T/C	T	C	SNV	HASPIN,ITGAE	ITGAE:intronic:NM_002208.4, HASPIN:exonic:NM_031965.2	1	45.45	1	missense	NM_00220	c.3085-11	p.?, p.Ile328Thr	-2.29		89		0.03(COSM44	FG-GAP repe	rs220461	0.319	YES	3.261E-1 (pos.3628212)	acute_myeloic	Integrin, al	ATP bindin	Attention Deficit Disorder with Hyperactivity			T=12, C=10	T=0.5455, C=0.4545	0.00001	81.7605	22	T=4/8, C=9/1
chr16:55531000	C/T	C	T	SNV	MMP2	MMP2:intronic:NM_004530.5	1	53.33		intronic	NM_00453	c.1609+26	p.?	0.73					Fibronectin t	rs17859974	0.0079	YES	0.0 (pos.55531000)		Matrix meta	angiogene	Abnormalities, Mul	AE-941,Captopril,Halofu		C=14, T=16	C=0.4667, T=0.5333	0.00001	159.925	30	C=6/8, T=6/10
chr16:55562257	A/A	G	A	SNV	LPCAT2	LPCAT2:intronic:NM_017839.4	1	100		intronic	NM_01783	c.312-32G	p.?	0.35					Acyltransfera	rs837549	1.00E-04	YES	9.999E-1 (pos.55562257)			1-acylglyc	Adnexal Diseases;Cysts;Endocrine System Di			G=0, A=32	G=0.0, A=1.0	0.00001	485.141	32	G=0/0, A=17/1
chr16:55728081	G/G	T	G	SNV	SLC6A2	SLC6A2:intronic:NM_001172504.1	1	100		intronic	NM_00117	c.1022+56	p.?	-1.42					Sodium:neur	rs28613651		YES			Solute carri	alpha-tub	Autonomic Nervous	3,4-Methylenedioxymet		T=0, G=22	T=0.0, G=1.0	0.00001	298.785	22	T=0/0, G=3/19
chr16:55735912	T/T	C	T	SNV	SLC6A2	SLC6A2:intronic:NM_001172504.1	1	100		intronic	NM_00117	c.1830+66	p.?	-1.35					Sodium:neur	rs2242447	0.3441	YES			Solute carri	alpha-tub	Autonomic Nervous	3,4-Methylenedioxymet		C=0, T=24	C=0.0, T=1.0	0.00001	329.32	24	C=0/0, T=11/1
chr16:55844609	T/T	G	T	SNV	CES1	CES1:intronic:NM_001025195.1	1	100		intronic	NM_00102	c.1171-33	p.?	-1.6					Carboxyleste	rs147989545,rs2244	0.2	YES	4.016E-1 (pos.55844609)		Carboxylest	carboxylic	Cocaine-Related Di	(1R)-1,2,2-trimethylprop		G=0, T=24	G=0.0, T=1.0	0.00001	334.73	24	G=0/0, T=21/3
chr16:55844617	A/A	G	A	SNV	CES1	CES1:intronic:NM_001025195.1	1	100		intronic	NM_00102	c.1171-41	p.?	0.3					Carboxyleste	rs146930283,rs2244	0.3907	YES	2.037E-1 (pos.55844617)		Carboxylest	carboxylic	Cocaine-Related Di	(1R)-1,2,2-trimethylprop		G=0, A=23	G=0.0, A=1.0	0.00001	316.747	23	G=0/0, A=20/3
chr9:19093287	A/G	A	G	SNV	HAUS6	HAUS6:exonic:NM_017645.4	1	46.43	4	synonymous	NM_01764	c.318T>C	p.(=)	0.33				0.08(COSM15	HAUS augmin	rs41269011	0.137	YES	8.462E-2 (pos.19093287)	adenocarcino	HAUS, augm	G2/M transition of mitotic cell cycle,HAUS complex,cell c				A=15, G=13	A=0.5357, G=0.4643	0.00001	117.927	28	A=10/5, G=11/
chr16:223706	G/G	A	G	SNV	HBA2	HBA2:utr_3:NM_000517.4	1	100	3	utr_3	NM_00051	c.536A>G	p.?	-1.2					Globin	rs2541640					Hemoglobin	bicarbona	Acneiform Eruptions;Anemia;A		Benign	A=0, G=21	A=0.0, G=1.0	0.00001	281.235	21	A=0/0, G=9/12
chr6:135323972	C/T	C	T	SNV	HBS1L	HBS1L:exonic:NM_006620.3	1	45.83	5	missense	NM_00662	c.439G>A	p.Val147Ile	-1.23	0.28	29	0		Elongation fa	rs111758776	0.0084		0.0 (pos.135323972)		HBS1-like p	GTP binding,GTPase activity,cytosol,exonucleolytic nucl				C=13, T=11	C=0.5417, T=0.4583	0.00001	93.4243	24	C=7/6, T=8/3
chr12:123200247	C/C	G	C	SNV	HCAR3	HCAR3:exonic:NM_006018.2	1	100	1	missense	NM_00601	c.1038C>G	p.Ile346Met	-0.39		10		0(COSM1476	7 transmemb	rs1696351	0.0735	YES	8.45E-1 (pos.123200247)	adenocarcino	Hydroxycar	G-protein	Carcinoma;Carcino	Inositol nicotinate,Niaci		G=0, C=43	G=0.0, C=1.0	0.00001	705.842	43	G=0/0, C=35/8
chr6:29760188	G/A	G	A	SNV	HCG4,HLA-V	HCG4:exonic_nc:NR_002139.2, HLA-V:intronic_nc:NR_13232	1	57.14	1	exonic_nc	NR_002139.2, NR_132323.1			-0.15					Class I Histoc	rs1610718		YES	3.628E-1 (pos.29760188)							G=39, A=52	G=0.4286, A=0.5714	0.00001	611.328	91	G=2/37, A=7/4
chr6:29759996	T/C	T	C	SNV	HCG4,HLA-V	HCG4:exonic_nc:NR_002139.2, HLA-V:exonic_nc:NR_13232	1	50	1	exonic_nc	NR_002139.2, NR_132323.1			-1.46				0(COSM3928	Class I Histoc	rs1610645		YES	3.032E-1 (pos.29759996)	carcinoma,glioma,papillary_renal_cell_carcinoma,squamous_cell_carcinoma						T=64, C=64	T=0.5, C=0.5	0.00001	681.108	128	T=44/20, C=47
chr16:55883412	T/T	G	T	SNV	CES5A	CES5A:intronic:NM_001190158.1	1	100		intronic	NM_00119	c.1510+12	p.?	1.03					Carboxyleste	rs2110815		YES				carboxylic ester hydrolase activity,extracellular region,I				G=0, T=32	G=0.0, T=1.0	0.00001	485.053	32	G=0/0, T=19/1
chr1:155260383	G/T	G	T	SNV	HCN3,PKLR	HCN3:downstream:NM_020897.2, PKLR:exonic:NM_00029	1	50	11	synonymous	NM_02089	c.*2129G>	p.?, p.(=)	3.38					Pyruvate kin	rs1052176	0.2673	YES	7.052E-1;1.166E-4 (pos.155260383)		Hyperpolar	ATP bindin	Anemia;Anemia, He	2-Phosphc	Benign	G=21, T=21	G=0.5, T=0.5	0.00001	228.879	42	G=3/18, T=11/
chr16:55883761	C/C	A	C	SNV	CES5A	CES5A:intronic:NM_001190158.1	1	100		intronic	NM_00119	c.1361-76	p.?	-0.75					Carboxyleste	rs7186875		YES				carboxylic ester hydrolase activity,extracellular region,I				A=0, C=39	A=0.0, C=1.0	0.00001	623.809	39	A=0/0, C=13/2
chr16:56362725	C/C	T	C	SNV	GNAO1	GNAO1:intronic:NM_138736.2	1	100		intronic	NM_13873	c.464+22T	p.?	-0.83					ADP-ribosyla	rs2241952	0.3286	YES	7.741E-1 (pos.56362725)		Guanine nu	G-protein	Mental Disorders;Substance Withdrawal Syn			T=0, C=141	T=0.0, C=1.0	0.00001	2362.46	141	T=0/0, C=62/7
chr16:56504724	G/C	G	C	SNV	OGFOD1	OGFOD1:intronic:NM_018233.3	1	57.14		intronic	NM_01823	c.1285+21	p.?	-0.13					2OG-Fe(II) oxy	rs13337155,rs75696	0.2376	YES	0.0;8.031E-2 (pos.56504724)		2-oxoglutar	L-ascorbic acid binding,cell prc		Vitamin C		G=15, C=2	G=0.4286, C=0.5714	0.00001	218.714	35	G=9/6, C=17/3
chr5:141014494	C/C	T	C	SNV	HDAC3	HDAC3:exonic:NM_003883.3	1	100	3	synonymous	NM_00388	c.165A>G	p.(=)	1.68				0.16(COSM37	Histone deac	rs2530223	0.3995	YES	3.071E-1 (pos.14101449	adenocarcino	Histone dea	Golgi appa	Cardiovascular Dise	Belinostat,Mocetinostat		T=0, C=31	T=0.0, C=1.0	0.00001	465.819	31	T=0/0, C=19/1
chr2:240003870	A/A	G	A	SNV	HDAC4	HDAC4:exonic:NM_006037.3	1	100	21	synonymous	NM_00603	c.2565C>T	p.(=)	0.04				0.16(COSM41	Glutamine ric	rs1063639	0.4748	YES	6.877E-1 (pos.24000387	neoplasm	Histone dea	A band,B c	Brachydactyly;Brair	2,2,2-TRIFLUORO-1-(5-(3-		G=0, A=22	G=0.0, A=1.0	0.00001	298.892	22	G=0/0, A=11/1
chr19:4472322	C/T	C	T	SNV	HDGFL2	HDGFL2:utr_5:NM_001001520.2	1	45.45	1	utr_5	NM_00100	c.-26C>T	p.?	0.11					Lens epitheli	rs61606737	0.1831	YES	1.694E-1;0.0 (pos.4472322)			nucleus,positive regulation of cell growth,protein bindin				C=12, T=10	C=0.5455, T=0.4545	0.00001	81.7625	22	C=6/6, T=2/8
chr16:56686471	G/T	G	T	SNV	MT1B	MT1B:intronic:NM_005947.2	1	50		intronic	NM_00594	c.29-12G>	p.?	-1.26					Metallothion	rs964374	0.4771	YES	3.126E-1;0.0 (pos.56686471)		Metallothic	cellular response to zinc ion,cy		Copper		G=10, T=10	G=0.5, T=0.5	0.00001	82.7692	20	G=9/1, T=7/3
chr18:44635048	T/T	C	T	SNV	HDHD2	HDHD2:utr_3:NM_032124.4	1	100	7	utr_3	NM_03212	c.785G>A	p.?	-1.81					HAD-hydrolase	rs2635058	0		9.999E-1 (pos.44635048)			biological_process,dephosphorylation,enzyme binding,e				C=0, T=23	C=0.0, T=1.0	0.00001	316.745	23	C=0/0, T=10/1
chr2:242192848	C/C	T	C	SNV	HDLBP	HDLBP:exonic:NM_203346.4	1	100	10	missense	NM_20334	c.1253A>G	p.Asn418Ser	0.02	0.22	46	0	0.19(COSM37	KH domain	rs7578199	0.2463	YES	6.938E-4;0.0 (pos.24219	malignant_me	High-densit	RNA bindi	Autistic Disorder;Child Development Disorde			T=0, C=24	T=0.0, C=1.0	0.00001	334.791	24	T=0/0, C=13/1

chr16:570454	C/C	T	C	SNV	RAB11FIP3	RAB11FIP3:intronic:NM 014700.3	1	100		intronic	NM 01470	c.2017-5T:	p.?	-0.12					EF hand,FIP d	rs11863276	0.4619	YES	9.946E-1 (pos.570454)		RAB11 fami	ADP-ribos Cardiovascular Diseases;Embolism and Thro	T=0, C=86	T=0.0, C=1.0	0.00001	1409.54	86	T=0/0, C=49/3
chr1:236719193	G/G	A	G	SNV	HEATR1	HEATR1:exonic:NM 018072.5	1	100	39	missense	NM 01807	c.5561T>C	p.Val1854Ala	1.28	0.49	64	0.002	0.26(COSM37	BP28CT (NUC	rs1885533	0.3173	YES	4.902E-1 (pos.23671919:	acute_myeloid leukaemia		90S preribosome.RNA binding.fibrillar center.maturatio	A=0, G=113	A=0.0, G=1.0	0.00001	1795.6	113	A=0/0, G=73/4
chr16:570623	A/A	G	A	SNV	RAB11FIP3	RAB11FIP3:intronic:NM 014700.3	1	100		intronic	NM 01470	c.2157+24	p.?	-0.87					EF hand,FIP d	rs2071978	0.4622	YES	6.17E-1 (pos.570623)		RAB11 fami	ADP-ribos Cardiovascular Diseases;Embolism and Thro	G=0, A=59	G=0.0, A=1.0	0.00001	885.367	59	G=0/0, A=22/3
chr16:57073683	C/C	G	C	SNV	NLRC5	NLRC5:intronic:NM 032206.4	1	100		intronic	NM 03220	c.2802-10	p.?	-1.31					Leucine Rich	rs864750	0.1848	YES	9.397E-1 (pos.57073683)		NLR family,	ATP binding.RNA polymerase II core promoter sequence-	G=0, C=34	G=0.0, C=1.0	0.00001	524.186	34	G=0/0, C=20/1
chr16:57255363	C/C	T	C	SNV	RSPRY1	RSPRY1:intronic:NM 133368.2	1	100		intronic	NM 13336	c.1161+36	p.?	-0.53					SPRY domain	rs7206038	0.0516	YES	9.815E-1 (pos.57255363)		RING finger-	extracellular region,metal ion binding	T=0, C=56	T=0.0, C=1.0	0.00001	832.082	56	T=0/0, C=34/2
chr16:57687714	A/C	A	C	SNV	ADGRG1	ADGRG1:intronic:NM 005682.6	1	50		intronic	NM 00568	c.621-184	p.?	-1.01					7 transmemb	rs8054093		YES			Adhesion G	G-protein coupled receptor activity,G-protein coupled re	A=17, C=17	A=0.5, C=0.5	0.00001	172.009	34	A=7/10, C=15/
chr1:236721660	C/C	T	C	SNV	HEATR1	HEATR1:exonic:NM 018072.5	1	100	36	missense	NM 01807	c.5081A>G	p.Asn1694Ser	-1.03	0.82	46	0	0.08(COSM44	BP28CT (NUC	rs2275689	0.2471	YES	6.533E-1 (pos.23672166(	acute_myeloid leukaemia		90S preribosome.RNA binding.fibrillar center.maturatio	T=0, C=42	T=0.0, C=1.0	0.00001	685.163	42	T=0/0, C=20/2
chr16:57695604	C/T	C	T	SNV	ADGRG1	ADGRG1:intronic:NM 005682.6	1	52.38		intronic	NM 00568	c.1683-5C:	p.?	-1.29					7 transmemb	rs2290177	0.1958	YES	5.278E-1 (pos.57695604)		Adhesion G	G-protein coupled receptor activity,G-prot Benign	C=10, T=11	C=0.4762, T=0.5238	0.00001	95.3003	21	C=5/5, T=10/1
chr1:236721731	C/C	T	C	SNV	HEATR1	HEATR1:exonic:NM 018072.5	1	100	36	synonymous	NM 01807	c.5010A>G	p.(=)	-1.36					BP28CT (NUC	rs819431	0		9.999E-1 (pos.236721731)			90S preribosome.RNA binding.fibrillar center.maturatio	T=0, C=33	T=0.0, C=1.0	0.00001	504.597	33	T=0/0, C=16/1
chr16:57736047	G/A	G	A	SNV	DRC7	DRC7:intronic:NM 032269.5	1	57.14		intronic	NM 03226	c.699+5G>	p.?	1.49				0.27(COSM5021889)		rs72795521	0.1373	YES	2.886E-1 (pos.57736047)	osteosarcoma		cytoplasm,cytoskeleton,motile cilium,protein binding	G=9, A=12	G=0.4286, A=0.5714	0.00001	109.009	21	G=2/7, A=7/5
chr16:57786517	T/T	C	T	SNV	KATNB1	KATNB1:intronic:NM 005886.2	1	100		intronic	NM 00588	c.704+4C>	p.?	-4.72					Anaphase-pr	rs2965797	0.2866	YES	3.171E-1 (pos.57786517)		Katanin, p8	axon.cell cycle.cell division.centrosome.cytoplasm.cyto	C=0, T=26	C=0.0, T=1.0	0.00001	371.42	26	C=0/0, T=21/5
chr1:236744712	A/A	C	A	SNV	HEATR1	HEATR1:exonic:NM 018072.5	1	100	20	synonymous	NM 01807	c.2565G>T	p.(=)	-0.21				0.78(COSM14	BP28CT (NUC	rs4518892	0.3227	YES	4.913E-1:0.0 (pos.23674	acute_myeloid leukaemia		90S preribosome.RNA binding.fibrillar center.maturatio	C=0, A=43	C=0.0, A=1.0	0.00001	705.809	43	C=0/0, A=24/1
chr16:57937895	A/A	G	A	SNV	CNGB1	CNGB1:intronic:NM 001297.4	1	100		intronic	NM 00129	c.2635-10	p.?	-0.26				0.03(COSM63	Cyclic nucleo	rs437920.rs7779490:	0.1507	YES	0.0 (pos.57937894).6.52	carcinoma	Cyclic nucle	Golgi mem Congenital, Hereditary, and Ne Benign	G=0, A=25	G=0.0, A=1.0	0.00001	353.014	25	G=0/0, A=11/1
chr16:57950178	G/C	G	C	SNV	CNGB1	CNGB1:intronic:NM 001297.4	1	51.85		intronic	NM 00129	c.2167-95	p.?	-1.34					Cyclic nucleo	rs440565		YES			Cyclic nucle	Golgi mem Congenital, Hereditary, and Neonatal Diseas	G=13, C=14	G=0.4815, C=0.5185	0.00001	132.666	27	G=5/8, C=6/8
chr16:58592278	C/T	C	T	SNV	CNOT1	CNOT1:intronic:NM 016284.4	1	57.14		intronic	NM 01628	c.2332+99	p.?	-0.15					CCR4-NOT tra	rs41445346		YES			CCR4-NOT t	CCR4-NOT complex,CCR4-NOT core complex,DNA damage	C=9, T=12	C=0.4286, T=0.5714	0.00001	109.102	21	C=4/5, T=2/10
chr16:58703966	G/G	A	G	SNV	SLC38A7	SLC38A7:intronic:NM 018231.2	1	100		intronic	NM 01823	c.1286+51	p.?	-1.48					Transmembr	rs9923128	0.4922	YES	7.192E-1 (pos.58703966)		Solute carri	L-alanine transmembrane transporter activity,L-alanine	A=0, G=45	A=0.0, G=1.0	0.00001	747.365	45	A=0/0, G=21/2
chr16:58704861	G/G	A	G	SNV	SLC38A7	SLC38A7:intronic:NM 018231.2	1	100		intronic	NM 01823	c.1231+88	p.?	0.06					Transmembr	rs9806843		YES			Solute carri	L-alanine transmembrane transporter activity,L-alanine	A=0, G=22	A=0.0, G=1.0	0.00001	298.892	22	A=0/0, G=9/13
chr1:236746443	T/T	C	T	SNV	HEATR1	HEATR1:exonic:NM 018072.5	1	100	18	synonymous	NM 01807	c.2295G>A	p.(=)	-0.13				0.07(COSM37	BP28CT (NUC	rs2794768	0.3109	YES	6.307E-1 (pos.23674644:	acute_myeloid leukaemia		90S preribosome.RNA binding.fibrillar center.maturatio	C=0, T=20	C=0.0, T=1.0	0.00001	263.786	20	C=0/0, T=16/4
chr1:236767330	G/G	A	G	SNV	HEATR1	HEATR1:exonic:NM 018072.5	1	100	2	synonymous	NM 01807	c.66T>C	p.(=)	-1.46					BP28CT (NUC	rs10802551	0.2948	YES	7.505E-1 (pos.236767330)			90S preribosome.RNA binding.fibrillar center.maturatio	A=0, G=362	A=0.0, G=1.0	0.00001	6009	362	A=0/0, G=192/
chr1:236716959	T/T	C	T	SNV	HEATR1,LGALS8	HEATR1:exonic:NM 018072.5. LGALS8:downstream:NM 00(	1	100	43	synonymous	NM 00649	c.*5498C>	p.?, p.(=)	-2.21				0.01(COSM14	BP28CT (NUC	rs2275685	0.3176	YES	4.906E-1 (pos.23671695	acute_myeloic	Lectin, gala	90S preribosome.RNA binding.carbohydrate binding.cell	C=0, T=28	C=0.0, T=1.0	0.00001	408.718	28	C=0/0, T=18/1
chr14:73958375	T/C	T	C	SNV	HEATR4,RIOX1	RIOX1:exonic:NM 024644.4, HEATR4:intronic:NM 203309.2	1	97.56	2	synonymous	NM 20330	c.2844+13	p.?, p.(=)	1.01					Cupin superf	rs758109	0.2483	YES	8.484E-1 (pos.73958375)			chromatin remodeling,histone H3-K36 demethylation,hi	T=2, C=80	T=0.0244, C=0.9756	0.00001	1262.03	82	T=2/0, C=43/3
chr14:31864485	C/C	G	C	SNV	HEATR5A	HEATR5A:exonic:NM 015473.3	1	100	5	missense	NM 01547	c.517C>G	p.Pro173Ala	0.99	0.25	27	0.112			rs7141532		YES	8.8E-1 (pos.31864485)				G=0, C=50	G=0.0, C=1.0	0.00001	852.711	50	G=0/0, C=20/3
chr2:37306343	A/A	G	A	SNV	HEATR5B	HEATR5B:exonic:NM 019024.2	1	100	3	synonymous	NM 01902	c.258C>T	p.(=)	-0.03						rs6743906.rs751105!	0.0013	YES	0.0 (pos.37306324),1.0 (pos.37306343)			extracellular exosome,membrane	G=0, A=28	G=0.0, A=1.0	0.00001	408.716	28	G=0/0, A=10/1
chr14:31582380	G/G	A	G	SNV	HECTD1	HECTD1:exonic:NM 015382.3	1	100	34	missense	NM 01538	c.6080T>C	p.Leu2027Pro	3.13	0.51	98	0	0.68(COSM45	Ankyrin repe	rs1315794			9.998E-1 (pos.31582380)	squamous_cell_carcinoma		cytoplasm.metal ion binding.protein binding.protein ubi	A=0, G=46	A=0.0, G=1.0	0.00001	768.264	46	A=0/0, G=4/42
chr14:31583512	C/T	C	T	SNV	HECTD1	HECTD1:exonic:NM 015382.3	1	57.14	31	missense	NM 01538	c.5653G>A	p.Glu1885Lys	5.23	0.05	56	0.043	0.98(COSM39	Ankyrin repe	rs61976859	0.0964	YES	1.656E-2 (pos.31583512)	acute_myeloid leukaemia		cytoplasm.metal ion binding.protein binding.protein ubi	C=21, T=28	C=0.4286, T=0.5714	0.00001	341.221	49	C=10/11, T=18
chr16:66793226	C/C	T	C	SNV	TERB1	TERB1:intronic:NM 001136505.1	1	100		intronic	NM 00113	c.1781-33	p.?	2.44					Myb-like DNA	rs363157	0.3617	YES	5.997E-1 (pos.66793226)		Telomere re	DNA binding,chromosome, telomeric region,meiotic atta	T=0, C=33	T=0.0, C=1.0	0.00001	504.596	33	T=0/0, C=14/1
chr16:66820340	T/T	C	T	SNV	TERB1	TERB1:intronic:NM 001136505.1	1	100		intronic	NM 00113	c.401-152	p.?	0.19					Myb-like DNA	rs363163		YES			Telomere re	DNA binding,chromosome, telomeric region,meiotic atta	C=0, T=68	C=0.0, T=1.0	0.00001	1053.54	68	C=0/0, T=10/5
chr16:66861966	A/A	G	A	SNV	NAE1	NAE1:intronic:NM 003905.3	1	100		intronic	NM 00390	c.54-1283	p.?	0.52					ThiF family	rs152164	0.3582	YES	6.015E-1 (pos.66861966)		NEDD8 acti	NEDD8 activating enzyme activ ATP	G=0, A=34	G=0.0, A=1.0	0.00001	524.177	34	G=0/0, A=20/1
chr16:66995333	G/A	G	A	SNV	CES3	CES3:intronic:NM 024922.5	1	47.62		intronic	NM 02492	c.82+41G>	p.?	0.13					Carboxyleste	rs71647884	0.0141	YES	0.0 (pos.66995333)		Carboxyles	carboxylic Cardiovascular Diseases;Digestive System Di	G=11, A=10	G=0.5238, A=0.4762	0.00001	82.2779	21	G=0/11, A=4/6
chr16:67221050	G/G	A	G	SNV	EXOC3L1	EXOC3L1:intronic:NM 178516.3	1	100		intronic	NM 17851	c.1041-9T:	p.?	-0.64					Exocyst comp	rs9924876	0.0235	YES	1.0 (pos.67221050)		Exocyst con	SNARE binding.exocyst.exocyst localization.exocytosis.n	A=0, G=41	A=0.0, G=1.0	0.00001	664.7	41	A=0/0, G=11/3
chr16:67316600	A/A	G	A	SNV	PLEKHG4	PLEKHG4:intronic:NM 001129728.1	1	100		intronic	NM 00112	c.1393-45	p.?	-0.17					RhoGEF doma	rs7200919	0.4193	YES	1.794E-1 (pos.67316600)		Pleckstrin h	Rho guany Ataxia;Brain Diseases;Central Nervous Syste	G=0, A=85	G=0.0, A=1.0	0.00001	1390.45	85	G=0/0, A=46/3
chr14:31647241	G/A	G	A	SNV	HECTD1	HECTD1:exonic:NM 015382.3	1	45.45	3	synonymous	NM 01538	c.360C>T	p.(=)	0.41				0.33(COSM37	Ankyrin repe	rs2274201	0.3944	YES	4.345E-1 (pos.31647241)	acute_myeloid leukaemia		cytoplasm,metal ion binding,protein binding,protein ubi	G=12, A=10	G=0.5455, A=0.4545	0.00001	81.7625	22	G=8/4, A=6/4
chr2:197118729	T/T	C	T	SNV	HECW2	HECW2:exonic:NM 020760.3	1	100	19	synonymous	NM 02076	c.3444G>A	p.(=)	-1.43					C2 domain.H	rs4850690	3.00E-04		9.999E-1 (pos.197118729)		HECT, C2, ar	cytoplasm.mitotic spindle.proteasome-mediated ubiqu	C=0, T=26	C=0.0, T=1.0	0.00001	371.418	26	C=0/0, T=4/22
chr16:68712217	C/C	T	C	SNV	CDH3	CDH3:intronic:NM 001793.5	1	100		intronic	NM 00179	c.390+37T	p.?	0.62					Cadherin cyt	rs2296410	0.1109	YES	7.668E-1 (pos.68712217)		Cadherin-1!	adherens Abnormalities, Multiple;Adnexal Diseases;Cc	T=0, C=27	T=0.0, C=1.0	0.00001	389.989	27	T=0/0, C=13/1
chr16:68718750	G/G	A	G	SNV	CDH3	CDH3:intronic:NM 001793.5	1	100		intronic	NM 00179	c.1424+23	p.?	-0.51					Cadherin cyt	rs2281850	0.3815	YES	5.509E-1 (pos.68718750)		Cadherin-1!	adherens Abnormalities, Multiple;Adnexal Diseases;Cc	A=0, G=42	A=0.0, G=1.0	0.00001	684.912	42	A=0/0, G=17/2
chr3:124692689	T/T	C	T	SNV	HEG1	HEG1:exonic:NM 020733.1	1	100	16	synonymous	NM 02073	c.3882G>A	p.(=)	-2.39					Calcium-bind	rs2270778	0.4032	YES	7.889E-1 (pos.124692689)		Heart of gla	calcium io Ear Diseases;Hearing Disorders;Hearing Loss	C=0, T=72	C=0.0, T=1.0	0.00001	1130.95	72	C=0/0, T=22/5
chr16:68725486	G/G	A	G	SNV	CDH3	CDH3:intronic:NM 001793.5	1	100		intronic	NM 00179	c.1796-13	p.?	-1.3					Cadherin cyt	rs2274240		YES			Cadherin-1!	adherens Abnormalities, Multiple;Adnexal Diseases;Cc	A=0, G=26	A=0.0, G=1.0	0.00001	371.42	26	A=0/0, G=12/1
chr16:69395434	A/G	A	G	SNV	TERF2	TERF2:intronic:NM 005652.4	1	50		intronic	NM 00565	c.1341-42	p.?	1.04					Myb-like DNA	rs251796	0.2976	YES	3.473E-1 (pos.69395434)		Telomeric r	G-rich stra Hemic and Lymphatic Diseases;Immune Syst	A=15, G=15	A=0.5, G=0.5	0.00001	145.02	30	A=7/8, G=5/10
chr16:69752464	G/G	C	G	SNV	NQO1	NQO1:intronic:NM 000903.2	1	100		intronic	NM 00090	c.8-27G>C	p.?	-0.41					Flavodoxin-li	rs689452	0.1231	YES	6.286E-1;1.166E-4 (pos.69752464)		NAD(P)H de	NAD(P)H d Anemia;Anemia, He 3-(HYDROXYMETHYL)-1-M	C=0, G=30	C=0.0, G=1.0	0.00001	446.635	30	C=0/0, G=11/1
chr16:70514828	A/C	A	C	SNV	COG4,FUK	COG4:utr 3:NM 015386.2, FUK:downstream:NM 145059.2	1	57.89		intronic	NM 14505	c.*1245A>	p.?, p.?	0.11					COG4 transpo	rs7919		YES			Component	ATP bindin Neoplasms;Precancerous Con Benign	A=16, C=22	A=0.4211, C=0.5789	0.00001	249.047	38	A=9/7, C=17/5
chr3:124746049	G/G	A	G	SNV	HEG1	HEG1:exonic:NM 020733.1	1	100	3	missense	NM 02073	c.913T>C	p.Ser305Pro	-1.91	0	74	0.002	0.01(COSM54	Calcium-bind	rs2981546	0.4953	YES	4.696E-1;1.375E-4 (pos.1	adenocarcinoı	Heart of gla	calcium io Ear Diseases;Hearing Disorders;Hearing Loss	A=0, G=27	A=0.0, G=1.0	0.00001	389.985	27	A=0/0, G=19/8
chr3:124746182	A/A	C	A	SNV	HEG1	HEG1:exonic:NM 020733.1	1	100	3	synonymous	NM 02073	c.780G>T	p.(=)	-0.25				0.04(COSM37	Calcium-bind	rs2333041	0.4941	YES	4.332E-1;3.495E-4 (pos.1	acute_myeloic	Heart of gla	calcium io Ear Diseases;Hearing Disorders;Hearing Loss	C=0, A=28	C=0.0, A=1.0	0.00001	408.718	28	C=0/0, A=19/9
chr16:70816893	T/T	C	T	SNV	VAC14	VAC14:intronic:NM 018052.4	1	100		intronic	NM 01805	c.811+43G	p.?	-0.5					Vacuolar 14 F	rs1875941.rs7649060	0.1631	YES	6.759E-1;0.0;0.0 (pos.70816893)		Vac14, S. ce	Golgi membrane.PAS complex.early endosome membra	C=0, T=28	C=0.0, T=1.0	0.00001	408.704	28	C=0/0, T=17/1
chr16:70891778	C/G	C	G	SNV	HYDIN	HYDIN:intronic:NM 001270974.2	1	51.85		intronic	NM 00127	c.12130-5	p.?	0.28					Abnormal spi	rs1774417		YES			Hydin, mou	axonemal Brain Diseases;Bronchial Diseases;Bronchie	C=39, G=42	C=0.4815, G=0.5185	0.00001	453.404	81	C=10/29, G=1
chr16:70908684	T/C	T	C	SNV	HYDIN	HYDIN:intronic:NM 001270974.2	1	53.13		intronic	NM 00127	c.10660+3	p.?	-0.54					Abnormal spi	rs1354549		YES			Hydin, mou	axonemal Brain Diseases;Bronchial Diseases;Bronchie	T=15, C=17	T=0.4688, C=0.5313	0.00001	170.343	32	T=9/6, C=13/4
chr3:124746347	C/C	T	C	SNV	HEG1	HEG1:exonic:NM 020733.1	1	100	3	synonymous	NM 02073	c.615A>G	p.(=)	-0.11				0(COSM3759	Calcium-bind	rs4404487	0.4035	YES	8.822E-1 (pos.12474634	adenocarcinoı	Heart of gla	calcium io Ear Diseases;Hearing Disorders;Hearing Loss	T=0, C=44	T=0.0, C=1.0	0.00001	726.061	44	T=0/0, C=20/2
chr16:70913467	T/C	T	C	SNV	HYDIN	HYDIN:intronic:NM 001270974.2	1	50		intronic	NM 00127	c.10367+4	p.?	-1.25					Abnormal spi	rs1774324	1.00E-04		0.0 (pos.70913467)		Hydin, mou	axonemal Brain Diseases;Bronchial Diseases;Bronchie	T=10, C=10	T=0.5, C=0.5	0.00001	77.1598	20	T=5/5, C=8/2
chr16:71127850	A/G	A	G	SNV	HYDIN	HYDIN:intronic:NM 001270974.2	1	46.51		intronic	NM 00127	c.1328-12	p.?	0.73					Abnormal spi	rs4315334		YES			Hydin, mou	axonemal Brain Diseases;Bronchial Diseases;Bronchie	A=23, G=20	A=0.5349, G=0.4651	0.00001	208.023	43	A=11/12, G=9/
chr4:84376743	T/T	A	T	SNV	HELQ,MRPS18C	HELQ:exonic:NM 133636.3, MRPS18C:upstream:NM 01606	1	100	1	missense	NM 13363	c.104T>A,	p.Val35Glu, p.?	-1.04	1	121	0		DEAD/DEAH b	rs6831595	1.00E-04		1.0 (pos.84376743)		Helicase, P	ATP bindin Nervous System Diseases;Neuromuscular Di	A=0, T=31	A=0.0, T=1.0	0.00001	470.2	31	A=0/0, T=18/1
chr16:71406024	T/T	C	T	SNV	CALB2	CALB2:intronic:NM 001740.4	1	100		intronic	NM 00174	c.95-32C>	p.?	0.03					EF hand,EF-ha	rs7203012	0.3819	YES	3.253E-1 (pos.71406024)		Calbindin 2	calcium io Amyotrophic Latera Calcium Citrate,Calcium	C=0, T=54	C=0.0, T=1.0	0.00001	798.139	54	C=0/0, T=35/1
chr16:71417783	T/T	C	T	SNV	CALB2	CALB2:intronic:NM 001740.4	1	100		intronic	NM 00174	c.478-90C	p.?	-0.08					EF hand,EF-ha	rs35885273		YES			Calbindin 2	calcium io Amyotrophic Latera Calcium Citrate,Calcium	C=0, T=52	C=0.0, T=1.0	0.00001	766.988	52	C=0/0, T=45/7
chr17:65104743	A/A	G	A	SNV	HELZ	HELZ:exonic:NM 014877.3	1	100	30	missense	NM 01487	c.4589C>T	p.Ala1530Val	4.77	0.44	64	0	0.04(COSM41	AAA domain,;	rs11653020	0.0665	YES	1.0 (pos.65104743)	neoplasm	Helicase wi	ATP binding.RNA binding.helicase activity.integral compo	G=0, A=38	G=0.0, A=1.0	0.00001	602.156	38	G=0/0, A=21/1
chr20:62196807	C/C	T	C	SNV	HELZ2	HELZ2:exonic:NM 001037335.2	1	100	9	missense	NM 00103	c.3368A>G	p.His1123Arg	-0.7	1	29	0	0.01(COSM44	AAA domain,I	rs310632	0.0474	YES	9.994E-1 (pos.62196807)	adenocarcinoma,neoplasn		ATP binding,DNA binding,RNA binding,helicase activity,li	T=0, C=23	T=0.0, C=1.0	0.00001	316.747	23	T=0/0, C=18/5
chr16:71607384	G/G	T	G	SNV	TAT	TAT:intronic:NM 000353.2	1	100		intronic	NM 00035	c.408+58A	p.?	0.89					Aminotransfe	rs2432520	0.3482	YES			Tyrosine an	2-oxogluta Amino Acid Disorde Glutamic Acid,L-Phenyla	T=0, G=121	T=0.0, G=1.0	0.00001	1953.68	121	T=0/0, G=73/4
chr20:62198686	G/A	G	A	SNV	HELZ2	HELZ2:exonic:NM 001037335.2	1	53.33	7	synonymous	NM 00103	c.2025C>T	p.(=)	-1.82				0.05(COSM37	AAA domain,I	rs3827025	0.0739	YES	1.985E-2 (pos.62198686)	adenocarcinoma		ATP binding.DNA binding.RNA binding.helicase activity.li	G=70, A=80	G=0.4667, A=0.5333	0.00001	864.841	150	G=23/47, A=2
chr16:71987044	CAA/C	CAA	C	INDEL	PKD1L3	PKD1L3:intronic:NM 181536.1	2	47.37		intronic	NM 18153	c.2810-52	p.?	-0.57, -0.14, -0.2					GPCR proteol	rs1044582619.rs356(	0.2677	YES			Polycystin 1	calcium channel activity.calciu Calcium Citrate,Calcium	CAA=40, C=	CAA=0.5263, C=0.47	0.00001	363.691	76	CAA=8/32, C=1
chr3:50614547	GC/G	GC	G	INDEL	HEMK1	HEMK1:exonic:NM 016173.4	1	52.38	5	frameshiftDeletion	NM 01617	c.484delC	p.Leu162fs	3.65, -1.03					Conserved hy	rs369125363			0.0 (pos.50614548)			DNA binding.DNA methylation.N-methyltransferase acti	GC=10, G=!	GC=0.4762, G=0.523	0.00001	55.5895	21	GC=6/4, G=9/2
chr11:124793682	C/C	T	C	SNV	HEPACAM	HEPACAM:exonic:NM 152722.4	1	100	3	missense	NM 15272	c.652A>G	p.Met218Val	0.21	1	21	0		Immunoglob	rs10790715	0.3125	YES	8.923E-1 (pos.124793682)		Hepatocyte	axon,cell a Brain Diseases;Brain Diseases Benign	T=0, C=36	T=0.0, C=1.0	0.00001	563.744	36	T=0/0, C=21/1
chr16:72050885	C/C	G	C	SNV	DHODH	DHODH:intronic:NM 001361.4	1	100		intronic	NM 00136	c.435-38G	p.?	-0.95					Dihydroorota	rs11075914	2.00E-04		1.0 (pos.72050885)		Dihydroord	'de novo' L Abnormalities, Mul (2Z)-2-cyano-N-(2,2'-dichl	G=0, C=25	G=0.0, C=1.0	0.00001	351.154	25	G=0/0, C=16/9
chr16:72139537	C/C	T	C	SNV	DHX38	DHX38:intronic:NM 014003.3	1	100		intronic	NM 01400	c.2487+14	p.?	-1.08					DEAD/DEAH b	rs42544.rs77649551	0.0364	YES	0.0 (pos.72139535).9.999E-1 (pos.72139		DEAH (Asp-C	ATP binding.ATP-dependent RNA helicase activity.RNA bi	T=0, C=27	T=0.0, C=1.0	0.00001	389.986	27	T=0/0, C=6/21
chr11:124790922	A/A	C	A	SNV	HEPACAM,HEPN1	HEPN1:downstream:NM 001037558.2. HEPACAM:utr 3:NM	1	100	7	utr 3	NM 15272	c.1363G>T	p.?, p.?	-0.59					Immunoglob	rs10893303		YES			Cancer susc	axon.cell a Brain Diseases:Brain Diseases Benign	C=0, A=20	C=0.0, A=1.0	0.00001	263.786	20	C=0/0, A=10/1
chrX:65382685	C/C	T	C	SNV	HEPH	HEPH:exonic:NM 138737.4	1	100	1	missense	NM 13873	c.116T>C	p.Val39Ala	-0.2	0.77	64	0	0.01(COSM46	Multicopper	rs5919015	0.3112	YES	1.0 (pos.65382685)	squamous_cel	Hephaestin	basolateral plasma membrane Copper	T=0, C=24	T=0.0, C=1.0	0.00001	327.847	24	T=0/0, C=16/8
chr11:93797619	A/G	A	G	SNV	HEPHL1	HEPHL1:exonic:NM 001098672.1	1	95.65	4	missense	NM 00109	c.751A>G	p.Asn251Asp	0.88	1	23	0		Multicopper	rs1945783	9.00E-04	YES	9.997E-1 (pos.93797619)			copper ion binding,copper ion transport,ferroxidase acti	A=1, G=22	A=0.0435, G=0.9565	0.00001	288.98	23	A=1/0, G=17/5
chr15:63922752	A/A	T	A	SNV	HERC1	HERC1:exonic:NM 003922.3	1	100	69	synonymous	NM 00392	c.12879A>	p.(=)	-0.46				0.13(COSM14	HECT-domain	rs10851731	0.1196	YES	8.631E-2 (pos.63922752)	adenocarcinoı	HECT domai	ARF guanyl-nucleotide exchange factor activity.Golgi apc	T=0, A=55	T=0.0, A=1.0	0.00001	814.907	55	T=0/0, A=21/3
chr16:74709206	G/A	G	A	SNV	MLKL	MLKL:intronic:NM 152649.3	1	46.88		intronic	NM 15264	c.1240+47	p.?	0.17					Protein kinas	rs11862413	0.1278	YES	5.084E-3 (pos.74709206)		Mixed linea	ATP binding.cytoplasm.cytosol.identical protein binding	G=17, A=15	G=0.5313, A=0.4688	0.00001	143.158	32	G=14/3, A=14/
chr15:63937209	G/G	C	G	SNV	HERC1	HERC1:exonic:NM 003922.3	1	100	57	missense	NM 00392	c.11166G>	p.Glu3722Asp	-0.22	1	45	0	0.25(COSM14	HECT-domain	rs2229749	0.1198	YES	8.699E-2:0.0 (pos.63937	adenocarcinoı	HECT domai	ARF guanyl-nucleotide exchange factor activity.Golgi apc	C=0, G=52	C=0.0, G=1.0	0.00001	767.016	52	C=0/0, G=29/2
chr16:75148604	A/A	G	A	SNV	LDHD	LDHD:intronic:NM 153486.3	1	100		intronic	NM 15348	c.470-21C	p.?	-0.21					FAD binding c	rs755099	0.2543	YES	3.043E-1 (pos.75148604)		D-lactate de	D-lactate dehydrogenase (cytochrome) activity,flavin ad	G=0, A=38	G=0.0, A=1.0	0.00001	596.648	38	G=0/0, A=20/1
chr15:63988357	G/G	C	G	SNV	HERC1	HERC1:exonic:NM 003922.3	1	100	27	missense	NM 00392	c.5087G>C	p.Gly1696Ala	3.88	1	60	0	0.16(COSM14	HECT-domain	rs2255243	0.1206	YES	8.766E-2;0.0 (pos.63988	adenocarcinoı	HECT domai	ARF guanyl-nucleotide exchange factor activity,Golgi apc	C=0, G=38	C=0.0, G=1.0	0.00001	607.957	38	C=0/0, G=19/1
chr16:76461519	A/A	C	A	SNV	CNTNAP4	CNTNAP4:intronic:NM 138994.4	1	100		intronic	NM 13899	c.463+23C	p.?	-0.06					F5/8 type C d	rs9927638	0.4405	YES	4.175E-1 (pos.76461519)		Contactin-a	cell adhesion.cell junction.dendrite.integral component	C=0, A=37	C=0.0, A=1.0	0.00001	583.707	37	C=0/0, A=14/2
chr16:76481824	C/A	C	A	SNV	CNTNAP4	CNTNAP4:intronic:NM 138994.4	1	96.67		intronic	NM 13899	c.464-85C	p.?	-0.03					F5/8 type C d	rs35839511		YES			Contactin-a	cell adhesion.cell junction.dendrite.integral component	C=1, A=29	C=0.0333, A=0.9667	0.00001	416.515	30	C=1/0, A=18/1
chr15:64066986	G/A	G	A	SNV	HERC1	HERC1:exonic:NM 003922.3	1	51.85	2	synonymous	NM 00392	c.837C>T	p.(=)	0.88				0.77(COSM14	HECT-domain	rs116967176	0.0059	YES	5.226E-3 (pos.64066986)	adenocarcinoı	HECT domai	ARF guanyl-nucleotide exchange factor activity,Golgi apc	G=13, A=14	G=0.4815, A=0.5185	0.00001	132.665	27	G=6/7, A=13/1
chr16:772840	G/GC	G	GC	INDEL	CCDC78,FAM173A	CCDC78:intronic:NM 001031737.2, FAM173A:downstream:	1	57.89		intronic	NM 02393	c.*283G>G	p.?, p.?	0.65					Domain of unknown function (DUF4472)						Coiled-coil	cell projection organization,centriole,de novo centriole	G=24, GC=:	G=0.4211, GC=0.578	0.00001	257.595	57	G=19/5, GC=2
chr15:28414665	C/C	T	C	SNV	HERC2	HERC2:exonic:NM 004667.5	1	100	66	synonymous	NM 00466	c.10194A>	p.(=)	0.32					Anaphase-pr	rs7494786	0.0086	YES	9.861E-1 (pos.28414665)		HECT domai	SUMO binc Asthma;Asthma, Occupational;Bronchial Dis	T=0, C=27	T=0.0, C=1.0	0.00001	389.988	27	T=0/0, C=8/19
chr16:7759206	T/T	G	T	SNV	RBFOX1	RBFOX1:intronic:NM 145891.2	1	100		intronic	NM 14589	c.1134+73	p.?	2.6					Calcitonin ge	rs62011897		YES			RNA-bindin	RNA bindir Autistic Disorder;Brain Diseases;Cardiovascu	G=0, T=31	G=0.0, T=1.0	0.00001	464.944	31	G=0/0, T=16/1
chr16:77822901	G/G	C	G	SNV	VAT1L	VAT1L:intronic:NM 020927.2	1	100		intronic	NM 02092	c.233+89C	p.?	0.02					Alcohol dehy	rs12444404		YES				oxidation- Alzheimer Disease;Brain Diseases;Central Ne	C=0, G=38	C=0.0, G=1.0	0.00001	593.901	38	C=0/0, G=25/1
chr16:78312489	G/T	G	T	SNV	WWOX	WWOX:intronic:NM 016373.3	1	58.33		intronic	NM 01637	c.517-108	p.?	-0.12					Enoyl-(Acyl ca	rs8048830	0.0699	YES	1.379E-1 (pos.78312489)		WW domai	Golgi appa Arthritis;Arthritis, Juvenile;Autoimmune Dise	G=10, T=14	G=0.4167, T=0.5833	0.00001	133.756	24	G=8/2, T=9/5
chr16:78312497	C/A	C	A	SNV	WWOX	WWOX:intronic:NM 016373.3	1	58.33		intronic	NM 01637	c.517-108	p.?	0.72				0(COSM4423	Enoyl-(Acyl ca	rs776357380,rs8050	0.4462	YES	0.0;0.0 (pos.78312495),4	astrocytoma	WW domai	Golgi appa Arthritis;Arthritis, Juvenile;Autoimmune Dise	C=10, A=14	C=0.4167, A=0.5833	0.00001	135.339	24	C=8/2, A=9/5
chr16:80666886	T/C	T	C	SNV	CDYL2	CDYL2:intronic:NM 152342.3	1	51.85		intronic	NM 15234	c.834+30A	p.?	-0.05					Chromo (CHR	rs59675671	0.3127	YES	2.64E-1 (pos.80666886)			catalytic a Breast Diseases;Breast Neoplasms;Neoplasr	T=26, C=28	T=0.4815, C=0.5185	0.00001	277.083	54	T=12/14, C=16
chr15:72638892	C/C	T	C	SNV	HEXA	HEXA:exonic:NM 000520.5	1	100	11	missense	NM 00052	c.1306A>G	p.Ile436Val	-0.2	0.22	29	0		Glycosyl hydr	rs1800431	0.002	YES	9.999E-1 (pos.72638892)		Hexosamin	N-acetyl-b Brain Diseases;Brain Diseases Benign/Likely	T=0, C=27	T=0.0, C=1.0	0.00001	389.988	27	T=0/0, C=14/1
chr6:126070789	G/G	T	G	SNV	HEY2	HEY2:utr 5:NM 012259.2	1	100	1	utr 5	NM 01225	c.-134T>G	p.?	-1.16					Hairy Orange	rs7764016		YES			Hairy/enha	DNA bindi Adenocarcinoma;Aortic Stenosis, Subvalvula	T=0, G=44	T=0.0, G=1.0	0.00001	726.569	44	T=0/0, G=41/3
chr1:91728053	A/A	G	A	SNV	HFM1	HFM1:exonic:NM 001017975.4	1	100	37	synonymous	NM 00101	c.4071C>T	p.(=)	2.15					DEAD/DEAH b	rs585898	0		1.0;0.0 (pos.91728053)		Hfm1, ATP-c	ATP binding,ATP-dependent RNA helicase activity,RNA se	G=0, A=28	G=0.0, A=1.0	0.00001	408.718	28	G=0/0, A=12/1
chr16:81145737	G/G	T	G	SNV	PKD1L2	PKD1L2:intronic:NM 052892.3	1	100		intronic	NM 05289	c.6983+31	p.?	0.03					Ion transport	rs8059318	0.0055	YES	7.822E-1;0.0 (pos.81145737)		Polycystin 1	calcium channel activity.calcium ion binding.calcium ior	T=0, G=61	T=0.0, G=1.0	0.00001	921.852	61	T=0/0, G=27/3
chr16:81145755	T/T	A	T	SNV	PKD1L2	PKD1L2:intronic:NM 052892.3	1	100		intronic	NM 05289	c.6983+13	p.?	2.8					Ion transport	rs11647962	0.4874	YES	6.196E-1 (pos.81145755)		Polycystin 1	calcium channel activity.calcium ion binding.calcium ior	A=0, T=61	A=0.0, T=1.0	0.00001	921.852	61	A=0/0, T=27/3
chr1:91859795	C/C	T	C	SNV	HFM1	HFM1:exonic:NM 001017975.4	1	100	4	missense	NM 00101	c.349A>G	p.Ile117Val	-0.29	1	29	0		DEAD/DEAH b	rs282009	0		1.0 (pos.91859795)		Hfm1, ATP-c	ATP binding,ATP-dependent RNA helicase activity,RNA se	T=0, C=88	T=0.0, C=1.0	0.00001	1426.63	88	T=0/0, C=44/4
chr3:120389316	A/A	T	A	SNV	HGD	HGD:exonic:NM 000187.3	1	100	4	missense	NM 00018	c.240A>T	p.Gln80His	-0.24	1	24	0		homogentisa	rs2255543	0.2635	YES	6.559E-1 (pos.120389316)		Homogenti:	L-phenylal Alkaptonuria;Amino Acid Meta Benign	T=0, A=84	T=0.0, A=1.0	0.00001	1378.49	84	T=0/0, A=38/4
chr16:81151163	T/T	C	T	SNV	PKD1L2	PKD1L2:intronic:NM 052892.3	1	100		intronic	NM 05289	c.6612-27	p.?	0.65					Ion transport	rs8053191	0.0746	YES	7.396E-1 (pos.81151163)		Polycystin 1	calcium channel activity,calcium ion binding,calcium ior	C=0, T=30	C=0.0, T=1.0	0.00001	446.611	30	C=0/0, T=17/1
chr4:3446132	G/G	C	G	SNV	HGFAC	HGFAC:exonic:NM 001528.3	1	100	6	missense	NM 00152	c.693C>G	p.Phe231Leu	-3.77	0.79	22	0	0(COSM3760	EGF-like dom	rs111368413.rs1987	0.3216	YES	9.309E-1 (pos.3446132)	adenocarcinoı	Hepatocyte	cytosol.ex Adenocarcinoma:Carcinoma:Carcinoma, Hec	C=0, G=76	C=0.0, G=1.0	0.00001	1175.35	76	C=0/0, G=28/4
chr16:81164274	G/G	C	G	SNV	PKD1L2	PKD1L2:intronic:NM 052892.3	1	100		intronic	NM 05289	c.5858-25	p.?	0.47					Ion transport	rs11150345	0		1.0 (pos.81164274)		Polycystin 1	calcium channel activity,calcium ion binding,calcium ior	C=0, G=44	C=0.0, G=1.0	0.00001	726.569	44	C=0/0, G=13/3
chr16:81164343	C/T	C	T	SNV	PKD1L2	PKD1L2:intronic:NM 052892.3	1	97.5		intronic	NM 05289	c.5858-94	p.?	-2.5					Ion transport	rs1530959		YES			Polycystin 1	calcium channel activity,calcium ion binding,calcium ior	C=1, T=39	C=0.025, T=0.975	0.00001	611.484	40	C=1/0, T=8/31
chr8:43054553	C/C	T	C	SNV	HGSNAT	HGSNAT:exonic:NM 152419.2	1	100	18	synonymous	NM 15241	c.1749T>C	p.(=)	-1.13					Domain of un	rs1126058	4.00E-04		1.0 (pos.43054553)		Heparan-al	glycosami Carbohydrate Metabolism, Inb Benign	T=0, C=64	T=0.0, C=1.0	0.00001	977.635	64	T=0/0, C=21/4
chr16:81185326	A/A	G	A	SNV	PKD1L2	PKD1L2:intronic:NM 052892.3	1	100		intronic	NM 05289	c.4553+46	p.?	-0.35						rs11649028	0.0759	YES	7.5E-1 (pos.81185326)		Polycystin 1	calcium channel activity,calcium ion binding,calcium ior	G=0, A=57	G=0.0, A=1.0	0.00001	849.615	57	G=0/0, A=31/2
chr1:210577873	C/C	G	C	SNV	HHAT	HHAT:exonic:NM 001122834.3	1	100	6	synonymous	NM 00112	c.534G>C	p.(=)	-0.47				0.27(COSM44	MBOAT, mem	rs2066721	0.2376	YES	6.207E-1 (pos.21057787	acute_myeloic	Hedgehog a	GTP bindir 46, XY Disorders of Sex Development;Bone Di	G=0, C=31	G=0.0, C=1.0	0.00001	465.819	31	G=0/0, C=20/1
chr16:81219329	T/T	G	T	SNV	PKD1L2	PKD1L2:intronic:NM 052892.3	1	100		intronic	NM 05289	c.1790-25	p.?	-0.75					Galactose bir	rs9319563	0.0011	YES	1.0 (pos.81219329)		Polycystin 1	calcium channel activity,calcium ion binding,calcium ior	G=0, T=43	G=0.0, T=1.0	0.00001	705.844	43	G=0/0, T=19/2
chr1:210847742	T/A	T	A	SNV	HHAT	HHAT:utr 3:NM 001122834.3	1	50	12	utr 3	NM 00112	c.1503T>A	p.?	0.18					MBOAT, mem	rs191482026	2.00E-04		0.0;0.0 (pos.210847742)		Hedgehog a	GTP bindir 46, XY Disorders of Sex Development;Bone Di	T=15, A=15	T=0.5, A=0.5	0.00001	144.99	30	T=6/9, A=7/8
chr1:222713502	G/A	G	A	SNV	HHIPL2	HHIPL2:exonic:NM 024746.3	1	55.77	4	missense	NM 02474	c.1300C>T	p.Arg434Cys	1.19	0.03	180	0.96		Folate recept	rs141478102	7.00E-04		0.0 (pos.222713502)			catalytic activity,extracellular region,protein binding	G=23, A=29	G=0.4423, A=0.5577	0.00001	299.622	52	G=10/13, A=1
chr16:81301460	G/C	G	C	SNV	BCO1	BCO1:intronic:NM 017429.2	1	50		intronic	NM 01742	c.620-53G	p.?	0.03					Retinal pigm	rs34500060		YES			Beta-carote	beta-carot Avitaminosis;Deficiency Diseases;Malnutriti	G=11, C=11	G=0.5, C=0.5	0.00001	94.6076	22	G=3/8, C=4/7
chr16:81303759	C/C	T	C	SNV	BCO1	BCO1:intronic:NM 017429.2	1	100		intronic	NM 01742	c.844-5T>C	p.?	1.66				0.12(COSM63	Retinal pigm	rs4889294	0.4569	YES	1.291E-1 (pos.81303759)	neoplasm	Beta-carote	beta-carot Avitaminosis;Deficiency Diseases;Malnutriti	T=0, C=47	T=0.0, C=1.0	0.00001	789.328	47	T=0/0, C=12/3
chr16:81320830	G/G	A	G	SNV	BCO1	BCO1:intronic:NM 017429.2	1	100		intronic	NM 01742	c.1303-70	p.?	-0.14					Retinal pigm	rs8048342		YES			Beta-carote	beta-carot Avitaminosis;Deficiency Diseases;Malnutriti	A=0, G=74	A=0.0, G=1.0	0.00001	1170.12	74	A=0/0, G=54/2
chr16:815628	G/G	A	G	SNV	MSLN	MSLN:intronic:NM 013404.4	1	100		intronic	NM 01340	c.795+11A	p.?	-0.49					Pre-pro-mega	rs1737340	0.0259	YES	1.0 (pos.815628)		Mesothelin	C-termina Adenocarcinoma;Adenoma;Adnexal Disease	A=0, G=28	A=0.0, G=1.0	0.00001	408.671	28	A=0/0, G=5/23
chr8:133089975	G/G	A	G	SNV	HHLA1	HHLA1:exonic:NM 001145095.1	1	100	11	missense	NM 00114	c.1169T>C	p.Leu390Ser	0.79	1	145	0			rs2280851	0.2398	YES	6.339E-1 (pos.133089975)		Human end	extracellular region	A=0, G=20	A=0.0, G=1.0	0.00001	263.785	20	A=0/0, G=11/9
chr16:81891998	C/C	G	C	SNV	PLCG2	PLCG2:intronic:NM 002661.4	1	100		intronic	NM 00266	c.431+37G	p.?	-1.26					C2 domain,Pl	rs4341734	0.1747	YES	7.058E-1 (pos.81891998)		Phospholip	B cell differentiation,B cell receptor signaling pathway,F	G=0, C=42	G=0.0, C=1.0	0.00001	685.22	42	G=0/0, C=22/2
chr16:81934047	C/C	T	C	SNV	PLCG2	PLCG2:intronic:NM 002661.4	1	100		intronic	NM 00266	c.1194-17	p.?	-0.74					C2 domain,Pl	rs6564933		YES			Phospholip	B cell differentiation,B cell receptor signaling pathway,F	T=0, C=22	T=0.0, C=1.0	0.00001	298.801	22	T=0/0, C=19/3
chr16:81973474	A/A	C	A	SNV	PLCG2	PLCG2:intronic:NM 002661.4	1	100		intronic	NM 00266	c.3314-23	p.?	-1.03					C2 domain,Pl	rs4405546	0.0704	YES	9.991E-1 (pos.81973474)		Phospholip	B cell differentiation,B cell receptor signaling pathway,F	C=0, A=26	C=0.0, A=1.0	0.00001	371.418	26	C=0/0, A=11/1
chr16:82197662	T/C	T	C	SNV	MPHOSPH6	MPHOSPH6:intronic:NM 005792.2	1	45.45		intronic	NM 00579	c.164+25A	p.?	-1.17					M-phase pho	rs6564992	0.0286	YES	2.677E-4 (pos.82197662)		M-phase ph	RNA binding,cytoplasm,exosome (RNase complex),matu	T=30, C=25	T=0.5455, C=0.4545	0.00001	232.695	55	T=16/14, C=22
chr16:828844	G/G	A	G	SNV			1	100		intronic				-3.45						rs1737342		YES					A=0, G=27	A=0.0, G=1.0	0.00001	389.988	27	A=0/0, G=15/1
chr8:133101818	A/A	G	A	SNV	HHLA1	HHLA1:exonic:NM 001145095.1	1	100	7	synonymous	NM 00114	c.477C>T	p.(=)	0.13				0.2(COSM4162673)		rs6471038	0.06	YES	6.689E-1 (pos.13310181	neoplasm	Human end	extracellular region	G=0, A=20	G=0.0, A=1.0	0.00001	263.786	20	G=0/0, A=12/8
chr16:83816827	CG/CG	C	CG	INDEL	CDH13	CDH13:intronic:NM 001220488.1	1	100		intronic	NM 00122	c.2057-32	p.?	-0.24					Cadherin dor	rs756427380,rs763149926,rs768404633			0.0 (pos.83816827),0.0:0.0 (pos.838168		Cadherin-13	(H-cadher Adenocarcinoma;Amphetamine-Related Disc	C=0, CG=47	C=0.0, CG=1.0	0.00001	442.399	47	C=0/0, CG=24/
chr16:83816828	G/G	A	G	SNV	CDH13	CDH13:intronic:NM 001220488.1	1	100		intronic	NM 00122	c.2057-31	p.?	-2.46					Cadherin dor	rs153654,rs7564273	0.1397	YES	0.0;0.0 (pos.83816819),0.0;0.0 (pos.838		Cadherin-13	(H-cadher Adenocarcinoma;Amphetamine-Related Disc	A=0, G=56	A=0.0, G=1.0	0.00001	790.891	56	A=0/0, G=33/2
chr8:133107830	C/C	T	C	SNV	HHLA1	HHLA1:exonic:NM 001145095.1	1	100	6	synonymous	NM 00114	c.384A>G	p.(=)	0.54						rs7833441	0.0484	YES	6.667E-1 (pos.133107830)		Human end	extracellular region	T=0, C=20	T=0.0, C=1.0	0.00001	263.786	20	T=0/0, C=7/13
chr16:84228612	C/C	CT	C	INDEL	ADAD2,LOC654780	LOC654780:intronic nc:NR 147175.1. ADAD2:intronic:NM	1	100		intronic	NM 00114	c.607+26C	p.?	-0.0, 0.0					Adenosine-de	rs35353940	0.3637	YES	5.272E-1 (pos.84228612)			RNA binding.RNA processing.adenosine deaminase activ	CT=0, C=20	CT=0.0, C=1.0	0.00001	261.646	20	CT=0/0, C=13/
chr3:108072298	C/C	T	C	SNV	HHLA2	HHLA2:exonic:NM 007072.3	1	100	4	missense	NM 00707	c.89T>C	p.Ile30Thr	-2.27	0.82	89	0	0.01(COSM40	Immunoglob	rs6779254	0.3232	YES	3.421E-1 (pos.10807229	acute_myeloic	Human end	T cell costimulation.cellular_component.integral compo	T=0, C=26	T=0.0, C=1.0	0.00001	371.382	26	T=0/0, C=6/20
chr14:62207557	C/T	C	T	SNV	HIF1A,LOC105370526	LOC105370526:intronic nc:NR 144368.1, HIF1A:exonic:NM	1	98.36	12	missense	NM 00153	c.1744C>T	p.Pro582Ser	2.44	0.14	74	0.243	0.89(COSM50	HIF-1 alpha C	rs11549465	0.1114	YES	4.368E-2 (pos.62207557)	acute_myeloic	Hypoxia-inc	B-1 B cell h Adenocarcinoma;A 2-Methoxyestradiol,Carv	C=1, T=60	C=0.0164, T=0.9836	0.00001	888.589	61	C=0/1, T=27/3
chr16:84271158	C/A	C	A	SNV	KCNG4	KCNG4:intronic:NM 172347.2	1	50		intronic	NM 17234	c.-39-27G	p.?	-0.08					BTB/POZ dom	rs3803640		YES			Potassium	delayed rectifier potassium channel activity,ion channe	C=11, A=11	C=0.5, A=0.5	0.00001	94.7093	22	C=7/4, A=6/5
chr16:84328752	T/T	G	T	SNV	WFDC1	WFDC1:intronic:NM 021197.3	1	100		intronic	NM 02119	c.144+31G	p.?	0.28					WAP-type (W	rs369797	0.002	YES	1.0 (pos.84328752)		WAP four-di	extracellu Melanoma;Neoplasms;Neoplasms by Histolc	G=0, T=42	G=0.0, T=1.0	0.00001	685.218	42	G=0/0, T=9/33
chr19:46823776	GC/GC	GCC	GC,G	INDEL	HIF3A	HIF3A:exonic:NM 152795.3	1	100	9	frameshiftDeletion	NM 15279	c.1107del	p.Ser370fs	-1.09, 0.47, 0.73				0(COSM1728	Hypoxia-indu	rs146803876,rs1502(	2.00E-04		0.0 (pos.46823776),0.0 (	hepatocellula	Hypoxia-inc	DNA binding,RNA polymerase II transcription factor activ	GCC=0, GC=	GCC=0.0, GC=1.0, G=	0.00001	294.723	28	GCC=0/0, GC=
chr19:46811984	A/G	A	G	SNV	HIF3A	HIF3A:exonic:NM 152795.3	1	56	5	synonymous	NM 15279	c.513A>G	p.(=)	-4.71				0.03(COSM14	Hypoxia-indu	rs2311054	0.1415	YES	7.599E-1 (pos.46811984)	adenocarcinoı	Hypoxia-inc	DNA binding,RNA polymerase II transcription factor activ	A=11, G=14	A=0.44, G=0.56	0.00001	134.35	25	A=2/9, G=11/3
chr16:84487056	C/T	C	T	SNV	ATP2C2	ATP2C2:intronic:NM 014861.3	1	45.83		intronic	NM 01486	c.1980+79	p.?	-1.07					Cation transp	rs247884		YES			ATPase, Ca(	ATP bindin Attention Deficit Disorder with Hyperactivity	C=26, T=22	C=0.5417, T=0.4583	0.00001	240.971	48	C=16/10, T=12
chr16:84516090	G/G	A	G	SNV	TLDC1	TLDC1:intronic:NM 020947.3	1	100		intronic	NM 02094	c.1077+10	p.?	-1.5					TLD	rs392199		YES				cytoplasm.lysosomal membrane.membrane.negative re	A=0, G=32	A=0.0, G=1.0	0.00001	464.315	32	A=0/0, G=16/1
chr11:119005088	T/T	C	T	SNV	HINFP	HINFP:exonic:NM 198971.2	1	100	10	synonymous	NM 19897	c.1434C>T	p.(=)	-2.1					C2H2-type zir	rs512703	0.0383	YES	1.0 (pos.119005088)			Cajal body.DNA binding.DNA binding transcription factor	C=0, T=57	C=0.0, T=1.0	0.00001	849.589	57	C=0/0, T=23/3
chr6:126278230	C/C	G	C	SNV	HINT3	HINT3:exonic:NM 138571.4	1	100	1	missense	NM 13857	c.107G>C	p.Gly36Ala	0.45	0.78	60	0	0(COSM4504	HIT domain,S	rs2295005	0.4787	YES	9.495E-1 (pos.12627823(	adenocarcinoı	Histidine tr	cytoplasm,extracellular exosome,hydrolase activity,nuc	G=0, C=69	G=0.0, C=1.0	0.00001	1072.62	69	G=0/0, C=41/2
chr16:845901	C/C	T	C	SNV	CHTF18	CHTF18:intronic:NM 022092.2	1	100		intronic	NM 02209	c.2326-46	p.?	-0.71					AAA domain (	rs2982244,rs773293	0.007		0.0;0.0 (pos.845899),9.999E-1 (pos.8459		Chromosom	ATP binding,Ctf18 RFC-like complex,DNA clamp loader ac	T=0, C=27	T=0.0, C=1.0	0.00001	389.988	27	T=0/0, C=14/1
chr16:84882911	C/C	T	C	SNV	CRISPLD2	CRISPLD2:intronic:NM 031476.3	1	100		intronic	NM 03147	c.360-80T	p.?	-3.63					Cysteine-rich	rs4783086		YES			Cysteine-ric	extracellu Digestive System Diseases;Liver Diseases;Lu	T=0, C=38	T=0.0, C=1.0	0.00001	601.781	38	T=0/0, C=13/2
chr16:84882974	A/A	C	A	SNV	CRISPLD2	CRISPLD2:intronic:NM 031476.3	1	100		intronic	NM 03147	c.360-17C	p.?	-0.52					Cysteine-rich	rs4783087,rs750132	0.3442	YES	0.0 (pos.84882972),6.169E-1 (pos.84882		Cysteine-ric	extracellu Digestive System Diseases;Liver Diseases;Lu	C=0, A=39	C=0.0, A=1.0	0.00001	612.526	39	C=0/0, A=11/2
chr16:84884072	G/G	T	G	SNV	CRISPLD2	CRISPLD2:intronic:NM 031476.3	1	100		intronic	NM 03147	c.493-102	p.?	-1.41					Cysteine-rich	rs4782673		YES			Cysteine-ric	extracellu Digestive System Diseases;Liver Diseases;Lu	T=0, G=86	T=0.0, G=1.0	0.00001	1409.76	86	T=0/0, G=60/2
chr16:84884116	G/G	A	G	SNV	CRISPLD2	CRISPLD2:intronic:NM 031476.3	1	100		intronic	NM 03147	c.493-58A	p.?	-2.04					Cysteine-rich	rs4783090		YES			Cysteine-ric	extracellu Digestive System Diseases;Liver Diseases;Lu	A=0, G=129	A=0.0, G=1.0	0.00001	2111.52	129	A=0/0, G=59/7
chr16:84907670	C/C	G	C	SNV	CRISPLD2	CRISPLD2:intronic:NM 031476.3	1	100		intronic	NM 03147	c.1156+10	p.?	-0.34					Cysteine-rich	rs2934486		YES			Cysteine-ric	extracellu Digestive System Diseases;Liver Diseases;Lu	G=0, C=29	G=0.0, C=1.0	0.00001	427.601	29	G=0/0, C=18/1
chr16:85112068	C/G	C	G	SNV	KIAA0513	KIAA0513:intronic:NM 014732.3	1	45.83		intronic	NM 01473	c.823+7C>	p.?	-1.27						rs2291966	0.0314	YES	8.762E-3 (pos.85112068)		KIAA0513 ge	cytoplasm Amyotrophic Lateral Sclerosis;Central Nervo	C=13, G=11	C=0.5417, G=0.4583	0.00001	93.4299	24	C=2/11, G=4/7
chr16:85112097	C/G	C	G	SNV	KIAA0513	KIAA0513:intronic:NM 014732.3	1	50		intronic	NM 01473	c.823+36C	p.?	-0.82						rs12932461	0.3888	YES	1.62E-1 (pos.85112097)		KIAA0513 ge	cytoplasm Amyotrophic Lateral Sclerosis;Central Nervo	C=12, G=12	C=0.5, G=0.5	0.00001	106.713	24	C=4/8, G=2/10

chr16:85768606	G/G	A	G	SNV	C16orf74	C16orf74:intronic:NM_206967.2	1	100		intronic	NM_20696	c.28+190T	p.?	1.79					Domain of un	rs409342		YES				protein binding	A=0, G=29	A=0.0, G=1.0	0.00001	427.601	29	A=0/0, G=18/1
chr16:85813963	T/T	C	T	SNV	EMC8	EMC8:intronic:NM_006067.4	1	100		intronic	NM_00606	c.473+22G	p.?	-0.23					Uncharacteri	rs301165	6.00E-04	YES	9.999E-1 (pos.85813963)		ER membra	ER membrane protein complex,cytoplasm,cytosol,meml	C=0, T=44	C=0.0, T=1.0	0.00001	726.551	44	C=0/0, T=19/2
chr16:85814901	G/GTCAG	G	GTCAG	INDEL	EMC8	EMC8:intronic:NM_006067.4	4	95.45		intronic	NM_00606	c.309-15C	p.?	0.15					Uncharacteri	rs5818555			0.0 (pos.85814901)		ER membra	ER membrane protein complex,cytoplasm,cytosol,meml	G=1, GTCA(	G=0.0455, GTCAG=0.	0.00001	271.51	22	G=0/1, GTCAG
chr12:123333065	T/T	C	T	SNV	HIP1R	HIP1R:exonic:NM_003959.2	1	100	3	synonymous	NM_00395	c.210C>T	p.(=)	-2.42					ANTH domair	rs2135014	0.1064	YES	7.394E-1 (pos.123333065)		Huntingtin-	SH3 domain binding,actin binding,actin filament binding	C=0, T=52	C=0.0, T=1.0	0.00001	767.011	52	C=0/0, T=42/1
chr16:85839543	G/G	C	G	SNV	COX4I1	COX4I1:intronic:NM_001861.4	1	100		intronic	NM_00186	c.373+73C	p.?	-1.37					Cytochrome (	rs2641797		YES			Cytochrome	cytochrome-c oxidase activity, Cholic Acid,N-Formylmet	C=0, G=114	C=0.0, G=1.0	0.00001	1795	114	C=0/0, G=48/6
chr16:86575460	G/G	C	G	SNV	MTHFSD	MTHFSD:intronic:NM_001159378.1	1	100		intronic	NM_00115	c.556-32G	p.?	-0.42					5-formyltetra	rs3213418	0.1021	YES	8.347E-1 (pos.86575460)		Methenylte	RNA binding,cytoplasm,intracellular mRNA localization	C=0, G=22	C=0.0, G=1.0	0.00001	298.094	22	C=0/0, G=4/18
chr16:86588655	C/C	G	C	SNV	FLJ30679,MTHFSD	MTHFSD:intronic:NM_001159378.1, FLJ30679:upstream:NR	1	100		intronic	NM_00115	c.16+120C	p.?	-1.98					5-formyltetra	rs2354022		YES			Methenylte	RNA binding,cytoplasm,intracellular mRNA localization	G=0, C=30	G=0.0, C=1.0	0.00001	446.622	30	G=0/0, C=14/1
chr16:8736246	A/A	G	A	SNV	METTL22	METTL22:intronic:NM_024109.3	1	100		intronic	NM_02410	c.908-74G	p.?	-0.1					Lysine methy	rs1731007		YES			Methyltran	heat shock protein binding,nucleolus,nucleoplasm,nucl	G=0, A=29	G=0.0, A=1.0	0.00001	427.603	29	G=0/0, A=20/9
chr12:123345736	G/G	C	G	SNV	HIP1R	HIP1R:exonic:NM_003959.2	1	100	30	synonymous	NM_00395	c.2949C>G	p.(=)	-0.31				0.41(COSM41	ANTH domair	rs10847910	0.0492	YES	9.895E-1 (pos.12334573(	neoplasm	Huntingtin-	SH3 domain binding,actin binding,actin filament binding	C=0, G=52	C=0.0, G=1.0	0.00001	767.015	52	C=0/0, G=38/1
chr16:8738622	T/T	C	T	SNV	METTL22	METTL22:intronic:NM_024109.3	1	100		intronic	NM_02410	c.1179+40	p.?	-2.65					Lysine methy	rs9933132	0		1.0 (pos.8738622)		Methyltran	heat shock protein binding,nucleolus,nucleoplasm,nucl	C=0, T=32	C=0.0, T=1.0	0.00001	485.104	32	C=0/0, T=11/2
chr16:8738641	G/G	A	G	SNV	METTL22	METTL22:intronic:NM_024109.3	1	100		intronic	NM_02410	c.1179+59	p.?	-2.66					Lysine methy	rs1730999			9.998E-1 (pos.8738641)		Methyltran	heat shock protein binding,nucleolus,nucleoplasm,nucl	A=0, G=32	A=0.0, G=1.0	0.00001	483.687	32	A=0/0, G=11/2
chr16:8738708	T/T	C	T	SNV	METTL22	METTL22:intronic:NM_024109.3	1	100		intronic	NM_02410	c.1179+12	p.?	-0.51					Lysine methy	rs1730998		YES			Methyltran	heat shock protein binding,nucleolus,nucleoplasm,nucl	C=0, T=34	C=0.0, T=1.0	0.00001	502.381	34	C=0/0, T=9/25
chr16:8739954	A/A	G	A	SNV	METTL22	METTL22:intronic:NM_024109.3	1	100		intronic	NM_02410	c.1180-11	p.?	-0.68					Lysine methy	rs1641070	0		9.999E-1;0.0 (pos.8739954)		Methyltran	heat shock protein binding,nucleolus,nucleoplasm,nucl	G=0, A=146	G=0.0, A=1.0	0.00001	2467.34	146	G=0/0, A=78/6
chr22:19420109	T/C	T	C	SNV	HIRA,MRPL40	HIRA:upstream:NM_003325.3, MRPL40:exonic:NM_003776	1	53.13	1	missense	NM_00332	c.-1110A>(	p.?, p.Leu11Pro	0.79	0.15	98	0.261	0.01(COSM37	Mitochondria	rs1128399	0.0727	YES	1.101E-1 (pos.19420109)	adenocarcinoi	Histone cel	DNA bindi 22q11 Deletion Syndrome:Abnormalities, Mu	T=15, C=17	T=0.4688, C=0.5313	0.00001	173.382	32	T=10/5, C=13/
chr6:26156843	G/A	G	A	SNV	HIST1H1E	HIST1H1E:exonic:NM_005321.2	1	57.14	1	synonymous	NM_00532	c.225G>A	p.(=)	1.91				0.22(COSM49	linker histone	rs150948103,rs7453	0.0057		2.89E-3;0.0;0.0 (pos.261!	adenoid_cysti	Histone 1, H	ADP bindir Adenocarcinoma:Carcinoma:Carcinoma, Ade	G=9, A=12	G=0.4286, A=0.5714	0.00001	109.098	21	G=7/2, A=8/4
chr6:25726675	A/A	G	A	SNV	HIST1H2AA,HIST1H2BA	HIST1H2AA:exonic:NM_170745.3, HIST1H2BA:upstream:NM	1	100	1	synonymous	NM_17074	c.81C>T, c.	p.(=), p.?	-7.09				0.03(COSM49	C-terminus o	rs9358871	0.2781	YES	7.062E-1 (pos.25726675)	adenocarcinoi	Histone 1, H	DNA binding,chromatin silencing,extracellular exosome	G=0, A=58	G=0.0, A=1.0	0.00001	867.385	58	G=0/0, A=25/3
chr16:87788756	G/G	A	G	SNV	KLHDC4	KLHDC4:intronic:NM_017566.3	1	100		intronic	NM_01756	c.369+44T	p.?	-1.14					Galactose ox	rs2303770	0.4235	YES	7.841E-1 (pos.87788756)			protein binding	A=0, G=42	A=0.0, G=1.0	0.00001	685.202	42	A=0/0, G=9/33
chr16:87870379	A/G	A	G	SNV	SLC7A5	SLC7A5:intronic:NM_003486.6	1	95.83		intronic	NM_00348	c.1141-12(	p.?	-0.4					Amino acid p	rs16943320		YES			Solute carri	L-alpha-ar Autoimmune Disea D-Leucine,D-Phenylalani	A=3, G=69	A=0.0417, G=0.9583	0.00001	1032.93	72	A=2/1, G=15/5
chr16:87871366	C/C	G	C	SNV	SLC7A5	SLC7A5:intronic:NM_003486.6	1	100		intronic	NM_00348	c.1140+85	p.?	-1.32					Amino acid p	rs3815559		YES			Solute carri	L-alpha-ar Autoimmune Disea D-Leucine,D-Phenylalani	G=0, C=31	G=0.0, C=1.0	0.00001	465.819	31	G=0/0, C=14/1
chr16:87871582	A/A	G	A	SNV	SLC7A5	SLC7A5:intronic:NM_003486.6	1	100		intronic	NM_00348	c.1044-35(	p.?	-2.45					Amino acid p	rs2287120	0.2177	YES	7.485E-2 (pos.87871582)		Solute carri	L-alpha-ar Autoimmune Disea D-Leucine,D-Phenylalani	G=0, A=21	G=0.0, A=1.0	0.00001	281.235	21	G=0/0, A=11/1
chr16:87871714	G/G	T	G	SNV	SLC7A5	SLC7A5:intronic:NM_003486.6	1	100		intronic	NM_00348	c.1044-16	p.?	-1.63					Amino acid p	rs2287121		YES			Solute carri	L-alpha-ar Autoimmune Disea D-Leucine,D-Phenylalani	T=0, G=21	T=0.0, G=1.0	0.00001	281.112	21	T=0/0, G=9/12
chr6:25726774	C/C	A	C	SNV	HIST1H2AA,HIST1H2BA	HIST1H2AA:utr_5:NM_170745.3, HIST1H2BA:upstream:NM	1	100	1	utr_5	NM_17074	c.-19T>G, c	p.?, p.?	-0.84					C-terminus o	rs4711095	0.2778	YES	7.065E-1;0.0;0.0 (pos.25726774)		Histone 1, H	DNA binding,chromatin silencing,extracellular exosome	A=0, C=48	A=0.0, C=1.0	0.00001	814.806	48	A=0/0, C=17/3
chr6:25727334	T/T	C	T	SNV	HIST1H2AA,HIST1H2BA	HIST1H2AA:upstream:NM_170745.3, HIST1H2BA:exonic:NM	1	100	1	synonymous	NM_17074	c.-579G>A,	p.?, p.(=)	-4.44					Core histone	rs4712961	0.278	YES	7.048E-1 (pos.25727334)		Histone 1, H	DNA binding,chromatin silencing,extracellular exosome	C=0, T=20	C=0.0, T=1.0	0.00001	263.716	20	C=0/0, T=13/7
chr6:26031868	A/A	G	A	SNV	HIST1H3B	HIST1H3B:utr_3:NM_003537.3	1	100	1	utr_3	NM_00353	c.421C>T	p.?	-0.63					Core histone	rs2213284,rs386698	0.3126	YES	8.329E-3;0.0 (pos.26031864),8.791E-1 (p		Histone 1, H	DNA bindi Astrocytoma;Glioblastoma;Glioma;Neoplasr	G=0, A=33	G=0.0, A=1.0	0.00001	504.446	33	G=0/0, A=21/1
chr6:26027135	A/A	G	A	SNV	HIST1H4B	HIST1H4B:utr_3:NM_003544.2	1	100	1	utr_3	NM_00354	c.346C>T	p.?	-0.08					Centromere I	rs3752420	0.4036	YES	7.311E-1 (pos.26027135)		Histone 1, H	CENP-A coi Intellectual Disability:Mental Disorders:Nerv	G=0, A=21	G=0.0, A=1.0	0.00001	279.426	21	G=0/0, A=12/9
chr1:228612838	T/T	G	T	SNV	HIST3H3	HIST3H3:exonic:NM_003493.2	1	100	1	synonymous	NM_00349	c.189C>A	p.(=)	2.31				0.89(COSM41	Core histone	rs2230656	0.1148	YES	6.616E-1 (pos.22861283!	neoplasm	Histone 3, H	double-str Intellectual Disability:Lung Diseases:Lung Di	G=0, T=42	G=0.0, T=1.0	0.00001	684.917	42	G=0/0, T=27/1
chr6:12120588	T/T	C	T	SNV	HIVEP1	HIVEP1:exonic:NM_002114.3	1	100	4	missense	NM_00211	c.560C>T	p.Thr187Met	0.64	0.02	81	0.982	0.44(COSM63	Zinc finger, C	rs2228209	0.3484	YES	2.313E-1 (pos.12120588)	carcinoma	Human imn	DNA binding,RNA polymerase II distal enhancer sequenc	C=0, T=66	C=0.0, T=1.0	0.00001	1015.4	66	C=0/0, T=19/4
chr16:88705804	C/C	T	C	SNV	IL17C	IL17C:intronic:NM_013278.3	1	100		intronic	NM_01327	c.335+87T	p.?	-0.51					Interleukin-1	rs2254073		YES			Interleukin	cell surface receptor signaling pathway,cell-cell signalin	T=0, C=68	T=0.0, C=1.0	0.00001	1043.66	68	T=0/0, C=3/65
chr6:12124855	A/A	G	A	SNV	HIVEP1	HIVEP1:exonic:NM_002114.3	1	100	4	missense	NM_00211	c.4827G>A	p.Met1609Ile	0.63	0.05	10	0.004	0.18(COSM11	Zinc finger, C	rs2228213	0.3431	YES	2.305E-1 (pos.12124855)	adenocarcinoi	Human imn	DNA binding,RNA polymerase II distal enhancer sequenc	G=0, A=29	G=0.0, A=1.0	0.00001	427.603	29	G=0/0, A=19/1
chr16:88714559	C/T	C	T	SNV	CYBA	CYBA:intronic:NM_000101.3	1	47.62		intronic	NM_00010	c.59-37G>	p.?	-0.35					Cytochrome (	rs2306422	0.3462	YES	3.585E-1 (pos.88714559)		Cytochrome	Golgi appa Arterial Occlusive D Dextromethorphan	C=11, T=10	C=0.5238, T=0.4762	0.00001	82.2957	21	C=5/6, T=5/5
chr6:143091263	G/G	A	G	SNV	HIVEP2	HIVEP2:exonic:NM_006734.3	1	100	5	missense	NM_00673	c.4613T>C	p.Leu1538Pro	0.55	0.93	98	0		Zinc finger, C	rs109836	0.0318	YES	9.997E-1 (pos.143091263)		Human imn	DNA bindi Cardiovascular Diseases:Heart Diseases:My	A=0, G=57	A=0.0, G=1.0	0.00001	849.615	57	A=0/0, G=16/4
chr16:88780685	T/T	C	T	SNV	CTU2	CTU2:intronic:NM_001012759.2	1	100		intronic	NM_00101	c.1097+50	p.?	-0.69					Cytoplasmic	rs145565756,rs3693	0.0973	YES	0.0 (pos.88780683),0.0;1.17E-4 (pos.88780678),1.16		cytoplasm	Autism Spectrum Disorder:Child Developmer	C=0, T=34	C=0.0, T=1.0	0.00001	529.661	34	C=0/0, T=21/1
chr16:88780923	G/G	C	G	SNV	CTU2,PIEZO1	CTU2:intronic:NM_001012759.2, PIEZO1:downstream:NM_(	1	100		intronic	NM_00101	c.1201+29	p.?, p.?	-0.94					Cytoplasmic	rs745811284,rs7740	0.0872	YES	0.0 (pos.88780921),2.317E-4;0.0 (pos.88		PIEZO1 ion (	cation cha Anemia;Anemia, Hemolytic;Anemia, Hemolyt	C=0, G=30	C=0.0, G=1.0	0.00001	443.243	30	C=0/0, G=20/1
chr1:41978566	G/G	T	G	SNV	HIVEP3	HIVEP3:exonic:NM_001127714.2	1	100	7	missense	NM_00112	c.6326A>C	p.Asp2109Ala	0.93		126	0		Zinc finger, C	rs2991344	0		1.0 (pos.41978566)		Human imn	DNA binding transcription factor activity,cytoplasm,met	T=0, G=27	T=0.0, G=1.0	0.00001	338.05	27	T=0/0, G=15/1
chr16:88783374	G/G	A	G	SNV	MIR4722,PIEZO1	MIR4722:upstream:NR_039873.1, PIEZO1:intronic:NM_001	1	100		intronic	NM_00114	c.6660+57	p.?	-0.95					Piezo,Piezo n	rs386794035,rs8044367		YES			PIEZO1 ion (	cation cha Anemia;Anemia, Hemolytic;Anemia, Hemolyt	A=0, G=31	A=0.0, G=1.0	0.00001	465.812	31	A=0/0, G=9/22
chr1:41978825	G/G	C	G	SNV	HIVEP3	HIVEP3:exonic:NM_001127714.2	1	100	7	missense	NM_00112	c.6067G>C	p.Ala2023Pro	4.05		27	0		Zinc finger, C	rs2483689	0		1.0 (pos.41978825)		Human imn	DNA binding transcription factor activity,cytoplasm,met	C=0, G=60	C=0.0, G=1.0	0.00001	903.527	60	C=0/0, G=14/4
chr1:42046812	T/T	C	T	SNV	HIVEP3	HIVEP3:exonic:NM_001127714.2	1	100	3	synonymous	NM_00112	c.3657G>A	p.(=)	0.18					Zinc finger, C	rs2810565	1.00E-04		1.0 (pos.42046812)		Human imn	DNA binding transcription factor activity,c Benign	C=0, T=27	C=0.0, T=1.0	0.00001	378.79	27	C=0/0, T=8/19
chr1:42048745	C/C	T	C	SNV	HIVEP3	HIVEP3:exonic:NM_001127714.2	1	100	3	missense	NM_00112	c.1724A>G	p.His575Arg	1.67		29	0	0.17(COSM41	Zinc finger, C	rs2810566	0		9.998E-1;1.156E-4 (pos.4	neoplasm	Human imn	DNA binding transcription factor activity,cytoplasm,met	T=0, C=65	T=0.0, C=1.0	0.00001	995.524	65	T=0/0, C=33/3
chr16:88792097	G/G	A	G	SNV	PIEZO1	PIEZO1:intronic:NM_001142864.3	1	100		intronic	NM_00114	c.3969-5T	p.?	-1.04				0.03(COSM37	Piezo,Piezo n	rs4782429,rs893432	0.1125	YES	9.407E-1 (pos.88792097)	hepatocellula	PIEZO1 ion (	cation cha Anemia;Anemia, Hemolytic;An Benign	A=0, G=87	A=0.0, G=1.0	0.00001	1429.57	87	A=0/0, G=43/4
chr16:88799238	C/C	T	C	SNV	LOC100289580,PIEZO1	PIEZO1:intronic:NM_001142864.3, LOC100289580:intronic	1	100		intronic	NM_00114	c.2665-98	p.?	-1.41					Piezo,Piezo n	rs3214053		YES			PIEZO1 ion (	cation cha Anemia;Anemia, Hemolytic;Anemia, Hemolyt	T=0, C=22	T=0.0, C=1.0	0.00001	298.892	22	T=0/0, C=10/1
chr16:88804618	G/A	G	A	SNV	LOC100289580,PIEZO1	PIEZO1:intronic:NM_001142864.3, LOC100289580:exonic_r	1	58.33		intronic	NM_00114	c.848+17C	p.?	-1.23					Piezo,Piezo n	rs8060643		YES	5.533E-1 (pos.88804618)		PIEZO1 ion (	cation cha Anemia;Anemia, Hemolytic;Anemia, Hemolyt	G=10, A=14	G=0.4167, A=0.5833	0.00001	135.326	24	G=2/8, A=5/9
chr16:88804924	T/T	TC	T	INDEL	LOC100289580,PIEZO1	PIEZO1:intronic:NM_001142864.3, LOC100289580:exonic_r	1	100		intronic	NM_00114	c.634+52G	p.?	1.75, 0.0					Piezo,Piezo n	rs34600315		YES			PIEZO1 ion (	cation cha Anemia;Anemia, Hemolytic;Anemia, Hemolyt	TC=0, T=66	TC=0.0, T=1.0	0.00001	949.676	66	TC=0/0, T=37/
chr1:42049032	A/A	G	A	SNV	HIVEP3	HIVEP3:exonic:NM_001127714.2	1	100	3	synonymous	NM_00112	c.1437C>T	p.(=)	0.32				0.54(COSM41	Zinc finger, C	rs2984694	5.00E-04	YES	1.0 (pos.42049032)	neoplasm	Human imn	DNA binding transcription factor activity,c Benign	G=0, A=46	G=0.0, A=1.0	0.00001	768.246	46	G=0/0, A=21/2
chr16:88808862	T/T	C	T	SNV	LOC339059,PIEZO1	LOC339059:upstream:NR_120387.1, PIEZO1:intronic:NM_0(	1	100		intronic	NM_00114	c.161-32G	p.?	-1.99					Piezo,Piezo n	rs7201439	0.1198	YES	9.604E-1 (pos.88808862)		PIEZO1 ion (	cation cha Anemia;Anemia, Hemolytic;Anemia, Hemolyt	C=0, T=43	C=0.0, T=1.0	0.00001	704.902	43	C=0/0, T=12/3
chr2:234750831	C/C	T	C	SNV	HJURP	HJURP:exonic:NM_018410.4	1	100	8	missense	NM_01841	c.595A>G	p.Arg199Gly	-1.03	1	125	0	0.01(COSM40	Centromere	rs3806589	0.3941	YES	7.487E-1 (pos.23475083:	acute_myeloic	Holliday jur	CENP-A coi Autoimmune Diseases;Female Urogenital Di:	T=0, C=46	T=0.0, C=1.0	0.00001	768.313	46	T=0/0, C=30/1
chr6:29913042	T/T	C	T	SNV	HLA-A	HLA-A:exonic:NM_001242758.1	1	100	7	synonymous	NM_00124	c.1077C>T	p.(=)	0.42					Class I Histoc	rs14288	0.1799	YES	9.899E-1 (pos.29913042)		Major histo	compatibili Ankylosis;Arthritis;, 3-Indolebutyric Acid,Coc	C=0, T=76	C=0.0, T=1.0	0.00001	1209.6	76	C=0/0, T=35/4
chr16:8904877	T/TATG	T	TATG	INDEL	PMM2	PMM2:intronic:NM_000303.2	3	95.45		intronic	NM_00030	c.348-59T	p.?	0.34					Eukaryotic pl	rs1054074206,rs1799772,rs347		YES			Phosphoma	GDP-manr Carbohydrate Metabolism, Inborn Errors:Con	T=1, TATG=	T=0.0455, TATG=0.9!	0.00001	271.51	22	T=1/0, TATG=1
chr6:31237124	C/C	T	C	SNV	HLA-C	HLA-C:exonic:NM_001243042.1	1	100	7	missense	NM_00124	c.1087A>G	p.Thr363Ala	-2.37	1	58	0	0(COSM4160	Class I Histoc	rs1130838	0.3127	YES	8.068E-1 (pos.31237124)	neoplasm	Major histo	compatibili Arthritis:Arthritis, Psoriatic:Bone Diseases:D	T=0, C=21	T=0.0, C=1.0	0.00001	281.234	21	T=0/0, C=10/1
chr16:89167792	T/T	C	T	SNV	ACSF3	ACSF3:intronic:NM_001127214.3	1	100		intronic	NM_00112	c.666+37C	p.?	-2.47					AMP-binding	rs8060043	0.2113	YES	4.888E-1 (pos.89167792)		Acyl-CoA sy	ATP bindin Amino Acid Metabolism, Inborn Errors:Conge	C=0, T=55	C=0.0, T=1.0	0.00001	811.249	55	C=0/0, T=11/4
chr16:89168911	C/C	T	C	SNV	ACSF3	ACSF3:intronic:NM_001127214.3	1	100		intronic	NM_00112	c.667-101	p.?	-1.19					AMP-binding	rs7200676		YES			Acyl-CoA sy	ATP bindin Amino Acid Metabolism, Inborn Errors:Conge	T=0, C=72	T=0.0, C=1.0	0.00001	1099.65	72	T=0/0, C=34/3
chr16:89168935	C/C	G	C	SNV	ACSF3	ACSF3:intronic:NM_001127214.3	1	100		intronic	NM_00112	c.667-77G	p.?	-2.53					AMP-binding	rs4782458		YES			Acyl-CoA sy	ATP bindin Amino Acid Metabolism, Inborn Errors:Conge	G=0, C=88	G=0.0, C=1.0	0.00001	1423.76	88	G=0/0, C=34/5
chr16:89168981	C/C	T	C	SNV	ACSF3	ACSF3:intronic:NM_001127214.3	1	100		intronic	NM_00112	c.667-31T	p.?	-0.02					AMP-binding	rs4782457	0.2324	YES	4.695E-1 (pos.89168981)		Acyl-CoA sy	ATP bindin Amino Acid Metabolism, Inborn Errors:Conge	T=0, C=88	T=0.0, C=1.0	0.00001	1447.34	88	T=0/0, C=26/6
chr16:89187378	C/C	G	C	SNV	ACSF3	ACSF3:intronic:NM_001127214.3	1	100		intronic	NM_00112	c.1239+57	p.?	0.67					AMP-binding	rs3743983		YES			Acyl-CoA sy	ATP bindin Amino Acid Metabolism, Inborn Errors:Conge	G=0, C=25	G=0.0, C=1.0	0.00001	351.684	25	G=0/0, C=3/22
chr6:31237162	G/G	C	G	SNV	HLA-C	HLA-C:exonic:NM_001243042.1	1	100	7	missense	NM_00124	c.1049G>C	p.Cys350Ser	-1.02	0.94	112	0	0.05(COSM41	Class I Histoc	rs35708511	0.313	YES	8.069E-1 (pos.31237162)	neoplasm	Major histo	compatibili Arthritis:Arthritis, Psoriatic:Bone Diseases:D	C=0, G=22	C=0.0, G=1.0	0.00001	298.874	22	C=0/0, G=10/1
chr6:31238135	A/A	G	A	SNV	HLA-C	HLA-C:exonic:NM_001243042.1	1	100	4	synonymous	NM_00124	c.747C>T	p.(=)	-0.72				0.05(COSM41	Class I Histoc	rs1050317		YES	7.586E-1 (pos.31238135)	adenocarcinoi	Major histo	compatibili Arthritis;Arthritis, Psoriatic;Bone Diseases;D	G=0, A=46	G=0.0, A=1.0	0.00001	768.313	46	G=0/0, A=19/2
chr6:31238138	T/T	C	T	SNV	HLA-C	HLA-C:exonic:NM_001243042.1	1	100	4	synonymous	NM_00124	c.744G>A	p.(=)	-0.11				0.08(COSM41	Class I Histoc	rs1050320		YES	7.604E-1 (pos.31238138)	adenocarcinoi	Major histo	compatibili Arthritis;Arthritis, Psoriatic;Bone Diseases;D	C=0, T=45	C=0.0, T=1.0	0.00001	750.308	45	C=0/0, T=19/2
chr6:31238147	G/G	C	G	SNV	HLA-C	HLA-C:exonic:NM_001243042.1	1	100	4	synonymous	NM_00124	c.735G>C	p.(=)	-0.14				0.1(COSM376	Class I Histoc	rs1050326,rs867627342		YES	2.178E-1;0.0 (pos.31238	acute_myeloic	Major histo	compatibili Arthritis;Arthritis, Psoriatic;Bone Diseases;D	C=0, G=47	C=0.0, G=1.0	0.00001	789.328	47	C=0/0, G=19/2
chr6:31238230	CGGGA/CGGG	GGGGG	CGGGA	MNV	HLA-C	HLA-C:exonic:NM_001243042.1	5	100	4	missense	NM_00124	c.648_652	p.Leu218Val	-5.19, -0.2	0.84	32	0	0(COSM4160	Class I Histoc	rs1050343,rs105034	0.3231	YES	1.04E-3 (pos.31238232),8	neoplasm	Major histo	compatibili Arthritis:Arthritis, Psoriatic:Bone Diseases:D	GGGGG=0,	GGGGG=0.0, CGGGA	0.00001	761.514	52	GGGGG=0/0, (
chr6:31238259	T/T	G	T	SNV	HLA-C	HLA-C:exonic:NM_001243042.1	1	100	4	missense	NM_00124	c.623C>A	p.Pro208His	0.63	0.73	77	0	0.03(COSM32	Class I Histoc	rs1131096	0.325	YES	8.036E-1;0.0 (pos.31238	basal_cell_car	Major histo	compatibili Arthritis:Arthritis, Psoriatic:Bone Diseases:D	G=0, T=53	G=0.0, T=1.0	0.00001	782.03	53	G=0/0, T=21/3
chr6:32904981	A/A	G	A	SNV	HLA-DMB	HLA-DMB:exonic:NM_002118.4	1	100	3	missense	NM_00211	c.590C>T	p.Thr197Ile	-0.79	0.71	89	0		Class II histo	rs1042337	0.2262	YES	6.737E-1 (pos.32904981)		Major histo	MHC class II protein complex,MHC class II protein compl	G=0, A=36	G=0.0, A=1.0	0.00001	563.744	36	G=0/0, A=24/1
chr16:89371827	A/A	G	A	SNV	ANKRD11	ANKRD11:intronic:NM_001256183.1	1	100		intronic	NM_00125	c.88-75C>	p.?	1.36					Ankyrin repe	rs3114912		YES			Ankyrin rep	bone deve Abnormalities, Multiple;Bone Diseases;Bone	G=0, A=39	G=0.0, A=1.0	0.00001	623.98	39	G=0/0, A=30/9
chr16:89611199	G/A	G	A	SNV	SPG7	SPG7:intronic:NM_003119.3	1	57.89		intronic	NM_00311	c.1449+19	p.?	-0.67					AAA domain (	rs79201073	0.0626	YES	3.139E-1 (pos.89611199)		Paraplegin	ATP bindin Congenital Abnormalities:Con Benign	G=32, A=44	G=0.4211, A=0.5789	0.00001	490.987	76	G=12/20, A=1
chr16:89613233	C/C	A	C	SNV	SPG7	SPG7:intronic:NM_003119.3	1	100		intronic	NM_00311	c.1552+65	p.?	-0.75					AAA domain (	rs461405		YES			Paraplegin	ATP bindin Congenital Abnormalities:Congenital, Heredi	A=0, C=36	A=0.0, C=1.0	0.00001	563.468	36	A=0/0, C=12/2
chr6:32713030	C/C	T	C	SNV	HLA-DQA2	HLA-DQA2:exonic:NM_020056.4	1	100	2	synonymous	NM_02005	c.177T>C	p.(=)	-1.84				0.01(COSM63	Class II histo	rs3208181	0.43	YES	8.209E-1 (pos.32713030)	carcinoma	Major histo	ER to Golgi Arthritis;Arthritis, F Insulin Pork	T=0, C=26	T=0.0, C=1.0	0.00001	371.408	26	T=0/0, C=17/9
chr16:89720409	A/A	T	A	SNV	CHMP1A	CHMP1A:intronic:NM_002768.4	1	100		intronic	NM_00276	c.8-78A>T	p.?	-0.08					Snf7	rs258338	0.0013	YES			CHMP famil	ESCRT III complex,ESCRT III complex disassembly,cell divi	T=0, A=44	T=0.0, A=1.0	0.00001	726.568	44	T=0/0, A=30/1
chr16:89836507	C/C	T	C	SNV	FANCA	FANCA:intronic:NM_000135.3	1	100		intronic	NM_00013	c.2316+67	p.?	-0.16					Fanconi anae	rs386794147,rs7200990		YES	9.997E-1 (pos.89836507)		Fanconi ane	DNA repai Adenocarcinoma;Anemia;Anemia, Aplastic;A	T=0, C=30	T=0.0, C=1.0	0.00001	446.321	30	T=0/0, C=8/22
chr6:32714175	C/T	C	T	SNV	HLA-DQA2	HLA-DQA2:utr_3:NM_020056.4	1	95.38	4	utr_3	NM_02005	c.772C>T	p.?	-2.85					Class II histo	rs2071799	0.479	YES	6.336E-1 (pos.32714175)		Major histo	ER to Golgi Arthritis:Arthritis, F Insulin Pork	C=3, T=62	C=0.0462, T=0.9538	0.00001	904.77	65	C=0/3, T=21/4
chr16:90075412	C/C	T	C	SNV	DBNDD1	DBNDD1:intronic:NM_024043.3	1	100		intronic	NM_02404	c.239-80A	p.?	0.38					Dysbindin (D	rs2241084		YES				cytoplasm,neuron projection development,synaptic ves	T=0, C=29	T=0.0, C=1.0	0.00001	427.543	29	T=0/0, C=24/5
chr6:32629802	A/G	A	G	SNV	HLA-DQB1	HLA-DQB1:exonic:NM_002123.4	1	53.33	3	synonymous	NM_00212	c.603T>C	p.(=)	-1.52					Class II histo	rs1049092	0.4038	YES	7.327E-1 (pos.32629802)		Major histo	ER to Golgi Angioedema;Asthm Insulin Pork	A=21, G=24	A=0.4667, G=0.5333	0.00001	276.154	45	A=21/0, G=20/
chr16:90141477	G/G	T	G	SNV	PRDM7	PRDM7:intronic:NM_001098173.1	1	100		intronic	NM_00109	c.194-46A	p.?	-0.42					KRAB box,SSX	rs7196459	0.081	YES	1.0 (pos.90141477)			chromosome,histone lysine methylation,histone-lysine	T=0, G=68	T=0.0, G=1.0	0.00001	1057.45	68	T=0/0, G=30/3
chr16:9014350	C/C	T	C	SNV	USP7	USP7:intronic:NM_003470.2	1	100		intronic	NM_00347	c.523-46A	p.?	0.17					ICP0-binding	rs1677470	2.00E-04		1.0 (pos.9014350)		Ubiquitin-s	PML body, Female Urogenital Diseases;Female Urogeni	T=0, C=46	T=0.0, C=1.0	0.00001	728.053	46	T=0/0, C=19/2
chr17:10216442	A/A	C	A	SNV	MYH13	MYH13:intronic:NM_003802.2	1	100		intronic	NM_00380	c.4181+33	p.?	-0.13					IQ calmoduli	rs4239113	0.2906	YES	5.191E-1 (pos.10216442)		Myosin, hea	ATP bindin Arthritis:Joint Diseases:Musculoskeletal Dis	C=0, A=37	C=0.0, A=1.0	0.00001	558.289	37	C=0/0, A=28/9
chr17:10219366	T/T	C	T	SNV	MYH13	MYH13:intronic:NM_003802.2	1	100		intronic	NM_00380	c.3739-24	p.?	-1.24					IQ calmoduli	rs2240577	0.2174	YES	8.345E-1 (pos.10219366)		Myosin, hea	ATP bindin Arthritis:Joint Diseases:Musculoskeletal Dis	C=0, T=37	C=0.0, T=1.0	0.00001	583.704	37	C=0/0, T=12/2
chr6:32725062	A/A	T	A	SNV	HLA-DQB2	HLA-DQB2:exonic:NM_001198858.1	1	100	4	synonymous	NM_00119	c.666A>T	p.(=)	-1.38				0.04(COSM37	CD80-like C2-	rs3213484		YES	8.004E-1 (pos.32725062)	adenocarcinoi	Major histo	ER to Golgi Autoimmune Diseases:Connective Tissue Dis	T=0, A=45	T=0.0, A=1.0	0.00001	743.481	45	T=0/0, A=27/1
chr17:10249888	GA/GA	G	GA	INDEL	MYH13	MYH13:intronic:NM_003802.2	1	100		intronic	NM_00380	c.1263+10	p.?	-0.84					IQ calmoduli	rs34168540,rs397767538,rs869		YES			Myosin, hea	ATP bindin Arthritis;Joint Diseases;Musculoskeletal Dis	G=0, GA=4(	G=0.0, GA=1.0	0.00001	546.611	46	G=0/0, GA=27/
chr6:32726838	T/T	C	T	SNV	HLA-DQB2	HLA-DQB2:exonic:NM_001198858.1	1	100	3	synonymous	NM_00119	c.435G>A	p.(=)	-4.54				0.01(COSM41	CD80-like C2-	rs17850863		YES	8.281E-1 (pos.32726838)	neoplasm,squ	Major histo	ER to Golgi Autoimmune Diseases:Connective Tissue Dis	C=0, T=52	C=0.0, T=1.0	0.00001	766.962	52	C=0/0, T=31/2
chr6:32731258	A/A	G	A	SNV	HLA-DQB2	HLA-DQB2:utr_5:NM_001198858.1	1	100	1	utr_5	NM_00119	c.-11C>T	p.?	-0.64					CD80-like C2-	rs1573649		YES	7.408E-1;0.0 (pos.32731258)		Major histo	ER to Golgi Autoimmune Diseases:Connective Tissue Dis	G=0, A=20	G=0.0, A=1.0	0.00001	263.786	20	G=0/0, A=10/1
chr6:30458064	A/A	G	A	SNV	HLA-E	HLA-E:exonic:NM_005516.5	1	100	3	missense	NM_00551	c.382G>A	p.Gly128Arg	0.81	0.05	125	0.552		CD80-like C2-	rs1264457	0.4323	YES	3.904E-1 (pos.30458064)		Major histo	CD8-positi Parapsoriasis;Skin Diseases;Skin Diseases, P	G=0, A=26	G=0.0, A=1.0	0.00001	371.419	26	G=0/0, A=11/1
chr17:10426343	C/T	C	T	SNV	MYH2,MYHAS	MYH2:intronic:NM_001100112.1, MYHAS:intronic_nc:NR_12	1	45.45		intronic	NR_12536	c.5673+64	p.?	-0.25					IQ calmoduli	rs7223755		YES			Myosin, hea	A band,ATI Contracture;Cranial Nerve Diseases;Eye Dise	C=12, T=10	C=0.5455, T=0.4545	0.00001	81.7619	22	C=8/4, T=7/3
chr6:29693011	T/T	C	T	SNV	HLA-F	HLA-F:exonic:NM_001098479.1	1	100	4	missense	NM_00109	c.814C>T	p.Pro272Ser	-0.02	1	74	0		Class I Histoc	rs1736924	0.1839	YES	9.86E-1 (pos.29693011)		Major histo	ER to Golgi Autoimmune Diseases:Autoimmune Disease	C=0, T=63	C=0.0, T=1.0	0.00001	958.767	63	C=0/0, T=12/5
chr17:10595024	C/T	C	T	SNV	SCO1	SCO1:intronic:NM_004589.3	1	50		intronic	NM_00458	c.655+165	p.?	-0.81					AhpC/TSA fan	rs2254730		YES			SCO1 cytoc	cellular co Brain Diseases:Cardiovascular Diseases:Cen	C=12, T=12	C=0.5, T=0.5	0.00001	106.709	24	C=8/4, T=9/3
chr6:29797696	A/G	A	G	SNV	HLA-G	HLA-G:exonic:NM_002127.5	1	53.49	6	synonymous	NM_00212	c.999A>G	p.(=)	0.03				0(COSM4003	Class I Histoc	rs1130363	0.4926	YES	6.083E-1 (pos.29797696)	acute_myeloic	HLA-G histo	ER to Golgi Asthma;Bronchial Diseases;Hypersensitivity	A=20, G=23	A=0.4651, G=0.5349	0.00001	264.514	43	A=17/3, G=15/
chr17:11511412	G/G	A	G	SNV	DNAH9	DNAH9:intronic:NM_001372.3	1	100		intronic	NM_00137	c.418-34A	p.?	-0.77					AAA domain (	rs386795400,rs4792	1.00E-04		9.999E-1 (pos.11511412)		Dynein, axo	ATP binding,ATP-dependent microtubule motor activity,	A=0, G=60	A=0.0, G=1.0	0.00001	903.519	60	A=0/0, G=26/3
chr3:148786108	T/T	C	T	SNV	HLTF	HLTF:exonic:NM_003071.3	1	100	8	synonymous	NM_00307	c.909G>A	p.(=)	-0.14				0.25(COSM11	HIRAN domai	rs812249	0.2877	YES	7.047E-1 (pos.14878610!	adenocarcinoma		ATP bindin Autoimmune Diseases:Female Urogenital Di:	C=0, T=23	C=0.0, T=1.0	0.00001	316.748	23	C=0/0, T=11/1
chr1:221057646	T/T	C	T	SNV	HLX	HLX:exonic:NM_021958.3	1	100	4	missense	NM_02195	c.1067C>T	p.Pro356Leu	0.96	0	98	0.069	0.6(COSM374	Homeobox d	rs2738755	0.3508	YES	6.578E-2;0.0 (pos.22105	adenocarcinoma,carcinom		cell differentiation,embryonic digestive tract morphoge	C=0, T=34	C=0.0, T=1.0	0.00001	528.784	34	C=0/0, T=16/1
chr1:221057739	G/G	C	G	SNV	HLX	HLX:exonic:NM_021958.3	1	100	4	missense	NM_02195	c.1160C>G	p.Ala387Gly	1.08	0	60	0.012	0.83(COSM41	Homeobox d	rs11578466	0.1497	YES	3.475E-4 (pos.22105773!	neoplasm,rhabdomyosarc		cell differentiation,embryonic digestive tract morphoge	C=0, G=95	C=0.0, G=1.0	0.00001	1342.36	95	C=0/0, G=50/4
chr1:186101539	G/G	A	G	SNV	HMCN1	HMCN1:exonic:NM_031935.2	1	100	86	missense	NM_03193	c.13310A>	p.Gln4437Arg	8.54		43	0.735	1(COSM3750	Calcium-bind	rs10911825	0.2626	YES	6.292E-1 (pos.18610153!	adenocarcinoi	Hemicentin	basement Eye Diseases;Macular Degenei Likely benign	A=0, G=24	A=0.0, G=1.0	0.00001	334.785	24	A=0/0, G=9/15
chr1:186113727	G/A	G	A	SNV	HMCN1	HMCN1:exonic:NM_031935.2	1	50	91	missense	NM_03193	c.14158G:	p.Ala4720Thr	0.5		58	0	0.03(COSM36	Calcium-bind	rs6693069	0.0158	YES	1.702E-2 (pos.18611372	adenocarcinoi	Hemicentin	basement Eye Diseases;Macular Degenei Likely benign	G=26, A=26	G=0.5, A=0.5	0.00001	256.251	52	G=20/6, A=13/
chr17:11894195	T/T	C	T	SNV	ZNF18	ZNF18:intronic:NM_144680.3	1	100		intronic	NM_14468	c.577+96G	p.?	-0.95					C2H2-type zir	rs35075542		YES			Zinc finger (	DNA binding,DNA binding transcription factor activity,RN	C=0, T=32	C=0.0, T=1.0	0.00001	485.14	32	C=0/0, T=28/4
chr1:185958737	A/G	A	G	SNV	HMCN1	HMCN1:exonic:NM_031935.2	1	45.45	21	missense	NM_03193	c.3166A>G	p.Thr1056Ala	3.5		58	0	0.09(COSM36	Calcium-bind	rs7539719	0.0214	YES	5.113E-3 (pos.18595873	adenocarcinoi	Hemicentin	basement Eye Diseases:Macular Degenei Likely benign	A=12, G=10	A=0.5455, G=0.4545	0.00001	81.7625	22	A=9/3, G=5/5
chr17:12905743	G/A	G	A	SNV	ELAC2	ELAC2:intronic:NM_001165962.1	1	51.85		intronic	NM_00116	c.1098+15	p.?	-1.54					Beta-lactama	rs55825263	0.297	YES	3.687E-2 (pos.12905743)		elaC, E. coli	3'-tRNA pr Genital Diseases, Male;Genita Benign	G=13, A=14	G=0.4815, A=0.5185	0.00001	132.666	27	G=7/6, A=5/9
chr17:12916606	A/G	A	G	SNV	ELAC2	ELAC2:intronic:NM_001165962.1	1	45.83		intronic	NM_00116	c.491-14T	p.?	0.26					Beta-lactama	rs74556312	0.1257	YES	2.417E-4 (pos.12916606)		elaC, E. coli	3'-tRNA pr Genital Diseases, Male;Genita Benign	A=26, G=22	A=0.5417, G=0.4583	0.00001	240.97	48	A=17/9, G=4/1
chr17:13905	A/A	G	A	SNV	DOC2B	DOC2B:intronic:NM_003585.4	1	100		intronic	NM_00358	c.528+16C	p.?	0.62					C2 domain	rs6565705	0.2121	YES	8.578E-1 (pos.13905)		Double C2-l	SNARE complex,calcium ion binding,calcium ion-regulate	G=0, A=41	G=0.0, A=1.0	0.00001	655.094	41	G=0/0, A=28/1
chr17:1410441	C/T	C	T	SNV	INPP5K	INPP5K:intronic:NM_016532.3	1	59.18		intronic	NM_01653	c.667-58G	p.?	-0.1					Endonucleas	rs35417891		YES			Inositol pol	G-protein coupled receptor signaling pathway,actin cyto	C=20, T=29	C=0.4082, T=0.5918	0.00001	360.78	49	C=6/14, T=13/
chr17:1489088	G/G	C	G	SNV	SLC43A2	SLC43A2:intronic:NM_152346.2	1	100		intronic	NM_15234	c.1217+11	p.?	-2.69					Major Facilit	rs4790208		YES	9.707E-1;0.0 (pos.1489088)		Solute carri	L-alpha-amino acid transmembrane transport,L-amino a	C=0, G=58	C=0.0, G=1.0	0.00001	867.254	58	C=0/0, G=46/1
chr17:1516480	C/C	T	C	SNV	SLC43A2	SLC43A2:intronic:NM_152346.2	1	100		intronic	NM_15234	c.501+9A>	p.?	-2.8					Major Facilit	rs386794447,rs7219	2.00E-04	YES	9.997E-1 (pos.1516480)		Solute carri	L-alpha-amino acid transmembrane transport,L-amino a	T=0, C=27	T=0.0, C=1.0	0.00001	389.45	27	T=0/0, C=17/1
chr17:1519662	A/A	G	A	SNV	SLC43A2	SLC43A2:intronic:NM_152346.2	1	100		intronic	NM_15234	c.368+194	p.?	0.12					Major Facilit	rs8065481		YES			Solute carri	L-alpha-amino acid transmembrane transport,L-amino a	G=0, A=33	G=0.0, A=1.0	0.00001	504.598	33	G=0/0, A=18/1
chr13:31036681	G/G	A	G	SNV	HMGB1	HMGB1:exonic:NM_002128.5	1	100	4	synonymous	NM_00212	c.465T>C	p.(=)	-0.41					HMG (high m	rs1929606	2.00E-04		1.0 (pos.31036681)		High-mobili	C-X-C chen Brain Diseases;Car Ethyl pyruvate	A=0, G=39	A=0.0, G=1.0	0.00001	623.963	39	A=0/0, G=27/1
chr17:15604444	G/G	A	G	SNV	ZNF286A	ZNF286A:intronic:NM_001130842.1	1	100		intronic	NM_00113	c.38-22A>(	p.?	0.1					C2H2-type zir	rs4792655	0.4845	YES	6.371E-1 (pos.15604444)			DNA binding,DNA binding transcription factor activity,int	A=0, G=30	A=0.0, G=1.0	0.00001	446.639	30	A=0/0, G=14/1
chr6:79944355	G/G	T	G	SNV	HMGN3,HMGN3-AS1	HMGN3-AS1:exonic_nc:NR_040671.1, HMGN3:utr_5:NM_00	1	100	1	utr_5	NM_00120	c.-78A>C	p.?	1.59					HMG14 and H	rs9361504	0.0402	YES			High-mobili	biological_process,chromatin,covalent chromatin modif	T=0, G=37	T=0.0, G=1.0	0.00001	583.679	37	T=0/0, G=14/2
chr17:15905190	G/G	A	G	SNV	TTC19	TTC19:intronic:NM_017775.3	1	100		intronic	NM_01777	c.313-39A	p.?	-0.07					Anaphase-pr	rs3760298	0.4641	YES	1.531E-1 (pos.15905190)		Tetratricop	centrosom Female Urogenital Diseases;Female Urogeni	A=0, G=32	A=0.0, G=1.0	0.00001	474.154	32	A=0/0, G=24/8
chr17:15961951	G/G	A	G	SNV	NCOR1	NCOR1:intronic:NM_006311.3	1	100		intronic	NM_00631	c.5882-38	p.?	-0.48					G-protein pat	rs2157991	0.435	YES	2.136E-1 (pos.15961951)		Nuclear rec	DNA bindi Astrocytoma;Breast Diseases;Breast Neopla	A=0, G=49	A=0.0, G=1.0	0.00001	831.633	49	A=0/0, G=26/2
chr17:15961993	A/A	T	A	SNV	NCOR1	NCOR1:intronic:NM_006311.3	1	100		intronic	NM_00631	c.5882-80	p.?	-0.17					G-protein pat	rs2157990		YES			Nuclear rec	DNA bindi Astrocytoma;Breast Diseases;Breast Neopla	T=0, A=47	T=0.0, A=1.0	0.00001	789.328	47	T=0/0, A=24/2
chr5:149406271	T/T	C	T	SNV	HMGXB3	HMGXB3:exonic:NM_014983.2	1	100	8	missense	NM_01498	c.1463C>T	p.Ala488Val	0.41	1	64	0	0.4(COSM440	HMG (high m	rs6579767	0.1977	YES	7.403E-1 (pos.14940627	hepatocellular_carcinoma		DNA binding,biological_process,cellular_component,kin	C=0, T=29	C=0.0, T=1.0	0.00001	427.592	29	C=0/0, T=23/6
chr22:35660875	T/T	G	T	SNV	HMGXB4	HMGXB4:exonic:NM_001003681.2	1	100	5	missense	NM_00100	c.494G>T	p.Gly165Val	1.07	0.15	109	0.012		Domain of un	rs1053593	0.3445	YES	5.431E-1;0.0 (pos.35660875)		HMG box dc	DNA binding,NURF complex,Wnt signaling pathway,endo	G=0, T=70	G=0.0, T=1.0	0.00001	1096.29	70	G=0/0, T=48/2
chr5:162890953	A/A	G	A	SNV	HMMR	HMMR:exonic:NM_001142556.1	1	100	2	synonymous	NM_00114	c.138G>A	p.(=)	-0.96				0.01(COSM45	Hyaluronan r	rs380101,rs758820243			0.0 (pos.162890950),1.0	squamous_cel	Hyaluronan	cell surfac Adnexal Diseases;/ Hyaluronic acid	G=0, A=29	G=0.0, A=1.0	0.00001	427.603	29	G=0/0, A=12/1
chr12:121435342	C/T	C	T	SNV	HNF1A	HNF1A:exonic:NM_000545.6	1	50	7	synonymous	NM_00054	c.1375C>T	p.(=)	3.94				0.97(COSM39	Hepatocyte r	rs2259820,rs544842	0.3111	YES	0.0 (pos.121435341),4.9	adenoma,carc	HNF1 home	DNA bindi Adenocarcinoma:A Norleucin Benign/Likely	C=11, T=11	C=0.5, T=0.5	0.00001	94.6062	22	C=4/7, T=7/4
chr17:1715435	A/A	G	A	SNV	SMYD4	SMYD4:intronic:NM_052928.2	1	100		intronic	NM_05292	c.135-26C	p.?	-0.05					MYND finger,	rs7503422	0.2083	YES	4.764E-1 (pos.1715435)			metal ion Glioma:Medulloblastoma:Neoplasms:Neopl	G=0, A=21	G=0.0, A=1.0	0.00001	281.202	21	G=0/0, A=11/1
chr2:138771760	G/G	A	G	SNV	HNMT	HNMT:utr_3:NM_006895.2	1	100	6	utr_3	NM_00689	c.939A>G	p.?	0.5					Methyltransf	rs1050891		YES			Histamine I	brain deve Asthma;Bronchial D 4-(DIMETHYLAMINO)BUTY	A=0, G=21	A=0.0, G=1.0	0.00001	281.235	21	A=0/0, G=5/16
chr1:13183439	C/C	T	C	SNV	HNRNPCL2	HNRNPCL2:exonic:NM_001136561.3	1	100	2	missense	NM_00113	c.434A>G	p.His145Arg	2.17		29			RNA recognit	rs28713604		YES	7.79E-1 (pos.13183439)			RNA binding,intracellular ribonucleoprotein complex,nu	T=0, C=180	T=0.0, C=1.0	0.00001	2983.47	180	T=0/0, C=117/
chr17:1756351	G/G	A	G	SNV	RPA1	RPA1:intronic:NM_002945.3	1	100		intronic	NM_00294	c.273-44A	p.?	-0.12					OB-fold nucle	rs2287321	0.3872	YES	5.335E-1 (pos.1756351)		Replication	DNA bindi Acneiform Eruptions;Chloracne;Skin Disease	A=0, G=23	A=0.0, G=1.0	0.00001	316.746	23	A=0/0, G=4/19
chr19:8551094	C/C	T	C	SNV	HNRNPM	HNRNPM:exonic:NM_005968.4	1	100	14	synonymous	NM_00596	c.1782T>C	p.(=)	-2.62				0.08(COSM37	Heterogeneo	rs2230647,rs531807	0.0264	YES	0.0 (pos.8551084),0.0 (p	adenocarcinoma,neoplasn		RNA binding,RNA metabolic process,alternative mRNA s	T=0, C=50	T=0.0, C=1.0	0.00001	852.747	50	T=0/0, C=12/3
chr17:1783812	C/C	T	C	SNV	RPA1	RPA1:intronic:NM_002945.3	1	100		intronic	NM_00294	c.1093-25	p.?	0.04					OB-fold nucle	rs2277694	0.232	YES	1.466E-1 (pos.1783812)		Replication	DNA bindi Acneiform Eruptions;Chloracne;Skin Disease	T=0, C=46	T=0.0, C=1.0	0.00001	768.312	46	T=0/0, C=23/2
chr14:23744826	T/T	TTCC	T	INDEL	HOMEZ	HOMEZ:exonic:NM_020834.2	3	100	2	nonframeshiftDeletion	NM_02083	c.1608_16	p.Glu537del	-2.59, 0.25, 0.25, -5.11				0.04(COSM37	Homeobox KI	rs35076736,rs67447	0.3686	YES	0.0 (pos.23744829),0.0;0	T_cell_large_g	Homeodom	DNA binding,DNA binding transcription factor activity,cyt	TTCC=0, T=	TTCC=0.0, T=1.0	0.00001	432.494	29	TTCC=0/0, T=1
chr17:17897739	T/C	T	C	SNV	DRC3	DRC3:intronic:NM_031294.3	1	57.14		intronic	NM_03129	c.711+7T>	p.?	-1.88					Leucine rich	rs4924832	0.2999	YES	8.914E-1 (pos.17897739)			axoneme,cytoplasm	T=12, C=16	T=0.4286, C=0.5714	0.00001	161.768	28	T=9/3, C=11/5
chr12:54367061	G/G	T	G	SNV	HOTAIR,HOXC11	HOTAIR:intronic_nc:NR_047517.1, HOXC11:exonic:NM_014	1	100	1	synonymous	NR_04751	c.36T>G	p.(=)	0.99				0.22(COSM41	Homeobox d	rs4759315	0		1.0 (pos.54367061)	neoplasm	Homeo box	RNA polymerase II core promoter proximal region seque	T=0, G=31	T=0.0, G=1.0	0.00001	465.819	31	T=0/0, G=8/23
chr17:18043766	C/G	C	G	SNV	MYO15A	MYO15A:intronic:NM_016239.3	1	58.33		intronic	NM_01623	c.5212-65	p.?	-1.34					FERM central	rs854780		YES			Myosin XVA	ATP bindin Ear Diseases:Hearing Disorders:Hearing Loss	C=25, G=35	C=0.4167, G=0.5833	0.00001	370.43	60	C=11/14, G=2
chr17:18044270	T/C	T	C	SNV	MYO15A	MYO15A:intronic:NM_016239.3	1	52.38		intronic	NM_01623	c.5407-63	p.?	-1.1					FERM central	rs854779		YES			Myosin XVA	ATP bindin Ear Diseases:Hearing Disorders:Hearing Loss	T=20, C=22	T=0.4762, C=0.5238	0.00001	245.207	42	T=11/9, C=13/
chr7:27135314	T/T	C	T	SNV	HOTAIRM1,HOXA1	HOXA1:exonic:NM_005522.4, HOTAIRM1:upstream:NR_038	1	100	1	missense	NM_00552	c.218G>A	p.Arg73His	1.33	0.77	29	0	0.13(COSM41	Homeobox d	rs10951154,rs36842	0.1437	YES	0.0;0.0;0.0 (pos.2713531	neoplasm	Homeo box	abducens Autistic Disorder:Cardiovascul Benign	C=0, T=26	C=0.0, T=1.0	0.00001	371.42	26	C=0/0, T=18/8
chr17:18291434	A/A	ACAGCCA	A	INDEL	EVPLL	EVPLL:intronic:NM_001145127.1	6	100		intronic	NM_00114	c.877-99A	p.?	0.21, 0.19, 0.21, 0.22, 0.19, 0.19, 0.21						rs140944304		YES				cornified envelope,epidermis development,intermediat	ACAGCCA=(	ACAGCCA=0.0, A=1.0	0.00001	281.235	21	ACAGCCA=0/0

chr17:18291456	G/G	C	G	SNV	EVPLL	EVPLL:intronic:NM 001145127.1	1	100		intronic	NM 00114	c.877-77C	p.?	-0.92						rs578670		YES				cornified envelope,epidermis development,intermediat	C=0, G=21	C=0.0, G=1.0	0.00001	281.235	21	C=0/0, G=11/1
chr17:18291470	G/G	C	G	SNV	EVPLL	EVPLL:intronic:NM 001145127.1	1	100		intronic	NM 00114	c.877-63C	p.?	0.19						rs586214		YES				cornified envelope,epidermis development,intermediat	C=0, G=21	C=0.0, G=1.0	0.00001	281.235	21	C=0/0, G=11/1
chr7:27196069	C/C	A	C	SNV	HOXA-AS3.HOXA7	HOXA-AS3:downstream:NR 038832.1. HOXA7:exonic:NM 0(	1	100	1	synonymous	NR 03883	c.96T>G	p.(=)	0.83				0.9(COSM400	Homeobox d	rs2301720	0.1271	YES	8.622E-1 (pos.27196069)	acute myeloic	Homeo box	RNA polymerase II core promoter proximal region seque	A=0, C=57	A=0.0, C=1.0	0.00001	849.595	57	A=0/0, C=15/4
chr17:18291618	T/T	C	T	SNV	EVPLL	EVPLL:intronic:NM 001145127.1	1	100		intronic	NM 00114	c.*946+16	p.?	0.19						rs586757		YES				cornified envelope,epidermis development,intermediat	C=0, T=21	C=0.0, T=1.0	0.00001	281.235	21	C=0/0, T=11/1
chr17:46607021	C/C	T	C	SNV	HOXB1	HOXB1:exonic:NM 002144.3	1	100	2	missense	NM 00214	c.794A>G	p.Glu265Gly	1.96	0.33	98	0	0.1(COSM413	Homeobox d	rs7226137,rs759784	0		1.0;0.0 (pos.46607021)	neoplasm	Homeo box	DNA bindi Congenital Abnormalities;Congenital, Heredi	T=0, C=20	T=0.0, C=1.0	0.00001	263.761	20	T=0/0, C=5/15
chr17:46670520	C/C	T	C	SNV	HOXB-AS3.HOXB5	HOXB-AS3:intronic nc:NR 110331.1. HOXB5:exonic:NM 002	1	100	1	synonymous	NR 11033	c.525A>G	p.(=)	0.42				0.2(COSM413	Homeobox d	rs7406798	5.00E-04	YES	1.0 (pos.46670520)	neoplasm	Homeo box	RNA polym Nutrition Disorders:Nutritional and Metaboli	T=0, C=34	T=0.0, C=1.0	0.00001	524.186	34	T=0/0, C=12/2
chr17:18776020	A/T	A	T	SNV	PRPSAP2	PRPSAP2:intronic:NM 002767.3	1	52.38		intronic	NM 00276	c.239+58A	p.?	1.07					N-terminal d	rs7503678	0.4462	YES			Phosphorib	bone development,enzyme inhibitor activity,magnesium	A=9, T=11	A=0.45, T=0.55	0.00001	98.5642	21	A=4/5, T=6/5
chr17:18863105	T/T	C	T	SNV	SLC5A10	SLC5A10:intronic:NM 152351.4	1	100		intronic	NM 15235	c.348+129	p.?	-0.31					Sodium:solut	rs2074276,rs371611689,rs5636		YES				extracellular exosome,glucose transmembrane transpo	C=0, T=24	C=0.0, T=1.0	0.00001	334.588	24	C=0/0, T=9/15
chr2:177054769	A/A	G	A	SNV	HOXD1	HOXD1:exonic:NM 024501.2	1	100	2	missense	NM 02450	c.886G>A	p.Ala296Thr	0.92	0.4	58	0.009		Homeobox d	rs6710142	0.2349	YES	2.196E-2 (pos.177054769)		Homeo box	embryonic Adnexal Diseases:Colonic Diseases:Colorect	G=0, A=23	G=0.0, A=1.0	0.00001	316.726	23	G=0/0, A=11/1
chr17:19240972	A/A	G	A	SNV	B9D1.EPN2	B9D1:utr 3:NM 001321218.1. EPN2:downstream:NM 0149	1	100		intronic	NM 01496	c.*3405G>	p.?, p.?	-1.94						rs11652712		YES	8.214E-1;3.571E-2 (pos.19240972)		B9 domain-	MKS complex,cadherin binding,camera-type eye develop	G=0, A=23	G=0.0, A=1.0	0.00001	316.747	23	G=0/0, A=12/1
chr2:177036754	C/C	T	C	SNV	HOXD3	HOXD3:exonic:NM 006898.4	1	100	3	synonymous	NM 00689	c.1051T>C	p.(=)	1.02				0.5(COSM329	Domain of un	rs1051929	0.3154	YES	7.727E-1 (pos.17703675	adenocarcinoi	Homeo box	Notch sign Genital Diseases, Male:Genital Neoplasms, I	T=0, C=73	T=0.0, C=1.0	0.00001	1149.53	73	T=0/0, C=40/3
chr17:20000130	AT/AT	A	AT	INDEL	SPECC1	SPECC1:intronic:NM 001243439.1	1	100		intronic	NM 00124	c.147+19A	p.?	-1.43					CAMSAP CH d	rs761589578			0.0 (pos.20000130)		Sperm antig	membrane,nucleus	A=0, AT=22	A=0.0, AT=1.0	0.00001	254.346	22	A=0/0, AT=15/
chr17:20000131	G/T	G	T	SNV	SPECC1	SPECC1:intronic:NM 001243439.1	1	95.45		intronic	NM 00124	c.147+20G	p.?	2.69					CAMSAP CH d	rs4925085	0.4351	YES	1.973E-1;1.163E-4 (pos.20000131)		Sperm antig	membrane,nucleus	G=1, T=21	G=0.0455, T=0.9545	0.00001	271.287	22	G=1/0, T=14/7
chr1:40149642	T/T	G	T	SNV	HPCAL4	HPCAL4:exonic:NM 016257.3	1	100	3	synonymous	NM 01625	c.345C>A	p.(=)	1.99				0.75(COSM42	EF hand,EF-ha	rs784634	0.1935	YES	7.934E-1 (pos.40149642)	neoplasm		calcium channel regulator activity,calcium ion binding,c	G=0, T=21	G=0.0, T=1.0	0.00001	281.235	21	G=0/0, T=9/12
chr4:170678993	C/C	T	C	SNV	HPF1	HPF1:exonic:NM 017867.2	1	100	1	synonymous	NM 01786	c.36A>G	p.(=)	0.65				0(COSM3749	Uncharacteri	rs11540095	0.1897	YES	8.909E-1 (pos.17067899	adenocarcinoi	Histone par	cellular response to DNA damage stimulus,histone bindi	T=0, C=47	T=0.0, C=1.0	0.00001	789.328	47	T=0/0, C=26/2
chr17:20160925	T/T	C	T	SNV	SPECC1	SPECC1:intronic:NM 001243439.1	1	100		intronic	NM 00124	c.2837+5C	p.?	0.97					CAMSAP CH d	rs3751979	0.1805	YES	1.095E-1 (pos.20160925)		Sperm antig	membrane,nucleus	C=0, T=126	C=0.0, T=1.0	0.00001	2052.57	126	C=0/0, T=66/6
chr3:148872987	G/A	G	A	SNV	HPS3	HPS3:exonic:NM 032383.4	1	57.58	8	synonymous	NM 03238	c.1494G>A	p.(=)	-0.26					Hermansky-P	rs6440589	0.4385	YES	3.695E-1 (pos.148872987)		HPS gene 3	BLOC-2 coi Albinism;Albinism, Oculocutan Benign	G=14, A=19	G=0.4242, A=0.5758	0.00001	204.385	33	G=8/6, A=7/12
chr17:20163616	T/T	C	T	SNV	SPECC1	SPECC1:intronic:NM 001243439.1	1	100		intronic	NM 00124	c.2940+9C	p.?	-0.13					CAMSAP CH d	rs11868429	0.18	YES	1.093E-1 (pos.20163616)		Sperm antig	membrane,nucleus	C=0, T=28	C=0.0, T=1.0	0.00001	408.718	28	C=0/0, T=10/1
chr3:148863151	A/G	A	G	SNV	HPS3	HPS3:exonic:NM 032383.4	1	50	5	synonymous	NM 03238	c.981A>G	p.(=)	-0.05				0.07(COSM37	Hermansky-P	rs1005049091,rs117	0.215	YES	1.524E-1;0.0 (pos.14886	adenocarcinoi	HPS gene 3	BLOC-2 coi Albinism:Albinism, Oculocutan Benign	A=13, G=13	A=0.5, G=0.5	0.00001	119.163	26	A=12/1, G=7/6
chr22:26854441	A/A	G	A	SNV	HPS4	HPS4:exonic:NM 152841.2	1	100	10	missense	NM 15284	c.1801C>T	p.His601Tyr	8.28	0.03	83	1	0.99(COSM4156096),0.99(C		rs1894706	0.0722	YES	7.952E-1 (pos.26854441)	neoplasm	HPS gene 4	BLOC-3 coi Albinism:Albinism, Oculocutan Benign	G=0, A=26	G=0.0, A=1.0	0.00001	371.416	26	G=0/0, A=12/1
chr17:20908325	C/C	T	C	SNV	USP22	USP22:intronic:NM 015276.1	1	100		intronic	NM 01527	c.1323-22	p.?	-0.67					Ubiquitin car	rs9797279	0.2047	YES	4.464E-1 (pos.20908325)		Ubiquitin-s	H4 histone acetyltransferase activity,SAGA complex,SAG	T=0, C=28	T=0.0, C=1.0	0.00001	408.717	28	T=0/0, C=11/1
chr22:26859942	T/T	C	T	SNV	HPS4	HPS4:exonic:NM 152841.2	1	100	9	missense	NM 15284	c.1639G>A	p.Val547Met	0.72	0.07	21	0.763			rs5752330	0.1119	YES	7.943E-1;0.0 (pos.26859942)		HPS gene 4	BLOC-3 coi Albinism;Albinism, Oculocutan Benign	C=0, T=40	C=0.0, T=1.0	0.00001	644.286	40	C=0/0, T=26/1
chr11:18327684	T/T	G	T	SNV	HPS5	HPS5:exonic:NM 181507.1	1	100	7	synonymous	NM 18150	c.822C>A	p.(=)	1.29						rs1140047	0.315	YES	9.748E-1 (pos.18327684)		HPS gene 5	BLOC-2 coi Albinism;Albinism, Oculocutan Benign	G=0, T=30	G=0.0, T=1.0	0.00001	446.639	30	G=0/0, T=16/1
chr17:21205354	GA/AG	GA	AG	MNV	MAP2K3	MAP2K3:intronic:NM 145109.2	2	47.37		intronic	NM 14510	c.400-101	p.?	-0.15, -0.69					Protein kinas	rs73982738,rs73982739,rs796900370,rs879150039						ATP binding,MAP kinase kinase activity,MAPK cascade,ac	GA=17, AG:	GA=0.4857, AG=0.51	0.00001	218.809	38	GA=16/1, AG=
chr17:21206556	T/T	C	T	SNV	MAP2K3	MAP2K3:intronic:NM 145109.2	1	100		intronic	NM 14510	c.568+10C	p.?	-2.83				0.01(COSM54	Protein kinas	rs736103	0.4764	YES	5.236E-1 (pos.21206556)	squamous cell carcinoma		ATP binding,MAP kinase kinase activity,MAPK cascade,ac	C=0, T=67	C=0.0, T=1.0	0.00001	1034.41	67	C=0/0, T=30/3
chr4:84230619	C/C	T	C	SNV	HPSE	HPSE:exonic:NM 001098540.2	1	100	7	missense	NM 00109	c.920A>G	p.Lys307Arg	3.93	0.81	26	0.004	0.78(COSM11	Glycosyl hydr	rs11099592	0.2267	YES	8.695E-1 (pos.84230619)	adenocarcinoi	Heparanas	angiogene Autoimmune Disea Dalteparin,Heparin	T=0, C=31	T=0.0, C=1.0	0.00001	465.817	31	T=0/0, C=7/24
chr10:100219374	A/A	T	A	SNV	HPSE2	HPSE2:exonic:NM 021828.4	1	100	12	missense	NM 02182	c.1736A>T	p.Tyr579Phe	7.56	0.69	22	0.001	0.97(COSM63	Glycosyl hydr	rs10883100	0.4866	YES	5.443E-1 (pos.10021937	carcinoma	Heparanas	biological Asthma;Autoimmune Diseases;Bronchial Dis	T=0, A=84	T=0.0, A=1.0	0.00001	1348.34	84	T=0/0, A=70/1
chr17:2237614	G/A	G	A	SNV	TSR1	TSR1:intronic:NM 018128.4	1	50		intronic	NM 01812	c.981+152	p.?	-1.91					40S ribosome	rs3785558		YES			TSR1, 20S r	GTP binding,GTPase activity,RNA binding,U3 snoRNA binc	G=11, A=11	G=0.5, A=0.5	0.00001	94.6067	22	G=3/8, A=7/4
chr17:2268681	T/C	T	C	SNV	SGSM2	SGSM2:intronic:NM 014853.2	1	57.14		intronic	NM 01485	c.1288+46	p.?	-0.54					RUN domain,	rs2429908	0.4417	YES	7.683E-1 (pos.2268681)		Small G pro	GTPase activator activity,Rab GTPase binding,activation	T=9, C=12	T=0.4286, C=0.5714	0.00001	109.102	21	T=3/6, C=4/8
chr17:2278644	T/T	C	T	SNV	SGSM2	SGSM2:intronic:NM 014853.2	1	100		intronic	NM 01485	c.2101-14	p.?	-0.48					RUN domain,	rs2252910		YES			Small G pro	GTPase activator activity,Rab GTPase binding,activation	C=0, T=21	C=0.0, T=1.0	0.00001	281.235	21	C=0/0, T=5/16
chr17:2573652	C/T	C	T	SNV	PAFAH1B1	PAFAH1B1:intronic:NM 000430.3	1	45.45		intronic	NM 00043	c.568+27C	p.?	-0.21					LisH,WD dom	rs3213696	0.0693	YES	9.386E-1 (pos.2573652)		Platelet-act	G2/M tran Carcinogenesis;Classical Lisse Benign/Likely	C=30, T=25	C=0.5455, T=0.4545	0.00001	232.694	55	C=11/19, T=9/
chr17:25938676	G/A	G	A	SNV	KSR1	KSR1:intronic:NM 014238.1	1	50		intronic	NM 01423	c.2148+21	p.?	-0.17					Kinase-like,P	rs7207500,rs752484	0.0054	YES	0.0;0.0 (pos.25938676)		Kinase supp	ATP bindin Crohn Disease;Digestive System Diseases;Ga	G=10, A=10	G=0.5, A=0.5	0.00001	82.8849	20	G=8/2, A=2/8
chr17:2595535	G/T	G	T	SNV	CLUH,MIR6776	CLUH:intronic:NM 015229.3. MIR6776:downstream:NR 10	1	52.38		intronic	NM 01522	c.3443+12	p.?	-1.29					Clustered mi	rs2240732		YES			Clustered m	cytoplasm,intracellular distribution of mitochondria,mR	G=10, T=11	G=0.4762, T=0.5238	0.00001	97.5868	21	G=3/7, T=1/10
chr8:21984650	G/G	A	G	SNV	HR	HR:exonic:NM 005144.4	1	100	3	synonymous	NM 00514	c.1305T>C	p.(=)	0.31				0.07(COSM45	JmjC domain	rs12675745	0.3758	YES	3.95E-1 (pos.21984650)	acute myeloic	Hairless, m	DNA bindi Alopecia:Hair Diseases:Hypotr Benign	A=0, G=49	A=0.0, G=1.0	0.00001	801.694	49	A=0/0, G=30/1
chr17:2598627	C/C	T	C	SNV	CLUH	CLUH:intronic:NM 015229.3	1	100		intronic	NM 01522	c.2427+41	p.?	-0.21					Clustered mi	rs1003398	2.00E-04		1.0;0.0;0.0 (pos.2598627)		Clustered m	cytoplasm,intracellular distribution of mitochondria,mR	T=0, C=49	T=0.0, C=1.0	0.00001	830.403	49	T=0/0, C=19/3
chr19:49658367	T/T	C	T	SNV	HRC	HRC:exonic:NM 002152.2	1	100	1	missense	NM 00215	c.128G>A	p.Ser43Asn	0.53	1	46	0	0.01(COSM41	Histidine-rich	rs3745298,rs776241	0.4515	YES	0.0 (pos.49658364),3.09	ductal carcinc	Histidine-ri	ATPase bir Cardiomegaly:Cardiovascular Diseases:Hear	C=0, T=60	C=0.0, T=1.0	0.00001	901.507	60	C=0/0, T=31/2
chr17:26091227	T/T	C	T	SNV	NOS2	NOS2:intronic:NM 000625.4	1	100		intronic	NM 00062	c.2429-57	p.?	-0.23					FAD binding d	rs2297511		YES			Nanos, Dro	FMN bindi Abnormalities, Mul (2S)-2-methyl-2,3-dihydr	C=0, T=34	C=0.0, T=1.0	0.00001	524.186	34	C=0/0, T=14/2
chr3:186395572	T/T	A	T	SNV	HRG	HRG:exonic:NM 000412.4	1	100	7	missense	NM 00041	c.1478A>T	p.Asn493Ile	1.92	1	149	0	0.01(COSM41	Cystatin dom	rs1042464	0.485	YES	7.974E-1 (pos.18639557	basal cell car	Histidine-ri	angiogene Breast Diseases:Breast Neoplasms:Hematol	A=0, T=49	A=0.0, T=1.0	0.00001	831.609	49	A=0/0, T=25/2
chr17:26669245	AG/AG	A	AG	INDEL	TNFAIP1	TNFAIP1:intronic:NM 021137.4	1	100		intronic	NM 02113	c.519-28A	p.?	0.2					BTB/POZ dom	rs5819838,rs869180	0		1.0 (pos.26669245)		Tumor necr	Cul3-RING Diabetes Complications;Diabetes Mellitus;D	A=0, AG=27	A=0.0, AG=1.0	0.00001	389.163	27	A=0/0, AG=21/
chr3:186395113	A/G	A	G	SNV	HRG	HRG:exonic:NM 000412.4	1	52.38	7	missense	NM 00041	c.1019A>G	p.His340Arg	-1.01	0.09	29	0.652	0.01(COSM14	Cystatin dom	rs2228243	0.2037	YES	3.872E-1;0.0 (pos.18639	adenocarcinoi	Histidine-ri	angiogene Breast Diseases;Breast Neoplasms;Hematol	A=50, G=55	A=0.4762, G=0.5238	0.00001	574.019	105	A=40/10, G=4
chr17:26695832	G/G	A	G	SNV	VTN	VTN:intronic:NM 000638.3	1	100		intronic	NM 00063	c.826+61T	p.?	-0.65					Hemopexin,S	rs2071379		YES			Vitronectin	Golgi lume Crohn Disease:Diat Abciximab	A=0, G=29	A=0.0, G=1.0	0.00001	427.603	29	A=0/0, G=13/1
chr1:152191709	G/G	C	G	SNV	HRNR	HRNR:exonic:NM 001009931.2	1	100	3	missense	NM 00100	c.2396G>C	p.Ser799Thr	0.56		58	0.334	0.12(COSM11	S-100/ICaBP	rs6662450,rs971526	0.1698	YES	3.894E-1;0.0 (pos.15219	acute myeloic	Hornerin	azurophil granule lumen,calcium ion binding,cell envelo	C=0, G=115	C=0.0, G=1.0	0.00001	1834.76	115	C=0/0, G=51/6
chr1:152191879	A/A	G	A	SNV	HRNR	HRNR:exonic:NM 001009931.2	1	100	3	synonymous	NM 00100	c.2226C>T	p.(=)	-2.45				0(COSM5425	S-100/ICaBP	rs10788828,rs75921	0.1691	YES	4.128E-1;2.32E-4 (pos.15	acute myeloic	Hornerin	azurophil granule lumen,calcium ion binding,cell envelo	G=0, A=23	G=0.0, A=1.0	0.00001	316.724	23	G=0/0, A=14/9
chr1:152192114	T/T	C	T	SNV	HRNR	HRNR:exonic:NM 001009931.2	1	100	3	missense	NM 00100	c.1991G>A	p.Arg664Gln	-1.65		43	0.016	0(COSM1334	S-100/ICaBP	rs7520249		YES	4.131E-1 (pos.15219211	acute myeloic	Hornerin	azurophil granule lumen,calcium ion binding,cell envelo	C=0, T=25	C=0.0, T=1.0	0.00001	353.018	25	C=0/0, T=11/1
chr17:26856261	G/G	A	G	SNV	FOXN1	FOXN1:intronic:NM 003593.2	1	100		intronic	NM 00359	c.830+19A	p.?	-1.51					Forkhead dor	rs548973	0.3516	YES	3.304E-1 (pos.26856261)		Forkhead b	RNA polym Alopecia;Autistic Disorder;Chil Benign	A=0, G=30	A=0.0, G=1.0	0.00001	438.108	30	A=0/0, G=13/1
chr1:152192631	T/T	C	T	SNV	HRNR	HRNR:exonic:NM 001009931.2	1	100	3	missense	NM 00100	c.1474G>A	p.Gly492Arg	-3.73		125	0.105	0(COSM3996	S-100/ICaBP	rs6587647	0.169	YES	4.129E-1 (pos.15219263	acute myeloic	Hornerin	azurophil granule lumen,calcium ion binding,cell envelo	C=0, T=27	C=0.0, T=1.0	0.00001	389.985	27	C=0/0, T=8/19
chr17:27290969	TG/TG	T	TG	INDEL	SEZ6	SEZ6:intronic:NM 178860.4	1	100		intronic	NM 17886	c.1240+11	p.?	-0.35					CUB domain,S	rs11450637,rs39774	0.4994	YES	4.139E-1 (pos.27290969)		Seizure-rela	adult loco Mental Disorders:Neurodevelopmental Diso	T=0, TG=39	T=0.0, TG=1.0	0.00001	442.781	39	T=0/0, TG=27/
chr17:27437134	A/G	A	G	SNV	MYO18A	MYO18A:intronic:NM 078471.3	1	47.62		intronic	NM 07847	c.3088-15	p.?	0.08					IQ calmodulin	rs2279958	0.4895	YES	8.815E-1 (pos.27437134)		Myosin XVII	ADP binding,ATP binding,ATPase activity,DNA binding,DN	A=22, G=20	A=0.5238, G=0.4762	0.00001	212.953	42	A=7/15, G=9/1
chr17:27449059	C/A	C	A	SNV	MYO18A	MYO18A:intronic:NM 078471.3	1	57.14		intronic	NM 07847	c.1088-84	p.?	0.29					IQ calmodulin	rs877492		YES			Myosin XVII	ADP binding,ATP binding,ATPase activity,DNA binding,DN	C=9, A=12	C=0.4286, A=0.5714	0.00001	110.304	21	C=5/4, A=9/3
chr17:27898828	C/C	T	C	SNV	TP53I13	TP53I13:intronic:NM 138349.3	1	100		intronic	NM 13834	c.313-47T	p.?	-0.34						rs565977	0.3002	YES	7.51E-1 (pos.27898828)			cytoplasm,integral component of membrane,molecular	T=0, C=34	T=0.0, C=1.0	0.00001	524.186	34	T=0/0, C=24/1
chr17:27901975	G/G	A	G	SNV	GIT1	GIT1:intronic:NM 001085454.1	1	100		intronic	NM 00108	c.2101-43	p.?	-0.35					Ankyrin repea	rs550818	0.2604	YES	9.178E-1 (pos.27901975)		G protein-c	GTPase ac Attention Deficit Disorder with Hyperactivity	A=0, G=85	A=0.0, G=1.0	0.00001	1390.46	85	A=0/0, G=38/4
chr17:28380152	T/C	T	C	SNV	EFCAB5	EFCAB5:intronic:NM 198529.3	1	59.18		intronic	NM 19852	c.1301-12	p.?	0.6						rs149229469						calcium ion binding	T=20, C=29	T=0.4082, C=0.5918	0.00001	360.526	49	T=9/11, C=12/
chr17:28386526	G/G	A	G	SNV	EFCAB5	EFCAB5:intronic:NM 198529.3	1	100		intronic	NM 19852	c.2581-37	p.?	-1.57						rs4075623	0.4552	YES	3.222E-1 (pos.28386526)			calcium ion binding	A=0, G=33	A=0.0, G=1.0	0.00001	504.595	33	A=0/0, G=13/2
chr1:152192687	C/C	T	C	SNV	HRNR	HRNR:exonic:NM 001009931.2	1	100	3	missense	NM 00100	c.1418A>G	p.Glu473Gly	-1.27		98	0		S-100/ICaBP	rs6587648	3.00E-04	YES	9.997E-1 (pos.152192687)		Hornerin	azurophil granule lumen,calcium ion binding,cell envelo	T=0, C=29	T=0.0, C=1.0	0.00001	427.604	29	T=0/0, C=8/21
chr17:29221227	T/T	A	T	SNV	ATAD5	ATAD5:intronic:NM 024857.4	1	100		intronic	NM 02485	c.5297+59	p.?	-0.57					ATPase famil	rs752936350,rs9898911		YES			ATPase fam	ATP bindin Adnexal Diseases:Endocrine Gland Neoplasm	A=0, T=48	A=0.0, T=1.0	0.00001	810.35	48	A=0/0, T=15/3
chr17:2923767	G/G	A	G	SNV	RAP1GAP2	RAP1GAP2:intronic:NM 015085.4	1	100		intronic	NM 01508	c.1633-4A	p.?	-0.12					Rap/ran-GAP	rs2304982	0.0528	YES	8.58E-1 (pos.2923767)			GTPase ac Asthma:Bronchial Diseases:Hypersensitivity	A=0, G=46	A=0.0, G=1.0	0.00001	768.313	46	A=0/0, G=35/1
chr1:152192825	T/T	C	T	SNV	HRNR	HRNR:exonic:NM 001009931.2	1	100	3	missense	NM 00100	c.1280G>A	p.Gly427Asp	-0.07		94	0.895	0.01(COSM54	S-100/ICaBP	rs6666097	0.409	YES	3.5E-1 (pos.152192825)	acute myeloic	Hornerin	azurophil granule lumen,calcium ion binding,cell envelo	C=0, T=73	C=0.0, T=1.0	0.00001	1120.25	73	C=0/0, T=39/3
chr1:152192978	C/C	T	C	SNV	HRNR	HRNR:exonic:NM 001009931.2	1	100	3	missense	NM 00100	c.1127A>G	p.Gln376Arg	-1.54		43	0		S-100/ICaBP	rs6587649	3.00E-04	YES	1.0 (pos.152192978)		Hornerin	azurophil granule lumen,calcium ion binding,cell envelo	T=0, C=22	T=0.0, C=1.0	0.00001	298.888	22	T=0/0, C=5/17
chr17:2930223	C/C	T	C	SNV	RAP1GAP2	RAP1GAP2:intronic:NM 015085.4	1	100		intronic	NM 01508	c.1981-15	p.?	-3.68					Rap/ran-GAP	rs4790114	0.3701	YES	5.507E-1 (pos.2930223)			GTPase ac Asthma;Bronchial Diseases;Hypersensitivity	T=0, C=51	T=0.0, C=1.0	0.00001	754.126	51	T=0/0, C=19/3
chr1:152193052	G/G	A	G	SNV	HRNR	HRNR:exonic:NM 001009931.2	1	100	3	synonymous	NM 00100	c.1053T>C	p.(=)	-3.03					S-100/ICaBP	rs6587650	6.00E-04	YES	9.986E-1;0.0 (pos.152193052)		Hornerin	azurophil granule lumen,calcium ion binding,cell envelo	A=0, G=130	A=0.0, G=1.0	0.00001	2135.55	130	A=0/0, G=74/5
chr17:29670190	G/G	C	G	SNV	NF1	NF1:intronic:NM 001042492.2	1	100		intronic	NM 00104	c.7189+37	p.?	-1.43					Divergent CR	rs7405740	0.0809	YES	8.881E-1 (pos.29670190)		Neurofibror	GTPase ac Adenoma:Astrocytoma:Autisti Benign	C=0, G=20	C=0.0, G=1.0	0.00001	263.786	20	C=0/0, G=9/11
chr17:29844658	C/G	C	G	SNV	RAB11FIP4	RAB11FIP4:intronic:NM 032932.5	1	58.33		intronic	NM 03293	c.337-11C	p.?	-0.08					EF-hand dom	rs3744619	0.2551	YES	6.491E-1 (pos.29844658)		RAB11 fami	ADP-ribosylation factor binding,Rab GTPase binding,calc	C=10, G=14	C=0.4167, G=0.5833	0.00001	135.338	24	C=8/2, G=13/1
chr17:29850698	A/A	G	A	SNV	RAB11FIP4	RAB11FIP4:intronic:NM 032932.5	1	100		intronic	NM 03293	c.1029+69	p.?	0.73					EF-hand dom	rs2074147		YES			RAB11 fami	ADP-ribosylation factor binding,Rab GTPase binding,calc	G=0, A=42	G=0.0, A=1.0	0.00001	685.16	42	G=0/0, A=8/34
chr1:152193121	T/T	G	T	SNV	HRNR	HRNR:exonic:NM 001009931.2	1	100	3	synonymous	NM 00100	c.984C>A	p.(=)	-2.74				0.01(COSM14	S-100/ICaBP	rs6587651	0.169	YES	3.897E-1 (pos.15219312	acute myeloic	Hornerin	azurophil granule lumen,calcium ion binding,cell envelo	G=0, T=132	G=0.0, T=1.0	0.00001	2176.52	132	G=0/0, T=73/5
chr17:30509939	G/G	C	G	SNV	RHOT1	RHOT1:intronic:NM 001033568.2	1	100		intronic	NM 00103	c.438+55C	p.?	-0.05					EF hand asso	rs6505286					Ras homolo	GTP binding,GTPase activity,calcium ion binding,cellular	C=0, G=100	C=0.0, G=1.0	0.00001	1679.91	100	C=0/0, G=27/7
chr17:30643361	G/G	A	G	SNV	RHBDL3	RHBDL3:intronic:NM 138328.2	1	100		intronic	NM 13832	c.943+50A	p.?	0.24					EF-hand dom	rs2344973	0.2208	YES	5.997E-1 (pos.30643361)			calcium ion binding,integral component of membrane,p	A=0, G=61	A=0.0, G=1.0	0.00001	921.854	61	A=0/0, G=38/2
chr17:30665005	C/C	T	C	SNV	C17orf75	C17orf75:intronic:NM 022344.3	1	100		intronic	NM 02234	c.492-32A	p.?	0.59					Mimu-R1-like	rs35677406	0.448	YES	3.849E-2 (pos.30665005)			cellular_component,molecular_function	T=0, C=39	T=0.0, C=1.0	0.00001	623.974	39	T=0/0, C=17/2
chr17:30696292	T/T	C	T	SNV	ZNF207	ZNF207:intronic:NM 001098507.1	1	100		intronic	NM 00109	c.1165-22	p.?	1.43						rs2286645	0.393	YES	7.288E-2 (pos.30696292)		Zinc finger p	DNA binding,DNA binding transcription factor activity,RN	C=0, T=95	C=0.0, T=1.0	0.00001	1597.04	95	C=0/0, T=67/2
chr1:152193286	T/T	G	T	SNV	HRNR	HRNR:exonic:NM 001009931.2	1	100	3	missense	NM 00100	c.819C>A	p.His273Gln	-5.14		24	0.001	0(COSM1466	S-100/ICaBP	rs7545406	0.409	YES	3.485E-1 (pos.15219328	acute myeloic	Hornerin	azurophil granule lumen,calcium ion binding,cell envelo	G=0, T=45	G=0.0, T=1.0	0.00001	745.845	45	G=0/0, T=18/2
chr17:31319024	C/C	A	C	SNV	SPACA3	SPACA3:intronic:NM 173847.4	1	100		intronic	NM 17384	c.34+34A>	p.?	-0.03					C-type lysozy	rs2188953	0.3437	YES	5.471E-1 (pos.31319024)		Sperm acro	acrosomal matrix,acrosomal membrane,cell wall macro	A=0, C=70	A=0.0, C=1.0	0.00001	1095.97	70	A=0/0, C=14/5
chr17:31319033	C/C	G	C	SNV	SPACA3	SPACA3:intronic:NM 173847.4	1	100		intronic	NM 17384	c.34+43G>	p.?	0.92					C-type lysozy	rs3922357	0.3653	YES	4.986E-1;0.0 (pos.31319033)		Sperm acro	acrosomal matrix,acrosomal membrane,cell wall macro	G=0, C=63	G=0.0, C=1.0	0.00001	958.917	63	G=0/0, C=8/55
chr1:152193291	T/T	G	T	SNV	HRNR	HRNR:exonic:NM 001009931.2	1	100	3	synonymous	NM 00100	c.814C>A	p.(=)	-2.72				0.01(COSM14	S-100/ICaBP	rs6587652	0.169	YES	4.11E-1 (pos.152193291)	acute myeloic	Hornerin	azurophil granule lumen,calcium ion binding,cell envelo	G=0, T=46	G=0.0, T=1.0	0.00001	768.3	46	G=0/0, T=18/2
chr1:152193544	G/G	A	G	SNV	HRNR	HRNR:exonic:NM 001009931.2	1	100	3	synonymous	NM 00100	c.561T>C	p.(=)	0.16				0(COSM1466	S-100/ICaBP	rs7535536	0.1698	YES	3.89E-1 (pos.152193544)	acute myeloic	Hornerin	azurophil granule lumen,calcium ion binding,cell envelo	A=0, G=35	A=0.0, G=1.0	0.00001	543.873	35	A=0/0, G=7/28
chr1:152193547	A/A	G	A	SNV	HRNR	HRNR:exonic:NM 001009931.2	1	100	3	synonymous	NM 00100	c.558C>T	p.(=)	-1.98					S-100/ICaBP	rs6587653	3.00E-04	YES	1.0 (pos.152193547)		Hornerin	azurophil granule lumen,calcium ion binding,cell envelo	G=0, A=35	G=0.0, A=1.0	0.00001	543.903	35	G=0/0, A=7/28
chr2:20823724	C/C	T	C	SNV	HS1BP3	HS1BP3:exonic:NM 022460.3	1	100	6	synonymous	NM 02246	c.852A>G	p.(=)	-4.22				0.01(COSM41	PX domain	rs6531248	1.00E-04		9.999E-1;0.0 (pos.20823	neoplasm	HS1-bindin	endoplasmic reticulum,mitochondrion,phosphatidylino	T=0, C=26	T=0.0, C=1.0	0.00001	371.419	26	T=0/0, C=10/1
chr2:20824559	T/T	C	T	SNV	HS1BP3	HS1BP3:exonic:NM 022460.3	1	100	5	synonymous	NM 02246	c.717G>A	p.(=)	-0.44				0.02(COSM41	PX domain	rs2305457	0.259	YES	4.979E-1 (pos.20824559)	carcinoma,ne	HS1-bindin	endoplasmic reticulum,mitochondrion,phosphatidylino	C=0, T=33	C=0.0, T=1.0	0.00001	498.122	33	C=0/0, T=16/1
chr16:1962046	TA/TA	CG	TA	MNV	HS3ST6	HS3ST6:exonic:NM 001009606.3	2	100	2	missense	NM 00100	c.573 574	p.Ala192Thr	3.32, 0.22	1	58	0.085	0.54(COSM39	Sulfotransfer	rs1657140,rs174239	0.3418	YES	4.718E-1 (pos.1962047),	carcinoma,ductal carcinor		Golgi mem Lung Diseases;Lung Diseases, Interstitial;Lur	CG=0, TA=6	CG=0.0, TA=1.0	0.00001	925.467	61	CG=0/0, TA=24
chr1:120057158	T/T	C	T	SNV	HSD3B1	HSD3B1:exonic:NM 000862.2	1	100	4	synonymous	NM 00086	c.1012C>T	p.(=)	1.56				0.48(COSM37	3-beta hydro	rs6203	0.4337	YES	6.688E-1 (pos.12005715	adenocarcinoi	Hydroxy-de	3-beta-hy Adnexal Diseases;(Androstenedione,NADH,	C=0, T=62	C=0.0, T=1.0	0.00001	940.32	62	C=0/0, T=31/3
chr1:120057246	A/A	C	A	SNV	HSD3B1	HSD3B1:exonic:NM 000862.2	1	100	4	missense	NM 00086	c.1100C>A	p.Thr367Asn	-0.09	0.32	65	0.021	0.02(COSM37	3-beta hydro	rs1047303,rs456093	0.3183	YES	9.376E-1 (pos.12005724	adenocarcinoi	Hydroxy-de	3-beta-hy Adnexal Diseases;(Androstenedione,NADH,	A=0, C=57	A=0.0, C=1.0	0.00001	849.007	57	C=0/0, A=24/3
chr17:33807250	A/A	T	A	SNV	SLFN12L	SLFN12L:intronic:NM 001195790.1	1	100		intronic	NM 00119	c.15-36A>	p.?	-0.58					Putative DNA	rs9905892	0.3277	YES	6.794E-1 (pos.33807250)		Schlafen far	ATP binding,integral component of membrane	T=0, A=61	T=0.0, A=1.0	0.00001	921.854	61	T=0/0, A=35/2
chr17:3419689	TG/TG	CA	TG	MNV	TRPV3	TRPV3:intronic:NM 001258205.1	2	100		intronic	NM 00125	c.2198+62	p.?	-1.37, -1.65					Ankyrin repea	rs386794632,rs8081785,rs8082		YES			Transient receptor potential cation channe	Menthol	CA=0, TG=2	CA=0.0, TG=1.0	0.00001	411.88	28	CA=0/0, TG=19
chr17:3424183	G/G	A	G	SNV	TRPV3	TRPV3:intronic:NM 001258205.1	1	100		intronic	NM 00125	c.1810+84	p.?	-0.32					Ankyrin repea	rs8079980		YES			Transient receptor potential cation channe	Menthol	A=0, G=22	A=0.0, G=1.0	0.00001	303.278	22	A=0/0, G=7/15
chr21:45078035	T/T	C	T	SNV	HSF2BP	HSF2BP:utr 5:NM 007031.1	1	100	2	utr 5	NM 00703	c.-58G>A	p.?	0.12						rs2838343		YES			Heat-shock	cytosol,pr Digestive System Diseases;Eosinophilia;Eosi	C=0, T=104	C=0.0, T=1.0	0.00001	1626.72	104	C=0/0, T=38/6
chr17:56544280	G/G	T	G	SNV	HSF5	HSF5:exonic:NM 001080439.2	1	100	3	missense	NM 00108	c.986A>C	p.Asn329Thr	3.24	1	65	0		HSF-type DNA	rs1017089	3.00E-04		1.0;0.0 (pos.56544280)			DNA binding transcription factor activity,nucleus,regulat	T=0, G=70	T=0.0, G=1.0	0.00001	1092.07	70	T=0/0, G=27/4
chr5:137892170	A/A	G	A	SNV	HSPA9	HSPA9:exonic:NM 004134.6	1	100	16	synonymous	NM 00413	c.1933C>T	p.(=)	0.89				0.69(COSM37	FGGY family c	rs10117	0.3997	YES	7.866E-1 (pos.13789217	adenocarcinoi	Heat-shock	ATP bindin Adenocarcinoma:Autoimmune Diseases:Bas	G=0, A=36	G=0.0, A=1.0	0.00001	563.743	36	G=0/0, A=21/1
chr17:34416665	G/G	A	G	SNV	CCL3	CCL3:intronic:NM 002983.2	1	100		intronic	NM 00298	c.74-22T>	p.?	-0.01					Small cytokin	rs1719133	9.00E-04		1.0 (pos.34416665)		Chemokine	CCR1 chen Adenocarcinoma;A ROX-888	A=0, G=32	A=0.0, G=1.0	0.00001	485.14	32	A=0/0, G=19/1
chr2:198362018	C/C	T	C	SNV	HSPD1	HSPD1:exonic:NM 002156.4	1	100	3	synonymous	NM 00215	c.273A>G	p.(=)	0.79				0.65(COSM37	TCP-1/cpn60	rs8539	0.3158	YES	5.216E-1 (pos.19836201	acute myeloic	Heat-shock	'de novo' p Acute Coronary Syndrome;Ade Benign/Likely	T=0, C=50	T=0.0, C=1.0	0.00001	852.747	50	T=0/0, C=26/2
chr1:22154845	G/G	A	G	SNV	HSPG2	HSPG2:exonic:NM 005529.6	1	100	89	synonymous	NM 00552	c.12312T>	p.(=)	1.35				0.11(COSM37	EGF-like dom	rs2228347	0.3063	YES	1.607E-1 (pos.22154845)	ductal carcinc	Heparan su	Golgi lume Bone Diseases;Bon Palifermin Benign	A=0, G=88	A=0.0, G=1.0	0.00001	1451.91	88	A=0/0, G=35/5
chr1:22165901	G/G	A	G	SNV	HSPG2	HSPG2:exonic:NM 005529.6	1	100	73	synonymous	NM 00552	c.9852T>C	p.(=)	-3.41				0.02(COSM54	EGF-like dom	rs2291826	0.2571	YES	1.621E-1 (pos.22165901)	acute myeloic	Heparan su	Golgi lume Bone Diseases;Bon Palifermin Benign	A=0, G=74	A=0.0, G=1.0	0.00001	1159.96	74	A=0/0, G=32/4
chr17:34869150	A/A	C	A	SNV	MYO19	MYO19:intronic:NM 001163735.1	1	100		intronic	NM 00116	c.894+122	p.?	-1.61					IQ calmodulin	rs6607296		YES			Myosin XIX	ATP binding,ATPase activity,actin binding,mitochondrial	C=0, A=53	C=0.0, A=1.0	0.00001	782.03	53	C=0/0, A=32/2
chr17:34869155	A/A	G	A	SNV	MYO19	MYO19:intronic:NM 001163735.1	1	100		intronic	NM 00116	c.894+117	p.?	-0.51					IQ calmodulin	rs8882		YES			Myosin XIX	ATP binding,ATPase activity,actin binding,mitochondrial	G=0, A=52	G=0.0, A=1.0	0.00001	760.746	52	G=0/0, A=31/2
chr17:35549275	C/C	T	C	SNV	ACACA	ACACA:intronic:NM 198834.2	1	100		intronic	NM 19883	c.4201-29	p.?	-1.64					ATP-grasp do	rs2305099	0.1829	YES	7.595E-1 (pos.35549275)		Acetyl-Coer	ATP bindin Adenocarcinoma;C Biotin	T=0, C=32	T=0.0, C=1.0	0.00001	485.141	32	T=0/0, C=13/1
chr1:22216574	A/A	C	A	SNV	HSPG2	HSPG2:exonic:NM 005529.6	1	100	6	synonymous	NM 00552	c.474G>T	p.(=)	-0.63					EGF-like dom	rs2254358	0.323	YES	3.736E-1 (pos.22216574)		Heparan su	Golgi lume Bone Diseases;Bon Palifermin Benign	C=0, A=44	C=0.0, A=1.0	0.00001	726.569	44	C=0/0, A=22/2
chr17:35608889	A/A	G	A	SNV	ACACA	ACACA:intronic:NM 198834.2	1	100		intronic	NM 19883	c.2081+42	p.?	0.26					ATP-grasp do	rs1519091	0.1862	YES	7.579E-1 (pos.35608889)		Acetyl-Coer	ATP bindin Adenocarcinoma;C Biotin	G=0, A=109	G=0.0, A=1.0	0.00001	1718.88	109	G=0/0, A=69/4
chr1:22216604	G/G	C	G	SNV	HSPG2	HSPG2:exonic:NM 005529.6	1	100	6	synonymous	NM 00552	c.444G>C	p.(=)	1.47				0.91(COSM39	EGF-like dom	rs2254357	0.3229	YES	3.732E-1;8.104E-4 (pos.2	acute myeloic	Heparan su	Golgi lume Bone Diseases;Bon Palifermin Benign	C=0, G=42	C=0.0, G=1.0	0.00001	685.035	42	C=0/0, G=19/2
chr1:22163390	G/A	G	A	SNV	HSPG2	HSPG2:exonic:NM 005529.6	1	59.09	75	synonymous	NM 00552	c.10260C>	p.(=)	2.38				0.8(COSM375	EGF-like dom	rs35444472	0.0737	YES	0.0 (pos.22163390)	adenocarcinoi	Heparan su	Golgi lume Bone Diseases;Bon Palifermin Likely benign	G=18, A=26	G=0.4091, A=0.5909	0.00001	311.151	44	G=10/8, A=20/
chr1:22169290	G/A	G	A	SNV	HSPG2	HSPG2:exonic:NM 005529.6	1	50	67	synonymous	NM 00552	c.8883C>T	p.(=)	-3.54					EGF-like dom	rs754291860			1.159E-4 (pos.22169290)		Heparan su	Golgi lume Bone Diseases;Bon Palifermin	G=11, A=11	G=0.5, A=0.5	0.00001	94.4797	22	G=9/2, A=10/1
chr17:3577146	C/T	C	T	SNV	P2RX5,P2RX5-TAX1BP3	P2RX5:utr 3:NM 002561.3. P2RX5-TAX1BP3:intronic nc:NR	1	45.45		intronic	NR 03792	c.1285G>A	p.?	-1.18					ATP P2X rece	rs1063497	0.1036	YES	2.261E-1;0.0 (pos.3577146)			ATP binding,blood coagulation,cation transmembrane tr	C=12, T=10	C=0.5455, T=0.4545	0.00001	81.7626	22	C=5/7, T=5/5
chr1:22149935	C/C	T	C	SNV	HSPG2,LDLRAD2	LDLRAD2:utr 3:NM 001013693.2. HSPG2:exonic:NM 0055	1	100	97	synonymous	NM 00101	c.2046T>C	p.?, p.(=)	-3.57				0.04(COSM37	EGF-like dom	rs897467	0.1719	YES	8.206E-1;0.0 (pos.22149	adenocarcinoi	Heparan su	Golgi lume Bone Diseases;Bon Palifermin Benign	T=0, C=32	T=0.0, C=1.0	0.00001	485.083	32	T=0/0, C=17/1
chr17:35956263	C/C	T	C	SNV	SYNRG	SYNRG:intronic:NM 001163544.2	1	100		intronic	NM 00116	c.240+7A>	p.?	-0.35						rs4795207	1.00E-04		1.0 (pos.35956263)			AP-1 adaptor complex,Golgi apparatus,cytoplasm,endoc	T=0, C=26	T=0.0, C=1.0	0.00001	371.419	26	T=0/0, C=15/1
chr1:22150160	G/T	G	T	SNV	HSPG2,LDLRAD2	LDLRAD2:utr 3:NM 001013693.2. HSPG2:exonic:NM 0055	1	59.09	96	synonymous	NM 00101	c.2271G>T	p.?, p.(=)	3.51				0.92(COSM37	EGF-like dom	rs3736358	0.1755	YES	1.613E-1;0.0 (pos.22150	adenocarcinoi	Heparan su	Golgi lume Bone Diseases;Bon Palifermin Benign	G=9, T=13	G=0.4091, T=0.5909	0.00001	122.568	22	G=9/0, T=9/4
chr1:22148817	G/A	G	A	SNV	HSPG2,LDLRAD2	LDLRAD2:utr 3:NM 001013693.2. HSPG2:utr 3:NM 00552	1	46.43	5	utr_3	NM 00101	c.928G>A,	p.?, p.?	-0.57					EGF-like dom	rs1049675		YES			Heparan su	Golgi lume Bone Diseases;Bon Palifermin Likely benign	G=15, A=13	G=0.5357, A=0.4643	0.00001	117.927	28	G=9/6, A=6/7
chr17:3635603	A/G	A	G	SNV	ITGAE	ITGAE:intronic:NM 002208.4	1	47.62		intronic	NM 00220	c.2754+59	p.?	0.3					FG-GAP repea	rs56163349		YES			Integrin, alp	cell adhes Attention Deficit Disorder with Hyperactivity	A=11, G=10	A=0.5238, G=0.4762	0.00001	81.7863	21	A=5/6, G=8/2
chr13:31725260	G/G	A	G	SNV	HSPH1	HSPH1:exonic:NM 006644.3	1	100	7	synonymous	NM 00664	c.732T>C	p.(=)	1.28				0.72(COSM37	Hsp70 protei	rs11556147	0.4722	YES	1.088E-2 (pos.31725260)	adenocarcinoi	Heat-shock	ATP bindin HIV Infections:Immune System Diseases:Imr	A=0, G=20	A=0.0, G=1.0	0.00001	263.786	20	A=0/0, G=9/11
chr13:31729729	G/G	A	G	SNV	HSPH1	HSPH1:exonic:NM 006644.3	1	100	3	synonymous	NM 00664	c.228T>C	p.(=)	1.48					Hsp70 protei	rs1047086	0.4724	YES	1.086E-2 (pos.31729729)		Heat-shock	ATP bindin HIV Infections;Immune System Diseases;Imr	A=0, G=28	A=0.0, G=1.0	0.00001	408.718	28	A=0/0, G=14/1
chr17:36638948	G/G	A	G	SNV	ARHGAP23	ARHGAP23:intronic:NM 001199417.1	1	100		intronic	NM 00119	c.2907+29	p.?	-0.19					PDZ domain,I	rs2303992		YES	9.221E-1 (pos.36638948)		RHO GTPase	GTPase ac Adnexal Diseases;Cysts;Endocrine System Di	A=0, G=36	A=0.0, G=1.0	0.00001	563.743	36	A=0/0, G=18/1
chr11:20404613	G/G	T	G	SNV	HTATIP2	HTATIP2:exonic:NM 001098520.1	1	100	6	missense	NM 00109	c.693T>G	p.Ser231Arg	-0.59	0.12	110	0.003		NAD(P)H-binc	rs3824886	0.0498	YES	4.978E-1 (pos.20404613)		HIV-1 TAT-in	angiogene Adenocarcinoma;Carcinoma;Carcinoma, Hep	T=0, G=23	T=0.0, G=1.0	0.00001	316.747	23	T=0/0, G=17/6
chr17:3665160	G/G	C	G	SNV	ITGAE	ITGAE:intronic:NM 002208.4	1	100		intronic	NM 00220	c.315+49G	p.?	-0.16					FG-GAP repea	rs220471	0.2879	YES	1.804E-1 (pos.3665160)		Integrin, alp	cell adhes Attention Deficit Disorder with Hyperactivity	C=0, G=56	C=0.0, G=1.0	0.00001	832.1	56	C=0/0, G=36/2
chr17:36734700	C/C	T	C	SNV	SRCIN1	SRCIN1:intronic:NM 025248.2	1	100		intronic	NM 02524	c.324+43A	p.?	-0.23					Actin interac	rs746355030,rs7503	0.1782	YES	0.0;0.0 (pos.36734699),1.451E-3 (pos.36		Src kinase s	actin cytoskeleton,axon,cytoplasm,dendrite,exocytosis	T=0, C=36	T=0.0, C=1.0	0.00001	570.387	36	T=0/0, C=22/1
chr11:20404757	C/C	T	C	SNV	HTATIP2	HTATIP2:utr 3:NM 001098520.1	1	100	6	utr 3	NM 00109	c.837T>C	p.?	-0.88					NAD(P)H-binc	rs3781679	0.0517	YES	5.232E-1 (pos.20404757)		HIV-1 TAT-in	angiogene Adenocarcinoma;Carcinoma;Carcinoma, Hep	T=0, C=23	T=0.0, C=1.0	0.00001	316.741	23	T=0/0, C=16/7
chr17:37070737	G/G	A	G	SNV	LASP1,MIR6779	LASP1:intronic nc:NR 073384.1. MIR6779:upstream:NR 10	1	100		intronic	NR 073384.1, NR 106837.1			-2.18					LIM domain,I	rs525767	0.3459	YES	6.121E-1 (pos.37070737)		LIM and SH3 protein 1	Autistic Disorder:Child Development Disorde	A=0, G=53	A=0.0, G=1.0	0.00001	781.317	53	A=0/0, G=20/3
chr17:37317672	G/G	C	G	SNV	ARL5C	ARL5C:intronic:NM 001143968.1	1	100		intronic	NM 00114	c.256-68G	p.?	0.06					ADP-ribosyla	rs511163		YES				GTP binding,intracellular,small GTPase mediated signal	C=0, G=24	C=0.0, G=1.0	0.00001	334.791	24	C=0/0, G=9/15
chr17:37453294	C/A	C	A	SNV	FBXL20	FBXL20:intronic:NM 032875.2	1	50		intronic	NM 03287	c.398+86G	p.?	0.94					F-box domain	rs632202		YES			F-box and le	behaviora Bile Duct Diseases;Biliary Tract Diseases;Chc	C=15, A=15	C=0.5, A=0.5	0.00001	144.158	30	C=12/3, A=11/
chr17:3769357	G/A	G	A	SNV	CAMKK1	CAMKK1:intronic:NM 172206.1	1	50		intronic	NM 17220	c.1423-52	p.?	-0.62					Kinase-like,P	rs2567875	0.3337	YES	4.092E-1 (pos.3769357)			ATP bindin Nervous System Diseases	G=18, A=18	G=0.5, A=0.5	0.00001	185.885	36	G=5/13, A=13/
chr13:47469940	A/A	G	A	SNV	HTR2A	HTR2A:exonic:NM 000621.4	1	100	2	synonymous	NM 00062	c.102C>T	p.(=)	-1.38				0.01(COSM37	7 transmemb	rs6313,rs755545262	0.4101	YES	0.0 (pos.47469927),5.82	adenocarcinoi	5-hydroxytr	1-(4-iodo-2 Alcohol-Related Dis 3,4-Methylenedioxymet	G=0, A=69	G=0.0, A=1.0	0.00001	1029.48	69	G=0/0, A=26/4
chr17:38140524	C/C	T	C	SNV	PSMD3	PSMD3:intronic:NM 002809.3	1	100		intronic	NM 00280	c.221-23T	p.?	0.48					PCI domain,P	rs3816471	0.072	YES	8.971E-1 (pos.38140524)		Proteasome	Fc-epsilon HIV Infections:Immune System Diseases:Imr	T=0, C=23	T=0.0, C=1.0	0.00001	316.747	23	T=0/0, C=8/15
chr17:38176476	G/G	A	G	SNV	MED24	MED24:intronic:NM 014815.3	1	100		intronic	NM 01481	c.2704+50	p.?	0.04					Mediator cor	rs9899955	0.0697	YES	8.71E-1 (pos.38176476)		Mediator co	RNA polym Blood Protein Disorders:Cardiovascular Dise	A=0, G=59	A=0.0, G=1.0	0.00001	884.849	59	A=0/0, G=35/2
chr13:47470824	T/T	C	T	SNV	HTR2A	HTR2A:utr 5:NM 000621.4	1	100	1	utr 5	NM 00062	c.-783G>A	p.?	4.84					7 transmemb	rs6312	0.0663	YES	9.97E-1 (pos.47470824)		5-hydroxytr	1-(4-iodo-2 Alcohol-Related Dis 3,4-Methylenedioxymet	C=0, T=24	C=0.0, T=1.0	0.00001	334.782	24	C=0/0, T=13/1
chr17:38179667	G/G	A	G	SNV	MED24	MED24:intronic:NM 014815.3	1	100		intronic	NM 01481	c.1986-19	p.?	-0.26					Mediator cor	rs2302778	0.2289	YES	8.833E-1 (pos.38179667)		Mediator co	RNA polym Blood Protein Disorders;Cardiovascular Dise	A=0, G=26	A=0.0, G=1.0	0.00001	371.39	26	A=0/0, G=10/1
chr17:38252660	G/A	G	A	SNV	NR1D1	NR1D1:intronic:NM 021724.4	1	50		intronic	NM 02172	c.604+36C	p.?	0.58					Ligand-bindir	rs883871	0.1312	YES	5.712E-1 (pos.38252660)		Nuclear rec	RNA polym Endocrine System Diseases;Thyroid Diseases	G=22, A=22	G=0.5, A=0.5	0.00001	243.63	44	G=10/12, A=8/
chr17:38253228	C/C	T	C	SNV	NR1D1	NR1D1:intronic:NM 021724.4	1	100		intronic	NM 02172	c.370+90A	p.?	-0.65					Ligand-bindir	rs2102928		YES			Nuclear rec	RNA polym Endocrine System Diseases;Thyroid Diseases	T=0, C=50	T=0.0, C=1.0	0.00001	740.59	50	T=0/0, C=25/2
chr17:38556770	C/C	T	C	SNV	TOP2A	TOP2A:intronic:NM 001067.3	1	100		intronic	NM 00106	c.2799+11	p.?	-0.15					C-terminal as	rs471692	0.1824	YES	7.315E-1 (pos.38556770)		Topoisomer	ATP bindin Adenocarcinoma;A Amonafide,Amrubicin,Ar	T=0, C=62	T=0.0, C=1.0	0.00001	940.316	62	T=0/0, C=31/3
chr3:183754294	C/C	G	C	SNV	HTR3D	HTR3D:splicesite 5:NM 001163646.1	1	100	4	splicesite	NM 00116	c.511+1G>	p.?	0.51				0.02(COSM14	Neurotransm	rs6443930	0.4491	YES	4.962E-1;0.0 (pos.18375	adenocarcinoi	5-hydroxytr	cation transmembrane transp Ergoloid mesylate,Ethan	G=0, C=36	G=0.0, C=1.0	0.00001	563.638	36	G=0/0, C=24/1
chr4:3219613	C/C	A	C	SNV	HTT	HTT:exonic:NM 002111.8	1	100	52	synonymous	NM 00211	c.7176A>C	p.(=)	-2.36					Huntingtin pr	rs2857790	5.00E-04		1.0 (pos.3219613)		Huntingtin,	Golgi appa Basal Ganglia Diseases;Brain Diseases;Centr	A=0, C=81	A=0.0, C=1.0	0.00001	1309.48	81	A=0/0, C=52/2
chr21:33368188	T/C	T	C	SNV	HUNK	HUNK:exonic:NM 014586.1	1	45.45	10	synonymous	NM 01458	c.1413T>C	p.(=)	0.17				0.03(COSM44	Kinase-like,L	rs2070371	0.2271	YES	6.781E-1 (pos.33368188)	acute myeloic	Hormonally	ATP bindin Abnormalities, Multiple:Chromosome Disord	T=24, C=20	T=0.5455, C=0.4545	0.00001	211.733	44	T=16/8, C=14/
chr7:48004962	T/T	C	T	SNV	HUS1	HUS1:exonic:NM 004507.3	1	100	8	synonymous	NM 00450	c.834G>A	p.(=)	-3.15				0.02(COSM37	Hus1-like pro	rs1056663	0.4902	YES	5.499E-1 (pos.48004962)	adenocarcinoi	Hydroxyure	DNA dama Nervous System Diseases:Neuromuscular Di	C=0, T=54	C=0.0, T=1.0	0.00001	798.148	54	C=0/0, T=35/1

chr3:50357869	C/C	A	C	SNV	HYAL2	HYAL2:exonic:NM 003773.4	1	100	2	missense	NM 00377	c.52T>G	p.Ser18Ala	0.17	1	99	0	0(COSM1162	Hyaluronidas	rs709210	0.3555	YES	7.665E-1;0.0 (pos.50357	adenocarcino	Hyaluronog	Golgi membrane,RNA polymerase II transcription coacti				A=0, C=40	A=0.0, C=1.0	0.00001	637.902	40	A=0/0, C=28/1
chr16:70908736	A/G	A	G	SNV	HYDIN	HYDIN:exonic:NM 001270974.2	1	46.88	63	synonymous	NM 00127	c.10644T>	p.(=)	0.68					Abnormal spi	rs1354550		YES			Hydin, mou	axonemal	Brain Diseases:Bronchial Diseases:Bronchie			A=17, G=15	A=0.5313, G=0.4688	0.00001	143.733	32	A=9/8, G=11/4
chr11:118919206	C/C	T	C	SNV	HYOU1	HYOU1:exonic:NM 001130991.2	1	100	19	synonymous	NM 00113	c.2235A>G	p.(=)	-5.01					Hsp70 protei	rs568922	0.1454	YES	9.531E-1 (pos.118919206)		Hypoxia-up	ATP bindin	Ataxia:Brain Diseases:Central Nervous Syste			T=0, C=36	T=0.0, C=1.0	0.00001	563.678	36	T=0/0, C=10/2
chr4:88732746	G/G	A	G	SNV	IBSP	IBSP:exonic:NM 004967.3	1	100	7	missense	NM 00496	c.638A>G	p.Asp213Gly	-0.54	1	94	0		Bone sialopro	rs13144371	0.2337	YES	5.868E-1 (pos.88732746)		Integrin-bin	bone mine	Bone Diseases:Bone Resorption:Musculoske			A=0, G=63	A=0.0, G=1.0	0.00001	958.917	63	A=0/0, G=35/2
chr4:88732763	G/G	A	G	SNV	IBSP	IBSP:exonic:NM 004967.3	1	100	7	missense	NM 00496	c.655A>G	p.Arg219Gly	0.41	0.51	125	0	0(COSM3760	Bone sialopro	rs17013181	0.2198	YES	2.117E-1 (pos.88732763)	adenocarcino	Integrin-bin	bone mine	Bone Diseases:Bone Resorption:Musculoske			A=0, G=61	A=0.0, G=1.0	0.00001	920.793	61	A=0/0, G=33/2
chr1:23885498	C/C	T	C	SNV	ID3	ID3:exonic:NM 002167.4	1	100	2	missense	NM 00216	c.313A>G	p.Thr105Ala	-0.62	0.52	58	0		Helix-loop-he	rs11574	0.2385	YES	9.754E-1 (pos.23885498)		Inhibitor of	DNA bindi	Adenocarcinoma;Burkitt Lymphoma;Carcino			T=0, C=34	T=0.0, C=1.0	0.00001	528.519	34	T=0/0, C=27/7
chr8:39872866	C/C	A	C	SNV	IDO2	IDO2:exonic:NM 194294.2	1	100	11	synonymous	NM 19429	c.1008A>C	p.(=)	0.11					Indoleamine	rs2981161	8.00E-04		1.0 (pos.39872866)		Indoleamin	cytoplasm,cytosol,heme binding,immune system proces				A=0, C=31	A=0.0, C=1.0	0.00001	465.813	31	A=0/0, C=20/1
chr17:39594185	C/C	T	C	SNV	KRT38	KRT38:intronic:NM 006771.3	1	100		intronic	NM 00677	c.1241+16	p.?	0.07					Intermediate	rs4796789,rs745806840		YES			Keratin 38	cornification,cytosol,extracellular exosome,intermedia				T=0, C=30	T=0.0, C=1.0	0.00001	446.614	30	T=0/0, C=19/1
chr17:39594204	A/A	T	A	SNV	KRT38	KRT38:intronic:NM 006771.3	1	100		intronic	NM 00677	c.1241+14	p.?	-2.79					Intermediate	rs2071613		YES			Keratin 38	cornification,cytosol,extracellular exosome,intermedia				T=0, A=30	T=0.0, A=1.0	0.00001	446.639	30	T=0/0, A=19/1
chr17:39594934	A/A	AC	A	INDEL	KRT38	KRT38:intronic:NM 006771.3	1	100		intronic	NM 00677	c.894+15G	p.?	1.52,-0.89					Intermediate	rs372006715,rs398030861,rs55		YES	8.287E-1;1.735E-2;0.0;0.0 (pos.3959493		Keratin 38	cornification,cytosol,extracellular exosome,intermedia				AC=0, A=20	AC=0.0, A=1.0	0.00001	165.573	20	AC=0/0, A=7/1
chr17:39596538	T/T	C	T	SNV	KRT38	KRT38:intronic:NM 006771.3	1	100		intronic	NM 00677	c.493-43G	p.?	-2.44					Intermediate	rs2071609	0.4015	YES	5.277E-1 (pos.39596538)		Keratin 38	cornification,cytosol,extracellular exosome,intermedia				C=0, T=52	C=0.0, T=1.0	0.00001	767.016	52	C=0/0, T=32/2
chr17:39596611	C/C	T	C	SNV	KRT38	KRT38:intronic:NM 006771.3	1	100		intronic	NM 00677	c.492+71A	p.?	-0.27					Intermediate	rs1350046		YES			Keratin 38	cornification,cytosol,extracellular exosome,intermedia				T=0, C=52	T=0.0, C=1.0	0.00001	766.942	52	T=0/0, C=32/2
chr17:39636044	A/A	G	A	SNV	KRT35	KRT35:intronic:NM 002280.4	1	100		intronic	NM 00228	c.472-11C	p.?	0.59					Intermediate	rs2011243	0.0893	YES	9.542E-1 (pos.39636044)		Keratin 35	anatomical structure morphogenesis,cornification,cytos				G=0, A=61	G=0.0, A=1.0	0.00001	921.845	61	G=0/0, A=36/2
chr17:39645003	C/G	C	G	SNV	KRT36	KRT36:intronic:NM 003771.4	1	53.33		intronic	NM 00377	c.460-27G	p.?	0.34					Intermediate	rs60125824	0.0492	YES	2.195E-1 (pos.39645003)		Keratin 36	biological_process,cornification,cytosol,extracellular ex				C=21, G=24	C=0.4667, G=0.5333	0.00001	276.154	45	C=8/13, G=5/1
chr4:996165	G/A	G	A	SNV	IDUA	IDUA:exonic:NM 000203.4	1	58.33	8	missense	NM 00020	c.1081G>A	p.Ala361Thr	0.9	1	58	0.004	0.52(COSM41	Glycosyl hydr	rs6831280	0.1583	YES	2.383E-1 (pos.996165)	neoplasm	Iduronidase	L-iduronid	Carbohydrate Metabolism, Inb		Benign	G=15, A=21	G=0.4167, A=0.5833	0.00001	234.326	36	G=7/8, A=5/16
chr17:39674540	A/G	A	G	SNV	KRT15	KRT15:intronic:NM 002275.3	1	45.83		intronic	NM 00227	c.498+42T	p.?	-0.53					Intermediate	rs3744784	0.3624	YES	8.179E-2 (pos.39674540)		Keratin 15	cornificati	Adenocarcinoma:Carcinogenesis:Carcinoma			A=13, G=11	A=0.5417, G=0.4583	0.00001	93.4399	24	A=7/6, G=5/6
chr17:39680580	C/C	A	C	SNV	KRT19	KRT19:intronic:NM 002276.4	1	100		intronic	NM 00227	c.822+51T	p.?	-2.51						rs1869719	0	YES	1.0 (pos.39680580)		Keratin 19	Notch sign	Asthma:Basal Ganglia Diseases:Brain Diseas			A=0, C=22	A=0.0, C=1.0	0.00001	298.661	22	A=0/0, C=9/13
chr17:39680796	A/A	T	A	SNV	KRT19	KRT19:intronic:NM 002276.4	1	100		intronic	NM 00227	c.661-4A>	p.?	-1.27						rs1869720	0	YES	1.0 (pos.39680796)		Keratin 19	Notch sign	Asthma:Basal Ganglia Diseases:Brain Diseas			T=0, A=26	T=0.0, A=1.0	0.00001	369.538	26	T=0/0, A=9/17
chr17:39766504	G/G	A	G	SNV	KRT16	KRT16:intronic:NM 005557.3	1	100		intronic	NM 00555	c.1281-16	p.?	-1.58					Intermediate	rs386797103,rs7406	0.0458	YES	1.0 (pos.39766504)		Keratin 16	aging,cell	Congenital Abnormalities;Congenital, Heredi			A=0, G=68	A=0.0, G=1.0	0.00001	1050.51	68	A=0/0, G=16/5
chr4:995305	T/C	T	C	SNV	IDUA	IDUA:exonic:NM 000203.4	1	46.51	5	synonymous	NM 00020	c.543T>C	p.(=)	-1.16				0.13(COSM40	Glycosyl hydr	rs6815946	0.1612	YES	2.12E-1 (pos.995305)	acute_myeloic	Iduronidase	L-iduronid	Carbohydrate Metabolism, Inb		Benign	T=23, C=20	T=0.5349, C=0.4651	0.00001	212.324	43	T=15/8, C=14/
chr1:181058313	G/A	G	A	SNV	IER5	IER5:exonic:NM 016545.4	1	50	1	missense	NM 01654	c.275G>A	p.Arg92His	0.17		29		0.07(COSM37	Immediate e	rs3747955	0.3098	YES	6.774E-1 (pos.181058313	adenocarcino	Immediate-	cellular response to heat,cytoplasm,identical protein bi				G=12, A=12	G=0.5, A=0.5	0.00001	106.707	24	G=7/5, A=5/7
chr17:41165493	G/G	A	G	SNV	IFI35	IFI35:exonic:NM 005533.4	1	100	5	missense	NM 00553	c.382A>G	p.Met128Val	1.2	0.5	21	0		Interferon-in	rs588703	2.00E-04		1.0 (pos.41165493)		Interferon-i	cytosol,nu	Abortion, Spontaneous:Female Urogenital Di			A=0, G=45	A=0.0, G=1.0	0.00001	747.391	45	A=0/0, G=20/2
chr17:41165878	T/T	C	T	SNV	IFI35,VAT1	VAT1:downstream:NM 006373.3, IFI35:exonic:NM 005533	1	100	6	synonymous	NM 00553	c.654C>T,	p.(=), p.?	-1.27					Interferon-in	rs13229	2.00E-04		1.0;0.0 (pos.41165878)		Interferon-i	azurophil	Abortion, Spontaneous:Female Urogenital Di			C=0, T=23	C=0.0, T=1.0	0.00001	312.754	23	C=0/0, T=15/8
chr10:91066460	A/A	T	A	SNV	IFIT2	IFIT2:exonic:NM 001547.4	1	100	2	synonymous	NM 00154	c.747T>A	p.(=)	-0.31					Tetratricoper	rs954439	0.4853	YES	8.762E-1 (pos.91066460)		Interferon-i	RNA bindir	Influenza, Human;Respiratory Tract Diseases			T=0, A=37	T=0.0, A=1.0	0.00001	583.706	37	T=0/0, A=26/1
chr10:91066769	A/A	C	A	SNV	IFIT2	IFIT2:exonic:NM 001547.4	1	100	2	missense	NM 00154	c.1056C>A	p.Asp352Glu	0.64	1	45	0	0.62(COSM54	Tetratricoper	rs1727	0.2517	YES	9.898E-1;0.0 (pos.91066	adenocarcino	Interferon-i	RNA bindir	Influenza, Human;Respiratory Tract Diseases			C=0, A=33	C=0.0, A=1.0	0.00001	504.598	33	C=0/0, A=20/1
chr10:91178000	T/T	C	T	SNV	IFIT5	IFIT5:exonic:NM 012420.2	1	100	2	synonymous	NM 01242	c.1044C>T	p.(=)	-0.45				0.03(COSM54	Tetratricoper	rs304448	0.4943	YES	8.656E-1 (pos.91178000)	acute_myeloic	Interferon-i	IkappaB ki	Influenza, Human;Respiratory Tract Diseases			C=0, T=32	C=0.0, T=1.0	0.00001	485.137	32	C=0/0, T=19/1
chr6:137519588	A/C	A	C	SNV	IFNGR1	IFNGR1:exonic:NM 000416.2	1	57.89	7	synonymous	NM 00041	c.1050T>G	p.(=)	-0.74				0.04(COSM37	Interferon ga	rs11914	0.1635	YES	1.201E-1;0.0 (pos.137519	adenocarcino	Immune int	cytokine b	Autoimmune Disea	Interferon	Benign/Likely	A=16, C=22	A=0.4211, C=0.5789	0.00001	249.053	38	A=6/10, C=12/
chr9:27524731	G/G	A	G	SNV	IFNK,MOB3B	IFNK:exonic:NM 020124.2, MOB3B:intronic:NM 024761.4	1	100	1	missense	NM 02476	c.-198+48	p.?, p.Lys133Glu	-0.06		56			Interferon al	rs700785	0.0091	YES	9.976E-1;0.0 (pos.27524731)		Interferon,	B cell differentiation,B cell proliferation,T cell activation				A=0, G=61	A=0.0, G=1.0	0.00001	921.763	61	A=0/0, G=35/2
chr17:40025264	T/T	G	T	SNV	ACLY	ACLY:intronic:NM 001096.2	1	100		intronic	NM 00109	c.3134+32	p.?	-0.56					ATP citrate ly	rs782183056,rs9912	0.23	YES	0.0 (pos.40025263),8.235E-1 (pos.40025		ATP citrate	ATP bindin	Adenocarcinoma:Carcinoma;Carcinoma, Hep			G=0, T=62	G=0.0, T=1.0	0.00001	940.287	62	G=0/0, T=32/3
chr17:40025285	G/G	A	G	SNV	ACLY	ACLY:intronic:NM 001096.2	1	100		intronic	NM 00109	c.3134+11	p.?	-0.58					ATP citrate ly	rs9914829	0.081	YES	9.999E-1 (pos.40025285)		ATP citrate	ATP bindin	Adenocarcinoma:Carcinoma;Carcinoma, Hep			A=0, G=64	A=0.0, G=1.0	0.00001	977.635	64	A=0/0, G=31/3
chr17:40025453	C/C	T	C	SNV	ACLY	ACLY:intronic:NM 001096.2	1	100		intronic	NM 00109	c.3052-75	p.?	0.31					ATP citrate ly	rs3744779		YES			ATP citrate	ATP bindin	Adenocarcinoma:Carcinoma;Carcinoma, Hep			T=0, C=22	T=0.0, C=1.0	0.00001	298.892	22	T=0/0, C=15/7
chr17:40028450	T/T	A	T	SNV	ACLY	ACLY:intronic:NM 001096.2	1	100		intronic	NM 00109	c.2643-15	p.?	0.86					ATP citrate ly	rs8071627	0.2299	YES	8.23E-1 (pos.40028450)		ATP citrate	ATP bindin	Adenocarcinoma:Carcinoma;Carcinoma, Hep			A=0, T=36	A=0.0, T=1.0	0.00001	563.744	36	A=0/0, T=17/1
chr17:4007875	T/T	C	T	SNV	ZZEF1	ZZEF1:intronic:NM 015113.3	1	100		intronic	NM 01511	c.1573+52	p.?	-1.05					Anaphase-pro	rs9907500	3.00E-04	YES				calcium ion binding,zinc ion binding				C=0, T=104	C=0.0, T=1.0	0.00001	1626.75	104	C=0/0, T=41/6
chr17:40146970	A/A	G	A	SNV	DNAJC7	DNAJC7:intronic:NM 003315.3	1	100		intronic	NM 00331	c.406-8C>	p.?	-0.59					DnaJ domain	rs6503679	0.1477	YES	8.636E-1 (pos.40146970)			ATPase ac	HIV Infections:Immune System Diseases:Imm			G=0, A=35	G=0.0, A=1.0	0.00001	543.901	35	G=0/0, A=22/1
chr17:40642452	C/C	T	C	SNV	ATP6V0A1	ATP6V0A1:intronic:NM 001130021.1	1	100		intronic	NM 00113	c.1024-53	p.?	-0.44					V-type ATPas	rs9899168		YES			ATPase, H+	ATP hydrolysis coupled proton transport,ATP synthesis c				T=0, C=22	T=0.0, C=1.0	0.00001	298.892	22	T=0/0, C=7/15
chr17:4074261	C/C	A	C	SNV	ANKFY1	ANKFY1:intronic:NM 001257999.2	1	100		intronic	NM 00125	c.3266-10	p.?	-0.65					Ankyrin repea	rs897030					Ankyrin rep	Golgi to lys	Female Urogenital Diseases:Female Urogeni			A=0, C=39	A=0.0, C=1.0	0.00001	623.98	39	A=0/0, C=15/2
chr17:40842084	A/A	G	A	SNV	CNTNAP1	CNTNAP1:intronic:NM 003632.2	1	100		intronic	NM 00363	c.1736-22	p.?	-0.41					F5/8 type C d	rs3760386	0.4916	YES	4.611E-1 (pos.40842084)		Contactin-a	SH3 domain binding,SH3/SH2 adaptor activity,cell adhes				G=0, A=86	G=0.0, A=1.0	0.00001	1410.92	86	G=0/0, A=52/3
chr15:65667724	G/G	A	G	SNV	IGDCC3	IGDCC3:exonic:NM 004884.3	1	100	2	synonymous	NM 00488	c.120T>C	p.(=)	-5.85				0.02(COSM41	Fibronectin t	rs666623	0.1054	YES	8.387E-1 (pos.65667724)	neoplasm		integral component of membrane,integral component o				A=0, G=64	A=0.0, G=1.0	0.00001	968.638	64	A=0/0, G=25/3
chr6:160505199	G/G	C	G	SNV	IGF2R	IGF2R:exonic:NM 000876.3	1	100	40	synonymous	NM 00087	c.6051C>G	p.(=)	-0.94					Autophagy-re	rs614754	0.0131		1.0 (pos.160505199)		Insulin-like	G-protein	Adenocarcinoma:A	Cerliponase alfa,Mecase		C=0, G=47	C=0.0, G=1.0	0.00001	789.203	47	C=0/0, G=17/3
chr19:46664400	G/G	A	G	SNV	IGFL2	IGFL2:utr 3:NM 001135113.1	1	100	4	utr 3	NM 00113	c.415A>G	p.?	-0.49					Insulin growt	rs10408162	0.3492	YES			IGF-like fam	biological_process,extracellular space,receptor binding				A=0, G=222	A=0.0, G=1.0	0.00001	3666.41	222	A=0/0, G=124/
chr1:201187764	C/C	T	C	SNV	IGFN1	IGFN1:exonic:NM 001164586.1	1	100	18	synonymous	NM 00116	c.9876T>C	p.(=)	-2.24				0.04(COSM37	Fibronectin t	rs10920144	0.1236	YES	9.993E-1 (pos.201187764	adenocarcino	Immunoglo	Z disc,biological_process,nucleus,protein binding				T=0, C=53	T=0.0, C=1.0	0.00001	770.887	53	T=0/0, C=30/2
chr1:201176018	A/G	A	G	SNV	IGFN1	IGFN1:exonic:NM 001164586.1	1	50	12	missense	NM 00116	c.1997A>G	p.Asp666Gly	-0.01		94			Fibronectin t	rs10753895	0.2866	YES	6.988E-1 (pos.201176018)		Immunoglo	Z disc,biological_process,nucleus,protein binding				A=11, G=11	A=0.5, G=0.5	0.00001	94.6064	22	A=6/5, G=4/7
chr3:151165678	C/C	T	C	SNV	IGSF10	IGSF10:exonic:NM 178822.4	1	100	4	synonymous	NM 17882	c.2091A>G	p.(=)	-1.2				0.04(COSM37	Immunoglob	rs9871952	0.2403	YES	7.737E-1 (pos.151165678	adenocarcino	Immunoglo	extracellular region,ossificatic		Pyridoxal Phosphate		T=0, C=52	T=0.0, C=1.0	0.00001	767.016	52	T=0/0, C=33/1
chr3:151171439	C/C	A	C	SNV	IGSF10	IGSF10:exonic:NM 178822.4	1	100	3	missense	NM 17882	c.448T>G	p.Tyr150Asp	2.41	0.09	160	0.938	0.89(COSM37	Immunoglob	rs7619322	0.2719	YES	7.407E-1 (pos.151171439	acute_myeloic	Immunoglo	extracellular region,ossificatic		Pyridoxal Phosphate		A=0, C=145	A=0.0, C=1.0	0.00001	2078.06	145	A=0/0, C=57/8
chr3:151171542	G/G	A	G	SNV	IGSF10	IGSF10:exonic:NM 178822.4	1	100	3	synonymous	NM 17882	c.345T>C	p.(=)	1.25				0.16(COSM37	Immunoglob	rs7621591	0.2296	YES	7.787E-1 (pos.151171542	adenocarcino	Immunoglo	extracellular region,ossificatic		Pyridoxal Phosphate		A=0, G=49	A=0.0, G=1.0	0.00001	831.63	49	A=0/0, G=16/3
chr3:151154666	C/C	T	C	SNV	IGSF10,MED12L	IGSF10:exonic:NM 178822.4, MED12L:downstream:NM 05	1	100	6	synonymous	NM 05300	c.*4074T>	p.?, p.(=)	-1.48					Immunoglob	rs2172250	0.1526	YES	8.995E-1 (pos.151154666)		Immunoglo	RNA polymerase II transcriptio		Pyridoxal Phosphate		T=0, C=51	T=0.0, C=1.0	0.00001	754.584	51	T=0/0, C=18/3
chr17:42083840	C/C	T	C	SNV	NAGS	NAGS:intronic:NM 153006.2	1	100		intronic	NM 15300	c.916-57T	p.?	-0.86					NAT, N-acetyl	rs228770		YES			N-acetylglu	acetyl-CoA	Amino Acid Metabo	Ammonia N-13,Glutamic		T=0, C=24	T=0.0, C=1.0	0.00001	334.792	24	T=0/0, C=7/17
chr1:18704862	G/G	A	G	SNV	IGSF21	IGSF21:utr 3:NM 032880.4	1	100	10	utr 3	NM 03288	c.1446A>G	p.?	0.09					Immunoglob	rs1043459,rs386629	0.4201	YES	6.415E-1 (pos.18704862)			extracellu	Female Urogenital Diseases:Female Urogeni			A=0, G=46	A=0.0, G=1.0	0.00001	768.137	46	A=0/0, G=27/1
chr21:41137507	C/C	G	C	SNV	IGSF5	IGSF5:exonic:NM 001080444.1	1	100	3	missense	NM 00108	c.146G>C	p.Arg49Thr	2.83	1	71	0	0.01(COSM41	Immunoglob	rs2205204	0.261	YES	6.899E-1 (pos.41137507)	neoplasm		apical pla	Abnormalities, Multiple:Chromosome Disord			G=0, C=21	G=0.0, C=1.0	0.00001	281.222	21	G=0/0, C=8/13
chr21:41165462	A/A	C	A	SNV	IGSF5	IGSF5:exonic:NM 001080444.1	1	100	8	missense	NM 00108	c.1050C>A	p.Asp350Glu	-0.7	1	45	0	0.01(COSM14	Immunoglob	rs2837225	0.2909	YES	4.597E-1 (pos.41165462)	adenocarcinoma		apical pla	Abnormalities, Multiple:Chromosome Disord			C=0, A=72	C=0.0, A=1.0	0.00001	1127.73	72	C=0/0, A=47/2
chr17:42290015	G/A	G	A	SNV	UBTF	UBTF:intronic:NM 014233.3	1	98.72		intronic	NM 01423	c.660+172	p.?	-1.3					HMG (high mo	rs2855818,rs965802607		YES			Upstream b	DNA binding,RNA binding,RNA polymerase I CORE elemer				G=1, A=77	G=0.0128, A=0.9872	0.00001	1213.94	78	G=0/1, A=42/3
chr1:159897126	C/T	C	T	SNV	IGSF9	IGSF9:utr 3:NM 001135050.1	1	96.67	21	utr 3	NM 00113	c.3549G>A	p.?	-2.86					Fibronectin t	rs13052	0.4679	YES	7.255E-1 (pos.159897126)		Immunoglo	axon,cell junction,cell-cell adhesion mediator activity,d				C=1, T=29	C=0.0333, T=0.9667	0.00001	418.875	30	C=0/1, T=20/9
chr2:219920037	G/G	A	G	SNV	IHH	IHH:exonic:NM 002181.3	1	100	3	synonymous	NM 00218	c.1128T>C	p.(=)	0.4				0.19(COSM44	Hedgehog am	rs394452	0.2033	YES	9.134E-1 (pos.219920037	neoplasm	Indian hede	bone reso	Adnexal Diseases:Bone Diseases:Bone Disea			A=0, G=55	A=0.0, G=1.0	0.00001	818.91	55	A=0/0, G=33/2
chr17:42824648	C/C	T	C	SNV	DBF4B	DBF4B:intronic:NM 145663.2	1	100		intronic	NM 14566	c.939+89T	p.?	-1.12					DBF zinc finge	rs9893320		YES			DBF4, S. cer	activation of protein kinase activity,aggresome,cell cycle				T=0, C=40	T=0.0, C=1.0	0.00001	644.286	40	T=0/0, C=16/2
chr17:42934372	G/G	A	G	SNV	EFTUD2	EFTUD2:intronic:NM 004247.3	1	100		intronic	NM 00424	c.2045+71	p.?	-0.26					116 kDa U5 s	rs6503405		YES			Elongation f	Cajal body	Bone Diseases;Bone Diseases, Development			A=0, G=33	A=0.0, G=1.0	0.00001	508.976	33	A=0/0, G=8/25
chr17:42961009	T/T	C	T	SNV	EFTUD2	EFTUD2:intronic:NM 004247.3	1	100		intronic	NM 00424	c.426+8G>	p.?	-0.21				0(COSM4000	116 kDa U5 s	rs2289677	0.4017	YES	4.919E-1 (pos.42961009)	acute_myeloic	Elongation f	Cajal body	Bone Diseases:Bone Diseases,		Benign/Likely	C=0, T=34	C=0.0, T=1.0	0.00001	524.186	34	C=0/0, T=14/2
chr17:42990810	G/A	G	A	SNV	GFAP	GFAP:intronic:NM 002055.4	1	47.37		intronic	NM 00205	c.619-12C	p.?	0.28					Intermediate	rs3744468	0.122	YES	1.005E-1 (pos.42990810)		Glial fibrilla	Bergmann	Alexander Disease:Amyotroph		Benign	G=20, A=18	G=0.5263, A=0.4737	0.00001	183.596	38	G=6/14, A=3/1
chr5:140027216	C/C	T	C	SNV	IK,MIR3655,NDUFA2	IK:upstream:NM 006083.3, MIR3655:upstream:NR 037428	1	100	1	utr 5	NM 00248	c.-48A>G,	p.?, p.?	-0.09					Mitochondria	rs778593	0.4112	YES	5.11E-1 (pos.140027216)		IK cytokine,	NADH deh	Brain Diseases;Brai	NADH	Likely benign	T=0, C=48	T=0.0, C=1.0	0.00001	814.808	48	T=0/0, C=20/2
chr5:140027304	C/C	T	C	SNV	IK,MIR3655,NDUFA2	IK:upstream:NM 006083.3, MIR3655:upstream:NR 037428	1	100	1	utr 5	NM 00248	c.-136A>G,	p.?, p.?	-0.67					Mitochondria	rs778594		YES	5.409E-1 (pos.140027304)		IK cytokine,	NADH deh	Brain Diseases;Brai	NADH	Likely benign	T=0, C=47	T=0.0, C=1.0	0.00001	789.318	47	T=0/0, C=18/2
chr17:43195981	T/C	T	C	SNV	PLCD3	PLCD3:intronic:NM 133373.4	1	50		intronic	NM 13337	c.914-122	p.?	-5.76					C2 domain,EF	rs3744760		YES				angiogenesis,cleavage furrow,cytosol,inositol phosphat				T=11, C=11	T=0.5, C=0.5	0.00001	94.6017	22	T=7/4, C=3/8
chr1:206651107	A/A	G	A	SNV	IKBKE	IKBKE:exonic:NM 014002.3	1	100	8	synonymous	NM 01400	c.717G>A	p.(=)	-5.16				0.01(COSM42	Protein kinas	rs2297545	0.2096	YES	6.8E-1 (pos.206651107)	haemangiobla	I-kappa-B ki	ATP binding,I-kappaB phosphorylation,IkappaB kinase ac				G=0, A=38	G=0.0, A=1.0	0.00001	603.228	38	G=0/0, A=16/2
chr17:4350182	T/C	T	C	SNV	SPNS3	SPNS3:intronic:NM 182538.4	1	50		intronic	NM 18253	c.555-9T>C	p.?	0.04				0(COSM5425	Major Facilita	rs2047233	0.0986	YES	2.176E-1 (pos.4350182)	acute_myeloic	Spinster, Dr	integral component of membrane,lipid transport,locom				T=18, C=18	T=0.5, C=0.5	0.00001	185.845	36	T=6/12, C=3/1
chr1:206647742	G/G	A	G	SNV	IKBKE,MIR6769B	MIR6769B:upstream:NR 106919.1, IKBKE:exonic:NM 0140	1	100	4	synonymous	NM 01400	c.156A>G	p.(=)	0.96				0.16(COSM41	Protein kinas	rs1539242	0.0024		1.0 (pos.206647742)	neoplasm	I-kappa-B ki	ATP binding,I-kappaB phosphorylation,IkappaB kinase ac				A=0, G=22	A=0.0, G=1.0	0.00001	298.891	22	A=0/0, G=11/1
chr17:44077044	C/C	T	C	SNV	MAPT,STH	STH:utr 3:NM 001007532.2, MAPT:intronic:NM 00112306	1	100		intronic	NM 00112	c.1827+30	p.?, p.?	-5.11					Tau and MAP	rs71375329	0.0014	YES	9.981E-1 (pos.44077044)		Microtubul	DNA bindi	Alzheimer Disease:	Docetaxel,Paclitaxel		T=0, C=21	T=0.0, C=1.0	0.00001	281.23	21	T=0/0, C=7/14
chr17:4436005	C/C	T	C	SNV	SPNS2	SPNS2:intronic:NM 001124758.2	1	100		intronic	NM 00112	c.935+26T	p.?	-1.97					Major Facilita	rs764028868,rs9892	0		0.0;5.81E-4 (pos.4435990),9.999E-1 (pos		Spinster, Dr	B cell homeostasis,T cell homeostasis,bone developmer				T=0, C=55	T=0.0, C=1.0	0.00001	817.408	55	T=0/0, C=12/4
chr1:206647787	C/C	T	C	SNV	IKBKE,MIR6769B	MIR6769B:upstream:NR 106919.1, IKBKE:exonic:NM 0140	1	100	4	synonymous	NM 01400	c.201T>C	p.(=)	-1.32					Protein kinas	rs1539243	0.1664	YES	9.764E-1 (pos.206647787)		I-kappa-B ki	ATP binding,I-kappaB phosphorylation,IkappaB kinase ac				T=0, C=22	T=0.0, C=1.0	0.00001	298.888	22	T=0/0, C=11/1
chr2:214012508	C/T	C	T	SNV	IKZF2	IKZF2:exonic:NM 016260.2	1	56	3	synonymous	NM 01626	c.63G>A	p.(=)	0.27				0.49(COSM37	C2H2-type zir	rs6709554	0.3748	YES	9.244E-4 (pos.214012508	adenocarcino	Ikaros famil	DNA bindi	Hemic and Lymphatic Diseases:Immune Syst			C=11, T=14	C=0.44, T=0.56	0.00001	134.35	25	C=8/3, T=7/7
chr1:67852335	G/A	G	A	SNV	IL12RB2	IL12RB2:exonic:NM 001559.2	1	50	14	synonymous	NM 00155	c.1929G>A	p.(=)	-0.62				0.02(COSM37	Fibronectin t	rs2228420	0.4293	YES	3.391E-1 (pos.67852335)	acute_myeloic	Interleukin	cell surfac	Behcet Syndrome:Bile Duct Diseases:Biliary T			G=10, A=10	G=0.5, A=0.5	0.00001	82.8805	20	G=3/7, A=1/9
chr17:45385012	T/T	C	T	SNV	ITGB3,THCAT158	THCAT158:intronic_nc:NR 110880.1, ITGB3:intronic:NM 00	1	100		intronic	NM 00021	c.2301+9C	p.?	-0.25					EGF-like dom	rs3809863	0.4758	YES	2.511E-1 (pos.45385012)		Integrin, be	C-X3-C che	Acute Coronary Syn	Abciximab	Benign	C=0, T=24	C=0.0, T=1.0	0.00001	334.792	24	C=0/0, T=19/5
chr17:45425144	T/T	C	T	SNV	EFCAB13	EFCAB13:intronic:NM 152347.4	1	100		intronic	NM 15234	c.518-30C	p.?	-1.59						rs3851808	0.3784	YES	6.054E-1 (pos.45425144)							C=0, T=48	C=0.0, T=1.0	0.00001	810.418	48	C=0/0, T=38/1
chr5:131995964	G/G	A	G	SNV	IL13	IL13:exonic:NM 002188.2	1	100	4	missense	NM 00218	c.431A>G	p.Gln144Arg	-2.74	1	43	0		Interleukin-1	rs20541	0.195	YES	6.723E-1 (pos.131995964)		Interleukin-	cell-cell si	Adenocarcinoma:A	Cintredeki	risk factor	A=0, G=47	A=0.0, G=1.0	0.00001	789.328	47	A=0/0, G=22/2
chr15:81582868	A/A	G	A	SNV	IL16	IL16:exonic:NM 172217.4	1	100	11	synonymous	NM 17221	c.1407G>A	p.(=)	1				0.64(COSM41	PDZ domain (	rs8031107	0.4456	YES	5.373E-1 (pos.81582868)	neoplasm	Interleukin	CD4 recep	Abortion, Spontaneous:Female Urogenital Di			G=0, A=76	G=0.0, A=1.0	0.00001	1209.6	76	G=0/0, A=32/4
chr17:45438952	T/T	C	T	SNV	EFCAB13	EFCAB13:intronic:NM 152347.4	1	100		intronic	NM 15234	c.805+65C	p.?	0.1						rs6504872		YES								C=0, T=42	C=0.0, T=1.0	0.00001	685.212	42	C=0/0, T=23/1
chr17:4544983	GAAG/GAAG	G	GAAG	INDEL	ALOX15	ALOX15:upstream:NM 001140.4	3	100		intronic	NM 00114	c.-37C>CT	p.?	0.51						rs4053416			0.0 (pos.4544983)		Arachidona	apoptotic	Arterial Occlusive D	(2E)-3-(2-OCT-1-YN-1-YLP		G=0, GAAG	G=0.0, GAAG=1.0	0.00001	445.621	30	G=0/0, GAAG=
chr15:81600995	G/G	A	G	SNV	IL16	IL16:exonic:NM 172217.4	1	100	19	synonymous	NM 17221	c.3855A>G	p.(=)	1.39					PDZ domain (	rs4238526	1.00E-04		9.689E-1 (pos.81600995)		Interleukin	CD4 recep	Abortion, Spontaneous;Female Urogenital Di			A=0, G=32	A=0.0, G=1.0	0.00001	485.085	32	A=0/0, G=19/1
chr17:4579638	G/A	G	A	SNV	PELP1	PELP1:intronic:NM 014389.2	1	47.62		intronic	NM 01438	c.1003+14	p.?	-0.7					NUC202 dom	rs386794753,rs8071	0.2968	YES	9.986E-1 (pos.4579638)		Proline-, glu	MLL1 complex,RNA binding,cellular response to estroger				G=22, A=20	G=0.5238, A=0.4762	0.00001	212.855	42	G=14/8, A=5/1
chr22:17565974	C/C	G	C	SNV	IL17RA	IL17RA:utr 5:NM 014339.6	1	100	1	utr 5	NM 01433	c.-8G>C	p.?	0.65					Interleukin 1	rs917865		YES	1.0 (pos.17565974)		Interleukin	cell surfac	Brain Diseases:Brain Ischemia		Benign	G=0, C=20	G=0.0, C=1.0	0.00001	263.781	20	G=0/0, C=11/9
chr17:45905850	T/T	C	T	SNV	MRPL10	MRPL10:intronic:NM 148887.2	1	100		intronic	NM 14888	c.252+17G	p.?	-0.38					Ribosomal pr	rs4793671	0.02	YES	9.981E-1 (pos.45905850)		Mitochondr	RNA binding,intracellular ribonucleoprotein complex,mi				C=0, T=32	C=0.0, T=1.0	0.00001	485.122	32	C=0/0, T=26/6
chr2:103001402	C/T	C	T	SNV	IL18R1	IL18R1:exonic:NM 003855.3	1	50	7	synonymous	NM 00385	c.753C>T	p.(=)	0.15				0.01(COSM40	TIR domain	rs1035130	0.2724	YES	4.028E-1 (pos.103001402	acute_myeloic	Interleukin	T-helper 1	Asthma;Bronchial Diseases;Celiac Disease;C			C=31, T=31	C=0.5, T=0.5	0.00001	305.02	62	C=18/13, T=6/
chr17:46048920	A/G	A	G	SNV	CDK5RAP3	CDK5RAP3:intronic:NM 001278197.1	1	51.92		intronic	NM 00127	c.127+147	p.?	-0.3					Protein of unl	rs72823513,rs982144347		YES			CDK5 regula	MDM2/MDM4 family protein binding,NF-kappaB binding,				A=25, G=27	A=0.4808, G=0.5192	0.00001	270.322	52	A=11/14, G=1
chr17:4621773	T/G	T	G	SNV	ARRB2	ARRB2:intronic:NM 001257330.1	1	50		intronic	NM 00125	c.706+133	p.?	0.68					Arrestin (or S	rs4522461		YES			Arrestin, be	14-3-3 pro	Amphetamine-Related Disorders:Mental Dis			T=10, G=10	T=0.5, G=0.5	0.00001	82.8833	20	T=1/9, G=3/7
chr2:113832312	C/C	T	C	SNV	IL1F10	IL1F10:exonic:NM 173161.2	1	100	4	missense	NM 17316	c.131T>C	p.Ile44Thr	1.59	0.01	89	0.002	0.24(COSM50	Interleukin-1	rs6761276	0.4147	YES	1.671E-1 (pos.113832312	carcinoma,duc	Interleukin	cytokine activity,cytokine-mediated signaling pathway,e				T=0, C=20	T=0.0, C=1.0	0.00001	263.784	20	T=0/0, C=8/12
chr17:4638010	G/G	T	G	SNV	CXCL16	CXCL16:intronic:NM 022059.3	1	100		intronic	NM 02205	c.776-63A	p.?	-0.81						rs3744700	0.3444	YES			CXC chemol	G-protein coupled receptor signaling pathway,T cell che				T=0, G=44	T=0.0, G=1.0	0.00001	726.559	44	T=0/0, G=15/2
chr17:4644491	G/G	A	G	SNV	ZMYND15	ZMYND15:intronic:NM 001267822.1	1	100		intronic	NM 00126	c.592+56A	p.?	-0.12					MYND finger	rs9903643		YES			Zinc finger M	cytoplasm,histone deacetylase binding,metal ion bindir				A=0, G=24	A=0.0, G=1.0	0.00001	334.21	24	A=0/0, G=5/19
chr6:137325847	T/T	C	T	SNV	IL20RA	IL20RA:exonic:NM 014432.3	1	100	6	missense	NM 01443	c.775G>A	p.Val259Ile	-0.4	1	29	0.001		Interferon-al	rs1555498	0.0066	YES	9.991E-1 (pos.137325847)		Interleukin	cytokine r	Abortion, Spontaneous:Female Urogenital Di			C=0, T=30	C=0.0, T=1.0	0.00001	446.609	30	C=0/0, T=9/21
chr17:4647002	G/G	A	G	SNV	ZMYND15	ZMYND15:intronic:NM 001267822.1	1	100		intronic	NM 00126	c.1298-60	p.?	-0.01					MYND finger	rs770417222,rs8082	0.3583	YES			Zinc finger M	cytoplasm,histone deacetylase binding,metal ion bindir				A=0, G=32	A=0.0, G=1.0	0.00001	485.108	32	A=0/0, G=10/2
chr1:24447214	A/A	G	A	SNV	IL22RA1	IL22RA1:utr 3:NM 021258.3	1	100	7	utr 3	NM 02125	c.1806C>T	p.?	-1.4					Interferon-al	rs3795300		YES				biological_process,cytokine-mediated signaling pathwa				G=0, A=96	G=0.0, A=1.0	0.00001	1617.87	96	G=0/0, A=54/4
chr1:67685387	C/C	T	C	SNV	IL23R	IL23R:exonic:NM 144701.2	1	100	7	missense	NM 14470	c.929T>C	p.Leu310Pro	2.35	1	98	0			rs7530511	0.1295	YES	9.831E-1 (pos.67685387)		Interleukin	defense re	Ankylosis;Arthritis;Autoimmune Diseases;Au			T=0, C=21	T=0.0, C=1.0	0.00001	281.234	21	T=0/0, C=13/8
chr2:113763575	G/G	A	G	SNV	IL36A	IL36A:exonic:NM 014440.2	1	100	2	missense	NM 01444	c.35A>G	p.Gln12Arg	-2.46	0.35	43	0	0(COSM4410	Interleukin-1	rs895497	0.2962	YES	9.933E-1;0.0 (pos.113763	hepatocellula	Interleukin	cytokine activity,cytokine-mediated signaling pathway,e				A=0, G=22	A=0.0, G=1.0	0.00001	298.891	22	A=0/0, G=13/9
chr2:113789316	T/T	C	T	SNV	IL36B	IL36B:utr 5:NM 014438.4	1	100	2	utr 5	NM 01443	c.-30G>A	p.?	-0.19						rs2305150	0.4377	YES	6.854E-1 (pos.113789316)		Interleukin	cytokine activity,cytokine-mediated signaling pathway,e				C=0, T=73	C=0.0, T=1.0	0.00001	1150.5	73	C=0/0, T=42/3
chr17:46973139	TG/T	TG	T	INDEL	ATP5G1,LOC105371814	ATP5G1:utr 3:NM 001002027.1, LOC105371814:intronic n	1	100		intronic	NR 13567	c.420delG	p.?	0.6,-0.37					ATP synthase	rs35074390,rs397715546,rs398		YES	(pos.46973138), (pos.46973140),1.0;0.0		ATP synthas	ATP biosynthetic process,ATP hydrolysis coupled proton				TG=0, T=22	TG=0.0, T=1.0	0.00001	154.655	22	TG=0/0, T=3/1
chr2:113671410	G/G	A	G	SNV	IL37	IL37:exonic:NM 014439.3	1	100	2	missense	NM 01443	c.124A>G	p.Thr42Ala	-0.67	0.3	58	0	0(COSM4133	Interleukin-1	rs3811047	0.2986	YES	8.233E-1 (pos.11367141	neoplasm	Interleukin	cytokine activity,cytokine-mediated signaling pathway,c				A=0, G=20	A=0.0, G=1.0	0.00001	262.506	20	A=0/0, G=10/1
chr17:47044463	T/T	C	T	SNV	GIP	GIP:intronic:NM 004123.2	1	100		intronic	NM 00412	c.86+46G>	p.?	-0.08					Peptide horm	rs62078384	0.4698	YES	7.045E-1 (pos.47044463)		C1q- and tu	G-protein	Diabetes Mellitus:Endocrine System Disease			C=0, T=21	C=0.0, T=1.0	0.00001	281.235	21	C=0/0, T=15/6
chr17:47238028	T/T	C	T	SNV	B4GALNT2	B4GALNT2:intronic:NM 153446.2	1	100		intronic	NM 15344	c.946+25C	p.?	0.01					Glycosyl tran	rs8071836	0		1.0 (pos.47238028)			Golgi membrane,UDP-N-acetylgalactosamine metabolic				C=0, T=68	C=0.0, T=1.0	0.00001	1053.45	68	C=0/0, T=22/4
chr17:47785064	A/A	C	A	SNV	SLC35B1	SLC35B1:intronic:NM 005827.2	1	100		intronic	NM 00582	c.215+28G	p.?	-0.14					EamA-like tra	rs9908959	0		9.999E-1 (pos.47785064)		Solute carri	UDP-galactose transmembrane transport,UDP-galactos				C=0, A=74	C=0.0, A=1.0	0.00001	1174.12	74	C=0/0, A=30/4
chr17:4796656	C/C	T	C	SNV	MINK1	MINK1:intronic:NM 153827.4	1	100		intronic	NM 15382	c.2401-73	p.?	-0.46					CNH domain,	rs2302320	0.2381	YES			Misshapen,	ATP binding,Golgi apparatus,JNK cascade,MAP kinase kir				T=0, C=23	T=0.0, C=1.0	0.00001	316.746	23	T=0/0, C=9/14
chrX:1497644	G/C	G	C	SNV	IL3RA	IL3RA:exonic:NM 002183.3	1	59.09	10	missense	NM 00218	c.967G>C	p.Val323Leu	-0.99	1	32	0	0(COSM1331	Interleukin-6	rs17883366	0.1839	YES	9.248E-2;0.0 (pos.14976	serous_carcin	Interleukin-	MAPK cascade,Ras guanyl-nucl		Sargramostim		G=9, C=13	G=0.4091, C=0.5909	0.00001	122.568	22	G=6/3, C=7/6
chr3:121712051	C/C	A	C	SNV	ILDR1	ILDR1:exonic:NM 001199799.1	1	100	7	synonymous	NM 00119	c.1545T>G	p.(=)	0.29				0.18(COSM41	Lipolysis stim	rs2877561	0.2876	YES	9.314E-1 (pos.121712051	neoplasm	Immunoglo	cellular re	Ear Diseases:Hearing Disorder		Benign	A=0, C=23	A=0.0, C=1.0	0.00001	316.736	23	A=0/0, C=6/17
chr17:48070979	G/A	G	A	SNV	DLX3	DLX3:intronic:NM 005220.2	1	50		intronic	NM 00522	c.326-25C	p.?	0.51					Homeobox do	rs116948493	0.0193	YES	0.0 (pos.48070979)		Distal-less l	DNA bindi	Amelogenesis Imperfecta:Congenital Abnorm			G=10, A=10	G=0.5, A=0.5	0.00001	82.8646	20	G=2/8, A=4/6
chr11:6629665	C/T	C	T	SNV	ILK	ILK:exonic:NM 004517.3	1	55.77	4	synonymous	NM 00451	c.297C>T	p.(=)	0.11				0.34(COSM37	Ankyrin repea	rs1043388	0.25	YES	3.436E-1 (pos.6629665)	adenocarcino	Integrin-linl	ATP bindin	Brain Diseases;Brain Ischemia		Benign	C=23, T=29	C=0.4423, T=0.5577	0.00001	299.668	52	C=6/17, T=12/
chr19:15226970	A/A	G	A	SNV	ILVBL	ILVBL:exonic:NM 006844.4	1	100	12	synonymous	NM 00684	c.1464C>T	p.(=)	-3.01				0.05(COSM37	Thiamine pyr	rs2074262	0.4944	YES	4.658E-1 (pos.15226970)	acute_myeloic	IlvB-like (ac	biological_process,catalytic activity,integral component				G=0, A=32	G=0.0, A=1.0	0.00001	485.111	32	G=0/0, A=15/1
chr17:48263021	C/T	C	T	SNV	COL1A1	COL1A1:intronic:NM 000088.3	1	51.85		intronic	NM 00008	c.4249-12	p.?	0.06					Collagen tripl	rs2249492	0.3757	YES	5.189E-1 (pos.48263021)		Collagen I, a	Golgi appa	Abortion, Spontane	Collagena	Benign/Likely	C=13, T=14	C=0.4815, T=0.5185	0.00001	132.669	27	C=7/6, T=10/4
chr19:15227030	A/A	G	A	SNV	ILVBL	ILVBL:exonic:NM 006844.4	1	100	12	synonymous	NM 00684	c.1404C>T	p.(=)	-1.19				0.09(COSM37	Thiamine pyr	rs2074263	0.2821	YES	5.376E-1 (pos.15227030)	acute_myeloic	IlvB-like (ac	biological_process,catalytic activity,integral component				G=0, A=42	G=0.0, A=1.0	0.00001	685.204	42	G=0/0, A=14/2
chr17:48274309	C/C	T	C	SNV	COL1A1	COL1A1:intronic:NM 000088.3	1	100		intronic	NM 00008	c.804+62A	p.?	-2.88					Collagen tripl	rs2075554	0.1326	YES			Collagen I, a	Golgi appa	Abortion, Spontane	Collagenase clostridium		T=0, C=48	T=0.0, C=1.0	0.00001	783.624	48	T=0/0, C=30/1
chr11:61906374	C/C	T	C	SNV	INCENP	INCENP:exonic:NM 001040694.1	1	100	7	synonymous	NM 00104	c.1188T>C	p.(=)	-1.68				0(COSM1472	Chromosome	rs1675126	0.0822	YES	4.549E-1 (pos.61906374)	NS,acute_mye	Inner centr	central ele	Melanoma;Neoplas	N~6~-cyclohexyl-N~2~-(4-		T=0, C=54	T=0.0, C=1.0	0.00001	798.135	54	T=0/0, C=36/1
chr14:105167807	T/T	C	T	SNV	INF2	INF2:exonic:NM 022489.3	1	100	2	synonymous	NM 02248	c.105C>T	p.(=)	-1.72					Diaphanous I	rs4983530	0.0247	YES	1.0 (pos.105167807)		Inverted for	Rho GTPas	Female Urogenital Diseases;Fe		Benign	C=0, T=25	C=0.0, T=1.0	0.00001	353.019	25	C=0/0, T=11/1
chr14:105179194	C/C	T	C	SNV	INF2	INF2:exonic:NM 022489.3	1	100	18	synonymous	NM 02248	c.2640T>C	p.(=)	-2.31				0.01(COSM37	Diaphanous I	rs10133301	0.1633	YES	9.439E-1 (pos.105179194	adenocarcino	Inverted for	Rho GTPas	Female Urogenital Diseases;Fe		Benign	T=0, C=34	T=0.0, C=1.0	0.00001	530.255	34	T=0/0, C=25/9
chr17:4849384	T/T	C	T	SNV	PFN1,RNF167	RNF167:downstream:NM 015528.2, PFN1:intronic:NM 005	1	100		intronic	NM 01552	c.*1073C>	p.?, p.?	-0.06					Profilin	rs2233656		YES			Profilin-1,RI	RNA bindir	Amyotrophic Latera	7-HYDROXY-4-METHYL-3-(		C=0, T=24	C=0.0, T=1.0	0.00001	334.792	24	C=0/0, T=13/1
chr17:48539997	C/C	T	C	SNV	ACSF2	ACSF2:intronic:NM 025149.5	1	100		intronic	NM 02514	c.792+51T	p.?	-1.06					AMP-binding	rs8082089	0.4453	YES	2.154E-1 (pos.48539997)		Acyl-CoA syn	ATP binding,acyl-CoA ligase activity,acyl-CoA metabolic p				T=0, C=28	T=0.0, C=1.0	0.00001	409.64	28	T=0/0, C=12/1
chr17:48545248	T/T	C	T	SNV	ACSF2,CHAD	CHAD:intronic:NM 001267.2, ACSF2:intronic:NM 025149.5	1	100		intronic	NM 02514	c.1216-31	p.?, p.?	-1.04					AMP-binding	rs736911		YES			Acyl-CoA syn	ATP binding,acyl-CoA ligase activity,acyl-CoA metabolic p				C=0, T=32	C=0.0, T=1.0	0.00001	485.136	32	C=0/0, T=20/1
chr14:105180706	G/G	A	G	SNV	INF2	INF2:exonic:NM 022489.3	1	100	21	synonymous	NM 02248	c.3207A>G	p.(=)	-1.57				0(COSM3765	Diaphanous I	rs1128840	0.3638	YES	9.14E-1;2.715E-2 (pos.10	carcinoma,he	Inverted for	Rho GTPas	Female Urogenital Diseases;Fe		Benign	A=0, G=42	A=0.0, G=1.0	0.00001	685.219	42	A=0/0, G=27/1
chr13:111368164	G/G	T	G	SNV	ING1	ING1:exonic:NM 005537.5	1	100	1	missense	NM 00553	c.374T>G	p.Leu125Arg	-0.03	1	102	0	0.11(COSM37	Inhibitor of gr	rs7338333,rs756193	1.00E-04		0.0 (pos.111368160),1.0;	adenocarcino	Inhibitor of	cell cycle,(	Carcinoma;Carcinoma, Squamous Cell;Derma			T=0, G=27	T=0.0, G=1.0	0.00001	389.982	27	T=0/0, G=5/22
chr17:48599450	T/T	A	T	SNV	MYCBPAP	MYCBPAP:intronic:NM 032133.4	1	100		intronic	NM 03213	c.1386+8A	p.?	-2.6					MYCBP-assoc	rs7216625	0.2724	YES	8.783E-1 (pos.48599450)		MYCBP-asso	cell differentiation,chemical synaptic transmission,cyto				A=0, T=32	A=0.0, T=1.0	0.00001	485.141	32	A=0/0, T=11/2
chr17:48606313	A/A	G	A	SNV	MYCBPAP	MYCBPAP:intronic:NM 032133.4	1	100		intronic	NM 03213	c.2683-66	p.?	-0.6					MYCBP-assoc	rs2290864		YES			MYCBP-asso	cell differentiation,chemical synaptic transmission,cyto				G=0, A=33	G=0.0, A=1.0	0.00001	504.585	33	G=0/0, A=19/1
chr13:111368316	T/T	C	T	SNV	ING1	ING1:exonic:NM 005537.5	1	100	1	synonymous	NM 00553	c.526C>T	p.(=)	-2.32				0.16(COSM37	Inhibitor of gr	rs9555726	0.2489	YES	7.407E-1 (pos.111368316	adenocarcino	Inhibitor of	cell cycle,(	Carcinoma:Carcinoma, Squamous Cell;Derma			C=0, T=26	C=0.0, T=1.0	0.00001	371.418	26	C=0/0, T=18/8
chr7:30795436	G/G	T	G	SNV	INMT,INMT-MINDY4	INMT-MINDY4:intronic_nc:NR 037598.1, INMT:exonic:NM 0	1	100	3	missense	NM 00677	c.761T>G	p.Phe254Cys	0.72	0.02	205	1	0.15(COSM37	NNMT/PNMT,	rs4720015	0.1153	YES	8.396E-1 (pos.30795436)	adenocarcino	Indolethyla	S-adenosyl-L-methionine:beta-alanine N-methyltransfer				T=0, G=23	T=0.0, G=1.0	0.00001	316.747	23	T=0/0, G=14/9
chr2:74687781	G/A	G	A	SNV	INO80B-WBP1,MOGS,WBP1	WBP1:exonic:NM 012477.3, MOGS:downstream:NM 0063	1	56	4	synonymous	NR 03784	c.783G>A,	p.(=), p.?	1.27					WW domain-	rs61733470,rs75650	0.0533	YES	0.0 (pos.74687780),3.501E-4 (pos.74687		Mannosyl-o	WW doma	Carbohydrate Metabolism, Inborn Errors:Con			G=11, A=14	G=0.44, A=0.56	0.00001	134.35	25	G=5/6, A=11/3
chr17:4862962	A/A	G	A	SNV	SPAG7	SPAG7:intronic:NM 004890.2	1	100		intronic	NM 00489	c.575-24C	p.?	0.23					R3H domain	rs12451097	0.0482	YES	2.877E-1 (pos.4862962)		Sperm-asso	nucleic acid binding,nucleus				G=0, A=25	G=0.0, A=1.0	0.00001	351.432	25	G=0/0, A=14/1
chr17:4863006	A/A	G	A	SNV	SPAG7	SPAG7:intronic:NM 004890.2	1	100		intronic	NM 00489	c.574+49C	p.?	-0.85					R3H domain	rs73343381	0.0505	YES	2.59E-1 (pos.4863006)		Sperm-asso	nucleic acid binding,nucleus				G=0, A=32	G=0.0, A=1.0	0.00001	485.133	32	G=0/0, A=14/1
chr17:48647011	G/G	T	G	SNV	CACNA1G	CACNA1G:intronic:NM 018896.4	1	100		intronic	NM 01889	c.489-56T	p.?	0.26					Ion transport	rs12946808		YES			Calcium cha	AV node ce	Alzheimer Disease:	Calcium Citrate,Calcium		T=0, G=51	T=0.0, G=1.0	0.00001	752.924	51	T=0/0, G=28/2
chr17:48678325	T/T	A	T	SNV	CACNA1G	CACNA1G:intronic:NM 018896.4	1	100		intronic	NM 01889	c.3792-87	p.?	-1.58					Ion transport	rs198530					Calcium cha	AV node ce	Alzheimer Disease:	Calcium Citrate,Calcium		A=0, T=39	A=0.0, T=1.0	0.00001	623.98	39	A=0/0, T=35/4
chr17:48680657	C/C	T	C	SNV	CACNA1G	CACNA1G:intronic:NM 018896.4	1	100		intronic	NM 01889	c.4169+97	p.?	-0.14					Ion transport	rs2158408		YES			Calcium cha	AV node ce	Alzheimer Disease:	Calcium Citrate,Calcium		T=0, C=22	T=0.0, C=1.0	0.00001	298.857	22	T=0/0, C=3/19

chr17:4881776	G/G	C	G	SNV	CAMTA2	CAMTA2:intronic:NM_001171167.1	1	100		intronic	NM_00117	c.1834+23	p.?	0.65					CG-1 domain	rs376516	0.3885	YES	4.01E-1;1.16E-4 (pos.4881776)		Calmodulin	DNA binding,cardiac muscle hypertrophy in response to	C=0, G=26	C=0.0, G=1.0	0.00001	371.42	26	C=0/0, G=16/1
chr17:49197654	AC/AC	A	AC	INDEL	SPAG9	SPAG9:intronic:NM_001130528.2	1	100		intronic	NM_00113	c.303+61T	p.?	1.66					JNK-interacti	rs397805057,rs5820842,rs991104456					Sperm-asso	JUN kinase binding,MAP-kinase scaffold activity,acrosom	A=0, AC=22	A=0.0, AC=1.0	0.00001	236.499	22	A=0/0, AC=17/
chr17:49279952	A/C	A	C	SNV	MBTD1	MBTD1:intronic:NM_017643.2	1	50		intronic	NM_01764	c.1063+11	p.?	0.12					mbt repeat	rs968622319,rs985322710						covalent chromatin modification,embryonic skeletal sys	A=15, C=15	A=0.5, C=0.5	0.00001	145.839	30	A=13/2, C=3/1
chr17:49713205	T/T	G	T	SNV	CA10	CA10:intronic:NM_001082533.1	1	100		intronic	NM_00108	c.789+11C	p.?	0.93					Eukaryotic-ty	rs10514986	0.4835	YES	3.149E-1 (pos.49713205)		Carbonic ar	brain development,cellular_cc Zonisamide	G=0, T=30	G=0.0, T=1.0	0.00001	446.639	30	G=0/0, T=18/1
chr2:191231458	A/A	G	A	SNV	INPP1	INPP1:exonic:NM_002194.3	1	100	4	synonymous	NM_00219	c.303G>A	p.(=)	0.03					Inositol mono	rs11544940	0.423	YES	7.663E-1 (pos.191231458)		Inositol pol	cytosol,de Autistic Disorder;Child Development Disorde	G=0, A=70	G=0.0, A=1.0	0.00001	1092.09	70	G=0/0, A=31/3
chr2:191231503	A/A	G	A	SNV	INPP1	INPP1:exonic:NM_002194.3	1	100	4	synonymous	NM_00219	c.348G>A	p.(=)	0.03				0.11(COSM37	Inositol mono	rs4940	0.2384	YES	4.717E-1 (pos.19123150:	adenocarcinoi	Inositol pol	cytosol,de Autistic Disorder;Child Development Disorde	G=0, A=73	G=0.0, A=1.0	0.00001	1150.5	73	G=0/0, A=32/4
chr2:191235901	C/C	A	C	SNV	INPP1	INPP1:exonic:NM_002194.3	1	100	6	synonymous	NM_00219	c.973A>C	p.(=)	0.43					Inositol mono	rs1882891	0.1397	YES	9.115E-1;0.0 (pos.191235901)		Inositol pol	cytosol,de Autistic Disorder;Child Development Disorde	A=0, C=44	A=0.0, C=1.0	0.00001	730.646	44	A=0/0, C=23/2
chr1:38327982	T/T	G	T	SNV	INPP5B	INPP5B:utr_3:NM_005540.2	1	100	24	utr_3	NM_00554	c.2747C>A	p.?	-0.86					Endonucleas	rs3843	0.2964	YES	5.007E-1 (pos.38327982)		Inositol pol	GTPase activator activity,Golgi D-Myo-Inositol-1,4-Bisph	G=0, T=30	G=0.0, T=1.0	0.00001	442.93	30	G=0/0, T=13/1
chr17:53076986	A/A	G	A	SNV	STXBP4	STXBP4:intronic:NM_178509.5	1	100		intronic	NM_17850	c.288-7G>	p.?	0.79					PDZ domain (	rs11658717	0.2851	YES	8.973E-1 (pos.53076986)		Syntaxin-bi	cellular re Breast Diseases;Breast Neoplasms;Neoplasr	G=0, A=35	G=0.0, A=1.0	0.00001	543.763	35	G=0/0, A=7/28
chr17:5326162	G/A	G	A	SNV	RPAIN	RPAIN:intronic:NM_001160243.1	1	57.14		intronic	NM_00116	c.313+13G	p.?	-0.07					Replication p	rs2189336	0.4878	YES	9.725E-1 (pos.5326162)		RPA-interac	DNA recombination,DNA repair,DNA-dependent DNA rep	G=21, A=28	G=0.4286, A=0.5714	0.00001	342.309	49	G=6/15, A=14/
chr17:53851071	G/G	A	G	SNV	PCTP	PCTP:intronic:NM_021213.3	1	100		intronic	NM_02121	c.340-14A	p.?	1.52					START domai	rs2960063	0.0301	YES	9.365E-1 (pos.53851071)		Phosphatid	cytosol,lipid binding,lipid trans Di-Linoleoyl-3-Sn-Phosph	A=0, G=35	A=0.0, G=1.0	0.00001	543.903	35	A=0/0, G=7/28
chr17:53851078	C/C	T	C	SNV	PCTP	PCTP:intronic:NM_021213.3	1	100		intronic	NM_02121	c.340-7T>C	p.?	-2.31				0.03(COSM63	START domai	rs2912558	0.0301	YES	9.362E-1 (pos.53851078)	neoplasm	Phosphatid	cytosol,lipid binding,lipid trans Di-Linoleoyl-3-Sn-Phosph	T=0, C=35	T=0.0, C=1.0	0.00001	543.886	35	T=0/0, C=7/28
chr2:234066969	G/G	C	G	SNV	INPP5D	INPP5D:exonic:NM_005541.4	1	100	9	synonymous	NM_00554	c.953C>G	p.(=)	0.06					Endonucleas	rs36127492	0.4899	YES	9.822E-1 (pos.234066969)		Inositol pol	PTB domai Alzheimer Disease;Brain Diseases;Cardiovas	C=0, G=24	C=0.0, G=1.0	0.00001	334.78	24	C=0/0, G=13/1
chr17:54428436	G/G	A	G	SNV	ANKFN1	ANKFN1:intronic:NM_153228.2	1	100		intronic	NM_15322	c.399+108	p.?	-0.48					Ankyrin repe	rs17759670		YES				Marijuana Abuse;Mental Disorders;Substanc	A=0, G=29	A=0.0, G=1.0	0.00001	427.578	29	A=0/0, G=14/1
chr11:15199957	C/C	T	C	SNV	INSC	INSC:exonic:NM_001031853.4	1	100	5	synonymous	NM_00103	c.684T>C	p.(=)	0.65					Inscuteable l	rs1714376	0.158	YES	9.858E-1 (pos.15199957)		Inscuteable	cell cortex,cell differentiation,nervous system developm	T=0, C=31	T=0.0, C=1.0	0.00001	465.8	31	T=0/0, C=10/2
chr14:36003547	G/GG	GGC	G,GG	INDEL	INSM2	INSM2:exonic:NM_032594.3	2	94.12	1	frameshiftDeletion, frameshiftDel	NM_03259	c.90_91de	p.Gln32fs, p.Gln32fs	0.28, 0.06, 3.66					C2H2-type zir	rs372506238	1.00E-04		0.0 (pos.36003547)		Insulinoma	DNA binding,cytoplasm,metal ion binding,nucleus,regul	GGC=0, G=:	GGC=0.0, G=0.9412,	0.00001	460.158	34	GGC=0/0, G=1
chr1:1254841	G/G	C	G	SNV	INTS11	INTS11:exonic:NM_017871.5	1	100	4	synonymous	NM_01787	c.264G>C	p.(=)	-0.15					Beta-Casp do	rs10907179	0.0678	YES	8.258E-1 (pos.1254841)		Cleavage ar	blood microparticle,cytoplasm,cytosol,hydrolase activit	C=0, G=154	C=0.0, G=1.0	0.00001	2422.67	154	C=0/0, G=95/5
chr4:106604364	A/G	A	G	SNV	INTS12	INTS12:exonic:NM_001142471.1	1	53.13	7	synonymous	NM_00114	c.915T>C	p.(=)	-0.41					PHD-finger	rs34072732	0.0683	YES	2.312E-4 (pos.106604364)		Integrator c	integrator complex,metal ion binding,nucleoplasm,nucl	A=30, G=34	A=0.4688, G=0.5313	0.00001	347.733	64	A=16/14, G=1!
chr12:27064232	C/T	C	T	SNV	INTS13	INTS13:exonic:NM_018164.2	1	50	15	synonymous	NM_01816	c.1824G>A	p.(=)	1.03				0.39(COSM41	Cell cycle anc	rs3210635	0.4734	YES	7.885E-1 (pos.27064232)	neoplasm		cell division,centrosome localization,cytoplasm,flagella	C=23, T=23	C=0.5, T=0.5	0.00001	258.573	46	C=10/13, T=5/
chr4:128565000	G/G	A	G	SNV	INTU	INTU:exonic:NM_015693.3	1	100	2	synonymous	NM_01569	c.471A>G	p.(=)	1				0.06(COSM4002934),0.06(C		rs3749507	0.475	YES	5.27E-1;0.0 (pos.128565(	acute_myeloic	Inturned, D	cell surface,ciliary basal body,cilium assembly,cytoplasi	A=0, G=122	A=0.0, G=1.0	0.00001	1973.68	122	A=0/0, G=48/7
chr4:128584580	A/A	G	A	SNV	INTU	INTU:exonic:NM_015693.3	1	100	4	synonymous	NM_01569	c.813G>A	p.(=)	-0.23				0.01(COSM4002936),0.01(C		rs4833380	0.4455	YES	5.332E-1 (pos.12858458(	acute_myeloic	Inturned, D	cell surface,ciliary basal body,cilium assembly,cytoplasi	G=0, A=49	G=0.0, A=1.0	0.00001	831.623	49	G=0/0, A=18/3
chr3:48727112	A/A	G	A	SNV	IP6K2	IP6K2:exonic:NM_001005909.2	1	100	5	synonymous	NM_00100	c.639C>T	p.(=)	-0.42					Inositol polyp	rs4858798	0.3658	YES	9.563E-1 (pos.48727112)		Inositol hex	ATP bindin Basal Ganglia Diseases;Brain Diseases;Centr	G=0, A=56	G=0.0, A=1.0	0.00001	832.087	56	G=0/0, A=21/3
chr17:56327999	G/G	A	G	SNV	LPO	LPO:intronic:NM_006151.2	1	100		intronic	NM_00615	c.780+17A	p.?	-0.49					Animal haem	rs7208924	0.341	YES	2.352E-1 (pos.56327999)		Lactoperox	basolateral plasma membrane,cellular oxidant detoxific	A=0, G=30	A=0.0, G=1.0	0.00001	446.639	30	A=0/0, G=13/1
chr14:24654489	T/C	T	C	SNV	IPO4	IPO4:exonic:NM_024658.3	1	50	14	synonymous	NM_02465	c.1308A>G	p.(=)	1.71				0.1(COSM375	HEAT repeat,l	rs2025258	0.3419	YES	8.025E-1 (pos.24654489)	adenocarcinoma		DNA replication-dependent nucleosome assembly,DNA	T=11, C=11	T=0.5, C=0.5	0.00001	94.6046	22	T=7/4, C=5/6
chr11:9406349	C/C	T	C	SNV	IPO7	IPO7:exonic:NM_006391.2	1	100	1	synonymous	NM_00639	c.39T>C	p.(=)	1.71					Cse1,Importi	rs4606477	1.00E-04		9.999E-1 (pos.9406349)		Importin 7	GTPase re Autoimmune Diseases;Female Urogenital Di:	T=0, C=38	T=0.0, C=1.0	0.00001	603.784	38	T=0/0, C=30/8
chr3:121526204	A/A	G	A	SNV	IQCB1	IQCB1:exonic:NM_001023570.3	1	100	7	synonymous	NM_00102	c.574C>T	p.(=)	1.66					IQ calmoduli	rs4543051	0.3757	YES	3.038E-1 (pos.121526204)		IQ motif-cor	calmoduli Congenital, Hereditary, and Ne Benign	G=0, A=44	G=0.0, A=1.0	0.00001	725.713	44	G=0/0, A=14/3
chr7:2611878	C/C	T	C	SNV	IQCE	IQCE:exonic:NM_152558.4	1	100	5	synonymous	NM_15255	c.312T>C	p.(=)	0.39				0(COSM4162:	IQ calmoduli	rs2917751	0.2434	YES	5.051E-1;3.491E-4 (pos.2	acute_myeloic	IQ domain-c	ciliary membrane,cilium,extrinsic component of membr	T=0, C=82	T=0.0, C=1.0	0.00001	1327.39	82	T=0/0, C=49/3
chr17:57247249	A/A	T	A	SNV	PRR11	PRR11:intronic:NM_018304.3	1	100		intronic	NM_01830	c.128+8T>	p.?	-0.42				0(COSM5425392)		rs9914425	0.1451	YES	1.578E-1 (pos.57247249)	acute_myeloic	Proline-rich	cell cycle arrest,cytoplasm,membrane,nucleus,regulatic	T=0, A=75	T=0.0, A=1.0	0.00001	1189.32	75	T=0/0, A=6/69
chr7:2613062	T/C	T	C	SNV	IQCE	IQCE:exonic:NM_152558.4	1	50	6	synonymous	NM_15255	c.405T>C	p.(=)	-1.91					IQ calmoduli	rs61739566	0.1618	YES	1.507E-3 (pos.2613062)		IQ domain-c	ciliary membrane,cilium,extrinsic component of membr	T=17, C=17	T=0.5, C=0.5	0.00001	172.009	34	T=11/6, C=12/
chr3:51930850	T/C	T	C	SNV	IQCF1	IQCF1:exonic:NM_152397.2	1	50	3	missense	NM_15239	c.169A>G	p.Lys57Glu	-1.75	0.07	56	0	0.01(COSM11	IQ calmoduli	rs57859638	0.1915	YES	9.824E-3;0.0 (pos.51930!	ductal_carcinoma,squamo		acrosomal vesicle,calmodulin binding,positive regulatio	T=19, C=19	T=0.5, C=0.5	0.00001	200.004	38	T=11/8, C=16/
chr15:67768151	C/C	G	C	SNV	IQCH,IQCH-AS1	IQCH-AS1:intronic_nc:NR_040051.1, IQCH:exonic:NM_0010:	1	100	18	missense	NM_00103	c.2794G>C	p.Val932Leu	0.2	0.89	32	0		IQ calmoduli	rs3985641	0		1.0 (pos.67768151)		IQ motif-cor	biological Melanoma;Neoplasms;Neoplasms by Histolc	G=0, C=67	G=0.0, C=1.0	0.00001	1034.43	67	G=0/0, C=38/2
chr17:59544863	G/A	G	A	SNV	TBX4	TBX4:intronic:NM_018488.3	1	53.33		intronic	NM_01848	c.402-8G>	p.?	2.32				0.82(COSM40	T-box	rs758596	0.263	YES	3.677E-1 (pos.59544863)	acute_myeloic	T-box 4	DNA bindi Bone Anteversion;Bone Diseas Benign	G=14, A=16	G=0.4667, A=0.5333	0.00001	159.925	30	G=10/4, A=9/7
chr5:75923294	G/G	T	G	SNV	IQGAP2	IQGAP2:exonic:NM_006633.3	1	100	14	missense	NM_00663	c.1581T>G	p.Asp527Glu	0.67	0.67	45	0		Calponin hon	rs2431352	0.0463	YES	8.848E-1;0.0 (pos.75923294)		IQ motif-cor	Arp2/3 cor Adenocarcinoma;Carcinoma;Carcinoma, Hep	T=0, G=45	T=0.0, G=1.0	0.00001	746.231	45	T=0/0, G=30/1
chr5:75923307	G/G	A	G	SNV	IQGAP2	IQGAP2:exonic:NM_006633.3	1	100	14	missense	NM_00663	c.1594A>G	p.Lys532Glu	-0.3	0.45	56	0		Calponin hon	rs2909888	3.00E-04		1.0;0.0 (pos.75923307)		IQ motif-cor	Arp2/3 cor Adenocarcinoma;Carcinoma;Carcinoma, Hec	A=0, G=45	A=0.0, G=1.0	0.00001	747.382	45	A=0/0, G=29/1
chr17:60753904	C/C	A	C	SNV	MRC2	MRC2:intronic:NM_006039.4	1	100		intronic	NM_00603	c.1834+12	p.?	-0.83					Fibronectin t	rs4968617	0.3512	YES	6.115E-1 (pos.60753904)		Mannose re	carbohydr Mammary Neoplasms, Animal;Mammary Nec	A=0, C=30	A=0.0, C=1.0	0.00001	451.013	30	A=0/0, C=8/22
chr5:75960865	C/C	T	C	SNV	IQGAP2	IQGAP2:exonic:NM_006633.3	1	100	22	synonymous	NM_00663	c.2544T>C	p.(=)	0.42					Calponin hon	rs253093	0.0038	YES	9.169E-1 (pos.75960865)		IQ motif-cor	Arp2/3 cor Adenocarcinoma;Carcinoma;Carcinoma, Hec	T=0, C=32	T=0.0, C=1.0	0.00001	485.134	32	T=0/0, C=9/23
chr1:156532977	C/C	T	C	SNV	IQGAP3	IQGAP3:exonic:NM_178229.4	1	100	8	synonymous	NM_17822	c.747A>G	p.(=)	1.3					Calponin hon	rs1171547	3.00E-04	YES	1.0 (pos.156532977)			ERK1 and ERK2 cascade,G1/S transition of mitotic cell cy	T=0, C=33	T=0.0, C=1.0	0.00001	504.592	33	T=0/0, C=20/1
chr17:60767461	G/G	C	G	SNV	MRC2	MRC2:intronic:NM_006039.4	1	100		intronic	NM_00603	c.3742+38	p.?	1.14					Fibronectin t	rs2465426	0.0161	YES	9.375E-1 (pos.60767461)		Mannose re	carbohydr Mammary Neoplasms, Animal;Mammary Nec	C=0, G=30	C=0.0, G=1.0	0.00001	446.637	30	C=0/0, G=19/1
chr17:60768243	G/G	A	G	SNV	MRC2	MRC2:intronic:NM_006039.4	1	100		intronic	NM_00603	c.4013-67	p.?	-0.34					Fibronectin t	rs2465425		YES			Mannose re	carbohydr Mammary Neoplasms, Animal;Mammary Nec	A=0, G=45	A=0.0, G=1.0	0.00001	747.39	45	A=0/0, G=24/2
chr17:60769406	C/C	T	C	SNV	MRC2	MRC2:intronic:NM_006039.4	1	100		intronic	NM_00603	c.4193-67	p.?	-0.28					Fibronectin t	rs35688424	0.4085	YES			Mannose re	carbohydr Mammary Neoplasms, Animal;Mammary Nec	T=0, C=42	T=0.0, C=1.0	0.00001	685.22	42	T=0/0, C=26/1
chr1:156536251	C/C	T	C	SNV	IQGAP3	IQGAP3:exonic:NM_178229.4	1	100	3	synonymous	NM_17822	c.213A>G	p.(=)	0.41					Calponin hon	rs947662	3.00E-04	YES	1.0 (pos.156536251)			ERK1 and ERK2 cascade,G1/S transition of mitotic cell cy	T=0, C=236	T=0.0, C=1.0	0.00001	3956.29	236	T=0/0, C=135/
chr1:156536221	C/C	G	C	SNV	IQGAP3	IQGAP3:exonic:NM_178229.4	1	99.58	3	synonymous	NM_17822	c.243C>G	p.(=)	0.62					Calponin hon	rs2066377	3.00E-04	YES	1.0 (pos.156536221)			ERK1 and ERK2 cascade,G1/S transition of mitotic cell cy	G=1, C=239	G=0.0042, C=0.9958	0.00001	4010.83	240	G=1/0, C=135/
chr17:61473325	A/A	C	A	SNV	TANC2	TANC2:intronic:NM_025185.3	1	100		intronic	NM_02518	c.3015+36	p.?	-0.77					Ankyrin repe	rs2270133	0.4971	YES	2.257E-1 (pos.61473325)		Tetratricop	in utero embryonic development	C=0, A=49	C=0.0, A=1.0	0.00001	831.629	49	C=0/0, A=25/2
chr3:10276163	A/A	T	A	SNV	IRAK2	IRAK2:exonic:NM_001570.3	1	100	11	missense	NM_00157	c.1293T>A	p.Asp431Glu	0.37	1	45	0	0.13(COSM44	Death domai	rs708035	0.3665	YES	9.96E-1 (pos.10276163)	ductal_carcinc	Interleukin	ATP binding,I-kappaB kinase/NF-kappaB signaling,JNK ca	T=0, A=79	T=0.0, A=1.0	0.00001	1269.33	79	T=0/0, A=44/3
chr17:61910167	A/A	G	A	SNV	PSMC5,SMARCD2	PSMC5:downstream:NM_002805.5, SMARCD2:utr_3:NM_00	1	100		intronic	NM_00280	c.*838G>A	p.?, p.?	1.38					SWIB/MDM2	rs2584622		YES			Proteasom	ATP bindin Dermatitis;Drug Eruptions;Drug Hypersensiti	G=0, A=94	G=0.0, A=1.0	0.00001	1561.95	94	G=0/0, A=55/3
chr12:66605228	G/G	A	G	SNV	IRAK3	IRAK3:exonic:NM_007199.2	1	100	5	missense	NM_00719	c.439A>G	p.Ile147Val	-0.34	0.75	29	0.001	0.05(COSM37	Death domai	rs1152888	0.0668	YES	5.895E-1 (pos.66605228)	acute_myeloic	Interleukin	ATP bindin Cardiovascular Diseases;Reperfusion Injury;\	A=0, G=37	A=0.0, G=1.0	0.00001	583.699	37	A=0/0, G=21/1
chr15:78758677	C/C	G	C	SNV	IREB2	IREB2:exonic:NM_004136.3	1	100	5	missense	NM_00413	c.475G>C	p.Val159Leu	0.87	0.53	32	0		Aconitase C-t	rs2958720	2.00E-04		1.0 (pos.78758677)		Iron-respon	4 iron, 4 su Lung Diseases;Lung Diseases, Obstructive;Ne	G=0, C=38	G=0.0, C=1.0	0.00001	603.775	38	G=0/0, C=18/2
chr17:61988014	A/A	G	A	SNV	CSHL1	CSHL1:intronic:NM_022579.2	1	100		intronic	NM_02257	c.190+91C	p.?	-2.52				0(COSM4590	Somatotropi	rs2006208		YES	3.965E-1 (pos.61988014)	acute_myeloic	Chorionic s	biological_process,cellular_component,extracellular re	G=0, A=24	G=0.0, A=1.0	0.00001	334.789	24	G=0/0, A=12/1
chr13:110435953	A/G	A	G	SNV	IRS2	IRS2:exonic:NM_003749.2	1	47.62	1	synonymous	NM_00374	c.2448T>C	p.(=)	-0.81				0.64(COSM43	PH domain,P	rs4773092	0.403	YES	5.394E-1 (pos.11043595:	adenocarcinoi	Insulin rece	1-phospha Adenocarcinoma;Carcinoma;Carcinoma, Hec	A=22, G=20	A=0.5238, G=0.4762	0.00001	212.952	42	A=12/10, G=1!
chr17:61996255	C/C	A	C	SNV	GH1	GH1:upstream:NM_000515.4	1	100		intronic	NM_00051	c.-119T>G	p.?	0.22						rs2005172,rs370359091,rs7635		YES			Growth hor	JAK-STAT c Acromegaly;Adenoma;Azotemia;Bone Diseas	A=0, C=61	A=0.0, C=1.0	0.00001	915.115	61	A=0/0, C=30/3
chr16:54318528	G/G	A	G	SNV	IRX3	IRX3:exonic:NM_024336.2	1	100	2	missense	NM_02433	c.1265T>C	p.Leu422Pro	1.46	0.07	98	0	0.12(COSM41	Homeobox KI	rs1450355	7.00E-04	YES	1.0 (pos.54318528)	neoplasm	Iroquois ho	axon,cyto Lung Diseases;Respiratory Tract Diseases	A=0, G=29	A=0.0, G=1.0	0.00001	424.302	29	A=0/0, G=10/1
chr1:948921	C/C	T	C	SNV	ISG15	ISG15:utr_5:NM_005101.3	1	100	1	utr_5	NM_00510	c.-33T>C	p.?	-0.97					Ubiquitin fan	rs15842	0.0436	YES	9.998E-1;0.0 (pos.948921)		ISG15 ubiqu	ISG15-prot Influenza, Human;Respiratory Tract Diseases	T=0, C=34	T=0.0, C=1.0	0.00001	524.186	34	T=0/0, C=27/7
chr1:949654	G/G	A	G	SNV	ISG15	ISG15:exonic:NM_005101.3	1	100	2	synonymous	NM_00510	c.294A>G	p.(=)	-0.85				0.04(COSM37	Ubiquitin fan	rs8997	0.053	YES	9.254E-1;0.0 (pos.94965	clear_cell_ren	ISG15 ubiqu	ISG15-prot Influenza, Human;Respiratory Tract Diseases	A=0, G=23	A=0.0, G=1.0	0.00001	316.747	23	A=0/0, G=14/9
chr15:89182596	A/G	A	G	SNV	ISG20	ISG20:utr_5:NM_002201.5	1	53.49	2	utr_5	NM_00220	c.-2A>G	p.?	-2.38					Exonuclease	rs3200942	0.4955	YES	2.573E-1 (pos.89182596)		Interferon-s	3'-5'-exoribonuclease activity,(Uridine monophosphate	A=20, G=23	A=0.4651, G=0.5349	0.00001	261.022	43	A=10/10, G=1!
chr17:62122677	A/A	G	A	SNV	ERN1	ERN1:intronic:NM_001433.3	1	100		intronic	NM_00143	c.2653+42	p.?	-0.75					Protein kinas	rs196917	0.0013	YES	1.0 (pos.62122677)		Endoplasmi	ADP bindir Brain Diseases;Cen N~2~-1H-benzimidazol-5-	G=0, A=45	G=0.0, A=1.0	0.00001	747.138	45	G=0/0, A=21/2
chr15:74467856	A/A	G	A	SNV	ISLR	ISLR:exonic:NM_201526.1	1	100	2	synonymous	NM_20152	c.657G>A	p.(=)	-6.32				0.02(COSM11	Immunoglob	rs1052622	0.3181	YES	5.855E-1 (pos.74467856)	acute_myeloic	Immunoglo	cell adhesion,extracellular exosome,extracellular regio	G=0, A=23	G=0.0, A=1.0	0.00001	316.698	23	G=0/0, A=15/8
chr5:128430757	T/C	T	C	SNV	ISOC1	ISOC1:exonic:NM_016048.2	1	50	1	synonymous	NM_01604	c.298T>C	p.(=)	0.55				0.15(COSM37	Isochorismat	rs1127827	0.391	YES	8.736E-1 (pos.12843075:	adenocarcinoma,neoplasn		biological_process,catalytic activity,cytoplasm,extracel	T=14, C=14	T=0.5, C=0.5	0.00001	131.941	28	T=8/6, C=9/5
chr17:62145672	A/G	A	G	SNV	ERN1	ERN1:intronic:NM_001433.3	1	46.43		intronic	NM_00143	c.479-21T	p.?	0.77					Protein kinas	rs196939	0.2402	YES	6.683E-1 (pos.62145672)		Endoplasmi	ADP bindir Brain Diseases;Cen N~2~-1H-benzimidazol-5-	A=15, G=13	A=0.5357, G=0.4643	0.00001	117.927	28	A=7/8, G=1/12
chr5:52096889	A/A	C	A	SNV	ITGA1,PELO	ITGA1:intronic:NM_181501.1, PELO:exonic:NM_015946.4	1	100	2	missense	NM_01594	c.661C>A,	p.Leu221Met, p.?	2.87	1	15	0.09	0.23(COSM44	FG-GAP repe	rs1499280	0.0847	YES	8.299E-1 (pos.52096889)	hepatocellulai	Integrin, alp	RNA surveillance,acrosomal vesicle,activation of MAPK a	C=0, A=45	C=0.0, A=1.0	0.00001	747.392	45	C=0/0, A=25/2
chr17:6331836	G/G	T	G	SNV	AIPL1	AIPL1:intronic:NM_014336.4	1	100		intronic	NM_01433	c.277-10A	p.?	-0.12				0(COSM3766	FKBP-type pe	rs12453262,rs77999!	0.4212	YES	4.973E-1;0.0 (pos.63318:	hepatocellulai	Arylhydroca	cytoplasm Congenital, Hereditary, and Ne Benign/Likely	T=0, G=35	T=0.0, G=1.0	0.00001	543.899	35	T=0/0, G=21/1
chr17:6371738	A/G	A	G	SNV	PITPNM3	PITPNM3:intronic:NM_031220.3	1	50		intronic	NM_03122	c.1774-77	p.?	-0.35					DDHD domai	rs35021251		YES			Phosphatid	calcium io Congenital, Hereditary, and Neonatal Diseas	A=24, G=24	A=0.5, G=0.5	0.00001	275.991	48	A=6/18, G=12/
chr17:6373737	C/T	C	T	SNV	PITPNM3	PITPNM3:intronic:NM_031220.3	1	50		intronic	NM_03122	c.1625-9G	p.?	-0.36				0.02(COSM37	DDHD domai	rs11654175	0.4701	YES	1.904E-1 (pos.6373737)	carcinoma,hei	Phosphatid	calcium io Congenital, Hereditary, and Ne Benign	C=14, T=14	C=0.5, T=0.5	0.00001	131.578	28	C=2/12, T=5/9
chr15:68624396	C/C	A	C	SNV	ITGA11	ITGA11:exonic:NM_001004439.1	1	100	14	missense	NM_00100	c.1571T>G	p.Leu524Arg	-0.13	0.8	102	0		FG-GAP repe	rs7168069	0.1773	YES	9.191E-1;1.165E-3 (pos.68624396)		Integrin, alp	cell adhes Attention Deficit Disorder with Hyperactivity	A=0, C=24	A=0.0, C=1.0	0.00001	334.792	24	A=0/0, C=15/9
chr17:64023781	T/T	C	T	SNV	CEP112	CEP112:intronic:NM_001199165.3	1	100		intronic	NM_00119	c.1597-10	p.?	0.34					Domain of un	rs11079629		YES				centroson Attention Deficit Disorder with Hyperactivity	C=0, T=35	C=0.0, T=1.0	0.00001	543.903	35	C=0/0, T=16/1
chr15:68649587	A/A	G	A	SNV	ITGA11	ITGA11:exonic:NM_001004439.1	1	100	7	synonymous	NM_00100	c.651C>T	p.(=)	0.41				0.33(COSM37	FG-GAP repe	rs12907890	0.2456	YES	9.988E-1 (pos.68649587)	adenocarcinoi	Integrin, alp	cell adhes Attention Deficit Disorder with Hyperactivity	G=0, A=27	G=0.0, A=1.0	0.00001	389.988	27	G=0/0, A=14/1
chr5:52347369	C/T	C	T	SNV	ITGA2	ITGA2:exonic:NM_002203.3	1	57.14	7	synonymous	NM_00220	c.759C>T	p.(=)	1.53				0.97(COSM40	FG-GAP repe	rs1126643	0.397	YES	3.018E-1 (pos.52347369)	acute_myeloic	Integrin, alp	amyloid-b Autoimmune Diseases;Cardiov Benign	C=12, T=16	C=0.4286, T=0.5714	0.00001	161.764	28	C=9/3, T=9/7
chr12:54799450	G/G	A	G	SNV	ITGA5,LOC102724050	LOC102724050:intronic_nc:NR_120486.1, ITGA5:exonic:NM	1	100	11	synonymous	NR_12048	c.1014T>C	p.(=)	-0.54					FG-GAP repe	rs1249378,rs796775!	2.00E-04		9.999E-1 (pos.54799450)		Integrin, alp	Golgi appa Carcinoma;Carcinoma, Squamous Cell;Cardic	A=0, G=22	A=0.0, G=1.0	0.00001	298.889	22	A=0/0, G=8/14
chr17:6493056	C/C	T	C	SNV	KIAA0753	KIAA0753:intronic:NM_014804.2	1	100		intronic	NM_01480	c.2786+43	p.?	-1.97					Protein moor	rs367918	0.0067	YES	8.409E-1 (pos.6493056)		KIAA0753 gi	centriolar satellite,centriole,centriole replication,centr	T=0, C=23	T=0.0, C=1.0	0.00001	314.546	23	T=0/0, C=9/14
chr12:56077964	C/C	A	C	SNV	ITGA7,METTL7B	ITGA7:downstream:NM_001144996.1, METTL7B:utr_3:NM_1	1	100	2	utr_3	NM_15263	c.866A>C,	p.?, p.?	-1.16					Hypothetical	rs2293409		YES			Integrin, alp	cell adhes Colonic Diseases;Colonic Neoplasms;Colorec	A=0, C=22	A=0.0, C=1.0	0.00001	298.89	22	A=0/0, C=10/1
chr17:3657159	T/T	C	T	SNV	ITGAE	ITGAE:exonic:NM_002208.4	1	100	13	missense	NM_00220	c.1445G>A	p.Arg482Gln	-0.22	0	43	0.783	0.02(COSM37	FG-GAP repe	rs2272606	0.2829	YES	2.146E-1 (pos.3657159)	acute_myeloic	Integrin, alp	cell adhes Attention Deficit Disorder with Hyperactivity	C=0, T=78	C=0.0, T=1.0	0.00001	1249.36	78	C=0/0, T=54/2
chr17:3657175	C/C	T	C	SNV	ITGAE	ITGAE:exonic:NM_002208.4	1	100	13	missense	NM_00220	c.1429A>G	p.Ile477Val	-0.54	1	29	0.001	0(COSM3766	FG-GAP repe	rs220479	0.1835	YES	3.869E-1 (pos.3657175)	acute_myeloic	Integrin, alp	cell adhes Attention Deficit Disorder with Hyperactivity	T=0, C=78	T=0.0, C=1.0	0.00001	1253.43	78	T=0/0, C=52/2
chr2:187532417	A/A	G	A	SNV	ITGAV	ITGAV:exonic:NM_002210.4	1	100	24	missense	NM_00221	c.2347G>A	p.Val783Ile	2.74	0.77	29	0.002		FG-GAP repe	rs2230616	2.00E-04		1.0 (pos.187532417)		Integrin, alp	C-X3-C che Brain Diseases;Car Antithymocyte immunog	G=0, A=33	G=0.0, A=1.0	0.00001	504.594	33	G=0/0, A=20/1
chr10:33209266	T/T	G	T	SNV	ITGB1	ITGB1:exonic:NM_002211.3	1	100	10	synonymous	NM_00221	c.1176C>A	p.(=)	0.57					EGF-like dom	rs2230396	0.1013	YES	6.762E-1;0.0 (pos.33209266)		Integrin, be	B cell diffe Bronchial Neoplasr Antithymocyte immunog	G=0, T=32	G=0.0, T=1.0	0.00001	485.14	32	G=0/0, T=21/1
chr17:6546259	T/C	T	C	SNV	MED31,TXNDC17	MED31:downstream:NM_016060.2, TXNDC17:intronic:NM_(	1	52.38		intronic	NM_03273	c.304-12T	p.?, p.?	-0.8					Eukaryotic pr	rs2285999	0.3769	YES	6.669E-1 (pos.6546259)		Thioredoxir	RNA polym Carcinoma;Carcinoma, Squamous Cell;Digest	T=20, C=22	T=0.4762, C=0.5238	0.00001	245.209	42	T=12/8, C=6/1
chr17:65610	T/T	G	T	SNV	RPH3AL	RPH3AL:intronic:NM_001190411.1	1	100		intronic	NM_00119	c.728-17C	p.?	0.94					FYVE-type zin	rs62057050	0.2063	YES	7.042E-3 (pos.65610)		Rabphilin 3	LIM domai Digestive System Diseases;Liver Cirrhosis;Liv	G=0, T=86	G=0.0, T=1.0	0.00001	1410.93	86	G=0/0, T=27/5
chr17:65739789	G/G	A	G	SNV	NOL11	NOL11:intronic:NM_015462.4	1	100		intronic	NM_01546	c.2044-70	p.?	0.28					NUC205 dom	rs2286917		YES			Nucleolar p	RNA binding,maturation of SSU-rRNA,nucleolus,nucleopl	A=0, G=24	A=0.0, G=1.0	0.00001	334.792	24	A=0/0, G=15/9
chr17:65887896	A/G	A	G	SNV	BPTF	BPTF:intronic:NM_182641.3	1	46.88		intronic	NM_18264	c.1865-64	p.?	4.21					Bromodomai	rs8079754		YES			Bromodomi	DNA-depe Adenocarcinoma;Carcinoma;Female Urogeni	A=51, G=45	A=0.5313, G=0.4688	0.00001	487.624	96	A=30/21, G=2
chr17:65989048	C/C	T	C	SNV	C17orf58	C17orf58:intronic:NM_181655.3	1	100		intronic	NM_18165	c.103+112	p.?	-1.32						rs200385109,rs2836	4.00E-04	YES	1.0 (pos.65989048)			extracellular region	T=0, C=31	T=0.0, C=1.0	0.00001	449.036	31	T=0/0, C=17/1
chr7:20449546	C/T	C	T	SNV	ITGB8	ITGB8:exonic:NM_002214.2	1	45.83	14	missense	NM_00221	c.2236C>T	p.Arg746Cys	5.36	0	180	1		EGF-like dom	rs747343754			0.0 (pos.20449546)			cartilage c Endometriosis;Female Urogenital Diseases;F	C=13, T=11	C=0.5417, T=0.4583	0.00001	93.4398	24	C=3/10, T=3/8
chr13:102368056	T/T	G	T	SNV	ITGBL1	ITGBL1:utr_3:NM_004791.2	1	100	11	utr_3	NM_00479	c.1537G>T	p.?	-0.37					EGF-like dom	rs1436266,rs769646!	0		9.993E-1;6.941E-4;0.0 (pos.102368056)		Integrin, be	cell adhes Nervous System Diseases;Neuromuscular Di:	G=0, T=61	G=0.0, T=1.0	0.00001	921.854	61	G=0/0, T=13/4
chr1:226923505	T/T	G	T	SNV	ITPKB	ITPKB:exonic:NM_002221.3	1	100	2	missense	NM_00222	c.1655C>A	p.Pro552Gln	-0.9	1	76	0		Inositol polyp	rs708776	0.0579	YES	9.999E-1;0.0 (pos.226923505)		Inositol 1,4	ATP binding,MAPK cascade,calmodulin binding,cell surfa	G=0, T=20	G=0.0, T=1.0	0.00001	263.786	20	G=0/0, T=13/7
chr3:4817057	C/C	T	C	SNV	ITPR1	ITPR1:exonic:NM_001168272.1	1	100	46	synonymous	NM_00116	c.6066T>C	p.(=)	-3.11					Inositol 1,4,5	rs6442905	0.0297	YES	9.87E-1 (pos.4817057)		Inositol 1,4	calcineuri Ataxia;Brain Diseas 1D-myo-inositol 1,4,5-tri	T=0, C=40	T=0.0, C=1.0	0.00001	644.283	40	T=0/0, C=14/2
chr21:35260481	C/C	T	C	SNV	ITSN1	ITSN1:exonic:NM_003024.2	1	100	40	synonymous	NM_00302	c.5043T>C	p.(=)	-2.76				0.03(COSM37	C2 domain,C	rs2073370	0.4278	YES	6.721E-1 (pos.35260481)	acute_myeloic	Intersectin	ATP bindin Abnormalities, Multiple;Chromosome Disord	T=0, C=27	T=0.0, C=1.0	0.00001	389.982	27	T=0/0, C=21/6
chr19:50666403	CG/C	CG	C	INDEL	IZUMO2	IZUMO2:exonic:NM_152358.2	1	100	1	frameshiftDeletion	NM_15235	c.48delC	p.Gly17fs	1.11, -1.97				0.09(COSM53	Izumo sperm-egg fusion, Ig domain-associated					adenocarcinoma		integral component of membrane,nucleus	CG=0, C=24	CG=0.0, C=1.0	0.00001	222.27	24	CG=0/0, C=10/
chr17:66925116	A/A	G	A	SNV	ABCA8	ABCA8:intronic:NM_007168.3	1	100		intronic	NM_00716	c.1125+74	p.?	0.02					ABC transpor	rs4147980		YES			ATP-binding	ATP bindin Colonic Diseases;Colorectal Neoplasms;Dige	G=0, A=24	G=0.0, A=1.0	0.00001	334.788	24	G=0/0, A=13/1
chr17:66972250	T/T	A	T	SNV	ABCA9	ABCA9:intronic:NM_080283.3	1	100		intronic	NM_08028	c.4776+26	p.?	-0.13					AAA domain,	rs2191212	0.3602	YES	2.98E-1 (pos.66972250)		ATP-binding	ATP bindin Colonic Diseases;Colorectal Neoplasms;Dige	A=0, T=22	A=0.0, T=1.0	0.00001	298.892	22	A=0/0, T=11/1
chr20:10620275	G/A	G	A	SNV	JAG1	JAG1:exonic:NM_000214.2	1	45.83	26	synonymous	NM_00021	c.3528C>T	p.(=)	5.36				0.93(COSM14	Calcium-bind	rs1051421	0.2836	YES	1.389E-1 (pos.10620275)	acute_myeloic	Jagged 1	Notch binc Abnormalities, Multiple;Adenc Benign	G=26, A=22	G=0.5417, A=0.4583	0.00001	240.962	48	G=13/13, A=1:
chr17:67023755	A/G	A	G	SNV	ABCA9	ABCA9:intronic:NM_080283.3	1	97.92		intronic	NM_08028	c.1791+26	p.?	-0.45					AAA domain,	rs6501947	0.3031	YES	3.08E-1 (pos.67023755)		ATP-binding	ATP bindin Colonic Diseases;Colorectal Neoplasms;Dige	A=1, G=47	A=0.0208, G=0.9792	0.00001	776.313	48	A=0/1, G=29/1
chr14:105611282	A/G	A	G	SNV	JAG2	JAG2:exonic:NM_002226.4	1	58.33	24	synonymous	NM_00222	c.3069T>C	p.(=)	-10.74				0(COSM3753	Calcium-bind	rs2272591	0.3419	YES	5.543E-1 (pos.10561128:	adenocarcinoi	Jagged 2	Notch binc Adenocarcinoma;Astrocytoma;Bone Disease	A=15, G=21	A=0.4167, G=0.5833	0.00001	234.328	36	A=6/9, G=15/6
chr17:67099129	G/G	A	G	SNV	ABCA6	ABCA6:intronic:NM_080284.2	1	100		intronic	NM_08028	c.2741-20	p.?	2.76					ABC transpor	rs2364315	0.3784	YES	2.747E-1 (pos.67099129)		ATP-binding	ATP bindin Colonic Diseases;Colorectal Neoplasms;Derr	A=0, G=25	A=0.0, G=1.0	0.00001	353.019	25	A=0/0, G=14/1
chr19:17937429	G/G	A	G	SNV	JAK3	JAK3:utr_3:NM_000215.3	1	100	24	utr_3	NM_00021	c.3498T>C	p.?	-0.39					Protein kinas	rs3008		YES			Janus kinas	ATP bindin Amyotrophic Latera 2-(1,1-DIM Benign	A=0, G=22	A=0.0, G=1.0	0.00001	298.891	22	A=0/0, G=19/3
chr6:15496662	T/T	C	T	SNV	JARID2	JARID2:exonic:NM_004973.3	1	100	7	synonymous	NM_00497	c.1206C>T	p.(=)	0.19				0.43(COSM37	ARID/BRIGHT	rs742099,rs7721479	0.2544	YES	0.0 (pos.15496657),3.05!	adenocarcinoma,osteosar		ESC/E(Z) cc Child Development Disorders, Pervasive;Mer	C=0, T=31	C=0.0, T=1.0	0.00001	465.819	31	C=0/0, T=10/2
chr17:67152893	C/C	CAAAT	C	INDEL	ABCA10	ABCA10:intronic:NM_080282.3	4	100		intronic	NM_08028	c.3455+80	p.?	-1.85, 0.81, 1.23, -2.75, 0.96					AAA domain,	rs10605889,rs397817739		YES			ATP-binding	ATP bindin Colonic Diseases;Colorectal Neoplasms;Dige	CAAAT=0, C	CAAAT=0.0, C=1.0	0.00001	625.016	37	CAAAT=0/0, C=
chr17:67160186	C/C	T	C	SNV	ABCA10	ABCA10:intronic:NM_080282.3	1	100		intronic	NM_08028	c.3363+29	p.?	1.62					AAA domain,	rs7208382	0.3392	YES	3.347E-1 (pos.67160186)		ATP-binding	ATP bindin Colonic Diseases;Colorectal Neoplasms;Dige	T=0, C=83	T=0.0, C=1.0	0.00001	1323.46	83	T=0/0, C=49/3
chr17:67188576	G/G	A	G	SNV	ABCA10	ABCA10:intronic:NM_080282.3	1	100		intronic	NM_08028	c.1871+12	p.?	-0.76					AAA domain,	rs8070232		YES			ATP-binding	ATP bindin Colonic Diseases;Colorectal Neoplasms;Dige	A=0, G=38	A=0.0, G=1.0	0.00001	603.786	38	A=0/0, G=20/1
chr17:67188668	C/C	T	C	SNV	ABCA10	ABCA10:intronic:NM_080282.3	1	100		intronic	NM_08028	c.1871+36	p.?	-0.47					AAA domain,	rs8074259	0.0056		1.0;0.0 (pos.67188668)		ATP-binding	ATP bindin Colonic Diseases;Colorectal Neoplasms;Dige	T=0, C=37	T=0.0, C=1.0	0.00001	583.706	37	T=0/0, C=18/1
chr17:67197475	C/C	T	C	SNV	ABCA10	ABCA10:intronic:NM_080282.3	1	100		intronic	NM_08028	c.1175+16	p.?	-0.39					AAA domain,	rs2364991		YES			ATP-binding	ATP bindin Colonic Diseases;Colorectal Neoplasms;Dige	T=0, C=89	T=0.0, C=1.0	0.00001	1472.09	89	T=0/0, C=50/3
chr17:67197491	C/C	T	C	SNV	ABCA10	ABCA10:intronic:NM_080282.3	1	100		intronic	NM_08028	c.1175+15	p.?	-0.34					AAA domain,	rs2364992		YES			ATP-binding	ATP bindin Colonic Diseases;Colorectal Neoplasms;Dige	T=0, C=90	T=0.0, C=1.0	0.00001	1496.07	90	T=0/0, C=49/4
chr6:15513482	A/A	G	A	SNV	JARID2	JARID2:exonic:NM_004973.3	1	100	16	synonymous	NM_00497	c.3279G>A	p.(=)	5.38				0.89(COSM45	ARID/BRIGHT	rs2235258	0.2312	YES	6.973E-1 (pos.15513482)	adenocarcinoma		ESC/E(Z) cc Child Development Disorders, Pervasive;Mer	G=0, A=25	G=0.0, A=1.0	0.00001	353.018	25	G=0/0, A=9/16
chr10:30306804	A/A	G	A	SNV	JCAD	JCAD:utr_3:NM_020848.3	1	100	4	utr_3	NM_02084	c.4088C>T	p.?	-0.19					Junctional pr	rs2478839	0.4326	YES	1.919E-1 (pos.30306804)		KIAA1462 gi	adherens junction,cell adhesion,cell-cell junction,perini	G=0, A=27	G=0.0, A=1.0	0.00001	389.988	27	G=0/0, A=17/1
chr17:67274054	A/A	C	A	SNV	ABCA5	ABCA5:intronic:NM_172232.3	1	100		intronic	NM_17223	c.2393-71	p.?	0.22					ABC transpor	rs12452340		YES			ATP-binding	ATP bindin Colonic Diseases;C Tacrolimus	C=0, A=31	C=0.0, A=1.0	0.00001	465.819	31	C=0/0, A=24/7
chr17:680299	T/C	T	C	SNV	GLOD4	GLOD4:intronic:NM_016080.3	1	45.45		intronic	NM_01608	c.141-71A	p.?	-1.4						rs2273453		YES				cadherin binding,extracellular exosome,mitochondrion	T=30, C=25	T=0.5455, C=0.4545	0.00001	233.906	55	T=6/24, C=9/1
chr14:75904660	A/G	A	G	SNV	JDP2	JDP2:exonic:NM_001135049.1	1	58.33	2	missense	NM_00113	c.70A>G	p.Thr24Ala	7.14	1	58	0	0.19(COSM41	Basic region l	rs3625	0.4715	YES	6.455E-1 (pos.75904660)	neoplasm	JUN dimeriz	DNA bindi Adenocarcinoma;C Pseudoephedrine	A=10, G=14	A=0.4167, G=0.5833	0.00001	135.339	24	A=9/1, G=9/5
chr10:64945364	A/A	G	A	SNV	JMJD1C	JMJD1C:exonic:NM_032776.2	1	100	20	synonymous	NM_03277	c.6789C>T	p.(=)	-0.38					JmjC domain,	rs3211105	0.1966	YES	6.201E-1 (pos.64945364)		Jumonji dor	blood coa Adenocarcinoma;Arthritis;Arthritis, Juvenile;	G=0, A=27	G=0.0, A=1.0	0.00001	389.972	27	G=0/0, A=22/5
chr10:64974537	T/T	A	T	SNV	JMJD1C	JMJD1C:exonic:NM_032776.2	1	100	8	missense	NM_03277	c.1390T>A	p.Ser464Thr	0.76	0.06	58	0.116		JmjC domain,	rs10761725	0.2209	YES	3.609E-1 (pos.64974537)		Jumonji dor	blood coa Adenocarcinoma;Arthritis;Arthritis, Juvenile;	A=0, T=58	A=0.0, T=1.0	0.00001	867.385	58	A=0/0, T=28/3
chr17:6902179	A/A	G	A	SNV	ALOX12,ALOX12-AS1	ALOX12:intronic:NM_000697.2, ALOX12-AS1:intronic_nc:NR	1	100		intronic	NR_04008	c.542+23G	p.?	-0.08					Lipoxygenase	rs312470	0.0712	YES	8.772E-1 (pos.6902179)		Arachidona	aging,arac Arthritis;Arthritis, Juvenile;Autoimmune Dise	G=0, A=38	G=0.0, A=1.0	0.00001	603.771	38	G=0/0, A=17/2
chr17:6927350	G/G	A	G	SNV	BCL6B	BCL6B:intronic:NM_181844.3	1	100		intronic	NM_18184	c.180-52A	p.?	-0.24					BTB/POZ dom	rs312461	8.00E-04	YES			B-cell lymph	RNA polymerase II regulatory region sequence-specific D	A=0, G=21	A=0.0, G=1.0	0.00001	281.235	21	A=0/0, G=11/1
chr10:65225450	T/T	C	T	SNV	JMJD1C,JMJD1C-AS1	JMJD1C-AS1:exonic_nc:NR_027182.1, JMJD1C:utr_5:NM_03	1	100	1	utr_5	NM_03277	c.-28G>A	p.?	-0.33					JmjC domain,	rs1054693	0.2761	YES	1.792E-2 (pos.65225450)		Jumonji dor	blood coa Adenocarcinoma;Arthritis;Arthritis, Juvenile;	C=0, T=28	C=0.0, T=1.0	0.00001	408.681	28	C=0/0, T=11/1
chr1:227923081	A/A	G	A	SNV	JMJD4,SNAP47	JMJD4:exonic:NM_023007.2, SNAP47:utr_5:NM_053052.3	1	100	1	missense	NM_02300	c.32C>T, c.	p.Ala11Val, p.?	-0.1	1	64	0	0(COSM3749	Cupin-like do	rs7419238	0		1.0;0.0 (pos.227923081)	adenocarcinoma,neoplasn		BLOC-1 complex,SNAP receptor activity,SNARE complex,c	G=0, A=79	G=0.0, A=1.0	0.00001	1269.57	79	G=0/0, A=58/2
chr15:42135988	T/T	C	T	SNV	JMJD7-PLA2G4B,PLA2G4B	JMJD7-PLA2G4B:exonic:NM_005090.3, PLA2G4B:exonic:NM	1	100	11	synonymous	NM_00509	c.1551C>T	p.(=), p.(=)	-1.31				0.01(COSM37	C2 domain,C	rs1672466	0.1881	YES	8.717E-1 (pos.42135988)	Wilms_tumou	Phospholip	IRE1-mediated unfolded protein response,arachidonic a	C=0, T=24	C=0.0, T=1.0	0.00001	334.792	24	C=0/0, T=19/5
chr17:7111620	GC/GC	G	GC	INDEL	DLG4	DLG4:intronic:NM_001128827.2	1	100		intronic	NM_00112	c.31-62C>	p.?	-0.17					Guanylate kinase,PDZ domain,PDZ domain (Also known as DHR or GLGF),PDZ-associated domain of N						Discs large,	AMPA glut Adnexal Diseases;A Guanidine,Guanosine-5'	G=0, GC=72	G=0.0, GC=1.0	0.00001	705.371	72	G=0/0, GC=18/
chr17:71192955	T/T	C	T	SNV	COG1	COG1:intronic:NM_018714.2	1	100		intronic	NM_01871	c.560+65C	p.?	0.07					Vps51/Vps67	rs1010442		YES			Component	ER to Golgi Carbohydrate Metabolism, Inborn Errors;Con	C=0, T=28	C=0.0, T=1.0	0.00001	408.718	28	C=0/0, T=12/1
chr19:2255336	G/G	A	G	SNV	JSRP1	JSRP1:utr_5:NM_144616.3	1	100	2	utr_5	NM_14461	c.-23T>C	p.?	-1.59					Junctional sa	rs886363	0.1978	YES	9.114E-1;0.0 (pos.2255336)		Junctional s	regulation Adnexal Diseases;Cysts;Endocrine System Di	A=0, G=22	A=0.0, G=1.0	0.00001	298.892	22	A=0/0, G=5/17
chr17:71197200	C/C	T	C	SNV	COG1	COG1:intronic:NM_018714.2	1	100		intronic	NM_01871	c.1282-48	p.?	-1.68					Vps51/Vps67	rs1026130	1.00E-04		9.994E-1 (pos.71197200)		Component	ER to Golgi Carbohydrate Metabolism, Inborn Errors;Con	T=0, C=28	T=0.0, C=1.0	0.00001	408.718	28	T=0/0, C=13/1
chr1:59248813	C/C	G	C	SNV	JUN	JUN:utr_5:NM_002228.3	1	100	1	utr_5	NM_00222	c.-71C>G	p.?	-0.17					Basic region l	rs2760499		YES			Avian sarco	DNA bindi Adenocarcinoma;A Arsenic trioxide,Irbesart	G=0, C=30	G=0.0, C=1.0	0.00001	426.216	30	G=0/0, C=11/1
chr17:39925925	A/G	A	G	SNV	JUP	JUP:exonic:NM_002230.3	1	100	3	synonymous	NM_00223	c.213T>C	p.(=)	0.58				0.28(COSM63	Armadillo/be	rs7405731	0.2448	YES	5.325E-1;0.0 (pos.39925!	carcinoma	Junction pla	Z disc,acti Arrhythmogenic Right Ventricu Benign/Likely	A=0, G=39	A=0.0, G=1.0	0.00001	614.224	39	A=0/0, G=25/1
chr9:742342	G/G	A	G	SNV	KANK1	KANK1:exonic:NM_015158.3	1	100	10	synonymous	NM_01515	c.3834A>G	p.(=)	-0.28				0.24(COSM37	Ankyrin repe	rs10733535,rs75693!	0.009		0.0;0.0 (pos.742341),1.0;	adenocarcinoi	KN motif-ar	beta-cate Brain Damage, Chronic;Brain Diseases;Centr	A=0, G=40	A=0.0, G=1.0	0.00001	636.749	40	A=0/0, G=20/2
chr19:11303943	A/G	A	G	SNV	KANK2	KANK2:exonic:NM_015493.6	1	57.58	2	synonymous	NM_01549	c.813T>C	p.(=)	-0.17				0.03(COSM11	Ankyrin repe	rs3745681	0.3879	YES	4.249E-1 (pos.11303943)	adenocarcinoi	KN motif-ar	apoptotic process,cytoplasm,mitochondrion,negative re	A=14, G=19	A=0.4242, G=0.5758	0.00001	204.392	33	A=1/13, G=14/
chr1:62713224	G/G	C	G	SNV	KANK4	KANK4:exonic:NM_181712.4	1	100	9	missense	NM_18171	c.2803G>C	p.Val935Leu	0.7	1	32	0		Ankyrin repe	rs2941679	0.0402	YES	9.998E-1 (pos.62713224)		KN motif-ar	cytosol,microtubule cytoskeleton	C=0, G=24	C=0.0, G=1.0	0.00001	334.792	24	C=0/0, G=15/9
chr1:62713246	A/A	G	A	SNV	KANK4	KANK4:exonic:NM_181712.4	1	100	9	synonymous	NM_18171	c.2781C>T	p.(=)	-0.52				0.15(COSM42	Ankyrin repe	rs10889315	0.4209	YES	1.617E-1 (pos.62713246)	acute_myeloic	KN motif-ar	cytosol,microtubule cytoskeleton	G=0, A=26	G=0.0, A=1.0	0.00001	371.361	26	G=0/0, A=15/1
chr17:71344889	T/T	C	T	SNV	SDK2	SDK2:intronic:NM_001144952.1	1	100		intronic	NM_00114	c.6039-25	p.?	-0.29					Fibronectin t	rs9905883	0.002	YES	8.77E-1 (pos.71344889)		Sidekick, Dr	camera-ty Anxiety Disorders;Mental Disorders;Panic Di	C=0, T=34	C=0.0, T=1.0	0.00001	524.173	34	C=0/0, T=14/2
chr17:71346750	G/G	C	G	SNV	SDK2	SDK2:intronic:NM_001144952.1	1	100		intronic	NM_00114	c.5899+39	p.?	0.04					Fibronectin t	rs1995163	0.0019	YES	8.772E-1 (pos.71346750)		Sidekick, Dr	camera-ty Anxiety Disorders;Mental Disorders;Panic Di	C=0, G=27	C=0.0, G=1.0	0.00001	389.83	27	C=0/0, G=24/3
chr17:71346943	G/G	A	G	SNV	SDK2	SDK2:intronic:NM_001144952.1	1	100		intronic	NM_00114	c.5759-14	p.?	-1.74					Fibronectin t	rs4969107	0.0019	YES	8.777E-1 (pos.71346943)		Sidekick, Dr	camera-ty Anxiety Disorders;Mental Disorders;Panic Di	A=0, G=51	A=0.0, G=1.0	0.00001	754.584	51	A=0/0, G=18/3
chr1:62728838	C/C	T	C	SNV	KANK4	KANK4:exonic:NM_181712.4	1	100	7	missense	NM_18171	c.2465A>G	p.His822Arg	3.31	0.23	29	0		Ankyrin repe	rs2258470	0.266	YES	8.4E-1;0.0 (pos.62728838)		KN motif-ar	cytosol,microtubule cytoskeleton	T=0, C=20	T=0.0, C=1.0	0.00001	263.786	20	T=0/0, C=10/1
chr17:7150552	G/G	A	G	SNV	CTDNEP1	CTDNEP1:intronic:NM_001143775.1	1	100		intronic	NM_00114	c.169+44T	p.?	-1.14					NLI interactir	rs2074217	0.3837	YES	3.852E-1 (pos.7150552)		C-terminal (	Nem1-Spo7 phosphatase complex,cytoplasm,dephosph	A=0, G=37	A=0.0, G=1.0	0.00001	583.706	37	A=0/0, G=12/2

chr1:62728861	C/C	T	C	SNV	KANK4	KANK4:exonic:NM_181712.4	1	100	7	synonymous	NM_18171	c.2442A>G	p.(=)	0.57					Ankyrin repea	rs2260581	0.2662	YES	8.404E-1 (pos.62728861)		KN motif- ar	cytosol,microtubule cytoskeleton				T=0, C=20	T=0.0, C=1.0	0.00001	269.284	20	T=0/0, C=10/1
chr17:7156186	G/G	A	G	SNV	ELP5	ELP5:intronic:NM_203414.2	1	100		intronic	NM_20341	c.155+37A	p.?	-0.19						rs2074218	0.3899	YES	3.798E-1 (pos.7156186)		Elongator a	Elongator holoenzyme complex,cytoplasm,cytosol,nucle				A=0, G=48	A=0.0, G=1.0	0.00001	809.857	48	A=0/0, G=8/40
chr17:7158203	G/G	T	G	SNV	ELP5	ELP5:intronic:NM_203414.2	1	100		intronic	NM_20341	c.457+81T	p.?	-2.31				0.01(COSM3820465)		rs402514		YES	6.739E-1 (pos.7158203)	carcinoma,squ	Elongator a	Elongator holoenzyme complex,cytoplasm,cytosol,nucle				T=0, G=22	T=0.0, G=1.0	0.00001	303.287	22	T=0/0, G=4/18
chr17:7192091	T/T	C	T	SNV	SLC2A4,YBX2	YBX2:utr_3:NM_015982.3, SLC2A4:downstream:NM_00104	1	100		intronic	NM_00104	c.*2143C>	p.?, p.?	-0.71					'Cold-shock' D	rs222842,rs7488910	0.2756	YES	0.0 (pos.7192090),3.385E-1 (pos.719209		Solute carri	D-glucose	Alzheimer Disease;	Fludeoxyglucose F-18,Gl		C=0, T=105	C=0.0, T=1.0	0.00001	1644.34	105	C=0/0, T=41/6
chr17:7193055	G/G	GA	G	INDEL	YBX2	YBX2:intronic:NM_015982.3	1	100		intronic	NM_01598	c.849-11T	p.?	0.03, 0.68					'Cold-shock' D	rs3833134,rs397840	0.2711	YES	0.0 (pos.7193056),3.354E-1 (pos.719305		Y box-bindir	DNA binding,RNA binding,cytoplasm,fibrillar center,nucl				GA=0, G=24	GA=0.0, G=1.0	0.00001	336.247	24	GA=0/0, G=9/1
chr17:7221544	T/C	T	C	SNV	NEURL4	NEURL4:intronic:NM_032442.2	1	45.83		intronic	NM_03244	c.3952+44	p.?	-2.43					Neuralized	rs7222233	0.4143	YES	9.364E-1 (pos.7221544)		Neuralized	centriole,protein binding				T=26, C=22	T=0.5417, C=0.4583	0.00001	240.97	48	T=4/22, C=12/
chr17:72281367	G/G	A	G	SNV	DNAI2	DNAI2:intronic:NM_023036.5	1	100		intronic	NM_02303	c.345+27A	p.?	-0.36						rs6501706	0.0012	YES	1.0 (pos.72281367)		Dynein, axo	ATP-deper	Bronchial Diseases;Bronchiect		Benign	A=0, G=23	A=0.0, G=1.0	0.00001	316.747	23	A=0/0, G=14/9
chr1:62728918	A/A	G	A	SNV	KANK4	KANK4:exonic:NM_181712.4	1	100	7	synonymous	NM_18171	c.2385C>T	p.(=)	-1.76				0.04(COSM54	Ankyrin repea	rs2262110	0.2662	YES	8.428E-1 (pos.62728918)	acute_myeloic	KN motif- ar	cytosol,microtubule cytoskeleton				G=0, A=21	G=0.0, A=1.0	0.00001	281.235	21	G=0/0, A=10/1
chr17:72308403	A/A	G	A	SNV	DNAI2	DNAI2:intronic:NM_023036.5	1	100		intronic	NM_02303	c.1722+34	p.?	0.09						rs4239008	0.083	YES	8.769E-1 (pos.72308403)		Dynein, axo	ATP-deper	Bronchial Diseases;Bronchiect		Benign	G=0, A=32	G=0.0, A=1.0	0.00001	484.981	32	G=0/0, A=17/1
chr17:72308410	T/T	C	T	SNV	DNAI2	DNAI2:intronic:NM_023036.5	1	100		intronic	NM_02303	c.1722+41	p.?	-2.51						rs4239009	0.0554	YES	9.244E-1 (pos.72308410)		Dynein, axo	ATP-deper	Bronchial Diseases;Bronchiect		Benign	C=0, T=32	C=0.0, T=1.0	0.00001	485.117	32	C=0/0, T=17/1
chr17:72308447	A/A	G	A	SNV	DNAI2	DNAI2:intronic:NM_023036.5	1	100		intronic	NM_02303	c.1722+78	p.?	0.59						rs1979371		YES			Dynein, axo	ATP-deper	Bronchial Diseases;Bronchiectasis;Cardiovas			G=0, A=31	G=0.0, A=1.0	0.00001	465.754	31	G=0/0, A=16/1
chr1:62740449	C/C	T	C	SNV	KANK4	KANK4:exonic:NM_181712.4	1	100	3	synonymous	NM_18171	c.327A>G	p.(=)	-1.6					Ankyrin repea	rs2666507	0.3266	YES	8.635E-1 (pos.62740449)		KN motif- ar	cytosol,microtubule cytoskeleton				T=0, C=46	T=0.0, C=1.0	0.00001	768.279	46	T=0/0, C=28/1
chr20:18143117	G/G	T	G	SNV	KAT14	KAT14:exonic:NM_020536.4	1	100	6	missense	NM_02053	c.1199T>G	p.Val400Gly	3.96	0.69	109	0		Acetyltransfe	rs1205193	0.0264	YES	1.0;0.0 (pos.18143117)		Lysine acet	Ada2/Gcn5/Ada3 transcription activator complex,G2/M				T=0, G=47	T=0.0, G=1.0	0.00001	779.912	47	T=0/0, G=27/2
chr17:72350277	G/G	T	G	SNV	KIF19	KIF19:intronic:NM_153209.3	1	100		intronic	NM_15320	c.2309-24	p.?	-0.02					Kinesin moto	rs2382642	0.4324	YES	4.077E-1 (pos.72350277)			ATP binding,ATP-dependent microtubule motor activity,				T=0, G=40	T=0.0, G=1.0	0.00001	644.272	40	T=0/0, G=30/1
chr16:31141826	G/G	A	G	SNV	KAT8,PRSS8	KAT8:exonic:NM_182958.2, PRSS8:downstream:NM_00277	1	100	9	synonymous	NM_18295	c.1056A>G	p.(=), p.?	-3.94				0.03(COSM41	MOZ/SAS fam	rs1549295	1.00E-04		9.994E-1 (pos.31141826)	neoplasm	K(lysine) ac	MLL1 com	Congenital Abnorm	1-4-(hydroxymethyl)pher		A=0, G=113	A=0.0, G=1.0	0.00001	1795.51	113	A=0/0, G=40/7
chr16:31141880	G/G	A	G	SNV	KAT8,PRSS8	KAT8:exonic:NM_182958.2, PRSS8:downstream:NM_00277	1	100	9	synonymous	NM_18295	c.1110A>G	p.(=), p.?	1.3				0.12(COSM62	MOZ/SAS fam	rs1549294	3.00E-04	YES	1.0 (pos.31141880)	neoplasm,non	K(lysine) ac	MLL1 com	Congenital Abnorm	1-4-(hydroxymethyl)pher		A=0, G=107	A=0.0, G=1.0	0.00001	1684.31	107	A=0/0, G=35/7
chr16:31142381	G/G	A	G	SNV	KAT8,PRSS8	KAT8:utr_3:NM_182958.2, PRSS8:downstream:NM_002773.	1	100	10	utr_3	NM_18295	c.1472A>G	p.?, p.?	0.73					MOZ/SAS fam	rs2855479		YES			K(lysine) ac	MLL1 com	Congenital Abnorm	1-4-(hydroxymethyl)pher		A=0, G=23	A=0.0, G=1.0	0.00001	316.746	23	A=0/0, G=19/4
chr17:72608926	A/A	G	A	SNV	CD300E	CD300E:intronic:NM_181449.2	1	100		intronic	NM_18144	c.498-14C	p.?	-0.74					Immunoglobi	rs509574	0.2863	YES	9.157E-2;0.0 (pos.72608926)		CD300E ant	extracellular exosome,innate immune response,integra				G=0, A=44	G=0.0, A=1.0	0.00001	726.31	44	G=0/0, A=31/1
chr13:41705495	G/G	A	G	SNV	KBTBD6	KBTBD6:exonic:NM_152903.4	1	100	1	synonymous	NM_15290	c.1153T>C	p.(=)	2.89					BTB And C-ter	rs584594	0.0021	YES	9.808E-1 (pos.41705495)		Kelch repea	Cul3-RING ubiquitin ligase complex,biological_process,c				A=0, G=45	A=0.0, G=1.0	0.00001	747.393	45	A=0/0, G=18/2
chr1:111217504	GC/G	GC	G	INDEL	KCNA3	KCNA3:utr_5:NM_002232.4	1	100	1	utr_5	NM_00223	c.-73GC>C	p.?	0.54, 1.02					BTB/POZ dom	rs997445914					Potassium	axon,delayed rectifier potassi	Dalfampridine			GC=0, G=28	GC=0.0, G=1.0	0.00001	203.845	28	GC=0/0, G=6/2
chr17:72791266	A/A	G	A	SNV	TMEM104	TMEM104:intronic:NM_017728.3	1	100		intronic	NM_01772	c.527+16G	p.?	-4					Transmembra	rs9910463	0.1738	YES	9.91E-1;0.0 (pos.72791266)			biological_process,cellular_component,integral compor				G=0, A=38	G=0.0, A=1.0	0.00001	603.777	38	G=0/0, A=20/1
chr11:30033191	A/A	G	A	SNV	KCNA4	KCNA4:exonic:NM_002233.3	1	100	2	synonymous	NM_00223	c.1035C>T	p.(=)	-4.73				0.02(COSM37	BTB/POZ dom	rs3802914	0.1631	YES	3.397E-2;0.0 (pos.30033	adenocarcino	Potassium	axon,integral component of me	Arginineamide,Dalfampr			G=0, A=36	G=0.0, A=1.0	0.00001	563.743	36	G=0/0, A=23/1
chr8:73850203	A/A	G	A	SNV	KCNB2	KCNB2:exonic:NM_004770.2	1	100	3	synonymous	NM_00477	c.2613G>A	p.(=)	2.19				0.83(COSM37	BTB/POZ dom	rs11782118	0.412	YES	9.754E-2 (pos.73850203)	adenocarcino	Potassium	delayed rectifier potassium ch	Dalfampridine			G=0, A=29	G=0.0, A=1.0	0.00001	427.603	29	G=0/0, A=13/1
chr1:210857457	G/G	A	G	SNV	KCNH1	KCNH1:exonic:NM_172362.2	1	100	11	synonymous	NM_17236	c.2136T>C	p.(=)	-1.21				0.01(COSM50	Cyclic nucleo	rs1135317	0.2863	YES	6.478E-1 (pos.21085745	acute_myeloic	Potassium	axon,calm	Abnormalities, Multiple;Brain Diseases;Brea			A=0, G=129	A=0.0, G=1.0	0.00001	2115.2	129	A=0/0, G=33/9
chr14:63174960	T/T	C	T	SNV	KCNH5	KCNH5:exonic:NM_139318.4	1	100	11	missense	NM_13931	c.2233G>A	p.Ala745Thr	3.75	0.42	58	0.017	0.86(COSM37	Cyclic nucleo	rs4902176	0.3471	YES	1.378E-1;0.0 (pos.63174	acute_myeloic	Potassium	calmodulin binding,cell surface,integral component of p				C=0, T=31	C=0.0, T=1.0	0.00001	465.819	31	C=0/0, T=13/1
chr17:72955227	C/G	C	G	SNV	HID1	HID1:intronic:NM_030630.2	1	95.35		intronic	NM_03063	c.1009-81	p.?	0.05					Dyggve-Melcl	rs373116696,rs902727,rs95945		YES			HID1 domai	Golgi apparatus,Golgi medial cisterna,Golgi trans cister				C=2, G=41	C=0.0465, G=0.9535	0.00001	534.203	43	C=1/1, G=8/33
chr17:61611423	C/C	T	C	SNV	KCNH6	KCNH6:exonic:NM_030779.3	1	100	5	synonymous	NM_03077	c.852T>C	p.(=)	-0.21				0.24(COSM63	Cyclic nucleo	rs7225568	0.4052	YES	2.596E-1 (pos.61611423)	carcinoma	Potassium	integral component of membra	Doxazosin,Ibutilide,Micc			T=0, C=62	T=0.0, C=1.0	0.00001	937.9	62	T=0/0, C=13/4
chr21:39671476	A/A	G	A	SNV	KCNJ15	KCNJ15:exonic:NM_170737.2	1	100	3	missense	NM_17073	c.293G>A	p.Gly98Asp	1.18		94		0.16(COSM37	Inward rectif	rs2230033	0.4527	YES	2.929E-1 (pos.39671476)	acute_myeloic	Potassium	G-protein	Abnormalities, Mul	Yohimbine		G=0, A=53	G=0.0, A=1.0	0.00001	782.03	53	G=0/0, A=33/2
chr17:73102269	C/C	G	C	SNV	SLC16A5	SLC16A5:downstream:NM_001271765.1	1	100		intronic	NM_00127	c.*141G>C	p.?	-0.12						rs4789141		YES				integral component of membra	Pyruvic acid			G=0, C=51	G=0.0, C=1.0	0.00001	754.584	51	G=0/0, C=12/3
chr17:73126746	G/G	GT	G	INDEL	ARMC7,NT5C	NT5C:intronic:NM_014595.2, ARMC7:downstream:NM_024	1	100		intronic	NM_02458	c.*1613GT	p.?, p.?	0.22, -0.61					5' nucleotida	rs10715821,rs74871	0.0015	YES	1.0 (pos.73126746)		5', 3' nucleo	5'-nucleot	Colonic Diseases:C	Lamivudine		GT=0, G=28	GT=0.0, G=1.0	0.00001	408.718	28	GT=0/0, G=12/
chr17:73228101	C/C	T	C	SNV	NUP85	NUP85:intronic:NM_024844.4	1	100		intronic	NM_02484	c.1396+27	p.?	-1.44					Nup85 Nucle	rs753871803,rs8993	3.00E-04		0.0 (pos.73228097),9.999E-1;1.16E-4 (po		Nucleopori	condensed chromosome kinetochore,cytosol,host cell,i				T=0, C=28	T=0.0, C=1.0	0.00001	408.716	28	T=0/0, C=15/1
chr2:155711357	T/C	T	C	SNV	KCNJ3	KCNJ3:exonic:NM_002239.3	1	45.45	3	synonymous	NM_00223	c.1038T>C	p.(=)	0.2				0.66(COSM56	Inward rectif	rs17642086	0.3435	YES	4.089E-2 (pos.15571135	carcinoma,dif	Potassium	G-protein activated inward rec	Ethanol,Halothane			T=18, C=15	T=0.5455, C=0.4545	0.00001	143.158	33	T=6/12, C=6/9
chr17:73257830	T/T	C	T	SNV	GGA3,MRPS7	GGA3:upstream:NM_138619.3, MRPS7:utr_5:NM_015971.3	1	100		intronic	NM_13861	c.-162G>A	p.?, p.?	1.26					Ribosomal pr	rs2279161		YES			Golgi assoc	ADP-ribos	Breast Diseases;Breast Neoplasms;Neoplas			C=0, T=32	C=0.0, T=1.0	0.00001	483.933	32	C=0/0, T=17/1
chr11:128781978	G/G	T	G	SNV	KCNJ5	KCNJ5:exonic:NM_000890.3	1	100	2	synonymous	NM_00089	c.810T>G	p.(=)	2.36					Inward rectif	rs7118824	0.1784	YES	8.791E-1 (pos.128781978)		Potassium	G-protein	Adenoma;Anderser	Ethanol,Gl	Benign	T=0, G=30	T=0.0, G=1.0	0.00001	446.632	30	T=0/0, G=12/1
chr11:128782002	C/C	T	C	SNV	KCNJ5	KCNJ5:exonic:NM_000890.3	1	100	2	synonymous	NM_00089	c.834T>C	p.(=)	-1.2					Inward rectif	rs7118833	0.1784	YES	8.79E-1 (pos.128782002)		Potassium	G-protein	Adenoma;Anderser	Ethanol,Gl	Benign	T=0, C=30	T=0.0, C=1.0	0.00001	446.639	30	T=0/0, C=12/1
chr11:128782012	C/G	C	G	SNV	KCNJ5	KCNJ5:exonic:NM_000890.3	1	96.67	2	missense	NM_00089	c.844C>G	p.Gln282Glu	6.85	1	29	0	0.29(COSM38	Inward rectif	rs1060503834,rs710	0.018	YES	1.0 (pos.128782012)	adenocarcino	Potassium	G-protein	Adenoma;Anderser	Ethanol,Gl	Benign	C=1, G=29	C=0.0333, G=0.9667	0.00001	416.511	30	C=0/1, G=12/1
chr17:7357864	C/A	C	A	SNV	CHRNB1	CHRNB1:intronic:NM_000747.2	1	57.14		intronic	NM_00074	c.1044+25	p.?	0.67					Neurotransm	rs7210231	0.207	YES	4.271E-2 (pos.7357864)		Cholinergic	acetylchol	Congenital, Heredit	Galantam	Benign	C=9, A=12	C=0.4286, A=0.5714	0.00001	109.102	21	C=4/5, A=4/8
chr17:7358803	A/A	G	A	SNV	CHRNB1	CHRNB1:intronic:NM_000747.2	1	100		intronic	NM_00074	c.1217+28	p.?	0.01					Neurotransm	rs7214344	2.00E-04	YES	1.0 (pos.7358803)		Cholinergic	acetylchol	Congenital, Heredit	Galantam	Benign	G=0, A=90	G=0.0, A=1.0	0.00001	1493.12	90	G=0/0, A=52/3
chr17:73626829	G/G	C	G	SNV	RECQL5	RECQL5:intronic:NM_004259.6	1	100		intronic	NM_00425	c.1644+30	p.?	-0.49					DEAD/DEAH b	rs781016037,rs8201	2.00E-04		1.472E-4 (pos.73626828),9.988E-1;0.0 (p		DNA helicas	ATP binding,ATP-dependent 3'-5' DNA helicase activity,A				C=0, G=24	C=0.0, G=1.0	0.00001	334.792	24	C=0/0, G=10/1
chr17:73720900	G/G	A	G	SNV	ITGB4	ITGB4:intronic:NM_000213.4	1	100		intronic	NM_00021	c.79+38A>	p.?	0.09					Calx-beta doi	rs820167	5.00E-04		1.0 (pos.73720900)		Integrin, be	G-protein	Abnormalities, Mul	R1295		A=0, G=38	A=0.0, G=1.0	0.00001	603.761	38	A=0/0, G=14/2
chr17:73732810	C/C	A	C	SNV	ITGB4	ITGB4:intronic:NM_000213.4	1	100		intronic	NM_00021	c.1990+35	p.?	0.1					Calx-beta doi	rs820177	0.2177	YES	5.616E-1;0.0 (pos.73732810)		Integrin, be	G-protein	Abnormalities, Mul	R1295		A=0, C=31	A=0.0, C=1.0	0.00001	465.819	31	A=0/0, C=17/1
chr17:73759304	T/T	G	T	SNV	GALK1	GALK1:intronic:NM_000154.1	1	100		intronic	NM_00015	c.476-74C	p.?	-0.31					GHMP kinase	rs820386		YES	9.999E-1 (pos.73759304)		Galactokina	ATP bindin	Brain Diseases;Brai	Phosphoaminophosphor		G=0, T=29	G=0.0, T=1.0	0.00001	427.59	29	G=0/0, T=12/1
chr17:73809442	A/G	A	G	SNV	UNK	UNK:intronic:NM_001080419.2	1	50		intronic	NM_00108	c.758+148	p.?	-0.09					Zinc finger C-	rs3803733		YES			Unkempt fa	RNA binding,cell morphogenesis involved in neuron diffe				A=22, G=22	A=0.5, G=0.5	0.00001	243.63	44	A=11/11, G=4/
chr21:39086965	C/C	T	C	SNV	KCNJ6	KCNJ6:exonic:NM_002240.4	1	100	3	synonymous	NM_00224	c.495A>G	p.(=)	-3.52				0.03(COSM36	Inward rectif	rs2070995	0.2134	YES	6.169E-1 (pos.39086965)	acute_myeloic	Potassium	G-protein	Abnormalities, Mul	Ethanol,Halothane		T=0, C=55	T=0.0, C=1.0	0.00001	814.907	55	T=0/0, C=28/2
chr2:26951449	A/A	G	A	SNV	KCNK3	KCNK3:utr_3:NM_002246.2	1	100	2	utr_3	NM_00224	c.1198G>A	p.?	2.42					Ion channel	rs1663002	0.001	YES	1.0 (pos.26951449)		Potassium	S100 prote	Nervous System Dis	Doxapram	Benign	G=0, A=49	G=0.0, A=1.0	0.00001	831.579	49	G=0/0, A=28/2
chr17:73987373	T/C	T	C	SNV	TEN1,TEN1-CDK3	TEN1-CDK3:intronic_nc:NR_037709.1, TEN1:intronic:NM_00	1	47.62		intronic	NM_00111	c.93-174T	p.?	-0.24						rs6501847		YES			TEN1, CST co	CST complex,negative regulation of telomere maintenan				T=11, C=10	T=0.5238, C=0.4762	0.00001	82.2898	21	T=2/9, C=3/7
chr11:64066859	A/A	G	A	SNV	KCNK4,KCNK4-TEX40	KCNK4-TEX40:exonic_nc:NR_133662.1, KCNK4:exonic:NM_0	1	100	7	synonymous	NM_03331	c.843G>A	p.(=)	-2.55				0.03(COSM17	Ion channel	rs2232417	0.0035	YES	9.27E-1 (pos.64066859)	carcinoma,tra	Potassium	cellular response to alkaline pH,cellular response to fat				G=0, A=44	G=0.0, A=1.0	0.00001	726.569	44	G=0/0, A=39/5
chr17:7400041	C/T	C	T	SNV	POLR2A	POLR2A:intronic:NM_000937.4	1	58.33		intronic	NM_00093	c.538-42C	p.?	0.31					RNA polymer	rs9890920	0.2453	YES	9.093E-1 (pos.7400041)		Polymerase	7-methylguanosine mRNA capping,DNA binding,DNA-dire				C=20, T=28	C=0.4167, T=0.5833	0.00001	334.141	48	C=10/10, T=16
chr5:113698544	C/C	G	C	SNV	KCNN2	KCNN2:exonic:NM_021614.3	1	100	1	synonymous	NM_02161	c.72G>C	p.(=)	0.98				0.71(COSM37	Calcium-acti	rs40179	0.4454	YES	8.605E-1 (pos.11369854	acute_myeloic	Potassium	Z disc,alph	Cardiovascular Dis	Miconazole,Procaine,Tri		G=0, C=94	G=0.0, C=1.0	0.00001	1576.17	94	G=0/0, C=70/2
chr1:154744807	G/G	C	G	SNV	KCNN3	KCNN3:exonic:NM_001204087.1	1	100	3	synonymous	NM_00120	c.1092G>C	p.(=)	-0.21				0.1(COSM414	Calcium-acti	rs1051614	0.385	YES	5.098E-1;0.0 (pos.154744	neoplasm	Potassium	calmoduli	Arrhythmias, Cardi	Miconazol	Benign	C=0, G=40	C=0.0, G=1.0	0.00001	644.22	40	C=0/0, G=23/1
chr17:7404209	A/A	G	A	SNV	POLR2A	POLR2A:intronic:NM_000937.4	1	100		intronic	NM_00093	c.1881+42	p.?	-2.12					RNA polymer	rs6503030	3.00E-04		1.0 (pos.7404209)		Polymerase	7-methylguanosine mRNA capping,DNA binding,DNA-dire				G=0, A=25	G=0.0, A=1.0	0.00001	353.019	25	G=0/0, A=15/1
chr1:154744852	G/G	A	G	SNV	KCNN3	KCNN3:exonic:NM_001204087.1	1	100	3	synonymous	NM_00120	c.1047T>C	p.(=)	-1.98				0.05(COSM41	Calcium-acti	rs1131820	0.2617	YES	9.863E-1 (pos.15474485	neoplasm	Potassium	calmoduli	Arrhythmias, Cardi	Miconazol	Benign	A=0, G=37	A=0.0, G=1.0	0.00001	572.835	37	A=0/0, G=20/1
chr17:7406343	C/C	T	C	SNV	POLR2A	POLR2A:intronic:NM_000937.4	1	100		intronic	NM_00093	c.2757-97	p.?	-0.54					RNA polymer	rs9890028		YES			Polymerase	7-methylguanosine mRNA capping,DNA binding,DNA-dire				T=0, C=38	T=0.0, C=1.0	0.00001	601.661	38	T=0/0, C=31/7
chr17:74066447	T/A	T	A	SNV	SRP68	SRP68:intronic:NM_014230.3	1	50		intronic	NM_01423	c.251+12A	p.?	-1.36					RNA-binding	rs2443168	0.4063	YES	5.368E-1 (pos.74066447)		Signal reco	7S RNA bin	Arsenic Poisoning;Congenital Abnormalities;			T=25, A=25	T=0.5, A=0.5	0.00001	288.837	50	T=10/15, A=18
chr1:41285087	C/C	T	C	SNV	KCNQ4	KCNQ4:exonic:NM_004700.3	1	100	5	synonymous	NM_00470	c.777T>C	p.(=)	-4.23				0.03(COSM37	Ion channel,I	rs4660468	0.2731	YES	4.488E-1 (pos.41285087)	acute_myeloic	Potassium	basal plas	Ear Diseases:Heari	Ezogabine	Benign	T=0, C=53	T=0.0, C=1.0	0.00001	783.871	53	T=0/0, C=14/3
chr2:18112473	A/A	G	A	SNV	KCNS3	KCNS3:exonic:NM_002252.4	1	100	3	synonymous	NM_00225	c.198G>A	p.(=)	2.4					BTB/POZ dom	rs4240213	0.0317	YES	1.0 (pos.18112473)		Potassium	Golgi apparatus,cytosol,delayed rectifier potassium cha				G=0, A=43	G=0.0, A=1.0	0.00001	705.842	43	G=0/0, A=26/1
chr2:18113623	G/G	A	G	SNV	KCNS3	KCNS3:exonic:NM_002252.4	1	100	3	missense	NM_00225	c.1348A>G	p.Thr450Ala	3.6		58		0.66(COSM37	BTB/POZ dom	rs4832524	0.3631	YES	9.794E-1 (pos.18113623)	adenocarcino	Potassium	Golgi apparatus,cytosol,delayed rectifier potassium cha				A=0, G=24	A=0.0, G=1.0	0.00001	334.792	24	A=0/0, G=11/1
chr17:74533347	C/C	T	C	SNV	CYGB	CYGB:intronic:NM_134268.4	1	100		intronic	NM_13426	c.143+135	p.?	-0.18					Globin	rs9914096		YES			Cytoglobin	catalase a	Congenital, Hereditary, and Neonatal Diseas			T=0, C=40	T=0.0, C=1.0	0.00001	644.284	40	T=0/0, C=30/1
chr1:196227526	G/G	A	G	SNV	KCNT2	KCNT2:exonic:NM_198503.4	1	100	26	synonymous	NM_19850	c.3009T>C	p.(=)	1.55					Calcium-acti	rs977157	2.00E-04		1.0 (pos.196227526)		Potassium	ATP binding,calcium-activated potassium channel activi				A=0, G=33	A=0.0, G=1.0	0.00001	504.598	33	A=0/0, G=6/27
chr7:128550680	G/G	GC	G	INDEL	KCP	KCP:splicesite_3:NM_001135914.1	1	100	2	splicesite	NM_001135914.1			0.0, 1.4					von Willebra	rs112748667,rs1133	5.00E-04	YES	1.0 (pos.128550680)	astrocytoma	Kielin/chor	extracellular region,extracellular space,hematopoietic				GC=0, G=41	GC=0.0, G=1.0	0.00001	531.309	41	GC=0/0, G=36/
chr18:24035675	T/C	T	C	SNV	KCTD1	KCTD1:utr_3:NM_001142730.2	1	52.27	5	utr_3	NM_00114	c.2630A>G	p.?	-0.01					BTB/POZ dom	rs3753	0.1063	YES	4.74E-2 (pos.24035675)		Potassium	identical p	Abnormalities, Multiple;Cardiovascular Dise			T=21, C=23	T=0.4773, C=0.5227	0.00001	259.938	44	T=21/0, C=20/
chr7:6505901	C/C	T	C	SNV	KDELR2	KDELR2:exonic:NM_006854.3	1	100	4	synonymous	NM_00685	c.405A>G	p.(=)	-0.48					ER lumen pro	rs2230263	0.066	YES	5.87E-1 (pos.6505901)			ER retention sequence binding,Golgi apparatus,Golgi me				T=0, C=61	T=0.0, C=1.0	0.00001	921.832	61	T=0/0, C=19/4
chr17:74734598	T/T	C	T	SNV	MFSD11	MFSD11:intronic:NM_001242532.2	1	100		intronic	NM_00124	c.96+68C>	p.?	0.35					Ion channel r	rs237053		YES	9.956E-1 (pos.74734598)			biological	Autoimmune Diseases;Female Urogenital Di			C=0, T=61	C=0.0, T=1.0	0.00001	925.766	61	C=0/0, T=26/3
chr17:7474277	T/T	C	T	SNV	SENP3,SENP3-EIF4A1	SENP3-EIF4A1:intronic_nc:NR_037926.1, SENP3:intronic:NM	1	100		intronic	NM_01567	c.1614+26	p.?	0.08					Ulp1 proteas	rs4968213,rs775980	0.0496	YES	0.0 (pos.7474274),5.743E-1 (pos.747427		Sentrin-spe	MLL1 complex,cysteine-type peptidase activity,cytoplas				C=0, T=21	C=0.0, T=1.0	0.00001	280.815	21	C=0/0, T=16/5
chr17:74750215	CT/CT	C	CT	INDEL	MFSD11	MFSD11:intronic:NM_001242532.2	1	100		intronic	NM_00124	c.682+46C	p.?	1.66					Ion channel r	rs11399997,rs20134	0.0011		9.998E-1;2.37E-4 (pos.74750215)			biological	Autoimmune Diseases;Female Urogenital Di			C=0, CT=33	C=0.0, CT=1.0	0.00001	465.766	33	C=0/0, CT=9/2
chr1:27278521	A/A	G	A	SNV	KDF1	KDF1:exonic:NM_152365.2	1	100	2	synonymous	NM_15236	c.351C>T	p.(=)	3.71				0.93(COSM41	Domain of un	rs3010110	0.2621	YES	3.726E-2 (pos.27278521)	neoplasm	Keratinocyt	cell cortex,cell junction,cell leading edge,cytoplasm,dev				G=0, A=36	G=0.0, A=1.0	0.00001	562.955	36	G=0/0, A=32/4
chr17:74920957	G/T	G	T	SNV	MGAT5B	MGAT5B:intronic:NM_144677.2	1	46.88		intronic	NM_14467	c.1026-91	p.?	-0.37					Glycosyltrans	rs3889146		YES			Alpha-1,6-m	Golgi appa	Adnexal Diseases;Cysts;Endocrine System Di			G=17, T=15	G=0.5313, T=0.4688	0.00001	143.733	32	G=9/8, T=7/8
chr11:66995603	C/C	T	C	SNV	KDM2A	KDM2A:exonic:NM_012308.2	1	100	11	synonymous	NM_01230	c.1053T>C	p.(=)	-0.03					CXXC zinc fing	rs3741189	0.0104	YES	9.529E-1 (pos.66995603)			DNA binding,double-strand break repair via nonhomolog				T=0, C=38	T=0.0, C=1.0	0.00001	603.786	38	T=0/0, C=17/2
chr5:137717265	A/A	G	A	SNV	KDM3B	KDM3B:exonic:NM_016604.3	1	100	6	missense	NM_01660	c.766G>A	p.Ala256Thr	-0.67	1	58	0		JmjC domain	rs6865472	0		1.0 (pos.137717265)		Lysine-spec	antioxidant activity,cellular oxidant detoxification,chro				G=0, A=37	G=0.0, A=1.0	0.00001	583.706	37	G=0/0, A=23/1
chr19:5041178	C/C	T	C	SNV	KDM4B	KDM4B:exonic:NM_015015.2	1	100	5	synonymous	NM_01501	c.348T>C	p.(=)	-2.45					JmjC domain	rs2240678	0.0958	YES	7.348E-1 (pos.5041178)		Lysine-spec	cytosol,his	Glioma;Medulloblastoma;Neoplasms;Neopl			T=0, C=44	T=0.0, C=1.0	0.00001	713.943	44	T=0/0, C=29/1
chr17:76118834	C/C	G	C	SNV	TMC6	TMC6:intronic:NM_001127198.2	1	100		intronic	NM_00112	c.1083-4C	p.?	0.79					TMC domain	rs2057188	0.1007	YES	1.248E-1 (pos.76118834)		Transmemb	Golgi appa	Epidermodysplasia Verruciformis;Skin Diseas			G=0, C=40	G=0.0, C=1.0	0.00001	646.039	40	G=0/0, C=8/32
chr17:76120065	G/G	A	G	SNV	TMC6	TMC6:intronic:NM_001127198.2	1	100		intronic	NM_00112	c.1082+5T	p.?	-2.61					TMC domain	rs2613522	0.2838	YES	4.864E-1 (pos.76120065)		Transmemb	Golgi appa	Epidermodysplasia Verruciformis;Skin Diseas			A=0, G=34	A=0.0, G=1.0	0.00001	524.186	34	A=0/0, G=20/1
chr1:202705455	C/C	G	C	SNV	KDM5B	KDM5B:exonic:NM_006618.4	1	100	21	synonymous	NM_00661	c.3150C>G	p.(=)	-4.02					ARID/BRIGHT	rs1141109	0.2226	YES	7.737E-1;0.0 (pos.202705455)		Lysine-spec	DNA binding,DNA binding transcription factor activity,br				G=0, C=49	G=0.0, C=1.0	0.00001	837.985	49	G=0/0, C=21/2
chr1:202733238	T/T	C	T	SNV	KDM5B	KDM5B:exonic:NM_006618.4	1	100	6	synonymous	NM_00661	c.747G>A	p.(=)	0.47					ARID/BRIGHT	rs3196669	0.222	YES	7.551E-1 (pos.202733238)		Lysine-spec	DNA binding,DNA binding transcription factor activity,br				C=0, T=30	C=0.0, T=1.0	0.00001	446.639	30	C=0/0, T=13/1
chr17:76198754	C/C	T	C	SNV	AFMID	AFMID:intronic:NM_001145526.2	1	100		intronic	NM_00114	c.260-30T	p.?	-0.41					Carboxyleste	rs35822441	0.3953	YES	7.581E-1 (pos.76198754)			arylformamidase activity,cytoplasm,cytosol,hydrolase a				T=0, C=117	T=0.0, C=1.0	0.00001	1874.05	117	T=0/0, C=47/7
chr17:76396610	A/A	G	A	SNV	PGS1	PGS1:intronic:NM_024419.4	1	100		intronic	NM_02441	c.702-148	p.?	-2.16						rs2292640		YES			Phosphatid	ATP binding,CDP-alcohol phosphatidyltransferase activi				G=0, A=25	G=0.0, A=1.0	0.00001	353.011	25	G=0/0, A=15/1
chr17:76400236	A/A	G	A	SNV	PGS1	PGS1:intronic:NM_024419.4	1	100		intronic	NM_02441	c.1402+66	p.?	-0.85						rs3744219		YES			Phosphatid	ATP binding,CDP-alcohol phosphatidyltransferase activi				G=0, A=44	G=0.0, A=1.0	0.00001	730.649	44	G=0/0, A=19/2
chr17:76400258	A/A	G	A	SNV	PGS1	PGS1:intronic:NM_024419.4	1	100		intronic	NM_02441	c.1402+88	p.?	0.26						rs3744217		YES			Phosphatid	ATP binding,CDP-alcohol phosphatidyltransferase activi				G=0, A=41	G=0.0, A=1.0	0.00001	664.655	41	G=0/0, A=16/2
chr17:76400268	G/G	C	G	SNV	PGS1	PGS1:intronic:NM_024419.4	1	100		intronic	NM_02441	c.1402+98	p.?	-1.08						rs3826323		YES			Phosphatid	ATP binding,CDP-alcohol phosphatidyltransferase activi				C=0, G=39	C=0.0, G=1.0	0.00001	623.973	39	C=0/0, G=14/2
chr17:76457910	C/C	G	C	SNV	DNAH17	DNAH17:intronic:NM_173628.3	1	100		intronic	NM_17362	c.9175-12	p.?	-0.21					AAA domain (	rs640292		YES			Dynein, axonemal, heavy chain 17					G=0, C=45	G=0.0, C=1.0	0.00001	747.392	45	G=0/0, C=27/1
chr17:76482256	G/G	A	G	SNV	DNAH17,DNAH17-AS1	DNAH17:intronic:NM_173628.3, DNAH17-AS1:intronic_nc:N	1	100		intronic	NM_17362	c.7102-41	p.?	-0.24					AAA domain (	rs691142	0.265	YES	7.095E-1;0.0 (pos.76482256)		Dynein, axonemal, heavy chain 17					A=0, G=20	A=0.0, G=1.0	0.00001	263.78	20	A=0/0, G=6/14
chr7:139797431	G/T	G	T	SNV	KDM7A	KDM7A:exonic:NM_030647.1	1	50	15	missense	NM_03064	c.1930C>A	p.Arg644Ser	2.47	0.93	110	0	0.17(COSM32	Cupin-like do	rs6950119	0.3398	YES	2.571E-1;0.0 (pos.13979	adenocarcinoma,ductal_ca		histone H3-K27 demethylation,histone H3-K36 demethy				G=16, T=16	G=0.5, T=0.5	0.00001	158.247	32	G=9/7, T=4/12
chr17:76525475	G/G	T	G	SNV	DNAH17	DNAH17:intronic:NM_173628.3	1	100		intronic	NM_17362	c.3507+79	p.?	-1.49					AAA domain (	rs884714		YES			Dynein, axonemal, heavy chain 17					T=0, G=29	T=0.0, G=1.0	0.00001	427.603	29	T=0/0, G=15/1
chr16:27231740	C/C	G	C	SNV	KDM8	KDM8:exonic:NM_001145348.1	1	100	7	synonymous	NM_00114	c.1134G>C	p.(=)	0.39					Cupin superfa	rs34577370	0.1443	YES	2.427E-3;0.0 (pos.27231740)			G2/M transition of mitotic cell cycle,chromatin binding,c				G=0, C=32	G=0.0, C=1.0	0.00001	484.904	32	G=0/0, C=25/7
chr17:76525805	G/G	C	G	SNV	DNAH17	DNAH17:intronic:NM_173628.3	1	100		intronic	NM_17362	c.3285-29	p.?	0.93					AAA domain (	rs4969212	0.3776	YES	6.076E-1 (pos.76525805)		Dynein, axonemal, heavy chain 17					C=0, G=25	C=0.0, G=1.0	0.00001	352.334	25	C=0/0, G=9/16
chr4:55946081	G/G	A	G	SNV	KDR	KDR:utr_3:NM_002253.2	1	100	30	utr_3	NM_00225	c.4098T>C	p.?	0.54					Immunoglob	rs4421048	2.00E-04		1.0 (pos.55946081)		Kinase inse	ATP bindin	Adenocarcinoma;B	3-(2-aminoquinazolin-6-		A=0, G=40	A=0.0, G=1.0	0.00001	644.266	40	A=0/0, G=20/2
chr17:76558159	C/C	T	C	SNV	DNAH17	DNAH17:intronic:NM_173628.3	1	100		intronic	NM_17362	c.1570-97	p.?	-2.94					AAA domain (	rs2376587					Dynein, axonemal, heavy chain 17					T=0, C=60	T=0.0, C=1.0	0.00001	907.068	60	T=0/0, C=28/3
chr12:91449990	T/T	C	T	SNV	KERA	KERA:exonic:NM_007035.3	1	100	2	synonymous	NM_00703	c.69G>A	p.(=)	0.37					Leucine Rich	rs2735333	0.221	YES	7.859E-1 (pos.91449990)		Keratocan	Golgi lume	Congenital, Hereditary, and Neonatal Diseas			C=0, T=43	C=0.0, T=1.0	0.00001	705.844	43	C=0/0, T=18/2
chr14:24909281	T/T	C	T	SNV	KHNYN,SDR39U1	KHNYN:utr_3:NM_015299.2, SDR39U1:utr_3:NM_020195.2	1	100	8	utr_3	NM_01529	c.4827C>T	p.?, p.?	-0.8					Domain of un	rs1043839	0.0873	YES	8.196E-1 (pos.24909281)		Short-chain	RNA binding,biological_process,cellular_component,mo				C=0, T=26	C=0.0, T=1.0	0.00001	371.41	26	C=0/0, T=19/7
chr14:24909362	C/C	T	C	SNV	KHNYN,SDR39U1	KHNYN:utr_3:NM_015299.2, SDR39U1:exonic:NM_020195.2	1	100	6	missense	NM_01529	c.4908T>C	p.?, p.Gln270Arg	3.58	0.37	43	0	0.45(COSM41	Domain of un	rs1043831	0.0875	YES	8.232E-1 (pos.24909362)	neoplasm	Short-chain	RNA binding,biological_process,cellular_component,mo				T=0, C=28	T=0.0, C=1.0	0.00001	408.718	28	T=0/0, C=15/1
chr17:7702357	T/T	C	T	SNV	DNAH2	DNAH2:intronic:NM_020877.3	1	100		intronic	NM_02087	c.8560-64	p.?	-1.6					AAA domain (	rs12949280		YES			Dynein, axo	ATP bindin	Autoimmune Diseases;Diabetes Mellitus;Dia			C=0, T=26	C=0.0, T=1.0	0.00001	371.42	26	C=0/0, T=21/5
chr14:24909475	G/G	C	G	SNV	KHNYN,SDR39U1	KHNYN:utr_3:NM_015299.2, SDR39U1:exonic:NM_020195.2	1	100	6	missense	NM_01529	c.5021C>G	p.?, p.Leu232Phe	0.69	1	22	0.016	0.83(COSM41	Domain of un	rs3211056	0.087	YES	8.208E-1;2.324E-4 (pos.2	acute_myeloic	Short-chain	RNA binding,biological_process,cellular_component,mo				C=0, G=80	C=0.0, G=1.0	0.00001	1289.3	80	C=0/0, G=49/3
chr17:77811523	A/A	AG	A	INDEL	CBX4	CBX4:intronic:NM_003655.2	1	100		intronic	NM_00365	c.179+113	p.?	0.16, 0.17					CBX family C-	rs577335150					Chromobox	PRC1 complex,PcG protein complex,SUMO binding,SUMC				AG=0, A=57	AG=0.0, A=1.0	0.00001	607.967	57	AG=0/0, A=33/
chr14:24910973	GA/GA	TT	GA	MNV	KHNYN,SDR39U1	KHNYN:downstream:NM_015299.2, SDR39U1:exonic:NM_0	2	100	4	missense	NM_01529	c.*4482TT	p.?, p.Ile79Leu	0.62, 0.05	0.6	5	0	0.18(COSM41	Domain of un	rs11625819,rs11625	0.0957	YES	0.0 (pos.24910972),8.15	neoplasm	Short-chain	RNA binding,biological_process,cellular_component,mo				TT=0, GA=44	TT=0.0, GA=1.0	0.00001	725.697	44	TT=0/0, GA=15
chr17:78061979	C/C	A	C	SNV	CCDC40	CCDC40:intronic:NM_017950.3	1	100		intronic	NM_01795	c.2711+78	p.?	-1.26					BRE1 E3 ubiqu	rs12942049		YES			Coiled-coil	axonemal	Bronchial Diseases;Bronchiectasis;Cardiovas			A=0, C=20	A=0.0, C=1.0	0.00001	263.785	20	A=0/0, C=3/17
chr16:27761040	T/T	C	T	SNV	KIAA0556	KIAA0556:exonic:NM_015202.3	1	100	16	missense	NM_01520	c.2759C>T	p.Pro920Leu	5.47	0.02	98	0.572	0.89(COSM39	Domain of un	rs61734902	0.0723	YES	3.389E-2 (pos.27761040)	acute_myeloic	Katanin-int	cell projection,cytoplasm,cytoskeleton,extracellular sp				C=0, T=26	C=0.0, T=1.0	0.00001	371.407	26	C=0/0, T=14/1
chr17:78081707	A/A	G	A	SNV	GAA	GAA:intronic:NM_001079804.2	1	100		intronic	NM_00107	c.955+12G	p.?	-1.32					Galactose mu	rs2252455	0.2455	YES	6.317E-1 (pos.78081707)		Glucosidase	alpha-1,4-	Aortic Stenosis, Sub	AT2220,Ac	Benign	G=0, A=44	G=0.0, A=1.0	0.00001	726.544	44	G=0/0, A=15/2
chr17:78081982	A/A	G	A	SNV	GAA	GAA:intronic:NM_001079804.2	1	100		intronic	NM_00107	c.956-107	p.?	-0.05					Galactose mu	rs2241888		YES			Glucosidase	alpha-1,4-	Aortic Stenosis, Sub	AT2220,Acarbose,Miglito		G=0, A=73	G=0.0, A=1.0	0.00001	1150.49	73	G=0/0, A=40/3
chr17:78082005	T/T	C	T	SNV	GAA	GAA:intronic:NM_001079804.2	1	100		intronic	NM_00107	c.956-84C	p.?	-1.71					Galactose mu	rs2241887		YES			Glucosidase	alpha-1,4-	Aortic Stenosis, Sub	AT2220,Acarbose,Miglito		C=0, T=72	C=0.0, T=1.0	0.00001	1130.68	72	C=0/0, T=39/3
chr17:78084418	A/A	G	A	SNV	GAA	GAA:intronic:NM_001079804.2	1	100		intronic	NM_00107	c.1438-10	p.?	-1.82					Galactose mu	rs12944802		YES			Glucosidase	alpha-1,4-	Aortic Stenosis, Sub	AT2220,Acarbose,Miglito		G=0, A=20	G=0.0, A=1.0	0.00001	263.783	20	G=0/0, A=10/1
chr17:78084507	C/C	G	C	SNV	GAA	GAA:intronic:NM_001079804.2	1	100		intronic	NM_00107	c.1438-19	p.?	-0.15					Galactose mu	rs2304844	0.2621	YES	5.284E-1 (pos.78084507)		Glucosidase	alpha-1,4-	Aortic Stenosis, Sub	AT2220,Ac	Benign	G=0, C=21	G=0.0, C=1.0	0.00001	281.235	21	G=0/0, C=8/13
chr17:78086846	G/G	A	G	SNV	GAA	GAA:intronic:NM_001079804.2	1	100		intronic	NM_00107	c.2040+20	p.?	0.09					Galactose mu	rs2304836,rs386799	0.2487	YES	6.412E-1 (pos.78086846)		Glucosidase	alpha-1,4-	Aortic Stenosis, Sub	AT2220,Ac	Benign	A=0, G=30	A=0.0, G=1.0	0.00001	440.025	30	A=0/0, G=6/24
chr17:6483046	C/C	T	C	SNV	KIAA0753	KIAA0753:utr_3:NM_014804.2	1	100	19	utr_3	NM_01480	c.2925A>G	p.?	0.16					Protein moor	rs3744720,rs773844	0.4253	YES	6.972E-1;0.0 (pos.6483046)		KIAA0753 ge	centriolar satellite,centriole,centriole replication,centr				T=0, C=36	T=0.0, C=1.0	0.00001	563.743	36	T=0/0, C=22/1
chr17:7811998	C/C	T	C	SNV	CHD3	CHD3:intronic:NM_001005271.2	1	100		intronic	NM_00100	c.5439-17	p.?	0.92					CHDCT2 (NUC	rs4239111	0.1657	YES	9.994E-1 (pos.7811998)		Chromodom	ATP bindin	Hemic and Lymphatic Diseases;Immune Syst			T=0, C=32	T=0.0, C=1.0	0.00001	488.671	32	T=0/0, C=17/1
chr17:6515387	G/G	A	G	SNV	KIAA0753	KIAA0753:exonic:NM_014804.2	1	100	8	missense	NM_01480	c.1397T>C	p.Leu466Pro	-0.78	0.17	98	0	0(COSM3755	Protein moor	rs2289642	0.4263	YES	6.97E-1 (pos.6515387)	acute_myeloic	KIAA0753 ge	centriolar satellite,centriole,centriole replication,centr				A=0, G=40	A=0.0, G=1.0	0.00001	644.286	40	A=0/0, G=27/1
chr17:78178182	T/C	T	C	SNV	CARD14	CARD14:intronic:NM_024110.4	1	57.14		intronic	NM_02411	c.2398+42	p.?	-2.77					Caspase recr	rs4889836	0.4803	YES	4.842E-1 (pos.78178182)		Caspase rec	CARD dom	Pityriasis;Pityriasis Rubra Pilaris;Psoriasis;S			T=15, C=20	T=0.4286, C=0.5714	0.00001	218.716	35	T=8/7, C=2/18
chr17:78178189	G/A	G	A	SNV	CARD14	CARD14:intronic:NM_024110.4	1	57.14		intronic	NM_02411	c.2398+49	p.?	0.55					Caspase recr	rs4889997	0.4637	YES	4.576E-1 (pos.78178189)		Caspase rec	CARD dom	Pityriasis;Pityriasis Rubra Pilaris;Psoriasis;S			G=15, A=20	G=0.4286, A=0.5714	0.00001	218.588	35	G=8/7, A=2/18
chr17:78324048	T/A	T	A	SNV	RNF213	RNF213:intronic:NM_001256071.2	1	52.27		intronic	NM_00125	c.10089-5	p.?	-0.31					Zinc finger, C	rs9944443	0.4739	YES			Ring finger	ATPase ac	Arterial Occlusive Diseases;Brain Diseases;C			T=21, A=23	T=0.4773, A=0.5227	0.00001	260.157	44	T=16/5, A=16/
chr17:6515454	T/T	C	T	SNV	KIAA0753	KIAA0753:exonic:NM_014804.2	1	100	8	missense	NM_01480	c.1330G>A	p.Asp444Asn	1.42	0.33	23	0.025	0.22(COSM11	Protein moor	rs2289643,rs386794	0.4631	YES	6.974E-1 (pos.6515454)	acute_myeloic	KIAA0753 ge	centriolar satellite,centriole,centriole replication,centr				C=0, T=42	C=0.0, T=1.0	0.00001	685.22	42	C=0/0, T=27/1
chr17:78599655	G/T	G	T	SNV	RPTOR	RPTOR:intronic:NM_020761.2	1	45.45		intronic	NM_02076	c.265+62G	p.?	0.14					HEAT repeat,	rs901065		YES			Regulatory-	14-3-3 pro	Mental Disorders;Nutrition Disorders;Nutriti			G=24, T=20	G=0.5455, T=0.4545	0.00001	213.744	44	G=6/18, T=12/
chr17:78765219	G/G	A	G	SNV	RPTOR	RPTOR:intronic:NM_020761.2	1	100		intronic	NM_02076	c.831-31A	p.?	-0.54					HEAT repeat,	rs4969429	0.2674	YES	7.376E-1 (pos.78765219)		Regulatory-	14-3-3 pro	Mental Disorders;Nutrition Disorders;Nutriti			A=0, G=21	A=0.0, G=1.0	0.00001	281.235	21	A=0/0, G=11/1
chr17:6531648	G/G	A	G	SNV	KIAA0753	KIAA0753:exonic:NM_014804.2	1	100	3	synonymous	NM_01480	c.507T>C	p.(=)	-1.67				0.05(COSM37	Protein moor	rs2072149	0.423	YES	6.971E-1;1.159E-4 (pos.6	acute_myeloic	KIAA0753 ge	centriolar satellite,centriole,centriole replication,centr				A=0, G=69	A=0.0, G=1.0	0.00001	1072.78	69	A=0/0, G=24/4
chr17:78896768	C/C	T	C	SNV	RPTOR	RPTOR:intronic:NM_020761.2	1	100		intronic	NM_02076	c.2624+14	p.?	-4.73					HEAT repeat,	rs2271606		YES			Regulatory-	14-3-3 pro	Mental Disorders;Nutrition Disorders;Nutriti			T=0, C=44	T=0.0, C=1.0	0.00001	726.569	44	T=0/0, C=17/2
chr17:79077957	C/C	T	C	SNV	BAIAP2	BAIAP2:intronic:NM_017451.2	1	100		intronic	NM_01745	c.1066+49	p.?	0.33					IRSp53/MIM	rs34057293	0.2631	YES	8.668E-1 (pos.79077957)		BAI1-associ	Fc-gamma receptor signaling pathway involved in phago				T=0, C=20	T=0.0, C=1.0	0.00001	263.786	20	T=0/0, C=12/8
chr1:155904177	C/C	T	C	SNV	KIAA0907	KIAA0907:utr_5:NM_014949.3	1	100	1	utr_5	NM_01494	c.-13A>G	p.?	-0.37						rs2275079	0.0811	YES	9.353E-1 (pos.155904177)			RNA binding,cytoplasm,mRNA splice site selection,nucle				T=0, C=45	T=0.0, C=1.0	0.00001	747.288	45	T=0/0, C=23/2
chr17:79163649	A/A	G	A	SNV	CEP131	CEP131:intronic:NM_014984.3	1	100		intronic	NM_01498	c.3169-6C	p.?	-0.06				0.07(COSM4000342),0.07(C		rs2292182	0.2576	YES	1.565E-1 (pos.79163649)	acute_myeloid_leukaemia		G2/M transition of mitotic cell cycle,acrosomal vesicle,c				G=0, A=36	G=0.0, A=1.0	0.00001	568.103	36	G=0/0, A=19/1
chr17:79166230	C/G	C	G	SNV	CEP131	CEP131:intronic:NM_014984.3	1	50		intronic	NM_01498	c.2548-38	p.?	-1.04						rs545348813			4.909E-4 (pos.79166230)			G2/M transition of mitotic cell cycle,acrosomal vesicle,c				C=13, G=13	C=0.5, G=0.5	0.00001	119.114	26	C=9/4, G=9/4
chr15:79755604	A/G	A	G	SNV	KIAA1024	KIAA1024:exonic:NM_015206.2	1	46.88	3	missense	NM_01520	c.2494A>G	p.Ile832Val	1.63	0.55	29	0	0.34(COSM41	Uncharacteri	rs2297773	0.1979	YES	1.481E-1;0.0 (pos.79755	neoplasm		integral component of membrane				A=17, G=15	A=0.5313, G=0.4688	0.00001	143.732	32	A=8/9, G=9/6
chr1:92647222	A/AG	A	AG	INDEL	KIAA1107	KIAA1107:exonic:NM_015237.3	1	100	6	frameshiftInsertion	NM_01523	c.2443_24	p.Asn815fs	1.85					Domain of unknown function (DUF4596)											A=0, AG=30	A=0.0, AG=1.0	0.00001	378.763	30	A=0/0, AG=18/
chr1:109704618	G/G	A	G	SNV	KIAA1324	KIAA1324:exonic:NM_020775.4	1	100	2	missense	NM_02077	c.256A>G	p.Ile86Val	3.16	1	29	0			rs678238	1.00E-04		9.986E-1 (pos.109704618)		KIAA1324 ge	RNA bindir	Nervous System Diseases;Neuromuscular Di			A=0, G=25	A=0.0, G=1.0	0.00001	353.019	25	A=0/0, G=21/4
chr1:109745618	C/C	T	C	SNV	KIAA1324	KIAA1324:exonic:NM_020775.4	1	100	22	missense	NM_02077	c.3026T>C	p.Leu1009Pro	1.26	0.34	98	0	0.08(COSM4142097)		rs1052878	0.0971	YES	9.682E-1 (pos.10974561	neoplasm	KIAA1324 ge	RNA bindir	Nervous System Diseases;Neuromuscular Di			T=0, C=20	T=0.0, C=1.0	0.00001	263.786	20	T=0/0, C=12/8

chr1:109737063	C/T	C	T	SNV	KIAA1324	KIAA1324:exonic:NM 020775.4	1	50	15	synonymous	NM 02077	c.1968C>T	p.(=)	-2.33				0.13(COSM5618807)		rs2359246	0.241	YES	9.731E-1 (pos.10973706:	ductal carcinc	KIAA1324 g	RNA bindir Nervous System Diseases;Neuromuscular Di	C=130, T=1	C=0.5, T=0.5	0.00001	1391.06	260	C=59/71, T=75
chr8:12879596	CT/CT	C	CT	INDEL	KIAA1456	KIAA1456:utr 3:NM 020844.2	1	100	5	utr 3	NM 02084	c.1408 14	p.?	0.08					Hypothetical methyltransferase,Methyltransferase domain,ubiE/COQ5 methyltransferase family						KIAA1456 g	cytoplasm,ferrous iron binding,nucleus,oxidation-reduc	C=0, CT=73	C=0.0, CT=1.0	0.00001	566.841	73	C=0/0, CT=37/
chr17:79422289	C/T	C	T	SNV	BAHCC1	BAHCC1:intronic:NM 001291324.1	1	57.89		intronic	NM 00129	c.4374-4C	p.?	-0.35				0.11(COSM62	BAH domain	rs1842041	0.3544	YES	1.758E-1 (pos.79422289)	neoplasm	BAH domair	chromatin,chromatin binding,chromatin silencing,chron	C=16, T=22	C=0.4211, T=0.5789	0.00001	248.656	38	C=5/11, T=11/
chr8:12879600	T/T	C	T	SNV	KIAA1456	KIAA1456:utr 3:NM 020844.2	1	100	5	utr 3	NM 02084	c.1412C>T	p.?	0.06					Hypothetical	rs498852	0		1.0 (pos.12879600)		KIAA1456 g	cytoplasm,ferrous iron binding,nucleus,oxidation-reduc	C=0, T=73	C=0.0, T=1.0	0.00001	1143.47	73	C=0/0, T=37/3
chr17:79424631	G/A	G	A	SNV	BAHCC1	BAHCC1:intronic:NM 001291324.1	1	57.14		intronic	NM 00129	c.4969+13	p.?	0.1					BAH domain	rs117681222	0.0844	YES	7.206E-3 (pos.79424631)		BAH domair	chromatin,chromatin binding,chromatin silencing,chron	G=9, A=12	G=0.4286, A=0.5714	0.00001	109.052	21	G=1/8, A=6/6
chr1:33235626	G/A	G	A	SNV	KIAA1522	KIAA1522:exonic:NM 020888.2	1	97.56	6	synonymous	NM 02088	c.846G>A	p.(=)	-2.8				0.01(COSM3751144),0.01(C		rs3737995	0.3536	YES	4.686E-1 (pos.33235626)	adenocarcinoma,neoplasn		cell differentiation	G=2, A=80	G=0.0244, A=0.9756	0.00001	1262.78	82	G=1/1, A=28/5
chr12:32134240	T/T	C	T	SNV	KIAA1551	KIAA1551:exonic:NM 018169.3	1	100	4	synonymous	NM 01816	c.351C>T	p.(=)	0.12					Domain of un	rs2388982	0.3065	YES	8.002E-1 (pos.32134240)				C=0, T=34	C=0.0, T=1.0	0.00001	524.186	34	C=0/0, T=25/9
chr12:32134638	C/C	T	C	SNV	KIAA1551	KIAA1551:exonic:NM 018169.3	1	100	4	missense	NM 01816	c.749T>C	p.Leu250Pro	0.96	1	98	0		Domain of un	rs2166807,rs762184(	0.2156	YES	8.32E-1;0.0;0.0 (pos.32134638)				T=0, C=29	T=0.0, C=1.0	0.00001	427.603	29	T=0/0, C=15/1
chr12:32135441	C/C	T	C	SNV	KIAA1551	KIAA1551:exonic:NM 018169.3	1	100	4	missense	NM 01816	c.1552T>C	p.Ser518Pro	-0.63	0.33	74	0		Domain of un	rs3759301	0.0579	YES	8.921E-1 (pos.32135441)				T=0, C=34	T=0.0, C=1.0	0.00001	524.186	34	T=0/0, C=17/1
chr22:25436904	C/C	T	C	SNV	KIAA1671	KIAA1671:exonic:NM 001145206.1	1	100	3	synonymous	NM 00114	c.3807T>C	p.(=)	-0.14				0(COSM5505(	Tankyrase bi	rs1040421		YES		carcinoma			T=0, C=23	T=0.0, C=1.0	0.00001	316.747	23	T=0/0, C=10/1
chr22:25577733	C/C	T	C	SNV	KIAA1671	KIAA1671:exonic:NM 001145206.1	1	100	8	synonymous	NM 00114	c.5142T>C	p.(=)	-4.09				0.01(COSM14	Tankyrase bi	rs9612887	0.4494	YES	7.622E-1 (pos.25577733)	adenocarcinoma			T=0, C=37	T=0.0, C=1.0	0.00001	583.698	37	T=0/0, C=24/1
chr22:25435028	G/T	G	T	SNV	KIAA1671	KIAA1671:exonic:NM 001145206.1	1	57.14	3	missense	NM 00114	c.1931G>T	p.Arg644Leu	-1.34	0.22	102	0.177		Tankyrase bi	rs73157984							G=12, T=16	G=0.4286, T=0.5714	0.00001	164.277	28	G=4/8, T=9/7
chr17:79862882	G/G	C	G	SNV	PCYT2	PCYT2:intronic:NM 001184917.2	1	100		intronic	NM 00118	c.1112+60	p.?	-0.65					Cytidylyltran:	rs146889521		YES				biosynthetic process,catalytic Lamivudine	C=0, G=31	C=0.0, G=1.0	0.00001	470.167	31	C=0/0, G=12/1
chr17:79893962	A/A	G	A	SNV	PYCR1	PYCR1:intronic:NM 006907.3	1	100		intronic	NM 0069C	c.138+37C	p.?	-0.35					NADP oxidore	rs34419046,rs75161:	0.0234	YES	0.0 (pos.79893960),7.13E-1 (pos.798939		Pyrroline-5-	L-proline b Bone Diseases;Bon L-Proline,NADH	G=0, A=35	G=0.0, A=1.0	0.00001	543.903	35	G=0/0, A=8/27
chr22:25435047	G/A	G	A	SNV	KIAA1671	KIAA1671:exonic:NM 001145206.1	1	53.33	3	synonymous	NM 00114	c.1950G>A	p.(=)	-0.89					Tankyrase bi	rs80228367							G=14, A=16	G=0.4667, A=0.5333	0.00001	159.924	30	G=5/9, A=8/8
chr17:80050272	A/A	G	A	SNV	FASN	FASN:intronic:NM 004104.4	1	100		intronic	NM 0041C	c.1029+59	p.?	-0.25					Acyl transfer:	rs17848925		YES			Fatty acid s	3-hydroxy Adenocarcinoma;A Cerulenin,Orlistat	G=0, A=26	G=0.0, A=1.0	0.00001	371.42	26	G=0/0, A=15/1
chr19:18368810	T/T	G	T	SNV	KIAA1683	KIAA1683:exonic:NM 001145304.1	1	100	4	missense	NM 00114	c.3284C>A	p.Pro1095His	-0.45	0.21	77	0	0(COSM1480	IQ calmodulir	rs999813	0.4548	YES	5.684E-1 (pos.18368810)	acute myeloid leukaemia		mitochondrion,nucleus,protein binding	G=0, T=21	G=0.0, T=1.0	0.00001	281.235	21	G=0/0, T=9/12
chr17:80356019	G/G	C	G	SNV	OGFOD3	OGFOD3:intronic:NM 024648.2	1	100		intronic	NM 02464	c.823+53G	p.?	-0.79					2OG-Fe(II) oxy	rs9905077		YES				L-ascorbic acid binding,dioxygenase activity,extracellula	C=0, G=37	C=0.0, G=1.0	0.00001	583.526	37	C=0/0, G=34/3
chr17:80402296	A/A	G	A	SNV	C17orf62	C17orf62:intronic:NM 001100407.2	1	100		intronic	NM 0011C	c.443+27C	p.?	-0.01					Domain of un	rs7221125	0.282	YES	8.598E-1 (pos.80402296)			integral cc Hemic and Lymphatic Diseases;Immune Syst	G=0, A=25	G=0.0, A=1.0	0.00001	353.019	25	G=0/0, A=14/1
chr19:18375659	T/T	C	T	SNV	KIAA1683	KIAA1683:exonic:NM 001145304.1	1	100	3	synonymous	NM 00114	c.2691G>A	p.(=)	0.24				0.04(COSM37	IQ calmodulir	rs12608843	0.2681	YES	3.88E-1;0.0;0.0 (pos.1837	carcinoma,hepatocellular		mitochondrion,nucleus,protein binding	C=0, T=25	C=0.0, T=1.0	0.00001	352.552	25	C=0/0, T=14/1
chr17:80545156	G/G	A	G	SNV	FOXK2	FOXK2:intronic:NM 004514.3	1	100		intronic	NM 00451	c.1786+8A	p.?	0.31					FHA domain,I	rs6502124	0.0135	YES	9.994E-1;0.0 (pos.80545156)		Forkhead bi	DNA binding transcription factor activity,RNA polymeras	A=0, G=62	A=0.0, G=1.0	0.00001	940.32	62	A=0/0, G=44/1
chr17:80574082	G/G	T	G	SNV	WDR45B	WDR45B:intronic:NM 019613.3	1	100		intronic	NM 01961	c.929-181	p.?	-2.44						rs753569		YES			WD repeat	autophagy Intellectual Disability;Mental Disorders;Nerv	T=0, G=40	T=0.0, G=1.0	0.00001	644.216	40	T=0/0, G=10/3
chr17:80585033	A/A	T	A	SNV	WDR45B	WDR45B:intronic:NM 019613.3	1	100		intronic	NM 01961	c.332+47A	p.?	-0.97						rs2291392	0.2145	YES	7.809E-1 (pos.80585033)		WD repeat	autophagy Intellectual Disability;Mental Disorders;Nerv	T=0, A=76	T=0.0, A=1.0	0.00001	1199.73	76	T=0/0, A=31/4
chr17:80616306	C/T	C	T	SNV	RAB40B	RAB40B:intronic:NM 006822.2	1	45.83		intronic	NM 00682	c.565+61G	p.?	-0.2					ADP-ribosylat	rs2306756		YES				GTP binding,GTPase activity,endosome,intracellular,intr	C=13, T=11	C=0.5417, T=0.4583	0.00001	93.4399	24	C=7/6, T=3/8
chr17:80693701	TG/TG	T	TG	INDEL	FN3K	FN3K:intronic:NM 022158.3	1	100		intronic	NM 02215	c.141+48T	p.?	-0.19					Fructosamine	rs753669266,rs761503033			0.0 (pos.80693695),0.0 (pos.80693701)		Fructosami	cellular component,cytosol,epithelial cell differentiatic	T=0, TG=35	T=0.0, TG=1.0	0.00001	322.907	35	T=0/0, TG=19/
chr17:80693705	G/G	A	G	SNV	FN3K	FN3K:intronic:NM 022158.3	1	100		intronic	NM 02215	c.141+52A	p.?	-1.41					Fructosamine	rs2256322,rs753669266			0.0 (pos.80693695),1.0 (pos.80693705)		Fructosami	cellular component,cytosol,epithelial cell differentiatic	A=0, G=35	A=0.0, G=1.0	0.00001	535.811	35	A=0/0, G=19/1
chr19:18376407	A/A	T	A	SNV	KIAA1683	KIAA1683:exonic:NM 001145304.1	1	100	3	missense	NM 00114	c.1943A>T	p.Tyr648Phe	-0.55	0	22	0.002	0.01(COSM36	IQ calmodulir	rs8110972	0.4258	YES	5.604E-1 (pos.18376407)	acute myeloid leukaemia		mitochondrion,nucleus,protein binding	T=0, A=47	T=0.0, A=1.0	0.00001	789.328	47	T=0/0, A=27/2
chr17:80827903	T/T	C	T	SNV	TBCD	TBCD:intronic:NM 005993.4	1	100		intronic	NM 00599	c.1319-19	p.?	-1.69					Tubulin foldir	rs9892064		YES			Tubulin-spe	GTPase activator activity,adherens junction,adherens ju	C=0, T=27	C=0.0, T=1.0	0.00001	389.859	27	C=0/0, T=19/8
chr17:80827955	G/G	A	G	SNV	TBCD	TBCD:intronic:NM 005993.4	1	100		intronic	NM 00599	c.1319-14!	p.?	-0.14					Tubulin foldir	rs7222931					Tubulin-spe	GTPase activator activity,adherens junction,adherens ju	A=0, G=29	A=0.0, G=1.0	0.00001	427.603	29	A=0/0, G=19/1
chr17:80828057	A/A	G	A	SNV	TBCD	TBCD:intronic:NM 005993.4	1	100		intronic	NM 00599	c.1319-43(	p.?	-0.07					Tubulin foldir	rs3744161	0.4229	YES	7.252E-1;0.0 (pos.80828057)		Tubulin-spe	GTPase activator activity,adherens junction,adherens ju	G=0, A=29	G=0.0, A=1.0	0.00001	427.564	29	G=0/0, A=16/1
chr17:80828303	A/A	G	A	SNV	TBCD	TBCD:intronic:NM 005993.4	1	100		intronic	NM 00599	c.1475+47	p.?	-1.68					Tubulin foldir	rs3744160	0.2521	YES	4.623E-1 (pos.80828303)		Tubulin-spe	GTPase activator activity,adherens junction,adherens ju	G=0, A=27	G=0.0, A=1.0	0.00001	389.983	27	G=0/0, A=9/18
chr17:80878389	G/G	A	G	SNV	TBCD	TBCD:intronic:NM 005993.4	1	100		intronic	NM 00599	c.2039-43	p.?	0.13					Tubulin foldir	rs1471197	0		1.0 (pos.80878389)		Tubulin-spe	GTPase activator activity,adherens junction,adherens ju	A=0, G=45	A=0.0, G=1.0	0.00001	747.338	45	A=0/0, G=31/1
chr17:80890638	C/C	T	C	SNV	TBCD	TBCD:intronic:NM 005993.4	1	100		intronic	NM 00599	c.3191+27	p.?	0.3					Tubulin foldir	rs898095	0.4844	YES	6.398E-1 (pos.80890638)		Tubulin-spe	GTPase activator activity,adherens junction,adherens ju	T=0, C=25	T=0.0, C=1.0	0.00001	350.246	25	T=0/0, C=4/21
chr17:80895253	A/A	G	A	SNV	TBCD	TBCD:intronic:NM 005993.4	1	100		intronic	NM 00599	c.3281+17	p.?	-3.27					Tubulin foldir	rs8079398	0.4239	YES	5.607E-1 (pos.80895253)		Tubulin-spe	GTPase activator activity,adherens junction,adherens ju	G=0, A=37	G=0.0, A=1.0	0.00001	583.706	37	G=0/0, A=20/1
chr19:18377274	G/G	A	G	SNV	KIAA1683	KIAA1683:exonic:NM 001145304.1	1	100	3	missense	NM 00114	c.1076T>C	p.Met359Thr	-0.49	0.46	81	0	0.01(COSM40	IQ calmodulir	rs3746186	0.4262	YES	5.612E-1 (pos.18377274)	acute myeloid leukaemia		mitochondrion,nucleus,protein binding	A=0, G=30	A=0.0, G=1.0	0.00001	446.638	30	A=0/0, G=10/2
chr17:81006286	A/A	G	A	SNV	B3GNTL1	B3GNTL1:intronic:NM 001009905.2	1	100		intronic	NM 0010C	c.277+61C	p.?	-0.16					Glycosyl tran	rs10852789		YES			Beat-1,3-N-	transferase activity, transferring glycosyl groups	G=0, A=94	G=0.0, A=1.0	0.00001	1571.14	94	G=0/0, A=61/3
chr19:18377496	G/G	C	G	SNV	KIAA1683	KIAA1683:exonic:NM 001145304.1	1	100	3	missense	NM 00114	c.854G>C	p.Ser285Thr	-0.13	1	58	0	0(COSM4000!	IQ calmodulir	rs8104533	0.4256	YES	5.604E-1 (pos.18377496)	acute myeloid leukaemia		mitochondrion,nucleus,protein binding	C=0, G=26	C=0.0, G=1.0	0.00001	371.419	26	C=0/0, G=9/17
chr19:18378219	G/G	A	G	SNV	KIAA1683	KIAA1683:exonic:NM 001145304.1	1	100	3	missense	NM 00114	c.131T>C	p.Leu44Pro	-1.3	0.22	98	0	0.01(COSM40	IQ calmodulir	rs1469023	0.4455	YES	5.609E-1 (pos.18378219)	acute myeloid leukaemia		mitochondrion,nucleus,protein binding	A=0, G=23	A=0.0, G=1.0	0.00001	316.747	23	A=0/0, G=7/16
chr20:36850940	G/C	G	C	SNV	KIAA1755	KIAA1755:exonic:NM 001029864.1	1	50	10	synonymous	NM 00102	c.2328C>G	p.(=)	-3.84						rs41310010	0.0677	YES	1.161E-1;0.0 (pos.36850940)				G=13, C=13	G=0.5, C=0.5	0.00001	119.613	26	G=9/4, C=1/12
chr2:8870814	G/G	A	G	SNV	KIDINS220	KIDINS220:utr 3:NM 020738.3	1	100	30	utr 3	NM 02073	c.5352T>C	p.?	0.57					Ankyrin repea	rs6431959	0.0114	YES	9.406E-1 (pos.8870814)		Kinase D-in	PDZ domain binding,activation of MAPKK activity,cellula	A=0, G=24	A=0.0, G=1.0	0.00001	334.756	24	A=0/0, G=18/6
chr17:8158273	G/G	A	G	SNV	PFAS	PFAS:intronic:NM 012393.2	1	100		intronic	NM 01239	c.279-72A	p.?	0.1					AIR synthase	rs6503094		YES			phosphorib	'de novo' IMP biosynthetic proc Glutamic Acid,L-Glutami	A=0, G=34	A=0.0, G=1.0	0.00001	523.622	34	A=0/0, G=22/1
chr6:17764279	C/C	A	C	SNV	KIF13A	KIF13A:utr 3:NM 022113.5	1	100	39	utr 3	NM 02211	c.5480T>G	p.?	0.65					FHA domain,I	rs1044654		YES			Kinesin fam	ATP binding,ATPase activity,Golgi to plasma membrane	A=0, C=31	A=0.0, C=1.0	0.00001	465.819	31	A=0/0, C=15/1
chr17:8169006	C/C	T	C	SNV	PFAS	PFAS:intronic:NM 012393.2	1	100		intronic	NM 01239	c.2545+49	p.?	-0.03					AIR synthase	rs11655563,rs74917	0.1819	YES	3.838E-1;0.0 (pos.8169006)		phosphorib	'de novo' IMP biosynthetic proc Glutamic Acid,L-Glutami	T=0, C=39	T=0.0, C=1.0	0.00001	623.98	39	T=0/0, C=34/5
chr17:8387439	ATGAA/ATGAA	A	ATGAA	INDEL	MYH10	MYH10:intronic:NM 001256012.1	4	100		intronic	NM 00125	c.5175+17	p.?	0.24					IQ calmodulin-binding motif,Myosin N-terminal SH3-like domain,Myosin head (motor domain),Myosi						Myosin, hea	ADP bindir Female Urogenital Diseases;Female Urogeni	A=0, ATGAA	A=0.0, ATGAA=1.0	0.00001	281.235	21	A=0/0, ATGAA=
chr17:8449767	G/G	A	G	SNV	MYH10	MYH10:intronic:NM 001256012.1	1	100		intronic	NM 00125	c.1278+95	p.?	-1.35					IQ calmodulir	rs4791724		YES			Myosin, hea	ADP bindir Female Urogenital Diseases;Female Urogeni	A=0, G=187	A=0.0, G=1.0	0.00001	3128.02	187	A=0/0, G=101/
chr6:17781450	A/A	G	A	SNV	KIF13A	KIF13A:exonic:NM 022113.5	1	100	30	synonymous	NM 02211	c.3627C>T	p.(=)	1.53				0.91(COSM37	FHA domain,I	rs942376	0.1901	YES	2.01E-1 (pos.17781450)	adenocarcinor	Kinesin fam	ATP binding,ATPase activity,Golgi to plasma membrane	G=0, A=24	G=0.0, A=1.0	0.00001	334.792	24	G=0/0, A=13/1
chr8:29005093	G/G	A	G	SNV	KIF13B	KIF13B:exonic:NM 015254.3	1	100	17	synonymous	NM 01525	c.1840T>C	p.(=)	2.31				0.19(COSM62	CAP-Gly doma	rs6986672	0.134	YES	4.282E-1 (pos.29005093)	neoplasm	Kinesin fam	14-3-3 protein binding,ATP binding,ATPase activity,T cell	A=0, G=30	A=0.0, G=1.0	0.00001	446.552	30	A=0/0, G=20/1
chr1:21011346	A/G	A	G	SNV	KIF17	KIF17:exonic:NM 020816.3	1	51.85	10	synonymous	NM 02081	c.2187T>C	p.(=)	-5.85					Kinesin moto	rs12028811	0.088	YES	3.818E-1 (pos.21011346)		Kinesin fam	ATP bindin Digestive System Diseases;Eosinophilia;Eosi	A=13, G=14	A=0.4815, G=0.5185	0.00001	132.664	27	A=5/8, G=6/8
chr11:28057957	C/C	T	C	SNV	KIF18A	KIF18A:exonic:NM 031217.3	1	100	14	missense	NM 03121	c.2203A>G	p.Ile735Val	-0.36	1	29	0	0.04(COSM44	Kinesin moto	rs10458896	0.2916	YES	4.442E-1 (pos.28057957)	acute myeloic	Kinesin fam	ATP bindin Autoimmune Diseases;Female Urogenital Di:	T=0, C=53	T=0.0, C=1.0	0.00001	782.027	53	T=0/0, C=37/1
chr17:9820695	C/T	C	T	SNV	GAS7	GAS7:intronic:NM 201433.1	1	56		intronic	NM 20143	c.1318-37	p.?	-0.62					Fes/CIP4, anc	rs4791910	0.4305	YES	5.75E-1 (pos.9820695)		Growth arre	DNA bindi Autoimmune Diseases;Female Urogenital Di:	C=11, T=14	C=0.44, T=0.56	0.00001	134.316	25	C=9/2, T=9/5
chr17:9820739	A/G	A	G	SNV	GAS7	GAS7:intronic:NM 201433.1	1	52.38		intronic	NM 20143	c.1318-81	p.?	-1.53					Fes/CIP4, anc	rs11078821		YES			Growth arre	DNA bindi Autoimmune Diseases;Female Urogenital Di:	A=10, G=11	A=0.4762, G=0.5238	0.00001	95.2548	21	A=8/2, G=6/5
chr18:10680017	C/C	T	C	SNV	PIEZO2	PIEZO2:intronic:NM 022068.3	1	100		intronic	NM 02206	c.7613+17	p.?	0.28					Piezo,Piezo n	rs386801177,rs688714		YES			PIEZO-type (	cation cha Abnormalities, Multiple;Arachnodactyly;Arth	T=0, C=40	T=0.0, C=1.0	0.00001	644.279	40	T=0/0, C=17/2
chr18:10680024	A/A	C	A	SNV	PIEZO2	PIEZO2:intronic:NM 022068.3	1	100		intronic	NM 02206	c.7613+17	p.?	0.2					Piezo,Piezo n	rs386801177,rs486001		YES			PIEZO-type (	cation cha Abnormalities, Multiple;Arachnodactyly;Arth	C=0, A=40	C=0.0, A=1.0	0.00001	644.286	40	C=0/0, A=17/2
chr18:10680104	T/T	C	T	SNV	PIEZO2	PIEZO2:intronic:NM 022068.3	1	100		intronic	NM 02206	c.7613+92	p.?	-1.78					Piezo,Piezo n	rs1944383		YES			PIEZO-type (	cation cha Abnormalities, Multiple;Arachnodactyly;Arth	C=0, T=39	C=0.0, T=1.0	0.00001	623.952	39	C=0/0, T=16/2
chr18:10680132	T/T	C	T	SNV	PIEZO2	PIEZO2:intronic:NM 022068.3	1	100		intronic	NM 02206	c.7613+64	p.?	-0.13					Piezo,Piezo n	rs688237		YES			PIEZO-type (	cation cha Abnormalities, Multiple;Arachnodactyly;Arth	C=0, T=46	C=0.0, T=1.0	0.00001	768.262	46	C=0/0, T=22/2
chr18:10680142	A/A	T	A	SNV	PIEZO2	PIEZO2:intronic:NM 022068.3	1	100		intronic	NM 02206	c.7613+54	p.?	0.43					Piezo,Piezo n	rs688222		YES			PIEZO-type (	cation cha Abnormalities, Multiple;Arachnodactyly;Arth	T=0, A=48	T=0.0, A=1.0	0.00001	810.434	48	T=0/0, A=24/2
chr18:10680163	A/A	G	A	SNV	PIEZO2	PIEZO2:intronic:NM 022068.3	1	100		intronic	NM 02206	c.7613+33	p.?	-0.17					Piezo,Piezo n	rs487037	0.1771	YES	6.714E-1 (pos.10680163)		PIEZO-type (	cation cha Abnormalities, Multiple;Arachnodactyly;Arth	G=0, A=48	G=0.0, A=1.0	0.00001	798.197	48	G=0/0, A=24/2
chr18:10741011	A/A	G	A	SNV	PIEZO2	PIEZO2:intronic:NM 022068.3	1	100		intronic	NM 02206	c.4633+18	p.?	4.12					Piezo,Piezo n	rs5024299	0.2087	YES	9.277E-1 (pos.10741011)		PIEZO-type (	cation cha Abnormalities, Multiple;Arach Benign	G=0, A=40	G=0.0, A=1.0	0.00001	644.286	40	G=0/0, A=31/9
chr17:72346855	C/C	T	C	SNV	KIF19	KIF19:exonic:NM 153209.3	1	100	12	synonymous	NM 15320	c.1398T>C	p.(=)	-2.52				0.01(COSM37	Kinesin moto	rs11077744	0.2037	YES	7.426E-1 (pos.72346855)	adenocarcinoma,squamou		ATP binding,ATP-dependent microtubule motor activity,	T=0, C=20	T=0.0, C=1.0	0.00001	263.778	20	T=0/0, C=11/9
chr17:72349067	T/T	C	T	SNV	KIF19	KIF19:exonic:NM 153209.3	1	100	15	synonymous	NM 15320	c.2088C>T	p.(=)	-0.05				0.12(COSM37	Kinesin moto	rs2280108	0.4909	YES	3.982E-1 (pos.72349067)	acute myeloid leukaemia		ATP binding,ATP-dependent microtubule motor activity,	C=0, T=26	C=0.0, T=1.0	0.00001	371.418	26	C=0/0, T=17/9
chr17:72350412	A/A	G	A	SNV	KIF19	KIF19:exonic:NM 153209.3	1	100	18	missense	NM 15320	c.2420G>A	p.Arg807His	1.29	0.02	29	0.008	0.07(COSM43	Kinesin moto	rs2271535	0.4672	YES	4.035E-1 (pos.72350412)	adenocarcinoma,neoplasn		ATP binding,ATP-dependent microtubule motor activity,	G=0, A=159	G=0.0, A=1.0	0.00001	2529.88	159	G=0/0, A=99/6
chr18:10763132	T/T	C	T	SNV	PIEZO2	PIEZO2:intronic:NM 022068.3	1	100		intronic	NM 02206	c.2872-36	p.?	-0.24					Piezo,Piezo n	rs7507093	0.2508	YES	2.721E-1 (pos.10763132)		PIEZO-type (	cation cha Abnormalities, Multiple;Arachnodactyly;Arth	C=0, T=65	C=0.0, T=1.0	0.00001	996.464	65	C=0/0, T=17/4
chr15:69728949	G/G	A	G	SNV	KIF23	KIF23:exonic:NM 138555.3	1	100	14	synonymous	NM 13855	c.1443A>G	p.(=)	-3.62					Arf6-interact	rs3825858	0.0312	YES	6.979E-1 (pos.69728949)		Kinesin fam	ATP bindin Anemia;Anemia, Dyserythropoietic, Congenit	A=0, G=22	A=0.0, G=1.0	0.00001	298.892	22	A=0/0, G=6/16
chr18:10789014	A/A	G	A	SNV	PIEZO2	PIEZO2:intronic:NM 022068.3	1	100		intronic	NM 02206	c.2169+63	p.?	0.44					Piezo,Piezo n	rs2870106		YES			PIEZO-type (	cation cha Abnormalities, Multiple;Arachnodactyly;Arth	G=0, A=201	G=0.0, A=1.0	0.00001	3249.74	201	G=0/0, A=134/
chr18:12014411	G/G	T	G	SNV	IMPA2	IMPA2:intronic:NM 014214.2	1	100		intronic	NM 01421	c.490+39T	p.?	0.08					Inositol monc	rs643107	1.00E-04		1.0 (pos.12014411)		Myo-inositc	cytoplasm Brain Diseases;Car Lithium	T=0, G=153	T=0.0, G=1.0	0.00001	2453.33	153	T=0/0, G=59/9
chr14:104641792	T/T	C	T	SNV	KIF26A	KIF26A:exonic:NM 015656.1	1	100	12	synonymous	NM 01565	c.2667C>T	p.(=)	-2.9				0.04(COSM43	Kinesin moto	rs2487305	0.338	YES	3.818E-1 (pos.10464179:	acute myeloic	Kinesin fam	ATP binding,ATPase activity,antigen processing and pres	C=0, T=24	C=0.0, T=1.0	0.00001	334.791	24	C=0/0, T=10/1
chr18:12353178	T/T	A	T	SNV	AFG3L2	AFG3L2:intronic:NM 006796.2	1	100		intronic	NM 00679	c.1165-21	p.?	0.89					ATPase famil	rs775166242,rs9966	0.116	YES	0.0;1.161E-4 (pos.12353173),1.615E-1 (p		ATPase fam	ATP bindin Ataxia;Brain Diseas ATP	A=0, T=41	A=0.0, T=1.0	0.00001	664.634	41	A=0/0, T=18/2
chr18:12512559	A/A	C	A	SNV	SPIRE1	SPIRE1:intronic:NM 001128626.1	1	100		intronic	NM 00112	c.730-29G	p.?	0.92					Kinase non-c	rs550613	0.1808	YES	7.521E-1;0.0 (pos.12512559)			actin binding,actin cytoskeleton organization,actin nucl	C=0, A=29	C=0.0, A=1.0	0.00001	426.041	29	C=0/0, A=12/1
chr18:12512574	C/C	T	C	SNV	SPIRE1	SPIRE1:intronic:NM 001128626.1	1	100		intronic	NM 00112	c.730-44A	p.?	-1.08					Kinase non-c	rs567709	0.005		9.999E-1 (pos.12512574)			actin binding,actin cytoskeleton organization,actin nucl	T=0, C=20	T=0.0, C=1.0	0.00001	263.786	20	T=0/0, C=3/17
chr14:104642341	G/G	A	G	SNV	KIF26A	KIF26A:exonic:NM 015656.1	1	100	12	synonymous	NM 01565	c.3216A>G	p.(=)	-4.82				0.02(COSM43	Kinesin moto	rs2487304	0.2716	YES	7.811E-1 (pos.10464234:	adenocarcinor	Kinesin fam	ATP binding,ATPase activity,antigen processing and pres	A=0, G=56	A=0.0, G=1.0	0.00001	832.1	56	A=0/0, G=19/3
chr18:13030383	A/A	G	A	SNV	CEP192	CEP192:intronic:NM 032142.3	1	100		intronic	NM 03214	c.1391-81	p.?	0.52						rs9964466		YES			Centrosom:	G2/M transition of mitotic cell cycle,centriole,centroson	G=0, A=24	G=0.0, A=1.0	0.00001	334.743	24	G=0/0, A=9/15
chr18:13049948	G/GA	G	GA	INDEL	CEP192	CEP192:intronic:NM 032142.3	1	100		intronic	NM 03214	c.3017+58	p.?	-0.32						rs11441028,rs397736454,rs869		YES			Centrosom:	G2/M transition of mitotic cell cycle,centriole,centroson	G=0, GA=37	G=0.0, GA=1.0	0.00001	265.418	37	G=0/0, GA=15/
chr14:104643859	G/G	A	G	SNV	KIF26A	KIF26A:exonic:NM 015656.1	1	100	12	synonymous	NM 01565	c.4734A>G	p.(=)	0.71				0.05(COSM37	Kinesin moto	rs2487300	0.3526	YES	4.83E-1 (pos.104643859)	adenocarcinor	Kinesin fam	ATP binding,ATPase activity,antigen processing and pres	A=0, G=21	A=0.0, G=1.0	0.00001	280.221	21	A=0/0, G=9/12
chr14:104644099	C/C	T	C	SNV	KIF26A	KIF26A:exonic:NM 015656.1	1	100	12	synonymous	NM 01565	c.4974T>C	p.(=)	-1.64				0.02(COSM63	Kinesin moto	rs2497297	0.3071	YES	3.977E-1 (pos.10464409!	carcinoma	Kinesin fam	ATP binding,ATPase activity,antigen processing and pres	T=0, C=28	T=0.0, C=1.0	0.00001	408.718	28	T=0/0, C=11/1
chr18:13732053	G/G	A	G	SNV	RNMT	RNMT:intronic:NM 003799.2	1	100		intronic	NM 00379	c.417+120	p.?	-0.66					Methyltransf	rs4797806		YES			RNA guanin	7-methylguanosine mRNA capping,RNA 5'-cap (guanine-N	A=0, G=45	A=0.0, G=1.0	0.00001	751.889	45	A=0/0, G=24/2
chr18:13732059	T/T	C	T	SNV	RNMT	RNMT:intronic:NM 003799.2	1	100		intronic	NM 00379	c.417+126	p.?	0.08					Methyltransf	rs4797807		YES			RNA guanin	7-methylguanosine mRNA capping,RNA 5'-cap (guanine-N	C=0, T=43	C=0.0, T=1.0	0.00001	705.163	43	C=0/0, T=22/2
chr1:245850261	G/A	G	A	SNV	KIF26B	KIF26B:exonic:NM 018012.4	1	51.92	12	missense	NM 01801	c.3976G>A	p.Ala1326Thr	0.31	0.15	58	0.002	0.08(COSM20	Kinesin moto	rs375160223	1.00E-04		0.0 (pos.245850261)	adenocarcinor	Kinesin fam	ATP binding,ATPase activity,cytoplasm,establishment of	G=75, A=81	G=0.4808, A=0.5192	0.00001	860.099	156	G=44/31, A=3
chr18:14538010	C/C	T	C	SNV	POTEC	POTEC:intronic:NM 001137671.1	1	100		intronic	NM 00113	c.637-37A	p.?	-1.03					Ankyrin repe	rs6505912	0.3498	YES	1.34E-1 (pos.14538010)				T=0, C=35	T=0.0, C=1.0	0.00001	533.33	35	T=0/0, C=28/7
chr9:86468715	T/T	A	T	SNV	KIF27	KIF27:exonic:NM 017576.3	1	100	15	synonymous	NM 01757	c.3186T>A	p.(=)	0.8				0.62(COSM37	Kinesin moto	rs295274	0.3585	YES	5.14E-1;0.0 (pos.864687:	adenocarcinor	Kinesin fam	ATP binding,ATP-dependent microtubule n Benign	A=0, T=22	A=0.0, T=1.0	0.00001	301.859	22	A=0/0, T=12/1
chr17:51901084	G/G	A	G	SNV	KIF2B	KIF2B:exonic:NM 032559.4	1	100	1	synonymous	NM 03255	c.690A>G	p.(=)	2.8				0.11(COSM41	Kinesin moto	rs3803825	0.1467	YES	8.066E-1 (pos.51901084)	neoplasm	Kinesin fam	ATP binding,ATPase activity,antigen processing and pres	A=0, G=37	A=0.0, G=1.0	0.00001	583.706	37	A=0/0, G=22/1
chr15:90176432	G/G	T	G	SNV	KIF7	KIF7:exonic:NM 198525.2	1	100	13	synonymous	NM 19852	c.2658A>C	p.(=)	-1.09				0.04(COSM14	Kinesin moto	rs3803531	0.0876	YES	7.193E-1;0.0 (pos.90176	adenocarcinor	Kinesin fam	ATP bindin Abnormalities, Multiple;Acroc: Benign/Likely	T=0, G=22	T=0.0, G=1.0	0.00001	298.663	22	T=0/0, G=19/3
chr18:19115896	T/T	A	T	SNV	ESCO1	ESCO1:intronic:NM 052911.2	1	100		intronic	NM 05291	c.2187+10	p.?	0.98					ESCO1/2 acet	rs4800780		YES			Establishm	N-acetyltransferase activity,chromatin,identical proteir	A=0, T=24	A=0.0, T=1.0	0.00001	334.792	24	A=0/0, T=17/7
chr15:90191827	C/C	T	C	SNV	KIF7	KIF7:exonic:NM 198525.2	1	100	5	missense	NM 19852	c.1102A>G	p.Thr368Ala	-0.48	0.56	58	0	0.01(COSM41	Kinesin moto	rs8037349	0.0955	YES	8.137E-1 (pos.90191827)	neoplasm	Kinesin fam	ATP bindin Abnormalities, Multiple;Acroc: Benign/Likely	T=0, C=164	T=0.0, C=1.0	0.00001	2659.26	164	T=0/0, C=78/8
chr18:203081	C/C	A	C	SNV	USP14	USP14:intronic:NM 005151.3	1	100		intronic	NM 00515	c.943-17A	p.?	-0.16					Ubiquitin car	rs471274	0.3887	YES	5.653E-1 (pos.203081)		Ubiquitin-s	cell surface,chemical synaptic transmission,cysteine-tv	A=0, C=28	A=0.0, C=1.0	0.00001	408.686	28	A=0/0, C=16/1
chr18:21088081	C/C	T	C	SNV	C18orf8	C18orf8:intronic:NM 013326.4	1	100		intronic	NM 01332	c.321+76T	p.?	-1.04					Colon cancer	rs1652381		YES				lysosomal membrane	T=0, C=28	T=0.0, C=1.0	0.00001	408.709	28	T=0/0, C=18/1
chr8:145692385	T/T	C	T	SNV	KIFC2	KIFC2:exonic:NM 145754.3	1	100	3	synonymous	NM 14575	c.222C>T	p.(=)	-0.18				0.52(COSM37	Kinesin moto	rs2250891,rs762058:	0.0036	YES	0.0 (pos.145692381),7.9:	adenocarcinor	Kinesin fam	ATP binding,ATPase activity,cytoplasm,kinesin complex,	C=0, T=26	C=0.0, T=1.0	0.00001	371.418	26	C=0/0, T=19/7
chr18:21103971	G/G	T	G	SNV	C18orf8	C18orf8:intronic:NM 013326.4	1	100		intronic	NM 01332	c.962-123	p.?	1.14					Colon cancer	rs891386		YES				lysosomal membrane	T=0, G=28	T=0.0, G=1.0	0.00001	408.699	28	T=0/0, G=23/5
chr18:21119291	G/G	A	G	SNV	NPC1	NPC1:intronic:NM 000271.4	1	100		intronic	NM 00027	c.2911+28	p.?	-1.35					MMPL family	rs6507720,rs981021	0.4719	YES	6.376E-1 (pos.21119291)		NPC1 gene,	Golgi appa Arterial Occlusive Diseases;Ar Benign	A=0, G=31	A=0.0, G=1.0	0.00001	465.819	31	A=0/0, G=16/1
chr8:145693720	G/G	A	G	SNV	KIFC2	KIFC2:exonic:NM 145754.3	1	100	8	synonymous	NM 14575	c.819A>G	p.(=)	-0.08				0.13(COSM41	Kinesin moto	rs2250657	0.001	YES	7.895E-1 (pos.14569372(	neoplasm	Kinesin fam	ATP binding,ATPase activity,cytoplasm,kinesin complex,	A=0, G=23	A=0.0, G=1.0	0.00001	316.747	23	A=0/0, G=14/9
chr19:55328993	G/C	G	C	SNV	KIR3DL1	KIR3DL1:exonic:NM 001322168.1	1	57.14	2	missense	NM 00132	c.39G>C	p.Leu13Phe	-0.24	1	22	0	0(COSM4132545),0(COSM4:		rs1142881	0.2869	YES	5.878E-1 (pos.55328993)	carcinoma,nec	Killer cell in	HLA-B spe HIV Infections;Immune System Diseases;Imm	G=15, C=20	G=0.4286, C=0.5714	0.00001	218.067	35	G=3/12, C=3/1
chr19:55241107	G/A	G	A	SNV	KIR3DL3	KIR3DL3:exonic:NM 153443.4	1	50	5	synonymous	NM 15344	c.804G>A	p.(=)	-1.93				0(COSM4132!	Immunoglobı	rs62132678			(pos.55241107)	neoplasm			G=41, A=41	G=0.5, A=0.5	0.00001	436.025	82	G=26/15, A=2
chr18:21511172	C/C	G	C	SNV	LAMA3	LAMA3:intronic:NM 198129.2	1	100		intronic	NM 19812	c.8576+7G	p.?	-0.56					Concanavalir	rs1258107	0.007	YES	9.999E-1;0.0 (pos.21511172)		Laminin, alı	basement Amyotrophic Lateral Sclerosis; Benign	G=0, C=32	G=0.0, C=1.0	0.00001	485.139	32	G=0/0, C=7/25
chr18:21694618	A/A	G	A	SNV	TTC39C	TTC39C:intronic:NM 001135993.1	1	100		intronic	NM 00113	c.1078+7G	p.?	-0.05					Cohesin load	rs4800176	0		1.0 (pos.21694618)				G=0, A=28	G=0.0, A=1.0	0.00001	408.718	28	G=0/0, A=13/1
chr19:55247614	T/C	T	C	SNV	KIR3DL3	KIR3DL3:utr 3:NM 153443.4	1	45.45	8	utr 3	NM 15344	c.1284T>C	p.?	-0.28					Immunoglobı	rs676354			0.0 (pos.55247614)				T=15, C=15	T=0.5, C=0.5	0.00001	188.284	33	T=11/4, C=8/7
chr1:158045965	G/A	G	A	SNV	KIRREL1	KIRREL1:exonic:NM 018240.6	1	52.27	2	missense	NM 01824	c.115G>A	p.Val39Met	1.11	0.01	21	0.993		CD80-like C2-	rs191487619	0.0066		0.0 (pos.158045965)			T cell costimulation,axon guidance,cell projection memt	G=21, A=23	G=0.4773, A=0.5227	0.00001	259.857	44	G=13/8, A=17/
chr18:21894438	T/T	C	T	SNV	OSBPL1A	OSBPL1A:intronic:NM 080597.3	1	100		intronic	NM 08059	c.871-127	p.?	-2.2					Ankyrin repe	rs7240306		YES			Oxysterol-b	antigen pr Alzheimer Disease;Bone Diseases;Bone Dise	C=0, T=28	C=0.0, T=1.0	0.00001	408.718	28	C=0/0, T=11/1
chr18:22008913	T/T	C	T	SNV	IMPACT	IMPACT:intronic:NM 018439.3	1	100		intronic	NM 01843	c.218+28C	p.?	-0.1					RWD domain	rs582358	0.0781	YES	6.627E-1 (pos.22008913)		Impact RWI	actin binding,biological process,cellular response to UV	C=0, T=45	C=0.0, T=1.0	0.00001	747.393	45	C=0/0, T=19/2
chr18:22010421	T/T	C	T	SNV	IMPACT	IMPACT:intronic:NM 018439.3	1	100		intronic	NM 01843	c.281+73C	p.?	0.03					RWD domain	rs9783924		YES			Impact RWI	actin binding,biological process,cellular response to UV	C=0, T=39	C=0.0, T=1.0	0.00001	623.98	39	C=0/0, T=19/2
chr18:22017752	A/A	C	A	SNV	IMPACT	IMPACT:intronic:NM 018439.3	1	100		intronic	NM 01843	c.282-167	p.?	-0.11					RWD domain	rs1941814		YES			Impact RWI	actin binding,biological process,cellular response to UV	C=0, A=21	C=0.0, A=1.0	0.00001	281.206	21	C=0/0, A=16/5
chr11:126294817	A/A	G	A	SNV	KIRREL3	KIRREL3:exonic:NM 032531.3	1	100	17	synonymous	NM 03253	c.1995C>T	p.(=)	5.06				0.94(COSM56	CD80-like C2-	rs948052	0.3665	YES	9.021E-1 (pos.12629481:	ductal carcinc	Kin of IRRE-l	axon,denc Intellectual Disability;Mental I Likely benign	G=0, A=74	G=0.0, A=1.0	0.00001	1170.13	74	G=0/0, A=24/5
chr20:21142813	T/C	T	C	SNV	KIZ	KIZ:exonic:NM 018474.5	1	57.14	5	missense	NM 01847	c.707T>C	p.Met236Thr	-0.8		81				rs2236178	0.3125	YES	5.699E-1 (pos.21142813)		Kizuna cent	cell projec Congenital, Hereditary, and Neonatal Diseas	T=9, C=12	T=0.4286, C=0.5714	0.00001	109.102	21	T=5/4, C=10/2
chr13:33590851	C/C	T	C	SNV	KL	KL:exonic:NM 004795.3	1	100	1	synonymous	NM 00479	c.273T>C	p.(=)	2.28				0.04(COSM37	Glycosyl hydr	rs2772364	5.00E-04	YES	1.0 (pos.33590851)	adenocarcinor	Klotho	1-phospha Arterial Occlusive Diseases;Ar Benign	T=0, C=82	T=0.0, C=1.0	0.00001	1329.63	82	T=0/0, C=64/1
chr13:33635835	C/C	T	C	SNV	KL	KL:exonic:NM 004795.3	1	100	4	synonymous	NM 00479	c.2619T>C	p.(=)	-0.17					Glycosyl hydr	rs649964	2.00E-04		9.998E-1 (pos.33635835)		Klotho	1-phospha Arterial Occlusive Diseases;Ar Benign	T=0, C=155	T=0.0, C=1.0	0.00001	2484.81	155	T=0/0, C=79/7
chr18:2606596	C/C	CTT	C	INDEL	NDC80	NDC80:intronic:NM 006101.2	2	100		intronic	NM 0061C	c.1557+90	p.?	-0.77, 0.21, 1.97					HEC/Ndc80p	rs200139374,rs201494035,rs40		YES			Ndc80 con	Autoimmune Diseases;Female Urogenital Di:	CTT=0, C=2	CTT=0.0, C=1.0	0.00001	427.534	29	CTT=0/0, C=19
chr4:39448542	G/G	C	G	SNV	KLB	KLB:exonic:NM 175737.3	1	100	4	synonymous	NM 17573	c.2196C>G	p.(=)	-3.27				0.01(COSM37	Glycosyl hydr	rs7685429	0.2325	YES	7.289E-1;0.0 (pos.39448!	adenocarcinor	Klotho, bet:	1-phosphatidylinositol-3-kinase activity,MAPK cascade,I	C=0, G=25	C=0.0, G=1.0	0.00001	353.019	25	C=0/0, G=6/19
chr18:2740625	G/A	G	A	SNV	SMCHD1	SMCHD1:intronic:NM 015295.2	1	50		intronic	NM 01529	c.3515-76	p.?	-0.11					Histidine kin:	rs521992		YES			Structural n	ATP bindin Autoimmune Diseases;Congenital, Hereditar	G=17, A=17	G=0.5, A=0.5	0.00001	169.734	34	G=15/2, A=8/9
chr18:2751475	G/G	A	G	SNV	SMCHD1	SMCHD1:intronic:NM 015295.2	1	100		intronic	NM 01529	c.4281+84	p.?	0.09					Histidine kin:	rs300293		YES			Structural n	ATP bindin Autoimmune Diseases;Congenital, Hereditar	A=0, G=55	A=0.0, G=1.0	0.00001	813.042	55	A=0/0, G=19/3
chr4:39448586	C/T	C	T	SNV	KLB	KLB:exonic:NM 175737.3	1	45.83	4	missense	NM 17573	c.2240C>T	p.Ala747Val	1.81	0.46	64	0.959		Glycosyl hydr	rs35372803	0.052	YES	6.018E-4 (pos.39448586)		Klotho, bet:	1-phosphatidylinositol-3-kinase activity,MAPK cascade,I	C=13, T=11	C=0.5417, T=0.4583	0.00001	93.4333	24	C=1/12, T=2/9
chr18:28721882	C/C	T	C	SNV	DSC1,DSCAS	DSCAS:intronic nc:NR 110785.1, DSC1:intronic:NM 024421	1	100		intronic	NR 11078!	c.1260+80	p.?	0.39					Cadherin cyt	rs12455973		YES			Desmocolli	calcium io Melanoma;Neoplasms;Neoplasms by Histolc	T=0, C=21	T=0.0, C=1.0	0.00001	281.234	21	T=0/0, C=8/13
chr18:28721913	T/T	C	T	SNV	DSC1,DSCAS	DSCAS:intronic nc:NR 110785.1, DSC1:intronic:NM 024421	1	100		intronic	NR 11078!	c.1260+49	p.?	-0.31					Cadherin cyt	rs12458870	0.1872	YES	5.563E-1 (pos.28721913)		Desmocolli	calcium io Melanoma;Neoplasms;Neoplasms by Histolc	C=0, T=21	C=0.0, T=1.0	0.00001	281.235	21	C=0/0, T=8/13
chr18:28721922	A/A	G	A	SNV	DSC1,DSCAS	DSCAS:intronic nc:NR 110785.1, DSC1:intronic:NM 024421	1	100		intronic	NR 11078!	c.1260+40	p.?	0.82					Cadherin cyt	rs12458919	0.1843	YES	5.571E-1 (pos.28721922)		Desmocolli	calcium io Melanoma;Neoplasms;Neoplasms by Histolc	G=0, A=21	G=0.0, A=1.0	0.00001	281.086	21	G=0/0, A=8/13
chr18:28737198	T/T	C	T	SNV	DSC1,DSCAS	DSCAS:intronic nc:NR 110785.1, DSC1:intronic:NM 024421	1	100		intronic	NR 11078!	c.351+136	p.?	-0.01					Cadherin cyt	rs2246736,rs71383017		YES			Desmocolli	calcium io Melanoma;Neoplasms;Neoplasms by Histolc	C=0, T=33	C=0.0, T=1.0	0.00001	504.593	33	C=0/0, T=29/4
chr18:28737299	G/G	T	G	SNV	DSC1,DSCAS	DSCAS:intronic nc:NR 110785.1, DSC1:intronic:NM 024421	1	100		intronic	NR 11078!	c.351+35A	p.?	0.07					Cadherin cyt	rs2118342	0.3548	YES	8.041E-1 (pos.28737299)		Desmocolli	calcium io Melanoma;Neoplasms;Neoplasms by Histolc	T=0, G=43	T=0.0, G=1.0	0.00001	705.844	43	T=0/0, G=29/1
chr14:104167564	G/G	A	G	SNV	KLC1,XRCC3	XRCC3:intronic:NM 001100118.1, KLC1:utr 3:NM 0011301	1	100	16	utr 3	NM 00113	c.1881A>G	p.?, p.?	-1.24					AAA domain,I	rs861536	0.3803	YES	5.705E-2 (pos.104167564)		Kinesin, ligh	ATP bindin Adenoma;Breast Diseases;Breast Neoplasm:	A=0, G=23	A=0.0, G=1.0	0.00001	316.353	23	A=0/0, G=20/3
chr2:10188649	T/T	A	T	SNV	KLF11	KLF11:exonic:NM 003597.4	1	100	3	synonymous	NM 00359	c.1185A>T	p.(=)	-1.33				0.16(COSM62	C2H2-type zir	rs11687357	0.1937	YES	8.467E-1 (pos.10188649)	neoplasm	Kruppel-like	DNA bindi Diabetes Mellitus;Endocrine System Disease	A=0, T=46	A=0.0, T=1.0	0.00001	768.313	46	A=0/0, T=20/2
chr1:44595113	C/C	G	C	SNV	KLF17	KLF17:exonic:NM 173484.3	1	100	2	missense	NM 17348	c.170G>C	p.Ser57Thr	-0.37	0.08	58	0.183	0.01(COSM37	C2H2-type zir	rs2429051	0.336	YES	6.68E-1;0.0 (pos.445951:	adenocarcinoma,ductal ca		DNA binding transcription factor activity,RNA polymeras	G=0, C=62	G=0.0, C=1.0	0.00001	940.21	62	G=0/0, C=25/3
chr2:207988613	T/T	C	T	SNV	KLF7	KLF7:exonic:NM 001270944.1	1	100	2	synonymous	NM 00127	c.534G>A	p.(=)	-1.3					Zinc finger, C:	rs2700166	1.00E-04	YES	9.999E-1 (pos.207988613)		Kruppel-like	DNA binding transcription factor activity,RNA polymeras	C=0, T=44	C=0.0, T=1.0	0.00001	726.505	44	C=0/0, T=17/2
chr18:2920725	T/T	A	T	SNV	LPIN2	LPIN2:intronic:NM 014646.2	1	100		intronic	NM 01464	c.2546+51	p.?	-1.22					LNS2 (Lipin/N	rs3737514	0.1127	YES	5.971E-1;0.0 (pos.2920725)		Lipin 2	cytosol,de Anemia;Anemia, Dyserythropoietic, Congenit	A=0, T=23	A=0.0, T=1.0	0.00001	316.746	23	A=0/0, T=17/6
chr14:50195679	G/G	A	G	SNV	KLHDC1	KLHDC1:exonic:NM 172193.2	1	100	7	synonymous	NM 17219	c.603A>G	p.(=)	-5.16					Galactose ox	rs4301946	0.0017	YES	9.995E-1;0.0 (pos.50195679)		KELCH dom:	cytosol	A=0, G=56	A=0.0, G=1.0	0.00001	832.093	56	A=0/0, G=27/2
chr1:18808292	A/A	C	A	SNV	KLHDC7A	KLHDC7A:exonic:NM 152375.2	1	100	1	missense	NM 15237	c.817C>A	p.His273Asn	2.56		68			Kelch motif	rs2992753,rs372156!	0.3713	YES	8.996E-1 (pos.18808292)			Cul3-RING ubiquitin ligase complex,integral component	C=0, A=97	C=0.0, A=1.0	0.00001	1638.96	97	C=0/0, A=48/4
chr1:18808465	A/A	T	A	SNV	KLHDC7A	KLHDC7A:exonic:NM 152375.2	1	100	1	synonymous	NM 15237	c.990T>A	p.(=)	-0.93				0.01(COSM37	Kelch motif	rs3007718	0.3714	YES	8.988E-1 (pos.18808465)	squamous cell carcinoma		Cul3-RING ubiquitin ligase complex,integral component	T=0, A=26	T=0.0, A=1.0	0.00001	371.42	26	T=0/0, A=15/1
chr18:29797958	C/C	T	C	SNV	MEP1B	MEP1B:intronic:NM 005925.2	1	100		intronic	NM 00592	c.2091+30	p.?	0.3					Astacin (Pept	rs680321	0.4644	YES	2.466E-1 (pos.29797958)		Meprin A, b	digestion,extracellular space,identical protein binding,i	T=0, C=45	T=0.0, C=1.0	0.00001	747.374	45	T=0/0, C=34/1
chr18:30672631	A/A	G	A	SNV	CCDC178	CCDC178:intronic:NM 001105528.1	1	100		intronic	NM 0011C	c.2388+94	p.?	-1.11						rs1029706001,rs1995096		YES					G=0, A=32	G=0.0, A=1.0	0.00001	485.141	32	G=0/0, A=6/26
chr18:3075503	CAAA/CAAA	C	CAAA	INDEL	MYOM1	MYOM1:intronic:NM 003803.3	3	100		intronic	NM 0038C	c.4686-29	p.?	0.25					Fibronectin t	rs10667835,rs20005!	0.0067	YES	1.0;0.0 (pos.3075503)		Myomesin :	M band,Z c Congenital, Hereditary, and Neonatal Diseas	C=0, CAAA=	C=0.0, CAAA=1.0	0.00001	353.019	25	C=0/0, CAAA=1
chr1:18808526	C/C	A	C	SNV	KLHDC7A	KLHDC7A:exonic:NM 152375.2	1	100	1	missense	NM 15237	c.1051A>C	p.Thr351Pro	-0.79		38		0.02(COSM42	Kelch motif	rs2992752,rs776045	0.3704	YES	0.0;0.0 (pos.18808514),8.	adenocarcinoma		Cul3-RING ubiquitin ligase complex,integral component	A=0, C=45	A=0.0, C=1.0	0.00001	747.392	45	A=0/0, C=22/2
chr1:18807851	C/T	C	T	SNV	KLHDC7A	KLHDC7A:exonic:NM 152375.2	1	46.88	1	missense	NM 15237	c.376C>T	p.Arg126Trp	-0.2		101			Kelch motif	rs72944731	0		0.0 (pos.18807851)			Cul3-RING ubiquitin ligase complex,integral component	C=17, T=15	C=0.5313, T=0.4688	0.00001	144.343	32	C=13/4, T=11/
chr18:31226058	C/C	T	C	SNV	ASXL3	ASXL3:intronic:NM 030632.2	1	100		intronic	NM 03063	c.247-151	p.?	-1.46					Asx homolog	rs9964810		YES			Additional s	DNA bindi Hemic and Lymphatic Diseases;Immune Syst	T=0, C=25	T=0.0, C=1.0	0.00001	353.016	25	T=0/0, C=13/1
chr22:50988193	G/G	A	G	SNV	KLHDC7B	KLHDC7B:exonic:NM 138433.3	1	100	1	missense	NM 13843	c.1598A>G	p.His533Arg	3.91		29		0.09(COSM41	Kelch motif	rs131779	0.3537	YES	6.603E-1 (pos.50988193)	neoplasm		Cul3-RING ubiquitin ligase complex,protein ubiquitinatic	A=0, G=20	A=0.0, G=1.0	0.00001	263.749	20	A=0/0, G=3/17
chr18:3272798	TA/TA	AG	TA	MNV	MYL12B	MYL12B:intronic:NM 033546.3	2	100		intronic	NM 03354	c.-14-84AG	p.?	-0.14, -0.12					EF hand,EF-h	rs368704794,rs535975900,rs65		YES			Myosin, ligh	Z disc,apical part of cell,brush border,calcium ion bindin	AG=0, TA=2	AG=0.0, TA=1.0	0.00001	298.892	22	AG=0/0, TA=13

chr18:3272855	T/T	C	T	SNV	MYL12B	MYL12B:intronic:NM_033546.3	1	100		intronic	NM_03354	c.-14-27C>	p.?	-0.24					EF hand,EF-ha	rs6506099	0.0195	YES	8.638E-1 (pos.3272855)		Myosin, ligh Z disc,apical part of cell,brush border,calcium ion bindin	C=0, T=23	C=0.0, T=1.0	0.00001	316.746	23	C=0/0, T=12/1
chr18:3273153	T/T	C	T	SNV	MYL12B	MYL12B:intronic:NM_033546.3	1	100		intronic	NM_03354	c.184+73C	p.?	0.62					EF hand,EF-ha	rs7407045		YES			Myosin, ligh Z disc,apical part of cell,brush border,calcium ion bindin	C=0, T=27	C=0.0, T=1.0	0.00001	389.988	27	C=0/0, T=16/1
chr18:32948445	T/TTTTTA	T	TTTTTA	INDEL	ZNF396	ZNF396:intronic:NM_145756.2	5	57.89		intronic	NM_14575	c.908-36A	p.?	0.09					C2H2-type zir	rs368068594,rs757057331,rs796097221			0.0 (pos.32948433),0.0 (pos.32948445)		Zinc finger p DNA binding,DNA binding transcription factor activity,cyt	T=16, TTTTT	T=0.4211, TTTTTA=0.5	0.00001	181.019	38	T=12/4, TTTTTA
chr1:161068836	C/C	A	C	SNV	KLHDC9	KLHDC9:exonic:NM_152366.4	1	100	1	missense	NM_15236	c.511A>C	p.Ser171Arg	-1.09	0.45	110	0	0.1(COSM424	Galactose ox	rs11576830	0.2823	YES	3.87E-1 (pos.161068836)	carcinoma	Kelch doma biological_process,cellular_component,cyclin binding	A=0, C=25	A=0.0, C=1.0	0.00001	352.87	25	A=0/0, C=13/1
chr18:335316	C/C	A	C	SNV	COLEC12	COLEC12:intronic:NM_130386.2	1	100		intronic	NM_13038	c.1328-86	p.?	-0.02					Collagen tripl	rs2012143		YES			Collectin 12 carbohydr Mental Disorders;Substance-Related Disorde	A=0, C=42	A=0.0, C=1.0	0.00001	688.51	42	A=0/0, C=18/2
chr17:39998141	T/T	C	T	SNV	KLHL10	KLHL10:exonic:NM_152467.4	1	100	2	synonymous	NM_15246	c.261C>T	p.(=)	-3.27					BTB And C-ter	rs1529933	0.2288	YES	8.242E-1 (pos.39998141)		Kelch-like 1 Cul3-RING ubiquitin ligase complex,cell morphogenesis,	C=0, T=57	C=0.0, T=1.0	0.00001	849.615	57	C=0/0, T=28/2
chr17:40010979	A/A	G	A	SNV	KLHL11	KLHL11:exonic:NM_018143.2	1	100	2	synonymous	NM_01814	c.1140C>T	p.(=)	1.25					BTB And C-ter	rs3744782	0.2302	YES	8.24E-1 (pos.40010979)		SCF ubiquitin ligase complex,cytoplasm,cytosol,nucleus	G=0, A=26	G=0.0, A=1.0	0.00001	371.42	26	G=0/0, A=21/5
chr18:33713287	A/A	T	A	SNV	ELP2	ELP2:intronic:NM_018255.3	1	100		intronic	NM_01825	c.217+8T>	p.?	1.08					WD domain,	rs1785905	0.371	YES	1.889E-1 (pos.33713287)		Elongator a Elongator Intellectual Disability;Mental Disorders;Nerv	T=0, A=50	T=0.0, A=1.0	0.00001	852.623	50	T=0/0, A=38/1
chrX:117119241	G/G	C	G	SNV	KLHL13	KLHL13:exonic:NM_001168299.1	1	100	1	missense	NM_00116	c.3G>C	p.Met1Ile	1.85	0	10		0.53(COSM57	BTB And C-terminal Kelch,BTB/POZ domain,Galactose oxidase, central domain,Kelch					adult_T_cell_l	Kelch-like 1 Cul3-RING ubiquitin ligase complex,cytokinesis,cytosol,r	C=0, G=25	C=0.0, G=1.0	0.00001	353.019	25	C=0/0, G=21/4
chr2:170605981	T/T	C	T	SNV	KLHL23,PHOSPHO2-KLHL23	PHOSPHO2-KLHL23:exonic:NM_001199290.2, KLHL23:exoni	1	100	4	synonymous	NM_00119	c.1416C>T	p.(=), p.(=)	-0.35					BTB And C-ter	rs4667607	0		1.0 (pos.170605981)		Cul3-RING ubiquitin ligase complex,protein binding,prot	C=0, T=22	C=0.0, T=1.0	0.00001	298.892	22	C=0/0, T=10/1
chr15:86313048	T/C	T	C	SNV	KLHL25,MIR1276	KLHL25:utr_5:NM_022480.3, MIR1276:downstream:NR_031	1	50	2	utr_5	NM_02248	c.-7A>G	p.?	-0.55					BTB And C-ter	rs2614657	0.1695	YES	7.927E-1 (pos.86313048)		Cul3-RING ubiquitin ligase complex,cytoplasm,cytosol,p	T=13, C=13	T=0.5, C=0.5	0.00001	119.162	26	T=8/5, C=6/7
chr2:23926659	C/C	T	C	SNV	KLHL29	KLHL29:exonic:NM_052920.1	1	100	13	synonymous	NM_05292	c.2370T>C	p.(=)	0.99					BTB And C-ter	rs3795948	0.4969	YES	7.262E-1 (pos.23926659)		Cul3-RING Cardiovascular Diseases;Hypertension;Vasc	T=0, C=50	T=0.0, C=1.0	0.00001	852.748	50	T=0/0, C=9/41
chr6:53519062	G/G	A	G	SNV	KLHL31	KLHL31:exonic:NM_001003760.4	1	100	2	synonymous	NM_00100	c.1009T>C	p.(=)	5.09					BTB And C-ter	rs10948758	0.2487	YES	7.085E-1 (pos.53519062)		Kelch-like 3 Cul3-RING ubiquitin ligase complex,protein ubiquitinatic	A=0, G=58	A=0.0, G=1.0	0.00001	867.239	58	A=0/0, G=31/2
chr18:33795425	A/A	G	A	SNV	MOCOS	MOCOS:intronic:NM_017947.3	1	100		intronic	NM_01794	c.1336-54	p.?	-1.73					Aminotransfe	rs631108		YES			Molybdenu Mo-molyb Congenital, Heredit Pyridoxal Phosphate	G=0, A=165	G=0.0, A=1.0	0.00001	2677.92	165	G=0/0, A=107/
chr6:53519605	T/T	C	T	SNV	KLHL31	KLHL31:exonic:NM_001003760.4	1	100	2	missense	NM_00100	c.466G>A	p.Val156Ile	4.9	0.15	29	0.309		BTB And C-ter	rs3799260	0.1835	YES	9.082E-1 (pos.53519605)		Kelch-like 3 Cul3-RING ubiquitin ligase complex,protein ubiquitinatic	C=0, T=49	C=0.0, T=1.0	0.00001	831.631	49	C=0/0, T=18/3
chr14:20897064	T/T	C	T	SNV	KLHL33	KLHL33:exonic:NM_001109997.2	1	100	4	missense	NM_00110	c.1546G>A	p.Ala516Thr	-1.45	1	58	0.001	0.02(COSM44	BTB And C-ter	rs7145318	0.4233	YES	6.286E-1 (pos.20897064)	acute_myeloid_leukaemia	Cul3-RING ubiquitin ligase complex,protein ubiquitinatic	C=0, T=33	C=0.0, T=1.0	0.00001	504.584	33	C=0/0, T=12/2
chr14:20898442	T/T	C	T	SNV	KLHL33	KLHL33:exonic:NM_001109997.2	1	100	2	synonymous	NM_00110	c.393G>A	p.(=)	-0.1				0.16(COSM41	BTB And C-ter	rs11160645	0.121	YES	8.987E-2 (pos.20898442)	neoplasm	Cul3-RING ubiquitin ligase complex,protein ubiquitinatic	C=0, T=26	C=0.0, T=1.0	0.00001	371.4	26	C=0/0, T=17/9
chr8:124664130	T/T	C	T	SNV	KLHL38	KLHL38:exonic:NM_001081675.2	1	100	1	missense	NM_00108	c.1037G>A	p.Arg346Lys	1.27	0.97	26	0	0.05(COSM41	BTB And C-ter	rs11780509	0.2113	YES	7.012E-1 (pos.12466413(	neoplasm	Cul3-RING ubiquitin ligase complex,protein binding,prot	C=0, T=73	C=0.0, T=1.0	0.00001	1150.02	73	C=0/0, T=48/2
chr18:43243679	G/G	A	G	SNV	SLC14A2	SLC14A2:intronic:NM_001242692.1	1	100		intronic	NM_00124	c.1352-71	p.?	-1.75					Urea transpo	rs8092227		YES			Solute carri apical plas Alopecia;Hair Disea Urea	A=0, G=21	A=0.0, G=1.0	0.00001	281.235	21	A=0/0, G=14/7
chr8:124664167	C/C	T	C	SNV	KLHL38	KLHL38:exonic:NM_001081675.2	1	100	1	missense	NM_00108	c.1000A>G	p.Ile334Val	-1.02	0.23	29	0		BTB And C-ter	rs11784192	0.1529	YES	7.007E-1 (pos.124664167)		Cul3-RING ubiquitin ligase complex,protein binding,prot	C=0, T=72	T=0.0, C=1.0	0.00001	1130.95	72	T=0/0, C=46/2
chr18:43446734	C/C	T	C	SNV	EPG5	EPG5:intronic:NM_020964.2	1	100		intronic	NM_02096	c.6621+29	p.?	-0.24						rs3826633	0.2726	YES	9.017E-1 (pos.43446734)		Ectopic P-gr autophagc Agenesis of Corpus Callosum;Cardiomyopath	T=0, C=26	T=0.0, C=1.0	0.00001	371.42	26	T=0/0, C=8/18
chr18:43678035	T/C	T	C	SNV	ATP5A1	ATP5A1:intronic:NM_004046.5	1	57.58		intronic	NM_00404	c.60+103A	p.?	0.94					ATP synthase	rs3753069		YES	6.15E-1 (pos.43678035)		ATP synthas ATP bindin Alzheimer Disease; 1-ACETYL-2-CARBOXYPIPE	T=14, C=19	T=0.4242, C=0.5758	0.00001	204.348	33	T=10/4, C=13/
chr18:44109033	C/C	T	C	SNV	LOXHD1	LOXHD1:intronic:NM_144612.6	1	100		intronic	NM_14461	c.4530+10	p.?	-1.89					PLAT/LH2 dor	rs1450425		YES			Lipoxygena calcium ch Ear Diseases;Hearing Disorder Likely benign	T=0, C=25	T=0.0, C=1.0	0.00001	353.015	25	T=0/0, C=13/1
chr8:124664792	T/T	C	T	SNV	KLHL38	KLHL38:exonic:NM_001081675.2	1	100	1	synonymous	NM_00108	c.375G>A	p.(=)	-1.49					BTB And C-ter	rs7387544	0.2115	YES	7E-1 (pos.124664792)		Cul3-RING ubiquitin ligase complex,protein binding,prot	C=0, T=21	C=0.0, T=1.0	0.00001	281.235	21	C=0/0, T=7/14
chr19:51323501	G/G	A	G	SNV	KLK1	KLK1:exonic:NM_002257.3	1	100	3	synonymous	NM_00225	c.405T>C	p.(=)	-4.49				0.01(COSM41	Trypsin,Tryps	rs1054713	0.3321	YES	7.97E-1 (pos.51323501)	neoplasm	Kallikrein 1 extracellu Acute Kidney Injury Aprotinin,Nafamostat	A=0, G=59	A=0.0, G=1.0	0.00001	885.282	59	A=0/0, G=24/3
chr19:51379893	T/T	C	T	SNV	KLK2	KLK2:exonic:NM_005551.4	1	100	3	synonymous	NM_00555	c.372C>T	p.(=)	-2.91				0.01(COSM37	Trypsin,Tryps	rs198972	0.3123	YES	3.02E-1 (pos.51379893)	acute_myeloic	Kallikrein-r extracellu Genital Diseases, Male;Genital Neoplasms, I	C=0, T=67	C=0.0, T=1.0	0.00001	1034.25	67	C=0/0, T=44/2
chr18:47088826	C/T	C	T	SNV	LIPG	LIPG:intronic:NM_006033.3	1	50		intronic	NM_00603	c.97+51C>	p.?	0.68					Lipase,PLAT/	rs35978968	0.3224	YES	3.227E-1 (pos.47088826)		Lipase G, er Golgi apparatus,cell proliferation,cell surface,cholester	C=14, T=14	C=0.5, T=0.5	0.00001	136.153	28	C=3/11, T=6/8
chr18:47088830	C/G	C	G	SNV	LIPG	LIPG:intronic:NM_006033.3	1	50		intronic	NM_00603	c.97+55C>	p.?	-0.01					Lipase,PLAT/	rs35816125		YES			Lipase G, er Golgi apparatus,cell proliferation,cell surface,cholester	C=14, G=14	C=0.5, G=0.5	0.00001	131.043	28	C=3/11, G=6/8
chr18:47093790	T/T	C	T	SNV	LIPG	LIPG:intronic:NM_006033.3	1	100		intronic	NM_00603	c.280-22C	p.?	-0.28					Lipase,PLAT/	rs2000812	0.1892	YES	5.493E-1 (pos.47093790)		Lipase G, er Golgi apparatus,cell proliferation,cell surface,cholester	C=0, T=63	C=0.0, T=1.0	0.00001	948.73	63	C=0/0, T=25/3
chr18:47463587	G/G	A	G	SNV	MYO5B	MYO5B:intronic:NM_001080467.2	1	100		intronic	NM_00108	c.1905+28	p.?	-0.18					DIL domain,I(	rs1790797	0.2784	YES	3.994E-1 (pos.47463587)		Myosin VB ATP bindin Amphetamine-Related Disorders;Bone Disea	A=0, G=35	A=0.0, G=1.0	0.00001	543.904	35	A=0/0, G=26/9
chr19:51452250	C/C	T	C	SNV	KLK5	KLK5:exonic:NM_001077492.1	1	100	4	missense	NM_00107	c.457A>G	p.Asn153Asp	5.04	1	23	0	0.02(COSM41	Trypsin	rs183854	3.00E-04		1.0 (pos.51452250)	neoplasm	Kallikrein-r cornificati Genital Diseases, Male;Genital Neoplasms, I	T=0, C=22	T=0.0, C=1.0	0.00001	298.85	22	T=0/0, C=10/1
chr4:187173012	T/G	T	G	SNV	KLKB1	KLKB1:exonic:NM_000892.4	1	58.33	10	missense	NM_00089	c.1141T>G	p.Ser381Ala	0.69	0.23	99	0.004	0.23(COSM44	PAN domain,!	rs4253301	0.1167	YES	6.234E-2 (pos.18717301	acute_myeloic	Kallikrein B Factor XII ; Blood Coagulation I Conestat alfa,Ecallantide	T=15, G=21	T=0.4167, G=0.5833	0.00001	234.324	36	T=8/7, G=13/8
chr12:10603669	GC/GC	G	GC	INDEL	KLRC1	KLRC1:exonic:NM_002259.4	1	100	2	frameshiftInsertion	NM_00225	c.86_87ins	p.Asn29fs	-0.28					Lectin C-type domain,UL45 protein, carbohydrate-binding C-type lectin-like						Killer cell le MHC class I protein complex binding,carbohydrate bindi	G=0, GC=49	G=0.0, GC=1.0	0.00001	725.355	49	G=0/0, GC=32/
chr12:10570965	G/G	C	G	SNV	KLRC3	KLRC3:exonic:NM_007333.2	1	100	4	missense	NM_00733	c.464G>C	p.Cys155Ser	-0.47	1	112	0		Lectin C-type	rs2682495,rs771023	0		0.0 (pos.10570964),1.0 (pos.10570965)		Killer cell le carbohydrate binding,cellular defense response,integra	C=0, G=25	C=0.0, G=1.0	0.00001	353.006	25	C=0/0, G=12/1
chr18:51900472	A/A	G	A	SNV	C18orf54	C18orf54:intronic:NM_173529.5	1	100		intronic	NM_17352	c.976-112	p.?	-1.62					Lung adenom	rs4940327		YES			Chromosom extracellular region,negative regulation of cell proliferat	G=0, A=96	G=0.0, A=1.0	0.00001	1618.01	96	G=0/0, A=43/5
chr18:52575002	A/A	G	A	SNV	CCDC68	CCDC68:intronic:NM_025214.2	1	100		intronic	NM_02521	c.950+15C	p.?	-1.13						rs1944382	2.00E-04		1.0 (pos.52575002)		Coiled-coil centriolar subdistal appendage,centriole,microtubule a	G=0, A=47	G=0.0, A=1.0	0.00001	789.296	47	G=0/0, A=28/1
chr18:53070914	G/A	G	A	SNV	TCF4	TCF4:intronic:NM_001243226.2	1	53.33		intronic	NM_00124	c.611-165	p.?	2.98				0.8(COSM459	Helix-loop-he	rs17522826		YES	5.26E-1 (pos.53070914)	astrocytoma_(	Transcriptic DNA bindi Bile Duct Diseases;Biliary Trac Likely benign	G=21, A=24	G=0.4667, A=0.5333	0.00001	276.154	45	G=9/12, A=14/
chr18:5407839	T/T	C	T	SNV	EPB41L3	EPB41L3:intronic:NM_012307.3	1	100		intronic	NM_01230	c.2122-10	p.?	-0.72					4.1 protein C	rs2874686		YES			Erythrocyte actin bind Breast Diseases;Breast Neoplasms;Neoplasr	C=0, T=23	C=0.0, T=1.0	0.00001	316.747	23	C=0/0, T=9/14
chr12:10571042	G/G	A	G	SNV	KLRC3	KLRC3:exonic:NM_007333.2	1	100	4	synonymous	NM_00733	c.387T>C	p.(=)	0.44				0.26(COSM45	Lectin C-type	rs2741882			1.0 (pos.10571042)	squamous_cel	Killer cell le carbohydrate binding,cellular defense response,integra	A=0, G=38	A=0.0, G=1.0	0.00001	603.778	38	A=0/0, G=19/1
chr12:10571091	G/G	T	G	SNV	KLRC3	KLRC3:exonic:NM_007333.2	1	100	4	missense	NM_00733	c.338A>C	p.His113Pro	-0.82	0.24	77	0.02	0.01(COSM45	Lectin C-type	rs2682494			9.997E-1 (pos.10571091)	squamous_cel	Killer cell le carbohydrate binding,cellular defense response,integra	T=0, G=36	T=0.0, G=1.0	0.00001	563.744	36	T=0/0, G=17/1
chr18:54339977	G/G	A	G	SNV	WDR7	WDR7:intronic:NM_015285.2	1	100		intronic	NM_01528	c.159+72A	p.?	0.17					WD domain,	rs566956		YES			WD repeat- cytoplasm,hematopoietic progenitor cell differentiation	A=0, G=25	A=0.0, G=1.0	0.00001	344.097	25	A=0/0, G=9/16
chr18:54346482	G/G	T	G	SNV	WDR7	WDR7:intronic:NM_015285.2	1	100		intronic	NM_01528	c.160-81T	p.?	-0.69					WD domain,	rs683248		YES			WD repeat- cytoplasm,hematopoietic progenitor cell differentiation	T=0, G=54	T=0.0, G=1.0	0.00001	798.148	54	T=0/0, G=28/2
chr18:54346510	A/A	G	A	SNV	WDR7	WDR7:intronic:NM_015285.2	1	100		intronic	NM_01528	c.160-53G	p.?	-0.29					WD domain,	rs597585		YES			WD repeat- cytoplasm,hematopoietic progenitor cell differentiation	G=0, A=54	G=0.0, A=1.0	0.00001	800.578	54	G=0/0, A=27/2
chr18:54348514	T/T	C	T	SNV	WDR7	WDR7:intronic:NM_015285.2	1	100		intronic	NM_01528	c.267-30C	p.?	0.78					WD domain,	rs605067	0.0035	YES	1.0 (pos.54348514)		WD repeat- cytoplasm,hematopoietic progenitor cell differentiation	C=0, T=22	C=0.0, T=1.0	0.00001	298.892	22	C=0/0, T=15/7
chr18:54348650	C/C	T	C	SNV	WDR7	WDR7:intronic:NM_015285.2	1	100		intronic	NM_01528	c.345+28T	p.?	-0.1					WD domain,	rs474607	0.0034	YES	1.0;0.0 (pos.54348650)		WD repeat- cytoplasm,hematopoietic progenitor cell differentiation	T=0, C=26	T=0.0, C=1.0	0.00001	376.36	26	T=0/0, C=15/1
chr18:54359062	A/A	G	A	SNV	WDR7	WDR7:intronic:NM_015285.2	1	100		intronic	NM_01528	c.966+14G	p.?	0.16					WD domain,	rs2110414	0.1452	YES	9.634E-1 (pos.54359062)		WD repeat- cytoplasm,hematopoietic progenitor cell differentiation	G=0, A=24	G=0.0, A=1.0	0.00001	334.79	24	G=0/0, A=10/1
chr18:55269777	A/A	G	A	SNV	NARS	NARS:intronic:NM_004539.3	1	100		intronic	NM_00453	c.1384-59	p.?	-0.26					OB-fold nucle	rs2276193		YES			Asparaginyl ATP binding,asparagine-tRNA li L-Asparagine	G=0, A=29	G=0.0, A=1.0	0.00001	427.603	29	G=0/0, A=15/1
chr18:55287739	C/C	A	C	SNV	NARS	NARS:intronic:NM_004539.3	1	100		intronic	NM_00453	c.93+62T>	p.?	-2.68					OB-fold nucle	rs388644		YES			Asparaginyl ATP binding,asparagine-tRNA li L-Asparagine	A=0, C=64	A=0.0, C=1.0	0.00001	977.493	64	A=0/0, C=41/2
chr18:55322367	A/T	A	T	SNV	ATP8B1,LOC100505549	LOC100505549:intronic:NM_001242804.1, ATP8B1:intronic	1	47.37		intronic	NM_00124	c.140-128	p.?, p.?	-0.9					Cation transp	rs17753176		YES			ATPase, cla ATP bindin Bile Duct Diseases;Biliary Tract Diseases;Chc	A=19, T=18	A=0.5135, T=0.4865	0.00001	177.132	38	A=10/9, T=10/
chr12:10572982	G/G	A	G	SNV	KLRC3	KLRC3:exonic:NM_007333.2	1	100	1	synonymous	NM_00733	c.168T>C	p.(=)	-2.34					Lectin C-type	rs2741881	2.00E-04		1.0 (pos.10572982)		Killer cell le carbohydrate binding,cellular defense response,integra	A=0, G=44	A=0.0, G=1.0	0.00001	726.555	44	A=0/0, G=28/1
chr18:55352436	A/A	G	A	SNV	ATP8B1	ATP8B1:intronic:NM_005603.5	1	100		intronic	NM_00560	c.1430-89	p.?	-0.92					Cation transp	rs319459		YES			ATPase, cla ATP bindin Bile Duct Diseases;Biliary Tract Diseases;Chc	G=0, A=58	G=0.0, A=1.0	0.00001	867.336	58	G=0/0, A=19/3
chr18:55362816	A/A	G	A	SNV	ATP8B1	ATP8B1:intronic:NM_005603.5	1	100		intronic	NM_00560	c.699-52C	p.?	-0.27					Cation transp	rs319442	2.00E-04		9.999E-1 (pos.55362816)		ATPase, cla ATP bindin Bile Duct Diseases;Biliary Tract Diseases;Chc	G=0, A=38	G=0.0, A=1.0	0.00001	598.198	38	G=0/0, A=18/2
chr12:10462052	G/G	T	G	SNV	KLRD1	KLRD1:exonic:NM_002262.4	1	100	2	missense	NM_00226	c.73T>G	p.Ser25Ala	-0.12	1	99	0		Lectin C-type	rs10772256	5.00E-04	YES	1.0 (pos.10462052)		Killer cell le MHC class I protein complex binding,MHC class Ib protei	T=0, G=27	T=0.0, G=1.0	0.00001	389.988	27	T=0/0, G=9/18
chr7:151873853	C/T	C	T	SNV	KMT2C	KMT2C:exonic:NM_170606.2	1	50	38	synonymous	NM_17060	c.8685G>A	p.(=)	0.17				0.02(COSM37	AT hook moti	rs6464211	0.1637	YES	3.157E-1 (pos.15187385	adenocarcinoi	Lysine (K)-s DNA bindi Adenocarcinoma;Carcinoma;Carcinoma, Ade	C=20, T=20	C=0.5, T=0.5	0.00001	214.326	40	C=10/10, T=8/
chr11:67957518	T/T	A	T	SNV	KMT5B	KMT5B:exonic:NM_017635.4	1	100	2	missense	NM_01763	c.26T>A	p.Ile9Asn	5.81	1	149	0	0.16(COSM45	SET domain	rs2512606		YES	1.0 (pos.67957518)	squamous_cel	Lysine meth condensed nuclear chromosome, centromeric region,his	A=0, T=33	A=0.0, T=1.0	0.00001	504.589	33	A=0/0, T=17/1
chr10:134999602	C/C	G	C	SNV	KNDC1	KNDC1:exonic:NM_152643.7	1	100	6	synonymous	NM_15264	c.750G>C	p.(=)	-0.34				0(COSM1745	Kinase non-c	rs3008393	0.1129	YES	7.041E-1 (pos.13499960	adenocarcinoi	Kinase non Ras guanyl-nucleotide exchange factor activity,biologica	G=0, C=43	G=0.0, C=1.0	0.00001	705.785	43	G=0/0, C=13/3
chr10:135012652	T/C	T	C	SNV	KNDC1	KNDC1:exonic:NM_152643.7	1	46.43	14	synonymous	NM_15264	c.2640T>C	p.(=)	-3.67				0(COSM3751	Kinase non-c	rs3008388		YES	5.345E-1 (pos.13501265	adenocarcinoi	Kinase non Ras guanyl-nucleotide exchange factor activity,biologica	T=15, C=13	T=0.5357, C=0.4643	0.00001	117.927	28	T=8/7, C=9/4
chr15:40898643	C/C	G	C	SNV	KNL1	KNL1:exonic:NM_144508.4	1	100	4	missense	NM_14450	c.128G>C	p.Arg43Thr	-0.48	1	71	0	0(COSM147880),0(COSM43		rs7177192	0.1559	YES	3.222E-1 (pos.40898643)	adenocarcinoi	Kinetochore CENP-A containing nucleosome assembly, Benign	G=0, C=27	G=0.0, C=1.0	0.00001	389.988	27	G=0/0, C=11/1
chr18:57134152	G/G	A	G	SNV	CCBE1	CCBE1:intronic:NM_133459.3	1	100		intronic	NM_13345	c.401-29T	p.?	-1.01					Calcium-bind	rs4940876	0.0015	YES	1.0 (pos.57134152)		Collagen an calcium io Congenital Abnormalities;Con Benign	A=0, G=39	A=0.0, G=1.0	0.00001	623.975	39	A=0/0, G=20/1
chr18:57147351	G/A	G	A	SNV	CCBE1	CCBE1:intronic:NM_133459.3	1	56		intronic	NM_13345	c.265+67C	p.?	-0.16					Calcium-bind	rs1663548		YES			Collagen an calcium io Congenital Abnormalities;Congenital, Heredi	G=11, A=14	G=0.44, A=0.56	0.00001	134.333	25	G=6/5, A=7/7
chr15:40913840	T/T	G	T	SNV	KNL1	KNL1:exonic:NM_144508.4	1	100	10	missense	NM_14450	c.1378G>T	p.Ala460Ser	-0.05	1	99	0			rs2412541	0.1563	YES	3.223E-1 (pos.40913840)		Kinetochore CENP-A containing nucleosome assembly, Benign	G=0, T=162	G=0.0, T=1.0	0.00001	2615.72	162	G=0/0, T=67/9
chr18:59777011	T/C	T	C	SNV	PIGN	PIGN:intronic:NM_176787.4	1	50		intronic	NM_17678	c.1574+56	p.?	0.11					Phosphatidyl	rs2163518		YES			Phosphatid GPI anchor biosynthetic process,cytosol,endoplasmic re	T=17, C=17	T=0.5, C=0.5	0.00001	171.999	34	T=5/12, C=8/9
chr15:40915190	G/G	A	G	SNV	KNL1	KNL1:exonic:NM_144508.4	1	100	10	missense	NM_14450	c.2728A>G	p.Arg910Gly	0.61	1	125	0	0(COSM147882),0(COSM43		rs8040502	0.1558	YES	3.228E-1 (pos.40915190)	adenocarcinoi	Kinetochore CENP-A containing nucleosome assembly, Benign	A=0, G=40	A=0.0, G=1.0	0.00001	630.227	40	A=0/0, G=25/1
chr13:50296115	T/T	C	T	SNV	KPNA3	KPNA3:exonic:NM_002267.3	1	100	9	synonymous	NM_00226	c.702G>A	p.(=)	0.47				0.45(COSM41	Armadillo/be	rs1127021	0.1313	YES	9.98E-1 (pos.50296115)	neoplasm	Karyopheri NLS-bearing protein import into nucleus,cytosol,host cel	C=0, T=84	C=0.0, T=1.0	0.00001	1375.98	84	C=0/0, T=40/4
chr12:25368462	T/T	C	T	SNV	KRAS	KRAS:exonic:NM_033360.3	1	100	5	synonymous	NM_03336	c.483G>A	p.(=)	4.7				0.43(COSM64	50S ribosome	rs4362222	0		1.0 (pos.25368462)	adenocarcinoi	Kirsten rat ERBB2 sign Abnormalities, Mul (3.7.11-TR Benign	C=0, T=50	C=0.0, T=1.0	0.00001	851.813	50	C=0/0, T=30/2
chr18:59821949	A/A	G	A	SNV	PIGN	PIGN:intronic:NM_176787.4	1	100		intronic	NM_17678	c.443-65C	p.?	-0.2					Phosphatidyl	rs11659731		YES			Phosphatid GPI anchor biosynthetic process,cytosol,endoplasmic re	G=0, A=66	G=0.0, A=1.0	0.00001	1015.27	66	G=0/0, A=42/2
chr18:61067122	T/T	C	T	SNV	VPS4B	VPS4B:intronic:NM_004869.3	1	100		intronic	NM_00486	c.790+159	p.?	0.15					AAA domain (	rs9965766		YES			Vacuolar pr ATP binding,ATPase activity,ATPase activity, coupled,ESC	C=0, T=25	C=0.0, T=1.0	0.00001	353.016	25	C=0/0, T=14/1
chr17:39661366	G/C	G	C	SNV	KRT13	KRT13:exonic:NM_153490.2	1	52.38	1	missense	NM_15349	c.437C>G	p.Ala146Gly	0.38	1	60	0.367		Intermediate	rs760134	0.0536	YES	3.179E-2 (pos.39661366)		Keratin 13 cellular re Carcinoma;Carcinoma, Squam Benign	G=10, C=11	G=0.4762, C=0.5238	0.00001	95.0412	21	G=5/5, C=6/5
chr18:61325905	T/G	T	G	SNV	SERPINB3	SERPINB3:intronic:NM_006919.2	1	47.62		intronic	NM_00691	c.352-41A	p.?	-0.16					Serpin (serin	rs7228687	0.4792	YES	2.782E-1 (pos.61325905)		Serpin pept autocrine Abortion, Spontane D-Serine,Dexfosfoserine	T=22, G=20	T=0.5238, G=0.4762	0.00001	212.953	42	T=5/17, G=3/1
chr17:38855772	T/C	T	C	SNV	KRT24	KRT24:exonic:NM_019016.2	1	50	6	missense	NM_01901	c.1285A>G	p.Lys429Glu	0.04	1	56	0	0.18(COSM37	Intermediate	rs2462961	0.4443	YES	5.982E-1 (pos.38855772)	acute_myeloic	Keratin 24 biological_process,cornification,cytosol,extracellular ex	T=44, C=44	T=0.5, C=0.5	0.00001	478.658	88	T=23/21, C=22
chr12:53185492	C/T	C	T	SNV	KRT3	KRT3:exonic:NM_057088.2	1	52.38	6	missense	NM_05708	c.1297G>A	p.Glu433Lys	4.74	0	56	0.999	0.99(COSM39	Intermediate	rs61929765	0.1278	YES	7.732E-2 (pos.53185492)	acute_myeloic	Keratin 3 cornificati Adnexal Diseases;Congenital, Hereditary, an	C=30, T=33	C=0.4762, T=0.5238	0.00001	333.931	63	C=18/12, T=20
chr17:39535859	A/G	A	G	SNV	KRT34	KRT34:exonic:NM_021013.3	1	45.83	4	missense	NM_02101	c.839T>C	p.Ile280Thr	2.52	1	89	0		Intermediate	rs2239710	0.349	YES	6.055E-1 (pos.39535859)		Keratin 34 cornification,cytosol,epidermis development,extracellu	A=52, G=44	A=0.5417, G=0.4583	0.00001	471.53	96	A=16/36, G=2
chr12:52911401	T/G	T	G	SNV	KRT5	KRT5:exonic:NM_000424.3	1	46.51	5	synonymous	NM_00042	c.1065A>C	p.(=)	-5.57				0.04(COSM39	Intermediate	rs4761924	0.1401	YES	2.785E-2 (pos.52911401)	acute_myeloic	Keratin 5 cornificati Adenocarcinoma;Carcinogene Benign	T=46, G=40	T=0.5349, G=0.4651	0.00001	416.494	86	T=37/9, G=35/
chr18:66513471	G/G	C	G	SNV	CCDC102B	CCDC102B:intronic:NM_001093729.1	1	100		intronic	NM_00109	c.828-79C	p.?	-0.01						rs1954878		YES			protein binding	C=0, G=20	C=0.0, G=1.0	0.00001	263.785	20	C=0/0, G=12/8
chr18:66513662	C/C	T	C	SNV	CCDC102B	CCDC102B:intronic:NM_001093729.1	1	100		intronic	NM_00109	c.936+4T>	p.?	-0.21						rs1977942,rs767992(	0.3881	YES	1.21E-4 (pos.66513661),3.528E-1;0.0 (pos.66513662)		protein binding	T=0, C=129	T=0.0, C=1.0	0.00001	2115.18	129	T=0/0, C=50/7
chr18:66513749	C/C	T	C	SNV	CCDC102B	CCDC102B:intronic:NM_001093729.1	1	100		intronic	NM_00109	c.936+91T	p.?	-2.59						rs1970675		YES			protein binding	T=0, C=127	T=0.0, C=1.0	0.00001	2074.54	127	T=0/0, C=47/8
chr18:66678131	G/G	A	G	SNV	CCDC102B	CCDC102B:intronic:NM_001093729.1	1	100		intronic	NM_00109	c.1264-40	p.?	-0.43						rs7234663	0		1.0 (pos.66678131)		protein binding	A=0, G=313	A=0.0, G=1.0	0.00001	5180.86	313	A=0/0, G=138/
chr12:52912768	C/T	C	T	SNV	KRT5	KRT5:exonic:NM_000424.3	1	45.45	2	synonymous	NM_00042	c.732G>A	p.(=)	0.48				0.73(COSM39	Intermediate	rs1132948	0.1406	YES	2.775E-2;0.0 (pos.52912	acute_myeloic	Keratin 5 cornificati Adenocarcinoma;Carcinogene Benign	C=12, T=10	C=0.5455, T=0.4545	0.00001	81.7628	22	C=8/4, T=4/6
chr18:674440	A/C	A	C	SNV	ENOSF1,TYMS	ENOSF1:intronic:NM_017512.5, TYMS:downstream:NM_001	1	50		intronic	NM_00107	c.*1443A>	p.?, p.?	-0.06					Enolase C-ter	rs11081251	0.309	YES	6.947E-1;0.0 (pos.674440)		Enolase suc DNA biosy Adenocarcinoma;A 10-Propargyl-5,8-Dideaz	A=10, C=10	A=0.5, C=0.5	0.00001	82.8791	20	A=4/6, C=8/2
chr12:52886490	G/G	A	G	SNV	KRT6A	KRT6A:exonic:NM_005554.3	1	100	1	synonymous	NM_00555	c.483T>C	p.(=)	-2.92				0.02(COSM11	Intermediate	rs711317	0.4164	YES	6.52E-1 (pos.52886490)	adenocarcinoi	Keratin 6A antimicrol Congenital Abnormalities;Congenital, Heredi	A=0, G=32	A=0.0, G=1.0	0.00001	485.141	32	A=0/0, G=17/1
chr12:52862770	C/C	T	C	SNV	KRT6C	KRT6C:utr_3:NM_173086.4	1	100	9	utr_3	NM_17308	c.1771A>G	p.?	-1.84					Intermediate	rs1053796		YES			Keratin 6C cornification,cytosol,extracellular exosome,intermedia	T=0, C=31	T=0.0, C=1.0	0.00001	465.819	31	T=0/0, C=24/7
chr18:6870524	C/C	T	C	SNV	ARHGAP28	ARHGAP28:intronic:NM_001010000.2	1	100		intronic	NM_00101	c.335-65T	p.?	-1.99					RhoGAP dom	rs62082833		YES			RHO GTPase-activating Endometriosis;Female Urogenital Diseases;F	T=0, C=79	T=0.0, C=1.0	0.00001	1267.25	79	T=0/0, C=25/5
chr12:52863621	G/G	T	G	SNV	KRT6C	KRT6C:exonic:NM_173086.4	1	100	7	synonymous	NM_17308	c.1257A>C	p.(=)	-0.01				0.19(COSM37	Intermediate	rs382367	0.425	YES	6.57E-1 (pos.52863621)	adenocarcinoi	Keratin 6C cornification,cytosol,extracellular exosome,intermedia	T=0, G=25	T=0.0, G=1.0	0.00001	352.939	25	T=0/0, G=14/1
chr18:6912004	A/A	G	A	SNV	ARHGAP28	ARHGAP28:intronic:NM_001010000.2	1	100		intronic	NM_00101	c.1619-55	p.?	-0.66					RhoGAP dom	rs1346931		YES			RHO GTPase-activating Endometriosis;Female Urogenital Diseases;F	G=0, A=41	G=0.0, A=1.0	0.00001	664.655	41	G=0/0, A=17/2
chr12:52863213	C/T	C	T	SNV	KRT6C	KRT6C:exonic:NM_173086.4	1	50	8	missense	NM_17308	c.1441G>A	p.Val481Ile	0.86	0	29	0.824	0.28(COSM37	Intermediate	rs412533	0.3678	YES	5.051E-1 (pos.52863213)	adenocarcinoi	Keratin 6C cornification,cytosol,extracellular exosome,intermedia	C=10, T=10	C=0.5, T=0.5	0.00001	82.8751	20	C=5/5, T=4/6
chr18:6950999	A/G	A	G	SNV	LAMA1	LAMA1:intronic:NM_005559.3	1	57.14		intronic	NM_00555	c.8208-29	p.?	0.88					Laminin B (Dc	rs62081480	0.393	YES	2.468E-1 (pos.6950999)		Laminin, alı basement Colonic Diseases;Colorectal Neoplasms;Diab	A=15, G=20	A=0.4286, G=0.5714	0.00001	218.708	35	A=5/10, G=9/1
chr12:52627215	G/G	A	G	SNV	KRT7	KRT7:exonic:NM_005556.3	1	100	1	synonymous	NM_00555	c.135A>G	p.(=)	-2.32				0.01(COSM37	Intermediate	rs7308888	0.1784	YES	8.967E-1 (pos.52627215)	adenocarcinoi	Keratin 7 cornificati Adenocarcinoma;A HspE7,Primaquine	A=0, G=31	A=0.0, G=1.0	0.00001	465.816	31	A=0/0, G=4/27
chr18:7035915	A/C	A	C	SNV	LAMA1	LAMA1:intronic:NM_005559.3	1	57.58		intronic	NM_00555	c.1839+71	p.?	0.02					Laminin B (Dc	rs677136		YES			Laminin, alı basement Colonic Diseases;Colorectal Neoplasms;Diab	A=14, C=19	A=0.4242, C=0.5758	0.00001	204.389	33	A=7/7, C=13/6
chr12:52946498	T/T	C	T	SNV	KRT71	KRT71:exonic:NM_033448.2	1	100	1	missense	NM_03344	c.364G>A	p.Glu122Lys	-0.13	0.04	56	0.703	0.14(COSM37	Intermediate	rs665470	0.237	YES	2.346E-2 (pos.52946498)	acute_myeloic	Keratin 71 cornificati Hair Diseases;Mammary Neoplasms, Animal	C=0, T=42	C=0.0, T=1.0	0.00001	682.938	42	C=0/0, T=10/3
chr18:71920966	C/C	T	C	SNV	CYB5A	CYB5A:intronic:NM_148923.3	1	100		intronic	NM_14892	c.324-66A	p.?	-0.13					Cytochrome I	rs1790871		YES			Cytochrome L-ascorbic Adrenal Gland Dise Dimethyl Propionate Est	T=0, C=20	T=0.0, C=1.0	0.00001	263.786	20	T=0/0, C=9/11
chr18:71930734	C/C	T	C	SNV	CYB5A	CYB5A:intronic:NM_148923.3	1	100		intronic	NM_14892	c.130-22A	p.?	-0.75					Cytochrome I	rs2032262,rs753203(	0.0064	YES	9.968E-1;0.0 (pos.71930734)		Cytochrome L-ascorbic Adrenal Gland Dise Dimethyl Propionate Est	T=0, C=115	T=0.0, C=1.0	0.00001	1720.34	115	T=0/0, C=52/6
chr18:72011050	C/C	A	C	SNV	C18orf63	C18orf63:intronic:NM_001174123.1	1	100		intronic	NM_00117	c.978+113	p.?	-0.17					Domain of un	rs11151947		YES				A=0, C=63	A=0.0, C=1.0	0.00001	958.915	63	A=0/0, C=22/4
chr12:52946543	T/T	C	T	SNV	KRT71	KRT71:exonic:NM_033448.2	1	100	1	missense	NM_03344	c.319G>A	p.Val107Ile	-0.11	0.3	29	0.143	0.11(COSM37	Intermediate	rs665522	0.2369	YES	2.358E-2 (pos.52946543)	acute_myeloic	Keratin 71 cornificati Hair Diseases;Mammary Neoplasms, Animal	C=0, T=40	C=0.0, T=1.0	0.00001	644.273	40	C=0/0, T=8/32
chr12:52940168	C/T	C	T	SNV	KRT71	KRT71:exonic:NM_033448.2	1	50	7	synonymous	NM_03344	c.1227G>A	p.(=)	-0.3					Intermediate	rs603193	0.0237	YES	0.0 (pos.52940168)		Keratin 71 cornificati Hair Diseases;Mammary Neoplasms, Animal	C=13, T=13	C=0.5, T=0.5	0.00001	119.162	26	C=11/2, T=9/4
chr18:72113730	G/G	A	G	SNV	FAM69C	FAM69C:intronic:NM_001044369.2	1	100		intronic	NM_00104	c.876+111	p.?	-0.25					N-term cyste	rs3861310		YES			Family with endoplasmic reticulum membrane,integral component	A=0, G=33	A=0.0, G=1.0	0.00001	504.458	33	A=0/0, G=24/9
chr12:52818504	G/G	T	G	SNV	KRT75	KRT75:exonic:NM_004693.2	1	100	9	missense	NM_00469	c.1453A>C	p.Ser485Arg	-0.32	0.04	110	0.799	0.05(COSM37	Intermediate	rs298104	0.3487	YES	6.927E-1;0.0 (pos.52818	adenocarcinoi	Keratin 75 cornificati Alopecia;Hair Diseases;Hypotrichosis;Loose	T=0, G=46	T=0.0, G=1.0	0.00001	768.277	46	T=0/0, G=22/2
chr12:52822258	G/G	A	G	SNV	KRT75	KRT75:exonic:NM_004693.2	1	100	7	synonymous	NM_00469	c.1164T>C	p.(=)	-2.25					Intermediate	rs401926	0.402	YES	6.979E-1 (pos.52822258)		Keratin 75 cornificati Alopecia;Hair Diseases;Hypotrichosis;Loose	A=0, G=83	A=0.0, G=1.0	0.00001	1349.88	83	A=0/0, G=45/3
chr12:52827818	C/C	T	C	SNV	KRT75	KRT75:exonic:NM_004693.2	1	100	1	missense	NM_00469	c.271A>G	p.Arg91Gly	0.97	1	125	0	0.01(COSM49	Intermediate	rs298109	0.4022	YES	6.957E-1;0.0 (pos.52827	ductal_carcinc	Keratin 75 cornificati Alopecia;Hair Diseases;Hypotrichosis;Loose	T=0, C=63	T=0.0, C=1.0	0.00001	962.612	63	T=0/0, C=30/3
chr12:53162773	G/G	A	G	SNV	KRT76	KRT76:exonic:NM_015848.4	1	100	9	synonymous	NM_01584	c.1641T>C	p.(=)	-1.3				0.01(COSM37	Intermediate	rs1464423,rs201384	0.125	YES	7.495E-3;0.0;0.0;0.0;0.0;	clear_cell_ren	Keratin 76, cornification,cytoskeleton organization,cytosol,extracel	A=0, G=21	A=0.0, G=1.0	0.00001	273.724	21	A=0/0, G=8/13
chr12:53165940	T/T	C	T	SNV	KRT76	KRT76:exonic:NM_015848.4	1	100	5	missense	NM_01584	c.1075G>A	p.Ala359Thr	0.65	0.02	58	0.986	0.31(COSM11	Intermediate	rs6580904,rs770706	0.1709	YES	0.0 (pos.53165937),5.31	acute_myeloic	Keratin 76, cornification,cytoskeleton organization,cytosol,extracel	C=0, T=38	C=0.0, T=1.0	0.00001	603.786	38	C=0/0, T=16/2
chr12:53085089	C/C	T	C	SNV	KRT77	KRT77:exonic:NM_175078.2	1	100	9	missense	NM_17507	c.1597A>G	p.Arg533Gly	-0.45	0.31	125	0	0(COSM3753	Intermediate	rs636127,rs7651554	0.0372	YES	9.998E-1;0.0;0.0 (pos.53(	adenocarcinoi	Keratin 77 biological_process,cornification,cytoskeleton,cytosol,e	T=0, C=22	T=0.0, C=1.0	0.00001	298.888	22	T=0/0, C=10/1
chr12:53096913	A/A	G	A	SNV	KRT77	KRT77:exonic:NM_175078.2	1	100	1	synonymous	NM_17507	c.306C>T	p.(=)	1.31					Intermediate	rs1625862	5.00E-04		1.0 (pos.53096913)		Keratin 77 biological_process,cornification,cytoskeleton,cytosol,e	G=0, A=35	G=0.0, A=1.0	0.00001	508.786	35	G=0/0, A=16/1
chr21:46057393	A/A	T	A	SNV	KRTAP10-10,TSPEAR	KRTAP10-10:exonic:NM_181688.2, TSPEAR:intronic:NM_144	1	100	1	missense	NM_14499	c.83-6950	p.?, p.Val20Asp	0.97		152		0(COSM4135	EPTP domain	rs2838602,rs797028	0.2929	YES	6.336E-1 (pos.46057393)	neoplasm,oste	Thrombosp cell surface,ciliary membrane,cytosol,extracellular regi	T=0, A=35	T=0.0, A=1.0	0.00001	543.881	35	T=0/0, A=19/1
chr21:46066764	T/T	C	T	SNV	KRTAP10-11,TSPEAR	KRTAP10-11:exonic:NM_198692.2, TSPEAR:intronic:NM_144	1	100	1	missense	NM_14499	c.82+6458	p.?, p.Ser130Phe	0.27		155		0.17(COSM50	EPTP domain	rs4818952,rs782508	0.2772	YES	0.0;0.0 (pos.46066763),6	osteosarcoma	Thrombosp cell surface,ciliary membrane,cytosol,extracellular regi	C=0, T=25	C=0.0, T=1.0	0.00001	353.019	25	C=0/0, T=9/16
chr18:77132755	T/G	T	G	SNV	ATP9B	ATP9B:intronic:NM_198531.4	1	50		intronic	NM_19853	c.3013-70	p.?	-2.27					Cation transp	rs2271392	0.2775	YES			ATPase, cla ATP bindin Cocaine-Related Disorders;Mental Disorders	T=13, G=13	T=0.5, G=0.5	0.00001	115.793	26	T=11/2, G=9/4
chr18:77132793	G/A	G	A	SNV	ATP9B	ATP9B:intronic:NM_198531.4	1	50		intronic	NM_19853	c.3013-32	p.?	-0.02					Cation transp	rs3817178	0.2721	YES	5.203E-1 (pos.77132793)		ATPase, cla ATP bindin Cocaine-Related Disorders;Mental Disorders	G=13, A=13	G=0.5, A=0.5	0.00001	119.159	26	G=11/2, A=9/4
chr21:45971018	C/G	C	G	SNV	KRTAP10-2,TSPEAR	KRTAP10-2:exonic:NM_198693.3, TSPEAR:intronic:NM_1449	1	53.33	1	synonymous	NM_14499	c.303+166	p.?, p.(=)	-1.65				0(COSM3766	EPTP domain	rs478947	0.1953	YES	3.428E-1 (pos.45971018)	acute_myeloic	Thrombosp cell surface,ciliary membrane,cytosol,extracellular regi	C=21, G=24	C=0.4667, G=0.5333	0.00001	276.152	45	C=10/11, G=1
chr21:45970759	ACTTGCAGCA(	ACTTGCAG	A	INDEL	KRTAP10-2,TSPEAR	KRTAP10-2:exonic:NM_198693.3, TSPEAR:intronic:NM_1449	15	50	1	nonframeshiftDeletion	NM_14499	c.303+169	p.?, p.Pro190_Lys194de	0.17, -2.0, -8.41, -0.46, -0.78, 1.53, 1.02, 2.31, 0.09, 2.48, -0.01,					EPTP domain	rs113146284,rs2002	0.1769	YES	0.0 (pos.45970760),0.0 ((	astrocytoma_(	Thrombosp cell surface,ciliary membrane,cytosol,extracellular regi	ACTTGCAG(	ACTTGCAGCAGACAG(	0.00001	82.8861	20	ACTTGCAGCAG
chr21:46021205	T/T	C	T	SNV	KRTAP10-7,TSPEAR	KRTAP10-7:exonic:NM_198689.2, TSPEAR:intronic:NM_1449	1	100	2	synonymous	NM_14499	c.83-3331	p.?, p.(=)	2.45				0.88(COSM41	EPTP domain	rs389167	0.0633	YES	8.069E-1;0.0 (pos.46021	neoplasm	Thrombosp cell surface,ciliary membrane,cytosol,extracellular regi	C=0, T=20	C=0.0, T=1.0	0.00001	254.474	20	C=0/0, T=14/6
chr18:7774104	C/C	T	C	SNV	PTPRM	PTPRM:intronic:NM_001105244.1	1	100		intronic	NM_00110	c.74-43T>	p.?	0.04					Fibronectin t	rs875530	0.4867	YES	8.487E-1 (pos.7774104)		Protein tyrc cadherin binding,cell-cell adherens junction,cell-cell jun	T=0, C=35	T=0.0, C=1.0	0.00001	543.9	35	T=0/0, C=19/1
chr18:77797523	A/C	A	C	SNV	RBFA	RBFA:intronic:NM_024805.2	1	52.38		intronic	NM_02480	c.378+17A	p.?	-1.02					Ribosome-binding factor A						biological_process,cellular_component,mitochondrion,	A=10, C=11	A=0.4762, C=0.5238	0.00001	95.3003	21	A=6/4, C=6/5
chr21:46021607	G/G	A	G	SNV	KRTAP10-7,TSPEAR	KRTAP10-7:exonic:NM_198689.2, TSPEAR:intronic:NM_1449	1	100	2	synonymous	NM_14499	c.83-3371	p.?, p.(=)	-10.06				0(COSM4135	EPTP domain	rs370092	0.0619	YES	8.066E-1 (pos.46021607)	neoplasm	Thrombosp cell surface,ciliary membrane,cytosol,extracellular regi	A=0, G=23	A=0.0, G=1.0	0.00001	314.227	23	A=0/0, G=3/20
chr21:46032094	G/G	A	G	SNV	KRTAP10-8,TSPEAR	KRTAP10-8:exonic:NM_198695.2, TSPEAR:intronic:NM_1449	1	100	1	missense	NM_14499	c.83-4420	p.?, p.His26Arg	-0.59		29		0(COSM4135	EPTP domain	rs411254	0.0553	YES	8.096E-1 (pos.46032094)	neoplasm	Thrombosp cell surface,ciliary membrane,cytosol,extracellular regi	A=0, G=36	A=0.0, G=1.0	0.00001	565.662	36	A=0/0, G=20/1
chr21:46047418	T/T	C	T	SNV	KRTAP10-9,TSPEAR	KRTAP10-9:exonic:NM_198690.2, TSPEAR:intronic:NM_1449	1	100	1	synonymous	NM_14499	c.83-5952	p.?, p.(=)	-0.28				0(COSM4135	EPTP domain	rs782237778,rs8126	0.2767	YES	1.156E-4 (pos.46047411)	neoplasm	Thrombosp cell surface,ciliary membrane,cytosol,extracellular regi	C=0, T=22	C=0.0, T=1.0	0.00001	298.892	22	C=0/0, T=15/7
chr21:46086377	G/G	A	G	SNV	KRTAP12-2,TSPEAR	KRTAP12-2:exonic:NM_181684.2, TSPEAR:intronic:NM_1449	1	100	1	missense	NM_14499	c.82+4497	p.?, p.Ser143Pro	-0.3		74		0(COSM5022	EPTP domain	rs2838622	0.4562	YES	5.596E-1;0.0;0.0 (pos.46(	osteosarcoma	Thrombosp cell surface,ciliary membrane,cytosol,extracellular regi	A=0, G=67	A=0.0, G=1.0	0.00001	1034.43	67	A=0/0, G=32/3
chr21:46086718	CG/CG	GA	CG	MNV	KRTAP12-2,TSPEAR	KRTAP12-2:exonic:NM_181684.2, TSPEAR:intronic:NM_1449	2	100	1	missense	NM_14499	c.82+4463	p.?, p.Ser29Arg	-0.39, -4.33		110		0.03(COSM41	EPTP domain	rs368303093,rs7275	0.1796	YES	5.542E-1;2.353E-4 (pos.4	neoplasm	Thrombosp cell surface,ciliary membrane,cytosol,extracellular regi	GA=0, CG=4	GA=0.0, CG=1.0	0.00001	664.7	41	GA=0/0, CG=1(
chr21:46086757	AT/AT	GC	AT	MNV	KRTAP12-2,TSPEAR	KRTAP12-2:exonic:NM_181684.2, TSPEAR:intronic:NM_1449	2	100	1	missense	NM_14499	c.82+4459	p.?, p.Ala16Met	-0.53, -4.56		84		0.01(COSM41	EPTP domain	rs13046903,rs35163	0.2944	YES	6.478E-1 (pos.46086758)	carcinoma,ne	Thrombosp cell surface,ciliary membrane,cytosol,extracellular regi	GC=0, AT=3	GC=0.0, AT=1.0	0.00001	563.744	36	GC=0/0, AT=15
chr21:46077946	G/G	A	G	SNV	KRTAP12-3,TSPEAR	KRTAP12-3:exonic:NM_198697.2, TSPEAR:intronic:NM_1449	1	100	1	missense	NM_14499	c.82+5340	p.?, p.His17Arg	-0.36		29		0.02(COSM37	EPTP domain	rs9306111	0.2875	YES	6.582E-1 (pos.46077946)	acute_myeloic	Thrombosp cell surface,ciliary membrane,cytosol,extracellular regi	A=0, G=63	A=0.0, G=1.0	0.00001	958.869	63	A=0/0, G=47/1

chr21:31802768	A/A	G	A	SNV	KRTAP13-4	KRTAP13-4:exonic:NM 181600.2	1	100	1	missense	NM 18160	c.175G>A	p.Ala59Thr	-0.66		[illegible]
chr18:9396597	G/G	A	G	SNV	TWSG1	TWSG1:intronic:NM 020648.5	1	100		intronic	NM 02064	c.490+53A	p.?	-2.83		[illegible]
chr18:9522606	G/A	G	A	SNV	RALBP1	RALBP1:intronic:NM 006788.3	1	53.33		intronic	NM 00678	c.1053+99	p.?	-0.29		[illegible]
chr21:31812772	A/A	C	A	SNV	KRTAP15-1	KRTAP15-1:exonic:NM 181623.1	1	100	1	missense	NM 18162	c.127C>A	p.Leu43Met	0.04		[illegible]
chr18:9944749	C/C	T	C	SNV	VAPA	VAPA:intronic:NM 003574.5	1	100		intronic	NM 00357	c.418-172	p.?	1.42		[illegible]
chr21:31973516	A/A	T	A	SNV	KRTAP22-1	KRTAP22-1:exonic:NM 181620.1	1	100	1	missense	NM 18162	c.77T>A	p.Leu26His	-1.11	0.83	[illegible]
chr21:31655212	C/C	A	C	SNV	KRTAP24-1	KRTAP24-1:exonic:NM 001085455.2	1	100	1	synonymous	NM 00108	c.39T>G	p.(=)	-1.8		[illegible]
chr19:10079013	C/C	G	C	SNV	COL5A3	COL5A3:intronic:NM 015719.3	1	100		intronic	NM 01571	c.4314+48	p.?	-1.53		[illegible]
chr17:39340597	A/A	G	A	SNV	KRTAP4-1	KRTAP4-1:utr 3:NM 033060.2	1	100	2	utr 3	NM 03306	c.453C>T	p.?	-0.01		[illegible]
chr19:10084422	T/T	C	T	SNV	COL5A3	COL5A3:intronic:NM 015719.3	1	100		intronic	NM 01571	c.3612+10	p.?	-1.1		[illegible]
chr19:10084551	G/G	T	G	SNV	COL5A3	COL5A3:intronic:NM 015719.3	1	100		intronic	NM 01571	c.3558+44	p.?	-0.18		[illegible]
chr17:39340707	T/T	C	T	SNV	KRTAP4-1	KRTAP4-1:exonic:NM 033060.2	1	100	2	missense	NM 03306	c.343G>A	p.Ala115Thr	-0.13	0.12	[illegible]
chr17:39274518	T/T	C	T	SNV	KRTAP4-11	KRTAP4-11:exonic:NM 033059.3	1	100	1	missense	NM 03305	c.50G>A	p.Arg17Gln	0.01	0.71	[illegible]
chr19:10097187	G/GC	G	GC	INDEL	COL5A3	COL5A3:intronic:NM 015719.3	1	100		intronic	NM 01571	c.2244+42	p.?	-0.06		[illegible]
chr19:10099910	T/T	C	T	SNV	COL5A3	COL5A3:intronic:NM 015719.3	1	100		intronic	NM 01571	c.2092-57(	p.?	-0.23		[illegible]
chr19:10100061	A/A	C	A	SNV	COL5A3	COL5A3:intronic:NM 015719.3	1	100		intronic	NM 01571	c.2047-19(	p.?	-0.18		[illegible]
chr19:10102567	G/A	G	A	SNV	COL5A3	COL5A3:intronic:NM 015719.3	1	97.5		intronic	NM 01571	c.1894-50(	p.?	1.35		[illegible]
chr19:10102676	G/G	A	G	SNV	COL5A3	COL5A3:intronic:NM 015719.3	1	100		intronic	NM 01571	c.1893+4T	p.?	-5.05		[illegible]
chr17:39240504	G/G	A	G	SNV	KRTAP4-7	KRTAP4-7:exonic:NM 033061.3	1	100	1	missense	NM 03306	c.46A>G	p.Ser16Gly	0.32		[illegible]
chr19:10116076	C/C	G	C	SNV	COL5A3	COL5A3:intronic:NM 015719.3	1	100		intronic	NM 01571	c.594+158	p.?	-0.51		[illegible]
chr17:39240511	T/T	A	T	SNV	KRTAP4-7	KRTAP4-7:exonic:NM 033061.3	1	100	1	missense	NM 03306	c.53A>T	p.Asp18Val	0.06		[illegible]
chr17:39253749	C/C	T	C	SNV	KRTAP4-8	KRTAP4-8:utr 3:NM 031960.2	1	100	1	utr 3	NM 03196	c.588A>G	p.?	0.01		[illegible]
chr17:39253835	T/T	C	T	SNV	KRTAP4-8	KRTAP4-8:exonic:NM 031960.2	1	100	1	missense	NM 03196	c.502G>A	p.Ala168Thr	0.1	1	[illegible]
chr17:39253886	C/C	G	C	SNV	KRTAP4-8	KRTAP4-8:exonic:NM 031960.2	1	100	1	missense	NM 03196	c.451C>G	p.Leu151Val	-0.15	0.28	[illegible]
chr21:32201969	G/G	GA	G	INDEL	KRTAP7-1	KRTAP7-1:splicesite 3:NM 181606.2	1	100	2	splicesite	NM 181606.2			3.07, 0.0		[illegible]
chr19:1053299	C/T	C	T	SNV	ABCA7	ABCA7:intronic:NM 019112.3	1	50		intronic	NM 01911	c.3221-29(	p.?	0.01		[illegible]
chr19:1059004	A/G	A	G	SNV	ABCA7	ABCA7:intronic:NM 019112.3	1	57.14		intronic	NM 01911	c.5401-18	p.?	-0.64		[illegible]
chr19:10602180	C/C	A	C	SNV	KEAP1	KEAP1:intronic:NM 203500.1	1	100		intronic	NM 20350	c.1325+73	p.?	-0.67		[illegible]
chr17:39346518	C/C	T	C	SNV	KRTAP9-1	KRTAP9-1:exonic:NM 001190460.1	1	100	1	missense	NM 00119	c.380T>C	p.Ile127Thr	-4.36	1	[illegible]
chr19:1065197	G/C	G	C	SNV	ABCA7,ARH GAP45	ABCA7:intronic:NM 019112.3, ARHGAP45:upstream:NM 00	1	95.45		intronic	NM 01911	c.6285+27	p.?, p.?	0.1		[illegible]
chr19:10665691	C/C	T	C	SNV	KRI1	KRI1:intronic:NM 023008.3	1	100		intronic	NM 02300	c.1799+72	p.?	-0.19		[illegible]
chr19:10665695	T/T	C	T	SNV	KRI1	KRI1:intronic:NM 023008.3	1	100		intronic	NM 02300	c.1799+68	p.?	1.53		[illegible]
chr19:10676343	T/T	A	T	SNV	CDKN2D,KRI 1	CDKN2D:downstream:NM 001800.3, KRI1:intronic:NM 023	1	98.99		intronic	NM 02300	c.186+50T	p.?, p.?	-2.22		[illegible]
chr19:35978299	T/T	C	T	SNV	KRTDAP	KRTDAP:utr 3:NM 207392.2	1	100	6	utr 3	NM 20739	c.331G>A	p.?	-1.26		[illegible]
chr12:118199286	G/G	C	G	SNV	KSR2	KSR2:exonic:NM 173598.4	1	100	4	synonymous	NM 17359	c.429G>C	p.(=)	0.12		[illegible]
chr3:134323156	G/G	A	G	SNV	KY	KY:exonic:NM 178554.5	1	100	11	synonymous	NM 17855	c.1251T>C	p.(=)	-0.29		[illegible]
chr1:89426902	A/A	G	A	SNV	KYAT3	KYAT3:exonic:NM 001008661.2	1	100	8	synonymous	NM 00100	c.735C>T	p.(=)	0.6		[illegible]
chr1:62676284	C/T	C	T	SNV	L1TD1	L1TD1:exonic:NM 019079.4	1	50	4	missense	NM 01907	c.1838C>T	p.Thr613Ile	0.4	0.21	[illegible]
chr19:1110656	C/T	C	T	SNV	SBNO2	SBNO2:intronic:NM 014963.2	1	45.45		intronic	NM 01496	c.3028+88	p.?	0.27		[illegible]
chr19:11107133	T/C	T	C	SNV	SMARCA4	SMARCA4:intronic:NM 001128849.1	1	50		intronic	NM 00112	c.1762-37	p.?	-2.96		[illegible]
chr1:201355522	C/C	T	C	SNV	LAD1	LAD1:exonic:NM 005558.3	1	100	3	missense	NM 00555	c.967A>G	p.Lys323Glu	1.12	1	[illegible]
chr19:11224181	T/T	C	T	SNV	LDLR,MIR68 86	MIR6886:exonic nc:NR 106946.1, LDLR:intronic:NM 00052	1	100		intronic	NM 00052	c.1359-30(	p.?	-0.13		[illegible]
chr18:6999628	T/C	T	C	SNV	LAMA1	LAMA1:exonic:NM 005559.3	1	50	32	synonymous	NM 00555	c.4479A>G	p.(=)	-4.6		[illegible]
chr6:129381026	A/A	C	A	SNV	LAMA2	LAMA2:exonic:NM 000426.3	1	100	3	synonymous	NM 00042	c.381C>A	p.(=)	-2.68		[illegible]
chr19:11266584	G/G	A	G	SNV	SPC24	SPC24:upstream:NM 182513.3	1	100		intronic	NM 18251	c.-132T>C	p.?	-0.49		[illegible]
chr18:21511089	G/G	A	G	SNV	LAMA3	LAMA3:exonic:NM 198129.2	1	100	65	missense	NM 19812	c.8500A>G	p.Ser2834Gly	-1.26	0.5	[illegible]
chr6:112457390	T/T	C	T	SNV	LAMA4	LAMA4:exonic:NM 001105206.2	1	100	25	missense	NM 00110	c.3349G>A	p.Gly1117Ser	3.71	0.88	[illegible]
chr19:11436302	AG/AG	A	AG	INDEL	RAB3D,TSPA N16	TSPAN16:intronic:NM 012466.3, RAB3D:intronic:NM 0042(	1	100		intronic	NM 01246	c.688-116	p.?, p.?	-1.62		[illegible]
chr19:11541712	C/C	T	C	SNV	CCDC151	CCDC151:intronic:NM 145045.4	1	100		intronic	NM 14504	c.366+7A>	p.?	-0.64		[illegible]
chr19:11727384	G/C	G	C	SNV	ZNF627	ZNF627:intronic:NM 145295.3	1	57.14		intronic	NM 14529	c.192-126(	p.?	0.03		[illegible]
chr6:112493872	G/G	A	G	SNV	LAMA4	LAMA4:exonic:NM 001105206.2	1	100	12	missense	NM 00110	c.1492T>C	p.Tyr498His	2.17	0.71	[illegible]
chr20:60887581	A/A	G	A	SNV	LAMA5	LAMA5:exonic:NM 005560.4	1	100	68	missense	NM 00556	c.9235C>T	p.Arg3079Trp	-0.61	0.01	[illegible]
chr20:60897487	T/T	C	T	SNV	LAMA5	LAMA5:exonic:NM 005560.4	1	100	47	missense	NM 00556	c.6184G>A	p.Asp2062Asn	0.84	0.29	[illegible]
chr20:60899206	T/T	C	T	SNV	LAMA5	LAMA5:exonic:NM 005560.4	1	100	43	missense	NM 00556	c.5698G>A	p.Val1900Met	0.49	0.12	[illegible]
chr19:1271529	T/T	C	T	SNV	CIRBP	CIRBP:intronic:NM 001280.2	1	100		intronic	NM 00128	c.350-21C	p.?	-0.14		[illegible]
chr19:12760664	T/C	T	C	SNV	MAN2B1	MAN2B1:intronic:NM 000528.3	1	57.14		intronic	NM 00052	c.2267+63	p.?	0		[illegible]
chr20:60905878	G/G	A	G	SNV	LAMA5	LAMA5:exonic:NM 005560.4	1	100	30	missense	NM 00556	c.3773T>C	p.Met1258Thr	1.19	0.85	[illegible]
chr20:60912683	C/C	T	C	SNV	LAMA5	LAMA5:exonic:NM 005560.4	1	100	16	synonymous	NM 00556	c.2127A>G	p.(=)	-1.77		[illegible]
chr19:13255536	C/C	T	C	SNV	STX10	STX10:intronic:NM 003765.2	1	100		intronic	NM 00376	c.578+42A	p.?	-2.46		[illegible]
chr20:60921643	C/C	T	C	SNV	LAMA5	LAMA5:exonic:NM 005560.4	1	100	9	missense	NM 00556	c.1201A>G	p.Thr401Ala	1.06	0.46	[illegible]
chr19:1370526	G/G	T	G	SNV	MUM1	MUM1:intronic:NM 032853.3	1	100		intronic	NM 03285	c.1553-11	p.?	-0.1		[illegible]
chr19:14065488	A/G	A	G	SNV	DCAF15	DCAF15:intronic:NM 138353.3	1	47.62		intronic	NM 13835	c.366+15A	p.?	-0.08		[illegible]
chr19:14162676	C/C	A	C	SNV	IL27RA	IL27RA:intronic:NM 004843.3	1	100		intronic	NM 00484	c.1623-16	p.?	-0.74		[illegible]
chr19:1422299	T/C	T	C	SNV	DAZAP1	DAZAP1:intronic:NM 018959.3	1	50		intronic	NM 01895	c.415-48T	p.?	-0.21		[illegible]
chr19:14508964	C/C	T	C	SNV	ADGRE5	ADGRE5:intronic:NM 078481.3	1	100		intronic	NM 07848	c.897+13T	p.?	-3.08		[illegible]
chr20:60907675	A/A	G	A	SNV	LAMA5,MIR 4758	LAMA5:exonic:NM 005560.4, MIR4758:upstream:NR 0399:	1	100	27	synonymous	NM 00556	c.3381C>T	p.(=)	-1.51		[illegible]
chr19:14744032	G/A	G	A	SNV	ADGRE3	ADGRE3:intronic:NM 032571.4	1	46.51		intronic	NM 03257	c.1551+16	p.?	-0.7		[illegible]
chr7:107593989	T/C	T	C	SNV	LAMB1	LAMB1:exonic:NM 002291.2	1	95.45	22	missense	NM 00229	c.3065A>G	p.Gln1022Arg	-1.28	1	[illegible]
chr1:209788687	G/A	G	A	SNV	LAMB3	LAMB3:exonic:NM 000228.2	1	57.58	23	synonymous	NM 00022	c.3448C>T	p.(=)	1.6		[illegible]
chr19:14779550	A/A	C	A	SNV	ADGRE3	ADGRE3:intronic:NM 032571.4	1	100		intronic	NM 03257	c.76+64G>	p.?	-0.1		[illegible]
chr1:209788703	T/C	T	C	SNV	LAMB3	LAMB3:exonic:NM 000228.2	1	57.58	23	synonymous	NM 00022	c.3432A>G	p.(=)	-4.71		[illegible]
chr1:183085755	A/G	A	G	SNV	LAMC1	LAMC1:exonic:NM 002293.3	1	50	7	missense	NM 00229	c.1372A>G	p.Ile458Val	-0.03	1	[illegible]
chr19:14866974	C/C	T	C	SNV	ADGRE2	ADGRE2:intronic:NM 013447.3	1	100		intronic	NM 01344	c.1192+76	p.?	-1.34		[illegible]
chr9:133884820	G/G	T	G	SNV	LAMC3	LAMC3:exonic:NM 006059.3	1	100	1	missense	NM 00605	c.219T>G	p.His73Gln	1.59	0.17	[illegible]
chr9:133948049	G/G	A	G	SNV	LAMC3	LAMC3:exonic:NM 006059.3	1	100	19	missense	NM 00605	c.3244A>G	p.Ser1082Gly	0.72	0.48	[illegible]
chr19:15064858	C/C	A	C	SNV	SLC1A6	SLC1A6:intronic:NM 005071.2	1	100		intronic	NM 00507	c.1364+89	p.?	-3.06		[illegible]
chr19:15065169	C/C	T	C	SNV	SLC1A6	SLC1A6:intronic:NM 005071.2	1	100		intronic	NM 00507	c.1170-28	p.?	-2.9		[illegible]
chr19:15072787	T/T	A	T	SNV	SLC1A6	SLC1A6:intronic:NM 005071.2	1	100		intronic	NM 00507	c.935+27T	p.?	-0.58		[illegible]
chr19:15075020	G/G	A	G	SNV	SLC1A6	SLC1A6:intronic:NM 005071.2	1	100		intronic	NM 00507	c.591+111	p.?	-0.3		[illegible]
chr9:133967240	C/C	T	C	SNV	LAMC3	LAMC3:utr 3:NM 006059.3	1	100	28	unknown	NM 00605	c.4794T>C	p.?	-1.05		[illegible]
chr19:15124474	T/T	C	T	SNV	CCDC105	CCDC105:intronic:NM 173482.2	1	100		intronic	NM 17348	c.571-10C	p.?	-0.03		[illegible]
chr19:15132574	G/G	C	G	SNV	CCDC105	CCDC105:intronic:NM 173482.2	1	100		intronic	NM 17348	c.1138-44(	p.?	-2.9		[illegible]
chr9:133936571	C/G	C	G	SNV	LAMC3	LAMC3:exonic:NM 006059.3	1	97.06	13	missense	NM 00605	c.2308C>G	p.Arg770Gly	-0.71	0.93	[illegible]
chrX:119590533	A/A	T	A	SNV	LAMP2	LAMP2:exonic:NM 001122606.1	1	100	2	synonymous	NM 00112	c.156A>T	p.(=)	2.19		[illegible]
chr3:182871962	T/T	C	T	SNV	LAMP3	LAMP3:exonic:NM 014398.3	1	100	2	synonymous	NM 01439	c.267G>A	p.(=)	0.62		[illegible]
chr4:17586703	C/C	T	C	SNV	LAP3	LAP3:exonic:NM 015907.2	1	100	6	synonymous	NM 01590	c.648T>C	p.(=)	-4.54		[illegible]
chr22:34022284	A/A	G	A	SNV	LARGE1	LARGE1:exonic:NM 133642.3	1	100	4	synonymous	NM 13364	c.435C>T	p.(=)	-0.23		[illegible]
chr22:33670584	G/A	G	A	SNV	LARGE1	LARGE1:exonic:NM 133642.3	1	51.85	15	synonymous	NM 13364	c.2100C>T	p.(=)	-0.18		[illegible]
chr4:129012181	A/A	G	A	SNV	LARP1B	LARP1B:exonic:NM 018078.3	1	100	6	synonymous	NM 01807	c.384G>A	p.(=)	2.01		[illegible]
chr19:15739315	C/C	T	C	SNV	CYP4F8	CYP4F8:intronic:NM 007253.3	1	100		intronic	NM 00725	c.1249-26	p.?	-1.08		[illegible]
chr4:129012638	G/G	A	G	SNV	LARP1B	LARP1B:exonic:NM 018078.3	1	100	7	synonymous	NM 01807	c.639A>G	p.(=)	0.19		[illegible]
chr19:15769449	G/G	A	G	SNV	CYP4F3	CYP4F3:intronic:NM 001199209.1	1	100		intronic	NM 00119	c.1314+84	p.?	-0.78		[illegible]
chr19:15769529	TT/TT	GC	TT	MNV	CYP4F3	CYP4F3:intronic:NM 001199209.1	2	100		intronic	NM 00119	c.1315-8G	p.?	-2.81, -0.78		[illegible]
chr19:15784285	T/T	C	T	SNV	CYP4F12	CYP4F12:intronic:NM 023944.3	1	100		intronic	NM 02394	c.0-54C>T	p.?	-2.12		[illegible]
chr19:15784319	T/T	G	T	SNV	CYP4F12	CYP4F12:intronic:NM 023944.3	1	100		intronic	NM 02394	c.0-20G>T	p.?	-1.55		[illegible]
chr4:129043204	G/G	C	G	SNV	LARP1B	LARP1B:exonic:NM 018078.3	1	100	11	missense	NM 01807	c.1385C>G	p.Pro462Arg	1.49	0	[illegible]
chr19:15791180	T/T	C	T	SNV	CYP4F12	CYP4F12:intronic:NM 023944.3	1	100		intronic	NM 02394	c.398-22C	p.?	-0.11		[illegible]
chr4:129043286	T/T	C	T	SNV	LARP1B	LARP1B:exonic:NM 018078.3	1	100	11	synonymous	NM 01807	c.1467C>T	p.(=)	2.11		[illegible]
chr19:15795842	T/T	C	T	SNV	CYP4F12	CYP4F12:intronic:NM 023944.3	1	100		intronic	NM 02394	c.986-36C	p.?	0.62		[illegible]
chr19:15806703	A/C	A	C	SNV	CYP4F12	CYP4F12:intronic:NM 023944.3	1	57.89		intronic	NM 02394	c.1116-43	p.?	-0.5		[illegible]
chr4:129100643	A/A	G	A	SNV	LARP1B	LARP1B:exonic:NM 018078.3	1	100	15	missense	NM 01807	c.1979G>A	p.Arg660His	5.07	1	[illegible]
chr3:45565554	G/G	A	G	SNV	LARS2	LARS2:exonic:NM 015340.3	1	100	20	synonymous	NM 01534	c.2358A>G	p.(=)	0.51		[illegible]
chr17:37070658	G/G	A	G	SNV	LASP1,MIR6 779	LASP1:exonic nc:NR 073384.1, MIR6779:upstream:NR 106	1	100	6	exonic nc	NR 073384.1, NR 106837.1			-0.55		[illegible]
chr13:21562832	T/T	C	T	SNV	LATS2	LATS2:exonic:NM 014572.2	1	100	4	missense	NM 01457	c.1087G>A	p.Gly363Ser	0.77	0.43	[illegible]
chr11:111420364	G/G	A	G	SNV	LAYN	LAYN:exonic:NM 001258390.1	1	100	4	synonymous	NM 00125	c.429A>G	p.(=)	-3.53		[illegible]
chr1:225611661	T/T	C	T	SNV	LBR	LBR:exonic:NM 002296.3	1	100	2	synonymous	NM 00229	c.117G>A	p.(=)	-2.98		[illegible]
chr2:74725144	C/C	T	C	SNV	LBX2	LBX2:exonic:NM 001009812.1	1	100	2	synonymous	NM 00100	c.495A>G	p.(=)	-1.44		[illegible]
chr6:80228541	G/G	A	G	SNV	LCA5	LCA5:exonic:NM 001122769.2	1	100	2	missense	NM 00112	c.71T>C	p.Leu24Ser	2.72	0.47	[illegible]
chr19:16178587	C/C	A	C	SNV	TPM4	TPM4:intronic:NM 001145160.1	1	100		intronic	NM 00114	c.114+39A	p.?	-0.77		[illegible]
chr19:16232508	G/G	A	G	SNV	RAB8A	RAB8A:intronic:NM 005370.4	1	100		intronic	NM 00537	c.186-52A	p.?	1.34		[illegible]
chr6:80228673	C/C	T	C	SNV	LCA5	LCA5:utr 5:NM 001122769.2	1	100	2	utr 5	NM 00112	c.-62A>G	p.?	-0.01		[illegible]
chr2:136546110	G/G	A	G	SNV	LCT	LCT:exonic:NM 002299.3	1	100	17	synonymous	NM 00229	c.5568T>C	p.(=)	0.05		[illegible]
chr19:16275515	A/G	A	G	SNV	CIB3	CIB3:intronic:NM 054113.3	1	98.72		intronic	NM 05411	c.542+14T	p.?	0.99		[illegible]
chr2:136594158	G/A	G	A	SNV	LCT	LCT:exonic:NM 002299.3	1	45.83	1	synonymous	NM 00229	c.582C>T	p.(=)	-0.11		[illegible]
chr19:16338532	G/A	G	A	SNV	AP1M1	AP1M1:intronic:NM 001130524.1	1	50		intronic	NM 00113	c.852+31G	p.?	0.65		[illegible]
chr19:11233941	G/G	A	G	SNV	LDLR	LDLR:exonic:NM 000527.4	1	100	15	synonymous	NM 00052	c.2232A>G	p.(=)	-0.59		[illegible]
chr19:11224265	G/G	A	G	SNV	LDLR,MIR68 86	MIR6886:downstream:NR 106946.1, LDLR:exonic:NM 000!	1	100	10	synonymous	NM 00052	c.1413A>G	p.(=)	1.22		[illegible]
chr19:16639121	C/G	C	G	SNV	CHERP	CHERP:intronic:NM 006387.5	1	50		intronic	NM 00638	c.1130-55(	p.?	-1.94		[illegible]
chr1:25889632	C/C	T	C	SNV	LDLRAP1	LDLRAP1:exonic:NM 015627.2	1	100	6	missense	NM 01562	c.604T>C	p.Ser202Pro	-0.62	0.1	[illegible]
chr1:25890189	G/G	A	G	SNV	LDLRAP1	LDLRAP1:exonic:NM 015627.2	1	100	7	synonymous	NM 01562	c.654A>G	p.(=)	-0.97		[illegible]
chr19:54962467	A/A	G	A	SNV	LENG8	LENG8:utr 5:NM 052925.3	1	100	2	utr 5	NM 05292	c.-55G>A	p.?	0.14		[illegible]
chr19:54965860	A/A	G	A	SNV	LENG8	LENG8:exonic:NM 052925.3	1	100	6	synonymous	NM 05292	c.678G>A	p.(=)	0.87		[illegible]
chr19:17088121	T/C	T	C	SNV	CPAMD8	CPAMD8:intronic:NM 015692.3	1	45.45		intronic	NM 01569	c.1899+57	p.?	0.12		[illegible]
chr19:17091141	G/T	G	T	SNV	CPAMD8	CPAMD8:intronic:NM 015692.3	1	51.85		intronic	NM 01569	c.1726+16	p.?	0.47		[illegible]
chr19:17137290	C/C	A	C	SNV	CPAMD8	CPAMD8:intronic:NM 015692.3	1	100		intronic	NM 01569	c.233+71T	p.?	0.16		[illegible]
chr19:17298679	C/C	G	C	SNV	MYO9B	MYO9B:intronic:NM 004145.3	1	100		intronic	NM 00414	c.2578-65(	p.?	-1.64		[illegible]
chr19:17298724	C/C	G	C	SNV	MYO9B	MYO9B:intronic:NM 004145.3	1	100		intronic	NM 00414	c.2578-20(	p.?	-4.65		[illegible]
chr19:17298893	G/T	G	T	SNV	MYO9B	MYO9B:intronic:NM 004145.3	1	96.67		intronic	NM 00414	c.2688+39	p.?	-2.09		[illegible]

chr19:17301863	A/A	G	A	SNV	MYO9B	MYO9B:intronic:NM_004145.3	1	100		intronic	NM_00414	c.2689-43(	p.?	-1.66					IQ calmoduli	rs962919	0.3971	YES	7.463E-1 (pos.17301863)		Myosin IXB	ADP bindir	Celiac Disease;Digestive System Diseases;Ga		G=0, A=25	G=0.0, A=1.0	0.00001	352.941	25	G=0/0, A=13/1
chr19:17311442	C/G	C	G	SNV	MYO9B	MYO9B:intronic:NM_004145.3	1	46.88		intronic	NM_00414	c.4373-6C	p.?	-0.02				0.27(COSM59	IQ calmoduli	rs2305766	0.359	YES	7.886E-1;1.371E-4 (pos.1	basal_cell_car	Myosin IXB	ADP bindir	Celiac Disease;Digestive System Diseases;Ga		C=16, G=15	C=0.5161, G=0.4839	0.00001	150.163	32	C=5/11, G=11/
chr19:54966579	G/G	C	G	SNV	LENG8	LENG8:exonic:NM_052925.3	1	100	8	synonymous	NM_05292	c.858C>G	p.(=)	-9.28				0.05(COSM37	SAC3/GANP fa	rs889147	0.3677	YES	3.833E-1;1.161E-4 (pos.5	acute_myeloic	Leukocyte r	protein binding			C=0, G=24	C=0.0, G=1.0	0.00001	333.432	24	C=0/0, G=14/1
chr19:17336962	T/T	C	T	SNV	OCEL1	OCEL1:upstream:NM_024578.2	1	100		intronic	NM_02457	c.-95C>T	p.?	1.36						rs7255142		YES				integral component of membrane			C=0, T=20	C=0.0, T=1.0	0.00001	263.426	20	C=0/0, T=16/4
chr19:17337223	A/A	G	A	SNV	OCEL1	OCEL1:intronic:NM_024578.2	1	100		intronic	NM_02457	c.69+98G>	p.?	-0.51					Occludin hom	rs2288542		YES				integral component of membrane			G=0, A=26	G=0.0, A=1.0	0.00001	352.545	26	G=0/0, A=5/21
chr19:17337733	T/T	C	T	SNV	OCEL1	OCEL1:intronic:NM_024578.2	1	100		intronic	NM_02457	c.246+55C	p.?	-2.59					Occludin hom	rs3745164		YES				integral component of membrane			C=0, T=22	C=0.0, T=1.0	0.00001	278.929	22	C=0/0, T=11/1
chr19:54973988	G/G	GCCT	G	INDEL	LENG8,LENG9	LENG9:exonic:NM_198988.1, LENG8:downstream:NM_0529	3	100	1	nonframeshiftDeletion	NM_05292	c.*1880GC	p.?, p.Glu262del	-3.19, -0.29, -0.36, -6.4					AKAP7 2'5' RN	rs115301361,rs1400	0.445	YES	0.0 (pos.54973990),0.0 (	acute_myeloic	Leukocyte r	metal ion binding,protein binding			GCCT=0, G=	GCCT=0.0, G=1.0	0.00001	419.449	29	GCCT=0/0, G=
chr19:54974318	C/C	T	C	SNV	LENG9	LENG9:exonic:NM_198988.1	1	100	1	missense	NM_19898	c.458A>G	p.His153Arg	3.18		29		0.01(COSM17	AKAP7 2'5' RN	rs10406453	0.3379	YES	4.29E-1 (pos.54974318)	adenocarcinoma,carcinom		metal ion binding			T=0, C=92	T=0.0, C=1.0	0.00001	1541.51	92	T=0/0, C=53/3
chr19:17367585	C/C	G	C	SNV	USHBP1	USHBP1:intronic:NM_031941.3	1	100		intronic	NM_03194	c.1221-56(	p.?	-0.24					PDZ domain c	rs4464206		YES			USH1C-bind	PDZ domain binding,protein binding			G=0, C=24	G=0.0, C=1.0	0.00001	334.792	24	G=0/0, C=13/1
chr19:17382505	A/T	A	T	SNV	BABAM1	BABAM1:intronic:NM_001033549.2	1	45.45		intronic	NM_00103	c.344+41A	p.?	-0.29						rs10420922	0.4337	YES	3.188E-1 (pos.17382505)			BRCA1-A c	Adnexal Diseases;Breast Diseases;Breast Ne		A=12, T=10	A=0.5455, T=0.4545	0.00001	82.1426	22	A=5/7, T=7/3
chr19:54974774	T/T	A	T	SNV	LENG9	LENG9:exonic:NM_198988.1	1	100	1	missense	NM_19898	c.2T>A	p.Met1Lys	0.31		95		0(COSM3749!	AKAP7 2'5' RN	rs1981829,rs774378227		YES	0.0;0.0;0.0 (pos.5497475	adenocarcinoma,neoplasn		metal ion binding			A=0, T=89	A=0.0, T=1.0	0.00001	1471.56	89	A=0/0, T=44/4
chr17:76970879	G/G	A	G	SNV	LGALS3BP	LGALS3BP:exonic:NM_005567.3	1	100	4	synonymous	NM_00556	c.267T>C	p.(=)	-2.75				0.02(COSM41	BTB And C-ter	rs3744166	0.0153	YES	8.247E-1 (pos.76970879)	neoplasm	Lectin, gala	blood microparticle,cell adhesion,cellular defense resp			A=0, G=39	A=0.0, G=1.0	0.00001	623.98	39	A=0/0, G=31/8
chr1:236702209	TG/TG	CA	TG	MNV	LGALS8	LGALS8:exonic:NM_006499.4	2	100	5	missense	NM_00649	c.165_166	p.Met56Val	-0.56, -0.0	1	21	0	0.29(COSM14	Galactoside-	rs1041936,rs104193	0.417	YES	4.81E-1 (pos.236702209)	acute_myeloic	Lectin, gala	carbohydrate binding,cellular response to virus,cytoplas			CA=0, TG=8	CA=0.0, TG=1.0	0.00001	1451.91	88	CA=0/0, TG=41
chr19:17450445	GG/CA	GG	CA	MNV	GTPBP3	GTPBP3:intronic:NM_001195422.1	2	52.38		intronic	NM_00119	c.1040+37	p.?	-0.2, -0.07					50S ribosome	rs11669718,rs38680	0.222	YES	1.482E-1 (pos.17450445),3.126E-1 (pos.1		GTP-binding	GTP binding,GTPase activity,mitochondrion,nucleus,pro			GG=10, CA	GG=0.4762, CA=0.52	0.00001	103.985	21	GG=3/7, CA=5/
chr19:17531450	A/A	G	A	SNV	MVB12A	MVB12A:intronic:NM_138401.3	1	100		intronic	NM_13840	c.286+7G>	p.?	-0.32				0.01(COSM37	Multivesicula	rs2303680	0.4201	YES	4.59E-1 (pos.17531450)	hepatocellular_carcinoma		ESCRT I complex,Golgi apparatus,SH3 domain binding,ce			G=0, A=30	G=0.0, A=1.0	0.00001	446.639	30	G=0/0, A=27/3
chr19:17533126	G/G	A	G	SNV	MVB12A	MVB12A:intronic:NM_138401.3	1	100		intronic	NM_13840	c.287-15A	p.?	-0.59					Multivesicula	rs2303678	0.1908	YES	3.516E-1;0.0 (pos.17533126)			ESCRT I complex,Golgi apparatus,SH3 domain binding,ce			A=0, G=40	A=0.0, G=1.0	0.00001	644.261	40	A=0/0, G=23/1
chr19:17534693	C/C	T	C	SNV	MVB12A	MVB12A:intronic:NM_138401.3	1	100		intronic	NM_13840	c.640+85T	p.?	-2.49					Multivesicula	rs2288408		YES				ESCRT I complex,Golgi apparatus,SH3 domain binding,ce			T=0, C=28	T=0.0, C=1.0	0.00001	408.436	28	T=0/0, C=13/1
chr10:95552653	C/C	T	C	SNV	LGI1	LGI1:exonic:NM_005097.3	1	100	6	synonymous	NM_00509	c.657T>C	p.(=)	2.21					EPTP domain	rs1111820	0.0017		1.0 (pos.95552653)		Leucine-ricl	axon guida	Brain Diseases;Central Nervous System Disea		T=0, C=21	T=0.0, C=1.0	0.00001	281.162	21	T=0/0, C=8/13
chr19:35616315	AT/AT	GC	AT	MNV	LGI4	LGI4:exonic:NM_139284.2	2	100	9	synonymous	NM_13928	c.1395_13	p.(=)	1.72, 1.75				0.37(COSM37	EPTP domain	rs12610146,rs12610	0.3547	YES	2.774E-1;1.44E-4 (pos.35	adenocarcino	Leucine-ricl	adult locomotory behavior,extracellular region,extracel			GC=0, AT=6	GC=0.0, AT=1.0	0.00001	1075.47	69	GC=0/0, AT=28
chr19:35617270	C/C	T	C	SNV	LGI4	LGI4:exonic:NM_139284.2	1	100	8	synonymous	NM_13928	c.1203A>G	p.(=)	-4.58				0.04(COSM43	EPTP domain	rs1319969	0.3565	YES	2.56E-1 (pos.35617270)	neoplasm	Leucine-ricl	adult locomotory behavior,extracellular region,extracel			T=0, C=21	T=0.0, C=1.0	0.00001	281.235	21	T=0/0, C=10/1
chr19:17737435	G/G	A	G	SNV	UNC13A	UNC13A:intronic:NM_001080421.2	1	100		intronic	NM_00108	c.4073+7T	p.?	-0.12				0.01(COSM36	C2 domain,D	rs12610816	0.1123	YES	1.526E-1 (pos.17737435)	acute_myeloic	UNC13, C. e	amyloid-b	Amyotrophic Lateral Sclerosis;Central Nervo		A=0, G=25	A=0.0, G=1.0	0.00001	353.019	25	A=0/0, G=19/6
chr19:35617513	G/C	G	C	SNV	LGI4	LGI4:exonic:NM_139284.2	1	50	8	synonymous	NM_13928	c.960C>G	p.(=)	-3.21					EPTP domain	rs77635806	0.0187	YES	0.0 (pos.35617513)		Leucine-ricl	adult locomotory behavior,extracellular region,extracel			G=11, C=11	G=0.5, C=0.5	0.00001	94.5774	22	G=8/3, C=7/4
chr19:1785772	T/C	T	C	SNV	ATP8B3	ATP8B3:intronic:NM_138813.3	1	57.89		intronic	NM_13881	c.3154-65	p.?	-0.61					Cation trans	rs8110462		YES			ATPase, cla	ATP binding,Golgi apparatus,Golgi organization,acrosom			T=16, C=22	T=0.4211, C=0.5789	0.00001	249.054	38	T=6/10, C=14/
chr19:17892478	G/G	A	G	SNV	FCHO1	FCHO1:intronic:NM_001161357.1	1	100		intronic	NM_00116	c.1829-43	p.?	-1.83					Fes/CIP4, anc	rs2042299	0.0555	YES	8.112E-1 (pos.17892478)		FCH domair	AP-2 adaptor complex binding,clathrin coat assembly,cl			A=0, G=68	A=0.0, G=1.0	0.00001	1053.56	68	A=0/0, G=59/9
chr14:93178191	G/G	A	G	SNV	LGMN	LGMN:exonic:NM_005606.6	1	100	9	synonymous	NM_00560	c.720T>C	p.(=)	1.24					Peptidase C1	rs1951708	0		1.0 (pos.93178191)		Legumain (	antigen processing and presentation of exogenous pepti			A=0, G=37	A=0.0, G=1.0	0.00001	583.704	37	A=0/0, G=16/2
chr19:49519230	G/G	T	G	SNV	LHB,RUVBL2	RUVBL2:utr_3:NM_006666.2, LHB:downstream:NM_000894	1	100	15	utr_3	NM_00666	c.1523T>G	p.?, p.?	-2.35						rs2013039		YES	2.604E-1;0.0 (pos.49519230)		Luteinizing	ADP bindir	Adnexal Diseases;Alcohol-Related Disorders		T=0, G=23	T=0.0, G=1.0	0.00001	316.747	23	T=0/0, G=15/8
chr5:77784738	G/G	A	G	SNV	LHFPL2	LHFPL2:exonic:NM_005779.2	1	100	5	synonymous	NM_00577	c.669T>C	p.(=)	1.51					Lipoma HMG	rs2241566	0.2335	YES	6.756E-1 (pos.77784738)	adenocarcino	LHFP-like pr	biological	Autoimmune Diseases;Female Urogenital Dis		A=0, G=24	A=0.0, G=1.0	0.00001	334.792	24	A=0/0, G=10/1
chr10:126172863	G/G	A	G	SNV	LHPP	LHPP:exonic:NM_022126.3	1	100	2	missense	NM_02212	c.281A>G	p.Gln94Arg	5.16	1	43	0		HAD-hydrolase	rs6597801	0.1142	YES	9.435E-1 (pos.126172863)		Phospholys	cytosol,dephosphorylation,inorganic diphosphatase act			A=0, G=31	A=0.0, G=1.0	0.00001	465.819	31	A=0/0, G=12/1
chr9:139094805	T/T	C	T	SNV	LHX3	LHX3:exonic:NM_014564.4	1	100	1	synonymous	NM_01456	c.81G>A	p.(=)	-0.83				0.09(COSM41	Homeobox do	rs2274116	0.2124	YES	1.471E-1 (pos.139094805	neoplasm	LIM/homeo	RNA polym	Bone Diseases;Bone Diseases,	Benign/Likely	C=0, T=36	C=0.0, T=1.0	0.00001	563.74	36	C=0/0, T=12/2
chr1:180243524	G/G	A	G	SNV	LHX4,LHX4-AS1	LHX4:exonic:NM_033343.3, LHX4-AS1:intronic_nc:NR_0376	1	100	6	missense	NM_03334	c.983A>G	p.Asn328Ser	2.53	1	46	0	0.92(COSM37	Homeobox do	rs7536561	0.484	YES	5.837E-1 (pos.18024352	adenocarcino	LIM homeo	animal or	Abnormalities, Multiple;Bone I	Benign	A=0, G=34	A=0.0, G=1.0	0.00001	524.185	34	A=0/0, G=23/1
chr19:18305578	C/C	G	C	SNV	MPV17L2	MPV17L2:intronic:NM_032683.2	1	100		intronic	NM_03268	c.359-9G>	p.?	0.16					Mpv17 / PMP	rs16982263	0.3103	YES	2.164E-1;0.0 (pos.18305578)		MPV17-like	integral cc	Autoimmune Diseases;Autoimmune Disease		G=0, C=23	G=0.0, C=1.0	0.00001	310.997	23	G=0/0, C=10/1
chr19:18308508	T/T	C	T	SNV	MPV17L2,RAB3A	RAB3A:intronic:NM_002866.4, MPV17L2:downstream:NM_(	1	100		intronic	NM_03268	c.*1643C>	p.?, p.?	-0.36					ADP-ribosyla	rs8109807	0.3419	YES	9.101E-2 (pos.18308508)		MPV17-like	ATPase ac	Autoimmune Diseases;Autoimmune Disease		C=0, T=43	C=0.0, T=1.0	0.00001	705.84	43	C=0/0, T=19/2
chr1:197889107	G/G	GC	G	INDEL	LHX9	LHX9:exonic:NM_020204.2	1	100	2	frameshiftDeletion	NM_02020	c.185delC	p.Pro62fs	9.16, 5.64					Homeobox do	rs752652822			0.0;0.0 (pos.197889108)		LIM homeo	DNA binding,cell proliferation,female gonad developmer			GC=0, G=2	GC=0.0, G=1.0	0.00001	238.75	23	GC=0/0, G=12/
chr19:54744387	C/T	C	T	SNV	LILRA6,LILRB3	LILRA6:exonic_nc:NR_104098.1, LILRB3:exonic:NM_00108	1	46.51	6	missense	NM_00108	c.1021G>A	p.Val341Met	0.79	0.03	21	1	0.04(COSM3757190)		rs71263238	0.3618	YES	4.057E-1 (pos.54744387)	adenocarcino	Leukocyte immunoglobulin-like receptor, subfamily B, member 3				C=23, T=20	C=0.5349, T=0.4651	0.00001	212.324	43	C=2/21, T=5/1
chr19:54783923	A/G	A	G	SNV	LILRB2	LILRB2:exonic:NM_005874.4	1	95.83	4	synonymous	NM_00587	c.78T>C	p.(=)	0.07					Immunoglobu	rs366337	0.0614	YES	9.998E-1 (pos.54783923)		Leukocyte immunoglot		Autoimmune Diseases;Female Urogenital Dis		A=1, G=23	A=0.0417, G=0.9583	0.00001	306.564	24	A=0/1, G=12/1
chr19:36243813	A/A	G	A	SNV	LIN37	LIN37:splicesite_3:NM_019104.2	1	100	5	splicesite	NM_01910	c.192-2G>	p.?	5.41					LIN37	rs231243		YES	1.0 (pos.36243813)			cell cycle,nucleoplasm,protein binding,regulation of cell			G=0, A=20	G=0.0, A=1.0	0.00001	268.111	20	G=0/0, A=8/12
chr4:83900043	G/G	A	G	SNV	LIN54	LIN54:exonic:NM_194282.3	1	100	3	synonymous	NM_19428	c.801T>C	p.(=)	2.7					Tesmin/TSO1	rs6535422	1.00E-04		1.0 (pos.83900043)		Lin54, C. ele	DNA binding,cell cycle,metal ion binding,nucleoplasm,p			A=0, G=181	A=0.0, G=1.0	0.00001	3004.11	181	A=0/0, G=80/1
chr18:6511473	C/T	C	T	SNV	LINC01387	LINC01387:exonic_nc:NR_120518.1	1	46.43	1	exonic_nc	NR_120518.1			0.03						rs4798453		YES	8.636E-1 (pos.6511473)						C=15, T=13	C=0.5357, T=0.4643	0.00001	117.973	28	C=6/9, T=8/5
chr15:22368862	A/A	G	A	SNV	LINC02203, LOC101927079,OR4M2	OR4M2:exonic:NM_001004719.2, LOC101927079:intronic_	1	100	1	missense	NR_11048(	c.287G>A	p.Gly96Glu	0.4		98		0.11(COSM44	7 transmemb	rs1835183	0.3648	YES	5.643E-1 (pos.22368862)	acute_myeloid_leukaemia		G-protein coupled receptor activity,G-protein coupled re			G=0, A=151	G=0.0, A=1.0	0.00001	2418.37	151	G=0/0, A=87/6
chr15:22368905	A/A	G	A	SNV	LINC02203, LOC101927079,OR4M2	OR4M2:exonic:NM_001004719.2, LOC101927079:intronic_	1	100	1	synonymous	NR_11048(	c.330G>A	p.(=)	-3.08				0.02(COSM54	7 transmemb	rs1835182	0.3721	YES	4.963E-1 (pos.22368905)	acute_myeloid_leukaemia		G-protein coupled receptor activity,G-protein coupled re			G=0, A=113	G=0.0, A=1.0	0.00001	1795.83	113	G=0/0, A=63/5
chr19:18650278	C/C	T	C	SNV	FKBP8	FKBP8:intronic:NM_012181.4	1	100		intronic	NM_01218	c.463-9A>(	p.?	-1.12					FKBP-type pe	rs7247733	0.0463	YES	9.999E-1 (pos.18650278)		FK506-bind	FK506 binding,apoptotic process,camera-type eye devel			T=0, C=28	T=0.0, C=1.0	0.00001	402.811	28	T=0/0, C=14/1
chr19:18778445	C/G	C	G	SNV	KLHL26	KLHL26:intronic:NM_018316.2	1	46.43		intronic	NM_01831	c.267-29C:	p.?	0.07					BTB And C-ter	rs10421503	0.3751	YES	6.754E-1 (pos.18778445)			Cul3-RING ubiquitin ligase complex,cytoplasm,fertilizati			C=15, G=13	C=0.5357, G=0.4643	0.00001	117.924	28	C=7/8, G=10/3
chr15:22369248	C/C	T	C	SNV	LINC02203, LOC101927079,OR4M2	OR4M2:exonic:NM_001004719.2, LOC101927079:intronic_	1	100	1	missense	NR_11048(	c.673T>C	p.Phe225Leu	-1.17		22		0.02(COSM45	7 transmemb	rs491208		YES	9.535E-1 (pos.22369248)	squamous_cell_carcinoma		G-protein coupled receptor activity,G-protein coupled re			T=0, C=21	T=0.0, C=1.0	0.00001	281.203	21	T=0/0, C=17/4
chr19:18971543	C/T	C	T	SNV	UPF1	UPF1:intronic:NM_002911.3	1	59.09		intronic	NM_00291	c.2301-92(	p.?	-0.15					AAA domain,I	rs529756587					Upf1, yeast	3'-UTR-mediated mRNA destabilization,ATP binding,ATP-(			C=9, T=13	C=0.4091, T=0.5909	0.00001	122.568	22	C=5/4, T=7/6
chr19:18980213	G/G	A	G	SNV	GDF1	GDF1:intronic:NM_001492.5	1	100		intronic	NM_00149	c.326-14T:	p.?	-0.06						rs4808864	2.00E-04		0.0 (pos.18980208),1.0 (pos.18980213)		Growth/diff	BMP signa	Abnormalities, Multiple;Cardi(	Benign	A=0, G=58	A=0.0, G=1.0	0.00001	867.078	58	A=0/0, G=25/3
chr15:22369290	G/G	A	G	SNV	LINC02203, LOC101927079,OR4M2	OR4M2:exonic:NM_001004719.2, LOC101927079:intronic_	1	100	1	missense	NR_11048(	c.715A>G	p.Met239Val	-1.35		21		0.08(COSM14	7 transmemb	rs12593418		YES	3.285E-1 (pos.22369290)	acute_myeloid_leukaemia		G-protein coupled receptor activity,G-protein coupled re			A=0, G=22	A=0.0, G=1.0	0.00001	298.89	22	A=0/0, G=16/6
chr15:22369426	A/A	G	A	SNV	LINC02203, LOC101927079,OR4M2	OR4M2:exonic:NM_001004719.2, LOC101927079:intronic_	1	100	1	missense	NR_11048(	c.851G>A	p.Arg284His	5.49		29		0.65(COSM13	7 transmemb	rs4087943	0.4416	YES	4.49E-1 (pos.22369426)	acute_myeloid_leukaemia		G-protein coupled receptor activity,G-protein coupled re			G=0, A=30	G=0.0, A=1.0	0.00001	446.639	30	G=0/0, A=16/1
chr3:191179193	G/G	A	G	SNV	LINCR-0002,PYDC2	LINCR-0002:intronic_nc:NR_120606.1, PYDC2:exonic:NM_0(	1	100	1	missense	NR_12060(	c.242A>G	p.Gln81Arg	0.93	1	43	0	0.01(COSM62	PAAD/DAPIN/	rs293833	0.256	YES	4.094E-1 (pos.19117919:	carcinoma,ne(	Pyrin doma	cytoplasm,inflammatory response,innate immune resp(			A=0, G=31	A=0.0, G=1.0	0.00001	465.796	31	A=0/0, G=19/1
chr15:101113862	T/T	C	T	SNV	LINS1	LINS1:exonic:NM_001040616.2	1	100	5	missense	NM_00104	c.1216G>A	p.Val406Met	2.09	0.65	21	0	0.02(COSM63	Lines C-termi	rs12719734	0.0066	YES	6.076E-1 (pos.10111386:	carcinoma	Lines, Dros(	cognition			C=0, T=26	C=0.0, T=1.0	0.00001	371.417	26	C=0/0, T=19/7
chr19:19646272	C/C	T	C	SNV	YJEFN3	YJEFN3:intronic:NM_198537.3	1	100		intronic	NM_19853	c.543+13T	p.?	-0.96					YjeF-related p	rs2304098	0.3473	YES	2.956E-1 (pos.19646272)			NADH met	Female Urogenital Diseases;Female Urogeni		T=0, C=34	T=0.0, C=1.0	0.00001	524.18	34	T=0/0, C=27/7
chr15:58834741	T/T	G	T	SNV	LIPC	LIPC:exonic:NM_000236.2	1	100	4	synonymous	NM_00023	c.465G>T	p.(=)	-0.02				0(COSM4419!	Lipase,PLAT/	rs690	0.412	YES	2.469E-1;0.0 (pos.58834)	acute_myeloic	Lipase C, he	apolipopro	Cardiovascular Diseases;Cong	Benign	G=0, T=75	G=0.0, T=1.0	0.00001	1187.61	75	G=0/0, T=62/1
chr15:58853079	A/A	C	A	SNV	LIPC	LIPC:exonic:NM_000236.2	1	100	7	missense	NM_00023	c.1068C>A	p.Phe356Leu	3.45	0.34	22	0.127		Lipase,PLAT/	rs3829462	0.0147	YES	9.375E-1 (pos.58853079)		Lipase C, he	apolipopro	Cardiovascular Diseases;Cong	Benign	C=0, A=52	C=0.0, A=1.0	0.00001	767.015	52	C=0/0, A=17/3
chr19:2042886	G/G	A	G	SNV	MKNK2	MKNK2:intronic:NM_199054.2	1	100		intronic	NM_19905	c.494-17T:	p.?	0.11					Lipopolysacc	rs7253052,rs770094!	0.4475	YES	7.069E-1;0.0 (pos.2042886)		Mitogen-ac	ATP binding,PML body,calcium-dependent protein serine			A=0, G=31	A=0.0, G=1.0	0.00001	457.138	31	A=0/0, G=25/6
chr15:58853109	G/G	A	G	SNV	LIPC	LIPC:exonic:NM_000236.2	1	100	7	synonymous	NM_00023	c.1098A>G	p.(=)	-0.15					Lipase,PLAT/	rs3829461	0.0147	YES	9.376E-1 (pos.58853109)		Lipase C, he	apolipopro	Cardiovascular Diseases;Cong	Benign	A=0, G=80	A=0.0, G=1.0	0.00001	1293.14	80	A=0/0, G=28/5
chr10:90356598	G/G	A	G	SNV	LIPJ	LIPJ:exonic:NM_001010939.2	1	100	8	missense	NM_00101	c.628A>G	p.Ile210Val	-6.31	0.26	29	0		Partial alpha	rs1409136	0.2807	YES	9.875E-1 (pos.90356598)		Lipase J	hydrolase activity, acting on ester bonds,lipid catabolic			A=0, G=22	A=0.0, G=1.0	0.00001	298.883	22	A=0/0, G=9/13
chr19:20808515	A/A	T	A	SNV	ZNF626	ZNF626:intronic:NM_001076675.2	1	100		intronic	NM_00107	c.227-59A:	p.?	-2.19					C2H2-type zin	rs1968385,rs782173227		YES				DNA binding,intracellular,metal ion binding,nucleic acid			T=0, A=45	T=0.0, A=1.0	0.00001	747.393	45	T=0/0, A=21/2
chr10:90350418	T/C	T	C	SNV	LIPJ	LIPJ:utr_5:NM_001010939.2	1	46.43	3	utr_5	NM_00101	c.-27T>C	p.?	-1.59					Partial alpha	rs17109533	0.0052	YES	1.987E-2 (pos.90350418)		Lipase J	hydrolase activity, acting on ester bonds,lipid catabolic			T=15, C=13	T=0.5357, C=0.4643	0.00001	117.926	28	T=6/9, C=9/4
chr2:99778985	T/C	T	C	SNV	LIPT1	LIPT1:exonic:NM_145199.2	1	53.49	2	synonymous	NM_14519	c.565T>C	p.(=)	0.72						rs2632277	0.364	YES	8.751E-1 (pos.99778985)		Lipoyltrans	cellular nitrogen compound m(	Lipoic Aci(	Benign	T=20, C=23	T=0.4651, C=0.5349	0.00001	260.981	43	T=9/11, C=9/1
chr11:74204727	G/G	A	G	SNV	LIPT2,LOC100287896	LIPT2:exonic:NM_001144869.2, LOC100287896:utr_5:NM_(	1	100	1	synonymous	NM_00114	c.22T>C, c.	p.(=), p.?	0.88				0(COSM4146:	Biotin/lipoat	rs4944895		YES	1.0 (pos.74204727)	neoplasm	Lipoyl(octa	cellular nitrogen compound metabolic process,cellular (			A=0, G=46	A=0.0, G=1.0	0.00001	768.313	46	A=0/0, G=32/1
chr19:2208859	C/C	T	C	SNV	DOT1L	DOT1L:intronic:NM_032482.2	1	100		intronic	NM_03248	c.964-75T:	p.?	0.17					Histone meth	rs2301799	0.4657	YES			Dot1, yeast	DNA bindi	Arthritis;Joint Diseases;Musculoskeletal Dis(		T=0, C=106	T=0.0, C=1.0	0.00001	1662.79	106	T=0/0, C=69/3
chr19:2214057	T/T	C	T	SNV	DOT1L	DOT1L:intronic:NM_032482.2	1	100		intronic	NM_03248	c.1797+72	p.?	-2.34					Histone meth	rs2074552		YES			Dot1, yeast	DNA bindi	Arthritis;Joint Diseases;Musculoskeletal Dis(		C=0, T=34	C=0.0, T=1.0	0.00001	524.184	34	C=0/0, T=14/2
chr16:11647492	C/C	T	C	SNV	LITAF	LITAF:exonic:NM_001136472.1	1	100	3	missense	NM_00113	c.274A>G	p.Ile92Val	0.24	1	29	0	0.01(COSM49	LITAF-like zinc	rs4280262	0.2129	YES	6.835E-2 (pos.11647492)	ductal_carcinc	LPS-induced	Golgi appa	Autoimmune Diseases;Charco	Benign	T=0, C=48	T=0.0, C=1.0	0.00001	810.434	48	T=0/0, C=15/3
chr19:22486535	G/G	A	G	SNV	ZNF729	ZNF729:intronic:NM_001242680.1	1	100		intronic	NM_00124	c.31-25A>(	p.?	-0.85					C2H2-type zin	rs9304996		YES	7.08E-1 (pos.22486535)			DNA binding,metal ion binding,nucleus,regulation of tra			A=0, G=62	A=0.0, G=1.0	0.00001	940.312	62	A=0/0, G=36/2
chr20:62714783	G/G	C	G	SNV	LKAAEAR1	LKAAEAR1:exonic:NM_001007125.1	1	100	2	synonymous	NM_00100	c.678G>C	p.(=)	0.67				0.08(COSM44	Family of unk	rs6010717	0.3752	YES	6.754E-1;1.207E-4 (pos.6	carcinoma,neoplasm					C=0, G=30	C=0.0, G=1.0	0.00001	446.585	30	C=0/0, G=15/1
chr7:142636666	C/T	C	T	SNV	LLCFC1	LLCFC1:exonic:NM_178829.4	1	57.58	1	missense	NM_17882	c.23C>T	p.Ser8Phe	0.71	0	155	0.681		Domain of un	rs200663678	0		0.0 (pos.142636666)			extracellular region			C=14, T=19	C=0.4242, T=0.5758	0.00001	204.363	33	C=8/6, T=15/4
chr19:2253590	T/T	C	T	SNV	JSRP1	JSRP1:intronic:NM_144616.3	1	100		intronic	NM_14461	c.436+29G	p.?	-1.76					Junctional sa	rs3746158	0.2988	YES	6.901E-1 (pos.2253590)		Junctional s	regulation	Adnexal Diseases;Cysts;Endocrine System Di		C=0, T=53	C=0.0, T=1.0	0.00001	782.03	53	C=0/0, T=15/3
chr17:73552185	A/A	G	A	SNV	LLGL2	LLGL2:exonic:NM_001031803.1	1	100	3	missense	NM_00103	c.134G>A	p.Arg45His	1.33	0.12	29	0.003	0.58(COSM37	LLGL2,WD do	rs1671036	0.4497	YES	4.534E-1 (pos.73552185)	acute_myeloid_leukaemia		GTPase activator activity,PDZ domain binding,Rab GTPas			G=0, A=50	G=0.0, A=1.0	0.00001	852.367	50	G=0/0, A=10/4
chr22:50944163	A/A	G	A	SNV	LMF2	LMF2:exonic:NM_033200.2	1	100	6	synonymous	NM_03320	c.856C>T	p.(=)	8.74					Lipase matur	rs128941	0.3984	YES	7.494E-1 (pos.50944163)			endoplasmic reticulum membrane,integral component (			G=0, A=25	G=0.0, A=1.0	0.00001	351.323	25	G=0/0, A=8/17
chr1:156107534	T/T	C	T	SNV	LMNA	LMNA:exonic:NM_170707.3	1	100	10	synonymous	NM_17070	c.1698C>T	p.(=)	-1.24				0.31(COSM37	Intermediate	rs4641	0.2531	YES	3.081E-1 (pos.15610753:	adenocarcino	Lamin A/C	IRE1-medi	ACMG Recommendations...;Ac	Benign/Likely	C=0, T=92	C=0.0, T=1.0	0.00001	1533.55	92	C=0/0, T=42/5
chr11:556857	C/C	G	C	SNV	LMNTD2	LMNTD2:exonic:NM_173573.2	1	100	8	synonymous	NM_17357	c.954C>G	p.(=)	0.01				0(COSM4145:	Lamin Tail Do	rs7938269	0.0603	YES	9.093E-1 (pos.556857)	neoplasm		positive regulation of mRNA splicing, via spliceosome			G=0, C=40	G=0.0, C=1.0	0.00001	644.285	40	G=0/0, C=16/2
chr11:554166	G/G	C	G	SNV	LMNTD2,LRRC56	LRRC56:exonic:NM_198075.3, LMNTD2:downstream:NM_17	1	100	14	missense	NM_19807	c.1519C>G	p.Arg507Gly, p.?	0.15	1	125	0	0(COSM4145815)		rs10902170	0.1769	YES	8.787E-1 (pos.554166)	acute_myeloid_leukaemia		positive regulation of mRNA splicing, via spliceosome			C=0, G=28	C=0.0, G=1.0	0.00001	412.747	28	C=0/0, G=3/25
chr13:76427403	G/A	G	A	SNV	LMO7	LMO7:exonic:NM_005358.5	1	52.38	27	missense	NM_00535	c.3694G>A	p.Val1232Met	-1.4	0.04	21	0.93		Calponin hon	rs71434812	0.0838	YES	4.66E-4;0.0 (pos.76427403)		LIM domain	actinin bir	Autoimmune Diseases;Diabetes Mellitus;Dia		G=50, A=55	G=0.4762, A=0.5238	0.00001	574.007	105	G=28/22, A=3(
chr7:123295964	C/T	C	T	SNV	LMOD2	LMOD2:utr_5:NM_207163.2	1	98.85	1	utr_5	NM_20716	c.-54C>T	p.?	-0.2					Tropomoduli	rs7779099		YES			Leiomodin	M band,actin binding,actin filament,actin filament polyr			C=1, T=86	C=0.0115, T=0.9885	0.00001	1394.96	87	C=0/1, T=54/3
chr19:23869823	G/G	T	G	SNV	ZNF675	ZNF675:intronic:NM_138330.2	1	100		intronic	NM_13833	c.3+10A>C	p.?	-0.58				0(COSM4000(	C2H2-type zin	rs2364624	0.1873	YES	4.74E-1 (pos.23869823)	acute_myeloid_leukaemia		DNA binding,I-kappaB kinase/NF-kappaB signaling,bone (			T=0, G=36	T=0.0, G=1.0	0.00001	563.57	36	T=0/0, G=28/8
chr1:165173216	G/G	A	G	SNV	LMX1A	LMX1A:exonic:NM_001174069.1	1	100	9	synonymous	NM_00117	c.1050T>C	p.(=)	3.46					Homeobox KI	rs1354509	0		1.0 (pos.165173216)		LIM homeo	RNA polymerase II regulatory region sequence-specific D			A=0, G=32	A=0.0, G=1.0	0.00001	485.114	32	A=0/0, G=18/1
chr3:100174722	A/A	G	A	SNV	LNP1	LNP1:exonic:NM_001085451.1	1	100	4	synonymous	NM_00108	c.489G>A	p.(=)	-1.06					Leukemia NU	rs1132022	0.405	YES	7.231E-1 (pos.100174722)						G=0, A=51	G=0.0, A=1.0	0.00001	754.584	51	G=0/0, A=21/3
chr20:44642833	C/C	A	C	SNV	LOC100128028,MMP9	MMP9:exonic:NM_004994.2, LOC100128028:exonic_nc:NR	1	100	11	synonymous	NM_00499	c.1821A>C	p.(=)	-2.13				0.01(COSM37	Fibronectin t	rs13969	0.3557	YES	3.086E-1 (pos.44642833)	adenocarcino	Matrix meta	cellular re	Abnormalities, Mul_(2R)-2-AMI	Benign	A=0, C=54	A=0.0, C=1.0	0.00001	803.433	54	A=0/0, C=41/1
chr20:44645010	T/T	C	T	SNV	LOC100128028,MMP9	MMP9:utr_3:NM_004994.2, LOC100128028:intronic_nc:NR	1	100	13	utr_3	NM_00499	c.2127C>T	p.?	1.8					Fibronectin t	rs20544	0.4186	YES	2.428E-3 (pos.44645010)		Matrix meta	cellular re	Abnormalities, Mul_(2R)-2-AMI	Benign	C=0, T=23	C=0.0, T=1.0	0.00001	323.476	23	C=0/0, T=8/15
chr19:23941457	CA/CA	C	CA	INDEL	ZNF681	ZNF681:intronic:NM_138286.2	1	100		intronic	NM_13828	c.3+92G>T	p.?	-0.4					C2H2-type zinc finger,KRAB box,Zinc finger, C2H2 type							DNA binding,DNA binding transcription factor activity,me			C=0, CA=52	C=0.0, CA=1.0	0.00001	514.379	52	C=0/0, CA=20/
chr19:51920613	T/T	C	T	SNV	LOC100129083,SIGLEC10	SIGLEC10:exonic:NM_033130.4, LOC100129083:utr_3:NM_(	1	100	2	synonymous	NM_03313	c.144G>A,	p.(=), p.?	-5.72					Immunoglobu	rs1010425	0.1209	YES	8.933E-1 (pos.51920613)		Sialic acid-b	adaptive immune response,carbohydrate binding,cell a(			C=0, T=50	C=0.0, T=1.0	0.00001	850.978	50	C=0/0, T=20/3
chr19:2477277	T/T	C	T	SNV	GADD45B	GADD45B:intronic:NM_015675.3	1	100		intronic	NM_01567	c.369+28C	p.?	3.12					Ribosomal pr	rs2024144	0.2345	YES	5.338E-1 (pos.2477277)		Growth arre	activation	Arsenic Poisoning;Heavy Metal Poisoning, Ne		C=0, T=21	C=0.0, T=1.0	0.00001	281.129	21	C=0/0, T=6/15
chr19:2717880	A/G	A	G	SNV	DIRAS1	DIRAS1:intronic:NM_145173.3	1	57.14		intronic	NM_14517	c.-68-7T>C	p.?	-3.35					ADP-ribosyla	rs7343093		YES	6.746E-1 (pos.2717880)		DIRAS famil	GTP binding,GTPase activity,membrane,mitogen-activat			A=18, G=24	A=0.4286, G=0.5714	0.00001	279.035	42	A=8/10, G=11/
chr8:146033347	C/C	T	C	SNV	LOC100130027,ZNF517	LOC100130027:downstream:NR_147501.1, ZNF517:exonic:	1	100	5	missense	NM_21360	c.1046T>C	p.Val349Ala	-0.4	0.26	64	0	0(COSM3750:	C2H2-type zin	rs2976653	0.0065		1.0 (pos.146033347)	adenocarcinoma,neoplasn		DNA binding,intracellular,metal ion binding,nucleic acid			T=0, C=34	T=0.0, C=1.0	0.00001	524.163	34	T=0/0, C=14/2
chr20:19261623	A/A	G	A	SNV	LOC100130264,SLC24A3	SLC24A3:exonic:NM_020689.3, LOC100130264:intronic_nc:	1	100	2	missense	NM_02068	c.163G>A	p.Val55Ile	-0.06	0.23	29	0	0(COSM5618:	Sodium/calci	rs1569767	0.4331	YES	4.996E-1 (pos.19261623)	ductal_carcinc	Solute carri	calcium ch	Adenocarcinoma;Carcinoma;Carcinoma, Ade		G=0, A=22	G=0.0, A=1.0	0.00001	298.89	22	G=0/0, A=7/15
chr19:2901114	C/C	CGCCGAA(	C	INDEL	ZNF57	ZNF57:intronic:NM_173480.2	10	100		intronic	NM_17348	c.3+68CGC	p.?	-1.53, -0.62, -1.56, -0.36, 0.31, 0.03, -0.38, 1.19, -0.12, -0.01, -0.					C2H2-type zir	rs1035342501,rs111:	0.1663	YES				DNA binding,intracellular,metal ion binding,nucleic acid			CGCCGAAG	CGCCGAAGTCT=0.0, (	0.00001	503.342	33	CGCCGAAGTC
chr6:13295515	C/C	A	C	SNV	LOC100130357,TBC1D7-LOC100130357	LOC100130357:exonic:NM_001242698.1, TBC1D7-LOC1001	1	100	2	missense	NM_00131	c.*921+97	p.?, p.Leu10Arg	0.36		102	0.974			rs1550526		YES	6.6E-1 (pos.13295515)			GTPase activator activity,Rab GTPase binding,activation			A=0, C=34	A=0.0, C=1.0	0.00001	524.107	34	A=0/0, C=22/1
chr6:13295536	T/T	C	T	SNV	LOC100130357,TBC1D7-LOC100130357	LOC100130357:exonic:NM_001242698.1, TBC1D7-LOC1001	1	100	2	missense	NM_00131	c.*921+97	p.?, p.Arg3Lys	-0.59		26	0			rs454563		YES	6.6E-1 (pos.13295536)			GTPase activator activity,Rab GTPase binding,activation			C=0, T=34	C=0.0, T=1.0	0.00001	524.048	34	C=0/0, T=22/1
chr17:73269530	G/G	A	G	SNV	LOC100287042,SLC25A19	SLC25A19:utr_3:NM_001126122.1, LOC100287042:exonic_	1	100	1	utr_3	NR_03652(	c.965T>C	p.?	-0.88					Mitochondria	rs1809352	0.0834	YES	9.996E-1 (pos.73269530)		Solute carri	deoxynucl	Congenital Abnormalities;Con	Benign/Likely	A=0, G=46	A=0.0, G=1.0	0.00001	768.302	46	A=0/0, G=20/2
chr19:30934385	C/C	G	C	SNV	ZNF536	ZNF536:intronic:NM_014717.2	1	100		intronic	NM_01471	c.-1-83G>C	p.?	0.78					C2H2-type zir	rs10411126		YES				RNA polym	Adenocarcinoma;Carcinoma;Carcinoma, Ren		G=0, C=25	G=0.0, C=1.0	0.00001	353.019	25	G=0/0, C=18/7
chr19:3113305	C/C	G	C	SNV	GNA11	GNA11:intronic:NM_002067.4	1	100		intronic	NM_00206	c.322-23G	p.?	-0.14					ADP-ribosyla	rs1682811,rs765768:	0.1388	YES	7.66E-1;0.0;2.325E-4 (pos.3113305)		Guanine nu	G-protein	Calcium Metabolism Disorders;Cardiovascul		G=0, C=111	G=0.0, C=1.0	0.00001	1755.98	111	G=0/0, C=41/7
chr19:3149990	C/C	G	C	SNV	GNA15,LOC100996351	GNA15:intronic:NM_002068.3, LOC100996351:intronic_nc:	1	100		intronic	NM_00206	c.331-139(	p.?	-2.2					ADP-ribosyla	rs170159,rs764262269		YES			Guanine nu	G-protein beta/gamma-subunit complex binding,G-prote			G=0, C=34	G=0.0, C=1.0	0.00001	524.119	34	G=0/0, C=30/4
chr17:73269676	T/T	C	T	SNV	LOC100287042,SLC25A19	SLC25A19:exonic:NM_001126122.1, LOC100287042:exonic	1	100	7	synonymous	NR_03652(	c.819G>A	p.(=)	0.26					Mitochondria	rs4789164	0.3222	YES	8.673E-1 (pos.73269676)		Solute carri	deoxynucl	Congenital Abnormalities;Con	Likely benign	C=0, T=59	C=0.0, T=1.0	0.00001	885.364	59	C=0/0, T=19/4
chr12:106903321	T/C	T	C	SNV	LOC100287944,POLR3B	POLR3B:exonic:NM_018082.5, LOC100287944:intronic_nc:N	1	46.43	28	synonymous	NM_01808	c.3396T>C	p.(=)	-0.06				0.58(COSM37	RNA polymera	rs13561	0.2741	YES	6.749E-1 (pos.10690332:	acute_myeloic	Polymerase	DNA bindi	Intellectual Disability;Mental I	Benign/Likely	T=15, C=13	T=0.5357, C=0.4643	0.00001	117.927	28	T=10/5, C=1/1
chr19:32954725	G/A	G	A	SNV	DPY19L3	DPY19L3:intronic:NM_001172774.1	1	45.45		intronic	NM_00117	c.1446-50(	p.?	-0.07					Q-cell neurob	rs1544511	0.3774	YES	6.312E-1 (pos.32954725)		DPY19-like	integral component of membrane,mannosyltransferase			G=12, A=10	G=0.5455, A=0.4545	0.00001	81.7599	22	G=8/4, A=6/4
chr8:143808951	T/T	C	T	SNV	LOC100288181,THEM6	THEM6:exonic:NM_016647.2, LOC100288181:upstream:NR	1	100	1	synonymous	NR_03892!	c.187C>T	p.(=)	-0.13				0.25(COSM37	Thioesterase	rs2585138	0.4737	YES	4.955E-1 (pos.14380895:	adenocarcinoma,neoplasn		extracellular region			C=0, T=38	C=0.0, T=1.0	0.00001	603.785	38	C=0/0, T=18/2

chr16:88808743	G/G	A	G	SNV	LOC100289580,LOC339059,PIEZO1	LOC339059:upstream:NR_120387.1, PIEZO1:exonic:NM_001	1	100	3	missense	NM_00114	c.248T>C	p.Ile83Thr	4.11	1	89	0		Piezo,Piezo n	rs6500495	0.1189	YES	9.568E-1 (pos.88808743)		PIEZO1 ion c	cation cha	Anemia:Anemia, Hemolytic:An		Benign	A=0, G=65	A=0.0, G=1.0	0.00001	996.401	65	A=0/0, G=26/3
chr19:33133936	C/C	CG	C	INDEL	ANKRD27	ANKRD27:intronic:NM_032139.2	1	100		intronic	NM_03213	c.782+93C	p.?	-3.1, -2.52					Ankyrin repea	rs3841621,rs758462615		YES				GTPase ac	Depressive Disorder;Depressive Disorder, M			CG=0, C=53	CG=0.0, C=1.0	0.00001	781.868	53	CG=0/0, C=32/
chr19:33149768	A/A	G	A	SNV	ANKRD27	ANKRD27:intronic:NM_032139.2	1	100		intronic	NM_03213	c.102+52C	p.?	1.24					Ankyrin repea	rs259251	0.3488	YES				GTPase ac	Depressive Disorder;Depressive Disorder, M			G=0, A=66	G=0.0, A=1.0	0.00001	1015.4	66	G=0/0, A=30/3
chr19:44610665	G/G	A	G	SNV	LOC100379224,ZNF224	ZNF224:exonic:NM_013398.3, LOC100379224:exonic_nc:NF	1	100	6	missense	NM_01339	c.352A>G	p.Met118Val	-1.46	0.58	21	0	0.03(COSM54	C2H2-type zir	rs2068061	0.1295	YES	5.816E-1 (pos.44610665)	acute_myeloic	Zinc finger p	DNA binding transcription factor activity,intracellular,m				A=0, G=85	A=0.0, G=1.0	0.00001	1390.52	85	A=0/0, G=37/4
chr19:44610798	T/T	A	T	SNV	LOC100379224,ZNF224	ZNF224:exonic:NM_013398.3, LOC100379224:exonic_nc:NF	1	100	6	missense	NM_01339	c.485A>T	p.His162Leu	-1.08	0.58	99	0	0.09(COSM13	C2H2-type zir	rs4239529	0.129	YES	6.051E-1 (pos.44610798)	acute_myeloic	Zinc finger p	DNA binding transcription factor activity,intracellular,m				A=0, T=53	A=0.0, T=1.0	0.00001	782.029	53	A=0/0, T=26/2
chr19:44611420	G/G	A	G	SNV	LOC100379224,ZNF224	ZNF224:exonic:NM_013398.3, LOC100379224:exonic_nc:NF	1	100	6	synonymous	NM_01339	c.1107A>G	p.(=)	-0.47				0.01(COSM56	C2H2-type zir	rs4508518	0.1799	YES	5.379E-1;0.0 (pos.446114	ductal_carcinc	Zinc finger p	DNA binding transcription factor activity,intracellular,m				A=0, G=61	A=0.0, G=1.0	0.00001	921.367	61	A=0/0, G=20/4
chr19:44612230	TG/TG	T	TG	INDEL	LOC100379224,ZNF224	ZNF224:exonic:NM_013398.3, LOC100379224:exonic_nc:NF	1	100	6	frameshiftInsertion	NM_01339	c.1917_19	p.Lys640fs	-0.03					C2H2-type zir	rs766242445			5.779E-4 (pos.44612230)		Zinc finger p	DNA binding transcription factor activity,intracellular,m				T=0, TG=45	T=0.0, TG=1.0	0.00001	657.069	45	T=0/0, TG=22/
chr6:75994151	G/G	A	G	SNV	LOC100506804,TMEM30A	TMEM30A:exonic:NM_018247.3, LOC100506804:upstream:	1	100	1	synonymous	NM_01824	c.204T>C	p.(=)	0.86				0.18(COSM41	LEM3 (ligand-	rs240374	0.0884	YES	1.106E-1 (pos.75994151)	ductal_carcinc	Transmemb	Golgi appa	Autoimmune Diseases:Female Urogenital Dis			A=0, G=55	A=0.0, G=1.0	0.00001	814.907	55	A=0/0, G=24/3
chr19:33490685	C/C	T	C	SNV	RHPN2	RHPN2:intronic:NM_033103.4	1	100		intronic	NM_03310	c.1106-74	p.?	-1.77					BRO1-like dor	rs8109247		YES				cytosol,pe	Colonic Diseases:Colorectal Neoplasms:Dige			T=0, C=39	T=0.0, C=1.0	0.00001	625.024	39	T=0/0, C=10/2
chr19:33620993	T/TCC	T	TCC	INDEL	GPATCH1	GPATCH1:intronic:NM_018025.2	2	47.62		intronic	NM_01802	c.2766-43	p.?	0.47					G-patch dom	rs35506888,rs77575	0.3647	YES	6.429E-1;0.0 (pos.33620993)			catalytic step 2 spliceosome,mRNA processing,mRNA sp				T=11, TCC=	T=0.5238, TCC=0.476	0.00001	81.9805	21	T=8/3, TCC=2/
chr1:144912153	G/G	A	G	SNV	LOC100996724,NBPF19,NBPF20,NBPF9,PDE4DIP	LOC100996724:exonic_nc:NR_144516.1, NBPF19:intronic:N	1	100	15	missense	NM_00127	c.5357+87	p.?, p.?, p.?, p.?, p.Cys708Ar	2.04		180	0		Centrosomin	rs1628172	0.2077	YES	9.281E-1 (pos.144912153)		Neuroblast	Golgi apparatus,cellular protein complex assembly,cent				A=0, G=87	A=0.0, G=1.0	0.00001	1431.39	87	A=0/0, G=42/4
chr22:38046695	A/A	C	A	SNV	LOC101927051,SH3BP1	SH3BP1:exonic:NM_018957.4, LOC101927051:exonic_nc:NI	1	100	16	missense	NM_01895	c.1561C>A	p.Pro521Thr	-0.29	0.01	38	0.022		BAR domain,I	rs12170939	0.1548	YES	5.353E-2 (pos.38046695)		SH3 domair	GTPase activator activity,SH3 domain binding,actin filan				C=0, A=55	C=0.0, A=1.0	0.00001	814.854	55	C=0/0, A=28/2
chr19:33700445	T/T	C	T	SNV	LRP3,SLC7A10	LRP3:downstream:NM_002333.3, SLC7A10:intronic:NM_01	1	100		intronic	NM_00233	c.*1964C>	p.?, p.?	-0.07					Amino acid p	rs2303093,rs766093223		YES	0.0 (pos.33700443)		Low density	D-alanine transport,D-serine transport,L-serine transme				C=0, T=21	C=0.0, T=1.0	0.00001	239.137	21	C=0/0, T=8/13
chr19:33702245	G/G	A	G	SNV	SLC7A10	SLC7A10:intronic:NM_019849.2	1	100		intronic	NM_01984	c.913-11T	p.?	3.25					Amino acid p	rs2241378	0.4648	YES	2.84E-1 (pos.33702245)		Solute carri	D-alanine transport,D-serine transport,L-serine transme				A=0, G=35	A=0.0, G=1.0	0.00001	543.874	35	A=0/0, G=22/1
chr19:33703103	T/T	C	T	SNV	SLC7A10	SLC7A10:intronic:NM_019849.2	1	100		intronic	NM_01984	c.788+95G	p.?	-2.98					Amino acid p	rs11669156		YES			Solute carri	D-alanine transport,D-serine transport,L-serine transme				C=0, T=27	C=0.0, T=1.0	0.00001	389.987	27	C=0/0, T=17/1
chr19:33703570	C/T	C	T	SNV	SLC7A10	SLC7A10:intronic:NM_019849.2	1	50		intronic	NM_01984	c.509-25G	p.?	-0.45					Amino acid p	rs56100350	0.024	YES	0.0 (pos.33703570)		Solute carri	D-alanine transport,D-serine transport,L-serine transme				C=35, T=46	C=0.4321, T=0.5679	0.00001	623.816	92	C=33/2, T=32/
chr15:22383482	G/G	GA	G	INDEL	LOC101927079,OR4N4	OR4N4:utr_3:NM_001005241.3, LOC101927079:exonic_nc:	1	100	9	utr_3	NR_11048	c.1011del	p.?	1.33, 0.41					7 transmemb	rs199664214,rs3841636,rs7544		YES				G-protein coupled receptor activity,G-protein coupled re				GA=0, G=48	GA=0.0, G=1.0	0.00001	810.435	48	GA=0/0, G=23/
chr2:101591304	G/G	A	G	SNV	LOC101927142,NPAS2	LOC101927142:intronic_nc:NR_110213.1, NPAS2:exonic:NM	1	100	13	missense	NM_00251	c.1180A>G	p.Thr394Ala	0.56	0.84	58	0	0.01(COSM41	Helix-loop-he	rs2305160	0.327	YES	8.45E-1 (pos.101591304)	neoplasm,oste	Neuronal P	DNA bindi	Autistic Disorder;Child Development Disorde			A=0, G=49	A=0.0, G=1.0	0.00001	831.579	49	A=0/0, G=32/1
chr6:57012930	C/T	C	T	SNV	LOC101927211,ZNF451	ZNF451:exonic:NM_001031623.2, LOC101927211:intronic	1	47.62	10	synonymous	NM_00103	c.2047C>T	p.(=)	0.05				0.45(COSM3762149),0.45(C		rs3734738	0.227	YES	1.285E-1 (pos.57012930)	adenocarcinoi	Zinc finger p	PML body,RNA polymerase II transcription corepressor a				C=33, T=30	C=0.5238, T=0.4762	0.00001	292.226	63	C=14/19, T=12
chr12:124069213	C/C	T	C	SNV	LOC101927415,TMED2	LOC101927415:upstream:NR_110049.2, TMED2:exonic:NM	1	100	1	synonymous	NR_11004	c.30T>C	p.(=)	-0.43					emp24/gp25	rs786446,rs8794125	1.00E-04		1.0 (pos.124069213)			COPI coati	Autoimmune Diseases;Cardiovascular Disea			T=0, C=70	T=0.0, C=1.0	0.00001	1092.06	70	T=0/0, C=28/4
chr19:35608148	T/T	C	T	SNV	FXYD3	FXYD3:intronic:NM_001136007.1	1	100		intronic	NM_00113	c.-48-13C>	p.?	-1.08					ATP1G1/PLM	rs1688021	0.4145	YES			FXYD domai	chloride cl	Digestive System Diseases:Digestive System			C=0, T=21	C=0.0, T=1.0	0.00001	281.233	21	C=0/0, T=13/8
chr19:35612168	A/A	T	A	SNV	FXYD3	FXYD3:intronic:NM_001136007.1	1	100		intronic	NM_00113	c.268+19T	p.?	-0.06				0.01(COSM47	ATP1G1/PLM	rs1688016	0.3565	YES	2.406E-1 (pos.35612168)	adenocarcinoi	FXYD domai	chloride cl	Digestive System Diseases:Digestive System			T=0, A=21	T=0.0, A=1.0	0.00001	281.231	21	T=0/0, A=5/16
chr19:35614336	C/C	T	C	SNV	FXYD3,MIR6887	FXYD3:intronic:NM_001136007.1, MIR6887:downstream:NI	1	100		intronic	NM_00113	c.419-9T>C	p.?	0.16					ATP1G1/PLM	rs1672974	0.3898	YES	5.521E-1 (pos.35614336)		FXYD domai	chloride cl	Digestive System Diseases;Digestive System			T=0, C=130	T=0.0, C=1.0	0.00001	2135.49	130	T=0/0, C=56/7
chr7:20180717	G/G	C	G	SNV	LOC101927668,MACC1,MACC1-AS1	MACC1-AS1:upstream:NR_046756.1, LOC101927668:upstre	1	100	7	missense	NR_11011	c.2411G>C	p.Arg804Thr	3.2	0	71	0.999	0.96(COSM11	ZU5 domain	rs3735615	0.4184	YES	8.501E-1 (pos.20180717)	squamous_cel	Metastasis	RNA polymerase II regulatory region sequence-specific D				C=0, G=25	C=0.0, G=1.0	0.00001	353.019	25	C=0/0, G=11/1
chr19:35617122	G/G	A	G	SNV	LGI4	LGI4:intronic:NM_139284.2	1	100		intronic	NM_13928	c.1299+52	p.?	-0.1					EPTP domain	rs937087	0.3398	YES	3.06E-1 (pos.35617122)		Leucine-ricl	adult locomotory behavior,extracellular region,extracel				A=0, G=23	A=0.0, G=1.0	0.00001	296.577	23	A=0/0, G=12/1
chr19:44500761	A/A	G	A	SNV	LOC101928063,ZNF155	ZNF155:exonic:NM_001260488.1, LOC101928063:downstre	1	100	6	missense	NM_00126	c.785G>A	p.Arg262His	-2.88	0.27	29	0.001		C2H2-type zir	rs386809623,rs4489	0.0081	YES	9.812E-1;1.156E-4 (pos.44500761)		Zinc finger p	DNA binding,DNA binding transcription factor activity,int				G=0, A=55	G=0.0, A=1.0	0.00001	814.907	55	G=0/0, A=31/2
chr19:44501116	G/G	A	G	SNV	LOC101928063,ZNF155	ZNF155:exonic:NM_001260488.1, LOC101928063:exonic_n	1	100	6	synonymous	NM_00126	c.1140A>G	p.(=)	-1.24					C2H2-type zir	rs388812	0.0066	YES	1.0 (pos.44501116)		Zinc finger p	DNA binding,DNA binding transcription factor activity,int				A=0, G=141	A=0.0, G=1.0	0.00001	2362.68	141	A=0/0, G=58/8
chr19:35622492	C/T	C	T	SNV	LGI4	LGI4:intronic:NM_139284.2	1	95.45		intronic	NM_13928	c.459-33G	p.?	-3.62					EPTP domain	rs1688001	0.3103	YES	4.099E-1 (pos.35622492)		Leucine-ricl	adult locomotory behavior,extracellular region,extracel				C=2, T=42	C=0.0455, T=0.9545	0.00001	663.774	44	C=0/2, T=15/2
chr19:35655151	C/C	T	C	SNV	FXYD5	FXYD5:intronic:NM_014164.5	1	100		intronic	NM_01416	c.382+3T>	p.?	-0.47					ATP1G1/PLM	rs1672994	0.2188	YES	7.91E-1;0.0 (pos.35655151)		FXYD domai	actin binding,cadherin binding,integral component of m				T=0, C=51	T=0.0, C=1.0	0.00001	754.545	51	T=0/0, C=24/2
chr19:44501227	G/G	A	G	SNV	LOC101928063,ZNF155	ZNF155:exonic:NM_001260488.1, LOC101928063:exonic_n	1	100	6	synonymous	NM_00126	c.1251A>G	p.(=)	-5.96					C2H2-type zir	rs388471	0.0067	YES	1.0 (pos.44501227)		Zinc finger p	DNA binding,DNA binding transcription factor activity,int				A=0, G=85	A=0.0, G=1.0	0.00001	1385.96	85	A=0/0, G=37/4
chr6:128403745	G/G	A	G	SNV	LOC101928140,PTPRK	LOC101928140:intronic_nc:NR_125849.1, PTPRK:exonic:NM	1	100	10	synonymous	NM_00113	c.1614T>C	p.(=)	-0.14					Fibronectin t	rs17828130	0.3621	YES	5.704E-1 (pos.128403745)		Protein-tyro	adherens	Genital Diseases, Male;Genital Neoplasms, I			A=0, G=53	A=0.0, G=1.0	0.00001	782.018	53	A=0/0, G=26/2
chr6:169637763	G/C	G	C	SNV	LOC101929523,THBS2	LOC101929523:intronic_nc:NR_134621.1, THBS2:exonic:NM	1	50	9	synonymous	NR_13462	c.1257C>G	p.(=)	1.07				0.71(COSM45	Calcium-bind	rs9766671	0.1862	YES	2.517E-1;0.0 (pos.16963	adenocarcinoi	Thrombosp	basement	Bone Diseases;Diabetes Mellitus;Endocrine S			G=18, C=18	G=0.5, C=0.5	0.00001	185.875	36	G=10/8, C=12/
chr2:167099158	G/G	A	G	SNV	LOC101929680,SCN9A	SCN9A:exonic:NM_002977.3, LOC101929680:intronic_nc:NI	1	100	19	missense	NR_11026	c.3448T>C	p.Trp1150Arg	0.95	1	101	0	0.01(COSM50	Cytoplasmic	rs6746030	0.1286	YES	9.543E-1 (pos.16709915	ductal_carcinc	Sodium cha	behaviora	Brain Diseases;Car	Brivaracet	Benign	A=0, G=56	A=0.0, G=1.0	0.00001	832.1	56	A=0/0, G=17/3
chr19:35835582	A/A	G	A	SNV	CD22,MIR5196	MIR5196:upstream:NR_049828.1, CD22:intronic:NM_0017	1	100		intronic	NM_00177	c.2036-15	p.?	-0.9					CD80-like C2-	rs3746250		YES			CD22 antige	carbohydrate binding,cell adhe		Epratuzumab		G=0, A=65	G=0.0, A=1.0	0.00001	996.461	65	G=0/0, A=16/4
chr19:35835940	C/T	C	T	SNV	CD22,MIR5196	MIR5196:upstream:NR_049828.1, CD22:intronic:NM_0017	1	57.14		intronic	NM_00177	c.2133-14	p.?	-1.75					CD80-like C2-	rs45453699	0.2683	YES	2.665E-1 (pos.35835940)		CD22 antige	carbohydrate binding,cell adhe		Epratuzumab		C=9, T=12	C=0.4286, T=0.5714	0.00001	108.879	21	C=7/2, T=11/1
chr2:167145142	G/G	A	G	SNV	LOC101929680,SCN9A	SCN9A:exonic:NM_002977.3, LOC101929680:intronic_nc:NI	1	100	10	synonymous	NR_11026	c.1119T>C	p.(=)	1.61				0.9(COSM400	Cytoplasmic	rs13414203	0.3879	YES	3.228E-1 (pos.16714514	acute_myeloic	Sodium cha	behaviora	Brain Diseases;Car	Brivaracet	Benign/Likely	A=0, G=40	A=0.0, G=1.0	0.00001	644.261	40	A=0/0, G=26/1
chr14:21467913	G/G	T	G	SNV	LOC101929718,SLC39A2	LOC101929718:upstream:NR_110139.1, SLC39A2:exonic:NI	1	100	2	missense	NR_11013	c.128T>G	p.Leu43Arg	0.39	1	102	0	0(COSM5425	ZIP Zinc trans	rs2234632,rs766129	0.3248	YES	0.0;0.0 (pos.21467911),7	acute_myeloic	Solute carri	cytoplasmic vesicle,integral component of plasma mem				T=0, G=74	T=0.0, G=1.0	0.00001	1170.09	74	T=0/0, G=36/3
chr19:35978458	A/A	G	A	SNV	KRTDAP	KRTDAP:intronic:NM_207392.2	1	100		intronic	NM_20739	c.262-90C	p.?	-0.37					Keratinocyte	rs17638216		YES				biological_process,cell differentiation,extracellular spa				G=0, A=49	G=0.0, A=1.0	0.00001	831.625	49	G=0/0, A=18/3
chr19:35979862	G/G	A	G	SNV	KRTDAP	KRTDAP:intronic:NM_207392.2	1	100		intronic	NM_20739	c.88-120T	p.?	-3.88					Keratinocyte	rs1006767		YES				biological_process,cell differentiation,extracellular spa				A=0, G=100	A=0.0, G=1.0	0.00001	1565.98	100	A=0/0, G=51/4
chr19:35991373	T/T	C	T	SNV	DMKN	DMKN:intronic:NM_033317.4	1	100		intronic	NM_03331	c.1287+62	p.?	0.29						rs2293695	0.4956	YES	6.837E-1 (pos.35991373)		Dermokine	cornified envelope assembly,extracellular exosome,ext				C=0, T=24	C=0.0, T=1.0	0.00001	340.084	24	C=0/0, T=14/1
chr14:64988924	C/C	T	C	SNV	LOC102723809,ZBTB1	ZBTB1:exonic:NM_001123329.1, LOC102723809:intronic_n	1	100	2	synonymous	NM_00112	c.702T>C	p.(=)	-0.07				0.61(COSM17	BTB/POZ dom	rs10133411	0.219	YES	1.972E-1 (pos.64988924)	adrenal_cortic	Zinc finger-	B cell differentiation,DNA binding,DNA repair,K63-linked				T=0, C=30	T=0.0, C=1.0	0.00001	446.629	30	T=0/0, C=8/22
chr2:166892788	C/T	C	T	SNV	LOC102724058,SCN1A	LOC102724058:exonic_nc:NR_110598.1, SCN1A:exonic:NM	1	50	19	missense	NR_11059	c.3166G>A	p.Ala1056Thr	-0.56	0.83	58	0	0.01(COSM37	Cytoplasmic	rs2298771	0.3245	YES	8.979E-1 (pos.16689278	adenocarcinoi	Sodium cha	T-tubule,Z	Brain Diseases;Cen	Brivaracet	Benign/Likely	C=53, T=53	C=0.5, T=0.5	0.00001	540.551	106	C=29/24, T=31
chr21:44837551	AGGGA/AGGG	GGGGG	AGGGA	MNV	LOC102724428,SIK1	LOC102724428:exonic:NM_001320643.2, SIK1:exonic:NM_	5	100	13	missense	NM_00132	c.1844_18	p.Ala615Val, p.Ala615V	-0.2, 5.29,	0.05, 0.05	64.0, 64.0	0.281, 0.281	0.4(COSM375	Kinase-like,P	rs430552,rs430554,r	0.1946	YES	0.0;0.0 (pos.44837554),8	adenocarcinoi	Salt-inducit	14-3-3 pro	Arthritis:Arthritis, J	Dabrafenib		GGGGG=0,	GGGGG=0.0, AGGGA	0.00001	1009.45	66	GGGGG=0/0, A
chr20:1099523	G/G	T	G	SNV	LOC105372493,PSMF1	PSMF1:exonic:NM_006814.4, LOC105372493:upstream:NR	1	100	1	missense	NR_13653	c.107T>G	p.Phe36Cys	-0.12	0.03	205	0	0.03(COSM37	PI31 proteas	rs1803415	0.1708	YES	4.701E-1 (pos.1099523)	acute_myeloid_leukaemia		Fc-epsilon receptor signaling pathway,MAPK cascade,NI				T=0, G=20	T=0.0, G=1.0	0.00001	263.786	20	T=0/0, G=9/11
chr19:36642310	C/C	T	C	SNV	COX7A1	COX7A1:intronic:NM_001864.3	1	100		intronic	NM_00186	c.187+54A	p.?	-0.23					Cytochrome	rs2008683	0.0179				Cytochrome	cytochrome-c oxidase activity,		Cholic Acid,N-Formylmet		T=0, C=27	T=0.0, C=1.0	0.00001	389.988	27	T=0/0, C=9/18
chr2:128321770	G/A	G	A	SNV	LOC105373609,MYO7B	MYO7B:exonic:NM_001080527.1, LOC105373609:intronic_	1	58.33	3	missense	NM_00108	c.61G>A	p.Gly21Ser	0.45		56	0.027	0.09(COSM49	FERM central	rs2404991	0.4992	YES	6.185E-1 (pos.12832177	ductal_carcinc	Myosin VIIb	ATP binding,actin binding,apical cytoplasm,brush borde				G=25, A=35	G=0.4167, A=0.5833	0.00001	370.434	60	G=13/12, A=1
chr19:36940965	A/G	A	G	SNV	ZNF566	ZNF566:intronic:NM_001145343.1	1	46.43		intronic	NM_00114	c.236-62T	p.?	0.01					C2H2-type zir	rs2912441		YES				DNA binding,integral component of membrane,intracell				A=15, G=13	A=0.5357, G=0.4643	0.00001	117.924	28	A=10/5, G=6/7
chr1:46521517	A/A	G	A	SNV	LOC110117498-PIK3R3,PIK3R3	LOC110117498-PIK3R3:exonic:NM_001303427.1, PIK3R3:e	1	100	7	synonymous	NM_00362	c.891C>T,	p.(=), p.(=)	1.08				0.53(COSM37	Phosphatidyl	rs785468	0.2923	YES	6.729E-1 (pos.46521517)	acute_myeloic	Phosphatid	1-phosphatidylinositol-3-kinas		Isoprenaline,SF1126		G=0, A=77	G=0.0, A=1.0	0.00001	1229.44	77	G=0/0, A=50/2
chr1:46521559	T/T	A	T	SNV	LOC110117498-PIK3R3,PIK3R3	LOC110117498-PIK3R3:exonic:NM_001303427.1, PIK3R3:e	1	100	7	missense	NM_00362	c.849T>A,	p.Asn283Lys, p.Asn329L	1.16	1.0, 1.0	94.0, 94.0	0.0, 0.0	0.38(COSM37	Phosphatidyl	rs785467	0.2919	YES	6.723E-1 (pos.46521559)	adenocarcinoi	Phosphatid	1-phosphatidylinositol-3-kinas		Isoprenaline,SF1126		A=0, T=78	A=0.0, T=1.0	0.00001	1249.34	78	A=0/0, T=50/2
chr8:22960704	C/C	G	C	SNV	LOC254896,TNFRSF10C	LOC254896:exonic_nc:NR_046173.1, TNFRSF10C:intronic:NI	1	100	4	exonic_nc	NR_04617	c.60+10G>	p.?	-1.51					TNFR/NGFR c	rs6557616	0.2245	YES	8.005E-1;0.0 (pos.22960704)		Tumor necr	TRAIL bind	Arthritis;Arthritis, Juvenile;Autoimmune Dise			G=0, C=58	G=0.0, C=1.0	0.00001	841.103	58	G=0/0, C=39/1
chr2:95537572	T/T	C	T	SNV	LOC442028,TEKT4	TEKT4:exonic:NM_144705.3, LOC442028:intronic_nc:NR_03	1	100	1	missense	NR_03759	c.248C>T	p.Thr83Met	3.63	0	81	0.973	0.74(COSM37	Tektin family	rs4854235	0.2952	YES	5.586E-1 (pos.95537572)	adenocarcinoma,neoplasn		cilium assembly,cilium movement involved in cell motili				C=0, T=51	C=0.0, T=1.0	0.00001	754.584	51	C=0/0, T=37/1
chr19:37399412	T/T	C	T	SNV	ZNF829	ZNF829:intronic:NM_001171979.1	1	100		intronic	NM_00117	c.340-51G	p.?	-0.18					C2H2-type zir	rs10418144	0.0987	YES	1.71E-1 (pos.37399412)			DNA binding,DNA binding transcription factor activity,me				C=0, T=35	C=0.0, T=1.0	0.00001	543.902	35	C=0/0, T=11/2
chr19:37441111	T/T	C	T	SNV	ZNF568	ZNF568:intronic:NM_001204838.1	1	100		intronic	NM_00120	c.358+129	p.?	0.2				0.01(COSM40	C2H2-type zir	rs571294	0.4262	YES	3.732E-1 (pos.37441111)	acute_myeloic	Zinc finger p	DNA binding transcription factor activity,embryonic plac				C=0, T=34	C=0.0, T=1.0	0.00001	524.186	34	C=0/0, T=9/25
chr19:37441462	C/C	T	C	SNV	ZNF568	ZNF568:intronic:NM_001204838.1	1	100		intronic	NM_00120	c.358+133	p.?	-4.72				0.04(COSM40	C2H2-type zir	rs546589	0.428	YES	3.177E-1 (pos.37441462)	acute_myeloic	Zinc finger p	DNA binding transcription factor activity,embryonic plac				T=0, C=119	T=0.0, C=1.0	0.00001	1899.78	119	T=0/0, C=71/4
chr19:374453	T/C	T	C	SNV	THEG	THEG:intronic:NM_016585.4	1	50		intronic	NM_01658	c.309-32A	p.?	-2.43					Testicular ha	rs3815960	0.2175	YES	5.331E-1 (pos.374453)		Testicular h	cell differentiation,chaperone-mediated protein comple				T=20, C=20	T=0.5, C=0.5	0.00001	214.33	40	T=10/10, C=10
chr19:374492	G/C	G	C	SNV	THEG	THEG:intronic:NM_016585.4	1	47.37		intronic	NM_01658	c.309-71C	p.?	-0.73					Testicular ha	rs10410345		YES	7.677E-1;0.0 (pos.374492)		Testicular h	cell differentiation,chaperone-mediated protein comple				G=20, C=18	G=0.5263, C=0.4737	0.00001	184.534	38	G=10/10, C=8/
chr2:95537622	G/G	A	G	SNV	LOC442028,TEKT4	TEKT4:exonic:NM_144705.3, LOC442028:intronic_nc:NR_03	1	100	1	missense	NR_03759	c.298A>G	p.Ser100Gly	0.39	0.15	56	0	0.04(COSM37	Tektin family	rs11164112	0.3656	YES	5.668E-1 (pos.95537622)	adenocarcinoma,ductal_ca		cilium assembly,cilium movement involved in cell motili				A=0, G=55	A=0.0, G=1.0	0.00001	814.772	55	A=0/0, G=39/1
chr2:100916306	A/G	A	G	SNV	LONRF2	LONRF2:exonic:NM_198461.3	1	97.3	5	synonymous	NM_19846	c.1140T>C	p.(=)	0.9					ATP-depende	rs2033748	0.3555	YES	9.571E-1 (pos.100916306)			intracellular,metal ion binding,positive regulation of prc				A=1, G=36	A=0.027, G=0.973	0.00001	551.778	37	A=1/0, G=18/1
chr8:23167353	G/G	T	G	SNV	LOXL2	LOXL2:exonic:NM_002318.2	1	100	10	missense	NM_00231	c.1708A>C	p.Met570Leu	0.01	0.49	15	0		Lysyl oxidase	rs1063582	0.2283	YES	7.429E-1 (pos.23167353)		Lysyl oxidas	aging,base	Basal Ganglia Diseases:Bile Duct Diseases:Bi			T=0, G=30	T=0.0, G=1.0	0.00001	446.639	30	T=0/0, G=10/2
chr3:188590446	G/G	A	G	SNV	LPP	LPP:exonic:NM_005578.4	1	100	10	synonymous	NM_00557	c.1605A>G	p.(=)	-0.11				0.57(COSM41	LIM domain	rs1136644	0.493	YES	4.56E-1 (pos.188590446)	neoplasm	Lipoma-pre	biological	Arthritis:Arthritis, Juvenile:Autoimmune Dise			A=0, G=47	A=0.0, G=1.0	0.00001	789.328	47	A=0/0, G=24/2
chr14:42360633	C/C	T	C	SNV	LRFN5	LRFN5:exonic:NM_152447.4	1	100	4	synonymous	NM_15244	c.1566T>C	p.(=)	0.55					Immunoglob	rs6572117	0.292	YES	7.476E-1 (pos.42360633)		Leucine-ricl	axonogenesis,cell surface,extracellular space,integral c				T=0, C=36	T=0.0, C=1.0	0.00001	563.745	36	T=0/0, C=16/2
chr7:133848257	G/T	G	T	SNV	LRGUK	LRGUK:exonic:NM_144648.2	1	50	7	missense	NM_14464	c.904G>T	p.Asp302Tyr	1.52	0.5	160	0.902	0.16(COSM37	Guanylate kir	rs17167553	0.1014	YES	3.168E-1 (pos.13384825	acute_myeloic	Leucine-ricl	ATP binding,acrosomal vesicle,axoneme assembly,cell d				G=15, T=15	G=0.5, T=0.5	0.00001	144.994	30	G=7/8, T=8/7
chr3:66433676	A/G	A	G	SNV	LRIG1	LRIG1:exonic:NM_015541.2	1	59.09	15	synonymous	NM_01554	c.2221T>C	p.(=)	7.53				0.97(COSM41	Immunoglob	rs900171	0.4386	YES	8.243E-1 (pos.66433676)	ductal_carcinc	Leucine-ricl	hair cycle process,innervation,integral co			Benign	A=9, G=13	A=0.4091, G=0.5909	0.00001	122.568	22	A=6/3, G=7/6
chr1:113657092	C/C	A	C	SNV	LRIG2	LRIG2:exonic:NM_014813.2	1	100	15	synonymous	NM_01481	c.2124A>C	p.(=)	-0.48					Immunoglob	rs1216793	0.0133	YES	9.677E-1 (pos.113657092)		Leucine-ricl	cytoplasm	Female Urogenital Diseases:Female Urogeni			A=0, C=40	A=0.0, C=1.0	0.00001	644.286	40	A=0/0, C=16/2
chr4:110791413	C/T	C	T	SNV	LRIT3	LRIT3:exonic:NM_198506.4	1	50	4	missense	NM_19850	c.1508C>T	p.Thr503Met	-0.36	0.09	81	0.004	0.03(COSM37	Immunoglob	rs2347132	0.1378	YES	2.821E-1 (pos.11079141	acute_myeloic	Leucine-ricl	dendrite,e	Congenital, Hereditary, and Ne		Benign	C=18, T=18	C=0.5, T=0.5	0.00001	185.884	36	C=12/6, T=11/
chr19:38027715	GT/GT	G	GT	INDEL	ZNF793	ZNF793:intronic:NM_001013659.2	1	100		intronic	NM_00101	c.239-84G	p.?	-0.23					C2H2-type zir	rs34919776,rs398034519		YES				DNA binding,intracellular,metal ion binding,nucleic acid				G=0, GT=35	G=0.0, GT=1.0	0.00001	516.204	35	G=0/0, GT=22/
chr12:25242595	G/G	C	G	SNV	LRMP	LRMP:exonic:NM_001204126.1	1	100	10	missense	NM_00120	c.421C>G	p.Leu141Val	0.93	0.21	32	0.009	0.77(COSM41	MRVI1 protei	rs7969931	0.2365	YES	8.797E-1;0.0 (pos.25242	neoplasm	Lymphoid-r	azurophil granule membrane,chromosome,endoplasmic				C=0, G=45	C=0.0, G=1.0	0.00001	747.375	45	C=0/0, G=16/2
chr12:57593100	CA/CA	C	CA	INDEL	LRP1	LRP1:exonic:NM_002332.2	1	100	61	frameshiftInsertion	NM_00233	c.9782_97	p.Thr3263fs	2.04					Calcium-bind	rs753856435			0.0 (pos.57593100)		Low density	RNA bindir	Alzheimer Disease;	Antihemophilic factor, hu		C=0, CA=51	C=0.0, CA=1.0	0.00001	635.587	51	C=0/0, CA=30/
chr2:141130695	T/T	C	T	SNV	LRP1B	LRP1B:exonic:NM_018557.2	1	100	69	synonymous	NM_01855	c.10650G>	p.(=)	0.49				0.09(COSM37	Calcium-bind	rs16843864	0.1293	YES	1.414E-2 (pos.14113069	adenocarcinoma,haemang		calcium io	Adenocarcinoma:Carcinoma:Carcinoma, Ren			C=0, T=58	C=0.0, T=1.0	0.00001	871.934	58	C=0/0, T=36/2
chr19:3819275	T/T	A	T	SNV	MIR1268A,ZFR2	MIR1268A:intronic_nc:NR_031672.1, ZFR2:intronic:NM_015	1	100		intronic	NR_03167	c.1741-42	p.?	-2.89					DZF domain,2	rs2301840	0.4326	YES	8.066E-1 (pos.3819275)			nucleic acid binding,zinc ion binding				A=0, T=36	A=0.0, T=1.0	0.00001	563.54	36	A=0/0, T=33/3
chr19:3819403	G/G	A	G	SNV	MIR1268A,ZFR2	MIR1268A:intronic_nc:NR_031672.1, ZFR2:intronic:NM_015	1	100		intronic	NR_03167	c.1741-17	p.?	-0.2					DZF domain,2	rs2301841		YES				nucleic acid binding,zinc ion binding				A=0, G=25	A=0.0, G=1.0	0.00001	352.993	25	A=0/0, G=9/16
chr2:141274576	C/C	T	C	SNV	LRP1B	LRP1B:exonic:NM_018557.2	1	100	50	synonymous	NM_01855	c.8031A>G	p.(=)	2.95				0.94(COSM40	Calcium-bind	rs4954672	0.2271	YES	4.041E-1 (pos.14127457	acute_myeloid_leukaemia		calcium io	Adenocarcinoma:Carcinoma:Carcinoma, Ren			T=0, C=52	T=0.0, C=1.0	0.00001	767.015	52	T=0/0, C=32/2
chr19:3820293	G/G	A	G	SNV	MIR1268A,ZFR2	MIR1268A:intronic_nc:NR_031672.1, ZFR2:intronic:NM_015	1	100		intronic	NR_03167	c.1632-5T	p.?	-0.99					DZF domain,2	rs2301844	0.4268	YES	7.287E-1 (pos.3820293)			nucleic acid binding,zinc ion binding				A=0, G=32	A=0.0, G=1.0	0.00001	477.601	32	A=0/0, G=7/25
chr19:3820305	G/G	T	G	SNV	MIR1268A,ZFR2	MIR1268A:intronic_nc:NR_031672.1, ZFR2:intronic:NM_015	1	100		intronic	NR_03167	c.1632-17	p.?	-0.01					DZF domain,2	rs2301845	0.4207	YES	7.179E-1 (pos.3820305)			nucleic acid binding,zinc ion binding				T=0, G=32	T=0.0, G=1.0	0.00001	485.141	32	T=0/0, G=7/25
chr19:3822008	G/G	C	G	SNV	MIR1268A,ZFR2	MIR1268A:intronic_nc:NR_031672.1, ZFR2:intronic:NM_015	1	100		intronic	NR_03167	c.1491+71	p.?	-0.74					DZF domain,2	rs28510805		YES				nucleic acid binding,zinc ion binding				C=0, G=57	C=0.0, G=1.0	0.00001	849.593	57	C=0/0, G=53/4
chr2:141571329	C/C	T	C	SNV	LRP1B	LRP1B:exonic:NM_018557.2	1	100	32	synonymous	NM_01855	c.5256A>G	p.(=)	-1.43				0.04(COSM41	Calcium-bind	rs3749010	0.1122	YES	9.888E-1;0.0 (pos.14157	neoplasm		calcium io	Adenocarcinoma;Carcinoma;Carcinoma, Ren			T=0, C=42	T=0.0, C=1.0	0.00001	681.63	42	T=0/0, C=14/2
chr2:141816621	G/G	A	G	SNV	LRP1B	LRP1B:exonic:NM_018557.2	1	100	9	synonymous	NM_01855	c.1239T>C	p.(=)	-0.2					Calcium-bind	rs1525579	2.00E-04	YES	1.0 (pos.141816621)			calcium io	Adenocarcinoma;Carcinoma;Carcinoma, Ren			A=0, G=20	A=0.0, G=1.0	0.00001	263.786	20	A=0/0, G=10/1
chr2:142567910	C/C	T	C	SNV	LRP1B	LRP1B:exonic:NM_018557.2	1	100	2	missense	NM_01855	c.143A>G	p.Gln48Arg	3.52	0.33	43	0.001	0.82(COSM50	Calcium-bind	rs12990449	0.2153	YES	4.024E-1 (pos.14256791	acute_myeloid_leukaemia		calcium io	Adenocarcinoma:Carcinoma:Carcinoma, Ren			T=0, C=30	T=0.0, C=1.0	0.00001	446.624	30	T=0/0, C=22/8
chr19:38943728	A/A	G	A	SNV	RYR1	RYR1:intronic:NM_000540.2	1	100		intronic	NM_00054	c.1440+74	p.?	-2.86					EF-hand dom	rs12460768,rs931450562		YES			Ryanodine	ATP bindin	ACMG Recommend	Caffeine,C	not provided	G=0, A=20	G=0.0, A=1.0	0.00001	263.754	20	G=0/0, A=10/1
chr19:38949729	G/G	T	G	SNV	RYR1	RYR1:intronic:NM_000540.2	1	100		intronic	NM_00054	c.2168-57	p.?	0.13					EF-hand dom	rs4802474		YES			Ryanodine	ATP bindin	ACMG Recommend	Caffeine,C	not provided	T=0, G=66	T=0.0, G=1.0	0.00001	1009.19	66	T=0/0, G=47/1
chr2:142888376	G/C	G	C	SNV	LRP1B	LRP1B:utr_5:NM_018557.2	1	46.43	1	utr_5	NM_01855	c.-78C>G	p.?	0.85					Calcium-bind	rs1375610,rs767585425		YES	2.942E-1;0.0 (pos.142888376)			calcium io	Adenocarcinoma;Carcinoma;Carcinoma, Ren			G=7, C=13	G=0.35, C=0.65	0.00001	238.215	28	G=7/0, C=5/8
chr19:38950947	C/T	C	T	SNV	RYR1	RYR1:intronic:NM_000540.2	1	95.83		intronic	NM_00054	c.2361-68	p.?	-0.18					EF-hand dom	rs2304147		YES			Ryanodine	ATP bindin	ACMG Recommend	Caffeine,C	not provided	C=1, T=23	C=0.0417, T=0.9583	0.00001	293.605	24	C=0/1, T=12/1
chr2:170003432	G/G	T	G	SNV	LRP2	LRP2:exonic:NM_004525.2	1	100	69	missense	NM_00452	c.12628A>	p.Ile4210Leu	6.29	0.01	5	0.995	0.98(COSM14	Calcium-bind	rs4667591	0.2099	YES	4.631E-1 (pos.17000343	acute_myeloic	Low density	Golgi appa	Agenesis of Corpus	Gentamici	Benign/Likely	T=0, G=21	T=0.0, G=1.0	0.00001	281.231	21	T=0/0, G=9/12

chr2:170032989	T/T	C	T	SNV	LRP2	LRP2:exonic:NM 004525.2	1	100	54	synonymous	NM 00452	c.10503G:	p.(=)	-1.79				0.05(COSM41	Calcium-bind	rs2229265	0.4824	YES	1.025E-1 (pos.17003298!	adenocarcinoi	Low density	Golgi appa	Agenesis of Corpus	Gentamici	Benign/Likely	C=0, T=48	C=0.0, T=1.0	0.00001	810.424	48	C=0/0, T=26/2
chr2:170099473	C/C	T	C	SNV	LRP2	LRP2:exonic:NM 004525.2	1	100	24	synonymous	NM 00452	c.3660A>G	p.(=)	-0.75				0.07(COSM50	Calcium-bind	rs386652562,rs8310-	0.4493	YES	4.705E-1 (pos.17009947:	adenocarcinoi	Low density	Golgi appa	Agenesis of Corpus	Gentamici	Benign/Likely	T=0, C=46	T=0.0, C=1.0	0.00001	768.294	46	T=0/0, C=24/2
chr2:170103336	C/C	T	C	SNV	LRP2	LRP2:exonic:NM 004525.2	1	100	21	synonymous	NM 00452	c.3069A>G	p.(=)	-0.99				0.03(COSM50	Calcium-bind	rs831043	0.4559	YES	4.72E-1 (pos.170103336)	adenocarcinoi	Low density	Golgi appa	Agenesis of Corpus	Gentamici	Benign/Likely	T=0, C=81	T=0.0, C=1.0	0.00001	1309.48	81	T=0/0, C=52/2
chr2:170115588	C/C	T	C	SNV	LRP2	LRP2:exonic:NM 004525.2	1	100	17	synonymous	NM 00452	c.2460A>G	p.(=)	0.41				0.55(COSM50	Calcium-bind	rs2241190	0.4677	YES	4.714E-1 (pos.17011558!	adenocarcinoi	Low density	Golgi appa	Agenesis of Corpus	Gentamici	Benign/Likely	T=0, C=131	T=0.0, C=1.0	0.00001	2161.94	131	T=0/0, C=46/8
chr19:38996620	T/T	C	T	SNV	RYR1	RYR1:intronic:NM 000540.2	1	100		intronic	NM 00054	c.8541+34	p.?	-0.22					EF-hand dom	rs2960342	0.2355	YES	3.388E-1 (pos.38996620)		Ryanodine	ATP bindin	ACMG Recommend	Caffeine,C	Benign	C=0, T=24	C=0.0, T=1.0	0.00001	334.792	24	C=0/0, T=9/15
chr19:38996623	G/G	T	G	SNV	RYR1	RYR1:intronic:NM 000540.2	1	100		intronic	NM 00054	c.8541+37	p.?	-0.38					EF-hand dom	rs2960343,rs747073!	0.2081	YES	0.0 (pos.38996622),3.38E-1;0.0;0.0 (pos.		Ryanodine	ATP bindin	ACMG Recommend	Caffeine,C	Benign	T=0, G=24	T=0.0, G=1.0	0.00001	334.793	24	T=0/0, G=9/15
chr19:38996640	G/G	A	G	SNV	RYR1	RYR1:intronic:NM 000540.2	1	100		intronic	NM 00054	c.8541+54	p.?	-2.23					EF-hand dom	rs2915954		YES			Ryanodine	ATP bindin	ACMG Recommend	Caffeine,C	not provided	A=0, G=27	A=0.0, G=1.0	0.00001	389.961	27	A=0/0, G=9/18
chr19:38997214	G/G	T	G	SNV	RYR1	RYR1:intronic:NM 000540.2	1	100		intronic	NM 00054	c.8692+28	p.?	-1.9					EF-hand dom	rs2960345	0.2483	YES	3.479E-1;0.0 (pos.38997214)		Ryanodine	ATP bindin	ACMG Recommend	Caffeine,C	Benign	T=0, G=78	T=0.0, G=1.0	0.00001	1253.5	78	T=0/0, G=35/4
chr19:38997323	C/C	T	C	SNV	RYR1	RYR1:intronic:NM 000540.2	1	100		intronic	NM 00054	c.8692+13	p.?	-0.98					EF-hand dom	rs2915957		YES			Ryanodine	ATP bindin	ACMG Recommend	Caffeine,Calcium Citrate		T=0, C=73	T=0.0, C=1.0	0.00001	1154.5	73	T=0/0, C=28/4
chr19:38997609	A/A	T	A	SNV	RYR1	RYR1:intronic:NM 000540.2	1	100		intronic	NM 00054	c.8816+17	p.?	-3.9					EF-hand dom	rs2915959	0.2466	YES	3.4E-1 (pos.38997609)		Ryanodine	ATP bindin	ACMG Recommend	Caffeine,C	Benign	T=0, A=43	T=0.0, A=1.0	0.00001	705.822	43	T=0/0, A=28/1
chr19:38997635	C/C	A	C	SNV	RYR1	RYR1:intronic:NM 000540.2	1	100		intronic	NM 00054	c.8816+43	p.?	-0.32					EF-hand dom	rs2960346	0.2458	YES	3.372E-1 (pos.38997635)		Ryanodine	ATP bindin	ACMG Recommend	Caffeine,C	Benign	A=0, C=43	A=0.0, C=1.0	0.00001	704.342	43	A=0/0, C=27/1
chr19:38997679	G/T	G	T	SNV	RYR1	RYR1:intronic:NM 000540.2	1	97.56		intronic	NM 00054	c.8816+87	p.?	-3.04					EF-hand dom	rs2960347		YES			Ryanodine	ATP bindin	ACMG Recommend	Caffeine,C	not provided	G=1, T=40	G=0.0244, T=0.9756	0.00001	634.906	41	G=1/0, T=24/1
chr2:170218847	G/G	C	G	SNV	LRP2	LRP2:exonic:NM 004525.2	1	100	1	synonymous	NM 00452	c.63G>C	p.(=)	0.05				0.13(COSM37	Calcium-bind	rs1559014	1.00E-04		1.0 (pos.170218847)	adenocarcinoi	Low density	Golgi appa	Agenesis of Corpus	Gentamici	Benign/Likely	C=0, G=23	C=0.0, G=1.0	0.00001	308.841	23	C=0/0, G=18/5
chr19:39191380	GC/GC	G	GC	INDEL	ACTN4	ACTN4:intronic:NM 004924.5	1	100		intronic	NM 00492	c.277+26G	p.?	-0.61					CAMSAP CH domain,Ca2+insensitive EF hand,Calponin homology (CH) domain,EF-hand domain pair,S						Actinin, alp	RNA bindir	Female Urogenital Diseases;Female Urogeni			G=0, GC=34	G=0.0, GC=1.0	0.00001	315.303	34	G=0/0, GC=13,
chr19:39191383	C/C	G	C	SNV	ACTN4	ACTN4:intronic:NM 004924.5	1	100		intronic	NM 00492	c.277+29G	p.?	0.12					CAMSAP CH d	rs2112649	0.2544	YES	6.269E-1;0.0 (pos.39191383)		Actinin, alp	RNA bindir	Female Urogenital Diseases;Female Urogeni			G=0, C=33	G=0.0, C=1.0	0.00001	505.716	33	G=0/0, C=13/2
chr19:33696708	C/C	T	C	SNV	LRP3	LRP3:exonic:NM 002333.3	1	100	5	synonymous	NM 00233	c.1032T>C	p.(=)	-3.49				0.01(COSM37	CUB domain,l	rs2112800	0.1616	YES	4.001E-1 (pos.33696708)	adenocarcinoi	Low density	clathrin-coated pit,integral component of membrane,re				T=0, C=81	T=0.0, C=1.0	0.00001	1307.96	81	T=0/0, C=35/4
chr11:46914583	C/C	T	C	SNV	LRP4	LRP4:exonic:NM 002334.3	1	100	13	synonymous	NM 00233	c.1638A>G	p.(=)	0.38					Calcium-bind	rs10838631	0.013	YES	9.999E-1 (pos.46914583)		Low density	BMP signa	Bone Diseases:Bone Diseases,		Benign	T=0, C=25	T=0.0, C=1.0	0.00001	344.671	25	T=0/0, C=11/1
chr19:39334811	A/A	T	A	SNV	HNRNPL	HNRNPL:intronic:NM 001533.2	1	100		intronic	NM 00153	c.713-69A	p.?	-0.3					RNA recogniti	rs2278011		YES			Heterogene	RNA bindir	Breast Diseases;Breast Neoplasms;Digestive			T=0, A=22	T=0.0, A=1.0	0.00001	298.892	22	T=0/0, A=5/17
chr19:39334830	T/T	C	T	SNV	HNRNPL	HNRNPL:intronic:NM 001533.2	1	100		intronic	NM 00153	c.713-88G	p.?	-1.17					RNA recogniti	rs2278012		YES			Heterogene	RNA bindir	Breast Diseases;Breast Neoplasms;Digestive			C=0, T=22	C=0.0, T=1.0	0.00001	298.892	22	C=0/0, T=5/17
chr19:39408688	G/G	C	G	SNV	SARS2	SARS2:intronic:NM 001145901.1	1	100		intronic	NM 00114	c.969-40G	p.?	-0.15					tRNA synthet	rs4803034	0.3794	YES	9.722E-1 (pos.39408688)		Seryl-tRNA	ATP binding,RNA binding,cytoplasm,ligase activity,mitoc				C=0, G=37	C=0.0, G=1.0	0.00001	583.696	37	C=0/0, G=26/1
chr19:39409236	A/A	C	A	SNV	SARS2	SARS2:intronic:NM 001145901.1	1	100		intronic	NM 00114	c.814-66G	p.?	1.58					tRNA synthet	rs11083489		YES			Seryl-tRNA	ATP binding,RNA binding,cytoplasm,ligase activity,mitoc				C=0, A=25	C=0.0, A=1.0	0.00001	353.019	25	C=0/0, A=10/1
chr11:46914598	G/G	A	G	SNV	LRP4	LRP4:exonic:NM 002334.3	1	100	13	synonymous	NM 00233	c.1623T>C	p.(=)	-0.26					Calcium-bind	rs10769215	0		1.0 (pos.46914598)		Low density	BMP signa	Bone Diseases:Bone Diseases,		Benign	A=0, G=24	A=0.0, G=1.0	0.00001	334.792	24	A=0/0, G=10/1
chr19:39435485	A/A	G	A	SNV	FBXO17	FBXO17:intronic:NM 148169.2	1	100		intronic	NM 14816	c.720+124	p.?	-0.6						rs10416055		YES			F-box only p	SCF ubiquitin ligase complex,cytosol,post-translational				G=0, A=41	G=0.0, A=1.0	0.00001	664.694	41	G=0/0, A=23/1
chr19:39517715	C/T	C	T	SNV	FBXO27	FBXO27:intronic:NM 178820.4	1	50		intronic	NM 17882	c.573-70G	p.?	-1.81					F-box associa	rs57724551		YES			F-box only p	SCF ubiquitin ligase complex,cytosol,post-translational				C=10, T=10	C=0.5, T=0.5	0.00001	82.8861	20	C=7/3, T=1/9
chr11:68171013	C/C	T	C	SNV	LRP5	LRP5:exonic:NM 002335.3	1	100	8	synonymous	NM 00233	c.1647T>C	p.(=)	-0.01				0.48(COSM41	Coagulation I	rs545382	0.093	YES	9.884E-1 (pos.68171013)	neoplasm	Low density	Wnt signa	Bone Diseases:Bone Diseases,		Benign	T=0, C=51	T=0.0, C=1.0	0.00001	754.51	51	T=0/0, C=25/2
chr8:145745182	C/C	T	C	SNV	LRRC14	LRRC14:exonic:NM 014665.3	1	100	2	synonymous	NM 01466	c.73T>C	p.(=)	0.21				0.06(COSM41	Leucine Rich	rs2721172	9.00E-04	YES	7.908E-1 (pos.14574518:	neoplasm		cytoplasm,kinase binding,negative regula			Benign	T=0, C=41	T=0.0, C=1.0	0.00001	640.021	41	T=0/0, C=23/1
chr8:145747920	T/G	T	G	SNV	LRRC14,LRRC24	LRRC14:utr 3:NM 014665.3, LRRC24:exonic:NM 00102467	1	58.33	5	missense	NM 01466	c.2540T>G	p.?, p.Glu494Ala	0.07	0.92	107	0	0.01(COSM37	Immunoglobi	rs13277542	0.4407	YES	5.29E-1;0.0 (pos.145747!	adenocarcinoma,neoplasn		cytoplasm,integral component of membrane,kinase bin				T=10, G=14	T=0.4167, G=0.5833	0.00001	135.339	24	T=6/4, G=8/6
chr19:39949764	T/T	C	T	SNV	SUPT5H	SUPT5H:intronic:NM 001130824.1	1	100		intronic	NM 00113	c.458+51C	p.?	-2.53					Early transcri	rs1529733	0.3517	YES	4.61E-1 (pos.39949764)		Suppressor	7-methylguanosine mRNA capping,DNA binding,DNA-tem				C=0, T=41	C=0.0, T=1.0	0.00001	664.686	41	C=0/0, T=20/2
chr19:39957030	T/T	C	T	SNV	SUPT5H	SUPT5H:intronic:NM 001130824.1	1	100		intronic	NM 00113	c.877-90C	p.?	-0.36					Early transcri	rs4802030		YES			Suppressor	7-methylguanosine mRNA capping,DNA binding,DNA-tem				C=0, T=24	C=0.0, T=1.0	0.00001	334.718	24	C=0/0, T=8/16
chr19:39963828	A/A	T	A	SNV	SUPT5H	SUPT5H:intronic:NM 001130824.1	1	100		intronic	NM 00113	c.2259-15	p.?	0.47					Early transcri	rs45568533	0.3496	YES	4.557E-1;0.0 (pos.39963828)		Suppressor	7-methylguanosine mRNA capping,DNA binding,DNA-tem				T=0, A=23	T=0.0, A=1.0	0.00001	316.745	23	T=0/0, A=5/18
chr5:195507	T/T	C	T	SNV	LRRC14B	LRRC14B:utr 3:NM 001080478.2	1	100	2	utr 3	NM 00108	c.1584C>T	p.?	1.77						rs13167067	0.4609	YES	2.942E-1 (pos.195507)							C=0, T=47	C=0.0, T=1.0	0.00001	789.307	47	C=0/0, T=29/1
chr19:40009683	C/T	C	T	SNV	SELENOV	SELENOV:intronic:NM 182704.1	1	97.5		intronic	NM 18270	c.964-28C	p.?	-0.47					Rdx family	rs4802034	0.2323	YES	3.495E-1 (pos.40009683)		Selenoprot	protein binding				C=1, T=39	C=0.025, T=0.975	0.00001	616.043	40	C=0/1, T=8/31
chr11:76370792	G/G	A	G	SNV	LRRC32	LRRC32:exonic:NM 005512.2	1	100	3	synonymous	NM 00551	c.1845T>C	p.(=)	-5.19				0.03(COSM41	Leucine Rich	rs4304809		YES	1.0 (pos.76370792)	neoplasm,squ	Leucine rich	integral component of plasma membrane,negative regu				A=0, G=40	A=0.0, G=1.0	0.00001	644.286	40	A=0/0, G=16/2
chr19:40224901	G/A	G	A	SNV	CLC	CLC:intronic:NM 001828.5	1	50		intronic	NM 00182	c.303+22C	p.?	-2.38					Galactoside-	rs453827	0.3337	YES	3.239E-1 (pos.40224901)		Cardiotropl	T cell apoptotic process,carbol		N-Ethylmaleimide,Para-I		G=23, A=23	G=0.5, A=0.5	0.00001	258.521	46	G=8/15, A=12/
chr11:76372418	C/C	T	C	SNV	LRRC32	LRRC32:exonic:NM 005512.2	1	100	3	synonymous	NM 00551	c.219A>G	p.(=)	-1.99				0.03(COSM63	Leucine Rich	rs3740778	0.1292	YES	7.847E-1 (pos.76372418)	carcinoma	Leucine rich	integral component of plasma membrane,negative regu				T=0, C=59	T=0.0, C=1.0	0.00001	885.163	59	T=0/0, C=55/4
chr17:45915788	C/C	T	C	SNV	LRRC46,SCRN2	LRRC46:downstream:NM 033413.3, SCRN2:exonic:NM 138	1	100	7	missense	NM 03341	c.*1302T>	p.?, p.Met323Val	-0.39	0.22	21	0		Peptidase fai	rs7350974	0.0199	YES	9.981E-1 (pos.45915788)		Secernin 2	biological process,dipeptidase activity,exocytosis,extra				T=0, C=34	T=0.0, C=1.0	0.00001	538.034	34	T=0/0, C=20/1
chr19:40408857	C/C	T	C	SNV	FCGBP	FCGBP:intronic:NM 003890.2	1	100		intronic	NM 00389	c.3991-9A	p.?	-4.06					C8 domain,Ig	rs4802064	0.2569	YES	4.895E-1 (pos.40408857)		Fc fragment	extracellu	Digestive System Diseases:Digestive System			T=0, C=31	T=0.0, C=1.0	0.00001	465.818	31	T=0/0, C=14/1
chr9:131670919	C/C	T	C	SNV	LRRC8A	LRRC8A:exonic:NM 001127245.1	1	100	2	synonymous	NM 00112	c.1476T>C	p.(=)	-2.48					Leucine Rich	rs3750319	0.2752	YES	6.903E-1 (pos.131670919)		Leucine-ricl	anion chai	Agammaglobulinemia:Blood Pi		Benign	T=0, C=49	T=0.0, C=1.0	0.00001	831.548	49	T=0/0, C=30/1
chr1:90178743	G/G	A	G	SNV	LRRC8C	LRRC8C:exonic:NM 032270.4	1	100	3	missense	NM 03227	c.614A>G	p.Asp205Gly	3.26	0.54	94	0		Leucine Rich	rs474536	3.00E-04		1.0 (pos.90178743)		Leucine-ricl	anion transmembrane transport,cytoplasm,endoplasmi				A=0, G=23	A=0.0, G=1.0	0.00001	316.683	23	A=0/0, G=6/17
chr19:41083444	C/T	C	T	SNV	SHKBP1	SHKBP1:intronic:NM 138392.3	1	50		intronic	NM 13839	c.187-19C	p.?	0.24					BTB/POZ dom	rs2290682	0.3036	YES	2.532E-1 (pos.41083444)		SH3KBP1-bi	lysosome,positive regulation of epidermal growth factor				C=11, T=11	C=0.5, T=0.5	0.00001	94.6063	22	C=11/0, T=5/6
chr15:101606889	AA/AA	GC	AA	MNV	LRRK1	LRRK1:exonic:NM 024652.5	2	100	33	missense	NM 02465	c.5813 58	p.Gly1938Glu	3.32,-2.72	0.05	98	0.216	0.66(COSM37	ADP-ribosyla	rs2924835,rs295919	0.3015	YES	3.611E-1;1.16E-4 (pos.10	carcinoma,duc	Leucine-ricl	ATP binding,GTP binding,bone resorption,cytoplasm,cyt				GC=0, AA=4	GC=0.0, AA=1.0	0.00001	810.435	48	GC=0/0, AA=3
chr15:101591923	A/G	A	G	SNV	LRRK1	LRRK1:exonic:NM 024652.5	1	57.58	24	synonymous	NM 02465	c.3447A>G	p.(=)	-2.59				0.04(COSM37	ADP-ribosyla	rs4965778	0.3866	YES	8.068E-1 (pos.10159192:	adenocarcinoi	Leucine-ricl	ATP binding,GTP binding,bone resorption,cytoplasm,cyt				A=14, G=19	A=0.4242, G=0.5758	0.00001	204.387	33	A=11/3, G=16/
chr12:40631791	C/C	T	C	SNV	LRRK2	LRRK2:exonic:NM 198578.3	1	100	5	synonymous	NM 19857	c.457T>C	p.(=)	-0.73				0.03(COSM11	ADP-ribosyla	rs10878245	0.3969	YES	3.466E-1 (pos.40631791)	adenocarcinoi	Leucine-ricl	ATP bindin	Adenocarcinoma;Basal Gangli		Conflicting in	T=0, C=67	T=0.0, C=1.0	0.00001	1034.43	67	T=0/0, C=41/2
chr19:41206208	A/G	A	G	SNV	COQ8B	COQ8B:intronic:NM 024876.3	1	45.83		intronic	NM 02487	c.1035+7T	p.?	-1.17				0.05(COSM63	ABC1 family	rs1064794994,rs224(	0.4536	YES	5.203E-1 (pos.41206208)	carcinoma	Coenzyme (	ATP binding,cerebellar Purkinje cell layer			Benign	A=13, G=11	A=0.5417, G=0.4583	0.00001	92.9839	24	A=8/5, G=4/7
chr12:40713834	A/A	C	A	SNV	LRRK2	LRRK2:exonic:NM 198578.3	1	100	34	synonymous	NM 19857	c.4872C>A	p.(=)	0.69				0.17(COSM37	ADP-ribosyla	rs1427263	0.3517	YES	5.005E-1 (pos.40713834)	adenocarcinoi	Leucine-ricl	ATP bindin	Adenocarcinoma;Basal Gangli		Conflicting in	C=0, A=53	C=0.0, A=1.0	0.00001	782.03	53	C=0/0, A=25/2
chr19:41313202	C/A	C	A	SNV	EGLN2,RAB4B-EGLN2	EGLN2:intronic:NM 053046.3, RAB4B-EGLN2:intronic nc:NR	1	50		intronic	NR 03779	c.1100+23	p.?	-0.07					2OG-Fe(II) ox	rs3736329	0.3692	YES	3.652E-1;0.0 (pos.41313202)		Egl9, C. eleg	L-ascorbic acid binding,cell rec		Roxadustat,Vitamin C		C=10, A=10	C=0.5, A=0.5	0.00001	82.8861	20	C=6/4, A=1/9
chr12:40716260	C/C	T	C	SNV	LRRK2	LRRK2:exonic:NM 198578.3	1	100	37	synonymous	NM 19857	c.5457T>C	p.(=)	-0.51				0.19(COSM56	ADP-ribosyla	rs10878371	0.4585	YES	4.756E-1 (pos.40716260)	diffuse large	Leucine-ricl	ATP bindin	Adenocarcinoma;Basal Gangli		Conflicting in	T=0, C=31	T=0.0, C=1.0	0.00001	465.819	31	T=0/0, C=17/1
chr19:41704832	G/G	A	G	SNV	CYP2S1	CYP2S1:intronic:NM 030622.7	1	100		intronic	NM 03062	c.834+39A	p.?	-0.59					Cytochrome I	rs338592			9.998E-1 (pos.41704832)		Cytochrome	arachidon	Psoriasis;Skin Diseases;Skin Diseases, Papul			A=0, G=37	A=0.0, G=1.0	0.00001	583.706	37	A=0/0, G=28/9
chr19:41707094	G/G	A	G	SNV	CYP2S1	CYP2S1:intronic:NM 030622.7	1	100		intronic	NM 03062	c.835-42A	p.?	-1.45					Cytochrome I	rs1628289,rs756037(	0.4967	YES	5.472E-1;0.0 (pos.41707094)		Cytochrome	arachidon	Psoriasis;Skin Diseases;Skin Diseases, Papul			A=0, G=122	A=0.0, G=1.0	0.00001	1973.22	122	A=0/0, G=68/5
chr12:40758652	C/C	T	C	SNV	LRRK2	LRRK2:exonic:NM 198578.3	1	100	49	missense	NM 19857	c.7190T>C	p.Met2397Thr	0.38	0.44	81	0	0.08(COSM37	ADP-ribosyla	rs3761863	0.3449	YES	4.731E-1 (pos.40758652)	adenocarcinoi	Leucine-ricl	ATP bindin	Adenocarcinoma;Basal Gangli		Benign	T=0, C=148	T=0.0, C=1.0	0.00001	2509.49	148	T=0/0, C=71/7
chr19:41928701	C/T	C	T	SNV	BCKDHA	BCKDHA:intronic:NM 000709.3	1	57.14		intronic	NM 00070	c.995+26C	p.?	-0.27					Dehydrogena	rs284653	0.4431	YES	7.942E-2 (pos.41928701)		Branched cl	3-methyl-2	Amino Acid Disorder:Amino Aci		Benign/Likely	C=12, T=16	C=0.4286, T=0.5714	0.00001	161.771	28	C=8/4, T=5/11
chr19:41945837	T/A	T	A	SNV	DMAC2	DMAC2:upstream:NM 001167867.1	1	50		intronic	NM 00116	c.-423A>T	p.?	-0.5						rs13346603	0.3959	YES	5.62E-1 (pos.41945837)			mitochondrial respiratory chain complex I,mitochondria				T=30, A=30	T=0.5, A=0.5	0.00001	293.697	60	T=16/14, A=19
chr19:4229635	G/G	A	G	SNV	EBI3	EBI3:intronic:NM 005755.2	1	100		intronic	NM 00575	c.67+21A>	p.?	-2.52					Fibronectin t	rs400089,rs7543133!	0.4602	YES	0.0 (pos.4229634),5.365E-1 (pos.422963		Epstein-Bar	T cell proli	Digestive System Diseases;Digestive System			A=0, G=32	A=0.0, G=1.0	0.00001	484.39	32	A=0/0, G=17/1
chr19:42373893	T/T	C	T	SNV	MIR6797,RPS19	RPS19:intronic:NM 001022.3, MIR6797:downstream:NR 1(	1	100		intronic	NM 00102	c.411+70C	p.?	0					Ribosomal pr	rs2075751	0.3394	YES			Ribosomal	Notch sign	Anemia:Anemia, Aplastic:Anemia, Diamond-I			C=0, T=27	C=0.0, T=1.0	0.00001	388.818	27	C=0/0, T=8/19
chr12:40713901	T/A	T	A	SNV	LRRK2	LRRK2:exonic:NM 198578.3	1	57.14	34	missense	NM 19857	c.4939T>A	p.Ser1647Thr	0.83	0.74	58	0	0.69(COSM44	ADP-ribosyla	rs11564148	0.2981	YES	3.341E-1 (pos.40713901)	adenocarcinoi	Leucine-ricl	ATP bindin	Adenocarcinoma;Basal Gangli		Conflicting in	T=24, A=32	T=0.4286, A=0.5714	0.00001	331.823	56	T=8/16, A=15/
chr3:3886580	G/G	A	G	SNV	LRRN1	LRRN1:exonic:NM 020873.6	1	100	2	synonymous	NM 02087	c.255A>G	p.(=)	-0.42					Immunoglobi	rs4685689	2.00E-04		9.876E-1 (pos.3886580)			axonogenesis,extracellular space,integral component o				A=0, G=20	A=0.0, G=1.0	0.00001	263.786	20	A=0/0, G=10/1
chr1:204587240	A/A	G	A	SNV	LRRN2	LRRN2:exonic:NM 201630.1	1	100	2	synonymous	NM 20163	c.1881C>T	p.(=)	1.72				0.92(COSM17	Immunoglobi	rs3747632	0.4519	YES	3.044E-1 (pos.20458724(	adenocarcinoma,carcinom		axonogenesis,cell adhesion,extracellular space,integra				G=0, A=22	G=0.0, A=1.0	0.00001	298.892	22	G=0/0, A=13/9
chr2:77745725	T/C	T	C	SNV	LRRTM4	LRRTM4:exonic:NM 001134745.2	1	46.43	3	missense	NM 00113	c.1270A>G	p.Lys424Glu	8.04	0	56	0.995		Leucine Rich	rs374627195	0		0.0 (pos.77745725)		Leucine-ricl	cell junction,cytokine-mediated signaling pathway,cyto				T=15, C=13	T=0.5357, C=0.4643	0.00001	117.972	28	T=3/12, C=2/1
chr19:35741456	C/C	T	C	SNV	LSR	LSR:exonic:NM 205834.3	1	100	2	synonymous	NM 20583	c.492T>C	p.(=)	1.01				0.59(COSM54	Lipolysis stir	rs2073900	0.2564	YES	4.642E-1 (pos.35741456)	acute myeloic	Lipolysis-st	chylomicr	Mammary Neoplasms, Animal:Mammary Nec			T=0, C=46	T=0.0, C=1.0	0.00001	767.436	46	T=0/0, C=23/2
chr19:35758275	GGGA/GGGA	G	GGGA	INDEL	LSR	LSR:exonic:NM 205834.3	3	100	9	nonframeshiftInsertion	NM 20583	c.1556 15	p.Gly518 Arg519insArg	0.34					Lipolysis stir	rs142507475,rs3977!	0.2399	YES	5.113E-1;0.0 (pos.35758275)		Lipolysis-st	chylomicr	Mammary Neoplasms, Animal:Mammary Nec			G=0, GGGA	G=0.0, GGGA=1.0	0.00001	730.942	44	G=0/0, GGGA=
chr2:33585796	T/C	T	C	SNV	LTBP1	LTBP1:exonic:NM 206943.2	1	100	27	missense	NM 20694	c.4133T>C	p.Val1378Ala	3.9	1	64	0		Calcium-bind	rs4422143,rs752348:	0		0.0 (pos.33585791),1.0 (pos.33585796)		Latent tran	aorta development,calcium ion binding,cellular protein				T=0, C=35	T=0.0, C=1.0	0.00001	516.809	35	T=0/0, C=22/1
chr19:43439793	A/A	G	A	SNV	PSG7	PSG7:intronic:NM 001206650.1	1	100		intronic	NM 00120	c.64+1372	p.?	-0.39					Immunoglobi	rs7245978			1.0 (pos.43439793)		Pregnancy-specific beta-1-glycoprotein-7					G=0, A=28	G=0.0, A=1.0	0.00001	408.718	28	G=0/0, A=10/1
chr19:41123093	AG/G	A	AG	INDEL	LTBP4	LTBP4:exonic:NM 001042544.1	1	95.56	24	missense	NM 00104	c.3233A>G	p.Gln1078Arg	3.37		43			Calcium-bind	rs201314196,rs3977:	3.00E-04		1.0 (pos.41123093)	osteosarcoma	Latent tran	calcium io	Cartilage Diseases;Congenital		Benign	A=0, AG=86	A=0.0, AG=0.9556, G	0.00001	1318.8	90	A=0/0, AG=43/
chr3:46501284	CCTT/CCTT	C	CCTT	INDEL	LTF	LTF:exonic:NM 002343.5	3	100	2	nonframeshiftInsertion	NM 00234	c.68 69ins	p.Arg23dup	-2.03					Transferrin	rs10662431,rs55665:	0.0275	YES	9.998E-1 (pos.46501284)		Lactotransf	DNA bindi	Bone Diseases:Bon	2-(4-(2-HYDROXY-3-(ISOPI		C=0, CCTT=	C=0.0, CCTT=1.0	0.00001	543.903	35	C=0/0, CCTT=2
chr19:43688898	TC/T	TC	T	INDEL	PSG5	PSG5:intronic:NM 001130014.1	1	50		intronic	NM 00113	c.430+36G	p.?	-0.19, 0.0					Immunoglobi	rs71336844	0.2589	YES	3.467E-4 (pos.43688898)		Pregnancy-	extracellu	Arthritis:Arthritis, Rheumatoid:Autoimmune			TC=11, T=1	TC=0.5, T=0.5	0.00001	69.1319	22	TC=1/10, T=4/
chr1:23418153	T/T	C	T	SNV	LUZP1	LUZP1:exonic:NM 001142546.1	1	100	3	missense	NM 00114	c.2602G>A	p.Asp868Asn	0.8	1	23	0	0(COSM5002336)		rs10799790	0.1707	YES	8.123E-1 (pos.23418153)	ductal carcinc	Leucine zip	artery development,extracellular exosome,membrane,i				C=0, T=80	C=0.0, T=1.0	0.00001	1289.34	80	C=0/0, T=45/3
chr11:25100229	G/A	G	A	SNV	LUZP2	LUZP2:utr 3:NM 001009909.3	1	56	12	utr 3	NM 00100	c.1066G>A	p.?	-0.34						rs10500994	0.421	YES	7.979E-1 (pos.25100229)		Leucine zip	extracellu	Alzheimer Disease;Brain Diseases;Central N			G=11, A=14	G=0.44, A=0.56	0.00001	134.349	25	G=7/4, A=8/6
chr5:115298378	C/T	C	T	SNV	LVRN	LVRN:exonic:NM 173800.4	1	53.13	1	synonymous	NM 17380	c.64C>T	p.(=)	0.23				0.1(COSM113	ERAP1-like C-	rs10062297	0.1758	YES	7.527E-1 (pos.11529837!	adenocarcinoi	Laeverin	aminopeptidase activity,cell-cell signaling,cytoplasm,in				C=30, T=34	C=0.4688, T=0.5313	0.00001	347.787	64	C=16/14, T=17
chr5:115298475	T/C	T	C	SNV	LVRN	LVRN:exonic:NM 173800.4	1	50	1	missense	NM 17380	c.161T>C	p.Leu54Ser	-0.55	0.75	145	0	0.01(COSM62	ERAP1-like C-	rs12520255	0.1887	YES	7.467E-1 (pos.11529847!	neoplasm	Laeverin	aminopeptidase activity,cell-cell signaling,cytoplasm,in				T=29, C=29	T=0.5, C=0.5	0.00001	282.583	58	T=15/14, C=13
chr19:43922300	G/A	G	A	SNV	TEX101	TEX101:intronic:NM 001130011.1	1	50		intronic	NM 00113	c.521-20G	p.?	-0.18					u-PAR/Ly-6 dc	rs2278834	0.2264	YES	5.882E-1 (pos.43922300)		Testis-expr	C-terminal protein lipidation,acrosomal membrane,acr				G=11, A=11	G=0.5, A=0.5	0.00001	94.6067	22	G=4/7, A=8/3
chr5:115298977	C/T	C	T	SNV	LVRN	LVRN:exonic:NM 173800.4	1	50	1	synonymous	NM 17380	c.663C>T	p.(=)	1.09				0.66(COSM44	ERAP1-like C-	rs1445708	0.39	YES	4.356E-1 (pos.11529897:	acute myeloic	Laeverin	aminopeptidase activity,cell-cell signaling,cytoplasm,in				C=18, T=18	C=0.5, T=0.5	0.00001	185.082	36	C=4/14, T=5/1
chr4:4276131	GC/GC	G	GC	INDEL	LYAR	LYAR:exonic:NM 017816.2	1	100	7	frameshiftInsertion	NM 01781	c.794 795	p.His265fs	-0.52					LYAR-type C2HC zinc finger						Ly1 antibod	RNA binding,metal ion binding,nucleolus,nucleus,protei				G=0, GC=62	G=0.0, GC=1.0	0.00001	601.638	62	G=0/0, GC=33,
chr4:4276132	C/C	T	C	SNV	LYAR	LYAR:exonic:NM 017816.2	1	100	7	missense	NM 01781	c.794A>G	p.His265Arg	0.57	0.37	29	0		LYAR-type C2	rs7376390	0		9.997E-1 (pos.4276132)		Ly1 antibod	RNA binding,metal ion binding,nucleolus,nucleus,protei				T=0, C=62	T=0.0, C=1.0	0.00001	913.749	62	T=0/0, C=33/2
chr19:44131297	C/C	T	C	SNV	CADM4	CADM4:intronic:NM 145296.1	1	100		intronic	NM 14529	c.364+21A	p.?	-3.6					CD80-like C2-	rs407049	0.0109		1.0 (pos.44131297)		Cell adhesi	cell adhesion molecule binding,cell recognition,cell-cell				T=0, C=35	T=0.0, C=1.0	0.00001	543.901	35	T=0/0, C=24/1
chr19:4429007	T/T	C	T	SNV	CHAF1A	CHAF1A:intronic:NM 005483.2	1	100		intronic	NM 00548	c.1604+12	p.?	-1.7					CAF1 comple	rs62130978		YES			Chromatin	CAF-1 com	Mouth Diseases;Stomatognathic Diseases			C=0, T=39	C=0.0, T=1.0	0.00001	605.325	39	C=0/0, T=7/32
chr19:4432973	A/A	G	A	SNV	CHAF1A	CHAF1A:intronic:NM 005483.2	1	100		intronic	NM 00548	c.2204-94(	p.?	-1.52					CAF1 comple	rs243372		YES			Chromatin	CAF-1 com	Mouth Diseases;Stomatognathic Diseases			G=0, A=28	G=0.0, A=1.0	0.00001	408.718	28	G=0/0, A=19/9
chr1:219383905	G/G	A	G	SNV	LYPLAL1	LYPLAL1:exonic:NM 138794.4	1	100	4	missense	NM 13879	c.393A>G	p.Ile131Met	1.71	0.92	10	0		Dienelactone	rs940570	2.00E-04	YES	1.0 (pos.219383905)		Lysophosph	biological process,carboxylic ester hydrolase activity,c				A=0, G=161	A=0.0, G=1.0	0.00001	2600.12	161	A=0/0, G=72/8
chr6:5086070	G/G	A	G	SNV	LYRM4-AS1,PPP1R3G	PPP1R3G:exonic:NM 001145115.2, LYRM4-AS1:intronic nc:	1	100	1	synonymous	NR 12601	c.351A>G	p.(=)	1.86				0(COSM3762	Carbohydrat	rs667752,rs796297442		YES	1.0 (pos.5086070)	adenocarcinoma,neoplasm						A=0, G=43	A=0.0, G=1.0	0.00001	705.823	43	A=0/0, G=18/2
chr15:100269309	G/G	T	G	SNV	LYSMD4	LYSMD4:utr 3:NM 152449.3	1	100	6	utr 3	NM 15244	c.913A>C	p.?	0.52						rs6598263	9.00E-04	YES	9.998E-1;1.156E-4;0.0 (pos.100269309)			integral component of membrane				T=0, G=33	T=0.0, G=1.0	0.00001	504.595	33	T=0/0, G=9/24
chr15:100269796	G/G	A	G	SNV	LYSMD4	LYSMD4:exonic:NM 152449.3	1	100	6	synonymous	NM 15244	c.426T>C	p.(=)	-0.99				0.01(COSM4148337),0.01(C		rs6598264	8.00E-04	YES	9.999E-1 (pos.10026979(	neoplasm		integral component of membrane				A=0, G=255	A=0.0, G=1.0	0.00001	4181.37	255	A=0/0, G=119/
chr19:4446995	T/T	C	T	SNV	MIR4746,UBXN6	MIR4746:downstream:NR 039901.1, UBXN6:intronic:NM 0	1	100		intronic	NM 02524	c.616-78G	p.?	-1.65					PUB domain,l	rs243389		YES			UBX domain	ERAD pathway,cytoplasm,cytosol,early endosome meml				C=0, T=24	C=0.0, T=1.0	0.00001	334.792	24	C=0/0, T=13/1
chr1:235972435	C/C	T	C	SNV	LYST	LYST:exonic:NM 000081.3	1	100	5	synonymous	NM 00008	c.1683A>G	p.(=)	-0.45				0.04(COSM37	Beige/BEACH	rs3820553	0.3997	YES	6.02E-2 (pos.235972435)	ductal carcinc	Lysosomal t	cytoplasm	Chediak-Higashi Syndrome;He		Benign	T=0, C=37	T=0.0, C=1.0	0.00001	583.706	37	T=0/0, C=16/2
chr1:235972867	C/C	T	C	SNV	LYST	LYST:exonic:NM 000081.3	1	100	5	synonymous	NM 00008	c.1251A>G	p.(=)	-0.06				0.52(COSM37	Beige/BEACH	rs1063128	0.3994	YES	6.009E-2 (pos.23597286:	hepatocellula	Lysosomal t	cytoplasm	Chediak-Higashi Syndrome;He		Benign	T=0, C=73	T=0.0, C=1.0	0.00001	1150.51	73	T=0/0, C=22/5
chr3:42438754	C/C	T	C	SNV	LYZL4	LYZL4:utr 3:NM 144634.3	1	100	5	utr 3	NM 14463	c.444A>G	p.?	-4.51					C-type lysozy	rs13054	0.4002	YES	5.507E-1;0.0;0.0 (pos.42438754)		Lysozyme-li	acrosomal vesicle,cell wall macromolecule catabolic pr				T=0, C=61	T=0.0, C=1.0	0.00001	921.792	61	T=0/0, C=32/2
chr10:102766574	A/G	A	G	SNV	LZTS2,PDZD7	LZTS2:exonic:NM 032429.3, PDZD7:downstream:NM 0011!	1	57.14	5	unknown	NM 03242	c.1659A>G	p.(=), p.?	-1.36				0.03(COSM37	Fez1	rs386747052,rs8070:	0.2431	YES	5.555E-1 (pos.10276657	neoplasm,squ	Leucine zip	USH2 com	Abnormalities, Multiple:Blindness:Congenita			A=45, G=60	A=0.4286, G=0.5714	0.00001	649.332	105	A=32/13, G=4
chr20:3147762	G/G	A	G	SNV	LZTS3	LZTS3:exonic:NM 001282533.1	1	100	3	synonymous	NM 00128	c.48T>C	p.(=)	0.07					Fez1	rs6051612	0.0153	YES	9.543E-1 (pos.3147762)			cell junction,cytoplasm,cytoskeleton,postsynaptic dens				A=0, G=41	A=0.0, G=1.0	0.00001	664.7	41	A=0/0, G=25/1
chr3:119434527	C/C	G	C	SNV	MAATS1	MAATS1:exonic:NM 033364.3	1	100	6	missense	NM 03336	c.619G>C	p.Ala207Pro	3.13	1	27	0	0.64(COSM59	Solute carrie	rs6438544	9.00E-04	YES	1.0 (pos.119434527)	basal cell car	MYCBP-ass	axoneme,cilium movement,integral component of mem				G=0, C=23	G=0.0, C=1.0	0.00001	321.75	23	G=0/0, C=13/1
chr1:39908506	A/A	G	A	SNV	MACF1	MACF1:exonic:NM 012090.5	1	100	73	missense	NM 01209	c.13048G>	p.Ala4350Thr	1.86	0.68	58	0	0.53(COSM37	Calponin hon	rs587404	0.3066	YES	3.609E-1 (pos.39908506)	adenocarcinoi	Microtubul	ATPase activity,Golgi apparatus,Golgi to plasma membr				G=0, A=31	G=0.0, A=1.0	0.00001	472.58	31	G=0/0, A=19/1
chr4:120981214	C/C	T	C	SNV	MAD2L1	MAD2L1:utr 3:NM 002358.3	1	100	5	utr 3	NM 00235	c.677A>G	p.?	-0.25					HORMA doma	rs2908990		YES			Mitotic arre	anaphase	Adnexal Diseases:Autoimmune Diseases:Car			T=0, C=38	T=0.0, C=1.0	0.00001	603.78	38	T=0/0, C=27/1
chr11:47298360	A/A	G	A	SNV	MADD	MADD:exonic:NM 003682.3	1	100	5	synonymous	NM 00368	c.1041G>A	p.(=)	4.67				0.93(COSM44	DENN (AEX-3)	rs326214	0.3076	YES	2.976E-1 (pos.47298360)	acute myeloic	Electron tra	Rab guanyl-nucleotide exchange factor activity,activatic				G=0, A=23	G=0.0, A=1.0	0.00001	316.676	23	G=0/0, A=15/8
chr19:4453324	CC/CC	TG	CC	MNV	UBXN6	UBXN6:intronic:NM 025241.2	2	100		intronic	NM 02524	c.312+131	p.?	-0.67, -0.1					PUB domain,l	rs376965,rs411833,rs70947751		YES			UBX domain	ERAD pathway,cytoplasm,cytosol,early endosome meml				TG=0, CC=4	TG=0.0, CC=1.0	0.00001	664.042	41	TG=0/0, CC=28
chr11:47303275	C/C	T	C	SNV	MADD	MADD:exonic:NM 003682.3	1	100	8	synonymous	NM 00368	c.1440T>C	p.(=)	-0.01				0.81(COSM41	DENN (AEX-3)	rs326217	0.3065	YES	2.742E-1 (pos.47303275)	acute myeloic	Electron tra	Rab guanyl-nucleotide exchange factor activity,activatic				T=0, C=82	T=0.0, C=1.0	0.00001	1333.74	82	T=0/0, C=27/5
chr8:145160646	C/C	T	C	SNV	MAF1	MAF1:exonic:NM 032272.4	1	100	2	synonymous	NM 03227	c.60T>C	p.(=)	-4.7				0.16(COSM41	Maf1 regulat	rs11136256	0.0012	YES	9.999E-1 (pos.14516064(	neoplasm	Maf1, S. cer	GABA receptor binding,RNA polymerase III			Benign	T=0, C=21	T=0.0, C=1.0	0.00001	281.235	21	T=0/0, C=17/4
chrX:27839572	C/C	T	C	SNV	MAGEB10	MAGEB10:exonic:NM 182506.3	1	100	3	missense	NM 18250	c.149T>C	p.Phe50Ser	-0.59		155		0(COSM4998:	MAGE family,	rs1368769	0.4679	YES	9.662E-2 (pos.27839572)	Merkel cell c	Melanoma antigen, family B, 10					T=0, C=40	T=0.0, C=1.0	0.00001	625.037	40	T=0/0, C=15/2
chrX:27839616	CA/CA	C	CA	INDEL	MAGEB10	MAGEB10:exonic:NM 182506.3	1	100	3	frameshiftInsertion	NM 18250	c.193 194	p.Arg65fs	-1.1				0.01(COSM55	MAGE family,	rs777281486			0.0 (pos.27839616)	malignant me	Melanoma antigen, family B, 10					C=0, CA=42	C=0.0, CA=1.0	0.00001	540.78	42	C=0/0, CA=11/
chr19:44585159	A/A	T	A	SNV	ZNF284	ZNF284:intronic:NM 001037813.3	1	100		intronic	NM 00103	c.16-9T>A	p.?	-1.34					C2H2-type zir	rs4803674	0.1236	YES	5.886E-1 (pos.44585159)			DNA binding,DNA binding transcription factor activity,me				T=0, A=33	T=0.0, A=1.0	0.00001	504.583	33	T=0/0, A=13/2
chrX:35820696	A/A	G	A	SNV	MAGEB16	MAGEB16:exonic:NM 001099921.1	1	100	2	missense	NM 00109	c.383G>A	p.Cys128Tyr	1.39		194		0(COSM5905	MAGE family,	rs1410962	0.4639	YES	7.666E-1 (pos.35820696)	basal cell car	Melanoma antigen, family B, 16					G=0, A=29	G=0.0, A=1.0	0.00001	427.602	29	G=0/0, A=16/1
chrX:35820795	G/G	A	G	SNV	MAGEB16	MAGEB16:exonic:NM 001099921.1	1	100	2	missense	NM 00109	c.482A>G	p.His161Arg	-1.03		29		0(COSM1507	MAGE family,	rs5973488	0.4647	YES	7.646E-1 (pos.35820795)	adenocarcinoi	Melanoma antigen, family B, 16					A=0, G=30	A=0.0, G=1.0	0.00001	446.639	30	A=0/0, G=15/1
chr19:44604914	C/C	T	C	SNV	ZNF224	ZNF224:intronic:NM 013398.3	1	100		intronic	NM 01339	c.16-40T>(	p.?	-3.04					C2H2-type zir	rs62115526	0.1824	YES	5.34E-1 (pos.44604914)		Zinc finger	DNA binding transcription factor activity,intracellular,m				T=0, C=46	T=0.0, C=1.0	0.00001	768.041	46	T=0/0, C=29/1
chrX:35821304	AT/AT	A	AT	INDEL	MAGEB16	MAGEB16:utr 3:NM 001099921.1	1	100	2	utr 3	NM 00109	c.991 992	p.?	-0.02					MAGE family,Melanoma associated antigen family N terminal						Melanoma antigen, family B, 16					A=0, AT=79	A=0.0, AT=1.0	0.00001	487.374	79	A=0/0, AT=38/
chrX:30254361	A/A	G	A	SNV	MAGEB3	MAGEB3:exonic:NM 002365.4	1	100	5	missense	NM 00236	c.320G>A	p.Arg107His	-3.47		29		0(COSM1132	MAGE family,	rs2071308	0.4466	YES	5.49E-1 (pos.30254361)	adenocarcinoi	Melanoma antigen, family B, 3					G=0, A=50	G=0.0, A=1.0	0.00001	852.739	50	G=0/0, A=28/2
chrX:30254376	C/C	T	C	SNV	MAGEB3	MAGEB3:exonic:NM 002365.4	1	100	5	missense	NM 00236	c.335T>C	p.Ile112Thr	-2.95		89		0(COSM1467	MAGE family,	rs2071309	0.1029	YES	5.708E-1 (pos.30254376)	neoplasm	Melanoma antigen, family B, 3					T=0, C=47	T=0.0, C=1.0	0.00001	789.328	47	T=0/0, C=25/2
chrX:30261002	G/G	A	G	SNV	MAGEB4	MAGEB4:exonic:NM 002367.3	1	100	1	synonymous	NM 00236	c.750A>G	p.(=)	-0.87				0(COSM1132	MAGE family,	rs2071311	0.103	YES	5.664E-1 (pos.30261002)	acute myeloic	Melanoma	cytoplasm,protein binding				A=0, G=21	A=0.0, G=1.0	0.00001	281.235	21	A=0/0, G=12/9
chrX:30261158	C/C	G	C	SNV	MAGEB4	MAGEB4:exonic:NM 002367.3	1	100	1	synonymous	NM 00236	c.906G>C	p.(=)	-1.32				0(COSM4002	MAGE family,	rs2856733	0.103	YES	5.649E-1 (pos.30261158)	acute myeloic	Melanoma	cytoplasm,protein binding				G=0, C=50	G=0.0, C=1.0	0.00001	743.032	50	G=0/0, C=39/1
chrX:140983127	A/A	G	A	SNV	MAGEC3	MAGEC3:exonic:NM 138702.1	1	100	5	missense	NM 13870	c.982G>A	p.Ala328Thr	0.47	0	58	0.98	0.01(COSM1466088),0.01(C		rs176026	0.0709	YES	4.74E-1 (pos.140983127)	acute myeloic	Melanoma antigen, family C, 3					G=0, A=53	G=0.0, A=1.0	0.00001	782.03	53	G=0/0, A=27/2
chr7:78119109	C/T	C	T	SNV	MAGI2	MAGI2:exonic:NM 012301.3	1	50	6	synonymous	NM 01230	c.1014G>A	p.(=)	1.82				0.7(COSM576	Guanylate kir	rs3735442	0.3036	YES	2.36E-1 (pos.78119109)	adenocarcinoi	Membrane-	SMAD binc	Adenocarcinoma;Carcinoma;C		Likely benign	C=11, T=11	C=0.5, T=0.5	0.00001	94.586	22	C=10/1, T=6/5
chrX:49021537	C/C	G	C	SNV	MAGIX	MAGIX:exonic:NM 024859.3	1	100	5	missense	NM 02485	c.517G>C	p.Val173Leu	2.46	1	32	0.001	0.01(COSM41	PDZ domain (	rs5905720,rs782156-	0.0017		0.0 (pos.49021533),9.99!	neoplasm						G=0, C=53	G=0.0, C=1.0	0.00001	782.025	53	G=0/0, C=25/2
chr6:10764727	G/G	A	G	SNV	MAK	MAK:exonic:NM 001242957.2	1	100	14	synonymous	NM 00124	c.1905T>C	p.(=)	-0.31					Protein kinas	rs126405	0.2309	YES	9.245E-1 (pos.10764727)		Male germ	ATP bindin	Congenital, Hereditary, and Ne		Benign	A=0, G=49	A=0.0, G=1.0	0.00001	831.633	49	A=0/0, G=38/1
chr19:44896487	C/C	G	C	SNV	ZNF285	ZNF285:intronic:NM 001291488.1	1	100		intronic	NM 00129	c.163+17C	p.?	0.09						rs2571123	0.1522	YES	2.346E-1 (pos.44896487)			DNA binding,intracellular,metal ion binding,nucleic acid				G=0, C=25	G=0.0, C=1.0	0.00001	353.018	25	G=0/0, C=10/1
chr19:4491476	C/C	G	C	SNV	HDGFL2	HDGFL2:intronic:NM 001001520.2	1	100		intronic	NM 00100	c.490-87G	p.?	-0.89					Lens epitheli	rs11878416		YES				nucleus,positive regulation of cell growth,protein bindin				G=0, C=31	G=0.0, C=1.0	0.00001	465.785	31	G=0/0, C=15/1
chr9:72741312	A/A	G	A	SNV	MAMDC2	MAMDC2:exonic:NM 153267.4	1	100	6	missense	NM 15326	c.881G>A	p.Ser294Asn	0.77	0.9	46	0.011	0.22(COSM41	MAM domain	rs1998972	0.0628	YES	9.998E-1 (pos.72741312)	neoplasm	MAM doma	endoplasmic reticulum,membrane,proteinaceous extra				G=0, A=33	G=0.0, A=1.0	0.00001	504.597	33	G=0/0, A=18/1
chr11:95825797	A/G	A	G	SNV	MAML2	MAML2:exonic:NM 032427.3	1	98.41	2	synonymous	NM 03242	c.1398T>C	p.(=)	-0.74				0.04(COSM37	MamL-1 dom	rs7931870	0.4217	YES	1.942E-1 (pos.95825797)	clear cell ren	Mastermin	Notch sign	Arthritis:Arthritis, Juvenile:Autoimmune Dise			A=1, G=62	A=0.0159, G=0.9841	0.00001	925.354	63	A=0/1, G=27/3
chrX:149680554	T/T	C	T	SNV	MAMLD1	MAMLD1:exonic:NM 001177465.2	1	100	5	synonymous	NM 00117	c.2208C>T	p.(=)	0.04				0.01(COSM456959)		rs2070779		YES	5.447E-1 (pos.14968055	neoplasm,squ	Mastermin	Golgi appa	Congenital Abnormalities:Congenital, Heredi			C=0, T=28	C=0.0, T=1.0	0.00001	407.776	28	C=0/0, T=15/1
chr19:45162189	C/A	C	A	SNV	PVR	PVR:intronic:NM 006505.4	1	50		intronic	NM 00650	c.1150+21	p.?	0.66				0.09(COSM54	CD80-like C2-	rs35385129	0.1607	YES	3.67E-1 (pos.45162189)	adenocarcinoi	Polio virus	adherens	Autoimmune Disea	MYRISTIC ACID,Sphingosi		C=10, A=10	C=0.5, A=0.5	0.00001	82.886	20	C=5/5, A=5/5
chr4:6623032	G/T	G	T	SNV	MAN2B2	MAN2B2:utr 3:NM 015274.2	1	96.77	19	utr 3	NM 01527	c.3050G>T	p.?	-0.31					Alpha manno	rs4234768	3.00E-04	YES	9.988E-1 (pos.6623032)			alpha-mannosidase activity,carbohydrate binding,extra				G=1, T=30	G=0.0323, T=0.9677	0.00001	435.399	31	G=1/0, T=15/1
chr12:12483713	A/G	A	G	SNV	MANSC1	MANSC1:exonic:NM 018050.2	1	57.14	4	synonymous	NM 01805	c.544T>C	p.(=)	0.27				0.04(COSM14	MANEC doma	rs4763282	0.245	YES	3.708E-1 (pos.12483713)	adenocarcinoma		integral component of membrane				A=30, G=40	A=0.4286, G=0.5714	0.00001	434.763	70	A=15/15, G=1
chr19:45488567	C/C	T	C	SNV	CLPTM1	CLPTM1:intronic:NM 001294.3	1	100		intronic	NM 00129	c.672+6T>	p.?	-1.38					Cleft lip and	rs204471	0.0443	YES	9.288E-1 (pos.45488567)		Cleft lip and	cell differe	Digestive System Diseases:Digestive System			T=0, C=20	T=0.0, C=1.0	0.00001	263.785	20	T=0/0, C=6/14
chr19:45573405	A/C	A	C	SNV	CLASRP	CLASRP:intronic:NM 007056.2	1	46.43		intronic	NM 00705	c.1957+43	p.?	-1.09					Alternative s	rs117782853	0.0222	YES	1.187E-4;0.0 (pos.45573405)			RNA splicing,mRNA processing,nucleus,protein binding				A=15, C=13	A=0.5357, C=0.4643	0.00001	117.993	28	A=6/9, C=6/7
chr1:232940974	G/A	G	A	SNV	MAP10	MAP10:exonic:NM 019090.2	1	47.37	1	missense	NM 01909	c.205G>A	p.Gly69Ser	0.18		56		0.07(COSM49	Domain of un	rs201625156	0		0.0 (pos.232940974)	rhabdomyosarcoma		cell division,centrosome,cytoplasmic microtubule,cytor				G=110, A=9	G=0.5263, A=0.4737	0.00001	1018.92	209	G=56/54, A=6
chr19:45681560	C/C	T	C	SNV	BLOC1S3,TRAPPC6A	TRAPPC6A:upstream:NM 024108.2, BLOC1S3:upstream:NM	1	100		intronic	NM 02410	c.-84A>G, (	p.?, p.?	-1.62						rs7254936					Biogenesis	BLOC-1 col	Adnexal Diseases;/	S-PALMITOYL-L-CYSTEINE		T=0, C=20	T=0.0, C=1.0	0.00001	263.781	20	T=0/0, C=12/8
chr1:232942028	A/G	A	G	SNV	MAP10	MAP10:exonic:NM 019090.2	1	45.83	1	missense	NM 01909	c.1259A>G	p.Asn420Ser	0.33		46			Domain of un	rs61739198	0.0012	YES	3.508E-2 (pos.232942028)			cell division,centrosome,cytoplasmic microtubule,cytor				A=13, G=11	A=0.5417, G=0.4583	0.00001	93.44	24	A=10/3, G=10/
chr19:46027752	T/T	C	T	SNV	VASP	VASP:intronic:NM 003370.3	1	100		intronic	NM 00337	c.957-76C	p.?	-1.15					VASP tetrame	rs4802260		YES			Vasodilator	SH3 domain binding,actin binding,actin cytoskeleton,act				C=0, T=54	C=0.0, T=1.0	0.00001	800.067	54	C=0/0, T=35/1
chr19:46121020	T/C	T	C	SNV	EML2	EML2:intronic:NM 001193268.2	1	45.45		intronic	NM 00119	c.1858-65	p.?	-0.17					HELP motif,W	rs12985660	0.4774	YES			Echinoderm	cytoplasm,integral component of membrane,microtubu				T=18, C=15	T=0.5455, C=0.4545	0.00001	143.132	33	T=7/11, C=6/9
chr19:46180150	G/G	A	G	SNV	GIPR	GIPR:intronic:NM 000164.3	1	100		intronic	NM 00016	c.634-57A	p.?	-0.35					7 transmemb	rs4803846		YES			Gastric inhi	G-protein	Bronchial Neoplasms;Carcinoma, Bronchoge			A=0, G=73	A=0.0, G=1.0	0.00001	1152.71	73	A=0/0, G=26/4
chr16:87436720	T/T	G	T	SNV	MAP1LC3B	MAP1LC3B:utr 3:NM 022818.4	1	100	4	utr 3	NM 02281	c.395G>T	p.?	-0.11					Autophagy pr	rs1054521	0.0614	YES	9.999E-1;0.0 (pos.87436720)		Microtubul	autophagosome,autophagosome assembly,autophagos				G=0, T=31	G=0.0, T=1.0	0.00001	465.819	31	G=0/0, T=19/1
chr19:46205050	C/C	T	C	SNV	QPCTL	QPCTL:intronic:NM 017659.3	1	100		intronic	NM 01765	c.887-6T>(	p.?	-0.43				0.01(COSM54	Peptidase fai	rs4803848	0.348	YES	7.915E-1 (pos.46205050)	squamous cell carcinoma		Golgi apparatus,Golgi membrane,glutaminyl-peptide cy(				T=0, C=26	T=0.0, C=1.0	0.00001	371.377	26	T=0/0, C=12/1

chr19:46332194	T/T	C	T	SNV	SYMPK	SYMPK:intronic:NM 004819.2	1	100		intronic	NM 00481	c.1985+34	p.?	-0.57					Domain of un	rs3745929	0.147	YES	8.984E-1 (pos.46332194)			bicellular Digestive System Diseases;Digestive System	C=0, T=31	C=0.0, T=1.0	0.00001	465.819	31	C=0/0, T=21/1
chr19:17837425	C/G	C	G	SNV	MAP1S	MAP1S:exonic:NM 018174.5	1	52.38	5	missense	NM 01817	c.1232C>G	p.Ser411Cys	7.63	0.01	112	1	0.99(COSM3749486)		rs17710707,rs78125	0.1553	YES	7.113E-2:0.0 (pos.17837	adenocarcino	Microtubul	DNA binding,actin filament binding,apoptotic process,a	C=40, G=44	C=0.4762, G=0.5238	0.00001	482.866	84	C=27/13, G=18
chr19:4117621	G/C	G	C	SNV	MAP2K2	MAP2K2:exonic:NM 030662.3	1	47.62	2	missense	NM 03066	c.99C>G	p.Asn33Lys	2.26	0	94	0.469		Protein kinas	rs940667388					Mitogen-ac	ATP bindin Abnormalities, Mul Bosutinib,Trametinib	G=11, C=1	G=0.5238, C=0.4762	0.00001	82.2792	21	G=3/8, C=6/4
chr17:21204266	C/C	T	C	SNV	MAP2K3	MAP2K3:exonic:NM 145109.2	1	100	5	synonymous	NM 14510	c.360T>C	p.(=)	0.27				0.31(COSM54	Protein kinas	rs2230435	0.4869	YES	5.23E-1 (pos.21204266)	squamous_cell_carcinoma		ATP binding,MAP kinase kinase activity,MAPK cascade,a	T=0, C=65	T=0.0, C=1.0	0.00001	993.535	65	T=0/0, C=43/2
chr5:56177443	A/A	G	A	SNV	MAP3K1	MAP3K1:exonic:NM 005921.1	1	100	14	missense	NM 00592	c.2416G>A	p.Asp806Asn	8.1	0	23	0.999	0.98(COSM40	Protein kinas	rs702689	0.2792	YES	2.066E-1 (pos.56177443)	acute_myeloid	Mitogen-ac	ATP bindin 46, XY Disorders of AZD-8330	G=0, A=90	G=0.0, A=1.0	0.00001	1493.12	90	G=0/0, A=46/4
chr5:56178217	C/C	A	C	SNV	MAP3K1	MAP3K1:exonic:NM 005921.1	1	100	14	synonymous	NM 00592	c.3190A>C	p.(=)	3.42				0.18(COSM41	Protein kinas	rs832583	0.1767	YES	5.712E-1;0.0 (pos.56178	acute_myeloid	Mitogen-ac	ATP bindin 46, XY Disorders of AZD-8330	A=0, C=53	A=0.0, C=1.0	0.00001	782.029	53	A=0/0, C=21/3
chr19:47030044	A/G	A	G	SNV	PPP5D1	PPP5D1:intronic:NM 001205281.1	1	50		intronic	NM 00120	c.235+138	p.?	-2.46					PPP5 TPR rep	rs3745793		YES					A=100, G=1	A=0.5, G=0.5	0.00001	1121.03	200	A=60/40, G=41
chr19:47111663	C/C	T	C	SNV	CALM3	CALM3:intronic:NM 005184.3	1	100		intronic	NM 00518	c.178+66T	p.?	-1.22					Ca2+ insensit	rs3729760		YES			Calmodulin	Fc-epsilon receptor signaling pathway,G2/M transition o	T=0, C=21	T=0.0, C=1.0	0.00001	281.235	21	T=0/0, C=8/13
chr17:43342141	C/C	G	C	SNV	MAP3K14,MAP3K14-AS1	MAP3K14-AS1:intronic_nc:NR 024435.2, MAP3K14:exonic:N	1	100	16	synonymous	NR 02443	c.2705C>G	p.(=)	-0.07					Protein kinas	rs1047833	0.2765	YES	7.308E-1 (pos.43342141)		Mitogen-ac	ATP binding,I-kappaB kinase/NF-kappaB signaling,MAP ki	G=0, C=55	G=0.0, C=1.0	0.00001	814.906	55	G=0/0, C=19/3
chr19:47193850	C/C	T	C	SNV	PRKD2	PRKD2:intronic:NM 016457.4	1	100		intronic	NM 01645	c.1809+7A	p.?	-1.04				0.03(COSM14	Lipopolysacc	rs314671,rs3868098	0.2608	YES	5.638E-1:0.0 (pos.47193	adenocarcino	Protein kin	ATP bindin Adnexal Diseases:Autoimmune Diseases:Cys	T=0, C=30	T=0.0, C=1.0	0.00001	446.615	30	T=0/0, C=17/1
chr19:47935352	C/C	T	C	SNV	SLC8A2	SLC8A2:intronic:NM 015063.2	1	100		intronic	NM 01506	c.2389+72	p.?	-0.17					C-terminal ex	rs4801722			1.0 (pos.47935352)		Solute carri	basolatera Brain Diseases:Cardiovascular Diseases:Cen	T=0, C=42	T=0.0, C=1.0	0.00001	684.496	42	T=0/0, C=32/1
chr2:135745545	C/T	C	T	SNV	MAP3K19	MAP3K19:exonic:NM 025052.4	1	57.14	7	synonymous	NM 02505	c.897G>A	p.(=)	0.2				0(COSM4415	Protein kinas	rs11899779,rs76486	0.2299	YES	0.0 (pos.135745539),5.3	acute_myeloid_leukaemia		ATP binding,activation of protein kinase activity,cytopla	C=18, T=24	C=0.4286, T=0.5714	0.00001	278.976	42	C=16/2, T=15/
chr2:174128513	C/T	C	T	SNV	MAP3K20,MAP3K20-AS1	MAP3K20:exonic:NM 016653.2, MAP3K20-AS1:intronic_nc:	1	50	19	missense	NM 01665	c.1592C>T	p.Ser531Leu	0.55	0.15	145	0.002	0.17(COSM37	Protein kinas	rs3769148	0.4924	YES	2.524E-1:0.0 (pos.174128	adenocarcinoma		ATP binding,DNA damage checkpoint,MAP kinase kinase	C=11, T=11	C=0.5, T=0.5	0.00001	94.6048	22	C=3/8, T=8/3
chr6:137113137	A/A	G	A	SNV	MAP3K5	MAP3K5:exonic:NM 005923.3	1	100	1	synonymous	NM 00592	c.159C>T	p.(=)	0.18				0.17(COSM37	Domain of un	rs55666429	0.403	YES	5.615E-1 (pos.137113137	NS,adenocarci	Mitogen-ac	ATP bindin Basal Ganglia Diseases;Brain Diseases;Brain	G=0, A=36	G=0.0, A=1.0	0.00001	563.71	36	G=0/0, A=16/2
chr19:48254392	T/C	T	C	SNV	NOP53	NOP53:intronic:NM 015710.4	1	59.09		intronic	NM 01571	c.598+28T	p.?	-3.49					Nop53 (60S ri	rs10402957	0.2936	YES	5.615E-1 (pos.48254392)			5S rRNA binding,DNA repair,RNA binding,cellular respons	T=17, C=26	T=0.3953, C=0.6047	0.00001	325.458	44	T=2/15, C=3/2
chr6:137026266	T/G	T	G	SNV	MAP3K5	MAP3K5:exonic:NM 005923.3	1	97.92	3	synonymous	NM 00592	c.594A>C	p.(=)	0.32				0.11(COSM44	Domain of un	rs9321567	0.488	YES	5.615E-1 (pos.13702626	hepatocellula	Mitogen-ac	ATP bindin Basal Ganglia Diseases;Brain Diseases;Brain	T=1, G=47	T=0.0208, G=0.9792	0.00001	780.598	48	T=0/1, G=17/3
chr19:48258551	T/C	T	C	SNV	NOP53,SNORD23	SNORD23:upstream:NR 003048.1, NOP53:intronic:NM 015	1	50		intronic	NM 01571	c.1054-54	p.?	0.41					Nop53 (60S ri	rs10406879		YES				5S rRNA binding,DNA repair,RNA binding,cellular respons	T=13, C=13	T=0.5, C=0.5	0.00001	119.166	26	T=2/11, C=8/5
chr19:48282077	C/CGCAGCGG	C	CGCAGCGG	INDEL	SELENOW	SELENOW:intronic:NM 003009.3	7	50		intronic	NM 00300	c.29+6C>C	p.?	0.16					Rdx family	rs111841672,rs8670	0.3334	YES	4.933E-1;0.0;0.0 (pos.48282077)		Selenoprot	antioxidant activity,cellular oxidant detoxification,cytop	C=13, CGC	C=0.5, CGCAGCGG=0	0.00001	129.442	26	C=4/9, CGCAG
chr1:27687466	G/T	G	T	SNV	MAP3K6	MAP3K6:exonic:NM 004672.4	1	54.46	14	missense	NM 00467	c.1866C>A	p.Asn622Lys	0.03	0.03	94	0.001	0.01(COSM15	Domain of un	rs35659744	0.2959	YES	1.118E-1 (pos.27687466)	adenocarcino	Mitogen-ac	ATP bindin Digestive System Diseases;Dig Benign	G=51, T=61	G=0.4554, T=0.5446	0.00001	655.753	112	G=17/34, T=27
chr3:47956424	C/T	C	T	SNV	MAP4	MAP4:exonic:NM 002375.4	1	58.33	8	missense	NM 00237	c.1882G>A	p.Val628Ile	-0.08	0.21	29	0.49	0.04(COSM48	Tau and MAP	rs1137524	0.3135	YES	2.847E-1 (pos.47956424)	squamous_cel	Microtubul	RNA binding,axoneme,cell divi Docetaxel,Paclitaxel	C=10, T=14	C=0.4167, T=0.5833	0.00001	135.334	24	C=5/5, T=10/4
chr3:47958037	G/T	G	T	SNV	MAP4	MAP4:exonic:NM 002375.4	1	50	7	missense	NM 00237	c.1280C>A	p.Ser427Tyr	1.87	0	144	0.319	0.45(COSM41	Tau and MAP	rs1060407	0.3093	YES	2.647E-1 (pos.47958037)	neoplasm	Microtubul	RNA binding,axoneme,cell divi Docetaxel,Paclitaxel	G=16, T=16	G=0.5, T=0.5	0.00001	158.383	32	G=7/9, T=9/7
chr19:48544753	A/A	G	A	SNV	CABP5	CABP5:intronic:NM 019855.4	1	100		intronic	NM 01985	c.94+77C>	p.?	-1.24					EF hand,EF-ha	rs3745745	0.0739	YES			Calcium-bir	calcium ion binding,cytosol,protein binding,signal transc	G=0, A=37	G=0.0, A=1.0	0.00001	583.706	37	G=0/0, A=32/5
chrX:20134980	A/A	G	A	SNV	MAP7D2	MAP7D2:exonic:NM 001168465.1	1	100	1	synonymous	NM 00116	c.18C>T	p.(=)	-2.04					MAP7 (E-MAP	rs17523345	0.2436	YES	2.481E-2 (pos.20134980)			microtubule cytoskeleton,microtubule cytoskeleton org	G=0, A=20	G=0.0, A=1.0	0.00001	263.736	20	G=0/0, A=8/12
chr22:50705466	A/G	A	G	SNV	MAPK11	MAPK11:exonic:NM 002751.6	1	96.67	7	synonymous	NM 00275	c.507T>C	p.(=)	-1.02					Lipopolysacc	rs760748	0.0056		1.0;0.0 (pos.50705466)			ATP bindin Cardiomyopathies; KC706,Regorafenib	A=1, G=29	A=0.0333, G=0.9667	0.00001	416.07	30	A=1/0, G=12/1
chr19:48601350	C/C	T	C	SNV	PLA2G4C	PLA2G4C:intronic:NM 003706.2	1	100		intronic	NM 00370	c.568+46A	p.?	0.13					Lysophospho	rs251685	0.3617	YES	7.349E-1;0.0 (pos.48601350)		Phospholip	arachidonic acid metabolic process,calcium-independe	T=0, C=23	T=0.0, C=1.0	0.00001	316.737	23	T=0/0, C=13/1
chr22:51042513	CG/CG	C	CG	INDEL	MAPK8IP2	MAPK8IP2:exonic:NM 012324.5	1	100	5	unknown	NM 012324.5			1.81				0.75(COSM11	Phosphotyro	rs140511	0.0062	YES	1.0 (pos.51042513)	small_cell_car	Mitogen-ac	JNK cascade,MAP-kinase scaffold activity,MAPK cascade	C=0, CG=29	C=0.0, CG=1.0	0.00001	427.603	29	C=0/0, CG=12/
chr19:48601528	T/T	G	T	SNV	PLA2G4C	PLA2G4C:intronic:NM 003706.2	1	100		intronic	NM 00370	c.448-12C	p.?	-0.58					Lysophospho	rs251683	0.3735	YES	7.336E-1 (pos.48601528)		Phospholip	arachidonic acid metabolic process,calcium-independe	G=0, T=23	G=0.0, T=1.0	0.00001	316.746	23	G=0/0, T=11/1
chr19:48640234	C/G	C	G	SNV	LIG1	LIG1:intronic:NM 000234.2	1	50		intronic	NM 00023	c.1331+31	p.?	0.13					ATP depende	rs2288880	0.0536	YES	2.231E-1 (pos.48640234)		Leucine-ric	ATP bindin Female Urogenital Bleomycin	C=17, G=17	C=0.5, G=0.5	0.00001	172.014	34	C=10/7, G=12/
chr20:31427635	T/T	C	T	SNV	MAPRE1	MAPRE1:exonic:NM 012325.2	1	100	5	synonymous	NM 01232	c.570C>T	p.(=)	-0.01				0.69(COSM44	Calponin hon	rs2070090	0.1671	YES	3.475E-1 (pos.31427635)	acute_myeloid	Microtubul	G2/M tran Cardiovascular Diseases;Heart Diseases;My	C=0, T=41	C=0.0, T=1.0	0.00001	664.699	41	C=0/0, T=24/1
chr2:27246285	T/T	C	T	SNV	MAPRE3	MAPRE3:exonic:NM 012326.3	1	100	3	synonymous	NM 01232	c.207C>T	p.(=)	-1.69				0.02(COSM54	Calponin hon	rs10865456	0.072	YES	8.358E-1 (pos.27246285)	acute_myeloid	Microtubul	cell cycle, Abortion, Spontaneous;Adnexal Diseases:Cy	C=0, T=38	C=0.0, T=1.0	0.00001	603.785	38	C=0/0, T=16/2
chr14:103934488	C/C	T	C	SNV	MARK3	MARK3:exonic:NM 001128918.2	1	100	12	missense	NM 00112	c.1229T>C	p.Phe410Ser	5.87	1	155	0	0.26(COSM45	Kinase assoc	rs10137161		YES	1.0 (pos.103934488)	squamous_cel	MAP/micro	ATP binding,MAPK cascade,cytosol,establishment of cell	T=0, C=82	T=0.0, C=1.0	0.00001	1323.13	82	T=0/0, C=42/4
chr3:186954285	G/G	A	G	SNV	MASP1	MASP1:exonic:NM 139125.3	1	100	11	synonymous	NM 13912	c.1374T>C	p.(=)	-4.53					CUB domain,	rs710452	0.0012	YES	9.997E-1 (pos.186954285)		Mannan-bir	calcium io Abnormalities, Multiple:Amyotrophic Lateral	A=0, G=37	A=0.0, G=1.0	0.00001	582.352	37	A=0/0, G=24/1
chr1:46496709	G/G	A	G	SNV	MAST2	MAST2:exonic:NM 015112.2	1	100	23	synonymous	NM 01511	c.2739A>G	p.(=)	-3.13				0.05(COSM37	Domain of un	rs925524	0.2813	YES	6.837E-1 (pos.46496709)	carcinoma,he	Microtubul	ATP binding,cytoplasm,cytoskeleton organization,intrac	A=0, G=58	A=0.0, G=1.0	0.00001	867.375	58	A=0/0, G=35/2
chr1:46500613	C/C	T	C	SNV	MAST2	MAST2:exonic:NM 015112.2	1	100	29	synonymous	NM 01511	c.4272T>C	p.(=)	0.2				0.43(COSM42	Domain of un	rs17855317	0.2603	YES	1.772E-2 (pos.46500613)	neoplasm	Microtubul	ATP binding,cytoplasm,cytoskeleton organization,intrac	T=0, C=51	T=0.0, C=1.0	0.00001	754.579	51	T=0/0, C=19/3
chr19:48954571	T/T	A	T	SNV	GRWD1	GRWD1:intronic:NM 031485.3	1	100		intronic	NM 03148	c.1023+83	p.?	-2.61					Histone-bind	rs1799274		YES			Glutamate-	DNA replication,DNA replication origin binding,RNA bind	A=0, T=25	A=0.0, T=1.0	0.00001	353.001	25	A=0/0, T=17/8
chr19:49116134	G/G	T	G	SNV	FAM83E	FAM83E:intronic:NM 017708.3	1	100		intronic	NM 01770	c.465+31A	p.?	-0.14					Protein of un	rs2617800	0.0444	YES	9.97E-1 (pos.49116134)			protein kinase binding	T=0, G=31	T=0.0, G=1.0	0.00001	464.829	31	T=0/0, G=14/1
chr19:18248146	C/C	T	C	SNV	MAST3	MAST3:exonic:NM 015016.1	1	100	18	synonymous	NM 01501	c.1983T>C	p.(=)	-6.11					Domain of un	rs541225	0.0645	YES	9.653E-1 (pos.18248146)		Microtubul	ATP binding,cytoskeleton organization,intracellular,intra	T=0, C=58	T=0.0, C=1.0	0.00001	870.728	58	T=0/0, C=19/3
chr19:49216926	G/G	A	G	SNV	MAMSTR	MAMSTR:intronic:NM 001130915.1	1	100		intronic	NM 00113	c.964+47T	p.?	-0.11					SAP domain	rs281384	0.057	YES	8.803E-1 (pos.49216926)		MEF2-activa	nucleus,positive regulation of myotube differentiation,p	A=0, G=29	A=0.0, G=1.0	0.00001	427.603	29	A=0/0, G=22/7
chr19:49217605	G/G	T	G	SNV	MAMSTR	MAMSTR:intronic:NM 001130915.1	1	100		intronic	NM 00113	c.723+18A	p.?	-2.42					SAP domain	rs281388	0.0427	YES	8.669E-1 (pos.49217605)		MEF2-activa	nucleus,positive regulation of myotube differentiation,p	T=0, G=23	T=0.0, G=1.0	0.00001	316.747	23	T=0/0, G=20/3
chr19:18234441	C/T	C	T	SNV	MAST3	MAST3:exonic:NM 015016.1	1	50	7	synonymous	NM 01501	c.522C>T	p.(=)	-2.44				0.02(COSM37	Domain of un	rs740691	0.4674	YES	6.805E-1 (pos.18234441)	adenocarcino	Microtubul	ATP binding,cytoskeleton organization,intracellular,intra	C=25, T=25	C=0.5, T=0.5	0.00001	288.837	50	C=10/15, T=17
chr19:49300605	C/C	T	C	SNV	BCAT2	BCAT2:intronic:NM 001190.3	1	100		intronic	NM 00119	c.696-15A	p.?	0.15					Amino-transf	rs526744	0.2488	YES	5.421E-1 (pos.49300605)		Branched cl	L-isoleucir Amino Acid Metabo Alpha-ketoisovalerate,G	T=0, C=37	T=0.0, C=1.0	0.00001	583.705	37	T=0/0, C=26/1
chr19:4944555	A/A	G	A	SNV	UHRF1	UHRF1:intronic:NM 001048201.2	1	100		intronic	NM 00104	c.1305+93	p.?	0.02					PHD-finger,S	rs2602730		YES			Ubiquitin-li	DNA bindi Adenocarcinoma;Carcinoma;Carcinoma, Ade	G=0, A=21	G=0.0, A=1.0	0.00001	281.211	21	G=0/0, A=14/7
chr19:49459104	G/G	A	G	SNV	BAX	BAX:intronic:NM 004324.3	1	100		intronic	NM 00432	c.233+14A	p.?	1					Apoptosis re	rs1805419	0.2797	YES	6.076E-1 (pos.49459104)		BCL2-assoc	B cell apo Acute Kidney Injury;Adnexal Diseases;Alzheir	A=0, G=29	A=0.0, G=1.0	0.00001	427.604	29	A=0/0, G=14/1
chr19:49464971	G/A	G	A	SNV	BAX	BAX:downstream:NM 004324.3	1	95.65		intronic	NM 00432	c.*617G>A	p.?	-0.53						rs704243		YES			BCL2-assoc	B cell apo Acute Kidney Injury:Adnexal Diseases:Alzheir	G=2, A=44	G=0.0435, A=0.9565	0.00001	707.349	46	G=1/1, A=16/2
chr5:66459878	G/C	G	C	SNV	MAST4	MAST4:exonic:NM 001164664.1	1	96.67	29	missense	NM 00116	c.4871G>C	p.Arg1624Pro	0.08	0.35	103	0	0.03(COSM37	Domain of un	rs1705399	0.0327	YES	9.035E-1:0.0 (pos.66459	adenocarcinoma		Brain Diseases:Central Nervous System Dise	G=1, C=29	G=0.0333, C=0.9667	0.00001	414.749	30	G=1/0, C=20/9
chr19:49643404	C/C	T	C	SNV	PPFIA3	PPFIA3:intronic:NM 003660.3	1	100		intronic	NM 00366	c.2368+59	p.?	-1.08					SAM domain	rs3764624		YES			Protein tyro	acrosomal vesicle,cytosol,epididymosome,glutamate se	T=0, C=48	T=0.0, C=1.0	0.00001	805.65	48	T=0/0, C=21/2
chr19:49644593	C/T	C	T	SNV	PPFIA3	PPFIA3:intronic:NM 003660.3	1	45.83		intronic	NM 00366	c.2369-84	p.?	0.06					SAM domain	rs7250958		YES			Protein tyro	acrosomal vesicle,cytosol,epididymosome,glutamate se	C=13, T=11	C=0.5417, T=0.4583	0.00001	93.44	24	C=11/2, T=7/4
chr8:98954012	C/C	T	C	SNV	MATN2	MATN2:exonic:NM 002380.4	1	100	4	synonymous	NM 00238	c.720T>C	p.(=)	0.96					Calcium-bind	rs2513845	0.0021	YES	9.219E-1 (pos.98954012)		Matrilin 2	axon guidance,basement membrane,biological_process	T=0, C=28	T=0.0, C=1.0	0.00001	408.715	28	T=0/0, C=20/8
chr19:49671771	C/T	C	T	SNV	TRPM4	TRPM4:intronic:NM 017636.3	1	50		intronic	NM 01763	c.613-39C	p.?	-0.99					Ion transport	rs113159745	0.0186	YES	6.938E-4 (pos.49671771)		Transient re	ATP bindin Arrhythmias, Cardiac:Brugada Syndrome:Car	C=19, T=19	C=0.5, T=0.5	0.00001	199.992	38	C=10/9, T=5/1
chr8:98991221	G/G	A	G	SNV	MATN2	MATN2:exonic:NM 002380.4	1	100	6	missense	NM 00238	c.1066A>G	p.Lys356Glu	0.08	0.29	56	0.005	0.07(COSM63	Calcium-bind	rs1869609	0.3418	YES	2.717E-1 (pos.98991221)	carcinoma	Matrilin 2	axon guidance,basement membrane,biological_process	A=0, G=60	A=0.0, G=1.0	0.00001	888.206	60	A=0/0, G=35/2
chr8:99006736	T/T	C	T	SNV	MATN2	MATN2:exonic:NM 002380.4	1	100	7	synonymous	NM 00238	c.1110C>T	p.(=)	-2.23				0.01(COSM44	Calcium-bind	rs11559202	0.1732	YES	7.837E-2 (pos.99006736)	acute_myeloid	Matrilin 2	axon guidance,basement membrane,biological_process	C=0, T=47	C=0.0, T=1.0	0.00001	789.328	47	C=0/0, T=40/7
chr8:99006748	T/T	C	T	SNV	MATN2	MATN2:exonic:NM 002380.4	1	100	7	synonymous	NM 00238	c.1122C>T	p.(=)	-2.72				0.01(COSM44	Calcium-bind	rs11559201	0.1734	YES	7.837E-2 (pos.99006748)	acute_myeloid	Matrilin 2	axon guidance,basement membrane,biological_process	C=0, T=47	C=0.0, T=1.0	0.00001	789.327	47	C=0/0, T=40/7
chr19:49935927	AG/AG	A	AG	INDEL	SLC17A7	SLC17A7:intronic:NM 020309.3	1	100		intronic	NM 02030	c.1018-19	p.?	-1.3					Major Facilit	rs398034942,rs5684	5.00E-04		1.0 (pos.49935927)		Solute carri	L-glutama Brain Diseases:Central Nervous System Dise	A=0, AG=31	A=0.0, AG=1.0	0.00001	416.42	31	A=0/0, AG=21/
chr8:99030280	C/C	T	C	SNV	MATN2	MATN2:exonic:NM 002380.4	1	100	12	synonymous	NM 00238	c.1755T>C	p.(=)	-5.38					Calcium-bind	rs2248014,rs386728	0.2429	YES	3.88E-1 (pos.99030280)		Matrilin 2	axon guidance,basement membrane,biological_process	T=0, C=40	T=0.0, C=1.0	0.00001	644.28	40	T=0/0, C=23/1
chr19:19446936	A/A	G	A	SNV	MAU2	MAU2:exonic:NM 015329.3	1	100	3	synonymous	NM 01532	c.300G>A	p.(=)	-0.71				0.04(COSM44	Cohesin load	rs769267	0.3337	YES	6.893E-1 (pos.19446936)	acute_myeloid	MAU2 chro	SMC loadi Bipolar Disorder:Bipolar and Related Disorde	G=0, A=31	G=0.0, A=1.0	0.00001	465.819	31	G=0/0, A=18/1
chr19:8953801	C/C	T	C	SNV	MBD3L1	MBD3L1:exonic:NM 145208.2	1	100	1	synonymous	NM 14520	c.447T>C	p.(=)	-0.16					C-terminal dc	rs2969292	0.0702	YES	9.024E-1 (pos.8953801)		Methyl-CpG	methyl-CpG binding,methylation-dependent chromatin	T=0, C=39	T=0.0, C=1.0	0.00001	623.977	39	T=0/0, C=17/2
chr19:49950413	G/G	C	G	SNV	PIH1D1	PIH1D1:intronic:NM 017916.2	1	100		intronic	NM 01791	c.612-57G	p.?	-0.52					pre-RNA proc	rs2335090	0.3388	YES			PIH1 domai	ATPase binding,R2TP complex,RNA polymerase I CORE ele	C=0, G=21	C=0.0, G=1.0	0.00001	281.235	21	C=0/0, G=14/7
chr19:8953922	T/T	C	T	SNV	MBD3L1	MBD3L1:exonic:NM 145208.2	1	100	1	missense	NM 14520	c.568C>T	p.Arg190Cys	-1.99		180			C-terminal dc	rs2972588	0.0705	YES	8.991E-1 (pos.8953922)		Methyl-CpG	methyl-CpG binding,methylation-dependent chromatin	C=0, T=39	C=0.0, T=1.0	0.00001	623.978	39	C=0/0, T=17/2
chr10:54528266	C/C	G	C	SNV	MBL2	MBL2:exonic:NM 000242.2	1	100	4	synonymous	NM 00024	c.378C>G	p.(=)	-1.29				0.01(COSM36	Collagen trip	rs930507	0.175	YES	7.616E-1 (pos.54528266)	acute_myeloid	Mannose-b	acute-pha Autoimmune Disea Alpha-Met Benign/Likely	G=0, C=29	G=0.0, C=1.0	0.00001	427.603	29	G=0/0, C=7/22
chr19:50140209	T/T	C	T	SNV	RRAS	RRAS:intronic:NM 006270.4	1	100		intronic	NM 00627	c.242-26G	p.?	-3.1					ADP-ribosyla	rs1368463	0.3367	YES	8.179E-1;0.0 (pos.50140209)		Oncogene R	GDP bindi Bone Marrow Diseases;Genital Diseases, Ma	C=0, T=57	C=0.0, T=1.0	0.00001	849.612	57	C=0/0, T=34/2
chr19:50140246	A/A	C	A	SNV	RRAS	RRAS:intronic:NM 006270.4	1	100		intronic	NM 00627	c.241+54G	p.?	0.32					ADP-ribosyla	rs1865076	0.3378	YES			Oncogene R	GDP bindi Bone Marrow Diseases;Genital Diseases, Ma	C=0, A=74	C=0.0, A=1.0	0.00001	1170.13	74	C=0/0, A=31/4
chr16:84115393	A/A	G	A	SNV	MBTPS1	MBTPS1:exonic:NM 003791.3	1	100	11	synonymous	NM 00379	c.1407C>T	p.(=)	-0.72				0.18(COSM39	Subtilase fan	rs12933523	0.1807	YES	9.29E-2 (pos.84115393)	acute_myeloid	Membrane-	ATF6-medi Adenocarcinoma;Carcinoma;Carcinoma, Hep	G=0, A=68	G=0.0, A=1.0	0.00001	1053.56	68	G=0/0, A=32/3
chr16:89985844	G/T	G	T	SNV	MC1R	MC1R:exonic:NM 002386.3	1	50	1	missense	NM 00238	c.178G>T	p.Val60Leu	2.42		32		0.81(COSM3765964)		rs1805005	0.1334	YES	1.164E-4 (pos.89985844)	hepatocellula	Melanocort	G-protein Albinism:Albinism, Afamelanc Benign, assoc	G=12, T=12	G=0.5, T=0.5	0.00001	106.704	24	G=0/12, T=4/8
chr19:50309621	TC/T	TC	T	INDEL	AP2A1,FUZ	AP2A1:intronic:NM 014203.2, FUZ:downstream:NM 02512	1	50		intronic	NM 01420	c.2856+11	p.?, p.?	-1.1, -1.0					Adaptin C-ter	rs3217109					Adaptor-rel	AP-2 adap Abnormalities, Multiple;Congenital Abnorma	TC=11, T=1	TC=0.5, T=0.5	0.00001	87.7574	22	TC=0/11, T=4/
chr5:112384802	A/A	G	A	SNV	MCC	MCC:exonic:NM 001085377.1	1	100	16	synonymous	NM 00108	c.2643C>T	p.(=)	1.21				0.76(COSM41	EF-hand dom	rs6594664	0.0256	YES	9.481E-1:0.0 (pos.11238	neoplasm	Mutated in	Wnt signa Adenoma;Adenomatous Polyposis Coli;Aden	G=0, A=29	G=0.0, A=1.0	0.00001	427.6	29	G=0/0, A=12/1
chr5:112479004	C/C	T	C	SNV	MCC	MCC:exonic:NM 001085377.1	1	100	5	synonymous	NM 00108	c.795A>G	p.(=)	-4.67					EF-hand dom	rs2227949	1.00E-04		9.997E-1 (pos.112479004)		Mutated in	Wnt signa Adenoma;Adenomatous Polyposis Coli;Aden	T=0, C=40	T=0.0, C=1.0	0.00001	644.277	40	T=0/0, C=22/1
chr5:112439941	T/C	T	C	SNV	MCC	MCC:exonic:NM 001085377.1	1	98.36	7	missense	NM 00108	c.1139A>G	p.Lys380Arg	4.55	1	26	0		EF-hand dom	rs6594681	0		1.0 (pos.112439941)		Mutated in	Wnt signa Adenoma;Adenomatous Polyposis Coli;Aden	T=1, C=60	T=0.0164, C=0.9836	0.00001	891.193	61	T=1/0, C=28/3
chr19:5041316	T/G	T	G	SNV	KDM4B	KDM4B:intronic:NM 015015.2	1	52.38		intronic	NM 01501	c.432+54T	p.?	-1.55					JmjC domain	rs2240679		YES			Lysine-spec	cytosol,hi Glioma;Medulloblastoma;Neoplasms;Neopla	T=20, G=22	T=0.4762, G=0.5238	0.00001	235.84	42	T=13/7, G=14/
chr19:50462244	A/A	G	A	SNV	SIGLEC11	SIGLEC11:intronic:NM 001135163.1	1	100		intronic	NM 00113	c.1105+43	p.?	-2.71					Immunoglobu	rs1811375	0.3962	YES	5.074E-1 (pos.50462244)		Sialic acid-b	carbohydrate binding,cell adhesion,integral component	G=0, A=26	G=0.0, A=1.0	0.00001	375.746	26	G=0/0, A=13/1
chr3:182790249	A/A	G	A	SNV	MCCC1	MCCC1:exonic:NM 020166.4	1	100	5	synonymous	NM 02016	c.396C>T	p.(=)	0.85					ATP-grasp do	rs7622479	0.0692	YES	9.997E-1 (pos.182790249)		3-Methylcro	3-methylc 3-Methylcrotonyl-C Biotin Benign	G=0, A=21	G=0.0, A=1.0	0.00001	281.235	21	G=0/0, A=7/14
chr19:50750419	T/T	C	T	SNV	MYH14	MYH14:intronic:NM 001145809.1	1	100		intronic	NM 00114	c.1329+40	p.?	-0.34					IQ calmodulin	rs607227	0.1892	YES	7.944E-1 (pos.50750419)		Myosin, hea	ATP bindin Ear Diseases;Genit (3aS)-3a-hydroxy-1-phen	C=0, T=61	C=0.0, T=1.0	0.00001	921.839	61	C=0/0, T=34/2
chr5:70945075	G/G	A	G	SNV	MCCC2	MCCC2:exonic:NM 022132.4	1	100	14	synonymous	NM 02213	c.1368A>G	p.(=)	-1.22				0.07(COSM44	Carboxyl trar	rs10064079	0.1321	YES	9.838E-1 (pos.70945075)	hepatocellula	3-Methylcro	3-methylc 3-Methylcrotonyl-C Biotin Benign	A=0, G=39	A=0.0, G=1.0	0.00001	623.957	39	A=0/0, G=16/2
chr19:50766707	C/C	T	C	SNV	MYH14	MYH14:intronic:NM 001145809.1	1	100		intronic	NM 00114	c.2694+30	p.?	-0.31					IQ calmodulin	rs1529492	0.2479	YES	8.539E-1 (pos.50766707)		Myosin, hea	ATP bindin Ear Diseases;Genit (3aS)-3a-hydroxy-1-phen	T=0, C=26	T=0.0, C=1.0	0.00001	371.417	26	T=0/0, C=13/1
chr19:50771635	C/C	T	C	SNV	MYH14	MYH14:intronic:NM 001145809.1	1	100		intronic	NM 00114	c.3033+11	p.?	-1.03					IQ calmodulin	rs930086	0.1194	YES	8.518E-1 (pos.50771635)		Myosin, hea	ATP bindin Ear Diseases;Genit (3aS)-3a-h Benign	T=0, C=21	T=0.0, C=1.0	0.00001	281.14	21	T=0/0, C=7/14
chr19:50789967	A/A	G	A	SNV	MYH14	MYH14:intronic:NM 001145809.1	1	100		intronic	NM 00114	c.4752+16	p.?	-0.35					IQ calmodulin	rs10406788	0.1045	YES	2.783E-2 (pos.50789967)		Myosin, hea	ATP bindin Ear Diseases;Genit (3aS)-3a-h Benign	G=0, A=51	G=0.0, A=1.0	0.00001	754.584	51	G=0/0, A=8/43
chr6:31496776	G/G	T	G	SNV	MCCD1	MCCD1:utr_5:NM 001011700.2	1	100	1	utr_5	NM 00101	c.-16T>G	p.?	-0.8					Mitochondria	rs3130054,rs751443	0.18	YES	0.0;0.0;0.0 (pos.31496762),9.269E-1;2.3		Mitochondr	mitochondrion	T=0, G=75	T=0.0, G=1.0	0.00001	1189.83	75	T=0/0, G=35/4
chr19:51129064	C/C	A	C	SNV	SYT3	SYT3:intronic:NM 001160329.1	1	100		intronic	NM 00116	c.1402+90	p.?	0.17					C2 domain	rs7260283					Synaptotag	calcium ion binding,calcium ion regulated exocytosis,ca	A=0, C=89	A=0.0, C=1.0	0.00001	1472.48	89	A=0/0, C=53/3
chr3:182925404	C/C	T	C	SNV	MCF2L2	MCF2L2:exonic:NM 015078.3	1	100	23	missense	NM 01507	c.2704A>G	p.Thr902Ala	3.13	0.09	58	0		Divergent CR	rs6804951	0.0793	YES	1.0 (pos.182925404)			Rho guanyl-nucleotide exchange factor activity,regulatic	T=0, C=123	T=0.0, C=1.0	0.00001	1993.61	123	T=0/0, C=72/5
chr6:100368759	A/A	G	A	SNV	MCHR2	MCHR2:utr_3:NM 032503.2	1	100	6	utr_3	NM 03250	c.1080C>T	p.?	0					7 transmemb	rs4839764		YES			Melanin-co	G-protein coupled peptide receptor activity,G-protein cc	G=0, A=38	G=0.0, A=1.0	0.00001	600.886	38	G=0/0, A=22/1
chr5:54516521	A/A	G	A	SNV	MCIDAS	MCIDAS:exonic:NM 001190787.1	1	100	7	synonymous	NM 00119	c.831C>T	p.(=)	1.72					Geminin	rs6879219		YES	4.848E-1 (pos.54516521)			cell cycle, Bronchial Diseases;Bronchiect Benign	G=0, A=67	G=0.0, A=1.0	0.00001	1038.29	67	G=0/0, A=32/3
chr19:51361644	G/G	A	G	SNV	KLK3	KLK3:intronic:NM 001648.2	1	100		intronic	NM 00164	c.494-71A	p.?	-0.16					Trypsin,Tryps	rs1810020		YES			Kallikrein B	antibacte Genital Diseases, Male;Genital Neoplasms,	A=0, G=34	A=0.0, G=1.0	0.00001	524.177	34	A=0/0, G=16/1
chr19:5137846	A/A	G	A	SNV	KDM4B	KDM4B:intronic:NM 015015.2	1	100		intronic	NM 01501	c.2442-12	p.?	-0.41					JmjC domain	rs2613786		YES			Lysine-spec	cytosol,hi Glioma;Medulloblastoma;Neoplasms;Neopla	G=0, A=36	G=0.0, A=1.0	0.00001	563.744	36	G=0/0, A=23/1
chr21:47703649	A/A	G	A	SNV	MCM3AP	MCM3AP:exonic:NM 003906.4	1	100	2	synonymous	NM 00390	c.1323C>T	p.(=)	1.57				0.59(COSM37	Binding regio	rs11702450	0.3928	YES	1.379E-1 (pos.47703649)	adenocarcino	Minichrom	DNA bindi Endocrine Gland Neoplasms;Endocrine Syste	G=0, A=46	G=0.0, A=1.0	0.00001	768.291	46	G=0/0, A=26/2
chr19:51380109	G/GA	G	GA	INDEL	KLK2	KLK2:intronic:NM 005551.4	1	53.33		intronic	NM 00555	c.494-19G	p.?	-1.64					Trypsin,Tryps	rs112421583					Kallikrein-r	extracellu Genital Diseases, Male;Genital Neoplasms,	G=21, GA=2	G=0.4667, GA=0.533	0.00001	219.621	45	G=12/9, GA=1
chr19:51380110	T/A	T	A	SNV	KLK2	KLK2:intronic:NM 005551.4	1	50		intronic	NM 00555	c.494-18T	p.?	-0.13					Trypsin,Tryps	rs6070	0.3374	YES	2.163E-1 (pos.51380110)		Kallikrein-r	extracellu Genital Diseases, Male;Genital Neoplasms,	T=22, A=22	T=0.5, A=0.5	0.00001	245.53	44	T=14/8, A=13/
chr8:6302153	A/AT	A	AT	INDEL	MCPH1	MCPH1:exonic:NM 024596.4	1	95.45	8	frameshiftInsertion	NM 02459	c.910_911	p.Arg304fs	-1.14					BRCA1 C Term	rs747489687			0.0 (pos.6302146)		Microcepha	bone deve Congenital Abnormalities;Congenital, Heredi	A=1, AT=21	A=0.0455, AT=0.954	0.00001	147.689	22	A=1/0, AT=14/
chr19:51466516	G/G	A	G	SNV	KLK6	KLK6:intronic:NM 001012964.2	1	100		intronic	NM 00101	c.445+42T	p.?	-1.29					Domain of un	rs1654537	0.3347	YES	3.374E-1 (pos.51466516)		Kallikrein-r	amyloid precursor protein met Benzamidine	A=0, G=29	A=0.0, G=1.0	0.00001	427.581	29	A=0/0, G=8/21
chr19:51485194	T/T	C	T	SNV	KLK7	KLK7:intronic:NM 001243126.1	1	100		intronic	NM 00124	c.200+389	p.?	-4.42					Trypsin	rs1654523	0.0929	YES	9.993E-1 (pos.51485194)			epidermal lamellar body,epide L-alanyl-N-(1S,2R)-1-benz	C=0, T=23	C=0.0, T=1.0	0.00001	316.744	23	C=0/0, T=17/6
chr19:51518222	G/A	G	A	SNV	KLK10	KLK10:intronic:NM 001077500.1	1	52.38		intronic	NM 00107	c.679-14C	p.?	0.2					Trypsin	rs2075696	0.3307	YES	2.734E-1 (pos.51518222)		Kallikrein-r	cell cycle, Abortion, Spontaneous;Adnexal Diseases;Bre	G=10, A=11	G=0.4762, A=0.5238	0.00001	93.9071	21	G=6/4, A=7/4
chr6:37623626	C/T	C	T	SNV	MDGA1	MDGA1:exonic:NM 153487.3	1	53.33	4	synonymous	NM 15348	c.429G>A	p.(=)	-2.29				0.05(COSM41	Immunoglobu	rs13204070	0.1939	YES	1.374E-1 (pos.37623626)	neoplasm	MAM doma	C-terminal protein lipidation,anchored component of pl	C=21, T=24	C=0.4667, T=0.5333	0.00001	276.153	45	C=8/13, T=18/
chr2:207621759	G/G	A	G	SNV	MDH1B	MDH1B:exonic:NM 001039845.2	1	100	4	synonymous	NM 00103	c.276T>C	p.(=)	2.57						rs1990849	0		1.0 (pos.207621759)			L-malate dehydrogenase activity,NADH metabolic proce	A=0, G=27	A=0.0, G=1.0	0.00001	389.967	27	A=0/0, G=22/5
chr12:68708761	T/T	C	T	SNV	MDM1	MDM1:exonic:NM 017440.4	1	100	9	missense	NM 01744	c.1466G>A	p.Arg489His	-0.04	0.65	29	0	0(COSM3753	Nuclear prot	rs2306393	0.2858	YES	4.202E-1 (pos.68708761)	acute_myeloid	Mouse doul	centriole,centrosome,cytosol,microtubule,microtubule	C=0, T=29	C=0.0, T=1.0	0.00001	427.603	29	C=0/0, T=20/9
chr19:52004551	T/T	C	T	SNV	SIGLEC12	SIGLEC12:intronic:NM 053003.3	1	100		intronic	NM 05300	c.427+10G	p.?	-0.9				0(COSM1487	Immunoglobu	rs3810109	0.1691	YES	4.053E-1 (pos.52004551)	acute_myeloid	Sialic acid-b	carbohydrate binding,cell adhesion,integral component	C=0, T=26	C=0.0, T=1.0	0.00001	371.419	26	C=0/0, T=12/1
chr1:204518842	A/A	C	A	SNV	MDM4	MDM4:utr_3:NM 002393.4	1	100	11	utr_3	NM 00239	c.1505C>A	p.?	-0.96					SWIB/MDM2	rs4245739	0.2459	YES	9.673E-1 (pos.204518842)		Mouse doul	DNA dama Astrocytoma;Breast Diseases;Breast Neopla	C=0, A=26	C=0.0, A=1.0	0.00001	371.42	26	C=0/0, A=17/9
chr19:52031148	T/G	T	G	SNV	SIGLEC6	SIGLEC6:intronic:NM 001245.6	1	47.62		intronic	NM 00124	c.1107-66	p.?	-0.31					Immunoglobu	rs77020082		YES			Sialic acid-b	carbohydrate binding,cell adhesion,cell-cell signaling,e	T=33, G=30	T=0.5238, G=0.4762	0.00001	289.407	63	T=7/26, G=22/
chr19:5211568	G/A	G	A	SNV	PTPRS	PTPRS:intronic:NM 002850.3	1	57.14		intronic	NM 00285	c.5234+33	p.?	-0.13					Fibronectin t	rs55858361	0.453	YES	5.726E-1 (pos.5211568)		Protein tyro	axon,cell adhesion,cell junctio Alendronic acid,Etidroni	G=9, A=12	G=0.4286, A=0.5714	0.00001	111.551	21	G=2/7, A=8/4
chr6:90402840	G/G	C	G	SNV	MDN1	MDN1:exonic:NM 014611.2	1	100	63	synonymous	NM 01461	c.9909G>C	p.(=)	0.14				0.96(COSM45	AAA domain (	rs9351212	0.1993	YES	9.915E-1:7.641E-3 (pos.9	squamous_cell_carcinoma		ATP binding,ATPase activity,cytosol,intermediate filame	C=0, G=61	C=0.0, G=1.0	0.00001	921.853	61	C=0/0, G=33/2
chr6:90418262	T/T	C	T	SNV	MDN1	MDN1:exonic:NM 014611.2	1	100	51	synonymous	NM 01461	c.7851G>A	p.(=)	-1.72					AAA domain (	rs2026634,rs748431	0.169	YES	0.0 (pos.90418257),9.99E-1 (pos.90418262)			ATP binding,ATPase activity,cytosol,intermediate filame	C=0, T=42	C=0.0, T=1.0	0.00001	685.22	42	C=0/0, T=13/2
chr6:90455084	G/G	A	G	SNV	MDN1	MDN1:exonic:NM 014611.2	1	100	29	synonymous	NM 01461	c.4086T>C	p.(=)	-0.03					AAA domain (	rs9359861	0.1687	YES	9.992E-1 (pos.90455084)			ATP binding,ATPase activity,cytosol,intermediate filame	A=0, G=28	A=0.0, G=1.0	0.00001	408.52	28	A=0/0, G=25/3
chr6:90448092	C/T	C	T	SNV	MDN1	MDN1:exonic:NM 014611.2	1	96.67	33	missense	NM 01461	c.4676G>A	p.Ser1559Asn	0.52	0.74	46	0.001	0.19(COSM41	AAA domain (	rs4140446	0.1692	YES	9.992E-1 (pos.90448092)	neoplasm		ATP binding,ATPase activity,cytosol,intermediate filame	C=1, T=29	C=0.0333, T=0.9667	0.00001	416.457	30	C=0/1, T=13/1
chr5:6378609	TC/TC	T	TC	INDEL	MED10	MED10:utr_5:NM 032286.2	1	100	1	utr_5	NM 03228	c.-13A>GA	p.?	-2.26					Transcriptior	rs539093516					Mediator cc	RNA polymerase II transcription cofactor activity,core m	T=0, TC=38	T=0.0, TC=1.0	0.00001	418.675	38	T=0/0, TC=4/3
chr5:6378614	C/C	G	C	SNV	MED10	MED10:utr_5:NM 032286.2	1	100	1	utr_5	NM 03228	c.-18C>G	p.?	-1.33					Transcriptior	rs764668946,rs7723	0		1.043E-3 (pos.6378613),9.99E-1:0.0 (pos		Mediator cc	RNA polymerase II transcription cofactor activity,core m	G=0, C=38	G=0.0, C=1.0	0.00001	606.842	38	G=0/0, C=4/34
chr3:150881774	C/C	A	C	SNV	MED12L	MED12L:exonic:NM 053002.5	1	100	8	missense	NM 05300	c.1202A>C	p.Gln401Pro	2.54	1	76	0.002		Eukaryotic M	rs17290219	0.1408	YES	2.313E-4 (pos.150881774)		Mediator cc	RNA polymerase II transcription coactivator activity,RNA	A=0, C=45	A=0.0, C=1.0	0.00001	747.387	45	A=0/0, C=12/3
chr3:151056598	C/C	A	C	SNV	MED12L,P2RY12	P2RY12:exonic:NM 176876.2, MED12L:intronic:NM 05300	1	100	2	synonymous	NM 05300	c.2146-11	p.?, p.(=)	-0.26				0(COSM5653	7 transmemb	rs6809699,rs775742	0.1641	YES	0.0 (pos.151056597),8.8	diffuse_large	Mediator cc	ADP recep Autoimmune Disea Cangrelor, Benign	A=0, C=62	A=0.0, C=1.0	0.00001	939.474	62	A=0/0, C=25/3
chr3:151046308	A/A	G	A	SNV	MED12L,P2RY13	P2RY13:exonic:NM 176894.2, MED12L:intronic:NM 05300	1	100	2	missense	NM 05300	c.2146-21	p.?, p.Thr179Met	5.05	1	81	0.001	0.06(COSM56	7 transmemb	rs1466684	0.1674	YES	8.397E-1 (pos.15104630	diffuse_large	Mediator cc	G-protein coupled purinergic nucleotide receptor activit	G=0, A=312	G=0.0, A=1.0	0.00001	5160.72	312	G=0/0, A=152/
chr19:52521192	C/C	A	C	SNV	ZNF614	ZNF614:intronic:NM 025040.3	1	100		intronic	NM 02504	c.238+69T	p.?	-0.14					C2H2-type zir	rs61287503		YES				DNA binding,biological_process,cellular_component,int	A=0, C=43	A=0.0, C=1.0	0.00001	705.822	43	A=0/0, C=20/2
chr17:60043884	A/A	G	A	SNV	MED13	MED13:exonic:NM 005121.2	1	100	19	synonymous	NM 00512	c.4320C>T	p.(=)	2.02					Mediator cor	rs671347	0		1.0 (pos.60043884)		Mediator cc	RNA polymerase II transcription cofactor activity,androg	G=0, A=26	G=0.0, A=1.0	0.00001	371.42	26	G=0/0, A=18/8
chr19:5264967	G/G	A	G	SNV	PTPRS	PTPRS:intronic:NM 002850.3	1	100		intronic	NM 00285	c.568+52T	p.?	-1.83					Fibronectin t	rs4807017	0.3026	YES			Protein tyro	axon,cell adhesion,cell junctio Alendronic acid,Etidroni	A=0, G=23	A=0.0, G=1.0	0.00001	321.107	23	A=0/0, G=8/15
chr19:871135	A/G	A	G	SNV	MED16	MED16:exonic:NM 005481.2	1	45.45	13	synonymous	NM 00548	c.2217T>C	p.(=)	2.18				0.1(COSM375	Mediator cor	rs1683569	0.2389	YES	4.305E-2 (pos.871135)	adenocarcinoma,neoplasn		androgen Endometriosis:Female Urogenital Diseases:F	A=12, G=10	A=0.5455, G=0.4545	0.00001	81.7566	22	A=5/7, G=2/8
chr19:52938648	G/G	C	G	SNV	ZNF534	ZNF534:intronic:NM 001143939.2	1	100		intronic	NM 00114	c.310+186	p.?	-0.94					C2H2-type zir	rs10853848		YES				DNA binding,DNA binding transcription factor activity,int	C=0, G=53	C=0.0, G=1.0	0.00001	782.022	53	C=0/0, G=23/3
chr19:52938655	A/A	G	A	SNV	ZNF534	ZNF534:intronic:NM 001143939.2	1	100		intronic	NM 00114	c.310+193	p.?	-0.83					C2H2-type zir	rs10853849		YES				DNA binding,DNA binding transcription factor activity,int	G=0, A=52	G=0.0, A=1.0	0.00001	767.016	52	G=0/0, A=22/3
chr19:52940931	T/T	A	T	SNV	ZNF534	ZNF534:intronic:NM 001143939.2	1	100		intronic	NM 00114	c.311-54A	p.?	2.85					C2H2-type zir	rs1428179		YES				DNA binding,DNA binding transcription factor activity,int	A=0, T=40	A=0.0, T=1.0	0.00001	644.283	40	A=0/0, T=23/1
chr11:93517874	G/G	C	G	SNV	MED17	MED17:exonic:NM 004268.4	1	100	1	synonymous	NM 00426	c.195C>G	p.(=)	0.22				0.76(COSM37	Subunit 17 of	rs7116967,rs775571	0.3632	YES	0.0 (pos.93517873),7.75	adenocarcino	Mediator cc	RNA polym Endometriosis:Female Urogen Benign	C=0, G=44	C=0.0, G=1.0	0.00001	726.567	44	C=0/0, G=39/5
chr11:93517886	C/C	G	C	SNV	MED17	MED17:exonic:NM 004268.4	1	100	1	missense	NM 00426	c.207G>C	p.Glu69Asp	0.19	1	45	0	0.27(COSM37	Subunit 17 of	rs2848477	0.3582	YES	7.758E-1:0.0 (pos.93517	adenocarcino	Mediator cc	RNA polym Endometriosis:Female Urogen Benign	G=0, C=43	G=0.0, C=1.0	0.00001	705.725	43	G=0/0, C=38/5
chr12:27175494	C/C	T	C	SNV	MED21	MED21:utr_5:NM 004264.4	1	100	1	utr_5	NM 00426	c.-19T>C	p.?	-2.82					Subunit 21 of	rs3963548	0.0673	YES	9.996E-1 (pos.27175494)		Mediator cc	DNA-directed 5'-3' RNA polymerase activity,RNA polymer	T=0, C=41	T=0.0, C=1.0	0.00001	664.696	41	T=0/0, C=21/2
chr17:38179492	A/G	A	G	SNV	MED24	MED24:exonic:NM 014815.3	1	53.13	20	synonymous	NM 01481	c.2142T>C	p.(=)	-0.44				0.09(COSM37	Mediator cor	rs2302777	0.375	YES	4.088E-1 (pos.38179492)	adenocarcino	Mediator cc	RNA polym Blood Protein Disorders;Cardiovascular Dise	A=15, G=17	A=0.4688, G=0.5313	0.00001	173.65	32	A=9/6, G=12/5
chr15:100246942	A/A	G	A	SNV	MEF2A	MEF2A:exonic:NM 005587.3	1	100	9	synonymous	NM 00558	c.873G>A	p.(=)	-0.14					Holliday junc	rs325407	0.0025	YES	9.999E-1 (pos.100246942)		MADS box tr	DNA bindi Arterial Occlusive Diseases;Arteriosclerosis;	G=0, A=28	G=0.0, A=1.0	0.00001	408.199	28	G=0/0, A=19/9
chr15:100246936	T/C	T	C	SNV	MEF2A	MEF2A:exonic:NM 005587.3	1	46.43	9	synonymous	NM 00558	c.867T>C	p.(=)	0.35				0.43(COSM39	Holliday junc	rs325408	0.2086	YES	7.319E-1 (pos.10024693	acute_myeloid	MADS box tr	DNA bindi Arterial Occlusive Diseases:Arteriosclerosis:	T=15, C=13	T=0.5357, C=0.4643	0.00001	117.927	28	T=15/0, C=4/9
chr1:156446903	T/C	T	C	SNV	MEF2D	MEF2D:exonic:NM 001271629.1	1	50	7	synonymous	NM 00127	c.756A>G	p.(=)	-4.5				0.11(COSM41	Holliday junc	rs2274317	0.3429	YES	7.224E-1 (pos.15644690	acute_myeloid	MADS box tr	DNA bindi Brain Diseases:Central Nervous System Dise	T=14, C=14	T=0.5, C=0.5	0.00001	131.75	28	T=9/5, C=7/7
chr5:126792974	A/A	T	A	SNV	MEGF10	MEGF10:exonic:NM 001256545.1	1	100	25	synonymous	NM 00125	c.3387T>A	p.(=)	-2.78				0.12(COSM40	Human growt	rs3812052	0.3771	YES	2.906E-1 (pos.12679297	acute_myeloid	Multiple ep	Notch binc Alzheimer Disease;Brain Disea Benign/Likely	T=0, A=25	T=0.0, A=1.0	0.00001	353.019	25	T=0/0, A=18/7
chr15:66274690	C/C	T	C	SNV	MEGF11	MEGF11:exonic:NM 032445.2	1	100	6	synonymous	NM 03244	c.531A>G	p.(=)	-3.08				0.01(COSM37	Human growt	rs551474	6.00E-04	YES	1.0 (pos.66274690)	adenocarcino	Multiple ep	basolateral plasma membrane,homotypic cell-cell adhe	T=0, C=35	T=0.0, C=1.0	0.00001	543.899	35	T=0/0, C=17/1
chr19:536900	A/A	G	A	SNV	CDC34	CDC34:intronic:NM 004359.1	1	100		intronic	NM 00435	c.363-113	p.?	-0.9					Ubiquitin-cor	rs892204		YES			Cell divisior	ATP binding,DNA replication initiation,G1/S transition of	G=0, A=21	G=0.0, A=1.0	0.00001	281.235	21	G=0/0, A=12/9
chr19:53784788	G/A	G	A	SNV	FAM90A27P	FAM90A27P:upstream:NR 046365.1	1	50		intronic	NR 046365.1			0.24						rs28669322		YES	4.646E-1 (pos.53784788)				G=10, A=1	G=0.5, A=0.5	0.00001	82.8861	20	G=8/2, A=9/1
chr1:3496479	C/C	T	C	SNV	MEGF6	MEGF6:exonic:NM 001409.3	1	100	4	missense	NM 00140	c.391A>G	p.Ser131Gly	0.21	1	56	0	0(COSM1162	Calcium-bind	rs2794340	0.084	YES	6.875E-1 (pos.3496479)	ductal_carcino	Multiple ep	biological_process,calcium ion binding,cellular_compon	T=0, C=21	T=0.0, C=1.0	0.00001	281.235	21	T=0/0, C=3/18
chr19:42874901	G/A	G	A	SNV	MEGF8	MEGF8:exonic:NM 001271938.1	1	52.38	40	missense	NM 00127	c.7054G>A	p.Val2352Met	7.34	0.05	21	1		CUB domain,	rs112167630	0.0248	YES	0.0 (pos.42874901)		Multiple ep	BMP signa Acrocephalosyndactylia;Bone Benign	G=20, A=22	G=0.4762, A=0.5238	0.00001	245.024	42	G=12/8, A=12/
chr22:42159229	T/T	G	T	SNV	MEI1	MEI1:exonic:NM 152513.3	1	100	19	synonymous	NM 15251	c.2172G>T	p.(=)	-0.6				0.02(COSM3759223),0.02(C		rs2050033	0.1868	YES	9.411E-1 (pos.42159229)	adenocarcinoma		cell,male meiosis I,meiosis I,meiotic telomere clusterin	G=0, T=33	G=0.0, T=1.0	0.00001	504.598	33	G=0/0, T=12/2
chr16:1912021	T/G	T	G	SNV	MEIOB	MEIOB:exonic:NM 001163560.2	1	59.18	4	missense	NM 00116	c.224A>C	p.Lys75Thr	0.45	0.14	78	0.027	0.78(COSM6351572)		rs1657125	0.1273	YES	2.846E-1 (pos.1912021)	carcinoma	Meiosis-spe	chromatin binding,chromosome,cytoplasm,double-stra	T=20, G=29	T=0.4082, G=0.5918	0.00001	360.52	49	T=12/8, G=14/

chr19:54606209	G/G	T	G	SNV	NDUFA3,OSCAR	NDUFA3:intronic:NM 004542.3, OSCAR:upstream:NM 2068	1	100		intronic	NM 20681	c.-2126A>(	p.?, p.?	-0.21					NADH dehydr	rs254260	0.0135	YES	7.089E-1 (pos.54606209)		NADH-ubiqu	NADH dehydrogenase (ubiquin	NADH			T=0, G=154	T=0.0, G=1.0	0.00001	2470.23	154	T=0/0, G=75/7
chr19:54627097	C/C	T	C	SNV	PRPF31	PRPF31:intronic:NM 015629.3	1	100		intronic	NM 01562	c.528-31T:	p.?	-1.54					Pro31 C term	rs2303557	0.0081	YES	7.212E-1 (pos.54627097)		Precursor R	Cajal body	Congenital, Hereditary, and Neonatal Diseas			T=0, C=24	T=0.0, C=1.0	0.00001	334.79	24	T=0/0, C=14/1
chr9:36674842	C/T	C	T	SNV	MELK	MELK:exonic:NM 014791.3	1	57.14	17	synonymous	NM 01479	c.1686C>T	p.(=)	-0.02					Kinase assoc	rs2274473	0.1018	YES	9.021E-1 (pos.36674842)		Maternal e	ATP bindin	Autoimmune Diseases;Female Urogenital Di			C=12, T=16	C=0.4286, T=0.5714	0.00001	161.747	28	C=4/8, T=6/10
chr19:54711258	G/G	T	G	SNV	RPS9	RPS9:intronic:NM 001013.3	1	100		intronic	NM 00101	c.408-8T>(	p.?	0.82					Ribosomal pr	rs2304523	0.157	YES	5.066E-1 (pos.54711258)		Ribosomal	RNA binding,SRP-dependent cotranslational protein targ				T=0, G=21	T=0.0, G=1.0	0.00001	281.227	21	T=0/0, G=15/6
chr11:64572557	G/G	A	G	SNV	MEN1	MEN1:exonic:NM 000244.3	1	100	9	synonymous	NM 00024	c.1314T>C	p.(=)	-0.3					Menin	rs540012	3.00E-04		1.0 (pos.64572557)		Menin	DNA repai	ACMG Recommendations...;Ad		Benign	A=0, G=67	A=0.0, G=1.0	0.00001	1034.42	67	A=0/0, G=35/3
chr6:46801071	G/T	G	T	SNV	MEP1A	MEP1A:exonic:NM 005588.2	1	51.92	11	missense	NM 00558	c.1405G>T	p.Val469Leu	0.34	0.55	32	0.004	0.16(COSM37	Astacin (Pept	rs2274658	0.3972	YES	1.738E-1 (pos.46801071)	acute myeloid	Meprin A, a	digestion,	Colonic Diseases;Colonic Neoplasms;Colorec			G=25, T=27	G=0.4808, T=0.5192	0.00001	270.53	52	G=7/18, T=8/1
chr19:54756657	T/T	C	T	SNV	LILRB5	LILRB5:intronic:NM 001081442.2	1	100		intronic	NM 00108	c.1474+77	p.?	-0.91					Immunoglobu	rs434755		YES			Leukocyte i	adaptive i	Autoimmune Diseases;Female Urogenital Di			C=0, T=64	C=0.0, T=1.0	0.00001	977.635	64	C=0/0, T=37/2
chr19:54759102	A/A	G	A	SNV	LILRB5	LILRB5:intronic:NM 001081442.2	1	100		intronic	NM 00108	c.952+47C	p.?	-1.88					Immunoglobu	rs393600	0.2486	YES	9.001E-1 (pos.54759102)		Leukocyte i	adaptive i	Autoimmune Diseases;Female Urogenital Di			G=0, A=21	G=0.0, A=1.0	0.00001	281.235	21	G=0/0, A=14/7
chr19:54759110	A/A	G	A	SNV	LILRB5	LILRB5:intronic:NM 001081442.2	1	100		intronic	NM 00108	c.952+39C	p.?	-0.04					Immunoglobu	rs440352	0.2483	YES	8.998E-1 (pos.54759110)		Leukocyte i	adaptive i	Autoimmune Diseases;Female Urogenital Di			G=0, A=21	G=0.0, A=1.0	0.00001	281.235	21	G=0/0, A=14/7
chr6:46793175	G/A	G	A	SNV	MEP1A	MEP1A:exonic:NM 005588.2	1	47.37	8	synonymous	NM 00558	c.723G>A	p.(=)	1.08				0.83(COSM37	Astacin (Pept	rs6920863	0.3973	YES	1.75E-1 (pos.46793175)	adenocarcinoı	Meprin A, a	digestion,	Colonic Diseases;Colonic Neoplasms;Colorec			G=20, A=18	G=0.5263, A=0.4737	0.00001	184.681	38	G=8/12, A=10/
chr18:29797921	T/T	C	T	SNV	MEP1B	MEP1B:exonic:NM 005925.2	1	100	14	missense	NM 00592	c.2084C>T	p.Pro695Leu	0.92	1	98	0	0(COSM3756:	Astacin (Pept	rs616114	0.3898	YES	2.429E-1 (pos.29797921)	acute myeloid	Meprin A, b	digestion,extracellular space,identical protein binding,i				C=0, T=45	C=0.0, T=1.0	0.00001	747.393	45	C=0/0, T=34/1
chr19:54962579	T/T	C	T	SNV	LENG8	LENG8:intronic:NM 052925.3	1	100		intronic	NM 05292	c.38+20C>	p.?	-0.01					SAC3/GANP fa	rs1035452	0.4379	YES	2.632E-1 (pos.54962579)		Leukocyte r	protein binding				C=0, T=21	C=0.0, T=1.0	0.00001	281.235	21	C=0/0, T=18/3
chr19:54963813	CCT/C	CCT	C	INDEL	LENG8	LENG8:intronic:NM 052925.3	2	58.33		intronic	NM 05292	c.214-17C	p.?	-2.53, 0.08, -0.4					SAC3/GANP fa	rs3841263,rs778432(	0.3329	YES	2.373E-1 (pos.54963813),4.002E-4 (pos.5		Leukocyte r	protein binding				CCT=15, C=	CCT=0.4167, C=0.583	0.00001	229.598	36	CCT=9/6, C=14
chr19:54964019	T/T	C	T	SNV	LENG8	LENG8:intronic:NM 052925.3	1	100		intronic	NM 05292	c.315+88C	p.?	-0.68					SAC3/GANP fa	rs963352		YES			Leukocyte r	protein binding				C=0, T=27	C=0.0, T=1.0	0.00001	389.988	27	C=0/0, T=14/1
chr1:170135760	T/C	T	C	SNV	METTL11B	METTL11B:exonic:NM 001136107.1	1	53.49	3	missense	NM 00113	c.448T>C	p.Ser150Pro	0.42	0.33	74	0.001	0.33(COSM39	AdoMet depe	rs6427235	0.2781	YES	1.561E-1 (pos.17013576(	acute myeloid	leukaemia	N-terminal protein N-methyltransferase activity,N-termi				T=40, C=46	T=0.4651, C=0.5349	0.00001	511.567	86	T=14/26, C=20
chr19:54965889	C/C	G	C	SNV	LENG8	LENG8:intronic:NM 052925.3	1	100		intronic	NM 05292	c.679+28G	p.?	-3.46					SAC3/GANP fa	rs3813147	0.3576	YES	3.822E-1;0.0;0.0 (pos.54965889)		Leukocyte r	protein binding				G=0, C=38	G=0.0, C=1.0	0.00001	603.095	38	G=0/0, C=22/1
chr1:171755071	G/G	A	G	SNV	METTL13	METTL13:exonic:NM 015935.4	1	100	3	synonymous	NM 01593	c.966A>G	p.(=)	3.19					Methyltransf	rs17650204	0.1052	YES	4.724E-4 (pos.171755071)			methylation,methyltransferase activity,protein binding				A=0, G=41	A=0.0, G=1.0	0.00001	664.698	41	A=0/0, G=25/1
chr1:171755182	A/A	G	A	SNV	METTL13	METTL13:exonic:NM 015935.4	1	100	3	missense	NM 01593	c.1077G>A	p.Met359Ile	9.47	0.15	10	0.722	1(COSM1466:	Methyltransf	rs2232819	0.195	YES	1.098E-1;0.0 (pos.171755	adenocarcinoma,ductal_ca		methylation,methyltransferase activity,protein binding				G=0, A=47	G=0.0, A=1.0	0.00001	789.328	47	G=0/0, A=23/2
chr2:208477852	A/A	G	A	SNV	METTL21A	METTL21A:exonic:NM 001127395.3	1	100	4	missense	NM 00112	c.575C>T	p.Thr192Ile	3.05	0.15	89	0.047	0.92(COSM41	Lysine methy	rs2551949	0.1831	YES	9.998E-1 (pos.20847785:	neoplasm	Methyltran	ATPase bir	Amyotrophic Lateral Sclerosis;Central Nervoı			G=0, A=24	G=0.0, A=1.0	0.00001	333.681	24	G=0/0, A=16/8
chr13:103339365	G/G	A	G	SNV	METTL21C	METTL21C:exonic:NM 001010977.2	1	100	3	synonymous	NM 00101	c.325T>C	p.(=)	-0.43				0.03(COSM41	Lysine methy	rs660207	0.48	YES	4.567E-1 (pos.10333936!	acute myeloid	Methyltran	cellular response to dexamethasone stimulus,heat shoc				A=0, G=39	A=0.0, G=1.0	0.00001	623.977	39	A=0/0, G=13/2
chr16:8738579	G/G	A	G	SNV	METTL22	METTL22:exonic:NM 024109.3	1	100	10	synonymous	NM 02410	c.1176A>G	p.(=)	1.37				0.09(COSM37	Lysine methy	rs1641067	0		1.0 (pos.8738579)	adenocarcinoı	Methyltran	heat shock protein binding,nucleolus,nucleoplasm,nucl				A=0, G=29	A=0.0, G=1.0	0.00001	427.602	29	A=0/0, G=11/1
chr16:8740015	C/C	A	C	SNV	METTL22	METTL22:utr 3:NM 024109.3	1	100	11	utr 3	NM 02410	c.1230A>C	p.?	-2.7					Lysine methy	rs1730994	0		1.0;0.0 (pos.8740015)		Methyltran	heat shock protein binding,nucleolus,nucleoplasm,nucl				A=0, C=148	A=0.0, C=1.0	0.00001	2508.53	148	A=0/0, C=78/7
chr19:55315307	G/G	C	G	SNV	KIR2DL4	KIR2DL4:intronic:NM 002255.5	1	100		intronic	NM 00225	c.41-39C>(	p.?	-5.27						rs2295802	0.4466	YES	6.438E-1 (pos.55315307)		Killer cell in	cellular defense response,integral component of memb				C=0, G=40	C=0.0, G=1.0	0.00001	644.277	40	C=0/0, G=19/2
chr19:55328959	T/T	G	T	SNV	KIR3DL1	KIR3DL1:intronic:NM 001322168.1	1	100		intronic	NM 00132	c.35-30G>	p.?	-0.65						rs620031		YES	8.919E-1;0.0 (pos.55328959)		Killer cell in	HLA-B spec	HIV Infections;Immune System Diseases;Imm			G=0, T=36	G=0.0, T=1.0	0.00001	563.744	36	G=0/0, T=7/29
chr12:82783668	T/T	C	T	SNV	METTL25	METTL25:exonic:NM 032230.2	1	100	3	synonymous	NM 03223	c.498C>T	p.(=)	0.26					Methyltransf	rs10778908	0.0438	YES	1.0;0.0 (pos.82783668)			methylation,methyltransferase activity				C=0, T=45	C=0.0, T=1.0	0.00001	747.392	45	C=0/0, T=24/2
chr19:55396613	A/A	G	A	SNV	FCAR	FCAR:intronic:NM 002000.3	1	100		intronic	NM 00200	c.71-34G>	p.?	-1.27						rs10402324	0.3017	YES	6.524E-1 (pos.55396613)		Fc fragment	Fc receptor signaling pathway,IgA binding,IgA receptor a				G=0, A=74	G=0.0, A=1.0	0.00001	1170.13	74	G=0/0, A=43/3
chr19:55441744	C/T	C	T	SNV	NLRP7	NLRP7:intronic:NM 001127255.1	1	47.62		intronic	NM 00112	c.2810+12	p.?	-0.43					Leucine Rich	rs647845		YES				ATP bindin	Female Urogenital Diseases ar		not provided	C=11, T=10	C=0.5238, T=0.4762	0.00001	82.2899	21	C=9/2, T=1/9
chr19:55441769	G/A	G	A	SNV	NLRP7	NLRP7:intronic:NM 001127255.1	1	47.62		intronic	NM 00112	c.2810+98	p.?	-2.86					Leucine Rich	rs269949		YES				ATP bindin	Female Urogenital Diseases and Pregnancy C			G=11, A=10	G=0.5238, A=0.4762	0.00001	82.2046	21	G=9/2, A=1/9
chr12:82792787	A/A	C	A	SNV	METTL25	METTL25:exonic:NM 032230.2	1	100	4	missense	NM 03223	c.745C>A	p.Gln249Lys	1.28	0.03	53	0.009		Methyltransf	rs4296098,rs750272:	0.0437	YES	1.196E-4 (pos.82792786),9.999E-1 (pos.82792787)			methylation,methyltransferase activity				C=0, A=43	C=0.0, A=1.0	0.00001	705.844	43	C=0/0, A=20/2
chr7:73249299	C/C	G	C	SNV	METTL27	METTL27:exonic:NM 152559.2	1	100	6	missense	NM 15255	c.512C>G	p.Ser171Trp	1.65	0.18	177	0.993		Methyltransf	rs13232463	0.2578	YES	6.039E-1 (pos.73249299)			protein binding				G=0, C=22	G=0.0, C=1.0	0.00001	297.164	22	G=0/0, C=9/13
chr7:128141865	C/C	T	C	SNV	METTL2B	METTL2B:exonic:NM 018396.2	1	100	9	synonymous	NM 01839	c.1032T>C	p.(=)	0.61				0.15(COSM63	Methyltransf	rs1053124	0.1971	YES	7.469E-1 (pos.12814186!	carcinoma		integral component of membrane,tRNA (cytosine) methy				T=0, C=25	T=0.0, C=1.0	0.00001	353.018	25	T=0/0, C=12/1
chr2:172180771	G/G	A	G	SNV	METTL8	METTL8:exonic:NM 024770.4	1	100	10	stoploss	NM 02477	c.1135T>C	p.Ter379Arg	5.33					Methyltransf	rs10205459	0		1.0 (pos.172180771)							A=0, G=62	A=0.0, G=1.0	0.00001	940.32	62	A=0/0, G=39/2
chr19:55529933	G/G	A	G	SNV	GP6	GP6:intronic:NM 001083899.2	1	100		intronic	NM 00108	c.724+87T	p.?	-0.03					Immunoglobu	rs1671192		YES			Glycoprotei	blood coagulation,cell surface,collagen binding,enzyme				A=0, G=33	A=0.0, G=1.0	0.00001	504.524	33	A=0/0, G=22/1
chr15:89450587	T/T	G	T	SNV	MFGE8	MFGE8:exonic:NM 005928.3	1	100	3	missense	NM 00592	c.226C>A	p.Leu76Met	2.74	1	15	0.001	0.12(COSM14	EGF-like dom	rs1878326	0.3476	YES	4.405E-1 (pos.89450587)	adenocarcinoı	Milk fat glob	angiogene	Breast Diseases;Breast Neoplasms;Diabetes			G=0, T=122	G=0.0, T=1.0	0.00001	1973.69	122	G=0/0, T=49/7
chr19:55665584	C/C	A	C	SNV	TNNI3	TNNI3:intronic:NM 000363.4	1	100		intronic	NM 00036	c.372-10T:	p.?	-3.34					Troponin,Tro	rs7252610			1.0 (pos.55665584)		Troponin-I,	actin bind	ACMG Recommend;	N-(6-Amin	Benign/Likely	A=0, C=23	A=0.0, C=1.0	0.00001	321.188	23	A=0/0, C=9/14
chr17:74733099	A/A	G	A	SNV	MFSD11,MIR636,SRSF2	SRSF2:exonic:NM 003016.4, MIR636:upstream:NR 030366	1	100	1	synonymous	NM 00301	c.144C>T, (	p.(=), p.?	2.03					RNA recogniti	rs237057	0.0676	YES	9.218E-1 (pos.74733099)		Serine/argi	PML body,	Autoimmune Diseases;Female Urogenital Di			G=0, A=31	G=0.0, A=1.0	0.00001	465.819	31	G=0/0, A=10/2
chr10:104231054	A/G	A	G	SNV	MFSD13A	MFSD13A:exonic:NM 024789.3	1	96.77	6	synonymous	NM 02478	c.729A>G	p.(=)	-0.74				0(COSM4144:	MFS/sugar tra	rs11191295	0.4466	YES	3.124E-1 (pos.10423105	carcinoma,neoplasm		integral component of membrane				A=1, G=30	A=0.0323, G=0.9677	0.00001	435.409	31	A=0/1, G=16/1
chr15:42026764	T/T	C	T	SNV	MGA	MGA:exonic:NM 001164273.1	1	100	12	synonymous	NM 00116	c.3888C>T	p.(=)	0.28				0.14(COSM54	Domain of un	rs1918314	0.1897	YES	8.874E-1 (pos.42026764)	ductal_carcinc	MAX dimeri	DNA bindi	Adenocarcinoma;Carcinoma;Carcinoma, Ade			C=0, T=106	C=0.0, T=1.0	0.00001	1662.39	106	C=0/0, T=41/6
chr19:55751123	A/A	G	A	SNV	MIR6802,PPP6R1	MIR6802:downstream:NR 106860.1, PPP6R1:intronic:NM (	1	100		intronic	NM 01493	c.1563+71	p.?	-0.28					SIT4 phospha	rs8104509		YES				COPII vesicle coating,Golgi membrane,cytosol,protein bi				G=0, A=38	G=0.0, A=1.0	0.00001	603.779	38	G=0/0, A=19/1
chr15:42041712	A/A	G	A	SNV	MGA	MGA:exonic:NM 001164273.1	1	100	17	synonymous	NM 00116	c.5907G>A	p.(=)	0.65				0.84(COSM50	Domain of un	rs2695163	0.1893	YES	8.868E-1 (pos.42041712)	osteosarcoma	MAX dimeri	DNA bindi	Adenocarcinoma;Carcinoma;Carcinoma, Ade			G=0, A=26	G=0.0, A=1.0	0.00001	371.297	26	G=0/0, A=11/1
chr15:42059521	T/T	A	T	SNV	MGA	MGA:utr 3:NM 001164273.1	1	100	24	utr 3	NM 00116	c.9241A>T	p.?	-0.07					Domain of un	rs2605570	0.1638	YES	8.876E-1 (pos.42059521)		MAX dimeri	DNA bindi	Adenocarcinoma;Carcinoma;Carcinoma, Ade			A=0, T=50	A=0.0, T=1.0	0.00001	852.745	50	A=0/0, T=21/2
chr19:41283365	G/G	C	G	SNV	MIA,MIA-RAB4B,RAB4B,RAB4B-EGLN2	RAB4B:upstream:NM 016154.4, MIA:utr 3:NM 001202553	1	100	5	utr_3	NM 00120	c.436C>G,	p.?, p.?	0.27					Variant SH3 c	rs2233159	0.068	YES	7.92E-1 (pos.41283365)		Melanoma	GTP bindir	Cardiovascular Diseases;Digestive System Di			C=0, G=75	C=0.0, G=1.0	0.00001	1196.79	75	C=0/0, G=12/6
chr19:55872191	G/A	G	A	SNV	FAM71E2	FAM71E2:intronic:NM 001145402.1	1	47.62		intronic	NM 00114	c.811+30C	p.?	-0.18					Protein of unl	rs897799	0.2206	YES	8.73E-1 (pos.55872191)			nucleus				G=20, A=2(	G=0.5, A=0.5	0.00001	245.212	42	G=9/11, A=13/
chr1:1558792	C/C	T	C	SNV	MIB2	MIB2:exonic:NM 080875.2	1	100	3	missense	NM 08087	c.305T>C	p.Met102Thr	-1.13	0.98	81	0.006		Ankyrin repe	rs12755088	0.0599	YES	6.241E-1;6.096E-3 (pos.1558792)		Mindbomb,	Notch signaling pathway,actin binding,cytoplasm,cytosc				T=0, C=24	T=0.0, C=1.0	0.00001	334.787	24	T=0/0, C=18/6
chr19:5595354	C/C	T	C	SNV	SAFB2	SAFB2:intronic:NM 014649.2	1	100		intronic	NM 01464	c.1919+18	p.?	-2.1					RNA recogniti	rs10413286	0.3501	YES	8.851E-1 (pos.5595354)		Scaffold att	RNA binding,Sertoli cell differentiation,cytoplasm,doubl				T=0, C=26	T=0.0, C=1.0	0.00001	370.931	26	T=0/0, C=10/1
chr11:12225946	G/G	T	G	SNV	MICAL2	MICAL2:exonic:NM 014632.3	1	100	4	synonymous	NM 01463	c.414T>G	p.(=)	0.12				0.32(COSM13	CAMSAP CH d	rs3763823	0.4406	YES	6.716E-1 (pos.12225946)	acute myeloid	Microtubul	FAD binding,NADPH:sulfur oxidoreductase activity,actin				T=0, G=36	T=0.0, G=1.0	0.00001	563.104	36	T=0/0, G=7/29
chr11:12246233	C/C	G	C	SNV	MICAL2	MICAL2:exonic:NM 014632.3	1	100	13	synonymous	NM 01463	c.1554G>C	p.(=)	0.3				0.62(COSM44	CAMSAP CH d	rs3816921	0.133	YES	5.305E-1;0.0 (pos.12246;	acute myeloid	Microtubul	FAD binding,NADPH:sulfur oxidoreductase activity,actin				G=0, C=40	G=0.0, C=1.0	0.00001	640.359	40	G=0/0, C=27/1
chr11:12315407	C/C	G	C	SNV	MICALCL	MICALCL:exonic:NM 032867.2	1	100	3	synonymous	NM 03286	c.429G>C	p.(=)	-1.26				0(COSM4145	Protein of unl	rs10741579	0.0707	YES	9.432E-1 (pos.12315407)	neoplasm	MICAL C ter	biological_process,cell differentiation,cytoplasm,mitog				G=0, C=28	G=0.0, C=1.0	0.00001	408.715	28	G=0/0, C=8/20
chr11:12315891	A/A	G	A	SNV	MICALCL	MICALCL:exonic:NM 032867.2	1	100	3	missense	NM 03286	c.913G>A	p.Ala305Thr	-0.09	1	58	0		Protein of unl	rs1493953	0.0707	YES	9.44E-1 (pos.12315891)		MICAL C ter	biological_process,cell differentiation,cytoplasm,mitog				G=0, A=33	G=0.0, A=1.0	0.00001	504.598	33	G=0/0, A=19/1
chr7:1482009	G/C	G	C	SNV	MICALL2	MICALL2:exonic:NM 182924.3	1	50	7	synonymous	NM 18292	c.1530C>G	p.(=)	1.2				0.12(COSM40	CAMSAP CH d	rs61744261	0.1123	YES	5.136E-1 (pos.1482009)	acute myeloid	leukaemia	Rab GTPase binding,actin cytoskeleton reorganization,a				G=14, C=14	G=0.5, C=0.5	0.00001	131.943	28	G=12/2, C=7/7
chr6:31473546	G/G	A	G	SNV	MICB	MICB:exonic:NM 005931.4	1	100	2	missense	NM 00593	c.223A>G	p.Asn75Asp	-0.49	1	23	0	0(COSM5021!	Class I Histoc	rs3131639,rs775573:	0.2176	YES	0.0;0.0 (pos.31473543),9	osteosarcoma	MHC class I	T cell mediated cytotoxicity,cell surface,cytolysis,gamm				A=0, G=56	A=0.0, G=1.0	0.00001	832.1	56	A=0/0, G=30/2
chr13:22178308	A/A	G	A	SNV	MICU2	MICU2:utr 5:NM 152726.2	1	100	1	utr 5	NM 15272	c.-21C>T	p.?	-1.55					EF-hand dom	rs2761924,rs796167:	9.00E-04		1.0 (pos.22178308)		Mitochondr	calcium channel complex,calcium ion binding,mitochon				G=0, A=38	G=0.0, A=1.0	0.00001	608.127	38	G=0/0, A=27/1
chr1:67390416	C/C	G	C	SNV	MIER1,WDR78	MIER1:upstream:NM 001077700.2, WDR78:exonic:NM 024	1	100	1	missense	NM 02476	c.99C>G, c	p.Cys33Trp, p.?	-2.35	0.27	215	0		WD domain, (	rs3008858	0.268	YES	8.752E-1 (pos.67390416)			ATP-dependent microtubule motor activity, plus-end-dir				G=0, C=39	G=0.0, C=1.0	0.00001	623.929	39	G=0/0, C=10/2
chr19:56511217	A/A	G	A	SNV	NLRP5	NLRP5:intronic:NM 153447.4	1	100		intronic	NM 15344	c.62+64G>	p.?	0					Leucine Rich	rs12462690	0.3224	YES				ATP binding,animal organ morphogenesis,apical cortex,(				G=0, A=34	G=0.0, A=1.0	0.00001	524.186	34	G=0/0, A=12/2
chr1:67390468	G/G	C	G	SNV	MIER1,WDR78	MIER1:upstream:NM 001077700.2, WDR78:exonic:NM 024	1	100	1	missense	NM 02476	c.47G>C, c	p.Gly16Ala, p.?	0.93	1	60	0		WD domain, (	rs1886686	0.268	YES	8.757E-1 (pos.67390468)			ATP-dependent microtubule motor activity, plus-end-dir				C=0, G=23	C=0.0, G=1.0	0.00001	316.747	23	C=0/0, G=8/15
chr1:12082334	A/T	A	T	SNV	MIIP	MIIP:exonic:NM 021933.3	1	50	3	missense	NM 02193	c.297A>T	p.Lys99Asn	-0.47	0.03	94	0.952	0.03(COSM16	Migration an	rs11553925	0.2501	YES	5.061E-2 (pos.12082334)	adenocarcinoma,astrocytc		negative regulation of G2/M transition of mitotic cell cyc				A=21, T=21	A=0.5, T=0.5	0.00001	228.746	42	A=17/4, T=10/
chr19:56693643	C/C	T	C	SNV	GALP	GALP:intronic:NM 033106.3	1	100		intronic	NM 03310	c.217+22T	p.?	-0.58					Galanin	rs274174,rs7571985	0		0.0 (pos.56693641),9.983E-1 (pos.56693		Galanin-like	antimicrol	Nutritional and Metabolic Diseases			T=0, C=39	T=0.0, C=1.0	0.00001	623.98	39	T=0/0, C=13/2
chr19:56918560	A/A	T	A	SNV	ZNF583	ZNF583:intronic:NM 001159860.1	1	100		intronic	NM 00115	c.9+68T>A	p.?	-1.75					C2H2-type zir	rs4801159	0.1961	YES				DNA binding,intracellular,metal ion binding,nucleic acid				T=0, A=46	T=0.0, A=1.0	0.00001	722.648	46	T=0/0, A=22/2
chr19:56918574	C/C	T	C	SNV	ZNF583	ZNF583:intronic:NM 001159860.1	1	100		intronic	NM 00115	c.9+82T>C	p.?	0.32					C2H2-type zir	rs2288862	0.1961	YES				DNA binding,intracellular,metal ion binding,nucleic acid				T=0, C=40	T=0.0, C=1.0	0.00001	643.505	40	T=0/0, C=16/2
chr19:56918598	C/C	T	C	SNV	ZNF583	ZNF583:intronic:NM 001159860.1	1	100		intronic	NM 00115	c.9+106T>	p.?	-0.06					C2H2-type zir	rs2288863		YES				DNA binding,intracellular,metal ion binding,nucleic acid				T=0, C=35	T=0.0, C=1.0	0.00001	543.042	35	T=0/0, C=12/2
chr1:150975108	C/C	T	C	SNV	MINDY1	MINDY1:exonic:NM 001163258.1	1	100	3	missense	NM 00116	c.130A>G	p.Lys44Glu	0.11	1	56	0	0(COSM6310!	MINDY deubi	rs771205	0.0201	YES	8.052E-1 (pos.15097510	neoplasm		K48-linked polyubiquitin modification-dependent protei				T=0, C=35	T=0.0, C=1.0	0.00001	543.898	35	T=0/0, C=21/1
chr20:2633936	C/C	T	C	SNV	MIR1292,NOP56,SNORD110	NOP56:exonic:NM 006392.3, SNORD110:upstream:NR 003	1	100	3	synonymous	NM 00639	c.105T>C	p.(=)	-0.03				0.07(COSM41	NOP5NT (NUC	rs2073194	0.205	YES	8.421E-1;0.0 (pos.26339:	neoplasm	Micro RNA 1	RNA bindir	Digestive System Diseases;Dig		Likely benign	T=0, C=26	T=0.0, C=1.0	0.00001	371.42	26	T=0/0, C=21/5
chr2:220414016	T/T	C	T	SNV	MIR3132,TMEM198	MIR3132:upstream:NR 036082.1, TMEM198:exonic:NM 00	1	100	4	synonymous	NM 00100	c.885C>T	p.(=)	0.49					Domain of un	rs6436156	0.0112	YES	1.0 (pos.220414016)			Wnt signaling pathway,cytoplasmic vesicle,integral com				C=0, T=51	C=0.0, T=1.0	0.00001	754.584	51	C=0/0, T=32/1
chr11:68851414	C/C	T	C	SNV	MIR3164,TPCN2	TPCN2:exonic:NM 139075.3, MIR3164:downstream:NR 03(	1	100	19	missense	NM 13907	c.1691T>C	p.Leu564Pro	3.57	0.1	98	0.001	0.7(COSM623	Ion transport	rs2376558	0.0091	YES	5.256E-1 (pos.68851414)	neoplasm	Two-pore se	NAADP-sensitive calcium-release channel activity,calciu				T=0, C=22	T=0.0, C=1.0	0.00001	298.891	22	T=0/0, C=7/15
chr3:75787726	A/A	G	A	SNV	MIR4273,ZNF717	ZNF717:exonic:NM 001128223.2, MIR4273:downstream:NI	1	100	5	missense	NM 00112	c.1048C>T	p.Arg350Cys	-0.87	0.01	180	0.997	0.03(COSM41	C2H2-type zir	rs1962893		YES		neoplasm,osteosarcoma						G=0, A=45	G=0.0, A=1.0	0.00001	747.391	45	G=0/0, A=17/2
chr3:75787739	T/T	A	T	SNV	MIR4273,ZNF717	ZNF717:exonic:NM 001128223.2, MIR4273:downstream:NI	1	100	5	synonymous	NM 00112	c.1035T>A	p.(=)	-0.78					C2H2-type zir	rs1962894,rs766372827		YES	0.0 (pos.75787738),5.263E-2;0.0;0.0 (pos.75787739)							A=0, T=45	A=0.0, T=1.0	0.00001	747.374	45	A=0/0, T=17/2
chr3:75788010	C/C	A	C	SNV	MIR4273,ZNF717	ZNF717:exonic:NM 001128223.2, MIR4273:downstream:NI	1	100	5	missense	NM 00112	c.764T>G	p.Val255Gly	0.06	0.37	109	0		C2H2-type zir	rs3009006		YES	0.0 (pos.75788010)							A=0, C=40	A=0.0, C=1.0	0.00001	644.286	40	A=0/0, C=22/1
chr3:75787794	A/G	A	G	SNV	MIR4273,ZNF717	ZNF717:exonic:NM 001128223.2, MIR4273:downstream:NI	1	59.09	5	missense	NM 00112	c.980T>C	p.Ile327Thr	-0.25	0.49	89	0	0.01(COSM45	C2H2-type zir	rs75467043			0.0 (pos.75787794)	carcinoma,neoplasm,squamous_cell_carcinoma						A=18, G=26	A=0.4091, G=0.5909	0.00001	312.071	44	A=7/11, G=8/1
chr3:75787880	A/G	A	G	SNV	MIR4273,ZNF717	ZNF717:exonic:NM 001128223.2, MIR4273:downstream:NI	1	57.14	5	synonymous	NM 00112	c.894T>C	p.(=)	-4.17				0(COSM5766	C2H2-type zir	rs78071099		YES		adenocarcinoma						A=18, G=24	A=0.4286, G=0.5714	0.00001	279.085	42	A=8/10, G=18/
chr9:139726239	G/G	A	G	SNV	MIR4292,RABL6	MIR4292:downstream:NR 036251.1, RABL6:exonic:NM 00:	1	100	6	synonymous	NM 00117	c.525A>G	p.(=)	-1.13				0.17(COSM41	Ras family	rs2811748	0.1359	YES	7.711E-1 (pos.13972623!	neoplasm	RAB-like prc	GTP bindir	Intellectual Disability;Mental Disorders;Nerv			A=0, G=25	A=0.0, G=1.0	0.00001	347.544	25	A=0/0, G=14/1
chr19:57752326	C/G	C	G	SNV	ZNF805	ZNF805:intronic:NM 001023563.3	1	45.83		intronic	NM 00102	c.30+37C>	p.?	0.9					C2H2-type zir	rs8111661	0.478	YES	1.932E-1 (pos.57752326)			DNA binding,metal ion binding,nucleus,regulation of trai				C=13, G=11	C=0.5417, G=0.4583	0.00001	93.4399	24	C=9/4, G=3/8
chr16:88783449	G/G	C	G	SNV	MIR4722,PIEZO1	MIR4722:upstream:NR 039873.1, PIEZO1:exonic:NM 0011	1	100	45	synonymous	NM 00114	c.6642G>C	p.(=)	0.98				0.37(COSM41	Piezo,Piezo n	rs8057031	0.4192	YES	8.235E-1 (pos.88783449)	neoplasm	PIEZO1 ion (	cation cha	Anemia;Anemia, Hemolytic;An		Benign	C=0, G=64	C=0.0, G=1.0	0.00001	964.347	64	C=0/0, G=17/4
chr16:88783521	C/C	T	C	SNV	MIR4722,PIEZO1	MIR4722:upstream:NR 039873.1, PIEZO1:exonic:NM 0011	1	100	45	synonymous	NM 00114	c.6570A>G	p.(=)	-11.5				0.04(COSM41	Piezo,Piezo n	rs6500491	0.0864	YES	9.067E-1 (pos.88783521)	neoplasm	PIEZO1 ion (	cation cha	Anemia;Anemia, Hemolytic;An		Benign	T=0, C=48	T=0.0, C=1.0	0.00001	809.11	48	T=0/0, C=13/3
chr20:33578899	C/C	T	C	SNV	MIR499A,MIR499B,MYH7B	MYH7B:exonic:NM 020884.4, MIR499B:upstream:NR 0399	1	100	24	synonymous	NM 02088	c.2284T>C	p.(=)	1.9					Myosin N-ter	rs2425009	0.2699	YES	7.641E-1 (pos.33578899)		Myosin, hea	ATP binding,actin filament binding,cardiac myofibril,mer				T=0, C=34	T=0.0, C=1.0	0.00001	524.186	34	T=0/0, C=14/2
chr6:97677118	A/A	G	A	SNV	MIR548H3,MMS22L	MMS22L:exonic:NM 198468.3, MIR548H3:intronic_nc:NR (	1	100	14	missense	NR 03167!	c.1691C>T	p.Thr564Met	0.4	0.02	81	0.985	0.11(COSM40	S-phase geno	rs9481410	0.2452	YES	2.136E-1 (pos.97677118)	acute myeloid	MMS22-like	FACT complex,MCM complex,cytosol,double-strand brea				G=0, A=33	G=0.0, A=1.0	0.00001	504.597	33	G=0/0, A=16/1
chr15:69327825	C/T	C	T	SNV	MIR548H4,NOX5	NOX5:exonic:NM 024505.3, MIR548H4:intronic_nc:NR 031	1	96.67	6	synonymous	NR 03168	c.987C>T	p.(=)	-4.31					EF hand,EF-ha	rs311889,rs7755315	0.0426	YES	0.0 (pos.69327824),7.53E-1 (pos.693278		NADPH oxid	NADP binding,angiogenesis,apoptotic process,calcium i				C=1, T=29	C=0.0333, T=0.9667	0.00001	417.42	30	C=0/1, T=20/9
chr19:58001512	A/A	T	A	SNV	ZNF419	ZNF419:intronic:NM 001098491.1	1	100		intronic	NM 00109	c.34-3T>A	p.?	-0.89					C2H2-type zir	rs11672136	0.3402	YES	6.233E-1;0.0 (pos.58001512)		Zinc finger p	DNA binding,intracellular,metal ion binding,nucleic acid				T=0, A=52	T=0.0, A=1.0	0.00001	756.513	52	T=0/0, A=22/3
chr19:58002819	A/A	T	A	SNV	ZNF419	ZNF419:intronic:NM 001098491.1	1	100		intronic	NM 00109	c.73-17T>A	p.?	0.03					C2H2-type zir	rs6510085	0.3056	YES	7.211E-1 (pos.58002819)		Zinc finger p	DNA binding,intracellular,metal ion binding,nucleic acid				T=0, A=36	T=0.0, A=1.0	0.00001	563.726	36	T=0/0, A=18/1
chr2:179498042	T/C	T	C	SNV	MIR548N,TTN	MIR548N:intronic_nc:NR 031666.1, TTN:exonic:NM 00125(	1	59.09	183	missense	NR 03166(	c.38035A>	p.Lys12679Glu	6.24		56		0.98(COSM37	Fibronectin t	rs6723526	0.1098	YES	7.027E-3 (pos.17949804;	adenocarcinoı	Titin	ATP bindin	Aortic Stenosis, Subvalvular;Ac		Benign/Likely	T=18, C=26	T=0.4091, C=0.5909	0.00001	312.072	44	T=14/4, C=19/
chr7:127721507	C/C	T	C	SNV	MIR593,SND1	MIR593:upstream:NR 030324.1, SND1:exonic:NM 014390.	1	100	18	synonymous	NM 01439	c.2064T>C	p.(=)	-4.96				0.01(COSM41	Staphylococc	rs322825	0.3327	YES	3.308E-1;0.0 (pos.12772:	neoplasm	EBNA-2 coa	RISC comp	Autistic Disorder;Child Development Disorde			T=0, C=52	T=0.0, C=1.0	0.00001	767.015	52	T=0/0, C=28/2
chr19:58003613	T/T	C	T	SNV	ZNF419	ZNF419:intronic:NM 001098491.1	1	100		intronic	NM 00109	c.301+34C	p.?	-0.38					C2H2-type zir	rs2074072	0.3057	YES	7.205E-1 (pos.58003613)		Zinc finger p	DNA binding,intracellular,metal ion binding,nucleic acid				C=0, T=36	C=0.0, T=1.0	0.00001	563.684	36	C=0/0, T=10/2
chr2:219144815	C/C	T	C	SNV	MIR6513,PNKD,TMBIM1	PNKD:intronic:NM 015488.4, TMBIM1:exonic:NM 022152.!	1	100	3	synonymous	NM 01548	c.236+854	p.?, p.(=)	-1.27					Hydroxyacylg	rs2045435	0		1.0 (pos.219144815)		Transmemb	Golgi appa	Adenocarcinoma;Carcinoma;Carcinoma, Ren			T=0, C=61	T=0.0, C=1.0	0.00001	921.854	61	T=0/0, C=29/3
chr19:580575	C/T	C	T	SNV	BSG	BSG:intronic:NM 001728.3	1	58.33		intronic	NM 00172	c.656-71C:	p.?	-1.19					Immunoglobu	rs28992474		YES			Basigin (blo	Golgi mem	Amyotrophic Lateral Sclerosis;Central Nervoı			C=15, T=21	C=0.4167, T=0.5833	0.00001	234.308	36	C=7/8, T=13/8
chr11:71184678	C/C	A	C	SNV	MIR6754,NADSYN1	MIR6754:downstream:NR 106812.1, NADSYN1:exonic:NM (	1	100	8	missense	NM 01816	c.612A>C	p.Gln204His	-0.01	1	24	0	0.18(COSM41	Carbon-nitro	rs7950441		YES	1.0;0.0 (pos.71184678)	neoplasm,squ	NAD synthe	ATP bindin	Avitaminosis;Defici	Glutamic Acid,L-Glutami		A=0, C=35	A=0.0, C=1.0	0.00001	543.903	35	A=0/0, C=22/1
chr3:39180099	C/C	T	C	SNV	MIR6822,TTC21A	MIR6822:downstream:NR 106880.1, TTC21A:exonic:NM 14	1	100	28	synonymous	NM 14575	c.3870T>C	p.(=)	-3.04				0.03(COSM63	Tetratricoper	rs784498	0.2487	YES	7.714E-1 (pos.39180099)	carcinoma		cilium,intraciliary retrograde transport,intraciliary tran:				T=0, C=54	T=0.0, C=1.0	0.00001	798.136	54	T=0/0, C=31/2
chr3:39180263	G/A	G	A	SNV	MIR6822,TTC21A	MIR6822:downstream:NR 106880.1, TTC21A:exonic:NM 14	1	97.3	29	missense	NM 14575	c.3947G>A	p.Arg1316Lys	0.88	0.74	26	0		Tetratricoper	rs704959	0.341	YES	7.464E-1 (pos.39180263)			cilium,intraciliary retrograde transport,intraciliary tran:				G=1, A=36	G=0.027, A=0.973	0.00001	551.578	37	G=1/0, A=29/7
chr6:31601344	T/C	T	C	SNV	MIR6832,PRRC2A	MIR6832:upstream:NR 106890.1, PRRC2A:exonic:NM 004(	1	45.83	17	missense	NM 00463	c.4508T>C	p.Leu1503Pro	1.66	1	98	0		BAT2 N-termi	rs2272593	0.2571	YES	9.79E-1 (pos.31601344)		Proline-rich	RNA bindir	Autoimmune Diseases;Female Urogenital Di			T=13, C=11	T=0.5417, C=0.4583	0.00001	92.4309	24	T=7/6, C=5/6
chr7:100466441	G/A	G	A	SNV	MIR6875,TRIP6	TRIP6:exonic:NM 003302.2, MIR6875:downstream:NR 106	1	50	4	missense	NM 00330	c.688G>A	p.Val230Ile	0.54	0.46	29	0	0.32(COSM37	LIM domain	rs2075756	0.269	YES	4.568E-1 (pos.10046644:	adenocarcinoı	Thyroid hor	RNA binding,cytoskeleton,cytosol,focal adhesion,focal a				G=18, A=18	G=0.5, A=0.5	0.00001	185.886	36	G=5/13, A=1/1
chr17:8048169	C/C	T	C	SNV	MIR6883,PER1	PER1:exonic:NM 002616.2, MIR6883:downstream:NR 106!	1	100	18	synonymous	NM 00261	c.2361A>G	p.(=)	-2.79				0.17(COSM41	PAS domain,F	rs2253820	0.164	YES	3.685E-1 (pos.8048169)	haemangioblastoma,neop		E-box bind	Arthritis;Arthritis, Juvenile;Autistic Disorder;			T=0, C=43	T=0.0, C=1.0	0.00001	705.844	43	T=0/0, C=40/3
chr8:144894496	A/A	G	A	SNV	MIR937,SCRIB	MIR937:downstream:NR 030633.1, SCRIB:exonic:NM 1827	1	100	9	synonymous	NM 18270	c.846C>T	p.(=)	-6.1					Leucine rich r	rs4875060	0.0922	YES	9.746E-1 (pos.144894496)		Scribble, Dr	GDP metabolic process,GMP metabolic process,Scrib-AP				G=0, A=29	G=0.0, A=1.0	0.00001	427.603	29	G=0/0, A=20/9
chr19:58483954	A/G	A	G	SNV	C19orf18	C19orf18:intronic:NM 152474.4	1	57.14		intronic	NM 15247	c.227-82T:	p.?	-0.04						rs11668548		YES				biological	Mental Disorders;Substance-Related Disorde			A=18, G=24	A=0.4286, G=0.5714	0.00001	279.077	42	A=12/6, G=10/
chr19:58485439	T/T	C	T	SNV	C19orf18	C19orf18:intronic:NM 152474.4	1	100		intronic	NM 15247	c.226+28G	p.?	-0.06						rs257676	0.3656	YES	7.544E-1 (pos.58485439)			biological	Mental Disorders;Substance-Related Disorde			C=0, T=22	C=0.0, T=1.0	0.00001	298.882	22	C=0/0, T=18/4
chr14:75483812	C/C	T	C	SNV	MLH3	MLH3:exonic:NM 001040108.1	1	100	13	synonymous	NM 00104	c.4335A>G	p.(=)	-0.84					DNA mismatc	rs13712	0.4697	YES	8.291E-1 (pos.75483812)		Mismatch r	ATP bindin	Colonic Diseases;Colorectal N		Benign/Likely	T=0, C=25	T=0.0, C=1.0	0.00001	353.019	25	T=0/0, C=10/1
chr19:58598784	G/G	A	G	SNV	ZSCAN18	ZSCAN18:intronic:NM 001145542.1	1	100		intronic	NM 00114	c.722-13T:	p.?	-0.34					SCAN domain	rs2260914	0.2237	YES	6.139E-1 (pos.58598784)			DNA binding,DNA binding transcription factor activity,RN				A=0, G=86	A=0.0, G=1.0	0.00001	1408.99	86	A=0/0, G=43/4
chr14:75513883	C/C	T	C	SNV	MLH3	MLH3:exonic:NM 001040108.1	1	100	2	missense	NM 00104	c.2476A>G	p.Asn826Asp	0.34	0.84	23	0		DNA mismatc	rs175081	0		1.0 (pos.75513883)		Mismatch r	ATP bindin	Colonic Diseases;Colorectal N		Benign/Likely	T=0, C=29	T=0.0, C=1.0	0.00001	426.384	29	T=0/0, C=13/1
chr6:53989526	A/A	G	A	SNV	MLIP	MLIP:exonic:NM 138569.2	1	100	3	missense	NM 13856	c.475G>A	p.Val159Ile	-0.71	0.21	29	0.022		Muscular LM	rs4712056	0.3599	YES	6.974E-1 (pos.53989526)		Muscular LI	PML body,negative regulation of cardiac muscle hypertr				G=0, A=37	G=0.0, A=1.0	0.00001	583.566	37	G=0/0, A=22/1
chr6:54054686	A/A	T	A	SNV	MLIP	MLIP:exonic:NM 138569.2	1	100	9	missense	NM 13856	c.958T>A	p.Ser320Thr	0.79	0.01	58	0.42		Muscular LM	rs6934690	0.0603	YES	7.754E-1 (pos.54054686)		Muscular LI	PML body,negative regulation of cardiac muscle hypertr				T=0, A=37	T=0.0, A=1.0	0.00001	583.684	37	T=0/0, A=21/1
chr11:102650389	T/T	C	T	SNV	MMP10	MMP10:exonic:NM 002425.2	1	100	2	missense	NM 00242	c.193G>A	p.Gly65Arg	2.17	0.03	125	0.015	0.97(COSM37	Hemopexin,N	rs17293607	0.1454	YES	9.038E-3 (pos.10265038!	malignant_me	Matrix meta	collagen c	Arthritis;Arthritis, F	Marimastat,N-ISOBUTYL-		C=0, T=33	C=0.0, T=1.0	0.00001	504.543	33	C=0/0, T=15/1
chr11:102738795	T/T	G	T	SNV	MMP12	MMP12:exonic:NM 002426.5	1	100	5	synonymous	NM 00242	c.630C>A	p.(=)	0.6					Hemopexin,N	rs5003690,rs797025(	6.00E-04	YES	1.0 (pos.102738795)		Matrix meta	calcium io	Abortion, Spontane	(1S,5S,7R)-N~7~-(BIPHENY		G=0, T=77	G=0.0, T=1.0	0.00001	1228.52	77	G=0/0, T=34/4
chr16:55523705	C/C	T	C	SNV	MMP2	MMP2:exonic:NM 004530.5	1	100	7	synonymous	NM 00453	c.1149T>C	p.(=)	-2.55				0.04(COSM41	Fibronectin t	rs201812551,rs2438	0.1544	YES	7.892E-1 (pos.55523705)	neoplasm	Matrix meta	angiogene	Abnormalities, Mul	AE-941,Ca	Benign	T=0, C=33	T=0.0, C=1.0	0.00001	504.589	33	T=0/0, C=15/1

chr11:102495998	G/G	T	G	SNV	MMP20	MMP20:exonic:NM 004771.3	1	100	1	missense	NM 00477	c.53A>C	p.Lys18Thr	2	0.34	78	0		Hemopexin,M	rs2245803	0.3559	YES	8.301E-1;1.158E-4;0.0 (pos.102495998)		Matrix meta	amelogen	Amelogenesis Impe	Marimast	Benign	T=0, G=42	T=0.0, G=1.0	0.00001	685.123	42	T=0/0, G=18/2
chr11:102576375	A/A	G	A	SNV	MMP27	MMP27:exonic:NM 022122.2	1	100	1	missense	NM 02212	c.71C>T	p.Thr24Met	-0.96	0.28	81	0.002	0(COSM1579	Hemopexin,M	rs1939015	0.165	YES	7.878E-1 (pos.10257637	serous_carcinoma		collagen catabolic process,ext		Marimastat		G=0, A=244	G=0.0, A=1.0	0.00001	4124.11	244	G=0/0, A=114/
chr11:102576358	C/C	T	C	SNV	MMP27	MMP27:exonic:NM 022122.2	1	99.58	1	missense	NM 02212	c.88A>G	p.Met30Val	-0.32	0.41	21	0.002		Hemopexin,M	rs2846707	0.3402	YES	9.071E-1 (pos.102576358)			collagen catabolic process,ext		Marimastat		T=1, C=239	T=0.0042, C=0.9958	0.00001	3999.86	240	T=0/1, C=115/
chr4:90872862	C/A	C	A	SNV	MMRN1	MMRN1:exonic:NM 007351.2	1	50	7	missense	NM 00735	c.3225C>A	p.Asn1075Lys	0.1	0	94	0.993		C1q domain,I	rs61734879	0.0048		0.0 (pos.90872862)		Multimerin	blood coagulation,calcium ion binding,cell adhesion,ext				C=16, A=16	C=0.5, A=0.5	0.00001	158.383	32	C=9/7, A=5/11
chr19:59025685	C/C	A	C	SNV	ZBTB45	ZBTB45:intronic:NM 032792.3	1	100		intronic	NM 03279	c.1280-8T	p.?	-0.56					BTB/POZ dom	rs4801602	0.0368	YES	9.195E-1 (pos.59025685)			DNA binding,metal ion binding,nervous system developr				A=0, C=27	A=0.0, C=1.0	0.00001	389.977	27	A=0/0, C=5/22
chr19:618624	G/A	G	A	SNV	POLRMT	POLRMT:intronic:NM 005035.3	1	50		intronic	NM 00503	c.3324-38	p.?	0					DNA-depende	rs111923638					Polymerase	DNA binding,DNA-directed 5'-3' RNA polymerase activity,				G=17, A=17	G=0.5, A=0.5	0.00001	171.982	34	G=13/4, A=13/
chr19:619772	G/C	G	C	SNV	POLRMT	POLRMT:intronic:NM 005035.3	1	50		intronic	NM 00503	c.2887-7C	p.?	-2.16				0(COSM3960	DNA-depende	rs11669381	0.4851	YES	5.906E-1;0.0 (pos.61977	basal_cell_car	Polymerase	DNA binding,DNA-directed 5'-3' RNA polymerase activity,				G=19, C=19	G=0.5, C=0.5	0.00001	199.562	38	G=9/10, C=9/1
chr10:88696621	AG/AG	A	AG	INDEL	MMRN2	MMRN2:exonic:NM 024756.2	1	100	7	frameshiftInsertion	NM 02475	c.2728_27	p.Val910fs	3.38					C1q domain,EMI domain							angiogenesis,basement membrane,extracellular exosor				A=0, AG=37	A=0.0, AG=1.0	0.00001	373.86	37	A=0/0, AG=23/
chr19:652205	CA/CA	C	CA	INDEL	RNF126	RNF126:intronic:NM 194460.2	1	100		intronic	NM 19446	c.198+28G	p.?	-0.37					Anaphase-promoting complex subunit 11 RING-H2 finger,RING-like zinc finger,RING-type zinc-finger,R						RING finger	cytoplasm,cytoplasm protein quality control by the ubiq				C=0, CA=49	C=0.0, CA=1.0	0.00001	695.476	49	C=0/0, CA=7/4
chr10:88696622	G/G	C	G	SNV	MMRN2	MMRN2:exonic:NM 024756.2	1	100	7	missense	NM 02475	c.2728G>C	p.Val910Leu	0.35	0.07	32	0.016	0.08(COSM44	C1q domain,I	rs34587013	0.082	YES	7.164E-2 (pos.88696622)	acute_myeloid leukaemia		angiogenesis,basement membrane,extracellular exosor				C=0, G=37	C=0.0, G=1.0	0.00001	518.698	37	C=0/0, G=23/1
chr10:88717140	A/A	T	A	SNV	MMRN2	MMRN2:exonic:NM 024756.2	1	100	1	synonymous	NM 02475	c.159A>T	p.(=)	-0.23					C1q domain,I	rs4244973	0.0315	YES	9.999E-1 (pos.88717140)			angiogenesis,basement membrane,extracellular exosor				T=0, A=27	T=0.0, A=1.0	0.00001	389.988	27	T=0/0, A=14/1
chr19:6677799	G/A	G	A	SNV	C3	C3:downstream:NM 000064.3	1	51.85		intronic	NM 00006	c.*94C>T	p.?	-1.6						rs11569585		YES			Complemer	C5L2 anap	Anemia:Anemia, He	Cyclo-Tetrametavanadat		G=13, A=14	G=0.4815, A=0.5185	0.00001	132.667	27	G=12/1, A=5/9
chr19:6690613	T/G	T	G	SNV	C3	C3:intronic:NM 000064.3	1	46.43		intronic	NM 00006	c.3489+27	p.?	-1.16					A-macroglobi	rs3745568	0.116	YES	8.171E-2 (pos.6690613)		Complemer	C5L2 anap	Anemia:Anemia, He	Cyclo-Tetrametavanadat		T=12, G=13	T=0.48, G=0.52	0.00001	154.928	28	T=7/5, G=8/5
chr9:27455585	G/A	G	A	SNV	MOB3B	MOB3B:utr 5:NM 024761.4	1	50	2	utr 5	NM 02476	c.-37C>T	p.?	1.07					Mob1/phocei	rs10757661,rs38673	0.4431	YES	6.334E-1 (pos.27455585)		MOB kinase	metal ion binding,protein binding				G=19, A=19	G=0.5, A=0.5	0.00001	199.458	38	G=14/5, A=10/
chr18:33779821	A/A	C	A	SNV	MOCOS	MOCOS:exonic:NM 017947.3	1	100	4	synonymous	NM 01794	c.475C>A	p.(=)	1.21					Aminotransfe	rs646424	0		1.0 (pos.33779821)		Molybdenu	Mo-molyb	Congenital, Heredit	Pyridoxal Phosphate		C=0, A=55	C=0.0, A=1.0	0.00001	814.889	55	C=0/0, A=19/3
chr18:33779896	G/G	A	G	SNV	MOCOS	MOCOS:exonic:NM 017947.3	1	100	4	missense	NM 01794	c.550A>G	p.Ser184Gly	-0.01	0.48	56	0		Aminotransfe	rs540967	0		1.0 (pos.33779896)		Molybdenu	Mo-molyb	Congenital, Heredit	Pyridoxal Phosphate		A=0, G=57	A=0.0, G=1.0	0.00001	849.615	57	A=0/0, G=18/3
chr18:33785185	G/G	A	G	SNV	MOCOS	MOCOS:exonic:NM 017947.3	1	100	6	synonymous	NM 01794	c.1164A>G	p.(=)	-2.53					Aminotransfe	rs667667	1.00E-04		1.0 (pos.33785185)		Molybdenu	Mo-molyb	Congenital, Heredit	Pyridoxal Phosphate		A=0, G=25	A=0.0, G=1.0	0.00001	353.019	25	A=0/0, G=6/19
chr6:29625001	G/G	A	G	SNV	MOG	MOG:exonic:NM 002433.4	1	100	1	synonymous	NM 00243	c.15A>G	p.(=)	-0.5				0(COSM5021	Immunoglobu	rs3130250	0.1626	YES	9.866E-1 (pos.29625001)	osteosarcoma	Myelin-olig	cell adhes	Autoimmune Diseases;Autoimmune Disease			A=0, G=28	A=0.0, G=1.0	0.00001	408.717	28	A=0/0, G=8/20
chr19:6833777	G/G	A	G	SNV	VAV1	VAV1:intronic:NM 001258207.1	1	100		intronic	NM 00125	c.1635+33	p.?	-0.24					CAMSAP CH d	rs186295	0.1265	YES	7.402E-1 (pos.6833777)		Oncogene V	DNA bindi	Glioma;Hemic and Lymphatic Diseases;Immu			A=0, G=21	A=0.0, G=1.0	0.00001	281.174	21	A=0/0, G=8/13
chr6:29634012	G/G	A	G	SNV	MOG	MOG:exonic:NM 002433.4	1	100	3	missense	NM 00243	c.520A>G	p.Ile174Val	-0.34	1	29	0		Immunoglobu	rs3130253	0.0888	YES	9.981E-1;0.0 (pos.29634012)		Myelin-olig	cell adhes	Autoimmune Diseases;Autoimmune Disease			A=0, G=62	A=0.0, G=1.0	0.00001	944.989	62	A=0/0, G=17/4
chr19:6937683	G/G	A	G	SNV	ADGRE1	ADGRE1:intronic:NM 001974.4	1	100		intronic	NM 00197	c.2655+24	p.?	-0.26					7 transmemb	rs388711	0.2598	YES	6.803E-1 (pos.6937683)		Adhesion G	G-protein coupled receptor activity,G-protein coupled re				A=0, G=50	A=0.0, G=1.0	0.00001	852.713	50	A=0/0, G=25/2
chr19:740560	C/C	T	C	SNV	PALM	PALM:intronic:NM 002579.2	1	100		intronic	NM 00257	c.634+77T	p.?	-1.82					Paralemmin	rs10419493		YES			Paralemmi	D3 dopamine receptor binding,apicolateral plasma men				T=0, C=28	T=0.0, C=1.0	0.00001	408.705	28	T=0/0, C=6/22
chr19:7506668	G/G	T	G	SNV	ARHGEF18	ARHGEF18:intronic:NM 001130955.1	1	100		intronic	NM 00113	c.655+17T	p.?	-0.16					PH domain,R	rs2287909	0.3538	YES	4.277E-1 (pos.7506668)		Rho guanin	G-protein coupled receptor signaling pathway,Rho guan				T=0, G=42	T=0.0, G=1.0	0.00001	685.214	42	T=0/0, G=24/1
chr19:7518268	TC/TC	T	TC	INDEL	ARHGEF18	ARHGEF18:intronic:NM 001130955.1	1	100		intronic	NM 00113	c.1292-85	p.?	0.37					PH domain,R	rs11463454,rs397711639		YES			Rho guanin	G-protein coupled receptor signaling pathway,Rho guan				T=0, TC=30	T=0.0, TC=1.0	0.00001	409.788	30	T=0/0, TC=18/
chr19:7622226	A/A	G	A	SNV	PNPLA6	PNPLA6:intronic:NM 001166111.1	1	100		intronic	NM 00116	c.3427+56	p.?	-0.33					Cyclic nucleo	rs1645800,rs552023867,rs7465		YES	0.0 (pos.7622221),0.0 (pos.7622222)		Patatin-like	developm	Abnormalities, Multiple:Ataxia:Blepharoptos			G=0, A=84	G=0.0, A=1.0	0.00001	1374.44	84	G=0/0, A=40/4
chr19:7623700	A/A	G	A	SNV	PNPLA6	PNPLA6:intronic:NM 001166111.1	1	100		intronic	NM 00116	c.3428-36	p.?	-0.13					Cyclic nucleo	rs535208	0.3923	YES	3.945E-1 (pos.7623700)		Patatin-like	developm	Abnormalities, Multiple:Ataxia:Blepharoptos			G=0, A=34	G=0.0, A=1.0	0.00001	503.735	34	G=0/0, A=16/1
chr16:77233394	G/G	A	G	SNV	MON1B,SYCE1L	SYCE1L:exonic:NM 001129979.2, MON1B:utr 3:NM 01494(	1	100	1	missense	NM 01494	c.2833A>G	p.?, p.Thr16Ala	-0.61	1	58	0		Synaptonema	rs4888607	3.00E-04		1.0 (pos.77233394)		Mon1, S. ce	cytoplasm,early viral transcription,endomembrane syst				A=0, G=30	A=0.0, G=1.0	0.00001	446.627	30	A=0/0, G=14/1
chr19:7755213	C/T	C	T	SNV	FCER2	FCER2:intronic:NM 002002.4	1	50		intronic	NM 00200	c.622-62G	p.?	1.41					Lectin C-type	rs4996972		YES			Fc fragment	IgE binding	Brain Diseases;Central Nervous System Dise			C=39, T=39	C=0.5, T=0.5	0.00001	400.403	78	C=19/20, T=18
chr19:7796497	G/G	C	G	SNV	CLEC4G	CLEC4G:intronic:NM 198492.3	1	100		intronic	NM 19849	c.166+50G	p.?	-1.44					Lectin C-type	rs11260026	0.0249	YES	7.023E-1 (pos.7796497)		C-type lecti	integral component of membrane,negative regulation of				C=0, G=56	C=0.0, G=1.0	0.00001	832.064	56	C=0/0, G=25/3
chr3:108690221	G/A	G	A	SNV	MORC1	MORC1:exonic:NM 014429.3	1	58.33	25	missense	NM 01442	c.2506C>T	p.His836Tyr	1.07	1	83	0	0.01(COSM40	CW-type Zinc	rs2593943	0.4215	YES	8.52E-2 (pos.108690221)	acute_myeloid	MORC famil	DNA hypermethylation,DNA methylation involved in gam				G=15, A=21	G=0.4167, A=0.5833	0.00001	234.327	36	G=6/9, A=14/7
chr19:7916475	C/C	T	C	SNV	EVI5L	EVI5L:intronic:NM 001159944.2	1	100		intronic	NM 00115	c.897+12T	p.?	-2.99					Rab-GTPase-1	rs2452012	0.1499	YES	7.001E-1 (pos.7916475)			GTPase activator activity,Rab GTPase binding,activation				T=0, C=30	T=0.0, C=1.0	0.00001	446.637	30	T=0/0, C=13/1
chr15:79185898	C/A	C	A	SNV	MORF4L1	MORF4L1:exonic:NM 206839.2	1	57.89	10	synonymous	NM 20683	c.675C>A	p.(=)	-0.29				0.43(COSM39	MRG,RNA bin	rs1435163	0.1267	YES	2.738E-1 (pos.79185898)	carcinoma	Mortality fa	NuA4 histo	Adenocarcinoma;Carcinoma;Carcinoma, Ade			C=24, A=33	C=0.4211, A=0.5789	0.00001	345.043	57	C=10/14, A=17
chr22:50555619	T/T	C	T	SNV	MOV10L1	MOV10L1:exonic:NM 018995.2	1	100	9	synonymous	NM 01899	c.1293C>T	p.(=)	-2.6					AAA domain	rs138222	0.0819	YES	8.929E-1 (pos.50555619)		Mov10-like	ATP binding,ATP-dependent RNA helicase activity,DNA m				C=0, T=63	C=0.0, T=1.0	0.00001	958.917	63	C=0/0, T=34/2
chr22:50555686	A/C	A	C	SNV	MOV10L1	MOV10L1:exonic:NM 018995.2	1	57.14	9	missense	NM 01899	c.1360A>C	p.Ile454Leu	0.75	0.81	5	0.059	0.48(COSM41	AAA domain	rs760749	0.1615	YES	2.255E-1 (pos.50555686)	acute_myeloid	Mov10-like	ATP binding,ATP-dependent RNA helicase activity,DNA m				A=27, C=36	A=0.4286, C=0.5714	0.00001	380.915	63	A=15/12, C=19
chr9:13112116	G/G	A	G	SNV	MPDZ	MPDZ:exonic:NM 003829.4	1	100	42	synonymous	NM 00382	c.5544T>C	p.(=)	1.05					L27_2,PDZ do	rs10809904	0		1.0;0.0 (pos.13112116)		Multiple PD	Schmidt-La	Brain Diseases;Central Nervous System Dise			A=0, G=32	A=0.0, G=1.0	0.00001	485.141	32	A=0/0, G=11/2
chr19:8137902	T/T	C	T	SNV	FBN3	FBN3:intronic:NM 032447.4	1	100		intronic	NM 03244	c.7937+45	p.?	-2.12					Calcium-bind	rs7254691	0.4603	YES	3.694E-1 (pos.8137902)			anatomical structure morphog		Calcium Citrate,Calcium		C=0, T=30	C=0.0, T=1.0	0.00001	446.364	30	C=0/0, T=6/24
chr19:8137908	T/T	C	T	SNV	FBN3	FBN3:intronic:NM 032447.4	1	100		intronic	NM 03244	c.7937+39	p.?	-0.84					Calcium-bind	rs28657412,rs38680	0.4795	YES	3.698E-1 (pos.8137908)			anatomical structure morphog		Calcium Citrate,Calcium		C=0, T=28	C=0.0, T=1.0	0.00001	413.153	28	C=0/0, T=4/24
chr17:41960343	C/C	T	C	SNV	MPP2	MPP2:exonic:NM 005374.4	1	100	5	synonymous	NM 00537	c.381A>G	p.(=)	-5.18					Guanylate kin	rs124719	0.1438	YES	9.997E-1 (pos.41960343)		Membrane	GDP metabolic process,GMP metabolic process,PDZ don				T=0, C=28	T=0.0, C=1.0	0.00001	408.718	28	T=0/0, C=22/6
chr19:815086	A/G	A	G	SNV	PLPPR3	PLPPR3:intronic:NM 024888.2	1	51.85		intronic	NM 02488	c.404-5T>C	p.?	-0.28				0.03(COSM37	PAP2 superfa	rs351988	0.4294	YES	4.367E-1 (pos.815086)	hepatocellula	Phospholip	integral component of membrane,integral component o				A=12, G=14	A=0.4615, G=0.5385	0.00001	143.549	27	A=8/4, G=10/4
chr22:43830996	T/T	C	T	SNV	MPPED1	MPPED1:exonic:NM 001044370.1	1	100	3	synonymous	NM 00104	c.267C>T	p.(=)	2.28					Calcineurin-l	rs2413761	0.4767	YES	9.125E-2 (pos.43830996)		Metallopho	biological_process,cellular_component,hydrolase activi				C=0, T=27	C=0.0, T=1.0	0.00001	389.988	27	C=0/0, T=19/8
chr22:43831056	C/T	C	T	SNV	MPPED1	MPPED1:exonic:NM 001044370.1	1	95.83	3	synonymous	NM 00104	c.327C>T	p.(=)	-0.3				0.12(COSM37	Calcineurin-l	rs4331615	0.479	YES	9.097E-2 (pos.43831056)	hepatocellula	Metallopho	biological_process,cellular_component,hydrolase activi				C=1, T=23	C=0.0417, T=0.9583	0.00001	306.637	24	C=1/0, T=14/9
chr11:118107805	G/G	A	G	SNV	MPZL3	MPZL3:exonic:NM 198275.2	1	100	3	synonymous	NM 19827	c.411T>C	p.(=)	0.17				0.06(COSM14	Immunoglobu	rs1793140	0.2601	YES	6.895E-1 (pos.11810780	acute_myeloid	Myelin prot	cell adhesion,extracellular matrix organization,hair cycl				A=0, G=24	A=0.0, G=1.0	0.00001	334.791	24	A=0/0, G=8/16
chr19:8190750	T/C	T	C	SNV	FBN3	FBN3:intronic:NM 032447.4	1	56.36		intronic	NM 03244	c.2731+26	p.?	-1.91					Calcium-bind	rs35988441	0.1618	YES	3.414E-1 (pos.8190750)			anatomical structure morphog		Calcium Citrate,Calcium		T=24, C=31	T=0.4364, C=0.5636	0.00001	312.079	55	T=19/5, C=19/
chr19:8191238	T/T	C	T	SNV	FBN3	FBN3:intronic:NM 032447.4	1	100		intronic	NM 03244	c.2555-7G	p.?	-1.21					Calcium-bind	rs10906993	0.0015		9.803E-1 (pos.8191238)			anatomical structure morphog		Calcium Citrate,Calcium		C=0, T=83	C=0.0, T=1.0	0.00001	1349.86	83	C=0/0, T=37/4
chr19:8191247	CG/CG	C	CG	INDEL	FBN3	FBN3:intronic:NM 032447.4	1	100		intronic	NM 03244	c.2555-16	p.?	-1.86					Calcium-bind	rs756551083			0.0 (pos.8191247)			anatomical structure morphog		Calcium Citrate,Calcium		C=0, CG=83	C=0.0, CG=1.0	0.00001	1025.57	83	C=0/0, CG=37/
chr19:8191248	G/G	A	G	SNV	FBN3	FBN3:intronic:NM 032447.4	1	100		intronic	NM 03244	c.2555-17	p.?	-0.88					Calcium-bind	rs10906994	0.0021	YES	9.615E-1 (pos.8191248)			anatomical structure morphog		Calcium Citrate,Calcium		A=0, G=83	A=0.0, G=1.0	0.00001	1349.55	83	A=0/0, G=37/4
chr19:8197786	G/A	G	A	SNV	FBN3	FBN3:intronic:NM 032447.4	1	45.83		intronic	NM 03244	c.1714+82	p.?	-1.46					Calcium-bind	rs11671264		YES				anatomical structure morphog		Calcium Citrate,Calcium		G=13, A=11	G=0.5417, A=0.4583	0.00001	95.5282	24	G=11/2, A=8/3
chr19:846548	C/T	C	T	SNV	PRTN3	PRTN3:intronic:NM 002777.3	1	95.65		intronic	NM 00277	c.600+171	p.?	-1.74					Domain of un	rs631822		YES			Proteinase-	antimicrol	Anti-Neutrophil Cyt	Elafin		C=2, T=44	C=0.0435, T=0.9565	0.00001	707.102	46	C=1/1, T=8/36
chr1:181024404	A/A	G	A	SNV	MR1	MR1:utr 3:NM 001531.2	1	100	7	utr 3	NM 00153	c.1029G>A	p.?	0.06					Class I Histoc	rs3747956	0.2922	YES	7.447E-1 (pos.181024404)		Major histo	MHC class I protein complex,M	Antithymocyte immunog			G=0, A=50	G=0.0, A=1.0	0.00001	852.746	50	G=0/0, A=30/2
chr19:8555886	C/C	G	C	SNV	PRAM1	PRAM1:intronic:NM 032152.4	1	100		intronic	NM 03215	c.1635-7C	p.?	-0.74					Helically-exte	rs2303110	0.275	YES	4.799E-1;0.0 (pos.8555886)			integrin-mediated signaling pathway,lipid binding,prote				G=0, C=24	G=0.0, C=1.0	0.00001	330.524	24	G=0/0, C=11/1
chr17:60766224	G/G	A	G	SNV	MRC2	MRC2:exonic:NM 006039.4	1	100	23	synonymous	NM 00603	c.3237A>G	p.(=)	-2.4				0.03(COSM41	Fibronectin t	rs2465430	0.0134	YES	9.357E-1 (pos.60766224)	neoplasm	Mannose re	carbohydr	Mammary Neoplasms, Animal;Mammary Nec			A=0, G=30	A=0.0, G=1.0	0.00001	446.63	30	A=0/0, G=8/22
chr17:60767015	A/A	G	A	SNV	MRC2	MRC2:exonic:NM 006039.4	1	100	24	missense	NM 00603	c.3467G>A	p.Arg1156His	0.99	0.59	29	0.005	0.88(COSM37	Fibronectin t	rs2429387	0.0159	YES	9.387E-1 (pos.60767015)	adenocarcinor	Mannose re	carbohydr	Mammary Neoplasms, Animal;Mammary Nec			G=0, A=27	G=0.0, A=1.0	0.00001	389.988	27	G=0/0, A=15/1
chr17:60769803	G/G	A	G	SNV	MRC2	MRC2:exonic:NM 006039.4	1	100	30	synonymous	NM 00603	c.4431A>G	p.(=)	3.74				0.08(COSM37	Fibronectin t	rs3826537	0.4004	YES	3.972E-1 (pos.60769803)	adenocarcinor	Mannose re	carbohydr	Mammary Neoplasms, Animal;Mammary Nec			A=0, G=36	A=0.0, G=1.0	0.00001	563.725	36	A=0/0, G=18/1
chr11:18159169	G/G	A	G	SNV	MRGPRX3	MRGPRX3:exonic:NM 054031.3	1	100	3	synonymous	NM 05403	c.420A>G	p.(=)	-2.37				0.01(COSM41	7 transmemb	rs2468839,rs760632	9.00E-04	YES	2.427E-3 (pos.18159162)	neoplasm	Mas-relate	G-protein coupled receptor activity,G-protein coupled re				A=0, G=35	A=0.0, G=1.0	0.00001	543.903	35	A=0/0, G=17/1
chr19:899567	G/G	C	G	SNV	R3HDM4	R3HDM4:intronic:NM 138774.3	1	100		intronic	NM 13877	c.647+34G	p.?	-1.57					R3H domain,I	rs2930885,rs768197	0.35	YES	0.0 (pos.899550),3.08E-1;0.0 (pos.899567)			nucleic acid binding,nucleus				C=0, G=22	C=0.0, G=1.0	0.00001	298.887	22	C=0/0, G=13/9
chr19:8995853	C/C	T	C	SNV	MUC16	MUC16:intronic:NM 024690.2	1	100		intronic	NM 02469	c.41287+1	p.?	-1.21					SEA domain	rs11672574		YES			Mucin 16	Golgi lume	Adnexal Diseases;E	Oregovomab		T=0, C=40	T=0.0, C=1.0	0.00001	615.879	40	T=0/0, C=25/1
chr19:9007748	A/G	A	G	SNV	MUC16	MUC16:intronic:NM 024690.2	1	50		intronic	NM 02469	c.39415+6	p.?	-0.28					SEA domain	rs11673136	0.4683	YES			Mucin 16	Golgi lume	Adnexal Diseases;E	Oregovomab		A=11, G=11	A=0.5, G=0.5	0.00001	94.6068	22	A=10/1, G=9/2
chr19:9019429	T/C	T	C	SNV	MUC16	MUC16:intronic:NM 024690.2	1	96.67		intronic	NM 02469	c.37547-8	p.?	-0.93					SEA domain	rs881877		YES			Mucin 16	Golgi lume	Adnexal Diseases;E	Oregovomab		T=1, C=29	T=0.0333, C=0.9667	0.00001	416.515	30	T=1/0, C=15/1
chr11:18195051	C/C	T	C	SNV	MRGPRX4	MRGPRX4:exonic:NM 054032.3	1	100	1	missense	NM 05403	c.248T>C	p.Leu83Ser	-2.96		145			7 transmemb	rs2445179	9.00E-04	YES	1.0 (pos.18195051)		Mas-relate	G-protein coupled receptor activity,G-protein coupled re				T=0, C=28	T=0.0, C=1.0	0.00001	408.678	28	T=0/0, C=16/1
chr11:18195448	C/T	C	T	SNV	MRGPRX4	MRGPRX4:exonic:NM 054032.3	1	50	1	synonymous	NM 05403	c.645C>T	p.(=)	-2.94				0(COSM1178	7 transmemb	rs4630269	0.285	YES	4.324E-1;0.0 (pos.18195	adenoma	Mas-relate	G-protein coupled receptor activity,G-protein coupled re				C=11, T=11	C=0.5, T=0.5	0.00001	94.6067	22	C=6/5, T=3/8
chr18:48327815	A/A	G	A	SNV	MRO	MRO:exonic:NM 001127176.1	1	100	5	synonymous	NM 00112	c.531C>T	p.(=)	0.1				0.11(COSM1130061),0.11(C		rs2276186	0.4976	YES	3.471E-1 (pos.48327815)	acute_myeloid	Maestro	nucleolus				G=0, A=25	G=0.0, A=1.0	0.00001	353.019	25	G=0/0, A=6/19
chr8:145223288	T/T	C	T	SNV	MROH1	MROH1:exonic:NM 032450.2	1	100	4	missense	NM 03245	c.113C>T	p.Ala38Val	0.13	0.4	64	0	0.03(COSM41	HEAT repeat,I	rs34819224	0.0035	YES	9.999E-1;0.0 (pos.14522	neoplasm						C=0, T=36	C=0.0, T=1.0	0.00001	554.792	36	C=0/0, T=19/1
chr5:41008780	G/G	A	G	SNV	MROH2B	MROH2B:exonic:NM 173489.4	1	100	33	missense	NM 17348	c.3536T>C	p.Leu1179Pro	1.51	1	98	0			rs2271704	0.1824	YES	6.166E-1;0.0 (pos.41008780)			acrosomal vesicle,cell differentiation,cytoplasm,proteir				A=0, G=41	A=0.0, G=1.0	0.00001	660.376	41	A=0/0, G=18/2
chr5:41042284	T/T	G	T	SNV	MROH2B	MROH2B:exonic:NM 173489.4	1	100	19	synonymous	NM 17348	c.1863C>A	p.(=)	-0.49				0.52(COSM4159875)		rs325854	0.1564	YES	6.879E-1 (pos.41042284)	neoplasm		acrosomal vesicle,cell differentiation,cytoplasm,proteir				G=0, T=28	G=0.0, T=1.0	0.00001	407.671	28	G=0/0, T=7/21
chr5:41049389	C/C	T	C	SNV	MROH2B	MROH2B:exonic:NM 173489.4	1	100	14	synonymous	NM 17348	c.1494A>G	p.(=)	-0.98						rs325863	0.1061	YES	6.644E-1 (pos.41049389)			acrosomal vesicle,cell differentiation,cytoplasm,proteir				T=0, C=43	T=0.0, C=1.0	0.00001	709.766	43	T=0/0, C=26/1
chr8:142459814	T/T	C	T	SNV	MROH5	MROH5:exonic:NM 207414.2	1	100	20	missense	NM 20741	c.2513G>A	p.Gly838Glu	0.97		98		0(COSM4162699),0(COSM4		rs7003306			9.644E-1 (pos.14245981	neoplasm,squamous_cell_carcinoma						C=0, T=40	C=0.0, T=1.0	0.00001	644.286	40	C=0/0, T=14/2
chr8:142476555	A/G	A	G	SNV	MROH5	MROH5:exonic:NM 207414.2	1	50	19	missense	NM 20741	c.2431T>C	p.Cys811Arg	-0.76		180				rs28439396	0.1825	YES	1.39E-1 (pos.142476555)							A=35, G=35	A=0.5, G=0.5	0.00001	355.118	70	A=10/25, G=8/
chr1:55118566	G/G	C	G	SNV	MROH7,MROH7-TTC4	MROH7-TTC4:exonic_nc:NR 037639.2, MROH7:utr 5:NM 0C	1	100	3	utr 5	NR 03763	c.-34C>G	p.?	0.11						rs1655520	0.0018	YES	1.0;0.0 (pos.55118566)			extracellular space,integral component of membrane				C=0, G=27	C=0.0, G=1.0	0.00001	389.988	27	C=0/0, G=19/8
chr1:55167773	A/A	T	A	SNV	MROH7,MROH7-TTC4	MROH7-TTC4:exonic_nc:NR 037639.2, MROH7:exonic:NM C	1	100	20	missense	NR 03763	c.3296T>A	p.Val1099Asp	0.57	1	152	0			rs646356	0.1655	YES	9.364E-1 (pos.55167773)			extracellular space,integral component of membrane				T=0, A=25	T=0.0, A=1.0	0.00001	353.018	25	T=0/0, A=12/1
chr1:55182300	T/A	T	A	SNV	MROH7-TTC4,TTC4	TTC4:exonic:NM 004623.4, MROH7-TTC4:exonic_nc:NR 037	1	47.37	2	missense	NR 03763	c.139T>A	p.Ser47Thr	5.93	0.15	58	0	0.15(COSM3751310)		rs1147990	0.489	YES	9.636E-1 (pos.55182300)	adenocarcinor	Tetratricopeptide repeat domain 4					T=40, A=36	T=0.5263, A=0.4737	0.00001	363.985	76	T=20/20, A=21
chr20:35740794	C/C	T	C	SNV	MROH8	MROH8:exonic:NM 152503.5	1	100	21	missense	NM 15250	c.2748A>G	p.Gln916Arg	-0.24		43		0.15(COSM5425295)		rs1744760	0.4802	YES	4.662E-1 (pos.35740794)	acute_myeloid leukaemia						T=0, C=93	T=0.0, C=1.0	0.00001	1555.01	93	T=0/0, C=7/86
chr19:9449149	G/G	A	G	SNV	ZNF559,ZNF559-ZNF177	ZNF559-ZNF177:intronic:NM 001172650.2, ZNF559:intronic	1	100		intronic	NM 00120	c.137-21A	p.?, p.?	-0.3					C2H2-type zir	rs8110197	0.407	YES	4.327E-1 (pos.9449149)			DNA bindi	Digestive System Diseases:Digestive System			A=0, G=25	A=0.0, G=1.0	0.00001	353.018	25	A=0/0, G=10/1
chr19:9491210	G/G	C	G	SNV	ZNF177,ZNF559-ZNF177	ZNF177:intronic:NM 001172651.1, ZNF559-ZNF177:intronic	1	100		intronic	NM 00117	c.336+395	p.?, p.?	-0.67					C2H2-type zir	rs1347165		YES			Zinc finger p	DNA bindi	Digestive System Diseases;Digestive System			C=0, G=36	C=0.0, G=1.0	0.00001	563.744	36	C=0/0, G=24/1
chr19:960187	C/C	G	C	SNV	ARID3A	ARID3A:intronic:NM 005224.2	1	100		intronic	NM 00522	c.766+23G	p.?	-0.28					ARID/BRIGHT	rs1637993	0.0247	YES	9.853E-1;0.0;0.0 (pos.960187)			DNA binding,DNA damage response, signal transduction				G=0, C=23	G=0.0, C=1.0	0.00001	316.748	23	G=0/0, C=16/7
chr19:960266	T/T	C	T	SNV	ARID3A	ARID3A:intronic:NM 005224.2	1	100		intronic	NM 00522	c.766+102	p.?	-1.88					ARID/BRIGHT	rs1683584		YES				DNA binding,DNA damage response, signal transduction				C=0, T=21	C=0.0, T=1.0	0.00001	281.235	21	C=0/0, T=14/7
chr20:35769592	C/C	T	C	SNV	MROH8	MROH8:exonic:NM 152503.5	1	100	12	synonymous	NM 15250	c.1462A>G	p.(=)	-0.22				0.04(COSM5426224)		rs1744769	0.1808	YES	5.061E-1 (pos.35769592)	acute_myeloid leukaemia						T=0, C=37	T=0.0, C=1.0	0.00001	581.249	37	T=0/0, C=30/7
chr19:991197	C/C	G	C	SNV	WDR18	WDR18:intronic:NM 024100.3	1	100		intronic	NM 02410	c.807-30G	p.?	0					Alternative W	rs138855476,rs201259019,rs60416441,rs7			0.0 (pos.991197),0.0;0.0 (pos.991195),0.0;0.0;0.0;0.0			cytoplasm,multicellular organism development,nucleol				G=0, C=58	G=0.0, C=1.0	0.00001	871.863	58	G=0/0, C=6/52
chr20:35769647	T/T	G	T	SNV	MROH8	MROH8:exonic:NM 152503.5	1	100	12	missense	NM 15250	c.1407C>A	p.Thr469Asn	0.02		65		0(COSM5425296)		rs1780680	0.4117	YES	4.856E-1;0.0 (pos.35769	acute_myeloid leukaemia						G=0, T=36	G=0.0, T=1.0	0.00001	560.312	36	G=0/0, T=28/8
chr2:100081317	T/C	T	C	SNV	REV1	REV1:intronic:NM 016316.3	1	56		intronic	NM 01631	c.54+67A>	p.?	0.23					BRCA1 C Term	rs28369944	0.1233	YES			Rev1, S. cer	DNA repair,DNA replication,damaged DNA binding,deox				T=11, C=14	T=0.44, C=0.56	0.00001	134.35	25	T=9/2, C=10/4
chr2:100081323	T/T	C	T	SNV	REV1	REV1:intronic:NM 016316.3	1	100		intronic	NM 01631	c.54+61G>	p.?	0.64					BRCA1 C Term	rs28369943	0.4588	YES			Rev1, S. cer	DNA repair,DNA replication,damaged DNA binding,deox				C=0, T=25	C=0.0, T=1.0	0.00001	353.019	25	C=0/0, T=19/6
chr2:99804642	C/C	T	C	SNV	MRPL30	MRPL30:exonic:NM 145212.3	1	100	3	synonymous	NM 14521	c.54T>C	p.(=)	-1.1					Ribosomal pr	rs10865030	0.1195	YES	9.7E-1 (pos.99804642)		Mitochondr	large ribosomal subunit,mitochondrial inner membrane				T=0, C=70	T=0.0, C=1.0	0.00001	1092.09	70	T=0/0, C=37/3
chr2:86437898	T/T	C	T	SNV	MRPL35	MRPL35:utr 3:NM 016622.3	1	100	4	utr 3	NM 01662	c.674C>T	p.?	-0.01					Ribosomal pr	rs13353		YES			Mitochondr	mitochondrial inner membrane,mitochondrial large ribc				C=0, T=72	C=0.0, T=1.0	0.00001	1130.94	72	C=0/0, T=24/4
chr5:1799001	T/G	T	G	SNV	MRPL36	MRPL36:exonic:NM 032479.3	1	45.45	2	missense	NM 03247	c.49A>C	p.Ser17Arg	0.01	0	110	0.987		Ribosomal pr	rs114776273	0.021	YES	1.16E-4;0.0 (pos.1799001)		Mitochondr	mitochondrial inner membrane,mitochondrial large ribc				T=12, G=10	T=0.5455, G=0.4545	0.00001	81.7603	22	T=9/3, G=6/4
chr2:10045147	G/G	A	G	SNV	TAF1B	TAF1B:intronic:NM 005680.2	1	100		intronic	NM 00568	c.955+12A	p.?	-1.42					Zinc-finger of	rs448327	0.0021	YES	1.0 (pos.10045147)			DNA binding transcription factor activity,RNA polymeras				A=0, G=20	A=0.0, G=1.0	0.00001	263.782	20	A=0/0, G=8/12
chr17:73900688	T/C	T	C	SNV	MRPL38	MRPL38:exonic:NM 032478.3	1	51.85	2	synonymous	NM 03247	c.180A>G	p.(=)	-0.43				0.01(COSM37	Phosphatidyl	rs3744015	0.3332	YES	2.607E-1 (pos.73900688)	adenocarcinor	Mitochondr	mitochondrial inner membrane,mitochondrial large ribc				T=26, C=28	T=0.4815, C=0.5185	0.00001	277.174	54	T=3/23, C=2/2
chr2:100925803	G/G	A	G	SNV	LONRF2	LONRF2:intronic:NM 198461.3	1	100		intronic	NM 19846	c.680-116	p.?	0.44					ATP-depende	rs10195793		YES				intracellular,metal ion binding,positive regulation of prc				A=0, G=22	A=0.0, G=1.0	0.00001	298.892	22	A=0/0, G=7/15
chr21:26965148	A/A	G	A	SNV	MRPL39	MRPL39:exonic:NM 080794.3	1	100	8	synonymous	NM 08079	c.897C>T	p.(=)	0.01				0.14(COSM5618718),0.14(C		rs1135638	0.1731	YES	7.258E-1 (pos.26965148)	carcinoma,duc	Mitochondr	RNA bindir	Abnormalities, Multiple;Chromosome Disord			G=0, A=66	G=0.0, A=1.0	0.00001	1015.4	66	G=0/0, A=27/3
chr2:101179341	GA/GA	AG	GA	MNV	PDCL3	PDCL3:upstream:NM 024065.4	2	100		intronic	NM 02406	c.-189AG>	p.?	-1.98, -0.2						rs2309848,rs2970988,rs353391		YES			Phosducin-l	angiogenesis,apoptotic process,cytoplasm,cytosol,nega				AG=0, GA=3	AG=0.0, GA=1.0	0.00001	489.574	32	AG=0/0, GA=2
chr2:101541798	T/T	C	T	SNV	NPAS2	NPAS2:intronic:NM 002518.3	1	100		intronic	NM 00251	c.181+42C	p.?	1.59					Helix-loop-he	rs356651	0.1766	YES	9.991E-1 (pos.101541798)		Neuronal P	DNA bindi	Autistic Disorder:Child Development Disorde			C=0, T=44	C=0.0, T=1.0	0.00001	726.36	44	C=0/0, T=7/37
chr19:39421388	G/G	A	G	SNV	MRPS12,SARS2	SARS2:utr 5:NM 001145901.1, MRPS12:upstream:NM 021	1	100	1	utr 5	NM 00114	c.-12T>C, c	p.?, p.?	0.33					tRNA synthet	rs730078	0.3791	YES	9.683E-1 (pos.39421388)		Mitochondr	ATP binding,RNA binding,cytoplasm,ligase			Benign	A=0, G=26	A=0.0, G=1.0	0.00001	371.27	26	A=0/0, G=12/1
chr1:36921408	G/A	G	A	SNV	MRPS15	MRPS15:exonic:NM 031280.3	1	45.83	8	missense	NM 03128	c.755C>T	p.Thr252Ile	0.35	0.25	89	0.001		Ribosomal pr	rs35544556	0.0123		0.0 (pos.36921408)		Mitochondr	RNA binding,mitochondrial inner membrane,mitochondi				G=13, A=11	G=0.5417, A=0.4583	0.00001	93.4399	24	G=7/6, A=6/5
chr21:35467472	A/A	AG	A	INDEL	MRPS6,SLC5A3	SLC5A3:utr 5:NM 006933.6, MRPS6:intronic:NM 032476.3	1	100	2	utr 5	NM 00693	c.-26AG>A	p.?, p.?	3.9, 0.0					Ribosomal pr	rs11295803,rs369490054,rs776		YES	1.0 (pos.35467472)		Mitochondr	mitochonc	Abnormalities, Multiple:Adenocarcinoma:Ar			AG=0, A=55	AG=0.0, A=1.0	0.00001	814.028	55	AG=0/0, A=17/
chr11:10655609	T/T	C	T	SNV	MRVI1	MRVI1:exonic:NM 001098579.2	1	100	2	missense	NM 00109	c.208G>A	p.Ala70Thr	-1.01	1	58	0		MRVI1 protei	rs2162044	0.0141	YES	9.995E-1 (pos.10655609)		Murine retr	endoplasr	Brain Diseases;Central Nervous System Dise			C=0, T=57	C=0.0, T=1.0	0.00001	849.611	57	C=0/0, T=21/3
chr2:102632574	A/G	A	G	SNV	IL1R2	IL1R2:intronic:NM 004633.3	1	57.14		intronic	NM 00463	c.513+61A	p.?	-0.74					Immunoglobu	rs3819370		YES			Interleukin-	cytokine-n	Ankylosis;Arthritis;Bone Diseases;Colitis;Col			A=96, G=12	A=0.4286, G=0.5714	0.00001	1480.59	224	A=50/46, G=6
chr2:102788409	A/A	G	A	SNV	IL1R1	IL1R1:intronic:NM 000877.3	1	100		intronic	NM 00087	c.839+31G	p.?	-0.3					Immunoglobu	rs2041747	0.0438	YES	9.986E-1 (pos.102788409)		Interleukin-	cell surfac	Autoimmune Disea	Anakinra,OMS-103HP,SD		G=0, A=75	G=0.0, A=1.0	0.00001	1189.79	75	G=0/0, A=38/3
chr11:60285575	G/G	A	G	SNV	MS4A13	MS4A13:exonic:NM 001012417.2	1	100	3	missense	NM 00101	c.19A>G	p.Ile7Val	0.02	1	29	0			rs10736706	8.00E-04	YES	1.0 (pos.60285575)			integral component of membrane				A=0, G=125	A=0.0, G=1.0	0.00001	2034.06	125	A=0/0, G=69/5
chr2:103040177	A/T	A	T	SNV	IL18RAP	IL18RAP:intronic:NM 003853.3	1	53.13		intronic	NM 00385	c.71-94A>	p.?	0.13					TIR domain	rs6543133		YES			Interleukin	T-helper 1	Colitis;Colitis, Ulcerative;Colonic Diseases;C			A=15, T=17	A=0.4688, T=0.5313	0.00001	173.56	32	A=8/7, T=7/10
chr2:103040182	T/G	T	G	SNV	IL18RAP	IL18RAP:intronic:NM 003853.3	1	53.13		intronic	NM 00385	c.71-89T>	p.?	-0.34					TIR domain	rs9807934		YES			Interleukin	T-helper 1	Colitis;Colitis, Ulcerative;Colonic Diseases;C			T=15, G=17	T=0.4688, G=0.5313	0.00001	173.559	32	T=8/7, G=7/10
chr2:103040249	G/G	C	G	SNV	IL18RAP	IL18RAP:intronic:NM 003853.3	1	100		intronic	NM 00385	c.71-22C>	p.?	-1.74					TIR domain	rs201039117,rs2272129,rs3215		YES	5.535E-1;0.0;0.0 (pos.103040249)		Interleukin	T-helper 1	Colitis;Colitis, Ulcerative;Colonic Diseases;C			C=0, G=32	C=0.0, G=1.0	0.00001	485.14	32	C=0/0, G=15/1
chr2:103125457	G/G	A	G	SNV	SLC9A4	SLC9A4:intronic:NM 001011552.3	1	100		intronic	NM 00101	c.1488+65	p.?	-2.22					Regulatory re	rs6739301,rs941727499		YES			Solute carri	apical pla	Acid-Base Imbalance;Acidosis;Metabolic Dis			A=0, G=47	A=0.0, G=1.0	0.00001	789.111	47	A=0/0, G=17/3
chr2:103125466	C/C	T	C	SNV	SLC9A4	SLC9A4:intronic:NM 001011552.3	1	100		intronic	NM 00101	c.1488+74	p.?	-0.7					Regulatory re	rs6742381		YES			Solute carri	apical pla	Acid-Base Imbalance;Acidosis;Metabolic Dis			T=0, C=46	T=0.0, C=1.0	0.00001	768.242	46	T=0/0, C=16/3
chr2:103136309	C/C	T	C	SNV	SLC9A4	SLC9A4:intronic:NM 001011552.3	1	100		intronic	NM 00101	c.1722-9T	p.?	-0.12				0.16(COSM37	Regulatory re	rs1403550	0.2303	YES	5.379E-1 (pos.10313630	hepatocellula	Solute carri	apical pla	Acid-Base Imbalance;Acidosis;Metabolic Dis			T=0, C=52	T=0.0, C=1.0	0.00001	767.009	52	T=0/0, C=37/1
chr2:103141447	A/G	A	G	SNV	SLC9A4	SLC9A4:intronic:NM 001011552.3	1	59.09		intronic	NM 00101	c.1819-36	p.?	-0.06					Regulatory re	rs2015478	0.3652	YES	5.031E-1 (pos.103141447)		Solute carri	apical pla	Acid-Base Imbalance;Acidosis;Metabolic Dis			A=9, G=13	A=0.4091, G=0.5909	0.00001	122.568	22	A=7/2, G=4/9
chr2:103141651	C/C	G	C	SNV	SLC9A4	SLC9A4:intronic:NM 001011552.3	1	100		intronic	NM 00101	c.1950+37	p.?	-2.04					Regulatory re	rs1024798	0.2308	YES	5.275E-1;0.0 (pos.103141651)		Solute carri	apical pla	Acid-Base Imbalance;Acidosis;Metabolic Dis			G=0, C=25	G=0.0, C=1.0	0.00001	351.604	25	G=0/0, C=8/17
chr2:106690542	A/A	G	A	SNV	C2orf40	C2orf40:intronic:NM 032411.2	1	100		intronic	NM 03241	c.285+43G	p.?	0.2					Oesophageal	rs35317173	0.3906	YES	4.265E-2 (pos.106690542)		Esophageal	G1 to G0 transition,anaphase-promoting complex-deper				G=0, A=20	G=0.0, A=1.0	0.00001	263.713	20	G=0/0, A=7/13
chr2:106782398	G/G	C	G	SNV	UXS1	UXS1:intronic:NM 001253875.1	1	100		intronic	NM 00125	c.122+114	p.?	-0.11					3-beta hydro	rs4851115		YES			UDP-glucur	Golgi ciste	Congenital Abnormalities;Congenital, Heredi			C=0, G=24	C=0.0, G=1.0	0.00001	334.787	24	C=0/0, G=7/17
chr2:10729897	T/T	C	T	SNV	NOL10	NOL10:intronic:NM 024894.3	1	100		intronic	NM 02489	c.1423-20	p.?	-0.59					NUC153 dom	rs759403	0.4943	YES	6.217E-1 (pos.10729897)		Nucleolar p	RNA bindir	Genital Diseases, Male;Genital Neoplasms, I			C=0, T=22	C=0.0, T=1.0	0.00001	298.892	22	C=0/0, T=12/1
chr11:60291413	T/T	C	T	SNV	MS4A13	MS4A13:exonic:NM 001012417.2	1	100	4	missense	NM 00101	c.173C>T	p.Pro58Leu	-0.21	0	98	0.962	0.06(COSM5766925)		rs6591595	0.1326	YES	7.197E-1 (pos.60291413)	adenocarcinoma,carcinom		integral component of membrane				C=0, T=31	C=0.0, T=1.0	0.00001	465.819	31	C=0/0, T=20/1
chr11:60165352	ATT/A	ATT	A	INDEL	MS4A14	MS4A14:exonic:NM 001261828.1	2	53.33	2	frameshiftDeletion	NM 00126	c.167_168	p.Ile56fs	3.93, 1.27, -0.79					CD20-like fan	rs3217518,rs747337	0.35	YES	0.0 (pos.60165350),0.0 (	adenocarcinoma,astrocyto		integral component of membrane				ATT=21, A=	ATT=0.4667, A=0.533	0.00001	275.658	45	ATT=9/12, A=2
chr2:10747207	G/G	A	G	SNV	NOL10	NOL10:intronic:NM 024894.3	1	100		intronic	NM 02489	c.1153+10	p.?	-0.2					NUC153 dom	rs6759714		YES			Nucleolar p	RNA bindir	Genital Diseases, Male;Genital Neoplasms, I			A=0, G=72	A=0.0, G=1.0	0.00001	1130.95	72	A=0/0, G=33/3
chr2:10747246	G/G	C	G	SNV	NOL10	NOL10:intronic:NM 024894.3	1	100		intronic	NM 02489	c.1153+65	p.?	-0.91					NUC153 dom	rs6744854		YES			Nucleolar p	RNA bindir	Genital Diseases, Male;Genital Neoplasms, I			C=0, G=71	C=0.0, G=1.0	0.00001	1120.2	71	C=0/0, G=32/3
chr8:72966002	A/A	G	A	SNV	MSC-AS1,TRPA1	TRPA1:exonic:NM 007332.2, MSC-AS1:exonic_nc:NR 03365	1	100	13	synonymous	NR 03365	c.1630C>T	p.(=)	1.25					Ankyrin repe	rs3735943	0.4923	YES	6.776E-1 (pos.72966002)		Transient re	calcium ch	Asthma;Bronchial D	Menthol		G=0, A=28	G=0.0, A=1.0	0.00001	408.711	28	G=0/0, A=21/7
chr8:72936145	T/C	T	C	SNV	MSC-AS1,TRPA1	TRPA1:exonic:NM 007332.2, MSC-AS1:intronic_nc:NR 0336	1	57.14	26	missense	NR 03365	c.3053A>G	p.His1018Arg	0.33	0.6	29	0	0(COSM3736	Ankyrin repe	rs959976	0.1776	YES	3.762E-1 (pos.72936145)	acute_myeloid	Transient re	calcium ch	Asthma;Bronchial D	Menthol		T=42, C=56	T=0.4286, C=0.5714	0.00001	673.832	98	T=21/21, C=35
chr8:15978063	T/T	C	T	SNV	MSR1	MSR1:exonic:NM 138715.2	1	100	9	synonymous	NM 13871	c.1086G>A	p.(=)	-0.38					Collagen trip	rs4338104	0		1.0 (pos.15978063)		Macrophag	amyloid-b	Autoimmune Diseases;Female Urogenital Dis			C=0, T=62	C=0.0, T=1.0	0.00001	940.32	62	C=0/0, T=27/3
chr2:10929133	G/G	A	G	SNV	PDIA6	PDIA6:intronic:NM 005742.3	1	100		intronic	NM 00574	c.841-26T	p.?	0.01					Thioredoxin	rs1734430	0.404	YES	4.566E-1 (pos.10929133)		Protein disu	IRE1-mediated unfolded protein response,apoptotic cell				A=0, G=92	A=0.0, G=1.0	0.00001	1534.54	92	A=0/0, G=18/7
chr2:10929867	T/C	T	C	SNV	PDIA6	PDIA6:intronic:NM 005742.3	1	96.67		intronic	NM 00574	c.840+8A>	p.?	-2.99				0.02(COSM40	Thioredoxin	rs11904084	0.4036	YES	4.555E-1;0.0 (pos.10929	acute_myeloid	Protein disu	IRE1-mediated unfolded protein response,apoptotic cell				T=1, C=29	T=0.0333, C=0.9667	0.00001	417.482	30	T=0/1, C=17/1
chr2:10930050	T/T	A	T	SNV	PDIA6	PDIA6:intronic:NM 005742.3	1	100		intronic	NM 00574	c.700-35T	p.?	0.6					Thioredoxin	rs1686514	0.4076	YES	4.567E-1 (pos.10930050)		Protein disu	IRE1-mediated unfolded protein response,apoptotic cell				A=0, T=29	A=0.0, T=1.0	0.00001	427.536	29	A=0/0, T=10/1
chr2:10931001	G/G	A	G	SNV	PDIA6	PDIA6:intronic:NM 005742.3	1	100		intronic	NM 00574	c.585-42T	p.?	-1.58					Thioredoxin	rs1614107	0.4049	YES	4.582E-1 (pos.10931001)		Protein disu	IRE1-mediated unfolded protein response,apoptotic cell				A=0, G=21	A=0.0, G=1.0	0.00001	281.235	21	A=0/0, G=15/6
chr2:10931046	A/A	G	A	SNV	PDIA6	PDIA6:intronic:NM 005742.3	1	100		intronic	NM 00574	c.585-87C	p.?	0.56					Thioredoxin	rs1734419		YES			Protein disu	IRE1-mediated unfolded protein response,apoptotic cell				G=0, A=20	G=0.0, A=1.0	0.00001	263.749	20	G=0/0, A=14/6
chr2:10932082	C/C	T	C	SNV	PDIA6	PDIA6:intronic:NM 005742.3	1	100		intronic	NM 00574	c.454-31A	p.?	-0.05					Thioredoxin	rs1734421	0.4051	YES	4.56E-1 (pos.10932082)		Protein disu	IRE1-mediated unfolded protein response,apoptotic cell				T=0, C=38	T=0.0, C=1.0	0.00001	603.786	38	T=0/0, C=22/1
chr2:10933346	T/TAGGC	T	TAGGC	INDEL	PDIA6	PDIA6:intronic:NM 005742.3	4	50		intronic	NM 00574	c.347-18A	p.?	0					Thioredoxin	rs11887543,rs14377	0.2852	YES	3.671E-1;0.0 (pos.10933346)		Protein disu	IRE1-mediated unfolded protein response,apoptotic cell				T=14, TAGC	T=0.5, TAGGC=0.5	0.00001	130.443	28	T=2/12, TAGG
chr2:10933421	A/A	G	A	SNV	PDIA6	PDIA6:intronic:NM 005742.3	1	100		intronic	NM 00574	c.347-93C	p.?	-0.13					Thioredoxin	rs1686517,rs781573813		YES			Protein disu	IRE1-mediated unfolded protein response,apoptotic cell				G=0, A=23	G=0.0, A=1.0	0.00001	316.747	23	G=0/0, A=6/17
chr3:49724808	T/C	T	C	SNV	MST1	MST1:exonic:NM 020998.3	1	97.5	4	synonymous	NM 02099	c.459A>G	p.(=)	-1.62					Kringle doma	rs3020779	0.1839	YES	9.91E-1 (pos.49724808)		Macrophag	cellular re	Bile Duct Diseases;Biliary Tract Diseases;Chc			T=1, C=39	T=0.025, C=0.975	0.00001	611.627	40	T=1/0, C=19/2

chr2:1093965	A/A	G	A	SNV	SNTG2	SNTG2:intronic:NM_018968.3	1	100		intronic	NM_01896	c.267+27G	p.?	-0.84					PDZ domain (	rs11127460	0.4889	YES	2.811E-1;0.0 (pos.1093965)			PDZ doma	Autistic Disorder;Child Development Disorde			G=0, A=57	G=0.0, A=1.0	0.00001	849.615	57	G=0/0, A=17/4
chr3:49928691	C/C	T	C	SNV	MST1R	MST1R:exonic:NM_002447.3	1	100	17	missense	NM_00244	c.3583A>G	p.Ser1195Gly	5.97	1	56	0	0.1(COSM459	IPT/TIG doma	rs7433231			1.0 (pos.49928691)	squamous_cel	Macrophag	ATP bindin	Digestive System Diseases:Drug-Induced Live			T=0, C=33	T=0.0, C=1.0	0.00001	504.541	33	T=0/0, C=22/1
chr5:174156168	C/C	T	C	SNV	MSX2	MSX2:exonic:NM_002449.4	1	100	2	missense	NM_00244	c.386T>C	p.Met129Thr	4.55	0.05	81	0	0.99(COSM56	Homeobox do	rs4242182	0.0986	YES	9.832E-1;0.0;0.0 (pos.174	diffuse_large	msh, Droso	BMP signa	Abortion, Spontaneous;Bone D		Benign	T=0, C=52	T=0.0, C=1.0	0.00001	767.015	52	T=0/0, C=38/1
chr16:56601720	G/G	A	G	SNV	MT4	MT4:exonic:NM_032935.2	1	100	2	missense	NM_03293	c.89A>G	p.Tyr30Cys	4.77	1	194	0		Metallothion	rs386791069,rs6666	0.0241	YES	7.132E-1 (pos.56601720)		Metallothic	biological	Female Urogenital	Copper		A=0, G=64	A=0.0, G=1.0	0.00001	977.61	64	A=0/0, G=33/3
chr2:109522720	A/A	G	A	SNV	EDAR	EDAR:intronic:NM_022336.3	1	100		intronic	NM_02233	c.1024+44	p.?	-0.83						rs260639	0.1707	YES	9.111E-1 (pos.109522720)		Ectodysplas	apical par	Abnormalities, Multiple;Conge		Uncertain sig	G=0, A=79	G=0.0, A=1.0	0.00001	1269.33	79	G=0/0, A=30/4
chr2:109527373	G/G	A	G	SNV	EDAR	EDAR:intronic:NM_022336.3	1	100		intronic	NM_02233	c.655+30T	p.?	-0.9				0(COSM3766487)		rs260630	0.0978	YES	9.983E-1 (pos.10952737	hepatocellula	Ectodysplas	apical par	Abnormalities, Multiple;Conge		Uncertain sig	A=0, G=29	A=0.0, G=1.0	0.00001	427.554	29	A=0/0, G=21/8
chr2:110053209	G/G	T	G	SNV	SH3RF3	SH3RF3:intronic:NM_001099289.2	1	100		intronic	NM_00109	c.1575-14	p.?	-0.4					RING-type zin	rs7370446		YES				metal ion	Amphetamine-Related Disorders:Mental Dis			T=0, G=30	T=0.0, G=1.0	0.00001	446.624	30	T=0/0, G=20/1
chr2:113300330	G/A	G	A	SNV	LOC105373562,POLR1B	LOC105373562:upstream:NR_136321.1, POLR1B:intronic:N	1	47.62		intronic	NR_13632	c.177+82G	p.?	-0.72					RNA polymer	rs4849071		YES			Polymerase	DNA binding,DNA-directed 5'-3' RNA polymerase activity,				G=11, A=10	G=0.5238, A=0.4762	0.00001	82.29	21	G=3/8, A=3/7
chr16:56601722	C/C	T	C	SNV	MT4	MT4:exonic:NM_032935.2	1	100	2	missense	NM_03293	c.91T>C	p.Trp31Arg	0.1	0.31	101	0		Metallothion	rs386791069,rs6666	0.0241	YES	7.129E-1 (pos.56601722)		Metallothic	biological	Female Urogenital	Copper		T=0, C=64	T=0.0, C=1.0	0.00001	977.634	64	T=0/0, C=33/3
chr2:113308622	T/T	C	T	SNV	POLR1B	POLR1B:intronic:NM_019014.5	1	100		intronic	NM_01901	c.762+43C	p.?	-1.09					RNA polymer	rs3816140	0.26	YES	3.507E-1 (pos.113308622)		Polymerase	DNA binding,DNA-directed 5'-3' RNA polymerase activity,				C=0, T=48	C=0.0, T=1.0	0.00001	810.431	48	C=0/0, T=19/2
chr2:113321900	T/C	T	C	SNV	POLR1B	POLR1B:intronic:NM_019014.5	1	46.43		intronic	NM_01901	c.1613-43	p.?	-0.73					RNA polymer	rs41280572,rs74910	0.4914	YES	3.672E-1;0.0 (pos.113321900)		Polymerase	DNA binding,DNA-directed 5'-3' RNA polymerase activity,				T=15, C=13	T=0.5357, C=0.4643	0.00001	117.927	28	T=11/4, C=7/6
chr2:113326502	T/T	C	T	SNV	POLR1B	POLR1B:intronic:NM_019014.5	1	100		intronic	NM_01901	c.2074+23	p.?	-2.3					RNA polymer	rs13010814	0.1701	YES	6.904E-1 (pos.113326502)		Polymerase	DNA binding,DNA-directed 5'-3' RNA polymerase activity,				C=0, T=40	C=0.0, T=1.0	0.00001	644.282	40	C=0/0, T=12/2
chr2:113532885	T/T	G	T	SNV	IL1A	IL1A:intronic:NM_000575.4	1	100		intronic	NM_00057	c.616-41C	p.?	-1.18					Interleukin-1	rs3783550	0.3001	YES	2.958E-1 (pos.113532885)		Interleukin-	apoptotic	Amnesia;Anemia;A	Rilonacept		G=0, T=30	G=0.0, T=1.0	0.00001	446.626	30	G=0/0, T=7/23
chr2:11359031	T/T	G	T	SNV	ROCK2	ROCK2:intronic:NM_004850.4	1	100		intronic	NM_00485	c.1320+61	p.?	-0.09					Protein kinas	rs9808228		YES			RHO-associ	ATP binding,G-protein coupled		(R)-TRANS-4-(1-AMINOETH		G=0, T=69	G=0.0, T=1.0	0.00001	1072.78	69	G=0/0, T=50/1
chr14:105935824	A/A	G	A	SNV	MTA1	MTA1:exonic:NM_004689.3	1	100	19	missense	NM_00468	c.1834G>A	p.Ala612Thr	1.78	1	58	0	0.1(COSM414	BAH domain,	rs13707	0.0016	YES	9.999E-1 (pos.10593582	neoplasm	Metastasis	DNA bindi	Endometriosis:Female Urogenital Diseases:F			G=0, A=27	G=0.0, A=1.0	0.00001	389.986	27	G=0/0, A=20/7
chr9:21816758	A/A	G	A	SNV	MTAP	MTAP:exonic:NM_002451.3	1	100	3	missense	NM_00245	c.166G>A	p.Val56Ile	6.62	0.05	29	0.006	0.98(COSM37	Phosphorylas	rs7023954	0.415	YES	4.49E-1 (pos.21816758)	adenocarcinor	Methylthio	L-methion	Bone Diseases:Bon	(1s)-1-(9-D	Benign	G=0, A=31	G=0.0, A=1.0	0.00001	465.815	31	G=0/0, A=23/8
chr2:113785727	G/G	A	G	SNV	IL36B	IL36B:intronic:NM_014438.4	1	100		intronic	NM_01443	c.261+789	p.?	-0.47						rs1562304	0.0712	YES	1.0 (pos.113785727)		Interleukin	cytokine activity,cytokine-mediated signaling pathway,e				A=0, G=20	A=0.0, G=1.0	0.00001	263.786	20	A=0/0, G=10/1
chr11:47640429	C/C	G	C	SNV	MTCH2	MTCH2:exonic:NM_014342.3	1	100	13	missense	NM_01434	c.868C>G	p.Pro290Ala	6.71	0.02	27	0.98	0.97(COSM14	Mitochondria	rs1064608,rs764352	0.3517	YES	2.904E-1;0.0;0.0 (pos.47	adenocarcinor	Mitochondr	extracellu	Nutrition Disorders;Nutritional and Metaboli			G=0, C=43	G=0.0, C=1.0	0.00001	705.844	43	G=0/0, C=26/1
chr18:8825192	G/G	A	G	SNV	MTCL1	MTCL1:exonic:NM_015210.3	1	100	15	synonymous	NM_01521	c.3684A>G	p.(=)	-0.33				0.03(COSM37	Domain of un	rs581894,rs7486587	0.4051	YES	0.0 (pos.8825191),3.786	acute_myeloi	Microtubul	RNA binding,apical plasma membrane,apicolateral plas				A=0, G=59	A=0.0, G=1.0	0.00001	885.366	59	A=0/0, G=26/3
chr22:30823196	C/C	T	C	SNV	MTFP1	MTFP1:exonic:NM_016498.4	1	100	3	synonymous	NM_01649	c.234T>C	p.(=)	-5.12				0.03(COSM4002140)		rs5753130	0.448	YES	6.84E-1 (pos.30823196)	acute_myeloi	Mitochondr	apoptotic process,integral component of membrane,mi				T=0, C=30	T=0.0, C=1.0	0.00001	446.192	30	T=0/0, C=17/1
chr10:135233541	G/G	A	G	SNV	MTG1,SPRN	MTG1:exonic:NM_138384.2, SPRN:downstream:NM_00101	1	100	11	missense	NM_13838	c.877A>G,	p.Ile293Val, p.?	0.79	1	29	0	0(COSM4144	50S ribosome	rs2255246	0		1.0 (pos.135233541)	neoplasm	Shadow of p	C-terminal protein lipidation,GTP binding,GTPase activit				A=0, G=30	A=0.0, G=1.0	0.00001	446.455	30	A=0/0, G=17/1
chr2:55470115	T/T	C	T	SNV	MTIF2	MTIF2:exonic:NM_002453.2	1	100	13	missense	NM_00245	c.1666G>A	p.Val556Ile	0.85	0.75	29	0.002		50S ribosome	rs11357	0.2218	YES	6.581E-1 (pos.55470115)		Mitochondr	GTP binding,GTPase activity,RNA binding,mitochondrial				C=0, T=29	C=0.0, T=1.0	0.00001	427.592	29	C=0/0, T=20/9
chr2:55490819	T/T	G	T	SNV	MTIF2	MTIF2:exonic:NM_002453.2	1	100	4	missense	NM_00245	c.176C>A	p.Thr59Asn	0.03	0.19	65	0.028		50S ribosome	rs1056445	0.2231	YES	6.6E-1;0.0 (pos.55490819)		Mitochondr	GTP binding,GTPase activity,RNA binding,mitochondrial				G=0, T=32	G=0.0, T=1.0	0.00001	485.104	32	G=0/0, T=3/29
chr2:113956821	C/G	C	G	SNV	PSD4	PSD4:intronic:NM_012455.2	1	57.14		intronic	NM_01245	c.2913+18	p.?	-0.85					PH domain,Pl	rs2305133	0.3945	YES	6.937E-1 (pos.113956821)		Pleckstrin a	ARF guanyl-nucleotide exchange factor activity,cleavage				C=12, G=16	C=0.4286, G=0.5714	0.00001	161.769	28	C=0/12, G=9/7
chr2:116497479	G/G	A	G	SNV	DPP10	DPP10:intronic:NM_001178034.1	1	100		intronic	NM_00117	c.864+10A	p.?	-0.32					Dienelactone	rs1550854	2.00E-04	YES	1.0 (pos.116497479)		Dipeptidyl p	dipeptidyl	Asthma;Bronchial Diseases;Child Developme			A=0, G=25	A=0.0, G=1.0	0.00001	353.019	25	A=0/0, G=12/1
chr2:116534922	G/G	A	G	SNV	DPP10	DPP10:intronic:NM_001178034.1	1	100		intronic	NM_00117	c.1325+47	p.?	1.57					Dienelactone	rs1374271	0.2723	YES	2.787E-1 (pos.116534922)		Dipeptidyl p	dipeptidyl	Asthma;Bronchial Diseases;Child Developme			A=0, G=40	A=0.0, G=1.0	0.00001	644.286	40	A=0/0, G=18/2
chr2:116548820	T/T	C	T	SNV	DPP10	DPP10:intronic:NM_001178034.1	1	100		intronic	NM_00117	c.1643-43	p.?	1.01					Dienelactone	rs3738879	0.2073	YES	9.244E-1 (pos.116548820)		Dipeptidyl p	dipeptidyl	Asthma;Bronchial Diseases;Child Developme			C=0, T=20	C=0.0, T=1.0	0.00001	263.786	20	C=0/0, T=12/8
chr2:11718591	T/T	C	T	SNV	GREB1	GREB1:intronic:NM_014668.3	1	100		intronic	NM_01466	c.772+34C	p.?	-4.47					Gene regulat	rs1529868	0.415	YES	5.483E-1 (pos.11718591)		GREB1 prot	extracellu	Endometriosis;Female Urogenital Diseases;F			C=0, T=31	C=0.0, T=1.0	0.00001	465.819	31	C=0/0, T=16/1
chrX:149931106	T/T	C	T	SNV	MTMR1	MTMR1:exonic:NM_003828.3	1	100	15	synonymous	NM_00382	c.1902C>T	p.(=)	-0.84				0.09(COSM14	GRAM domai	rs16995747	0.1204	YES	9.956E-1 (pos.14993110	adenocarcinor	Myotubular	cytoplasm,cytosol,peptidyl-tyrosine dephosphorylation				C=0, T=136	C=0.0, T=1.0	0.00001	2258.85	136	C=0/0, T=59/7
chr2:11741193	A/A	C	A	SNV	GREB1	GREB1:intronic:NM_014668.3	1	100		intronic	NM_01466	c.2529+72	p.?	-0.29					Gene regulat	rs11681838		YES			GREB1 prot	extracellu	Endometriosis;Female Urogenital Diseases;F			C=0, A=27	C=0.0, A=1.0	0.00001	389.988	27	C=0/0, A=14/1
chr2:11755443	G/G	T	G	SNV	GREB1	GREB1:intronic:NM_014668.3	1	100		intronic	NM_01466	c.3322+27	p.?	-3.2					Gene regulat	rs2252958	0.1848	YES	9.796E-1;0.0 (pos.11755443)		GREB1 prot	extracellu	Endometriosis;Female Urogenital Diseases;F			T=0, G=30	T=0.0, G=1.0	0.00001	446.636	30	T=0/0, G=10/2
chr8:17270787	G/G	A	G	SNV	MTMR7	MTMR7:exonic:NM_004686.4	1	100	1	synonymous	NM_00468	c.15T>C	p.(=)	0.77				0.01(COSM41	Myotubularin	rs67972978	1.00E-04		1.0 (pos.17270787)	neoplasm	Myotubular	cytosol,in	Brain Diseases:Central Nervous System Dise			A=0, G=28	A=0.0, G=1.0	0.00001	413.424	28	A=0/0, G=3/25
chr2:118704477	CG/CG	C	CG	INDEL	CCDC93	CCDC93:intronic:NM_019044.4	1	100		intronic	NM_01904	c.1225-19	p.?	-1.28					Coiled-coil do	rs752261577			5.795E-4 (pos.118704477)			Golgi to plasma membrane transport,early endosome,p				C=0, CG=71	C=0.0, CG=1.0	0.00001	1004.35	71	C=0/0, CG=48/
chr2:118706879	G/G	A	G	SNV	CCDC93	CCDC93:intronic:NM_019044.4	1	100		intronic	NM_01904	c.1142+19	p.?	-0.26					Coiled-coil do	rs1035598	2.00E-04	YES	9.999E-1;0.0 (pos.118706879)			Golgi to plasma membrane transport,early endosome,p				A=0, G=28	A=0.0, G=1.0	0.00001	408.717	28	A=0/0, G=14/1
chr2:11905876	C/T	C	T	SNV	LPIN1	LPIN1:intronic:NM_001261428.2	1	45.45		intronic	NM_00126	c.339+17C	p.?	-3.25					LNS2 (Lipin/N	rs10209969	0.3441	YES	1.982E-1 (pos.11905876)		Lipin 1	animal org	Muscular Diseases;Musculosk		Benign	C=18, T=15	C=0.5455, T=0.4545	0.00001	143.162	33	C=11/7, T=4/1
chr2:119732042	C/C	T	C	SNV	MARCO	MARCO:intronic:NM_006770.3	1	100		intronic	NM_00677	c.568+26T	p.?	-4.11					Collagen tripl	rs11693199	0.4128	YES	6.284E-2 (pos.119732042)		Macrophag	G-protein coupled receptor binding,amyloid-beta bindin				T=0, C=22	T=0.0, C=1.0	0.00001	298.88	22	T=0/0, C=14/8
chr8:11162496	C/C	A	C	SNV	MTMR9	MTMR9:exonic:NM_015458.3	1	100	4	synonymous	NM_01545	c.564A>C	p.(=)	-0.69				0.19(COSM37	Myotubularin	rs2164272	0.4049	YES	7.324E-1 (pos.11162496)	adenocarcinor	Myotubular	cytosol,dephosphorylation,enzyme regulator activity,ph				A=0, C=87	A=0.0, C=1.0	0.00001	1431.04	87	A=0/0, C=37/5
chr2:120362407	T/T	C	T	SNV	CFAP221	CFAP221:intronic:NM_001271049.1	1	100		intronic	NM_00127	c.912+39C	p.?	-0.86					Flagellar-ass	rs1568540	0.0448	YES	9.194E-1 (pos.120362407)			axoneme,	Attention Deficit Disorder with Hyperactivity			C=0, T=139	C=0.0, T=1.0	0.00001	2325.1	139	C=0/0, T=66/7
chr6:74171467	G/G	C	G	SNV	MTO1	MTO1:utr_5:NM_133645.2	1	100	1	utr_5	NM_13364	c.-111C>G	p.?	0.63					FAD binding c	rs1713862		YES			Mitochondr	RNA binding,flavin adenine dinucleotide binding,mitoch				C=0, G=70	C=0.0, G=1.0	0.00001	1067.28	70	C=0/0, G=41/2
chr6:153311001	C/C	A	C	SNV	MTRF1L	MTRF1L:utr_3:NM_019041.6	1	100	7	utr_3	NM_01904	c.1172T>G	p.?	-0.28					PCRF domain	rs3860814	3.00E-04		9.995E-1 (pos.153311001)			mitochondrial matrix,mitochondrial translational termi				A=0, C=25	A=0.0, C=1.0	0.00001	357.408	25	A=0/0, C=13/1
chr5:7870973	G/G	A	G	SNV	MTRR	MTRR:exonic:NM_024010.2	1	100	2	missense	NM_02401	c.147A>G	p.Ile49Met	0.18	0	10	1	0.1(COSM500	FAD binding c	rs1801394	0.454	YES	2.811E-1 (pos.7870973)	ductal_carcinc	Methionine	DNA meth	Abnormalities, Seve	Cyanocob	drug respons	A=0, G=34	A=0.0, G=1.0	0.00001	524.16	34	A=0/0, G=14/2
chr8:17612456	C/T	C	T	SNV	MTUS1	MTUS1:exonic:NM_001001924.2	1	95.83	2	synonymous	NM_00100	c.861G>A	p.(=)	-0.15						rs11203910	0.0604	YES	8.169E-1 (pos.17612456)			Golgi appa	Adenocarcinoma:Carcinoma:Carcinoma, Hec			C=3, T=69	C=0.0417, T=0.9583	0.00001	1036.01	72	C=3/0, T=37/3
chr2:120918585	C/C	A	C	SNV	EPB41L5	EPB41L5:intronic:NM_020909.3	1	100		intronic	NM_02090	c.1887+35	p.?	-0.65					FERM C-termi	rs12617570,rs749840	0.2784	YES	0.0 (pos.120918583),7.113E-1 (pos.1209		Erythrocyte	actomyosin structure organization,apical constriction,a				A=0, C=35	A=0.0, C=1.0	0.00001	535.881	35	A=0/0, C=13/2
chr2:121036385	GA/GA	TT	GA	MNV	RALB	RALB:intronic:NM_002881.2	2	100		intronic	NM_00288	c.114+31T	p.?	-0.25, -1.21					ADP-ribosylat	rs12711939,rs12711	0.3017	YES	0.0 (pos.121036383),3.996E-1;0.0 (pos.1		RAS-like pro	ATPase binding,GDP binding,GTP binding,GTPase activity				TT=0, GA=2	TT=0.0, GA=1.0	0.00001	281.235	21	TT=0/0, GA=7/
chr2:121043403	A/A	G	A	SNV	RALB	RALB:intronic:NM_002881.2	1	100		intronic	NM_00288	c.115-47G	p.?	-2.35					ADP-ribosylat	rs4091515	0.2892	YES	4.008E-1 (pos.121043403)		RAS-like pro	ATPase binding,GDP binding,GTP binding,GTPase activity				G=0, A=38	G=0.0, A=1.0	0.00001	603.785	38	G=0/0, A=16/2
chr2:121729490	C/T	C	T	SNV	GLI2	GLI2:intronic:NM_005270.4	1	96.67		intronic	NM_00527	c.1060-27	p.?	-0.17					Zinc finger, C2	rs2592591	0.0156	YES	9.997E-1 (pos.121729490)		GLI-Kruppel	DNA bindi	Abnormalities, Multiple:Agene		Benign	C=1, T=29	C=0.0333, T=0.9667	0.00001	416.535	30	C=1/0, T=9/20
chr2:121744225	G/G	A	G	SNV	GLI2	GLI2:intronic:NM_005270.4	1	100		intronic	NM_00527	c.2293+35	p.?	0.68					Zinc finger, C2	rs2276553	0.2654	YES	5.156E-1 (pos.121744225)		GLI-Kruppel	DNA bindi	Abnormalities, Multiple:Agene		Benign	A=0, G=20	A=0.0, G=1.0	0.00001	263.764	20	A=0/0, G=6/14
chr2:122125104	C/T	C	T	SNV	CLASP1	CLASP1:intronic:NM_015282.2	1	45.83		intronic	NM_01528	c.3886+60	p.?	-0.16					CLASP N term	rs2304663,rs994974732		YES			CLIP-associ	G2/M transition of mitotic cell cycle,Golgi apparatus,Gol				C=13, T=11	C=0.5417, T=0.4583	0.00001	93.4396	24	C=7/6, T=10/1
chr7:100635661	C/C	T	C	SNV	MUC12	MUC12:exonic:NM_001164462.1	1	100	2	missense	NM_00116	c.1817T>C	p.Met606Thr	-0.11	1	81	0.002		SEA domain	rs10953313	0.2722	YES	8.315E-1 (pos.100635661)		Mucin 12	Golgi lumen,O-glycan processing,integral component of				T=0, C=37	T=0.0, C=1.0	0.00001	589.244	37	T=0/0, C=15/2
chr2:125671544	A/A	G	A	SNV	CNTNAP5	CNTNAP5:intronic:NM_130773.3	1	100		intronic	NM_13077	c.3725-12	p.?	0.24					F5/8 type C d	rs1840205		YES			Contactin-a	cell adhesion,integral component of membrane				G=0, A=68	G=0.0, A=1.0	0.00001	1053.56	68	G=0/0, A=40/2
chr2:127447763	A/A	G	A	SNV	GYPC	GYPC:intronic:NM_002101.4	1	100		intronic	NM_00210	c.50-68G>	p.?	-2.45						rs6709803	0.456	YES			Glycophori	cell adhes	Anemia:Anemia, Hemolytic:Anemia, Hemolyt			G=0, A=27	G=0.0, A=1.0	0.00001	397.094	27	G=0/0, A=14/1
chr2:127951458	AT/AT	GC	AT	MNV	CYP27C1	CYP27C1:intronic:NM_001001665.3	2	100		intronic	NM_00100	c.720-15G	p.?	-0.92, -2.27					Cytochrome P	rs12476709,rs20891	0.4109	YES	5.636E-1 (pos.127951458),5.642E-1;0.0 (pos.127951			11-cis retinal binding,11-cis-retinal 3,4-desaturase activ				GC=0, AT=6	GC=0.0, AT=1.0	0.00001	977.32	64	GC=0/0, AT=33
chr7:100638856	G/G	A	G	SNV	MUC12	MUC12:exonic:NM_001164462.1	1	100	2	missense	NM_00116	c.5012A>G	p.His1671Arg	-0.97	0	29	0.663	0(COSM1503	SEA domain	rs28463186		YES	8.294E-1 (pos.10063885	adenocarcinor	Mucin 12	Golgi lumen,O-glycan processing,integral component of				A=0, G=70	A=0.0, G=1.0	0.00001	1092.09	70	A=0/0, G=33/3
chr2:128350578	TG/TG	T	TG	INDEL	MYO7B	MYO7B:intronic:NM_001080527.1	1	100		intronic	NM_00108	c.2145+57	p.?	-2.06					FERM central	rs397717927,rs58867441,rs869		YES			Myosin VIIb	ATP binding,actin binding,apical cytoplasm,brush border				T=0, TG=20	T=0.0, TG=1.0	0.00001	242.07	20	T=0/0, TG=11/
chr2:128400783	A/A	G	A	SNV	LIMS2	LIMS2:intronic:NM_017980.4	1	100		intronic	NM_01798	c.432-136	p.?	-0.7					LIM domain	rs2244612		YES			LIM and ser	cell junction assembly,cell-cell adhesion,cell-cell junctic				G=0, A=23	G=0.0, A=1.0	0.00001	316.501	23	G=0/0, A=7/16
chr2:128411934	G/G	A	G	SNV	LIMS2	LIMS2:intronic:NM_017980.4	1	100		intronic	NM_01798	c.431+64T	p.?	-2.73					LIM domain	rs6707277		YES			LIM and ser	cell junction assembly,cell-cell adhesion,cell-cell junctic				A=0, G=42	A=0.0, G=1.0	0.00001	685.22	42	A=0/0, G=18/2
chr2:128411950	G/G	A	G	SNV	LIMS2	LIMS2:intronic:NM_017980.4	1	100		intronic	NM_01798	c.431+48T	p.?	-0.76					LIM domain	rs6707282	0.1356	YES	9.875E-1 (pos.128411950)		LIM and ser	cell junction assembly,cell-cell adhesion,cell-cell junctic				A=0, G=43	A=0.0, G=1.0	0.00001	705.844	43	A=0/0, G=18/2
chr2:128495645	A/A	G	A	SNV	WDR33	WDR33:intronic:NM_018383.4	1	100		intronic	NM_01838	c.725-112	p.?	0.05					WD domain,	rs6754215	0.4002	YES	4.052E-1;0.0 (pos.128495645)			RNA binding,collagen trimer,fibrillar center,mRNA 3'-end				G=0, A=25	G=0.0, A=1.0	0.00001	353.019	25	G=0/0, A=17/8
chr2:128615390	T/T	G	T	SNV	POLR2D	POLR2D:intronic:NM_004805.3	1	100		intronic	NM_00480	c.73+212C	p.?	-0.65					RNA polymer	rs13031513		YES			Polymerase	7-methylguanosine mRNA capping,DNA-directed 5'-3' RN				G=0, T=132	G=0.0, T=1.0	0.00001	2173.6	132	G=0/0, T=101/
chr2:128880899	A/A	T	A	SNV	UGGT1	UGGT1:intronic:NM_020120.3	1	100		intronic	NM_02012	c.1134+79	p.?	0.9					UDP-glucose:	rs4480941		YES			UDP-glucos	'de novo' posttranslational protein folding,ER-associatec				T=0, A=37	T=0.0, A=1.0	0.00001	583.696	37	T=0/0, A=19/1
chr2:128936175	ATGA/A	ATGA	A	INDEL	UGGT1	UGGT1:intronic:NM_020120.3	3	47.37		intronic	NM_02012	c.3815+32	p.?	-0.52, 0.38, 0.04, -0.03					UDP-glucose:Glycoprotein Glucosyltransferase						UDP-glucos	'de novo' posttranslational protein folding,ER-associatec				ATGA=30, A	ATGA=0.5263, A=0.4	0.00001	256	57	ATGA=13/17, A
chr7:100639012	T/T	C	T	SNV	MUC12	MUC12:exonic:NM_001164462.1	1	100	2	missense	NM_00116	c.5168C>T	p.Thr1723Ile	0.89	0	89	0.915	0.01(COSM41	SEA domain	rs55638317		YES	8.304E-1 (pos.10063901	carcinoma,ne	Mucin 12	Golgi lumen,O-glycan processing,integral component of				C=0, T=29	C=0.0, T=1.0	0.00001	427.567	29	C=0/0, T=14/1
chr2:130930819	C/T	C	T	SNV	SMPD4	SMPD4:intronic:NM_017751.4	1	47.62		intronic	NM_01775	c.462+33G	p.?	0					Mitochondria	rs2290295	0.3599		4.64E-1 (pos.130930819)		Sphingomye	Golgi apparatus,Golgi membra		Phosphatidyl serine		C=11, T=10	C=0.5238, T=0.4762	0.00001	82.29	21	C=3/8, T=2/8
chr7:100639040	T/T	C	T	SNV	MUC12	MUC12:exonic:NM_001164462.1	1	100	2	synonymous	NM_00116	c.5196C>T	p.(=)	-0.36				0(COSM4161	SEA domain	rs28459498		YES	8.171E-1 (pos.10063904	neoplasm	Mucin 12	Golgi lumen,O-glycan processing,integral component of				C=0, T=20	C=0.0, T=1.0	0.00001	263.786	20	C=0/0, T=5/15
chr2:132249372	T/T	C	T	SNV	MIR4784,MZT2A	MZT2A:intronic:NM_001085365.1, MIR4784:upstream:NR_(	1	100		intronic	NM_00108	c.319+77G	p.?	-0.18					Mitotic-spinc	rs6706952		YES				centrosome,cytosol,gamma-tubulin ring complex,microt				C=0, T=41	C=0.0, T=1.0	0.00001	664.678	41	C=0/0, T=14/2
chr7:100639049	C/C	G	C	SNV	MUC12	MUC12:exonic:NM_001164462.1	1	100	2	missense	NM_00116	c.5205G>C	p.Leu1735Phe	0.14	0.25	22	0.16	0.01(COSM41	SEA domain	rs56357244		YES	8.171E-1;0.0 (pos.10063	carcinoma,ne	Mucin 12	Golgi lumen,O-glycan processing,integral component of				G=0, C=20	G=0.0, C=1.0	0.00001	263.785	20	G=0/0, C=5/15
chr7:100639066	T/T	C	T	SNV	MUC12	MUC12:exonic:NM_001164462.1	1	100	2	missense	NM_00116	c.5222C>T	p.Ser1741Leu	-0.18	0	145	0.663	0(COSM4592	SEA domain	rs28483629		YES	8.171E-1 (pos.10063906	adenocarcinor	Mucin 12	Golgi lumen,O-glycan processing,integral component of				C=0, T=20	C=0.0, T=1.0	0.00001	263.785	20	C=0/0, T=5/15
chr7:100647875	A/A	C	A	SNV	MUC12	MUC12:exonic:NM_001164462.1	1	100	2	synonymous	NM_00116	c.14031C>	p.(=)	-0.88				0(COSM1503	SEA domain	rs11766048	0.34	YES	0.0 (pos.100647869),6.6	adenocarcinor	Mucin 12	Golgi lumen,O-glycan processing,integral component of				C=0, A=72	C=0.0, A=1.0	0.00001	1130.96	72	C=0/0, A=36/3
chr2:135700151	T/T	A	T	SNV	CCNT2	CCNT2:intronic:NM_058241.2	1	100		intronic	NM_05824	c.431-31A	p.?	1.27					Cyclin, N-term	rs3816339	0.2675	YES	4.781E-1 (pos.135700151)		Cyclin T2	7SK snRNA binding,RNA polymerase binding,cell cycle,ce				A=0, T=33	A=0.0, T=1.0	0.00001	504.453	33	A=0/0, T=21/1
chr2:135710322	G/G	A	G	SNV	CCNT2	CCNT2:intronic:NM_058241.2	1	100		intronic	NM_05824	c.774+41A	p.?	-0.04					Cyclin, N-term	rs10166142	0.4686	YES	8.763E-1 (pos.135710322)		Cyclin T2	7SK snRNA binding,RNA polymerase binding,cell cycle,ce				A=0, G=37	A=0.0, G=1.0	0.00001	583.704	37	A=0/0, G=10/2
chr7:100648029	A/A	G	A	SNV	MUC12	MUC12:exonic:NM_001164462.1	1	100	2	missense	NM_00116	c.14185G>	p.Ala4729Thr	-1.78	0.08	58	0.005	0(COSM1503	SEA domain	rs10953315	0.2725	YES	8.314E-1 (pos.10064802	adenocarcinor	Mucin 12	Golgi lumen,O-glycan processing,integral component of				G=0, A=30	G=0.0, A=1.0	0.00001	446.638	30	G=0/0, A=11/1
chr2:135743234	TCAAA/T	TCAAA	T	INDEL	MAP3K19	MAP3K19:intronic:NM_025052.4	4	57.14		intronic	NM_02505	c.3072+13	p.?	-0.99, -0.11, 0.03, 1.98, 0.51					Protein kinas	rs1009979658,rs10582468,rs20		YES				ATP binding,activation of protein kinase activity,cytoplas				TCAAA=15,	TCAAA=0.4286, T=0.5	0.00001	219.944	35	TCAAA=12/3, T
chr7:100647819	C/T	C	T	SNV	MUC12	MUC12:exonic:NM_001164462.1	1	47.62	2	missense	NM_00116	c.13975C>	p.His4659Tyr	-0.5	0	83	0.462	0(COSM1503	SEA domain	rs11766024		YES	6.192E-1 (pos.10064781	adenocarcinor	Mucin 12	Golgi lumen,O-glycan processing,integral component of				C=22, T=20	C=0.5238, T=0.4762	0.00001	212.952	42	C=18/4, T=7/1
chr3:124646705	AAG/AAG	A	AAG	INDEL	MUC13	MUC13:exonic:NM_033049.3	2	100	2	frameshiftInsertion	NM_03304	c.184_185	p.Phe62fs	-1.9					SEA domain	rs10630030,rs39775	4.00E-04		1.0 (pos.124646705)	adenocarcinor	Mucin 13, c	Golgi lumen,O-glycan processing,apical plasma membra				A=0, AAG=2	A=0.0, AAG=1.0	0.00001	218.442	20	A=0/0, AAG=9/
chr2:136548116	C/C	G	C	SNV	LCT	LCT:intronic:NM_002299.3	1	100		intronic	NM_00229	c.5335+11	p.?	-0.19					Glycosyl hydr	rs892717		YES			Lactase (lac	apical pla	Carbohydrate Meta	(1S,2S,3R,4S,5S)-2,3,4-TR		G=0, C=84	G=0.0, C=1.0	0.00001	1370.13	84	G=0/0, C=43/4
chr2:136548169	G/G	A	G	SNV	LCT	LCT:intronic:NM_002299.3	1	100		intronic	NM_00229	c.5335+59	p.?	1.07					Glycosyl hydr	rs892716		YES			Lactase (lac	apical pla	Carbohydrate Meta	(1S,2S,3R,4S,5S)-2,3,4-TR		A=0, G=84	A=0.0, G=1.0	0.00001	1370.17	84	A=0/0, G=41/4
chr2:136565028	G/G	C	G	SNV	LCT	LCT:intronic:NM_002299.3	1	100		intronic	NM_00229	c.3905-62	p.?	-0.56					Glycosyl hydr	rs10928549		YES			Lactase (lac	apical pla	Carbohydrate Meta	(1S,2S,3R,4S,5S)-2,3,4-TR		C=0, G=100	C=0.0, G=1.0	0.00001	1702.15	100	C=0/0, G=30/7
chr2:136569848	C/A	C	A	SNV	LCT	LCT:intronic:NM_002299.3	1	50		intronic	NM_00229	c.2353+33	p.?	-0.35					Glycosyl hydr	rs12988076	0.3034	YES	3.927E-1 (pos.136569848)		Lactase (lac	apical pla	Carbohydrate Meta	(1S,2S,3R,4S,5S)-2,3,4-TR		C=19, A=19	C=0.5, A=0.5	0.00001	198.357	38	C=9/10, A=15/
chr19:9057408	G/G	A	G	SNV	MUC16	MUC16:exonic:NM_024690.2	1	100	3	missense	NM_02469	c.30038T>	p.Leu10013Pro	0		98	0	0(COSM5438	SEA domain	rs1423051	0.0793	YES	8.401E-1 (pos.9057408)	squamous_cel	Mucin 16	Golgi lume	Adnexal Diseases:E	Oregovomab		A=0, G=43	A=0.0, G=1.0	0.00001	705.844	43	A=0/0, G=25/1
chr19:9057687	G/G	A	G	SNV	MUC16	MUC16:exonic:NM_024690.2	1	100	3	missense	NM_02469	c.29759T>	p.Ile9920Thr	-0.53		89	0	0(COSM5438	SEA domain	rs1895364	0.1018	YES	8.41E-1 (pos.9057687)	squamous_cel	Mucin 16	Golgi lume	Adnexal Diseases:E	Oregovomab		A=0, G=118	A=0.0, G=1.0	0.00001	1893.93	118	A=0/0, G=68/5
chr19:9059808	G/G	A	G	SNV	MUC16	MUC16:exonic:NM_024690.2	1	100	3	missense	NM_02469	c.27638T>	p.Ile9213Thr	-0.73		89	0		SEA domain	rs7508222	0.1011	YES	8.38E-1;0.0 (pos.9059808)		Mucin 16	Golgi lume	Adnexal Diseases:E	Oregovomab		A=0, G=88	A=0.0, G=1.0	0.00001	1451.91	88	A=0/0, G=36/5
chr2:138163391	C/C	T	C	SNV	THSD7B	THSD7B:intronic:NM_001316349.1	1	100		intronic	NM_00131	c.2695+14	p.?	0.26					Thrombospor	rs1551281	0.051	YES	9.703E-1 (pos.138163391)							T=0, C=31	T=0.0, C=1.0	0.00001	465.819	31	T=0/0, C=17/1
chr2:138169158	C/C	T	C	SNV	THSD7B	THSD7B:intronic:NM_001316349.1	1	100		intronic	NM_00131	c.2696-21	p.?	-0.17					Thrombospor	rs1463285	0		9.97E-1 (pos.138169158)							T=0, C=49	T=0.0, C=1.0	0.00001	805.861	49	T=0/0, C=30/1
chr19:9062415	G/G	T	G	SNV	MUC16	MUC16:exonic:NM_024690.2	1	100	3	missense	NM_02469	c.25031A>	p.Asp8344Ala	-0.63		126	0		SEA domain	rs12978757	0.3884	YES	5.745E-1;0.0 (pos.9062415)		Mucin 16	Golgi lume	Adnexal Diseases:E	Oregovomab		T=0, G=45	T=0.0, G=1.0	0.00001	747.376	45	T=0/0, G=24/2
chr2:140992337	C/A	C	A	SNV	LRP1B	LRP1B:intronic:NM_018557.2	1	57.58		intronic	NM_01855	c.13659+1	p.?	0.52					Calcium-bind	rs13387241	0.4065	YES	5.209E-1 (pos.140992337)			calcium io	Adenocarcinoma;Carcinoma;Carcinoma, Ren			C=14, A=19	C=0.4242, A=0.5758	0.00001	204.392	33	C=9/5, A=16/3
chr2:141110440	A/A	G	A	SNV	LRP1B	LRP1B:intronic:NM_018557.2	1	100		intronic	NM_01855	c.11650+8	p.?	1.8					Calcium-bind	rs16843835		YES				calcium io	Adenocarcinoma;Carcinoma;Carcinoma, Ren			G=0, A=25	G=0.0, A=1.0	0.00001	353.006	25	G=0/0, A=17/8
chr19:9071763	A/A	T	A	SNV	MUC16	MUC16:exonic:NM_024690.2	1	100	3	missense	NM_02469	c.15683A>	p.Asn5228Ile	-3.19		149	0		SEA domain	rs10422567	0.1483	YES	8.376E-1 (pos.9071763)		Mucin 16	Golgi lume	Adnexal Diseases:E	Oregovomab		T=0, A=42	T=0.0, A=1.0	0.00001	685.22	42	T=0/0, A=12/3
chr2:141201763	C/C	T	C	SNV	LRP1B	LRP1B:intronic:NM_018557.2	1	100		intronic	NM_01855	c.10294+1	p.?	-4.01					Calcium-bind	rs4992954		YES				calcium io	Adenocarcinoma;Carcinoma;Carcinoma, Ren			T=0, C=79	T=0.0, C=1.0	0.00001	1269.32	79	T=0/0, C=76/3
chr2:141274295	T/T	C	T	SNV	LRP1B	LRP1B:intronic:NM_018557.2	1	100		intronic	NM_01855	c.8149+16	p.?	-1.09					Calcium-bind	rs1542655		YES				calcium io	Adenocarcinoma;Carcinoma;Carcinoma, Ren			C=0, T=88	C=0.0, T=1.0	0.00001	1445.25	88	C=0/0, T=47/4
chr19:9087615	A/A	T	A	SNV	MUC16	MUC16:exonic:NM_024690.2	1	100	1	missense	NM_02469	c.4200A>T	p.Lys1400Asn	-1.44		94	0		SEA domain	rs1596798	0		9.853E-1 (pos.9087615)		Mucin 16	Golgi lume	Adnexal Diseases:E	Oregovomab		T=0, A=71	T=0.0, A=1.0	0.00001	1097.56	71	T=0/0, A=40/3
chr2:141473786	T/T	C	T	SNV	LRP1B	LRP1B:intronic:NM_018557.2	1	100		intronic	NM_01855	c.5894-11	p.?	0.23					Calcium-bind	rs17513410		YES				calcium io	Adenocarcinoma;Carcinoma;Carcinoma, Ren			C=0, T=32	C=0.0, T=1.0	0.00001	485.12	32	C=0/0, T=13/1
chr2:141528592	C/C	T	C	SNV	LRP1B	LRP1B:intronic:NM_018557.2	1	100		intronic	NM_01855	c.5501-17	p.?	0.09					Calcium-bind	rs7599219	0.1287	YES	9.994E-1;0.0 (pos.141528592)			calcium io	Adenocarcinoma;Carcinoma;Carcinoma, Ren			T=0, C=102	T=0.0, C=1.0	0.00001	1593.36	102	T=0/0, C=43/5
chr19:9088017	G/G	T	G	SNV	MUC16	MUC16:exonic:NM_024690.2	1	100	1	missense	NM_02469	c.3798A>C	p.Lys1266Asn	-1.69		94	0	0(COSM5021	SEA domain	rs1596797	0.3014	YES	7.223E-1;0.0 (pos.90880	neoplasm,oste	Mucin 16	Golgi lume	Adnexal Diseases:E	Oregovomab		T=0, G=40	T=0.0, G=1.0	0.00001	644.28	40	T=0/0, G=13/2
chr2:141777763	T/T	G	T	SNV	LRP1B	LRP1B:intronic:NM_018557.2	1	100		intronic	NM_01855	c.1790-92	p.?	-0.13					Calcium-bind	rs12615322		YES				calcium io	Adenocarcinoma;Carcinoma;Carcinoma, Ren			G=0, T=21	G=0.0, T=1.0	0.00001	281.232	21	G=0/0, T=16/5
chr19:9070402	G/T	G	T	SNV	MUC16	MUC16:exonic:NM_024690.2	1	59.09	3	missense	NM_02469	c.17044C>	p.Pro5682Thr	-2.31		38	0.998	0(COSM4998	SEA domain	rs12150888	0.2916	YES	3.966E-3 (pos.9070402)	ductal_carcinc	Mucin 16	Golgi lume	Adnexal Diseases:E	Oregovomab		G=9, T=13	G=0.4091, T=0.5909	0.00001	122.567	22	G=2/7, T=5/8
chr19:9057896	G/C	G	C	SNV	MUC16	MUC16:exonic:NM_024690.2	1	58.33	3	missense	NM_02469	c.29550C>	p.Asp9850Glu	-4.11		45	0	0(COSM5057	SEA domain	rs12984471	0.3372	YES	2.262E-2;0.0 (pos.90578	intestinal_ade	Mucin 16	Golgi lume	Adnexal Diseases:E	Oregovomab		G=20, C=28	G=0.4167, C=0.5833	0.00001	345.946	48	G=10/10, C=1
chr19:9084183	G/A	G	A	SNV	MUC16	MUC16:exonic:NM_024690.2	1	57.14	1	synonymous	NM_02469	c.7632C>T	p.(=)	-0.11				0(COSM4415	SEA domain	rs1609460	0.4926	YES	3.232E-1 (pos.9084183)	acute_myeloi	Mucin 16	Golgi lume	Adnexal Diseases:E	Oregovomab		G=9, A=12	G=0.4286, A=0.5714	0.00001	109.102	21	G=5/4, A=6/6
chr2:143787274	T/T	C	T	SNV	KYNU	KYNU:intronic:NM_003937.2	1	100		intronic	NM_00393	c.955+26C	p.?	0.22					Aminotransfe	rs386651341,rs6430	8.00E-04	YES	9.625E-1 (pos.143787274)		Kynurenina	L-kynureni	Amino Acid Metabo	3-Hydroxyhippuric acid,L		C=0, T=88	C=0.0, T=1.0	0.00001	1451.86	88	C=0/0, T=56/3
chr2:144193293	C/T	C	T	SNV	ARHGAP15	ARHGAP15:intronic:NM_018460.3	1	46.88		intronic	NM_01846	c.573+25C	p.?	0.13					PH domain,R	rs12621011	0.019	YES	2.112E-1 (pos.144193293)		Rho GTPase	GTPase activator activity,cytosol,membrane,positive re				C=17, T=15	C=0.5313, T=0.4688	0.00001	143.455	32	C=7/10, T=11/
chr2:149529083	C/C	T	C	SNV	EPC2	EPC2:intronic:NM_015630.3	1	100		intronic	NM_01563	c.1720+12	p.?	-1.39					Enhancer of P	rs4972266		YES			Enhancer of	DNA repair,Piccolo NuA4 histone acetyltransferase com				T=0, C=50	T=0.0, C=1.0	0.00001	852.582	50	T=0/0, C=34/1
chr2:152303171	A/A	G	A	SNV	RIF1	RIF1:intronic:NM_018151.4	1	100		intronic	NM_01815	c.2244+82	p.?	-1.5					Rap1-interac	rs2432941		YES			Ligand-depe	cell cycle,cellular response to DNA damage stimulus,cel				G=0, A=21	G=0.0, A=1.0	0.00001	281.235	21	G=0/0, A=10/1
chr19:9084216	T/C	T	C	SNV	MUC16	MUC16:exonic:NM_024690.2	1	50	1	synonymous	NM_02469	c.7599A>G	p.(=)	-0.04				0(COSM4132	SEA domain	rs1609459	0.3273	YES	2.296E-2 (pos.9084216)	adenocarcinor	Mucin 16	Golgi lume	Adnexal Diseases:E	Oregovomab		T=12, C=12	T=0.5, C=0.5	0.00001	106.709	24	T=5/7, C=6/6
chr7:100678086	C/C	T	C	SNV	MUC17	MUC17:exonic:NM_001040105.1	1	100	3	missense	NM_00104	c.3389T>C	p.Ile1130Thr	-0.52	0	89	0		SEA domain	rs4729647,rs755100	0.1047	YES	1.156E-4 (pos.100678085),9.177E-1;5.45		Mucin 17	Golgi lumen,O-glycan processing,PDZ domain binding,ap				T=0, C=37	T=0.0, C=1.0	0.00001	583.704	37	T=0/0, C=17/2
chr7:100675512	G/A	G	A	SNV	MUC17	MUC17:exonic:NM_001040105.1	1	59.09	3	missense	NM_00104	c.815G>A	p.Gly272Glu	-2.65	0	98	0	0(COSM6331	SEA domain	rs10259584	0.1147	YES	4.997E-2 (pos.10067551	neoplasm	Mucin 17	Golgi lumen,O-glycan processing,PDZ domain binding,ap				G=9, A=13	G=0.4091, A=0.5909	0.00001	122.562	22	G=9/0, A=9/4
chr2:152349026	A/A	G	A	SNV	NEB	NEB:intronic:NM_004543.4	1	100		intronic	NM_00454	c.19162-1	p.?	0.08					Nebulin repe	rs2288195	0.3336	YES	7.801E-1 (pos.152349026)		Nebulin	Z disc,acti	Congenital, Hereditary, and Ne		Benign	G=0, A=40	G=0.0, A=1.0	0.00001	644.286	40	G=0/0, A=10/3
chr7:100680370	C/T	C	T	SNV	MUC17	MUC17:exonic:NM_001040105.1	1	50	3	synonymous	NM_00104	c.5673C>T	p.(=)	-3.56				0(COSM5766	SEA domain	rs10257974,rs20176	0.1148	YES	5.887E-2 (pos.10068037	adenocarcinor	Mucin 17	Golgi lumen,O-glycan processing,PDZ domain binding,ap				C=14, T=14	C=0.5, T=0.5	0.00001	129.092	28	C=7/7, T=9/5
chr7:100695138	G/A	G	A	SNV	MUC17	MUC17:exonic:NM_001040105.1	1	50	9	missense	NM_00104	c.12998G>	p.Arg4333Gln	-0.05	0.1	43	0.36	0.01(COSM37	SEA domain	rs73168398	0.1088	YES	1.047E-1 (pos.10069513	adenocarcinor	Mucin 17	Golgi lumen,O-glycan processing,PDZ domain binding,ap				G=37, A=37	G=0.5, A=0.5	0.00001	383.419	74	G=15/22, A=1
chr7:100679809	C/T	C	T	SNV	MUC17	MUC17:exonic:NM_001040105.1	1	45.45	3	synonymous	NM_00104	c.5112C>T	p.(=)	-4.23				0(COSM3762	SEA domain	rs4992074	0.1162	YES	2.102E-1 (pos.10067980	acute_myeloi	Mucin 17	Golgi lumen,O-glycan processing,PDZ domain binding,ap				C=29, T=25	C=0.537, T=0.463	0.00001	247.553	55	C=21/8, T=14/
chr11:1094690	T/T	C	T	SNV	MUC2	MUC2:exonic:NM_002457.4	1	100	32	synonymous	NM_00245	c.5778C>T	p.(=)	-4.19				0(COSM3752	C8 domain,M	rs10794291	0.4995	YES	9.245E-1 (pos.1094690)	adenocarcinor	Mucin 2, intestinal/tra		Adenocarcinoma;A	Pranlukast		C=0, T=30	C=0.0, T=1.0	0.00001	446.639	30	C=0/0, T=22/8
chr2:153492910	T/T	G	T	SNV	FMNL2	FMNL2:intronic:NM_052905.3	1	100		intronic	NM_05290	c.2681-31	p.?	-1.94					Diaphanous F	rs1561267	0.1	YES	8.602E-1 (pos.153492910)		Formin-like	Rho GTPase binding,actin binding,cadherin binding,corti				G=0, T=20	G=0.0, T=1.0	0.00001	258.088	20	G=0/0, T=16/4
chr2:153519524	G/G	A	G	SNV	PRPF40A	PRPF40A:intronic:NM_017892.3	1	100		intronic	NM_01789	c.2226+25	p.?	-0.43					FF domain,W	rs1036526	0.1823	YES	7.423E-1 (pos.153519524)		Precursor m	RNA binding,U1 snRNP,U2-type prespliceosome,cell cycl				A=0, G=47	A=0.0, G=1.0	0.00001	793.7	47	A=0/0, G=26/2
chr2:153572438	T/T	C	T	SNV	PRPF40A	PRPF40A:intronic:NM_017892.3	1	100		intronic	NM_01789	c.216+71G	p.?	0.32					FF domain,W	rs2346548,rs386651723					Precursor m	RNA binding,U1 snRNP,U2-type prespliceosome,cell cycl				C=0, T=38	C=0.0, T=1.0	0.00001	603.786	38	C=0/0, T=23/1
chr2:153577013	A/A	G	A	SNV	ARL6IP6	ARL6IP6:intronic:NM_152522.5	1	100		intronic	NM_15252	c.401-34G	p.?	-0.9					Haemopoieti	rs9646744	0.1802	YES	7.434E-1 (pos.153577013)		ADP-ribosyl	integral component of membrane				G=0, A=28	G=0.0, A=1.0	0.00001	413.531	28	G=0/0, A=10/1
chr2:155099146	G/A	G	A	SNV	GALNT13	GALNT13:intronic:NM_052917.3	1	57.14		intronic	NM_05291	c.479-65G	p.?	-0.07					Glycosyl tran	rs9288324		YES			UDP-N-acet	Golgi membrane,O-glycan processing,carbohydrate bind				G=42, A=56	G=0.4286, A=0.5714	0.00001	673.784	98	G=16/26, A=30
chr2:15519992	T/T	G	T	SNV	NBAS	NBAS:intronic:NM_015909.3	1	100		intronic	NM_01590	c.3361-37	p.?	-0.39					Neuroblastor	rs2058866	0.3157	YES	2.253E-1;0.0 (pos.15519992)		Neuroblast	Dsl1/NZR c	Digestive System Diseases;Digestive System			G=0, T=37	G=0.0, T=1.0	0.00001	583.447	37	G=0/0, T=18/1
chr2:15523266	A/T	A	T	SNV	NBAS	NBAS:intronic:NM_015909.3	1	95.45		intronic	NM_01590	c.3360+73	p.?	-2.19					Neuroblastor	rs6756934		YES			Neuroblast	Dsl1/NZR c	Digestive System Diseases;Digestive System			A=1, T=21	A=0.0455, T=0.9545	0.00001	269.34	22	A=1/0, T=15/6
chr2:155295380	C/C	T	C	SNV	GALNT13,LOC100144595	GALNT13:intronic:NM_052917.3, LOC100144595:intronic:N	1	100		intronic	NM_05291	c.1530+14	p.?, p.?	-3.52					Glycosyl tran	rs697647					UDP-N-acet	Golgi membrane,O-glycan processing,carbohydrate bind				T=0, C=88	T=0.0, C=1.0	0.00001	1451.89	88	T=0/0, C=39/4
chr11:1094753	C/C	A	C	SNV	MUC2	MUC2:exonic:NM_002457.4	1	100	32	synonymous	NM_00245	c.5841A>C	p.(=)	-1.34				0(COSM3752	C8 domain,M	rs10794292,rs38674	0.4168	YES	9.932E-1;0.0 (pos.10947	adenocarcinor	Mucin 2, intestinal/tra		Adenocarcinoma;A	Pranlukast		A=0, C=35	A=0.0, C=1.0	0.00001	530.484	35	A=0/0, C=21/1
chr11:1087972	C/T	C	T	SNV	MUC2	MUC2:exonic:NM_002457.4	1	45.45	25	synonymous	NM_00245	c.3447C>T	p.(=)	-1.94				0.01(COSM41	C8 domain,M	rs10902088	0.1996	YES	4.185E-1 (pos.1087972)	neoplasm	Mucin 2, intestinal/tra		Adenocarcinoma;A	Pranlukast		C=18, T=15	C=0.5455, T=0.4545	0.00001	143.162	33	C=13/5, T=12/
chr2:15601574	T/T	C	T	SNV	NBAS	NBAS:intronic:NM_015909.3	1	100		intronic	NM_01590	c.2203-11	p.?	0.95					Neuroblastor	rs7590458		YES			Neuroblast	Dsl1/NZR c	Digestive System Diseases;Digestive System			C=0, T=42	C=0.0, T=1.0	0.00001	681.03	42	C=0/0, T=15/2
chr6:30993958	G/G	A	G	SNV	MUC22	MUC22:exonic:NM_001198815.1	1	100	3	synonymous	NM_00119	c.750A>G	p.(=)	-1.07				0(COSM5766	Mucin, cataly	rs2523897		YES	7.771E-1 (pos.30993958)	adenocarcinor	Mucin 22	integral cc	Autoimmune Diseases;Endocrine System Dis			A=0, G=40	A=0.0, G=1.0	0.00001	644.285	40	A=0/0, G=29/1
chr6:30997692	C/C	G	C	SNV	MUC22	MUC22:exonic:NM_001198815.1	1	100	3	missense	NM_00119	c.4484G>C	p.Ser1495Thr	-3.6	1	58	0.009	0(COSM4594	Mucin, cataly	rs9262549		YES	6.714E-1 (pos.30997692)	squamous_cel	Mucin 22	integral cc	Autoimmune Diseases;Endocrine System Dis			G=0, C=82	G=0.0, C=1.0	0.00001	1328.33	82	G=0/0, C=55/2
chr2:15616013	G/G	C	G	SNV	NBAS	NBAS:intronic:NM_015909.3	1	100		intronic	NM_01590	c.1148-9G	p.?	-0.67				0.01(COSM44	Neuroblastor	rs1990755	0.3176	YES	1.588E-1;0.0 (pos.15616	acute_myeloi	Neuroblast	Dsl1/NZR c	Digestive System Diseases;Digestive System			C=0, G=93	C=0.0, G=1.0	0.00001	1555.33	93	C=0/0, G=34/5
chr2:15616030	T/T	C	T	SNV	NBAS	NBAS:intronic:NM_015909.3	1	100		intronic	NM_01590	c.1148-26	p.?	-0.19					Neuroblastor	rs1990756	0.3181	YES	1.612E-1 (pos.15616030)		Neuroblast	Dsl1/NZR c	Digestive System Diseases;Digestive System			C=0, T=94	C=0.0, T=1.0	0.00001	1576.17	94	C=0/0, T=34/6
chr2:15618339	A/A	G	A	SNV	NBAS	NBAS:intronic:NM_015909.3	1	100		intronic	NM_01590	c.1147+11	p.?	0.38					Neuroblastor	rs1125534	0.3165	YES	2.14E-1 (pos.15618339)		Neuroblast	Dsl1/NZR c	Digestive System Diseases;Digestive System			G=0, A=46	G=0.0, A=1.0	0.00001	768.313	46	G=0/0, A=17/2
chr3:195512186	C/C	T	C	SNV	MUC4	MUC4:exonic:NM_018406.6	1	100	2	missense	NM_01840	c.6265A>G	p.Ile2089Val	-4.83	0.8	29	0.029	0(COSM1617	Nidogen-like,	rs202060675,rs751858617,rs75		YES	1.721E-3;0.0 (pos.19551	astrocytoma_(	Mucin 4, tra	ErbB-2 cla	Abortion, Spontaneous;Adenocarcinoma;Car			T=0, C=34	T=0.0, C=1.0	0.00001	524.184	34	T=0/0, C=25/9
chr2:15676684	GA/GA	G	GA	INDEL	NBAS	NBAS:intronic:NM_015909.3	1	100		intronic	NM_01590	c.514-9C>	p.?	0.8					Neuroblastor	rs781067753			0.0 (pos.15676684)		Neuroblast	Dsl1/NZR c	Digestive System Diseases;Digestive System			G=0, GA=31	G=0.0, GA=1.0	0.00001	431.321	31	G=0/0, GA=12/
chr2:15737343	G/G	A	G	SNV	DDX1	DDX1:intronic:NM_004939.2	1	100		intronic	NM_00493	c.163-156	p.?	1.03					DEAD/DEAH b	rs2302930		YES			DEAD/H box	ATP bindin	Congenital, Hereditary, and Neonatal Diseas			A=0, G=22	A=0.0, G=1.0	0.00001	298.884	22	A=0/0, G=15/7
chr2:15737480	A/A	C	A	SNV	DDX1	DDX1:intronic:NM_004939.2	1	100		intronic	NM_00493	c.163-19C	p.?	-0.05					DEAD/DEAH b	rs2302931	0.1564	YES	1.947E-1 (pos.15737480)		DEAD/H box	ATP bindin	Congenital, Hereditary, and Neonatal Diseas			C=0, A=21	C=0.0, A=1.0	0.00001	281.216	21	C=0/0, A=9/12

chr2:157427593	C/C	T	C	SNV	GPD2	GPD2:intronic:NM_000408.4	1	100		intronic	NM_00040	c.1609-53	p.?	-1.09					C-terminal dc	rs1469652		YES			Glycerol-3-	calcium io	Amino Acid Metabolism, Inborn Errors;Bone D			T=0, C=71	T=0.0, C=1.0	0.00001	1109.16	71	T=0/0, C=14/5
chr2:15753482	G/G	C	G	SNV	DDX1	DDX1:intronic:NM_004939.2	1	100		intronic	NM_00493	c.1017+74	p.?	-0.23					DEAD/DEAH b	rs2302935		YES			DEAD/H box	ATP bindin	Congenital, Hereditary, and Neonatal Diseas			C=0, G=89	C=0.0, G=1.0	0.00001	1469.16	89	C=0/0, G=46/4
chr2:158401137	C/C	T	C	SNV	ACVR1C	ACVR1C:intronic:NM_145259.2	1	100		intronic	NM_14525	c.776-13A	p.?	0.46					Protein kinas	rs10432556	0.1915	YES	9.131E-1 (pos.158401137)		Activin A re	ATP binding,activin receptor activity, type I,activin recep				T=0, C=94	T=0.0, C=1.0	0.00001	1576.15	94	T=0/0, C=48/4
chr3:195497143	C/T	C	T	SNV	MUC4	MUC4:exonic:NM_018406.6	1	46.51	6	missense	NM_01840	c.13342G>	p.Ala4448Thr	1.86	0.56	58	0		Nidogen-like	rs2293232	0.1259	YES	1.984E-1 (pos.195497143)		Mucin 4, tra	ErbB-2 cla	Abortion, Spontaneous;Adenocarcinoma;Car			C=23, T=20	C=0.5349, T=0.4651	0.00001	212.325	43	C=9/14, T=7/1
chr11:1252708	C/C	G	C	SNV	MUC5B	MUC5B:exonic:NM_002458.2	1	100	14	synonymous	NM_00245	c.1596G>C	p.(=)	-1.72				0.02(COSM41	C8 domain,M	rs2735709	0.4981	YES	6.575E-1;0.0 (pos.12527(	carcinoma,ne	Mucin 5, su	Golgi lume	Bronchial Diseases;Bronchiolit		Benign	G=0, C=55	G=0.0, C=1.0	0.00001	814.907	55	G=0/0, C=28/2
chr2:159526461	T/T	C	T	SNV	PKP4,PKP4-AS1	PKP4-AS1:intronic_nc:NR_135242.1, PKP4:intronic:NM_003	1	100		intronic	NM_00362	c.2924+34	p.?	-1.15					Armadillo/be	rs13396528	0.1186	YES	3.965E-2 (pos.159526461)		Plakophilin	cell junction,cell-cell adhesion,cell-cell contact zone,cel				C=0, T=44	C=0.0, T=1.0	0.00001	726.569	44	C=0/0, T=15/2
chr11:1253942	C/C	T	C	SNV	MUC5B	MUC5B:exonic:NM_002458.2	1	100	17	synonymous	NM_00245	c.2007T>C	p.(=)	-1.09				0.02(COSM37	C8 domain,M	rs908229	0.3896	YES	7.178E-1 (pos.1253942)	neoplasm,squ	Mucin 5, su	Golgi lume	Bronchial Diseases;Bronchiolit		Benign	T=0, C=21	T=0.0, C=1.0	0.00001	281.235	21	T=0/0, C=15/6
chr2:160033174	G/G	A	G	SNV	TANC1	TANC1:intronic:NM_033394.2	1	100		intronic	NM_03339	c.1902+14	p.?	-0.79					Ankyrin repe	rs759034		YES			Tetratricop	axon term	Cardiovascular Diseases;Death, Sudden, Car			A=0, G=48	A=0.0, G=1.0	0.00001	810.435	48	A=0/0, G=23/2
chr11:1264823	C/C	T	C	SNV	MUC5B	MUC5B:exonic:NM_002458.2	1	100	31	missense	NM_00245	c.6713T>C	p.Leu2238Pro	-2.46	0.16	98	0.028	0(COSM1600	C8 domain,M	rs200832980,rs4963(	0.4364	YES	9.824E-1 (pos.1264823)	adenocarcino	Mucin 5, su	Golgi lume	Bronchial Diseases;Bronchiolit		Benign	T=0, C=220	T=0.0, C=1.0	0.00001	3590.13	220	T=0/0, C=107/
chr11:1268608	G/G	A	G	SNV	MUC5B	MUC5B:exonic:NM_002458.2	1	100	31	missense	NM_00245	c.10498A>	p.Ser3500Gly	-0.72	1	56	0	0(COSM3765	C8 domain,M	rs2943527	0.4251	YES	6.562E-1 (pos.1268608)	hepatocellula	Mucin 5, su	Golgi lume	Bronchial Diseases;Bronchiolit		Benign	A=0, G=114	A=0.0, G=1.0	0.00001	1803.17	114	A=0/0, G=65/4
chr11:1280238	G/A	G	A	SNV	MUC5B	MUC5B:exonic:NM_002458.2	1	96.77	44	missense	NM_00245	c.16660G>	p.Asp5554Asn	-0.37	0.15	23	0.22		C8 domain,M	rs55856616	0.0301	YES	6.25E-3 (pos.1280238)		Mucin 5, su	Golgi lume	Bronchial Diseases;Bronchiolit		Benign	G=1, A=30	G=0.0323, A=0.9677	0.00001	434.776	31	G=0/1, A=16/1
chr2:160128202	C/C	A	C	SNV	WDSUB1	WDSUB1:intronic:NM_001128212.2	1	100		intronic	NM_00112	c.770+11T	p.?	0.48					SAM domain	rs11891627	0.0436	YES	2.002E-1 (pos.160128202)			protein ubiquitination,ubiquitin-protein transferase act				A=0, C=24	A=0.0, C=1.0	0.00001	334.792	24	A=0/0, C=11/1
chr11:1019375	T/T	C	T	SNV	MUC6	MUC6:exonic:NM_005961.2	1	100	30	synonymous	NM_00596	c.3930G>A	p.(=)	-1.54				0(COSM4145	C8 domain,Tr	rs9704308	0.0699	YES	8.68E-1 (pos.1019375)	neoplasm	Mucin 6, ga	Golgi lume	Digestive System Diseases;Digestive System			C=0, T=22	C=0.0, T=1.0	0.00001	298.892	22	C=0/0, T=13/9
chr2:160139048	C/T	C	T	SNV	WDSUB1	WDSUB1:intronic:NM_001128212.2	1	98.36		intronic	NM_00112	c.398+135	p.?	0					SAM domain	rs10490001		YES				protein ubiquitination,ubiquitin-protein transferase act				C=1, T=60	C=0.0164, T=0.9836	0.00001	888.34	61	C=1/0, T=38/2
chr2:160176943	C/C	A	C	SNV	BAZ2B	BAZ2B:intronic:NM_013450.3	1	100		intronic	NM_01345	c.6354-14	p.?	0.45					Bromodomai	rs174229	4.00E-04	YES	1.0 (pos.160176943)		Bromodoma	DNA bindi	Cardiovascular Diseases;Death, Sudden, Car			A=0, C=35	A=0.0, C=1.0	0.00001	543.903	35	A=0/0, C=19/1
chr2:160243183	AT/AT	TA	AT	MNV	BAZ2B	BAZ2B:intronic:NM_013450.3	2	100		intronic	NM_01345	c.3217-65	p.?	4.07, 0.28					Bromodomai	rs35227840,rs879258048,rs918		YES			Bromodoma	DNA bindi	Cardiovascular Diseases;Death, Sudden, Car			TA=0, AT=5	TA=0.0, AT=1.0	0.00001	784.587	53	TA=0/0, AT=16
chr11:1028379	C/T	C	T	SNV	MUC6	MUC6:exonic:NM_005961.2	1	58.33	14	missense	NM_00596	c.1600G>A	p.Gly534Ser	4.52	0	56	1		C8 domain,Tr	rs116256283	0.0135		2.373E-4 (pos.1028379)		Mucin 6, ga	Golgi lume	Digestive System Diseases;Digestive System			C=15, T=21	C=0.4167, T=0.5833	0.00001	234.325	36	C=3/12, T=15/
chr2:160585398	C/C	G	C	SNV	MARCH7	MARCH7:intronic:NM_022826.3	1	100		intronic	NM_02282	c.-13-122C	p.?	-2.58					RING-variant	rs3732289		YES			Membrane-	negative r	Autoimmune Diseases;Female Urogenital Di			G=0, C=67	G=0.0, C=1.0	0.00001	1034.38	67	G=0/0, C=35/3
chr2:160585740	A/A	C	A	SNV	MARCH7	MARCH7:intronic:NM_022826.3	1	100		intronic	NM_02282	c.153+54C	p.?	0.1					RING-variant	rs3821299		YES			Membrane-	negative r	Autoimmune Diseases;Female Urogenital Di			C=0, A=61	C=0.0, A=1.0	0.00001	921.851	61	C=0/0, A=23/3
chr11:1017173	G/A	G	A	SNV	MUC6	MUC6:exonic:NM_005961.2	1	50	31	synonymous	NM_00596	c.5628C>T	p.(=)	0.11				0.07(COSM41	C8 domain,Tr	rs61869004		YES	4.992E-1 (pos.1017173)	acute_myeloic	Mucin 6, ga	Golgi lume	Digestive System Diseases;Digestive System			G=178, A=1	G=0.5, A=0.5	0.00001	1905.22	356	G=73/105, A=
chr19:1360575	G/A	G	A	SNV	MUM1	MUM1:exonic:NM_032853.3	1	95.65	5	missense	NM_03285	c.658G>A	p.Gly220Arg	0.39	1	125	0	0(COSM5021975)		rs3826942	0.243	YES	4.876E-1 (pos.1360575)	carcinoma,osteosarcoma		DNA repair,chromatin organization,cytosol,nucleosome				G=1, A=22	G=0.0435, A=0.9565	0.00001	288.89	23	G=0/1, A=4/18
chr6:49425521	T/T	C	T	SNV	MUT	MUT:exonic:NM_000255.3	1	100	3	synonymous	NM_00025	c.636G>A	p.(=)	0.67				0.39(COSM63	B12 binding c	rs2229384	0.3836	YES	4.905E-1 (pos.49425521)	carcinoma	Methylmalc	cobalamir	Amino Acid Metabo	Cyanocob	Benign	C=0, T=58	C=0.0, T=1.0	0.00001	867.385	58	C=0/0, T=21/3
chr2:160729134	C/C	A	C	SNV	LY75,LY75-CD302	LY75:intronic:NM_002349.3, LY75-CD302:intronic:NM_0011	1	100		intronic	NM_00119	c.1975-30	p.?, p.?	0.56					Fibronectin t	rs1397705	0.0336	YES	1.0 (pos.160729134)			carbohydrate binding,endocytosis,extracellular exosom				A=0, C=213	A=0.0, C=1.0	0.00001	3485.62	213	A=0/0, C=146/
chr2:160729186	C/C	T	C	SNV	LY75,LY75-CD302	LY75:intronic:NM_002349.3, LY75-CD302:intronic:NM_0011	1	100		intronic	NM_00119	c.1975-82	p.?, p.?	0.63					Fibronectin t	rs10168271		YES				carbohydrate binding,endocytosis,extracellular exosom				T=0, C=212	T=0.0, C=1.0	0.00001	3465.69	212	T=0/0, C=142/
chr2:160737857	T/TGTA	T	TGTA	INDEL	LY75,LY75-CD302	LY75:intronic:NM_002349.3, LY75-CD302:intronic:NM_0011	3	96.77		intronic	NM_00119	c.1247-10	p.?, p.?	-0.19					Fibronectin t	rs3835165,rs397826060		YES				carbohydrate binding,endocytosis,extracellular exosom				T=1, TGTA=	T=0.0323, TGTA=0.96	0.00001	435.409	31	T=1/0, TGTA=1
chr2:160805993	G/G	C	G	SNV	PLA2R1	PLA2R1:intronic:NM_007366.4	1	100		intronic	NM_00736	c.3709+12	p.?	-0.05					Fibronectin t	rs6726923		YES			Phospholip	carbohydr	Autoimmune Diseases;Female Urogenital Di			C=0, G=20	C=0.0, G=1.0	0.00001	263.786	20	C=0/0, G=10/1
chr2:160807742	T/T	A	T	SNV	PLA2R1	PLA2R1:intronic:NM_007366.4	1	100		intronic	NM_00736	c.3540+10	p.?	0.44					Fibronectin t	rs952184		YES			Phospholip	carbohydr	Autoimmune Diseases;Female Urogenital Di			A=0, T=25	A=0.0, T=1.0	0.00001	352.954	25	A=0/0, T=14/1
chr16:88724347	T/T	G	T	SNV	MVD	MVD:exonic:NM_002461.2	1	100	3	synonymous	NM_00246	c.232C>A	p.(=)	4.33				0.89(COSM37	GHMP kinase	rs2279258,rs751341	0.34	YES	0.0;0.0 (pos.88724340),6	acute_myeloic	Mevalonate	ATP bindin	Congenital, Hereditary, and Neonatal Diseas			G=0, T=22	G=0.0, T=1.0	0.00001	298.892	22	G=0/0, T=15/7
chrX:3228891	G/G	A	G	SNV	MXRA5	MXRA5:exonic:NM_015419.3	1	100	7	synonymous	NM_01541	c.7353T>C	p.(=)	-2.83				0.01(COSM37	Immunoglobu	rs1635235	0.424	YES	7.654E-1 (pos.3228891)	adenocarcino	Matrix remo	extracellu	Bronchial Neoplasms;Carcinoma, Bronchoge			A=0, G=23	A=0.0, G=1.0	0.00001	316.747	23	A=0/0, G=18/5
chr2:165358655	A/A	T	A	SNV	GRB14	GRB14:intronic:NM_004490.2	1	100		intronic	NM_00449	c.1104+11	p.?	-0.01					BPS (Betweer	rs7584035		YES			Growth fact	SH3/SH2 a	Diabetes Mellitus;Diabetes Mellitus, Type 2;			T=0, A=34	T=0.0, A=1.0	0.00001	523.911	34	T=0/0, A=21/1
chr2:165358672	G/G	A	G	SNV	GRB14	GRB14:intronic:NM_004490.2	1	100		intronic	NM_00449	c.1104+93	p.?	-0.63					BPS (Betweer	rs4667463		YES			Growth fact	SH3/SH2 a	Diabetes Mellitus;Diabetes Mellitus, Type 2;			A=0, G=36	A=0.0, G=1.0	0.00001	563.744	36	A=0/0, G=21/1
chrX:3235724	T/T	C	T	SNV	MXRA5	MXRA5:exonic:NM_015419.3	1	100	6	missense	NM_01541	c.5998G>A	p.Gly2000Ser	0.84	0.55	56	0.117	0.2(COSM375	Immunoglobu	rs1635242	0.3548	YES	7.704E-1 (pos.3235724)	adenocarcino	Matrix remo	extracellu	Bronchial Neoplasms;Carcinoma, Bronchoge			C=0, T=25	C=0.0, T=1.0	0.00001	352.895	25	C=0/0, T=11/1
chr2:166153499	G/G	A	G	SNV	SCN2A	SCN2A:intronic:NM_001040143.1	1	100		intronic	NM_00104	c.268-28A	p.?	-0.21					Cytoplasmic	rs7593568	0.1562	YES	8.162E-1 (pos.166153499)		Sodium cha	T-tubule,a	Brain Diseases;Cen	Brivaracetam,Lamotrigi		A=0, G=21	A=0.0, G=1.0	0.00001	281.228	21	A=0/0, G=9/12
chr2:166170127	C/C	T	C	SNV	SCN2A	SCN2A:intronic:NM_001040143.1	1	100		intronic	NM_00104	c.1035-3T	p.?	0.56					Cytoplasmic	rs2121371	0.159	YES	7.75E-1 (pos.166170127)		Sodium cha	T-tubule,a	Brain Diseases;Cen	Brivaracet	Benign	T=0, C=25	T=0.0, C=1.0	0.00001	353.019	25	T=0/0, C=8/17
chr2:166172317	G/G	A	G	SNV	SCN2A	SCN2A:intronic:NM_001040143.1	1	100		intronic	NM_00104	c.1671+49	p.?	-1.94					Cytoplasmic	rs1838846	0.1498	YES	7.78E-1 (pos.166172317)		Sodium cha	T-tubule,a	Brain Diseases;Cen	Brivaracetam,Lamotrigi		A=0, G=69	A=0.0, G=1.0	0.00001	1072.78	69	A=0/0, G=32/3
chrX:3239545	T/T	C	T	SNV	MXRA5	MXRA5:exonic:NM_015419.3	1	100	5	missense	NM_01541	c.4181G>A	p.Gly1394Asp	-0.02	1	94	0		Immunoglobu	rs1726199	0.3549	YES	7.763E-1;0.0 (pos.3239545)		Matrix remo	extracellu	Bronchial Neoplasms;Carcinoma, Bronchoge			C=0, T=25	C=0.0, T=1.0	0.00001	353.017	25	C=0/0, T=13/1
chrX:3241791	A/A	G	A	SNV	MXRA5	MXRA5:exonic:NM_015419.3	1	100	5	synonymous	NM_01541	c.1935C>T	p.(=)	0.13				0.18(COSM37	Immunoglobu	rs1635250	0.4297	YES	3.93E-1;0.0 (pos.324179	adenocarcino	Matrix remo	extracellu	Bronchial Neoplasms;Carcinoma, Bronchoge			G=0, A=32	G=0.0, A=1.0	0.00001	485.14	32	G=0/0, A=24/8
chr17:79899495	G/A	G	A	SNV	MYADML2	MYADML2:exonic:NM_001145113.2	1	96.67	3	synonymous	NM_00114	c.123C>T	p.(=)	0.06				0.69(COSM43	Membrane-a	rs4796854	0.3853	YES	3.448E-1 (pos.79899495)	adenocarcinoma,carcinom		cytoplasm,integral component of membrane				G=2, A=58	G=0.0333, A=0.9667	0.00001	841.439	60	G=0/2, A=20/3
chr2:166799893	A/A	G	A	SNV	TTC21B,TTC21B-AS1	TTC21B:intronic:NM_024753.4, TTC21B-AS1:intronic_nc:NR	1	100		intronic	NM_02475	c.430-42C	p.?	-0.59					Tetratricoper	rs10930190,rs76914	0		0.0 (pos.166799890),0.0 (pos.16679989		Tetratricop	ciliary tip,	Abnormalities, Multiple;Bardet-Biedl Syndro			G=0, A=97	G=0.0, A=1.0	0.00001	1638.1	97	G=0/0, A=40/5
chr17:4455500	C/C	T	C	SNV	MYBBP1A	MYBBP1A:exonic:NM_001105538.1	1	100	7	synonymous	NM_00110	c.825A>G	p.(=)	-4.36				0.01(COSM41	DNA polymer	rs7206974	2.00E-04		1.0 (pos.4455500)	neoplasm	MYB-bindin	DNA binding,DNA biosynthetic process,DNA-directed DN				T=0, C=30	T=0.0, C=1.0	0.00001	446.634	30	T=0/0, C=18/1
chr2:166905375	A/G	A	G	SNV	SCN1A	SCN1A:intronic:NM_006920.5	1	50		intronic	NM_00692	c.1028+21	p.?	0.32					Cytoplasmic	rs1542484	0.4575	YES	5.75E-1 (pos.166905375)		Sodium cha	T-tubule,Z	Brain Diseases;Cen	Brivaracetam,Dronedarc		A=24, G=24	A=0.5, G=0.5	0.00001	273.703	48	A=6/18, G=12/
chr2:166930214	A/A	T	A	SNV	SCN1A	SCN1A:intronic:NM_006920.5	1	100		intronic	NM_00692	c.-48-34A>	p.?	-0.2						rs566839		YES			Sodium cha	T-tubule,Z	Brain Diseases;Cen	Brivaracetam,Dronedarc		T=0, A=37	T=0.0, A=1.0	0.00001	583.706	37	T=0/0, A=12/2
chr1:109838918	T/T	C	T	SNV	MYBPHL	MYBPHL:exonic:NM_001010985.2	1	100	6	missense	NM_00101	c.805G>A	p.Asp269Asn	3.46	0.56	23	0.01		Fibronectin t	rs629001	0.0655	YES	9.999E-1 (pos.109838918)			M band,Z disc,actin filament binding,actin filament orga				C=0, T=49	C=0.0, T=1.0	0.00001	831.632	49	C=0/0, T=35/1
chr1:109839433	G/G	A	G	SNV	MYBPHL	MYBPHL:exonic:NM_001010985.2	1	100	5	synonymous	NM_00101	c.702T>C	p.(=)	-1.24					Fibronectin t	rs1278285	0.0014	YES	1.0 (pos.109839433)			M band,Z disc,actin filament binding,actin filament orga				A=0, G=25	A=0.0, G=1.0	0.00001	353.019	25	A=0/0, G=10/1
chr2:167328750	T/T	C	T	SNV	SCN7A	SCN7A:intronic:NM_002976.3	1	100		intronic	NM_00297	c.572+77G	p.?	-2.16					Ion transport	rs2293567		YES			Sodium cha	glial cell p	Autistic Disorder;Cl	Brivaracetam,Valproic A		C=0, T=52	C=0.0, T=1.0	0.00001	767.015	52	C=0/0, T=24/2
chr13:77738664	G/G	A	G	SNV	MYCBP2	MYCBP2:exonic:NM_015057.4	1	100	43	synonymous	NM_01505	c.6472T>C	p.(=)	0.91				0.43(COSM39	Anaphase-pr	rs2274547	0.1697	YES	3.461E-1 (pos.77738664)	acute_myeloic	Myc-bindin	membran	Female Urogenital Diseases and Pregnancy C			A=0, G=38	A=0.0, G=1.0	0.00001	603.706	38	A=0/0, G=18/2
chr17:48606537	G/G	C	G	SNV	MYCBPAP	MYCBPAP:exonic:NM_032133.4	1	100	18	missense	NM_03213	c.2841C>G	p.Ser947Arg	0.85	1	110	0	0.02(COSM41	MYCBP-assoc	rs1133818	0.271	YES	8.726E-1 (pos.48606537)	neoplasm	MYCBP-ass	cell differentiation,chemical synaptic transmission,cyto				C=0, G=45	C=0.0, G=1.0	0.00001	746.795	45	C=0/0, G=20/2
chr2:169487668	C/C	A	C	SNV	CERS6	CERS6:intronic:NM_001256126.1	1	100		intronic	NM_00125	c.465+116	p.?	0.17					Homeobox dc	rs4667585		YES			Ceramide s	DNA bindi	Mental Disorders;Substance-Related Disorde			A=0, C=20	A=0.0, C=1.0	0.00001	263.786	20	A=0/0, C=9/11
chr2:169801488	A/G	A	G	SNV	ABCB11	ABCB11:intronic:NM_003742.2	1	98.41		intronic	NM_00374	c.2344-17	p.?	0.26					ABC transpor	rs853789	0.3138	YES	9.923E-1 (pos.169801488)		ATP-binding	ATP bindin	Afibrinogenemia;Bi	ATP,Boser	Benign	A=1, G=62	A=0.0159, G=0.9841	0.00001	925.373	63	A=0/1, G=21/4
chr17:48608717	C/C	T	C	SNV	MYCBPAP	MYCBPAP:exonic:NM_032133.4	1	100	19	synonymous	NM_03213	c.2883T>C	p.(=)	-5.29				0(COSM5424	MYCBP-assoc	rs1064055	0.2714	YES	8.771E-1;0.0 (pos.48608	acute_myeloic	MYCBP-ass	cell differentiation,chemical synaptic transmission,cyto				T=0, C=79	T=0.0, C=1.0	0.00001	1268.89	79	T=0/0, C=57/2
chr1:40363054	C/C	G	C	SNV	MYCL	MYCL:exonic:NM_001033082.2	1	100	3	missense	NM_00103	c.1175C>G	p.Thr392Ser	0.98	1	58	0	0.11(COSM39	Helix-loop-he	rs3134614	0.1328	YES	9.994E-1 (pos.40363054)	adenocarcino	Oncogene M	DNA bindi	Bronchial Neoplasms;Carcinoma, Bronchoge			G=0, C=32	G=0.0, C=1.0	0.00001	485.133	32	G=0/0, C=11/2
chr17:10247199	G/A	G	A	SNV	MYH13	MYH13:exonic:NM_003802.2	1	50	16	synonymous	NM_00380	c.1812C>T	p.(=)	-0.79				0.01(COSM50	IQ calmoduli	rs12938754	0.2921	YES	2.312E-4;0.0 (pos.10247	osteosarcoma	Myosin, hea	ATP bindin	Arthritis;Joint Diseases;Musculoskeletal Dis			G=13, A=13	G=0.5, A=0.5	0.00001	119.163	26	G=8/5, A=9/4
chr2:170082773	C/A	C	A	SNV	LRP2	LRP2:intronic:NM_004525.2	1	58.33		intronic	NM_00452	c.5394+15	p.?	-0.19					Calcium-bind	rs13383183		YES			Low density	Golgi appa	Agenesis of Corpus	Gentamicin,Insulin Hum		C=10, A=14	C=0.4167, A=0.5833	0.00001	135.339	24	C=10/0, A=5/9
chr2:170082776	A/T	A	T	SNV	LRP2	LRP2:intronic:NM_004525.2	1	58.33		intronic	NM_00452	c.5394+15	p.?	0.68					Calcium-bind	rs35836996		YES			Low density	Golgi appa	Agenesis of Corpus	Gentamicin,Insulin Hum		A=10, T=14	A=0.4167, T=0.5833	0.00001	135.339	24	A=10/0, T=5/9
chr2:170099446	G/G	T	G	SNV	LRP2	LRP2:intronic:NM_004525.2	1	100		intronic	NM_00452	c.3667+20	p.?	-2.4					Calcium-bind	rs831041	0.4557	YES	4.71E-1 (pos.170099446)		Low density	Golgi appa	Agenesis of Corpus	Gentamici	Benign	T=0, G=45	T=0.0, G=1.0	0.00001	747.392	45	T=0/0, G=24/2
chr19:50762418	G/G	A	G	SNV	MYH14	MYH14:exonic:NM_001145809.1	1	100	19	synonymous	NM_00114	c.2250A>G	p.(=)	-10.43				0(COSM3722	IQ calmoduli	rs1651553	0.2492	YES	8.551E-1 (pos.50762418)	squamous_cel	Myosin, hea	ATP bindin	Ear Diseases;Genit	(3aS)-3a-h	Benign	A=0, G=22	A=0.0, G=1.0	0.00001	298.884	22	A=0/0, G=11/1
chr17:10448769	A/T	A	T	SNV	MYH2,MYHAS	MYH2:exonic:NM_001100112.1, MYHAS:intronic_nc:NR_125	1	50	5	synonymous	NR_12536	c.399T>A	p.(=)	-6.08					IQ calmoduli	rs11078850	0.4083	YES	8.526E-1;0.0 (pos.10448769)		Myosin, hea	A band,ATI	Contracture;Cranial Nerve Dis		Benign	A=10, T=10	A=0.5, T=0.5	0.00001	82.8867	20	A=9/1, T=7/3
chr17:10348354	T/C	T	C	SNV	MYH4,MYHAS	MYHAS:intronic_nc:NR_125367.1, MYH4:exonic:NM_017533	1	50	37	missense	NR_12536	c.5405A>G	p.Asp1802Gly	6.26	0	94	0.993	0.98(COSM41	IQ calmoduli	rs2277649	0.3547	YES	8.417E-1 (pos.10348354)	diffuse_large	Myosin, hea	ATP binding,ATP metabolic process,ATPase activity,actin				T=11, C=11	T=0.5, C=0.5	0.00001	94.6067	22	T=3/8, C=6/5
chr14:23855569	G/G	A	G	SNV	MYH6	MYH6:exonic:NM_002471.3	1	100	33	synonymous	NM_00247	c.4914T>C	p.(=)	-4.34				0.01(COSM55	Myosin N-ter	rs178640	0.4855	YES	2.608E-1 (pos.23855569)	carcinoma	Myosin, hea	ATP bindin	Aortic Stenosis, Subvalvular;Ac		Benign/Likely	A=0, G=39	A=0.0, G=1.0	0.00001	623.939	39	A=0/0, G=19/2
chr2:170428127	T/G	T	G	SNV	FASTKD1	FASTKD1:intronic:NM_024622.5	1	47.62		intronic	NM_02462	c.377+36A	p.?	-2.43					FAST kinase-l	rs4668146	0.3867	YES	5.907E-1 (pos.170428127)		Fast kinase	RNA binding,cellular respiration,mitochondrion,protein				T=11, G=10	T=0.5238, G=0.4762	0.00001	82.2898	21	T=7/4, G=9/1
chr17:10318636	G/G	A	G	SNV	MYH8,MYHAS	MYHAS:intronic_nc:NR_125367.1, MYH8:exonic:NM_002472	1	100	8	synonymous	NR_12536	c.714T>C	p.(=)	-5.42					IQ calmoduli	rs8068729	0.0543	YES	9.998E-1 (pos.10318636)		Myosin, hea	ATP bindin	Abnormalities, Multiple;Arthrc		Benign/Likely	A=0, G=70	A=0.0, G=1.0	0.00001	1092.09	70	A=0/0, G=39/3
chr2:170676227	C/C	T	C	SNV	METTL5	METTL5:intronic:NM_014168.3	1	100		intronic	NM_01416	c.407-74A	p.?	-0.93					Conserved hy	rs1541776		YES				methylation,methyltransferase activity,nucleic acid bin				T=0, C=96	T=0.0, C=1.0	0.00001	1618.35	96	T=0/0, C=22/7
chr2:170762487	T/T	C	T	SNV	UBR3	UBR3:intronic:NM_172070.3	1	100		intronic	NM_17207	c.1646-54	p.?	-0.06					Putative zinc	rs10171268		YES			Ubiquitin pr	cytoplasm,embryo development,in utero embryonic dev				C=0, T=97	C=0.0, T=1.0	0.00001	1639	97	C=0/0, T=58/3
chr2:170843469	C/C	T	C	SNV	UBR3	UBR3:intronic:NM_172070.3	1	100		intronic	NM_17207	c.3784+16	p.?	-2.2					Putative zinc	rs2061620		YES			Ubiquitin pr	cytoplasm,embryo development,in utero embryonic dev				T=0, C=58	T=0.0, C=1.0	0.00001	866.71	58	T=0/0, C=42/1
chr2:171070810	T/T	G	T	SNV	MYO3B	MYO3B:intronic:NM_138995.4	1	100		intronic	NM_13899	c.322-79G	p.?	-0.06					IQ calmoduli	rs67994603		YES			Myosin IIIB	ATP binding,actin binding,cochlea morphogenesis,cytop				G=0, T=59	G=0.0, T=1.0	0.00001	885.362	59	G=0/0, T=40/1
chr2:171071369	T/T	C	T	SNV	MYO3B	MYO3B:intronic:NM_138995.4	1	100		intronic	NM_13899	c.526+31C	p.?	1.27					IQ calmoduli	rs4667626	0.1005	YES	9.957E-1 (pos.171071369)		Myosin IIIB	ATP binding,actin binding,cochlea morphogenesis,cytop				C=0, T=64	C=0.0, T=1.0	0.00001	977.629	64	C=0/0, T=29/3
chr17:10304261	G/A	G	A	SNV	MYH8,MYHAS	MYHAS:intronic_nc:NR_125367.1, MYH8:exonic:NM_002472	1	50	26	synonymous	NR_12536	c.3270C>T	p.(=)	0.04				0.79(COSM37	IQ calmoduli	rs3744552	0.419	YES	1.133E-1 (pos.10304261)	acute_myeloic	Myosin, hea	ATP bindin	Abnormalities, Multiple;Arthrc		Benign/Likely	G=63, A=63	G=0.5, A=0.5	0.00001	670.061	126	G=37/26, A=3
chr6:16143390	C/C	A	C	SNV	MYLIP	MYLIP:exonic:NM_013262.3	1	100	4	missense	NM_01326	c.604A>C	p.Ile202Leu	5	0.07	5	0.001		FERM C-termi	rs79992066	0.0199	YES	0.0 (pos.16143390)		Myosin regu	actomyosi	Endometriosis;Female Urogenital Diseases;F			A=0, C=20	A=0.0, C=1.0	0.00001	263.786	20	A=0/0, C=6/14
chr2:171358211	A/A	G	A	SNV	MYO3B	MYO3B:intronic:NM_138995.4	1	100		intronic	NM_13899	c.3290-84	p.?	0.98					IQ calmoduli	rs7593940		YES			Myosin IIIB	ATP binding,actin binding,cochlea morphogenesis,cytop				G=0, A=32	G=0.0, A=1.0	0.00001	485.136	32	G=0/0, A=19/1
chr2:171902604	T/T	A	T	SNV	TLK1	TLK1:intronic:NM_001136554.1	1	100		intronic	NM_00113	c.1025+80	p.?	1.13					Protein kinas	rs6751462		YES				ATP bindin	Adenocarcinoma;Autoimmune Diseases;Car			A=0, T=28	A=0.0, T=1.0	0.00001	408.639	28	A=0/0, T=15/1
chr3:123418913	A/A	G	A	SNV	MYLK	MYLK:exonic:NM_053025.3	1	100	18	synonymous	NM_05302	c.3402C>T	p.(=)	2.44					Fibronectin t	rs865358	0.0036	YES	9.998E-1 (pos.123418913)		Myosin-ligh	ATP bindin	Acute Lung Injury;Brain Diseas		Benign/Likely	G=0, A=29	G=0.0, A=1.0	0.00001	427.603	29	G=0/0, A=12/1
chr3:123451773	C/C	G	C	SNV	MYLK	MYLK:exonic:NM_053025.3	1	100	11	missense	NM_05302	c.1486C>G	p.Leu496Val	2.15	1	32	0		Fibronectin t	rs9833275	0		1.0 (pos.123451773)		Myosin-ligh	ATP bindin	Acute Lung Injury;Brain Diseas		Benign/Likely	G=0, C=61	G=0.0, C=1.0	0.00001	926.372	61	G=0/0, C=29/3
chr2:172712480	C/C	T	C	SNV	SLC25A12	SLC25A12:intronic:NM_003705.4	1	100		intronic	NM_00370	c.210-21A	p.?	2.99					Mitochondria	rs2056202	0.1341	YES	8.871E-1 (pos.172712480)		Solute carri	L-aspartat	Amyotrophic Latera	L-Aspartic Acid		T=0, C=20	T=0.0, C=1.0	0.00001	263.786	20	T=0/0, C=16/4
chr2:172821739	T/T	G	T	SNV	HAT1	HAT1:intronic:NM_003642.3	1	100		intronic	NM_00364	c.310-120	p.?	-0.96					Histone acet	rs1017285794,rs1982288		YES			Histone ace	DNA packaging,DNA replication-dependent nucleosome				G=0, T=39	G=0.0, T=1.0	0.00001	623.979	39	G=0/0, T=22/1
chr2:173600783	CG/CG	C	CG	INDEL	RAPGEF4,RAPGEF4-AS1	RAPGEF4-AS1:intronic_nc:NR_026995.1, RAPGEF4:intronic:N	1	100		intronic	NR_02699	c.65+7C>C	p.?	0.36					Cyclic nucleo	rs111546206,rs3457	0.0056	YES	1.0 (pos.173600783)			G-protein coupled receptor signaling pathway,Ras GTPa				C=0, CG=23	C=0.0, CG=1.0	0.00001	284.923	23	C=0/0, CG=20/
chr2:173659913	C/C	T	C	SNV	RAPGEF4	RAPGEF4:intronic:NM_007023.3	1	100		intronic	NM_00702	c.208+18T	p.?	-0.14					Cyclic nucleo	rs7585729	5.00E-04		1.0 (pos.173659913)			G-protein coupled receptor signaling pathway,Ras GTPa				T=0, C=24	T=0.0, C=1.0	0.00001	334.792	24	T=0/0, C=13/1
chr3:123457893	A/A	G	A	SNV	MYLK	MYLK:exonic:NM_053025.3	1	100	7	missense	NM_05302	c.439C>T	p.Pro147Ser	2.8	0.15	74	0.058		Fibronectin t	rs9840993	0.0071	YES	9.383E-1 (pos.123457893)		Myosin-ligh	ATP bindin	Acute Lung Injury;Brain Diseas		Benign/Likely	G=0, A=24	G=0.0, A=1.0	0.00001	334.778	24	G=0/0, A=9/15
chr2:174230153	C/G	C	G	SNV	CDCA7	CDCA7:intronic:NM_031942.4	1	50		intronic	NM_03194	c.895-27C	p.?	0.62					Zinc-finger dc	rs2288993	0.1528	YES	9.822E-1 (pos.174230153)		Cell divisior	apoptotic process,cytosol,nucleoplasm,nucleus,regulat				C=29, G=29	C=0.5, G=0.5	0.00001	282.437	58	C=12/17, G=1
chr2:174943685	C/T	C	T	SNV	OLA1	OLA1:intronic:NM_013341.4	1	52.38		intronic	NM_01334	c.1089+11	p.?	-0.6					50S ribosome	rs11675247	0.203	YES	2.053E-2 (pos.174943685)		OBG-like AT	ATP binding,ATP metabolic process,ATPase activity,GTP				C=50, T=55	C=0.4762, T=0.5238	0.00001	563.006	105	C=22/28, T=28
chr5:16769273	G/A	G	A	SNV	MYO10	MYO10:exonic:NM_012334.2	1	50	10	missense	NM_01233	c.970C>T	p.Arg324Trp	2.85	0.04	101	0.989	0.92(COSM37	FERM central	rs11750538	0.4017	YES	5.736E-1 (pos.16769273)	adenocarcino	Myosin X	ATP bindin	Depressive Disorder;Depressive Disorder, M			G=28, A=28	G=0.5, A=0.5	0.00001	272.133	56	G=13/15, A=1
chr2:175311194	A/A	T	A	SNV	GPR155	GPR155:intronic:NM_152529.6	1	100		intronic	NM_15252	c.2013+14	p.?	-0.22					Domain foun	rs1562756		YES				cognition,	Autistic Disorder;Child Development Disorde			T=0, A=20	T=0.0, A=1.0	0.00001	263.631	20	T=0/0, A=3/17
chr2:175335076	T/T	G	T	SNV	GPR155	GPR155:intronic:NM_152529.6	1	100		intronic	NM_15252	c.1026+42	p.?	1.14					Domain foun	rs2358637	1.00E-04		1.0 (pos.175335076)			cognition,	Autistic Disorder;Child Development Disorde			G=0, T=39	G=0.0, T=1.0	0.00001	625.511	39	G=0/0, T=21/1
chr13:109661359	G/G	C	G	SNV	MYO16	MYO16:exonic:NM_015011.2	1	100	22	missense	NM_01501	c.2491C>G	p.Pro831Ala	4.69	0.07	27	0.97		Ankyrin repe	rs3825491	0.3757	YES	6.604E-1 (pos.109661359)		Myosin XVI	ATP bindin	Alzheimer Disease;Brain Diseases;Central Ne			C=0, G=32	C=0.0, G=1.0	0.00001	485.137	32	C=0/0, G=8/24
chr2:175613600	C/C	T	C	SNV	CHRNA1	CHRNA1:intronic:NM_000079.3	1	100		intronic	NM_00007	c.1003-53	p.?	0.41					Neurotransm	rs2646159		YES			Cholinergic	acetylchol	Abnormalities, Mul	Agmatine,Galantamine		T=0, C=21	T=0.0, C=1.0	0.00001	281.233	21	T=0/0, C=8/13
chr2:175677022	T/T	C	T	SNV	CHN1	CHN1:intronic:NM_001822.5	1	100		intronic	NM_00182	c.886+15G	p.?	0.02					Phorbol este	rs12613075	0.4821	YES	4.525E-1 (pos.175677022)		Chimerin 1	GTPase ac	Congenital, Hereditary, and Ne		Likely benign	C=0, T=62	C=0.0, T=1.0	0.00001	940.32	62	C=0/0, T=48/1
chr13:109793720	C/C	A	C	SNV	MYO16	MYO16:exonic:NM_015011.2	1	100	32	synonymous	NM_01501	c.5094A>C	p.(=)	-1.55				0.02(COSM41	Ankyrin repe	rs7986595	9.00E-04	YES	1.0;0.0 (pos.109793720)	neoplasm	Myosin XVI	ATP bindin	Alzheimer Disease;Brain Diseases;Central Ne			A=0, C=26	A=0.0, C=1.0	0.00001	371.186	26	A=0/0, C=12/1
chr22:26166900	C/C	G	C	SNV	MYO18B	MYO18B:exonic:NM_032608.6	1	100	6	missense	NM_03260	c.1641G>C	p.Trp547Cys	-0.26	0.03	215	0	0(COSM5428	IQ calmoduli	rs3859866	0.0135	YES	9.023E-1 (pos.26166900)	acute_myeloic	Myosin XVII	ATP bindin	Adenoma;Mesothelioma;Neoplasms;Neopla			G=0, C=26	G=0.0, C=1.0	0.00001	371.42	26	G=0/0, C=18/8
chr2:17773524	G/A	G	A	SNV	VSNL1	VSNL1:intronic:NM_003385.4	1	50		intronic	NM_00338	c.162+21G	p.?	-1.64					EF hand,EF-ha	rs2680827	0.3013	YES	9.829E-1 (pos.17773524)		Visinin-like	calcium io	Alzheimer Disease;Brain Diseases;Central Ne			G=23, A=23	G=0.5, A=0.5	0.00001	254.092	46	G=13/10, A=5/
chr2:178301428	C/T	C	T	SNV	AGPS	AGPS:intronic:NM_003659.3	1	57.63		intronic	NM_00365	c.442-64C	p.?	-0.64					FAD binding c	rs3754989		YES			Alkylglycerc	FAD bindir	Autoimmune Diseases;Bone Diseases;Bone			C=25, T=34	C=0.4237, T=0.5763	0.00001	356.5	59	C=16/9, T=22/
chr22:26173661	C/C	T	C	SNV	MYO18B	MYO18B:exonic:NM_032608.6	1	100	8	missense	NM_03260	c.1981T>C	p.Trp661Arg	0.71	1	101	0		IQ calmoduli	rs5761170,rs758330	0.0148	YES	0.0 (pos.26173660),8.795E-1 (pos.26173		Myosin XVII	ATP bindin	Adenoma;Mesothelioma;Neoplasms;Neopla			T=0, C=27	T=0.0, C=1.0	0.00001	381.794	27	T=0/0, C=11/1
chr2:17895975	T/T	C	T	SNV	SMC6	SMC6:intronic:NM_024624.5	1	100		intronic	NM_02462	c.1730+15	p.?	-0.29					AAA domain,l	rs2344691		YES			Structural n	ATP binding,PML body,Smc5-Smc6 complex,cellular resp				C=0, T=39	C=0.0, T=1.0	0.00001	623.98	39	C=0/0, T=20/1
chr2:17927266	T/C	T	C	SNV	SMC6	SMC6:intronic:NM_024624.5	1	52.38		intronic	NM_02462	c.-4-48A>G	p.?	-0.77					AAA domain,l	rs72768644					Structural n	ATP binding,PML body,Smc5-Smc6 complex,cellular resp				T=50, C=55	T=0.4762, C=0.5238	0.00001	574.02	105	T=29/21, C=22
chr2:17927275	C/T	C	T	SNV	SMC6	SMC6:intronic:NM_024624.5	1	47.62		intronic	NM_02462	c.-4-57G>A	p.?	0.13					AAA domain,l	rs12474069		YES			Structural n	ATP binding,PML body,Smc5-Smc6 complex,cellular resp				C=55, T=50	C=0.5238, T=0.4762	0.00001	497.413	105	C=22/33, T=29
chr22:26194045	C/C	T	C	SNV	MYO18B	MYO18B:exonic:NM_032608.6	1	100	12	synonymous	NM_03260	c.2502T>C	p.(=)	-0.57					IQ calmoduli	rs738643	0.2933	YES	6.794E-1 (pos.26194045)		Myosin XVII	ATP bindin	Adenoma;Mesothelioma;Neoplasms;Neopla			T=0, C=21	T=0.0, C=1.0	0.00001	281.23	21	T=0/0, C=10/1
chr2:179615931	C/G	C	G	SNV	TTN	TTN:intronic:NM_001256850.1	1	95.83		intronic	NM_00125	c.10360+1	p.?	-0.15				0.09(COSM50	Fibronectin t	rs922985	8.00E-04		1.0 (pos.179615931)	osteosarcoma	Titin	ATP bindin	Aortic Stenosis, Subvalvular;Ac		Benign	C=1, G=23	C=0.0417, G=0.9583	0.00001	306.638	24	C=1/0, G=13/1
chr2:179618019	A/A	T	A	SNV	TTN	TTN:intronic:NM_001256850.1	1	100		intronic	NM_00125	c.10304-1	p.?	0.12					Fibronectin t	rs3754953		YES			Titin	ATP bindin	Aortic Stenosis, Subvalvular;Aortic Valve Ster			T=0, A=46	T=0.0, A=1.0	0.00001	767.949	46	T=0/0, A=30/1
chr2:179620951	T/T	C	T	SNV	TTN	TTN:intronic:NM_001256850.1	1	100		intronic	NM_00125	c.10303+2	p.?	3.71					Fibronectin t	rs7585334	0.0679	YES	7.527E-1 (pos.179620951)		Titin	ATP bindin	Aortic Stenosis, Subvalvular;Ac		Benign	C=0, T=31	C=0.0, T=1.0	0.00001	465.486	31	C=0/0, T=17/1
chr22:26239850	A/A	C	A	SNV	MYO18B	MYO18B:exonic:NM_032608.6	1	100	18	missense	NM_03260	c.3357C>A	p.His1119Gln	0.89	1	24	0	0.4(COSM415	IQ calmoduli	rs5761268	0.2864	YES	9.967E-1 (pos.26239850)	neoplasm	Myosin XVII	ATP bindin	Adenoma;Mesothelioma;Neoplasms;Neopla			C=0, A=42	C=0.0, A=1.0	0.00001	685.203	42	C=0/0, A=29/1
chr2:179623939	C/C	T	C	SNV	TTN	TTN:intronic:NM_001256850.1	1	100		intronic	NM_00125	c.10115-4	p.?	-2.64					Fibronectin t	rs6705594	0		9.998E-1 (pos.179623939)		Titin	ATP bindin	Aortic Stenosis, Subvalvular;Aortic Valve Ster			T=0, C=40	T=0.0, C=1.0	0.00001	648.659	40	T=0/0, C=9/31
chr7:45004063	C/C	T	C	SNV	MYO1G	MYO1G:exonic:NM_033054.2	1	100	19	missense	NM_03305	c.2582A>G	p.Gln861Arg	0.37	0.44	43	0		Myosin head	rs7792760	0.1581	YES	9.132E-1 (pos.45004063)		Myosin IG	ATP binding,Fc-gamma receptor signaling pathway invol				T=0, C=33	T=0.0, C=1.0	0.00001	504.598	33	T=0/0, C=18/1
chr7:45009341	G/G	A	G	SNV	MYO1G	MYO1G:exonic:NM_033054.2	1	100	11	missense	NM_03305	c.1466T>C	p.Met489Thr	3.67	0.34	81	0	0.05(COSM41	Myosin head	rs3735485	0.1591	YES	9.147E-1 (pos.45009341)	neoplasm	Myosin IG	ATP binding,Fc-gamma receptor signaling pathway invol				A=0, G=33	A=0.0, G=1.0	0.00001	504.532	33	A=0/0, G=9/24
chr2:179635882	T/T	C	T	SNV	TTN	TTN:intronic:NM_001256850.1	1	100		intronic	NM_00125	c.8116+56	p.?	-1.51					Fibronectin t	rs4894047		YES			Titin	ATP bindin	Aortic Stenosis, Subvalvular;Aortic Valve Ster			C=0, T=22	C=0.0, T=1.0	0.00001	298.892	22	C=0/0, T=18/4
chr2:179642425	A/A	G	A	SNV	LOC101927055,TTN	LOC101927055:exonic_nc:NR_120594.1, TTN:intronic:NM_0	1	100		intronic	NM_00125	c.4480+6C	p.?	-0.04					Fibronectin t	rs719201	0.0086	YES	8.757E-1 (pos.179642425)		Titin	ATP bindin	Aortic Stenosis, Subvalvular;Ac		Benign/Likely	G=0, A=106	G=0.0, A=1.0	0.00001	1662.79	106	G=0/0, A=48/5
chr2:179643886	G/G	A	G	SNV	LOC101927055,TTN	LOC101927055:intronic_nc:NR_120594.1, TTN:intronic:NM	1	100		intronic	NM_00125	c.3964-41	p.?	0.3					Fibronectin t	rs2291302	0.0087	YES	8.775E-1 (pos.179643886)		Titin	ATP bindin	Aortic Stenosis, Subvalvular;Aortic Valve Ster			A=0, G=21	A=0.0, G=1.0	0.00001	281.186	21	A=0/0, G=9/12
chr2:179714675	T/T	C	T	SNV	CCDC141	CCDC141:intronic:NM_173648.3	1	100		intronic	NM_17364	c.3357+10	p.?	0.49					Immunoglobu	rs4894055		YES			Coiled-coil	cytoplasm,microtubule organizing center,protein bindin				C=0, T=97	C=0.0, T=1.0	0.00001	1638.67	97	C=0/0, T=47/5
chr2:179753245	T/T	C	T	SNV	CCDC141	CCDC141:intronic:NM_173648.3	1	100		intronic	NM_17364	c.1407+9G	p.?	0.72				0(COSM4001	Immunoglobu	rs7573293		YES	2.942E-1 (pos.17975324	acute_myeloic	Coiled-coil	cytoplasm,microtubule organizing center,protein bindin				C=0, T=63	C=0.0, T=1.0	0.00001	958.913	63	C=0/0, T=33/3
chr10:26286159	T/T	G	T	SNV	MYO3A	MYO3A:exonic:NM_017433.4	1	100	6	synonymous	NM_01743	c.480G>T	p.(=)	-1.67					IQ calmoduli	rs12257119	0.0546	YES	2.09E-3;0.0 (pos.26286159)		Myosin IIIA	ADP bindir	Ear Diseases;Hearing Disorder		Benign/Likely	G=0, T=189	G=0.0, T=1.0	0.00001	3173.1	189	G=0/0, T=79/1
chr2:180815528	T/T	A	T	SNV	CWC22	CWC22:intronic:NM_020943.2	1	100		intronic	NM_02094	c.1919+24	p.?	0.5					MA3 domain	rs2304858	0.2958	YES	5.737E-1 (pos.180815528)			RNA binding,U2-type catalytic step 1 spliceosome,cataly				A=0, T=42	A=0.0, T=1.0	0.00001	685.218	42	A=0/0, T=16/2
chr2:180843126	T/T	C	T	SNV	CWC22	CWC22:intronic:NM_020943.2	1	100		intronic	NM_02094	c.453-81G	p.?	-1.6					MA3 domain	rs2367990		YES				RNA binding,U2-type catalytic step 1 spliceosome,cataly				C=0, T=56	C=0.0, T=1.0	0.00001	832.1	56	C=0/0, T=28/2
chr10:26357747	TA/TA	CG	TA	MNV	MYO3A	MYO3A:exonic:NM_017433.4	2	100	12	missense	NM_01743	c.1104_11	p.Val369Ile	-0.14, 4.23	0.17	29	0.001	0.41(COSM15	IQ calmoduli	rs35379457,rs38174	0.4856	YES	2.772E-1 (pos.26357747)	astrocytoma_	Myosin IIIA	ADP bindir	Ear Diseases;Hearing Disorder		Benign	CG=0, TA=3	CG=0.0, TA=1.0	0.00001	485.141	32	CG=0/0, TA=14
chr10:26446312	A/A	G	A	SNV	MYO3A	MYO3A:exonic:NM_017433.4	1	100	26	missense	NM_01743	c.2867G>A	p.Ser956Asn	5.33	0.97	46	0	0.22(COSM14	IQ calmoduli	rs3758449	0.4508	YES	3.357E-1 (pos.26446312)	acute_myeloic	Myosin IIIA	ADP bindir	Ear Diseases;Hearing Disorder		Benign/Likely	G=0, A=51	G=0.0, A=1.0	0.00001	754.584	51	G=0/0, A=25/2
chr2:182359613	A/A	G	A	SNV	ITGA4	ITGA4:intronic:NM_000885.5	1	100		intronic	NM_00088	c.1339+74	p.?	-1.91					FG-GAP repea	rs165419		YES			Integrin, alp	B cell diffe	Autistic Disorder;A	ATL1102,CDP323,Natali		G=0, A=42	G=0.0, A=1.0	0.00001	685.219	42	G=0/0, A=17/2
chr2:182363613	A/A	T	A	SNV	ITGA4	ITGA4:intronic:NM_000885.5	1	100		intronic	NM_00088	c.1695+10	p.?	-0.57					FG-GAP repea	rs1349196		YES			Integrin, alp	B cell diffe	Autistic Disorder;A	ATL1102,CDP323,Natali		T=0, A=51	T=0.0, A=1.0	0.00001	754.555	51	T=0/0, A=11/4
chr2:182376546	G/G	T	G	SNV	ITGA4	ITGA4:intronic:NM_000885.5	1	100		intronic	NM_00088	c.1922+44	p.?	-0.72					FG-GAP repea	rs1466109	7.00E-04		1.0 (pos.182376546)		Integrin, alp	B cell diffe	Autistic Disorder;A	ATL1102,CDP323,Natali		T=0, G=135	T=0.0, G=1.0	0.00001	2238.22	135	T=0/0, G=60/7
chr2:182403792	AT/AT	A	AT	INDEL	CERKL,ITGA4	CERKL:intronic:NM_201548.4, ITGA4:downstream:NM_0008	1	100		intronic	NM_00088	c.*3538A>	p.?, p.?	0.67					Diacylglycerc	rs57234957			1.0;0.0 (pos.182403792)		Ceramide k	B cell diffe	Autistic Disorder;A	ATL1102,CDP323,Natali		A=0, AT=20	A=0.0, AT=1.0	0.00001	263.786	20	A=0/0, AT=13/
chr10:26305773	C/T	C	T	SNV	MYO3A	MYO3A:exonic:NM_017433.4	1	58.33	7	missense	NM_01743	c.533C>T	p.Thr178Ile	6.03	0	89	0.999		IQ calmoduli	rs33968748	3.00E-04		0.0 (pos.26305773)		Myosin IIIA	ADP bindir	Ear Diseases;Hearing Disorder		Benign	C=10, T=14	C=0.4167, T=0.5833	0.00001	135.323	24	C=6/4, T=8/6
chr2:182404139	C/G	C	G	SNV	CERKL,ITGA4	CERKL:intronic:NM_201548.4, ITGA4:downstream:NM_0008	1	56		intronic	NM_00088	c.*3885C>	p.?, p.?	-7.9					Diacylglycerc	rs17226490		YES			Ceramide k	B cell diffe	Autistic Disorder;A	ATL1102,CDP323,Natali		C=22, G=28	C=0.44, G=0.56	0.00001	293.35	50	C=14/8, G=22/
chr2:182928555	C/C	G	C	SNV	PPP1R1C	PPP1R1C:intronic:NM_001261424.1	1	100		intronic	NM_00126	c.262+37G	p.?	-0.56					Protein phos	rs1196161	0.0418	YES	8.063E-1 (pos.182928555)		Protein pho	cell cycle,cell division,cytoplasm,intracellular signal tra				G=0, C=48	G=0.0, C=1.0	0.00001	810.435	48	G=0/0, C=15/3
chr2:183088584	A/A	G	A	SNV	PDE1A	PDE1A:intronic:NM_001258312.1	1	100		intronic	NM_00125	c.836+7C>	p.?	-0.03					3'5'-cyclic nu	rs3754929	0.3214	YES	7.178E-1 (pos.183088584)		Phosphodiesterase-1A			Bepridil,Caffeine,Felodic		G=0, A=22	G=0.0, A=1.0	0.00001	298.892	22	G=0/0, A=7/15
chr2:183851058	T/T	C	T	SNV	NCKAP1	NCKAP1:intronic:NM_205842.2	1	100		intronic	NM_20584	c.966-103	p.?	-0.21					Membrane-a	rs10185562		YES			NCK-associ	Fc-gamma receptor signaling pathway involved in phago				C=0, T=42	C=0.0, T=1.0	0.00001	685.22	42	C=0/0, T=11/3

chr2:171260787	A/A	G	A	SNV	MYO3B	MYO3B:exonic:NM 138995.4	1	100	20	missense	NM 13899	c.2308G>A	p.Val770Ile	-0.02	0.25	29	0	0.02(COSM63	IQ calmodulii	rs6736609	0.1001	YES	6.937E-1 (pos.17126078	carcinoma	Myosin IIIB	ATP binding,actin binding,cochlea morphogenesis,cytop			G=0, A=56	G=0.0, A=1.0	0.00001	832.088	56	G=0/0, A=28/2
chr2:171260820	C/C	T	C	SNV	MYO3B	MYO3B:exonic:NM 138995.4	1	100	20	synonymous	NM 13899	c.2341T>C	p.(=)	-0.24				0.06(COSM63	IQ calmodulii	rs6751520	0.1003	YES	6.951E-1 (pos.17126082(	carcinoma	Myosin IIIB	ATP binding,actin binding,cochlea morphogenesis,cytop			T=0, C=56	T=0.0, C=1.0	0.00001	832.094	56	T=0/0, C=28/2
chr15:52667552	A/A	G	A	SNV	MYO5A	MYO5A:exonic:NM 000259.3	1	100	20	synonymous	NM 00025	c.2526C>T	p.(=)	-0.51					DIL domain,I(	rs2414145	0.0047	YES	9.735E-1 (pos.52667552)		Myosin Va	ATP bindin	Abnormalities, Multiple;Albinism;Amino Acid		G=0, A=57	G=0.0, A=1.0	0.00001	848.582	57	G=0/0, A=24/3
chr15:52689526	C/C	T	C	SNV	MYO5A	MYO5A:exonic:NM 000259.3	1	100	10	synonymous	NM 00025	c.1191A>G	p.(=)	-0.54					DIL domain,I(	rs2924130	2.00E-04	YES	1.0;0.0 (pos.52689526)		Myosin Va	ATP bindin	Abnormalities, Multiple;Albinism;Amino Acid		T=0, C=29	T=0.0, C=1.0	0.00001	427.572	29	T=0/0, C=10/1
chr15:52689631	G/G	T	G	SNV	MYO5A	MYO5A:exonic:NM 000259.3	1	100	10	missense	NM 00025	c.1086A>C	p.Glu362Asp	-0.11	0.7	45	0	0.07(COSM41	DIL domain,I(	rs1724577	0.0042	YES	9.824E-1 (pos.52689631)	neoplasm	Myosin Va	ATP bindin	Abnormalities, Multiple;Albinism;Amino Acid		T=0, G=36	T=0.0, G=1.0	0.00001	559.347	36	T=0/0, G=17/1
chr15:52643564	G/A	G	A	SNV	MYO5A	MYO5A:exonic:NM 000259.3	1	52.38	28	missense	NM 00025	c.3736C>T	p.Arg1246Cys	2.5	0.01	180	0.592	0.82(COSM44	DIL domain,I(	rs1058219	0.1675	YES	1.833E-1 (pos.52643564)	acute_myeloic	Myosin Va	ATP bindin	Abnormalities, Multiple;Albini	Benign	G=10, A=11	G=0.4762, A=0.5238	0.00001	95.3	21	G=7/3, A=7/4
chr18:47563299	C/C	T	C	SNV	MYO5B	MYO5B:exonic:NM 001080467.2	1	100	4	missense	NM 00108	c.376A>G	p.Thr126Ala	6.06	1	58	0		DIL domain,I(	rs1815930	7.00E-04		1.0 (pos.47563299)		Myosin VB	ATP bindin	Amphetamine-Related Disorde	Benign	T=0, C=31	T=0.0, C=1.0	0.00001	465.819	31	T=0/0, C=9/22
chr15:52531941	G/G	A	G	SNV	MYO5C	MYO5C:exonic:NM 018728.3	1	100	21	synonymous	NM 01872	c.2692T>C	p.(=)	0.57				0.41(COSM56	DIL domain,I(	rs11635028	0.0609	YES	2.867E-1 (pos.52531941)	ductal_carcinc	Myosin Vc	ATP bindin	Mental Disorders;Substance-Related Disorde		A=0, G=70	A=0.0, G=1.0	0.00001	1092.09	70	A=0/0, G=34/3
chr15:52534344	A/A	G	A	SNV	MYO5C	MYO5C:exonic:NM 018728.3	1	100	20	synonymous	NM 01872	c.2457C>T	p.(=)	-0.96					DIL domain,I(	rs3751631	0.1864	YES	3.455E-2 (pos.52534344)		Myosin Vc	ATP bindin	Mental Disorders;Substance-Related Disorde		G=0, A=24	G=0.0, A=1.0	0.00001	334.707	24	G=0/0, A=8/16
chr2:187611748	A/A	G	A	SNV	FAM171B	FAM171B:intronic:NM 177454.3	1	100		intronic	NM 17745	c.566-71G	p.?	-0.12					Uncharacteri	rs2247924		YES				integral component of membrane			G=0, A=51	G=0.0, A=1.0	0.00001	754.584	51	G=0/0, A=35/1
chr2:189866199	A/A	T	A	SNV	COL3A1	COL3A1:intronic:NM 000090.3	1	100		intronic	NM 00009	c.2337+23	p.?	0.41					Collagen tripl	rs2271682	0.2921	YES	9.273E-1 (pos.189866199)		Collagen III,	SMAD binc	ACMG Recommend: Collagena	Benign	T=0, A=21	T=0.0, A=1.0	0.00001	280.876	21	T=0/0, A=11/1
chr15:52556429	G/G	A	G	SNV	MYO5C	MYO5C:exonic:NM 018728.3	1	100	9	synonymous	NM 01872	c.1005T>C	p.(=)	-1.1					DIL domain,I(	rs4776032	2.00E-04		1.0 (pos.52556429)		Myosin Vc	ATP bindin	Mental Disorders;Substance-Related Disorde		A=0, G=23	A=0.0, G=1.0	0.00001	316.746	23	A=0/0, G=3/20
chr2:189899541	C/C	T	C	SNV	COL5A2	COL5A2:intronic:NM 000393.4	1	100		intronic	NM 00039	c.4353+10	p.?	-0.48					Collagen tripl	rs4666770		YES			Collagen V,	SMAD binc	Abortion, Spontaneous;Cardiovascular Disea		T=0, C=50	T=0.0, C=1.0	0.00001	852.738	50	T=0/0, C=33/1
chr2:189899572	C/C	T	C	SNV	COL5A2	COL5A2:intronic:NM 000393.4	1	100		intronic	NM 00039	c.4353+70	p.?	-0.37					Collagen tripl	rs7420715,rs796842690		YES			Collagen V,	SMAD binc	Abortion, Spontaneous;Cardiovascular Disea		T=0, C=50	T=0.0, C=1.0	0.00001	852.71	50	T=0/0, C=33/1
chr15:52539673	C/T	C	T	SNV	MYO5C	MYO5C:exonic:NM 018728.3	1	97.06	15	synonymous	NM 01872	c.1863G>A	p.(=)	0.43					DIL domain,I(	rs10163109	0.0104	YES	8.774E-1 (pos.52539673)		Myosin Vc	ATP bindin	Mental Disorders;Substance-Related Disorde		C=1, T=33	C=0.0294, T=0.9706	0.00001	492.966	34	C=0/1, T=17/1
chr11:76912636	T/T	A	T	SNV	MYO7A	MYO7A:exonic:NM 000260.3	1	100	36	missense	NM 00026	c.4996A>T	p.Ser1666Cys	2.92	1	112	0	0.23(COSM37	FERM central	rs2276288	0.4575	YES	4.93E-1 (pos.76912636)	adenocarcinoı	Myosin VIIA	ADP bindir	Abnormalities, Multiple;Blindr	Benign	A=0, T=39	A=0.0, T=1.0	0.00001	623.979	39	A=0/0, T=16/2
chr2:189923104	A/G	A	G	SNV	COL5A2	COL5A2:intronic:NM 000393.4	1	96.77		intronic	NM 00039	c.2229+51	p.?	-3.28					Collagen tripl	rs7556733	0.0179	YES	9.662E-1 (pos.189923104)		Collagen V,	SMAD binc	Abortion, Spontaneous;Cardiovascular Disea		A=1, G=30	A=0.0323, G=0.9677	0.00001	434.767	31	A=0/1, G=25/5
chr11:76917220	G/G	A	G	SNV	MYO7A	MYO7A:exonic:NM 000260.3	1	100	41	synonymous	NM 00026	c.5715A>G	p.(=)	1.45				0.32(COSM37	FERM central	rs2276293	0.4817	YES	5.229E-1 (pos.76917220)	acute_myeloic	Myosin VIIA	ADP bindir	Abnormalities, Multiple;Blindr	Benign	A=0, G=31	A=0.0, G=1.0	0.00001	465.796	31	A=0/0, G=19/1
chr2:189949759	T/T	C	T	SNV	COL5A2	COL5A2:intronic:NM 000393.4	1	100		intronic	NM 00039	c.798+127	p.?	-0.31					Collagen tripl	rs58532741		YES			Collagen V,	SMAD binc	Abortion, Spontaneous;Cardiovascular Disea		C=0, T=31	C=0.0, T=1.0	0.00001	465.801	31	C=0/0, T=18/1
chr2:189949786	AT/AT	TG	AT	MNV	COL5A2	COL5A2:intronic:NM 000393.4	2	100		intronic	NM 00039	c.798+100	p.?	-1.48, -0.02					Collagen tripl	rs386653665,rs57796506,rs613		YES			Collagen V,	SMAD binc	Abortion, Spontaneous;Cardiovascular Disea		TG=0, AT=3	TG=0.0, AT=1.0	0.00001	459.289	32	TG=0/0, AT=18
chr2:189950556	A/A	G	A	SNV	COL5A2	COL5A2:intronic:NM 000393.4	1	100		intronic	NM 00039	c.691-58C	p.?	-0.27					Collagen tripl	rs60355473	0.1468	YES			Collagen V,	SMAD binc	Abortion, Spontaneous;Cardiovascular Disea		G=0, A=37	G=0.0, A=1.0	0.00001	583.704	37	G=0/0, A=10/2
chr2:189953285	A/AAATG	A	AAATG	INDEL	COL5A2	COL5A2:intronic:NM 000393.4	4	97.3		intronic	NM 00039	c.645+136	p.?	-0.41					Collagen tripl	rs1054970548,rs201479630,rs3		YES			Collagen V,	SMAD binc	Abortion, Spontaneous;Cardiovascular Disea		A=1, AAATG	A=0.027, AAATG=0.9	0.00001	548.133	37	A=1/0, AAATG=
chr2:189953508	C/T	C	T	SNV	COL5A2	COL5A2:intronic:NM 000393.4	1	97.06		intronic	NM 00039	c.568-10G	p.?	-2.13				0.01(COSM44	Collagen tripl	rs58106884	0.1486	YES	9.618E-2 (pos.18995350	acute_myeloic	Collagen V,	SMAD binc	Abortion, Spontaneous;Cardio	Benign/Likely	C=1, T=33	C=0.0294, T=0.9706	0.00001	492.999	34	C=1/0, T=15/1
chr2:189953551	A/A	G	A	SNV	COL5A2	COL5A2:intronic:NM 000393.4	1	100		intronic	NM 00039	c.568-53C	p.?	0.21					Collagen tripl	rs58243388	0.1471	YES			Collagen V,	SMAD binc	Abortion, Spontaneous;Cardiovascular Disea		G=0, A=33	G=0.0, A=1.0	0.00001	504.598	33	G=0/0, A=15/1
chr2:190327400	C/C	T	C	SNV	WDR75	WDR75:intronic:NM 032168.2	1	100		intronic	NM 03216	c.937+32T	p.?	-3.11					WD domain,	rs2304701	0.1914	YES	8.523E-2 (pos.190327400)			RNA binding,nucleolus,nucleoplasm,positive regulation			T=0, C=51	T=0.0, C=1.0	0.00001	754.583	51	T=0/0, C=12/3
chr2:190445076	G/G	T	G	SNV	SLC40A1	SLC40A1:intronic:NM 014585.5	1	100		intronic	NM 01458	c.43+68A>	p.?	-0.49					Ferroportin1	rs4287798	0.3652	YES			Solute carri	basolater:	Anemia:Autistic Disorder:Carcinogenesis:Cel		T=0, G=32	T=0.0, G=1.0	0.00001	485.141	32	T=0/0, G=12/2
chr15:72191073	C/C	T	C	SNV	MYO9A	MYO9A:exonic:NM 006901.3	1	100	25	synonymous	NM 00690	c.3771A>G	p.(=)	0.26					IQ calmodulii	rs2415128	0.2662	YES	3.784E-1 (pos.72191073)		Myosin IXa	ATP binding,GTPase activator activity,actin binding,cell i			T=0, C=157	T=0.0, C=1.0	0.00001	2521.02	157	T=0/0, C=93/6
chr15:72252406	C/T	C	T	SNV	MYO9A	MYO9A:exonic:NM 006901.3	1	53.49	14	missense	NM 00690	c.2018G>A	p.Arg673His	7.75	0	29	1		IQ calmodulii	rs201188859	1.00E-04		0.0 (pos.72252406)		Myosin IXa	ATP binding,GTPase activator activity,actin binding,cell i			C=18, T=23	C=0.439, T=0.561	0.00001	282.343	43	C=13/5, T=9/1
chr2:191109533	T/T	C	T	SNV	HIBCH	HIBCH:intronic:NM 014362.3	1	100		intronic	NM 01436	c.891+80G	p.?	-1.33					Enoyl-CoA hyc	rs11883568		YES			3-hydroxyis	3-hydroxyi	Abnormalities, Mul	Quercetin	C=0, T=42	C=0.0, T=1.0	0.00001	685.22	42	C=0/0, T=18/2
chr2:191117206	A/A	G	A	SNV	HIBCH	HIBCH:intronic:NM 014362.3	1	100		intronic	NM 01436	c.518-173	p.?	0.04					Enoyl-CoA hyc	rs291402		YES			3-hydroxyis	3-hydroxyi	Abnormalities, Mul	Quercetin	G=0, A=27	G=0.0, A=1.0	0.00001	389.987	27	G=0/0, A=10/1
chr2:191227492	G/G	A	G	SNV	INPP1	INPP1:intronic:NM 002194.3	1	100		intronic	NM 00219	c.265+79A	p.?	-0.43					Inositol monc	rs972689		YES			Inositol pol	cytosol,de	Autistic Disorder;Child Development Disorde		A=0, G=42	A=0.0, G=1.0	0.00001	680.454	42	A=0/0, G=23/1
chr18:3164385	C/T	C	T	SNV	MYOM1	MYOM1:exonic:NM 003803.3	1	50	10	synonymous	NM 00380	c.1392G>A	p.(=)	0.48				0.6(COSM375	Fibronectin t	rs11659820	0.2072	YES	8.04E-3 (pos.3164385)	adenocarcinoı	Myomesin 1	M band,Z (	Congenital, Hereditary, and Ne	Benign	C=12, T=12	C=0.5, T=0.5	0.00001	106.71	24	C=7/5, T=6/6
chr8:2040337	C/C	T	C	SNV	MYOM2	MYOM2:exonic:NM 003970.3	1	100	16	synonymous	NM 00397	c.1992T>C	p.(=)	-2.3					Fibronectin t	rs6420202	0		1.0 (pos.2040337)		Myomesin 2	M band,Z disc,actin filament binding,actin filament orga			T=0, C=50	T=0.0, C=1.0	0.00001	852.593	50	T=0/0, C=18/3
chr8:2071174	T/C	T	C	SNV	MYOM2	MYOM2:exonic:NM 003970.3	1	57.14	29	missense	NM 00397	c.3503T>C	p.Val1168Ala	0.33	0.75	64	0.002	0.04(COSM63	Fibronectin t	rs17854780	0.1164	YES	1.993E-1 (pos.2071174)	carcinoma	Myomesin 2	M band,Z disc,actin filament binding,actin filament orga			T=9, C=12	T=0.4286, C=0.5714	0.00001	109.102	21	T=4/5, C=4/8
chr2:191922841	A/A	G	A	SNV	STAT4	STAT4:intronic:NM 001243835.1	1	100		intronic	NM 00124	c.1113-4C	p.?	-0.08				0.09(COSM44	SH2 domain,S	rs3024866	0.2558	YES	5.188E-1 (pos.19192284	acute_myeloic	Signal trans	DNA bindi	Adenocarcinoma;Arthritis;Arth	Benign	G=0, A=65	G=0.0, A=1.0	0.00001	996.402	65	G=0/0, A=31/3
chr2:192250543	G/G	A	G	SNV	MYO1B	MYO1B:intronic:NM 001130158.2	1	100		intronic	NM 00113	c.1354-67	p.?	1.03					IQ calmodulii	rs13000398		YES			Myosin IB	ATP binding,actin filament,actin filament binding,actin fi			A=0, G=31	A=0.0, G=1.0	0.00001	465.684	31	A=0/0, G=26/5
chr2:192250568	C/C	T	C	SNV	MYO1B	MYO1B:intronic:NM 001130158.2	1	100		intronic	NM 00113	c.1354-42	p.?	-0.18					IQ calmodulii	rs13026888	0.0285	YES	9.433E-1 (pos.192250568)		Myosin IB	ATP binding,actin filament,actin filament binding,actin fi			T=0, C=22	T=0.0, C=1.0	0.00001	298.22	22	T=0/0, C=17/5
chr8:2046700	C/T	C	T	SNV	MYOM2	MYOM2:exonic:NM 003970.3	1	50	19	missense	NM 00397	c.2327C>T	p.Thr776Met	0.03	0.03	81	0.999	0.14(COSM44	Fibronectin t	rs2294066	0.1442	YES	2.501E-1 (pos.2046700)	acute_myeloic	Myomesin 2	M band,Z disc,actin filament binding,actin filament orga			C=21, T=21	C=0.5, T=0.5	0.00001	231.338	42	C=10/11, T=10
chr2:192711162	C/C	A	C	SNV	CAVIN2	CAVIN2:intronic:NM 004657.5	1	100		intronic	NM 00465	c.483+7T>	p.?	0.68					PTRF/SDPR fa	rs4471838	0.0016	YES	9.995E-1;0.0 (pos.192711162)		Caveolae-a	caveola,cytoplasm,cytosol,membrane raft,phosphatidy			A=0, C=32	A=0.0, C=1.0	0.00001	485.14	32	A=0/0, C=21/1
chr2:192820811	TGTGTGAATG(	T	TGTGTGAATGCTT	INDEL	TMEFF2	TMEFF2:intronic:NM 016192.3	12	100		intronic	NM 01619	c.869+170	p.?	0.31					Kazal-type serine protease inhibitor domain						Transmemt	extracellu	Adnexal Diseases;Cysts;Diabetes Mellitus;Er		T=0, TGTGT	T=0.0, TGTGTGAATG(	0.00001	500.725	33	T=0/0, TGTGTG
chr2:192820898	G/G	T	G	SNV	TMEFF2	TMEFF2:intronic:NM 016192.3	1	100		intronic	NM 01619	c.869+83A	p.?	-0.86					Kazal-type se	rs7606017		YES			Transmemt	extracellu	Adnexal Diseases;Cysts;Diabetes Mellitus;Er		T=0, G=42	T=0.0, G=1.0	0.00001	685.22	42	T=0/0, G=26/1
chr1:24393563	C/T	C	T	SNV	MYOM3	MYOM3:exonic:NM 152372.3	1	95.45	28	missense	NM 15237	c.3371G>A	p.Arg1124Gln	5.36	0.08	43	0	0.01(COSM37	Fibronectin t	rs16829071	0.1388	YES	3.267E-1 (pos.24393563)	adenocarcinoı	Myomesin 3	M band,Z disc,actin filament binding,actin filament orga			C=1, T=21	C=0.0455, T=0.9545	0.00001	271.51	22	C=1/0, T=8/13
chr9:34372873	C/G	C	G	SNV	MYORG	MYORG:exonic:NM 020702.4	1	53.33	2	stoploss	NM 02070	c.69G>C	p.Ter23Tyr	0.3					Glycosyl hydr	rs4879782	0.2152	YES	9.467E-1 (pos.34372873)			carbohydrate metabolic process,endoplasmic reticulum			C=14, G=16	C=0.4667, G=0.5333	0.00001	159.924	30	C=7/7, G=10/6
chr2:197010823	T/T	C	T	SNV	STK17B	STK17B:intronic:NM 004226.3	1	100		intronic	NM 00422	c.336-44G	p.?	0.02					Protein kinas	rs2271888	0.4164	YES	5.598E-1 (pos.197010823)		Serine/thre	ATP binding,actin cytoskeleton		Quercetin	C=0, T=30	C=0.0, T=1.0	0.00001	446.517	30	C=0/0, T=13/1
chr2:197021086	C/T	C	T	SNV	STK17B	STK17B:intronic:NM 004226.3	1	97.06		intronic	NM 00422	c.335+77G	p.?	0.24					Protein kinas	rs6712631		YES			Serine/thre	ATP binding,actin cytoskeleton		Quercetin	C=1, T=33	C=0.0294, T=0.9706	0.00001	492.867	34	C=1/0, T=21/1
chr2:197021141	A/A	G	A	SNV	STK17B	STK17B:intronic:NM 004226.3	1	100		intronic	NM 00422	c.335+22C	p.?	0.57					Protein kinas	rs6712829	0.417	YES	5.894E-1 (pos.197021141)		Serine/thre	ATP binding,actin cytoskeleton		Quercetin	G=0, A=34	G=0.0, A=1.0	0.00001	524.186	34	G=0/0, A=22/1
chr5:137206560	C/C	A	C	SNV	MYOT	MYOT:exonic:NM 006790.2	1	100	2	missense	NM 00679	c.220A>C	p.Lys74Gln	2.81	1	53	0		Immunoglobı	rs386352828,rs6890(	0		1.0 (pos.137206560)		Myotilin (tit	Z disc,acti	Cardiomyopathies;Cardiovasc	Benign	A=0, C=29	A=0.0, C=1.0	0.00001	426.447	29	A=0/0, C=17/1
chr2:197298232	T/T	C	T	SNV	HECW2	HECW2:intronic:NM 020760.3	1	100		intronic	NM 02076	c.-34-50G>	p.?	-1.89					C2 domain,H	rs2204640		YES			HECT, C2, ar	cytoplasm,mitotic spindle,proteasome-mediated ubiqu			C=0, T=58	C=0.0, T=1.0	0.00001	867.326	58	C=0/0, T=6/52
chr2:197954780	G/G	A	G	SNV	ANKRD44	ANKRD44:intronic:NM 001195144.1	1	100		intronic	NM 00119	c.1101-24	p.?	0.33					Ankyrin repe:	rs4300850	0.0394	YES	9.997E-1 (pos.197954780)			protein binding			A=0, G=22	A=0.0, G=1.0	0.00001	298.874	22	A=0/0, G=5/17
chr2:1926437	T/T	G	T	SNV	MYT1L	MYT1L:exonic:NM 015025.3	1	100	10	synonymous	NM 01502	c.1104C>A	p.(=)	-0.8					Myelin trans(	rs1529667	0.031	YES	1.0 (pos.1926437)		Myelin tran	DNA binding,DNA binding transcription factor activity,ch			G=0, T=29	G=0.0, T=1.0	0.00001	427.603	29	G=0/0, T=15/1
chr4:40121562	A/A	G	A	SNV	N4BP2	N4BP2:exonic:NM 018177.5	1	100	9	missense	NM 01817	c.1831G>A	p.Asp611Asn	0.56	1	23	0	0(COSM4478	AAA domain,I	rs794001	0.2272	YES	9.661E-1 (pos.40121562)	carcinoma		ATP binding,ATP-dependent polydeoxyribonucleotide 5'-			G=0, A=90	G=0.0, A=1.0	0.00001	1493.12	90	G=0/0, A=57/3
chr4:40103767	G/T	G	T	SNV	N4BP2	N4BP2:exonic:NM 018177.5	1	47.37	4	missense	NM 01817	c.302G>T	p.Ser101Ile	-0.14	0	142	0.335		AAA domain,I	rs17511668	0.23	YES	3.321E-2 (pos.40103767)			ATP binding,ATP-dependent polydeoxyribonucleotide 5'-			G=80, T=72	G=0.5263, T=0.4737	0.00001	719.579	152	G=53/27, T=33
chr2:200173684	G/G	T	G	SNV	SATB2	SATB2:intronic:NM 001172517.1	1	100		intronic	NM 00117	c.1543-4A	p.?	0.56					CUT domain,(	rs10153730	5.00E-04	YES	1.0 (pos.200173684)		Special AT-r	DNA bindi	Abnormalities, Multiple;Bronchial Neoplasm		T=0, G=35	T=0.0, G=1.0	0.00001	543.745	35	T=0/0, G=7/28
chr2:200245245	C/G	C	G	SNV	SATB2	SATB2:intronic:NM 001172517.1	1	55.77		intronic	NM 00117	c.474-35G	p.?	0.07					CUT domain,(	rs1348813	0.2465	YES	3.504E-1 (pos.200245245)		Special AT-r	DNA bindi	Abnormalities, Multiple;Bronchial Neoplasm		C=23, G=29	C=0.4423, G=0.5577	0.00001	299.659	52	C=15/8, G=15/
chr2:201488758	G/G	A	G	SNV	AOX1	AOX1:intronic:NM 001159.3	1	100		intronic	NM 00115	c.2124+52	p.?	-1.94					2Fe-2S bindir	rs2348021	0.3324	YES	5.836E-1 (pos.201488758)		Aldehyde o:	2 iron, 2 su	Genital Diseases, N	Allopurinol,Aminocaproi	A=0, G=28	A=0.0, G=1.0	0.00001	408.718	28	A=0/0, G=16/1
chr2:202025131	A/A	G	A	SNV	CFLAR	CFLAR:intronic:NM 003879.6	1	100		intronic	NM 00387	c.794-24G	p.?	-1.97					Caspase dom	rs2518142	0.0913	YES	8.633E-1 (pos.202025131)		CASP8- and	CD95 deat	Bone Marrow Diseases;Digestive System Dis		G=0, A=25	G=0.0, A=1.0	0.00001	353.017	25	G=0/0, A=12/1
chr14:57876265	A/A	G	A	SNV	NAA30	NAA30:utr 3:NM 001011713.2	1	100	5	utr 3	NM 00101	c.1120G>A	p.?	0.26					Acetyltransfe	rs11620639	0.0421	YES	8.607E-1 (pos.57876265)			N-acetyltransferase activity,N-terminal peptidyl-methio			G=0, A=71	G=0.0, A=1.0	0.00001	1111.48	71	G=0/0, A=34/3
chr2:202154397	C/G	C	G	SNV	ALS2CR12	ALS2CR12:intronic:NM 001127391.2	1	50		intronic	NM 00112	c.1032+22	p.?	-0.4						rs6715284	0.0928	YES	1.145E-2 (pos.202154397)			cell projec	Arthritis;Arthritis, Rheumatoid;Autoimmune		C=20, G=20	C=0.5, G=0.5	0.00001	218.862	40	C=11/9, G=15/
chr2:202402250	A/A	AAC	A	INDEL	C2CD6	C2CD6:intronic:NM 001168221.1	2	100		intronic	NM 00116	c.1163-48	p.?	-1.64, 0.16, -1.19					Amyotrophic	rs202074378,rs3083	0.329	YES	0.0 (pos.202402252),9.407E-1 (pos.202402250)			protein binding			AAC=0, A=1	AAC=0.0, A=1.0	0.00001	3077.13	184	AAC=0/0, A=96
chr2:202410147	A/A	C	A	SNV	C2CD6	C2CD6:intronic:NM 001168221.1	1	100		intronic	NM 00116	c.1162+11	p.?	0.74					Amyotrophic	rs10931955		YES				protein binding			C=0, A=21	C=0.0, A=1.0	0.00001	281.235	21	C=0/0, A=11/1
chr4:76857309	T/T	C	T	SNV	NAAA	NAAA:exonic:NM 014435.3	1	100	3	missense	NM 01443	c.451G>A	p.Val151Ile	-0.82	0.79	29	0	0.01(COSM36	Linear amide	rs4859571	0.2317	YES	3.575E-1 (pos.76857309)	acute_myeloic	N-acylethar	biological_process,cytoplasm,extracellular exosome,hy			C=0, T=25	C=0.0, T=1.0	0.00001	352.977	25	C=0/0, T=20/5
chr2:202580370	A/A	G	A	SNV	ALS2	ALS2:intronic:NM 020919.3	1	100		intronic	NM 02091	c.4004+25	p.?	-1.35					MORN repeat	rs3219167	0.1638	YES	7.474E-1 (pos.202580370)		Alsin	Rab GTPas	Amyotrophic Lateral Sclerosis;	Benign	G=0, A=24	G=0.0, A=1.0	0.00001	334.792	24	G=0/0, A=15/9
chr2:202608877	A/A	C	A	SNV	ALS2	ALS2:intronic:NM 020919.3	1	100		intronic	NM 02091	c.2170+10	p.?	0.57					MORN repeat	rs4673207		YES			Alsin	Rab GTPas	Amyotrophic Lateral Sclerosis;Central Nervoı		C=0, A=31	C=0.0, A=1.0	0.00001	465.819	31	C=0/0, A=19/1
chr4:76857388	T/T	C	T	SNV	NAAA	NAAA:exonic:NM 014435.3	1	100	3	synonymous	NM 01443	c.372G>A	p.(=)	-0.24					Linear amide	rs4859572	0.2323	YES	3.562E-1 (pos.76857388)		N-acylethar	biological_process,cytoplasm,extracellular exosome,hy			C=0, T=22	C=0.0, T=1.0	0.00001	298.892	22	C=0/0, T=17/5
chr2:203690387	A/A	G	A	SNV	ICA1L	ICA1L:intronic:NM 138468.5	1	100		intronic	NM 13846	c.235+31C	p.?	0.1					Arfaptin-like	rs1344959	0.0507	YES	9.28E-1 (pos.203690387)			acrosomal vesicle,protein binding,protein domain speci			G=0, A=38	G=0.0, A=1.0	0.00001	603.705	38	G=0/0, A=22/1
chr2:203693559	G/G	C	G	SNV	ICA1L	ICA1L:intronic:NM 138468.5	1	100		intronic	NM 13846	c.162+12G	p.?	-0.21					Arfaptin-like	rs4675296	0.0502	YES	9.259E-1 (pos.203693559)			acrosomal vesicle,protein binding,protein domain speci			C=0, G=32	C=0.0, G=1.0	0.00001	485.091	32	C=0/0, G=18/1
chr2:204002809	C/C	A	C	SNV	NBEAL1	NBEAL1:intronic:NM 001114132.1	1	100		intronic	NM 00111	c.4479-76	p.?	-0.15					Beige/BEACH	rs10180815		YES			Neurobeachin-like 1		Nutrition Disorders;Nutritional and Metaboli		A=0, C=64	A=0.0, C=1.0	0.00001	948.58	64	A=0/0, C=40/2
chr3:174951756	C/C	T	C	SNV	NAALADL2	NAALADL2:exonic:NM 207015.2	1	100	3	missense	NM 20701	c.581T>C	p.Met194Thr	-1.11	0.23	81	0	0.02(COSM14	Peptidase fai	rs4371530	0.266	YES	6.729E-1 (pos.17495175	adenocarcinoı	N-acetylate	integral cc	Cardiovascular Diseases;Embolism and Thro		T=0, C=109	T=0.0, C=1.0	0.00001	1718.93	109	T=0/0, C=74/3
chr3:175473047	C/C	T	C	SNV	NAALADL2	NAALADL2:exonic:NM 207015.2	1	100	13	missense	NM 20701	c.2030T>C	p.Leu677Ser	2.21	0.8	145	0		Peptidase fai	rs765439957,rs9826	0.0635	YES	0.0 (pos.175473037),1.0 (pos.17547304		N-acetylate	integral cc	Cardiovascular Diseases;Embolism and Thro		T=0, C=29	T=0.0, C=1.0	0.00001	427.525	29	T=0/0, C=22/7
chr3:175165079	C/T	C	T	SNV	NAALADL2	NAALADL2:exonic:NM 207015.2	1	98.17	6	missense	NM 20701	c.1153C>T	p.Pro385Ser	0.2	0.5	74	0		Peptidase fai	rs6802937,rs772640(	7.00E-04		0.0 (pos.175165078),1.0 (pos.17516507		N-acetylate	integral cc	Cardiovascular Diseases;Embolism and Thro		C=2, T=107	C=0.0183, T=0.9817	0.00001	1650.13	109	C=2/0, T=69/3
chr12:57108203	G/G	A	G	SNV	NACA	NACA:exonic:NM 001113203.2	1	100	7	synonymous	NM 00111	c.2307T>C	p.(=)	-1.16				0.16(COSM11	NAC domain	rs4902	0.3785	YES	7.434E-1 (pos.57108203)	adenocarcinoı	Nascent-polypeptide-associated complex alpha polypeptide				A=0, G=20	A=0.0, G=1.0	0.00001	263.786	20	A=0/0, G=4/16
chr2:204360201	G/G	A	G	SNV	RAPH1	RAPH1:intronic:NM 213589.2	1	100		intronic	NM 21358	c.1-125T>(	p.?	-0.01					PH domain,R	rs2305417		YES				axon exte	Nervous System Diseases;Neuromuscular Di		A=0, G=86	A=0.0, G=1.0	0.00001	1415.05	86	A=0/0, G=32/5
chr2:206036913	GT/GT	G	GT	INDEL	PARD3B	PARD3B:intronic:NM 152526.5	1	100		intronic	NM 15252	c.1435-22	p.?	-1.81					N-terminal of Par3 and HAL proteins,PDZ domain,PDZ domain (Also known as DHR or GLGF)							bicellular	Acquired Immunodeficiency Syndrome;Autist		G=0, GT=36	G=0.0, GT=1.0	0.00001	350.285	36	G=0/0, GT=17/
chr2:206036918	T/T	C	T	SNV	PARD3B	PARD3B:intronic:NM 152526.5	1	100		intronic	NM 15252	c.1435-17	p.?	0.15					N-terminal ol	rs736320	0.2314	YES	6.372E-1 (pos.206036918)			bicellular	Acquired Immunodeficiency Syndrome;Autist		C=0, T=36	C=0.0, T=1.0	0.00001	550.582	36	C=0/0, T=17/1
chr2:206364791	G/G	C	G	SNV	PARD3B	PARD3B:intronic:NM 152526.5	1	100		intronic	NM 15252	c.2994+36	p.?	-0.39					N-terminal ol	rs11683289	0.4132	YES	3.888E-1 (pos.206364791)			bicellular	Acquired Immunodeficiency Syndrome;Autist		C=0, G=39	C=0.0, G=1.0	0.00001	623.975	39	C=0/0, G=18/2
chr2:206418032	A/A	G	A	SNV	PARD3B	PARD3B:intronic:NM 152526.5	1	100		intronic	NM 15252	c.2995-16	p.?	2.37					N-terminal ol	rs6730102,rs771674	7.00E-04	YES	0.0 (pos.206418028),1.0 (pos.206418032)			bicellular	Acquired Immunodeficiency Syndrome;Autist		G=0, A=52	G=0.0, A=1.0	0.00001	767.013	52	G=0/0, A=30/2
chr12:57109792	G/G	A	G	SNV	NACA	NACA:exonic:NM 001113203.2	1	100	5	missense	NM 00111	c.2063T>C	p.Leu688Pro	3.59	1	98	0	0.28(COSM41	NAC domain	rs2958149	0.2789	YES	7.48E-1 (pos.57109792)	neoplasm	Nascent-polypeptide-associated complex alpha polypeptide				A=0, G=20	A=0.0, G=1.0	0.00001	263.786	20	A=0/0, G=15/5
chr12:57109931	T/T	A	T	SNV	NACA	NACA:exonic:NM 001113203.2	1	100	5	missense	NM 00111	c.1924T>A	p.Ser642Thr	-0.59	1	58	0	0.06(COSM41	NAC domain	rs2926747	0.3784	YES	7.399E-1 (pos.57109931)	neoplasm	Nascent-polypeptide-associated complex alpha polypeptide				A=0, T=25	A=0.0, T=1.0	0.00001	353.016	25	A=0/0, T=14/1
chr12:57114100	G/G	A	G	SNV	NACA	NACA:exonic:NM 001113203.2	1	100	3	missense	NM 00111	c.1214T>C	p.Phe405Ser	0.06	1	155	0	0(COSM5453(	NAC domain	rs2926743	0.2782	YES	7.469E-1 (pos.57114100)	adenocarcinoı	Nascent-polypeptide-associated complex alpha polypeptide				A=0, G=29	A=0.0, G=1.0	0.00001	427.603	29	A=0/0, G=9/20
chr12:57114307	T/T	A	T	SNV	NACA	NACA:exonic:NM 001113203.2	1	100	3	missense	NM 00111	c.1007T>A	p.Val336Glu	-0.11	0.3	121	0.002		NAC domain	rs2958127	0.3787	YES	7.436E-1 (pos.57114307)		Nascent-polypeptide-associated complex alpha polypeptide				A=0, T=75	A=0.0, T=1.0	0.00001	1187.82	75	A=0/0, T=40/3
chr7:45122464	C/C	A	C	SNV	NACAD	NACAD:exonic:NM 001146334.1	1	100	2	missense	NM 00114	c.3315T>G	p.Asp1105Glu	-1.09	1	45	0	0.01(COSM37	NAC domain	rs10243185	0.4082	YES	3.241E-1 (pos.45122464)	adenocarcinoma		nascent polypeptide-associated complex,nucleus,prote			A=0, C=40	A=0.0, C=1.0	0.00001	644.246	40	A=0/0, C=21/1
chr9:130829039	G/G	A	G	SNV	NAIF1	NAIF1:exonic:NM 197956.3	1	100	1	synonymous	NM 19795	c.342T>C	p.(=)	-2.65				0.02(COSM41	Myb/SANT-lik	rs1539350	0		1.0 (pos.130829039)	neoplasm	Nuclear apo	mitochondrion,molecular_function,negative regulation			A=0, G=31	A=0.0, G=1.0	0.00001	465.815	31	A=0/0, G=13/1
chr7:105903904	C/C	T	C	SNV	NAMPT	NAMPT:exonic:NM 005746.2	1	100	7	synonymous	NM 00574	c.903A>G	p.(=)	-0.15					Nicotinate pl	rs2302559	0.3748	YES	9.157E-1;0.0 (pos.105903904)		Nicotinami	NAD biosy	Autoimmune Disea	Daporinad,GMX1777	T=0, C=49	T=0.0, C=1.0	0.00001	831.63	49	T=0/0, C=17/3
chr2:207625567	C/C	T	C	SNV	MDH1B	MDH1B:intronic:NM 001039845.2	1	100		intronic	NM 00103	c.135+58A	p.?	0.18						rs1862087		YES				L-malate dehydrogenase activity,NADH metabolic proce			T=0, C=22	T=0.0, C=1.0	0.00001	298.831	22	T=0/0, C=7/15
chr9:100819070	T/G	T	G	SNV	NANS	NANS:utr 5:NM 018946.3	1	46.88	1	utr 5	NM 01894	c.-21T>G	p.?	-0.44					NeuB family	rs3199064,rs766298	0.4446	YES	3.417E-1;0.0 (pos.100819070)		N-acetylneu	CMP-N-acetylneuraminate biosynthetic process,N-acety			T=17, G=15	T=0.5313, G=0.4688	0.00001	142.085	32	T=9/8, G=2/13
chr12:76449898	TC/TC	T	TC	INDEL	NAP1L1	NAP1L1:exonic:NM 004537.6	1	100	7	frameshiftInsertion	NM 00453	c.472 473	p.Asp158fs	8.04					Nucleosome assembly protein (NAP)						Nucleosom	DNA replic	Nutrition Disorders;Nutritional and Metaboli		T=0, TC=46	T=0.0, TC=1.0	0.00001	616.294	46	T=0/0, TC=16/
chr12:76453966	A/A	G	A	SNV	NAP1L1	NAP1L1:exonic:NM 004537.6	1	100	5	synonymous	NM 00453	c.300C>T	p.(=)	3.02				0.44(COSM49	Nucleosome	rs4663	0.3975	YES	9.583E-1 (pos.76453966)	adenocarcinoı	Nucleosom	DNA replic	Nutrition Disorders;Nutritional and Metaboli		G=0, A=44	G=0.0, A=1.0	0.00001	726.569	44	G=0/0, A=17/2
chr2:20824708	C/C	T	C	SNV	HS1BP3	HS1BP3:intronic:NM 022460.3	1	100		intronic	NM 02246	c.624-56A	p.?	-0.24					PX domain	rs10191266		YES			HS1-binding	endoplasmic reticulum,mitochondrion,phosphatidylino			T=0, C=20	T=0.0, C=1.0	0.00001	263.786	20	T=0/0, C=12/8
chr2:20845273	A/A	G	A	SNV	HS1BP3	HS1BP3:intronic:NM 022460.3	1	100		intronic	NM 02246	c.33-8C>T	p.?	0.84				0(COSM6353	PX domain	rs2304423	0.3508	YES	7.357E-1 (pos.20845273)	carcinoma	HS1-binding	endoplasmic reticulum,mitochondrion,phosphatidylino			G=0, A=23	G=0.0, A=1.0	0.00001	316.748	23	G=0/0, A=9/14
chr12:76449900	T/C	T	C	SNV	NAP1L1	NAP1L1:exonic:NM 004537.6	1	97.92	7	synonymous	NM 00453	c.471A>G	p.(=)	-0.43					Nucleosome	rs1059143	0.1862	YES	9.995E-1;0.0 (pos.76449900)		Nucleosom	DNA replic	Nutrition Disorders;Nutritional and Metaboli		T=1, C=47	T=0.0208, C=0.9792	0.00001	754.783	48	T=0/1, C=18/2
chr2:73867897	G/G	A	G	SNV	NAT8	NAT8:utr 3:NM 003960.3	1	100	2	utr 3	NM 00396	c.859T>C	p.?	0.39					Acetyltransfe	rs1879662		YES			N-acetyltra	amyloid-b	Female Urogenital Diseases;Female Urogeni		A=0, G=33	A=0.0, G=1.0	0.00001	504.598	33	A=0/0, G=10/2
chr11:20101704	T/T	C	T	SNV	NAV2	NAV2:exonic:NM 001244963.1	1	100	27	synonymous	NM 00124	c.5442C>T	p.(=)	-1.42					Calponin hon	rs1867114	0.1395	YES	9.512E-1 (pos.20101704)		Neuron nav	ATP bindin	Digestive System Diseases;Hepatitis;Liver Di		C=0, T=72	C=0.0, T=1.0	0.00001	1130.95	72	C=0/0, T=47/2
chr11:20101744	A/A	C	A	SNV	NAV2	NAV2:exonic:NM 001244963.1	1	100	27	synonymous	NM 00124	c.5482C>A	p.(=)	7.53					Calponin hon	rs1867115,rs777669	0.1391	YES	3.516E-4 (pos.20101741),9.525E-1 (pos.2		Neuron nav	ATP bindin	Digestive System Diseases;Hepatitis;Liver Di		C=0, A=96	C=0.0, A=1.0	0.00001	1622.3	96	C=0/0, A=64/3
chr11:20104669	A/A	G	A	SNV	NAV2	NAV2:exonic:NM 001244963.1	1	100	28	synonymous	NM 00124	c.5619G>A	p.(=)	-0.7				0.07(COSM44	Calponin hon	rs2028570	0.4399	YES	2.823E-1 (pos.20104669)	acute_myeloic	Neuron nav	ATP bindin	Digestive System Diseases;Hepatitis;Liver Di		G=0, A=52	G=0.0, A=1.0	0.00001	767.012	52	G=0/0, A=38/1
chr11:20112492	A/A	G	A	SNV	NAV2	NAV2:exonic:NM 001244963.1	1	100	29	synonymous	NM 00124	c.5757G>A	p.(=)	-4.33				0.04(COSM39	Calponin hon	rs1837971	0.4902	YES	2.982E-1 (pos.20112492)	acute_myeloic	Neuron nav	ATP bindin	Digestive System Diseases;Hepatitis;Liver Di		G=0, A=65	G=0.0, A=1.0	0.00001	996.464	65	G=0/0, A=36/2
chr11:20124914	C/C	A	C	SNV	NAV2	NAV2:exonic:NM 001244963.1	1	100	35	synonymous	NM 00124	c.6708A>C	p.(=)	0.03					Calponin hon	rs2243411	0.0108	YES	9.452E-1;0.0 (pos.20124914)		Neuron nav	ATP bindin	Digestive System Diseases;Hepatitis;Liver Di		A=0, C=46	A=0.0, C=1.0	0.00001	768.31	46	A=0/0, C=14/3
chr12:78225374	G/G	A	G	SNV	NAV3	NAV3:exonic:NM 014903.5	1	100	1	missense	NM 01490	c.133A>G	p.Thr45Ala	0.98	1	58	0		ATPase famil	rs10735309	5.00E-04		1.0;0.0 (pos.78225374)		Neuron nav	ATP bindin	Adenocarcinoma;Carcinoma;Carcinoma, Ren		A=0, G=92	A=0.0, G=1.0	0.00001	1534.51	92	A=0/0, G=46/4
chr2:209010891	G/A	G	A	SNV	CRYGB,LOC100507443	CRYGB:upstream:NM 005210.3, LOC100507443:intronic nc	1	50		intronic	NR 03843	c.-47C>T	p.?	1.08						rs2289917	0.3986	YES	1.243E-1;0.0 (pos.209010891)		Crystallin, g	cellular_c	Cataract;Eye Diseases;Lens Diseases		G=17, A=17	G=0.5, A=0.5	0.00001	171.407	34	G=11/6, A=5/1
chr2:209035658	T/T	C	T	SNV	C2orf80	C2orf80:intronic:NM 001099334.2	1	100		intronic	NM 00109	c.573+11G	p.?	-0.04						rs6748431	0		1.0 (pos.209035658)		Chromosome 2 open reading frame 80				C=0, T=26	C=0.0, T=1.0	0.00001	371.42	26	C=0/0, T=13/1
chr2:15601441	T/T	C	T	SNV	NBAS	NBAS:exonic:NM 015909.3	1	100	21	synonymous	NM 01590	c.2223G>A	p.(=)	-0.71				0.1(COSM375	Neuroblastoı	rs7590340	0.316	YES	2.117E-1 (pos.15601441)	acute_myeloic	Neuroblast	Dsl1/NZR (	Digestive System Diseases;Digestive System		C=0, T=37	C=0.0, T=1.0	0.00001	583.707	37	C=0/0, T=17/2
chr2:15614404	C/C	T	C	SNV	NBAS	NBAS:exonic:NM 015909.3	1	100	15	synonymous	NM 01590	c.1386A>G	p.(=)	-0.3				0.03(COSM37	Neuroblastoı	rs13014906	0.3162	YES	2.167E-1 (pos.15614404)	acute_myeloic	Neuroblast	Dsl1/NZR (	Digestive System Diseases;Digestive System		T=0, C=51	T=0.0, C=1.0	0.00001	753.599	51	T=0/0, C=23/2
chr2:209194696	C/C	G	C	SNV	PIKFYVE	PIKFYVE:intronic:NM 015040.3	1	100		intronic	NM 01504	c.3791+59	p.?	-0.69					Domain foun	rs1437414	0.0186	YES			Phosphatid	'de novo' p	Congenital, Hereditary, and Neonatal Diseas		G=0, C=31	G=0.0, C=1.0	0.00001	465.81	31	G=0/0, C=17/1
chr2:209201739	C/C	T	C	SNV	PIKFYVE	PIKFYVE:intronic:NM 015040.3	1	100		intronic	NM 01504	c.4611+87	p.?	-2.13					Domain foun	rs7596571		YES			Phosphatid	'de novo' p	Congenital, Hereditary, and Neonatal Diseas		T=0, C=31	T=0.0, C=1.0	0.00001	465.819	31	T=0/0, C=13/1
chr2:209209997	T/T	C	T	SNV	PIKFYVE	PIKFYVE:intronic:NM 015040.3	1	100		intronic	NM 01504	c.5111+79	p.?	-0.38					Domain foun	rs2043718					Phosphatid	'de novo' p	Congenital, Hereditary, and Neonatal Diseas		C=0, T=49	C=0.0, T=1.0	0.00001	831.633	49	C=0/0, T=16/3
chr2:15615910	G/G	A	G	SNV	NBAS	NBAS:exonic:NM 015909.3	1	100	14	synonymous	NM 01590	c.1242T>C	p.(=)	-4.65				0.02(COSM14	Neuroblastoı	rs1990754	0.3164	YES	1.558E-1 (pos.15615910)	acute_myeloic	Neuroblast	Dsl1/NZR (	Digestive System Diseases;Digestive System		A=0, G=94	A=0.0, G=1.0	0.00001	1575.51	94	A=0/0, G=39/5
chr2:209215448	G/G	A	G	SNV	PIKFYVE	PIKFYVE:intronic:NM 015040.3	1	100		intronic	NM 01504	c.5435-47	p.?	1.79					Domain foun	rs994696	0.0183	YES	9.434E-1 (pos.209215448)		Phosphatid	'de novo' p	Congenital, Hereditary, and Neonatal Diseas		A=0, G=62	A=0.0, G=1.0	0.00001	940.32	62	A=0/0, G=21/4
chr2:15674686	C/C	T	C	SNV	NBAS	NBAS:exonic:NM 015909.3	1	100	9	missense	NM 01590	c.727A>G	p.Ile243Val	-0.12	1	29	0.001	0.08(COSM37	Neuroblastoı	rs13029846	0.319	YES	1.51E-1 (pos.15674686)	acute_myeloic	Neuroblast	Dsl1/NZR (	Digestive System Diseases;Digestive System		T=0, C=41	T=0.0, C=1.0	0.00001	664.7	41	T=0/0, C=16/2
chr2:20939706	T/C	T	C	SNV	LDAH	LDAH:intronic:NM 021925.3	1	50		intronic	NM 02192	c.703+25A	p.?	-0.36					Lipid-droplet	rs199600420,rs2046	0.0044,0.	YES	4.54E-1;0.0 (pos.20939706)			endoplasmic reticulum,lipase activity,lipid catabolic prc			T=14, C=14	T=0.5, C=0.5	0.00001	131.939	28	T=7/7, C=9/5
chr2:20939708	C/T	C	T	SNV	LDAH	LDAH:intronic:NM 021925.3	1	50		intronic	NM 02192	c.703+23G	p.?	-1.79					Lipid-droplet	rs199600420,rs3866	0.2416	YES	4.54E-1;0.0 (pos.20939706),4.936E-1 (pos.20939708)			endoplasmic reticulum,lipase activity,lipid catabolic prc			C=14, T=14	C=0.5, T=0.5	0.00001	131.941	28	C=7/7, T=9/5
chr2:210517785	T/T	C	T	SNV	MAP2	MAP2:intronic:NM 002374.3	1	100		intronic	NM 00237	c.-28-81C>	p.?	-0.64					MAP2/Tau pr	rs2254169					Microtubul	CA3 pyram	Abortion, Spontane	Docetaxel,Estramustine	C=0, T=51	C=0.0, T=1.0	0.00001	754.584	51	C=0/0, T=29/2
chr2:210594540	G/G	A	G	SNV	MAP2	MAP2:intronic:NM 002374.3	1	100		intronic	NM 00237	c.5156-34	p.?	0.72					MAP2/Tau pr	rs4673486	0.107	YES	7.714E-1;0.0 (pos.210594540)		Microtubul	CA3 pyram	Abortion, Spontane	Docetaxel,Estramustine	A=0, G=32	A=0.0, G=1.0	0.00001	485.141	32	A=0/0, G=15/1
chr2:210594768	C/C	T	C	SNV	MAP2	MAP2:intronic:NM 002374.3	1	100		intronic	NM 00237	c.5268+82	p.?	-0.14					MAP2/Tau pr	rs7594601		YES			Microtubul	CA3 pyram	Abortion, Spontane	Docetaxel,Estramustine	T=0, C=69	T=0.0, C=1.0	0.00001	1072.6	69	T=0/0, C=34/3
chr2:204062061	G/G	A	G	SNV	NBEAL1	NBEAL1:exonic:NM 001114132.1	1	100	47	missense	NM 00111	c.6988A>G	p.Ile2330Val	4.88	1	29	0		Beige/BEACH	rs4675323	0.0353	YES	9.24E-1 (pos.204062061)		Neurobeachin-like 1		Nutrition Disorders;Nutritional and Metaboli		A=0, G=42	A=0.0, G=1.0	0.00001	685.219	42	A=0/0, G=31/1
chr2:210889759	A/G	A	G	SNV	KANSL1L	KANSL1L:intronic:NM 152519.3	1	58.33		intronic	NM 15251	c.2564+69	p.?	0.04					PEHE domain	rs6435550		YES			KAT8 regula	histone acetyltransferase complex			A=10, G=14	A=0.4167, G=0.5833	0.00001	135.339	24	A=7/3, G=13/1
chr2:211069438	G/A	G	A	SNV	ACADL	ACADL:intronic:NM 001608.3	1	98.41		intronic	NM 00160	c.769-32C	p.?	1.32					Acyl-CoA deh	rs263677	1.00E-04		9.999E-1 (pos.211069438)		Acyl-Coenzy	acyl-CoA d	Congenital, Hereditary, and Neonatal Diseas		G=1, A=62	G=0.0159, A=0.9841	0.00001	925.364	63	G=0/1, A=41/2
chr2:211465113	A/A	G	A	SNV	CPS1	CPS1:intronic:NM 001875.4	1	100		intronic	NM 00187	c.1550-16	p.?	-1.44					ATP-grasp do	rs9288418		YES			Carbamoyl-	'de novo' p	Amino Acid Metabo	Carglumic Acid	G=0, A=30	G=0.0, A=1.0	0.00001	446.635	30	G=0/0, A=21/9
chr1:148594412	GA/G	GA	G	INDEL	NBPF15,NBPF25P	NBPF25P:intronic nc:NR 104217.1, NBPF15:exonic:NM 17	1	45.45	19	frameshiftDeletion	NR 10421	c.1786del	p.Met596fs	-0.52, -0.07					Repeat of unl	rs782044118			1.162E-4 (pos.148594412)		Neuroblast	cytoplasm			GA=24, G=2	GA=0.5455, G=0.454	0.00001	211.734	44	GA=9/15, G=2/
chr2:211533070	G/G	A	G	SNV	CPS1	CPS1:intronic:NM 001875.4	1	100		intronic	NM 00187	c.4101+62	p.?	-4					ATP-grasp do	rs10932347		YES			Carbamoyl-	'de novo' p	Amino Acid Metabo	Carglumic Acid	A=0, G=71	A=0.0, G=1.0	0.00001	1111.04	71	A=0/0, G=46/2
chr1:21795388	G/G	A	G	SNV	NBPF3	NBPF3:exonic:NM 032264.4	1	100	3	missense	NM 03226	c.341A>G	p.Tyr114Cys	-0.06	0	194	0.999	0.01(COSM37	Repeat of unl	rs1827293	0.447	YES	5.618E-1 (pos.21795388)	adenocarcinoı	Neuroblast	cytoplasm,positive regulation of protein targeting to mit			A=0, G=83	A=0.0, G=1.0	0.00001	1349.87	83	A=0/0, G=27/5
chr2:212285103	C/C	T	C	SNV	ERBB4	ERBB4:intronic:NM 005235.2	1	100		intronic	NM 00523	c.3135+63	p.?	-1.38					Furin-like cys	rs2289086		YES			Avian eryth	ATP bindin	Amyotrophic Latera	Afatinib	T=0, C=42	T=0.0, C=1.0	0.00001	685.22	42	T=0/0, C=29/1
chr1:120387120	G/A	G	A	SNV	NBPF7	NBPF7:exonic:NM 001047980.2	1	95.45	1	synonymous	NM 00104	c.39C>T	p.(=)	0.6				0.04(COSM44	Repeat of unl	rs12405228,rs75824(	0.4327	YES	0.0 (pos.120387117),3.7(	acute_myeloic	Neuroblast	cytoplasm			G=1, A=21	G=0.0455, A=0.9545	0.00001	275.761	22	G=1/0, A=15/6
chr22:50962782	C/G	C	G	SNV	NCAPH2,SCO2	SCO2:exonic:NM 001169111.1, NCAPH2:utr 3:NM 001185(	1	96.77	2	missense	NM 00118	c.2799C>G	p.?, p.Arg20Pro	-3.57		103		0.02(COSM14	Condensin II	rs140523	0.383	YES	6.989E-1;0.0;0.0 (pos.50	adenocarcinoı	Non-SMC cc	cell juncti	Aortic Stenosis, Subvalvular;Ac	Benign	C=1, G=30	C=0.0323, G=0.9677	0.00001	436.02	31	C=1/0, G=10/2
chr2:212615516	T/C	T	C	SNV	ERBB4	ERBB4:intronic:NM 005235.2	1	57.14		intronic	NM 00523	c.557-87A	p.?	0.7					Furin-like cys	rs6757068		YES			Avian eryth	ATP bindin	Amyotrophic Latera	Afatinib	T=18, C=24	T=0.4286, C=0.5714	0.00001	279.085	42	T=8/10, C=9/1
chr2:214012405	A/C	A	C	SNV	IKZF2	IKZF2:intronic:NM 016260.2	1	95.83		intronic	NM 01626	c.139+27T	p.?	1.08					C2H2-type zir	rs547307585,rs6738(	0.0621	YES	3.628E-1;0.0;0.0 (pos.214012403),5.916		Ikaros famil	DNA bindi	Hemic and Lymphatic Diseases;Immune Syst		A=1, C=23	A=0.0417, C=0.9583	0.00001	306.728	24	A=0/1, C=14/9
chr12:54905815	G/G	C	G	SNV	NCKAP1L	NCKAP1L:exonic:NM 005337.4	1	100	9	synonymous	NM 00533	c.867C>G	p.(=)	0.75					Membrane-a	rs2458409	0.2027	YES	9.658E-1;0.0 (pos.54905815)		B cell hom	Autoimmune Diseases;Female Urogenital Di			C=0, G=84	C=0.0, G=1.0	0.00001	1369.5	84	C=0/0, G=43/4
chr2:214174767	T/T	C	T	SNV	SPAG16	SPAG16:intronic:NM 024532.4	1	100		intronic	NM 02453	c.280-16C	p.?	-0.6					WD domain,	rs10498003	0.2985	YES	1.166E-3 (pos.214174767)		Sperm-asso	axonemal central apparatus,axoneme,axoneme assem			C=0, T=63	C=0.0, T=1.0	0.00001	958.885	63	C=0/0, T=21/4
chr2:214182100	T/T	A	T	SNV	SPAG16	SPAG16:intronic:NM 024532.4	1	100		intronic	NM 02453	c.536+20A	p.?	0.29					WD domain,	rs10183630	0.3166	YES	3.672E-3;1.266E-4 (pos.214182100)		Sperm-asso	axonemal central apparatus,axoneme,axoneme assem			A=0, T=33	A=0.0, T=1.0	0.00001	508.69	33	A=0/0, T=10/2
chr2:214228792	T/T	C	T	SNV	SPAG16	SPAG16:intronic:NM 024532.4	1	100		intronic	NM 02453	c.763-8C>	p.?	0.04					WD domain,	rs12469417	5.00E-04		1.0 (pos.214228792)		Sperm-asso	axonemal central apparatus,axoneme,axoneme assem			C=0, T=101	C=0.0, T=1.0	0.00001	1572.33	101	C=0/0, T=59/4
chr2:215812297	A/A	G	A	SNV	ABCA12,LOC101928103	ABCA12:intronic:NM 173076.2, LOC101928103:intronic nc	1	100		intronic	NR 11029	c.7105-17	p.?	1.41					AAA domain,	rs2274412	0.4321	YES	3.424E-1 (pos.215812297)		ATP-binding	ATP bindin	Colonic Diseases;Colorectal N	Benign	G=0, A=26	G=0.0, A=1.0	0.00001	371.42	26	G=0/0, A=10/1
chr2:133489401	G/G	A	G	SNV	NCKAP5	NCKAP5:exonic:NM 207363.2	1	100	17	synonymous	NM 20736	c.5352T>C	p.(=)	-1.01					Nck-associat	rs17397142	0.1641	YES	1.626E-1 (pos.133489401)		NCK-associ	biological	Alzheimer Disease;Brain Diseases;Central Ne		A=0, G=108	A=0.0, G=1.0	0.00001	1699.93	108	A=0/0, G=58/5
chr2:215855780	A/A	G	A	SNV	ABCA12	ABCA12:intronic:NM 173076.2	1	100		intronic	NM 17307	c.3295-25	p.?	-0.76					AAA domain,	rs4533467	0.498	YES	2.712E-1 (pos.215855780)		ATP-binding	ATP bindin	Colonic Diseases;Colorectal Neoplasms;Con		G=0, A=176	G=0.0, A=1.0	0.00001	2901.42	176	G=0/0, A=95/8

chr2:215855897	G/G	T	G	SNV	ABCA12	ABCA12:intronic:NM_173076.2	1	100		intronic	NM_17307	c.3295-14	p.?	-0.13					AAA domain,	rs1880134		YES			ATP-binding	ATP bindin	Colonic Diseases;Colorectal Neoplasms;Con			T=0, G=166	T=0.0, G=1.0	0.00001	2699.16	166	T=0/0, G=81/8
chr2:215862383	A/A	G	A	SNV	ABCA12	ABCA12:intronic:NM_173076.2	1	100		intronic	NM_17307	c.3294+36	p.?	0.42					AAA domain,	rs1980844,rs764638	0.0999	YES	0.0 (pos.215862374),9.597E-1 (pos.2158		ATP-binding	ATP bindin	Colonic Diseases;Colorectal Neoplasms;Con			G=0, A=28	G=0.0, A=1.0	0.00001	408.715	28	G=0/0, A=15/1
chr2:215874918	C/C	T	C	SNV	ABCA12	ABCA12:intronic:NM_173076.2	1	100		intronic	NM_17307	c.2472+13	p.?	-0.62					AAA domain,	rs4673929					ATP-binding	ATP bindin	Colonic Diseases;Colorectal Neoplasms;Con			T=0, C=24	T=0.0, C=1.0	0.00001	334.789	24	T=0/0, C=10/1
chr2:215874965	T/T	G	T	SNV	ABCA12	ABCA12:intronic:NM_173076.2	1	100		intronic	NM_17307	c.2472+90	p.?	-0.92					AAA domain,	rs6733264		YES			ATP-binding	ATP bindin	Colonic Diseases;Colorectal Neoplasms;Con			G=0, T=25	G=0.0, T=1.0	0.00001	352.986	25	G=0/0, T=10/1
chr2:215875251	T/T	A	T	SNV	ABCA12	ABCA12:intronic:NM_173076.2	1	100		intronic	NM_17307	c.2333-57	p.?	0.51					AAA domain,	rs1409607					ATP-binding	ATP bindin	Colonic Diseases;Colorectal Neoplasms;Con			A=0, T=43	A=0.0, T=1.0	0.00001	705.842	43	A=0/0, T=9/34
chr2:133489555	C/T	C	T	SNV	NCKAP5	NCKAP5:exonic:NM_207363.2	1	55.14	17	missense	NM_20736	c.5198G>	p.Arg1733His	6.14	0	29	1	0.98(COSM40	Nck-associat	rs371367810	1.00E-04		1.168E-4 (pos.13348955	adenocarcinoi	NCK-associ	biological	Alzheimer Disease;Brain Diseases;Central Ne			C=96, T=11	C=0.4486, T=0.5514	0.00001	1322.31	214	C=52/44, T=31
chr2:215883988	C/C	T	C	SNV	ABCA12	ABCA12:intronic:NM_173076.2	1	100		intronic	NM_17307	c.1657+72	p.?	-0.73					AAA domain,	rs1403835					ATP-binding	ATP bindin	Colonic Diseases;Colorectal Neoplasms;Con			T=0, C=50	T=0.0, C=1.0	0.00001	829.585	50	T=0/0, C=35/1
chr2:215928972	C/C	G	C	SNV	ABCA12	ABCA12:intronic:NM_173076.2	1	100		intronic	NM_17307	c.164-30C	p.?	-0.9					AAA domain,	rs1523721	0.4123	YES	6.904E-1 (pos.215928972)		ATP-binding	ATP bindin	Colonic Diseases;Colorectal Neoplasms;Con			G=0, C=24	G=0.0, C=1.0	0.00001	334.683	24	G=0/0, C=7/17
chr2:215976231	A/AT	A	AT	INDEL	ABCA12	ABCA12:intronic:NM_173076.2	1	100		intronic	NM_17307	c.163+89T	p.?	-0.2					AAA domain,	rs2948978		YES			ATP-binding	ATP bindin	Colonic Diseases;Colorectal Neoplasms;Con			A=0, AT=27	A=0.0, AT=1.0	0.00001	151.519	27	A=0/0, AT=15/
chr2:216190691	C/C	A	C	SNV	ATIC	ATIC:intronic:NM_004044.6	1	100		intronic	NM_00404	c.380-19A	p.?	-4.01					AICARFT/IMP	rs2177737	0		9.999E-1;1.156E-4 (pos.216190691)		5-aminoimi	'de novo' II	Arthritis;Arthritis, F	2-5-Hydroxy-3-Methyl-1-		A=0, C=22	A=0.0, C=1.0	0.00001	298.883	22	A=0/0, C=8/14
chr2:216199881	G/G	T	G	SNV	ATIC	ATIC:intronic:NM_004044.6	1	100		intronic	NM_00404	c.1008+15	p.?	-1.57					AICARFT/IMP	rs7594036		YES			5-aminoimi	'de novo' II	Arthritis;Arthritis, F	2-5-Hydroxy-3-Methyl-1-		T=0, G=33	T=0.0, G=1.0	0.00001	504.536	33	T=0/0, G=17/1
chr20:33337751	C/C	T	C	SNV	NCOA6	NCOA6:exonic:NM_014071.4	1	100	9	synonymous	NM_01407	c.2247A>	p.(=)	-0.62				0.4(COSM375	Putative nucl	rs3787220	0.1638	YES	7.616E-1 (pos.33337751)	adenocarcinoi	Nuclear rec	DNA recon	Endometriosis;Female Urogenital Diseases;F			T=0, C=29	T=0.0, C=1.0	0.00001	427.6	29	T=0/0, C=22/7
chr2:216240128	A/A	C	A	SNV	FN1	FN1:intronic:NM_212482.2	1	100		intronic	NM_21248	c.5978-12	p.?	-0.11					Fibronectin t	rs3214672,rs71044591,rs78972		YES	7.828E-1 (pos.216240126)		Fibronectin	acute-pha	Abortion, Spontane	Ocriplasmin		C=0, A=23	C=0.0, A=1.0	0.00001	316.747	23	C=0/0, A=10/1
chr12:124856660	A/A	T	A	SNV	NCOR2	NCOR2:exonic:NM_006312.5	1	100	22	synonymous	NM_00631	c.2715A>T	p.(=)	-4.94				0.09(COSM41	G-protein pat	rs3741513	0.391	YES	1.977E-1 (pos.12485666(	carcinoma,ne	Nuclear rec	DNA bindi	Cocaine-Related Disorders;Mental Disorders			T=0, A=109	T=0.0, A=1.0	0.00001	1718.87	109	T=0/0, A=82/2
chr2:216269031	TTG/TTG	GTT	TTG	MNV	FN1	FN1:intronic:NM_212482.2	3	100		intronic	NM_21248	c.3253+81	p.?	-1.03, -5.2, -0.06					Fibronectin t	rs1004625238,rs199752385,rs3		YES			Fibronectin	acute-pha	Abortion, Spontane	Ocriplasmin		GTT=0, TTG	GTT=0.0, TTG=1.0	0.00001	596.694	39	GTT=0/0, TTG=
chr2:216283958	A/A	T	A	SNV	FN1	FN1:intronic:NM_212482.2	1	100		intronic	NM_21248	c.1819+7A	p.?	2.52				0.72(COSM50	Fibronectin t	rs3796123	0.2377	YES	9.223E-1;0.0 (pos.21628	osteosarcoma	Fibronectin	acute-pha	Abortion, Spontane	Ocriplasmin		T=0, A=55	T=0.0, A=1.0	0.00001	787.24	55	T=0/0, A=33/2
chr6:41309552	C/C	T	C	SNV	NCR2	NCR2:exonic:NM_004828.3	1	100	3	missense	NM_00482	c.415T>C	p.Ser139Pro	-0.19	0.11	74	0.497	0.01(COSM14	Immunoglobu	rs2236369	0.4063	YES	5.442E-1 (pos.41309552)	adenocarcinoi	Natural cyt	cellular defense response,innate immune response,inte				T=0, C=22	T=0.0, C=1.0	0.00001	296.288	22	T=0/0, C=11/1
chrX:119005705	G/G	C	G	SNV	NDUFA1,RNF113A	RNF113A:utr_5:NM_006978.2, NDUFA1:upstream:NM_0045	1	100	1	utr_5	NM_00697	c.-129G>C	p.?, p.?	0.04					RING-type zin	rs708463		YES			NADH dehy	NADH deh	Abnormalities, Mul	NADH		C=0, G=20	C=0.0, G=1.0	0.00001	263.786	20	C=0/0, G=15/5
chr15:41689166	A/A	C	A	SNV	NDUFAF1	NDUFAF1:exonic:NM_016013.3	1	100	2	missense	NM_01601	c.92G>T	p.Arg31Leu	-0.95	0.48	102	0	0.01(COSM54	Complex I int	rs3204853	0.2608	YES	1.342E-2;0.0 (pos.41689	acute_myeloic	NADH-ubiq	cytosol,mi	Metabolic Diseases;Mitochon		Likely benign	C=0, A=159	C=0.0, A=1.0	0.00001	2530.8	159	C=0/0, A=93/6
chr2:219209393	G/G	A	G	SNV	CATIP-AS2,PNKD	PNKD:intronic:NM_015488.4, CATIP-AS2:intronic_nc:NR_125	1	100		intronic	NM_01548	c.984+100	p.?	0.12					Hydroxyacylg	rs1350336						hydroxyac	Adenocarcinoma;Carcinoma;Carcinoma, Ren			A=0, G=21	A=0.0, G=1.0	0.00001	281.224	21	A=0/0, G=13/8
chr15:41689232	T/T	C	T	SNV	NDUFAF1	NDUFAF1:exonic:NM_016013.3	1	100	2	missense	NM_01601	c.26G>A	p.Arg9His	-0.81	0.03	29	0.056	0(COSM5021	Complex I int	rs1899	0.2597	YES	1.328E-2 (pos.41689232)	acute_myeloic	NADH-ubiq	cytosol,mi	Metabolic Diseases;Mitochon		Likely benign	C=0, T=116	C=0.0, T=1.0	0.00001	1857.62	116	C=0/0, T=67/4
chr2:219257688	C/C	T	C	SNV	SLC11A1	SLC11A1:intronic:NM_000578.3	1	100		intronic	NM_00057	c.1165-16	p.?	0.05					Natural resis	rs2045433	5.00E-04		1.0 (pos.219257688)		Solute carri	L-arginine	Acute Kidney Injury;Arthritis;Arthritis, Juveni			T=0, C=34	T=0.0, C=1.0	0.00001	524.155	34	T=0/0, C=24/1
chr2:219266108	G/G	C	G	SNV	CTDSP1	CTDSP1:intronic:NM_001206878.1	1	100		intronic	NM_00120	c.68-179C	p.?	-0.14					NLI interactir	rs2607726		YES			C-terminal	CTD phosphatase activity,depl	Aspartate Beryllium Trifl			C=0, G=28	C=0.0, G=1.0	0.00001	408.712	28	C=0/0, G=19/9
chr14:92582472	A/A	G	A	SNV	NDUFB1	NDUFB1:utr_3:NM_004545.3	1	100	3	utr_3	NM_00454	c.383C>T	p.?	0.63					MNLL subuni	rs13350	0.3391	YES			NADH-ubiq	NADH dehydrogenase (ubiquin		NADH		G=0, A=132	G=0.0, A=1.0	0.00001	2171.55	132	G=0/0, A=113/
chr5:52942197	G/G	A	G	SNV	NDUFS4	NDUFS4:exonic:NM_002495.3	1	100	3	synonymous	NM_00249	c.312A>G	p.(=)	0.67				0.34(COSM41	ETC complex I	rs31303	0.2377	YES	7.631E-1 (pos.52942197)	neoplasm	NADH dehy	NADH deh	Brain Diseases:Brai	NADH	Benign/Likely	A=0, G=43	A=0.0, G=1.0	0.00001	705.844	43	A=0/0, G=18/2
chr18:9117867	C/C	T	C	SNV	NDUFV2	NDUFV2:exonic:NM_021074.4	1	100	2	missense	NM_02107	c.86T>C	p.Val29Ala	1.54	1	64	0	0.04(COSM37	Thioredoxin-l	rs906807	0.1806	YES	7.098E-1 (pos.9117867)	acute_myeloic	NADH dehy	2 iron, 2 su	Bipolar Disorder;Bi	NADH	Conflicting in	T=0, C=84	T=0.0, C=1.0	0.00001	1370.13	84	T=0/0, C=7/77
chr21:44323590	G/G	T	G	SNV	NDUFV3	NDUFV3:exonic:NM_021075.3	1	100	3	synonymous	NM_02107	c.468T>G	p.(=)	-1.35				0.02(COSM41	NADH dehydr	rs4148973	0.3869	YES	3.29E-1 (pos.44323590)	carcinoma,ne	NADH-ubiq	NADH dehydrogenase (ubiquin		NADH		T=0, G=52	T=0.0, G=1.0	0.00001	765.952	52	T=0/0, G=34/1
chr2:152422076	G/G	C	G	SNV	NEB	NEB:exonic:NM_004543.4	1	100	88	missense	NM_00454	c.13166G	p.Arg4389Thr	5.72		71	0	0.17(COSM41	Nebulin repe	rs2288210	0.3367	YES	8.002E-1 (pos.15242207	NS.diffuse_lar	Nebulin	Z disc,acti	Congenital, Hereditary, and Ne		Benign/Likely	C=0, G=20	C=0.0, G=1.0	0.00001	263.739	20	C=0/0, G=5/15
chr2:220033830	G/G	A	G	SNV	SLC23A3	SLC23A3:intronic:NM_001144890.1	1	100		intronic	NM_00114	c.443-6T>	p.?	0.18				0.04(COSM63	Permease fa	rs3795986	0.3579	YES	2.722E-1 (pos.22003383(	carcinoma		integral component of membrane,membrane,transmem				A=0, G=20	A=0.0, G=1.0	0.00001	263.689	20	A=0/0, G=12/8
chr2:152527572	T/T	C	T	SNV	NEB	NEB:exonic:NM_004543.4	1	100	38	missense	NM_00454	c.4471G>	p.Val1491Met	2.7		21	0.997	0.93(COSM40	Nebulin repe	rs7426114	0.1675	YES	4.071E-1 (pos.15252757	acute_myeloic	Nebulin	Z disc,acti	Congenital, Hereditary, and Ne		Benign/Likely	C=0, T=232	C=0.0, T=1.0	0.00001	3868.14	232	C=0/0, T=124/
chr2:220041090	CA/CA	C	CA	INDEL	CNPPD1	CNPPD1:intronic:NM_015680.5	1	100		intronic	NM_01568	c.70-37G>	p.?	0.3					Cyclin							integral component of membrane,protein kinase bindin				C=0, CA=30	C=0.0, CA=1.0	0.00001	315.261	30	C=0/0, CA=11/
chr2:220041433	A/A	C	A	SNV	CNPPD1	CNPPD1:intronic:NM_015680.5	1	100		intronic	NM_01568	c.69+49G>	p.?	-0.09					Cyclin	rs200450802,rs6436	0.2387,0.	YES	3.482E-1;0.0 (pos.220041433)			integral component of membrane,protein kinase bindin				C=0, A=33	C=0.0, A=1.0	0.00001	504.598	33	C=0/0, A=29/4
chr2:152573981	G/G	A	G	SNV	NEB	NEB:exonic:NM_004543.4	1	100	10	synonymous	NM_00454	c.771T>C	p.(=)	-0.44				0.11(COSM40	Nebulin repe	rs4611637	0.1729	YES	4.735E-1 (pos.15257398	acute_myeloic	Nebulin	Z disc,acti	Congenital, Hereditary, and Ne		Benign/Likely	A=0, G=176	A=0.0, G=1.0	0.00001	2901.44	176	A=0/0, G=104/
chr2:220072766	T/T	G	T	SNV	ZFAND2B	ZFAND2B:intronic:NM_138802.2	1	100		intronic	NM_13880	c.434+6G>	p.?	0.02					AN1-like Zinc	rs4674366	6.00E-04		1.0 (pos.220072766)		Zinc finger A	SRP-dependent cotranslational protein targeting to men				G=0, T=22	G=0.0, T=1.0	0.00001	298.869	22	G=0/0, T=9/13
chr2:220078496	C/C	T	C	SNV	ABCB6	ABCB6:intronic:NM_005689.3	1	100		intronic	NM_00568	c.1578+52	p.?	-0.74					ABC transpor	rs2139823	0.001	YES			ATP-binding	ATP bindin	Adnexal Diseases;Coloboma;Colonic Disease			T=0, C=29	T=0.0, C=1.0	0.00001	424.626	29	T=0/0, C=22/7
chr2:152411495	C/T	C	T	SNV	NEB	NEB:exonic:NM_004543.4	1	50	97	missense	NM_00454	c.14239G	p.Asp4747Asn	6.87		23	1	0.99(COSM10	Nebulin repe	rs562175196			0.0 (pos.152411495)	endometrioid	Nebulin	Z disc,acti	Congenital, Hereditary, and Neonatal Diseas			C=10, T=10	C=0.5, T=0.5	0.00001	82.8861	20	C=7/3, T=4/6
chr2:220147732	C/T	C	T	SNV	DNAJB2	DNAJB2:intronic:NM_006736.5	1	50		intronic	NM_00673	c.445+81C	p.?	-0.15					A repeated d	rs2276639		YES			DnaJ, E. coli	ATPase ac	Charcot-Marie-Tooth Disease;Congenital Abr			C=17, T=17	C=0.5, T=0.5	0.00001	172.01	34	C=12/5, T=13/
chr19:45389224	A/G	A	G	SNV	NECTIN2	NECTIN2:exonic:NM_001042724.1	1	47.62	7	synonymous	NM_00104	c.1227A>	p.(=)	-4.11					CD80-like C2-	rs283814	0.0749	YES	9.999E-1 (pos.45389224)		Nectin 2	acrosome assembly,adherens junction organization,adh				A=22, G=20	A=0.5238, G=0.4762	0.00001	212.948	42	A=13/9, G=10/
chr15:56152872	T/T	C	T	SNV	NEDD4	NEDD4:exonic:NM_198400.3	1	100	3	missense	NM_19840	c.1820G>	p.Arg607Gln	1.87	0.11	43	0.744	0.85(COSM39	HECT-domain	rs2303580	0.2044	YES	5.85E-1;0.0;0.0 (pos.561	acute_myeloic	Neural prec	Golgi appa	Cardiovascular Diseases;Collagen Diseases:			C=0, T=20	C=0.0, T=1.0	0.00001	263.784	20	C=0/0, T=15/5
chr2:220239534	G/A	G	A	SNV	DNPEP	DNPEP:intronic:NM_012100.3	1	50		intronic	NM_01210	c.1407+43	p.?	0.43					Aminopeptid	rs3731904,rs772014	0.1304	YES	0.0 (pos.220239531),7.267E-1 (pos.2202		Aspartyl am	aminopeptidase activity,blooc		Glutamic Acid		G=16, A=16	G=0.5, A=0.5	0.00001	158.383	32	G=12/4, A=14/
chr2:220246218	C/C	T	C	SNV	DNPEP	DNPEP:intronic:NM_012100.3	1	100		intronic	NM_01210	c.1137+49	p.?	-0.54					Aminopeptid	rs6436148,rs925246	0.0024	YES	9.116E-1 (pos.220246218)		Aspartyl am	aminopeptidase activity,blooc		Glutamic Acid		T=0, C=43	T=0.0, C=1.0	0.00001	705.844	43	T=0/0, C=24/1
chr1:72058552	A/A	G	A	SNV	NEGR1	NEGR1:exonic:NM_173808.2	1	100	6	synonymous	NM_17380	c.888C>T	p.(=)	-1.27					Immunoglobu	rs1413368	0.0031	YES	8.724E-1 (pos.72058552)		Neuronal gr	C-termina	Nutrition Disorders;Nutritional and Metaboli			G=0, A=109	G=0.0, A=1.0	0.00001	1718.9	109	G=0/0, A=29/8
chr2:220250897	A/A	AG	A	INDEL	DNPEP	DNPEP:intronic:NM_012100.3	1	100		intronic	NM_01210	c.460-77C	p.?	0.53, 0.15					Aminopeptid	rs11352428,rs796431130		YES			Aspartyl am	aminopeptidase activity,blooc		Glutamic Acid		AG=0, A=34	AG=0.0, A=1.0	0.00001	502.456	34	AG=0/0, A=12/
chr2:220316577	CT/CT	C	CT	INDEL	SPEG	SPEG:intronic:NM_005876.4	1	100		intronic	NM_00587	c.2440+48	p.?	-5.12					Immunoglobulin I-set domain,Immunoglobulin domain,Protein kinase domain,Protein tyrosine kina						SPEG compl	ATP bindin	Muscular Diseases;Musculoskeletal Disease			C=0, CT=21	C=0.0, CT=1.0	0.00001	249.986	21	C=0/0, CT=11/
chr4:178274694	G/G	T	G	SNV	NEIL3	NEIL3:exonic:NM_018248.2	1	100	8	synonymous	NM_01824	c.1272T>G	p.(=)	-1.05				0.03(COSM44	Formamidop	rs10007075	0.2458	YES	4.006E-1;0.0 (pos.17827	acute_myeloid leukaemia		DNA N-glyc	Autoimmune Diseases;Female Urogenital Di			T=0, G=26	T=0.0, G=1.0	0.00001	371.156	26	T=0/0, G=12/1
chr4:178274750	T/T	C	T	SNV	NEIL3	NEIL3:exonic:NM_018248.2	1	100	8	missense	NM_01824	c.1328C>T	p.Pro443Leu	-0.51	0.77	98	0	0.01(COSM37	Formamidop	rs13112358	0.2369	YES	3.993E-1 (pos.17827475(	acute_myeloid leukaemia		DNA N-glyc	Autoimmune Diseases;Female Urogenital Di			C=0, T=25	C=0.0, T=1.0	0.00001	353.019	25	C=0/0, T=15/1
chr4:178274835	C/C	A	C	SNV	NEIL3	NEIL3:exonic:NM_018248.2	1	100	8	missense	NM_01824	c.1413A>C	p.Gln471His	-0.18	1	24	0	0(COSM1131	Formamidop	rs13112390	0.2056	YES	4.553E-1 (pos.17827483	acute_myeloid leukaemia		DNA N-glyc	Autoimmune Diseases;Female Urogenital Di			A=0, C=21	A=0.0, C=1.0	0.00001	281.196	21	A=0/0, C=10/1
chr2:220356806	T/T	C	T	SNV	SPEG	SPEG:intronic:NM_005876.4	1	100		intronic	NM_00587	c.9462-27	p.?	0.86					Immunoglobu	rs907146	6.00E-04	YES	1.0 (pos.220356806)		SPEG compl	ATP bindin	Muscular Diseases;Musculoskeletal Disease			C=0, T=25	C=0.0, T=1.0	0.00001	353.019	25	C=0/0, T=11/1
chr4:178281754	A/A	G	A	SNV	NEIL3	NEIL3:exonic:NM_018248.2	1	100	9	missense	NM_01824	c.1558G>	p.Gly520Arg	2.95	1	125	0		Formamidop	rs1876268	0.0328	YES	9.78E-1 (pos.178281754)			DNA N-glyc	Autoimmune Diseases;Female Urogenital Di			G=0, A=35	G=0.0, A=1.0	0.00001	543.903	35	G=0/0, A=18/1
chr3:27326451	A/G	A	G	SNV	NEK10	NEK10:exonic:NM_199347.3	1	50	22	synonymous	NM_19934	c.1791T>C	p.(=)	3.86				0.97(COSM11	Phosphotran	rs10510594	0.2785	YES	1.062E-1 (pos.27326451)	acute_myeloid leukaemia		ATP bindin	Breast Diseases;Breast Neoplasms;Neoplasr			A=18, G=18	A=0.5, G=0.5	0.00001	185.885	36	A=5/13, G=4/1
chr3:130947435	T/T	A	T	SNV	NEK11	NEK11:exonic:NM_024800.4	1	100	15	missense	NM_02480	c.1463A>T	p.Glu488Val	5.12	0.05	121	1	0.84(COSM40	Protein kinas	rs3738000,rs760488	0.2916	YES	0.0 (pos.130947434),3.9(	acute_myeloic	Never in mi	ATP binding,histone phosphory		Dabrafenib		A=0, T=27	A=0.0, T=1.0	0.00001	389.988	27	A=0/0, T=17/1
chr2:220428381	A/T	A	T	SNV	OBSL1	OBSL1:intronic:NM_015311.2	1	95.65		intronic	NM_01531	c.2408-32	p.?	0.8					Immunoglobu	rs2303543	0.3222	YES	6.88E-1;0.0 (pos.220428381)		Obscurin-lik	3M compl	Bone Diseases;Bone Diseases, Development			A=1, T=22	A=0.0435, T=0.9565	0.00001	288.977	23	A=1/0, T=13/9
chr3:52797634	G/C	G	C	SNV	NEK4	NEK4:exonic:NM_003157.5	1	57.45	5	missense	NM_00315	c.673C>G	p.Pro225Ala	7.53	0.02	27	0.88	0.84(COSM40	Protein kinas	rs1029871	0.4034	YES	4.286E-1 (pos.52797634)	acute_myeloic	NIMA (neve	ATP bindin	Autoimmune Diseases;Female Urogenital Di			G=20, C=27	G=0.4255, C=0.5745	0.00001	326.535	47	G=9/11, C=8/1
chr2:220466199	T/T	G	T	SNV	STK11IP	STK11IP:intronic:NM_052902.3	1	100		intronic	NM_05290	c.267+37G	p.?	0					LKB1 serine/t	rs620698	0.0268	YES	9.964E-1 (pos.220466199)		Serine/thre	azurophil granule lumen,cytoplasm,extracellular region				G=0, T=121	G=0.0, T=1.0	0.00001	1935.52	121	G=0/0, T=40/8
chr2:220472596	T/T	G	T	SNV	STK11IP	STK11IP:intronic:NM_052902.3	1	100		intronic	NM_05290	c.1220-17	p.?	-1.18					LKB1 serine/t	rs630063		YES			Serine/thre	azurophil granule lumen,cytoplasm,extracellular region				G=0, T=20	G=0.0, T=1.0	0.00001	263.786	20	G=0/0, T=14/6
chr14:50251369	G/G	A	G	SNV	NEMF	NEMF:exonic:NM_004713.5	1	100	33	synonymous	NM_00471	c.3216T>C	p.(=)	1.04					Domain of un	rs10143621	5.00E-04	YES	1.0 (pos.50251369)		Nuclear exc	nuclear export,nucleus				A=0, G=47	A=0.0, G=1.0	0.00001	789.328	47	A=0/0, G=23/2
chr2:220479312	C/C	T	C	SNV	STK11IP	STK11IP:intronic:NM_052902.3	1	100		intronic	NM_05290	c.2869+44	p.?	-2.06					LKB1 serine/t	rs624773	2.00E-04		1.0;0.0 (pos.220479312)		Serine/thre	azurophil granule lumen,cytoplasm,extracellular region				T=0, C=23	T=0.0, C=1.0	0.00001	316.745	23	T=0/0, C=10/1
chr15:73564881	T/T	C	T	SNV	NEO1	NEO1:exonic:NM_002499.3	1	100	19	synonymous	NM_00249	c.2943C>T	p.(=)	-2.45				0.02(COSM37	Fibronectin t	rs1131854	0.475	YES	2.928E-1 (pos.73564881)	acute_myeloic	Neogenin, c	Golgi apparatus,axon guidance,cell adhesion,co-recepto				C=0, T=31	C=0.0, T=1.0	0.00001	465.819	31	C=0/0, T=13/1
chr2:220498349	AC/AC	A	AC	INDEL	SLC4A3	SLC4A3:intronic:NM_201574.2	1	100		intronic	NM_20157	c.1542+17	p.?	-0.08					Band 3 cytoplasmic domain,HCO3- transporter family							anion tran	Cardiovascular Diseases;Heart Diseases			A=0, AC=29	A=0.0, AC=1.0	0.00001	361.559	29	A=0/0, AC=15/
chr2:220498350	C/C	G	C	SNV	SLC4A3	SLC4A3:intronic:NM_201574.2	1	100		intronic	NM_20157	c.1542+17	p.?	2.91					Band 3 cytopl	rs2289782		YES				anion tran	Cardiovascular Diseases;Heart Diseases			G=0, C=40	G=0.0, C=1.0	0.00001	612.178	40	G=0/0, C=26/1
chr2:222301357	C/C	T	C	SNV	EPHA4	EPHA4:intronic:NM_004438.4	1	100		intronic	NM_00443	c.2137-29	p.?	-0.19					Ephrin recept	rs3213844	0.2829	YES	6.548E-1 (pos.222301357)		Ephrin rece	ATP bindin	Nervous System Diseases			T=0, C=108	T=0.0, C=1.0	0.00001	1700.02	108	T=0/0, C=19/8
chr2:223159119	A/A	C	A	SNV	PAX3	PAX3:intronic:NM_181459.3	1	100		intronic	NM_18145	c.452-99G	p.?	-1.39					'Paired box' d	rs2303948		YES			Paired box	DNA bindi	Abnormalities, Multiple;Carcinoma;Congenit			C=0, A=20	C=0.0, A=1.0	0.00001	263.786	20	C=0/0, A=5/15
chr2:223161624	G/G	A	G	SNV	PAX3	PAX3:intronic:NM_181459.3	1	100		intronic	NM_18145	c.321+73T	p.?	-1.4					'Paired box' d	rs12694578		YES			Paired box	DNA bindi	Abnormalities, Multiple;Carcinoma;Congenit			A=0, G=33	A=0.0, G=1.0	0.00001	504.598	33	A=0/0, G=12/2
chr15:73595089	TA/TA	T	TA	INDEL	NEO1	NEO1:utr_3:NM_002499.3	1	100	29	utr_3	NM_00249	c.4438_44	p.?	-0.49					Fibronectin t	rs3833036,rs397715	0.4935	YES	2.661E-1 (pos.73595089)		Neogenin, c	Golgi apparatus,axon guidance,cell adhesion,co-recepto				T=0, TA=46	T=0.0, TA=1.0	0.00001	745.677	46	T=0/0, TA=16/
chr3:112727184	A/T	A	T	SNV	NEPRO	NEPRO:exonic:NM_015412.3	1	58.33	8	missense	NM_01541	c.1069T>A	p.Phe357Ile	0.29	0.17	21	0.005	0.13(COSM41	Domain of un	rs2306857	0.2252	YES	3.484E-1 (pos.11272718	neoplasm	Nucleolus a	negative regulation of neuron differentiation,nucleolus,				A=20, T=28	A=0.4167, T=0.5833	0.00001	343.434	48	A=8/12, T=9/1
chr2:223787559	A/A	G	A	SNV	ACSL3	ACSL3:intronic:NM_203372.1	1	100		intronic	NM_20337	c.1080+10	p.?	-1.19					AMP-binding	rs11891410	0.2235	YES	9.762E-1 (pos.223787559)		Acyl-CoA sy	ATP binding,Golgi apparatus,bı		Icosapent		G=0, A=57	G=0.0, A=1.0	0.00001	849.268	57	G=0/0, A=8/49
chr2:224765894	A/A	G	A	SNV	WDFY1	WDFY1:intronic:NM_020830.4	1	100		intronic	NM_02083	c.485+6C>	p.?	7.39				0.99(COSM54	FYVE zinc fing	rs604990	0.0438	YES	8.097E-1 (pos.22476589	acute_myeloid leukaemia		1-phosphatidylinositol binding,cytosol,early endosome,				G=0, A=41	G=0.0, A=1.0	0.00001	655.658	41	G=0/0, A=27/1
chr2:224770780	T/T	C	T	SNV	WDFY1	WDFY1:intronic:NM_020830.4	1	100		intronic	NM_02083	c.280-62G	p.?	0					FYVE zinc fing	rs622896	0.0421	YES				1-phosphatidylinositol binding,cytosol,early endosome,				C=0, T=156	C=0.0, T=1.0	0.00001	2502.28	156	C=0/0, T=68/8
chr2:224862821	C/T	C	T	SNV	SERPINE2	SERPINE2:intronic:NM_006216.3	1	52.38		intronic	NM_00621	c.487+11G	p.?	-2.77					Serpin (serine	rs3795879	0.203	YES	1.282E-1 (pos.224862821)		Protease in	blood coa	Cardiovascular Diseases;Vascular Diseases			C=10, T=11	C=0.4762, T=0.5238	0.00001	95.2903	21	C=3/7, T=6/5
chr1:156640503	C/T	C	T	SNV	NES	NES:exonic:NM_006617.1	1	47.37	4	synonymous	NM_00661	c.3477G>	p.(=)	-0.54				0.05(COSM37	Intermediate	rs3828043	0.2858	YES	2.687E-1 (pos.15664050	acute_myeloic	Nestin	G2/M tran	Brain Diseases;Brain Ischemia;Cardiovascul			C=20, T=18	C=0.5263, T=0.4737	0.00001	186.197	38	C=6/14, T=7/1
chr2:224970	T/T	C	T	SNV	SH3YL1	SH3YL1:intronic:NM_015677.3	1	100		intronic	NM_01567	c.782-50G	p.?	-2.51					Las17-bindin	rs2290912	0.3087	YES	8.188E-1 (pos.224970)		SH3 domair	phosphatase binding,phosphatidylinositol binding,phos				C=0, T=90	C=0.0, T=1.0	0.00001	1482.01	90	C=0/0, T=47/4
chr10:5495218	C/C	T	C	SNV	NET1	NET1:exonic:NM_001047160.2	1	100	7	synonymous	NM_00104	c.600T>C	p.(=)	0.99					PH domain,Pl	rs4567355	2.00E-04		1.0 (pos.5495218)		Solute carri	G-protein coupled receptor signaling pathway,GTP-Rho				T=0, C=62	T=0.0, C=1.0	0.00001	940.32	62	T=0/0, C=52/1
chr2:226273490	C/C	T	C	SNV	NYAP2	NYAP2:intronic:NM_020864.1	1	100		intronic	NM_02086	c.-16-90T>	p.?	-0.3					Neuronal tyr	rs2048935		YES				intracellular,neuron projection morphogenesis,phospha				T=0, C=24	T=0.0, C=1.0	0.00001	334.788	24	T=0/0, C=18/6
chr10:105344756	C/C	T	C	SNV	NEURL1	NEURL1:exonic:NM_004210.4	1	100	4	synonymous	NM_00421	c.1113T>C	p.(=)	-0.79				0.11(COSM37	Neuralized,Zi	rs2236209	0.4947	YES	6.855E-1 (pos.10534475	hepatocellula	Neuralized	Notch sign	Colonic Diseases;Colorectal Neoplasms;Dige			T=0, C=187	T=0.0, C=1.0	0.00001	3047.34	187	T=0/0, C=39/1
chr2:227912331	G/A	G	A	SNV	COL4A4	COL4A4:intronic:NM_000092.4	1	50		intronic	NM_00009	c.3215-66	p.?	-1.15					C-terminal ta	rs2272200		YES			Collagen IV,	basal lami	Collagen Diseases;Congenital Abnormalities			G=15, A=15	G=0.5, A=0.5	0.00001	145.021	30	G=13/2, A=10/
chr2:228109784	T/T	C	T	SNV	COL4A3,LOC654841	COL4A3:intronic:NM_000091.4, LOC654841:intronic_nc:NR	1	100		intronic	NM_00009	c.324+73C	p.?	0.23					C-terminal ta	rs6750210	0.1837	YES			Collagen IV,	activation	Collagen Diseases;Congenital Abnormalities			C=0, T=47	C=0.0, T=1.0	0.00001	789.242	47	C=0/0, T=18/2
chr2:228149107	G/G	A	G	SNV	COL4A3,LOC654841	COL4A3:intronic:NM_000091.4, LOC654841:intronic_nc:NR	1	100		intronic	NM_00009	c.2881+46	p.?	-1.19					C-terminal ta	rs6436672	0.0864	YES	7.515E-1 (pos.228149107)		Collagen IV,	activation	Collagen Diseases;Congenital Abnormalities			A=0, G=61	A=0.0, G=1.0	0.00001	921.835	61	A=0/0, G=48/1
chr2:228194570	A/A	G	A	SNV	MFF	MFF:intronic:NM_020194.5	1	100		intronic	NM_02019	c.38+71G>	p.?	-1.26					Mitochondria	rs6706656	0.0396	YES			Mitochondr	cell juncti	Brain Diseases;Brain Diseases, Metabolic;Ce			G=0, A=66	G=0.0, A=1.0	0.00001	1015.38	66	G=0/0, A=40/2
chr2:228197368	G/G	A	G	SNV	MFF	MFF:intronic:NM_020194.5	1	100		intronic	NM_02019	c.429+64A	p.?	-1.66					Mitochondria	rs4500938		YES			Mitochondr	cell juncti	Brain Diseases;Brain Diseases, Metabolic;Ce			A=0, G=65	A=0.0, G=1.0	0.00001	996.452	65	A=0/0, G=24/4
chr1:204943362	C/T	C	T	SNV	NFASC	NFASC:exonic:NM_001005388.2	1	57.14	13	synonymous	NM_00100	c.1335C>T	p.(=)	1.97					Bravo-like int	rs147248519	0.0166	YES	1.164E-4 (pos.204943362)		Neurofascir	Schwann cell microvillus,axon guidance,axon initial segr				C=12, T=16	C=0.4286, T=0.5714	0.00001	161.768	28	C=4/8, T=6/10
chr2:228477814	G/C	G	C	SNV	C2orf83	C2orf83:intronic:NM_020161.3	1	46.51		intronic	NM_02016	c.191-144	p.?	-0.7					Reduced fola	rs7586824		YES				membrane,thiamine transmembrane transport				G=23, C=20	G=0.5349, C=0.4651	0.00001	208.411	43	G=13/10, C=9/
chr2:228973660	G/G	A	G	SNV	SPHKAP	SPHKAP:intronic:NM_001142644.1	1	100		intronic	NM_00114	c.139-5T>	p.?	0.83				0.04(COSM44	A-kinase ancl	rs7561798	0.4851	YES	6.835E-1 (pos.22897366(	acute_myeloic	SPHK1 inter	extracellular exosome,mitochondrion,protein binding,p				A=0, G=94	A=0.0, G=1.0	0.00001	1569.14	94	A=0/0, G=24/7
chr2:230223451	G/A	G	A	SNV	DNER	DNER:intronic:NM_139072.3	1	45.45		intronic	NM_13907	c.2103-84	p.?	-0.34					Calcium-bind	rs57056068		YES			Delta-and n	Notch binc	Diabetes Mellitus;Diabetes Mellitus, Type 2;			G=24, A=20	G=0.5455, A=0.4545	0.00001	211.735	44	G=6/18, A=5/1
chr2:230663576	T/T	C	T	SNV	TRIP12	TRIP12:intronic:NM_004238.2	1	100		intronic	NM_00423	c.3257+15	p.?	-2.54					HECT-domain	rs6720868	0.3262	YES	2.238E-1 (pos.230663576)		Thyroid hor	DNA repair,cellular response to DNA damage stimulus,c				C=0, T=27	C=0.0, T=1.0	0.00001	389.988	27	C=0/0, T=6/21
chr1:204948659	A/G	A	G	SNV	NFASC	NFASC:exonic:NM_001005388.2	1	50	19	synonymous	NM_00100	c.2148A>	p.(=)	-2.6					Bravo-like int	rs6667532	0.1157	YES	8.239E-3 (pos.204948659)		Neurofascir	Schwann cell microvillus,axon guidance,axon initial segr				A=20, G=20	A=0.5, G=0.5	0.00001	214.328	40	A=6/14, G=8/1
chr18:77171061	G/G	T	G	SNV	NFATC1	NFATC1:exonic:NM_172387.2	1	100	2	synonymous	NM_17238	c.747T>G	p.(=)	-1.19				0.07(COSM37	IPT/TIG doma	rs2230112	0.3014	YES	8.468E-1;0.0 (pos.77171(	adenocarcinoi	Nuclear fac	DNA binding,DNA binding trans		Pseudoephedrine		T=0, G=58	T=0.0, G=1.0	0.00001	867.382	58	T=0/0, G=33/2
chr14:24839083	C/C	G	C	SNV	NFATC4	NFATC4:exonic:NM_001136022.2	1	100	3	missense	NM_00113	c.668G>C	p.Gly223Ala	2.03	1	60	0.001	0.83(COSM41	IPT/TIG doma	rs2229309	0.414	YES	1.608E-1 (pos.24839083)	neoplasm	Nuclear fac	DNA binding,DNA binding transcription factor activity,RN				G=0, C=55	G=0.0, C=1.0	0.00001	759.849	55	G=0/0, C=29/2
chr14:24843620	C/C	T	C	SNV	NFATC4	NFATC4:exonic:NM_001136022.2	1	100	7	synonymous	NM_00113	c.2010T>C	p.(=)	-0.49				0.28(COSM41	IPT/TIG doma	rs2295298	0.3951	YES	1.441E-1 (pos.24843620)	neoplasm	Nuclear fac	DNA binding,DNA binding transcription factor activity,RN				T=0, C=41	T=0.0, C=1.0	0.00001	652.72	41	T=0/0, C=17/2
chr2:231328696	T/T	C	T	SNV	SP100	SP100:intronic:NM_001080391.1	1	100		intronic	NM_00108	c.1058-86	p.?	-0.85					Bromodomai	rs2306274		YES			Nuclear ant	DNA binding,DNA damage response, signal transduction				C=0, T=71	C=0.0, T=1.0	0.00001	1111.44	71	C=0/0, T=59/1
chr2:231334679	A/G	A	G	SNV	SP100	SP100:intronic:NM_001080391.1	1	57.45		intronic	NM_00108	c.1346-51	p.?	-1.43					Bromodomai	rs1678162	0.3245	YES	8.036E-2;0.0 (pos.231334679)		Nuclear ant	DNA binding,DNA damage response, signal transduction				A=20, G=27	A=0.4255, G=0.5745	0.00001	326.534	47	A=14/6, G=16/
chr4:103514658	C/C	T	C	SNV	NFKB1	NFKB1:exonic:NM_003998.3	1	100	12	synonymous	NM_00399	c.1143T>C	p.(=)	-7.54					Ankyrin repe	rs1609993	0.0808	YES	9.99E-1;1.156E-4 (pos.103514658)		Nuclear fac	DNA bindi	Adenocarcinoma;A	Acetylsalicylic acid,Andr		T=0, C=56	T=0.0, C=1.0	0.00001	832.088	56	T=0/0, C=23/3
chr10:104160434	G/G	A	G	SNV	NFKB2	NFKB2:exonic:NM_001077494.3	1	100	17	synonymous	NM_00107	c.1821A>	p.(=)	-2.27				0.08(COSM41	Ankyrin repe	rs4919634	1.00E-04		1.0 (pos.104160434)	neoplasm	Nuclear fac	Bcl3/NF-ka	Brain Diseases:Cen	Acetylsali	Benign	A=0, G=43	A=0.0, G=1.0	0.00001	705.844	43	A=0/0, G=19/2
chr2:231860838	T/T	C	T	SNV	SPATA3,SPATA3-AS1	SPATA3:upstream:NM_139073.3, SPATA3-AS1:upstream:NR	1	100		intronic	NR_03387	c.-111C>T	p.?	-1.74						rs2271369		YES				cellular_component,integral component of membrane,r				C=0, T=77	C=0.0, T=1.0	0.00001	1229.42	77	C=0/0, T=53/2
chr2:231860848	G/G	A	G	SNV	SPATA3,SPATA3-AS1	SPATA3:utr_5:NM_139073.3, SPATA3-AS1:upstream:NR_033	1	100		intronic	NR_03387	c.-101A>G	p.?	2.3					Spermatoger	rs6722863		YES				cellular_component,integral component of membrane,r				A=0, G=76	A=0.0, G=1.0	0.00001	1209.53	76	A=0/0, G=51/2
chr2:232324780	G/G	A	G	SNV	NCL,SNORD82	NCL:intronic:NM_005381.2, SNORD82:downstream:NR_004	1	100		intronic	NM_00538	c.1040+83	p.?	0.56					RNA recogniti	rs2442322		YES			Nucleolin,S	DNA topoi	Cardiovascular Diseases;Heart Diseases;My			A=0, G=37	A=0.0, G=1.0	0.00001	582.912	37	A=0/0, G=28/9
chr2:232326801	T/T	A	T	SNV	NCL	NCL:intronic:NM_005381.2	1	100		intronic	NM_00538	c.136-73T	p.?	-2.06					RNA recogniti	rs2595514	0.3368	YES	8.076E-1 (pos.232326801)		Nucleolin	DNA topoi	Cardiovascular Diseases;Heart Diseases;My			A=0, T=25	A=0.0, T=1.0	0.00001	353.019	25	A=0/0, T=14/1
chr14:35871217	A/A	G	A	SNV	NFKBIA	NFKBIA:utr_3:NM_020529.2	1	100	6	utr_3	NM_02052	c.956C>T	p.?	-0.36					Ankyrin repe	rs8904	0.3692	YES	4.222E-1 (pos.35871217)		Nuclear fac	Fc-epsilon	Abnormalities, Mul	Acetylsali	Benign	G=0, A=27	G=0.0, A=1.0	0.00001	389.988	27	G=0/0, A=17/1
chr2:233757697	G/G	A	G	SNV	NGEF	NGEF:exonic:NM_019850.2	1	100	7	synonymous	NM_01985	c.1053T>C	p.(=)	0.37					PH domain,R	rs895432	0.0435	YES	8.894E-1 (pos.233757697)		Neuronal gu	G-protein	Mental Disorders;Schizophrenia;Schizophren			A=0, G=27	A=0.0, G=1.0	0.00001	389.988	27	A=0/0, G=6/21
chr2:233243660	C/C	T	C	SNV	ALPP	ALPP:intronic:NM_001632.4	1	100		intronic	NM_00163	c.77-21T>	p.?	0.05					Alkaline phos	rs2014448	0.0711	YES	9.192E-1 (pos.233243660)		Alkaline ph	alkaline phosphatase activity,		METHYL-PHOSPHONIC AC		T=0, C=21	T=0.0, C=1.0	0.00001	281.235	21	T=0/0, C=8/13
chr2:233245090	G/G	A	G	SNV	ALPP	ALPP:intronic:NM_001632.4	1	100		intronic	NM_00163	c.793-40A	p.?	-3.29					Alkaline phos	rs2260307		YES	7.799E-1;0.0 (pos.233245090)		Alkaline ph	alkaline phosphatase activity,		METHYL-PHOSPHONIC AC		A=0, G=62	A=0.0, G=1.0	0.00001	940.32	62	A=0/0, G=37/2
chr5:177577919	AT/AT	A	AT	INDEL	NHP2	NHP2:exonic:NM_017838.3	1	100	3	frameshiftInsertion	NM_01783	c.305_306	p.Asn102fs	0.08					Ribosomal protein L7Ae/L30e/S12e/Gadd45 family						Nucleolar p	RNA bindir	Congenital Abnormalities;Congenital, Heredi			A=0, AT=47	A=0.0, AT=1.0	0.00001	518.267	47	A=0/0, AT=31/
chr2:233322875	C/T	C	T	SNV	ALPI	ALPI:intronic:NM_001631.4	1	50		intronic	NM_00163	c.991+33C	p.?	-4.76					Alkaline phos	rs2272421	0.163	YES	4.962E-2;0.0 (pos.233322875)		Alkaline ph	C-terminal protein lipidation,a		Monopotassium phosph		C=8, T=13	C=0.381, T=0.619	0.00001	171.512	26	C=2/6, T=9/4
chr5:177577923	T/T	C	T	SNV	NHP2	NHP2:exonic:NM_017838.3	1	100	3	missense	NM_01783	c.302G>A	p.Arg101Gln	2.6	0.15	43	0.004	0.88(COSM58	Ribosomal pr	rs145890370	0.0101		0.0 (pos.177577923)	rhabdomyosar	Nucleolar p	RNA bindir	Congenital Abnormalities;Con		Benign/Likely	C=0, T=51	C=0.0, T=1.0	0.00001	754.353	51	C=0/0, T=31/2
chr2:233350548	G/A	G	A	SNV	ECEL1	ECEL1:intronic:NM_004826.3	1	50		intronic	NM_00482	c.786+30C	p.?	-0.64					Peptidase fa	rs113660447	0.1276	YES	1.088E-1 (pos.233350548)		Endothelin-	integral component of plasma membrane,metal ion binc				G=19, A=19	G=0.5, A=0.5	0.00001	192.147	38	G=7/12, A=1/1
chr2:233470843	A/A	G	A	SNV	EFHD1		1	100		intronic				-0.8						rs7571133		YES	2.5E-1 (pos.233470843)		EF-hand domain family		Adenocarcinoma;Carcinoma;Carcinoma, Ade			G=0, A=102	G=0.0, A=1.0	0.00001	1583.98	102	G=0/0, A=69/3
chr2:233470900	T/T	TC	T	INDEL	EFHD1		1	100		intronic				0.2, 0.1						rs200998017,rs55750809,rs796		YES	6.173E-3 (pos.233470900)		EF-hand domain family		Adenocarcinoma;Carcinoma;Carcinoma, Ade			TC=0, T=10	TC=0.0, T=1.0	0.00001	1484.28	103	TC=0/0, T=68/
chr2:233470931	G/G	C	G	SNV	EFHD1		1	100		intronic				-0.6						rs10171498		YES	6.173E-3 (pos.233470931)		EF-hand domain family		Adenocarcinoma;Carcinoma;Carcinoma, Ade			C=0, G=107	C=0.0, G=1.0	0.00001	1681.26	107	C=0/0, G=68/3
chr2:233641083	C/C	G	C	SNV	GIGYF2,KCNJ13	KCNJ13:intronic:NM_002242.4, GIGYF2:intronic:NM_00110	1	100		intronic	NM_00110	c.533-107	p.?, p.?	0.02					GYF domain,I	rs737027	0.3686	YES	6.616E-1 (pos.233641083)		GRB10-inte	Golgi appa	Basal Ganglia Diseases;Brain Diseases;Centr			G=0, C=34	G=0.0, C=1.0	0.00001	524.186	34	G=0/0, C=17/1
chr6:138754323	G/G	A	G	SNV	NHSL1	NHSL1:exonic:NM_001144060.1	1	100	6	synonymous	NM_00114	c.1159T>C	p.(=)	0.99					NHS-like	rs2327891	0.2093	YES	9.244E-1 (pos.138754323)			membrane,motor neuron migration				A=0, G=79	A=0.0, G=1.0	0.00001	1269.33	79	A=0/0, G=39/4
chr2:233925406	T/G	T	G	SNV	INPP5D	INPP5D:intronic:NM_005541.4	1	46.88		intronic	NM_00554	c.134+84T	p.?	-0.62					Endonucleas	rs34218488		YES			Inositol pol	PTB domai	Alzheimer Disease;Brain Diseases;Cardiovas			T=34, G=30	T=0.5313, G=0.4688	0.00001	288.133	64	T=11/23, G=16
chr2:234054989	G/G	T	G	SNV	INPP5D	INPP5D:intronic:NM_005541.4	1	100		intronic	NM_00554	c.747-24T	p.?	-0.95					Endonucleas	rs36180605	0.4507	YES	6.89E-1 (pos.234054989)		Inositol pol	PTB domai	Alzheimer Disease;Brain Diseases;Cardiovas			T=0, G=54	T=0.0, G=1.0	0.00001	798.148	54	T=0/0, G=31/2
chr2:234055004	C/C	T	C	SNV	INPP5D	INPP5D:intronic:NM_005541.4	1	100		intronic	NM_00554	c.747-9T>	p.?	-1.56					Endonucleas	rs36189208	0.4868	YES	8.135E-1 (pos.234055004)		Inositol pol	PTB domai	Alzheimer Disease;Brain Diseases;Cardiovas			T=0, C=54	T=0.0, C=1.0	0.00001	798.136	54	T=0/0, C=31/2
chr14:52478315	G/G	A	G	SNV	NID2	NID2:exonic:NM_007361.3	1	100	17	synonymous	NM_00736	c.3507T>C	p.(=)	-0.73					Calcium-bind	rs1051069	0.4581	YES	7.595E-1;0.0 (pos.52478315)		Nidogen 2	basement membrane,basement membrane organizatio				A=0, G=52	A=0.0, G=1.0	0.00001	766.36	52	A=0/0, G=31/2
chr2:234130	G/G	T	G	SNV	SH3YL1	SH3YL1:intronic:NM_015677.3	1	100		intronic	NM_01567	c.404+30A	p.?	0.22					Las17-bindin	rs3748872	0.3439	YES	5.877E-1 (pos.234130)		SH3 domair	phosphatase binding,phosphatidylinositol binding,phos				T=0, G=28	T=0.0, G=1.0	0.00001	408.717	28	T=0/0, G=17/1
chr2:234173503	A/A	G	A	SNV	ATG16L1	ATG16L1:intronic:NM_030803.6	1	100		intronic	NM_03080	c.390-35G	p.?	-0.69					Autophagy pr	rs12994997,rs76752	0.4771	YES	0.0 (pos.234173490),3.325E-1 (pos.2341		Autophagy	GTPase bi	Crohn Disease;Digestive System Diseases;Ga			G=0, A=70	G=0.0, A=1.0	0.00001	1092.07	70	G=0/0, A=19/5
chr2:234235749	G/C	G	C	SNV	SAG	SAG:intronic:NM_000541.4	1	59.09		intronic	NM_00054	c.436-18G	p.?	-2.32					Arrestin (or S	rs2304774	0.4923	YES	3.927E-1 (pos.234235749)		RING finger	cell surfac	Congenital, Hereditary, and Ne		Benign	G=9, C=13	G=0.4091, C=0.5909	0.00001	122.568	22	G=3/6, C=5/8
chr2:234375944	C/T	C	T	SNV	DGKD	DGKD:intronic:NM_152879.2	1	50		intronic	NM_15287	c.3424+95	p.?	0.04					Diacylglycerc	rs2305540		YES			Diacylglyce	ATP binding,NAD+ kinase activi		Phosphatidyl serine		C=14, T=14	C=0.5, T=0.5	0.00001	130.633	28	C=8/6, T=9/5
chr14:52496407	T/T	C	T	SNV	NID2	NID2:exonic:NM_007361.3	1	100	10	synonymous	NM_00736	c.2259G>	p.(=)	-2.62					Calcium-bind	rs2273431	0.0871	YES	9.776E-1 (pos.52496407)		Nidogen 2	basement membrane,basement membrane organizatio				C=0, T=43	C=0.0, T=1.0	0.00001	705.768	43	C=0/0, T=30/1
chr2:234449462	C/C	T	C	SNV	USP40	USP40:intronic:NM_018218.2	1	100		intronic	NM_01821	c.1099-50	p.?	-0.74					Ubiquitin car	rs1545525,rs758027	0.213	YES	8.701E-1;0.0 (pos.234449462)		Ubiquitin-s	protein deubiquitination,thiol-dependent ubiquitinyl hy				T=0, C=63	T=0.0, C=1.0	0.00001	958.913	63	T=0/0, C=22/4
chr14:52520853	G/G	A	G	SNV	NID2	NID2:exonic:NM_007361.3	1	100	4	synonymous	NM_00736	c.954T>C	p.(=)	-0.44					Calcium-bind	rs904059	1.00E-04		1.0 (pos.52520853)		Nidogen 2	basement membrane,basement membrane organizatio				A=0, G=33	A=0.0, G=1.0	0.00001	504.41	33	A=0/0, G=25/8
chr14:52535648	T/T	G	T	SNV	NID2	NID2:exonic:NM_007361.3	1	100	1	missense	NM_00736	c.65C>A	p.Pro22Gln	0.36	0.31	76	0.002	0.14(COSM41	Calcium-bind	rs3920038,rs748077	0.1346	YES	0.0;0.0 (pos.52535646),9	neoplasm	Nidogen 2	basement membrane,basement membrane organizatio				G=0, T=25	G=0.0, T=1.0	0.00001	353.019	25	G=0/0, T=10/1
chr2:234753008	A/A	G	A	SNV	HJURP	HJURP:intronic:NM_018410.4	1	100		intronic	NM_01841	c.496-79C	p.?	-1.08					Centromere	rs169925		YES			Holliday jur	CENP-A co	Autoimmune Diseases;Female Urogenital Di			G=0, A=24	G=0.0, A=1.0	0.00001	334.497	24	G=0/0, A=8/16
chr2:234754506	G/A	G	A	SNV	HJURP	HJURP:intronic:NM_018410.4	1	58.33		intronic	NM_01841	c.403-40C	p.?	0.51					Centromere	rs2302155	0.4893	YES	3.763E-1 (pos.234754506)		Holliday jur	CENP-A co	Autoimmune Diseases;Female Urogenital Di			G=20, A=28	G=0.4167, A=0.5833	0.00001	342.458	48	G=8/12, A=10/
chr2:234890391	G/T	G	T	SNV	TRPM8	TRPM8:intronic:NM_024080.4	1	45.45		intronic	NM_02408	c.2448-27	p.?	-0.96					Ion transport	rs6708995	0.4827	YES	5.127E-1 (pos.234890391)		Transient re	calcium ch	Brain Diseases;Cen	Menthol		G=36, T=30	G=0.5455, T=0.4545	0.00001	285.779	66	G=21/15, T=17
chr14:51204996	T/T	C	T	SNV	NIN	NIN:exonic:NM_020921.3	1	100	27	synonymous	NM_02092	c.5637G>	p.(=)	-0.73						rs11376	0.4827	YES	3.323E-1 (pos.51204996)		Ninein	GTP bindir	Autoimmune Diseases;Connec		Likely benign	C=0, T=44	C=0.0, T=1.0	0.00001	726.506	44	C=0/0, T=19/2
chr14:51223789	T/T	C	T	SNV	NIN	NIN:exonic:NM_020921.3	1	100	18	missense	NM_02092	c.3959G>	p.Gly1320Glu	4	1	98	0	0.07(COSM5428255),0.07(C		rs2073347,rs766808	0.2384	YES	0.0 (pos.51223787),6.68	acute_myeloic	Ninein	GTP bindir	Autoimmune Diseases;Connec		Benign	C=0, T=33	C=0.0, T=1.0	0.00001	504.598	33	C=0/0, T=23/1
chr3:52510592	A/A	G	A	SNV	NISCH	NISCH:exonic:NM_007184.3	1	100	8	missense	NM_00718	c.895G>A	p.Val299Ile	5.75	1	29	0.001	0.18(COSM41	PX domain	rs9856575	0.0207	YES	1.0 (pos.52510592)	neoplasm	Nischarin	Rac protei	Arrhythmias, Cardi	Agmatine,Moxonidine,Ti		G=0, A=26	G=0.0, A=1.0	0.00001	371.42	26	G=0/0, A=15/1

chr6:28227436	A/G	A	G	SNV	NKAPL	NKAPL:exonic:NM_001007531.2	1	51.85	1	missense	NM_00100	c.287A>G	p.Tyr96Cys	-0.09		194		0.09(COSM37	NF-kappa-B-a	rs12000	0.2043	YES	4.365E-1 (pos.28227436)	adenocarcinoma,carcinom		Notch sign Mental Disorders;Schizophrenia;Schizophren	A=13, G=14	A=0.4815, G=0.5185	0.00001	132.665	27	A=8/5, G=8/6
chr10:134598628	G/G	A	G	SNV	NKX6-2	NKX6-2:exonic:NM_177400.2	1	100	3	missense	NM_17740	c.626T>C	p.Val209Ala	4.86	1	64	0	0.08(COSM41	Homeobox do	rs2804003	0.0624	YES	9.999E-1 (pos.13459862	neoplasm	NK6 transcr	DNA binding,DNA binding transcription factor activity,RN	A=0, G=36	A=0.0, G=1.0	0.00001	563.744	36	A=0/0, G=10/2
chr16:3602227	A/A	AG	A	INDEL	NLRC3	NLRC3:splicesite_3:NM_178844.3	1	100	11	splicesite	NM_178844.3			3.84, 0.61					Leucine Rich	rs201180592,rs3980	3.00E-04	YES	1.0 (pos.3602227)	astrocytoma (	NLR family,	ATP binding,I-kappaB kinase/NF-kappaB signaling,T cell a	AG=0, A=68	AG=0.0, A=1.0	0.00001	1030.3	68	AG=0/0, A=41/
chr16:3613150	G/G	A	G	SNV	NLRC3	NLRC3:exonic:NM_178844.3	1	100	5	synonymous	NM_17884	c.1788T>C	p.(=)	-1.04				0.01(COSM37	Leucine Rich	rs8044743	0.4921	YES	8.447E-1 (pos.3613150)	adenocarcinoı	NLR family,	ATP binding,I-kappaB kinase/NF-kappaB signaling,T cell a	A=0, G=41	A=0.0, G=1.0	0.00001	664.7	41	A=0/0, G=21/2
chr19:56321414	A/A	C	A	SNV	NLRP11	NLRP11:exonic:NM_145007.3	1	100	5	missense	NM_14500	c.562G>T	p.Ala188Ser	0.83	0.51	99	0		Leucine Rich	rs299163	0.0658	YES	1.0 (pos.56321414)			ATP binding,RNA binding,oxidation-reduction process,ox	C=0, A=31	C=0.0, A=1.0	0.00001	465.818	31	C=0/0, A=17/1
chr11:7059960	C/C	A	C	SNV	NLRP14	NLRP14:exonic:NM_176822.3	1	100	2	missense	NM_17682	c.143A>C	p.Asn48Thr	-0.13	1	65	0		Leucine Rich	rs12801277	5.00E-04	YES	1.0 (pos.7059960)			ATP binding,cell differentiation,multicellular organism d	A=0, C=40	A=0.0, C=1.0	0.00001	644.286	40	A=0/0, C=25/1
chr11:7063755	T/T	C	T	SNV	NLRP14	NLRP14:exonic:NM_176822.3	1	100	4	synonymous	NM_17682	c.498C>T	p.(=)	0.13					Leucine Rich	rs7123944	0.0023	YES	1.0 (pos.7063755)			ATP binding,cell differentiation,multicellular organism d	C=0, T=73	C=0.0, T=1.0	0.00001	1140.19	73	C=0/0, T=40/3
chr11:7071031	A/A	G	A	SNV	NLRP14	NLRP14:exonic:NM_176822.3	1	100	6	synonymous	NM_17682	c.2253G>	p.(=)	0.47					Leucine Rich	rs1552726	0.0012	YES	1.0 (pos.7071031)			ATP binding,cell differentiation,multicellular organism d	G=0, A=23	G=0.0, A=1.0	0.00001	316.746	23	G=0/0, A=10/1
chr19:55494881	C/G	C	G	SNV	NLRP2	NLRP2:exonic:NM_001174081.2	1	50	6	synonymous	NM_00117	c.1815C>G	p.(=)	-5.36				0(COSM3757	Leucine Rich	rs11672113	0.3744	YES	2.44E-1;0.0;0.0 (pos.5549	acute_myeloid_leukaemia		ATP binding,Golgi apparatus,Pyrin domain binding,apopt	C=35, G=35	C=0.5, G=0.5	0.00001	355.229	70	C=24/11, G=24
chr2:241452198	T/C	T	C	SNV	ANKMY1	ANKMY1:intronic:NM_016552.4	1	50		intronic	NM_01655	c.1878+21	p.?	0.39					Ankyrin repea	rs12695025	0.338	YES	7.702E-1 (pos.241452198)			metal ion binding	T=13, C=13	T=0.5, C=0.5	0.00001	117.269	26	T=8/5, C=5/8
chr2:241680802	G/A	G	A	SNV	KIF1A	KIF1A:intronic:NM_001244008.1	1	57.14		intronic	NM_00124	c.3641-8C	p.?	1.96				0.08(COSM57	FHA domain,I	rs56024577	0.2506	YES	5.409E-1;0.0 (pos.24168(	adenocarcinoı	Kinesin fam	ATP bindin Charcot-Marie-Toot Adenosine Likely benign	G=9, A=12	G=0.4286, A=0.5714	0.00001	109.094	21	G=1/8, A=2/10
chr2:242007541	C/T	C	T	SNV	SNED1	SNED1:intronic:NM_001080437.2	1	45.83		intronic	NM_00108	c.3194+17	p.?	-0.63					Calcium-bind	rs62186648		YES			SUSHI, NIDC	calcium ion binding,cell-matrix adhesion,extracellular e	C=13, T=11	C=0.5417, T=0.4583	0.00001	93.4446	24	C=8/5, T=1/10
chr2:242142737	C/C	T	C	SNV	ANO7	ANO7:intronic:NM_001001891.3	1	100		intronic	NM_00100	c.886-11T	p.?	0.57					Calcium-acti	rs1179530	5.00E-04		1.0 (pos.242142737)			calcium activated galactosylceramide scrambling,calciu	T=0, C=54	T=0.0, C=1.0	0.00001	798.043	54	T=0/0, C=29/2
chr2:242142921	C/T	C	T	SNV	ANO7	ANO7:intronic:NM_001001891.3	1	50		intronic	NM_00100	c.1051+8C	p.?	-1.18					Calcium-acti	rs77640154	0.0061	YES	3.326E-1 (pos.242142921)			calcium activated galactosylceramide scrambling,calciu	C=11, T=12	C=0.4783, T=0.5217	0.00001	114.918	24	C=10/1, T=4/8
chr2:242143012	G/A	G	A	SNV	ANO7	ANO7:intronic:NM_001001891.3	1	59.09		intronic	NM_00100	c.1051+99	p.?	-2.18					Calcium-acti	rs190698845						calcium activated galactosylceramide scrambling,calciu	G=9, A=13	G=0.4091, A=0.5909	0.00001	122.554	22	G=7/2, A=4/9
chr2:242149073	A/A	G	A	SNV	ANO7	ANO7:intronic:NM_001001891.3	1	100		intronic	NM_00100	c.1521+23	p.?	-0.19					Calcium-acti	rs10192730,rs10556	0.2713	YES	0.0 (pos.242149071),4.594E-1 (pos.242149073)			calcium activated galactosylceramide scrambling,calciu	G=0, A=27	G=0.0, A=1.0	0.00001	389.984	27	G=0/0, A=18/9
chr1:247587531	G/G	A	G	SNV	NLRP3	NLRP3:exonic:NM_001079821.2	1	100	5	synonymous	NM_00107	c.786A>G	p.(=)	-0.16					Fish-specific	rs4925543	0.0464	YES	9.986E-1 (pos.247587531)		NLR family,	ATP bindin Anaphylaxis;Autoimmune Dise Benign	A=0, G=72	A=0.0, G=1.0	0.00001	1130.05	72	A=0/0, G=53/1
chr1:247587408	C/T	C	T	SNV	NLRP3	NLRP3:exonic:NM_001079821.2	1	57.58	5	synonymous	NM_00107	c.663C>T	p.(=)	-10.31				0(COSM5426	Fish-specific	rs7525979	0.0617	YES	1.469E-1;0.0 (pos.24758	acute_myeloic	NLR family,	ATP bindin Anaphylaxis;Autoimmune Dise Benign	C=14, T=19	C=0.4242, T=0.5758	0.00001	204.394	33	C=13/1, T=16/
chr2:242206956	T/T	C	T	SNV	HDLBP	HDLBP:intronic:NM_203346.4	1	100		intronic	NM_20334	c.-36-635	p.?	-0.01					KH domain	rs35334418		YES	9.924E-1 (pos.242206956)		High-densit	RNA bindir Autistic Disorder;Child Development Disorde	C=0, T=31	C=0.0, T=1.0	0.00001	465.818	31	C=0/0, T=24/7
chr19:56565164	A/A	G	A	SNV	NLRP5	NLRP5:exonic:NM_153447.4	1	100	13	missense	NM_15344	c.3289G>	p.Ala1097Thr	-0.25	0.5	58	0	0(COSM5445	Leucine Rich	rs3103057	0.0208	YES	7.987E-1 (pos.56565164)	squamous_cell_carcinoma		ATP binding,animal organ morphogenesis,apical cortex,(	G=0, A=53	G=0.0, A=1.0	0.00001	782.011	53	G=0/0, A=28/2
chr2:242593114	A/G	A	G	SNV	ATG4B	ATG4B:intronic:NM_013325.4	1	45.45		intronic	NM_01332	c.283+89A	p.?	-0.59					Peptidase fai	rs4567973		YES			Autophagy-	C-terminal protein lipidation,autophagosome assembly	A=34, G=3C	A=0.5313, G=0.4688	0.00001	314.756	66	A=2/32, G=3/2
chr2:242606036	C/C	G	C	SNV	ATG4B	ATG4B:intronic:NM_013325.4	1	100		intronic	NM_01332	c.539-24G	p.?	-0.86					Peptidase fai	rs6709768	0.419	YES	2.478E-1;0.0 (pos.242606036)		Autophagy-	C-terminal protein lipidation,autophagosome assembly	G=0, C=25	G=0.0, C=1.0	0.00001	352.932	25	G=0/0, C=8/17
chr19:56539240	C/T	C	T	SNV	NLRP5	NLRP5:exonic:NM_153447.4	1	98.36	7	synonymous	NM_15344	c.1641C>T	p.(=)	-0.42				0.01(COSM14	Leucine Rich	rs397977	0.451	YES	3.499E-1 (pos.56539240)	acute_myeloid_leukaemia		ATP binding,animal organ morphogenesis,apical cortex,(	C=1, T=60	C=0.0164, T=0.9836	0.00001	889.16	61	C=1/0, T=29/3
chr11:280221	C/C	A	C	SNV	NLRP6	NLRP6:exonic:NM_138329.2	1	100	4	missense	NM_13832	c.487A>C	p.Met163Leu	1.17	1	15	0	0(COSM3752	NACHT domai	rs6421985	0.1024	YES	2.39E-1 (pos.280221)	adenocarcinoma,carcinom		ATP binding,G-protein coupled receptor signaling pathwa	A=0, C=51	A=0.0, C=1.0	0.00001	752.93	51	A=0/0, C=44/7
chr11:280673	T/T	C	T	SNV	NLRP6	NLRP6:exonic:NM_138329.2	1	100	4	synonymous	NM_13832	c.939C>T	p.(=)	-0.74				0.07(COSM41	NACHT domai	rs12807092	0.1418	YES	3.929E-1 (pos.280673)	neoplasm		ATP binding,G-protein coupled receptor signaling pathwa	C=0, T=21	C=0.0, T=1.0	0.00001	281.218	21	C=0/0, T=15/6
chr2:24432667	G/G	C	G	SNV	ITSN2	ITSN2:intronic:NM_006277.2	1	100		intronic	NM_00627	c.4473+20	p.?	0.27					C2 domain,C	rs2303295	0.0836	YES	9.172E-1 (pos.24432667)		Intersectin	Rho guany Arthritis;Arthritis, Rheumatoid;Autoimmune	C=0, G=98	C=0.0, G=1.0	0.00001	1660.04	98	C=0/0, G=27/7
chr2:24475205	C/C	T	C	SNV	ITSN2	ITSN2:intronic:NM_006277.2	1	100		intronic	NM_00627	c.3120+9A	p.?	-0.74					C2 domain,C	rs4450560	0.0019		1.0 (pos.24475205)		Intersectin	Rho guany Arthritis;Arthritis, Rheumatoid;Autoimmune	T=0, C=20	T=0.0, C=1.0	0.00001	263.786	20	T=0/0, C=8/12
chr11:280816	TC/TC	AT	TC	MNV	NLRP6	NLRP6:exonic:NM_138329.2	2	100	4	missense	NM_13832	c.1082_10	p.Tyr361Phe	-0.17, -0.3	0.69	22	0	0.01(COSM39	NACHT domai	rs113686637,rs3701	0.2988	YES	2.521E-1 (pos.280816),2.	acute_myeloid_leukaemia		ATP binding,G-protein coupled receptor signaling pathwa	AT=0, TC=2	AT=0.0, TC=1.0	0.00001	371.42	26	AT=0/0, TC=5/
chr19:55451232	C/T	C	T	SNV	NLRP7	NLRP7:exonic:NM_001127255.1	1	56	4	missense	NM_00112	c.955G>A	p.Val319Ile	-1.18	1	29	0.01	0.01(COSM44	Leucine Rich	rs775882	0.2102	YES	1.527E-1 (pos.55451232)	acute_myeloid_leukaemia		ATP bindin Female Urogenital Diseases ar Benign/Likely	C=11, T=14	C=0.44, T=0.56	0.00001	134.35	25	C=8/3, T=9/5
chr2:25458546	T/T	C	T	SNV	DNMT3A	DNMT3A:intronic:NM_022552.4	1	100		intronic	NM_02255	c.2597+30	p.?	-3.79					C-5 cytosine-	rs2304429	0.4126	YES	2.892E-1 (pos.25458546)		DNA methyl	C-5 methy Bone Marrow Diseases;Brain Diseases;Brain	C=0, T=100	C=0.0, T=1.0	0.00001	1702.26	100	C=0/0, T=49/5
chr2:25466888	T/T	G	T	SNV	DNMT3A	DNMT3A:intronic:NM_022552.4	1	100		intronic	NM_02255	c.1852-37	p.?	0.88					C-5 cytosine-	rs2289093	0.2492	YES	6.506E-1 (pos.25466888)		DNA methyl	C-5 methy Bone Marrow Diseases;Brain Diseases;Brain	G=0, T=31	G=0.0, T=1.0	0.00001	465.819	31	G=0/0, T=22/9
chr19:55451797	C/T	C	T	SNV	NLRP7	NLRP7:exonic:NM_001127255.1	1	50	4	synonymous	NM_00112	c.390G>A	p.(=)	-2.88				0(COSM3757	Leucine Rich	rs775883	0.3338	YES	1.603E-1 (pos.55451797)	acute_myeloid_leukaemia		ATP bindin Female Urogenital Diseases ar Benign/Likely	C=35, T=35	C=0.5, T=0.5	0.00001	355.233	70	C=23/12, T=20
chr2:26420708	T/G	T	G	SNV	HADHA	HADHA:intronic:NM_000182.4	1	97.92		intronic	NM_00018	c.1393-62	p.?	2.44					3-hydroxyacy	rs13387855		YES			Hydroxyacy	3-hydroxy Arthritis;Cardiomyo NADH	T=1, G=47	T=0.0208, G=0.9792	0.00001	780.862	48	T=0/1, G=8/39
chr2:26537499	C/C	A	C	SNV	ADGRF3	ADGRF3:intronic:NM_001145168.1	1	100		intronic	NM_00114	c.923-8T>	p.?	0.02					7 transmemb	rs10187359	0.0191	YES	9.516E-1 (pos.26537499)			G-protein coupled receptor activity,G-protein coupled re	A=0, C=43	A=0.0, C=1.0	0.00001	705.844	43	A=0/0, C=27/1
chr19:56477687	G/G	A	G	SNV	NLRP8	NLRP8:exonic:NM_176811.2	1	100	5	synonymous	NM_17681	c.2322A>G	p.(=)	-0.72					Leucine Rich	rs306497	0.2295	YES	8.941E-1 (pos.56477687)			ATP binding,cellular_component,cytoplasm,molecular_f	A=0, G=36	A=0.0, G=1.0	0.00001	563.679	36	A=0/0, G=27/9
chr19:56477710	C/C	T	C	SNV	NLRP8	NLRP8:exonic:NM_176811.2	1	100	5	missense	NM_17681	c.2345T>C	p.Val782Ala	0.24	1	64	0	0(COSM3757	Leucine Rich	rs306496	0.487	YES	6.936E-1;0.0 (pos.56477	adenocarcinoma		ATP binding,cellular_component,cytoplasm,molecular_f	T=0, C=36	T=0.0, C=1.0	0.00001	563.73	36	T=0/0, C=27/9
chr2:26671544	G/G	A	G	SNV	DRC1	DRC1:intronic:NM_145038.4	1	100		intronic	NM_14503	c.1397-15	p.?	-3.14					Sperm tail,Sp	rs386644168,rs4072	0.1742	YES	2.674E-1 (pos.26671544)		Dynein regu	axonemal Bronchial Diseases;Bronchiect Benign	A=0, G=112	A=0.0, G=1.0	0.00001	1783.78	112	A=0/0, G=32/8
chr19:56487603	G/G	A	G	SNV	NLRP8	NLRP8:exonic:NM_176811.2	1	100	8	missense	NM_17681	c.2810A>G	p.Lys937Arg	-0.43	0.84	26	0.018	0(COSM3757	Leucine Rich	rs306481	0.4444	YES	6.66E-1 (pos.56487603)	adenocarcinoma		ATP binding,cellular_component,cytoplasm,molecular_f	A=0, G=30	A=0.0, G=1.0	0.00001	433.445	30	A=0/0, G=17/1
chr19:56459342	C/T	C	T	SNV	NLRP8	NLRP8:exonic:NM_176811.2	1	47.62	1	missense	NM_17681	c.74C>T	p.Pro25Leu	-0.44	0	98	0.026	0.01(COSM37	Leucine Rich	rs306507	0.4157	YES	8.006E-1 (pos.56459342)	adenocarcinoma		ATP binding,cellular_component,cytoplasm,molecular_f	C=33, T=30	C=0.5238, T=0.4762	0.00001	289.406	63	C=20/13, T=16
chr15:85201419	T/C	T	C	SNV	NMB	NMB:utr_5:NM_205858.1	1	50	1	utr_5	NM_20585	c.-36A>G	p.?	-0.65					Bombesin-lik	rs12904605		YES	6.522E-2 (pos.85201419)		Neuromedi	G-protein coupled receptor signaling pathway,arachidor	T=18, C=18	T=0.5, C=0.5	0.00001	185.818	36	T=3/15, C=1/1
chr3:48342882	T/T	C	T	SNV	NME6	NME6:utr_5:NM_005793.4	1	100	1	utr_5	NM_00579	c.-80G>A	p.?	-0.18						rs35297395	0.2942	YES			Nonmetast	ATP binding,CTP biosynthetic process,GTP biosynthetic p	C=0, T=26	C=0.0, T=1.0	0.00001	371.408	26	C=0/0, T=12/1
chr2:27263122	C/C	T	C	SNV	TMEM214	TMEM214:intronic:NM_017727.4	1	100		intronic	NM_01772	c.1791+56	p.?	0.01					TMEM214, C-	rs1866654	0.3721	YES			Transmemb	Golgi apparatus,apoptotic process,cytoplasmic microtu	T=0, C=54	T=0.0, C=1.0	0.00001	798.148	54	T=0/0, C=17/3
chr7:37907304	C/C	T	C	SNV	NME8	NME8:exonic:NM_016616.4	1	100	11	missense	NM_01661	c.622T>C	p.Cys208Arg	0.99	0.13	180	0.001	0.01(COSM41	Nucleoside d	rs10250905	0.2521	YES	5.651E-1 (pos.37907304)	neoplasm	NME/NM23	CTP biosyr Ciliary Motility Disorders;Otor Benign	T=0, C=25	T=0.0, C=1.0	0.00001	353.019	25	T=0/0, C=4/21
chr2:27324036	A/A	G	A	SNV	CGREF1,KHK	CGREF1:utr_3:NM_001166239.1, KHK:downstream:NM_006	1	100		intronic	NM_00648	c.*1418G>	p.?, p.?	0.22					EF-hand dom	rs1057394,rs386644208,rs7746		YES			Cell growth	ATP bindin Carbohydrate Metabolism, Inborn Errors:Con	G=0, A=114	G=0.0, A=1.0	0.00001	1815.31	114	G=0/0, A=63/5
chr17:43183028	G/A	G	A	SNV	NMT1	NMT1:utr_3:NM_021079.4	1	50	12	utr_3	NM_02107	c.1512G>	p.?	2.45					Myristoyl-Co	rs1053733	0.4308	YES	7.698E-1 (pos.43183028)		N-myristoyl	N-terminal peptidyl-glycine N-r (1-Methyl-1h-Imidazol-2-	G=14, A=14	G=0.5, A=0.5	0.00001	131.941	28	G=1/13, A=7/7
chr5:151771817	T/T	C	T	SNV	NMUR2	NMUR2:exonic:NM_020167.4	1	100	4	missense	NM_02016	c.1183G>	p.Ala395Thr	0.98	1	58	0		7 transmemb	rs1363422	0.0028		1.0 (pos.151771817)		Neuromedi	G-protein coupled receptor activity,G-protein coupled re	C=0, T=39	C=0.0, T=1.0	0.00001	623.98	39	C=0/0, T=14/2
chr2:27428815	G/G	C	G	SNV	SLC5A6	SLC5A6:intronic:NM_021095.2	1	100		intronic	NM_02109	c.579+79G	p.?	-0.91					Sodium:solut	rs1275526		YES			Solute carri	biotin metabolic process,bioti Biotin,Gabapentin Enaca	C=0, G=60	C=0.0, G=1.0	0.00001	900.288	60	C=0/0, G=22/3
chr2:27428877	G/G	C	G	SNV	SLC5A6	SLC5A6:intronic:NM_021095.2	1	100		intronic	NM_02109	c.579+17G	p.?	-0.36					Sodium:solut	rs1275527	0.0651	YES	9.986E-1 (pos.27428877)		Solute carri	biotin metabolic process,bioti Biotin,Gabapentin Enaca	C=0, G=54	C=0.0, G=1.0	0.00001	794.893	54	C=0/0, G=16/3
chr1:881627	G/A	G	A	SNV	NOC2L	NOC2L:exonic:NM_015658.3	1	50	16	synonymous	NM_01565	c.1843C>T	p.(=)	4.09				0.79(COSM13	Noc2p family	rs2272757	0.3621	YES	6.56E-1 (pos.881627)	adenocarcinoı	Nucleolar c	Noc1p-Noc2p complex,Noc2p-Noc3p complex,RNA bindi	G=14, A=14	G=0.5, A=0.5	0.00001	131.95	28	G=4/10, A=2/1
chr2:27435374	A/A	G	A	SNV	ATRAID,SLC5A6	SLC5A6:upstream:NM_021095.2, ATRAID:intronic:NM_0011	1	100		intronic	NM_02109	c.-4856C>	p.?, p.?	0.33					Human growt	rs11608	0.2879	YES	8.459E-1 (pos.27435374)		Solute carri	biotin metabolic process,bioti Biotin,Gabapentin Enaca	G=0, A=20	G=0.0, A=1.0	0.00001	263.785	20	G=0/0, A=11/9
chr2:27606098	G/G	A	G	SNV	PPM1G	PPM1G:intronic:NM_177983.2	1	100		intronic	NM_17798	c.1201+13	p.?	0.53					Protein phos	rs2384629					Protein pho	cell cycle arrest,cytoplasm,membrane,metal ion binding	A=0, G=26	A=0.0, G=1.0	0.00001	370.975	26	A=0/0, G=16/1
chr2:276908	G/G	C	G	SNV	ACP1	ACP1:intronic:NM_004300.3	1	100		intronic	NM_00430	c.294-72C	p.?	0.51					Low molecul	rs7568288		YES			Acid phospl	acid phosphatase activity,cyto 4-Nitrophenyl Phosphate	C=0, G=117	C=0.0, G=1.0	0.00001	1874.17	117	C=0/0, G=65/5
chr2:276942	G/G	A	G	SNV	ACP1	ACP1:intronic:NM_004300.3	1	100		intronic	NM_00430	c.294-38A	p.?	0.63					Low molecul	rs7573495	0.339	YES	5.95E-1 (pos.276942)		Acid phospl	acid phosphatase activity,cyto 4-Nitrophenyl Phosphate	A=0, G=117	A=0.0, G=1.0	0.00001	1874.22	117	A=0/0, G=63/5
chr16:50745199	T/T	C	T	SNV	NOD2	NOD2:exonic:NM_022162.2	1	100	4	synonymous	NM_02216	c.1377C>T	p.(=)	-0.04				0.04(COSM37	Caspase recr	rs2066843	0.271	YES	6.626E-3;0.0 (pos.50745	hepatocellula	Nucleotide-	ATP bindin Arthritis;Arthritis, Psoriatic;Au Benign	C=0, T=21	C=0.0, T=1.0	0.00001	281.206	21	C=0/0, T=11/1
chr7:156755862	T/C	T	C	SNV	NOM1	NOM1:exonic:NM_138400.1	1	57.45	6	missense	NM_13840	c.1904T>C	p.Val635Ala	-0.91	0.75	64	0		MA3 domain,	rs61738590	1.00E-04		0.0 (pos.156755862)		Nucleolar p	RNA bindir Hemic and Lymphatic Diseases;Immune Syst	T=20, C=27	T=0.4255, C=0.5745	0.00001	326.52	47	T=11/9, C=11/
chr19:48255804	A/G	A	G	SNV	NOP53	NOP53:exonic:NM_015710.4	1	50	6	synonymous	NM_01571	c.705A>G	p.(=)	-2.15				0.03(COSM54	Nop53 (60S ri	rs1042558	0.4455	YES	5.137E-1 (pos.48255804)	acute_myeloid_leukaemia		5S rRNA binding,DNA repair,RNA binding,cellular respons	A=19, G=19	A=0.5, G=0.5	0.00001	199.884	38	A=4/15, G=10/
chr2:27839107	C/C	T	C	SNV	ZNF512	ZNF512:intronic:NM_032434.3	1	100		intronic	NM_03243	c.1234-22	p.?	-0.44					Zinc finger, C	rs6737921	0.4947	YES	8.468E-1 (pos.27839107)			DNA binding,metal ion binding,nucleic acid binding,nucl	T=0, C=52	T=0.0, C=1.0	0.00001	766.992	52	T=0/0, C=32/2
chr2:28718897	G/G	A	G	SNV	PLB1	PLB1:upstream:NM_153021.4	1	100		intronic	NM_15302	c.-85A>G	p.?	0.28						rs4665423		YES			Phospholip	apical pla Arthritis;Arthritis, Rheumatoid;Autoimmune	A=0, G=77	A=0.0, G=1.0	0.00001	1229.45	77	A=0/0, G=45/3
chr2:28748857	C/C	T	C	SNV	PLB1	PLB1:intronic:NM_153021.4	1	100		intronic	NM_15302	c.325+45T	p.?	0.26					GDSL-like Lip	rs4666085	0.0299	YES	9.943E-1 (pos.28748857)		Phospholip	apical pla Arthritis;Arthritis, Rheumatoid;Autoimmune	T=0, C=26	T=0.0, C=1.0	0.00001	368.149	26	T=0/0, C=13/1
chr2:28752555	C/C	T	C	SNV	PLB1	PLB1:intronic:NM_153021.4	1	100		intronic	NM_15302	c.417-40T	p.?	-1.35					GDSL-like Lip	rs4473343	0.0674	YES	9.941E-1 (pos.28752555)		Phospholip	apical pla Arthritis;Arthritis, Rheumatoid;Autoimmune	T=0, C=20	T=0.0, C=1.0	0.00001	263.785	20	T=0/0, C=10/1
chr2:28752727	G/G	C	G	SNV	PLB1	PLB1:intronic:NM_153021.4	1	100		intronic	NM_15302	c.468+81C	p.?	-0.22					GDSL-like Lip	rs6710800		YES			Phospholip	apical pla Arthritis;Arthritis, Rheumatoid;Autoimmune	C=0, G=21	C=0.0, G=1.0	0.00001	281.214	21	C=0/0, G=8/13
chr2:28752741	G/G	A	G	SNV	PLB1	PLB1:intronic:NM_153021.4	1	100		intronic	NM_15302	c.468+95A	p.?	-1.13					GDSL-like Lip	rs6725577		YES			Phospholip	apical pla Arthritis;Arthritis, Rheumatoid;Autoimmune	A=0, G=21	A=0.0, G=1.0	0.00001	281.234	21	A=0/0, G=8/13
chr14:24771256	C/C	T	C	SNV	NOP9	NOP9:exonic:NM_174913.2	1	100	4	synonymous	NM_17491	c.894T>C	p.(=)	0.54				0.62(COSM4148039)		rs4247001	0.0352	YES	8.565E-1 (pos.24771256)	neoplasm		RNA bindir Breast Diseases;Breast Neoplasms;Neoplasr	T=0, C=23	T=0.0, C=1.0	0.00001	315.369	23	T=0/0, C=11/1
chr12:117701714	G/G	A	G	SNV	NOS1	NOS1:exonic:NM_001204218.1	1	100	13	synonymous	NM_00120	c.2202T>C	p.(=)	-0.38				0.02(COSM41	FAD binding c	rs2293054	0.2866	YES	7.729E-1;0.0 (pos.11770	neoplasm	NANOS, Dro	FMN bindi Amphetamine-Rela 2'-Monophosphoadenos	A=0, G=24	A=0.0, G=1.0	0.00001	334.792	24	A=0/0, G=17/7
chr2:29083850	A/A	C	A	SNV	TRMT61B	TRMT61B:intronic:NM_017910.3	1	100		intronic	NM_01791	c.993+134	p.?	-0.22					Protein-L-iso	rs13025081		YES				mitochondrial matrix,mitochondrial tRNA methylation,n	C=0, A=49	C=0.0, A=1.0	0.00001	726.564	49	C=0/0, A=26/2
chr17:26089867	C/C	T	C	SNV	NOS2	NOS2:exonic:NM_000625.4	1	100	22	synonymous	NM_00062	c.2757A>G	p.(=)	-0.63				0(COSM1243	FAD binding c	rs1060826	0.3859	YES	7.451E-1 (pos.26089867)	adenocarcinoı	Nanos, Dro	FMN bindi Abnormalities, Mul (2S)-2-methyl-2,3-dihydr	T=0, C=30	T=0.0, C=1.0	0.00001	446.639	30	T=0/0, C=18/1
chr17:26096597	G/A	G	A	SNV	NOS2	NOS2:exonic:NM_000625.4	1	50	16	missense	NM_00062	c.1823C>T	p.Ser608Leu	6.2	0.01	145	0.918	0.97(COSM40	FAD binding c	rs2297518	0.1953	YES	1.432E-1 (pos.26096597)	acute_myeloic	Nanos, Dro	FMN bindi Abnormalities, Mul (2S)-2-methyl-2,3-dihydr	G=14, A=14	G=0.5, A=0.5	0.00001	131.941	28	G=8/6, A=3/11
chr2:29287937	C/CTGC	C	CTGC	INDEL	C2orf71	C2orf71:intronic:NM_001029883.2	3	51.92		intronic	NM_00102	c.3669-4G	p.?	1.41					Retinal prote	rs59833221			0.0 (pos.29287937)		Chromosom	cilium,phc Congenital, Hereditary, and Neonatal Diseas	C=50, CTGC	C=0.4808, CTGC=0.5	0.00001	559.714	104	C=26/24, CTGC
chr6:32185796	C/T	C	T	SNV	NOTCH4	NOTCH4:exonic:NM_004557.3	1	59.09	9	missense	NM_00455	c.1600G>	p.Gly534Ser	-0.72	0.05	56	0.847	0.15(COSM50	Ankyrin repe	rs8192591	0.036	YES	6.115E-3 (pos.32185796)	haemangiobla	Notch, Dros	Golgi mem Adenocarcinoma;Asthma;Breast Diseases;Br	C=18, T=26	C=0.4091, T=0.5909	0.00001	312.072	44	C=7/11, T=15/
chr19:46443886	A/A	C	A	SNV	NOVA2	NOVA2:exonic:NM_002516.3	1	100	4	synonymous	NM_00251	c.714G>T	p.(=)	0.5				0.75(COSM37	KH domain	rs1047061	0.3244	YES	8.223E-1 (pos.46443886)	adenocarcinoı	NOVA alterr	RNA binding,mRNA splicing, via spliceosome,nucleus,reg	C=0, A=22	C=0.0, A=1.0	0.00001	298.888	22	C=0/0, A=12/1
chr2:29498210	G/G	A	G	SNV	ALK	ALK:intronic:NM_004304.4	1	100		intronic	NM_00430	c.1912+58	p.?	0.72					Glycine rich c	rs4589708		YES			Anaplastic l	ATP bindin Adenocarcinoma;A ATP,Alectinib,Ceritinib,C	A=0, G=48	A=0.0, G=1.0	0.00001	810.427	48	A=0/0, G=23/2
chr18:21119777	A/A	G	A	SNV	NPC1	NPC1:exonic:NM_000271.4	1	100	18	synonymous	NM_00027	c.2793C>T	p.(=)	4.74				0.98(COSM43	MMPL family	rs1140458	0.4698	YES	6.266E-1 (pos.21119777)	acute_myeloic	NPC1 gene,	Golgi appa Arterial Occlusive Diseases;Ar Benign/Likely	G=0, A=88	G=0.0, A=1.0	0.00001	1451.89	88	G=0/0, A=51/3
chr2:30790818	T/T	G	T	SNV	LCLAT1	LCLAT1:intronic:NM_001002257.2	1	100		intronic	NM_00100	c.512-108	p.?	-0.97					Acyltransfera	rs6759011		YES			Lysocardiol	1-acylglycerol-3-phosphate O-acyltransferase activity,CI	G=0, T=123	G=0.0, T=1.0	0.00001	1993.76	123	G=0/0, T=61/6
chr2:30791152	G/G	A	G	SNV	LCLAT1	LCLAT1:intronic:NM_001002257.2	1	100		intronic	NM_00100	c.628+110	p.?	0.09					Acyltransfera	rs6749074		YES			Lysocardiol	1-acylglycerol-3-phosphate O-acyltransferase activity,CI	A=0, G=37	A=0.0, G=1.0	0.00001	583.488	37	A=0/0, G=15/2
chr2:31133939	G/C	G	C	SNV	GALNT14	GALNT14:intronic:NM_001253826.1	1	57.14		intronic	NM_00125	c.1516-14	p.?	0.52					Glycosyl tran	rs2278079,rs750350	0.2433	YES	0.0 (pos.31133936),2.162E-1;0.0 (pos.31		UDP-N-acet	Golgi mem Congenital, Hereditary, and Neonatal Diseas	G=9, C=12	G=0.4286, C=0.5714	0.00001	112.836	21	G=8/1, C=6/6
chr2:31467351	T/A	T	A	SNV	EHD3	EHD3:intronic:NM_014600.2	1	50		intronic	NM_01460	c.404+35T	p.?	0.07					50S ribosome	rs2295473	0.4234	YES	8.088E-1 (pos.31467351)		EH domain-	ATP bindin Leukemia;Leukemia, Myeloid;Leukemia, Mye	T=15, A=15	T=0.5, A=0.5	0.00001	137.114	30	T=12/3, A=8/7
chr2:31571241	C/C	G	C	SNV	XDH	XDH:intronic:NM_000379.3	1	100		intronic	NM_00037	c.3052-12	p.?	0.23					2Fe-2S bindir	rs13415401	0.2405	YES	8.503E-1 (pos.31571241)		Xanthine de	2 iron, 2 su Autistic Disorder;Bl Aldesleuki Benign	G=0, C=60	G=0.0, C=1.0	0.00001	903.528	60	G=0/0, C=37/2
chr18:21140432	C/C	T	C	SNV	NPC1	NPC1:exonic:NM_000271.4	1	100	6	missense	NM_00027	c.644A>G	p.His215Arg	0.59	0.5	29	0		MMPL family	rs1805081	0.4026	YES	2.384E-1 (pos.21140432)		NPC1 gene,	Golgi appa Arterial Occlusive Diseases;Ar Benign/Likely	T=0, C=26	T=0.0, C=1.0	0.00001	371.415	26	T=0/0, C=11/1
chr2:32369901	C/C	T	C	SNV	SPAST	SPAST:intronic:NM_014946.3	1	100		intronic	NM_01494	c.1617-10	p.?	-1.15					ATPase famil	rs2365556		YES			Spastin	ATP bindin Congenital Abnormalities;Congenital, Heredi	T=0, C=20	T=0.0, C=1.0	0.00001	263.786	20	T=0/0, C=11/9
chr2:32620722	A/T	A	T	SNV	BIRC6	BIRC6:intronic:NM_016252.3	1	53.49		intronic	NM_01625	c.1034+61	p.?	0.53					Baculoviral I/	rs3731581	0.0943	YES			Baculoviral	Flemming body,Golgi apparatus,apoptotic process,cell c	A=40, T=46	A=0.4651, T=0.5349	0.00001	496.542	86	A=17/23, T=24
chr2:32712875	C/C	A	C	SNV	BIRC6	BIRC6:intronic:NM_016252.3	1	100		intronic	NM_01625	c.7968+7A	p.?	-0.71					Baculoviral I/	rs2366891		YES	1.0 (pos.32712875)		Baculoviral	Flemming body,Golgi apparatus,apoptotic process,cell c	A=0, C=73	A=0.0, C=1.0	0.00001	1150.04	73	A=0/0, C=30/4
chr7:44579826	C/T	C	T	SNV	NPC1L1	NPC1L1:exonic:NM_013389.2	1	57.89	2	missense	NM_01338	c.170G>A	p.Cys57Tyr	6.75	0	194	1		Niemann-Picl	rs150484803	1.00E-04		0.0;0.0 (pos.44579826)		NPC1-like 1	Rab GTPase binding,apical pla Ezetimibe	C=16, T=22	C=0.4211, T=0.5789	0.00001	249.054	38	C=5/11, T=12/
chr2:32961745	A/A	T	A	SNV	TTC27	TTC27:intronic:NM_017735.4	1	100		intronic	NM_01773	c.1330-16	p.?	-0.11					Tetratricope	rs10200333	3.00E-04		1.0;0.0 (pos.32961745)			biological_process,cellular_component,molecular_func	T=0, A=27	T=0.0, A=1.0	0.00001	388.547	27	T=0/0, A=12/1
chr1:5926507	T/C	T	C	SNV	NPHP4	NPHP4:exonic:NM_015102.4	1	45.45	26	synonymous	NM_01510	c.3570A>G	p.(=)	0.29				0.02(COSM1126976)		rs555164	0.4197	YES	3.327E-1 (pos.5926507)	adenocarcinoı	Nephrocyst	actin cyto Congenital, Hereditary, and Ne Benign	T=18, C=15	T=0.5455, C=0.4545	0.00001	143.084	33	T=14/4, C=12/
chr2:33413949	A/A	G	A	SNV	LTBP1	LTBP1:intronic:NM_206943.2	1	100		intronic	NM_20694	c.1701+31	p.?	-0.37					Calcium-bind	rs4542826	0.4374	YES	4.334E-1 (pos.33413949)		Latent tran	aorta development,calcium ion binding,cellular protein	G=0, A=67	G=0.0, A=1.0	0.00001	1034.43	67	G=0/0, A=37/3
chr1:179544898	C/C	T	C	SNV	NPHS2	NPHS2:exonic:NM_014625.3	1	100	1	synonymous	NM_01462	c.102A>G	p.(=)	-0.45				0.01(COSM37	SPFH domain	rs1079292	0.0067	YES	9.812E-1 (pos.17954489	adenocarcinoı	Podocin	actin cyto Albuminuria;Female Urogenit Benign	T=0, C=56	T=0.0, C=1.0	0.00001	832.101	56	T=0/0, C=15/4
chr2:3469506	C/C	T	C	SNV	TRAPPC12	TRAPPC12:intronic:NM_016030.5	1	100		intronic	NM_01603	c.1776+40	p.?	-3.06					Tetratricope	rs35561008	0.1148	YES	1.561E-2 (pos.3469506)		Trafficking p	ER to Golgi vesicle-mediated transport,Golgi organizatio	T=0, C=226	T=0.0, C=1.0	0.00001	3753.58	226	T=0/0, C=89/1
chr17:79596811	T/T	C	T	SNV	NPLOC4	NPLOC4:exonic:NM_017921.3	1	100	2	synonymous	NM_01792	c.36G>A	p.(=)	-3.1				0.01(COSM37	NPL4 family,l	rs9894429	0.4618	YES	7.705E-1 (pos.79596811)	adenocarcinoma		Golgi organization,UFD1-NPL4 complex,VCP-NPL4-UFD1	C=0, T=21	C=0.0, T=1.0	0.00001	281.235	21	C=0/0, T=15/6
chr4:106861730	G/G	A	G	SNV	NPNT	NPNT:exonic:NM_001184691.1	1	100	8	missense	NM_00118	c.790A>G	p.Ile264Val	0.59	0.12	29	0		Calcium-bind	rs4340795	0.0905	YES	9.994E-1 (pos.106861730)		Nephronect	basement membrane,branching involved in ureteric buc	A=0, G=34	A=0.0, G=1.0	0.00001	524.173	34	A=0/0, G=14/2
chr2:3504819	G/G	A	G	SNV	ADI1	ADI1:intronic:NM_018269.3	1	100		intronic	NM_01826	c.241-55T	p.?	-0.17					ARD/ARD' fan	rs34240449		YES			Acireductor	L-methion Genital Diseases, N Isopropyl Alcohol	A=0, G=94	A=0.0, G=1.0	0.00001	1576.12	94	A=0/0, G=54/4
chr2:3623540	G/G	A	G	SNV	RPS7	RPS7:intronic:NM_001011.3	1	100		intronic	NM_00101	c.147+62A	p.?	-1.57					Ribosomal pr	rs3087873	0.2348	YES			Ribosomal	90S prerib Anemia;Anemia, Aplastic;Anemia, Diamond-l	A=0, G=109	A=0.0, G=1.0	0.00001	1718.93	109	A=0/0, G=49/6
chr2:3625393	G/A	G	A	SNV	RPS7	RPS7:intronic:NM_001011.3	1	45.83		intronic	NM_00101	c.356+29G	p.?	0.12					Ribosomal pr	rs113127192	0.0126	YES	0.0 (pos.3625393)		Ribosomal	90S prerib Anemia;Anemia, Aplastic;Anemia, Diamond-l	G=13, A=11	G=0.5417, A=0.4583	0.00001	93.4399	24	G=9/4, A=5/6
chr2:37088083	T/T	C	T	SNV	STRN	STRN:intronic:NM_003162.3	1	100		intronic	NM_00316	c.1669+19	p.?	-1.37					Striatin famil	rs2540935		YES			Striatin, cal	Wnt signaling pathway,armadillo repeat domain binding	C=0, T=64	C=0.0, T=1.0	0.00001	958.435	64	C=0/0, T=41/2
chr2:37094945	G/G	A	G	SNV	STRN	STRN:intronic:NM_003162.3	1	100		intronic	NM_00316	c.1547+12	p.?	-1.38					Striatin famil	rs2540923	0.0129	YES	9.995E-1 (pos.37094945)		Striatin, cal	Wnt signaling pathway,armadillo repeat domain binding	A=0, G=33	A=0.0, G=1.0	0.00001	504.256	33	A=0/0, G=14/1
chr17:78444658	G/G	T	G	SNV	NPTX1	NPTX1:exonic:NM_002522.3	1	100	5	synonymous	NM_00252	c.1254A>C	p.(=)	-6.13				0.01(COSM11	Concanavalir	rs12943620	0.2181	YES	7.647E-1;0.0 (pos.78444(	adenocarcinoı	Pentraxin I,	axonogenesis involved in innervation,central nervous sy	T=0, G=38	T=0.0, G=1.0	0.00001	603.776	38	T=0/0, G=14/2
chr2:37317869	T/T	C	T	SNV	GPATCH11	GPATCH11:intronic:NM_174931.3	1	100		intronic	NM_17493	c.394+4C>	p.?	0.15					Domain of un	rs4670654		YES	1.0 (pos.37317869)			condensed chromosome kinetochore,kinetochore,nucle	C=0, T=21	C=0.0, T=1.0	0.00001	281.235	21	C=0/0, T=13/8
chr2:37402270	G/G	C	G	SNV	SULT6B1	SULT6B1:intronic:NM_001032377.1	1	100		intronic	NM_00103	c.510+5G>	p.?	3.19					Sulfotransfer	rs11569755	0.3629	YES	2.019E-1 (pos.37402270)		Sulfotransfe	3'-phosphoadenosine 5'-phosphosulfate metabolic proc	C=0, G=25	C=0.0, G=1.0	0.00001	353.018	25	C=0/0, G=15/1
chr2:37402373	G/G	A	G	SNV	SULT6B1	SULT6B1:intronic:NM_001032377.1	1	100		intronic	NM_00103	c.416-4T>	p.?	2.16					Sulfotransfer	rs11569758	0.3629	YES	2.045E-1;0.0 (pos.37402373)		Sulfotransfe	3'-phosphoadenosine 5'-phosphosulfate metabolic proc	A=0, G=27	A=0.0, G=1.0	0.00001	389.988	27	A=0/0, G=15/1
chr7:24325009	A/A	G	A	SNV	NPY	NPY:exonic:NM_000905.3	1	100	2	synonymous	NM_00090	c.150G>A	p.(=)	-0.34				0.14(COSM14	Pancreatic hc	rs5573	0.4999	YES	6.555E-1;0.0 (pos.24325(	adenocarcinoı	Neuropepti	G-protein Airway Obstruction Alpha-Aminoisobutyric A	G=0, A=99	G=0.0, A=1.0	0.00001	1681.1	99	G=0/0, A=63/3
chr7:24329133	T/T	C	T	SNV	NPY	NPY:exonic:NM_000905.3	1	100	3	synonymous	NM_00090	c.204C>T	p.(=)	-0.35				0.2(COSM376	Pancreatic hc	rs5574	0.4685	YES	3.558E-1 (pos.24329133)	acute_myeloic	Neuropepti	G-protein Airway Obstruction Alpha-Aminoisobutyric A	C=0, T=23	C=0.0, T=1.0	0.00001	316.747	23	C=0/0, T=15/8
chr2:3749943	T/TCTC	T	TCTC	INDEL	ALLC	ALLC:intronic:NM_018436.3	3	50		intronic	NM_01843	c.976-10T	p.?	0.67					Allantoicase repeat						Allantoicas	allantoicase activity,allantoin catabolic process	T=12, TCTC	T=0.5, TCTC=0.5	0.00001	106.71	24	T=12/0, TCTC=
chr2:38178362	T/C	T	C	SNV	RMDN2,RMDN2-AS1	RMDN2-AS1:intronic_nc:NR_102712.1, RMDN2:intronic:NM	1	50		intronic	NR_10271	c.18-14T>	p.?	1.1						rs2197561	0.2516	YES	6.944E-1 (pos.38178362)			cytosol,integral component of membrane,microtubule,p	T=11, C=11	T=0.5, C=0.5	0.00001	94.7066	22	T=10/1, C=8/3
chr3:24006477	A/A	T	A	SNV	NR1D2	NR1D2:exonic:NM_005126.4	1	100	6	missense	NM_00512	c.1156T>A	p.Leu386Met	6.02	1	15	0.001	0.49(COSM45	Ligand-bindir	rs4858097			1.0 (pos.24006477)	squamous_cel	Nuclear rec	DNA bindi Endometriosis;Female Urogenital Diseases;F	T=0, A=26	T=0.0, A=1.0	0.00001	370.75	26	T=0/0, A=7/19
chr2:39045885	T/T	C	T	SNV	DHX57	DHX57:intronic:NM_198963.2	1	100		intronic	NM_19896	c.3471+13	p.?	-1.41					DEAD/DEAH b	rs10176932		YES				ATP binding,ATP-dependent RNA helicase activity,RNA bi	C=0, T=25	C=0.0, T=1.0	0.00001	353.015	25	C=0/0, T=16/9
chr3:119533910	T/C	T	C	SNV	NR1I2	NR1I2:exonic:NM_022002.2	1	95.45	6	synonymous	NM_02200	c.996T>C	p.(=)	-0.46				0(COSM4157	Ligand-bindir	rs4058490	1.00E-04		1.0 (pos.119533910)	neoplasm	Nuclear rec	DNA bindi Adenocarcinoma;B Docetaxel,Erlotinib,Estra	T=1, C=21	T=0.0455, C=0.9545	0.00001	271.83	22	T=0/1, C=3/18
chr19:17351535	A/A	G	A	SNV	NR2F6	NR2F6:exonic:NM_005234.3	1	100	2	synonymous	NM_00523	c.318C>T	p.(=)	2.69				0.9(COSM375	Ligand-bindir	rs2288539	0.1081	YES	2.323E-1 (pos.17351535)	adenocarcinoı	Nuclear rec	DNA bindi Breast Diseases;Breast Neoplasms;Endomet	G=0, A=31	G=0.0, A=1.0	0.00001	465.816	31	G=0/0, A=20/1
chr4:149356516	G/G	A	G	SNV	NR3C2	NR3C2:exonic:NM_000901.4	1	100	2	synonymous	NM_00090	c.1497T>C	p.(=)	0.8					Ligand-bindir	rs5525	0.1091	YES	8.567E-1;0.0 (pos.149356516)		Nuclear rec	DNA bindi Cardiomegaly;Card (11-BETA)- Benign	A=0, G=70	A=0.0, G=1.0	0.00001	1092.09	70	A=0/0, G=37/3
chr10:115393929	A/A	G	A	SNV	NRAP	NRAP:exonic:NM_001261463.1	1	100	15	missense	NM_00126	c.1469C>T	p.Ser490Leu	2.95	0.02	145	0.008	0.89(COSM11	LIM domain,l	rs3189030	0.3084	YES	4.384E-1 (pos.11539392	acute_myeloic	Nebulin-rel	actin binding,biological_process,fascia adherens,metal	G=0, A=143	G=0.0, A=1.0	0.00001	2408.64	143	G=0/0, A=92/5
chr2:39224351	T/T	G	T	SNV	SOS1	SOS1:intronic:NM_005633.3	1	100		intronic	NM_00563	c.2964+43	p.?	-0.4					Core histone	rs1454223	0.0631	YES	8.246E-1 (pos.39224351)		Son of sevei	B cell hom Cardiovascular Abnormalities; Benign	G=0, T=187	G=0.0, T=1.0	0.00001	3128.38	187	G=0/0, T=94/9
chr2:39281730	C/C	G	C	SNV	SOS1	SOS1:intronic:NM_005633.3	1	100		intronic	NM_00563	c.720+25C	p.?	-1.31					Core histone	rs997344	0.0791	YES	8.3E-1 (pos.39281730)		Son of sevei	B cell hom Cardiovascular Abnormalities; Benign	G=0, C=39	G=0.0, C=1.0	0.00001	623.98	39	G=0/0, C=20/1
chr2:39282079	G/G	A	G	SNV	SOS1	SOS1:intronic:NM_005633.3	1	100		intronic	NM_00563	c.511-115	p.?	0.43					Core histone	rs2060987		YES			Son of sevei	B cell hom Cardiovascular Abnormalities;Cardiovascula	A=0, G=24	A=0.0, G=1.0	0.00001	334.792	24	A=0/0, G=4/20
chr10:115405615	C/C	T	C	SNV	NRAP	NRAP:exonic:NM_001261463.1	1	100	11	missense	NM_00126	c.1079A>G	p.Gln360Arg	1.74	0.54	43	0	0.43(COSM49	LIM domain,l	rs3127106	0.385	YES	7.177E-1 (pos.11540561	adenocarcinoı	Nebulin-rel	actin binding,biological_process,fascia adherens,metal	T=0, C=49	T=0.0, C=1.0	0.00001	835.802	49	T=0/0, C=27/2
chr2:43902511	C/C	G	C	SNV	C1GALT1C1L,PLEKHH2	PLEKHH2:intronic:NM_172069.3, C1GALT1C1L:utr_3:NM_00	1	100		intronic	NM_17206	c.124-349	p.?, p.?	-1.61					FERM central	rs755862	0.2792	YES	2.874E-1 (pos.43902511)		Pleckstrin h	actin binding,cortical actin cytoskeleton,cytoplasm,cyto	G=0, C=57	G=0.0, C=1.0	0.00001	829.6	57	G=0/0, C=43/1
chr10:115405664	T/T	C	T	SNV	NRAP	NRAP:exonic:NM_001261463.1	1	100	11	missense	NM_00126	c.1030G>	p.Ala344Thr	1.97	0.27	58	0.234	0.63(COSM49	LIM domain,l	rs3121478	0.3851	YES	7.176E-1 (pos.11540566	ductal_carcinc	Nebulin-rel	actin binding,biological_process,fascia adherens,metal	C=0, T=49	C=0.0, T=1.0	0.00001	831.616	49	C=0/0, T=27/2
chr10:115411614	G/G	A	G	SNV	NRAP	NRAP:exonic:NM_001261463.1	1	100	7	missense	NM_00126	c.623T>C	p.Val208Ala	2.21	1	64	0		LIM domain,l	rs2154028	0.1078	YES	8.991E-1 (pos.115411614)		Nebulin-rel	actin binding,biological_process,fascia adherens,metal	A=0, G=38	A=0.0, G=1.0	0.00001	603.784	38	A=0/0, G=26/1
chr10:115417289	A/A	G	A	SNV	NRAP	NRAP:exonic:NM_001261463.1	1	100	4	synonymous	NM_00126	c.300C>T	p.(=)	-0.28					LIM domain,l	rs1891762	0.1077	YES	8.994E-1;0.0 (pos.115417289)		Nebulin-rel	actin binding,biological_process,fascia adherens,metal	G=0, A=112	G=0.0, A=1.0	0.00001	1776.44	112	G=0/0, A=61/5
chr2:43953664	A/G	A	G	SNV	PLEKHH2	PLEKHH2:intronic:NM_172069.3	1	95.45		intronic	NM_17206	c.2721+74	p.?	3.01					FERM central	rs7574334		YES			Pleckstrin h	actin binding,cortical actin cytoskeleton,cytoplasm,cyto	A=1, G=21	A=0.0455, G=0.9545	0.00001	271.626	22	A=1/0, G=5/16
chr2:43991357	C/C	A	C	SNV	PLEKHH2	PLEKHH2:intronic:NM_172069.3	1	100		intronic	NM_17206	c.4159-10	p.?	0.67				0.21(COSM40	FERM central	rs7565517	0.2251	YES	4.49E-1 (pos.43991357)	acute_myeloic	Pleckstrin h	actin binding,cortical actin cytoskeleton,cytoplasm,cyto	A=0, C=58	A=0.0, C=1.0	0.00001	867.339	58	A=0/0, C=32/2
chr2:44016885	C/G	C	G	SNV	DYNC2LI1	DYNC2LI1:intronic:NM_001193464.1	1	57.14		intronic	NM_00119	c.320+25C	p.?	0.36					Dynein light i	rs3815994	0.0765	YES	1.755E-1 (pos.44016885)		Dynein, cyt	ATP-dependent microtubule motor activity, minus-end-d	C=12, G=16	C=0.4286, G=0.5714	0.00001	152.042	28	C=3/9, G=10/6
chr10:33552696	G/G	A	G	SNV	NRP1	NRP1:exonic:NM_003873.5	1	100	4	missense	NM_00387	c.536T>C	p.Val179Ala	8.04	1	64	0		C-terminal dc	rs386742804,rs7079	7.00E-04		1.0 (pos.33552696)		Neuropilin	GTPase ac Cardiovascular Abn Palifermin,Pegaptanib	A=0, G=49	A=0.0, G=1.0	0.00001	831.625	49	A=0/0, G=27/2
chr2:44101538	T/T	C	T	SNV	ABCG8	ABCG8:intronic:NM_022437.2	1	100		intronic	NM_02243	c.1412-8C	p.?	0.14					ABC transpor	rs112765285,rs2851	0.1504	YES	9.783E-1 (pos.44101538)		ATP-binding	ATP bindin Arterial Occlusive D Ezetimibe Benign	C=0, T=24	C=0.0, T=1.0	0.00001	327.837	24	C=0/0, T=18/6
chr2:206581033	A/A	G	A	SNV	NRP2	NRP2:exonic:NM_201266.1	1	100	3	missense	NM_20126	c.368G>A	p.Arg123Lys	1.61	1	26	0		C-terminal dc	rs849541	0.001	YES	1.0 (pos.206581033)		Neuropilin	angiogene Autistic Disorder;Brain Diseases;Central Ner	G=0, A=23	G=0.0, A=1.0	0.00001	316.612	23	G=0/0, A=9/14
chr2:206592695	T/C	T	C	SNV	NRP2	NRP2:exonic:NM_201266.1	1	58.33	7	synonymous	NM_20126	c.1071T>C	p.(=)	-0.67				0.21(COSM37	C-terminal dc	rs849526	0.4588	YES	8.125E-1 (pos.20659269	adenocarcinoı	Neuropilin	angiogene Autistic Disorder;Brain Diseases;Central Ner	T=10, C=14	T=0.4167, C=0.5833	0.00001	135.333	24	T=6/4, C=7/7
chr2:44550290	G/G	A	G	SNV	PREPL	PREPL:intronic:NM_001171603.1	1	100		intronic	NM_00117	c.1896+11	p.?	-0.73					Prolyl oligope	rs2053454		YES			Prolyl endo	Golgi appa Congenital Abnormalities;Congenital, Heredi	A=0, G=77	A=0.0, G=1.0	0.00001	1229.44	77	A=0/0, G=49/2
chr2:44573624	A/A	G	A	SNV	PREPL	PREPL:intronic:NM_001171603.1	1	100		intronic	NM_00117	c.220-95C	p.?	-0.12					Prolyl oligope	rs698763		YES			Prolyl endo	Golgi appa Congenital Abnormalities;Congenital, Heredi	G=0, A=47	G=0.0, A=1.0	0.00001	789.328	47	G=0/0, A=30/1
chr2:44573658	C/C	T	C	SNV	PREPL	PREPL:intronic:NM_001171603.1	1	100		intronic	NM_00117	c.220-129	p.?	-1.33					Prolyl oligope	rs698764		YES			Prolyl endo	Golgi appa Congenital Abnormalities;Congenital, Heredi	T=0, C=47	T=0.0, C=1.0	0.00001	789.327	47	T=0/0, C=30/1
chr2:44573671	T/T	G	T	SNV	PREPL	PREPL:intronic:NM_001171603.1	1	100		intronic	NM_00117	c.220-142	p.?	-0.12					Prolyl oligope	rs698765		YES			Prolyl endo	Golgi appa Congenital Abnormalities;Congenital, Heredi	G=0, T=47	G=0.0, T=1.0	0.00001	789.328	47	G=0/0, T=30/1
chr2:44617324	A/A	T	A	SNV	CAMKMT	CAMKMT:intronic:NM_024766.4	1	100		intronic	NM_02476	c.312-56T	p.?	-0.37					Lysine methy	rs1085486	0.2607	YES			Calmodulin	Golgi appa Congenital Abnormalities;Congenital, Heredi	T=0, A=42	T=0.0, A=1.0	0.00001	685.219	42	T=0/0, A=15/2
chr2:50148972	C/T	C	T	SNV	NRXN1	NRXN1:utr_3:NM_004801.5	1	50	22	utr_3	NM_00480	c.4544G>	p.?	4.36					EGF-like dom	rs1045881		YES			Neurexin 1	NMDA glut Autistic Disorder;Cl Calcium Ci Benign	C=71, T=71	C=0.5, T=0.5	0.00001	784.721	142	C=46/25, T=46
chr11:64418900	A/A	G	A	SNV	NRXN2	NRXN2:exonic:NM_015080.3	1	100	14	synonymous	NM_01508	c.2745C>T	p.(=)	-0.69				0.32(COSM41	Laminin G do	rs526338	0.2919	YES	2.295E-1;0.0 (pos.64418	carcinoma,du	Neurexin 2	adult beha Child Development Disorders, Likely benign	G=0, A=60	G=0.0, A=1.0	0.00001	903.098	60	G=0/0, A=32/2
chr4:40778146	T/T	C	T	SNV	NSUN7	NSUN7:exonic:NM_024677.5	1	100	7	synonymous	NM_02467	c.906C>T	p.(=)	-0.96				0.02(COSM14	16S rRNA me	rs2465570	0.197	YES	5.142E-1 (pos.40778146)	acute_myeloic	NOP2/SUN	RNA methyl Genital Diseases, Male;Infertility;Infertility, I	C=0, T=48	C=0.0, T=1.0	0.00001	810.435	48	C=0/0, T=22/2

chr2:47703379	T/T	C	T	SNV	MSH2	MSH2:intronic:NM_000251.2	1	100		intronic	NM_00025	c.2006-12	p.?	-0.18					MutS domain	rs2059521					mutS, E. col	ADP bindir	ACMG Recommendations...;Adnexal Disease	C=0, T=37	C=0.0, T=1.0	0.00001	583.641	37	C=0/0, T=25/1
chr4:40810747	G/G	A	G	SNV	NSUN7	NSUN7:exonic:NM_024677.5	1	100	12	missense	NM_02467	c.1948A>G	p.Lys650Glu	2.53	1	56	0		16S rRNA met	rs2261167	0.1072	YES	1.0 (pos.40810747)		NOP2/SUN	RNA methylt	Genital Diseases, Male:Infertility:Infertility, I	A=0, G=37	A=0.0, G=1.0	0.00001	583.707	37	A=0/0, G=21/1
chr4:40778162	T/G	T	G	SNV	NSUN7	NSUN7:exonic:NM_024677.5	1	95.83	7	missense	NM_02467	c.922T>G	p.Ser308Ala	5.38	1	99	0	0.05(COSM14	16S rRNA met	rs2437323	0.197	YES	5.148E-1 (pos.40778162)	adenocarcinor	NOP2/SUN	RNA methylt	Genital Diseases, Male:Infertility:Infertility, I	T=2, G=46	T=0.0417, G=0.9583	0.00001	738.421	48	T=2/0, G=20/2
chr17:39983808	C/C	G	C	SNV	NT5C3B	NT5C3B:exonic:NM_052935.4	1	100	8	missense	NM_05293	c.638C>G	p.Ser213Cys	7.75	0	112	0.991		Pyrimidine 5'	rs1046404,rs201647	0.2398	YES	8.302E-1 (pos.39983808)			5'-nucleotidase activity,cytoplasm,cytosol,dephosphory		G=0, C=35	G=0.0, C=1.0	0.00001	543.888	35	G=0/0, C=16/1
chr17:39983820	A/A	G	A	SNV	NT5C3B	NT5C3B:exonic:NM_052935.4	1	100	8	missense	NM_05293	c.626C>T	p.Ala209Val	2.79	0.68	64	0.001	0.51(COSM41	Pyrimidine 5'	rs1046403	0.2398	YES	8.298E-1 (pos.39983820)	neoplasm		5'-nucleotidase activity,cytoplasm,cytosol,dephosphory		G=0, A=35	G=0.0, A=1.0	0.00001	543.903	35	G=0/0, A=16/1
chr2:48844968	G/G	C	G	SNV	GTF2A1L,STON1-GTF2A1L	GTF2A1L:utr_5:NM_006872.4, STON1-GTF2A1L:intronic:NM	1	100		intronic	NM_00119	c.2134-30	p.?, p.?	-1.02					Adaptor com	rs2293269	0.3173	YES	9.223E-1 (pos.48844968)		General tra	DNA bindi	Adnexal Diseases:Cysts:Endocrine System Di	C=0, G=60	C=0.0, G=1.0	0.00001	903.53	60	C=0/0, G=30/3
chr17:39983849	C/C	G	C	SNV	NT5C3B	NT5C3B:exonic:NM_052935.4	1	100	8	synonymous	NM_05293	c.597C>G	p.(=)	1.81				0.88(COSM41	Pyrimidine 5'	rs1128966	0.2401	YES	8.289E-1 (pos.39983849)	neoplasm		5'-nucleotidase activity,cytoplasm,cytosol,dephosphory		G=0, C=36	G=0.0, C=1.0	0.00001	563.739	36	G=0/0, C=16/2
chr2:48925746	T/T	C	T	SNV	LHCGR	LHCGR:intronic:NM_000233.3	1	100		intronic	NM_00023	c.866+8G>	p.?	0.17				0.01(COSM40	7 transmemb	rs6755901	0.1567	YES	4.77E-1 (pos.48925746)	acute_myeloic	Luteinizing	G-protein	46, XY Disorders of; Buserelin, Benign	C=0, T=26	C=0.0, T=1.0	0.00001	371.42	26	C=0/0, T=11/1
chr3:52558133	T/C	T	C	SNV	NT5DC2,STAB1	NT5DC2:downstream:NM_001134231.1, STAB1:exonic:NM	1	95.65	68	synonymous	NM_01513	c.7560T>C	p.(=), p.?	-4.3				0.03(COSM40	EGF domain,E	rs13621,rs78035727	0.4683	YES	4.346E-1:0.0 (pos.52558	acute_myeloid_leukaemia		5'-nucleot	Arthritis:Arthritis, Juvenile:Autoimmune Dise	T=1, C=22	T=0.0435, C=0.9565	0.00001	288.971	23	T=1/0, C=12/1
chr6:86199233	G/G	A	G	SNV	NT5E	NT5E:exonic:NM_002526.3	1	100	6	missense	NM_00252	c.1126A>G	p.Thr376Ala	-0.07	0.91	58	0		5'-nucleotida	rs2229523	0.3076	YES	5.989E-1 (pos.86199233)		5' nucleotid	5'-nucleot	Calcinosis:Calcium_Cytarabine,Pentoxifyllin	A=0, G=30	A=0.0, G=1.0	0.00001	446.639	30	A=0/0, G=21/9
chr2:54135429	G/C	G	C	SNV	PSME4	PSME4:intronic:NM_014614.2	1	45.83		intronic	NM_01461	c.2784+28	p.?	0.57					Domain of un	rs805319	0.309	YES	4.631E-1 (pos.54135429)		Proteasom	DNA repai	HIV Infections;Immune System Diseases;Imm	G=13, C=11	G=0.5417, C=0.4583	0.00001	93.4391	24	G=1/12, C=8/3
chr2:55091029	T/T	C	T	SNV	EML6	EML6:intronic:NM_001039753.2	1	100		intronic	NM_00103	c.1932+3C	p.?	-0.01				0.58(COSM44	Anaphase-pr	rs2358155	0.2772	YES	1.994E-1 (pos.55091029)	acute_myeloid_leukaemia		cytoplasm	Nutrition Disorders:Nutritional and Metaboli	C=0, T=30	C=0.0, T=1.0	0.00001	446.639	30	C=0/0, T=11/1
chr2:55181351	C/C	T	C	SNV	EML6	EML6:intronic:NM_001039753.2	1	100		intronic	NM_00103	c.4486+58	p.?	0.08					Anaphase-pr	rs4671999		YES				cytoplasm	Nutrition Disorders:Nutritional and Metaboli	T=0, C=77	T=0.0, C=1.0	0.00001	1229.34	77	T=0/0, C=19/5
chr2:55181382	A/A	G	A	SNV	EML6	EML6:intronic:NM_001039753.2	1	100		intronic	NM_00103	c.4486+89	p.?	-1.53					Anaphase-pr	rs4672001		YES				cytoplasm	Nutrition Disorders:Nutritional and Metaboli	G=0, A=75	G=0.0, A=1.0	0.00001	1189.74	75	G=0/0, A=15/6
chr1:156846233	G/A	G	A	SNV	NTRK1	NTRK1:exonic:NM_002529.3	1	57.89	14	synonymous	NM_00252	c.1674G>A	p.(=)	0.28				0.84(COSM37	Leucine rich	rs6334	0.2194	YES	3.553E-1:0.0 (pos.15684	adenocarcinor	Neurotroph	ATP bindin	Adenocarcinoma:B; Amitriptyl_Benign	G=16, A=22	G=0.4211, A=0.5789	0.00001	246.276	38	G=5/11, A=8/1
chr2:55201038	T/T	C	T	SNV	RTN4	RTN4:intronic:NM_020532.4	1	100		intronic	NM_02053	c.3431-35	p.?	-0.52					Reticulon	rs2255026	0.165	YES	7.637E-1 (pos.55201038)		Neurite out	RNA bindir	Mental Disorders;Schizophrenia;Schizophren	C=0, T=92	C=0.0, T=1.0	0.00001	1534.54	92	C=0/0, T=45/4
chr2:55214618	C/C	A	C	SNV	RTN4	RTN4:intronic:NM_020532.4	1	100		intronic	NM_02053	c.3221+9T	p.?	2.35				0.91(COSM40	Reticulon	rs2580769	0.2399	YES	7.621E-1 (pos.55214618)	acute_myeloic	Neurite out	RNA bindir	Mental Disorders;Schizophrenia;Schizophren	A=0, C=59	A=0.0, C=1.0	0.00001	885.348	59	A=0/0, C=31/2
chr15:88679785	A/A	G	A	SNV	NTRK3	NTRK3:exonic:NM_001012338.2	1	100	8	synonymous	NM_00101	c.678C>T	p.(=)	-2.4				0.02(COSM41	Immunoglobu	rs1863480	0.0058		1.0 (pos.88679785)	neoplasm	Neurotroph	ATP bindin	Brain Diseases:Carcinoma:Central Nervous S	G=0, A=34	G=0.0, A=1.0	0.00001	524.186	34	G=0/0, A=17/1
chr2:55460605	T/T	A	T	SNV	MIR4426,RPS27A	MIR4426:upstream:NR_039624.1, RPS27A:intronic:NM_001	1	100		intronic	NM_00113	c.103+52A	p.?	-3.43					Ribosomal pr	rs386646301,rs6737227		YES	6.43E-1 (pos.55460605)		Ribosomal	DNA damage response, detection of DNA damage,DNA d		A=0, T=44	A=0.0, T=1.0	0.00001	724.667	44	A=0/0, T=19/2
chr2:11810095	A/A	G	A	SNV	NTSR2	NTSR2:exonic:NM_012344.3	1	100	1	missense	NM_01234	c.161C>T	p.Ala54Val	0.95	1	64	0	0.03(COSM37	7 transmemb	rs6432225	5.00E-04	YES	1.0 (pos.11810095)	adenocarcinor	Neurotensi	G-protein coupled neurotensin	Levocabastine	G=0, A=37	G=0.0, A=1.0	0.00001	583.701	37	G=0/0, A=19/1
chr2:55471407	C/C	T	C	SNV	MTIF2	MTIF2:intronic:NM_002453.2	1	100		intronic	NM_00245	c.1107-38	p.?	0.57					50S ribosome	rs2589083	0.0372	YES	9.811E-1 (pos.55471407)		Mitochondr	GTP binding,GTPase activity,RNA binding,mitochondrial t		T=0, C=291	T=0.0, C=1.0	0.00001	4910.51	291	T=0/0, C=143/
chr2:55490766	C/A	C	A	SNV	MTIF2	MTIF2:intronic:NM_002453.2	1	46.88		intronic	NM_00245	c.219+10G	p.?	-0.91					50S ribosome	rs769282337			0.0 (pos.55490766)		Mitochondr	GTP binding,GTPase activity,RNA binding,mitochondrial t		C=17, A=15	C=0.5313, A=0.4688	0.00001	140.7	32	C=1/16, A=3/1
chr12:106460938	C/C	G	C	SNV	NUAK1	NUAK1:exonic:NM_014840.2	1	100	7	missense	NM_01484	c.1628C>G	p.Pro543Arg	3.53	0.04	103	0.93	0.94(COSM14	Lipopolysacc	rs3741883	0.228	YES	4.208E-1:0.0 (pos.10646	acute_myeloic	NUAK famil	ATP bindin	Neoplasm Invasiveness:Neoplasms:Neoplas	G=0, C=26	G=0.0, C=1.0	0.00001	371.417	26	G=0/0, C=11/1
chr2:55599655	A/A	G	A	SNV	CCDC88A	CCDC88A:intronic:NM_001135597.1	1	100		intronic	NM_00113	c.344-9C>T	p.?	3.19				0.08(COSM14	HOOK protein	rs3111411	0.3865	YES	1.706E-1 (pos.55599655)	adenocarcinor	Coiled-coil	DNA replication,Golgi apparatus,TOR signaling,actin bin		G=0, A=36	G=0.0, A=1.0	0.00001	561.218	36	G=0/0, A=16/2
chr1:205275421	G/G	A	G	SNV	NUAK2	NUAK2:exonic:NM_030952.2	1	100	5	synonymous	NM_03095	c.717T>C	p.(=)	-0.23					Haspin like ki	rs4951197	9.00E-04		1.0 (pos.205275421)		NUAK famil	ATP binding,actin cytoskeleton organization,apoptotic p		A=0, G=30	A=0.0, G=1.0	0.00001	446.639	30	A=0/0, G=16/1
chr11:17333663	A/A	G	A	SNV	NUCB2	NUCB2:splicesite_5:NM_005013.3	1	100	10	splicesite	NM_00501	c.907+1G>	p.?	4.51					EF hand,EF-ha	rs2521998	0		1.0 (pos.17333663)		Nucleobind	DNA bindi	Genital Diseases, M; Calcium Citrate,Calcium	G=0, A=65	G=0.0, A=1.0	0.00001	996.354	65	G=0/0, A=24/4
chr2:55899228	T/T	A	T	SNV	PNPT1	PNPT1:intronic:NM_033109.4	1	100		intronic	NM_03310	c.867-47T	p.?	-0.2					3' exoribonuc	rs782631	0.0555	YES	5.138E-1 (pos.55899228)		Polyribonuc	3'-5'-exoril	Adenocarcinoma:Carcinoma:Carcinoma, Hep	A=0, T=52	A=0.0, T=1.0	0.00001	767.016	52	A=0/0, T=29/2
chr2:56098272	G/A	G	A	SNV	EFEMP1	EFEMP1:intronic:NM_001039348.2	1	56		intronic	NM_00103	c.1001-14	p.?	0.76					Calcium-bind	rs45535043	0.0621	YES	4.635E-4 (pos.56098272)		EGF-contair	calcium io	Adenocarcinoma:Breast Disea_Benign	G=11, A=14	G=0.44, A=0.56	0.00001	134.35	25	G=7/4, A=13/1
chr11:17351683	C/G	C	G	SNV	NUCB2	NUCB2:exonic:NM_005013.3	1	51.92	12	missense	NM_00501	c.1012C>G	p.Gln338Glu	3.99	0.43	29	0	0.95(COSM39	EF hand,EF-ha	rs757081	0.3205	YES	4.014E-1 (pos.17351683)	adenocarcinor	Nucleobind	DNA bindi	Genital Diseases, M; Calcium Citrate,Calcium	C=25, G=27	C=0.4808, G=0.5192	0.00001	270.394	52	C=17/8, G=14/
chr5:102891695	G/G	A	G	SNV	NUDT12	NUDT12:exonic:NM_031438.3	1	100	4	synonymous	NM_03143	c.901T>C	p.(=)	1.82				0.73(COSM41	Ankyrin repea	rs10045774	0.3054	YES	5.501E-1 (pos.10289169	adenocarcinoma,neoplasm		NAD biosynthesis via nicotinamide riboside salvage path		A=0, G=100	A=0.0, G=1.0	0.00001	1702.25	100	A=0/0, G=60/4
chr5:102894977	C/C	T	C	SNV	NUDT12	NUDT12:exonic:NM_031438.3	1	100	3	synonymous	NM_03143	c.399A>G	p.(=)	1.85				0.65(COSM37	Ankyrin repea	rs7734923	0.1681	YES	7.409E-3 (pos.10289497	adenocarcinoma		NAD biosynthesis via nicotinamide riboside salvage path		T=0, C=40	T=0.0, C=1.0	0.00001	627.789	40	T=0/0, C=10/3
chr8:21964802	T/T	C	T	SNV	NUDT18	NUDT18:utr_3:NM_024815.3	1	100	5	utr_3	NM_02481	c.981G>A	p.?	0.01					NUDIX domai	rs1552286	0.1318	YES	9.934E-1 (pos.21964802)		Nucleoside	8-hydroxy-dADP phosphatase activity,8-oxo-GDP phosph		C=0, T=32	C=0.0, T=1.0	0.00001	485.14	32	C=0/0, T=21/1
chr2:61605614	T/A	T	A	SNV	USP34	USP34:intronic:NM_014709.3	1	59.09		intronic	NM_01470	c.1015-55	p.?	0.22					Ubiquitin car	rs10208769	0.4918	YES			Ubiquitin-s	Wnt signaling pathway,cysteine-type endopeptidase act		T=36, A=52	T=0.4091, A=0.5909	0.00001	615.614	88	T=12/24, A=18
chr4:123843716	C/C	T	C	SNV	NUDT6,SPATA5	SPATA5:upstream:NM_145207.2, NUDT6:exonic:NM_00708	1	100	1	synonymous	NM_00708	c.12A>G, c	p.(=), p.?	-1.32				0(COSM1753	NUDIX domai	rs13134412,rs76518	0.1042	YES	0.0 (pos.123843709),9.5	adenocarcinor	Spermatoge	ATP bindin	Colonic Diseases:Colonic Diseases, Function	T=0, C=181	T=0.0, C=1.0	0.00001	3004.12	181	T=0/0, C=75/1
chr8:27918057	C/C	T	C	SNV	NUGGC	NUGGC:exonic:NM_001010906.1	1	100	8	missense	NM_00101	c.983A>G	p.Gln328Arg	0.51	0.97	43	0		50S ribosome	rs386723926,rs7817	0.0013	YES	1.0;0.0 (pos.27918057)	adenocarcinoma		GTP binding,GTPase activity,cellular response to lipopol		T=0, C=46	T=0.0, C=1.0	0.00001	768.313	46	T=0/0, C=18/2
chr2:62099156	T/C	T	C	SNV	CCT4	CCT4:intronic:NM_006430.3	1	58.33		intronic	NM_00643	c.1491+61	p.?	-0.02					TCP-1/cpn60	rs3828276		YES			Chaperonin	'de novo' protein folding,ATP binding,RNA binding,bindin		T=10, C=14	T=0.4167, C=0.5833	0.00001	135.342	24	T=8/2, C=6/8
chr19:41173880	C/T	C	T	SNV	NUMBL	NUMBL:exonic:NM_004756.4	1	46.43	10	synonymous	NM_00475	c.1323G>A	p.(=)	0.01				0.67(COSM37	NUMB domai	rs199622242,rs2604	0.384	YES	0.0 (pos.41173864),0.0 (	adenocarcinor	Numb, Dros	adherens junction organization,axonogenesis,cytokine-		C=15, T=13	C=0.5357, T=0.4643	0.00001	114.673	28	C=9/6, T=7/6
chr2:64126775	C/A	C	A	SNV	VPS54	VPS54:intronic:NM_016516.2	1	50		intronic	NM_01651	c.2626-60	p.?	-1.5					Vacuolar-sor	rs78227932		YES			Vacuolar pr	GARP com	Central Nervous System Diseases;Motor Neu	C=17, A=17	C=0.5, A=0.5	0.00001	173.556	34	C=8/9, A=5/12
chr2:64140301	T/T	A	T	SNV	VPS54	VPS54:intronic:NM_016516.2	1	100		intronic	NM_01651	c.2422+56	p.?	-0.36					Vacuolar-sor	rs10207847	0.2209	YES			Vacuolar pr	GARP com	Central Nervous System Diseases;Motor Neu	A=0, T=23	A=0.0, T=1.0	0.00001	316.673	23	A=0/0, T=18/5
chr2:65481008	C/C	A	C	SNV	ACTR2	ACTR2:intronic:NM_001005386.2	1	100		intronic	NM_00100	c.600+10A	p.?	0.81				0.23(COSM54	Actin	rs268871	0.1524	YES	7.886E-1:0.0 (pos.65481	adenocarcinor	Actin-relate	ATP bindin	Autoimmune Disea_(2S)-2-(3-bromophenyl)-3	A=0, C=20	A=0.0, C=1.0	0.00001	263.774	20	A=0/0, C=5/15
chr2:65488538	A/A	G	A	SNV	ACTR2	ACTR2:intronic:NM_001005386.2	1	100		intronic	NM_00100	c.896+12G	p.?	0.26					Actin	rs268862	0.1519	YES	7.887E-1 (pos.65488538)		Actin-relate	ATP bindin	Autoimmune Disea_(2S)-2-(3-bromophenyl)-3	G=0, A=76	G=0.0, A=1.0	0.00001	1209.61	76	G=0/0, A=47/2
chr2:65495570	CC/CC	AT	CC	MNV	ACTR2	ACTR2:intronic:NM_001005386.2	2	100		intronic	NM_00100	c.1030-12	p.?	-0.25, -2.43					Actin	rs386646815,rs55843720,rs609		YES			Actin-relate	ATP bindin	Autoimmune Disea_(2S)-2-(3-bromophenyl)-3	AT=0, CC=4	AT=0.0, CC=1.0	0.00001	685.22	42	AT=0/0, CC=33
chr2:65495573	AC/AC	A	AC	INDEL	ACTR2	ACTR2:intronic:NM_001005386.2	1	100		intronic	NM_00100	c.1030-12	p.?	-0.39					Actin	rs200763705,rs386646815,rs39		YES			Actin-relate	ATP bindin	Autoimmune Disea_(2S)-2-(3-bromophenyl)-3	A=0, AC=42	A=0.0, AC=1.0	0.00001	684.171	42	A=0/0, AC=33/
chr6:17629616	A/A	G	A	SNV	NUP153	NUP153:exonic:NM_005124.3	1	100	18	synonymous	NM_00512	c.2814C>T	p.(=)	0.01					Nucleoporin	rs942467	0.0044	YES	7.142E-1 (pos.17629616)		Nucleopori	DNA binding,cytosol,host cell,identical protein binding,i		G=0, A=49	G=0.0, A=1.0	0.00001	831.633	49	G=0/0, A=28/2
chr5:37364443	T/T	C	T	SNV	NUP155	NUP155:exonic:NM_153485.2	1	100	2	synonymous	NM_15348	c.201G>A	p.(=)	-1.72					Non-repetitiv	rs217852	1.00E-04	YES	1.0 (pos.37364443)		Nucleopori	RNA export from nucleus,atrial cardiac muscle cell actio		C=0, T=20	C=0.0, T=1.0	0.00001	263.786	20	C=0/0, T=8/12
chr11:47834592	A/A	G	A	SNV	NUP160	NUP160:exonic:NM_015231.2	1	100	15	synonymous	NM_01523	c.1794C>T	p.(=)	-0.06					Nucleoporin	rs7110289	0		1.0 (pos.47834592)		Nucleopori	cytosol,host cell,intracellular transport of virus,kinetoch		G=0, A=72	G=0.0, A=1.0	0.00001	1130.95	72	G=0/0, A=35/3
chr7:135304273	C/C	G	C	SNV	NUP205	NUP205:exonic:NM_015135.2	1	100	29	missense	NM_01513	c.4066G>C	p.Glu1356Gln	3.81	1	29	0		Nuclear pore	rs7810767	1.00E-04		1.0 (pos.135304273)		Nucleopori	host cell,i	Bone Diseases;Musculoskeletal Diseases;Os	G=0, C=41	G=0.0, C=1.0	0.00001	664.7	41	G=0/0, C=23/1
chr2:68511584	G/G	A	G	SNV	CNRIP1		1	100		intronic				1.11				0.07(COSM148871)		rs735815	0.4299	YES	5.35E-1 (pos.68511584)	acute_myeloid_leukaemia,adenocarcinoma				A=0, G=28	A=0.0, G=1.0	0.00001	408.718	28	A=0/0, G=7/21
chr2:68546304	T/C	T	C	SNV	CNRIP1	CNRIP1:intronic:NM_015463.2	1	50		intronic	NM_01546	c.179+50A	p.?	-0.96					CB1 cannabir	rs4671897	0.4798	YES	1.796E-1 (pos.68546304)			myelin sheath		T=14, C=14	T=0.5, C=0.5	0.00001	131.907	28	T=13/1, C=9/5
chr3:13368892	G/A	G	A	SNV	NUP210	NUP210:exonic:NM_024923.3	1	53.49	32	synonymous	NM_02492	c.4332C>T	p.(=)	-0.21				0.2(COSM445	Bacterial Ig-li	rs2271509	0.4881	YES	3.011E-1 (pos.13368892)	adenocarcinor	Nucleopori	endoplasmic reticulum membrane,host cell,integral cor		G=20, A=23	G=0.4651, A=0.5349	0.00001	260.78	43	G=7/13, A=6/1
chr1:154108387	A/A	G	A	SNV	NUP210L	NUP210L:exonic:NM_207308.2	1	100	7	synonymous	NM_20730	c.912C>T	p.(=)	-0.03					Bacterial Ig-li	rs3001361	4.00E-04		1.0;0.0 (pos.154108387)			Sertoli cell development,integral component of membra		G=0, A=45	G=0.0, A=1.0	0.00001	747.392	45	G=0/0, A=23/2
chr19:50412417	A/A	G	A	SNV	NUP62	NUP62:exonic:NM_001193357.1	1	100	2	synonymous	NM_00119	c.648C>T	p.(=)	-0.02				0.05(COSM37	Nsp1-like C-te	rs999583	0.2421	YES	9.643E-1 (pos.50412417)	adenocarcinor	Nucleopori	Flemming	Autonomic Nervous System Dis; Likely benign	G=0, A=80	G=0.0, A=1.0	0.00001	1289.36	80	G=0/0, A=50/3
chr2:69409029	A/A	C	A	SNV	ANTXR1	ANTXR1:intronic:NM_032208.2	1	100		intronic	NM_03220	c.1185+16	p.?	-0.56					Anthrax rece	rs6749826	0.4331	YES	7.607E-1 (pos.69409029)		Anthrax tox	actin cyto	Alopecia:Anodontia:Congenita_Benign	C=0, A=36	C=0.0, A=1.0	0.00001	563.744	36	C=0/0, A=20/1
chr17:73230856	T/T	C	T	SNV	NUP85	NUP85:exonic:NM_024844.4	1	100	17	synonymous	NM_02484	c.1740C>T	p.(=)	-0.75					Nup85 Nucle	rs9988	0.1843	YES	9.416E-1 (pos.73230856)		Nucleopori	condensed chromosome kinetochore,cytosol,host cell,i		C=0, T=28	C=0.0, T=1.0	0.00001	408.717	28	C=0/0, T=16/1
chr2:7005069	T/T	C	T	SNV	CMPK2	CMPK2:intronic:NM_207315.3	1	100		intronic	NM_20731	c.675+84G	p.?	-0.35					Deoxynucleo	rs2595172		YES			Cytidine mo	ATP binding,UMP kinase activity,cellular response to lipo		C=0, T=21	C=0.0, T=1.0	0.00001	280.785	21	C=0/0, T=12/9
chr17:5294976	T/A	T	A	SNV	NUP88	NUP88:exonic:NM_002532.5	1	50	10	synonymous	NM_00253	c.1389A>T	p.(=)	-1.68				0.13(COSM37	Nuclear pore	rs14231	0.3942	YES	8.444E-1 (pos.5294976)	carcinoma,he	Nucleopori	cytosol,ho	HIV Infections;Immune System Diseases;Imm	T=14, A=14	T=0.5, A=0.5	0.00001	131.941	28	T=2/12, A=10/
chr15:41634587	GA/GA	AC	GA	MNV	NUSAP1	NUSAP1:exonic:NM_016359.4	2	100	2	missense	NM_01635	c.97_98de	p.Thr33Asp	1.57, 1.04	1	85	0	0.03(COSM11	Nucleolar an	rs386783399,rs7178	0.0072	YES	2.215E-1 (pos.41634588)	acute_myeloic	Nucleolar a	DNA bindi	Autoimmune Diseases;Female Urogenital Dis	AC=0, GA=2	AC=0.0, GA=1.0	0.00001	399.403	28	AC=0/0, GA=21
chr15:34640378	C/C	T	C	SNV	NUTM1	NUTM1:exonic:NM_175741.2	1	100	3	synonymous	NM_17574	c.225T>C	p.(=)	-0.33				0.05(COSM41	NUT protein	rs1028971027,rs430	0.001	YES	9.999E-1 (pos.34640378)	neoplasm		cytoplasm,nucleus,protein binding		T=0, C=182	T=0.0, C=1.0	0.00001	3036.9	182	T=0/0, C=119/
chr1:224517842	T/T	C	T	SNV	NVL	NVL:utr_5:NM_002533.3	1	100	1	utr_5	NM_00253	c.-13G>A	p.?	-2.95					AAA domain (	rs2896990	0.0566	YES	8.985E-1 (pos.224517842)		Nuclear val	ATP binding,RNA binding,membrane,nucleolus,nucleopl		C=0, T=65	C=0.0, T=1.0	0.00001	996.139	65	C=0/0, T=39/2
chr19:16860105	A/A	G	A	SNV	NWD1	NWD1:exonic:NM_001007525.4	1	100	6	missense	NM_00100	c.652G>A	p.Ala218Thr	0.69	1	58	0		Anaphase-pr	rs706764	0		9.219E-1 (pos.16860105)		NACHT dom	ATP binding,RNA binding,RNA splicing,U5 snRNP,catalytic		G=0, A=38	G=0.0, A=1.0	0.00001	603.784	38	G=0/0, A=16/2
chr19:16899838	T/T	A	T	SNV	NWD1	NWD1:exonic:NM_001007525.4	1	100	13	missense	NM_00100	c.2777A>T	p.Asn926Ile	2.85	0.84	149	0		Anaphase-pr	rs2608737	0		9.954E-1 (pos.16899838)		NACHT dom	ATP binding,RNA binding,RNA splicing,U5 snRNP,catalytic		A=0, T=72	A=0.0, T=1.0	0.00001	1130.93	72	A=0/0, T=38/3
chr2:71360723	G/G	A	G	SNV	MPHOSPH10	MPHOSPH10:intronic:NM_005791.2	1	100		intronic	NM_00579	c.768+17A	p.?	0.04					Mpp10 prote	rs1813159	0.1466	YES	9.993E-1 (pos.71360723)			Mpp10 complex,RNA binding,RNA processing,RNA splicin		A=0, G=21	A=0.0, G=1.0	0.00001	281.235	21	A=0/0, G=5/16
chr11:62564009	C/C	T	C	SNV	NXF1	NXF1:exonic:NM_006362.4	1	100	14	synonymous	NM_00636	c.1209A>G	p.(=)	-0.1				0.09(COSM56	Nuclear trans	rs4693	0.3609	YES	5.342E-1 (pos.62564009)	ductal_carcinc	Nuclear RN	RNA binding,RNA export from nucleus,cytoplasm,cytosol		T=0, C=27	T=0.0, C=1.0	0.00001	389.981	27	T=0/0, C=17/1
chr2:71708825	TATCC/TATCC	T	TATCC	INDEL	DYSF	DYSF:intronic:NM_001130987.1	4	100		intronic	NM_00113	c.148-184	p.?	-1.58					C2 domain,Fe	rs71402983,rs75288288					Dysferlin	T-tubule,c	Arthritis;Arthritis, Juvenile:Autoimmune Dise	T=0, TATCC	T=0.0, TATCC=1.0	0.00001	809.168	55	T=0/0, TATCC=
chr2:71747432	T/T	G	T	SNV	DYSF	DYSF:intronic:NM_001130987.1	1	100		intronic	NM_00113	c.1033+94	p.?	0.41					C2 domain,Fe	rs11687223		YES			Dysferlin	T-tubule,c	Arthritis;Arthritis, Juvenile:Autoimmune Dise	G=0, T=21	G=0.0, T=1.0	0.00001	273.949	21	G=0/0, T=11/1
chr2:71747851	C/C	T	C	SNV	DYSF	DYSF:intronic:NM_001130987.1	1	100		intronic	NM_00113	c.1034-68	p.?	-1.01					C2 domain,Fe	rs12713755		YES			Dysferlin	T-tubule,c	Arthritis;Arthritis, Juvenile:Autoimmune Dise	T=0, C=26	T=0.0, C=1.0	0.00001	371.42	26	T=0/0, C=10/1
chr2:71871236	G/G	A	G	SNV	DYSF	DYSF:intronic:NM_001130987.1	1	100		intronic	NM_00113	c.4626+43	p.?	-1.63					C2 domain,Fe	rs84182	0.1179	YES	8.222E-1 (pos.71871236)		Dysferlin	T-tubule,c	Arthritis;Arthritis, Juvenile:Aut_Benign	A=0, G=34	A=0.0, G=1.0	0.00001	523.199	34	A=0/0, G=19/1
chr11:114393652	T/T	C	T	SNV	NXPE1	NXPE1:exonic:NM_152315.4	1	100	5	missense	NM_15231	c.631G>A	p.Gly211Arg	5.59	0	125	1	0.96(COSM11	Neurexophili	rs10891692	0.3449	YES	6.728E-1 (pos.11439365	acute_myeloid_leukaemia		extracellu	Colitis:Colitis, Ulcerative:Colonic Diseases:D	C=0, T=27	C=0.0, T=1.0	0.00001	389.988	27	C=0/0, T=13/1
chr2:226273689	C/C	T	C	SNV	NYAP2	NYAP2:exonic:NM_020864.1	1	100	2	synonymous	NM_02086	c.93T>C	p.(=)	-1.7				0.06(COSM39	Neuronal tyro	rs2048936	0.1761	YES	6.188E-1 (pos.22627368	acute_myeloid_leukaemia		intracellular,neuron projection morphogenesis,phospha		T=0, C=162	T=0.0, C=1.0	0.00001	2613.14	162	T=0/0, C=42/1
chr11:120099679	A/A	G	A	SNV	OAF	OAF:exonic:NM_178507.3	1	100	4	missense	NM_17850	c.650G>A	p.Arg217His	0.78	0.25	29	0.006	0.6(COSM414	Transcriptior	rs2508490	0.2071	YES	1.339E-1 (pos.12009967	carcinoma,neoplasm		extracellular exosome		G=0, A=28	G=0.0, A=1.0	0.00001	398.871	28	G=0/0, A=6/22
chr2:74129664	G/G	A	G	SNV	ACTG2	ACTG2:intronic:NM_001615.3	1	100		intronic	NM_00161	c.255+49A	p.?	-2.58					Actin	rs1653256	0.4203	YES	4.871E-1 (pos.74129664)		Actin, gamn	ATP bindin	Abnormalities, Multiple;Colonic Diseases;Co	A=0, G=64	A=0.0, G=1.0	0.00001	977.635	64	A=0/0, G=4/60
chr12:113357193	G/A	G	A	SNV	OAS1	OAS1:splicesite_3:NM_016816.3	1	50	6	splicesite	NM_01681	c.1039-1G	p.?	0.5					2'-5'-oligoade	rs10774671	0.3616	YES	7.837E-1 (pos.113357193)		2',5'-oligoac	2'-5'-oligo	Autoimmune Disea_Cysteine-S-Acetamide	G=14, A=14	G=0.5, A=0.5	0.00001	131.941	28	G=8/6, A=11/3
chr1:228402508	T/T	C	T	SNV	OBSCN	OBSCN:exonic:NM_001271223.2	1	100	5	synonymous	NM_00127	c.1537C>T	p.(=)	0.34				0.13(COSM47	Fibronectin t	rs2776853	0.39	YES	1.936E-1 (pos.22840250	adenocarcinoma				C=0, T=34	C=0.0, T=1.0	0.00001	523.746	34	C=0/0, T=22/1
chr2:75116627	T/T	C	T	SNV	HK2	HK2:intronic:NM_000189.4	1	100		intronic	NM_00018	c.2609+22	p.?	-0.48					Hexokinase	rs4853069	0.4413	YES	6.452E-1 (pos.75116627)		Hexokinase	ATP bindin	Cardiovascular Diseases;Heart Diseases;My	C=0, T=52	C=0.0, T=1.0	0.00001	744.737	52	C=0/0, T=19/3
chr1:228412308	A/A	G	A	SNV	OBSCN	OBSCN:exonic:NM_001271223.2	1	100	10	synonymous	NM_00127	c.3078G>A	p.(=)	-1.13				0.06(COSM44	Fibronectin t	rs1757152	0.3925	YES	4.144E-1 (pos.22841230	acute_myeloid_leukaemia,neoplasm				G=0, A=52	G=0.0, A=1.0	0.00001	762.227	52	G=0/0, A=30/2
chr1:228431095	G/G	A	G	SNV	OBSCN	OBSCN:exonic:NM_001271223.2	1	100	11	synonymous	NM_00127	c.3417A>G	p.(=)	-3.38					Fibronectin t	rs1771455	0.3172	YES	5.783E-1 (pos.228431095)					A=0, G=79	A=0.0, G=1.0	0.00001	1269.33	79	A=0/0, G=50/2
chr1:228444565	A/A	T	A	SNV	OBSCN	OBSCN:exonic:NM_001271223.2	1	100	16	missense	NM_00127	c.4799T>A	p.Val1600Asp	6.58	1	152		0.03(COSM45	Fibronectin t	rs7532342		YES	1.0 (pos.228444565)	squamous_cell_carcinoma				T=0, A=110	T=0.0, A=1.0	0.00001	1737.83	110	T=0/0, A=58/5
chr1:228464248	G/G	T	G	SNV	OBSCN	OBSCN:exonic:NM_001271223.2	1	100	26	missense	NM_00127	c.7443T>G	p.Asp2481Glu	-0.29	1	45		0.07(COSM13	Fibronectin t	rs1188721	0.3035	YES	7.28E-1 (pos.228464248)	adenocarcinoma,carcinoma				T=0, G=54	T=0.0, G=1.0	0.00001	798.147	54	T=0/0, G=23/3
chr2:84774893	G/A	G	A	SNV	DNAH6	DNAH6:intronic:NM_001370.1	1	53.33		intronic	NM_00137	c.1186+15	p.?	0.31					AAA domain (	rs12474026		YES			Dynein, axo	ATP bindin	Congenital Abnormalities;Congenital, Heredi	G=14, A=16	G=0.4667, A=0.5333	0.00001	153.177	30	G=5/9, A=8/8
chr1:228471379	C/C	G	C	SNV	OBSCN	OBSCN:exonic:NM_001271223.2	1	100	38	synonymous	NM_00127	c.10200G>	p.(=)	0.21				0.13(COSM39	Fibronectin t	rs56168609	0.3681	YES	1.522E-1 (pos.22847137	acute_myeloid_leukaemia				G=0, C=70	G=0.0, C=1.0	0.00001	1091.82	70	G=0/0, C=37/3
chr2:84806553	A/A	G	A	SNV	DNAH6	DNAH6:intronic:NM_001370.1	1	100		intronic	NM_00137	c.2077-98	p.?	-0.19					AAA domain (	rs6547569		YES			Dynein, axo	ATP bindin	Congenital Abnormalities;Congenital, Heredi	G=0, A=106	G=0.0, A=1.0	0.00001	1662.79	106	G=0/0, A=37/6
chr2:84852011	C/T	C	T	SNV	DNAH6	DNAH6:intronic:NM_001370.1	1	50		intronic	NM_00137	c.4354-15	p.?	0.28					AAA domain (	rs11676670	0.0801	YES	1.485E-2 (pos.84852011)		Dynein, axo	ATP bindin	Congenital Abnormalities;Con_Benign	C=12, T=12	C=0.5, T=0.5	0.00001	106.708	24	C=4/8, T=4/8
chr1:228482028	C/C	G	C	SNV	OBSCN	OBSCN:exonic:NM_001271223.2	1	100	47	synonymous	NM_00127	c.12594G>	p.(=)	1.39				0.88(COSM44	Fibronectin t	rs4653547	0.1766	YES	1.818E-1 (pos.22848202	acute_myeloid_leukaemia				G=0, C=42	G=0.0, C=1.0	0.00001	685.22	42	G=0/0, C=11/3
chr2:84899360	G/A	G	A	SNV	DNAH6	DNAH6:intronic:NM_001370.1	1	57.89		intronic	NM_00137	c.6455-91	p.?	-0.34					AAA domain (	rs4832106		YES			Dynein, axo	ATP bindin	Congenital Abnormalities;Congenital, Heredi	G=16, A=22	G=0.4211, A=0.5789	0.00001	249.038	38	G=8/8, A=15/7
chr1:228503677	G/G	A	G	SNV	OBSCN	OBSCN:exonic:NM_001271223.2	1	100	61	missense	NM_00127	c.16013A>	p.His5338Arg	-0.14	0.52	29			Fibronectin t	rs1150912	0.2944	YES	7.335E-1 (pos.228503677)					A=0, G=48	A=0.0, G=1.0	0.00001	810.265	48	A=0/0, G=30/1
chr1:228504472	C/C	T	C	SNV	OBSCN	OBSCN:exonic:NM_001271223.2	1	100	62	missense	NM_00127	c.16219T>	p.Cys5407Arg	0.55	0.55	180		0.02(COSM37	Fibronectin t	rs1188732	0.2992	YES	7.283E-1 (pos.22850447	adenocarcinoma,neoplasm				T=0, C=159	T=0.0, C=1.0	0.00001	2560.66	159	T=0/0, C=86/7
chr1:228504669	A/A	G	A	SNV	OBSCN	OBSCN:exonic:NM_001271223.2	1	100	62	synonymous	NM_00127	c.16416G>	p.(=)	0.01				0.31(COSM37	Fibronectin t	rs61825302	0.1453	YES	1.271E-1 (pos.22850466	adenocarcinoma,hepatocellular_carcinoma				G=0, A=76	G=0.0, A=1.0	0.00001	1209.41	76	G=0/0, A=48/2
chr2:85573033	A/A	G	A	SNV	RETSAT	RETSAT:intronic:NM_017750.3	1	100		intronic	NM_01775	c.1117+65	p.?	-0.96					FAD binding d	rs908302		YES	9.645E-1 (pos.85573033)		Retinol satu	all-trans-r	Arrhythmias, Cardi; Vitamin A	G=0, A=86	G=0.0, A=1.0	0.00001	1410.93	86	G=0/0, A=56/3
chr2:85573049	A/A	G	A	SNV	RETSAT	RETSAT:intronic:NM_017750.3	1	100		intronic	NM_01775	c.1117+49	p.?	-0.05					FAD binding d	rs908304	0.3867	YES	5.22E-1 (pos.85573049)		Retinol satu	all-trans-r	Arrhythmias, Cardi; Vitamin A	G=0, A=86	G=0.0, A=1.0	0.00001	1410.93	86	G=0/0, A=56/3
chr1:228505204	A/A	G	A	SNV	OBSCN	OBSCN:exonic:NM_001271223.2	1	100	63	missense	NM_00127	c.16472G>	p.Arg5491His	1.33	0.08	29		0.03(COSM49	Fibronectin t	rs4653942	0.1944	YES	5.245E-1 (pos.22850520	ductal_carcinoma,osteosarcoma				G=0, A=76	G=0.0, A=1.0	0.00001	1209.59	76	G=0/0, A=38/3
chr2:85890758	T/C	T	C	SNV	SFTPB	SFTPB:intronic:NM_198843.2	1	53.33		intronic	NM_19884	c.892+29A	p.?	-0.47					Saposin A-typ	rs2304566	0.2571	YES	9.039E-2 (pos.85890758)		Pulmonary	G-protein	Congenital, Hereditary, and Neonatal Diseas	T=14, C=16	T=0.4667, C=0.5333	0.00001	152.554	30	T=7/7, C=7/9
chr2:85925667	G/A	G	A	SNV	GNLY	GNLY:intronic:NM_006433.4	1	55.77		intronic	NM_00643	c.428-20G	p.?	0.9						rs4240201	0.3816	YES	5.819E-1 (pos.85925667)		Granulysin	antimicrobial humoral immune	3N-MorpholinoPropane	G=23, A=29	G=0.4423, A=0.5577	0.00001	299.667	52	G=21/2, A=28/
chr2:86305123	C/C	T	C	SNV	POLR1A	POLR1A:intronic:NM_015425.5	1	100		intronic	NM_01542	c.1258-19	p.?	0.54					RNA polymer	rs2288117	0.0623	YES	9.816E-1 (pos.86305123)		Polymerase	DNA binding,DNA-directed 5'-3' RNA polymerase activity,		T=0, C=23	T=0.0, C=1.0	0.00001	316.739	23	T=0/0, C=20/3
chr2:86359405	T/T	G	T	SNV	PTCD3	PTCD3:intronic:NM_017952.5	1	100		intronic	NM_01795	c.1267-36	p.?	-0.06					PPR repeat,P	rs1559515	0.1542	YES	6.574E-1 (pos.86359405)		Pentatricor	RNA binding,cytosol,mitochondrial inner membrane,mit		G=0, T=20	G=0.0, T=1.0	0.00001	242.557	20	G=0/0, T=13/7
chr2:86437551	G/G	T	G	SNV	MRPL35	MRPL35:intronic:NM_016622.3	1	100		intronic	NM_01662	c.379-52T	p.?	1.39					Ribosomal pr	rs7565645	0.0744	YES			Mitochondr	mitochondrial inner membrane,mitochondrial large ribo		T=0, G=25	T=0.0, G=1.0	0.00001	353.004	25	T=0/0, G=9/16
chr1:228505668	G/G	C	G	SNV	OBSCN	OBSCN:exonic:NM_001271223.2	1	100	64	missense	NM_00127	c.16796C>	p.Ser5599Cys	2.59	0.11	112			Fibronectin t	rs1188729	0.2984	YES	7.293E-1 (pos.228505668)					C=0, G=81	C=0.0, G=1.0	0.00001	1309.42	81	C=0/0, G=55/2
chr2:86850022	C/C	T	C	SNV	RNF103,RNF103-CHMP3	RNF103:intronic:NM_001198951.1, RNF103-CHMP3:intronic	1	100		intronic	NM_00119	c.-7-2430A	p.?, p.?	-0.04					RING-like zinc	rs396354,rs9700096	0.2743	YES	9.384E-1 (pos.86850022)			ESCRT III co	Arthritis;Arthritis, Juvenile;Autoimmune Dise	T=0, C=34	T=0.0, C=1.0	0.00001	524.186	34	T=0/0, C=3/31
chr1:228525008	A/A	G	A	SNV	OBSCN	OBSCN:exonic:NM_001271223.2	1	100	77	missense	NM_00127	c.19595G>	p.Arg6532His	6.88	0.66	29		0.95(COSM13	Fibronectin t	rs3795809	0.1356	YES	7.357E-2 (pos.22852500	adenocarcinoma				G=0, A=20	G=0.0, A=1.0	0.00001	263.784	20	G=0/0, A=12/8
chr1:228560139	G/G	A	G	SNV	OBSCN	OBSCN:exonic:NM_001271223.2	1	100	105	synonymous	NM_00127	c.24531A>	p.(=)	-3.08				0.02(COSM41	Fibronectin t	rs498382,rs7690236	0.0741	YES	0.0 (pos.228560129),0.0	neoplasm				A=0, G=41	A=0.0, G=1.0	0.00001	664.699	41	A=0/0, G=32/9
chr2:220430203	T/T	C	T	SNV	OBSL1	OBSL1:exonic:NM_015311.2	1	100	6	missense	NM_01531	c.2168G>A	p.Arg723Lys	-0.12	1	26	0	0.03(COSM41	Immunoglobu	rs1039898	0.0957	YES	9.11E-1 (pos.220430203)	carcinoma,neo	Obscurin-lik	3M compl	Bone Diseases;Bone Diseases,_Benign	C=0, T=58	C=0.0, T=1.0	0.00001	867.257	58	C=0/0, T=44/1
chr8:133067315	A/A	G	A	SNV	OC90	OC90:utr_5:NM_001080399.2	1	100	2	utr_5	NM_00108	c.-42C>T	p.?	-1.48				0(COSM3982	Phospholipas	rs4736538	0.3639	YES	3.825E-1 (pos.13306731	acute_myeloic	Otoconin 9	arachidon	Ear Diseases;Labyrinth Diseases;Otorhinolar	G=0, A=53	G=0.0, A=1.0	0.00001	782.03	53	G=0/0, A=17/3
chr2:9419411	A/A	G	A	SNV	ASAP2	ASAP2:intronic:NM_003887.2	1	100		intronic	NM_00388	c.127-35G	p.?	0.08					Ankyrin repea	rs10193043	0.0986	YES	7.304E-1;0.0 (pos.9419411)			GTPase activator activity,Golgi cisterna membrane,met		G=0, A=22	G=0.0, A=1.0	0.00001	298.892	22	G=0/0, A=8/14
chr15:28096538	G/G	A	G	SNV	OCA2	OCA2:exonic:NM_000275.2	1	100	22	synonymous	NM_00027	c.2328T>C	p.(=)	1.72				0.05(COSM17	Citrate trans	rs1800419	0.4298	YES	4.416E-1 (pos.28096538)	adenocarcinor	Pink-eye dil	L-tyrosine	Albinism:Albinism, Oculocutan_Benign	A=0, G=29	A=0.0, G=1.0	0.00001	427.094	29	A=0/0, G=10/1
chr15:28211921	A/A	G	A	SNV	OCA2	OCA2:exonic:NM_000275.2	1	100	15	synonymous	NM_00027	c.1551C>T	p.(=)	1.92				0.88(COSM44	Citrate trans	rs1800411	0.2938	YES	1.422E-1 (pos.28211921)	acute_myeloic	Pink-eye dil	L-tyrosine	Albinism:Albinism, Oculocutan_Benign	G=0, A=65	G=0.0, A=1.0	0.00001	996.427	65	G=0/0, A=37/2
chr15:28235773	T/T	C	T	SNV	OCA2	OCA2:exonic:NM_000275.2	1	100	10	synonymous	NM_00027	c.1065G>A	p.(=)	-1.27					Citrate trans	rs1800404	0.2102	YES	3.865E-1 (pos.28235773)		Pink-eye dil	L-tyrosine	Albinism;Albinism, Oculocutan_Benign	C=0, T=27	C=0.0, T=1.0	0.00001	389.988	27	C=0/0, T=18/9
chr8:103572992	G/G	C	G	SNV	ODF1	ODF1:exonic:NM_024410.3	1	100	2	synonymous	NM_02441	c.633C>G	p.(=)	-1.96					Hsp20/alpha	rs3018444,rs386728	0.1067	YES	0.0 (pos.103572988),0.0;0.0;0.0 (pos.10		Outer dens	cell differentiation,multicellular organism development		C=0, G=20	C=0.0, G=1.0	0.00001	263.786	20	C=0/0, G=9/11
chr2:96781986	C/C	G	C	SNV	ADRA2B	ADRA2B:upstream:NM_000682.6	1	100		intronic	NM_00068	c.-98C>G	p.?	-1.78						rs3111873		YES			Adrenergic,	G-protein	Brain Diseases;Car; Amoxapine,Amphetamir	G=0, C=22	G=0.0, C=1.0	0.00001	298.792	22	G=0/0, C=3/19
chr1:86818572	C/C	T	C	SNV	ODF2L	ODF2L:exonic:NM_001007022.2	1	100	17	synonymous	NM_00100	c.1863A>G	p.(=)	0.45						rs272496	0.3502	YES	8.324E-1 (pos.86818572)			centrosome		T=0, C=27	T=0.0, C=1.0	0.00001	389.967	27	T=0/0, C=24/3
chr1:86820267	T/T	C	T	SNV	ODF2L	ODF2L:exonic:NM_001007022.2	1	100	16	synonymous	NM_00100	c.1713G>A	p.(=)	-0.01				0.08(COSM3747087),0.08(C		rs1182098	0.3416	YES	8.313E-1 (pos.86820267)	hepatocellular_carcinoma		centrosome		C=0, T=111	C=0.0, T=1.0	0.00001	1757.15	111	C=0/0, T=61/5
chr1:86838158	T/T	C	T	SNV	ODF2L	ODF2L:exonic:NM_001007022.2	1	100	9	synonymous	NM_00100	c.876G>A	p.(=)	0.31				0.02(COSM3747090),0.02(C		rs421105	0.342	YES	8.35E-1 (pos.86838158)	carcinoma,hepatocellular		centrosome		C=0, T=62	C=0.0, T=1.0	0.00001	939.035	62	C=0/0, T=23/3
chr2:97867870	C/C	G	C	SNV	ANKRD36	ANKRD36:intronic:NM_001164315.1	1	100		intronic	NM_00116	c.2858-69	p.?	-0.48					Ankyrin repea	rs9324211		YES						G=0, C=67	G=0.0, C=1.0	0.00001	1034.43	67	G=0/0, C=31/3
chr2:97873630	T/T	A	T	SNV	ANKRD36	ANKRD36:intronic:NM_001164315.1	1	100		intronic	NM_00116	c.3193-41	p.?	-0.28					Ankyrin repea	rs12052928		YES	2.483E-1 (pos.97873630)					A=0, T=46	A=0.0, T=1.0	0.00001	772.685	46	A=0/0, T=24/2
chr19:9964823	G/G	A	G	SNV	OLFM2	OLFM2:utr_3:NM_058164.3	1	100	6	utr_3	NM_05816	c.1404T>C	p.?	-2.57					Neurogenesis	rs8111802	0.0102	YES	6.78E-1 (pos.9964823)		Olfactomed	AMPA glutamate receptor complex,cell junction,cytopla		A=0, G=24	A=0.0, G=1.0	0.00001	328.776	24	A=0/0, G=3/21
chr2:97877399	C/C	T	C	SNV	ANKRD36	ANKRD36:intronic:NM_001164315.1	1	100		intronic	NM_00116	c.3397-7T	p.?	0.14				0(COSM4001	Ankyrin repea	rs10186233	0.123	YES	6.506E-1 (pos.97877399)	acute_myeloid_leukaemia,carcinoma				T=0, C=470	T=0.0, C=1.0	0.00001	7875.06	470	T=0/0, C=213/
chr2:98169583	G/T	G	T	SNV	ANKRD36B	ANKRD36B:intronic:NM_025190.3	1	50		intronic	NM_02519	c.1273+48	p.?	-1.88					Ankyrin repea	rs9679293	0.3488	YES	3.281E-1;1.45E-4 (pos.98169583)			protein binding		G=14, T=14	G=0.5, T=0.5	0.00001	131.936	28	G=5/9, T=9/5
chr2:98376989	T/T	TA	T	INDEL	TMEM131	TMEM131:intronic:NM_015348.1	1	100		intronic	NM_01534	c.5108+67	p.?	-2.84, -0.25					Transmembra	rs3214760,rs397819712		YES			Transmemb	biological_process,cellular_component,integral compor		TA=0, T=31	TA=0.0, T=1.0	0.00001	401.112	31	TA=0/0, T=14/
chr2:98418812	C/C	CA	C	INDEL	TMEM131	TMEM131:intronic:NM_015348.1	1	100		intronic	NM_01534	c.2651+79	p.?	0.56, -0.62					Transmembra	rs11340992,rs397872143		YES			Transmemb	biological_process,cellular_component,integral compor		CA=0, C=23	CA=0.0, C=1.0	0.00001	307.533	23	CA=0/0, C=8/1
chr2:98449796	T/T	C	T	SNV	TMEM131	TMEM131:intronic:NM_015348.1	1	100		intronic	NM_01534	c.1074+32	p.?	-1.53					Transmembra	rs6721250			1.0 (pos.98449796)		Transmemb	biological_process,cellular_component,integral compor		C=0, T=54	C=0.0, T=1.0	0.00001	798.138	54	C=0/0, T=25/2
chr1:59004979	T/T	C	T	SNV	OMA1	OMA1:utr_5:NM_145243.4	1	100	2	utr_5	NM_14524	c.-13G>A	p.?	0.95					Peptidase fan	rs902925	6.00E-04		1.0 (pos.59004979)		OMA1 zinc r	cristae formation,diet induced thermogenesis,energy hc		C=0, T=30	C=0.0, T=1.0	0.00001	446.635	30	C=0/0, T=17/1
chr2:98906955	T/C	T	C	SNV	VWA3B	VWA3B:intronic:NM_144992.4	1	58.33		intronic	NM_14499	c.3046-19	p.?	0.11					Domain of un	rs62155282	0.0592	YES	2.583E-4 (pos.98906955)		von Willebr	cytoplasm		T=10, C=14	T=0.4167, C=0.5833	0.00001	135.31	24	T=9/1, C=11/3
chr6:74079041	C/C	A	C	SNV	OOEP	OOEP:exonic:NM_001080507.2	1	100	2	synonymous	NM_00108	c.258T>G	p.(=)	-1.26					KH-like RNA-b	rs497243	4.00E-04		1.0 (pos.74079041)		Oocyte-exp	RNA binding,apical part of cell,biological_process,cell co		A=0, C=79	A=0.0, C=1.0	0.00001	1268.61	79	A=0/0, C=49/3
chr7:128415195	T/G	T	G	SNV	OPN1SW	OPN1SW:exonic:NM_001708.2	1	56	2	synonymous	NM_00170	c.366A>C	p.(=)	-0.74				0.08(COSM15	7 transmemb	rs1799922	0.4036	YES	5.61E-2 (pos.128415195)	adenocarcinor	Blue cone p	G-protein	Color Vision Defects:Eye Diseases:Nervous Sy	T=11, G=14	T=0.44, G=0.56	0.00001	134.35	25	T=7/4, G=7/7
chr1:29138975	T/T	G	T	SNV	OPRD1	OPRD1:exonic:NM_000911.3	1	100	1	missense	NM_00091	c.80G>T	p.Cys27Phe	0.52	0.76	205	0	0.02(COSM37	7 transmemb	rs1042114,rs770681	0.1112	YES	1.0;0.0 (pos.29138975)	adenocarcinor	Opioid rece	G-protein	Autonomic Nervous; 3-Methylfentanyl,3-Meth	G=0, T=63	G=0.0, T=1.0	0.00001	947.487	63	G=0/0, T=35/2
chr6:154414446	T/T	A	T	SNV	OPRM1	OPRM1:exonic:NM_001008505.2	1	100	4	missense	NM_00100	c.1206A>T	p.Gln402His	-0.68	1	24	0	0(COSM3761	7 transmemb	rs540825	0.2377	YES	9.373E-1;0.0 (pos.15441	adenocarcinor	Opioid rece	G-protein	Alcohol-Related Dis; 3-Methylfentanyl,3-Meth	A=0, T=52	A=0.0, T=1.0	0.00001	767.009	52	A=0/0, T=22/3
chr2:9983716	C/C	A	C	SNV	TAF1B	TAF1B:intronic:NM_005680.2	1	100		intronic	NM_00568	c.18+28A>	p.?	-0.12					Zinc-finger of	rs385513	0.0054	YES	1.0;0.0 (pos.9983716)			DNA binding,transcription factor activity,RNA polymeras		A=0, C=55	A=0.0, C=1.0	0.00001	814.907	55	A=0/0, C=4/51
chr2:9991556	A/A	G	A	SNV	TAF1B	TAF1B:intronic:NM_005680.2	1	100		intronic	NM_00568	c.206-114	p.?	-1.71					Zinc-finger of	rs111708404,rs287991,rs869072018						DNA binding,transcription factor activity,RNA polymeras		G=0, A=23	G=0.0, A=1.0	0.00001	315.32	23	G=0/0, A=7/16
chr2:99998561	T/T	C	T	SNV	EIF5B	EIF5B:intronic:NM_015904.3	1	100		intronic	NM_01590	c.2062-61	p.?	0.47					50S ribosome	rs2290256	0.4547	YES				GTP binding,GTPase activity,RNA binding,cytoplasm,cyto		C=0, T=35	C=0.0, T=1.0	0.00001	543.901	35	C=0/0, T=15/2

chr20:10280083	G/A	G	A	SNV	SNAP25	SNAP25:intronic:NM_003081.4	1	52.38		intronic	NM_00308	c.552+23G	p.?	1.28					SNAP-25 fami	rs363006	0.172	YES	1.127E-1 (pos.10280083)		Synaptosom	BLOC-1 co	Bipolar Disorder;Bi	Botulinum Toxin Type A		G=10, A=11	G=0.4762, A=0.5238	0.00001	95.3001	21	G=1/9, A=4/7
chr6:154414563	G/G	A	G	SNV	OPRM1	OPRM1:exonic:NM_001008505.2	1	100	4	synonymous	NM_00100	c.1323A>G	p.(=)	-0.32				0.06(COSM14	7 transmemb	rs675026,rs7463899	0.3319	YES	0.0 (pos.154414558),8.8	adenocarcino	Opioid rece	G-protein	Alcohol-Related Dis	3-Methylfentanyl,3-Meth		A=0, G=33	A=0.0, G=1.0	0.00001	504.598	33	A=0/0, G=17/1
chr20:10633313	C/C	T	C	SNV	JAG1	JAG1:intronic:NM_000214.2	1	100		intronic	NM_00021	c.756-67A	p.?	-3.61					Calcium-bind	rs6040055		YES			Jagged 1	Notch binc	Abnormalities, Multiple;Adenocarcinoma;Ala			T=0, C=175	T=0.0, C=1.0	0.00001	2885.04	175	T=0/0, C=77/9
chr6:154414573	T/T	C	T	SNV	OPRM1	OPRM1:exonic:NM_001008505.2	1	100	4	synonymous	NM_00100	c.1333C>T	p.(=)	0.36				0.12(COSM37	7 transmemb	rs562859,rs7463899	0.3343	YES	0.0 (pos.154414558),8.8	adenocarcino	Opioid rece	G-protein	Alcohol-Related Dis	3-Methylfentanyl,3-Meth		C=0, T=33	C=0.0, T=1.0	0.00001	504.593	33	C=0/0, T=17/1
chr20:1277099	G/A	G	A	SNV	SNPH	SNPH:intronic:NM_014723.3	1	57.14		intronic	NM_01472	c.50+34G>	p.?	-4.18					Golgi-localise	rs6078493	0.1157	YES	6.709E-2 (pos.1277099)		Syntaphilin	brain development,cell junction,cytoplasm,cytoplasmic				G=36, A=48	G=0.4286, A=0.5714	0.00001	550.577	84	G=17/19, A=31
chr10:13166076	G/G	A	G	SNV	OPTN	OPTN:exonic:NM_021980.4	1	100	8	missense	NM_02198	c.964A>G	p.Lys322Glu	6.05	1	56	0		Leucine zippe	rs386741044,rs5237	0.0012		1.0 (pos.13166076)		Optineurin	G2/M tran	Amyotrophic Lateral Sclerosis;Bone Diseases			A=0, G=55	A=0.0, G=1.0	0.00001	814.876	55	A=0/0, G=29/2
chr20:12990044	C/C	T	C	SNV	SPTLC3	SPTLC3:intronic:NM_018327.3	1	100		intronic	NM_01832	c.117+12T	p.?	-0.15					Aminotransfe	rs3761895	0.2497	YES	5.382E-1 (pos.12990044)		Serine palm	biosynthe	Bipolar Disorder;Bi	Pyridoxal Phosphate		T=0, C=26	T=0.0, C=1.0	0.00001	371.42	26	T=0/0, C=7/19
chr11:7949350	G/G	A	G	SNV	OR10A6	OR10A6:exonic:NM_001004461.1	1	100	1	missense	NM_00100	c.860T>C	p.Leu287Pro	7.05	1	98	0		7 transmemb	rs4758258	0.2201	YES	7.989E-1 (pos.7949350)			G-protein coupled receptor activity,G-protein coupled re				A=0, G=73	A=0.0, G=1.0	0.00001	1150.51	73	A=0/0, G=35/3
chr11:7949791	C/C	A	C	SNV	OR10A6	OR10A6:exonic:NM_001004461.1	1	100	1	missense	NM_00100	c.419T>G	p.Val140Gly	1.16	0.02	109	0.979		7 transmemb	rs7933807	0.4006	YES	5.186E-1 (pos.7949791)			G-protein coupled receptor activity,G-protein coupled re				A=0, C=50	A=0.0, C=1.0	0.00001	733.515	50	A=0/0, C=20/3
chr20:13074235	A/A	G	A	SNV	SPTLC3	SPTLC3:intronic:NM_018327.3	1	100		intronic	NM_01832	c.826+11G	p.?	-0.9					Aminotransfe	rs708937	0.0637	YES	1.0 (pos.13074235)		Serine palm	biosynthe	Bipolar Disorder;Bi	Pyridoxal Phosphate		G=0, A=54	G=0.0, A=1.0	0.00001	798.142	54	G=0/0, A=32/2
chr20:13074243	A/A	T	A	SNV	SPTLC3	SPTLC3:intronic:NM_018327.3	1	100		intronic	NM_01832	c.826+19T	p.?	-0.13					Aminotransfe	rs1011290	0.4015	YES	3.118E-1 (pos.13074243)		Serine palm	biosynthe	Bipolar Disorder;Bi	Pyridoxal Phosphate		T=0, A=54	T=0.0, A=1.0	0.00001	798.06	54	T=0/0, A=32/2
chr11:7949860	A/A	G	A	SNV	OR10A6	OR10A6:exonic:NM_001004461.1	1	100	1	missense	NM_00100	c.350C>T	p.Ala117Val	0.55	0.61	64	0.006	0.16(COSM41	7 transmemb	rs7928451	0.403	YES	5.177E-1 (pos.7949860)	neoplasm		G-protein coupled receptor activity,G-protein coupled re				G=0, A=61	G=0.0, A=1.0	0.00001	921.484	61	G=0/0, A=22/3
chr12:48596641	A/G	A	G	SNV	OR10AD1	OR10AD1:exonic:NM_001004134.1	1	57.63	1	synonymous	NM_00100	c.435T>C	p.(=)	-2.28					7 transmemb	rs75539159	0.0829	YES	1.16E-4 (pos.48596641)			G-protein coupled receptor activity,G-protein coupled re				A=25, G=34	A=0.4237, G=0.5763	0.00001	356.5	59	A=11/14, G=1
chr12:48596579	C/T	C	T	SNV	OR10AD1	OR10AD1:exonic:NM_001004134.1	1	50	1	missense	NM_00100	c.497G>A	p.Arg166Gln	-2.92	0.16	43	0.068		7 transmemb	rs17224674	0.0829	YES	3.529E-4;0.0 (pos.48596579)			G-protein coupled receptor activity,G-protein coupled re				C=58, T=58	C=0.5, T=0.5	0.00001	602.299	116	C=25/33, T=22
chr20:13698006	C/C	T	C	SNV	ESF1	ESF1:intronic:NM_001276380.1	1	100		intronic	NM_00127	c.2262+9A	p.?	-1.18					NUC153 dom	rs4239710	9.00E-04		1.0 (pos.13698006)			RNA binding,extracellular space,nucleolus,nucleoplasm				T=0, C=28	T=0.0, C=1.0	0.00001	408.705	28	T=0/0, C=24/4
chr6:29407970	T/C	T	C	SNV	OR10C1	OR10C1:exonic:NM_013941.3	1	52.38	1	missense	NM_01394	c.178T>C	p.Phe60Leu	0.46		22		0.47(COSM37	7 transmemb	rs2074469	0.1878	YES	2.225E-1 (pos.29407970)	acute_myeloid_leukaemia		G-protein coupled receptor activity,G-protein coupled re				T=30, C=33	T=0.4762, C=0.5238	0.00001	333.903	63	T=19/11, C=19
chr20:13763897	ATTAA/ATTAA	A	ATTAA	INDEL	ESF1	ESF1:intronic:NM_001276380.1	4	100		intronic	NM_00127	c.-42-68T>	p.?	0.99					NUC153 domain							RNA binding,extracellular space,nucleolus,nucleoplasm				A=0, ATTAA	A=0.0, ATTAA=1.0	0.00001	281.235	21	A=0/0, ATTAA=
chr20:13782409	G/G	A	G	SNV	NDUFAF5	NDUFAF5:intronic:NM_024120.4	1	100		intronic	NM_02412	c.717+80A	p.?	0.37					Methyltransf	rs1015517		YES			NADH dehy	extrinsic c	Brain Diseases;Brain Diseases, Metabolic;Br			A=0, G=21	A=0.0, G=1.0	0.00001	281.235	21	A=0/0, G=6/15
chr14:22038659	C/C	T	C	SNV	OR10G3	OR10G3:exonic:NM_001005465.1	1	100	1	missense	NM_00100	c.217A>G	p.Ser73Gly	-0.08	0.48	56	0	0.04(COSM13	7 transmemb	rs17792778	0.3317	YES	7.159E-1 (pos.22038659)	acute_myeloid_leukaemia		G-protein coupled receptor activity,G-protein coupled re				T=0, C=87	T=0.0, C=1.0	0.00001	1433.71	87	T=0/0, C=59/2
chr20:1457045	T/G	T	G	SNV	SIRPB2	SIRPB2:intronic:NM_001122962.1	1	50		intronic	NM_00112	c.860-64A	p.?	0.02					Immunoglobu	rs6042499		YES			Signal regul	integral component of membrane				T=11, G=11	T=0.5, G=0.5	0.00001	94.6065	22	T=6/5, G=5/6
chr14:22038210	G/T	G	T	SNV	OR10G3	OR10G3:exonic:NM_001005465.1	1	50	1	synonymous	NM_00100	c.666C>A	p.(=)	-0.03				0.18(COSM41	7 transmemb	rs17792766	0.1509	YES	3.814E-3 (pos.22038210)	neoplasm		G-protein coupled receptor activity,G-protein coupled re				G=12, T=12	G=0.5, T=0.5	0.00001	106.706	24	G=9/3, T=8/4
chr11:123886865	A/A	T	A	SNV	OR10G4	OR10G4:exonic:NM_001004462.1	1	100	1	missense	NM_00100	c.584T>A	p.Val195Glu	0.61	1	121	0	0(COSM4145	7 transmemb	rs386758379,rs4084	0.2368	YES	7.304E-1 (pos.12388686	neoplasm		G-protein coupled receptor activity,G-protein coupled re				T=0, A=20	T=0.0, A=1.0	0.00001	263.786	20	T=0/0, A=13/7
chr11:123887184	G/G	T	G	SNV	OR10G4	OR10G4:exonic:NM_001004462.1	1	100	1	synonymous	NM_00100	c.903T>G	p.(=)	-7.41				0(COSM1473	7 transmemb	rs4936882	0.237	YES	7.229E-1 (pos.12388718	adenocarcinoma		G-protein coupled receptor activity,G-protein coupled re				T=0, G=31	T=0.0, G=1.0	0.00001	465.791	31	T=0/0, G=14/1
chr11:123909695	C/C	G	C	SNV	OR10G7	OR10G7:exonic:NM_001004463.1	1	100	1	missense	NM_00100	c.14C>G	p.Thr5Ser	-0.2	0.8	58	0		7 transmemb	rs3894197	0.2428	YES	7.386E-1 (pos.123909695)			G-protein coupled receptor activity,G-protein coupled re				G=0, C=63	G=0.0, C=1.0	0.00001	958.865	63	G=0/0, C=39/2
chr11:123894136	G/G	A	G	SNV	OR10G9	OR10G9:exonic:NM_001001953.1	1	100	1	synonymous	NM_00100	c.417A>G	p.(=)	-3.34				0(COSM4594	7 transmemb	rs28499345		YES	8.011E-1 (pos.12389413	squamous_cell_carcinoma		G-protein coupled receptor activity,G-protein coupled re				A=0, G=119	A=0.0, G=1.0	0.00001	1913.82	119	A=0/0, G=48/7
chr20:16729262	T/T	C	T	SNV	OTOR	OTOR:intronic:NM_020157.3	1	100		intronic	NM_02015	c.115+101	p.?	-0.47					Variant SH3 c	rs6080387		YES			Otoraplin	cartilage condensation,extracellular region,sensory per				C=0, T=112	C=0.0, T=1.0	0.00001	1776.36	112	C=0/0, T=59/5
chr20:17389830	C/C	T	C	SNV	PCSK2	PCSK2:intronic:NM_002594.4	1	100		intronic	NM_00259	c.544-78T	p.?	-0.32					Peptidase S8	rs2217182		YES			Proprotein	dendrite,e	Diabetes Mellitus;[	Insulin Human,Insulin Pc		T=0, C=45	T=0.0, C=1.0	0.00001	747.143	45	T=0/0, C=39/6
chr19:15918455	G/A	G	A	SNV	OR10H1	OR10H1:exonic:NM_013940.2	1	52.38	1	synonymous	NM_01394	c.393C>T	p.(=)	0.18				0.22(COSM41	7 transmemb	rs61739573	0.2897	YES	1.943E-1 (pos.15918455)	neoplasm		G-protein coupled receptor signaling pathway,G-protein				G=50, A=55	G=0.4762, A=0.5238	0.00001	571.444	105	G=18/32, A=2
chr19:15838972	T/A	T	A	SNV	OR10H2	OR10H2:exonic:NM_013939.2	1	56	1	missense	NM_01393	c.119T>A	p.Leu40Gln	0.64		113		0.42(COSM37	7 transmemb	rs4569397,rs776540	0.2875	YES	2.117E-2;0.0 (pos.15838	adenocarcinoma,ductal_ca		G-protein coupled receptor signaling pathway,G-protein				T=11, A=14	T=0.44, A=0.56	0.00001	134.35	25	T=11/0, A=6/8
chr19:15852223	C/C	A	C	SNV	OR10H3	OR10H3:exonic:NM_013938.1	1	100	1	missense	NM_01393	c.21A>C	p.Arg7Ser	-0.23	0.61	110	0	0(COSM1179	7 transmemb	rs1966357	0.265	YES	3.516E-1;1.159E-4 (pos.1	acute_myeloid_leukaemia		G-protein coupled receptor signaling pathway,G-protein				A=0, C=115	A=0.0, C=1.0	0.00001	1834.87	115	A=0/0, C=82/3
chr19:15852872	G/A	G	A	SNV	OR10H3	OR10H3:exonic:NM_013938.1	1	52.38	1	missense	NM_01393	c.670G>A	p.Val224Met	-1.72	0.03	21	0.581	0.03(COSM14	7 transmemb	rs2240228	0.2591	YES	1.953E-1 (pos.15852872)	adenocarcinoma		G-protein coupled receptor signaling pathway,G-protein				G=30, A=33	G=0.4762, A=0.5238	0.00001	333.933	63	G=16/14, A=2
chr19:16060658	A/G	A	G	SNV	OR10H4	OR10H4:exonic:NM_001004465.1	1	59.18	1	missense	NM_00100	c.841A>G	p.Thr281Ala	0.8	0.01	58	0.051	0.04(COSM40	7 transmemb	rs11880184	0.2381	YES	9.35E-2 (pos.16060658)	acute_myeloid_leukaemia		G-protein coupled receptor signaling pathway,G-protein				A=20, G=29	A=0.4082, G=0.5918	0.00001	360.526	49	A=9/11, G=13/
chr1:159409857	G/G	A	G	SNV	OR10J1	OR10J1:exonic:NM_012351.2	1	100	1	missense	NM_01235	c.309A>G	p.Ile103Met	-1.59		10		0.08(COSM14	7 transmemb	rs12048482	0.3635	YES	2.353E-1 (pos.15940985	adenocarcinoma,neoplasn		G-protein coupled receptor activity,G-protein coupled re				A=0, G=60	A=0.0, G=1.0	0.00001	903.531	60	A=0/0, G=15/4
chr20:18393499	T/T	C	T	SNV	DZANK1	DZANK1:intronic:NM_001099407.1	1	100		intronic	NM_00109	c.1243-20	p.?	1.98				0.01(COSM63	Chitobiase/b	rs6075338	0.2137	YES	2.905E-1 (pos.18393499)	carcinoma		metal ion binding				C=0, T=90	C=0.0, T=1.0	0.00001	1493.12	90	C=0/0, T=60/3
chr1:159505297	T/T	C	T	SNV	OR10J5	OR10J5:exonic:NM_001004469.1	1	100	1	synonymous	NM_00100	c.501G>A	p.(=)	-2.66				0.04(COSM14	7 transmemb	rs4656837	0.2873	YES	4.722E-1 (pos.15950529	adenocarcinoma,carcinom		G-protein coupled receptor signaling pathway,G-protein				C=0, T=38	C=0.0, T=1.0	0.00001	603.786	38	C=0/0, T=18/2
chr20:18464110	C/C	T	C	SNV	POLR3F	POLR3F:intronic:NM_006466.3	1	100		intronic	NM_00646	c.874-15T	p.?	0.53					RNA polymera	rs3827965,rs765100	0.131	YES	8.128E-1;0.0;0.0 (pos.18464110)		Polymerase	DNA-directed 5'-3' RNA polymerase activity,DNA-directe				T=0, C=118	T=0.0, C=1.0	0.00001	1797.77	118	T=0/0, C=70/4
chr20:18470664	G/G	A	G	SNV	RBBP9	RBBP9:intronic:NM_006606.2	1	100		intronic	NM_00660	c.335-30T	p.?	-0.48					Serine hydrol	rs3748450	0.1287	YES	8.218E-1 (pos.18470664)		Retinoma-b	cytoplasm,extracellular exosome,hydrolase activity,nuc				A=0, G=60	A=0.0, G=1.0	0.00001	903.531	60	A=0/0, G=29/3
chr1:158450382	C/T	C	T	SNV	OR10R2	OR10R2:exonic:NM_001004472.1	1	55.77	1	missense	NM_00100	c.715C>T	p.Leu239Phe	-6.67	0.03	22	0.336		7 transmemb	rs1418843	0.4331	YES	5.529E-1 (pos.158450382)			G-protein coupled receptor activity,G-protein coupled re				C=23, T=29	C=0.4423, T=0.5577	0.00001	299.667	52	C=14/9, T=24/
chr11:59480951	C/C	T	C	SNV	OR10V1	OR10V1:exonic:NM_001005324.1	1	100	1	missense	NM_00100	c.368A>G	p.Gln123Arg	3.05	1	43	0		7 transmemb	rs386754016,rs4990	0.0873	YES	1.0;0.0 (pos.59480951)			G-protein coupled receptor activity,G-protein coupled re				T=0, C=51	T=0.0, C=1.0	0.00001	754.581	51	T=0/0, C=20/3
chr14:20665790	A/A	T	A	SNV	OR11G2	OR11G2:exonic:NM_001005503.1	1	100	1	missense	NM_00100	c.296T>A	p.Ile99Asn	4.84	0	149	0.913	0.95(COSM39	7 transmemb	rs4981822	0.4817	YES	4.924E-1;3.472E-4;0.0 (pc	acute_myeloid_leukaemia		G-protein coupled receptor activity,G-protein coupled re				T=0, A=66	T=0.0, A=1.0	0.00001	1015.4	66	T=0/0, A=28/3
chr14:20665840	A/A	G	A	SNV	OR11G2	OR11G2:exonic:NM_001005503.1	1	100	1	missense	NM_00100	c.346G>A	p.Val116Ile	0.27	0.07	29	0.185	0.26(COSM39	7 transmemb	rs4981088	0.482	YES	5.214E-1 (pos.20665840)	acute_myeloid_leukaemia		G-protein coupled receptor activity,G-protein coupled re				G=0, A=68	G=0.0, A=1.0	0.00001	1051.15	68	G=0/0, A=28/4
chr14:20665887	C/C	G	C	SNV	OR11G2	OR11G2:exonic:NM_001005503.1	1	100	1	synonymous	NM_00100	c.393G>C	p.(=)	-2.65				0.07(COSM39	7 transmemb	rs4981823	0.0184	YES	7.033E-1 (pos.20665887)	acute_myeloid_leukaemia		G-protein coupled receptor activity,G-protein coupled re				G=0, C=70	G=0.0, C=1.0	0.00001	1091.99	70	G=0/0, C=29/4
chr14:20692453	G/T	G	T	SNV	OR11H6	OR11H6:exonic:NM_001004480.1	1	53.13	1	missense	NM_00100	c.585G>T	p.Leu195Phe	-2.55	1	22	0		7 transmemb	rs17211285	0.4831	YES	2.254E-1 (pos.20692453)			G-protein coupled receptor activity,G-protein coupled re				G=15, T=17	G=0.4688, T=0.5313	0.00001	173.557	32	G=3/12, T=6/1
chr20:20140196	C/C	T	C	SNV	CFAP61	CFAP61:intronic:NM_015585.3	1	100		intronic	NM_01558	c.1026+10	p.?	0.22					Domain of un	rs2179823		YES				axoneme,cilium movement,cilium organization,cytoplas				T=0, C=54	T=0.0, C=1.0	0.00001	798.146	54	T=0/0, C=33/2
chr20:20168497	G/G	A	G	SNV	CFAP61	CFAP61:intronic:NM_015585.3	1	100		intronic	NM_01558	c.1386-77	p.?	-1.71					Domain of un	rs2179825		YES				axoneme,cilium movement,cilium organization,cytoplas				A=0, G=56	A=0.0, G=1.0	0.00001	832.1	56	A=0/0, G=28/2
chr1:248004614	A/A	G	A	SNV	OR11L1	OR11L1:exonic:NM_001001959.1	1	100	1	synonymous	NM_00100	c.585C>T	p.(=)	-3.37				0.01(COSM41	7 transmemb	rs6667389	0.118	YES	9.975E-1 (pos.24800461	neoplasm		G-protein coupled receptor activity,G-protein coupled re				G=0, A=167	G=0.0, A=1.0	0.00001	2723.02	167	G=0/0, A=49/1
chr20:21321294	T/T	C	T	SNV	XRN2	XRN2:intronic:NM_012255.4	1	100		intronic	NM_01225	c.1279-65	p.?	-0.03					XRN 5'-3' exo	rs202845		YES			Exoribonucl	3'-5'-exoribonuclease activity,5'-3' exonuclease activity,				C=0, T=38	C=0.0, T=1.0	0.00001	599.729	38	C=0/0, T=24/1
chr1:248004687	G/G	C	G	SNV	OR11L1	OR11L1:exonic:NM_001001959.1	1	100	1	missense	NM_00100	c.512G>C	p.Arg171Pro	0.88	1	103	0	0(COSM4143	7 transmemb	rs10888255	0.1178	YES	9.975E-1 (pos.24800468	neoplasm		G-protein coupled receptor activity,G-protein coupled re				C=0, G=29	C=0.0, G=1.0	0.00001	427.603	29	C=0/0, G=18/1
chr1:248004848	C/C	G	C	SNV	OR11L1	OR11L1:exonic:NM_001001959.1	1	100	1	missense	NM_00100	c.351C>G	p.Phe117Leu	-1.81	0.04	22	0	0.01(COSM41	7 transmemb	rs4607924	0.1177	YES	9.973E-1 (pos.24800484	neoplasm		G-protein coupled receptor activity,G-protein coupled re				G=0, C=28	G=0.0, C=1.0	0.00001	408.716	28	G=0/0, C=11/1
chr20:23064810	T/T	C	T	SNV	CD93	CD93:intronic:NM_012072.3	1	100		intronic	NM_01207	c.1934+86	p.?	0.25					Calcium-bind	rs11087413		YES			Complemen	calcium ion binding,carbohydrate binding,cell surface,ce				C=0, T=33	C=0.0, T=1.0	0.00001	504.595	33	C=0/0, T=22/1
chr1:248004877	T/T	C	T	SNV	OR11L1	OR11L1:exonic:NM_001001959.1	1	100	1	missense	NM_00100	c.322G>A	p.Gly108Ser	0.21	0.02	56	0.998		7 transmemb	rs10888257	0.1179	YES	9.974E-1 (pos.248004877)			G-protein coupled receptor activity,G-protein coupled re				C=0, T=29	C=0.0, T=1.0	0.00001	427.603	29	C=0/0, T=11/1
chr1:248004884	C/C	T	C	SNV	OR11L1	OR11L1:exonic:NM_001001959.1	1	100	1	synonymous	NM_00100	c.315A>G	p.(=)	-2.77					7 transmemb	rs1339845,rs796179	0.0357	YES	1.0 (pos.248004884)			G-protein coupled receptor activity,G-protein coupled re				T=0, C=28	T=0.0, C=1.0	0.00001	413.1	28	T=0/0, C=11/1
chr6:29364787	T/T	C	T	SNV	OR12D2	OR12D2:exonic:NM_013936.3	1	100	1	missense	NM_01393	c.311C>T	p.Ser104Phe	-0.17		155			7 transmemb	rs3128853	0.0395	YES	9.998E-1 (pos.29364787)			G-protein coupled receptor activity,G-protein coupled re				C=0, T=171	C=0.0, T=1.0	0.00001	2751.91	171	C=0/0, T=119/
chr20:2361684	T/T	C	T	SNV	TGM6	TGM6:intronic:NM_198994.2	1	100		intronic	NM_19899	c.7+63C>T	p.?	-1.7					Transglutami	rs2422753	0.2498	YES			Transglutar	cytoplasm	Alzheimer Disease;	L-Glutamine		C=0, T=26	C=0.0, T=1.0	0.00001	371.419	26	C=0/0, T=6/20
chr6:29364815	G/G	C	G	SNV	OR12D2	OR12D2:exonic:NM_013936.3	1	100	1	missense	NM_01393	c.339C>G	p.Phe113Leu	-0.35		22		0(COSM4418	7 transmemb	rs2073154	0.4448	YES	3.204E-1;0.0 (pos.29364	acute_myeloid_leukaemia		G-protein coupled receptor activity,G-protein coupled re				C=0, G=159	C=0.0, G=1.0	0.00001	2561.13	159	C=0/0, G=112/
chr20:2376963	A/A	T	A	SNV	TGM6	TGM6:intronic:NM_198994.2	1	100		intronic	NM_19899	c.425-189	p.?	0.02					Transglutami	rs6048642		YES			Transglutar	cytoplasm	Alzheimer Disease;	L-Glutamine		T=0, A=43	T=0.0, A=1.0	0.00001	705.57	43	T=0/0, A=23/2
chr20:2380396	A/A	G	A	SNV	TGM6	TGM6:intronic:NM_198994.2	1	100		intronic	NM_19899	c.850+12G	p.?	0					Transglutami	rs2076404	0.2911	YES	6.665E-1 (pos.2380396)		Transglutar	cytoplasm	Alzheimer Disease;	L-Glutami	Benign	G=0, A=34	G=0.0, A=1.0	0.00001	523.618	34	G=0/0, A=25/9
chr20:23805832	A/A	G	A	SNV	CST2	CST2:intronic:NM_001322.2	1	100		intronic	NM_00132	c.342+15C	p.?	-1.52					Cystatin dom	rs6036548		YES	9.263E-1;0.0 (pos.23805832)		Cystatin SA	cysteine-type endopeptidase inhibitor activity,detectio				G=0, A=43	G=0.0, A=1.0	0.00001	705.823	43	G=0/0, A=15/2
chr20:23807028	C/C	A	C	SNV	CST2	CST2:intronic:NM_001322.2	1	100		intronic	NM_00132	c.228+42T	p.?	-0.3					Cystatin dom	rs4407312	0.251	YES	9.265E-1;0.0 (pos.23807028)		Cystatin SA	cysteine-type endopeptidase inhibitor activity,detectio				A=0, C=37	A=0.0, C=1.0	0.00001	583.483	37	A=0/0, C=15/2
chr6:29364835	G/G	T	G	SNV	OR12D2	OR12D2:exonic:NM_013936.3	1	100	1	missense	NM_01393	c.359T>G	p.Leu120Arg	4.86		102		0.02(COSM56	7 transmemb	rs2073153	0.4446	YES	3.192E-1;0.0 (pos.29364	acute_myeloid_leukaemia		G-protein coupled receptor activity,G-protein coupled re				T=0, G=161	T=0.0, G=1.0	0.00001	2599.29	161	T=0/0, G=112/
chr20:238507	C/C	A	C	SNV	DEFB132	DEFB132:intronic:NM_207469.2	1	100		intronic	NM_20746	c.58+30A>	p.?	0.17					Beta defensi	rs373225	0.4156	YES	6.787E-1 (pos.238507)			cell surface,defense response to Gram-negative bacteri				A=0, C=32	A=0.0, C=1.0	0.00001	484.407	32	A=0/0, C=9/23
chr20:238530	G/G	T	G	SNV	DEFB132	DEFB132:intronic:NM_207469.2	1	100		intronic	NM_20746	c.58+53T>	p.?	-0.06					Beta defensi	rs412143	0.4029	YES				cell surface,defense response to Gram-negative bacteri				T=0, G=30	T=0.0, G=1.0	0.00001	446.637	30	T=0/0, G=7/23
chr20:238540	T/T	C	T	SNV	DEFB132	DEFB132:intronic:NM_207469.2	1	100		intronic	NM_20746	c.58+63C>	p.?	0.68					Beta defensi	rs416360	0.4035	YES				cell surface,defense response to Gram-negative bacteri				C=0, T=30	C=0.0, T=1.0	0.00001	446.639	30	C=0/0, T=7/23
chr20:239664	C/C	T	C	SNV	DEFB132	DEFB132:intronic:NM_207469.2	1	100		intronic	NM_20746	c.59-54T>	p.?	-0.61					Beta defensi	rs420525		YES				cell surface,defense response to Gram-negative bacteri				T=0, C=20	T=0.0, C=1.0	0.00001	263.786	20	T=0/0, C=9/11
chr20:239688	A/A	G	A	SNV	DEFB132	DEFB132:intronic:NM_207469.2	1	100		intronic	NM_20746	c.59-30G>	p.?	0.36					Beta defensi	rs399083	0.3847	YES	6.791E-1 (pos.239688)			cell surface,defense response to Gram-negative bacteri				G=0, A=20	G=0.0, A=1.0	0.00001	263.785	20	G=0/0, A=9/11
chr20:239697	C/C	G	C	SNV	DEFB132	DEFB132:intronic:NM_207469.2	1	100		intronic	NM_20746	c.59-21G>	p.?	0.5					Beta defensi	rs399094,rs7782685	0.3843	YES	0.0 (pos.239695),6.795E-1 (pos.239697)			cell surface,defense response to Gram-negative bacteri				G=0, C=20	G=0.0, C=1.0	0.00001	263.785	20	G=0/0, C=9/11
chr20:24524218	G/G	C	G	SNV	SYNDIG1	SYNDIG1:intronic:NM_024893.2	1	100		intronic	NM_02489	c.480+5C>	p.?	3.38					Interferon-in	rs4815274		YES	1.0 (pos.24524218)		Synapse dif	cell body,cell junction,dendritic shaft,dendritic spine,ea				C=0, G=50	C=0.0, G=1.0	0.00001	852.73	50	C=0/0, G=26/2
chr20:24952037	C/T	C	T	SNV	APMAP	APMAP:intronic:NM_020531.2	1	50		intronic	NM_02053	c.538+59G	p.?	-1.53					SMP-30/Gluc	rs6050225		YES			Adipocyte p	arylesterase activity,biological_process,biosynthetic pr				C=46, T=46	C=0.5, T=0.5	0.00001	507.591	92	C=20/26, T=31
chr6:29364951	A/A	G	A	SNV	OR12D2	OR12D2:exonic:NM_013936.3	1	100	1	missense	NM_01393	c.475G>A	p.Val159Ile	-3.5		29		0.01(COSM37	7 transmemb	rs2073151	0.4452	YES	3.218E-1 (pos.29364951)	acute_myeloid_leukaemia		G-protein coupled receptor activity,G-protein coupled re				G=0, A=112	G=0.0, A=1.0	0.00001	1776.02	112	G=0/0, A=58/5
chr6:29365241	C/C	T	C	SNV	OR12D2	OR12D2:exonic:NM_013936.3	1	100	1	synonymous	NM_01393	c.765T>C	p.(=)	-2.42				0(COSM3761	7 transmemb	rs2073150	0.4466	YES	3.216E-1 (pos.29365241)	acute_myeloid_leukaemia		G-protein coupled receptor activity,G-protein coupled re				T=0, C=54	T=0.0, C=1.0	0.00001	798.148	54	T=0/0, C=27/2
chr20:24993414	C/C	T	C	SNV	ACSS1	ACSS1:intronic:NM_032501.3	1	100		intronic	NM_03250	c.1707+34	p.?	-0.8					AMP-binding	rs6132785	0.2837	YES	7.642E-2 (pos.24993414)		Acyl-CoA sy	AMP binding,ATP binding,aceta		ATP,Adenosine monophc		T=0, C=23	T=0.0, C=1.0	0.00001	321.542	23	T=0/0, C=14/9
chr9:107361439	G/C	G	C	SNV	OR13C5	OR13C5:exonic:NM_001004482.1	1	45.83	1	missense	NM_00100	c.256C>G	p.Leu86Val	-1.38	0.03	32	0.948	0.05(COSM41	7 transmemb	rs4629933		YES	8.616E-1;0.0;0.0 (pos.10	neoplasm,squamous_cell		G-protein coupled receptor activity,G-protein coupled re				G=13, C=11	G=0.5417, C=0.4583	0.00001	93.4398	24	G=10/3, C=7/4
chr9:107267304	C/T	C	T	SNV	OR13F1	OR13F1:exonic:NM_001004485.1	1	47.62	1	missense	NM_00100	c.761C>T	p.Thr254Met	1.93	0.01	81	0.999	0.37(COSM44	7 transmemb	rs7030820	0.3951	YES	4.457E-1 (pos.10726730	acute_myeloid_leukaemia		G-protein coupled receptor signaling pathway,adenylate				C=22, T=20	C=0.5238, T=0.4762	0.00001	212.952	42	C=14/8, T=7/1
chr20:25004071	T/T	G	T	SNV	ACSS1	ACSS1:intronic:NM_032501.3	1	100		intronic	NM_03250	c.807+31C	p.?	-0.76					AMP-binding	rs4813543	0.0426	YES	1.0 (pos.25004071)		Acyl-CoA sy	AMP binding,ATP binding,aceta		ATP,Adenosine monophc		G=0, T=90	G=0.0, T=1.0	0.00001	1492.98	90	G=0/0, T=53/3
chr1:247978319	G/G	A	G	SNV	OR14A16	OR14A16:exonic:NM_001001966.1	1	100	1	missense	NM_00100	c.713T>C	p.Ile238Thr	2.64	1	89	0.004		7 transmemb	rs6695283	1.00E-04		1.0 (pos.247978319)			G-protein coupled receptor activity,G-protein coupled re				A=0, G=20	A=0.0, G=1.0	0.00001	263.786	20	A=0/0, G=9/11
chr20:25288505	T/T	C	T	SNV	ABHD12	ABHD12:intronic:NM_015600.4	1	100		intronic	NM_01560	c.867+97G	p.?	-1.57					Alpha/beta h	rs1026382946,rs884613		YES			Abhydrolas	AMPA glut	Ataxia;Cataract;Congenital, Hereditary, and			C=0, T=21	C=0.0, T=1.0	0.00001	281.162	21	C=0/0, T=12/9
chr1:247978996	G/G	A	G	SNV	OR14A16	OR14A16:exonic:NM_001001966.1	1	100	1	synonymous	NM_00100	c.36T>C	p.(=)	-3.61					7 transmemb	rs10888249	0.0709	YES	9.995E-1 (pos.247978996)			G-protein coupled receptor activity,G-protein coupled re				A=0, G=89	A=0.0, G=1.0	0.00001	1471.51	89	A=0/0, G=45/4
chr20:2597978	A/A	G	A	SNV	TMC2	TMC2:intronic:NM_080751.2	1	100		intronic	NM_08075	c.2180+21	p.?	-0.56					TMC domain	rs4815428	0.3335	YES	5.348E-1 (pos.2597978)		Transmemb	calcium ion transmembrane transport,cellular_compon				G=0, A=20	G=0.0, A=1.0	0.00001	263.778	20	G=0/0, A=6/14
chr20:2616556	C/C	T	C	SNV	TMC2	TMC2:intronic:NM_080751.2	1	100		intronic	NM_08075	c.2307-16	p.?	-0.77					TMC domain	rs910271	0.2486	YES	8.621E-1 (pos.2616556)		Transmemb	calcium ion transmembrane transport,cellular_compon				T=0, C=83	T=0.0, C=1.0	0.00001	1349.73	83	T=0/0, C=43/4
chr20:2616679	G/G	C	G	SNV	TMC2	TMC2:intronic:NM_080751.2	1	100		intronic	NM_08075	c.2385+29	p.?	-0.56					TMC domain	rs2422808	0.249	YES	8.624E-1;0.0 (pos.2616679)		Transmemb	calcium ion transmembrane transport,cellular_compon				C=0, G=86	C=0.0, G=1.0	0.00001	1397.44	86	C=0/0, G=43/4
chr1:248512094	T/T	C	T	SNV	OR14C36	OR14C36:exonic:NM_001001918.1	1	100	1	synonymous	NM_00100	c.18C>T	p.(=)	-1.21				0(COSM3751	7 transmemb	rs28728105	0.4305	YES	4.223E-1 (pos.24851209	acute_myeloid_leukaemia		G-protein coupled receptor activity,G-protein coupled re				C=0, T=21	C=0.0, T=1.0	0.00001	281.235	21	C=0/0, T=8/13
chr1:248512637	T/T	C	T	SNV	OR14C36	OR14C36:exonic:NM_001001918.1	1	100	1	synonymous	NM_00100	c.561C>T	p.(=)	-0.71				0.01(COSM37	7 transmemb	rs2039824	0.4309	YES	4.21E-1 (pos.248512637)	acute_myeloid_leukaemia		G-protein coupled receptor activity,G-protein coupled re				C=0, T=37	C=0.0, T=1.0	0.00001	583.697	37	C=0/0, T=12/2
chr20:2640102	G/A	G	A	SNV	IDH3B	IDH3B:intronic:NM_006899.4	1	52.38		intronic	NM_00689	c.1071+69	p.?	-0.75					Isocitrate/isc	rs2235955	0.275	YES			Isocitrate d	2-oxogluta	Congenital, Heredit	NADH		G=10, A=11	G=0.4762, A=0.5238	0.00001	95.299	21	G=4/6, A=7/4
chr20:2644407	G/G	C	G	SNV	IDH3B	IDH3B:intronic:NM_006899.4	1	100		intronic	NM_00689	c.118-3G>	p.?	-0.74					Isocitrate/isc	rs2073193	0.2749	YES	6.049E-1 (pos.2644407)		Isocitrate d	2-oxogluta	Congenital, Heredit	NADH	Benign	C=0, G=41	C=0.0, G=1.0	0.00001	664.587	41	C=0/0, G=33/8
chr20:2845130	G/A	G	A	SNV	VPS16	VPS16:intronic:NM_022575.3	1	45.45		intronic	NM_02257	c.1818+22	p.?	0.02					Vps16, C-terr	rs730819	0.3909	YES	6.623E-1 (pos.2845130)		Vacuolar pr	HOPS complex,actin binding,actin filament binding,auto				G=24, A=20	G=0.5455, A=0.4545	0.00001	211.733	44	G=13/11, A=1
chr20:30037783	C/T	C	T	SNV	DEFB123	DEFB123:intronic:NM_153324.3	1	47.62		intronic	NM_15332	c.59-49C>	p.?	0.02					Beta defensi	rs2273468	0.472	YES	7.215E-1 (pos.30037783)		Defensin, b	cell surface,defense response to bacterium,extracellula				C=11, T=10	C=0.5238, T=0.4762	0.00001	82.2896	21	C=5/6, T=1/9
chr1:248845411	A/A	G	A	SNV	OR14I1	OR14I1:exonic:NM_001004734.1	1	100	1	synonymous	NM_00100	c.195C>T	p.(=)	-3.14					7 transmemb	rs11485825,rs75612	0.1195	YES	0.0 (pos.248845410),8.307E-1 (pos.248845411)			G-protein coupled receptor activity,G-protein coupled re				G=0, A=25	G=0.0, A=1.0	0.00001	353.019	25	G=0/0, A=12/1
chr1:248845458	T/T	C	T	SNV	OR14I1	OR14I1:exonic:NM_001004734.1	1	100	1	missense	NM_00100	c.148G>A	p.Asp50Asn	-1.44	0.04	23	0.975		7 transmemb	rs4509608	0.1164	YES	8.304E-1 (pos.248845458)			G-protein coupled receptor activity,G-protein coupled re				C=0, T=26	C=0.0, T=1.0	0.00001	371.411	26	C=0/0, T=12/1
chr20:31315862	G/A	G	A	SNV	COMMD7	COMMD7:intronic:NM_053041.2	1	57.14		intronic	NM_05304	c.138+34C	p.?	-0.28					COMM domai	rs2064936	0.1537	YES	4.158E-1 (pos.31315862)		COMM dom	NF-kappaB binding,cytoplasmic vesicle,extracellular exc				G=12, A=16	G=0.4286, A=0.5714	0.00001	161.77	28	G=9/3, A=12/4
chr20:31387954	G/G	C	G	SNV	DNMT3B	DNMT3B:intronic:NM_006892.3	1	100		intronic	NM_00689	c.1760-5C	p.?	-3.08					C-5 cytosine-	rs1997797	0.4484	YES	9.973E-1;0.0 (pos.31387954)		DNA methyl	C-5 methy	Breast Diseases;Breast Neopl		Benign	C=0, G=28	C=0.0, G=1.0	0.00001	408.714	28	C=0/0, G=20/8
chr20:31388636	C/C	T	C	SNV	DNMT3B	DNMT3B:intronic:NM_006892.3	1	100		intronic	NM_00689	c.1906-5T	p.?	-0.06					C-5 cytosine-	rs2424928,rs371416	0.449	YES	9.973E-1;0.0 (pos.31388636)		DNA methyl	C-5 methy	Breast Diseases;Breast Neopl		Benign	T=0, C=22	T=0.0, C=1.0	0.00001	298.892	22	T=0/0, C=6/16
chr1:248845471	C/C	T	C	SNV	OR14I1	OR14I1:exonic:NM_001004734.1	1	100	1	synonymous	NM_00100	c.135A>G	p.(=)	-6.82					7 transmemb	rs4575113	0.0057	YES	1.0 (pos.248845471)			G-protein coupled receptor activity,G-protein coupled re				T=0, C=25	T=0.0, C=1.0	0.00001	357.386	25	T=0/0, C=12/1
chr1:248845499	G/G	A	G	SNV	OR14I1	OR14I1:exonic:NM_001004734.1	1	100	1	missense	NM_00100	c.107T>C	p.Val36Ala	0.88	1	64	0		7 transmemb	rs4462184	0.0042	YES	9.999E-1 (pos.248845499)			G-protein coupled receptor activity,G-protein coupled re				A=0, G=26	A=0.0, G=1.0	0.00001	371.419	26	A=0/0, G=12/1
chr17:3101076	C/C	G	C	SNV	OR1A2	OR1A2:exonic:NM_012352.2	1	100	1	synonymous	NM_01235	c.264G>C	p.(=)	-4.63					7 transmemb	rs2241092	0.0591	YES	7.995E-1;0.0 (pos.3101076)			G-protein coupled receptor activity,G-protein coupled re				G=0, C=27	G=0.0, C=1.0	0.00001	380.258	27	G=0/0, C=18/9
chr20:31622768	C/A	C	A	SNV	BPIFB6	BPIFB6:intronic:NM_174897.2	1	45.45		intronic	NM_17489	c.452+50C	p.?	-0.39					LBP / BPI / CE	rs6059032	0.3747	YES	7.667E-1 (pos.31622768)		BPI fold-con	extracellular region,lipid binding				C=11, A=10	C=0.5238, A=0.4762	0.00001	85.7001	22	C=7/4, A=9/1
chr20:31622839	T/C	T	C	SNV	BPIFB6	BPIFB6:intronic:NM_174897.2	1	45.45		intronic	NM_17489	c.453-48T	p.?	1.15					LBP / BPI / CE	rs6059033,rs758479	0.3764	YES	0.0 (pos.31622838),7.751E-1 (pos.31622		BPI fold-con	extracellular region,lipid binding				T=12, C=10	T=0.5455, C=0.4545	0.00001	81.7626	22	T=8/4, C=9/1
chr20:3175742	A/G	A	G	SNV	DDRGK1	DDRGK1:intronic:NM_023935.2	1	52.38		intronic	NM_02393	c.633+135	p.?	-0.04					DDRGK doma	rs2295551		YES			DDRGK dom	RNA polymerase II repressing transcription factor bindin				A=10, G=11	A=0.4762, G=0.5238	0.00001	95.2894	21	A=5/5, G=9/2
chr20:31814348	A/A	G	A	SNV	BPIFA3	BPIFA3:intronic:NM_178466.4	1	100		intronic	NM_17846	c.621+52G	p.?	-0.01					LBP / BPI / CE	rs1570033,rs778217401		YES	0.0 (pos.31814347),4.325E-1 (pos.31814348)			extracellular region,lipid binding				G=0, A=34	G=0.0, A=1.0	0.00001	524.122	34	G=0/0, A=11/2
chr9:125390874	C/C	A	C	SNV	OR1B1	OR1B1:exonic:NM_001004450.1	1	100	1	missense	NM_00100	c.941T>G	p.Val314Gly	-2.04	0.41	109	0		7 transmemb	rs1556189	0.1935	YES	9.531E-1 (pos.125390874)			G-protein coupled receptor activity,G-protein coupled re				A=0, C=31	A=0.0, C=1.0	0.00001	465.819	31	A=0/0, C=12/1
chr20:32000351	G/G	A	G	SNV	SNTA1	SNTA1:intronic:NM_003098.2	1	100		intronic	NM_00309	c.909+30T	p.?	0.07					PDZ domain (	rs291694	0.3107	YES	6.286E-1 (pos.32000351)		Syntrophin,	ATPase bir	Arrhythmias, Cardiac;Brain Diseases;Cardiov			A=0, G=41	A=0.0, G=1.0	0.00001	664.552	41	A=0/0, G=20/2
chr9:125391127	T/T	C	T	SNV	OR1B1	OR1B1:exonic:NM_001004450.1	1	100	1	missense	NM_00100	c.688G>A	p.Ala230Thr	-1.67	0.65	58	0.031	0.01(COSM37	7 transmemb	rs1476859	0.2988	YES	8.126E-1;0.0 (pos.12539	adenocarcinoma,angioimn		G-protein coupled receptor activity,G-protein coupled re				C=0, T=52	C=0.0, T=1.0	0.00001	766.511	52	C=0/0, T=32/2
chr20:3236015	G/G	A	G	SNV	C20orf194	C20orf194:intronic:NM_001009984.2	1	100		intronic	NM_00100	c.3262-85	p.?	-0.11						rs1569942		YES			Chromosom	biological_process,cellular_component,protein binding				A=0, G=61	A=0.0, G=1.0	0.00001	921.838	61	A=0/0, G=26/3
chr9:125391241	A/A	G	A	SNV	OR1B1	OR1B1:exonic:NM_001004450.1	1	100	1	nonsense	NM_00100	c.574C>T	p.Arg192Ter	-1.27				0.04(COSM37	7 transmemb	rs1476860	0.297	YES	6.15E-1 (pos.125391241)	acute_myeloid_leukaemia		G-protein coupled receptor activity,G-protein coupled re				G=0, A=43	G=0.0, A=1.0	0.00001	705.844	43	G=0/0, A=26/1
chr1:247921100	G/G	T	G	SNV	OR1C1	OR1C1:exonic:NM_012353.2	1	100	1	synonymous	NM_01235	c.609A>C	p.(=)	-0.81				0(COSM5626	7 transmemb	rs1552812	0.1088	YES	7.571E-1;0.0 (pos.24792	squamous_cell_carcinoma		G-protein coupled receptor activity,G-protein coupled re				T=0, G=77	T=0.0, G=1.0	0.00001	1229.44	77	T=0/0, G=26/5
chr20:33334540	C/C	T	C	SNV	NCOA6	NCOA6:intronic:NM_014071.4	1	100		intronic	NM_01407	c.2914+71	p.?	-0.41					Putative nucl	rs910872		YES			Nuclear rec	DNA recon	Endometriosis;Female Urogenital Diseases;F			T=0, C=34	T=0.0, C=1.0	0.00001	524.185	34	T=0/0, C=20/1
chr19:15198263	G/G	A	G	SNV	OR1I1	OR1I1:exonic:NM_001004713.1	1	100	1	synonymous	NM_00100	c.387A>G	p.(=)	-2.97				0(COSM4131	7 transmemb	rs8105277	7.00E-04		9.987E-1;0.0 (pos.15198	neoplasm		G-protein coupled receptor activity,G-protein coupled re				A=0, G=139	A=0.0, G=1.0	0.00001	2321.04	139	A=0/0, G=75/6
chr20:33342439	C/C	T	C	SNV	NCOA6	NCOA6:intronic:NM_014071.4	1	100		intronic	NM_01407	c.1675+86	p.?	-0.64					Putative nucl	rs1884432		YES			Nuclear rec	DNA recon	Endometriosis;Female Urogenital Diseases;F			T=0, C=53	T=0.0, C=1.0	0.00001	782.019	53	T=0/0, C=18/3
chr20:33575347	CAT/CAT	C	CAT	INDEL	MYH7B	MYH7B:intronic:NM_020884.4	2	100		intronic	NM_02088	c.1274-13	p.?	-0.9					Myosin N-ter	rs3053708,rs762855	0.2748	YES	0.0 (pos.33575344),7.614E-1 (pos.33575		Myosin, hea	ATP binding,actin filament binding,cardiac myofibril,mer				C=0, CAT=2	C=0.0, CAT=1.0	0.00001	267.455	20	C=0/0, CAT=9/
chr19:15198631	C/C	A	C	SNV	OR1I1	OR1I1:exonic:NM_001004713.1	1	100	1	missense	NM_00100	c.755A>C	p.Tyr252Ser	1.27	0.02	144	0.999	0.74(COSM54	7 transmemb	rs8105737	0.3257	YES	3.826E-1 (pos.15198631)	acute_myeloid_leukaemia		G-protein coupled receptor activity,G-protein coupled re				A=0, C=49	A=0.0, C=1.0	0.00001	831.343	49	A=0/0, C=26/2
chr19:15198764	C/C	T	C	SNV	OR1I1	OR1I1:exonic:NM_001004713.1	1	100	1	synonymous	NM_00100	c.888T>C	p.(=)	-0.3				0.04(COSM56	7 transmemb	rs12975625	0.0919	YES	7.992E-1 (pos.15198764)	ductal_carcinoma		G-protein coupled receptor activity,G-protein coupled re				T=0, C=29	T=0.0, C=1.0	0.00001	427.587	29	T=0/0, C=10/1
chr20:33762489	T/T	C	T	SNV	PROCR	PROCR:intronic:NM_006404.4	1	100		intronic	NM_00640	c.71-16C>	p.?	0.4					Class I Histoc	rs2069948	0.4358	YES	3.645E-1 (pos.33762489)		Protein C re	blood coa	Cardiovascular Dis	Drotrecogin alfa,Phosph		C=0, T=23	C=0.0, T=1.0	0.00001	316.747	23	C=0/0, T=13/1
chr9:125486968	A/A	G	A	SNV	OR1L4	OR1L4:exonic:NM_001005235.1	1	100	1	missense	NM_00100	c.700G>A	p.Gly234Arg	0.95	0.01	125	0.344		7 transmemb	rs2215530	0.0357	YES	9.191E-1;0.0 (pos.125486968)			G-protein coupled receptor activity,G-protein coupled re				G=0, A=206	G=0.0, A=1.0	0.00001	3347.53	206	G=0/0, A=115/
chr20:33857711	A/C	A	C	SNV	MMP24	MMP24:intronic:NM_006690.3	1	50		intronic	NM_00669	c.1333+10	p.?	0.01					Domain of un	rs2254207	0.2185	YES	2.767E-1 (pos.33857711)			cadherin binding,cell-cell adhe		Marimastat		A=29, C=29	A=0.5, C=0.5	0.00001	282.584	58	A=7/22, C=17/
chr19:9204307	G/G	A	G	SNV	OR1M1	OR1M1:exonic:NM_001004456.1	1	100	1	synonymous	NM_00100	c.387A>G	p.(=)	-11.59					7 transmemb	rs4804097	0.3495	YES	8.943E-1 (pos.9204307)			G-protein coupled receptor activity,G-protein coupled re				A=0, G=40	A=0.0, G=1.0	0.00001	644.285	40	A=0/0, G=20/2
chr20:3553750	A/A	T	A	SNV	ATRN	ATRN:intronic:NM_139321.2	1	100		intronic	NM_13932	c.2092+15	p.?	-0.07					CUB domain,	rs464831		YES			Attractin (m	carbohydrate binding,cerebellum development,cytoplas				T=0, A=21	T=0.0, A=1.0	0.00001	281.234	21	T=0/0, A=13/8
chr11:57983194	A/G	A	G	SNV	OR1S1	OR1S1:exonic:NM_001004458.1	1	50	1	stoploss	NM_00100	c.978A>G	p.Ter326Trp	0.03					7 transmemb	rs7103033	0.3267	YES	7.563E-1;0.0 (pos.57983194)			G-protein coupled receptor activity,G-protein coupled re				A=24, G=24	A=0.5, G=0.5	0.00001	276.204	48	A=18/6, G=12/
chr20:3565546	G/G	C	G	SNV	ATRN	ATRN:intronic:NM_139321.2	1	100		intronic	NM_13932	c.3184+19	p.?	0.13					CUB domain,	rs235538	0.2615	YES	9.988E-1 (pos.3565546)		Attractin (m	carbohydrate binding,cerebellum development,cytoplas				C=0, G=29	C=0.0, G=1.0	0.00001	427.603	29	C=0/0, G=11/1
chr7:143747870	A/G	A	G	SNV	OR2A5	OR2A5:exonic:NM_012365.1	1	46.43	1	missense	NM_01236	c.376A>G	p.Ile126Val	5.95	0.01	29	0.965	0.99(COSM41	7 transmemb	rs2961144	0.3164	YES	6.49E-3 (pos.143747870)	neoplasm		G-protein coupled receptor activity,G-protein coupled re				A=15, G=13	A=0.5357, G=0.4643	0.00001	117.925	28	A=7/8, G=9/4
chr20:3575248	C/C	G	C	SNV	ATRN	ATRN:intronic:NM_139321.2	1	100		intronic	NM_13932	c.3416+29	p.?	0.29					CUB domain,	rs235532	0.2627	YES	9.987E-1;1.167E-4 (pos.3575248)		Attractin (m	carbohydrate binding,cerebellum development,cytoplas				G=0, C=24	G=0.0, C=1.0	0.00001	334.792	24	G=0/0, C=17/7
chr11:6806496	C/C	T	C	SNV	OR2AG1	OR2AG1:exonic:NM_001004489.2	1	100	1	synonymous	NM_00100	c.228T>C	p.(=)	-0.65				0.02(COSM37	7 transmemb	rs11041022	0.3359	YES	1.983E-1 (pos.6806496)	acute_myeloid_leukaemia		G-protein coupled receptor activity,G-protein coupled re				T=0, C=78	T=0.0, C=1.0	0.00001	1228.05	78	T=0/0, C=37/4
chr11:6806827	T/T	G	T	SNV	OR2AG1	OR2AG1:exonic:NM_001004489.2	1	100	1	missense	NM_00100	c.559G>T	p.Val187Leu	-0.26		32		0.01(COSM37	7 transmemb	rs2659880	0.3359	YES	1.982E-1 (pos.6806827)	adenocarcinoma		G-protein coupled receptor activity,G-protein coupled re				G=0, T=72	G=0.0, T=1.0	0.00001	1126.12	72	G=0/0, T=40/3
chr20:35835657	T/T	C	T	SNV	RPN2	RPN2:intronic:NM_002951.4	1	100		intronic	NM_00295	c.691-19C	p.?	0.03					Oligosacchar	rs11698281	0.2164	YES	4.695E-1 (pos.35835657)		Ribophorin	aging,autc	Genital Diseases, Male;Genital Neoplasms, I			C=0, T=31	C=0.0, T=1.0	0.00001	465.819	31	C=0/0, T=10/2
chr20:35835889	G/G	A	G	SNV	RPN2	RPN2:intronic:NM_002951.4	1	100		intronic	NM_00295	c.867+37A	p.?	0.05					Oligosacchar	rs1079657	0.1847	YES	4.713E-1;0.0 (pos.35835889)		Ribophorin	aging,autc	Genital Diseases, Male;Genital Neoplasms, I			A=0, G=24	A=0.0, G=1.0	0.00001	334.792	24	A=0/0, G=5/19
chr20:35838591	C/C	T	C	SNV	RPN2	RPN2:intronic:NM_002951.4	1	100		intronic	NM_00295	c.986+23T	p.?	-1.15					Oligosacchar	rs2050091	0.0443	YES	1.0 (pos.35838591)		Ribophorin	aging,autc	Genital Diseases, Male;Genital Neoplasms, I			T=0, C=27	T=0.0, C=1.0	0.00001	398.909	27	T=0/0, C=12/1
chr20:35852395	C/C	A	C	SNV	RPN2	RPN2:intronic:NM_002951.4	1	100		intronic	NM_00295	c.1184+23	p.?	-2.92					Oligosacchar	rs2076397	0.1823	YES	5.02E-1 (pos.35852395)		Ribophorin	aging,autc	Genital Diseases, Male;Genital Neoplasms, I			A=0, C=36	A=0.0, C=1.0	0.00001	563.659	36	A=0/0, C=21/1
chr11:6789929	A/A	C	A	SNV	OR2AG2	OR2AG2:exonic:NM_001004490.1	1	100	1	missense	NM_00100	c.260G>T	p.Arg87Leu	-1.75	1	102	0	0.02(COSM14	7 transmemb	rs11828782	0.3513	YES	1.716E-1;2.314E-4 (pos.6	acute_myeloid_leukaemia		G-protein coupled receptor activity,G-protein coupled re				C=0, A=35	C=0.0, A=1.0	0.00001	543.798	35	C=0/0, A=14/2
chr20:36031097	G/G	A	G	SNV	SRC	SRC:intronic:NM_198291.2	1	100		intronic	NM_19829	c.1271-55	p.?	-0.17					Protein kinas	rs2145792		YES			Protooncog	ATP bindin	Brain Diseases;Bre	(2E)-N-(4-(3-bromopheny		A=0, G=43	A=0.0, G=1.0	0.00001	705.842	43	A=0/0, G=21/2
chr1:247614693	T/T	C	T	SNV	OR2B11	OR2B11:exonic:NM_001004492.1	1	100	1	missense	NM_00100	c.592G>A	p.Val198Met	-3.17	0.15	21	0.007		7 transmemb	rs6695302	0.0026	YES	1.0 (pos.247614693)			G-protein coupled receptor activity,G-protein coupled re				C=0, T=79	C=0.0, T=1.0	0.00001	1269.21	79	C=0/0, T=36/4
chr1:247695612	G/A	G	A	SNV	OR2C3	OR2C3:exonic:NM_198074.4	1	95.45	2	missense	NM_19807	c.202C>T	p.Pro68Ser	1.4		74			7 transmemb	rs6657127	1.00E-04		1.0;0.0 (pos.247695612)			G-protein coupled receptor activity,G-protein coupled re				G=1, A=21	G=0.0455, A=0.9545	0.00001	272.182	22	G=0/1, A=12/9
chr11:6913127	G/G	A	G	SNV	OR2D2	OR2D2:exonic:NM_003700.1	1	100	1	missense	NM_00370	c.605T>C	p.Met202Thr	0.68	0.06	81	0.002	0.19(COSM49	7 transmemb	rs2741804	0.441	YES	4.32E-1 (pos.6913127)	ductal_carcinc	Olfactory re	G-protein coupled receptor activity,G-protein coupled re				A=0, G=29	A=0.0, G=1.0	0.00001	427.239	29	A=0/0, G=24/5
chr20:36959566	G/G	A	G	SNV	BPI	BPI:intronic:NM_001725.2	1	100		intronic	NM_00172	c.1284+74	p.?	-2.27					LBP / BPI / CE	rs2425357	0.0399	YES			Bactericida	antimicrobial humoral respon		Di-Stearoyl-3-Sn-Phosph		A=0, G=85	A=0.0, G=1.0	0.00001	1389.7	85	A=0/0, G=40/4
chr20:37274861	G/G	A	G	SNV	ARHGAP40	ARHGAP40:intronic:NM_001164431.1	1	100		intronic	NM_00116	c.1707+31	p.?	0.57					RhoGAP dom	rs117796		YES	5.45E-1 (pos.37274861)							A=0, G=34	A=0.0, G=1.0	0.00001	521.872	34	A=0/0, G=26/8
chr6:29141743	G/G	A	G	SNV	OR2J2	OR2J2:exonic:NM_030905.2	1	100	1	missense	NM_03090	c.331A>G	p.Thr111Ala	-0.82	0.18	58	0.01	0.01(COSM41	7 transmemb	rs3129157	0.033	YES	9.896E-1 (pos.29141743)	neoplasm		G-protein coupled receptor activity,G-protein coupled re				A=0, G=66	A=0.0, G=1.0	0.00001	1015.39	66	A=0/0, G=28/3
chr6:29141772	G/G	A	G	SNV	OR2J2	OR2J2:exonic:NM_030905.2	1	100	1	synonymous	NM_03090	c.360A>G	p.(=)	-2.52					7 transmemb	rs3129158	0.1483	YES	5.549E-1 (pos.29141772)			G-protein coupled receptor activity,G-protein coupled re				A=0, G=68	A=0.0, G=1.0	0.00001	1053.56	68	A=0/0, G=29/3
chr6:29141849	C/C	T	C	SNV	OR2J2	OR2J2:exonic:NM_030905.2	1	100	1	missense	NM_03090	c.437T>C	p.Val146Ala	-0.16	1	64	0	0(COSM5021	7 transmemb	rs3116856	0.4591	YES	3.467E-1 (pos.29141849)	acute_myeloid_leukaemia		G-protein coupled receptor activity,G-protein coupled re				T=0, C=31	T=0.0, C=1.0	0.00001	465.819	31	T=0/0, C=21/1

chr6:29080349	G/G	A	G	SNV	OR2J3	OR2J3:exonic:NM 001005216.3	1	100	1	missense	NM 00100	c.682A>G	p.Ile228Val	-1.13		29		0.01(COSM40	7 transmemb	rs3130764	0.1479	YES	5.649E-1 (pos.29080349)	acute_myeloic	Olfactory re	G-protein coupled receptor activity,G-protein coupled re	A=0, G=66	A=0.0, G=1.0	0.00001	1015.36	66	A=0/0, G=33/3
chr20:37635027	C/C	T	C	SNV	DHX35	DHX35:intronic:NM 021931.3	1	100		intronic	NM 02193	c.1222+28	p.?	0.08					Helicase ass	rs3752298	0.3156	YES	4.511E-1 (pos.37635027)			ATP binding,ATP-dependent RNA helicase activity,RNA bi	T=0, C=63	T=0.0, C=1.0	0.00001	958.679	63	T=0/0, C=31/3
chr20:37643659	C/C	T	C	SNV	DHX35	DHX35:intronic:NM 021931.3	1	100		intronic	NM 02193	c.1402+82	p.?	-0.57					Helicase ass	rs3764703		YES				ATP binding,ATP-dependent RNA helicase activity,RNA bi	T=0, C=28	T=0.0, C=1.0	0.00001	408.718	28	T=0/0, C=21/7
chr20:3783649	C/C	G	C	SNV	CDC25B	CDC25B:intronic:NM 021873.3	1	100		intronic	NM 02187	c.1257+30	p.?	0.13					M-phase indu	rs592687	0.0048		9.999E-1;1.156E-4 (pos.3783649)		Cell divisior	G2/M tran Bipolar Disorder:Bi Cysteinesulfonic Acid	G=0, C=22	G=0.0, C=1.0	0.00001	298.888	22	G=0/0, C=17/5
chr20:39788855	A/G	A	G	SNV	PLCG1	PLCG1:intronic:NM 002660.2	1	45.45		intronic	NM 00266	c.512+62A	p.?	-1.64					C2 domain,PI	rs2076148	0.4626	YES			Phospholip	COP9 signa Hemangiosarcoma;Hemic and Lymphatic Dis	A=12, G=10	A=0.5455, G=0.4545	0.00001	81.7625	22	A=7/5, G=4/6
chr6:29080450	A/A	G	A	SNV	OR2J3	OR2J3:exonic:NM 001005216.3	1	100	1	missense	NM 00100	c.783G>A	p.Met261Ile	-2.46		10			7 transmemb	rs3130765	0.159	YES	5.641E-1 (pos.29080450)		Olfactory re	G-protein coupled receptor activity,G-protein coupled re	G=0, A=76	G=0.0, A=1.0	0.00001	1209.58	76	G=0/0, A=29/4
chr20:40076509	C/C	T	C	SNV	CHD6	CHD6:intronic:NM 032221.4	1	100		intronic	NM 03222	c.3773+13	p.?	0.75					Chromo (CHR	rs909882	0.1271	YES	1.82E-1 (pos.40076509)		Chromodon	ATP bindin Carcinoma:Carcinoma, Transitional Cell:Fem	T=0, C=27	T=0.0, C=1.0	0.00001	389.878	27	T=0/0, C=18/9
chr20:40179909	A/A	G	A	SNV	CHD6	CHD6:intronic:NM 032221.4	1	100		intronic	NM 03222	c.33+35C>	p.?	-1.41					Chromo (CHR	rs386814214,rs6129	0.221	YES	5.078E-1 (pos.40179909)		Chromodon	ATP bindin Carcinoma:Carcinoma, Transitional Cell:Fem	G=0, A=50	G=0.0, A=1.0	0.00001	852.747	50	G=0/0, A=30/2
chr1:248112745	T/T	G	T	SNV	OR2L13,OR2L8	OR2L8:exonic:NM 001001963.1, OR2L13:intronic:NM 1759	1	100	1	missense	NM 17591	c.-143+12(	p.?, p.Gly196Cys	-5.09	0.1	159	0.008	0(COSM4591	7 transmemb	rs4925788		YES	7.444E-1 (pos.24811274	squamous_cell_carcinoma		G-protein coupled receptor activity,G-protein coupled re	G=0, T=100	G=0.0, T=1.0	0.00001	1702.26	100	G=0/0, T=55/4
chr20:44002665	G/G	C	G	SNV	SYS1-DBNDD2,TP53TG5	TP53TG5:intronic:NM 014477.2, SYS1-DBNDD2:intronic_nc:	1	100		intronic	NR 00318	c.769-14G	p.?	0.37					Cellular tumc	rs2231626	0.2224	YES	5.399E-1;3.479E-4 (pos.44002665)		TP53 target	cytoplasm,intracellular signal transduction,molecular_f	C=0, G=38	C=0.0, G=1.0	0.00001	603.779	38	C=0/0, G=17/2
chr20:44006078	C/G	C	G	SNV	SYS1-DBNDD2,TP53TG5	TP53TG5:intronic:NM 014477.2, SYS1-DBNDD2:intronic_nc:	1	97.5		intronic	NR 00318	c.124-96G	p.?	-0.73					Cellular tumc	rs2231614		YES			TP53 target	cytoplasm,intracellular signal transduction,molecular_f	C=1, G=39	C=0.025, G=0.975	0.00001	611.373	40	C=1/0, G=22/1
chr1:248112762	CA/CA	TG	CA	MNV	OR2L13,OR2L8	OR2L8:exonic:NM 001001963.1, OR2L13:intronic:NM 1759	2	100	1	missense	NM 17591	c.-143+12(	p.?, p.Ala202Thr	-4.76, -0.9	0.53	58	0	0(COSM3930	7 transmemb	rs34851853,rs49257	0.2195	YES	7.128E-1;7.059E-4 (pos.2	adenocarcinoma,carcinom		G-protein coupled receptor activity,G-protein coupled re	TG=0, CA=9	TG=0.0, CA=1.0	0.00001	1636.57	98	TG=0/0, CA=55
chr20:44053069	C/C	T	C	SNV	PIGT	PIGT:intronic:NM 015937.5	1	100		intronic	NM 01593	c.1400+48	p.?	-0.03					Gpi16 subuni	rs2741566	0.2384	YES	5.24E-1 (pos.44053069)		Phosphatid	GPI-ancho Anemia;Anemia, Hemolytic;Bone Marrow Dis	T=0, C=28	T=0.0, C=1.0	0.00001	408.718	28	T=0/0, C=9/19
chr20:44528388	C/A	C	A	SNV	CTSA,PLTP	CTSA:downstream:NM 000308.3, PLTP:intronic:NM 00622	1	50		intronic	NM 00030	c.*1299C>	p.?, p.?	-1.26					LBP / BPI / CE	rs56235473	0.0163				Cathepsin A	azurophil Congenital, Heredit Sofosbuvir	C=13, A=13	C=0.5, A=0.5	0.00001	119.163	26	C=8/5, A=7/6
chr20:44574533	T/T	C	T	SNV	PCIF1	PCIF1:intronic:NM 022104.3	1	100		intronic	NM 02210	c.1341+11	p.?	-2.57					Phosphorylat	rs11696196	0.4302	YES	1.274E-3 (pos.44574533)			intercellular bridge,microtubule cytoskeleton,negative	C=0, T=29	C=0.0, T=1.0	0.00001	427.603	29	C=0/0, T=8/21
chr20:44578776	G/G	A	G	SNV	ZNF335	ZNF335:intronic:NM 022095.3	1	100		intronic	NM 02209	c.3488-68	p.?	-0.06					C2H2-type zir	rs6065910		YES			Zinc finger	RNA polymerase II core promoter sequence-specific DNA	A=0, G=38	A=0.0, G=1.0	0.00001	603.785	38	A=0/0, G=11/2
chr1:248112809	G/G	A	G	SNV	OR2L13,OR2L8	OR2L8:exonic:NM 001001963.1, OR2L13:intronic:NM 1759	1	100	1	missense	NM 17591	c.-143+12	p.?, p.Tyr217Cys	6.76	0.01	194	1		7 transmemb	rs4925583	0.0553	YES	7.84E-1 (pos.248112809)			G-protein coupled receptor activity,G-protein coupled re	A=0, G=105	A=0.0, G=1.0	0.00001	1644.49	105	A=0/0, G=55/5
chr1:248112836	G/G	A	G	SNV	OR2L13,OR2L8	OR2L8:exonic:NM 001001963.1, OR2L13:intronic:NM 1759	1	100	1	missense	NM 17591	c.-143+12	p.?, p.His226Arg	-2.75	0.84	29	0		7 transmemb	rs4925792	0.0531	YES	8.696E-1;0.0 (pos.248112836)			G-protein coupled receptor activity,G-protein coupled re	A=0, G=123	A=0.0, G=1.0	0.00001	1993.67	123	A=0/0, G=59/6
chr20:44599839	C/C	G	C	SNV	ZNF335	ZNF335:intronic:NM 022095.3	1	100		intronic	NM 02209	c.201+10C	p.?	-1.31					C2H2-type zir	rs6017715	0.0409	YES	8.093E-1 (pos.44599839)		Zinc finger	RNA polymerase II core promoter sequenc Likely benign	G=0, C=42	G=0.0, C=1.0	0.00001	685.181	42	G=0/0, C=27/1
chr1:248112915	G/G	A	G	SNV	OR2L13,OR2L8	OR2L8:exonic:NM 001001963.1, OR2L13:intronic:NM 1759	1	100	1	synonymous	NM 17591	c.-143+12	p.?, p.(=)	-4.56					7 transmemb	rs10888280	0.305	YES	4.246E-1 (pos.248112915)			G-protein coupled receptor activity,G-protein coupled re	A=0, G=22	A=0.0, G=1.0	0.00001	298.876	22	A=0/0, G=7/15
chr1:248113026	A/A	T	A	SNV	OR2L13,OR2L8	OR2L8:exonic:NM 001001963.1, OR2L13:intronic:NM 1759	1	100	1	nonsense	NM 17591	c.-143+12	p.?, p.Tyr289Ter	-2.95					7 transmemb	rs10888281	0.0717	YES	7.812E-1 (pos.248113026)			G-protein coupled receptor activity,G-protein coupled re	T=0, A=27	T=0.0, A=1.0	0.00001	389.988	27	T=0/0, A=8/19
chr20:44675136	C/C	T	C	SNV	SLC12A5	SLC12A5:intronic:NM 001134771.1	1	100		intronic	NM 00113	c.1856+61	p.?	-0.72					Amino acid p	rs3848727		YES			Solute carri	cellular ch Brain Diseases:Cen Bumetanide,Potassium	T=0, C=24	T=0.0, C=1.0	0.00001	334.791	24	T=0/0, C=17/7
chr1:248343945	G/G	C	G	SNV	OR2M2	OR2M2:exonic:NM 001004688.1	1	100	1	missense	NM 00100	c.658C>G	p.Arg220Gly	-0.96	0.29	125	0.971	0.01(COSM11	7 transmemb	rs4244171	0.3669	YES	4.074E-1;0.0 (pos.24834	adenoma,basal_cell_carci		G-protein coupled receptor activity,G-protein coupled re	C=0, G=28	C=0.0, G=1.0	0.00001	408.709	28	C=0/0, G=16/1
chr20:44985307	G/G	T	G	SNV	SLC35C2	SLC35C2:intronic:NM 015945.11	1	100		intronic	NM 01594	c.272-31A	p.?	-0.09					Triose-phosp	rs911435	1.00E-04		9.999E-1;0.0 (pos.44985307)			Golgi apparatus,UDP-glucose transport,cis-Golgi networ	T=0, G=25	T=0.0, G=1.0	0.00001	353.001	25	T=0/0, G=7/18
chr20:45816639	G/G	A	G	SNV	EYA2	EYA2:intronic:NM 005244.4	1	100		intronic	NM 00524	c.1537-62	p.?	-1.08					haloacid deh	rs3818013		YES			Eyes absent	DNA repair,cytosol,extrinsic apoptotic signaling pathwa	A=0, G=21	A=0.0, G=1.0	0.00001	281.23	21	A=0/0, G=16/5
chr1:248366702	A/A	G	A	SNV	OR2M3	OR2M3:exonic:NM 001004689.1	1	100	1	synonymous	NM 00100	c.333G>A	p.(=)	-1.06				0(COSM5008	7 transmemb	rs4916112	0.3824	YES	4.304E-1 (pos.24836670	adenocarcinoma,carcinom		G-protein coupled receptor activity,G-protein coupled re	G=0, A=130	G=0.0, A=1.0	0.00001	2135.44	130	G=0/0, A=80/5
chr20:46267950	T/T	C	T	SNV	NCOA3	NCOA3:intronic:NM 181659.2	1	100		intronic	NM 18165	c.2707+4C	p.?	2.15					Domain of un	rs4810648	0.0809	YES	1.0;0.0 (pos.46267950)		Nuclear rec	RNA polym Adenocarcinoma;Astrocytoma;Bone Disease	C=0, T=119	C=0.0, T=1.0	0.00001	1913.83	119	C=0/0, T=45/7
chr20:46294725	T/T	C	T	SNV	SULF2	SULF2:intronic:NM 018837.3	1	100		intronic	NM 01883	c.1806-28	p.?	1.37					Sulfatase,Sul	rs2076545	0.4441	YES	5.564E-1 (pos.46294725)		Sulfatase 2	Golgi stacl Endometriosis;Female Urogenital Diseases;F	C=0, T=20	C=0.0, T=1.0	0.00001	263.785	20	C=0/0, T=8/12
chr20:46313135	T/T	C	T	SNV	SULF2	SULF2:intronic:NM 018837.3	1	100		intronic	NM 01883	c.888+40G	p.?	-2.44					Sulfatase,Sul	rs2297586	0.2974	YES	2.341E-1 (pos.46313135)		Sulfatase 2	Golgi stacl Endometriosis;Female Urogenital Diseases;F	C=0, T=40	C=0.0, T=1.0	0.00001	643.706	40	C=0/0, T=22/1
chr1:248487300	T/T	C	T	SNV	OR2M7	OR2M7:exonic:NM 001004691.1	1	100	1	missense	NM 00100	c.571G>A	p.Asp191Asn	-0.53	0.08	23	0.041	0.01(COSM37	7 transmemb	rs4916129	0.4497	YES	4.831E-1 (pos.24848730	adenocarcinoma,neoplasn		G-protein coupled receptor activity,G-protein coupled re	C=0, T=26	C=0.0, T=1.0	0.00001	371.398	26	C=0/0, T=9/17
chr20:47249189	C/C	T	C	SNV	PREX1	PREX1:intronic:NM 020820.3	1	100		intronic	NM 02082	c.4268-12	p.?	-1.28					Domain foun	rs4413212	0.4001	YES	3.256E-1 (pos.47249189)		Phosphatid	G-protein coupled receptor signaling pathway,GTPase a	T=0, C=32	T=0.0, C=1.0	0.00001	485.14	32	T=0/0, C=19/1
chr20:47256479	C/C	T	C	SNV	PREX1	PREX1:intronic:NM 020820.3	1	100		intronic	NM 02082	c.3776-47	p.?	-0.39					Domain foun	rs4611698	0.2601	YES	2.626E-1 (pos.47256479)		Phosphatid	G-protein coupled receptor signaling pathway,GTPase a	T=0, C=44	T=0.0, C=1.0	0.00001	726.569	44	T=0/0, C=17/2
chr20:47260936	G/G	A	G	SNV	PREX1	PREX1:intronic:NM 020820.3	1	100		intronic	NM 02082	c.3601+11	p.?	-1.86					Domain foun	rs6019344	0.4506	YES	4.153E-1 (pos.47260936)		Phosphatid	G-protein coupled receptor signaling pathway,GTPase a	A=0, G=48	A=0.0, G=1.0	0.00001	810.435	48	A=0/0, G=29/1
chr1:248487338	A/A	C	A	SNV	OR2M7	OR2M7:exonic:NM 001004691.1	1	100	1	missense	NM 00100	c.533G>T	p.Cys178Phe	0.04	1	205	0	0.06(COSM41	7 transmemb	rs4916130	0.2601	YES	8.603E-1 (pos.24848733	neoplasm		G-protein coupled receptor activity,G-protein coupled re	C=0, A=27	C=0.0, A=1.0	0.00001	389.988	27	C=0/0, A=10/1
chr20:47361519	C/C	T	C	SNV	PREX1	PREX1:intronic:NM 020820.3	1	100		intronic	NM 02082	c.414+43A	p.?	-0.28					Domain foun	rs3746816	0.4444	YES	2.989E-1 (pos.47361519)		Phosphatid	G-protein coupled receptor signaling pathway,GTPase a	T=0, C=42	T=0.0, C=1.0	0.00001	685.22	42	T=0/0, C=19/2
chr20:47364452	C/T	C	T	SNV	PREX1	PREX1:intronic:NM 020820.3	1	50		intronic	NM 02082	c.220-35G	p.?	-3.08					Domain foun	rs140209816	0.0147	YES	1.475E-4 (pos.47364452)		Phosphatid	G-protein coupled receptor signaling pathway,GTPase a	C=18, T=18	C=0.5, T=0.5	0.00001	185.885	36	C=8/10, T=6/1
chr20:47845220	T/A	T	A	SNV	DDX27	DDX27:intronic:NM 017895.7	1	50		intronic	NM 01789	c.800-32T	p.?	0.31					DEAD/DEAH b	rs238165	0.2302	YES	5.613E-1;0.0 (pos.47845220)		DEAD box p	ATP binding,ATP-dependent RNA helicase activity,RNA bi	T=11, A=11	T=0.5, A=0.5	0.00001	94.6067	22	T=7/4, A=7/4
chr1:248487768	G/G	A	G	SNV	OR2M7	OR2M7:exonic:NM 001004691.1	1	100	1	missense	NM 00100	c.103T>C	p.Phe35Leu	6.92	0.01	22	0.85	0.89(COSM16	7 transmemb	rs7555424	0.2536	YES	8.61E-1 (pos.248487768)	carcinoma		G-protein coupled receptor activity,G-protein coupled re	A=0, G=20	A=0.0, G=1.0	0.00001	263.773	20	A=0/0, G=5/15
chr1:248789504	C/C	T	C	SNV	OR2T11	OR2T11:exonic:NM 001001964.1	1	100	1	missense	NM 00100	c.926A>G	p.Gln309Arg	-0.65	0.18	43	0		7 transmemb	rs1892442	0.0091	YES	9.994E-1 (pos.248789504)			G-protein coupled receptor activity,G-protein coupled re	T=0, C=28	T=0.0, C=1.0	0.00001	408.718	28	T=0/0, C=16/1
chr20:48330044	G/GA	G	GA	INDEL	B4GALT5	B4GALT5:intronic:NM 004776.3	1	58.33		intronic	NM 00477	c.115+69C	p.?	1.41					N-terminal domain of galactosyltransferase,N-terminal region of glycosyl transferase group 7						Beta-1,4-ga	Golgi ciste Arthritis:Arthritis, Juvenile:Autoimmune Dise	G=10, GA=1	G=0.4167, GA=0.583	0.00001	85.3844	24	G=7/3, GA=9/5
chr1:248790075	G/G	A	G	SNV	OR2T11	OR2T11:exonic:NM 001001964.1	1	100	1	missense	NM 00100	c.355T>C	p.Cys119Arg	-0.03	1	180	0		7 transmemb	rs1892443	0.009	YES	1.0 (pos.248790075)			G-protein coupled receptor activity,G-protein coupled re	A=0, G=22	A=0.0, G=1.0	0.00001	298.892	22	A=0/0, G=10/1
chr20:50071258	G/G	A	G	SNV	NFATC2	NFATC2:intronic:NM 173091.3	1	100		intronic	NM 17309	c.1709-33	p.?	-1.23					IPT/TIG doma	rs228840	0.1638	YES	7.674E-1 (pos.50071258)		Nuclear fac	B cell rece Nervous System Diseases	A=0, G=212	A=0.0, G=1.0	0.00001	3458.12	212	A=0/0, G=110/
chr20:50244098	C/C	T	C	SNV	ATP9A	ATP9A:intronic:NM 006045.2	1	100		intronic	NM 00604	c.1845+41	p.?	-1.14					Cation trans	rs2426317	0.171	YES	8.035E-1;0.0 (pos.50244098)			ATP binding,early endosome,early endosome membrane	T=0, C=25	T=0.0, C=1.0	0.00001	352.853	25	T=0/0, C=16/9
chr1:248457979	A/A	C	A	SNV	OR2T12	OR2T12:exonic:NM 001004692.1	1	100	1	missense	NM 00100	c.902G>T	p.Arg301Leu	-1.84	0	102	0.01	0(COSM4143	7 transmemb	rs6667171	0.2519	YES	8.586E-1;0.0;0.0 (pos.24	ductal_carcinoma,neoplas		G-protein coupled receptor activity,G-protein coupled re	C=0, A=113	C=0.0, A=1.0	0.00001	1795.75	113	C=0/0, A=66/4
chr20:50705211	G/G	A	G	SNV	ZFP64		1	100		intronic				0.18						rs2273382	0.4673	YES	3.304E-1 (pos.50705211)				A=0, G=40	A=0.0, G=1.0	0.00001	618.271	40	A=0/0, G=19/2
chr20:52645534	C/C	T	C	SNV	BCAS1	BCAS1:intronic:NM 003657.3	1	100		intronic	NM 00365	c.143-23A	p.?	-0.93						rs466264	0.3517	YES	9E-1 (pos.52645534)		Breast carc	cytoplasm Genital Diseases, Male;Genital Neoplasms, I	T=0, C=100	T=0.0, C=1.0	0.00001	1702.25	100	T=0/0, C=57/4
chr1:248458676	C/C	T	C	SNV	OR2T12	OR2T12:exonic:NM 001004692.1	1	100	1	missense	NM 00100	c.205A>G	p.Met69Val	-2.79	0.09	21	0	0(COSM1600	7 transmemb	rs6678138,rs760727862		YES	0.0 (pos.248458675),2.8	acute_myeloid_leukaemia		G-protein coupled receptor activity,G-protein coupled re	T=0, C=35	T=0.0, C=1.0	0.00001	543.903	35	T=0/0, C=17/1
chr1:248458689	G/G	A	G	SNV	OR2T12	OR2T12:exonic:NM 001004692.1	1	100	1	synonymous	NM 00100	c.192T>C	p.(=)	-4.09				0(COSM1468	7 transmemb	rs6674940		YES	2.592E-1;2.621E-4;2.621	acute_myeloid_leukaemia		G-protein coupled receptor activity,G-protein coupled re	A=0, G=36	A=0.0, G=1.0	0.00001	563.744	36	A=0/0, G=17/1
chr1:248458717	G/G	C	G	SNV	OR2T12	OR2T12:exonic:NM 001004692.1	1	100	1	missense	NM 00100	c.164G>C	p.Arg55Thr	0.4	1	71	0	0(COSM3773	7 transmemb	rs12745228		YES	8.505E-1;0.0 (pos.24845	acute_myeloid_leukaemia		G-protein coupled receptor activity,G-protein coupled re	C=0, G=36	C=0.0, G=1.0	0.00001	563.744	36	C=0/0, G=17/1
chr1:248525638	C/C	CA	C	INDEL	OR2T4	OR2T4:exonic:NM 001004696.1	1	100	1	frameshiftDeletion	NM 00100	c.757delA	p.Ile253fs	-0.61, 7.33					7 transmemb	rs34079073,rs75001	0.4611	YES	0.0 (pos.248525639),3.0	astrocytoma_Grade_IV,sq		G-protein coupled receptor activity,G-protein coupled re	CA=0, C=21	CA=0.0, C=1.0	0.00001	281.059	21	CA=0/0, C=14/
chr20:5294496	T/T	C	T	SNV	PROKR2	PROKR2:intronic:NM 144773.3	1	100		intronic	NM 14477	c.458+62G	p.?	0.61					7 transmemb	rs8116897		YES			Prokineticin	G-protein 46, XY Disorders of Sex Development;Amphet	C=0, T=80	C=0.0, T=1.0	0.00001	1288.91	80	C=0/0, T=40/4
chr20:54578843	C/G	C	G	SNV	CBLN4	CBLN4:intronic:NM 080617.5	1	56		intronic	NM 08061	c.291+94G	p.?	0.06					C1q domain	rs422330		YES			Precerebell	cell junction,extracellular space,protein secretion,syna	C=11, G=14	C=0.44, G=0.56	0.00001	134.35	25	C=6/5, G=8/6
chr1:248550976	G/G	T	G	SNV	OR2T6	OR2T6:exonic:NM 001005471.1	1	100	1	missense	NM 00100	c.67T>G	p.Cys23Gly	-1.58	0.33	159	0		7 transmemb	rs6587467	0.2993	YES	7.115E-1 (pos.248550976)			G-protein coupled receptor activity,G-protein coupled re	T=0, G=53	T=0.0, G=1.0	0.00001	779.817	53	T=0/0, G=30/2
chr20:54974424	A/A	C	A	SNV	CSTF1	CSTF1:intronic:NM 001033521.1	1	100		intronic	NM 00103	c.1036+11	p.?	-0.51					Cleavage stir	rs2426617,rs753824	0.0099	YES	0.0 (pos.54974423),9.098E-1 (pos.54974		Cleavage st	RNA binding,mRNA 3'-end processing,mRNA splicing, via	C=0, A=21	C=0.0, A=1.0	0.00001	280.318	21	C=0/0, A=11/1
chr1:248084434	A/A	G	A	SNV	OR2T8	OR2T8:exonic:NM 001005522.1	1	100	1	missense	NM 00100	c.115G>A	p.Gly39Ser	1.83	0.14	56	0.026	0.27(COSM39	7 transmemb	rs11204563		YES	3.717E-1;2.333E-4 (pos.2	acute_myeloid_leukaemia		G-protein coupled receptor activity,G-protein coupled re	G=0, A=35	G=0.0, A=1.0	0.00001	543.903	35	G=0/0, A=18/1
chr1:248084464	C/C	T	C	SNV	OR2T8	OR2T8:exonic:NM 001005522.1	1	100	1	missense	NM 00100	c.145T>C	p.Trp49Arg	-5.96	0.25	101	0	0(COSM4591	7 transmemb	rs11204564		YES	7.2E-1 (pos.248084464)	squamous_cell_carcinoma		G-protein coupled receptor activity,G-protein coupled re	T=0, C=34	T=0.0, C=1.0	0.00001	524.186	34	T=0/0, C=17/1
chr5:180582604	G/G	A	G	SNV	OR2V2	OR2V2:exonic:NM 206880.1	1	100	1	missense	NM 20688	c.662A>G	p.His221Arg	-3.06	1	29	0		7 transmemb	rs2546423	0.4665	YES	5.639E-1 (pos.180582604)			G-protein coupled receptor activity,G-protein coupled re	A=0, G=27	A=0.0, G=1.0	0.00001	389.986	27	A=0/0, G=11/1
chr1:247654498	G/A	G	A	SNV	OR2W5	OR2W5:exonic:NM 001004698.2	1	53.49	1	synonymous	NM 00100	c.69G>A	p.(=)	-1.51					7 transmemb	rs61070846	0.2105	YES	2.776E-2 (pos.247654498)			G-protein coupled receptor activity,G-protein coupled re	G=20, A=23	G=0.4651, A=0.5349	0.00001	261.022	43	G=9/11, A=13/
chr5:180166461	G/G	C	G	SNV	OR2Y1	OR2Y1:exonic:NM 001001657.1	1	100	1	missense	NM 00100	c.598G>C	p.Val200Leu	1.81	0.07	32	0.985		7 transmemb	rs10464105	0.0199	YES	9.999E-1 (pos.180166461)			G-protein coupled receptor activity,G-protein coupled re	C=0, G=83	C=0.0, G=1.0	0.00001	1349.88	83	C=0/0, G=71/1
chr11:48510777	A/A	G	A	SNV	OR4A47	OR4A47:exonic:NM 001005512.2	1	100	1	missense	NM 00100	c.433G>A	p.Val145Met	-0.26	0.15	21	0.826	0.11(COSM49	7 transmemb	rs7103992	0.3767	YES	4.774E-1 (pos.48510777)	ductal_carcinoma		G-protein coupled receptor activity,G-protein coupled re	G=0, A=20	G=0.0, A=1.0	0.00001	263.786	20	G=0/0, A=13/7
chr20:56726107	T/T	C	T	SNV	C20orf85	C20orf85:intronic:NM 178456.2	1	100		intronic	NM 17845	c.68+19C>	p.?	-0.6					Normal lung	rs386815621,rs6070	2.00E-04	YES	9.993E-1 (pos.56726107)				C=0, T=47	C=0.0, T=1.0	0.00001	789.327	47	C=0/0, T=27/2
chr20:56886062	A/G	A	G	SNV	RAB22A	RAB22A:intronic:NM 020673.2	1	50		intronic	NM 02067	c.37-37A>	p.?	-0.07					50S ribosome	rs542165	0.4141	YES	4.684E-1 (pos.56886062)		Ras-associa	GDP binding,GTP binding,GTPase activity,actin cytoskele	A=11, G=11	A=0.5, G=0.5	0.00001	94.6067	22	A=5/6, G=6/5
chr11:48238421	G/G	T	G	SNV	OR4B1	OR4B1:exonic:NM 001005470.1	1	100	1	synonymous	NM 00100	c.60T>G	p.(=)	-2.33					7 transmemb	rs10769329	5.00E-04		1.0;0.0 (pos.48238421)			G-protein coupled receptor activity,G-protein coupled re	T=0, G=155	T=0.0, G=1.0	0.00001	2485.01	155	T=0/0, G=98/5
chr11:49974371	G/A	G	A	SNV	OR4C13	OR4C13:exonic:NM 001001955.2	1	97.3	1	missense	NM 00100	c.397G>A	p.Val133Ile	0.21		29			7 transmemb	rs28662375	2.00E-04		1.0 (pos.49974371)			G-protein Adenocarcinoma:Carcinoma:Carcinoma, Ren	G=1, A=36	G=0.027, A=0.973	0.00001	551.736	37	G=0/1, A=22/1
chr11:59271637	T/C	T	C	SNV	OR4D11	OR4D11:exonic:NM 001004706.1	1	50	1	missense	NM 00100	c.589T>C	p.Phe197Leu	-0.41	1	22	0	0.01(COSM14	7 transmemb	rs7120079	0.3021	YES	2.755E-1 (pos.59271637)	acute_myeloid_leukaemia		G-protein coupled receptor activity,G-protein coupled re	T=12, C=12	T=0.5, C=0.5	0.00001	106.71	24	T=8/4, C=7/5
chr11:59224720	G/G	A	G	SNV	OR4D6	OR4D6:exonic:NM 001004708.1	1	100	1	missense	NM 00100	c.287A>G	p.Asp96Gly	0.64	1	94	0		7 transmemb	rs1453543	0.0119	YES	9.418E-1 (pos.59224720)			G-protein coupled receptor activity,G-protein coupled re	A=0, G=24	A=0.0, G=1.0	0.00001	334.789	24	A=0/0, G=5/19
chr14:20502120	T/T	C	T	SNV	OR4K13	OR4K13:exonic:NM 001004714.1	1	100	1	synonymous	NM 00100	c.798G>A	p.(=)	-2.04				0(COSM3677	7 transmemb	rs17277032	0.4987	YES	2.491E-1 (pos.20502120)	acute_myeloid_leukaemia		G-protein coupled receptor activity,G-protein coupled re	C=0, T=22	C=0.0, T=1.0	0.00001	298.891	22	C=0/0, T=19/3
chr20:60712347	A/A	G	A	SNV	PSMA7	PSMA7:intronic:NM 002792.3	1	100		intronic	NM 00279	c.654+73C	p.?	-1.38					Proteasome	rs2057168		YES			Proteasom	Fc-epsilon receptor signaling p (3AR,6R,6AS)-6-((S)-((S)-C)	G=0, A=41	G=0.0, A=1.0	0.00001	646.589	41	G=0/0, A=22/1
chr20:60712511	G/G	A	G	SNV	PSMA7	PSMA7:intronic:NM 002792.3	1	100		intronic	NM 00279	c.592-29T	p.?	-0.06					Proteasome	rs2281740,rs746240	0.1988	YES	0.0 (pos.60712510),7.858E-1 (pos.60712		Proteasom	Fc-epsilon receptor signaling p (3AR,6R,6AS)-6-((S)-((S)-C)	A=0, G=40	A=0.0, G=1.0	0.00001	644.286	40	A=0/0, G=20/2
chr14:20482471	T/T	C	T	SNV	OR4K14	OR4K14:exonic:NM 001004712.1	1	100	1	synonymous	NM 00100	c.882G>A	p.(=)	-1.34					7 transmemb	rs12590785	0.1609	YES	9.828E-1 (pos.20482471)			G-protein coupled receptor activity,G-protein coupled re	C=0, T=61	C=0.0, T=1.0	0.00001	920.822	61	C=0/0, T=21/4
chr11:48285842	G/G	A	G	SNV	OR4X1	OR4X1:exonic:NM 001004726.1	1	100	1	missense	NM 00100	c.430A>G	p.Arg144Gly	0.79	0.73	125	0		7 transmemb	rs1503193	2.00E-04		1.0 (pos.48285842)			G-protein coupled receptor activity,G-protein coupled re	A=0, G=79	A=0.0, G=1.0	0.00001	1269.33	79	A=0/0, G=40/3
chr20:60773684	T/T	C	T	SNV	MTG2	MTG2:intronic:NM 015666.3	1	100		intronic	NM 01566	c.469-6C>	p.?	1.08					50S ribosome	rs4925355	0.4473	YES	8.776E-1 (pos.60773684)			GTP binding,GTPase activity,magnesium ion binding,mit	C=0, T=50	C=0.0, T=1.0	0.00001	852.066	50	C=0/0, T=32/1
chr20:60857034	A/A	G	A	SNV	OSBPL2	OSBPL2:intronic:NM 144498.2	1	100		intronic	NM 14449	c.872+99G	p.?	-0.58					Oxysterol-bir	rs2274938		YES			Oxysterol-b	bile acid biosynthetic process,cholesterol binding,cytos	G=0, A=109	G=0.0, A=1.0	0.00001	1713.07	109	G=0/0, A=47/6
chr11:48285856	C/T	C	T	SNV	OR4X1	OR4X1:exonic:NM 001004726.1	1	50	1	synonymous	NM 00100	c.444C>T	p.(=)	-6.51					7 transmemb	rs10838850	0.2455	YES	5.895E-1 (pos.48285856)			G-protein coupled receptor activity,G-protein coupled re	C=35, T=37	C=0.4861, T=0.5139	0.00001	392.577	74	C=20/15, T=13
chr11:4967468	A/A	G	A	SNV	OR51A4	OR51A4:exonic:NM 001005329.1	1	100	1	missense	NM 00100	c.863C>T	p.Thr288Met	5.39	1	81	0.005		7 transmemb	rs28698374	0.1495	YES	9.012E-1 (pos.4967468)			G-protein coupled receptor activity,G-protein coupled re	G=0, A=68	G=0.0, A=1.0	0.00001	1051.98	68	G=0/0, A=35/3
chr20:60895738	G/G	A	G	SNV	LAMA5	LAMA5:intronic:NM 005560.4	1	100		intronic	NM 00556	c.6649-13	p.?	0.09					Laminin B (Dc	rs6089346	0.0504	YES	9.837E-1 (pos.60895738)		Laminin, al	angiogene Colonic Diseases;Colorectal Neoplasms;Dige	A=0, G=28	A=0.0, G=1.0	0.00001	399.564	28	A=0/0, G=11/1
chr11:4928866	T/T	C	T	SNV	OR51A7	OR51A7:exonic:NM 001004749.1	1	100	1	synonymous	NM 00100	c.267C>T	p.(=)	-7.1				0.02(COSM15	7 transmemb	rs7941509	0.3832	YES	8.164E-2 (pos.4928866)	adenocarcinoma,neoplasn		G-protein coupled receptor activity,G-protein coupled re	C=0, T=81	C=0.0, T=1.0	0.00001	1309.48	81	C=0/0, T=49/3
chr11:5344552	T/T	C	T	SNV	OR51B2	OR51B2:utr 3:NM 033180.4	1	100	1	utr 3	NM 03318	c.976G>A	p.?	-0.5					7 transmemb	rs386750139,rs7934	0.0874	YES	5.209E-1 (pos.5344552)			G-protein coupled receptor activity,G-protein coupled re	C=0, T=34	C=0.0, T=1.0	0.00001	522.963	34	C=0/0, T=15/1
chr20:60902883	T/T	C	T	SNV	LAMA5	LAMA5:intronic:NM 005560.4	1	100		intronic	NM 00556	c.4797+39	p.?	-4.95					Laminin B (Dc	rs4925375	0.2993	YES	8.809E-1 (pos.60902883)		Laminin, al	angiogene Colonic Diseases;Colorectal Neoplasms;Dige	C=0, T=30	C=0.0, T=1.0	0.00001	451.016	30	C=0/0, T=17/1
chr11:5344561	A/A	G	A	SNV	OR51B2	OR51B2:utr 3:NM 033180.4	1	100	1	utr 3	NM 03318	c.967C>T	p.?	-0.67					7 transmemb	rs386750139,rs7933	0.0871	YES	5.195E-1;6.143E-4;1.229E-4 (pos.5344561)			G-protein coupled receptor activity,G-protein coupled re	G=0, A=35	G=0.0, A=1.0	0.00001	543.901	35	G=0/0, A=15/2
chr20:60906055	T/T	C	T	SNV	LAMA5	LAMA5:intronic:NM 005560.4	1	100		intronic	NM 00556	c.3650+33	p.?	-1.07					Laminin B (Dc	rs73613564	0.3574	YES	7.836E-1 (pos.60906055)		Laminin, al	angiogene Colonic Diseases;Colorectal Neoplasms;Dige	C=0, T=42	C=0.0, T=1.0	0.00001	685.211	42	C=0/0, T=9/33
chr11:5344847	G/G	A	G	SNV	OR51B2	OR51B2:exonic:NM 033180.4	1	100	1	synonymous	NM 03318	c.681T>C	p.(=)	-3.35				0.01(COSM39	7 transmemb	rs10768793	0.0867	YES	5.194E-1 (pos.5344847)	acute_myeloid_leukaemia		G-protein coupled receptor activity,G-protein coupled re	A=0, G=46	A=0.0, G=1.0	0.00001	768.31	46	A=0/0, G=26/2
chr20:60911958	A/A	G	A	SNV	LAMA5	LAMA5:intronic:NM 005560.4	1	100		intronic	NM 00556	c.2165-11	p.?	-1.35					Laminin B (Dc	rs2890083		YES	7.747E-1 (pos.60911958)		Laminin, al	angiogene Colonic Diseases;Colorectal Neoplasms;Dige	G=0, A=25	G=0.0, A=1.0	0.00001	353.019	25	G=0/0, A=11/1
chr11:5344902	G/G	C	G	SNV	OR51B2	OR51B2:exonic:NM 033180.4	1	100	1	missense	NM 03318	c.626G>C	p.Cys209Ser	-0.99		112		0(COSM3998	7 transmemb	rs7937237	0.0867	YES	5.192E-1 (pos.5344902)	acute_myeloid_leukaemia		G-protein coupled receptor activity,G-protein coupled re	C=0, G=48	C=0.0, G=1.0	0.00001	810.434	48	C=0/0, G=26/2
chr20:60913495	C/C	T	C	SNV	LAMA5	LAMA5:intronic:NM 005560.4	1	100		intronic	NM 00556	c.1618+29	p.?	-0.92					Laminin B (Dc	rs2151512	0.3044	YES	7.556E-1;0.0 (pos.60913495)		Laminin, al	angiogene Colonic Diseases;Colorectal Neoplasms;Dige	T=0, C=35	T=0.0, C=1.0	0.00001	548.26	35	T=0/0, C=19/1
chr11:5345170	G/G	A	G	SNV	OR51B2	OR51B2:exonic:NM 033180.4	1	100	1	missense	NM 03318	c.358T>C	p.Cys120Arg	1.82		180		0(COSM3998	7 transmemb	rs7952293	0.0867	YES	5.199E-1 (pos.5345170)	acute_myeloid_leukaemia		G-protein coupled receptor activity,G-protein coupled re	A=0, G=137	A=0.0, G=1.0	0.00001	2283.29	137	A=0/0, G=71/6
chr20:60927538	T/T	C	T	SNV	LAMA5,LAMA5-AS1	LAMA5:intronic:NM 005560.4, LAMA5-AS1:upstream:NR 10	1	100		intronic	NM 00556	c.569-122	p.?	-3.24					Laminin B (Dc	rs623834		YES			Laminin, al	angiogene Colonic Diseases;Colorectal Neoplasms;Dige	C=0, T=21	C=0.0, T=1.0	0.00001	281.16	21	C=0/0, T=12/9
chr20:60927551	G/G	A	G	SNV	LAMA5,LAMA5-AS1	LAMA5:intronic:NM 005560.4, LAMA5-AS1:upstream:NR 10	1	100		intronic	NM 00556	c.569-135	p.?	0.26					Laminin B (Dc	rs482010		YES			Laminin, al	angiogene Colonic Diseases;Colorectal Neoplasms;Dige	A=0, G=21	A=0.0, G=1.0	0.00001	281.165	21	A=0/0, G=12/9
chr20:61389569	G/G	C	G	SNV	NTSR1	NTSR1:intronic:NM 002531.2	1	100		intronic	NM 00253	c.917-49C	p.?	0.2					7 transmemb	rs112862855,rs62198382,rs750		YES	0.0 (pos.61389569),6.649E-4;0.0 (pos.61		Neurotensi	D-asparta Child Development Disorders, Pervasive;Mer	C=0, G=90	C=0.0, G=1.0	0.00001	1493.08	90	C=0/0, G=66/2
chr20:61443547	G/G	A	G	SNV	OGFR	OGFR:intronic:NM 007346.3	1	100		intronic	NM 00734	c.615-35A	p.?	-1.66					Opioid growt	rs2075776	0.4349	YES	4.807E-1 (pos.61443547)			cellular_component,cytoplasm,membrane,nucleus,opic	A=0, G=41	A=0.0, G=1.0	0.00001	664.7	41	A=0/0, G=18/2
chr11:5373114	A/A	G	A	SNV	OR51B5,OR51B6	OR51B6:exonic:NM 001004750.1, OR51B5:intronic:NM 00	1	100	1	missense	NM 00100	c.-358-497	p.?, p.Ser126Asn	2.39	1	46	0	0.01(COSM63	7 transmemb	rs5006888	0.4101	YES	5.25E-1 (pos.5373114)	carcinoma		G-protein coupled receptor activity,G-protein coupled re	G=0, A=21	G=0.0, A=1.0	0.00001	281.206	21	G=0/0, A=16/5
chr20:61595537	G/G	A	G	SNV	SLC17A9	SLC17A9:intronic:NM 022082.3	1	100		intronic	NM 02208	c.823-43A	p.?	-2.35					Major Facilit	rs2427464	1.00E-04		1.0;0.0 (pos.61595537)		Solute carri	anion tran Congenital, Hereditary, and Neonatal Diseas	A=0, G=57	A=0.0, G=1.0	0.00001	847.242	57	A=0/0, G=19/3
chr11:5373646	C/C	T	C	SNV	OR51B5,OR51B6	OR51B6:exonic:NM 001004750.1, OR51B5:intronic:NM 00	1	100	1	synonymous	NM 00100	c.-358-550	p.?, p.(=)	-1.66				0.01(COSM50	7 transmemb	rs5024041	0.4037	YES	5.294E-1 (pos.5373646)	acute_myeloid_leukaemia		G-protein coupled receptor activity,G-protein coupled re	T=0, C=51	T=0.0, C=1.0	0.00001	754.583	51	T=0/0, C=29/2
chr20:61869223	T/C	T	C	SNV	BIRC7,MIR3196	BIRC7:intronic:NM 139317.2, MIR3196:upstream:NR 0361	1	50		intronic	NM 13931	c.350-32T	p.?	-0.74					Inhibitor of A	rs6062855	0.4609	YES	5.554E-1 (pos.61869223)		Baculoviral	Golgi appa Adenocarcinoma;Carcinoma;Carcinoma, Ren	T=41, C=41	T=0.5, C=0.5	0.00001	435.939	82	T=24/17, C=27
chr20:61951789	T/T	C	T	SNV	COL20A1	COL20A1:intronic:NM 020882.2	1	100		intronic	NM 02088	c.3153+69	p.?	-1.05					Collagen tripl	rs6062891		YES				collagen trimer,endoplasmic reticulum lumen,extracell	C=0, T=22	C=0.0, T=1.0	0.00001	298.891	22	C=0/0, T=10/1
chr20:61980991	T/C	T	C	SNV	CHRNA4	CHRNA4:intronic:NM 000744.6	1	95.45		intronic	NM 00074	c.1758+14	p.?	-1.79					Neurotransm	rs3827020	0.1754	YES	4.657E-1 (pos.61980991)		Cholinergic	B cell activ Attention Deficit Di Amobarbit Benign	T=1, C=21	T=0.0455, C=0.9545	0.00001	271.51	22	T=0/1, C=15/6
chr11:5410934	G/G	T	G	SNV	OR51B5,OR51M1	OR51M1:exonic:NM 001004756.2, OR51B5:intronic:NM 00	1	100	1	missense	NM 00100	c.-358-427	p.?, p.His102Gln	-3.27		24		0.06(COSM63	7 transmemb	rs1498467	0.1732	YES	5.122E-1 (pos.5410934)	carcinoma		G-protein coupled receptor activity,G-protein coupled re	T=0, G=35	T=0.0, G=1.0	0.00001	535.697	35	T=0/0, G=15/2
chr11:5411579	AC/AC	GT	AC	MNV	OR51B5,OR51M1	OR51M1:exonic:NM 001004756.2, OR51B5:intronic:NM 00	2	100	1	missense	NM 00100	c.-358-434	p.?, p.Phe318Leu	-1.06, -1.08		22		0.11(COSM63	7 transmemb	rs10768906,rs10768	0.1745	YES	5.324E-1;0.0 (pos.54115	carcinoma		G-protein coupled receptor activity,G-protein coupled re	GT=0, AC=3	GT=0.0, AC=1.0	0.00001	583.706	37	GT=0/0, AC=11
chr20:62173029	G/G	A	G	SNV	SRMS	SRMS:intronic:NM 080823.3	1	100		intronic	NM 08082	c.947-56T	p.?	-1.9					Protein kinas	rs310656		YES				ATP bindin Digestive System Diseases;Digestive System	A=0, G=33	A=0.0, G=1.0	0.00001	504.587	33	A=0/0, G=18/1
chr11:5443867	T/T	C	T	SNV	OR51B5,OR51Q1	OR51Q1:exonic:NM 001004757.2, OR51B5:intronic:NM 00	1	100	1	missense	NM 00100	c.-358-757	p.?, p.Thr146Ile	0.02		89		0.01(COSM37	7 transmemb	rs10838092	0.3924	YES	7.255E-1 (pos.5443867)	adenocarcinoma		G-protein coupled receptor activity,G-protein coupled re	C=0, T=30	C=0.0, T=1.0	0.00001	446.598	30	C=0/0, T=17/1
chr11:5443887	C/C	T	C	SNV	OR51B5,OR51Q1	OR51Q1:exonic:NM 001004757.2, OR51B5:intronic:NM 00	1	100	1	missense	NM 00100	c.-358-757	p.?, p.Cys153Arg	-1.07		180		0.04(COSM37	7 transmemb	rs10838093	0.3921	YES	7.247E-1 (pos.5443887)	adenocarcinoma		G-protein coupled receptor activity,G-protein coupled re	T=0, C=32	T=0.0, C=1.0	0.00001	485.141	32	T=0/0, C=17/1
chr20:62223371	G/G	A	G	SNV	GMEB2	GMEB2:intronic:NM 012384.4	1	100		intronic	NM 01238	c.952+4T>	p.?	-1.71					SAND domair	rs311494	0.0971	YES	9.99E-1 (pos.62223371)		Glucocortic	DNA binding,RNA polymerase II core promoter proximal	A=0, G=75	A=0.0, G=1.0	0.00001	1193.93	75	A=0/0, G=17/5
chr20:62234241	G/G	A	G	SNV	GMEB2	GMEB2:intronic:NM 012384.4	1	100		intronic	NM 01238	c.357+77T	p.?	-5.74					SAND domair	rs2145262,rs879408133		YES			Glucocortic	DNA binding,RNA polymerase II core promoter proximal	A=0, G=25	A=0.0, G=1.0	0.00001	353.019	25	A=0/0, G=16/9
chr11:5443893	A/A	G	A	SNV	OR51B5,OR51Q1	OR51Q1:exonic:NM 001004757.2, OR51B5:intronic:NM 00	1	100	1	missense	NM 00100	c.-358-757	p.?, p.Val155Ile	-1.23		29		0.06(COSM37	7 transmemb	rs10838094	0.3922	YES	7.247E-1 (pos.5443893)	adenocarcinoma,neoplasn		G-protein coupled receptor activity,G-protein coupled re	G=0, A=31	G=0.0, A=1.0	0.00001	465.816	31	G=0/0, A=16/1
chr11:5443963	A/A	G	A	SNV	OR51B5,OR51Q1	OR51Q1:exonic:NM 001004757.2, OR51B5:intronic:NM 00	1	100	1	missense	NM 00100	c.-358-758	p.?, p.Arg178His	3.52		29		0.16(COSM37	7 transmemb	rs10838095	0.3922	YES	7.257E-1 (pos.5443963)	adenocarcinoma,ductal_ca		G-protein coupled receptor activity,G-protein coupled re	G=0, A=24	G=0.0, A=1.0	0.00001	334.79	24	G=0/0, A=7/17
chr11:5443739	T/C	T	C	SNV	OR51B5,OR51Q1	OR51Q1:exonic:NM 001004757.2, OR51B5:intronic:NM 00	1	98.41	1	synonymous	NM 00100	c.-358-755	p.?, p.(=)	-1.8				0.03(COSM37	7 transmemb	rs2736587	0.3925	YES	7.265E-1 (pos.5443739)	adenocarcinoma,neoplasn		G-protein coupled receptor activity,G-protein coupled re	T=1, C=62	T=0.0159, C=0.9841	0.00001	925.368	63	T=1/0, C=31/3
chr20:8713842	A/A	G	A	SNV	PLCB1	PLCB1:intronic:NM 015192.3	1	100		intronic	NM 01519	c.1889-43	p.?	-1.24					PLC-beta C te	rs993670	0.0243	YES	9.985E-1 (pos.8713842)		Phospholip	G-protein Bone Marrow Diseases;Brain Diseases;Centr	G=0, A=96	G=0.0, A=1.0	0.00001	1616.49	96	G=0/0, A=46/5
chr20:8769180	T/T	C	T	SNV	PLCB1	PLCB1:intronic:NM 015192.3	1	100		intronic	NM 01519	c.3188+8C	p.?	0.03					PLC-beta C te	rs2327089	0.0429	YES	9.984E-1 (pos.8769180)		Phospholip	G-protein Bone Marrow Diseases;Brain D Benign	C=0, T=71	C=0.0, T=1.0	0.00001	1111.28	71	C=0/0, T=40/3
chr20:8770108	C/C	A	C	SNV	PLCB1	PLCB1:intronic:NM 015192.3	1	100		intronic	NM 01519	c.3279-57	p.?	-0.26					PLC-beta C te	rs4633993	0.0412	YES			Phospholip	G-protein Bone Marrow Diseases;Brain Diseases;Centr	A=0, C=38	A=0.0, C=1.0	0.00001	603.786	38	A=0/0, C=17/2
chr20:8770130	G/G	A	G	SNV	PLCB1	PLCB1:intronic:NM 015192.3	1	100		intronic	NM 01519	c.3279-35	p.?	-0.18					PLC-beta C te	rs2327090	0.0277	YES	9.985E-1 (pos.8770130)		Phospholip	G-protein Bone Marrow Diseases;Brain Diseases;Centr	A=0, G=38	A=0.0, G=1.0	0.00001	603.785	38	A=0/0, G=17/2
chr11:5510284	G/G	A	G	SNV	OR51B5,OR52D1	OR52D1:exonic:NM 001005163.2, OR51B5:intronic:NM 00	1	100	1	synonymous	NM 00100	c.-359+16	p.?, p.(=)	-6.54					7 transmemb	rs417425	0.0296	YES	9.182E-1 (pos.5510284)			G-protein coupled receptor activity,G-protein coupled re	A=0, G=121	A=0.0, G=1.0	0.00001	1961.57	121	A=0/0, G=43/7
chr20:9543688	T/G	T	G	SNV	PAK5	PAK5:intronic:NM 177990.2	1	50		intronic	NM 17799	c.1483-17	p.?	0.09					Kinase-like P	rs2297347	0.2248	YES	2.788E-1 (pos.9543688)			ATP binding,Rac GTPase binding,Rho protein signal trans	T=13, G=13	T=0.5, G=0.5	0.00001	119.162	26	T=9/4, G=5/8

chr20:9625003	A/A	G	A	SNV	PAK5	PAK5:intronic:NM_177990.2	1	100		intronic	NM_17799	c.-10-16C>	p.?	2.09					Kinase-like,P	rs4816157	0.0608	YES	9.984E-1 (pos.9625003)			ATP binding,Rac GTPase binding,Rho protein signal trans				G=0, A=124	G=0.0, A=1.0	0.00001	2017.84	124	G=0/0, A=49/7
chr21:10910291	C/T	C	T	SNV	TPTE	TPTE:intronic:NM_199261.3	1	45.45		intronic	NM_19926	c.1449+16	p.?	-1.14					C2 domain of	rs150480		YES	5.492E-1 (pos.10910291)		Transmemb	integral component of membrane,peptidyl-tyrosine dep				C=36, T=30	C=0.5455, T=0.4545	0.00001	279.745	66	C=18/18, T=7/
chr11:5510497	C/C	T	C	SNV	OR51B5,OR52D1	OR52D1:exonic:NM_001005163.2, OR51B5:intronic:NM_00	1	100	1	synonymous	NM_00100	c.-359+16:	p.?, p.(=)	-1.57					7 transmemb	rs2467219,rs386750:	0.0297	YES	9.185E-1 (pos.5510497)			G-protein coupled receptor activity,G-protein coupled re				T=0, C=36	T=0.0, C=1.0	0.00001	563.732	36	T=0/0, C=23/1
chr21:15964920	G/G	C	G	SNV	LOC388813,SAMSN1-AS1	LOC388813:utr_3:NM_001256579.2, SAMSN1-AS1:intronic	1	100		intronic	NR_04651:	c.422G>C	p.?	-1.13						rs466913		YES	5.201E-1 (pos.15964920)							C=0, G=77	C=0.0, G=1.0	0.00001	1233.87	77	C=0/0, G=30/4
chr21:15966421	A/A	T	A	SNV	LOC388813,SAMSN1-AS1	LOC388813:intronic:NM_001256579.2, SAMSN1-AS1:introni	1	100		intronic	NR_04651:	c.316-22A:	p.?	0.03						rs460852		YES	5.455E-1 (pos.15966421)							T=0, A=97	T=0.0, A=1.0	0.00001	1626.99	97	T=0/0, A=42/5
chr11:5510626	C/C	T	C	SNV	OR51B5,OR52D1	OR52D1:exonic:NM_001005163.2, OR51B5:intronic:NM_00	1	100	1	synonymous	NM_00100	c.-359+16:	p.?, p.(=)	-4.85					7 transmemb	rs444878,rs7774971:	0.0297	YES	9.18E-1;0.0 (pos.5510626)			G-protein coupled receptor activity,G-protein coupled re				T=0, C=72	T=0.0, C=1.0	0.00001	1130.95	72	T=0/0, C=35/3
chr21:22656316	C/C	T	C	SNV	NCAM2	NCAM2:intronic:NM_004540.4	1	100		intronic	NM_00454	c.131-198'	p.?	-0.75					Fibronectin t	rs9980326		YES			Cell adhesi	axon,axon	Abnormalities, Multiple;Chromosome Disord			T=0, C=21	T=0.0, C=1.0	0.00001	281.147	21	T=0/0, C=13/8
chr21:22841123	A/A	G	A	SNV	NCAM2	NCAM2:intronic:NM_004540.4	1	100		intronic	NM_00454	c.1896+19	p.?	-0.36					Fibronectin t	rs2250508	0.4403	YES	4.274E-2 (pos.22841123)		Cell adhesi	axon,axon	Abnormalities, Multiple;Chromosome Disord			G=0, A=26	G=0.0, A=1.0	0.00001	371.418	26	G=0/0, A=9/17
chr21:26965100	A/A	C	A	SNV	MRPL39	MRPL39:intronic:NM_080794.3	1	100		intronic	NM_08079	c.921+24G	p.?	-0.26						rs2829809	0.1765	YES	5.587E-1 (pos.26965100)		Mitochondr	RNA bindir	Abnormalities, Multiple;Chromosome Disord			C=0, A=58	C=0.0, A=1.0	0.00001	870.086	58	C=0/0, A=24/3
chr11:4674575	A/A	G	A	SNV	OR51E1	OR51E1:exonic:NM_152430.3	1	100	2	synonymous	NM_15243	c.819G>A	p.(=)	-1.77				0.05(COSM37	7 transmemb	rs3817098	0.4577	YES	5.85E-2 (pos.4674575)	adenocarcino	Olfactory re	G-protein	Mental Disorders;Substance-Related Disorde			G=0, A=73	G=0.0, A=1.0	0.00001	1150.48	73	G=0/0, A=44/2
chr21:26976250	G/G	A	G	SNV	MRPL39	MRPL39:intronic:NM_080794.3	1	100		intronic	NM_08079	c.281-3T>(	p.?	1.8				0.85(COSM4417230),0.85(C		rs7278164	0.2422	YES	1.935E-1 (pos.26976250)	acute_myeloid	Mitochondr	RNA bindir	Abnormalities, Multiple;Chromosome Disord			A=0, G=31	A=0.0, G=1.0	0.00001	465.818	31	A=0/0, G=13/1
chr11:4791111	T/C	T	C	SNV	OR51F1	OR51F1:exonic:NM_001004752.1	1	59.09	1	missense	NM_00100	c.37A>G	p.Thr13Ala	-0.93	0.18	58	0.005	0.06(COSM37	7 transmemb	rs17324812	0.2688	YES	6.984E-2 (pos.4791111)	adenocarcinoma						T=9, C=13	T=0.4091, C=0.5909	0.00001	122.568	22	T=6/3, C=7/6
chr11:4944892	G/C	G	C	SNV	OR51G1	OR51G1:exonic:NM_001005237.1	1	57.58	1	synonymous	NM_00100	c.678C>G	p.(=)	-4.8				0.03(COSM50	7 transmemb	rs34583466	0.1326	YES	0.0;0.0 (pos.4944892)	adenocarcinoma,carcinom		G-protein coupled receptor activity,G-protein coupled re				G=14, C=19	G=0.4242, C=0.5758	0.00001	204.012	33	G=9/5, C=2/17
chr11:5020416	G/G	A	G	SNV	OR51L1	OR51L1:exonic:NM_001004755.1	1	100	1	synonymous	NM_00100	c.204A>G	p.(=)	2.7				0.04(COSM39	7 transmemb	rs2445290	0.1477	YES	6.893E-1 (pos.5020416)	acute_myeloid_leukaemia		G-protein coupled receptor activity,G-protein coupled re				A=0, G=89	A=0.0, G=1.0	0.00001	1472.49	89	A=0/0, G=48/4
chr21:30252088	A/A	T	A	SNV	N6AMT1	N6AMT1:intronic:NM_013240.5	1	100		intronic	NM_01324	c.396+104	p.?	0.92					Methyltransf	rs2205449		YES			N6 adenine	S-adenosy	Abnormalities, Multiple;Chromosome Disord			T=0, A=25	T=0.0, A=1.0	0.00001	352.996	25	T=0/0, A=7/18
chr21:30257729	G/G	A	G	SNV	N6AMT1	N6AMT1:upstream:NM_013240.5	1	100		intronic	NM_01324	c.-62T>C	p.?	0						rs2859734		YES			N6 adenine	S-adenosy	Abnormalities, Multiple;Chromosome Disord			A=0, G=26	A=0.0, G=1.0	0.00001	371.402	26	A=0/0, G=19/7
chr11:4869649	A/A	G	A	SNV	OR51S1	OR51S1:exonic:NM_001004758.1	1	100	1	missense	NM_00100	c.790C>T	p.Leu264Phe	0.71	0.04	22	0.003	0.02(COSM37	7 transmemb	rs12361955	0.4304	YES	5.673E-2 (pos.4869649)	adenocarcinoma		G-protein coupled receptor activity,G-protein coupled re				G=0, A=42	G=0.0, A=1.0	0.00001	685.127	42	G=0/0, A=25/1
chr11:4870261	C/C	G	C	SNV	OR51S1	OR51S1:exonic:NM_001004758.1	1	100	1	missense	NM_00100	c.178C>G	p.Gln60Glu	0.34	1	29	0	0(COSM3752	7 transmemb	rs11602499	0.4304	YES	5.683E-2 (pos.4870261)	adenocarcinoma,neoplasn		G-protein coupled receptor activity,G-protein coupled re				G=0, C=21	G=0.0, C=1.0	0.00001	281.235	21	G=0/0, C=3/18
chr11:5221645	C/A	C	A	SNV	OR51V1	OR51V1:exonic:NM_001004760.2	1	51.85	1	missense	NM_00100	c.286G>T	p.Gly96Trp	0.47	0.08	184	1	0.13(COSM41	7 transmemb	rs11512276	0.2947	YES	1.925E-1;3.188E-1 (pos.5	neoplasm,squamous_cell		G-protein coupled receptor activity,G-protein coupled re				C=13, A=14	C=0.4815, A=0.5185	0.00001	132.776	27	C=7/6, A=4/10
chr11:5602968	A/A	G	A	SNV	OR52B6	OR52B6:exonic:NM_001005162.2	1	100	1	missense	NM_00100	c.862G>A	p.Val288Ile	-1.78	1	29	0.002		7 transmemb	rs10769086	0.2465	YES	9.908E-1 (pos.5602968)			G-protein coupled receptor activity,G-protein coupled re				G=0, A=55	G=0.0, A=1.0	0.00001	814.907	55	G=0/0, A=29/2
chr11:5602438	T/A	T	A	SNV	OR52B6	OR52B6:exonic:NM_001005162.2	1	50	1	missense	NM_00100	c.332T>A	p.Leu111His	0.47	0.23	99	0	0.04(COSM49	7 transmemb	rs2341432,rs541562(	0.0025,0.4	YES	9.777E-1;0.0 (pos.56024:	ductal_carcinoma		G-protein coupled receptor activity,G-protein coupled re				T=20, A=20	T=0.5, A=0.5	0.00001	214.325	40	T=11/9, A=11/
chr21:33073985	C/C	T	C	SNV	SCAF4	SCAF4:intronic:NM_020706.2	1	100		intronic	NM_02070	c.600+104	p.?	-1.26					RNA polymer:	rs204734		YES			SR-related (	RNA bindir	Abnormalities, Multiple;Chromosome Disord			T=0, C=39	T=0.0, C=1.0	0.00001	623.872	39	T=0/0, C=31/8
chr11:5079946	T/T	C	T	SNV	OR52E2	OR52E2:exonic:NM_001005164.2	1	100	1	synonymous	NM_00100	c.912G>A	p.(=)	-0.76					7 transmemb	rs2445332	0.0383	YES	7.844E-1 (pos.5079946)			G-protein coupled receptor activity,G-protein coupled re				C=0, T=67	C=0.0, T=1.0	0.00001	1032.7	67	C=0/0, T=24/4
chr11:5906073	G/T	G	T	SNV	OR52E4	OR52E4:exonic:NM_001005165.1	1	59.09	1	missense	NM_00100	c.551G>T	p.Arg184Met	-0.29	1	91	0		7 transmemb	rs4757986	0.2123	YES	8.199E-1;0.0 (pos.5906073)			G-protein coupled receptor activity,G-protein coupled re				G=9, T=13	G=0.4091, T=0.5909	0.00001	122.954	22	G=6/3, T=8/5
chr11:5566030	G/G	A	G	SNV	OR52H1	OR52H1:exonic:NM_001005289.1	1	100	1	missense	NM_00100	c.724T>C	p.Cys242Arg	-0.39	1	180	0		7 transmemb	rs1995158	0.1923	YES	9.305E-1 (pos.5566030)			G-protein coupled receptor activity,G-protein coupled re				A=0, G=48	A=0.0, G=1.0	0.00001	810.436	48	A=0/0, G=24/2
chr21:34638859	A/A	G	A	SNV	IL10RB,IL10RB-AS1	IL10RB:intronic:NM_000628.4, IL10RB-AS1:upstream:NR_03	1	100		intronic	NR_03897	c.49+40G>	p.?	0.84						rs2239573	0.2573	YES	7.519E-1 (pos.34638859)			cytokine-n	Abnormalities, Multiple;Brain Diseases;Brair			G=0, A=27	G=0.0, A=1.0	0.00001	366.646	27	G=0/0, A=13/1
chr21:34726127	A/A	T	A	SNV	IFNAR1	IFNAR1:intronic:NM_000629.2	1	100		intronic	NM_00062	c.1440+38	p.?	0.07					Interferon-al	rs2856973	0.2611	YES	9.979E-1;0.0 (pos.34726127)		Interferon,	JAK-STAT c	Abnormalities, Mul	Interferon Alfa-2a, Recor		T=0, A=100	T=0.0, A=1.0	0.00001	1702.01	100	T=0/0, A=47/5
chr21:34799350	G/G	C	G	SNV	IFNGR2	IFNGR2:intronic:NM_005534.3	1	100		intronic	NM_00553	c.561+11C	p.?	-0.03					Interferon-al	rs11910627	0.3081	YES	9.931E-1 (pos.34799350)		Interferon g	Golgi mem	Abnormalities, Mul	Interferon_	Benign	C=0, G=23	C=0.0, G=1.0	0.00001	325.708	23	C=0/0, G=14/9
chr11:5566365	C/C	T	C	SNV	OR52H1	OR52H1:exonic:NM_001005289.1	1	100	1	missense	NM_00100	c.389A>G	p.His130Arg	2.57	1	29	0		7 transmemb	rs1566275	0.1922	YES	9.309E-1 (pos.5566365)			G-protein coupled receptor activity,G-protein coupled re				T=0, C=37	T=0.0, C=1.0	0.00001	583.604	37	T=0/0, C=11/2
chr21:34894623	C/C	T	C	SNV	GART	GART:intronic:NM_000819.4	1	100		intronic	NM_00081	c.1299-34	p.?	-1					AIR synthase	rs2834234	0.2393	YES	9.922E-1 (pos.34894623)		Phosphorib	'de novo' II	Abnormalities, Mul	10-CF3C(OH)2-DDACTHF.(		T=0, C=89	T=0.0, C=1.0	0.00001	1472.31	89	T=0/0, C=36/5
chr11:6007272	G/G	A	G	SNV	OR52L1	OR52L1:exonic:NM_001005173.3	1	100	1	missense	NM_00100	c.889T>C	p.Trp297Arg	-2.46		101			7 transmemb	rs4237768	0.1756	YES	8.709E-1 (pos.6007272)			G-protein coupled receptor activity,G-protein coupled re				A=0, G=158	A=0.0, G=1.0	0.00001	2541.83	158	A=0/0, G=53/1
chr21:34915324	GC/GC	CG	GC	MNV	GART,SON	SON:upstream:NM_138927.2, GART:upstream:NM_000819.	2	100		intronic	NM_00081	c.-3703CG	p.?, p.?	1.07, 0.68						rs1041745,rs1041746,rs355068		YES			Phosphorib	'de novo' II	Abnormalities, Mul	10-CF3C(OH)2-DDACTHF.(		CG=0, GC=3	CG=0.0, GC=1.0	0.00001	465.813	31	CG=0/0, GC=2
chr11:6007613	G/G	T	G	SNV	OR52L1	OR52L1:exonic:NM_001005173.3	1	100	1	missense	NM_00100	c.548A>C	p.Lys183Thr	-0.1		78			7 transmemb	rs4354673	0.208	YES	8.708E-1 (pos.6007613)			G-protein coupled receptor activity,G-protein coupled re				T=0, G=20	T=0.0, G=1.0	0.00001	263.786	20	T=0/0, G=5/15
chr11:6007680	G/G	A	G	SNV	OR52L1	OR52L1:exonic:NM_001005173.3	1	100	1	missense	NM_00100	c.481T>C	p.Cys161Arg	-4.14		180			7 transmemb	rs386750238,rs4436	0.2059	YES	8.668E-1;0.0 (pos.6007680)			G-protein coupled receptor activity,G-protein coupled re				A=0, G=41	A=0.0, G=1.0	0.00001	664.695	41	A=0/0, G=14/2
chr11:6007743	G/G	A	G	SNV	OR52L1	OR52L1:exonic:NM_001005173.3	1	100	1	missense	NM_00100	c.418T>C	p.Cys140Arg	1.85		180		0.6(COSM562	7 transmemb	rs4436525	0.236	YES	8.695E-1 (pos.6007743)	squamous_cell_carcinoma		G-protein coupled receptor activity,G-protein coupled re				A=0, G=26	A=0.0, G=1.0	0.00001	371.419	26	A=0/0, G=12/1
chr11:5809308	T/T	A	T	SNV	OR52N1	OR52N1:exonic:NM_001001913.1	1	100	1	missense	NM_00100	c.739T>A	p.Phe247Ile	-0.2	1	21	0		7 transmemb	rs7934670	0.0333	YES	8.961E-1 (pos.5809308)			G-protein coupled receptor activity,G-protein coupled re				A=0, T=36	A=0.0, T=1.0	0.00001	563.742	36	A=0/0, T=17/1
chr11:5809230	G/A	G	A	SNV	OR52N1	OR52N1:exonic:NM_001001913.1	1	58.33	1	synonymous	NM_00100	c.817C>T	p.(=)	0.71					7 transmemb	rs12794769	0.1488	YES	3.145E-1 (pos.5809230)			G-protein coupled receptor activity,G-protein coupled re				G=15, A=21	G=0.4167, A=0.5833	0.00001	234.34	36	G=5/10, A=12/
chr21:37518362	G/G	A	G	SNV	CBR3,CBR3-AS1	CBR3-AS1:intronic_nc:NR_038892.1, CBR3:intronic:NM_001	1	100		intronic	NR_03889	c.398-12A	p.?	-1.7					Enoyl-(Acyl ca	rs2835286	0.1821	YES	8.549E-1 (pos.37518362)		Carbonyl re	3-keto ste	Abnormalities, Mul	Deutetrabenazine,Doxo		A=0, G=62	A=0.0, G=1.0	0.00001	940.304	62	A=0/0, G=17/4
chr11:5776470	G/G	T	G	SNV	OR52N4	OR52N4:exonic:NM_001005175.3	1	100	1	missense	NM_00100	c.500T>G	p.Leu167Arg	-0.34		102			7 transmemb	rs7394584	0.0287	YES	8.937E-1;0.0;1.159E-4 (pos.5776470)			G-protein coupled receptor activity,G-protein coupled re				T=0, G=42	T=0.0, G=1.0	0.00001	685.219	42	T=0/0, G=16/2
chr21:38092039	A/C	A	C	SNV	SIM2	SIM2:intronic:NM_005069.5	1	50		intronic	NM_00506	c.349-83A	p.?	-2.75					Helix-loop-he	rs2070650		YES			Single-mind	DNA bindi	Abnormalities, Multiple;Chromosome Disord			A=12, C=12	A=0.5, C=0.5	0.00001	106.709	24	A=9/3, C=9/3
chr21:38113998	A/A	C	A	SNV	SIM2	SIM2:intronic:NM_005069.5	1	100		intronic	NM_00506	c.851-20C	p.?	-1					Helix-loop-he	rs2070519	0.2929	YES	2.38E-1 (pos.38113998)		Single-mind	DNA bindi	Abnormalities, Multiple;Chromosome Disord			C=0, A=21	C=0.0, A=1.0	0.00001	281.235	21	C=0/0, A=13/8
chr11:5776623	T/T	A	T	SNV	OR52N4	OR52N4:exonic:NM_001005175.3	1	100	1	missense	NM_00100	c.653A>T	p.Asn218Ile	2.63		149			7 transmemb	rs7396938	0.0286	YES	8.938E-1 (pos.5776623)			G-protein coupled receptor activity,G-protein coupled re				A=0, T=78	A=0.0, T=1.0	0.00001	1249.35	78	A=0/0, T=55/2
chr11:6221169	A/G	A	G	SNV	OR52W1	OR52W1:exonic:NM_001005178.1	1	57.14	1	missense	NM_00100	c.716A>G	p.His239Arg	2.89	1	29	0	0.25(COSM16	7 transmemb	rs10839531	0.2268	YES	9.659E-1;0.0 (pos.62211(	astrocytoma_Grade_IV,glic		G-protein coupled receptor activity,G-protein coupled re				A=15, G=20	A=0.4286, G=0.5714	0.00001	218.716	35	A=2/13, G=10/
chr21:38467616	A/A	C	A	SNV	TTC3	TTC3:intronic:NM_001001894.2	1	100		intronic	NM_00100	c.688-34C	p.?	0.05					RING-type zin	rs2006941	0.4702	YES	3.965E-1;0.0 (pos.38467616)		Tetratricop	cytosol,m	Abnormalities, Multiple;Chromosome Disord			C=0, A=80	C=0.0, A=1.0	0.00001	1288.58	80	C=0/0, A=63/1
chr21:38468958	G/G	A	G	SNV	TTC3	TTC3:intronic:NM_001001894.2	1	100		intronic	NM_00100	c.845+15A	p.?	0.04					RING-type zin	rs2835600	0.4702	YES	3.954E-1 (pos.38468958)		Tetratricop	cytosol,m	Abnormalities, Multiple;Chromosome Disord			A=0, G=21	A=0.0, G=1.0	0.00001	281.235	21	A=0/0, G=5/16
chr21:38525198	G/A	G	A	SNV	TTC3	TTC3:intronic:NM_001001894.2	1	51.85		intronic	NM_00100	c.2414-53	p.?	-2.98					RING-type zin	rs2051162		YES			Tetratricop	cytosol,m	Abnormalities, Multiple;Chromosome Disord			G=13, A=14	G=0.4815, A=0.5185	0.00001	132.662	27	G=10/3, A=7/7
chr11:5758118	C/C	A	C	SNV	OR56B1	OR56B1:exonic:NM_001005180.2	1	100	1	synonymous	NM_00100	c.372A>C	p.(=)	-2.57					7 transmemb	rs7396766	0.0447	YES	8.772E-1 (pos.5758118)			G-protein coupled receptor activity,G-protein coupled re				A=0, C=21	A=0.0, C=1.0	0.00001	281.234	21	A=0/0, C=9/12
chr21:38599934	T/T	TG	T	INDEL	DSCR3	DSCR3:intronic:NM_006052.1	1	100		intronic	NM_00605	c.811+21C	p.?	-0.19, 0.1					Arrestin (or S	rs11300896,rs397767793			1.0 (pos.38599934)		Down syndr	biological	Abnormalities, Multiple;Chromosome Disord			TG=0, T=77	TG=0.0, T=1.0	0.00001	1110.03	77	TG=0/0, T=32/
chr11:58190136	G/G	A	G	SNV	OR5B2	OR5B2:exonic:NM_001005566.2	1	100	1	missense	NM_00100	c.599T>C	p.Met200Thr	0.51		81		0.15(COSM37	7 transmemb	rs4298923	0.4183	YES	3.032E-1 (pos.58190136)	adenocarcinoma,carcinom		G-protein coupled receptor activity,G-protein coupled re				A=0, G=48	A=0.0, G=1.0	0.00001	810.413	48	A=0/0, G=19/2
chr11:55563776	G/G	T	G	SNV	OR5D14	OR5D14:exonic:NM_001004735.1	1	100	1	missense	NM_00100	c.745T>G	p.Ser249Ala	0.37	1	99	0		7 transmemb	rs297054	7.00E-04		1.0 (pos.55563776)			G-protein coupled receptor activity,G-protein coupled re				T=0, G=40	T=0.0, G=1.0	0.00001	644.279	40	T=0/0, G=22/1
chr21:39762883	T/C	T	C	SNV	ERG	ERG:intronic:NM_182918.3	1	47.62		intronic	NM_18291	c.919+34A	p.?	-1.46					Ets-domain,S	rs2246468	0.0965	YES	1.509E-1 (pos.39762883)		Erythroblas	DNA bindi	Abnormalities, Multiple;Chromosome Disord			T=33, C=30	T=0.5238, C=0.4762	0.00001	289.35	63	T=19/14, C=20
chr11:55606818	C/T	C	T	SNV	OR5D16	OR5D16:exonic:NM_001005496.1	1	58.33	1	synonymous	NM_00100	c.591C>T	p.(=)	-3.16				0.01(COSM11	7 transmemb	rs11231253	0.3694	YES	6.276E-1 (pos.55606818)	adenocarcinoma,adenoma		G-protein coupled receptor activity,G-protein coupled re				C=65, T=91	C=0.4167, T=0.5833	0.00001	1028.31	156	C=33/32, T=47
chr11:55761738	G/G	T	G	SNV	OR5F1	OR5F1:exonic:NM_003697.1	1	100	1	synonymous	NM_00369	c.364A>C	p.(=)	0.36					7 transmemb	rs2128152,rs759979(	0	YES	1.0;0.0 (pos.55761738)		Olfactory re	G-protein coupled receptor activity,G-protein coupled re				T=0, G=57	T=0.0, G=1.0	0.00001	849.615	57	T=0/0, G=19/3
chr3:98001777	C/C	G	C	SNV	OR5H2	OR5H2:exonic:NM_001005482.1	1	100	1	missense	NM_00100	c.46G>C	p.Glu16Gln	0.78	0.08	29	0.096	0.11(COSM37	7 transmemb	rs72487753	0.1205	YES	2.62E-1;0.0 (pos.980017	adenocarcinoma		G-protein coupled receptor activity,G-protein coupled re				G=0, C=24	G=0.0, C=1.0	0.00001	334.792	24	G=0/0, C=8/16
chr11:56344993	A/A	C	A	SNV	OR5M10	OR5M10:exonic:NM_001004741.1	1	100	1	missense	NM_00100	c.205G>T	p.Val69Leu	-1.75	0.15	32	0.09		7 transmemb	rs10792043	0.3346	YES	8.506E-1 (pos.56344993)			G-protein coupled receptor activity,G-protein coupled re				C=0, A=99	C=0.0, A=1.0	0.00001	1681.12	99	C=0/0, A=51/4
chr21:40642189	T/T	C	T	SNV	BRWD1	BRWD1:intronic:NM_018963.4	1	100		intronic	NM_01896	c.1395+20	p.?	-2.58					Bromodomai	rs6517532	0.066	YES	6.118E-1 (pos.40642189)			chromatin	Abnormalities, Multiple;Cardiovascular Dise			C=0, T=23	C=0.0, T=1.0	0.00001	316.747	23	C=0/0, T=15/8
chr11:56310222	T/T	C	T	SNV	OR5M11	OR5M11:exonic:NM_001005245.1	1	100	1	missense	NM_00100	c.512G>A	p.Ser171Asn	-0.11	1	46	0		7 transmemb	rs628524	0.2524	YES	8.511E-1 (pos.56310222)			G-protein coupled receptor activity,G-protein coupled re				C=0, T=46	C=0.0, T=1.0	0.00001	768.268	46	C=0/0, T=22/2
chr11:56237722	T/A	T	A	SNV	OR5M3	OR5M3:exonic:NM_001004742.2	1	57.14	1	missense	NM_00100	c.252A>T	p.Leu84Phe	-3.23		22		0.04(COSM37	7 transmemb	rs605734	0.2361	YES	1.174E-1;0.0 (pos.56237	adenocarcinoma		G-protein coupled receptor activity,G-protein coupled re				T=27, A=36	T=0.4286, A=0.5714	0.00001	382.747	63	T=9/18, A=20/
chr11:7847466	G/G	A	G	SNV	OR5P3	OR5P3:exonic:NM_153445.1	1	100	1	synonymous	NM_15344	c.54T>C	p.(=)	-2.7					7 transmemb	rs1482792	0.0431	YES	6.232E-1 (pos.7847466)			G-protein coupled receptor activity,G-protein coupled re				A=0, G=22	A=0.0, G=1.0	0.00001	298.892	22	A=0/0, G=12/1
chr11:7847471	AT/AT	A	AT	INDEL	OR5P3	OR5P3:exonic:NM_153445.1	1	100	1	frameshiftInsertion	NM_15344	c.48_49ins	p.Leu17fs	-0.14					7 transmembrane receptor (rhodopsin family),Olfactory receptor							G-protein coupled receptor activity,G-protein coupled re				A=0, AT=22	A=0.0, AT=1.0	0.00001	267.359	22	A=0/0, AT=12/
chr11:56043702	C/C	G	C	SNV	OR5T1	OR5T1:exonic:NM_001004745.1	1	100	1	synonymous	NM_00100	c.588G>C	p.(=)	-6.16					7 transmemb	rs7125697	0.3422	YES	8.521E-1 (pos.56043702)			G-protein coupled receptor signaling pathway,adenylate				G=0, C=25	G=0.0, C=1.0	0.00001	353.017	25	G=0/0, C=22/3
chr21:42767733	C/C	T	C	SNV	MX2	MX2:intronic:NM_002463.1	1	100		intronic	NM_00246	c.1070+21	p.?	-0.44					Dynamin GTP	rs440364	0.1055	YES	9.693E-1 (pos.42767733)		Myxovirus (	GTP bindir	Abnormalities, Multiple;Basal Ganglia Disea			T=0, C=50	T=0.0, C=1.0	0.00001	852.748	50	T=0/0, C=6/44
chr21:42775180	T/T	C	T	SNV	MX2	MX2:intronic:NM_002463.1	1	100		intronic	NM_00246	c.1574-14	p.?	-0.73					Dynamin GTP	rs111859659,rs4597(	0.1757	YES	0.0 (pos.42775178),0.0;0.0;0.0 (pos.427		Myxovirus (	GTP bindir	Abnormalities, Multiple;Basal Ganglia Disea			C=0, T=20	C=0.0, T=1.0	0.00001	286.175	20	C=0/0, T=15/5
chr11:56000403	G/G	C	G	SNV	OR5T2	OR5T2:exonic:NM_001004746.1	1	100	1	missense	NM_00100	c.259G>C	p.Val87Leu	-3.24	1	32	0		7 transmemb	rs10791893	0.124	YES	9.994E-1;0.0 (pos.56000403)			G-protein coupled receptor signaling pathway,G-protein				C=0, G=67	C=0.0, G=1.0	0.00001	1038.33	67	C=0/0, G=51/1
chr6:29323259	A/A	G	A	SNV	OR5V1	OR5V1:exonic:NM_030876.5	1	100	1	synonymous	NM_03087	c.714C>T	p.(=)	-1.48					7 transmemb	rs3117438	0.033	YES	9.998E-1 (pos.29323259)			G-protein	Barrett Esophagus;Digestive System Abnorm			G=0, A=119	G=0.0, A=1.0	0.00001	1902.37	119	G=0/0, A=62/5
chr21:43258000	C/C	T	C	SNV	PRDM15	PRDM15:intronic:NM_022115.4	1	100		intronic	NM_02211	c.2099+43	p.?	-0.13					C2H2-type zir	rs8127003	0.0874	YES	7.469E-1 (pos.43258000)		PR domain-	DNA bindi	Abnormalities, Multiple;Chromosome Disord			T=0, C=68	T=0.0, C=1.0	0.00001	1053.53	68	T=0/0, C=42/2
chr12:55945119	G/G	A	G	SNV	OR6C4	OR6C4:exonic:NM_001005494.1	1	100	1	missense	NM_00100	c.109A>G	p.Ile37Val	-2.97	1	29	0		7 transmemb	rs7313899	0.014		1.0 (pos.55945119)			G-protein coupled receptor activity,G-protein coupled re				A=0, G=23	A=0.0, G=1.0	0.00001	320.813	23	A=0/0, G=14/9
chr1:247875608	G/G	C	G	SNV	OR6F1	OR6F1:exonic:NM_001005286.1	1	100	1	synonymous	NM_00100	c.450G>C	p.(=)	-7.94				0(COSM1468	7 transmemb	rs6587382	0.256	YES	2.324E-1 (pos.24787560	acute_myeloid_leukaemia		G-protein coupled receptor activity,G-protein coupled re				C=0, G=33	C=0.0, G=1.0	0.00001	504.597	33	C=0/0, G=6/27
chr1:247875689	G/G	A	G	SNV	OR6F1	OR6F1:exonic:NM_001005286.1	1	100	1	synonymous	NM_00100	c.369T>C	p.(=)	0.07				0.01(COSM11	7 transmemb	rs7512807	0.121	YES	2.343E-1 (pos.24787568	acute_myeloid_leukaemia		G-protein coupled receptor activity,G-protein coupled re				A=0, G=60	A=0.0, G=1.0	0.00001	915.603	60	A=0/0, G=16/4
chr1:158736215	A/A	G	A	SNV	OR6N1	OR6N1:exonic:NM_001005185.1	1	100	1	synonymous	NM_00100	c.258C>T	p.(=)	-0.1				0.28(COSM63	7 transmemb	rs6666753	0.0716	YES	9.184E-1 (pos.15873621	carcinoma		G-protein coupled receptor activity,G-protein coupled re				G=0, A=20	G=0.0, A=1.0	0.00001	263.785	20	G=0/0, A=11/9
chr14:21109170	A/A	T	A	SNV	OR6S1	OR6S1:exonic:NM_001001968.1	1	100	1	synonymous	NM_00100	c.681A>T	p.(=)	0.09					7 transmemb	rs11628297	2.00E-04		9.999E-1 (pos.21109170)			G-protein coupled receptor activity,G-protein coupled re				T=0, A=35	T=0.0, A=1.0	0.00001	543.902	35	T=0/0, A=10/2
chr14:21109141	C/T	C	T	SNV	OR6S1	OR6S1:exonic:NM_001001968.1	1	96.67	1	missense	NM_00100	c.710G>A	p.Arg237His	0.1	0.03	29	0.993		7 transmemb	rs17277522	0.4434	YES	7.517E-1;0.0 (pos.21109141)			G-protein coupled receptor activity,G-protein coupled re				C=2, T=58	C=0.0333, T=0.9667	0.00001	841.442	60	C=1/1, T=24/3
chr21:43732425	C/T	C	T	SNV	TFF3	TFF3:intronic:NM_003226.3	1	57.14		intronic	NM_00322	c.272-46G	p.?	-0.16					Trefoil (P-typ	rs533093	0.403	YES	5.077E-2 (pos.43732425)		Trefoil factc	cytoplasm	Acute Kidney Injury;Digestive System Disease			C=15, T=20	C=0.4286, T=0.5714	0.00001	218.556	35	C=1/14, T=7/1
chr21:43829758	A/A	G	A	SNV	UBASH3A	UBASH3A:intronic:NM_001001895.2	1	100		intronic	NM_00100	c.354+41G	p.?	-0.04					Histidine phc	rs11702374	0.4547	YES	2.96E-1 (pos.43829758)		Ubiquitin-a	cytosol,ex	Arthritis;Arthritis, Rheumatoid;Autoimmune			G=0, A=62	G=0.0, A=1.0	0.00001	940.309	62	G=0/0, A=30/3
chr21:43959770	G/G	A	G	SNV	SLC37A1	SLC37A1:intronic:NM_018964.3	1	100		intronic	NM_01896	c.486+13A	p.?	-1.21					Major Facilit	rs228063	0.1947	YES	2.707E-1;0.0 (pos.43959770)		Solute carri	carbohydrate transport,endoplasmic reticulum membra				A=0, G=23	A=0.0, G=1.0	0.00001	316.456	23	A=0/0, G=9/14
chr21:43963759	G/G	A	G	SNV	SLC37A1	SLC37A1:intronic:NM_018964.3	1	100		intronic	NM_01896	c.730+47A	p.?	-0.07					Major Facilit	rs228075	0.1294	YES	4.67E-1 (pos.43963759)		Solute carri	carbohydrate transport,endoplasmic reticulum membra				A=0, G=34	A=0.0, G=1.0	0.00001	524.186	34	A=0/0, G=20/1
chr11:123813823	G/A	G	A	SNV	OR6T1	OR6T1:exonic:NM_001005187.1	1	47.62	1	synonymous	NM_00100	c.723C>T	p.(=)	0.25					7 transmemb	rs7129884	0.3337	YES	4E-1 (pos.123813823)			G-protein coupled receptor signaling pathway,G-protein				G=11, A=10	G=0.5238, A=0.4762	0.00001	82.2499	21	G=8/3, A=4/6
chr21:43999983	C/C	T	C	SNV	SLC37A1	SLC37A1:intronic:NM_018964.3	1	100		intronic	NM_01896	c.1586+73	p.?	-1.31					Major Facilit	rs453592		YES			Solute carri	carbohydrate transport,endoplasmic reticulum membra				T=0, C=26	T=0.0, C=1.0	0.00001	371.42	26	T=0/0, C=9/17
chr21:44000366	G/G	A	G	SNV	SLC37A1	SLC37A1:intronic:NM_018964.3	1	100		intronic	NM_01896	c.1587-89	p.?	-1.15					Major Facilit	rs388907	0.2634	YES			Solute carri	carbohydrate transport,endoplasmic reticulum membra				A=0, G=22	A=0.0, G=1.0	0.00001	303.261	22	A=0/0, G=7/15
chr21:44163839	G/G	A	G	SNV	PDE9A	PDE9A:intronic:NM_002606.2	1	100		intronic	NM_00260	c.569-47A	p.?	-2.68					3'5'-cyclic nu	rs386818962,rs6586	0.017	YES	9.46E-1 (pos.44163839)		Phosphodie	3',5'-cyclic-GMP phosphodieste		3-isobutyl-1-methyl-7H-x		A=0, G=20	A=0.0, G=1.0	0.00001	263.693	20	A=0/0, G=17/3
chr21:44180866	AC/A	AC	A	INDEL	PDE9A	PDE9A:intronic:NM_002606.2	1	100		intronic	NM_00260	c.1003-69	p.?	-0.01, 0.0					3'5'-cyclic nu	rs34765923,rs397867031,rs540		YES			Phosphodie	3',5'-cyclic-GMP phosphodieste		3-isobutyl-1-methyl-7H-x		AC=0, A=29	AC=0.0, A=1.0	0.00001	268.248	29	AC=0/0, A=13/
chr21:44195374	G/A	G	A	SNV	PDE9A	PDE9A:intronic:NM_002606.2	1	51.85		intronic	NM_00260	c.1769-16	p.?	-0.24					3'5'-cyclic nu	rs763665858,rs9637	0.1698	YES	1.058E-1;3.353E-3;0.0 (pos.44195374)		Phosphodie	3',5'-cyclic-GMP phosphodieste		3-isobutyl-1-methyl-7H-x		G=13, A=14	G=0.4815, A=0.5185	0.00001	134.88	27	G=6/7, A=11/3
chr19:14910438	T/T	C	T	SNV	OR7C1	OR7C1:exonic:NM_198944.1	1	100	1	missense	NM_19894	c.511G>A	p.Glu171Lys	-3.76	0.34	56	0.017		7 transmemb	rs10415312	0.0902	YES	1.0 (pos.14910438)			G-protein coupled receptor activity,G-protein coupled re				C=0, T=36	C=0.0, T=1.0	0.00001	567.775	36	C=0/0, T=8/28
chr19:14910573	T/T	C	T	SNV	OR7C1	OR7C1:exonic:NM_198944.1	1	100	1	missense	NM_19894	c.376G>A	p.Val126Ile	1.72	1	29	0.007	0.02(COSM63	7 transmemb	rs10415562	0.2393	YES	8.875E-1 (pos.14910573)	carcinoma		G-protein coupled receptor activity,G-protein coupled re				C=0, T=44	C=0.0, T=1.0	0.00001	712.84	44	C=0/0, T=9/35
chr19:9362443	C/T	C	T	SNV	OR7E24	OR7E24:exonic:NM_001079935.1	1	45.45	1	missense	NM_00107	c.724C>T	p.Pro242Ser	0.56	1	74	0	0(COSM5002	7 transmemb	rs2240928	0.2561	YES	5.661E-1;0.0 (pos.93624	acute_myeloid_leukaemia		G-protein coupled receptor activity,G-protein coupled re				C=30, T=25	C=0.5455, T=0.4545	0.00001	232.695	55	C=22/8, T=17/
chr11:124440362	C/G	C	G	SNV	OR8A1	OR8A1:exonic:NM_001005194.1	1	56	1	missense	NM_00100	c.398C>G	p.Thr133Arg	3.4	0.01	71	0.956	0.95(COSM37	7 transmemb	rs55861866	0.3472	YES	2.764E-1;0.0 (pos.12444(	adenocarcinoma		G-protein	Anxiety Disorders;Mental Disorders			C=11, G=14	C=0.44, G=0.56	0.00001	134.35	25	C=6/5, G=9/5
chr11:124179835	C/T	C	T	SNV	OR8D1	OR8D1:exonic:NM_001002917.1	1	53.33	1	synonymous	NM_00100	c.828G>A	p.(=)	-2.82				0.03(COSM39	7 transmemb	rs4936918	0.3936	YES	4.338E-1 (pos.12417983	acute_myeloid_leukaemia		G-protein coupled receptor activity,G-protein coupled re				C=14, T=16	C=0.4667, T=0.5333	0.00001	159.925	30	C=3/11, T=8/8
chr21:45089877	A/A	G	A	SNV	RRP1B	RRP1B:intronic:NM_015056.2	1	100		intronic	NM_01505	c.213+30G	p.?	0.59					Nucleolar prc	rs2838344	0.4135	YES	8.013E-1 (pos.45089877)		Ribosomal I	RNA bindir	Digestive System Diseases;Eosinophilia;Eosi			G=0, A=53	G=0.0, A=1.0	0.00001	782.03	53	G=0/0, A=28/2
chr11:124121199	G/C	G	C	SNV	OR8G1	OR8G1:exonic:NM_001002905.1	1	50	1	stoploss	NM_00100	c.777G>C	p.Ter259Tyr	-1.74					7 transmemb	rs4268525	0.4528	YES	5.858E-1 (pos.124121199)			G-protein coupled receptor activity,G-protein coupled re				G=49, C=49	G=0.5, C=0.5	0.00001	551.833	98	G=37/12, C=2
chr21:45107285	G/G	C	G	SNV	RRP1B	RRP1B:intronic:NM_015056.2	1	100		intronic	NM_01505	c.1142-11	p.?	0.09					Nucleolar prc	rs2838348		YES			Ribosomal I	RNA bindir	Digestive System Diseases;Eosinophilia;Eosi			C=0, G=21	C=0.0, G=1.0	0.00001	281.235	21	C=0/0, G=8/13
chr11:124120821	T/C	T	C	SNV	OR8G1	OR8G1:exonic:NM_001002905.1	1	45.45	1	synonymous	NM_00100	c.399T>C	p.(=)	-2.86					7 transmemb	rs12099154	0.2952	YES	2.62E-1 (pos.124120821)			G-protein coupled receptor activity,G-protein coupled re				T=18, C=15	T=0.5455, C=0.4545	0.00001	137.467	33	T=9/9, C=9/6
chr21:45215046	T/C	T	C	SNV	RRP1	RRP1:intronic:NM_003683.5	1	45.45		intronic	NM_00368	c.361-24T	p.?	-0.56					Nucleolar prc	rs2838371	0.4416	YES	7.481E-1 (pos.45215046)			RNA binding,nucleolus,nucleus,preribosome, large subu				T=18, C=15	T=0.5455, C=0.4545	0.00001	143.166	33	T=15/3, C=9/6
chr21:45215055	C/G	C	G	SNV	RRP1	RRP1:intronic:NM_003683.5	1	45.45		intronic	NM_00368	c.361-15C	p.?	0.56					Nucleolar prc	rs2838373	0.1776	YES	2.205E-1;0.0 (pos.45215055)			RNA binding,nucleolus,nucleus,preribosome, large subu				C=18, G=15	C=0.5455, G=0.4545	0.00001	143.173	33	C=15/3, G=9/6
chr11:55873368	A/A	G	A	SNV	OR8H2	OR8H2:exonic:NM_001005200.1	1	100	1	missense	NM_00100	c.850G>A	p.Val284Met	-0.15	1	21	0		7 transmemb	rs1842696	0		1.0 (pos.55873368)			G-protein coupled receptor activity,G-protein coupled re				G=0, A=90	G=0.0, A=1.0	0.00001	1493.12	90	G=0/0, A=47/4
chr21:45540143	A/A	G	A	SNV	PWP2	PWP2:intronic:NM_005049.2	1	100		intronic	NM_00504	c.1189-86	p.?	-2.17					Dip2/Utp12 F	rs2855653		YES			PWP2 perio	Pwp2p-containing subcomplex of 90S preribosome,RNA I				G=0, A=21	G=0.0, A=1.0	0.00001	281.235	21	G=0/0, A=17/4
chr11:56127746	G/G	A	G	SNV	OR8J1	OR8J1:exonic:NM_001005205.2	1	100	1	synonymous	NM_00100	c.24A>G	p.(=)	-3.2					7 transmemb	rs7942390	0.1177	YES	8.512E-1 (pos.56127746)			G-protein coupled receptor activity,G-protein coupled re				A=0, G=25	A=0.0, G=1.0	0.00001	352.734	25	A=0/0, G=10/1
chr21:45550460	T/T	TC	T	INDEL	PWP2	PWP2:intronic:NM_005049.2	1	100		intronic	NM_00504	c.2586-19	p.?	-1.13, -0.6					Dip2/Utp12 F	rs11311220,rs200724221,rs397		YES	1.0 (pos.45550460)		PWP2 perio	Pwp2p-containing subcomplex of 90S preribosome,RNA I				TC=0, T=74	TC=0.0, T=1.0	0.00001	1088.45	74	TC=0/0, T=50/
chr11:56128524	T/T	C	T	SNV	OR8J1	OR8J1:exonic:NM_001005205.2	1	100	1	synonymous	NM_00100	c.802C>T	p.(=)	-6.11					7 transmemb	rs7928704	0.1172	YES	8.511E-1 (pos.56128524)			G-protein coupled receptor activity,G-protein coupled re				C=0, T=188	C=0.0, T=1.0	0.00001	3149.15	188	C=0/0, T=89/9
chr21:45651413	G/G	A	G	SNV	ICOSLG	ICOSLG:intronic:NM_015259.5	1	100		intronic	NM_01525	c.698-86T	p.?	0.01					CD80-like C2-	rs3746962		YES			Inducible T-	B cell activ	Celiac Disease;Colitis;Colitis, Ulcerative;Colc			A=0, G=68	A=0.0, G=1.0	0.00001	1044.42	68	A=0/0, G=28/4
chr11:56113516	C/C	T	C	SNV	OR8K1	OR8K1:exonic:NM_001002907.1	1	100	1	missense	NM_00100	c.2T>C	p.Met1Thr	-0.02	0.01	81	0.009		7 transmemb	rs1905055	0.3417	YES	8.781E-1 (pos.56113516)			G-protein coupled receptor activity,G-protein coupled re				T=0, C=24	T=0.0, C=1.0	0.00001	334.79	24	T=0/0, C=16/8
chr21:45743594	T/T	C	T	SNV	PFKL	PFKL:intronic:NM_002626.5	1	100		intronic	NM_00262	c.1498-55	p.?	-5.78					Phosphofruct	rs12482028		YES			Phosphofru	6-phosphofructokinase activity,6-phosphofructokinase (				C=0, T=28	C=0.0, T=1.0	0.00001	408.871	28	C=0/0, T=9/19
chr21:45750248	A/A	G	A	SNV	C21orf2	C21orf2:intronic:NM_001271441.1	1	100		intronic	NM_00127	c.1000-39	p.?	-2.53						rs11552067	0.4164	YES	3.297E-1 (pos.45750248)		Chromoson	DNA damage response, detection of DNA damage,ciliary				G=0, A=51	G=0.0, A=1.0	0.00001	754.582	51	G=0/0, A=20/3
chr21:45942111	G/G	A	G	SNV	TSPEAR	TSPEAR:intronic:NM_144991.2	1	100		intronic	NM_14499	c.1337-11	p.?	-0.09					EPTP domain	rs233230,rs386819161		YES			Thrombosp	cell surface,ciliary membrane,extracellular region,mole				A=0, G=85	A=0.0, G=1.0	0.00001	1389.79	85	A=0/0, G=41/4
chr11:56086147	G/G	T	G	SNV	OR8K3	OR8K3:exonic:NM_001005202.1	1	100	1	missense	NM_00100	c.365T>G	p.Leu122Arg	4.74	1	102	0	0.75(COSM44	7 transmemb	rs960193	0.3423	YES	8.79E-1;0.0 (pos.560861	squamous_cell_carcinoma		G-protein coupled receptor activity,G-protein coupled re				T=0, G=21	T=0.0, G=1.0	0.00001	281.232	21	T=0/0, G=10/1
chr12:48919796	C/T	C	T	SNV	OR8S1	OR8S1:exonic:NM_001005203.2	1	50	1	missense	NM_00100	c.382C>T	p.Arg128Cys	-0.82	0.02	180	0.003		7 transmemb	rs12425460	0.4688	YES	7.244E-1 (pos.48919796)			G-protein	Female Urogenital Diseases;Female Urogeni			C=21, T=21	C=0.5, T=0.5	0.00001	228.879	42	C=9/12, T=14/
chr11:56468047	GT/GT	AC	GT	MNV	OR9G1,OR9G9	OR9G9:exonic:NM_001013358.2, OR9G1:exonic:NM_00100	2	100	1	missense	NM_00100	c.184_185	p.Thr62Val, p.Thr62Val	0.18, 2.73	0.15, 0.15	69.0, 69.0	0.0, 0.0	0.51(COSM45	7 transmemb	rs2865519,rs2865520,rs714582		YES	0.0 (pos.56468045),7.74	adenocarcinoma,squamou		G-protein coupled receptor activity,G-protein coupled re				AC=0, GT=5	AC=0.0, GT=1.0	0.00001	837.614	56	AC=0/0, GT=2
chr11:56468198	A/G	A	G	SNV	OR9G1,OR9G9	OR9G9:exonic:NM_001013358.2, OR9G1:exonic:NM_00100	1	50	1	missense	NM_00100	c.335A>G,	p.Tyr112Cys, p.Tyr112C	0.23	0.02, 0.02	194.0, 194	1.0, 1.0	0.18(COSM45	7 transmemb	rs4990194,rs775651567,rs7967		YES	0.0 (pos.56468195),4.99	adenocarcinoma,basal_ce		G-protein coupled receptor activity,G-protein coupled re				A=26, G=26	A=0.5, G=0.5	0.00001	256.249	52	A=13/13, G=1
chr11:56468200	C/T	C	T	SNV	OR9G1,OR9G9	OR9G9:exonic:NM_001013358.2, OR9G1:exonic:NM_00100	1	50	1	synonymous	NM_00100	c.337C>T,	p.(=), p.(=)	-0.28				0.08(COSM45	7 transmemb	rs4990195,rs796788592		YES	4.998E-1 (pos.56468200)	adenocarcinoma,squamou		G-protein coupled receptor activity,G-protein coupled re				C=26, T=26	C=0.5, T=0.5	0.00001	256.254	52	C=13/13, T=19
chr11:56468202	G/A	G	A	SNV	OR9G1,OR9G9	OR9G9:exonic:NM_001013358.2, OR9G1:exonic:NM_00100	1	50	1	synonymous	NM_00100	c.339G>A,	p.(=), p.(=)	-2.89				0.11(COSM45	7 transmemb	rs372282722,rs4990196,rs7967		YES	4.998E-1 (pos.56468202)	adenocarcinoma,squamou		G-protein coupled receptor activity,G-protein coupled re				G=26, A=26	G=0.5, A=0.5	0.00001	256.251	52	G=13/13, A=1
chr11:56468440	G/T	G	T	SNV	OR9G1,OR9G9	OR9G9:exonic:NM_001013358.2, OR9G1:exonic:NM_00100	1	50	1	missense	NM_00100	c.577G>T,	p.Gly193Cys, p.Gly193C	-1.08	0.1, 0.1	159.0, 15	0.885, 0.885	0.08(COSM45	7 transmemb	rs12421330		YES	3.999E-1;2.454E-4 (pos.5	acute_myeloid_leukaemia		G-protein coupled receptor activity,G-protein coupled re				G=170, T=1	G=0.5, T=0.5	0.00001	1876.62	340	G=102/68, T=
chr17:45885687	G/G	A	G	SNV	OSBPL7	OSBPL7:exonic:NM_145798.2	1	100	23	synonymous	NM_14579	c.2499T>C	p.(=)	-1.52				0.05(COSM17	Oxysterol-bir	rs9907142	0.053	YES	8.089E-1 (pos.45885687)	carcinoma,ne	Oxysterol-b	autophagosome,bile acid biosynthetic process,cellular r				A=0, G=37	A=0.0, G=1.0	0.00001	583.705	37	A=0/0, G=19/1
chr2:190617643	G/G	A	G	SNV	OSGEPL1	OSGEPL1:exonic:NM_022353.2	1	100	6	synonymous	NM_02235	c.1026T>C	p.(=)	-0.44					Glycoproteas	rs2243876	5.00E-04	YES	1.0 (pos.190617643)			EKC/KEOPS complex,N(6)-L-threonylcarbamoyladenine sy				A=0, G=61	A=0.0, G=1.0	0.00001	921.854	61	A=0/0, G=38/2
chr21:46078220	C/C	T	C	SNV	KRTAP12-3,TSPEAR	KRTAP12-3:utr_3:NM_198697.2, TSPEAR:intronic:NM_14499	1	100		intronic	NM_14499	c.82+5312	p.?, p.?	0.71					EPTP domain	rs9306112	0.3382	YES	6.591E-1 (pos.46078220)		Thrombosp	cell surface,ciliary membrane,cytosol,extracellular regi				T=0, C=204	T=0.0, C=1.0	0.00001	3310.73	204	T=0/0, C=81/1
chr2:26741961	A/A	G	A	SNV	OTOF	OTOF:exonic:NM_194248.2	1	100	4	missense	NM_19424	c.244C>T	p.Arg82Cys	1.15	0.04	180	0.002	0.7(COSM375	C2 domain,Fe	rs13031859	0.485	YES	1.07E-1 (pos.26741961)	adenocarcino	Otoferlin	basolater	Deafness;Ear Diseases;Hearin		Benign/Likely	G=0, A=63	G=0.0, A=1.0	0.00001	955.892	63	G=0/0, A=31/3
chr12:80712420	A/A	G	A	SNV	OTOGL	OTOGL:exonic:NM_173591.3	1	100	32	synonymous	NM_17359	c.3702G>A	p.(=)	0.52					Alpha-L-arabi	rs10778725	0.0191	YES	9.489E-1 (pos.80712420)		Otogelin-like protein				Benign	G=0, A=30	G=0.0, A=1.0	0.00001	446.637	30	G=0/0, A=17/1
chr12:80747242	G/G	A	G	SNV	OTOGL	OTOGL:exonic:NM_173591.3	1	100	45	missense	NM_17359	c.5518A>G	p.Ile1840Val	0.99	0.21	29	0.011	0.15(COSM54	Alpha-L-arabi	rs7297767	0.1402	YES	8.267E-1 (pos.80747242)	acute_myeloic	Otogelin-like protein				Benign	A=0, G=27	A=0.0, G=1.0	0.00001	389.988	27	A=0/0, G=9/18
chr21:46102090	C/C	T	C	SNV	KRTAP12-1,TSPEAR	KRTAP12-1:upstream:NM_181686.1, TSPEAR:intronic:NM_1	1	100		intronic	NM_14499	c.82+2925	p.?, p.?	-0.29					EPTP domain	rs8129858	0.3473	YES	6.071E-1 (pos.46102090)		Thrombosp	cell surface,ciliary membrane,cytosol,extracellular regi				T=0, C=31	T=0.0, C=1.0	0.00001	465.816	31	T=0/0, C=8/23
chr21:46321659	C/A	C	A	SNV	ITGB2	ITGB2:intronic:NM_000211.4	1	55.38		intronic	NM_00021	c.500-11G	p.?	0.11					Integrin beta	rs55865320	0.1887	YES	8.169E-2;1.172E-4 (pos.46321659)		Integrin, be	ICAM-3 rec	Acneiform Eruption	Simvastat	Likely benign	C=29, A=36	C=0.4462, A=0.5538	0.00001	372.452	65	C=15/14, A=24
chr12:80752660	G/G	A	G	SNV	OTOGL	OTOGL:exonic:NM_173591.3	1	100	51	missense	NM_17359	c.6220A>G	p.Ile2074Val	0.49	1	29	0		Alpha-L-arabi	rs2034528	0.0758	YES	1.0 (pos.80752660)		Otogelin-like protein				Benign,Uncer	A=0, G=42	A=0.0, G=1.0	0.00001	685.213	42	A=0/0, G=12/3
chr21:46925425	C/A	C	A	SNV	COL18A1	COL18A1:intronic:NM_030582.3	1	50		intronic	NM_03058	c.3618+80	p.?	0.12					Collagen tripl	rs62214274		YES			Collagen XV	angiogene	Calcinosis;Calcium Metabolism Disorders;Ca			C=22, A=22	C=0.5, A=0.5	0.00001	243.622	44	C=1/21, A=2/2

chr12:80761430	A/A	G	A	SNV	OTOGL	OTOGL:exonic:NM_173591.3	1	100	53	missense	NM_17359	c.6394G>A	p.Ala2132Thr	2.2	1	58	0	0.16(COSM54	Alpha-L-arab	rs1551118	0.1615	YES	7.672E-1 (pos.80761430)	acute_myeloid	Otogelin-like protein			Benign	G=0, A=183	G=0.0, A=1.0	0.00001	3045.42	183	G=0/0, A=73/1
chr21:47417975	G/G	A	G	SNV	COL6A1	COL6A1:intronic:NM_001848.2	1	100		intronic	NM_00184	c.1525-60	p.?	-1.69					Collagen tripl	rs3737437	0.1478	YES			Collagen VI	cell adhes	Abortion, Spontaneous;Bone D	Benign	A=0, G=21	A=0.0, G=1.0	0.00001	281.235	21	A=0/0, G=11/1
chr21:47420761	A/A	G	A	SNV	COL6A1	COL6A1:intronic:NM_001848.2	1	100		intronic	NM_00184	c.1822+80	p.?	-0.6					Collagen tripl	rs7283989	0.4541	YES			Collagen VI	cell adhes	Abortion, Spontaneous;Bone D	Benign	G=0, A=20	G=0.0, A=1.0	0.00001	263.741	20	G=0/0, A=14/6
chr21:47420769	G/G	A	G	SNV	COL6A1	COL6A1:intronic:NM_001848.2	1	100		intronic	NM_00184	c.1822+88	p.?	-1.14					Collagen tripl	rs7276782	0.1505	YES			Collagen VI	cell adhes	Abortion, Spontaneous;Bone D	Benign	A=0, G=20	A=0.0, G=1.0	0.00001	263.751	20	A=0/0, G=14/6
chr21:47422412	T/T	C	T	SNV	COL6A1	COL6A1:intronic:NM_001848.2	1	100		intronic	NM_00184	c.2251-29	p.?	-0.12					Collagen tripl	rs35796750	0.4555	YES	7.112E-1 (pos.47422412)		Collagen VI	cell adhes	Abortion, Spontaneous;Bone D	Benign	C=0, T=80	C=0.0, T=1.0	0.00001	1289.36	80	C=0/0, T=50/3
chr21:47423133	G/G	C	G	SNV	COL6A1	COL6A1:intronic:NM_001848.2	1	100		intronic	NM_00184	c.2464+70	p.?	-0.67					Collagen tripl	rs2236489	0.153	YES			Collagen VI	cell adhes	Abortion, Spontaneous;Bone Diseases;Conge		C=0, G=21	C=0.0, G=1.0	0.00001	284.422	21	C=0/0, G=3/18
chr21:47542861	G/G	A	G	SNV	COL6A2	COL6A2:intronic:NM_001849.3	1	100		intronic	NM_00184	c.1671+10	p.?	-3.34					Collagen tripl	rs915786	0.1829	YES	8.796E-1 (pos.47542861)		Collagen VI	cell adhes	Arthritis;Congenital, Hereditar	Benign/Likely	A=0, G=30	A=0.0, G=1.0	0.00001	446.625	30	A=0/0, G=11/1
chr12:80761465	A/A	G	A	SNV	OTOGL	OTOGL:exonic:NM_173591.3	1	100	53	synonymous	NM_17359	c.6429G>A	p.(=)	0.9				0.29(COSM41	Alpha-L-arab	rs2717482	0.0786	YES	9.999E-1 (pos.80761465)	neoplasm	Otogelin-like protein			Benign	G=0, A=181	G=0.0, A=1.0	0.00001	3004.1	181	G=0/0, A=71/1
chr12:80771813	A/A	G	A	SNV	OTOGL	OTOGL:exonic:NM_173591.3	1	100	58	synonymous	NM_17359	c.7020G>A	p.(=)	-1.55				0.02(COSM54	Alpha-L-arab	rs2717477	0.1609	YES	7.737E-1 (pos.80771813)	acute_myeloid	Otogelin-like protein			Benign	G=0, A=24	G=0.0, A=1.0	0.00001	334.792	24	G=0/0, A=11/1
chr21:47588646	G/A	G	A	SNV	SPATC1L	SPATC1L:intronic:NM_001142854.1	1	57.14		intronic	NM_00114	c.194-74C	p.?	-2.71					Speriolin C-te	rs11909543		YES				biological_process,cellular_component,centrosome,mo			G=33, A=44	G=0.4286, A=0.5714	0.00001	491.684	77	G=13/20, A=3
chr21:47633789	G/G	C	G	SNV	LSS	LSS:intronic:NM_001001438.2	1	100		intronic	NM_00100	c.1012-40	p.?	0.03					Prenyltransfe	rs9974665	0.393	YES	1.491E-1;0.0 (pos.47633789)		Lanosterol	cholester	Congenital, Heredit Lanosterol,R048-8071		C=0, G=100	C=0.0, G=1.0	0.00001	1702.26	100	C=0/0, G=67/3
chr21:47639492	G/G	C	G	SNV	LSS	LSS:intronic:NM_001001438.2	1	100		intronic	NM_00100	c.551-6G>	p.?	-0.5				0.02(COSM63	Prenyltransfe	rs2277826	0.4072	YES	7.031E-1;0.0 (pos.47639	carcinoma	Lanosterol	cholester	Congenital, Heredit Lanosterol,R048-8071		C=0, G=26	C=0.0, G=1.0	0.00001	370.85	26	C=0/0, G=6/20
chr21:47656712	C/C	T	C	SNV	MCM3AP,MCM3AP-AS1	MCM3AP:intronic:NM_003906.4, MCM3AP-AS1:intronic_nc:	1	100		intronic	NR_00277	c.5784+31	p.?	-0.22					Binding regio	rs2839166,rs908349	0.3999	YES	1.155E-1 (pos.47656712)		Minichrom	DNA bindi	Endocrine Gland Neoplasms;Endocrine Syste		T=0, C=65	T=0.0, C=1.0	0.00001	996.458	65	T=0/0, C=40/2
chr21:47681127	T/C	T	C	SNV	MCM3AP	MCM3AP:intronic:NM_003906.4	1	47.62		intronic	NM_00390	c.3467+67	p.?	-0.9					Binding regio	rs2839180		YES			Minichrom	DNA bindi	Endocrine Gland Neoplasms;Endocrine Syste		T=11, C=10	T=0.5238, C=0.4762	0.00001	82.29	21	T=5/6, C=2/8
chr12:80750659	G/A	G	A	SNV	OTOGL	OTOGL:exonic:NM_173591.3	1	50	48	missense	NM_17359	c.5957G>A	p.Arg1986Gln	3.25	0.27	43	0.003	0.14(COSM41	Alpha-L-arab	rs11836060	0.1965	YES	4.223E-2 (pos.80750659)	neoplasm	Otogelin-like protein			Benign	G=23, A=23	G=0.5, A=0.5	0.00001	258.573	46	G=14/9, A=9/1
chr12:80747209	C/G	C	G	SNV	OTOGL	OTOGL:exonic:NM_173591.3	1	46.43	45	missense	NM_17359	c.5485C>G	p.Leu1829Val	0.24	0.03	32	0.057		Alpha-L-arab	rs7312569	0.1954	YES	6.024E-2 (pos.80747209)		Otogelin-like protein			Benign	C=15, G=13	C=0.5357, G=0.4643	0.00001	117.925	28	C=7/8, G=3/10
chr21:47766650	A/A	G	A	SNV	PCNT	PCNT:intronic:NM_006031.5	1	100		intronic	NM_00603	c.721-7G>	p.?	-1.89				0.01(COSM63	Pericentrin-A	rs2839217	0.3777	YES	4.795E-1;0.0 (pos.47766	carcinoma,neo	Nucleopori	G2/M tran	Bone Diseases;Bone Diseases,	Benign	G=0, A=37	G=0.0, A=1.0	0.00001	583.699	37	G=0/0, A=15/2
chr4:4199634	T/T	A	T	SNV	OTOP1	OTOP1:exonic:NM_177998.2	1	100	5	missense	NM_17799	c.927T>A	p.Asp309Glu	-2.1	1	45	0.108		Otopetrin	rs2916414	0.2921	YES	6.525E-1 (pos.4199634)		Otopetrin 1	biomineral tissue development,extracellular space,inte			A=0, T=20	A=0.0, T=1.0	0.00001	262.538	20	A=0/0, T=15/5
chr4:4204299	C/C	T	C	SNV	OTOP1	OTOP1:exonic:NM_177998.2	1	100	4	synonymous	NM_17799	c.606A>G	p.(=)	-0.59					Otopetrin	rs2920182	0.2328	YES	6.859E-1 (pos.4204299)		Otopetrin 1	biomineral tissue development,extracellular space,inte			T=0, C=227	T=0.0, C=1.0	0.00001	3735.31	227	T=0/0, C=110/
chr21:47786494	T/T	C	T	SNV	PCNT	PCNT:intronic:NM_006031.5	1	100		intronic	NM_00603	c.2610-5C	p.?	0.15					Pericentrin-A	rs2839226	0.1948	YES	8.1E-1 (pos.47786494)		Nucleopori	G2/M tran	Bone Diseases;Bone Diseases,	Benign	C=0, T=30	C=0.0, T=1.0	0.00001	446.631	30	C=0/0, T=15/1
chr20:16729016	C/G	C	G	SNV	OTOR	OTOR:utr_5:NM_020157.3	1	46.58	1	utr_5	NM_02015	c.-31C>G	p.?	0.32					Variant SH3 c	rs17686413	0.0453	YES	2.315E-4 (pos.16729016)		Otoraplin	cartilage condensation,extracellular region,sensory per			C=39, G=34	C=0.5342, G=0.4658	0.00001	336.852	73	C=20/19, G=1
chr21:47847852	C/C	T	C	SNV	PCNT	PCNT:intronic:NM_006031.5	1	100		intronic	NM_00603	c.7494+14	p.?	-2.51					Pericentrin-A	rs2073374		YES			Nucleopori	G2/M tran	Bone Diseases;Bone Diseases, Development		T=0, C=104	T=0.0, C=1.0	0.00001	1603.35	104	T=0/0, C=35/6
chr21:47852151	AG/AG	A	AG	INDEL	PCNT	PCNT:intronic:NM_006031.5	1	100		intronic	NM_00603	c.8751+22	p.?	-0.12					Pericentrin-A	rs201904039,rs5844	0.0117	YES	1.0;0.0 (pos.47852151)		Nucleopori	G2/M tran	Bone Diseases;Bone Diseases, Development		A=0, AG=46	A=0.0, AG=1.0	0.00001	757.993	46	A=0/0, AG=16/
chr21:47917170	A/A	G	A	SNV	DIP2A	DIP2A:intronic:NM_015151.3	1	100		intronic	NM_01515	c.403+150	p.?	-0.99					AMP-binding	rs2839292		YES				catalytic activity,cell surface,metabolic process,multice			G=0, A=54	G=0.0, A=1.0	0.00001	800.56	54	G=0/0, A=24/3
chr21:47957332	A/A	G	A	SNV	DIP2A	DIP2A:intronic:NM_015151.3	1	100		intronic	NM_01515	c.1708-27	p.?	-2					AMP-binding	rs2070432	0.3124	YES	4.427E-1;2.335E-4 (pos.47957332)			catalytic activity,cell surface,metabolic process,multice			G=0, A=28	G=0.0, A=1.0	0.00001	408.718	28	G=0/0, A=17/1
chr21:47971539	A/A	G	A	SNV	DIP2A	DIP2A:intronic:NM_015151.3	1	100		intronic	NM_01515	c.2840-8G	p.?	-0.75					AMP-binding	rs2255526	0.278	YES	7.666E-1 (pos.47971539)			catalytic activity,cell surface,metabolic process,multice			G=0, A=31	G=0.0, A=1.0	0.00001	465.816	31	G=0/0, A=12/1
chr21:47978321	GC/GC	G	GC	INDEL	DIP2A	DIP2A:intronic:NM_015151.3	1	100		intronic	NM_01515	c.3969+15	p.?	-0.17					AMP-binding	rs201565223,rs3492	0.0073	YES	9.997E-1;0.0;2.719E-4 (pos.47978321)			catalytic activity,cell surface,metabolic process,multice			G=0, GC=73	G=0.0, GC=1.0	0.00001	1023.59	73	G=0/0, GC=35/
chr21:47980570	G/G	A	G	SNV	DIP2A	DIP2A:intronic:NM_015151.3	1	100		intronic	NM_01515	c.3970-65	p.?	-0.29					AMP-binding	rs2070429	0.3055	YES				catalytic activity,cell surface,metabolic process,multice			A=0, G=25	A=0.0, G=1.0	0.00001	353.019	25	A=0/0, G=11/1
chr21:47986633	A/A	C	A	SNV	DIP2A	DIP2A:intronic:NM_015151.3	1	100		intronic	NM_01515	c.4463+37	p.?	-4.26					AMP-binding	rs3819044	0.0914	YES	8.875E-1 (pos.47986633)			catalytic activity,cell surface,metabolic process,multice			C=0, A=31	C=0.0, A=1.0	0.00001	465.819	31	C=0/0, A=19/1
chr20:16729048	T/C	T	C	SNV	OTOR	OTOR:exonic:NM_020157.3	1	46.58	1	missense	NM_02015	c.2T>C	p.Met1Thr	5.17	0	81	0.956		Variant SH3 c	rs17686437	0.0453	YES	2.312E-4 (pos.16729048)		Otoraplin	cartilage condensation,extracellular regio		Benign	T=39, C=34	T=0.5342, C=0.4658	0.00001	341.106	73	T=20/19, C=13
chr12:29630081	A/A	G	A	SNV	OVCH1	OVCH1:exonic:NM_001353179.1	1	100	12	missense	NM_00135	c.1436C>T	p.Thr479Ile	-0.21	0.53	89	0		CUB domain,	rs7975356	0.1425	YES	9.222E-1;0.0 (pos.29630081)						G=0, A=25	G=0.0, A=1.0	0.00001	353.019	25	G=0/0, A=8/17
chr22:17488727	C/C	A	C	SNV	GAB4	GAB4:intronic:NM_001037814.1	1	100		intronic	NM_00103	c.174+104	p.?	0.04					PH domain	rs5748800,rs942441753		YES							A=0, C=54	A=0.0, C=1.0	0.00001	797.244	54	A=0/0, C=32/2
chr12:29639186	T/C	T	C	SNV	OVCH1	OVCH1:exonic:NM_001353179.1	1	50	8	missense	NM_00135	c.988A>G	p.Lys330Glu	-0.43	1	56	0		CUB domain,	rs3847680	0.4156	YES	7.682E-1 (pos.29639186)						T=30, C=30	T=0.5, C=0.5	0.00001	293.739	60	T=15/15, C=15
chr22:17586471	T/T	C	T	SNV	IL17RA	IL17RA:intronic:NM_014339.6	1	100		intronic	NM_01433	c.932-10C	p.?	-0.12					Interleukin 1	rs2241046,rs759512	0.2194	YES	0.0 (pos.17586470),8.248E-1 (pos.17586		Interleukin	cell surfac	Brain Diseases;Brain Ischemia	Benign	C=0, T=46	C=0.0, T=1.0	0.00001	768.312	46	C=0/0, T=16/3
chr22:17662591	G/G	C	G	SNV	ADA2	ADA2:intronic:NM_001282225.1	1	100		intronic	NM_00128	c.1442+11	p.?	-0.77					Adenosine/A	rs11703884		YES				adenosine catabolic process,adenosine deaminase acti			C=0, G=31	C=0.0, G=1.0	0.00001	465.739	31	C=0/0, G=15/1
chr22:17662679	T/T	C	T	SNV	ADA2	ADA2:intronic:NM_001282225.1	1	100		intronic	NM_00128	c.1442+31	p.?	-1.37					Adenosine/A	rs41282461	0.3245	YES	4.891E-2 (pos.17662679)			adenosine catabolic process,adenosine deaminase acti			C=0, T=32	C=0.0, T=1.0	0.00001	485.14	32	C=0/0, T=14/1
chr22:17662699	G/G	A	G	SNV	ADA2	ADA2:intronic:NM_001282225.1	1	100		intronic	NM_00128	c.1442+11	p.?	0.01					Adenosine/A	rs58754958	0.3349	YES	4.868E-2 (pos.17662699)			adenosine catabolic process,adenosine d		Benign	A=0, G=32	A=0.0, G=1.0	0.00001	485.141	32	A=0/0, G=14/1
chr11:7727886	T/T	C	T	SNV	OVCH2	OVCH2:exonic:NM_198185.4	1	100	1	missense	NM_19818	c.56G>A	p.Arg19Gln	0.18	0.91	43	0	0(COSM4299	CUB domain,	rs7927138	0.3667	YES	4.736E-1 (pos.7727886)	acute_myeloid_leukaemia,adenocarcinoma,carcinoma					C=0, T=40	C=0.0, T=1.0	0.00001	644.249	40	C=0/0, T=20/2
chr22:17662917	C/C	G	C	SNV	ADA2	ADA2:intronic:NM_001282225.1	1	100		intronic	NM_00128	c.1240-5C	p.?	-0.3				0(COSM6353	Adenosine/A	rs3764846,rs755716	0.3231	YES	4.907E-2;0.0;1.16E-4 (pos	carcinoma		adenosine catabolic process,adenosine d		Benign	G=0, C=37	G=0.0, C=1.0	0.00001	583.707	37	G=0/0, C=14/2
chr22:17684355	G/G	A	G	SNV	ADA2	ADA2:intronic:NM_001282225.1	1	100		intronic	NM_00128	c.753+98T	p.?	-0.3					Adenosine/A	rs5747022		YES				adenosine catabolic process,adenosine deaminase acti			A=0, G=24	A=0.0, G=1.0	0.00001	334.792	24	A=0/0, G=7/17
chr2:42990336	T/G	T	G	SNV	OXER1	OXER1:exonic:NM_148962.4	1	53.33	1	synonymous	NM_14896	c.984A>C	p.(=)	-1.76				0.01(COSM44	7 transmemb	rs1992286	0.4528	YES	7.796E-1 (pos.42990336)	adenocarcinoma		5(S)-hydroxyperoxy-6E,8Z,11Z,14Z-icosatetraenoic acid b			T=21, G=24	T=0.4667, G=0.5333	0.00001	276.154	45	T=5/16, G=6/1
chr22:18370066	A/A	C	A	SNV	MICAL3	MICAL3:intronic:NM_015241.2	1	100		intronic	NM_01524	c.2004+23	p.?	-1.97					CAMSAP CH d	rs4819472	0.0034		1.0 (pos.18370066)		Microtubul	FAD binding,actin binding,actin filament depolymerizatio			C=0, A=28	C=0.0, A=1.0	0.00001	408.711	28	C=0/0, A=10/1
chr13:97639414	T/T	C	T	SNV	OXGR1	OXGR1:exonic:NM_080818.4	1	100	4	synonymous	NM_08081	c.600G>A	p.(=)	-2.42				0.05(COSM37	7 transmemb	rs9284174	0.4657	YES	4.371E-1 (pos.97639414)	acute_myeloid_leukaemia		G-protein coupled receptor activity,G-protein coupled re			C=0, T=39	C=0.0, T=1.0	0.00001	628.353	39	C=0/0, T=14/2
chr22:19165216	G/A	G	A	SNV	SLC25A1	SLC25A1:intronic:NM_005984.4	1	57.14		intronic	NM_00598	c.441+24C	p.?	1.02					Mitochondria	rs2854642	0.0639	YES	5.251E-2;0.0 (pos.19165216)		Solute carri	citrate tra	Brain Diseases;Brai Sodium Citrate		G=12, A=16	G=0.4286, A=0.5714	0.00001	161.77	28	G=8/4, A=12/4
chr3:16327855	T/G	T	G	SNV	OXNAD1	OXNAD1:exonic:NM_138381.4	1	51.85	5	missense	NM_13838	c.190T>G	p.Ser64Ala	1.87	0.55	99	0.001	0.75(COSM37	Ferric reduct	rs842274	0.425	YES	5.131E-1 (pos.16327855)	acute_myeloid_leukaemia		biological_process,cellular_component,mitochondrion,			T=13, G=14	T=0.4815, G=0.5185	0.00001	133.034	27	T=7/6, G=9/5
chr17:3568012	A/G	A	G	SNV	P2RX5-TAX1BP3,TAX1BP3	P2RX5-TAX1BP3:exonic_nc:NR_037928.1, TAX1BP3:exonic:N	1	50	2	synonymous	NM_01460	c.120T>C	p.(=)	0.31					PDZ domain,I	rs8278	0.1069	YES	6.912E-1 (pos.3568012)		TAX1-bindir	Rho protein signal transduction,Wnt signaling pathway,a			A=10, G=10	A=0.5, G=0.5	0.00001	82.8865	20	A=4/6, G=3/7
chr22:19959955	G/G	A	G	SNV	ARVCF	ARVCF:intronic:NM_001670.2	1	100		intronic	NM_00167	c.2642-21	p.?	0.79					Armadillo/be	rs2073744,rs771720	0.0912	YES	0.0 (pos.19959952),5.42E-1 (pos.199599		Armadillo r	calcium-d	22q11 Deletion Syndrome;Abnormalities, Mu		A=0, G=30	A=0.0, G=1.0	0.00001	446.639	30	A=0/0, G=9/21
chr22:19960188	C/C	T	C	SNV	ARVCF	ARVCF:intronic:NM_001670.2	1	100		intronic	NM_00167	c.2641+72	p.?	-0.46					Armadillo/be	rs2518823		YES			Armadillo r	calcium-d	22q11 Deletion Syndrome;Abnormalities, Mu		T=0, C=54	T=0.0, C=1.0	0.00001	798.1	54	T=0/0, C=30/2
chr22:20129106	T/C	T	C	SNV	ZDHHC8	ZDHHC8:intronic:NM_001185024.1	1	50		intronic	NM_00118	c.1125+53	p.?	-2.9					DHHC palmit	rs77480122		YES			Zinc finger D	Golgi apparatus,cytoplasmic vesicle membrane,cytosol,			T=26, C=26	T=0.5, C=0.5	0.00001	256.251	52	T=12/14, C=11
chr22:21377301	A/A	C	A	SNV	P2RX6	P2RX6:exonic:NM_005446.4	1	100	5	synonymous	NM_00544	c.534C>A	p.(=)	-4.7				0.02(COSM41	ATP P2X rece	rs1548411	0.4762	YES	6.385E-1;0.0 (pos.21377	neoplasm		ATP bindin	Digestive System Diseases;Eosinophilia;Eosi		C=0, A=56	C=0.0, A=1.0	0.00001	831.504	56	C=0/0, A=41/1
chr22:20784908	TT/GA	TT	GA	MNV	SCARF2	SCARF2:intronic:NM_182895.4	2	57.14		intronic	NM_18289	c.1074-64	p.?	-0.31, 0.08					Human growt	rs35574298,rs56935344,rs8791		YES			Scavenger r	focal adhe	Abnormalities, Multiple;Arachnodactyly;Blec		TT=9, GA=1	TT=0.4286, GA=0.57	0.00001	109.102	21	TT=3/6, GA=5/
chr22:21213380	T/T	C	T	SNV	PI4KA,SNAP29	PI4KA:upstream:NM_058004.3, SNAP29:utr_5:NM_004782.	1	100		intronic	NM_05800	c.-367G>A	p.?, p.?	-1.49						rs1061063	0.4724	YES	4.213E-1 (pos.21213380)		Phosphatid	1-phospha	Abnormalities, Multiple;Conge	Benign	C=0, T=27	C=0.0, T=1.0	0.00001	389.988	27	C=0/0, T=24/3
chr22:21327834	G/G	C	G	SNV	AIFM3	AIFM3:intronic:NM_144704.2	1	100		intronic	NM_14470	c.245+25C	p.?	-1.73						rs178265,rs7962624	0.0345	YES	9.993E-1;0.0 (pos.21327834)		Apoptosis-i	2 iron, 2 sulfur cluster binding,cytosol,endoplasmic retic			C=0, G=31	C=0.0, G=1.0	0.00001	465.819	31	C=0/0, G=6/25
chr1:43212431	G/G	A	G	SNV	P3H1	P3H1:utr_3:NM_001243246.1	1	100	14	utr_3	NM_00124	c.2567T>C	p.?	-0.15				0(COSM4595	2OG-Fe(II) oxy	rs4660662,rs756267392			0.0;0.0 (pos.43212430),1	squamous_cel	Prolyl 3-hyd	L-ascorbic	Bone Diseases;Bon L-Proline,S	Likely benign	A=0, G=50	A=0.0, G=1.0	0.00001	828.087	50	A=0/0, G=20/3
chr22:21332122	A/A	G	A	SNV	AIFM3	AIFM3:intronic:NM_144704.2	1	100		intronic	NM_14470	c.1360-55	p.?	-0.17						rs178277	0.4362	YES			Apoptosis-i	2 iron, 2 sulfur cluster binding,cytosol,endoplasmic retic			G=0, A=29	G=0.0, A=1.0	0.00001	427.601	29	G=0/0, A=17/1
chr22:21349037	G/G	A	G	SNV	LZTR1	LZTR1:intronic:NM_006767.3	1	100		intronic	NM_00676	c.1785+21	p.?	-0.76					BTB/POZ dom	rs178292	0.2342	YES	7.275E-1;4.674E-4 (pos.21349037)		Leucine-zip	DNA bindi	Astrocytoma;Autistic Disorder;Cardiovascula		A=0, G=55	A=0.0, G=1.0	0.00001	815.642	55	A=0/0, G=10/4
chr12:6939850	G/G	A	G	SNV	P3H3	P3H3:exonic:NM_014262.4	1	100	5	missense	NM_01426	c.900A>G	p.Thr300Ala	0.35		58				rs10744716	0.4726	YES	6.964E-1 (pos.6939850)		Prolyl 3-hyd	L-ascorbic	Arthritis;Joint Dise L-Proline,Succinic acid,V		A=0, G=52	A=0.0, G=1.0	0.00001	766.954	52	A=0/0, G=24/2
chr17:39959189	G/G	C	G	SNV	P3H4	P3H4:utr_3:NM_006455.2	1	100	8	utr_3	NM_00645	c.1334G>C	p.?	-1.77						rs1065029	0.1115	YES	9.999E-1 (pos.39959189)		Prolyl 3-hyd	bone remodeling,catalytic complex,collagen biosynthet			C=0, G=24	C=0.0, G=1.0	0.00001	334.111	24	C=0/0, G=13/1
chr4:135121066	T/G	T	G	SNV	PABPC4L	PABPC4L:exonic:NM_001114734.1	1	56	2	missense	NM_00111	c.1283A>C	p.His428Pro	2.59		77		0.51(COSM38	RNA recogniti	rs11099273		YES	9.739E-1 (pos.13512106	carcinoma,ductal_carcinoma,squamous_cell_carcinoma					T=11, G=14	T=0.44, G=0.56	0.00001	134.35	25	T=3/8, G=4/10
chr22:43289473	G/A	G	A	SNV	PACSIN2	PACSIN2:exonic:NM_001184970.2	1	47.37	3	synonymous	NM_00118	c.207C>T	p.(=)	-2.88				0.04(COSM37	Fes/CIP4, anc	rs5759013	0.4498	YES	9.575E-2 (pos.43289473)	adenocarcinor	Protein kina	actin cyto	Arthritis;Arthritis, Juvenile;Autoimmune Dise		G=20, A=18	G=0.5263, A=0.4737	0.00001	179.803	38	G=7/13, A=6/1
chrX:55116466	G/G	C	G	SNV	PAGE2	PAGE2:exonic:NM_207339.3	1	100	2	missense	NM_20733	c.13C>G	p.Leu5Val	-0.3	1	32	0			rs1845444	0.0057	YES	1.0 (pos.55116466)		P antigen fa	protein binding			C=0, G=113	C=0.0, G=1.0	0.00001	1795.68	113	C=0/0, G=61/5
chr12:103237468	C/C	G	C	SNV	PAH	PAH:exonic:NM_000277.1	1	100	11	synonymous	NM_00027	c.1155C>G	p.(=)	-0.18					ACT domain,E	rs772897	0.161	YES	8.414E-1;0.0 (pos.103237468)		Phenylalan	L-phenylal	Amino Acid Disorde Beta(2-Thi	Benign	G=0, C=25	G=0.0, C=1.0	0.00001	352.956	25	G=0/0, C=11/1
chr3:196555296	T/C	T	C	SNV	PAK2	PAK2:utr_3:NM_002577.4	1	57.14	15	utr_3	NM_00257	c.1595T>C	p.?	0.48					P21-Rho-binc	rs200028313	0.0027		0.0 (pos.196555296)		Protein kina	ATP bindin	Female Urogenital Diseases;Female Urogeni		T=21, C=28	T=0.4286, C=0.5714	0.00001	335.938	49	T=10/11, C=19
chr5:102345546	A/C	A	C	SNV	PAM	PAM:exonic:NM_000919.3	1	50	20	synonymous	NM_00091	c.2307A>C	p.(=)	0.74				0.78(COSM44	Copper type I	rs2230457	0.2907	YES	4.216E-1 (pos.10234554	hepatocellula	Myc-bindin	L-ascorbic	Anxiety Disorders;E Copper,N-Alpha-Acetyl-3		A=26, C=26	A=0.5, C=0.5	0.00001	256.251	52	A=14/12, C=1
chr12:56722060	G/G	T	G	SNV	PAN2	PAN2:exonic:NM_001127460.2	1	100	4	missense	NM_00112	c.535A>C	p.Ile179Leu	-2.65	0.45	5	0.001	0.02(COSM41	Exonuclease,	rs1918496	0.0038	YES	9.999E-1 (pos.56722060)	neoplasm	NACHT dom	3'-5'-exoribonuclease activity,P-body,PAN complex,RNA			T=0, G=30	T=0.0, G=1.0	0.00001	446.639	30	T=0/0, G=15/1
chr10:91404448	C/G	C	G	SNV	PANK1	PANK1:exonic:NM_148977.2	1	53.33	1	synonymous	NM_14897	c.612G>C	p.(=)	0.17				0.12(COSM37	Fumble	rs11185826	0.2118	YES	3.649E-1 (pos.91404448)	Wilms_tumou	Pantothena	ATP binding,cell periphery,clathrin coat,coenzyme A bios			C=28, G=32	C=0.4667, G=0.5333	0.00001	323.557	60	C=17/11, G=2
chr10:135204950	G/G	C	G	SNV	PAOX	PAOX:exonic:NM_152911.3	1	100	7	synonymous	NM_15291	c.1527C>G	p.(=)	-1.22				0.04(COSM42	Flavin contai	rs1046175	0.1243	YES	9.694E-1 (pos.13520495	osteosarcoma	Polyamine	N(1),N(12)	Brain Diseases;Cardiovascular Diseases;Cen		C=0, G=75	C=0.0, G=1.0	0.00001	1192.76	75	C=0/0, G=20/5
chr10:135205009	G/G	C	G	SNV	PAOX	PAOX:utr_3:NM_152911.3	1	100	7	utr_3	NM_15291	c.1586C>G	p.?	-0.29				0.01(COSM41	Flavin contai	rs1046178	0.1212	YES	9.686E-1;0.0 (pos.13520	neoplasm	Polyamine	N(1),N(12)	Brain Diseases;Cardiovascular Diseases;Cen		C=0, G=76	C=0.0, G=1.0	0.00001	1206.23	76	C=0/0, G=17/5
chr5:6748659	C/C	T	C	SNV	PAPD7	PAPD7:exonic:NM_006999.4	1	100	8	synonymous	NM_00699	c.792T>C	p.(=)	-2.3				0.05(COSM14	Cid1 family p	rs274681	0.2958	YES	6.062E-1 (pos.6748659)	adenocarcinoma,carcinom		ATP binding,Golgi apparatus,SMC family protein binding,			T=0, C=34	T=0.0, C=1.0	0.00001	524.17	34	T=0/0, C=9/25
chr10:34688287	A/A	G	A	SNV	PARD3	PARD3:exonic:NM_019619.3	1	100	7	synonymous	NM_01961	c.861C>T	p.(=)	2.96					N-terminal of	rs2796011	0.0022		1.0 (pos.34688287)		Partitioning	adherens junction,asymmetric cell division,axonal grow			G=0, A=24	G=0.0, A=1.0	0.00001	334.791	24	G=0/0, A=13/1
chr2:206480486	T/C	T	C	SNV	PARD3B	PARD3B:exonic:NM_152526.5	1	53.33	22	synonymous	NM_15252	c.3381T>C	p.(=)	-0.53					N-terminal of	rs56272619	0.0274	YES	0.0 (pos.206480486)			bicellular	Acquired Immunodeficiency Syndrome;Autist		T=14, C=16	T=0.4667, C=0.5333	0.00001	159.775	30	T=10/4, C=3/1
chr22:22899360	GAAAG/GAAA(	GAAGA	GAAAG	MNV	PRAME	PRAME:intronic:NM_206954.2	2	100		intronic	NM_20695	c.-76-34TC	p.?	-0.7, -1.01, -0.1, 0.28, -1.51						rs200263167,rs2283799,rs3710		YES			Preferentia	apoptotic	Adenocarcinoma;Blood Protein Disorders;Bo		GAAGA=0, (	GAAGA=0.0, GAAAG=	0.00001	581.76	37	GAAGA=0/0, G
chr22:24109550	C/C	T	C	SNV	CHCHD10	CHCHD10:intronic:NM_213720.2	1	100		intronic	NM_21372	c.261+11A	p.?	0.22					CHCH domair	rs131444	0.0987	YES	7.191E-1 (pos.24109550)		Coiled-coil-	ATP biosyr	Amyotrophic Lateral Sclerosis;	Benign	T=0, C=38	T=0.0, C=1.0	0.00001	603.015	38	T=0/0, C=29/9
chr22:24125565	A/A	G	A	SNV	MMP11	MMP11:intronic:NM_005940.4	1	100		intronic	NM_00594	c.1334-33	p.?	-1.44					Hemopexin,I	rs2877173	0.071	YES	7.177E-1;0.0 (pos.24125565)		Matrix met	Golgi lume	Abortion, Spontane 1-Benzyloxycarbonylami		G=0, A=34	G=0.0, A=1.0	0.00001	524.186	34	G=0/0, A=9/25
chr22:24176287	G/A	G	A	SNV	DERL3,SMARCB1	SMARCB1:intronic:NM_003073.4, DERL3:downstream:NM_(	1	50		intronic	NM_00307	c.1119-41	p.?, p.?	-0.76				0.03(COSM10	SNF5 / SMARC	rs5030613	0.1284	YES	1.716E-1 (pos.24176287)	acute_lympho	DER1-like d	ATP-deper	Abnormalities, Multiple;Brain Diseases;Brair		G=21, A=21	G=0.5, A=0.5	0.00001	228.437	42	G=12/9, A=14/
chr22:24472270	G/G	A	G	SNV	CABIN1	CABIN1:intronic:NM_012295.3	1	100		intronic	NM_01229	c.2748+37	p.?	-0.12					MEF2 binding	rs2283807	0.1791	YES	6.097E-1 (pos.24472270)		Calcineurin	DNA replic	Cardiomegaly;Cardiovascular Diseases;Hear		A=0, G=38	A=0.0, G=1.0	0.00001	603.785	38	A=0/0, G=17/2
chr22:24480503	A/A	G	A	SNV	CABIN1	CABIN1:intronic:NM_012295.3	1	100		intronic	NM_01229	c.2911-29	p.?	-2.71					MEF2 binding	rs4820581	0.0633	YES	9.063E-1 (pos.24480503)		Calcineurin	DNA replic	Cardiomegaly;Cardiovascular Diseases;Hear		G=0, A=39	G=0.0, A=1.0	0.00001	623.978	39	G=0/0, A=13/2
chr13:25009099	C/C	A	C	SNV	PARP4	PARP4:exonic:NM_006437.3	1	100	31	missense	NM_00643	c.4180T>G	p.Ser1394Ala	-3.58	1	99	0	0(COSM5021	BRCA1 C Term	rs9511259	0.0448	YES	9.983E-1 (pos.25009099)	osteosarcoma	Poly(ADP-ri	DNA bindi	Cardiovascular Diseases;Colonic Diseases;C		A=0, C=40	A=0.0, C=1.0	0.00001	644.261	40	A=0/0, C=4/36
chr22:24944053	T/T	C	T	SNV	GUCD1	GUCD1:intronic:NM_031444.3	1	100		intronic	NM_03144	c.129-12G	p.?	-3.41					Guanylylate	rs4822506		YES	1.0 (pos.24944053)			protein bi	Genital Diseases, Male;Genital Neoplasms, I		C=0, T=26	C=0.0, T=1.0	0.00001	371.42	26	C=0/0, T=4/22
chr13:25020863	A/A	G	A	SNV	PARP4	PARP4:exonic:NM_006437.3	1	100	27	missense	NM_00643	c.3322C>T	p.Arg1108Cys	-0.22	0.42	180	0	0.01(COSM50	BRCA1 C Term	rs9318554	0.0551	YES	9.811E-1 (pos.25020863)	osteosarcoma	Poly(ADP-ri	DNA bindi	Cardiovascular Diseases;Colonic Diseases;C		G=0, A=32	G=0.0, A=1.0	0.00001	485.136	32	G=0/0, A=17/1
chr13:25021200	C/C	A	C	SNV	PARP4	PARP4:exonic:NM_006437.3	1	100	26	missense	NM_00643	c.3239T>G	p.Leu1080Arg	-0.02	0.86	102	0		BRCA1 C Term	rs9318558	0.0458	YES	9.981E-1;0.0 (pos.25021200)		Poly(ADP-ri	DNA bindi	Cardiovascular Diseases;Colonic Diseases;C		A=0, C=31	A=0.0, C=1.0	0.00001	465.814	31	A=0/0, C=6/25
chr13:25021211	T/T	C	T	SNV	PARP4	PARP4:exonic:NM_006437.3	1	100	26	synonymous	NM_00643	c.3228G>A	p.(=)	-0.27				0.05(COSM45	BRCA1 C Term	rs7333856		YES	8.127E-1 (pos.25021211)	squamous_cel	Poly(ADP-ri	DNA bindi	Cardiovascular Diseases;Colonic Diseases;C		C=0, T=31	C=0.0, T=1.0	0.00001	465.819	31	C=0/0, T=6/25
chr13:25021245	G/G	A	G	SNV	PARP4	PARP4:exonic:NM_006437.3	1	100	26	missense	NM_00643	c.3194T>C	p.Val1065Ala	0.48	1	64	0	0(COSM4591	BRCA1 C Term	rs7334587		YES	5.708E-1 (pos.25021245)	squamous_cel	Poly(ADP-ri	DNA bindi	Cardiovascular Diseases;Colonic Diseases;C		A=0, G=34	A=0.0, G=1.0	0.00001	524.181	34	A=0/0, G=9/25
chr13:25029218	T/T	C	T	SNV	PARP4	PARP4:exonic:NM_006437.3	1	100	22	missense	NM_00643	c.2695G>A	p.Ala899Thr	5.87	0.03	58	1	0.96(COSM41	BRCA1 C Term	rs2275660	0.2299	YES	6.84E-1;0.0 (pos.250292	neoplasm	Poly(ADP-ri	DNA bindi	Cardiovascular Diseases;Colonic Diseases;C		C=0, T=27	C=0.0, T=1.0	0.00001	389.985	27	C=0/0, T=24/3
chr1:62261168	G/G	A	G	SNV	PATJ	PATJ:exonic:NM_176877.3	1	100	10	missense	NM_17687	c.1198A>G	p.Ile400Val	0.53	0.2	29	0.008	0.61(COSM11	L27_2,PDZ dc	rs7516332	0.4848	YES	4.165E-1 (pos.62261168)	acute_myeloid	INAD, Drosophila, homolog of				A=0, G=81	A=0.0, G=1.0	0.00001	1281.61	81	A=0/0, G=37/4
chr1:62330080	G/G	T	G	SNV	PATJ	PATJ:exonic:NM_176877.3	1	100	20	missense	NM_17687	c.2610T>G	p.Ile870Met	4.73	1	10	0		L27_2,PDZ dc	rs2799627	1.00E-04		1.0;0.0 (pos.62330080)		INAD, Drosophila, homolog of				T=0, G=27	T=0.0, G=1.0	0.00001	389.988	27	T=0/0, G=4/23
chr15:44966389	C/C	T	C	SNV	PATL2	PATL2:exonic:NM_001145112.1	1	100	4	missense	NM_00114	c.262A>G	p.Met88Val	0.15	1	21	0		Topoisomera	rs8026845	3.00E-04		1.0 (pos.44966389)		Protein ass	P-body,RNA binding,cytoplasm,cytoplasmic mRNA proce			T=0, C=51	T=0.0, C=1.0	0.00001	754.572	51	T=0/0, C=20/3
chr22:26222505	A/A	G	A	SNV	MYO18B	MYO18B:intronic:NM_032608.6	1	100		intronic	NM_03260	c.2786+39	p.?	-0.17					IQ calmoduli	rs2331162	0.272	YES	6.758E-1 (pos.26222505)		Myosin XVII	ATP bindin	Adenoma;Mesothelioma;Neoplasms;Neopla		G=0, A=20	G=0.0, A=1.0	0.00001	263.786	20	G=0/0, A=12/8
chr22:26228797	G/G	A	G	SNV	MYO18B	MYO18B:intronic:NM_032608.6	1	100		intronic	NM_03260	c.2980-87	p.?	-0.49					IQ calmoduli	rs3859879		YES			Myosin XVII	ATP bindin	Adenoma;Mesothelioma;Neoplasms;Neopla		A=0, G=31	A=0.0, G=1.0	0.00001	465.549	31	A=0/0, G=14/1
chr7:127251188	G/G	T	G	SNV	PAX4	PAX4:exonic:NM_006193.2	1	100	9	missense	NM_00619	c.962A>C	p.His321Pro	-1.29	0.7	77	0		'Paired box' d	rs712701	0.2004	YES	4.202E-1 (pos.127251188)		Paired box	DNA bindi	Autoimmune Diseases;Diabet	Benign	T=0, G=93	T=0.0, G=1.0	0.00001	1555.33	93	T=0/0, G=41/5
chr22:26239906	G/G	A	G	SNV	MYO18B	MYO18B:intronic:NM_032608.6	1	100		intronic	NM_03260	c.3368+45	p.?	-1.73					IQ calmoduli	rs5761269	0.2872	YES	9.964E-1 (pos.26239906)		Myosin XVII	ATP bindin	Adenoma;Mesothelioma;Neoplasms;Neopla		A=0, G=35	A=0.0, G=1.0	0.00001	543.903	35	A=0/0, G=22/1
chr21:47329352	C/C	T	C	SNV	PCBP3	PCBP3:exonic:NM_020528.2	1	100	6	synonymous	NM_02052	c.423T>C	p.(=)	-0.14				0.2(COSM375	KH domain	rs2277812	0.3065	YES	9.964E-1 (pos.47329352)	adenocarcinor	Poly(rC)-bin	C-rich single-stranded DNA binding,RNA binding,cytosol,(			T=0, C=74	T=0.0, C=1.0	0.00001	1170.13	74	T=0/0, C=27/4
chr5:141336264	T/T	G	T	SNV	PCDH12	PCDH12:exonic:NM_016580.3	1	100	1	missense	NM_01658	c.1153C>A	p.His385Asn	0.36	0.55	68	0	0.02(COSM59	Cadherin dor	rs164075	0.484	YES	8.187E-1;0.0 (pos.14133	carcinoma	Protocadhe	calcium ion binding,calcium-dependent cell-cell adhesic			G=0, T=146	G=0.0, T=1.0	0.00001	2463.61	146	G=0/0, T=71/7
chr22:26879823	A/A	C	A	SNV	SRRD	SRRD:upstream:NM_001013694.2	1	100		intronic	NM_00101	c.-34C>A	p.?	-0.85						rs71322613,rs968425		YES	8.5E-1 (pos.26879823)		SRR1 domai	rhythmic process		Benign	C=0, A=24	C=0.0, A=1.0	0.00001	334.789	24	C=0/0, A=9/15
chr5:141335241	A/G	A	G	SNV	PCDH12	PCDH12:exonic:NM_016580.3	1	97.5	1	synonymous	NM_01658	c.2176T>C	p.(=)	0.18					Cadherin dor	rs164073,rs7548361	0.4409	YES	0.0 (pos.141335227),8.253E-1 (pos.1413		Protocadhe	calcium ion binding,calcium-dependent cell-cell adhesic			A=1, G=39	A=0.025, G=0.975	0.00001	611.634	40	A=0/1, G=17/2
chr13:61986918	C/A	C	A	SNV	PCDH20	PCDH20:exonic:NM_022843.3	1	97.06	2	synonymous	NM_02284	c.1314G>T	p.(=)	-0.38					Cadherin dor	rs3812872	0.2931	YES	9.28E-1 (pos.61986918)		Protocadhe	RNA binding,calcium ion binding,homophilic cell adhesic			C=1, A=33	C=0.0294, A=0.9706	0.00001	492.834	34	C=1/0, A=19/1
chr13:67802339	T/T	A	T	SNV	PCDH9	PCDH9:exonic:NM_203487.2	1	100	2	synonymous	NM_20348	c.234T>A	p.(=)	-0.05					Cadherin dor	rs8000556	0.2019	YES	7.06E-1 (pos.67802339)		Protocadhe	calcium io	Child Development Disorders, Pervasive;Mer		A=0, T=96	A=0.0, T=1.0	0.00001	1618.01	96	A=0/0, T=36/6
chr5:140263290	G/G	T	G	SNV	PCDHA1,PCDHA10,PCDHA11,PCDHA12,PCDHA13,PCDHA2,PCDHA3,PCDHA4,PCDHA5,PCDHA6,PCDHA7,PCDHA8,PCDHA9	PCDHA13:exonic:NM_018904.2, PCDHA12:intronic:NM_018	1	100	1	synonymous	NM_01890	c.2394+95	p.?, p.?, p.?, p.?, p.?, p.?,	-4.79				0.03(COSM63	Cadherin C-te	rs251380	0.3284	YES	4.899E-1 (pos.14026329	carcinoma	Protocadhe	calcium io	Abortion, Spontaneous;Female Urogenital Di		T=0, G=93	T=0.0, G=1.0	0.00001	1555.33	93	T=0/0, G=55/3
chr22:29877165	G/G	GC	G	INDEL	NEFH	NEFH:intronic:NM_021076.3	1	100		intronic	NM_02107	c.883+31G	p.?	0.24, -0.22					Intermediate filament protein,Repeat of unknown function (DUF1388)						Neurofilam	axon,axon	Amyotrophic Lateral Sclerosis;Brain Disease		GC=0, G=23	GC=0.0, G=1.0	0.00001	274.536	23	GC=0/0, G=18/
chr22:29881884	A/A	G	A	SNV	NEFH	NEFH:intronic:NM_021076.3	1	100		intronic	NM_02107	c.1208+48	p.?	-0.16					Intermediate	rs4823040	0.051	YES	1.0 (pos.29881884)		Neurofilam	axon,axon	Amyotrophic Lateral Sclerosis;Brain Disease		G=0, A=30	G=0.0, A=1.0	0.00001	446.639	30	G=0/0, A=9/21
chr22:29904519	C/C	T	C	SNV	THOC5	THOC5:intronic:NM_001002879.1	1	100		intronic	NM_00100	c.1989-10	p.?	-1.16					Fms-interact	rs2283859,rs781723	0.2553	YES	2.08E-1;0.0 (pos.29904519)		THO comple	RNA export from nucleus,RNA splicing,THO complex,THO			T=0, C=37	T=0.0, C=1.0	0.00001	583.706	37	T=0/0, C=17/2
chr5:140255792	A/A	C	A	SNV	PCDHA1,PCDHA10,PCDHA11,PCDHA12,PCDHA2,PCDHA3,PCDHA4,PCDHA5,PCDHA6,PCDHA7,PCDHA8,PCDHA9	PCDHA1:intronic:NM_018900.3, PCDHA5:intronic:NM_0189	1	100	1	synonymous	NM_01890	c.2394+87	p.?, p.?, p.?, p.?, p.?, p.?,	-4.7				0.04(COSM40	Cadherin C-te	rs251377	0.4913	YES	4.5E-1 (pos.140255792)	acute_myeloid	Protocadhe	calcium io	Abortion, Spontaneous;Female Urogenital Di		C=0, A=48	C=0.0, A=1.0	0.00001	810.427	48	C=0/0, A=30/1
chr22:29924212	C/C	T	C	SNV	THOC5	THOC5:intronic:NM_001002879.1	1	100		intronic	NM_00100	c.967-46A	p.?	-0.66					Fms-interact	rs5763297	0.2316	YES	4.345E-1;0.0 (pos.29924212)		THO comple	RNA export from nucleus,RNA splicing,THO complex,THO			T=0, C=30	T=0.0, C=1.0	0.00001	446.628	30	T=0/0, C=10/2
chr22:29925284	C/C	T	C	SNV	THOC5	THOC5:intronic:NM_001002879.1	1	100		intronic	NM_00100	c.848-56A	p.?	-0.41					Fms-interact	rs2302141	0.2266	YES			THO comple	RNA export from nucleus,RNA splicing,THO complex,THO			T=0, C=31	T=0.0, C=1.0	0.00001	465.782	31	T=0/0, C=15/1
chr22:30738737	A/A	G	A	SNV	SF3A1	SF3A1:intronic:NM_005877.5	1	100		intronic	NM_00587	c.726+57C	p.?	0.25					Pre-mRNA spl	rs5753080	0.1754	YES			Splicing fact	RNA binding,RNA processing,U2 snRNP,U2-type presplice			G=0, A=34	G=0.0, A=1.0	0.00001	524.186	34	G=0/0, A=24/1

chr5:140236950	G/G	C	G	SNV	PCDHA1,PCDHA10,PCDHA2,PCDHA3,PCDHA4,PCDHA5,PCDHA6,PCDHA7,PCDHA8,PCDHA9	PCDHA1:intronic:NM_018900.3, PCDHA5:intronic:NM_0189	1	100	1	missense	NM_01890	c.2394+68	p.?, p.?, p.?, p.?, p.?, p.?, p.?,	1.21	0.15	110	0.059	0.25(COSM5805041),0.25(C		rs251362	0.4534	YES	4.599E-1:0.0 (pos.14023(	neoplasm	Protocadhe	calcium io	Abortion, Spontaneous;Female Urogenital Di			C=0, G=68	C=0.0, G=1.0	0.00001	1052.09	68	C=0/0, G=37/3
chr22:30795742	G/C	G	C	SNV	SEC14L2	SEC14L2:intronic:NM_012429.4	1	50		intronic	NM_01242	c.130+35G	p.?	0.55					CRAL/TRIO do	rs142517473	0.0144	YES	1.172E-4 (pos.30795742)		Sec14-like 2	cytoplasm,cytosol,extracellula		Palmitic Acid,Vitamin E		G=12, C=14	G=0.4615, C=0.5385	0.00001	161.093	28	G=5/7, C=9/5
chr22:30812549	G/G	C	G	SNV	SEC14L2	SEC14L2:intronic:NM_012429.4	1	100		intronic	NM_01242	c.1081+15	p.?	-0.14					CRAL/TRIO do	rs5997642		YES			Sec14-like 2	cytoplasm,cytosol,extracellula		Palmitic Acid,Vitamin E		C=0, G=51	C=0.0, G=1.0	0.00001	754.582	51	C=0/0, G=25/2
chr5:140237109	G/G	T	G	SNV	PCDHA1,PCDHA10,PCDHA2,PCDHA3,PCDHA4,PCDHA5,PCDHA6,PCDHA7,PCDHA8,PCDHA9	PCDHA1:intronic:NM_018900.3, PCDHA5:intronic:NM_0189	1	100	1	synonymous	NM_01890	c.2394+68	p.?, p.?, p.?, p.?, p.?, p.?, p.?,	-0.38				0.01(COSM1744050),0.01(C		rs251363	0.3691	YES	4.66E-1 (pos.140237109)	adenocarcino	Protocadhe	calcium io	Abortion, Spontaneous;Female Urogenital Di			T=0, G=35	T=0.0, G=1.0	0.00001	543.894	35	T=0/0, G=17/1
chr22:30862146	A/A	C	A	SNV	SEC14L3	SEC14L3:intronic:NM_174975.4	1	100		intronic	NM_17497	c.580+175	p.?	1.02					CRAL/TRIO do	rs1047917		YES			Sec14-like 3	extracellular exosome,integra		Vitamin E		C=0, A=20	C=0.0, A=1.0	0.00001	263.786	20	C=0/0, A=9/11
chr22:30862400	A/A	T	A	SNV	SEC14L3	SEC14L3:intronic:NM_174975.4	1	100		intronic	NM_17497	c.520-19A	p.?	-0.35					CRAL/TRIO do	rs1057995	0.3916	YES	8.498E-1 (pos.30862400)		Sec14-like 3	extracellular exosome,integra		Vitamin E		T=0, A=70	T=0.0, A=1.0	0.00001	1092.09	70	T=0/0, A=56/1
chr5:140237387	G/G	T	G	SNV	PCDHA1,PCDHA10,PCDHA2,PCDHA3,PCDHA4,PCDHA5,PCDHA6,PCDHA7,PCDHA8,PCDHA9	PCDHA1:intronic:NM_018900.3, PCDHA5:intronic:NM_0189	1	100	1	missense	NM_01890	c.2394+69	p.?, p.?, p.?, p.?, p.?, p.?, p.?,	0.5	1	109	0	0.01(COSM3722956),0.01(C		rs251364	0.3767	YES	4.686E-1 (pos.14023738	adenocarcino	Protocadhe	calcium io	Abortion, Spontaneous;Female Urogenital Di			T=0, G=68	T=0.0, G=1.0	0.00001	1051.21	68	T=0/0, G=27/4
chr5:140237548	G/G	A	G	SNV	PCDHA1,PCDHA10,PCDHA2,PCDHA3,PCDHA4,PCDHA5,PCDHA6,PCDHA7,PCDHA8,PCDHA9	PCDHA1:intronic:NM_018900.3, PCDHA5:intronic:NM_0189	1	100	1	missense	NM_01890	c.2394+69	p.?, p.?, p.?, p.?, p.?, p.?, p.?,	-0.61	1	58	0	0.02(COSM4999253),0.02(C		rs630162	0.3762	YES	4.704E-1 (pos.14023754	adenocarcino	Protocadhe	calcium io	Abortion, Spontaneous;Female Urogenital Di			A=0, G=83	A=0.0, G=1.0	0.00001	1349.38	83	A=0/0, G=50/3
chr22:31266777	C/C	T	C	SNV	OSBP2	OSBP2:intronic:NM_030758.3	1	100		intronic	NM_03075	c.1107+10	p.?	-2.62					Oxysterol-bir	rs4820909					Oxysterol-b	apical dendrite,cholesterol binding,lipid transport,mem				T=0, C=32	T=0.0, C=1.0	0.00001	485.14	32	T=0/0, C=12/2
chr22:31266830	C/C	T	C	SNV	OSBP2	OSBP2:intronic:NM_030758.3	1	100		intronic	NM_03075	c.1107+16	p.?	-2.71					Oxysterol-bir	rs4820030		YES			Oxysterol-b	apical dendrite,cholesterol binding,lipid transport,mem				T=0, C=28	T=0.0, C=1.0	0.00001	411.139	28	T=0/0, C=7/21
chr22:31285680	G/A	G	A	SNV	LOC107985544,OSBP2	LOC107985544:exonic_nc:NR_146603.1, OSBP2:intronic:NN	1	57.14		intronic	NM_03075	c.1623+57	p.?	-0.71					Oxysterol-bir	rs5749186		YES			Oxysterol-b	apical dendrite,cholesterol binding,lipid transport,mem				G=9, A=12	G=0.4286, A=0.5714	0.00001	109.102	21	G=6/3, A=7/5
chr5:140228164	A/A	C	A	SNV	PCDHA1,PCDHA2,PCDHA3,PCDHA4,PCDHA5,PCDHA6,PCDHA7,PCDHA8,PCDHA9	PCDHA1:intronic:NM_018900.3, PCDHA5:intronic:NM_0189	1	100	1	missense	NM_01890	c.2394+59	p.?, p.?, p.?, p.?, p.?, p.?, p.?,	0.18	0.05	110	0.006	0.54(COSM4003324),0.54(C		rs251353	0.4635	YES	4.78E-1 (pos.140228164)	acute_myeloic	Protocadhe	calcium io	Abortion, Spontaneous;Female Urogenital Di			C=0, A=81	C=0.0, A=1.0	0.00001	1313.57	81	C=0/0, A=52/2
chr5:140229086	G/G	C	G	SNV	PCDHA1,PCDHA2,PCDHA3,PCDHA4,PCDHA5,PCDHA6,PCDHA7,PCDHA8,PCDHA9	PCDHA1:intronic:NM_018900.3, PCDHA5:intronic:NM_0189	1	100	1	missense	NM_01890	c.2394+60	p.?, p.?, p.?, p.?, p.?, p.?, p.?,	-0.78	1	32	0	0(COSM4003326),0(COSM4(		rs251354	0.3856	YES	4.837E-1 (pos.14022908(	acute_myeloic	Protocadhe	calcium io	Abortion, Spontaneous;Female Urogenital Di			C=0, G=26	C=0.0, G=1.0	0.00001	371.42	26	C=0/0, G=10/1
chr22:31687142	C/C	G	C	SNV	PIK3IP1	PIK3IP1:intronic:NM_052880.4	1	100		intronic	NM_05288	c.187+24C	p.?	0.51					Kringle doma	rs5753544		YES	1.0 (pos.31687142)			extracellular exosome,integral component of membran				G=0, C=75	G=0.0, C=1.0	0.00001	1169.72	75	G=0/0, C=33/4
chr22:31904170	G/G	A	G	SNV	SFI1	SFI1:intronic:NM_001007467.2	1	100		intronic	NM_00100	c.-29-71A>	p.?	-0.71						rs2413054		YES			SFI1, yeast.	G2/M transition of mitotic cell cycle,centriole,centroson				A=0, G=41	A=0.0, G=1.0	0.00001	664.609	41	A=0/0, G=15/2
chr22:31971173	TCTT/TCTT	T	TCTT	INDEL	SFI1	SFI1:intronic:NM_001007467.2	3	100		intronic	NM_00100	c.923-44T:	p.?	-0.11											SFI1, yeast.	G2/M transition of mitotic cell cycle,centriole,centroson				T=0, TCTT=:	T=0.0, TCTT=1.0	0.00001	353.019	25	T=0/0, TCTT=1:
chr5:140230370	CC/CC	AA	CC	MNV	PCDHA1,PCDHA2,PCDHA3,PCDHA4,PCDHA5,PCDHA6,PCDHA7,PCDHA8,PCDHA9	PCDHA1:intronic:NM_018900.3, PCDHA5:intronic:NM_0189	2	100	1	missense	NM_01890	c.2394+62	p.?, p.?, p.?, p.?, p.?, p.?, p.?,	-1.32, -0.6	1	103	0	0.02(COSM3927817),0.02(C		rs35021536,rs36963(	0.376	YES	4.806E-1 (pos.14023037	adenocarcino	Protocadhe	calcium io	Abortion, Spontaneous;Female Urogenital Di			AA=0, CC=5	AA=0.0, CC=1.0	0.00001	885.368	59	AA=0/0, CC=2!
chr22:32002294	T/C	T	C	SNV	SFI1	SFI1:intronic:NM_001007467.2	1	57.14		intronic	NM_00100	c.2055-20	p.?	-0.47						rs5753723	0.2111	YES	9.295E-1 (pos.32002294)		SFI1, yeast.	G2/M transition of mitotic cell cycle,centriole,centroson				T=12, C=16	T=0.4286, C=0.5714	0.00001	157.699	28	T=6/6, C=8/8
chr22:32179850	C/C	G	C	SNV	DEPDC5	DEPDC5:intronic:NM_001242896.1	1	100		intronic	NM_00124	c.484-43G	p.?	-0.51					Domain foun	rs138286,rs7646724(	0.0404	YES	0.0 (pos.32179848),9.999E-1 (pos.32179		DEP domair	GATOR1 cc	Adenocarcinoma:Brain Diseases:Carcinoma:			G=0, C=28	G=0.0, C=1.0	0.00001	408.718	28	G=0/0, C=12/1
chr5:140559914	C/C	T	C	SNV	PCDHB8	PCDHB8:exonic:NM_019120.4	1	100	1	missense	NM_01912	c.2299T>C	p.Phe767Leu	3.38		22		0.8(COSM400	Cadherin cyt	rs2740583	0.3483	YES	1.351E-1:0.0 (pos.14055	acute_myeloic	Protocadhe	calcium io	Melanoma:Neoplasms:Neoplasms by Histolc			T=0, C=70	T=0.0, C=1.0	0.00001	1092.09	70	T=0/0, C=31/3
chr22:32804313	A/A	G	A	SNV	RTCB	RTCB:intronic:NM_014306.4	1	100		intronic	NM_01430	c.173-84C	p.?	0.09					tRNA-splicing	rs5754073		YES			RNA 2-prim	ATP binding,RNA binding,RNA li		Sucrose		G=0, A=71	G=0.0, A=1.0	0.00001	1111.45	71	G=0/0, A=19/5
chr5:140568034	CA/CA	C	CA	INDEL	PCDHB9	PCDHB9:exonic:NM_019119.4	1	100	1	unknown	NM_019119.4			-0.66					Cadherin cyt	rs33974901,rs39778	1.00E-04	YES	1.0;0.0 (pos.140568034)		Protocadhe	calcium ion binding,calcium-dependent cell-cell adhesic				C=0, CA=23	C=0.0, CA=1.0	0.00001	289.507	23	C=0/0, CA=8/1
chr5:140710705	A/A	G	A	SNV	PCDHGA1	PCDHGA1:exonic:NM_018912.2	1	100	1	missense	NM_01891	c.454G>A	p.Val152Ile	2.37	0.21	29	0			rs2472647	0.025	YES	8.765E-1 (pos.140710705)		Protocadhe	calcium ion binding,cell adhesion,cell-cell signaling,hor				G=0, A=20	G=0.0, A=1.0	0.00001	263.786	20	G=0/0, A=9/11
chr5:140798639	G/C	G	C	SNV	PCDHGA1,PCDHGA10,PCDHGA2,PCDHGA3,PCDHGA4,PCDHGA5,PCDHGA6,PCDHGA7,PCDHGA8,PCDHGA9,PCDHGB1,PCDHGB2,PCDHGB3,PCDHGB4,PCDHGB5,PCDHGB6,PCDHGB7	PCDHGA8:intronic:NM_032088.1, PCDHGA7:intronic:NM_01	1	50	1	missense	NM_01891	c.2422-75	p.?, p.?, p.?, p.?, p.?, p.?, p.?,	0.66	0.38	32	0.18			rs17208397	0.1577	YES	3.551E-2 (pos.140798639)		Protocadhe	RNA bindir	Breast Diseases;Breast Neoplasms;Neoplasr			G=12, C=12	G=0.5, C=0.5	0.00001	106.708	24	G=2/10, C=5/7
chr5:140719090	G/A	G	A	SNV	PCDHGA1,PCDHGA2	PCDHGA2:exonic:NM_018915.3, PCDHGA1:intronic:NM_01{	1	52.27	1	synonymous	NM_01891	c.2421+64	p.?, p.(=)	-3.99				0.03(COSM6351853),0.03(C		rs6861047	0.1587	YES	6.461E-2 (pos.14071909(	carcinoma	Protocadhe	calcium ion binding,cell adhesion,cell-cell signaling,hor				G=42, A=4(	G=0.4773, A=0.5227	0.00001	511.718	88	G=25/17, A=1!
chr5:140774288	C/C	T	C	SNV	PCDHGA1,PCDHGA2,PCDHGA3,PCDHGA4,PCDHGA5,PCDHGA6,PCDHGA7,PCDHGA8,PCDHGB1,PCDHGB2,PCDHGB3,PCDHGB4	PCDHGA8:exonic:NM_032088.1, PCDHGA7:intronic:NM_01{	1	100	1	synonymous	NM_01891	c.2421+61	p.?, p.?, p.?, p.?, p.?, p.?, p.?,	-6.4						rs4912749	1.00E-04		9.999E-1 (pos.140774288)		Protocadhe	calcium ion binding,calcium-dependent cell-cell adhesic				T=0, C=36	T=0.0, C=1.0	0.00001	563.744	36	T=0/0, C=11/2
chr5:140750044	C/C	T	C	SNV	PCDHGA1,PCDHGA2,PCDHGA3,PCDHGA4,PCDHGA5,PCDHGB1,PCDHGB2,PCDHGB3	PCDHGA5:intronic:NM_018918.2, PCDHGA4:intronic:NM_01	1	100	1	missense	NM_01891	c.2421+37	p.?, p.?, p.?, p.?, p.?, p.?, p.?,	-0.88	1	64	0	0.02(COSM4591948),0.02(C		rs6860609		YES	1.0 (pos.140750044)	squamous_cel	Protocadhe	calcium ion binding,cell adhesion,cell-cell signaling,end				T=0, C=39	T=0.0, C=1.0	0.00001	623.977	39	T=0/0, C=23/1
chr5:140732220	C/A	C	A	SNV	PCDHGA1,PCDHGA2,PCDHGA3,PCDHGB1	PCDHGA3:intronic:NM_018916.3, PCDHGA2:intronic:NM_01	1	47.62	1	missense	NM_01891	c.2421+19	p.?, p.?, p.?, p.Pro798His	0.06	0.12	77	0.717			rs200777796	7.00E-04		0.0 (pos.140732220)		Protocadhe	calcium ion binding,cell adhesion,cell-cell signaling,end				C=11, A=10	C=0.5238, A=0.4762	0.00001	82.29	21	C=7/4, A=8/2
chr20:56137895	G/C	G	C	SNV	PCK1	PCK1:exonic:NM_002591.3	1	95.45	4	missense	NM_00259	c.550G>C	p.Val184Leu	4.39	1	32	0	0.26(COSM37	Phosphoenol	rs707555	0.1343	YES	7.835E-1 (pos.56137895)	acute_myeloic	Phosphoen	GDP bindi	Adenocarcinoma:C	1-(2-Fluor	Benign	G=1, C=21	G=0.0455, C=0.9545	0.00001	271.545	22	G=0/1, C=13/8
chr20:56137866	C/T	C	T	SNV	PCK1	PCK1:exonic:NM_002591.3	1	50	4	missense	NM_00259	c.521C>T	p.Thr174Met	7.13	0	81	1	0.93(COSM25	Phosphoenol	rs199822253	1.00E-04		0.0 (pos.56137866)	carcinoma	Phosphoen	GDP bindi	Adenocarcinoma:C	1-(2-Fluorobenzyl)-3-Buty		C=11, T=11	C=0.5, T=0.5	0.00001	94.596	22	C=11/0, T=4/7
chr20:56137834	A/G	A	G	SNV	PCK1	PCK1:exonic:NM_002591.3	1	47.62	4	synonymous	NM_00259	c.489A>G	p.(=)	-4.04				0.04(COSM37	Phosphoenol	rs1062601	0.4951	YES	3.614E-2 (pos.56137834)	diffuse_large_	Phosphoen	GDP bindi	Adenocarcinoma:C	1-(2-Fluor	Benign	A=11, G=1(	A=0.5238, G=0.4762	0.00001	82.2558	21	A=11/0, G=4/6
chr14:24567498	C/C	A	C	SNV	PCK2	PCK2:exonic:NM_004563.3	1	100	3	missense	NM_00456	c.362A>C	p.Gln121Pro	5.79	1	76	0	0.27(COSM45	Phosphoenol	rs3021119		YES	1.0 (pos.24567498)	squamous_cel	Phosphoen	GTP bindir	Brain Diseases;Brain Diseases, Metabolic;Br			A=0, C=31	A=0.0, C=1.0	0.00001	465.746	31	A=0/0, C=15/1
chr21:47773103	A/A	C	A	SNV	PCNT	PCNT:exonic:NM_006031.5	1	100	10	synonymous	NM_00603	c.1542C>A	p.(=)	-5.28				0.02(COSM11	Pericentrin-A	rs2249057	0.3307	YES	4.264E-1:0.0 (pos.47773	acute_myeloic	Nucleopori	G2/M tran	Bone Diseases;Bone Diseases,		Benign	C=0, A=39	C=0.0, A=1.0	0.00001	623.955	39	C=0/0, A=23/1
chr22:36708049	C/C	CTCCTGTG	C	INDEL	MYH9	MYH9:intronic:NM_002473.5	8	100		intronic	NM_00247	c.1728+45	p.?	-0.33, -1.16, -0.76, -0.85, -0.21, -4.13, -4.72, -0.46, -1.27					IQ calmoduli	rs1014580671,rs3842715,rs766		YES	6.114E-1 (pos.36708049)		Myosin, hea	ADP bindir	Blood Platelet Disorders;Cardi		Benign	CTCCTGTG/	CTCCTGTGA=0.0, C=1	0.00001	371.42	26	CTCCTGTGA=0,
chr22:36708084	T/T	C	T	SNV	MYH9	MYH9:intronic:NM_002473.5	1	100		intronic	NM_00247	c.1728+10	p.?	-1.97					IQ calmoduli	rs2413396	0.0878	YES	8.899E-1 (pos.36708084)		Myosin, hea	ADP bindir	Blood Platelet Disorders;Cardi		Benign	C=0, T=28	C=0.0, T=1.0	0.00001	408.717	28	C=0/0, T=8/20
chr22:36872750	G/G	T	G	SNV	TXN2	TXN2:intronic:NM_012473.3	1	100		intronic	NM_01247	c.387+30A	p.?	-0.58					Thioredoxin,	rs2281082,rs768831	0.1957	YES	0.0 (pos.36872749),2.666E-1 (pos.36872		Thioredoxir	cell,cell re	Cardiovascular Diseases;Congenital Abnorm.			T=0, G=57	T=0.0, G=1.0	0.00001	849.603	57	T=0/0, G=49/8
chr21:47777063	A/A	G	A	SNV	PCNT	PCNT:exonic:NM_006031.5	1	100	13	missense	NM_00603	c.2111G>A	p.Gly704Glu	3.84	1	98	0	0.04(COSM41	Pericentrin-A	rs2839223	0.1243	YES	8.131E-1 (pos.47777063)	neoplasm	Nucleopori	G2/M tran	Bone Diseases;Bone Diseases,		Benign	G=0, A=78	G=0.0, A=1.0	0.00001	1249.36	78	G=0/0, A=41/3
chr21:47836653	T/T	C	T	SNV	PCNT	PCNT:exonic:NM_006031.5	1	100	30	missense	NM_00603	c.6821C>T	p.Pro2274Leu	0.04	0.45	98	0.001	0.08(COSM37	Pericentrin-A	rs2070425	0.2562	YES	4.011E-1 (pos.47836653)	adenocarcino	Nucleopori	G2/M tran	Bone Diseases;Bone Diseases,		Benign	C=0, T=33	C=0.0, T=1.0	0.00001	504.583	33	C=0/0, T=10/2
chr22:36907098	C/C	T	C	SNV	EIF3D	EIF3D:intronic:NM_003753.3	1	100		intronic	NM_00375	c.1634-51	p.?	-2.68					Eukaryotic tr	rs728785	0.2977	YES	2.502E-1 (pos.36907098)		Eukaryotic t	IRES-dependent viral translational initiation,RNA bindin				T=0, C=33	T=0.0, C=1.0	0.00001	504.577	33	T=0/0, C=11/2
chr22:37163329	T/T	C	T	SNV	IFT27,LOC105373021	IFT27:intronic:NM_001177701.2, LOC105373021:downstre	1	100		intronic	NR_13462	c.174+21G	p.?	-2					Ras family,Ra	rs1048012	0.191	YES	9.099E-1 (pos.37163329)		Intraflagell	GTP bindir	Abnormalities, Multiple;Bardet-Biedl Syndror			C=0, T=41	C=0.0, T=1.0	0.00001	653.105	41	C=0/0, T=22/1
chr1:233134086	T/T	C	T	SNV	PCNX2	PCNX2:exonic:NM_014801.3	1	100	32	missense	NM_01480	c.5702G>A	p.Ser1901Asn	1.9	0.09	46	0	0.42(COSM37	Pecanex prot	rs56231757	0.2287	YES	1.491E-1 (pos.23313408(	adenocarcino	Pecanex, Di	biological_process,cellular_component,integral compor				C=0, T=61	C=0.0, T=1.0	0.00001	916.073	61	C=0/0, T=37/2
chr11:65383896	C/C	G	C	SNV	PCNX3	PCNX3:exonic:NM_032223.3	1	100	1	synonymous	NM_03222	c.114G>C	p.(=)	0.02				0.31(COSM45	Pecanex prot	rs7947140		YES	1.0;0.0 (pos.65383896)	squamous_cel	Pecanex, Di	integral component of membrane				G=0, C=11(	G=0.0, C=1.0	0.00001	1737.98	110	G=0/0, C=52/5
chr19:1482396	G/A	G	A	SNV	PCSK4	PCSK4:exonic:NM_017573.4	1	50	14	missense	NM_01757	c.1775C>T	p.Thr592Ile	0.2	0.2	89	0.483		Peptidase S8	rs61733913	0.0085		0.0 (pos.1482396)		Proprotein	acrosomal membrane,acrosomal vesicle,acrosome reac				G=27, A=27	G=0.5, A=0.5	0.00001	262.864	54	G=17/10, A=1!
chr1:55523033	G/G	A	G	SNV	PCSK9	PCSK9:exonic:NM_174936.3	1	100	7	synonymous	NM_17493	c.1026A>G	p.(=)	0.94					Peptidase inl	rs509504	2.00E-04		1.0 (pos.55523033)		Proprotein	COPII-coat	ACMG Recommend.	Alirocuma	Conflicting in	A=0, G=71	A=0.0, G=1.0	0.00001	1110.89	71	A=0/0, G=45/2
chr1:55529187	A/A	G	A	SNV	PCSK9	PCSK9:exonic:NM_174936.3	1	100	12	missense	NM_17493	c.2009G>A	p.Gly670Glu	0.51	1	98	0		Peptidase inl	rs505151	0.0353	YES	9.46E-1 (pos.55529187)		Proprotein	COPII-coat	ACMG Recommend.	Alirocuma	Benign/Likely	G=0, A=21	G=0.0, A=1.0	0.00001	281.235	21	G=0/0, A=3/18
chr17:53851228	T/T	C	T	SNV	PCTP	PCTP:exonic:NM_021213.3	1	100	4	synonymous	NM_02121	c.483C>T	p.(=)	-0.14				0.25(COSM41	START domair	rs2960062	0.0305	YES	9.38E-1 (pos.53851228)	neoplasm	Phosphatid	cytosol,lipid binding,lipid trans	Di-Linoleoyl-3-Sn-Phosph			C=0, T=39	C=0.0, T=1.0	0.00001	623.746	39	C=0/0, T=22/1
chr6:165832227	T/T	C	T	SNV	PDE10A	PDE10A:exonic:NM_001130690.2	1	100	11	synonymous	NM_00113	c.894G>A	p.(=)	-0.75				0.07(COSM37	3'5'-cyclic nu	rs220740	0.2917	YES	9.298E-2 (pos.16583222	acute_myeloic	Phosphodie	3',5'-cyclic	Attention Deficit ar	2-((4-4-(pyridin-4-ylmeth		C=0, T=31	C=0.0, T=1.0	0.00001	465.819	31	C=0/0, T=23/8

chr22:38025375	C/C	T	C	SNV	GGA1	GGA1:intronic:NM_013365.4	1	100		intronic	NM_01336	c.1094-94	p.?	-0.18					Adaptin C-ter	rs2071766		YES			Golgi assoc	ADP-ribosylation factor binding,Golgi apparatus,cellular				T=0, C=24	T=0.0, C=1.0	0.00001	334.792	24	T=0/0, C=8/16
chr22:38043444	C/A	C	A	SNV	LOC101927051.SH3BP1	SH3BP1:intronic:NM_018957.4, LOC101927051:intronic_nc	1	95.83		intronic	NM_01895	c.1119-4C	p.?	-1.81				0.11(COSM55	BAR domain,I	rs3213528	0.4679	YES	2.54E-1:0.0 (pos.380434	carcinoma	SH3 domair	GTPase activator activity,SH3 domain binding,actin filan				C=2, A=46	C=0.0417, A=0.9583	0.00001	746.121	48	C=2/0, A=29/1
chr7:32209425	G/G	A	G	SNV	PDE1C	PDE1C:exonic:NM_001191058.3	1	100	3	missense	NM_00119	c.280T>C	p.Ser94Pro	6.42	1	74	0		3'5'-cyclic nuc	rs386711852,rs977625			1.0 (pos.32209425)		Phosphodie	3',5'-cyclic	Endometriosis:Fem	Caffeine,Trapidil		A=0, G=62	A=0.0, G=1.0	0.00001	930.378	62	A=0/0, G=32/3
chr12:20766559	T/T	C	T	SNV	PDE3A	PDE3A:exonic:NM_000921.4	1	100	3	synonymous	NM_00092	c.1194C>T	p.(=)	0.13					3'5'-cyclic nuc	rs10743380	0.0663	YES	9.972E-1;0.0 (pos.20766559)		Phosphodie	3',5'-cyclic	Brachydactyly;Card	Aminophylline,Amrinone		C=0, T=34	C=0.0, T=1.0	0.00001	524.139	34	C=0/0, T=19/1
chr22:38142468	G/G	A	G	SNV	TRIOBP	TRIOBP:intronic:NM_001039141.2	1	100		intronic	NM_00103	c.5323-53	p.?	0.4					PH domain	rs5756803,rs745576759,rs9189		YES	0.0 (pos.38142449),1.0 (pos.38142468)		Trio- and F-a	GTP-Rho b	Ear Diseases;Hearing Disorders;Hearing Loss			A=0, G=41	A=0.0, G=1.0	0.00001	664.693	41	A=0/0, G=18/2
chr19:18329240	C/C	T	C	SNV	PDE4C	PDE4C:exonic:NM_000923.5	1	100	11	synonymous	NM_00092	c.1134A>G	p.(=)	0.94				0.09(COSM14	3'5'-cyclic nuc	rs1042050	0.382	YES	2.035E-1 (pos.18329240)	acute_myeloic	Phosphodie	3',5'-cyclic-AMP phosphodieste		Caffeine,Crisaborole,Dyr		T=0, C=26	T=0.0, C=1.0	0.00001	383.787	26	T=0/0, C=12/1
chr22:38503847	T/T	C	T	SNV	BAIAP2L2	BAIAP2L2:intronic:NM_025045.5	1	100		intronic	NM_02504	c.276+12G	p.?	-1.34					IRSp53/MIM I	rs6001006	0.2502	YES	6.722E-1 (pos.38503847)		Bai1-associ	actin crosslink formation,actin cytoskeleton,actin filame				C=0, T=36	C=0.0, T=1.0	0.00001	563.734	36	C=0/0, T=29/7
chr4:120528327	A/A	G	A	SNV	PDE5A	PDE5A:exonic:NM_001083.3	1	100	2	missense	NM_00108	c.278C>T	p.Ala93Val	1.02	0.13	64	0		3'5'-cyclic nuc	rs3733526	0.1808	YES	8.952E-1 (pos.120528327)		Phosphodie	3',5'-cyclic	Cardiovascular Dise	3-isobutyl-1-methyl-7H-x		G=0, A=58	G=0.0, A=1.0	0.00001	863.358	58	G=0/0, A=27/3
chr4:120474859	A/G	A	G	SNV	PDE5A	PDE5A:exonic:NM_001083.3	1	47.37	8	synonymous	NM_00108	c.1242T>C	p.(=)	1.84				0.83(COSM40	3'5'-cyclic nuc	rs11947234	0.2863	YES	3.9E-1 (pos.120474859)	acute_myeloic	Phosphodie	3',5'-cyclic	Cardiovascular Dise	3-isobutyl-1-methyl-7H-x		A=50, G=45	A=0.5263, G=0.4737	0.00001	488.471	95	A=31/19, G=28
chr22:40641980	A/A	G	A	SNV	TNRC6B	TNRC6B:intronic:NM_001162501.1	1	100		intronic	NM_00116	c.6-39G>A	p.?	0.28					Argonaute hc	rs3752513	0.3999	YES	5.438E-1 (pos.40641980)		Trinucleotic	P-body,RN	Adnexal Diseases:Cysts:Endocrine System Di			G=0, A=42	G=0.0, A=1.0	0.00001	685.218	42	G=0/0, A=35/7
chr22:40800544	C/T	C	T	SNV	SGSM3	SGSM3:intronic:NM_015705.5	1	52.38		intronic	NM_01570	c.367-17C	p.?	-1.99					RUN domain,	rs2235318	0.1695	YES	1.998E-1;0.0 (pos.40800544)		Small G pro	GTPase ac	Breast Diseases:Breast Neoplasms:Neoplasr			C=10, T=11	C=0.4762, T=0.5238	0.00001	95.2532	21	C=5/5, T=2/9
chr22:40801986	C/T	C	T	SNV	SGSM3	SGSM3:intronic:NM_015705.5	1	50		intronic	NM_01570	c.815-96C	p.?	-1.55					RUN domain,	rs9607716		YES			Small G pro	GTPase ac	Breast Diseases:Breast Neoplasms:Neoplasr			C=15, T=15	C=0.5, T=0.5	0.00001	145.565	30	C=8/7, T=8/7
chr22:40803367	C/T	C	T	SNV	SGSM3	SGSM3:intronic:NM_015705.5	1	45.45		intronic	NM_01570	c.1368+35	p.?	-0.68					RUN domain,	rs77222252	0.0509	YES	4.78E-3;0.0 (pos.40803367)		Small G pro	GTPase ac	Breast Diseases;Breast Neoplasms;Neoplasr			C=17, T=15	C=0.5313, T=0.4688	0.00001	157.052	33	C=7/10, T=5/1
chr22:41244398	C/G	C	G	SNV	ST13	ST13:intronic:NM_003932.4	1	47.62		intronic	NM_00393	c.169-25G	p.?	-1.03					Tetratricope	rs138344	0.4662	YES	5.765E-2 (pos.41244398)		Suppressio	Hsp70 protein binding,chaperone binding,chaperone co				C=11, G=10	C=0.5238, G=0.4762	0.00001	82.29	21	C=3/8, G=6/4
chr22:41545611	C/C	G	C	SNV	EP300	EP300:intronic:NM_001429.3	1	100		intronic	NM_00142	c.2380-15	p.?	0.21					Bromodomai	rs4479527		YES			E1A-binding	B cell diffe	Abnormalities, Multiple:Adenocarcinoma:Bo			G=0, C=63	G=0.0, C=1.0	0.00001	958.865	63	G=0/0, C=32/3
chr22:41864216	C/C	A	C	SNV	ACO2,PHF5A	PHF5A:intronic:NM_032758.3, ACO2:upstream:NM_001098	1	100		intronic	NM_03275	c.53-65T>C	p.?, p.?	0.57					PHF5-like pro	rs505533	0.2109	YES			Aconitase, i	3 iron, 4 su	Adenocarcinoma:C	4-Hydroxy-Aconitate Ion		A=0, C=51	A=0.0, C=1.0	0.00001	753.962	51	A=0/0, C=19/3
chr22:42016699	C/G	C	G	SNV	DESI1,XRCC6	XRCC6:upstream:NM_001469.4, DESI1:intronic:NM_015704	1	52.38		intronic	NM_01570	c.88+57G>	p.?, p.?	-0.44					PPPDE putati	rs2267437	0.4415	YES			Desumoyla	5'-deoxyribose-5-phosphate lyase activity,ATP binding,A				C=10, G=11	C=0.4762, G=0.5238	0.00001	95.3095	21	C=2/8, G=7/4
chr4:648643	A/A	G	A	SNV	PDE6B	PDE6B:exonic:NM_000283.3	1	100	6	missense	NM_00028	c.958G>A	p.Val320Ile	2.67	1	29	0	0.03(COSM41	3'5'-cyclic nuc	rs10902758	0		1.0 (pos.648643)	neoplasm	Phosphodie	3',5'-cyclic	Congenital, Heredit	Caffeine,T	Benign	G=0, A=29	G=0.0, A=1.0	0.00001	427.559	29	G=0/0, A=3/26
chr22:42109917	G/G	A	G	SNV	MEI1	MEI1:intronic:NM_152513.3	1	100		intronic	NM_15251	c.350-89A	p.?	0.51						rs5758431		YES				cell,male meiosis I,meiosis I,meiotic telomere clusterin				A=0, G=35	A=0.0, G=1.0	0.00001	543.903	35	A=0/0, G=22/1
chr10:95372764	C/T	C	T	SNV	PDE6C	PDE6C:exonic:NM_006204.3	1	53.33	1	synonymous	NM_00620	c.282C>T	p.(=)	1.1				0.87(COSM37	3'5'-cyclic nuc	rs12781149	0.0967	YES	4.632E-4 (pos.95372764)	adenocarcinor	Phosphodie	3',5'-cyclic	Amphetamine-Rela	Caffeine,T	Benign/Likely	C=14, T=16	C=0.4667, T=0.5333	0.00001	158.949	30	C=6/8, T=7/9
chr5:76704849	C/C	T	C	SNV	PDE8B	PDE8B:exonic:NM_003719.3	1	100	14	synonymous	NM_00371	c.1497T>C	p.(=)	-0.36				0.13(COSM45	3'5'-cyclic nuc	rs186753		YES	1.0 (pos.76704849)	squamous_cel	Phosphodie	3',5'-cyclic	Adrenal Gland Dise	Caffeine,Trapidil		T=0, C=55	T=0.0, C=1.0	0.00001	814.907	55	T=0/0, C=16/3
chr4:186423637	A/A	G	A	SNV	PDLIM3	PDLIM3:exonic:NM_014476.5	1	100	8	synonymous	NM_01447	c.906C>T	p.(=)	-3.19				0.03(COSM48	Domain of un	rs4635850	0.1465	YES	8.066E-1 (pos.186423637	ductal_carcinoma		Z disc,actin cytoskeleton,actin filament or			Benign	G=0, A=28	G=0.0, A=1.0	0.00001	408.714	28	G=0/0, A=13/1
chr5:131602166	C/C	T	C	SNV	PDLIM4	PDLIM4:exonic:NM_003687.3	1	100	3	synonymous	NM_00368	c.255T>C	p.(=)	0.04				0.11(COSM63	Domain of un	rs1007602	0.3512	YES	3.299E-1 (pos.131602166	carcinoma		actin cyto:	Adnexal Diseases:Bone Diseases:Bone Disea			T=0, C=109	T=0.0, C=1.0	0.00001	1718.89	109	T=0/0, C=73/3
chr22:43564963	G/G	C	G	SNV	TTLL12	TTLL12:intronic:NM_015140.3	1	100		intronic	NM_01514	c.1645-45	p.?	-0.24					Tubulin-tyros	rs138931	0.3199	YES	9.808E-1;0.0 (pos.43564963)			ATP bindin	Congenital Abnormalities:Congenital, Heredi			C=0, G=24	C=0.0, G=1.0	0.00001	334.776	24	C=0/0, G=13/1
chr4:95531417	G/G	A	G	SNV	PDLIM5	PDLIM5:exonic:NM_001256426.1	1	100	10	synonymous	NM_00125	c.837A>G	p.(=)	-0.29					Domain of un	rs10017461		YES	9.697E-1 (pos.95531417)				Cardiovascular Diseases;Genital Diseases, N			A=0, G=22	A=0.0, G=1.0	0.00001	298.892	22	A=0/0, G=13/9
chr4:95578588	A/A	G	A	SNV	PDLIM5	PDLIM5:exonic:NM_001256426.1	1	100	15	missense	NM_00125	c.1562G>A	p.Ser521Asn	6.27	1	46	0.001		Domain of un	rs13107595	0.0063	YES	1.0 (pos.95578588)				Cardiovascular Diseases;Genital Diseases, N			G=0, A=52	G=0.0, A=1.0	0.00001	767.002	52	G=0/0, A=36/1
chr1:13940864	G/G	C	G	SNV	PDPN	PDPN:exonic:NM_006474.4	1	100	5	missense	NM_00647	c.668C>G	p.Ala223Gly	0.52	1	60	0		Podoplanin	rs2486188	0.0452	YES	9.999E-1 (pos.13940864)		Podoplanin	Rho protei	Female Urogenital Diseases:Female Urogeni			C=0, G=47	C=0.0, G=1.0	0.00001	789.29	47	C=0/0, G=15/3
chr22:44582499	C/C	T	C	SNV	PARVG	PARVG:intronic:NM_022141.6	1	100		intronic	NM_02214	c.144+747	p.?	-0.54				0(COSM6245	Calponin hon	rs79478		YES	7.683E-1 (pos.44582499)	neoplasm	Parvin, gam	actin binding,actin cytoskeleton reorganization,cell-mat				T=0, C=68	T=0.0, C=1.0	0.00001	1050.94	68	T=0/0, C=33/3
chr22:45218223	T/T	C	T	SNV	ARHGAP8,PRR5-ARHGAP8	ARHGAP8:intronic:NM_001017526.1, PRR5-ARHGAP8:intron	1	100		intronic	NM_18133	c.780-28C	p.?, p.?	-0.21					CRAL/TRIO do	rs136662	0.0394	YES	9.206E-1 (pos.45218223)		GTPase-act	GTPase ac	Colonic Diseases;Colonic Neoplasms;Colorec			C=0, T=50	C=0.0, T=1.0	0.00001	852.743	50	C=0/0, T=38/1
chr22:45682975	A/G	A	G	SNV	UPK3A	UPK3A:intronic:NM_006953.3	1	56		intronic	NM_00695	c.209-78A	p.?	-3.12						rs2673087		YES			Uroplakin 3	apical pla:	Congenital Abnormalities:Congenital, Heredi			A=11, G=14	A=0.44, G=0.56	0.00001	134.35	25	A=9/2, G=3/11
chr10:119042761	C/T	C	T	SNV	PDZD8	PDZD8:utr_3:NM_173791.4	1	47.37	5	utr_3	NM_17379	c.3483G>A	p.?	-0.64					PDZ domain,I	rs363293	0.0022	YES	1.0 (pos.119042761)		PDZ domair	cytoskeleton organization,intracellular,intracellular sig				C=40, T=36	C=0.5263, T=0.4737	0.00001	364.05	76	C=18/22, T=24
chr1:156877457	T/C	T	C	SNV	PEAR1	PEAR1:exonic:NM_001080471.1	1	97.5	7	missense	NM_00108	c.700T>C	p.Ser234Pro	2.07	1	74	0		Human growt	rs1952294	5.00E-04		1.0 (pos.156877457)		Platelet end	integral component of membrane,negative regulation of				T=1, C=39	T=0.025, C=0.975	0.00001	611.389	40	T=1/0, C=21/1
chr22:45794925	A/A	C	A	SNV	SMC1B	SMC1B:intronic:NM_148674.4	1	100		intronic	NM_14867	c.1113+50	p.?	0.75					AAA ATPase d	rs79154	0.4791	YES	7.078E-1;0.0 (pos.45794925)			ATP bindin	Female Urogenital Diseases;Female Urogeni			C=0, A=20	C=0.0, A=1.0	0.00001	263.786	20	C=0/0, A=17/3
chr22:45810278	AC/AC	A	AC	INDEL	RIBC2,SMC1B	SMC1B:upstream:NM_148674.4, RIBC2:exonic:NM_015653.	1	100		intronic	NM_14867	c.-830T>G	p.?	0.62					RIB43A	rs111381981,rs3978	0.0022		1.0 (pos.45810278)	neoplasm		ATP bindin	Breast Diseases:Breast Neoplasms:Female L			A=0, AC=40	A=0.0, AC=1.0	0.00001	630.396	40	A=0/0, AC=20/
chr17:4575283	G/G	A	G	SNV	PELP1	PELP1:exonic:NM_014389.2	1	100	16	synonymous	NM_01438	c.3153T>C	p.(=)	-4.51					NUC202 dom	rs3862485,rs745599	0.0611	YES	0.0:0.0 (pos.4575277),9.989E-1 (pos.457		Proline-, glu	MLL1 complex,RNA binding,cellular response to estroger				A=0, G=63	A=0.0, G=1.0	0.00001	958.917	63	A=0/0, G=42/2
chr19:33878198	G/G	A	G	SNV	PEPD	PEPD:utr_3:NM_000285.3	1	100	15	utr_3	NM_00028	c.1534T>C	p.?	-1.15					Aminopeptid	rs1061338	0.2479	YES			Peptidase D	aminopep	Abnormalities, Multiple;Aminc		Benign	A=0, G=27	A=0.0, G=1.0	0.00001	385.406	27	A=0/0, G=20/7
chr22:46724558	C/C	T	C	SNV	GTSE1	GTSE1:intronic:NM_016426.6	1	100		intronic	NM_01642	c.1725-27	p.?	-0.97					G-2 and S-pha	rs140053	0.0755	YES	9.999E-1 (pos.46724558)			DNA damage response, signal transduction by p53 class				T=0, C=65	T=0.0, C=1.0	0.00001	991.316	65	T=0/0, C=48/1
chr19:33878837	A/A	G	A	SNV	PEPD	PEPD:exonic:NM_000285.3	1	100	14	missense	NM_00028	c.1303C>T	p.Leu435Phe	2.27	0.96	22	0.001	0.59(COSM37	Aminopeptid	rs17570,rs74964002	0.2398	YES	0.0 (pos.33878834),0.0 (p	adenocarcinor	Peptidase D	aminopep	Abnormalities, Multiple;Aminc		Benign	G=0, A=62	G=0.0, A=1.0	0.00001	938.191	62	G=0/0, A=39/2
chr2:239161957	C/T	C	T	SNV	PER2	PER2:exonic:NM_022817.2	1	57.14	19	missense	NM_02281	c.2707G>A	p.Val903Ile	0.08	0.19	29	0.984		PAS domain,F	rs35333999	0.0456	YES	0.0 (pos.239161957)		Period, Dro	circadian r	Alcohol-Related Disorders:Alcoholic Intoxica			C=12, T=16	C=0.4286, T=0.5714	0.00001	161.747	28	C=5/7, T=7/9
chr22:47064164	A/A	C	A	SNV	GRAMD4	GRAMD4:intronic:NM_015124.4	1	100		intronic	NM_01512	c.930+100	p.?	-0.29					GRAM domai	rs2748338		YES			Gram doma	apoptotic process,integral component of membrane,mit				C=0, A=26	C=0.0, A=1.0	0.00001	371.417	26	C=0/0, A=11/1
chr22:47064665	T/T	C	T	SNV	GRAMD4	GRAMD4:intronic:NM_015124.4	1	100		intronic	NM_01512	c.975-31C	p.?	-1.01					GRAM domai	rs9616088	0.3972	YES	3.509E-1 (pos.47064665)		Gram doma	apoptotic process,integral component of membrane,mit				C=0, T=72	C=0.0, T=1.0	0.00001	1130.34	72	C=0/0, T=48/2
chr22:47064837	C/C	A	C	SNV	GRAMD4	GRAMD4:intronic:NM_015124.4	1	100		intronic	NM_01512	c.1084+32	p.?	-0.68					GRAM domai	rs2076711	0.2964	YES	3.66E-1;0.0 (pos.47064837)		Gram doma	apoptotic process,integral component of membrane,mit				A=0, C=42	A=0.0, C=1.0	0.00001	685.217	42	A=0/0, C=17/2
chr22:47070432	C/C	G	C	SNV	GRAMD4	GRAMD4:intronic:NM_015124.4	1	100		intronic	NM_01512	c.1385-12	p.?	-0.91					GRAM domai	rs2073275		YES			Gram doma	apoptotic process,integral component of membrane,mit				G=0, C=24	G=0.0, C=1.0	0.00001	329.653	24	G=0/0, C=11/1
chr1:7890055	T/A	T	A	SNV	PER3	PER3:exonic:NM_016831.2	1	50	19	synonymous	NM_01683	c.3021T>A	p.(=)	-1				0(COSM2156	PAS domain,F	rs11121034,rs747122763,rs778116536			0.0 (pos.7890047),0.0 (pc	adenocarcinor	Period, Dro	circadian r	Bone Marrow Diseases:Breast Diseases:Brea			T=15, A=15	T=0.5, A=0.5	0.00001	145.014	30	T=0/15, A=4/1
chr7:92131289	T/T	G	T	SNV	PEX1	PEX1:exonic:NM_000466.2	1	100	14	synonymous	NM_00046	c.2331C>A	p.(=)	2.29					ATPase famil	rs10278857	0.0876	YES	9.676E-1 (pos.92131289)		Peroxisome	ATP bindin	Abnormalities, Multiple:Amelc		Benign/Likely	G=0, T=42	G=0.0, T=1.0	0.00001	685.22	42	G=0/0, T=19/2
chr22:47097450	T/T	C	T	SNV	CERK	CERK:intronic:NM_022766.5	1	100		intronic	NM_02276	c.790+83G	p.?	-1.01					Diacylglycerc	rs135688		YES			Ceramide k	ATP binding,NAD+ kinase activity,ceramide kinase activit				C=0, T=42	C=0.0, T=1.0	0.00001	685.219	42	C=0/0, T=24/1
chr22:49103660	C/C	T	C	SNV	FAM19A5	FAM19A5:intronic:NM_001082967.2	1	100		intronic	NM_00108	c.390+4T>	p.?	-1.67				0(COSM4407	TAFA family	rs132234	0.2615	YES	6.851E-1;0.0 (pos.491036	hepatocellula	Family with	extracellu	Digestive System Diseases;Digestive System			T=0, C=24	T=0.0, C=1.0	0.00001	334.792	24	T=0/0, C=17/7
chr15:90226947	A/A	C	A	SNV	PEX11A	PEX11A:exonic:NM_003847.2	1	100	3	synonymous	NM_00384	c.405G>T	p.(=)	0.74				0.75(COSM39	Peroxisomal	rs7169981	0.2917	YES	3.031E-1 (pos.90226947)	acute_myeloic	Peroxisome	brown fat cell differentiation,integral component of perc				C=0, A=47	C=0.0, A=1.0	0.00001	789.135	47	C=0/0, A=25/2
chr17:8168224	C/C	T	C	SNV	PFAS	PFAS:exonic:NM_012393.2	1	100	18	synonymous	NM_01239	c.2061T>C	p.(=)	-3.17				0.02(COSM43	AIR synthase	rs9902252	0.2439	YES	4.058E-1 (pos.8168224)	neoplasm	phosphorib	'de novo' IMP biosynthetic proc	Glutamic Acid,L-Glutami			T=0, C=43	T=0.0, C=1.0	0.00001	705.844	43	T=0/0, C=4/39
chr22:50518723	G/G	A	G	SNV	MLC1	MLC1:intronic:NM_139202.2	1	100		intronic	NM_13920	c.321+50T	p.?	-2.57						rs79301	0.0727	YES	9.962E-1 (pos.50518723)		MLC1 gene	apical pla:	Adenocarcinoma;Brain Diseas		Benign	A=0, G=135	A=0.0, G=1.0	0.00001	2248.34	135	A=0/0, G=108/
chr22:50528916	C/T	C	T	SNV	MOV10L1	MOV10L1:intronic:NM_018995.2	1	50		intronic	NM_01899	c.97+302C	p.?	-0.57				0(COSM5426	AAA domain	rs9617018	0.1474	YES	2.597E-1 (pos.50528916)	acute_myeloic	Mov10-like	ATP binding,ATP-dependent RNA helicase activity,DNA m				C=16, T=16	C=0.5, T=0.5	0.00001	158.382	32	C=8/8, T=15/1
chr6:33256471	C/C	T	C	SNV	PFDN6,WDR46	WDR46:exonic:NM_005452.5, PFDN6:upstream:NM_00126	1	100	3	missense	NM_00545	c.280A>G,	p.Thr94Ala, p.?	1.18	1	58	0		BING4CT (NU	rs3130257	0.1086	YES	9.978E-1 (pos.33256471)		WD repeat-	90S prerib	Digestive System Diseases:Digestive System			T=0, C=42	T=0.0, C=1.0	0.00001	685.206	42	T=0/0, C=18/2
chr21:45732116	T/T	C	T	SNV	PFKL	PFKL:exonic:NM_002626.5	1	100	4	synonymous	NM_00262	c.366C>T	p.(=)	-0.27				0.42(COSM41	Phosphofruct	rs1057034,rs754247	0.4263	YES	2.965E-3:0.0 (pos.45732	neoplasm	Phosphofru	6-phosphofructokinase activity,6-phosphc			Benign	C=0, T=40	C=0.0, T=1.0	0.00001	644.276	40	C=0/0, T=19/2
chr10:3143643	C/C	A	C	SNV	PFKP	PFKP:exonic:NM_002627.4	1	100	4	synonymous	NM_00262	c.351A>C	p.(=)	-0.86				0.09(COSM41	Phosphofruct	rs4881080	1.00E-04		9.998E-1;2.381E-4;0.0 (pc	neoplasm	Phosphofru	6-phosphofructokinase activity,ATP binding,cadherin bir				A=0, C=39	A=0.0, C=1.0	0.00001	623.403	39	A=0/0, C=9/30
chr22:50757348	A/A	G	A	SNV	DENND6B	DENND6B:intronic:NM_001001794.3	1	100		intronic	NM_00100	c.217-30C	p.?	-1.89					DENN (AEX-3)	rs62241232	0.4216	YES	5.673E-1 (pos.50757348)			Rab guanyl-nucleotide exchange factor activity,cytosol,r				G=0, A=24	G=0.0, A=1.0	0.00001	334.763	24	G=0/0, A=13/1
chr22:50876235	C/C	T	C	SNV	PPP6R2	PPP6R2:intronic:NM_001242898.1	1	100		intronic	NM_00124	c.1782-37	p.?	-3.54					SIT4 phospha	rs5770779	0.2576	YES	9.474E-1 (pos.50876235)			cytosol,intracellular membrane-bounded organelle,prot				T=0, C=30	T=0.0, C=1.0	0.00001	446.639	30	T=0/0, C=16/1
chr17:4849284	A/A	G	A	SNV	PFN1,RNF167	RNF167:downstream:NM_015528.2, PFN1:exonic:NM_0050	1	100	3	synonymous	NM_01552	c.*973G>A	p.?, p.(=)	0.29				0.87(COSM44	Profilin	rs13204	0.0402	YES	2.054E-1 (pos.4849284)	acute_myeloic	Profilin-1,RI	RNA bindir	Amyotrophic Latera	7-HYDROX	Benign	G=0, A=26	G=0.0, A=1.0	0.00001	371.409	26	G=0/0, A=14/1
chr1:153314153	A/A	C	A	SNV	PGLYRP4	PGLYRP4:exonic:NM_020393.3	1	100	6	missense	NM_02039	c.575G>T	p.Gly192Val	0.9	0.57	109	0		N-acetylmura	rs3006448	0.1485	YES	8.741E-1;0.0 (pos.153314153)			N-acetylmuramoyl-L-alanine amidase activity,antimicro				C=0, A=107	C=0.0, A=1.0	0.00001	1681.3	107	C=0/0, A=37/7
chr22:50886557	G/G	A	G	SNV	SBF1	SBF1:intronic:NM_002972.3	1	100		intronic	NM_00297	c.5363+10	p.?	0					DENN (AEX-3)	rs6010041		YES			SET-binding	Rab guany	Charcot-Marie-Tooth Disease;Congenital Abr			A=0, G=33	A=0.0, G=1.0	0.00001	504.572	33	A=0/0, G=24/9
chr11:74053547	T/T	C	T	SNV	PGM2L1	PGM2L1:exonic:NM_173582.4	1	100	12	missense	NM_17358	c.1591G>A	p.Val531Ile	-2.75	0.18	29	0.001		Phosphogluc	rs592644	0.3107	YES	8.064E-1 (pos.74053547)		Phosphoglu	canonical glycolysis,cytosol,galactose catabolic process				C=0, T=176	C=0.0, T=1.0	0.00001	2901.45	176	C=0/0, T=49/1
chr11:74047743	T/A	T	A	SNV	PGM2L1	PGM2L1:exonic:NM_173582.4	1	45.45	14	missense	NM_17358	c.1823A>T	p.Asn608Ile	7.23	0	149	0.009		Phosphogluc	rs36014178	0.0412	YES	1.156E-4 (pos.74047743)		Phosphoglu	canonical glycolysis,cytosol,galactose catabolic process				T=48, A=40	T=0.5455, A=0.4545	0.00001	406.846	88	T=22/26, A=18
chr12:9070366	A/A	T	A	SNV	PHC1	PHC1:exonic:NM_004426.2	1	100	2	synonymous	NM_00442	c.93T>A	p.(=)	-0.12					SAM domain	rs4883198	0.2747	YES	7.566E-1 (pos.9070366)		Polyhomeo	DNA bindi	Neoplasms:Neoplasms by Histologic Type:Ne			T=0, A=50	T=0.0, A=1.0	0.00001	852.748	50	T=0/0, A=10/4
chr22:51020666	CA/CA	C	CA	INDEL	CHKB,CHKB-AS1,CHKB-CPT1B	CHKB:intronic:NM_005198.4, CHKB-CPT1B:intronic_nc:NR_0	1	100		intronic	NR_02792	c.333+12G	p.?	-0.06					Choline/ethanolamine kinase,Phosphotransferase enzyme family						Choline kin	ATP bindin	Congenital, Heredit	Choline		C=0, CA=45	C=0.0, CA=1.0	0.00001	651.663	45	C=0/0, CA=33/
chr12:9086898	G/G	A	G	SNV	PHC1	PHC1:exonic:NM_004426.2	1	100	10	missense	NM_00442	c.2077A>G	p.Thr693Ala	-0.53	0.51	58	0	0.09(COSM39	SAM domain	rs1049925	0.4426	YES	4.002E-1 (pos.9086898)	acute_myeloic	Polyhomeo	DNA bindi	Neoplasms:Neoplasms by Histologic Type:Ne			A=0, G=102	A=0.0, G=1.0	0.00001	1593.33	102	A=0/0, G=37/6
chr22:51045433	T/T	C	T	SNV	MAPK8IP2	MAPK8IP2:intronic:NM_012324.5	1	100		intronic	NM_01232	c.2302+14	p.?	0.59					Phosphotyro:	rs131736,rs7497870	0.3631	YES	0.0 (pos.51045429),8.178E-1 (pos.51045		Mitogen-ac	JNK cascade,MAP-kinase scaffold activity,MAPK cascade				C=0, T=25	C=0.0, T=1.0	0.00001	353.008	25	C=0/0, T=17/8
chr3:100086861	C/C	T	C	SNV	TOMM70	TOMM70:intronic:NM_014820.4	1	100		intronic	NM_01482	c.1673+27	p.?	-0.55					TPR repeat,T	rs277640,rs7759280	0.3366	YES	1.161E-4;0.0 (pos.100086856),9.373E-1 (pos.100086			extracellular exosome,integral component of membran				T=0, C=89	T=0.0, C=1.0	0.00001	1472.49	89	T=0/0, C=61/2
chr6:33382288	A/A	G	A	SNV	PHF1	PHF1:exonic:NM_024165.2	1	100	10	missense	NM_02416	c.911G>A	p.Arg304Lys	5.16	0.38	26	0		PHD-finger,P	rs3116713	0.0872	YES	9.994E-1 (pos.33382288)		PHD finger p	DNA binding transcription factor activity,ESC/E(Z) comple				G=0, A=26	G=0.0, A=1.0	0.00001	371.182	26	G=0/0, A=9/17
chr3:100354683	A/A	G	A	SNV	ADGRG7	ADGRG7:intronic:NM_032787.2	1	100		intronic	NM_03278	c.597+13G	p.?	-1.18					7 transmemb	rs1520652	0.3486	YES	9.891E-1 (pos.100354683)		Adhesion G	G-protein coupled receptor activity,G-protein coupled re				G=0, A=26	G=0.0, A=1.0	0.00001	371.42	26	G=0/0, A=17/9
chr3:100368692	G/G	A	G	SNV	ADGRG7	ADGRG7:intronic:NM_032787.2	1	100		intronic	NM_03278	c.1379+41	p.?	-0.17					7 transmemb	rs1144118	0.0067	YES	9.974E-1 (pos.100368692)		Adhesion G	G-protein coupled receptor activity,G-protein coupled re				A=0, G=34	A=0.0, G=1.0	0.00001	524.178	34	A=0/0, G=19/1
chr1:6681744	T/T	C	T	SNV	PHF13	PHF13:utr_3:NM_153812.2	1	100	4	utr_3	NM_15381	c.950C>T	p.?	-2.04					PHD-finger	rs3747994	0.2884	YES	6.144E-1 (pos.6681744)			cell division,chromatin binding,chromosome segregatio				C=0, T=20	C=0.0, T=1.0	0.00001	263.786	20	C=0/0, T=7/13
chr3:100594326	C/C	CGGCATA	C	INDEL	ABI3BP	ABI3BP:intronic:NM_015429.3	6	100		intronic	NM_01542	c.838+26T	p.?	0.67, 0.08, -0.38, -0.46, 0.23, -3.63, 1.23					Fibronectin t	rs11278619,rs20116	0.4248	YES	0.0 (pos.100594324),0.0 (pos.100594325),0.0 (pos.1(			collagen b	Angioedema;Asthma;Bronchial Diseases;Car			CGGCATA=(	CGGCATA=0.0, C=1.0	0.00001	285.632	21	CGGCATA=0/0
chr3:10081545	C/C	G	C	SNV	FANCD2	FANCD2:intronic:NM_033084.4	1	100		intronic	NM_03308	c.695+16G	p.?	0.05					Fanconi anae	rs17032283,rs75360	0.1636	YES	0.0 (pos.10081543),6.564E-2 (pos.10081		Fanconi ane	DNA polyr	Anemia;Anemia, Aplastic;Aner		Benign	G=0, C=30	G=0.0, C=1.0	0.00001	446.639	30	G=0/0, C=8/22
chr3:10084224	T/T	C	T	SNV	FANCD2	FANCD2:intronic:NM_033084.4	1	100		intronic	NM_03308	c.784-19C	p.?	0.89					Fanconi anae	rs9879080	0.1643	YES	6.542E-2 (pos.10084224)		Fanconi ane	DNA polyr	Anemia;Anemia, Aplastic;Aner		Benign	C=0, T=34	C=0.0, T=1.0	0.00001	524.185	34	C=0/0, T=26/8
chr3:10085035	T/T	G	T	SNV	FANCD2	FANCD2:intronic:NM_033084.4	1	100		intronic	NM_03308	c.990-133	p.?	0.18					Fanconi anae	rs803335		YES			Fanconi ane	DNA polyr	Anemia;Anemia, Aplastic;Anemia, Hypoplast			G=0, T=84	G=0.0, T=1.0	0.00001	1370.17	84	G=0/0, T=41/4
chr3:10085130	G/G	C	G	SNV	FANCD2	FANCD2:intronic:NM_033084.4	1	100		intronic	NM_03308	c.990-38C	p.?	-0.34					Fanconi anae	rs9809061	0.1612	YES	6.758E-2;1.247E-4 (pos.10085130)		Fanconi ane	DNA polyr	Anemia;Anemia, Aplastic;Aner		Benign	C=0, G=86	C=0.0, G=1.0	0.00001	1410.93	86	C=0/0, G=41/4
chr3:10119917	C/C	T	C	SNV	FANCD2	FANCD2:intronic:NM_033084.4	1	100		intronic	NM_03308	c.2976+36	p.?	0.04					Fanconi anae	rs6805869	0.164	YES	6.593E-2 (pos.10119917)		Fanconi ane	DNA polyr	Anemia;Anemia, Aplastic;Aner		Benign	T=0, C=21	T=0.0, C=1.0	0.00001	281.235	21	T=0/0, C=3/18
chr8:133844508	T/T	C	T	SNV	PHF20L1	PHF20L1:exonic:NM_016018.4	1	100	15	synonymous	NM_01601	c.1773C>T	p.(=)	-0.06				0.45(COSM15	PHD finger pr	rs16904746	0.416	YES	3.336E-1 (pos.13384450	acute_myeloid leukaemia		metal ion binding,nucleus,protein binding,regulation of				C=0, T=123	C=0.0, T=1.0	0.00001	1982.15	123	C=0/0, T=66/5
chr3:10188340	T/T	TA	T	INDEL	VHL	VHL:intronic:NM_000551.3	1	100		intronic	NM_00055	c.463+20T	p.?	0.55, 1.73					VHL beta domain,VHL box domain						VHL gene	VCB compl	ACMG Recommendations...:Adenocarcinoma			TA=0, T=63	TA=0.0, T=1.0	0.00001	666.518	63	TA=0/0, T=13/
chr3:10219729	A/G	A	G	SNV	IRAK2	IRAK2:intronic:NM_001570.3	1	45.83		intronic	NM_00157	c.277+25A	p.?	-1.19					Death domai	rs1681663,rs200498	0.2253	YES	2.598E-1 (pos.10219729)		Interleukin	ATP binding,I-kappaB kinase/NF-kappaB signaling,JNK ca				A=13, G=11	A=0.5417, G=0.4583	0.00001	95.4894	24	A=3/10, G=9/2
chr3:10258762	C/C	G	C	SNV	IRAK2	IRAK2:intronic:NM_001570.3	1	100		intronic	NM_00157	c.903+30G	p.?	-0.25					Death domai	rs2302860	0.2258	YES	1.288E-1;0.0 (pos.10258762)		Interleukin	ATP binding,I-kappaB kinase/NF-kappaB signaling,JNK ca				G=0, C=32	G=0.0, C=1.0	0.00001	485.141	32	G=0/0, C=11/2
chr1:120285546	A/A	G	A	SNV	PHGDH	PHGDH:exonic:NM_006623.3	1	100	11	synonymous	NM_00662	c.1326G>A	p.(=)	-5.17				0.02(COSM17	D-isomer spe	rs543703	0.3174	YES	6.049E-1 (pos.12028554	carcinoma,nec	Phosphogly	L-malate c	Abnormalities, Mul	NADH	Benign	G=0, A=22	G=0.0, A=1.0	0.00001	298.891	22	G=0/0, A=12/1
chr3:10401856	T/T	G	T	SNV	ATP2B2	ATP2B2:intronic:NM_001001331.2	1	100		intronic	NM_00100	c.1660-49	p.?	0.22					Cation transi	rs2278554	0.3519	YES	5.445E-1 (pos.10401856)		ATPase, Ca+	ATP bindin	Ear Diseases;Hearing Disorders;Hearing Loss			G=0, T=20	G=0.0, T=1.0	0.00001	263.683	20	G=0/0, T=11/9
chr6:79708000	C/C	A	C	SNV	PHIP	PHIP:exonic:NM_017934.6	1	100	18	missense	NM_01793	c.1988T>G	p.Val663Gly	0.22	1	109	0		Bromodomai	rs7747479	0.0481	YES	9.993E-1;0.0 (pos.79708000)		Pleckstrin h	cytoskelet	Arthritis:Arthritis, Rheumatoid:Autoimmune			A=0, C=43	A=0.0, C=1.0	0.00001	705.695	43	A=0/0, C=17/2
chr3:105495432	T/T	G	T	SNV	CBLB	CBLB:intronic:NM_170662.4	1	100		intronic	NM_17066	c.420-46C	p.?	-1.01					CBL proto-on	rs9809911	1.00E-04		1.0;0.0 (pos.105495432)		CAS-BR-M m	NLS-bearir	Autoimmune Diseases;Autoimmune Disease			G=0, T=27	G=0.0, T=1.0	0.00001	389.988	27	G=0/0, T=14/1
chr3:107776511	T/T	C	T	SNV	CD47	CD47:intronic:NM_001777.3	1	100		intronic	NM_00177	c.785-95G	p.?	-0.17					CD47 immun	rs709436					CD47 antige	cell adhesion,extracellular exosome,extracellular matri				C=0, T=23	C=0.0, T=1.0	0.00001	316.747	23	C=0/0, T=14/9
chr3:107884211	C/C	T	C	SNV	IFT57	IFT57:intronic:NM_018010.3	1	100		intronic	NM_01801	c.1044+67	p.?	-1.3					Intra-flagella	rs327167	0				Intraflagell	DNA binding,Golgi apparatus,activation of cysteine-type				T=0, C=24	T=0.0, C=1.0	0.00001	334.792	24	T=0/0, C=12/1
chr3:107884269	A/G	A	G	SNV	IFT57	IFT57:intronic:NM_018010.3	1	46.43		intronic	NM_01801	c.1044+9T	p.?	-1.19					Intra-flagella	rs62264150	0.1059	YES	1.175E-1 (pos.107884269)		Intraflagell	DNA binding,Golgi apparatus,activation of cysteine-type				A=15, G=13	A=0.5357, G=0.4643	0.00001	117.927	28	A=8/7, G=4/9
chr1:114240319	TA/TA	T	TA	INDEL	PHTF1	PHTF1:utr_3:NM_006608.2	1	100	18	utr_3	NM_00660	c.2296_22	p.?	-0.49					Male germ-ce	rs113145080,rs3835	0.244	YES	3.555E-1 (pos.114240319)		Putative ho	DNA bindi	Arthritis:Arthritis, Rheumatoid:Autoimmune			T=0, TA=66	T=0.0, TA=1.0	0.00001	997.636	66	T=0/0, TA=36/
chr3:108117694	G/A	G	A	SNV	MYH15	MYH15:intronic:NM_014981.1	1	58.33		intronic	NM_01498	c.4990-7C	p.?	-0.14				0.07(COSM57	Myosin N-ter	rs13087507	0.2885	YES	7.773E-3 (pos.10811769	adenocarcinor	Myosin, hea	ATP binding,actin filament binding,calmodulin binding,e				G=25, A=35	G=0.4167, A=0.5833	0.00001	370.434	60	G=14/11, A=2(
chr3:108147295	A/A	C	A	SNV	MYH15	MYH15:intronic:NM_014981.1	1	100		intronic	NM_01498	c.3762+44	p.?	-0.05					Myosin N-ter	rs1350235	0.1335	YES	4.641E-1 (pos.108147295)		Myosin, hea	ATP binding,actin filament binding,calmodulin binding,e				C=0, A=32	C=0.0, A=1.0	0.00001	485.141	32	C=0/0, A=19/1
chr3:108678002	G/A	G	A	SNV	MORC1	MORC1:intronic:NM_014429.3	1	50		intronic	NM_01442	c.2800-35	p.?	0.37					CW-type Zinc	rs2197738,rs371614	0.349	YES	8.593E-2 (pos.108678002)		MORC famil	DNA hypermethylation,DNA methylation involved in gam				G=12, A=12	G=0.5, A=0.5	0.00001	106.71	24	G=4/8, A=6/6
chr1:151288391	G/G	A	G	SNV	PI4KB	PI4KB:exonic:NM_001198774.1	1	100	2	synonymous	NM_00119	c.567T>C	p.(=)	-0.73					Phosphatidyl	rs1752379	2.00E-04		1.0 (pos.151288391)			1-phosphatidylinositol 4-kinase activity,14-3-3 protein b				A=0, G=26	A=0.0, G=1.0	0.00001	371.417	26	A=0/0, G=18/8
chr3:108776343	T/T	C	T	SNV	MORC1	MORC1:intronic:NM_014429.3	1	100		intronic	NM_01442	c.1032-10	p.?	-0.88					CW-type Zinc	rs1031252	0.0215	YES	9.999E-1 (pos.108776343)		MORC famil	DNA hypermethylation,DNA methylation involved in gam				C=0, T=24	C=0.0, T=1.0	0.00001	334.754	24	C=0/0, T=6/18
chr3:109027157	T/T	C	T	SNV	DPPA2	DPPA2:intronic:NM_138815.3	1	100		intronic	NM_13881	c.397-17G	p.?	-0.64					Dppa2/4 con:	rs7611135	0.2735	YES	9.052E-1 (pos.109027157)		Developme	chromatin binding,lung-associated mesenchyme develo				C=0, T=75	C=0.0, T=1.0	0.00001	1189.39	75	C=0/0, T=28/4
chr3:109027820	A/A	G	A	SNV	DPPA2	DPPA2:intronic:NM_138815.3	1	100		intronic	NM_13881	c.396+53C	p.?	-0.27					Dppa2/4 con:	rs7611871	0.3231	YES			Developme	chromatin binding,lung-associated mesenchyme develo				G=0, A=103	G=0.0, A=1.0	0.00001	1581.87	103	G=0/0, A=52/5
chr3:110791113	G/G	A	G	SNV	NECTIN3	NECTIN3:intronic:NM_015480.2	1	100		intronic	NM_01548	c.160+89A	p.?	0.1					CD80-like C2-	rs4682228		YES			Nectin 3	adherens junction organization,apical junction complex				A=0, G=46	A=0.0, G=1.0	0.00001	768.262	46	A=0/0, G=10/3
chr16:88792047	G/G	A	G	SNV	PIEZO1	PIEZO1:exonic:NM_001142864.3	1	100	28	synonymous	NM_00114	c.4014T>C	p.(=)	0.32					Piezo,Piezo n	rs4782430	0.1119	YES	9.353E-1 (pos.88792047)		PIEZO1 ion c	cation cha	Anemia:Anemia, Hemolytic:An		Benign	A=0, G=82	A=0.0, G=1.0	0.00001	1327.45	82	A=0/0, G=46/3
chr3:111451603	C/C	G	C	SNV	PLCXD2	PLCXD2:intronic:NM_153268.3	1	100		intronic	NM_15326	c.866+186	p.?	-1.77						rs3821919		YES				lipid catabolic process,lipid metabolic process,nucleus,				G=0, C=23	G=0.0, C=1.0	0.00001	316.337	23	G=0/0, C=11/1
chr18:10758023	A/A	G	A	SNV	PIEZO2	PIEZO2:exonic:NM_022068.3	1	100	25	synonymous	NM_02206	c.3792C>T	p.(=)	2.22					Piezo,Piezo n	rs7407105	0.2087	YES	9.32E-1 (pos.10758023)		PIEZO-type i	cation cha	Abnormalities, Multiple;Arach		Benign	G=0, A=42	G=0.0, A=1.0	0.00001	685.214	42	G=0/0, A=13/2
chr3:111766559	G/G	T	G	SNV	TMPRSS7	TMPRSS7:intronic:NM_001042575.2	1	100		intronic	NM_00104	c.353-27T	p.?	-1					CUB domain,l	rs1688304	0.0393	YES	1.0 (pos.111766559)			integral cc	Mental Disorders;Substance-Related Disorde			T=0, G=24	T=0.0, G=1.0	0.00001	328.908	24	T=0/0, G=11/1
chr18:10762962	A/A	G	A	SNV	PIEZO2	PIEZO2:exonic:NM_022068.3	1	100	20	synonymous	NM_02206	c.3006C>T	p.(=)	-0.32					Piezo,Piezo n	rs7241380	0.2093	YES	9.132E-1 (pos.10762962)		PIEZO-type i	cation cha	Abnormalities, Multiple;Arach		Benign	G=0, A=66	G=0.0, A=1.0	0.00001	1015.37	66	G=0/0, A=23/4
chr18:10763007	C/C	T	C	SNV	PIEZO2	PIEZO2:exonic:NM_022068.3	1	100	20	synonymous	NM_02206	c.2961A>G	p.(=)	-0.22					Piezo,Piezo n	rs7227167	0.2068	YES	9.341E-1 (pos.10763007)		PIEZO-type i	cation cha	Abnormalities, Multiple;Arach		Benign	T=0, C=71	T=0.0, C=1.0	0.00001	1111.48	71	T=0/0, C=23/4
chr18:10787047	A/A	G	A	SNV	PIEZO2	PIEZO2:exonic:NM_022068.3	1	100	16	synonymous	NM_02206	c.2305C>T	p.(=)	-1.39					Piezo,Piezo n	rs6505592	0.15	YES	9.556E-1 (pos.10787047)		PIEZO-type i	cation cha	Abnormalities, Multiple;Arach		Benign	G=0, A=27	G=0.0, A=1.0	0.00001	389.987	27	G=0/0, A=14/1
chr3:111958862	A/A	C	A	SNV	SLC9C1	SLC9C1:intronic:NM_183061.2	1	100		intronic	NM_18306	c.1280-9G	p.?	-0.59				0.03(COSM40	Cyclic nucleo	rs13089635	0.3466	YES	4.16E-1 (pos.111958862)	acute_myeloid leukaemia		cell differentiation,flagellated sperm motility,hydrogen				C=0, A=143	C=0.0, A=1.0	0.00001	2383.51	143	C=0/0, A=55/8
chr3:111981704	T/T	C	T	SNV	SLC9C1	SLC9C1:intronic:NM_183061.2	1	100		intronic	NM_18306	c.1197+67	p.?	-1.49					Cyclic nucleo	rs201088102,rs5003791,rs6689		YES				cell differentiation,flagellated sperm motility,hydrogen				C=0, T=113	C=0.0, T=1.0	0.00001	1799.51	113	C=0/0, T=47/6
chr3:111993716	A/A	G	A	SNV	SLC9C1	SLC9C1:intronic:NM_183061.2	1	100		intronic	NM_18306	c.613+28C	p.?	0.14					Cyclic nucleo	rs6805112	0.2438	YES	9.883E-1 (pos.111993716)			cell differentiation,flagellated sperm motility,hydrogen				G=0, A=34	G=0.0, A=1.0	0.00001	524.185	34	G=0/0, A=24/1
chr3:111997732	T/TCATTTATT	T	TCATTTATT	INDEL	SLC9C1	SLC9C1:intronic:NM_183061.2	8	50		intronic	NM_18306	c.190-28A	p.?	0.24					Cyclic nucleotide-binding domain,Ion transport protein,Sodium/hydrogen exchanger family							cell differentiation,flagellated sperm motility,hydrogen				T=18, TCAT	T=0.5, TCATTTATT=0.!	0.00001	185.885	36	T=8/10, TCATT
chr3:112052173	C/C	T	C	SNV	CD200,LOC105374042	CD200:intronic:NM_001004196.3, LOC105374042:downstr	1	100		intronic	NR_14741	c.12+102T	p.?	-0.23					Immunoglobu	rs9883527		YES				cell adhesion molecule binding,cell recognition,cell-cell				T=0, C=51	T=0.0, C=1.0	0.00001	754.584	51	T=0/0, C=29/2
chr14:68066949	A/A	G	A	SNV	PIGH	PIGH:utr_5:NM_004569.3	1	100	1	utr_5	NM_00456	c.-29C>T	p.?	-0.46					GPI-GlcNAc tr	rs11547225	0.2618	YES	2.736E-1 (pos.68066949)		Phosphatid	GPI ancho	Breast Diseases:Breast Neoplasms:Neoplasr			G=0, A=45	G=0.0, A=1.0	0.00001	747.392	45	G=0/0, A=24/2
chr3:112267269	C/C	T	C	SNV	ATG3	ATG3:intronic:NM_022488.4	1	100		intronic	NM_02248	c.343+111	p.?	-0.27					Autophagocy	rs7625366		YES				Atg12 transferase activity,Atg8 ligase activity,autophagc				T=0, C=20	T=0.0, C=1.0	0.00001	263.786	20	T=0/0, C=13/7
chr3:112267523	A/A	G	A	SNV	ATG3	ATG3:intronic:NM_022488.4	1	100		intronic	NM_02248	c.236-36C	p.?	0.26					Autophagocy	rs768872909,rs9809	0.0841	YES	0.0 (pos.112267522),7.501E-1 (pos.112267523)			Atg12 transferase activity,Atg8 ligase activity,autophagc				G=0, A=89	G=0.0, A=1.0	0.00001	1472.49	89	G=0/0, A=45/4
chr3:112280218	A/A	G	A	SNV	ATG3,SLC35A5	ATG3:intronic:NM_022488.4, SLC35A5:upstream:NM_0179	1	100		intronic	NM_02248	c.72+86C>	p.?, p.?	-0.8					Autophagocy	rs2279532		YES				Atg12 transferase activity,Atg8 ligase activity,Golgi mem				G=0, A=22	G=0.0, A=1.0	0.00001	298.889	22	G=0/0, A=9/13
chr14:68066979	T/T	C	T	SNV	PIGH	PIGH:utr_5:NM_004569.3	1	100	1	utr_5	NM_00456	c.-59G>A	p.?	-0.99					GPI-GlcNAc tr	rs7154825		YES			Phosphatid	GPI ancho	Breast Diseases:Breast Neoplasms:Neoplasr			C=0, T=44	C=0.0, T=1.0	0.00001	726.569	44	C=0/0, T=23/2
chr1:77634948	A/A	G	A	SNV	PIGK	PIGK:exonic:NM_005482.2	1	100	4	synonymous	NM_00548	c.372C>T	p.(=)	1.59					Peptidase C1	rs1779199	1.00E-04		1.0 (pos.77634948)		Phosphatid	GPI anchor binding,GPI-anchor transamidase activity,GP				G=0, A=21	G=0.0, A=1.0	0.00001	281.181	21	G=0/0, A=8/13
chr18:59805532	C/C	T	C	SNV	PIGN	PIGN:splicesite_3:NM_176787.4	1	100	14	splicesite	NM_17678	c.1117-1A	p.?	4.13					Phosphatidyl	rs9959632		YES	1.0 (pos.59805532)		Phosphatid	GPI anchor biosynthetic process,cytosol,endoplasmic re				T=0, C=20	T=0.0, C=1.0	0.00001	263.711	20	T=0/0, C=9/11
chr3:112666859	T/T	C	T	SNV	CD200R1	CD200R1:intronic:NM_138806.3	1	100		intronic	NM_13880	c.68-88G>	p.?	-0.23					CD80-like C2-	rs2133946		YES			CD200 rece	external side of plasma membrane,extracellular region,				C=0, T=20	C=0.0, T=1.0	0.00001	263.786	20	C=0/0, T=3/17
chr18:59814268	A/A	G	A	SNV	PIGN	PIGN:exonic:NM_176787.4	1	100	9	synonymous	NM_17678	c.741C>T	p.(=)	-0.86				0.01(COSM37	Phosphatidyl	rs9320000	0.3784	YES	6.685E-1 (pos.59814268)	adenocarcinor	Phosphatid	GPI anchor biosynthetic process,cytosol,e			Benign	G=0, A=40	G=0.0, A=1.0	0.00001	641.864	40	G=0/0, A=10/3
chr18:59814324	C/C	G	C	SNV	PIGN	PIGN:exonic:NM_176787.4	1	100	9	missense	NM_17678	c.685C>G	p.His229Asp	1.77	1	81	0	0.03(COSM54	Phosphatidyl	rs9320001	0.2473	YES	9.052E-1 (pos.59814324)	acute_myeloic	Phosphatid	GPI anchor biosynthetic process,cytosol,e			Benign	G=0, C=20	G=0.0, C=1.0	0.00001	263.785	20	G=0/0, C=8/12
chr21:38444863	T/T	C	T	SNV	PIGP,TTC3	PIGP:exonic:NM_153681.2, TTC3:upstream:NM_001001894	1	100	1	missense	NM_15368	c.25G>A, c	p.Ala9Thr, p.?	0.65	1	58	0	0(COSM4591	PIG-P	rs2507733		YES	1.0 (pos.38444863)	squamous_cel	Phosphatid	GPI ancho	Abnormalities, Multiple;Chromosome Disord			C=0, T=38	C=0.0, T=1.0	0.00001	603.78	38	C=0/0, T=17/2
chr21:38444890	G/G	A	G	SNV	PIGP,TTC3	PIGP:utr_5:NM_153681.2, TTC3:upstream:NM_001001894.	1	100	1	utr_5	NM_15368	c.-3T>C, c.-	p.?, p.?	0.67					PIG-P	rs2298682	0.4969	YES	4.007E-1 (pos.38444890)		Phosphatid	GPI ancho	Abnormalities, Multiple;Chromosome Disord			A=0, G=38	A=0.0, G=1.0	0.00001	603.656	38	A=0/0, G=17/2
chr3:113720624	T/T	C	T	SNV	CCDC191	CCDC191:intronic:NM_020817.1	1	100		intronic	NM_02081	c.2062-81	p.?	-0.53						rs6438178										C=0, T=48	C=0.0, T=1.0	0.00001	810.415	48	C=0/0, T=18/3
chr1:207110936	C/C	T	C	SNV	PIGR	PIGR:exonic:NM_002644.3	1	100	4	synonymous	NM_00264	c.549A>G	p.(=)	-1.4				0.02(COSM55	Immunoglobu	rs291096	0.4677	YES	4.369E-1 (pos.20711093	carcinoma	Polymeric in	Fc recepto	Autoimmune Diseases:Female Urogenital Dis			T=0, C=34	T=0.0, C=1.0	0.00001	524.186	34	T=0/0, C=22/1
chr20:44052992	A/A	G	A	SNV	PIGT	PIGT:exonic:NM_015937.5	1	100	10	synonymous	NM_01593	c.1371G>A	p.(=)	-1.28				0.05(COSM14	Gpi16 subuni	rs13217	0.4836	YES	2.536E-1 (pos.44052992)	adenocarcinor	Phosphatid	GPI-ancho	Anemia:Anemia, Hemolytic:Bone Marrow Dis			G=0, A=25	G=0.0, A=1.0	0.00001	353.019	25	G=0/0, A=9/16

chr3:196674749	T/T	C	T	SNV	PIGZ	PIGZ:exonic:NM_025163.3	1	100	3	missense	NM_02516	c.1019G>	p.Arg340Gln	1.39	0.29	43	0	0.09(COSM41	Alg9-like mar	rs4916589	0.4155	YES	8.062E-1 (pos.19667474	ductal_carcinc	Phosphatid	GPI anchor biosynthetic process,alpha-1,2-mannosyltra				C=0, T=34	C=0.0, T=1.0	0.00001	524.185	34	C=0/0, T=24/1
chr19:49949652	A/A	G	A	SNV	PIH1D1	PIH1D1:exonic:NM_017916.2	1	100	9	missense	NM_01791	c.860C>T	p.Pro287Leu	1.15	0.1	98	0		pre-RNA proc	rs7462	0.3366	YES	2.429E-3 (pos.49949652)		PIH1 domai	ATPase binding,R2TP complex,RNA polymerase I CORE ele				G=0, A=26	G=0.0, A=1.0	0.00001	371.415	26	G=0/0, A=8/18
chr19:49950298	T/T	C	T	SNV	PIH1D1	PIH1D1:exonic:NM_017916.2	1	100	7	missense	NM_01791	c.670G>A	p.Val224Ile	0.16	1	29	0	0.08(COSM17	pre-RNA proc	rs13394	0.2329	YES	3.985E-1 (pos.49950298)	neoplasm,trar	PIH1 domai	ATPase binding,R2TP complex,RNA polymerase I CORE ele				C=0, T=23	C=0.0, T=1.0	0.00001	316.746	23	C=0/0, T=16/7
chr3:11744584	T/T	C	T	SNV	VGLL4		1	100		intronic				0.1						rs6781438		YES								C=0, T=30	C=0.0, T=1.0	0.00001	446.635	30	C=0/0, T=9/21
chr19:49950356	G/G	C	G	SNV	PIH1D1	PIH1D1:exonic:NM_017916.2	1	100	7	synonymous	NM_01791	c.612G>C	p.(=)	0.16				0.04(COSM11	pre-RNA proc	rs12443	0.2331	YES	3.856E-1 (pos.49950356)	ductal_carcinc	PIH1 domai	ATPase binding,R2TP complex,RNA polymerase I CORE ele				C=0, G=24	C=0.0, G=1.0	0.00001	334.657	24	C=0/0, G=15/9
chr3:118909962	C/C	T	C	SNV	UPK1B	UPK1B:intronic:NM_006952.3	1	100		intronic	NM_00695	c.468+11T	p.?	-2.92					Tetraspanin f	rs3796356	0.033	YES	9.217E-1 (pos.118909962)		Uroplakin 1	apical plasma membrane,cell surface receptor signaling				T=0, C=30	T=0.0, C=1.0	0.00001	446.638	30	T=0/0, C=9/21
chr1:204403659	G/G	A	G	SNV	PIK3C2B	PIK3C2B:exonic:NM_002646.3	1	100	25	synonymous	NM_00264	c.3594T>C	p.(=)	-1.02				0.05(COSM37	C2 domain,PI	rs3747636	0.2181	YES	5.213E-1 (pos.20440365	adenocarcinoi	Phosphatid	1-phospha	Neoplasm Metastasis:Neoplasms:Neoplastic			A=0, G=285	A=0.0, G=1.0	0.00001	4784.93	285	A=0/0, G=136/
chr12:18435398	G/G	GCCC	G	INDEL	PIK3C2G	PIK3C2G:exonic:NM_004570.5	3	100	2	nonframeshiftDeletion	NM_00457	c.385_387	p.Pro129del	1.09, -0.5, 0.76, 1.24					C2 domain,PI	rs35277916,rs75736	0.3992	YES	0.0 (pos.18435397),0.0 (	astrocytoma_(	Phosphatid	1-phosphatidylinositol-3-kinase activity,1-phosphatidyli				GCCC=0, G	GCCC=0.0, G=1.0	0.00001	629.632	39	GCCC=0/0, G=
chr12:18435452	T/T	C	T	SNV	PIK3C2G	PIK3C2G:exonic:NM_004570.5	1	100	2	missense	NM_00457	c.437C>T	p.Pro146Leu	0.4	0.2	98	0.002	0.08(COSM39	C2 domain,PI	rs11044004	0.3993	YES	2.717E-1 (pos.18435452)	acute_myeloic	Phosphatid	1-phosphatidylinositol-3-kinase activity,1-phosphatidyli				C=0, T=40	C=0.0, T=1.0	0.00001	644.278	40	C=0/0, T=17/2
chr3:119529113	T/T	G	T	SNV	NR1I2	NR1I2:intronic:NM_022002.2	1	100		intronic	NM_02200	c.448+72G	p.?	-1.19					Ligand-bindir	rs3732356		YES			Nuclear rec	DNA bindi	Adenocarcinoma;B	Docetaxel,Erlotinib,Estra		G=0, T=70	G=0.0, T=1.0	0.00001	1092.09	70	G=0/0, T=22/4
chr3:119533773	C/C	T	C	SNV	NR1I2	NR1I2:intronic:NM_022002.2	1	100		intronic	NM_02200	c.912-53T	p.?	-0.04					Ligand-bindir	rs6797879		YES			Nuclear rec	DNA bindi	Adenocarcinoma;B	Docetaxel,Erlotinib,Estra		T=0, C=20	T=0.0, C=1.0	0.00001	263.786	20	T=0/0, C=5/15
chr3:178927410	A/G	A	G	SNV	PIK3CA	PIK3CA:exonic:NM_006218.3	1	47.62	7	missense	NM_00621	c.1173A>G	p.Ile391Met	1.3	0.5	10	0.011	0.56(COSM32	PI3-kinase fa	rs2230461	0.068	YES	0.0 (pos.178927410)	adenocarcinoi	Phosphatid	1-phospha	Abnormalities, Mul	(1S,6BR,9/	Benign	A=22, G=20	A=0.5238, G=0.4762	0.00001	212.953	42	A=17/5, G=18/
chr7:106508057	C/T	C	T	SNV	PIK3CG	PIK3CG:exonic:NM_002649.3	1	58.33	2	synonymous	NM_00264	c.51C>T	p.(=)	0.22				0.14(COSM50	PI3-kinase fa	rs3729673	0.0451	YES	0.0 (pos.106508057)	haemangiobla	Phosphatid	1-phospha	Autistic Disorder;C	(1S,6BR,9AS,11R,11BR)-9		C=10, T=14	C=0.4167, T=0.5833	0.00001	135.314	24	C=5/5, T=6/8
chr19:18279638	C/C	T	C	SNV	PIK3R2	PIK3R2:exonic:NM_005027.3	1	100	15	synonymous	NM_00502	c.1911T>C	p.(=)	-2.83				0.08(COSM41	Phosphatidyl	rs273269	0.1137	YES	9.309E-1 (pos.18279638)	neoplasm	Phosphatid	1-phospha	Brain Diseases;Cen	Isoprenaline,SF1126		T=0, C=33	T=0.0, C=1.0	0.00001	491.996	33	T=0/0, C=7/26
chr3:119904125	G/G	A	G	SNV	GPR156	GPR156:intronic:NM_153002.2	1	100		intronic	NM_15300	c.696+21T	p.?	-1.7					7 transmemb	rs902791	0.0834	YES	9.161E-1 (pos.119904125)		G protein-c	G-protein coupled GABA receptor activity,G-protein coup				A=0, G=39	A=0.0, G=1.0	0.00001	623.98	39	A=0/0, G=15/2
chr3:119904315	C/C	G	C	SNV	GPR156	GPR156:intronic:NM_153002.2	1	100		intronic	NM_15300	c.601-74C	p.?	-1.27					7 transmemb	rs902792		YES			G protein-c	G-protein coupled GABA receptor activity,G-protein coup				G=0, C=35	G=0.0, C=1.0	0.00001	543.902	35	G=0/0, C=4/31
chr3:119905641	A/A	G	A	SNV	GPR156	GPR156:intronic:NM_153002.2	1	100		intronic	NM_15300	c.475-11C	p.?	0.84					7 transmemb	rs2319542	0.0835	YES	9.161E-1 (pos.119905641)		G protein-c	G-protein coupled GABA receptor activity,G-protein coup				G=0, A=41	G=0.0, A=1.0	0.00001	664.677	41	G=0/0, A=18/2
chr3:120371414	A/A	G	A	SNV	HGD	HGD:intronic:NM_000187.3	1	100		intronic	NM_00018	c.342+25C	p.?	0.3					homogentisa	rs2551607	0.1029	YES	9.827E-1 (pos.120371414)		Homogenti	L-phenylal	Alkaptonuria:Amino Acid Metabolism, Inborn			G=0, A=46	G=0.0, A=1.0	0.00001	767.914	46	G=0/0, A=29/1
chr17:8725214	AG/AG	A	AG	INDEL	PIK3R6	PIK3R6:exonic:NM_001010855.3	1	100	18	unknown	NM_001010855.3			4.81					Phosphoinos	rs397775164,rs56632	9.00E-04		1.0 (pos.8725214)	basal_cell_car	Phosphatid	1-phosphatidylinositol-3-kinase regulator activity,G-pro				A=0, AG=94	A=0.0, AG=1.0	0.00001	916.913	94	A=0/0, AG=46/
chr3:120833993	C/T	C	T	SNV	STXBP5L	STXBP5L:intronic:NM_014980.2	1	56		intronic	NM_01498	c.605+87C	p.?	-0.79					LLGL2,Lethal	rs16832111		YES			Syntaxin-bi	GTPase activator activity,Rab GTPase binding,cytoplasm				C=11, T=14	C=0.44, T=0.56	0.00001	134.35	25	C=9/2, T=4/10
chr3:121132268	A/A	T	A	SNV	STXBP5L	STXBP5L:intronic:NM_014980.2	1	100		intronic	NM_01498	c.3186+98	p.?	0.7					LLGL2,Lethal	rs9849118		YES			Syntaxin-bi	GTPase activator activity,Rab GTPase binding,cytoplasm				T=0, A=23	T=0.0, A=1.0	0.00001	316.744	23	T=0/0, A=7/16
chr2:209191082	A/A	C	A	SNV	PIKFYVE	PIKFYVE:exonic:NM_015040.3	1	100	20	missense	NM_01504	c.3547C>A	p.Gln1183Lys	2.57	1	53	0		Domain foun	rs1529979	0.0184	YES	9.43E-1 (pos.209191082)		Phosphatid	'de novo' p	Congenital, Hereditary, and Ne		Benign	C=0, A=43	C=0.0, A=1.0	0.00001	705.678	43	C=0/0, A=21/2
chr2:209191099	C/C	T	C	SNV	PIKFYVE	PIKFYVE:exonic:NM_015040.3	1	100	20	synonymous	NM_01504	c.3564T>C	p.(=)	-0.63					Domain foun	rs1529978	1.00E-04		1.0 (pos.209191099)		Phosphatid	'de novo' p	Congenital, Hereditary, and Ne		Benign	T=0, C=46	T=0.0, C=1.0	0.00001	768.29	46	T=0/0, C=21/2
chr3:121353289	T/TA	T	TA	INDEL	HCLS1	HCLS1:intronic:NM_005335.5	1	57.14		intronic	NM_00533	c.692-24A	p.?	0.45					Repeat in HS1/Cortactin,SH3 domain,Variant SH3 domain						Hematopoi	RNA polym	Arthritis;Arthritis, Rheumatoid;Autoimmune			T=15, TA=2	T=0.4286, TA=0.5714	0.00001	164.228	35	T=3/12, TA=2/
chr3:121526037	T/G	T	G	SNV	IQCB1	IQCB1:intronic:NM_001023570.3	1	57.14		intronic	NM_00102	c.587+154	p.?	-1.02					IQ calmoduli	rs4298081		YES			IQ motif-cor	calmoduli	Congenital, Hereditary, and Neonatal Diseas			T=9, G=12	T=0.4286, G=0.5714	0.00001	109.102	21	T=5/4, G=3/9
chr2:209214770	G/G	A	G	SNV	PIKFYVE	PIKFYVE:exonic:NM_015040.3	1	100	36	synonymous	NM_01504	c.5397A>G	p.(=)	1.69					Domain foun	rs2118297	0.0184	YES	9.433E-1 (pos.209214770)		Phosphatid	'de novo' p	Congenital, Hereditary, and Ne		Benign	A=0, G=23	A=0.0, G=1.0	0.00001	316.724	23	A=0/0, G=9/14
chr2:209215586	G/G	A	G	SNV	PIKFYVE	PIKFYVE:exonic:NM_015040.3	1	100	37	synonymous	NM_01504	c.5526A>G	p.(=)	-0.07					Domain foun	rs994697	0.0184	YES	9.435E-1 (pos.209215586)		Phosphatid	'de novo' p	Congenital, Hereditary, and Ne		Benign	A=0, G=81	A=0.0, G=1.0	0.00001	1309.48	81	A=0/0, G=34/4
chr7:99971834	G/G	A	G	SNV	PILRA	PILRA:exonic:NM_013439.2	1	100	2	missense	NM_01343	c.232A>G	p.Arg78Gly	1.32	1	125	0		Immunoglob	rs1859788	0.3097	YES	3.842E-1;0.0 (pos.99971834)		Paired imm	MHC class I protein binding,extracellular exosome,integ				A=0, G=59	A=0.0, G=1.0	0.00001	883.967	59	A=0/0, G=23/3
chr3:121836973	G/G	GAG	G	INDEL	CD86	CD86:intronic:NM_175862.4	2	100		intronic	NM_17586	c.893+33G	p.?	0.42, 2.06, 0.24					Immunoglob	rs932089800					CD86 antige	B cell activ	Autoimmune Disea	Abatacept,Antithymocyt		GAG=0, G=	GAG=0.0, G=1.0	0.00001	298.892	22	GAG=0/0, G=8,
chr1:20977221	T/T	A	T	SNV	PINK1,PINK1-AS	PINK1:utr_3:NM_032409.2, PINK1-AS:exonic_nc:NR_046507	1	100	8	utr_3	NM_03240	c.1783A>T	p.?	-1.14					Protein kinas	rs686658	0.1207	YES	8.094E-1 (pos.20977221)		PTEN-induc	ATP bindin	Basal Ganglia Diseases:Brain D		Benign	A=0, T=47	A=0.0, T=1.0	0.00001	760.335	47	A=0/0, T=27/2
chr11:67263767	G/G	A	G	SNV	PITPNM1	PITPNM1:exonic:NM_004910.2	1	100	15	synonymous	NM_00491	c.2199T>C	p.(=)	-3.65				0.03(COSM41	DDHD domai	rs618633	0.0024	YES	9.799E-1 (pos.67263767)	neoplasm		Golgi ciste	Mental Disorders:Schizophrenia:Schizophren			A=0, G=38	A=0.0, G=1.0	0.00001	603.786	38	A=0/0, G=15/2
chr10:3200292	A/A	G	A	SNV	PITRM1	PITRM1:exonic:NM_001242307.1	1	100	11	missense	NM_00124	c.1190C>T	p.Ala397Val	5.41	0.14	64	0.005		Insulinase (P	rs3182535	0.2944	YES	5.921E-1 (pos.3200292)			catalytic activity,enzyme activator activity,metal ion bin				G=0, A=26	G=0.0, A=1.0	0.00001	371.417	26	G=0/0, A=13/1
chr10:3206027	G/G	A	G	SNV	PITRM1	PITRM1:exonic:NM_001242307.1	1	100	7	synonymous	NM_00124	c.681T>C	p.(=)	-0.62				0.2(COSM576	Insulinase (P	rs4609511	0.2787	YES	5.946E-1;0.0 (pos.320602	adenocarcinoma		catalytic activity,enzyme activator activity,metal ion bin				A=0, G=20	A=0.0, G=1.0	0.00001	263.786	20	A=0/0, G=14/6
chr3:122446642	C/T	C	T	SNV	PARP14	PARP14:intronic:NM_017554.2	1	56		intronic	NM_01755	c.4942-17	p.?	-0.09					Macro domai	rs2303883	0.1506	YES	1.226E-1 (pos.122446642)		Poly(ADP-ril	NAD+ ADP-ribosyltransferase activity,NAD+ binding,RNA				C=11, T=14	C=0.44, T=0.56	0.00001	134.318	25	C=11/0, T=8/6
chr3:122807995	A/A	G	A	SNV	PDIA5	PDIA5:intronic:NM_006810.3	1	100		intronic	NM_00681	c.43-20G>	p.?	-0.35					AhpC/TSA fan	rs772829092,rs8368	0.3915	YES	0.0;0.0 (pos.122807994),4.848E-1 (pos.1		Protein dis	IRE1-mediated unfolded protein response,cell,cell redox				G=0, A=57	G=0.0, A=1.0	0.00001	849.507	57	G=0/0, A=26/3
chr3:122843110	T/T	G	T	SNV	PDIA5	PDIA5:intronic:NM_006810.3	1	100		intronic	NM_00681	c.702-9G>	p.?	-4.5				0.03(COSM44	AhpC/TSA fan	rs861375	0.2129	YES	5.042E-1 (pos.12284311	acute_myeloic	Protein dis	IRE1-mediated unfolded protein response,cell,cell redox				G=0, T=91	G=0.0, T=1.0	0.00001	1499.08	91	G=0/0, T=50/4
chr3:122843212	T/T	C	T	SNV	PDIA5	PDIA5:intronic:NM_006810.3	1	100		intronic	NM_00681	c.773+22C	p.?	-1.65					AhpC/TSA fan	rs2305051	0.4167	YES	4.118E-1;0.0 (pos.122843212)		Protein dis	IRE1-mediated unfolded protein response,cell,cell redox				C=0, T=101	C=0.0, T=1.0	0.00001	1579.75	101	C=0/0, T=42/5
chr3:122869041	T/T	C	T	SNV	PDIA5	PDIA5:intronic:NM_006810.3	1	100		intronic	NM_00681	c.1143-40	p.?	-0.64					AhpC/TSA fan	rs11720822	0.0734	YES	3.577E-4 (pos.122869041)		Protein dis	IRE1-mediated unfolded protein response,cell,cell redox				C=0, T=22	C=0.0, T=1.0	0.00001	294.669	22	C=0/0, T=15/7
chr3:122869053	A/A	G	A	SNV	PDIA5	PDIA5:intronic:NM_006810.3	1	100		intronic	NM_00681	c.1143-28	p.?	-0.09					AhpC/TSA fan	rs11720885	0.0754	YES	3.577E-4;0.0 (pos.122869053)		Protein dis	IRE1-mediated unfolded protein response,cell,cell redox				G=0, A=24	G=0.0, A=1.0	0.00001	334.759	24	G=0/0, A=15/9
chr3:123037047	C/C	A	C	SNV	ADCY5	ADCY5:intronic:NM_183357.2	1	100		intronic	NM_18335	c.2257-83	p.?	-0.34					Adenylate an	rs4327330					Adenylate c	ATP bindin	Bone Diseases:Bon	2',5'-DIDEOXY-ADENOSINE		A=0, C=36	A=0.0, C=1.0	0.00001	563.744	36	A=0/0, C=24/1
chr10:3180227	T/C	T	C	SNV	PITRM1	PITRM1:exonic:NM_001242307.1	1	97.06	27	missense	NM_00124	c.3113A>G	p.Gln1038Arg	0.77	1	43	0	0.09(COSM41	Insulinase (P	rs6901	0.2898	YES	7.596E-1 (pos.3180227)	acute_myeloid_leukaemia		catalytic activity,enzyme activator activity,metal ion bin				T=1, C=33	T=0.0294, C=0.9706	0.00001	492.881	34	T=1/0, C=23/1
chr10:3180298	C/T	C	T	SNV	PITRM1	PITRM1:exonic:NM_001242307.1	1	57.58	27	synonymous	NM_00124	c.3042G>	p.(=)	2.02				0.87(COSM56	Insulinase (P	rs10175,rs75042885	0.2717	YES	1.812E-1 (pos.3180298)	ductal_carcinoma		catalytic activity,enzyme activator activity,metal ion bin				C=14, T=19	C=0.4242, T=0.5758	0.00001	204.394	33	C=10/4, T=12/
chr10:3189380	T/T	C	T	SNV	PITRM1,PITRM1-AS1	PITRM1:exonic:NM_001242307.1, PITRM1-AS1:exonic_nc:NI	1	100	20	synonymous	NM_00124	c.2301G>	p.(=)	-0.49				0.1(COSM399	Insulinase (P	rs3740607	0.4921	YES	6.891E-1 (pos.3189380)	acute_myeloid_leukaemia		catalytic activity,enzyme activator activity,metal ion bin				C=0, T=22	C=0.0, T=1.0	0.00001	298.892	22	C=0/0, T=7/15
chr12:130839165	G/A	G	A	SNV	PIWIL1	PIWIL1:exonic:NM_004764.4	1	50	10	synonymous	NM_00476	c.1128G>	p.(=)	-0.03				0.06(COSM37	Argonaute lir	rs10848087	0.1929	YES	1.069E-1 (pos.13083916	acute_myeloic	Piwi. Droso	P granule,	Mental Disorders:Substance-Related Disorde			G=13, A=13	G=0.5, A=0.5	0.00001	119.162	26	G=7/6, A=9/4
chr22:25145453	T/T	C	T	SNV	PIWIL3	PIWIL3:exonic:NM_001008496.3	1	100	11	missense	NM_00100	c.1252G>	p.Val418Met	1.78	1	21	0		PAZ domain,F	rs1892723	0.085	YES	9.369E-1 (pos.25145453)		PIWI-like 3	RNA bindir	Genital Diseases, Male;Infertility;Infertility, I			C=0, T=108	C=0.0, T=1.0	0.00001	1700.01	108	C=0/0, T=76/3
chr3:123652476	A/A	G	A	SNV	CCDC14	CCDC14:intronic:NM_022757.4	1	100		intronic	NM_02275	c.1447+44	p.?	0.92					Coiled-coil dc	rs6784695	0.2379	YES	9.041E-1 (pos.123652476)		Coiled-coil	centriolar satellite,centrosome,protein binding,protein				G=0, A=23	G=0.0, A=1.0	0.00001	313.857	23	G=0/0, A=10/1
chr3:124113929	T/T	A	T	SNV	KALRN	KALRN:intronic:NM_001024660.4	1	100		intronic	NM_00102	c.1957-53	p.?	-0.03					CRAL/TRIO do	rs482285	0.1622	YES			Kalirin	ATP binding,G-protein coupled receptor signaling pathwa				A=0, T=35	A=0.0, T=1.0	0.00001	543.903	35	A=0/0, T=17/1
chr7:47913579	C/C	T	C	SNV	PKD1L1	PKD1L1:exonic:NM_138295.4	1	100	24	missense	NM_13829	c.3814A>G	p.Lys1272Glu	-1.96	1	56	0		PKD domain,	rs1470859,rs386712	0.1722	YES	9.813E-1 (pos.47913579)		Polycystin 1	calcium channel activity,calcium channel complex,calciu				T=0, C=26	T=0.0, C=1.0	0.00001	370.783	26	T=0/0, C=10/1
chr16:81142257	C/C	T	C	SNV	PKD1L2	PKD1L2:exonic:NM_052892.3	1	100	43	synonymous	NM_05289	c.7127A>G	p.(=)	-0.05					Ion transport	rs1869348	0.4581	YES	6.427E-1 (pos.81142257)		Polycystin 1	calcium channel activity,calcium ion binding,calcium ior				T=0, C=26	T=0.0, C=1.0	0.00001	371.409	26	T=0/0, C=17/9
chr3:124692785	T/T	C	T	SNV	HEG1	HEG1:intronic:NM_020733.1	1	100		intronic	NM_02073	c.3822-36	p.?	1.57					Calcium-bind	rs2270779	0.4053	YES	7.888E-1 (pos.124692785)		Heart of gla	calcium io	Ear Diseases;Hearing Disorders;Hearing Loss			C=0, T=72	C=0.0, T=1.0	0.00001	1130.95	72	C=0/0, T=21/5
chr16:81145807	G/G	C	G	SNV	PKD1L2	PKD1L2:exonic:NM_052892.3	1	100	41	missense	NM_05289	c.6944G>C	p.Met2314Ile	0.11		10			Ion transport	rs8054182	0.0055	YES	7.827E-1 (pos.81145807)		Polycystin 1	calcium channel activity,calcium ion binding,calcium ior				C=0, G=63	C=0.0, G=1.0	0.00001	958.911	63	C=0/0, G=27/3
chr16:81151122	C/C	A	C	SNV	PKD1L2	PKD1L2:exonic:NM_052892.3	1	100	40	missense	NM_05289	c.6626T>G	p.Ser2208Arg	1.91		110			Ion transport	rs4889238	0		1.0 (pos.81151122)		Polycystin 1	calcium channel activity,calcium ion binding,calcium ior				A=0, C=32	A=0.0, C=1.0	0.00001	485.09	32	A=0/0, C=20/1
chr16:81161578	A/A	G	A	SNV	PKD1L2	PKD1L2:exonic:NM_052892.3	1	100	36	missense	NM_05289	c.6137C>T	p.Pro2046Leu	6.63		98			Ion transport	rs7206183	0.3083	YES	5.296E-1 (pos.81161578)		Polycystin 1	calcium channel activity,calcium ion binding,calcium ior				G=0, A=67	G=0.0, A=1.0	0.00001	1005.2	67	G=0/0, A=28/3
chr16:81185416	G/G	A	G	SNV	PKD1L2	PKD1L2:exonic:NM_052892.3	1	100	27	synonymous	NM_05289	c.4509T>C	p.(=)	0.92						rs8061038	0.0011	YES	9.998E-1 (pos.81185416)		Polycystin 1	calcium channel activity,calcium ion binding,calcium ior				A=0, G=85	A=0.0, G=1.0	0.00001	1360.69	85	A=0/0, G=48/3
chr16:81181821	T/C	T	C	SNV	PKD1L2	PKD1L2:exonic:NM_052892.3	1	56	29	missense	NM_05289	c.4895A>G	p.Asp1632Gly	0.15		94	0(COSM4129390)			rs9921812	0.0565	YES	1.303E-1 (pos.81181821)	neoplasm	Polycystin 1	calcium channel activity,calcium ion binding,calcium ior				T=11, C=14	T=0.44, C=0.56	0.00001	133.957	25	T=3/8, C=6/8
chr16:81232275	G/A	G	A	SNV	PKD1L2	PKD1L2:exonic:NM_052892.3	1	50	7	missense	NM_05289	c.1535C>T	p.Pro512Leu	1.93		98			Galactose bii	rs7205673	0.2073	YES	9.566E-1 (pos.81232275)		Polycystin 1	calcium channel activity,calcium ion binding,calcium ior				G=38, A=38	G=0.5, A=0.5	0.00001	394.963	76	G=19/19, A=2
chr16:72011181	T/T	G	T	SNV	PKD1L3	PKD1L3:exonic:NM_181536.1	1	100	11	missense	NM_18153	c.1713C>A	p.His571Gln	0.74		24		0.11(COSM97	GPCR proteol	rs1559401	0.2153	YES	6.977E-1;0.0 (pos.72011	endometrioid	Polycystin 1	calcium channel activity,calciu		Calcium Citrate,Calcium		G=0, T=94	G=0.0, T=1.0	0.00001	1576.09	94	G=0/0, T=55/3
chr16:72011193	C/C	A	C	SNV	PKD1L3	PKD1L3:exonic:NM_181536.1	1	100	11	synonymous	NM_18153	c.1701T>G	p.(=)	-1.76				0.01(COSM97	GPCR proteol	rs1559400	0.2153	YES	6.994E-1 (pos.72011193)	adenocarcinoi	Polycystin 1	calcium channel activity,calciu		Calcium Citrate,Calcium		A=0, C=96	A=0.0, C=1.0	0.00001	1615.78	96	A=0/0, C=55/4
chr16:71967927	C/T	C	T	SNV	PKD1L3	PKD1L3:exonic:NM_181536.1	1	53.49	28	missense	NM_18153	c.4711G>	p.Arg1570His	0.14		29		0.04(COSM44	GPCR proteol	rs17358402	0.2747	YES	4.47E-2 (pos.71967927)	acute_myeloic	Polycystin 1	calcium channel activity,calciu		Calcium Citrate,Calcium		C=20, T=23	C=0.4651, T=0.5349	0.00001	261.021	43	C=13/7, T=8/1
chr3:126138778	T/C	T	C	SNV	CFAP100	CFAP100:intronic:NM_182628.2	1	97.56		intronic	NM_18262	c.914-45T	p.?	-0.25					Domain of un	rs2271630	0.0737	YES	5.97E-1 (pos.126138778)			axonemal outer doublet,cilium movement,cytoplasm,dy				T=2, C=80	T=0.0244, C=0.9756	0.00001	1259.88	82	T=2/0, C=43/3
chr3:126158622	A/C	A	C	SNV	ZXDC	ZXDC:intronic:NM_025112.4	1	47.37		intronic	NM_02511	c.2395-52	p.?	-1.74					Zinc finger, C	rs1799404	0.4942	YES	5.211E-1 (pos.126158622)		ZCD family	C2H2 zinc finger domain binding,DNA binding transcripti				A=20, C=18	A=0.5263, C=0.4737	0.00001	184.53	38	A=2/18, C=2/1
chr3:126218167	A/A	G	A	SNV	UROC1	UROC1:intronic:NM_001165974.1	1	100		intronic	NM_00116	c.1496+13	p.?	-2.25					Urocanase C-	rs1168595	0.2983	YES	2.617E-1;0.0 (pos.126218167)		Urocanase	cytosol,his	Amino Acid Metabolism, Inborn Errors:Ataxia			G=0, A=35	G=0.0, A=1.0	0.00001	543.903	35	G=0/0, A=27/8
chr5:137244517	A/A	G	A	SNV	PKD2L2	PKD2L2:exonic:NM_014386.3	1	100	8	missense	NM_01438	c.1210G>	p.Val404Ile	4.45	0.14	29	0	0.83(COSM44	Ion transport	rs1880458	0.2222	YES	9.671E-1 (pos.13724451	hepatocellula	Polycystin 2	biological_process,calcium channel activity,calcium ion				G=0, A=124	G=0.0, A=1.0	0.00001	2013.87	124	G=0/0, A=68/5
chr3:126365127	CT/CT	C	CT	INDEL	TXNRD3	TXNRD3:intronic:NM_052883.1	1	100		intronic	NM_05288	c.305-64G	p.?	0.11					FAD binding c	rs11453652,rs199565566,rs896		YES			Thioredoxir	cell,cell differentiation,cell redox homeostasis,cellular				C=0, CT=26	C=0.0, CT=1.0	0.00001	376.421	26	C=0/0, CT=6/2
chr3:12660291	T/T	C	T	SNV	RAF1	RAF1:intronic:NM_002880.3	1	100		intronic	NM_00288	c.-25-45G	p.?	-0.27					Kinase-like,P	rs3729926		YES			Oncogene R	ATP bindin	Abnormalities, Mul	Dabrafenib,LErafAON,Re		C=0, T=42	C=0.0, T=1.0	0.00001	684.888	42	C=0/0, T=21/2
chr3:126739291	C/C	T	C	SNV	PLXNA1	PLXNA1:intronic:NM_032242.3	1	100		intronic	NM_03224	c.4038+10	p.?	-1.34					IPT/TIG doma	rs6439030		YES			Plexin A1 (N	branchiomotor neuron axon guidance,dichotomous sub				T=0, C=24	T=0.0, C=1.0	0.00001	334.792	24	T=0/0, C=12/1
chr3:1269763	A/G	A	G	SNV	CNTN6	CNTN6:intronic:NM_014461.3	1	50		intronic	NM_01446	c.358+86A	p.?	-0.4					Fibronectin t	rs60719807		YES			Contactin 6	Notch bin	Adenocarcinoma:Carcinoma:Carcinoma, Ade			A=73, G=73	A=0.5, G=0.5	0.00001	814.241	146	A=44/29, G=3
chr3:127339736	C/T	C	T	SNV	MCM2	MCM2:intronic:NM_004526.3	1	50		intronic	NM_00452	c.2448+13	p.?	-3.41					AAA domain (	rs369690515	2.00E-04		3.486E-4 (pos.127339736)		Minichrom	ATP bindin	Colonic Diseases:Colonic Neoplasms:Colorec			C=59, T=59	C=0.5, T=0.5	0.00001	615.48	118	C=17/42, T=27
chr5:137259179	C/C	T	C	SNV	PKD2L2	PKD2L2:exonic:NM_014386.3	1	100	10	missense	NM_01438	c.1520T>C	p.Leu507Pro	1.12	0.5	98	0		Ion transport	rs12187140	2.00E-04		1.0 (pos.137259179)		Polycystin 2	biological_process,calcium channel activity,calcium ion				T=0, C=30	T=0.0, C=1.0	0.00001	445.048	30	T=0/0, C=18/1
chr6:51924774	G/G	A	G	SNV	PKHD1	PKHD1:exonic:NM_138694.3	1	100	15	synonymous	NM_13869	c.1185T>C	p.(=)	0.39					G8 domain,IF	rs1896976	9.00E-04	YES	1.0 (pos.51924774)		Fibrocystin	anchored	Congenital, Hereditary, and Ne		Benign	A=0, G=157	A=0.0, G=1.0	0.00001	2522.71	157	A=0/0, G=80/7
chr6:51875250	A/C	A	C	SNV	PKHD1	PKHD1:exonic:NM_138694.3	1	97.06	35	missense	NM_13869	c.5608T>G	p.Leu1870Val	5.05	1	32	0		G8 domain,IF	rs2435322	9.00E-04	YES	1.0 (pos.51875250)		Fibrocystin	anchored	Congenital, Hereditary, and Ne		Benign	A=1, C=33	A=0.0294, C=0.9706	0.00001	498.046	34	A=1/0, C=16/1
chr3:128840034	C/C	T	C	SNV	ISY1-RAB43,RAB43	RAB43:intronic:NM_001204886.1, ISY1-RAB43:intronic:NM	1	100		intronic	NM_00120	c.204+95A	p.?, p.?	-0.52					ADP-ribosyla	rs9865992		YES				GTP binding,GTPase activity,Golgi apparatus,Golgi organ				T=0, C=39	T=0.0, C=1.0	0.00001	623.968	39	T=0/0, C=15/2
chr3:128877521	T/T	C	T	SNV	ISY1,ISY1-RAB43	ISY1-RAB43:intronic:NM_001204890.1, ISY1:intronic:NM_00	1	100		intronic	NM_00120	c.27-119G	p.?, p.?	-1.43					Isy1-like splic	rs4452346		YES			ISY1 splicin	Prp19 complex,RNA binding,catalytic step 1 spliceosome				C=0, T=22	C=0.0, T=1.0	0.00001	298.892	22	C=0/0, T=12/1
chr6:51732807	C/T	C	T	SNV	PKHD1	PKHD1:exonic:NM_138694.3	1	53.33	48	synonymous	NM_13869	c.7587G>	p.(=)	-1.56				0.06(COSM11	G8 domain,IF	rs12210295	0.3685	YES	1.22E-1;0.0 (pos.517328	adenoma,hae	Fibrocystin	anchored	Congenital, Hereditary, and Ne		Benign	C=28, T=32	C=0.4667, T=0.5333	0.00001	323.557	60	C=20/8, T=14/
chr3:128971113	T/C	T	C	SNV	COPG1	COPG1:intronic:NM_016128.3	1	50		intronic	NM_01612	c.91-11T>	p.?	0.53					Adaptin N ter	rs4927953	0.1047	YES	1.225E-1 (pos.128971113)		Coatomer p	COPI vesicle coat,ER to Golgi ve		S-(Dimethylarsenic)Cyste		T=11, C=11	T=0.5, C=0.5	0.00001	94.5896	22	T=5/6, C=6/5
chr3:129303197	C/C	T	C	SNV	PLXND1	PLXND1:intronic:NM_015103.2	1	100		intronic	NM_01510	c.2029+31	p.?	-2.73					IPT/TIG doma	rs2811463	0		9.998E-1 (pos.129303197)		Plexin D1	angiogene	Abnormalities, Multiple;Cardiovascular Abnc			T=0, C=43	T=0.0, C=1.0	0.00001	711.501	43	T=0/0, C=7/36
chr9:131483749	A/A	T	A	SNV	PKN3,ZDHHC12	PKN3:downstream:NM_013355.4, ZDHHC12:exonic:NM_03	1	100	5	missense	NM_01335	c.*864T>A	p.?, p.Gln172Leu	-0.13	0.49	113	0	0.04(COSM41	DHHC palmit	rs2900268	0		1.0 (pos.131483749)	neoplasm	Protein kina	ATP binding,Golgi apparatus,endoplasmic reticulum,epi				T=0, A=35	T=0.0, A=1.0	0.00001	543.903	35	T=0/0, A=17/1
chr3:12976863	C/C	T	C	SNV	IQSEC1	IQSEC1:intronic:NM_001134382.2	1	100		intronic	NM_00113	c.1568+85	p.?	-1.74					PH domain,Se	rs2600206		YES			IQ motif- an	ARF guanyl-nucleotide exchange factor activity,actin cyt				T=0, C=24	T=0.0, C=1.0	0.00001	334.766	24	T=0/0, C=8/16
chr1:20490518	C/C	T	C	SNV	PLA2G2C	PLA2G2C:exonic:NM_001316722.1	1	100	4	missense	NM_00131	c.410A>G	p.Gln137Arg	0.1	1	43	0	0.02(COSM37	Phospholipa	rs6426616	0.4058	YES	7.346E-1 (pos.20490518)	acute_myeloid_leukaemia,hepatocellular_carcinoma						T=0, C=29	T=0.0, C=1.0	0.00001	427.603	29	T=0/0, C=7/22
chr19:48551546	T/T	A	T	SNV	PLA2G4C	PLA2G4C:utr_3:NM_003706.2	1	100	17	utr_3	NM_00370	c.1680T>A	p.?	-0.54					Lysophospho	rs9226	0.3294	YES			Phospholip	arachidonic acid metabolic process,calcium-independe				A=0, T=23	A=0.0, T=1.0	0.00001	316.747	23	A=0/0, T=15/8
chr19:48601454	C/C	T	C	SNV	PLA2G4C	PLA2G4C:exonic:NM_003706.2	1	100	6	synonymous	NM_00370	c.510A>G	p.(=)	-2.36					Lysophospho	rs251684	0.3709	YES	7.357E-1 (pos.48601454)		Phospholip	arachidonic acid metabolic process,calcium-independe				T=0, C=25	T=0.0, C=1.0	0.00001	353.018	25	T=0/0, C=13/1
chr3:130132302	A/A	T	A	SNV	COL6A5	COL6A5:intronic:NM_001278298.1	1	100		intronic	NM_00127	c.4663-87	p.?	0.96					Collagen trip	rs11717623,rs71639084		YES			Collagen, ty	cell adhesion,collagen catabolic process,collagen trime				T=0, A=26	T=0.0, A=1.0	0.00001	371.353	26	T=0/0, A=17/9
chr3:130132304	T/T	G	T	SNV	COL6A5	COL6A5:intronic:NM_001278298.1	1	100		intronic	NM_00127	c.4663-85	p.?	0.48					Collagen trip	rs11713542,rs71639084		YES			Collagen, ty	cell adhesion,collagen catabolic process,collagen trime				G=0, T=26	G=0.0, T=1.0	0.00001	371.4	26	G=0/0, T=17/9
chr3:130132509	G/G	A	G	SNV	COL6A5	COL6A5:intronic:NM_001278298.1	1	100		intronic	NM_00127	c.4698+85	p.?	-1.21					Collagen trip	rs2219026	0.2708	YES			Collagen, ty	cell adhesion,collagen catabolic process,collagen trime				A=0, G=24	A=0.0, G=1.0	0.00001	334.792	24	A=0/0, G=9/15
chr3:130132851	G/G	A	G	SNV	COL6A5	COL6A5:intronic:NM_001278298.1	1	100		intronic	NM_00127	c.4699-62	p.?	-0.52					Collagen trip	rs10512767	0.1267	YES			Collagen, ty	cell adhesion,collagen catabolic process,collagen trime				A=0, G=23	A=0.0, G=1.0	0.00001	299.073	23	A=0/0, G=8/15
chr15:42342824	T/G	T	G	SNV	PLA2G4E	PLA2G4E:exonic:NM_001206670.1	1	59.09	1	missense	NM_00120	c.78A>C	p.Glu26Asp	0.69	0	45	0		C2 domain,Ly	rs1668546		YES	1.029E-1 (pos.42342824)			cytosol,lysophospholipase activity,lysosomal membran				T=9, G=13	T=0.4091, G=0.5909	0.00001	123.219	22	T=6/3, G=6/7
chr15:42287578	A/A	G	A	SNV	PLA2G4E,PLA2G4E-AS1	PLA2G4E-AS1:intronic_nc:NR_120334.1, PLA2G4E:exonic:NM	1	100	12	synonymous	NR_12033	c.1227C>T	p.(=)	-0.97				0.01(COSM39	C2 domain,Ly	rs1668586	0.1268	YES	5.577E-1 (pos.42287578)	acute_myeloid_leukaemia		cytosol,lysophospholipase activity,lysosomal membran				G=0, A=25	G=0.0, A=1.0	0.00001	353.008	25	G=0/0, A=13/1
chr15:42439376	A/A	G	A	SNV	PLA2G4F	PLA2G4F:exonic:NM_213600.3	1	100	13	synonymous	NM_21360	c.1365C>T	p.(=)	-2.8				0.02(COSM43	C2 domain,Ly	rs4923929	0.475	YES	2.542E-1 (pos.42439376)	carcinoma,squamous_cell		arachidonic acid secretion,calcium-dependent phospho				G=0, A=27	G=0.0, A=1.0	0.00001	389.988	27	G=0/0, A=17/1
chr3:130748733	A/G	A	G	SNV	NEK11	NEK11:intronic:NM_024800.4	1	95.83		intronic	NM_02480	c.170+11A	p.?	0.32					Protein kinas	rs9864242	0.4159	YES	2.389E-1 (pos.130748733)		Never in mi	ATP binding,histone phosphory		Dabrafenib		A=1, G=23	A=0.0417, G=0.9583	0.00001	306.433	24	A=1/0, G=13/1
chr3:130881409	C/C	T	C	SNV	NEK11	NEK11:intronic:NM_024800.4	1	100		intronic	NM_02480	c.1082+38	p.?	-0.19					Protein kinas	rs1913898	0.425	YES	2.614E-1 (pos.130881409)		Never in mi	ATP binding,histone phosphory		Dabrafenib		T=0, C=52	T=0.0, C=1.0	0.00001	767.006	52	T=0/0, C=23/2
chr15:42437824	C/T	C	T	SNV	PLA2G4F	PLA2G4F:exonic:NM_213600.3	1	46.43	16	missense	NM_21360	c.1729G>	p.Gly577Ser	2.44	0.34	56	1	0.71(COSM37	C2 domain,Ly	rs781426338			0.0 (pos.42437824)	primitive_neuroectoderma		arachidonic acid secretion,calcium-dependent phospho				C=15, T=13	C=0.5357, T=0.4643	0.00001	117.927	28	C=2/13, T=3/1
chr3:131404826	A/G	A	G	SNV	CPNE4	CPNE4:intronic:NM_130808.2	1	50		intronic	NM_13080	c.508-24T	p.?	-0.27					C2 domain,C	rs11716477	0.1087	YES	2.2E-2 (pos.131404826)		Copine IV	extracellular exosome,protein binding				A=10, G=10	A=0.5, G=0.5	0.00001	82.8858	20	A=2/8, G=4/6
chr3:131624097	G/A	G	A	SNV	CPNE4	CPNE4:intronic:NM_130808.2	1	53.13		intronic	NM_13080	c.180+11C	p.?	2.55					C2 domain,C	rs3749262	0.27	YES	1.049E-1 (pos.131624097)		Copine IV	extracellular exosome,protein binding				G=45, A=51	G=0.4688, A=0.5313	0.00001	587.464	96	G=28/17, A=3
chr3:132075417	C/C	A	C	SNV	ACPP	ACPP:intronic:NM_001134194.1	1	100		intronic	NM_00113	c.969-113	p.?	-0.23					Histidine ph	rs3853148		YES			Acid phosph	5'-nucleot	Arthritis;Arthritis, J	Alpha-Benzyl-Aminobenz		A=0, C=50	A=0.0, C=1.0	0.00001	852.687	50	A=0/0, C=14/3
chr3:132075759	T/C	T	C	SNV	ACPP	ACPP:intronic:NM_001134194.1	1	57.63		intronic	NM_00113	c.1138+60	p.?	0.37					Histidine ph	rs73215937	0.1302	YES	3.598E-2 (pos.132075759)		Acid phosph	5'-nucleot	Arthritis;Arthritis, J	Alpha-Benzyl-Aminobenz		T=50, C=68	T=0.4237, C=0.5763	0.00001	755.61	118	T=29/21, C=29
chr3:132218196	C/T	C	T	SNV	DNAJC13	DNAJC13:intronic:NM_015268.3	1	47.37		intronic	NM_01526	c.4341+42	p.?	-0.5					DnaJ domain	rs2088712	0.349	YES	7.696E-1;0.0 (pos.132218196)		DNAJ/HSP4	WASH com	Central Nervous System Diseases;Essential T			C=20, T=18	C=0.5263, T=0.4737	0.00001	184.536	38	C=13/7, T=13/
chr3:132244313	C/T	C	T	SNV	DNAJC13	DNAJC13:intronic:NM_015268.3	1	57.14		intronic	NM_01526	c.6061-14	p.?	-0.41					DnaJ domain	rs9843883		YES			DNAJ/HSP4	WASH com	Central Nervous System Diseases;Essential T			C=12, T=16	C=0.4286, T=0.5714	0.00001	160.95	28	C=7/5, T=8/8
chr2:160901517	A/G	A	G	SNV	PLA2R1	PLA2R1:exonic:NM_007366.4	1	52.38	2	synonymous	NM_00736	c.261T>C	p.(=)	-0.21				0.03(COSM54	Fibronectin t	rs4665143	0.3657	YES	5.788E-1 (pos.16090151	acute_myeloic	Phospholip	carbohydr	Autoimmune Diseases;Female Urogenital Di			A=10, G=11	A=0.4762, G=0.5238	0.00001	95.2994	21	A=5/5, G=10/1
chr3:133553527	T/T	C	T	SNV	RAB6B	RAB6B:intronic:NM_016577.3	1	100		intronic	NM_01657	c.496-42G	p.?	0.2					50S ribosome	rs2280672,rs756559	0.3624	YES	0.0;0.0;0.0 (pos.133553521),4.123E-1 (p		Ras-associa	GTP binding,GTPase activity,Golgi apparatus,Golgi meml				C=0, T=22	C=0.0, T=1.0	0.00001	298.883	22	C=0/0, T=7/15
chr3:13361238	C/T	C	T	SNV	NUP210	NUP210:intronic:NM_024923.3	1	53.49		intronic	NM_02492	c.5383+25	p.?	0.75					Bacterial Ig-li	rs2276748	0.4884	YES	6.948E-1 (pos.13361238)		Nucleopori	endoplasmic reticulum membrane,host cell,integral cor				C=20, T=23	C=0.4651, T=0.5349	0.00001	261.023	43	C=10/10, T=6/
chr2:28865760	T/C	T	C	SNV	PLB1	PLB1:exonic:NM_153021.4	1	50	58	synonymous	NM_15302	c.4210T>C	p.(=)	-0.36				0(COSM6310	GDSL-like Lip	rs2272387	0.3871	YES	4.343E-1 (pos.28865760)	carcinoma,ne	Phospholip	apical pla	Arthritis;Arthritis, Rheumatoid;Autoimmune			T=14, C=14	T=0.5, C=0.5	0.00001	131.941	28	T=12/2, C=10/
chr3:133692497	G/G	A	G	SNV	SLCO2A1	SLCO2A1:intronic:NM_005630.2	1	100		intronic	NM_00563	c.397+10T	p.?	1.86					Kazal-type se	rs4634113	0.1531	YES	9.908E-1 (pos.133692497)		Solute carri	integral component of membra		Alprostadil,Arachidonic		A=0, G=30	A=0.0, G=1.0	0.00001	446.18	30	A=0/0, G=22/8
chr3:133698280	G/G	C	G	SNV	SLCO2A1	SLCO2A1:intronic:NM_005630.2	1	100		intronic	NM_00563	c.234+45G	p.?	-0.41					Kazal-type se	rs4640563	0.1191	YES	9.364E-1 (pos.133698280)		Solute carri	integral component of membra		Alprostadil,Arachidonic		C=0, G=167	C=0.0, G=1.0	0.00001	2711.95	167	C=0/0, G=87/8
chr3:1337162	A/G	A	G	SNV	CNTN6	CNTN6:intronic:NM_014461.3	1	50		intronic	NM_01446	c.455-123	p.?	-0.71					Fibronectin t	rs2063243		YES			Contactin 6	Notch bin	Adenocarcinoma:Carcinoma:Carcinoma, Ade			A=68, G=68	A=0.5, G=0.5	0.00001	745.345	136	A=33/35, G=3
chr3:1337203	G/G	A	G	SNV	CNTN6	CNTN6:intronic:NM_014461.3	1	100		intronic	NM_01446	c.455-82A	p.?	-0.56					Fibronectin t	rs565499		YES			Contactin 6	Notch bin	Adenocarcinoma:Carcinoma:Carcinoma, Ade			A=0, G=140	A=0.0, G=1.0	0.00001	2341.31	140	A=0/0, G=64/7
chr3:133941504	G/A	G	A	SNV	RYK	RYK:intronic:NM_001005861.2	1	52.38		intronic	NM_00100	c.231-121	p.?	-0.97					Protein kinas	rs9865808		YES			RYK recept	ATP bindin	Cleft Palate;Congenital Abnormalities;Conge			G=20, A=22	G=0.4762, A=0.5238	0.00001	245.18	42	G=8/12, A=13/
chr12:14664595	G/G	A	G	SNV	PLBD1	PLBD1:exonic:NM_024829.5	1	100	7	synonymous	NM_02482	c.895T>C	p.(=)	-0.36				0.06(COSM39	Phospholipa	rs10846013	0.3045	YES	1.999E-1 (pos.14664595)	acute_myeloid_leukaemia		cytosol,ex	Bronchial Neoplasms;Carcinoma, Bronchoge			A=0, G=22	A=0.0, G=1.0	0.00001	298.891	22	A=0/0, G=11/1
chr12:113825679	T/T	C	T	SNV	PLBD2	PLBD2:exonic:NM_173542.3	1	100	11	missense	NM_17354	c.1570C>T	p.Arg524Cys	-0.1	0.04	180	0.971	0.3(COSM375	Phospholipa	rs12425042	0.1478	YES	2.311E-4 (pos.11382567	adenocarcinoma,neoplasm		extracellular exosome,hydrolase activity,lipid catabolic				C=0, T=30	C=0.0, T=1.0	0.00001	446.635	30	C=0/0, T=10/2
chr3:134852051	A/A	C	A	SNV	EPHB1	EPHB1:intronic:NM_004441.4	1	100		intronic	NM_00444	c.1297+16	p.?	-2.47					Ephrin recept	rs1525014					eph tyrosin	ATP binding,angiogenesis,axon,axon guidance,axon guid				C=0, A=73	C=0.0, A=1.0	0.00001	1150.51	73	C=0/0, A=37/3
chr3:134967368	G/A	G	A	SNV	EPHB1	EPHB1:intronic:NM_004441.4	1	50		intronic	NM_00444	c.2690+17	p.?	0.26					Ephrin recept	rs11717042	0.2177	YES	3.691E-1;0.0 (pos.134967368)		eph tyrosin	ATP binding,angiogenesis,axon,axon guidance,axon guid				G=11, A=11	G=0.5, A=0.5	0.00001	94.5977	22	G=1/10, A=4/7
chr3:13679044	G/A	G	A	SNV	FBLN2	FBLN2:intronic:NM_001004019.1	1	50		intronic	NM_00100	c.3339-18	p.?	-0.43					Anaphylotoxi	rs4684148	0.2995	YES	7.049E-1 (pos.13679044)		Fibulin-2	calcium io	Colonic Diseases;Colorectal Neoplasms;Dige			G=21, A=21	G=0.5, A=0.5	0.00001	228.814	42	G=16/5, A=10/
chr3:137717991	C/C	G	C	SNV	CLDN18	CLDN18:intronic:NM_001002026.2	1	100		intronic	NM_00100	c.220+61G	p.?	-1.68					PMP-22/EMP,	rs6764398		YES				TNFSF11-mediated signaling pathway,bicellular tight jun				G=0, C=22	G=0.0, C=1.0	0.00001	298.395	22	G=0/0, C=11/1
chr12:113826442	G/G	C	G	SNV	PLBD2	PLBD2:utr_3:NM_173542.3	1	100	12	utr_3	NM_17354	c.1781C>G	p.?	0.07					Phospholipa	rs78927955	0.1484	YES	2.338E-4 (pos.113826442)			extracellular exosome,hydrolase activity,lipid catabolic				C=0, G=27	C=0.0, G=1.0	0.00001	381.852	27	C=0/0, G=5/22
chr3:137958438	C/T	C	T	SNV	ARMC8	ARMC8:intronic:NM_015396.5	1	56.36		intronic	NM_01539	c.795+68C	p.?	0.27					Armadillo/be	rs6778511	0.4531	YES				extracellular region,neutrophil degranulation,protein bi				C=24, T=31	C=0.4364, T=0.5636	0.00001	319.023	55	C=15/9, T=10/
chr3:138091701	T/T	G	T	SNV	MRAS	MRAS:intronic:NM_001085049.2	1	100		intronic	NM_00108	c.-17-7G>T	p.?	0.58					ADP-ribosyla	rs1199333	0.186	YES	9.692E-1 (pos.138091701)		Muscle Ras	GTP bindir	Arterial Occlusive Diseases;Arteriosclerosis;			G=0, T=24	G=0.0, T=1.0	0.00001	334.786	24	G=0/0, T=12/1
chr3:138403390	T/T	C	T	SNV	PIK3CB	PIK3CB:intronic:NM_006219.2	1	100		intronic	NM_00621	c.2315+77	p.?	0					PI3-kinase fa	rs625468		YES			Phosphatid	1-phospha	Hemic and Lympha	Caffeine,XL765		C=0, T=20	C=0.0, T=1.0	0.00001	263.784	20	C=0/0, T=14/6
chr11:64022459	G/G	A	G	SNV	PLCB3	PLCB3:exonic:NM_000932.2	1	100	4	synonymous	NM_00093	c.336A>G	p.(=)	-0.54					C2 domain,PI	rs632439	7.00E-04	YES	1.0 (pos.64022459)		Phospholip	G-protein coupled receptor signaling pathway,Wnt signa				A=0, G=26	A=0.0, G=1.0	0.00001	371.379	26	A=0/0, G=18/8
chr3:139075644	A/A	C	A	SNV	COPB2,MRPS22	COPB2:downstream:NM_004766.2, MRPS22:intronic:NM_0	1	100		intronic	NM_02019	c.988-117	p.?, p.?	0.13					Mitochondria	rs9878548		YES			Coatomer p	COPI vesic	Breast Diseases;Breast Neoplasms;Congenit			C=0, A=255	C=0.0, A=1.0	0.00001	4140.45	255	C=0/0, A=109/
chr3:139075938	C/T	C	T	SNV	COPB2,MRPS22	COPB2:downstream:NM_004766.2, MRPS22:downstream:N	1	57.14		intronic	NM_02019	c.*82C>T,	p.?, p.?	-0.07						rs7833		YES			Coatomer p	COPI vesic	Breast Diseases;Breast Neoplasms;Congenit			C=9, T=12	C=0.4286, T=0.5714	0.00001	109.106	21	C=3/6, T=6/6
chr3:139102120	A/A	C	A	SNV	COPB2	COPB2:intronic:NM_004766.2	1	100		intronic	NM_00476	c.141+20G	p.?	0.09					Coatomer WI	rs7373302	0.1395	YES	9.352E-1 (pos.139102120)		Coatomer p	COPI vesicle coat,ER to Golgi vesicle-mediated transport				C=0, A=33	C=0.0, A=1.0	0.00001	504.598	33	C=0/0, A=16/1
chr3:13916407	T/T	G	T	SNV	WNT7A	WNT7A:intronic:NM_004625.3	1	100		intronic	NM_00462	c.298+37C	p.?	-0.99					wnt family	rs3749319	0.448	YES	7.788E-1 (pos.13916407)		Wingless-ty	Golgi lume	Amyotrophic Lateral Sclerosis;Central Nervo			G=0, T=21	G=0.0, T=1.0	0.00001	281.221	21	G=0/0, T=10/1
chr20:9288522	A/A	G	A	SNV	PLCB4	PLCB4:exonic:NM_000933.3	1	100	1	missense	NM_00093	c.61G>A	p.Ala21Thr	7.07	0.34	58	0.928	0.96(COSM44	C2 domain,PI	rs6077510	0.3269	YES	4.39E-1 (pos.9288522)	acute_myeloic	Phospholip	G-protein	Ear Diseases;Melanoma;Neop		Benign	G=0, A=46	G=0.0, A=1.0	0.00001	768.313	46	G=0/0, A=16/3
chr17:43192821	G/G	A	G	SNV	PLCD3	PLCD3:exonic:NM_133373.4	1	100	9	synonymous	NM_13337	c.1450T>C	p.(=)	1.91				0.02(COSM43	C2 domain,EF	rs713101	0.4284	YES	6.94E-1 (pos.43192821)	acute_myeloid_leukaemia		angiogenesis,cleavage furrow,cytosol,inositol phosphat				A=0, G=88	A=0.0, G=1.0	0.00001	1450.61	88	A=0/0, G=53/3
chr3:140692486	T/T	G	T	SNV	SLC25A36	SLC25A36:intronic:NM_001104647.1	1	100		intronic	NM_00110	c.453-72G	p.?	-0.81					Mitochondria	rs2555707		YES			Solute carri	integral component of membrane,mitochondrial genom				G=0, T=38	G=0.0, T=1.0	0.00001	603.785	38	G=0/0, T=12/2
chr3:140998214	A/A	G	A	SNV	PXYLP1	PXYLP1:intronic:NM_001037172.2	1	100		intronic	NM_00103	c.239-6G>	p.?	-1.43					Histidine ph	rs13087169	0.3613	YES	5.209E-1 (pos.140998214)			Golgi apparatus,Golgi membrane,chondroitin sulfate pr				G=0, A=77	G=0.0, A=1.0	0.00001	1229.44	77	G=0/0, A=46/3
chr2:219492924	T/C	T	C	SNV	PLCD4	PLCD4:exonic:NM_032726.3	1	46.88	7	synonymous	NM_03272	c.945T>C	p.(=)	0.01					C2 domain,EF	rs612874	0.4275	YES	7.696E-1 (pos.219492924)			acrosome reaction,calcium ion binding,cytosol,endopla				T=34, C=30	T=0.5313, C=0.4688	0.00001	288.058	64	T=8/26, C=14/

chr16:81819768	C/C	T	C	SNV	PLCG2	PLCG2:exonic:NM_002661.4	1	100	2 synonymous	NM_00266 c.174T>C p.(=)	-3.14		C2 domain;Pl rs1143685	0.3128 YES	8.731E-1 (pos.81819768)	Phospholip B cell differentiation,B cell receptor signal Benign	T=0, C=42 T=0.0, C=1.0	0.00001	684.563	42 T=0/0, C=21/2
chr1:2430617	T/T	C	T	SNV	PLCH2	PLCH2:exonic:NM_014638.3	1	100	19 synonymous	NM_01463 c.2529C>T p.(=)	-1.57	0.09(COSM37	C2 domain;El rs2296442	0.4737 YES	5.221E-1 (pos.2430617) acute_myeloi	Phospholip biological Mental Disorders;Schizophrenia;Schizophrer	C=0, T=33 C=0.0, T=1.0	0.00001	504.597	33 C=0/0, T=28/5
chr3:142567327	A/A	G	A	SNV	PCOLCE2	PCOLCE2:intronic:NM_013363.3	1	100	intronic	NM_0133€ c.193-13C p.?	-0.06		CUB domain; rs893883	0.3365 YES	3.368E-1 (pos.142567327)	Procollager cellular re Amphetamine-Related Disorders;Mental Dis	G=0, A=69 G=0.0, A=1.0	0.00001	1072.78	69 G=0/0, A=38/3
chr3:142746988	A/G	A	G	SNV	U2SURP	U2SURP:intronic:NM_001080415.1	1	95.83	intronic	NM_00108 c.1380-19 p.?	-0.35		RNA recognit rs1839406	YES		RNA binding,RNA processing,mRNA splicing, via spliceos	A=1, G=23 A=0.0417, G=0.9583	0.00001	306.434	24 A=0/1, G=11/
chr3:146307635	A/A	C	A	SNV	PLSCR5	PLSCR5:intronic:NM_001085420.1	1	100	intronic	NM_00108 c.616-34G p.?	-0.01		Scramblase rs343289	0.2882 YES	7.36E-1 (pos.146307635)	phospholipid scramblase activity,phospholipid scrambl	C=0, A=121 C=0.0, A=1.0	0.00001	1953.69	121 C=0/0, A=48/7
chr3:17131421	T/T	C	T	SNV	PLCL2	PLCL2:utr_3:NM_015184.5	1	100	6 utr_3	NM_01518 c.3023C>T p.?	-0.01		C2 domain;Pl rs35185988	0.1778 YES	1.282E-2 (pos.17131421)	Phospholip B cell proli Arthritis;Arthritis, Rheumatoid;Autoimmune	C=0, T=23 C=0.0, T=1.0	0.00001	316.747	23 C=0/0, T=15/8
chrX:200860	T/C	T	C	SNV	PLCXD1	PLCXD1:exonic:NM_018390.3	1	45.45	2 synonymous	NM_01839 c.67>C p.(=)	-0.28		Phosphatidyl rs9785927	0.2612 YES	8.003E-1;0.0 (pos.200860)	Phospholip cytoplasm,lipid metabolic process,phosphoric diester h	T=36, C=30 T=0.5455, C=0.4545	0.00001	285.787	66 T=20/16, C=1
chr8:144992361	C/C	T	C	SNV	PLEC	PLEC:exonic:NM_201380.3	1	100	32 synonymous	NM_20138 c.12039A> p.(=)	-3.57	0.07(COSM41	Calponin hon rs7822511	0.4439 YES	1.971E-1 (pos.14499236: neoplasm	Plectin 1 RNA bindi Congenital Abnormalities;Con Benign/Likely	T=0, C=33 T=0.0, C=1.0	0.00001	504.573	33 T=0/0, C=23/1
chr8:144996029	A/G	A	G	SNV	PLEC	PLEC:exonic:NM_201380.3	1	57.14	32 missense	NM_20138 c.8371T>C p.Ser2791Pro	0.44 1 74	0.07(COSM14	Calponin hon rs2833924	0.4309 YES	1.473E-1 (pos.14499602! adenocarcinoi	Plectin 1 RNA bindi Congenital Abnormalities;Con Benign/Likely	A=9, G=12 A=0.4286, G=0.5714	0.00001	161.77	28 A=9/3, G=6/10
chr8:144998169	C/T	C	T	SNV	PLEC	PLEC:exonic:NM_201380.3	1	50	31 synonymous	NM_20138 c.6339G> p.(=)	-7.44	0.03(COSM37	Calponin hon rs1140522	0.3675 YES	1.651E-1 (pos.14499816! adenocarcinoi	Plectin 1 RNA bindi Congenital Abnormalities;Con Benign/Likely	C=15, T=15 C=0.5, T=0.5	0.00001	144.623	30 C=14/1, T=11/
chr3:149192851	GC/GC	G	GC	INDEL	TM4SF4	TM4SF4:intronic:NM_004617.3	1	100	intronic	NM_00461 c.174+13C p.?	-0.16		L6 membran: rs143312618,rs3981:	0.178 YES	1.015E-1 (pos.149192851)	Transmemt integral cc Digestive System Diseases;Liver Diseases	G=0, GC=3: G=0.0, GC=1.0	0.00001	486.452	33 G=0/0, GC=20/
chr8:144999642	A/C	A	C	SNV	PLEC	PLEC:exonic:NM_201380.3	1	50	31 synonymous	NM_20138 c.4866T>G p.(=)	-5.09	0.12(COSM14	Calponin hon rs56117011	0.3747 YES	1.475E-1;0.0 (pos.14499! adenocarcinoi	Plectin 1 RNA bindi Congenital Abnormalities;Con Benign/Likely	A=14, C=14 A=0.5, C=0.5	0.00001	131.941	28 A=10/4, C=5/9
chr3:150384807	G/G	T	G	SNV	ERICH6	ERICH6:intronic:NM_152394.4	1	100	intronic	NM_15239 c.1500-5A p.?	0.5	0(COSM5352	FAM194 prot rs6440691	0.1115 YES	9.999E-1 (pos.15038480: Wilms_tumour		T=0, G=56 T=0.0, G=1.0	0.00001	832.084	56 T=0/0, G=24/3
chr8:68622914	A/A	G	A	SNV	PLEK	PLEK:exonic:NM_002664.2	1	100	9 missense	NM_00266 c.1019G>A p.Arg340Lys	4.58 1 26	0 0.28(COSM37	Domain foun rs1063479	0.2753 YES	9.334E-1 (pos.68622914) adenocarcinoi	Pleckstrin actin cytoi Arthritis;Arthritis, Rheumatoid;Autoimmune	G=0, A=60 G=0.0, A=1.0	0.00001	903.531	60 G=0/0, A=22/3
chr2:68622952	C/C	G	C	SNV	PLEK	PLEK:utr_3:NM_002664.2	1	100	9 utr_3	NM_00266 c.1057G>C p.?	-0.84		Domain foun rs1050181	0.4572 YES	1.995E-1 (pos.68622952)	Pleckstrin actin cytoi Arthritis;Arthritis, Rheumatoid;Autoimmune	G=0, C=60 G=0.0, C=1.0	0.00001	903.531	60 G=0/0, C=22/3
chr10:124189197	G/G	A	G	SNV	PLEKHA1	PLEKHA1:exonic:NM_001001974.2	1	100	12 missense	NM_0010C c.958A>G p.Thr320Ala	1.19 0.7 58	0 0.24(COSM37	PH domain rs1045216	0.3973 YES	7.978E-1 (pos.12418919: adenocarcinoi	Pleckstrin cell rece Eye Diseases;Macu Citric Acid	A=0, G=33 A=0.0, G=1.0	0.00001	504.447	33 A=0/0, G=16/1
chr4:204198209	G/G	A	G	SNV	PLEKHA6	PLEKHA6:exonic:NM_014935.4	1	100	19 synonymous	NM_01493 c.2607T>C p.(=)	-2.84		PH domain;P: rs3795571	0.4579 YES	5.02E-1 (pos.204198209)		A=0, G=23 A=0.0, G=1.0	0.00001	316.747	23 A=0/0, G=10/1
chr1:204199614	T/T	C	T	SNV	PLEKHA6	PLEKHA6:exonic:NM_014935.4	1	100	18 missense	NM_01493 c.2510G>A p.Arg837Lys	3.2 0.57 26	0 0.38(COSM13	PH domain;P rs10900562,rs77374:	0.4556 YES	0.0 (pos.20419961 1).5.4: adenocarcinoma,carcinoma,ductal_carcinoma		C=0, T=55 C=0.0, T=1.0	0.00001	814.891	55 C=0/0, T=30/2
chr19:151103070	G/G	T	G	SNV	MED12L	MED12L:intronic:NM_053002.5	1	100	intronic	NM_0530C c.4983+91 p.?	-0.23		Eukaryotic M rs4603933	YES		Mediator cc RNA polymerase II transcription coactivator activity,RNA	T=0, G=29 T=0.0, G=1.0	0.00001	427.602	29 T=0/0, G=9/20
chr19:39913558	A/G	A	G	SNV	PLEKHG2	PLEKHG2:exonic:NM_022835.2	1	50	18 missense	NM_02283 c.1864A>G p.Ile622Val	0.1 0.26 29	0.002 0.04(COSM13	PH domain,R: rs16973407	0.0558 YES	1.158E-4 (pos.39913558) ductal_carcinc	Pleckstrin h G-protein coupled receptor signaling pathway,Rho guan	A=23, G=23 A=0.5, G=0.5	0.00001	261.262	46 A=12/11, G=4/
chr3:151558265	A/A	G	A	SNV	IGSF10	IGSF10:intronic:NM_178822.4	1	100	intronic	NM_17882 c.5964-18 p.?	-0.04		Immunoglobi rs937125	YES		Immunoglo extracellular region,ossificatic Pyridoxal Phosphate	G=0, A=60 G=0.0, A=1.0	0.00001	903.531	60 G=0/0, A=39/2
chr5:163205	C/C	G	C	SNV	PLEKHG4B	PLEKHG4B:exonic:NM_052909.4	1	100	13 synonymous	NM_0529C c.3018G>C p.(=)	-1.5	0(COSM1132	RhoGEF dom: rs3810870	0.3068 YES	3.851E-1;0.0 (pos.163205 adenocarcinoma,carcinom	Rho guanyl-nucleotide exchange factor activity,protein E	G=0, C=84 G=0.0, C=1.0	0.00001	1368.62	84 G=0/0, C=50/3
chr5:163266	G/G	C	G	SNV	PLEKHG4B	PLEKHG4B:exonic:NM_052909.4	1	100	13 missense	NM_0529C c.3079C>G p.Arg1027Gly	-1.04 0.64 125	0 0(COSM3761	RhoGEF dom: rs3810867	0.394 YES	1.757E-1;0.0 (pos.16326t adenocarcinoma,carcinom	Rho guanyl-nucleotide exchange factor activity,protein E	C=0, G=50 C=0.0, G=1.0	0.00001	849.455	50 C=0/0, G=28/2
chr5:181730	G/G	C	G	SNV	PLEKHG4B	PLEKHG4B:exonic:NM_052909.4	1	100	19 missense	NM_0529C c.4504C>G p.Arg1502Gly	0.66 0.01 125	0.998 0.84(COSM37	RhoGEF dom: rs12519352	0.454 YES	2.409E-1;0.0 (pos.18: acute_myeloid_leukaemia	Rho guanyl-nucleotide exchange factor activity,protein E	C=0, G=44 C=0.0, G=1.0	0.00001	720.607	44 C=0/0, G=21/2
chr3:151542737	A/A	G	A	SNV	AADAC,AADACL2-AS1,MIR548H2	AADAC:intronic:NM_001086.2, AADACL2-AS1:intronic_nc:NR	1	100	intronic	NR_03167: c.603+115 p.?	-0.15		Carboxyleste rs13098550,rs386667108,rs869	YES		Arylacetam catabolic process,catalytic activity,deacetylase activity	G=0, A=29 G=0.0, A=1.0	0.00001	427.499	29 G=0/0, A=14/1
chr3:151542749	C/C	CTATT	C	INDEL	AADAC,AADACL2-AS1,MIR548H2	AADAC:intronic:NM_001086.2, AADACL2-AS1:intronic_nc:NR	4	100	intronic	NR_03167: c.603+127 p.?	1.03,-0.21,-0.34,0.78,-0.61		Carboxyleste rs151253803,rs200299661,rs20	YES		Arylacetam catabolic process,catalytic activity,deacetylase activity	CTATT=0,C CTATT=0.0, C=1.0	0.00001	389.942	27 CTATT=0/0, C=
chr12:6421495	A/A	G	A	SNV	PLEKHG6	PLEKHG6:exonic:NM_018173.3	1	100	2 missense	NM_01817 c.103G>A p.Ala35Thr	-0.15 0.09 58	0.099 0.02(COSM53	RhoGEF dom: rs740842	0.4273 YES	2.556E-1 (pos.6421495) Wilms_tumou	Pleckstrin h GTPase activator activity,Rho guanyl-nucleotide exchang	G=0, A=68 G=0.0, A=1.0	0.00001	1053.56	68 G=0/0, A=45/2
chr3:153870511	A/A	G	A	SNV	ARHGEF26	ARHGEF26:intronic:NM_001251962.1	1	100	intronic	NM_00125 c.1327-50 p.?	-0.4		RhoGEF dom: rs1727956	YES	5.725E-1 (pos.153870511)	Rho guanin G-protein coupled receptor signaling pathway,Rho guan	G=0, A=95 G=0.0, A=1.0	0.00001	1601.28	95 G=0/0, A=61/3
chr3:153972500	C/C	T	C	SNV	ARHGEF26	ARHGEF26:intronic:NM_001251962.1	1	100	intronic	NM_00125 c.2369-97 p.?	-0.1		RhoGEF dom: rs355749	0.1432 YES	9.992E-1 (pos.153972500)	Rho guanin G-protein coupled receptor signaling pathway,Rho guan	T=0, C=46 T=0.0, C=1.0	0.00001	768.3	46 T=0/0, C=25/2
chr3:154021063	A/A	G	A	SNV	DHX36	DHX36:intronic:NM_020865.2	1	100	intronic	NM_0208€ c.1217+55 p.?	0.06		DEAD/DEAH b rs355756	YES		DEAH (Asp-C ATP binding,ATP-dependent RNA helicase activity,DNA-d	G=0, A=153 G=0.0, A=1.0	0.00001	2448.91	153 G=0/0, A=63/9
chr2:43931176	C/C	T	C	SNV	PLEKHH2	PLEKHH2:exonic:NM_172069.3	1	100	9 synonymous	NM_17206 c.1707T>C p.(=)	0.4	0.01(COSM44	FERM central rs4953002	0.469 YES	1.562E-1 (pos.43931176) adenocarcinoi	Pleckstrin h actin bindir cortical actin cytoskeleton,cytoplasm,cyto	T=0, C=191 T=0.0, C=1.0	0.00001	3223.37	191 T=0/0, C=56/1
chr3:155271825	G/A	G	A	SNV	PLCH1	PLCH1:intronic:NM_001130960.1	1	45.83	intronic	NM_00113 c.1154+40 p.?	-0.63		C2 domain;El rs359549	0.3458 YES	2.914E-1 (pos.155271825)	Phospholip calcium ion binding,calcium-dependent phospholipase C	G=13, A=11 G=0.5417, A=0.4583	0.00001	93.4399	24 G=7/6, A=1/10
chr2:43927459	C/G	C	G	SNV	PLEKHH2	PLEKHH2:exonic:NM_172069.3	1	45.45	8 synonymous	NM_17206 c.1362C>G p.(=)	-1.31	0.9(COSM413	FERM central rs12623703	0.1485 YES	9.512E-2 (pos.43927459) anaplastic_ca	Pleckstrin h actin bindir cortical actin cytoskeleton,cytoplasm,cyto	C=24, G=2C C=0.5455, G=0.4545	0.00001	212.417	44 C=16/8, G=15/
chr3:157004323	C/C	A	C	SNV	VEPH1	VEPH1:intronic:NM_001167912.1	1	100	intronic	NM_0011€ c.2128+23 p.?	0.44		PH domain rs4680353	0.4853 YES	4.218E-1;0.0 (pos.157004323)	Ventricular plasma membrane	A=0, C=165 A=0.0, C=1.0	0.00001	2682.97	165 A=0/0, C=77/8
chr3:157146307	T/T	A	T	SNV	VEPH1	VEPH1:intronic:NM_001167912.1	1	100	intronic	NM_0011€ c.530-30T: p.?	0.02		PH domain rs1456099	0.4915 YES	4.7E-1 (pos.157146307)	Ventricular plasma membrane	A=0, T=53 A=0.0, T=1.0	0.00001	782.015	53 A=0/0, T=10/4
chr2:208811097	T/T	C	T	SNV	PLEKHM3	PLEKHM3:exonic:NM_001080475.2	1	100	4 synonymous	NM_00108 c.1686G>A p.(=)	0.06	0.21(COSM37	PH domain;R rs10932215	0.3697 YES	3.89E-1 (pos.208811097) acute_myeloid_leukaemia	Pleckstrin h metal ion binding	C=0, T=30 C=0.0, T=1.0	0.00001	445.614	30 C=0/0, T=17/1
chr3:157160061	C/C	A	C	SNV	PTX3,VEPH1	VEPH1:intronic:NM_001167912.1, PTX3:intronic:NM_00285	1	100	intronic	NM_0011€ c.530-137: p.?, p.?	-0.26		Concanavalir rs2316706	YES		Pentraxin-3 (1->3)-beta-I Arsenic Poisoning;Autoimmune Diseases;Fer	A=0, C=40 A=0.0, C=1.0	0.00001	644.285	40 A=0/0, C=17/2
chr3:15752830	C/C	T	C	SNV	ANKRD28	ANKRD28:intronic:NM_015199.3	1	100	intronic	NM_01519 c.1184-49 p.?	1.78		Ankyrin repe rs2455833	0.4056 YES	3.741E-1 (pos.15752830)	Ankyrin rep COPII vesicle coating,Golgi membrane,cytosol,nucleopla	T=0, C=44 T=0.0, C=1.0	0.00001	730.942	44 T=0/0, C=13/3
chrX:157820423	T/T	C	T	SNV	SHOX2	SHOX2:intronic:NM_003030.4	1	100	intronic	NM_00303 c.627+48 p.?	0.25		Homeobox dc rs4462902	0.1355 YES	9.957E-1 (pos.157820423)	Short statu cardiac atrium morphogenesis,cartilage development ir	C=0, T=20 C=0.0, T=1.0	0.00001	263.741	20 C=0/0, T=9/11
chr3:15807725	A/A	T	A	SNV	ANKRD28	ANKRD28:intronic:NM_015199.3	1	100	intronic	NM_01519 c.190+16A p.?	0.02		Ankyrin repe rs6789076	0.4094 YES	3.577E-1 (pos.15807725)	Ankyrin rep COPII vesicle coating,Golgi membrane,cytosol,nucleopla	T=0, A=55 T=0.0, A=1.0	0.00001	814.907	55 T=0/0, A=30/2
chr3:158300732	G/G	A	G	SNV	MLF1	MLF1:intronic:NM_022443.4	1	100	intronic	NM_02244 c.48-9491: p.?	-2.11		Myelodyspla rs2566341	YES	9.949E-1 (pos.158300732)	Myeloid lei DNA bindi Digestive System Diseases;Digestive System	A=0, G=104 A=0.0, G=1.0	0.00001	1626.73	104 A=0/0, G=16/8
chr2:208841964	G/G	A	G	SNV	PLEKHM3	PLEKHM3:exonic:NM_001080475.2	1	100	3 synonymous	NM_00108 c.957T>C p.(=)	-0.49	0.1(COSM40C	PH domain;P rs4441448	0.285 YES	4.489E-1;0.0 (pos.20884: acute_myeloid_leukaemia	metal ion binding	A=0, G=69 A=0.0, G=1.0	0.00001	1072.78	69 A=0/0, G=37/3
chr3:158431508	A/G	A	G	SNV	RARRES1	RARRES1:intronic:NM_206963.1	1	53.13	intronic	NM_20696 c.339+78T p.?	-0.97		Latexin rs2279562	YES		Retinoic aci extracellula Digestive System D Tretinoin	A=15, G=17 A=0.4688, G=0.5313	0.00001	173.545	32 A=9/6, G=14/3
chr3:158431533	G/A	G	A	SNV	RARRES1	RARRES1:intronic:NM_206963.1	1	50	intronic	NM_20696 c.339+53C p.?	-0.49		Latexin rs184390017	YES		Retinoic aci extracellula Digestive System D Tretinoin	G=17, A=17 G=0.5, A=0.5	0.00001	170.582	34 G=9/8, A=14/3
chr3:160679744	T/T	A	T	SNV	PPM1L	PPM1L:intronic:NM_139245.3	1	100	intronic	NM_13924 c.574+46A p.?	-0.24		Protein phos rs111355383,rs7629	0.1214 YES	7.578E-1 (pos.160679744)	Protein phc MAPK casc Nutrition Disorders;Nutritional and Metaboli	A=0, T=21 A=0.0, T=1.0	0.00001	276.512	21 A=0/0, T=4/17
chr3:161216845	T/T	C	T	SNV	OTOL1	OTOL1:intronic:NM_001080440.1	1	100	intronic	NM_00108 c.365-114 p.?	-0.88		C1q domain,(rs1506482	YES		collagen trimer,extracellular region	C=0, T=24 C=0.0, T=1.0	0.00001	334.791	24 C=0/0, T=13/1
chr3:161216860	C/C	T	C	SNV	OTOL1	OTOL1:intronic:NM_001080440.1	1	100	intronic	NM_00108 c.365-99T: p.?	-0.13		C1q domain,(rs1506483	YES		collagen trimer,extracellular region	T=0, C=24 T=0.0, C=1.0	0.00001	334.79	24 T=0/0, C=13/1
chr3:161216912	C/C	T	C	SNV	OTOL1	OTOL1:intronic:NM_001080440.1	1	100	intronic	NM_00108 c.365-47T: p.?	-0.77		C1q domain,(rs1506484	0.3123 YES	1.845E-1 (pos.161216912)	collagen trimer,extracellular region	T=0, C=26 T=0.0, C=1.0	0.00001	371.403	26 T=0/0, C=12/1
chr6:161152240	A/A	G	A	SNV	PLG	PLG:exonic:NM_000301.3	1	100	11 missense	NM_0003C c.1414G>A p.Asp472Asn	0.06 0.67 23	0	Domain of un rs4252125	0.3028 YES	6.941E-4 (pos.161152240)	Plasminoge apolipopro Blood Coagulation L Alteplase,Aminocaproic	G=0, A=29 G=0.0, A=1.0	0.00001	427.603	29 G=0/0, A=8/21
chr19:4859937	C/C	T	C	SNV	PLIN3	PLIN3:exonic:NM_005817.4	1	100	3 missense	NM_00581 c.166A>G p.Ile56Val	-0.51 0.6 29	0 0.01(COSM63	Apolipoprote rs6289	0.2719 YES	4.994E-1 (pos.4859937) carcinoma	Perilipin 3 Golgi apparatus,cadherin bind Galsulfase,Idursulfase	T=0, C=77 T=0.0, C=1.0	0.00001	1229.43	77 T=0/0, C=13/6
chr19:4511350	A/A	T	A	SNV	PLIN4	PLIN4:exonic:NM_001080400.1	1	100	3 missense	NM_00108 c.2580A>T p.Lys860Asn	-6.6 1 94	0 0(COSM3766	Apolipoprote rs7259721,rs762135146	YES	0.0 (pos.4511348).4.076(hepatocellula	Perilipin 4 cytosol,intracellular membrane-bounded organelle,lipic	T=0, A=21 T=0.0, A=1.0	0.00001	278.999	21 T=0/0, A=9/12
chr19:4525046	G/G	A	G	SNV	PLIN5	PLIN5:exonic:NM_001013706.2	1	100	7 missense	NM_00101 c.763T>C p.Cys255Arg	-0.35 0.39 180	0 0(COSM3766	Perilipin fam rs1630090,rs961578!	0.1015 YES	6.818E-1 (pos.4525046) adenocarcinoi	Perilipin 5 cytoplasm,cytosol identical protein binding,lipase bindi	A=0, G=35 A=0.0, G=1.0	0.00001	548.282	35 A=0/0, G=14/2
chr4:128814964	T/T	G	T	SNV	PLK4	PLK4:exonic:NM_014264.4	1	100	13 missense	NM_0142€ c.2490G>T p.Glu830Asp	-2.33 0.79 45	0 0.01(COSM4C	POLO box du rs17012739	0.33 YES	6.525E-1;0.0 (pos.12881: acute_myeloid_leukaemia	Polo-like kin ATP bindir Bone Diseases;Bone Diseases, Development	G=0, T=46 G=0.0, T=1.0	0.00001	768.313	46 G=0/0, T=18/2
chr19:281360	T/T	C	T	SNV	PLPP2	PLPP2:utr_3:NM_177543.2	1	100	6 utr_3	NM_17754 c.958G>A p.?	-0.27		PAP2 superfa rs7815	0.3385 YES	8.68E-2 (pos.281360)	Phospholip dephosphorylation,integral component of membrane,ir	C=0, T=39 C=0.0, T=1.0	0.00001	623.98	39 C=0/0, T=28/1
chr3:17051081	A/A	T	A	SNV	PLCL2	PLCL2:intronic:NM_015184.5	1	100	intronic	NM_01518 c.-50-85T> p.?	0.01		C2 domain,Pl rs28498854	YES		Phospholip B cell proli Arthritis;Arthritis, Rheumatoid;Autoimmune	T=0, A=128 T=0.0, A=1.0	0.00001	2093.98	128 T=0/0, A=81/4
chr10:122348969	T/T	C	T	SNV	PLPP4	PLPP4:exonic:NM_001030059.2	1	100	7 synonymous	NM_00103 c.771C>T p.(=)	0.27(COSM56		PAP2 superfa rs1047369	0.2362 YES	5.693E-2 (pos.12234896! squamous_cell_carcinoma	Fc-gamma receptor signaling pathway involved in phago	C=0, T=122 C=0.0, T=1.0	0.00001	1973.55	122 C=0/0, T=63/5
chr17:7292107	A/A	G	A	SNV	PLSCR3,TMEM256-PLSCR3,TNK1	PLSCR3:downstream:NM_001201576.1, TMEM256-PLSCR3:c	1	100	12 missense	NM_00125 c.1792G>A p.Val598Met, p.?	2.24 0 21	0.132 0.94(COSM41	Protein kinas rs6503018	0.0457 YES	7.625E-1 (pos.7292107) neoplasm	Phospholip ATP binding,SH3 domain binding,apoptotic process,calc	G=0, A=162 G=0.0, A=1.0	0.00001	2617.55	162 G=0/0, A=98/6
chr17:7293715	T/T	C	T	SNV	PLSCR3,TMEM256-PLSCR3,TNK1	PLSCR3:exonic:NM_001201576.1, TMEM256-PLSCR3:exonic	1	100	8 missense	NM_00125 c.*1312C> p.?, p.Val293Ile	-1.71 1 29	0	Scramblase rs3744549	0.1502 YES	3.269E-1 (pos.7293715)	Phospholip ATP binding,SH3 domain binding,apoptotic process,calci	C=0, T=23 C=0.0, T=1.0	0.00001	316.747	23 C=0/0, T=11/1
chr10:20534333	G/G	A	G	SNV	PLXDC2	PLXDC2:exonic:NM_032812.8	1	100	13 missense	NM_03281 c.1372A>G p.Ile458Val	2.1 1 29	0 0.22(COSM54	Plexin repeat rs2778979	0.0959 YES	5.535E-1 (pos.20534333) acute_myeloi	Plexin dom: extracellu Cardiovascular Diseases;Diabetes Complicat	A=0, G=27 A=0.0, G=1.0	0.00001	389.988	27 A=0/0, G=21/6
chr3:208207839	A/A	G	A	SNV	PLXNA2	PLXNA2:exonic:NM_025179.3	1	100	27 synonymous	NM_02517 c.4863C>T p.(=)	-0.69	0.14(COSM37	IPT/TIG doma rs3736963	0.4255 YES	2.59E-1 (pos.208207839) hepatocellula	Plexin A2 (C branchion Mental Disorders;Schizophrenia;Schizophrer	G=0, A=74 G=0.0, A=1.0	0.00001	1170.13	74 G=0/0, A=19/5
chr3:176765253	A/A	G	A	SNV	TBL1XR1	TBL1XR1:intronic:NM_024665.5	1	100	intronic	NM_0246€ c.766+12C p.?	-0.07		Anaphase-pr rs1201300	0 YES	1.0 (pos.176765253)	Transducin beta-cater Autoimmune Diseases;Female Urogenital Di	G=0, A=25 G=0.0, A=1.0	0.00001	353.019	25 G=0/0, A=3/22
chr3:176765269	T/C	T	C	SNV	TBL1XR1	TBL1XR1:intronic:NM_024665.5	1	56	intronic	NM_0246€ c.766+5A> p.?	-0.51	0.07(COSM4C	Anaphase-pr rs3749234	0.205 YES	5.235E-1 (pos.17676526! acute_myeloi	Transducin beta-cater Autoimmune Diseases;Female Urogenital Di	T=11, C=14 T=0.44, C=0.56	0.00001	134.319	25 T=2/9, C=1/13
chr3:176769608	G/A	G	A	SNV	TBL1XR1	TBL1XR1:intronic:NM_024665.5	1	47.62	intronic	NM_0246€ c.205-94C p.?	0.43		Anaphase-pr rs1881979	YES		Transducin beta-cater Autoimmune Diseases;Female Urogenital Di	G=11, A=1C G=0.5238, A=0.4762	0.00001	82.29	21 G=4/7, A=3/7
chr3:208390469	T/T	C	T	SNV	PLXNA2	PLXNA2:exonic:NM_025179.3	1	100	2 missense	NM_02517 c.799G>A p.Ala267Thr	3.53 0.12 58	0.053 0.79(COSM37	IPT/TIG doma rs3748735	0.196 YES	1.37E-1 (pos.208390469) acute_myeloi	Plexin A2 (C branchion Mental Disorders;Schizophrenia;Schizophrer	C=0, T=29 C=0.0, T=1.0	0.00001	427.603	29 C=0/0, T=14/1
chrX:153694334	G/G	C	G	SNV	PLXNA3	PLXNA3:exonic:NM_017514.4	1	100	14 missense	NM_01751 c.2589C>G p.Asp863Glu	2.84 1 45	0 0.35(COSM37	IPT/TIG doma rs5945430	0.133 YES	1.384E-1 (pos.15369433! adenocarcinoma	branchion Endometrial Neoplasms;Female Urogenital D	C=0, G=29 C=0.0, G=1.0	0.00001	427.603	29 C=0/0, G=24/5
chr12:94603406	G/C	C	G	SNV	PLXNC1	PLXNC1:exonic:NM_005761.2	1	50	5 missense	NM_00576 c.1480G>C p.Glu494Gln	0.1 0.24 29	0	IPT/TIG doma rs34565975	0.0128 YES	0.0 (pos.94603406)	cell adhes Endometriosis;Female Urogenital Diseases;Fe	G=9, C=9 G=0.5, C=0.5	0.00001	121.346	18 G=4/5, C=11/2
chr3:205801872	G/G	A	G	SNV	PM20D1	PM20D1:exonic:NM_152491.4	1	100	11 missense	NM_15249 c.1137T>G p.Ile380Thr	0.91 1 89	0.016 0.19(COSM37	Peptidase di rs1361754	0.4771 YES	2.315E-3 (pos.20580187; acute_myeloid_leukaemia	Peptidase M adaptive thermogenesis,amide biosynthetic process,ce	A=0, G=40 A=0.0, G=1.0	0.00001	644.279	40 A=0/0, G=19/2
chr3:179448383	C/T	C	T	SNV	USP13	USP13:intronic:NM_003940.2	1	52.27	intronic	NM_00394 c.1161-23 p.?	0.26		UBA/TS-N dor rs3213761,rs762393:	0.2767 YES	0.0 (pos.17944837).7.51E-1 (pos.179448	Ubiquitin-si BAT3 comp Arsenic Poisoning;Heavy Metal Poisoning, Ne	C=20, T=23 C=0.4651, T=0.5349	0.00001	251.804	44 C=15/5, T=9/1
chr3:205814497	C/C	T	C	SNV	PM20D1	PM20D1:exonic:NM_152491.4	1	100	3 missense	NM_15249 c.445A>G p.Ile149Val	4.73 0.04 29	0	Peptidase di rs1891460	0.0541 YES	7.406E-1 (pos.205814497)	Peptidase M adaptive thermogenesis,amide biosynthetic process,ce	T=0, C=27 T=0.0, C=1.0	0.00001	389.969	27 T=0/0, C=17/1
chr18:57567451	C/T	C	T	SNV	PMAIP1	PMAIP1:intronic:NM_021127.2	1	45.83	1 synonymous	NM_02112 c.42C>T p.(=)	-3.43	0(COSM3756	Phorbol-12-n rs61736947	0.0415 YES	0.0 (pos.57567451) adenocarcinoi	Phorbol-12- T cell hom Hemic and Lymphatic Diseases;Immune Syst	C=13, T=11 C=0.5417, T=0.4583	0.00001	95.5703	24 C=6/7, T=7/4
chr20:56227532	T/T	C	T	SNV	PMEPA1	PMEPA1:intronic:NM_020182.4	1	100	intronic	NM_02018 c.441A>G p.(=)	-8.38	0.02(COSM1412695)	rs6025699	0.2364 YES	8.417E-1 (pos.56227532) neoplasm	Prostate tr: Golgi membrane,R-SMAD binding,WW domain binding,a	C=0, T=28 C=0.0, T=1.0	0.00001	408.699	28 C=0/0, T=10/1
chr3:182590354	T/T	C	T	SNV	ATP11B	ATP11B:intronic:NM_014616.2	1	100	intronic	NM_01461 c.2048+29 p.?	-0.69		Cation transı rs771156	0.2422 YES	4.588E-1 (pos.182590354)	ATPase, cla ATP binding,Golgi apparatus,aminophospholipid transpc	C=0, T=44 C=0.0, T=1.0	0.00001	725.665	44 C=0/0, T=18/2
chr3:182681853	C/C	T	C	SNV	DCUN1D1	DCUN1D1:intronic:NM_020640.3	1	100	intronic	NM_02064 c.221-16A p.?	0.02		Cullin bindin rs4859147,rs772799:	0.1373 YES	2.49E-1 (pos.182681853)	DCN1 doma biological_process,cullin family protein binding,cytosol,	T=0, C=21 T=0.0, C=1.0	0.00001	281.235	21 T=0/0, C=12/9
chr16:72170408	C/A	C	A	SNV	PMFBP1	PMFBP1:exonic:NM_031293.2	1	46.51	9 missense	NM_03129 c.1142G>T p.Arg381Leu	-0.1 0.01 102	0.901	rs35781168	0.0151 YES	0.0;3.467E-4 (pos.72170408)	biological_process,cytoplasm,molecular_function	C=23, A=20 C=0.5349, A=0.4651	0.00001	212.321	43 C=11/12, A=6/
chr3:182871299	G/G	T	G	SNV	LAMP3	LAMP3:intronic:NM_014398.3	1	100	intronic	NM_01439 c.759+171 p.?	-1.83		Lysosome-as rs6414500	YES		adaptive i Bipolar Disorder;Bipolar and Related Disord	T=0, G=50 T=0.0, G=1.0	0.00001	838.265	50 T=0/0, G=39/1
chr3:182871464	A/G	A	G	SNV	LAMP3	LAMP3:intronic:NM_014398.3	1	53.33	intronic	NM_01439 c.759+6T> p.?	1.41		Lysosome-as rs500288	0.0802 YES	3.533E-2 (pos.182871464)	adaptive i Bipolar Disorder;Bipolar and Related Disord	A=21, G=24 A=0.4667, G=0.5333	0.00001	276.147	45 A=16/5, G=16/
chr5:75003678	T/C	T	C	SNV	POC5	POC5:exonic:NM_001099271.1	1	50	3 missense	NM_00109 c.107A>G p.His36Arg	2.17 1 29	0 0.04(COSM4159986).0.04(C	rs2307111	0.4033 YES	6.157E-1;0.0 (pos.75003€ neoplasm	cell cycle, Nutrition Disorders;Nutritional and Metaboli	T=13, C=13 T=0.5, C=0.5	0.00001	119.163	26 T=11/2, C=5/8
chr3:53544439	A/A	G	A	SNV	PODN	PODN:intronic:NM_001199081.2	1	100	9 synonymous	NM_0011€ c.1344G>A p.(=)	0.01	0.14(COSM39	Leucine Rich rs1288388	0.272 YES	3.015E-1;0.0 (pos.53544€ acute_myeloid	Podocan collagen binding,cytokine-mediated signaling pathway,e	G=0, A=24 G=0.0, A=1.0	0.00001	334.792	24 G=0/0, A=12/1
chr3:53544597	C/C	T	C	SNV	PODN	PODN:exonic:NM_001199081.2	1	100	10 missense	NM_0011€ c.1502C>T p.Val501Ala	2.27 1 64	0	Leucine Rich rs1288389	0.1119 YES	3.871E-1 (pos.53544597)	Podocan collagen binding,cytokine-mediated signaling pathway,e	T=0, C=69 T=0.0, C=1.0	0.00001	1072.78	69 T=0/0, C=35/3
chr3:183493991	A/A	G	A	SNV	YEATS2	YEATS2:intronic:NM_018023.4	1	100	intronic	NM_01802 c.2577+80 p.?	-0.45		YEATS family rs2606220	YES		YEATS doma Ada2/Gcn5/Ada3 transcription activator complex,TBP-cl	G=0, A=30 G=0.0, A=1.0	0.00001	446.483	30 G=0/0, A=18/1
chr3:183669396	T/T	C	T	SNV	ABCC5	ABCC5:intronic:NM_005688.3	1	100	intronic	NM_00568 c.2815-38 p.?	0.07		ABC transpor rs4148586	0.3179 YES	2.56E-1;0.0 (pos.183669396)	ATP-bindin ATP binding Arthritis;Arthritis, F Adefovir Dipivoxil,Alpros	C=0, T=29 C=0.0, T=1.0	0.00001	427.603	29 C=0/0, T=9/20
chr7:131191461	A/G	A	G	SNV	PODXL	PODXL:exonic:NM_005397.3	1	57.45	5 synonymous	NM_00539 c.1030T>C p.(=)	-0.34		CD34/Podoc: rs3212300	0.0764 YES	3.467E-4 (pos.131191461)	Podocalyxir apical pla: Brain Diseases;Cardiovascular Diseases;Car	A=20, G=27 A=0.4255, G=0.5745	0.00001	326.535	47 A=7/13, G=10/
chr3:183689332	C/CG	C	CG	INDEL	ABCC5	ABCC5:intronic:NM_005688.3	1	50	intronic	NM_00568 c.1761+19 p.?	-0.64		ABC transpor rs142294585	1.00E-04	0.0 (pos.183689332)	ATP-bindin ATP binding Arthritis;Arthritis, F Adefovir Dipivoxil,Alpros	C=10, CG=1 C=0.5, CG=0.5	0.00001	53.7449	20 C=0/1, CG=1/9
chr3:84534383	C/C	A	C	SNV	POF1B	POF1B:utr_3:NM_024921.3	1	100	17 utr_3	NM_02492 c.1814T>G p.?	-0.4		rs2227090,rs762909	0.0024 YES	2.528E-3;0.0;0.0;0.0 (pos.84534383;1.9.8	FLJ22792 pr actin cytoi Adnexal Diseases;Endocrine S: Benign	A=0, C=23 A=0.0, C=1.0	0.00001	296.279	23 A=0/0, C=13/1
chr3:183756426	C/C	T	C	SNV	HTR3D	HTR3D:intronic:NM_001163646.1	1	100	intronic	NM_0011€ c.1135+14 p.?	0.05		Neurotransn rs6799766	0.4098 YES	7.685E-1 (pos.183756426)	5-hydroxytr cation transmembrane transpc Ergoloid mesylate,Ethan	T=0, C=20 T=0.0, C=1.0	0.00001	263.738	20 T=0/0, C=10/1
chr3:18392819	C/T	C	T	SNV	SATB1	SATB1:intronic:NM_001131010.3	1	50	intronic	NM_00113 c.779+46 p.?	-0.08		Special AT-r rs116044839	YES	7.212E-3 (pos.18392819)	Special AT-r CD4-positive, alpha-beta T cell differentiation,CD8-posit	C=16, T=16 C=0.5, T=0.5	0.00001	158.383	32 C=11/5, T=7/9
chr3:84563135	A/A	T	A	SNV	POF1B	POF1B:exonic:NM_024921.3	1	100	10 missense	NM_02492 c.1045A>T p.Met349Leu	1.11 1 15	0 0.06(COSM4157042).0.06(C	rs36774	0.2277 YES	9.765E-1 (pos.84563135) neoplasm	FLJ22792 pr actin cytoi Adnexal Diseases;Endocrine S: Benign	T=0, A=34 T=0.0, A=1.0	0.00001	523.649	34 T=0/0, A=7/27
chr17:26676135	T/T	C	T	SNV	POLDIP2	POLDIP2:exonic:NM_015584.4	1	100	11 synonymous	NM_01558 c.965G>A p.(=)	0.13		ApaG domair rs13469	0.4802 YES	7.187E-1 (pos.26676135)	Polymerase DNA binding,mitochondrial nucleoid,mitochondrion,mit	C=0, T=47 C=0.0, T=1.0	0.00001	788.736	47 C=0/0, T=21/2
chr3:121154974	C/C	G	C	SNV	POLQ	POLQ:exonic:NM_199420.3	1	100	28 missense	NM_19942 c.7538A>G p.Gln2513Arg	-0.47 1 43	0	DEAD/DEAH b rs1381057	0.3176 YES	8.976E-1 (pos.121154974)	Polymerase 5'-3' exonuclease activity,5'-deoxyribose-5-phosphate ly	G=0, C=42 G=0.0, C=1.0	0.00001	685.219	42 G=0/0, C=25/1
chr3:121208833	C/C	G	C	SNV	POLQ	POLQ:exonic:NM_199420.3	1	100	16 missense	NM_19942 c.2945C>G p.Thr982Arg	-2.24 0.51 71	0 0.02(COSM37	DEAD/DEAH b rs3218649	0.3714 YES	8.964E-1 (pos.12120883: adenocarcinoi	Polymerase 5'-3' exonuclease activity,5'-deoxyribose-5-phosphate ly	G=0, C=42 G=0.0, C=1.0	0.00001	685.22	42 G=0/0, C=28/1
chr3:183960252	C/C	A	C	SNV	ALG3,MIR1224,VWA5B2	MIR1224:downstream:NR_030410.1, ALG3:utr_3:NM_0057	1	100		NM_13834 c.*426A>C p.?, p.?	0.22		ALG3 protein rs706584	0.2031 YES	2.738E-1 (pos.183960252)	Alg3, S. cere alpha-1,3- Carbohydrate Metabolism, Inb Benign/Likely	A=0, C=23 A=0.0, C=1.0	0.00001	316.747	23 A=0/0, C=16/7
chr12:111308516	A/A	T	A	SNV	POLR1B	POLR1B:exonic:NM_019014.5	1	100	5 synonymous	NM_01901 c.699T>A p.(=)	-4.04	0.04(COSM4C	RNA polymer rs2304555	0.2593 YES	3.504E-1 (pos.11130851€ acute_myeloid_leukaemia	Polymerase DNA binding,DNA-directed 5'-3' RNA polymerase activity,	T=0, A=95 T=0.0, A=1.0	0.00001	1596.53	95 T=0/0, A=41/5
chr3:183996213	C/T	C	T	SNV	ECE2,EEF1AKMT4-ECE2,EEF1AKMT4-ECE2	EEF1AKMT4-ECE2:intronic:NM_014693.3, ECE2:intronic:NM	1	97.3	intronic	NM_01465 c.1105-67l p.?, p.?	-1.8		Methyltransf rs843372	0.2288 YES		Endothelin- Golgi membrane,brain development,cardioblast differer	C=1, T=36 C=0.027, T=0.973	0.00001	551.735	37 C=0/1, T=11/2
chr3:184003194	T/T	C	T	SNV	ECE2,EEF1A KMT4-ECE2	EEF1AKMT4-ECE2:intronic:NM_014693.3, ECE2:intronic:NM	1	100	intronic	NM_01465 c.1503-72(p.?, p.?	-0.46		Methyltransf rs1687230	YES		Endothelin- Golgi membrane,brain development,cardioblast differer	C=0, T=52 C=0.0, T=1.0	0.00001	766.909	52 C=0/0, T=24/2
chr17:7400137	C/C	T	C	SNV	POLR2A	POLR2A:exonic:NM_000937.4	1	100	5 synonymous	NM_00093 c.597T>C p.(=)	1.74	0.44(COSM41	RNA polymer rs9898024	3.00E-04	1.0 (pos.7400137) neoplasm	Polymerase 7-methylguanosine mRNA capping,DNA binding,DNA-dire	T=0, C=31 T=0.0, C=1.0	0.00001	465.819	31 T=0/0, C=7/24
chr3:184045799	G/G	A	G	SNV	EIF4G1	EIF4G1:intronic:NM_001194947.1	1	100	intronic	NM_00119 c.3974+9A p.?	-2.13		MA3 domain, rs939317	0.2426 YES	7.233E-1 (pos.184045799)	Eukaryotic translation initiation factor 4 gamma, 1	A=0, G=29 A=0.0, G=1.0	0.00001	427.603	29 A=0/0, G=14/1
chr3:184070675	G/G	A	G	SNV	CLCN2	CLCN2:intronic:NM_004366.5	1	100	intronic	NM_0043€ c.2144-77 p.?	-0.4		Voltage gate rs3749223	YES		Chloride ch cell differe Brain Diseases;Cen Lubiprostone	A=0, G=26 A=0.0, G=1.0	0.00001	371.417	26 A=0/0, G=11/1
chr3:184654166	G/G	A	G	SNV	VPS8	VPS8:intronic:NM_015303.3	1	100	intronic	NM_0153C c.2982+43 p.?	-0.28		Golgi CORVET rs6444004	0 YES	1.0 (pos.184654166)	CORVET complex,early endosome,endosomal vesicle fus	A=0, G=27 A=0.0, G=1.0	0.00001	389.988	27 A=0/0, G=10/1
chr3:184682214	C/C	G	C	SNV	VPS8	VPS8:intronic:NM_015303.3	1	100	intronic	NM_0153C c.3178-42 p.?	-1.89		Golgi CORVET rs6799328	0.2342 YES	3.764E-1 (pos.184682214)	CORVET complex,early endosome,endosomal vesicle fus	G=0, C=83 G=0.0, C=1.0	0.00001	1349.87	83 G=0/0, C=40/4
chr17:7404107	C/C	T	C	SNV	POLR2A	POLR2A:exonic:NM_000937.4	1	100	11 synonymous	NM_00093 c.1821T>C p.(=)	1.11		RNA polymer rs7217707	3.00E-04	1.0 (pos.7404107)	Polymerase 7-methylguanosine mRNA capping,DNA binding,DNA-dire	T=0, C=37 T=0.0, C=1.0	0.00001	583.666	37 T=0/0, C=8/29
chr3:186276393	T/T	C	T	SNV	TBCCD1	TBCCD1:intronic:NM_018138.4	1	100	intronic	NM_01813 c.337-32G p.?	-1.94		Tubulin bindi rs9854418	0.4548 YES	7.175E-1;0.0 (pos.186276393)	cytoplasm,maintenance of Golgi location,maintenance c	C=0, T=27 C=0.0, T=1.0	0.00001	389.88	27 C=0/0, T=10/1
chr17:7417086	A/A	G	A	SNV	POLR2A	POLR2A:exonic:NM_000937.4	1	100	29 missense	NM_00093 c.5503G>A p.Ala1835Thr	-4.21 0.9 58	0	RNA polymer rs4344809	0 YES	9.999E-1 (pos.7417086)	Polymerase 7-methylguanosine mRNA capping,DNA binding,DNA-dire	G=0, A=38 G=0.0, A=1.0	0.00001	603.725	38 G=0/0, A=14/2
chr17:7399866	G/A	G	A	SNV	POLR2A	POLR2A:exonic:NM_000937.4	1	59.18	4 synonymous	NM_00093 c.471G>A p.(=)	-1.61	0.32(COSM4C	RNA polymer rs2301609	0.2091 YES	3.559E-2 (pos.7399866) acute_myeloi	Polymerase 7-methylguanosine mRNA capping,DNA binding,DNA-dire	G=20, A=29 G=0.4082, A=0.5918	0.00001	360.526	49 G=13/7, A=8/2
chr17:7411770	C/T	C	T	SNV	POLR2A	POLR2A:exonic:NM_000937.4	1	50	20 synonymous	NM_00093 c.3441C>T p.(=)	-1.55		RNA polymer rs2228132	0.2088 YES	5.354E-2 (pos.7411770)	Polymerase 7-methylguanosine mRNA capping,DNA binding,DNA-dire	C=12, T=12 C=0.5, T=0.5	0.00001	106.709	24 C=5/7, T=6/6
chr3:186445186	GA/GA	G	GA	INDEL	KNG1	KNG1:intronic:NM_001102416.2	1	100	intronic	NM_0011€ c.672+53C p.?	-0.2		Cathelicidin rs3841557	YES		Kininogen 1 G-protein Acute Kidney Injury;Anaphylaxis;Angioedema	G=0, GA=3: G=0.0, GA=1.0	0.00001	528.564	34 G=0/0, GA=11/
chr17:7404426	G/A	G	A	SNV	POLR2A	POLR2A:exonic:NM_000937.4	1	47.62	12 synonymous	NM_00093 c.2049G>A p.(=)	1.81		RNA polymerase Rpb1 C-terminal repeat,RNA polymerase Rpb1, domain 1,RNA polymerase Rpb1, do			Polymerase 7-methylguanosine mRNA capping,DNA binding,DNA-dire	G=11, A=1C G=0.5238, A=0.4762	0.00001	82.2866	21 G=9/2, A=7/3
chr3:187003495	G/G	A	G	SNV	MASP1	MASP1:intronic:NM_139125.3	1	100	intronic	NM_13912 c.237+118 p.?	-0.9		CUB domain; rs698105	YES		Mannan-bir calcium io Abnormalities, Multiple;Amyotrophic Lateral	A=0, G=20 A=0.0, G=1.0	0.00001	263.785	20 A=0/0, G=11/9
chr5:140251	G/G	C	G	SNV	PORL2	POLR2L:utr_3:NM_021128.4	1	100	2 utr_3	NM_02112 c.*325G>C p.?	-1.59		RNA polymer rs11629	YES		Polymerase 7-methylguanosine mRNA capping,DNA binding,DNA-dire	C=0, G=58 C=0.0, G=1.0	0.00001	867.278	58 C=0/0, G=29/2
chr20:18462421	G/G	A	G	SNV	POLR3F	POLR3F:exonic:NM_006466.3	1	100	8 synonymous	NM_00646 c.840A>G p.(=)	-1.22		RNA polymer rs1055171	0.1284 YES	8.056E-1 (pos.18462421)	Polymerase DNA-directed 5'-3' RNA polymerase activity,DNA-directe	A=0, G=53 A=0.0, G=1.0	0.00001	781.924	53 A=0/0, G=38/1
chr3:188202550	T/T	C	T	SNV	LPP	LPP:intronic:NM_005578.4	1	100	intronic	NM_00557 c.306+58A p.?	0.27		LIM domain rs2067078	YES		Lipoma-pre biological Arthritis;Arthritis, Juvenile;Autoimmune Dise	C=0, T=24 C=0.0, T=1.0	0.00001	334.753	24 C=0/0, T=12/1
chr3:188590358	T/T	C	T	SNV	LPP	LPP:intronic:NM_005578.4	1	100	intronic	NM_00557 c.1590-73l p.?	-2.59		LIM domain rs4687721	YES		Lipoma-pre biological Arthritis;Arthritis, Juvenile;Autoimmune Dise	C=0, T=41 C=0.0, T=1.0	0.00001	664.7	41 C=0/0, T=23/1
chr3:53103945	GC/TC	GG	GC	SNV	POM121L12	POM121L12:exonic:NM_182595.3	2	51.72	1 synonymous	NM_18259 c.581_582 p.Gly194Val, p.(=)	-0.19,-1.72	109	POM121 fam rs12113170,rs20058	0.001 YES	0.0 (pos.53103945).2.807E-1;0.0 (pos.53103946)	RNA export from nucleus,nuclear localization sequence I	GG=2, CG=: GG=0.0345, CG=0.03	0.00001	804.306	58 GG=0/2, CG=2
chr3:189604149	G/G	T	G	SNV	TP63	TP63:intronic:NM_003722.4	1	100	intronic	NM_00372 c.1350-34 p.?	-1.82		P53 DNA-binc rs1554131	0.2055 YES	5.912E-1;2.326E-4 (pos.189604149)	Tumor prot DNA bindi Abnormalities, Multiple;Adenc Benign	T=0, G=31 T=0.0, G=1.0	0.00001	465.818	31 T=0/0, G=19/1
chr3:189604160	C/C	T	C	SNV	TP63	TP63:intronic:NM_003722.4	1	100	intronic	NM_00372 c.1350-23 p.?	0.15		P53 DNA-binc rs1345186	0.2055 YES	5.958E-1 (pos.189604160)	Tumor prot DNA bindi Abnormalities, Multiple;Adenc Benign	T=0, C=33 T=0.0, C=1.0	0.00001	504.597	33 T=0/0, C=19/1
chr3:190106242	C/C	T	C	SNV	CLDN16	CLDN16:intronic:NM_006580.3	1	100	intronic	NM_00658 c.324+10T p.?	-0.15		PMP-22/EMP rs1491994	0.248 YES	2.151E-2 (pos.190106242)	Claudin 16 : bicellular Calcinosis;Calcium Metabolisr Benign	T=0, C=42 T=0.0, C=1.0	0.00001	685.219	42 T=0/0, C=14/2
chr3:190282197	G/A	A	G	SNV	IL1RAP	IL1RAP:intronic:NM_001167931.1	1	58.33	intronic	NM_0011€ c.64+55G> p.?	-0.33		Immunoglobi rs6764468	0.3246 YES		Interleukin cytokine-n Digestive System Diseases;Liver Diseases	G=35, A=4! G=0.4167, A=0.5833	0.00001	568.124	84 G=23/12, A=2
chr3:190995847	C/C	T	C	SNV	UTS2B	UTS2B:intronic:NM_198152.3	1	100	intronic	NM_19815 c.202+14A p.?	0.63		Urotensin II rs3214008	0.3971 YES	6.039E-1 (pos.190995847)	G-protein coupled receptor binding,G-protein coupled re	T=0, C=70 T=0.0, C=1.0	0.00001	1089.03	70 T=0/0, C=25/4
chr6:27277402	T/T	C	T	SNV	POM121L2	POM121L2:exonic:NM_033482.3	1	100	1 missense	NM_03348 c.2548G>A p.Gly850Ser	-0.5 56		POM121 fam rs6456773	0.0217 YES	9.936E-1 (pos.27277402)	RNA expor Mental Disorders;Schizophrenia;Schizophrer	C=0, T=45 C=0.0, T=1.0	0.00001	747.393	45 C=0/0, T=29/1

chr3:191178925	A/A	G	A	SNV	LINCR-0002.PYDC2	LINCR-0002:intronic nc:NR 120606.1, PYDC2:upstream:NM	1	100		intronic	NR 12060(	c.-27G>A	p.?	-0.24						rs539897	0.4624	YES	6.022E-2 (pos.191178925)		Pyrin doma	cytoplasm,inflammatory response,innate immune resp(			G=0, A=20	G=0.0, A=1.0	0.00001	263.779	20	G=0/0, A=11/9
chr1:46655158	C/C	T	C	SNV	POMGNT1	POMGNT1:exonic:NM 001243766.1	1	100	21	missense	NM 00124	c.1867A>G	p.Met623Val	5.76	1	21	0		GNT-I family,I	rs6659553	6.00E-04	YES	1.0 (pos.46655158)		Protein 0-mannose bet		Cobblestone Lissencephaly;Cc	Benign	T=0, C=62	T=0.0, C=1.0	0.00001	940.319	62	T=0/0, C=17/4
chr3:192444899	C/A	C	A	SNV	FGF12		1	45.83		intronic				3.73						rs13060864	0.1898	YES			Fibroblast growth factor-12				C=26, A=22	C=0.5417, A=0.4583	0.00001	222.895	48	C=20/6, A=8/1
chr3:193016895	C/C	T	C	SNV	ATP13A5	ATP13A5:intronic:NM 198505.3	1	100		intronic	NM 19850	c.2848+25	p.?	-0.94					Cation trans	rs1878716	0.1571	YES	9.858E-1 (pos.193016895)			ATP binding,ATP hydrolysis coupled cation transmembra			T=0, C=28	T=0.0, C=1.0	0.00001	389.493	28	T=0/0, C=18/1
chr3:193062906	A/A	G	A	SNV	ATP13A5	ATP13A5:intronic:NM 198505.3	1	100		intronic	NM 19850	c.742-42C	p.?	0.21					Cation trans	rs6444706	0.0524	YES	9.62E-1 (pos.193062906)			ATP binding,ATP hydrolysis coupled cation transmembra			G=0, A=25	G=0.0, A=1.0	0.00001	353.019	25	G=0/0, A=13/1
chr3:193081864	G/A	G	A	SNV	ATP13A5	ATP13A5:intronic:NM 198505.3	1	57.89		intronic	NM 19850	c.237+32C	p.?	0.07					Cation trans	rs71632258,rs92349!	0.26	YES	2.07E-1 (pos.193081864)			ATP binding,ATP hydrolysis coupled cation transmembra			G=16, A=22	G=0.4211, A=0.5789	0.00001	249.051	38	G=9/7, A=13/9
chr3:193355074	C/C	T	C	SNV	OPA1	OPA1:intronic:NM 130837.2	1	100		intronic	NM 13083	c.1035+4T	p.?	0.9					Dynamin fam	rs166850	0.1737	YES	9.609E-1 (pos.193355074)		Optic atrop	GTP bindir	Arthritis:Ataxia:Brain Diseases	Benign	T=0, C=98	T=0.0, C=1.0	0.00001	1660.04	98	T=0/0, C=55/4
chr3:43121559	T/T	C	T	SNV	POMGNT2	POMGNT2:exonic:NM 032806.5	1	100	2	synonymous	NM 03280	c.1365G>A	p.(=)	-3.25					Protein of unl	rs604033	0.2009	YES	7.126E-1 (pos.43121559)		Protein O-m	acetylgluc	Cobblestone Lissencephaly;Cc	Benign	C=0, T=59	C=0.0, T=1.0	0.00001	885.363	59	C=0/0, T=23/3
chr3:19436587	C/C	T	C	SNV	KCNH8	KCNH8:intronic:NM 144633.2	1	100		intronic	NM 14463	c.970-9T>C	p.?	0.11					Cyclic nucleo	rs10470642	0.2998	YES	6.391E-1 (pos.19436587)			integral component of plasma membrane,intracellular,p			T=0, C=70	T=0.0, C=1.0	0.00001	1088.97	70	T=0/0, C=24/4
chr9:134385436	G/G	A	G	SNV	POMT1	POMT1:exonic:NM 007171.3	1	100	8	missense	NM 00717	c.752A>G	p.Gln251Arg	-0.83	0.62	43	0		C-terminal fo	rs2296949,rs386738!	0.0485	YES	9.156E-1 (pos.134385436)		Protein O-m	ER-associa	Cobblestone Lissencephaly;Cc	Benign	A=0, G=90	A=0.0, G=1.0	0.00001	1492.7	90	A=0/0, G=39/5
chr9:134386744	C/C	T	C	SNV	POMT1	POMT1:exonic:NM 007171.3	1	100	10	synonymous	NM 00717	c.942T>C	p.(=)	-1.73					C-terminal fo	rs10901065	0.0478	YES	9.162E-1 (pos.134386744)		Protein O-m	ER-associa	Cobblestone Lissencephaly;Cc	Benign	T=0, C=28	T=0.0, C=1.0	0.00001	408.718	28	T=0/0, C=14/1
chr3:195595632	G/G	A	G	SNV	TNK2	TNK2:intronic:NM 001010938.1	1	100		intronic	NM 00101	c.1778-52	p.?	0.15					EGFR recepto	rs13087251	0.1873	YES	9.448E-1 (pos.195595632)		Tyrosine kir	ATP bindin	Digestive System Di	ATP,Debromohymenialdi	A=0, G=25	A=0.0, G=1.0	0.00001	352.969	25	A=0/0, G=13/1
chr3:195789708	T/T	G	T	SNV	TFRC	TFRC:intronic:NM 001128148.2	1	100		intronic	NM 00112	c.1404+17	p.?	-1.02					PA domain,P(	rs2239640	0.399	YES	1.52E-1;0.0 (pos.195789708)		Transferrin	HFE-transf	Abortion, Spontane	Gallium citrate Ga 67,Ga	G=0, T=20	G=0.0, T=1.0	0.00001	263.785	20	G=0/0, T=5/15
chr3:195798258	C/C	T	C	SNV	TFRC	TFRC:intronic:NM 001128148.2	1	100		intronic	NM 00112	c.687+9A>	p.?	-0.06					PA domain,P(	rs480760	0.035	YES	1.0 (pos.195798258)		Transferrin	HFE-transf	Abortion, Spontane	Gallium citrate Ga 67,Ga	T=0, C=31	T=0.0, C=1.0	0.00001	465.808	31	T=0/0, C=14/1
chr3:195925419	C/C	T	C	SNV	ZDHHC19	ZDHHC19:intronic:NM 001039617.1	1	100		intronic	NM 00103	c.774-97A	p.?	0.06					DHHC palmit	rs56699931		YES	0.0 (pos.195925419)			endoplasmic reticulum,integral component of membran			T=0, C=27	T=0.0, C=1.0	0.00001	389.988	27	T=0/0, C=3/24
chr9:134387488	C/C	T	C	SNV	POMT1	POMT1:exonic:NM 007171.3	1	100	11	synonymous	NM 00717	c.1113T>C	p.(=)	-1.58					C-terminal fo	rs3739494	0.0478	YES	9.157E-1 (pos.134387488)		Protein O-m	ER-associa	Cobblestone Lissencephaly;Cc	Benign	T=0, C=23	T=0.0, C=1.0	0.00001	316.747	23	T=0/0, C=15/8
chr9:134398534	C/C	T	C	SNV	POMT1,UCK1	UCK1:downstream:NM 001261451.2, POMT1:utr 3:NM 00	1	100	20	utr 3	NM 00717	c.2285T>C	p.?, p.?	-1.48					C-terminal fo	rs3739495	0.0479	YES	9.175E-1 (pos.134398534)		Protein O-m	ATP bindin	Cobblestone Lissencephaly;Cc	Benign	T=0, C=64	T=0.0, C=1.0	0.00001	977.635	64	T=0/0, C=24/4
chr14:77745193	C/C	A	C	SNV	POMT2	POMT2:exonic:NM 013382.5	1	100	19	synonymous	NM 01338	c.1911T>G	p.(=)	-0.78				0.06(COSM41	C-terminal fo	rs3209079	0.1903	YES	9.65E-1 (pos.77745193)	astrocytoma (	Putative pr	ER-associa	Cobblestone Lissencephaly;Cc	Benign	A=0, C=34	A=0.0, C=1.0	0.00001	524.186	34	A=0/0, C=25/9
chr3:196746629	C/C	T	C	SNV	MELTF	MELTF:intronic:NM 005929.5	1	100		intronic	NM 00592	c.712+164	p.?	-1.9				0(COSM3760(	ABC transpor	rs1056664,rs749674!	0.2223	YES	0.0 (pos.196746619),2.9	acute_myeloid	leukaemia	C-terminal protein lipidation,anchored component of pl			T=0, C=89	T=0.0, C=1.0	0.00001	1472.21	89	T=0/0, C=13/7
chr3:196778438	C/T	C	T	SNV	DLG1	DLG1:intronic:NM 004087.2	1	46.51		intronic	NM 00408	c.2674+10	p.?	-0.24				0.02(COSM47	Guanylate kir	rs7629533	0.28	YES	7.41E-2 (pos.196778438)	adenocarcinoı	Discs, large	GDP meta	Cleft Palate:Congenital Abnormalities:Conge		C=23, T=20	C=0.5349, T=0.4651	0.00001	212.351	43	C=11/12, T=6/
chr3:197483279	G/G	A	G	SNV	FYTTD1	FYTTD1:intronic:NM 032288.6	1	100		intronic	NM 03228	c.104-18A	p.?	-1.09					Forty-two-thr	rs6794391	0.1362	YES	9.961E-1 (pos.197483279)		Forty-two-tl	RNA binding,intracellular,mRNA binding,mRNA export frc			A=0, G=42	A=0.0, G=1.0	0.00001	684.943	42	A=0/0, G=27/1
chr14:77751925	T/T	C	T	SNV	POMT2	POMT2:exonic:NM 013382.5	1	100	13	synonymous	NM 01338	c.1383G>A	p.(=)	0.02				0.4(COSM414	C-terminal fo	rs2270419	0.1935	YES	9.651E-1 (pos.77751925)	neoplasm	Putative pr	ER-associa	Cobblestone Lissencephaly;Cc	Benign	C=0, T=20	C=0.0, T=1.0	0.00001	263.785	20	C=0/0, T=7/13
chr3:197496984	G/G	A	G	SNV	FYTTD1	FYTTD1:intronic:NM 032288.6	1	100		intronic	NM 03228	c.385-19A	p.?	-0.42					Forty-two-thr	rs1708460	0.0204	YES	1.0 (pos.197496984)		Forty-two-tl	RNA binding,intracellular,mRNA binding,mRNA export frc			A=0, G=21	A=0.0, G=1.0	0.00001	281.234	21	A=0/0, G=18/3
chr7:94937446	T/C	T	C	SNV	PON1	PON1:exonic:NM 000446.5	1	59.09	6	missense	NM 00044	c.575A>G	p.Gln192Arg	-1.3	0.61	43	0	0.03(COSM15	Arylesterase	rs662	0.285	YES	6.614E-1 (pos.94937446)	adenocarcinoı	Paraoxonas	acyl-L-hon	Acute Coronary Syn	Cefazolin association,ı	T=18, C=26	T=0.4091, C=0.5909	0.00001	312.072	44	T=8/10, C=22/
chr3:20141265	G/C	G	C	SNV	KAT2B	KAT2B:intronic:NM 003884.4	1	57.58		intronic	NM 00388	c.577-89G	p.?	0.38					Acetyltransfe	rs41285057,rs554683099		YES				A band,Ada2/Gcn5/Ada3 trans	(3E)-4-(1-METHYL-1H-IND(		G=14, C=19	G=0.4242, C=0.5758	0.00001	204.379	33	G=4/10, C=12/
chr3:20156302	T/C	T	C	SNV	KAT2B	KAT2B:intronic:NM 003884.4	1	53.13		intronic	NM 00388	c.1044-72	p.?	-0.06					Acetyltransfe	rs2365794		YES				A band,Ada2/Gcn5/Ada3 trans	(3E)-4-(1-METHYL-1H-IND(		T=15, C=17	T=0.4688, C=0.5313	0.00001	173.553	32	T=11/4, C=13/
chr3:20212189	G/G	A	G	SNV	SGO1	SGO1:intronic:NM 001012410.4	1	100		intronic	NM 00101	c.1564+27	p.?	0.94					Shugoshin C t	rs1052930,rs796405	0.4006	YES	9.617E-1 (pos.20212189)		Shugoshin-l	attachment of spindle microtubules to kinetochore,cell			A=0, G=44	A=0.0, G=1.0	0.00001	726.567	44	A=0/0, G=29/1
chr3:119367390	C/C	T	C	SNV	POPDC2	POPDC2:exonic:NM 022135.3	1	100	3	synonymous	NM 02213	c.726A>G	p.(=)	-8.25					Popeye prote	rs2688643	9.00E-04	YES	9.997E-1 (pos.119367390)		Popeye don	biological_process,integral component of membrane,m(			T=0, C=182	T=0.0, C=1.0	0.00001	3024.65	182	T=0/0, C=106/
chr7:75614953	C/C	T	C	SNV	POR	POR:exonic:NM 000941.2	1	100	13	synonymous	NM 00094	c.1455T>C	p.(=)	-6.59				0.05(COSM41	FAD binding c	rs2228104	0.0336	YES	8.582E-1:0.0 (pos.75614!	neoplasm	Cytochrome	FMN bindi	Adrenal Gland Dise	2'-Monoph Benign	T=0, C=45	T=0.0, C=1.0	0.00001	747.142	45	T=0/0, C=31/1
chr3:24009279	C/C	A	C	SNV	NR1D2	NR1D2:intronic:NM 005126.4	1	100		intronic	NM 00512	c.1333-25	p.?	1.47					Ligand-bindir	rs67657770	0.4286	YES	4.803E-1 (pos.24009279)		Nuclear rec	DNA bindi	Endometriosis:Female Urogenital Diseases:F		A=0, C=54	A=0.0, C=1.0	0.00001	798.148	54	A=0/0, C=31/2
chr18:14543139	C/C	T	C	SNV	POTEC	POTEC:exonic:NM 001137671.1	1	100	1	missense	NM 00113	c.7A>G	p.Thr3Ala	-1.08	1	58	0	0(COSM1522!	Ankyrin repe	rs28535987		YES	7.111E-1;0.0 (pos.14543	adenocarcinoma,astrocytoma_Grade_IV,neoplasm,squamous_cell_carcinoma					T=0, C=31	T=0.0, C=1.0	0.00001	465.811	31	T=0/0, C=19/1
chr3:27332282	C/T	C	T	SNV	NEK10	NEK10:intronic:NM 199347.3	1	57.14		intronic	NM 19934	c.1606-37(	p.?	-0.21					Phosphotran	rs62255594	0.2681	YES	1.011E-1 (pos.27332282)			ATP bindin	Breast Diseases;Breast Neoplasms;Neoplası		C=18, T=24	C=0.4286, T=0.5714	0.00001	274.323	42	C=9/9, T=4/20
chr3:28381886	AT/AT	A	AT	INDEL	AZI2	AZI2:intronic:NM 022461.4	1	100		intronic	NM 02246	c.216+7T>	p.?	-0.03					TBD domain						5-azacytidiı	I-kappaB kinase/NF-kappaB signaling,T cell activation,cy			A=0, AT=46	A=0.0, AT=1.0	0.00001	619.765	46	A=0/0, AT=25/
chr11:120107411	G/A	G	A	SNV	POU2F3	POU2F3:exonic:NM 001244682.1	1	95.83	1	missense	NM 00124	c.29G>A	p.Gly10Glu	-1.06	0.02	98			Homeobox d(	rs882856		YES	0.0 (pos.120107411)		POU domai	DNA binding,DNA binding transcription factor activity,RN			G=1, A=23	G=0.0417, A=0.9583	0.00001	306.073	24	G=0/1, A=11/1
chr3:2925027	C/T	C	T	SNV	CNTN4	CNTN4:intronic:NM 175607.2	1	50		intronic	NM 17560	c.755+96C	p.?	0.1					Fibronectin t	rs113735808					Contactin 4	C-termina	Autistic Disorder:Biliary Tract Diseases:Biliar		C=17, T=17	C=0.5, T=0.5	0.00001	172.009	34	C=10/7, T=6/1
chr3:29977739	C/C	T	C	SNV	RBMS3	RBMS3:intronic:NM 001003793.2	1	100		intronic	NM 00100	c.1050+52	p.?	-0.23					RNA recogniti	rs1449287	0.0922	YES	9.615E-1 (pos.29977739)		RNA-bindinı	RNA binding,cytoplasm,defense response to tumor cell,r			T=0, C=75	T=0.0, C=1.0	0.00001	1193.81	75	T=0/0, C=53/2
chr3:3030223	C/A	C	A	SNV	CNTN4	CNTN4:intronic:NM 175607.2	1	52.27		intronic	NM 17560	c.1486+67	p.?	-0.44					Fibronectin t	rs13068264		YES			Contactin 4	C-termina	Autistic Disorder:Biliary Tract Diseases:Biliar		C=21, A=23	C=0.4773, A=0.5227	0.00001	260.154	44	C=12/9, A=10/
chrX:82764040	G/G	A	G	SNV	POU3F4	POU3F4:exonic:NM 000307.4	1	100	1	synonymous	NM 00030	c.708A>G	p.(=)	2.27				0.18(COSM41	Homeobox d(	rs386826000,rs5921978			1.0 (pos.82764040)	neoplasm,squ	POU domai	AT DNA bir	Congenital, Hereditary, and Ne	Benign	A=0, G=29	A=0.0, G=1.0	0.00001	427.57	29	A=0/0, G=21/8
chrX:82764042	C/C	G	C	SNV	POU3F4	POU3F4:exonic:NM 000307.4	1	100	1	missense	NM 00030	c.710G>C	p.Gly237Ala	5.87		60		0.23(COSM11	Homeobox d(	rs113043342,rs386826000,rs5921979			1.0 (pos.82764042)	neoplasm,squ	POU domai	AT DNA bir	Congenital, Hereditary, and Ne	Benign	G=0, C=29	G=0.0, C=1.0	0.00001	427.496	29	G=0/0, C=21/8
chr3:3170910	G/G	A	G	SNV	TRNT1	TRNT1:intronic:NM 182916.2	1	100		intronic	NM 18291	c.148+38A	p.?	-0.41					Poly A polym(	rs334772	0.0016	YES	1.0 (pos.3170910)		tRNA nucle(	5'-3' RNA polymerase activity,ATP binding,ATP:3'-cytidine			A=0, G=119	A=0.0, G=1.0	0.00001	1913.51	119	A=0/0, G=38/8
chr4:23815924	C/C	T	C	SNV	PPARGC1A	PPARGC1A:exonic:NM 013261.4	1	100	8	synonymous	NM 01326	c.1182A>G	p.(=)	-1.91				0(COSM3760!	RNA recogniti	rs2970847	0.2023	YES	7.709E-1 (pos.23815924)	adenocarcinoı	Peroxisome	DNA bindi	Amyotrophic Lateral Sclerosis;Bowen's Disea		T=0, C=34	T=0.0, C=1.0	0.00001	524.17	34	T=0/0, C=20/1
chr3:3197871	C/C	T	C	SNV	CRBN	CRBN:intronic:NM 016302.3	1	100		intronic	NM 01630	c.750+32A	p.?	-1.09					ATP-depende	rs1705789,rs756831!	0.0015	YES	0.0 (pos.3197869),1.0 (pos.3197871)		Cereblon	Cul4A-RIN(	Intellectual Disabil	Lenalidomide,Pomalido	T=0, C=43	T=0.0, C=1.0	0.00001	705.841	43	T=0/0, C=18/2
chr3:3215954	G/G	A	G	SNV	CRBN	CRBN:intronic:NM 016302.3	1	100		intronic	NM 01630	c.175-9T>C	p.?	-0.31					ATP-depende	rs1669321	0.0023	YES	1.0 (pos.3215954)		Cereblon	Cul4A-RIN(	Intellectual Disabil	Lenalidom Benign	A=0, G=63	A=0.0, G=1.0	0.00001	958.917	63	A=0/0, G=32/3
chr4:57273840	C/G	C	G	SNV	PPAT	PPAT:exonic:NM 002703.4	1	45.45	2	synonymous	NM 00270	c.171G>C	p.(=)	0.34				0.6(COSM113	Glutamine ar	rs11538098	0.4169	YES	7.407E-2:1.156E-4 (pos.5	acute_myeloid	Phosphorib	'de novo' II	Cardiovascular Dis(	Fluorouracil,L-Glutamine	C=18, G=15	C=0.5455, G=0.4545	0.00001	143.161	33	C=12/6, G=12/
chr3:33414923	G/A	G	A	SNV	FBXL2	FBXL2:intronic:NM 012157.4	1	50		intronic	NM 01215	c.455+76G	p.?	-0.27					F-box domair	rs2291898		YES			F-box and le	SCF ubiquitin ligase complex,SCF-dependent proteasom			G=13, A=13	G=0.5, A=0.5	0.00001	119.06	26	G=0/13, A=3/1
chr4:76793228	G/G	A	G	SNV	PPEF2	PPEF2:exonic:NM 006239.2	1	100	13	synonymous	NM 00623	c.1599T>C	p.(=)	-0.92					Calcineurin-l	rs1394916	2.00E-04		1.0 (pos.76793228)		Protein pho	Hsp70 protein binding,Hsp90 protein binding,calcium io			A=0, G=33	A=0.0, G=1.0	0.00001	504.598	33	A=0/0, G=18/1
chr3:35756778	T/T	A	T	SNV	ARPP21	ARPP21:intronic:NM 001267619.1	1	100		intronic	NM 00126	c.796-153	p.?	-0.23					R3H domain	rs2305233		YES				biological_process,calmodulin binding,cellular response			A=0, T=28	A=0.0, T=1.0	0.00001	408.715	28	A=0/0, T=25/3
chr3:36484745	CT/CT	C	CT	INDEL	STAC	STAC:intronic:NM 003149.2	1	100		intronic	NM 00314	c.112-111(	p.?	-0.04					Phorbol esters/diacylglycerol binding domain (C1 domain),SH3 domain,Unstructured on SH3 and cys						SRC homolo	T-tubule,c	Dermatitis;Dermatitis, Allergic Contact;Derm		C=0, CT=28	C=0.0, CT=1.0	0.00001	301.806	28	C=0/0, CT=20/
chr4:76805902	T/T	C	T	SNV	PPEF2	PPEF2:exonic:NM 006239.2	1	100	8	synonymous	NM 00623	c.591G>A	p.(=)	-4.23					Calcineurin-l	rs1976518	0.0171	YES	9.934E-1;0.0 (pos.76805902)		Protein pho	Hsp70 protein binding,Hsp90 protein binding,calcium io			C=0, T=69	C=0.0, T=1.0	0.00001	1072.75	69	C=0/0, T=41/2
chr3:36896653	C/C	G	C	SNV	TRANK1	TRANK1:intronic:NM 014831.2	1	100		intronic	NM 01483	c.4417+11	p.?	0.12						rs9818599	1.00E-04		1.0;0.0 (pos.36896653)						G=0, C=22	G=0.0, C=1.0	0.00001	298.892	22	G=0/0, C=13/9
chr3:36915814	T/G	T	G	SNV	TRANK1	TRANK1:intronic:NM 014831.2	1	50		intronic	NM 01483	c.776-27A	p.?	0.77						rs4296548		YES	6.647E-1 (pos.36915814)						T=24, G=24	T=0.5, G=0.5	0.00001	270.871	48	T=14/10, G=9/
chr3:37163039	A/A	G	A	SNV	LRRFIP2	LRRFIP2:intronic:NM 006309.3	1	100		intronic	NM 00630	c.286-12C	p.?	1.29					LRRFIP family	rs336595,rs7618099!	0		0.0 (pos.37163038),1.0 (pos.37163039)		Leucine-ricl	LRR domai	Colonic Diseases:Colorectal Neoplasms:Colo		G=0, A=28	G=0.0, A=1.0	0.00001	413.098	28	G=0/0, A=15/1
chr3:37285152	C/T	C	T	SNV	GOLGA4	GOLGA4:intronic:NM 001172713.1	1	50		intronic	NM 00117	c.72+39C>	p.?	-0.06					GRIP domain	rs2271548	0.3857	YES	6.328E-2 (pos.37285152)		Golgi autoa	GTPase bi(	Arsenic Poisoning:Genital Diseases, Male:Ge		C=17, T=17	C=0.5, T=0.5	0.00001	172.005	34	C=3/14, T=10/
chr3:37695192	A/A	G	A	SNV	ITGA9	ITGA9:intronic:NM 002207.2	1	100		intronic	NM 00220	c.1840-13(	p.?	-0.41					FG-GAP repe	rs3821909	0.2083	YES	2.389E-1 (pos.37695192)		Integrin, alp	basal plas	Amyotrophic Lateral Sclerosis;Carcinoma;Ce		G=0, A=23	G=0.0, A=1.0	0.00001	316.747	23	G=0/0, A=12/1
chr3:38390020	C/A	C	A	SNV	XYLB	XYLB:intronic:NM 005108.3	1	95.83		intronic	NM 00510	c.58-21C>	p.?	-1.17					FGGY family c	rs2615631	0.0012	YES	1.0 (pos.38390020)		Xylulokinas	ATP binding,D-xylose metabolic process,carbohydrate m			C=1, A=23	C=0.0417, A=0.9583	0.00001	306.605	24	C=0/1, A=18/5
chr3:38442490	G/A	G	A	SNV	XYLB	XYLB:intronic:NM 005108.3	1	45.45		intronic	NM 00510	c.1533+14	p.?	-0.96					FGGY family c	rs2070488	0.4371	YES	2.729E-1 (pos.38442490)		Xylulokinas	ATP binding,D-xylose metabolic process,carbohydrate m			G=12, A=10	G=0.5455, A=0.4545	0.00001	81.7626	22	G=3/9, A=5/5
chr3:38646423	C/T	C	T	SNV	SCN5A	SCN5A:intronic:NM 001160161.1	1	52.27		intronic	NM 00116	c.1339-24(	p.?	-2.11					Cytoplasmic	rs7428779	0.1935	YES	1.18E-1 (pos.38646423)		Sodium cha	AV node c(	ACMG Recommend	Ajmaline,/ Benign	C=21, T=23	C=0.4773, T=0.5227	0.00001	260.157	44	C=5/16, T=1/2
chr11:7662732	T/T	C	T	SNV	PPFIBP2	PPFIBP2:exonic:NM 003621.3	1	100	16	synonymous	NM 00362	c.1398C>T	p.(=)	-0.81				0.01(COSM54	SAM domain	rs4643071	0.3337	YES	5.539E-1 (pos.7662732)	acute_myeloid	leukaemia	cytosol,ex	Genital Diseases, Male;Genital Neoplasms, I		C=0, T=24	C=0.0, T=1.0	0.00001	334.791	24	C=0/0, T=11/1
chr11:7673015	A/A	G	A	SNV	PPFIBP2	PPFIBP2:exonic:NM 003621.3	1	100	23	synonymous	NM 00362	c.2376G>A	p.(=)	2.06				0.59(COSM41	SAM domain	rs5864,rs745820945	0.3331	YES	0.0 (pos.7673014),5.785	acute_myeloid	leukaemia	cytosol,ex	Genital Diseases, Male;Genital Neoplasms, I		G=0, A=68	G=0.0, A=1.0	0.00001	1059.35	68	G=0/0, A=33/3
chr16:4933939	C/C	G	C	SNV	PPL	PPL:exonic:NM 002705.4	1	100	22	missense	NM 00270	c.4717C>G	p.Gln1573Glu	9.43	0.4	29	0.994	1(COSM4416794)		rs2037912,rs780840!	0.4479	YES	0.0 (pos.4933920),6.586	acute_myeloid	Periplakin	cadherin binding,cornification,cornified envelope,cytosl			G=0, C=35	G=0.0, C=1.0	0.00001	543.744	35	G=0/0, C=18/1
chr16:4934549	A/A	G	A	SNV	PPL	PPL:exonic:NM 002705.4	1	100	22	synonymous	NM 00270	c.4107C>T	p.(=)	1.23				0.77(COSM4419969)		rs1049208	0.4472	YES	6.424E-1 (pos.4934549)	acute_myeloid	Periplakin	cadherin binding,cornification,cornified envelope,cytosl			G=0, A=27	G=0.0, A=1.0	0.00001	389.988	27	G=0/0, A=8/19
chr3:38798836	C/T	C	T	SNV	SCN10A	SCN10A:intronic:NM 006514.3	1	53.33		intronic	NM 00651	c.951-186(	p.?	-0.26					Ion transport	rs6798015		YES			Sodium cha	AV node c(	Arrhythmias, Cardi	Benzocaine,Brivaracetar	C=14, T=16	C=0.4667, T=0.5333	0.00001	159.925	30	C=4/10, T=10/
chr16:4940809	G/G	A	G	SNV	PPL	PPL:exonic:NM 002705.4	1	100	17	synonymous	NM 00270	c.2082T>C	p.(=)	2.06						rs2471844	0.0013	YES	1.0 (pos.4940809)		Periplakin	cadherin binding,cornification,cornified envelope,cytosl			A=0, G=21	A=0.0, G=1.0	0.00001	276.391	21	A=0/0, G=15/6
chr16:4944518	G/G	C	G	SNV	PPL	PPL:exonic:NM 002705.4	1	100	12	synonymous	NM 00270	c.1344G>C	p.(=)	1.86						rs1876363	0.0019	YES	1.0 (pos.4944518)		Periplakin	cadherin binding,cornification,cornified envelope,cytosl			C=0, G=22	C=0.0, G=1.0	0.00001	298.892	22	C=0/0, G=13/9
chr3:39166484	T/T	C	T	SNV	TTC21A	TTC21A:intronic:NM 145755.2	1	100		intronic	NM 14575	c.1118-70(	p.?	-0.28					Tetratricope	rs784508		YES				cilium,intraciliary retrograde transport,intraciliary tran:			C=0, T=28	C=0.0, T=1.0	0.00001	408.718	28	C=0/0, T=14/1
chr3:39166509	G/G	A	G	SNV	TTC21A	TTC21A:intronic:NM 145755.2	1	100		intronic	NM 14575	c.1118-45	p.?	-0.14					Tetratricope	rs784507	0.128	YES	8.966E-1 (pos.39166509)			cilium,intraciliary retrograde transport,intraciliary tran:			A=0, G=27	A=0.0, G=1.0	0.00001	389.988	27	A=0/0, G=13/1
chr3:39167905	A/A	G	A	SNV	TTC21A	TTC21A:intronic:NM 145755.2	1	100		intronic	NM 14575	c.1546+24	p.?	-1.97					Tetratricope	rs784506	0.1258	YES	8.965E-1 (pos.39167905)			cilium,intraciliary retrograde transport,intraciliary tran:			G=0, A=43	G=0.0, A=1.0	0.00001	705.844	43	G=0/0, A=6/37
chr2:29001691	G/G	A	G	SNV	PPP1CB	PPP1CB:exonic:NM 206876.1	1	100	4	synonymous	NM 20687	c.201A>G	p.(=)	0.09					Calcineurin-l	rs1128416	0.3982	YES	2.144E-1 (pos.29001691)		Protein pho	G2/M transition of mitotic cell cycle,PTW/PP1 phosphata			A=0, G=122	A=0.0, G=1.0	0.00001	1973.69	122	A=0/0, G=43/7
chr3:39180162	C/C	A	C	SNV	MIR6822,TTC21A	MIR6822:downstream:NR 106880.1, TTC21A:intronic:NM 1	1	100		intronic	NM 14575	c.3886-40	p.?	-0.05					Tetratricope	rs811969	0.3226	YES	7.582E-1 (pos.39180162)			cilium,intraciliary retrograde transport,intraciliary tran:			A=0, C=53	A=0.0, C=1.0	0.00001	761.059	53	A=0/0, C=29/2
chr1:204379617	C/C	T	C	SNV	PPP1R15B	PPP1R15B:exonic:NM 032833.4	1	100	1	missense	NM 03283	c.923A>G	p.Asn308Ser	0.02	1	46	0		Phosphatase	rs3014626	8.00E-04		1.0 (pos.204379617)		Protein phosphatase 1, regulatory subunit 15B				T=0, C=44	T=0.0, C=1.0	0.00001	726.569	44	T=0/0, C=24/2
chr19:45649838	G/G	T	G	SNV	PPP1R37	PPP1R37:utr 3:NM 019121.1	1	100	13	utr 3	NM 01912	c.2094T>G	p.?	1.15					Leucine Rich	rs78073763		YES				negative regulation of phosphatase activity,phosphatas			T=0, G=27	T=0.0, G=1.0	0.00001	389.988	27	T=0/0, G=14/1
chr17:48227293	G/A	G	A	SNV	PPP1R9B	PPP1R9B:exonic:NM 032595.4	1	47.62	3	synonymous	NM 03259	c.582C>T	p.(=)	2.72					PDZ domain (	rs111354487	0.0315	YES	0.0 (pos.48227293)		Protein pho	D2 dopam	Nose Diseases;Otorhinolaryngologic Disease		G=11, A=10	G=0.5238, A=0.4762	0.00001	82.2896	21	G=1/10, A=2/8
chr3:39185968	G/G	A	G	SNV	CSRNP1	CSRNP1:intronic:NM 033027.3	1	100		intronic	NM 03302	c.466-26T	p.?	2.47					Cysteine/seri	rs810743	0.2478	YES	7.71E-1 (pos.39185968)			DNA bindi	Cardiomegaly;Cardiomyopathies;Cardiomyo		A=0, G=25	A=0.0, G=1.0	0.00001	353.019	25	A=0/0, G=10/1
chr4:6325018	T/T	A	T	SNV	PPP2R2C	PPP2R2C:utr 3:NM 181876.2	1	100	9	utr 3	NM 18187	c.1355T>A	p.?	0.12						rs3796402	0.4073	YES	9.197E-1 (pos.6325018)		Protein pho	cytosol,developmental process,mitotic cell cycle,peptid			A=0, T=64	A=0.0, T=1.0	0.00001	981.433	64	A=0/0, T=37/2
chr4:6325086	A/A	G	A	SNV	PPP2R2C	PPP2R2C:exonic:NM 181876.2	1	100	9	synonymous	NM 18187	c.1287C>T	p.(=)	0.43				0.67(COSM3760689),0.67(C		rs3796403	0.4112	YES	9.167E-1 (pos.6325086)	adenocarcinoı	Protein pho	cytosol,developmental process,mitotic cell cycle,peptid			G=0, A=62	G=0.0, A=1.0	0.00001	940.32	62	G=0/0, A=35/2
chr10:75265154	T/C	T	C	SNV	PPP3CB-AS1,USP54	USP54:exonic:NM 152586.3, PPP3CB-AS1:exonic nc:NR 13	1	52.38	19	missense	NR 13210	c.4082A>G	p.His1361Arg	2.61	0.01	29	0.875		Ubiquitin car	rs150962177	2.00E-04		0.0 (pos.75265154)			protein binding,protein deubiquitination,thiol-depende			T=10, C=11	T=0.4762, C=0.5238	0.00001	95.2975	21	T=1/9, C=4/7
chr3:39433147	A/A	G	A	SNV	SLC25A38	SLC25A38:intronic:NM 017875.2	1	100		intronic	NM 01787	c.456+36G	p.?	-0.06					Mitochondri	rs1995236	0.0056	YES	9.185E-1 (pos.39433147)		Solute carri	erythrocyt	Anemia;Anemia, Sideroblastic;Bone Marrow		G=0, A=72	G=0.0, A=1.0	0.00001	1130.95	72	G=0/0, A=40/3
chr3:403288	A/A	G	A	SNV	CHL1	CHL1:intronic:NM 006614.3	1	100		intronic	NM 00661	c.1307-94(	p.?	-1.81					Bravo-like int	rs9824311		YES			CHL1, mous	adult loco	Intellectual Disability;Mental Disorders;Ner		G=0, A=64	G=0.0, A=1.0	0.00001	977.606	64	G=0/0, A=38/2
chr3:403297	A/A	C	A	SNV	CHL1	CHL1:intronic:NM 006614.3	1	100		intronic	NM 00661	c.1307-85(	p.?	-0.31					Bravo-like int	rs9839027		YES			CHL1, mous	adult loco	Intellectual Disability;Mental Disorders;Ner		C=0, A=66	C=0.0, A=1.0	0.00001	1015.4	66	C=0/0, A=38/2
chr3:403308	A/A	G	A	SNV	CHL1	CHL1:intronic:NM 006614.3	1	100		intronic	NM 00661	c.1307-74(	p.?	0.57					Bravo-like int	rs9824442		YES			CHL1, mous	adult loco	Intellectual Disability;Mental Disorders;Ner		G=0, A=66	G=0.0, A=1.0	0.00001	1015.29	66	G=0/0, A=38/2
chr3:403344	CG/CG	C	CG	INDEL	CHL1	CHL1:intronic:NM 006614.3	1	100		intronic	NM 00661	c.1307-38(	p.?	-3.57					Bravo-like int	rs752529188			0.0 (pos.403344)		CHL1, mous	adult loco	Intellectual Disability;Mental Disorders;Ner		C=0, CG=67	C=0.0, CG=1.0	0.00001	862.678	67	C=0/0, CG=38/
chr2:68415767	G/A	G	A	SNV	PPP3R1	PPP3R1:exonic:NM 000945.3	1	57.14	3	synonymous	NM 00094	c.99C>T	p.(=)	5.08				0.92(COSM37	EF hand,EF-ha	rs687	0.3946	YES	5.133E-1 (pos.68415767)	acute_myeloid	Protein pho	Fc-epsilon	Cardiomegaly;Card	MYRISTIC ACID,Voclospor	G=12, A=16	G=0.4286, A=0.5714	0.00001	161.769	28	G=10/2, A=12/
chr2:55805440	C/C	T	C	SNV	PPP4R3B	PPP4R3B:exonic:NM 001122964.2	1	100	10	missense	NM 00112	c.1507A>G	p.Ile503Val	-0.32	0.71	29	0		Component c	rs2903704		YES	1.0 (pos.55805440)		Protein pho	centrosome,cytoplasm,nuclear speck,nucleoplasm,posi			T=0, C=25	T=0.0, C=1.0	0.00001	353.018	25	T=0/0, C=9/16
chr2:55844445	C/C	T	C	SNV	PPP4R3B	PPP4R3B:utr 5:NM 001122964.2	1	100	1	utr 5	NM 00112	c.-24A>G	p.?	0.91					Component c	rs782594	0.4877	YES	1.129E-1 (pos.55844445)		Protein pho	centrosome,cytoplasm,nuclear speck,nucleoplasm,posi			T=0, C=53	T=0.0, C=1.0	0.00001	782.029	53	T=0/0, C=13/4
chr3:41938500	C/C	G	C	SNV	ULK4	ULK4:intronic:NM 017886.3	1	100		intronic	NM 01788	c.1349-5C	p.?	-1.97					Protein kinas	rs1717005	0.0059	YES	1.0 (pos.41938500)		Unc51-like	ATP bindin	Blood Protein Disorders;Cardic	Benign	G=0, C=39	G=0.0, C=1.0	0.00001	628.187	39	G=0/0, C=5/34
chr3:42234758	A/A	C	A	SNV	TRAK1	TRAK1:intronic:NM 001042646.2	1	100		intronic	NM 00104	c.900+61C	p.?	0.15					HAP1 N-term	rs3816397		YES			Trafficking	GABA rece	Mental Disorders;Schizophrenia;Schizophren		C=0, A=49	C=0.0, A=1.0	0.00001	831.629	49	C=0/0, A=25/2
chr19:46878989	G/A	G	A	SNV	PPP5C	PPP5C:exonic:NM 006247.3	1	50	3	synonymous	NM 00624	c.492G>A	p.(=)	-1.38				0.03(COSM37	Calcineurin-l	rs4239538	0.2877	YES	6.253E-2 (pos.46878989)	adenocarcinoı	Protein pho	ADP bindir	Crohn Disease;Digestive System Diseases;Ga		G=17, A=17	G=0.5, A=0.5	0.00001	172	34	G=4/13, A=3/1
chr22:50877166	C/C	G	C	SNV	PPP6R2	PPP6R2:exonic:NM 001242898.1	1	100	19	synonymous	NM 00124	c.2103G>C	p.(=)	-1.03				0.01(COSM37	SIT4 phospha	rs4824131	0.3595	YES	9.386E-1:0.0 (pos.50877	adenocarcinoma		cytosol,intracellular membrane-bounded organelle,prot			G=0, C=20	G=0.0, C=1.0	0.00001	263.786	20	G=0/0, C=12/8
chr3:42448296	C/C	T	C	SNV	LYZL4	LYZL4:intronic:NM 144634.3	1	100		intronic	NM 14463	c.292+42A	p.?	-0.16					C-type lysozy	rs33518	0.4065	YES	4.305E-1 (pos.42448296)		Lysozyme-li	acrosomal vesicle,cell wall macromolecule catabolic pr(			T=0, C=38	T=0.0, C=1.0	0.00001	603.776	38	T=0/0, C=23/1
chr5:64883217	C/G	C	G	SNV	PPWD1	PPWD1:exonic:NM 015342.3	1	57.45	11	synonymous	NM 01534	c.1935C>G	p.(=)	0.77					Cyclophilin ty	rs1024899432						catalytic step 2 spliceosome,mRNA splicing, via spliceos			C=40, G=54	C=0.4255, G=0.5745	0.00001	643.072	94	C=18/22, G=2
chr18:77663940	A/A	G	A	SNV	PQLC1	PQLC1:utr 3:NM 025078.4	1	100	6	utr 3	NM 02507	c.852C>T	p.?	-0.65					PQ loop repe	rs3744881	0.3864	YES	3.489E-1 (pos.77663940)			integral component of membrane			G=0, A=29	G=0.0, A=1.0	0.00001	427.594	29	G=0/0, A=22/7
chr8:8185889	G/A	G	A	SNV	PRAG1	PRAG1:exonic:NM 001080826.2	1	56	5	synonymous	NM 00108	c.2403C>T	p.(=)	-0.06					Protein kinas	rs201882445	6.00E-04		0.0 (pos.8185889)		PEAK1-relat	ATP binding,non-membrane spanning protein tyrosine ki			G=11, A=14	G=0.44, A=0.56	0.00001	134.35	25	G=6/5, A=8/6
chr8:8234077	G/C	G	C	SNV	PRAG1	PRAG1:exonic:NM 001080826.2	1	55.38	3	synonymous	NM 00108	c.1842C>G	p.(=)	-2.8				0.14(COSM37	Protein kinas	rs386721946,rs4840!	0.2746	YES	6.363E-1:1.163E-4 (pos.8	adenocarcinoı	PEAK1-relat	ATP binding,non-membrane spanning protein tyrosine ki			G=29, C=36	G=0.4462, C=0.5538	0.00001	372.808	65	G=6/23, C=32/
chr22:22899234	G/G	A	G	SNV	PRAME	PRAME:exonic:NM 206954.2	1	100	3	missense	NM 20695	c.19T>C	p.Trp7Arg	0.67	0.9	101	0	0.01(COSM85675)		rs1129172	0.4277	YES	7.489E-1 (pos.22899234)	adenocarcinoı	Preferentia	apoptotic	Adenocarcinoma:Blood Protein Disorders:Bo		A=0, G=44	A=0.0, G=1.0	0.00001	726.569	44	A=0/0, G=32/1
chr3:43414318	G/G	A	G	SNV	ANO10	ANO10:intronic:NM 018075.4	1	100		intronic	NM 01807	c.1915-58	p.?	-1.06					Calcium-acti	rs6441771			1.0 (pos.43414318)		Anoctamin	calcium ac	Nutrition Disorders;Nutritional and Metaboli		A=0, G=71	A=0.0, G=1.0	0.00001	1111.48	71	A=0/0, G=39/3
chr3:43607136	T/C	T	C	SNV	ANO10	ANO10:intronic:NM 018075.4	1	50		intronic	NM 01807	c.1293+9A	p.?	-0.08					Calcium-acti	rs7616708	0.4147	YES	8.982E-1 (pos.43607136)		Anoctamin	calcium ac	Nutrition Disorders;Nutritiona	Benign	T=13, C=13	T=0.5, C=0.5	0.00001	119.163	26	T=8/5, C=6/7
chr1:12855835	G/G	C	G	SNV	PRAMEF1	PRAMEF1:exonic:NM 023013.4	1	100	4	missense	NM 02301	c.1115C>G	p.Ala372Gly	-0.72	0.14	60	0.005	0(COSM4142311),0(COSM4		rs1063795			1.0;0.0 (pos.12855835)	neoplasm,squamous_cell		negative regulation of apoptotic process,negative regula			C=0, G=32	C=0.0, G=1.0	0.00001	471.799	32	C=0/0, G=21/1
chr1:12855845	C/C	G	C	SNV	PRAMEF1	PRAMEF1:exonic:NM 023013.4	1	100	4	synonymous	NM 02301	c.1125G>C	p.(=)	0.35				0(COSM4142314),0(COSM4		rs1063797			1.0 (pos.12855845)	neoplasm,squamous_cell		negative regulation of apoptotic process,negative regula			G=0, C=32	G=0.0, C=1.0	0.00001	462.378	32	G=0/0, C=21/1
chr3:44362408	A/A	G	A	SNV	TOPAZ1	TOPAZ1:intronic:NM 001145030.1	1	100		intronic	NM 00114	c.4307-11	p.?	0.12					Putative asp	rs9857957		YES			Testis-and	apoptotic process,cytosol,metal ion binding,ncRNA tran			G=0, A=183	G=0.0, A=1.0	0.00001	3045.4	183	G=0/0, A=120/
chr1:12835868	C/C	T	C	SNV	PRAMEF12	PRAMEF12:exonic:NM 001080830.4	1	100	2	missense	NM 00108	c.470T>C	p.Met157Thr	-1.54	1	81	0			rs1812242,rs386628!	0.1965	YES	7.309E-1 (pos.12835868)			negative regulation of apoptotic process,negative regula			T=0, C=69	T=0.0, C=1.0	0.00001	1072.78	69	T=0/0, C=39/3
chr3:4461698	T/T	C	T	SNV	SUMF1	SUMF1:intronic:NM 182760.3	1	100		intronic	NM 18276	c.602+50G	p.?	-0.46					Sulfatase-mo	rs711666	0.0022	YES	9.809E-1 (pos.4461698)		Sulfatase-m	endoplasr	Autoimmune Diseases;Autoim	Benign	C=0, T=31	C=0.0, T=1.0	0.00001	465.814	31	C=0/0, T=23/8
chr3:447069	T/T	C	T	SNV	CHL1	CHL1:intronic:NM 006614.3	1	100		intronic	NM 00661	c.3459-10	p.?	-0.69					Bravo-like int	rs11705894		YES			CHL1, mous	adult loco	Intellectual Disability;Mental Disorders;Ner		C=0, T=26	C=0.0, T=1.0	0.00001	371.42	26	C=0/0, T=22/4
chr3:44869026	T/T	A	T	SNV	KIF15	KIF15:intronic:NM 020242.2	1	100		intronic	NM 02024	c.2856+6A	p.?	-1.21					Hyaluronan r	rs4682999	0		1.0 (pos.44869026)		Kinesin fam	ATP bindin	Autoimmune Diseases:Female Urogenital Di:		A=0, T=21	A=0.0, T=1.0	0.00001	281.12	21	A=0/0, T=9/12
chr3:45017904	AG/TT	AG	TT	MNV	EXOSC7,ZDHHC3	EXOSC7:intronic:NM 015004.3, ZDHHC3:upstream:NM 016	2	50		intronic	NM 01659	c.-16976C	p.?, p.?	-0.9, 0.64					3' exoribonuc	rs6441873,rs6441874,rs879308		YES			Exosome co	3'-5'-exoribonuclease activity,AU-rich element binding,G			AG=30, TT=	AG=0.5, TT=0.5	0.00001	293.739	60	AG=15/15, TT=
chr8:70964399	A/G	A	G	SNV	PRDM14	PRDM14:exonic:NM 024504.3	1	57.14	8	synonymous	NM 02450	c.1629T>C	p.(=)	-0.54				0.03(COSM41	C2H2-type zir	rs10089937	0.3722	YES	7.306E-1 (pos.70964399)	adenocarcinoı	PR domain-	RNA bindir	Endocrine Gland Neoplasms;Endocrine Syste		A=9, G=12	A=0.4286, G=0.5714	0.00001	109.102	21	A=7/2, G=10/2
chr3:45461102	GT/GT	G	GT	INDEL	LARS2	LARS2:intronic:NM 015340.3	1	100		intronic	NM 01534	c.456-59G	p.?	0.44					Anticodon-bi	rs11383389,rs39772	0.2642	YES			Leucyl-tRN/	ATP bindin	46, XX Disorders of	L-Leucine	G=0, GT=10	G=0.0, GT=1.0	0.00001	1512.3	105	G=0/0, GT=69/
chr21:43239972	G/G	A	G	SNV	PRDM15	PRDM15:exonic:NM 022115.4	1	100	25	synonymous	NM 02211	c.3324T>C	p.(=)	3.78					C2H2-type zir	rs28477638	0.1026	YES	7.121E-1 (pos.43239972)		PR domain-	DNA bindi	Abnormalities, Multiple:Chromosome Disord		A=0, G=50	A=0.0, G=1.0	0.00001	852.748	50	A=0/0, G=22/2
chr3:45677637	TGC/TGC	GCA	TGC	MNV	LIMD1	LIMD1:intronic:NM 014240.2	3	100		intronic	NM 01424	c.1409-5G	p.?	1.08, -2.25, 0.69					LIM domain	rs5848762,rs62242177,rs62242		YES	1.0 (pos.45677638),9.773E-1 (pos.45677		LIM domain	P-body,RISC complex,adherens junction,cell migration,c			GCA=0, TGC	GCA=0.0, TGC=1.0	0.00001	298.844	22	GCA=0/0, TGC=
chr3:45748427	CA/CA	C	CA	INDEL	SACM1L	SACM1L:intronic:NM 014016.4	1	100		intronic	NM 01401	c.333+28C	p.?	-0.44					SacI homolog	rs775119610			0.0 (pos.45748427)		SAC1 suppr(	AMPA glutamate receptor complex,Golgi apparatus,Golg			C=0, CA=22	C=0.0, CA=1.0	0.00001	147.878	22	C=0/0, CA=6/1
chr3:45748429	A/A	G	A	SNV	SACM1L	SACM1L:intronic:NM 014016.4	1	100		intronic	NM 01401	c.333+30G	p.?	-1.27					SacI homolog	rs2742441	0.465	YES	2.344E-1 (pos.45748429)		SAC1 suppr(	AMPA glutamate receptor complex,Golgi apparatus,Golg			G=0, A=22	G=0.0, A=1.0	0.00001	298.388	22	G=0/0, A=6/16
chr3:45748435	G/G	A	G	SNV	SACM1L	SACM1L:intronic:NM 014016.4	1	100		intronic	NM 01401	c.333+36A	p.?	-0.09					SacI homolog	rs2673039	0.4681	YES	1.869E-1 (pos.45748435)		SAC1 suppr(	AMPA glutamate receptor complex,Golgi apparatus,Golg			A=0, G=23	A=0.0, G=1.0	0.00001	316.739	23	A=0/0, G=6/17
chr3:45772742	C/C	T	C	SNV	SACM1L	SACM1L:intronic:NM 014016.4	1	100		intronic	NM 01401	c.922-51T	p.?	-0.33					SacI homolog	rs2531755,rs796640(	0.3857	YES	3.743E-1;0.0 (pos.45772742)		SAC1 suppr(	AMPA glutamate receptor complex,Golgi apparatus,Golg			T=0, C=76	T=0.0, C=1.0	0.00001	1202.05	76	T=0/0, C=47/2
chr1:14143003	G/G	A	G	SNV	PRDM2	PRDM2:exonic:NM 012231.4	1	100	9	synonymous	NM 01223	c.5118A>G	p.(=)	-1.88				0(COSM5021	C2H2-type zir	rs1046331	0.1564	YES	7.162E-1 (pos.14143003)	osteosarcoma	PR domain-	DNA bindi	Amphetamine-Related Disorders;Endocrine (		A=0, G=20	A=0.0, G=1.0	0.00001	263.786	20	A=0/0, G=6/14
chr3:45977899	C/C	T	C	SNV	FYCO1	FYCO1:intronic:NM 024513.3	1	100		intronic	NM 02451	c.4040+41	p.?	-4.1					FYVE zinc fing	rs1873002	2.00E-04		9.992E-1 (pos.45977899)		FYVE and co	'de novo' p	Cataract;Eye Diseases;Lens Diseases		T=0, C=20	T=0.0, C=1.0	0.00001	263.785	20	T=0/0, C=13/7
chr5:176733116	G/G	A	G	SNV	PRELID1	PRELID1:exonic:NM 013237.3	1	100	4	synonymous	NM 01323	c.450A>G	p.(=)	0.35				0.31(COSM15	PRELI-like fan	rs4631	0.1142	YES	4.99E-1 (pos.176733116)	acute_myeloid	PRELI doma	apoptotic process,immune response,mitochondrial inte			A=0, G=44	A=0.0, G=1.0	0.00001	726.569	44	A=0/0, G=20/2
chr2:44547574	A/A	G	A	SNV	PREPL,SLC3A1	PREPL:utr 3:NM 001171603.1, SLC3A1:exonic:NM 000341.	1	100	10	missense	NM 00034	c.1854G>A	p.Met618Ile, p.?	-0.16	0.31	10	0.001	0.23(COSM54	Alpha amylas	rs698761,rs8860560	0.3236	YES	3.221E-1;0.0 (pos.44547!	acute_myeloid	Prolyl endo	Golgi appa	Congenital Abnorm	Cystine Benign	G=0, A=90	G=0.0, A=1.0	0.00001	1492.77	90	G=0/0, A=36/5
chr2:44547555	C/T	C	T	SNV	PREPL,SLC3A1	PREPL:utr 3:NM 001171603.1, SLC3A1:exonic:NM 000341.	1	53.33	10	missense	NM 00034	c.1835C>T	p.Ser612Leu, p.?	1.92	0.04	145	0.002	0.36(COSM35	Alpha amylas	rs759732153			0.0 (pos.44547555)	malignant_me	Prolyl endo	Golgi appa	Congenital Abnorm	Cystine	C=42, T=48	C=0.4667, T=0.5333	0.00001	543.964	90	C=20/22, T=16
chr20:47261017	C/C	T	C	SNV	PREX1	PREX1:exonic:NM 020820.3	1	100	27	synonymous	NM 02082	c.3531A>G	p.(=)	0.32				0.41(COSM14	Domain foun	rs6066802	0.2607	YES	3.409E-1 (pos.47261017)	acute_myeloid	Phosphatid	G-protein coupled receptor signaling pathway,GTPase a(			T=0, C=53	T=0.0, C=1.0	0.00001	782.03	53	T=0/0, C=34/1
chr20:47246127	G/A	G	A	SNV	PREX1	PREX1:exonic:NM 020820.3	1	57.63	37	synonymous	NM 02082	c.4626C>T	p.(=)	-0.1				0.56(COSM41	Domain foun	rs3936193	0.0943	YES	1.401E-1 (pos.47246127)	neoplasm	Phosphatid	G-protein coupled receptor signaling pathway,GTPase a(			G=25, A=34	G=0.4237, A=0.5763	0.00001	356.592	59	G=6/19, A=30/
chr3:46574256	T/T	C	T	SNV	LRRC2	LRRC2:intronic:NM 024512.4	1	100		intronic	NM 02451	c.627+7G>	p.?	-1.2				0.01(COSM54	Leucine Rich	rs9849400	0.3367	YES	2.997E-1 (pos.46574256)	ductal_carcinc	Leucine-rich repeat-containing 2				C=0, T=69	C=0.0, T=1.0	0.00001	1071.73	69	C=0/0, T=43/2
chr11:57157405	G/G	A	G	SNV	PRG2	PRG2:exonic:NM 001243245.2	1	100	2	synonymous	NM 00124	c.13T>C	p.(=)	0.63						rs490358	0.269	YES	7.765E-1 (pos.57157405)		Peroxidasir	carbohydrate binding,defense		Sargramostim	A=0, G=42	A=0.0, G=1.0	0.00001	685.084	42	A=0/0, G=18/2
chr3:46658872	T/T	G	T	SNV	FAM240A	FAM240A:intronic:NM 001195442.1	1	100		intronic	NM 00119	c.179+54G	p.?	-0.76						rs9870935		YES							G=0, T=216	G=0.0, T=1.0	0.00001	3547.48	216	G=0/0, T=113/
chr3:47035900	T/T	C	T	SNV	NBEAL2	NBEAL2:intronic:NM 015175.2	1	100		intronic	NM 01517	c.1198-45(	p.?	0.09					Beige/BEACH	rs749512	0.3641	YES	3.704E-1 (pos.47035900)		Neurobeacl	endoplasr	Blood Coagulation Disorders, I	Benign	C=0, T=43	C=0.0, T=1.0	0.00001	705.844	43	C=0/0, T=19/2
chr3:47043386	T/T	C	T	SNV	NBEAL2	NBEAL2:intronic:NM 015175.2	1	100		intronic	NM 01517	c.4783-24(	p.?	0.88					Beige/BEACH	rs73075647	0.2003	YES	3.5E-4 (pos.47043386)		Neurobeacl	endoplasr	Blood Coagulation Disorders, I	Benign	C=0, T=21	C=0.0, T=1.0	0.00001	281.235	21	C=0/0, T=6/15
chr3:47277458	G/G	A	G	SNV	KIF9,KIF9-AS1	KIF9:intronic:NM 182902.3, KIF9-AS1:intronic nc:NR 0333	1	100		intronic	NR 03337	c.2217+66	p.?	-1.03					Kinesin moto	rs1874264		YES			Kinesin fam	ATP binding,ATPase activity,cytoplasm,extracellular mat			A=0, G=34	A=0.0, G=1.0	0.00001	524.168	34	A=0/0, G=21/1
chr3:47286227	T/T	G	T	SNV	KIF9,KIF9-AS1	KIF9:intronic:NM 182902.3, KIF9-AS1:downstream:NR 033	1	100		intronic	NR 03337	c.1514+54	p.?	-0.2					Kinesin moto	rs6772449		YES			Kinesin fam	ATP binding,ATPase activity,cytoplasm,extracellular mat			G=0, T=23	G=0.0, T=1.0	0.00001	316.748	23	G=0/0, T=16/7

chr3:47456225	C/C	T	C	SNV	SCAP	SCAP:intronic:NM_012235.3	1	100		intronic	NM_01223	c.3307-83	p.?	-0.84					Patched fami	rs6442066		YES			SREBP cleav	ER to Golgi	Adenocarcinoma;Carcinoma;Carcinoma, Hep			T=0, C=73	T=0.0, C=1.0	0.00001	1149.07	73	T=0/0, C=39/3
chr3:47462587	G/G	A	G	SNV	SCAP	SCAP:intronic:NM_012235.3	1	100		intronic	NM_01223	c.1246-68	p.?	-0.68					Patched fami	rs900691		YES			SREBP cleav	ER to Golgi	Adenocarcinoma;Carcinoma;Carcinoma, Hec			A=0, G=23	A=0.0, G=1.0	0.00001	316.741	23	A=0/0, G=7/16
chr3:47604299	T/T	C	T	SNV	CSPG5	CSPG5:intronic:NM_001206943.1	1	100		intronic	NM_00120	c.1540-48	p.?	-1.57					Chondroitin s	rs4858839	0.0014	YES	1.0 (pos.47604299)			Golgi apparatus,Golgi lumen,Golgi membrane,Golgi-ass				C=0, T=37	C=0.0, T=1.0	0.00001	583.707	37	C=0/0, T=16/2
chr3:47882727	C/T	C	T	SNV	DHX30	DHX30:intronic:NM_138615.2	1	53.49		intronic	NM_13861	c.668+59C	p.?	-2.22					DEAD/DEAH b	rs17290490		YES			DEAH box p	ATP binding,ATP-dependent RNA helicase activity,RNA bi				C=20, T=23	C=0.4651, T=0.5349	0.00001	261.022	43	C=12/8, T=5/1
chr11:57148175	G/G	A	G	SNV	PRG3	PRG3:exonic:NM_006093.3	1	100	2	missense	NM_00609	c.7T>C	p.Cys3Arg	-1.02	0.82	180	0		Lectin C-type	rs669661,rs7628806	0.001	YES	0.0;0.0;0.0 (pos.57148174),9.983E-1 (pos		Proteoglyca	basophil activation,carbohydrate binding,extracellular				A=0, G=31	A=0.0, G=1.0	0.00001	465.761	31	A=0/0, G=14/1
chr6:57512510	G/G	T	G	SNV	PRIM2	PRIM2:exonic:NM_000947.4	1	100	14	missense	NM_00094	c.1338T>G	p.His446Gln	0.99		24			Eukaryotic ar	rs4294007		YES	6.188E-1 (pos.57512510)			4 iron, 4 sulfur cluster binding,DNA biosynthetic process				T=0, G=57	T=0.0, G=1.0	0.00001	853.361	57	T=0/0, G=29/2
chr4:185587165	G/G	A	G	SNV	PRIMPOL	PRIMPOL:exonic:NM_152683.3	1	100	6	missense	NM_15268	c.503A>G	p.Gln168Arg	1.88	1	43	0		Herpesvirida	rs2463447	0.0029	YES	9.998E-1 (pos.185587165)			DNA primase activity,DNA replication, synthesis of RNA				A=0, G=48	A=0.0, G=1.0	0.00001	810.429	48	A=0/0, G=26/2
chr3:48209486	T/T	C	T	SNV	CDC25A	CDC25A:intronic:NM_001789.2	1	100		intronic	NM_00178	c.931-52G	p.?	-1.68					M-phase indu	rs3731539	4.00E-04		1.0 (pos.48209486)		Cell divisio	DNA replication,G1/S transition of mitotic cell cycle,G2/				C=0, T=39	C=0.0, T=1.0	0.00001	623.979	39	C=0/0, T=9/30
chr7:106799997	A/A	G	A	SNV	PRKAR2B	PRKAR2B:exonic:NM_002736.2	1	100	11	synonymous	NM_00273	c.1227G>A	p.(=)	0.81				0.31(COSM40	Cyclic nucleo	rs257376	0.462	YES	4.507E-1 (pos.10679999	acute_myeloic	Protein kina	G2/M tran	Nutrition Disorders	Cyclic Adenosine Monop		G=0, A=49	G=0.0, A=1.0	0.00001	831.535	49	G=0/0, A=18/3
chr3:48338173	T/T	C	T	SNV	NME6	NME6:intronic:NM_005793.4	1	100		intronic	NM_00579	c.217+44G	p.?	0.84					Nucleoside d	rs4479622	0.3073	YES	1.217E-1 (pos.48338173)		Nonmetast	ATP binding,CTP biosynthetic process,GTP biosynthetic p				C=0, T=23	C=0.0, T=1.0	0.00001	316.748	23	C=0/0, T=17/6
chr7:106685448	G/T	G	T	SNV	PRKAR2B	PRKAR2B:exonic:NM_002736.2	1	52.38	1	synonymous	NM_00273	c.96G>T	p.(=)	0.76				0.86(COSM37	Cyclic nucleo	rs75385144	0.2995	YES	4.412E-1 (pos.10668544	adenocarcinoi	Protein kina	G2/M tran	Nutrition Disorders	Cyclic Adenosine Monop		G=50, T=55	G=0.4762, T=0.5238	0.00001	573.952	105	G=30/20, T=40
chr3:48414197	T/T	A	T	SNV	FBXW12	FBXW12:intronic:NM_001159929.1	1	100		intronic	NM_00115	c.33+387A	p.?	1.21						rs9852211		YES			F-box and W	cytosol,post-translational protein modification,protein				A=0, T=25	A=0.0, T=1.0	0.00001	352.868	25	A=0/0, T=13/1
chr3:48415007	G/G	C	G	SNV	FBXW12	FBXW12:intronic:NM_001159929.1	1	100		intronic	NM_00115	c.72-31C>	p.?	-0.41						rs4858793	0.4752	YES	6.594E-1 (pos.48415007)		F-box and W	cytosol,post-translational protein modification,protein				C=0, G=78	C=0.0, G=1.0	0.00001	1253.29	78	C=0/0, G=23/5
chr3:48602523	GGGC/GGGC	G	GGGC	INDEL	COL7A1	COL7A1:intronic:NM_000094.3	3	100		intronic	NM_00009	c.8620+19	p.?	-0.32					Collagen tripl	rs138595277,rs7624(	0	YES	1.0;0.0;0.0;0.0 (pos.48602523)		Collagen VII	COPII vesi	Breast Diseases;Breast Neopl		Likely benign	G=0, GGGC	G=0.0, GGGC=1.0	0.00001	523.278	34	G=0/0, GGGC=
chr3:48624611	C/T	C	T	SNV	COL7A1	COL7A1:intronic:NM_000094.3	1	45.45		intronic	NM_00009	c.3139+12	p.?	-2.92					Collagen tripl	rs2255532	0.3285	YES	1.525E-1 (pos.48624611)		Collagen VII	COPII vesi	Breast Diseases;Breast Neopl		Benign	C=12, T=10	C=0.5455, T=0.4545	0.00001	81.7624	22	C=7/5, T=4/6
chr3:48719638	C/C	T	C	SNV	NCKIPSD	NCKIPSD:intronic:NM_016453.3	1	100		intronic	NM_01645	c.487-49A	p.?	0.03					Protein of unl	rs34577723,rs76338	0.3584	YES	9.547E-1 (pos.48719638)		NCK-interac	COP9 signalosome,Fc-gamma receptor signaling pathwa				T=0, C=25	T=0.0, C=1.0	0.00001	352.986	25	T=0/0, C=9/16
chr19:47177913	G/G	A	G	SNV	PRKD2	PRKD2:exonic:NM_016457.4	1	100	18	missense	NM_01645	c.2504T>C	p.Val835Ala	3.96	1	64	0	0.16(COSM41	Lipopolysacc	rs314665	9.00E-04		1.0 (pos.47177913)	neoplasm	Protein kina	ATP bindin	Adnexal Diseases;Autoimmune Diseases;Cys			A=0, G=40	A=0.0, G=1.0	0.00001	644.286	40	A=0/0, G=21/1
chr3:48897078	G/G	A	G	SNV	SLC25A20	SLC25A20:intronic:NM_000387.5	1	100		intronic	NM_00038	c.536-18T	p.?	0.32					Mitochondria	rs6414612	0.0091		9.971E-1 (pos.48897078)		Solute carri	acyl carnit	Carnitine-acylcarni	L-Carnitin	Benign	A=0, G=30	A=0.0, G=1.0	0.00001	446.639	30	A=0/0, G=18/1
chr8:48805816	AG/G	A	AG	INDEL	PRKDC	PRKDC:exonic:NM_006904.6	1	85.42	31	frameshiftInsertion	NM_00690	c.3730T>C	p.Phe1244Leu, p.Phe12	8.38		22		0.98(COSM41	FAT domain,F	rs11411516,rs370101	4.00E-04		1.0;0.0 (pos.48805816)	mixed_adenos	Protein kina	ATP bindin	Adenocarcinoma;B	Caffeine,SF1126		A=0, AG=41	A=0.0, AG=0.8542, A	0.00001	742.95	48	A=0/0, AG=24/
chr10:120353910	C/C	T	C	SNV	PRLHR	PRLHR:exonic:NM_004248.2	1	100	2	missense	NM_00424	c.847A>G	p.Ile283Val	3.87	1	29	0	0(COSM3749(	7 transmemb	rs1613448	1.00E-04		1.0 (pos.120353910)	adenocarcinoma		G-protein coupled receptor activity,G-protein coupled re				T=0, C=29	T=0.0, C=1.0	0.00001	427.602	29	T=0/0, C=16/1
chr16:11367143	A/A	G	A	SNV	PRM3	PRM3:exonic:NM_021247.2	1	100	1	nonsense	NM_02124	c.310C>T	p.Arg104Ter	2.19						rs438289	0.0054	YES	1.0 (pos.11367143)								G=0, A=33	G=0.0, A=1.0	0.00001	504.598	33	G=0/0, A=13/2
chr16:11367154	T/T	C	T	SNV	PRM3	PRM3:exonic:NM_021247.2	1	100	1	missense	NM_02124	c.299G>A	p.Arg100Gln	0.55	1	43	0	0.02(COSM5468287)		rs429744	0.0614	YES	4.952E-1 (pos.11367154)	adenocarcinoma							C=0, T=34	C=0.0, T=1.0	0.00001	524.182	34	C=0/0, T=13/2
chr21:48063476	G/G	C	G	SNV	PRMT2	PRMT2:exonic:NM_001535.4	1	100	3	synonymous	NM_00153	c.69C>G	p.(=)	-0.26					Methyltransf	rs10854485	0.2156	YES	8.903E-1;0.0;1.157E-4 (pos.48063476)		HMT1 (hnRN	Rb-E2F complex,androgen receptor binding,cytoplasm,c				C=0, G=47	C=0.0, G=1.0	0.00001	789.274	47	C=0/0, G=17/3
chr3:49463287	A/A	G	A	SNV	NICN1	NICN1:intronic:NM_032316.3	1	100		intronic	NM_03231	c.423+29C	p.?	0.12						rs6446272	0.3137	YES	6.71E-2 (pos.49463287)		Nicolin 1	microtubule,nucleoplasm				G=0, A=23	G=0.0, A=1.0	0.00001	316.602	23	G=0/0, A=16/7
chr11:20529886	A/A	G	A	SNV	PRMT3	PRMT3:exonic:NM_005788.3	1	100	16	missense	NM_00578	c.1523G>A	p.Ser508Asn	3.9	1	46	0		C2H2 type zir	rs6483700	0		9.556E-1 (pos.20529886)			cytoplasm,cytosol,histone arg		S-adenosyl-L-homocyste		G=0, A=39	G=0.0, A=1.0	0.00001	623.98	39	G=0/0, A=28/1
chr3:49548294	C/C	T	C	SNV	DAG1	DAG1:intronic:NM_001177639.2	1	100		intronic	NM_00117	c.285+42T	p.?	-0.46					Dystroglycan	rs2329024	6.00E-04		1.0 (pos.49548294)		Dystrophin-	Golgi lume	Cobblestone Lissencephaly;Cc		Benign	T=0, C=32	T=0.0, C=1.0	0.00001	485.141	32	T=0/0, C=8/24
chr3:49568095	A/A	G	A	SNV	DAG1	DAG1:intronic:NM_001177639.2	1	100		intronic	NM_00117	c.286-135	p.?	-2.28					Dystroglycan	rs2311801		YES			Dystrophin-	Golgi lume	Cobblestone Lissencephaly;Cc		not provided	G=0, A=20	G=0.0, A=1.0	0.00001	263.786	20	G=0/0, A=11/9
chr20:33764632	G/G	C	G	SNV	PROCR	PROCR:utr_3:NM_006404.4	1	100	4	utr_3	NM_00640	c.733C>G	p.?	0.56					Class I Histoc	rs9574	0.4366	YES	3.766E-1;1.258E-4 (pos.33764632)		Protein C re	blood coa	Cardiovascular Dis	Drotrecogin alfa,Phosph		C=0, G=35	C=0.0, G=1.0	0.00001	543.903	35	C=0/0, G=29/6
chr22:18904414	G/G	A	G	SNV	PRODH	PRODH:exonic:NM_016335.4	1	100	13	synonymous	NM_01633	c.1515T>C	p.(=)	1.57				0.05(COSM41	Proline dehy	rs455072		YES	8.988E-1;0.0 (pos.18904	neoplasm,squ	Proline deh	4-hydroxy	Amino Acid Metabo	Glycine betaine,L-Proline		A=0, G=37	A=0.0, G=1.0	0.00001	583.705	37	A=0/0, G=13/2
chr20:5283256	G/G	C	G	SNV	PROKR2	PROKR2:exonic:NM_144773.3	1	100	3	synonymous	NM_14477	c.585G>C	p.(=)	-2.51				0.01(COSM37	7 transmemb	rs3746682	0.2557	YES	6.675E-1 (pos.5283256)	adenocarcinoi	Prokineticir	G-protein	46, XY Disorders of Sex Develo		Benign	C=0, G=29	C=0.0, G=1.0	0.00001	427.576	29	C=0/0, G=9/20
chr20:5283316	G/C	G	C	SNV	PROKR2	PROKR2:exonic:NM_144773.3	1	50	3	synonymous	NM_14477	c.525C>G	p.(=)	-2.97				0.03(COSM44	7 transmemb	rs3746683	0.1158	YES	3.525E-2;0.0 (pos.52833	acute_myeloic	Prokineticir	G-protein	46, XY Disorders of Sex Develo		Benign/Likely	G=11, C=11	G=0.5, C=0.5	0.00001	94.6959	22	G=4/7, C=6/5
chr19:36258937	A/A	AG	A	INDEL	PROSER3	PROSER3:splicesite_5:NM_001039887.2	1	100	9	splicesite	NM_001039887.2			1.17, 0.91						rs398034467,rs5827939,rs781793184,rs78			(pos.36258938),0.0 (pos	adenocarcinoma,astrocytoma_Grade_IV,endometrioid_carcinoma,squamous_cell						AG=0, A=21	AG=0.0, A=1.0	0.00001	268.447	21	AG=0/0, A=5/1
chr3:49739507	C/C	G	C	SNV	RNF123	RNF123:intronic:NM_022064.4	1	100		intronic	NM_02206	c.1496-9G	p.?	-1.49					SPRY domain	rs1491985	0.1835	YES	9.95E-1 (pos.49739507)		Ring finger	cytoplasm,cytosol,metal ion binding,protein deubiquitir				G=0, C=22	G=0.0, C=1.0	0.00001	298.891	22	G=0/0, C=8/14
chr19:54627915	C/T	C	T	SNV	PRPF31	PRPF31:exonic:NM_015629.3	1	47.62	8	synonymous	NM_01562	c.735C>T	p.(=)	-3.1				0.06(COSM37	Prp31 C term	rs11556769	0.0934	YES	4.899E-3 (pos.54627915)	adenocarcinoi	Precursor R	Cajal body	Congenital, Hereditary, and Ne		Likely benign	C=22, T=20	C=0.5238, T=0.4762	0.00001	208.876	42	C=6/16, T=6/1
chr9:116044997	A/AG	A	AG	INDEL	PRPF4	PRPF4:exonic:NM_004697.4	1	46.43	4	frameshiftInsertion	NM_00469	c.467_468	p.Ser158fs	6.41					WD domain, G-beta repeat,pre-mRNA processing factor 4 (PRP4) like						Precursor m	Cajal body	Congenital, Hereditary, and Neonatal Diseas			A=15, AG=1	A=0.5357, AG=0.464	0.00001	82.6641	28	A=3/12, AG=1/
chr6:42689755	G/G	A	G	SNV	PRPH2	PRPH2:exonic:NM_000322.4	1	100	1	synonymous	NM_00032	c.318T>C	p.(=)	0.97				0.01(COSM41	Tetraspanin f	rs7764439	0.4169	YES	5.822E-1 (pos.42689755)	acute_myeloic	Peripherin	cell adhes	Choroid Diseases;Congenital, I		Benign/Likely	A=0, G=26	A=0.0, G=1.0	0.00001	371.414	26	A=0/0, G=7/19
chr3:50645635	G/G	T	G	SNV	CISH	CISH:intronic:NM_145071.2	1	100		intronic	NM_14507	c.242-62A	p.?	0.12					SH2 domain,S	rs621975		YES			Cytokine in	cellular_c	Dermatitis;Dermatitis, Allergic Contact;Derm			T=0, G=61	T=0.0, G=1.0	0.00001	921.854	61	T=0/0, G=30/3
chr3:50683809	C/C	T	C	SNV	MAPKAPK3	MAPKAPK3:intronic:NM_001243925.1	1	100		intronic	NM_00124	c.829+114	p.?	0.05					Lipopolysacc	rs876105		YES				ATP binding,MAP kinase kinase		2-(2-QUINOLIN-3-YLPYRID		T=0, C=25	T=0.0, C=1.0	0.00001	352.947	25	T=0/0, C=11/1
chr3:50684489	G/G	A	G	SNV	MAPKAPK3	MAPKAPK3:intronic:NM_001243925.1	1	100		intronic	NM_00124	c.916-65A	p.?	-1.89					Lipopolysacc	rs113444263,rs4468971						ATP binding,MAP kinase kinase		2-(2-QUINOLIN-3-YLPYRID		A=0, G=26	A=0.0, G=1.0	0.00001	362.145	26	A=0/0, G=16/1
chr17:18775900	A/G	A	G	SNV	PRPSAP2	PRPSAP2:exonic:NM_002767.3	1	52.38	5	synonymous	NM_00276	c.177A>G	p.(=)	-0.36				0.09(COSM43	N-terminal d	rs4393623	0.4408	YES	3.318E-1 (pos.18775900)	acute_myeloic	Phosphorib	bone development,enzyme inhibitor activity,magnesium				A=10, G=11	A=0.4762, G=0.5238	0.00001	95.2996	21	A=4/6, G=6/5
chr19:50097784	T/C	T	C	SNV	PRR12	PRR12:exonic:NM_020719.2	1	50	3	synonymous	NM_02071	c.273T>C	p.(=)	1.36				0.43(COSM43	Domain of un	rs10414643	0.2398	YES	2.037E-1 (pos.50097784)	hepatocellula	Proline-rich	cell junction,neuron projection,nucleus,postsynaptic de				T=15, C=15	T=0.5, C=0.5	0.00001	145.021	30	T=7/8, C=5/10
chr3:52238656	C/C	T	C	SNV	ALAS1	ALAS1:intronic:NM_199166.2	1	100		intronic	NM_19916	c.578-53T	p.?	-1.83					5-aminolevul	rs352167		YES			Aminolevul	5-aminole	Metabolic Disease	Glycine,Pyridoxal Phospl		T=0, C=85	T=0.0, C=1.0	0.00001	1390.52	85	T=0/0, C=55/3
chr3:52238677	T/T	C	T	SNV	ALAS1	ALAS1:intronic:NM_199166.2	1	100		intronic	NM_19916	c.578-32C	p.?	-0.42					5-aminolevul	rs352166	0.4486	YES	4.223E-1 (pos.52238677)		Aminolevul	5-aminole	Metabolic Disease	Glycine,Pyridoxal Phospl		C=0, T=84	C=0.0, T=1.0	0.00001	1361.37	84	C=0/0, T=54/3
chr3:52258372	C/C	T	C	SNV	TLR9	TLR9:intronic:NM_017442.3	1	100		intronic	NM_01744	c.4-44A>G	p.?	-0.34					Cofilin/tropo	rs201426920,rs3521	0.418	YES	3.696E-1 (pos.52258372)		Toll-like rec	1-phospha	Crohn Disease;Dige	Chloroquine,Golotimod,		T=0, C=61	T=0.0, C=1.0	0.00001	921.854	61	T=0/0, C=26/3
chr3:52404111	G/A	G	A	SNV	DNAH1	DNAH1:intronic:NM_015512.4	1	50		intronic	NM_01551	c.6139-15	p.?	0.76					AAA domain (	rs746239	0.1218	YES	4.641E-4 (pos.52404111)		Dynein, axo	ATP bindin	Bronchial Diseases;Bronchiect		Benign	G=11, A=11	G=0.5, A=0.5	0.00001	94.692	22	G=5/6, A=10/1
chr12:53839956	G/G	C	G	SNV	PRR13	PRR13:utr_3:NM_018457.3	1	100	4	utr_3	NM_01845	c.560C>G	p.?	0.86						rs8707		YES			Proline-rich	cytosol,nu	Bronchial Neoplasms;Carcinoma, Bronchoge			C=0, G=29	C=0.0, G=1.0	0.00001	427.603	29	C=0/0, G=16/1
chr3:52506282	G/G	A	G	SNV	NISCH	NISCH:intronic:NM_007184.3	1	100		intronic	NM_00718	c.574-37A	p.?	-0.35					PX domain	rs386660926,rs4312(	0.0526	YES	9.832E-1 (pos.52506282)		Nischarin	Rac protei	Arrhythmias, Cardi	Agmatine,Moxonidine,Ti		A=0, G=27	A=0.0, G=1.0	0.00001	389.986	27	A=0/0, G=18/9
chr3:52506426	T/T	C	T	SNV	NISCH	NISCH:intronic:NM_007184.3	1	100		intronic	NM_00718	c.669+12C	p.?	-0.09					PX domain	rs6784615	0.0569	YES	9.816E-1 (pos.52506426)		Nischarin	Rac protei	Arrhythmias, Cardi	Agmatine,Moxonidine,Ti		C=0, T=28	C=0.0, T=1.0	0.00001	408.718	28	C=0/0, T=20/8
chr7:29606287	CT/CT	C	CT	INDEL	PRR15	PRR15:exonic:NM_175887.2	1	100	2	frameshiftInsertion	NM_17588	c.342_343	p.Pro115fs	0.38					ATPase family AAA domain containing 4							multicellular organism development				C=0, CT=25	C=0.0, CT=1.0	0.00001	261.563	25	C=0/0, CT=12/
chr3:52513940	C/C	T	C	SNV	NISCH	NISCH:intronic:NM_007184.3	1	100		intronic	NM_00718	c.1416+62	p.?	-1.89					PX domain	rs4687618		YES			Nischarin	Rac protei	Arrhythmias, Cardi	Agmatine,Moxonidine,Ti		T=0, C=32	T=0.0, C=1.0	0.00001	489.253	32	T=0/0, C=17/1
chr3:52522659	C/C	T	C	SNV	NISCH	NISCH:intronic:NM_007184.3	1	100		intronic	NM_00718	c.3113+38	p.?	-0.13					PX domain	rs887514	0.0512	YES	9.81E-1 (pos.52522659)		Nischarin	Rac protei	Arrhythmias, Cardi	Agmatine,Moxonidine,Ti		T=0, C=51	T=0.0, C=1.0	0.00001	751.949	51	T=0/0, C=17/3
chr6:166720806	C/C	G	C	SNV	PRR18	PRR18:exonic:NM_175922.3	1	100	1	synonymous	NM_17592	c.825C>G	p.(=)	-0.37				0.01(COSM37	Proline-rich	rs911203	0.2513	YES	9.17E-1 (pos.166720806)	adenocarcinoma							G=0, C=28	G=0.0, C=1.0	0.00001	408.346	28	G=0/0, C=11/1
chr3:52551265	T/C	T	C	SNV	STAB1	STAB1:intronic:NM_015136.2	1	46.43		intronic	NM_01513	c.4490-71	p.?	-1.63					EGF domain,E	rs66824127		YES				calcium io	Arthritis;Arthritis, Juvenile;Autoimmune Dise			T=15, C=13	T=0.5357, C=0.4643	0.00001	117.921	28	T=14/1, C=6/7
chr3:52557038	C/T	C	T	SNV	STAB1	STAB1:intronic:NM_015136.2	1	50		intronic	NM_01513	c.6911-3C	p.?	2.51				0.75(COSM40	EGF domain,E	rs4234633	0.459	YES	4.375E-1;0.0 (pos.52557(	acute_myeloid_leukaemia		calcium io	Arthritis;Arthritis, Juvenile;Autoimmune Dise			C=16, T=16	C=0.5, T=0.5	0.00001	158.376	32	C=8/8, T=11/5
chr3:138724757	G/G	C	G	SNV	PRR23A	PRR23A:exonic:NM_001134659.1	1	100	1	synonymous	NM_00113	c.354G>C	p.(=)	-0.77				0.01(COSM14	Protein of unl	rs11924188,rs74922	0.2322	YES	0.0 (pos.138724754),6.4	acute_myeloid_leukaemia,adenocarcinoma,clear_cell_renal_cell_carcinoma,neopl						C=0, G=33	C=0.0, G=1.0	0.00001	504.584	33	C=0/0, G=17/1
chr3:52558904	A/T	A	T	SNV	NT5DC2,STAB1	NT5DC2:intronic:NM_001134231.1, STAB1:downstream:NM	1	56		intronic	NM_01513	c.*532A>T,	p.?, p.?	0.36				0.08(COSM44	5' nucleotida	rs34005367	0.4578	YES	1.17E-4 (pos.52558897),	hepatocellular_carcinoma	5'-nucleot		Arthritis;Arthritis, Juvenile;Autoimmune Dise			A=22, T=28	A=0.44, T=0.56	0.00001	341.079	50	A=11/11, T=12
chr3:52643307	A/G	A	G	SNV	PBRM1	PBRM1:intronic:NM_018313.4	1	50		intronic	NM_01831	c.2567+22	p.?	0.1					BAH domain,l	rs3733045	0.4306	YES	4.339E-1 (pos.52643307)		Polybromo	DNA bindi	Adenocarcinoma;Arthritis;Carcinoma;Carcin			A=13, G=13	A=0.5, G=0.5	0.00001	119.397	26	A=10/3, G=6/7
chr19:7938306	AG/AG	A	AG	INDEL	PRR36	PRR36:exonic:NM_001190467.1	1	100	3	unknown	NM_001190467.1			0.95					Domain of un	rs201708318,rs58971992			1.0 (pos.7938306)							A=0, AG=44	A=0.0, AG=1.0	0.00001	690.296	44	A=0/0, AG=7/3
chr3:52821177	A/C	A	C	SNV	ITIH1	ITIH1:intronic:NM_002215.3	1	51.92		intronic	NM_00221	c.1889-27	p.?	3.94					Inter-alpha-t	rs2286798	0.3815	YES	3.644E-1 (pos.52821177)		Inter-alpha	blood mic	Bipolar Disorder;Bipolar and Related Disorde			A=25, C=27	A=0.4808, C=0.5192	0.00001	270.376	52	A=15/10, C=18
chr3:52878721	C/C	G	C	SNV	TMEM110,TMEM110-MUSTN1	TMEM110-MUSTN1:intronic:NM_001198974.2, TMEM110:in	1	100		intronic	NM_00119	c.540+124	p.?, p.?	0.71					Musculoskel	rs7650966		YES			Transmemb	activation of store-operated calcium channel activity,ca				G=0, C=20	G=0.0, C=1.0	0.00001	263.786	20	G=0/0, C=9/11
chr3:52878825	A/A	C	A	SNV	TMEM110,TMEM110-MUSTN1	TMEM110-MUSTN1:intronic:NM_001198974.2, TMEM110:in	1	100		intronic	NM_00119	c.540+20G	p.?, p.?	-0.18					Musculoskel	rs7629528	0.0014	YES	1.0 (pos.52878825)		Transmemb	activation of store-operated calcium channel activity,ca				C=0, A=20	C=0.0, A=1.0	0.00001	263.784	20	C=0/0, A=8/12
chr3:52968801	C/C	T	C	SNV	SFMBT1	SFMBT1:intronic:NM_016329.3	1	100		intronic	NM_01632	c.453+28A	p.?	-1.68					SAM domain	rs2710315	3.00E-04		1.0 (pos.52968801)		SCM-like pr	cell differe	Colitis;Colitis, Ulcerative;Colonic Diseases;D			T=0, C=28	T=0.0, C=1.0	0.00001	408.718	28	T=0/0, C=14/1
chr3:53003021	C/C	G	C	SNV	SFMBT1	SFMBT1:intronic:NM_016329.3	1	100		intronic	NM_01632	c.28+96C>	p.?	0.19					SAM domain	rs2245424		YES			SCM-like pr	cell differe	Colitis;Colitis, Ulcerative;Colonic Diseases;D			G=0, C=53	G=0.0, C=1.0	0.00001	781.993	53	G=0/0, C=25/2
chr3:53212383	C/T	C	T	SNV	PRKCD	PRKCD:intronic:NM_212539.1	1	59.09		intronic	NM_21253	c.-18-37C>	p.?	-0.51					Phorbol este	rs2306570		YES			Protein kina	ATP bindin	Autoimmune Disea	13-Acetylphorbol,Ingeno		C=9, T=13	C=0.4091, T=0.5909	0.00001	122.613	22	C=4/5, T=10/3
chr3:53213538	G/G	C	G	SNV	PRKCD	PRKCD:intronic:NM_212539.1	1	100		intronic	NM_21253	c.116-55C	p.?	-0.11					Phorbol este	rs2035451	0.0429	YES	9.965E-1 (pos.53213538)		Protein kina	ATP bindin	Autoimmune Disea	13-Acetylphorbol,Ingeno		C=0, G=108	C=0.0, G=1.0	0.00001	1700.02	108	C=0/0, G=65/4
chr3:53260716	T/T	C	T	SNV	TKT	TKT:intronic:NM_001258028.1	1	100		intronic	NM_00125	c.1720+56	p.?	-0.05					Dehydrogena	rs2279322		YES			Discoidin d	catalytic a	Alcohol Amnestic Disorder;Alcohol-Induced D			C=0, T=20	C=0.0, T=1.0	0.00001	263.786	20	C=0/0, T=7/13
chr3:53267155	G/A	G	A	SNV	TKT	TKT:intronic:NM_001258028.1	1	95.83		intronic	NM_00125	c.772+17C	p.?	0.15					Dehydrogena	rs2242301	0.4299	YES	5.799E-1 (pos.53267155)		Discoidin d	catalytic a	Alcohol Amnestic Disorder;Alcohol-Induced D			G=1, A=23	G=0.0417, A=0.9583	0.00001	306.63	24	G=1/0, A=13/1
chr3:53766026	C/C	T	C	SNV	CACNA1D	CACNA1D:intronic:NM_000720.3	1	100		intronic	NM_00072	c.2467-17	p.?	-0.39					Ion transport	rs2612033	0.1402	YES	9.999E-1 (pos.53766026)		Calcium cha	L-type volt	Adenoma;Adrenal (	Amlodipine,Calcium Citr		T=0, C=23	T=0.0, C=1.0	0.00001	316.745	23	T=0/0, C=10/1
chr6:31604044	G/G	T	G	SNV	PRRC2A	PRRC2A:exonic:NM_004638.3	1	100	26	missense	NM_00463	c.5683T>G	p.Leu1895Val	1.34	1	32	0		BAT2 N-termi	rs3132453	0.067	YES	1.0 (pos.31604044)		Proline-rich	RNA bindir	Autoimmune Diseases;Female Urogenital Dis			T=0, G=25	T=0.0, G=1.0	0.00001	352.478	25	T=0/0, G=6/19
chr9:134346315	C/C	T	C	SNV	PRRC2B	PRRC2B:exonic:NM_013318.3	1	100	13	synonymous	NM_01331	c.2052T>C	p.(=)	-2.29				0.06(COSM41	BAT2 N-termi	rs4310250	0.057	YES	9.249E-1 (pos.13434631	neoplasm		RNA binding,cell differentiation,protein binding				T=0, C=26	T=0.0, C=1.0	0.00001	371.397	26	T=0/0, C=6/20
chr9:134350647	C/C	T	C	SNV	PRRC2B	PRRC2B:exonic:NM_013318.3	1	100	15	missense	NM_01331	c.3131T>C	p.Met1044Thr	4.35	1	81	0		BAT2 N-termi	rs10793873	6.00E-04		9.998E-1;2.324E-4 (pos.134350647)			RNA binding,cell differentiation,protein binding				T=0, C=21	T=0.0, C=1.0	0.00001	281.227	21	T=0/0, C=3/18
chr3:56591178	G/G	A	G	SNV	CCDC66	CCDC66:upstream:NM_001141947.2	1	100		intronic	NM_00114	c.-93A>G	p.?	-0.4						rs73079891	0.4786	YES								A=0, G=33	A=0.0, G=1.0	0.00001	508.973	33	A=0/0, G=21/1
chr9:134351065	G/T	G	T	SNV	PRRC2B	PRRC2B:exonic:NM_013318.3	1	95.65	15	synonymous	NM_01331	c.3549G>T	p.(=)	0.36					BAT2 N-termi	rs7858488	0.0564	YES	9.403E-1 (pos.134351065)			RNA binding,cell differentiation,protein binding				G=1, T=22	G=0.0435, T=0.9565	0.00001	277.669	23	G=1/0, T=10/1
chr1:171509327	A/A	G	A	SNV	PRRC2C	PRRC2C:exonic:NM_015172.3	1	100	16	missense	NM_01517	c.2716G>A	p.Ala906Thr	0.11	1	58	0.001	0.03(COSM11	BAT2 N-termi	rs760644	0.1877	YES	8.896E-1 (pos.17150932	carcinoma,duc	Proline-rich	RNA bindir	Alzheimer Disease;Brain Diseases;Central Ne			G=0, A=29	G=0.0, A=1.0	0.00001	427.603	29	G=0/0, A=19/1
chr1:171511039	G/G	C	G	SNV	PRRC2C	PRRC2C:exonic:NM_015172.3	1	100	16	synonymous	NM_01517	c.4428C>G	p.(=)	-0.71				0.26(COSM63	BAT2 N-termi	rs1687064	0.1895	YES	8.893E-1;0.0 (pos.17151	carcinoma	Proline-rich	RNA bindir	Alzheimer Disease;Brain Diseases;Central Ne			C=0, G=28	C=0.0, G=1.0	0.00001	408.214	28	C=0/0, G=13/1
chr3:56674190	C/C	A	C	SNV	FAM208A	FAM208A:intronic:NM_001112736.1	1	100		intronic	NM_00111	c.2620-32	p.?	-0.46					Protein of unl	rs1489949	0.0635	YES	1.0 (pos.56674190)		Family with	RNA binding,chromosome,nucleoplasm,protein binding,				A=0, C=65	A=0.0, C=1.0	0.00001	996.462	65	A=0/0, C=22/4
chr3:57294085	G/G	C	G	SNV	APPL1	APPL1:intronic:NM_012096.2	1	100		intronic	NM_01209	c.1658+38	p.?	-0.1					BAR domain	rs10510791,rs77279	0.3906	YES	0.0 (pos.57294082),8.604E-1 (pos.57294		Adaptor pro	NuRD complex,cell cycle,cell proliferation,cytoplasm,cy				C=0, G=23	C=0.0, G=1.0	0.00001	316.747	23	C=0/0, G=9/14
chr3:57388114	T/T	C	T	SNV	DNAH12	DNAH12:intronic:NM_001291661.1	1	100		intronic	NM_00129	c.6961+92	p.?	-0.22					AAA domain (	rs6445873	0.4635	YES	4.265E-1 (pos.57388114)		Dynein, heavy chain-5		Genital Diseases, Male;Genita		Benign	C=0, T=65	C=0.0, T=1.0	0.00001	996.464	65	C=0/0, T=28/3
chr3:57422824	A/G	A	G	SNV	DNAH12	DNAH12:intronic:NM_001291661.1	1	50		intronic	NM_00129	c.4546-37	p.?	-3.44					AAA domain (	rs4234394	0.492	YES	4.337E-1 (pos.57422824)		Dynein, heavy chain-5		Genital Diseases, Male;Genital Neoplasms, I			A=15, G=15	A=0.5, G=0.5	0.00001	145.021	30	A=8/7, G=9/6
chr3:58089877	G/G	A	G	SNV	FLNB	FLNB:intronic:NM_001164317.1	1	100		intronic	NM_00116	c.1610+65	p.?	-1.1					Calponin hon	rs1522386		YES			Filamin B	RNA bindir	Abnormalities, Multiple;Bone Diseases;Bone			A=0, G=33	A=0.0, G=1.0	0.00001	504.595	33	A=0/0, G=3/30
chr3:58094895	C/C	CT	C	INDEL	FLNB	FLNB:intronic:NM_001164317.1	1	100		intronic	NM_00116	c.2056-11	p.?	-0.05, 0.87					Calponin homology (CH) domain,Filamin/ABP280 repeat						Filamin B	RNA bindir	Abnormalities, Multiple;Bone Diseases;Bone			CT=0, C=27	CT=0.0, C=1.0	0.00001	358.369	27	CT=0/0, C=21/
chr16:29825126	C/C	T	C	SNV	PRRT2	PRRT2:exonic:NM_001256442.1	1	100	2	synonymous	NM_00125	c.751T>C	p.(=)	0.34					Interferon-in	rs11150573	0		1.0 (pos.29825126)		Proline-rich	SH3 doma	Brain Diseases;Central Nervou		Benign	T=0, C=31	T=0.0, C=1.0	0.00001	464.43	31	T=0/0, C=9/22
chr3:9991388	C/C	G	C	SNV	PRRT3,PRRT3-AS1	PRRT3:exonic:NM_207351.4, PRRT3-AS1:intronic_nc:NR_04(	1	100	2	missense	NM_20735	c.412C>G	p.Gln138Glu	0.5	1	29	0			rs279601	0.0264	YES	9.652E-1 (pos.9991388)			integral component of membrane				G=0, C=25	G=0.0, C=1.0	0.00001	342.502	25	G=0/0, C=18/7
chr3:58118700	G/G	A	G	SNV	FLNB	FLNB:intronic:NM_001164317.1	1	100		intronic	NM_00116	c.4607+42	p.?	-1.38					Calponin hon	rs12638356	0.25	YES	9.207E-1 (pos.58118700)		Filamin B	RNA bindir	Abnormalities, Multiple;Bone Diseases;Bone			A=0, G=59	A=0.0, G=1.0	0.00001	883.951	59	A=0/0, G=20/3
chr7:142460865	C/C	T	C	SNV	PRSS1	PRSS1:exonic:NM_002769.4	1	100	5	synonymous	NM_00276	c.738T>C	p.(=)	0.22				0.01(COSM45	Immunoglob	rs6667		YES	2.309E-1 (pos.14246086	carcinoma,squ	Protease, s	blood mic	Digestive System D	(1-{2(4-Car	Benign	T=0, C=52	T=0.0, C=1.0	0.00001	767.864	52	T=0/0, C=36/1
chr3:58186719	A/A	C	A	SNV	DNASE1L3	DNASE1L3:intronic:NM_004944.3	1	100		intronic	NM_00494	c.546+5G>	p.?	-1.12					Endonucleas	rs3772985	0.1099	YES	2.982E-1;0.0 (pos.58186719)		Deoxyribon	DNA bindi	Arthritis;Arthritis, Rheumatoid;Autoimmune			C=0, A=41	C=0.0, A=1.0	0.00001	664.7	41	C=0/0, A=24/1
chr3:58194051	A/A	C	A	SNV	DNASE1L3	DNASE1L3:intronic:NM_004944.3	1	100		intronic	NM_00494	c.142-18G	p.?	-1.01					Endonucleas	rs3732631	0.3347	YES	3.716E-1 (pos.58194051)		Deoxyribon	DNA bindi	Arthritis;Arthritis, Rheumatoid;Autoimmune			C=0, A=35	C=0.0, A=1.0	0.00001	543.903	35	C=0/0, A=21/1
chr3:58520306	C/C	G	C	SNV	ACOX2	ACOX2:intronic:NM_003500.3	1	100		intronic	NM_00350	c.161-57C	p.?	0.12					Acyl-CoA deh	rs7611026		YES	9.999E-1;0.0 (pos.58520306)		Acyl-Coenzy	3alpha,7alpha,12alpha-trihydroxy-5beta-cholestanoyl-(				G=0, C=43	G=0.0, C=1.0	0.00001	705.793	43	G=0/0, C=28/1
chr16:2871555	A/A	G	A	SNV	PRSS21	PRSS21:exonic:NM_006799.3	1	100	6	synonymous	NM_00679	c.894G>A	p.(=)	-0.38				0.04(COSM37	Trypsin	rs10641	0.3069	YES	6.803E-1 (pos.2871555)	hepatocellula	Protease, s	C-terminal protein lipidation,anchored component of m				G=0, A=20	G=0.0, A=1.0	0.00001	263.786	20	G=0/0, A=8/12
chr3:58625975	C/G	C	G	SNV	FAM3D	FAM3D:intronic:NM_138805.2	1	50		intronic	NM_13880	c.323-71G	p.?	0.71					Interleukin-li	rs3749287	0.4843	YES			Family with	cytokine a	Mental Disorders;Schizophrenia;Schizophren			C=31, G=31	C=0.5, G=0.5	0.00001	305.339	62	C=17/14, G=18
chr3:62431415	C/C	T	C	SNV	CADPS	CADPS:intronic:NM_003716.3	1	100		intronic	NM_00371	c.3669+25	p.?	-0.42					Domain of Ur	rs6791296,rs757338	0.1305	YES	0.0;0.0;0.0 (pos.62431409),8.256E-1;0.0		Ca(2+)-depe	catechola	Nervous System Dis	Calcium Citrate,Calcium		T=0, C=63	T=0.0, C=1.0	0.00001	958.917	63	T=0/0, C=11/5
chr3:62498378	A/A	G	A	SNV	CADPS	CADPS:intronic:NM_003716.3	1	100		intronic	NM_00371	c.2599+48	p.?	1.32					Domain of Ur	rs1376917	0.0798	YES	6.965E-1 (pos.62498378)		Ca(2+)-depe	catechola	Nervous System Dis	Calcium Citrate,Calcium		G=0, A=28	G=0.0, A=1.0	0.00001	407.998	28	G=0/0, A=17/1
chr3:62647904	C/C	T	C	SNV	CADPS	CADPS:intronic:NM_003716.3	1	100		intronic	NM_00371	c.969+85A	p.?	0.16					Domain of Ur	rs11712073		YES			Ca(2+)-depe	catechola	Nervous System Dis	Calcium Citrate,Calcium		T=0, C=91	T=0.0, C=1.0	0.00001	1518.33	91	T=0/0, C=52/3
chr1:228003839	C/T	C	T	SNV	PRSS38	PRSS38:exonic:NM_183062.2	1	50	2	missense	NM_18306	c.197C>T	p.Ala66Val	1.94	0.09	64	0.892	0.09(COSM37	Domain of un	rs79840641	0.1462	YES	2.282E-1;0.0 (pos.22800	adenocarcinoma		extracellular region,proteolysis,serine-type endopeptid				C=13, T=13	C=0.5, T=0.5	0.00001	119.163	26	C=6/7, T=8/5
chr3:64526942	C/C	A	C	SNV	ADAMTS9	ADAMTS9:intronic:NM_182920.1	1	100		intronic	NM_18292	c.5388-38	p.?	0.23					ADAM-TS Spa	rs3796383,rs879850	0.1497	YES	3.962E-1;0.0 (pos.64526942)		A disintegri	aorta mor	Diabetes Mellitus;Diabetes Mellitus, Type 2;			A=0, C=78	A=0.0, C=1.0	0.00001	1235.01	78	A=0/0, C=40/3
chr3:64527465	T/T	C	T	SNV	ADAMTS9	ADAMTS9:intronic:NM_182920.1	1	100		intronic	NM_18292	c.5197+49	p.?	-1.32					ADAM-TS Spa	rs1036917	0.0417	YES	4.726E-1;3.052E-1 (pos.64527465)		A disintegri	aorta mor	Diabetes Mellitus;Diabetes Mellitus, Type 2;			C=0, T=33	C=0.0, T=1.0	0.00001	504.553	33	C=0/0, T=17/1
chr4:152212603	G/G	T	G	SNV	PRSS48	PRSS48:exonic:NM_183375.4	1	100	5	stoploss	NM_18337	c.985T>G	p.Ter329Glu	-0.34					Domain of un	rs2407221	0.0331	YES	7.601E-1 (pos.152212603)			extracellular region,proteolysis,serine-type endopeptid				T=0, G=37	T=0.0, G=1.0	0.00001	583.705	37	T=0/0, G=20/1
chr8:10383226	C/G	C	G	SNV	PRSS55	PRSS55:exonic:NM_198464.3	1	59.09	1	missense	NM_19846	c.131C>G	p.Pro44Arg	-0.65	0.1	103	0	0.01(COSM41	Domain of un	rs4521726	0.2006	YES	7.36E-1;0.0 (pos.103832	neoplasm	Protease, s	cytosol,integral component of membrane,proteolysis,se				C=36, G=52	C=0.4091, G=0.5909	0.00001	617.884	88	C=4/32, G=28/
chr3:67411166	A/G	A	G	SNV	SUCLG2		1	53.49		intronic				1.3				0.13(COSM3824580)		rs902321		YES	4.747E-1 (pos.67411166)	carcinoma	Succinate-CoA ligase, G		Amino Acid Metabo	Succinic a	Benign	A=20, G=23	A=0.4651, G=0.5349	0.00001	260.956	43	A=7/13, G=7/1
chr3:68788363	G/T	G	T	SNV	FAM19A4	FAM19A4:intronic:NM_001005527.2	1	50		intronic	NM_00100	c.287-13C	p.?	0.41					TAFA family	rs1504301	0.3763	YES	9.153E-1 (pos.68788363)		Family with	extracellu	Breast Diseases;Breast Neoplasms;Neoplasr			G=24, T=24	G=0.5, T=0.5	0.00001	273.699	48	G=9/15, T=9/1
chr1:151006539	A/A	C	A	SNV	PRUNE1	PRUNE1:exonic:NM_021222.2	1	100	8	synonymous	NM_02122	c.1191C>A	p.(=)	0.48					DHH family,D	rs3738476	0.1302	YES	8.703E-1;1.411E-2 (pos.151006539)		Prune exop	cytoplasm,cytosol,dephosphorylation,focal adhesion,in				C=0, A=75	C=0.0, A=1.0	0.00001	1189.48	75	C=0/0, A=40/3
chr9:79318375	G/G	GGTGACA(	G	INDEL	PRUNE2	PRUNE2:exonic:NM_015225.2	15	100	9	nonframeshiftDeletion	NM_01522	c.8139_81	p.Leu2714_Thr2718del	2.7, 1.4, -0.78, 1.61, 1.48, 2.66, -0.02, 1.31, -1.25, 0.6, 0.84, 3.6					Bcl2-/adenov	rs11267615,rs113471142,rs132		YES	0.0 (pos.79318376),0.0 (	acute_myeloic	PRUNE, Dro	apoptotic process,cytoplasm,integral component of me				GGTGACAG	GGTGACAGCCTGCAA	0.00001	780.658	53	GGTGACAGCC
chr3:69244162	A/A	G	A	SNV	FRMD4B	FRMD4B:intronic:NM_015123.2	1	100		intronic	NM_01512	c.1488+11	p.?	0.56					Domain of un	rs9853587	0.0102	YES	6.186E-1 (pos.69244162)		FERM doma	adherens	Celiac Disease;Digestive System Diseases;G			G=0, A=39	G=0.0, A=1.0	0.00001	623.978	39	G=0/0, A=19/2
chr3:69265371	C/C	G	C	SNV	FRMD4B	FRMD4B:intronic:NM_015123.2	1	100		intronic	NM_01512	c.876+43C	p.?	-1.19					Domain of un	rs62254460	0.1076	YES	9.923E-1;0.0 (pos.69265371)		FERM doma	adherens	Celiac Disease;Digestive System Diseases;G			G=0, C=48	G=0.0, C=1.0	0.00001	810.435	48	G=0/0, C=27/2
chr3:69267578	T/T	A	T	SNV	FRMD4B	FRMD4B:intronic:NM_015123.2	1	100		intronic	NM_01512	c.732-48T	p.?	-1.34					Domain of un	rs4855301	0.1079	YES	9.912E-1 (pos.69267578)		FERM doma	adherens	Celiac Disease;Digestive System Diseases;G			A=0, T=86	A=0.0, T=1.0	0.00001	1405.38	86	A=0/0, T=27/5
chr3:71015021	T/T	G	T	SNV	FOXP1	FOXP1:intronic:NM_001244815.1	1	100		intronic	NM_00124	c.1895+20	p.?	-1.46					FOXP coiled-c	rs7638391	0.08	YES	9.999E-1 (pos.71015021)		Forkhead b	DNA bindi	Adenocarcinoma;Arthritis;Artt		Benign	G=0, T=35	G=0.0, T=1.0	0.00001	543.527	35	G=0/0, T=20/1
chr9:79318471	T/T	C	T	SNV	PRUNE2	PRUNE2:exonic:NM_015225.2	1	100	9	synonymous	NM_01522	c.8058G>A	p.(=)	-0.01					Bcl2-/adenov	rs560094,rs7813711	0.2374	YES	0.0 (pos.79318469),9.402E-1 (pos.79318		PRUNE, Dro	apoptotic process,cytoplasm,integral component of me				C=0, T=58	C=0.0, T=1.0	0.00001	867.385	58	C=0/0, T=39/1
chr3:74344134	A/G	A	G	SNV	CNTN3	CNTN3:intronic:NM_020872.2	1	57.89		intronic	NM_02087	c.2517+13	p.?	0.26					Fibronectin t	rs1352475		YES			Contactin 3	C-termina	Autistic Disorder;Child Development Disorde			A=24, G=33	A=0.4211, G=0.5789	0.00001	345.067	57	A=10/14, G=9/
chr9:79318677	C/C	T	C	SNV	PRUNE2	PRUNE2:exonic:NM_015225.2	1	100	9	missense	NM_01522	c.7852A>G	p.Ser2618Gly	0.11	0.44	56	0	0(COSM4164(	Bcl2-/adenov	rs561970	0.237	YES	9.407E-1 (pos.79318677)	neoplasm	PRUNE, Dro	apoptotic process,cytoplasm,integral component of me				T=0, C=58	T=0.0, C=1.0	0.00001	863.046	58	T=0/0, C=20/3
chr9:79320640	T/T	C	T	SNV	PRUNE2	PRUNE2:exonic:NM_015225.2	1	100	8	missense	NM_01522	c.6550G>A	p.Ala2184Thr	-2.08	0.61	58	0		Bcl2-/adenov	rs512110	0.2373	YES	9.404E-1 (pos.79320640)		PRUNE, Dro	apoptotic process,cytoplasm,integral component of me				C=0, T=59	C=0.0, T=1.0	0.00001	888.756	59	C=0/0, T=28/3
chr9:79321871	T/T	C	T	SNV	PRUNE2	PRUNE2:exonic:NM_015225.2	1	100	8	synonymous	NM_01522	c.5319G>A	p.(=)	-0.65				0.02(COSM63	Bcl2-/adenov	rs577993	0.3606	YES	4.489E-1 (pos.79321871)	carcinoma	PRUNE, Dro	apoptotic process,cytoplasm,integral component of me				C=0, T=24	C=0.0, T=1.0	0.00001	334.792	24	C=0/0, T=10/1
chr9:79322068	T/T	G	T	SNV	PRUNE2	PRUNE2:exonic:NM_015225.2	1	100	8	missense	NM_01522	c.5122C>A	p.His1708Asn	0.85	0.19	68	0.005	0.2(COSM500	Bcl2-/adenov	rs683866	0.3837	YES	4.498E-1 (pos.79322068)	carcinoma,duc	PRUNE, Dro	apoptotic process,cytoplasm,integral component of me				G=0, T=38	G=0.0, T=1.0	0.00001	601.091	38	G=0/0, T=14/2
chr9:79322674	G/G	A	G	SNV	PRUNE2	PRUNE2:exonic:NM_015225.2	1	100	8	missense	NM_01522	c.4516T>C	p.Cys1506Arg	1.03	0.28	180	0		Bcl2-/adenov	rs680775	0.2356	YES	9.404E-1 (pos.79322674)		PRUNE, Dro	apoptotic process,cytoplasm,integral component of me				A=0, G=175	A=0.0, G=1.0	0.00001	2875.79	175	A=0/0, G=99/7
chr9:79325558	C/C	G	C	SNV	PRUNE2	PRUNE2:exonic:NM_015225.2	1	100	8	synonymous	NM_01522	c.1632C>G	p.(=)	1.68				0.06(COSM63	Bcl2-/adenov	rs600859	0.3836	YES	4.556E-1;0.0;0.0 (pos.79	carcinoma	PRUNE, Dro	apoptotic process,cytoplasm,integral component of me				G=0, C=29	G=0.0, C=1.0	0.00001	427.592	29	G=0/0, C=18/1
chr9:79325615	G/G	A	G	SNV	PRUNE2	PRUNE2:exonic:NM_015225.2	1	100	8	synonymous	NM_01522	c.1575T>C	p.(=)	-0.18					Bcl2-/adenov	rs611461	0.2359	YES	9.392E-1 (pos.79325615)		PRUNE, Dro	apoptotic process,cytoplasm,integral component of me				A=0, G=32	A=0.0, G=1.0	0.00001	485.126	32	A=0/0, G=18/1
chr19:40900865	T/T	C	T	SNV	PRX	PRX:exonic:NM_181882.2	1	100	7	missense	NM_18188	c.3394G>A	p.Gly1132Arg	-0.1	0.85	125	0	0.01(COSM3756794)		rs268674	0.0643	YES	9.79E-1 (pos.40900865)	adenocarcinoi	Periaxin	axon ensh	Congenital Abnormalities;Con		Benign/Likely	C=0, T=24	C=0.0, T=1.0	0.00001	334.788	24	C=0/0, T=19/5
chr2:113940681	C/C	T	C	SNV	PSD4	PSD4:exonic:NM_012455.2	1	100	2	synonymous	NM_01245	c.648T>C	p.(=)	-1.19				0.02(COSM44	PH domain,Pl	rs3748914	0.4085	YES	6.134E-1 (pos.11394068	neoplasm	Pleckstrin a	ARF guanyl-nucleotide exchange factor activity,cleavage				T=0, C=46	T=0.0, C=1.0	0.00001	768.311	46	T=0/0, C=24/2
chr3:75788565	G/G	A	G	SNV	ZNF717	ZNF717:intronic:NM_001128223.2	1	100		intronic	NM_00112	c.278-69T	p.?	0.47					C2H2-type zir	rs371099657,rs75435225		YES								A=0, G=61	A=0.0, G=1.0	0.00001	895.758	61	A=0/0, G=34/2
chr2:113940839	C/C	G	C	SNV	PSD4	PSD4:exonic:NM_012455.2	1	100	2	missense	NM_01245	c.806G>C	p.Gly269Ala	-0.26	0.02	60	0.639	0.16(COSM41	PH domain,Pl	rs4849167	0.4078	YES	6.109E-1;8.104E-4 (pos.1	neoplasm	Pleckstrin a	ARF guanyl-nucleotide exchange factor activity,cleavage				G=0, C=32	G=0.0, C=1.0	0.00001	485.141	32	G=0/0, C=18/1
chr2:113943470	G/G	A	G	SNV	PSD4	PSD4:exonic:NM_012455.2	1	100	5	synonymous	NM_01245	c.1266A>G	p.(=)	0.14				0(COSM4133(	PH domain,Pl	rs2241976	0.418	YES	6.365E-1;0.0 (pos.11394	neoplasm	Pleckstrin a	ARF guanyl-nucleotide exchange factor activity,cleavage				A=0, G=51	A=0.0, G=1.0	0.00001	744.73	51	A=0/0, G=31/2
chr2:113956371	G/C	G	C	SNV	PSD4	PSD4:exonic:NM_012455.2	1	57.14	15	synonymous	NM_01245	c.2679G>C	p.(=)	-0.59				0.15(COSM37	PH domain,Pl	rs2276561	0.4583	YES	6.548E-1 (pos.11395637	adenocarcinoi	Pleckstrin a	ARF guanyl-nucleotide exchange factor activity,cleavage				G=12, C=16	G=0.4286, C=0.5714	0.00001	155.148	28	G=4/8, C=10/6
chr3:75790972	T/T	C	T	SNV	ZNF717	ZNF717:intronic:NM_001128223.2	1	100		intronic	NM_00112	c.58-85G>	p.?	-1.44					C2H2-type zir	rs11710621		YES	3.108E-1 (pos.75790972)							C=0, T=26	C=0.0, T=1.0	0.00001	371.379	26	C=0/0, T=16/1
chr3:77666642	T/C	T	C	SNV	ROBO2	ROBO2:intronic:NM_002942.4	1	57.14		intronic	NM_00294	c.3294-22	p.?	-0.28					Fibronectin type III domain,Immunoglobulin I-set domain,Immunoglobulin V-set domain,Immunoglo						Roundabo	Roundabo	Adenocarcinoma;Autistic Disorder;Carcinom			T=24, C=32	T=0.4286, C=0.5714	0.00001	331.834	56	T=8/16, C=17/
chr3:78676405	T/C	T	C	SNV	ROBO1	ROBO1:intronic:NM_002941.3	1	56		intronic	NM_00294	c.3875+66	p.?	-0.49					Fibronectin t	rs7625555		YES			Roundabou	LRR domai	Adenocarcinoma;Autistic Disorder;Carcinom			T=11, C=14	T=0.44, C=0.56	0.00001	134.35	25	T=5/6, C=4/10
chr3:78676467	G/G	A	G	SNV	ROBO1	ROBO1:intronic:NM_002941.3	1	100		intronic	NM_00294	c.3875+4T	p.?	-2.35					Fibronectin t	rs386662758,rs7636(	0.0281	YES	9.919E-1 (pos.78676467)		Roundabou	LRR domai	Adenocarcinoma;Autistic Disorder;Carcinom			A=0, G=26	A=0.0, G=1.0	0.00001	371.42	26	A=0/0, G=9/17
chr3:78700779	A/A	C	A	SNV	ROBO1	ROBO1:intronic:NM_002941.3	1	100		intronic	NM_00294	c.2812+10	p.?	-1.33					Fibronectin t	rs3925684,rs752530728		YES			Roundabou	LRR domai	Adenocarcinoma;Autistic Disorder;Carcinom			C=0, A=32	C=0.0, A=1.0	0.00001	485.139	32	C=0/0, A=19/1
chr3:78717508	A/A	G	A	SNV	ROBO1	ROBO1:intronic:NM_002941.3	1	100		intronic	NM_00294	c.1631-56	p.?	0.58					Fibronectin t	rs2255164		YES			Roundabou	LRR domai	Adenocarcinoma;Autistic Disorder;Carcinom			G=0, A=44	G=0.0, A=1.0	0.00001	726.527	44	G=0/0, A=11/3
chr3:78796078	A/A	C	A	SNV	ROBO1	ROBO1:intronic:NM_002941.3	1	100		intronic	NM_00294	c.500-28G	p.?	-0.87					Fibronectin t	rs2304503	0.4729	YES	2.437E-1 (pos.78796078)		Roundabou	LRR domai	Adenocarcinoma;Autistic Disorder;Carcinom			C=0, A=122	C=0.0, A=1.0	0.00001	1969.93	122	C=0/0, A=50/7

chr3:8543713	G/GT	G	GT	INDEL	LMCD1,LMCD1-AS1	LMCD1:intronic:NM_014583.3, LMCD1-AS1:upstream:NR_0:	1	97.92		intronic	NR_03337	c.42+47G>	p.?	-0.68					LIM domain,F	rs397766983,rs5846	2.00E-04		1.0 (pos.8543713)		LIM and cys	cellular pr	Cardiovascular Diseases;Heart Diseases;He			G=1, GT=47	G=0.0208, GT=0.979	0.00001	704.679	48	G=0/1, GT=26/
chr3:8543757	C/C	A	C	SNV	LMCD1,LMCD1-AS1	LMCD1:intronic:NM_014583.3, LMCD1-AS1:upstream:NR_0:	1	100		intronic	NR_03337	c.42+91A>	p.?	-0.31					LIM domain,F	rs2324625		YES			LIM and cys	cellular pr	Cardiovascular Diseases;Heart Diseases;He			A=0, C=46	A=0.0, C=1.0	0.00001	768.308	46	A=0/0, C=24/2
chr3:8667896	C/C	T	C	SNV	SSUH2	SSUH2:intronic:NM_001256748.1	1	100		intronic	NM_00125	c.767+19A	p.?	-3.52						rs355058	0.2201	YES	9.998E-1 (pos.8667896)		Ssu2, C. ele	cytoplasm,nucleus,odontogenesis				T=0, C=30	T=0.0, C=1.0	0.00001	446.634	30	T=0/0, C=26/4
chr19:43709647	A/A	G	A	SNV	PSG4	PSG4:exonic:NM_002780.4	1	100	1	synonymous	NM_00278	c.42C>T	p.(=)	0.5				0.04(COSM45	Immunoglobi	rs12985206		YES	8.434E-1;0.0 (pos.43709	squamous_cel	Pregnancy-	extracellular exosome,female pregnancy				G=0, A=25	G=0.0, A=1.0	0.00001	357.26	25	G=0/0, A=13/1
chr19:43709654	A/A	C	A	SNV	PSG4	PSG4:exonic:NM_002780.4	1	100	1	missense	NM_00278	c.35G>T	p.Arg12Leu	-5.43	0.33	102	0.001	0(COSM4591	Immunoglobi	rs11883277,rs796129150		YES	9.342E-1;1.386E-4 (pos.4	basal_cell_car	Pregnancy-	extracellular exosome,female pregnancy				C=0, A=25	C=0.0, A=1.0	0.00001	353.016	25	C=0/0, A=12/1
chr3:8943988	A/A	C	A	SNV	RAD18	RAD18:intronic:NM_020165.3	1	100		intronic	NM_02016	c.1168+76	p.?	-0.01					RING-type zin	rs464302		YES			Rad18, S. ce	DNA binding,DNA damage response, detection of DNA da				C=0, A=120	C=0.0, A=1.0	0.00001	1938.62	120	C=0/0, A=86/3
chr3:8955240	C/C	T	C	SNV	RAD18	RAD18:intronic:NM_020165.3	1	100		intronic	NM_02016	c.966+88A	p.?	-0.52					RING-type zin	rs430781		YES			Rad18, S. ce	DNA binding,DNA damage response, detection of DNA da				T=0, C=35	T=0.0, C=1.0	0.00001	543.903	35	T=0/0, C=21/1
chr19:43709656	G/G	C	G	SNV	PSG4	PSG4:exonic:NM_002780.4	1	100	1	missense	NM_00278	c.33G>C	p.Gln11His	-1.21	0.55	24	0.01	0.01(COSM11	Immunoglobi	rs11883278,rs796129150		YES	9.339E-1 (pos.43709656)	astrocytoma_(	Pregnancy-	extracellular exosome,female pregnancy				C=0, G=25	C=0.0, G=1.0	0.00001	353.019	25	C=0/0, G=12/1
chr3:9069747	G/G	A	G	SNV	SRGAP3	SRGAP3:intronic:NM_014850.3	1	100		intronic	NM_01485	c.1540-10	p.?	-0.28					Fes/CIP4, anc	rs416685	0.2341	YES	6.909E-1 (pos.9069747)		Slit-robo GT	GTPase ac	Astrocytoma;Glioma;Intellectual Disability;N			A=0, G=42	A=0.0, G=1.0	0.00001	685.214	42	A=0/0, G=23/1
chr19:43680051	T/T	C	T	SNV	PSG5	PSG5:exonic:NM_001130014.1	1	100	3	missense	NM_00113	c.680G>A	p.Arg227His	-0.27	0.11	29	0.029		Immunoglobi	rs1058285	0.3983	YES	9.835E-1 (pos.43680051)		Pregnancy-	extracellu	Arthritis;Arthritis, Rheumatoid;Autoimmune			C=0, T=63	C=0.0, T=1.0	0.00001	958.894	63	C=0/0, T=24/3
chr3:97466432	G/G	T	G	SNV	EPHA6	EPHA6:intronic:NM_001080448.2	1	100		intronic	NM_00108	c.3278+16	p.?	-3.1					Ephrin recept	rs79617067	0.2397	YES	4.1E-1 (pos.97466432)		Ephrin receptor EphA6					T=0, G=26	T=0.0, G=1.0	0.00001	362.329	26	T=0/0, G=13/1
chr19:43690506	C/C	G	C	SNV	PSG5	PSG5:exonic:NM_001130014.1	1	100	1	missense	NM_00113	c.52C>G	p.Leu18Val	0.01	0.1	32	0.14	0.01(COSM15	Immunoglobi	rs8107936	0.399	YES	9.741E-1 (pos.43690506)	ductal_carcinc	Pregnancy-	extracellu	Arthritis;Arthritis, Rheumatoid;Autoimmune			G=0, C=40	G=0.0, C=1.0	0.00001	632.324	40	G=0/0, C=30/1
chr19:43680037	T/A	T	A	SNV	PSG5	PSG5:exonic:NM_001130014.1	1	50	3	missense	NM_00113	c.694A>T	p.Thr232Ser	-0.02	0.09	58	0.422	0(COSM1394	Immunoglobi	rs1135901	0.259	YES	3.467E-4 (pos.43680037)	adenocarcinor	Pregnancy-	extracellu	Arthritis;Arthritis, Rheumatoid;Autoimmune			T=32, A=32	T=0.5, A=0.5	0.00001	317.426	64	T=9/23, A=16/
chr3:9803126	G/G	A	G	SNV	CAMK1	CAMK1:intronic:NM_003656.4	1	100		intronic	NM_00365	c.632+19T	p.?	-2.82					Kinase-like,P	rs17252807	0.468	YES	1.473E-1 (pos.9803126)		Calcium/ca	ATP binding,calmodulin binding,calmodulin-dependent				A=0, G=23	A=0.0, G=1.0	0.00001	316.747	23	A=0/0, G=12/1
chr3:9822275	T/T	C	T	SNV	TADA3	TADA3:intronic:NM_001278270.1	1	100		intronic	NM_00127	c.1107-42	p.?	0.04					Histone acet	rs3868897	0.2792	YES	7.251E-1 (pos.9822275)		Transcriptic	Ada2/Gcn5/Ada3 transcription activator complex,DNA b				C=0, T=85	C=0.0, T=1.0	0.00001	1390.11	85	C=0/0, T=38/4
chr19:43420451	G/C	G	C	SNV	PSG6	PSG6:exonic:NM_001031850.3	1	46.58	2	missense	NM_00103	c.253C>G	p.His85Asp	-1.74	1	81	0	0(COSM3756895),0(COSM3		rs3198831,rs386809	0.2596	YES	4.35E-1;0.0 (pos.434204	acute_myeloic	Pregnancy-	extracellular region,female pregnancy,molecular_functi				G=39, C=34	G=0.5342, C=0.4658	0.00001	336.87	73	G=32/7, C=16/
chr3:98541012	A/A	G	A	SNV	DCBLD2	DCBLD2:intronic:NM_080927.3	1	100		intronic	NM_08092	c.830+60C	p.?	0.28					CUB domain,	rs866873	0.0013	YES	9.805E-1;0.0 (pos.98541012)		Discoidin, C	cell surfac	Adnexal Diseases;Cysts;Digestive System Dis			G=0, A=119	G=0.0, A=1.0	0.00001	1913.83	119	G=0/0, A=63/5
chr19:43433805	A/G	A	G	SNV	PSG7	PSG7:exonic:NM_001206650.1	1	50	2	synonymous	NM_00120	c.132T>C	p.(=)	-2.94					Immunoglobi	rs1065178	0.2815	YES	4.31E-1 (pos.43433805)		Pregnancy-specific beta-1-glycoprotein-7					A=11, G=11	A=0.5, G=0.5	0.00001	94.6067	22	A=6/5, G=7/4
chr20:60713311	A/A	G	A	SNV	PSMA7	PSMA7:exonic:NM_002792.3	1	100	5	synonymous	NM_00279	c.507C>T	p.(=)	-0.35					Proteasome	rs7076	0.203	YES	7.851E-1 (pos.60713311)		Proteasom	Fc-epsilon receptor signaling p		(3AR,6R,6AS)-6-((S)-((S)-C)		G=0, A=23	G=0.0, A=1.0	0.00001	316.742	23	G=0/0, A=14/9
chr20:60713332	G/G	A	G	SNV	PSMA7	PSMA7:exonic:NM_002792.3	1	100	5	synonymous	NM_00279	c.486T>C	p.(=)	0.35					Proteasome	rs1135961	0.1438	YES	8.336E-1;0.0 (pos.60713332)		Proteasom	Fc-epsilon receptor signaling p		(3AR,6R,6AS)-6-((S)-((S)-C)		A=0, G=23	A=0.0, G=1.0	0.00001	316.747	23	A=0/0, G=14/9
chr3:9979660	G/G	A	G	SNV	CRELD1	CRELD1:intronic:NM_001031717.3	1	100		intronic	NM_00103	c.369-39A	p.?	-1.66					Calcium-bind	rs2302786	0.2553	YES	2.042E-1 (pos.9979660)		Cysteine-ric	calcium io	Abnormalities, Multiple;Cardiovascular Abnc			A=0, G=58	A=0.0, G=1.0	0.00001	867.373	58	A=0/0, G=45/1
chr7:103007949	C/C	A	C	SNV	PSMC2	PSMC2:exonic:NM_002803.3	1	100	10	synonymous	NM_00280	c.934A>C	p.(=)	2					ATPase famil	rs4520097	6.00E-04	YES	1.0 (pos.103007949)		Proteasom	ATP binding,ATPase activity,Fc-epsilon receptor signalin				A=0, C=36	A=0.0, C=1.0	0.00001	563.744	36	A=0/0, C=18/1
chr4:100052539	A/C	A	C	SNV	ADH4,LOC100507053	ADH4:intronic:NM_000670.4, LOC100507053:intronic_nc:N	1	45.45		intronic	NR_03788	c.843+116	p.?	-0.84					Alcohol dehy	rs29001208		YES			Alcohol deh	NAD bindi	Digestive System D	Cyclohexylformamide,Et		A=24, C=20	A=0.5455, C=0.4545	0.00001	207.29	44	A=19/5, C=14/
chr4:10020480	T/T	C	T	SNV	SLC2A9	SLC2A9:intronic:NM_020041.2	1	100		intronic	NM_02004	c.249+119	p.?	-0.9					Major Facilit	rs2240720		YES			Solute carri	apical pla	Arthritis;Congenita	Fludeoxyglucose F-18,Gl		C=0, T=38	C=0.0, T=1.0	0.00001	603.786	38	C=0/0, T=30/8
chr4:100205819	C/A	C	A	SNV	ADH1A,LOC100507053	ADH1A:intronic:NM_000667.3, LOC100507053:intronic_nc:	1	50		intronic	NR_03788	c.348-44G	p.?	-0.81					Alcohol dehy	rs28364312	0.3836	YES	2.314E-2;0.0 (pos.100205819)		Alcohol deh	alcohol dehydrogenase (NAD) a		4-Iodopyrazole,Ethanol,I		C=19, A=19	C=0.5, A=0.5	0.00001	199.992	38	C=11/8, A=4/1
chr19:40478362	T/C	T	C	SNV	PSMC4	PSMC4:exonic:NM_006503.3	1	52.38	3	synonymous	NM_00650	c.222T>C	p.(=)	-1.05					AAA domain (	rs35555615	0.0345	YES	3.474E-4 (pos.40478362)		Proteasom	ATP binding,ATPase activity,Fc-epsilon receptor signalin				T=10, C=11	T=0.4762, C=0.5238	0.00001	95.3003	21	T=3/7, C=3/8
chr11:244106	G/G	A	G	SNV	PSMD13	PSMD13:exonic:NM_175932.2	1	100	2	missense	NM_17593	c.161A>G	p.Glu54Gly	-0.33	0.3	98	0.003	0.1(COSM375	PCI domain	rs10902112,rs37390	0.2902	YES	8.106E-1 (pos.244106)	acute_myeloic	Proteasom	Fc-epsilon	HIV Infections;Immune System Diseases;Imr			A=0, G=20	A=0.0, G=1.0	0.00001	263.784	20	A=0/0, G=9/11
chr11:244108	G/G	A	G	SNV	PSMD13	PSMD13:exonic:NM_175932.2	1	100	2	missense	NM_17593	c.163A>G	p.Asn55Asp	-0.24	0.19	23	0.048	0.06(COSM37	PCI domain	rs373900649,rs7107	0.2893	YES	8.105E-1 (pos.244108)	adenocarcinor	Proteasom	Fc-epsilon	HIV Infections;Immune System Diseases;Imr			A=0, G=20	A=0.0, G=1.0	0.00001	263.786	20	A=0/0, G=9/11
chr11:244115	A/A	G	A	SNV	PSMD13	PSMD13:exonic:NM_175932.2	1	100	2	missense	NM_17593	c.170G>A	p.Cys57Tyr	-2.48	0.35	194	0	0.02(COSM85	PCI domain	rs7128044	0.2767	YES	8.105E-1 (pos.244115)	astrocytoma_(	Proteasom	Fc-epsilon	HIV Infections;Immune System Diseases;Imr			G=0, A=20	G=0.0, A=1.0	0.00001	263.786	20	G=0/0, A=9/11
chr4:101338217	C/C	T	C	SNV	EMCN	EMCN:intronic:NM_016242.3	1	100		intronic	NM_01624	c.689+57A	p.?	0.01					Endomucin	rs3733437	0.362	YES				extracellular region,integral component of membrane,p				T=0, C=27	T=0.0, C=1.0	0.00001	389.988	27	T=0/0, C=24/3
chr11:244129	T/T	C	T	SNV	PSMD13	PSMD13:exonic:NM_175932.2	1	100	2	missense	NM_17593	c.184C>T	p.Arg62Trp	0.16	0.12	101	0		PCI domain	rs7116130	0.2716	YES	8.157E-1 (pos.244129)		Proteasom	Fc-epsilon	HIV Infections;Immune System Diseases;Imr			C=0, T=20	C=0.0, T=1.0	0.00001	263.786	20	C=0/0, T=9/11
chr11:244141	A/A	G	A	SNV	PSMD13	PSMD13:exonic:NM_175932.2	1	100	2	missense	NM_17593	c.196G>A	p.Ala66Thr	-2.2	0.11	58	0	0.02(COSM39	PCI domain	rs7128029	0.2711	YES	8.153E-1 (pos.244141)	acute_myeloic	Proteasom	Fc-epsilon	HIV Infections;Immune System Diseases;Imr			G=0, A=20	G=0.0, A=1.0	0.00001	263.785	20	G=0/0, A=9/11
chr4:104000973	C/C	G	C	SNV	BDH2	BDH2:intronic:NM_020139.3	1	100		intronic	NM_02013	c.685-61C	p.?	-1.52					Enoyl-(Acyl ca	rs12504262	0.2027	YES				2,3-dihydro-2,3-dihydroxybenzoate dehydrogenase activ				G=0, C=31	G=0.0, C=1.0	0.00001	465.806	31	G=0/0, C=22/9
chr4:104061266	A/A	G	A	SNV	CENPE	CENPE:intronic:NM_001813.2	1	100		intronic	NM_00181	c.5914-30	p.?	-0.21					Kinesin moto	rs7686105	0.1784	YES	1.346E-1 (pos.104061266)		Centromer	ATP bindin	Bone Diseases;Bon	GSK-923295		G=0, A=27	G=0.0, A=1.0	0.00001	389.988	27	G=0/0, A=16/1
chr11:244167	T/T	C	T	SNV	PSMD13	PSMD13:exonic:NM_175932.2	1	100	2	synonymous	NM_17593	c.222C>T	p.(=)	-0.82				0.24(COSM29	PCI domain	rs1128320	0.266	YES	8.138E-1 (pos.244167)	adenocarcinor	Proteasom	Fc-epsilon	HIV Infections;Immune System Diseases;Imr			C=0, T=20	C=0.0, T=1.0	0.00001	263.783	20	C=0/0, T=9/11
chr4:104063071	T/T	C	T	SNV	CENPE	CENPE:intronic:NM_001813.2	1	100		intronic	NM_00181	c.5305-6G	p.?	-0.04				0(COSM1497	Kinesin moto	rs2711898	0.3925	YES	2.865E-1 (pos.10406307	acute_myeloic	Centromer	ATP bindin	Bone Diseases;Bon	GSK-923295		C=0, T=24	C=0.0, T=1.0	0.00001	334.792	24	C=0/0, T=12/1
chr4:104066049	T/T	C	T	SNV	CENPE	CENPE:intronic:NM_001813.2	1	100		intronic	NM_00181	c.4857+15	p.?	-0.39					Kinesin moto	rs1031804		YES			Centromer	ATP bindin	Bone Diseases;Bon	GSK-923295		C=0, T=36	C=0.0, T=1.0	0.00001	563.744	36	C=0/0, T=16/2
chr4:104066890	A/A	T	A	SNV	CENPE	CENPE:intronic:NM_001813.2	1	100		intronic	NM_00181	c.4414-52	p.?	0.47					Kinesin moto	rs2169508	0.3896	YES	2.884E-1 (pos.104066890)		Centromer	ATP bindin	Bone Diseases;Bon	GSK-923295		T=0, A=34	T=0.0, A=1.0	0.00001	524.186	34	T=0/0, A=24/1
chr4:104079424	T/T	C	T	SNV	CENPE	CENPE:intronic:NM_001813.2	1	100		intronic	NM_00181	c.3033+33	p.?	-1.17					Kinesin moto	rs2251322	0.3904	YES	2.823E-1 (pos.104079424)		Centromer	ATP bindin	Bone Diseases;Bon	GSK-923295		C=0, T=22	C=0.0, T=1.0	0.00001	298.891	22	C=0/0, T=11/1
chr11:244171	C/C	T	C	SNV	PSMD13	PSMD13:exonic:NM_175932.2	1	100	2	synonymous	NM_17593	c.226T>C	p.(=)	-0.62				0.1(COSM375	PCI domain	rs1128321	0.2666	YES	8.137E-1 (pos.244171)	adenocarcinor	Proteasom	Fc-epsilon	HIV Infections;Immune System Diseases;Imr			T=0, C=20	T=0.0, C=1.0	0.00001	263.786	20	T=0/0, C=9/11
chr11:244197	C/C	T	C	SNV	PSMD13	PSMD13:exonic:NM_175932.2	1	100	2	synonymous	NM_17593	c.252T>C	p.(=)	-0.16				0.59(COSM37	PCI domain	rs1128322	0.2579	YES	8.162E-1 (pos.244197)	acute_myeloic	Proteasom	Fc-epsilon	HIV Infections;Immune System Diseases;Imr			T=0, C=20	T=0.0, C=1.0	0.00001	263.786	20	T=0/0, C=9/11
chr1:151239051	C/C	T	C	SNV	PSMD4	PSMD4:exonic:NM_002810.2	1	100	9	synonymous	NM_00281	c.951T>C	p.(=)	-1.54					Ssl1-like,Ubic	rs7489	0.2951	YES	6.288E-1 (pos.151239051)		Proteasom	Fc-epsilon receptor signaling pathway,MAPK cascade,NI				T=0, C=20	T=0.0, C=1.0	0.00001	263.786	20	T=0/0, C=6/14
chr4:10509699	T/C	T	C	SNV	CLNK	CLNK:intronic:NM_052964.3	1	51.85		intronic	NM_05296	c.907-39A	p.?	-0.29					SH2 domain	rs2868941	0.2701	YES	8.178E-1 (pos.10509699)		Cytokine-de	SH3/SH2 a	Hypopigmentation;Pigmentation Disorders;S			T=13, C=14	T=0.4815, C=0.5185	0.00001	132.664	27	T=10/3, C=5/9
chr4:10522474	A/C	A	C	SNV	CLNK	CLNK:intronic:NM_052964.3	1	58.33		intronic	NM_05296	c.732-19T	p.?	0.92					SH2 domain	rs201621066			0.0 (pos.10522474)		Cytokine-de	SH3/SH2 a	Hypopigmentation;Pigmentation Disorders;S			A=25, C=35	A=0.4167, C=0.5833	0.00001	370.434	60	A=19/6, C=11/
chr21:40552307	G/G	A	G	SNV	PSMG1	PSMG1:exonic:NM_003720.3	1	100	3	synonymous	NM_00372	c.297T>C	p.(=)	0.01					Proteasome	rs3171465	0.4541	YES	4.072E-1 (pos.40552307)		Proteasom	Golgi appa	Abnormalities, Multiple;Chromosome Disord			A=0, G=34	A=0.0, G=1.0	0.00001	524.186	34	A=0/0, G=20/1
chr4:106861557	T/T	A	T	SNV	NPNT	NPNT:intronic:NM_001184691.1	1	100		intronic	NM_00118	c.731-114	p.?	0.61					Calcium-bind	rs4627867		YES			Nephronect	basement membrane,branching involved in ureteric buc				A=0, T=26	A=0.0, T=1.0	0.00001	371.42	26	A=0/0, T=16/1
chr6:3264487	C/C	T	C	SNV	PSMG4	PSMG4:exonic:NM_001128592.1	1	100	3	missense	NM_00112	c.295T>C	p.Trp99Arg	-1.3	0.93	101	0.024		Proteasome	rs4959786	0.1964	YES	6.817E-1 (pos.3264487)		Proteasom	proteasome assembly				T=0, C=20	T=0.0, C=1.0	0.00001	263.785	20	T=0/0, C=12/8
chr6:3264502	C/C	G	C	SNV	PSMG4	PSMG4:exonic:NM_001128592.1	1	100	3	missense	NM_00112	c.310G>C	p.Gly104Arg	-0.02	0	125	0.078		Proteasome	rs4959787	0.1967	YES	6.801E-1 (pos.3264502)		Proteasom	proteasome assembly				G=0, C=20	G=0.0, C=1.0	0.00001	263.75	20	G=0/0, C=12/8
chr4:108866008	A/A	T	A	SNV	CYP2U1	CYP2U1:intronic:NM_183075.2	1	100		intronic	NM_18307	c.491-118	p.?	-3.33					Cytochrome	rs13104867,rs536313224		YES			Cytochrom	aromatas	Nervous System Diseases;Neurologic Manife			T=0, A=21	T=0.0, A=1.0	0.00001	281.235	21	T=0/0, A=6/15
chr4:108866058	A/A	G	A	SNV	CYP2U1	CYP2U1:intronic:NM_183075.2	1	100		intronic	NM_18307	c.491-68G	p.?	-3.27					Cytochrome	rs10002671		YES			Cytochrom	aromatas	Nervous System Diseases;Neurologic Manife			G=0, A=22	G=0.0, A=1.0	0.00001	298.854	22	G=0/0, A=6/16
chr6:31105891	G/G	A	G	SNV	PSORS1C1,PSORS1C2	PSORS1C1:intronic:NM_014068.2, PSORS1C2:exonic:NM_01	1	100	2	missense	NM_01406	c.14-316A	p.?, p.Leu83Pro	2.76	1	98	0	0.01(COSM41	Psoriasis sus	rs2233952,rs386698	0.1135	YES	0.0 (pos.31105885),9.40	neoplasm	SEEK1 gene	biological	Autoimmune Diseases;Behcet Syndrome;Blo			A=0, G=33	A=0.0, G=1.0	0.00001	504.598	33	A=0/0, G=17/1
chr4:108944579	T/T	C	T	SNV	HADH	HADH:intronic:NM_001184705.2	1	100		intronic	NM_00118	c.547-51C	p.?	-0.95					3-hydroxyacy	rs4956032	0.0759	YES	9.733E-1 (pos.108944579)			3-hydroxy	3-hydroxyacyl-CoA (	3-Hydroxybutyryl-Coenzy		C=0, T=45	C=0.0, T=1.0	0.00001	747.393	45	C=0/0, T=15/3
chr4:109578572	A/A	G	A	SNV	OSTC	OSTC:intronic:NM_001267818.1	1	100		intronic	NM_00126	c.234-34G	p.?	-0.16					OST3 / OST6 f	rs2851379	0.0062		1.0 (pos.109578572)			dolichyl-diphosphooligosaccharide-protein glycotransfe				G=0, A=21	G=0.0, A=1.0	0.00001	281.234	21	G=0/0, A=12/9
chr4:109766290	G/G	T	G	SNV	COL25A1	COL25A1:intronic:NM_198721.3	1	100		intronic	NM_19872	c.1578+55	p.?	0.33					Collagen tripl	rs11098004	0.1395	YES			Collagen, ty	amyloid-beta binding,axonogenesis involved in innervat				T=0, G=21	T=0.0, G=1.0	0.00001	281.235	21	T=0/0, G=17/4
chr4:110384049	TTTTC/TTTTC	T	TTTTC	INDEL	SEC24B	SEC24B:intronic:NM_006323.4	4	100		intronic	NM_00632	c.134-8T>T	p.?	1.19					Gelsolin repe	rs869153737					Sec24-relat	COPII vesicle coat,COPII vesicle coating,ER to Golgi trans				T=0, TTTTC	T=0.0, TTTTC=1.0	0.00001	754.615	57	T=0/0, TTTTC=
chr4:110670807	C/C	G	C	SNV	CFI	CFI:intronic:NM_000204.4	1	100		intronic	NM_00020	c.941-49C	p.?	-0.24					Low-density l	rs7441380	6.00E-04	YES	1.0 (pos.110670807)		Complemer	compleme	Afibrinogenemia;Anemia;Anemia, Hemolytic			G=0, C=65	G=0.0, C=1.0	0.00001	987.213	65	G=0/0, C=38/2
chr6:31105857	AG/A	AG	A	INDEL	PSORS1C1,PSORS1C2	PSORS1C1:intronic:NM_014068.2, PSORS1C2:exonic:NM_01	1	50	2	frameshiftDeletion	NM_01406	c.14-350A	p.?, p.Pro94fs	2.51, 4.11					Psoriasis sus	rs373592714,rs7668	0.0953	YES	1.276E-2;0.0 (pos.31105	astrocytoma_(	SEEK1 gene	biological	Autoimmune Diseases;Behcet Syndrome;Blo			AG=11, A=1	AG=0.5, A=0.5	0.00001	94.2097	22	AG=4/7, A=4/7
chr4:110679002	T/T	G	T	SNV	CFI	CFI:intronic:NM_000204.4	1	100		intronic	NM_00020	c.884-63C	p.?	-0.58					Low-density l	rs7437142		YES			Complemer	compleme	Afibrinogenemia;Anemia;Anemia, Hemolytic			G=0, T=67	G=0.0, T=1.0	0.00001	1034.43	67	G=0/0, T=26/4
chr4:110737549	A/A	G	A	SNV	GAR1	GAR1:intronic:NM_032993.2	1	100		intronic	NM_03299	c.214+15G	p.?	-1.11					Gar1/Naf1 RN	rs6828254	0.002		1.0 (pos.110737549)		Nucleolar p	RNA bindi	Congenital Abnormalities;Congenital, Heredi			G=0, A=31	G=0.0, A=1.0	0.00001	469.427	31	G=0/0, A=6/25
chr1:45292866	A/A	G	A	SNV	PTCH2	PTCH2:exonic:NM_003738.4	1	100	16	synonymous	NM_00373	c.2487C>T	p.(=)	0.8				0.7(COSM375	Patched fami	rs2295996	0.167	YES	2.22E-1;0.0 (pos.452928	acute_myeloic	Patched, Dr	cell fate d	Abnormalities, Multiple;Basal Cell Nevus Syn			G=0, A=40	G=0.0, A=1.0	0.00001	644.286	40	G=0/0, A=26/1
chr1:45293518	G/G	A	G	SNV	PTCH2	PTCH2:exonic:NM_003738.4	1	100	14	synonymous	NM_00373	c.2055T>C	p.(=)	2.1				0.06(COSM37	Patched fami	rs7525308	0.3423	YES	4.844E-1 (pos.45293518)	adenocarcinor	Patched, Dr	cell fate d	Abnormalities, Multiple;Basal Cell Nevus Syn			A=0, G=20	A=0.0, G=1.0	0.00001	263.776	20	A=0/0, G=10/1
chr10:27702169	T/T	C	T	SNV	PTCHD3	PTCHD3:exonic:NM_001034842.3	1	100	1	synonymous	NM_00103	c.1011G>A	p.(=)	-0.22					Patched fami	rs6482625	0.2264	YES	1.098E-2 (pos.27702169)		Patched do	cellular_component,integral component of membrane,r				C=0, T=27	C=0.0, T=1.0	0.00001	389.963	27	C=0/0, T=14/1
chr10:27702688	T/T	C	T	SNV	PTCHD3	PTCHD3:exonic:NM_001034842.3	1	100	1	synonymous	NM_00103	c.492G>A	p.(=)	-3.94				0.02(COSM41	Patched fami	rs7087552	0.2251	YES	1.099E-2 (pos.27702688)	neoplasm	Patched do	cellular_component,integral component of membrane,r				C=0, T=21	C=0.0, T=1.0	0.00001	281.229	21	C=0/0, T=9/12
chr10:16547058	A/G	A	G	SNV	PTER	PTER:exonic:NM_001261836.1	1	52.27	4	synonymous	NM_00126	c.738A>G	p.(=)	0.05				0.14(COSM37	Phosphotries	rs7904014	0.4648	YES	8.419E-1 (pos.16547058)	adenocarcinor	Phosphotri	catabolic	Digestive System Diseases;Digestive System			A=21, G=23	A=0.4773, G=0.5227	0.00001	260.157	44	A=14/7, G=17/
chr14:52781195	A/A	G	A	SNV	PTGER2	PTGER2:utr_5:NM_000956.3	1	100	1	utr_5	NM_00095	c.-72G>A	p.?	-0.98					7 transmemb	rs1254598		YES			Prostaglanc	G-protein	Asthma;Asthma, As	Alprostadil,Dinoproston		G=0, A=29	G=0.0, A=1.0	0.00001	427.603	29	G=0/0, A=11/1
chr4:114267023	A/A	G	A	SNV	ANK2	ANK2:intronic:NM_001148.4	1	100		intronic	NM_00114	c.4249-33	p.?	-1.28					Ankyrin repe	rs9968405	3.00E-04		1.0 (pos.114267023)		Ankyrin-2, n	A band,ATI	Arrhythmias, Cardiac;Cardiovascular Abnorm			G=0, A=137	G=0.0, A=1.0	0.00001	2279.55	137	G=0/0, A=62/7
chr17:41133071	C/C	T	C	SNV	PTGES3L,PTGES3L-AARSD1,RUNDC1	PTGES3L:upstream:NM_001261430.1, RUNDC1:exonic:NM	1	100	1	missense	NM_00113	c.-872A>G	p.?, p.?, p.Trp160Arg	-0.23	0.47	101	0	0(COSM3755	RUN domain	rs1708875	0.0014	YES	1.0;0.0 (pos.41133071)	adenocarcinoma,neoplasn		ATP binding,Ser-tRNA(Ala) hydrolase activity,alanine-tRN				T=0, C=27	T=0.0, C=1.0	0.00001	389.652	27	T=0/0, C=8/19
chr9:114359624	A/A	C	A	SNV	PTGR1	PTGR1:exonic:NM_012212.3	1	100	2	missense	NM_01221	c.79G>T	p.Ala27Ser	0.09	0.41	99	0.004	0.07(COSM37	N-terminal dc	rs1053959	0.3985	YES	6.427E-1 (pos.11435962	acute_myeloic	Prostaglanc	13-prostaglandin reductase ac		(5E,13E)-11-HYDROXY-9,1		C=0, A=28	C=0.0, A=1.0	0.00001	408.718	28	C=0/0, A=18/1
chr14:74340797	T/T	A	T	SNV	PTGR2	PTGR2:exonic:NM_001146154.1	1	100	4	synonymous	NM_00114	c.228A>T	p.(=)	0.4					N-terminal dc	rs11159042	0.4864	YES	5.37E-1 (pos.74340797)			13-prostaglandin reductase ac		(5E,13E)-11-HYDROXY-9,1		A=0, T=54	A=0.0, T=1.0	0.00001	798.148	54	A=0/0, T=32/2
chr4:119161619	G/G	A	G	SNV	NDST3	NDST3:intronic:NM_004784.2	1	100		intronic	NM_00478	c.2119-60	p.?	-0.17					Sulfotransfer	rs641962		YES				Golgi membrane,cellular_component,heparan sulfate N				A=0, G=95	A=0.0, G=1.0	0.00001	1596.74	95	A=0/0, G=43/5
chr4:119736598	T/T	C	T	SNV	SEC24D	SEC24D:intronic:NM_014822.3	1	100		intronic	NM_01482	c.673+8G>	p.?	-0.83					Gelsolin repe	rs6843524	0.1159	YES	6.953E-1 (pos.119736598)		Sec24-relat	COPII vesi	Bone Diseases, Development			C=0, T=27	C=0.0, T=1.0	0.00001	389.978	27	C=0/0, T=17/1
chr4:120106982	T/C	T	C	SNV	MYOZ2	MYOZ2:intronic:NM_016599.4	1	50		intronic	NM_01659	c.561-139	p.?	0.8					Calcineurin-b	rs7661020		YES			Myozenin 2	FATZ binding,Z disc,actin binding,actin cytoskeleton,biol				T=20, C=20	T=0.5, C=0.5	0.00001	213.766	40	T=6/14, C=5/1
chr8:27311703	C/C	T	C	SNV	PTK2B	PTK2B:exonic:NM_173176.2	1	100	29	synonymous	NM_17317	c.2628T>C	p.(=)	-4.27				0.05(COSM44	FERM central	rs1879184	0.1347	YES	5.318E-1;2.321E-4 (pos.2	acute_myeloic	Protein tyrc	3-phospho	Alzheimer Disease;	4-(4-((1R,2R)-2-(dimethyl		T=0, C=25	T=0.0, C=1.0	0.00001	353.019	25	T=0/0, C=19/6
chr4:120486402	A/G	A	G	SNV	PDE5A	PDE5A:intronic:NM_001083.3	1	50		intronic	NM_00108	c.993+74T	p.?	-0.39					3'5'-cyclic nuc	rs55825515		YES			Phosphodie	3',5'-cyclic	Cardiovascular Dise	3-isobutyl-1-methyl-7H-x		A=10, G=10	A=0.5, G=0.5	0.00001	82.8861	20	A=1/9, G=4/6
chr8:27315876	G/G	A	G	SNV	PTK2B	PTK2B:exonic:NM_173176.2	1	100	31	synonymous	NM_17317	c.2880A>G	p.(=)	-3.06					FERM central	rs1879182	0.1347	YES	5.247E-1 (pos.27315876)		Protein tyrc	3-phospho	Alzheimer Disease;	4-(4-((1R,2R)-2-(dimethyl		A=0, G=28	A=0.0, G=1.0	0.00001	384.393	28	A=0/0, G=23/5
chr8:27315900	G/G	A	G	SNV	PTK2B	PTK2B:exonic:NM_173176.2	1	100	31	synonymous	NM_17317	c.2904A>G	p.(=)	3.24					FERM central	rs1879181	0.1348	YES	5.265E-1;0.0 (pos.27315900)		Protein tyrc	3-phospho	Alzheimer Disease;	4-(4-((1R,2R)-2-(dimethyl		A=0, G=28	A=0.0, G=1.0	0.00001	408.654	28	A=0/0, G=23/5
chr4:122137662	G/G	T	G	SNV	TNIP3	TNIP3:intronic:NM_001244764.1	1	100		intronic	NM_00124	c.4-7A>C	p.?	-0.66					Leucine zippe	rs7663440		YES	8.737E-1 (pos.122137662)			MyD88-inc	Breast Diseases;Breast Neoplasms;Neoplasr			T=0, G=21	T=0.0, G=1.0	0.00001	281.235	21	T=0/0, G=8/13
chr4:122257942	C/C	A	C	SNV	QRFPR	QRFPR:intronic:NM_198179.2	1	100		intronic	NM_19817	c.561+20T	p.?	-0.44					7 transmemb	rs4558890	0.0188	YES	1.0 (pos.122257942)			G-protein coupled receptor activity,G-protein coupled re				A=0, C=93	A=0.0, C=1.0	0.00001	1555.3	93	A=0/0, C=40/5
chr8:27255263	A/G	A	G	SNV	PTK2B	PTK2B:exonic:NM_173176.2	1	45.45	2	synonymous	NM_17317	c.162A>G	p.(=)	3.61				0.12(COSM15	FERM central	rs1045512	0.4465	YES	2.589E-1 (pos.27255263)	adenocarcinor	Protein tyrc	3-phospho	Alzheimer Disease;	4-(4-((1R,2R)-2-(dimethyl		A=24, G=20	A=0.5455, G=0.4545	0.00001	213.089	44	A=7/17, G=9/1
chr4:123097080	AT/A	AT	A	INDEL	KIAA1109	KIAA1109:intronic:NM_015312.3	1	45.45		intronic	NM_01531	c.358+11A	p.?	-0.3, 0.38					Fragile site-associated protein C-terminus						KIAA1109 ge	integral cc	Arthritis;Arthritis, Rheumatoid;Autoimmune			AT=12, A=1	AT=0.5455, A=0.4545	0.00004	44.1029	22	AT=5/7, A=1/9
chr4:123140739	C/C	G	C	SNV	KIAA1109	KIAA1109:intronic:NM_015312.3	1	100		intronic	NM_01531	c.2475+17	p.?	-0.22					Fragile site-a	rs6534345	2.00E-04	YES	1.0 (pos.123140739)		KIAA1109 ge	integral cc	Arthritis;Arthritis, Rheumatoid;Autoimmune			G=0, C=52	G=0.0, C=1.0	0.00001	767.015	52	G=0/0, C=25/2
chr1:114377568	G/G	A	G	SNV	PTPN22	PTPN22:exonic:NM_015967.6	1	100	14	missense	NM_01596	c.1858T>C	p.Trp620Arg	0.65	1	101	0		Protein-tyros	rs2476601	0.0941	YES	1.0 (pos.114377568)		Protein tyrc	SH3 doma	Arteritis;Arthritis;Arthritis, Rheumatoid;Auto			A=0, G=24	A=0.0, G=1.0	0.00001	334.792	24	A=0/0, G=14/1
chr4:123868279	T/T	G	T	SNV	SPATA5	SPATA5:intronic:NM_145207.2	1	100		intronic	NM_14520	c.1459-10	p.?	0.03					ATPase famil	rs11098682		YES			Spermatoge	ATP bindin	Colonic Diseases;Colonic Diseases, Function			G=0, T=36	G=0.0, T=1.0	0.00001	563.744	36	G=0/0, T=16/2
chr1:44083507	C/C	T	C	SNV	PTPRF	PTPRF:exonic:NM_002840.4	1	100	25	synonymous	NM_00284	c.4296T>C	p.(=)	-4.01				0.03(COSM63	Fibronectin t	rs641365	0.2521	YES	7.858E-1 (pos.44083507)	neoplasm	Protein tyrc	cell adhes	Digestive System Diseases;Digestive System			T=0, C=50	T=0.0, C=1.0	0.00001	852.492	50	T=0/0, C=39/1
chr19:55699454	C/C	T	C	SNV	PTPRH	PTPRH:exonic:NM_002842.4	1	100	13	missense	NM_00284	c.2467A>G	p.Lys823Glu	2.03	1	56	0	0(COSM4590	Fibronectin t	rs890870			9.988E-1;0.0 (pos.55699	squamous_cel	Protein tyrosine phosphatase, receptor type, H					T=0, C=33	T=0.0, C=1.0	0.00001	504.593	33	T=0/0, C=17/1
chr4:126329526	C/G	C	G	SNV	FAT4	FAT4:intronic:NM_024582.4	1	46.51		intronic	NM_02458	c.5570-73	p.?	-0.62					Cadherin dor	rs10518464		YES			FAT tumor s	Notch sign	Abnormalities, Multiple;Adenocarcinoma;Ca			C=23, G=20	C=0.5349, G=0.4651	0.00001	212.324	43	C=9/14, G=12/
chr18:8069868	T/T	C	T	SNV	PTPRM	PTPRM:exonic:NM_001105244.1	1	100	8	synonymous	NM_00110	c.1317C>T	p.(=)	-0.07				0.08(COSM40	Fibronectin t	rs2230601	0.4824	YES	2.699E-1 (pos.8069868)	acute_myeloic	Protein tyrc	cadherin binding,cell-cell adherens junction,cell-cell jun				C=0, T=21	C=0.0, T=1.0	0.00001	281.225	21	C=0/0, T=13/8
chr4:126355609	G/G	T	G	SNV	FAT4	FAT4:intronic:NM_024582.4	1	100		intronic	NM_02458	c.7199+29	p.?	-0.95					Cadherin dor	rs7656135	0		1.0;0.0 (pos.126355609)		FAT tumor s	Notch sign	Abnormalities, Multiple;Adenocarcinoma;Ca			T=0, G=72	T=0.0, G=1.0	0.00001	1130.95	72	T=0/0, G=26/4
chr7:157985149	C/G	C	G	SNV	PTPRN2	PTPRN2:exonic:NM_002847.4	1	98.41	5	missense	NM_00284	c.419G>C	p.Ser140Thr	-0.67	0.8	58	0.001		Protein-tyros	rs3800855	0.1488	YES	2.149E-1 (pos.157985149)		Protein-tyrc	cell juncti	Eye Diseases;Myopia;Nervous System Diseas			C=1, G=62	C=0.0159, G=0.9841	0.00001	925.297	63	C=1/0, G=25/3
chr12:80889829	A/A	T	A	SNV	PTPRQ	PTPRQ:exonic:NM_001145026.1	1	100	10	missense	NM_00114	c.1418T>A	p.Val473Asp	4.44	0.01	152	0.999	0.96(COSM56	Fibronectin t	rs10778752	0.0635	YES	4.644E-1 (pos.80889829)	ductal_carcinc	Protein-tyrosine phosphatase, receptor-type, Q					T=0, A=40	T=0.0, A=1.0	0.00001	648.659	40	T=0/0, A=17/2
chr12:80900397	C/G	C	G	SNV	PTPRQ	PTPRQ:exonic:NM_001145026.1	1	51.85	13	synonymous	NM_00114	c.1989C>G	p.(=)	-1.7					Fibronectin t	rs12824064	0.3353	YES	4.129E-1 (pos.80900397)		Protein-tyrosine phosphatase, receptor-type, Q					C=13, G=14	C=0.4815, G=0.5185	0.00001	132.665	27	C=7/6, G=9/5
chr12:80900348	T/G	T	G	SNV	PTPRQ	PTPRQ:exonic:NM_001145026.1	1	50	13	missense	NM_00114	c.1940T>G	p.Val647Gly	2.39	1	109	0	0.02(COSM11	Fibronectin t	rs7965277	0.2731	YES	5.39E-2;0.0 (pos.809003	clear_cell_ren	Protein-tyrosine phosphatase, receptor-type, Q					T=14, G=14	T=0.5, G=0.5	0.00001	131.941	28	T=9/5, G=8/6
chr4:128621272	C/C	A	C	SNV	INTU	INTU:intronic:NM_015693.3	1	100		intronic	NM_01569	c.1503+54	p.?	0.2						rs12331481	0.4418	YES			Inturned, D	cell surface,ciliary basal body,cilium assembly,cytoplasi				A=0, C=21	A=0.0, C=1.0	0.00001	281.229	21	A=0/0, C=7/14
chr4:128635022	T/T	C	T	SNV	INTU	INTU:intronic:NM_015693.3	1	100		intronic	NM_01569	c.2560-69	p.?	-3.7						rs34240473		YES			Inturned, D	cell surface,ciliary basal body,cilium assembly,cytoplasi				C=0, T=69	C=0.0, T=1.0	0.00001	1072.78	69	C=0/0, T=32/3
chr19:5214697	G/G	A	G	SNV	PTPRS	PTPRS:exonic:NM_002850.3	1	100	29	missense	NM_00285	c.4369T>C	p.Cys1457Arg	3.03	0.19	180	0		Fibronectin t	rs4807697	0.0015		1.0 (pos.5214697)		Protein tyrc	axon,cell adhesion,cell junctio		Alendronic acid,Etidroni		A=0, G=44	A=0.0, G=1.0	0.00001	726.567	44	A=0/0, G=23/2
chr4:37962186	G/C	G	C	SNV	PTTG2,TBC1D1	TBC1D1:intronic:NM_015173.3, PTTG2:exonic:NM_006607.:	1	50	1	missense	NM_01517	c.418-539	p.?, p.Arg44Pro	-0.04	1	103	0		Domain of un	rs6811863	0.4355	YES	9.572E-1 (pos.37962186)		Pituitary tu	GTPase ac	Nutrition Disorders;Nutritional and Metaboli			G=27, C=27	G=0.5, C=0.5	0.00001	264.277	54	G=8/19, C=15/
chr3:157155314	A/A	C	A	SNV	PTX3,VEPH1	VEPH1:intronic:NM_001167912.1, PTX3:exonic:NM_002852	1	100	2	missense	NM_00116	c.530-903	p.?, p.Ala48Asp	3.67	1	126	0	0.15(COSM63	Concanavalir	rs3816527	0.4212	YES	7.911E-1 (pos.15715531	carcinoma	Pentraxin-3	(1->3)-beta	Arsenic Poisoning;Autoimmune Diseases;Fer			C=0, A=29	C=0.0, A=1.0	0.00001	427.591	29	C=0/0, A=17/1
chr19:45162038	G/G	A	G	SNV	PVR	PVR:exonic:NM_006505.4	1	100	6	missense	NM_00650	c.1020A>G	p.Ile340Met	-2.56	1	10	0		CD80-like C2-	rs203710	0.0247	YES	1.0 (pos.45162038)		Polio virus r	adherens	Autoimmune Disea	MYRISTIC ACID,Sphingosi		A=0, G=22	A=0.0, G=1.0	0.00001	298.88	22	A=0/0, G=13/9
chr21:45528919	A/A	G	A	SNV	PWP2	PWP2:exonic:NM_005049.2	1	100	2	missense	NM_00504	c.73G>A	p.Asp25Asn	7.22	0.09	23	0.696	0.98(COSM56	Dip2/Utp12 F	rs2020945	0.1921	YES	7.115E-1 (pos.45528919)	ductal_carcinc	PWP2 perio	Pwp2p-containing subcomplex of 90S preribosome,RNA				G=0, A=172	G=0.0, A=1.0	0.00001	2815.58	172	G=0/0, A=97/7
chr21:45544594	C/C	T	C	SNV	PWP2	PWP2:exonic:NM_005049.2	1	100	15	synonymous	NM_00504	c.1951T>C	p.(=)	0.78					Dip2/Utp12 F	rs2187313	0.1827	YES	8.054E-1 (pos.45544594)		PWP2 perio	Pwp2p-containing subcomplex of 90S preribosome,RNA				T=0, C=34	T=0.0, C=1.0	0.00001	524.186	34	T=0/0, C=19/1
chr2:1664654	G/G	A	G	SNV	PXDN	PXDN:exonic:NM_012293.2	1	100	14	synonymous	NM_01229	c.1836T>C	p.(=)	-0.06					Animal haem	rs17841813	0.2999	YES	9.799E-1 (pos.1664654)		Peroxidasir	cellular ox	Corneal Diseases;Eye Disease:		Benign	A=0, G=25	A=0.0, G=1.0	0.00001	353.016	25	A=0/0, G=14/1
chr8:52366200	A/A	G	A	SNV	PXDNL	PXDNL:exonic:NM_144651.4	1	100	10	synonymous	NM_14465	c.1128C>T	p.(=)	1.98					Animal haem	rs6473599	0.0591	YES	6.883E-1 (pos.52366200)		Peroxidasir	cellular oxidant detoxification,cytoplasm,			Benign	G=0, A=211	G=0.0, A=1.0	0.00001	3445.86	211	G=0/0, A=129/
chr20:32295541	C/T	C	T	SNV	PXMP4	PXMP4:exonic:NM_007238.4	1	56	4	missense	NM_00723	c.610G>A	p.Val204Ile	0.2	1	29	0	0.29(COSM37	Tim17/Tim22	rs910397	0.4797	YES	7.84E-1 (pos.32295541)	adenocarcinor	Peroxisoma	biological_process,integral component of membrane,pe				C=11, T=14	C=0.44, T=0.56	0.00001	134.35	25	C=2/9, T=8/6
chr4:13615293	A/A	C	A	SNV	BOD1L1	BOD1L1:intronic:NM_148894.2	1	100		intronic	NM_14889	c.1175-8G	p.?	-0.15					COMPASS (Co	rs2109917	0.1875	YES	9.618E-1;0.0 (pos.13615293)		BOD1-like p	DNA repair,cellular response to DNA damage stimulus,cl				C=0, A=93	C=0.0, A=1.0	0.00001	1554.97	93	C=0/0, A=56/3
chr12:120661977	C/C	T	C	SNV	PXN	PXN:exonic:NM_001243756.1	1	100	2	missense	NM_00124	c.217A>G	p.Ser73Gly	-0.28	0.46	56	0.001		LIM domain,F	rs4767884	0.2226	YES	9.863E-1 (pos.120661977)		Paxillin,Per	beta-cate	Bronchial Neoplasms;Carcinoma, Bronchoge			T=0, C=25	T=0.0, C=1.0	0.00001	351.904	25	T=0/0, C=13/1
chr4:139153317	A/A	T	A	SNV	SLC7A11	SLC7A11:intronic:NM_014331.3	1	100		intronic	NM_01433	c.520+104	p.?	-0.93					Amino acid p	rs6823642		YES			Solute carri	L-glutama	Dermatitis;Dermat	Acetylcysteine,Cystine,C		T=0, A=83	T=0.0, A=1.0	0.00001	1349.87	83	T=0/0, A=44/3
chr4:141311960	C/C	T	C	SNV	CLGN	CLGN:intronic:NM_004362.2	1	100		intronic	NM_00436	c.1652-78	p.?	-1.17					Calreticulin f	rs358329		YES			Calmegin	binding of sperm to zona pellucida,calcium ion binding,e				T=0, C=109	T=0.0, C=1.0	0.00001	1722.35	109	T=0/0, C=59/5
chr4:141549393	A/A	C	A	SNV	TBC1D9	TBC1D9:intronic:NM_015130.2	1	100		intronic	NM_01513	c.2812+61	p.?	-2.24					GRAM domai	rs2291711	0.3843	YES				GTPase activator activity,Rab GTPase binding,activation				C=0, A=29	C=0.0, A=1.0	0.00001	427.473	29	C=0/0, A=16/1
chr4:141555062	T/T	G	T	SNV	TBC1D9	TBC1D9:intronic:NM_015130.2	1	100		intronic	NM_01513	c.2746+40	p.?	-1.81					GRAM domai	rs6851737	0.3826	YES	6.476E-1 (pos.141555062)			GTPase activator activity,Rab GTPase binding,activation				G=0, T=49	G=0.0, T=1.0	0.00001	831.632	49	G=0/0, T=20/2
chr4:141598290	A/G	A	G	SNV	TBC1D9	TBC1D9:intronic:NM_015130.2	1	57.14		intronic	NM_01513	c.852-35T	p.?	1.69					GRAM domai	rs2303912	0.1508	YES	8.702E-2 (pos.141598290)			GTPase activator activity,Rab GTPase binding,activation				A=20, G=28	A=0.4167, G=0.5833	0.00001	358.125	49	A=7/13, G=22/
chr12:56295734	G/G	A	G	SNV	PYM1	PYM1:exonic:NM_032345.2	1	100	3	synonymous	NM_03234	c.537T>C	p.(=)	-0.76					Mago bindin	rs7302270	1.00E-04	YES	1.0;0.0 (pos.56295734)			RNA binding,cell junction,cytosol,exon-exon junction cor				A=0, G=44	A=0.0, G=1.0	0.00001	726.569	44	A=0/0, G=29/1
chr4:143130033	GT/GT	G	GT	INDEL	INPP4B	INPP4B:intronic:NM_001101669.2	1	100		intronic	NM_00110	c.967+16C	p.?	-0.54						rs761349112			0.0 (pos.143130033)			cytoplasm	Amyotrophic Lateral Sclerosis;Central Nervo			G=0, GT=77	G=0.0, GT=1.0	0.00001	1054.48	77	G=0/0, GT=46/
chr17:42030531	G/C	G	C	SNV	PYY	PYY:exonic:NM_004160.5	1	47.37	6	missense	NM_00416	c.215C>G	p.Thr72Arg	0.14	0.05	71	0.089	0.11(COSM37	Pancreatic hc	rs1058046	0.3352	YES	3.262E-1 (pos.42030531)	hepatocellula	Peptide YY	G-protein	Alzheimer Disease;Brain Diseases;Central Ne			G=20, C=18	G=0.5263, C=0.4737	0.00001	184.539	38	G=15/5, C=14/
chr12:9345273	G/G	A	G	SNV	PZP	PZP:exonic:NM_002864.2	1	100	12	synonymous	NM_00286	c.1317T>C	p.(=)	-2.41					A-macroglobi	rs10771407	0.0808	YES	9.754E-1;0.0 (pos.9345273)		Pregnancy zone protei		Digestive System Diseases;Drug-Induced Live			A=0, G=179	A=0.0, G=1.0	0.00001	2929.69	179	A=0/0, G=80/9
chr12:9346792	C/C	G	C	SNV	PZP	PZP:exonic:NM_002864.2	1	100	11	missense	NM_00286	c.1135C>G	p.Leu379Val	-1	1	32	0	0.03(COSM41	A-macroglobi	rs12230214	0.2791	YES	4.223E-1 (pos.9346792)	acute_myeloic	Pregnancy zone protei		Digestive System Diseases;Drug-Induced Live			G=0, C=38	G=0.0, C=1.0	0.00001	593.558	38	G=0/0, C=18/2
chr4:144920638	T/T	A	T	SNV	GYPB	GYPB:intronic:NM_001304382.1	1	100		intronic	NM_00130	c.59-36T>	p.?	-0.36						rs760637131,rs7662	0.3112	YES	0.0 (pos.144920634),4.298E-2 (pos.1449		Glycophorir	integral component of membrane,integral component o				A=0, T=45	A=0.0, T=1.0	0.00001	747.268	45	A=0/0, T=25/2
chr4:145040784	T/C	T	C	SNV	GYPA	GYPA:intronic:NM_002099.7	1	46.88		intronic	NM_00209	c.232+55A	p.?	0.43					Glycophorin	rs62334651		YES			Glycophorir	biological	Neoplasms;Neoplasms by Site;Skin Diseases			T=34, C=30	T=0.5313, C=0.4688	0.00001	288.133	64	T=23/11, C=24
chr4:146046136	T/TC	T	TC	INDEL	ABCE1	ABCE1:intronic:NM_002940.2	1	100		intronic	NM_00294	c.1641-6T	p.?	0.2					4Fe-4S binding domain,ABC transporter,Possible Fer4-like domain in RNase L inhibitor, RLI						ATP-binding	ATP bindin	Autoimmune Diseases;Colonic Diseases;Colc			T=0, TC=46	T=0.0, TC=1.0	0.00001	363.475	46	T=0/0, TC=42/
chr4:148591769	T/T	G	T	SNV	PRMT9	PRMT9:intronic:NM_138364.3	1	100		intronic	NM_13836	c.846+23C	p.?	-0.49					Ribosomal pr	rs2036502	3.00E-04		1.0 (pos.148591769)		F-box only p	cytoplasm,mRNA processing,peptidyl-arginine methylat				G=0, T=86	G=0.0, T=1.0	0.00001	1410.93	86	G=0/0, T=50/3
chr4:148985720	C/C	T	C	SNV	ARHGAP10	ARHGAP10:intronic:NM_024605.3	1	100		intronic	NM_02460	c.2272+62	p.?	-2.21					BAR domain (	rs3775327		YES			RHO GTPase	GTPase activator activity,cytoskeleton organization,cytc				T=0, C=46	T=0.0, C=1.0	0.00001	768.309	46	T=0/0, C=28/1
chr12:9315209	A/G	A	G	SNV	PZP	PZP:exonic:NM_002864.2	1	51.85	22	synonymous	NM_00286	c.2772T>C	p.(=)	-2.09				0(COSM6353	A-macroglobi	rs10771381	0.43	YES	4.119E-1 (pos.9315209)	carcinoma	Pregnancy zone protei		Digestive System Diseases;Drug-Induced Live			A=52, G=56	A=0.4815, G=0.5185	0.00001	583.135	108	A=22/30, G=30
chr4:151829659	C/C	T	C	SNV	LRBA	LRBA:intronic:NM_006726.4	1	100		intronic	NM_00672	c.1360-40	p.?	-0.97					Beige/BEACH	rs1201207	8.00E-04	YES	9.995E-1 (pos.151829659)		Lipopolysac	Golgi appa	Common Variable Immunodeficiency;Immun			T=0, C=51	T=0.0, C=1.0	0.00001	752.671	51	T=0/0, C=20/3
chr4:17506042	G/A	G	A	SNV	QDPR	QDPR:exonic:NM_000320.2	1	59.09	3	synonymous	NM_00032	c.255C>T	p.(=)	0.33				0.32(COSM44	short chain d	rs12645938	0.0463	YES	5.662E-2 (pos.17506042)	acute_myeloic	Quinoid dih	6,7-dihydr	Amino Acid Disorde	2-(N-Morp	Benign/Likely	G=9, A=13	G=0.4091, A=0.5909	0.00001	122.568	22	G=2/7, A=5/8
chr9:139110654	T/T	C	T	SNV	QSOX2	QSOX2:exonic:NM_181701.3	1	100	8	synonymous	NM_18170	c.957G>A	p.(=)	1.65				0.77(COSM39	Erv1 / Alr fam	rs12684650	0.2967	YES	1.411E-1 (pos.13911065	acute_myeloic	Quiescin QS	Golgi apparatus,cell redox homeostasis,extracellular sp				C=0, T=33	C=0.0, T=1.0	0.00001	504.598	33	C=0/0, T=20/1

chr10:99991408	G/G	A	G	SNV	R3HCC1L	R3HCC1L:exonic:NM_001256619.1	1	100	7	missense	NM_00125	c.1967A>G	p.His656Arg	4.42	1	29	0	0.25(COSM6243636),0.25(C		rs1952061	5.00E-04		1.0;0.0 (pos.99991408)	neoplasm		exon-exon junction complex,protein binding			A=0, G=20	A=0.0, G=1.0	0.00001	263.785	20	A=0/0, G=6/14
chr10:119768551	T/T	C	T	SNV	RAB11FIP2	RAB11FIP2:exonic:NM_014904.2	1	100	5	synonymous	NM_01490	c.1497G>A	p.(=)	-0.95					C2 domain.FI	rs7923321	0.035	YES	5.84E-1 (pos.119768551)		RAB11 fami	Rab GTPas	Autoimmune Diseases:Female Urogenital Di:		C=0, T=41	C=0.0, T=1.0	0.00001	664.7	41	C=0/0, T=19/2
chr4:155161666	T/T	C	T	SNV	DCHS2	DCHS2:intronic:NM_017639.3	1	100		intronic	NM_01763	c.5994+23	p.?	0.52					Cadherin dor	rs6535989	0.0795	YES	7.883E-1 (pos.155161666)		Dachsous. [	biological	Alzheimer Disease:Brain Diseases:Central Ne		C=0, T=26	C=0.0, T=1.0	0.00001	368.163	26	C=0/0, T=19/7
chr4:155244475	G/T	G	T	SNV	DCHS2	DCHS2:intronic:NM_017639.3	1	53.49		intronic	NM_01763	c.2654-83!	p.?	-0.3					Cadherin dor	rs12500437		YES	8.931E-1 (pos.155244475)		Dachsous. [	biological	Alzheimer Disease:Brain Diseases:Central Ne		G=20. T=23	G=0.4651, T=0.5349	0.00001	261.022	43	G=12/8, T=13/
chr16:570557	C/C	T	C	SNV	RAB11FIP3	RAB11FIP3:exonic:NM_014700.3	1	100	13	synonymous	NM_01470	c.2115T>C	p.(=)	-9.03				0.02(COSM41	EF hand,FIP d	rs11537754	0.4605	YES	9.945E-1;0.0 (pos.57055;	neoplasm	RAB11 fami	ADP-ribos	Cardiovascular Diseases;Embolism and Thror		T=0, C=50	T=0.0, C=1.0	0.00001	740.947	50	T=0/0, C=23/2
chr4:155287317	G/C	G	C	SNV	DCHS2	DCHS2:intronic:NM_017639.3	1	53.33		intronic	NM_01763	c.694+45C	p.?	0.53					Cadherin dor	rs10005849	0.464	YES	4.497E-1 (pos.155287317)		Dachsous. [	biological	Alzheimer Disease;Brain Diseases;Central Ne		G=14, C=16	G=0.4667, C=0.5333	0.00001	159.925	30	G=7/7, C=6/10
chr4:155410822	A/A	G	A	SNV	DCHS2		1	100		intronic				-0.47				0.43(COSM4407056).0.43(C		rs4696593	0.4469	YES	8.629E-1 (pos.15541082;	hepatocellula	Dachsous. Drosophila.		Alzheimer Disease:Brain Diseases:Central Ne		G=0, A=41	G=0.0, A=1.0	0.00001	664.698	41	G=0/0, A=16/2
chr4:155411104	C/C	A	C	SNV	DCHS2		1	100		intronic				-1.48				0.04(COSM4158878).0.04(C		rs6536016	0.0022	YES	1.0 (pos.155411104)	neoplasm	Dachsous. Drosophila.		Alzheimer Disease:Brain Diseases:Central Ne		A=0. C=195	A=0.0, C=1.0	0.00001	3295.66	195	A=0/0, C=102/
chr4:155510224	T/T	A	T	SNV	FGA	FGA:intronic:NM_000508.4	1	100		intronic	NM_00050	c.181-96T:	p.?	-0.76					Fibrinogen al	rs7656433		YES			Fibrinogen,	adaptive i	Afibrinogenemia;Ar	Alfimeprase,Alteplase,A	A=0, T=22	A=0.0, T=1.0	0.00001	298.893	22	A=0/0, T=10/1
chr4:15597984	T/T	C	T	SNV	CC2D2A	CC2D2A:intronic:NM_001080522.2	1	100		intronic	NM_00108	c.4437+15	p.?	-0.45					C2 domain,C(	rs10212939		YES			Coiled-coil ;	MKS comp	Abnormalities, Multiple;Ataxia;Bile Duct Dise		C=0, T=81	C=0.0, T=1.0	0.00001	1309.07	81	C=0/0, T=37/4
chr16:2203456	C/C	T	C	SNV	RAB26	RAB26:utr_3:NM_014353.4	1	100	9	utr_3	NM_01435	c.805T>C	p.?	-0.04					ADP-ribosylat	rs26931	0.0093		1.0;0.0 (pos.2203456)		RAS-associa	GMP binding.GTP binding.GTPase activity.Golgi membrai			T=0, C=25	T=0.0, C=1.0	0.00001	353.011	25	T=0/0, C=22/3
chr4:156696105	G/A	G	A	SNV	GUCY1B3	GUCY1B3:intronic:NM_000857.3	1	50		intronic	NM_00085	c.78-15G>,	p.?	-0.97					Adenylate an	rs34237400	0.0216	YES	7.091E-4 (pos.156696105)		Guanylate (	GTP binding.Hsp90 protein binding.adenylate cyclase ac			G=51. A=51	G=0.5, A=0.5	0.00001	519.096	102	G=31/20, A=2
chr4:156826166	C/G	C	G	SNV	TDO2	TDO2:intronic:NM_005651.3	1	47.62		intronic	NM_00565	c.142-53C:	p.?	-0.98					Tryptophan 2	rs2137778	0.0177	YES			Tryptophan	amino aci	Autistic Disorder:Cl	L-Tryptophan.Nalidixic A	C=44. G=40	C=0.5238. G=0.4762	0.00001	418.358	84	C=28/16, G=1
chr3:19992432	T/T	G	T	SNV	RAB5A	RAB5A:exonic:NM_004162.4	1	100	2	synonymous	NM_00416	c.42G>T	p.(=)	0.98				0.49(COSM56	50S ribosome	rs6773437	0.2422	YES	3.112E-1;0.0 (pos.19992;	ductal_carcinc	RAS-associa	GDP bindi	Cardiovascular Dise	Guanosine-5'-Diphospha	G=0, T=154	G=0.0, T=1.0	0.00001	2431.61	154	G=0/0, T=64/9
chr2:130738163	A/A	G	A	SNV	RAB6C,RAB6C-AS1	RAB6C:exonic:NM_032144.2. RAB6C-AS1:upstream:NR_036!	1	100	1	missense	NR_03653	c.475G>A	p.Ala159Thr	0.59		58		0.52(COSM37	ADP-ribosylat	rs4662674		YES	6.547E-1 (pos.13073816:	acute_myeloic	Ras-associa	GTP binding.GTPase activity.Golgi apparatus.centrosom			G=0, A=32	G=0.0, A=1.0	0.00001	484.226	32	G=0/0, A=6/26
chr4:159568383	G/G	A	G	SNV	RXFP1	RXFP1:intronic:NM_021634.3	1	100		intronic	NM_02163	c.1756+30	p.?	-1.24					7 transmemb	rs13111939	0.002	YES	9.999E-1;0.0 (pos.159568383)		Relaxin/ins	G-protein	Endometriosis:Female Urogenital Diseases:F		A=0, G=44	A=0.0, G=1.0	0.00001	726.569	44	A=0/0, G=18/2
chr19:16238277	C/CT	C	CT	INDEL	RAB8A	RAB8A:exonic:NM_005370.4	1	100	5	frameshiftInsertion	NM_00537	c.356_357	p.Gly120fs	3.94					ADP-ribosylation factor family.Gtr1/RagA G protein conserved region.Ras family.Ras of Complex. Roc						Ras-associa	G2/M tran	Arthritis:Arthritis. Rheumatoid:Autoimmune		C=0. CT=33	C=0.0, CT=1.0	0.00001	256.149	33	C=0/0, CT=12/
chr19:16238279	T/T	C	T	SNV	RAB8A	RAB8A:exonic:NM_005370.4	1	100	5	synonymous	NM_00537	c.357C>T	p.(=)	-2.55				0.07(COSM37	ADP-ribosylat	rs1043308	0.3243	YES	3.198E-1 (pos.16238279)	adenocarcinoi	Ras-associa	G2/M tran	Arthritis;Arthritis, Rheumatoid;Autoimmune		C=0, T=51	C=0.0, T=1.0	0.00001	752.502	51	C=0/0, T=30/2
chr4:15991275	A/A	G	A	SNV	PROM1	PROM1:intronic:NM_006017.2	1	100		intronic	NM_00601	c.2076+80	p.?	-1.11					Apolipoprote	rs2240685		YES			Prominin 1	actinin bir	Astrocytoma;Colonic Diseases;Colonic Neopl		G=0, A=33	G=0.0, A=1.0	0.00001	504.598	33	G=0/0, A=18/1
chr4:164048199	C/C	G	C	SNV	NAF1		1	100		intronic				-1.43				0(COSM5654012)		rs4691895	0.2307	YES	7.548E-1 (pos.16404819!	diffuse_large_	TNFAIP3-interacting protein 1				G=0, C=33	G=0.0, C=1.0	0.00001	509.068	33	G=0/0, C=15/1
chr19:42463447	T/T	C	T	SNV	RABAC1	RABAC1:utr_5:NM_006423.2	1	100	1	utr_5	NM_00642	c.-3G>A	p.?	3.65					PRA1 family r	rs735571	0.3634	YES	8.141E-1 (pos.42463447)			Golgi apparatus.cell junction.identical protein binding.i			C=0, T=58	C=0.0, T=1.0	0.00001	867.386	58	C=0/0, T=28/3
chr4:164438523	A/A	G	A	SNV	TMA16	TMA16:intronic:NM_018352.2	1	100		intronic	NM_01835	c.389-63G	p.?	-1.36					Translation n	rs12643109	0.4827	YES				nucleolus,nucleus,protein binding,ribosome			G=0, A=34	G=0.0, A=1.0	0.00001	524.186	34	G=0/0, A=11/2
chr9:139732331	C/C	G	C	SNV	RABL6	RABL6:exonic:NM_001173988.1	1	100	10	missense	NM_00117	c.1147G>C	p.Glu383Gln	3.69	0.54	29	0.998	0.58(COSM16	Ras family	rs2811741	0.1793	YES	8.71E-1 (pos.139732331)	carcinoma	RAB-like prc	GTP bindir	Intellectual Disability;Mental Disorders;Nerv		G=0, C=33	G=0.0, C=1.0	0.00001	504.58	33	G=0/0, C=23/1
chr3:8955389	T/T	C	T	SNV	RAD18	RAD18:exonic:NM_020165.3	1	100	8	missense	NM_02016	c.905G>A	p.Arg302Gln	2.42	0.62	43	0.001	0.02(COSM56	RING-type zin	rs373572	0.2758	YES	3.532E-1 (pos.8955389)	diffuse_large_	Rad18. S. ce	DNA binding.DNA damage response. detection of DNA da			C=0, T=30	C=0.0, T=1.0	0.00001	446.601	30	C=0/0, T=15/1
chr3:51697493	G/G	A	G	SNV	RAD54L2	RAD54L2:utr_3:NM_015106.3	1	100	23	utr_3	NM_01510	c.4461A>G	p.?	0.05					Helicase con:	rs904145		YES				ATP binding.DNA binding.helicase activity.nucleus.prote			A=0, G=28	A=0.0, G=1.0	0.00001	406.441	28	A=0/0, G=16/1
chr7:4841470	C/C	T	C	SNV	RADIL	RADIL:exonic:NM_018059.4	1	100	12	missense	NM_01805	c.2656A>G	p.Ser886Gly	-0.57	0.45	56	0	0(COSM3762	DIL domain.P	rs414035	0.0072		1.0 (pos.4841470)	adenocarcinoi	RAP GTPase	microtubule.multicellular organism development.prote			T=0, C=52	T=0.0, C=1.0	0.00001	767.015	52	T=0/0, C=24/2
chr17:17696755	A/A	C	A	SNV	RAI1	RAI1:exonic:NM_030665.3	1	100	3	missense	NM_03066	c.493C>A	p.Pro165Thr	0.68	0	38	1	0.83(COSM41	PHD-like zinc	rs11649804	0.3073	YES	8.511E-1 (pos.17696755)	diffuse_large_	Retinoic aci	DNA bindi	Abnormalities, Multiple;Anxiet	Benign/Likely	C=0, A=29	C=0.0, A=1.0	0.00001	427.603	29	C=0/0, A=21/8
chr9:135974079	C/C	G	C	SNV	RALGDS	RALGDS:exonic:NM_001271775.1	1	100	18	synonymous	NM_00127	c.2637C>G	p.(=)	0.03				0.57(COSM44	Ras associati	rs1062356	0.1471	YES	7.657E-1 (pos.13597407!	acute_myeloic	ral guanine	GTPase re	Intellectual Disability;Mental Disorders;Nerv		G=0, C=21	G=0.0, C=1.0	0.00001	281.234	21	G=0/0, C=5/16
chr4:1659128	C/C	T	C	SNV	FAM53A	FAM53A:intronic:NM_001174070.2	1	100		intronic	NM_00117	c.136+7A>	p.?	0.98					Family of FAN	rs2895461	0.4119	YES	3.456E-1 (pos.1659128)		Family with	nucleus			T=0, C=25	T=0.0, C=1.0	0.00001	353.014	25	T=0/0, C=15/1
chr20:32664864	CCAG/CCAG	C	CCAG	INDEL	RALY	RALY:exonic:NM_016732.2	3	100	8	nonframeshiftInsertion	NM_01673	c.690_691	p.Ala230_Gly231insSer	0.2					RNA recogniti	rs10649600.rs75692(	0.337	YES	0.0:0.0 (pos.32664859).0	adenocarcinoma.ductal_ca		RNA bindir	Amphetamine-Related Disorders:Breast Dise		C=0. CCAG:	C=0.0, CCAG=1.0	0.00001	1585.29	102	C=0/0, CCAG=(
chr4:167012506	G/G	A	G	SNV	TLL1	TLL1:intronic:NM_012464.4	1	100		intronic	NM_01246	c.2656+13	p.?	0.01					Astacin (Pept	rs2254420	0.0349	YES	9.968E-1 (pos.167012506)		Tolloid-like	calcium io	Cardiovascular Abnormalities:Cardiovascula		A=0, G=93	A=0.0, G=1.0	0.00001	1555.02	93	A=0/0, G=39/5
chr20:32664926	G/A	G	A	SNV	RALY	RALY:exonic:NM_016732.2	1	50	8	missense	NM_01673	c.751G>A	p.Gly251Ser	-0.3	0.43	56	0		RNA recogniti	rs2281209	0.0507	YES	1.031E-1 (pos.32664926)			RNA bindir	Amphetamine-Related Disorders;Breast Dise		G=68, A=68	G=0.5, A=0.5	0.00001	741.026	136	G=23/45, A=4
chr5:170626735	A/G	A	G	SNV	RANBP17	RANBP17:exonic:NM_022897.4	1	50	19	synonymous	NM_02289	c.2100A>G	p.(=)	-0.01					Importin-bet:	rs35724654	0.1626	YES	5.782E-4 (pos.170626735)		RAN-bindin	GTP binding,Ran GTPase binding,cytoplasm,intracellular			A=11, G=11	A=0.5, G=0.5	0.00001	94.6063	22	A=5/6, G=3/8
chr4:169301059	C/C	T	C	SNV	DDX60L	DDX60L:intronic:NM_001012967.2	1	100		intronic	NM_00101	c.4117-78,	p.?	-0.54					DEAD/DEAH b	rs1489232		YES			DEAD box p	ATP binding.RNA binding.helicase activity.nucleic acid bi			T=0, C=79	T=0.0, C=1.0	0.00001	1269.26	79	T=0/0, C=29/5
chr4:169315571	T/T	TA	T	INDEL	DDX60L	DDX60L:intronic:NM_001012967.2	1	100		intronic	NM_00101	c.3814+41	p.?	0.63, 0.61					DEAD/DEAH b	rs11337868.rs20204!	0		5E-1 (pos.169315570).5E-1 (pos.169315!		DEAD box p	ATP binding.RNA binding.helicase activity.nucleic acid bi			TA=0, T=43	TA=0.0, T=1.0	0.00001	684.062	43	TA=0/0, T=17/
chr2:109389463	A/A	G	A	SNV	RANBP2	RANBP2:exonic:NM_006267.4	1	100	23	synonymous	NM_00626	c.8253G>A	p.(=)	1.41					Cyclophilin ty	rs826580	3.00E-04		1.0 (pos.109389463)		RAN-bindin	G1/S transition of mitotic cell cycle.GTPase activator act			G=0, A=33	G=0.0, A=1.0	0.00001	504.465	33	G=0/0, A=15/1
chr4:169327905	G/T	G	T	SNV	DDX60L	DDX60L:intronic:NM_001012967.2	1	59.09		intronic	NM_00101	c.2980-48(	p.?	-3.22					DEAD/DEAH b	rs62334119	0.0987	YES	1.143E-1 (pos.169327905)		DEAD box p	ATP binding,RNA binding,helicase activity,nucleic acid bi			G=79, T=11	G=0.4031, T=0.5969	0.00001	1460.91	198	G=28/51, T=66
chr4:169340431	A/C	A	C	SNV	DDX60L	DDX60L:intronic:NM_001012967.2	1	47.37		intronic	NM_00101	c.2610+22	p.?	0.1					DEAD/DEAH b	rs2018241	0.123	YES	2.544E-1 (pos.169340431)		DEAD box p	ATP binding.RNA binding.helicase activity.nucleic acid bi			A=20. C=18	A=0.5263, C=0.4737	0.00001	184.537	38	A=12/8, C=6/1
chr9:6012734	A/C	A	C	SNV	RANBP6	RANBP6:exonic:NM_012416.3	1	50	1	synonymous	NM_01241	c.2874T>G	p.(=)	1.81					HEAT repeat.I	rs106033	0.2322	YES	4.02E-1 (pos.6012734)			NLS-bearing protein import into nucleus.cytoplasm.nucl			A=28. C=28	A=0.5, C=0.5	0.00001	272.137	56	A=16/12, C=2
chr4:169348476	A/A	G	A	SNV	DDX60L	DDX60L:intronic:NM_001012967.2	1	100		intronic	NM_00101	c.1678-3C:	p.?	0.29					DEAD/DEAH b	rs74346802	0.1791	YES	1.115E-2 (pos.169348476)		DEAD box p	ATP binding.RNA binding.helicase activity.nucleic acid bi			G=0, A=58	G=0.0, A=1.0	0.00001	867.385	58	G=0/0, A=22/3
chr1:21943820	G/A	G	A	SNV	RAP1GAP	RAP1GAP:exonic:NM_001145658.2	1	47.62	7	synonymous	NM_00114	c.462C>T	p.(=)	7.25				0.99(COSM41	GoLoco motif	rs2275360	0.2591	YES	6.099E-1 (pos.21943820)	neoplasm		GTPase ac	Digestive System Diseases;Digestive System		G=22, A=20	G=0.5238, A=0.4762	0.00001	212.881	42	G=3/19, A=6/1
chr4:170017860	G/A	G	A	SNV	SH3RF1	SH3RF1:intronic:NM_020870.3	1	56		intronic	NM_02087	c.2499-22(	p.?	0.49					RING-type zin	rs1471172	0.3447	YES	6.327E-1 (pos.170017860)			Golgi apparatus,apoptotic process,cytosol,lamellipodiu			G=11, A=14	G=0.44, A=0.56	0.00001	134.35	25	G=7/4, A=4/10
chr4:170601172	A/G	A	G	SNV	CLCN3	CLCN3:intronic:NM_173872.3	1	45.83		intronic	NM_17387	c.161-29A:	p.?	-0.36					CBS domain.\	rs33965812	0.2399	YES	4.028E-2 (pos.170601172)		Chloride ch	ATP bindin	Digestive System Diseases:Digestive System		A=13. G=11	A=0.5417. G=0.4583	0.00001	93.4397	24	A=9/4, G=7/4
chr4:170650607	A/A	AGAA	A	INDEL	HPF1	HPF1:downstream:NM_017867.2	3	100		intronic	NM_01786	c.*122TTC	p.?	0.61, 0.17, 0.56, 0.68						rs199658190.rs3074610.rs6095(		YES			Histone par	cellular response to DNA damage stimulus.histone bindi			AGAA=0. A:	AGAA=0.0, A=1.0	0.00001	464.848	31	AGAA=0/0, A=
chr17:2929392	A/A	G	A	SNV	RAP1GAP2	RAP1GAP2:exonic:NM_015085.4	1	100	20	synonymous	NM_01508	c.1842G>A	p.(=)	-0.57				0.12(COSM14	Rap/ran-GAP	rs55904912	0.4085	YES	5.425E-1 (pos.2929392)	adenocarcinoma.carcinom		GTPase ac	Asthma:Bronchial Diseases:Hypersensitivity		G=0, A=23	G=0.0, A=1.0	0.00001	316.747	23	G=0/0, A=17/6
chr17:2929674	C/C	T	C	SNV	RAP1GAP2	RAP1GAP2:exonic:NM_015085.4	1	100	21	synonymous	NM_01508	c.1896T>C	p.(=)	-1.61				0.04(COSM32	Rap/ran-GAP	rs12941934	0.3933	YES	6.55E-1 (pos.2929674)	neoplasm,plasma_cell_my		GTPase ac	Asthma:Bronchial Diseases:Hypersensitivity		T=0, C=41	T=0.0, C=1.0	0.00001	664.597	41	T=0/0, C=24/1
chr4:172735897	G/G	C	G	SNV	GALNTL6	GALNTL6:intronic:NM_001034845.2	1	100		intronic	NM_00103	c.138+28C	p.?	0.92					Glycosyl tran	rs3796654	0.0584	YES	8.289E-1 (pos.172735897)		UDP-N-acet	Golgi membrane,carbohydrate binding,integral compon			C=0, G=22	C=0.0, G=1.0	0.00001	298.724	22	C=0/0, G=9/13
chr17:2930339	A/A	G	A	SNV	RAP1GAP2	RAP1GAP2:exonic:NM_015085.4	1	100	22	synonymous	NM_01508	c.2082G>A	p.(=)	-2.31				0.05(COSM13	Rap/ran-GAP	rs4790115	0.4074	YES	6.069E-1 (pos.2930339)	adenocarcinoma.hepatoce		GTPase ac	Asthma:Bronchial Diseases:Hypersensitivity		G=0, A=50	G=0.0, A=1.0	0.00001	852.748	50	G=0/0, A=4/46
chr4:17516771	T/T	C	T	SNV	CLRN2	CLRN2:upstream:NM_001079827.2	1	100		intronic	NM_00107	c.-119C>T	p.?	-2						rs2597784		YES				integral component of membrane			C=0, T=31	C=0.0, T=1.0	0.00001	465.819	31	C=0/0, T=19/1
chr12:48144925	C/C	T	C	SNV	RAPGEF3	RAPGEF3:exonic:NM_001098531.2	1	100	6	missense	NM_00109	c.577A>G	p.Arg193Gly	1.85	0.47	125	0	0.02(COSM41	Cyclic nucleo	rs2016123	4.00E-04		1.0 (pos.48144925)	neoplasm			Acute Kidney Injury:Female Urogenital Disea:		T=0, C=27	T=0.0, C=1.0	0.00001	389.988	27	T=0/0, C=18/9
chr4:175414202	T/T	C	T	SNV	HPGD	HPGD:intronic:NM_000860.5	1	100		intronic	NM_00086	c.662+100	p.?	-0.38					Enoyl-(Acyl ca	rs41279511		YES			Hydroxypro	15-hydrox	Adenocarcinoma;B	NADH	C=0, T=36	C=0.0, T=1.0	0.00001	563.494	36	C=0/0, T=16/2
chr4:175414246	G/G	A	G	SNV	HPGD	HPGD:intronic:NM_000860.5	1	100		intronic	NM_00086	c.662+56T	p.?	-0.28					Enoyl-(Acyl ca	rs2612694		YES			Hydroxypro	15-hydrox	Adenocarcinoma;B	NADH	A=0, G=36	A=0.0, G=1.0	0.00001	563.74	36	A=0/0, G=16/2
chr7:22202117	A/A	G	A	SNV	RAPGEF5	RAPGEF5:exonic:NM_012294.3	1	100	13	synonymous	NM_01229	c.867C>T	p.(=)	-0.02				0.19(COSM40	RasGEF N-ter	rs12592	0.4491	YES	6.131E-1 (pos.22202117)	acute_myeloid_leukaemia		GTP-deper	Amphetamine-Related Disorders:Mental Dis		G=0, A=63	G=0.0, A=1.0	0.00001	958.917	63	G=0/0, A=28/3
chr7:22202046	G/T	G	T	SNV	RAPGEF5	RAPGEF5:exonic:NM_012294.3	1	47.62	13	missense	NM_01229	c.938C>A	p.Pro313His	1.82	0.49	77	0		RasGEF N-ter	rs28716840	0.0226	YES	5.93E-3 (pos.22202046)			GTP-deper	Amphetamine-Related Disorders:Mental Dis		G=22. T=20	G=0.5238, T=0.4762	0.00001	212.949	42	G=14/8, T=3/1
chr4:17616214	A/A	C	A	SNV	MED28	MED28:upstream:NM_025205.4	1	100		intronic	NM_02520	c.-64C>A	p.?	-1.36						rs2286773		YES			Mediator co	actin bind	Breast Diseases;Breast Neoplasms;Neoplasr		C=0, A=23	C=0.0, A=1.0	0.00001	316.747	23	C=0/0, A=10/1
chr4:17625209	C/C	G	C	SNV	MED28	MED28:intronic:NM_025205.4	1	100		intronic	NM_02520	c.340-15G	p.?	-2.64					Mediator cor	rs11733730	0.395	YES	8.951E-1 (pos.17625209)		Mediator co	actin bind	Breast Diseases;Breast Neoplasms;Neoplasr		G=0, C=64	G=0.0, C=1.0	0.00001	977.625	64	G=0/0, C=35/2
chr5:130766662	C/C	T	C	SNV	RAPGEF6	RAPGEF6:exonic:NM_001164386.1	1	100	27	missense	NM_00116	c.4379A>G	p.Gln1460Arg	5.2	1	43	0		Cyclic nucleo	rs1291602	0.1581	YES	8.893E-1 (pos.130766662)		RAP guanin	GTP-dependent protein binding.Ras GTPase binding.Ras			T=0, C=79	T=0.0, C=1.0	0.00001	1269.32	79	T=0/0, C=40/3
chr2:204322298	C/C	T	C	SNV	RAPH1	RAPH1:exonic:NM_213589.2	1	100	8	synonymous	NM_21358	c.1113A>G	p.(=)	1.14				0.09(COSM44	PH domain.R:	rs2469953	0.3988	YES	3.827E-1 (pos.20432229!	acute_myeloid_leukaemia		axon exte	Nervous System Diseases:Neuromuscular Di:		T=0, C=55	T=0.0, C=1.0	0.00001	797.289	55	T=0/0, C=32/2
chr11:47460306	G/G	A	G	SNV	RAPSN	RAPSN:exonic:NM_005055.4	1	100	7	synonymous	NM_00505	c.1143T>C	p.(=)	0.19				0.3(COSM393	Rapsyn N-ter	rs7126210	0.2905	YES	6.249E-1 (pos.47460306)	carcinoma	Receptor-a:	Golgi appa	Abnormalities. Multiple:Arthrc	Benign/Likely	A=0, G=30	A=0.0, G=1.0	0.00001	446.596	30	A=0/0, G=15/1
chr4:177049854	T/T	C	T	SNV	WDR17	WDR17:intronic:NM_170710.4	1	100		intronic	NM_17071	c.863-35C:	p.?	-0.29					Anaphase-pr	rs28698547	0.2464	YES	1.452E-1 (pos.177049854)		WD repeat-	cytoplasm	Digestive System Diseases;Digestive System		C=0, T=40	C=0.0, T=1.0	0.00001	644.281	40	C=0/0, T=26/1
chr4:177049880	C/C	T	C	SNV	WDR17	WDR17:intronic:NM_170710.4	1	100		intronic	NM_17071	c.863-9T>(	p.?	1.05					Anaphase-pr	rs28607147	0.2473	YES	1.452E-1 (pos.177049880)		WD repeat-	cytoplasm	Digestive System Diseases;Digestive System		T=0, C=39	T=0.0, C=1.0	0.00001	623.977	39	T=0/0, C=25/1
chr4:177050057	A/A	G	A	SNV	WDR17	WDR17:intronic:NM_170710.4	1	100		intronic	NM_17071	c.985+46G	p.?	0.04					Anaphase-pr	rs71602424.rs763994414.rs786		YES	0.0 (pos.177050054).1.602E-1 (pos.1770		WD repeat-	cytoplasm	Digestive System Diseases:Digestive System		G=0, A=52	G=0.0, A=1.0	0.00001	767.015	52	G=0/0, A=26/2
chr4:177050062	C/C	CTATCAAA'	C	INDEL	WDR17	WDR17:intronic:NM_170710.4	12	100		intronic	NM_17071	c.985+51C	p.?	-0.78. 0.98. -1.74. 0.57. -1.58. 0.8. 1.29. 0.14. 3.32. -0.03. 0.46.					Anaphase-pr	rs71602424.rs71830(	0.1839	YES	0.0 (pos.177050063).1.558E-1 (pos.1770		WD repeat-	cytoplasm	Digestive System Diseases:Digestive System		CTATCAAAT	CTATCAAATAAAA=0.0	0.00001	717.751	50	CTATCAAATAA
chr4:177082032	G/G	A	G	SNV	WDR17	WDR17:intronic:NM_170710.4	1	100		intronic	NM_17071	c.2731-30,	p.?	0.34					Anaphase-pr	rs10520338	0.2449	YES	1.441E-1 (pos.177082032)		WD repeat-	cytoplasm	Digestive System Diseases:Digestive System		A=0, G=83	A=0.0, G=1.0	0.00001	1269.77	83	A=0/0, G=43/4
chr11:47469439	G/G	A	G	SNV	RAPSN	RAPSN:exonic:NM_005055.4	1	100	2	synonymous	NM_00505	c.456T>C	p.(=)	-2.04				0.04(COSM63	Rapsyn N-ter	rs7111873	0.2809	YES	6.45E-1 (pos.47469439)	carcinoma,ne(	Receptor-a:	Golgi appa	Abnormalities. Multiple;Arthrc	Benign/Likely	A=0, G=55	A=0.0, G=1.0	0.00001	814.907	55	A=0/0, G=32/2
chr4:177083166	G/G	A	G	SNV	WDR17	WDR17:intronic:NM_170710.4	1	100		intronic	NM_17071	c.2823-60,	p.?	-0.9					Anaphase-pr	rs2877924		YES			WD repeat-	cytoplasm	Digestive System Diseases;Digestive System		A=0, G=60	A=0.0, G=1.0	0.00001	903.529	60	A=0/0, G=22/3
chr4:177087322	T/T	C	T	SNV	WDR17	WDR17:intronic:NM_170710.4	1	100		intronic	NM_17071	c.3083+27	p.?	-0.04					Anaphase-pr	rs4388031	0.0173	YES	1.0 (pos.177087322)		WD repeat-	cytoplasm	Digestive System Diseases;Digestive System		C=0, T=28	C=0.0, T=1.0	0.00001	408.716	28	C=0/0, T=9/19
chr11:63313644	T/C	T	C	SNV	RARRES3	RARRES3:exonic:NM_004585.4	1	95.83	4	synonymous	NM_00458	c.411T>C	p.(=)	-0.81					Lecithin retir	rs584905	0		1.0 (pos.63313644)		Retinoic aci	cytosol.integral component of membrane.lipid catabolic			T=1, C=23	T=0.0417, C=0.9583	0.00001	306.621	24	T=1/0, C=8/15
chr12:113553481	T/T	C	T	SNV	RASAL1	RASAL1:exonic:NM_001193521.1	1	100	10	missense	NM_00119	c.962G>A	p.Arg321His	3.62	0.01	29	0.838		BTK motif.C2	rs1284879	0.2276	YES	7.854E-1 (pos.113553481)		Ras protein	GTPase ac	Female Urogenital Diseases:Female Urogeni		C=0, T=37	C=0.0, T=1.0	0.00001	574.588	37	C=0/0, T=18/1
chr15:79296172	A/A	G	A	SNV	RASGRF1	RASGRF1:exonic:NM_002891.4	1	100	16	synonymous	NM_00289	c.2469C>T	p.(=)	-3.46				0.03(COSM54	IQ calmodulir	rs1562008	0.2362	YES	9.651E-2 (pos.79296172)	acute_myeloid_leukaemia		MAPK casc	Central Nervous System Diseases;Dyskinesia		G=0, A=45	G=0.0, A=1.0	0.00001	747.393	45	G=0/0, A=11/3
chr4:74451073	G/G	A	G	SNV	RASSF6	RASSF6:exonic:NM_201431.2	1	100	6	missense	NM_20143	c.487T>C	p.Ser163Pro	1.49	0.17	74	0.009	0.82(COSM37	Novel Ras eff	rs12507775	0.2761	YES	1.053E-1 (pos.74451073)	acute_myeloic	Ras associa	apoptotic process,protein binding,regulation of apoptot			A=0, G=23	A=0.0, G=1.0	0.00001	316.747	23	A=0/0, G=9/14
chr19:10431799	G/T	G	T	SNV	RAVER1	RAVER1:exonic:NM_133452.2	1	58.33	8	synonymous	NM_13345	c.1449C>A	p.(=)	-1.7				0.01(COSM33	RNA recogniti	rs281425	0.1819	YES	2.676E-1;0.0 (pos.10431;	adenocarcinoi	Raver1. mo	RNA binding.cytoplasm.mRNA splicing. via spliceosome.i			G=10. T=14	G=0.4167, T=0.5833	0.00001	135.412	24	G=2/8, T=6/8
chr4:178355668	G/G	C	G	SNV	AGA	AGA:intronic:NM_000027.3	1	100		intronic	NM_00002	c.699-25G	p.?	-0.31					Asparaginase	rs4690521.rs749471!	1.00E-04		0.0 (pos.178355667).1.0 (pos.17835566:		Aspartylglu	N4-(beta-N	Aspartylglucosaminuria;Congenital. Heredit:		C=0, G=45	C=0.0, G=1.0	0.00001	751.489	45	C=0/0, G=19/2
chr1:33133968	C/C	T	C	SNV	RBBP4	RBBP4:exonic:NM_005610.2	1	100	4	synonymous	NM_00561	c.453T>C	p.(=)	-0.12					Histone-bind	rs2762904	6.00E-04		1.0 (pos.33133968)			ATP-dependent chromatin remodeling,CAF-1 complex,CE			T=0, C=20	T=0.0, C=1.0	0.00001	263.786	20	T=0/0, C=5/15
chr4:178363378	A/A	G	A	SNV	AGA	AGA:intronic:NM_000027.3	1	100		intronic	NM_00002	c.127+25C	p.?	-2.07					Asparaginase	rs11131799	0.3391	YES	6.317E-1 (pos.178363378)		Aspartylglu	N4-(beta-N	Aspartylglucosaminuria;Congenital, Heredit:		G=0, A=105	G=0.0, A=1.0	0.00001	1644.46	105	G=0/0, A=29/7
chr12:114377899	C/C	G	C	SNV	RBM19	RBM19:exonic:NM_001146699.1	1	100	15	missense	NM_00114	c.1804C>G	p.Gln602Glu	5.43	1	29	0		RNA recogniti	rs7299217,rs796776:	3.00E-04		1.0 (pos.114377899)		RNA-bindin	RNA binding,chromosome,cytoplasm,membrane,multic(			G=0, C=106	G=0.0, C=1.0	0.00001	1662.79	106	G=0/0, C=54/5
chr7:155534653	G/G	C	G	SNV	RBM33	RBM33:exonic:NM_053043.2	1	100	13	synonymous	NM_05304	c.2190C>G	p.(=)	-1.07				0.16(COSM6350848).0.16(C		rs73167165	0.0359	YES	1.814E-2:0.0 (pos.15553:	carcinoma		RNA binding,nucleic acid binding			C=0, G=36	C=0.0, G=1.0	0.00001	563.741	36	C=0/0, G=16/2
chr4:184170025	A/A	G	A	SNV	WWC2	WWC2:intronic:NM_024949.5	1	100		intronic	NM_02494	c.879+12G	p.?	-1.95					WW domain	rs907357	2.00E-04		1.0 (pos.184170025)			cytosol.kinase binding.negative regulation of hippo sign:			G=0, A=37	G=0.0, A=1.0	0.00001	583.696	37	G=0/0, A=14/2
chr4:184201909	A/A	G	A	SNV	WWC2	WWC2:intronic:NM_024949.5	1	100		intronic	NM_02494	c.2563-20(	p.?	-6.28					WW domain	rs11132173	0.4019	YES	3.238E-1 (pos.184201909)			cytosol,kinase binding,negative regulation of hippo sign:			G=0, A=70	G=0.0, A=1.0	0.00001	1092.09	70	G=0/0, A=41/2
chr4:184204099	T/T	C	T	SNV	WWC2	WWC2:intronic:NM_024949.5	1	100		intronic	NM_02494	c.2883+40	p.?	-1.79					WW domain	rs3814421	0.3995	YES	2.751E-1 (pos.184204099)			cytosol,kinase binding,negative regulation of hippo sign:			C=0, T=56	C=0.0, T=1.0	0.00001	832.1	56	C=0/0, T=24/3
chr4:40428010	C/C	T	C	SNV	RBM47	RBM47:exonic:NM_001098634.1	1	100	7	missense	NM_00109	c.1693A>G	p.Met565Val	2.48	0.47	21	0	0.03(COSM37	RNA recogniti	rs278981	0.2357	YES	5.823E-1 (pos.40428010)	neoplasm,squamous_cell		RNA bindir	Arthritis;Arthritis, Juvenile;Autoimmune Dise		T=0, C=66	T=0.0, C=1.0	0.00001	1015.19	66	T=0/0, C=26/4
chr4:40438576	G/G	A	G	SNV	RBM47	RBM47:exonic:NM_001098634.1	1	100	5	synonymous	NM_00109	c.1212T>C	p.(=)	2.38				0.23(COSM41	RNA recogniti	rs2307046	0.1182	YES	8.124E-1 (pos.40438576)	neoplasm		RNA bindir	Arthritis:Arthritis. Juvenile:Autoimmune Dise		A=0, G=20	A=0.0, G=1.0	0.00001	263.786	20	A=0/0, G=11/9
chr4:184572036	T/T	C	T	SNV	RWDD4	RWDD4:intronic:NM_152682.3	1	100		intronic	NM_15268	c.363+101	p.?	-1.75					RWD domain	rs4547833		YES							C=0, T=84	C=0.0, T=1.0	0.00001	1364.85	84	C=0/0, T=52/3
chr4:40440215	C/C	T	C	SNV	RBM47	RBM47:exonic:NM_001098634.1	1	100	4	synonymous	NM_00109	c.696A>G	p.(=)	1					RNA recogniti	rs2291580	0.1765	YES	7.609E-1 (pos.40440215)			RNA bindir	Arthritis;Arthritis, Juvenile;Autoimmune Dise		T=0, C=84	T=0.0, C=1.0	0.00001	1346.52	84	T=0/0, C=53/3
chr4:185553353	A/T	A	T	SNV	CASP3	CASP3:intronic:NM_032991.2	1	50		intronic	NM_03299	c.307+69T	p.?	-1.81					Caspase dom	rs1405944		YES			Caspase 3,	B cell hom	Alzheimer Disease;	(1S)-1-(3-chlorophenyl)-2	A=16, T=16	A=0.5, T=0.5	0.00001	157.389	32	A=4/12, T=9/7
chr4:40440854	C/C	G	C	SNV	RBM47	RBM47:exonic:NM_001098634.1	1	100	4	synonymous	NM_00109	c.57C>G	p.(=)	-1.14				0.07(COSM37	RNA recogniti	rs1052153.rs770840(	0.1093	YES	0.0 (pos.40440850).8.12:	adenocarcinoma		RNA bindir	Arthritis:Arthritis. Juvenile:Autoimmune Dise		G=0, C=38	G=0.0, C=1.0	0.00001	603.321	38	G=0/0, C=5/33
chr4:185622046	T/T	C	T	SNV	CENPU	CENPU:intronic:NM_024629.3	1	100		intronic	NM_02462	c.925-11G	p.?	-0.23					CENP-A nucle	rs11728389	0.3274	YES	2.945E-1 (pos.185622046)		MLF1-intera	CENP-A co	Autoimmune Diseases:Cardiovascular Disea:		C=0, T=28	C=0.0, T=1.0	0.00001	407.918	28	C=0/0, T=9/19
chr4:185681475	C/C	G	C	SNV	ACSL1	ACSL1:intronic:NM_001995.3	1	100		intronic	NM_00199	c.1782+36	p.?	-2.16					AMP-binding	rs6825554	0		1.0;0.0 (pos.185681475)		Acyl-CoA sy	ATP bindin	Arthritis;Arthritis, E	ATP,Adenosine monophc	G=0, C=25	G=0.0, C=1.0	0.00001	352.995	25	G=0/0, C=16/9
chr12:56956213	C/C	T	C	SNV	RBMS2	RBMS2:exonic:NM_002898.3	1	100	2	synonymous	NM_00289	c.79T>C	p.(=)	-0.66					RNA recogniti	rs3759091	0.175	YES	8.294E-1 (pos.56956213)		RNA-bindin	RNA binding,RNA processing,nucleic acid binding,nucleu			T=0, C=56	T=0.0, C=1.0	0.00001	832.073	56	T=0/0, C=32/2
chr4:185940461	T/T	C	T	SNV	HELT	HELT:intronic:NM_001300781.1	1	100		intronic	NM_00130	c.28-80C>T	p.?	-1.04					Hairy Orange	rs4862491		YES			HELT basic I	DNA binding,GABAergic neuron differentiation in basal g:			C=0, T=78	C=0.0, T=1.0	0.00001	1249.32	78	C=0/0, T=33/4
chr4:186180257	AT/A	AT	A	INDEL	SNX25	SNX25:intronic:NM_001317781.1	1	50		intronic	NM_00131	c.239+39A	p.?	0.99, -1.71					PX domain.P:	rs754673843,rs757745173			0.0;0.0 (pos.186180257)			cellular_component.endosome membrane.intracellular			AT=12. A=1	AT=0.5, A=0.5	0.00009	40.6677	24	AT=5/7, A=4/8
chr4:186366315	C/C	T	C	SNV	C4orf47,CCDC110	CCDC110:downstream:NM_152775.3, C4orf47:intronic:NM	1	100		intronic	NM_00111	c.881+31T	p.?, p.?	0.26					Domain of un	rs2120414	0.0896	YES	9.936E-1 (pos.186366315)			centrosome,cytoplasm,nucleus,protein binding			T=0, C=84	T=0.0, C=1.0	0.00001	1370.17	84	T=0/0, C=57/2
chr12:56975001	C/T	C	T	SNV	RBMS2	RBMS2:exonic:NM_002898.3	1	52.38	6	synonymous	NM_00289	c.576C>T	p.(=)	0.14					RNA recogniti	rs147815692	8.00E-04		0.0 (pos.56975001)		RNA-bindin	RNA binding,RNA processing,nucleic acid binding,nucleu			C=10, T=11	C=0.4762, T=0.5238	0.00001	94.7439	21	C=2/8, T=10/1
chr4:186423655	A/A	G	A	SNV	PDLIM3	PDLIM3:intronic:NM_014476.5	1	100		intronic	NM_01447	c.906-18C:	p.?	0.05					Domain of un	rs12644280	0.1602	YES	3.016E-1 (pos.186423655)			Z disc.actin cytoskeleton.actin filament or		Benign	G=0, A=30	G=0.0, A=1.0	0.00001	451.058	30	G=0/0, A=12/1
chr4:186423677	A/A	G	A	SNV	PDLIM3	PDLIM3:intronic:NM_014476.5	1	100		intronic	NM_01447	c.906-40C:	p.?	0.72					Domain of un	rs10866276	0.1458	YES	8.076E-1 (pos.186423677)			Z disc.actin cytoskeleton.actin filament organization.cyt			G=0, A=33	G=0.0, A=1.0	0.00001	504.597	33	G=0/0, A=12/2
chr4:186427841	C/C	T	C	SNV	PDLIM3	PDLIM3:intronic:NM_014476.5	1	100		intronic	NM_01447	c.663-35A:	p.?	-3.71					Domain of un	rs2306705	0.1451	YES	7.961E-1 (pos.186427841)			Z disc.actin cytoskeleton.actin filament organization.cyt			T=0, C=54	T=0.0, C=1.0	0.00001	798.138	54	T=0/0, C=33/2
chr1:24861704	G/A	G	A	SNV	RCAN3	RCAN3:exonic:NM_013441.3	1	95.45	5	synonymous	NM_01344	c.663G>A	p.(=)	-3.59				0.04(COSM13	Calcipressin	rs196432	0.4612	YES	7.088E-1 (pos.24861704)	adenocarcinoi	Regulator o	RNA binding,anatomical structure morphogenesis,calciu			G=2, A=42	G=0.0455, A=0.9545	0.00001	659.952	44	G=1/1, A=19/2
chr1:24859617	G/A	G	A	SNV	RCAN3	RCAN3:exonic:NM_013441.3	1	52.38	4	synonymous	NM_01344	c.414G>A	p.(=)	-2.35				0.02(COSM37	Calcipressin	rs196429	0.3194	YES	6.5E-1 (pos.24859617)	adenocarcinoi	Regulator o	RNA binding,anatomical structure morphogenesis,calciu			G=9, A=11	G=0.45, A=0.55	0.00001	108.176	21	G=5/4, A=8/3
chr13:50126382	A/A	G	A	SNV	RCBTB1	RCBTB1:exonic:NM_018191.3	1	100	7	synonymous	NM_01819	c.643C>T	p.(=)	0.57					BTB/POZ dom	rs2274278	0.166	YES	8.936E-1 (pos.50126382)		RCC1 doma	cell cycle.(	Alcohol-Related Disorders:Alcoholism:Menta		G=0, A=20	G=0.0, A=1.0	0.00001	263.781	20	G=0/0, A=10/1
chr13:50141345	A/A	G	A	SNV	RCBTB1	RCBTB1:exonic:NM_018191.3	1	100	3	missense	NM_01819	c.71C>T	p.Ala24Val	9.36	0.16	64	1		BTB/POZ dom	rs4942848	0.3155	YES	6.354E-1 (pos.50141345)		RCC1 doma	cell cycle.(	Alcohol-Related Disorders:Alcoholism:Menta		G=0, A=21	G=0.0, A=1.0	0.00001	281.235	21	G=0/0, A=8/13
chr4:187131504	A/A	G	A	SNV	CYP4V2	CYP4V2:intronic:NM_207352.3	1	100		intronic	NM_20735	c.1406-11!	p.?	-0.76					Cytochrome I	rs2102575		YES			Cytochrome	endoplasr	Congenital, Hereditary, and Neonatal Diseas		G=0, A=128	G=0.0, A=1.0	0.00001	2094.85	128	G=0/0, A=53/7
chr1:17739586	A/A	G	A	SNV	RCC2	RCC2:exonic:NM_001136204.2	1	100	9	synonymous	NM_00113	c.1296C>T	p.(=)	3.05					Regulator of (	rs942457	0.3514	YES	1.624E-1 (pos.17739586)			RNA binding,Rac GTPase binding,activation of GTPase ac			G=0, A=20	G=0.0, A=1.0	0.00001	263.747	20	G=0/0, A=11/9
chr4:187455864	A/A	AG	A	INDEL	MTNR1A	MTNR1A:intronic:NM_005958.4	1	100		intronic	NM_00595	c.185-153(	p.?	1.02, 0.0					7 transmemb	rs200182002,rs377703433,rs60(		YES			Melatonin r	G-protein	Child Development	Agomelatine,Melatonin,	AG=0, A=37	AG=0.0, A=1.0	0.00001	575.137	37	AG=0/0, A=10/
chr4:187518980	C/C	T	C	SNV	FAT1	FAT1:intronic:NM_005245.3	1	100		intronic	NM_00524	c.12258-3:	p.?	-2.01					Cadherin dor	rs1280096	0.2399	YES	9.878E-1 (pos.187518980)		FAT tumor s	actin filar	Adenocarcinoma:Astrocytoma:Carcinoma:Ca		T=0, C=60	T=0.0, C=1.0	0.00001	903.531	60	T=0/0, C=24/3
chr9:4860300	G/G	A	G	SNV	RCL1	RCL1:utr_3:NM_005772.4	1	100	9	utr_3	NM_00577	c.1147A>G	p.?	-5.34					RNA 3'-termir	rs15583	0.3049	YES	4.537E-1 (pos.4860300)		RNA termin	RNA processing.RNA-3'-phosphate cyclase activity.biolo(			A=0, G=53	A=0.0, G=1.0	0.00001	782.027	53	A=0/0, G=20/3
chr11:63681513	C/C	T	C	SNV	RCOR2	RCOR2:exonic:NM_173587.3	1	100	8	synonymous	NM_17358	c.804A>G	p.(=)	-0.43				0.05(COSM41	ELM2 domair	rs10897461	3.00E-04		1.0;0.0 (pos.63681513)	neoplasm	Rest corepr	DNA binding transcription factor activity,enzyme binding			T=0, C=41	T=0.0, C=1.0	0.00001	664.549	41	T=0/0, C=16/2
chr19:10129552	G/G	C	G	SNV	RDH8	RDH8:exonic:NM_015725.2	1	100	3	missense	NM_01572	c.468C>G	p.His156Gln	0.61	0.03	24	1	0.86(COSM11	Enoyl-(Acyl ca	rs1122206	0.1341	YES	6.86E-2 (pos.10129552)	adenocarcinoi	Retinol deh	NADP-retinol dehydrogenase a		Vitamin A	C=0, G=38	C=0.0, G=1.0	0.00001	603.76	38	C=0/0, G=8/30
chr4:187534542	C/C	T	C	SNV	FAT1	FAT1:intronic:NM_005245.3	1	100		intronic	NM_00524	c.9230-46,	p.?	0.27					Cadherin dor	rs2249926	0.4792	YES	4.023E-1;2.853E-1 (pos.187534542)		FAT tumor s	actin filar	Adenocarcinoma;Astrocytoma;Carcinoma;Ca		T=0, C=90	T=0.0, C=1.0	0.00001	1480.81	90	T=0/0, C=49/4
chr4:187535588	C/C	T	C	SNV	FAT1	FAT1:intronic:NM_005245.3	1	100		intronic	NM_00524	c.9076-90,	p.?	-0.76					Cadherin dor	rs56242383		YES			FAT tumor s	actin filar	Adenocarcinoma:Astrocytoma:Carcinoma:Ca		T=0, C=27	T=0.0, C=1.0	0.00001	389.988	27	T=0/0, C=13/1
chr19:10131999	C/C	T	C	SNV	RDH8	RDH8:exonic:NM_015725.2	1	100	5	missense	NM_01572	c.665T>C	p.Met222Thr	-0.46	0.69	81	0	0.02(COSM37	Enoyl-(Acyl ca	rs1644731.rs386806'	0.4543	YES	5.495E-1:0.0 (pos.10131!	adenocarcinoi	Retinol deh	NADP-retinol dehydrogenase a		Vitamin A	T=0, C=60	T=0.0, C=1.0	0.00001	903.456	60	T=0/0, C=38/2
chr4:187584771	G/G	C	G	SNV	FAT1	FAT1:intronic:NM_005245.3	1	100		intronic	NM_00524	c.3266-4G	p.?	-0.68					Cadherin dor	rs172903	0.0502	YES	9.999E-1 (pos.187584771)		FAT tumor s	actin filar	Adenocarcinoma;Astrocytoma;Carcinoma;Ca		C=0, G=65	C=0.0, G=1.0	0.00001	996.463	65	C=0/0, G=31/3
chr8:145737636	G/G	A	G	SNV	RECQL4	RECQL4:exonic:NM_004260.3	1	100	19	synonymous	NM_00426	c.3127T>C	p.(=)	3.29					DEAD/DEAH b	rs4925828	0.001	YES	7.806E-1 (pos.145737636)		DNA helicas	ATP bindin	Bone Diseases;Bone Diseases,	Benign	A=0, G=58	A=0.0, G=1.0	0.00001	867.343	58	A=0/0, G=25/3
chr8:21997738	A/T	A	T	SNV	REEP4	REEP4:exonic:NM_025232.3	1	52.38	3	synonymous	NM_02523	c.129T>A	p.(=)	-0.63				0.15(COSM45	TB2/DP1, HV/	rs35574275	0.1131	YES	3.946E-2 (pos.21997738)	neoplasm	Receptor ex	cell division,endoplasmic reticulum,endoplasmic reticul			A=10, T=11	A=0.4762, T=0.5238	0.00001	95.3017	21	A=8/2, T=5/6
chr2:79254261	C/C	T	C	SNV	REG3G	REG3G:exonic:NM_001008387.2	1	100	4	synonymous	NM_00100	c.297T>C	p.(=)	-1.06					Lectin C-type	rs452267	0.1872	YES	9.102E-1 (pos.79254261)		Regenerati	MyD88-dependent toll-like receptor signaling pathway.a			T=0, C=32	T=0.0, C=1.0	0.00001	485.14	32	T=0/0, C=10/2
chr1:8418650	C/C	T	C	SNV	RERE	RERE:exonic:NM_001042681.1	1	100	20	synonymous	NM_00104	c.3945A>G	p.(=)	-0.91				0.13(COSM37	Atrophin-1 fa	rs1058766.rs386628:	0.321	YES	0.0 (pos.8418638).0.0:1.:	adenocarcinoi	RE repeats-	DNA bindi	Arthritis:Arthritis. Juvenile:Autoimmune Dise		T=0, C=27	T=0.0, C=1.0	0.00001	389.988	27	T=0/0, C=6/21
chr4:189065104	G/G	T	G	SNV	TRIML1	TRIML1:intronic:NM_178556.4	1	100		intronic	NM_17855	c.759-86T:	p.?	-0.53					RING-type zin	rs9784566	0.1025	YES				metal ion binding,multicellular organism development,(			T=0, G=47	T=0.0, G=1.0	0.00001	789.282	47	T=0/0, G=30/1
chr1:8421203	C/C	T	C	SNV	RERE	RERE:exonic:NM_001042681.1	1	100	18	synonymous	NM_00104	c.2364A>G	p.(=)	-3.73				0.03(COSM42	Atrophin-1 fa	rs13596	0.316	YES	9.204E-1 (pos.8421203)	adenocarcinoi	RE repeats-	DNA bindi	Arthritis;Arthritis, Juvenile;Autoimmune Dise		T=0, C=29	T=0.0, C=1.0	0.00001	427.599	29	T=0/0, C=22/7
chr1:8425900	C/C	T	C	SNV	RERE	RERE:exonic:NM_001042681.1	1	100	13	synonymous	NM_00104	c.1419A>G	p.(=)	-0.81				0.14(COSM50	Atrophin-1 fa	rs3753275	0.1912	YES	4.694E-2 (pos.8425900)	osteosarcoma	RE repeats-	DNA bindi	Arthritis:Arthritis. Juvenile:Autoimmune Dise		T=0. C=211	T=0.0, C=1.0	0.00001	3453.06	211	T=0/0, C=108/
chr4:2087202	G/G	A	G	SNV	POLN	POLN:intronic:NM_181808.3	1	100		intronic	NM_18180	c.2197+13	p.?	0.24					DNA polymer	rs3135157		YES			Polymerase	5'-3' exonuclease activity.DNA binding.DNA biosynthetic			A=0, G=73	A=0.0, G=1.0	0.00001	1150.5	73	A=0/0, G=34/3
chr2:220193932	C/A	C	A	SNV	RESP18	RESP18:exonic:NM_001007089.3	1	53.13	5	nonsense	NM_00100	c.508G>T	p.Glu170Ter	1.37				0(COSM5592:	RESP18 doma	rs183914238	3.00E-04		0.0;0.0;0.0 (pos.2201939	malignant_me	Regulated e	Golgi apparatus,endoplasmic reticulum,extracellular re			C=15, A=17	C=0.4688, A=0.5313	0.00001	173.559	32	C=11/4, A=6/1
chr2:220197424	C/T	C	T	SNV	RESP18	RESP18:exonic:NM_001007089.3	1	50	2	synonymous	NM_00100	c.54G>A	p.(=)	-0.82				0.01(COSM37	RESP18 doma	rs2385403		YES	6.379E-2 (pos.22019742:	adenocarcinoi	Regulated e	Golgi apparatus,endoplasmic reticulum,extracellular re			C=24, T=24	C=0.5, T=0.5	0.00001	277.234	48	C=9/15, T=8/1
chr4:25391983	C/T	C	T	SNV	ANAPC4	ANAPC4:intronic:NM_013367.2	1	57.14		intronic	NM_01336	c.600+141	p.?	-0.08					Anaphase-pr	rs10001307		YES			Anaphase-p	anaphase-promoting complex,anaphase-promoting com			C=15, T=20	C=0.4286, T=0.5714	0.00001	218.683	35	C=1/14, T=3/1
chr4:26364300	G/G	C	G	SNV	RBPJ	RBPJ:intronic:NM_015874.4	1	100		intronic	NM_01587	c.21-2367!	p.?	-2.73					Beta-trefoil C	rs13116873	0.4397	YES	5.517E-1;0.0 (pos.26364300)		Recombina	B cell diffe	Abnormalities. Multiple:Arthritis:Arthritis. Rl		C=0, G=24	C=0.0, G=1.0	0.00001	334.79	24	C=0/0, G=14/1
chr4:2829077	T/C	T	C	SNV	SH3BP2	SH3BP2:intronic:NM_001145856.1	1	95.83		intronic	NM_00114	c.688+32T	p.?	-1.05					PH domain.SI	rs2269485	0.2835	YES	5.312E-1 (pos.2829077)		SH3-domair	SH3 doma	Bone Diseases:Bone Diseases. Development		T=1, C=23	T=0.0417, C=0.9583	0.00001	306.643	24	T=1/0, C=3/20
chr4:2933031	C/T	C	T	SNV	MFSD10	MFSD10:intronic:NM_001146069.1	1	57.14		intronic	NM_00114	c.1115-30(	p.?	0.18					Major Facilit:	rs55754437	0.2343	YES	3.177E-2 (pos.2933031)			apoptotic process,brush border membrane,cytoplasmic			C=12, T=16	C=0.4286, T=0.5714	0.00001	161.757	28	C=5/7, T=14/2
chr4:3029588	A/A	G	A	SNV	GRK4	GRK4:intronic:NM_182982.2	1	100		intronic	NM_18298	c.971-51G	p.?	0.01					Kinase-like,P	rs2857844	0.3933	YES	6.804E-1 (pos.3029588)		G protein-c	ATP binding,G-protein coupled receptor internalization,(			G=0, A=40	G=0.0, A=1.0	0.00001	644.253	40	G=0/0, A=32/8
chr4:3116847	A/G	A	G	SNV	HTT	HTT:intronic:NM_002111.8	1	45.45		intronic	NM_00211	c.748-184,	p.?	-1.74					Huntingtin pr	rs1419036		YES			Huntingtin,	Golgi appa	Basal Ganglia Diseases;Brain Diseases;Centr		A=18, G=15	A=0.5455, G=0.4545	0.00001	141.438	33	A=16/2, G=14/
chr10:43613843	T/T	G	T	SNV	RET	RET:exonic:NM_020975.4	1	100	13	synonymous	NM_02097	c.2307G>T	p.(=)	1.23				0.79(COSM44	Cadherin dor	rs1800861	0.2347	YES	4.999E-1 (pos.43613843)	acute_myeloic	RET transfo	ATP bindin	ACMG Recommend:	1-Ter-Buty Benign	G=0, T=44	G=0.0, T=1.0	0.00001	726.569	44	G=0/0, T=18/2
chr4:3235184	G/G	GC	G	INDEL	HTT	HTT:intronic:NM_002111.8	1	100		intronic	NM_00211	c.8456+10	p.?	-0.06, -1.2					Huntingtin pr	rs3216820,rs796542024		YES			Huntingtin,	Golgi appa	Basal Ganglia Diseases:Brain Diseases:Centr		GC=0, G=15	GC=0.0, G=1.0	0.00001	2404.44	156	GC=0/0, G=56/
chr4:3235241	G/G	A	G	SNV	HTT	HTT:intronic:NM_002111.8	1	100		intronic	NM_00211	c.8456+16	p.?	0.15					Huntingtin pr	rs362314		YES			Huntingtin,	Golgi appa	Basal Ganglia Diseases;Brain Diseases;Centr		A=0, G=159	A=0.0, G=1.0	0.00001	2563.4	159	A=0/0, G=55/1
chr2:220047393	T/T	C	T	SNV	RETREG2	RETREG2:utr_3:NM_024293.5	1	100	9	utr_3	NM_02429	c.1674C>T	p.?	0.26						rs3731901	0.3753	YES	2.842E-1 (pos.220047393)			integral component of membrane,protein binding			T=0, C=50	T=0.0, C=1.0	0.00001	836.868	50	T=0/0, C=28/2
chr4:3446279	AC/AC	A	AC	INDEL	HGFAC	HGFAC:intronic:NM_001528.3	1	100		intronic	NM_00152	c.731-71A	p.?	-1.51					EGF-like domain.Fibronectin type I domain.Fibronectin type II domain.Kringle domain.Growth factor-like EGF.						Hepatocyte	cytosol.ex	Adenocarcinoma;Carcinoma, Hep		AC=0, A=25	AC=0.0, A=1.0	0.00001	271.498	25	AC=0/0, A=13/1
chr4:3516742	A/G	A	G	SNV	LRPAP1	LRPAP1:intronic:NM_002337.3	1	50		intronic	NM_00233	c.835-87T	p.?	0.12					Alpha-2-macr	rs1794442		YES			Low densit	Golgi lumen,amyloid-beta binding,cell s			A=12, G=12	A=0.5, G=0.5	0.00001	106.706	24	A=2/10, G=6/6

chr6:111688682	G/A	G	A	SNV	REV3L	REV3L:exonic:NM 002912.4	1	50	15	synonymous	NM 00291	c.6309C>T	p.(=)	0.66					C4-type zinc-f	rs3218590	3.00E-04	YES	0.0 (pos.111688682)		Rev-3, yeas	3'-5' exonu	Abnormalities, Multiple;Congenital Abnorma		G=10, A=1C	G=0.5, A=0.5	0.00001	82.8861	20	G=3/7, A=4/6
chr4:36149025	A/A	G	A	SNV	ARAP2	ARAP2:intronic:NM 015230.3	1	100		intronic	NM 01523	c.3200-44	p.?	0.18					PH domain.P	rs1567483	0.0648	YES	9.102E-1 (pos.36149025)		ArfGAP with	GTPase ac	Amyotrophic Lateral Sclerosis;Central Nervo		G=0, A=25	G=0.0, A=1.0	0.00001	353.019	25	G=0/0, A=6/19
chr4:36149113	T/T	C	T	SNV	ARAP2	ARAP2:intronic:NM 015230.3	1	100		intronic	NM 01523	c.3199+57	p.?	-0.8					PH domain.P	rs1567484		YES			ArfGAP with	GTPase ac	Amyotrophic Lateral Sclerosis;Central Nervo		C=0, T=75	C=0.0, T=1.0	0.00001	1189.76	75	C=0/0, T=35/4
chr4:36160340	T/T	G	T	SNV	ARAP2	ARAP2:intronic:NM 015230.3	1	100		intronic	NM 01523	c.2752+12	p.?	-0.36					PH domain.P	rs1546495	0.1823	YES	9.079E-1;0.0 (pos.36160340)		ArfGAP with	GTPase ac	Amyotrophic Lateral Sclerosis;Central Nervo		G=0, T=20	G=0.0, T=1.0	0.00001	268.183	20	G=0/0, T=10/1
chr4:36168706	CAAAA/C	CAAAA	C	INDEL	ARAP2	ARAP2:intronic:NM 015230.3	4	47.62		intronic	NM 01523	c.1858-37	p.?	0.0, -2.54, 0.03, 0.13, 0.24					PH domain.P	rs146070746,rs2001	0.3943	YES	0.0 (pos.36168710),0.0;0.0 (pos.361687(		ArfGAP with	GTPase ac	Amyotrophic Lateral Sclerosis;Central Nervo		CAAAA=33,	CAAAA=0.5238, C=0.	0.00001	50	63	
chr16:20855309	G/G	A	G	SNV	REXO5	REXO5:exonic:NM 030941.2	1	100	16	synonymous	NM 03094	c.1680A>G	p.(=)	0.02					Exonuclease,	rs9929443	0.1013	YES	8.752E-1 (pos.20855309)			RNA binding,exonuclease activity,extracellular exosome			A=0, G=44	A=0.0, G=1.0	0.00001	726.412	44	A=0/0, G=37/7
chr4:37828175	T/C	T	C	SNV	PGM2	PGM2:upstream:NM 018290.3	1	57.58		intronic	NM 01829	c.-180T>C	p.?	-2.27						rs2059484,rs527878678		YES			Phosphoglu	carbohydrate metabolic process,cytosol,deoxyribose ph			T=14, C=19	T=0.4242, C=0.5758	0.00001	204.396	33	T=12/2, C=12/
chr4:39302029	C/C	T	C	SNV	RFC1	RFC1:exonic:NM 001204747.1	1	100	20	synonymous	NM 00120	c.2544A>G	p.(=)	-1.97				0.11(COSM37	ATPase famil	rs2066786	0.4349	YES	6.623E-1 (pos.39302029)	adenocarcinoi	Replication	ATP bindin	Colonic Diseases;Colonic Neoplasms;Colorec		T=0, C=32	T=0.0, C=1.0	0.00001	485.138	32	T=0/0, C=15/1
chr4:38933690	A/C	A	C	SNV	FAM114A1	FAM114A1:intronic:NM 138389.3	1	95.45		intronic	NM 13838	c.1324-16	p.?	0.28					Protein of unl	rs4832792		YES				Golgi apparatus,cytosol,nucleoplasm,nucleus,protein b			A=1, C=21	A=0.0455, C=0.9545	0.00001	271.086	22	A=1/0, C=11/1
chr4:38933726	G/G	A	G	SNV	FAM114A1	FAM114A1:intronic:NM 138389.3	1	100		intronic	NM 13838	c.1324-13	p.?	0.01					Protein of unl	rs4833106		YES				Golgi apparatus,cytosol,nucleoplasm,nucleus,protein b			A=0, G=22	A=0.0, G=1.0	0.00001	298.891	22	A=0/0, G=12/1
chr1:151316161	A/A	G	A	SNV	RFX5	RFX5:exonic:NM 000449.3	1	100	9	synonymous	NM 00044	c.753C>T	p.(=)	0.23					AT hook moti	rs1752386	2.00E-04		1.0 (pos.151316161)		Regulatory	DNA bindi	Congenital, Hereditary, and Ne	Benign	G=0, A=26	G=0.0, A=1.0	0.00001	371.42	26	G=0/0, A=20/6
chr4:39064126	C/C	G	C	SNV	KLHL5	KLHL5:intronic:NM 001007075.2	1	100		intronic	NM 00100	c.-93-53G>	p.?	2.37					BTB And C-ter	rs2711942	0.2525	YES	8.354E-1;0.0 (pos.39064126)		Kelch-like 5	Cul3-RING	Asthma;Bronchial Diseases;Cocaine-Related		G=0, C=37	G=0.0, C=1.0	0.00001	583.706	37	G=0/0, C=17/2
chr4:39064162	C/C	A	C	SNV	KLHL5	KLHL5:intronic:NM 001007075.2	1	100		intronic	NM 00100	c.-93-17A>	p.?	0.82					BTB And C-ter	rs2711941	0.253	YES	8.352E-1;0.0 (pos.39064162)		Kelch-like 5	Cul3-RING	Asthma;Bronchial Diseases;Cocaine-Related		A=0, C=38	A=0.0, C=1.0	0.00001	603.765	38	A=0/0, C=16/2
chr4:39104772	T/T	G	T	SNV	KLHL5	KLHL5:intronic:NM 001007075.2	1	100		intronic	NM 00100	c.1301-13	p.?	-0.98					BTB And C-ter	rs12506723		YES			Kelch-like 5	Cul3-RING	Asthma;Bronchial Diseases;Cocaine-Related		G=0, T=53	G=0.0, T=1.0	0.00001	783.856	53	G=0/0, T=14/3
chr4:39109399	G/G	A	G	SNV	KLHL5	KLHL5:intronic:NM 001007075.2	1	100		intronic	NM 00100	c.1688+48	p.?	-1.05					BTB And C-ter	rs12508577	0.3931	YES	5.279E-1 (pos.39109399)		Kelch-like 5	Cul3-RING	Asthma;Bronchial Diseases;Cocaine-Related		A=0, G=39	A=0.0, G=1.0	0.00001	623.98	39	A=0/0, G=15/2
chr4:39205365	T/T	C	T	SNV	WDR19	WDR19:intronic:NM 025132.3	1	100		intronic	NM 02513	c.603+23C	p.?	-0.11					WD domain.	rs1451817	7.00E-04	YES	1.0 (pos.39205365)		WD repeat-	cell morph	Abnormalities, Multiple;Bone Diseases;Bone		C=0, T=59	C=0.0, T=1.0	0.00001	885.367	59	C=0/0, T=28/3
chr15:56386577	G/G	T	G	SNV	RFX7	RFX7:exonic:NM 022841.5	1	100	9	missense	NM 02284	c.3349A>C	p.Asn1117His	6.31	1	68	0		RFX DNA-binc	rs7170589	0	YES	1.0 (pos.56386577)		Regulatory	DNA bindi	Hemic and Lymphatic Diseases;Immune Syst		T=0, G=67	T=0.0, G=1.0	0.00001	1034.43	67	T=0/0, G=23/4
chr2:108496435	A/A	T	A	SNV	RGPD4	RGPD4:exonic:NM 182588.2	1	100	21	missense	NM 18258	c.4936T>A	p.Trp1646Arg	3.94	0	101	0.994	0.79(COSM11	GRIP domain	rs832352		YES	3.259E-1 (pos.10849643!	acute myeloic	RANBP2-like	G1/S transition of mitotic cell cycle,GTPase activator act			T=0, A=23	T=0.0, A=1.0	0.00001	316.747	23	T=0/0, A=7/16
chr2:108443511	G/G	C	G	SNV	RGPD4,RGPD4-AS1	RGPD4-AS1:upstream:NR 024439.1, RGPD4:exonic:NM 182	1	99.26	1	synonymous	NR 02443	c.42C>G	p.(=)	-0.14				0.01(COSM41	GRIP domain,	rs7576738		YES	3.991E-1;0.0;0.0 (pos.10{	neoplasm,squ	RANBP2-like	G1/S transition of mitotic cell cycle,GTPase activator act			C=1, G=135	C=0.0074, G=0.9926	0.00001	2219.74	136	C=0/1, G=57/7
chr4:39456635	C/C	T	C	SNV	RPL9	RPL9:intronic:NM 001024921.3	1	100		intronic	NM 00102	c.392-71A	p.?	-0.23					Ribosomal pr	rs10025155		YES			Ribosomal	RNA binding,SRP-dependent cotranslational protein targ			T=0, C=39	T=0.0, C=1.0	0.00001	623.977	39	T=0/0, C=11/2
chr4:39474868	A/A	G	A	SNV	LIAS	LIAS:intronic:NM 006859.3	1	100		intronic	NM 00685	c.1066+37	p.?	-1.3					N-terminal dc	rs749915	0.2126	YES	7.25E-2 (pos.39474868)		Lipoic acid	4 iron, 4 sulfur cluster binding,	Lipoic Acid		G=0, A=36	G=0.0, A=1.0	0.00001	563.744	36	G=0/0, A=16/2
chr10:86012713	T/T	C	T	SNV	RGR	RGR:exonic:NM 002921.3	1	100	4	synonymous	NM 00292	c.471C>T	p.(=)	1.7				0.12(COSM14	7 transmemb	rs1042454	0.3643	YES	2.974E-1;0.0 (pos.860127	adenocarcinoi	Ral guanine	G-protein	Congenital, Hereditary, and Ne	Benign	C=0, T=29	C=0.0, T=1.0	0.00001	427.601	29	C=0/0, T=18/1
chr10:86004865	C/T	C	T	SNV	RGR	RGR:exonic:NM 002921.3	1	50	1	synonymous	NM 00292	c.19C>T	p.(=)	0.79					7 transmemb	rs11200938	0.0784	YES	2.814E-2 (pos.86004865)		Ral guanine	G-protein	Congenital, Hereditary, and Ne	Benign/Likely	C=34, T=34	C=0.5, T=0.5	0.00001	344.073	68	C=8/26, T=15/
chr1:192127637	G/G	T	G	SNV	RGS18	RGS18:utr 5:NM 130782.2	1	100	1	utr 5	NM 13078	c.-131T>G	p.?	0.02					Regulator of	rs4495675		YES				G-protein coupled receptor signaling pathway,GTPase ac			T=0, G=84	T=0.0, G=1.0	0.00001	1370.17	84	T=0/0, G=66/1
chr14:73029178	G/A	G	A	SNV	RGS6	RGS6:utr 3:NM 001204424.1	1	50	18	utr 3	NM 00120	c.1476G>A	p.?	2.58					Domain foun	rs2074647	0.0917	YES	6.345E-2 (pos.73029178)		Regulator o	G-protein	Mental Disorders;Schizophrenia;Schizophren		G=28, A=28	G=0.5, A=0.5	0.00001	272.138	56	G=23/5, A=5/2
chr1:241519098	A/A	T	A	SNV	RGS7	RGS7:utr 5:NM 002924.6	1	100	2	utr 5	NM 00292	c.-22A>T	p.?	1.25					Domain foun	rs4659599	0.0136	YES	9.965E-1 (pos.241519098)		Regulator o	G-protein	Diabetes Mellitus;Diabetes Mellitus, Type 2:		T=0, A=77	T=0.0, A=1.0	0.00001	1229.44	77	T=0/0, A=46/3
chr5:63905068	A/G	A	G	SNV	RGS7BP	RGS7BP:exonic:NM 001029875.2	1	52.38	6	missense	NM 00102	c.763A>G	p.Ile255Val	3.93	1	29	0	0.06(COSM149845)		rs889248	0.1383	YES	5.921E-1 (pos.63905068)	adenocarcinoi	Regulator o	G-protein coupled receptor signaling pathway,cytoplasn			A=10, G=11	A=0.4762, G=0.5238	0.00001	95.3	21	A=5/5, G=4/7
chr19:33167455	T/T	G	T	SNV	RGS9BP	RGS9BP:exonic:NM 207391.2	1	100	1	missense	NM 20739	c.286G>T	p.Ala96Ser	4.82		99		0.85(COSM3749494)		rs259290	0.2862	YES	2.772E-1 (pos.33167455)	adenocarcinoi	Regulator o	detection	Congenital, Hereditary, and Ne	Benign	G=0, T=40	G=0.0, T=1.0	0.00001	644.272	40	G=0/0, T=33/7
chr19:33167879	A/A	G	A	SNV	RGS9BP	RGS9BP:utr 3:NM 207391.2	1	100	1	utr 3	NM 20739	c.710G>A	p.?	-0.86						rs259291,rs7715750	0.2937	YES	1.56E-4:0.0 (pos.33167875),3.47E-1 (pos		Regulator o	detection	Congenital, Hereditary, and Ne	Benign	G=0, A=32	G=0.0, A=1.0	0.00001	485.14	32	G=0/0, A=4/28
chr12:2997397	A/A	G	A	SNV	RHNO1	RHNO1:exonic:NM 001257098.1	1	100	3	synonymous	NM 00125	c.489G>A	p.(=)	-3.58				0.03(COSM37	RAD9, RAD1,	rs2907608	0.3448	YES	6.318E-1 (pos.2997397)	adenocarcinoi	Rad9-, Rad1	DNA damage checkpoint,DNA replication,cell cycle,cellu			G=0, A=21	G=0.0, A=1.0	0.00001	278.289	21	G=0/0, A=11/1
chr11:66837965	C/C	T	C	SNV	RHOD	RHOD:exonic:NM 014578.3	1	100	4	missense	NM 01457	c.400T>C	p.Cys134Arg	1.43	1	180	0		ADP-ribosyla	rs4930409	0		1.0 (pos.66837965)		Ras homolo	GTP binding,GTPase activity,Rho protein signal transduc			T=0, C=36	T=0.0, C=1.0	0.00001	563.624	36	T=0/0, C=21/1
chr4:41615690	A/A	T	A	SNV	LIMCH1	LIMCH1:intronic:NM 014988.4	1	100		intronic	NM 01498	c.682+12T	p.?	0.54					Domain of un	rs13146318	0.2709	YES	7.398E-1 (pos.41615690)		LIM and cal	actin bind	Mental Disorders;Substance-Related Disorde		T=0, A=49	T=0.0, A=1.0	0.00001	831.632	49	T=0/0, A=18/3
chr2:152311570	A/A	G	A	SNV	RIF1	RIF1:exonic:NM 018151.4	1	100	22	missense	NM 01815	c.2506G>A	p.Gly836Ser	1.09		56	0	0.02(COSM59	Rap1-interac	rs2444263	0.3371	YES	7.801E-1 (pos.15231157(	basal cell car	Ligand-depe	cell cycle,cellular response to DNA damage stimulus,cel			G=0, A=60	G=0.0, A=1.0	0.00001	902.856	60	G=0/0, A=42/1
chr2:152320118	A/A	G	A	SNV	RIF1	RIF1:exonic:NM 018151.4	1	100	30	missense	NM 01815	c.4084G>A	p.Val1362Met	-0.42		21	0		Rap1-interac	rs2123465	0.3374	YES	7.809E-1 (pos.152320118)		Ligand-depe	cell cycle,cellular response to DNA damage stimulus,cel			G=0, A=98	G=0.0, A=1.0	0.00001	1659.06	98	G=0/0, A=59/3
chr4:42003835	G/G	C	G	SNV	SLC30A9	SLC30A9:intronic:NM 006345.3	1	100		intronic	NM 00634	c.274+38C	p.?	-0.99					Cation efflux	rs2581453	0.2246	YES	9.695E-1 (pos.42003835)		Solute carri	DNA binding transcription factor activity,cation transme			C=0, G=40	C=0.0, G=1.0	0.00001	644.282	40	C=0/0, G=16/2
chr4:42040947	T/T	C	T	SNV	SLC30A9	SLC30A9:intronic:NM 006345.3	1	100		intronic	NM 00634	c.670-56C	p.?	-1.3					Cation efflux	rs12641900	0.2068	YES			Solute carri	DNA binding transcription factor activity,cation transme			C=0, T=49	C=0.0, T=1.0	0.00001	831.578	49	C=0/0, T=33/1
chr2:152322095	T/T	A	T	SNV	RIF1	RIF1:exonic:NM 018151.4	1	100	30	missense	NM 01815	c.6061A>T	p.Asn2021Tyr	-1.09		143	0		Rap1-interac	rs2444257	0.271	YES	7.826E-1 (pos.152322095)		Ligand-depe	cell cycle,cellular response to DNA damage stimulus,cel			A=0, T=30	A=0.0, T=1.0	0.00001	442.855	30	A=0/0, T=15/1
chr4:42065269	G/G	A	G	SNV	SLC30A9	SLC30A9:intronic:NM 006345.3	1	100		intronic	NM 00634	c.1032+13	p.?	-0.88					Cation efflux	rs3804190		YES			Solute carri	DNA binding transcription factor activity,cation transme			A=0, G=32	A=0.0, G=1.0	0.00001	485.141	32	A=0/0, G=11/2
chr4:42065293	T/T	C	T	SNV	SLC30A9	SLC30A9:intronic:NM 006345.3	1	100		intronic	NM 00634	c.1032+15	p.?	-1.42					Cation efflux	rs3804191		YES			Solute carri	DNA binding transcription factor activity,cation transme			C=0, T=31	C=0.0, T=1.0	0.00001	470.138	31	C=0/0, T=11/2
chr4:42466641	T/T	C	T	SNV	ATP8A1	ATP8A1:intronic:NM 006095.2	1	100		intronic	NM 00609	c.2619+66	p.?	0.18					Cation transı	rs12647640		YES				ATP binding,ATP hydrolysis cou	Phosphatidyl serine		C=0, T=37	C=0.0, T=1.0	0.00001	571.722	37	C=0/0, T=20/1
chr4:42582047	C/C	A	C	SNV	ATP8A1	ATP8A1:intronic:NM 006095.2	1	100		intronic	NM 00609	c.835-52T	p.?	1.78					Cation transı	rs6447171	0.0715	YES	9.765E-1 (pos.42582047)			ATP binding,ATP hydrolysis cou	Phosphatidyl serine		A=0, C=21	A=0.0, C=1.0	0.00001	281.235	21	A=0/0, C=6/15
chr11:66099987	T/T	C	T	SNV	RIN1	RIN1:exonic:NM 004292.2	1	100	10	synonymous	NM 00429	c.2112G>A	p.(=)	0.53				0.23(COSM37	Ras associati	rs1784029	0.3094	YES	5.242E-1 (pos.66099987)	acute myeloid	leukaemia	GTPase ac	Melanoma;Neoplasms;Neoplasms by Histolc		C=0, T=29	C=0.0, T=1.0	0.00001	427.602	29	C=0/0, T=24/5
chr11:66100152	C/C	T	C	SNV	RIN1	RIN1:exonic:NM 004292.2	1	100	10	synonymous	NM 00429	c.1947A>G	p.(=)	-0.77				0.02(COSM41	Ras associati	rs1784030	0.0013	YES	1.0 (pos.66100152)	neoplasm		GTPase ac	Melanoma;Neoplasms;Neoplasms by Histolc		T=0, C=34	T=0.0, C=1.0	0.00001	524.003	34	T=0/0, C=18/1
chr4:44637736	C/C	T	C	SNV	YIPF7	YIPF7:intronic:NM 182592.2	1	100		intronic	NM 18259	c.352+203	p.?	-0.46					Yip1 domain	rs1347902		YES				Golgi apparatus,endoplasmic reticulum membrane,inte			T=0, C=44	T=0.0, C=1.0	0.00001	726.569	44	T=0/0, C=24/2
chr4:44696538	G/G	C	G	SNV	GUF1	GUF1:intronic:NM 021927.2	1	100		intronic	NM 02192	c.1715+8C	p.?	-0.69					50S ribosome	rs12641137	3.00E-04		1.0 (pos.44696538)		GTPase, S. c	GTP binding,GTPase activity,mitochondrial inner membr			C=0, G=25	C=0.0, G=1.0	0.00001	353.019	25	C=0/0, G=10/1
chr4:44699484	T/G	T	G	SNV	GUF1	GUF1:intronic:NM 021927.2	1	46.43		intronic	NM 02192	c.1872+23	p.?	3.27					50S ribosome	rs6851576	0.3637	YES	4.004E-1 (pos.44699484)		GTPase, S. c	GTP binding,GTPase activity,mitochondrial inner membr			T=15, G=13	T=0.5357, G=0.4643	0.00001	117.927	28	T=11/4, G=3/1
chr20:19955862	GGC/GGC	GGGCC	GC,GG,GGC	INDEL	RIN2	RIN2:exonic:NM 001242581.1	2	100	8	frameshiftDeletion	NM 00124	c.1342 13	p.Ala448fs	1.72, -0.8, 2.87, 1.59, 1.92					Ras associati	rs376231790,rs7761	1.00E-04		0.0 (pos.19955862),0.0 (pos.19955865)		RAS and RAI	GTPase ac	Alopecia;Bone Diseases;Congenital Abnorma		GGGCC=0,	GGGCC=0.0, GC=0.0,	0.00001	471.091	34	GGGCC=0/0, G
chr14:93118038	G/G	A	G	SNV	RIN3	RIN3:exonic:NM 024832.4	1	100	6	missense	NM 02483	c.644A>G	p.His215Arg	-0.2	0.15	29	0	0.08(COSM39	Vacuolar sort	rs3829947	0.43	YES	2.729E-1 (pos.93118038)	acute myeloid	leukaemia	GTPase ac	Bone Diseases;Lung Diseases;Lung Diseases		A=0, G=39	A=0.0, G=1.0	0.00001	592.336	39	A=0/0, G=9/30
chr6:33179689	C/T	C	T	SNV	RING1	RING1:exonic:NM 002931.3	1	46.43	6	synonymous	NM 00293	c.1029C>T	p.(=)	-1.25				0.16(COSM37	RAWUL doma	rs2854028	0.2368	YES	2.08E-1:0.0 (pos.3317968	adenocarcinoi	RING finger	PRC1 complex,PcG protein complex,anterior/posterior p			C=15, T=13	C=0.5357, T=0.4643	0.00001	117.927	28	C=7/8, T=4/9
chr4:48073501	T/T	G	T	SNV	TXK	TXK:intronic:NM 003328.2	1	100		intronic	NM 00332	c.1515+33	p.?	-0.48					Protein kinas	rs906486	0.0953	YES	9.998E-1 (pos.48073501)		TXK tyrosine	ATP binding,RNA polymerase II core promoter proximal r			G=0, T=39	G=0.0, T=1.0	0.00001	623.98	39	G=0/0, T=23/1
chr4:48147612	G/G	T	G	SNV	TEC	TEC:intronic:NM 003215.2	1	100		intronic	NM 00321	c.1082-16	p.?	-0.1					BTK motif,PH	rs2661547	0.2121	YES	9.158E-1 (pos.48147612)		Transient e	ATP binding,B cell receptor signaling pathway,Fc-epsilon			T=0, G=114	T=0.0, G=1.0	0.00001	1815.31	114	T=0/0, G=82/3
chr4:48169769	G/G	A	G	SNV	TEC	TEC:intronic:NM 003215.2	1	100		intronic	NM 00321	c.671+26T	p.?	-0.81					BTK motif,PH	rs7689953	0.2145	YES	9.158E-1 (pos.48169769)		Transient e	ATP binding,B cell receptor signaling pathway,Fc-epsilon			A=0, G=38	A=0.0, G=1.0	0.00001	603.762	38	A=0/0, G=24/1
chr4:48372085	G/G	T	G	SNV	SLAIN2	SLAIN2:intronic:NM 020846.1	1	100		intronic	NM 02084	c.538+71T	p.?	-0.72					SLAIN motif-c	rs1466631		YES			SLAIN motif	centroson	Crohn Disease;Digestive System Diseases;Ga		T=0, G=31	T=0.0, G=1.0	0.00001	465.812	31	T=0/0, G=21/1
chr4:48492135	C/C	T	C	SNV	SLC10A4,ZAR1	SLC10A4:downstream:NM 152679.3, ZAR1:upstream:NM 1	1	100		intronic	NM 15267	c.*1179T>	p.?, p.?	-0.03						rs10755182		YES			Zygote arre	bile acid and bile salt transport,bile acid:sodium sympo			T=0, C=20	T=0.0, C=1.0	0.00001	263.766	20	T=0/0, C=17/3
chr4:48492188	T/T	A	T	SNV	SLC10A4,ZAR1	SLC10A4:downstream:NM 152679.3, ZAR1:upstream:NM 1	1	100		intronic	NM 15267	c.*1232A>	p.?, p.?	-0.11						rs9997932		YES			Zygote arre	bile acid and bile salt transport,bile acid:sodium sympo			A=0, T=24	A=0.0, T=1.0	0.00001	334.792	24	A=0/0, T=15/9
chr6:7393452	T/T	C	T	SNV	RIOK1	RIOK1:exonic:NM 031480.2	1	100	2	synonymous	NM 03148	c.192C>T	p.(=)	-0.86					RIO1 family	rs2274212,rs368069	0.4363	YES	1.511E-3 (pos.7393449),4.789E-1:0.0 (po		RIO kinase	ATP binding,cytosol,hydrolase activity,maturation of SSU			C=0, T=56	C=0.0, T=1.0	0.00001	832.097	56	C=0/0, T=14/4
chr4:48577121	T/T	C	T	SNV	FRYL	FRYL:intronic:NM 015030.1	1	100		intronic	NM 01503	c.2846+13	p.?	-1.61					Cell morphog	rs28558226	0		1.0 (pos.48577121)			actin filament reorganization,cell cortex,cell morphoger			C=0, T=31	C=0.0, T=1.0	0.00001	465.819	31	C=0/0, T=20/1
chr4:48894981	A/A	G	A	SNV	OCIAD2	OCIAD2:intronic:NM 001014446.2	1	100		intronic	NM 00101	c.266-75C	p.?	-0.14					Ovarian carci	rs2289065		YES				endosome,mitochondrial inner membrane			G=0, A=66	G=0.0, A=1.0	0.00001	1015.34	66	G=0/0, A=32/3
chr4:48994138	T/T	G	T	SNV	CWH43	CWH43:intronic:NM 025087.2	1	100		intronic	NM 02508	c.511+31G	p.?	-0.25						rs11410065	0.3051	YES	2.995E-1 (pos.48994138)			GPI anchor biosynthetic process,integral component of r			G=0, T=62	G=0.0, T=1.0	0.00001	929.807	62	G=0/0, T=25/3
chr3:97673268	C/C	A	C	SNV	RIOX2	RIOX2:exonic:NM 153182.3	1	100	5	synonymous	NM 15318	c.753T>G	p.(=)	-0.75					Cupin superf	rs832079	3.00E-04		9.753E-1 (pos.97673268)			chromatin remodeling,cytosol,histone H3-K36 demethyl			A=0, C=29	A=0.0, C=1.0	0.00001	427.604	29	A=0/0, C=9/20
chr21:43161022	C/T	C	T	SNV	RIPK4	RIPK4:exonic:NM 020639.2	1	53.33	8	synonymous	NM 02063	c.2331G>A	p.(=)	-2.57				0.02(COSM17	Ankyrin repe	rs7276592	0.4282	YES	6.074E-1 (pos.43161022)	adenocarcinoi	Receptor-interacting s		Abnormalities, Multiple;Bone I	Benign	C=35, T=40	C=0.4667, T=0.5333	0.00001	427.994	75	C=22/13, T=28
chr4:52926931	G/A	G	A	SNV	SPATA18	SPATA18:intronic:NM 145263.3	1	45.45		intronic	NM 14526	c.194-17G	p.?	-0.56					Mitochondria	rs371430744	8.00E-04		2.313E-4 (pos.52926931)		Spermatog	cellular response to DNA damage stimulus,cellular resp			G=54, A=45	G=0.5455, A=0.4545	0.00001	485.133	99	G=28/26, A=2!
chr4:52927113	A/A	C	A	SNV	SPATA18	SPATA18:intronic:NM 145263.3	1	100		intronic	NM 14526	c.309+50C	p.?	-1.03					Mitochondria	rs999634	0.4185	YES	3.55E-1;0.0 (pos.52927113)		Spermatog	cellular response to DNA damage stimulus,cellular resp			C=0, A=63	C=0.0, A=1.0	0.00001	954.256	63	C=0/0, A=23/4
chr4:53428	A/A	G	A	SNV	ZNF595,ZNF718	ZNF595:intronic:NM 001286054.1, ZNF718:intronic:NM 00	1	100		intronic	NM 00128	c.-647+43	p.?, p.?	-1.69					C2H2-type zir	rs1004384489,rs285	8.00E-04		9.918E-1;2.319E-4 (pos.53428)			DNA binding,intracellular,metal ion binding,nucleic acid			G=0, A=24	G=0.0, A=1.0	0.00001	334.792	24	G=0/0, A=4/20
chr4:54310188	G/G	A	G	SNV	FIP1L1	FIP1L1:intronic:NM 001134937.1	1	100		intronic	NM 00113	c.1212-27	p.?	-0.07					Neuroendocr	rs11722866	0.319	YES	4.402E-1 (pos.54310188)		Fip1-like 1	RNA bindir	Eosinophilia;Hematologic Diseases;Hemic ar		A=0, G=106	A=0.0, G=1.0	0.00001	1662.79	106	A=0/0, G=72/3
chr4:54310281	G/G	T	G	SNV	FIP1L1	FIP1L1:intronic:NM 001134937.1	1	100		intronic	NM 00113	c.1267+11	p.?	-0.93					Neuroendocr	rs11723755	0.3191	YES	4.401E-1 (pos.54310281)		Fip1-like 1	RNA bindir	Eosinophilia;Hematologic Dise	Benign	T=0, G=105	T=0.0, G=1.0	0.00001	1644.56	105	T=0/0, G=67/3
chr4:54319011	A/A	C	A	SNV	FIP1L1	FIP1L1:intronic:NM 001134937.1	1	100		intronic	NM 00113	c.1268-76	p.?	-0.13					Neuroendocr	rs6850629		YES			Fip1-like 1	RNA bindir	Eosinophilia;Hematologic Diseases;Hemic ar		C=0, A=35	C=0.0, A=1.0	0.00001	543.903	35	C=0/0, A=20/1
chr18:40323497	G/T	G	T	SNV	RIT2	RIT2:exonic:NM 002930.3	1	50	5	synonymous	NM 00293	c.615C>A	p.(=)	-0.02					ADP-ribosyla	rs1804730	0.3612	YES	7.215E-1 (pos.40323497)		Ric-like prot	GTP bindir	Basal Ganglia Diseases;Brain Diseases;Centr		G=14, T=14	G=0.5, T=0.5	0.00001	131.941	28	G=9/5, T=8/6
chr2:38178406	A/G	A	G	SNV	RMDN2,RMDN2-AS1	RMDN2-AS1:intronic nc:NR 102712.1, RMDN2:exonic:NM 1	1	50	2	synonymous	NR 10271	c.48A>G	p.(=)	1.22				0.04(COSM4133833)		rs4670799	0.48	YES	1.008E-1 (pos.38178406)	neoplasm		cytosol,integral component of membrane,microtubule,c			A=13, G=13	A=0.5, G=0.5	0.00001	119.151	26	A=8/5, G=10/3
chr2:86979091	GA/GA	G	GA	INDEL	RMND5A	RMND5A:exonic:NM 022780.3	1	100	3	frameshiftInsertion	NM 02278	c.359 360	p.Val121fs	8.6					CTLH/CRA C-terminal to LisH motif domain,RING-type zinc-finger							GID complex,cytoplasm,nucleus,proteasome-mediated			G=0, GA=47	G=0.0, GA=1.0	0.00001	274.979	47	G=0/0, GA=24/
chr4:55151711	A/A	C	A	SNV	PDGFRA	PDGFRA:intronic:NM 006206.5	1	100		intronic	NM 00620	c.2439+58	p.?	-0.23					Immunoglob	rs2412559,rs962123697		YES			Platelet-de	ATP bindin	Chondrosarcoma;C	Amuvatinib,Becaplermir	C=0, A=26	C=0.0, A=1.0	0.00001	371.417	26	C=0/0, A=7/19
chr4:55156380	A/A	G	A	SNV	PDGFRA	PDGFRA:intronic:NM 006206.5	1	100		intronic	NM 00620	c.2881-10	p.?	0.29					Immunoglob	rs4864506		YES			Platelet-de	ATP bindin	Chondrosarcoma;C	Amuvatinib,Becaplermir	G=0, A=27	G=0.0, A=1.0	0.00001	389.707	27	G=0/0, A=13/1
chr4:55156400	G/G	A	G	SNV	PDGFRA	PDGFRA:intronic:NM 006206.5	1	100		intronic	NM 00620	c.2881-80	p.?	-0.06					Immunoglob	rs2276948		YES			Platelet-de	ATP bindin	Chondrosarcoma;C	Amuvatinib,Becaplermir	A=0, G=29	A=0.0, G=1.0	0.00001	427.603	29	A=0/0, G=13/1
chr4:5577884	C/C	T	C	SNV	EVC2	EVC2:intronic:NM 147127.4	1	100		intronic	NM 14712	c.3272+83	p.?	-3.92					Ellis van Crev	rs6840591		YES			EVC2 (limbi	ciliary mer	Abnormalities, Multiple;Bone Diseases;Bone		T=0, C=32	T=0.0, C=1.0	0.00001	485.128	32	T=0/0, C=11/2
chr2:86979093	A/A	G	A	SNV	RMND5A	RMND5A:exonic:NM 022780.3	1	100	3	synonymous	NM 02278	c.360G>A	p.(=)	3.76				0.92(COSM50	CTLH/CRA C-t	rs10186663	0.3498	YES	9.154E-1 (pos.86979093)	ductal carcinoma,osteosa		GID complex,cytoplasm,nucleus,proteasome-mediated			G=0, A=47	G=0.0, A=1.0	0.00001	788.279	47	G=0/0, A=24/2
chr4:56291479	T/T	C	T	SNV	TMEM165	TMEM165:intronic:NM 018475.4	1	100		intronic	NM 01847	c.899-64C	p.?	0.07					Uncharacteri	rs7677952		YES			Transmemb	Golgi apparatus,Golgi calcium ion transport,cellular calc			C=0, T=23	C=0.0, T=1.0	0.00001	321.141	23	C=0/0, T=8/15
chr4:56291528	C/C	T	C	SNV	TMEM165	TMEM165:intronic:NM 018475.4	1	100		intronic	NM 01847	c.899-15T	p.?	1.08					Uncharacteri	rs1064794924,rs563	0.3422	YES	0.0;1.156E-4 (pos.56291526),6.016E-1;0.		Transmemb	Golgi apparatus,Golgi calcium ion transpc		Benign	T=0, C=24	T=0.0, C=1.0	0.00001	326.663	24	T=0/0, C=8/16
chr4:56319088	C/C	A	C	SNV	CLOCK	CLOCK:intronic:NM 004898.3	1	100		intronic	NM 00489	c.1206+13	p.?	1.22					Helix-loop-he	rs4864996		YES			Circadian lc	DNA bindi	Cardiovascular Diseases;Vascular Diseases		A=0, C=36	A=0.0, C=1.0	0.00001	563.744	36	A=0/0, C=25/1
chr4:56319105	T/T	C	T	SNV	CLOCK	CLOCK:intronic:NM 004898.3	1	100		intronic	NM 00489	c.1206+11	p.?	1.6					Helix-loop-he	rs2412647		YES			Circadian lc	DNA bindi	Cardiovascular Diseases;Vascular Diseases		C=0, T=36	C=0.0, T=1.0	0.00001	547.029	36	C=0/0, T=25/1
chr4:56734701	T/T	C	T	SNV	EXOC1	EXOC1:intronic:NM 001024924.1	1	100		intronic	NM 00102	c.603+12C	p.?	-0.03					Exocyst comp	rs3213948	0.4273	YES	6.699E-1 (pos.56734701)		Exocyst con	Flemming body,GTP-Rho binding,Golgi to plasma membr			C=0, T=39	C=0.0, T=1.0	0.00001	623.96	39	C=0/0, T=27/1
chr4:56736699	G/A	G	A	SNV	EXOC1	EXOC1:intronic:NM 001024924.1	1	53.13		intronic	NM 00102	c.604-145	p.?	-0.04					Exocyst comp	rs11935692		YES			Exocyst con	Flemming body,GTP-Rho binding,Golgi to plasma membr			G=15, A=17	G=0.4688, A=0.5313	0.00001	173.559	32	G=10/5, A=8/9
chr4:56875829	A/G	A	G	SNV	CEP135	CEP135:intronic:NM 025009.4	1	52.38		intronic	NM 02500	c.2337-72	p.?	-0.04						rs2611824		YES			Centrosom	G2/M transition of mitotic cell cycle,centriole,centriole r			A=10, G=11	A=0.4762, G=0.5238	0.00001	95.2985	21	A=10/0, G=7/4
chr4:57179649	T/T	A	T	SNV	KIAA1211	KIAA1211:intronic:NM 020722.1	1	100		intronic	NM 02072	c.537+104	p.?	-0.2					Domain of un	rs6554344		YES							A=0, T=27	A=0.0, T=1.0	0.00001	394.376	27	A=0/0, T=10/1
chr14:21511497	C/T	C	T	SNV	RNASE7	RNASE7:exonic:NM 032572.3	1	96.67	2	missense	NM 03257	c.346C>T	p.His116Tyr	0.17		83		0(COSM1180	Pancreatic ri	rs1243469	0.1153	YES	8.697E-1 (pos.21511497)	squamous cel	Ribonuclea	RNA phosphodiester bond hydrolysis,antibacterial humc			C=2, T=58	C=0.0333, T=0.9667	0.00001	841.416	60	C=0/2, T=32/2
chr4:5747131	A/A	C	A	SNV	EVC	EVC:intronic:NM 153717.2	1	100		intronic	NM 15371	c.939+63C	p.?	0.42						rs13145958		YES			EVC gene	cartilage c	Abnormalities, Multiple;Bone Diseases;Bone		C=0, A=21	C=0.0, A=1.0	0.00001	281.235	21	C=0/0, A=10/1
chr4:57883175	C/C	G	C	SNV	POLR2B	POLR2B:intronic:NM 000938.2	1	100		intronic	NM 00093	c.1956-34	p.?	-1.58					RNA polymera	rs1718866	0.1294	YES	9.742E-1 (pos.57883175)			7-methylguanosine mRNA capping,DNA binding,DNA-dire			G=0, C=20	G=0.0, C=1.0	0.00001	263.776	20	G=0/0, C=4/16
chr4:5830414	C/C	T	C	SNV	CRMP1	CRMP1:intronic:NM 001014809.2	1	100		intronic	NM 00101	c.1624-19	p.?	-0.11					Amidohydrol	rs6818391	0.0699	YES	9.997E-1 (pos.5830414)		Collapsin re	axon guidance,cytosol,filamin binding,hydrolase activity			T=0, C=31	T=0.0, C=1.0	0.00001	465.814	31	T=0/0, C=11/2
chr4:6087057	T/T	C	T	SNV	JAKMIP1	JAKMIP1:intronic:NM 001099433.1	1	100		intronic	NM 00109	c.834+90G	p.?	-0.51					JAKMIP CC3 d	rs7666743		YES			Janus kinas	GABA rece	Autistic Disorder;Child Development Disorde		C=0, T=86	C=0.0, T=1.0	0.00001	1410.92	86	C=0/0, T=64/2
chr4:62453209	G/G	A	G	SNV	ADGRL3	ADGRL3:intronic:NM 015236.5	1	100		intronic	NM 01523	c.269+51A	p.?	-2.75					7 transmemb	rs2172802	0.247	YES			Adhesion G	G-protein coupled receptor activity,G-protein coupled re			A=0, G=236	A=0.0, G=1.0	0.00001	3956.64	236	A=0/0, G=124/
chr14:21024786	G/G	A	G	SNV	RNASE9	RNASE9:exonic:NM 001001673.3	1	100	2	missense	NM 00100	c.443T>C	p.Phe148Ser	-1.91		155		0(COSM5002!	Pancreatic ri	rs12590446	0.2198	YES	6.563E-1 (pos.21024786)	ductal carcinoma		endonucle	Genital Diseases, Male;Genital Neoplasms, I		A=0, G=38	A=0.0, G=1.0	0.00001	603.786	38	A=0/0, G=29/9
chr19:12924157	C/T	C	T	SNV	RNASEH2A	RNASEH2A:exonic:NM 006397.2	1	47.37	8	synonymous	NM 00639	c.777C>T	p.(=)	-1.44					Ribonuclease	rs76634951	0.0178	YES	1.479E-2 (pos.12924157)		Ribonuclea	DNA replic	Autoimmune Diseases;Autoim	Benign/Likely	C=30, T=27	C=0.5263, T=0.4737	0.00001	256	57	C=7/23, T=13/
chr4:6292915	G/G	A	G	SNV	WFS1	WFS1:intronic:NM 006005.3	1	100		intronic	NM 00600	c.461-9A>	p.?	0.64						rs10010131	0.3909	YES	9.348E-1 (pos.6292915)		Wolframin	ATPase bir	Abnormalities, Multiple;Blindr	Benign/Likely	A=0, G=20	A=0.0, G=1.0	0.00001	263.776	20	A=0/0, G=9/11
chr18:29645930	A/T	A	T	SNV	RNF125	RNF125:exonic:NM 017831.3	1	50	5	missense	NM 01783	c.570A>T	p.Arg190Ser	2.21	0.06	110	0.981	0.64(COSM41	Drought indu	rs150663325	0.0014		0.0 (pos.29645930)	neoplasm	RING finger	Golgi membrane,VCP-NPL4-UFD1 AAA ATPa		Benign	A=13, T=13	A=0.5, T=0.5	0.00001	119.163	26	A=6/7, T=12/1
chr19:652238	G/G	A	G	SNV	RNF126	RNF126:exonic:NM 194460.2	1	100	3	synonymous	NM 19446	c.193T>C	p.(=)	1.35				0.72(COSM37	Anaphase-pr	rs11554788	0.3787	YES	1.184E-1 (pos.652238)	adenocarcinoi	RING finger	cytoplasm,cytoplasm protein quality control by the ubiq			A=0, G=65	A=0.0, G=1.0	0.00001	996.464	65	A=0/0, G=4/61
chr2:7154632	C/C	T	C	SNV	RNF144A	RNF144A:exonic:NM 014746.5	1	100	4	synonymous	NM 01474	c.183T>C	p.(=)	-0.16				0.36(COSM45	IBR domain,	rs309325			9.934E-1 (pos.7154632)	squamous cell carcinoma		Golgi apparatus,cytoplasmic vesicle membrane,endoso			T=0, C=48	T=0.0, C=1.0	0.00001	809.162	48	T=0/0, C=26/2
chr4:141789457	T/A	T	A	SNV	RNF150	RNF150:exonic:NM 020724.1	1	47.37	7	synonymous	NM 02072	c.1275A>T	p.(=)	0.84				0.74(COSM56	PA domain,RI	rs61744098	0.0464	YES	1.159E-4 (pos.14178945)	ductal carcinoma		integral cc	Lung Diseases;Lung Diseases, Obstructive;Pu		T=20, A=18	T=0.5263, A=0.4737	0.00001	184.537	38	T=8/12, A=12/
chr13:25367282	A/C	A	C	SNV	RNF17	RNF17:exonic:NM 031277.2	1	97.5	10	missense	NM 03127	c.1038A>C	p.Lys346Asn	0.33	0.23	94	0.001	0.19(COSM37	Tudor domair	rs1451568	0.1591	YES	2.311E-4 (pos.25367282)	malignant me	RING finger	cytoplasm,metal ion binding,multicellular organism dev			A=2, C=78	A=0.025, C=0.975	0.00001	1198.9	80	A=0/2, C=63/1
chr4:6374189	G/G	A	G	SNV	PPP2R2C	PPP2R2C:intronic:NM 181876.2	1	100		intronic	NM 18187	c.625+61T	p.?	-1.07						rs6837903		YES			Protein pho	cytosol,developmental process,mitotic cell cycle,peptid			A=0, G=44	A=0.0, G=1.0	0.00001	726.553	44	A=0/0, G=27/1
chr4:6374230	CA/CA	C	CA	INDEL	PPP2R2C	PPP2R2C:intronic:NM 181876.2	1	100		intronic	NM 18187	c.625+20G	p.?	-2.83						rs201247014,rs3552(	0.2676	YES	9.907E-1 (pos.6374230)		Protein pho	cytosol,developmental process,mitotic cell cycle,peptid			C=0, CA=45	C=0.0, CA=1.0	0.00001	643.116	45	C=0/0, CA=26/
chr4:6374468	C/C	T	C	SNV	PPP2R2C	PPP2R2C:intronic:NM 181876.2	1	100		intronic	NM 18187	c.448-41A	p.?	-0.96						rs12511721	0.268	YES	3.581E-1 (pos.6374468)		Protein pho	cytosol,developmental process,mitotic cell cycle,peptid			T=0, C=36	T=0.0, C=1.0	0.00001	563.743	36	T=0/0, C=20/1
chr4:6377530	G/G	C	G	SNV	PPP2R2C	PPP2R2C:intronic:NM 181876.2	1	100		intronic	NM 18187	c.447+16G	p.?	-1.71						rs4688997	0.2831	YES	9.909E-1;0.0 (pos.6377530)		Protein pho	cytosol,developmental process,mitotic cell cycle,peptid			C=0, G=55	C=0.0, G=1.0	0.00001	814.905	55	C=0/0, G=37/1
chr4:6377534	G/G	A	G	SNV	PPP2R2C	PPP2R2C:intronic:NM 181876.2	1	100		intronic	NM 18187	c.447+12T	p.?	-2.36						rs4688998	0.0487	YES	9.999E-1 (pos.6377534)		Protein pho	cytosol,developmental process,mitotic cell cycle,peptid			A=0, G=55	A=0.0, G=1.0	0.00001	814.904	55	A=0/0, G=37/1
chr4:154631543	T/T	A	T	SNV	RNF175	RNF175:exonic:NM 173662.2	1	100	9	missense	NM 17366	c.965T>A	p.Ile322Asn	2.3	1	149	0			rs2405432	0.0058	YES	1.0 (pos.154631543)			Golgi membrane,endoplasmic reticulum membrane,enc			A=0, T=63	A=0.0, T=1.0	0.00001	958.912	63	A=0/0, T=41/2
chr4:65165661	A/A	G	A	SNV	TECRL	TECRL:intronic:NM 001010874.4	1	100		intronic	NM 00101	c.774+31C	p.?	0.24					3-oxo-5-alpha	rs11935568	0.0522	YES	9.972E-1 (pos.65165661)		Trans-2,3-e	cytoplasm	Cardiovascular Diseases;Hemic and Lymphat		G=0, A=34	G=0.0, A=1.0	0.00001	524.157	34	G=0/0, A=16/1
chr4:6594827	G/G	A	G	SNV	MAN2B2	MAN2B2:intronic:NM 015274.2	1	100		intronic	NM 01527	c.681-73A	p.?	-0.69					Alpha manno	rs10937747		YES				alpha-mannosidase activity,carbohydrate binding,extra			A=0, G=21	A=0.0, G=1.0	0.00001	281.234	21	A=0/0, G=6/15
chr4:6596476	G/A	G	A	SNV	MAN2B2	MAN2B2:intronic:NM 015274.2	1	50		intronic	NM 01527	c.1057+17	p.?	-0.06					Alpha manno	rs2301794	0.3566	YES	5.323E-1 (pos.6596476)			alpha-mannosidase activity,carbohydrate binding,extra			G=26, A=26	G=0.5, A=0.5	0.00001	256.223	52	G=8/18, A=14/
chr4:6596515	T/T	C	T	SNV	MAN2B2	MAN2B2:intronic:NM 015274.2	1	100		intronic	NM 01527	c.1057+56	p.?	-1.08					Alpha manno	rs4234761		YES				alpha-mannosidase activity,carbohydrate binding,extra			C=0, T=47	C=0.0, T=1.0	0.00001	789.298	47	C=0/0, T=17/3
chr7:156469190	G/G	A	G	SNV	RNF32	RNF32:exonic:NM 001184997.1	1	100	9	synonymous	NM 00118	c.930A>G	p.(=)	-1.06				0.01(COSM37	Anaphase-pr	rs2302145	0.3774	YES	5.336E-1 (pos.15646919(	adenocarcinoi	RING finger	aggresome,endosome,metal ion binding,protein binding			A=0, G=63	A=0.0, G=1.0	0.00001	956.632	63	A=0/0, G=34/2
chr4:66286088	A/A	G	A	SNV	EPHA5	EPHA5:intronic:NM 004439.7	1	100		intronic	NM 00443	c.1527+71	p.?	0.12					Ephrin recept	rs6551921		YES			Ephrin rece	ATP binding,GPI-linked ephrin receptor activity,adheren			G=0, A=26	G=0.0, A=1.0	0.00001	371.42	26	G=0/0, A=18/8
chr7:156469123	G/A	G	A	SNV	RNF32	RNF32:exonic:NM 001184997.1	1	50	9	missense	NM 00118	c.863G>A	p.Arg288Gln	2.31	0.26	43	0.231	0.46(COSM44	Anaphase-pr	rs2302148	0.0305	YES	1.193E-1 (pos.15646912	acute myeloic	RING finger	aggresome,endosome,metal ion binding,protein binding			G=34, A=34	G=0.5, A=0.5	0.00001	344.636	68	G=17/17, A=26
chr7:156469133	C/G	C	G	SNV	RNF32	RNF32:exonic:NM 001184997.1	1	50	9	missense	NM 00118	c.873C>G	p.His291Gln	-1.45	0.3	24	0.033	0.14(COSM37	Anaphase-pr	rs2302147,rs765232	0.3453	YES	4.161E-1:0.0:0.0 (pos.15(	adenocarcinoi	RING finger	aggresome,endosome,metal ion binding,protein binding			C=35, G=35	C=0.5, G=0.5	0.00001	355.191	70	C=26/9, G=17/
chr4:68396525	G/C	G	C	SNV	CENPC	CENPC:intronic:NM 001812.2	1	58.33		intronic	NM 00181	c.331+8C>	p.?	-0.34				0(COSM4415	Centromere a	rs355477	0.4012	YES	8.008E-1 (pos.68396525)	acute myeloic	Centromere	CENP-A containing nucleosome assembly,DNA binding,at			G=25, C=35	G=0.4167, C=0.5833	0.00001	370.185	60	G=12/13, C=17
chr4:68436883	C/C	T	C	SNV	STAP1	STAP1:intronic:NM 012108.3	1	100		intronic	NM 01210	c.192+10T	p.?	-2.07				0(COSM6351!	PH domain,SI	rs13111555	0.0952	YES	4.294E-1 (pos.68436883)	carcinoma	Signal trans	SH3/SH2 a	Congenital, Hereditary, and Neonatal Diseas		T=0, C=29	T=0.0, C=1.0	0.00001	427.603	29	T=0/0, C=21/8
chr4:68490593	A/C	A	C	SNV	UBA6	UBA6:intronic:NM 018227.5	1	45.45		intronic	NM 01822	c.2712+11	p.?	0.02					ThiF family,U	rs10010078		YES			Ubiquitin a	ATP binding,FAT10 activating enzyme activity,amygdala c			A=36, C=30	A=0.5455, C=0.4545	0.00001	285.721	66	A=16/20, C=20
chr4:68515054	A/G	A	G	SNV	UBA6	UBA6:intronic:NM 018227.5	1	50		intronic	NM 01822	c.1105-12	p.?	0.15					ThiF family,U	rs6840867		YES			Ubiquitin a	ATP binding,FAT10 activating enzyme activity,amygdala c			A=27, G=27	A=0.5, G=0.5	0.00001	262.859	54	A=19/8, G=8/1
chr4:69083563	T/T	C	T	SNV	TMPRSS11BNL	TMPRSS11BNL:intronic nc:NR 104048.1	1	100		intronic	NR 104048.1			-2.41						rs10008636	0.2156	YES							C=0, T=97	C=0.0, T=1.0	0.00001	1639	97	C=0/0, T=42/5
chr11:124747837	A/A	G	A	SNV	ROBO3	ROBO3:exonic:NM 022370.3	1	100	21	synonymous	NM 02237	c.2991G>A	p.(=)	-1.17					Fibronectin t	rs7933204	0.3172	YES	5.047E-1 (pos.124747837)		Roundabou	Roundabo	Autistic Disorder;Bone Disease	Benign	G=0, A=37	G=0.0, A=1.0	0.00001	583.695	37	G=0/0, A=24/1
chr9:94495608	C/C	T	C	SNV	ROR2	ROR2:exonic:NM 004560.3	1	100	6	missense	NM 00456	c.733A>G	p.Thr245Ala	0.86	0.77	58	0	0.05(COSM15	Fz domain,Im	rs10820900	0.3542	YES	4.762E-1 (pos.94495608)	adenocarcinoi	Receptor ty	ATP bindin	Brachydactyly;Congenital Abn	Benign/Likely	T=0, C=21	T=0.0, C=1.0	0.00001	281.162	21	T=0/0, C=6/15
chr4:6925009	A/C	A	C	SNV	TBC1D14	TBC1D14:intronic:NM 020773.2	1	55.38		intronic	NM 02077	c.-16-91A>	p.?	0.21					Rab-GTPase-	rs386671131,rs41516949		YES			TBC1 domai	GTPase activator activity,Golgi apparatus,Rab GTPase bi			A=29, C=36	A=0.4462, C=0.5538	0.00001	377.635	65	A=19/10, C=23
chr4:69343353	G/G	A	G	SNV	TMPRSS11E	TMPRSS11E:intronic:NM 014058.3	1	100		intronic	NM 01405	c.967+7A>	p.?	0.78					Domain of un	rs1026341	0.3557	YES	8.64E-1;0.0 (pos.69343353)		Transmemb	cognition,extracellular region,integral component of me			A=0, G=24	A=0.0, G=1.0	0.00001	334.792	24	A=0/0, G=7/17
chr4:69344733	G/T	G	T	SNV	TMPRSS11E	TMPRSS11E:intronic:NM 014058.3	1	98.36		intronic	NM 01405	c.1110+24	p.?	0.48					Domain of un	rs2603180	0.3557	YES	8.645E-1 (pos.69344733)		Transmemb	cognition,extracellular region,integral component of me			G=1, T=60	G=0.0164, T=0.9836	0.00001	890.61	61	G=1/0, T=27/3
chr8:55539057	G/A	G	A	SNV	RP1	RP1:exonic:NM 006269.1	1	53.33	4	missense	NM 00626	c.2615G>A	p.Arg872His	1.8	0.04	29	0.239	0.04(COSM14	Doublecortin	rs444772	0.2712	YES	4.381E-1 (pos.55539057)	squamous cel	Microtubul	axoneme,	Congenital, Hereditary, and Ne	Benign/Likely	G=14, A=16	G=0.4667, A=0.5333	0.00001	159.925	30	G=11/3, A=8/8
chr4:69682471	G/G	A	G	SNV	UGT2B10	UGT2B10:intronic:NM 001075.5	1	100		intronic	NM 00107	c.718+16A	p.?	-2.04					Glycosyltrans	rs294777	0.0017	YES	1.0 (pos.69682471)		Uridine dipl	endoplasr	Melanoma;Neoplasms;Neoplasms by Histolc		A=0, G=20	A=0.0, G=1.0	0.00001	263.776	20	A=0/0, G=9/11
chr4:69687964	A/A	G	A	SNV	UGT2B10	UGT2B10:intronic:NM 001075.5	1	100		intronic	NM 00107	c.868-25G	p.?	-0.39					Glycosyltrans	rs861340	0.1001	YES	6.905E-1 (pos.69687964)		Uridine dipl	endoplasr	Melanoma;Neoplasms;Neoplasms by Histolc		G=0, A=45	G=0.0, A=1.0	0.00001	743.419	45	G=0/0, A=20/2
chr8:55541513	T/C	T	C	SNV	RP1	RP1:exonic:NM 006269.1	1	52.38	4	missense	NM 00626	c.5071T>C	p.Ser1691Pro	-0.23	0.15	74	0	0.02(COSM41	Doublecortin	rs414352	0.2722	YES	4.395E-1 (pos.55541513)	acute myeloic	Microtubul	axoneme,	Congenital, Hereditary, and Ne	Benign/Likely	T=10, C=11	T=0.4762, C=0.5238	0.00001	95.3003	21	T=9/1, C=5/6
chr8:10467207	A/A	C	A	SNV	RP1L1	RP1L1:exonic:NM 178857.5	1	100	4	missense	NM 17885	c.4401G>T	p.Arg1467Ser	0.46	0.01	110	0		Doublecortin	rs4840498	0.0013	YES	9.999E-1 (pos.10467207)		RP1-like protein 1		Congenital, Hereditary, and Ne	Benign	C=0, A=50	C=0.0, A=1.0	0.00001	852.271	50	C=0/0, A=17/3
chr4:69796548	A/A	G	A	SNV	UGT2A3	UGT2A3:intronic:NM 024743.3	1	100		intronic	NM 02474	c.1085-65	p.?	-0.47					Glycosyltrans	rs2017125		YES			Uridine dipl	cellular glucuronidation,glucuronosyltransferase activit			G=0, A=26	G=0.0, A=1.0	0.00001	371.42	26	G=0/0, A=9/17
chr4:69879718	A/G	A	G	SNV	UGT2B10	UGT2B10:intronic:NM 001075.5	1	45.45		intronic	NM 00107	c.968+23T	p.?	-0.07					Glycosyltrans	rs2292524		YES	4.312E-1 (pos.69879718)		Uridine dipl	endoplasr	Melanoma;Neoplasms;Neoplasms by Histolc		A=24, G=20	A=0.5455, G=0.4545	0.00001	211.726	44	A=12/12, G=18
chr4:70620708	G/A	G	A	SNV	SULT1B1	SULT1B1:intronic:NM 014465.3	1	50		intronic	NM 01446	c.148+80C	p.?	-0.99					Sulfotransfer	rs2134668		YES			Sulfotransfe	3'-phosphoadenosine 5'-phosp	Adenosine-3'-5'-Diphosp		G=14, A=14	G=0.5, A=0.5	0.00001	131.941	28	G=9/5, A=6/8

chr8:10469817	A/A	G	A	SNV	RP1L1	RP1L1:exonic:NM 178857.5	1	100	4	synonymous	NM 17885	c.1791C>T	p.(=)	-0.04				0.01(COSM44	Doublecortin	rs6996950	0.1391	YES	6.435E-1 (pos.10469817)	hepatocellula	RP1-like protein 1		Congenital, Hereditary, and Ne	Benign	G=0, A=20	G=0.0, A=1.0	0.00001	263.786	20	G=0/0, A=17/3
chr8:10465097	C/T	C	T	SNV	RP1L1	RP1L1:exonic:NM 178857.5	1	57.63	4	missense	NM 17885	c.6511G>A	p.Glu2171Lys	0.04	0	56	0.007	0.02(COSM37	Doublecortin	rs4354268,rs777494	0.4672	YES	2.589E-2;0.0 (pos.10465(	acute_myeloic	RP1-like protein 1		Congenital, Hereditary, and Ne	Benign	C=25, T=34	C=0.4237, T=0.5763	0.00001	356.982	59	C=18/7, T=26/
chr4:71021888	T/T	C	T	SNV	PRR27	PRR27:intronic:NM 214711.3	1	100		intronic	NM 21471	c.75+94C>	p.?	-0.15						rs1709274		YES				extracellular exosome			C=0, T=38	C=0.0, T=1.0	0.00001	603.713	38	C=0/0, T=26/1
chr17:1795180	C/C	T	C	SNV	RPA1	RPA1:exonic:NM 002945.3	1	100	15	synonymous	NM 00294	c.1605T>C	p.(=)	0.44				0.82(COSM40	OB-fold nucle	rs2230931	0.1783	YES	2.598E-1 (pos.1795180)	acute_myeloic	Replication	DNA bindi	Acneiform Eruptions;Chloracne;Skin Disease:		T=0, C=29	T=0.0, C=1.0	0.00001	427.603	29	T=0/0, C=14/1
chr17:1733399	G/G	A	G	SNV	RPA1,SMYD4	RPA1:exonic:NM 002945.3, SMYD4:upstream:NM 052928.	1	100	1	synonymous	NM 05292	c.-2111T>(	p.?, p.(=)	-1.77				0.15(COSM37	OB-fold nucle	rs5030749	0.2171	YES	4.413E-1 (pos.1733399)	adenocarcinoi	Replication	DNA bindi	Acneiform Eruptions;Chloracne;Glioma;Medu		A=0, G=32	A=0.0, G=1.0	0.00001	485.138	32	A=0/0, G=21/1
chr15:41815516	C/C	G	C	SNV	RPAP1	RPAP1:exonic:NM 015540.3	1	100	18	missense	NM 01554	c.2473C>G	p.Gln825Glu	3.63	1	29	0	0.01(COSM41	RPAP1-like, C	rs8027526	0.001		1.0 (pos.41815516)	neoplasm	RNA polyme	DNA binding,DNA-directed 5'-3' RNA polymerase activity,			G=0, C=22	G=0.0, C=1.0	0.00001	298.892	22	G=0/0, C=10/1
chr4:71250308	A/A	T	A	SNV	SMR3B	SMR3B:intronic:NM 006685.3	1	100		intronic	NM 00668	c.54+27T>	p.?	-1.31						rs2233586,rs745526657		YES	0.0;0.0 (pos.71250307),9.999E-1 (pos.71		Submaxillai	biological_process,extracellular exosome,extracellular			T=0, A=25	T=0.0, A=1.0	0.00001	353.018	25	T=0/0, A=17/8
chr4:71250399	G/G	A	G	SNV	SMR3B	SMR3B:intronic:NM 006685.3	1	100		intronic	NM 00668	c.54+118A	p.?	-0.11						rs7676909		YES			Submaxillai	biological_process,extracellular exosome,extracellular			A=0, G=25	A=0.0, G=1.0	0.00001	353.018	25	A=0/0, G=17/8
chr4:71265172	C/C	A	C	SNV	OPRPN	OPRPN:intronic:NM 021225.4	1	100		intronic	NM 02122	c.51+119A	p.?	0.09					Proline-rich s	rs1844635		YES				endopeptidase inhibitor activity,extracellular region,ex			A=0, C=24	A=0.0, C=1.0	0.00001	334.788	24	A=0/0, C=12/1
chr4:71275012	C/C	T	C	SNV	OPRPN	OPRPN:intronic:NM 021225.4	1	100		intronic	NM 02122	c.52-85T>(	p.?	-0.63					Proline-rich s	rs1352689		YES				endopeptidase inhibitor activity,extracellular region,ex			T=0, C=88	T=0.0, C=1.0	0.00001	1451.91	88	T=0/0, C=57/3
chr15:41819716	T/T	C	T	SNV	RPAP1	RPAP1:exonic:NM 015540.3	1	100	12	missense	NM 01554	c.1516G>A	p.Glu506Lys	5.65	0.01	56	0.995	0.99(COSM14	RPAP1-like, C	rs1200345	0.4963	YES	3.256E-1 (pos.41819716)	acute_myeloic	RNA polyme	DNA binding,DNA-directed 5'-3' RNA polymerase activity,			C=0, T=23	C=0.0, T=1.0	0.00001	316.014	23	C=0/0, T=9/14
chr4:71588498	C/T	C	T	SNV	RUFY3	RUFY3:intronic:NM 001037442.3	1	50		intronic	NM 00103	c.178+30C	p.?	0.73					RUN domain	rs28435678,rs76778	0.0336	YES	0.0 (pos.71588497),4.632E-4 (pos.71588		FUN and FY	actin filar	Arsenic Poisoning;Heavy Metal Poisoning, Ne		C=17, T=17	C=0.5, T=0.5	0.00001	172.009	34	C=8/9, T=11/6
chr4:72423624	T/T	C	T	SNV	SLC4A4	SLC4A4:intronic:NM 001134742.1	1	100		intronic	NM 00113	c.2937+22	p.?	-0.75					Band 3 cytopl	rs1453474	0.0443	YES	7.539E-1 (pos.72423624)		Solute carrier family 4,		Acid-Base Imbalanc	Sodium bicarbonate	C=0, T=212	C=0.0, T=1.0	0.00001	3465.69	212	C=0/0, T=93/1
chr14:21770681	G/G	A	G	SNV	RPGRIP1	RPGRIP1:exonic:NM 020366.3	1	100	4	synonymous	NM 02036	c.525A>G	p.(=)	-1.36				0.03(COSM37	C2 domain,Fi	rs17792599	0.189	YES	1.535E-1 (pos.21770681)	adenocarcinoi	Retinitis pi	axoneme,	Congenital, Hereditary, and Ne	Benign/Likely	A=0, G=22	A=0.0, G=1.0	0.00001	298.892	22	A=0/0, G=5/17
chr3:40503809	A/A	G	A	SNV	RPL14	RPL14:utr 3:NM 003973.4	1	100	6	utr 3	NM 00397	c.734G>A	p.?	-2.77					Ribosomal pr	rs11539046		YES			Ribosomal	RNA bindir	Adenocarcinoma;Basal Ganglia Diseases;Bra		G=0, A=58	G=0.0, A=1.0	0.00001	861.516	58	G=0/0, A=16/4
chr4:74448079	T/T	TCTT	T	INDEL	RASSF6	RASSF6:intronic:NM 201431.2	3	100		intronic	NM 20143	c.664-72A	p.?	-0.51, 0.38, 2.31, 0.17					Novel Ras eff	rs148589925		YES			Ras associa	apoptotic process,protein binding,regulation of apoptot			TCTT=0, T=6	TCTT=0.0, T=1.0	0.00001	976.61	64	TCTT=0/0, T=3
chr2:101635538	T/T	C	T	SNV	RPL31,TBC1D8	RPL31:utr 3:NM 001098577.2, TBC1D8:intronic:NM 00110	1	100	5	utr 3	NM 00109	c.425C>T,	p.?, p.?	-1.9					GRAM domai	rs13007048	0.2969	YES	1.536E-1 (pos.101635538)		Ribosomal	GTPase ac	Bone Diseases;Bone Diseases, Metabolic;Bre		C=0, T=53	C=0.0, T=1.0	0.00001	782.012	53	C=0/0, T=24/2
chr3:128356851	AT/AT	A	AT	INDEL	RPN1	RPN1:exonic:NM 002950.3	1	100	3	frameshiftInsertion	NM 00295	c.423 424	p.Tyr142fs	8.83					Ribophorin I						Ribophorin	RNA binding,cellular protein modification process,cytos			A=0, AT=53	A=0.0, AT=1.0	0.00001	354.147	53	A=0/0, AT=24/
chr3:128356852	T/T	C	T	SNV	RPN1	RPN1:exonic:NM 002950.3	1	100	3	synonymous	NM 00295	c.423G>A	p.(=)	-3.83				0.04(COSM37	Ribophorin I	rs1697	0.386	YES	7.493E-1 (pos.12835685	adenocarcinoi	Ribophorin	RNA binding,cellular protein modification process,cytos			C=0, T=53	C=0.0, T=1.0	0.00001	780.459	53	C=0/0, T=24/2
chr4:76721459	G/A	G	A	SNV	USO1	USO1:intronic:NM 003715.3	1	59.09		intronic	NM 00371	c.1413-45(	p.?	0.64					Uso1 / p115 l	rs4859409	0.39	YES	8.006E-1 (pos.76721459)		USO1 vesicl	COPII vesi	Arsenic Poisoning;Genital Diseases, Male;Ge		G=9, A=13	G=0.4091, A=0.5909	0.00001	122.568	22	G=5/4, A=4/9
chr4:76725245	T/T	G	T	SNV	USO1	USO1:intronic:NM 003715.3	1	100		intronic	NM 00371	c.1836-42(	p.?	-0.01					Uso1 / p115 l	rs381067	0.0011	YES	1.0 (pos.76725245)		USO1 vesicl	COPII vesi	Arsenic Poisoning;Genital Diseases, Male;Ge		G=0, T=46	G=0.0, T=1.0	0.00001	768.313	46	G=0/0, T=31/1
chr20:35865054	T/T	C	T	SNV	RPN2	RPN2:exonic:NM 002951.4	1	100	16	synonymous	NM 00295	c.1825C>T	p.(=)	0.41				0.53(COSM41	Oligosacchar	rs4608	0.1847	YES	4.697E-1 (pos.35865054)	acute_myeloic	Ribophorin	aging,autc	Genital Diseases, Male;Genital Neoplasms, I		C=0, T=36	C=0.0, T=1.0	0.00001	563.67	36	C=0/0, T=20/1
chr4:76804067	G/G	A	G	SNV	PPEF2	PPEF2:intronic:NM 006239.2	1	100		intronic	NM 00623	c.933+12T	p.?	-0.67					Calcineurin-l	rs4859564	0.0077	YES	9.948E-1 (pos.76804067)		Protein pho	Hsp70 protein binding,Hsp90 protein binding,calcium io			A=0, G=61	A=0.0, G=1.0	0.00001	921.854	61	A=0/0, G=30/3
chr4:76805692	G/G	A	G	SNV	PPEF2	PPEF2:intronic:NM 006239.2	1	100		intronic	NM 00623	c.746+55T	p.?	-0.58					Calcineurin-l	rs7666135		YES			Protein pho	Hsp70 protein binding,Hsp90 protein binding,calcium io			A=0, G=92	A=0.0, G=1.0	0.00001	1538.94	92	A=0/0, G=58/3
chr18:33647254	C/C	A	C	SNV	RPRD1A	RPRD1A:exonic:NM 018170.4	1	100	1	synonymous	NM 01817	c.114T>G	p.(=)	0.52					Cell-cycle alt	rs1789517	0.0669	YES	1.0 (pos.33647254)		Regulation	DNA-direc	Autoimmune Diseases;Female Urogenital Di:		A=0, C=77	A=0.0, C=1.0	0.00001	1229.42	77	A=0/0, C=32/4
chr4:76805962	A/A	G	A	SNV	PPEF2	PPEF2:intronic:NM 006239.2	1	100		intronic	NM 00623	c.580-49C	p.?	1.14					Calcineurin-l	rs1976517	0.0176	YES	9.934E-1 (pos.76805962)		Protein pho	Hsp70 protein binding,Hsp90 protein binding,calcium io			G=0, A=72	G=0.0, A=1.0	0.00001	1130.92	72	G=0/0, A=41/3
chr4:76805978	T/T	C	T	SNV	PPEF2	PPEF2:intronic:NM 006239.2	1	100		intronic	NM 00623	c.580-65G	p.?	0.04					Calcineurin-l	rs1566975		YES			Protein pho	Hsp70 protein binding,Hsp90 protein binding,calcium io			C=0, T=72	C=0.0, T=1.0	0.00001	1130.95	72	C=0/0, T=41/3
chr1:26879920	C/C	T	C	SNV	RPS6KA1	RPS6KA1:exonic:NM 001006665.1	1	100	6	synonymous	NM 00100	c.534T>C	p.(=)	-2.31					Kinase-like,Li	rs4970490	1.00E-04		1.0;0.0 (pos.26879920)		Ribosomal	ATP bindin	Hemic and Lympha	Purvalanol A	T=0, C=34	T=0.0, C=1.0	0.00001	524.186	34	T=0/0, C=16/1
chr6:166826304	C/C	T	C	SNV	RPS6KA2	RPS6KA2:exonic:NM 001006932.2	1	100	22	synonymous	NM 00100	c.2172A>G	p.(=)	-0.54				0.06(COSM37	Kinase-like,Li	rs760670	0.3043	YES	7.991E-1 (pos.16682630	adenocarcinoi	Ribosomal	ATP bindin	Dental Caries;Stomatognathic Diseases;Toot		T=0, C=24	T=0.0, C=1.0	0.00001	334.791	24	T=0/0, C=12/1
chr4:76857395	G/G	A	G	SNV	NAAA	NAAA:intronic:NM 014435.3	1	100		intronic	NM 01443	c.372-7T>(	p.?	1.08				0.1(COSM368	Linear amide	rs4859573	0.2309	YES	3.568E-1 (pos.76857395)	acute_myeloic	N-acylethar	biological_process,cytoplasm,extracellular exosome,hy			A=0, G=22	A=0.0, G=1.0	0.00001	298.833	22	A=0/0, G=17/5
chr6:167271711	C/C	T	C	SNV	RPS6KA2	RPS6KA2:exonic:NM 001006932.2	1	100	2	missense	NM 00100	c.100A>G	p.Thr34Ala	-3.09	0.77	58	0		Kinase-like,Li	rs943687	0.0117	YES	9.56E-1;0.0 (pos.167271711)		Ribosomal	ATP bindin	Dental Caries;Stomatognathic Diseases;Toot		T=0, C=20	T=0.0, C=1.0	0.00001	263.786	20	T=0/0, C=11/9
chr4:76943677	A/A	G	A	SNV	CXCL10	CXCL10:intronic:NM 001565.3	1	100		intronic	NM 00156	c.189-69C	p.?	0.48					Small cytokin	rs4859588		YES			Chemokine	CXCR3 che	Abortion, Spontane	N-Methylleucine	G=0, A=22	G=0.0, A=1.0	0.00001	298.829	22	G=0/0, A=9/13
chr4:76944491	C/C	T	C	SNV	CXCL10	CXCL10:intronic:NM 001565.3	1	100		intronic	NM 00156	c.61+33A>	p.?	-0.07					Small cytokin	rs4241578	0.4604	YES	9.494E-1 (pos.76944491)		Chemokine	CXCR3 che	Abortion, Spontane	N-Methylleucine	T=0, C=38	T=0.0, C=1.0	0.00001	603.786	38	T=0/0, C=31/7
chr6:167271716	C/C	T	C	SNV	RPS6KA2	RPS6KA2:exonic:NM 001006932.2	1	100	2	missense	NM 00100	c.95A>G	p.Glu32Gly	0.97	0.04	98	0.122		Kinase-like,Li	rs9347162	0.248	YES	8.758E-1;0.0 (pos.167271716)		Ribosomal	ATP bindin	Dental Caries;Stomatognathic Diseases;Toot		T=0, C=20	T=0.0, C=1.0	0.00001	263.786	20	T=0/0, C=11/9
chr4:77021288	C/C	G	C	SNV	ART3	ART3:intronic:NM 001130016.2	1	100		intronic	NM 00113	c.848-156(	p.?	0.19					NAD:arginine	rs7654988		YES			ADP-ribosyl	C-terminal protein lipidation,NAD(P)+-protein-arginine A			G=0, C=27	G=0.0, C=1.0	0.00001	389.988	27	G=0/0, C=15/1
chr4:77021433	T/T	C	T	SNV	ART3	ART3:intronic:NM 001130016.2	1	100		intronic	NM 00113	c.848-11C	p.?	0.13					NAD:arginine	rs6532189	0.2677	YES	3.752E-1 (pos.77021433)		ADP-ribosyl	C-terminal protein lipidation,NAD(P)+-protein-arginine A			C=0, T=27	C=0.0, T=1.0	0.00001	389.988	27	C=0/0, T=12/1
chr14:75386576	A/A	G	A	SNV	RPS6KL1	RPS6KL1:exonic:NM 031464.4	1	100	3	missense	NM 03146	c.362C>T	p.Pro121Leu	3.05	0.07	98	0.106		MIT (microtul	rs2286913	0.3662	YES	5.339E-1 (pos.75386576)			ATP binding,cytoplasm,protein kinase activity,protein pl			G=0, A=26	G=0.0, A=1.0	0.00001	371.42	26	G=0/0, A=15/1
chr4:77039352	G/G	A	G	SNV	NUP54	NUP54:intronic:NM 017426.3	1	100		intronic	NM 01742	c.1165-5T:	p.?	0.23				0.07(COSM40	Nucleoporin	rs13115827	0.178	YES	3.75E-1 (pos.77039352)	acute_myeloic	Nucleopori	NLS-bearing protein import into nucleus,host cell,intrac			A=0, G=28	A=0.0, G=1.0	0.00001	408.717	28	A=0/0, G=14/1
chr4:77065264	T/T	C	T	SNV	NUP54	NUP54:intronic:NM 017426.3	1	100		intronic	NM 01742	c.295+38G	p.?	-1.23					Nucleoporin	rs11736522	0.3903	YES	8.92E-1 (pos.77065264)		Nucleopori	NLS-bearing protein import into nucleus,host cell,intrac			C=0, T=138	C=0.0, T=1.0	0.00001	2300.28	138	C=0/0, T=86/5
chr14:75388183	T/T	C	T	SNV	RPS6KL1	RPS6KL1:exonic:NM 031464.4	1	100	2	missense	NM 03146	c.62G>A	p.Arg21Gln	1.32	0.01	43	0.462	0.11(COSM50	MIT (microtul	rs7156590	0.3753	YES	5.416E-1 (pos.75388183)	ductal_carcinoma		ATP binding,cytoplasm,protein kinase activity,protein pl			C=0, T=28	C=0.0, T=1.0	0.00001	408.718	28	C=0/0, T=22/6
chr19:54711329	C/T	C	T	SNV	RPS9	RPS9:exonic:NM 001013.3	1	52.38	5	synonymous	NM 00101	c.471C>T	p.(=)	-0.48				0.4(COSM619	Ribosomal pr	rs4838	0.0409	YES	2.196E-3;0.0 (pos.54711:	carcinoma	Ribosomal	RNA binding,SRP-dependent cotranslational protein targ			C=10, T=11	C=0.4762, T=0.5238	0.00001	95.2686	21	C=8/2, T=7/4
chr17:78820329	T/T	C	T	SNV	RPTOR	RPTOR:exonic:NM 020761.2	1	100	11	synonymous	NM 02076	c.1269C>T	p.(=)	-2.21				0.07(COSM37	HEAT repeat,	rs3751945	0.3703	YES	4.448E-1 (pos.78820329)	adenocarcinoi	Regulatory-	14-3-3 pro	Mental Disorders;Nutrition Disorders;Nutriti		C=0, T=29	C=0.0, T=1.0	0.00001	427.603	29	C=0/0, T=15/1
chr20:17606220	C/T	C	T	SNV	RRBP1	RRBP1:exonic:NM 004587.2	1	57.14	12	synonymous	NM 00458	c.1692G>A	p.(=)	-2.8					Ribosome rec	rs145483952	0.0074		3.474E-4 (pos.17606220)		Ribosome b	RNA binding,endoplasmic reticulum,integral component			C=9, T=12	C=0.4286, T=0.5714	0.00001	109.102	21	C=3/6, T=4/8
chr4:77987351	G/G	A	G	SNV	CCNI	CCNI:intronic:NM 006835.2	1	100		intronic	NM 00683	c.114+51T	p.?	0.57					Cyclin, N-terr	rs4241602	0.0891	YES	9.993E-1 (pos.77987351)			regulation of cell cycle,spermatogenesis			A=0, G=40	A=0.0, G=1.0	0.00001	644.285	40	A=0/0, G=25/1
chr4:786401	C/C	T	C	SNV	CPLX1	CPLX1:intronic:NM 006651.3	1	100		intronic	NM 00665	c.32-5A>G	p.?	-0.96				0.03(COSM40	Synaphin pro	rs2306251	0.3694	YES	2.792E-1 (pos.786401)	acute_myeloic	Complexin	chemical s	Bipolar Disorder;Bipolar and Related Disorde		T=0, C=24	T=0.0, C=1.0	0.00001	334.792	24	T=0/0, C=10/1
chr4:78870938	TA/TA	T	TA	INDEL	MRPL1	MRPL1:intronic:NM 020236.3	1	100		intronic	NM 02023	c.778-13T:	p.?	-0.32					Ribosomal protein L1p/L10e family						Mitochondr	RNA binding,box H/ACA snoRNA binding,box H/ACA snoRN			T=0, TA=24	T=0.0, TA=1.0	0.00001	305.354	24	T=0/0, TA=14/
chr4:79186171	A/A	G	A	SNV	FRAS1	FRAS1:intronic:NM 025074.6	1	100		intronic	NM 02507	c.604-8G>	p.?	0.1				0.08(COSM40	Cadherin-like	rs2867014	0.0058	YES	9.509E-1 (pos.79186171)	acute_myeloic	FRAS1 gene	basement	Abnormalities, Multiple;Conge	Benign	G=0, A=25	G=0.0, A=1.0	0.00001	353.011	25	G=0/0, A=18/7
chr4:110765361	G/G	T	G	SNV	RRH	RRH:utr 3:NM 006583.2	1	100	7	utr 3	NM 00658	c.1022T>G	p.?	0.93					7 transmemb	rs7681770	0.0022	YES	1.0 (pos.110765361)		Rhodopsin l	G-protein coupled photoreceptor activity,G-protein coup			T=0, G=58	T=0.0, G=1.0	0.00001	867.385	58	T=0/0, G=32/2
chr11:4159466	A/A	G	A	SNV	RRM1	RRM1:exonic:NM 001033.4	1	100	19	synonymous	NM 00103	c.2232G>A	p.(=)	-1.27				0.12(COSM39	ATP cone don	rs1042858	0.07	YES	7.412E-1;0.0 (pos.41594(	acute_myeloic	Ribonucleo	ATP bindin	Adenocarcinoma;A	Cladribine,Clofarabine,F	G=0, A=39	G=0.0, A=1.0	0.00001	623.98	39	G=0/0, A=30/9
chr4:8034497	A/A	G	A	SNV	ABLIM2	ABLIM2:intronic:NM 001130083.1	1	100		intronic	NM 00113	c.1048-29	p.?	-2.88					LIM domain,F	rs13151663		YES			Actin-bindir	actin binding,actin cytoskeleton,actin cytoskeleton orga			G=0, A=128	G=0.0, A=1.0	0.00001	2060.85	128	G=0/0, A=55/7
chr11:4141132	C/A	C	A	SNV	RRM1	RRM1:exonic:NM 001033.4	1	50	9	synonymous	NM 00103	c.850C>A	p.(=)	5.75				0.99(COSM54	ATP cone don	rs183484	0.4665	YES	3.812E-1 (pos.4141132)	acute_myeloic	Ribonucleo	ATP bindin	Adenocarcinoma;A	Cladribine,Clofarabine,F	C=11, A=11	C=0.5, A=0.5	0.00001	94.6067	22	C=2/9, A=7/4
chr4:80993378	AC/AC	A	AC	INDEL	ANTXR2	ANTXR2:intronic:NM 001145794.1	1	100		intronic	NM 00114	c.152+185	p.?	-0.28					Anthrax receptor C-terminus region,Anthrax receptor extracellular domain,von Willebrand factor ty						Anthrax tox	endoplasr	Ankylosis;Arthritis;Bone Diseases;Bone Dise		A=0, AC=31	A=0.0, AC=1.0	0.00001	252.949	31	A=0/0, AC=21/
chr4:80993382	G/C	G	C	SNV	ANTXR2	ANTXR2:intronic:NM 001145794.1	1	96.77		intronic	NM 00114	c.152+181	p.?	-1.96					Anthrax rece	rs6818557		YES			Anthrax tox	endoplasr	Ankylosis;Arthritis;Bone Diseases;Bone Dise		G=1, C=30	G=0.0323, C=0.9677	0.00001	423.125	31	G=0/1, C=21/9
chr8:103220351	G/G	A	G	SNV	RRM2B	RRM2B:utr 3:NM 001172477.1	1	100	9	utr 3	NM 00117	c.1282T>C	p.?	-2.2					Ribonucleoti	rs1265117	0		9.992E-1;0.0 (pos.103220351)		Ribonucleo	DNA repai	Acid-Base Imbalanc	Cladribine	A=0, G=26	A=0.0, G=1.0	0.00001	371.418	26	A=0/0, G=15/1
chr10:99160965	G/G	A	G	SNV	RRP12	RRP12:exonic:NM 015179.3	1	100	1	synonymous	NM 01517	c.24T>C	p.(=)	-3.64				0.01(COSM54	NUC173 dom	rs2275091	0.0203	YES	8.932E-1;0.0 (pos.9916(	squamous_cel	Ribosomal l	RNA binding,integral component of membrane,nuclear n			A=0, G=53	A=0.0, G=1.0	0.00001	775.155	53	A=0/0, G=25/2
chr21:45096206	A/A	G	A	SNV	RRP1B	RRP1B:exonic:NM 015056.2	1	100	7	synonymous	NM 01505	c.603G>A	p.(=)	0.79				0.69(COSM14	Nucleolar prc	rs2155722	0.3969	YES	7.984E-1 (pos.45096206)	adenocarcinoi	Ribosomal l	RNA bindir	Digestive System Diseases;Eosinophilia;Eosi		G=0, A=20	G=0.0, A=1.0	0.00001	263.785	20	G=0/0, A=11/9
chr21:45107562	C/C	T	C	SNV	RRP1B	RRP1B:exonic:NM 015056.2	1	100	13	missense	NM 01505	c.1307T>C	p.Leu436Pro	-0.26	0.26	98	0		Nucleolar prc	rs9306160	0.4091	YES	8.022E-1 (pos.45107562)		Ribosomal l	RNA bindir	Digestive System Diseases;Eosinophilia;Eosi		T=0, C=50	T=0.0, C=1.0	0.00001	852.743	50	T=0/0, C=34/1
chr11:6622714	A/A	G	A	SNV	RRP8	RRP8:exonic:NM 015324.3	1	100	3	synonymous	NM 01532	c.582C>T	p.(=)	0.12					Hypothetical	rs2288283	0.1404	YES	8.76E-1 (pos.6622714)		Ribosomal l	RNA binding,S-adenosylmethionine-dependent methyltr			G=0, A=73	G=0.0, A=1.0	0.00001	1154.5	73	G=0/0, A=37/3
chr4:8237096	C/C	G	C	SNV	SH3TC1	SH3TC1:intronic:NM 018986.4	1	100		intronic	NM 01898	c.3283-64(	p.?	-0.01					SH3 domain	rs1678260		YES				biological_	Colonic Diseases;Colorectal Neoplasms;Dige		G=0, C=24	G=0.0, C=1.0	0.00001	324.271	24	G=0/0, C=10/1
chr4:8304038	C/C	T	C	SNV	HTRA3	HTRA3:intronic:NM 053044.4	1	100		intronic	NM 05304	c.1052-15:	p.?	-1.6					Insulin-like gr	rs4696797		YES			Calpain 5,H	endopeptidase activity,extracellular region,insulin-like			T=0, C=24	T=0.0, C=1.0	0.00001	334.759	24	T=0/0, C=18/6
chr4:8304137	A/A	G	A	SNV	HTRA3	HTRA3:intronic:NM 053044.4	1	100		intronic	NM 05304	c.1052-53(	p.?	-1.3					Insulin-like gr	rs13144236	0.2788	YES			Calpain 5,H	endopeptidase activity,extracellular region,insulin-like			G=0, A=37	G=0.0, A=1.0	0.00001	583.701	37	G=0/0, A=19/1
chr4:83582211	C/G	C	G	SNV	SCD5	SCD5:intronic:NM 001037582.2	1	53.49		intronic	NM 00103	c.569+196	p.?	1.37				0(COSM6383:	Fatty acid de	rs3733227	0.3417	YES	6.072E-1 (pos.83582211)	carcinoma		endoplasmic reticulum membrane,fatty-acyl-CoA biosyn			C=60, G=69	C=0.4651, G=0.5349	0.00001	765.488	129	C=30/30, G=3
chr4:8376596	T/C	T	C	SNV	ACOX3	ACOX3:intronic:NM 003501.2	1	57.14		intronic	NM 00350	c.1828+10	p.?	-0.04					Acyl-CoA deh	rs1010760502					Acyl-coenzy	FAD binding,acyl-CoA dehydrogenase activity,fatty acid b			T=9, C=12	T=0.4286, C=0.5714	0.00001	109.102	21	T=5/4, C=5/7
chr4:83774687	G/G	C	G	SNV	SEC31A	SEC31A:intronic:NM 001318120.1	1	100		intronic	NM 00131	c.2154+36	p.?	1.74					Sec23-bindin	rs13112559	0.2153	YES	2.687E-1 (pos.83774687)		Sec31, yeas	COPII vesi	Hemic and Lymphatic Diseases;Immune Syst		C=0, G=44	C=0.0, G=1.0	0.00001	726.567	44	C=0/0, G=29/1
chr17:48557326	A/A	G	A	SNV	RSAD1	RSAD1:exonic:NM 018346.2	1	100	3	missense	NM 01834	c.355G>A	p.Ala119Thr	0.28	0.56	58	0	0.07(COSM40	HemN C-term	rs2290862	0.3734	YES	2.129E-1 (pos.48557326)	acute_myeloid leukaemia		4 iron, 4 sulfur cluster binding,catalytic activity,copropo			G=0, A=20	G=0.0, A=1.0	0.00001	263.784	20	G=0/0, A=11/9
chr4:83788562	C/C	T	C	SNV	SEC31A	SEC31A:intronic:NM 001318120.1	1	100		intronic	NM 00131	c.883-93A	p.?	-0.67					Sec23-bindin	rs2045479		YES			Sec31, yeas	COPII vesi	Hemic and Lymphatic Diseases;Immune Syst		T=0, C=74	T=0.0, C=1.0	0.00001	1170.13	74	T=0/0, C=37/3
chr17:48557348	C/C	T	C	SNV	RSAD1	RSAD1:exonic:NM 018346.2	1	100	3	missense	NM 01834	c.377T>C	p.Leu126Ser	4.39	0.39	145	0	0.04(COSM40	HemN C-term	rs2290861	0.373	YES	2.132E-1 (pos.48557348)	acute_myeloid leukaemia		4 iron, 4 sulfur cluster binding,catalytic activity,copropo			T=0, C=20	T=0.0, C=1.0	0.00001	263.786	20	T=0/0, C=11/9
chr4:83852065	A/A	G	A	SNV	LIN54	LIN54:intronic:NM 194282.3	1	100		intronic	NM 19428	c.2048+31	p.?	-0.11					Tesmin/TSO1	rs985899	1.00E-04		1.0 (pos.83852065)		Lin54, C. ele	DNA binding,cell cycle,metal ion binding,nucleoplasm,pı			G=0, A=30	G=0.0, A=1.0	0.00001	440.336	30	G=0/0, A=21/9
chr2:7017903	G/G	A	G	SNV	RSAD2	RSAD2:utr 5:NM 080657.4	1	100	1	utr 5	NM 08065	c.-29A>G	p.?	-0.03					4Fe-4S single	rs10182815	9.00E-04	YES	9.995E-1 (pos.7017903)		Radical S-ac	4 iron, 4 su	Cardiovascular Diseases;Embolism and Thro		A=0, G=28	A=0.0, G=1.0	0.00001	407.347	28	A=0/0, G=8/20
chr7:77365770	A/G	A	G	SNV	RSBN1L	RSBN1L:exonic:NM 198467.2	1	50	2	synonymous	NM 19846	c.630A>G	p.(=)	0.47				0.02(COSM3762841)		rs3764824,rs760502	0.4927	YES	2.119E-3;0.0 (pos.77365	adenocarcinoma,carcinom	nucleus				A=13, G=13	A=0.5, G=0.5	0.00001	120.854	26	A=6/7, G=8/5
chr22:32795641	T/T	C	T	SNV	RTCB	RTCB:exonic:NM 014306.4	1	100	6	synonymous	NM 01430	c.603G>A	p.(=)	0.52					tRNA-splicing	rs5749426	0.3486	YES	5.366E-1 (pos.32795641)		RNA 2-prim	ATP binding,RNA binding,RNA li		Sucrose	C=0, T=63	C=0.0, T=1.0	0.00001	958.907	63	C=0/0, T=41/2
chr4:84232104	C/C	T	C	SNV	HPSE	HPSE:intronic:NM 001098540.2	1	100		intronic	NM 00109	c.674-61A	p.?	-1.73					Glycosyl hydr	rs6535455		YES			Heparanas	angiogene	Autoimmune Disea	Dalteparin,Heparin	T=0, C=31	T=0.0, C=1.0	0.00001	465.814	31	T=0/0, C=7/24
chr4:84367050	G/A	G	A	SNV	HELQ	HELQ:intronic:NM 133636.3	1	53.49		intronic	NM 13363	c.1465+11	p.?	0.48					DEAD/DEAH b	rs11099599		YES			Helicase, P	ATP bindin	Nervous System Diseases;Neuromuscular Di:		G=40, A=46	G=0.4651, A=0.5349	0.00001	513.968	86	G=19/21, A=2
chr22:32808078	G/A	G	A	SNV	RTCB	RTCB:exonic:NM 014306.4	1	59.09	1	synonymous	NM 01430	c.66C>T	p.(=)	3.38				0.91(COSM37	tRNA-splicing	rs11545749	0.0433	YES	1.082E-2;0.0 (pos.32808(	adenocarcinoi	RNA 2-prim	ATP binding,RNA binding,RNA li		Sucrose	G=27, A=39	G=0.4091, A=0.5909	0.00001	424.767	66	G=9/18, A=16/
chr10:63958112	T/C	T	C	SNV	RTKN2	RTKN2:exonic:NM 145307.3	1	95.83	12	missense	NM 14530	c.1385A>G	p.His462Arg	1.67	0.13	29	0		Cell division	rs3125734	0.3588	YES	9.2E-1 (pos.63958112)			hemopoie	Arthritis;Arthritis, Rheumatoid;Autoimmune		T=2, C=46	T=0.0417, C=0.9583	0.00001	745.25	48	T=2/0, C=22/2
chr4:845772	C/C	T	C	SNV	GAK	GAK:intronic:NM 005255.3	1	100		intronic	NM 00525	c.3284-10	p.?	0.37					C2 domain of	rs10031922	0.04	YES	1.0 (pos.845772)		Cyclin G ass	ATP bindin	Basal Ganglia Diseases;Brain Diseases;Centr		T=0, C=25	T=0.0, C=1.0	0.00001	353.018	25	T=0/0, C=14/1
chr4:85625701	G/G	A	G	SNV	WDFY3	WDFY3:intronic:NM 014991.4	1	100		intronic	NM 01499	c.8336-10	p.?	0.72					Beige/BEACH	rs1838039		YES			WD repeat-	1-phosphatidylinositol binding,Atg12-Atg5-Atg16 comple			A=0, G=28	A=0.0, G=1.0	0.00001	408.718	28	A=0/0, G=11/1
chr3:187088926	A/A	G	A	SNV	RTP4	RTP4:exonic:NM 022147.2	1	100	2	missense	NM 02214	c.506G>A	p.Cys169Tyr	-1.11	1	194	0		Zinc-binding	rs1533594	0.1758	YES	9.572E-1 (pos.187088926)		Receptor-tr	cell surfac	Influenza, Human;Respiratory Tract Diseases		G=0, A=51	G=0.0, A=1.0	0.00001	754.583	51	G=0/0, A=24/2
chr18:67718688	A/G	A	G	SNV	RTTN	RTTN:exonic:NM 173630.3	1	52.38	39	missense	NM 17363	c.5282T>C	p.Phe1761Ser	-1.4	1	155	0		Rotatin, an a	rs4891392	0.0376	YES	9.994E-1 (pos.67718688)		Rotatin	centrosome,ciliary basal body,cilium orga		Benign/Likely	A=40, G=44	A=0.4762, G=0.5238	0.00001	482.875	84	A=20/20, G=1
chr4:860146	G/G	A	G	SNV	GAK	GAK:intronic:NM 005255.3	1	100		intronic	NM 00525	c.3043+6T	p.?	0.15					C2 domain of	rs2306248	0.3679	YES	6.574E-1 (pos.860146)		Cyclin G ass	ATP bindin	Basal Ganglia Diseases;Brain Diseases;Centr		A=0, G=31	A=0.0, G=1.0	0.00001	465.81	31	A=0/0, G=10/2
chr5:179020622	T/C	T	C	SNV	RUFY1	RUFY1:exonic:NM 025158.4	1	50	11	synonymous	NM 02515	c.1389T>C	p.(=)	-0.57				0.29(COSM37	FYVE zinc fing	rs4701135	0.1114	YES	8.768E-2 (pos.17902062	adenocarcinoi	RUN and FY	SH2 domain binding,SH3 domain binding,cytosol,early er			T=92, C=92	T=0.5, C=0.5	0.00001	1003.08	184	T=47/45, C=54
chr4:8602793	C/C	T	C	SNV	CPZ	CPZ:intronic:NM 001014447.2	1	100		intronic	NM 00101	c.122-57T:	p.?	-2.66					Carboxypepti	rs4299567		YES			Carboxypep	Wnt signaling pathway,extracellular exosome,extracell			T=0, C=23	T=0.0, C=1.0	0.00001	316.747	23	T=0/0, C=8/15
chr4:8602798	C/C	G	C	SNV	CPZ	CPZ:intronic:NM 001014447.2	1	100		intronic	NM 00101	c.122-52G	p.?	-1.5					Carboxypepti	rs2241069	0.4415	YES			Carboxypep	Wnt signaling pathway,extracellular exosome,extracell			G=0, C=23	G=0.0, C=1.0	0.00001	316.746	23	G=0/0, C=8/15
chr4:8602816	G/G	A	G	SNV	CPZ	CPZ:intronic:NM 001014447.2	1	100		intronic	NM 00101	c.122-34A	p.?	-0.31					Carboxypepti	rs386671388,rs4594	1.00E-04		9.998E-1 (pos.8602816)		Carboxypep	Wnt signaling pathway,extracellular exosome,extracell			A=0, G=24	A=0.0, G=1.0	0.00001	334.783	24	A=0/0, G=8/16
chr10:70105560	A/A	G	A	SNV	RUFY2	RUFY2:exonic:NM 017987.4	1	100	18	synonymous	NM 01798	c.1891C>T	p.(=)	0.1					FYVE zinc fing	rs1162753	0.1474	YES	8.487E-1 (pos.70105560)		RUN and FY	SH3 domain binding,biological_process,cytoplasm,endo			G=0, A=36	G=0.0, A=1.0	0.00001	563.727	36	G=0/0, A=20/1
chr1:95709821	C/C	T	C	SNV	RWDD3,TMEM56-RWDD3	RWDD3:exonic:NM 015485.4, TMEM56-RWDD3:utr 3:NM (	1	100	2	missense	NM 00119	c.621T>C,	p.?, p.Val47Ala	-0.51	0.96	64	0		RWD domain	rs259358	5.00E-04		1.0 (pos.95709821)		RWD domai	integral component of membrane			T=0, C=101	T=0.0, C=1.0	0.00001	1579.75	101	T=0/0, C=44/5
chr4:8608634	T/T	C	T	SNV	CPZ	CPZ:intronic:NM 001014447.2	1	100		intronic	NM 00101	c.1068+9C	p.?	-0.22					Carboxypepti	rs2302580	0.4308	YES	5.239E-1 (pos.8608634)		Carboxypep	Wnt signaling pathway,extracellular exosome,extracell			C=0, T=21	C=0.0, T=1.0	0.00001	285.726	21	C=0/0, T=6/15
chr4:8608934	T/T	C	T	SNV	CPZ	CPZ:intronic:NM 001014447.2	1	100		intronic	NM 00101	c.1069-60(	p.?	-0.68					Carboxypepti	rs2302579		YES			Carboxypep	Wnt signaling pathway,extracellular exosome,extracell			C=0, T=42	C=0.0, T=1.0	0.00001	684.459	42	C=0/0, T=27/1
chr4:8608979	A/A	G	A	SNV	CPZ	CPZ:intronic:NM 001014447.2	1	100		intronic	NM 00101	c.1069-15(	p.?	-1.42					Carboxypepti	rs2302578	0.4912	YES	5.091E-1 (pos.8608979)		Carboxypep	Wnt signaling pathway,extracellular exosome,extracell			G=0, A=44	G=0.0, A=1.0	0.00001	726.569	44	G=0/0, A=17/2
chr4:184570715	G/G	T	G	SNV	RWDD4	RWDD4:exonic:NM 152682.3	1	100	5	missense	NM 15268	c.370A>C	p.Ile124Leu	1.57		5		0.61(COSM44	RWD domain	rs10015804	0.2354	YES	1.314E-1 (pos.18457071	acute_myeloid leukaemia,carcinoma					T=0, G=143	T=0.0, G=1.0	0.00001	2334.29	143	T=0/0, G=61/8
chr13:32360547	G/G	A	G	SNV	RXFP2	RXFP2:exonic:NM 130806.4	1	100	12	synonymous	NM 13080	c.957A>G	p.(=)	0.23					7 transmemb	rs7325513	0.4008	YES	7.767E-1 (pos.32360547)			G-protein	Congenital Abnormalities;Congenital, Heredi		A=0, G=56	A=0.0, G=1.0	0.00001	832.1	56	A=0/0, G=32/2
chr19:38995438	C/C	T	C	SNV	RYR1	RYR1:exonic:NM 000540.2	1	100	51	synonymous	NM 00054	c.8118T>C	p.(=)	-1.89				0.02(COSM14	EF-hand dom	rs2960340	0.2492	YES	3.426E-1 (pos.38995438)	adenocarcinoi	Ryanodine	ATP bindin	ACMG Recommend	Caffeine,C Benign	T=0, C=28	T=0.0, C=1.0	0.00001	408.717	28	T=0/0, C=11/1
chr19:38995510	C/C	T	C	SNV	RYR1	RYR1:exonic:NM 000540.2	1	100	51	synonymous	NM 00054	c.8190T>C	p.(=)	-3.21				0.03(COSM14	EF-hand dom	rs2915951	0.2545	YES	3.434E-1 (pos.38995510)	acute_myeloic	Ryanodine	ATP bindin	ACMG Recommend	Caffeine,C Benign	T=0, C=38	T=0.0, C=1.0	0.00001	603.786	38	T=0/0, C=21/1
chr19:38995975	A/A	G	A	SNV	RYR1	RYR1:exonic:NM 000540.2	1	100	53	synonymous	NM 00054	c.8337G>A	p.(=)	3.16				0.91(COSM14	EF-hand dom	rs2915952	0.2476	YES	3.422E-1 (pos.38995975)	adenocarcinoi	Ryanodine	ATP bindin	ACMG Recommend	Caffeine,C Benign	G=0, A=111	G=0.0, A=1.0	0.00001	1762.71	111	G=0/0, A=9/10
chr4:87731238	A/A	G	A	SNV	PTPN13	PTPN13:intronic:NM 080685.2	1	100		intronic	NM 08068	c.7314+10	p.?	-0.09					FERM C-termi	rs11930396		YES			Protein tyrc	bicellular	Nervous System Diseases;Neuromuscular Di:		G=0, A=67	G=0.0, A=1.0	0.00001	1034.38	67	G=0/0, A=32/3
chr19:39002725	G/G	A	G	SNV	RYR1	RYR1:exonic:NM 000540.2	1	100	62	synonymous	NM 00054	c.9186A>G	p.(=)	-3.83				0.03(COSM37	EF-hand dom	rs2071089	0.2513	YES	3.409E-1 (pos.39002725)	adenocarcinoi	Ryanodine	ATP bindin	ACMG Recommend	Caffeine,C Benign	A=0, G=37	A=0.0, G=1.0	0.00001	583.706	37	A=0/0, G=20/1
chr4:87812564	C/C	G	C	SNV	C4orf36	C4orf36:intronic:NM 144645.3	1	100		intronic	NM 14464	c.65+45C>	p.?	-0.65					Protein of unl	rs10011798	0.4544	YES	3.152E-1 (pos.87812564)				Amyotrophic Lateral Sclerosis;Central Nervo		G=0, C=48	G=0.0, C=1.0	0.00001	810.435	48	G=0/0, C=26/2
chr19:38949904	C/T	C	T	SNV	RYR1	RYR1:exonic:NM 000540.2	1	97.3	19	synonymous	NM 00054	c.2286C>T	p.(=)	-6.73				0.01(COSM41	EF-hand dom	rs3745847	0.3421	YES	6.159E-1 (pos.38949904)	neoplasm	Ryanodine	ATP bindin	ACMG Recommend	Caffeine,C Benign	C=2, T=72	C=0.027, T=0.973	0.00001	1108.29	74	C=0/2, T=41/3
chr4:88401737	A/A	ATA	A	INDEL	SPARCL1	SPARCL1:intronic:NM 004684.5	2	100		intronic	NM 00468	c.1669-65	p.?	0.33, -0.04, 0.38					Follistatin/O:	rs112085933,rs34452129,rs558		YES				anatomic:	Brain Diseases;Central Nervous System Dise:		ATA=0, A=3	ATA=0.0, A=1.0	0.00001	446.639	30	ATA=0/0, A=13
chr19:38976655	C/T	C	T	SNV	RYR1	RYR1:exonic:NM 000540.2	1	57.14	34	missense	NM 00054	c.5360C>T	p.Pro1787Leu	0.74	0.01	98	0.009		EF-hand dom	rs34934920	0.0232	YES	1.183E-4 (pos.38976655)		Ryanodine	ATP bindin	ACMG Recommend	Caffeine,C Benign/Likely	C=15, T=20	C=0.4286, T=0.5714	0.00001	218.694	35	C=12/3, T=7/1
chr19:38991543	G/A	G	A	SNV	RYR1	RYR1:exonic:NM 000540.2	1	52.38	47	synonymous	NM 00054	c.7527G>A	p.(=)	0.38				0.72(COSM50	EF-hand dom	rs2071088	0.0862	YES	1.595E-1 (pos.38991543)	adenocarcinoi	Ryanodine	ATP bindin	ACMG Recommend	Caffeine,C Benign	G=10, A=11	G=0.4762, A=0.5238	0.00001	95.3159	21	G=7/3, A=4/7
chr1:237711797	G/G	A	G	SNV	RYR2	RYR2:exonic:NM 001035.2	1	100	26	synonymous	NM 00103	c.2973A>G	p.(=)	-0.49				0.08(COSM41	EF-hand dom	rs2253273	0.0415	YES	8.849E-1;0.0;0.0 (pos.23	neoplasm	Ryanodine	BMP signa	ACMG Recommend	Calcium Ci Benign/Likely	A=0, G=63	A=0.0, G=1.0	0.00001	958.777	63	A=0/0, G=21/4
chr1:237890437	T/T	C	T	SNV	RYR2	RYR2:exonic:NM 001035.2	1	100	76	synonymous	NM 00103	c.10776C>	p.(=)	-3.35					EF-hand dom	rs2685301	5.00E-04	YES	1.0 (pos.237890437)		Ryanodine	BMP signa	ACMG Recommend	Calcium Ci Benign/Likely	C=0, T=33	C=0.0, T=1.0	0.00001	504.598	33	C=0/0, T=11/2
chr15:33954652	T/T	C	T	SNV	RYR3	RYR3:exonic:NM 001036.4	1	100	35	missense	NM 00103	c.4921C>T	p.Arg1641Cys	-0.11	0.03	180	0.99		EF hand,EF-ha	rs4780144	0.0359	YES	9.282E-1 (pos.33954652)		Ryanodine	calcium ion binding,calcium io	Calcium Citrate,Calcium		C=0, T=32	C=0.0, T=1.0	0.00001	485.13	32	C=0/0, T=28/4
chr15:34016274	A/A	G	A	SNV	RYR3	RYR3:exonic:NM 001036.4	1	100	45	missense	NM 00103	c.6809G>A	p.Gly2270Glu	2.1	0.48	98	0		EF hand,EF-ha	rs6495228	0.1481	YES	9.814E-1 (pos.34016274)		Ryanodine	calcium ion binding,calcium io	Calcium Citrate,Calcium		G=0, A=33	G=0.0, A=1.0	0.00001	504.598	33	G=0/0, A=13/2
chr1:153587308	T/T	C	T	SNV	S100A14	S100A14:utr 3:NM 020672.2	1	100	4	utr 3	NM 02067	c.368G>A	p.?	0.59					S-100/ICaBP t	rs11548104		YES			S100 Calciu	apoptotic process,calcium ion binding,calcium ion home			C=0, T=48	C=0.0, T=1.0	0.00001	810.435	48	C=0/0, T=28/2
chr1:153431406	G/G	C	G	SNV	S100A7	S100A7:exonic:NM 002963.3	1	100	2	missense	NM 00296	c.84G>C	p.Glu28Asp	0.24	1	45	0	0(COSM5003:	S-100/ICaBP t	rs3014837	0.056	YES	1.0 (pos.153431406)	ductal_carcinc	S100 calciu	RAGE rece	Psoriasis;Skin Dise:	1-Anilino-8-Naphthalene	C=0, G=33	C=0.0, G=1.0	0.00001	504.592	33	C=0/0, G=16/1
chr4:89015857	T/T	C	T	SNV	ABCG2	ABCG2:intronic:NM 004827.2	1	100		intronic	NM 00482	c.1738-46(	p.?	-0.08					ABC transpor	rs2231164	0.1348	YES	5.366E-1;0.0 (pos.89015857)		ATP-binding	ATP bindin	Adenoma;Arthritis;	Afatinib,Alectinib,Alvoci	C=0, T=100	C=0.0, T=1.0	0.00001	1695.23	100	C=0/0, T=16/8
chr4:89016782	G/G	A	G	SNV	ABCG2	ABCG2:intronic:NM 004827.2	1	100		intronic	NM 00482	c.1648-21	p.?	0.34					ABC transpor	rs2231162	0.0355	YES	7.94E-1 (pos.89016782)		ATP-binding	ATP bindin	Adenoma;Arthritis;	Afatinib,Alectinib,Alvoci	A=0, G=22	A=0.0, G=1.0	0.00001	298.848	22	A=0/0, G=6/16
chr4:89018565	A/G	A	G	SNV	ABCG2	ABCG2:intronic:NM 004827.2	1	58.33		intronic	NM 00482	c.1647+40	p.?	-0.47					ABC transpor	rs2231157	0.4645	YES	2.965E-1 (pos.89018565)		ATP-binding	ATP bindin	Adenoma;Arthritis;	Afatinib,Alectinib,Alvoci	A=9, G=14	A=0.3913, G=0.6087	0.00001	149.899	24	A=6/3, G=7/7
chr4:89389640	A/A	C	A	SNV	HERC5	HERC5:intronic:NM 016323.3	1	100		intronic	NM 01632	c.1134+67	p.?	0.41					HECT-domain	rs4585264		YES			HECT domai	ISG15 tran	Endometriosis;Female Urogenital Diseases;F		C=0, A=22	C=0.0, A=1.0	0.00001	298.759	22	C=0/0, A=5/17
chr4:8951816	G/G	A	G	SNV			1	100		intronic				-0.65					7 transmemb	rs3952877	0.0057		1.0;0.0;0.0 (pos.8951816)						A=0, G=32	A=0.0, G=1.0	0.00001	485.14	32	A=0/0, G=19/1
chr4:89575324	A/A	T	A	SNV	HERC3	HERC3:intronic:NM 014606.2	1	100		intronic	NM 01460	c.777+40T	p.?	0.12					HECT-domain	rs2860427	0.2598	YES	5.275E-1;0.0 (pos.89575324)		HECT domai	cytoplasm	Digestive System Diseases;Digestive System		T=0, A=42	T=0.0, A=1.0	0.00001	685.217	42	T=0/0, A=23/1
chr4:89585235	G/G	A	G	SNV	HERC3	HERC3:intronic:NM 014606.2	1	100		intronic	NM 01460	c.1272-69	p.?	0.23					HECT-domain	rs2972018	0.2429	YES			HECT domai	cytoplasm	Digestive System Diseases;Digestive System		A=0, G=93	A=0.0, G=1.0	0.00001	1555.33	93	A=0/0, G=59/3
chr4:89588689	T/T	C	T	SNV	HERC3	HERC3:intronic:NM 014606.2	1	100		intronic	NM 01460	c.1443+50	p.?	-0.31					HECT-domain	rs3017921	0.2834	YES	5.247E-1 (pos.89588689)		HECT domai	cytoplasm	Digestive System Diseases;Digestive System		C=0, T=257	C=0.0, T=1.0	0.00001	4208.66	257	C=0/0, T=101/
chr4:89660161	C/T	C	T	SNV	FAM13A	FAM13A:intronic:NM 014883.3	1	45.83		intronic	NM 01488	c.2562+20	p.?	-0.7					RhoGAP dom	rs116320485	3.00E-04		2.313E-4 (pos.89660161)		Family with	GTPase ac	Bronchial Diseases;Bronchitis;Bronchitis, Ch		C=26, T=22	C=0.5417, T=0.4583	0.00001	240.97	48	C=10/16, T=14
chr1:153410822	A/A	C	A	SNV	S100A7L2	S100A7L2:exonic:NM 001045479.1	1	100	2	missense	NM 00104	c.17G>T	p.Gly6Val	-0.07	0.01	109	0.159		S-100/ICaBP t	rs10888561	0.2638	YES	5.647E-1;0.0 (pos.153410822)						C=0, A=48	C=0.0, A=1.0	0.00001	810.435	48	C=0/0, A=17/3
chr4:6698706	C/C	A	C	SNV	S100P	S100P:exonic:NM 005980.2	1	100	2	synonymous	NM 00598	c.225A>C	p.(=)	-1.42					EF-hand dom	rs11733989	2.00E-04		1.0;0.0 (pos.6698706)		S100 calciu	RAGE rece	Adnexal Diseases;C	Cromoglicic acid	A=0, C=147	A=0.0, C=1.0	0.00001	2488.41	147	A=0/0, C=74/7
chr4:942006	G/A	G	A	SNV	TMEM175	TMEM175:intronic:NM 032326.3	1	57.14		intronic	NM 03232	c.192+64G	p.?	-0.84					Protein of unl	rs2290403	0.3671	YES	2.085E-1 (pos.942006)		Transmemb	endosome	Basal Ganglia Diseases;Brain Diseases;Centr		G=9, A=12	G=0.4286, A=0.5714	0.00001	109.08	21	G=2/7, A=5/7
chr4:95255637	G/G	T	G	SNV	HPGDS	HPGDS:intronic:NM 014485.2	1	100		intronic	NM 01448	c.133+11A	p.?	-0.46					Glutathione S	rs2289186	0.3649	YES	3.019E-1 (pos.95255637)		Prostaglanc	calcium io	Digestive System Di	1-PHENYL-1H-PYRAZOLE-4	T=0, G=26	T=0.0, G=1.0	0.00001	371.42	26	T=0/0, G=18/8
chr4:95529430	T/T	C	T	SNV	PDLIM5	PDLIM5:intronic:NM 001256426.1	1	100		intronic	NM 00125	c.731+101	p.?	0.79					Domain of un	rs10033359		YES					Cardiovascular Diseases;Genital Diseases, M		C=0, T=54	C=0.0, T=1.0	0.00001	798.11	54	C=0/0, T=10/4
chr4:95529444	A/A	C	A	SNV	PDLIM5	PDLIM5:intronic:NM 001256426.1	1	100		intronic	NM 00125	c.731+115	p.?	0.54					Domain of un	rs9307146		YES					Cardiovascular Diseases;Genital Diseases, M		C=0, A=52	C=0.0, A=1.0	0.00001	767.866	52	C=0/0, A=9/43
chr1:101704532	T/T	G	T	SNV	S1PR1	S1PR1:utr 5:NM 001400.4	1	100	2	utr 5	NM 00140	c.-9G>T	p.?	-0.34					7 transmemb	rs3737577,rs773052	0.3281	YES	1.581E-1;0.0 (pos.101704532)		Sphingosine	G-protein	Mammary Neoplas	Asfotase Alfa	G=0, T=90	G=0.0, T=1.0	0.00001	1476.02	90	G=0/0, T=56/3
chr11:18253176	T/T	C	T	SNV	SAA2-SAA4,SAA4	SAA4:exonic:NM 006512.3, SAA2-SAA4:exonic:NM 001199	1	100	6	missense	NM 00651	c.266G>A,	p.Cys89Tyr, p.Cys167Tyr	-1.54	1.0, 1.0	194.0, 194	0.0, 0.0		Serum amylo	rs2460827	0.0278	YES	9.02E-1 (pos.18253176)		Serum amyl	acute-phase response,cell chemotaxis,chemoattractan			C=0, T=38	C=0.0, T=1.0	0.00001	603.785	38	C=0/0, T=22/1
chr13:23907677	T/T	C	T	SNV	SACS	SACS:exonic:NM 014363.5	1	100	10	synonymous	NM 01436	c.10338G>	p.(=)	-1.17					HEPN domain	rs2737701	1.00E-04		1.0 (pos.23907677)		Sacsin	Hsp70 pro	Ataxia;Brain Diseases;Central	Benign/Likely	C=0, T=69	C=0.0, T=1.0	0.00001	1078.6	69	C=0/0, T=34/3
chr13:23985391	C/C	T	C	SNV	SACS	SACS:utr 5:NM 014363.5	1	100	2	utr 5	NM 01436	c.-13A>G	p.?	0.43					HEPN domain	rs17078720	0.3268	YES	4.782E-1 (pos.23985391)		Sacsin	Hsp70 pro	Ataxia;Brain Diseases;Central	Benign/Likely	T=0, C=62	T=0.0, C=1.0	0.00001	938.65	62	T=0/0, C=42/2
chr16:51173559	A/A	G	A	SNV	SALL1	SALL1:exonic:NM 002968.2	1	100	2	synonymous	NM 00296	c.2574C>T	p.(=)	-2.43				0.06(COSM11	C2H2-type zir	rs1965024	0.3601	YES	7.091E-1;0.0 (pos.51173	acute_myeloic	Sal-like 1	DNA bindi	Abnormalities, Multiple;Anus,	Benign	G=0, A=94	G=0.0, A=1.0	0.00001	1575.02	94	G=0/0, A=48/4
chr4:995422	G/C	G	C	SNV	IDUA	IDUA:intronic:NM 000203.4	1	50		intronic	NM 00020	c.590-45G	p.?	-0.01					Glycosyl hydr	rs6829789	0.1603	YES	2.252E-1;0.0 (pos.995422)		Iduronidase	L-iduronid	Carbohydrate Metabolism, Inb	Benign	G=21, C=21	G=0.5, C=0.5	0.00001	228.667	42	G=11/10, C=1
chr4:996012	G/C	G	C	SNV	IDUA	IDUA:intronic:NM 000203.4	1	50		intronic	NM 00020	c.973-45G	p.?	-1.53				0.02(COSM41	Glycosyl hydr	rs6831021	0.1436	YES	3.543E-1;0.0 (pos.99601	adenocarcinoi	Iduronidas	L-iduronid	Carbohydrate Metabolism, Inb	Benign	G=41, C=41	G=0.5, C=0.5	0.00001	436.176	82	G=4/37, C=14
chr16:51175658	T/C	T	C	SNV	SALL1	SALL1:exonic:NM 002968.2	1	45.45	2	missense	NM 00296	c.475A>G	p.Ser159Gly	-0.08	0.44	56	0.034	0.33(COSM11	C2H2-type zir	rs13336129,rs74799	0.063	YES	0.0 (pos.51175657),0.0:1	adenocarcinoi	Sal-like 1	DNA bindi	Abnormalities, Multiple;Anus,	Benign	T=18, C=15	T=0.5455, C=0.4545	0.00001	143.15	33	T=11/7, C=8/7

chr14:21993498	A/A	G	A	SNV	SALL2	SALL2:exonic:NM 005407.2	1	100	2	missense	NM 00540	c.364C>T	p.Pro122Ser	0.02	1	74	0		C2H2-type zir	rs1263811	0.1355	YES	7.949E-1 (pos.21993498)		Sal-like 2	DNA bindi	Coloboma;Congenital Abnormalities;Congen	G=0, A=23	G=0.0, A=1.0	0.00001	316.745	23	G=0/0, A=7/16
chr5:101724307	T/T	A	T	SNV	SLCO6A1	SLCO6A1:intronic:NM 173488.4	1	100		intronic	NM 17348	c.2017+85	p.?	-0.93					Kazal-type se	rs6873305		YES			Solute carri	integral cc	Mental Disorders:Schizophrenia:Schizophren	A=0, T=78	A=0.0, T=1.0	0.00001	1249.36	78	A=0/0, T=37/4
chr5:101755879	T/T	C	T	SNV	SLCO6A1	SLCO6A1:intronic:NM 173488.4	1	100		intronic	NM 17348	c.1277-15	p.?	-0.4					Kazal-type se	rs896546		YES			Solute carri	integral cc	Mental Disorders:Schizophrenia:Schizophren	C=0, T=24	C=0.0, T=1.0	0.00001	334.79	24	C=0/0, T=14/1
chr18:76753768	G/G	C	G	SNV	SALL3	SALL3:exonic:NM 171999.3	1	100	2	missense	NM 17199	c.1777C>G	p.Leu593Val	0.29	0.28	32	0.006	0.12(COSM11	C2H2-type zir	rs2447437	0.1269	YES	9.261E-1 (pos.76753768)	adenocarcinoı	Sal-like 3	DNA bindi	Burkitt Lymphoma;Congenital Abnormalities	C=0, G=110	C=0.0, G=1.0	0.00001	1755.95	110	C=0/0, G=65/4
chr18:76754466	T/T	C	T	SNV	SALL3	SALL3:exonic:NM 171999.3	1	100	2	synonymous	NM 17199	c.2475C>T	p.(=)	1.08				0.46(COSM37	C2H2-type zir	rs2472644	0.1463	YES	9.225E-1 (pos.76754466)	adenocarcinoı	Sal-like 3	DNA bindi	Burkitt Lymphoma;Congenital Abnormalities	C=0, T=90	C=0.0, T=1.0	0.00001	1479.7	90	C=0/0, T=34/5
chr8:29927300	G/G	C	G	SNV	SARAF	SARAF:exonic:NM 016127.5	1	100	3	synonymous	NM 01612	c.558G>C	p.(=)	0.65				0.07(COSM62	SOCE-associa	rs6468093	0		1.0 (pos.29927300)	neoplasm	Transmemb	calcium ion transport,endoplasmic reticulum,integral cc		C=0, G=39	C=0.0, G=1.0	0.00001	623.98	39	C=0/0, G=26/1
chr5:102489511	T/G	T	G	SNV	PPIP5K2	PPIP5K2:intronic:NM 015216.4	1	50		intronic	NM 01521	c.1131-41	p.?	0.96					Histidine phc	rs34767	0.2894	YES	4.655E-1;0.0 (pos.102489511)		Histidine ac	ATP binding,cytosol,diphosphoinositol-pentakisphospha		T=16, G=18	T=0.4706, G=0.5294	0.00001	188.385	36	T=4/12, G=4/1
chr8:29927321	G/G	A	G	SNV	SARAF	SARAF:exonic:NM 016127.5	1	100	3	synonymous	NM 01612	c.537T>C	p.(=)	-2.6					SOCE-associa	rs6468094	1.00E-04		1.0;0.0 (pos.29927321)		Transmemb	calcium ion transport,endoplasmic reticulum,integral cc		A=0, G=39	A=0.0, G=1.0	0.00001	623.98	39	A=0/0, G=26/1
chr17:26699367	GC/GC	G	GC	INDEL	SARM1	SARM1:exonic:NM 015077.3	1	100	4	unknown	NM 015077.3			1.01					SAM domain	rs11437594	0.0042	YES	1.0 (pos.26699367)			axon,cell junction,cytoplasm,cytosol,dendrite,extrinsic		G=0, GC=8(	G=0.0, GC=1.0	0.00001	1232.38	80	G=0/0, GC=53/
chr17:26727721	G/G	GA	G	INDEL	SARM1,SLC46A1	SLC46A1:splicesite 3:NM 080669.5, SARM1:utr 3:NM 015(	1	100	13	utr 3	NM 01507	c.6589del	p.?	2.72, 0.94					Major Facilit	rs199895119,rs2005(	2.00E-04	YES	1.0 (pos.26727721)		Solute carri	apical pla:	Amino Acid Metabo Aminopte Benign	GA=0, G=8!	GA=0.0, G=1.0	0.00001	1365.98	85	GA=0/0, G=28/
chr5:102895209	A/A	AG	A	INDEL	NUDT12	NUDT12:intronic:NM 031438.3	1	100		intronic	NM 03143	c.207-40C	p.?	0.52, 0.66					Ankyrin repe:	rs11312358,rs79636:	0.1616	YES	7.196E-3 (pos.102895209)			NAD biosynthesis via nicotinamide riboside salvage path		AG=0, A=25	AG=0.0, A=1.0	0.00001	351.003	25	AG=0/0, A=9/1
chr5:102895241	A/A	C	A	SNV	NUDT12	NUDT12:intronic:NM 031438.3	1	100		intronic	NM 03143	c.207-72G	p.?	-0.79					Ankyrin repe:	rs7725090		YES				NAD biosynthesis via nicotinamide riboside salvage path		C=0, A=26	C=0.0, A=1.0	0.00001	371.42	26	C=0/0, A=9/17
chr5:1053341	A/G	A	G	SNV	SLC12A7	SLC12A7:intronic:NM 006598.2	1	50		intronic	NM 00659	c.3160+12	p.?	-0.12					Amino acid p	rs2241602		YES			Solute carri	cell volum	Female Urogenital Potassium Chloride	A=12, G=12	A=0.5, G=0.5	0.00001	100.706	24	A=7/5, G=10/2
chr5:1053396	C/G	C	G	SNV	SLC12A7	SLC12A7:intronic:NM 006598.2	1	50		intronic	NM 00659	c.3160+68	p.?	1.46					Amino acid p	rs2241603		YES			Solute carri	cell volum	Female Urogenital Potassium Chloride	C=16, G=16	C=0.5, G=0.5	0.00001	158.383	32	C=5/11, G=10/
chr5:10748231	T/T	C	T	SNV	DAP	DAP:intronic:NM 004394.2	1	100		intronic	NM 00439	c.152+56G	p.?	0.73					Death-associ	rs267944		YES			Aspartyl am	activation	Colitis:Colitis, Ulcerative:Colonic Diseases:D	C=0, T=36	C=0.0, T=1.0	0.00001	563.744	36	C=0/0, T=24/1
chr5:107684324	T/T	C	T	SNV	FBXL17	FBXL17:intronic:NM 001163315.2	1	100		intronic	NM 00116	c.1375-93	p.?	0.53					F-box domair	rs1862195		YES			F-box and leucine-rich repeat protein 17			C=0, T=22	C=0.0, T=1.0	0.00001	303.287	22	C=0/0, T=12/1
chr5:109090931	T/TA	T	TA	INDEL	MAN2A1	MAN2A1:intronic:NM 002372.3	1	58.33		intronic	NM 00237	c.708-99T	p.?	1.83					Alpha manno	rs148653396		YES			Mannosida	Golgi med	Mental Disorders:S (1R,2R,3R,4S,5R)-4-(BENZ	T=10, TA=1	T=0.4167, TA=0.583	0.00001	135.171	24	T=4/6, TA=8/6
chr12:108918146	T/T	C	T	SNV	SART3	SART3:exonic:NM 014706.3	1	100	18	synonymous	NM 01470	c.2661G>A	p.(=)	-2.21					LSM-interact	rs2303633	0.1656	YES	9.266E-1 (pos.108918146)		Squamous c	Cajal body	Congenital, Hereditary, and Neonatal Diseas	C=0, T=171	C=0.0, T=1.0	0.00001	2799.74	171	C=0/0, T=115/
chr22:50885775	G/G	A	G	SNV	SBF1	SBF1:exonic:NM 002972.3	1	100	40	synonymous	NM 00297	c.5559T>C	p.(=)	0.01				0.1(COSM440	DENN (AEX-3)	rs1053744	0.4576	YES	4.513E-1 (pos.50885775)	hepatocellula	SET-binding	Rab guany	Charcot-Marie-Tooth Disease; Benign	A=0, G=59	A=0.0, G=1.0	0.00001	885.363	59	A=0/0, G=41/1
chr5:110784822	G/G	A	G	SNV	CAMK4	CAMK4:intronic:NM 001744.5	1	100		intronic	NM 00174	c.551-5A>	p.?	-2.76					Protein kinas	rs2116824	0.2044	YES	5.529E-1 (pos.110784822)		Ca(2+)-calm	ATP bindin	Cocaine-Related Di 3-(((3S)-3,4-dihydroxybut	A=0, G=21	A=0.0, G=1.0	0.00001	281.235	21	A=0/0, G=11/1
chr19:56052770	T/T	C	T	SNV	SBK3	SBK3:exonic:NM 001199824.2	1	100	4	synonymous	NM 00119	c.522G>A	p.(=)	-4.17					Protein kinas	rs654439		YES	6.649E-1 (pos.56052770)			ATP binding,protein phosphorylation,protein serine/thre		C=0, T=68	C=0.0, T=1.0	0.00001	1053.52	68	C=0/0, T=16/5
chr5:11082762	C/C	T	C	SNV	CTNND2	CTNND2:intronic:NM 001332.3	1	100		intronic	NM 00133	c.2788+46	p.?	-1.91					Armadillo/be	rs2277053	0.2909	YES	9.248E-1 (pos.11082762)		Catenin, de	Wnt signa	Abnormalities, Multiple;Amyotrophic Lateral	T=0, C=25	T=0.0, C=1.0	0.00001	349.857	25	T=0/0, C=22/3
chr8:73982161	G/G	A	G	SNV	SBSPON	SBSPON:exonic:NM 153225.3	1	100	4	missense	NM 15322	c.556T>C	p.Trp186Arg	2.57	1	101	0	0.15(COSM63	Thrombospoı	rs2291219	0.2943	YES	3.039E-1 (pos.73982161)	carcinoma		extracellu	Crohn Disease;Digestive System Diseases;Ga	A=0, G=48	A=0.0, G=1.0	0.00001	809.377	48	A=0/0, G=18/3
chr5:111506069	A/A	G	A	SNV	EPB41L4A	EPB41L4A:intronic:NM 022140.4	1	100		intronic	NM 02214	c.1671-3C	p.?	-0.22					FERM C-termi	rs10040749	0.3675	YES	9.987E-1 (pos.111506069)		Erythrocyte	actomyosin structure organization,biological process,c		G=0, A=66	G=0.0, A=1.0	0.00001	1015.39	66	G=0/0, A=30/3
chr19:50154904	C/C	A	C	SNV	SCAF1	SCAF1:exonic:NM 021228.2	1	100	7	missense	NM 02122	c.1258A>C	p.Thr420Pro	-0.02	1	38	0	0(COSM3757044)		rs7251334			1.0 (pos.50154904)	adenocarcinoı	SR-related (	RNA binding,RNA splicing,mRNA processing,nucleus,prot		A=0, C=58	A=0.0, C=1.0	0.00001	867.38	58	A=0/0, C=23/3
chr19:50156896	C/C	T	C	SNV	SCAF1	SCAF1:exonic:NM 021228.2	1	100	7	synonymous	NM 02122	c.3250T>C	p.(=)	1.44						rs10401743	0.0749	YES	1.0 (pos.50156896)		SR-related (	RNA binding,RNA splicing,mRNA processing,nucleus,prot		T=0, C=51	T=0.0, C=1.0	0.00001	754.567	51	T=0/0, C=25/2
chr8:27737198	G/G	A	G	SNV	SCARA5	SCARA5:exonic:NM 173833.5	1	100	8	synonymous	NM 17383	c.1239T>C	p.(=)	0.21				0.23(COSM37	Collagen tripl	rs4732610	0.137	YES	8.025E-1 (pos.27737198)	adenocarcinoı	Scavenger r	cell surface,cellular iron ion homeostasis,cellular respo		A=0, G=64	A=0.0, G=1.0	0.00001	977.631	64	A=0/0, G=30/3
chr5:111595745	C/A	C	A	SNV	EPB41L4A	EPB41L4A:intronic:NM 022140.4	1	45.45		intronic	NM 02214	c.643-69G	p.?	-0.39					FERM C-termi	rs28620667		YES			Erythrocyte	actomyosin structure organization,biological process,c		C=18, A=15	C=0.5455, A=0.4545	0.00001	142.581	33	C=8/10, A=7/8
chr12:125284748	G/G	A	G	SNV	SCARB1	SCARB1:exonic:NM 005505.4	1	100	8	synonymous	NM 00550	c.1050T>C	p.(=)	-6.33				0.01(COSM37	CD36 family	rs5888	0.4828	YES	7.465E-1 (pos.12528474&	adenocarcinoı	Scavenger r	1-phospha	Adenocarcinoma:C Phosphatidyl serine	A=0, G=21	A=0.0, G=1.0	0.00001	281.235	21	A=0/0, G=6/15
chr10:102116311	A/C	A	C	SNV	SCD	SCD:exonic:NM 005063.4	1	59.09	5	missense	NM 00506	c.670A>C	p.Met224Leu	-3.82	1	15	0	0.1(COSM3997733)		rs2234970	0.4217	YES	3.946E-1 (pos.10211631:	acute myeloic	Solute carri	endoplasr	Adenocarcinoma:Autoimmune Diseases:Carı	A=9, C=13	A=0.4091, C=0.5909	0.00001	122.568	22	A=6/3, C=9/4
chr13:78146301	T/T	C	T	SNV	SCEL	SCEL:exonic:NM 144777.2	1	100	9	synonymous	NM 14477	c.522C>T	p.(=)	-1.55						rs678047	5.00E-04		1.0 (pos.78146301)		Sciellin	cornified envelope,cytoplasm,embryo development,epi		C=0, T=74	C=0.0, T=1.0	0.00001	1170.12	74	C=0/0, T=28/4
chr5:112385004	GC/GC	CA	GC	MNV	MCC	MCC:intronic:NM 001085377.1	2	100		intronic	NM 00108	c.2449-8T(	p.?	-0.28, -0.79					EF-hand dom:	rs202036404,rs7159(	0.0019	YES	6.226E-1 (pos.112385005),6.255E-1;0.0 (		Mutated in	Wnt signa	Adenoma;Adenomatous Polyposis Coli;Aden	CA=0, GC=9	CA=0.0, GC=1.0	0.00001	1618.52	97	CA=0/0, GC=4!
chr5:112385035	G/G	T	G	SNV	MCC	MCC:intronic:NM 001085377.1	1	100		intronic	NM 00108	c.2449-39,	p.?	-1.65					EF-hand dom:	rs7708603	0.4899	YES	6.156E-1;0.0 (pos.112385035)		Mutated in	Wnt signa	Adenoma;Adenomatous Polyposis Coli;Aden	T=0, G=96	T=0.0, G=1.0	0.00001	1618.01	96	T=0/0, G=42/5
chr14:31099738	G/G	A	G	SNV	SCFD1	SCFD1:exonic:NM 016106.3	1	100	3	missense	NM 01610	c.188A>G	p.Lys63Arg	8.72	0.08	26	0.023	0.95(COSM43	Sec1 family	rs229150	0.3419	YES	6.801E-1 (pos.31099738)	carcinoma,diffuse large B		COPII vesicle coating,Golgi cisterna membrane,Golgi tra		A=0, G=31	A=0.0, G=1.0	0.00001	462.166	31	A=0/0, G=9/22
chr4:129867280	A/A	T	A	SNV	SCLT1	SCLT1:exonic:NM 144643.3	1	100	16	missense	NM 14464	c.1321A>T	p.Ser441Cys	0.01	0.06	112	0.973	0.09(COSM5983423),0.09(C		rs10028124	0.3453	YES	6.882E-1 (pos.12986728(	squamous cel	Sodium cha	centriole,(	Lung Diseases:Lung Diseases, Obstructive:Pu	T=0, A=104	T=0.0, A=1.0	0.00001	1626.75	104	T=0/0, A=57/4
chr5:112926982	A/G	A	G	SNV	YTHDC2	YTHDC2:intronic:NM 022828.4	1	50		intronic	NM 02282	c.4029+41	p.?	-1.61					DEAD/DEAH b	rs2303717	0.4847	YES	3.05E-1 (pos.112926982)		YTH domain	ATP binding,ATP-dependent RNA helicase activity,N6-me		A=13, G=13	A=0.5, G=0.5	0.00001	119.121	26	A=6/7, G=8/5
chr2:238999883	T/C	T	C	SNV	SCLY,UBE2F-SCLY	SCLY:exonic:NM 016510.5, UBE2F-SCLY:exonic nc:NR 0379(	1	50	8	synonymous	NR 03790	c.933T>C	p.(=)	-3.36				0.05(COSM14	Aminotransfe	rs1128552	0.2241	YES	6.228E-2 (pos.23899988:	adenocarcinoı	Selenocyste	amino acid binding,catalytic ac	Pyridoxal Phosphate,Sel	T=20, C=20	T=0.5, C=0.5	0.00001	213.782	40	T=10/10, C=17
chr5:114462589	C/C	A	C	SNV	TRIM36	TRIM36:intronic:NM 018700.3	1	100		intronic	NM 01870	c.1833-35	p.?	-0.18					B-box zinc fin	rs2921623	2.00E-04		1.0 (pos.114462589)		Tripartite m	acrosomal vesicle,acrosome reaction,alpha-tubulin bin		A=0, C=20	A=0.0, C=1.0	0.00001	263.785	20	A=0/0, C=11/9
chr5:114598204	T/T	C	T	SNV	PGGT1B	PGGT1B:intronic:NM 005023.3	1	100		intronic	NM 00502	c.140+205	p.?	-0.08					Prenyltransfe	rs267307		YES			Protein ger	CAAX-prot	Autoimmune Disea 2-METHYL-(5-GERANYL-4-	C=0, T=136	C=0.0, T=1.0	0.00001	2258.86	136	C=0/0, T=87/4
chr3:38739845	G/G	A	G	SNV	SCN10A	SCN10A:exonic:NM 006514.3	1	100	27	synonymous	NM 00651	c.4866T>C	p.(=)	1.31				0.84(COSM60	Ion transport	rs6599242	0.0849	YES	9.703E-1 (pos.38739845)	adenocarcinoı	Sodium cha	AV node ce	Arrhythmias, Cardi Benzocain Benign	A=0, G=40	A=0.0, G=1.0	0.00001	644.234	40	A=0/0, G=22/1
chr3:38793989	G/A	G	A	SNV	SCN10A	SCN10A:exonic:NM 006514.3	1	58.33	11	synonymous	NM 00651	c.1476C>T	p.(=)	-1.29				0.01(COSM37	Ion transport	rs7617919	0.2523	YES	4.016E-1 (pos.38793989)	acute myeloic	Sodium cha	AV node ce	Arrhythmias, Cardi Benzocain Benign	G=30, A=42	G=0.4167, A=0.5833	0.00001	464.858	72	G=10/20, A=1!
chr3:38768300	T/C	T	C	SNV	SCN10A	SCN10A:exonic:NM 006514.3	1	50	16	missense	NM 00651	c.2884A>G	p.Ile962Val	0.05	0.29	29	0.437	0.06(COSM11	Ion transport	rs57326399	0.262	YES	3.919E-1 (pos.38768300)	adenocarcinoı	Sodium cha	AV node ce	Arrhythmias, Cardi Benzocain Benign	T=12, C=12	T=0.5, C=0.5	0.00001	106.709	24	T=4/8, C=6/6
chr5:115338732	A/A	G	A	SNV	LVRN	LVRN:intronic:NM 173800.4	1	100		intronic	NM 17380	c.1897+12	p.?	-0.18					ERAP1-like C-	rs1560080		YES			Laeverin	aminopeptidase activity,cell-cell signaling,cytoplasm,in		G=0, A=81	G=0.0, A=1.0	0.00001	1309.48	81	G=0/0, A=32/4
chr5:115339086	G/G	A	G	SNV	LVRN	LVRN:intronic:NM 173800.4	1	100		intronic	NM 17380	c.2037+9A	p.?	-1.02					ERAP1-like C-	rs6594929	0.168	YES	7.825E-1 (pos.115339086)		Laeverin	aminopeptidase activity,cell-cell signaling,cytoplasm,in		A=0, G=22	A=0.0, G=1.0	0.00001	298.892	22	A=0/0, G=15/7
chr5:115351150	G/G	T	G	SNV	LVRN	LVRN:intronic:NM 173800.4	1	100		intronic	NM 17380	c.2618+34	p.?	-0.17					ERAP1-like C-	rs10214076	0.3981	YES	7.018E-1 (pos.115351150)		Laeverin	aminopeptidase activity,cell-cell signaling,cytoplasm,in		T=0, G=50	T=0.0, G=1.0	0.00001	852.739	50	T=0/0, G=33/1
chr5:115389626	A/C	A	C	SNV	ARL14EPL	ARL14EPL:intronic:NM 001195581.1	1	58.33		intronic	NM 00119	c.97-85A>	p.?	0.31					ARF7 effector	rs954763		YES						A=20, C=28	A=0.4167, C=0.5833	0.00001	343.434	48	A=9/11, C=19/
chr5:115394357	C/C	T	C	SNV	ARL14EPL	ARL14EPL:intronic:NM 001195581.1	1	100		intronic	NM 00119	c.237-65T	p.?	-3.51					ARF7 effector	rs6860908								T=0, C=58	T=0.0, C=1.0	0.00001	867.367	58	T=0/0, C=33/2
chr3:38945560	G/G	A	G	SNV	SCN11A	SCN11A:exonic:NM 014139.2	1	100	12	synonymous	NM 01413	c.1638T>C	p.(=)	-0.52				0.1(COSM400	Ion transport	rs4073113	0.3495	YES	6.34E-1 (pos.38945560)	acute myeloic	Sodium cha	C-fiber,ext	Cardiovascular Dis Brivaracet Benign	A=0, G=61	A=0.0, G=1.0	0.00001	921.849	61	A=0/0, G=25/3
chr17:62018773	C/C	T	C	SNV	SCN4A	SCN4A:exonic:NM 000334.4	1	100	24	synonymous	NM 00033	c.4869A>G	p.(=)	-8.06				0.01(COSM54	IQ calmodulii	rs2070720	0.3529	YES	4.229E-1 (pos.62018773)	acute myeloic	Sodium cha	integral cc	Congenital, Heredit Brivaracet Benign	T=0, C=77	T=0.0, C=1.0	0.00001	1229.44	77	T=0/0, C=27/5
chr5:115827356	C/T	C	T	SNV	SEMA6A	SEMA6A:intronic:NM 020796.4	1	45.83		intronic	NM 02079	c.535+80G	p.?	-2.63					Plexin repeat	rs154599		YES			Semaphorir	animal organ morphogenesis,apoptotic process,axon,a>		C=13, T=11	C=0.5417, T=0.4583	0.00001	93.4319	24	C=5/8, T=2/9
chr5:118556350	T/T	C	T	SNV	DMXL1	DMXL1:intronic:NM 005509.5	1	100		intronic	NM 00550	c.8072+62	p.?	0.01					RAVE protein	rs2271059		YES			DMX-like 1	RAVE comı	Alzheimer Disease;Brain Diseases;Central Ne	C=0, T=72	C=0.0, T=1.0	0.00001	1130.87	72	C=0/0, T=47/2
chr17:62020348	C/C	T	C	SNV	SCN4A	SCN4A:exonic:NM 000334.4	1	100	23	missense	NM 00033	c.4126A>G	p.Asn1376Asp	4.8	1	23	0	0.28(COSM14	IQ calmodulii	rs2058194	0.4681	YES	5.118E-1 (pos.62020348)	acute myeloic	Sodium cha	integral cc	Congenital, Heredit Brivaracet Benign	T=0, C=45	T=0.0, C=1.0	0.00001	747.354	45	T=0/0, C=25/2
chr3:38674712	C/C	T	C	SNV	SCN5A	SCN5A:exonic:NM 001160161.1	1	100	2	synonymous	NM 00116	c.87A>G	p.(=)	-4.41					Cytoplasmic	rs6599230,rs794728!	0.1928	YES	6.902E-1 (pos.38674712)		Sodium cha	AV node ce	ACMG Recommend Ajmaline,/ Benign/Likely	T=0, C=31	T=0.0, C=1.0	0.00001	464.11	31	T=0/0, C=15/1
chr5:1212615	G/A	G	A	SNV	SLC6A19	SLC6A19:intronic:NM 001003841.2	1	57.89		intronic	NM 00100	c.663+16G	p.?	-1.72					Sodium:neur	rs75252137	0.0726	YES	1.12E-1;0.0 (pos.1212615)		Solute carri	amino aci	Amino Acid Metabolism, Inborn Errors;Amino	G=24, A=33	G=0.4211, A=0.5789	0.00001	342.316	57	G=15/9, A=17/
chr5:121726714	A/A	G	A	SNV	SNCAIP	SNCAIP:intronic:NM 005460.3	1	100		intronic	NM 00546	c.-45-70G>	p.?	-0.24					Ankyrin repe:	rs1946649		YES			Synuclein-a	cell death,cellular protein metabolic process,cytoplasm		G=0, A=43	G=0.0, A=1.0	0.00001	705.839	43	G=0/0, A=27/1
chr5:1225564	C/C	T	C	SNV	SLC6A18,SLC6A19	SLC6A19:downstream:NM 001003841.2, SLC6A18:utr 5:NM	1	100		intronic	NM 00100	c.*3545T>	p.?, p.?	-4.83					Sodium:neur	rs7728646	0.2209	YES	5.893E-1 (pos.1225564)		Solute carri	amino aci	Amino Acid Metabolism, Inborn Errors;Amino	T=0, C=38	T=0.0, C=1.0	0.00001	603.78	38	T=0/0, C=28/1
chr2:167334085	T/T	G	T	SNV	SCN7A	SCN7A:exonic:NM 002976.3	1	100	2	missense	NM 00297	c.122C>A	p.Thr41Asn	-0.62	1	65	0	0.01(COSM40	Ion transport	rs7565062	0.3211	YES	7.236E-1 (pos.16733408!	acute myeloic	Sodium cha	glial cell p	Autistic Disorder:Cl Brivaracetam,Valproic A	G=0, T=21	G=0.0, T=1.0	0.00001	281.172	21	G=0/0, T=12/9
chr12:52184271	C/C	T	C	SNV	SCN8A	SCN8A:exonic:NM 014191.3	1	100	25	synonymous	NM 01419	c.4509T>C	p.(=)	-0.85				0.08(COSM41	Cytoplasmic	rs303815	0.2528	YES	5.779E-1 (pos.52184271)	neoplasm	Sodium cha	ATP bindin	Ataxia;Brain Diseas Brivaracet Benign/Likely	T=0, C=22	T=0.0, C=1.0	0.00001	297.499	22	T=0/0, C=10/1
chr8:144885580	G/G	A	G	SNV	SCRIB	SCRIB:exonic:NM 182706.4	1	100	24	synonymous	NM 18270	c.3576T>C	p.(=)	-0.36				0.09(COSM37	Leucine rich	rs13281371	0.0882	YES	9.743E-1 (pos.14488558(	adenocarcinoı	Scribble, Dr	GDP metabolic process,GMP metabolic process,Scrib-AP		A=0, G=38	A=0.0, G=1.0	0.00001	603.776	38	A=0/0, G=21/1
chr2:175292580	TTCAAATTTAT(	TTCAAATTI	T	INDEL	SCRN3	SCRN3:exonic:NM 024583.4	13	50	8	frameshiftDeletion	NM 02458	c.1251 12	p.Asn417fs	6.47, 0.85, 2.45, 0.44, 1.22, 3.59, 2.58, 2.68, 4.36, 5.15, 0.85, -(					Peptidase fai	rs145699077,rs7607!	0.329	YES	0.0 (pos.175292591),0.0	acute myeloic	Secernin 3	dipeptidase activity,exocytosis,proteolysis		TTCAAATTT,	TTCAAATTTATCAG=0.	0.00001	258.01	46	TTCAAATTTATC
chr2:120199140	A/G	A	G	SNV	SCTR	SCTR:exonic:NM 002980.2	1	45.83	12	synonymous	NM 00298	c.1176T>C	p.(=)	-1.52					7 transmemb	rs1126839	0.1286	YES	9.106E-1 (pos.120199140)		Secretin rec	G-protein coupled receptor act	Secretin	A=13, G=11	A=0.5417, G=0.4583	0.00001	93.4361	24	A=10/3, G=10/
chr5:122685801	A/A	G	A	SNV	CEP120	CEP120:intronic:NM 153223.3	1	100		intronic	NM 15322	c.2581-18	p.?	0.52					C2 domain,C	rs485847		YES	1.0 (pos.122685801)		Centrosom:	astral mic	Abnormalities, Multiple;Bone Diseases:Bone	G=0, A=93	G=0.0, A=1.0	0.00001	1555.33	93	G=0/0, A=61/3
chr5:1232491	C/C	G	C	SNV	SLC6A18	SLC6A18:intronic:NM 182632.2	1	100		intronic	NM 18263	c.301+17G	p.?	-0.21					Sodium:neur	rs4975623	0.0789	YES	6.33E-1;0.0 (pos.1232491)		Solute carri	amino aci	Cardiovascular Diseases;Congenital, Heredit	G=0, C=32	G=0.0, C=1.0	0.00001	485.141	32	G=0/0, C=11/2
chr11:9052423	C/C	A	C	SNV	SCUBE2	SCUBE2:exonic:NM 020974.2	1	100	17	missense	NM 02097	c.2052T>G	p.His684Gln	0.31	1	24	0		CUB domain,	rs7395988	1.00E-04		1.0 (pos.9052423)		Signal pepti	calcium ion binding,cell differentiation,cell surface,extr:		A=0, C=29	A=0.0, C=1.0	0.00001	427.6	29	A=0/0, C=6/23
chr4:76878688	A/A	G	A	SNV	SDAD1	SDAD1:exonic:NM 018115.3	1	100	19	synonymous	NM 01811	c.1752C>T	p.(=)	2.57				0.93(COSM54	NUC130/3NT	rs2242470	0.2487	YES	3.803E-1 (pos.76878688)	acute myeloid leukaemia		actin cytoskeleton organization,intracellular,molecular		G=0, A=50	G=0.0, A=1.0	0.00001	852.748	50	G=0/0, A=26/2
chr2:20403794	T/T	A	T	SNV	SDC1	SDC1:exonic:NM 002997.4	1	100	3	missense	NM 00299	c.407T>A	p.Leu136Gln	-0.84	0.48	113	0		Syndecan dor	rs10205485	2.00E-04	YES	1.0 (pos.20403794)		Syndecan 1	Golgi lume	Nutritional and Metabolic Diseases	A=0, T=20	A=0.0, T=1.0	0.00001	263.783	20	A=0/0, T=11/9
chr5:231111	C/C	T	C	SNV	SDHA	SDHA:exonic:NM 004168.3	1	100	7	synonymous	NM 00416	c.891T>C	p.(=)	1.29				0.24(COSM41	FAD binding c	rs1126417	0.2418	YES	3.281E-1 (pos.231111)	neoplasm	Succinate d	anaerobic	Arthritis:Brain Dise 2-Hexylox Benign/Likely	T=0, C=75	T=0.0, C=1.0	0.00001	1181.48	75	T=0/0, C=46/2
chr1:17380497	T/T	G	T	SNV	SDHB	SDHB:exonic:NM 003000.2	1	100	1	synonymous	NM 00300	c.18C>A	p.(=)	-1.75					2Fe-2S iron-s	rs2746462	0.027	YES	9.986E-1;0.0 (pos.17380497)		Succinate d	2 iron, 2 su	ACMG Recommend 2-Hexylox Benign	G=0, T=27	G=0.0, T=1.0	0.00001	389.988	27	G=0/0, T=11/1
chr7:4116665	G/A	G	A	SNV	SDK1	SDK1:exonic:NM 152744.3	1	53.49	21	missense	NM 15274	c.3046G>A	p.Asp1016Asn	2	1	23	0.778		Fibronectin t	rs11978101	0.2826	YES	7.785E-2 (pos.4116665)		Sidekick, Dr	behavioral response to cocaine,cell junction,cell-cell jur		G=20, A=23	G=0.4651, A=0.5349	0.00001	260.993	43	G=9/11, A=16/
chr17:71503636	C/C	T	C	SNV	SDK2	SDK2:exonic:NM 001144952.1	1	100	2	synonymous	NM 00114	c.165A>G	p.(=)	-0.53				0.17(COSM63	Fibronectin t	rs9895895	0.3347	YES	4.758E-1 (pos.71503636)	carcinoma	Sidekick, Dr	camera-ty	Anxiety Disorders:Mental Disorders:Panic Di	T=0, C=53	T=0.0, C=1.0	0.00001	782.028	53	T=0/0, C=25/2
chr17:75190846	C/C	A	C	SNV	SEC14L1	SEC14L1:exonic:NM 003003.3	1	100	7	missense	NM 00300	c.562A>C	p.Thr188Pro	0.84	0.43	38	0		CRAL/TRIO do	rs673918	0		1.0 (pos.75190846)			Golgi apparatus,RIG-I binding,choline transport,cytoplas		A=0, C=25	A=0.0, C=1.0	0.00001	350.064	25	A=0/0, C=12/1
chr22:30864610	G/G	A	G	SNV	SEC14L3	SEC14L3:exonic:NM 174975.4	1	100	5	missense	NM 17497	c.308T>C	p.Ile103Thr	7.4	0.06	89	0.001	0.98(COSM40	CRAL/TRIO do	rs4820853	0.3345	YES	4.469E-1 (pos.30864610)	acute myeloic	Sec14-like 3	extracellular exosome,integra	Vitamin E	A=0, G=48	A=0.0, G=1.0	0.00001	810.223	48	A=0/0, G=22/2
chr22:30934816	T/C	T	C	SNV	SEC14L6	SEC14L6:exonic:NM 001193336.3	1	59.18	2	missense	NM 00119	c.128A>G	p.Gln43Arg	0.39	1	43	0	0.07(COSM41	CRAL/TRIO do	rs5753190		YES	6.303E-1 (pos.30934816)	neoplasm,squamous cell		integral component of membrane,intracellular,transpor		T=20, C=29	T=0.4082, C=0.5918	0.00001	360.521	49	T=7/13, C=18/
chr4:83788344	T/T	A	T	SNV	SEC31A	SEC31A:exonic:NM 001318120.1	1	100	11	synonymous	NM 00131	c.1008T>A	p.(=)	-0.3					Sec23-bindin	rs2045478	1.00E-04		1.0 (pos.83788344)		Sec31, yeas	COPII vesi	Hemic and Lymphatic Diseases;Immune Syst	A=0, T=59	A=0.0, T=1.0	0.00001	885.368	59	A=0/0, T=32/2
chr5:131822072	A/G	A	G	SNV	IRF1	IRF1:intronic:NM 002198.2	1	56		intronic	NM 00219	c.545-7T>	p.?	0.12					Interferon re	rs2070724	0.3203	YES	3.431E-1 (pos.131822072)		Interferon r	CD8-positi	Anemia:Anemia, Macrocytic:Brain Diseases:I	A=11, G=14	A=0.44, G=0.56	0.00001	134.331	25	A=6/5, G=7/7
chr5:131822133	T/C	T	C	SNV	IRF1	IRF1:intronic:NM 002198.2	1	50		intronic	NM 00219	c.545-68A	p.?	-0.64					Interferon re	rs765689655,rs9282763		YES	0.0 (pos.131822132),3.439E-1 (pos.1318		Interferon r	CD8-positi	Anemia:Anemia, Macrocytic:Brain Diseases:I	T=15, C=15	T=0.5, C=0.5	0.00001	145.021	30	T=4/11, C=6/9
chr5:131822224	T/C	T	C	SNV	IRF1	IRF1:intronic:NM 002198.2	1	50		intronic	NM 00219	c.544+25A	p.?	-0.83					Interferon re	rs9282761	0.3281	YES	3.488E-1 (pos.131822224)		Interferon r	CD8-positi	Anemia;Anemia, Macrocytic;Brain Diseases;I	T=15, C=15	T=0.5, C=0.5	0.00001	145.021	30	T=3/12, C=5/1
chr5:131995843	C/C	T	C	SNV	IL13	IL13:intronic:NM 002188.2	1	100		intronic	NM 00218	c.334-24T	p.?	-0.82					Interleukin-1	rs1295686	0.2005	YES	6.752E-1 (pos.131995843)		Interleukin-	cell-cell si	Adenocarcinoma:A Cintredekin Besudotox,L	T=0, C=32	T=0.0, C=1.0	0.00001	485.14	32	T=0/0, C=24/8
chr6:108233929	A/A	G	A	SNV	SEC63	SEC63:exonic:NM 007214.4	1	100	6	synonymous	NM 00721	c.564C>T	p.(=)	0.51					DnaJ domain	rs675117	0.1373	YES	7.208E-1 (pos.108233929)		Sec63, S. ce	IRE1-medi	Cysts;Digestive System Diseas Benign	G=0, A=23	G=0.0, A=1.0	0.00001	316.747	23	G=0/0, A=17/6
chr5:132039132	A/A	C	A	SNV	KIF3A	KIF3A:intronic:NM 007054.6	1	100		intronic	NM 00705	c.1385+23	p.?	-0.39					Kinesin moto	rs2285700	0.2593	YES	1.484E-1 (pos.132039132)		Kinesin fam	ATP bindin	Asthma:Bone Diseases:Bone Diseases, Deve	C=0, A=67	C=0.0, A=1.0	0.00001	1038.86	67	C=0/0, A=25/4
chr6:108243115	T/TGGG	T	TGGG	INDEL	SEC63	SEC63:splicesite 3:NM 007214.4	3	100	4	splicesite	NM 00721	c.340-2A>	p.?	7.34					DnaJ domain	rs142388422,rs866934133		YES	4.879E-1;1.066E-3;1.333E-4;0.0 (pos.108		Sec63, S. ce	IRE1-medi	Cysts;Digestive System Diseases;Female Uro	T=0, TGGG:	T=0.0, TGGG=1.0	0.00001	333.902	21	T=0/0, TGGG=
chr5:132198287	G/G	C	G	SNV	GDF9	GDF9:intronic:NM 005260.5	1	100		intronic	NM 00526	c.398-39G	p.?	-0.92					Transforming	rs254285	0.0959	YES	5.567E-1 (pos.132198287)		Growth/diff	BMP signa	Adnexal Diseases;Cysts;Endocrine System Di	C=0, G=42	C=0.0, G=1.0	0.00001	685.019	42	C=0/0, G=15/2
chr5:132202744	C/T	C	T	SNV	UQCRQ	UQCRQ:intronic:NM 014402.4	1	50		intronic	NM 01440	c.154+17C	p.?	-2.98					UcrQ family	rs145649482	0.0187	YES	0.0 (pos.132202744)		Ubiquinol-c	cerebellar	Metabolic Disease: (5S)-3-ANII Benign	C=15, T=15	C=0.5, T=0.5	0.00001	147.213	30	C=12/3, T=8/7
chr5:132227677	G/G	A	G	SNV	AFF4	AFF4:intronic:NM 014423.3	1	100		intronic	NM 01442	c.2637+17	p.?	-0.13					AF-4 proto-or	rs6596093		YES			AF4/FMR2 f:	DNA bindi	Bone Diseases:Bone Diseases, Development	A=0, G=24	A=0.0, G=1.0	0.00001	339.186	24	A=0/0, G=18/6
chr5:134343469	A/A	G	A	SNV	CATSPER3	CATSPER3:intronic:NM 178019.2	1	100		intronic	NM 17801	c.493-178	p.?	-1.48					Ion transport	rs177251		YES			Cation char	CatSper complex,acrosomal vesicle,calcium ion transme		G=0, A=25	G=0.0, A=1.0	0.00001	353.019	25	G=0/0, A=7/18
chr6:108279290	A/A	G	A	SNV	SEC63	SEC63:utr 5:NM 007214.4	1	100	1	utr 5	NM 00721	c.-77C>T	p.?	-0.1					DnaJ domain	rs2272884		YES			Sec63, S. ce	IRE1-medi	Cysts;Digestive System Diseas Benign	G=0, A=20	G=0.0, A=1.0	0.00001	268.146	20	G=0/0, A=7/13
chr15:49325289	C/C	A	C	SNV	SECISBP2L	SECISBP2L:exonic:NM 001193489.1	1	100	4	synonymous	NM 00119	c.537T>G	p.(=)	2.71				0.71(COSM37	Ribosomal pr	rs11638215	0.3736	YES	3.817E-1 (pos.49325289)	adenocarcinoı	Selenocyste	RNA binding,intracellular ribonucleoprotein complex,mF		A=0, C=22	A=0.0, C=1.0	0.00001	298.892	22	A=0/0, C=19/3
chr18:12984144	A/A	C	A	SNV	SEH1L	SEH1L:exonic:NM 001013437.1	1	100	8	missense	NM 00101	c.1025C>A	p.Thr342Asn	1.69	0.64	65	0		WD domain,	rs6505776	0.3793	YES	7.916E-1 (pos.12984144)		Seh1-like pr	GATOR2 complex,attachment of mitotic spindle microtul		C=0, A=24	C=0.0, A=1.0	0.00001	334.774	24	C=0/0, A=12/1
chr20:13847420	G/G	A	G	SNV	SEL1L2	SEL1L2:exonic:NM 001271539.1	1	100	15	synonymous	NM 00127	c.1332T>C	p.(=)	-1.19					Sel1 repeat	rs660710	0.0154	YES	9.999E-1 (pos.13847420)		Suppressor	integral component of membrane		A=0, G=55	A=0.0, G=1.0	0.00001	814.905	55	A=0/0, G=19/3
chr22:31503560	T/T	C	T	SNV	SELENOM	SELENOM:utr 5:NM 080430.3	1	100	1	utr 5	NM 08043	c.-69G>A	p.?	-3.15						rs13053829		YES			Selenoprot	Golgi apparatus,adipose tissue development,corticoste		C=0, T=31	C=0.0, T=1.0	0.00001	465.819	31	C=0/0, T=7/24
chr5:137257288	G/G	A	G	SNV	PKD2L2	PKD2L2:intronic:NM 014386.3	1	100		intronic	NM 01438	c.1329-37	p.?	-1.62					Ion transport	rs4304074	2.00E-04		1.0 (pos.137257288)		Polycystin 2	biological process,calcium channel activity,calcium ion		A=0, G=41	A=0.0, G=1.0	0.00001	664.372	41	A=0/0, G=22/1
chr7:83590852	C/C	T	C	SNV	SEMA3A	SEMA3A:exonic:NM 006080.2	1	100	17	synonymous	NM 00608	c.2151A>G	p.(=)	-1.14					Sema domair	rs797821	0.3708	YES	7.03E-1 (pos.83590852)		Semaphorir	apoptotic	46, XY Disorders of Sex Develoı Benign	T=0, C=61	T=0.0, C=1.0	0.00001	921.766	61	T=0/0, C=22/3
chr5:137259251	T/T	C	T	SNV	PKD2L2	PKD2L2:intronic:NM 014386.3	1	100		intronic	NM 01438	c.1551+41	p.?	-0.74					Ion transport	rs13188503	1.00E-04		1.0;0.0 (pos.137259251)		Polycystin 2	biological process,calcium channel activity,calcium ion		C=0, T=30	C=0.0, T=1.0	0.00001	446.639	30	C=0/0, T=17/1
chr5:137281708	C/C	A	C	SNV	FAM13B	FAM13B:intronic:NM 016603.3	1	100		intronic	NM 01660	c.2179-22	p.?	1.88					RhoGAP dom:	rs1125979	0.2179	YES	9.673E-1 (pos.137281708)		Family with	GTPase activator activity,cytosol,positive regulation of (		A=0, C=75	A=0.0, C=1.0	0.00001	1193.74	75	A=0/0, C=32/4
chr5:13729714	G/G	A	G	SNV	DNAH5	DNAH5:intronic:NM 001369.2	1	100		intronic	NM 00136	c.11762-4	p.?	-2.84					AAA domain (	rs3777093	0.4189	YES	6.463E-1 (pos.13729714)		Dynein, axo	ATP bindin	Asthma:Bronchial Diseases:Br Benign	A=0, G=34	A=0.0, G=1.0	0.00001	524.186	34	A=0/0, G=16/1
chr5:137297923	T/T	C	T	SNV	FAM13B	FAM13B:intronic:NM 016603.3	1	100		intronic	NM 01660	c.1244+17	p.?	0.36					RhoGAP dom:	rs4835738		YES			Family with	GTPase activator activity,cytosol,positive regulation of (		C=0, T=38	C=0.0, T=1.0	0.00001	603.785	38	C=0/0, T=14/2
chr5:137323117	G/G	A	G	SNV	FAM13B	FAM13B:intronic:NM 016603.3	1	100		intronic	NM 01660	c.1046+33	p.?	1.88					RhoGAP dom:	rs7714536	0.2169	YES	9.695E-1 (pos.137323117)		Family with	GTPase activator activity,cytosol,positive regulation of (		A=0, G=133	A=0.0, G=1.0	0.00001	2197.06	133	A=0/0, G=79/5
chr7:80374438	C/C	T	C	SNV	SEMA3C	SEMA3C:exonic:NM 006379.4	1	100	18	synonymous	NM 00637	c.2028A>G	p.(=)	-0.96					Immunoglobi	rs1949972	0.0095	YES	7.667E-1 (pos.80374438)		Semaphorir	axon guid:	Aortopulmonary Septal Defect:Cardiovascula	T=0, C=48	T=0.0, C=1.0	0.00001	814.805	48	T=0/0, C=16/3
chr5:137506434	A/A	G	A	SNV	BRD8	BRD8:intronic:NM 139199.1	1	100		intronic	NM 13919	c.440+87C	p.?	0.09					Bromodomai	rs13155519		YES			Bromodom:	DNA bindi	Endometriosis:Female Urogenital Diseases:F	G=0, A=21	G=0.0, A=1.0	0.00001	281.235	21	G=0/0, A=11/1
chr7:80378319	C/C	G	C	SNV	SEMA3C	SEMA3C:exonic:NM 006379.4	1	100	17	synonymous	NM 00637	c.1737C>G	p.(=)	0.61					Immunoglobi	rs2272351	0.0085	YES	7.652E-1 (pos.80378319)		Semaphorir	axon guid:	Aortopulmonary Septal Defect;Cardiovascula	G=0, C=41	G=0.0, C=1.0	0.00001	664.7	41	G=0/0, C=8/33
chr7:80427495	C/A	C	A	SNV	SEMA3C	SEMA3C:exonic:NM 006379.4	1	50	11	synonymous	NM 00637	c.1044G>T	p.(=)	0.27					Immunoglobi	rs1880959	0.0942	YES	1.275E-3;0.0 (pos.80427495)		Semaphorir	axon guid:	Aortopulmonary Septal Defect;Cardiovascula	C=24, A=24	C=0.5, A=0.5	0.00001	272.293	48	C=16/8, A=13/
chr7:82996831	A/T	A	T	SNV	SEMA3E	SEMA3E:utr 3:NM 012431.2	1	46.88	17	utr 3	NM 01243	c.2399T>A	p.?	1.19					Immunoglobi	rs75164403		YES			Semaphorir	branching	Abnormalities, Multiple;CHARGE Syndrome;C	A=17, T=15	A=0.5313, T=0.4688	0.00001	143.733	32	A=15/2, T=14/
chr1:156146218	C/T	C	T	SNV	SEMA4A	SEMA4A:exonic:NM 001193300.1	1	52.27	15	synonymous	NM 00119	c.1716C>T	p.(=)	0.49				0.18(COSM54	Plexin repeat	rs12401573	0.4121	YES	2.381E-1 (pos.15614621&	carcinoma,duc	Semaphorir	T-helper 1	Congenital, Hereditary, and Ne Benign	C=21, T=23	C=0.4773, T=0.5227	0.00001	261.769	44	C=17/4, T=22/
chr5:137909675	A/A	AGAA	A	INDEL	HSPA9	HSPA9:intronic:NM 004134.6	3	100		intronic	NM 00413	c.140+82T	p.?	-0.92, -0.83, -0.55, -0.73					FGGY family c	rs34472800,rs386692811,rs412		YES			Heat-shock	ATP bindin	Adenocarcinoma:Autoimmune Diseases:Bas	AGAA=0, A:	AGAA=0.0, A=1.0	0.00001	1128.85	72	AGAA=0/0, A=:
chr15:90768271	C/T	C	T	SNV	SEMA4B	SEMA4B:exonic:NM 198925.2	1	50	10	synonymous	NM 19892	c.1266C>T	p.(=)	0.24				0.74(COSM37	Plexin repeat	rs11547966	0.2358	YES	5.556E-2 (pos.90768271)	acute myeloic	Semaphorir	cell differentiation,extracellular space,integral compon		C=24, T=24	C=0.5, T=0.5	0.00001	273.123	48	C=18/6, T=8/1
chr9:92020433	A/A	G	A	SNV	SEMA4D	SEMA4D:utr 5:NM 006378.3	1	100	5	utr 5	NM 00637	c.-62C>T	p.?	-1.83					Immunoglobi	rs7856677		YES			Semaphorir	cell adhesion,extracellular space,identical protein bindi		G=0, A=48	G=0.0, A=1.0	0.00001	810.178	48	G=0/0, A=18/3
chr5:115813726	G/G	A	G	SNV	SEMA6A	SEMA6A:exonic:NM 020796.4	1	100	14	missense	NM 02079	c.1552T>C	p.Tyr518His	6.74	1	83	0	0.24(COSM45	Plexin repeat	rs34966			1.0 (pos.115813726)	squamous cel	Semaphorir	animal organ morphogenesis,apoptotic process,axon,a>		A=0, G=27	A=0.0, G=1.0	0.00001	389.973	27	A=0/0, G=17/1
chr1:151105824	G/G	C	G	SNV	SEMA6C	SEMA6C:exonic:NM 001178061.1	1	100	20	synonymous	NM 00117	c.2025G>C	p.(=)	-0.78				0.13(COSM37	Sema domair	rs4971006	0.1933	YES	9.228E-1 (pos.15110582‹	adenocarcinoı	Semaphorir	axon guidance,integral component of plasma membrane		C=0, G=55	C=0.0, G=1.0	0.00001	813.684	55	C=0/0, G=18/3
chr1:151108137	G/G	T	G	SNV	SEMA6C	SEMA6C:exonic:NM 001178061.1	1	100	14	missense	NM 00117	c.1363A>C	p.Thr455Pro	0.9	0.4	38	0		Sema domair	rs4971007	0.1907	YES	9.196E-1 (pos.151108137)		Semaphorir	axon guidance,integral component of plasma membrane		T=0, G=57	T=0.0, G=1.0	0.00001	846.584	57	T=0/0, G=23/3
chr15:48063076	C/C	T	C	SNV	SEMA6D	SEMA6D:exonic:NM 153618.1	1	100	19	synonymous	NM 15361	c.2316T>C	p.(=)	-0.7					Plexin repeat	rs568215	0.3922	YES	8.363E-1 (pos.48063076)		Semaphorir	Golgi appa	Mental Disorders;Substance-Related Disorde	T=0, C=59	T=0.0, C=1.0	0.00001	885.299	59	T=0/0, C=32/2
chr17:7470285	C/C	CA	C	INDEL	SENP3,SENP3-EIF4A1	SENP3-EIF4A1:exonic nc:NR 037926.1, SENP3:splicesite 5:I	1	100	7	splicesite	NM_015670.5, NR_037926.1			1.13, 5.71					Ulp1 proteas	rs111576270,rs398030265,rs58		YES	1.0 (pos.7470285)	astrocytoma (	Sentrin-spe	MLL1 complex,cysteine-type peptidase activity,cytoplas		CA=0, C=27	CA=0.0, C=1.0	0.00001	308.568	27	CA=0/0, C=12/
chr5:140057216	A/A	G	A	SNV	HARS	HARS:intronic:NM 002109.5	1	100		intronic	NM 00210	c.729+24C	p.?	0					Anticodon bi	rs761595983,rs8113:	0.2041	YES	0.0 (pos.140057215),1.463E-1 (pos.1400		Histidyl-tRN	ATP bindin	Abnormalities, Mul L-Histidine	G=0, A=10(	G=0.0, A=1.0	0.00001	1562.7	100	G=0/0, A=40/6
chr6:76369054	G/G	A	G	SNV	SENP6	SENP6:exonic:NM 015571.3	1	100	8	synonymous	NM 01557	c.627A>G	p.(=)	0.79					Ulp1 proteas	rs507662	0.0191	YES	8.889E-1 (pos.76369054)		Sentrin-spe	SUMO-specific protease activity,cysteine-type peptidase		A=0, G=53	A=0.0, G=1.0	0.00001	782.03	53	A=0/0, G=26/2
chr15:72432583	G/G	A	G	SNV	SENP8	SENP8:exonic:NM 001172111.1	1	100	3	missense	NM 00117	c.619A>G	p.Thr207Ala	0.81		58			Ulp1 proteas	rs930871	8.00E-04	YES	1.0 (pos.72432583)		Sentrin-spe	NEDD8-specific protease activity,cysteine-type peptidas		A=0, G=60	A=0.0, G=1.0	0.00001	903.528	60	A=0/0, G=17/4
chr16:30393660	G/A	G	A	SNV	SEPT1,ZNF48	SEPT1:exonic:NM 052838.4, ZNF48:intronic:NM 00121490(	1	95.83	3	synonymous	NM 05283	c.171C>T,(	p.(=), p.?	-1.41					50S ribosome	rs4787643	0.3253	YES	9.228E-1 (pos.30393660)		Septin 1	DNA binding,GTP binding,cell cycle,cell division,cytoplas		G=1, A=23	G=0.0417, A=0.9583	0.00001	306.64	24	G=0/1, A=6/17
chr11:18034590	AC/AC	A	AC	INDEL	SERGEF	SERGEF:utr 5:NM 012139.3	1	100	1	utr 5	NM 01213	c.-33T>GT	p.?	-0.41					Regulator of	rs1002927610					Secretion-r	Ran guanyl-nucleotide exchange factor activity,aggresor		A=0, AC=43	A=0.0, AC=1.0	0.00001	578.344	43	A=0/0, AC=16/
chr11:18034605	G/G	C	G	SNV	SERGEF	SERGEF:utr 5:NM 012139.3	1	100	1	utr 5	NM 01213	c.-48G>C	p.?	-0.42					Regulator of	rs3741201		YES	(pos.18034605)		Secretion-r	Ran guanyl-nucleotide exchange factor activity,aggresor		C=0, G=42	C=0.0, G=1.0	0.00001	689.6	42	C=0/0, G=16/2
chr5:79551867	G/G	A	G	SNV	SERINC5	SERINC5:utr 5:NM 001174072.2	1	100	1	utr 5	NM 00117	c.-126T>C	p.?	-0.06					Serine incorp	rs1966470					Serine incoı	Golgi apparatus,L-serine biosynthetic process,L-serine t		A=0, G=48	A=0.0, G=1.0	0.00001	798.455	48	A=0/0, G=20/2
chr14:94847415	A/G	A	G	SNV	SERPINA1	SERPINA1:exonic:NM 001127700.1	1	50	3	missense	NM 00112	c.710T>C	p.Val237Ala	-0.45	0.31	64	0	0.09(COSM37	Serpin (serin	rs6647	0.2117	YES	2.051E-2 (pos.94847415)	adenocarcinoı	Alpha-1-ant	COPII vesi	Adenocarcinoma;A 2-3,4-Dihy Benign/Likely	A=14, G=14	A=0.5, G=0.5	0.00001	131.94	28	A=8/6, G=7/7
chr14:94750292	G/A	G	A	SNV	SERPINA10	SERPINA10:utr 3:NM 016186.2	1	58.33	5	utr 3	NM 01618	c.1345C>T	p.?	-3.79					Serpin (serin	rs1950641	0.168	YES	5.91E-1 (pos.94750292)		Serine (or c	biolumine	Cardiovascular Diseases:Embolism and Thro	G=10, A=14	G=0.4167, A=0.5833	0.00001	135.259	24	G=1/9, A=6/8
chr5:140257591	T/T	C	T	SNV	PCDHA1,PCDHA10,PCDHA11,PCDHA12,PCDHA2,PCDHA3,PCDHA4,PCDHA5,PCDHA6,PCDHA7,PCDHA8,PCDHA9	PCDHA1:intronic:NM 018900.3, PCDHA5:intronic:NM 0189	1	100		intronic	NM 01890	c.2394+89	p.?, p.?, p.?, p.?, p.?, p.?, p.?, p.?,	-1.79					Cadherin C-te	rs251378		YES			Protocadhe	calcium io	Abortion, Spontaneous:Female Urogenital Di	C=0, T=59	C=0.0, T=1.0	0.00001	885.295	59	C=0/0, T=29/3

chr14:95053890	A/A	G	A	SNV	SERPINA5	SERPINA5:exonic:NM_000624.5	1	100	3	missense	NM_00062	c.191G>A	p.Ser64Asn	6.68	1	46	0	0.08(COSM41	Serpin (serine	rs386780205,rs6115,	0.3387	YES	4.345E-1 (pos.95053890)	neoplasm		acrosin bi	Amphetamine-Rela	Drotrecogin alfa,T-1249,		G=0, A=28	G=0.0, A=1.0	0.00001	408.718	28	G=0/0, A=12/1
chr14:95054176	C/C	T	C	SNV	SERPINA5	SERPINA5:exonic:NM_000624.5	1	100	3	synonymous	NM_00062	c.477T>C	p.(=)	0.1					Serpin (serine	rs6112	0.3371	YES	4.243E-1 (pos.95054176)			acrosin bi	Amphetamine-Rela	Drotrecogin alfa,T-1249.		T=0, C=22	T=0.0, C=1.0	0.00001	297.451	22	T=0/0, C=19/3
chr14:94935566	A/A	G	A	SNV	SERPINA9	SERPINA9:exonic:NM_175739.3	1	100	2	synonymous	NM_17573	c.666C>T	p.(=)	2.13				0.86(COSM39	Serpin (serine	rs4900231	0.1923	YES	1.553E-1 (pos.94935566)	acute_myeloid	Serpin pept	cytoplasm,extracellular space,membrane,negative regu				G=0, A=25	G=0.0, A=1.0	0.00001	353.019	25	G=0/0, A=4/21
chr14:94933640	A/T	A	T	SNV	SERPINA9	SERPINA9:exonic:NM_175739.3	1	57.14	3	missense	NM_17573	c.762T>A	p.His254Gln	-0.06	0.35	24	0		Serpin (serine	rs28583900	0.3004	YES	4.392E-1 (pos.94933640)		Serpin pept	cytoplasm,extracellular space,membrane,negative regu				A=27, T=36	A=0.4286, T=0.5714	0.00001	380.915	63	A=9/18, T=22/
chr18:61600384	T/T	C	T	SNV	SERPINB10	SERPINB10:exonic:NM_005024.2	1	100	7	missense	NM_00502	c.736C>T	p.Arg246Cys	-0.16	0.06	180	0.068	0.11(COSM1389512)		rs963075	0.2586	YES	4.268E-1;0.0 (pos.61600	acute_myeloid	Protease in	cytosol,ex	Genital Diseases, Male;Genital Neoplasms, I			C=0, T=24	C=0.0, T=1.0	0.00001	339.632	24	C=0/0, T=18/6
chr18:61170925	C/C	T	C	SNV	SERPINB5	SERPINB5:exonic:NM_002639.4	1	100	7	synonymous	NM_00263	c.1098T>C	p.(=)	1.43					Serpin (serine	rs8093128	0.0044		1.0 (pos.61170925)		Protease in	cytoplasm	Adenocarcinoma;B	S,S-(2-Hydroxyethyl)Thio		T=0, C=87	T=0.0, C=1.0	0.00001	1431.39	87	T=0/0, C=52/3
chr6:2959513	T/T	C	T	SNV	SERPINB6	SERPINB6:exonic:NM_001271822.1	1	100	2	synonymous	NM_00127	c.96G>A	p.(=)	-0.63				0.04(COSM15	Serpin (serine	rs2236277	0.321	YES	4.973E-1 (pos.2959513)	adenocarcinoma	Protease in	cellular response to osmotic st		Drotrecog	Benign	C=0, T=33	C=0.0, T=1.0	0.00001	504.597	33	C=0/0, T=14/1
chr18:61654329	T/T	C	T	SNV	SERPINB8	SERPINB8:exonic:NM_002640.3	1	100	7	synonymous	NM_00264	c.942C>T	p.(=)	-1.84					Serpin (serine	rs3744958	0.0409	YES	7.681E-2 (pos.61654329)		Serpin fami	cytosol,ep	Psoriasis;Skin Diseases;Skin Diseases, Papul			C=0, T=75	C=0.0, T=1.0	0.00001	1189.83	75	C=0/0, T=58/1
chr2:224862842	A/G	A	G	SNV	SERPINE2	SERPINE2:exonic:NM_006216.3	1	50	3	synonymous	NM_00621	c.477T>C	p.(=)	0.34				0.62(COSM37	Serpin (serine	rs12457	0.1952	YES	1.287E-1 (pos.22486284	adenocarcinoma	Protease in	blood coa	Cardiovascular Diseases;Vascular Diseases			A=10, G=10	A=0.5, G=0.5	0.00001	82.8861	20	A=3/7, G=5/5
chr5:140955777	T/T	C	T	SNV	DIAPH1	DIAPH1:intronic:NM_005219.4	1	100		intronic	NM_00521	c.1461+20	p.?	0.58					DRF Autoregu	rs2302103	0.3936	YES	3.054E-1 (pos.140955777)		Diaphanous	RNA bindi	Ear Diseases;Hearing Disorder		Benign	C=0, T=32	C=0.0, T=1.0	0.00001	485.141	32	C=0/0, T=11/2
chr17:1673276	T/T	C	T	SNV	SERPINF1	SERPINF1:exonic:NM_002615.6	1	100	3	missense	NM_00261	c.215C>T	p.Thr72Met	-0.75	0.25	81	0.005	0.01(COSM13	Serpin (serine	rs1136287	0.3481	YES	5.376E-1 (pos.1673276)	basal_cell_car	Serpin pept	aging,axon	Adenocarcinoma;Bone Disease		Benign	C=0, T=28	C=0.0, T=1.0	0.00001	408.713	28	C=0/0, T=24/4
chr5:141035955	G/A	G	A	SNV	ARAP3	ARAP3:intronic:NM_022481.5	1	50		intronic	NM_02248	c.3790-11	p.?	-0.75					PH domain,P	rs2306339		YES			ArfGAP with	GTPase ac	HIV Infections;Hemic and Lymphatic Disease			G=20, A=21	G=0.4878, A=0.5122	0.00001	239.791	42	G=6/14, A=6/1
chr5:141049407	A/A	G	A	SNV	ARAP3	ARAP3:intronic:NM_022481.5	1	100		intronic	NM_02248	c.2250-29	p.?	0.11					PH domain,P	rs3763120	0.4444	YES	4.392E-1 (pos.141049407)		ArfGAP with	GTPase ac	HIV Infections;Hemic and Lymphatic Disease			G=0, A=28	G=0.0, A=1.0	0.00001	408.716	28	G=0/0, A=12/1
chr5:141049656	A/G	A	G	SNV	ARAP3	ARAP3:intronic:NM_022481.5	1	57.89		intronic	NM_02248	c.2108-26	p.?	1.7					PH domain,P	rs73285857	0.0669	YES	3.925E-2 (pos.141049656)		ArfGAP with	GTPase ac	HIV Infections;Hemic and Lymphatic Disease			A=31, G=44	A=0.4133, G=0.5867	0.00001	508.669	76	A=22/9, G=23/
chr17:1680002	T/C	T	C	SNV	SERPINF1	SERPINF1:exonic:NM_002615.6	1	47.37	7	synonymous	NM_00261	c.963T>C	p.(=)	-3.69				0.04(COSM37	Serpin (serine	rs6828	0.2921	YES	7.931E-1 (pos.1680002)	hepatocellular	Serpin pept	aging,axon	Adenocarcinoma;Bone Disease		Benign	T=20, C=18	T=0.5263, C=0.4737	0.00001	184.537	38	T=18/2, C=15/
chr11:75277628	G/G	A	G	SNV	SERPINH1	SERPINH1:exonic:NM_001235.3	1	100	2	synonymous	NM_00123	c.234A>G	p.(=)	0.92				0.1(COSM375	Serpin (serine	rs584961	0.1035	YES	7.041E-1 (pos.75277628)	adenocarcinoma	Serpin pept	RNA bindi	Bone Diseases;Bone Diseases,		Benign	A=0, G=37	A=0.0, G=1.0	0.00001	583.707	37	A=0/0, G=23/1
chr11:75277757	G/G	C	G	SNV	SERPINH1	SERPINH1:exonic:NM_001235.3	1	100	2	synonymous	NM_00123	c.363C>G	p.(=)	1.14				0.91(COSM42	Serpin (serine	rs650241	0.3016	YES	2.13E-1 (pos.75277757)	adenocarcinoma	Serpin pept	RNA bindi	Bone Diseases;Bone Diseases,		Benign	C=0, G=54	C=0.0, G=1.0	0.00001	797.717	54	C=0/0, G=23/3
chr11:75282882	A/A	G	A	SNV	SERPINH1	SERPINH1:exonic:NM_001235.3	1	100	5	synonymous	NM_00123	c.1011G>A	p.(=)	1.35					Serpin (serine	rs585821	0.3039	YES	1.943E-1 (pos.75282882)		Serpin pept	RNA bindi	Bone Diseases;Bone Diseases,		Benign	G=0, A=39	G=0.0, A=1.0	0.00001	623.946	39	G=0/0, A=13/2
chr2:180047908	T/C	T	C	SNV	SESTD1	SESTD1:exonic:NM_178123.4	1	96.67	3	synonymous	NM_17812	c.63A>G	p.(=)	0.47					Divergent CR	rs6757681,rs770918	0.2324	YES	0.0 (pos.180047895),9.138E-1 (pos.180047908)		1-phosphatidylinositol binding,calcium channel comple					T=1, C=29	T=0.0333, C=0.9667	0.00001	416.713	30	T=0/1, C=11/1
chr18:42533130	G/G	A	G	SNV	SETBP1	SETBP1:exonic:NM_015559.2	1	100	4	synonymous	NM_01555	c.3825A>G	p.(=)	-2.19					AT hook moti	rs8096662	0.1362	YES	9.578E-1 (pos.42533130)		SET-binding	DNA bindi	Abnormalities, Multiple;Bone I		Benign	A=0, G=22	A=0.0, G=1.0	0.00001	298.892	22	A=0/0, G=13/9
chr5:14377962	C/C	A	C	SNV	TRIO	TRIO:intronic:NM_007118.3	1	100		intronic	NM_00711	c.3332-15	p.?	-1.57					CRAL/TRIO do	rs30769		YES			Triple funct	ATP bindin	Digestive System Diseases:Digestive System			A=0, C=27	A=0.0, C=1.0	0.00001	389.941	27	A=0/0, C=15/1
chr5:14387489	G/C	G	C	SNV	TRIO	TRIO:intronic:NM_007118.3	1	59.09		intronic	NM_00711	c.3571-58	p.?	-1.15					CRAL/TRIO do	rs30794		YES			Triple funct	ATP bindin	Digestive System Diseases:Digestive System			G=9, C=13	G=0.4091, C=0.5909	0.00001	122.567	22	G=3/6, C=8/5
chr5:145252574	T/T	C	T	SNV	GRXCR2	GRXCR2:upstream:NM_001080516.1	1	100		intronic	NM_00108	c.-43G>A	p.?	0.07						rs10037373	0.4528	YES	7.388E-1 (pos.145252574)		Glutaredox	microvillus,sensory perception of sound,stereocilium				C=0, T=27	C=0.0, T=1.0	0.00001	389.969	27	C=0/0, T=7/20
chr18:42529996	G/C	G	C	SNV	SETBP1	SETBP1:exonic:NM_015559.2	1	50	4	missense	NM_01555	c.691G>C	p.Val231Leu	-0.12	0.06	32	0.006		AT hook moti	rs11082414	0.2044	YES	1.301E-2;0.0 (pos.42529996)		SET-binding	DNA bindi	Abnormalities, Multiple;Bone I		Benign	G=17, C=17	G=0.5, C=0.5	0.00001	171.964	34	G=9/8, C=13/4
chr12:122261456	A/A	G	A	SNV	SETD1B	SETD1B:exonic:NM_001353345.1	1	100	11	synonymous	NM_00135	c.4971G>A	p.(=)	-3.45				0.05(COSM37	COMPASS (Co	rs3741593	0.4497	YES	5.976E-1 (pos.12226145	hepatocellular	SET domain-containing protein 1B					G=0, A=34	G=0.0, A=1.0	0.00001	529.092	34	G=0/0, A=19/1
chr5:145838440	A/A	G	A	SNV	TCERG1	TCERG1:intronic:NM_006706.3	1	100		intronic	NM_00670	c.439-7G>A	p.?	0.82					FF domain,W	rs11743333	0.354	YES	1.429E-1 (pos.145838440)		Transcriptio	RNA binding,RNA polymerase II repressing transcription				G=0, A=32	G=0.0, A=1.0	0.00001	485.14	32	G=0/0, A=22/1
chr9:135202829	C/C	T	C	SNV	SETX	SETX:exonic:NM_015046.6	1	100	10	missense	NM_01504	c.4156A>G	p.Ile1386Val	-0.66	0.78	29	0	0.06(COSM15	AAA domain,I	rs543573	0.1543	YES	2.984E-1 (pos.13520282	acute_myeloid	Senataxin	ATP bindin	Amyotrophic Lateral Sclerosis;		Benign/Likely	T=0, C=46	T=0.0, C=1.0	0.00001	768.312	46	T=0/0, C=30/1
chr5:147480868	G/A	G	A	SNV	SPINK5	SPINK5:intronic:NM_001127698.1	1	50		intronic	NM_00112	c.1221-50	p.?	-1.36					Kazal-type se	rs2303066	0.4885	YES	4.809E-1 (pos.147480868)		Serine prot	anagen,ce	Abnormalities, Multiple;Congenital Abnorma			G=22, A=22	G=0.5, A=0.5	0.00001	243.623	44	G=12/10, A=7/
chr9:135203408	GC/GC	G	GC	INDEL	SETX	SETX:exonic:NM_015046.6	1	100	10	frameshiftInsertion	NM_01504	c.3576_35	p.Leu1193fs	-0.11					AAA domain,UvrD/REP helicase N-terminal domain						Senataxin	ATP bindin	Amyotrophic Lateral Sclerosis;Ataxia;Brain D			G=0, GC=44	G=0.0, GC=1.0	0.00001	462.853	44	G=0/0, GC=13/
chr9:135203409	C/C	A	C	SNV	SETX	SETX:exonic:NM_015046.6	1	100	10	missense	NM_01504	c.3576T>G	p.Asp1192Glu	-0.99	0.14	45	0.001		AAA domain,I	rs1185193	0.1383	YES	3.062E-1;0.0 (pos.135203409)		Senataxin	ATP bindin	Amyotrophic Lateral Sclerosis;		Benign/Likely	A=0, C=45	A=0.0, C=1.0	0.00001	741.918	45	A=0/0, C=13/3
chr9:135206460	G/G	A	G	SNV	SETX	SETX:exonic:NM_015046.6	1	100	9	synonymous	NM_01504	c.1077T>C	p.(=)	0.15				0.2(COSM150	AAA domain,I	rs9411449	0.1534	YES	3.022E-1 (pos.13520646	acute_myeloid	Senataxin	ATP bindin	Amyotrophic Lateral Sclerosis;		Benign/Likely	A=0, G=42	A=0.0, G=1.0	0.00001	684.985	42	A=0/0, G=11/3
chr2:198265526	G/G	A	G	SNV	SF3B1	SF3B1:exonic:NM_012433.3	1	100	18	synonymous	NM_01243	c.2631T>C	p.(=)	0.47				0.52(COSM37	Splicing facto	rs788018	0.3149	YES	5.361E-1 (pos.19826552	acute_myeloid	Splicing fact	RNA bindi	Eye Diseases;Eye Neoplasms;Hemic and Lym			A=0, G=39	A=0.0, G=1.0	0.00001	623.98	39	A=0/0, G=20/1
chr5:1479678	C/C	G	C	SNV	LPCAT1	LPCAT1:intronic:NM_024830.4	1	100		intronic	NM_02483	c.816+58C	p.?	-0.33					Acyltransfera	rs27059	0.2571	YES			Acyltransfe	1-acylglycerol-3-phosphate O-acyltransferase activity,1-				G=0, C=35	G=0.0, C=1.0	0.00001	543.903	35	G=0/0, C=17/1
chr5:148406386	T/C	T	C	SNV	SH3TC2	SH3TC2:intronic:NM_024577.3	1	58.33		intronic	NM_02457	c.2872+37	p.?	0.36				0.08(COSM14	SH3 domain	rs17722209	0.1887	YES	2.433E-1 (pos.14840638	acute_myeloid	SH3 domain	periphera	Charcot-Marie-Tooth Disease;Congenital Abr			T=15, C=21	T=0.4167, C=0.5833	0.00001	234.328	36	T=0/15, C=9/1
chr2:198283305	C/C	T	C	SNV	SF3B1	SF3B1:exonic:NM_012433.3	1	100	5	synonymous	NM_01243	c.423A>G	p.(=)	3.84				0.6(COSM112	Splicing facto	rs788023	0.3153	YES	5.384E-1 (pos.19828330	acute_myeloid	Splicing fact	RNA bindi	Eye Diseases;Eye Neoplasms;Hemic and Lym			T=0, C=28	T=0.0, C=1.0	0.00001	408.718	28	T=0/0, C=14/1
chr22:31971258	C/C	T	C	SNV	SFI1	SFI1:exonic:NM_001007467.2	1	100	10	missense	NM_00100	c.964T>C	p.Tyr322His	0.09	0.61	83	0			rs5753700	0.0495	YES	9.864E-1 (pos.31971258)		SFI1, yeast,	G2/M transition of mitotic cell cycle,centriole,centroson				T=0, C=26	T=0.0, C=1.0	0.00001	371.415	26	T=0/0, C=16/1
chr12:132237848	C/C	T	C	SNV	SFSWAP	SFSWAP:exonic:NM_001261411.1	1	100	8	missense	NM_00126	c.1262T>C	p.Leu421Pro	2.76	1	98	0		Alternative s	rs1982528	0.0217	YES	9.935E-1 (pos.132237848)		Splicing fact	RNA bindi	Child Development Disorders, Pervasive;Mer			T=0, C=32	T=0.0, C=1.0	0.00001	485.09	32	T=0/0, C=12/2
chr5:149417138	G/T	G	T	SNV	HMGXB3	HMGXB3:intronic:NM_014983.2	1	46.43		intronic	NM_01498	c.2413+48	p.?	0.35					HMG (high m	rs6887999	0.1144	YES	1.006E-1 (pos.149417138)			DNA binding,biological_process,cellular_component,kin				G=30, T=26	G=0.5357, T=0.4643	0.00001	248.026	56	G=11/19, T=6/
chr12:132271113	C/C	T	C	SNV	SFSWAP	SFSWAP:exonic:NM_001261411.1	1	100	16	synonymous	NM_00126	c.2670T>C	p.(=)	0.33				0.11(COSM37	Alternative s	rs7136643	3.00E-04		1.0 (pos.132271113)	adenocarcinoma	Splicing fact	RNA bindi	Child Development Disorders, Pervasive;Mer			T=0, C=33	T=0.0, C=1.0	0.00001	504.598	33	T=0/0, C=21/1
chr5:149644597	G/G	T	G	SNV	CAMK2A	CAMK2A:intronic:NM_015981.3	1	100		intronic	NM_01598	c.158-19A	p.?	0.62					Calcium/caln	rs891938	0.0078		9.997E-1 (pos.149644597)		Calcium/calmodulin-de		Basal Ganglia Disea	(2Z,3E)-2,3'-BIINDOLE-2',3		T=0, G=22	T=0.0, G=1.0	0.00001	298.795	22	T=0/0, G=8/14
chr5:150407456	C/C	T	C	SNV	GPX3	GPX3:intronic:NM_002084.4	1	100		intronic	NM_00208	c.460-14T	p.?	0.01					Glutathione	rs8177447	0.1604	YES	9.753E-1;0.0 (pos.150407456)		Glutathione	cellular ox	Adenocarcinoma;C	Glutathione		T=0, C=54	T=0.0, C=1.0	0.00001	797.907	54	T=0/0, C=40/1
chr5:150413406	C/C	A	C	SNV	TNIP1	TNIP1:intronic:NM_001252391.1	1	100		intronic	NM_00125	c.1588-46	p.?	-0.37						rs2233305	0.1633	YES	9.651E-1 (pos.150413406)		TNFAIP3-int	MyD88-de	Arthritis;Arthritis, Rheumatoid;Autoimmune			A=0, C=32	A=0.0, C=1.0	0.00001	485.141	32	A=0/0, C=11/2
chr5:150413528	A/G	A	G	SNV	TNIP1	TNIP1:intronic:NM_001252391.1	1	50		intronic	NM_00125	c.1588-16	p.?	-0.16						rs60602178		YES			TNFAIP3-int	MyD88-de	Arthritis;Arthritis, Rheumatoid;Autoimmune			A=16, G=16	A=0.5, G=0.5	0.00001	158.383	32	A=6/10, G=4/1
chr6:30899233	T/T	C	T	SNV	SFTA2	SFTA2:utr_3:NM_205854.2	1	100	3	utr_3	NM_20585	c.269G>A	p.?	-0.73					Surfactant-as	rs4713402	0.1536	YES	5.346E-1 (pos.30899233)			Golgi apparatus,extracellular region,transport vesicle				C=0, T=26	C=0.0, T=1.0	0.00001	371.42	26	C=0/0, T=13/1
chr6:30899571	T/T	C	T	SNV	SFTA2	SFTA2:exonic:NM_205854.2	1	100	2	synonymous	NM_20585	c.63G>A	p.(=)	0.36				0.49(COSM40	Surfactant-as	rs2286656	0.1495	YES	3.385E-1;0.0 (pos.30899	acute_myeloid_leukaemia		Golgi apparatus,extracellular region,transport vesicle				C=0, T=31	C=0.0, T=1.0	0.00001	465.819	31	C=0/0, T=18/1
chr5:150704724	T/C	T	C	SNV	SLC36A2	SLC36A2:intronic:NM_181776.2	1	52.38		intronic	NM_18177	c.1010+12	p.?	-0.02					Transmembr	rs246497		YES			Solute carri	L-alanine t	Amino Acid Metabo	Cycloserine		T=10, C=11	T=0.4762, C=0.5238	0.00001	95.3022	21	T=2/8, C=5/6
chr5:150704758	A/G	A	G	SNV	SLC36A2	SLC36A2:intronic:NM_181776.2	1	52.38		intronic	NM_18177	c.1010+89	p.?	-2.9					Transmembr	rs246496,rs386693612		YES	9.827E-1 (pos.150704758)		Solute carri	L-alanine t	Amino Acid Metabo	Cycloserine		A=10, G=11	A=0.4762, G=0.5238	0.00001	95.3003	21	A=2/8, G=5/6
chr8:22021517	G/A	G	A	SNV	SFTPC	SFTPC:exonic:NM_001172410.1	1	45.83	5	missense	NM_00117	c.557G>A	p.Ser186Asn	0.61	0.06	46	0.005	0.27(COSM37	BRICHOS dom	rs1124	0.3349	YES	3.265E-1 (pos.22021517)	adenocarcinoma	Surfactant,	cellular pr	Adnexal Diseases;Congenital,		Benign	G=13, A=11	G=0.5417, A=0.4583	0.00001	93.4399	24	G=2/11, A=2/9
chr5:150887200	G/G	A	G	SNV	FAT2	FAT2:intronic:NM_001447.2	1	100		intronic	NM_00144	c.12060-2	p.?	-1.22					Cadherin dor	rs41290577	0.1804	YES	1.507E-1 (pos.150887200)		Fat tumor s	calcium io	Carcinoma;Carcinoma, Squamous Cell;Digest			A=0, G=49	A=0.0, G=1.0	0.00001	831.627	49	A=0/0, G=23/2
chr17:48252804	C/C	T	C	SNV	SGCA	SGCA:utr_3:NM_000023.3	1	100	9	utr_3	NM_00002	c.1170T>C	p.?	-3.61					Sarcoglycan a	rs2696288	0.0411	YES	9.998E-1 (pos.48252804)		Sarcoglycar	calcium io	Cardiomyopathies;Cardiovasc		Benign	T=0, C=21	T=0.0, C=1.0	0.00001	281.231	21	T=0/0, C=7/14
chr5:155771579	C/C	T	C	SNV	SGCD	SGCD:exonic:NM_001128209.1	1	100	2	synonymous	NM_00112	c.81T>C	p.(=)	-0.35				0.17(COSM41	Sarcoglycan c	rs1801193	0.4316	YES	7.835E-1 (pos.15577157	neoplasm	Sarcoglycar	cardiac mi	Cardiomegaly;Cardiomyopathi		Benign	T=0, C=119	T=0.0, C=1.0	0.00001	1913.83	119	T=0/0, C=50/6
chr13:23898509	T/C	T	C	SNV	SGCG	SGCG:exonic:NM_000231.2	1	50	8	synonymous	NM_00023	c.705T>C	p.(=)	0.04					Sarcoglycan c	rs1800353	0.3347	YES	7.69E-1 (pos.23898509)		Sarcoglycar	cardiac mi	Arthritis;Cardiomyopathies;Ca		Benign/Likely	T=16, C=16	T=0.5, C=0.5	0.00001	158.383	32	T=11/5, C=12/
chr2:223423362	G/G	A	G	SNV	SGPP2	SGPP2:exonic:NM_152386.3	1	100	5	synonymous	NM_15238	c.945A>G	p.(=)	-2.47					PAP2 superfa	rs4674664	0.0669	YES	9.999E-1 (pos.223423362)		Sphingosine	dephosphorylation,dihydrosphingosine-1-phosphate ph				A=0, G=121	A=0.0, G=1.0	0.00001	1953.69	121	A=0/0, G=62/5
chr7:101928495	G/C	G	C	SNV	SH2B2	SH2B2:exonic:NM_020979.4	1	47.62	1	missense	NM_02097	c.46G>C	p.Ala16Pro	-0.84		27			PH domain,P	rs803074	0.369	YES	2.984E-1 (pos.101928495)			B-1 B cell homeostasis,JAK pathway signal transduction				G=22, C=20	G=0.5238, C=0.4762	0.00001	212.953	42	G=16/6, C=6/1
chr19:6751281	C/T	C	T	SNV	SH2D3A,TRIP10	SH2D3A:downstream:NM_005490.2, TRIP10:utr_3:NM_004	1	50	14	utr_3	NM_00424	c.1697C>T	p.?, p.?	1.8				0.65(COSM45	Fes/CIP4, and	rs1049230,rs386806353,rs6773		YES	2.542E-1 (pos.6751281)	carcinoma,neo	Thyroid hor	GTPase activator activity,Golgi apparatus			Benign	C=14, T=14	C=0.5, T=0.5	0.00001	113.847	28	C=10/4, T=5/9
chr8:19221700	G/A	G	A	SNV	SH2D4A	SH2D4A:exonic:NM_001174159.1	1	47.62	7	missense	NM_00117	c.824G>A	p.Ser275Asn	0.09	0.51	46	0.001	0.05(COSM50	SH2 domain	rs34608771	0.3103	YES	5.162E-2;0.0 (pos.19221	carcinoma	SH2 domain	cytosol,negative regulation of phosphatase activity,pho				G=22, A=20	G=0.5238, A=0.4762	0.00001	214.147	42	G=3/19, A=8/1
chr1:21050958	T/T	C	T	SNV	SH2D5	SH2D5:exonic:NM_001103161.1	1	100	6	synonymous	NM_00110	c.561G>A	p.(=)	0.63						rs592060	0.244	YES	9.932E-1 (pos.21050958)			cell junction,postsynaptic density,postsynaptic membra				C=0, T=35	C=0.0, T=1.0	0.00001	543.903	35	C=0/0, T=23/1
chr2:235950187	C/C	T	C	SNV	SH3BP4	SH3BP4:exonic:NM_014521.2	1	100	4	synonymous	NM_01452	c.774T>C	p.(=)	-3.69				0.05(COSM41	SH3 domain,\	rs1469375,rs386656	0.45	YES	9.226E-1 (pos.23595018	neoplasm	SH3 domain	GDP-dissociation inhibitor activity,Ras GTPase binding,a				T=0, C=79	T=0.0, C=1.0	0.00001	1269.33	79	T=0/0, C=37/4
chr2:235962513	C/T	C	T	SNV	SH3BP4	SH3BP4:utr_3:NM_014521.2	1	47.62	6	utr_3	NM_01452	c.2945C>T	p.?	-0.02					SH3 domain,\	rs10929083		YES			SH3 domain	GDP-dissociation inhibitor activity,Ras GTPase binding,a				C=11, T=10	C=0.5238, T=0.4762	0.00001	81.1084	21	C=6/5, T=6/4
chr3:15311325	A/G	A	G	SNV	SH3BP5	SH3BP5:exonic:NM_004844.4	1	47.62	4	synonymous	NM_00484	c.390T>C	p.(=)	-3.24				0.01(COSM40	SH3 domain-I	rs1287467	0.3148	YES	1.621E-1 (pos.15311325)	acute_myeloid	SH3 domain	SH3 doma	Mental Disorders;Substance-Related Disorde			A=11, G=10	A=0.5238, G=0.4762	0.00001	82.29	21	A=8/3, G=8/2
chr9:17579189	CG/CG	C	CG	INDEL	SH3GL2	SH3GL2:utr_5:NM_003026.3	1	100	1	utr_5	NM_00302	c.-52C>CG	p.?	0.59					BAR domain,I	rs201727474					SH3 domain	Golgi mem	Basal Ganglia Diseases;Brain Diseases;Centr			C=0, CG=39	C=0.0, CG=1.0	0.00001	379.396	39	C=0/0, CG=14/
chr9:17579198	G/G	T	G	SNV	SH3GL2	SH3GL2:utr_5:NM_003026.3	1	100	1	utr_5	NM_00302	c.-43T>G	p.?	-0.07					BAR domain,I	rs4961574	0		1.0 (pos.17579198)		SH3 domain	Golgi mem	Basal Ganglia Diseases;Brain Diseases;Centr			T=0, G=39	T=0.0, G=1.0	0.00001	613.399	39	T=0/0, G=14/2
chr10:105361816	G/G	T	G	SNV	SH3PXD2A	SH3PXD2A:exonic:NM_014631.2	1	100	14	synonymous	NM_01463	c.3075A>C	p.(=)	0.1				0.18(COSM41	PX domain,SI	rs4918031	0.2294	YES	9.992E-1 (pos.10536181	Wilms_tumour,neoplasm		cell junction,cell projection,cytosol,extracellular matrix				T=0, G=22	T=0.0, G=1.0	0.00001	298.879	22	T=0/0, G=11/1
chr5:145393364	T/T	C	T	SNV	SH3RF2	SH3RF2:exonic:NM_152550.3	1	100	5	missense	NM_15255	c.799C>T	p.Arg267Cys	0.65	0.05	180	0.004	0.07(COSM41	RING-type zin	rs758037	0.3901	YES	7.029E-1 (pos.14539336	ductal_carcinoma	SH3 domain	metal ion binding,negative regulation of phosphatase ac				C=0, T=22	C=0.0, T=1.0	0.00001	298.892	22	C=0/0, T=16/6
chr5:158139856	T/C	T	C	SNV	EBF1	EBF1:intronic:NM_024007.4	1	59.09		intronic	NM_02400	c.1369+12	p.?	-0.68					IPT/TIG doma	rs12520521		YES			C2H2 zinc		Breast Diseases;Breast Neoplasms;Carcinog			T=9, C=13	T=0.4091, C=0.5909	0.00001	122.563	22	T=4/5, C=10/3
chr5:158697481	G/G	C	G	SNV	UBLCP1	UBLCP1:intronic:NM_145049.4	1	100		intronic	NM_14504	c.332+28C	p.?	-0.13					NLI interactir	rs3734104	0.3869	YES	5.437E-1 (pos.158697481)		Ubiquitin-li	nucleolus,nucleus,protein binding,protein dephosphory				C=0, G=47	C=0.0, G=1.0	0.00001	789.319	47	C=0/0, G=12/3
chr5:145439647	C/C	T	C	SNV	SH3RF2	SH3RF2:exonic:NM_152550.3	1	100	9	missense	NM_15255	c.1774T>C	p.Trp592Arg	0.69	1	101	0	0.22(COSM41	RING-type zin	rs2962525	1.00E-04		1.0 (pos.145439647)	neoplasm	SH3 domain	metal ion binding,negative regulation of phosphatase ac				T=0, C=60	T=0.0, C=1.0	0.00001	874.962	60	T=0/0, C=22/3
chr5:160044855	A/A	G	A	SNV	ATP10B	ATP10B:intronic:NM_025153.2	1	100		intronic	NM_02515	c.2526+16	p.?	0.48					Cation transp	rs1363199	0.2015	YES	8.424E-1;0.0 (pos.160044855)			ATP binding,cytoplasmic vesicle membrane,endoplasmi				G=0, A=33	G=0.0, A=1.0	0.00001	504.591	33	G=0/0, A=26/7
chr5:160047265	G/G	C	G	SNV	ATP10B	ATP10B:intronic:NM_025153.2	1	100		intronic	NM_02515	c.2416+89	p.?	0.3					Cation transp	rs6862674		YES				ATP binding,cytoplasmic vesicle membrane,endoplasmi				C=0, G=34	C=0.0, G=1.0	0.00001	524.184	34	C=0/0, G=24/1
chr4:8228894	C/C	T	C	SNV	SH3TC1	SH3TC1:exonic:NM_018986.4	1	100	12	synonymous	NM_01898	c.1473T>C	p.(=)	-4.13				0.01(COSM41	SH3 domain	rs1292647	3.00E-04	YES	9.999E-1 (pos.8228894)	neoplasm		biological	Colonic Diseases;Colorectal Neoplasms;Dige			T=0, C=22	T=0.0, C=1.0	0.00001	298.887	22	T=0/0, C=19/3
chr4:8229774	C/T	C	T	SNV	SH3TC1	SH3TC1:exonic:NM_018986.4	1	57.14	12	missense	NM_01898	c.2353C>T	p.Arg785Cys	0.55	0.15	180	0.017	0.06(COSM41	SH3 domain	rs1281149	0.2326	YES	4.729E-1 (pos.8229774)	neoplasm		biological	Colonic Diseases;Colorectal Neoplasms;Dige			C=9, T=12	C=0.4286, T=0.5714	0.00001	109.102	21	C=5/4, T=5/7
chr5:161322889	A/A	G	A	SNV	GABRA1	GABRA1:intronic:NM_001127645.1	1	100		intronic	NM_00112	c.1059+15	p.?	0.16					Neurotransm	rs2279020	0.3409	YES	4.337E-1 (pos.161322889)		Gamma-am	GABA rece	Adnexal Diseases;A	Acampros	Benign/Likely	G=0, A=22	G=0.0, A=1.0	0.00001	298.885	22	G=0/0, A=10/1
chr4:8229713	A/G	A	G	SNV	SH3TC1	SH3TC1:exonic:NM_018986.4	1	50	12	synonymous	NM_01898	c.2292A>G	p.(=)	1.36				0.17(COSM54	SH3 domain	rs1281148,rs769062	0.2391	YES	1.178E-4;0.0 (pos.82296	acute_myeloid_leukaemia		biological	Colonic Diseases;Colorectal Neoplasms;Dige			A=10, G=10	A=0.5, G=0.5	0.00001	82.8861	20	A=6/4, G=7/3
chr5:148407708	C/C	A	C	SNV	SH3TC2	SH3TC2:exonic:NM_024577.3	1	100	11	synonymous	NM_02457	c.1587T>G	p.(=)	0.03					SH3 domain	rs1432794,rs863224	0.0309	YES	9.998E-1 (pos.148407708)		SH3 domain	periphera	Charcot-Marie-Tooth Disease;		Benign/Likely	A=0, C=89	A=0.0, C=1.0	0.00001	1472.21	89	A=0/0, C=64/2
chr9:37974743	C/C	T	C	SNV	SHB	SHB:exonic:NM_003028.2	1	100	3	synonymous	NM_00302	c.930A>G	p.(=)	-6.17				0.01(COSM41	SH2 domain	rs1138374	0.4838	YES	7.848E-1 (pos.37974743)	hepatocellular	SHB adapto	B cell proliferation,SH3/SH2 adaptor activity,T cell recep				T=0, C=24	T=0.0, C=1.0	0.00001	334.792	24	T=0/0, C=13/1
chr5:16508674	G/G	A	G	SNV	RETREG1	RETREG1:intronic:NM_001034850.2	1	100		intronic	NM_00103	c.459-250	p.?	-0.22						rs332811	0.3785	YES	3.611E-1 (pos.16508674)			Golgi apparatus,cis-Golgi network,endopl			Benign	A=0, G=23	A=0.0, G=1.0	0.00001	316.747	23	A=0/0, G=11/1
chr5:16762646	C/C	T	C	SNV	MYO10	MYO10:intronic:NM_012334.2	1	100		intronic	NM_01233	c.1587+8A	p.?	-0.82				0.07(COSM54	FERM central	rs31511	0.0168	YES	8.006E-1 (pos.16762646)	acute_myeloid	Myosin X	ATP bindin	Depressive Disorder;Depressive Disorder, M			T=0, C=66	T=0.0, C=1.0	0.00001	1015.4	66	T=0/0, C=49/1
chr5:16763908	G/A	G	A	SNV	MYO10	MYO10:intronic:NM_012334.2	1	50		intronic	NM_01233	c.1327-44	p.?	-1.49					FERM central	rs39900	0.1439	YES	1.527E-1 (pos.16763908)		Myosin X	ATP bindin	Depressive Disorder;Depressive Disorder, M			G=18, A=18	G=0.5, A=0.5	0.00001	185.885	36	G=7/11, A=6/1
chr9:38068532	G/G	A	G	SNV	SHB	SHB:exonic:NM_003028.2	1	100	1	synonymous	NM_00302	c.111T>C	p.(=)	0.21				0.01(COSM63	SH2 domain	rs3750444		YES	4.801E-1 (pos.38068532)	carcinoma	SHB adapto	B cell proliferation,SH3/SH2 adaptor activity,T cell recep				A=0, G=34	A=0.0, G=1.0	0.00001	518.026	34	A=0/0, G=6/28
chr16:46655210	G/G	A	G	SNV	SHCBP1	SHCBP1:exonic:NM_024745.4	1	100	1	missense	NM_02474	c.62T>C	p.Met21Thr	-0.33	1	81	0	0(COSM3754	Periplasmic c	rs6598679	1.00E-04		1.0;0.0 (pos.46655210)	adenocarcinoma	SHC SH2 do	SH2 domain binding,cytoplasm,fibroblast growth factor				A=0, G=30	A=0.0, G=1.0	0.00001	446.638	30	A=0/0, G=12/1
chr5:167798585	C/T	C	T	SNV	WWC1	WWC1:intronic:NM_001161661.1	1	97.56		intronic	NM_00116	c.229+47C	p.?	-1.33					C2 domain,W	rs2287703	0.4272	YES	7.286E-1 (pos.167798585)		WW, C2, an	cell migration,cytoplasm,cytosol,establishment of cell p				C=1, T=40	C=0.0244, T=0.9756	0.00001	631.841	41	C=0/1, T=26/1
chr5:167920779	T/A	T	A	SNV	RARS	RARS:intronic:NM_002887.3	1	50		intronic	NM_00288	c.370-120	p.?	-0.79					Arginyl tRNA	rs74936344		YES			Arginyl-tRN	ATP bindin	Autoimmune Diseases;Female Urogenital Di			T=23, A=23	T=0.5, A=0.5	0.00001	258.572	46	T=11/12, A=16
chr1:154474131	G/G	C	G	SNV	SHE,TDRD10	SHE:exonic:NM_001010846.2, TDRD10:upstream:NM_0010	1	100	1	synonymous	NM_00101	c.372G>C,	p.(=), p.?	0.12				0.39(COSM37	SH2 domain	rs4845635	0.1683	YES	9.128E-1 (pos.15447413	adenocarcinoma	SH2 domain	RNA binding,SH3/SH2 adaptor activity,positive regulatio				C=0, G=50	C=0.0, G=1.0	0.00001	852.748	50	C=0/0, G=45/5
chr4:77660731	C/G	C	G	SNV	SHROOM3	SHROOM3:exonic:NM_020859.3	1	59.09	5	missense	NM_02085	c.1405C>G	p.Pro469Ala	3.26	0.02	27	0.668	0.9(COSM143	Apx/Shroom	rs344141	0.3858	YES	2.508E-1;0.0 (pos.77660	acute_myeloid	Shroom fam	actin filam	Digestive System Diseases;Eos		Benign	C=9, G=13	C=0.4091, G=0.5909	0.00001	122.403	22	C=6/3, G=8/5
chr4:77662309	C/T	C	T	SNV	SHROOM3	SHROOM3:exonic:NM_020859.3	1	55.77	5	synonymous	NM_02085	c.2983C>T	p.(=)	0.1				0.14(COSM37	Apx/Shroom	rs344143	0.4161	YES	4.261E-1 (pos.77662309)	adenocarcinoma	Shroom fam	actin filam	Digestive System Diseases;Eos		Benign	C=22, T=29	C=0.4314, T=0.5686	0.00001	308.166	52	C=12/10, T=10
chr3:164714532	T/T	C	T	SNV	SI	SI:exonic:NM_001041.3	1	100	39	missense	NM_00104	c.4569G>A	p.Met1523Ile	-1.35	1	10	0		Glycosyl hydr	rs4855271	0.0712	YES	9.189E-1 (pos.164714532)		Sucrase-iso	Golgi appa	Carbohydrate Meta	Acarbose,	Benign	C=0, T=248	C=0.0, T=1.0	0.00001	4208.41	248	C=0/0, T=119/
chr5:169361	T/T	C	T	SNV	PLEKHG4B	PLEKHG4B:intronic:NM_052909.4	1	100		intronic	NM_05290	c.3477-94	p.?	-2.06					RhoGEF doma	rs13159868		YES				Rho guanyl-nucleotide exchange factor activity,protein k				C=0, T=34	C=0.0, T=1.0	0.00001	524.186	34	C=0/0, T=23/1
chr5:169702390	A/A	C	A	SNV	LCP2	LCP2:intronic:NM_005565.4	1	100		intronic	NM_00556	c.255-35G	p.?	-1.41					SAM domain	rs2271146	0.4723	YES	7.948E-2 (pos.169702390)		Lymphocyte	Fc-epsilon receptor signaling pathway,T cell receptor sig				C=0, A=21	C=0.0, A=1.0	0.00001	281.235	21	C=0/0, A=14/7
chr5:169724471	G/A	G	A	SNV	LCP2	LCP2:intronic:NM_005565.4	1	59.18		intronic	NM_00556	c.78+67C>	p.?	-0.65					SAM domain	rs554080947					Lymphocyte	Fc-epsilon receptor signaling pathway,T cell receptor sig				G=40, A=58	G=0.4082, A=0.5918	0.00001	710.289	98	G=26/14, A=40
chr3:164764719	A/A	C	A	SNV	SI	SI:exonic:NM_001041.3	1	100	16	synonymous	NM_00104	c.1797G>T	p.(=)	-0.99				0.04(COSM11	Glycosyl hydr	rs9838509	0.4024	YES	8.189E-1;0.0 (pos.16476	adenocarcinoma	Sucrase-iso	Golgi appa	Carbohydrate Meta	Acarbose,	Benign	C=0, A=35	C=0.0, A=1.0	0.00001	543.903	35	C=0/0, A=20/1
chr5:171480055	A/A	G	A	SNV	STK10	STK10:intronic:NM_005990.3	1	100		intronic	NM_00599	c.2653-9C	p.?	-0.41					Polo kinase k	rs4569891	0.3998	YES	7.423E-1;0.0 (pos.171480055)		Serine/thre	ATP bindin	Endocrine Gland Neoplasms;Endocrine Syste			G=0, A=79	G=0.0, A=1.0	0.00001	1269.33	79	G=0/0, A=40/3
chr5:171491650	G/G	C	G	SNV	STK10	STK10:intronic:NM_005990.3	1	100		intronic	NM_00599	c.2082+74	p.?	-0.41					Polo kinase k	rs3812022		YES			Serine/thre	ATP bindin	Endocrine Gland Neoplasms;Endocrine Syste			C=0, G=35	C=0.0, G=1.0	0.00001	543.903	35	C=0/0, G=20/1
chr5:171491680	CT/CT	C	CT	INDEL	STK10	STK10:intronic:NM_005990.3	1	100		intronic	NM_00599	c.2082+44	p.?	1.32					Polo kinase kinase,Protein kinase domain,Protein tyrosine kinase						Serine/thre	ATP bindin	Endocrine Gland Neoplasms;Endocrine Syste			C=0, CT=54	C=0.0, CT=1.0	0.00001	395.43	54	C=0/0, CT=19/
chr5:171491682	T/T	C	T	SNV	STK10	STK10:intronic:NM_005990.3	1	100		intronic	NM_00599	c.2082+42	p.?	0.28					Polo kinase k	rs3812021	0.0499	YES	2.985E-2 (pos.171491682)		Serine/thre	ATP bindin	Endocrine Gland Neoplasms;Endocrine Syste			C=0, T=54	C=0.0, T=1.0	0.00001	797.318	54	C=0/0, T=19/3
chr3:164793758	C/A	C	A	SNV	SI	SI:exonic:NM_001041.3	1	47.62	2	missense	NM_00104	c.43G>T	p.Val15Phe	2.19	0.01	50	0.955	0.87(COSM37	Glycosyl hydr	rs9290264	0.2969	YES	1.137E-1 (pos.16479375	adenocarcinoma	Sucrase-iso	Golgi appa	Carbohydrate Meta	Acarbose,	Likely benign	C=11, A=10	C=0.5238, A=0.4762	0.00001	82.2902	21	C=3/8, A=6/4
chr5:172395408	A/A	T	A	SNV	RPL26L1	RPL26L1:intronic:NM_016093.3	1	100		intronic	NM_01609	c.169-54T	p.?	-2.31					KOW motif,Ri	rs475936		YES				RNA binding,cytoplasmic trans		Alpha-Hydroxy-Beta-Phe		T=0, A=27	T=0.0, A=1.0	0.00001	389.845	27	T=0/0, A=21/6
chr5:172396394	T/T	C	T	SNV	RPL26L1	RPL26L1:intronic:NM_016093.3	1	100		intronic	NM_01609	c.310-22C	p.?	-0.77					KOW motif,Ri	rs505788,rs7628748	0.0044		0.0 (pos.172396393),1.0 (pos.172396394)			RNA binding,cytoplasmic trans		Alpha-Hydroxy-Beta-Phe		C=0, T=34	C=0.0, T=1.0	0.00001	524.175	34	C=0/0, T=13/2
chr5:173200	T/T	C	T	SNV	PLEKHG4B	PLEKHG4B:intronic:NM_052909.4	1	100		intronic	NM_05290	c.4221+18	p.?	0.29					RhoGEF doma	rs6885136	0.4072	YES	2.612E-1;0.0 (pos.173200)			Rho guanyl-nucleotide exchange factor activity,protein k				C=0, T=24	C=0.0, T=1.0	0.00001	334.792	24	C=0/0, T=7/17
chr5:173258	G/G	A	G	SNV	PLEKHG4B	PLEKHG4B:intronic:NM_052909.4	1	100		intronic	NM_05290	c.4221+76	p.?	-0.32					RhoGEF doma	rs6893369		YES				Rho guanyl-nucleotide exchange factor activity,protein k				A=0, G=21	A=0.0, G=1.0	0.00001	281.235	21	A=0/0, G=6/15
chr3:113320477	C/T	C	T	SNV	SIDT1	SIDT1:exonic:NM_017699.2	1	50	11	missense	NM_01769	c.1088C>T	p.Thr363Ile	5.08	0.45	89	0.292	0.97(COSM63	dsRNA-gated	rs33990195	0.1014	YES	3.777E-2 (pos.11332047	carcinoma		RNA bindir	Genital Diseases, Male;Genital Neoplasms, I			C=27, T=27	C=0.5, T=0.5	0.00001	262.866	54	C=9/18, T=11/
chr5:175992258	G/G	A	G	SNV	CDHR2	CDHR2:intronic:NM_017675.4	1	100		intronic	NM_01767	c.-14-81A>	p.?	0.1					Cadherin dor	rs6556275		YES				apical plasma membrane,brush border,brush border me				A=0, G=21	A=0.0, G=1.0	0.00001	281.235	21	A=0/0, G=12/9
chr5:175995571	G/G	C	G	SNV	CDHR2	CDHR2:intronic:NM_017675.4	1	100		intronic	NM_01767	c.125-108	p.?	0.38					Cadherin dor	rs12716317		YES				apical plasma membrane,brush border,brush border me				C=0, G=21	C=0.0, G=1.0	0.00001	274.503	21	C=0/0, G=15/6
chr5:176004312	G/G	A	G	SNV	CDHR2	CDHR2:intronic:NM_017675.4	1	100		intronic	NM_01767	c.1195-88	p.?	-0.31					Cadherin dor	rs11749537						apical plasma membrane,brush border,brush border me				A=0, G=30	A=0.0, G=1.0	0.00001	451.019	30	A=0/0, G=26/4
chr5:176004846	T/T	C	T	SNV	CDHR2	CDHR2:intronic:NM_017675.4	1	100		intronic	NM_01767	c.1512+47	p.?	-1.29					Cadherin dor	rs1565149,rs756396	0.0726	YES	0.0 (pos.176004844),7.819E-1 (pos.176004846)			apical plasma membrane,brush border,brush border me				C=0, T=35	C=0.0, T=1.0	0.00001	543.895	35	C=0/0, T=17/1
chr5:176004903	C/C	T	C	SNV	CDHR2	CDHR2:intronic:NM_017675.4	1	100		intronic	NM_01767	c.1512+10	p.?	-2.57					Cadherin dor	rs11747451		YES				apical plasma membrane,brush border,brush border me				T=0, C=35	T=0.0, C=1.0	0.00001	543.903	35	T=0/0, C=13/2
chr11:408174	G/T	G	T	SNV	SIGIRR	SIGIRR:exonic:NM_021805.2	1	52.38	4	missense	NM_02180	c.239C>A	p.Ser80Tyr	2.56	0	144	0.999		TIR domain	rs117739035	0.0309	YES	3.485E-4 (pos.408174)		Single immu	acute-phase response,integral component of membrane				G=10, T=11	G=0.4762, T=0.5238	0.00001	95.3003	21	G=6/4, T=2/9
chr20:3677357	C/C	T	C	SNV	SIGLEC1	SIGLEC1:exonic:NM_023068.3	1	100	10	synonymous	NM_02306	c.2559A>G	p.(=)	0.22				0.04(COSM41	CD80-like C2-	rs501538	0.0144	YES	1.0 (pos.3677357)	neoplasm	Sialic acid-b	carbohydr	Influenza, Human;R	Beta-D-Glucose,N-acetyl		T=0, C=27	T=0.0, C=1.0	0.00001	389.976	27	T=0/0, C=15/1
chr5:176017549	T/T	C	T	SNV	CDHR2	CDHR2:intronic:NM_017675.4	1	100		intronic	NM_01767	c.3415-15	p.?	-0.27					Cadherin dor	rs4868662	0.4482	YES	2.358E-2 (pos.176017549)			apical plasma membrane,brush border,brush border me				C=0, T=90	C=0.0, T=1.0	0.00001	1493.12	90	C=0/0, T=47/4
chr19:52004743	G/A	G	A	SNV	SIGLEC12	SIGLEC12:exonic:NM_053003.3	1	57.14	1	missense	NM_05300	c.245C>T	p.Ala82Val	-1.32	0.23	64	0.118	0(COSM1487	Immunoglob	rs3810110	0.3598	YES	3.509E-1 (pos.52004743)	acute_myeloid	Sialic acid-b	carbohydrate binding,cell adhesion,integral component				G=21, A=28	G=0.4286, A=0.5714	0.00001	342.279	49	G=13/8, A=14/
chr5:176305466	A/A	T	A	SNV	UNC5A	UNC5A:intronic:NM_133369.2	1	100		intronic	NM_13336	c.2020-10	p.?	-0.73				0.06(COSM54	Death domai	rs2304516	0.1859	YES	6.04E-1 (pos.176305466)	acute_myeloid	UNC5, C. ele	anterior/posterior axon guidance,apoptotic process,axc				T=0, A=50	T=0.0, A=1.0	0.00001	852.207	50	T=0/0, A=20/3
chr5:176306676	A/A	G	A	SNV	UNC5A	UNC5A:intronic:NM_133369.2	1	100		intronic	NM_13336	c.2364-46	p.?	0.1					Death domai	rs2292257	0.1907	YES	4.732E-1;1.197E-4 (pos.176306676)		UNC5, C. ele	anterior/posterior axon guidance,apoptotic process,axc				G=0, A=22	G=0.0, A=1.0	0.00001	298.892	22	G=0/0, A=16/6
chr19:51628529	G/G	A	G	SNV	SIGLEC9	SIGLEC9:exonic:NM_014441.2	1	100	1	missense	NM_01444	c.298A>G	p.Lys100Glu	-1.31	0.23	56	0.076	0(COSM4000	Immunoglob	rs2075803	0.4322	YES	5.599E-1 (pos.51628529)	acute_myeloid	Sialic acid-b	carbohydrate binding,cell adhesion,cell surface recepto				A=0, G=114	A=0.0, G=1.0	0.00001	1815.3	114	A=0/0, G=39/7
chr19:51630482	A/A	C	A	SNV	SIGLEC9	SIGLEC9:exonic:NM_014441.2	1	100	4	missense	NM_01444	c.944C>A	p.Ala315Glu	-0.53	1	107	0	0(COSM4000	Immunoglob	rs2258983	0.4256	YES	5.595E-1 (pos.51630482)	acute_myeloid	Sialic acid-b	carbohydrate binding,cell adhesion,cell surface recepto				C=0, A=29	C=0.0, A=1.0	0.00001	427.603	29	C=0/0, A=10/1
chr5:176831980	C/C	T	C	SNV	F12	F12:intronic:NM_000505.3	1	100		intronic	NM_00050	c.530-65A	p.?	-4.14					EGF-like dom	rs17876029		YES			Coagulation	Factor XII	Angioedema;Angio	Conestat alfa,Ethanolam		T=0, C=20	T=0.0, C=1.0	0.00001	263.784	20	T=0/0, C=4/16
chr9:34635598	C/C	T	C	SNV	SIGMAR1	SIGMAR1:utr_3:NM_005866.3	1	100	4	utr_3	NM_00586	c.703A>G	p.?	0.79					ERG2 and Sig	rs4879809	6.00E-04		1.0;0.0 (pos.34635598)		Sigma nono	cell juncti	Amnesia;Amyotrop	Amitriptyl	Uncertain sig	T=0, C=39	T=0.0, C=1.0	0.00001	623.978	39	T=0/0, C=20/1
chr19:16976286	G/G	A	G	SNV	SIN3B	SIN3B:exonic:NM_015260.3	1	100	12	synonymous	NM_01526	c.1545A>G	p.(=)	-1.92				0.03(COSM16	C-terminal do	rs773911	0		9.957E-1 (pos.16976286)	carcinoma	Sin3, yeast,	RNA polymerase II transcription corepressor activity,Sin				A=0, G=38	A=0.0, G=1.0	0.00001	603.785	38	A=0/0, G=27/1
chr5:176930135	A/A	G	A	SNV	DOK3	DOK3:downstream:NM_024872.2	1	100		intronic	NM_02487	c.*849C>T	p.?	-0.25						rs28376231	3.00E-04		1.0 (pos.176930135)		Docking pro	ficolin-1-ri	Lung Diseases;Lung Neoplasms;Neoplasms;			G=0, A=39	G=0.0, A=1.0	0.00001	623.98	39	G=0/0, A=20/1
chr5:176936272	G/T	G	T	SNV	DOK3	DOK3:intronic:NM_024872.2	1	50		intronic	NM_02487	c.234+204	p.?	0.43					PTB domain (	rs2161110		YES			Docking pro	ficolin-1-ri	Lung Diseases;Lung Neoplasms;Neoplasms;			G=19, T=19	G=0.5, T=0.5	0.00001	200.223	38	G=11/8, T=10/
chr5:176939051	C/C	T	C	SNV	DDX41	DDX41:intronic:NM_016222.3	1	100		intronic	NM_01622	c.1732+46	p.?	-1.69					DEAD/DEAH b	rs28464438,rs75778	1.00E-04		0.0 (pos.176939050),1.0 (pos.17693905		DEAD/H box	ATP bindin	Leukemia;Leukemia, Myeloid;Leukemia, Mye			T=0, C=62	T=0.0, C=1.0	0.00001	939.123	62	T=0/0, C=58/4
chr5:176939688	T/T	C	T	SNV	DDX41	DDX41:intronic:NM_016222.3	1	100		intronic	NM_01622	c.1400-42	p.?	0.06					DEAD/DEAH b	rs28544348	2.00E-04		1.0 (pos.176939688)		DEAD/H box	ATP bindin	Leukemia;Leukemia, Myeloid;Leukemia, Mye			C=0, T=42	C=0.0, T=1.0	0.00001	685.199	42	C=0/0, T=35/7
chr5:176939735	G/G	A	G	SNV	DDX41	DDX41:intronic:NM_016222.3	1	100		intronic	NM_01622	c.1399+46	p.?	-0.41					DEAD/DEAH b	rs335436	0.001	YES	1.0 (pos.176939735)		DEAD/H box	ATP bindin	Leukemia;Leukemia, Myeloid;Leukemia, Mye			A=0, G=64	A=0.0, G=1.0	0.00001	977.6	64	A=0/0, G=34/3
chr5:176940871	C/C	G	C	SNV	DDX41	DDX41:intronic:NM_016222.3	1	100		intronic	NM_01622	c.936-23C	p.?	0.5					DEAD/DEAH b	rs335439	2.00E-04	YES	1.0 (pos.176940871)		DEAD/H box	ATP bindin	Leukemia;Leukemia, Myeloid;Leukemia, Mye			G=0, C=21	G=0.0, C=1.0	0.00001	281.212	21	G=0/0, C=11/1
chr19:16976289	T/C	T	C	SNV	SIN3B	SIN3B:exonic:NM_015260.3	1	47.37	12	synonymous	NM_01526	c.1548T>C	p.(=)	-4.72				0.03(COSM37	C-terminal do	rs2303091	0.1576	YES	2.588E-1 (pos.16976289)	adenocarcinoma	Sin3, yeast,	RNA polymerase II transcription corepressor activity,Sin				T=20, C=18	T=0.5263, C=0.4737	0.00001	184.537	38	T=16/4, C=11/
chr5:177019688	C/C	G	C	SNV	TMED9	TMED9:intronic:NM_017510.5	1	100		intronic	NM_01751	c.285+12G	p.?	0.42					emp24/gp25	rs4607363,rs757348392,rs7639		YES	1.334E-2 (pos.177019687),5.612E-3;1.459E-2;0.0;0.0			COPI coating of Golgi vesicle,ER to Golgi vesicle-mediate				G=0, C=80	G=0.0, C=1.0	0.00001	1279.7	80	G=0/0, C=36/4
chr11:65415028	G/G	T	G	SNV	SIPA1	SIPA1:exonic:NM_006747.3	1	100	9	synonymous	NM_00674	c.2205T>G	p.(=)	-1.97				0.02(COSM41	PDZ domain (	rs4930157,rs774696	8.00E-04		0.0 (pos.65415026),1.0 (	neoplasm	Signal-indu	GTPase ac	Melanoma;Neoplasms;Neoplasms by Histolo			T=0, G=39	T=0.0, G=1.0	0.00001	623.977	39	T=0/0, G=9/30
chr5:177036043	G/G	A	G	SNV	B4GALT7	B4GALT7:intronic:NM_007255.2	1	100		intronic	NM_00725	c.828+28A	p.?	-1.86					N-terminal do	rs6600953	3.00E-04		1.0 (pos.177036043)		Xylosylprot	Golgi appa	Cardiovascular Diseases;Collagen Diseases;			A=0, G=25	A=0.0, G=1.0	0.00001	353.019	25	A=0/0, G=16/9
chr20:1551485	T/T	C	T	SNV	SIRPB1	SIRPB1:exonic:NM_006065.4	1	100	4	synonymous	NM_00606	c.1050G>A	p.(=)	-1.35				0.01(COSM37	CD80-like C2-	rs2254458	0.3476	YES	8.153E-1 (pos.1551485)	adenocarcinoma		cell surface receptor signaling pathway,extracellular ex				C=0, T=97	C=0.0, T=1.0	0.00001	1634.89	97	C=0/0, T=45/5
chr20:1552430	C/C	T	C	SNV	SIRPB1	SIRPB1:exonic:NM_006065.4	1	100	3	missense	NM_00606	c.687A>G	p.Ile229Met	-0.01	0.01	10	0	0(COSM1130	CD80-like C2-	rs2253427,rs386811	0.2153	YES	8.159E-1 (pos.1552430)	adenocarcinoma,neoplasm		cell surface receptor signaling pathway,extracellular ex				T=0, C=27	T=0.0, C=1.0	0.00001	389.984	27	T=0/0, C=13/1
chr20:1532595	C/C	T	C	SNV	SIRPD	SIRPD:exonic:NM_178460.2	1	100	2	missense	NM_17846	c.163A>G	p.Asn55Asp	-0.44	0.52	23	0		Immunoglob	rs2249317	0.1533	YES	8.127E-1 (pos.1532595)			extracellular region				T=0, C=28	T=0.0, C=1.0	0.00001	408.714	28	T=0/0, C=20/8
chr19:4180836	T/T	C	T	SNV	SIRT6	SIRT6:exonic:NM_016539.3	1	100	2	missense	NM_01653	c.137G>A	p.Ser46Asn	1.56	0.45	46	0		Sir2 family	rs352493	0.0527	YES	7.181E-1 (pos.4180836)		Sirtuin 6 (Si	NAD(P)+-pr	Cardiomegaly;Cardiovascular Diseases;Hear			C=0, T=51	C=0.0, T=1.0	0.00001	754.584	51	C=0/0, T=24/2
chr18:47908556	A/A	G	A	SNV	SKA1	SKA1:exonic:NM_001039535.2	1	100	4	missense	NM_00103	c.271G>A	p.Val91Ile	1.08	1	29	0	0.05(COSM37	Spindle and k	rs6507992	0.3936	YES	9.026E-1 (pos.47908556)	adenocarcinoma	Spindle- and	cell division,chromosome segregation,condensed chron				G=0, A=86	G=0.0, A=1.0	0.00001	1410.93	86	G=0/0, A=47/3
chr13:21732094	T/T	C	T	SNV	SKA3	SKA3:exonic:NM_145061.5	1	100	7	synonymous	NM_14506	c.1086G>A	p.(=)	0.17						rs4770121	0.0556	YES	9.995E-1 (pos.21732094)			cell division,chromosome segregation,condensed chron				C=0, T=31	C=0.0, T=1.0	0.00001	465.819	31	C=0/0, T=16/1
chr5:178421269	T/T	G	T	SNV	GRM6	GRM6:intronic:NM_000843.3	1	100		intronic	NM_00084	c.504+173	p.?	-1.53					7 transmemb	rs2856359,rs386695560		YES			Glutamate	G-protein	Congenital, Hereditary, and Neonatal Diseas			G=0, T=24	G=0.0, T=1.0	0.00001	334.749	24	G=0/0, T=10/1

chr17:46262171	T/T	C	T	SNV	SKAP1	SKAP1:exonic:NM 003726.3	1	100	7	missense	NM 00372	c.481G>A	p.Gly161Ser	1.84	1	56	0	0.11(COSM11	PH domain,SI	rs2278868,rs767650	0.3966	YES	7.593E-1;0.0 (pos.46262	adenocarcino	SRC kinase-	SH2 doma	Adnexal Diseases;Endocrine Gland Neoplasm			C=0, T=41	C=0.0, T=1.0	0.00001	664.7	41	C=0/0, T=12/2
chr4:48492434	C/C	G	C	SNV	SLC10A4,ZAR1	SLC10A4:downstream:NM 152679.3, ZAR1:exonic:NM 175	1	100	1	missense	NM 15267	c.*1478G>	p.?, p.Gln42His	0.05	0.74	24	0	0.01(COSM37	Zinc-binding	rs10008444,rs78008	0	YES	1.0;0.0 (pos.48492434)	adenocarcino	Zygote arre	bile acid and bile salt transport,bile acid:sodium sympo				G=0, C=37	G=0.0, C=1.0	0.00001	585.159	37	G=0/0, C=12/2
chr4:87769929	C/C	T	C	SNV	SLC10A6	SLC10A6:exonic:NM 197965.2	1	100	1	missense	NM 19796	c.340A>G	p.Ile114Val	1.26	0.04	29	0.119	0.8(COSM376	Sodium Bile a	rs13106574	0.2007	YES	9.309E-4 (pos.87769929)	adenocarcino	Solute carri	bile acid and bile salt transpor		Prasterone sulfate,Pregr		T=0, C=46	T=0.0, C=1.0	0.00001	766.357	46	T=0/0, C=20/2
chr2:219254622	G/G	A	G	SNV	SLC11A1	SLC11A1:exonic:NM 000578.3	1	100	9	synonymous	NM 00057	c.825A>G	p.(=)	-1.3					Natural resis	rs2695342,rs770766	5.00E-04		1.0 (pos.219254622),4.634E-4;0.0 (pos.2		Solute carri	L-arginine	Acute Kidney Injury;Arthritis;Arthritis, Juveni			A=0, G=40	A=0.0, G=1.0	0.00001	643.942	40	A=0/0, G=20/2
chr15:48580713	C/C	T	C	SNV	SLC12A1	SLC12A1:exonic:NM 001184832.1	1	100	23	missense	NM 00118	c.2873T>C	p.Val958Ala	1.08	1	64	0		Amino acid p	rs1552311	0		1.0 (pos.48580713)		Solute carri	apical pla	Adrenal Gland Dise	Bumetani	Benign	T=0, C=112	T=0.0, C=1.0	0.00001	1776.44	112	T=0/0, C=44/6
chr15:48595192	T/T	G	T	SNV	SLC12A1	SLC12A1:utr 3:NM 001184832.1	1	100	27	utr 3	NM 00118	c.3410G>T	p.?	-0.38					Amino acid p	rs8025278		YES			Solute carri	apical pla	Adrenal Gland Dise	Bumetani	Benign	G=0, T=23	G=0.0, T=1.0	0.00001	316.747	23	G=0/0, T=7/16
chr5:179069309	C/C	G	C	SNV	C5orf60	C5orf60:intronic:NM 001142306.2	1	100		intronic	NM 00114	c.*847+18	p.?	-1.67						rs6887611	6.00E-04	YES	1.0 (pos.179069309)			integral component of membrane,protein binding				G=0, C=23	G=0.0, C=1.0	0.00001	316.748	23	G=0/0, C=9/14
chr5:179563633	G/G	T	G	SNV	RASGEF1C	RASGEF1C:intronic:NM 175062.3	1	100		intronic	NM 17506	c.301-118	p.?	-0.6					RasGEF N-ter	rs1867454		YES				guanyl-nucleotide exchange factor activity,intracellular,				T=0, G=43	T=0.0, G=1.0	0.00001	705.844	43	T=0/0, G=7/36
chr5:179565071	C/C	G	C	SNV	RASGEF1C	RASGEF1C:intronic:NM 175062.3	1	100		intronic	NM 17506	c.-5-13C>G	p.?	0.88					RasGEF N-ter	rs4700878	9.00E-04	YES	9.999E-1;0.0 (pos.179565071)			guanyl-nucleotide exchange factor activity,intracellular,				G=0, C=27	G=0.0, C=1.0	0.00001	389.949	27	G=0/0, C=19/8
chr5:179565136	C/A	C	A	SNV	RASGEF1C	RASGEF1C:intronic:NM 175062.3	1	97.06		intronic	NM 17506	c.-5-78G>T	p.?	1.12					RasGEF N-ter	rs4700715		YES				guanyl-nucleotide exchange factor activity,intracellular,				C=1, A=33	C=0.0294, A=0.9706	0.00001	492.963	34	C=0/1, A=19/1
chr5:179728707	C/C	T	C	SNV	GFPT2	GFPT2:intronic:NM 005110.3	1	100		intronic	NM 00511	c.2005-99	p.?	-2.15					Glutamine ar	rs4700931		YES			Glutamine:	UDP-N-acetylglucosamine bios		L-Glutamine		T=0, C=21	T=0.0, C=1.0	0.00001	281.229	21	T=0/0, C=12/9
chr16:56904587	G/G	C	G	SNV	SLC12A3	SLC12A3:exonic:NM 000339.2	1	100	6	missense	NM 00033	c.791C>G	p.Ala264Gly	7.97	1	60	0	0.06(COSM41	Amino acid p	rs1529927,rs753631	0.0367	YES	9.997E-1;3.467E-4 (pos.5	neoplasm	Solute carri	apical pla	Cardiovascular Dis	Bendroflumethiazide,Be		C=0, G=37	C=0.0, G=1.0	0.00001	583.597	37	C=0/0, G=7/30
chr5:179765467	T/T	C	T	SNV	GFPT2	GFPT2:intronic:NM 005110.3	1	100		intronic	NM 00511	c.115+26G	p.?	-1.48					Glutamine ar	rs55946431	0.3653	YES	5.269E-1 (pos.179765467)		Glutamine:	UDP-N-acetylglucosamine bios		L-Glutamine		C=0, T=37	C=0.0, T=1.0	0.00001	583.701	37	C=0/0, T=30/7
chr20:44678347	CA/CA	C	CA	INDEL	SLC12A5	SLC12A5:exonic:NM 001134771.1	1	100	17	frameshiftInsertion	NM 00113	c.2168 21	p.Lys725fs	7.65					Amino acid permease,Solute carrier family 12						Solute carri	cellular ch	Brain Diseases;Cen	Bumetanide,Potassium (		C=0, CA=14	C=0.0, CA=1.0	0.00001	2115.52	142	C=0/0, CA=57/
chr5:180041008	C/C	T	C	SNV	FLT4	FLT4:intronic:NM 182925.4	1	100		intronic	NM 18292	c.3331+60	p.?	-0.29					Immunoglobi	rs366388		YES			fms-related	ATP bindin	Adnexal Diseases:(	ABT-869,Axitinib,IMC-1C		T=0, C=38	T=0.0, C=1.0	0.00001	603.786	38	T=0/0, C=20/1
chr5:180056620	A/A	G	A	SNV	FLT4	FLT4:intronic:NM 182925.4	1	100		intronic	NM 18292	c.816+76C	p.?	-0.31					Immunoglobi	rs414348		YES			fms-related	ATP bindin	Adnexal Diseases:(	ABT-869,Axitinib,IMC-1C		G=0, A=22	G=0.0, A=1.0	0.00001	298.892	22	G=0/0, A=13/9
chr3:124802881	G/G	A	G	SNV	SLC12A8	SLC12A8:exonic:NM 024628.5	1	100	14	synonymous	NM 02462	c.1998T>C	p.(=)	-0.36				0.08(COSM54	Amino acid p	rs2981483	0.4816	YES	5.732E-1;0.0 (pos.12480	acute_myeloid	leukaemia	chloride transmembrane transport,integral component				A=0, G=44	A=0.0, G=1.0	0.00001	726.569	44	A=0/0, G=12/3
chr5:180486224	G/G	T	G	SNV	BTNL9	BTNL9:intronic:NM 152547.4	1	100		intronic	NM 15254	c.983-13T	p.?	0.01					Immunoglobi	rs7379015	0.158	YES	8.011E-1 (pos.180486224)			integral component of membrane,plasma membrane				T=0, G=79	T=0.0, G=1.0	0.00001	1269.33	79	T=0/0, G=49/3
chr3:124802888	T/T	C	T	SNV	SLC12A8	SLC12A8:exonic:NM 024628.5	1	100	14	missense	NM 02462	c.1991G>	p.Arg664Gln	-0.95	0.59	43	0.022		Amino acid p	rs2981482	0.4793	YES	5.708E-1 (pos.124802888)			chloride transmembrane transport,integral component				C=0, T=45	C=0.0, T=1.0	0.00001	747.388	45	C=0/0, T=12/3
chr5:180627224	G/A	G	A	SNV	TRIM7	TRIM7:intronic:NM 203293.2	1	50		intronic	NM 20329	c.619-143	p.?	-0.1					B-box zinc fin	rs2546397		YES			Tripartite m	cytoplasm,nucleus,protein binding,protein ubiquitinatic				G=15, A=15	G=0.5, A=0.5	0.00001	145.018	30	G=6/9, A=8/7
chr3:124837684	C/C	T	C	SNV	SLC12A8	SLC12A8:exonic:NM 024628.5	1	100	8	missense	NM 02462	c.841A>G	p.Ile281Val	0.67	1	29	0		Amino acid p	rs621383	1.00E-04		9.999E-1 (pos.124837684)			chloride transmembrane transport,integral component				T=0, C=54	T=0.0, C=1.0	0.00001	798.148	54	T=0/0, C=12/4
chr3:124839470	G/A	G	A	SNV	SLC12A8	SLC12A8:exonic:NM 024628.5	1	95.35	7	missense	NM 02462	c.797C>T	p.Pro266Leu	6.07	1	98	0		Amino acid p	rs863642	2.00E-04		1.0 (pos.124839470)			chloride transmembrane transport,integral component				G=2, A=41	G=0.0465, A=0.9535	0.00001	649.298	43	G=1/1, A=23/1
chr5:230980	G/G	A	G	SNV	SDHA	SDHA:intronic:NM 004168.3	1	100		intronic	NM 00416	c.771-11A	p.?	-0.49					FAD binding c	rs2288461	0.1183	YES	6.643E-1;0.0 (pos.230980)		Succinate d	anaerobic	Arthritis;Brain Dise	2-Hexylox	Benign/Likely	A=0, G=83	A=0.0, G=1.0	0.00001	1349.13	83	A=0/0, G=60/2
chr7:100458795	C/C	T	C	SNV	SLC12A9	SLC12A9:exonic:NM 020246.3	1	100	10	synonymous	NM 02024	c.1254T>C	p.(=)	-5.47				0.06(COSM40	Amino acid p	rs314378	0.4763	YES	8.18E-1 (pos.100458795)	acute_myeloi	Solute carri	cation:chl	Cardiovascular Diseases:Hypertension:Vasc			T=0, C=25	T=0.0, C=1.0	0.00001	353.019	25	T=0/0, C=16/9
chr7:122809234	C/C	T	C	SNV	SLC13A1	SLC13A1:exonic:NM 022444.3	1	100	4	missense	NM 02244	c.521A>G	p.Asn174Ser	-0.43	0.34	46	0	0.03(COSM15	Citrate trans	rs2140516	0.3151	YES	3.525E-1 (pos.12280923	acute_myeloi	Solute carri	integral component of membra		Succinic acid		T=0, C=24	T=0.0, C=1.0	0.00001	334.792	24	T=0/0, C=10/1
chr17:26817537	A/A	G	A	SNV	SLC13A2	SLC13A2:exonic:NM 001145975.1	1	100	3	synonymous	NM 00114	c.444G>A	p.(=)	-5.35				0.01(COSM37	Citrate trans	rs11568466	0.2103	YES	3.446E-2;0.0 (pos.26817	adenocarcino	Solute carri	anion transmembrane transpo		Sodium Citrate,Succinic		G=0, A=29	G=0.0, A=1.0	0.00001	427.601	29	G=0/0, A=12/1
chr7:135406176	G/G	A	G	SNV	SLC13A4	SLC13A4:exonic:NM 012450.3	1	100	2	synonymous	NM 01245	c.195T>C	p.(=)	0.66				0.14(COSM15	Citrate trans	rs4596594	0.4584	YES	4.625E-1 (pos.13540617	adenocarcino	Solute carri	anion transmembrane transport,integral component of				A=0, G=27	A=0.0, G=1.0	0.00001	389.988	27	A=0/0, G=21/6
chr18:43206985	G/G	A	G	SNV	SLC14A2	SLC14A2:exonic:NM 001242692.1	1	100	5	missense	NM 00124	c.394A>G	p.Ile132Val	7.11	1	29	0		Urea transpo	rs1484873	0.0516	YES	8.58E-1 (pos.43206985)		Solute carri	apical pla	Alopecia;Hair Disea	Urea		A=0, G=94	A=0.0, G=1.0	0.00001	1576.17	94	A=0/0, G=17/7
chr18:43247001	G/G	C	G	SNV	SLC14A2	SLC14A2:exonic:NM 001242692.1	1	100	14	synonymous	NM 00124	c.1659C>G	p.(=)	0.72				0.89(COSM14	Urea transpo	rs2156609	0.1664	YES	4.042E-1 (pos.43247001)	acute_myeloi	Solute carri	apical pla	Alopecia;Hair Disea	Urea		C=0, G=20	C=0.0, G=1.0	0.00001	263.784	20	C=0/0, G=11/9
chr5:33643617	T/T	C	T	SNV	ADAMTS12	ADAMTS12:intronic:NM 030955.3	1	100		intronic	NM 03095	c.1480-42	p.?	-0.6					ADAM-TS Spa	rs4866409	0.357	YES	6.702E-1;0.0 (pos.33643617)		A disintegri	cell migration,cell-matrix adhesion,cellular response to				C=0, T=55	C=0.0, T=1.0	0.00001	814.907	55	C=0/0, T=10/4
chr5:34811734	C/T	C	T	SNV	RAI14	RAI14:intronic:NM 001145520.1	1	97.3		intronic	NM 00114	c.558-138	p.?	-1.64					Ankyrin repe	rs17522578		YES			Retinoic aci	apoptotic signaling pathway,cell cortex,cell differentiati				C=1, T=36	C=0.027, T=0.973	0.00001	553.019	37	C=0/1, T=24/1
chr12:16369828	T/T	A	T	SNV	SLC15A5	SLC15A5:exonic:NM 001170798.1	1	100	7	missense	NM 00117	c.1482T>A	p.Asp494Glu	3.66	1	45	0		POT family	rs1671511	0		1.0 (pos.16369828)			integral component of membrane,protein transport,sym				A=0, T=24	A=0.0, T=1.0	0.00001	334.791	24	A=0/0, T=12/1
chr12:16430284	A/A	G	A	SNV	SLC15A5	SLC15A5:exonic:NM 001170798.1	1	100	1	synonymous	NM 00117	c.336C>T	p.(=)	0.1					POT family	rs1671487	0		1.0 (pos.16430284)			integral component of membrane,protein transport,sym				G=0, A=34	G=0.0, A=1.0	0.00001	524.185	34	G=0/0, A=18/1
chr5:354051	C/C	T	C	SNV	AHRR	AHRR:intronic:NM 001242412.1	1	100		intronic	NM 00124	c.256+25T	p.?	-0.92					Helix-loop-he	rs2013782	0.3802	YES	2.795E-1 (pos.354051)		Arylhydroca	DNA binding,cytosol,negative regulation of transcription				T=0, C=32	T=0.0, C=1.0	0.00001	485.102	32	T=0/0, C=21/1
chr17:6942108	T/T	C	T	SNV	SLC16A13	SLC16A13:exonic:NM 201566.2	1	100	3	synonymous	NM 20156	c.981C>T	p.(=)	-1.14					Major Facilit	rs11078663	0.3987	YES	5.089E-1 (pos.6942108)			Golgi appa	Diabetes Mellitus;Diabetes Mellitus, Type 2:			C=0, T=25	C=0.0, T=1.0	0.00001	353.019	25	C=0/0, T=13/1
chr5:35753715	C/C	T	C	SNV	SPEF2	SPEF2:intronic:NM 024867.3	1	100		intronic	NM 02486	c.3331-11	p.?	0.84					Adenylate kir	rs11957963	0.3336	YES	6.618E-1 (pos.35753715)		Sperm flagellar 2		Autoimmune Diseases;Autoim		Benign	T=0, C=32	T=0.0, C=1.0	0.00001	485.141	32	T=0/0, C=15/1
chr5:35776544	G/A	G	A	SNV	SPEF2	SPEF2:intronic:NM 024867.3	1	47.62		intronic	NM 02486	c.4217+47	p.?	-0.37					Adenylate kir	rs374151576	2.00E-04		0.0 (pos.35776544)		Sperm flagellar 2		Autoimmune Diseases;Autoimmune Disease			G=11, A=10	G=0.5238, A=0.4762	0.00001	82.2886	21	G=4/7, A=5/5
chr5:35792356	C/C	A	C	SNV	SPEF2	SPEF2:intronic:NM 024867.3	1	100		intronic	NM 02486	c.4448-86	p.?	-1.77					Adenylate kir	rs7730148		YES			Sperm flagellar 2		Autoimmune Diseases;Autoimmune Disease			A=0, C=29	A=0.0, C=1.0	0.00001	427.486	29	A=0/0, C=21/8
chr5:35807070	T/C	T	C	SNV	SPEF2	SPEF2:intronic:NM 024867.3	1	47.62		intronic	NM 02486	c.5256+16	p.?	-0.09					Adenylate kir	rs36093359	0.2875	YES	2.213E-1 (pos.35807070)		Sperm flagellar 2		Autoimmune Diseases;Autoimmune Disease			T=11, C=10	T=0.5238, C=0.4762	0.00001	80.9533	21	T=9/2, C=8/2
chr5:35957202	A/G	A	G	SNV	UGT3A1	UGT3A1:intronic:NM 152404.3	1	45.83		intronic	NM 15240	c.1075+88	p.?	-1.02					Glycosyltrans	rs1478449		YES			Uridine dipl	UDP-N-acetylglucosamine tran		Kaempherol,Quercetin		A=13, G=11	A=0.5417, G=0.4583	0.00001	93.3594	24	A=8/5, G=5/6
chr5:36137620	G/G	A	G	SNV	LMBRD2	LMBRD2:intronic:NM 001007527.1	1	100		intronic	NM 00100	c.369-77T	p.?	0.27					LMBR1-like m	rs267759		YES				integral component of membrane,membrane				A=0, G=58	A=0.0, G=1.0	0.00001	867.376	58	A=0/0, G=32/2
chr5:36201336	T/T	C	T	SNV	NADK2	NADK2:intronic:NM 001085411.2	1	100		intronic	NM 00108	c.957-73G	p.?	-0.1					ATP-NAD kina	rs7705396		YES			NAD kinase	ATP bindin	Congenital, Hereditary, and Neonatal Diseas			C=0, T=69	C=0.0, T=1.0	0.00001	1072.74	69	C=0/0, T=34/3
chr5:36217764	G/GTTAATCA	G	GTTAATCA	INDEL	NADK2	NADK2:intronic:NM 001085411.2	7	95.65		intronic	NM 00108	c.781+86C	p.?	0.64					ATP-NAD kina	rs111441491,rs892856171		YES			NAD kinase	ATP bindin	Congenital, Hereditary, and Neonatal Diseas			G=1, GTTAA	G=0.0435, GTTAATCA	0.00001	288.42	23	G=1/0, GTTAAT
chr5:36268217	AG/AG	A	AG	INDEL	RANBP3L	RANBP3L:intronic:NM 001161429.2	1	100		intronic	NM 00116	c.343+78T	p.?	2.03					RanBP1 domain						RAN-bindin	G1/S transition of mitotic cell cycle,GTPase activator act				A=0, AG=71	A=0.0, AG=1.0	0.00001	953.287	71	A=0/0, AG=42/
chr17:73089852	C/C	T	C	SNV	SLC16A5	SLC16A5:exonic:NM 001271765.1	1	100	3	synonymous	NM 00127	c.121T>C	p.(=)	2.52				0.21(COSM14	Major Facilit	rs4788863	0.2727	YES	3.527E-1 (pos.73089852)	neoplasm		integral component of membra		Pyruvic acid		T=0, C=27	T=0.0, C=1.0	0.00001	389.974	27	T=0/0, C=12/1
chr17:73089706	G/A	G	A	SNV	SLC16A5	SLC16A5:utr 5:NM 001271765.1	1	57.14	3	utr 5	NM 00127	c.-26G>A	p.?	2.67					Major Facilit	rs4789134	0.3445	YES	2.442E-1 (pos.73089706)			integral component of membra		Pyruvic acid		G=15, A=20	G=0.4286, A=0.5714	0.00001	218.655	35	G=10/5, A=14/
chr5:38527228	T/G	T	G	SNV	LIFR	LIFR:intronic:NM 002310.5	1	50		intronic	NM 00231	c.397+29A	p.?	-0.41					Fibronectin t	rs2289779	0.4519	YES	5.834E-1 (pos.38527228)		Leukemia in	cell surfac	Adnexal Diseases;Bone Diseases;Bone Disea			T=12, G=12	T=0.5, G=0.5	0.00001	106.71	24	T=5/7, G=7/5
chr5:38876573	C/C	T	C	SNV	OSMR	OSMR:intronic:NM 003999.2	1	100		intronic	NM 00399	c.246+98T	p.?	-0.62					Fibronectin t	rs357271		YES			Oncostatin	apical pla	Amyloidosis;Amyloidosis, Familial;Autoimmu			T=0, C=44	T=0.0, C=1.0	0.00001	726.565	44	T=0/0, C=26/1
chr5:38953427	C/C	T	C	SNV	RICTOR	RICTOR:intronic:NM 152756.4	1	100		intronic	NM 15275	c.2790+13	p.?	0.56					Rapamycin-ir	rs2115948		YES				TOR signal	Nutrition Disorders;Nutritional and Metaboli			T=0, C=68	T=0.0, C=1.0	0.00001	1053.45	68	T=0/0, C=34/3
chr5:38953447	T/A	T	A	SNV	RICTOR	RICTOR:intronic:NM 152756.4	1	97.06		intronic	NM 15275	c.2790+11	p.?	1.26					Rapamycin-ir	rs2115947		YES				TOR signal	Nutrition Disorders;Nutritional and Metaboli			T=2, A=66	T=0.0294, A=0.9706	0.00001	988.813	68	T=2/0, A=32/3
chr5:38954994	C/C	T	C	SNV	RICTOR	RICTOR:intronic:NM 152756.4	1	100		intronic	NM 15275	c.2610-31	p.?	-4.17					Rapamycin-ir	rs9292726	0.0214	YES	9.921E-1 (pos.38954994)			TOR signal	Nutrition Disorders;Nutritional and Metaboli			T=0, C=33	T=0.0, C=1.0	0.00001	490.24	33	T=0/0, C=7/26
chr5:38962543	G/G	A	G	SNV	RICTOR	RICTOR:intronic:NM 152756.4	1	100		intronic	NM 15275	c.1668+44	p.?	0.6					Rapamycin-ir	rs7729745	0.0212	YES	9.923E-1 (pos.38962543)			TOR signal	Nutrition Disorders;Nutritional and Metaboli			A=0, G=56	A=0.0, G=1.0	0.00001	832.055	56	A=0/0, G=35/2
chr5:39108309	G/G	A	G	SNV	FYB1	FYB1:intronic:NM 001243093.1	1	100		intronic	NM 00124	c.2497+24	p.?	0.13					Helically-exte	rs377022	0.21	YES	6.215E-1 (pos.39108309)			NLS-bearing protein import into nucleus,T cell receptor s				A=0, G=145	A=0.0, G=1.0	0.00001	2433.76	145	A=0/0, G=66/7
chr5:39108322	G/G	A	G	SNV	FYB1	FYB1:intronic:NM 001243093.1	1	100		intronic	NM 00124	c.2497+11	p.?	-0.76					Helically-exte	rs430630	0.1159	YES	8.401E-1 (pos.39108322)			NLS-bearing protein import into nucleus,T cell receptor s				A=0, G=146	A=0.0, G=1.0	0.00001	2463.83	146	A=0/0, G=67/7
chr20:61598899	G/G	A	G	SNV	SLC17A9	SLC17A9:utr 3:NM 022082.3	1	100	13	utr 3	NM 02208	c.1358A>G	p.?	-0.96					Major Facilit	rs3746750	0.384	YES	7.569E-1 (pos.61598899)		Solute carri	anion tran	Congenital, Hereditary, and Neonatal Diseas			A=0, G=22	A=0.0, G=1.0	0.00001	297.885	22	A=0/0, G=11/1
chr5:39390061	A/A	C	A	SNV	DAB2	DAB2:intronic:NM 001343.3	1	100		intronic	NM 00134	c.463-27G	p.?	0.4					Phosphotyro	rs2855512	0.0034	YES	6.403E-1 (pos.39390061)		Disabled, D	SMAD binc	Digestive System Diseases;Digestive System			C=0, A=41	C=0.0, A=1.0	0.00001	664.662	41	C=0/0, A=27/1
chr8:20036713	G/G	A	G	SNV	SLC18A1	SLC18A1:exonic:NM 003053.3	1	100	3	missense	NM 00305	c.407T>C	p.Ile136Thr	5.54	0.42	89	0		Major Facilit	rs1390938	0.2387	YES	7.224E-1 (pos.20036713)		Solute carri	aminergic neurotransmitter lo		Ephedra,MMDA,Metham		A=0, G=21	A=0.0, G=1.0	0.00001	281.235	21	A=0/0, G=9/12
chr5:40949997	C/T	C	T	SNV	C7	C7:intronic:NM 000587.3	1	53.49		intronic	NM 00058	c.983-9C>T	p.?	-0.67				0(COSM4418	Low-density l	rs1450656	0.354	YES	7.4E-1 (pos.40949997)	acute_myeloi	Complemer	cellular sc	Immune System Diseases;Immunologic Defic			C=20, T=23	C=0.4651, T=0.5349	0.00001	265.073	43	C=5/15, T=8/1
chr5:40958027	A/A	G	A	SNV	C7	C7:intronic:NM 000587.3	1	100		intronic	NM 00058	c.1261-10	p.?	-0.42					Low-density l	rs2652323		YES			Complemer	cellular sc	Immune System Diseases;Immunologic Defic			G=0, A=20	G=0.0, A=1.0	0.00001	263.786	20	G=0/0, A=6/14
chr5:40964816	A/A	C	A	SNV	C7	C7:intronic:NM 000587.3	1	100		intronic	NM 00058	c.1750-27	p.?	0.11					Low-density l	rs7713884	0.2295	YES	3.417E-1 (pos.40964816)		Complemer	cellular sc	Immune System Diseases;Immunologic Defic			C=0, A=35	C=0.0, A=1.0	0.00001	543.902	35	C=0/0, A=18/1
chr5:40980102	T/T	C	T	SNV	C7	C7:intronic:NM 000587.3	1	100		intronic	NM 00058	c.2350+91	p.?	0.07					Low-density l	rs2292727		YES			Complemer	cellular sc	Immune System Diseases;Immunologic Defic			C=0, T=21	C=0.0, T=1.0	0.00001	281.235	21	C=0/0, T=5/16
chr8:20036827	C/G	C	G	SNV	SLC18A1	SLC18A1:exonic:NM 003053.3	1	50	3	missense	NM 00305	c.293G>C	p.Ser98Thr	-0.75	0.74	58	0.033	0.12(COSM14	Major Facilit	rs2270637,rs757551	0.1907	YES	0.0 (pos.20036823),1.45	acute_myeloi	Solute carri	aminergic neurotransmitter lo		Ephedra,MMDA,Metham		C=28, G=28	C=0.5, G=0.5	0.00001	272.091	56	C=18/10, G=1
chr5:41032998	G/G	A	G	SNV	MROH2B	MROH2B:intronic:NM 173489.4	1	100		intronic	NM 17348	c.2362-75	p.?	-0.39						rs325831		YES				acrosomal vesicle,cell differentiation,cytoplasm,proteir				A=0, G=62	A=0.0, G=1.0	0.00001	940.32	62	A=0/0, G=26/3
chr10:50818762	T/T	C	T	SNV	SLC18A3	SLC18A3:utr 5:NM 003055.2	1	100	1	utr 5	NM 00305	c.-25C>T	p.?	0.21					Major Facilit	rs1880675	0.0056	YES	6.77E-1 (pos.50818762)		Solute carri	AP-1 adaptor complex,AP-2 adaptor complex,acetylcholi				C=0, T=24	C=0.0, T=1.0	0.00001	334.762	24	C=0/0, T=21/3
chr1:169435027	T/T	C	T	SNV	SLC19A2	SLC19A2:utr 3:NM 006996.2	1	100	6	utr 3	NM 00699	c.1554G>	p.?	0.35					Major Facilit	rs2678166					Solute carri	folic acid t	Anemia;Anemia, M	Thiamine	Benign	C=0, T=33	C=0.0, T=1.0	0.00001	508.97	33	C=0/0, T=21/1
chr1:53553702	G/G	A	G	SNV	SLC1A7	SLC1A7:exonic:NM 006671.5	1	100	11	synonymous	NM 00667	c.1662T>C	p.(=)	-2.17					Sodium:dicar	rs1288400	0.0012	YES	9.998E-1 (pos.53553702)			L-glutamate transmembrane t		Glutamic Acid		A=0, G=42	A=0.0, G=1.0	0.00001	685.21	42	A=0/0, G=24/1
chr1:53558270	G/G	A	G	SNV	SLC1A7	SLC1A7:exonic:NM 006671.5	1	100	7	synonymous	NM 00667	c.987T>C	p.(=)	-2.92				0.02(COSM41	Sodium:dicar	rs1288406	0.1598	YES	9.595E-1 (pos.53558270)	neoplasm		L-glutamate transmembrane t		Glutamic Acid		A=0, G=28	A=0.0, G=1.0	0.00001	408.697	28	A=0/0, G=12/1
chr5:43292908	C/A	C	A	SNV	HMGCS1	HMGCS1:intronic:NM 002130.7	1	50		intronic	NM 00213	c.1309+42	p.?	-0.15					Hydroxymeth	rs55794239	0.48	YES	1.44E-1 (pos.43292908)		3-hydroxy-3	brain deve	Nutritional and Me	(3R,5S,7R,12S,13R)-13-F(		C=28, A=28	C=0.5, A=0.5	0.00001	272.138	56	C=12/16, A=1
chr5:466811	C/C	T	C	SNV	EXOC3	EXOC3:intronic:NM 007277.4	1	100		intronic	NM 00727	c.2067-31	p.?	-6.35					Exocyst comp	rs2434697	0.1151	YES	5.682E-1 (pos.466811)		Exocyst con	Golgi appa	Congenital Abnormalities;Congenital, Heredi			T=0, C=34	T=0.0, C=1.0	0.00001	524.186	34	T=0/0, C=20/1
chr5:476530	G/G	C	G	SNV	SLC9A3,SLC9A3-AS1	SLC9A3:intronic:NM 004174.3, SLC9A3-AS1:exonic nc:NR 1	1	100		intronic	NM 00417	c.1891-37	p.?	-0.3					Sodium/hydr	rs2244240	0.1297	YES	5.257E-1 (pos.476530)		Solute carri	PDZ doma	Cardiovascular Diseases;Diabetes Mellitus;[			C=0, G=32	C=0.0, G=1.0	0.00001	484.753	32	C=0/0, G=17/1
chr5:479879	GC/GC	G	GC	INDEL	SLC9A3,SLC9A3-AS1	SLC9A3:intronic:NM 004174.3, SLC9A3-AS1:intronic nc:NR	1	100		intronic	NM 00417	c.1647+72	p.?	0.08					Sodium/hydr	rs376525752,rs5865334		YES			Solute carri	PDZ doma	Cardiovascular Diseases;Diabetes Mellitus;[			G=0, GC=34	G=0.0, GC=1.0	0.00001	381.24	34	G=0/0, GC=23,
chr5:479905	C/C	A	C	SNV	SLC9A3,SLC9A3-AS1	SLC9A3:intronic:NM 004174.3, SLC9A3-AS1:exonic nc:NR 1	1	100		intronic	NM 00417	c.1647+46	p.?	-1.12					Sodium/hydr	rs890986	0.1163	YES	5.636E-1 (pos.479905)		Solute carri	PDZ doma	Cardiovascular Diseases;Diabetes Mellitus;[			A=0, C=34	A=0.0, C=1.0	0.00001	523.6	34	A=0/0, C=23/1
chr5:49698180	A/A	G	A	SNV	EMB	EMB:intronic:NM 198449.2	1	100		intronic	NM 19844	c.878-27C	p.?	-0.14					Immunoglobi	rs13172025	0.4538	YES	4.906E-1 (pos.49698180)		Embigin	cell juncti	Dermatitis;Dermatitis, Allergic Contact;Derm			G=0, A=74	G=0.0, A=1.0	0.00001	1170.13	74	G=0/0, A=42/3
chr5:5146223	C/C	G	C	SNV	ADAMTS16	ADAMTS16:intronic:NM 139056.3	1	100		intronic	NM 13905	c.176-20G	p.?	-0.27					ADAM-TS Spa	rs1871468	0.1427	YES	6.407E-1 (pos.5146223)		A disintegri	branching involved in ureteric bud morphogenesis,male				G=0, C=29	G=0.0, C=1.0	0.00001	427.603	29	G=0/0, C=14/1
chr6:160551204	C/C	G	C	SNV	SLC22A1	SLC22A1:exonic:NM 003057.2	1	100	2	missense	NM 00305	c.480G>C	p.Leu160Phe	0.21	0.69	22	0.004		Major Facilit	rs683369,rs76305211	0.2213	YES	0.0 (pos.160551201),8.593E-1 (pos.1605		Solute carri	acetate es	Bile Duct Diseases;	Acebutolol,Acepromazin		G=0, C=31	G=0.0, C=1.0	0.00001	465.752	31	G=0/0, C=16/1
chr5:52193414	CA/CA	C	CA	INDEL	ITGA1	ITGA1:intronic:NM 181501.1	1	100		intronic	NM 18150	c.1164+55	p.?	-0.81					FG-GAP repea	rs201620281,rs3771	2.00E-04	YES			Integrin, al	acrosomal vesicle,activation of MAPK activity,basal part				C=0, CA=33	C=0.0, CA=1.0	0.00001	388.628	33	C=0/0, CA=16/
chr5:52322721	G/T	G	T	SNV	ITGA2	ITGA2:intronic:NM 002203.3	1	53.49		intronic	NM 00220	c.185+23G	p.?	-1.3					FG-GAP repea	rs26678	0.3129	YES	2.548E-1 (pos.52322721)		Integrin, al	amyloid-b	Autoimmune Diseases;Cardiovascular Disea			G=20, T=23	G=0.4651, T=0.5349	0.00001	261.02	43	G=14/6, T=13/
chr1:116534852	T/T	C	T	SNV	SLC22A15	SLC22A15:exonic:NM 018420.2	1	100	2	synonymous	NM 01842	c.288C>T	p.(=)	-0.52				0.18(COSM54	Major Facilit	rs4046190	0.3771	YES	4.712E-1 (pos.11653485	acute_myeloi	Solute carri	integral cc	Colonic Diseases;Colorectal Neoplasms;Dige			C=0, T=119	C=0.0, T=1.0	0.00001	1909.41	119	C=0/0, T=45/7
chr5:52351746	G/A	G	A	SNV	ITGA2	ITGA2:intronic:NM 002203.3	1	57.58		intronic	NM 00220	c.931-68G	p.?	-0.94					FG-GAP repea	rs3212522		YES			Integrin, al	amyloid-b	Autoimmune Diseases;Cardiovascular Disea			G=14, A=19	G=0.4242, A=0.5758	0.00001	204.373	33	G=11/3, A=9/1
chr5:5262766	A/A	G	A	SNV	ADAMTS16	ADAMTS16:intronic:NM 139056.3	1	100		intronic	NM 13905	c.2663-4G	p.?	0.16				0(COSM6351	ADAM-TS Spa	rs34433845	0.2382	YES	1.024E-1 (pos.5262766)	carcinoma	A disintegri	branching involved in ureteric bud morphogenesis,male				G=0, A=23	G=0.0, A=1.0	0.00001	322.73	23	G=0/0, A=14/9
chr1:116569530	C/C	G	C	SNV	SLC22A15	SLC22A15:exonic:NM 018420.2	1	100	5	synonymous	NM 01842	c.615G>C	p.(=)	1.01					Major Facilit	rs2488429	0.0496	YES	8.953E-1 (pos.116569530)		Solute carri	integral cc	Colonic Diseases;Colorectal Neoplasms;Dige			G=0, C=46	G=0.0, C=1.0	0.00001	768.307	46	G=0/0, C=27/1
chr11:2920666	T/T	C	T	SNV	SLC22A18,SLC22A18AS	SLC22A18AS:exonic:NM 007105.3, SLC22A18:upstream:NM	1	100	3	missense	NM 00710	c.266G>A.	p.Gly89Asp, p.?	-0.38	0.97	94	0	0(COSM4145646)		rs365605	0.3407	YES	3.662E-1 (pos.2920666)	neoplasm		apical pla	Breast Diseases;Breast Neoplasms;Lung Dise			C=0, T=20	C=0.0, T=1.0	0.00001	263.781	20	C=0/0, T=12/8
chr6:160679400	C/A	C	A	SNV	SLC22A2	SLC22A2:exonic:NM 003058.3	1	46.43	1	synonymous	NM 00305	c.390G>T	p.(=)	-0.57				0.21(COSM37	Major Facilit	rs624249	0.4065	YES	1.749E-1 (pos.16067940	acute_myeloi	Solute carri	acetate es	Bile Duct Diseases;	Agmatine,Amantadine,A		C=15, A=13	C=0.5357, A=0.4643	0.00001	117.927	28	C=4/11, A=9/4
chr11:64981853	T/T	G	T	SNV	SLC22A20	SLC22A20:exonic nc:NR 033396.1	1	100	2	exonic nc	NR 033396.1			9.24				0.96(COSM42	Sugar (and ot	rs239259	0.4269	YES	3.891E-1 (pos.64981853)	adenocarcino	Solute carrier family 22, member 20					G=0, T=108	G=0.0, T=1.0	0.00001	1699.89	108	G=0/0, T=56/5
chr5:54706325	T/TTTTAAA	T	TTTTAAA	INDEL	SKIV2L2	SKIV2L2:intronic:NM 015360.4	6	95.38		intronic	NM 01536	c.2646-27	p.?	1.16					DEAD/DEAH b	rs139320115,rs8667	0.1103	YES	7.315E-1;0.0 (pos.54706325)			ATP binding,ATP-dependent RNA helicase activity,RNA bi				T=3, TTTTA	T=0.0462, TTTTAAA=(	0.00001	894.52	65	T=0/3, TTTTAA
chr5:54709943	A/A	G	A	SNV	SKIV2L2	SKIV2L2:intronic:NM 015360.4	1	100		intronic	NM 01536	c.2752-67	p.?	0.21					DEAD/DEAH b	rs230766	0.1009	YES				ATP binding,ATP-dependent RNA helicase activity,RNA bi				G=0, A=46	G=0.0, A=1.0	0.00001	749.733	46	G=0/0, A=35/1
chr5:54721642	C/C	A	C	SNV	PLPP1,SKIV2L2	SKIV2L2:downstream:NM 015360.4, PLPP1:intronic:NM 17	1	100		intronic	NM 01536	c.*1042A>	p.?, p.?	-0.47					PAP2 superfa	rs230750	0.0991	YES	7.366E-1 (pos.54721642)		Phospholip	ATP binding,ATP-dependent RNA helicase activity,RNA bi				A=0, C=75	A=0.0, C=1.0	0.00001	1193.86	75	A=0/0, C=43/3
chr5:54944976	C/C	G	C	SNV	SLC38A9	SLC38A9:intronic:NM 173514.3	1	100		intronic	NM 17351	c.1060+59	p.?	-0.41					Transmembr	rs11738633		YES			Solute carri	Ragulator	Digestive System Diseases;Digestive System			G=0, C=154	G=0.0, C=1.0	0.00001	2466.85	154	G=0/0, C=97/5
chr6:3287118	C/C	T	C	SNV	SLC22A23	SLC22A23:exonic:NM 015482.1	1	100	7	synonymous	NM 01548	c.1521A>G	p.(=)	-5.04				0.02(COSM41	Major Facilit	rs6910086	0.0631	YES	7.262E-1 (pos.3287118)	hepatocellula	Solute carri	integral cc	Crohn Disease;Digestive System Diseases;Ga			T=0, C=52	T=0.0, C=1.0	0.00001	766.998	52	T=0/0, C=35/1
chr11:62847453	C/C	T	C	SNV	SLC22A24	SLC22A24:exonic:NM 001136506.2	1	100	10	missense	NM 00113	c.1618A>G	p.Ile540Val	-1.32	0.33	29	0		Major Facilit	rs7945121	0.198	YES	8.232E-1 (pos.62847453)		Solute carrier family 22		Acute Kidney Injury;Female Urogenital Disea			T=0, C=50	T=0.0, C=1.0	0.00001	852.722	50	T=0/0, C=30/2
chr5:54993829	G/G	A	G	SNV	SLC38A9	SLC38A9:intronic:NM 173514.3	1	100		intronic	NM 17351	c.-33-9T>C	p.?	3.07					Transmembr	rs3761766	0.3503	YES	4.617E-1 (pos.54993829)		Solute carri	Ragulator	Digestive System Diseases;Digestive System			A=0, G=33	A=0.0, G=1.0	0.00001	504.24	33	A=0/0, G=18/1
chr5:55212379	G/G	A	G	SNV	IL31RA	IL31RA:intronic:NM 139017.5	1	100		intronic	NM 13901	c.1819-93	p.?	-0.09					Fibronectin t	rs161700		YES			Interleukin	JAK-STAT cascade,MAPK cascade,T-helper 2 cell cytokine				A=0, G=46	A=0.0, G=1.0	0.00001	772.683	46	A=0/0, G=28/1
chr11:62848445	G/G	A	G	SNV	SLC22A24	SLC22A24:exonic:NM 001136506.2	1	100	9	synonymous	NM 00113	c.1545T>C	p.(=)	-1.03				0.1(COSM467	Major Facilit	rs11231340	0.4683	YES	4.426E-1 (pos.62848445)	adenocarcino	Solute carrier family 22		Acute Kidney Injury;Female Urogenital Disea			A=0, G=85	A=0.0, G=1.0	0.00001	1383.77	85	A=0/0, G=46/3
chr11:62848487	C/C	A	C	SNV	SLC22A24	SLC22A24:exonic:NM 001136506.2	1	100	9	nonsense	NM 00113	c.1503T>G	p.Tyr501Ter	0.02					Major Facilit	rs11231341	0.1986	YES	8.219E-1 (pos.62848487)		Solute carrier family 22		Acute Kidney Injury;Female Urogenital Disea			A=0, C=84	A=0.0, C=1.0	0.00001	1369.97	84	A=0/0, C=42/4
chr11:62848568	G/G	C	G	SNV	SLC22A24	SLC22A24:exonic:NM 001136506.2	1	100	9	synonymous	NM 00113	c.1422G>C	p.(=)	-0.42				0.03(COSM63	Major Facilit	rs11231342	0.4683	YES	4.281E-1 (pos.62848568)	carcinoma	Solute carrier family 22		Acute Kidney Injury;Female Urogenital Disea			C=0, G=55	C=0.0, G=1.0	0.00001	814.906	55	C=0/0, G=6/49
chr5:56526819	G/G	GT	G	INDEL	GPBP1	GPBP1:intronic:NM 001127236.2	1	100		intronic	NM 00112	c.208+23G	p.?	-0.01, 0.24					Vascular prot	rs202155609,rs3214	0.2632	YES	7.013E-1 (pos.56526819)		GC-rich pro	DNA binding,DNA binding transcription factor activity,cyt				GT=0, G=57	GT=0.0, G=1.0	0.00001	829.626	57	GT=0/0, G=32/
chr5:57753456	T/T	C	T	SNV	PLK2	PLK2:intronic:NM 006622.3	1	100		intronic	NM 00662	c.714-46G	p.?	-0.3					POLO box du	rs702723	0.4037	YES	9.029E-1 (pos.57753456)		Polo-like kir	ATP bindin	Arthritis;Arthritis, Juvenile;Autoimmune Dise			C=0, T=20	C=0.0, T=1.0	0.00001	263.523	20	C=0/0, T=9/11
chr5:57754808	A/A	G	A	SNV	PLK2	PLK2:intronic:NM 006622.3	1	100		intronic	NM 00662	c.378+4C>	p.?	0				0.19(COSM44	POLO box du	rs697133	0.4023	YES	9.039E-1 (pos.57754808)	hepatocellula	Polo-like kir	ATP bindin	Arthritis;Arthritis, Juvenile;Autoimmune Dise			G=0, A=60	G=0.0, A=1.0	0.00001	903.531	60	G=0/0, A=27/3
chr5:58271696	T/T	C	T	SNV	PDE4D	PDE4D:intronic:NM 001104631.1	1	100		intronic	NM 00110	c.1831-30	p.?	0.21					3'5'-cyclic nu	rs10051847	0.1549	YES	1.741E-1 (pos.58271696)		Phosphodie	3',5'-cyclic	Amphetamine-Rela	(4R)-4-(3-butoxy-4-methc		C=0, T=29	C=0.0, T=1.0	0.00001	427.603	29	C=0/0, T=21/8
chr5:58272361	T/T	C	T	SNV	PDE4D	PDE4D:intronic:NM 001104631.1	1	100		intronic	NM 00110	c.1708-62	p.?	-1.48					3'5'-cyclic nu	rs3763083		YES			Phosphodie	3',5'-cyclic	Amphetamine-Rela	(4R)-4-(3-butoxy-4-methc		C=0, T=50	C=0.0, T=1.0	0.00001	852.747	50	C=0/0, T=20/3
chr5:58285422	C/C	CTTGA	C	INDEL	PDE4D	PDE4D:intronic:NM 001104631.1	4	100		intronic	NM 00110	c.1452+16	p.?	-1.75, -0.23, -0.41, -0.47, 0.3					3'5'-cyclic nu	rs149962845,rs80307266		YES			Phosphodie	3',5'-cyclic	Amphetamine-Rela	(4R)-4-(3-butoxy-4-methc		CTTGA=0, C	CTTGA=0.0, C=1.0	0.00001	570.235	36	CTTGA=0/0, C=
chr5:59940502	T/T	A	T	SNV	DEPDC1B	DEPDC1B:intronic:NM 018369.2	1	100		intronic	NM 01836	c.709+70T	p.?	-4.28					Domain foun	rs6877561		YES			DEP domair	GTPase activator activity,cell migration,cytosol,intracel				A=0, T=47	A=0.0, T=1.0	0.00001	789.328	47	A=0/0, T=26/2
chr5:60083275	A/A	C	A	SNV	ELOVL7	ELOVL7:intronic:NM 001104558.1	1	100		intronic	NM 00110	c.-33-17G	p.?	-0.33					GNS1/SUR4 fa	rs10072745	0.4787	YES	8.983E-1;0.0 (pos.60083275)		Elongation	3-oxo-arachidoyl-CoA synthase activity,3-oxo-cerotoyl-C				C=0, A=60	C=0.0, A=1.0	0.00001	903.529	60	C=0/0, A=13/4
chr11:62910849	C/C	T	C	SNV	SLC22A24	SLC22A24:splicesite 5:NM 001136506.2	1	100	1	splicesite	NM 00113	c.402+1A>	p.?	2.51					Major Facilit	rs1939749	0.1719	YES	9.184E-1 (pos.62910849)		Solute carrier family 22		Acute Kidney Injury;Female Urogenital Disea			T=0, C=69	T=0.0, C=1.0	0.00001	1072.78	69	T=0/0, C=32/3
chr5:60448947	A/A	G	A	SNV	NDUFAF2	NDUFAF2:downstream:NM 174889.4	1	100		intronic	NM 17488	c.*165G>A	p.?	0.04						rs810883		YES			NADH dehy	NADH deh	Brain Diseases;Brain Diseases, Metabolic;Br			G=0, A=83	G=0.0, A=1.0	0.00001	1349.74	83	G=0/0, A=33/5
chr5:61733348	A/A	G	A	SNV	IPO11	IPO11:intronic:NM 001134779.1	1	100		intronic	NM 00113	c.258+104	p.?	-1.39					Importin-bet	rs153857		YES			Importin 11	Ran GTPase binding,cytosol,fibrillar center,intracellular				G=0, A=28	G=0.0, A=1.0	0.00001	408.718	28	G=0/0, A=17/1
chr5:61762864	T/G	T	G	SNV	IPO11	IPO11:intronic:NM 001134779.1	1	45.83		intronic	NM 00113	c.637-94T	p.?	-0.6					Importin-bet	rs75500070		YES			Importin 11	Ran GTPase binding,cytosol,fibrillar center,intracellular				T=13, G=11	T=0.5417, G=0.4583	0.00001	93.44	24	T=9/4, G=10/1
chr5:61762880	A/G	A	G	SNV	IPO11	IPO11:intronic:NM 001134779.1	1	45.83		intronic	NM 00113	c.637-78A	p.?	0.51					Importin-bet	rs35119		YES			Importin 11	Ran GTPase binding,cytosol,fibrillar center,intracellular				A=13, G=11	A=0.5417, G=0.4583	0.00001	93.4398	24	A=9/4, G=10/1
chr5:61781127	A/A	C	A	SNV	IPO11	IPO11:intronic:NM 001134779.1	1	100		intronic	NM 00113	c.1295-11	p.?	-0.33					Importin-bet	rs26640		YES			Importin 11	Ran GTPase binding,cytosol,fibrillar center,intracellular				C=0, A=42	C=0.0, A=1.0	0.00001	685.22	42	C=0/0, A=20/2
chr5:61785936	C/C	T	C	SNV	IPO11	IPO11:intronic:NM 001134779.1	1	100		intronic	NM 00113	c.1478-6T	p.?	0.22				0.05(COSM44	Importin-bet	rs26644	0.4082	YES	3.97E-1 (pos.61785936)	acute_myeloi	Importin 11	Ran GTPase binding,cytosol,fibrillar center,intracellular				T=0, C=34	T=0.0, C=1.0	0.00001	524.178	34	T=0/0, C=19/1
chr11:62984868	C/C	T	C	SNV	SLC22A25	SLC22A25:exonic:NM 199352.4	1	100	4	missense	NM 19935	c.748A>G	p.Ser250Gly	0.21	1	56	0.001	0(COSM3998	Major Facilit	rs11231409	0.3791	YES	5.761E-1 (pos.62984868)	acute_myeloid	leukaemia	inorganic anion exchanger activity,inorganic anion trans				T=0, C=52	T=0.0, C=1.0	0.00001	765.08	52	T=0/0, C=13/3
chr20:4854682	A/G	A	G	SNV	SLC23A2	SLC23A2:exonic:NM 203327.1	1	50	11	synonymous	NM 20332	c.1002T>C	p.(=)	-0.85				0.02(COSM11	Permease fa	rs1110277	0.3337	YES	2.31E-1 (pos.4854682)	acute_myeloi	Solute carri	L-ascorbat	Bile Duct Diseases;Biliary Tract Diseases;Ch			A=12, G=12	A=0.5, G=0.5	0.00001	106.709	24	A=5/7, G=5/7
chr20:19560664	A/A	G	A	SNV	SLC24A3	SLC24A3:exonic:NM 020689.3	1	100	4	synonymous	NM 02068	c.369G>A	p.(=)	-2.31				0.02(COSM40	Sodium/calci	rs3790261	0.2415	YES	6.564E-1 (pos.19560664)	acute_myeloi	Solute carri	calcium ch	Adenocarcinoma;Carcinoma;Carcinoma, Ade			G=0, A=84	G=0.0, A=1.0	0.00001	1370.16	84	G=0/0, A=49/3
chr20:19634732	A/A	G	A	SNV	SLC24A3	SLC24A3:exonic:NM 020689.3	1	100	7	synonymous	NM 02068	c.639G>A	p.(=)	0.78				0.59(COSM37	Sodium/calci	rs3790278	0.3753	YES	2.534E-1 (pos.19634732)	acute_myeloi	Solute carri	calcium ch	Adenocarcinoma;Carcinoma;Carcinoma, Ade			G=0, A=112	G=0.0, A=1.0	0.00001	1776.44	112	G=0/0, A=86/2
chr5:64447777	T/T	C	T	SNV	ADAMTS6	ADAMTS6:intronic:NM 197941.3	1	100		intronic	NM 19794	c.3245-5G	p.?	-0.2					ADAM-TS Spa	rs17206779	0.4862	YES	3.593E-1 (pos.64447777)		A disintegri	metalloen	Neoplasms;Neoplasms by Histologic Type;Ne			C=0, T=349	C=0.0, T=1.0	0.00001	5928.51	349	C=0/0, T=164/
chr5:64556640	C/C	T	C	SNV	ADAMTS6	ADAMTS6:intronic:NM 197941.3	1	100		intronic	NM 19794	c.1767-15	p.?	0.01					ADAM-TS Spa	rs10940023		YES			A disintegri	metalloen	Neoplasms;Neoplasms by Histologic Type;Ne			T=0, C=65	T=0.0, C=1.0	0.00001	996.419	65	T=0/0, C=25/4
chr20:19634747	C/C	T	C	SNV	SLC24A3	SLC24A3:exonic:NM 020689.3	1	100	7	synonymous	NM 02068	c.654T>C	p.(=)	0.03				0.33(COSM37	Sodium/calci	rs3790279	0.3752	YES	2.539E-1 (pos.19634747)	acute_myeloi	Solute carri	calcium ch	Adenocarcinoma;Carcinoma;Carcinoma, Ade			T=0, C=112	T=0.0, C=1.0	0.00001	1775.9	112	T=0/0, C=85/2
chr5:64825102	T/T	C	T	SNV	CENPK	CENPK:intronic:NM 001267038.1	1	100		intronic	NM 00126	c.289-76G	p.?	-0.23					Centromere-	rs1017576		YES				CENP-A containing nucleosome assembly,condensed chr				C=0, T=57	C=0.0, T=1.0	0.00001	849.615	57	C=0/0, T=24/3
chr5:64838584	A/A	G	A	SNV	CENPK	CENPK:intronic:NM 001267038.1	1	100		intronic	NM 00126	c.288+45C	p.?	-2.21					Centromere-	rs6891597	0.3714	YES	6.284E-1 (pos.64838584)			CENP-A containing nucleosome assembly,condensed chr				G=0, A=40	G=0.0, A=1.0	0.00001	644.284	40	G=0/0, A=22/1
chr5:64881688	GT/G	GT	G	INDEL	PPWD1	PPWD1:intronic:NM 015342.3	1	45.83		intronic	NM 01534	c.1615-13	p.?	0.01, -1.39					Cyclophilin ty	rs35368186		YES				catalytic step 2 spliceosome,mRNA splicing, via spliceos				GT=26, G=2	GT=0.5417, G=0.458	0.00001	221.688	48	GT=13/13, G=8
chr14:92792255	A/A	G	A	SNV	SLC24A4	SLC24A4:exonic:NM 153646.3	1	100	2	synonymous	NM 15364	c.174G>A	p.(=)	-1.49					Sodium/calci	rs941646	9.00E-04	YES	1.0 (pos.92792255)		Solute carri	amelogen	Alzheimer Disease;Amelogenesis Imperfecta			G=0, A=128	G=0.0, A=1.0	0.00001	2094.83	128	G=0/0, A=52/7
chr5:65016452	G/G	A	G	SNV	SGTB	SGTB:intronic:NM 019072.2	1	100		intronic	NM 01907	c.100+83T	p.?	0.81					Homodimeris	rs9291846		YES				protein binding				A=0, G=80	A=0.0, G=1.0	0.00001	1289.37	80	A=0/0, G=42/3
chr5:6602544	C/C	T	C	SNV	NSUN2	NSUN2:intronic:NM 017755.5	1	100		intronic	NM 01775	c.1997+30	p.?	-0.14					16S rRNA met	rs2303711	0.3152	YES	6.584E-1 (pos.6602544)		NOP2/SUN f	RNA bindir	Congenital Abnormalities;Congenital, Heredi			T=0, C=37	T=0.0, C=1.0	0.00001	583.706	37	T=0/0, C=21/1
chr5:6611757	T/T	A	T	SNV	NSUN2	NSUN2:intronic:NM 017755.5	1	100		intronic	NM 01775	c.1095+81	p.?	-0.78					16S rRNA met	rs10454287,rs777200149		YES			NOP2/SUN f	RNA bindir	Congenital Abnormalities;Congenital, Heredi			A=0, T=29	A=0.0, T=1.0	0.00001	427.6	29	A=0/0, T=16/1
chr5:66195701	T/T	C	T	SNV	MAST4	MAST4:intronic:NM 001164664.1	1	100		intronic	NM 00116	c.643-78C	p.?	-0.88					Domain of un	rs458115	0.4525	YES					Brain Diseases;Central Nervous System Dise			C=0, T=30	C=0.0, T=1.0	0.00001	446.639	30	C=0/0, T=23/7
chr14:92920371	T/T	C	T	SNV	SLC24A4	SLC24A4:exonic:NM 153646.3	1	100	11	synonymous	NM 15364	c.1008C>T	p.(=)	0.99				0.84(COSM13	Sodium/calci	rs7144273	0.4941	YES	5.448E-1 (pos.92920371)	adenocarcino	Solute carri	amelogen	Alzheimer Disease;Amelogenesis Imperfecta			C=0, T=65	C=0.0, T=1.0	0.00001	996.464	65	C=0/0, T=42/2
chr17:79682051	C/T	C	T	SNV	SLC25A10	SLC25A10:exonic:NM 001270953.1	1	59.09	11	synonymous	NM 00127	c.162C>T	p.(=)	-4.09				0.05(COSM37	Mitochondri	rs3204270	0.1032	YES	3.544E-1 (pos.79682051)		Solute carri	antiporter	Mammary Neoplas	Succinic acid		C=11, T=10	C=0.4091, T=0.5909	0.00001	122.568	22	C=3/6, T=12/1
chr9:130864676	C/C	T	C	SNV	SLC25A25	SLC25A25:exonic:NM 001006641.3	1	100	3	synonymous	NM 00100	c.504T>C	p.(=)	-0.26					EF hand,EF-h	rs7859709	7.00E-04	YES	1.0 (pos.130864676)			ADP transmembrane transporter activity,ADP transport,S				[illegible]	[illegible]	0.00001	767.919	46	[illegible]
chr3:66287056	A/A	G	A	SNV	SLC25A26	SLC25A26:exonic:NM 173471.3	1	100	3	missense	NM 17347	c.122G>A	p.Ser41Asn	0.88	0.41	46	0	0.09(COSM17	Mitochondri	rs146159281	0.2872	YES	4.102E-1 (pos.66287056)	acute_myeloi	Solute carri	S-adenosyl-L-methionine transmembrane transport,S-ad				G=0, A=23	G=0.0, A=1.0	0.00001	316.746	23	G=0/0, A=10/1

chr5:68412048	AT/AT	A	AT	INDEL	SLC30A5	SLC30A5:intronic:NM_022902.4	1	100		intronic	NM_0229C c.1072+7A	p.?	0.66					Cation efflux	rs34215592,rs38640-	0.3876	YES	8.57E-1 (pos.68412048)	osteosarcoma	Solute carri	Golgi appa	Autoimmune Diseases;Female Urogenital Di	A=0, AT=22	A=0.0, AT=1.0	0.00001	240.69	22	A=0/0, AT=15/
chr5:68412051	C/T	C	T	SNV	SLC30A5	SLC30A5:intronic:NM_022902.4	1	45.45		intronic	NM_0229C c.1072+10	p.?	0.18				0(COSM3784	Cation efflux	rs17330153			0.0;0.0 (pos.68412051)	adenocarcinoma	Solute carri	Golgi appa	Autoimmune Diseases;Female Urogenital Di	C=12, T=10	C=0.5455, T=0.4545	0.00001	77.5974	22	C=11/1, T=4/6
chr3:39431081	A/A	G	A	SNV	SLC25A38	SLC25A38:exonic:NM_017875.2	1	100	2	synonymous	NM_01787 c.165G>A	p.(=)	0.94				0.85(COSM56	Mitochondri	rs2270770	0.485	YES	6.216E-1 (pos.39431081)	ductal_carcinoma	Solute carri	erythrocyt	Anemia;Anemia, Sideroblastic Benign	G=0, A=48	G=0.0, A=1.0	0.00001	810.431	48	G=0/0, A=20/2
chr14:100795139	T/T	G	T	SNV	SLC25A47	SLC25A47:exonic:NM_207117.3	1	100	5	missense	NM_20711 c.404G>T	p.Arg135Leu	1.6	0.41	102	0.936	0.92(COSM43	Mitochondri	rs35007880	0.48	YES	2.26E-1;1.182E-4 (pos.10	ductal_carcinoma		carnitine transmembrane transporter activity,carnitine		G=0, T=45	G=0.0, T=1.0	0.00001	747.388	45	G=0/0, T=18/2
chr5:135178124	T/C	T	C	SNV	SLC25A48	SLC25A48:exonic:NM_145282.4	1	97.56	2	synonymous	NM_14528 c.667>C	p.(=)	-2.39					Mitochondri	rs2304075	0.177	YES	7.069E-1 (pos.135178124)		Solute carri	L-ornithine transmembrane transporter activity,integra		T=1, C=40	T=0.0244, C=0.9756	0.00001	631.844	41	T=0/1, C=12/2
chr18:29339910	G/T	G	T	SNV	SLC25A52	SLC25A52:exonic:NM_001034172.2	1	96.67	1	missense	NM_00103 c.745C>A	p.Leu249Ile	2.33		5		0.04(COSM41	Mitochondri	rs3859364	1.00E-04		1.0 (pos.29339910)	neoplasm	Solute carri	integral component of membrane,mitochondrial inner n		G=1, T=29	G=0.0333, T=0.9667	0.00001	420.834	30	G=1/0, T=19/1
chr7:107414419	G/G	A	G	SNV	SLC26A3	SLC26A3:exonic:NM_000111.2	1	100	17	synonymous	NM_00011 c.1953T>C	p.(=)	-1.64					STAS domain	rs41669	0.0119		9.998E-1 (pos.107414419)		Solute carri	DNA bindi	Colitis;Colitis, Ulcerative;Colo: Benign	A=0, G=50	A=0.0, G=1.0	0.00001	852.737	50	A=0/0, G=8/4
chr5:72112430	G/G	C	G	SNV	TNPO1		1	100		intronic			0.8						rs34647		YES			Transportin 1			C=0, G=29	C=0.0, G=1.0	0.00001	426.426	29	C=0/0, G=19/1
chr6:35980121	T/T	C	T	SNV	SLC26A8	SLC26A8:exonic:NM_001193476.1	1	100	3	missense	NM_00119 c.217G>A	p.Val73Met	0.03	0.38	21	0.003		STAS domain	rs743923	0.2464	YES	1.068E-1 (pos.35980121)		Solute carri	anion tran	Azoospermia;Genital Diseases, Male;Infertili	C=0, T=27	C=0.0, T=1.0	0.00001	389.987	27	C=0/0, T=14/1
chr5:72347090	A/T	A	T	SNV	FCHO2	FCHO2:intronic:NM_138782.2	1	50		intronic	NM_13878 c.940-86A	p.?	-1.58					Fes/CIP4, anc	rs7712673		YES			FCH domair	clathrin coat assembly,clathrin-coated pit,clathrin-coat		A=15, T=15	A=0.5, T=0.5	0.00001	145.021	30	A=10/5, T=6/9
chr15:50474766	C/C	A	C	SNV	SLC27A2	SLC27A2:exonic:NM_003645.3	1	100	1	missense	NM_00364 c.142A>C	p.Lys48Gln	0.03	0.56	53	0	0.02(COSM37	AMP-binding	rs1648348	0.335	YES	8.166E-1 (pos.50474766)	adenocarcinoma	Solute carri	ATP binding,bile acid biosynthetic process,decanoate--C		A=0, C=28	A=0.0, C=1.0	0.00001	408.718	28	A=0/0, C=14/1
chr5:128362830	A/G	A	G	SNV	SLC27A6	SLC27A6:exonic:NM_014031.4	1	50	8	synonymous	NM_01403 c.1260A>G	p.(=)	1.48					AMP-binding	rs2304578	0.0171	YES	1.203E-2 (pos.128362830)			fatty acid transport,fatty acid transporter activity,integr		A=13, G=13	A=0.5, G=0.5	0.00001	121.363	26	A=5/8, G=12/1
chr10:73115941	CA/CA	TG	CA	MNV	SLC29A3	SLC29A3:exonic:NM_018344.5	2	100	5	missense	NM_01834 c.714_715	p.Val239Ile	-0.48, 0.14	0.44	29	0.002		Nucleoside tr	rs2252996,rs225299	0.111	YES	6.838E-1 (pos.73115941);6.857E-1 (pos.7		Solute carri	Golgi appa	Autoimmune Diseases;Bone D Conflicting in	TG=0, CA=6	TG=0.0, CA=1.0	0.00001	1060.88	69	TG=0/0, CA=26
chr10:73121945	C/C	T	C	SNV	SLC29A3	SLC29A3:exonic:NM_018344.5	1	100	6	synonymous	NM_01834 c.1008T>C	p.(=)	0.32					Nucleoside tr	rs1084004	0.0823	YES	7.219E-1 (pos.73121945)		Solute carri	Golgi appa	Autoimmune Diseases;Bone D Benign/Likely	T=0, C=43	T=0.0, C=1.0	0.00001	705.837	43	T=0/0, C=36/7
chr10:73122468	G/T	G	T	SNV	SLC29A3	SLC29A3:utr_3:NM_018344.5	1	57.58	6	utr_3	NM_01834 c.1531G>T	p.?	1.53					Nucleoside tr	rs780680		YES	1.313E-1;0.0 (pos.73122468)		Solute carri	Golgi appa	Autoimmune Diseases;Bone D Benign	G=14, T=19	G=0.4242, T=0.5758	0.00001	204.397	33	G=8/6, T=5/14
chr5:74526489	GT/G	GT	G	INDEL	ANKRD31	ANKRD31:intronic:NM_001164443.1	1	45.45		intronic	NM_0011€ c.105-29A	p.?	-0.79, 0.75					Ankyrin repe	rs11295265,rs14730	0.373	YES	4.756E-1 (pos.74526489)					GT=12, G=1	GT=0.5455, G=0.454	0.00001	81.3096	22	GT=5/7, G=6/4
chr5:74988369	A/G	A	G	SNV	POC5	POC5:intronic:NM_001099271.1	1	47.37		intronic	NM_00109 c.691-44T	p.?	1.92						rs888789	0.4607	YES	5.359E-1 (pos.74988369)			cell cycle,n	Nutrition Disorders;Nutritional and Metaboli	A=40, G=36	A=0.5263, G=0.4737	0.00001	364.013	76	A=14/26, G=1
chr4:10022981	T/T	C	T	SNV	SLC2A9	SLC2A9:exonic:NM_020041.2	1	100	1	missense	NM_02004 c.73G>A	p.Gly25Arg	-0.13	0.03	125	0.013	0.07(COSM14	Major Faciliti	rs2276961	0.463	YES	4.246E-1 (pos.10022981)	adenocarcinoma	Solute carri	apical pla	Arthritis;Congenita Fludeoxyg Benign	C=0, T=33	C=0.0, T=1.0	0.00001	499.444	33	C=0/0, T=16/1
chr5:75888788	A/A	G	A	SNV	IQGAP2	IQGAP2:intronic:NM_006633.3	1	100		intronic	NM_00663 c.907+38G	p.?	-2.75					Calponin hom	rs3736394	0.4415	YES	4.691E-1 (pos.75888788)		IQ motif-co	Arp2/3 cor	Adenocarcinoma;Carcinoma;Carcinoma, Hep	G=0, A=41	G=0.0, A=1.0	0.00001	664.696	41	G=0/0, A=24/1
chr4:9909923	A/A	G	A	SNV	SLC2A9	SLC2A9:exonic:NM_020041.2	1	100	8	missense	NM_02004 c.1049C>T	p.Pro350Leu	-0.07	0.31	98	0.064	0.04(COSM49	Major Faciliti	rs2280205	0.4788	YES	2.383E-1;0.0 (pos.99099	ductal_carcinoma	Solute carri	apical pla	Arthritis;Congenita Fludeoxyg Benign	G=0, A=28	G=0.0, A=1.0	0.00001	408.718	28	G=0/0, A=15/1
chr5:68414385	C/A	C	A	SNV	SLC30A5	SLC30A5:exonic:NM_022902.4	1	57.14	12	missense	NM_0229C c.1499C>A	p.Ala500Glu	7.8	0	107	1	0.99(COSM34	Cation efflux	rs768665809			0.0;0.0 (pos.68414385)	adenocarcinoma	Solute carri	Golgi appa	Autoimmune Diseases;Female Urogenital Di	C=57, A=76	C=0.4286, A=0.5714	0.00001	885.065	133	C=28/29, A=3
chr5:75960825	C/C	G	C	SNV	IQGAP2	IQGAP2:intronic:NM_006633.3	1	100		intronic	NM_00663 c.2530-26	p.?	-0.33					Calponin hom	rs253092,rs3866892	0.13,0.26	YES	9.116E-1 (pos.75960825)		IQ motif-co	Arp2/3 cor	Adenocarcinoma;Carcinoma;Carcinoma, Hec	G=0, C=30	G=0.0, C=1.0	0.00001	446.602	30	G=0/0, C=9/21
chr11:45832935	A/A	G	A	SNV	SLC35C1	SLC35C1:utr_3:NM_001145266.1	1	100	3	utr_3	NM_00114 c.1105G>A	p.?	-0.23					Triose-phosp	rs1139266	0.2084	YES	3.562E-1 (pos.45832935)		Solute carri	GDP-fucos	Carbohydrate Metabolism, Inb Benign	G=0, A=28	G=0.0, A=1.0	0.00001	419.051	28	G=0/0, A=9/19
chr17:7386055	C/T	C	T	SNV	SLC35G6,ZBTB4	SLC35G6:exonic:NM_001102614.1, ZBTB4:intronic:NM_020	1	95.65	2	missense	NM_02089 c.-80+126I	p.?, p.Pro251Leu	2.17	0.09	98	0	0.01(COSM41	BTB/POZ dom	rs4491591	0.001	YES	1.0 (pos.7386055)	neoplasm	Zinc finger a	RNA polymerase II regulatory region sequence-specific D		C=1, T=22	C=0.0435, T=0.9565	0.00001	289.745	23	C=1/0, T=13/9
chr5:76259350	T/C	T	C	SNV	CRHBP	CRHBP:intronic:NM_001882.3	1	45.83		intronic	NM_00188 c.811+65T	p.?	-1.16					Corticotropir	rs7728378		YES			Corticotrop	Golgi appa	Cocaine-Related Disorders;Mental Disorders	T=26, C=22	T=0.5417, C=0.4583	0.00001	242.99	48	T=6/20, C=2/2
chr21:43985955	A/A	G	A	SNV	SLC37A1	SLC37A1:exonic:NM_018964.3	1	100	16	missense	NM_01896 c.1240G>A	p.Val414Ile	0.32	1	29	0.001	0.01(COSM4C	Major Faciliti	rs228104	0.1042	YES	5.044E-1 (pos.43985955)	acute_myeloid	Solute carri	carbohydrate transport,endoplasmic reticulum membra		G=0, A=25	G=0.0, A=1.0	0.00001	353.019	25	G=0/0, A=10/1
chr7:140082363	G/A	G	A	SNV	SLC37A3	SLC37A3:utr_5:NM_207113.2	1	95.65	2	utr_5	NM_20711 c.-37C>T	p.?	-1.68					Major Faciliti	rs6968618	0.2952	YES	8.066E-1 (pos.140082363)			carbohydrate transport,cytosol,glucose 6-phosphate:ino		G=1, A=22	G=0.0435, A=0.9565	0.00001	288.952	23	G=1/0, A=15/7
chr5:76734222	G/A	G	A	SNV	WDR41	WDR41:intronic:NM_018268.3	1	53.13		intronic	NM_01826 c.883-36C	p.?	0.58					WD domain,	rs7720548	0.0228	YES	1.194E-1 (pos.76734222)		WD repeat-	ATG1/ULK1 kinase complex,Rab guanyl-nucleotide excha		G=15, A=17	G=0.4688, A=0.5313	0.00001	173.56	32	G=3/12, A=7/1
chr5:76926651	C/C	A	C	SNV	OTP	OTP:intronic:NM_032109.2	1	100		intronic	NM_0321C c.448-32T	p.?	0.56					Homeobox do	rs62362480	0.4426	YES	3.331E-1 (pos.76926651)		Orthopedia	forebrain neuron differentiation,hypothalamus cell diffe		A=0, C=62	A=0.0, C=1.0	0.00001	940.248	62	A=0/0, C=21/4
chr5:7695845	A/A	C	A	SNV	ADCY2	ADCY2:intronic:NM_020546.2	1	100		intronic	NM_02054 c.870-20C	p.?	-1.9					Adenylate an	rs11134251	0.4524	YES	8.154E-1 (pos.7695845)		Adenylyl cy	ATP bindin	Asthma;Bipolar Dis 2',5'-DIDEOXY-ADENOSINE	C=0, A=31	C=0.0, A=1.0	0.00001	465.161	31	C=0/0, A=17/1
chr5:77412068	C/T	C	T	SNV	AP3B1	AP3B1:intronic:NM_003664.4	1	45.83		intronic	NM_00366 c.1969-10	p.?	0.51					Adaptin N ter	rs77009095	0.0747	YES	4.703E-4 (pos.77412068)		Adaptor-rel	AP-3 adap	Albinism;Albinism, Oculocutar Benign/Likely	C=13, T=11	C=0.5417, T=0.4583	0.00001	93.44	24	C=6/7, T=1/10
chr5:77755035	C/C	T	C	SNV	SCAMP1	SCAMP1:intronic:NM_004866.5	1	100		intronic	NM_00486 c.734-33T	p.?	1.41					SCAMP family	rs6453391	0.2014	YES	9.261E-1 (pos.77755035)		Secretory c	clathrin-coated vesicle,integral component of membran		T=0, C=77	T=0.0, C=1.0	0.00001	1229.38	77	T=0/0, C=58/1
chr17:79220224	G/C	G	C	SNV	SLC38A10	SLC38A10:exonic:NM_001037984.2	1	57.14	16	missense	NM_00103 c.2492C>G	p.Ala831Gly	0.37	0	60	0.666	0.15(COSM37	Transmembr	rs2725405	0.4377	YES	7.394E-1 (pos.79220224)	adenocarcinoma	Solute carri	Golgi apparatus,amino acid transmembrane transport,a		G=12, C=16	G=0.4286, C=0.5714	0.00001	160.36	28	G=7/5, C=10/6
chr12:47168898	A/A	G	A	SNV	SLC38A4	SLC38A4:exonic:NM_001143824.1	1	100	13	synonymous	NM_00114 c.1233C>T	p.(=)	0.11					Transmembr	rs180414	0		1.0 (pos.47168898)		Solute carri	amino acid transmembrane transport,amino acid transr		G=0, A=68	G=0.0, A=1.0	0.00001	1053.56	68	G=0/0, A=12/5
chr5:54960673	G/G	C	G	SNV	SLC38A9	SLC38A9:exonic:NM_173514.3	1	100	8	missense	NM_17351 c.545G>C	p.Ser182Thr	1.2	0.81	58	0	0.12(COSM12	Transmembr	rs4865615	0.3469	YES	4.55E-1 (pos.54960673)	adenocarcinoma	Solute carri	Regulator	Digestive System Diseases;Digestive System	C=0, G=46	C=0.0, G=1.0	0.00001	755.107	46	C=0/0, G=20/2
chr5:78340411	C/C	T	C	SNV	DMGDH	DMGDH:intronic:NM_013391.3	1	100		intronic	NM_01339 c.746-36A	p.?	-0.7					Aminomethyl	rs531982	0.4908	YES	6.525E-1;0.0 (pos.78340411)		Dimethylgly	RNA bindi	Amino Acid Metabolism, Inborn Errors;Conge	T=0, C=81	T=0.0, C=1.0	0.00001	1309.45	81	T=0/0, C=38/4
chr5:7866135	T/T	TTGAGG	T	INDEL	FASTKD3	FASTKD3:intronic:NM_024091.3	5	100		intronic	NM_02409 c.1439-39	p.?	-2.29, -0.16, -0.6, -2.09, 0.53, 0.27					FAST kinase-l	rs10059339,rs14193	0.3105	YES	0.0 (pos.7866139);0.0 (pos.7866140);2.7		Fast kinase	RNA binding,cellular respiration,mitochondrion,nucleus		TTGAGG=0,	TTGAGG=0.0, T=1.0	0.00001	1149.27	73	TTGAGG=0/0,
chr5:54960687	T/T	C	T	SNV	SLC38A9	SLC38A9:exonic:NM_173514.3	1	100	8	synonymous	NM_17351 c.531G>A	p.(=)	0.13				0.16(COSM63	Transmembr	rs4865616	0.3469	YES	4.579E-1 (pos.54960687)	carcinoma	Solute carri	Regulator	Digestive System Diseases;Digestive System	C=0, T=44	C=0.0, T=1.0	0.00001	730.942	44	C=0/0, T=19/2
chr10:18242304	C/C	T	C	SNV	SLC39A12	SLC39A12:exonic:NM_001145195.1	1	100	2	synonymous	NM_00114 c.99T>C	p.(=)	-0.45					ZIP Zinc trans	rs691112	0.0886	YES	9.236E-1;0.0 (pos.18242304)			cellular zir	Melanoma;Neoplasms;Neoplasms by Histolo	T=0, C=25	T=0.0, C=1.0	0.00001	353.019	25	T=0/0, C=6/19
chr10:18266989	A/A	G	A	SNV	SLC39A12	SLC39A12:exonic:NM_001145195.1	1	100	5	missense	NM_00114 c.910G>A	p.Val304Ile	-4.1	0.41	29	0		ZIP Zinc trans	rs2478568	0.3685	YES	6.74E-1 (pos.18266989)			cellular zir	Melanoma;Neoplasms;Neoplasms by Histolo	G=0, A=157	G=0.0, A=1.0	0.00001	2522.48	157	G=0/0, A=74/8
chr11:47431728	G/G	A	G	SNV	SLC39A13	SLC39A13:exonic:NM_001128225.2	1	100	2	missense	NM_00112 c.83A>G	p.Glu28Gly	0.64	1	98	0		ZIP Zinc trans	rs2010519	8.00E-04	YES	1.0 (pos.47431728)		Solute carri	Golgi appa	Bone Diseases;Bone Diseases, Benign	A=0, G=60	A=0.0, G=1.0	0.00001	892.789	60	A=0/0, G=32/2
chr19:2732986	C/C	T	C	SNV	SLC39A3	SLC39A3:exonic:NM_144564.4	1	100	3	synonymous	NM_1445€ c.708A>G	p.(=)	0.72				0.4(COSM439	ZIP Zinc trans	rs759073	0.4517	YES	6.471E-1;0.0 (pos.27329	NS,adenocarci	Solute carri	T cell homeostasis,cell morphogenesis,embryonic crani		T=0, C=29	T=0.0, C=1.0	0.00001	427.563	29	T=0/0, C=17/1
chr18:33706614	G/G	C	G	SNV	SLC39A6	SLC39A6:exonic:NM_012319.3	1	100	2	missense	NM_01231 c.357G>C	p.Glu119Asp	-0.03	1	45	0		ZIP Zinc trans	rs1789528,rs370143	4.00E-04	YES	2.425E-4;1.213E-4 (pos.33706599);2.485E-4;0.0;0.0;(		cell surfac	Breast Diseases;Breast Neoplasms;Carcinom		C=0, G=128	C=0.0, G=1.0	0.00001	2092.64	128	C=0/0, G=93/3
chr5:79735858	G/G	A	G	SNV	ZFYVE16	ZFYVE16:intronic:NM_014733.5	1	100		intronic	NM_01473 c.2419+7A	p.?	-0.33				0.05(COSM44	Domain of un	rs168939	0.0946	YES	9.316E-1 (pos.79735858)	hepatocellular	Zinc finger	FYVE domain-containing protein 16		A=0, G=58	A=0.0, G=1.0	0.00001	867.38	58	A=0/0, G=16/4
chr5:79741018	T/A	T	A	SNV	ZFYVE16	ZFYVE16:intronic:NM_014733.5	1	96.77		intronic	NM_01473 c.2582-64	p.?	0.07					Domain of un	rs259034		YES			Zinc finger	FYVE domain-containing protein 16		T=1, A=30	T=0.0323, A=0.9677	0.00001	435.431	31	T=1/0, A=18/1
chr5:79743819	TA/TA	T	TA	INDEL	ZFYVE16	ZFYVE16:intronic:NM_014733.5	1	100		intronic	NM_01473 c.2725-26	p.?	-0.43					Domain of unknown function (DUF3480),FYVE zinc finger						Zinc finger	FYVE domain-containing protein 16		T=0, TA=26	T=0.0, TA=1.0	0.00001	378.971	26	T=0/0, TA=19/
chr1:75681511	C/C	T	C	SNV	SLC44A5	SLC44A5:exonic:NM_152697.4	1	100	19	synonymous	NM_15269 c.1656A>G	p.(=)	-0.86					Plasma-mem	rs595009	0.1564	YES	9.945E-1 (pos.75681511)			choline transmembrane transporter activity,choline tra		T=0, C=55	T=0.0, C=1.0	0.00001	814.905	55	T=0/0, C=16/3
chr5:79974646	C/C	T	C	SNV	MSH3	MSH3:intronic:NM_002439.4	1	100		intronic	NM_00243 c.1174-10	p.?	-0.87					MutS domain	rs836807					mutS, E. col	ATP bindin	Adenocarcinoma;Carcinoma;Endometrial Ne	T=0, C=42	T=0.0, C=1.0	0.00001	682.507	42	T=0/0, C=26/1
chr5:80256909	T/C	T	C	SNV	RASGRF2,RASGRF2-AS1	RASGRF2:intronic:NM_006909.2, RASGRF2-AS1:upstream:NF	1	45.45		intronic	NR_10501 c.288+64T	p.?	-0.21					IQ calmoduli	rs888821		YES			Ras protein	G-protein	Mammary Neoplasms, Animal;Mammary Ne	T=24, C=20	T=0.5455, C=0.4545	0.00001	207.664	44	T=10/14, C=6/
chr5:80382806	T/T	G	T	SNV	RASGRF2	RASGRF2:intronic:NM_006909.2	1	100		intronic	NM_0069C c.1390+34	p.?	0.18					IQ calmoduli	rs7733625	0.2881	YES	2.708E-1 (pos.80382806)		Ras protein	G-protein	Mammary Neoplasms, Animal;Mammary Ne	G=0, T=37	G=0.0, T=1.0	0.00001	583.707	37	G=0/0, T=13/2
chr5:80390225	T/T	C	T	SNV	RASGRF2	RASGRF2:intronic:NM_006909.2	1	100		intronic	NM_0069C c.1618+44	p.?	0.02					IQ calmoduli	rs505748	0.4708	YES	4.508E-1 (pos.80390225)		Ras protein	G-protein	Mammary Neoplasms, Animal;Mammary Ne	C=0, T=20	C=0.0, T=1.0	0.00001	263.786	20	C=0/0, T=4/16
chr5:80562120	A/A	G	A	SNV	CKMT2,CKMT2-AS1,RNU5D-1,RNU5E-1	CKMT2:utr_3:NM_001099735.1, RNU5E-1:intronic_nc:NR_0(	1	100		intronic	NR_00275 c.1303G>A	p.?	1.47					ATP:guanido	rs545	0.283	YES	8.407E-2 (pos.80562120)		Creatine kir	ATP binding,creatine kinase ac	Creatine	G=0, A=31	G=0.0, A=1.0	0.00001	460.638	31	G=0/0, A=19/1
chr5:80631511	A/A	G	A	SNV	ACOT12,RNU5D-1,RNU5E-1	RNU5E-1:intronic_nc:NR_002754.2, RNU5D-1:intronic_nc:NF	1	100		intronic	NR_00275 c.1262+76	p.?	2.33					START domai	rs6452399		YES			Acyl-CoA thi	ATP binding,acetyl-CoA hydrolase activity,acetyl-CoA me		G=0, A=64	G=0.0, A=1.0	0.00001	977.635	64	G=0/0, A=27/3
chr5:82360783	C/C	T	C	SNV	SCARNA18,TMEM167A	TMEM167A:intronic:NM_174909.4, SCARNA18:upstream:NR	1	100		intronic	NM_1749C c.113+44A	p.?	-2.87					Protein of un	rs2306336		YES	1.835E-1 (pos.82360783)		Small cajal	Golgi membrane,endomembrane system,integral compo		T=0, C=22	T=0.0, C=1.0	0.00001	298.892	22	T=0/0, C=7/15
chr5:82407098	G/A	G	A	SNV	XRCC4	XRCC4:intronic:NM_022406.3	1	58.33		intronic	NM_0224C c.315+76G	p.?	0.39					DNA double-s	rs1478482		YES			X-ray repair	DNA bindi	Bone Diseases;Bon S-(Dimethylarsenic)Cyste	G=10, A=14	G=0.4167, A=0.5833	0.00001	135.339	24	G=5/5, A=5/9
chr5:82407105	A/G	A	G	SNV	XRCC4	XRCC4:intronic:NM_022406.3	1	58.33		intronic	NM_0224C c.315+83A	p.?	0.05					DNA double-s	rs1478481		YES			X-ray repair	DNA bindi	Bone Diseases;Bon S-(Dimethylarsenic)Cyste	A=10, G=14	A=0.4167, G=0.5833	0.00001	135.336	24	A=5/5, G=5/9
chr1:8404169	A/A	T	A	SNV	SLC45A1	SLC45A1:utr_3:NM_001080397.2	1	100	9	utr_3	NM_00108 c.2445T>A	p.?	-0.29					MFS/sugar tra	rs6685844					Solute carrier family 45, member 1			T=0, A=26	T=0.0, A=1.0	0.00001	371.418	26	T=0/0, A=17/9
chr17:42333071	C/T	C	T	SNV	SLC4A1	SLC4A1:exonic:NM_000342.3	1	45.83	14	synonymous	NM_00034 c.1770G>A	p.(=)	0.72					Band 3 cytop	rs35807245	0.0219	YES	1.156E-4 (pos.42333071)		Solute carri	2 disc,anic	Acid-Base Imbalance;Acidosis; Benign/Likely	C=13, T=11	C=0.5417, T=0.4583	0.00001	93.3332	24	C=8/5, T=6/5
chr7:150765065	C/C	T	C	SNV	SLC4A2	SLC4A2:exonic:NM_003040.3	1	100	8	synonymous	NM_00304 c.1071T>C	p.(=)	-7.95				0.02(COSM41	Band 3 cytop	rs2229550	0.1738	YES	9.51E-1 (pos.150765065)	neoplasm	Solute carri	anion tran	Bile Duct Diseases;Biliary Tract Diseases;Che	T=0, C=50	T=0.0, C=1.0	0.00001	838.891	50	T=0/0, C=42/8
chr2:220494118	C/C	A	C	SNV	SLC4A3	SLC4A3:exonic:NM_201574.2	1	100	4	missense	NM_20157 c.470A>C	p.His157Pro	0.9	1	77	0	0(COSM133	Band 3 cytop	rs597306,rs7598838	9.00E-04	YES	2.088E-3;0.0;0.0;0.0 (pos.	neoplasm		anion tran	Cardiovascular Diseases;Heart Diseases	A=0, C=34	A=0.0, C=1.0	0.00001	524.185	34	A=0/0, C=23/1
chr4:72433759	T/T	C	T	SNV	SLC4A4	SLC4A4:utr_3:NM_001134742.1	1	100	25	utr_3	NM_00113 c.3434C>T	p.?	1.35					Band 3 cytop	rs1453453		YES			Solute carrier family 4,		Acid-Base Imbalanc Sodium bicarbonate	C=0, T=35	C=0.0, T=1.0	0.00001	543.903	35	C=0/0, T=20/1
chr3:195955762	G/A	G	A	SNV	SLC51A	SLC51A:exonic:NM_152672.5	1	50	6	missense	NM_15267 c.604G>A	p.Val202Ile	-0.1	0.8	29	0	0.01(COSM37	Organic solut	rs939885	0.4777	YES	7.716E-1 (pos.195955762)	adenocarcinoma,ductal_c		bile acid a	Bile Duct Diseases; Conjugated estrogens,Di	G=21, A=21	G=0.5, A=0.5	0.00001	231.07	42	G=13/8, A=6/1
chr16:24902194	C/C	T	C	SNV	SLC5A11	SLC5A11:exonic:NM_052944.4	1	100	9	synonymous	NM_05294 c.669T>C	p.(=)	0.35				0.58(COSM45	Sodium:solut	rs274081,rs386789922			9.993E-1 (pos.24902194)	squamous_cel	Solute carri	apoptotic	Autoimmune Diseases;Connective Tissue Dis	T=0, C=28	T=0.0, C=1.0	0.00001	407.998	28	T=0/0, C=17/1
chr5:870555	A/G	A	G	SNV	BRD9	BRD9:intronic:NM_023924.4	1	57.14		intronic	NM_02392 c.1525+33	p.?	-1.7					Bromodomai	rs28497180	0.0567	YES	1.517E-3 (pos.870555)			biological	Hemic and Lymphatic Diseases;Immune Syst	A=9, G=12	A=0.4286, G=0.5714	0.00001	109.101	21	A=4/5, G=9/3
chr11:26702664	T/T	C	T	SNV	SLC5A12	SLC5A12:exonic:NM_178498.3	1	100	12	synonymous	NM_17849 c.1413G>A	p.(=)	-0.26					Sodium:solut	rs10734383	1.00E-04	YES	1.0 (pos.26702664)		Solute carri	apical plasma membrane,extracellular exosome,integra		C=0, T=84	C=0.0, T=1.0	0.00001	1369.83	84	C=0/0, T=43/4
chr11:26734209	T/T	C	T	SNV	SLC5A12	SLC5A12:exonic:NM_178498.3	1	100	2	synonymous	NM_17849 c.384G>A	p.(=)	-2.12					Sodium:solut	rs10742156	0.0433	YES	8.707E-1 (pos.26734209)		Solute carri	apical plasma membrane,extracellular exosome,integra		C=0, T=48	C=0.0, T=1.0	0.00001	810.401	48	C=0/0, T=14/3
chr2:27424636	A/A	G	A	SNV	SLC5A6	SLC5A6:exonic:NM_021095.2	1	100	14	missense	NM_02109 c.1442C>T	p.Ser481Phe	2.02	0.04	155	0.012	0.7(COSM413	Sodium:solut	rs1395	0.3101	YES	8.526E-1 (pos.27424636)	ductal_carcinoma	Solute carri	biotin metabolic process,bioti	Biotin,Gabapentin Enaca	G=0, A=39	G=0.0, A=1.0	0.00001	616.614	39	G=0/0, A=13/2
chr5:89924976	A/A	C	A	SNV	ADGRV1	ADGRV1:intronic:NM_032119.3	1	100		intronic	NM_03211 c.1510-51	p.?	-1.2					7 transmemb	rs2366771,rs386690	0.3281	YES			Adhesion G	G-protein coupled receptor activity,G-protein coupled re		C=0, A=22	C=0.0, A=1.0	0.00001	298.892	22	C=0/0, A=8/14
chr5:1225613	A/A	G	A	SNV	SLC6A18,SLC6A19	SLC6A19:downstream:NM_001003841.2, SLC6A18:exonic:N	1	100	1	synonymous	NM_0010C c.*3594G>	p.?, p.(=)	-2.75				0(COSM1497	Sodium:neur	rs7704058	0.2205	YES	5.904E-1 (pos.1225613)	adenocarcinoma	Solute carri	amino aci	Amino Acid Metabolism, Inborn Errors;Amino	G=0, A=54	G=0.0, A=1.0	0.00001	774.187	54	G=0/0, A=28/2
chr5:90049238	G/G	A	G	SNV	ADGRV1	ADGRV1:intronic:NM_032119.3	1	100		intronic	NM_03211 c.11122-1	p.?	-2.96					7 transmemb	rs2438339		YES			Adhesion G	G-protein coupled receptor activity,G-protein coupled re		A=0, G=44	A=0.0, G=1.0	0.00001	725.44	44	A=0/0, G=37/7
chr5:1225626	A/A	T	A	SNV	SLC6A18,SLC6A19	SLC6A19:downstream:NM_001003841.2, SLC6A18:exonic:N	1	100	1	missense	NM_0010C c.*3607T>	p.?, p.Cys12Ser	0.82	0.82	112	0	0.01(COSM54	Sodium:neur	rs7728667	0.22	YES	5.893E-1 (pos.1225626)	adenocarcinoma	Solute carri	amino aci	Amino Acid Metabolism, Inborn Errors;Amino	T=0, A=53	T=0.0, A=1.0	0.00001	786.518	53	T=0/0, A=28/2
chr5:94619545	GC/GC	G	GC	INDEL	MCTP1	MCTP1:intronic:NM_024717.5	1	100		intronic	NM_02471 c.720+15C	p.?	0.36					C2 domain,Pl	rs5869672,rs774776	0.0075	YES	1.0;0.0 (pos.94619545)		Multiple C2	calcium io	Bipolar Disorder;Bipolar and Related Disorde	G=0, GC=26	G=0.0, GC=1.0	0.00001	317.269	26	G=0/0, GC=20/
chr5:1225687	TC/TC	CT	TC	MNV	SLC6A18,SLC6A19	SLC6A19:downstream:NM_001003841.2, SLC6A18:exonic:N	2	100	1	missense	NM_0010C c.*3668CT	p.?, p.Thr32Ile	6.07, -1.81	1	89	0	0.07(COSM14	Sodium:neur	rs386684404,rs7705	0.0945	YES	5.889E-1;1.16E-4 (pos.12	adenocarcinoma	Solute carri	amino aci	Amino Acid Metabolism, Inborn Errors;Amino	CT=0, TC=5	CT=0.0, TC=1.0	0.00001	849.61	57	CT=0/0, TC=26
chr5:94749903	C/CT	C	CT	INDEL	FAM81B	FAM81B:intronic:NM_152548.2	1	100		intronic	NM_15254 c.537+9C>	p.?	-1.41						rs11418250,rs39770	0.297	YES	0.0;0.0 (pos.94749901),1.263E-1;0.0 (pos.94749903)		nucleus,protein binding			C=0, CT=24	C=0.0, CT=1.0	0.00001	132.719	24	C=0/0, CT=7/1
chr5:1225703	G/G	A	G	SNV	SLC6A18,SLC6A19	SLC6A19:downstream:NM_001003841.2, SLC6A18:exonic:N	1	100	1	synonymous	NM_0010C c.*3684A>	p.?, p.(=)	-1.06				0.05(COSM14	Sodium:neur	rs199774515,rs77248	0.05	YES	6.266E-1 (pos.1225703)	adenocarcinoma	Solute carri	amino aci	Amino Acid Metabolism, Inborn Errors;Amino	A=0, G=58	A=0.0, G=1.0	0.00001	852.515	58	A=0/0, G=27/3
chr3:45817418	G/G	A	G	SNV	SLC6A20	SLC6A20:exonic:NM_020208.3	1	100	4	synonymous	NM_0202C c.417T>C	p.(=)	-0.19					Sodium:neur	rs758386	0.0431	YES	9.148E-1 (pos.45817418)		X transport	amino aci	Adenoma;Amino Acid Metabol Likely benign	A=0, G=29	A=0.0, G=1.0	0.00001	427.603	29	A=0/0, G=9/20
chr5:96122260	G/G	T	G	SNV	ERAP1	ERAP1:intronic:NM_016442.4	1	100		intronic	NM_01644 c.1680-7A	p.?	-0.08				0(COSM4416	ERAP1-like C-	rs30379	0.3565	YES	4.787E-1 (pos.96122260)	acute_myeloid	Endoplasmi	adaptive i	Ankylosis;Arthritis;Behcet Syndrome;Bone Di	T=0, G=23	T=0.0, G=1.0	0.00001	316.746	23	T=0/0, G=11/1
chr5:96122281	C/C	A	C	SNV	ERAP1	ERAP1:intronic:NM_016442.4	1	100		intronic	NM_01644 c.1680-28	p.?	1.28					ERAP1-like C-	rs30380	0.3567	YES	4.818E-1 (pos.96122281)		Endoplasmi	adaptive i	Ankylosis;Arthritis;Behcet Syndrome;Bone Di	A=0, C=24	A=0.0, C=1.0	0.00001	334.791	24	A=0/0, C=11/1
chr5:96126197	G/G	A	G	SNV	ERAP1	ERAP1:intronic:NM_016442.4	1	100		intronic	NM_01644 c.1452+18	p.?	0.34					ERAP1-like C-	rs27710	0.3563	YES	4.761E-1 (pos.96126197)		Endoplasmi	adaptive i	Ankylosis;Arthritis;Behcet Syndrome;Bone Di	A=0, G=32	A=0.0, G=1.0	0.00001	485.13	32	A=0/0, G=19/1
chr5:96127905	C/C	T	C	SNV	ERAP1	ERAP1:intronic:NM_016442.4	1	100		intronic	NM_01644 c.1189-10	p.?	-1.68				0.01(COSM4C	ERAP1-like C-	rs27640	0.2199	YES	4.801E-1 (pos.96127905)	acute_myeloid	Endoplasmi	adaptive i	Ankylosis;Arthritis;Behcet Syndrome;Bone Di	T=0, C=69	T=0.0, C=1.0	0.00001	1072.77	69	T=0/0, C=35/3
chr11:20639400	G/G	A	G	SNV	SLC6A5	SLC6A5:exonic:NM_004211.4	1	100	7	synonymous	NM_00421 c.1230A>G	p.(=)	0.05					Sodium:neur	rs7925597	1.00E-04		1.0;0.0 (pos.20639400)		Solute carri	chemical s	Apnea;Brain Diseas Glycine Benign	A=0, G=54	A=0.0, G=1.0	0.00001	798.131	54	A=0/0, G=43/1
chr13:30091714	A/A	G	A	SNV	SLC7A1	SLC7A1:exonic:NM_003045.4	1	100	10	synonymous	NM_00304 c.1506C>T	p.(=)	2					Amino acid p	rs2482089	0.0152	YES	9.466E-1 (pos.30091714)		Solute carri	amino aci	Genital Diseases, N L-Arginine,L-Lysine,Ornit	G=0, A=20	G=0.0, A=1.0	0.00001	263.783	20	G=0/0, A=5/15
chr4:139140494	G/C	G	C	SNV	SLC7A11	SLC7A11:exonic:NM_014331.3	1	95.45	5	synonymous	NM_01433 c.672C>G	p.(=)	-0.02				0.86(COSM54	Amino acid p	rs6838248	0.4856	YES	8.219E-2;0.0 (pos.13914(	acute_myeloid	Solute carri	L-glutama	Dermatitis;Dermat Acetylcysteine,Cystine,G	G=1, C=21	G=0.0455, C=0.9545	0.00001	271.51	22	G=1/0, C=8/13
chr5:96322419	T/T	C	T	SNV	LNPEP	LNPEP:intronic:NM_175920.3	1	100		intronic	NM_17592 c.1089+45	p.?	0.48					ERAP1-like C-	rs38034	0.4178	YES	3.45E-1 (pos.96322419)		Leucyl-cysti	SMAD prot	Chorioretinitis;Choroid Diseases;Choroiditis	C=0, T=21	C=0.0, T=1.0	0.00001	285.63	21	C=0/0, T=3/18
chr5:98207937	T/G	T	G	SNV	CHD1	CHD1:intronic:NM_001270.2	1	45.45		intronic	NM_00127 c.3711-32	p.?	2.52					Chromo (CHR	rs374718074	3.00E-04		0.0 (pos.98207937)		Chromodon	ATP binding,ATP-dependent DN	Epirubicin	T=12, G=10	T=0.5455, G=0.4545	0.00001	81.7626	22	T=10/2, G=7/3
chr5:99921841	G/C	G	C	SNV	FAM174A	FAM174A:intronic:NM_198507.2	1	50		intronic	NM_1985C c.570-44G	p.?	-1.14					Protein of un	rs2370612	0.4977	YES	1.03E-1;0.0 (pos.99921841)			integral component of membrane		G=13, C=13	G=0.5, C=0.5	0.00001	119.163	26	G=9/4, C=4/9
chr14:23282110	G/G	A	G	SNV	SLC7A7	SLC7A7:exonic:NM_001126105.2	1	100	3	synonymous	NM_00112 c.498T>C	p.(=)	0.17				0.36(COSM54	Amino acid p	rs8018462	0.4717	YES	7.935E-1 (pos.23282110)	adenocarcinoma	Solute carri	L-alpha-ar	Amino Acid Metabo Agmatine Benign	A=0, G=33	A=0.0, G=1.0	0.00001	504.529	33	A=0/0, G=15/1
chr1:27427041	C/C	T	C	SNV	SLC9A1	SLC9A1:exonic:NM_003047.4	1	100	12	synonymous	NM_00304 c.2205A>G	p.(=)	1.28					Regulatory re	rs4418629	0.0401	YES	1.0 (pos.27427041)		Solute carri	T-tubule,a	Aortic Stenosis, Sut Amiloride,Homoserine L	T=0, C=30	T=0.0, C=1.0	0.00001	446.615	30	T=0/0, C=9/21
chr3:111921225	T/T	G	T	SNV	SLC9C1	SLC9C1:exonic:NM_183061.2	1	100	18	missense	NM_1830€ c.2194C>A	p.Gln732Lys	1.61	0.95	53	0.524		Cyclic nucleo	rs6781844	0.2456	YES	9.888E-1 (pos.111921225)			cell differentiation,flagellated sperm motility,hydrogen		G=0, T=31	G=0.0, T=1.0	0.00001	465.819	31	G=0/0, T=15/1
chr6:105244526	A/T	A	T	SNV	HACE1	HACE1:intronic:NM_020771.3	1	50		intronic	NM_02077 c.816+47	p.?	1.19				0.06(COSM44	Ankyrin repe	rs6918700	0.4738	YES	4.34E-1 (pos.105244526)	hepatocellular	HECT domai	Golgi ciste	Adrenal Gland Diseases;Adrenal Gland Neop	A=32, T=32	A=0.5, T=0.5	0.00001	317.411	64	A=25/7, T=27/
chr6:105577110	T/T	TA	T	INDEL	BVES	BVES:intronic:NM_007073.4	1	100		intronic	NM_00707 c.351+144	p.?	-0.97, 0.0					Popeye prote	rs36095052,rs397888091		YES			Blood vesse	bicellular tight junction,cAMP binding,caveola,cell junct		TA=0, T=21	TA=0.0, T=1.0	0.00001	284.267	21	TA=0/0, T=12/
chr6:105577172	G/G	A	G	SNV	BVES	BVES:intronic:NM_007073.4	1	100		intronic	NM_00707 c.351+82T	p.?	-0.06					Popeye prote	rs9404603		YES			Blood vesse	bicellular tight junction,cAMP binding,caveola,cell junct		A=0, G=26	A=0.0, G=1.0	0.00001	371.42	26	A=0/0, G=12/1
chr6:105577224	C/C	A	C	SNV	BVES	BVES:intronic:NM_007073.4	1	100		intronic	NM_00707 c.351+30T	p.?	-0.14					Popeye prote	rs778602457,rs9404604		YES	0.0 (pos.105577223);9.061E-1 (pos.1055		Blood vesse	bicellular tight junction,cAMP binding,caveola,cell junct		A=0, C=27	A=0.0, C=1.0	0.00001	389.988	27	A=0/0, C=12/1
chr6:105577401	T/T	C	T	SNV	BVES	BVES:intronic:NM_007073.4	1	100		intronic	NM_00707 c.227-23G	p.?	-0.69					Popeye prote	rs4946656	0.0423	YES	9.254E-1 (pos.105577401)		Blood vesse	bicellular tight junction,cAMP binding,caveola,cell junct		C=0, T=85	C=0.0, T=1.0	0.00001	1390.52	85	C=0/0, T=39/4
chr3:111923123	A/A	G	A	SNV	SLC9C1	SLC9C1:exonic:NM_183061.2	1	100	17	missense	NM_1830€ c.2114C>T	p.Thr705Ile	-1.17	1	89	0.001		Cyclic nucleo	rs4434123	0.2455	YES	9.887E-1 (pos.111923123)			cell differentiation,flagellated sperm motility,hydrogen		G=0, A=38	G=0.0, A=1.0	0.00001	603.785	38	G=0/0, A=20/1
chr6:106992592	A/T	A	T	SNV	CRYBG1	CRYBG1:intronic:NM_001624.3	1	58.33		intronic	NM_00162 c.3942+20	p.?	0.09					Beta/Gamma	rs2247335	0.1391	YES	9.463E-1 (pos.106992592)			biological_process,carbohydrate binding,cellular_comp		A=20, T=28	A=0.4167, T=0.5833	0.00001	343.413	48	A=13/7, T=18/
chr6:107088585	T/A	T	A	SNV	QRSL1	QRSL1:intronic:NM_018292.4	1	98.41		intronic	NM_01829 c.185-113	p.?	1.26					Amidase	rs2306098		YES			Glutaminyl-	ATP binding,amidase activity,glutaminyl-tRNA synthase		T=2, A=124	T=0.0159, A=0.9841	0.00001	1982.67	126	T=1/1, A=85/3
chr6:107351457	T/T	G	T	SNV	C6orf203	C6orf203:intronic:NM_016487.4	1	100		intronic	NM_01648 c.-12+198	p.?	-1.16						rs1665924		YES				mitochondrion		G=0, T=40	G=0.0, T=1.0	0.00001	644.286	40	G=0/0, T=13/2
chr12:21028208	C/C	G	C	SNV	SLCO1B3	SLCO1B3:exonic:NM_019844.3	1	100	9	missense	NM_01984 c.767G>C	p.Gly256Ala	7.37	0	60	0.998	0.96(COSM5C	Kazal-type se	rs60140950	0.1563	YES	1.156E-4 (pos.21028208)	haemangioblastoma	Solute carri	basolater	Colonic Diseases;C Ambrisent Benign/Likely	G=0, C=21	G=0.0, C=1.0	0.00001	271.925	21	G=0/0, C=11/1
chr12:21036411	G/G	A	G	SNV	SLCO1B3	SLCO1B3:exonic:NM_019844.3	1	100	13	synonymous	NM_01984 c.1557A>G	p.(=)	-2.73				0.01(COSM39	Kazal-type se	rs2053098	0.1364	YES	7.258E-1 (pos.21036411)	acute_myeloid	Solute carri	basolater	Colonic Diseases;C Ambrisent Benign	A=0, G=57	A=0.0, G=1.0	0.00001	849.601	57	A=0/0, G=29/2
chr15:92647645	C/C	G	C	SNV	SLCO3A1	SLCO3A1:exonic:NM_013272.3	1	100	4	missense	NM_01327 c.882G>C	p.Glu294Asp	1.06	1	45	0		Kazal-type se	rs1517618	0.1682	YES	9.629E-1 (pos.92647645)		Solute carri	integral cc	Hemic and Lympha Alprostadil,Aminohippur	G=0, C=46	G=0.0, C=1.0	0.00001	768.313	46	G=0/0, C=32/1
chr5:101816104	A/A	G	A	SNV	SLCO6A1	SLCO6A1:exonic:NM_173488.4	1	100	2	synonymous	NM_17348 c.393C>T	p.(=)	-0.71				0.01(COSM41	Kazal-type se	rs6884141	0.2709	YES	2.851E-1 (pos.101816104)	acute_myeloid	Solute carri	integral cc	Mental Disorders;Schizophrenia;Schizophren	G=0, A=22	G=0.0, A=1.0	0.00001	298.892	22	G=0/0, A=14/8
chr6:10882100	A/A	G	A	SNV	GCM2	GCM2:upstream:NM_004752.3	1	100		intronic	NM_00475 c.-74C>T	p.?	-2.04						rs2153156		YES			Glial cells m	DNA bindi	Congenital, Hereditary, and Ne Benign	G=0, A=48	G=0.0, A=1.0	0.00001	809.238	48	G=0/0, A=27/2
chr6:10894267	CA/TG	CA	TG	MNV	SYCP2L	SYCP2L:intronic:NM_001040274.2	2	46.51		intronic	NM_00104 c.216+30C	p.?	-1.24, -0.27						rs386697066,rs6456	0.0541	YES	1.338E-1 (pos.10894267);3.41E-1 (pos.1(		Synaptone	condense	Ear Diseases;Hearing Disorders;Hearing Loss	CA=46, TG=	CA=0.5349, TG=0.46	0.00001	418.486	86	CA=35/11, TG
chr17:33689926	C/C	T	C	SNV	SLFN11	SLFN11:exonic:NM_001104589.1	1	100	3	missense	NM_0011C c.901A>G	p.Asn301Asp	-1.81	0.94	23	0		Putative DNA	rs4796077	0.0327	YES	9.797E-1 (pos.33689926)		Schlafen fa	ATP binding,aggresome,cytosol,defense response to viru		T=0, C=40	T=0.0, C=1.0	0.00001	644.05	40	T=0/0, C=16/2
chr6:109993291	G/A	G	A	SNV	AK9	AK9:intronic:NM_001145128.2	1	57.45		intronic	NM_00114 c.234+27C	p.?	-0.29					AAA domain,	rs12197838	0.3959	YES	4.066E-1 (pos.109993291)		Adenylate k	AMP phosphorylation,ATP binding,ATP generation from A		G=20, A=27	G=0.4255, A=0.5745	0.00001	326.535	47	G=8/12, A=8/1
chr6:110059510	C/A	C	A	SNV	FIG4	FIG4:intronic:NM_014845.5	1	45.45		intronic	NM_01484 c.647-18C	p.?	0.52					Sac1 homolog	rs2273752	0.39	YES	3.777E-1 (pos.110059510)		Fig4, S. cere	Golgi mem	Abnormalities, Multiple;Amyo Benign	C=12, A=10	C=0.5455, A=0.4545	0.00001	81.7625	22	C=4/8, A=3/7
chr6:110752289	G/A	G	A	SNV	SLC22A16	SLC22A16:intronic:NM_033125.3	1	50		intronic	NM_03312 c.1521+85	p.?	-0.05					Major Faciliti	rs1033572		YES			Solute carri	acid secre	Arthritis;Arthritis, J Doxorubicin,L-Carnitine	G=16, A=16	G=0.5, A=0.5	0.00001	158.362	32	G=5/11, A=7/9
chr6:111679869	G/T	G	T	SNV	REV3L	REV3L:intronic:NM_002912.4	1	47.62		intronic	NM_00291 c.7072+15	p.?	1.19					C4-type zinc-f	rs110732		YES			Rev-3, yeas	3'-5' exonu	Abnormalities, Multiple;Congenital Abnorma	G=11, T=10	G=0.5238, T=0.4762	0.00001	82.2591	21	G=8/3, T=5/5
chr17:33806863	A/A	G	A	SNV	SLFN12L	SLFN12L:exonic:NM_001195790.1	1	100	2	synonymous	NM_00119 c.366C>T	p.(=)	-3.07				0(COSM3680	Putative DNA	rs12451746	0.4258	YES	2.938E-1 (pos.33806863)	acute_myeloid	Schlafen fa	ATP binding,integral component of membrane		G=0, A=38	G=0.0, A=1.0	0.00001	603.785	38	G=0/0, A=29/9
chr4:20552510	A/C	A	C	SNV	SLIT2	SLIT2:exonic:NM_004787.3	1	53.13	25	synonymous	NM_00478 c.2550A>C	p.(=)	-0.91					EGF-like dom	rs17542486	0.1085	YES	1.277E-3 (pos.20552510)		Slit, Drosop	GTPase inl	Adenocarcinoma;Brain Diseases;Bronchial N	A=15, C=17	A=0.4688, C=0.5313	0.00001	173.559	32	A=5/10, C=4/1
chr5:168098463	G/G	A	G	SNV	SLIT3	SLIT3:exonic:NM_001271946.1	1	100	34	synonymous	NM_00127 c.3888T>C	p.(=)	-4.44					EGF-like dom	rs292486	0.0409	YES	1.0 (pos.168098463)		Slit, Drosop	Roundabout binding,Roundabout signaling pathway,ani		A=0, G=48	A=0.0, G=1.0	0.00001	810.435	48	A=0/0, G=26/2
chr6:116751950	C/CA	C	CA	INDEL	DSE	DSE:intronic:NM_001080976.2	1	53.13		intronic	NM_00108 c.671-167	p.?	-0.68					Domain of un	rs11389417,rs397823302		YES			Dermatan s	Golgi apparatus,Golgi membrane,chondroitin sulfate bic		C=15, CA=1	C=0.4688, CA=0.531	0.00001	142.797	32	C=12/3, CA=1C
chr6:116864570	A/C	A	C	SNV	CALHM4,TRAPPC3L	TRAPPC3L:intronic:NM_001139444.2, CALHM4:intronic:NM	1	50		intronic	NM_00113 c.43-7T>G	p.?, p.?	0.95				0.33(COSM4C	Calcium hom	rs4142087		YES	4.904E-1 (pos.11686457(	acute_myeloid	Bet3, S. cer	Golgi apparatus,Golgi vesicle transport,TRAPP complex,I		A=14, C=14	A=0.5, C=0.5	0.00001	127.978	28	A=9/5, C=9/5
chr5:168180081	T/T	C	T	SNV	SLIT3	SLIT3:exonic:NM_001271946.1	1	100	18	missense	NM_00127 c.1852G>A	p.Gly618Ser	6.3	1	56	0	0.29(COSM41	EGF-like dom	rs10036727	0.4871	YES	5.232E-1 (pos.16818008:	acute_myeloid	Slit, Drosop	Roundabout binding,Roundabout signaling pathway,ani		C=0, T=38	C=0.0, T=1.0	0.00001	603.786	38	C=0/0, T=22/1
chr6:118832320	C/C	T	C	SNV	CEP85L	CEP85L:intronic:NM_001178035.1	1	100		intronic	NM_00117 c.1266+14	p.?	-0.21						rs9489424		YES				centrosome,cytoplasm		T=0, C=67	T=0.0, C=1.0	0.00001	1034.43	67	T=0/0, C=18/4
chrX:144904882	C/C	T	C	SNV	SLITRK2	SLITRK2:exonic:NM_001144009.2	1	100	2	synonymous	NM_00114 c.939T>C	p.(=)	-0.59					Leucine Rich	rs2748588,rs868910	0.1356	YES	9.994E-1 (pos.144904882)		SLIT- and NT	axonogenesis,integral component of membrane,plasma		T=0, C=82	T=0.0, C=1.0	0.00001	1329.65	82	T=0/0, C=66/1
chr13:86369120	T/T	C	T	SNV	SLITRK6	SLITRK6:exonic:NM_032229.2	1	100	2	synonymous	NM_03222 c.1524G>A	p.(=)	0.04				0.27(COSM13	Leucine Rich	rs3825413	0.279	YES	6.961E-1 (pos.86369120)	haemangioblastoma	SLIT- and NT	adult loco	Ear Diseases;Eye Diseases;Hearing Disorders	C=0, T=47	C=0.0, T=1.0	0.00001	789.328	47	C=0/0, T=25/2
chr10:105762591	G/A	G	A	SNV	SLK	SLK:exonic:NM_014720.3	1	46.43	9	missense	NM_01472 c.1655G>A	p.Cys552Tyr	-0.1	0.32	194	0.001	0.01(COSM37	Polo kinase k	rs805657	0.167	YES	7.913E-2 (pos.10576259:	adenocarcinoma	STE20-like k	ATP bindin	Mental Disorders;S 4-(4-(5-CYCLOPROPYL-1H	G=15, A=13	G=0.5357, A=0.4643	0.00001	117.929	28	G=3/12, A=7/6
chr6:121436422	T/T	G	T	SNV	TBC1D32	TBC1D32:intronic:NM_152730.5	1	100		intronic	NM_15273 c.2984-35	p.?	-0.19					Broad-minde	rs386705309,rs7772	0		1.0 (pos.121436422)		TBC1 domai	cilium,cyto	Congenital Abnormalities;Congenital, Heredi	G=0, T=39	G=0.0, T=1.0	0.00001	623.971	39	G=0/0, T=20/1
chr5:159835658	G/G	A	G	SNV	SLU7	SLU7:exonic:NM_006425.4	1	100	7	missense	NM_00642 c.686T>C	p.Met229Thr	0.56	0.76	81	0	0.04(COSM44	Pre-mRNA sp	rs2961944	0.2627	YES	5.359E-1 (pos.159835658)	adenocarcinoma,hepatoc	RNA export from nucleus,RNA splicing, via transesterific			A=0, G=78	A=0.0, G=1.0	0.00001	1249.35	78	A=0/0, G=33/4

chr6:123369910	C/C	T	C	SNV	CLVS2	CLVS2:intronic:NM 001010852.3	1	100		intronic	NM 00101	c.675+33T	p.?	-1.17					CRAL/TRIO do	rs6900862	0.1269	YES	9.999E-1 (pos.123369910)		Clavesin 2	clathrin-coated endocytic vesicle membrane,clathrin-co				T=0, C=227	T=0.0, C=1.0	0.00001	3765.51	227	T=0/0, C=99/1
chr6:123600100	C/C	CA	C	INDEL	TRDN	TRDN:intronic:NM 006073.3	1	100		intronic	NM 00607	c.1537+10	p.?	0.17, 0.48					Aspartyl beta	rs143535631					Triadin	cellular calcium ion homeostasis,cytoplasmic microtubu				CA=0, C=33	CA=0.0, C=1.0	0.00001	488.27	33	CA=0/0, C=24/
chr6:123658680	G/G	A	G	SNV	TRDN	TRDN:intronic:NM 006073.3	1	100		intronic	NM 00607	c.1420+84	p.?	1					Aspartyl beta	rs12216080		YES			Triadin	cellular calcium ion homeostasis,cytoplasmic microtubu				A=0, G=24	A=0.0, G=1.0	0.00001	321.984	24	A=0/0, G=13/1
chr15:67457335	G/G	A	G	SNV	SMAD3	SMAD3:exonic:NM 005902.3	1	100	2	synonymous	NM 00590	c.309A>G	p.(=)	1.54					MH1 domain	rs1065080	0.1163	YES	7.586E-1 (pos.67457335)		Mothers ag	DEAD/H-bc	ACMG Recommendations...:Ab		Benign	A=0, G=42	A=0.0, G=1.0	0.00001	685.214	42	A=0/0, G=27/1
chr6:125402711	C/T	C	T	SNV	RNF217	RNF217:intronic:NM 152553.4	1	50		intronic	NM 15255	c.679+58C	p.?	-0.72					IBR domain, a	rs492800	0.4881	YES				cytoplasm,cytosol,integral component of membrane,me				C=19, T=19	C=0.5, T=0.5	0.00001	199.992	38	C=5/14, T=8/1
chr5:135513085	TC/TC	T	TC	INDEL	SMAD5	SMAD5:exonic:NM 001001420.2	1	100	7	unknown	NM 001001420.2			0.43					MH1 domain,	rs112079663,rs5576	2.00E-04	YES	1.0 (pos.135513085)		Mothers ag	BMP signaling pathway,DEAD/H-box RNA helicase bindin				T=0, TC=13	T=0.0, TC=1.0	0.00001	2167.64	134	T=0/0, TC=72/
chr6:126072853	G/G	C	G	SNV	HEY2	HEY2:intronic:NM 012259.2	1	100		intronic	NM 01225	c.84-94C>	p.?	-0.11					Hairy Orange	rs2875881		YES			Hairy/enha	DNA bindi	Adenocarcinoma:Aortic Stenosis, Subvalvula			C=0, G=11C	C=0.0, G=1.0	0.00001	1737.1	110	C=0/0, G=45/6
chr19:11170839	T/C	T	C	SNV	SMARCA4	SMARCA4:exonic:NM 001128849.1	1	51.85	35	synonymous	NM 00112	c.4983T>C	p.(=)	-2.76				0.02(COSM13	BRK domain,I	rs7275	0.255	YES	8.727E-2 (pos.11170839)	adenocarcinoı	SWI/SNF-related, matri		Abnormalities, Multiple:Adnex		Benign	T=13, C=14	T=0.4815, C=0.5185	0.00001	132.666	27	T=3/10, C=6/8
chr6:126288023	T/C	T	C	SNV	HINT3	HINT3:intronic:NM 138571.4	1	57.14		intronic	NM 13857	c.202-10T	p.?	-0.07					HIT domain,S	rs3757212	0.3556	YES	9.489E-1 (pos.126288023)		Histidine tr	cytoplasm,extracellular exosome,hydrolase activity,nuc				T=9, C=12	T=0.4286, C=0.5714	0.00001	109.612	21	T=5/4, C=5/7
chr4:144467104	C/C	T	C	SNV	SMARCA5	SMARCA5:exonic:NM 003601.3	1	100	19	synonymous	NM 00360	c.2424T>C	p.(=)	0.57					DEAD/DEAH b	rs13139128	0		1.0 (pos.144467104)		SWI/SNF-rel	ATP binding,ATP-dependent ch		4-Deoxy-Alpha-D-Glucos		T=0, C=20	T=0.0, C=1.0	0.00001	263.786	20	T=0/0, C=5/15
chr22:45767455	G/G	A	G	SNV	SMC1B	SMC1B:exonic:NM 148674.4	1	100	14	synonymous	NM 14867	c.2209T>C	p.(=)	3.05				0.03(COSM37	AAA ATPase d	rs136569	0.4803	YES	7.076E-1 (pos.45767455)	adenocarcinoma		ATP bindin	Female Urogenital Diseases;Female Urogeni			A=0, G=42	A=0.0, G=1.0	0.00001	685.088	42	A=0/0, G=29/1
chr6:128330249	C/C	T	C	SNV	PTPRK	PTPRK:intronic:NM 001135648.2	1	100		intronic	NM 00113	c.2333+26	p.?	0.14					Fibronectin t	rs761556884,rs9321	0.097	YES	0.0 (pos.128330245),9.935E-1 (pos.1283		Protein-tyrc	adherens	Genital Diseases, Male;Genital Neoplasms, I			T=0, C=47	T=0.0, C=1.0	0.00001	789.325	47	T=0/0, C=23/2
chr6:128385998	G/G	A	G	SNV	LOC101928140,PTPRK	LOC101928140:intronic_nc:NR 125849.1, PTPRK:intronic:N	1	100		intronic	NM 00113	c.2158-59	p.?	0.96					Fibronectin t	rs4897241	0.1464	YES			Protein-tyrc	adherens	Genital Diseases, Male;Genital Neoplasms, I			A=0, G=65	A=0.0, G=1.0	0.00001	996.463	65	A=0/0, G=9/56
chr22:45789642	C/C	A	C	SNV	SMC1B	SMC1B:exonic:NM 148674.4	1	100	9	missense	NM 14867	c.1417T>G	p.Phe473Val	1.82	1	50	0	0.06(COSM45	AAA ATPase d	rs136603,rs767793963		YES	1.0;0.0 (pos.45789642)	squamous_cell_carcinoma		ATP bindin	Female Urogenital Diseases;Female Urogeni			A=0, C=25	A=0.0, C=1.0	0.00001	353.018	25	A=0/0, C=19/6
chr10:112361870	G/G	A	G	SNV	SMC3	SMC3:exonic:NM 005445.3	1	100	25	synonymous	NM 00544	c.3039A>G	p.(=)	0.08					AAA ATPase d	rs2419565	7.00E-04		1.0;0.0 (pos.112361870)		Structural n	ATP bindin	Abnormalities, Multiple:Chron		Benign	A=0, G=36	A=0.0, G=1.0	0.00001	563.3	36	A=0/0, G=17/1
chr6:129802302	C/C	G	C	SNV	LAMA2	LAMA2:intronic:NM 000426.3	1	100		intronic	NM 00042	c.7573-10	p.?	-0.93					Laminin B (Dc	rs2275214		YES			Laminin, al	Schwann c	Charcot-Marie-Tooth Disease:Congenital Abr			G=0, C=77	G=0.0, C=1.0	0.00001	1229.44	77	G=0/0, C=57/2
chr6:129835370	A/A	T	A	SNV	LAMA2	LAMA2:intronic:NM 000426.3	1	100		intronic	NM 00042	c.8989-14	p.?	1.2					Laminin B (Dc	rs13196682,rs902102778		YES			Laminin, al	Schwann c	Charcot-Marie-Tooth Disease:Congenital Abr			T=0, A=24	T=0.0, A=1.0	0.00001	334.108	24	T=0/0, A=19/5
chr9:72897434	A/A	G	A	SNV	SMC5	SMC5:exonic:NM 015110.3	1	100	7	missense	NM 01511	c.916G>A	p.Val306Ile	-0.06	0.63	29	0	0.01(COSM50	AAA ATPase d	rs1180116	0.0962	YES	8.613E-1 (pos.72897434)	ductal_carcinc	Structural n	ATP binding,PML body,Smc5-Smc6 complex,cell division,				G=0, A=60	G=0.0, A=1.0	0.00001	903.278	60	G=0/0, A=36/2
chr18:2707619	G/A	G	A	SNV	SMCHD1	SMCHD1:exonic:NM 015295.2	1	50	16	missense	NM 01529	c.2122G>A	p.Val708Ile	0.28	1	29	0	0.03(COSM39	Histidine kin	rs2276092	0.2969	YES	6.097E-1 (pos.2707619)	NS,acute_mye	Structural n	ATP bindin	Autoimmune Diseases;Congen		Benign	G=27, A=27	G=0.5, A=0.5	0.00001	262.866	54	G=16/11, A=1
chr12:27627805	A/A	G	A	SNV	SMCO2	SMCO2:exonic:NM 001145010.1	1	100	4	synonymous	NM 00114	c.321G>A	p.(=)	-0.72					TMCO5 famil	rs411816	0.198	YES	7.219E-1 (pos.27627805)			integral component of membrane				G=0, A=93	G=0.0, A=1.0	0.00001	1555.33	93	G=0/0, A=33/6
chr6:130530815	T/T	C	T	SNV	SAMD3	SAMD3:intronic:NM 001277185.1	1	100		intronic	NM 00127	c.342-62G	p.?	-3.13					SAM domain	rs1932100		YES				protein binding				C=0, T=33	C=0.0, T=1.0	0.00001	504.597	33	C=0/0, T=17/1
chr6:131940890	A/C	A	C	SNV	MED23	MED23:intronic:NM 004830.3	1	59.09		intronic	NM 00483	c.667+77T	p.?	-0.35					Mediator cor	rs2297638		YES			Mediator cc	nucleoplasm,protein binding,regulation of transcription				A=9, C=13	A=0.4091, C=0.5909	0.00001	122.516	22	A=5/4, C=7/6
chr6:131995209	T/C	T	C	SNV	ENPP3	ENPP3:intronic:NM 005021.4	1	58.33		intronic	NM 00502	c.643-93T	p.?	-0.59					DNA/RNA nor	rs6930385		YES			Ectonucleo	NADH pyrophosphatase activity,catalytic activity,extrac				T=10, C=14	T=0.4167, C=0.5833	0.00001	135.276	24	T=6/4, C=12/2
chr16:18823379	C/C	T	C	SNV	SMG1	SMG1:exonic:NM 015092.4	1	100	61	synonymous	NM 01509	c.10692A>	p.(=)	0.91					FATC domain	rs2606556	1.00E-04	YES	9.992E-1 (pos.18823379)		SMG1, C. el	ATP binding,DNA repair,RNA binding,cytoplasm,cytosol,r				T=0, C=67	T=0.0, C=1.0	0.00001	1034.43	67	T=0/0, C=20/4
chr6:131997798	A/C	A	C	SNV	ENPP3	ENPP3:intronic:NM 005021.4	1	58.33		intronic	NM 00502	c.873-78A	p.?	-1.41					DNA/RNA nor	rs2286453		YES			Ectonucleo	NADH pyrophosphatase activity,catalytic activity,extrac				A=10, C=14	A=0.4167, C=0.5833	0.00001	135.331	24	A=6/4, C=6/8
chr6:13228008	G/C	G	C	SNV	PHACTR1	PHACTR1:intronic:NM 001242648.2	1	56		intronic	NM 00124	c.987-40G	p.?	0.67					RPEL repeat	rs9395550	0.0893	YES	9.069E-2 (pos.13228008)		Phosphatas	actin bind	Arterial Occlusive Diseases:Arteriosclerosis:			G=11, C=14	G=0.44, C=0.56	0.00001	134.349	25	G=8/3, C=6/8
chr6:13278648	G/G	A	G	SNV	LOC100130357,PHACTR1,TBC1D7-LOC100130357	LOC100130357:downstream:NM 001242698.1, TBC1D7-LO	1	100		intronic	NM 00124	c.1509+87	p.?, p.?, p.?	-1					RPEL repeat	rs3817735		YES			Phosphatas	GTPase ac	Arterial Occlusive Diseases:Arteriosclerosis:			A=0, G=59	A=0.0, G=1.0	0.00001	885.346	59	A=0/0, G=26/3
chr6:13281412	G/G	A	G	SNV	LOC100130357,PHACTR1,TBC1D7-LOC100130357	LOC100130357:intronic:NM 001242698.1, TBC1D7-LOC100	1	100		intronic	NM 00124	c.1510-22	p.?, p.?, p.?	-0.15					RPEL repeat	rs3846903		YES	2.742E-1 (pos.13281412)		Phosphatas	GTPase ac	Arterial Occlusive Diseases:Arteriosclerosis:			A=0, G=51	A=0.0, G=1.0	0.00001	754.584	51	A=0/0, G=14/3
chr16:18861335	G/G	A	G	SNV	SMG1	SMG1:exonic:NM 015092.4	1	100	35	synonymous	NM 01509	c.5397T>C	p.(=)	0.07				0.77(COSM37	FATC domain,	rs2650613	0.4361	YES	2.198E-1 (pos.18861335)	acute_myeloic	SMG1, C. el	ATP binding,DNA repair,RNA binding,cytoplasm,cytosol,r				A=0, G=30	A=0.0, G=1.0	0.00001	446.638	30	A=0/0, G=8/22
chr6:132860522	C/G	C	G	SNV	TAAR9	TAAR9:downstream:NM 175057.3	1	50		intronic	NM 17505	c.*47C>G	p.?	-1.03						rs2842898	0.271	YES	8.986E-1 (pos.132860522)			G-protein coupled receptor activity,G-protein coupled re				C=11, G=11	C=0.5, G=0.5	0.00001	94.6067	22	C=7/4, G=9/2
chr6:13295502	C/C	T	C	SNV	LOC100130357,TBC1D7-LOC100130357	LOC100130357:intronic:NM 001242698.1, TBC1D7-LOC100	1	100		intronic	NM 00131	c.*921+97	p.?, p.?	-0.84						rs4602719		YES	6.566E-1 (pos.13295502)			GTPase activator activity,Rab GTPase binding,activation				T=0, C=34	T=0.0, C=1.0	0.00001	524.186	34	T=0/0, C=22/1
chr17:2203023	T/TG	T	TG	INDEL	SMG6	SMG6:exonic:NM 017575.4	1	100	2	frameshiftInsertion	NM 01757	c.1023_10	p.Ser342fs	1.51					Est1 DNA/RNA binding domain,PIN domain,Telomerase activating protein Est1						SMG6, C. el	DNA polym	Arterial Occlusive D	Grn163l		T=0, TG=31	T=0.0, TG=1.0	0.00001	459.227	31	T=0/0, TG=13/
chr17:2203025	T/G	T	G	SNV	SMG6	SMG6:exonic:NM 017575.4	1	95.65	2	missense	NM 01757	c.1022A>C	p.Asn341Thr	3.3	0	65	0.001	0.97(COSM41	Est1 DNA/RN	rs1885987	0.3866	YES	1.648E-1 (pos.2203025)	basal_cell_car	SMG6, C. el	DNA polym	Arterial Occlusive D	Grn163l		T=2, G=44	T=0.0435, G=0.9565	0.00001	648.418	46	T=0/2, G=28/1
chr7:128846328	C/C	G	C	SNV	SMO	SMO:exonic:NM 005631.4	1	100	6	synonymous	NM 00563	c.1164G>C	p.(=)	1.13				0.42(COSM40	Frizzled/Smo	rs2228617	0.1609	YES	7.695E-1 (pos.12884632	acute_myeloic	Smoothene	G-protein	Ameloblastoma:Bo	Fluocinoni	Benign	G=0, C=22	G=0.0, C=1.0	0.00001	302.178	22	G=0/0, C=5/17
chr6:133138749	T/C	T	C	SNV	RPS12,SNORA33,SNORD100	SNORD100:downstream:NR 002435.1, SNORA33:downstrea	1	53.33		intronic	NM 00101	c.*68T>C	p.?	-0.14						rs2236382	0.3909	YES			Ribosomal	RNA binding,SRP-dependent cotranslational protein targ				T=12, C=16	T=0.4286, C=0.5714	0.00001	182.947	30	T=7/5, C=7/9
chr6:13365913	G/G	C	G	SNV	GFOD1	GFOD1:intronic:NM 018988.3	1	100		intronic	NM 01898	c.254-19G	p.?	1.51					Oxidoreducta	rs9367440	0.4935	YES	6.775E-1 (pos.13365913)			extracellular region,oxidation-reduction process,oxidor				C=0, G=21	C=0.0, G=1.0	0.00001	281.235	21	C=0/0, G=7/14
chr6:133836430	G/A	G	A	SNV	EYA4,TARID	EYA4:intronic:NM 172105.3, TARID:intronic_nc:NR 109982.	1	46.43		intronic	NM 17210	c.1502-29	p.?	0.32						rs56378005	0.0164	YES	1.16E-4 (pos.133836430)		Eyes absent	DNA repai	Cardiomegaly;Cardiomyopathies;Cardiomyo			G=15, A=13	G=0.5357, A=0.4643	0.00001	117.924	28	G=9/6, A=6/7
chr6:134493947	A/G	A	G	SNV	SGK1	SGK1:intronic:NM 001143676.1	1	50		intronic	NM 00114	c.835-35T	p.?	1.89					Kinase-like,P	rs1743966	0.207	YES	2.531E-1 (pos.134493947)		Serum/gluc	ATP bindin	Diabetes Complica	4-(5-naphthalen-2-yl-1H-		A=19, G=19	A=0.5, G=0.5	0.00001	199.996	38	A=7/12, G=15/
chr11:6411935	C/C	T	C	SNV	SMPD1	SMPD1:exonic:NM 000543.4	1	100	1	missense	NM 00054	c.107T>C	p.Val36Ala	0.5	0.31	64	0	0.05(COSM15	Calcineurin-l	rs1050228,rs383878	0.4351	YES	0.0 (pos.6411932),0.0 (pc	Overgrowth_s	Sphingomye	acid sphin	Brain Diseases;Bra	Amlodipin	Benign	T=0, C=32	T=0.0, C=1.0	0.00001	485.139	32	T=0/0, C=14/1
chr6:135357790	C/T	C	T	SNV	HBS1L	HBS1L:intronic:NM 006620.3	1	53.33		intronic	NM 00662	c.430+292	p.?	1.63					Elongation fa	rs186634007	0.0082		0.0 (pos.135357790)		HBS1-like p	GTP binding,GTPase activity,cytosol,exonucleolytic nucl				C=91, T=10	C=0.4667, T=0.5333	0.00001	1189.04	195	C=58/33, T=59
chr6:135748200	G/G	A	G	SNV	AHI1	AHI1:intronic:NM 001134830.1	1	100		intronic	NM 00113	c.2764+10	p.?	0.12					SH3 domain,\	rs2757639		YES			Abelson hel	Kupffer's v	Autistic Disorder:Autoimmune Diseases:Aut			A=0, G=25	A=0.0, G=1.0	0.00001	353.016	25	A=0/0, G=9/16
chr6:13659079	T/G	T	G	SNV	RANBP9	RANBP9:intronic:NM 005493.2	1	100		intronic	NM 00549	c.684-15A	p.?	-0.34					CTLH/CRA C-t	rs204232	0.4317	YES	4.886E-1;0.0 (pos.13659079)		RAN-bindin	MAPK cascade,Ran GTPase binding,Ras guanyl-nucleotid				T=0, G=26	T=0.0, G=1.0	0.00001	422.399	26	T=0/0, G=4/22
chr4:71232430	C/C	T	C	SNV	SMR3A	SMR3A:exonic:NM 012390.3	1	100	3	missense	NM 01239	c.124T>C	p.Cys42Arg	-0.44	0.34	180	0		Proline-rich s	rs10024123,rs75517	0.0472	YES	1.0;0.0 (pos.71232430)			extracellular region				T=0, C=27	T=0.0, C=1.0	0.00001	389.986	27	T=0/0, C=8/19
chr4:71232701	T/T	C	T	SNV	SMR3A	SMR3A:exonic:NM 012390.3	1	100	3	missense	NM 01239	c.395C>T	p.Pro132Leu	0.01	1	98	0.033	0.04(COSM11	Proline-rich s	rs6853742	0.3295	YES	6.247E-1 (pos.71232701)	adenocarcinoma		extracellular region				C=0, T=59	C=0.0, T=1.0	0.00001	885.334	59	C=0/0, T=24/3
chr22:31491295	C/C	G	C	SNV	SMTN	SMTN:exonic:NM 001207017.1	1	100	13	missense	NM 00120	c.1894G>C	p.Ala632Pro	1.08	1	27	0	0.01(COSM44	CAMSAP CH d	rs3205187	0.3364	YES	1.147E-1 (pos.31491295)	acute_myeloic	Smoothelin	actin binding,actin cytoskeleton,cytoplasm,cytoskeleto				G=0, C=29	G=0.0, C=1.0	0.00001	427.59	29	G=0/0, C=15/1
chr6:137477823	T/T	C	T	SNV	IL22RA2	IL22RA2:intronic:NM 052962.2	1	100		intronic	NM 05296	c.293+73G	p.?	-1.27					Interferon-al	rs2064501		YES			Interleukin	cytokine-mediated signaling pathway,cytosol,extracellu				C=0, T=59	C=0.0, T=1.0	0.00001	885.365	59	C=0/0, T=44/1
chr2:88387557	C/C	A	C	SNV	SMYD1	SMYD1:exonic:NM 198274.3	1	100	3	missense	NM 19827	c.491A>C	p.Gln164Pro	3.83	0.28	76	0.001		MYND finger,	rs1542087	0.1049	YES	2.458E-2 (pos.88387557)			DNA binding,chromatin remodeling,cytoplasm,heart dev				A=0, C=34	A=0.0, C=1.0	0.00001	524.186	34	A=0/0, C=9/25
chr1:214492286	A/A	G	A	SNV	SMYD2	SMYD2:exonic:NM 020197.2	1	100	5	missense	NM 02019	c.494G>A	p.Gly165Glu	4.96	1	98	0		MYND finger,	rs1134647	0.0826	YES	6.172E-1 (pos.214492286)		SET and MY	RNA polymerase II core binding,cytoplasm,cytosol,heart				G=0, A=29	G=0.0, A=1.0	0.00001	427.599	29	G=0/0, A=22/7
chr6:142491463	CG/CG	C	CG	INDEL	VTA1	VTA1:intronic:NM 016485.4	1	100		intronic	NM 01648	c.336-20C	p.?	-0.41					Vta1 like	rs11446143,rs39780	2.00E-04	YES	1.0;0.0;0.0 (pos.142491463)		SKD1-bindi	ESCRT III complex disassembly,cytosol,endosomal transı				C=0, CG=52	C=0.0, CG=1.0	0.00001	756.148	52	C=0/0, CG=30/
chr17:1731268	C/C	T	C	SNV	SMYD4	SMYD4:exonic:NM 052928.2	1	100	2	synonymous	NM 05292	c.21A>G	p.(=)	-0.33				0.02(COSM37	MYND finger,	rs776969022,rs8067	0.2116	YES	0.0 (pos.1731267),4.771	adenocarcinoma,hepatoce		metal ion	Glioma:Medulloblastoma:Neoplasms:Neopl			T=0, C=21	T=0.0, C=1.0	0.00001	281.235	21	T=0/0, C=14/7
chr17:1686410	T/C	T	C	SNV	SMYD4	SMYD4:exonic:NM 052928.2	1	51.85	10	missense	NM 05292	c.2180A>G	p.Tyr727Cys	-3.37	0.09	194	0.037	0.02(COSM37	MYND finger,	rs777761760,rs9902	0.2924	YES	0.0;0.0 (pos.1686409),7.	hepatocellular_carcinoma		metal ion	Glioma:Medulloblastoma:Neoplasms:Neopl			T=13, C=14	T=0.4815, C=0.5185	0.00001	132.665	27	T=5/8, C=2/12
chr6:145167978	C/G	C	G	SNV	UTRN	UTRN:intronic:NM 007124.2	1	57.14		intronic	NM 00712	c.10293+1	p.?	-0.97					Calponin hor	rs9403617	0.0986	YES	2.241E-1 (pos.145167978)		Utrophin (h	actin binding,actin filament binding,cell junction,contra				C=9, G=12	C=0.4286, G=0.5714	0.00001	109.102	21	C=6/3, G=7/5
chr6:146216184	C/T	C	T	SNV	SHPRH	SHPRH:intronic:NM 001042683.2	1	45.45		intronic	NM 00104	c.4516-71	p.?	-0.23					Helicase con	rs2247206		YES				ATP binding,DNA binding,DNA repair,cellular response to				C=18, T=15	C=0.5455, T=0.4545	0.00001	143.162	33	C=0/18, T=3/1
chr6:146268623	T/A	T	A	SNV	SHPRH	SHPRH:intronic:NM 001042683.2	1	50		intronic	NM 00104	c.1212+6A	p.?	1.95					Helicase con	rs9386141	0.4158	YES	6.477E-1 (pos.146268623)			ATP binding,DNA binding,DNA repair,cellular response to				T=22, A=22	T=0.5, A=0.5	0.00001	243.629	44	T=14/8, A=10/
chr6:147054450	A/C	A	C	SNV	ADGB	ADGB:intronic:NM 024694.3	1	50		intronic	NM 02469	c.2656+59	p.?	0.04					Calpain famil	rs3761782		YES			Androglobir	calcium-dependent cysteine-type endopeptidase activit				A=13, C=13	A=0.5, C=0.5	0.00001	119.163	26	A=4/9, C=9/4
chr6:147684318	T/T	C	T	SNV	STXBP5	STXBP5:intronic:NM 001127715.2	1	100		intronic	NM 00112	c.2546-15	p.?	-1.34					LLGL2,Lethal	rs9403852		YES			Syntaxin-bi	GTPase ac	Bronchopulmonary Dysplasia;Congenital, He			C=0, T=30	C=0.0, T=1.0	0.00001	446.628	30	C=0/0, T=22/8
chr6:148761456	C/C	G	C	SNV	SASH1	SASH1:intronic:NM 015278.4	1	100		intronic	NM 01527	c.337-38G	p.?	1.57					Lymphocyte	rs2272998	0.4066	YES	4.235E-1;0.0 (pos.148761456)		Sterile alph	mitogen-a	Alopecia;Diabetes Complications;Diabetes N			G=0, C=33	G=0.0, C=1.0	0.00001	504.598	33	G=0/0, C=11/2
chr6:148761603	A/A	C	A	SNV	SASH1	SASH1:intronic:NM 015278.4	1	100		intronic	NM 01527	c.386+60C	p.?	0.13					Lymphocyte	rs9498038	0.3947	YES			Sterile alph	mitogen-a	Alopecia;Diabetes Complications;Diabetes N			C=0, A=30	C=0.0, A=1.0	0.00001	446.639	30	C=0/0, A=8/22
chr6:148869296	A/ACACT	A	ACACT	INDEL	SASH1	SASH1:intronic:NM 015278.4	4	56.36		intronic	NM 01527	c.3481-13	p.?	0.09					Lymphocyte	rs150196011,rs58403589		YES			Sterile alph	mitogen-a	Alopecia;Diabetes Complications;Diabetes N			A=24, ACAC	A=0.4364, ACACT=0.	0.00001	317.932	55	A=10/14, ACAC
chr6:84303342	C/C	T	C	SNV	SNAP91	SNAP91:exonic:NM 001242792.1	1	100	17	synonymous	NM 00124	c.1551A>G	p.(=)	0.48					ANTH domain	rs1033655	0.2939	YES	8.859E-1;2.675E-4 (pos.84303342)		Synaptoson	1-phosphatidylinositol binding,clathrin binding,clathrin				T=0, C=71	T=0.0, C=1.0	0.00001	1100.37	71	T=0/0, C=32/3
chr6:149953835	G/G	A	G	SNV	KATNA1	KATNA1:intronic:NM 007044.3	1	100		intronic	NM 00704	c.320+60T	p.?	0.22					AAA domain (	rs9322202		YES			Katanin, p6	ATP binding,cell cycle,cell division,cytoplasm,microtubu				A=0, G=21	A=0.0, G=1.0	0.00001	281.235	21	A=0/0, G=8/13
chr19:7986638	G/G	C	G	SNV	SNAPC2	SNAPC2:exonic:NM 003083.3	1	100	3	missense	NM 00308	c.352C>G	p.Leu118Val	-0.32	0.18	32	0.992		Small nuclea	rs475002	0.4408	YES	5.101E-1 (pos.7986638)		Small nucle	DNA binding transcription factor activity,cytosol,nuclear				C=0, G=21	C=0.0, G=1.0	0.00001	281.231	21	C=0/0, G=15/6
chr16:11773662	G/G	C	G	SNV	SNN,TXNDC11	SNN:downstream:NM 003498.5, TXNDC11:exonic:NM 015	1	100	12	missense	NM 00349	c.*3480C>	p.?, p.Val756Leu	1.52	1	32	0	0.3(COSM375	Thioredoxin	rs3190321	0.458	YES	4.226E-1;1.162E-4 (pos.1	adenocarcinoı	Stannin,Thi	cell,cell redox homeostasis,cytoplasm,endoplasmic reti				C=0, G=30	C=0.0, G=1.0	0.00001	446.635	30	C=0/0, G=4/26
chr6:151258127	A/A	G	A	SNV	MTHFD1L	MTHFD1L:intronic:NM 001242767.1	1	100		intronic	NM 00124	c.1396+51	p.?	-0.33					Formate--tet	rs742830	0.2956	YES	5.019E-1 (pos.151258127)		Methylenet	10-formylt	Alzheimer Disease:Brain Diseases:Cardiovas			G=0, A=23	G=0.0, A=1.0	0.00001	314.554	23	G=0/0, A=9/14
chr20:1286711	C/C	T	C	SNV	SNPH	SNPH:utr_3:NM 014723.3	1	100	6	utr_3	NM 01472	c.1498T>C	p.?	-1.48					Golgi-localise	rs4813082	2.00E-04		1.0 (pos.1286711)		Syntaphilin	brain development,cell junction,cytoplasm,cytoplasmic				T=0, C=32	T=0.0, C=1.0	0.00001	485.141	32	T=0/0, C=12/2
chr6:7590623	CT/CT	C	CT	INDEL	SNRNP48	SNRNP48:exonic:NM 152551.3	1	100	1	frameshiftInsertion	NM 15255	c.133_134	p.Pro45fs	0.45					U11-48K-like CHHC zinc finger							RNA splicing,U12-type spliceosomal complex,cytosol,mF				C=0, CT=47	C=0.0, CT=1.0	0.00001	477.394	47	C=0/0, CT=18/
chr16:50709723	A/A	G	A	SNV	SNX20	SNX20:exonic:NM 182854.3	1	100	3	synonymous	NM 18285	c.240C>T	p.(=)	0.09				0.78(COSM37	PX domain	rs6596	0.1688	YES	1.62E-3;0.0 (pos.507097	adenocarcinoı	Sorting nex	early endosome membrane,nucleoplasm,phosphatidyli				G=0, A=56	G=0.0, A=1.0	0.00001	832.076	56	G=0/0, A=22/3
chr6:152461049	T/T	TGTTT	T	INDEL	SYNE1	SYNE1:intronic:NM 182961.3	4	100		intronic	NM 18296	c.25458+3	p.?	0.27, 0.98, -0.27, -0.77, 0.44					Calponin hor	rs147380321,rs745510943,rs78		YES	0.0 (pos.152461051),0.0 (pos.15246105		Spectrin re	Golgi appa	Ataxia:Bipolar Disorder:Bipolar and Related I			TGTTT=0, T	TGTTT=0.0, T=1.0	0.00001	431.199	29	TGTTT=0/0, T=
chr6:152461061	G/A	G	A	SNV	SYNE1	SYNE1:intronic:NM 182961.3	1	96.67		intronic	NM 18296	c.25458+2	p.?	-0.3					Calponin hor	rs3818110	0.3941	YES	2.926E-1 (pos.152461061)		Spectrin re	Golgi appa	Ataxia:Bipolar Disorder:Bipola		Benign	G=1, A=29	G=0.0333, A=0.9667	0.00001	416.506	30	G=0/1, A=15/1
chr1:99127392	C/C	T	C	SNV	SNX7	SNX7:exonic:NM 015976.4	1	100	1	synonymous	NM 01597	c.105T>C	p.(=)	-0.09				0(COSM4144	PX domain	rs9728148			1.0 (pos.99127392)	neoplasm,squ	Sorting nex	cytoplasmic vesicle membrane,endocytosis,endosome,				T=0, C=24	T=0.0, C=1.0	0.00001	334.761	24	T=0/0, C=13/1
chr7:2303986	C/C	T	C	SNV	SNX8	SNX8:exonic:NM 013321.3	1	100	6	synonymous	NM 01332	c.729A>G	p.(=)	-5.59					PX domain	rs2286206	0.0398	YES	8.161E-1 (pos.2303986)		Sorting nex	cytosol,early endosome membrane,early endosome to				T=0, C=43	T=0.0, C=1.0	0.00001	705.816	43	T=0/0, C=19/2
chr6:152653037	T/C	T	C	SNV	SYNE1	SYNE1:intronic:NM 182961.3	1	47.37		intronic	NM 18296	c.12795-1	p.?	-0.01					Calponin hor	rs9478314	0.4607	YES	1.624E-1 (pos.152653037)		Spectrin re	Golgi appa	Ataxia:Bipolar Disorder:Bipola		Benign	T=40, C=36	T=0.5263, C=0.4737	0.00001	363.952	76	T=25/15, C=18
chr2:46986416	G/G	A	G	SNV	SOCS5	SOCS5:exonic:NM 014011.4	1	100	2	synonymous	NM 01401	c.747A>G	p.(=)	-0.21				0.39(COSM37	SH2 domain,	rs6738426	0.3817	YES	1.105E-1 (pos.46986416)	adenocarcinoı	Suppressor	JAK-STAT cascade,cell growth,cellular response to low-d				A=0, G=58	A=0.0, G=1.0	0.00001	867.376	58	A=0/0, G=38/2
chr6:152722228	A/G	A	G	SNV	SYNE1	SYNE1:intronic:NM 182961.3	1	50		intronic	NM 18296	c.7029+45	p.?	0.33					Calponin hor	rs177330	0.3803	YES	7.64E-1 (pos.152722228)		Spectrin re	Golgi appa	Ataxia:Bipolar Disorder:Bipola		Benign	A=11, G=11	A=0.5, G=0.5	0.00001	94.6068	22	A=6/5, G=7/4
chr6:152776750	G/A	G	A	SNV	SYNE1	SYNE1:intronic:NM 182961.3	1	50		intronic	NM 18296	c.2728-25	p.?	-0.11				0.03(COSM50	Calponin hor	rs214968	0.2298	YES	3.315E-1 (pos.15277675	carcinoma	Spectrin re	Golgi appa	Ataxia:Bipolar Disorder:Bipola		Benign	G=31, A=31	G=0.5, A=0.5	0.00001	305.384	62	G=15/16, A=1
chr6:152793412	A/A	C	A	SNV	SYNE1	SYNE1:intronic:NM 182961.3	1	100		intronic	NM 18296	c.1463+24	p.?	0.08					Calponin hor	rs4523096	0.2956	YES	9.668E-1 (pos.152793412)		Spectrin re	Golgi appa	Ataxia:Bipolar Disorder:Bipola		Benign	C=0, A=31	C=0.0, A=1.0	0.00001	465.819	31	C=0/0, A=15/1
chr6:152805889	T/T	C	T	SNV	SYNE1	SYNE1:intronic:NM 182961.3	1	100		intronic	NM 18296	c.1185+81	p.?	-0.56					Calponin hor	rs9397512		YES			Spectrin re	Golgi appa	Ataxia:Bipolar Disorder:Bipolar and Related I			C=0, T=207	C=0.0, T=1.0	0.00001	3367.37	207	C=0/0, T=128/
chr6:152809527	T/T	A	T	SNV	SYNE1	SYNE1:intronic:NM 182961.3	1	100		intronic	NM 18296	c.1047+4T	p.?	1.62					Calponin hor	rs777848987,rs9397	0.4328	YES	0.0 (pos.152809523),7.564E-1 (pos.1528		Spectrin re	Golgi appa	Ataxia:Bipolar Disorder:Bipola		Benign/Likely	A=0, T=157	A=0.0, T=1.0	0.00001	2522.67	157	A=0/0, T=101/
chr2:46987300	G/G	T	G	SNV	SOCS5	SOCS5:utr_3:NM 014011.4	1	100	2	utr_3	NM 01401	c.1631T>G	p.?	1.91					SH2 domain,	rs3768720	0.3613	YES	5.543E-1 (pos.46987300)		Suppressor	JAK-STAT cascade,cell growth,cellular response to low-d				T=0, G=21	T=0.0, G=1.0	0.00001	281.175	21	T=0/0, G=13/8
chr6:154414375	C/C	A	C	SNV	OPRM1	OPRM1:intronic:NM 001008505.2	1	100		intronic	NM 00100	c.1165-30	p.?	0.07					7 transmemb	rs9282821	0.4066	YES	7.862E-1 (pos.154414375)		Opioid rece	G-protein	Alcohol-Related Dis	3-Methylfentanyl,3-Met		A=0, C=24	A=0.0, C=1.0	0.00001	334.44	24	A=0/0, C=10/1
chr21:34925142	T/T	C	T	SNV	SON	SON:exonic:NM 138927.2	1	100	3	missense	NM 13892	c.3605C>T	p.Ser1202Leu	2.22	1	145	0		Arginine/Seri	rs13433428	1.00E-04		1.0 (pos.34925142)		SON DNA-bi	DNA bindi	Abnormalities, Multiple:Adenocarcinoma:Ca			C=0, T=59	C=0.0, T=1.0	0.00001	885.311	59	C=0/0, T=38/2
chr21:34926260	T/T	C	T	SNV	SON	SON:exonic:NM 138927.2	1	100	3	missense	NM 13892	c.4723C>T	p.Arg1575Cys	-0.04	1	180	0		Arginine/Seri	rs13047599	0.3266	YES	7.46E-1 (pos.34926260)		SON DNA-bi	DNA bindi	Abnormalities, Multiple:Adenocarcinoma:Ca			C=0, T=20	C=0.0, T=1.0	0.00001	263.786	20	C=0/0, T=7/13
chr10:97141523	A/A	G	A	SNV	SORBS1	SORBS1:exonic:NM 001034954.1	1	100	16	synonymous	NM 00103	c.1572C>T	p.(=)	3.15				0.76(COSM54	SH3 domain,	rs2274490	0.4098	YES	6.356E-1 (pos.97141523)	acute_myeloic	Sorbin and	SH3/SH2 a	Diabetes Complications:Diabetes Mellitus:D			G=0, A=41	G=0.0, A=1.0	0.00001	664.699	41	G=0/0, A=23/1
chr6:154431393	G/G	C	G	SNV	OPRM1		1	100		intronic				0.2						rs497332		YES			Opioid receptor, mu-1		Alcohol-Related Dis	3-Methylfentanyl,3-Met		C=0, G=59	C=0.0, G=1.0	0.00001	885.303	59	C=0/0, G=30/2
chr6:154749225	A/T	A	T	SNV	CNKSR3	CNKSR3:intronic:NM 173515.3	1	45.45		intronic	NM 17351	c.729+37T	p.?	0.02					Connector er	rs9384217	0.48	YES	4.014E-1 (pos.154749225)		CNKSR fami	cytoplasm	Basal Ganglia Diseases;Brain Diseases;Centr			A=24, T=20	A=0.5455, T=0.4545	0.00001	211.733	44	A=10/14, T=13
chr8:22428658	C/C	T	C	SNV	SORBS3	SORBS3:exonic:NM 005775.4	1	100	18	missense	NM 00577	c.1667T>C	p.Ile556Thr	1.48	0.89	89	0	0.01(COSM37	SH3 domain,	rs2449331	0.0476	YES	1.0 (pos.22428658)	adenocarcinoı	Sorbin and	actin filament organization,cell adhesion,cell-substrate				T=0, C=73	T=0.0, C=1.0	0.00001	1150.15	73	T=0/0, C=26/4
chr8:22432377	A/A	C	A	SNV	SORBS3	SORBS3:utr_3:NM 005775.4	1	100	21	utr_3	NM 00577	c.2152C>A	p.?	0.37					SH3 domain,	rs710098		YES	2.677E-1 (pos.22432377)		Sorbin and	actin filament organization,cell adhesion,cell-substrate				C=0, A=42	C=0.0, A=1.0	0.00001	685.217	42	C=0/0, A=15/2
chr4:7716932	C/C	T	C	SNV	SORCS2	SORCS2:exonic:NM 020777.2	1	100	17	missense	NM 02077	c.2146T>C	p.Ser716Pro	0.39	0.3	74	0		BNR-Asp box	rs6816604	0.0584	YES	9.995E-1 (pos.7716932)		SORCS rece	integral component of membrane,membrane,neuropep				T=0, C=27	T=0.0, C=1.0	0.00001	383.082	27	T=0/0, C=17/1
chr6:155571113	G/G	A	G	SNV	TIAM2	TIAM2:intronic:NM 012454.3	1	100		intronic	NM 01245	c.3951+10	p.?	0.11					PDZ domain (	rs3011902	0.0051	YES	9.997E-1 (pos.155571113)		T-cell lymph	G-protein	Mental Disorders;Substance-Related Disorde			A=0, G=26	A=0.0, G=1.0	0.00001	371.42	26	A=0/0, G=12/1
chr6:155572986	A/A	G	A	SNV	TIAM2	TIAM2:intronic:NM 012454.3	1	100		intronic	NM 01245	c.4061-93	p.?	0.65					PDZ domain (	rs3011899		YES			T-cell lymph	G-protein	Mental Disorders;Substance-Related Disorde			G=0, A=20	G=0.0, A=1.0	0.00001	263.748	20	G=0/0, A=11/9
chr6:155574207	C/C	T	C	SNV	TIAM2	TIAM2:intronic:NM 012454.3	1	100		intronic	NM 01245	c.4225+20	p.?	-1.01					PDZ domain (	rs2274824,rs386707	0.2542	YES	3.428E-1 (pos.155574207)		T-cell lymph	G-protein	Mental Disorders;Substance-Related Disorde			T=0, C=27	T=0.0, C=1.0	0.00001	389.988	27	T=0/0, C=19/8
chr4:7717012	G/A	G	A	SNV	SORCS2	SORCS2:exonic:NM 020777.2	1	57.14	17	synonymous	NM 02077	c.2226G>A	p.(=)	0.54				0.7(COSM400	BNR-Asp box	rs2285781	0.4231	YES	2.381E-1 (pos.7717012)	acute_myeloic	SORCS rece	integral component of membrane,membrane,neuropep				G=15, A=20	G=0.4286, A=0.5714	0.00001	218.71	35	G=8/7, A=8/12
chr11:121403229	C/C	T	C	SNV	SORL1	SORL1:exonic:NM 003105.5	1	100	12	synonymous	NM 00310	c.1653T>C	p.(=)	-0.55					Fibronectin t	rs636588	0		1.0 (pos.121403229)		Sortilin-rela	ADP-ribos	Alzheimer Disease;Brain Diseases;Central Ne			T=0, C=27	T=0.0, C=1.0	0.00001	379.493	27	T=0/0, C=18/9
chr11:121437819	G/G	C	G	SNV	SORL1	SORL1:exonic:NM 003105.5	1	100	22	missense	NM 00310	c.3220C>G	p.Gln1074Glu	3.17	1	29	0		Fibronectin t	rs1699107	1.00E-04		1.0 (pos.121437819)		Sortilin-rela	ADP-ribos	Alzheimer Disease;Brain Diseases;Central Ne			C=0, G=53	C=0.0, G=1.0	0.00001	782.014	53	C=0/0, G=30/2
chr6:158516610	T/C	T	C	SNV	SYNJ2	SYNJ2:intronic:NM 003898.3	1	57.58		intronic	NM 00389	c.3745-40	p.?	0.21					Domain of un	rs142715550	0.0031		0.0 (pos.158516610)		Synaptojan	RNA bindi	Breast Diseases;Breast Neoplasms;Neoplası			T=14, C=19	T=0.4242, C=0.5758	0.00001	204.391	33	T=9/5, C=10/9
chr11:121491782	A/A	G	A	SNV	SORL1	SORL1:exonic:NM 003105.5	1	100	44	missense	NM 00310	c.5899G>A	p.Val1967Ile	1.66	1	29	0		Fibronectin t	rs1792120	1.00E-04		1.0 (pos.121491782)		Sortilin-rela	ADP-ribos	Alzheimer Disease;Brain Diseases;Central Ne			G=0, A=31	G=0.0, A=1.0	0.00001	469.76	31	G=0/0, A=16/1
chr7:16505341	C/C	T	C	SNV	SOSTDC1	SOSTDC1:utr_5:NM 015464.2	1	100	1	utr_5	NM 01546	c.-48A>G	p.?	-0.14					Cystine-knot	rs6945425	0.1356	YES	9.112E-1 (pos.16505341)		Sclerostin d	BMP binding,BMP receptor activity,BMP signaling pathw				T=0, C=32	T=0.0, C=1.0	0.00001	485.14	32	T=0/0, C=12/2
chr13:95364337	G/G	T	G	SNV	SOX21,SOX21-AS1	SOX21-AS1:upstream:NR 046514.1, SOX21:utr_5:NM 0070	1	100	1	utr_5	NM 00708	c.-34A>C	p.?	-0.86					HMG (high m	rs2253604	0.1736	YES	6.504E-1 (pos.95364337)		SRY-box 21	DNA binding,DNA binding transcription factor activity,RN				T=0, G=28	T=0.0, G=1.0	0.00001	408.711	28	T=0/0, G=10/1
chr6:158994577	G/G	T	G	SNV	TMEM181	TMEM181:intronic:NM 020823.1	1	100		intronic	NM 02082	c.523+22T	p.?	0.07					Wnt-binding	rs4709217	0.0064		1.0 (pos.158994577)		Transmemb	integral component of membrane,pathogenesis,toxic su				T=0, G=20	T=0.0, G=1.0	0.00001	249.085	20	T=0/0, G=9/11
chr6:159139270	C/T	C	T	SNV	SYTL3	SYTL3:intronic:NM 001242384.1	1	50		intronic	NM 00124	c.720+27C	p.?	-2.54					C2 domain,F	rs41267057		YES	4.167E-3 (pos.159139270)			Rab GTPase binding,calcium ion binding,calcium ion-regı				C=21, T=21	C=0.5, T=0.5	0.00001	228.862	42	C=9/12, T=5/1
chr6:159188282	T/G	T	G	SNV	EZR	EZR:intronic:NM 003379.4	1	50		intronic	NM 00337	c.1596+11	p.?	0.42					Ezrin/radixin	rs9364994	0.194	YES	6.84E-2 (pos.159188282)		Ezrin	ATPase bir	Arthritis;Genital Diseases, Male;Genital Neo			T=14, G=16	T=0.4667, G=0.5333	0.00001	172.219	32	T=9/5, G=15/1
chr17:70120551	C/C	A	C	SNV	SOX9	SOX9:utr_3:NM 000346.3	1	100	3	utr_3	NM 00034	c.1553A>C	p.?	-3.25					HMG (high m	rs1042667	0.4445	YES	5.343E-1 (pos.70120551)		SRY (sex-de	DNA bindi	46, XX Disorders of Sex Develo		Benign	A=0, C=57	A=0.0, C=1.0	0.00001	849.584	57	A=0/0, C=30/2
chr12:53777171	G/G	A	G	SNV	SP1	SP1:exonic:NM 138473.2	1	100	3	synonymous	NM 13847	c.1440A>G	p.(=)	2.46				0.48(COSM39	C2H2-type zi	rs3741651	0.1751	YES	1.996E-1 (pos.53777171)	acute_myeloic	Pregnancy-	DNA bindi	Carcinogenesis;Cell Transformation, Neoplas			A=0, G=37	A=0.0, G=1.0	0.00001	583.707	37	A=0/0, G=17/2
chr2:231406680	C/C	T	C	SNV	SP100	SP100:exonic:NM 001080391.1	1	100	28	missense	NM 00108	c.2477T>C	p.Met826Thr	-2.34	0.58	81	0.004	0.02(COSM40	Bromodomai	rs836237	0.145	YES	4.564E-1 (pos.23140668	acute_myeloic	Nuclear ant	DNA binding,DNA damage response, signal transduction				T=0, C=21	T=0.0, C=1.0	0.00001	281.235	21	T=0/0, C=10/1
chr2:231407663	T/T	C	T	SNV	SP100	SP100:utr_3:NM 001080391.1	1	100	29	utr_3	NM 00108	c.2660C>T	p.?	-0.04					Bromodomai	rs9288668	0.4555	YES	2.355E-1 (pos.231407663)		Nuclear ant	DNA binding,DNA damage response, signal transduction				C=0, T=25	C=0.0, T=1.0	0.00001	353.018	25	C=0/0, T=13/1
chr2:231050715	G/G	A	G	SNV	SP110	SP110:exonic:NM 080424.2	1	100	11	missense	NM 08042	c.1274T>C	p.Leu425Ser	-0.36	0.21	145	0		HSR domain,	rs3948464	0.129	YES	9.968E-1;0.0 (pos.231050715)		SP110 nucl	DNA bindi	Cardiovascular Diseases;Dige		Conflicting in	A=0, G=24	A=0.0, G=1.0	0.00001	334.787	24	A=0/0, G=15/9
chr2:231077725	G/G	A	G	SNV	SP110	SP110:exonic:NM 080424.2	1	100	4	missense	NM 08042	c.334T>C	p.Trp112Arg	-0.19	0.91	101	0.003		HSR domain,	rs1129411	0.1165	YES	9.977E-1 (pos.231077725)		SP110 nucl	DNA bindi	Cardiovascular Diseases;Dige		Benign	A=0, G=20	A=0.0, G=1.0	0.00001	263.785	20	A=0/0, G=15/5
chr6:159659523	G/G	A	G	SNV	FNDC1	FNDC1:intronic:NM 032532.2	1	100		intronic	NM 03253	c.4069-63	p.?	0.01					Fibronectin t	rs450538		YES			Fibronectin	extracellu	Cardiovascular Diseases;Coronary Disease;H			A=0, G=25	A=0.0, G=1.0	0.00001	339.451	25	A=0/0, G=14/1
chr6:159682206	T/T	C	T	SNV	FNDC1	FNDC1:intronic:NM 032532.2	1	100		intronic	NM 03253	c.5175-16	p.?	1.12					Fibronectin t	rs294883,rs7690069	0.2648	YES	0.0;0.0 (pos.159682198),5.935E-1 (pos.1		Fibronectin	extracellu	Cardiovascular Diseases;Coronary Disease;H			C=0, T=28	C=0.0, T=1.0	0.00001	408.463	28	C=0/0, T=15/1
chr2:231149108	A/A	G	A	SNV	SP140	SP140:exonic:NM 007237.4	1	100	16	missense	NM 00723	c.1546G>A	p.Glu516Lys	-0.45	1	56	0		Bromodomai	rs4972946	0.0801	YES	9.986E-1 (pos.231149108)			DNA bindi	Autoimmune Diseases:Autoimmune Disease			G=0, A=27	G=0.0, A=1.0	0.00001	387.181	27	G=0/0, A=14/1
chr12:53722098	G/G	A	G	SNV	SP7	SP7:exonic:NM 152860.1	1	100	2	synonymous	NM 15286	c.1128T>C	p.(=)	-0.47				0.02(COSM41	Zinc finger, C	rs7138938	7.00E-04	YES	9.995E-1 (pos.53722098)	neoplasm	Transcriptic	DEAD/H-bc	Bone Diseases;Bone Diseases,		Benign	A=0, G=104	A=0.0, G=1.0	0.00001	1626.74	104	A=0/0, G=42/6
chr6:160677614	C/C	G	C	SNV	SLC22A2	SLC22A2:intronic:NM 003058.3	1	100		intronic	NM 00305	c.518+32C	p.?	-1.07					Major Facilit	rs2774230	0.2372	YES	8.662E-1;0.0 (pos.160677614)		Solute carri	acetate es	Bile Duct Diseases;	Agmatine,Amantadine,A		G=0, C=327	G=0.0, C=1.0	0.00001	5467.72	327	G=0/0, C=158/
chr12:53722128	A/A	G	A	SNV	SP7	SP7:exonic:NM 152860.1	1	100	2	synonymous	NM 15286	c.1098C>T	p.(=)	0.24				0.73(COSM41	Zinc finger, C	rs7139272	0.0157	YES	9.629E-1 (pos.53722128)	neoplasm	Transcriptic	DEAD/H-bc	Bone Diseases;Bone Diseases,		Benign	G=0, A=105	G=0.0, A=1.0	0.00001	1644.56	105	G=0/0, A=42/6
chr6:161137663	G/G	T	G	SNV	PLG	PLG:intronic:NM 000301.3	1	100		intronic	NM 00030	c.669-14T	p.?	3.53					Domain of un	rs4252109	0.3009	YES	6.998E-4 (pos.161137663)		Plasminoge	apolipopro	Blood Coagulation I	Alteplase,Aminocaproic		T=0, G=35	T=0.0, G=1.0	0.00001	543.827	35	T=0/0, G=22/1
chr6:161143376	G/G	A	G	SNV	PLG	PLG:intronic:NM 000301.3	1	100		intronic	NM 00030	c.1097-64	p.?	0.37					Domain of un	rs4252117		YES			Plasminoge	apolipopro	Blood Coagulation I	Alteplase,Aminocaproic		A=0, G=40	A=0.0, G=1.0	0.00001	644.286	40	A=0/0, G=17/2
chr6:161143608	C/C	T	C	SNV	PLG	PLG:intronic:NM 000301.3	1	100		intronic	NM 00030	c.1256+9T	p.?	1.98					Domain of un	rs4252120	0.303	YES	6.933E-4 (pos.161143608)		Plasminoge	apolipopro	Blood Coagulation I	Alteplase,Aminocaproic		T=0, C=25	T=0.0, C=1.0	0.00001	353.019	25	T=0/0, C=15/1
chr8:7308386	C/C	T	C	SNV	SPAG11B	SPAG11B:exonic:NM 058200.2	1	100	3	missense	NM 05820	c.251A>G	p.His84Arg	0.26	0.54	29	0	0.01(COSM45	Beta defensi	rs1042797		YES	5.616E-1;0.0 (pos.73083	squamous_cell_carcinoma		antimicrobial humoral immune response mediated by a				T=0, C=35	T=0.0, C=1.0	0.00001	543.902	35	T=0/0, C=16/1
chr6:161152294	G/G	A	G	SNV	PLG	PLG:intronic:NM 000301.3	1	100		intronic	NM 00030	c.1438+30	p.?	-1.36					Domain of un	rs4252126	0.3026	YES	7.004E-4 (pos.161152294)		Plasminoge	apolipopro	Blood Coagulation I	Alteplase,Aminocaproic		A=0, G=30	A=0.0, G=1.0	0.00001	446.638	30	A=0/0, G=8/22
chr1:118693217	C/C	T	C	SNV	SPAG17	SPAG17:exonic:NM 206996.3	1	100	3	synonymous	NM 20699	c.264A>G	p.(=)	-0.49				0.06(COSM42	Flagellar-ass	rs1935992	0.218	YES	3.825E-1 (pos.11869321	acute_myeloic	Sperm-asso	axonemal central apparatus,axonemal central apparatu				T=0, C=35	T=0.0, C=1.0	0.00001	525.249	35	T=0/0, C=9/26

chr6:161502094	C/T	C	T	SNV	MAP3K4	MAP3K4:intronic:NM_005922.3	1	96.77		intronic	NM_00592	c.2255+24	p.?	-3.08					Kinase-like,P	rs9347479	0.2572	YES	9.957E-1 (pos.161502094)		Mitogen-ac	ATP bindin	Endometrial Neoplasms;Female Urogenital D	C=1, T=30	C=0.0323, T=0.9677	0.00001	416.541	31	C=1/0, T=15/1
chr6:161531006	C/C	G	C	SNV	MAP3K4	MAP3K4:intronic:NM_005922.3	1	100		intronic	NM_00592	c.4396+60	p.?	0.87					Kinase-like,P	rs4709493		YES			Mitogen-ac	ATP bindin	Endometrial Neoplasms;Female Urogenital D	G=0, C=38	G=0.0, C=1.0	0.00001	603.785	38	G=0/0, C=8/30
chr6:161532855	C/C	CT	C	INDEL	MAP3K4	MAP3K4:intronic:NM_005922.3	1	100		intronic	NM_00592	c.4397-13(	p.?	-3.69, 0.15					Kinase-like,P	rs139755069,rs35961625,rs397		YES	0.0 (pos.161532852),9.939E-1 (pos.1615		Mitogen-ac	ATP bindin	Endometrial Neoplasms;Female Urogenital D	CT=0, C=20	CT=0.0, C=1.0	0.00001	224.407	20	CT=0/0, C=9/1
chrX:142795438	T/T	G	T	SNV	SPANXN2	SPANXN2:exonic:NM_001009615.2	1	100	2	synonymous	NM_00100	c.240C>A	p.(=)	-3.53					Sperm protei	rs5908761	0.1183	YES	2.042E-1;0.0 (pos.142795438)		SPANX family, member N2			G=0, T=59	G=0.0, T=1.0	0.00001	856.607	59	G=0/0, T=11/4
chr6:161570203	T/T	A	T	SNV	AGPAT4	AGPAT4:intronic:NM_020133.2	1	100		intronic	NM_02013	c.767+16T	p.?	0.9					Acyltransfera	rs749013	0.0477	YES	0.0;0.0 (pos.161570203)		1-acylglycei	1-acylglycerol-3-phosphate O-acyltransferase activity,C(		A=0, T=24	A=0.0, T=1.0	0.00001	334.767	24	A=0/0, T=8/16
chrX:52825471	G/G	A	G	SNV	SPANXN5	SPANXN5:utr_3:NM_001009616.3	1	100	2	utr_3	NM_00100	c.276T>C	p.?	-3.1					Sperm protei	rs2806837		YES			SPANX family, member N5			A=0, G=39	A=0.0, G=1.0	0.00001	623.98	39	A=0/0, G=26/1
chr6:165693624	T/T	C	T	SNV	C6orf118	C6orf118:intronic:NM_144980.3	1	100		intronic	NM_14498	c.1357-25(	p.?	-2.25					Translin-assc	rs1413100	0.3989	YES	5.429E-1 (pos.165693624)					C=0, T=43	C=0.0, T=1.0	0.00001	705.844	43	C=0/0, T=14/2
chrX:52825546	G/G	C	G	SNV	SPANXN5	SPANXN5:exonic:NM_001009616.3	1	100	2	synonymous	NM_00100	c.201G>C	p.(=)	0.19					Sperm protei	rs2806838	0.0296	YES	1.0;0.0 (pos.52825546)		SPANX family, member N5			C=0, G=37	C=0.0, G=1.0	0.00001	583.688	37	C=0/0, G=23/1
chr6:165845027	A/A	AATATACT/	A	INDEL	PDE10A	PDE10A:intronic:NM_001130690.2	14	100		intronic	NM_00113	c.694-67G	p.?	2.92, -0.24, 0.53, 0.88, -0.06, -0.3, 0.08, 0.08, 0.92, 0.95, 0.05, (					3'5'-cyclic nu	rs1021936260,rs103	0.3409	YES			Phosphodie	3',5'-cyclic	Attention Deficit ar 2-((4-4-(pyridin-4-ylmeth	AATATACTA	AATATACTATATATC=0	0.00001	446.639	30	AATATACTATAT
chr6:165845139	A/A	T	A	SNV	PDE10A	PDE10A:intronic:NM_001130690.2	1	100		intronic	NM_00113	c.694-179	p.?	-0.28					3'5'-cyclic nu	rs13211782		YES			Phosphodie	3',5'-cyclic	Attention Deficit ar 2-((4-4-(pyridin-4-ylmeth	T=0, A=30	T=0.0, A=1.0	0.00001	446.639	30	T=0/0, A=16/1
chr4:88401616	G/G	A	G	SNV	SPARCL1	SPARCL1:exonic:NM_004684.5	1	100	9	synonymous	NM_00468	c.1725T>C	p.(=)	-0.09				0.35(COSM54	Follistatin/O	rs9933	0.3914	YES	6.499E-1 (pos.88401616)	adenocarcinoma		anatomica	Brain Diseases:Central Nervous System Dise	A=0, G=141	A=0.0, G=1.0	0.00001	2362.67	141	A=0/0, G=40/1
chr6:166739684	A/A	G	A	SNV	SFT2D1	SFT2D1:intronic:NM_145169.2	1	100		intronic	NM_14516	c.316-29C	p.?	-0.8					Got1/Sft2-like	rs10806847	0.2435	YES	9.34E-1 (pos.166739684)			integral component of membrane,membrane,protein tr		G=0, A=21	G=0.0, A=1.0	0.00001	281.205	21	G=0/0, A=11/1
chr4:88415636	C/C	G	C	SNV	SPARCL1	SPARCL1:exonic:NM_004684.5	1	100	4	missense	NM_00468	c.316C>G	p.His106Asp	0.94	1	81	0	0(COSM1497(	Follistatin/O	rs1049544	0.3717	YES	4.353E-1 (pos.88415636)	acute_myeloid_leukaemia		anatomica	Brain Diseases:Central Nervous System Dise	G=0, C=45	G=0.0, C=1.0	0.00001	747.392	45	G=0/0, C=22/2
chr6:166827436	C/C	T	C	SNV	RPS6KA2	RPS6KA2:intronic:NM_001006932.2	1	100		intronic	NM_00100	c.1963-17	p.?	-0.87					Kinase-like,L	rs2235295	0.3192	YES	7.844E-1 (pos.166827436)		Ribosomal	ATP bindin	Dental Caries;Stomatognathic Diseases;Toot	T=0, C=58	T=0.0, C=1.0	0.00001	867.385	58	T=0/0, C=27/3
chr6:166831694	T/T	C	T	SNV	RPS6KA2	RPS6KA2:intronic:NM_001006932.2	1	100		intronic	NM_00100	c.1962+19	p.?	-1.72					Kinase-like,L	rs10946164	0.4558	YES	2.05E-1 (pos.166831694)		Ribosomal	ATP bindin	Dental Caries;Stomatognathic Diseases;Toot	C=0, T=66	C=0.0, T=1.0	0.00001	1015.35	66	C=0/0, T=37/2
chr4:88416188	T/T	G	T	SNV	SPARCL1	SPARCL1:exonic:NM_004684.5	1	100	3	missense	NM_00468	c.146C>A	p.Ala49Asp	0.9	1	126	0	0.04(COSM14	Follistatin/O	rs13051,rs76455482	0.3716	YES	0.0 (pos.88416182),4.34(	NS,acute_myeloid_leukaer		anatomica	Brain Diseases:Central Nervous System Dise	G=0, T=42	G=0.0, T=1.0	0.00001	685.22	42	G=0/0, T=21/2
chr4:88416223	G/G	A	G	SNV	SPARCL1	SPARCL1:exonic:NM_004684.5	1	100	3	synonymous	NM_00468	c.111T>C	p.(=)	-2					Follistatin/O	rs8342	0.3914	YES	6.504E-1 (pos.88416223)			anatomica	Brain Diseases:Central Nervous System Dise	A=0, G=42	A=0.0, G=1.0	0.00001	685.181	42	A=0/0, G=21/2
chr1:16731510	G/G	C	G	SNV	SPATA21	SPATA21:exonic:NM_198546.1	1	100	8	missense	NM_19854	c.763G>C	p.Val255Leu	0.06	0.9	32	0.001		EF hand,EF-ha	rs525409	0.2415	YES	1.339E-1 (pos.16731510)			calcium io	Adnexal Diseases:Cysts:Digestive System Dis	C=0, G=21	C=0.0, G=1.0	0.00001	281.235	21	C=0/0, G=9/12
chr17:3352294	A/G	A	G	SNV	SPATA22	SPATA22:exonic:NM_001170697.1	1	50	6	missense	NM_00117	c.479T>C	p.Ile160Thr	0.74	0.2	89	0.001	0.07(COSM4416982)		rs1488689	0.1844	YES	4.24E-1 (pos.3352294)	acute_myeloi	Spermatoge	chromoso	Eye Diseases;Myopia;Refractive Errors	A=12, G=12	A=0.5, G=0.5	0.00001	106.709	24	A=5/7, G=8/4
chr17:3352309	T/C	T	C	SNV	SPATA22	SPATA22:exonic:NM_001170697.1	1	50	6	missense	NM_00117	c.464A>G	p.Gln155Arg	0.2	0.08	43	0.718	0.03(COSM4129845)		rs11556563	0.4828	YES	9.244E-4 (pos.3352309)	neoplasm	Spermatoge	chromoso	Eye Diseases;Myopia;Refractive Errors	T=12, C=12	T=0.5, C=0.5	0.00001	106.709	24	T=8/4, C=5/7
chr17:3352331	C/T	C	T	SNV	SPATA22	SPATA22:exonic:NM_001170697.1	1	50	6	missense	NM_00117	c.442G>A	p.Val148Met	-1.65	0.68	21	0.001	0(COSM4418808)		rs1488690	0.1843	YES	4.238E-1 (pos.3352331)	acute_myeloi	Spermatoge	chromoso	Eye Diseases;Myopia;Refractive Errors	C=12, T=12	C=0.5, T=0.5	0.00001	106.709	24	C=5/7, T=8/4
chr1:213009469	G/G	A	G	SNV	SPATA45	SPATA45:exonic:NM_001024601.2	1	100	2	missense	NM_00102	c.23T>C	p.Ile8Thr	-5.8	1	89	0	0(COSM3746778)		rs10864004	0.2928	YES	5.389E-1 (pos.21300946	hepatocellular_carcinoma				A=0, G=21	A=0.0, G=1.0	0.00001	281.235	21	A=0/0, G=6/15
chr6:168458074	G/A	G	A	SNV	FRMD1	FRMD1:intronic:NM_024919.4	1	50		intronic	NM_02491	c.1408-55(	p.?	-0.53					FERM N-term	rs3734900		YES				cytoskeleton		G=20, A=2(	G=0.5, A=0.5	0.00001	214.331	40	G=9/11, A=6/1
chr14:88852031	G/G	A	G	SNV	SPATA7	SPATA7:utr_5:NM_018418.4	1	100	1	utr_5	NM_01841	c.-132A>G	p.?	-0.4					Spermatoger	rs709886		YES			Spermatoge	axoneme,	Congenital, Hereditary, and Neonatal Diseas	A=0, G=22	A=0.0, G=1.0	0.00001	303.332	22	A=0/0, G=13/9
chr6:168475822	G/G	A	G	SNV	FRMD1	FRMD1:intronic:NM_024919.4	1	100		intronic	NM_02491	c.304+103	p.?	0.25					FERM N-term	rs3816859		YES				cytoskeleton		A=0, G=36	A=0.0, G=1.0	0.00001	563.695	36	A=0/0, G=23/1
chr6:169620247	C/C	T	C	SNV	LOC101929523,THBS2	LOC101929523:intronic_nc:NR_134621.1,THBS2:intronic:N	1	100		intronic	NR_13462	c.3511+46	p.?	-2.36					Calcium-bind	rs9393165	0.3231	YES	4.954E-1 (pos.169620247)		Thrombosp	basement	Bone Diseases:Diabetes Mellitus:Endocrine	T=0, C=38	T=0.0, C=1.0	0.00001	603.769	38	T=0/0, C=33/5
chr5:169021610	T/T	C	T	SNV	SPDL1	SPDL1:exonic:NM_017785.4	1	100	7	synonymous	NM_01778	c.816C>T	p.(=)	-0.1						rs4493692	0.31	YES	7.333E-1 (pos.169021610)		Spindly app	cell divisic	Female Urogenital Diseases and Pregnancy C	C=0, T=49	C=0.0, T=1.0	0.00001	835.437	49	C=0/0, T=24/2
chr5:169028481	C/C	T	C	SNV	SPDL1	SPDL1:exonic:NM_017785.4	1	100	11	missense	NM_01778	c.1522T>C	p.Tyr508His	-0.23	0.17	83	0			rs3797713	0.3097	YES	7.351E-1 (pos.169028481)		Spindly app	cell divisic	Female Urogenital Diseases and Pregnancy C	T=0, C=37	T=0.0, C=1.0	0.00001	583.33	37	T=0/0, C=27/1
chr17:20108184	G/G	C	G	SNV	SPECC1	SPECC1:exonic:NM_001243439.1	1	100	4	missense	NM_00124	c.822C>G	p.Ser274Arg	3.07	0.04	110	0.999	0.97(COSM40	CAMSAP CH d	rs9908032	0.1342	YES	1.063E-1 (pos.20108184)	acute_myeloi	Sperm anti	membrane,nucleus		C=0, G=27	C=0.0, G=1.0	0.00001	389.986	27	C=0/0, G=18/9
chr6:170118085	T/T	C	T	SNV	PHF10	PHF10:intronic:NM_018288.3	1	100		intronic	NM_01828	c.326-83G	p.?	-0.43					PHD-finger	rs112292948,rs7760142		YES			PHD finger	metal ion binding,nervous system development,npBAF c		C=0, T=25	C=0.0, T=1.0	0.00001	349.673	25	C=0/0, T=11/1
chr6:170151766	C/T	C	T	SNV	ERMARD,TCTE3	ERMARD:intronic:NM_018341.2,TCTE3:upstream:NM_1749	1	47.37		intronic	NM_17491	c.-212G>A	p.?, p.?	-3.16					Domain of un	rs3749882	0.1088	YES	3.45E-1 (pos.170151766)		Endoplasmi	biological	Congenital Abnormalities;Con Benign	C=30, T=27	C=0.5263, T=0.4737	0.00001	257.915	57	C=4/26, T=10/
chr17:20108239	T/T	A	T	SNV	SPECC1	SPECC1:exonic:NM_001243439.1	1	100	4	missense	NM_00124	c.877A>T	p.Met293Leu	-1.38	1	15	0		CAMSAP CH d	rs2703806	0.4227	YES	9.898E-1 (pos.20108239)		Sperm anti	membrane,nucleus		A=0, T=28	A=0.0, T=1.0	0.00001	408.718	28	A=0/0, T=18/1
chr6:170597276	C/T	C	T	SNV	DLL1	DLL1:intronic:NM_005618.3	1	47.62		intronic	NM_00561	c.670+51G	p.?	-0.39					Calcium-bind	rs2738822	0.3928	YES	6.739E-2 (pos.170597276)			Notch binc	Abnormalities, Multiple:Agenesis of Corpus C	C=22, T=20	C=0.5238, T=0.4762	0.00001	212.952	42	C=20/2, T=16/
chr17:20163529	G/G	A	G	SNV	SPECC1	SPECC1:exonic:NM_001243439.1	1	100	12	synonymous	NM_00124	c.2862A>G	p.(=)	-1.74				0.04(COSM56	CAMSAP CH d	rs2703791	0.423	YES	9.897E-1 (pos.20163529)	ductal_carcinc	Sperm anti	membrane,nucleus		A=0, G=29	A=0.0, G=1.0	0.00001	427.591	29	A=0/0, G=11/1
chr22:24761467	A/A	G	A	SNV	SPECC1L,SPECC1L-ADORA2A	SPECC1L:exonic:NM_001145468.3,SPECC1L-ADORA2A:exoni	1	100	12	missense	NR_10354	c.2851G>A	p.Val951Met	1.83	0.61	21	0	0.12(COSM45	CAMSAP CH d	rs204718			9.991E-1 (pos.24761467)	squamous_cel	Sperm antigen with cal		Bone Diseases:Bone Diseases, Development	G=0, A=65	G=0.0, A=1.0	0.00001	994.868	65	G=0/0, A=21/4
chr5:35709184	C/C	G	C	SNV	SPEF2	SPEF2:exonic:NM_024867.3	1	100	19	missense	NM_02486	c.2800G>C	p.Ala934Pro	-2.5	0.43	27	0	0(COSM3761	Adenylate kir	rs13170390	0.2056	YES	7.775E-1 (pos.35709184)	adenocarcino	Sperm flagellar 2		Autoimmune Diseases:Autoim Benign	G=0, C=39	G=0.0, C=1.0	0.00001	623.975	39	G=0/0, C=23/1
chr6:17817519	T/T	C	T	SNV	KIF13A	KIF13A:intronic:NM_022113.5	1	100		intronic	NM_02211	c.1787-55(	p.?	-0.45					FHA domain,I	rs41267720		YES			Kinesin fam	ATP binding,ATPase activity,Golgi to plasma membrane		C=0, T=24	C=0.0, T=1.0	0.00001	334.792	24	C=0/0, T=8/16
chr6:18222102	CTT/CTT	C	CTT	INDEL	KDM1B	KDM1B:intronic:NM_153042.3	2	100		intronic	NM_15304	c.1690-38(	p.?	-0.14					CW-type Zinc	rs140834,rs3977235	0.2217	YES	9.923E-2;0.0;0.0 (pos.18222102)		Lysine-spec	DNA binding,DNA methylation involved in gamete genera		C=0, CTT=2	C=0.0, CTT=1.0	0.00001	279.229	21	C=0/0, CTT=17
chr2:220333950	C/C	T	C	SNV	SPEG	SPEG:exonic:NM_005876.4	1	100	13	synonymous	NM_00587	c.3564T>C	p.(=)	-6.83					Immunoglobu	rs10167209	1.00E-04		1.0;0.0 (pos.220333950)		SPEG compl	ATP bindin	Muscular Diseases;Musculoskeletal Disease	T=0, C=21	T=0.0, C=1.0	0.00001	281.235	21	T=0/0, C=12/9
chr6:24416593	A/G	A	G	SNV	MRS2	MRS2:intronic:NM_020662.3	1	50		intronic	NM_02066	c.720-32A	p.?	-0.09						rs75878386	0.1419	YES	1.758E-1;0.0 (pos.24416593)			integral component of membrane,lactate metabolic pro		A=14, G=14	A=0.5, G=0.5	0.00001	131.936	28	A=6/8, G=8/6
chr2:220348619	CG/C	CG	C	INDEL	SPEG	SPEG:exonic:NM_005876.4	1	95.45	30	frameshiftDeletion	NM_00587	c.6439del	p.Ala2147fs	1.45, -8.2				0.16(COSM60	Immunoglobulin I-set domain,Immunoglobulin domain,Protein kinase domain,Protei					adenocarcino	SPEG compl	ATP bindin	Muscular Diseases:Musculoskeletal Disease	CG=1, C=21	CG=0.0455, C=0.954	0.00001	179.483	22	CG=0/1, C=14/
chr6:24448061	C/C	A	C	SNV	GPLD1	GPLD1:intronic:NM_001503.3	1	100		intronic	NM_00150	c.1678+44	p.?	-0.93					FG-GAP repea	rs796985	0.1498	YES	4.382E-1;0.0 (pos.24448061)		Phospholip	C-terminal protein lipidation,GPI anchor release,cell mi		A=0, C=42	A=0.0, C=1.0	0.00001	685.22	42	A=0/0, C=23/1
chr6:24463055	G/G	A	G	SNV	GPLD1	GPLD1:intronic:NM_001503.3	1	100		intronic	NM_00150	c.822-32T	p.?	-1.49					FG-GAP repea	rs2793442		YES	5.642E-1 (pos.24463055)		Phospholip	C-terminal protein lipidation,GPI anchor release,cell mi		A=0, G=183	A=0.0, G=1.0	0.00001	3049.51	183	A=0/0, G=98/8
chr6:24476553	G/A	G	A	SNV	GPLD1	GPLD1:intronic:NM_001503.3	1	50		intronic	NM_00150	c.233-47C	p.?	0.91					FG-GAP repea	rs760904	0.1812	YES	4.637E-1 (pos.24476553)		Phospholip	C-terminal protein lipidation,GPI anchor release,cell mi		G=12, A=12	G=0.5, A=0.5	0.00001	106.71	24	G=7/5, A=8/4
chr6:24828318	T/T	C	T	SNV	RIPOR2	RIPOR2:intronic:NM_014722.4	1	100		intronic	NM_01472	c.2728+47	p.?	-2.23					Filopodia upr	rs35374726	0.3498	YES	7.643E-1 (pos.24828318)			14-3-3 protein binding,apical plasma membrane,cell adl		C=0, T=44	C=0.0, T=1.0	0.00001	731.083	44	C=0/0, T=26/1
chr2:220352984	C/T	C	T	SNV	SPEG	SPEG:exonic:NM_005876.4	1	50	32	missense	NM_00587	c.7810C>T	p.Leu2604Phe	4.98	0.09	22	0.959	0.85(COSM35	Immunoglobu	rs77314619	0.0057		0.0 (pos.220352984)	malignant_me	SPEG compl	ATP bindin	Muscular Diseases:Musculosk Uncertain sig	C=11, T=11	C=0.5, T=0.5	0.00001	94.6067	22	C=5/6, T=3/8
chr6:25689487	C/C	G	C	SNV	SCGN	SCGN:intronic:NM_006998.3	1	100		intronic	NM_00699	c.573+42G	p.?	-0.17					EF hand,EF-ha	rs2294346	0.4109	YES	7.246E-1 (pos.25689487)			biological_process,calcium ion binding,cytoplasm,cytos		G=0, C=28	G=0.0, C=1.0	0.00001	408.645	28	G=0/0, C=11/1
chr1:16259813	G/G	A	G	SNV	SPEN	SPEN:exonic:NM_015001.2	1	100	11	missense	NM_01500	c.7078A>G	p.Asn2360Asp	2.2		23	0	0.09(COSM41	RNA recogniti	rs848210	0.4238	YES	3.278E-1;0.0 (pos.16259	acute_myeloi	One-twenty	DNA binding,Notch signaling pathway,RNA binding,RNA p		A=0, G=30	A=0.0, G=1.0	0.00001	446.639	30	A=0/0, G=6/24
chr17:74381555	G/A	G	A	SNV	SPHK1	SPHK1:exonic:NM_182965.2	1	47.37	2	missense	NM_18296	c.88G>A	p.Ala30Thr	0.09	0.15	58	0.025	0.03(COSM13	Diacylglycerc	rs2247856	0.4114	YES	4.069E-1 (pos.74381555)	adenocarcino	Sphingosine	ATP bindin	Bone Marrow Disea Fingolimod	G=20, A=18	G=0.5263, A=0.4737	0.00001	184.58	38	G=9/11, A=9/9
chr5:147555029	T/T	C	T	SNV	SPINK14	SPINK14:utr_3:NM_001001325.2	1	100	5	utr_3	NM_00100	c.362C>T	p.?	-2.46						rs10074328		YES				acrosomal vesicle,extracellular space,negative regulati		C=0, T=23	C=0.0, T=1.0	0.00001	316.747	23	C=0/0, T=14/9
chr6:25770239	G/G	A	G	SNV	SLC17A4	SLC17A4:intronic:NM_005495.2	1	100		intronic	NM_00549	c.298-56A	p.?	1.78					Major Facilita	rs2328893		YES			Solute carri	apical pla	Cardiovascular Diseases	A=0, G=28	A=0.0, G=1.0	0.00001	408.692	28	A=0/0, G=12/1
chr6:25770707	A/A	G	A	SNV	SLC17A4	SLC17A4:intronic:NM_005495.2	1	100		intronic	NM_00549	c.619+8G>	p.?	-0.37				0(COSM4003	Major Facilita	rs4712970	0.2751	YES	7.606E-1 (pos.25770707)	acute_myeloi	Solute carri	apical pla	Cardiovascular Diseases	G=0, A=21	G=0.0, A=1.0	0.00001	281.22	21	G=0/0, A=9/12
chr6:25801057	C/C	T	C	SNV	SLC17A1	SLC17A1:intronic:NM_005074.3	1	100		intronic	NM_00507	c.1269+61	p.?	-1.73					Major Facilita	rs1165210		YES			Solute carri	apical pla	Digestive System Diseases;Liver Cirrhosis;Liv	T=0, C=88	T=0.0, C=1.0	0.00001	1451.89	88	T=0/0, C=42/4
chr6:25918688	A/A	G	A	SNV	SLC17A2	SLC17A2:intronic:NM_005835.3	1	100		intronic	NM_00583	c.649+27C	p.?	-1.94					Major Facilita	rs3752421	0.3899	YES	7.244E-1 (pos.25918688)		Solute carri	integral component of plasma membrane,membrane,pl		G=0, A=27	G=0.0, A=1.0	0.00001	372.612	27	G=0/0, A=13/1
chr9:33240165	G/C	G	C	SNV	SPINK4	SPINK4:utr_5:NM_014471.2	1	50	1	utr_5	NM_01447	c.-42G>C	p.?	-0.65						rs555445	0.2322	YES	2.749E-1 (pos.33240165)		Serine prote	extracellular region,negative regulation of endopeptida		G=32, C=32	G=0.5, C=0.5	0.00001	317.425	64	G=23/9, C=18/
chr9:33240225	G/A	G	A	SNV	SPINK4	SPINK4:exonic:NM_014471.2	1	46.88	1	missense	NM_01447	c.19G>A	p.Val7Ile	-0.67	0.6	29	0.01	0.02(COSM49	Kazal-type se	rs706107,rs7614734	0.2338	YES	0.0 (pos.33240206),2.89	adenocarcino	Serine prote	extracellular region,negative regulation of endopeptida		G=34, A=30	G=0.5313, A=0.4688	0.00001	290.031	64	G=18/16, A=1
chr5:147480955	A/G	A	G	SNV	SPINK5	SPINK5:exonic:NM_001127698.1	1	57.89	14	missense	NM_00112	c.1258A>G	p.Lys420Glu	0.15	0.69	56	0	0(COSM4003	Kazal-type se	rs2303067	0.4867	YES	4.843E-1 (pos.14748095	acute_myeloi	Serine prote	anagen,ce	Abnormalities, Multiple;Conge Benign	A=16, G=22	A=0.4211, G=0.5789	0.00001	249.053	38	A=5/11, G=4/1
chr5:147510866	T/C	T	C	SNV	SPINK5	SPINK5:exonic:NM_001127698.1	1	57.14	32	synonymous	NM_00112	c.3099T>C	p.(=)	0.49				0.4(COSM149	Kazal-type se	rs2400478	0.383	YES	4.692E-1;1.162E-4 (pos.1	acute_myeloi	Serine prote	anagen,ce	Abnormalities, Multiple;Conge Benign	T=15, C=20	T=0.4286, C=0.5714	0.00001	218.715	35	T=11/4, C=8/1
chr5:147593497	A/A	C	A	SNV	SPINK6	SPINK6:exonic:NM_001195290.1	1	100	4	missense	NM_00119	c.106C>A	p.Pro36Thr	1.36	0.43	38	1	0.17(COSM40	Kazal-type se	rs12186491	0.077	YES	7.007E-1;0.0 (pos.14759	acute_myeloi	Serine pept	cornification,extracellular regi	Leucine - Reduced Carbo	C=0, A=98	C=0.0, A=1.0	0.00001	1660.04	98	C=0/0, A=48/5
chr17:4352539	C/T	C	T	SNV	SPNS3	SPNS3:exonic:NM_182538.4	1	53.33	7	synonymous	NM_18253	c.780C>T	p.(=)	0.21				0.2(COSM375	Major Facilita	rs7224464	0.2845	YES	1.573E-1 (pos.4352539)	adenocarcino	Spinster, Dr	integral component of membrane,lipid transport,locom		C=14, T=16	C=0.4667, T=0.5333	0.00001	159.216	30	C=14/0, T=9/7
chr6:26466225	C/C	A	C	SNV	BTN2A1	BTN2A1:intronic:NM_007049.4	1	100		intronic	NM_00704	c.955+24A	p.?	0					Immunoglobu	rs1977198	0.4848	YES	6.901E-1 (pos.26466225)		Butyrophili	extracellular exosome,integral component of plasma m		A=0, C=77	A=0.0, C=1.0	0.00001	1229.44	77	A=0/0, C=49/2
chr1:32280610	C/C	T	C	SNV	SPOCD1	SPOCD1:exonic:NM_144569.5	1	100	2	missense	NM_14456	c.325A>G	p.Thr109Ala	0.36	1	58	0	0(COSM4143	SPOC domain	rs6664445	0.3105	YES	7.016E-1 (pos.32280610)	neoplasm		negative regulation of phosphatase activity,transcriptio		T=0, C=41	T=0.0, C=1.0	0.00001	664.398	41	T=0/0, C=15/2
chr4:1165741	G/G	A	G	SNV	SPON2	SPON2:exonic:NM_012445.3	1	100	2	missense	NM_01244	c.119T>C	p.Leu40Pro	0.33	0.2	98	0.174	0.15(COSM41	Spondin_N,Tl	rs922697	8.00E-04	YES	9.999E-1 (pos.1165741)	neoplasm	Spondin 2	antigen bi	Bronchial Diseases;Bronchial Hyperreactivity	A=0, G=28	A=0.0, G=1.0	0.00001	406.861	28	A=0/0, G=24/4
chr6:2685677	C/C	T	C	SNV	MYLK4	MYLK4:intronic:NM_001012418.4	1	100		intronic	NM_00101	c.436-38A	p.?	-1.98					Lipopolysacc	rs966232	0.3101	YES	9.093E-1 (pos.2685677)			ATP binding,extracellular exosome,intracellular,intrace		T=0, C=39	T=0.0, C=1.0	0.00001	623.98	39	T=0/0, C=22/1
chr4:1165748	C/C	T	C	SNV	SPON2	SPON2:exonic:NM_012445.3	1	100	2	missense	NM_01244	c.112A>G	p.Arg38Gly	1.46	0.25	125	0.523	0.12(COSM37	Spondin_N,Tl	rs6836335	0.1211	YES	9.374E-4;0.0 (pos.11657	adenocarcino	Spondin 2	antigen bi	Bronchial Diseases;Bronchial Hyperreactivity	T=0, C=28	T=0.0, C=1.0	0.00001	408.576	28	T=0/0, C=24/4
chr4:88904005	G/A	G	A	SNV	SPP1	SPP1:exonic:NM_001040058.1	1	53.33	7	missense	NM_00104	c.902G>A	p.Arg301His	0.29	0.17	29	0.132		Osteopontin	rs4660	9.00E-04		2.327E-4 (pos.88904005)		Secreted ph	Golgi appa	Acute Kidney Injury:Adenocarcinoma:Adenor	G=49, A=56	G=0.4667, A=0.5333	0.00001	589.828	105	G=21/28, A=2
chr6:28239932	TG/TG	T	TG	INDEL	ZSCAN26	ZSCAN26:intronic:NM_152736.5	1	100		intronic	NM_15273	c.15+220T	p.?	4.07					C2H2-type zir	rs61622742,rs868069968			9.998E-1 (pos.28239932)		Zinc finger-	DNA binding transcription factor activity,cytosol,metal i		T=0, TG=53	T=0.0, TG=1.0	0.00001	777.626	53	T=0/0, TG=26/
chr6:2834265	T/T	C	T	SNV	SERPINB1	SERPINB1:intronic:NM_030666.3	1	100		intronic	NM_03066	c.736-19G	p.?	-2.15					Serpin (serine	rs386915	0.277	YES	7.404E-1 (pos.2834265)			extracellular exosome,extracellular matrix,extracellula		C=0, T=34	C=0.0, T=1.0	0.00001	524.181	34	C=0/0, T=16/1
chr6:2836266	A/A	T	A	SNV	SERPINB1	SERPINB1:intronic:NM_030666.3	1	100		intronic	NM_03066	c.568-9A>	p.?	0.34					Serpin (serine	rs385955,rs7156095	0.4248	YES	5.604E-1;1.156E-4 (pos.2836266)			extracellular exosome,extracellular matrix,extracellula		T=0, A=23	T=0.0, A=1.0	0.00001	310.879	23	T=0/0, A=19/4
chr6:28366262	G/A	G	A	SNV	ZSCAN12	ZSCAN12:intronic:NM_001163391.1	1	46.51		intronic	NM_00116	c.-67-12C>	p.?	-0.03					C2H2-type zir	rs2232422		YES			Zinc finger- and SCAN domain-containing protein 12			G=23, A=20	G=0.5349, A=0.4651	0.00001	209.878	43	G=14/9, A=9/1
chr6:28403603	T/C	T	C	SNV	ZSCAN23	ZSCAN23:intronic:NM_001012455.1	1	58.33		intronic	NM_00101	c.408+33A	p.?	-1.74					C2H2-type zir	rs11752919	0.3831	YES	3.106E-1 (pos.28403603)			DNA binding,DNA binding transcription factor activity,ex		T=10, C=14	T=0.4167, C=0.5833	0.00001	135.31	24	T=5/5, C=11/3
chr2:234967539	A/A	T	A	SNV	SPP2	SPP2:exonic:NM_006944.2	1	100	3	synonymous	NM_00694	c.270T>A	p.(=)	-2.69				0.01(COSM50	Cathelicidin,	rs593668	0.2788	YES	6.132E-1 (pos.23496753	carcinoma	Secreted ph	bone remodeling,cellular protein metabolic process,enc		T=0, A=56	T=0.0, A=1.0	0.00001	832.1	56	T=0/0, A=33/2
chr19:2339949	C/C	T	C	SNV	SPPL2B	SPPL2B:exonic:NM_152988.2	1	100	6	synonymous	NM_15298	c.726T>C	p.(=)	1.71				0.03(COSM50	PA domain,Si	rs3746285	0.2407	YES	3.734E-1 (pos.2339949)	osteosarcoma		Golgi membrane,Golgi-associated vesicle membrane,ac		T=0, C=64	T=0.0, C=1.0	0.00001	977.599	64	T=0/0, C=45/1
chr19:2339958	C/C	T	C	SNV	SPPL2B	SPPL2B:exonic:NM_152988.2	1	100	6	synonymous	NM_15298	c.735T>C	p.(=)	1.44					PA domain,Si	rs1609752	0.0371	YES	9.754E-1 (pos.2339958)			Golgi membrane,Golgi-associated vesicle membrane,ac		T=0, C=65	T=0.0, C=1.0	0.00001	994.502	65	T=0/0, C=45/2
chr19:2340154	GC/GC	G	GC	INDEL	SPPL2B	SPPL2B:exonic:NM_152988.2	1	100	7	unknown	NM_152988.2			0.61					PA domain,Si	rs5826760	0.0014		1.0 (pos.2340154)			Golgi membrane,Golgi-associated vesicle membrane,ac		G=0, GC=58	G=0.0, GC=1.0	0.00001	733.531	58	G=0/0, GC=38/
chr1:153005156	T/T	C	T	SNV	SPRR1B	SPRR1B:utr_3:NM_003125.2	1	100	2	utr_3	NM_00312	c.335C>T	p.?	-1.44					Cornifin (SPRI	rs2711		YES			Small prolir	cornification,cornified envelope,cytoplasm,cytosol,epid		C=0, T=45	C=0.0, T=1.0	0.00001	747.298	45	C=0/0, T=24/2
chr1:153122310	A/A	G	A	SNV	SPRR2G	SPRR2G:utr_3:NM_001014291.3	1	100	2	utr_3	NM_00101	c.277C>T	p.?	-1.48					Small proline	rs510277		YES			Small prolir	cornification,cornified envelope,cytosol,epidermis deve		G=0, A=35	G=0.0, A=1.0	0.00001	543.875	35	G=0/0, A=12/2
chr1:152975495	A/A	G	A	SNV	SPRR3	SPRR3:utr_5:NM_001097589.1	1	100	2	utr_5	NM_00109	c.-2G>A	p.?	0.18					Cornifin (SPRI	rs2075739	0.4565	YES	6.746E-1 (pos.152975495)		Small prolir	Golgi apparatus,cornification,cornified envelope,cytopl		G=0, A=142	G=0.0, A=1.0	0.00001	2382.22	142	G=0/0, A=95/4
chr1:152975941	G/G	C	G	SNV	SPRR3	SPRR3:exonic:NM_001097589.1	1	100	2	missense	NM_00109	c.445C>G	p.Leu149Val	0.42		32		0(COSM3750	Cornifin (SPRI	rs1055935	0.4557	YES	6.71E-1 (pos.152975941)	adenocarcino	Small prolir	Golgi apparatus,cornification,cornified envelope,cytopl		C=0, G=41	C=0.0, G=1.0	0.00001	664.7	41	C=0/0, G=19/2
chr16:1827836	C/C	T	C	SNV	SPSB3	SPSB3:exonic:NM_080861.3	1	100	6	synonymous	NM_08086	c.633A>G	p.(=)	-2.39				0.37(COSM41	SPRY domain	rs1178432	0.127	YES	7.61E-1 (pos.1827836)	acute_myeloi	SPRY domai	cytosol,post-translational protein modification,protein		T=0, C=86	T=0.0, C=1.0	0.00001	1410.92	86	T=0/0, C=55/3
chr1:158647495	T/T	A	T	SNV	SPTA1	SPTA1:exonic:NM_003126.2	1	100	7	synonymous	NM_00312	c.942T>A	p.(=)	-0.52					Ca2+insensit	rs325996	0.0029	YES	1.0 (pos.158647495)		Spectrin, al	ER to Golgi	Anemia;Anemia, Hemolytic;An Benign/Likely	A=0, T=88	A=0.0, T=1.0	0.00001	1451.91	88	A=0/0, T=53/3
chr1:158655036	A/A	G	A	SNV	SPTA1	SPTA1:exonic:NM_003126.2	1	100	2	synonymous	NM_00312	c.126C>T	p.(=)	-0.15					Ca2+insensit	rs435080	4.00E-04		1.0 (pos.158655036)		Spectrin, al	ER to Golgi	Anemia;Anemia, Hemolytic;An Benign/Likely	G=0, A=66	G=0.0, A=1.0	0.00001	1015.02	66	G=0/0, A=52/1
chr14:65260227	T/G	T	G	SNV	SPTB	SPTB:exonic:NM_001024858.2	1	47.37	13	synonymous	NM_00102	c.2154A>C	p.(=)	-1.1				0.01(COSM41	CAMSAP CH d	rs229591	0.3659	YES	1.835E-1 (pos.65260227)	neoplasm	Spectrin, be	ER to Golgi	Anemia;Anemia, Hemolytic;An Benign/Likely	T=50, G=45	T=0.5263, G=0.4737	0.00001	488.07	95	T=25/25, G=22
chr6:2953445	G/G	A	G	SNV	SERPINB6	SERPINB6:intronic:NM_001271822.1	1	100		intronic	NM_00127	c.473-25T	p.?	0.26					Serpin (serine	rs3818276	0.1706	YES	9.721E-1;1.157E-4 (pos.2953445)		Protease in	cellular response to osmotic st	Drotrecogin alfa	A=0, G=24	A=0.0, G=1.0	0.00001	334.792	24	A=0/0, G=15/9
chr6:29574595	A/AC	A	AC	INDEL	GABBR1	GABBR1:intronic:NM_001470.3	1	58.33		intronic	NM_00147	c.2217+79	p.?	0.01					7 transmemb	rs36073154,rs5875197		YES				G-protein	Alcohol-Related Dis Arbaclofen,Arbaclofen Pl	A=20, AC=2	A=0.4167, AC=0.583	0.00001	317.42	48	A=10/10, AC=
chr11:66472274	C/C	T	C	SNV	SPTBN2	SPTBN2:exonic:NM_006946.2	1	100	14	missense	NM_00694	c.2473A>G	p.Ser825Gly	2.02		56	0	0.03(COSM37	CAMSAP CH d	rs4930388	4.00E-04		1.0 (pos.66472274)	adenocarcino	Spectrin, be	ER to Golgi	Ataxia;Brain Diseases;Central Benign	T=0, C=168	T=0.0, C=1.0	0.00001	2739.23	168	T=0/0, C=76/9
chr15:42143533	G/G	T	G	SNV	SPTBN5	SPTBN5:exonic:NM_016642.3	1	100	64	missense	NM_01664	c.10691A>	p.Asn3564Thr	-1.74	0.31	65	0	0(COSM1478	Calponin hon	rs1197665	0.0799	YES	4.465E-1;0.0 (pos.42143	adenocarcino	Spectrin, be	ER to Golgi	vesicle-mediated transport,Golgi organizatio	T=0, G=28	T=0.0, G=1.0	0.00001	408.705	28	T=0/0, G=8/20
chr15:42171537	G/G	A	G	SNV	SPTBN5	SPTBN5:exonic:NM_016642.3	1	100	16	missense	NM_01664	c.3103T>C	p.Cys1035Arg	0.74	0.2	180	0	0(COSM4148	Calponin hon	rs1197683	0.1363	YES	9.843E-1 (pos.42171537)	neoplasm	Spectrin, be	ER to Golgi	vesicle-mediated transport,Golgi organizatio	A=0, G=61	A=0.0, G=1.0	0.00001	908.241	61	A=0/0, G=23/3
chr6:29635508	G/A	G	A	SNV	MOG	MOG:intronic:NM_002433.4	1	59.09		intronic	NM_00243	c.571+68G	p.?	-1.13					Immunoglobu	rs1318631	0.2168	YES			Myelin-olig	cell adhes	Autoimmune Diseases:Autoimmune Disease	G=9, A=13	G=0.4091, A=0.5909	0.00001	122.565	22	G=8/1, A=10/3
chr6:29692634	G/G	T	G	SNV	HLA-F	HLA-F:intronic:NM_001098479.1	1	100		intronic	NM_00109	c.611-174	p.?	-0.41					Class I Histoc	rs1736925		YES			Major histo	ER to Golgi	Autoimmune Diseases:Autoimmune Disease	T=0, G=36	T=0.0, G=1.0	0.00001	568.08	36	T=0/0, G=17/1
chr14:78028803	G/G	A	G	SNV	SPTLC2	SPTLC2:exonic:NM_004863.3	1	100	6	synonymous	NM_00486	c.786T>C	p.(=)	1.11					Aminotransfe	rs2364602	0.0659	YES	9.945E-1 (pos.78028803)		Serine palr	biosynthe	Congenital Abnorm L-Serine,P Benign	A=0, G=138	A=0.0, G=1.0	0.00001	2300.28	138	A=0/0, G=92/4
chr20:13053017	TG/TG	T	TG	INDEL	SPTLC3	SPTLC3:exonic:NM_018327.3	1	100	3	frameshiftInsertion	NM_01832	c.417_418	p.Leu140fs	-0.45					Aminotransferase class I and II,Aminotransferase class-V						Serine palr	biosynthe	Bipolar Disorder:Bi Pyridoxal Phosphate	T=0, TG=21	T=0.0, TG=1.0	0.00001	220.275	21	T=0/0, TG=6/1
chr20:13053035	G/G	A	G	SNV	SPTLC3	SPTLC3:exonic:NM_018327.3	1	100	3	synonymous	NM_01832	c.435A>G	p.(=)	-0.56					Aminotransfe	rs243888	0.3782	YES	5.77E-1 (pos.13053035)		Serine palr	biosynthe	Bipolar Disorder:Bi Pyridoxal Phosphate	A=0, G=21	A=0.0, G=1.0	0.00001	281.235	21	A=0/0, G=5/16
chr16:30724070	T/T	G	T	SNV	SRCAP	SRCAP:exonic:NM_006662.2	1	100	14	synonymous	NM_00666	c.2064G>T	p.(=)	-0.41					AT hook moti	rs4889500	3.00E-04	YES	1.0 (pos.30724070)		SNF2-relate	ATP bindin	Abnormalities, Multiple:Adenocarcinoma:Ca	G=0, T=26	G=0.0, T=1.0	0.00001	371.391	26	G=0/0, T=14/1
chr6:29912830	C/C	T	C	SNV	HLA-A	HLA-A:intronic:NM_001242758.1	1	100		intronic	NM_00124	c.1013-6T	p.?	-0.55					Class I Histoc	rs2571381	0.1759	YES	9.665E-1 (pos.29912830)		Major histocompatibili		Ankylosis;Arthritis;. 3-Indolebutyric Acid,Coc	T=0, C=38	T=0.0, C=1.0	0.00001	603.786	38	T=0/0, C=11/2
chr6:29912935	T/T	C	T	SNV	HLA-A	HLA-A:intronic:NM_001242758.1	1	100		intronic	NM_00124	c.1045+67	p.?	-0.7					Class I Histoc	rs2916802		YES			Major histocompatibili		Ankylosis;Arthritis;. 3-Indolebutyric Acid,Coc	C=0, T=87	C=0.0, T=1.0	0.00001	1431.39	87	C=0/0, T=42/4
chr6:29912944	C/C	T	C	SNV	HLA-A	HLA-A:intronic:NM_001242758.1	1	100		intronic	NM_00124	c.1046-67	p.?	-1.28					Class I Histoc	rs2916803		YES			Major histocompatibili		Ankylosis;Arthritis;. 3-Indolebutyric Acid,Coc	T=0, C=87	T=0.0, C=1.0	0.00001	1431.39	87	T=0/0, C=42/4
chr12:64472803	T/T	A	T	SNV	SRGAP1	SRGAP1:exonic:NM_020762.3	1	100	9	synonymous	NM_02076	c.1230A>T	p.(=)	-0.65					Fes/CIP4, anc	rs789710	0		1.0 (pos.64472803)		Slit-robo GT	GTPase ac	Adenocarcinoma:Adenocarcinoma, Follicula	A=0, T=41	A=0.0, T=1.0	0.00001	662.854	41	A=0/0, T=26/1
chr6:29913074	G/G	A	G	SNV	HLA-A	HLA-A:intronic:NM_001242758.1	1	100		intronic	NM_00124	c.1093+16	p.?	-1.6					Class I Histoc	rs9260205		YES	9.662E-1 (pos.29913074)		Major histocompatibili		Ankylosis;Arthritis;. 3-Indolebutyric Acid,Coc	A=0, G=76	A=0.0, G=1.0	0.00001	1209.59	76	A=0/0, G=35/4
chr6:29913078	G/G	A	G	SNV	HLA-A	HLA-A:intronic:NM_001242758.1	1	100		intronic	NM_00124	c.1093+20	p.?	-0.04					Class I Histoc	rs41546815	0.1999	YES	9.661E-1 (pos.29913078)		Major histocompatibili		Ankylosis;Arthritis;. 3-Indolebutyric Acid,Coc	A=0, G=76	A=0.0, G=1.0	0.00001	1209.58	76	A=0/0, G=35/4
chr6:30029651	G/G	C	G	SNV	ZNRD1,ZNRD1ASP	ZNRD1ASP:upstream:NR_026751.2,ZNRD1:intronic:NM_17(	1	100		intronic	NR_02675	c.146-43C	p.?	-0.16					Topoisomera	rs1150741	0.2754	YES	2.809E-1 (pos.30029651)		Zinc ribbon	DNA-direc	Acquired Immunodeficiency Syndrome:Diges	C=0, G=87	C=0.0, G=1.0	0.00001	1431.39	87	C=0/0, G=42/4
chr6:30030114	G/G	A	G	SNV	ZNRD1	ZNRD1:intronic:NM_170783.3	1	100		intronic	NM_17078	c.356+4A>	p.?	-0.11					Topoisomera	rs9261269	0.1285	YES	9.905E-1 (pos.30030114)		Zinc ribbon	DNA-direc	Acquired Immunodeficiency Syndrome:Diges	A=0, G=37	A=0.0, G=1.0	0.00001	583.706	37	A=0/0, G=24/1
chr6:30042955	T/T	C	T	SNV	RNF39	RNF39:intronic:NM_025236.3	1	100		intronic	NM_02523	c.567+45G	p.?	0.29					RING-type zin	rs1150736	0.2767	YES	3.562E-1 (pos.30042955)		Ring finger	biological_process,cellular_component,cytoplasm,meta		C=0, T=28	C=0.0, T=1.0	0.00001	408.718	28	C=0/0, T=22/6
chr16:2812939	C/A	C	A	SNV	SRRM2	SRRM2:exonic:NM_016333.3	1	50	11	missense	NM_01633	c.2410C>A	p.Pro804Thr	0.75	0.09	38	0	0.05(COSM39	cwf21 domai	rs2240140	0.39	YES	3.76E-1 (pos.2812939)	acute_myeloi	Serine/argi	C2H2 zinc finger domain binding,Cajal body,RNA binding,		C=35, A=35	C=0.5, A=0.5	0.00001	346.074	70	C=21/14, A=24
chr12:119588962	A/A	G	A	SNV	SRRM4	SRRM4:exonic:NM_194286.3	1	100	10	missense	NM_19428	c.1217G>A	p.Arg406Gln	0.69	0.01	43	0.098	0.23(COSM37	Serine/argini	rs2723880	0.3864	YES	1.307E-1;0.0 (pos.11958	adenocarcino	Serine/argi	RNA splicing,cell differentiation,mRNA binding,mRNA prc		G=0, A=30	G=0.0, A=1.0	0.00001	446.637	30	G=0/0, A=16/1
chr19:44118353	G/A	G	A	SNV	SRRM5,ZNF428	SRRM5:exonic:NM_001145641.1,ZNF428:intronic:NM_1824	1	46.43	1	missense	NM_18249	c.76+28C>	p.?, p.Asp694Asn	-0.13		23		0(COSM4000775),0(COSM6		rs2302421	0.1574	YES	2.125E-1 (pos.44118353)	acute_myeloid_leukaemia		metal ion binding,nucleic acid binding		G=15, A=13	G=0.5357, A=0.4643	0.00001	117.927	28	G=9/6, A=12/1
chr6:30553871	T/T	C	T	SNV	ABCF1	ABCF1:intronic:NM_001025091.1	1	100		intronic	NM_00102	c.1714-40	p.?	-0.76					ABC transpor	rs1264437,rs386698	0.3003	YES	6.944E-1 (pos.30553871)		ATP-binding	ATP bindin	Autoimmune Diseases;Endocrine System Dis	C=0, T=42	C=0.0, T=1.0	0.00001	685.191	42	C=0/0, T=28/1
chr7:76033805	T/T	A	T	SNV	SSC4D	SSC4D:utr_5:NM_080744.1	1	100	2	utr_5	NM_08074	c.-49T>A	p.?	0.19					Scavenger re	rs11773653	0.2128	YES	5.955E-2 (pos.76033805)			extracellu	Basal Ganglia Diseases;Brain Diseases;Centr	A=0, T=25	A=0.0, T=1.0	0.00001	357.61	25	A=0/0, T=16/9
chr6:30576781	C/C	G	C	SNV	PPP1R10	PPP1R10:intronic:NM_002714.3	1	100		intronic	NM_00271	c.194+73C	p.?	-2.81					TFIIS helical b	rs1264419		YES			Protein pho	DNA binding,PTW/PP1 phosphatase complex,RNA bindin		G=0, C=110	G=0.0, C=1.0	0.00001	1737.26	110	G=0/0, C=55/5
chr6:30679628	A/A	T	A	SNV	MDC1,MDC1-AS1	MDC1:intronic:NM_014641.2,MDC1-AS1:intronic_nc:NR_13	1	100		intronic	NM_01464	c.2068+23	p.?	0.33					FHA domain,I	rs3094093,rs751838	0.0655	YES	0.0 (pos.30679627),1.0 (pos.30679628)		Mediator of	FHA domain binding,chromosome,double-strand break r		T=0, A=29	T=0.0, A=1.0	0.00001	432.317	29	T=0/0, A=12/1
chr7:149476666	GC/GC	G	GC	INDEL	SSPO	SSPO:splicesite_5:NM_198455.2	1	100	9	splicesite	NM_19845	c.1117-1G	p.?	0.42					C8 domain,F	rs397826817,rs5755	0.0019		1.0;0.0 (pos.149476666)		Subcommis	cell adhesion,cell differentiation,extracellular space,ne		G=0, GC=34	G=0.0, GC=1.0	0.00001	318.211	34	G=0/0, GC=12/
chr7:149506211	GC/GC	G	GC	INDEL	SSPO	SSPO:exonic:NM_198455.2	1	100	63	frameshiftInsertion	NM_19845	c.9204_92	p.Ser3068fs	1.74					C8 domain,F	rs397815440,rs7119	3.00E-04		0.0;0.0 (pos.149506193),0.0;0.0 (pos.14		Subcommis	cell adhesion,cell differentiation,extracellular space,ne		G=0, GC=38	G=0.0, GC=1.0	0.00001	585.398	38	G=0/0, GC=24/
chr6:30877202	C/C	T	C	SNV	GTF2H4	GTF2H4:intronic:NM_001517.4	1	100		intronic	NM_00151	c.138-10T	p.?	-0.47					Transcriptior	rs916920	0.1772	YES	5.772E-1 (pos.30877202)		General tra	7-methylguanosine mRNA capping,ATP-dependent DNA h		T=0, C=20	T=0.0, C=1.0	0.00001	268.067	20	T=0/0, C=11/9
chr6:30883878	A/A	AAAGCAAC	A	INDEL	VARS2	VARS2:intronic:NM_001167734.1	8	100		intronic	NM_00116	c.664-51A	p.?	-0.23, 0.12, 0.07, 0.51, -0.34, -0.41, -0.01, 0.23, 0.78					Anticodon-bi	rs112873231,rs1999	0.2432	YES	0.0 (pos.30883882),0.0 (pos.30883884),		Valyl-tRNA	ATP binding,aminoacyl-tRNA editing activity,aminoacyl-t		AAAGCAAC	AAAGCAACC=0.0, A=1	0.00001	482.65	32	AAAGCAACC=0
chr7:149480069	C/T	C	T	SNV	SSPO	SSPO:exonic:NM_198455.2	1	56	15	missense	NM_19845	c.2035C>T	p.Pro679Ser	0.5	0.25	74	0.033		C8 domain,F	rs113627612	2.00E-04		2.038E-3 (pos.149480069)		Subcommis	cell adhesion,cell differentiation,extracellular space,ne		C=11, T=14	C=0.44, T=0.56	0.00001	134.35	25	C=8/3, T=8/6
chr6:30899077	C/T	C	T	SNV	SFTA2	SFTA2:downstream:NM_205854.2	1	95.45		intronic	NM_20585	c.*188G>A	p.?	-0.33						rs9368646		YES				Golgi apparatus,extracellular region,transport vesicle		C=1, T=21	C=0.0455, T=0.9545	0.00001	275.812	22	C=1/0, T=12/9
chr7:149480042	G/A	G	A	SNV	SSPO	SSPO:exonic:NM_198455.2	1	50	15	missense	NM_19845	c.2008G>A	p.Ala670Thr	7.81	0.02	58	0.898		C8 domain,F	rs111458363	2.00E-04		2.161E-3 (pos.149480042)		Subcommis	cell adhesion,cell differentiation,extracellular space,ne		G=17, A=17	G=0.5, A=0.5	0.00001	172.135	34	G=10/7, A=11/
chr2:107423188	G/A	G	A	SNV	ST6GAL2	ST6GAL2:exonic:NM_032528.2	1	50	6	synonymous	NM_03252	c.1536C>T	p.(=)	-5.7				0.02(COSM37	Glycosyltrans	rs1470591,rs775787	0.4614	YES	0.0;0.0 (pos.107423187),	adenocarcinoma,hepatoce		Golgi cisterna membrane,Golgi membrane,beta-galacto		G=40, A=40	G=0.5, A=0.5	0.00001	422.245	80	G=26/14, A=1
chr6:30993236	G/G	A	G	SNV	MUC22	MUC22:intronic:NM_001198815.1	1	100		intronic	NM_00119	c.71-43A>	p.?	-0.04					Mucin, cataly	rs4713419		YES	4.5E-1 (pos.30993236)		Mucin 22	integral cc	Autoimmune Diseases:Endocrine System Dis	A=0, G=47	A=0.0, G=1.0	0.00001	763.103	47	A=0/0, G=28/1
chr2:107460474	T/G	T	G	SNV	ST6GAL2	ST6GAL2:utr_5:NM_032528.2	1	50	2	utr_5	NM_03252	c.-41A>C	p.?	-0.53					Glycosyltrans	rs12473361	0.4408	YES	1.147E-1 (pos.107460474)			Golgi cisterna membrane,Golgi membrane,beta-galacto		T=18, G=18	T=0.5, G=0.5	0.00001	182.455	36	T=11/7, G=9/9
chr3:52544470	G/G	A	G	SNV	STAB1	STAB1:exonic:NM_015136.2	1	100	25	missense	NM_01513	c.2734A>G	p.Met912Val	0.38	0.45	21	0		EGF domain,E	rs9835659	0.0223	YES	1.0 (pos.52544470)			calcium io	Arthritis;Arthritis, Juvenile;Autoimmune Dise	A=0, G=25	A=0.0, G=1.0	0.00001	353.019	25	A=0/0, G=17/8
chr7:89861832	A/A	G	A	SNV	STEAP2	STEAP2:exonic:NM_152999.3	1	100	5	missense	NM_15299	c.1367G>A	p.Arg456Gln	1.92	0.2	43	0.003	0.86(COSM37	Ferric reduct	rs194524	0.4717	YES	1.919E-1 (pos.89861832)	acute_myeloi	Six-transme	Golgi to plasma membrane transport,copper ion import,		G=0, A=44	G=0.0, A=1.0	0.00001	726.284	44	G=0/0, A=28/1
chr7:89861888	AT/AT	A	AT	INDEL	STEAP2	STEAP2:exonic:NM_152999.3	1	100	5	frameshiftInsertion	NM_15299	c.1424_14	p.Met475fs	0.15					Ferric reductase like transmembrane component,NADP oxidoreductase coenzyme F420-dependent						Six-transme	Golgi to plasma membrane transport,copper ion import,		A=0, AT=50	A=0.0, AT=1.0	0.00001	664.284	50	A=0/0, AT=26/
chr7:89861942	T/T	C	T	SNV	STEAP2	STEAP2:utr_3:NM_152999.3	1	100	5	utr_3	NM_15299	c.1477C>T	p.?	-0.97					Ferric reduct	rs194526	0.1538	YES	9.991E-1 (pos.89861942)		Six-transme	Golgi to plasma membrane transport,copper ion import,		C=0, T=48	C=0.0, T=1.0	0.00001	810.435	48	C=0/0, T=25/2
chr6:31097453	G/G	T	G	SNV	PSORS1C1	PSORS1C1:intronic:NM_014068.2	1	100		intronic	NM_01406	c.13+31T>	p.?	-1.46					Psoriasis sus	rs3130560	0.273	YES	8.946E-1 (pos.31097453)		SEEK1 gene		Autoimmune Diseases;Behcet Syndrome;Blo	T=0, G=59	T=0.0, G=1.0	0.00001	885.353	59	T=0/0, G=36/2
chr1:47717189	A/G	A	G	SNV	STIL	STIL:exonic:NM_001048166.1	1	47.62	17	synonymous	NM_00104	c.3486T>C	p.(=)	0.02					SCL-interrupt	rs2758735	0.475	YES	4.494E-1 (pos.47717189)		SCL/TAL1-in	cell prolife	Congenital Abnormalities;Con Benign	A=11, G=10	A=0.5238, G=0.4762	0.00001	82.29	21	A=5/6, G=6/4
chr2:220476443	T/T	C	T	SNV	STK11IP	STK11IP:exonic:NM_052902.3	1	100	18	missense	NM_05290	c.2222C>T	p.Ser741Phe	-0.08	0.03	155	0.004		LKB1 serine/t	rs627530	0.0267	YES	9.965E-1 (pos.220476443)		Serine/thre	azurophil granule lumen,cytoplasm,extracellular region		C=0, T=238	C=0.0, T=1.0	0.00001	3996.05	238	C=0/0, T=115/
chr2:220113242	G/G	T	G	SNV	STK16	STK16:exonic:NM_001008910.3	1	100	8	synonymous	NM_00100	c.879T>G	p.(=)	1.52				0.05(COSM41	Protein kinas	rs7601549	2.00E-04		1.0 (pos.220113242)	neoplasm		ATP binding,Golgi-associated vesicle,cellular response t		T=0, G=50	T=0.0, G=1.0	0.00001	743.035	50	T=0/0, G=17/3

chr6:31112899	T/T	C	T	SNV	CCHCR1	CCHCR1:intronic:NM 001105563.1	1	100		intronic	NM 00110	c.1768+44	p.?	-0.57					Alpha helical	rs3094226	0.2438	YES	7.143E-1 (pos.31112899)		Coiled-coil	cell differe	Amphetamine-Related Disorders;Colitis;Coli		C=0, T=42	C=0.0, T=1.0	0.00001	685.171	42	C=0/0, T=26/1
chr7:43664280	G/G	A	G	SNV	STK17A	STK17A:exonic:NM 004760.2	1	100	7	missense	NM 00476	c.1084A>G	p.Lys362Glu	1.08	1	56	0		Protein kinas	rs1044141	0.2349	YES	6.913E-1 (pos.43664280)		Serine/thre	ATP binding,apoptotic process,intracellular signal transd			A=0, G=29	A=0.0, G=1.0	0.00001	427.603	29	A=0/0, G=11/1
chr6:31113106	G/G	A	G	SNV	CCHCR1	CCHCR1:intronic:NM 001105563.1	1	100		intronic	NM 00110	c.1632-27	p.?	-0.11					Alpha helical	rs3130575	0.2422	YES	7.138E-1 (pos.31113106)		Coiled-coil	cell differe	Amphetamine-Related Disorders;Colitis;Coli		A=0, G=38	A=0.0, G=1.0	0.00001	603.779	38	A=0/0, G=25/1
chr7:43664198	A/G	A	G	SNV	STK17A	STK17A:exonic:NM 004760.2	1	46.43	7	synonymous	NM 00476	c.1002A>G	p.(=)	1.29					Protein kinas	rs10233697	0.2116	YES	6.964E-3 (pos.43664198)		Serine/thre	ATP binding,apoptotic process,intracellular signal transd			A=15, G=13	A=0.5357, G=0.4643	0.00001	117.927	28	A=3/12, G=8/5
chr6:31125158	C/T	C	T	SNV	CCHCR1	CCHCR1:intronic:NM 001105563.1	1	46.51		intronic	NM 00110	c.216+4G>	p.?	0.99				0.03(COSM63	Alpha helical	rs743401	0.3967	YES	6.021E-1 (pos.31125158)	carcinoma	Coiled-coil	cell differe	Amphetamine-Related Disorders;Colitis;Coli		C=23, T=20	C=0.5349, T=0.4651	0.00001	212.329	43	C=12/11, T=8/
chr2:197004439	G/G	A	G	SNV	STK17B	STK17B:exonic:NM 004226.3	1	100	7	synonymous	NM 00422	c.741T>C	p.(=)	0.32					Protein kinas	rs1054537	0.3773	YES	9.053E-1 (pos.197004439)		Serine/thre	ATP binding,actin cytoskeleton		Quercetin	A=0, G=31	A=0.0, G=1.0	0.00001	465.819	31	A=0/0, G=19/1
chr5:146619206	C/C	G	C	SNV	STK32A	STK32A:exonic:NM 001112724.1	1	100	2	synonymous	NM 00111	c.9G>C	p.(=)	0.22				0.05(COSM44	Kinase-like,P	rs4705132	0.4407	YES	3.595E-1;0.0 (pos.14661	carcinoma,hepatocellular		ATP binding,intracellular,intracellular signal transductic			G=0, C=41	G=0.0, C=1.0	0.00001	664.696	41	G=0/0, C=25/1
chr6:31132649	C/C	A	C	SNV	POU5F1,TCF 19	TCF19:downstream:NM 001077511.1, POU5F1:intronic:NM	1	100		intronic	NM 00107	c.*2155A>	p.?, p.?	0.08					Homeobox do	rs2394882	0.3416	YES	6.636E-1 (pos.31132649)		Pou domair	BMP signa	Amphetamine-Rela	Dopamine,Norepinephri	A=0, C=42	A=0.0, C=1.0	0.00001	685.207	42	A=0/0, C=17/2
chr6:31236998	T/T	C	T	SNV	HLA-C	HLA-C:intronic:NM 001243042.1	1	100		intronic	NM 00124	c.1097-48	p.?	-0.68					Class I Histoc	rs2001181	0.1418	YES	8.252E-1 (pos.31236998)		Major histocompatibili		Arthritis;Arthritis, Psoriatic;Bone Diseases;D		C=0, T=21	C=0.0, T=1.0	0.00001	281.235	21	C=0/0, T=14/7
chr6:31237014	A/A	G	A	SNV	HLA-C	HLA-C:intronic:NM 001243042.1	1	100		intronic	NM 00124	c.1097-64	p.?	-0.77					Class I Histoc	rs386698954,rs386698955,rs39		YES			Major histocompatibili		Arthritis;Arthritis, Psoriatic;Bone Diseases;D		G=0, A=22	G=0.0, A=1.0	0.00001	298.884	22	G=0/0, A=14/8
chr6:31237017	T/T	C	T	SNV	HLA-C	HLA-C:intronic:NM 001243042.1	1	100		intronic	NM 00124	c.1097-67	p.?	-0.5					Class I Histoc	rs2394960,rs386698955		YES			Major histocompatibili		Arthritis;Arthritis, Psoriatic;Bone Diseases;D		C=0, T=22	C=0.0, T=1.0	0.00001	298.892	22	C=0/0, T=14/8
chr6:31237027	T/T	C	T	SNV	HLA-C	HLA-C:intronic:NM 001243042.1	1	100		intronic	NM 00124	c.1097-77	p.?	-1.33					Class I Histoc	rs2394961		YES			Major histocompatibili		Arthritis;Arthritis, Psoriatic;Bone Diseases;D		C=0, T=22	C=0.0, T=1.0	0.00001	298.892	22	C=0/0, T=13/9
chr6:31237048	G/G	A	G	SNV	HLA-C	HLA-C:intronic:NM 001243042.1	1	100		intronic	NM 00124	c.1096+67	p.?	-0.46					Class I Histoc	rs9264594		YES			Major histocompatibili		Arthritis;Arthritis, Psoriatic;Bone Diseases;D		A=0, G=21	A=0.0, G=1.0	0.00001	281.174	21	A=0/0, G=12/9
chr4:5448429	A/G	A	G	SNV	STK32B	STK32B:exonic:NM 018401.2	1	51.85	7	missense	NM 01840	c.592A>G	p.Arg198Gly	4.29	1	125	0	0.02(COSM63	Kinase-like,P	rs3733182	0.113	YES	5.241E-1 (pos.5448429)	carcinoma		ATP bindin	Alzheimer Disease;Brain Diseases;Cardiovas		A=26, G=28	A=0.4815, G=0.5185	0.00001	278.618	54	A=14/12, G=15
chr11:8486310	A/A	C	A	SNV	STK33	STK33:exonic:NM 030906.3	1	100	5	synonymous	NM 03090	c.399G>T	p.(=)	-0.05				0.21(COSM14	Lipopolysacc	rs1446464	0.4095	YES	5.317E-1 (pos.8486310)	acute_myeloi	Serine/thre	ATP binding,intracellular,intracellular signal transductic			C=0, A=80	C=0.0, A=1.0	0.00001	1289.34	80	C=0/0, A=43/3
chr12:27470866	A/A	G	A	SNV	STK38L	STK38L:exonic:NM 015000.3	1	100	11	synonymous	NM 01500	c.993G>A	p.(=)	-1.12					Protein kinas	rs6487601	0.0278	YES	1.0 (pos.27470866)		Serine/thre	ATP binding,actin binding,actin cytoskeleton,cytoplasm,			G=0, A=47	G=0.0, A=1.0	0.00001	789.321	47	G=0/0, A=24/2
chr10:105659826	C/C	T	C	SNV	STN1	STN1:exonic:NM 024928.4	1	100	5	missense	NM 02492	c.451A>G	p.Thr151Ala	-1.84	0.25	58	0		CST, Suppress	rs2487999	0.103	YES	1.0 (pos.105659826)		STN1, CST cc	CST complex,fibrillar center,intermediate filament cytos			T=0, C=45	T=0.0, C=1.0	0.00001	747.393	45	T=0/0, C=24/2
chr2:48808594	C/T	C	T	SNV	STON1,STON 1-GTF2A1L	STON1:exonic:NM 001198595.1, STON1-GTF2A1L:exonic:NM	1	50	1	synonymous	NM 00119	c.822C>T,	p.(=), p.(=)	-1.97				0.01(COSM37	Adaptor com	rs3749142	0.4951	YES	4.173E-1 (pos.48808594)	acute_myeloi	Stonin 1	cytoplasm	Adnexal Diseases;Cysts;Endocrine System Di		C=16, T=16	C=0.5, T=0.5	0.00001	160.567	32	C=9/7, T=6/10
chr2:48808882	T/A	T	A	SNV	STON1,STON 1-GTF2A1L	STON1:exonic:NM 001198595.1, STON1-GTF2A1L:exonic:NM	1	45.83	1	synonymous	NM 00119	c.1110T>A	p.(=), p.(=)	-0.28				0.07(COSM37	Adaptor com	rs3749144	0.3198	YES	4.171E-1 (pos.48808882)	acute_myeloi	Stonin 1	cytoplasm	Adnexal Diseases;Cysts;Endocrine System Di		T=13, A=11	T=0.5417, A=0.4583	0.00001	93.4395	24	T=6/7, A=6/5
chr2:48808898	C/T	C	T	SNV	STON1,STON 1-GTF2A1L	STON1:exonic:NM 001198595.1, STON1-GTF2A1L:exonic:NM	1	45.83	1	synonymous	NM 00119	c.1126C>T	p.(=), p.(=)	2.58				0.88(COSM37	Adaptor com	rs3749145	0.3199	YES	4.154E-1 (pos.48808898)	acute_myeloi	Stonin 1	cytoplasm	Adnexal Diseases;Cysts;Endocrine System Di		C=13, T=11	C=0.5417, T=0.4583	0.00001	93.693	24	C=6/7, T=6/5
chr6:31322470	G/G	C	G	SNV	HLA-B,MIR6891	HLA-B:intronic:NM 005514.7, MIR6891:downstream:NR 10	1	100		intronic	NM 00551	c.1013-28	p.?	-1.25						rs2442717	0.1867	YES	8.07E-1 (pos.31322470)		Major histocompatibili		Ankylosis;Aortic Arc	Coccidioides immitis sph	C=0, G=78	C=0.0, G=1.0	0.00001	1245.55	78	C=0/0, G=48/3
chr6:31378257	G/A	G	A	SNV	MICA	MICA:intronic:NM 000247.2	1	51.92		intronic	NM 00024	c.71-63G>	p.?	-0.28					Class I Histoc	rs144888775		YES			MHC class I	T cell med	Adenocarcinoma;Aneurysm;Ankylosis;Arthrit		G=25, A=27	G=0.4808, A=0.5192	0.00001	270.388	52	G=15/10, A=2
chr14:81744736	G/G	A	G	SNV	STON2	STON2:exonic:NM 001256430.1	1	100	6	missense	NM 00125	c.919T>C	p.Ser307Pro	4.03	1	74	0	0.04(COSM39	Adaptor com	rs3813535	0.1479	YES	5.598E-1 (pos.81744736)	acute_myeloi	Stonin 2	cell junction,clathrin-coated vesicle,cytosol,endocytosis			A=0, G=44	A=0.0, G=1.0	0.00001	725.916	44	A=0/0, G=24/2
chr4:184931818	A/A	G	A	SNV	STOX2	STOX2:exonic:NM 020225.2	1	100	3	synonymous	NM 02022	c.1827G>A	p.(=)	-1.79				0.03(COSM37	Winged helix	rs4861597	0.2687	YES	6.228E-1 (pos.18493181	acute_myeloi	Storkhead b	cellular_component,embryo development,maternal pla			G=0, A=26	G=0.0, A=1.0	0.00001	371.42	26	G=0/0, A=15/1
chr1:24718075	T/C	T	C	SNV	STPG1	STPG1:exonic:NM 001199013.1	1	95.83	3	synonymous	NM 00119	c.165A>G	p.(=)	3.95				0.73(COSM37	Sperm-tail PC	rs2294520		YES	4.239E-1 (pos.24718075)	acute_myeloi	Sperm-tail	intracellular,mitochondrion,molecular_function,nucleu			T=1, C=23	T=0.0417, C=0.9583	0.00001	306.614	24	T=0/1, C=7/16
chr1:24706269	A/G	A	G	SNV	STPG1	STPG1:exonic:NM 001199013.1	1	45.45	5	synonymous	NM 00119	c.336T>C	p.(=)	-1.2				0.02(COSM13	Sperm-tail PC	rs1064842	0.3414	YES	4.38E-1 (pos.24706269)	acute_myeloi	Sperm-tail	intracellular,mitochondrion,molecular_function,nucleu			A=18, G=15	A=0.5455, G=0.4545	0.00001	143.162	33	A=2/16, G=7/8
chr6:31498497	A/A	C	A	SNV	ATP6V1G2-DDX39B,DDX39B,MCCD1	DDX39B:intronic:NM 080598.5, MCCD1:downstream:NM 0	1	100		intronic	NM 00101	c.*785C>A	p.?, p.?	-0.38					DEAD/DEAH b	rs3093978		YES			DEAD box p	ATP binding,ATP-dependent RN		Isopropyl Alcohol	C=0, A=26	C=0.0, A=1.0	0.00001	371.417	26	C=0/0, A=8/18
chr1:24706292	T/C	T	C	SNV	STPG1	STPG1:exonic:NM 001199013.1	1	45.45	5	missense	NM 00119	c.313A>G	p.Ile105Val	-0.06	0.93	29	0.002	0.04(COSM39	Sperm-tail PC	rs1142057	0.3328	YES	4.372E-1 (pos.24706292)	acute_myeloi	Sperm-tail	intracellular,mitochondrion,molecular_function,nucleu			T=18, C=15	T=0.5455, C=0.4545	0.00001	143.162	33	T=2/16, C=7/8
chr6:31506648	T/T	G	T	SNV	ATP6V1G2-DDX39B,DDX39B	DDX39B:intronic:NM 080598.5, ATP6V1G2-DDX39B:intronic	1	100		intronic	NR 03785	c.340-16C	p.?	1.47				0.83(COSM50	DEAD/DEAH b	rs933208	0.2566	YES	8.034E-1 (pos.31506648)	osteosarcoma	DEAD box p	ATP binding,ATP-dependent RN		Isopropyl Alcohol	G=0, T=27	G=0.0, T=1.0	0.00001	389.961	27	G=0/0, T=13/1
chr6:31555130	G/G	A	G	SNV	LST1	LST1:intronic:NM 205839.2	1	100		intronic	NM 20583	c.19+35A>	p.?	0.13					LST-1 protein	rs2256965	0.4234	YES	7.445E-1 (pos.31555130)		Leukocyte-s	Golgi apparatus,Golgi membrane,anatomical structure			A=0, G=24	A=0.0, G=1.0	0.00001	334.792	24	A=0/0, G=10/1
chr7:73122923	A/G	A	G	SNV	STX1A	STX1A:exonic:NM 004603.3	1	50	3	synonymous	NM 00460	c.204T>C	p.(=)	-3.98					SNARE domai	rs2228607	0.4337	YES	4.218E-1 (pos.73122923)		Syntaxin 1A	ATP-deper	Autistic Disorder;Child Development Disorde		A=12, G=12	A=0.5, G=0.5	0.00001	106.71	24	A=6/6, G=5/7
chr12:131276448	T/T	G	T	SNV	STX2	STX2:utr 3:NM 194356.3	1	100	11	utr 3	NM 19435	c.987C>A	p.?	-0.04					SNARE domai	rs4759786	1.00E-04		1.0 (pos.131276448)		Epimorphin	SNAP rece	Genital Diseases, Male;Infertility;Infertility,		G=0, T=39	G=0.0, T=1.0	0.00001	623.979	39	G=0/0, T=18/2
chr6:31605199	G/C	G	C	SNV	PRRC2A	PRRC2A:intronic:NM 004638.3	1	45.45		intronic	NM 00463	c.6334-24	p.?	-0.91					BAT2 N-termi	rs2736155,rs757634	0.4917	YES	0.0 (pos.31605197),5.879E-1 (pos.31605		Proline-rich	RNA bindir	Autoimmune Diseases;Female Urogenital Dis		G=12, C=10	G=0.5455, C=0.4545	0.00001	81.7627	22	G=7/5, C=5/5
chr6:31607035	C/G	C	G	SNV	BAG6	BAG6:intronic:NM 001098534.1	1	51.85		intronic	NM 00109	c.3286-32	p.?	0.2					Domain of un	rs28732153	0.04	YES	5.055E-3;0.0 (pos.31607035)		BCL2-assoc	BAT3 comp	Adenocarcinoma;Arthritis;Arthritis, Rheumat		C=13, G=14	C=0.4815, G=0.5185	0.00001	132.662	27	C=1/12, G=3/1
chr6:31607050	C/T	C	T	SNV	BAG6	BAG6:intronic:NM 001098534.1	1	51.85		intronic	NM 00109	c.3286-47	p.?	-0.5					Domain of un	rs750332,rs7654461	0.175	YES	0.0 (pos.31607045),8.576E-1 (pos.31607		BCL2-assoc	BAT3 comp	Adenocarcinoma;Arthritis;Arthritis, Rheumat		C=13, T=14	C=0.4815, T=0.5185	0.00001	132.762	27	C=1/12, T=3/1
chr6:31922254	A/C	A	C	SNV	NELFE	NELFE:intronic:NM 002904.5	1	53.13		intronic	NM 00290	c.743-35T	p.?	-1.23					RNA recogniti	rs630379	0.281	YES	7.125E-1 (pos.31922254)			NELF comp	Eye Diseases;Macular Degeneration;Retinal		A=30, C=34	A=0.4688, C=0.5313	0.00001	347.409	64	A=18/12, C=17
chr6:31977493	C/T	C	T	SNV	CYP21A1P,CYP21A2,TNXA	CYP21A1P:downstream:NR 040090.1, TNXA:intronic nc:NR	1	50		intronic	NM 00050	c.*1317C>	p.?	5.06					Fibrinogen be	rs79770049		YES	3.207E-1 (pos.31977493)		Cytochrome	endoplasr	46, XX Disorders of	Ketoconazole	C=17, T=17	C=0.5, T=0.5	0.00001	172.008	34	C=5/12, T=9/8
chr6:32134656	T/C	T	C	SNV	EGFL8,PPT2-EGFL8	PPT2-EGFL8:intronic nc:NR 037861.1, EGFL8:intronic:NM 0	1	45.45		intronic	NR 03786	c.335-31T	p.?	-1.45					Calcium-bind	rs3130347	0.2121	YES	4.185E-2 (pos.32134656)		Epidermal g	calcium io	Autoimmune Diseases;Female Urogenital Dis		T=12, C=10	T=0.5455, C=0.4545	0.00001	81.7626	22	T=2/10, C=4/6
chr12:131306314	G/G	C	G	SNV	STX2	STX2:exonic:NM 194356.3	1	100	3	missense	NM 19435	c.125G>C	p.Ser42Thr	-0.07	0.39	58	0.389		SNARE domai	rs17564	0.3429	YES	8.844E-1 (pos.131306314)		Epimorphin	SNAP rece	Genital Diseases, Male;Infertility;Infertility,		C=0, G=26	C=0.0, G=1.0	0.00001	371.311	26	C=0/0, G=15/1
chr6:32362416	T/G	T	G	SNV	BTNL2,HCG 23	HCG23:downstream:NR 044996.1, BTNL2:intronic:NM 001	1	51.85		intronic	NR 04499	c.1360+10	p.?	-1.1						rs2076534		YES			Butyrophili	integral cc	Adenocarcinoma;Arthritis;Arthritis, Rheumat		T=13, G=14	T=0.4815, G=0.5185	0.00001	132.666	27	T=10/3, G=13/
chr6:32362447	C/T	C	T	SNV	BTNL2,HCG 23	HCG23:downstream:NR 044996.1, BTNL2:intronic:NM 001	1	51.85		intronic	NR 04499	c.1360+74	p.?	-0.27						rs28732203		YES			Butyrophili	integral cc	Adenocarcinoma;Arthritis;Arthritis, Rheumat		C=13, T=14	C=0.4815, T=0.5185	0.00001	132.664	27	C=10/3, T=13/
chr17:9395231	A/A	G	A	SNV	STX8	STX8:exonic:NM 004853.2	1	100	6	synonymous	NM 00485	c.456C>T	p.(=)	-0.36					SNARE domai	rs9893664	0.1556	YES	8.332E-1 (pos.9395231)		Syntaxin 8	SNAP receptor activity,SNARE complex,chloride channel			G=0, A=73	G=0.0, A=1.0	0.00001	1148.63	73	G=0/0, A=35/3
chr17:53076799	A/A	G	A	SNV	STXBP4	STXBP4:exonic:NM 178509.5	1	100	5	missense	NM 17850	c.274G>A	p.Gly92Arg	2	1	125	0	0.02(COSM59	PDZ domain (	rs1156287	0.2833	YES	8.976E-1 (pos.53076799)	basal_cell_car	Syntaxin-bi	cellular re	Breast Diseases;Breast Neoplasms;Neoplasr		G=0, A=63	G=0.0, A=1.0	0.00001	958.913	63	G=0/0, A=20/4
chr7:75659815	C/C	T	C	SNV	STYXL1	STYXL1:exonic:NM 016086.2	1	100	2	synonymous	NM 01608	c.27A>G	p.(=)	-0.45					Dual specifici	rs1044483	0.302	YES	4.009E-1 (pos.75659815)		Serine/thre	intracellular,intracellular signal transduction,protein de			T=0, C=31	T=0.0, C=1.0	0.00001	462.595	31	T=0/0, C=11/2
chr19:19387356	C/T	C	T	SNV	SUGP1	SUGP1:utr 3:NM 172231.3	1	50	14	utr 3	NM 17223	c.2039G>A	p.?	-0.27					G-patch doma	rs2010506		YES			SURP and G	RNA binding,RNA processing,mRNA splicing, via spliceos			C=11, T=11	C=0.5, T=0.5	0.00001	94.5872	22	C=4/7, T=6/5
chr6:32713188	T/T	C	T	SNV	HLA-DQA2	HLA-DQA2:intronic:NM 020056.4	1	100		intronic	NM 02005	c.331+4C>	p.?	-2.81					Class II histo	rs2213565	0.2302	YES	9.047E-1 (pos.32713188)		Major histo	ER to Golgi	Arthritis;Arthritis, F	Insulin Pork	C=0, T=41	C=0.0, T=1.0	0.00001	664.696	41	C=0/0, T=26/1
chr6:32713888	C/C	T	C	SNV	HLA-DQA2	HLA-DQA2:intronic:NM 020056.4	1	100		intronic	NM 02005	c.613+39T	p.?	-2.61					Class II histo	rs17213337,rs78117	0.4941	YES	1.174E-4 (pos.32713886),8.213E-1;0.0 (p		Major histo	ER to Golgi	Arthritis;Arthritis, F	Insulin Pork	T=0, C=86	T=0.0, C=1.0	0.00001	1409.29	86	T=0/0, C=41/4
chr16:28617485	T/T	C	T	SNV	SULT1A1	SULT1A1:exonic:NM 177530.2	1	100	7	missense	NM 17753	c.667G>A	p.Val223Met	2.12	0.14	21	0.261		Nuclear pore	rs1801030	0.0021	YES	1.0 (pos.28617485)		Sulfotransfe	3'-phosph	Cardiovascular Dise	Acetaminophen,Adenosi	C=0, T=24	C=0.0, T=1.0	0.00001	334.788	24	C=0/0, T=15/9
chr6:32724258	T/T	C	T	SNV	HLA-DQB2	HLA-DQB2:intronic:NM 001198858.1	1	100		intronic	NM 00119	c.671-15G	p.?	-6.97					CD80-like C2-	rs3213490		YES	8.206E-1;0.0 (pos.32724258)		Major histo	ER to Golgi	Autoimmune Diseases;Connective Tissue Dis		C=0, T=33	C=0.0, T=1.0	0.00001	504.596	33	C=0/0, T=20/1
chr6:32724312	G/G	C	G	SNV	HLA-DQB2	HLA-DQB2:intronic:NM 001198858.1	1	100		intronic	NM 00119	c.671-69G	p.?	-0.6					CD80-like C2-	rs3213488		YES			Major histo	ER to Golgi	Autoimmune Diseases;Connective Tissue Dis		C=0, G=34	C=0.0, G=1.0	0.00001	523.937	34	C=0/0, G=14/2
chr6:32724314	GT/GT	G	GT	INDEL	HLA-DQB2	HLA-DQB2:intronic:NM 001198858.1	1	100		intronic	NM 00119	c.671-71C	p.?	-1.15					CD80-like C2-set immunoglobulin domain,Class II histocompatibility antigen, beta domain,Immunog						Major histo	ER to Golgi	Autoimmune Diseases;Connective Tissue Dis		G=0, GT=34	G=0.0, GT=1.0	0.00001	206.68	34	G=0/0, GT=14/
chr6:32724324	C/C	T	C	SNV	HLA-DQB2	HLA-DQB2:intronic:NM 001198858.1	1	100		intronic	NM 00119	c.671-81A	p.?	-1.75					CD80-like C2-	rs3213486		YES			Major histo	ER to Golgi	Autoimmune Diseases;Connective Tissue Dis		T=0, C=34	T=0.0, C=1.0	0.00001	523.28	34	T=0/0, C=14/2
chr2:108863767	C/C	A	C	SNV	SULT1C3	SULT1C3:exonic:NM 001008743.2	1	100	2	synonymous	NM 00100	c.117A>C	p.(=)	-0.81					Sulfotransfer	rs9308806	0.3006	YES	9.09E-1 (pos.108863767)		Sulfotransfe	alcohol sulfotransferase activity,aryl sulfotransferase ac			A=0, C=24	A=0.0, C=1.0	0.00001	334.785	24	A=0/0, C=7/17
chr6:32725193	G/G	A	G	SNV	HLA-DQB2	HLA-DQB2:intronic:NM 001198858.1	1	100		intronic	NM 00119	c.647-112	p.?	0.3					CD80-like C2-	rs2301271		YES			Major histo	ER to Golgi	Autoimmune Diseases;Connective Tissue Dis		A=0, G=52	A=0.0, G=1.0	0.00001	767.015	52	A=0/0, G=22/3
chr19:48389425	G/G	A	G	SNV	SULT2A1	SULT2A1:exonic:NM 003167.3	1	100	1	synonymous	NM 00316	c.90T>C	p.(=)	0.22					Sulfotransfer	rs11083907	7.00E-04		1.0 (pos.48389425)		Sulfotransfe	3'-phosph	Digestive System D	Abiraterone,Acetaminoc	A=0, G=20	A=0.0, G=1.0	0.00001	263.782	20	A=0/0, G=9/11
chr7:56136194	T/T	C	T	SNV	SUMF2	SUMF2:exonic:NM 001130069.2	1	100	2	synonymous	NM 00113	c.144C>T	p.(=)	1.63				0.56(COSM37	Sulfatase-mo	rs7808729	0.2235	YES	5.402E-2 (pos.56136194)	adenocarcinor	Sulfatase-m	endoplasmic reticulum,endoplasmic reticulum lumen,m			C=0, T=20	C=0.0, T=1.0	0.00001	263.786	20	C=0/0, T=4/16
chr6:32798711	T/C	T	C	SNV	TAP2	TAP2:intronic:NM 018833.2	1	45.45		intronic	NM 01883	c.1273-12	p.?	-0.74					ABC transpor	rs117821		YES			Sec14-like 3	ATP bindin	Congenital, Hereditary, and Neonatal Diseas		T=24, C=20	T=0.5455, C=0.4545	0.00001	213.284	44	T=5/19, C=5/1
chr7:56136260	A/A	C	A	SNV	SUMF2	SUMF2:exonic:NM 001130069.2	1	100	2	missense	NM 00113	c.210C>A	p.Asp70Glu	0.63	1	45	0		Sulfatase-mo	rs4245575	0.0016		1.0;0.0 (pos.56136260)		Sulfatase-m	endoplasmic reticulum,endoplasmic reticulum lumen,m			C=0, A=22	C=0.0, A=1.0	0.00001	298.892	22	C=0/0, A=4/18
chr6:149721690	A/A	G	A	SNV	SUMO4,TAB 2	SUMO4:exonic:NM 001002255.1, TAB2:intronic:NM 01509	1	100	1	missense	NM 01509	c.1939+13	p.?, p.Val55Met	1.18		21		0.11(COSM63	CUE domain,I	rs237025	0.4853	YES	7.181E-1 (pos.14972169	acute_myeloi	Small ubiqu	Fc-epsilon	Autoimmune Diseases;Diabet	risk factor	G=0, A=63	G=0.0, A=1.0	0.00001	958.861	63	G=0/0, A=30/3
chr6:32916699	T/T	C	T	SNV	HLA-DMA	HLA-DMA:intronic:NM 006120.3	1	100		intronic	NM 00612	c.782-54G	p.?	-0.55					Class II histo	rs129654,rs7714800	0.2263	YES			Major histocompatibili		Dermatitis;Dermatitis, Contact;Dermatitis, (		C=0, T=197	C=0.0, T=1.0	0.00001	3329.34	197	C=0/0, T=96/1
chr6:32945530	C/C	G	C	SNV	BRD2	BRD2:intronic:NM 001291986.1	1	100		intronic	NM 00129	c.970-4G>	p.?	-0.85					Bromodomai	rs3097644	0.0829	YES	9.949E-1;0.0 (pos.32945530)		Bromodom	chromatin	Glioma;Medulloblastoma;Neoplasms;Neopl		G=0, C=22	G=0.0, C=1.0	0.00001	298.891	22	G=0/0, C=8/14
chr6:32975381	A/G	A	G	SNV	HLA-DOA	HLA-DOA:intronic:NM 002119.3	1	52.38		intronic	NM 00211	c.332-12T	p.?	-1.55					Class II histo	rs453779	0.4438	YES	7.045E-1 (pos.32975381)			MHC class	Digestive System Diseases;Hepatitis;Hepatit		A=10, G=11	A=0.4762, G=0.5238	0.00001	95.3003	21	A=4/6, G=2/9
chr6:33132338	A/A	G	A	SNV	COL11A2	COL11A2:intronic:NM 080680.2	1	100		intronic	NM 08068	c.4864-88	p.?	-0.04					Collagen tripl	rs2744514		YES			Collagen XI,	cartilage c	Abnormalities, Multiple;Arthritis;Bone Disea		G=0, A=20	G=0.0, A=1.0	0.00001	263.786	20	G=0/0, A=6/14
chr6:33135391	TG/TG	CA	TG	MNV	COL11A2	COL11A2:intronic:NM 080680.2	2	100		intronic	NM 08068	c.4015-81	p.?	0.14, -0.01					Collagen tripl	rs2855449,rs2855450,rs344382		YES			Collagen XI,	cartilage c	Abnormalities, Multiple;Arthritis;Bone Disea		CA=0, TG=3	CA=0.0, TG=1.0	0.00001	443.911	30	CA=0/0, TG=11
chr6:33136575	C/T	C	T	SNV	COL11A2	COL11A2:intronic:NM 080680.2	1	95.45		intronic	NM 08068	c.3853-39	p.?	-0.26					Collagen tripl	rs2855448	0.3388	YES	9.697E-1 (pos.33136575)		Collagen XI,	cartilage c	Abnormalities, Multiple;Arthritis;Bone Disea		C=1, T=21	C=0.0455, T=0.9545	0.00001	253.452	22	C=1/0, T=10/1
chr6:33138746	C/C	CA	C	INDEL	COL11A2	COL11A2:intronic:NM 080680.2	1	100		intronic	NM 08068	c.3367-52	p.?	-0.42, -0.57					Collagen tripl	rs373222267,rs7254	0.3188	YES	9.126E-1 (pos.33138746)		Collagen XI,	cartilage c	Abnormalities, Multiple;Arthritis;Bone Disea		CA=0, C=85	CA=0.0, C=1.0	0.00001	1388.18	85	CA=0/0, C=29/
chr14:21831052	C/C	T	C	SNV	SUPT16H	SUPT16H:exonic:NM 007192.3	1	100	14	synonymous	NM 00719	c.1566A>G	p.(=)	0.28					FACT comple	rs10138699	0.0922	YES	8.792E-1;0.0 (pos.21831052)		Suppressor	DNA repair,DNA replication,DNA replication-independer			T=0, C=24	T=0.0, C=1.0	0.00001	334.775	24	T=0/0, C=15/9
chr6:33141920	C/C	G	C	SNV	COL11A2	COL11A2:intronic:NM 080680.2	1	100		intronic	NM 08068	c.2484+22	p.?	-0.01					Collagen tripl	rs2744512	0.2819	YES	9.693E-1 (pos.33141920)		Collagen XI,	cartilage c	Abnormalities, Multiple;Arthri	Benign	G=0, C=37	G=0.0, C=1.0	0.00001	583.707	37	G=0/0, C=11/2
chr6:33152835	A/T	A	T	SNV	COL11A2	COL11A2:intronic:NM 080680.2	1	50		intronic	NM 08068	c.877-4T>	p.?	0.64					Collagen tripl	rs1799907	0.2817	YES	2.077E-1 (pos.33152835)		Collagen XI,	cartilage c	Abnormalities, Multiple;Arthri	Benign	A=14, T=14	A=0.5, T=0.5	0.00001	134.169	28	A=7/7, T=7/7
chr9:113169126	G/A	G	A	SNV	SVEP1	SVEP1:exonic:NM 153366.3	1	47.37	38	synonymous	NM 15336	c.8754C>T	p.(=)	-1.45				0.03(COSM37	Calcium-bind	rs2281937	0.4267	YES	1.005E-1;0.0 (pos.11316	acute_myeloi	Sushi, von W	calcium ion binding,cell adhesion,chromatin binding,cyt			G=20, A=18	G=0.5263, A=0.4737	0.00001	184.537	38	G=14/6, A=11/
chr10:29783885	G/G	A	G	SNV	SVIL	SVIL:exonic:NM 021738.2	1	100	20	synonymous	NM 02173	c.3799T>C	p.(=)	0.38				0.35(COSM37	Gelsolin repe	rs1056782	0.3981	YES	3.402E-1 (pos.29783885)	acute_myeloi	Supervillin	actin cyto	Leukemia;Leukemia, Myeloid;Leukemia, Mye		A=0, G=66	A=0.0, G=1.0	0.00001	1015.4	66	A=0/0, G=39/2
chr6:33261871	A/G	A	G	SNV	RGL2	RGL2:intronic:NM 004761.4	1	50		intronic	NM 00476	c.1354-28	p.?	0.04					Ras associati	rs2239842	0.3384	YES	2.415E-1 (pos.33261871)			Ras guanyl-nucleotide exchange factor activity,Ras prote			A=15, G=15	A=0.5, G=0.5	0.00001	145.012	30	A=9/6, G=9/6
chr7:138363767	A/G	A	G	SNV	SVOPL	SVOPL:exonic:NM 001139456.1	1	50	1	synonymous	NM 00113	c.24T>C	p.(=)	-6.35				0(COSM4419	Major Facilit	rs10250576	0.4051	YES	6.19E-1 (pos.138363767)	acute_myeloi	SV2-related	integral component of membrane,substrate-specific tra			A=42, G=42	A=0.5, G=0.5	0.00001	450.209	84	A=24/18, G=1
chr6:33382943	T/T	G	T	SNV	PHF1	PHF1:intronic:NM 024165.2	1	100		intronic	NM 02416	c.1239+22	p.?	-1.95					PHD-finger,P	rs3106196	0.0872	YES	9.994E-1 (pos.33382943)		PHD finger	DNA binding,transcription factor activity,ESC/E(Z) comple			G=0, T=23	G=0.0, T=1.0	0.00001	316.747	23	G=0/0, T=16/7
chr6:33403422	C/T	C	T	SNV	SYNGAP1	SYNGAP1:intronic:NM 006772.2	1	97.5		intronic	NM 00677	c.762+32C	p.?	0.03					C2 domain,D	rs453590	0.4	YES	6.21E-1 (pos.33403422)		Synaptic Ra	GTPase ac	Autistic Disorder;Brain Diseases;Central Ner		C=1, T=39	C=0.025, T=0.975	0.00001	611.634	40	C=1/0, T=23/1
chr1:85624549	T/T	C	T	SNV	SYDE2	SYDE2:exonic:NM 032184.1	1	100	7	missense	NM 03218	c.3469G>A	p.Val1157Ile	2.15	1	29	0		RhoGAP dom	rs709786,rs7724810	5.00E-04		0.0 (pos.85624548),1.0 (pos.85624549)			GTPase activator activity,activation of GTPase activity,c			C=0, T=53	C=0.0, T=1.0	0.00001	782.013	53	C=0/0, T=17/3
chr6:335175	T/T	A	T	SNV	DUSP22	DUSP22:intronic:NM 020185.4	1	100		intronic	NM 02018	c.188+12A	p.?	0.94					Dual specifici	rs2671431,rs756790	1.00E-04		0.0 (pos.335174),1.0 (pos.335175)		Dual-specif	apoptotic process,cell proliferation,cytoplasm,inactivat			A=0, T=51	A=0.0, T=1.0	0.00001	754.583	51	A=0/0, T=13/3
chr6:33644768	C/C	T	C	SNV	ITPR3	ITPR3:intronic:NM 002224.3	1	100		intronic	NM 00222	c.3448-24	p.?	-0.28					Inositol 1,4,5	rs2068236	0.0123	YES	9.977E-1 (pos.33644768)		Inositol 1,4	G-protein	Autoimmune Disea	Caffeine	T=0, C=23	T=0.0, C=1.0	0.00001	316.747	23	T=0/0, C=11/1
chr6:33644773	A/A	T	A	SNV	ITPR3	ITPR3:intronic:NM 002224.3	1	100		intronic	NM 00222	c.3448-19	p.?	-2.99					Inositol 1,4,5	rs4713652	0.0123	YES	9.977E-1 (pos.33644773)		Inositol 1,4	G-protein	Autoimmune Disea	Caffeine	T=0, A=23	T=0.0, A=1.0	0.00001	316.747	23	T=0/0, A=11/1
chr6:33647651	G/G	A	G	SNV	ITPR3	ITPR3:intronic:NM 002224.3	1	100		intronic	NM 00222	c.3973-8A	p.?	-3.72				0.02(COSM54	Inositol 1,4,5	rs4713654	0.3302	YES	8.922E-1;1.168E-4 (pos.3	acute_myeloi	Inositol 1,4	G-protein	Autoimmune Disea	Caffeine	A=0, G=28	A=0.0, G=1.0	0.00001	402.772	28	A=0/0, G=22/6
chr6:33654378	G/G	A	G	SNV	ITPR3	ITPR3:intronic:NM 002224.3	1	100		intronic	NM 00222	c.5979+82	p.?	1.27					Inositol 1,4,5	rs4640882		YES			Inositol 1,4	G-protein	Autoimmune Disea	Caffeine	A=0, G=102	A=0.0, G=1.0	0.00001	1594.2	102	A=0/0, G=53/4
chr6:33656654	C/G	C	G	SNV	ITPR3	ITPR3:intronic:NM 002224.3	1	47.62		intronic	NM 00222	c.6694+96	p.?	-0.39					Inositol 1,4,5	rs11759396		YES			Inositol 1,4	G-protein	Autoimmune Disea	Caffeine	C=22, G=20	C=0.5238, G=0.4762	0.00001	212.82	42	C=9/13, G=10/
chr6:34664207	G/G	T	G	SNV	C6orf106,LOC101929243	LOC101929243:exonic nc:NR 134625.1, C6orf106:intronic:	1	100		intronic	NM 02429	c.158+16A	p.?	0.14					Ig-like domai	rs3800456	0.2415	YES	6.977E-1 (pos.34664207)		Chromosom	autophag	Cardiomyopathies;Cardiovascular Diseases;		T=0, G=28	T=0.0, G=1.0	0.00001	408.715	28	T=0/0, G=12/1
chr6:34725658	A/A	G	A	SNV	SNRPC	SNRPC:intronic:NM 003093.2	1	100		intronic	NM 00309	c.9-31G>A	p.?	0.3				0.18(COSM40	U1 zinc finger	rs140215092,rs2839	0.3458	YES	4.434E-1;0.0 (pos.34725	adenocarcinor	Small nucle	Cajal body,RNA binding,U1 snRNA binding,U1 snRNP,U1 s			G=0, A=40	G=0.0, A=1.0	0.00001	644.125	40	G=0/0, A=29/1
chr1:85624864	A/A	G	A	SNV	SYDE2	SYDE2:exonic:NM 032184.1	1	100	7	synonymous	NM 03218	c.3154C>T	p.(=)	1.22					RhoGAP dom	rs817412	2.00E-04		1.0 (pos.85624864)			GTPase activator activity,activation of GTPase activity,c			G=0, A=115	G=0.0, A=1.0	0.00001	1834.87	115	G=0/0, A=49/6
chr6:348080	G/G	A	G	SNV	DUSP22	DUSP22:intronic:NM 020185.4	1	100		intronic	NM 02018	c.264-23A	p.?	-3.05					Dual specifici	rs2797333	1.00E-04		1.0;0.0 (pos.348080)		Dual-specif	apoptotic process,cell proliferation,cytoplasm,inactivat			A=0, G=69	A=0.0, G=1.0	0.00001	1072.42	69	A=0/0, G=47/2
chr1:85648620	T/T	C	T	SNV	SYDE2	SYDE2:exonic:NM 032184.1	1	100	3	missense	NM 03218	c.1705G>A	p.Val569Ile	0.01	1	29	0		RhoGAP dom	rs817443	4.00E-04		1.0 (pos.85648620)			GTPase activator activity,activation of GTPase activity,c			C=0, T=52	C=0.0, T=1.0	0.00001	766.994	52	C=0/0, T=27/2
chr3:12232074	G/G	A	G	SNV	SYN2	SYN2:exonic:NM 133625.4	1	100	14	synonymous	NM 13362	c.1695A>G	p.(=)	-0.72					Synapsin N-te	rs310764	0.0984	YES	9.999E-1 (pos.12232074)		Synapsin II		Brain Diseases;Central Nervous System Dise		A=0, G=47	A=0.0, G=1.0	0.00001	789.328	47	A=0/0, G=25/2
chr6:152647681	T/T	A	T	SNV	SYNE1	SYNE1:exonic:NM 182961.3	1	100	79	missense	NM 18296	c.15043T>	p.Leu5015Met	2.94	0.51	15	0.002	0.73(COSM50	Calponin hon	rs2306916	0.2506	YES	8.384E-1 (pos.15264768	adenocarcinor	Spectrin re	Golgi appa	Ataxia;Bipolar Disorder;Bipola	Benign/Likely	A=0, T=25	A=0.0, T=1.0	0.00001	352.999	25	A=0/0, T=22/3
chr6:34962019	C/C	G	C	SNV	ANKS1A	ANKS1A:intronic:NM 015245.2	1	100		intronic	NM 01524	c.1303-60	p.?	-0.42					Ankyrin repea	rs11755713		YES				cytosol,ep	Arterial Occlusive Diseases;Arteriosclerosis;		G=0, C=24	G=0.0, C=1.0	0.00001	334.792	24	G=0/0, C=9/15
chr6:34962060	A/A	G	A	SNV	ANKS1A	ANKS1A:intronic:NM 015245.2	1	100		intronic	NM 01524	c.1303-19	p.?	0.17					Ankyrin repea	rs3823438	0.4331	YES	2.91E-1 (pos.34962060)			cytosol,ep	Arterial Occlusive Diseases;Arteriosclerosis;		G=0, A=24	G=0.0, A=1.0	0.00001	334.792	24	G=0/0, A=9/15
chr6:152652034	T/T	A	T	SNV	SYNE1	SYNE1:exonic:NM 182961.3	1	100	78	missense	NM 18296	c.13786T>	p.Ser4596Thr	-2.51	0.4	58	0	0.02(COSM14	Calponin hon	rs6911096	0.251	YES	8.461E-1 (pos.15265203	adenocarcinor	Spectrin re	Golgi appa	Ataxia;Bipolar Disorder;Bipola	Benign/Likely	A=0, T=31	A=0.0, T=1.0	0.00001	465.818	31	A=0/0, T=20/1
chr6:152658142	T/C	T	C	SNV	SYNE1	SYNE1:exonic:NM 182961.3	1	56	76	missense	NM 18296	c.12362A>	p.Lys4121Arg	1.43	1	26	0	0.18(COSM50	Calponin hon	rs386707192,rs8788	0.2806	YES	8.544E-1 (pos.15265814	adenocarcinor	Spectrin re	Golgi appa	Ataxia;Bipolar Disorder;Bipola	Benign/Likely	T=11, C=14	T=0.44, C=0.56	0.00001	126.827	25	T=5/6, C=2/12
chr14:64497930	A/A	G	A	SNV	SYNE2	SYNE2:exonic:NM 182914.2	1	100	45	missense	NM 18291	c.7076G>A	p.Ser2359Asn	0.98	0.27	46	0.004		CAMSAP CH d	rs4027404	0.1218	YES	7.824E-1 (pos.64497930)		Spectrin re	LINC comp	Adenocarcinoma;Arrhythmias,	Benign	G=0, A=45	G=0.0, A=1.0	0.00001	751.843	45	G=0/0, A=22/2
chr6:35088855	G/G	C	G	SNV	TCP11	TCP11:intronic:NM 001093728.2	1	100		intronic	NM 00109	c.618-33G	p.?	-3.17					T-complex pr	rs13203612	0.1552	YES	9.817E-2 (pos.35088855)		T-complex	acrosomal vesicle,cell differentiation,integral compone			C=0, G=61	C=0.0, G=1.0	0.00001	921.853	61	C=0/0, G=29/3
chr6:35258539	A/A	C	A	SNV	ZNF76	ZNF76:intronic:NM 003427.4	1	100		intronic	NM 00342	c.625+46C	p.?	0.01					C2H2-type zir	rs763157	0.1203	YES	9.947E-1 (pos.35258539)		Zinc finger	metal ion binding,nucleic acid binding,nucleus,positive			C=0, A=25	C=0.0, A=1.0	0.00001	353.017	25	C=0/0, A=3/22
chr14:64498037	A/A	G	A	SNV	SYNE2	SYNE2:exonic:NM 182914.2	1	100	45	missense	NM 18291	c.7183G>A	p.Ala2395Thr	1.82	0.01	58	0.009		CAMSAP CH d	rs4027405	0.0773	YES	8.479E-1 (pos.64498037)		Spectrin re	LINC comp	Adenocarcinoma;Arrhythmias,	Benign	G=0, A=42	G=0.0, A=1.0	0.00001	685.22	42	G=0/0, A=18/2
chr6:35280697	T/T	C	T	SNV	DEF6	DEF6:intronic:NM 022047.3	1	100		intronic	NM 02204	c.807+148	p.?	0.22					PH domain	rs6938946		YES			Def6, mous	cytoskeleton,cytosol,filopodium,membrane,nucleoplas			C=0, T=20	C=0.0, T=1.0	0.00001	263.785	20	C=0/0, T=9/11
chr14:64519455	G/G	A	G	SNV	SYNE2	SYNE2:exonic:NM 182914.2	1	100	48	missense	NM 18291	c.8824A>G	p.Ile2942Val	-0.84	0	29	0.002		CAMSAP CH d	rs3829767	0.1363	YES	7.874E-1;0.0 (pos.64519455)		Spectrin re	LINC comp	Adenocarcinoma;Arrhythmias,	Benign	A=0, G=59	A=0.0, G=1.0	0.00001	885.357	59	A=0/0, G=21/3
chr6:35704831	C/G	C	G	SNV	ARMC12,LOC285847	LOC285847:upstream:NR 027117.2, ARMC12:utr 5:NM 14	1	53.13		intronic	NR 02711	c.-55C>G	p.?	-0.33						rs56354119		YES				nucleus			C=15, G=17	C=0.4688, G=0.5313	0.00001	173.56	32	C=10/5, G=8/9
chr14:64522674	C/C	G	C	SNV	SYNE2	SYNE2:exonic:NM 182914.2	1	100	49	missense	NM 18291	c.9757G>C	p.Asp3253His	2.76	0.01	81	0.343	0.92(COSM54	CAMSAP CH d	rs8010911	0.1365	YES	7.868E-1 (pos.64522674)	acute_myeloi	Spectrin re	LINC comp	Adenocarcinoma;Arrhythmias,	Benign	G=0, C=45	G=0.0, C=1.0	0.00001	747.391	45	G=0/0, C=16/2
chr6:35803283	T/T	C	T	SNV	SRPK1	SRPK1:intronic:NM 003137.4	1	100		intronic	NM 00313	c.1784-18	p.?	-0.03					Protein kinas	rs2859420	0.3535	YES	2.181E-1 (pos.35803283)		Protein kina	ATP binding,RNA binding,RNA splicing,chromosome segre			C=0, T=61	C=0.0, T=1.0	0.00001	921.851	61	C=0/0, T=20/4
chr6:158505088	G/G	A	G	SNV	SYNJ2	SYNJ2:exonic:NM 003898.3	1	100	22	synonymous	NM 00389	c.3090A>G	p.(=)	0.13				0(COSM1131	Domain of un	rs1744173	0.1401	YES	5.878E-1 (pos.15850508	adenocarcinor	Synaptojan	RNA bindir	Breast Diseases;Breast Neoplasms;Neoplasr		A=0, G=69	A=0.0, G=1.0	0.00001	1064.7	69	A=0/0, G=22/4
chr6:36292007	G/A	G	A	SNV	C6orf222	C6orf222:intronic:NM 001010903.4	1	50		intronic	NM 00101	c.1169-40	p.?	-1.86					C6orf222, un	rs749612412,rs7751	0.1219	YES	0.0;0.0 (pos.36292006),4.238E-1 (pos.36292007)						G=14, A=14	G=0.5, A=0.5	0.00001	131.944	28	G=5/9, A=4/10
chr6:36353472	C/T	C	T	SNV	ETV7	ETV7:intronic:NM 016135.3	1	47.37		intronic	NM 01613	c.7-26G>A	p.?	-0.1					Ets-domain,S	rs2234072	0.0673	YES	4.467E-3 (pos.36353472)		ETS variant	RNA polymerase II regulatory region sequence-specific D			C=20, T=18	C=0.5263, T=0.4737	0.00001	184.537	38	C=7/13, T=8/1
chr1:155837865	C/C	G	C	SNV	SYT11	SYT11:exonic:NM 152280.4	1	100	2	missense	NM 15228	c.144G>C	p.Gln48His	1.15	0.8	24	0		C2 domain	rs822522	3.00E-04	YES	1.0 (pos.155837865)			axon,beta-tubulin binding,calcium ion binding,calcium i			G=0, C=29	G=0.0, C=1.0	0.00001	427.589	29	G=0/0, C=14/1
chr6:37447075	A/A	G	A	SNV	CMTR1	CMTR1:intronic:NM 015050.2	1	100		intronic	NM 01505	c.2375+44	p.?	-0.62					FtsJ-like metl	rs2797790	0.3359	YES	4.425E-1 (pos.37447075)		CAP methyl	7-methylguanosine mRNA capping,cap1 mRNA methylati			G=0, A=51	G=0.0, A=1.0	0.00001	751.049	51	G=0/0, A=20/3
chr19:51128661	C/C	T	C	SNV	SYT3	SYT3:exonic:NM 001160329.1	1	100	8	synonymous	NM 00116	c.1563A>G	p.(=)	0.03				0.01(COSM41	C2 domain	rs16986142	0.153	YES	3.75E-1 (pos.51128661)	carcinoma,ne	Synaptotag	calcium ion binding,calcium ion regulated exocytosis,ca			T=0, C=43	T=0.0, C=1.0	0.00001	705.09	43	T=0/0, C=8/35
chr19:51133356	G/G	C	G	SNV	SYT3	SYT3:exonic:NM 001160329.1	1	100	5	synonymous	NM 00116	c.747G>C	p.(=)	0.45				0.72(COSM41	C2 domain	rs10407511	0.3334	YES	1.418E-1 (pos.51133356)	neoplasm	Synaptotag	calcium ion binding,calcium ion regulated exocytosis,ca			C=0, G=56	C=0.0, G=1.0	0.00001	836.752	56	C=0/0, G=46/1
chr11:1857173	C/C	T	C	SNV	SYT8	SYT8:exonic:NM 138567.4	1	100	4	missense	NM 13856	c.358T>C	p.Cys120Arg	-1.04	0.42	180	0		C2 domain	rs386749726,rs5642	2.00E-04		1.0 (pos.1857173)		Synaptotag	acrosomal vesicle,acrosome reaction,calcium ion bindir			T=0, C=24	T=0.0, C=1.0	0.00001	334.789	24	T=0/0, C=3/21
chr6:39046655	C/T	C	T	SNV	GLP1R	GLP1R:intronic:NM 002062.4	1	46.43		intronic	NM 00206	c.885-78C	p.?	-1.56					7 transmemb	rs742761		YES			Glucagon-li	G-protein	Cardiovascular Dise	Albiglutide,Dulaglutide,E	C=15, T=13	C=0.5357, T=0.4643	0.00001	117.927	28	C=5/10, T=4/9
chr17:39845937	T/A	T	A	SNV	DAAM2	DAAM2:intronic:NM 001201427.1	1	58.33		intronic	NM 0012	c.102+23	p.?	-3.65					Diaphanous I	rs113729828	0.0113		2.275E-2 (pos.39845937)		Dishevellec	Rho GTPase binding,actin binding,actin cytoskeleton org			T=10, A=14	T=0.4167, A=0.5833	0.00001	135.339	24	T=2/8, A=2/12
chr6:39846137	A/G	A	G	SNV	DAAM2	DAAM2:intronic:NM 001201427.1	1	50		intronic	NM 0012	c.1361-43	p.?	-1.41					Diaphanous I	rs2504086	0.4281	YES	7.979E-1 (pos.39846137)		Dishevellec	Rho GTPase binding,actin binding,actin cytoskeleton org			A=11, G=11	A=0.5, G=0.5	0.00001	94.6067	22	A=7/4, G=3/8

chr6:4057233	T/T	C	T	SNV	PRPF4B	PRPF4B:intronic:NM_003913.4	1	100		intronic	NM_00391	c.2582-37	p.?	-0.21					Protein kinas	rs778353194,rs8534	0.3233	YES	0.0 (pos.4057229),7.68E-1 (pos.4057233		Pre-mRNA p	ATP binding,RNA binding,RNA splicing,catalytic step 2 spl	C=0, T=71	C=0.0, T=1.0	0.00001	1111.48	71	C=0/0, T=48/2
chr6:4057429	G/G	A	G	SNV	PRPF4B	PRPF4B:intronic:NM_003913.4	1	100		intronic	NM_00391	c.2733+8A	p.?	0.55					Protein kinas	rs853408	0.324	YES	7.685E-1 (pos.4057429)		Pre-mRNA p	ATP binding,RNA binding,RNA splicing,catalytic step 2 spl	A=0, G=31	A=0.0, G=1.0	0.00001	465.819	31	A=0/0, G=11/2
chr11:1857762	C/T	C	T	SNV	SYT8	SYT8:exonic:NM_138567.4	1	47.62	6	synonymous	NM_13856	c.666C>T	p.(=)	-2.53				0.01(COSM37	C2 domain	rs3741230	0.1164	YES	7.397E-2 (pos.1857762)	adenocarcinoi	Synaptotag	acrosomal vesicle,acrosome reaction,calcium ion bindi	C=11, T=10	C=0.5238, T=0.4762	0.00001	84.386	21	C=3/8, T=4/6
chrX:37932886	G/G	A	G	SNV	SYTL5	SYTL5:exonic:NM_001163334.1	1	100	4	synonymous	NM_00116	c.489A>G	p.(=)	-0.2				0.02(COSM63	C2 domain,F\	rs4827330	0.2469	YES	1.281E-1 (pos.37932886)	carcinoma		Rab GTPase binding,calcium ion binding,calcium ion-reg	A=0, G=37	A=0.0, G=1.0	0.00001	582.615	37	A=0/0, G=20/1
chr6:132859609	T/A	T	A	SNV	TAAR9	TAAR9:exonic:NM_175057.3	1	53.13	1	stoploss	NM_17505	c.181T>A	p.Ter61Lys	3.97				0.05(COSM44	7 transmemb	rs2842899	0.2701	YES	8.949E-1 (pos.13285960	basal_cell_carcinoma,hep		G-protein coupled receptor activity,G-protein coupled re	T=15, A=17	T=0.4688, A=0.5313	0.00001	171.89	32	T=10/5, A=5/1
chr10:123845322	C/C	T	C	SNV	TACC2	TACC2:exonic:NM_206862.3	1	100	4	missense	NM_20686	c.3307T>C	p.Trp1103Arg	0.43	0.76	101	0		Transforming	rs7073433	5.00E-04		9.999E-1 (pos.123845322)		Transformir	cell prolife Eye Diseases;Myopia;Refractive Errors	T=0, C=36	T=0.0, C=1.0	0.00001	563.74	36	T=0/0, C=14/2
chr17:35771468	T/T	C	T	SNV	TADA2A	TADA2A:exonic:NM_001166105.2	1	100	2	missense	NM_00116	c.16C>T	p.Pro6Ser	1.97	1	74	0		Myb-like DNA	rs112855394,rs7211	0.1417	YES	7.384E-1 (pos.35771468)		Transcriptic	Ada2/Gcn5/Ada3 transcription activator complex,DNA b	C=0, T=74	C=0.0, T=1.0	0.00001	1170.12	74	C=0/0, T=44/3
chr6:41309698	C/C	G	C	SNV	NCR2	NCR2:intronic:NM_004828.3	1	100		intronic	NM_00482	c.530+31G	p.?	-1.63					Immunoglobi	rs2236370	0.3605	YES	5.453E-1;0.0 (pos.41309698)		Natural cyt	cellular defense response,innate immune response,inte	G=0, C=54	G=0.0, C=1.0	0.00001	781.454	54	G=0/0, C=21/3
chr6:34855748	G/C	G	C	SNV	TAF11	TAF11:utr_5:NM_005643.3	1	95.45	1	utr_5	NM_00564	c.-14C>G	p.?	-1.86					Histone-like t	rs1051115	0.1187	YES	1.15E-1 (pos.34855748)		TAF11 RNA p	Golgi apparatus,RNA polymerase II transcriptional prein	G=1, C=21	G=0.0455, C=0.9545	0.00001	271.419	22	G=1/0, C=10/1
chr2:10045014	T/T	C	T	SNV	TAF1B	TAF1B:exonic:NM_005680.2	1	100	9	synonymous	NM_00568	c.834C>T	p.(=)	0.31					Zinc-finger of	rs400917	0.0672	YES	8.281E-1;2.312E-4 (pos.10045014)			DNA binding transcription factor activity,RNA polymeras	C=0, T=20	C=0.0, T=1.0	0.00001	263.786	20	C=0/0, T=8/12
chr6:41905174	A/A	T	A	SNV	CCND3	CCND3:intronic:NM_001760.4	1	100		intronic	NM_00176	c.415-42A	p.?	0.06					Cyclin, C-tern	rs2479717	0.2715	YES	9.084E-1 (pos.41905174)		Cyclin D3	T cell proliferation,cell cycle,cell division,cyclin-depende	T=0, A=41	T=0.0, A=1.0	0.00001	673.361	41	T=0/0, A=16/2
chr6:42036395	C/C	A	C	SNV	TAF8	TAF8:intronic:NM_138572.2	1	100		intronic	NM_13857	c.780+50A	p.?	-0.76					Bromodomai	rs2492926	0.2568	YES	8.277E-1 (pos.42036395)			cell differentiation,inner cell mass cell proliferation,mai	A=0, C=29	A=0.0, C=1.0	0.00001	427.603	29	A=0/0, C=5/24
chr6:42045026	A/A	G	A	SNV	TAF8	TAF8:intronic:NM_138572.2	1	100		intronic	NM_13857	c.920+49G	p.?	-0.51					Bromodomai	rs2493839	0.0978	YES	1.39E-2 (pos.42045026)			cell differentiation,inner cell mass cell proliferation,mai	G=0, A=25	G=0.0, A=1.0	0.00001	355.818	25	G=0/0, A=13/1
chr6:42045171	G/G	A	G	SNV	TAF8	TAF8:intronic:NM_138572.2	1	100		intronic	NM_13857	c.921-100	p.?	-0.68					Bromodomai	rs2492931		YES				cell differentiation,inner cell mass cell proliferation,mai	A=0, G=23	A=0.0, G=1.0	0.00001	316.224	23	A=0/0, G=13/1
chr2:10045024	A/A	G	A	SNV	TAF1B	TAF1B:exonic:NM_005680.2	1	100	9	missense	NM_00568	c.844G>A	p.Val282Ile	0.42	0.61	29	0		Zinc-finger of	rs396190	0.0021	YES	1.0 (pos.10045024)			DNA binding transcription factor activity,RNA polymeras	G=0, A=20	G=0.0, A=1.0	0.00001	263.786	20	G=0/0, A=8/12
chr2:9983686	T/T	G	T	SNV	TAF1B	TAF1B:exonic:NM_005680.2	1	100	1	missense	NM_00568	c.16G>T	p.Ala6Ser	0.2	0.08	99	0.021	0.57(COSM37	Zinc-finger of	rs2303914	0.4782	YES	6.83E-1 (pos.9983686)	adenocarcinoma,hepatoce		DNA binding transcription factor activity,RNA polymeras	G=0, T=53	G=0.0, T=1.0	0.00001	781.55	53	G=0/0, T=4/49
chr6:42182183	T/T	C	T	SNV	MRPS10	MRPS10:intronic:NM_018141.3	1	100		intronic	NM_01814	c.49-101G	p.?	-0.31					Ribosomal pr	rs1884317		YES			Mitochondr	biological_process,mitochondrial inner membrane,mito	C=0, T=28	C=0.0, T=1.0	0.00001	408.718	28	C=0/0, T=9/19
chr8:120790304	T/C	T	C	SNV	TAF2	TAF2:exonic:NM_003184.3	1	45.45	18	synonymous	NM_00318	c.2319A>G	p.(=)	-0.27						rs1509348	0.4354	YES	4.361E-1 (pos.120790304)		TAF2 RAN pc	G2/M tran Intellectual Disability;Mental Disorders:Nerv	T=12, C=10	T=0.5455, C=0.4545	0.00001	81.7625	22	T=6/6, C=3/7
chr6:42612312	T/T	C	T	SNV	UBR2	UBR2:intronic:NM_015255.2	1	100		intronic	NM_01525	c.2284+38	p.?	-0.32					ATP-depende	rs7767888	0.1009	YES	9.981E-1 (pos.42612312)		Ubiquitin-p	cellular response to leucine,chromatin,chromatin silenc	C=0, T=20	C=0.0, T=1.0	0.00001	263.78	20	C=0/0, T=11/9
chr10:8006798	G/G	A	G	SNV	TAF3	TAF3:exonic:NM_031923.3	1	100	3	missense	NM_03192	c.1325A>G	p.Asn442Ser	-0.16	0.15	46	0.003		Bromodomai	rs4747647	0.3913	YES	7.033E-1 (pos.8006798)		TAF3 RNA pc	maintenance of protein location in nucleus,metal ion bi	A=0, G=34	A=0.0, G=1.0	0.00001	524.186	34	A=0/0, G=20/1
chr1:229738170	G/G	T	G	SNV	TAF5L	TAF5L:exonic:NM_014409.3	1	100	4	synonymous	NM_01440	c.744A>C	p.(=)	-4.91				0.03(COSM41	WD domain,	rs3753886	0.4876	YES	7.638E-1;1.157E-4 (pos.2	neoplasm		DNA binding transcription factor activity,STAGA complex	T=0, G=70	T=0.0, G=1.0	0.00001	1092.09	70	T=0/0, G=25/4
chr1:229730452	G/A	G	A	SNV	TAF5L	TAF5L:exonic:NM_014409.3	1	45.83	5	synonymous	NM_01440	c.1362C>T	p.(=)	0.12				0.84(COSM41	WD domain,	rs2295625	0.4636	YES	3.293E-1 (pos.22973045	carcinoma,neoplasm		DNA binding transcription factor activity,STAGA complex	G=13, A=11	G=0.5417, A=0.4583	0.00001	93.4399	24	G=7/6, A=10/1
chrX:100547933	G/G	A	G	SNV	TAF7L	TAF7L:exonic:NM_024885.3	1	100	1	missense	NM_02488	c.101T>C	p.Leu34Pro	-1.29	1	98	0		TAFII55 prote	rs5951328	0.0114		1.0 (pos.100547933)		TAF7-like RN	RNA polymerase II transcriptional preinitiation complex	A=0, G=24	A=0.0, G=1.0	0.00001	334.777	24	A=0/0, G=16/8
chr6:43399837	G/G	A	G	SNV	ABCC10	ABCC10:intronic:NM_001198934.1	1	100		intronic	NM_00119	c.162-43A	p.?	1.02					ABC transpor	rs700008	0.114	YES	9.459E-1 (pos.43399837)		ATP-binding	ATP binding,ATP hydrolysis cou Cyclosporine,Cytarabine	A=0, G=20	A=0.0, G=1.0	0.00001	263.786	20	A=0/0, G=12/8
chr6:43745095	A/A	C	A	SNV	VEGFA	VEGFA:intronic:NM_001171623.1	1	100		intronic	NM_00117	c.119-111	p.?	0.39					PDGF/VEGF d	rs2146323		YES				VEGF-activ Acute Kidney Injury ABT-510,Aflibercept,Bev	C=0, A=37	C=0.0, A=1.0	0.00001	583.706	37	C=0/0, A=28/9
chr6:44268806	T/TTGTC	T	TTGTC	INDEL	AARS2	AARS2:intronic:NM_020745.3	4	45.83		intronic	NM_02074	c.2793+87	p.?	0.23					Threonyl and	rs10627820,rs34468603		YES			Alanyl-tRNA	ATP binding,alanine-tRNA ligas L-Alanine	T=13, TTGT	T=0.5417, TTGTC=0.4	0.00001	93.44	24	T=7/6, TTGTC=
chr6:44273546	A/C	A	C	SNV	AARS2	AARS2:intronic:NM_020745.3	1	47.37		intronic	NM_02074	c.1301-23	p.?	-0.3					Threonyl and	rs324137	0.4344	YES	4.111E-1 (pos.44273546)		Alanyl-tRNA	ATP binding,alanine-tRNA ligas L-Alanine	A=50, C=45	A=0.5263, C=0.4737	0.00001	488.469	95	A=20/30, C=22
chr6:45922849	T/T	C	T	SNV	CLIC5	CLIC5:intronic:NM_001114086.1	1	100		intronic	NM_00111	c.650+23G	p.?	0.35					Glutathione S	rs3734206	0.1048	YES	4.672E-1 (pos.45922849)		Chloride int	Golgi apparatus,actin cytoskeleton,auditory receptor ce	C=0, T=41	C=0.0, T=1.0	0.00001	664.557	41	C=0/0, T=25/1
chr6:46630068	G/A	G	A	SNV	SLC25A27	SLC25A27:intronic:NM_004277.4	1	50		intronic	NM_00427	c.384-45G	p.?	-0.44					Mitochondria	rs9381469	0.4866	YES	5.798E-1 (pos.46630068)		Solute carri	apical part of cell,cellular triglyceride homeostasis,inne	G=11, A=11	G=0.5, A=0.5	0.00001	94.61	22	G=3/8, A=10/1
chr1:47685455	C/C	T	C	SNV	TAL1	TAL1:exonic:NM_003189.5	1	100	6	synonymous	NM_00318	c.933A>G	p.(=)	1.38				0.13(COSM37	Helix-loop-he	rs927462		YES	1.0;0.0 (pos.47685455)	adenocarcinoi	T-cell acute	DNA bindi Hemic and Lymphatic Diseases:Immune Syst	T=0, C=67	T=0.0, C=1.0	0.00001	1034.42	67	T=0/0, C=32/3
chr3:11887991	G/G	C	G	SNV	TAMM41	TAMM41:exonic:NM_138807.3	1	100	1	synonymous	NM_13880	c.120G>C	p.(=)	2.01				0.02(COSM40	Mitochondria	rs408600	0.4595	YES	2.548E-1 (pos.11887991)	acute_myeloic	Translocatc	CDP-diacylglycerol biosynthetic process,cardiolipin bios	C=0, G=32	C=0.0, G=1.0	0.00001	475.946	32	C=0/0, G=12/2
chr2:160035207	T/C	T	C	SNV	TANC1	TANC1:exonic:NM_033394.2	1	57.14	14	synonymous	NM_03339	c.2043T>C	p.(=)	-1.25					Ankyrin repea	rs6748288	0.0024	YES	9.999E-1;0.0 (pos.160035207)		Tetratricop	axon term Cardiovascular Diseases:Death, Sudden, Car	T=30, C=40	T=0.4286, C=0.5714	0.00001	434.875	70	T=11/19, C=22
chr17:61315272	A/A	G	A	SNV	TANC2	TANC2:exonic:NM_025185.3	1	100	6	synonymous	NM_02518	c.645G>A	p.(=)	1.78				0.87(COSM33	Ankyrin repea	rs2460111	0.4879	YES	3.145E-1 (pos.61315272)	plasma_cell_n	Tetratricop	in utero embryonic development	G=0, A=47	G=0.0, A=1.0	0.00001	792.335	47	G=0/0, A=30/1
chr12:118682751	T/T	C	T	SNV	TAOK3	TAOK3:exonic:NM_016281.3	1	100	4	missense	NM_01628	c.140G>A	p.Ser47Asn	3.28	1	46	0		Protein kinas	rs428073	0.3073	YES	7.238E-1 (pos.118682751)		TAO kinase	ATP binding,DNA repair,MAP kinase kinase kinase activit	C=0, T=37	C=0.0, T=1.0	0.00001	583.705	37	C=0/0, T=20/1
chr12:6562285	T/T	C	T	SNV	TAPBPL	TAPBPL:exonic:NM_018009.4	1	100	2	synonymous	NM_01800	c.117C>T	p.(=)	1.35				0.61(COSM37	Immunoglobi	rs2041384	0.2866	YES	4E-1 (pos.6562285)	adenocarcinoma,neoplasn		Golgi membrane,endoplasmic reticulum membrane,imr	C=0, T=25	C=0.0, T=1.0	0.00001	353.019	25	C=0/0, T=10/1
chr12:6562293	T/T	C	T	SNV	TAPBPL	TAPBPL:exonic:NM_018009.4	1	100	2	missense	NM_01800	c.125C>T	p.Ala42Val	0.29	1	64	0.001	0.03(COSM37	Immunoglobi	rs2041385	0.2879	YES	3.96E-1 (pos.6562293)	adenocarcinoma,neoplasn		Golgi membrane,endoplasmic reticulum membrane,imr	C=0, T=25	C=0.0, T=1.0	0.00001	353.007	25	C=0/0, T=10/1
chr6:47549883	C/C	T	C	SNV	CD2AP	CD2AP:intronic:NM_012120.2	1	100		intronic	NM_01212	c.1108+82	p.?	-0.27					SH3 domain,\	rs2275446		YES			CD2-associ	SH3 doma Alzheimer Disease;Brain Diseases:Central Ne	T=0, C=156	T=0.0, C=1.0	0.00001	2503.58	156	T=0/0, C=61/9
chr15:102224341	A/A	G	A	SNV	TARSL2	TARSL2:exonic:NM_152334.2	1	100	12	synonymous	NM_15233	c.1587C>T	p.(=)	-1.18					Anticodon bii	rs12901450	0.2571	YES	6.929E-1 (pos.102224341)			ATP binding,biological_process,cellular_component,cytc	G=0, A=35	G=0.0, A=1.0	0.00001	543.901	35	G=0/0, A=26/9
chr6:47577064	A/A	G	A	SNV	CD2AP	CD2AP:intronic:NM_012120.2	1	100		intronic	NM_01212	c.1814+24	p.?	-0.86					SH3 domain,\	rs9395287	0.3122	YES	2.958E-1 (pos.47577064)		CD2-associ	SH3 doma Alzheimer Disease;Brain Diseases:Central Ne	G=0, A=27	G=0.0, A=1.0	0.00001	394.382	27	G=0/0, A=12/1
chr6:47641307	C/C	G	C	SNV	ADGRF2	ADGRF2:intronic:NM_153839.6	1	100		intronic	NM_15383	c.72+80G>	p.?	-0.8					7 transmemb	rs1411049		YES				G-protein coupled receptor activity,G-protein coupled re	G=0, C=30	G=0.0, C=1.0	0.00001	451.025	30	G=0/0, C=10/2
chr15:102264304	G/C	G	C	SNV	TARSL2	TARSL2:exonic:NM_152334.2	1	97.92	1	missense	NM_15233	c.287C>G	p.Ala96Gly	0.09	0.25	60	0	0.04(COSM37	Anticodon bii	rs1143138	0.1976	YES	8.277E-1 (pos.1022643	adenocarcinoma,neoplasn		ATP binding,biological_process,cellular_component,cytc	G=1, C=47	G=0.0208, C=0.9792	0.00001	773.104	48	G=0/1, C=36/1
chr1:6635231	G/G	A	G	SNV	TAS1R1	TAS1R1:exonic:NM_138697.3	1	100	3	missense	NM_13869	c.1039A>G	p.Lys347Glu	0.44	0.97	56	0.017	0.01(COSM41	7 transmemb	rs10864628	7.00E-04	YES	1.0 (pos.6635231)	neoplasm	Taste recep	G-protein coupled receptor activity,G-protein coupled re	A=0, G=27	A=0.0, G=1.0	0.00001	389.987	27	A=0/0, G=12/1
chr1:19166294	A/A	G	A	SNV	TAS1R2	TAS1R2:exonic:NM_152232.2	1	100	6	synonymous	NM_15223	c.2319C>T	p.(=)	0.17				0.12(COSM37	7 transmemb	rs12033832	0.3358	YES	4.544E-1 (pos.19166294)	acute_myeloic	Taste recep	G-protein coupled receptor act Aspartame	G=0, A=95	G=0.0, A=1.0	0.00001	1597.06	95	G=0/0, A=42/5
chr1:19184077	A/G	A	G	SNV	TAS1R2	TAS1R2:exonic:NM_152232.2	1	50	2	synonymous	NM_15223	c.231T>C	p.(=)	-1.97					7 transmemb	rs68081213	0.3357	YES	1.123E-1 (pos.19184077)		Taste recep	G-protein coupled receptor act Aspartame	A=17, G=17	A=0.5, G=0.5	0.00001	172.009	34	A=2/15, G=3/1
chr6:47654817	A/A	C	A	SNV	ADGRF2	ADGRF2:intronic:NM_153839.6	1	100		intronic	NM_15383	c.1905+48	p.?	0.75					7 transmemb	rs1556133	0.3539	YES	2.984E-1 (pos.47654817)			G-protein coupled receptor activity,G-protein coupled re	C=0, A=179	C=0.0, A=1.0	0.00001	2962.73	179	C=0/0, A=95/8
chr6:47654864	G/G	T	G	SNV	ADGRF2	ADGRF2:intronic:NM_153839.6	1	100		intronic	NM_15383	c.1905+95	p.?	0.72					7 transmemb	rs1556131		YES				G-protein coupled receptor activity,G-protein coupled re	T=0, G=173	T=0.0, G=1.0	0.00001	2840.29	173	T=0/0, G=89/8
chr6:47681494	C/C	T	C	SNV	ADGRF4	ADGRF4:intronic:NM_153838.4	1	100		intronic	NM_15383	c.553-40T	p.?	-0.43					7 transmemb	rs6916562	0.1137	YES	8.685E-1 (pos.47681494)		Adhesion G	G-protein coupled receptor activity,G-protein coupled re	T=0, C=35	T=0.0, C=1.0	0.00001	543.903	35	T=0/0, C=16/1
chr6:4891925	AT/A	AT	A	INDEL	CDYL	CDYL:intronic:NM_004824.3	1	100		intronic	NM_00482	c.25-22AT	p.?	0.06, 0.09					Chromo (CHR	rs747676317,rs7537	0.0013		3.077E-4;0.0 (pos.4891925)		Chromodon	catalytic a Bronchial Neoplasms;Carcinoma, Bronchoge	AT=0, A=20	AT=0.0, A=1.0	0.00001	131.014	20	AT=0/0, A=16/
chr5:9629529	G/A	G	A	SNV	TAS2R1	TAS2R1:exonic:NM_019599.2	1	50	1	missense	NM_01959	c.616C>T	p.Arg206Trp	-0.2		101		0.02(COSM14	Taste recept	rs2234233	0.1653	YES	1.167E-1 (pos.9629529)	acute_myeloid_leukaemia		G-protein coupled receptor activity,G-protein coupled re	G=13, A=13	G=0.5, A=0.5	0.00001	111.392	26	G=2/11, A=5/8
chr12:10978402	A/A	G	A	SNV	TAS2R10	TAS2R10:exonic:NM_023921.1	1	100	1	missense	NM_02392	c.467C>T	p.Thr156Met	-3.53	0.2	81	0		Taste recept	rs597468	0.0047	YES	1.0 (pos.10978402)			G-protein coupled receptor activity,G-protein coupled re	G=0, A=36	G=0.0, A=1.0	0.00001	568.121	36	G=0/0, A=12/2
chr6:49663465	A/A	G	A	SNV	CRISP2	CRISP2:intronic:NM_001142407.2	1	100		intronic	NM_00114	c.604+84C	p.?	-0.62					Crisp,Cystein	rs2248263		YES			Cysteine-ric	extracellular space	G=0, A=20	G=0.0, A=1.0	0.00001	263.781	20	G=0/0, A=12/8
chr6:49665750	T/T	A	T	SNV	CRISP2	CRISP2:intronic:NM_001142407.2	1	100		intronic	NM_00114	c.418-80T	p.?	0.09					Crisp,Cystein	rs575562		YES			Cysteine-ric	extracellular space	A=0, T=25	A=0.0, T=1.0	0.00001	353.013	25	A=0/0, T=13/1
chr12:11338983	T/C	T	C	SNV	TAS2R42	TAS2R42:exonic:NM_181429.2	1	57.89	1	synonymous	NM_18142	c.561A>G	p.(=)	0.03					Taste recept	rs1650019	0.1609	YES	9.66E-1 (pos.11338983)		Taste receptor, type 2, member 42		T=24, C=33	T=0.4211, C=0.5789	0.00001	345.07	57	T=8/16, C=9/2
chr12:11339020	T/A	T	A	SNV	TAS2R42	TAS2R42:exonic:NM_181429.2	1	57.14	1	missense	NM_18142	c.524A>T	p.Tyr175Phe	0.33	0.19	22	0.997		Taste recept	rs35969491	0.405	YES	7.531E-1 (pos.11339020)		Taste receptor, type 2, member 42		T=24, A=32	T=0.4286, A=0.5714	0.00001	331.279	56	T=8/16, A=8/2
chr12:10962115	G/G	A	G	SNV	TAS2R9	TAS2R9:exonic:NM_023917.2	1	100	1	missense	NM_02391	c.560T>C	p.Val187Ala	-3.11		64		0(COSM4770	Taste recept	rs3741845	0.3874	YES	7.22E-1 (pos.10962115)	adenocarcinoma,ductal_ca		G-protein coupled receptor activity,G-protein coupled re	A=0, G=81	A=0.0, G=1.0	0.00001	1309.36	81	A=0/0, G=40/4
chr6:51798753	A/A	G	A	SNV	PKHD1	PKHD1:intronic:NM_138694.3	1	100		intronic	NM_13869	c.6121+15	p.?	-0.11					G8 domain,IF	rs1266888		YES			Fibrocystin	anchored Congenital, Hereditary, and Neonatal Diseas	G=0, A=98	G=0.0, A=1.0	0.00001	1647.36	98	G=0/0, A=58/4
chr10:96163039	G/G	C	G	SNV	TBC1D12	TBC1D12:exonic:NM_015188.1	1	100	1	synonymous	NM_01518	c.669C>G	p.(=)	-0.4				0(COSM3749	Rab-GTPase-T	rs2477534	0.2994	YES	8.594E-1 (pos.96163039)	adenocarcinoma,neoplasn		GTPase activator activity,Rab GTPase binding,activation	C=0, G=21	C=0.0, G=1.0	0.00001	281.168	21	C=0/0, G=11/1
chr10:96269925	A/T	A	T	SNV	TBC1D12	TBC1D12:exonic:NM_015188.1	1	57.63	8	missense	NM_01518	c.1678A>T	p.Ile560Phe	8.12	0	21	0.333		Rab-GTPase-T	rs376682556	1.00E-04		0.0;1.159E-4 (pos.96269925)			GTPase activator activity,Rab GTPase binding,activation	A=25, T=34	A=0.4237, T=0.5763	0.00001	356.467	59	A=16/9, T=25/
chr6:52847551	C/C	G	C	SNV	GSTA4	GSTA4:intronic:NM_001512.3	1	100		intronic	NM_00151	c.415-48C	p.?	-0.52					Glutathione S	rs316133	0.3866	YES	7.387E-1;0.0 (pos.52847551)		Glutathione	cytosol,glu Arterial Occlusive D Glutathione	G=0, C=47	G=0.0, C=1.0	0.00001	789.305	47	G=0/0, C=17/3
chr12:72278779	GAA/GA	GAA	GA,G	INDEL	TBC1D15	TBC1D15:exonic:NM_022771.4	1	83.87	5	frameshiftDeletion	NM_02277	c.537delA	p.Lys179fs	4.07, 2.6, 1.81				0.97(COSM56	Domain of un	rs373705972	1.00E-04			diffuse_large_	TBC1 domai	GTPase activator activity,Rab GTPase binding,activation	GAA=5, GA	GAA=0.1613, GA=0.8	0.00001	196.415	31	GAA=3/2, GA=
chr19:50383591	G/G	A	G	SNV	TBC1D17	TBC1D17:exonic:NM_024682.2	1	100	4	missense	NM_02468	c.251A>G	p.Asp84Gly	6.99	1	94	0	0.12(COSM45	Domain of un	rs8109661			1.0 (pos.50383591)	squamous_cel	TBC1 domai	GTPase activator activity,Rab GTPase binding,activation	A=0, G=63	A=0.0, G=1.0	0.00001	958.537	63	A=0/0, G=22/4
chr6:53986503	T/T	A	T	SNV	MLIP	MLIP:intronic:NM_138569.2	1	100		intronic	NM_13856	c.219+103	p.?	-0.04					Muscular LM	rs2145754		YES			Muscular LI	PML body,negative regulation of cardiac muscle hypertr	A=0, T=31	A=0.0, T=1.0	0.00001	465.759	31	A=0/0, T=14/1
chrX:48418659	A/A	G	A	SNV	TBC1D25	TBC1D25:exonic:NM_002536.3	1	100	6	missense	NM_00253	c.1363G>A	p.Ala455Thr	-1.13	1	58	0	0.01(COSM37	Rab-GTPase-T	rs235836	0.4275	YES	5.581E-1 (pos.48418659)	adenocarcinoi	TBC1 domai	GTPase activator activity,Rab GTPase binding,activation	G=0, A=39	G=0.0, A=1.0	0.00001	623.978	39	G=0/0, A=17/2
chr8:124142489	C/C	T	C	SNV	TBC1D31	TBC1D31:exonic:NM_145647.3	1	100	16	synonymous	NM_14564	c.2322T>C	p.(=)	0.33				0.79(COSM39	Rab-GTPase-T	rs2272724	0.346	YES	2.015E-1 (pos.12414248	acute_myeloid_leukaemia		centrosome	T=0, C=36	T=0.0, C=1.0	0.00001	568.761	36	T=0/0, C=21/1
chr6:54735606	A/A	G	A	SNV	FAM83B	FAM83B:intronic:NM_001010872.2	1	100		intronic	NM_00101	c.444+118	p.?	0.64					PLD-like dom	rs995852		YES				cell proliferation,cytoplasm,epidermal growth factor rec	G=0, A=103	G=0.0, A=1.0	0.00001	1609.43	103	G=0/0, A=38/6
chr6:55360086	G/G	A	G	SNV	HMGCLL1	HMGCLL1:intronic:NM_019036.2	1	100		intronic	NM_01903	c.885+131	p.?	0.13					HMGL-like	rs4538708		YES				cytosol,en Nutrition Disorders;Nutritional and Metaboli	A=0, G=29	A=0.0, G=1.0	0.00001	427.603	29	A=0/0, G=9/20
chr6:55381262	A/A	G	A	SNV	HMGCLL1	HMGCLL1:intronic:NM_019036.2	1	100		intronic	NM_01903	c.483+44C	p.?	1.33					HMGL-like	rs6459082	0.2923	YES	5.584E-1 (pos.55381262)			cytosol,en Nutrition Disorders;Nutritional and Metaboli	G=0, A=31	G=0.0, A=1.0	0.00001	465.819	31	G=0/0, A=8/23
chr6:56340803	A/A	G	A	SNV	DST	DST:intronic:NM_001144769.2	1	100		intronic	NM_00114	c.15507+1	p.?	-1.87					Calponin hon	rs6928601		YES			Dystonin (b	H zone,Z disc,actin binding,actin cytoskeleton,axon,axor	G=0, A=55	G=0.0, A=1.0	0.00001	814.874	55	G=0/0, A=21/3
chr6:56381912	G/A	G	A	SNV	DST	DST:intronic:NM_001144769.2	1	58.33		intronic	NM_00114	c.12000+8	p.?	-0.74					Calponin hon	rs4715626		YES			Dystonin (b	H zone,Z disc,actin binding,actin cytoskeleton,axon,axor	G=15, A=21	G=0.4167, A=0.5833	0.00001	234.327	36	G=13/2, A=11/
chr6:121620608	T/T	G	T	SNV	TBC1D32	TBC1D32:exonic:NM_152730.5	1	100	10	missense	NM_15273	c.1124C>A	p.Thr375Lys	2.2	1	78	0		Broad-minde	rs9387944	0		1.0 (pos.121620608)		TBC1 domai	cilium,cyto Congenital Abnormalities;Congenital, Heredi	G=0, T=123	G=0.0, T=1.0	0.00001	1987.74	123	G=0/0, T=68/5
chr13:75936519	G/G	C	G	SNV	TBC1D4	TBC1D4:exonic:NM_014832.4	1	100	2	synonymous	NM_01483	c.723G>C	p.(=)	-0.53					Domain of un	rs7983969	0		9.999E-1;1.159E-4 (pos.75936519)		TPC1 domai	GTPase activator activity,Rab GTPase binc Likely benign	C=0, G=49	C=0.0, G=1.0	0.00001	831.633	49	C=0/0, G=10/3
chr2:101656726	C/C	T	C	SNV	TBC1D8	TBC1D8:exonic:NM_001102426.2	1	100	6	missense	NM_00110	c.949A>G	p.Thr317Ala	1.19	0.59	58	0	0.02(COSM41	GRAM domai	rs2289953	0.3364	YES	3.201E-1 (pos.1016567	neoplasm		GTPase ac Bone Diseases;Bone Diseases, Metabolic;Mu	T=0, C=28	T=0.0, C=1.0	0.00001	408.452	28	T=0/0, C=6/22
chr6:64694620	C/C	T	C	SNV	EYS	EYS:intronic:NM_001142800.1	1	100		intronic	NM_00114	c.6835-12	p.?	-0.04					EGF-like dom	rs1482456		YES			Eyes shut, C	calcium io Congenital, Hereditary, and Neonatal Diseas	T=0, C=61	T=0.0, C=1.0	0.00001	921.825	61	T=0/0, C=22/3
chr6:65098768	G/G	A	G	SNV	EYS	EYS:intronic:NM_001142800.1	1	100		intronic	NM_00114	c.5928-35	p.?	0.09					EGF-like dom	rs587278	0.2872	YES	5.526E-1 (pos.65098768)		Eyes shut, C	calcium io Congenital, Hereditary, and Neonatal Diseas	A=0, G=42	A=0.0, G=1.0	0.00001	685.179	42	A=0/0, G=15/2
chr6:42713618	G/G	A	G	SNV	TBCC	TBCC:exonic:NM_003192.2	1	100	1	missense	NM_00319	c.194T>C	p.Val65Ala	1.85		64			Tubulin bindi	rs2234026	0.0131	YES	1.0 (pos.42713618)		Tubulin-spe	GTPase activity,chaperone binding,cytoplasm,cytoskele	A=0, G=23	A=0.0, G=1.0	0.00001	316.747	23	A=0/0, G=11/1
chr1:235600741	A/G	A	G	SNV	TBCE	TBCE:exonic:NM_001079515.2	1	50	12	synonymous	NM_00107	c.1068A>G	p.(=)	0.52					CAP-Gly dom	rs6666168	0.019	YES	0.0 (pos.235600741)		Tubulin-spe	adult loco Abnormalities, Multiple;Bone I Likely benign	A=11, G=11	A=0.5, G=0.5	0.00001	94.6061	22	A=5/6, G=4/7
chr4:107168431	C/C	G	C	SNV	TBCK	TBCK:exonic:NM_001163435.2	1	100	10	missense	NM_00116	c.796C>G	p.Gln266Glu	2.66	1	29	0		Protein kinas	rs3775091	0		9.733E-1 (pos.107168431)		TBC1 domai	ATP binding,GTPase activator activity,Rab GTPase bindin	G=0, C=22	G=0.0, C=1.0	0.00001	298.886	22	G=0/0, C=9/13
chr6:69728522	T/T	C	T	SNV	ADGRB3	ADGRB3:intronic:NM_001704.2	1	100		intronic	NM_00170	c.2107+13	p.?	-0.33					7 transmemb	rs2785573		YES			Adhesion G	G-protein coupled receptor activity,G-protein coupled re	C=0, T=53	C=0.0, T=1.0	0.00001	784.234	53	C=0/0, T=20/3
chr6:69772809	T/C	T	C	SNV	ADGRB3	ADGRB3:intronic:NM_001704.2	1	45.45		intronic	NM_00170	c.2334-17	p.?	0.29					7 transmemb	rs13207626,rs77313	0.1397	YES	0.0 (pos.69772804),3.703E-4 (pos.69772		Adhesion G	G-protein coupled receptor activity,G-protein coupled re	T=12, C=10	T=0.5455, C=0.4545	0.00001	81.7805	22	T=5/7, C=3/7
chr6:70423739	G/G	A	G	SNV	LMBRD1	LMBRD1:intronic:NM_018368.3	1	100		intronic	NM_01836	c.763-50T	p.?	2.58					LMBR1-like m	rs2305837	0.3995	YES	5.507E-1 (pos.70423739)		LMBR1 dom	clathrin-cc Amino Acid Metabolism, Inbor Likely benign	A=0, G=25	A=0.0, G=1.0	0.00001	352.954	25	A=0/0, G=8/17
chr6:70500120	A/A	G	A	SNV	LMBRD1	LMBRD1:intronic:NM_018368.3	1	100		intronic	NM_01836	c.246+68C	p.?	-0.58					LMBR1-like m	rs2125024,rs386702447		YES			LMBR1 dom	clathrin-cc Amino Acid Metabolism, Inborn Errors;Conge	G=0, A=35	G=0.0, A=1.0	0.00001	543.891	35	G=0/0, A=10/2
chr6:70500451	A/A	C	A	SNV	LMBRD1	LMBRD1:intronic:NM_018368.3	1	100		intronic	NM_01836	c.70-87G>	p.?	-2.97					LMBR1-like m	rs2305838		YES			LMBR1 dom	clathrin-cc Amino Acid Metabolism, Inborn Errors;Conge	C=0, A=38	C=0.0, A=1.0	0.00001	601.851	38	C=0/0, A=11/2
chr6:70500463	G/G	T	G	SNV	LMBRD1	LMBRD1:intronic:NM_018368.3	1	100		intronic	NM_01836	c.70-99A>	p.?	0.02					LMBR1-like m	rs2305839		YES			LMBR1 dom	clathrin-cc Amino Acid Metabolism, Inborn Errors;Conge	T=0, G=36	T=0.0, G=1.0	0.00001	563.741	36	T=0/0, G=10/2
chr19:3595794	G/G	A	G	SNV	TBXA2R	TBXA2R:exonic:NM_201636.2	1	100	3	synonymous	NM_20163	c.924T>C	p.(=)	3.93				0.05(COSM37	7 transmemb	rs4523	0.3452	YES	2.214E-1 (pos.3595794)	adenocarcinoi	Thromboxa	G-protein Asthma;Asthma, As Ridogrel Benign	A=0, G=28	A=0.0, G=1.0	0.00001	408.718	28	A=0/0, G=14/1
chr6:70894909	T/T	A	T	SNV	COL19A1	COL19A1:intronic:NM_001858.5	1	100		intronic	NM_00185	c.2856+10	p.?	0.65					Collagen tripl	rs2296005		YES			Collagen XI	cell adhesion,cell differentiation,cell-cell adhesion,colla	A=0, T=31	A=0.0, T=1.0	0.00001	465.819	31	A=0/0, T=17/1
chr6:70962044	A/G	A	G	SNV	COL9A1	COL9A1:intronic:NM_001851.4	1	46.88		intronic	NM_00185	c.1765-26	p.?	0.23					Collagen tripl	rs519068	0.4945	YES	2.895E-1 (pos.70962044)		Collagen IX,	animal or Bone Diseases;Bone Diseases, Benign	A=17, G=15	A=0.5313, G=0.4688	0.00001	143.698	32	A=8/9, G=2/13
chr6:70965125	C/C	A	C	SNV	COL9A1	COL9A1:intronic:NM_001851.4	1	100		intronic	NM_00185	c.1504-32	p.?	-0.02					Collagen tripl	rs633762	0.2257	YES	9.233E-1;0.0 (pos.70965125)		Collagen IX,	animal or Bone Diseases;Bone Diseases, Benign	A=0, C=47	A=0.0, C=1.0	0.00001	759.375	47	A=0/0, C=29/1
chr14:92268615	G/G	T	G	SNV	TC2N	TC2N:exonic:NM_152332.5	1	100	4	missense	NM_15233	c.452A>C	p.Lys151Thr	0.04	0.68	78	0	0.02(COSM16	C2 domain	rs2402073	1.00E-04	YES	9.999E-1;1.156E-4 (pos.9	acute_lymphoblastic_leuk		calcium ion binding,calcium ion-regulated exocytosis of	T=0, G=39	T=0.0, G=1.0	0.00001	623.963	39	T=0/0, G=19/2
chr6:71010101	A/G	A	G	SNV	COL9A1	COL9A1:intronic:NM_001851.4	1	50		intronic	NM_00185	c.89-12T>C	p.?	0.3					Collagen tripl	rs12210123	0.1916	YES	4.63E-4 (pos.71010101)		Collagen IX,	animal or Bone Diseases;Bone Diseases, Benign/Likely	A=11, G=11	A=0.5, G=0.5	0.00001	94.6067	22	A=10/1, G=3/8
chr6:71190577	A/G	A	G	SNV	FAM135A	FAM135A:intronic:NM_001162529.2	1	50		intronic	NM_00116	c.543-27A	p.?	-1.11					Protein FAM1	rs2273210	0.1614	YES	8.394E-2 (pos.71190577)			carboxylic ester hydrolase activity,cellular lipid metabo	A=42, G=42	A=0.5, G=0.5	0.00001	450.211	84	A=25/17, G=2
chr20:590542	GC/GC	CG	GC	MNV	TCF15	TCF15:exonic:NM_004609.3	2	100	1	missense	NM_00460	c.339_340	p.Val114Leu	3.79, -1.27	1	32	0	0.14(COSM37	Helix-loop-he	rs2665781,rs2754502,rs71212728,rs87926			1.0 (pos.590542),1.0 (pos	adenocarcinoi	Transcriptic	DNA bindi Adnexal Diseases;Cysts;Endocrine System Di	CG=0, GC=4	CG=0.0, GC=1.0	0.00001	685.202	42	CG=0/0, GC=2
chr6:73951987	G/G	T	G	SNV	KHDC1	KHDC1:intronic:NM_030568.4	1	100		intronic	NM_03056	c.113-27A	p.?	-0.29					KH-like RNA-b	rs6453659,rs755490	0.2347	YES	0.0;0.0 (pos.73951974),3.208E-1 (pos.73		KH homolog	RNA binding,integral component of membrane	T=0, G=47	T=0.0, G=1.0	0.00001	786.636	47	T=0/0, G=25/2
chr6:73952103	C/C	A	C	SNV	KHDC1	KHDC1:intronic:NM_030568.4	1	100		intronic	NM_03056	c.112+26T	p.?	-0.91					KH-like RNA-b	rs6453660	0.1921	YES	2.544E-1 (pos.73952103)		KH homolog	RNA binding,integral component of membrane	A=0, C=39	A=0.0, C=1.0	0.00001	623.961	39	A=0/0, C=12/2
chr6:73952109	A/A	C	A	SNV	KHDC1	KHDC1:intronic:NM_030568.4	1	100		intronic	NM_03056	c.112+20G	p.?	-2.58					KH-like RNA-b	rs76146370	0.1903	YES	2.535E-1;1.17E-4 (pos.73952109)		KH homolog	RNA binding,integral component of membrane	C=0, A=39	C=0.0, A=1.0	0.00001	623.737	39	C=0/0, A=12/2
chr6:73952112	TA/TA	GG	TA	MNV	KHDC1	KHDC1:intronic:NM_030568.4	2	100		intronic	NM_03056	c.112+17C	p.?	-0.16, -0.48					KH-like RNA-b	rs373630974,rs74562538,rs792		YES	2.529E-1 (pos.73952112),2.538E-1 (pos.7		KH homolog	RNA binding,integral component of membrane	GG=0, TA=3	GG=0.0, TA=1.0	0.00001	623.98	39	GG=0/0, TA=1
chr6:73952118	A/G	A	G	SNV	KHDC1	KHDC1:intronic:NM_030568.4	1	97.5		intronic	NM_03056	c.112+11T	p.?	-1.1					KH-like RNA-b	rs6453661	0.1804	YES	2.536E-1 (pos.73952118)		KH homolog	RNA binding,integral component of membrane	A=1, G=39	A=0.025, G=0.975	0.00001	612.018	40	A=0/1, G=12/2
chr6:7396836	A/A	G	A	SNV	RIOK1	RIOK1:intronic:NM_031480.2	1	100		intronic	NM_03148	c.368-100	p.?	-0.27					RIO1 family	rs12212766		YES			RIO kinase 1	ATP binding,cytosol,hydrolase activity,maturation of SSL	G=0, A=63	G=0.0, A=1.0	0.00001	958.914	63	G=0/0, A=26/3
chr6:7396887	A/A	G	A	SNV	RIOK1	RIOK1:intronic:NM_031480.2	1	100		intronic	NM_03148	c.368-49G	p.?	0.79					RIO1 family	rs12212772	0.4382	YES	4.843E-1 (pos.7396887)		RIO kinase 1	ATP binding,cytosol,hydrolase activity,maturation of SSL	G=0, A=65	G=0.0, A=1.0	0.00001	1000.27	65	G=0/0, A=26/3
chr6:31129616	G/G	A	G	SNV	TCF19	TCF19:exonic:NM_001077511.1	1	100	3	missense	NM_00107	c.631A>G	p.Met211Val	1.11	1	21	0		FHA domain	rs2073721	0.2432	YES	6.97E-1 (pos.31129616)		Transcriptic	DNA bindi Autoimmune Diseases;Colonic Diseases;Colc	A=0, G=43	A=0.0, G=1.0	0.00001	705.741	43	A=0/0, G=17/2
chr6:74119084	C/C	T	C	SNV	DDX43	DDX43:intronic:NM_018665.2	1	100		intronic	NM_01866	c.1280+13	p.?	-1.2					DEAD/DEAH b	rs4708053	0.0012	YES	1.0 (pos.74119084)		DEAD/H box	ATP binding,ATP-dependent RNA helicase activity,RNA bi	T=0, C=82	T=0.0, C=1.0	0.00001	1329.6	82	T=0/0, C=27/5
chr6:31129642	C/C	A	C	SNV	TCF19	TCF19:exonic:NM_001077511.1	1	100	3	synonymous	NM_00107	c.657A>C	p.(=)	0.3				0(COSM4160	FHA domain	rs2073722		YES	6.979E-1;2.195E-2 (pos.3	acute_myeloic	Transcriptic	DNA bindi Autoimmune Diseases;Colonic Diseases;Colc	A=0, C=44	A=0.0, C=1.0	0.00001	726.567	44	A=0/0, C=17/2
chr6:74189574	A/A	G	A	SNV	MTO1	MTO1:intronic:NM_133645.2	1	100		intronic	NM_13364	c.938+7G>	p.?	1.35					FAD binding c	rs7766570		YES	1.0 (pos.74189574)		Mitochondr	RNA binding,flavin adenine dinucleotide b Benign	G=0, A=27	G=0.0, A=1.0	0.00001	389.982	27	G=0/0, A=7/20
chr6:74228006	G/G	A	G	SNV	EEF1A1,SNORD141A,SNORD141B	EEF1A1:intronic:NM_001402.5, SNORD141B:intronic_nc:NR	1	100		intronic	NM_00140	c.1030-19	p.?	-0.87					Elongation fa	rs1874229	0.2837	YES	5.448E-1 (pos.74228006)		Eukaryotic t	GTP bindir Digestive System D Guanosine-5'-Diphospha	A=0, G=60	A=0.0, G=1.0	0.00001	903.515	60	A=0/0, G=15/4
chr6:74228049	G/G	A	G	SNV	EEF1A1,SNORD141A,SNORD141B	EEF1A1:intronic:NM_001402.5, SNORD141B:intronic_nc:NR	1	100		intronic	NM_00140	c.1029+28	p.?	-1.11					Elongation fa	rs1874230	0.3217	YES	4.43E-1 (pos.74228049)		Eukaryotic t	GTP bindir Digestive System D Guanosine-5'-Diphospha	A=0, G=189	A=0.0, G=1.0	0.00001	3169.99	189	A=0/0, G=103/
chr6:74490344	C/C	A	C	SNV	CD109	CD109:intronic:NM_133493.4	1	100		intronic	NM_13349	c.1902+12	p.?	0.22					A-macroglobi	rs9293941		YES			CD109 anti	C-termina Breast Diseases;Breast Neoplasms;Colonic D	A=0, C=25	A=0.0, C=1.0	0.00001	353.019	25	A=0/0, C=6/19
chr1:152085505	C/C	A	C	SNV	TCHH	TCHH:exonic:NM_007113.3	1	100	3	missense	NM_00711	c.188T>G	p.Leu63Arg	0.55		102	0.197		S-100/ICaBP t	rs2515663	2.00E-04		1.0;0.0 (pos.152085505)		Trichohyali	biological_process,calcium ion binding,cornification,cor	A=0, C=48	A=0.0, C=1.0	0.00001	810.432	48	A=0/0, C=32/1
chr6:74495257	G/G	A	G	SNV	CD109	CD109:intronic:NM_133493.4	1	100		intronic	NM_13349	c.2337+57	p.?	-3.33					A-macroglobi	rs6453700		YES			CD109 anti	C-termina Breast Diseases;Breast Neoplasms;Colonic D	A=0, G=23	A=0.0, G=1.0	0.00001	316.747	23	A=0/0, G=11/1
chr11:67814979	C/C	G	C	SNV	TCIRG1	TCIRG1:exonic:NM_006019.3	1	100	11	synonymous	NM_00601	c.1245G>C	p.(=)	0.47					V-type ATPase	rs2471829	5.00E-04		1.0 (pos.67814979)		T-cell immu	ATP hydrol Agranulocytosis;Bone Disease Benign	G=0, C=49	G=0.0, C=1.0	0.00001	831.633	49	G=0/0, C=22/2
chr6:7558318	C/C	T	C	SNV	DSP	DSP:intronic:NM_004415.3	1	100		intronic	NM_00441	c.274-31T	p.?	0.06					Plectin repea	rs10484326	0.2349	YES	4.521E-2 (pos.7558318)		Desmoplak	RNA bindir ACMG Recommendations...;Ar Benign	T=0, C=22	T=0.0, C=1.0	0.00001	298.892	22	T=0/0, C=12/1
chr6:7578819	G/G	T	G	SNV	DSP	DSP:intronic:NM_004415.3	1	100		intronic	NM_00441	c.3084+24	p.?	-0.04					Plectin repea	rs2806229	0.1977	YES	8.035E-1 (pos.7578819)		Desmoplak	RNA bindir ACMG Recommendations...;Ar Benign	T=0, G=31	T=0.0, G=1.0	0.00001	465.751	31	T=0/0, G=11/2
chr6:7578823	A/A	G	A	SNV	DSP	DSP:intronic:NM_004415.3	1	100		intronic	NM_00441	c.3084+28	p.?	0.77					Plectin repea	rs2842691	0.197	YES	8.037E-1 (pos.7578823)		Desmoplak	RNA bindir ACMG Recommendations...;Ar Benign	G=0, A=31	G=0.0, A=1.0	0.00001	465.819	31	G=0/0, A=11/2
chr6:167592524	C/C	T	C	SNV	TCP10L2	TCP10L2:exonic:NM_001145121.1	1	100	6	missense	NM_00114	c.683T>C	p.Leu228Pro	0.09	0.2	98	0	0.01(COSM3928224),0.01(C		rs2989545		YES	5.386E-1 (pos.16759252	acute_myeloid_leukaemia,adenocarcinoma,ductal_carcinoma,glioma,neoplasm,sq			T=0, C=54	T=0.0, C=1.0	0.00001	798.137	54	T=0/0, C=41/1
chr6:167592576	A/A	G	A	SNV	TCP10L2	TCP10L2:exonic:NM_001145121.1	1	100	6	synonymous	NM_00114	c.735G>A	p.(=)	-0.47						rs60976240		YES	5.44E-1 (pos.167592576)				G=0, A=60	G=0.0, A=1.0	0.00001	903.502	60	G=0/0, A=43/1
chr6:35088381	G/G	C	G	SNV	TCP11	TCP11:exonic:NM_001093728.2	1	100	7	missense	NM_00109	c.797G>C	p.Gly266Ala	1.46	0.64	60	0		T-complex pr	rs2234045	0.1552	YES	9.79E-2 (pos.35088381)		T-complex t	acrosomal vesicle,cell differentiation,integral compone	C=0, G=97	C=0.0, G=1.0	0.00001	1626.01	97	C=0/0, G=39/5
chr6:7590653	G/G	A	G	SNV	SNRNP48	SNRNP48:intronic:NM_152551.3	1	100		intronic	NM_15255	c.156+7A>	p.?	-0.02					U11-48K-like	rs2744384	0.3955	YES	3.273E-1 (pos.7590653)			RNA splicing,U12-type spliceosomal complex,cytosol,mF	A=0, G=43	A=0.0, G=1.0	0.00001	705.574	43	A=0/0, G=14/2
chr6:75968720	A/A	G	A	SNV	TMEM30A	TMEM30A:intronic:NM_018247.3	1	100		intronic	NM_01824	c.686-18C	p.?	0.09					LEM3 (ligand-	rs147523	0.0495	YES	9.796E-1 (pos.75968720)		Transmemb	Golgi appa Autoimmune Diseases;Female Urogenital Dis	G=0, A=136	G=0.0, A=1.0	0.00001	2244.01	136	G=0/0, A=83/5
chr12:111080137	A/A	AG	A	INDEL	TCTN1	TCTN1:exonic:NM_001082538.2	1	100	11	frameshiftDeletion	NM_00108	c.1279del	p.Val427fs	2.33, 0.8					Protein of unl	rs754890276			0.0 (pos.111080137)			MKS comp Abnormalities, Multiple;Brain Diseases:Cent	AG=0, A=21	AG=0.0, A=1.0	0.00001	222.849	21	AG=0/0, A=8/1
chr6:76022090	C/C	G	C	SNV	FILIP1	FILIP1:intronic:NM_015687.4	1	100		intronic	NM_01568	c.3435+23	p.?	0.38					Cortactin-bin	rs9343281	0.2998	YES	5.486E-1 (pos.76022090)			actin cyto Digestive System Diseases;Digestive System	G=0, C=37	G=0.0, C=1.0	0.00001	583.7	37	G=0/0, C=16/2
chr10:97444321	C/T	C	T	SNV	TCTN3	TCTN3:exonic:NM_015631.5	1	50	9	missense	NM_01563	c.1030G>A	p.Gly344Arg	-0.06	0.15	125	0.006		Protein of unl	rs141088838	0.0062		2.313E-4 (pos.97444321)		Tectonic far	apoptotic Abnormalities, Multiple;Bone I Benign/Likely	C=64, T=64	C=0.5, T=0.5	0.00001	684.057	128	C=41/23, T=29
chr6:76607983	T/A	T	A	SNV	MYO6	MYO6:intronic:NM_004999.3	1	50		intronic	NM_00499	c.3138-10	p.?	-1.68					IQ calmoduli	rs720862		YES			Myosin VI	ADP bindir Aortic Stenosis, Subvalvular;Aortic Valve Ster	T=15, A=15	T=0.5, A=0.5	0.00001	145.021	30	T=10/5, A=7/8
chr6:76735065	G/G	A	G	SNV	IMPG1	IMPG1:intronic:NM_001563.3	1	100		intronic	NM_00156	c.498-90T	p.?	-0.15					SEA domain	rs1341568		YES			Interphotor	extracellular matrix structural constituent,proteinaceo	A=0, G=43	A=0.0, G=1.0	0.00001	705.844	43	A=0/0, G=16/2
chr6:79577644	C/C	A	C	SNV	IRAK1BP1	IRAK1BP1:intronic:NM_001010844.3	1	100		intronic	NM_00101	c.315+36A	p.?	-2.19					Protein of unl	rs9350789	0.0983	YES	8.849E-1;0.0 (pos.79577644)		Interleukin	I-kappaB k Arthritis;Arthritis, Rheumatoid;Autoimmune	A=0, C=39	A=0.0, C=1.0	0.00001	623.89	39	A=0/0, C=17/2
chr1:154516571	C/C	T	C	SNV	TDRD10	TDRD10:exonic:NM_001098475.1	1	100	9	synonymous	NM_00109	c.636T>C	p.(=)	0.23					RNA recogniti	rs6678164	8.00E-04	YES	1.0 (pos.154516571)			RNA binding	T=0, C=47	T=0.0, C=1.0	0.00001	789.327	47	T=0/0, C=5/42
chr6:79713612	T/G	T	G	SNV	PHIP	PHIP:intronic:NM_017934.6	1	45.45		intronic	NM_01793	c.1525-37	p.?	0.41					Bromodomai	rs73464147	2.00E-04		0.0 (pos.79713612)		Pleckstrin h	cytoskelet Arthritis;Arthritis, Rheumatoid;Autoimmune	T=12, G=10	T=0.5455, G=0.4545	0.00001	81.7625	22	T=3/9, G=6/4
chr9:100190780	G/G	A	G	SNV	TDRD7	TDRD7:exonic:NM_014290.2	1	100	2	synonymous	NM_01429	c.33A>G	p.(=)	-1.64				0.02(COSM39	OST-HTH/LOT	rs1381532	0.4756	YES	5.583E-1 (pos.1001907	acute_myeloic	Tudor doma	chromatoi Cataract;Eye Diseases;Genital Benign	A=0, G=75	A=0.0, G=1.0	0.00001	1189.49	75	A=0/0, G=24/5
chr6:8016089	T/T	C	T	SNV	BLOC1S5,BLOC1S5-TXNDC5,EEF1E1-BLOC1S5	EEF1E1-BLOC1S5:intronic_nc:NR_037618.1, BLOC1S5:introni	1	100		intronic	NR_03761	c.193-28G	p.?	-0.63						rs2057185	0.2042	YES	9.607E-1 (pos.8016089)		Biogenesis	BLOC-1 complex,anterograde axonal transport,anterogr	C=0, T=35	C=0.0, T=1.0	0.00001	543.9	35	C=0/0, T=8/27
chr9:100194406	C/C	T	C	SNV	TDRD7	TDRD7:exonic:NM_014290.2	1	100	4	missense	NM_01429	c.449T>C	p.Val150Ala	0	0.58	64	0.01	0.13(COSM15	OST-HTH/LOT	rs2045732	0.4753	YES	5.579E-1 (pos.1001944	acute_myeloic	Tudor doma	chromatoi Cataract;Eye Diseases;Genital Benign	T=0, C=50	T=0.0, C=1.0	0.00001	852.747	50	T=0/0, C=22/2

chr7:97873994	G/A	G	A	SNV	TECPR1	TECPR1:exonic:NM_015395.2	1	50	5	synonymous	NM_01539	c.420C>T	p.(=)	-2.1				0.03(COSM45	Integral pero	rs1874344	0.2371	YES	4.808E-1 (pos.97873994)	adenocarcinoı	Tectonin be	autophagosome maturation,autophagosome membran				G=39, A=3!	G=0.5, A=0.5	0.00001	408.121	78	G=11/28, A=24
chr6:8026577	T/C	T	C	SNV	BLOC1S5,BLOC1S5-TXNDC5,EEF1E1-BLOC1S5	EEF1E1-BLOC1S5:intronic_nc:NR_037618.1, BLOC1S5:introni	1	57.14		intronic	NR_03761(	c.192+23A	p.?	0						rs9328452	0.373	YES	9.371E-1 (pos.8026577)		Biogenesis	BLOC-1 complex,anterograde axonal transport,anterogr				T=36, C=48	T=0.4286, C=0.5714	0.00001	549.544	84	T=13/23, C=30
chr6:80580038	G/G	T	G	SNV			1	100		intronic				-0.49						rs9361569			1.0 (pos.80580038)							T=0, G=22	T=0.0, G=1.0	0.00001	298.892	22	T=0/0, G=7/15
chr6:80721045	A/G	A	G	SNV	TTK	TTK:intronic:NM_003318.4	1	47.62		intronic	NM_00331	c.614-106/	p.?	-0.92					Kinase-like,P	rs239558,rs386703044		YES			TTK protein	ATP bindin	Adnexal Diseases:/	2,6-Dihydroanthra/1,9-C		A=11, G=1(	A=0.5238, G=0.4762	0.00001	82.2778	21	A=7/4, G=6/4
chr6:80724156	T/C	T	C	SNV	TTK	TTK:intronic:NM_003318.4	1	45.45		intronic	NM_00331	c.985-24T:	p.?	1.6					Kinase-like,P	rs2295840,rs760777(	0.3277	YES	0.0 (pos.80724153),6.3E-1 (pos.8072415		TTK protein	ATP bindin	Adnexal Diseases:/	2,6-Dihydroanthra/1,9-C		T=12, C=10	T=0.5455, C=0.4545	0.00001	81.7626	22	T=9/3, C=2/8
chr6:80745306	C/T	C	T	SNV	TTK	TTK:intronic:NM_003318.4	1	57.14		intronic	NM_00331	c.1924+17	p.?	-0.12					Kinase-like,P	rs642745		YES			TTK protein	ATP bindin	Adnexal Diseases:/	2,6-Dihydroanthra/1,9-C		C=9, T=12	C=0.4286, T=0.5714	0.00001	109.097	21	C=5/4, T=4/8
chr14:102901201	G/G	A	G	SNV	TECPR2	TECPR2:exonic:NM_014844.4	1	100	9	missense	NM_01484	c.2047A>G	p.Ile683Val	-0.84	0.77	29	0	0.03(COSM39	Propeller,WD	rs10149146	0.3379	YES	1.195E-1 (pos.10290120:	acute_myeloic	Tectonin be	autophag\	Brain Diseases:Central Nervou		Benign	A=0, G=61	A=0.0, G=1.0	0.00001	921.799	61	A=0/0, G=32/2
chr11:120996292	G/G	A	G	SNV	TECTA	TECTA:exonic:NM_005422.2	1	100	7	synonymous	NM_00542	c.1485A>G	p.(=)	-8.63				0.01(COSM41	C8 domain,N	rs536069	0.3064	YES	6.779E-1 (pos.12099629:	neoplasm	Tectorin, al	C-termina	Ear Diseases;Hearing Disorder		Benign	A=0, G=40	A=0.0, G=1.0	0.00001	644.286	40	A=0/0, G=15/2
chr6:83754094	C/C	T	C	SNV	UBE3D	UBE3D:intronic:NM_198920.2	1	100		intronic	NM_19892	c.597+53A	p.?	-0.74					HECT-like Ubi	rs4455637		YES				cyclin binding,cytoplasm,protein autoubiquitination,pro				T=0, C=26	T=0.0, C=1.0	0.00001	371.419	26	T=0/0, C=15/1
chr11:121000784	C/C	T	C	SNV	TECTA	TECTA:exonic:NM_005422.2	1	100	9	synonymous	NM_00542	c.2805T>C	p.(=)	3.87				0.09(COSM41	C8 domain,N	rs586473	0.3096	YES	6.799E-1 (pos.12100078-	neoplasm	Tectorin, al	C-termina	Ear Diseases;Hearing Disorder		Benign	T=0, C=21	T=0.0, C=1.0	0.00001	279.648	21	T=0/0, C=10/1
chr11:121032978	A/A	G	A	SNV	TECTA	TECTA:exonic:NM_005422.2	1	100	15	missense	NM_00542	c.5171G>A	p.Ser1724Asn	5.9		46	0		C8 domain,N	rs526433	0.0107		1.0 (pos.121032978)		Tectorin, al	C-termina	Ear Diseases;Hearing Disorder		Benign	G=0, A=45	G=0.0, A=1.0	0.00001	747.393	45	G=0/0, A=33/1
chr6:86199329	C/C	G	C	SNV	NT5E	NT5E:intronic:NM_002526.3	1	100		intronic	NM_00252	c.1210+12	p.?	-1.13					5'-nucleotida	rs9450284	0.4172	YES	5.473E-1;0.0 (pos.86199329)		5' nucleotid	5'-nucleot	Calcinosis:Calcium_	Cytarabine,Pentoxifyllin		G=0, C=30	G=0.0, C=1.0	0.00001	446.633	30	G=0/0, C=21/9
chr6:86248460	C/C	T	C	SNV	SNX14	SNX14:intronic:NM_153816.5	1	100		intronic	NM_15381	c.1475+96	p.?	2.01					PX domain,P>	rs9294337		YES			Sorting nexi	autophag(	Ataxia;Brain Diseases:Central Nervous Syste			T=0, C=101	T=0.0, C=1.0	0.00001	1578.92	101	T=0/0, C=51/5
chr6:88228524	G/A	G	A	SNV	RARS2	RARS2:intronic:NM_020320.4	1	47.62		intronic	NM_02032	c.1305+18	p.?	0.28					DALR anticod	rs3734908	0.0469	YES	1.902E-2 (pos.88228524)		Arginyl-tRN/	ATP bindin	Autonomic Nervous System Di:		Benign	G=11, A=1(	G=0.5238, A=0.4762	0.00001	82.29	21	G=6/5, A=5/5
chr9:27183463	C/C	A	C	SNV	TEK	TEK:exonic:NM_000459.4	1	100	8	missense	NM_00045	c.1037A>C	p.Gln346Pro	1.09	1	76	0		Fibronectin t	rs682632	0.038	YES	9.999E-1;0.0 (pos.27183463)		TEK tyrosine	ATP bindin	Arteriovenous Malf	Ampicillin,N-(4-METHYL-3		A=0, C=25	A=0.0, C=1.0	0.00001	353.017	25	A=0/0, C=15/1
chr9:27205021	G/A	G	A	SNV	TEK	TEK:exonic:NM_000459.4	1	51.92	14	synonymous	NM_00045	c.2322G>A	p.(=)	1.35					Fibronectin t	rs542913	0.2585	YES	1.213E-1 (pos.27205021)		TEK tyrosine	ATP bindin	Arteriovenous Malf	Ampicillin	Benign	G=25, A=27	G=0.4808, A=0.5192	0.00001	270.394	52	G=10/15, A=1;
chr17:15215660	C/C	T	C	SNV	TEKT3	TEKT3:exonic:NM_031898.2	1	100	7	synonymous	NM_03189	c.1017A>G	p.(=)	0.62				0.27(COSM37	Tektin family	rs2286516	0.2586	YES	2.523E-1 (pos.15215660)	adenocarcinoı	Tektin 3	acrosomal membrane,cilium assembly,cilium movemen				T=0, C=91	T=0.0, C=1.0	0.00001	1513.8	91	T=0/0, C=61/3
chr16:1545448	G/G	A	G	SNV	TELO2	TELO2:exonic:NM_016111.3	1	100	3	missense	NM_01611	c.437A>G	p.Gln146Arg	0.59	0.41	43	0.346	0.23(COSM37	Telomere len	rs2235624	0.4048	YES	6.25E-1 (pos.1545448)	acute_myeloic	Telomere m	Hsp90 protein binding,TORC1 complex,TORC2 complex,c				A=0, G=22	A=0.0, G=1.0	0.00001	298.889	22	A=0/0, G=9/13
chr6:90361811	A/A	G	A	SNV	LOC101929057,MDN1	MDN1:intronic:NM_014611.2, LOC101929057:intronic_nc:N	1	100		intronic	NR_11191!	c.15915+1	p.?	0.24					AAA domain (	rs761729		YES				ATP binding,ATPase activity,cytosol,intermediate filame				G=0, A=69	G=0.0, A=1.0	0.00001	1076.63	69	G=0/0, A=56/1
chr6:90381849	G/G	A	G	SNV	LOC101929057,MDN1	MDN1:intronic:NM_014611.2, LOC101929057:intronic_nc:N	1	100		intronic	NR_11191!	c.13794+7	p.?	0.09					AAA domain (	rs7764076		YES				ATP binding,ATPase activity,cytosol,intermediate filame				A=0, G=83	A=0.0, G=1.0	0.00001	1349.2	83	A=0/0, G=50/3
chrX:123554646	C/C	T	C	SNV	TENM1	TENM1:exonic:NM_001163278.1	1	100	25	synonymous	NM_00116	c.4497A>G	p.(=)	-0.04				0.75(COSM40	GHH signatur	rs12013090	0.1293	YES	9.849E-2 (pos.12355464(	acute_myeloic	Teneurin tr:	Golgi apparatus,cell adhesion molecule binding,cell mo				T=0, C=29	T=0.0, C=1.0	0.00001	427.603	29	T=0/0, C=22/7
chr5:167674370	T/T	C	T	SNV	TENM2	TENM2:exonic:NM_001122679.1	1	100	27	synonymous	NM_00112	c.6399C>T	p.(=)	0.94				0.68(COSM15	GHH signatur	rs3733988	0.4727	YES	1.622E-1 (pos.16767437(	acute_myeloic	Teneurin transmembra		Mental Disorders;Substance-Related Disord(			C=0, T=23	C=0.0, T=1.0	0.00001	316.743	23	C=0/0, T=7/16
chr6:90432662	T/T	C	T	SNV	MDN1	MDN1:intronic:NM_014611.2	1	100		intronic	NM_01461	c.5967+12	p.?	-1.74					AAA domain (	rs756363	0.1691	YES	9.992E-1 (pos.90432662)			ATP binding,ATPase activity,cytosol,intermediate filame				C=0, T=20	C=0.0, T=1.0	0.00001	270.213	20	C=0/0, T=11/9
chr4:183652175	C/C	T	C	SNV	TENM3	TENM3:exonic:NM_001080477.3	1	100	16	synonymous	NM_00108	c.2850T>C	p.(=)	1.09				0.38(COSM45	GHH signatur	rs10866256		YES	1.0 (pos.183652175)	squamous_cel	Teneurin tr:	axon,cam(	Asthma:Asthma, Occupational		Benign	T=0, C=29	T=0.0, C=1.0	0.00001	427.602	29	T=0/0, C=19/1(
chr4:183601847	T/C	T	C	SNV	TENM3	TENM3:exonic:NM_001080477.3	1	50	10	synonymous	NM_00108	c.1791T>C	p.(=)	0.54					GHH signatur	rs2871328	0.3546	YES	7.908E-1 (pos.183601847)		Teneurin tr:	axon,cam(	Asthma:Asthma, Occupational		Benign	T=53, C=53	T=0.5, C=0.5	0.00001	540.492	106	T=13/40, C=16
chr14:20847202	A/G	A	G	SNV	TEP1	TEP1:exonic:NM_007110.4	1	59.09	36	synonymous	NM_00711	c.5190T>C	p.(=)	-3.98				0.01(COSM37	Domain of un	rs938887	0.1963	YES	2.621E-1 (pos.20847202)	adenocarcinoı	Telomerase	ATP bindin	Lung Diseases;Lung Neoplasms;Neoplasms:N			A=9, G=13	A=0.4091, G=0.5909	0.00001	122.568	22	A=7/2, G=6/7
chr14:20872881	G/T	G	T	SNV	TEP1	TEP1:exonic:NM_007110.4	1	58.33	5	missense	NM_00711	c.921C>A	p.Asn307Lys	0.08	0.19	94	0	0.02(COSM39	Domain of un	rs1760898	0.2169	YES	4.372E-1 (pos.20872881)	acute_myeloic	Telomerase	ATP bindin	Lung Diseases;Lung Neoplasms;Neoplasms:N			G=10, T=14	G=0.4167, T=0.5833	0.00001	135.339	24	G=2/8, T=10/4
chr17:56659018	T/T	C	T	SNV	TEX14	TEX14:exonic:NM_001201457.1	1	100	20	missense	NM_00120	c.3263G>A	p.Gly1088Asp	0.44	0.85	94	0	0(COSM4000:	Ankyrin repea	rs6503870	0.3762	YES	4.814E-1 (pos.56659018)	acute_myeloic	Testis-expr(	ATP binding,attachment of spindle microtubules to kinet				C=0, T=52	C=0.0, T=1.0	0.00001	767.015	52	C=0/0, T=36/1(
chr6:96034881	ATATG/ATATG	A	ATATG	INDEL	MANEA	MANEA:intronic:NM_024641.3	4	100		intronic	NM_02464	c.544+22A	p.?	-0.76				0.03(COSM39	Glycosyl hydr	rs112818736,rs78175628			4.884E-2;3.728E-1;5.635(	squamous_cel	Mannosida	Golgi app:	Adnexal Diseases;Cocaine-Related Disorders			A=0, ATATG	A=0.0, ATATG=1.0	0.00001	332.961	24	A=0/0, ATATG=
chr17:80320561	A/A	G	A	SNV	TEX19	TEX19:utr_3:NM_207459.3	1	100	2	utr_3	NM_20745	c.535G>A	p.?	0.13					Testis-expres	rs7502620	0.2626	YES	3.299E-1 (pos.80320561)		Testis-expr(	cell differentiation,cytoplasm,male meiotic nuclear divi:				G=0, A=36	G=0.0, A=1.0	0.00001	563.744	36	G=0/0, A=11/2
chr6:99771197	T/C	T	C	SNV	FAXC	FAXC:intronic:NM_032511.3	1	58.33		intronic	NM_03251	c.823+123	p.?	0.72					Glutathione !	rs9389250		YES				integral component of membrane				T=10, C=14	T=0.4167, C=0.5833	0.00001	135.327	24	T=3/7, C=13/1
chr13:111980537	T/T	C	T	SNV	TEX29	TEX29:exonic:NM_152324.2	1	100	3	synonymous	NM_15232	c.66C>T	p.(=)	-0.01				0.28(COSM37	Testis-expres	rs1359428	0.4878	YES	8.213E-1 (pos.11198053;	adenocarcinoma		integral component of membrane				C=0, T=26	C=0.0, T=1.0	0.00001	371.42	26	C=0/0, T=20/6
chr6:99853866	A/A	G	A	SNV	PNISR	PNISR:intronic:NM_015491.2	1	100		intronic	NM_01549	c.1002+41	p.?	0.35					Arginine/seri	rs7744072	1.00E-04	YES	1.0 (pos.99853866)		PNN-intera(	RNA binding,cytosol,nuclear speck				G=0, A=68	G=0.0, A=1.0	0.00001	1041.41	68	G=0/0, A=49/1
chr2:232458443	C/C	T	C	SNV	TEX44	TEX44:exonic:NM_152614.2	1	100	1	missense	NM_15261	c.781T>C	p.Ser261Pro	-0.94		74		0.01(COSM37	Domain of un	rs10933378	0.0928	YES	9.988E-1;0.0 (pos.23245{	adenocarcinoma,neoplasn		cytoplasm,protein binding				T=0, C=36	T=0.0, C=1.0	0.00001	563.745	36	T=0/0, C=17/1
chr2:232457880	T/C	T	C	SNV	TEX44	TEX44:exonic:NM_152614.2	1	57.89	1	missense	NM_15261	c.218T>C	p.Val73Ala	-0.78		64			Domain of un	rs74460533	0.0774	YES	3.585E-3 (pos.232457880)			cytoplasm,protein binding				T=16, C=22	T=0.4211, C=0.5789	0.00001	249.054	38	T=8/8, C=9/13
chr7:88424115	G/G	C	G	SNV	TEX47,ZNF804B	ZNF804B:intronic:NM_181646.3, TEX47:exonic:NM_152706	1	100	2	missense	NM_18164	c.108+347	p.?, p.Asp48His	0.09		81		0(COSM4417:	Zinc-finger dc	rs2373396,rs778078	0.1481	YES	8.86E-2;0.0 (pos.884241:	acute_myeloid_leukaemia		metal ion binding,nucleic acid binding				C=0, G=153	C=0.0, G=1.0	0.00001	2448.66	153	C=0/0, G=85/6
chr6:99930593	C/T	C	T	SNV	USP45	USP45:intronic:NM_001080481.1	1	50		intronic	NM_00108	c.845+36G	p.?	-0.26					Ubiquitin car	rs117666915	0.0204	YES	0.0 (pos.99930593)			DNA repair,cytoplasm,global genome nucleotide-excisio				C=30, T=30	C=0.5, T=0.5	0.00001	293.738	60	C=16/14, T=20
chr6:99951750	A/A	G	A	SNV	USP45	USP45:intronic:NM_001080481.1	1	100		intronic	NM_00108	c.378-9C>1	p.?	1.15					Ubiquitin car	rs6934692,rs746507	0.2039	YES	0.0 (pos.99951747),9.993E-1 (pos.99951750)			DNA repair,cytoplasm,global genome nucleotide-excisio				G=0, A=50	G=0.0, A=1.0	0.00001	848.023	50	G=0/0, A=20/3
chr7:100224636	C/CTG	C	CTG	INDEL	TFR2	TFR2:intronic:NM_003227.3	2	52.38		intronic	NM_00322	c.1996-11(	p.?	1.71					PA domain,P(	rs35142847,rs66849551		YES			Transferrin	HFE-transf	Anemia:Anemia, He	Gallium citrate Ga 67,Ga		C=10, CTG=	C=0.4762, CTG=0.52	0.00001	95.3003	21	C=5/5, CTG=5/
chr7:100280896	C/C	A	C	SNV	GIGYF1	GIGYF1:intronic:NM_022574.5	1	100		intronic	NM_02257	c.2193+31	p.?	-0.91					GYF domain	rs221793	0.0933	YES	1.0 (pos.100280896)		GRB10-inte	biological_process,insulin-like growth factor receptor si				A=0, C=41	A=0.0, C=1.0	0.00001	664.701	41	A=0/0, C=26/1
chr9:117428960	C/C	T	C	SNV	TEX48	TEX48:exonic:NM_001199233.1	1	100	5	missense	NM_00119	c.326A>G	p.His109Arg	-2.68		29	0			rs1055207		YES	6.364E-1 (pos.117428960)							T=0, C=88	T=0.0, C=1.0	0.00001	1442.5	88	T=0/0, C=41/4
chr9:117430611	T/T	A	T	SNV	TEX48	TEX48:exonic:NM_001199233.1	1	100	4	missense	NM_00119	c.134T>A	p.Leu45Gln	0.67		113	0			rs1359992		YES	7.353E-1 (pos.117430611)							A=0, T=26	A=0.0, T=1.0	0.00001	371.392	26	A=0/0, T=6/20
chr16:4310468	G/G	C	G	SNV	TFAP4	TFAP4:exonic:NM_003223.2	1	100	5	missense	NM_00322	c.654G>C	p.Gln218His	0.6	0.34	24	0	0.1(COSM137	Helix-loop-he	rs251732	0.4992	YES	7.955E-1;0.0 (pos.43104(	squamous_cel	Transcriptic	DNA binding,DNA damage response, signal transduction				C=0, G=30	C=0.0, G=1.0	0.00001	441.368	30	C=0/0, G=14/1
chr6:41652604	C/C	T	C	SNV	TFEB	TFEB:exonic:NM_001167827.2	1	100	9	synonymous	NM_00116	c.1206A>G	p.(=)	0.09				0.05(COSM45	Domain of un	rs1062966	0.2826	YES	7.728E-1 (pos.41652604)	neoplasm	Transcriptic	DNA binding,transcription factor activity,adaptive immu				T=0, C=24	T=0.0, C=1.0	0.00001	334.776	24	T=0/0, C=8/16
chr6:41658889	T/T	C	T	SNV	TFEB	TFEB:exonic:NM_001167827.2	1	100	2	synonymous	NM_00116	c.105G>A	p.(=)	3.32				0.91(COSM15	Domain of un	rs1015149	0.4772	YES	4.705E-1 (pos.41658889)	adenocarcinoı	Transcriptic	DNA binding,transcription factor activity,adaptive immu				C=0, T=47	C=0.0, T=1.0	0.00001	787.959	47	C=0/0, T=12/3
chr3:100467018	C/C	T	C	SNV	TFG	TFG:exonic:NM_001195478.1	1	100	8	synonymous	NM_00119	c.846T>C	p.(=)	1.05				0.3(COSM415	PB1 domain	rs11353	0.3433	YES	6.366E-1 (pos.10046701{	acute_myeloic	TRK-fused g	COPII vesi(	Autoimmune Diseases;Carcinc		Benign	T=0, C=49	T=0.0, C=1.0	0.00001	831.632	49	T=0/0, C=33/1
chr7:100468103	G/A	G	A	SNV	TRIP6	TRIP6:intronic:NM_003302.2	1	50		intronic	NM_00330	c.829+18G	p.?	-0.29					LIM domain	rs7800241	0.2886	YES	4.57E-1 (pos.100468103)		Thyroid hor	RNA binding,cytoskeleton,cytosol,focal adhesion,focal a				G=10, A=1(	G=0.5, A=0.5	0.00001	82.8861	20	G=2/8, A=5/5
chr22:26895337	A/A	G	A	SNV	TFIP11	TFIP11:exonic:NM_001008697.2	1	100	10	synonymous	NM_00100	c.1062C>T	p.(=)	-0.93				0.09(COSM41	G-patch dom:	rs1008530	0.1266	YES	8.162E-1 (pos.26895337)	neoplasm	Tuftelin-int(	DNA binding,RNA processing,U2-type post-mRNA release				G=0, A=25	G=0.0, A=1.0	0.00001	353.016	25	G=0/0, A=9/16
chr8:133900252	G/G	T	G	SNV	TG	TG:exonic:NM_003235.4	1	100	10	missense	NM_00323	c.2200T>G	p.Ser734Ala	6.08	1	99	0	0.05(COSM41	Carboxyleste	rs180223	0.4828	YES	7.609E-1;0.0;0.0 (pos.13:	neoplasm	Thyroglobul	extracellu	Autoimmune Diseases;Bone D		Benign/Likely	T=0, G=88	T=0.0, G=1.0	0.00001	1457.02	88	T=0/0, G=40/4
chr8:133900386	C/C	T	C	SNV	TG	TG:exonic:NM_003235.4	1	100	10	synonymous	NM_00323	c.2334T>C	p.(=)	-7.78					Carboxyleste	rs2069550	0.4824	YES	7.609E-1 (pos.133900386)		Thyroglobul	extracellu	Autoimmune Diseases;Bone D		Benign/Likely	T=0, C=89	T=0.0, C=1.0	0.00001	1472.41	89	T=0/0, C=34/5
chr8:133909974	G/G	A	G	SNV	TG	TG:exonic:NM_003235.4	1	100	12	missense	NM_00323	c.3082A>G	p.Met1028Val	-0.45	1	21	0	0(COSM4162(	Carboxyleste	rs853326	0.4824	YES	7.598E-1 (pos.13390997	neoplasm	Thyroglobul	extracellu	Autoimmune Diseases;Bone D		Benign/Likely	A=0, G=43	A=0.0, G=1.0	0.00001	705.84	43	A=0/0, G=13/3
chr8:133980214	A/G	A	G	SNV	TG	TG:exonic:NM_003235.4	1	45.45	31	synonymous	NM_00323	c.5862A>G	p.(=)	1.35					Carboxyleste	rs16904799	0.0031	YES	0.0 (pos.133980214)		Thyroglobul	extracellu	Autoimmune Diseases;Bone D		Likely benign	A=24, G=2(	A=0.5455, G=0.4545	0.00001	207.513	44	A=16/8, G=8/1
chr5:135382989	C/C	G	C	SNV	TGFBI	TGFBI:exonic:NM_000358.2	1	100	6	synonymous	NM_00035	c.651G>C	p.(=)	-0.34				0.45(COSM40	Fasciclin dor	rs1442	0.4659	YES	3.71E-1 (pos.135382989)	acute_myeloic	Transformir	angiogene	Abortion, Spontaneous:Autoin		Benign/Likely	G=0, C=109	G=0.0, C=1.0	0.00001	1718.92	109	G=0/0, C=57/5
chr1:92174260	G/G	A	G	SNV	TGFBR3	TGFBR3:exonic:NM_003243.4	1	100	14	synonymous	NM_00324	c.2247T>C	p.(=)	2.56					Zona pellucic	rs284878	0.0585	YES	7.731E-1 (pos.92174260)		Transformir	BMP signaling pathway,PDZ domain binding,SMAD bindir				A=0, G=30	A=0.0, G=1.0	0.00001	446.639	30	A=0/0, G=19/1
chr14:24718515	A/A	G	A	SNV	TGM1	TGM1:utr_3:NM_000359.2	1	100	15	utr_3	NM_00035	c.2458C>T	p.?	0.33					Transglutami	rs2229463	0.1402	YES	5.814E-1 (pos.24718515)		Transglutar	cell envelc	Acneiform Eruption	L-Glutamine		G=0, A=67	G=0.0, A=1.0	0.00001	1038.85	67	G=0/0, A=31/3
chr20:2290333	A/A	C	A	SNV	TGM3	TGM3:exonic:NM_003245.3	1	100	2	missense	NM_00324	c.38C>A	p.Thr13Lys	-1.74	0.38	78	0.727	0(COSM5048:	Transglutami	rs214803	0.1793	YES	9.315E-1 (pos.2290333)	NS,adenocarci	Transglutar	calcium io	Carcinoma:Carcino	5'-Guanosine-Diphospha		C=0, A=25	C=0.0, A=1.0	0.00001	352.81	25	C=0/0, A=15/1
chr15:43571390	C/G	C	G	SNV	TGM7	TGM7:exonic:NM_052955.2	1	47.62	11	synonymous	NM_05295	c.1764G>C	p.(=)	-1.38				0(COSM3754:	Transglutami	rs567357	0.3271	YES	4.157E-1;0.0 (pos.43571:	adenocarcinoı	Transglutar	metal ion binding,peptide cros		L-Glutamine		C=44, G=4(	C=0.5238, G=0.4762	0.00001	418.276	84	C=22/22, G=2!
chr2:85549868	G/G	A	G	SNV	TGOLN2	TGOLN2:exonic:NM_001206840.1	1	100	4	stoploss	NM_00120	c.1363T>C	p.Ter455Gln	-2.37						rs4240199	0.3944	YES	9.643E-1 (pos.85549868)		Trans-golgi	Golgi app:	Endocrine System Diseases;Thyroid Diseases			A=0, G=45	A=0.0, G=1.0	0.00001	751.805	45	A=0/0, G=18/2
chr2:85553784	T/T	C	T	SNV	TGOLN2	TGOLN2:exonic:NM_001206840.1	1	100	2	synonymous	NM_00120	c.1071G>A	p.(=)	-1.44				0.01(COSM5653851),0.01(C		rs1044973	0.458	YES	8.845E-1 (pos.85553784)	diffuse_large_	Trans-golgi	Golgi app:	Endocrine System Diseases;Thyroid Diseases			C=0, T=20	C=0.0, T=1.0	0.00001	263.783	20	C=0/0, T=11/9
chr8:56686224	C/C	T	C	SNV	TGS1,TMEM68	TGS1:exonic:NM_024831.7, TMEM68:upstream:NM_152417	1	100	1	missense	NM_15241	c.-10706A:	p.?, p.Ile16Thr	1.99	0.06	89	0.09		Met-10+ like-	rs1818	0.1085	YES	9.921E-1 (pos.56686224)		Trimethylgu	7-methylguanosine RNA capping,7-methylguanosine cap				T=0, C=23	T=0.0, C=1.0	0.00001	316.747	23	T=0/0, C=14/9
chr22:21354970	G/G	C	G	SNV	THAP7	THAP7:exonic:NM_001008695.1	1	100	4	missense	NM_00100	c.343G>C	p.Ala115Pro	0.22	1	27	0	0.01(COSM14	THAP domain	rs426938	0.1416	YES	8.442E-1;1.156E-4 (pos.2	adenocarcinoma,squamou		C2H2 zinc finger domain binding,DNA binding,chromosor				C=0, G=22	C=0.0, G=1.0	0.00001	298.892	22	C=0/0, G=9/13
chr7:100692680	G/G	T	G	SNV	MUC17	MUC17:intronic:NM_001040105.1	1	100		intronic	NM_00104	c.12722+1	p.?	-0.21					SEA domain	rs10246021	0.3828	YES	8.097E-1;0.0 (pos.100692680)		Mucin 17	Golgi lumen,O-glycan processing,PDZ domain binding,ap				T=0, G=41	T=0.0, G=1.0	0.00001	660.405	41	T=0/0, G=22/1
chr4:83838262	G/T	G	T	SNV	THAP9	THAP9:exonic:NM_024672.5	1	50	5	missense	NM_02467	c.897G>T	p.Leu299Phe	0.29	0.16	22	0.906	0.72(COSM40	THAP domain	rs897945	0.442	YES	1.999E-1 (pos.83838262)	acute_myeloic	THAP doma	DNA binding,DNA integration,DNA recombination,metal				G=38, T=38	G=0.5, T=0.5	0.00001	391.319	76	G=21/17, T=3(
chr6:169641971	C/T	C	T	SNV	THBS2	THBS2:exonic:NM_003247.3	1	57.14	6	synonymous	NM_00324	c.777G>A	p.(=)	-0.52				0.06(COSM37	Calcium-bind	rs61730651	0.1858	YES	7.816E-3 (pos.16964197:	adenocarcinoı	Thrombosp	basement	Bone Diseases;Diabetes Mellitus;Endocrine !			C=21, T=28	C=0.4286, T=0.5714	0.00001	342.304	49	C=10/11, T=17
chr7:101265350	C/C	T	C	SNV	MYL10	MYL10:intronic:NM_138403.4	1	100		intronic	NM_13840	c.454+26A	p.?	0.89					EF hand,EF-h:	rs4263675	0.0327	YES	9.998E-1;0.0 (pos.101265350)		Myosin ligh	calcium ion binding,cytosol,mitochondrion,muscle cont				T=0, C=98	T=0.0, C=1.0	0.00001	1657.1	98	T=0/0, C=35/6
chr7:101747575	A/G	A	G	SNV	CUX1	CUX1:intronic:NM_181552.3	1	50		intronic	NM_18155	c.407-41A:	p.?	-0.19					CUT domain,I	rs201480,rs3765554	0.3898	YES	9.943E-2 (pos.101747575)			Golgi app:	Carcinogenesis;Neoplasms;Neoplastic Proce			A=12, G=12	A=0.5, G=0.5	0.00001	106.708	24	A=4/8, G=9/3
chr7:101870599	G/G	A	G	SNV	CUX1	CUX1:intronic:NM_181552.3	1	100		intronic	NM_18155	c.3131-48/	p.?	-0.66					CUT domain,I	rs430841	0.0034		1.0 (pos.101870599)			Golgi app:	Carcinogenesis;Neoplasms;Neoplastic Proce			A=0, G=20	A=0.0, G=1.0	0.00001	263.773	20	A=0/0, G=8/12
chr6:169648767	G/A	G	A	SNV	THBS2	THBS2:exonic:NM_003247.3	1	50	4	synonymous	NM_00324	c.354C>T	p.(=)	0.03				0.29(COSM40	Calcium-bind	rs12178180	0.2633	YES	1.656E-2 (pos.16964876:	acute_myeloic	Thrombosp	basement	Bone Diseases;Diabetes Mellitus;Endocrine !			G=16, A=16	G=0.5, A=0.5	0.00001	158.082	32	G=5/11, A=8/8
chr7:102109609	C/T	C	T	SNV	LRWD1	LRWD1:intronic:NM_152892.2	1	95.83		intronic	NM_15289	c.1228+10	p.?	-3.1					Leucine Rich	rs10953369		YES			Leucine-ricl	DNA replication initiation,chromatin binding,chromatin				C=1, T=23	C=0.0417, T=0.9583	0.00001	306.632	24	C=0/1, T=3/20
chr7:102110366	A/A	T	A	SNV	LRWD1	LRWD1:intronic:NM_152892.2	1	100		intronic	NM_15289	c.1442+37	p.?	-0.09					Leucine Rich	rs1044424608,rs132:	0.3262	YES	4.279E-1 (pos.102110366)		Leucine-ricl	DNA replication initiation,chromatin binding,chromatin				T=0, A=62	T=0.0, A=1.0	0.00001	940.314	62	T=0/0, A=6/56
chr7:102760722	G/G	A	G	SNV	NAPEPLD	NAPEPLD:intronic:NM_001122838.1	1	100		intronic	NM_00112	c.295-52T:	p.?	0.27					Beta-lactam:	rs1974715,rs772231(	0.0026	YES	0.0 (pos.102760721),1.0 (pos.10276072:		N-acyl phos	N-acetylphosphatidylethanolamine-hydrolysing phosph(				A=0, G=96	A=0.0, G=1.0	0.00001	1617.91	96	A=0/0, G=35/6
chr5:79351735	C/T	C	T	SNV	THBS4	THBS4:exonic:NM_003248.5	1	57.14	3	synonymous	NM_00324	c.420C>T	p.(=)	-2.66				0.02(COSM40	Calcium-bind	rs423906	0.1755	YES	2.243E-1 (pos.79351735)	acute_myeloic	Thrombosp	basement membrane,behavioral response to pain,calci				C=9, T=12	C=0.4286, T=0.5714	0.00001	109.102	21	C=2/7, T=6/6
chr7:103018016	G/G	A	G	SNV	SLC26A5	SLC26A5:intronic:NM_198999.2	1	100		intronic	NM_19899	c.1986+30	p.?	0.06					STAS domain,	rs10273883	0.0016	YES	1.0 (pos.103018016)		Solute carri	anion:anic	Deafness;Ear Diseases;Hearing Disorders;He			A=0, G=43	A=0.0, G=1.0	0.00001	705.784	43	A=0/0, G=20/2
chr7:103138517	G/G	C	G	SNV	LOC101927870,RELN	RELN:intronic:NM_173054.2, LOC101927870:intronic_nc:NF	1	100		intronic	NR_11014:	c.8843+7G	p.?	0.08					Human growt	rs2711885	0.0129	YES	9.999E-1 (pos.103138517)		Reelin	NMDA glut	Alzheimer Disease;Autistic Dis		Benign/Likely	C=0, G=28	C=0.0, G=1.0	0.00001	408.703	28	C=0/0, G=18/1
chr7:103175965	C/T	C	T	SNV	RELN	RELN:intronic:NM_173054.2	1	96.77		intronic	NM_17305	c.7181-34(	p.?	-0.22					Human growt	rs362706	0.2413	YES	6.005E-1;0.0 (pos.103175965)		Reelin	NMDA glut	Alzheimer Disease;Autistic Dis		Benign	C=2, T=60	C=0.0323, T=0.9677	0.00001	877.614	62	C=1/1, T=17/4
chr7:103275803	C/C	T	C	SNV	RELN	RELN:intronic:NM_173054.2	1	100		intronic	NM_17305	c.2465+69	p.?	-0.07					Human growt	rs362646		YES			Reelin	NMDA glut	Alzheimer Disease;Autistic Disorder;Bipolar I			T=0, C=51	T=0.0, C=1.0	0.00001	754.584	51	T=0/0, C=33/1
chr7:104800939	C/T	C	T	SNV	SRPK2	SRPK2:intronic:NM_182692.2	1	46.43		intronic	NM_18269	c.621+15G	p.?	-0.75					Protein kinas	rs4730071	0.1249	YES	3.622E-1 (pos.104800939)			14-3-3 pro	Eye Diseases;Ment:	Adenine,Phosphoamino		C=15, T=13	C=0.5357, T=0.4643	0.00001	117.926	28	C=7/8, T=6/7
chr19:32083696	C/C	T	C	SNV	THEG5	THEG5:exonic:NM_001205273.3	1	100	2	synonymous	NM_00120	c.198T>C	p.(=)	-0.93						rs7245697		YES	1.0 (pos.32083696)							T=0, C=23	T=0.0, C=1.0	0.00001	316.373	23	T=0/0, C=5/18
chr7:105733619	A/A	G	A	SNV	SYPL1	SYPL1:intronic:NM_006754.3	1	100		intronic	NM_00675	c.457-36C:	p.?	-1.01					Membrane-a	rs176486	0.0153	YES	1.0 (pos.105733619)		Synaptophy	chemical !	Autoimmune Diseases;Female Urogenital Di:			G=0, A=266	G=0.0, A=1.0	0.00001	4393.47	266	G=0/0, A=134/
chr7:105752555	G/A	G	A	SNV	SYPL1	SYPL1:intronic:NM_006754.3	1	100		intronic	NM_00675	c.123+31C	p.?	-0.04					Membrane-a	rs2240473	0.4819	YES	5.582E-1 (pos.105752555)		Synaptophy	chemical !	Autoimmune Diseases;Female Urogenital Di:			G=0, A=27	G=0.0, A=1.0	0.00001	345.238	27	G=0/0, A=9/18
chr6:128040839	C/C	T	C	SNV	THEMIS	THEMIS:exonic:NM_001164685.1	1	100	6	missense	NM_00116	c.2005A>G	p.Ile669Val	-0.55		29	0	0.01(COSM15	Cell-cycle sus	rs675531	0.3176	YES	9.84E-2 (pos.128040839)	adenocarcinoı	Thymocyte-	COP9 sign:	Celiac Disease;Digestive System Diseases;Ga			T=0, C=174	T=0.0, C=1.0	0.00001	2860.64	174	T=0/0, C=93/8
chr1:28209305	G/G	A	G	SNV	THEMIS2	THEMIS2:exonic:NM_001105556.2	1	100	4	synonymous	NM_00110	c.1470A>G	p.(=)	-0.26				0.03(COSM41	Cell-cycle sus	rs3766398	0.3496	YES	5.385E-1 (pos.28209305)	neoplasm		T cell rece	Breast Diseases;Breast Neoplasms;Neoplasr			A=0, G=48	A=0.0, G=1.0	0.00001	810.435	48	A=0/0, G=27/2
chr7:106519943	C/C	T	C	SNV	PIK3CG	PIK3CG:intronic:NM_002649.3	1	100		intronic	NM_00264	c.2392-21'	p.?	-0.01					PI3-kinase fa	rs849370	0.0537	YES	9.988E-1 (pos.106519943)		Phosphatid	1-phospha	Autistic Disorder;C:	(1S,6BR,9AS,11R,11BR)-9		T=0, C=45	T=0.0, C=1.0	0.00001	747.392	45	T=0/0, C=26/1
chr1:28209362	C/C	T	C	SNV	THEMIS2	THEMIS2:exonic:NM_001105556.2	1	100	4	synonymous	NM_00110	c.1527T>C	p.(=)	-3.59					Cell-cycle sus	rs3766399	0.3486	YES	5.26E-1 (pos.28209362)			T cell rece	Breast Diseases;Breast Neoplasms;Neoplasr			T=0, C=50	T=0.0, C=1.0	0.00001	852.745	50	T=0/0, C=27/2
chr1:28209366	G/G	A	G	SNV	THEMIS2	THEMIS2:exonic:NM_001105556.2	1	100	4	missense	NM_00110	c.1531A>G	p.Lys511Glu	1.85	0.6	56	0		Cell-cycle sus	rs3766400	0.3586	YES	5.262E-1 (pos.28209366)			T cell rece	Breast Diseases;Breast Neoplasms;Neoplasr			A=0, G=50	A=0.0, G=1.0	0.00001	852.73	50	A=0/0, G=27/2
chr7:106836242	C/C	T	C	SNV	HBP1	HBP1:intronic:NM_001244262.1	1	100		intronic	NM_00124	c.1098-37'	p.?	-0.28					Ataxin-1 and	rs3801964	0.2281	YES	4.473E-1 (pos.106836242)		HMG-box tr	DNA binding,RNA binding,Wnt signaling pathway,cell cyc				T=0, C=28	T=0.0, C=1.0	0.00001	408.711	28	T=0/0, C=20/8
chr7:106922004	A/A	T	A	SNV	COG5	COG5:intronic:NM_006348.3	1	100		intronic	NM_00634	c.1569-16(	p.?	2.3					Golgi transpc	rs6954422		YES			Component	ER to Golgi	Arthritis;Joint Diseases;Musculoskeletal Dis(			T=0, A=23	T=0.0, A=1.0	0.00001	316.737	23	T=0/0, A=10/1
chr7:106965009	G/G	A	G	SNV	COG5	COG5:intronic:NM_006348.3	1	100		intronic	NM_00634	c.1120-43'	p.?	1.48					Golgi transpc	rs62482495	0.2389	YES	3.692E-1 (pos.106965009)		Component	ER to Golgi	Arthritis;Joint Diseases;Musculoskeletal Dis(			A=0, G=44	A=0.0, G=1.0	0.00001	726.546	44	A=0/0, G=15/2
chr7:106965039	T/T	C	T	SNV	COG5	COG5:intronic:NM_006348.3	1	100		intronic	NM_00634	c.1120-73(	p.?	0.1					Golgi transpc	rs6466164		YES			Component	ER to Golgi	Arthritis;Joint Diseases;Musculoskeletal Dis(			C=0, T=38	C=0.0, T=1.0	0.00001	603.755	38	C=0/0, T=8/30
chr18:252559	A/A	G	A	SNV	THOC1	THOC1:exonic:NM_005131.2	1	100	9	synonymous	NM_00513	c.657C>T	p.(=)	3.42					Death domai	rs657138	4.00E-04	YES	1.0 (pos.252559)		THO comple	DNA binding,RNA binding,RNA export from nucleus,RNA p				G=0, A=23	G=0.0, A=1.0	0.00001	316.743	23	G=0/0, A=15/8
chr7:107188719	T/T	G	T	SNV	COG5	COG5:intronic:NM_006348.3	1	100		intronic	NM_00634	c.440+4C>	p.?	-0.12				0.07(COSM40	Golgi transpc	rs2066735	0.296	YES	3.298E-1 (pos.10718871!	acute_myeloic	Component	ER to Golgi	Arthritis;Joint Diseases;Muscu		Benign/Likely	G=0, T=51	G=0.0, T=1.0	0.00001	754.566	51	G=0/0, T=20/3
chr22:29913272	T/T	C	T	SNV	THOC5	THOC5:exonic:NM_001002879.1	1	100	16	missense	NM_00100	c.1573G>A	p.Val525Ile	0.25	1	29	0		Fms-interact	rs737976	0.4977	YES	7.584E-1 (pos.29913272)		THO comple	RNA export from nucleus,RNA splicing,THO complex,THO				C=0, T=31	C=0.0, T=1.0	0.00001	465.787	31	C=0/0, T=9/22
chr7:107545799	T/C	T	C	SNV	DLD	DLD:intronic:NM_000108.4	1	58.33		intronic	NM_00010	c.439-7T>(	p.?	0.54					FAD binding c	rs10263341	0.4216	YES	8.489E-1 (pos.107545799)		Dihydrolipo	2-oxogluta	Amino Acid Disorde	Flavin ade	Benign/Likely	T=10, C=14	T=0.4167, C=0.5833	0.00001	135.284	24	T=7/3, C=5/9
chr2:137852550	G/A	G	A	SNV	THSD7B	THSD7B:exonic:NM_001316349.1	1	50	4	missense	NM_00131	c.1058G>A	p.Arg353His	1.39	0.72	29	0.024		Thrombospoı	rs61741426	0.054	YES	1.162E-4 (pos.137852550)							G=13, A=13	G=0.5, A=0.5	0.00001	119.163	26	G=5/8, A=5/8
chr7:107746214	TG/GC	TG	GC	MNV	LAMB4	LAMB4:intronic:NM_007356.2	2	50		intronic	NM_00735	c.870+48C	p.?	0.09, -0.14					Laminin EGF (	rs370533,rs3867166!	0.0352	YES	2.186E-1 (pos.107746215),3.82E-1 (pos.1		Laminin, be	basement membrane,cell adhesion				TG=15, GC=	TG=0.5, GC=0.5	0.00001	145.021	30	TG=5/10, GC=
chr7:107838637	A/A	C	A	SNV	NRCAM	NRCAM:intronic:NM_001037132.2	1	100		intronic	NM_00103	c.1208-94(	p.?	-0.98					Bravo-like int	rs388557		YES			Neuronal ce	angiogene	Autistic Disorder;Breast Diseases;Breast Ne(			C=0, A=58	C=0.0, A=1.0	0.00001	865.817	58	C=0/0, A=30/2
chr7:111382324	T/T	C	T	SNV	DOCK4	DOCK4:intronic:NM_014705.3	1	100		intronic	NM_01470	c.4651-13(	p.?	-0.44					C2 domain in	rs3757648		YES			Dedicator c	GTPase ac	Autistic Disorder;Child Development Disorde			C=0, T=111	C=0.0, T=1.0	0.00001	1756.86	111	C=0/0, T=58/5
chr7:111629025	A/A	T	A	SNV	DOCK4	DOCK4:intronic:NM_014705.3	1	100		intronic	NM_01470	c.464+45A	p.?	-2.83					C2 domain in	rs17401943	0.3508	YES	5.61E-3 (pos.111629025)		Dedicator c	GTPase ac	Autistic Disorder;Child Development Disorde			T=0, A=25	T=0.0, A=1.0	0.00001	352.715	25	T=0/0, A=13/1
chr2:137917922	T/A	T	A	SNV	THSD7B	THSD7B:exonic:NM_001316349.1	1	50	6	missense	NM_00131	c.1509T>A	p.Asp503Glu	1.12	0.15	45	0.006		Thrombospoı	rs4954474	0.4801	YES	1.313E-1 (pos.137917922)							T=12, A=12	T=0.5, A=0.5	0.00001	106.697	24	T=3/9, A=8/4
chr3:9426224	G/A	G	A	SNV	THUMPD3	THUMPD3:exonic:NM_015453.2	1	50	10	missense	NM_01545	c.1376G>A	p.Arg459Gln	5.39	0.01	43	0.056	0.94(COSM14	Putative RNA	rs1129174	0.1481	YES	9.799E-2 (pos.9426224)	adenocarcinoma,carcinom		RNA binding,cytosol,nucleolus,tRNA (guanine) methyltra				G=29, A=29	G=0.5, A=0.5	0.00001	282.577	58	G=15/14, A=1
chr7:115608355	G/G	T	G	SNV	TFEC	TFEC:intronic:NM_012252.3	1	100		intronic	NM_01225	c.267+586	p.?	0.29					Domain of un	rs10248479		YES			Transcriptic	DNA bindi	Autoimmune Diseases;Female Urogenital Di:			T=0, G=21	T=0.0, G=1.0	0.00001	281.231	21	T=0/0, G=10/1
chr7:115874560	C/T	C	T	SNV	TES	TES:intronic:NM_015641.3	1	50		intronic	NM_01564	c.28-28C>1	p.?	-0.3					LIM domain,F	rs2030978	0.2652	YES	5.665E-1 (pos.115874560)		Testin	RNA bindi	Astrocytoma;Glioblastoma;Glioma;Neoplasr			C=29, T=29	C=0.5, T=0.5	0.00001	282.583	58	C=19/10, T=14
chr7:116555991	G/G	A	G	SNV	CAPZA2	CAPZA2:intronic:NM_006136.2	1	100		intronic	NM_00613	c.658-123/	p.?	-0.6					F-actin cappi	rs1858968					Capping prc	ER to Golgi	Autoimmune Diseases;Female Urogenital Di:			A=0, G=34	A=0.0, G=1.0	0.00001	524.186	34	A=0/0, G=31/3
chr6:155561760	C/C	T	C	SNV	TIAM2	TIAM2:exonic:NM_012454.3	1	100	15	missense	NM_01245	c.3265T>C	p.Ser1089Pro	1.52	1	74	0		PDZ domain (	rs4259257	0.0052	YES	9.995E-1 (pos.155561760)		T-cell lymph	G-protein	Mental Disorders;Substance-Related Disorde			T=0, C=85	T=0.0, C=1.0	0.00001	1390.49	85	T=0/0, C=55/3
chr7:120372823	A/G	A	G	SNV	KCND2	KCND2:intronic:NM_012281.2	1	56		intronic	NM_01228	c.1116-13:	p.?	-0.17					BTB/POZ dom	rs12673992		YES			Potassium	A-type (transient outward) pot:		Amitriptyline,Dalfampric		A=11, G=14	A=0.44, G=0.56	0.00001	134.349	25	A=10/1, G=11/
chr6:155575607	G/A	G	A	SNV	TIAM2	TIAM2:exonic:NM_012454.3	1	50	25	synonymous	NM_01245	c.4368G>A	p.(=)	-0.38					PDZ domain (	rs3011895	0.4156	YES	1.167E-2 (pos.155575607)		T-cell lymph	G-protein	Mental Disorders;Substance-Related Disorde			G=14, A=14	G=0.5, A=0.5	0.00001	128.21	28	G=8/6, A=6/8
chr7:120764573	A/A	C	A	SNV	CPED1	CPED1:intronic:NM_024913.4	1	100		intronic	NM_02491	c.1061+46	p.?	0.25					Cadherin-like	rs762046137,rs7645!	0.0856	YES	0.0 (pos.120764553),0.0;0.0 (pos.120764569),9.413E			endoplasr	Brain Diseases;Central Nervous System Dise:			C=0, A=34	C=0.0, A=1.0	0.00001	524.186	34	C=0/0, A=13/2
chr19:4818378	C/C	T	C	SNV	TICAM1	TICAM1:exonic:NM_182919.3	1	100	2	synonymous	NM_18291	c.12A>G	p.(=)	-4.53					RIP homotypi	rs7255265	0.3656	YES	5.743E-1 (pos.4818378)		TIR domain-	I-kappaB k	Cardiovascular Diseases;Hypo		Benign	T=0, C=21	T=0.0, C=1.0	0.00001	281.235	21	T=0/0, C=14/7
chr7:120969969	G/GCT	G	GCT	INDEL	WNT16	WNT16:intronic:NM_057168.1	2	50		intronic	NM_05716	c.346+98G	p.?	1.7					wnt family	rs142005327		YES			Wingless-ty	Wnt signaling pathway,bone remodeling,cardiac epithel				G=13, GCT=	G=0.5, GCT=0.5	0.00001	124.224	26	G=12/1, GCT=
chr7:122033379	A/G	A	G	SNV	CADPS2	CADPS2:intronic:NM_001009571.3	1	50		intronic	NM_00100	c.2870+11	p.?	5.62				0.41(COSM40	Domain of Ur	rs2074588	0.316	YES	3.911E-1 (pos.12203337!	acute_myeloic	Calcium-de	cell juncti(	Autistic Disorder;Cl	Calcium Citrate,Calcium		A=10, G=1(	A=0.5, G=0.5	0.00001	82.8861	20	A=4/6, G=1/9
chr15:90128966	T/T	C	T	SNV	TICRR	TICRR:exonic:NM_152259.3	1	100	4	missense	NM_15225	c.1204C>T	p.Arg402Trp	1.75	1	101	0	0.05(COSM14	Treslin N-terr	rs11629584	0.4973	YES	2.356E-1 (pos.90128966)	adenocarcinoma		DNA repai	Nervous System Diseases;Neuromuscular Di:			C=0, T=23	C=0.0, T=1.0	0.00001	316.733	23	C=0/0, T=10/1
chr15:90138670	T/T	C	T	SNV	TICRR	TICRR:exonic:NM_152259.3	1	100	7	synonymous	NM_15225	c.1728C>T	p.(=)	0.68				0.8(COSM147	Treslin N-terr	rs8042146	0.0893	YES	7.026E-1 (pos.90138670)	acute_myeloid_leukaemia		DNA repai	Nervous System Diseases;Neuromuscular Di:			C=0, T=23	C=0.0, T=1.0	0.00001	316.745	23	C=0/0, T=11/1
chr1:43771016	TA/T	TA	T	INDEL	TIE1	TIE1:splicesite_5:NM_005424.4	1	52.38	3	splicesite	NM_00542	c.484+2TA	p.?	3.1, 1.25					Fibronectin t	rs3831248,rs397840!	0.3864	YES	8.786E-1 (pos.43771016)		Tyrosine kir	ATP binding,angiogenesis,in utero embryonic developme				TA=20, T=2	TA=0.4762, T=0.523!	0.00001	236.696	42	TA=8/12, T=10
chr4:113199075	G/G	A	G	SNV	TIFA	TIFA:exonic:NM_052864.2	1	100	2	synonymous	NM_05286	c.498T>C	p.(=)	-0.66					FHA domain	rs386678484,rs7017!	0.4773	YES	7.4E-1 (pos.113199075)		TRAF-intera	I-kappaB kinase/NF-kappaB signaling,intracellular,prote				A=0, G=27	A=0.0, G=1.0	0.00001	388.935	27	A=0/0, G=22/5
chr5:149374932	C/C	T	C	SNV	TIGD6	TIGD6:exonic:NM_030953.3	1	100	2	missense	NM_03095	c.980A>G	p.Gln327Arg	2.15	1	43	0	0(COSM4407:	CENP-B N-terı	rs10875553	0.2045	YES	7.209E-1 (pos.14937493:	carcinoma,hepatocellular		DNA binding,nucleus				T=0, C=32	T=0.0, C=1.0	0.00001	477.509	32	T=0/0, C=15/1
chr12:56827974	T/T	C	T	SNV	TIMELESS	TIMELESS:utr_5:NM_003920.4	1	100	2	utr_5	NM_00392	c.-20G>A	p.?	-4.46					Timeless pro'	rs2638312,rs769376(	5.00E-04	YES	0.0 (pos.56827972),9.999E-1 (pos.56827		Timeless, D	DNA replic	Autoimmune Diseases;Female Urogenital Di:			C=0, T=45	C=0.0, T=1.0	0.00001	747.389	45	C=0/0, T=25/2
chr12:56827694	C/G	C	G	SNV	TIMELESS	TIMELESS:exonic:NM_003920.4	1	50	3	synonymous	NM_00392	c.114G>C	p.(=)	2.49				0.95(COSM14	Timeless pro'	rs2279665	0.4547	YES	2.603E-1 (pos.56827694)	adenocarcinoı	Timeless, D	DNA replic	Autoimmune Diseases;Female Urogenital Di:			C=13, G=13	C=0.5, G=0.5	0.00001	118.294	26	C=8/5, G=10/3
chrX:48751471	G/G	A	G	SNV	TIMM17B	TIMM17B:exonic:NM_001167947.2	1	100	6	synonymous	NM_00116	c.378T>C	p.(=)	-0.64				0.04(COSM41	Tim17/Tim22	rs1128363	0.4643	YES	7.729E-1 (pos.48751471)	neoplasm	Translocase	P-P-bond-hydrolysis-driven protein transmembrane tran				A=0, G=39	A=0.0, G=1.0	0.00001	623.954	39	A=0/0, G=28/1
chr18:71825719	G/G	A	G	SNV	TIMM21	TIMM21:utr_3:NM_014177.2	1	100	6	utr_3	NM_01417	c.751A>G	p.?	-0.18					Cytochrome (	rs370852314,rs3722:	0.1068	YES	1.208E-2 (pos.71825719)		Translocase	integral component of membrane,mitochondrial inner m				A=0, G=42	A=0.0, G=1.0	0.00001	689.505	42	A=0/0, G=25/1
chr7:12420092	C/C	G	C	SNV	VWDE	VWDE:intronic:NM_001135924.2	1	100		intronic	NM_00113	c.789+20C	p.?	-2.31					Human growt	rs1025787	0.2832	YES	6.035E-1 (pos.12420092)			calcium ion binding,extracellular region				G=0, C=31	G=0.0, C=1.0	0.00001	465.165	31	G=0/0, C=18/1
chr15:66644521	G/G	C	G	SNV	TIPIN	TIPIN:exonic:NM_017858.2	1	100	3	missense	NM_01785	c.158G>C	p.Arg53Pro	4.13	1	103	0		Replication F	rs9806123	1.00E-04		1.0 (pos.66644521)		Timeless-in	DNA binding,DNA replication,DNA replication checkpoint				C=0, G=50	C=0.0, G=1.0	0.00001	852.736	50	C=0/0, G=22/2
chr7:124465509	C/C	A	C	SNV	POT1	POT1:intronic:NM_015450.2	1	100		intronic	NM_01545	c.1687-98'	p.?	0.13					Telomeric sir	rs10250202		YES			Protection (	8-hydroxy-	Chromosome Disorders;Congenital Abnorma			A=0, C=66	A=0.0, C=1.0	0.00001	1015.35	66	A=0/0, C=40/2
chr15:66641417	T/C	T	C	SNV	TIPIN	TIPIN:exonic:NM_017858.2	1	57.89	6	synonymous	NM_01785	c.456A>G	p.(=)	1.1				0.87(COSM17	Replication F	rs11856320	0.0783	YES	6.22E-4 (pos.66641417)	adenocarcinoı	Timeless-in	DNA binding,DNA replication,DNA replication checkpoint				T=16, C=22	T=0.4211, C=0.5789	0.00001	249.047	38	T=8/8, C=15/7
chr15:30018627	T/C	T	C	SNV	TJP1	TJP1:exonic:NM_003257.4	1	50	18	missense	NM_00325	c.2368A>G	p.Ile790Val	2.83	0.08	29	0.73	0.92(COSM48	Guanylate kir	rs2229515	0.1101	YES	1.238E-1 (pos.30018627)	squamous_cel	Tight juncti(	apical jun(	Brain Diseases;Cardiovascular Diseases;Cen			T=10, C=10	T=0.5, C=0.5	0.00001	82.8861	20	T=5/5, C=8/2
chr19:3738649	C/C	A	C	SNV	TJP3	TJP3:exonic:NM_001267561.1	1	100	12	synonymous	NM_00126	c.1408A>C	p.(=)	0				0.08(COSM37	Guanylate kir	rs599587	0.3393	YES	6.44E-1;0.0 (pos.373864!	adenocarcinoı	Tight juncti(	bicellular	Genital Diseases, Male;Genital Neoplasms, I			A=0, C=22	A=0.0, C=1.0	0.00001	298.861	22	A=0/0, C=9/13
chr11:61106892	C/C	G	C	SNV	TKFC	TKFC:exonic:NM_015533.3	1	100	5	synonymous	NM_01553	c.471G>C	p.(=)	-7.75				0.01(COSM41	DAK2 domair	rs2513329	0.0085	YES	9.596E-1;2.336E-4 (pos.6	neoplasm		ATP binding,FAD-AMP lyase (cyclizing) activity,carbohydr:				G=0, C=90	G=0.0, C=1.0	0.00001	1454.54	90	G=0/0, C=15/7

chr11:61112802	T/T	C	T	SNV	TKFC	TKFC:exonic:NM 015533.3	1	100	14	synonymous	NM 01553	c.1311C>T	p.(=)	0.76				0.52(COSM54	DAK2 domair	rs2305465	0.0086	YES	9.626E-1 (pos.61112802)	acute myeloid leukaemia		ATP binding,FAD-AMP lyase (cyclizing) activity,carbohydr:	C=0, T=20	C=0.0, T=1.0	0.00001	263.785	20	C=0/0, T=11/9
chr16:84520396	G/G	A	G	SNV	TLDC1	TLDC1:exonic:NM 020947.3	1	100	5	missense	NM 02094	c.799T>C	p.Cys267Arg	0.84	1	180	0		TLD	rs422145	0		1.0 (pos.84520396)			cytoplasm,lysosomal membrane,membrane,negative re	A=0, G=23	A=0.0, G=1.0	0.00001	316.747	23	A=0/0, G=14/9
chr16:84520537	C/C	T	C	SNV	TLDC1	TLDC1:exonic:NM 020947.3	1	100	5	missense	NM 02094	c.658A>G	p.Ile220Val	-0.44	1	29	0		TLD	rs431818	0.0026		1.0 (pos.84520537)			cytoplasm,lysosomal membrane,membrane,negative re	T=0, C=56	T=0.0, C=1.0	0.00001	832.063	56	T=0/0, C=34/2
chr7:128415290	C/T	C	T	SNV	OPN1SW	OPN1SW:intronic:NM 001708.2	1	50		intronic	NM 00170	c.353-82G	p.?	-0.55					7 transmemb	rs17476081		YES			Blue cone p	G-protein Color Vision Defects:Eye Diseases:Nervous S\	C=12, T=12	C=0.5, T=0.5	0.00001	106.708	24	C=6/6, T=5/7
chr7:128444820	A/G	A	G	SNV	CCDC136	CCDC136:intronic:NM 022742.4	1	47.62		intronic	NM 02274	c.782+5A>	p.?	0.43						rs2291573	0.1373	YES	2.729E-1;0.0 (pos.128444820)		Coiled-coil	acrosomal membrane,acrosome assembly,integral com	A=11, G=10	A=0.5238, G=0.4762	0.00001	82.2898	21	A=9/2, G=8/2
chr4:167012381	A/A	G	A	SNV	TLL1	TLL1:exonic:NM 012464.4	1	100	19	synonymous	NM 01246	c.2544G>A	p.(=)	-2.52					Astacin (Pept	rs2654492	0.0347	YES	9.969E-1 (pos.167012381)		Tolloid-like	calcium io Cardiovascular Abnormalities;Cardiovascula	G=0, A=129	G=0.0, A=1.0	0.00001	2084.19	129	G=0/0, A=58/7
chr4:167020644	A/G	A	G	SNV	TLL1	TLL1:exonic:NM 012464.4	1	59.09	20	missense	NM 01246	c.2872A>G	p.Thr958Ala	1.31	0.05	58	0	0.3(COSM400	Astacin (Pept	rs2291822	0.0449	YES	4.399E-1 (pos.16702064	acute myeloic	Tolloid-like	calcium io Cardiovascular Abnormalities;Cardiovascula	A=9, G=13	A=0.4091, G=0.5909	0.00001	122.568	22	A=7/2, G=8/5
chr7:129094195	C/C	T	C	SNV	STRIP2	STRIP2:intronic:NM 020704.2	1	100		intronic	NM 02070	c.600-67T	p.?	-0.37					Domain of un	rs581829		YES				cell migration,cytoplasm,cytoskeleton organization,cytc	T=0, C=71	T=0.0, C=1.0	0.00001	1111.48	71	T=0/0, C=43/2
chr7:129142775	A/A	G	A	SNV	SMKR1	SMKR1:intronic:NM 001195243.1	1	100		intronic	NM 00119	c.3+107G>	p.?	-1.07						rs610591		YES					G=0, A=61	G=0.0, A=1.0	0.00001	921.769	61	G=0/0, A=28/3
chrX:12924826	G/G	A	G	SNV	TLR8,TLR8-AS1	TLR8-AS1:intronic nc:NR 030727.1. TLR8:exonic:NM 13863	1	100	1	missense	NR 03072	c.1A>G	p.Met1Val	-0.63	0.45	21	0	0(COSM3759	Leucine Rich	rs3764880	0.2473	YES	8.178E-1 (pos.12924826)	adenocarcinoi	Toll-like rec	DNA bindi Autoimmune Disea Imiquimod	A=0, G=29	A=0.0, G=1.0	0.00001	427.597	29	A=0/0, G=12/1
chr3:149093238	G/G	A	G	SNV	TM4SF1	TM4SF1:exonic:NM 014220.2	1	100	3	synonymous	NM 01422	c.405T>C	p.(=)	-2.16					L6 membrane	rs4771	0.1428	YES	8.801E-1;0.0 (pos.149093238)		Transmemb	biological process,integral component of membrane,int	A=0, G=28	A=0.0, G=1.0	0.00001	408.718	28	A=0/0, G=17/1
chr3:149193684	C/C	T	C	SNV	TM4SF4	TM4SF4:exonic:NM 004617.3	1	100	2	synonymous	NM 00461	c.249T>C	p.(=)	-1.38					L6 membrane	rs1802837	0.424	YES	8.347E-2 (pos.149193684)		Transmemb	integral cc Digestive System Diseases;Liver Diseases	T=0, C=27	T=0.0, C=1.0	0.00001	389.972	27	T=0/0, C=11/1
chr7:131191536	G/G	A	G	SNV	PODXL	PODXL:intronic:NM 005397.3	1	100		intronic	NM 00539	c.1006-51	p.?	-0.29					CD34/Podoca	rs3212303	0.4005	YES	5.019E-1 (pos.131191536)		Podocalyxi	apical pla: Brain Diseases;Cardiovascular Diseases;Car	A=0, G=43	A=0.0, G=1.0	0.00001	705.844	43	A=0/0, G=13/3
chr7:131191554	T/T	TC	T	INDEL	PODXL	PODXL:intronic:NM 005397.3	1	100		intronic	NM 00539	c.1006-69	p.?	-0.9, 0.56					CD34/Podoca	rs199558721,rs398095387,rs56		YES			Podocalyxi	apical pla: Brain Diseases;Cardiovascular Diseases;Car	TC=0, T=38	TC=0.0, T=1.0	0.00001	598.959	38	TC=0/0, T=8/3
chr7:132070054	T/C	T	C	SNV	PLXNA4	PLXNA4:intronic:NM 020911.1	1	57.58		intronic	NM 02091	c.1372-87	p.?	-0.47				0.02(COSM49	IPT/TIG doma	rs741664	0.4197	YES	1.331E-1 (pos.13207005	acute myeloic	Plexin A4	anterior c Cardiovascular Diseases:Embolism and Thro	T=28, C=38	T=0.4242, C=0.5758	0.00001	407.638	66	T=8/20, C=20/
chr7:133824418	C/C	G	C	SNV	LRGUK	LRGUK:intronic:NM 144648.2	1	100		intronic	NM 14464	c.487+148	p.?	-0.88					Guanylate kir	rs11981906		YES			Leucine-ricl	ATP binding,acrosomal vesicle,axoneme assembly,cell d	G=0, C=22	G=0.0, C=1.0	0.00001	298.755	22	G=0/0, C=16/6
chr17:4684095	G/A	G	A	SNV	TM4SF5	TM4SF5:exonic:NM 003963.2	1	50	2	synonymous	NM 00396	c.189G>A	p.(=)	-6.3				0(COSM3755	L6 membrane	rs13520	0.4831	YES	1.901E-1;0.0 (pos.46840	adenocarcinoi	Transmemb	integral component of plasma membrane	G=11, A=11	G=0.5, A=0.5	0.00001	94.6067	22	G=5/6, A=5/6
chr4:164435265	C/C	A	C	SNV	TMA16	TMA16:exonic:NM 018352.2	1	100	4	missense	NM 01835	c.194A>C	p.Gln65Pro	1.4	0.12	76	0	0.17(COSM14	Translation n	rs2304802	0.4692	YES	3.263E-1 (pos.16443526	acute myeloid leukaemia		nucleolus,nucleus,protein binding,ribosome	A=0, C=22	A=0.0, C=1.0	0.00001	255.653	22	A=0/0, C=13/9
chr4:164440581	C/C	T	C	SNV	TMA16	TMA16:exonic:NM 018352.2	1	100	7	missense	NM 01835	c.527T>C	p.Ile176Thr	-0.44	0.36	89	0	0.1(COSM149	Translation n	rs1561736	0.4794	YES	3.274E-1 (pos.16444058	acute myeloid leukaemia		nucleolus,nucleus,protein binding,ribosome	T=0, C=89	T=0.0, C=1.0	0.00001	1396.32	89	T=0/0, C=44/4
chr7:134819466	G/G	T	G	SNV	AGBL3	AGBL3:intronic:NM 178563.3	1	100		intronic	NM 17856	c.2343-12	p.?	-0.5					Zinc carboxyp	rs11765077		YES			ATP/GTP-bir	cytosol,metallocarboxypeptidase activity,protein side c	T=0, G=21	T=0.0, G=1.0	0.00001	281.234	21	T=0/0, G=13/8
chr4:164440743	A/A	T	A	SNV	TMA16	TMA16:utr 3:NM 018352.2	1	100	7	utr 3	NM 01835	c.689T>A	p.?	-0.06					Translation n	rs4362779		YES				nucleolus,nucleus,protein binding,ribosome	T=0, A=23	T=0.0, A=1.0	0.00001	316.744	23	T=0/0, A=9/14
chr7:134872003	G/G	C	G	SNV	WDR91	WDR91:intronic:NM 014149.3	1	100		intronic	NM 01414	c.1882-82	p.?	-2.24					WD domain,	rs292558		YES			WD repeat-	cytosol,early endosome membrane,early endosome to l	C=0, G=88	C=0.0, G=1.0	0.00001	1451.8	88	C=0/0, G=54/3
chr7:134873355	A/A	G	A	SNV	WDR91	WDR91:intronic:NM 014149.3	1	100		intronic	NM 01414	c.1769-58	p.?	-1.54					WD domain,	rs292564		YES			WD repeat-	cytosol,early endosome membrane,early endosome to l	G=0, A=27	G=0.0, A=1.0	0.00001	389.987	27	G=0/0, A=18/9
chr20:2621998	C/C	T	C	SNV	TMC2	TMC2:utr 3:NM 080751.2	1	100	20	utr 3	NM 08075	c.2722T>C	p.?	-1.13					TMC domain	rs6050798	0.4858	YES	5.547E-1 (pos.2621998)		Transmemb	calcium ion transmembrane transport,cellular compon	T=0, C=22	T=0.0, C=1.0	0.00001	298.892	22	T=0/0, C=15/7
chr15:81636267	C/T	C	T	SNV	TMC3,TMC3-AS1	TMC3-AS1:intronic nc:NR 120365.1. TMC3:exonic:NM 001(	1	57.58	14	synonymous	NR 12036	c.1638G>A	p.(=)	0.87					TMC domain	rs7161991	0.1409	YES	7.063E-1 (pos.81636267)		Transmemb	integral cc Basal Ganglia Diseases:Brain Diseases:Centr	C=14, T=19	C=0.4242, T=0.5758	0.00001	204.394	33	C=8/6, T=13/6
chr17:76121864	G/G	A	G	SNV	TMC6	TMC6:exonic:NM 001127198.2	1	100	5	missense	NM 00112	c.373T>C	p.Trp125Arg	2.02	1	101	0	0.07(COSM37	TMC domain	rs2748427	0.1959	YES	3.669E-1 (pos.76121864)	adenocarcinoi	Transmemb	Golgi appa Epidermodysplasia Verruciforr Benign	A=0, G=65	A=0.0, G=1.0	0.00001	995.721	65	A=0/0, G=27/3
chr7:135262533	T/C	T	C	SNV	NUP205	NUP205:intronic:NM 015135.2	1	59.09		intronic	NM 01513	c.649-11T	p.?	0.2					Nuclear pore	rs10252250	0.4481	YES	2.654E-1 (pos.135262533)		Nucleopori	host cell,i Bone Diseases;Musculoskeletal Diseases;Os	T=9, C=13	T=0.4091, C=0.5909	0.00001	126.413	22	T=7/2, C=6/7
chr3:129546729	C/C	T	C	SNV	TMCC1	TMCC1:exonic:NM 001017395.4	1	100	4	missense	NM 00101	c.493A>G	p.Ser165Gly	4.58	0.02	56	0		Predicted tra	rs784689	3.00E-04	YES	9.974E-1 (pos.129546729)		Transmemb	cytosol,endoplasmic reticulum membrane,endoplasmic	T=0, C=82	T=0.0, C=1.0	0.00001	1329.64	82	T=0/0, C=50/3
chr15:79606137	G/A	G	A	SNV	TMED3	TMED3:exonic:NM 007364.3	1	97.56	2	synonymous	NM 00736	c.207G>A	p.(=)	1.94					emp24/gp25	rs3825933	2.00E-04		9.787E-1 (pos.79606137)			COPI vesicle coat,ER to Golgi vesicle-mediated transport	G=1, A=40	G=0.0244, A=0.9756	0.00001	631.821	41	G=0/1, A=12/2
chr7:136939582	T/T	G	T	SNV	PTN	PTN:intronic:NM 002825.6	1	100		intronic	NM 00282	c.115+24C	p.?	-0.47					PTN/MK hepa	rs1162122		YES	1.0 (pos.136939582)		Pleiotrophi	basement Brain Diseases:Cardiovascular Diseases:Cen	G=0, T=56	G=0.0, T=1.0	0.00001	830.86	56	G=0/0, T=23/3
chr17:72784932	A/A	G	A	SNV	TMEM104	TMEM104:exonic:NM 017728.3	1	100	4	missense	NM 01772	c.172G>A	p.Val58Met	2.58	1	21	0.097	0.64(COSM37	Transmembra	rs2016126	0.3598	YES	4.083E-1 (pos.72784932)	acute myeloid leukaemia		biological process,cellular component,integral compor	G=0, A=40	G=0.0, A=1.0	0.00001	644.286	40	G=0/0, A=25/1
chr7:137773563	G/G	T	G	SNV	AKR1D1	AKR1D1:intronic:NM 005989.3	1	100		intronic	NM 00598	c.261+49T	p.?	-0.61					Aldo/keto red	rs2166188	0.2615	YES	6.627E-1 (pos.137773563)		Bile acid syi	C21-steroi Bile Duct Diseases; (5BETA)-PREGNANE-3,20-I	T=0, G=38	T=0.0, G=1.0	0.00001	603.763	38	T=0/0, G=17/2
chr7:138200117	T/T	A	T	SNV	TRIM24	TRIM24:intronic:NM 015905.2	1	100		intronic	NM 01590	c.631+13A	p.?	-0.25					B-box zinc fin	rs3757381	0.1741	YES	5.01E-1 (pos.138200117)		Tripartite m	calcium io Adenocarcinoma:Carcinoma:Carcinoma, Hec	A=0, T=93	A=0.0, T=1.0	0.00001	1555.02	93	A=0/0, T=56/3
chr7:138200210	T/C	T	C	SNV	TRIM24	TRIM24:intronic:NM 015905.2	1	50		intronic	NM 01590	c.631+106	p.?	-0.29					B-box zinc fin	rs68115887		YES			Tripartite m	calcium io Adenocarcinoma:Carcinoma:Carcinoma, Hec	T=48, C=48	T=0.5, C=0.5	0.00001	536.853	96	T=24/24, C=22
chr7:138356732	GCT/GCT	CTG	GCT	MNV	SVOPL	SVOPL:intronic:NM 001139456.1	3	100		intronic	NM 00113	c.273+32C	p.?	-0.43, -0.47, -1.26					Major Facilit	rs368333846,rs5887896,rs6248		YES	1.0 (pos.138356733)		SV2-related	integral component of membrane,substrate-specific tra	CTG=0, GCT	CTG=0.0, GCT=1.0	0.00001	741.983	51	CTG=0/0, GCT=
chr12:122186268	T/T	C	T	SNV	TMEM120B	TMEM120B:exonic:NM 001080825.2	1	100	3	synonymous	NM 00108	c.225C>T	p.(=)	-0.43				0.07(COSM41	TMPIT-like pr	rs28651018	0.4538	YES	5.236E-1 (pos.12218626	acute myeloic	Transmemb	biological process,cellular component,fat cell different	C=0, T=23	C=0.0, T=1.0	0.00001	316.608	23	C=0/0, T=10/1
chr7:138400796	T/T	C	T	SNV	ATP6V0A4	ATP6V0A4:intronic:NM 130841.2	1	100		intronic	NM 13084	c.2140-17	p.?	-0.6					V-type ATPas	rs999229		YES			ATPase, H+	ATP hydrol Acid-Base Imbalance:Acidosis:Acidosis, Rena	C=0, T=35	C=0.0, T=1.0	0.00001	543.896	35	C=0/0, T=17/1
chr12:122186317	A/A	G	A	SNV	TMEM120B	TMEM120B:exonic:NM 001080825.2	1	100	3	missense	NM 00108	c.274G>A	p.Asp92Asn	5.31	0.08	23	0.007	0.84(COSM15	TMPIT-like pr	rs28655666	0.4544	YES	5.234E-1 (pos.12218631	acute myeloic	Transmemb	biological process,cellular component,fat cell different	G=0, A=23	G=0.0, A=1.0	0.00001	316.651	23	G=0/0, A=9/14
chr7:138487620	A/A	G	A	SNV	TMEM213	TMEM213:intronic:NM 001085429.1	1	100		intronic	NM 00108	c.155-25G	p.?	-1.41					TMEM213 far	rs73465048	0.0107	YES	3.199E-3 (pos.138487620)			integral component of membrane	G=0, A=75	G=0.0, A=1.0	0.00001	1193.9	75	G=0/0, A=20/5
chr7:138565987	G/G	C	G	SNV	KIAA1549	KIAA1549:intronic:NM 001164665.1	1	100		intronic	NM 00116	c.4229+14	p.?	-4.7					Domain of un	rs6968792		YES			KIAA1549 g	integral cc Astrocytoma;Glioma;Neoplasms;Neoplasms	C=0, G=33	C=0.0, G=1.0	0.00001	504.595	33	C=0/0, G=22/1
chr7:138713715	C/C	G	C	SNV	ZC3HAV1L	ZC3HAV1L:intronic:NM 080660.3	1	100		intronic	NM 08066	c.502-9C>	p.?	2.17				0.44(COSM4416770)		rs11767739	0.479	YES	2.317E-1 (pos.13871371	acute myeloid leukaemia		cytosol,metal ion binding	G=0, C=54	G=0.0, C=1.0	0.00001	798.148	54	G=0/0, C=34/2
chr4:1718883	A/T	A	T	SNV	TMEM129	TMEM129:utr 3:NM 001127266.1	1	52.38	4	utr 3	NM 00112	c.1113T>A	p.?	0.37					Putative tran	rs2236787		YES	8.026E-1 (pos.1718883)		Transmemb	endoplasmic reticulum,endoplasmic reticulum membra	A=10, T=11	A=0.4762, T=0.5238	0.00001	95.3003	21	A=3/7, T=8/3
chr7:98460824	A/A	C	A	SNV	TMEM130	TMEM130:exonic:NM 001134450.1	1	100	2	synonymous	NM 00113	c.285G>T	p.(=)	0.08				0.03(COSM4006800),0.03(C		rs10238224	0.135	YES	8.702E-1 (pos.98460824)	carcinoma		Golgi apparatus,Golgi membrane,integral component of	C=0, A=27	C=0.0, A=1.0	0.00001	389.976	27	C=0/0, A=12/1
chr7:138768839	G/G	A	G	SNV	ZC3HAV1	ZC3HAV1:intronic:NM 020119.3	1	100		intronic	NM 02011	c.445-61T	p.?	1.1					Poly(ADP-ribc	rs6943128		YES			Zinc finger C	NAD+ ADP-ribosyltransferase activity,NAD+ binding,RNA	A=0, G=34	A=0.0, G=1.0	0.00001	524.183	34	A=0/0, G=17/1
chr7:138793677	C/C	G	C	SNV	ZC3HAV1	ZC3HAV1:intronic:NM 020119.3	1	100		intronic	NM 02011	c.308+93C	p.?	-1.05					Poly(ADP-ribc	rs1839606		YES			Zinc finger C	NAD+ ADP-ribosyltransferase activity,NAD+ binding,RNA	G=0, C=36	G=0.0, C=1.0	0.00001	555.887	36	G=0/0, C=20/1
chr7:138822762	A/G	A	G	SNV	TTC26	TTC26:intronic:NM 024926.3	1	52.38		intronic	NM 02492	c.234+77A	p.?	-0.66					Anaphase-pr	rs7809077		YES			Tetratricop	centrosome,ciliary basal body,ciliary tip,cilium,cilium as	A=10, G=11	A=0.4762, G=0.5238	0.00001	95.3002	21	A=6/4, G=1/10
chr7:139091806	C/T	C	T	SNV	C7orf55-LUC7L2,LUC7L2	C7orf55-LUC7L2:intronic:NM 001244584.2. LUC7L2:introni	1	50		intronic	NM 00124	c.502-114	p.?, p.?	-0.35					LUC7 N term	rs146301353					Luc7, S. cer	RNA binding,U1 snRNP,U2-type prespliceosome,enzyme	C=11, T=11	C=0.5, T=0.5	0.00001	94.6232	22	C=8/3, T=5/6
chr7:139102490	G/G	A	G	SNV	C7orf55-LUC7L2,LOC100129148,LUC7L2	LOC100129148:intronic nc:NR 033999.1. C7orf55-LUC7L2:	1	100		intronic	NM 00124	c.992+15A	p.?, p.?	-0.59					LUC7 N term	rs1011878231,rs377	0.2427	YES	2.229E-1 (pos.139102490)		Luc7, S. cer	RNA binding,U1 snRNP,U2-type prespliceosome,enzyme	A=0, G=169	A=0.0, G=1.0	0.00001	2759.35	169	A=0/0, G=88/8
chr2:98612266	G/G	A	G	SNV	TMEM131	TMEM131:utr 5:NM 015348.1	1	100	1	utr 5	NM 01534	c.-141T>C	p.?	0.32					Transmembra	rs3088327		YES			Transmemb	biological process,cellular component,integral compor	A=0, G=34	A=0.0, G=1.0	0.00001	524.186	34	A=0/0, G=17/1
chr12:125834395	C/C	T	C	SNV	TMEM132B	TMEM132B:exonic:NM 052907.3	1	100	2	synonymous	NM 05290	c.450T>C	p.(=)	-1.49					Mature oligo	rs3803155	0.2488	YES	8.199E-1 (pos.125834395)			biological Melanoma;Neoplasms;Neoplasms by Histolc	T=0, C=24	T=0.0, C=1.0	0.00001	334.787	24	T=0/0, C=6/18
chr12:126068417	C/C	T	C	SNV	TMEM132B	TMEM132B:exonic:NM 052907.3	1	100	5	synonymous	NM 05290	c.1299T>C	p.(=)	-4.38					Mature oligo	rs6489006	0.0591	YES	9.575E-1 (pos.126068417)			biological Melanoma;Neoplasms;Neoplasms by Histolc	T=0, C=34	T=0.0, C=1.0	0.00001	528.567	34	T=0/0, C=12/2
chr7:140244627	A/A	G	A	SNV	DENND2A	DENND2A:intronic:NM 015689.4	1	100		intronic	NM 01568	c.2179-61	p.?	-2.17					DENN (AEX-3)	rs2293176		YES				Rab guanyl-nucleotide exchange factor activity,actin cyt	G=0, A=42	G=0.0, A=1.0	0.00001	685.218	42	G=0/0, A=23/1
chr7:140449071	G/G	C	G	SNV	BRAF	BRAF:intronic:NM 004333.4	1	100		intronic	NM 00433	c.1992+16	p.?	0.29					Kinase-like,P	rs3789806	0.1436	YES	1.803E-1;3.484E-4 (pos.140449071)		Murine sarc	ATP bindin Abnormalities, Mul (1E)-5-(1-p Benign	C=0, G=136	C=0.0, G=1.0	0.00001	2258.87	136	C=0/0, G=75/6
chr12:129190474	A/A	G	A	SNV	TMEM132C	TMEM132C:exonic:NM 001136103.2	1	100	9	synonymous	NM 00113	c.2961G>A	p.(=)	-1.2				0.04(COSM43	Mature oligo	rs3812792	0.428	YES	5.424E-1 (pos.12919047	adenocarcinoma,hepatoce		biological process,cellular component,integral compor	G=0, A=22	G=0.0, A=1.0	0.00001	298.892	22	G=0/0, A=11/1
chr17:32965285	A/A	C	A	SNV	TMEM132E	TMEM132E:utr 3:NM 001304438.1	1	100	9	utr 3	NM 00130	c.3259C>A	p.?	-0.5					Mature oligo	rs11080288,rs36877	0.5021	YES	7.023E-2;2.809E-1;4.214E-1;0.0;0.0;0.0 (		Transmemb	integral component of membrane	C=0, A=23	C=0.0, A=1.0	0.00001	315.503	23	C=0/0, A=12/1
chr7:141759846	T/T	C	T	SNV	MGAM	MGAM:intronic:NM 004668.2	1	100		intronic	NM 00466	c.4059+80	p.?	-0.62					Glycosyl hydr	rs4341082		YES			Maltase-glu	alpha-1,4-glucosidase activity, Acarbose,Miglitol,Voglib	C=0, T=22	C=0.0, T=1.0	0.00001	298.892	22	C=0/0, T=8/14
chr11:87032287	G/C	G	C	SNV	TMEM135	TMEM135:exonic:NM 022918.3	1	47.62	15	missense	NM 02291	c.1289G>C	p.Gly430Ala	2.74	0.07	60	0.001	0.91(COSM39	N-terminal cy	rs11235097	0.0241	YES	1.782E-1 (pos.87032287)	acute myeloic	Transmemb	integral cc Autoimmune Diseases:Female Urogenital Di:	G=22, C=20	G=0.5238, C=0.4762	0.00001	212.948	42	G=13/9, C=14/
chr7:134849213	A/A	G	A	SNV	TMEM140	TMEM140:exonic:NM 018295.4	1	100	2	missense	NM 01829	c.20G>A	p.Arg7Gln	-0.04		43		0.07(COSM49	TM140 protei	rs3800592	0.4916	YES	2.495E-1 (pos.13484921	basal cell carcinoma,duct		integral component of membrane,protein binding	G=0, A=31	G=0.0, A=1.0	0.00001	465.819	31	G=0/0, A=15/1
chr5:87498849	A/A	G	A	SNV	TMEM161B	TMEM161B:exonic:NM 153354.4	1	100	9	synonymous	NM 15335	c.844C>T	p.(=)	4.21				0.93(COSM50	Predicted tra	rs3097146	0.3944	YES	7.674E-1;0.0 (pos.87498	osteosarcoma,squamous		DNA integration,integral component of membrane,nucle	G=0, A=42	G=0.0, A=1.0	0.00001	685.22	42	G=0/0, A=6/36
chr2:62729674	C/C	T	C	SNV	TMEM17	TMEM17:exonic:NM 198276.2	1	100	3	synonymous	NM 19827	c.216A>G	p.(=)	0.5					Predicted me	rs4672527	0.1117	YES	7.03E-1 (pos.62729674)		Transmemb	MKS comp Crohn Disease;Digestive System Diseases;Ga	T=0, C=28	T=0.0, C=1.0	0.00001	408.702	28	T=0/0, C=16/1
chr5:72419267	T/C	T	C	SNV	TMEM171	TMEM171:exonic:NM 173490.7	1	47.62	2	missense	NM 17349	c.67T>C	p.Phe23Leu	5.4	0	22	0.087	0.87(COSM40	Transmembra	rs638333	0.2834	YES	8.995E-2 (pos.72419267)	acute myeloid leukaemia		integral component of membrane,protein binding	T=11, C=10	T=0.5238, C=0.4762	0.00001	82.2861	21	T=6/5, C=7/3
chr7:143632265	A/G	A	G	SNV	OR2F2	OR2F2:upstream:NM 001004685.1	1	45.45		intronic	NM 00100	c.-61A>G	p.?	0.04						rs2240358		YES				G-protein coupled receptor activity,G-protein coupled re	A=12, G=10	A=0.5455, G=0.4545	0.00001	82.0959	22	A=1/11, G=8/2
chr7:150500729	G/G	A	G	SNV	TMEM176A	TMEM176A:exonic:NM 018487.2	1	100	5	missense	NM 01848	c.364A>G	p.Thr122Ala	-1.29	0.7	58	0	0(COSM1163	CD20-like fan	rs741064	0.2058	YES	9.49E-1 (pos.150500729)	ductal carcinoma		integral component of membrane,negative regulation of	A=0, G=71	A=0.0, G=1.0	0.00001	1111.48	71	A=0/0, G=19/5
chr7:144462951	T/T	C	T	SNV	TPK1	TPK1:intronic:NM 022445.3	1	100		intronic	NM 02244	c.115+22G	p.?	-1.93					Thiamin pyro	rs3779087	0.4138	YES	7.952E-1 (pos.144462951)		Thiamine p	ATP binding,cytosol,kinase acti Pyrithiamine Pyrophospl	C=0, T=22	C=0.0, T=1.0	0.00001	298.891	22	C=0/0, T=18/4
chr7:14775823	C/C	G	C	SNV	DGKB	DGKB:intronic:NM 001350705.1	1	100		intronic	NM 00135	c.169-4C>	p.?	-0.33				0.04(COSM39	Diacylglycerc	rs546131981,rs5628	0.1893	YES	1.027E-2 (pos.14775822)	acute myeloic	Diacylglycerol kinase, I	Diabetes Mellitus;Diabetes Mellitus, Type 2;	G=0, C=84	G=0.0, C=1.0	0.00001	1373.85	84	G=0/0, C=54/3
chr7:150490376	C/T	C	T	SNV	TMEM176B	TMEM176B:exonic:NM 001101312.1	1	53.33	5	missense	NM 00110	c.400G>A	p.Ala134Thr	0.4	0.12	58	0.999	0.3(COSM376	CD20-like fan	rs2072443	0.4174	YES	5.335E-1 (pos.15049037	adenocarcinoma		animal organ morphogenesis,cell differentiation,integra	C=14, T=16	C=0.4667, T=0.5333	0.00001	159.927	30	C=8/6, T=5/11
chr7:148463860	C/C	T	C	SNV	CUL1	CUL1:intronic:NM 003592.2	1	100		intronic	NM 00359	c.952+45T	p.?	-2.26					Cullin family,	rs1014095	0.2544	YES	6.709E-1 (pos.148463860)		Cullin 1	Fc-epsilon Dermatitis;Drug Eruptions;Drug Hypersensiti	T=0, C=33	T=0.0, C=1.0	0.00001	504.598	33	T=0/0, C=10/2
chr7:148480990	T/T	C	T	SNV	CUL1	CUL1:intronic:NM 003592.2	1	100		intronic	NM 00359	c.1191+8C	p.?	-2.5				0.01(COSM44	Cullin family,	rs10271133,rs75028	0.2278	YES	2.625E-1;0.0 (pos.14848	acute myeloic	Cullin 1	Fc-epsilon Dermatitis;Drug Eruptions;Drug Hypersensiti	C=0, T=36	C=0.0, T=1.0	0.00001	563.678	36	C=0/0, T=27/9
chr7:148543525	G/G	A	G	SNV	EZH2	EZH2:intronic:NM 004456.4	1	100		intronic	NM 00445	c.246+37T	p.?	0.2					SET domain,V	rs10274535,rs77926	0.3098	YES	0.0 (pos.148543524),6.651E-1 (pos.1485		Enhancer of	DNA bindi Abnormalities, Multiple;Autoimmune Diseas	A=0, G=30	A=0.0, G=1.0	0.00001	446.639	30	A=0/0, G=9/21
chr7:148705221	G/G	A	G	SNV	PDIA4	PDIA4:intronic:NM 004911.4	1	100		intronic	NM 00491	c.1131+30	p.?	-1.2					Calsequestri	rs2306172	0.0037	YES	7.619E-1;0.0 (pos.148705221)			RNA binding,cell,cell redox homeostasis,cell surface,cha	A=0, G=140	A=0.0, G=1.0	0.00001	2285.46	140	A=0/0, G=74/6
chr7:148705490	G/A	G	A	SNV	PDIA4	PDIA4:intronic:NM 004911.4	1	47.62		intronic	NM 00491	c.980-88C	p.?	0.42					Calsequestri	rs10272564		YES				RNA binding,cell,cell redox homeostasis,cell surface,cha	G=22, A=20	G=0.5238, A=0.4762	0.00001	212.953	42	G=10/12, A=1(
chr2:120438696	G/G	A	G	SNV	TMEM177	TMEM177:exonic:NM 001105199.1	1	100	2	synonymous	NM 00110	c.267A>G	p.(=)	-0.08						rs939777	0		1.0 (pos.120438696)			integral component of membrane	A=0, G=45	A=0.0, G=1.0	0.00001	747.379	45	A=0/0, G=19/2
chr2:120439230	G/G	C	G	SNV	TMEM177	TMEM177:exonic:NM 001105199.1	1	100	2	missense	NM 00110	c.801C>G	p.Asp267Glu	-1.93		45		0.04(COSM6352936)		rs1983406,rs769597	0.1562	YES	1.157E-4;0.0 (pos.12043	carcinoma		integral component of membrane	C=0, G=98	C=0.0, G=1.0	0.00001	1651.43	98	C=0/0, G=40/5
chr14:105070791	T/T	G	T	SNV	TMEM179	TMEM179:exonic:NM 001286389.1	1	100	1	synonymous	NM 00128	c.288C>A	p.(=)	0.73				0.29(COSM1562728),0.29(C		rs3803313	0.2199	YES	4.671E-1 (pos.10507079	adenocarcinoma,neoplasn		integral component of membrane	G=0, T=52	G=0.0, T=1.0	0.00001	767.832	52	G=0/0, T=31/2
chr7:149484891	G/G	A	G	SNV	SSPO	SSPO:intronic:NM 198455.2	1	100		intronic	NM 19845	c.3704+9A	p.?	0.13					C8 domain,F	rs855688	0.141	YES	1.759E-1 (pos.149484891)		Subcommis	cell adhesion,cell differentiation,extracellular space,ne	A=0, G=40	A=0.0, G=1.0	0.00001	644.286	40	A=0/0, G=13/2
chr7:149487019	A/G	A	G	SNV	SSPO	SSPO:intronic:NM 198455.2	1	50		intronic	NM 19845	c.4684+10	p.?	-0.04					C8 domain,F	rs855696		YES			Subcommis	cell adhesion,cell differentiation,extracellular space,ne	A=38, G=38	A=0.5, G=0.5	0.00001	394.919	76	A=16/22, G=14
chr7:149487319	G/G	C	G	SNV	SSPO	SSPO:intronic:NM 198455.2	1	100		intronic	NM 19845	c.4685-52	p.?	2.17					C8 domain,F	rs201175745,rs2579591,rs3867		YES			Subcommis	cell adhesion,cell differentiation,extracellular space,ne	C=0, G=39	C=0.0, G=1.0	0.00001	623.98	39	C=0/0, G=20/1
chr7:149500992	G/G	A	G	SNV	SSPO	SSPO:intronic:NM 198455.2	1	100		intronic	NM 19845	c.8219+91	p.?	-0.11					C8 domain,F	rs4725830		YES			Subcommis	cell adhesion,cell differentiation,extracellular space,ne	A=0, G=25	A=0.0, G=1.0	0.00001	352.854	25	A=0/0, G=5/20
chr9:74360234	C/T	C	T	SNV	TMEM2	TMEM2:exonic:NM 013390.2	1	50	4	missense	NM 01339	c.734G>A	p.Arg245Lys	1.75	1	26	0		G8 domain,Ir	rs25688	0.0778	YES	1.296E-2 (pos.74360234)		Transmemb	angiogenesis,cadherin binding,calcium ion binding,extra	C=55, T=55	C=0.5, T=0.5	0.00001	563.57	110	C=21/34, T=27
chr7:149981830	G/G	T	G	SNV	ACTR3C	ACTR3C:intronic:NM 001164458.1	1	100		intronic	NM 00116	c.564+12A	p.?	-1.08					Actin	rs4725334		YES	9.887E-1 (pos.149981830)			ATP binding,Arp2/3 complex-mediated actin nucleation,	T=0, G=35	T=0.0, G=1.0	0.00001	543.904	35	T=0/0, G=17/1
chr11:61165280	A/A	G	A	SNV	TMEM216	TMEM216:exonic:NM 001173991.2	1	100	4	synonymous	NM 00117	c.264G>A	p.(=)	-2.47					Predicted me	rs3741265	0.139	YES	9.66E-1 (pos.61165280)		Transmemb	MKS comp Abnormalities, Multiple;Bone I Benign/Likely	G=0, A=27	G=0.0, A=1.0	0.00001	389.988	27	G=0/0, A=19/8
chr11:61165731	CA/CA	C	CA	INDEL	TMEM216	TMEM216:splicesite 3:NM 001173991.2	1	100	5	splicesite	NM 00117	c.432-2C>	p.?	1.26					Predicted me	rs11382548,rs86666	0.1411	YES	9.625E-1 (pos.61165731)		Transmemb	MKS comp Abnormalities, Multiple;Bone I Benign	C=0, CA=34	C=0.0, CA=1.0	0.00001	524.186	34	C=0/0, CA=6/2
chr11:61165741	C/C	G	C	SNV	TMEM216	TMEM216:exonic:NM 001173991.2	1	100	5	missense	NM 00117	c.440G>C	p.Arg147Thr	0.79	0.01	71	0.002	0.69(COSM15	Predicted me	rs10897158	0.15	YES	9.588E-1;1.176E-4 (pos.6	secretory	Transmemb	MKS comp Abnormalities, Multiple;Bone I Benign	G=0, C=34	G=0.0, C=1.0	0.00001	524.183	34	G=0/0, C=6/28
chr17:10633181	A/G	A	G	SNV	TMEM220,TMEM220-AS1	TMEM220-AS1:exonic nc:NR 073458.1. TMEM220:utr 5:NM	1	50	1	utr 5	NM 00100	c.-13T>C	p.?	-1.54					Transmembra	rs3826448	0.3265	YES	4.219E-1;0.0 (pos.10633181)			integral component of membrane	A=30, G=30	A=0.5, G=0.5	0.00001	293.742	60	A=12/18, G=5/
chr7:150438089	C/C	T	C	SNV	GIMAP1-GIMAP5,GIMAP5	GIMAP1-GIMAP5:intronic:NM 001199577.1. GIMAP5:intron	1	100		intronic	NM 00119	c.655+74T	p.?, p.?	-0.09					50S ribosome	rs112619806,rs4725	0.4173	YES			GTPase, IM	GTP bindir Immune System Diseases	T=0, C=59	T=0.0, C=1.0	0.00001	885.368	59	T=0/0, C=13/4
chr19:17547292	G/G	A	G	SNV	TMEM221	TMEM221:exonic:NM 001190844.1	1	100	3	missense	NM 00119	c.851T>C	p.Met284Thr	0.34	0.53	81	0	0(COSM4131	Jiraiya	rs4808641		YES	8.182E-1 (pos.17547292)	neoplasm		integral component of membrane	A=0, G=28	A=0.0, G=1.0	0.00001	408.718	28	A=0/0, G=16/1
chr7:150490431	C/G	C	G	SNV	TMEM176B	TMEM176B:intronic:NM 001101312.1	1	53.33		intronic	NM 00110	c.373-28G	p.?	-0.02					CD20-like fan	rs2072442	0.4235	YES	6.179E-1;5.838E-4 (pos.150490431)			animal organ morphogenesis,cell differentiation,integra	C=14, G=16	C=0.4667, G=0.5333	0.00001	159.925	30	C=8/6, G=5/11
chr11:123754844	C/C	T	C	SNV	TMEM225	TMEM225:exonic:NM 001013743.2	1	100	3	missense	NM 00101	c.401A>G	p.Asn134Ser	-1.11	1	46	0			rs10893099	9.00E-04		1.0 (pos.123754844)			acrosomal membrane,integral component of membrane	T=0, C=37	T=0.0, C=1.0	0.00001	583.706	37	T=0/0, C=16/2
chr7:150558399	G/A	G	A	SNV	AOC1	AOC1:downstream:NM 001272072.1	1	51.92		intronic	NM 00127	c.*102G>A	p.?	-0.14						rs2071517		YES				amine me Cardiomegaly;Card Amiloride,Copper,Dimin	G=25, A=27	G=0.4808, A=0.5192	0.00001	267.711	52	G=15/10, A=1
chr7:150698879	G/G	A	G	SNV	NOS3	NOS3:intronic:NM 000603.4	1	100		intronic	NM 00060	c.1503-30	p.?	-0.34					FAD binding	rs1800780	0.4635	YES	6.127E-1 (pos.150698879)		Nanos, Dro	FMN bindi Abruptio Placentae (3S,5E)-3-propyl-3,4-dihy	A=0, G=103	A=0.0, G=1.0	0.00001	1609.5	103	A=0/0, G=44/5
chr7:150720902	G/G	A	G	SNV	ATG9B	ATG9B:intronic nc:NR 073169.1	1	100		intronic	NR 073169.1			-2.09					Autophagy pr	rs13307588		YES			Autophagy 9, S. cerevisiae, homolog of, B		A=0, G=24	A=0.0, G=1.0	0.00001	334.784	24	A=0/0, G=7/17
chr7:150747746	G/G	A	G	SNV	ASIC3	ASIC3:intronic:NM 020321.3	1	100		intronic	NM 02032	c.813+51A	p.?	-1.45					Amiloride-se	rs2288645	0.1965	YES	7.843E-1 (pos.150747746)		Cation char	PDZ domain binding,acid-sensi Agmatine	A=0, G=20	A=0.0, G=1.0	0.00001	263.786	20	A=0/0, G=10/1
chr7:150752066	C/C	T	C	SNV	CDK5	CDK5:intronic:NM 004935.3	1	100		intronic	NM 00493	c.650+48A	p.?	-5.49					Protein kinas	rs891507	0.1575	YES	9.637E-1 (pos.150752066)		Cyclin-depe	ATP bindin Nervous System Dis 6-PHENYL5HPYRROLO2,3	T=0, C=23	T=0.0, C=1.0	0.00001	316.747	23	T=0/0, C=9/14
chr11:123753937	A/G	A	G	SNV	TMEM225	TMEM225:exonic:NM 001013743.2	1	97.3	4	missense	NM 00101	c.586T>C	p.Cys196Arg	-2.35	0.36	180	0	0(COSM5765636)		rs1939927	0.1248	YES	8.318E-1 (pos.12375393	adenocarcinoma		acrosomal membrane,integral component of membrane	A=1, G=36	A=0.027, G=0.973	0.00001	551.739	37	A=1/0, G=19/1
chr7:150778524	G/A	G	A	SNV	FASTK,TMUB1	FASTK:upstream:NM 006712.4. TMUB1:utr 3:NM 0011360	1	95.83		intronic	NM 00671	c.-671C>T,	p.?, p.?	0.38					Ubiquitin fan	rs4725387		YES			Fas-activat	ATP binding,Fas-activated serine/threonine kinase activ	G=3, A=69	G=0.0417, A=0.9583	0.00001	1038.24	72	G=0/3, A=68/1
chr6:130154667	C/C	T	C	SNV	TMEM244	TMEM244:exonic:NM 001010876.1	1	100	4	missense	NM 00101	c.257A>G	p.Glu86Gly	4.3	0	98	0.996	0.89(COSM4160176)		rs9492393	0.3429	YES	1.982E-1 (pos.13015466	basal cell carcinoma,neo		integral component of membrane	T=0, C=95	T=0.0, C=1.0	0.00001	1597.02	95	T=0/0, C=45/5
chr7:150825839	G/A	G	A	SNV	AGAP3	AGAP3:intronic:NM 031946.6	1	50		intronic	NM 03194	c.1326+68	p.?	-0.51					Ankyrin repea	rs7808252		YES			ARF GTPase	GTP binding,GTPase activator activity,GTPase activity,cy	G=13, A=13	G=0.5, A=0.5	0.00001	119.162	26	G=4/9, A=2/11
chr6:130154686	G/G	A	G	SNV	TMEM244	TMEM244:exonic:NM 001010876.1	1	100	4	missense	NM 00101	c.238T>C	p.Phe80Leu	0.23	1	22	0	0.06(COSM4160177)		rs4629709	0.1808	YES	3.369E-1 (pos.13015468	acute myeloid leukaemia		integral component of membrane	A=0, G=96	A=0.0, G=1.0	0.00001	1530.44	96	A=0/0, G=45/5
chr6:130152479	T/C	T	C	SNV	TMEM244	TMEM244:exonic:NM 001010876.1	1	50	5	synonymous	NM 00101	c.372A>G	p.(=)	-1.95				0(COSM4416061)		rs7758540	0.3531	YES	1.408E-1 (pos.13015247	acute myeloid leukaemia		integral component of membrane	T=37, C=37	T=0.5, C=0.5	0.00001	381.508	74	T=19/18, C=15
chr7:151048484	A/A	G	A	SNV	NUB1	NUB1:intronic:NM 001243351.1	1	100		intronic	NM 00124	c.358-26G	p.?	-0.93					UBA/TS-N dor	rs449081	0.2868	YES	8.947E-1 (pos.151048484)		Negative re	cytosol,nucleolus,nucleus,positive regulation of proteas	G=0, A=38	G=0.0, A=1.0	0.00001	603.786	38	G=0/0, A=23/1
chr7:151053043	G/G	A	G	SNV	NUB1	NUB1:intronic:NM 001243351.1	1	100		intronic	NM 00124	c.670+7A>	p.?	-0.25					UBA/TS-N dor	rs446886	0.3312	YES	8.903E-1 (pos.151053043)		Negative re	cytosol,nucleolus,nucleus,positive regulation of proteas	A=0, G=70	A=0.0, G=1.0	0.00001	1088.16	70	A=0/0, G=41/2
chr9:111782670	A/A	G	A	SNV	TMEM245	TMEM245:utr 3:NM 032012.3	1	100	18	utr 3	NM 03201	c.2710C>T	p.?	0.56					AI-2E family t	rs10816767	0.4774	YES	4.069E-1 (pos.111782670)			biological Mental Disorders;Schizophrenia;Schizophren	G=0, A=26	G=0.0, A=1.0	0.00001	361.681	26	G=0/0, A=14/1
chr7:151668225	T/T	A	T	SNV	GALNTL5	GALNTL5:intronic:NM 145292.3	1	100		intronic	NM 14529	c.368+75A	p.?	-1					Glycosyl tran	rs1919521,rs59993749,rs75215		YES			UDP-N-acet	integral component of membrane,late endosome memb	A=0, T=41	A=0.0, T=1.0	0.00001	646.442	41	A=0/0, T=7/34
chr9:111868885	G/A	G	A	SNV	TMEM245	TMEM245:exonic:NM 032012.3	1	50	3	synonymous	NM 03201	c.702C>T	p.(=)	0.55				0.46(COSM41	AI-2E family t	rs2271878	0.2788	YES	2.888E-1 (pos.11186888	acute myeloid leukaemia		biological Mental Disorders;Schizophrenia;Schizophren	G=14, A=14	G=0.5, A=0.5	0.00001	131.941	28	G=6/8, A=9/5
chr14:57052511	T/T	G	T	SNV	TMEM260	TMEM260:exonic:NM 017799.3	1	100	3	synonymous	NM 01779	c.225G>T	p.(=)	-0.04				0.31(COSM39	Dolichyl-phos	rs3737171,rs772072	0.4101	YES	5.377E-1;0.0;0.0 (pos.57	acute myeloic	Transmemb	integral component of membrane	G=0, T=25	G=0.0, T=1.0	0.00001	353.019	25	G=0/0, T=11/1
chr7:1518336	G/A	G	A	SNV	INTS1	INTS1:intronic:NM 001080453.2	1	50		intronic	NM 00108	c.4489+26	p.?	0.04					Protein of unl	rs11977356	0.3966	YES	7.118E-1 (pos.1518336)		Integrator c	U2 snRNA 3'-end processing,integral component of mem	G=12, A=12	G=0.5, A=0.5	0.00001	106.709	24	G=5/7, A=7/5
chr11:64855986	A/G	A	G	SNV	TMEM262,ZFPL1	ZFPL1:downstream:NM 006782.3. TMEM262:exonic:NM 0C	1	97.3	3	missense	NM 00678	c.*400A>G	p.?, p.Val87Ala	4.72	1	64	0.002	0.93(COSM4146093)		rs2282498		YES	1.313E-1 (pos.64855986)	ductal carcinoma,neoplas		DNA binding,Golgi apparatus,integral component of mer	A=1, G=36	A=0.027, G=0.973	0.00001	551.745	37	A=1/0, G=12/2
chr7:1523240	G/C	G	C	SNV	INTS1	INTS1:intronic:NM 001080453.2	1	50		intronic	NM 00108	c.3541+13	p.?	-0.01					Protein of unl	rs550473125			0.0 (pos.1523240)		Integrator c	U2 snRNA 3'-end processing,integral component of mem	G=22, C=22	G=0.5, C=0.5	0.00001	243.012	44	G=9/13, C=15/
chr7:152511598	T/T	C	T	SNV	ACTR3B	ACTR3B:intronic:NM 020445.5	1	100		intronic	NM 02044	c.337-37C	p.?	0.71					Actin	rs34032313			8.686E-1 (pos.152511598)			ATP binding,Arp2/3 complex-mediated actin nucleation,	C=0, T=58	C=0.0, T=1.0	0.00001	860.045	58	C=0/0, T=40/1
chr7:15458033	G/G	C	G	SNV	AGMO	AGMO:intronic:NM 001004320.1	1	100		intronic	NM 00100	c.609+150	p.?	2.99					Fatty acid hy	rs4469360		YES			Alkylglycerc	endoplasr Nutrition Disorders;Nutritional and Metaboli	C=0, G=57	C=0.0, G=1.0	0.00001	852.806	57	C=0/0, G=38/1
chr7:154679447	A/A	G	A	SNV	DPP6	DPP6:intronic:NM 130797.3	1	100		intronic	NM 13079	c.2304+3G	p.?	0.63					Dipeptidyl pe	rs940378	0.0369	YES	9.998E-1 (pos.154679447)		Dipeptidylp	dipeptidyl Amyotrophic Lateral Sclerosis;Arrhythmias, C	G=0, A=63	G=0.0, A=1.0	0.00001	958.912	63	G=0/0, A=50/1
chr7:154748908	A/A	G	A	SNV	PAXIP1	PAXIP1:intronic:NM 007349.3	1	100		intronic	NM 00734	c.2549+26	p.?	-3.64					BRCA1 C Term	rs111310745,rs3817	0.4136	YES	6.034E-1 (pos.154748908)		PAX transcr	DNA dama Alzheimer Disease;Brain Diseases;Central Ne	G=0, A=59	G=0.0, A=1.0	0.00001	881.596	59	G=0/0, A=33/2
chr7:154760889	C/C	A	C	SNV	PAXIP1	PAXIP1:intronic:NM 007349.3	1	100		intronic	NM 00734	c.1075-53	p.?	0.39					BRCA1 C Term	rs3800580		YES			PAX transcr	DNA dama Alzheimer Disease;Brain Diseases;Central Ne	A=0, C=91	A=0.0, C=1.0	0.00001	1513.81	91	A=0/0, C=31/6
chr7:155093826	T/C	T	C	SNV	INSIG1	INSIG1:intronic:NM 005542.5	1	97.06		intronic	NM 00554	c.538-135	p.?	-0.77					Insulin-induc	rs77130377		YES			Insulin-indu	SREBP sign Mammary Neoplasms, Animal;Mammary Nec	T=2, C=66	T=0.0294, C=0.9706	0.00001	989.134	68	T=2/0, C=34/3
chr7:155094592	T/T	C	T	SNV	INSIG1	INSIG1:intronic:NM 005542.5	1	100		intronic	NM 00554	c.804+36C	p.?	-0.81					Insulin-induc	rs9768652	0.1384	YES	3.177E-1;0.0 (pos.155094592)		Insulin-indu	SREBP sign Mammary Neoplasms, Animal;Mammary Nec	C=0, T=44	C=0.0, T=1.0	0.00001	726.569	44	C=0/0, T=7/37
chr7:155530363	G/G	A	G	SNV	RBM33	RBM33:intronic:NM 053043.2	1	100		intronic	NM 05304	c.1393+7A	p.?	-0.29				0.01(COSM4417319),0.01(C		rs2249337	0.1928	YES	3.31E-1 (pos.155530363)	acute myeloid leukaemia		RNA binding,nucleic acid binding	A=0, G=112	A=0.0, G=1.0	0.00001	1767.96	112	A=0/0, G=61/5
chr7:73275501	C/C	T	C	SNV	TMEM270	TMEM270:utr 5:NM 182504.3	1	100	1	utr 5	NM 18250	c.-24T>C	p.?	-0.56					Williams-Beu	rs11770024	0.2518	YES	6.466E-1 (pos.73275501)			integral component of membrane	T=0, C=22	T=0.0, C=1.0	0.00001	298.891	22	T=0/0, C=17/5
chr7:73275565	A/A	T	A	SNV	TMEM270	TMEM270:exonic:NM 182504.3	1	100	1	missense	NM 18250	c.41T>A	p.Ile14Asn	2.01	0.01	149	0.835		Williams-Beu	rs11770052	0.2524	YES	6.471E-1 (pos.73275565)			integral component of membrane	T=0, A=21	T=0.0, A=1.0	0.00001	285.67	21	T=0/0, A=14/7
chr14:61747644	G/G	A	G	SNV	TMEM30B	TMEM30B:exonic:NM 001017970.2	1	100	1	synonymous	NM 00101	c.222T>C	p.(=)	0.76				0.03(COSM37	LEM3 (ligand-	rs3196765	0.1276	YES	8.579E-1 (pos.61747644)	adenocarcinoi	Transmemb	aminopho Melanoma;Neoplasms;Neoplasms by Histolc	A=0, G=24	A=0.0, G=1.0	0.00001	334.792	24	A=0/0, G=9/15
chr3:194349178	T/T	C	T	SNV	TMEM44	TMEM44:exonic:NM 001166305.1	1	100	2	synonymous	NM 00116	c.198G>A	p.(=)	-5.8						rs1563970	0.3393	YES	4.873E-1 (pos.194349178)			L-lysine transmembrane transporter activity,arginine tr	C=0, T=33	C=0.0, T=1.0	0.00001	504.597	33	C=0/0, T=14/1
chr1:15546077	A/A	G	A	SNV	TMEM51	TMEM51:exonic:NM 018022.2	1	100	3	synonymous	NM 01802	c.600G>A	p.(=)	-2.25				0.11(COSM37	Transmembra	rs3753313	0.3535	YES	5.687E-2 (pos.15546077)	hepatocellular carcinoma		biological process,cellular component,integral compor	G=0, A=40	G=0.0, A=1.0	0.00001	644.285	40	G=0/0, A=12/2
chr7:157873975	C/C	T	C	SNV	PTPRN2	PTPRN2:intronic:NM 002847.4	1	100		intronic	NM 00284	c.1723+15	p.?	-0.14					Protein-tyros	rs3800863,rs386720	0.3492	YES	1.157E-4;1.157E-4 (pos.157873961),9.33		Protein-tyro	cell juncti Eye Diseases;Myopia;Nervous System Diseas	T=0, C=31	T=0.0, C=1.0	0.00001	465.819	31	T=0/0, C=11/2

chr14:77697967	G/G	A	G	SNV	TMEM63C	TMEM63C:exonic:NM 020431.3	1	100	7	synonymous	NM 02043	c.387A>G	p.(=)	-3.14					Calcium-depe	rs375566	0		1.0 (pos.77697967)			calcium activated cation channel activity,cation transm				A=0, G=28	A=0.0, G=1.0	0.00001	408.714	28	A=0/0, G=17/1
chr7:158455089	A/A	G	A	SNV	NCAPG2	NCAPG2:intronic:NM 017760.6	1	100		intronic	NM 01776	c.1816-30	p.?	0.05					Condensin II	rs4484634	0.1146	YES	9.764E-1 (pos.158455089)		Non-SMC cc	cell cycle,cell division,chromosome condensation,conde				G=0, A=21	G=0.0, A=1.0	0.00001	281.154	21	G=0/0, A=14/7
chr7:158456710	T/T	C	T	SNV	NCAPG2	NCAPG2:intronic:NM 017760.6	1	100		intronic	NM 01776	c.1815+16	p.?	0.12					Condensin II	rs10227735		YES			Non-SMC cc	cell cycle,cell division,chromosome condensation,conde				C=0, T=24	C=0.0, T=1.0	0.00001	334.765	24	C=0/0, T=16/8
chr7:158457095	C/C	G	C	SNV	NCAPG2	NCAPG2:intronic:NM 017760.6	1	100		intronic	NM 01776	c.1703-10	p.?	0.39					Condensin II	rs2290392		YES			Non-SMC cc	cell cycle,cell division,chromosome condensation,conde				G=0, C=58	G=0.0, C=1.0	0.00001	914.745	58	G=0/0, C=3/55
chr7:158566138	C/C	T	C	SNV	ESYT2	ESYT2:intronic:NM 020728.2	1	100		intronic	NM 02072	c.892-51A	p.?	-0.29					C2 domain,Sy	rs842447	0.1192	YES	5.345E-1 (pos.158566138)		Extended sy	cadherin binding,calcium ion binding,calcium-dependen				T=0, C=60	T=0.0, C=1.0	0.00001	876.174	60	T=0/0, C=20/4
chr8:109797032	A/G	A	G	SNV	TMEM74	TMEM74:exonic:NM 153015.2	1	50	2	missense	NM 15301	c.296T>C	p.Val99Ala	4.46		64			Neurensin						Transmemb	autophagosome membrane,cytoplasmic vesicle,integra				A=14, G=14	A=0.5, G=0.5	0.00001	131.941	28	A=4/10, G=5/9
chr1:205052939	A/A	G	A	SNV	TMEM81	TMEM81:exonic:NM 203376.1	1	100	1	synonymous	NM 20337	c.510C>T	p.(=)	-0.55						rs4950979	0.1426	YES	9.711E-1 (pos.205052939)			integral component of membrane				G=0, A=60	G=0.0, A=1.0	0.00001	903.524	60	G=0/0, A=30/3
chr7:158719084	T/T	C	T	SNV	WDR60	WDR60:intronic:NM 018051.4	1	100		intronic	NM 01805	c.2372-9C	p.?	0.47						rs2244789	0.2086	YES	9.152E-1 (pos.158719084)		WD repeat	ATP-deper	Abnormalities, Multiple;Bone Diseases;Bone			C=0, T=25	C=0.0, T=1.0	0.00001	353.019	25	C=0/0, T=11/1
chr7:158726798	TC/TC	T	TC	INDEL	WDR60	WDR60:intronic:NM 018051.4	1	100		intronic	NM 01805	c.2547-22	p.?	0.04						rs34181207	0.1843	YES	9.477E-1 (pos.158726798)		WD repeat	ATP-deper	Abnormalities, Multiple;Bone Diseases;Bone			T=0, TC=32	T=0.0, TC=1.0	0.00001	485.141	32	T=0/0, TC=13/
chr7:158727015	CT/CT	C	CT	INDEL	WDR60	WDR60:intronic:NM 018051.4	1	100		intronic	NM 01805	c.2647-94	p.?	0.07						rs200977903,rs35962377,rs397		YES			WD repeat	ATP-deper	Abnormalities, Multiple;Bone Diseases;Bone			C=0, CT=12	C=0.0, CT=1.0	0.00001	1623.39	125	C=0/0, CT=88/
chr1:16069644	T/T	C	T	SNV	TMEM82	TMEM82:exonic:NM 001013641.2	1	100	3	synonymous	NM 00101	c.291C>T	p.(=)	-0.98					Transmembr	rs11584945	0.2774	YES	3.599E-2;0.0 (pos.16069644)			integral component of membrane				C=0, T=32	C=0.0, T=1.0	0.00001	485.136	32	C=0/0, T=14/1
chr7:16577376	A/A	G	A	SNV	LRRC72	LRRC72:intronic:NM 001195280.1	1	100		intronic	NM 00119	c.234+55G	p.?	-0.2					Leucine-rich	rs6961390		YES								G=0, A=39	G=0.0, A=1.0	0.00001	623.979	39	G=0/0, A=17/2
chr7:16598655	A/A	G	A	SNV	LRRC72	LRRC72:intronic:NM 001195280.1	1	100		intronic	NM 00119	c.427+31G	p.?	-0.31					Leucine-rich	rs6979816		YES	6.77E-1 (pos.16598655)							G=0, A=22	G=0.0, A=1.0	0.00001	298.89	22	G=0/0, A=8/14
chr7:16605818	A/A	G	A	SNV	LRRC72	LRRC72:intronic:NM 001195280.1	1	100		intronic	NM 00119	c.428-120	p.?	0.07					Leucine-rich	rs499608		YES								G=0, A=37	G=0.0, A=1.0	0.00001	583.548	37	G=0/0, A=22/1
chr19:55738634	C/C	T	C	SNV	TMEM86B	TMEM86B:exonic:NM 173804.4	1	100	3	missense	NM 17380	c.596A>G	p.His199Arg	1.07	1	29	0		YhhN family	rs4644955	0.0952	YES	9.116E-1;1.19E-4 (pos.55738634)			alkenylglycerophosphocholine hydrolase activity,alkeny				T=0, C=25	T=0.0, C=1.0	0.00001	353.004	25	T=0/0, C=21/4
chr7:16834551	A/A	C	A	SNV	AGR2	AGR2:intronic:NM 006408.3	1	100		intronic	NM 00640	c.478+9G>	p.?	-0.51					Thioredoxin-l	rs4719480	0.1514	YES	9.588E-1 (pos.16834551)		Anterior gra	cell differe	Breast Diseases;Breast Neoplasms;Genital D			C=0, A=64	C=0.0, A=1.0	0.00001	967.912	64	C=0/0, A=34/3
chr16:426432	C/C	T	C	SNV	TMEM8A	TMEM8A:exonic:NM 021259.2	1	100	6	missense	NM 02125	c.928A>G	p.Ile310Val	-2.1	0.74	29	0		Protein of unl	rs2071915	0.4333	YES	3.121E-1 (pos.426432)			biological_process,extracellular exosome,integral comp				T=0, C=77	T=0.0, C=1.0	0.00001	1229.44	77	T=0/0, C=43/3
chr16:427516	C/C	T	C	SNV	TMEM8A	TMEM8A:exonic:NM 021259.2	1	100	3	synonymous	NM 02125	c.369A>G	p.(=)	-0.02				0.03(COSM43	Protein of unl	rs11649031	0.4325	YES	3.772E-1 (pos.427516)	adenocarcinoma,neoplasn		biological_process,extracellular exosome,integral comp				T=0, C=142	T=0.0, C=1.0	0.00001	2383.53	142	T=0/0, C=65/7
chr3:69096614	A/A	G	A	SNV	TMF1	TMF1:exonic:NM 007114.2	1	100	2	synonymous	NM 00711	c.1242C>T	p.(=)	-0.14				0.04(COSM40	TATA element	rs2292199	0.1925	YES	6.47E-1 (pos.69096614)	acute_myeloic	TATA eleme	DNA bindii	Adnexal Diseases;Cysts;Endocrine System Di			G=0, A=28	G=0.0, A=1.0	0.00001	408.718	28	G=0/0, A=11/1
chr19:4292597	C/T	C	T	SNV	TMIGD2	TMIGD2:exonic:NM 144615.2	1	57.14	5	synonymous	NM 14461	c.848G>A	p.(=)	-0.35				0(COSM3756	Immunoglobu	rs758313911,rs8889	0.2763	YES	0.0 (pos.4292594),3.385	acute_myeloic	Transmemb	T cell costimulation,coreceptor activity,extracellular spa				C=21, T=28	C=0.4286, T=0.5714	0.00001	342.296	49	C=9/12, T=11/
chr4:69094507	A/A	T	A	SNV	TMPRSS11B	TMPRSS11B:exonic:NM 182502.3	1	100	9	missense	NM 18250	c.1042A>T	p.Thr348Ser	-0.05	0.14	58	0.316		SEA domain,T	rs2319797	0.2209	YES	9.956E-1 (pos.69094507)			biological_process,extracellular exosome,integral comp				T=0, A=34	T=0.0, A=1.0	0.00001	524.186	34	T=0/0, A=22/1
chr4:69094575	G/G	T	G	SNV	TMPRSS11B	TMPRSS11B:exonic:NM 182502.3	1	100	9	missense	NM 18250	c.974A>C	p.Asp325Ala	4.56	1	126	0		SEA domain,T	rs2319796	0		1.0 (pos.69094575)			biological_process,extracellular exosome,integral comp				T=0, G=34	T=0.0, G=1.0	0.00001	524.151	34	T=0/0, G=22/1
chr7:21695649	A/A	G	A	SNV	DNAH11	DNAH11:intronic:NM 001277115.1	1	100		intronic	NM 00127	c.5094+50	p.?	0.38					AAA domain (	rs4385378	0.4201	YES	6.346E-1 (pos.21695649)		Dynein, axo	ATP bindin	Blood Protein Disorders;Bronchial Diseases;I			G=0, A=27	G=0.0, A=1.0	0.00001	389.988	27	G=0/0, A=7/20
chr12:51236931	A/A	G	A	SNV	TMPRSS12	TMPRSS12:exonic:NM 182559.2	1	100	1	missense	NM 18255	c.184G>A	p.Glu62Lys	-1.25	1	56	0	0(COSM3753	Trypsin	rs829121	6.00E-04	YES	1.0 (pos.51236931)	adenocarcinoma		integral component of membrane,proteolysis,serine-typ				G=0, A=26	G=0.0, A=1.0	0.00001	371.42	26	G=0/0, A=12/1
chr11:117774394	A/G	A	G	SNV	TMPRSS13	TMPRSS13:exonic:NM 001077263.2	1	47.37	12	synonymous	NM 00107	c.1653T>C	p.(=)	-1.25				0.02(COSM44	Domain of un	rs59590191	0.1284	YES	1.729E-1 (pos.11777439	acute_myeloic	Transmembrane protein, serine 13					A=20, G=18	A=0.5263, G=0.4737	0.00001	184.393	38	A=8/12, G=8/1
chr21:19670118	G/A	G	A	SNV	TMPRSS15	TMPRSS15:exonic:NM 002772.2	1	50	19	missense	NM 00277	c.2194C>T	p.Pro732Ser	1.92	1	74	0	0(COSM4001	CUB domain,	rs2824721	0.217	YES	6.633E-1 (pos.19670118)	acute_myeloid leukaemia		brush borc	Congenital, Hereditary, and Neonatal Diseas			G=25, A=25	G=0.5, A=0.5	0.00001	288.837	50	G=8/17, A=16/
chr22:37480797	T/T	C	T	SNV	TMPRSS6	TMPRSS6:exonic:NM 153609.3	1	100	9	synonymous	NM 15360	c.1083G>A	p.(=)	-2.82					Domain of un	rs2111833	0.3352	YES	4.092E-1 (pos.37480797)		Transmemb	angiogene	Anemia;Anemia, Hypochromic		Benign	C=0, T=69	C=0.0, T=1.0	0.00001	1072.78	69	C=0/0, T=48/2
chr7:21943722	T/C	T	C	SNV	CDCA7L	CDCA7L:intronic:NM 018719.4	1	56		intronic	NM 01871	c.1197+6A	p.?	-0.5				0.07(COSM44	Zinc-finger dc	rs35898649	0.1265	YES	5.104E-2 (pos.21943722)	acute_myeloic	Cell divisior	cytosol,fibrillar center,integral component of membrane				T=22, C=28	T=0.44, C=0.56	0.00001	340.988	50	T=12/10, C=8/
chr22:37485724	C/C	T	C	SNV	TMPRSS6	TMPRSS6:exonic:NM 153609.3	1	100	7	missense	NM 15360	c.757A>G	p.Lys253Glu	-1.87	0.64	56	0	0.01(COSM37	Domain of un	rs2235324	0.3814	YES	4.124E-1 (pos.37485724)	hepatocellula	Transmemb	angiogene	Anemia;Anemia, Hypochromic		Benign	T=0, C=22	T=0.0, C=1.0	0.00001	298.892	22	T=0/0, C=10/1
chr3:111760771	G/G	A	G	SNV	TMPRSS7	TMPRSS7:utr 5:NM 001042575.2	1	100	2	utr 5	NM 00104	c.-34A>G	p.?	1.55					CUB domain,	rs386664649,rs774774		YES	7.556E-1 (pos.111760771)			integral cc	Mental Disorders;Substance-Related Disorde			A=0, G=26	A=0.0, G=1.0	0.00001	371.42	26	A=0/0, G=23/3
chr7:2258899	A/A	G	A	SNV	MAD1L1	MAD1L1:intronic:NM 001013837.1	1	100		intronic	NM 00101	c.596+68C	p.?	-0.85					Mitotic check	rs1639916		YES			Mitotic arre	actin cytos	Arthritis;Arthritis, Juvenile;Autoimmune Dise			G=0, A=23	G=0.0, A=1.0	0.00001	314.649	23	G=0/0, A=13/1
chr3:111799845	T/T	A	T	SNV	TMPRSS7	TMPRSS7:exonic:NM 001042575.2	1	100	16	missense	NM 00104	c.2068A>T	p.Ser690Cys	5.58	1	112	0		CUB domain,	rs340151	0.044	YES	9.854E-1 (pos.111799845)			integral cc	Mental Disorders;Substance-Related Disorde			A=0, T=37	A=0.0, T=1.0	0.00001	583.682	37	A=0/0, T=25/1
chr7:2304191	C/C	A	C	SNV	SNX8	SNX8:intronic:NM 013321.3	1	100		intronic	NM 01332	c.622-98T	p.?	-6.05					PX domain	rs2286205		YES			Sorting nexi	cytosol,early endosome membrane,early endosome to (				A=0, C=26	A=0.0, C=1.0	0.00001	371.081	26	A=0/0, C=8/18
chr7:23164526	G/G	C	G	SNV	KLHL7	KLHL7:intronic:NM 001031710.2	1	100		intronic	NM 00103	c.317+126	p.?	0.01					BTB And C-ter	rs2058275		YES	8.476E-1 (pos.23164526)		Kelch-like 7	Cul3-RING	Congenital, Hereditary, and Neonatal Diseas			C=0, G=38	C=0.0, G=1.0	0.00001	603.774	38	C=0/0, G=18/2
chr7:23810517	A/A	G	A	SNV	STK31	STK31:intronic:NM 031414.4	1	100		intronic	NM 03141	c.1714-10	p.?	0.98					Protein kinas	rs7785154		YES			Serine/thre	ATP bindin	Nervous System Diseases;Neuromuscular Dis			G=0, A=31	G=0.0, A=1.0	0.00001	465.819	31	G=0/0, A=17/1
chr7:23854717	T/T	C	T	SNV	STK31	STK31:intronic:NM 031414.4	1	100		intronic	NM 03141	c.2761-46	p.?	-0.05					Protein kinas	rs2390814	0.2697	YES	5.983E-1 (pos.23854717)		Serine/thre	ATP bindin	Nervous System Diseases;Neuromuscular Dis			C=0, T=24	C=0.0, T=1.0	0.00001	334.777	24	C=0/0, T=14/1
chr7:2414142	G/G	A	G	SNV	EIF3B	EIF3B:intronic:NM 001037283.1	1	100		intronic	NM 00103	c.1811-21	p.?	-4.01					Eukaryotic tr	rs886632	0.2707	YES	8.938E-1 (pos.2414142)		Eukaryotic t	IRES-depe	HIV Infections;Immune System Diseases;Imr			A=0, G=39	A=0.0, G=1.0	0.00001	622.913	39	A=0/0, G=23/1
chr13:101287365	C/C	T	C	SNV	TMTC4	TMTC4:exonic:NM 032813.4	1	100	11	synonymous	NM 03281	c.1287A>G	p.(=)	-3.58					Domain of un	rs946838	0.132	YES	9.903E-1 (pos.101287365)			integral component of membrane				T=0, C=30	T=0.0, C=1.0	0.00001	446.639	30	T=0/0, C=8/22
chr9:117792583	G/G	C	G	SNV	TNC	TNC:exonic:NM 002160.3	1	100	24	missense	NM 00216	c.6022G>C	p.Glu2008Gln	6.28	1	29	0	0.2(COSM500	EGF-like dom	rs13321	0.2952	YES	5.557E-1 (pos.11779258	adenocarcinoi	Tenascin C	basement	Asthma;Bronchial Diseases;Cardiovascular D			C=0, G=92	C=0.0, G=1.0	0.00001	1534.52	92	C=0/0, G=69/2
chr7:2552882	GATGG/GATG(	G	GATGG	INDEL	LFNG		4	100		intronic				-0.48				0.02(COSM5691550)		rs61564232,rs757595123,rs760021968,rs7			0.0 (pos.2552882),4.513	small_cell_car	Lunatic fringe		Abnormalities, Multiple;Bone Diseases;Bone			G=0, GATG(	G=0.0, GATGG=1.0	0.00001	568.045	37	G=0/0, GATGG
chr9:117846580	C/C	T	C	SNV	TNC	TNC:exonic:NM 002160.3	1	100	4	missense	NM 00216	c.2039A>G	p.Gln680Arg	-0.36	1	43	0	0.06(COSM15	EGF-like dom	rs1061494	0.4377	YES	2.609E-1 (pos.11784658	acute_myeloic	Tenascin C	basement	Asthma;Bronchial Diseases;Cardiovascular D			T=0, C=59	T=0.0, C=1.0	0.00001	885.365	59	T=0/0, C=24/3
chr7:2613008	A/G	A	G	SNV	IQCE	IQCE:intronic:NM 152558.4	1	50		intronic	NM 15255	c.395-44A	p.?	-0.74					IQ calmodulii	rs62442625	0.1621	YES	1.574E-3;0.0 (pos.2613008)		IQ domain-c	ciliary membrane,cilium,extrinsic component of membr				A=17, G=17	A=0.5, G=0.5	0.00001	172.009	34	A=11/6, G=12/
chr7:2613042	C/C	G	C	SNV	IQCE	IQCE:intronic:NM 152558.4	1	100		intronic	NM 15255	c.395-10G	p.?	-0.7				0(COSM4419	IQ calmodulii	rs2304540	0.2468	YES	4.828E-1 (pos.2613042)	acute_myeloic	IQ domain-c	ciliary membrane,cilium,extrinsic component of membr				G=0, C=34	G=0.0, C=1.0	0.00001	524.186	34	G=0/0, C=23/1
chr9:117848394	C/C	T	C	SNV	TNC	TNC:exonic:NM 002160.3	1	100	3	missense	NM 00216	c.1616A>G	p.Gln539Arg	-0.46	1	43	0		EGF-like dom	rs1757095	0.0681	YES	9.997E-1 (pos.117848394)		Tenascin C	basement	Asthma;Bronchial Diseases;Cardiovascular D			T=0, C=91	T=0.0, C=1.0	0.00001	1501.71	91	T=0/0, C=55/3
chr7:2618070	C/CA	C	CA	INDEL	IQCE	IQCE:intronic:NM 152558.4	1	50		intronic	NM 15255	c.580-40C	p.?	-1					IQ calmodulii	rs3214664,rs3977520	0.4434	YES	1.489E-1;1.162E-4 (pos.2618070)		IQ domain-c	ciliary membrane,cilium,extrinsic component of membr				C=48, CA=4	C=0.5, CA=0.5	0.00001	517.494	96	C=36/12, CA=
chr7:26223294	G/G	A	G	SNV	NFE2L3	NFE2L3:intronic:NM 004289.6	1	100		intronic	NM 00428	c.751-27A	p.?	-0.69					Basic region l	rs12113404,rs75375	0.1005	YES	0.0 (pos.26223288),0.0;0.0 (pos.26223		Nuclear fac	DNA binding transcription factor activity,RNA polymeras				A=0, G=43	A=0.0, G=1.0	0.00001	661.996	43	A=0/0, G=27/1
chr9:117849314	C/C	T	C	SNV	TNC	TNC:exonic:NM 002160.3	1	100	3	synonymous	NM 00216	c.696A>G	p.(=)	-0.2				0.01(COSM37	EGF-like dom	rs2992147	0.4379	YES	2.635E-1 (pos.11784931	adenocarcinoi	Tenascin C	basement	Asthma;Bronchial Diseases;Cardiovascular D			T=0, C=35	T=0.0, C=1.0	0.00001	543.902	35	T=0/0, C=14/2
chr7:27169617	C/C	T	C	SNV	HOXA4	HOXA4:intronic:NM 002141.4	1	100		intronic	NM 00214	c.616+120	p.?	3.01					Homeobox dc	rs6943542		YES			Homeo box	DNA bindir	Congenital Abnormalities;Congenital, Heredi			T=0, C=26	T=0.0, C=1.0	0.00001	371.395	26	T=0/0, C=12/1
chr9:117836056	G/A	G	A	SNV	TNC	TNC:exonic:NM 002160.3	1	57.58	10	missense	NM 00216	c.3040C>T	p.Arg1014Cys	2.36	0.03	180	0.921	0.9(COSM145	EGF-like dom	rs373842890			0.0 (pos.117836056)	adenocarcinoi	Tenascin C	basement	Asthma;Bronchial Diseases;Cardiovascular D			G=42, A=57	G=0.4242, A=0.5758	0.00001	690.632	99	G=23/19, A=3
chr7:2749565	A/A	G	A	SNV	AMZ1	AMZ1:intronic:NM 133463.3	1	100		intronic	NM 13346	c.948+115	p.?	-0.3						rs13224990		YES			Archaelysin	cellular_component,metallopeptidase activity,peptidas				G=0, A=22	G=0.0, A=1.0	0.00001	298.854	22	G=0/0, A=10/1
chr5:118691693	A/A	C	A	SNV	TNFAIP8	TNFAIP8:utr 5:NM 014350.3	1	100	1	utr 5	NM 01435	c.-93C>A	p.?	-0.59					Domain of un	rs3797339		YES			Tumor necr	apoptotic	Arthritis;Arthritis, Juvenile;Autoimmune Dise			C=0, A=26	C=0.0, A=1.0	0.00001	371.4	26	C=0/0, A=21/5
chr7:29103867	C/C	T	C	SNV	CPVL	CPVL:intronic:NM 019029.3	1	100		intronic	NM 01902	c.964-17A	p.?	-3.28					Serine carbo	rs644763	0.1612	YES	8.68E-1;0.0 (pos.29103867)		Carboxypep	extracellu	Mental Disorders;Psychotic Disorders;Schizo			T=0, C=36	T=0.0, C=1.0	0.00001	563.744	36	T=0/0, C=22/1
chr8:23049292	T/T	C	T	SNV	TNFRSF10A	TNFRSF10A:exonic:NM 003844.3	1	100	10	missense	NM 00384	c.1322G>A	p.Arg441Lys	-0.51	1	26	0		Death domai	rs2230229	0.1543	YES	9.807E-1 (pos.23049292)		Tumor necr	TRAIL bind	Adenocarcinoma;Agranulocytosis;Alopecia;A			C=0, T=33	C=0.0, T=1.0	0.00001	503.16	33	C=0/0, T=16/1
chr8:22974450	C/C	T	C	SNV	TNFRSF10C	TNFRSF10C:exonic:NM 003841.4	1	100	5	missense	NM 00384	c.686T>C	p.Ile229Thr	-2.8	1	89	0		TNFR/NGFR c	rs752447767,rs9644	0.1866	YES	3.876E-3;1.628E-1;5.426E-2;5.814E-3;0.0		Tumor necr	TRAIL bind	Arthritis;Arthritis, Juvenile;Autoimmune Dise			T=0, C=41	T=0.0, C=1.0	0.00001	664.627	41	T=0/0, C=10/3
chr17:16855878	T/T	C	T	SNV	TNFRSF13B	TNFRSF13B:exonic:NM 012452.2	1	100	2	synonymous	NM 01245	c.81G>A	p.(=)	-1.84					TACI, cysteine	rs8072293	0.2598	YES	9.993E-1 (pos.16855878)		Tumor necr	B cell hom	Blood Protein Disorders;Cardic		Benign	C=0, T=35	C=0.0, T=1.0	0.00001	543.773	35	C=0/0, T=14/2
chr16:12060163	G/G	A	G	SNV	TNFRSF17	TNFRSF17:exonic:NM 001192.2	1	100	2	missense	NM 00119	c.242A>G	p.Asn81Ser	-1.1	0.57	46	0		BCMA, TALL-1	rs373496,rs7686908	0.0117	YES	0.0 (pos.12060158),9.995E-1 (pos.12060		Tumor necr	adaptive immune response,cell proliferation,endomem				A=0, G=73	A=0.0, G=1.0	0.00001	1150.51	73	A=0/0, G=34/3
chr13:24243200	C/C	T	C	SNV	TNFRSF19	TNFRSF19:exonic:NM 018647.3	1	100	9	synonymous	NM 01864	c.1209T>C	p.(=)	-6.41				0.01(COSM41	TNFR/NGFR c	rs3751363	0.1208	YES	8.28E-1 (pos.24243200)	neoplasm		JNK cascac	Carcinoma;Head and Neck Neoplasms;Melar			T=0, C=134	T=0.0, C=1.0	0.00001	2217.58	134	T=0/0, C=68/6
chr13:24243133	C/T	C	T	SNV	TNFRSF19	TNFRSF19:exonic:NM 018647.3	1	45.52	9	missense	NM 01864	c.1142C>T	p.Thr381Ile	0.38	0	89	0.279		TNFR/NGFR c	rs369698575	2.00E-04		0.0 (pos.24243133)			JNK cascac	Carcinoma;Head and Neck Neoplasms;Melar			C=73, T=61	C=0.5448, T=0.4552	0.00001	630.548	134	C=33/40, T=38
chr1:12198423	CGGGGC/CGG	CGGGGGC	CGGGG,CGGGGC	INDEL	TNFRSF8	TNFRSF8:exonic:NM 001243.4	1	100	14	frameshiftDeletion	NM 00124	c.1478del	p.Gly493fs	-0.65, 0.63, 1.45, -0.24, 0.27, 0.23, -1.02					TNFR/NGFR c	rs370010968,rs3734	0		0.0 (pos.12198424),0.0 (pos.12198428),(		Tumor necr	apoptotic	Hemic and Lympha	Brentuximab vedotin		CGGGGGC=	CGGGGGC=0.0, CGG(	0.00001	316.656	26	CGGGGGC=0/(
chr3:172224303	G/G	A	G	SNV	TNFSF10	TNFSF10:exonic:NM 003810.3	1	100	5	synonymous	NM 00381	c.825T>C	p.(=)	-0.44					TNF(Tumour N	rs1131532	0.3243	YES	5.998E-1;0.0 (pos.172224303)		Tumor necr	activation	Abortion, Spontaneous;Adenocarcinoma;Adr			A=0, G=44	A=0.0, G=1.0	0.00001	722.977	44	A=0/0, G=27/1
chr17:7460517	C/C	G	C	SNV	TNFSF12,TNFSF12-TNFSF13	TNFSF12:exonic nc:NR 037146.1, TNFSF12-TNFSF13:intronic	1	100	8	exonic nc	NR 03714	c.498+298	p.?	-2.17				0(COSM2924	TNF(Tumour N	rs3803798	0.4751	YES	6.084E-1 (pos.7460517)	acute_myeloic	Tumor necr	angiogene	Autoimmune Diseases;Common Variable Imr			G=0, C=35	G=0.0, C=1.0	0.00001	543.9	35	G=0/0, C=20/1
chr17:7460559	G/G	T	G	SNV	TNFSF12,TNFSF12-TNFSF13	TNFSF12:exonic nc:NR 037146.1, TNFSF12-TNFSF13:intronic	1	100	8	exonic nc	NR 03714	c.498+340	p.?	1.02				0.05(COSM37	TNF(Tumour N	rs4968189	3.00E-04		9.999E-1;1.159E-4 (pos.7	adenocarcinoi	Tumor necr	angiogene	Autoimmune Diseases;Common Variable Imr			T=0, G=34	T=0.0, G=1.0	0.00001	523.973	34	T=0/0, G=19/1
chr19:6665020	C/C	T	C	SNV	TNFSF14	TNFSF14:exonic:NM 003807.4	1	100	5	missense	NM 00380	c.640A>G	p.Lys214Glu	0.13	1	56	0	0.01(COSM41	TNF(Tumour N	rs344560	0.0551	YES	9.208E-1 (pos.6665020)	neoplasm	Tumor necr	T cell activ	Arthritis;Arthritis, Rheumatoid;Autoimmune			T=0, C=63	T=0.0, C=1.0	0.00001	958.911	63	T=0/0, C=40/2
chr17:7286326	T/A	T	A	SNV	TNK1	TNK1:exonic:NM 001251902.1	1	46.88	2	synonymous	NM 00125	c.81T>A	p.(=)	-2.96				0.06(COSM40	Protein kinas	rs1554948,rs766030	0.461	YES	2.625E-1;0.0 (pos.72863	acute_myeloic	Tyrosine kir	ATP binding,cell differentiation,cell migration,cytoplasm				T=17, A=15	T=0.5313, A=0.4688	0.00001	143.728	32	T=7/10, A=10/
chr7:32591158	A/A	G	A	SNV	AVL9	AVL9:intronic:NM 015060.2	1	100		intronic	NM 01506	c.462+123	p.?	-2.35					Docking dom	rs3213973		YES			AVL9, S. cer	cell migra	Arthritis;Arthritis, Juvenile;Autoimmune Dise			G=0, A=24	G=0.0, A=1.0	0.00001	339.18	24	G=0/0, A=7/17
chr7:32609565	G/G	A	G	SNV	AVL9	AVL9:intronic:NM 015060.2	1	100		intronic	NM 01506	c.1216-67	p.?	-0.43					Docking dom	rs2305320		YES			AVL9, S. cer	cell migra	Arthritis;Arthritis, Juvenile;Autoimmune Dise			A=0, G=38	A=0.0, G=1.0	0.00001	603.659	38	A=0/0, G=21/1
chr11:57081145	T/TC	T	TC	INDEL	TNKS1BP1	TNKS1BP1:exonic:NM 033396.2	1	45.83	5	frameshiftInsertion	NM 03339	c.1016 10	p.Ser340fs	-1.88					Tankyrase binding protein C terminal domain						Tankyrase 1	CCR4-NOT complex,DNA damage response, signal transd				T=13, TC=1	T=0.5417, TC=0.4583	0.00001	79.656	24	T=6/7, TC=8/3
chr7:32615553	T/T	C	T	SNV	AVL9	AVL9:intronic:NM 015060.2	1	100		intronic	NM 01506	c.1571-14	p.?	-0.07					Docking dom	rs6462381,rs796558	0.258	YES	8.323E-1 (pos.32615553)		AVL9, S. cer	cell migra	Arthritis;Arthritis, Juvenile;Autoimmune Dise			C=0, T=69	C=0.0, T=1.0	0.00001	1072.77	69	C=0/0, T=40/2
chr7:33016120	T/T	G	T	SNV	FKBP9	FKBP9:intronic:NM 007270.4	1	100		intronic	NM 00727	c.703+9G>	p.?	-0.83				0.01(COSM54	EF hand,FKBP	rs3750116,rs780358	0.0799	YES	2.353E-4 (pos.33016118)	adenocarcinoi	FK506-bind	FK506 binding,calcium ion binding,chaperone-mediated				G=0, T=45	G=0.0, T=1.0	0.00001	747.258	45	G=0/0, T=29/1
chr7:331085	CA/CA	C	CA	INDEL			1	100		intronic				-0.41					Forkhead domain											C=0, CA=24	C=0.0, CA=1.0	0.00001	294.628	24	C=0/0, CA=7/1
chr7:33136983	A/A	G	A	SNV	RP9	RP9:intronic:NM 203288.1	1	100		intronic	NM 20328	c.314-9C>	p.?	1.17						rs6462460,rs760406	0.0085		9.311E-4 (pos.33136977),9.991E-1 (pos.3		RP9 gene (p	RNA bindir	Congenital, Hereditary, and Ne		Benign	G=0, A=37	G=0.0, A=1.0	0.00001	569.178	37	G=0/0, A=23/1
chr5:72184098	T/T	C	T	SNV	TNPO1	TNPO1:exonic:NM 153188.2	1	100	13	synonymous	NM 15318	c.1473C>T	p.(=)	0.5				0.35(COSM44	HEAT-like rep	rs17606	0.1474	YES	8.46E-1 (pos.72184098)	hepatocellula	Transportin	NLS-bearing protein import into nucleus,RNA binding,Ra				C=0, T=69	C=0.0, T=1.0	0.00001	1072.78	69	C=0/0, T=37/3
chr7:35930260	T/T	G	T	SNV	SEPT7	SEPT7:intronic:NM 001242956.1	1	100		intronic	NM 00124	c.765-18G	p.?	0.48					50S ribosome	rs28522781	0.3223	YES	4.094E-1 (pos.35930260)		Septin 7					G=0, T=25	G=0.0, T=1.0	0.00001	351.158	25	G=0/0, T=15/1
chr1:175299301	C/C	T	C	SNV	TNR	TNR:exonic:NM 003285.2	1	100	21	synonymous	NM 00328	c.3702A>G	p.(=)	1.1					EGF-like dom	rs2027867	0.2772	YES	8.202E-1 (pos.175299301)		Tenascin R	associativ	Abortion, Spontaneous;Female Urogenital Di			T=0, C=20	T=0.0, C=1.0	0.00001	263.565	20	T=0/0, C=11/9
chr7:36370499	G/G	C	G	SNV	KIAA0895	KIAA0895:intronic:NM 001100425.1	1	100		intronic	NM 00110	c.1404+92	p.?	-0.45					Domain of un	rs196659		YES								C=0, G=63	C=0.0, G=1.0	0.00001	958.916	63	C=0/0, G=28/3
chr7:36370555	T/T	G	T	SNV	KIAA0895	KIAA0895:intronic:NM 001100425.1	1	100		intronic	NM 00110	c.1404+36	p.?	-2.94					Domain of un	rs196660	0.0241	YES	9.797E-1 (pos.36370555)							G=0, T=61	G=0.0, T=1.0	0.00001	921.854	61	G=0/0, T=26/3
chr7:37901581	A/A	G	A	SNV	NME8	NME8:intronic:NM 016616.4	1	100		intronic	NM 01661	c.271-49G	p.?	0.61					Nucleoside d	rs2249451	0.0559	YES	6.945E-1 (pos.37901581)		NME/NM23	CTP biosyr	Ciliary Motility Disorders;Otorl		Benign	G=0, A=32	G=0.0, A=1.0	0.00001	485.118	32	G=0/0, A=17/1
chr7:37903904	T/T	C	T	SNV	NME8	NME8:intronic:NM 016616.4	1	100		intronic	NM 01661	c.455-46C	p.?	-0.57					Nucleoside d	rs3816418	0.2494	YES	5.825E-1;0.0 (pos.37903904)		NME/NM23	CTP biosyr	Ciliary Motility Disorders;Otorl		Benign	C=0, T=82	C=0.0, T=1.0	0.00001	1329.36	82	C=0/0, T=45/3
chr7:37904099	A/A	G	A	SNV	NME8	NME8:intronic:NM 016616.4	1	100		intronic	NM 01661	c.528+76G	p.?	0.35					Nucleoside d	rs3816417		YES			NME/NM23	CTP biosyr	Ciliary Motility Disorders;Otorhinolaryngolog			G=0, A=87	G=0.0, A=1.0	0.00001	1431.38	87	G=0/0, A=43/4
chr1:175335234	T/T	C	T	SNV	TNR	TNR:exonic:NM 003285.2	1	100	11	synonymous	NM 00328	c.2094G>A	p.(=)	-4.24				0.01(COSM37	EGF-like dom	rs1385541	0.2429	YES	9.457E-2 (pos.17533523	adenocarcinoi	Tenascin R	associativ	Abortion, Spontaneous;Female Urogenital Di			C=0, T=83	C=0.0, T=1.0	0.00001	1349.88	83	C=0/0, T=32/5
chr7:37923849	G/A	G	A	SNV	NME8	NME8:intronic:NM 016616.4	1	58.33		intronic	NM 01661	c.995-56G	p.?	0.34					Nucleoside d	rs1993052		YES			NME/NM23	CTP biosyr	Ciliary Motility Disorders;Otorhinolaryngolog			G=10, A=14	G=0.4167, A=0.5833	0.00001	135.339	24	G=8/2, A=6/8
chr7:37933983	G/G	C	G	SNV	NME8	NME8:intronic:NM 016616.4	1	100		intronic	NM 01661	c.1400-85	p.?	-0.23					Nucleoside d	rs10260487		YES			NME/NM23	CTP biosyr	Ciliary Motility Disorders;Otorhinolaryngolog			C=0, G=50	C=0.0, G=1.0	0.00001	849.514	50	C=0/0, G=35/1
chr7:38505025	A/A	G	A	SNV	AMPH	AMPH:intronic:NM 001635.3	1	100		intronic	NM 00163	c.749+42C	p.?	-0.06					BAR domain	rs722334	0.4366	YES	6.476E-1 (pos.38505025)		Amphiphysi	actin cytoskeleton,cell junction,chemical synaptic trans				G=0, A=90	G=0.0, A=1.0	0.00001	1493.12	90	G=0/0, A=47/4
chr7:38514851	A/T	A	T	SNV	AMPH	AMPH:intronic:NM 001635.3	1	57.14		intronic	NM 00163	c.590+80T	p.?	1.74					BAR domain	rs35367242		YES			Amphiphysi	actin cytoskeleton,cell junction,chemical synaptic trans				A=18, T=24	A=0.4286, T=0.5714	0.00001	279.004	42	A=10/8, T=10/
chr17:76060954	A/C	A	C	SNV	TNRC6C	TNRC6C:exonic:NM 001142640.1	1	58.33	5	synonymous	NM 00114	c.2538A>C	p.(=)	-1.78				0.03(COSM40	Argonaute hc	rs61730171	0.1029	YES	2.057E-1 (pos.76060954)	Wilms_tumou	Trinucleotic	RNA binding,Wnt signaling pathway, calcium modulating				A=20, C=28	A=0.4167, C=0.5833	0.00001	343.408	48	A=6/14, C=15/
chr7:38813711	C/T	C	T	SNV	VPS41	VPS41:intronic:NM 014396.3	1	97.56		intronic	NM 01439	c.1012+4G	p.?	2.34					Region in Cla	rs976350	0.008		9.946E-1 (pos.38813711)		Vacuolar pr	AP-3 adaptor complex,CORVET complex,GTPase binding,(				C=1, T=40	C=0.0244, T=0.9756	0.00001	631.828	41	C=1/0, T=27/1
chr7:38857364	A/A	G	A	SNV	VPS41	VPS41:intronic:NM 014396.3	1	100		intronic	NM 01439	c.450+53C	p.?	1.71					Region in Cla	rs2072248	0.4761	YES	8.869E-1 (pos.38857364)		Vacuolar pr	AP-3 adaptor complex,CORVET complex,GTPase binding,(				G=0, A=39	G=0.0, A=1.0	0.00001	623.98	39	G=0/0, A=21/1
chr7:40220507	C/G	C	G	SNV	SUGCT	SUGCT:intronic:NM 001193313.1	1	50		intronic	NM 00119	c.122-39C	p.?	-1.1					CoA-transfera	rs2036041	0.463	YES	4.356E-1 (pos.40220507)			formyl-Co/	Amino Acid Metabolism, Inborn Errors;Brain I			C=22, G=22	C=0.5, G=0.5	0.00001	243.628	44	C=9/13, G=12/
chr7:4051677	G/A	G	A	SNV	SDK1	SDK1:intronic:NM 152744.3	1	56		intronic	NM 15274	c.2280-50	p.?	-3.37					Fibronectin t	rs894776	0.1679	YES	5.92E-2 (pos.4051677)		Sidekick, Dr	behavioral response to cocaine,cell junction,cell-cell jur				G=11, A=14	G=0.44, A=0.56	0.00001	134.35	25	G=7/4, A=10/4
chr7:47408029	G/G	A	G	SNV	TNS3	TNS3:exonic:NM 022748.11	1	100	17	synonymous	NM 02274	c.2214T>C	p.(=)	-0.98					C2 domain of	rs2255744	0.1725	YES	4.643E-1 (pos.47408029)		Tensin 3	cell migra	Genital Diseases, Male;Genital Neoplasms, I			A=0, G=33	A=0.0, G=1.0	0.00001	504.597	33	A=0/0, G=16/1
chr3:44283808	A/A	C	A	SNV	TOPAZ1	TOPAZ1:exonic:NM 001145030.1	1	100	1	missense	NM 00114	c.263C>A	p.Pro88Gln	-1.82	0.57	76	0		Putative asp	rs7645375	0.1892	YES	8.355E-1 (pos.44283808)		Testis- and	apoptotic process,cytosol,metal ion binding,ncRNA tran				C=0, A=26	C=0.0, A=1.0	0.00001	371.42	26	C=0/0, A=8/18
chr3:133342218	C/T	C	T	SNV	TOPBP1	TOPBP1:exonic:NM 007027.3	1	55.38	18	synonymous	NM 00702	c.3012G>A	p.(=)	0.36				0.91(COSM40	BRCA1 C Term	rs4854738	0.0738	YES	2.47E-1 (pos.133342218)	acute_myeloic	DNA topois	DNA bindii	Hemic and Lymphatic Diseases;Immune Syst			C=29, T=36	C=0.4462, T=0.5538	0.00001	379.576	65	C=11/18, T=7/
chr1:179887125	A/A	G	A	SNV	TOR1AIP1	TOR1AIP1:exonic:NM 001267578.1	1	100	10	synonymous	NM 00126	c.1506G>A	p.(=)	-0.02					Lamina-asso	rs627897	0.0455	YES	9.008E-1 (pos.179887125)		Torsin A-int	ATPase activator activity,ATPase binding,(			Benign	G=0, A=29	G=0.0, A=1.0	0.00001	427.59	29	G=0/0, A=11/1
chr17:7579472	G/C	G	C	SNV	TP53	TP53:exonic:NM 000546.5	1	50	4	missense	NM 00054	c.215C>G	p.Pro72Arg	1.02	0.26	103	0.083	0.22(COSM45	P53 DNA-binc	rs1042522,rs730882	0.2548	YES	5.892E-1 (pos.7579472)	adenocarcinoi	Tumor prot	ATP bindin	ACMG Recommend:	1-(9-ethyl-	drug respons	G=23, C=23	G=0.5, C=0.5	0.00001	254.843	46	G=13/10, C=1
chr15:43748304	A/G	A	G	SNV	TP53BP1	TP53BP1:exonic:NM 001141980.1	1	51.85	12	synonymous	NM 00114	c.2502T>C	p.(=)	1.16				0.02(COSM37	Tumour supp	rs690367	0.3032	YES	4.163E-1;0.0 (pos.43748	acute_myeloic	Tumor prot	DNA damage checkpoint,DNA repair complex,RNA polym				A=13, G=14	A=0.4815, G=0.5185	0.00001	132.661	27	A=8/5, G=11/3
chr7:43810820	C/T	C	T	SNV	BLVRA	BLVRA:intronic:NM 000712.3	1	45.45		intronic	NM 00071	c.12+51C>	p.?	-0.27					Biliverdin rec	rs10234057	0.2095	YES	8.115E-3 (pos.43810820)		Biliverdin re	biliverdin	Bile Duct Diseases;	NADH		C=12, T=10	C=0.5455, T=0.4545	0.00001	81.7619	22	C=0/12, T=4/6
chr6:83075914	A/A	G	A	SNV	TPBG	TPBG:exonic:NM 001166392.1	1	100	2	synonymous	NM 00116	c.1236G>A	p.(=)	0.8				0.27(COSM41	Leucine Rich	rs700494	0.3217	YES	5.839E-1 (pos.83075914)	acute_myeloic	Trophoblas	cell adhes	Genital Diseases, Male;Genital Neoplasms, I			G=0, A=230	G=0.0, A=1.0	0.00001	3814.36	230	G=0/0, A=138/
chr11:68840397	C/C	T	C	SNV	TPCN2	TPCN2:exonic:NM 139075.3	1	100	13	synonymous	NM 13907	c.1158T>C	p.(=)	-4.86				0(COSM1162	Ion transport	rs1466221,rs386754	0.4981	YES	3.085E-1 (pos.68840397)	acute_myeloic	Two-pore se	NAADP-sensitive calcium-release channel activity,calciu				T=0, C=35	T=0.0, C=1.0	0.00001	543.902	35	T=0/0, C=19/1
chr11:68840412	G/G	C	G	SNV	TPCN2	TPCN2:exonic:NM 139075.3	1	100	13	synonymous	NM 13907	c.1173C>G	p.(=)	0.59				0.01(COSM37	Ion transport	rs1466220	0.4994	YES	3.087E-1 (pos.68840412)	acute_myeloic	Two-pore se	NAADP-sensitive calcium-release channel activity,calciu				C=0, G=35	C=0.0, G=1.0	0.00001	543.779	35	C=0/0, G=19/1
chr11:68816370	A/G	A	G	SNV	TPCN2	TPCN2:utr 5:NM 139075.3	1	95.65	1	utr 5	NM 13907	c.-96A>G	p.?	0.87					Ion transport	rs4930642		YES			Two-pore se	NAADP-sensitive calcium-release channel activity,calciu				A=1, G=22	A=0.0435, G=0.9565	0.00001	288.972	23	A=0/1, G=3/19
chr9:6328946	AC/AC	A	AC	INDEL	TPD52L3	TPD52L3:exonic:NM 033516.5	1	100	1	frameshiftInsertion	NM 03351	c.351 352	p.Phe118fs	-2.83					Tumour protein D52 family						Tumor prot	protein binding				A=0, AC=81	A=0.0, AC=1.0	0.00001	871.993	81	A=0/0, AC=45/
chr7:45753239	G/G	A	G	SNV	ADCY1	ADCY1:intronic:NM 021116.3	1	100		intronic	NM 02111	c.3058-53	p.?	-1.73					Adenylate an	rs2293107		YES			Adenylate c	ATP bindin	Bronchial Neoplasr	ATP,Adenosine monophc		A=0, G=97	A=0.0, G=1.0	0.00001	1639	97	A=0/0, G=37/6
chr15:63351840	C/A	C	A	SNV	TPM1	TPM1:exonic:NM 001018020.1	1	97.5	4	synonymous	NM 00101	c.453C>A	p.(=)	3.36				0.86(COSM37	Tropomyosin	rs1071646	0.3542	YES	9.575E-1 (pos.63351840)	adenocarcinoi	Tropomyosi	actin bind	ACMG Recommendations...;Ao		Benign/Likely	C=1, A=39	C=0.025, A=0.975	0.00001	610.647	40	C=0/1, A=15/2
chr7:47407888	A/A	G	A	SNV	TNS3	TNS3:intronic:NM 022748.11	1	100		intronic	NM 02274	c.2281+74	p.?	-0.1					C2 domain of	rs2255749		YES			Tensin 3	cell migra	Genital Diseases, Male;Genital Neoplasms, I			G=0, A=33	G=0.0, A=1.0	0.00001	503.999	33	G=0/0, A=24/9
chr9:35689949	AAA/TTT	AAA	TTT	MNV	TPM2	TPM2:utr 5:NM 213674.1	3	50	1	utr 5	NM 21367	c.-135TTT>	p.?	1.5, 0.54, 0.61					Tropomyosin	rs376121464,rs3793538,rs3793		YES			Tropomyosi	actin bind	Arthrogryposis;Carcinoma;Carcinoma, Squan			AAA=10, TT	AAA=0.5, TTT=0.5	0.00001	85.0884	20	AAA=6/4, TTT=
chr13:103275386	C/C	T	C	SNV	TPP2	TPP2:exonic:NM 003291.3	1	100	6	synonymous	NM 00329	c.780T>C	p.(=)	0.44				0.76(COSM39	Subtilase fan	rs2230854	0.483	YES	4.436E-1 (pos.10327538	acute_myeloic	Tripeptidyl	aminopeptidase activity,cytoplasm,cytosol,endopeptid				T=0, C=57	T=0.0, C=1.0	0.00001	849.614	57	T=0/0, C=21/3
chr7:47835116	C/C	T	C	SNV	C7orf69,PKD1L1	PKD1L1:intronic:NM 138295.4, C7orf69:intronic:NM 0250	1	100		intronic	NM 13829	c.8355+47	p.?, p.?	-1.41					PKD domain,	rs2348459		YES			Polycystin 1	calcium channel activity,calcium channel complex,calci				T=0, C=31	T=0.0, C=1.0	0.00001	465.813	31	T=0/0, C=19/1
chr5:665280	C/C	T	C	SNV	TPPP	TPPP:exonic:NM 007030.2	1	100	4	synonymous	NM 00703	c.597A>G	p.(=)	-5.35				0.02(COSM14	p25-alpha	rs1697963	0.394	YES	6.664E-1 (pos.665280)	adenocarcinoi	Tubulin poly	cytoplasm,cytosol,microtubule,microtubule binding,mic				T=0, C=36	T=0.0, C=1.0	0.00001	553.862	36	T=0/0, C=15/2
chr16:1273533	CGG/CG	CGG	C,CG	INDEL	TPSG1	TPSG1:exonic:NM 012467.3	1	39.13	3	frameshiftDeletion	NM 01246	c.134delC	p.Pro45fs	-6.17, -4.26, -0.52					Trypsin	rs377147956,rs7467	0		0.0 (pos.1273534),0.0;0.0 (pos.1273533		Tryptase, ga	integral component of plasma membrane,proteolysis,se				CGG=12, C	CGG=0.5217, C=0.08	0.00001	52.5747	23	CGG=10/2, C=
chr16:1272865	A/A	G	A	SNV	TPSG1	TPSG1:exonic:NM 012467.3	1	100	4	synonymous	NM 01246	c.298C>T	p.(=)	-0.41				0.01(COSM55	Trypsin	rs4984639,rs769682	0.2674	YES	0.0 (pos.1272863),9.139	carcinoma	Tryptase, ga	integral component of plasma membrane,proteolysis,se				G=0, A=30	G=0.0, A=1.0	0.00001	446.639	30	G=0/0, A=10/2
chr7:48335265	T/T	C	T	SNV	ABCA13	ABCA13:intronic:NM 152701.4	1	100		intronic	NM 15270	c.8956-32	p.?	-0.16					ABC transpor	rs10280046	0.36	YES	9.883E-1 (pos.48335265)		ATP-binding	ATP bindin	Colonic Diseases;Colorectal Neoplasms;Dige			C=0, T=104	C=0.0, T=1.0	0.00001	1626.74	104	C=0/0, T=32/7
chr21:10910347	G/G	A	G	SNV	TPTE	TPTE:exonic:NM 199261.3	1	100	22	missense	NM 19926	c.1409T>C	p.Leu470Pro	2.21	1	98	0	0(COSM1180	C2 domain of	rs150482		YES	9.969E-1 (pos.10910347)	astrocytoma (	Transmemb	integral component of membrane,peptidyl-tyrosine dep				A=0, G=68	A=0.0, G=1.0	0.00001	1053.56	68	A=0/0, G=24/4
chr9:139820421	A/A	G	A	SNV	TRAF2	TRAF2:utr 3:NM 021138.3	1	100	11	utr 3	NM 02113	c.1574G>A	p.?	-2.24					MATH domai	rs754006972,rs7852	0.4201	YES	0.0;2.737E-4 (pos.139820420),7.315E-1;		TNF recepto	AIP1-IRE1 complex,CD40 receptor binding,CD40 receptor				G=0, A=38	G=0.0, A=1.0	0.00001	602.413	38	G=0/0, A=14/2
chr7:5232881	A/A	C	A	SNV	WIPI2	WIPI2:intronic:NM 015610.3	1	100		intronic	NM 01561	c.128+79C	p.?	-0.05						rs6463314		YES			WD40 repe	autophagosome,autophagosome assembly,autophagos				C=0, A=125	C=0.0, A=1.0	0.00001	2032.55	125	C=0/0, A=73/5
chr7:53103267	T/G	T	G	SNV	POM121L12	POM121L12:upstream:NM 182595.3	1	97.06		intronic	NM 18259	c.-98T>G	p.?	-0.6						rs10248048		YES				RNA export from nucleus,nuclear localization sequence I				T=1, G=33	T=0.0294, G=0.9706	0.00001	492.963	34	T=0/1, G=30/3
chr1:209950760	G/G	C	G	SNV	TRAF3IP3	TRAF3IP3:exonic:NM 025228.3	1	100	12	missense	NM 02522	c.1117C>G	p.Gln373Glu	3.83	1	29	0	0.02(COSM1127153)		rs669694	0.2012	YES	8.086E-1 (pos.20995076	adenocarcinoi	TRAF3-inter	integral component of membrane,protein binding				C=0, G=31	C=0.0, G=1.0	0.00001	465.819	31	C=0/0, G=21/1
chr3:42236415	G/G	A	G	SNV	TRAK1	TRAK1:exonic:NM 001042646.2	1	100	10	synonymous	NM 00104	c.1095A>G	p.(=)	-7.49					HAP1 N-termi	rs9851455	0.2492	YES	9.863E-1 (pos.42236415)		Trafficking p	GABA rece	Mental Disorders;Schizophrenia;Schizophren			A=0, G=47	A=0.0, G=1.0	0.00001	789.303	47	A=0/0, G=13/3
chr7:55223790	C/C	T	C	SNV	EGFR	EGFR:intronic:NM 005228.4	1	100		intronic	NM 00522	c.1006+15	p.?	-0.73					Furin-like cys	rs3735059		YES			Epidermal g	AP-2 adap	Acneiform Eruption	Afatinib,Alvocidib,Caner		T=0, C=46	T=0.0, C=1.0	0.00001	760.1	46	T=0/0, C=20/2
chr10:17204187	A/A	G	A	SNV	TRDMT1	TRDMT1:exonic:NM 004412.6	1	100	4	missense	NM 00441	c.301C>T	p.His101Tyr	0.63	0.97	83	0.011	0.01(COSM37	C-5 cytosine-	rs11254413	0.4531	YES	2.434E-1 (pos.17204187)	NS,acute_mye	tRNA aspart	RNA binding,cytoplasm,methyl		Glycerin,Pentamidine,S-		G=0, A=23	G=0.0, A=1.0	0.00001	316.745	23	G=0/0, A=10/1
chr7:5567596	T/T	C	T	SNV	ACTB	ACTB:intronic:NM 001101.3	1	100		intronic	NM 00110	c.984+39G	p.?	0.12					Actin	rs852424	0.3327	YES	2.071E-1 (pos.5567596)		Actin, beta	ATP bindin	Adenocarcinoma;Arthritis;Blepharoptosis;Bc			C=0, T=34	C=0.0, T=1.0	0.00001	524.186	34	C=0/0, T=21/1
chr7:5568366	G/G	A	G	SNV	ACTB	ACTB:intronic:NM 001101.3	1	100		intronic	NM 00110	c.364-16T	p.?	-0.88					Actin	rs852423	0.4238	YES	3.02E-1 (pos.5568366)		Actin, beta	ATP bindin	Adenocarcinoma;Arthritis;Blei		Benign	A=0, G=88	A=0.0, G=1.0	0.00001	1451.82	88	A=0/0, G=34/5
chr7:56131910	G/G	C	G	SNV	CCT6A,SUMF2	CCT6A:downstream:NM 001762.3, SUMF2:upstream:NM 0	1	100		intronic	NM 00176	c.*1132C>	p.?, p.?	-0.3						rs62457279	0.1932	YES	7.609E-2;0.0 (pos.56131910)		Chaperonin	'de novo' protein folding,ATP binding,RNA binding,WD40-				C=0, G=127	C=0.0, G=1.0	0.00001	2074.26	127	C=0/0, G=88/3
chr6:123869607	C/C	G	C	SNV	TRDN	TRDN:exonic:NM 006073.3	1	100	3	missense	NM 00607	c.383C>G	p.Thr128Ser	-0.01	0.63	58	0.058		Aspartyl beta	rs9490809	0.4625	YES	1.014E-1 (pos.123869607)		Triadin	cellular calcium ion homeostasis,cytoplas			Benign	G=0, C=41	G=0.0, C=1.0	0.00001	664.696	41	G=0/0, C=20/2
chr6:41196222	GCC/GC	GCC	GCCG,GCG,GC	INDEL	TREML4	TREML4:exonic:NM 198153.2	1	51.49	1	frameshiftDeletion	NM 19815	c.59delC	p.Pro20fs	0.22, -1.47, -1.92					Immunoglobu	rs777257478	1.00E-04		0.0 (pos.41196222)		Triggering r	extracellular region,innate immune response,plasma m				GCC=49, G(	GCC=0.4851, GCCG=	0.00001	516.377	101	GCC=22/27, G
chr7:56144656	T/T	C	T	SNV	SUMF2	SUMF2:intronic:NM 001130069.2	1	100		intronic	NM 00113	c.648+74C	p.?	-2.79					Sulfatase-mo	rs62457286	0.1094	YES			Sulfatase-m	endoplasmic reticulum,endoplasmic reticulum lumen,r				C=0, T=26	C=0.0, T=1.0	0.00001	371.42	26	C=0/0, T=17/9
chr7:56155219	T/T	C	T	SNV	PHKG1	PHKG1:intronic:NM 001258459.1	1	100		intronic	NM 00125	c.262+72G	p.?	-0.02					Kinase-like,L	rs6945795		YES			Phosphoryl	ATP bindin	Carbohydrate Metabolism, Inborn Errors;Con			C=0, T=39	C=0.0, T=1.0	0.00001	627.543	39	C=0/0, T=32/7
chr7:5944651	T/G	T	G	SNV	CCZ1	CCZ1:intronic:NM 015622.5	1	58.33		intronic	NM 01562	c.523-74T	p.?	-0.12					Fungal domai	rs55794922		YES				aggresome,cytosol,guanyl-nucleotide exchange factor a				T=10, G=14	T=0.4167, G=0.5833	0.00001	135.339	24	T=1/9, G=2/12
chr6:41196156	T/T	C	T	SNV	TREML4	TREML4:utr 5:NM 198153.2	1	100	1	utr 5	NM 19815	c.-10C>T	p.?	-2.87					Immunoglobu	rs2803496	0.095	YES	9.522E-1 (pos.41196156)		Triggering r	extracellular region,innate immune response,plasma m				C=0, T=91	C=0.0, T=1.0	0.00001	1513.06	91	C=0/0, T=61/3
chr7:63673608	T/T	C	T	SNV	ZNF735	ZNF735:intronic:NM 001159524.1	1	100		intronic	NM 00115	c.166+12C	p.?	-0.08					C2H2-type zir	rs10252019	0.3325	YES	8.848E-1 (pos.63673608)			DNA binding,metal ion binding,nucleus,regulation of trai				C=0, T=34	C=0.0, T=1.0	0.00001	524.184	34	C=0/0, T=15/1

chr7:63796559	C/C	A	C	SNV	ZNF736	ZNF736:intronic:NM_001170905.2	1	100		intronic	NM_00117	c.4-78A>C	p.?	-1.15					C2H2-type zir	rs620631		YES				DNA binding,metal ion binding,nucleus,regulation of trai	A=0, C=25	A=0.0, C=1.0	0.00001	353.019	25	A=0/0, C=9/16
chr7:63796835	T/C	T	C	SNV	ZNF736	ZNF736:intronic:NM_001170905.2	1	45.45		intronic	NM_00117	c.130+72T	p.?	0.58					C2H2-type zir	rs7787036		YES				DNA binding,metal ion binding,nucleus,regulation of trai	T=18, C=15	T=0.5455, C=0.4545	0.00001	143.162	33	T=9/9, C=7/8
chr6:41196727	C/T	C	T	SNV	TREML4	TREML4:exonic:NM_198153.2	1	59.09	2	synonymous	NM_19815	c.339C>T	p.(=)	-1.51				0.06(COSM57	Immunoglobu	rs2101269	0.2091	YES	8.443E-1 (pos.41196727)	adenocarcinoi	Triggering r	extracellular region,innate immune response,plasma m	C=27, T=39	C=0.4091, T=0.5909	0.00001	417.112	66	C=14/13, T=16
chr7:28996944	C/C	T	C	SNV	TRIL	TRIL:exonic:NM_014817.3	1	100	3	missense	NM_01481	c.718A>G	p.Asn239Ser	-0.04		46			Leucine Rich	rs740250		YES	8.684E-1 (pos.28996944)		TLR4 intera	inflammatory response,innate immune response,lipopo	T=0, C=49	T=0.0, C=1.0	0.00001	831.633	49	T=0/0, C=12/3
chr7:64439938	T/T	C	T	SNV	ERV3-1-ZNF117,ZNF117	ERV3-1-ZNF117:intronic:NM_001348050.1, ZNF117:intronic	1	100		intronic	NM_01585	c.35-24G>	p.?, p.?	-1.42					C2H2-type zir	rs1852016	0.0014	YES	1.0 (pos.64439938)		Zinc finger p	DNA binding,DNA binding transcription factor activity,nu	C=0, T=30	C=0.0, T=1.0	0.00001	446.621	30	C=0/0, T=17/1
chr6:30122154	C/C	T	C	SNV	TRIM10	TRIM10:exonic:NM_006778.3	1	100	7	synonymous	NM_00677	c.1038A>G	p.(=)	-1.02					B-box zinc fin	rs3094134	0.0951	YES	9.977E-1 (pos.30122154)			biological_process,cytoplasm,erythrocyte differentiatio	T=0, C=50	T=0.0, C=1.0	0.00001	852.745	50	T=0/0, C=17/3
chr7:6541347	C/T	C	T	SNV	GRID2IP	GRID2IP:intronic:NM_001145118.1	1	57.14		intronic	NM_00114	c.3399+65	p.?	-3.54					Formin Homc	rs7791451		YES				cell junction,dendritic spine,long term synaptic depress	C=9, T=12	C=0.4286, T=0.5714	0.00001	102.86	21	C=2/7, T=7/5
chr7:6544247	T/T	C	T	SNV	GRID2IP	GRID2IP:intronic:NM_001145118.1	1	100		intronic	NM_00114	c.2710+17	p.?	-0.28					Formin Homc	rs12702519		YES				cell junction,dendritic spine,long term synaptic depress	C=0, T=26	C=0.0, T=1.0	0.00001	371.414	26	C=0/0, T=17/9
chr7:65554352	T/T	C	T	SNV	ASL	ASL:intronic:NM_000048.3	1	100		intronic	NM_00004	c.978+30C	p.?	-2.46					Argininosucci	rs160647	0.2567	YES	6.684E-1;1.071E-3 (pos.65554352)		Argininosuc	ammonia Amino Acid Disorde 2-(N-Morpholino)-Ethane	C=0, T=54	C=0.0, T=1.0	0.00001	798.088	54	C=0/0, T=25/2
chr7:65554385	T/T	C	T	SNV	ASL	ASL:intronic:NM_000048.3	1	100		intronic	NM_00004	c.978+63C	p.?	0.39					Argininosucci	rs2460432	0.3712	YES			Argininosuc	ammonia Amino Acid Disorde 2-(N-Morpholino)-Ethane	C=0, T=55	C=0.0, T=1.0	0.00001	814.907	55	C=0/0, T=24/3
chr7:66273850	G/A	G	A	SNV	RABGEF1	RABGEF1:intronic:NM_014504.2	1	57.58		intronic	NM_01450	c.1078-23	p.?	-1.34					A20-like zinc	rs77283486	0.0243	YES	0.0;0.0 (pos.66273850)		RAB guanin	DNA binding,Rab GTPase binding,Rab guanyl-nucleotide	G=14, A=19	G=0.4242, A=0.5758	0.00001	204.387	33	G=9/5, A=14/5
chr7:70229595	G/G	T	G	SNV	AUTS2	AUTS2:intronic:NM_015570.3	1	100		intronic	NM_01557	c.1215-14	p.?	0.36					Autism susce	rs1983487		YES			actin cyto	Brain Diseases;Central Nervous System Dise	T=0, G=20	T=0.0, G=1.0	0.00001	263.786	20	T=0/0, G=9/11
chr7:70252185	G/G	A	G	SNV	AUTS2	AUTS2:intronic:NM_015570.3	1	100		intronic	NM_01557	c.2309-10	p.?	0.65				0.78(COSM54	Autism susce	rs2293500	0.1734	YES	5.383E-1 (pos.70252185)	acute_myeloid_leukaemia	actin cyto	Brain Diseases;Central Nervous System Dise	A=0, G=74	A=0.0, G=1.0	0.00001	1170.11	74	A=0/0, G=38/3
chr7:70598132	T/T	C	T	SNV	GALNT17	GALNT17:intronic:NM_022479.2	1	100		intronic	NM_02247	c.238+106	p.?	0.04					Glycosyl tran	rs6976185		YES				Golgi membrane,carbohydrate binding,integral compon	C=0, T=64	C=0.0, T=1.0	0.00001	970.946	64	C=0/0, T=16/4
chr7:70885883	CT/CT	C	CT	INDEL	GALNT17	GALNT17:intronic:NM_022479.2	1	100		intronic	NM_02247	c.765-11C	p.?	0.28					Glycosyl tran	rs11455717,rs39793	0.3115	YES	4.166E-1;0.0 (pos.70885883)			Golgi membrane,carbohydrate binding,integral compon	C=0, CT=59	C=0.0, CT=1.0	0.00001	773.929	59	C=0/0, CT=32/
chr11:119999191	G/G	A	G	SNV	TRIM29	TRIM29:exonic:NM_012101.3	1	100	2	synonymous	NM_01210	c.817T>C	p.(=)	2.47				0.05(COSM41	B-box zinc fin	rs569044	0.1072	YES	9.676E-1 (pos.11999919	neoplasm	Tripartite m	DNA binding transcription factor activity,cadherin bindir	A=0, G=82	A=0.0, G=1.0	0.00001	1329.57	82	A=0/0, G=43/3
chr11:5664831	C/A	C	A	SNV	TRIM34,TRIM6-TRIM34	TRIM6-TRIM34:exonic:NM_001003819.3, TRIM34:exonic:NM	1	46.51	8	synonymous	NM_00100	c.2421C>A	p.(=), p.(=)	-0.99				0.01(COSM37	B-box zinc fin	rs3740998	0.2376	YES	6.193E-1 (pos.5664831)	adenocarcinoi	Tripartite m	cytoplasm,cytosol,defense response to virus,interferon-	C=23, A=20	C=0.5349, A=0.4651	0.00001	212.283	43	C=12/11, A=8/
chr7:7277807	A/A	G	A	SNV	C1GALT1	C1GALT1:intronic:NM_020156.4	1	100		intronic	NM_02015	c.221-79G	p.?	0.35					Fringe-like,G	rs3807862		YES			Core 1 syntl	Golgi membrane,O-glycan processing,O-glycan processir	G=0, A=43	G=0.0, A=1.0	0.00001	712.921	43	G=0/0, A=23/2
chr7:73019975	A/G	A	G	SNV	MLXIPL	MLXIPL:intronic:NM_032951.2	1	57.14		intronic	NM_03295	c.901+43T	p.?	-0.73					Helix-loop-he	rs61010704	0.2721	YES	8.409E-2 (pos.73019975)		MLX interac	DNA bindi Adenocarcinoma;Carcinoma;Carcinoma, Hec	A=9, G=12	A=0.4286, G=0.5714	0.00001	109.106	21	A=7/2, G=4/8
chr6:25967068	T/T	C	T	SNV	TRIM38	TRIM38:exonic:NM_006355.4	1	100	3	synonymous	NM_00635	c.318C>T	p.(=)	-1.55				0.03(COSM37	B-box zinc fin	rs2009610	0.2288	YES	7.968E-2 (pos.25967068)	adenocarcinoma,neoplasn		cellular_component,cytosol,interferon-gamma-mediate	C=0, T=46	C=0.0, T=1.0	0.00001	772.552	46	C=0/0, T=14/3
chr11:49055751	C/C	T	C	SNV	TRIM49B	TRIM49B:exonic:NM_001206626.1	1	100	3	synonymous	NM_00120	c.561T>C	p.(=)	-0.23				0(COSM4595	B-box zinc fin	rs2727042		YES	4.642E-1 (pos.49055751)	squamous_cell_carcinoma		intracellular,zinc ion binding	T=0, C=34	T=0.0, C=1.0	0.00001	518.921	34	T=0/0, C=5/29
chr11:49059362	A/A	C	A	SNV	TRIM49B	TRIM49B:exonic:NM_001206626.1	1	100	6	missense	NM_00120	c.1192C>A	p.Leu398Met	-2.25	0.24	15	0.004	0(COSM4590	B-box zinc fin	rs2696914		YES	6.297E-1 (pos.49059362)	squamous_cell_carcinoma		intracellular,zinc ion binding	C=0, A=43	C=0.0, A=1.0	0.00001	705.842	43	C=0/0, A=21/2
chr7:72738749	G/G	A	G	SNV	TRIM50	TRIM50:exonic:NM_178125.3	1	100	2	missense	NM_17812	c.37T>C	p.Trp13Arg	1.31	0.71	101	0	0.12(COSM41	B-box zinc fin	rs28626137			1.0;0.0 (pos.72738749)	neoplasm,squ	Tripartite m	cytosol,identical protein binding,transferase activity,zin	A=0, G=23	A=0.0, G=1.0	0.00001	316.747	23	A=0/0, G=6/17
chr7:73803644	A/A	T	A	SNV	CLIP2	CLIP2:intronic:NM_003388.4	1	100		intronic	NM_00338	c.2720+55	p.?	-1.12					CAP-Gly doma	rs35415684		YES			CAP-GLY do	cytoplasm Aortic Stenosis, Supravalvular;Aortic Valve St	T=0, A=48	T=0.0, A=1.0	0.00001	810.427	48	T=0/0, A=33/1
chr7:72738762	TG/TG	CA	TG	MNV	TRIM50	TRIM50:exonic:NM_178125.3	2	100	2	missense	NM_17812	c.23_24de	p.Leu8Pro	-7.09, 0.05	1	98	0	0.04(COSM41	B-box zinc fin	rs6980124,rs6980258,rs71517080			9.997E-1 (pos.72738762)	neoplasm,squ	Tripartite m	cytosol,identical protein binding,transferase activity,zin	CA=0, TG=2	CA=0.0, TG=1.0	0.00001	316.747	23	CA=0/0, TG=6/
chr7:73932683	C/C	T	C	SNV	GTF2IRD1	GTF2IRD1:intronic:NM_001199207.1	1	100		intronic	NM_00119	c.701+31T	p.?	-1.83					GTF2I-like rep	rs587649448,rs6143	0.1879	YES	0.0 (pos.73932682),0.0;0.0;0.0 (pos.739		GTF21 repe	DNA bindi Aortic Stenosis, Supravalvular;Aortic Valve St	T=0, C=34	T=0.0, C=1.0	0.00001	524.186	34	T=0/0, C=14/2
chr11:55652971	C/A	C	A	SNV	TRIM51	TRIM51:exonic:NM_032681.3	1	98.72	2	missense	NM_03268	c.67C>A	p.Leu23Ile	1.23	1	5	0	0(COSM3765	RING-type zin	rs2063277		YES	9.998E-1 (pos.55652971)	hepatocellular_carcinoma		intracellular,zinc ion binding	C=1, A=77	C=0.0128, A=0.9872	0.00001	1214.19	78	C=0/1, A=18/5
chr7:7415217	C/T	C	T	SNV	COL28A1	COL28A1:intronic:NM_001037763.2	1	58.33		intronic	NM_00103	c.2323-89	p.?	-1.53					Collagen tripl	rs1294651		YES			Collagen, ty	basement Amphetamine-Related Disorders;Mental Dis	C=55, T=77	C=0.4167, T=0.5833	0.00001	899.943	132	C=23/32, T=29
chr7:75441108	T/T	C	T	SNV	CCL24	CCL24:downstream:NM_002991.2	1	100		intronic	NM_00299	c.*6G>A	p.?	-2.61						rs11465315	0.4548	YES	7.769E-1 (pos.75441108)		Chemokine	CCR3 chen Congenital, Hereditary, and Neonatal Diseas	C=0, T=26	C=0.0, T=1.0	0.00001	371.42	26	C=0/0, T=12/1
chr7:75441111	G/T	G	T	SNV	CCL24	CCL24:downstream:NM_002991.2	1	100		intronic	NM_00299	c.*3C>A	p.?	-2.51						rs11465314	0.4548	YES	7.773E-1;0.0 (pos.75441111)		Chemokine	CCR3 chen Congenital, Hereditary, and Neonatal Diseas	G=0, T=26	G=0.0, T=1.0	0.00001	345.979	26	G=0/0, T=12/1
chr7:75613195	GCC/GC	GCC	GC,G	INDEL	POR	POR:intronic:NM_000941.2	1	GC=100.00, G=0.00		intronic	NM_00094	c.1066+21	p.?	-1.44, -0.35, -0.15					FAD binding c	rs562843415			8.787E-4 (pos.75613195)		Cytochrome	FMN bindi Adrenal Gland Dise 2'-Monophosphoadenos	GCC=0, GC=	GCC=0.0, GC=1.0, G=	0.00001	535.159	45	GCC=0/0, GC=
chr7:75614296	A/A	G	A	SNV	POR	POR:intronic:NM_000941.2	1	100		intronic	NM_00094	c.1248+20	p.?	-0.54					FAD binding c	rs2286823,rs782146	0.2986	YES	0.0;0.0 (pos.75614279),4.291E-1 (pos.75		Cytochrome	FMN bindi Adrenal Gland Dise 2'-Monoph Benign	G=0, A=21	G=0.0, A=1.0	0.00001	281.23	21	G=0/0, A=4/17
chr7:75614724	A/A	G	A	SNV	POR	POR:intronic:NM_000941.2	1	100		intronic	NM_00094	c.1399-17	p.?	0.07					FAD binding c	rs6961174,rs781836055		YES			Cytochrome	FMN bindi Adrenal Gland Dise 2'-Monophosphoadenos	G=0, A=22	G=0.0, A=1.0	0.00001	298.892	22	G=0/0, A=16/6
chr7:75614863	CT/CT	TG	CT	MNV	POR	POR:intronic:NM_000941.2	2	100		intronic	NM_00094	c.1399-34	p.?	-0.22, -0.78					FAD binding c	rs2302431,rs230243	0.4425	YES	8.364E-1;0.0 (pos.75614864),8.415E-1 (p		Cytochrome	FMN bindi Adrenal Gland Dise 2'-Monophosphoadenos	TG=0, CT=2	TG=0.0, CT=1.0	0.00001	408.718	28	TG=0/0, CT=13
chr1:248039451	C/T	C	T	SNV	TRIM58	TRIM58:exonic:NM_015431.3	1	50	6	missense	NM_01543	c.1121C>T	p.Thr374Met	0.17	0.23	81	0.999	0.08(COSM14	B-box zinc fin	rs3811444	0.3458	YES	2.429E-1 (pos.24803945	adenocarcinoma,ductal_ca		biological_process,dynein heavy chain binding,dynein in	C=12, T=12	C=0.5, T=0.5	0.00001	106.71	24	C=4/8, T=9/3
chr7:75621661	C/C	G	C	SNV	TMEM120A	TMEM120A:intronic:NM_031925.2	1	100		intronic	NM_03192	c.201-83C	p.?	-2.58					TMPIT-like pr	rs2286826,rs374842324		YES			Transmemb	cellular_component,fat cell differentiation,integral com	G=0, C=21	G=0.0, C=1.0	0.00001	281.233	21	G=0/0, C=16/5
chr7:75621979	C/C	T	C	SNV	TMEM120A	TMEM120A:intronic:NM_031925.2	1	100		intronic	NM_03192	c.82-104A	p.?	-0.79					TMPIT-like pr	rs6467956					Transmemb	cellular_component,fat cell differentiation,integral com	T=0, C=25	T=0.0, C=1.0	0.00001	353.019	25	T=0/0, C=7/18
chr1:33646950	G/G	A	G	SNV	TRIM62	TRIM62:exonic:NM_018207.2	1	100	1	synonymous	NM_01820	c.84T>C	p.(=)	4.62				0.1(COSM375	Apolipoprote	rs622407	0.2129	YES	7.854E-1 (pos.33646950)	adenocarcinoi	Tripartite m	cytoplasm,cytosol,innate immune response,interferon-(	A=0, G=24	A=0.0, G=1.0	0.00001	334.792	24	A=0/0, G=7/17
chr7:76019746	G/G	C	G	SNV	SSC4D	SSC4D:intronic:NM_080744.1	1	100		intronic	NM_08074	c.1412-54	p.?	1					Scavenger re	rs55920832		YES				extracellu Basal Ganglia Diseases;Brain Diseases;Centr	C=0, G=26	C=0.0, G=1.0	0.00001	371.407	26	C=0/0, G=12/1
chr7:76023131	AG/AG	A	AG	INDEL	SSC4D	SSC4D:intronic:NM_080744.1	1	100		intronic	NM_08074	c.1021+16	p.?	-2.19					Scavenger re	rs35508921,rs36844	0.0019		9.998E-1;0.0 (pos.76023131)			extracellu Basal Ganglia Diseases;Brain Diseases;Centr	A=0, AG=27	A=0.0, AG=1.0	0.00001	331.048	27	A=0/0, AG=18/
chr7:76024520	T/T	C	T	SNV	SSC4D	SSC4D:intronic:NM_080744.1	1	100		intronic	NM_08074	c.946+50G	p.?	-0.35					Scavenger re	rs2286427	0.1967	YES	5.45E-2 (pos.76024520)			extracellu Basal Ganglia Diseases;Brain Diseases;Centr	C=0, T=40	C=0.0, T=1.0	0.00001	644.28	40	C=0/0, T=28/1
chr7:76033544	C/C	T	C	SNV	SSC4D	SSC4D:intronic:NM_080744.1	1	100		intronic	NM_08074	c.133+80A	p.?	0.31					Scavenger re	rs11773646		YES				extracellu Basal Ganglia Diseases;Brain Diseases;Centr	T=0, C=42	T=0.0, C=1.0	0.00001	687.281	42	T=0/0, C=13/2
chr11:49075605	A/A	G	A	SNV	TRIM64C	TRIM64C:exonic:NM_001206631.1	1	100	6	synonymous	NM_00120	c.1014C>T	p.(=)	-5.93				0(COSM3931	SPRY domain	rs2851535		YES	5.514E-1 (pos.49075605)	carcinoma,squamous_cell_carcinoma			G=0, A=84	G=0.0, A=1.0	0.00001	1370.16	84	G=0/0, A=49/3
chr7:7622696	T/T	C	T	SNV	MIOS	MIOS:intronic:NM_019005.3	1	100		intronic	NM_01900	c.1394-53	p.?	-1.68					Zinc-ribbon li	rs4720741		YES			Missing ooc	GATOR2 complex,cellular protein complex localization,c	C=0, T=27	C=0.0, T=1.0	0.00001	386.869	27	C=0/0, T=6/21
chr7:7629343	T/C	T	C	SNV	MIOS	MIOS:intronic:NM_019005.3	1	50		intronic	NM_01900	c.2043+14	p.?	0.42					Zinc-ribbon li	rs76124801		YES			Missing ooc	GATOR2 complex,cellular protein complex localization,c	T=15, C=15	T=0.5, C=0.5	0.00001	145.021	30	T=7/8, C=11/4
chr7:7645535	G/G	A	G	SNV	MIOS	MIOS:intronic:NM_019005.3	1	100		intronic	NM_01900	c.2402-38	p.?	-1.7					Zinc-ribbon li	rs10276434	0.0251	YES	9.994E-1 (pos.7645535)		Missing ooc	GATOR2 complex,cellular protein complex localization,c	A=0, G=78	A=0.0, G=1.0	0.00001	1249.34	78	A=0/0, G=22/5
chr7:76908199	C/C	A	C	SNV	CCDC146	CCDC146:intronic:NM_020879.2	1	100		intronic	NM_02087	c.1529+42	p.?	0.35						rs3828947	0.3378	YES	1.475E-1;0.0 (pos.76908199)			centriole,protein binding	A=0, C=22	A=0.0, C=1.0	0.00001	288.907	22	A=0/0, C=9/13
chr16:31230463	T/T	C	T	SNV	TRIM72	TRIM72:exonic:NM_001008274.3	1	100	3	synonymous	NM_00100	c.438C>T	p.(=)	-0.25					B-box zinc fin	rs7186832	0.2979	YES	7.608E-1 (pos.31230463)		Tripartite m	cytoplasmic vesicle membrane,exocytosis,muscle contr	C=0, T=21	C=0.0, T=1.0	0.00001	281.235	21	C=0/0, T=10/1
chr7:77266238	T/C	T	C	SNV	PTPN12	PTPN12:intronic:NM_002835.3	1	59.09		intronic	NM_00283	c.2143-97	p.?	0.25					Protein-tyros	rs551750796					Protein tyrc	ERBB2 signaling pathway,SH3 domain binding,cell projec	T=9, C=13	T=0.4091, C=0.5909	0.00001	122.566	22	T=3/6, C=7/6
chr4:189061106	A/A	G	A	SNV	TRIML1	TRIML1:exonic:NM_178556.4	1	100	1	missense	NM_17855	c.394G>A	p.Glu132Lys	2.61	0.14	56	0.99	0.8(COSM376	RING-type zin	rs13131525	0.4994	YES	7.282E-2 (pos.18906110	acute_myeloid_leukaemia		metal ion binding,multicellular organism development,p	G=0, A=27	G=0.0, A=1.0	0.00001	389.974	27	G=0/0, A=17/1
chr7:77552127	A/A	G	A	SNV	PHTF2	PHTF2:intronic:NM_001127358.1	1	100		intronic	NM_00112	c.1005+32	p.?	-2.2					Male germ-ce	rs848486	0.4164	YES	7.796E-1;0.0 (pos.77552127)		Putative ho	DNA binding,endoplasmic reticulum,nucleus,regulation	G=0, A=38	G=0.0, A=1.0	0.00001	603.786	38	G=0/0, A=17/2
chr7:780980	T/G	T	G	SNV	DNAAF5	DNAAF5:intronic:NM_017802.3	1	50		intronic	NM_01780	c.906-4T>	p.?	-2.31					HEAT repeats	rs6943337	0.1741	YES	7.108E-1 (pos.780980)		Dynein axo	cilium movement,cytoplasm,dynein interi Benign	T=13, G=13	T=0.5, G=0.5	0.00001	119.097	26	T=1/12, G=4/9
chr22:38122462	G/G	A	G	SNV	TRIOBP	TRIOBP:exonic:NM_001039141.2	1	100	7	missense	NM_00103	c.3899A>G	p.His1300Arg	1.34	1	29	0	0.05(COSM37	PH domain	rs739138	0.241	YES	9.232E-1 (pos.38122462)	adenocarcinoi	Trio- and F-a	GTP-Rho b Ear Diseases;Hearing Disorder Benign	A=0, G=113	A=0.0, G=1.0	0.00001	1794.1	113	A=0/0, G=64/4
chr7:78119199	A/C	A	C	SNV	MAGI2	MAGI2:intronic:NM_012301.3	1	50		intronic	NM_01230	c.966-42T	p.?	4.57					Guanylate kir	rs1990577	0.4677	YES	7.646E-1;0.0 (pos.78119199)		Membrane-	SMAD binc Adenocarcinoma;Carcinoma;Carcinoma, Ade	A=11, C=11	A=0.5, C=0.5	0.00001	94.6054	22	A=6/5, C=10/1
chr14:92471229	C/T	C	T	SNV	TRIP11	TRIP11:exonic:NM_004239.4	1	50	11	missense	NM_00423	c.3091G>A	p.Glu1031Lys	7.78	0	56	1			rs766077430			0.0 (pos.92471229)		Thyroid hor	Golgi appa Achondroplasia;Bone Diseases;Bone Disease	C=13, T=13	C=0.5, T=0.5	0.00001	119.143	26	C=10/3, T=4/9
chr7:80378148	C/C	T	C	SNV	SEMA3C	SEMA3C:intronic:NM_006379.4	1	100		intronic	NM_00637	c.1842+66	p.?	-1.83					Immunoglobu	rs1405743		YES			Semaphorin	axon guid Aortopulmonary Septal Defect:Cardiovascula	T=0, C=42	T=0.0, C=1.0	0.00001	685.22	42	T=0/0, C=12/3
chr7:80378175	C/C	T	C	SNV	SEMA3C	SEMA3C:intronic:NM_006379.4	1	100		intronic	NM_00637	c.1842+39	p.?	-1.67					Immunoglobu	rs2886793	9.00E-04	YES	1.0 (pos.80378175)		Semaphorin	axon guid Aortopulmonary Septal Defect:Cardiovascula	T=0, C=43	T=0.0, C=1.0	0.00001	705.844	43	T=0/0, C=12/3
chr2:230668858	C/C	T	C	SNV	TRIP12	TRIP12:exonic:NM_004238.2	1	100	18	synonymous	NM_00423	c.2511A>G	p.(=)	-3.16				0.06(COSM14	HECT-domain	rs13018957	0.4808	YES	3.248E-1 (pos.23066885	acute_myeloid	Thyroid hor	DNA repair,cellular response to DNA damage stimulus,c	T=0, C=21	T=0.0, C=1.0	0.00001	281.235	21	T=0/0, C=13/8
chr3:3189279	G/G	A	G	SNV	TRNT1	TRNT1:exonic:NM_182916.2	1	100	7	synonymous	NM_18291	c.948A>G	p.(=)	0.88				0.82(COSM37	Poly A polym	rs1705805	0.1589	YES	9.402E-1 (pos.3189279)	adenocarcinoi	tRNA nuclec	5'-3' RNA polymerase activity,ATP binding, Benign	A=0, G=79	A=0.0, G=1.0	0.00001	1269.89	79	A=0/0, G=43/3
chr8:72981327	A/G	A	G	SNV	TRPA1	TRPA1:exonic:NM_007332.2	1	96.77	3	synonymous	NM_00733	c.375T>C	p.(=)	1.44					Ankyrin repea	rs1811457	0.3983	YES	9.881E-1 (pos.72981327)		Transient re	calcium ch Asthma;Bronchial D Menthol	A=1, G=30	A=0.0323, G=0.9677	0.00001	435.406	31	A=0/1, G=17/1
chr20:33657126	A/A	G	A	SNV	TRPC4AP	TRPC4AP:exonic:NM_015638.2	1	100	3	synonymous	NM_01563	c.387C>T	p.(=)	2.46					Protein of unl	rs1998233	0.274	YES	7.641E-1 (pos.33657126)		TRPC4-asso	Cul4A-RING E3 ubiquitin ligase complex,calcium channel	G=0, A=48	G=0.0, A=1.0	0.00001	810.414	48	G=0/0, A=27/2
chr7:81350208	G/G	A	G	SNV	HGF	HGF:intronic:NM_000601.5	1	100		intronic	NM_00060	c.1169-45	p.?	-1.32					Kringle doma	rs12536657	0.2003	YES	8.549E-1 (pos.81350208)		Hepatic gro	MAPK casc Adenocarcinoma;B ABT-510,Foretinib,N,O6-l	A=0, G=90	A=0.0, G=1.0	0.00001	1493.12	90	A=0/0, G=35/5
chr7:81350223	G/A	G	A	SNV	HGF	HGF:intronic:NM_000601.5	1	95.45		intronic	NM_00060	c.1169-60	p.?	-0.43					Kringle doma	rs10272030		YES			Hepatic gro	MAPK casc Adenocarcinoma;B ABT-510,Foretinib,N,O6-l	G=4, A=84	G=0.0455, A=0.9545	0.00001	1326.73	88	G=3/1, A=31/5
chr7:81372156	A/G	A	G	SNV	HGF	HGF:intronic:NM_000601.5	1	95.45		intronic	NM_00060	c.865+513	p.?	-1.01					Kringle doma	rs5745676		YES			Hepatic gro	MAPK casc Adenocarcinoma;B ABT-510,Foretinib,N,O6-l	A=1, G=21	A=0.0455, G=0.9545	0.00001	271.504	22	A=0/1, G=12/9
chr7:82784017	T/T	G	T	SNV	PCLO	PCLO:intronic:NM_033026.5	1	100		intronic	NM_03302	c.1893+47	p.?	-1.43					C2 domain,PI	rs6951239	0.3178	YES	3.834E-1 (pos.82784017)		Piccolo, mo	cAMP-mec Autonomic Nervous System Diseases;Basal G	G=0, T=85	G=0.0, T=1.0	0.00001	1392.09	85	G=0/0, T=59/2
chr15:31369123	G/G	A	G	SNV	TRPM1	TRPM1:exonic:NM_001252020.1	1	100	2	missense	NM_00125	c.119T>C	p.Met40Thr	0.04	1	81	0		Ion transport	rs4779816	0.1988	YES	9.815E-1 (pos.31369123)		Transient re	G-protein Congenital, Hereditary, and Ne Benign	A=0, G=87	A=0.0, G=1.0	0.00001	1402.99	87	A=0/0, G=44/4
chr7:83029280	G/G	T	G	SNV	SEMA3E	SEMA3E:intronic:NM_012431.2	1	100		intronic	NM_01243	c.1366+64	p.?	-0.03					Immunoglobu	rs2255906		YES			Semaphorin	branching Abnormalities, Multiple;CHARGE Syndrome;C	T=0, G=225	T=0.0, G=1.0	0.00001	3727.96	225	T=0/0, G=143/
chr7:83031907	C/C	T	C	SNV	SEMA3E	SEMA3E:intronic:NM_012431.2	1	100		intronic	NM_01243	c.1143+41	p.?	-5.87					Immunoglobu	rs2709930	2.00E-04		1.0 (pos.83031907)		Semaphorin	branching Abnormalities, Multiple;CHARGE Syndrome;C	T=0, C=72	T=0.0, C=1.0	0.00001	1130.93	72	T=0/0, C=30/4
chr7:83034754	T/A	T	A	SNV	SEMA3E	SEMA3E:intronic:NM_012431.2	1	50		intronic	NM_01243	c.998+12A	p.?	-0.2					Immunoglobu	rs2245441	0.4131	YES	8.785E-1 (pos.83034754)		Semaphorin	branching Abnormalities, Multiple;CHAR(Benign	T=54, A=54	T=0.5, A=0.5	0.00001	543.137	108	T=26/28, A=35
chr7:83034899	T/G	T	G	SNV	SEMA3E	SEMA3E:intronic:NM_012431.2	1	50		intronic	NM_01243	c.929-64A	p.?	-0.11					Immunoglobu	rs2709935	0.4136	YES			Semaphorin	branching Abnormalities, Multiple;CHARGE Syndrome;C	T=41, G=41	T=0.5, G=0.5	0.00001	436.184	82	T=12/29, G=19
chr7:83035177	G/A	G	A	SNV	SEMA3E	SEMA3E:intronic:NM_012431.2	1	50		intronic	NM_01243	c.928+84C	p.?	-0.4					Immunoglobu	rs3735512		YES			Semaphorin	branching Abnormalities, Multiple;CHARGE Syndrome;C	G=25, A=25	G=0.5, A=0.5	0.00001	288.837	50	G=21/4, A=24/
chr11:2435946	G/G	A	G	SNV	TRPM5	TRPM5:exonic:NM_014555.3	1	100	11	synonymous	NM_01455	c.1743T>C	p.(=)	0.02				0.67(COSM37	Ion transport	rs4929981	0.2552	YES	8.947E-1;0.0 (pos.24359	adenocarcinoi	Transient re	calcium activated cation channel activity,calcium ion tra	A=0, G=57	A=0.0, G=1.0	0.00001	849.607	57	A=0/0, G=11/4
chr7:83592491	C/C	T	C	SNV	SEMA3A	SEMA3A:intronic:NM_006080.2	1	100		intronic	NM_00608	c.1860+30	p.?	0.07					Sema domair	rs7809708	0.2735	YES	4.53E-2 (pos.83592491)		Semaphorin	apoptotic 46, XY Disorders of Sex Development;Congen	T=0, C=22	T=0.0, C=1.0	0.00001	298.892	22	T=0/0, C=14/8
chr7:84644346	C/G	C	G	SNV	SEMA3D	SEMA3D:intronic:NM_152754.2	1	50		intronic	NM_15275	c.1703+29	p.?	-0.38					Sema domair	rs6468008	0.455	YES	7.53E-1;0.0 (pos.84644346)		Semaphorin	cell differe Colonic Diseases;Congenital Abnormalities;C	C=11, G=11	C=0.5, G=0.5	0.00001	94.6067	22	C=9/2, G=1/10
chr7:86795947	T/T	TAG	T	INDEL	DMTF1	DMTF1:intronic:NM_001142327.1	2	100		intronic	NM_00114	c.232+26T	p.?	-3.39, -0.12, 0.89					Myb-like DNA	rs3058402,rs397781	0.2307	YES	0.0 (pos.86795948),0.0 (pos.86795949),		Cyclin D-bin	DNA binding,DNA binding transcription factor activity,RN	TAG=0, T=2	TAG=0.0, T=1.0	0.00001	289.688	21	TAG=0/0, T=9/
chr2:234915540	C/G	C	G	SNV	TRPM8	TRPM8:exonic:NM_024080.4	1	50	23	synonymous	NM_02408	c.3174C>G	p.(=)	1.45				0.72(COSM44	Ion transport	rs11563071	0.0966	YES	1.126E-1 (pos.23491554	acute_myeloid	Transient re	calcium ch Brain Diseases;Cen Menthol	C=18, G=18	C=0.5, G=0.5	0.00001	185.873	36	C=4/14, G=10/
chr7:87069758	T/C	T	C	SNV	ABCB4	ABCB4:intronic:NM_018849.2	1	53.13		intronic	NM_01884	c.1357-40	p.?	-0.01					ABC transpor	rs31675	0.0785	YES	7.185E-1 (pos.87069758)		ATP-binding	ATP bindin Adenocarcinoma;B Colchicine,Etravirine,Sil	T=15, C=17	T=0.4688, C=0.5313	0.00001	173.56	32	T=9/6, C=9/8
chr8:116635942	T/T	C	T	SNV	TRPS1	TRPS1:utr_5:NM_014112.4	1	100	2	utr_5	NM_01411	c.-78G>A	p.?	-0.87					GATA zinc fing	rs2721939		YES			Zinc finger t	DNA bindi Bone Diseases;Bone Diseases, Benign	C=0, T=60	C=0.0, T=1.0	0.00001	903.529	60	C=0/0, T=25/3
chr7:87215114	C/C	A	C	SNV	ABCB1	ABCB1:intronic:NM_000927.4	1	100		intronic	NM_00092	c.118-118	p.?	-1.45					ABC transpor	rs1211152		YES			ATP-binding	ATP bindin Adenocarcinoma;A ATP,Acebutolol,Acetami	A=0, C=91	A=0.0, C=1.0	0.00001	1513.77	91	A=0/0, C=48/4
chr7:87840263	C/C	T	C	SNV	SRI	SRI:intronic:NM_003130.3	1	100		intronic	NM_00313	c.206-23A	p.?	-0.45					EF hand,EF-ha	rs6964163	0.428	YES	1.84E-1 (pos.87840263)		Sorcin (clas	T-tubule,Z disc,action potentia Calcium Citrate,Calcium	T=0, C=26	T=0.0, C=1.0	0.00001	371.419	26	T=0/0, C=16/1
chr7:882895	C/G	C	G	SNV	SUN1	SUN1:intronic:NM_001130965.2	1	59.09		intronic	NM_00113	c.478+26C	p.?	-1.61					Apolipoprote	rs6970511	0.4367	YES	6.516E-1 (pos.882895)		UNC84, C. e	LINC complex,acrosomal membrane,centrosome localiz	C=18, G=26	C=0.4091, G=0.5909	0.00001	312.072	44	C=11/7, G=18/
chr17:3445901	G/G	T	G	SNV	TRPV3	TRPV3:exonic:NM_001258205.1	1	100	6	synonymous	NM_00125	c.558A>C	p.(=)	-3.85				0(COSM6339	Ankyrin repea	rs11078458	0.3936	YES	7.247E-1;0.0 (pos.34459	neoplasm	Transient receptor potential cation channe	Menthol Benign	T=0, G=51	T=0.0, G=1.0	0.00001	754.58	51	T=0/0, G=22/2
chr7:88424291	T/T	C	T	SNV	TEX47,ZNF804B	ZNF804B:intronic:NM_181646.3, TEX47:utr_5:NM_152706.3	1	100		intronic	NM_18164	c.108+348	p.?, p.?	-3.41					Zinc-finger dc	rs4363132	0.1437	YES	8.565E-2 (pos.88424291)			metal ion binding,nucleic acid binding	C=0, T=120	C=0.0, T=1.0	0.00001	1928.28	120	C=0/0, T=65/5
chr17:3458072	C/C	T	C	SNV	TRPV3	TRPV3:exonic:NM_001258205.1	1	100	2	missense	NM_00125	c.73A>G	p.Ile25Val	-2	0.57	29	0		Ankyrin repea	rs322965	0		1.0 (pos.3458072)		Transient receptor potential cation channe	Menthol Benign	T=0, C=35	T=0.0, C=1.0	0.00001	543.903	35	T=0/0, C=10/2
chr7:142572908	T/C	T	C	SNV	TRPV6	TRPV6:exonic:NM_018646.5	1	57.63	9	missense	NM_01864	c.1252A>G	p.Met418Val	-1.22	1	21	0	0.02(COSM49	Ankyrin repea	rs4987667	0.0784	YES	1.78E-2 (pos.142572908)	rhabdomyosar	Transient re	calcium ch Adnexal Diseases;C Calcium Citrate,Calcium	T=25, C=34	T=0.4237, C=0.5763	0.00001	356.496	59	T=7/18, C=10/
chr1:184023529	C/C	A	C	SNV	TSEN15	TSEN15:exonic:NM_052965.3	1	100	2	missense	NM_05296	c.177A>C	p.Gln59His	1.48	0	24	0.002	0.66(COSM37	Sen15 protei	rs1046934	0.3476	YES	4.861E-1;0.0 (pos.18402	adenocarcinoi	tRNA splicir	RNA phosphodiester bond hydrolysis, endonucleolytic,r	A=0, C=86	A=0.0, C=1.0	0.00001	1410.87	86	A=0/0, C=30/5
chr1:115576023	G/G	A	G	SNV	TSHB	TSHB:exonic:NM_000549.4	1	100	2	missense	NM_00054	c.40A>G	p.Thr14Ala	0.23	0.22	58	0		Cystine-knot	rs10776792	0.0345	YES	1.0 (pos.115576023)		Thyroid-stir	G-protein Bipolar Disorder;Bipolar and R Benign	A=0, G=80	A=0.0, G=1.0	0.00001	1289.35	80	A=0/0, G=40/4
chr7:89887587	T/T	G	T	SNV	CFAP69	CFAP69:intronic:NM_001039706.2	1	100		intronic	NM_00103	c.246+110	p.?	-0.03						rs11771963		YES					G=0, T=24	G=0.0, T=1.0	0.00001	334.768	24	G=0/0, T=16/8
chr14:81610583	C/C	G	C	SNV	TSHR	TSHR:exonic:NM_000369.2	1	100	10	missense	NM_00036	c.2181G>C	p.Glu727Asp	-0.13	1	45	0		7 transmemb	rs1991517	0.0866	YES	8.252E-1 (pos.81610583)		Thyroid-stir	B cell diffe Adenoma;Adrenal (Thyrotropi Benign/Likely	G=0, C=36	G=0.0, C=1.0	0.00001	563.74	36	G=0/0, C=18/1
chr17:79612397	G/G	A	G	SNV	TSPAN10	TSPAN10:exonic:NM_031945.4	1	100	2	missense	NM_03194	c.416A>G	p.Tyr139Cys	4.68		194		0.05(COSM15	Tetraspanin f	rs6420484	0.3474	YES	9.973E-1;0.0 (pos.79612	neoplasm		cell surface receptor signaling pathway,enzyme binding,	A=0, G=96	A=0.0, G=1.0	0.00001	1617.49	96	A=0/0, G=46/5
chr7:16823095	A/A	G	A	SNV	TSPAN13	TSPAN13:exonic:NM_014399.3	1	100	6	synonymous	NM_01439	c.594G>A	p.(=)	0.41				0.66(COSM15	Tetraspanin f	rs11491	0.4451	YES	3.096E-1 (pos.16823095)	NS,adenocarci	Tetraspanir	calcium channel regulator activity,cell surface receptor	G=0, A=21	G=0.0, A=1.0	0.00001	278.411	21	G=0/0, A=9/12
chr19:19625547	G/G	A	G	SNV	TSSK6	TSSK6:exonic:NM_032037.3	1	100	1	synonymous	NM_03203	c.690T>C	p.(=)	1.5				0.16(COSM43	Kinase-like,L	rs7250893	0.3495	YES	2.952E-1 (pos.19625547)	adenocarcinoi	Testis-spec	ATP binding,cytoplasm,intracellular signal transduction,	A=0, G=60	A=0.0, G=1.0	0.00001	898.024	60	A=0/0, G=35/2
chr3:180319981	T/T	G	T	SNV	TTC14	TTC14:utr_5:NM_133462.3	1	100	1	utr_5	NM_13346	c.-69G>T	p.?	-0.01					TPR repeat,T	rs1532485		YES				nucleic acid binding	G=0, T=69	G=0.0, T=1.0	0.00001	1077.03	69	G=0/0, T=28/4
chr7:91736794	T/A	T	A	SNV	AKAP9	AKAP9:intronic:NM_005751.4	1	50		intronic	NM_00575	c.11546+5	p.?	0.13					Pericentrin-A	rs7785971		YES			A-kinase an	DNA bindi Arrhythmias, Cardiac;Arsenic Poisoning;Card	T=16, A=16	T=0.5, A=0.5	0.00001	158.148	32	T=13/3, A=4/1
chr2:166788336	C/C	T	C	SNV	TTC21B	TTC21B:exonic:NM_024753.4	1	100	8	missense	NM_02475	c.826A>G	p.Thr276Ala	1.68	1	58	0		Tetratricope	rs7592429	0	YES	1.0 (pos.166788336)		Tetratricop	ciliary tip, Abnormalities, Multiple;Barde Benign/Likely	T=0, C=62	T=0.0, C=1.0	0.00001	940.32	62	T=0/0, C=33/2
chr2:166773971	G/A	G	A	SNV	TTC21B	TTC21B:exonic:NM_024753.4	1	53.13	14	synonymous	NM_02475	c.1695C>T	p.(=)	2.08				0.4(COSM400	Tetratricope	rs6750044	0.389	YES	1.982E-1 (pos.16677397	acute_myeloid	Tetratricop	ciliary tip, Abnormalities, Multiple;Barde Benign/Likely	G=15, A=17	G=0.4688, A=0.5313	0.00001	173.491	32	G=5/10, A=9/8
chr1:55251689	C/C	T	C	SNV	TTC22	TTC22:exonic:NM_001114108.1	1	100	5	synonymous	NM_00111	c.987A>G	p.(=)	0.35						rs586462	0		1.0 (pos.55251689)				T=0, C=82	T=0.0, C=1.0	0.00001	1329.65	82	T=0/0, C=53/2
chr2:32983526	C/T	C	T	SNV	TTC27	TTC27:exonic:NM_017735.4	1	50	13	synonymous	NM_01773	c.1620C>T	p.(=)	6.09				0.99(COSM14	Tetratricope	rs2273665	0.4102	YES	3.465E-1 (pos.32983526)	adenocarcinoma,neoplasn		biological_process,cellular_component,molecular_func	C=33, T=33	C=0.5, T=0.5	0.00001	329.772	66	C=22/11, T=10
chr21:38563639	C/T	C	T	SNV	TTC3,TTC3-AS1	TTC3-AS1:intronic_nc:NR_046651.1, TTC3:exonic:NM_00100	1	46.43	40	missense	NM_00100	c.5029C>T	p.Pro1677Ser	0.08	0.8	74	0		RING-type zin	rs61740519	0.0274	YES	0.0 (pos.38563639)		Tetratricop	cytosol,m Abnormalities, Multiple;Chromosome Disord	C=15, T=13	C=0.5357, T=0.4643	0.00001	117.927	28	C=11/4, T=7/6
chr2:178481818	T/T	G	T	SNV	TTC30A	TTC30A:exonic:NM_152275.3	1	100	1	synonymous	NM_15227	c.1612C>A	p.(=)	4.85					Tetratricope	rs10497497,rs74719	0.1323	YES	0.0 (pos.178481817),9.822E-1 (pos.178481818)			cilium,cilium assembly,intraciliary transport,intraciliary	G=0, T=89	G=0.0, T=1.0	0.00001	1472.46	89	G=0/0, T=43/4
chr7:97498535	G/G	A	G	SNV	ASNS,CZ1P-ASNS	ASNS:intronic:NM_001673.4, CZ1P-ASNS:intronic_nc:NR_147	1	100		intronic	NM_00167	c.-22-44T>	p.?	-0.69					Aluminium in	rs6465630		YES			Asparagine	ATP bindin Congenital, Heredit ATP,Glutamic Acid,L-Asp	A=0, G=120	A=0.0, G=1.0	0.00001	1906.97	120	A=0/0, G=63/5
chr7:97846857	G/G	GAGG	G	INDEL	TECPR1	TECPR1:intronic:NM_015395.2	3	100		intronic	NM_01539	c.3385-42	p.?	-1.65, 0.59, 2.71, 0.5					Integral pero	rs1029962951,rs11471393,rs35		YES	(pos.97846857),0.0 (pos.97846859),9.4		Tectonin be	autophagosome maturation,autophagosome membran	GAGG=0, G	GAGG=0.0, G=1.0	0.00001	307.497	22	GAGG=0/0, G=
chr7:97851109	C/C	T	C	SNV	TECPR1	TECPR1:intronic:NM_015395.2	1	100		intronic	NM_01539	c.3065-44	p.?	-0.33					Integral pero	rs4729416	0		1.0 (pos.97851109)		Tectonin be	autophagosome maturation,autophagosome membran	T=0, C=36	T=0.0, C=1.0	0.00001	563.726	36	T=0/0, C=9/27
chr22:46677607	C/C	T	C	SNV	TTC38	TTC38:exonic:NM_017931.2	1	100	7	missense	NM_01793	c.727T>C	p.Phe243Leu	1.16	0.02	22	0			rs130642	0		1.0;0.0 (pos.46677607)			extracellular exosome	T=0, C=36	T=0.0, C=1.0	0.00001	563.353	36	T=0/0, C=13/2
chr7:97874094	C/T	C	T	SNV	TECPR1	TECPR1:intronic:NM_015395.2	1	50		intronic	NM_01539	c.409-89G	p.?	-0.32					Integral pero	rs903592		YES			Tectonin be	autophagosome maturation,autophagosome membran	C=11, T=11	C=0.5, T=0.5	0.00001	94.6067	22	C=6/5, T=6/5
chr7:97874143	C/A	C	A	SNV	TECPR1	TECPR1:intronic:NM_015395.2	1	56		intronic	NM_01539	c.408+54G	p.?	-0.09					Integral pero	rs3816944		YES			Tectonin be	autophagosome maturation,autophagosome membran	C=11, A=14	C=0.44, A=0.56	0.00001	134.35	25	C=6/5, A=6/8
chr18:21710321	T/T	C	T	SNV	TTC39C	TTC39C:exonic:NM_001135993.1	1	100	12	synonymous	NM_00113	c.1563C>T	p.(=)	1.83					Cohesin load	rs12970083	0		1.0 (pos.21710321)				C=0, T=53	C=0.0, T=1.0	0.00001	781.65	53	C=0/0, T=23/3
chr14:20770036	C/C	T	C	SNV	TTC5	TTC5:exonic:NM_138376.2	1	100	2	missense	NM_13837	c.140A>G	p.Gln47Arg	-0.34	0.46	43	0	0.04(COSM11	Tetratricope	rs3742945	0.2681	YES	8.004E-1 (pos.20770036)	adenoma		DNA binding,DNA repair,chromatin binding,cytoplasm,nu	T=0, C=41	T=0.0, C=1.0	0.00001	664.497	41	T=0/0, C=21/2
chr7:98553739	TC/TC	T	TC	INDEL	TRRAP	TRRAP:intronic:NM_001244580.1	1	100		intronic	NM_00124	c.5917-30	p.?	-0.3					FAT domain,F	rs200160654,rs7865	0.0823	YES	7.577E-1;0.0 (pos.98553739)		Transforma	DNA repai Melanoma;Mental Disorders;Neoplasms;Ne	T=0, TC=11	T=0.0, TC=1.0	0.00001	1381.05	111	T=0/0, TC=34/
chr7:98645517	G/A	G	A	SNV	SMURF1	SMURF1:intronic:NM_020429.2	1	57.14		intronic	NM_02042	c.1032-12	p.?	-0.27					C2 domain,H	rs219824,rs7470091	0.3328	YES	1.165E-4;0.0 (pos.98645516),4.014E-1 (p		SMAD ubiqu	BMP signaling pathway,I-SMAD binding,R-SMAD binding,\	G=12, A=16	G=0.4286, A=0.5714	0.00001	161.77	28	G=3/9, A=2/14
chr9:135263573	T/T	G	T	SNV	TTF1	TTF1:exonic:NM_007344.3	1	100	8	synonymous	NM_00734	c.2265C>A	p.(=)	-3.98					Myb-like DNA	rs556386	0.006	YES	9.768E-1 (pos.135263573)		NK2 homeo	DNA-templated transcription, termination,RNA polymer	G=0, T=50	G=0.0, T=1.0	0.00001	852.739	50	G=0/0, T=25/2
chr7:99315276	T/T	C	T	SNV	CYP3A7,CYP3A7-CYP3A51P	CYP3A7-CYP3A51P:intronic:NM_001256497.2, CYP3A7:intro	1	100		intronic	NM_00125	c.319-14G	p.?, p.?	-0.05					Cytochrome P	rs41279866	0.0652	YES	3.134E-2 (pos.99315276)		Cytochrome	aromatase activity,endoplasm Alfentanil,Alprazolam,Ar	C=0, T=30	C=0.0, T=1.0	0.00001	446.63	30	C=0/0, T=21/9
chr7:99361466	T/T	C	T	SNV	CYP3A4	CYP3A4:intronic:NM_017460.5	1	100		intronic	NM_01746	c.1026+12	p.?	-2.54					Cytochrome P	rs2242480	0.0966	YES	2.639E-1 (pos.99361466)		Cytochrome	1,8-cineol Arrhythmias, Cardi Abiraterone,Acenocoum	C=0, T=79	C=0.0, T=1.0	0.00001	1269.33	79	C=0/0, T=41/3
chr7:99459144	T/C	T	C	SNV	CYP3A43	CYP3A43:intronic:NM_022820.4	1	50		intronic	NM_02282	c.1027-92	p.?	0.07					Cytochrome P	rs503115		YES			Cytochrome	aromatas Genital Diseases, N Cobimetinib,Daclatasvir	T=47, C=47	T=0.5, C=0.5	0.00001	522.231	94	T=24/23, C=22
chr7:99696370	G/G	C	G	SNV	MCM7	MCM7:intronic:NM_005916.4	1	100		intronic	NM_00591	c.583-32G	p.?	-0.95					AAA domain (	rs1534309	0.1835	YES	8.806E-1;0.0;0.0 (pos.99696370)		Minichrom	ATP binding,ATP-dependent DNA helicase activity,DNA bi	C=0, G=64	C=0.0, G=1.0	0.00001	977.63	64	C=0/0, G=30/3
chr7:99751017	G/G	A	G	SNV	LAMTOR4	LAMTOR4:intronic:NM_001008395.3	1	100		intronic	NM_00100	c.85-6A>G	p.?	-3.56						rs3736591	0.3928	YES	6.756E-1 (pos.99751017)			Ragulator complex,cell cycle arrest,cellular response to	A=0, G=26	A=0.0, G=1.0	0.00001	371.41	26	A=0/0, G=18/8
chr6:167754661	G/G	A	G	SNV	TTLL2	TTLL2:exonic:NM_031949.4	1	100	3	missense	NM_03194	c.1273A>G	p.Arg425Gly	0.32	0.08	125	0.005		Tubulin-tyros	rs909545	0.2272	YES	8.092E-1 (pos.167754661)			ATP binding,cellular protein modification process,ligase	A=0, G=123	A=0.0, G=1.0	0.00001	1993.76	123	A=0/0, G=61/6
chr14:76330181	G/A	G	A	SNV	TTLL5	TTLL5:exonic:NM_015072.4	1	56	29	synonymous	NM_01507	c.3498G>A	p.(=)	2.73				0.91(COSM54	Tubulin-tyros	rs3742764,rs752932	0.084	YES	0.0 (pos.76330180),4.15	adenocarcinoi	Tubulin tyrc	ATP bindin Arthritis;Arthritis, Juvenile;Autoimmune Dise	G=11, A=14	G=0.44, A=0.56	0.00001	134.35	25	G=7/4, A=2/12
chr7:99780283	G/A	G	A	SNV	STAG3	STAG3:intronic:NM_012447.3	1	53.33		intronic	NM_01244	c.220-63G	p.?	0.36					STAG domain	rs2056726		YES			Stromalin 3	chromosome, centromeric region,extracellular space,la	G=14, A=16	G=0.4667, A=0.5333	0.00001	159.925	30	G=13/1, A=12/
chr2:179623758	T/T	C	T	SNV	TTN	TTN:exonic:NM_001256850.1	1	100	44	missense	NM_00125	c.10256G>	p.Ser3419Asn	0.4		46			Fibronectin t	rs2291310	0.0676	YES	7.636E-1 (pos.179623758)		Titin	ATP bindin Aortic Stenosis, Subvalvular;Ac Benign/Likely	C=0, T=57	C=0.0, T=1.0	0.00001	849.612	57	C=0/0, T=21/3
chr2:179629363	C/C	T	C	SNV	TTN	TTN:exonic:NM_001256850.1	1	100	42	synonymous	NM_00125	c.9879A>G	p.(=)	0.4					Fibronectin t	rs4894043	8.00E-04		1.0 (pos.179629363)		Titin	ATP bindin Aortic Stenosis, Subvalvular;Ac Benign/Likely	T=0, C=78	T=0.0, C=1.0	0.00001	1247.31	78	T=0/0, C=21/5
chr7:99821381	G/G	C	G	SNV	GATS	GATS:intronic:NM_178831.6	1	100		intronic	NM_17883	c.412-60G	p.?	-1.08					ACT domain	rs858508	0.2637	YES					C=0, G=28	C=0.0, G=1.0	0.00001	408.718	28	C=0/0, G=15/1
chr7:99821470	G/G	A	G	SNV	GATS	GATS:intronic:NM_178831.6	1	100		intronic	NM_17883	c.411+35T	p.?	-2.99					ACT domain	rs858509	0.2594	YES	3.78E-1 (pos.99821470)				A=0, G=24	A=0.0, G=1.0	0.00001	334.756	24	A=0/0, G=12/1
chr2:130951548	A/A	G	A	SNV	TUBA3E	TUBA3E:exonic:NM_207312.2	1	100	4	synonymous	NM_20731	c.867C>T	p.(=)	-2.39				0.03(COSM41	Tubulin C-ter	rs1052319		YES	9.922E-1 (pos.13095154	neoplasm,squamous_cell		GTP binding,GTPase activity,bi CYT997	G=0, A=31	G=0.0, A=1.0	0.00001	465.819	31	G=0/0, A=10/2
chr10:5435918	A/A	G	A	SNV	TUBAL3	TUBAL3:exonic:NM_024803.2	1	100	4	synonymous	NM_02480	c.903C>T	p.(=)	-0.05				0.37(COSM11	Tubulin C-ter	rs7097775	0.3577	YES	6.377E-1 (pos.5435918)	adenocarcinoma		GTP binding,GTPase activity,cytoplasm,cytoskeleton org	G=0, A=21	G=0.0, A=1.0	0.00001	281.228	21	G=0/0, A=8/13
chr8:100133706	G/G	T	G	SNV	VPS13B	VPS13B:intronic:NM_017890.4	1	100		intronic	NM_01789	c.1206+33	p.?	0.04					N-terminal re	rs7460625,rs750619	0.2062	YES	0.0 (pos.100133690),5.476E-1 (pos.1001		Vacuolar pr	protein tra Congenital Abnormalities;Con Benign	T=0, G=25	T=0.0, G=1.0	0.00001	353.019	25	T=0/0, G=6/19
chr10:5436260	G/G	A	G	SNV	TUBAL3	TUBAL3:exonic:NM_024803.2	1	100	4	synonymous	NM_02480	c.561T>C	p.(=)	-2.56					Tubulin C-ter	rs7910290	0.3049	YES	8.305E-1 (pos.5436260)			GTP binding,GTPase activity,cytoplasm,cytoskeleton org	A=0, G=27	A=0.0, G=1.0	0.00001	389.983	27	A=0/0, G=11/1

chr19:6495732	A/AG	A	AG	INDEL	TUBB4A	TUBB4A:exonic:NM 006087.3	1	50	4	frameshiftInsertion	NM 00608	c.777 778	p.Phe260fs	9.08					Tubulin C-terminal domain,Tubulin/FtsZ family, GTPase domain						Tubulin, bet	G2/M tran	Basal Ganglia Disea	CYT997,Epothilone D,Pat		A=31, AG=3	A=0.5, AG=0.5	0.00001	87.0375	62	A=18/13, AG=
chr17:57963537	G/G	A	G	SNV	TUBD1	TUBD1:exonic:NM 016261.3	1	100	3	missense	NM 01626	c.227T>C	p.Met76Thr	4.17	1	81	0	0.46(COSM14	Tubulin/FtsZ	rs1292053	0.4362	YES	5.814E-1 (pos.57963537)	acute_myeloi	Tubulin, del	GTP bindir	Digestive System Di	Vinblastine		A=0, G=22	A=0.0, G=1.0	0.00001	298.892	22	A=0/0, G=10/1
chr10:135097434	G/G	A	G	SNV	TUBGCP2	TUBGCP2:exonic:NM 001256617.1	1	100	15	synonymous	NM 00125	c.2181T>C	p.(=)	0.36					Spc97 / Spc98	rs2995326	0.1088	YES	9.548E-1;0.0 (pos.135097434)			centrosome,centrosome duplication,cytoplasm,cytopla				A=0, G=26	A=0.0, G=1.0	0.00001	371.41	26	A=0/0, G=13/1
chr16:28857645	A/G	A	G	SNV	TUFM	TUFM:utr 5:NM 003321.4	1	59.09	1	utr 5	NM 00332	c.-55T>C	p.?	-0.35					Elongation fa	rs7187776		YES			Tu translati	GTP bindir	Acid-Base Imbalanc	Guanosine	Benign	A=9, G=13	A=0.4091, G=0.5909	0.00001	122.563	22	A=1/8, G=9/4
chr6:158735087	T/T	C	T	SNV	TULP4	TULP4:exonic:NM 020245.4	1	100	1	synonymous	NM 02024	c.39C>T	p.(=)	-0.25					Tub family	rs9364951	0.0991	YES	8.956E-1 (pos.158735087)			cilium,cytoplasm,cytosol,phosphatidylinositol binding,p				C=0, T=46	C=0.0, T=1.0	0.00001	768.307	46	C=0/0, T=18/2
chr6:158735090	C/C	T	C	SNV	TULP4	TULP4:exonic:NM 020245.4	1	100	1	synonymous	NM 02024	c.42T>C	p.(=)	0.93					Tub family	rs7756620	0.0991	YES	8.957E-1 (pos.158735090)			cilium,cytoplasm,cytosol,phosphatidylinositol binding,p				T=0, C=46	T=0.0, C=1.0	0.00001	768.312	46	T=0/0, C=18/2
chr6:158923434	C/C	G	C	SNV	TULP4	TULP4:exonic:NM 020245.4	1	100	13	synonymous	NM 02024	c.2739G>C	p.(=)	0				0.14(COSM41	Tub family	rs9364968	0.241	YES	8.9E-1 (pos.158923434)	ductal_carcinoma,neoplas		cilium,cytoplasm,cytosol,phosphatidylinositol binding,p				G=0, C=24	G=0.0, C=1.0	0.00001	330.928	24	G=0/0, C=15/9
chr9:25677698	C/C	A	C	SNV	TUSC1	TUSC1:exonic:NM 001004125.2	1	100	1	missense	NM 00100	c.622T>G	p.Ser208Ala	-0.23		99		0.02(COSM3763864)		rs72631815	0.2618	YES	2.906E-1;0.0 (pos.25677	adenocarcinoi	Tumor suppressor candidate 1					A=0, C=20	A=0.0, C=1.0	0.00001	263.785	20	A=0/0, C=13/7
chr9:25677933	C/C	T	C	SNV	TUSC1	TUSC1:exonic:NM 001004125.2	1	100	1	synonymous	NM 00100	c.387A>G	p.(=)	0.36				0(COSM3750169)		rs35110225	0.4192	YES	3.617E-1 (pos.25677933)	adenocarcinoi	Tumor suppressor candidate 1					T=0, C=57	T=0.0, C=1.0	0.00001	849.363	57	T=0/0, C=37/2
chr9:25677953	C/C	T	C	SNV	TUSC1	TUSC1:exonic:NM 001004125.2	1	100	1	missense	NM 00100	c.367A>G	p.Asn123Asp	0.36		23		0(COSM3750170)		rs34498078	0.055	YES	8.698E-1 (pos.25677953)	adenocarcinoi	Tumor suppressor candidate 1					T=0, C=60	T=0.0, C=1.0	0.00001	903.526	60	T=0/0, C=34/2
chr9:25678122	C/C	G	C	SNV	TUSC1	TUSC1:exonic:NM 001004125.2	1	100	1	synonymous	NM 00100	c.198C>G	p.(=)	0.44				0.13(COSM4163713)		rs72631814	0.2308	YES	1.646E-1;0.0 (pos.25678	carcinoma,ne	Tumor suppressor candidate 1					G=0, C=44	G=0.0, C=1.0	0.00001	726.465	44	G=0/0, C=23/2
chr8:105367096	T/C	T	C	SNV	DCSTAMP	DCSTAMP:intronic:NM 030788.3	1	97.56		intronic	NM 03078	c.1030-9T	p.?	0.42					DC-STAMP-lik	rs2458431	0.2843	YES	5.877E-1 (pos.105367096)		Transmemb	cell surfac	Amphetamine-Related Disorders:Bone Disea			T=1, C=40	T=0.0244, C=0.9756	0.00001	631.605	41	T=1/0, C=24/1
chr8:107754583	G/G	T	G	SNV	OXR1	OXR1:intronic:NM 001198532.1	1	100		intronic	NM 00119	c.2415+38	p.?	-0.13					LysM domain	rs1784468	0.1838	YES	9.571E-1 (pos.107754583)		Oxidation r	adult walking behavior,cellular response to hydroperoxi				T=0, G=32	T=0.0, G=1.0	0.00001	484.56	32	T=0/0, G=22/1
chr8:109455802	G/A	G	A	SNV	EMC2	EMC2:upstream:NM 014673.4	1	59.09		intronic	NM 01467	c.-86G>A	p.?	-0.31						rs3737501		YES			ER membra	ER membrane protein complex,cytoplasm,endoplasmic				G=9, A=13	G=0.4091, A=0.5909	0.00001	123.817	22	G=6/3, A=4/9
chr4:48069696	G/C	G	C	SNV	TXK	TXK:exonic:NM 003328.2	1	50	15	synonymous	NM 00332	c.1542C>G	p.(=)	-0.19					Protein kinas	rs2230594,rs752521	0.474	YES	0.0 (pos.48069695),4.36E-1 (pos.480696		TXK tyrosin	ATP binding,RNA polymerase II core promoter proximal r				G=23, C=23	G=0.5, C=0.5	0.00001	258.452	46	G=14/9, C=9/1
chr8:110505886	C/T	C	T	SNV	PKHD1L1	PKHD1L1:intronic:NM 177531.4	1	57.14		intronic	NM 17753	c.10237-4	p.?	0.3					G8 domain,IP	rs2607628	0.3667	YES	3.284E-1 (pos.110505886)		PKHD1-like	cilium,cytosol,extracellular space,immune response,int				C=9, T=12	C=0.4286, T=0.5714	0.00001	109.1	21	C=4/5, T=7/5
chr14:52907353	T/T	C	T	SNV	TXNDC16	TXNDC16:exonic:NM 020784.2	1	100	19	synonymous	NM 02078	c.1932G>A	p.(=)	-0.43					Thioredoxin,T	rs1952254	0.4104	YES	7.039E-1 (pos.52907353)		Thioredoxir	biological_process,cell,cell redox homeostasis,endopla				C=0, T=32	C=0.0, T=1.0	0.00001	485.015	32	C=0/0, T=16/1
chr8:11164012	C/C	T	C	SNV	MTMR9	MTMR9:intronic:NM 015458.3	1	100		intronic	NM 01545	c.809+96T	p.?	0.08					Myotubularir	rs17797443,rs370445200,rs529		YES			Myotubular	cytosol,dephosphorylation,enzyme regulator activity,ph				T=0, C=37	T=0.0, C=1.0	0.00001	583.654	37	T=0/0, C=12/2
chr18:9886890	G/G	A	G	SNV	TXNDC2	TXNDC2:exonic:NM 001098529.1	1	100	2	synonymous	NM 00109	c.414A>G	p.(=)	-0.37				0.01(COSM37	Thioredoxin	rs601874	0.2806	YES	8.089E-4 (pos.9886890)	adenocarcinoi	Thioredoxir	cell,cell differentiation,cell redox homeostasis,cellular				A=0, G=79	A=0.0, G=1.0	0.00001	1269.33	79	A=0/0, G=29/5
chr9:113091523	T/G	T	G	SNV	TXNDC8	TXNDC8:exonic:NM 001003936.3	1	50	3	missense	NM 00100	c.178A>C	p.Asn60His	0.38	0.05	68	0.988	0.26(COSM63	Thioredoxin	rs7041938	0.1243	YES	8.188E-2 (pos.11309152	carcinoma	Thioredoxir	Golgi apparatus,acrosomal vesicle,acrosome assembly,				T=11, G=11	T=0.5, G=0.5	0.00001	94.6067	22	T=4/7, G=4/7
chr8:113259132	T/T	A	T	SNV	CSMD3	CSMD3:intronic:NM 198123.1	1	100		intronic	NM 19812	c.10222+1	p.?	0.19					CUB domain,S	rs1895014		YES			Cub and Sus	integral cc	Adenocarcinoma:Autistic Disorder:Carcinom			A=0, T=20	A=0.0, T=1.0	0.00001	263.786	20	A=0/0, T=7/13
chr8:11629119	T/C	T	C	SNV	NEIL2	NEIL2:intronic:NM 001135746.2	1	47.62		intronic	NM 00113	c.138+25T	p.?	-1.32					Formamidop	rs804269	0.3279	YES	9.74E-1 (pos.11629119)		Endonuclea	DNA N-glycosylase activity,class I DNA-(apurinic or apyri				T=11, C=10	T=0.5238, C=0.4762	0.00001	82.2883	21	T=1/10, C=6/4
chr8:11629129	C/G	C	G	SNV	NEIL2	NEIL2:intronic:NM 001135746.2	1	47.62		intronic	NM 00113	c.138+35C	p.?	-0.25					Formamidop	rs804268	0.4043	YES	5.507E-1;0.0 (pos.11629129)		Endonuclea	DNA N-glycosylase activity,class I DNA-(apurinic or apyri				C=11, G=10	C=0.5238, G=0.4762	0.00001	82.2894	21	C=1/10, G=6/4
chr12:104645405	C/C	T	C	SNV	TXNRD1	TXNRD1:exonic:NM 001093771.2	1	100	2	synonymous	NM 00109	c.192T>C	p.(=)	-0.18					FAD binding c	rs7975161	0.1578	YES	8.884E-1 (pos.104645405)		Thioredoxir	cell,cell pr	Adenoma;Arthritis;	Arsenic trioxide,Flavin a		T=0, C=61	T=0.0, C=1.0	0.00001	919.888	61	T=0/0, C=34/2
chr22:19907099	C/A	C	A	SNV	TXNRD2	TXNRD2:exonic:NM 006440.4	1	57.14	3	missense	NM 00644	c.196G>T	p.Ala66Ser	2.56		99			FAD binding c	rs5748469	0.3312	YES	7.942E-1 (pos.19907099)		Thioredoxir	cell,cell re	Eye Diseases;Genit	motexafin	Benign	C=15, A=20	C=0.4286, A=0.5714	0.00001	218.712	35	C=8/7, A=14/6
chr3:126329910	G/G	A	G	SNV	TXNRD3	TXNRD3:exonic:NM 052883.1	1	100	14	synonymous	NM 05288	c.1698T>C	p.(=)	-1.17					FAD binding c	rs777241		YES	8.889E-1 (pos.126329910)		Thioredoxir	cell,cell differentiation,cell redox homeostasis,cellular				A=0, G=72	A=0.0, G=1.0	0.00001	1130.95	72	A=0/0, G=35/3
chr8:11695872	C/C	T	C	SNV	FDFT1	FDFT1:intronic:NM 004462.4	1	100		intronic	NM 00446	c.1033-25	p.?	-1.66					Squalene/ph	rs10903343,rs75503	0.4519	YES	0.0 (pos.11695871),9.6E-1;4.64E-4;0.0 (p		Farnesyl-di	biosynthe	Adenocarcinoma;C	TAK-475		T=0, C=21	T=0.0, C=1.0	0.00001	281.012	21	T=0/0, C=13/8
chr8:119938725	T/G	T	G	SNV	TNFRSF11B	TNFRSF11B:intronic:NM 002546.3	1	50		intronic	NM 00254	c.817+8A>	p.?	-0.17					Death domai	rs7844539	0.1324	YES	7.055E-3 (pos.119938725)		Tumor necr	apoptotic	Arthritis;Arthritis, Experimenta		Benign/Likely	T=19, G=19	T=0.5, G=0.5	0.00001	200.777	38	T=10/9, G=17/
chr8:119945166	G/A	G	A	SNV	TNFRSF11B	TNFRSF11B:intronic:NM 002546.3	1	50		intronic	NM 00254	c.400+4C>	p.?	-1.85					Death domai	rs1564858	0.1307	YES	7.086E-3 (pos.119945166)		Tumor necr	apoptotic	Arthritis;Arthritis, Experimenta		Benign/Likely	G=14, A=14	G=0.5, A=0.5	0.00001	131.941	28	G=6/8, A=4/10
chr15:41857303	C/C	T	C	SNV	TYRO3	TYRO3:exonic:NM 006293.3	1	100	6	synonymous	NM 00629	c.747T>C	p.(=)	-0.5					Fibronectin t	rs2277536	0.2855	YES	1.57E-1 (pos.41857303)		TYRO3 prote	ATP bindin	Cardiovascular Diseases:Embolism and Thro			T=0, C=71	T=0.0, C=1.0	0.00001	1111.48	71	T=0/0, C=31/4
chr8:120602908	T/T	C	T	SNV	ENPP2	ENPP2:intronic:NM 006209.4	1	100		intronic	NM 00620	c.1238-38	p.?	-0.51					DNA/RNA nor	rs935359	0.0878	YES	9.999E-1 (pos.120602908)		Ectonucleot	G-protein	Digestive System Diseases:Endocrine Gland I			C=0, T=39	C=0.0, T=1.0	0.00001	623.979	39	C=0/0, T=16/2
chr19:34959979	G/G	A	G	SNV	UBA2	UBA2:exonic:NM 005499.2	1	100	17	synonymous	NM 00549	c.1776A>G	p.(=)	-0.83					SUMO-activa	rs7259160	0.1228	YES	8.252E-1 (pos.34959979)		Ubiquitin-li	ATP binding,SUMO activating enzyme activity,SUMO acti				A=0, G=113	A=0.0, G=1.0	0.00001	1795.11	113	A=0/0, G=38/7
chr8:121534786	A/G	A	G	SNV	MTBP	MTBP:intronic:NM 022045.4	1	50		intronic	NM 02204	c.2611-10	p.?	0.33					MDM2-bindir	rs4285526		YES			Mouse doul	cell cycle arrest,chromatin,kinetochore,negative regula				A=10, G=10	A=0.5, G=0.5	0.00001	82.8861	20	A=4/6, G=2/8
chr9:34241742	TG/T	TG	T	INDEL	UBAP1	UBAP1:exonic:NM 001171201.1	1	45.83	3	frameshiftDeletion	NM 00117	c.914delG	p.Gly305fs	6.91, 2.61											Ubiquitin-a	ESCRT I complex,Golgi apparatus,cytoplasm,cytosol,end				TG=13, T=1	TG=0.5417, T=0.458	0.00005	42.8127	24	TG=4/9, T=2/9
chr15:65398658	A/AC	A	AC	INDEL	UBAP1L	UBAP1L:utr 5:NM 001163692.1	1	47.62	1	utr 5	NM 00116	c.-105T>G	p.?	0.48						rs201198440,rs397723277,rs58		YES				ESCRT I complex,ubiquitin binding,ubiquitin-dependent				A=11, AC=1	A=0.5238, AC=0.476	0.00001	63.223	21	A=6/5, AC=6/4
chr8:124140806	ATAA/ATAA	A	ATAA	INDEL	TBC1D31	TBC1D31:intronic:NM 145647.3	3	100		intronic	NM 14564	c.2117+53	p.?	1.12					Rab-GTPase-T	rs943079206						centrosome				A=0, ATAA=	A=0.0, ATAA=1.0	0.00001	316.747	23	A=0/0, ATAA=1
chr9:33941759	T/C	T	C	SNV	UBAP2	UBAP2:exonic:NM 018449.3	1	97.5	16	missense	NM 01844	c.1817A>G	p.Asn606Ser	2.12	1	46	0.02	0.14(COSM15	Ubiquitin-ass	rs307658	0.3827	YES	1.708E-1 (pos.33941759)	adenocarcinoma,ductal_ca		RNA binding,cadherin binding,cytoplasm,nucleus,positi				T=1, C=39	T=0.025, C=0.975	0.00001	611.635	40	T=1/0, C=12/2
chr12:125397750	G/G	A	G	SNV	UBC	UBC:exonic:NM 021009.6	1	100	2	missense	NM 02100	c.568T>C	p.Ser190Pro	3.18	1	74	0	0.03(COSM45	Ubiquitin fan	rs7137123		YES	1.0 (pos.125397750)	squamous_cel	Ubiquitin C	DNA damage response, detecti		N-Formylmethionine		A=0, G=36	A=0.0, G=1.0	0.00001	563.731	36	A=0/0, G=21/1
chr8:124206394	G/G	C	G	SNV	FAM83A	FAM83A:intronic:NM 032899.6	1	100		intronic	NM 03289	c.773+6C>	p.?	-0.43				0.14(COSM39	Protein of unl	rs7813460	0.2058	YES	2.379E-1 (pos.12420639	acute_myeloid_leukaemia		cell proliferation,cytoplasm,epidermal growth factor re				C=0, G=31	C=0.0, G=1.0	0.00001	465.793	31	C=0/0, G=12/1
chr8:124381525	T/T	C	T	SNV	ATAD2	ATAD2:intronic:NM 014109.3	1	100		intronic	NM 01410	c.932-110	p.?	0.01					AAA domain (	rs3857956		YES			ATPase fam	ATP bindin	Autoimmune Diseases:Female Urogenital Di			C=0, T=32	C=0.0, T=1.0	0.00001	485.138	32	C=0/0, T=8/24
chr16:1374818	G/G	A	G	SNV	UBE2I	UBE2I:utr 3:NM 194260.2	1	100	7	utr 3	NM 19426	c.501A>G	p.?	0.47					Ubiquitin-cor	rs770412443,rs8063	0.2769	YES	9.456E-1;1.158E-4 (pos.1374818)		Ubiquitin-c	ATP bindin	Mammary Neoplasms, Animal:Mammary Ne			A=0, G=27	A=0.0, G=1.0	0.00001	389.849	27	A=0/0, G=12/1
chr6:90039670	C/C	G	C	SNV	UBE2J1	UBE2J1:exonic:NM 016021.2	1	100	8	missense	NM 01602	c.685C>G	p.Leu229Val	-0.94	0.44	32	0		Ubiquitin-cor	rs10502	0.378	YES	6.987E-1 (pos.90039670)		Ubiquitin-c	ATP binding,cytoplasm,endoplasmic reticulum membra				G=0, C=22	G=0.0, C=1.0	0.00001	298.892	22	G=0/0, C=13/9
chr17:47000251	T/T	C	T	SNV	UBE2Z	UBE2Z:exonic:NM 023079.4	1	100	6	synonymous	NM 02307	c.846C>T	p.(=)	-0.09				0.3(COSM543	Ubiquitin-cor	rs1058018	0.4245	YES	7.471E-1 (pos.47000251)	adenocarcinoi	Ubiquitin-c	ATP bindin	Cardiovascular Diseases;Coronary Disease;H			C=0, T=36	C=0.0, T=1.0	0.00001	563.744	36	C=0/0, T=17/1
chr8:124665211	T/T	C	T	SNV	KLHL38	KLHL38:upstream:NM 001081675.2	1	100		intronic	NM 00108	c.-45G>A	p.?	-0.31						rs7387855	0.1529	YES	7.02E-1;0.0 (pos.124665211)			Cul3-RING ubiquitin ligase complex,protein binding,prot				C=0, T=40	C=0.0, T=1.0	0.00001	644.286	40	C=0/0, T=10/3
chr20:3102855	C/T	C	T	SNV	UBOX5,UBOX5-AS1	UBOX5:exonic:NM 014948.3, UBOX5-AS1:intronic_nc:NR 0	1	45.83	3	missense	NR 03839	c.430G>A	p.Ala144Thr	3.13	0.06	58	0.045		RING-type zin	rs146513940	0.001		0.0 (pos.3102855)			focal adhesion,metal ion binding,nuclear body,nucleopl				C=13, T=11	C=0.5417, T=0.4583	0.00001	93.4392	24	C=5/8, T=7/4
chr11:5536537	C/C	A	C	SNV	UBQLNL	UBQLNL:exonic:NM 145053.4	1	100	1	missense	NM 14505	c.1135T>G	p.Trp379Gly	0.09		184			Ubiquitin fan	rs393044	0.0014		1.0 (pos.5536537)							A=0, C=23	A=0.0, C=1.0	0.00001	320.842	23	A=0/0, C=10/1
chr8:124786259	A/G	A	G	SNV	FAM91A1	FAM91A1:intronic:NM 144963.3	1	45.83		intronic	NM 14496	c.73-61A>	p.?	-0.76					FAM91 C-terminus,FAM91 N-terminus											A=13, G=11	A=0.5417, G=0.4583	0.00001	63.0912	24	A=7/6, G=7/4
chr11:5536848	A/A	G	A	SNV	UBQLNL	UBQLNL:exonic:NM 145053.4	1	100	1	missense	NM 14505	c.824C>T	p.Ala275Val	0.59		64		0.05(COSM14	Ubiquitin fan	rs2017433	0.2879	YES	3.738E-1 (pos.5536848)	acute_myeloid_leukaemia,adenocarcinoma						G=0, A=32	G=0.0, A=1.0	0.00001	485.141	32	G=0/0, A=16/1
chr6:42559926	G/A	G	A	SNV	UBR2	UBR2:exonic:NM 015255.2	1	51.85	3	missense	NM 01525	c.376G>A	p.Glu126Lys	1.72	0.02	56	0.001	0.8(COSM376	ATP-depende	rs62414610	0.0326	YES	2.314E-4;0.0 (pos.42559	adenocarcinoi	Ubiquitin-p	cellular response to leucine,chromatin,chromatin silenc				G=52, A=56	G=0.4815, A=0.5185	0.00001	583.1	108	G=28/24, A=3
chr8:12592956	A/A	C	A	SNV	LONRF1	LONRF1:intronic:NM 152271.4	1	100		intronic	NM 15227	c.1452-47	p.?	-1.7					ATP-depende	rs13251315	0.0503	YES	9.514E-1 (pos.12592956)			cytosol,intracellular,metal ion binding,positive regulati				C=0, A=30	C=0.0, A=1.0	0.00001	446.625	30	C=0/0, A=14/1
chr8:126056215	GC/GC	G	GC	INDEL	WASHC5	WASHC5:intronic:NM 014846.3	1	100		intronic	NM 01484	c.2770+19	p.?	0.24					Hereditary s	rs11370883	0		1.0 (pos.126056215)			WASH complex,cytosol,early endosome,endoplasmic re				G=0, GC=1	G=0.0, GC=1.0	0.00001	2208.62	134	G=0/0, GC=58
chr1:19411129	G/G	A	G	SNV	UBR4	UBR4:exonic:NM 020765.2	1	100	102	synonymous	NM 02076	c.14877T>	p.(=)	-0.64				0.1(COSM375	E3 ubiquitin-	rs1043905	0.3978	YES	8.122E-1;0.0 (pos.19411	adenocarcinoi	Ubiquitin p	calmodulin binding,centrosom		HspE7,PCL-016		A=0, G=37	A=0.0, G=1.0	0.00001	583.706	37	A=0/0, G=19/1
chr1:19413261	A/A	T	A	SNV	UBR4	UBR4:exonic:NM 020765.2	1	100	100	missense	NM 02076	c.14599A>	p.Met4867Leu	2.62		15	0	0.75(COSM37	E3 ubiquitin-	rs12584	0.4342	YES	7.618E-1;0.0 (pos.19413	adenocarcinoi	Ubiquitin p	calmodulin binding,centrosom		HspE7,PCL-016		T=0, A=69	T=0.0, A=1.0	0.00001	1072.78	69	T=0/0, A=35/3
chr8:12990518	A/A	T	A	SNV	DLC1	DLC1:intronic:NM 182643.2	1	100		intronic	NM 18264	c.1349-17	p.?	-0.11					RhoGAP dom	rs13275331	0.2876	YES	4.226E-1 (pos.12990518)		Deleted in l	GTPase ac	Adnexal Diseases:Digestive System Diseases			T=0, A=51	T=0.0, A=1.0	0.00001	754.584	51	T=0/0, A=15/3
chr8:130764791	A/G	A	G	SNV	GSDMC	GSDMC:intronic:NM 031415.2	1	45.83		intronic	NM 03141	c.844+153	p.?	0.08					Gasdermin fa	rs4285452		YES			Gasdermin	biological_process,cytoplasm,cytosol,microtubule orga				A=26, G=22	A=0.5417, G=0.4583	0.00001	240.508	48	A=17/9, G=9/1
chr1:19433449	G/G	A	G	SNV	UBR4	UBR4:exonic:NM 020765.2	1	100	82	synonymous	NM 02076	c.12117T>	p.(=)	-2.56				0.08(COSM37	E3 ubiquitin-	rs6426677	0.3974	YES	8.107E-1 (pos.19433449)	acute_myeloi	Ubiquitin p	calmodulin binding,centrosom		HspE7,PCL-016		A=0, G=36	A=0.0, G=1.0	0.00001	563.715	36	A=0/0, G=15/2
chr1:19447843	G/G	C	G	SNV	UBR4	UBR4:exonic:NM 020765.2	1	100	68	synonymous	NM 02076	c.9981G>C	p.(=)	0.35				0.92(COSM13	E3 ubiquitin-	rs1044010	0.4342	YES	7.611E-1 (pos.19447843)	adenocarcinoi	Ubiquitin p	calmodulin binding,centrosom		HspE7,PCL-016		C=0, G=35	C=0.0, G=1.0	0.00001	543.903	35	C=0/0, G=9/26
chr8:132996359	T/T	C	T	SNV	EFR3A	EFR3A:intronic:NM 015137.5	1	100		intronic	NM 01513	c.1576-27	p.?	0.05						rs11783016	0.1557	YES	3.025E-2 (pos.132996359)		EFR3, S. cer	cytosol,ex	Autoimmune Diseases:Female Urogenital Di			C=0, T=20	C=0.0, T=1.0	0.00001	263.786	20	C=0/0, T=11/9
chr8:133008823	A/A	G	A	SNV	EFR3A	EFR3A:intronic:NM 015137.5	1	100		intronic	NM 01513	c.2157+79	p.?	0.22						rs2270873		YES			EFR3, S. cer	cytosol,ex	Autoimmune Diseases:Female Urogenital Di			G=0, A=29	G=0.0, A=1.0	0.00001	427.563	29	G=0/0, A=16/1
chr8:133046926	A/AC	A	AC	INDEL	OC90	OC90:intronic:NM 001080399.2	1	100		intronic	NM 00108	c.733+102	p.?	0.51					Phospholipase A2						Otoconin 9	arachidon	Ear Diseases;Labyrinth Diseases;Otorhinolar			A=0, AC=27	A=0.0, AC=1.0	0.00001	222.966	27	A=0/0, AC=4/2
chr8:133046977	G/G	A	G	SNV	OC90	OC90:intronic:NM 001080399.2	1	100		intronic	NM 00108	c.733+51T	p.?	-0.31					Phospholipa	rs7834841	0.2824	YES			Otoconin 9	arachidon	Ear Diseases;Labyrinth Diseases;Otorhinolar			A=0, G=37	A=0.0, G=1.0	0.00001	583.706	37	A=0/0, G=13/2
chr8:133049810	G/G	A	G	SNV	OC90	OC90:intronic:NM 001080399.2	1	100		intronic	NM 00108	c.629-75T	p.?	1.8					Phospholipa	rs6992506	0.2841	YES			Otoconin 9	arachidon	Ear Diseases;Labyrinth Diseases;Otorhinolar			A=0, G=45	A=0.0, G=1.0	0.00001	747.393	45	A=0/0, G=6/39
chr17:42287519	T/T	C	T	SNV	UBTF	UBTF:exonic:NM 014233.3	1	100	15	synonymous	NM 01423	c.1599G>A	p.(=)	0.74				0.8(COSM375	HMG (high m	rs2071167	0.2528	YES	7.153E-1 (pos.42287519)	adenocarcinoi	Upstream b	DNA binding,RNA binding,RNA polymerase I CORE elemer				C=0, T=60	C=0.0, T=1.0	0.00001	903.425	60	C=0/0, T=26/3
chr1:26610891	C/C	A	C	SNV	UBXN11	UBXN11:exonic:NM 183008.2	1	100	12	missense	NM 18300	c.935T>G	p.Leu312Arg	-0.44	1	102	0	0(COSM3997	SEP domain,U	rs4332350	0.0627	YES	4.478E-1;0.0 (pos.26610	acute_myeloi	UBX domair	cytoplasm,cytoskeleton,proteasome-mediated ubiquiti				A=0, C=45	A=0.0, C=1.0	0.00001	747.39	45	A=0/0, C=17/2
chr1:26609259	C/T	C	T	SNV	UBXN11	UBXN11:exonic:NM 183008.2	1	50	15	synonymous	NM 18300	c.1173G>A	p.(=)	-2.87				0.02(COSM41	SEP domain,U	rs7522905	0.3388	YES	2.9E-1 (pos.26609259)	carcinoma,ne	UBX domair	cytoplasm,cytoskeleton,proteasome-mediated ubiquiti				C=22, T=22	C=0.5, T=0.5	0.00001	243.598	44	C=16/6, T=18/
chr19:4454083	T/T	C	T	SNV	UBXN6	UBXN6:exonic:NM 025241.2	1	100	2	missense	NM 02524	c.91G>A	p.Ala31Thr	0.55	0.34	58	0.001		PUB domain,	rs1127888	0.2709	YES	5.419E-2 (pos.4454083)		UBX domair	ERAD pathway,cytoplasm,cytosol,early endosome meml				C=0, T=22	C=0.0, T=1.0	0.00001	298.729	22	C=0/0, T=6/16
chr8:133107893	G/G	A	G	SNV	HHLA1	HHLA1:intronic:NM 001145095.1	1	100		intronic	NM 00114	c.365-44T	p.?	-0.09						rs10956634	0.0484	YES	6.624E-1 (pos.133107893)		Human end	extracellular region				A=0, G=25	A=0.0, G=1.0	0.00001	357.408	25	A=0/0, G=10/1
chr19:4454002	C/T	C	T	SNV	UBXN6	UBXN6:exonic:NM 025241.2	1	57.14	2	missense	NM 02524	c.172G>A	p.Ala58Thr	6.86	0	58	0.978		PUB domain,UBX domain						UBX domair	ERAD pathway,cytoplasm,cytosol,early endosome meml				C=12, T=16	C=0.4286, T=0.5714	0.00001	161.804	28	C=6/6, T=5/11
chr8:133769560	A/A	AGA	A	INDEL	TMEM71	TMEM71:intronic:NM 144649.2	2	100		intronic	NM 14464	c.41-20TC	p.?	0.92, 0.04, 3.65					TMEM71 protein family							integral component of membrane,mitochondrion				AGA=0, A=5	AGA=0.0, A=1.0	0.00001	849.615	57	AGA=0/0, A=2
chr9:134401335	T/T	C	T	SNV	UCK1	UCK1:exonic:NM 001261451.2	1	100	6	synonymous	NM 00126	c.648G>A	p.(=)	-1.78				0.06(COSM16	Phosphoribul	rs2296957	0.0507	YES	9.127E-1;0.0 (pos.13440	bronchioloalv	Uridine/cyt	ATP binding,CTP salvage,UMP salvage,cytosol,nucleosid				C=0, T=27	C=0.0, T=1.0	0.00001	389.982	27	C=0/0, T=14/1
chr10:13275553	G/G	T	G	SNV	UCMA	UCMA:exonic:NM 145314.2	1	100	3	synonymous	NM 14531	c.205A>C	p.(=)	1.04					Unique cartil	rs3829925	0.0886	YES	8.193E-1;2.314E-4 (pos.13275553)			aggresome,extracellular space,negative regulation of os				T=0, G=27	T=0.0, G=1.0	0.00001	389.957	27	T=0/0, G=13/1
chr11:73715542	G/A	G	A	SNV	UCP3	UCP3:exonic:NM 003356.3	1	56	5	synonymous	NM 00335	c.630C>T	p.(=)	-1.16				0.04(COSM63	Mitochondria	rs2075577	0.4519	YES	4.16E-1 (pos.73715542)	carcinoma	Uncoupling	adaptive t	Cardiovascular Diseases:Diabetes Mellitus:I			G=33, A=42	G=0.44, A=0.56	0.00001	460.827	75	G=9/24, A=16/
chr2:234627937	C/C	T	C	SNV	UGT1A10,UGT1A4,UGT1A5,UGT1A6,UGT1A7,UGT1A8,UGT1A9	UGT1A10:intronic:NM 019075.2, UGT1A5:intronic:NM 019	1	100	1	synonymous	NM 01907	c.856-477	p.?, p.?, p.?, p.?, p.?, p.?,	-0.55					Glycosyltrans	rs2011404	0.1656	YES	9.88E-1;0.0 (pos.234627937)		UDP-glycos	bilirubin c	Acute Kidney Injury	Acetaminophen,Ambrise		T=0, C=56	T=0.0, C=1.0	0.00001	832.086	56	T=0/0, C=35/2
chr8:133960991	T/T	C	T	SNV	TG	TG:intronic:NM 003235.4	1	100		intronic	NM 00323	c.5234-30	p.?	-2.33					Carboxyleste	rs2246624	0.2148	YES	8.066E-1;0.0 (pos.133960991)		Thyroglobul	extracellu	Autoimmune Diseases;Bone Diseases;Bone I			C=0, T=53	C=0.0, T=1.0	0.00001	782.02	53	C=0/0, T=30/2
chr4:69795626	T/T	C	T	SNV	UGT2A3	UGT2A3:exonic:NM 024743.3	1	100	6	missense	NM 02474	c.1489G>A	p.Ala497Thr	-0.15	0.31	58	0.63	0.04(COSM54	Glycosyltrans	rs13128286	0.1414	YES	6.792E-1 (pos.69795626)	acute_myeloi	Uridine dipl	cellular glucuronidation,glucuronosyltransferase activit				C=0, T=20	C=0.0, T=1.0	0.00001	263.776	20	C=0/0, T=11/9
chr8:134225396	TC/TC	T	TC	INDEL	WISP1	WISP1:intronic:NM 003882.3	1	100		intronic	NM 00388	c.349+10T	p.?	0.81					Cystine-knot	rs55733525	1.00E-04		1.0 (pos.134225396)		Wnt-1 indu	Wnt signaling pathway,cell adhesion,cell-cell signaling,c				T=0, TC=29	T=0.0, TC=1.0	0.00001	398.6	29	T=0/0, TC=17/
chr4:69687987	A/A	C	A	SNV	UGT2B10	UGT2B10:splicesite 3:NM 001075.5	1	100	3	utr 3	NM 00107	c.868-2C>	p.?	4.54					Glycosyltrans	rs2942857	0.003	YES	9.127E-1 (pos.69687987)		Uridine dipl	endoplasr	Melanoma:Neoplasms:Neoplasms by Histolc			C=0, A=45	C=0.0, A=1.0	0.00001	747.393	45	C=0/0, A=19/2
chr8:135595944	T/C	T	C	SNV	ZFAT	ZFAT:intronic:NM 020863.3	1	50		intronic	NM 02086	c.2887+13	p.?	-0.71					C2H2-type zir	rs7825462		YES				DNA bindi	Autoimmune Diseases:Cardiovascular Disea			T=22, C=22	T=0.5, C=0.5	0.00001	243.629	44	T=15/7, C=13/
chr8:135612595	G/G	A	G	SNV	ZFAT,ZFAT-AS1	ZFAT:intronic:NM 020863.3, ZFAT-AS1:exonic_nc:NR 00243	1	100		intronic	NM 02086	c.2475+84	p.?	-0.34					C2H2-type zir	rs894343	0.2923	YES	9.142E-1 (pos.135612595)			DNA bindi	Autoimmune Diseases:Cardiovascular Disea			A=0, G=24	A=0.0, G=1.0	0.00001	334.792	24	A=0/0, G=7/17
chr8:136561153	G/G	A	G	SNV	KHDRBS3	KHDRBS3:intronic:NM 006558.2	1	100		intronic	NM 00655	c.471+10A	p.?	-1.87					KH domain,Q	rs11166602	0.3699	YES	7.945E-1;2.343E-3 (pos.136561153)		KH domain-	RNA binding,SH3 domain binding,identical protein bindir				A=0, G=25	A=0.0, G=1.0	0.00001	352.983	25	A=0/0, G=18/7
chr4:69513007	G/A	G	A	SNV	UGT2B15	UGT2B15:exonic:NM 001076.3	1	50	6	missense	NM 00107	c.1408C>T	p.Arg470Cys	0.15	0.01	180	0.862		Glycosyltrans	rs147164238	0.0066		0.0 (pos.69513007)		UDP-glucur	cellular gl	Genital Diseases, N	Acetaminophen,Dabigat		G=25, A=25	G=0.5, A=0.5	0.00001	288.801	50	G=14/11, A=1
chr8:139617046	C/C	A	C	SNV	COL22A1	COL22A1:intronic:NM 152888.2	1	100		intronic	NM 15288	c.4105-34	p.?	-0.74					Collagen tripl	rs12546923	0.2244	YES	6.633E-1;2.591E-4 (pos.139617046)		Collagen, ty	collagen t	Bronchial Neoplasms;Carcinoma, Bronchoge			A=0, C=92	A=0.0, C=1.0	0.00001	1537.97	92	A=0/0, C=52/4
chr8:139620049	G/G	T	G	SNV	COL22A1	COL22A1:intronic:NM 152888.2	1	100		intronic	NM 15288	c.4032+13	p.?	1.26					Collagen tripl	rs12681582		YES			Collagen, ty	collagen t	Bronchial Neoplasms;Carcinoma, Bronchoge			T=0, G=81	T=0.0, G=1.0	0.00001	1309.48	81	T=0/0, G=36/4
chr8:139626176	G/G	C	G	SNV	COL22A1	COL22A1:intronic:NM 152888.2	1	100		intronic	NM 15288	c.3925-13	p.?	-0.66					Collagen tripl	rs7000261,rs748431	0.0644	YES	0.0;0.0 (pos.139626172),7.948E-1;0.0 (p		Collagen, ty	collagen t	Bronchial Neoplasms;Carcinoma, Bronchoge			C=0, G=54	C=0.0, G=1.0	0.00001	798.129	54	C=0/0, G=32/2
chr8:139767349	T/C	T	C	SNV	COL22A1	COL22A1:intronic:NM 152888.2	1	57.89		intronic	NM 15288	c.2031+51	p.?	0.15					Collagen tripl	rs2318338	0.1844	YES	9.796E-1;0.0 (pos.139767349)		Collagen, ty	collagen t	Bronchial Neoplasms;Carcinoma, Bronchoge			T=16, C=22	T=0.4211, C=0.5789	0.00001	249.051	38	T=9/7, C=10/1
chr8:141549291	G/G	A	G	SNV	AGO2	AGO2:intronic:NM 012154.4	1	100		intronic	NM 01215	c.2169+12	p.?	-2.93					Argonaute lir	rs2977469		YES			Argonaute 2	P-body,RIS	Cocaine-Related Disorders;Leukemia:Leuker			A=0, G=38	A=0.0, G=1.0	0.00001	603.772	38	A=0/0, G=17/2
chr8:141549346	C/C	CT	C	INDEL	AGO2	AGO2:intronic:NM 012154.4	1	100		intronic	NM 01215	c.2169+73	p.?	-2.1, -1.03					Argonaute lir	rs35387146		YES			Argonaute 2	P-body,RIS	Cocaine-Related Disorders;Leukemia:Leuker			CT=0, C=41	CT=0.0, C=1.0	0.00001	642.557	41	CT=0/0, C=18/
chr8:141679817	C/C	T	C	SNV	PTK2	PTK2:intronic:NM 005607.4	1	100		intronic	NM 00560	c.2776-12	p.?	0.18				0.25(COSM54	FERM central	rs10283368		YES	3.646E-1 (pos.14167981	carcinoma	PTK2 protein tyrosine k		Adenocarcinoma;A	2-({5-CHLORO-2-(2-METH		T=0, C=40	T=0.0, C=1.0	0.00001	644.281	40	T=0/0, C=7/33
chr8:142202856	T/T	C	T	SNV	DENND3	DENND3:intronic:NM 014957.3	1	100		intronic	NM 01495	c.3396+94	p.?	-0.43					DENN (AEX-3)	rs6990186		YES	6.519E-1 (pos.142202856)		DENN doma	Rab guanyl-nucleotide exchange factor activity,cellular				C=0, T=20	C=0.0, T=1.0	0.00001	263.757	20	C=0/0, T=7/13
chr4:115544714	G/G	A	G	SNV	UGT8	UGT8:exonic:NM 001128174.2	1	100	2	synonymous	NM 00112	c.678A>G	p.(=)	-2.51					Glycosyltrans	rs6851610,rs878976	0.0014		9.997E-1 (pos.115544714)		UDP glycos	N-acylsphingosine galactosyltransferase activity,UDP-ga				A=0, G=43	A=0.0, G=1.0	0.00001	705.839	43	A=0/0, G=22/2
chr6:34803946	CT/CT	C	CT	INDEL	UHRF1BP1	UHRF1BP1:exonic:NM 017754.3	1	100	8	frameshiftInsertion	NM 01775	c.854 855	p.Ser287fs	5.36					N-terminal region of Chorein or VPS13							histone de	Autoimmune Diseases:Connective Tissue Dis			C=0, CT=31	C=0.0, CT=1.0	0.00001	397.908	31	C=0/0, CT=16/
chr6:34824636	G/G	A	G	SNV	UHRF1BP1	UHRF1BP1:exonic:NM 017754.3	1	100	11	missense	NM 01775	c.1361A>G	p.Gln454Arg	2.54	1	43	0	0.22(COSM37	N-terminal re	rs11755393	0.3499	YES	4.523E-1 (pos.34824636)	adenocarcinoma,neoplasn		histone de	Autoimmune Diseases:Connective Tissue Dis			A=0, G=76	A=0.0, G=1.0	0.00001	1209.6	76	A=0/0, G=50/2
chr8:142437815	G/A	G	A	SNV	PTP4A3	PTP4A3:intronic:NM 032611.2	1	50		intronic	NM 03261	c.330-35G	p.?	1.14					Dual specifici	rs7465455	0.1546	YES	1.424E-1 (pos.142437815)		Protein-tyr	Notch sign	Colonic Diseases;Colonic Neoplasms;Colore			G=12, A=12	G=0.5, A=0.5	0.00001	106.76	24	G=4/8, A=3/9
chr8:142437934	C/T	C	T	SNV	PTP4A3	PTP4A3:intronic:NM 032611.2	1	57.14		intronic	NM 03261	c.404+10C	p.?	-0.64					Dual specifici	rs7465654	0.1586	YES	1.483E-1 (pos.142437934)		Protein-tyr	Notch sign	Colonic Diseases:Colonic Neoplasms:Colore			C=9, T=12	C=0.4286, T=0.5714	0.00001	109.453	21	C=1/8, T=6/6
chr8:142444780	C/C	G	C	SNV	MROH5	MROH5:intronic:NM 207414.2	1	100		intronic	NM 20741	c.3782-55	p.?	-0.31						rs10104806	0.3862	YES								G=0, C=21	G=0.0, C=1.0	0.00001	281.231	21	G=0/0, C=17/4
chr6:34831856	C/C	T	C	SNV	UHRF1BP1	UHRF1BP1:exonic:NM 017754.3	1	100	15	missense	NM 01775	c.3293T>C	p.Met1098Thr	6.83	0	81	0.835		N-terminal re	rs13205210	0.1112	YES	1.144E-1 (pos.34831856)			histone de	Autoimmune Diseases:Connective Tissue Dis			T=0, C=49	T=0.0, C=1.0	0.00001	831.63	49	T=0/0, C=21/2
chr12:100452027	C/C	T	C	SNV	UHRF1BP1L	UHRF1BP1L:exonic:NM 015054.1	1	100	14	missense	NM 01505	c.3028A>G	p.Ile1010Val	0.53	1	29	0	0.18(COSM37	N-terminal re	rs60592197	0.067	YES	0.0 (pos.100452027)	adenocarcinoma			Arthritis;Arthritis, Juvenile;Autoimmune Dise			T=0, C=29	T=0.0, C=1.0	0.00001	427.601	29	T=0/0, C=13/1
chr8:143560895	A/A	G	A	SNV	ADGRB1	ADGRB1:intronic:NM 001702.2	1	100		intronic	NM 00170	c.1726+47	p.?	-1					7 transmemb	rs12682337	0.1818	YES	7.581E-1 (pos.143560895)		Adhesion G	G-protein coupled receptor activity,G-protein coupled re				G=0, A=23	G=0.0, A=1.0	0.00001	316.747	23	G=0/0, A=9/14
chr8:143560898	C/C	T	C	SNV	ADGRB1	ADGRB1:intronic:NM 001702.2	1	100		intronic	NM 00170	c.1726+50	p.?	-3.12					7 transmemb	rs12680973	0.1693	YES	7.941E-1 (pos.143560898)		Adhesion G	G-protein coupled receptor activity,G-protein coupled re				T=0, C=23	T=0.0, C=1.0	0.00001	316.737	23	T=0/0, C=9/14
chr8:143560996	A/A	AGCCAGCT	A	INDEL	ADGRB1	ADGRB1:intronic:NM 001702.2	7	100		intronic	NM 00170	c.1727-58	p.?	-0.55, 0.01, 0.5, -0.34, -0.11, 0.45, -1.02, 0.1					7 transmemb	rs113565610,rs373430969,rs60		YES			Adhesion G	G-protein coupled receptor activity,G-protein coupled re				AGCCAGCT	AGCCAGCT=0.0, A=1.	0.00001	298.245	22	AGCCAGCT=0/
chr6:150263186	C/C	A	C	SNV	ULBP2	ULBP2:utr 5:NM 025217.3	1	100	1	utr 5	NM 02521	c.-23A>C	p.?	-5.16					Class I Histoc	rs4343924	0.0324	YES	1.0 (pos.150263186)		UL16-bindir	C-termina	Digestive System Diseases;Digestive System			A=0, C=28	A=0.0, C=1.0	0.00001	408.701	28	A=0/0, C=18/1
chr6:150267527	C/C	A	C	SNV	ULBP2	ULBP2:exonic:NM 025217.3	1	100	3	synonymous	NM 02521	c.369A>C	p.(=)	-0.55				0(COSM3761	Class I Histoc	rs2282235		YES	8.237E-1 (pos.15026752	adenocarcinoi	UL16-bindir	C-termina	Digestive System Diseases;Digestive System			A=0, C=128	A=0.0, C=1.0	0.00001	2094.73	128	A=0/0, C=71/5
chr8:143857265	T/T	G	T	SNV	LYNX1	LYNX1:intronic:NM 177477.3	1	100		intronic	NM 17747	c.52+59C>	p.?	-2.09					Snake toxin a	rs2585125	0	YES			Lynx1, mou	acetylcholine receptor binding,acetylcholine receptor re				G=0, T=47	G=0.0, T=1.0	0.00001	788.844	47	G=0/0, T=22/2
chr8:143922788	C/T	C	T	SNV	GML	GML:intronic:NM 002066.2	1	56		intronic	NM 00206	c.181+147	p.?	0.02					u-PAR/Ly-6 dc	rs3802226		YES			Glycosylphc	DNA damage response, signal transduction by p53 class				C=11, T=14	C=0.44, T=0.56	0.00001	139.189	25	C=10/1, T=6/8
chr12:132401565	CT/CT	C	CT	INDEL	ULK1	ULK1:exonic:NM 003565.2	1	100	21	frameshiftInsertion	NM 00356	c.2140 21	p.Pro714fs	0.82					Domain of unknown function (DUF3543),Lipopolysaccharide kinase (Kdo/WaaP) family,Protein kinas						UNC51-like	ATG1/ULK1 kinase complex,ATP binding,GTPase binding,				C=0, CT=35	C=0.0, CT=1.0	0.00001	448.582	35	C=0/0, CT=5/3
chr12:132403161	G/G	A	G	SNV	ULK1	ULK1:exonic:NM 003565.2	1	100	23	missense	NM 00356	c.2446A>G	p.Thr816Ala	-0.28	0.44	58	0	0.03(COSM41	Domain of un	rs11609348	4.00E-04		1.0 (pos.132403161)	neoplasm	UNC51-like	ATG1/ULK1 kinase complex,ATP binding,GTPase binding,				A=0, G=39	A=0.0, G=1.0	0.00001	628.362	39	A=0/0, G=12/2
chr15:75131661	G/G	A	G	SNV	ULK3	ULK3:exonic:NM 001099436.3	1	100	8	synonymous	NM 00109	c.906T>C	p.(=)	-6.47				0.04(COSM11	MIT (microtul	rs4886615	0.286	YES	6.978E-1 (pos.75131661)	adenocarcinoi	UNC51-like	ATP binding,autophagy,cellular senescence,ciliary tip,cy				A=0, G=39	A=0.0, G=1.0	0.00001	623.616	39	A=0/0, G=25/1
chr8:144398029	G/G	A	G	SNV	TOP1MT	TOP1MT:intronic:NM 052963.2	1	100		intronic	NM 05296	c.1459-38	p.?	-1.83					C-terminal to	rs13275896	0.4351	YES	7.73E-1 (pos.144398029)		Topoisomer	DNA binding,DNA replication,D		Irinotecan,Topotecan		A=0, G=33	A=0.0, G=1.0	0.00001	508.881	33	A=0/0, G=15/1
chr8:144399885	CT/CT	AG	CT	MNV	TOP1MT	TOP1MT:intronic:NM 052963.2	2	100		intronic	NM 05296	c.1330+8C	p.?	-0.94, -0.92					C-terminal to	rs2272636,rs227263	0.4501	YES	0.0 (pos.144399880),7.582E-1 (pos.1443		Topoisomer	DNA binding,DNA replication,D		Irinotecan,Topotecan		AG=0, CT=4	AG=0.0, CT=1.0	0.00001	789.328	47	AG=0/0, CT=23
chr8:144406881	A/A	G	A	SNV	TOP1MT	TOP1MT:intronic:NM 052963.2	1	100		intronic	NM 05296	c.672-82C	p.?	-2.29					C-terminal to	rs12680498		YES			Topoisomer	DNA binding,DNA replication,D		Irinotecan,Topotecan		G=0, A=20	G=0.0, A=1.0	0.00001	263.786	20	G=0/0, A=5/15
chr8:144463686	T/T	C	T	SNV	RHPN1	RHPN1:intronic:NM 052924.2	1	100		intronic	NM 05292	c.1489-56	p.?	-0.72					BRO1-like do	rs3817720,rs533319215		YES			Rhophilin 1	cytosol,pr	Adnexal Diseases;Cysts;Endocrine System Di			C=0, T=31	C=0.0, T=1.0	0.00001	465.812	31	C=0/0, T=12/1
chr8:144657152	C/G	C	G	SNV	NAPRT	NAPRT:intronic:NM 145201.5	1	59.09		intronic	NM 14520	c.1554+4G	p.?	-0.45						rs77951814	0.0657	YES	1.938E-1 (pos.144657152)			NAD biosynthesis via nicotinamide riboside salvage path				C=45, G=65	C=0.4091, G=0.5909	0.00001	715.086	110	C=26/19, G=4
chr8:144660523	G/G	A	G	SNV	NAPRT	NAPRT:upstream:NM 145201.5	1	100		intronic	NM 14520	c.-35T>C	p.?	0.03						rs4475500,rs775807901,rs7968		YES	(pos.144660515), (pos.144660523)			NAD biosynthesis via nicotinamide riboside salvage path				A=0, G=25	A=0.0, G=1.0	0.00001	359.839	25	A=0/0, G=13/1
chr3:41925423	C/C	T	C	SNV	ULK4	ULK4:exonic:NM 017886.3	1	100	17	synonymous	NM 01788	c.1599A>G	p.(=)	1.81					Protein kinas	rs1716687	0.0046	YES	1.0 (pos.41925423)		Unc51-like	ATP bindin	Blood Protein Disorders:Cardic		Benign	T=0, C=31	T=0.0, C=1.0	0.00001	465.795	31	T=0/0, C=20/1
chr8:144695176	C/T	C	T	SNV	TSTA3	TSTA3:intronic:NM 003313.3	1	50		intronic	NM 00331	c.911-41G	p.?	0.21					GDP-mannos	rs2242087	0.1876	YES	5.678E-1 (pos.144695176)		Tissue-spec	'de novo' GDP-L-fucose biosynt		NADH		C=17, T=17	C=0.5, T=0.5	0.00001	172.085	34	C=1/16, T=11/
chr16:20360101	A/A	G	A	SNV	UMOD	UMOD:exonic:NM 001008389.2	1	100	3	synonymous	NM 00100	c.522C>T	p.(=)	2.47				0.85(COSM37	Calcium-bind	rs7193058	0.2322	YES	3.545E-1 (pos.20360101)	adenocarcinoi	Uromodulir	Golgi lume	Arthritis;Cardiovascular Disea		Benign	G=0, A=27	G=0.0, A=1.0	0.00001	389.988	27	G=0/0, A=19/8
chr21:43531008	C/C	T	C	SNV	UMODL1	UMODL1:exonic:NM 173568.3	1	100	11	missense	NM 17356	c.1676T>C	p.Met559Thr	-0.21	1	81	0		Calcium-bind	rs220126,rs7969960	0.2009	YES	8.798E-1;0.0 (pos.43531008)		Uromodulir	adipose ti	Nutrition Disorders;Nutritional and Metaboli			T=0, C=40	T=0.0, C=1.0	0.00001	644.268	40	T=0/0, C=17/2
chr21:43531632	C/C	T	C	SNV	UMODL1	UMODL1:exonic:NM 173568.3	1	100	11	missense	NM 17356	c.2300T>C	p.Ile767Thr	-0.31	1	89	0		Calcium-bind	rs220129	0.1854	YES	8.595E-1 (pos.43531632)		Uromodulir	adipose ti	Nutrition Disorders;Nutritional and Metaboli			T=0, C=45	T=0.0, C=1.0	0.00001	747.367	45	T=0/0, C=21/2
chr8:144873666	C/C	G	C	SNV	SCRIB	SCRIB:intronic:NM 182706.4	1	100		intronic	NM 18270	c.4771-56	p.?	-1.97					Leucine rich	rs9314416	0.0799	YES			Scribble, Dr	GDP metabolic process,GMP metabolic process,Scrib-AP				G=0, C=28	G=0.0, C=1.0	0.00001	398.261	28	G=0/0, C=16/1
chr8:144874366	C/C	T	C	SNV	SCRIB	SCRIB:intronic:NM 182706.4	1	100		intronic	NM 18270	c.4514+24	p.?	-4.1					Leucine rich	rs7461245	7.00E-04		9.999E-1 (pos.144874366)		Scribble, Dr	GDP metabolic process,GMP metabolic process,Scrib-AP				T=0, C=24	T=0.0, C=1.0	0.00001	334.751	24	T=0/0, C=15/9
chr21:43547788	C/C	T	C	SNV	UMODL1	UMODL1:exonic:NM 173568.3	1	100	19	synonymous	NM 17356	c.3921T>C	p.(=)	-4.49					Calcium-bind	rs220158	0.0816	YES	7.839E-1;0.0 (pos.43547788)		Uromodulir	adipose ti	Nutrition Disorders;Nutritional and Metaboli			T=0, C=49	T=0.0, C=1.0	0.00001	821.296	49	T=0/0, C=32/1
chr8:144887656	G/G	A	G	SNV	SCRIB	SCRIB:intronic:NM 182706.4	1	100		intronic	NM 18270	c.2347-51	p.?	-0.62					Leucine rich	rs7830442	0.1175	YES	6.818E-1 (pos.144887656)		Scribble, Dr	GDP metabolic process,GMP metabolic process,Scrib-AP				A=0, G=36	A=0.0, G=1.0	0.00001	563.741	36	A=0/0, G=9/27
chr21:43510437	A/G	A	G	SNV	UMODL1	UMODL1:exonic:NM 173568.3	1	50	6	missense	NM 17356	c.820A>G	p.Asn274Asp	1.86	0.01	23	0.983	0.16(COSM14	Calcium-bind	rs17114359	0.207	YES	3.492E-4;3.488E-1 (pos.4	acute_myeloi	Uromodulir	adipose ti	Nutrition Disorders;Nutritional and Metaboli			A=122, G=1	A=0.5, G=0.5	0.00001	1353.23	244	A=70/52, G=6
chr8:144894641	T/T	C	T	SNV	MIR937,SCRIB	MIR937:downstream:NR 030633.1, SCRIB:intronic:NM 182	1	100		intronic	NM 18270	c.788-87G	p.?	-6.14					Leucine rich	rs4875061		YES			Scribble, Dr	GDP metabolic process,GMP metabolic process,Scrib-AP				C=0, T=23	C=0.0, T=1.0	0.00001	316.747	23	C=0/0, T=12/1
chr2:210843383	A/A	G	A	SNV	UNC80	UNC80:exonic:NM 032504.1	1	100	58	synonymous	NM 03250	c.8871G>A	p.(=)	1.47				0.86(COSM56	Cation chann	rs2075117	0.3743	YES	6.205E-1 (pos.21084338	carcinoma,du	Unc-80 hor	integral component of membrane,ion transmembrane t				G=0, A=65	G=0.0, A=1.0	0.00001	1000.27	65	G=0/0, A=25/4
chr6:167728774	C/C	T	C	SNV	UNC93A	UNC93A:exonic:NM 018974.3	1	100	8	missense	NM 01897	c.1208T>C	p.Met403Thr	0.53	1	81	0	0.01(COSM37	Ion channel r	rs663606,rs71545154,rs879596		YES	7.755E-1 (pos.16772877	adenocarcinoi	UNC93, C. e	biological_process,integral component of membrane,m				T=0, C=37	T=0.0, C=1.0	0.00001	583.698	37	T=0/0, C=10/2
chr6:167717440	C/T	C	T	SNV	UNC93A	UNC93A:exonic:NM 018974.3	1	58.33	5	missense	NM 01897	c.659C>T	p.Ala220Val	1.98	1	64	0.001	0(COSM1442	Ion channel r	rs34838751	2.00E-04		0.0 (pos.167717440)	adenocarcinoi	UNC93, C. e	biological_process,integral component of membrane,m				C=10, T=14	C=0.4167, T=0.5833	0.00001	135.29	24	C=2/8, T=7/7

chr7:1275651	C/T	C	T	SNV	UNCX	UNCX:exonic:NM_001080461.2	1	45.45	3	synonymous	NM_00108	c.634C>T	p.(=)	1.66					Homeobox d	rs151119092	2.00E-04		0.0 (pos.1275651)			cartilage condensation,common myeloid progenitor cell				C=12, T=10	C=0.5455, T=0.4545	0.00001	81.7625	22	C=12/0, T=5/5
chr16:1447278	G/G	A	G	SNV	UNKL	UNKL:exonic:NM_001193388.3	1	100	6	synonymous	NM_00119	c.753T>C	p.(=)	0.26				0.41(COSM37	Zinc finger C-	rs61738727		YES	1.81E-1 (pos.1447264),8.	adenocarcino	Unkempt fa	cytosol,metal ion binding,nucleus,protein binding,prote				A=0, G=21	A=0.0, G=1.0	0.00001	281.234	21	A=0/0, G=8/13
chr8:145008342	C/A	C	A	SNV	PLEC	PLEC:intronic:NM_201380.3	1	52.38		intronic	NM_20138	c.1581-83	p.?	-1.75					Calponin hon	rs11782331		YES			Plectin 1	RNA bindir	Congenital Abnormalities;Congenital, Heredi			C=10, A=11	C=0.4762, A=0.5238	0.00001	97.0749	21	C=8/2, A=7/4
chr19:18942888	C/G	C	G	SNV	UPF1	UPF1:utr_5:NM_002911.3	1	50	1	utr_5	NM_00291	c.-131C>G	p.?	0.25					AAA domain,I	rs4808161		YES			Upf1, yeast	3'-UTR-mediated mRNA destabilization,ATP binding,ATP-				C=29, G=29	C=0.5, G=0.5	0.00001	281.645	58	C=19/10, G=19
chrX:74494470	G/G	T	G	SNV	UPRT	UPRT:exonic:NM_145052.3	1	100	1	synonymous	NM_14505	c.381T>G	p.(=)	1.14				0.07(COSM41	Uracil phospl	rs4892396	0.001	YES	1.0 (pos.74494470)	neoplasm	Uracil phos	GTP binding,UMP biosynthetic process,cytosol,female p				T=0, G=41	T=0.0, G=1.0	0.00001	664.7	41	T=0/0, G=19/2
chr8:145234508	C/C	G	C	SNV	MROH1	MROH1:intronic:NM_032450.2	1	100		intronic	NM_03245	c.300+19G	p.?	2.36					HEAT repeat,	rs7842771	0.0268	YES	9.922E-1;0.0 (pos.145234508)							G=0, C=39	G=0.0, C=1.0	0.00001	623.979	39	G=0/0, C=6/33
chr8:145649781	T/T	A	T	SNV	VPS28	VPS28:intronic:NM_183057.2	1	100		intronic	NM_18305	c.403-130	p.?	-2.52					VPS28 protei	rs2928377		YES			Vacuolar pr	ESCRT I complex,cytoplasm,cytosol,early endosome,end				A=0, T=32	A=0.0, T=1.0	0.00001	485.138	32	A=0/0, T=14/1
chr8:145690111	CCTCT/C	CCTCT	C	INDEL	CYHR1	CYHR1:intronic:NM_138496.1	4	53.13		intronic	NM_13849	c.93+81AG	p.?	-0.01, -0.23, 0.16, -0.35, 0.2						rs112453572,rs201276388,rs35		YES			Cysteine-ai	integral component of membrane,nucleoplasm,perinucl				CCTCT=15,	CCTCT=0.4688, C=0.5	0.00001	171.807	32	CCTCT=12/3, C
chr21:33694224	G/G	C	G	SNV	URB1	URB1:exonic:NM_014825.2	1	100	34	missense	NM_01482	c.5371G>C	p.Val1791Leu	-4.22	1	32	0	0.02(COSM14	Nucleolar pre	rs3761342	0.0723	YES	7.663E-1 (pos.33694224)	adenocarcino	URB1 ribos	RNA bindir	Abnormalities, Multiple;Chromosome Disord			C=0, G=20	C=0.0, G=1.0	0.00001	263.593	20	C=0/0, G=7/13
chr1:229772693	G/G	T	G	SNV	URB2	URB2:exonic:NM_014777.3	1	100	4	missense	NM_01477	c.2333T>G	p.Val778Gly	-0.57	0.82	109	0	0.03(COSM50	Urb2/Npa2 fa	rs3811473	0.4872	YES	7.64E-1 (pos.229772693)	ductal_carcinoma		aggresome,midbody,nucleolus,ribosome biogenesis				T=0, G=31	T=0.0, G=1.0	0.00001	465.564	31	T=0/0, G=9/22
chr1:229795070	G/G	T	G	SNV	URB2	URB2:utr_3:NM_014777.3	1	100	10	utr_3	NM_01477	c.4601T>G	p.?	-1.51					Urb2/Npa2 fa	rs1048306	0.4841	YES	8.567E-1 (pos.229795070)			aggresome,midbody,nucleolus,ribosome biogenesis				T=0, G=33	T=0.0, G=1.0	0.00001	504.598	33	T=0/0, G=7/26
chr8:145699601	C/C	T	C	SNV	FOXH1,KIFC2	KIFC2:downstream:NM_145754.3, FOXH1:utr_3:NM_00392	1	100		intronic	NM_14575	c.*768T>C	p.?, p.?	-1.52					Forkhead dor	rs2721176,rs763529	0.0011	YES	0.0 (pos.145699597),7.93E-1 (pos.14569		Kinesin fam	ATP bindin	Abnormalities, Multiple;Agene		Benign	T=0, C=20	T=0.0, C=1.0	0.00001	254.048	20	T=0/0, C=10/1
chr19:17330060	C/C	T	C	SNV	USE1	USE1:exonic:NM_018467.3	1	100	7	missense	NM_01846	c.461T>C	p.Leu154Ser	4.78	1	145	0		Membrane fu	rs414528	2.00E-04		9.246E-1 (pos.17330060)		Ubiquitin-c	ER to Golgi vesicle-mediated transport,endoplasmic reti				T=0, C=34	T=0.0, C=1.0	0.00001	524.184	34	T=0/0, C=17/1
chr11:17515833	G/G	A	G	SNV	USH1C	USH1C:utr_3:NM_153676.3	1	100	27	utr_3	NM_15367	c.2746T>C	p.?	-0.17					PDZ domain (	rs1055574	0.4718	YES	3.338E-1 (pos.17515833)		Harmonin (I	G2/M tran	Abnormalities, Multiple;Blindr		Benign/Likely	A=0, G=34	A=0.0, G=1.0	0.00001	515.181	34	A=0/0, G=27/7
chr1:216348764	T/T	C	T	SNV	USH2A	USH2A:exonic:NM_206933.2	1	100	21	missense	NM_20693	c.4457G>A	p.Arg1486Lys	-1.63	0.84	26	0.094	0.03(COSM37	Concanavalir	rs1805049	0.3883	YES	7.839E-1 (pos.21634876	adenocarcino	Usherin	USH2 com	Abnormalities, Multiple;Blindr		Benign	C=0, T=33	C=0.0, T=1.0	0.00001	504.597	33	C=0/0, T=18/1
chr1:216595306	T/T	C	T	SNV	USH2A	USH2A:exonic:NM_206933.2	1	100	2	missense	NM_20693	c.373G>A	p.Ala125Thr	0.35	0.86	58	0		Concanavalir	rs10779261	0.3009	YES	8.348E-1 (pos.216595306)		Usherin	USH2 com	Abnormalities, Multiple;Blindr		Benign	C=0, T=29	C=0.0, T=1.0	0.00001	432.444	29	C=0/0, T=15/1
chr1:215901492	C/T	C	T	SNV	USH2A	USH2A:exonic:NM_206933.2	1	50	61	synonymous	NM_20693	c.11946G>	p.(=)	1.01					Concanavalir	rs2820718	0.1845	YES	1.388E-3 (pos.215901492)		Usherin	USH2 com	Abnormalities, Multiple;Blindr		Benign	C=14, T=14	C=0.5, T=0.5	0.00001	131.938	28	C=8/6, T=3/11
chr19:17366278	C/C	T	C	SNV	USHBP1	USHBP1:exonic:NM_031941.3	1	100	10	synonymous	NM_03194	c.1608A>G	p.(=)	-3.37				0.02(COSM41	PDZ domain c	rs4430871	0.397	YES	8.2E-1 (pos.17366278)	acute_myeloid	USH1C-bind	PDZ domain binding,protein binding				T=0, C=27	T=0.0, C=1.0	0.00001	389.986	27	T=0/0, C=12/1
chr8:1514170	C/C	G	C	SNV	DLGAP2,DLGAP2-AS1	DLGAP2-AS1:intronic_nc:NR_103863.1, DLGAP2:intronic:NM	1	100		intronic	NM_00134	c.1442+11	p.?	-0.02					Guanylate-ki	rs2301964		YES			Discs large associated		Autistic Disorder;Child Development Disorde			G=0, C=53	G=0.0, C=1.0	0.00001	782.03	53	G=0/0, C=28/2
chr8:1575196	C/C	G	C	SNV	DLGAP2	DLGAP2:intronic:NM_001346810.1	1	100		intronic	NM_00134	c.1590+14	p.?	2.67					Guanylate-ki	rs35821406		YES			Discs large associated		Autistic Disorder;Child Development Disorde			G=0, C=46	G=0.0, C=1.0	0.00001	766.587	46	G=0/0, C=17/2
chr8:1581226	G/G	A	G	SNV	DLGAP2	DLGAP2:intronic:NM_001346810.1	1	100		intronic	NM_00134	c.1810+14	p.?	0					Guanylate-ki	rs6995760	0.2255	YES	1.743E-1 (pos.1581226)		Discs large associated		Autistic Disorder;Child Development Disorde			A=0, G=104	A=0.0, G=1.0	0.00001	1629.14	104	A=0/0, G=65/3
chr4:76649924	G/G	C	G	SNV	USO1	USO1:utr_5:NM_003715.3	1	100	1	utr_5	NM_00371	c.-80C>G	p.?	0.89					Uso1 / p115 l	rs2280095		YES			USO1 vesicl	COPII vesi	Arsenic Poisoning;Genital Diseases, Male;Ge			C=0, G=48	C=0.0, G=1.0	0.00001	818.104	48	C=0/0, G=45/3
chr8:17063335	G/G	A	G	SNV	ZDHHC2	ZDHHC2:intronic:NM_016353.4	1	100		intronic	NM_01635	c.597+51A	p.?	-1.28					DHHC palmit	rs2904674	0.276	YES				Golgi appa	Digestive System Diseases;Digestive System			A=0, G=37	A=0.0, G=1.0	0.00001	583.692	37	A=0/0, G=13/2
chr11:119229964	A/A	G	A	SNV	USP2	USP2:exonic:NM_004205.4	1	100	5	synonymous	NM_00420	c.1038C>T	p.(=)	-0.48					Ubiquitin car	rs2241646	0.2935	YES	8.375E-1 (pos.119229964)			cell cortex	Adenocarcinoma;Carcinoma;Carcinoma, Hec			G=0, A=72	G=0.0, A=1.0	0.00001	1130.96	72	G=0/0, A=33/3
chr8:17406409	G/A	G	A	SNV	SLC7A2	SLC7A2:intronic:NM_001164771.1	1	51.85		intronic	NM_00116	c.818+57G	p.?	0.01					Amino acid p	rs144868701		YES			Solute carri	L-arginine import across plasm		L-Lysine,Ornithine		G=13, A=14	G=0.4815, A=0.5185	0.00001	132.666	27	G=4/9, A=5/9
chr8:17417756	GT/GT	TA	GT	MNV	SLC7A2	SLC7A2:intronic:NM_001164771.1	2	100		intronic	NM_00116	c.1419-81	p.?	0.01, -2.02					Amino acid p	rs2301540,rs386723003,rs9792		YES			Solute carri	L-arginine import across plasm		L-Lysine,Ornithine		TA=0, GT=3	TA=0.0, GT=1.0	0.00001	504.598	33	TA=0/0, GT=12
chr11:119252199	T/G	T	G	SNV	USP2,USP2-AS1	USP2-AS1:upstream:NR_034160.1, USP2:utr_5:NM_004205.	1	57.14	1	utr_5	NM_00420	c.-8009A>	p.?	2.29					Ubiquitin car	rs670906		YES	9.646E-1 (pos.119252199)			cell cortex	Adenocarcinoma;Carcinoma;Carcinoma, Hec			T=15, G=20	T=0.4286, G=0.5714	0.00001	218.571	35	T=8/7, G=10/1
chr8:17917346	T/T	C	T	SNV	ASAH1	ASAH1:intronic:NM_004315.5	1	100		intronic	NM_00431	c.834-134	p.?	-1.25					Linear amide	rs10448130		YES			N-acylsphin	catalytic a	Brain Diseases;Brain Diseases, Metabolic;Br			C=0, T=79	C=0.0, T=1.0	0.00001	1268.05	79	C=0/0, T=31/4
chr9:132623868	C/C	T	C	SNV	USP20	USP20:exonic:NM_006676.7	1	100	8	synonymous	NM_00667	c.474T>C	p.(=)	-3.58					DUSP domair	rs10115249	0.0964	YES	9.968E-1 (pos.132623868)		Ubiquitin-s	G-protein coupled receptor binding,centrosome,cystein				T=0, C=34	T=0.0, C=1.0	0.00001	524.18	34	T=0/0, C=23/1
chr8:17920623	T/T	C	T	SNV	ASAH1	ASAH1:intronic:NM_004315.5	1	100		intronic	NM_00431	c.551+71G	p.?	-0.58					Linear amide	rs6586683	0.3504	YES			N-acylsphin	catalytic a	Brain Diseases;Brain Diseases, Metabolic;Br			C=0, T=20	C=0.0, T=1.0	0.00001	263.195	20	C=0/0, T=14/6
chr8:17927294	T/T	C	T	SNV	ASAH1	ASAH1:intronic:NM_004315.5	1	100		intronic	NM_00431	c.351+7G>	p.?	0.35					Linear amide	rs4921834	0.4901	YES	3.688E-1 (pos.17927294)		N-acylsphin	catalytic a	Brain Diseases;Brain Diseases		Benign	C=0, T=38	C=0.0, T=1.0	0.00001	603.786	38	C=0/0, T=14/2
chr17:20924523	A/A	G	A	SNV	USP22	USP22:exonic:NM_015276.1	1	100	3	synonymous	NM_01527	c.321C>T	p.(=)	1.42				0.52(COSM63	Ubiquitin car	rs2291037	0.2075	YES	4.305E-1 (pos.20924523)	carcinoma	Ubiquitin-s	H4 histone acetyltransferase activity,SAGA complex,SAG				G=0, A=36	G=0.0, A=1.0	0.00001	563.744	36	G=0/0, A=16/2
chr8:17930727	A/A	G	A	SNV	ASAH1	ASAH1:intronic:NM_004315.5	1	100		intronic	NM_00431	c.174-182	p.?	0.11					Linear amide	rs6586687	0.4859	YES	3.42E-1;0.0 (pos.17930727)		N-acylsphin	catalytic a	Brain Diseases;Brain Diseases, Metabolic;Br			G=0, A=25	G=0.0, A=1.0	0.00001	353.015	25	G=0/0, A=17/8
chr8:17930734	A/A	C	A	SNV	ASAH1	ASAH1:intronic:NM_004315.5	1	100		intronic	NM_00431	c.174-183	p.?	-0.23					Linear amide	rs7461977	0.48	YES	3.455E-1 (pos.17930734)		N-acylsphin	catalytic a	Brain Diseases;Brain Diseases, Metabolic;Br			C=0, A=25	C=0.0, A=1.0	0.00001	353.018	25	C=0/0, A=17/8
chr8:17930772	T/T	C	T	SNV	ASAH1	ASAH1:intronic:NM_004315.5	1	100		intronic	NM_00431	c.174-187	p.?	-1.05				0.01(COSM37	Linear amide	rs3753115,rs771458	0.4866	YES	0.0 (pos.17930769),3.51	adenocarcino	N-acylsphin	catalytic a	Brain Diseases;Brain Diseases		Benign	C=0, T=27	C=0.0, T=1.0	0.00001	389.988	27	C=0/0, T=17/1
chr8:18430201	T/A	T	A	SNV	PSD3	PSD3:intronic:NM_015310.3	1	50		intronic	NM_01531	c.2640-19	p.?	0.4					PH domain,Pl	rs2277137	0.1634	YES	4.407E-1 (pos.18430201)		Pleckstrin a	ARF guany	Amphetamine-Related Disorders;Glioma;Me			T=16, A=16	T=0.5, A=0.5	0.00001	158.383	32	T=10/6, A=10/
chr11:113679119	T/T	C	T	SNV	USP28	USP28:exonic:NM_020886.3	1	100	18	synonymous	NM_02088	c.2205G>A	p.(=)	-4.77					Tetratricoper	rs2465647	0.1818	YES	8.935E-1;0.0 (pos.113679119)		Ubiquitin-s	DNA damage checkpoint,DNA repair,Ras protein signal t				C=0, T=71	C=0.0, T=1.0	0.00001	1111.48	71	C=0/0, T=26/4
chr19:57642098	A/A	G	A	SNV	USP29	USP29:exonic:NM_020903.2	1	100	4	synonymous	NM_02090	c.2055G>A	p.(=)	0.17				0.02(COSM37	N-terminal of	rs3764574	0.4683	YES	3.374E-1 (pos.57642098)	acute_myeloid leukaemia		cellular_component,protein deubiquitination,thiol-dep				G=0, A=38	G=0.0, A=1.0	0.00001	603.786	38	G=0/0, A=16/2
chr8:20028553	T/C	T	C	SNV	SLC18A1	SLC18A1:intronic:NM_003053.3	1	50		intronic	NM_00305	c.858+61A	p.?	0.01					Major Facilit	rs7828538	0.1879	YES			Solute carri	aminergic neurotransmitter lo		Ephedra,MMDA,Metham		T=37, C=37	T=0.5, C=0.5	0.00001	375.36	74	T=24/13, C=19
chr12:109511256	C/C	T	C	SNV	USP30	USP30:exonic:NM_032663.4	1	100	7	synonymous	NM_03266	c.639T>C	p.(=)	-0.49					Ubiquitin car	rs7967322	0.199	YES	9.761E-1 (pos.109511256)		Ubiquitin-s	autophagy of mitochondrion,cysteine-type endopeptida				T=0, C=26	T=0.0, C=1.0	0.00001	371.056	26	T=0/0, C=15/1
chr12:109520768	CG/CG	C	CG	INDEL	USP30	USP30:exonic:NM_032663.4	1	100	11	frameshiftInsertion	NM_03266	c.1069_10	p.His357fs	4.19					Ubiquitin carboxyl-terminal hydrolase						Ubiquitin-s	autophagy of mitochondrion,cysteine-type endopeptida				C=0, CG=51	C=0.0, CG=1.0	0.00001	409.316	51	C=0/0, CG=29/
chr8:20068163	G/G	T	G	SNV	ATP6V1B2	ATP6V1B2:intronic:NM_001693.3	1	100		intronic	NM_00169	c.463+6T>	p.?	-0.54					ATP synthase	rs7460146	0.0274	YES	9.999E-1 (pos.20068163)		ATPase, H+	ATP bindin	Abnormalities, Mul	4-(2-AMINOETHYL)BENZEI		T=0, G=76	T=0.0, G=1.0	0.00001	1206.25	76	T=0/0, G=17/5
chr8:20110219	G/A	G	A	SNV	LZTS1	LZTS1:intronic:NM_021020.3	1	50		intronic	NM_02102	c.1149+74	p.?	-0.73					Fez1	rs2645385		YES			Leucine zip	DNA bindi	Digestive System Diseases;Digestive System			G=11, A=11	G=0.5, A=0.5	0.00001	94.6067	22	G=5/6, A=1/10
chr12:109520769	G/G	A	G	SNV	USP30	USP30:exonic:NM_032663.4	1	100	11	missense	NM_03266	c.1070A>G	p.His357Arg	-0.01	0.75	29	0	0.02(COSM44	Ubiquitin car	rs16939904	0.0829	YES	1.456E-1 (pos.10952076	acute_myeloid	Ubiquitin-s	autophagy of mitochondrion,cysteine-type endopeptida				A=0, G=51	A=0.0, G=1.0	0.00001	754.579	51	A=0/0, G=29/2
chr8:2046576	G/T	G	T	SNV	MYOM2	MYOM2:intronic:NM_003970.3	1	50		intronic	NM_00397	c.2314-11	p.?	-0.1					Fibronectin t	rs2294065		YES			Myomesin 2	M band,Z disc,actin filament binding,actin filament orga				G=16, T=16	G=0.5, T=0.5	0.00001	158.373	32	G=10/6, T=6/1
chr16:23079353	C/C	T	C	SNV	USP31	USP31:utr_3:NM_020718.3	1	100	16	utr_3	NM_02071	c.4073A>G	p.?	0.44					Ubiquitin car	rs8047644	0.0867	YES	9.134E-1 (pos.23079353)		Ubiquitin-s	nucleus,protein deubiquitination,thiol-dependent ubiqu				T=0, C=26	T=0.0, C=1.0	0.00001	371.391	26	T=0/0, C=12/1
chr8:2048518	G/G	A	G	SNV	MYOM2	MYOM2:intronic:NM_003970.3	1	100		intronic	NM_00397	c.2441-14	p.?	0.69					Fibronectin t	rs3779839		YES			Myomesin 2	M band,Z disc,actin filament binding,actin filament orga				A=0, G=49	A=0.0, G=1.0	0.00001	831.633	49	A=0/0, G=46/3
chr8:2048640	A/A	C	A	SNV	MYOM2	MYOM2:intronic:NM_003970.3	1	100		intronic	NM_00397	c.2441-26	p.?	-0.57					Fibronectin t	rs3779838	0.4313	YES	6.573E-1 (pos.2048640)		Myomesin 2	M band,Z disc,actin filament binding,actin filament orga				C=0, A=51	C=0.0, A=1.0	0.00001	751.577	51	C=0/0, A=42/9
chr8:2063920	CAT/C	CAT	C	INDEL	MYOM2	MYOM2:intronic:NM_003970.3	2	50		intronic	NM_00397	c.3325+24	p.?	-0.96, -3.0, -0.24					Fibronectin t	rs201826780,rs3437	0.4603	YES	0.0 (pos.2063921),2.831E-1 (pos.206392		Myomesin 2	M band,Z disc,actin filament binding,actin filament orga				CAT=16, C=	CAT=0.5, C=0.5	0.00001	158.383	32	CAT=6/10, C=4
chr2:61522123	C/C	T	C	SNV	USP34	USP34:exonic:NM_014709.3	1	100	32	synonymous	NM_01470	c.4422A>G	p.(=)	0.02					Ubiquitin car	rs13014347	0.0178	YES	9.997E-1 (pos.61522123)		Ubiquitin-s	Wnt signaling pathway,cysteine-type endopeptidase act				T=0, C=32	T=0.0, C=1.0	0.00001	485.137	32	T=0/0, C=17/1
chr8:21891968	G/G	C	G	SNV	NPM2	NPM2:intronic:NM_182795.1	1	100		intronic	NM_18279	c.532-53C	p.?	-0.14					Nucleoplasm	rs4871915	0.0248	YES			Nucleophos	chromatin binding,chromatin remodeling,covalent chro				C=0, G=100	C=0.0, G=1.0	0.00001	1702.22	100	C=0/0, G=57/4
chr8:21925214	G/G	C	G	SNV	DMTN	DMTN:intronic:NM_001114136.2	1	100		intronic	NM_00111	c.249+21C	p.?	-0.07					Putative adh	rs6997244	0.1663	YES	9.752E-1 (pos.21925214)			actin binding,actin cytoskeleton,actin cytoskeleton orga				C=0, G=21	C=0.0, G=1.0	0.00001	281.235	21	C=0/0, G=16/5
chr8:21927460	G/G	C	G	SNV	DMTN	DMTN:intronic:NM_001114136.2	1	100		intronic	NM_00111	c.451+12C	p.?	0.02					Putative adh	rs7386762	0.1783	YES	9.753E-1 (pos.21927460)			actin binding,actin cytoskeleton,actin cytoskeleton orga				C=0, G=28	C=0.0, G=1.0	0.00001	408.718	28	C=0/0, G=12/1
chr8:21937870	A/A	G	A	SNV	DMTN	DMTN:intronic:NM_001114136.2	1	100		intronic	NM_00111	c.901-53G	p.?	0.72					Putative adh	rs7835670	0.1565	YES				actin binding,actin cytoskeleton,actin cytoskeleton orga				G=0, A=26	G=0.0, A=1.0	0.00001	371.42	26	G=0/0, A=9/17
chr8:21959191	G/G	A	G	SNV	FAM160B2	FAM160B2:intronic:NM_022749.5	1	100		intronic	NM_02274	c.1708-28	p.?	-5.06					Retinoic acid	rs6557810	5.00E-04	YES	9.999E-1 (pos.21959191)							A=0, G=92	A=0.0, G=1.0	0.00001	1538.77	92	A=0/0, G=47/4
chr2:61575308	G/G	A	G	SNV	USP34	USP34:exonic:NM_014709.3	1	100	15	missense	NM_01470	c.1982T>C	p.Met661Thr	1.3	0.54	81	0		Ubiquitin car	rs6722430	0.0193	YES	9.995E-1 (pos.61575308)		Ubiquitin-s	Wnt signaling pathway,cysteine-type endopeptidase act				A=0, G=28	A=0.0, G=1.0	0.00001	408.717	28	A=0/0, G=13/1
chr8:21973970	A/A	C	A	SNV	HR	HR:intronic:NM_005144.4	1	100		intronic	NM_00514	c.3379-29	p.?	0.85					JmjC domain	rs6557829	0.4071	YES	3.779E-1 (pos.21973970)		Hairless, m	DNA bindi	Alopecia;Hair Diseases;Hypotrichosis;Skin Di			C=0, A=39	C=0.0, A=1.0	0.00001	628.17	39	C=0/0, A=8/31
chr2:61493225	T/A	T	A	SNV	USP34	USP34:exonic:NM_014709.3	1	56	42	synonymous	NM_01470	c.5511A>T	p.(=)	-0.82				0.24(COSM22	Ubiquitin car	rs113399084	0.0307	YES	5.798E-4 (pos.61493225)	diffuse_large	Ubiquitin-s	Wnt signaling pathway,cysteine-type endopeptidase act				T=11, A=14	T=0.44, A=0.56	0.00001	134.348	25	T=5/6, A=5/9
chr11:77921527	C/C	G	C	SNV	USP35	USP35:exonic:NM_020798.3	1	100	10	missense	NM_02079	c.2626G>C	p.Ala876Pro	-0.36	0.57	27	0	0.02(COSM44	Ubiquitin car	rs2512526	0.0391	YES	6.47E-1 (pos.77921527)	acute_myeloid leukaemia		protein deubiquitination,thiol-dependent ubiquitin-spe				G=0, C=33	G=0.0, C=1.0	0.00001	494.247	33	G=0/0, C=4/29
chr8:22020294	A/A	C	A	SNV	SFTPC	SFTPC:intronic:NM_001172410.1	1	100		intronic	NM_00117	c.201+49C	p.?	-0.37					BRICHOS dom	rs2070684	0.0837	YES	7.327E-1;1.278E-3 (pos.22020294)		Surfactant,	cellular pr	Adnexal Diseases;Congenital, Hereditary, an			C=0, A=21	C=0.0, A=1.0	0.00001	281.235	21	C=0/0, A=4/17
chr17:76799860	T/C	T	C	SNV	USP36	USP36:exonic:NM_001321291.1	1	57.14	16	missense	NM_00132	c.2417A>G	p.Gln806Arg	-1.81	0.96	43	0	0.02(COSM37	Ubiquitin car	rs3088040,rs748398	0.4553	YES	6.002E-1;0.0;0.0 (pos.76	adenocarcino	Ubiquitin-s	RNA binding,nuclear speck,nucleolus,positive regulatior				T=12, C=20	T=0.375, C=0.625	0.00001	244.877	35	T=2/10, C=11/
chr8:22424489	G/G	A	G	SNV	SORBS3	SORBS3:intronic:NM_005775.4	1	100		intronic	NM_00577	c.1191-85	p.?	0.05					SH3 domain,S	rs11779261		YES			Sorbin and S	actin filament organization,cell adhesion,cell-substrate				A=0, G=71	A=0.0, G=1.0	0.00001	1111.27	71	A=0/0, G=27/4
chr2:234449316	A/A	G	A	SNV	USP40	USP40:exonic:NM_018218.2	1	100	9	synonymous	NM_01821	c.1195C>T	p.(=)	2.71					Ubiquitin car	rs2167884	0.2123	YES	8.694E-1 (pos.234449316)		Ubiquitin-s	protein deubiquitination,thiol-dependent ubiquitinyl hy				G=0, A=62	G=0.0, A=1.0	0.00001	930.44	62	G=0/0, A=25/3
chr6:99883704	C/C	T	C	SNV	USP45	USP45:exonic:NM_001080481.1	1	100	18	missense	NM_00108	c.2333A>G	p.Asn778Ser	1.69	1	46	0		Ubiquitin car	rs6570065	1.00E-04		1.0;0.0 (pos.99883704)			DNA repair,cytoplasm,global genome nucleotide-excisio				T=0, C=63	T=0.0, C=1.0	0.00001	958.917	63	T=0/0, C=40/2
chr8:22460204	G/G	A	G	SNV	C8orf58	C8orf58:intronic:NM_001013842.2	1	100		intronic	NM_00101	c.986+48A	p.?	0.86					Domain of un	rs34268501	0.3384	YES	2.825E-1 (pos.22460204)							A=0, G=58	A=0.0, G=1.0	0.00001	867.385	58	A=0/0, G=17/4
chr8:22464064	C/C	T	C	SNV	CCAR2	CCAR2:intronic:NM_021174.5	1	100		intronic	NM_02117	c.151-56T	p.?	-2.03					DBC1,S1-like	rs2291232		YES	2.003E-1 (pos.22464064)		Cell divisior	DBIRD complex,RNA binding,RNA polymerase II core bind				T=0, C=25	T=0.0, C=1.0	0.00001	353.019	25	T=0/0, C=14/1
chr6:99883694	T/C	T	C	SNV	USP45	USP45:exonic:NM_001080481.1	1	57.14	18	synonymous	NM_00108	c.2343A>G	p.(=)	1.2				0.84(COSM15	Ubiquitin car	rs9402791	0.189	YES	5.109E-1 (pos.99883694)	acute_myeloid leukaemia		DNA repair,cytoplasm,global genome nucleotide-excisio				T=27, C=36	T=0.4286, C=0.5714	0.00001	379.937	63	T=22/5, C=18/
chr6:99894086	C/G	C	G	SNV	USP45	USP45:exonic:NM_001080481.1	1	50	14	missense	NM_00108	c.1562G>C	p.Arg521Thr	1.77	0.53	71	0.669	0.16(COSM37	Ubiquitin car	rs41288947	0.3294	YES	8.341E-2 (pos.99894086)	acute_myeloid leukaemia		DNA repair,cytoplasm,global genome nucleotide-excisio				C=15, G=15	C=0.5, G=0.5	0.00001	145.025	30	C=10/5, G=11/
chr1:22109370	A/A	G	A	SNV	USP48	USP48:exonic:NM_032236.7	1	100	1	synonymous	NM_03223	c.81C>T	p.(=)	1.73				0.62(COSM54	Ubiquitin car	rs10917042	0.2186	YES	6.977E-1 (pos.22109370)	adenocarcino	Ubiquitin-s	cytosol,mi	Amphetamine-Related Disorders;Mental Dis			G=0, A=68	G=0.0, A=1.0	0.00001	1053.55	68	G=0/0, A=16/5
chr15:50773755	A/A	G	A	SNV	USP8	USP8:exonic:NM_005154.4	1	100	11	synonymous	NM_00515	c.1296G>A	p.(=)	-0.81					Rhodanese-li	rs3131561	0		1.0 (pos.50773755)		Ubiquitin-s	SH3 doma	ACTH-Secreting Pituitary Adenoma;Adenoma			G=0, A=37	G=0.0, A=1.0	0.00001	583.705	37	G=0/0, A=25/1
chr15:50782703	A/G	A	G	SNV	USP8	USP8:exonic:NM_005154.4	1	50	14	missense	NM_00515	c.2215A>G	p.Thr739Ala	-1.08	1	58	0		Rhodanese-li	rs11638390	0.1829	YES	1.875E-3 (pos.50782703)		Ubiquitin-s	SH3 doma	ACTH-Secreting Pituitary Adenoma;Adenoma			A=16, G=16	A=0.5, G=0.5	0.00001	158.383	32	A=6/10, G=11/
chr8:23117643	C/C	T	C	SNV	CHMP7	CHMP7:intronic:NM_152272.4	1	100		intronic	NM_15227	c.1121-14	p.?	-0.08					FLYWCH-type	rs2294125	0.4721	YES	2.967E-2 (pos.23117643)		CHMP famil	ESCRT III complex,ESCRT III complex disassembly,cell sep				T=0, C=61	T=0.0, C=1.0	0.00001	921.853	61	T=0/0, C=35/2
chr12:101750814	A/A	T	A	SNV	UTP20	UTP20:exonic:NM_014503.2	1	100	43	missense	NM_01450	c.5645T>A	p.Leu1882Gln	4.45	0.88	113	0		Down-regula	rs10082778	0.011		9.998E-1 (pos.101750814)		UTP20, S. ce	90S prerib	Alcohol-Related Disorders;Alcoholism;Menta			T=0, A=114	T=0.0, A=1.0	0.00001	1815.31	114	T=0/0, A=71/4
chr8:23167560	C/C	T	C	SNV	LOXL2	LOXL2:intronic:NM_002318.2	1	100		intronic	NM_00231	c.1637-13	p.?	-0.56					Lysyl oxidase	rs12548642,rs934247956		YES			Lysyl oxidas	aging,base	Basal Ganglia Diseases;Bile Duct Diseases;Bi			T=0, C=68	T=0.0, C=1.0	0.00001	1053.56	68	T=0/0, C=31/3
chr8:23174748	A/G	A	G	SNV	LOXL2	LOXL2:intronic:NM_002318.2	1	97.06		intronic	NM_00231	c.1471-12	p.?	0.4					Lysyl oxidase	rs13250587		YES			Lysyl oxidas	aging,base	Basal Ganglia Diseases;Bile Duct Diseases;Bi			A=1, G=33	A=0.0294, G=0.9706	0.00001	492.963	34	A=1/0, G=25/8
chr8:23174823	CCA/CCA	GTG	CCA	MNV	LOXL2	LOXL2:intronic:NM_002318.2	3	100		intronic	NM_00231	c.1471-19	p.?	0.04, -0.61, -0.89					Lysyl oxidase	rs35951329,rs377292868,rs752		YES			Lysyl oxidas	aging,base	Basal Ganglia Diseases;Bile Duct Diseases;Bi			GTG=0, CC	GTG=0.0, CCA=1.0	0.00001	281.179	21	GTG=0/0, CCA
chr3:190999990	T/T	C	T	SNV	UTS2B	UTS2B:utr_5:NM_198152.3	1	100	5	utr_5	NM_19815	c.-12G>A	p.?	0.51					Urotensin II	rs6444535	0.3957	YES	5.715E-1 (pos.190999990)			G-protein coupled receptor binding,G-protein coupled re				C=0, T=119	C=0.0, T=1.0	0.00001	1879.9	119	C=0/0, T=42/7
chr6:30888161	C/C	T	C	SNV	VARS2	VARS2:exonic:NM_001167734.1	1	100	14	missense	NM_00116	c.1435T>C	p.Trp479Arg	1.3	1	101	0	0.26(COSM54	Anticodon-bi	rs2249464,rs759704	0.4599	YES	0.0 (pos.30888151),8.41	acute_myeloid	Valyl-tRNA s	ATP binding,aminoacyl-tRNA editing activi			Benign	T=0, C=23	T=0.0, C=1.0	0.00001	316.747	23	T=0/0, C=18/5
chr14:77236380	T/T	C	T	SNV	VASH1	VASH1:exonic:NM_014909.4	1	100	2	synonymous	NM_01490	c.384C>T	p.(=)	6.02				0.97(COSM37	Vasohibin	rs3815550	0.3829	YES	4.746E-1 (pos.77236380)	acute_myeloid leukaemia		angiogenesis,apical part of cell,cell cycle arrest,cytopla				C=0, T=21	C=0.0, T=1.0	0.00001	281.235	21	C=0/0, T=13/8
chr8:24358850	G/G	C	G	SNV	ADAM7,LOC101929294,LOC101929315	LOC101929315:intronic_nc:NR_125809.1, LOC101929294:i	1	100		intronic	NR_12580	c.2109-14	p.?	0.48					ADAM cysteir	rs1388819		YES			A disintegri	integral component of membrane,metal ion binding,me				C=0, G=40	C=0.0, G=1.0	0.00001	644.281	40	C=0/0, G=36/4
chr1:101203827	A/A	G	A	SNV	VCAM1	VCAM1:exonic:NM_001078.3	1	100	9	synonymous	NM_00107	c.2208G>A	p.(=)	0.87				0.06(COSM63	Immunoglobi	rs3176879	0.0313	YES	8.306E-1 (pos.10120382	carcinoma	Vascular ce	B cell diffe	Adenocarcinoma;A	Carvedilol,Ethanol,Succi		G=0, A=55	G=0.0, A=1.0	0.00001	814.906	55	G=0/0, A=33/2
chr8:25216620	A/T	A	T	SNV	DOCK5	DOCK5:intronic:NM_024940.7	1	51.85		intronic	NM_02494	c.2949+43	p.?	-0.37					C2 domain in	rs17053446	0.3369	YES	3.588E-1 (pos.25216620)		Dedicator o	cytoplasm,cytosol,guanyl-nucleotide exchange factor ac				A=13, T=14	A=0.4815, T=0.5185	0.00001	132.666	27	A=1/12, T=4/1
chr8:25231044	G/G	T	G	SNV	DOCK5	DOCK5:intronic:NM_024940.7	1	100		intronic	NM_02494	c.3685-90	p.?	-0.25					C2 domain in	rs12676152		YES			Dedicator o	cytoplasm,cytosol,guanyl-nucleotide exchange factor ac				T=0, G=26	T=0.0, G=1.0	0.00001	371.42	26	T=0/0, G=12/1
chr5:82875800	T/T	C	T	SNV	VCAN	VCAN:exonic:NM_004385.4	1	100	14	synonymous	NM_00438	c.9882C>T	p.(=)	0.79					EGF-like dom	rs308365	0.0347	YES	1.0 (pos.82875800)		Versican (cl	Golgi lume	Adenocarcinoma;C	Hyaluroni	Benign	C=0, T=49	C=0.0, T=1.0	0.00001	826.555	49	C=0/0, T=20/2
chr5:82833145	A/A	G	A	SNV	VCAN,VCAN-AS1	VCAN:exonic:NM_004385.4, VCAN-AS1:intronic_nc:NR_1362	1	100	8	synonymous	NM_00438	c.4323G>A	p.(=)	-0.65				0.02(COSM44	EGF-like dom	rs2548541	0.3898	YES	6.347E-1 (pos.82833145)	adenocarcino	Versican (cl	Golgi lume	Adenocarcinoma;C	Hyaluroni	Benign	G=0, A=47	G=0.0, A=1.0	0.00001	789.328	47	G=0/0, A=24/2
chr8:25317691	T/T	A	T	SNV	CDCA2	CDCA2:intronic:NM_152562.3	1	100		intronic	NM_15256	c.1-65A>T	p.?	1.06					Protein phos	rs6987936	0.0031	YES				cell cycle,	Nervous System Diseases;Neuromuscular Di			A=0, T=26	A=0.0, T=1.0	0.00001	371.419	26	A=0/0, T=20/6
chr8:25744257	A/A	G	A	SNV	EBF2	EBF2:intronic:NM_022659.3	1	100		intronic	NM_02265	c.1009+14	p.?	-0.67					IPT/TIG doma	rs6557865	0.1258	YES	9.99E-1 (pos.25744257)		Early B-cell	DNA bindi	Genital Diseases, Male;Genital Neoplasms, I			G=0, A=36	G=0.0, A=1.0	0.00001	563.744	36	G=0/0, A=20/1
chr8:26240646	A/G	A	G	SNV	BNIP3L,SDAD1P1	BNIP3L:utr_5:NM_004331.2, SDAD1P1:upstream:NR_14455	1	50		intronic	NR_14455	c.-1A>G	p.?	-2.81					BNIP3	rs1055479	0.3541	YES	8.853E-1 (pos.26240646)		BCL2/adenc	cellular response to hypoxia,cytosol,defense response t				A=17, G=17	A=0.5, G=0.5	0.00001	172.01	34	A=14/3, G=10/
chr5:82833368	AG/AG	A	AG	INDEL	VCAN,VCAN-AS1	VCAN:exonic:NM_004385.4, VCAN-AS1:intronic_nc:NR_1362	1	100	8	frameshiftInsertion	NM_00438	c.4546_45	p.Lys1516fs	-0.18					EGF-like dom	rs144610900			6.063E-3 (pos.82833368)		Versican (cl	Golgi lume	Adenocarcinoma;C	Hyaluronic acid		A=0, AG=46	A=0.0, AG=1.0	0.00001	626.873	46	A=0/0, AG=34/
chr8:26484064	T/G	T	G	SNV	DPYSL2	DPYSL2:intronic:NM_001197293.2	1	58.33		intronic	NM_00119	c.794-69T	p.?	0.61					Amidohydrol	rs4872457	0.423	YES			Dihydropyri	axon guida	Alzheimer Disease;Arthritis;Brain Diseases;C			T=10, G=14	T=0.4167, G=0.5833	0.00001	135.339	24	T=1/9, G=9/5
chr5:82834299	A/A	G	A	SNV	VCAN,VCAN-AS1	VCAN:exonic:NM_004385.4, VCAN-AS1:intronic_nc:NR_1362	1	100	8	missense	NM_00438	c.5477G>A	p.Arg1826His	0.79	0.02	29	0.235	0.13(COSM44	EGF-like dom	rs188703	0.4001	YES	3.522E-1 (pos.82834299)	adenocarcino	Versican (cl	Golgi lume	Adenocarcinoma;C	Hyaluroni	Benign	G=0, A=38	G=0.0, A=1.0	0.00001	603.784	38	G=0/0, A=19/1
chr8:27291557	C/C	T	C	SNV	MIR6842,PTK2B	MIR6842:downstream:NR_106901.1, PTK2B:intronic:NM_17	1	100		intronic	NM_17317	c.1106-53	p.?	-1.63					FERM central	rs919493	0.1392	YES			Protein tyrc	3-phosphc	Alzheimer Disease;	4-(4-((1R,2R)-2-(dimethyl		T=0, C=51	T=0.0, C=1.0	0.00001	752.926	51	T=0/0, C=33/1
chr5:82834630	C/C	T	C	SNV	VCAN,VCAN-AS1	VCAN:exonic:NM_004385.4, VCAN-AS1:intronic_nc:NR_1362	1	100	8	synonymous	NM_00438	c.5808T>C	p.(=)	0.03				0.57(COSM44	EGF-like dom	rs309557	0.4957	YES	4.091E-1 (pos.82834630)	adenocarcino	Versican (cl	Golgi lume	Adenocarcinoma;C	Hyaluroni	Benign	T=0, C=72	T=0.0, C=1.0	0.00001	1130.95	72	T=0/0, C=31/4
chr5:82835545	G/G	A	G	SNV	VCAN,VCAN-AS1	VCAN:exonic:NM_004385.4, VCAN-AS1:intronic_nc:NR_1362	1	100	8	synonymous	NM_00438	c.6723A>G	p.(=)	-0.44				0.01(COSM44	EGF-like dom	rs160279	0.4957	YES	4.092E-1 (pos.82835545)	adenocarcino	Versican (cl	Golgi lume	Adenocarcinoma;C	Hyaluroni	Benign	A=0, G=28	A=0.0, G=1.0	0.00001	408.718	28	A=0/0, G=18/1
chr5:82837631	T/T	G	T	SNV	VCAN,VCAN-AS1	VCAN:exonic:NM_004385.4, VCAN-AS1:intronic_nc:NR_1362	1	100	8	missense	NM_00438	c.8809G>T	p.Asp2937Tyr	0.64	0	160	0.979	0.07(COSM44	EGF-like dom	rs160277	0.3992	YES	3.514E-1 (pos.82837631)	adenocarcino	Versican (cl	Golgi lume	Adenocarcinoma;C	Hyaluroni	Benign	G=0, T=36	G=0.0, T=1.0	0.00001	563.731	36	G=0/0, T=21/1
chr8:27321597	C/C	G	C	SNV	CHRNA2	CHRNA2:intronic:NM_000742.3	1	100		intronic	NM_00074	c.450-87C	p.?	-1.09					Neurotransm	rs3735757		YES			Cholinergic	acetylchol	Brain Diseases;Cen	Atracurium besylate,Bip		G=0, C=29	G=0.0, C=1.0	0.00001	427.542	29	G=0/0, C=9/20
chr17:4689572	C/C	T	C	SNV	VMO1	VMO1:exonic:NM_182566.2	1	100	1	missense	NM_18256	c.76A>G	p.Thr26Ala	0.07	1	58	0	0(COSM4966	Vitelline mer	rs4790706,rs751491	0.4678	YES	0.0 (pos.4689565),5.387	acute_myeloid leukaemia		biological	Adenocarcinoma;Carcinoma;Carcinoma, Ren			T=0, C=160	T=0.0, C=1.0	0.00001	2580.47	160	T=0/0, C=72/8
chr6:133070995	T/T	G	T	SNV	VNN2	VNN2:exonic:NM_004665.4	1	100	6	missense	NM_00466	c.1210C>A	p.Leu404Met	-0.3	0.07	15	0.038		Carbon-nitro	rs4895944	8.00E-04		1.0 (pos.133070995)		Vanin 2	C-termina	Congenital, Hereditary, and Neonatal Diseas			G=0, T=70	G=0.0, T=1.0	0.00001	1092.08	70	G=0/0, T=46/2
chr8:27641609	A/A	G	A	SNV	ESCO2	ESCO2:intronic:NM_001017420.2	1	100		intronic	NM_00101	c.1013+35	p.?	-0.05					ESCO1/2 acet	rs1824449	0		1.0 (pos.27641609)		Establishm	Golgi appa	Bone Diseases;Bone Diseases,		Benign	G=0, A=55	G=0.0, A=1.0	0.00001	814.893	55	G=0/0, A=39/1
chr11:118939939	TC/TC	T	TC	INDEL	VPS11	VPS11:exonic:NM_021729.5	1	100	2	unknown	NM_021729.5			6.1					Region in Cla	rs199535207,rs5795	9.00E-04		9.819E-1 (pos.118939939)		Vacuolar pr	AP-3 adaptor complex,CORVET complex,HOPS complex,a				T=0, TC=22	T=0.0, TC=1.0	0.00001	292.618	22	T=0/0, TC=11/
chr8:100865836	G/A	G	A	SNV	VPS13B	VPS13B:exonic:NM_017890.4	1	47.37	56	missense	NM_01789	c.10294G>	p.Gly3432Arg	1.29	0.46	125	0	0.14(COSM37	N-terminal re	rs6468694	0.1258	YES	1.169E-1;0.0 (pos.10086	acute_myeloid	Vacuolar pr	protein tra	Congenital Abnormalities;Con		Benign/Likely	G=30, A=27	G=0.5263, A=0.4737	0.00001	256.51	57	G=15/15, A=1
chr15:62202482	C/T	C	T	SNV	VPS13C	VPS13C:exonic:NM_020821.2	1	51.85	64	missense	NM_02082	c.8738G>A	p.Ser2913Asn	0.85	1	46	0	0.19(COSM44	Autophagy-re	rs10851704	0.4648	YES	4.902E-1 (pos.62202482)	acute_myeloid	Vacuolar pr	Golgi to endosome transport,cytoplasm,cytosol,extrace				C=13, T=14	C=0.4815, T=0.5185	0.00001	133.848	27	C=10/3, T=13/
chr8:27957529	C/T	C	T	SNV	ELP3	ELP3:intronic:NM_018091.5	1	57.14		intronic	NM_01809	c.258+46C	p.?	-0.11					Acetyltransfe	rs2290370	0.0963	YES	3.065E-1;0.0 (pos.27957529)		Elongation	Elongator holoenzyme complex,H3 histone acetyltransfe				C=12, T=16	C=0.4286, T=0.5714	0.00001	161.77	28	C=7/5, T=6/10
chr8:2796277	T/T	C	T	SNV	CSMD1	CSMD1:intronic:NM_033225.5	1	100		intronic	NM_03322	c.10536-1	p.?	0.31					CUB domain,	rs585000	0.167	YES	8.99E-1;0.0 (pos.2796277)		Cub and Sus	glucose hc	Alzheimer Disease;Amphetamine-Related Di			C=0, T=26	C=0.0, T=1.0	0.00001	371.42	26	C=0/0, T=7/19
chr8:27967856	G/G	A	G	SNV	ELP3	ELP3:intronic:NM_018091.5	1	100		intronic	NM_01809	c.394-31A	p.?	-0.86					Acetyltransfe	rs3213997	0.451	YES	8.804E-1 (pos.27967856)		Elongation	Elongator holoenzyme complex,H3 histone acetyltransfe				A=0, G=39	A=0.0, G=1.0	0.00001	623.98	39	A=0/0, G=23/1
chr8:28016009	A/A	G	A	SNV	ELP3	ELP3:intronic:NM_018091.5	1	100		intronic	NM_01809	c.1192-76	p.?	0.07					Acetyltransfe	rs149274779,rs199656443,rs37		YES			Elongation	Elongator holoenzyme complex,H3 histone acetyltransfe				G=0, A=22	G=0.0, A=1.0	0.00001	298.892	22	G=0/0, A=12/1
chr15:91543761	T/T	C	T	SNV	VPS33B	VPS33B:exonic:NM_018668.4	1	100	20	missense	NM_01866	c.1540G>A	p.Gly514Ser	4.27	1	56	0.002	0.14(COSM37	Sec1 family	rs11073964	0.3981	YES	9.965E-1;0.0 (pos.91543	adenocarcino	Vacuolar pr	AP-3 adap	Arthrogryposis;Bile Duct Disea		Benign	C=0, T=21	C=0.0, T=1.0	0.00001	281.235	21	C=0/0, T=14/7
chr8:2820187	A/A	C	A	SNV	CSMD1	CSMD1:intronic:NM_033225.5	1	100		intronic	NM_03322	c.9455-26	p.?	0.19					CUB domain,	rs4875859,rs757780	0.1472	YES	0.0 (pos.2820183),2.475E-1 (pos.282018		Cub and Sus	glucose hc	Alzheimer Disease;Amphetamine-Related Di			C=0, A=21	C=0.0, A=1.0	0.00001	285.365	21	C=0/0, A=4/17
chr8:28210613	G/C	G	C	SNV	ZNF395	ZNF395:intronic:NM_018660.2	1	50		intronic	NM_01866	c.819+77C	p.?	-0.82					Domain of un	rs6558057		YES			Zinc finger p	DNA binding,RNA polymerase II core promoter proximal				G=35, C=35	G=0.5, C=0.5	0.00001	354.503	70	G=16/19, C=1
chr8:2824083	G/G	C	G	SNV	CSMD1	CSMD1:intronic:NM_033225.5	1	100		intronic	NM_03322	c.9100+9G	p.?	0.52				0.01(COSM39	CUB domain,	rs73657532	0.1142	YES	2.445E-1 (pos.2824083)	acute_myeloid	Cub and Sus	glucose hc	Alzheimer Disease;Amphetamine-Related Di			C=0, G=26	C=0.0, G=1.0	0.00001	371.42	26	C=0/0, G=21/5
chr16:46696284	A/A	G	A	SNV	VPS35	VPS35:exonic:NM_018206.5	1	100	15	synonymous	NM_01820	c.1938C>T	p.(=)	0.33					Vacuolar pro	rs168745	3.00E-04		1.0 (pos.46696284)		Vacuolar pr	D1 dopam	Basal Ganglia Diseases;Brain Diseases;Centr			G=0, A=24	G=0.0, A=1.0	0.00001	334.792	24	G=0/0, A=12/1
chr8:28984572	A/A	G	A	SNV	KIF13B	KIF13B:intronic:NM_015254.3	1	100		intronic	NM_01525	c.3222+67	p.?	0.04					CAP-Gly dom	rs3763566		YES			Kinesin fam	14-3-3 protein binding,ATP binding,ATPase activity,T cell				G=0, A=36	G=0.0, A=1.0	0.00001	563.739	36	G=0/0, A=5/31
chr7:38796570	A/A	G	A	SNV	VPS41	VPS41:exonic:NM_014396.3	1	100	19	synonymous	NM_01439	c.1563C>T	p.(=)	3.35					Region in Cla	rs2240350	0.358	YES	4.465E-1 (pos.38796570)		Vacuolar pr	AP-3 adaptor complex,CORVET complex,GTPase binding,				G=0, A=235	G=0.0, A=1.0	0.00001	3935.79	235	G=0/0, A=106/
chr8:2964003	C/C	T	C	SNV	CSMD1	CSMD1:intronic:NM_033225.5	1	100		intronic	NM_03322	c.6949+47	p.?	0.83					CUB domain,	rs10503192,rs20185	0.2027	YES	9.64E-1 (pos.2964003)		Cub and Sus	glucose hc	Alzheimer Disease;Amphetamine-Related Di			T=0, C=38	T=0.0, C=1.0	0.00001	598.597	38	T=0/0, C=18/2
chr10:50227785	C/C	T	C	SNV	VSTM4	VSTM4:exonic:NM_001031746.4	1	100	8	synonymous	NM_00103	c.873A>G	p.(=)	0.43					Immunoglobi	rs1913525	5.00E-04	YES	1.0;0.0 (pos.50227785)			extracellu	Brain Diseases;Central Nervous System Dise			T=0, C=30	T=0.0, C=1.0	0.00001	446.635	30	T=0/0, C=11/1
chr1:117690272	A/A	G	A	SNV	VTCN1	VTCN1:utr_3:NM_024626.3	1	100	5	utr_3	NM_02462	c.857C>T	p.?	-0.21					Immunoglobi	rs10754339	0.0765	YES	8.939E-1 (pos.117690272)			adaptive i	Arthritis;Joint Diseases;Musculoskeletal Dis			G=0, A=85	G=0.0, A=1.0	0.00001	1394.56	85	G=0/0, A=43/4
chr8:29996313	G/C	G	C	SNV	MBOAT4	MBOAT4:intronic:NM_001100916.1	1	50		intronic	NM_00110	c.120-41C	p.?	-0.68					MBOAT, mem	rs7823643	0.0409	YES	7.677E-1;0.0 (pos.29996313)		Membrane-	O-acyltransferase activity,endoplasmic reticulum memb				G=22, C=22	G=0.5, C=0.5	0.00001	243.625	44	G=5/17, C=8/1
chr8:3038763	G/C	G	C	SNV	CSMD1	CSMD1:intronic:NM_033225.5	1	47.37		intronic	NM_03322	c.5621-27	p.?	-0.34					CUB domain,	rs11785446	0.1781	YES	1.691E-1;0.0 (pos.3038763)		Cub and Sus	glucose hc	Alzheimer Disease;Amphetamine-Related Di			G=20, C=18	G=0.5263, C=0.4737	0.00001	183.977	38	G=5/15, C=11/
chr8:30601805	T/T	C	T	SNV	UBXN8	UBXN8:intronic:NM_005671.3	1	100		intronic	NM_00567	c.88+11C>	p.?	-1.3					UBX domain	rs2278092	0.1791	YES	8.122E-1 (pos.30601805)		UBX domair	RNA phosphodiester bond hydrolysis, endonucleolytic,R				C=0, T=39	C=0.0, T=1.0	0.00001	614.909	39	C=0/0, T=20/1
chr8:3087809	C/C	T	C	SNV	CSMD1	CSMD1:intronic:NM_033225.5	1	100		intronic	NM_03322	c.4154-56	p.?	-3.01					CUB domain,	rs1442403		YES			Cub and Sus	glucose hc	Alzheimer Disease;Amphetamine-Related Di			T=0, C=87	T=0.0, C=1.0	0.00001	1431.29	87	T=0/0, C=26/6
chr10:116038581	G/A	G	A	SNV	VWA2	VWA2:exonic:NM_001272046.1	1	50	8	synonymous	NM_00127	c.804G>A	p.(=)	-2.04					Calcium-bind	rs750698718			0.0 (pos.116038581)			basement membrane,calcium ion binding,calcium-indep				G=11, A=11	G=0.5, A=0.5	0.00001	94.6067	22	G=7/4, A=4/7

chr8:3141555	G/G	A	G	SNV	CSMD1	CSMD1:intronic:NM 033225.5	1	100		intronic	NM 03322	c.4153+11	p.?	0.75					CUB domain,:	rs10099968		YES		Cub and Su:	glucose hc	Alzheimer Disease;Amphetamine-Related Di			A=0, G=30	A=0.0, G=1.0	0.00001	446.633	30	A=0/0, G=19/1
chr8:3141586	A/A	T	A	SNV	CSMD1	CSMD1:intronic:NM 033225.5	1	100		intronic	NM 03322	c.4153+80	p.?	-1.55					CUB domain,:	rs4875508		YES		Cub and Su:	glucose hc	Alzheimer Disease;Amphetamine-Related Di			T=0, A=30	T=0.0, A=1.0	0.00001	446.629	30	T=0/0, A=19/1
chr8:3165207	G/G	A	G	SNV	CSMD1	CSMD1:intronic:NM 033225.5	1	100		intronic	NM 03322	c.3950+10	p.?	-0.84				0.05(COSM39	CUB domain,:	rs10103770	0.4417	YES	2.075E-1 (pos.3165207) acute_myeloid	Cub and Su:	glucose hc	Alzheimer Disease;Amphetamine-Related Di			A=0, G=76	A=0.0, G=1.0	0.00001	1209.6	76	A=0/0, G=55/2
chr8:3226919	T/T	C	T	SNV	CSMD1	CSMD1:intronic:NM 033225.5	1	100		intronic	NM 03322	c.2783-27(	p.?	-3.04					CUB domain,:	rs270096,rs7676006!	1.00E-04		0.0 (pos.3226911),9.998E-1 (pos.322691	Cub and Su:	glucose hc	Alzheimer Disease;Amphetamine-Related Di			C=0, T=55	C=0.0, T=1.0	0.00001	814.905	55	C=0/0, T=28/2
chr3:183948662	GA/GA	G	GA	INDEL	VWA5B2	VWA5B2:exonic:NM 138345.2	1	100	2	frameshiftInsertion	NM 13834	c.267_268	p.Gly90fs	0.9					Vault protein inter-alpha-trypsin domain,von Willebrand factor type A domain										G=0, GA=3!	G=0.0, GA=1.0	0.00001	226.358	35	G=0/0, GA=32/
chr8:3253942	G/G	A	G	SNV	CSMD1	CSMD1:intronic:NM 033225.5	1	100		intronic	NM 03322	c.2406-39'	p.?	-0.08					CUB domain,:	rs3802305	0.4931	YES	3.097E-1 (pos.3253942)	Cub and Su:	glucose hc	Alzheimer Disease;Amphetamine-Related Di			A=0, G=82	A=0.0, G=1.0	0.00001	1333.39	82	A=0/0, G=36/4
chr8:3253997	A/A	G	A	SNV	CSMD1	CSMD1:intronic:NM 033225.5	1	100		intronic	NM 03322	c.2406-94(	p.?	-0.63					CUB domain,:	rs3802306		YES		Cub and Su:	glucose hc	Alzheimer Disease;Amphetamine-Related Di			G=0, A=85	G=0.0, A=1.0	0.00001	1390.51	85	G=0/0, A=37/4
chr8:3263888	A/A	AGATA	A	INDEL	CSMD1	CSMD1:intronic:NM 033225.5	4	100		intronic	NM 03322	c.2072-14!	p.?	0.71, -0.58, 0.63, -1.07, 0.51					CUB domain,:	rs1012564936,rs71199572				Cub and Su:	glucose hc	Alzheimer Disease;Amphetamine-Related Di			AGATA=0, A	AGATA=0.0, A=1.0	0.00001	601.386	38	AGATA=0/0, A=
chr3:183948686	GC/G	GC	G	INDEL	VWA5B2	VWA5B2:exonic:NM 138345.2	1	100	2	frameshiftDeletion	NM 13834	c.295delC	p.Arg99fs	0.55, 3.25					Vault protein inter-alpha-trypsin domain,von Willebrand factor type A domain										GC=0, G=2!	GC=0.0, G=1.0	0.00001	231.63	29	GC=0/0, G=24/
chr8:35093427	C/CAGCT	C	CAGCT	INDEL	UNC5D	UNC5D:intronic:NM 080872.3	4	56		intronic	NM 08087	c.103+22C	p.?	-0.21					Death domai	rs10694226,rs54888!	0.2768	YES	(pos.35093427)	Unc5. C. ele	apoptotic process,axon guidance,cell surface,cell-cell a(				C=11, CAG(	C=0.44, CAGCT=0.56	0.00001	134.35	25	C=4/7, CAGCT=
chr8:3565900	T/T	C	T	SNV	CSMD1	CSMD1:intronic:NM 033225.5	1	100		intronic	NM 03322	c.1009+36	p.?	0.29					CUB domain,:	rs12674750	0.1674	YES	2.665E-1 (pos.3565900)	Cub and Su:	glucose hc	Alzheimer Disease;Amphetamine-Related Di			C=0, T=30	C=0.0, T=1.0	0.00001	446.638	30	C=0/0, T=19/1
chr3:183951135	T/T	C	T	SNV	VWA5B2	VWA5B2:exonic:NM 138345.2	1	100	3	synonymous	NM 13834	c.480C>T	p.(=)	2.56				0.92(COSM37	Vault protein	rs729377		YES	4.058E-1 (pos.18395113! adenocarcinoma,squamous_cell_carcinoma						C=0, T=35	C=0.0, T=1.0	0.00001	543.903	35	C=0/0, T=8/27
chr8:38385795	C/C	T	C	SNV	C8orf86	C8orf86:intronic:NM 207412.2	1	100		intronic	NM 20741	c.315+46A	p.?	-1.25						rs2445020	0.1374	YES	9.412E-1;0.0 (pos.38385795)						T=0, C=31	T=0.0, C=1.0	0.00001	465.819	31	T=0/0, C=26/5
chr8:3855658	G/A	G	A	SNV	CSMD1	CSMD1:intronic:NM 033225.5	1	57.14		intronic	NM 03322	c.611-26C:	p.?	-0.01					CUB domain,:	rs17068480	0.0718	YES	3.097E-1 (pos.3855658)	Cub and Su:	glucose hc	Alzheimer Disease;Amphetamine-Related Di			G=18, A=24	G=0.4286, A=0.5714	0.00001	279.102	42	G=6/12, A=13/
chr8:38809663	C/C	A	C	SNV	PLEKHA2	PLEKHA2:intronic:NM 021623.1	1	100		intronic	NM 02162	c.487-21A:	p.?	-0.29					Meiotic cell c	rs6984871	0.1254	YES	9.168E-1 (pos.38809663)	Pleckstrin h	PDZ domain binding,cytoplasm,fibronectin binding,lami				A=0, C=22	A=0.0, C=1.0	0.00001	298.889	22	A=0/0, C=17/5
chr8:38840591	T/T	C	T	SNV	HTRA4	HTRA4:intronic:NM 153692.3	1	100		intronic	NM 15369	c.1268+24	p.?	0.29					Insulin-like g	rs7814264	0.3884	YES	7.515E-1 (pos.38840591)	HTRA serine	endopeptidase activity,extracellular region,insulin-like				C=0, T=85	C=0.0, T=1.0	0.00001	1390.5	85	C=0/0, T=37/4
chr3:183953972	A/A	G	A	SNV	VWA5B2	VWA5B2:exonic:NM 138345.2	1	100	8	synonymous	NM 13834	c.1134G>A	p.(=)	-7.04				0.01(COSM41	Vault protein	rs1709657	0.2074	YES	2.572E-1 (pos.18395397; neoplasm						G=0, A=67	G=0.0, A=1.0	0.00001	1032.13	67	G=0/0, A=36/3
chr8:38884344	TCTTT/TCTTT	T	TCTTT	INDEL	ADAM9	ADAM9:intronic:NM 003816.2	4	100		intronic	NM 00381	c.1130+15	p.?	0.19					ADAM cysteine-rich,Disintegrin,Metallo-peptidase family M12,Metallo-peptidase family M12B Repr					A disintegri	PMA-induc	Autoimmune Disea	INCB7839		T=0, TCTTT=	T=0.0, TCTTT=1.0	0.00001	604.31	38	T=0/0, TCTTT=
chr8:38911953	A/A	C	A	SNV	ADAM9	ADAM9:intronic:NM 003816.2	1	100		intronic	NM 00381	c.1303-47(	p.?	-0.3					ADAM cysteir	rs9643905	0.2499	YES	9.48E-1 (pos.38911953)	A disintegri	PMA-induc	Autoimmune Disea	INCB7839		C=0, A=20	C=0.0, A=1.0	0.00001	263.785	20	C=0/0, A=6/14
chr8:39142264	G/G	A	G	SNV	ADAM32	ADAM32:intronic:NM 145004.6	1	100		intronic	NM 14500	c.2358-48,	p.?	-0.44					ADAM cysteir	rs7008808	0.3826	YES	1.318E-1 (pos.39142264)		integral component of membrane,integrin-mediated sig				A=0, G=24	A=0.0, G=1.0	0.00001	334.792	24	A=0/0, G=11/1
chr8:39463823	C/C	T	C	SNV	ADAM18	ADAM18:intronic:NM 014237.2	1	100		intronic	NM 01423	c.133-3T>C	p.?	-0.32					ADAM cysteir	rs13268577	0.4488	YES	1.604E-1 (pos.39463823)		cell differentiation,integral component of membrane,m(				T=0, C=55	T=0.0, C=1.0	0.00001	814.908	55	T=0/0, C=21/3
chr7:49815390	G/G	C	G	SNV	VWC2	VWC2:exonic:NM 198570.4	1	100	2	missense	NM 19857	c.359C>G	p.Ala120Gly	0.02	0.45	60	0	0.04(COSM37	RNA-binding,	rs769604	0.461	YES	4.375E-1 (pos.49815390) adenocarcinoi	von Willebr	AMPA glutamate receptor complex,basement membran				C=0, G=27	C=0.0, G=1.0	0.00001	389.987	27	C=0/0, G=12/1
chr8:39503079	A/A	G	A	SNV	ADAM18	ADAM18:intronic:NM 014237.2	1	100		intronic	NM 01423	c.1046+86	p.?	0.05					ADAM cysteir	rs6474161		YES			cell differentiation,integral component of membrane,m(				G=0, A=39	G=0.0, A=1.0	0.00001	623.978	39	G=0/0, A=20/1
chr8:39505826	A/A	C	A	SNV	ADAM18	ADAM18:intronic:NM 014237.2	1	100		intronic	NM 01423	c.1047-37(	p.?	-0.58					ADAM cysteir	rs3735880	0.2238	YES	2.665E-1 (pos.39505826)		cell differentiation,integral component of membrane,m(				C=0, A=26	C=0.0, A=1.0	0.00001	375.353	26	C=0/0, A=11/1
chr8:39525750	A/A	C	A	SNV	ADAM18	ADAM18:intronic:NM 014237.2	1	100		intronic	NM 01423	c.1525+35	p.?	0.09					ADAM cysteir	rs2305392	0.2386	YES	2.558E-1 (pos.39525750)		cell differentiation,integral component of membrane,m(				C=0, A=28	C=0.0, A=1.0	0.00001	408.718	28	C=0/0, A=11/1
chr7:12395813	C/C	T	C	SNV	VWDE	VWDE:exonic:NM 001135924.2	1	100	18	synonymous	NM 00113	c.3669A>G	p.(=)	-0.91					Human growt	rs2119141	0.2608	YES	8.248E-1 (pos.12395813)		calcium ion binding,extracellular region				T=0, C=27	T=0.0, C=1.0	0.00001	389.986	27	T=0/0, C=16/1
chr7:12400837	A/A	G	A	SNV	VWDE	VWDE:exonic:NM 001135924.2	1	100	15	missense	NM 00113	c.3095C>T	p.Thr1032Met	5.01	0.53	81	0	0.03(COSM41	Human growt	rs2053380	0.275	YES	7.314E-1 (pos.12400837) neoplasm		calcium ion binding,extracellular region				G=0, A=26	G=0.0, A=1.0	0.00001	371.42	26	G=0/0, A=20/6
chr8:40554742	C/T	C	T	SNV	ZMAT4	ZMAT4:intronic:NM 024645.2	1	57.14		intronic	NM 02464	c.349+22G	p.?	-2.42					Zinc-finger dc	rs11781516	0.472	YES	5.667E-1 (pos.40554742)		DNA bindi	Eye Diseases;Refractive Errors			C=9, T=12	C=0.4286, T=0.5714	0.00001	109.102	21	C=7/2, T=6/6
chr8:41393799	C/C	T	C	SNV	GINS4,LOC102723729	GINS4:intronic:NM 032336.2, LOC102723729:intronic nc:N	1	100		intronic	NM 03233	c.97-80T>C	p.?	1.23					DNA replicati	rs11779810		YES		GINS compl	3'-5' DNA helicase activity,DNA duplex unwinding,DNA re				T=0, C=25	T=0.0, C=1.0	0.00001	352.38	25	T=0/0, C=15/1
chr8:41519248	C/C	T	C	SNV	ANK1	ANK1:intronic:NM 001142446.1	1	100		intronic	NM 00114	c.5667+14	p.?	-0.01					Ankyrin repe:	rs516946		YES	8.711E-1 (pos.41519248)	Ankyrin-1, e	ATPase bir	Anemia:Anemia, Hemolytic:Anemia, Hemolyt			T=0, C=22	T=0.0, C=1.0	0.00001	298.839	22	T=0/0, C=18/4
chr8:41519462	G/G	A	G	SNV	ANK1	ANK1:intronic:NM 001142446.1	1	100		intronic	NM 00114	c.5602-3T:	p.?	0.87				0.1(COSM590	Ankyrin repe:	rs515071,rs7716786!	0.2434	YES	0.0 (pos.41519461),8.38! basal_cell_car	Ankyrin-1, e	ATPase bir	Anemia:Anemia, Hemolytic:An		Benign/Likely	A=0, G=25	A=0.0, G=1.0	0.00001	353.019	25	A=0/0, G=10/1
chr8:41529856	A/G	A	G	SNV	ANK1	ANK1:intronic:NM 001142446.1	1	96.67		intronic	NM 00114	c.5219+16	p.?	-0.34					Ankyrin repe:	rs508112	6.00E-04		1.0;0.0 (pos.41529856)	Ankyrin-1, e	ATPase bir	Anemia:Anemia, Hemolytic:An		Benign	A=1, G=29	A=0.0333, G=0.9667	0.00001	398.812	30	A=1/0, G=20/9
chr8:41553835	A/A	G	A	SNV	ANK1	ANK1:intronic:NM 001142446.1	1	100		intronic	NM 00114	c.3083+46	p.?	-0.74					Ankyrin repe:	rs2304883	0.3298	YES	2.781E-1 (pos.41553835)	Ankyrin-1, e	ATPase bir	Anemia;Anemia, Hemolytic;Anemia, Hemolyt			G=0, A=28	G=0.0, A=1.0	0.00001	408.718	28	G=0/0, A=12/1
chr7:12406989	G/G	C	G	SNV	VWDE	VWDE:exonic:NM 001135924.2	1	100	13	missense	NM 00113	c.2892G>C	p.Lys964Asn	0.22	0.03	94	0.997	0.36(COSM11	Human growt	rs6460939	0.4751	YES	5.487E-1 (pos.12406989) carcinoma,clear_cell_rena		calcium ion binding,extracellular region				C=0, G=21	C=0.0, G=1.0	0.00001	281.235	21	C=0/0, G=8/13
chr8:41585571	T/T	C	T	SNV	ANK1	ANK1:intronic:NM 001142446.1	1	100		intronic	NM 00114	c.328-47G	p.?	-1.75					Ankyrin repe:	rs1872877	0.3729	YES	2.949E-1;1.189E-4 (pos.41585571)	Ankyrin-1, e	ATPase bir	Anemia:Anemia, Hemolytic:Anemia, Hemolyt			C=0, T=74	C=0.0, T=1.0	0.00001	1170.1	74	C=0/0, T=37/3
chr8:41834902	T/C	T	C	SNV	KAT6A	KAT6A:intronic:NM 006766.4	1	57.58		intronic	NM 00676	c.1044-57,	p.?	1.26					MOZ/SAS fam	rs34473679		YES		K(lysine) ac	DNA bindi	Adenocarcinoma:Carcinoma:Carcinoma, Ade			T=13, C=19	T=0.4063, C=0.5938	0.00001	208.678	33	T=1/12, C=7/1
chr8:42175303	T/T	A	T	SNV	IKBKB	IKBKB:intronic:NM 001556.2	1	100		intronic	NM 00155	c.1240+14	p.?	-0.02					I-kappa-kinas	rs2294100	0.0736	YES	8.79E-1 (pos.42175303)	Inhibitor of	ATP bindin	Brain Diseases;Car	Acetylcyst	Benign	A=0, T=60	A=0.0, T=1.0	0.00001	907.071	60	A=0/0, T=28/3
chr8:42196441	T/T	C	T	SNV	POLB	POLB:intronic:NM 002690.2	1	100		intronic	NM 00269	c.62-89C>T	p.?	-0.12					DNA polymer	rs3136717		YES		Polymerase	DNA bindi	Female Urogenital	(1S)-1,2,3,4-TETRAHYDRO		C=0, T=42	C=0.0, T=1.0	0.00001	682.742	42	C=0/0, T=18/2
chr7:12410112	A/A	G	A	SNV	VWDE	VWDE:exonic:NM 001135924.2	1	100	12	missense	NM 00113	c.1820C>T	p.Ser607Phe	5.67	1	155	0	0.08(COSM37	Human growt	rs963323	0.1398	YES	8.19E-1 (pos.12410112) acute_myeloid_leukaemia		calcium ion binding,extracellular region				G=0, A=23	G=0.0, A=1.0	0.00001	316.747	23	G=0/0, A=15/8
chr8:442389	G/A	G	A	SNV	TDRP	TDRP:intronic:NM 001256113.1	1	58.33		intronic	NM 00125	c.498+70C	p.?	-1.38					Testis develo	rs3779745	0.1816	YES	4.086E-1 (pos.442389)		cytoplasm,cytosol,intracellular membrane-bounded org				G=10, A=14	G=0.4167, A=0.5833	0.00001	134.355	24	G=6/4, A=8/6
chr8:48196478	T/T	A	T	SNV	SPIDR	SPIDR:intronic:NM 001080394.3	1	100		intronic	NM 00108	c.190-139,	p.?	-0.34					Domain of un	rs3736254		YES		Scaffolding	cellular response to DNA damage stimulus,cellular resp				A=0, T=37	A=0.0, T=1.0	0.00001	583.701	37	A=0/0, T=25/1
chr7:12417407	T/T	C	T	SNV	VWDE	VWDE:exonic:NM 001135924.2	1	100	7	missense	NM 00113	c.1019G>A	p.Gly340Asp	3.62	1	94	0	0.19(COSM11	Human growt	rs7793993	0.2804	YES	9.337E-1 (pos.12417407) clear_cell_renal_cell_carci		calcium ion binding,extracellular region				C=0, T=22	C=0.0, T=1.0	0.00001	298.89	22	C=0/0, T=12/1
chr8:48846519	C/C	T	C	SNV	PRKDC	PRKDC:intronic:NM 006904.6	1	100		intronic	NM 00690	c.1623+6A	p.?	-0.64					FAT domain,F	rs1231202	0.0511	YES	9.997E-1 (pos.48846519)	Protein kin:	ATP bindin	Adenocarcinoma:B	Caffeine.S	Benign	T=0, C=30	T=0.0, C=1.0	0.00001	446.638	30	T=0/0, C=12/1
chr8:52287041	T/T	C	T	SNV	PXDNL	PXDNL:intronic:NM 144651.4	1	100		intronic	NM 14465	c.3692+11	p.?	-0.33					Animal haem	rs2977003		YES		Peroxidasir	cellular oxidant detoxification,cytoplasm,endonuclease				C=0, T=42	C=0.0, T=1.0	0.00001	689.581	42	C=0/0, T=26/1
chr12:6128443	C/C	T	C	SNV	VWF	VWF:exonic:NM 000552.4	1	100	28	missense	NM 00055	c.4141A>G	p.Thr1381Ala	1.83	1	58	0		C8 domain,Tr	rs216311	0.3801	YES	7.615E-1 (pos.6128443)	Coagulatior	Weibel-Pa	Arrhythmias, Cardi:	Antihemo	Benign/Likely	T=0, C=90	T=0.0, C=1.0	0.00001	1492.99	90	T=0/0, C=44/4
chr12:6143984	C/C	T	C	SNV	VWF	VWF:exonic:NM 000552.4	1	100	20	missense	NM 00055	c.2555A>G	p.Gln852Arg	-0.54	1	43	0	0.06(COSM41	C8 domain,Tr	rs216321	0.0893	YES	7.982E-1 (pos.6143984) neoplasm	Coagulatior	Weibel-Pa	Arrhythmias, Cardi:	Antihemophilic factor, h		T=0, C=39	T=0.0, C=1.0	0.00001	623.979	39	T=0/0, C=10/2
chr12:6172202	C/C	T	C	SNV	VWF	VWF:exonic:NM 000552.4	1	100	13	missense	NM 00055	c.1451A>G	p.His484Arg	0.27	1	29	0.017	0.24(COSM43	C8 domain,Tr	rs1800378	0.3431	YES	7.081E-1;0.0 (pos.61722(acute_myeloid	Coagulatior	Weibel-Pa	Arrhythmias, Cardi:	Antihemo	Benign/Likely	T=0, C=30	T=0.0, C=1.0	0.00001	446.636	30	T=0/0, C=13/1
chr12:105546172	A/A	G	A	SNV	WASHC4	WASHC4:exonic:NM 015275.2	1	100	26	missense	NM 01527	c.2701G>A	p.Val901Ile	2.8	0.85	29	0		WASH compl	rs1663564,rs759197'	0.0803	YES	0.0 (pos.105546170),1.0 (pos.10554617	WASH comp	BLOC-1 complex,WASH complex,early endosome,endosc				G=0, A=21	G=0.0, A=1.0	0.00001	281.235	21	G=0/0, A=10/1
chr8:61144790	T/T	G	T	SNV	CA8	CA8:intronic:NM 004056.5	1	100		intronic	NM 00405	c.513+53C	p.?	0.64					Eukaryotic-ty	rs73683396	0.2803	YES		Carbonic ar	carbonate	Ataxia;Brain Diseas	Zonisamide		G=0, T=29	G=0.0, T=1.0	0.00001	427.586	29	G=0/0, T=8/21
chr10:104572963	C/C	T	C	SNV	WBP1L	WBP1L:exonic:NM 001083913.1	1	100	4	missense	NM 00108	c.967T>C	p.Ser323Pro	0.61	0.63	74	0	0.03(COSM37	WW domain-	rs284860	0.4103	YES	4.209E-1 (pos.10457296: adenocarcinoma,carcinom		integral component of membrane,protein binding				T=0, C=42	T=0.0, C=1.0	0.00001	685.184	42	T=0/0, C=34/8
chr8:61732521	A/G	A	G	SNV	CHD7	CHD7:intronic:NM 017780.3	1	45.45		intronic	NM 01778	c.2614-45,	p.?	1.18					BRK domain,(	rs6471902	0.2256	YES	9.326E-1 (pos.61732521)	Chromodon	ATP bindin	46, XY Disorders of Sex Develop		Benign/Likely	A=18, G=1!	A=0.5455, G=0.4545	0.00001	143.149	33	A=4/14, G=3/1
chr8:62416074	A/A	G	A	SNV	ASPH	ASPH:intronic:NM 004318.3	1	100		intronic	NM 00431	c.2127-6C:	p.?	3.25					Aspartyl beta	rs7005617	0.0202	YES	1.0 (pos.62416074)	Aspartate b	activation	Congenital Abnorm	L-Aspartic Acid,Succinic a		G=0, A=26	G=0.0, A=1.0	0.00001	371.419	26	G=0/0, A=11/1
chr8:62438530	G/G	A	G	SNV	ASPH	ASPH:intronic:NM 004318.3	1	100		intronic	NM 00431	c.1900+6T	p.?	0.76					Aspartyl beta	rs4738904	0.0203	YES	1.0 (pos.62438530)	Aspartate b	activation	Congenital Abnorm	L-Aspartic Acid,Succinic a		A=0, G=45	A=0.0, G=1.0	0.00001	747.393	45	A=0/0, G=20/2
chr8:6272457	G/G	T	G	SNV	MCPH1	MCPH1:intronic:NM 024596.4	1	100		intronic	NM 02459	c.233+53T	p.?	-0.71					BRCA1 C Term	rs2305021		YES		Microcepha	bone deve	Congenital Abnormalities;Congenital, Heredi			T=0, G=60	T=0.0, G=1.0	0.00001	903.531	60	T=0/0, G=27/3
chr8:6272477	G/G	A	G	SNV	MCPH1	MCPH1:intronic:NM 024596.4	1	100		intronic	NM 02459	c.233+73A	p.?	0.05					BRCA1 C Term	rs2515415		YES		Microcepha	bone deve	Congenital Abnormalities;Congenital, Heredi			A=0, G=59	A=0.0, G=1.0	0.00001	885.363	59	A=0/0, G=26/3
chr8:6272481	G/G	C	G	SNV	MCPH1	MCPH1:intronic:NM 024596.4	1	100		intronic	NM 02459	c.233+77C	p.?	-0.15					BRCA1 C Term	rs2442545		YES		Microcepha	bone deve	Congenital Abnormalities;Congenital, Heredi			C=0, G=59	C=0.0, G=1.0	0.00001	885.368	59	C=0/0, G=26/3
chr22:42416056	G/G	A	G	SNV	WBP2NL	WBP2NL:exonic:NM 152613.2	1	100	4	missense	NM 15261	c.362A>G	p.Asp121Gly	5.33	1	94	0	0.03(COSM37	GRAM domai	rs133335	0.4642	YES	8.569E-1 (pos.42416056) adenocarcinoi	WWBP2 N-t	WW doma	Depressive Disorder;Depressive Disorder, M			A=0, G=36	A=0.0, G=1.0	0.00001	563.744	36	A=0/0, G=15/2
chr8:6335208	C/C	T	C	SNV	MCPH1	MCPH1:intronic:NM 024596.4	1	100		intronic	NM 02459	c.1973+56	p.?	0.02					BRCA1 C Term	rs2277136	0.128	YES		Microcepha	bone deve	Congenital Abnormalities;Congenital, Heredi			T=0, C=21	T=0.0, C=1.0	0.00001	281.11	21	T=0/0, C=11/1
chr8:6357307	C/C	T	C	SNV	ANGPT2,MCPH1	ANGPT2:utr 3:NM 001147.2, MCPH1:intronic:NM 024596.4	1	100		intronic	NM 02459	c.2137-66'	p.?, p.?	-0.11					BRCA1 C Term	rs2916715		YES		Angiopoieti	Tie signali	Brain Diseases;Cardiovascular Diseases;Cen			T=0, C=71	T=0.0, C=1.0	0.00001	1111.48	71	T=0/0, C=35/3
chr13:52313195	T/T	C	T	SNV	WDFY2	WDFY2:exonic:NM 052950.3	1	100	7	synonymous	NM 05295	c.609C>T	p.(=)	-2.06					FYVE zinc fing	rs1885854	0.157	YES	1.246E-1 (pos.52313195)	WD repeat-	early endosome,metal ion binding,positive regulation of				C=0, T=29	C=0.0, T=1.0	0.00001	427.603	29	C=0/0, T=16/1
chr4:177082069	C/C	G	C	SNV	WDR17	WDR17:exonic:NM 170710.4	1	100	21	missense	NM 17071	c.2738G>C	p.Cys913Ser	4.12	0.01	112	0.727	0.97(COSM37	Anaphase-pr	rs7693453	0.26	YES	1.447E-1 (pos.17708206! acute_myeloid	WD repeat-	cytoplasm	Digestive System Diseases:Digestive System			G=0, C=77	G=0.0, C=1.0	0.00001	1229.45	77	G=0/0, C=37/4
chr4:177100644	A/A	G	A	SNV	WDR17	WDR17:exonic:NM 170710.4	1	100	31	missense	NM 17071	c.3883G>A	p.Ala1295Thr	0.6	0.23	58	0.003		Anaphase-pr	rs11736872	0.2974	YES	1.215E-1 (pos.177100644)	WD repeat-	cytoplasm	Digestive System Diseases:Digestive System			G=0, A=42	G=0.0, A=1.0	0.00001	685.219	42	G=0/0, A=25/1
chr8:6614606	A/G	A	G	SNV	AGPAT5	AGPAT5:intronic:NM 018361.3	1	50		intronic	NM 01836	c.870-78A:	p.?	0.29					Acyltransfera	rs10102758		YES		1-acylglyce	1-acylglycerol-3-phosphate O-acyltransferase activity,Cl				A=10, G=10	A=0.5, G=0.5	0.00001	82.8861	20	A=4/6, G=2/8
chr14:102675350	C/C	G	C	SNV	WDR20	WDR20:exonic:NM 001242417.1	1	100	4	synonymous	NM 00124	c.936G>C	p.(=)	-2.3				0.09(COSM41	WD domain,	rs6575900	0.2048	YES	5.873E-2;0.0 (pos.10267! neoplasm	WD repeat-	nucleoplasm,protein binding,protein deubiquitination,t				G=0, C=54	G=0.0, C=1.0	0.00001	798.148	54	G=0/0, C=25/2
chr8:6735300	A/A	T	A	SNV	DEFB1	DEFB1:intronic:NM 005218.3	1	100		intronic	NM 00521	c.61+19A>	p.?	-2.85					Beta defensir	rs2293958	0.2052	YES	8.821E-1 (pos.6735300)	Defensin, b	CCR6 chen	Dermatitis;Dermatitis, Contact;Diabetes Me			T=0, A=29	T=0.0, A=1.0	0.00001	427.518	29	T=0/0, A=15/1
chr14:100847523	C/C	T	C	SNV	WDR25	WDR25:exonic:NM 024515.5	1	100	2	missense	NM 02451	c.262T>C	p.Trp88Arg	-0.21	0.82	101	0		WD domain,	rs2181170	0		1.0 (pos.100847523)		protein binding				T=0, C=23	T=0.0, C=1.0	0.00001	316.595	23	T=0/0, C=7/16
chr5:110439509	A/G	A	G	SNV	WDR36	WDR36:exonic:NM 139281.2	1	50	7	missense	NM 13928	c.790A>G	p.Ile264Val	0.23	1	29	0		Anaphase-pr	rs11241095	0.3086	YES	2.465E-1 (pos.110439509)	WD repeat-	Pwp2p-co	Asthma;Bronchial Diseases;Ev		Benign	A=11, G=11	A=0.5, G=0.5	0.00001	94.6065	22	A=6/5, G=5/6
chr8:67361018	C/C	G	C	SNV	ADHFE1	ADHFE1:intronic:NM 144650.2	1	100		intronic	NM 14465	c.629-82G	p.?	-0.16					Iron-containi	rs12544088		YES		Alcohol deh	2-oxoglutarate metabolic proc		Sodium oxybate		G=0, C=135	G=0.0, C=1.0	0.00001	2237.16	135	G=0/0, C=76/5
chr9:127618776	G/G	A	G	SNV	WDR38	WDR38:exonic:NM 001276374.1	1	100	6	missense	NM 00127	c.515A>G	p.His172Arg	0.53	0.75	29	0	0.03(COSM54	WD domain,	rs2274970	0.0043	YES	7.624E-1;0.0 (pos.12761! acute_myeloid_leukaemia						A=0, G=20	A=0.0, G=1.0	0.00001	263.785	20	A=0/0, G=10/1
chr9:127618839	G/G	C	G	SNV	WDR38	WDR38:exonic:NM 001276374.1	1	100	6	missense	NM 00127	c.578C>G	p.Ala193Gly	2.23	1	60	0		WD domain,	rs10760381	5.00E-04		1.0 (pos.127618839)						C=0, G=22	C=0.0, G=1.0	0.00001	298.892	22	C=0/0, G=10/1
chr8:67813633	G/G	A	G	SNV	MCMDC2	MCMDC2:intronic:NM 173518.4	1	100		intronic	NM 17351	c.1769+50	p.?	-0.44					MCM2/3/5 fa	rs2358008	0.1126	YES	1.0 (pos.67813633)	Minichrom	ATP binding,DNA binding,DNA replication initiation,doub				A=0, G=72	A=0.0, G=1.0	0.00001	1130.95	72	A=0/0, G=41/3
chr8:67922880	G/T	G	T	SNV	PPP1R42	PPP1R42:intronic:NM 001013626.3	1	58.33		intronic	NM 00101	c.552+70C	p.?	-0.67					Leucine Rich	rs117200428				Protein pho	actin binding,centrosome,cytoplasm,cytoskeletal prote				G=15, T=21	G=0.4167, T=0.5833	0.00001	234.328	36	G=8/7, T=11/1
chr2:29135468	G/G	C	G	SNV	WDR43	WDR43:exonic:NM 015131.2	1	100	4	synonymous	NM 01513	c.498C>G	p.(=)	-0.97					Dip2/Utp12 F	rs6715296	0.2793	YES	8.541E-1;0.0 (pos.29135468)	WD repeat-	RNA binding,fibrillar center,nucleolus,nucleoplasm,posi				C=0, G=82	C=0.0, G=1.0	0.00001	1329.65	82	C=0/0, G=53/2
chr9:137005023	T/T	C	T	SNV	WDR5	WDR5:exonic:NM 017588.2	1	100	2	synonymous	NM 01758	c.24C>T	p.(=)	-1.05				0.05(COSM41	Anaphase-pr	rs11556390	0.4429	YES	3.775E-1 (pos.13700502: neoplasm		Ada2/Gcn5/Ada3 transcription activator complex,MLL1 (				C=0, T=124	C=0.0, T=1.0	0.00001	2013.89	124	C=0/0, T=84/4
chr8:67976754	T/A	T	A	SNV	CSPP1	CSPP1:intronic:NM 024790.6	1	45.45		intronic	NM 02479	c.98+23T>,	p.?	-1						rs114350375	0.0268	YES	1.226E-2 (pos.67976754)	Centrosom	centrosome,cytoplasm,microtubule,positive regulation				T=12, A=10	T=0.5455, A=0.4545	0.00001	81.8928	22	T=6/6, A=8/2
chr8:69030962	A/G	A	G	SNV	PREX2	PREX2:intronic:NM 024870.3	1	50		intronic	NM 02487	c.3421+83	p.?	-2.56					Domain foun	rs879517172				Phosphatid	G-protein coupled receptor signaling pathway,GTPase a(				A=13, G=13	A=0.5, G=0.5	0.00001	119.163	26	A=7/6, G=4/9
chr8:69104521	G/G	A	G	SNV	PREX2	PREX2:intronic:NM 024870.3	1	100		intronic	NM 02487	c.4414-49,	p.?	0.23					Domain foun	rs6472389	0.1171	YES	9.995E-1 (pos.69104521)	Phosphatid	G-protein coupled receptor signaling pathway,GTPase a(				A=0, G=30	A=0.0, G=1.0	0.00001	446.599	30	A=0/0, G=20/1
chr5:140048209	C/C	T	C	SNV	WDR55	WDR55:exonic:NM 017706.4	1	100	4	synonymous	NM 01770	c.393T>C	p.(=)	0.64				0.11(COSM37	Anaphase-pr	rs2251860	0.4485	YES	4.567E-1 (pos.14004820! adenocarcinoma,carcinom		biological_process,cellular_component,cytoplasm,mole				T=0, C=20	T=0.0, C=1.0	0.00001	263.773	20	T=0/0, C=9/11
chr7:158672476	G/G	C	G	SNV	WDR60	WDR60:exonic:NM 018051.4	1	100	5	missense	NM 01805	c.675C>G	p.Asn225Lys	0.89	1	94	0			rs2709859	0.0116		1.0 (pos.158672476)	WD repeat	ATP-deper	Abnormalities, Multiple;Bone Diseases;Bone			C=0, G=60	C=0.0, G=1.0	0.00001	903.493	60	C=0/0, G=20/4
chr8:72942210	T/T	G	T	SNV	MSC-AS1,TRPA1	TRPA1:intronic:NM 007332.2, MSC-AS1:intronic nc:NR 033	1	100		intronic	NR 03365:	c.2869-6C:	p.?	-1.14					Ankyrin repe:	rs3824151,rs755916(	0.4095	YES	0.0 (pos.72942207),6.8E-1 (pos.7294221	Transient re	calcium ch	Asthma;Bronchial [	Menthol		G=0, T=20	G=0.0, T=1.0	0.00001	263.784	20	G=0/0, T=17/3
chr8:72952144	AT/AT	A	AT	INDEL	MSC-AS1,TRPA1	TRPA1:intronic:NM 007332.2, MSC-AS1:intronic nc:NR 033	1	100		intronic	NR 03365:	c.2062-11:	p.?	0.33					Ankyrin repeat,Ankyrin repeats (3 copies),Ankyrin repeats (many copies),Ion transport protein					Transient re	calcium ch	Asthma;Bronchial [	Menthol		A=0, AT=25	A=0.0, AT=1.0	0.00001	211.701	25	A=0/0, AT=11/
chr8:72952145	T/T	C	T	SNV	MSC-AS1,TRPA1	TRPA1:intronic:NM 007332.2, MSC-AS1:intronic nc:NR 033	1	100		intronic	NR 03365:	c.2062-11:	p.?	-1.27					Ankyrin repe:	rs2044271		YES		Transient re	calcium ch	Asthma;Bronchial [	Menthol		C=0, T=25	C=0.0, T=1.0	0.00001	353.005	25	C=0/0, T=11/1
chr8:72952185	A/A	G	A	SNV	MSC-AS1,TRPA1	TRPA1:intronic:NM 007332.2, MSC-AS1:intronic nc:NR 033	1	100		intronic	NR 03365:	c.2062-15:	p.?	1.22					Ankyrin repe:	rs2044272		YES		Transient re	calcium ch	Asthma;Bronchial [	Menthol		G=0, A=20	G=0.0, A=1.0	0.00001	263.786	20	G=0/0, A=6/14
chr7:158715219	G/G	A	G	SNV	WDR60	WDR60:exonic:NM 018051.4	1	100	16	synonymous	NM 01805	c.2073A>G	p.(=)	2.32						rs2730238	0.1793	YES	9.48E-1 (pos.158715219)	WD repeat	ATP-deper	Abnormalities, Multiple;Bone Diseases;Bone			A=0, G=74	A=0.0, G=1.0	0.00001	1143.08	74	A=0/0, G=36/3
chr8:72981221	G/A	G	A	SNV	TRPA1	TRPA1:intronic:NM 007332.2	1	46.43		intronic	NM 00733	c.444+37C	p.?	0.21					Ankyrin repe:	rs16937970	0.1677	YES	5.643E-2 (pos.72981221)	Transient re	calcium ch	Asthma;Bronchial [	Menthol		G=15, A=1!	G=0.5357, A=0.4643	0.00001	119.989	28	G=3/12, A=1/1
chr19:36590329	C/C	T	C	SNV	WDR62	WDR62:exonic:NM 001083961.1	1	100	22	missense	NM 00108	c.2549T>C	p.Leu850Ser	0.7	1	145	0	0(COSM4131'	Anaphase-pr	rs2285745	0.3507	YES	5.803E-1 (pos.36590329) acute_myeloid	WD repeat-	RNA bindir	Brain Diseases;Central Nervou		Benign/Likely	T=0, C=33	T=0.0, C=1.0	0.00001	504.598	33	T=0/0, C=25/8
chr1:241964559	T/T	C	T	SNV	WDR64	WDR64:utr 3:NM 144625.4	1	100	27	utr 3	NM 14462	c.3288C>T	p.?	0.03					WD domain,	rs4658661	0.4076	YES	4.375E-1;1.411E-1 (pos.241964559)						C=0, T=34	C=0.0, T=1.0	0.00001	524.185	34	C=0/0, T=13/2
chr18:54339818	G/G	C	G	SNV	WDR7	WDR7:exonic:NM 015285.2	1	100	2	synonymous	NM 01528	c.72C>G	p.(=)	-0.71					WD domain,	rs568078	0.0034	YES	1.0;0.0 (pos.54339818)	WD repeat-	cytoplasm,hematopoietic progenitor cell differentiation				C=0, G=82	C=0.0, G=1.0	0.00001	1326.74	82	C=0/0, G=43/3
chr8:73926235	TG/TG	T	TG	INDEL	TERF1	TERF1:intronic:NM 017489.2	1	100		intronic	NM 01748	c.415+10T	p.?	1.11					Myb-like DNA	rs111481301				Telomeric r	DNA binding,DNA binding, bending,G-rich strand telomer				T=0, TG=35	T=0.0, TG=1.0	0.00001	496.369	35	T=0/0, TG=16/
chr8:73951274	G/G	T	G	SNV	TERF1	TERF1:intronic:NM 017489.2	1	100		intronic	NM 01748	c.1040-77'	p.?	-2.55					Myb-like DNA	rs2306493		YES		Telomeric r	DNA binding,DNA binding, bending,G-rich strand telomer				T=0, G=20	T=0.0, G=1.0	0.00001	263.784	20	T=0/0, G=17/3
chr15:54008845	C/C	T	C	SNV	WDR72	WDR72:exonic:NM 182758.3	1	100	4	missense	NM 18275	c.298A>G	p.Met100Val	0.92	0.61	21	0		WD domain,	rs690346	1.00E-04	YES	1.0 (pos.54008845)	WD repeat-	cytoplasm	Amelogenesis Imperfecta;Con		Benign	T=0, C=21	T=0.0, C=1.0	0.00001	281.235	21	T=0/0, C=6/15
chr15:44120559	G/G	T	G	SNV	WDR76	WDR76:exonic:NM 024908.3	1	100	2	missense	NM 02490	c.457T>G	p.Ser153Ala	1.39	0.48	99	0	0.02(COSM3754339)		rs678084	0.1824	YES	9.985E-1 (pos.44120559) adenocarcinoma		DNA binding,cellular response to DNA damage stimulus,				T=0, G=64	T=0.0, G=1.0	0.00001	977.635	64	T=0/0, G=32/3
chr8:75262841	A/A	G	A	SNV	GDAP1	GDAP1:intronic:NM 018972.2	1	100		intronic	NM 01897	c.117+28G	p.?	-0.96					Glutathione !	rs4321999	1.00E-04		1.0 (pos.75262841)	Ganglioside	cellular re	Charcot-Marie-Tooth Disease;Congenital Abr			G=0, A=37	G=0.0, A=1.0	0.00001	583.706	37	G=0/0, A=6/31
chr8:75272219	T/T	TTGAA	T	INDEL	GDAP1	GDAP1:intronic:NM 018972.2	4	100		intronic	NM 01897	c.311-153'	p.?	0.85, -0.21, -0.39, 0.74, 0.36					Glutathione !	rs10557622,rs10593912,rs2006!		YES		Ganglioside	cellular re	Charcot-Marie-Tooth Disease;Congenital Abr			TTGAA=0, T	TTGAA=0.0, T=1.0	0.00001	298.892	22	TTGAA=0/0, T=
chr8:76472560	A/T	A	T	SNV	HNF4G	HNF4G:intronic:NM 004133.4	1	50		intronic	NM 00413	c.1094-19,	p.?	-0.41					Ligand-bindir	rs2941473	0.4759	YES	3.491E-1;0.0 (pos.76472560)	Hepatocyte	DNA binding transcription fact(		Palmitic Acid		A=26, T=26	A=0.5, T=0.5	0.00001	256.25	52	A=20/6, T=14/
chr8:7718266	T/T	A	T	SNV	SPAG11A	SPAG11A:intronic:NM 001081552.2	1	100		intronic	NM 00108	c.292+21A	p.?	1.46						rs3860876		YES	1.0 (pos.7718266)		defense response,extracellular region				A=0, T=32	A=0.0, T=1.0	0.00001	485.14	32	A=0/0, T=13/1
chr8:77690337	G/A	G	A	SNV	ZFHX4	ZFHX4:intronic:NM 024721.4	1	46.88		intronic	NM 02472	c.3094-10'	p.?	-0.72					C2H2-type zir	rs11782906		YES		Zinc finger h	nucleus,regulation of transcription, DNA-templated,seq				G=17, A=1!	G=0.5313, A=0.4688	0.00001	143.731	32	G=7/10, A=3/1
chr8:79601420	C/C	T	C	SNV	ZC2HC1A	ZC2HC1A:intronic:NM 016010.2	1	100		intronic	NM 01601	c.353-37T:	p.?	-0.28					zinc-finger of	rs1872072	0.2668	YES	9.127E-1 (pos.79601420)		metal ion binding,protein binding				T=0, C=20	T=0.0, C=1.0	0.00001	263.786	20	T=0/0, C=6/14
chr7:151097323	G/G	A	G	SNV	WDR86	WDR86:exonic:NM 198285.2	1	100	2	synonymous	NM 19828	c.168T>C	p.(=)	-0.69				0.01(COSM54	WD domain,	rs11767693	0.2749	YES	8.326E-1 (pos.15109732: acute_lymphoblastic_leukaemia						A=0, G=29	A=0.0, G=1.0	0.00001	427.603	29	A=0/0, G=10/1
chr8:81733554	A/G	A	G	SNV	ZNF704	ZNF704:intronic:NM 001033723.2	1	50		intronic	NM 00103	c.221+55T	p.?	-0.05						rs76946802		YES			enhancer sequence-specific DNA binding,metal ion bind				A=14, G=14	A=0.5, G=0.5	0.00001	131.94	28	A=6/8, G=5/9
chr19:38376485	A/A	T	A	SNV	WDR87	WDR87:exonic:NM 031951.4	1	100	6	missense	NM 03195	c.7709A>T	p.His2570Leu	1.72	1	99	0	0(COSM4131'	WD domain,	rs10408510	0.2508	YES	8.846E-1 (pos.38376485) neoplasm						T=0, A=32	T=0.0, A=1.0	0.00001	483.461	32	T=0/0, A=22/1
chr8:81892766	G/A	G	A	SNV	PAG1	PAG1:intronic:NM 018440.3	1	58.33		intronic	NM 01844	c.877-37C:	p.?	0.15					Phosphoprot	rs2241972	0.3327	YES	5.143E-1 (pos.81892766)		SH2 doma	Female Urogenital Diseases;Female Urogeni			G=15, A=21	G=0.4167, A=0.5833	0.00001	234.326	36	G=8/7, A=14/7
chr19:38378539	C/C	G	C	SNV	WDR87	WDR87:exonic:NM 031951.4	1	100	6	missense	NM 03195	c.5655C>G	p.Asn1885Lys	0.11	1	94	0	0(COSM4590!	WD domain,	rs10422056		YES	8.85E-1 (pos.38378539) squamous_cell_carcinoma						G=0, C=57	G=0.0, C=1.0	0.00001	849.613	57	G=0/0, C=28/2
chr8:85716963	A/A	C	A	SNV	RALYL	RALYL:intronic:NM 001100391.1	1	100		intronic	NM 00110	c.372-42C:	p.?	-1.41					RNA recognit	rs11784597,rs777590197		YES	0.0 (pos.85716960),8.365E-1 (pos.85716	RALY-like pr	RNA bindir	Breast Diseases;Breast Neoplasms;Neoplasr			C=0, A=26	C=0.0, A=1.0	0.00001	374.887	26	C=0/0, A=17/9
chr8:86121311	T/T	G	T	SNV	E2F5	E2F5:intronic:NM 001951.3	1	100		intronic	NM 00195	c.616-66G	p.?	1.01					E2F transcrip	rs3808538		YES		E2F transcri	DNA binding,DNA binding transcription factor activity,an				G=0, T=22	G=0.0, T=1.0	0.00001	298.892	22	G=0/0, T=16/6
chr8:86158249	T/T	C	T	SNV	CA13	CA13:intronic:NM 198584.2	1	100		intronic	NM 19858	c.37+155C	p.?	2.91					Eukaryotic-ty	rs17740658		YES			bicarbonate transport,carbon:		N-(4-chlorobenzyl)-N-met		C=0, T=65	C=0.0, T=1.0	0.00001	1003.8	65	C=0/0, T=20/4
chr8:86250709	C/C	T	C	SNV	CA1	CA1:intronic:NM 001128831.3	1	100		intronic	NM 00112	c.38-31A>C	p.?	-2.1					Eukaryotic-ty	rs4740049	0.2769	YES	4.698E-1 (pos.86250709)	Carbonic ar	arylestera	Adenocarcinoma;C	3-4-(AMINOSULFONYL)PH		T=0, C=28	T=0.0, C=1.0	0.00001	408.718	28	T=0/0, C=9/19
chr19:38379446	T/T	C	T	SNV	WDR87	WDR87:exonic:NM 031951.4	1	100	6	missense	NM 03195	c.4748G>A	p.Arg1583Gln	-1.56	0.45	43	0.001	0.01(COSM50	WD domain,	rs6508750	0.3919	YES	7.943E-1 (pos.38379446) ductal_carcinoma						C=0, T=33	C=0.0, T=1.0	0.00001	504.187	33	C=0/0, T=17/1
chr8:8643582	G/G	C	G	SNV	MFHAS1	MFHAS1:intronic:NM 004225.2	1	100		intronic	NM 00422	c.3126-17(	p.?	0.76					Leucine Rich	rs2271340	0.2408	YES	9.066E-1;0.0 (pos.8643582)	Malignant f	plasma m(	Nervous System Diseases;Neuromuscular Di:			C=0, G=21	C=0.0, G=1.0	0.00001	281.227	21	C=0/0, G=10/1
chr8:8655023	T/T	C	T	SNV	MFHAS1	MFHAS1:intronic:NM 004225.2	1	100		intronic	NM 00422	c.2999-22(	p.?	-1.5					Leucine Rich	rs3748144,rs762913!	0.2397	YES	0.0 (pos.8655022),9.044E-1 (pos.865502	Malignant f	plasma m(	Nervous System Diseases;Neuromuscular Di:			C=0, T=31	C=0.0, T=1.0	0.00001	447.432	31	C=0/0, T=16/1
chr8:87567305	C/C	A	C	SNV	CPNE3	CPNE3:intronic:NM 003909.4	1	100		intronic	NM 00390	c.1254+93	p.?	-0.12					C2 domain,C(	rs3816240		YES		Copine III	ERBB2 sigr	Genital Diseases, Male:Genital Neoplasms, I			A=0, C=38	A=0.0, C=1.0	0.00001	603.726	38	A=0/0, C=20/1
chr19:33666386	G/C	G	C	SNV	WDR88	WDR88:exonic:NM 173479.3	1	58.52	11	missense	NM 17347	c.1327G>C	p.Asp443His	0.4	0.09	81	0.838		PQQ enzyme	rs151252826	2.00E-04		0.0 (pos.33666386)						G=73, C=1(	G=0.4148, C=0.5852	0.00001	1201.52	176	G=36/37, C=41
chr8:89068596	T/T	C	T	SNV	MMP16	MMP16:intronic:NM 005941.4	1	100		intronic	NM 00594	c.1223-90(	p.?	-0.01					Domain of un	rs2616495		YES		Matrix meti	Golgi lumen,cell surface,chonc		Batimastat,Marimastat		C=0, T=31	C=0.0, T=1.0	0.00001	465.818	31	C=0/0, T=17/1
chr8:92021036	T/T	C	T	SNV	PIP4P2	PIP4P2:intronic:NM 018710.2	1	100		intronic	NM 01871	c.487-13G	p.?	-0.19					Transmembr	rs2054805,rs775043:	0.2716	YES	0.0 (pos.92021032),2.537E-1;0.0 (pos.92021036)		integral component of membrane,late endosome memb				C=0, T=48	C=0.0, T=1.0	0.00001	810.429	48	C=0/0, T=19/2
chr8:92139254	T/C	T	C	SNV	LRRC69	LRRC69:intronic:NM 001129890.1	1	56		intronic	NM 00112	c.311-62T:	p.?	0.95					Leucine Rich	rs12544236		YES							T=11, C=14	T=0.44, C=0.56	0.00001	134.35	25	T=6/5, C=3/11
chr16:706534	G/A	G	A	SNV	WDR90	WDR90:exonic:NM 145294.4	1	50	18	synonymous	NM 14529	c.2199G>A	p.(=)	1.14				0.91(COSM37	Protein of un	rs28485323	0.2164	YES	6.769E-1 (pos.706534) adenocarcinoma		protein binding				G=28, A=28	G=0.5, A=0.5	0.00001	270.965	56	G=14/14, A=1:
chr7:134879830	A/A	G	A	SNV	WDR91	WDR91:exonic:NM 014149.3	1	100	9	synonymous	NM 01414	c.1251C>T	p.(=)	0.76					WD domain,	rs292575	0.2767	YES	5.925E-1 (pos.134879830)	WD repeat-	cytosol,early endosome membrane,early endosome to l				G=0, A=27	G=0.0, A=1.0	0.00001	389.988	27	G=0/0, A=13/1
chr8:95186295	C/C	T	C	SNV	CDH17	CDH17:intronic:NM 004063.3	1	100		intronic	NM 00406	c.583+35A	p.?	-0.07					Cadherin dor	rs2514813	0.2316	YES	3.473E-1 (pos.95186295)	Cadherin-1'	adherens	Asthma;Asthma, Occupational;Bronchial Dis(			T=0, C=43	T=0.0, C=1.0	0.00001	710.205	43	T=0/0, C=22/2
chr7:134889141	A/A	G	A	SNV	WDR91	WDR91:exonic:NM 014149.3	1	100	6	missense	NM 01414	c.770C>T	p.Pro257Leu	6.47	0	98	0.899	0.95(COSM32	WD domain,	rs292592	0.2764	YES	5.861E-1 (pos.13488914: ductal_carcinc	WD repeat-	cytosol,early endosome membrane,early endosome to l				G=0, A=27	G=0.0, A=1.0	0.00001	389.953	27	G=0/0, A=9/18
chr8:95793515	G/A	G	A	SNV	DPY19L4	DPY19L4:intronic:NM 181787.2	1	95.65		intronic	NM 18178	c.1715+12	p.?	0.57					Q-cell neurob	rs4398879		YES		DPY19-like	integral component of membrane,mannosyltransferase				G=1, A=22	G=0.0435, A=0.9565	0.00001	288.986	23	G=1/0, A=16/6
chr8:96053784	T/T	TCT	T	INDEL	NDUFAF6	NDUFAF6:intronic:NM 152416.3	2	100		intronic	NM 15241	c.421-14T(	p.?	1.0, -0.95, 0.55					Squalene/ph	rs200535848,rs201518779,rs54		YES	9.417E-1 (pos.96053782)	NADH dehy	biosynthe	Alzheimer Disease;Brain Diseases;Brain Dise			TCT=0, T=3(	TCT=0.0, T=1.0	0.00001	446.639	30	TCT=0/0, T=18,
chr8:97258719	C/T	C	T	SNV	MTERF3	MTERF3:intronic:NM 015942.4	1	54.46		intronic	NM 01594	c.678-37G	p.?	0.71					mTERF	rs1878638	0.4615	YES	1.866E-1 (pos.97258719)	Transcriptic	RNA splicing,developmental process,double-stranded D				C=51, T=61	C=0.4554, T=0.5446	0.00001	658.464	112	C=35/16, T=26
chr15:90245174	A/A	T	A	SNV	WDR93	WDR93:exonic:NM 020212.1	1	100	2	missense	NM 02021	c.197T>A	p.Leu66His	-0.05	0.18	99	0.01			rs4287542	0.4475	YES	2.145E-1 (pos.90245174)		electron transport chain,oxidoreductase activity, acting				T=0, A=32	T=0.0, A=1.0	0.00001	485.137	32	T=0/0, A=16/1
chr2:160104896	A/A	C	A	SNV	WDSUB1	WDSUB1:exonic:NM 001128212.2	1	100	10	synonymous	NM 00112	c.1260G>T	p.(=)	0.25					SAM domain	rs73004952	0.0287	YES	2.312E-4 (pos.160104896)		protein ubiquitination,ubiquitin-protein transferase act				C=0, A=40	C=0.0, A=1.0	0.00001	644.286	40	C=0/0, A=18/2
chr2:160112881	T/T	G	T	SNV	WDSUB1	WDSUB1:exonic:NM 001128212.2	1	100	9	missense	NM 00112	c.958C>A	p.Arg320Ser	0.38	0.77	110	0	0(COSM1489(	SAM domain	rs7591849	0.4266	YES	2.466E-1;0.0 (pos.16011: acute_myeloid_leukaemia		protein ubiquitination,ubiquitin-protein transferase act				G=0, T=29	G=0.0, T=1.0	0.00001	427.602	29	G=0/0, T=8/21
chr2:160136337	A/A	G	A	SNV	WDSUB1	WDSUB1:exonic:NM 001128212.2	1	100	3	missense	NM 00112	c.518C>T	p.Pro173Leu	6.85	1	98	0		SAM domain	rs174264	0		1.0 (pos.160136337)		protein ubiquitination,ubiquitin-protein transferase act				G=0, A=20	G=0.0, A=1.0	0.00001	263.786	20	G=0/0, A=8/12
chr8:99016092	G/G	A	G	SNV	MATN2	MATN2:intronic:NM 002380.4	1	100		intronic	NM 00238	c.1327+81	p.?	-0.08					Calcium-bind	rs10955143		YES		Matrilin 2	axon guidance,basement membrane,biological_process				A=0, G=47	A=0.0, G=1.0	0.00001	789.315	47	A=0/0, G=7/40
chr7:141420768	G/G	A	G	SNV	WEE2,WEE2-AS1	WEE2:exonic:NM 001105558.1, WEE2-AS1:intronic nc:NR (	1	100	5	synonymous	NR 01539:	c.792A>G	p.(=)	-1.93				0.03(COSM40	Protein kinas	rs6967301	0.4957	YES	3.173E-1 (pos.14142076! acute_myeloid	Wee1, S. po	ATP binding,cytosol,female meiotic nuclear division,fem				A=0, G=85	A=0.0, G=1.0	0.00001	1389.74	85	A=0/0, G=54/3

chr8:99040154	C/C	A	C	SNV	MATN2	MATN2:intronic:NM 002380.4	1	100		intronic	NM 00238	c.2356+97	p.?	0.32					Calcium-bind	rs2290467		YES			Matrilin 2	axon guidance,basement membrane,biological process			A=0, C=53	A=0.0, C=1.0	0.00001	782.03	53	A=0/0, C=16/3
chr8:99895940	A/A	G	A	SNV	STK3		1	100		intronic				-0.38						rs16897117		YES	0.0 (pos.99895940)		Serine/threonine protein kinase 3				G=0, A=20	G=0.0, A=1.0	0.00001	263.786	20	G=0/0, A=10/1
chr4:6293696	G/G	C	G	SNV	WFS1	WFS1:exonic:NM 006005.3	1	100	6	synonymous	NM 00600	c.684C>G	p.(=)	-0.1						rs1801213	0.3173	YES	9.366E-1 (pos.6293696)		Wolframin	ATPase bir	Abnormalities, Multiple:Blindr	Benign/Likely	C=0, G=21	C=0.0, G=1.0	0.00001	281.235	21	C=0/0, G=11/1
chr4:6302707	T/T	C	T	SNV	WFS1	WFS1:exonic:NM 006005.3	1	100	8	synonymous	NM 00600	c.1185C>T	p.(=)	-1.44				0.14(COSM5021444)		rs1801206	0.4017	YES	9.358E-1 (pos.6302707)	osteosarcoma	Wolframin	ATPase bir	Abnormalities, Multiple:Blindr	Benign/Likely	C=0, T=36	C=0.0, T=1.0	0.00001	553.901	36	C=0/0, T=15/2
chr9:100226982	A/A	G	A	SNV	TDRD7	TDRD7:intronic:NM 014290.2	1	100		intronic	NM 01429	c.1443-14	p.?	-0.34					OST-HTH/LOT	rs4278253		YES			Tudor doma	chromatoi	Cataract;Eye Diseases;Genital Diseases, Mal		G=0, A=24	G=0.0, A=1.0	0.00001	334.792	24	G=0/0, A=14/1
chr9:100227011	C/C	T	C	SNV	TDRD7	TDRD7:intronic:NM 014290.2	1	100		intronic	NM 01429	c.1443-11	p.?	-0.25					OST-HTH/LOT	rs4397526		YES			Tudor doma	chromatoi	Cataract;Eye Diseases;Genital Diseases, Mal		T=0, C=24	T=0.0, C=1.0	0.00001	334.792	24	T=0/0, C=14/1
chr9:100366953	G/G	A	G	SNV	TSTD2	TSTD2:intronic:NM 139246.4	1	100		intronic	NM 13924	c.1252+56	p.?	-0.14					Rhodanase C	rs2805779		YES	5.031E-1 (pos.100366953)			biological process,cellular component,molecular func			A=0, G=20	A=0.0, G=1.0	0.00001	263.697	20	A=0/0, G=13/7
chr4:6304087	G/G	A	G	SNV	WFS1	WFS1:exonic:NM 006005.3	1	100	8	synonymous	NM 00600	c.2565A>G	p.(=)	-2.78				0.01(COSM448013)		rs1046316	0.313	YES	9.368E-1;3.49E-4 (pos.63	neoplasm	Wolframin	ATPase bir	Abnormalities, Multiple:Blindr	Benign/Likely	A=0, G=47	A=0.0, G=1.0	0.00001	789.328	47	A=0/0, G=29/1
chr9:117166338	C/C	G	C	SNV	WHRN	WHRN:exonic:NM 015404.3	1	100	10	missense	NM 01540	c.2256C>G	p.His752Gln	4.29	1	24	0		PDZ domain (	rs6478078	0		1.0 (pos.117166338)		Whirlin	USH2 complex,actin filament,auditory rec		Benign	G=0, C=56	G=0.0, C=1.0	0.00001	832.089	56	G=0/0, C=30/2
chr9:101006443	C/C	T	C	SNV	TBC1D2	TBC1D2:intronic:NM 001267571.1	1	100		intronic	NM 00126	c.512-32A	p.?	-1.77					PH domain,R	rs10818669	0.4973	YES	4.661E-1 (pos.101006443)		TBC1 domai	GTPase ac	Mental Disorders;Stress Disorders, Post-Trau		T=0, C=24	T=0.0, C=1.0	0.00001	334.792	24	T=0/0, C=17/7
chr9:101539601	A/A	G	A	SNV	ANKS6	ANKS6:intronic:NM 173551.4	1	100		intronic	NM 17355	c.1617+86	p.?	0.41					Ankyrin repe	rs4742741		YES			Ankyrin rep	cilium,cyt	Cardiovascular Abnormalities;Cardiovascula		G=0, A=23	G=0.0, A=1.0	0.00001	314.692	23	G=0/0, A=16/7
chr9:101785558	T/G	T	G	SNV	COL15A1	COL15A1:intronic:NM 001855.4	1	45.45		intronic	NM 00185	c.1762-81	p.?	-0.22					Collagen trip	rs7848837		YES			Collagen XV	angiogene	Adnexal Diseases;Endocrine Gland Neoplasm		T=12, G=10	T=0.5455, G=0.4545	0.00001	81.759	22	T=10/2, G=8/2
chr9:101785598	G/T	G	T	SNV	COL15A1	COL15A1:intronic:NM 001855.4	1	50		intronic	NM 00185	c.1762-41	p.?	0.03					Collagen trip	rs7861925	0.1251	YES	2.619E-1;0.0 (pos.101785598)		Collagen XV	angiogene	Adnexal Diseases;Endocrine Gland Neoplasm		G=13, T=13	G=0.5, T=0.5	0.00001	119.13	26	G=10/3, T=8/5
chr9:103091630	A/G	A	G	SNV	TEX10	TEX10:intronic:NM 017746.3	1	57.89		intronic	NM 01774	c.1490-73	p.?	0.97					Rix1 complex	rs10819759		YES			Testis-expr	MLL1 complex,Rix1 complex,cytoplasm,nucleolus,nucle			A=16, G=22	A=0.4211, G=0.5789	0.00001	249.054	38	A=11/5, G=10/
chr9:103111705	G/G	T	G	SNV	TEX10	TEX10:intronic:NM 017746.3	1	100		intronic	NM 01774	c.-8-51A>C	p.?	-0.07					Rix1 complex	rs914711		YES			Testis-expr	MLL1 complex,Rix1 complex,cytoplasm,nucleolus,nucle			T=0, G=72	T=0.0, G=1.0	0.00001	1130.95	72	T=0/0, G=49/2
chr9:103114793	C/C	G	C	SNV	TEX10	TEX10:intronic:NM 017746.3	1	100		intronic	NM 01774	c.-9+261C	p.?	0.78					Rix1 complex	rs1930244	0.2021	YES			Testis-expr	MLL1 complex,Rix1 complex,cytoplasm,nucleolus,nucle			G=0, C=51	G=0.0, C=1.0	0.00001	754.535	51	G=0/0, C=33/1
chr9:117188566	T/T	C	T	SNV	WHRN	WHRN:exonic:NM 015404.3	1	100	4	missense	NM 01540	c.1091G>A	p.Arg364His	4.78	1	29	0		PDZ domain (	rs10817610	1.00E-04		1.0 (pos.117188566)		Whirlin	USH2 complex,actin filament,auditory rec		Benign	C=0, T=35	C=0.0, T=1.0	0.00001	536.417	35	C=0/0, T=20/1
chr2:175436940	A/A	G	A	SNV	WIPF1	WIPF1:exonic:NM 003387.4	1	100	5	missense	NM 00338	c.593C>T	p.Pro198Leu	2.51	1	98	0		WH2 motif	rs4972450	0.0414	YES	9.906E-1 (pos.175436940)		WAS/WASL-	Fc-gamma	Autoimmune Diseases;Blood C	Benign	G=0, A=20	G=0.0, A=1.0	0.00001	263.783	20	G=0/0, A=12/8
chr12:987482	A/A	G	A	SNV	WNK1	WNK1:exonic:NM 001184985.1	1	100	11	synonymous	NM 00118	c.3567G>A	p.(=)	-0.87				0.08(COSM37	Oxidative-str	rs1012729	0.2594	YES	8.004E-1;0.0 (pos.98748	adenocarcinoi	WNK lysine	ATP bindin	Cardiovascular Diseases;Char	Benign	G=0, A=30	G=0.0, A=1.0	0.00001	446.637	30	G=0/0, A=24/6
chr12:968489	T/C	T	C	SNV	WNK1	WNK1:exonic:NM 001184985.1	1	57.14	6	synonymous	NM 00118	c.1479T>C	p.(=)	0.84				0.81(COSM37	Oxidative-str	rs2286006	0.1503	YES	6.722E-2 (pos.968489)	acute myeloic	WNK lysine	ATP bindin	Cardiovascular Diseases;Char	Benign	T=33, C=44	T=0.4286, C=0.5714	0.00001	492.244	77	T=23/10, C=21
chr9:107531361	C/C	T	C	SNV	NIPSNAP3B	NIPSNAP3B:intronic:NM 018376.3	1	100		intronic	NM 01837	c.430+59T	p.?	-0.46					NIPSNAP	rs2472478		YES				mitochondrion			T=0, C=53	T=0.0, C=1.0	0.00001	782.03	53	T=0/0, C=29/2
chr9:107547622	C/G	C	G	SNV	ABCA1	ABCA1:intronic:NM 005502.3	1	51.85		intronic	NM 00550	c.6645+55	p.?	-0.95					AAA domain,	rs1331924		YES			ATP-binding	ATP bindin	Arterial Occlusive D	ATP,Glyburide,Probucol	C=13, G=14	C=0.4815, G=0.5185	0.00001	132.623	27	C=9/4, G=9/5
chr9:107566877	A/A	C	A	SNV	ABCA1	ABCA1:intronic:NM 005502.3	1	100		intronic	NM 00550	c.4559+30	p.?	-0.02					AAA domain,	rs2777801	0.1159	YES	9.993E-1 (pos.107566877)		ATP-binding	ATP bindin	Arterial Occlusive D	ATP,Glyburide,Probucol	C=0, A=67	C=0.0, A=1.0	0.00001	1034.15	67	C=0/0, A=58/9
chr7:120969769	G/A	G	A	SNV	WNT16	WNT16:exonic:NM 057168.1	1	50	2	missense	NM 05716	c.244G>A	p.Gly82Arg	1.05	0.23	125	0	0.15(COSM15	wnt family	rs2908004	0.455	YES	1.779E-1;0.0 (pos.12096	acute myeloic	Wingless-ty	Wnt signaling pathway,bone remodeling,cardiac epithel			G=13, A=13	G=0.5, A=0.5	0.00001	119.148	26	G=7/6, A=7/6
chr9:107593182	A/T	A	T	SNV	ABCA1	ABCA1:intronic:NM 005502.3	1	50		intronic	NM 00550	c.1892+24	p.?	0.9					AAA domain,	rs4743763	0.2797	YES	8.148E-1 (pos.107593182)		ATP-binding	ATP bindin	Arterial Occlusive D	ATP,Glyburide,Probucol	A=26, T=26	A=0.5, T=0.5	0.00001	256.695	52	A=8/18, T=6/2
chr9:107651526	G/G	A	G	SNV	ABCA1	ABCA1:intronic:NM 005502.3	1	100		intronic	NM 00550	c.67-50T>C	p.?	-0.08					AAA domain,	rs2275545	0.1472	YES	7.247E-2 (pos.107651526)		ATP-binding	ATP bindin	Arterial Occlusive D	ATP,Glyburide,Probucol	A=0, G=61	A=0.0, G=1.0	0.00001	921.79	61	A=0/0, G=16/4
chr9:110047132	C/C	G	C	SNV	RAD23B	RAD23B:intronic:NM 002874.4	1	100		intronic	NM 00287	c.66+1156	p.?	0.02					UBA/TS-N dor	rs10733562		YES			RAD23 (Sac	XPC compl	Congenital Abnormalities;Congenital, Heredi		G=0, C=24	G=0.0, C=1.0	0.00001	334.792	24	G=0/0, C=12/1
chr5:137426447	A/A	G	A	SNV	WNT8A	WNT8A:exonic:NM 058244.3	1	100	6	synonymous	NM 05824	c.741G>A	p.(=)	-2.21				0.04(COSM37	wnt family	rs6596422	0.4741	YES	5.947E-1 (pos.13742644	adenocarcinoi	Wingless-ty	Wnt signa	Arrhythmias, Cardiac;Atrial Fibrillation;Cardi		G=0, A=31	G=0.0, A=1.0	0.00001	465.819	31	G=0/0, A=15/1
chr9:111637304	G/T	G	T	SNV	ELP1	ELP1:intronic:NM 003640.4	1	50		intronic	NM 00364	c.3856-54	p.?	-1					IKI3 family	rs2027433	0.2857	YES			Elongator holoenzyme complex,RNA polymerase II core b				G=21, T=21	G=0.5, T=0.5	0.00001	231.406	42	G=8/13, T=13/
chr9:111670707	T/C	T	C	SNV	ELP1	ELP1:intronic:NM 003640.4	1	57.45		intronic	NM 00364	c.1361-23	p.?	-0.11					IKI3 family	rs838824	0.3479	YES	7.041E-1 (pos.111670707)		Elongator holoenzyme complex,RNA polymerase II core b				T=20, C=27	T=0.4255, C=0.5745	0.00001	326.535	47	T=12/8, C=11/
chr8:30938704	G/A	G	A	SNV	WRN	WRN:exonic:NM 000553.4	1	50	9	missense	NM 00055	c.1161G>A	p.Met387Ile	0.11	0.43	10	0.204	0.03(COSM37	3'-5' exonucle	rs1800391	0.0792	YES	1.622E-3 (pos.30938704)	adenocarcinoi	DNA helicas	3'-5' DNA h	Congenital, Hereditary, and Ne	Benign/Likely	G=16, A=16	G=0.5, A=0.5	0.00001	157.858	32	G=14/2, A=15/
chr9:111843055	T/T	A	T	SNV	TMEM245	TMEM245:intronic:NM 032012.3	1	100		intronic	NM 03201	c.1449+64	p.?	0.27					AI-2E family t	rs497196		YES				biological	Mental Disorders;Schizophrenia;Schizophren		A=0, T=102	A=0.0, T=1.0	0.00001	1592.11	102	A=0/0, T=48/5
chr9:111849732	T/G	T	G	SNV	TMEM245	TMEM245:intronic:NM 032012.3	1	50		intronic	NM 03201	c.1151-11	p.?	-0.28					AI-2E family t	rs12344205		YES				biological	Mental Disorders;Schizophrenia;Schizophren		T=17, G=17	T=0.5, G=0.5	0.00001	172.006	34	T=9/8, G=6/11
chr17:6014176	G/G	A	G	SNV	WSCD1	WSCD1:exonic:NM 015253.1	1	100	7	synonymous	NM 01525	c.1095A>G	p.(=)	-1.43					Sulfotransfer	rs2302837	0.074	YES	9.539E-1 (pos.6014176)			integral cc	Digestive System Diseases;Digestive System		A=0, G=75	A=0.0, G=1.0	0.00001	1193.77	75	A=0/0, G=41/3
chr19:7685843	G/A	G	A	SNV	XAB2	XAB2:exonic:NM 020196.2	1	58.33	14	synonymous	NM 02019	c.1860C>T	p.(=)	0.01					PPR repeat,T	rs794078	0.1989	YES	1.681E-1 (pos.7685843)		XPA-binding	Prp19 complex,blastocyst development,catalytic step 1			G=10, A=14	G=0.4167, A=0.5833	0.00001	135.339	24	G=6/4, A=1/13
chr9:112025473	A/A	C	A	SNV	EPB41L4B	EPB41L4B:intronic:NM 019114.4	1	100		intronic	NM 01911	c.579-91G	p.?	-0.7					FERM C-termi	rs1982226		YES			Erythrocyte	actomyosin structure organization,apical part of cell,bic			C=0, A=26	C=0.0, A=1.0	0.00001	370.624	26	C=0/0, A=5/21
chr9:112225774	T/C	T	C	SNV	PTPN3	PTPN3:intronic:NM 002829.3	1	53.13		intronic	NM 00282	c.-16-43A>	p.?	-0.67					Dual specific	rs2274786		YES			Protein-tyro	ATPase bir	Adenocarcinoma;Bile Duct Diseases;Bile Duc		T=15, C=17	T=0.4688, C=0.5313	0.00001	173.555	32	T=6/9, C=8/9
chr17:6659324	A/G	A	G	SNV	XAF1	XAF1:utr 5:NM 017523.4	1	50	1	utr 5	NM 01752	c.-74A>G	p.?	-0.13						rs3785793	0.44	YES			XIAP-associ	apoptotic	Colonic Diseases;Colonic Neoplasms;Colorec		A=12, G=12	A=0.5, G=0.5	0.00001	108.793	24	A=8/4, G=8/4
chr2:31571786	G/G	A	G	SNV	XDH	XDH:exonic:NM 000379.3	1	100	27	synonymous	NM 00037	c.3030T>C	p.(=)	1.45					2Fe-2S bindir	rs1884725	0.24	YES	8.534E-1 (pos.31571786)		Xanthine de	2 iron, 2 su	Autistic Disorder;Bl	Aldesleuki Benign	A=0, G=21	A=0.0, G=1.0	0.00001	281.233	21	A=0/0, G=11/1
chr9:113141815	G/G	A	G	SNV	SVEP1	SVEP1:intronic:NM 153366.3	1	100		intronic	NM 15336	c.10238-1	p.?	0.75					Calcium-bind	rs992883	0.402	YES	2.807E-1 (pos.113141815)		Sushi, von v	calcium ion binding,cell adhesion,chromatin binding,cyt			A=0, G=38	A=0.0, G=1.0	0.00001	603.786	38	A=0/0, G=21/1
chrX:123041111	C/C	G	C	SNV	XIAP	XIAP:utr 3:NM 001204401.1	1	100	7	utr 3	NM 00120	c.1574G>C	p.?	0.35					Inhibitor of A	rs12838858		YES	0.0;0.0 (pos.123041111)		Inhibitor of	Wnt signa	Adnexal Diseases;/	1-3.3-Dim Benign	G=0, C=32	G=0.0, C=1.0	0.00001	485.124	32	G=0/0, C=15/1
chr9:113547322	C/C	T	C	SNV	MUSK	MUSK:intronic:NM 005592.3	1	100		intronic	NM 00559	c.1586+26	p.?	0.33					Fz domain,Im	rs484010	0.0782	YES	1.0 (pos.113547322)		Receptor ty	ATP bindin	Arthrogryposis;Autoimmune D	Benign	T=0, C=52	T=0.0, C=1.0	0.00001	767.015	52	T=0/0, C=42/1
chr9:114356367	C/C	T	C	SNV	PTGR1	PTGR1:intronic:NM 012212.3	1	100		intronic	NM 01221	c.152+135	p.?	0.05					N-terminal dc	rs3739704		YES			Prostaglanc	13-prostaglandin reductase ac	(5E,13E)-11-HYDROXY-9,1		T=0, C=40	T=0.0, C=1.0	0.00001	644.278	40	T=0/0, C=20/2
chr3:39229900	C/C	T	C	SNV	XIRP1	XIRP1:exonic:NM 194293.3	1	100	2	missense	NM 19429	c.1037A>G	p.Gln346Arg	1.56		43			Xin repeat	rs6805248	0		1.0;0.0 (pos.39229900)			RNA binding,actin binding,actin cytoskeleton organizatic			T=0, C=32	T=0.0, C=1.0	0.00001	484.786	32	T=0/0, C=15/1
chr2:168099738	T/C	T	C	SNV	XIRP2	XIRP2:exonic:NM 152381.5	1	57.58	9	synonymous	NM 15238	c.1836T>C	p.(=)	0.33				0.53(COSM40	Xin repeat	rs10497323	0.1568	YES	3.069E-1 (pos.16809973	acute myeloic	Cardiomyor	Z disc,acti	Congenital, Hereditary, and Neonatal Diseas		T=14, C=19	T=0.4242, C=0.5758	0.00001	204.39	33	T=7/7, C=8/11
chr22:17264565	G/T	G	T	SNV	XKR3	XKR3:exonic:NM 175878.4	1	50	4	missense	NM 17587	c.1324C>A	p.His442Asn	-0.21	0.13	68	0.898		XK-related pr	rs5748622		YES			X Kell blood	integral component of membrane,plasma membrane			G=14, T=14	G=0.5, T=0.5	0.00001	131.941	28	G=11/3, T=3/1
chr8:6673377	C/C	CA	C	INDEL	XKR5	XKR5:splicesite 3:NM 207411.4	1	100	7	splicesite	NM 207411.4			-1.0, 0.0					XK-related pr	rs11338621,rs200192065,rs753		YES	(pos.6673376), (pos.667	astrocytoma	Grade IV,ost	integral component of membrane			CA=0, C=29	CA=0.0, C=1.0	0.00001	430.649	29	CA=0/0, C=13/
chr9:100459578	C/C	T	C	SNV	XPA	XPA:utr 5:NM 000380.3	1	100	1	utr 5	NM 00038	c.-4A>G	p.?	-0.18					XPA protein C	rs1800975,rs747448	0.2902	YES	4.981E-1;0.0;1.243E-4 (pos.100459578)		XPA comple	DNA repai	Carcinoma;Carcinoma, Basal C	Benign	T=0, C=33	T=0.0, C=1.0	0.00001	504.574	33	T=0/0, C=14/1
chr9:114480602	A/A	C	A	SNV	C9orf84	C9orf84:intronic:NM 173521.4	1	100		intronic	NM 17352	c.1940-34	p.?	-1.21						rs2418174	0.4679	YES	8.749E-1 (pos.114480602)						C=0, A=43	C=0.0, A=1.0	0.00001	705.842	43	C=0/0, A=23/2
chr9:114489887	T/T	C	T	SNV	C9orf84	C9orf84:intronic:NM 173521.4	1	100		intronic	NM 17352	c.1642+26	p.?	-0.43						rs953536	0.467	YES	8.745E-1 (pos.114489887)						C=0, T=21	C=0.0, T=1.0	0.00001	281.235	21	C=0/0, T=11/1
chr3:14197987	T/T	A	T	SNV	XPC	XPC:exonic:NM 004628.4	1	100	10	synonymous	NM 00462	c.1881T>A	p.(=)	-2.39				0.02(COSM45	Rad4 beta-ha	rs2958057		YES	1.0 (pos.14197987)	squamous cel	XPC gene	DNA repai	Adenocarcinoma;Amino Acid N	Benign	A=0, T=44	A=0.0, T=1.0	0.00001	722.57	44	A=0/0, T=20/2
chr9:114989616	G/G	A	G	SNV	PTBP3	PTBP3:intronic:NM 001163790.2	1	100		intronic	NM 00116	c.1457+75	p.?	-0.69					RNA recogniti	rs6477900		YES				RNA binding,RNA splicing,anatomical structure morphog			A=0, G=34	A=0.0, G=1.0	0.00001	524.121	34	A=0/0, G=12/2
chr9:115060205	G/G	C	G	SNV	PTBP3	PTBP3:intronic:NM 001163790.2	1	100		intronic	NM 00116	c.43-9G>C	p.?	-0.09				0.16(COSM63	RNA recogniti	rs10981348	0.323	YES	3.747E-1 (pos.11506020	carcinoma		RNA binding,RNA splicing,anatomical structure morphog			C=0, G=301	C=0.0, G=1.0	0.00001	4934.42	301	C=0/0, G=149/
chr9:115092624	A/A	T	A	SNV	PTBP3	PTBP3:intronic:NM 001163790.2	1	100		intronic	NM 00116	c.42+97A>	p.?	2.08					RNA recogniti	rs2900539		YES				RNA binding,RNA splicing,anatomical structure morphog			T=0, A=34	T=0.0, A=1.0	0.00001	524.186	34	T=0/0, A=14/2
chr9:115142507	G/G	A	G	SNV	HSDL2	HSDL2:intronic:NM 032303.4	1	100		intronic	NM 03230	c.17+47A>	p.?	0.29					Enoyl-(Acyl ca	rs7867831	3.00E-04		1.0 (pos.115142507)			biological	Digestive System Diseases;Digestive System		A=0, G=91	A=0.0, G=1.0	0.00001	1503.27	91	A=0/0, G=54/3
chr9:115567018	TGT/TGT	T	TGT	INDEL	SNX30	SNX30:intronic:NM 001012994.1	2	100		intronic	NM 00101	c.157-38T	p.?	1.33					BAR domain,PX domain							endocytos	Ear Diseases;Hearing Disorders;Hearing Loss		T=0, TGT=3	T=0.0, TGT=1.0	0.00001	600.444	39	T=0/0, TGT=16
chr19:44047826	T/A	T	A	SNV	XRCC1	XRCC1:exonic:NM 006297.2	1	97.3	16	missense	NM 00629	c.1726A>T	p.Asn576Tyr	5.78	1	143	0		BRCA1 C Term	rs2682557,rs386809	1.00E-04		0.0 (pos.44047824),0.0;0.0 (pos.440478		X-ray-repair	3' overhan	Adenoma;Brain Diseases;Breast Diseases;Ca		T=1, A=36	T=0.027, A=0.973	0.00001	551.778	37	T=0/1, A=17/1
chr9:116356373	C/C	G	C	SNV	RGS3	RGS3:intronic:NM 144488.5	1	100		intronic	NM 14448	c.3081-33	p.?	1.5				0.08(COSM45	C2 domain,PI	rs12350531,rs77760	0.3179	YES	0.0 (pos.116356371),8.7	neoplasm	Regulator o	GTPase activator activity,cytoplasm,cytosol,inactivation			G=0, C=36	G=0.0, C=1.0	0.00001	563.742	36	G=0/0, C=17/1
chr9:116356516	G/G	A	G	SNV	RGS3	RGS3:intronic:NM 144488.5	1	100		intronic	NM 14448	c.3081-19	p.?	0.37				0(COSM4163	C2 domain,PI	rs12341266	0.1178	YES	1.0 (pos.116356516)	neoplasm	Regulator o	GTPase activator activity,cytoplasm,cytosol,inactivation			A=0, G=23	A=0.0, G=1.0	0.00001	316.638	23	A=0/0, G=9/14
chr9:116356529	G/G	C	G	SNV	RGS3	RGS3:intronic:NM 144488.5	1	100		intronic	NM 14448	c.3081-18	p.?	0.72				0(COSM4163	C2 domain,PI	rs10817493	0.2749	YES	9.929E-1;0.0 (pos.11635	neoplasm	Regulator o	GTPase activator activity,cytoplasm,cytosol,inactivation			C=0, G=22	C=0.0, G=1.0	0.00001	298.892	22	C=0/0, G=7/15
chr19:44057227	T/C	T	C	SNV	XRCC1	XRCC1:exonic:NM 006297.2	1	45.83	7	synonymous	NM 00629	c.618A>G	p.(=)	-3.22				0(COSM3756	BRCA1 C Term	rs915927	0.4286	YES	1.491E-1 (pos.44057227)	adenocarcinoi	X-ray-repair	3' overhan	Adenoma;Brain Diseases;Breast Diseases;Ca		T=52, C=44	T=0.5417, C=0.4583	0.00001	471.522	96	T=28/24, C=21
chr17:48437456	G/G	C	G	SNV	XYLT2	XYLT2:exonic:NM 022167.3	1	100	11	missense	NM 02216	c.2402C>G	p.Thr801Arg	3.02	0	71	0.025	0.46(COSM13	Core-2/I-Bran	rs6504649	0.3975	YES	8.388E-2;0.0 (pos.48437	adenocarcinoi	Xylosyltran	Golgi mem	Bone Diseases;Bone Diseases,	risk factor	C=0, G=26	C=0.0, G=1.0	0.00001	371.335	26	C=0/0, G=12/1
chr9:116858625	G/G	T	G	SNV	KIF12	KIF12:intronic:NM 138424.1	1	100		intronic	NM 13842	c.324+42A	p.?	0.63					Kinesin moto	rs6478062	0.3131	YES	6.946E-1 (pos.116858625)		Kinesin fam	ATP binding,ATPase activity,biological process,cytoplas			T=0, G=37	T=0.0, G=1.0	0.00001	583.438	37	T=0/0, G=22/1
chr21:47717616	A/A	G	A	SNV	YBEY	YBEY:utr 3:NM 058181.2	1	100	5	utr 3	NM 05818	c.572G>A	p.?	-0.61					Uncharacteri	rs8651		YES			YBEY metall	endonuclease activity,metal ion binding,metalloendope			G=0, A=27	G=0.0, A=1.0	0.00001	389.973	27	G=0/0, A=5/22
chr9:117054542	C/T	C	T	SNV	COL27A1	COL27A1:intronic:NM 032888.3	1	53.13		intronic	NM 03288	c.4584+52	p.?	-0.64					Collagen trip	rs751327595,rs7855	0.3272	YES	0.0 (pos.117054541),3.772E-1 (pos.1170		Collagen, tv	endoplasr	Basal Ganglia Diseases;Brain Diseases;Centr		C=15, T=17	C=0.4688, T=0.5313	0.00001	173.551	32	C=9/6, T=13/4
chr3:183432983	T/T	C	T	SNV	YEATS2	YEATS2:exonic:NM 018023.4	1	100	2	synonymous	NM 01802	c.33C>T	p.(=)	-3.87				0.01(COSM37	YEATS family	rs3736535	0.1602	YES	3.089E-1 (pos.18343298	acute myeloic	YEATS doma	Ada2/Gcn5/Ada3 transcription activator complex,TBP-cl			C=0, T=29	C=0.0, T=1.0	0.00001	427.599	29	C=0/0, T=5/24
chr12:69753595	T/T	C	T	SNV	YEATS4	YEATS4:utr 5:NM 006530.3	1	100	1	utr 5	NM 00653	c.-158C>T	p.?	-0.07					YEATS family	rs622656		YES			YEATS doma	DNA bindi	Cardiovascular Diseases;Embolism and Thro		C=0, T=26	C=0.0, T=1.0	0.00001	371.419	26	C=0/0, T=16/1
chr18:724612	G/G	A	G	SNV	YES1	YES1:exonic:NM 005433.3	1	100	12	synonymous	NM 00543	c.1444T>C	p.(=)	2.09					Protein kinas	rs537141	1.00E-04		1.0 (pos.724612)		Oncogene Y	ATP bindin	Autoimmune Disea	Dasatinib	A=0, G=89	A=0.0, G=1.0	0.00001	1470.39	89	A=0/0, G=44/4
chr14:75279395	A/A	T	A	SNV	YLPM1	YLPM1:exonic:NM 019589.2	1	100	11	synonymous	NM 01958	c.5412T>A	p.(=)	0.59				0.43(COSM41	AAA domain	rs2302834	0.3016	YES	7.604E-1 (pos.75279395)	neoplasm		RNA binding,biological process,cytosol,nuclear speck,nu			T=0, A=73	T=0.0, A=1.0	0.00001	1150.51	73	T=0/0, A=37/3
chr8:64098729	AG/AG	A	AG	INDEL	YTHDF3	YTHDF3:exonic:NM 001277817.1	1	100	4	unknown	NM 001277817.1			1.75					YT521-B-like	rs58419856,rs75212	0	YES	1.0;0.0 (pos.64098729)			N6-methyl	Autoimmune Diseases;Female Urogenital Di		A=0, AG=32	A=0.0, AG=1.0	0.00001	485.141	32	A=0/0, AG=19/
chr7:100371100	A/A	C	A	SNV	ZAN	ZAN:exonic:NM 173059.2	1	100	30	missense	NM 17305	c.5618C>A	p.Ala1873Glu	-0.75		107			C8 domain,M	rs2734880	0.4445	YES	1.648E-1;0.0 (pos.100371100)		Zonadhesin	binding of sperm to zona pellucida,cell-cell adhesion,int			C=0, A=51	C=0.0, A=1.0	0.00001	754.567	51	C=0/0, A=18/3
chr7:100371114	T/T	C	T	SNV	ZAN	ZAN:exonic:NM 173059.2	1	100	30	missense	NM 17305	c.5632C>T	p.Pro1878Ser	-0.51		74			C8 domain,M	rs314298	0.4444	YES	1.63E-1 (pos.100371114)		Zonadhesin	binding of sperm to zona pellucida,cell-cell adhesion,int			C=0, T=50	C=0.0, T=1.0	0.00001	852.748	50	C=0/0, T=17/3
chr7:100385561	G/G	GGCTTCAG	G	INDEL	ZAN	ZAN:exonic:NM 173059.2	35	100	39	frameshiftDeletion	NM 17305	c.7028 70	p.Phe2343fs	0.34, 4.09, -0.06, -0.26, 0.45, -0.73, -0.07, -1.38,				0.07(COSM62	C8 domain,M	rs1007604387,rs112	0.458	YES	0.0 (pos.100385559),0.0	ductal carcinc	Zonadhesin	binding of sperm to zona pellucida,cell-cell adhesion,int			GGCTTCAG	GGCTTCAGCTACCGCT	0.00001	1249.36	78	GGCTTCAGCTA
chr9:117848112	T/T	A	T	SNV	TNC	TNC:intronic:NM 002160.3	1	100		intronic	NM 00216	c.1867+31	p.?	-0.77					EGF-like dom	rs1617917	0.3153	YES	3.053E-1 (pos.117848112)		Tenascin C	basement	Asthma;Bronchial Diseases;Cardiovascular D		A=0, T=103	A=0.0, T=1.0	0.00001	1609.52	103	A=0/0, T=52/5
chr3:167077691	A/A	T	A	SNV	ZBBX	ZBBX:exonic:NM 001199201.1	1	100	8	synonymous	NM 00119	c.399A>T	p.(=)	-0.24					B-box zinc fin	rs4257551	0.0054		1.0 (pos.167077691)						T=0, A=96	T=0.0, A=1.0	0.00001	1618.01	96	T=0/0, A=56/4
chr22:50277835	C/C	T	C	SNV	ZBED4	ZBED4:exonic:NM 014838.2	1	100	2	synonymous	NM 01483	c.525T>C	p.(=)	-0.95					BED zinc finge	rs910796	0.1024	YES	9.657E-1 (pos.50277835)		Zinc finger E	DNA bindi	Autoimmune Diseases;Female Urogenital Di		T=0, C=66	T=0.0, C=1.0	0.00001	1015.4	66	T=0/0, C=46/2
chr9:119448979	C/T	C	T	SNV	ASTN2,TRIM 32	ASTN2:intronic:NM 014010.4, TRIM32:upstream:NM 0010	1	58.33		intronic	NM 01401	c.2654-34	p.?, p.?	0.36				0.16(COSM37	Coagulation I	rs10983304		YES	0.0 (pos.119448979)	adenocarcinoi	Astrotactin	RNA bindi	Abnormalities, Multiple:Amphetamine-Relat		C=15, T=21	C=0.4167, T=0.5833	0.00001	234.325	36	C=13/2, T=15/
chr22:50277883	G/G	A	G	SNV	ZBED4	ZBED4:exonic:NM 014838.2	1	100	2	synonymous	NM 01483	c.573A>G	p.(=)	-2.56					BED zinc finge	rs910797	0.1024	YES	9.657E-1 (pos.50277883)		Zinc finger E	DNA bindi	Autoimmune Diseases;Female Urogenital Di		A=0, G=86	A=0.0, G=1.0	0.00001	1410.83	86	A=0/0, G=48/3
chr9:119977051	G/T	G	T	SNV	ASTN2	ASTN2:intronic:NM 014010.4	1	50		intronic	NM 01401	c.631-30C	p.?	0.87					Coagulation I	rs7856971	0.3361	YES	4.095E-1 (pos.119977051)		Astrotactin	calcium io	Amphetamine-Related Disorders;Attention D		G=40, T=40	G=0.5, T=0.5	0.00001	424.157	80	G=25/15, T=23
chr20:56186884	C/C	T	C	SNV	ZBP1	ZBP1:exonic:NM 030776.2	1	100	6	missense	NM 03077	c.773A>G	p.Gln258Arg	-1.67	0.6	43	0		Adenosine de	rs2865394	0.0077		1.0;0.0 (pos.56186884)		Insulin-like	DNA binding,RNA binding,biological process,cytoplasm,			T=0, C=21	T=0.0, C=1.0	0.00001	281.216	21	T=0/0, C=15/6
chr9:123280950	C/C	T	C	SNV	CDK5RAP2	CDK5RAP2:intronic:NM 018249.5	1	100		intronic	NM 01824	c.1093-27	p.?	-0.34					Centrosomin	rs2095064	0.0723	YES	7.418E-1 (pos.123280950)		CDK5 regula	G2/M tran	Agenesis of Corpus Callosum;A	Likely benign	T=0, C=121	T=0.0, C=1.0	0.00001	1957.64	121	T=0/0, C=75/4
chr9:123550027	A/A	G	A	SNV	FBXW2	FBXW2:intronic:NM 012164.3	1	100		intronic	NM 01216	c.490+20C	p.?	-0.57					F-box domair	rs7026635	0.2595	YES	5.509E-1 (pos.123550027)		F-box and W	cellular protein modification process,cytosol,post-trans			G=0, A=26	G=0.0, A=1.0	0.00001	371.292	26	G=0/0, A=17/9
chr9:123632045	G/G	A	G	SNV	PHF19	PHF19:intronic:NM 015651.2	1	100		intronic	NM 01565	c.465+78T	p.?	-2.64				0.01(COSM45	PHD-finger,P	rs1056567	0.3222	YES	5.558E-1 (pos.12363204	adenocarcinoi	PHD finger p	ESC/E(Z) cc	Arthritis;Arthritis, Rheumatoid;Autoimmune		A=0, G=23	A=0.0, G=1.0	0.00001	316.251	23	A=0/0, G=14/9
chr9:123632829	C/C	G	C	SNV	PHF19	PHF19:intronic:NM 015651.2	1	100		intronic	NM 01565	c.269-13C	p.?	-1.25					PHD-finger,P	rs4836833	0.3224	YES	5.063E-1;5.818E-4 (pos.123632829)		PHD finger p	ESC/E(Z) cc	Arthritis;Arthritis, Rheumatoid;Autoimmune		G=0, C=26	G=0.0, C=1.0	0.00001	371.335	26	G=0/0, C=13/1
chr9:123888242	T/T	G	T	SNV	CNTRL	CNTRL:intronic:NM 007018.4	1	100		intronic	NM 00701	c.2025+28	p.?	-0.34					Leucine Rich	rs10760150	0.2946	YES	9.63E-1 (pos.123888242)			Flemming body,G2/M transition of mitotic cell cycle,cell			G=0, T=89	G=0.0, T=1.0	0.00001	1469.7	89	G=0/0, T=44/4
chr9:123908345	A/A	G	A	SNV	CNTRL	CNTRL:intronic:NM 007018.4	1	100		intronic	NM 00701	c.3311-41	p.?	0.6					Leucine Rich	rs7023214	0.3128	YES	7.861E-1;0.0 (pos.123908345)			Flemming body,G2/M transition of mitotic cell cycle,cell			G=0, A=42	G=0.0, A=1.0	0.00001	684.371	42	G=0/0, A=21/2
chr9:123929710	G/G	A	G	SNV	CNTRL	CNTRL:intronic:NM 007018.4	1	100		intronic	NM 00701	c.5656-57	p.?	-0.03					Leucine Rich	rs10818513		YES				Flemming body,G2/M transition of mitotic cell cycle,cell			A=0, G=50	A=0.0, G=1.0	0.00001	852.741	50	A=0/0, G=16/3
chr9:124091135	A/A	G	A	SNV	GSN	GSN:intronic:NM 000177.4	1	100		intronic	NM 00017	c.1916-34	p.?	-0.87					Gelsolin repe	rs306771	0.217	YES	9.909E-1 (pos.124091135)		Gelsolin	actin bind	Acute Coronary Syn	Latrunculin A	G=0, A=23	G=0.0, A=1.0	0.00001	316.747	23	G=0/0, A=19/4
chr9:125144145	G/G	A	G	SNV	PTGS1	PTGS1:intronic:NM 000962.3	1	100		intronic	NM 00096	c.762+119	p.?	0.25					Animal haem	rs1238420		YES			Prostaglanc	Golgi appa	Adnexal Diseases;/	(3-Chloro-4-Propoxy-Phe	A=0, G=37	A=0.0, G=1.0	0.00001	583.541	37	A=0/0, G=13/2
chr1:22835677	G/G	A	G	SNV	ZBTB40	ZBTB40:exonic:NM 014870.3	1	100	9	missense	NM 01487	c.1784A>G	p.Tyr595Cys	0.22	0.27	194	0	0.03(COSM37	BTB/POZ dom	rs209729	0.2808	YES	8.982E-1 (pos.22835677)	diffuse large	Zinc finger-	DNA bindi	Intellectual Disability;Mental Disorders;Nerv		A=0, G=20	A=0.0, G=1.0	0.00001	263.786	20	A=0/0, G=15/5
chr9:37441808	G/G	A	G	SNV	ZBTB5	ZBTB5:exonic:NM 014872.2	1	100	2	synonymous	NM 01487	c.741T>C	p.(=)	-0.44					BTB/POZ dom	rs309450	0		1.0 (pos.37441808)		Zinc finger-	RNA polymerase II regulatory region sequence-specific D			A=0, G=35	A=0.0, G=1.0	0.00001	543.903	35	A=0/0, G=12/2
chr9:125673051	G/G	T	G	SNV	ZBTB6	ZBTB6:utr 3:NM 006626.5	1	100	2	utr 3	NM 00662	c.1301A>C	p.?	-0.16					BTB/POZ dom	rs1147321	0.1434	YES	3.358E-1;0.0 (pos.125673051)			DNA binding,metal ion binding,mitochondrion,nucleus,p			T=0, G=37	T=0.0, G=1.0	0.00001	576.062	37	T=0/0, G=21/1
chr1:33065947	C/C	G	C	SNV	ZBTB8A	ZBTB8A:exonic:NM 001040441.2	1	100	5	missense	NM 00104	c.1253G>C	p.Gly418Ala	3.23	1	60	0		BTB/POZ dom	rs704886	1.00E-04		1.0 (pos.33065947)			DNA binding,biological process,cellular component,me			G=0, C=37	G=0.0, C=1.0	0.00001	583.706	37	G=0/0, C=16/2
chr6:33422976	G/G	T	G	SNV	ZBTB9	ZBTB9:exonic:NM 152735.3	1	100	2	synonymous	NM 15273	c.99T>G	p.(=)	-0.17					BTB/POZ dom	rs3119025	0.0765	YES	9.95E-1 (pos.33422976)			DNA binding,metal ion binding,nucleus,protein binding,r			T=0, G=21	T=0.0, G=1.0	0.00001	281.235	21	T=0/0, G=5/16
chr9:125760863	C/C	T	C	SNV	RABGAP1	RABGAP1:intronic:NM 012197.3	1	100		intronic	NM 01219	c.1205-13	p.?	0.26					Kinesin prote	rs587364,rs7605287	0.1367	YES	0.0 (pos.125760862),2.911E-1 (pos.1257		RAB GTPase	G-protein coupled receptor activity,G-protein coupled re			T=0, C=46	T=0.0, C=1.0	0.00001	760.466	46	T=0/0, C=30/1
chr9:125861695	C/C	T	C	SNV	RABGAP1	RABGAP1:intronic:NM 012197.3	1	100		intronic	NM 01219	c.2818-62	p.?	-2.73					Kinesin prote	rs700080	0.1285	YES			RAB GTPase	G-protein coupled receptor activity,G-protein coupled re			T=0, C=106	T=0.0, C=1.0	0.00001	1662.75	106	T=0/0, C=68/3
chr9:125861888	G/G	A	G	SNV	RABGAP1	RABGAP1:intronic:NM 012197.3	1	100		intronic	NM 01219	c.2889+60	p.?	-0.09					Kinesin prote	rs700081	0.1263	YES			RAB GTPase	G-protein coupled receptor activity,G-protein coupled re			A=0, G=104	A=0.0, G=1.0	0.00001	1626.75	104	A=0/0, G=64/4
chr8:79610710	G/G	A	G	SNV	ZC2HC1A	ZC2HC1A:exonic:NM 016010.2	1	100	7	synonymous	NM 01601	c.666A>G	p.(=)	0.37				0.8(COSM156	zinc-finger of	rs1054283	0.2655	YES	9.144E-1 (pos.79610710)	neoplasm		metal ion binding,protein binding			A=0, G=109	A=0.0, G=1.0	0.00001	1718.92	109	A=0/0, G=71/3
chr1:203787700	C/C	T	C	SNV	ZC3H11A	ZC3H11A:exonic:NM 014827.5	1	100	6	synonymous	NM 01482	c.57T>C	p.(=)	-0.2					RNA-binding,	rs4951260	8.00E-04		1.0 (pos.203787700)		Zinc finger C	RNA bindi	Breast Diseases;Breast Neoplasms;Neoplasr		T=0, C=101	T=0.0, C=1.0	0.00001	1579.75	101	T=0/0, C=42/5
chr9:126794648	G/A	G	A	SNV	LHX2	LHX2:intronic:NM 004789.3	1	46.43		intronic	NM 00478	c.934-51G	p.?	-1.71					Homeobox KI	rs10986179,rs38641	0.3336	YES	9.516E-2;0.0 (pos.126794648)		LIM/homeo	DNA bindi	Arthritis;Arthritis, Rheumatoid;Autoimmune		G=15, A=13	G=0.5357, A=0.4643	0.00001	114.441	28	G=11/4, A=4/9
chr13:46549722	C/C	CT	C	INDEL	ZC3H13	ZC3H13:exonic:NM 015070.5	1	100	12	frameshiftDeletion	NM 01507	c.2163del	p.Glu722fs	5.7, 0.01					Zinc finger C-	rs1007101582					Zinc finger C	RNA binding,metal ion binding,nuclear speck,nucleoplas			CT=0, C=62	CT=0.0, C=1.0	0.00001	796.296	62	CT=0/0, C=57/
chr7:138732457	A/A	G	A	SNV	ZC3HAV1	ZC3HAV1:exonic:NM 020119.3	1	100	13	synonymous	NM 02011	c.2592C>T	p.(=)	0.01				0.14(COSM37	Poly(ADP-ribc	rs3735008	0.4988	YES	3.114E-1 (pos.13873245	acute myeloic	Zinc finger C	NAD+ ADP-ribosyltransferase activity,NAD+ binding,RNA			G=0, A=22	G=0.0, A=1.0	0.00001	290.981	22	G=0/0, A=13/9
chr9:127637215	G/G	T	G	SNV	ARPC5L	ARPC5L:intronic:NM 030978.2	1	100		intronic	NM 03097	c.223-39T	p.?	-1.46					ARP2/3 comp	rs581558	0.0047	YES	8.069E-1 (pos.127637215)			Arp2/3 complex-mediated actin nucleation,Arp2/3 prote			T=0, G=39	T=0.0, G=1.0	0.00001	623.98	39	T=0/0, G=19/2
chr9:127644882	A/A	G	A	SNV	GOLGA1	GOLGA1:intronic:NM 002077.3	1	100		intronic	NM 00207	c.1906-34	p.?	-1.27					GRIP domain	rs596231	5.00E-04		1.0 (pos.127644882)		Golgi autoa	Golgi apparatus,Golgi cisterna membrane,cytosol,perin			G=0, A=60	G=0.0, A=1.0	0.00001	903.531	60	G=0/0, A=25/3
chr7:138732497	A/A	G	A	SNV	ZC3HAV1	ZC3HAV1:exonic:NM 020119.3	1	100	13	missense	NM 02011	c.2552C>T	p.Thr851Ile	-0.37	0.94	89	0	0.01(COSM37	Poly(ADP-ribc	rs3735007	0.4814	YES	3.506E-1 (pos.13873249	acute myeloic	Zinc finger C	NAD+ ADP-ribosyltransferase activity,NAD+ binding,RNA			G=0, A=21	G=0.0, A=1.0	0.00001	281.23	21	G=0/0, A=12/9
chr9:129158002	G/A	G	A	SNV	MVB12B	MVB12B:intronic:NM 033446.2	1	50		intronic	NM 03344	c.662+26G	p.?	-0.16					Multivesicula	rs722538	0.3172	YES	1.067E-1 (pos.129158002)			ESCRT I cor	Nutrition Disorders:Nutritional and Metaboli		G=27, A=27	G=0.5, A=0.5	0.00001	262.866	54	G=18/9, A=16/
chr16:87446053	A/A	G	A	SNV	ZCCHC14	ZCCHC14:exonic:NM 015144.2	1	100	12	synonymous	NM 01514	c.1863C>T	p.(=)	-2.33				0.03(COSM32	Zinc knuckle	rs2042395	0.2445	YES	3.027E-1 (pos.87446053)	acute myeloid leukaemia		nucleic acid binding,phosphatidylinositol binding,zinc io			G=0, A=58	G=0.0, A=1.0	0.00001	867.384	58	G=0/0, A=26/3
chr3:28520417	A/A	T	A	SNV	ZCWPW2	ZCWPW2:exonic:NM 001040432.3	1	100	5	missense	NM 00104	c.605T>A	p.Leu202Gln	-0.56	1	113	0	0(COSM5021	CW-type Zinc	rs1563656	0.4255	YES	6.247E-1 (pos.28520417)	osteosarcoma		zinc ion binding			T=0, A=21	T=0.0, A=1.0	0.00001	281.235	21	T=0/0, A=5/16
chr9:130652800	A/A	G	A	SNV	ST6GALNAC 6	ST6GALNAC6:intronic:NM 013443.4	1	100		intronic	NM 01344	c.704+116	p.?	-0.06					Glycosyltrans	rs2281897		YES			ST6 alpha-N	Golgi membrane,alpha-N-acetylgalactosaminide alpha-			G=0, A=25	G=0.0, A=1.0	0.00001	353.019	25	G=0/0, A=17/8
chr9:130670903	A/A	C	A	SNV	ST6GALNAC 4	ST6GALNAC4:intronic:NM 175039.3	1	100		intronic	NM 17503	c.720-43G	p.?	-1.63					Glycosyltrans	rs10760511	0.0015	YES	9.942E-1 (pos.130670903)			(alpha-N-acetylneuraminyl-2,3-beta-galactosyl-1,3)-N-ac			C=0, A=51	C=0.0, A=1.0	0.00001	754.577	51	C=0/0, A=34/1
chr2:207169731	A/A	G	A	SNV	ZDBF2	ZDBF2:exonic:NM 020923.2	1	100	5	missense	NM 02092	c.479G>A	p.Arg160Lys	1.26	1	26	0		DBF zinc finge	rs10932150	0.4512	YES	1.794E-1 (pos.207169731)		Zinc finger D	nucleic ac	Bronchial Neoplasms;Carcinoma, Bronchoge		G=0, A=36	G=0.0, A=1.0	0.00001	542.037	36	G=0/0, A=6/30
chr2:207174316	C/C	T	C	SNV	ZDBF2	ZDBF2:exonic:NM 020923.2	1	100	5	synonymous	NM 02092	c.5064T>C	p.(=)	-0.76					DBF zinc finge	rs3732084	0.395	YES	3.288E-1 (pos.207174316)		Zinc finger D	nucleic ac	Bronchial Neoplasms;Carcinoma, Bronchoge		T=0, C=21	T=0.0, C=1.0	0.00001	281.235	21	T=0/0, C=9/12
chr2:207173288	A/G	A	G	SNV	ZDBF2	ZDBF2:exonic:NM 020923.2	1	54.46	5	missense	NM 02092	c.4036A>G	p.Ile1346Val	-0.96	0.51	29	0		DBF zinc finge	rs367704091	2.00E-04		0.0 (pos.207173288)		Zinc finger D	nucleic ac	Bronchial Neoplasms;Carcinoma, Bronchoge		A=46, G=55	A=0.4554, G=0.5446	0.00001	591.632	101	A=23/23, G=2
chr11:19192006	C/G	C	G	SNV	ZDHHC13	ZDHHC13:exonic:NM 019028.2	1	46.58	15	missense	NM 01902	c.1523C>G	p.Thr508Ser	3.6	0.39	58	0.752	0.92(COSM57	Ankyrin repe	rs374897174	3.00E-04		0.0 (pos.19192006)	ductal carcinc	Zinc finger D	Golgi mem	Alopecia;Amyloidosis;Bone Diseases;Bone D		C=86, G=75	C=0.5342, G=0.4658	0.00001	758.075	161	C=57/29, G=6
chr9:130861039	G/G	A	G	SNV	SLC25A25	SLC25A25:intronic:NM 001006641.3	1	100		intronic	NM 00100	c.262-233	p.?	-4.42					EF hand,EF-ha	rs947623	0.0072	YES	9.987E-1 (pos.130861039)			ADP transmembrane transporter activity,ADP transport,			A=0, G=26	A=0.0, G=1.0	0.00001	371.42	26	A=0/0, G=11/1
chr6:157963743	A/A	G	A	SNV	ZDHHC14	ZDHHC14:exonic:NM 024630.2	1	100	2	synonymous	NM 02463	c.372G>A	p.(=)	-2.36				0.11(COSM41	DHHC palmit	rs2365611	0.3921	YES	3.765E-1 (pos.15796374	acute myeloid leukaemia		endoplasmic reticulum,integral component of membran			G=0, A=76	G=0.0, A=1.0	0.00001	1209.52	76	G=0/0, A=26/5
chr9:130914434	G/G	A	G	SNV	LCN2	LCN2:intronic:NM 005564.4	1	100		intronic	NM 00556	c.476-28A	p.?	-0.19					Lipocalin / cy	rs2232629	0.0302	YES	9.999E-1 (pos.130914434)		Lipocalin 2	antimicrol	Acute Kidney Injury	2,3,-Dihydroxybenzoylse	A=0, G=29	A=0.0, G=1.0	0.00001	426.931	29	A=0/0, G=10/1
chr9:130932396	A/A	AAC	A	INDEL	CIZ1	CIZ1:intronic:NM 001257975.1	2	100		intronic	NM 00125	c.2034-10	p.?	1.94, 0.4, -0.27					Zinc-finger dc	rs144699223,rs2005	0.3513	YES	5.174E-1 (pos.130932396)		CDKN1A-int	cyclin binc	Central Nervous System Diseases:Dyskinesia		AAC=0, A=3	AAC=0.0, A=1.0	0.00001	504.051	33	AAC=0/0, A=12
chr12:72013832	G/G	A	G	SNV	ZFC3H1	ZFC3H1:exonic:NM 144982.4	1	100	26	synonymous	NM 14498	c.4923T>C	p.(=)	0.06					Putative zinc	rs1298463	0.2484	YES	1.814E-1 (pos.72013832)			RNA bindi	Arthritis;Arthritis, Juvenile;Autoimmune Dise		A=0, G=38	A=0.0, G=1.0	0.00001	603.786	38	A=0/0, G=18/2
chr16:72827758	C/C	T	C	SNV	ZFHX3	ZFHX3:exonic:NM 006885.3	1	100	9	synonymous	NM 00688	c.8823A>G	p.(=)	2.47					Homeobox dc	rs699444	0.1624	YES	9.988E-1 (pos.72827758)		Zinc finger h	DNA bindi	Arrhythmias, Cardiac;Atrial Fibrillation;Brain		T=0, C=35	T=0.0, C=1.0	0.00001	543.054	35	T=0/0, C=19/1
chr16:72832135	A/A	T	A	SNV	ZFHX3	ZFHX3:exonic:NM 006885.3	1	100	9	synonymous	NM 00688	c.4446A>T	p.(=)	-4.2					Homeobox dc	rs740178	0.1988	YES	9.689E-1 (pos.72832135)		Zinc finger h	DNA bindi	Arrhythmias, Cardiac;Atrial Fibrillation;Brain		T=0, A=33	T=0.0, A=1.0	0.00001	504.598	33	T=0/0, A=24/9
chr9:13125201	C/C	T	C	SNV	MPDZ	MPDZ:intronic:NM 003829.4	1	100		intronic	NM 00382	c.4807+14	p.?	-0.45					L27 2,PDZ dc	rs2297003	0.3147	YES	5.431E-1 (pos.13125201)		Multiple PD	Schmidt-L	Brain Diseases;Central Nervou	Benign	T=0, C=25	T=0.0, C=1.0	0.00001	353.004	25	T=0/0, C=16/9
chr9:131267212	C/G	C	G	SNV	GLE1,MIR12 68A	MIR1268A:intronic nc:NR 031672.1, GLE1:intronic:NM 001	1	51.85		intronic	NR 03167	c.99+29C>	p.?	-0.17					GLE1-like pro	rs56164320	0.0435	YES	1.573E-1 (pos.131267212)		Gle1, RNA e	cytoplasm	Arthrogryposis;Congenital Abnormalities;Cor		C=13, G=14	C=0.4815, G=0.5185	0.00001	132.666	27	C=9/4, G=8/6
chr9:131374165	G/G	C	G	SNV	MIR1268A,S PTAN1	MIR1268A:intronic nc:NR 031672.1, SPTAN1:intronic:NM C	1	100		intronic	NR 03167	c.4905+41	p.?	-1.82					Ca2+ insensit	rs7024498	3.00E-04		1.0 (pos.131374165)		Spectrin, al	ER to Golgi	Brain Diseases;Central Nervou	Likely benign	C=0, G=40	C=0.0, G=1.0	0.00001	644.286	40	C=0/0, G=25/1
chr9:131394297	G/G	A	G	SNV	MIR1268A,S PTAN1	MIR1268A:intronic nc:NR 031672.1, SPTAN1:intronic:NM C	1	100		intronic	NR 03167	c.6763-10	p.?	0.01					Ca2+ insensit	rs10760569		YES			Spectrin, al	ER to Golgi	Brain Diseases;Central Nervous System Dise		A=0, G=45	A=0.0, G=1.0	0.00001	747.393	45	A=0/0, G=29/1
chr9:131454120	T/T	C	T	SNV	SET	SET:intronic:NM 001248000.1	1	100		intronic	NM 00124	c.105-17C	p.?	-1.1					Nucleosome	rs3750330	0.0063	YES	9.058E-1 (pos.131454120)		SET gene	DNA binding,DNA replication,cytoplasm,cytosol,endopla			C=0, T=24	C=0.0, T=1.0	0.00001	334.792	24	C=0/0, T=9/15
chr9:131455903	C/T	C	T	SNV	SET	SET:intronic:NM 001248000.1	1	98.17		intronic	NM 00124	c.466-14C	p.?	-0.63					Nucleosome	rs4836618	0.0066	YES	9.063E-1 (pos.131455903)		SET gene	DNA binding,DNA replication,cytoplasm,cytosol,endopla			C=2, T=107	C=0.0183, T=0.9817	0.00001	1649.94	109	C=2/0, T=95/1
chr9:131482467	C/C	T	C	SNV	PKN3,ZDHH C12	PKN3:intronic:NM 013355.4, ZDHHC12:downstream:NM 0	1	100		intronic	NM 01335	c.2377-15	p.?, p.?	-0.06					Hr1 repeat,P	rs3750325	0.0434	YES	9.089E-1 (pos.131482467)		Protein kina	ATP binding,Golgi apparatus,endoplasmic reticulum,epi			T=0, C=26	T=0.0, C=1.0	0.00001	371.415	26	T=0/0, C=7/19

chr19:57058998	C/G	C	G	SNV	ZFP28	ZFP28:exonic:NM_020828.1	1	50	3	missense	NM_02082	c.422C>G	p.Ser141Trp	1.4	0	177	0.99	0.26(COSM39	C2H2-type zir	rs34136271	0.189	YES	7.798E-2;0.0 (pos.57058	adenocarcino	Zinc finger r	DNA binding,DNA binding transcription factor activity,int	C=28, G=28	C=0.5, G=0.5	0.00001	272.136	56	C=19/9, G=24/
chr9:131553768	G/G	A	G	SNV	TBC1D13	TBC1D13:intronic:NM_018201.4	1	100		intronic	NM_01820	c.200+48A	p.?	-2.87					Rab-GTPase-	rs10760577	0.0014		9.985E-1 (pos.131553768)		TBC1 domai	GTPase activator activity,Rab GTPase binding,activation	A=0, G=36	A=0.0, G=1.0	0.00001	563.728	36	A=0/0, G=17/1
chr9:131585994	C/C	T	C	SNV	SPOUT1	SPOUT1:intronic:NM_016390.3	1	100		intronic	NM_01639	c.1062+32	p.?	-1.2					Putative RNA	rs2997918	4.00E-04		1.0;0.0 (pos.131585994)		SPOUT dom	RNA binding,cell cycle,cell division,condensed chromosc	T=0, C=50	T=0.0, C=1.0	0.00001	852.716	50	T=0/0, C=11/3
chr9:131587240	A/A	G	A	SNV	SPOUT1	SPOUT1:intronic:NM_016390.3	1	100		intronic	NM_01639	c.712+16C	p.?	0.49					Putative RNA	rs2293968	0.2907	YES	6.615E-1 (pos.131587240)		SPOUT dom	RNA binding,cell cycle,cell division,condensed chromosc	G=0, A=24	G=0.0, A=1.0	0.00001	334.792	24	G=0/0, A=14/1
chr8:144332082	T/C	T	C	SNV	ZFP41	ZFP41:exonic:NM_173832.5	1	95.65	2	synonymous	NM_17383	c.72T>C	p.(=)	0.41				0(COSM3763	C2H2-type zir	rs11136275	0.481	YES	7.561E-1 (pos.14433208	acute_myeloid_leukaemia		DNA binding,cell differentiation,metal ion binding,multi	T=2, C=44	T=0.0435, C=0.9565	0.00001	704.734	46	T=2/0, C=19/2
chr9:131882761	C/C	T	C	SNV	PTPA	PTPA:intronic:NM_178001.2	1	100		intronic	NM_17800	c.32-31T>	p.?	-0.45					Phosphotyro	rs9409311	0		1.0 (pos.131882761)		Protein pho	ATP binding,ATPase activity,calcium channel complex,cy	T=0, C=20	T=0.0, C=1.0	0.00001	263.786	20	T=0/0, C=15/5
chr9:131897221	T/T	C	T	SNV	PTPA	PTPA:intronic:NM_178001.2	1	100		intronic	NM_17800	c.665+48C	p.?	-0.1					Phosphotyro	rs7019373	0.31	YES	5.635E-1 (pos.131897221)		Protein pho	ATP binding,ATPase activity,calcium channel complex,cy	C=0, T=24	C=0.0, T=1.0	0.00001	334.792	24	C=0/0, T=9/15
chr9:13192320	G/G	C	G	SNV	MPDZ	MPDZ:intronic:NM_003829.4	1	100		intronic	NM_00382	c.1804-26	p.?	-0.11					L27_2,PDZ dc	rs1331676	0.0028	YES	9.966E-1 (pos.13192320)		Multiple PD	Schmidt-L Brain Diseases:Central Nervous System Disea	C=0, G=40	C=0.0, G=1.0	0.00001	644.286	40	C=0/0, G=25/1
chr9:132382253	T/T	C	T	SNV	C9orf50	C9orf50:intronic:NM_199350.3	1	100		intronic	NM_19935	c.508+93G	p.?	-0.19					Domain of un	rs7467760		YES					C=0, T=27	C=0.0, T=1.0	0.00001	389.521	27	C=0/0, T=14/1
chr14:73440864	C/T	C	T	SNV	ZFYVE1	ZFYVE1:exonic:NM_021260.3	1	50	11	synonymous	NM_02126	c.2025G>	p.(=)	-2.81				0.04(COSM37	FYVE zinc fing	rs12931	0.4855	YES	3.682E-1;1.16E-4 (pos.73	acute_myeloid	Zinc finger F	1-phosphatidylinositol binding,ER-mitochondrion memb	C=11, T=11	C=0.5, T=0.5	0.00001	94.5519	22	C=3/8, T=5/6
chr9:132623917	C/A	C	A	SNV	USP20	USP20:intronic:NM_006676.7	1	97.06		intronic	NM_00667	c.497+26C	p.?	-0.5					DUSP domair	rs10124224	0.0952	YES	9.967E-1 (pos.132623917)		Ubiquitin-s	G-protein coupled receptor binding,centrosome,cystein	C=1, A=33	C=0.0294, A=0.9706	0.00001	492.933	34	C=1/0, A=22/1
chr9:132625417	TG/TG	CA	TG	MNV	USP20	USP20:intronic:NM_006676.7	2	100		intronic	NM_00667	c.498-48C	p.?	-0.72, -1.66					DUSP domair	rs4836676,rs483741	0.1042	YES	9.742E-1 (pos.132625417),9.952E-1 (pos		Ubiquitin-s	G-protein coupled receptor binding,centrosome,cystein	CA=0, TG=2	CA=0.0, TG=1.0	0.00001	285.631	21	CA=0/0, TG=9/
chr9:132625602	C/C	T	C	SNV	USP20	USP20:intronic:NM_006676.7	1	100		intronic	NM_00667	c.611+24T	p.?	-5.33					DUSP domair	rs2900279	0.0916	YES	9.956E-1 (pos.132625602)		Ubiquitin-s	G-protein coupled receptor binding,centrosome,cystein	T=0, C=30	T=0.0, C=1.0	0.00001	446.638	30	T=0/0, C=8/22
chr9:132627723	A/A	G	A	SNV	USP20	USP20:intronic:NM_006676.7	1	100		intronic	NM_00667	c.690+63G	p.?	0.22					DUSP domair	rs913320	0.1172	YES			Ubiquitin-s	G-protein coupled receptor binding,centrosome,cystein	G=0, A=39	G=0.0, A=1.0	0.00001	623.963	39	G=0/0, A=20/1
chr9:132689671	G/G	A	G	SNV	FNBP1	FNBP1:intronic:NM_015033.2	1	100		intronic	NM_01503	c.643-51T	p.?	0.39					Fes/CIP4, anc	rs4837429,rs765351	0		1.16E-4;0.0 (pos.132689670)		Formin-binc	cell cortex,clathrin-coated pit,cytoplasmic vesicle,cytos	A=0, G=20	A=0.0, G=1.0	0.00001	263.739	20	A=0/0, G=3/17
chr9:132872686	G/G	A	G	SNV	GPR107	GPR107:intronic:NM_020960.4	1	100		intronic	NM_02096	c.1306+28	p.?	-0.71					Lung seven tr	rs4837460	0.0198	YES	1.0 (pos.132872686)			Golgi apparatus,clathrin heavy chain binding,clathrin-co	A=0, G=46	A=0.0, G=1.0	0.00001	771.615	46	A=0/0, G=8/38
chr9:133346983	G/G	A	G	SNV	ASS1	ASS1:intronic:NM_000050.4	1	100		intronic	NM_00005	c.597+81A	p.?	4.19					Argininosuccin	rs641510	0.0047	YES			Argininosuc	ATP bindin Amino Acid Disorde ATP,L-Arginine,L-Aspartic	A=0, G=31	A=0.0, G=1.0	0.00001	465.819	31	A=0/0, G=3/28
chr5:79734297	C/C	T	C	SNV	ZFYVE16	ZFYVE16:exonic:NM_014733.5	1	100	3	missense	NM_01473	c.1793T>C	p.Ile598Thr	0.38	1	89	0	0.01(COSM44	Domain of un	rs259028	0.0566	YES	9.33E-1 (pos.79734297)	hepatocellula	Zinc finger FYVE domain-containing protein 16		T=0, C=20	T=0.0, C=1.0	0.00001	263.767	20	T=0/0, C=10/1
chr5:79734409	A/A	G	A	SNV	ZFYVE16	ZFYVE16:exonic:NM_014733.5	1	100	3	synonymous	NM_01473	c.1905G>	p.(=)	-0.04				0(COSM4407	Domain of un	rs259029	0.0488	YES	9.329E-1 (pos.79734409)	hepatocellula	Zinc finger FYVE domain-containing protein 16		G=0, A=39	G=0.0, A=1.0	0.00001	623.98	39	G=0/0, A=22/1
chr10:99504630	T/T	G	T	SNV	ZFYVE27	ZFYVE27:exonic:NM_144588.6	1	100	4	missense	NM_14458	c.413G>T	p.Gly138Val	3.33	1	109	0		FYVE zinc fing	rs10882993	0.238	YES	5.47E-1 (pos.99504630)		Zinc finger F	axon,denc Congenital Abnormalities;Con Benign/Likely	G=0, T=42	G=0.0, T=1.0	0.00001	685.049	42	G=0/0, T=16/2
chr16:29791561	G/G	T	G	SNV	ZG16	ZG16:exonic:NM_152338.3	1	100	4	missense	NM_15233	c.325T>G	p.Leu109Val	1.15	1	32	0		Jacalin-like le	rs235637	0		1.0 (pos.29791561)		Zymogen gr	Golgi lumen,carbohydrate binding,cytoplasmic vesicle lu	T=0, G=20	T=0.0, G=1.0	0.00001	263.628	20	T=0/0, G=3/17
chr19:10419130	C/T	C	T	SNV	ZGLP1	ZGLP1:exonic:NM_001103167.1	1	52.27	1	synonymous	NM_00110	c.228G>A	p.(=)	0.11				0.02(COSM32	GATA zinc fing	rs281417,rs7575720	0.3183	YES	0.0 (pos.10419128),1.11	adenocarcino	Zinc finger C	DNA binding transcription factor activity,RNA polymeras	C=21, T=23	C=0.4773, T=0.5227	0.00001	260.157	44	C=4/17, T=10/
chr8:123966159	C/C	G	C	SNV	ZHX2	ZHX2:exonic:NM_014943.3	1	100	3	synonymous	NM_01494	c.2409G>C	p.(=)	-0.27					Homeobox dc	rs3802265	0.3517	YES	7.184E-1;0.0 (pos.123966159)			DNA binding,DNA binding transcription factor activity,ide	G=0, C=98	G=0.0, C=1.0	0.00001	1659.82	98	G=0/0, C=41/5
chr9:134067795	A/A	G	A	SNV	NUP214	NUP214:intronic:NM_005085.3	1	100		intronic	NM_00508	c.3659+11	p.?	-1.68						rs4602942		YES			Nucleopori	RNA expor Leukemia;Leukemia, Myeloid;Leukemia, Mye	G=0, A=21	G=0.0, A=1.0	0.00001	281.235	21	G=0/0, A=3/18
chr8:123965193	C/T	C	T	SNV	ZHX2	ZHX2:exonic:NM_014943.3	1	57.14	3	synonymous	NM_01494	c.1443C>T	p.(=)	-1.63				0.06(COSM14	Homeobox dc	rs111726812	0.0323	YES	0.0 (pos.123965193)	adenocarcinoma,neoplasn		DNA binding,DNA binding transcription factor activity,ide	C=12, T=16	C=0.4286, T=0.5714	0.00001	161.768	28	C=9/3, T=8/8
chr19:57647236	C/C	T	C	SNV	ZIM3	ZIM3:exonic:NM_052882.1	1	100	5	missense	NM_05288	c.469A>G	p.Asn157Asp	-2.39	0.63	23	0	0.01(COSM13	C2H2-type zir	rs7252632	0.3722	YES	9.411E-1;0.0;0.0 (pos.57	adenocarcinoma		DNA binding,DNA binding transcription factor activity,me	T=0, C=45	T=0.0, C=1.0	0.00001	747.383	45	T=0/0, C=15/3
chr3:44612050	T/T	C	T	SNV	ZKSCAN7	ZKSCAN7:exonic:NM_018651.3	1	100	6	missense	NM_01865	c.1448C>T	p.Thr483Ile	-2.31	1	89	0	0.03(COSM54	C2H2-type zir	rs9873604	0.2793	YES	8.421E-1;0.0 (pos.44612	ductal_carcinoma		DNA binding,DNA binding transcription factor activity,int	C=0, T=61	C=0.0, T=1.0	0.00001	921.577	61	C=0/0, T=20/4
chrX:101138792	C/C	T	C	SNV	ZMAT1	ZMAT1:exonic:NM_001011657.3	1	100	7	missense	NM_00101	c.1607A>G	p.Gln536Arg	-0.2	1	43	0	0(COSM4590	Zinc finger, C2	rs5944882			1.0 (pos.101138792)	squamous_cel	Zinc finger, matrin-type 1		T=0, C=44	T=0.0, C=1.0	0.00001	726.569	44	T=0/0, C=29/1
chr3:178742690	C/C	T	C	SNV	ZMAT3	ZMAT3:utr_3:NM_022470.3	1	100	6	utr_3	NM_02247	c.985A>G	p.?	-3.5					Zinc-finger dc	rs4607155		YES				RNA binding,cellular response to DNA damage stimulus,i	T=0, C=37	T=0.0, C=1.0	0.00001	583.696	37	T=0/0, C=10/2
chr3:178785549	A/A	G	A	SNV	ZMAT3	ZMAT3:utr_5:NM_022470.3	1	100	2	utr_5	NM_02247	c.-9C>T	p.?	3.44					Zinc-finger dc	rs6772028	0.2474	YES	9.433E-1 (pos.178785549)			RNA binding,cellular response to DNA damage stimulus,i	G=0, A=54	G=0.0, A=1.0	0.00001	792.791	54	G=0/0, A=16/3
chr9:134386903	A/A	G	A	SNV	POMT1	POMT1:intronic:NM_007171.3	1	100		intronic	NM_00717	c.1052+49	p.?	-0.55					C-terminal fo	rs10901066	0.0478	YES	9.154E-1 (pos.134386903)		Protein O-m	ER-associa Cobblestone Lissencephaly:Cc Benign	G=0, A=28	G=0.0, A=1.0	0.00001	406.525	28	G=0/0, A=12/1
chr17:4645324	C/C	T	C	SNV	ZMYND15	ZMYND15:exonic:NM_001267822.1	1	100	4	synonymous	NM_00126	c.942T>C	p.(=)	-0.73				0.1(COSM376	MYND finger	rs3826517	0.448	YES	1.183E-1 (pos.4645324)	hepatocellula	Zinc finger M	cytoplasm,histone deacetylase binding,metal ion bindir	T=0, C=52	T=0.0, C=1.0	0.00001	767.978	52	T=0/0, C=17/3
chr9:134398249	T/T	C	T	SNV	POMT1,UCK1	UCK1:downstream:NM_001261451.2, POMT1:intronic:NM	1	100		intronic	NM_00717	c.2070-70	p.?, p.?	-0.74					C-terminal fo	rs10122068		YES			Protein O-m	ATP bindin Cobblestone Lissencephaly;Congenital Abno	C=0, T=31	C=0.0, T=1.0	0.00001	465.751	31	C=0/0, T=23/8
chr20:45853037	G/G	A	G	SNV	ZMYND8	ZMYND8:exonic:NM_183047.3	1	100	19	synonymous	NM_18304	c.3051T>C	p.(=)	0.69					Bromodomai	rs2664544	0.1442	YES	9.361E-1 (pos.45853037)		Zinc finger M	RNA polymerase II regulatory region sequence-specific D	A=0, G=70	A=0.0, G=1.0	0.00001	1092.06	70	A=0/0, G=24/4
chr19:21909948	A/A	G	A	SNV	ZNF100	ZNF100:exonic:NM_173531.3	1	100	5	missense	NM_17353	c.1166C>T	p.Thr389Ile	0	0.05	89	0.719		C2H2-type zir	rs7246400	0.4351	YES	6.623E-1 (pos.21909948)		Zinc finger r	DNA binding,intracellular,metal ion binding,nucleic acid	G=0, A=61	G=0.0, A=1.0	0.00001	921.851	61	G=0/0, A=44/1
chr9:134465398	C/C	T	C	SNV	RAPGEF1	RAPGEF1:intronic:NM_198679.1	1	100		intronic	NM_19867	c.2273-35	p.?	-0.4					RasGEF N-ter	rs3818581	0.1697	YES	8.003E-1 (pos.134465398)		RAP guanin	Rap guanyl-nucleotide exchange factor activity,Rap prot	T=0, C=42	T=0.0, C=1.0	0.00001	684.46	42	T=0/0, C=20/2
chr9:134467633	G/C	G	C	SNV	RAPGEF1	RAPGEF1:intronic:NM_198679.1	1	50		intronic	NM_19867	c.2186-63	p.?	0.45					RasGEF N-ter	rs78837410		YES			RAP guanin	Rap guanyl-nucleotide exchange factor activity,Rap prot	G=12, C=12	G=0.5, C=0.5	0.00001	106.709	24	G=9/3, C=7/5
chr9:134952790	C/C	T	C	SNV	MED27	MED27:intronic:NM_004269.3	1	100		intronic	NM_00426	c.348+39A	p.?	-0.34					Mediator cor	rs4440729	0.0215	YES	1.0 (pos.134952790)		Mediator cc	cytosol,mediator complex,nucleolus,nucleoplasm,nucle	T=0, C=55	T=0.0, C=1.0	0.00001	830.49	55	T=0/0, C=34/2
chr9:135153668	C/T	C	T	SNV	SETX	SETX:intronic:NM_015046.6	1	50		intronic	NM_01504	c.6655-24	p.?	-1.06					AAA domain,	rs11243704	0.2956	YES	7.205E-1 (pos.135153668)		Senataxin	ATP bindin Amyotrophic Lateral Sclerosis: Benign	C=50, T=50	C=0.5, T=0.5	0.00001	566.788	100	C=22/28, T=11
chr9:135153673	T/C	T	C	SNV	SETX	SETX:intronic:NM_015046.6	1	50		intronic	NM_01504	c.6655-29	p.?	0.42					AAA domain,	rs11243705	0.1472	YES	2.923E-1 (pos.135153673)		Senataxin	ATP bindin Amyotrophic Lateral Sclerosis;Ataxia;Brain D	T=50, C=50	T=0.5, C=0.5	0.00001	567.037	100	T=22/28, C=11
chr19:58945962	T/T	C	T	SNV	ZNF132	ZNF132:exonic:NM_003433.3	1	100	3	synonymous	NM_00343	c.849G>A	p.(=)	-0.67					C2H2-type zir	rs9941466	0.0551	YES	9.807E-1 (pos.58945962)		Zinc finger r	DNA binding,DNA binding transcription factor activity,int	C=0, T=55	C=0.0, T=1.0	0.00001	806.567	55	C=0/0, T=28/2
chr19:58572979	A/A	G	A	SNV	ZNF135	ZNF135:exonic:NM_007134.1	1	100	2	missense	NM_00713	c.101G>A	p.Gly34Asp	1.11	1	94	0	0.01(COSM41	C2H2-type zir	rs1469087	0.1435	YES	4.874E-1 (pos.58572979)	neoplasm	Zinc finger r	DNA binding,DNA binding transcription factor activity,cyt	G=0, A=29	G=0.0, A=1.0	0.00001	427.587	29	G=0/0, A=8/21
chr11:9537904	C/C	G	C	SNV	ZNF143	ZNF143:exonic:NM_003442.5	1	100	14	missense	NM_00344	c.1681G>C	p.Glu561Gln	3.79	1	29	0	0.24(COSM63	C2H2-type zir	rs10743108	0.0221	YES	7.481E-1 (pos.9537904)	carcinoma	Zinc finger r	RNA polymerase II core promoter proximal region seque	G=0, C=36	G=0.0, C=1.0	0.00001	563.567	36	G=0/0, C=10/2
chr3:124951821	C/T	C	T	SNV	ZNF148	ZNF148:exonic:NM_021964.2	1	98.72	9	synonymous	NM_02196	c.1749G>	p.(=)	-0.12				0.2(COSM44	Zinc finger, C2	rs1053738	0.2389	YES	5.091E-1 (pos.12495182	carcinoma,hei	Zinc finger r	Golgi apparatus,RNA polymerase II core promoter proxir	C=1, T=77	C=0.0128, T=0.9872	0.00001	1213.72	78	C=1/0, T=24/5
chr16:3452193	G/G	T	G	SNV	ZNF174	ZNF174:exonic:NM_003450.2	1	100	1	synonymous	NM_00345	c.189T>G	p.(=)	-2.53					SCAN domain	rs37811	0.1355	YES	9.015E-1 (pos.3452193)		Zinc finger r	DNA binding transcription factor activity,RNA polymeras	T=0, G=88	T=0.0, G=1.0	0.00001	1451.91	88	T=0/0, G=28/6
chr9:135265875	AC/AC	A	AC	INDEL	TTF1	TTF1:intronic:NM_007344.3	1	100		intronic	NM_00734	c.2222+10	p.?	-0.16					Myb-like DNA-binding domain						NK2 homeo	DNA-templated transcription, termination,RNA polymer	A=0, AC=44	A=0.0, AC=1.0	0.00001	428.602	44	A=0/0, AC=19/
chr9:135265876	T/C	T	C	SNV	TTF1	TTF1:intronic:NM_007344.3	1	95.45		intronic	NM_00734	c.2222+10	p.?	-0.44					Myb-like DNA	rs471890		YES			NK2 homeo	DNA-templated transcription, termination,RNA polymer	T=2, C=42	T=0.0455, C=0.9545	0.00001	664.151	44	T=2/0, C=17/2
chr9:135601354	C/C	T	C	SNV	AK8	AK8:intronic:NM_152572.2	1	100		intronic	NM_15257	c.1203-42	p.?	-0.33					AAA domain,	rs396819	0.4315	YES	5.116E-1 (pos.135601354)		Adenylate k	ATP bindin Attention Deficit Di Bis(Adenosine)-5'-Pentac	T=0, C=41	T=0.0, C=1.0	0.00001	664.7	41	T=0/0, C=25/1
chrX:152083006	C/C	G	C	SNV	ZNF185	ZNF185:utr_5:NM_001178106.1	1	100	1	utr_5	NM_00117	c.-28G>C	p.?	-0.02						rs6526140	0.2906	YES	1.329E-1 (pos.152083006)		Zinc finger c	actin cytoskeleton,actin filament binding,cytoplasm,foc	G=0, C=46	G=0.0, C=1.0	0.00001	768.259	46	G=0/0, C=28/1
chrX:152083012	A/A	G	A	SNV	ZNF185	ZNF185:utr_5:NM_001178106.1	1	100	1	utr_5	NM_00117	c.-22G>A	p.?	-0.18						rs6526141	0.2875	YES	1.384E-1 (pos.152083012)		Zinc finger c	actin cytoskeleton,actin filament binding,cytoplasm,foc	G=0, A=46	G=0.0, A=1.0	0.00001	768.101	46	G=0/0, A=28/1
chr9:136305439	A/A	G	A	SNV	ADAMTS13	ADAMTS13:intronic:NM_139025.4	1	100		intronic	NM_13902	c.1787-26	p.?	-0.12					Metallo-pept	rs2073932	0.4444	YES	8.228E-1 (pos.136305439)		A disintegri	calcium io Autoimmune Diseases;Blood Coagulation Dis	G=0, A=48	G=0.0, A=1.0	0.00001	810.435	48	G=0/0, A=22/2
chrX:152083059	A/A	G	A	SNV	ZNF185	ZNF185:exonic:NM_001178106.1	1	100	1	missense	NM_00117	c.26G>A	p.Arg9His	1.26	0.04	29	0.799	0.06(COSM3759409)		rs6526142	0.293	YES	1.572E-1 (pos.15208305	adenocarcino	Zinc finger r	actin cytoskeleton,actin filament binding,cytoplasm,foc	G=0, A=45	G=0.0, A=1.0	0.00001	747.379	45	G=0/0, A=27/1
chr16:71509685	G/G	C	G	SNV	ZNF19	ZNF19:exonic:NM_006961.3	1	100	6	synonymous	NM_00696	c.765G>C	p.(=)	-2.09				0.01(COSM63	KRAB box,Zin	rs2288489	0.1651	YES	5.655E-1;1.507E-3;0.0 (pc	carcinoma	Zinc finger r	DNA binding,DNA binding transcription factor activity,int	C=0, G=116	C=0.0, G=1.0	0.00001	1855.61	116	C=0/0, G=21/9
chr2:95847041	G/G	GCGG	G	INDEL	ZNF2	ZNF2:exonic:NM_021088.3	3	100	5	nonframeshiftDeletion	NM_02108	c.474_476	p.Arg157del	0.0, 0.0, -0.36, -0.21				0.35(COSM63	C2H2-type zir	rs10578519,rs200455994,rs397802135			1.0 (pos.95847040)	carcinoma	Zinc finger c	DNA binding,DNA binding transcription factor activity,int	GCGG=0, G	GCGG=0.0, G=1.0	0.00001	548.623	35	GCGG=0/0, G=
chr19:12244026	G/C	G	C	SNV	ZNF20,ZNF625-ZNF20	ZNF625-ZNF20:exonic_nc:NR_037802.1, ZNF20:exonic:NM_(	1	57.63	4	missense	NM_02114	c.975C>G	p.His325Gln	-0.29	0.05	24	0.112		C2H2-type zir	rs767730766			0.0 (pos.12244026)		Zinc finger r	DNA binding,intracellular,metal ion binding,nucleic acid	G=25, C=34	G=0.4237, C=0.5763	0.00001	356.499	59	G=11/14, C=1
chr19:22154732	T/C	T	C	SNV	ZNF208	ZNF208:exonic:NM_007153.3	1	47.37	4	missense	NM_00715	c.3104A>G	p.Asp1035Gly	-0.55	1	94	0.002	0.02(COSM37	C2H2-type zir	rs8108957	0.4353	YES	0.0 (pos.22154715),6.9	acute_myeloid	Zinc finger c	DNA binding,intracellular,metal ion binding,nucleic acid	T=27, C=27	T=0.5, C=0.5	0.00001	299.392	57	T=3/24, C=7/2
chr9:136531839	C/G	C	G	SNV	SARDH	SARDH:intronic:NM_001134707.1	1	50		intronic	NM_00113	c.2631+18	p.?	-1.75					Aminomethyl	rs12235666	0.0314	YES	1.26E-1 (pos.136531839)		Sarcosine d	biological Amino Acid Metabolism, Inborn Errors:Conge	C=13, G=14	C=0.4815, G=0.5185	0.00001	129.926	28	C=7/6, G=7/7
chr9:136629256	C/C	CTG	C	INDEL	VAV2	VAV2:intronic:NM_001134398.1	2	100		intronic	NM_00113	c.2590-25	p.?	-1.29, -0.81, 0.79					CAMSAP CH d	rs34181788,rs375990263,rs398		YES	0.0 (pos.136629254),0.0 (pos.13662925		Oncogene V	Fc-epsilon Autoimmune Diseases;Autoimmune Disease	CTG=0, C=4	CTG=0.0, C=1.0	0.00001	771.519	46	CTG=0/0, C=18
chr9:136629298	G/G	A	G	SNV	VAV2	VAV2:intronic:NM_001134398.1	1	100		intronic	NM_00113	c.2590-67	p.?	-0.78					CAMSAP CH d	rs2797825	0.478	YES			Oncogene V	Fc-epsilon Autoimmune Diseases;Autoimmune Disease	A=0, G=47	A=0.0, G=1.0	0.00001	789.32	47	A=0/0, G=13/3
chr9:136804211	A/A	G	A	SNV	VAV2	VAV2:intronic:NM_001134398.1	1	100		intronic	NM_00113	c.321+14C	p.?	0.1					CAMSAP CH d	rs2073886	0.2279	YES	7.878E-1 (pos.136804211)		Oncogene V	Fc-epsilon Autoimmune Diseases;Autoimmune Disease	G=0, A=62	G=0.0, A=1.0	0.00001	936.551	62	G=0/0, A=38/2
chr11:7022160	G/G	A	G	SNV	ZNF214	ZNF214:exonic:NM_013249.2	1	100	3	missense	NM_01324	c.754T>C	p.Cys252Arg	2.14	0.27	180	0.001		KRAB box,Zin	rs2857919	0.0308	YES	9.994E-1 (pos.7022160)		Zinc finger c	DNA binding,nucleus,regulation of transcription, DNA-te	A=0, G=34	A=0.0, G=1.0	0.00001	524.185	34	A=0/0, G=23/1
chr9:137320855	G/T	G	T	SNV	RXRA	RXRA:intronic:NM_002957.5	1	57.14		intronic	NM_00295	c.911-99G	p.?	-1.41					Ligand-bindir	rs3118570		YES			Retinoid X r	9-cis retin Amyotrophic Latera (5BETA)-PREGNANE-3,20-	G=21, T=28	G=0.4286, T=0.5714	0.00001	344.26	49	G=19/2, T=22/
chr11:6976988	T/T	A	T	SNV	ZNF215	ZNF215:exonic:NM_013250.2	1	100	7	synonymous	NM_01325	c.780A>T	p.(=)	-0.09					C2H2-type zir	rs2239731	0.2585	YES	7.345E-1 (pos.6976988)		Zinc finger c	DNA binding,DNA binding transcription factor activity,me	A=0, T=20	A=0.0, T=1.0	0.00001	263.786	20	A=0/0, T=5/15
chr9:137620474	T/T	C	T	SNV	COL5A1	COL5A1:intronic:NM_001278074.1	1	100		intronic	NM_00127	c.787-42C	p.?	-3.56					Collagen tripl	rs3124302	0.4558	YES	4.746E-1;0.0 (pos.137620474)		Collagen V, alpha-1 pol	Abortion, Spontaneous;Cardio Benign	C=0, T=32	C=0.0, T=1.0	0.00001	485.137	32	C=0/0, T=12/2
chr9:137623193	T/T	C	T	SNV	COL5A1	COL5A1:intronic:NM_001278074.1	1	100		intronic	NM_00127	c.1165-14	p.?	-3.26					Collagen tripl	rs3124309		YES			Collagen V, alpha-1 pol	Abortion, Spontaneous;Cardiovascular Disea	C=0, T=23	C=0.0, T=1.0	0.00001	316.747	23	C=0/0, T=14/9
chr19:44470420	C/C	T	C	SNV	ZNF221	ZNF221:exonic:NM_013359.2	1	100	6	missense	NM_01335	c.766T>C	p.Cys256Arg	0.52	0	180	1		C2H2-type zir	rs439676	0.0306	YES	9.809E-1 (pos.44470420)			DNA binding,DNA binding transcription factor activity,int	T=0, C=28	T=0.0, C=1.0	0.00001	408.718	28	T=0/0, C=10/1
chr9:138586309	A/G	A	G	SNV	SOHLH1	SOHLH1:intronic:NM_001101677.1	1	52.38		intronic	NM_00110	c.876-6T>	p.?	-1.65					Helix-loop-he	rs484524	0.0767	YES	9.801E-1 (pos.138586309)		Spermatoge	RNA polymerase II regulatory region sequence-specific D	A=30, G=33	A=0.4762, G=0.5238	0.00001	333.907	63	A=10/20, G=5/
chr9:138586854	C/T	C	T	SNV	SOHLH1	SOHLH1:intronic:NM_001101677.1	1	59.09		intronic	NM_00110	c.875+42G	p.?	-0.76					Helix-loop-he	rs3118655	0.0752	YES	9.793E-1 (pos.138586854)		Spermatoge	RNA polymerase II regulatory region sequence-specific D	C=9, T=13	C=0.4091, T=0.5909	0.00001	122.568	22	C=7/2, T=9/4
chr9:138837113	C/C	T	C	SNV	UBAC1	UBAC1:intronic:NM_016172.2	1	100		intronic	NM_01617	c.654-17A	p.?	-0.61					UBA/TS-N dor	rs10776856	0.4813	YES	5.124E-1 (pos.138837113)		Ubiquitin-a	Golgi apparatus,cytosol,extracellular exosome,plasma r	T=0, C=52	T=0.0, C=1.0	0.00001	767.015	52	T=0/0, C=31/2
chr9:139094773	G/G	A	G	SNV	LHX3	LHX3:intronic:NM_014564.4	1	100		intronic	NM_01456	c.94+19T>	p.?	-1.6					Homeobox dc	rs2274115		YES	4.118E-1 (pos.139094773)		LIM/homeo	RNA polym Bone Diseases;Bone Diseases, Benign	A=0, G=32	A=0.0, G=1.0	0.00001	485.102	32	A=0/0, G=13/1
chr19:44470548	G/G	A	G	SNV	ZNF221	ZNF221:exonic:NM_013359.2	1	100	6	synonymous	NM_01335	c.894A>G	p.(=)	-6.93					C2H2-type zir	rs453640	0.0012	YES	1.0 (pos.44470548)			DNA binding,DNA binding transcription factor activity,int	A=0, G=51	A=0.0, G=1.0	0.00001	754.57	51	A=0/0, G=24/2
chr19:44470663	C/C	G	C	SNV	ZNF221	ZNF221:exonic:NM_013359.2	1	100	6	missense	NM_01335	c.1009G>C	p.Ala337Pro	2.95	1	27	0		C2H2-type zir	rs435590	0.0012	YES	1.0;0.0 (pos.44470663)			DNA binding,DNA binding transcription factor activity,int	G=0, C=150	G=0.0, C=1.0	0.00001	2551.75	150	G=0/0, C=60/9
chr19:44471209	A/A	T	A	SNV	ZNF221	ZNF221:exonic:NM_013359.2	1	100	6	missense	NM_01335	c.1555T>A	p.Ser519Thr	-0.32	0.07	58	0.038	0.18(COSM49	C2H2-type zir	rs365745	0.1406	YES	8.483E-1 (pos.44471209)	ductal_carcinoma		DNA binding,DNA binding transcription factor activity,int	T=0, A=74	T=0.0, A=1.0	0.00001	1170.13	74	T=0/0, A=24/5
chr9:139354437	G/A	G	A	SNV	SEC16A	SEC16A:intronic:NM_014866.1	1	50		intronic	NM_01486	c.5073+57	p.?	0.38					Sec23-bindin	rs45613441	0.0867	YES			Sec16, S. Ce	COPII vesicle coating,Golgi apparatus,Golgi membrane,c	G=60, A=60	G=0.5, A=0.5	0.00001	625.858	120	G=29/31, A=4
chr9:139360354	G/G	A	G	SNV	SEC16A	SEC16A:intronic:NM_014866.1	1	100		intronic	NM_01486	c.4303+60	p.?	-1.86					Sec23-bindin	rs3124590		YES			Sec16, S. Ce	COPII vesicle coating,Golgi apparatus,Golgi membrane,c	A=0, G=22	A=0.0, G=1.0	0.00001	298.885	22	A=0/0, G=7/15
chr9:139410424	G/G	A	G	SNV	NOTCH1	NOTCH1:intronic:NM_017617.4	1	100		intronic	NM_01761	c.1669+9T	p.?	-3.63					Ankyrin repe	rs3125006	0.1395	YES	9.382E-1 (pos.139410424)		Notch, Dros	DNA bindi Abnormalities, Multiple:Adenc Benign	A=0, G=23	A=0.0, G=1.0	0.00001	316.747	23	A=0/0, G=16/7
chr19:44570393	A/A	C	A	SNV	ZNF223	ZNF223:exonic:NM_013361.5	1	100	5	missense	NM_01336	c.412C>A	p.Leu138Ile	-1.56	0.04	5	0.182		C2H2-type zir	rs6509138	0.3537	YES	3.074E-1 (pos.44570393)			DNA binding,biological_process,intracellular,metal ion b	C=0, A=27	C=0.0, A=1.0	0.00001	389.987	27	C=0/0, A=8/19
chr19:44571154	C/C	T	C	SNV	ZNF223	ZNF223:exonic:NM_013361.5	1	100	5	synonymous	NM_01336	c.1173T>C	p.(=)	-2.76					C2H2-type zir	rs4433920	0.3193	YES	3.082E-1 (pos.44571154)			DNA binding,biological_process,intracellular,metal ion b	T=0, C=32	T=0.0, C=1.0	0.00001	485.127	32	T=0/0, C=3/29
chr19:44739303	C/C	T	C	SNV	ZNF227	ZNF227:exonic:NM_182490.2	1	100	6	synonymous	NM_18249	c.720T>C	p.(=)	0.34				0.05(COSM44	C2H2-type zir	rs2279073	0.4521	YES	4.284E-1 (pos.44739303)	acute_myeloid_leukaemia		DNA binding,DNA binding transcription factor activity,int	T=0, C=59	T=0.0, C=1.0	0.00001	885.365	59	T=0/0, C=13/4
chr19:44739399	C/C	T	C	SNV	ZNF227	ZNF227:exonic:NM_182490.2	1	100	6	synonymous	NM_18249	c.816T>C	p.(=)	0.08				0(COSM4419	C2H2-type zir	rs2279072	0.4365	YES	5.109E-1 (pos.44739399)	acute_myeloid_leukaemia		DNA binding,DNA binding transcription factor activity,int	T=0, C=161	T=0.0, C=1.0	0.00001	2573.27	161	T=0/0, C=91/7
chr19:44511202	T/T	A	T	SNV	ZNF230	ZNF230:utr_5:NM_006300.3	1	100	2	utr_5	NM_00630	c.-29A>T	p.?	0.21					C2H2-type zir	rs2356549	0.297	YES	3.009E-1 (pos.44511202)		RING finger	DNA bindi Hemic and Lymphatic Diseases:Immune Syst	A=0, T=54	A=0.0, T=1.0	0.00001	798.148	54	A=0/0, T=31/2
chr19:44511223	G/G	T	G	SNV	ZNF230	ZNF230:utr_5:NM_006300.3	1	100	2	utr_5	NM_00630	c.-8T>G	p.?	-1.78					C2H2-type zir	rs424147	0.004	YES	1.0 (pos.44511223)		RING finger	DNA bindi Hemic and Lymphatic Diseases:Immune Syst	T=0, G=54	T=0.0, G=1.0	0.00001	798.144	54	T=0/0, G=30/2
chr9:139815052	TA/TA	T	TA	INDEL	TRAF2	TRAF2:intronic:NM_021138.3	1	100		intronic	NM_02113	c.960+85T	p.?	-1.38					MATH domain,TNF receptor-associated factor BIRC3 binding domain,TRAF-type zinc finger,Zinc finger,						TNF recepto	AIP1-IRE1 complex,CD40 receptor binding,CD40 recepto	T=0, TA=65	T=0.0, TA=1.0	0.00001	856.332	65	T=0/0, TA=31/
chr9:139815053	G/A	G	A	SNV	TRAF2	TRAF2:intronic:NM_021138.3	1	95.38		intronic	NM_02113	c.960+86G	p.?	-0.74					MATH domai	rs10781522		YES			TNF recepto	AIP1-IRE1 complex,CD40 receptor binding,CD40 recepto	G=3, A=62	G=0.0462, A=0.9538	0.00001	905.754	65	G=3/0, A=28/3
chr18:74671768	C/C	T	C	SNV	ZNF236	ZNF236:exonic:NM_007345.3	1	100	29	synonymous	NM_00734	c.5232T>C	p.(=)	2.34				0.21(COSM37	C2H2-type zir	rs470403	0.3313	YES	6.531E-1 (pos.74671768)	adenocarcino	Zinc finger c	DNA binding,cellular response to glucose stimulus,meta	T=0, C=99	T=0.0, C=1.0	0.00001	1681.12	99	T=0/0, C=33/6
chr10:44052903	C/C	A	C	SNV	ZNF239	ZNF239:exonic:NM_001099283.1	1	100	2	missense	NM_00109	c.625T>G	p.Cys209Gly	4.46		159		0.75(COSM37	Zinc finger, C2	rs2230661	0.4654	YES	5.554E-1 (pos.44052903)	adenocarcino	Zinc finger c	DNA binding,DNA binding transcription factor activity,RN	A=0, C=29	A=0.0, C=1.0	0.00001	427.604	29	A=0/0, C=15/1
chr9:139840521	C/C	T	C	SNV	C8G	C8G:intronic:NM_000606.2	1	100		intronic	NM_00060	c.347-16T	p.?	-0.91					Lipocalin / cy	rs41307442	0.136	YES	2.452E-2 (pos.139840521)		Complemer	blood microparticle,compleme Citric Acid,Lauric Acid	T=0, C=36	T=0.0, C=1.0	0.00001	563.744	36	T=0/0, C=31/5
chr10:44053013	C/C	G	C	SNV	ZNF239	ZNF239:exonic:NM_001099283.1	1	100	2	missense	NM_00109	c.515C>G	p.Ala172Gly	-0.51		60		0.04(COSM37	Zinc finger, C2	rs2230660	0.4642	YES	5.56E-1;0.0 (pos.44053	adenocarcino	Zinc finger r	DNA binding,DNA binding transcription factor activity,RN	G=0, C=64	G=0.0, C=1.0	0.00001	977.635	64	G=0/0, C=44/2
chr19:58455381	T/T	C	T	SNV	ZNF256	ZNF256:exonic:NM_005773.2	1	100	2	synonymous	NM_00577	c.81G>A	p.(=)	-0.44					C2H2-type zir	rs257652	6.00E-04		9.841E-1 (pos.58455381)		Zinc finger r	DNA binding,DNA binding transcription factor activity,me	C=0, T=20	C=0.0, T=1.0	0.00001	254.311	20	C=0/0, T=10/1
chr9:139841212	C/C	T	C	SNV	C8G	C8G:intronic:NM_000606.2	1	100		intronic	NM_00060	c.596-8T>	p.?	0.08					Lipocalin / cy	rs41309980	0.1415	YES	2.883E-2 (pos.139841212)		Complemer	blood microparticle,compleme Citric Acid,Lauric Acid	T=0, C=72	T=0.0, C=1.0	0.00001	1130.95	72	T=0/0, C=44/2
chr9:139879067	C/C	T	C	SNV	LCNL1	LCNL1:intronic:NM_207510.3	1	100		intronic	NM_20751	c.196+52T	p.?	-3.96					Lipocalin / cy	rs2049042	0		1.0 (pos.139879067)				T=0, C=59	T=0.0, C=1.0	0.00001	884.62	59	T=0/0, C=28/3
chr19:58453342	T/C	T	C	SNV	ZNF256	ZNF256:exonic:NM_005773.2	1	58.33	3	synonymous	NM_00577	c.834A>G	p.(=)	-6.25					C2H2-type zir	rs959231	0.2964	YES	3.152E-1;0.0 (pos.58453342)		Zinc finger r	DNA binding,DNA binding transcription factor activity,me	T=25, C=35	T=0.4167, C=0.5833	0.00001	370.434	60	T=16/9, C=18/
chr19:53303527	G/G	A	G	SNV	ZNF28	ZNF28:exonic:NM_006969.3	1	100	4	missense	NM_00696	c.1571T>C	p.Met524Thr	-2.8	0.06	81	0		C2H2-type zir	rs8107444	0.0962	YES	9.197E-1;0.0 (pos.53303527)			DNA binding,DNA binding transcription factor activity,int	A=0, G=70	A=0.0, G=1.0	0.00001	1092.09	70	A=0/0, G=50/2
chr9:139906513	C/C	T	C	SNV	ABCA2	ABCA2:intronic:NM_001606.4	1	100		intronic	NM_00160	c.5360+41	p.?	-1.84					AAA domain,	rs2271863	0.2843	YES	8.072E-1 (pos.139906513)		ATP-binding	ATP binding,ATP-binding cassette (ABC) transporter comp	T=0, C=45	T=0.0, C=1.0	0.00001	747.381	45	T=0/0, C=22/2
chr9:139908260	AC/AC	A	AC	INDEL	ABCA2	ABCA2:intronic:NM_001606.4	1	100		intronic	NM_00160	c.4447+24	p.?	-0.65					AAA domain,	rs759791307			0.0 (pos.139908260)		ATP-binding	ATP binding,ATP-binding cassette (ABC) transporter comp	A=0, AC=39	A=0.0, AC=1.0	0.00001	471.575	39	A=0/0, AC=18/
chr9:139908265	C/C	T	C	SNV	ABCA2	ABCA2:intronic:NM_001606.4	1	100		intronic	NM_00160	c.4447+19	p.?	-0.88					AAA domain,	rs10781531	0.0359	YES	1.0 (pos.139908265)		ATP-binding	ATP binding,ATP-binding cassette (ABC) transporter comp	T=0, C=39	T=0.0, C=1.0	0.00001	623.767	39	T=0/0, C=18/2
chr9:139910648	C/C	T	C	SNV	ABCA2	ABCA2:intronic:NM_001606.4	1	100		intronic	NM_00160	c.3105-22	p.?	-4.52					AAA domain,	rs6560657	0.0303	YES	9.957E-1 (pos.139910648)		ATP-binding	ATP binding,ATP-binding cassette (ABC) transporter comp	T=0, C=23	T=0.0, C=1.0	0.00001	316.747	23	T=0/0, C=8/15
chr22:22868211	A/A	G	A	SNV	ZNF280A	ZNF280A:utr_3:NM_080740.4	1	100	2	utr_3	NM_08074	c.1744C>T	p.?	-0.67					C2H2-type zir	rs361558		YES				DNA binding,metal ion binding,nucleic acid binding,nucl	G=0, A=22	G=0.0, A=1.0	0.00001	288.442	22	G=0/0, A=12/1
chr9:139943549	A/A	G	A	SNV	ENTPD2	ENTPD2:intronic:NM_203468.2	1	100		intronic	NM_20346	c.1150-22	p.?	0.04					GDA1/CD39 (	rs7390981	0.0741	YES	8.737E-1 (pos.139943549)		Ectonucleo	ATP bindin Bile Duct Diseases:Biliary Tract Diseases:Chc	G=0, A=52	G=0.0, A=1.0	0.00001	767.013	52	G=0/0, A=28/2
chr9:140001978	A/A	C	A	SNV	MAN1B1	MAN1B1:intronic:NM_016219.4	1	100		intronic	NM_01621	c.1765-5C	p.?	-0.48				0.35(COSM50	Glycosyl hydr	rs12115325,rs77284	0.3603	YES	0.0;0.0 (pos.140001974),	haemangiobla	Mannosida	Golgi appa Intellectual Disabil 1,4-Butan Benign/Likely	C=0, A=30	C=0.0, A=1.0	0.00001	446.603	30	C=0/0, A=14/1
chr9:140002220	G/G	A	G	SNV	MAN1B1	MAN1B1:intronic:NM_016219.4	1	100		intronic	NM_01621	c.1896+10	p.?	-1.27					Glycosyl hydr	rs10117795		YES	7.278E-1 (pos.140002220)		Mannosida	Golgi appa Intellectual Disabil 1,4-Butanediol,Duvoglus	A=0, G=32	A=0.0, G=1.0	0.00001	485.139	32	A=0/0, G=16/1
chr9:140002261	G/G	A	G	SNV	MAN1B1	MAN1B1:intronic:NM_016219.4	1	100		intronic	NM_01621	c.1896+14	p.?	-3.74					Glycosyl hydr	rs11790092		YES	1.256E-2;0.0 (pos.140002261)		Mannosida	Golgi appa Intellectual Disabil 1,4-Butanediol,Duvoglus	A=0, G=23	A=0.0, G=1.0	0.00001	316.741	23	A=0/0, G=6/17
chr22:22868493	T/T	G	T	SNV	ZNF280A	ZNF280A:exonic:NM_080740.4	1	100	2	missense	NM_08074	c.1462C>A	p.Arg488Ser	4.89		110		0.98(COSM40	C2H2-type zir	rs361666	0.3191	YES	4.374E-1;7.869E-3 (pos.2	acute_myeloid_leukaemia		DNA binding,metal ion binding,nucleic acid binding,nucl	G=0, T=27	G=0.0, T=1.0	0.00001	389.988	27	G=0/0, T=8/19
chr22:22868497	C/C	G	C	SNV	ZNF280A	ZNF280A:exonic:NM_080740.4	1	100	2	missense	NM_08074	c.1458C>G	p.Phe486Leu	-0.22		22		0.01(COSM40	C2H2-type zir	rs361762	0.3193	YES	4.373E-1 (pos.22868497)	acute_myeloid_leukaemia		DNA binding,metal ion binding,nucleic acid binding,nucl	G=0, C=27	G=0.0, C=1.0	0.00001	389.988	27	G=0/0, C=8/19
chr22:22868773	A/A	G	A	SNV	ZNF280A	ZNF280A:exonic:NM_080740.4	1	100	2	synonymous	NM_08074	c.1182C>T	p.(=)	-1.42				0.02(COSM11	C2H2-type zir	rs361737	0.3195	YES	4.378E-1 (pos.22868773)	acute_myeloid_leukaemia		DNA binding,metal ion binding,nucleic acid binding,nucl	G=0, A=89	G=0.0, A=1.0	0.00001	1472.23	89	G=0/0, A=47/4
chr9:140146651	C/C	T	C	SNV	STPG3,STPG3-AS1	STPG3:intronic:NM_001256699.1, STPG3-AS1:intronic_nc:NI	1	100		intronic	NR_03838	c.403+64T	p.?	0.12					Sperm-tail PC	rs28567631		YES					T=0, C=36	T=0.0, C=1.0	0.00001	563.744	36	T=0/0, C=28/8
chr9:140158871	T/C	T	C	SNV	NELFB	NELFB:intronic:NM_015456.4	1	47.62		intronic	NM_01545	c.1040+62	p.?	-1.47					Cofactor of BI	rs13295537		YES			Cofactor of	NELF complex,RNA binding,cytoplasm,negative regulatio	T=11, C=10	T=0.5238, C=0.4762	0.00001	82.2896	21	T=4/7, C=4/6
chr22:22868776	A/A	G	A	SNV	ZNF280A	ZNF280A:exonic:NM_080740.4	1	100	2	synonymous	NM_08074	c.1179C>T	p.(=)	-0.25				0.72(COSM11	C2H2-type zir	rs362173	0.3198	YES	4.373E-1 (pos.22868776)	acute_myeloid_leukaemia		DNA binding,metal ion binding,nucleic acid binding,nucl	G=0, A=88	G=0.0, A=1.0	0.00001	1451.91	88	G=0/0, A=47/4
chr22:22869123	C/C	T	C	SNV	ZNF280A	ZNF280A:exonic:NM_080740.4	1	100	2	missense	NM_08074	c.832A>G	p.Asn278Asp	3.23		23		0.01(COSM40	C2H2-type zir	rs362003	0.3192	YES	4.392E-1;0.0 (pos.22869	acute_myeloid_leukaemia		DNA binding,metal ion binding,nucleic acid binding,nucl	T=0, C=67	T=0.0, C=1.0	0.00001	1034.41	67	T=0/0, C=17/5
chr9:140325898	A/A	G	A	SNV	NOXA1	NOXA1:intronic:NM_006647.1	1	100		intronic	NM_00664	c.804+105	p.?	-2.55					PB1 domain,	rs7467896		YES			NADPH oxid	NADPH oxidase complex,Rac GTPase binding,SH3 domai	G=0, A=26	G=0.0, A=1.0	0.00001	371.408	26	G=0/0, A=4/22
chr9:140693181	A/A	G	A	SNV	EHMT1	EHMT1:intronic:NM_024757.4	1	100		intronic	NM_02475	c.2506-84	p.?	-0.37					Ankyrin repe	rs4876947		YES			Euchromati	C2H2 zinc Cardiovascular Abnormalities;Cardiovascula	G=0, A=39	G=0.0, A=1.0	0.00001	602.501	39	G=0/0, A=29/1
chr22:22869538	G/G	A	G	SNV	ZNF280A	ZNF280A:exonic:NM_080740.4	1	100	2	synonymous	NM_08074	c.417T>C	p.(=)	-0.79				0.01(COSM40	C2H2-type zir	rs361611	0.3221	YES	4.373E-1;2.323E-4 (pos.2	acute_myeloid_leukaemia		DNA binding,metal ion binding,nucleic acid binding,nucl	A=0, G=40	A=0.0, G=1.0	0.00001	644.282	40	A=0/0, G=31/9
chr9:141009015	T/T	C	T	SNV	CACNA1B	CACNA1B:intronic:NM_000718.3	1	100		intronic	NM_00071	c.5649+73	p.?	-0.38					Ion transport	rs4876928		YES			Calcium cha	ATP bindin Nervous System Dis Agmatine,Amlodipine,G	C=0, T=42	C=0.0, T=1.0	0.00001	689.588	42	C=0/0, T=7/35
chr22:22869545	G/G	T	G	SNV	ZNF280A	ZNF280A:exonic:NM_080740.4	1	100	2	missense	NM_08074	c.410A>C	p.Tyr137Ser	0.14		144		0(COSM4002	C2H2-type zir	rs361580	0.3215	YES	4.375E-1 (pos.22869545)	acute_myeloid_leukaemia		DNA binding,metal ion binding,nucleic acid binding,nucl	T=0, G=41	T=0.0, G=1.0	0.00001	664.7	41	T=0/0, G=31/1
chr9:14306944	G/G	A	G	SNV	NFIB	NFIB:intronic:NM_001190737.1	1	100		intronic	NM_00119	c.562+44T	p.?	0.2					CTF/NF-I fami	rs4741362	1.00E-04		1.0 (pos.14306944)		Nuclear fac	Clara cell Adenocarcinoma;Carcinoma;Carcinoma, Ade	A=0, G=45	A=0.0, G=1.0	0.00001	747.386	45	A=0/0, G=27/1
chr9:14640055	T/T	C	T	SNV	ZDHHC21	ZDHHC21:intronic:NM_178566.4	1	100		intronic	NM_17856	c.505-45G	p.?	-0.16					DHHC palmit	rs6474849	0.0532	YES	9.211E-1 (pos.14640055)		Zinc finger D	Golgi apparatus,Golgi membrane,hair follicle developme	C=0, T=69	C=0.0, T=1.0	0.00001	1072.78	69	C=0/0, T=34/3
chr9:14770825	T/C	T	C	SNV	FREM1	FREM1:intronic:NM_144966.5	1	46.88		intronic	NM_14496	c.4858-21	p.?	-0.17					Cadherin-like	rs7860370	0.1834	YES	1.522E-4 (pos.14770812),2.667E-1 (pos.1		FRAS1-relat	basement Abnormalities, Multiple:Bone Diseases:Bone	T=17, C=15	T=0.5313, C=0.4688	0.00001	143.733	32	T=4/13, C=6/9
chr22:22869548	C/C	T	C	SNV	ZNF280A	ZNF280A:exonic:NM_080740.4	1	100	2	missense	NM_08074	c.407A>G	p.Asn136Ser	-0.34		46		0.01(COSM40	C2H2-type zir	rs362011	0.321	YES	4.377E-1 (pos.22869548)	acute_myeloid_leukaemia		DNA binding,metal ion binding,nucleic acid binding,nucl	T=0, C=41	T=0.0, C=1.0	0.00001	664.691	41	T=0/0, C=31/1
chr9:17309336	C/C	T	C	SNV	CNTLN	CNTLN:intronic:NM_017738.3	1	100		intronic	NM_01773	c.1341+86	p.?	1.23						rs10756860		YES			Centlein	centriole,centriole-centriole cohesion,centrosome,cyto	T=0, C=35	T=0.0, C=1.0	0.00001	543.873	35	T=0/0, C=15/2
chr9:17330558	A/A	T	A	SNV	CNTLN	CNTLN:intronic:NM_017738.3	1	100		intronic	NM_01773	c.1342-72	p.?	-0.43						rs3765040		YES			Centlein	centriole,centriole-centriole cohesion,centrosome,cyto	T=0, A=62	T=0.0, A=1.0	0.00001	940.32	62	T=0/0, A=31/3
chr9:17342517	G/G	A	G	SNV	CNTLN	CNTLN:intronic:NM_017738.3	1	100		intronic	NM_01773	c.1886+75	p.?	3.35						rs10756869		YES			Centlein	centriole,centriole-centriole cohesion,centrosome,cyto	A=0, G=28	A=0.0, G=1.0	0.00001	408.717	28	A=0/0, G=15/1
chr22:22869742	A/A	C	A	SNV	ZNF280A	ZNF280A:exonic:NM_080740.4	1	100	2	missense	NM_08074	c.213G>T	p.Lys71Asn	-0.01		94		0(COSM4002	C2H2-type zir	rs361959	0.3194	YES	4.398E-1 (pos.22869742)	acute_myeloid_leukaemia		DNA binding,metal ion binding,nucleic acid binding,nucl	C=0, A=24	C=0.0, A=1.0	0.00001	334.051	24	C=0/0, A=17/7
chr9:17395108	T/T	C	T	SNV	CNTLN	CNTLN:intronic:NM_017738.3	1	100		intronic	NM_01773	c.2615+41	p.?	3.83						rs3739488	0.1235	YES	8.128E-1 (pos.17395108)		Centlein	centriole,centriole-centriole cohesion,centrosome,cyto	C=0, T=79	C=0.0, T=1.0	0.00001	1269.32	79	C=0/0, T=34/4
chr22:22842206	C/C	T	C	SNV	ZNF280B	ZNF280B:exonic:NM_080764.3	1	100	4	synonymous	NM_08076	c.1518A>G	p.(=)	-1.26					C2H2-type zir	rs11109	0.103	YES	7.821E-1 (pos.22842206)			DNA binding,metal ion binding,nucleic acid binding,nucl	T=0, C=21	T=0.0, C=1.0	0.00001	281.235	21	T=0/0, C=11/1
chr22:22842007	G/A	G	A	SNV	ZNF280B	ZNF280B:utr_3:NM_080764.3	1	57.89	4	utr_3	NM_08076	c.1717C>T	p.?	-0.4					C2H2-type zir	rs117413130		YES				DNA binding,metal ion binding,nucleic acid binding,nucl	G=16, A=22	G=0.4211, A=0.5789	0.00001	249.051	38	G=11/5, A=17/
chr19:44351694	T/T	C	T	SNV	ZNF283	ZNF283:exonic:NM_181845.1	1	100	7	missense	NM_18184	c.941C>T	p.Thr314Ile	0.07	0.17	89	0	0.01(COSM41	C2H2-type zir	rs2195980	0.1425	YES	5.95E-1 (pos.44351694)	ductal_carcinoma,neoplas		DNA binding,DNA binding transcription factor activity,int	C=0, T=44	C=0.0, T=1.0	0.00001	726.449	44	C=0/0, T=27/1
chr9:17761379	T/T	C	T	SNV	SH3GL2	SH3GL2:intronic:NM_003026.3	1	100		intronic	NM_00302	c.115-56C	p.?	-2.21					BAR domain,I	rs1886589	0.4683	YES			SH3 domair	Golgi mem Basal Ganglia Diseases:Brain Diseases:Centr	C=0, T=83	C=0.0, T=1.0	0.00001	1349.88	83	C=0/0, T=49/3
chr19:44351836	A/A	G	A	SNV	ZNF283	ZNF283:exonic:NM_181845.1	1	100	7	synonymous	NM_18184	c.1083G>	p.(=)	-1.42				0(COSM4000	C2H2-type zir	rs957569	0.2359	YES	3.59E-1 (pos.44351836)	acute_myeloid_leukaemia		DNA binding,DNA binding transcription factor activity,int	G=0, A=38	G=0.0, A=1.0	0.00001	602.087	38	G=0/0, A=10/2
chr19:44590000	C/C	CTCC	C	INDEL	ZNF284	ZNF284:exonic:NM_001037813.3	3	100	5	nonframeshiftDeletion	NM_00103	c.370_372	p.Ser124del	-3.98, -1.52, -1.81, -4.3				0.01(COSM42	C2H2-type zir	rs139900131,rs3694	0.341	YES	2.36E-3 (pos.44590000),	adenocarcinoma,carcinom		DNA binding,DNA binding transcription factor activity,me	CTCC=0, C=	CTCC=0.0, C=1.0	0.00001	1154.49	73	CTCC=0/0, C=4
chr19:44590558	T/T	C	T	SNV	ZNF284	ZNF284:exonic:NM_001037813.3	1	100	5	synonymous	NM_00103	c.927C>T	p.(=)	-0.35					C2H2-type zir	rs7255701	0.3474	YES	3.112E-1 (pos.44590558)			DNA binding,DNA binding transcription factor activity,me	C=0, T=69	C=0.0, T=1.0	0.00001	1072.78	69	C=0/0, T=27/4
chr9:18817246	C/C	A	C	SNV	ADAMTSL1	ADAMTSL1:intronic:NM_001040272.5	1	100		intronic	NM_00104	c.3934+11	p.?	-0.53					Immunoglob	rs1433809	0.1015	YES	9.525E-1 (pos.18817246)		ADAMTS-like	endoplasmic reticulum lumen,metalloendopeptidase ac	A=0, C=65	A=0.0, C=1.0	0.00001	996.423	65	A=0/0, C=30/3
chr9:18817372	T/T	C	T	SNV	ADAMTSL1	ADAMTSL1:intronic:NM_001040272.5	1	100		intronic	NM_00104	c.3934+13	p.?	1.06					Immunoglob	rs7873880		YES			ADAMTS-like	endoplasmic reticulum lumen,metalloendopeptidase ac	C=0, T=57	C=0.0, T=1.0	0.00001	849.608	57	C=0/0, T=19/3

chr19:44891783	GC/GC	G	GC	INDEL	ZNF285	ZNF285:exonic:NM 001291488.1	1	100	5	frameshiftInsertion	NM 00129	c.644 645	p.Asn215fs	-1.96												DNA binding,intracellular,metal ion binding,nucleic acid	G=0, GC=2:	G=0.0, GC=1.0	0.00001	254.251	23	G=0/0, GC=13/
chr9:20720599	C/C	A	C	SNV	FOCAD	FOCAD:intronic:NM 017794.4	1	100		intronic	NM 01779	c.287+66A	p.?	-0.37					Focadhesin,F	rs4977898		YES			Focadhesin	focal adhesion,integral component of membrane,protei	A=0, C=65	A=0.0, C=1.0	0.00001	996.464	65	A=0/0, C=31/3
chr9:21026540	T/T	G	T	SNV	HACD4	HACD4:intronic:NM 001010915.4	1	100		intronic	NM 00101	c.270+55C	p.?	-4.23					Protein tyros	rs2275889		YES			Protein tyrc	3-hydroxy-arachidoyl-CoA dehydratase activity,3-hydrox	G=0, T=51	G=0.0, T=1.0	0.00001	754.578	51	G=0/0, T=18/3
chr19:44891784	C/C	T	C	SNV	ZNF285	ZNF285:exonic:NM 001291488.1	1	100	5	missense	NM 00129	c.644A>G	p.Asn215Ser	0.21	0.61	46	0.002	0.01(COSM5427149)		rs2571089	0.0964	YES	6.443E-1:0.0 (pos.44891:	acute_myeloid_leukaemia		DNA binding,intracellular,metal ion binding,nucleic acid	T=0, C=23	T=0.0, C=1.0	0.00001	309.927	23	T=0/0, C=13/1
chr17:15611495	C/C	T	C	SNV	ZNF286A	ZNF286A:exonic:NM 001130842.1	1	100	5	missense	NM 00113	c.268T>C	p.Tyr90His	1.34	0.04	83	0.001	0.21(COSM43	C2H2-type zir	rs3760299	0.3673	YES	6.348E-1 (pos.15611495)	adenocarcinoma,carcinom		DNA binding,DNA binding transcription factor activity,int	T=0, C=41	T=0.0, C=1.0	0.00001	670.014	41	T=0/0, C=22/1
chr19:53384185	T/T	C	T	SNV	ZNF320	ZNF320:exonic:NM 207333.3	1	100	5	synonymous	NM 20733	c.1194G>A	p.(=)	-2.39				0.01(COSM63	C2H2-type zir	rs6509701	0.3113	YES	3.805E-1 (pos.53384185)	carcinoma	Zinc finger p	DNA binding,DNA binding transcription factor activity,int	C=0, T=86	C=0.0, T=1.0	0.00001	1410.93	86	C=0/0, T=29/5
chr19:54080144	G/G	A	G	SNV	ZNF331	ZNF331:exonic:NM 001253799.1	1	100	6	synonymous	NM 00125	c.330A>G	p.(=)	-3.13				0.01(COSM14	C2H2-type zir	rs8109631	0.2865	YES	6.772E-1 (pos.54080144)	adenocarcinoi	Zinc finger p	DNA bindi Arsenic Poisoning:Heavy Metal Poisoning, Ne	A=0, G=32	A=0.0, G=1.0	0.00001	484.489	32	A=0/0, G=10/2
chr20:44596207	G/G	C	G	SNV	ZNF335	ZNF335:exonic:NM 022095.3	1	100	6	missense	NM 02209	c.881G>C	p.Ser294Thr	1.97	1	58	0	0.03(COSM40	C2H2-type zir	rs6032606	0.0535	YES	5.471E-1 (pos.44596207)	acute_myeloic	Zinc finger p	RNA polymerase II core promoter sequenc Likely benign	C=0, G=21	C=0.0, G=1.0	0.00001	281.234	21	C=0/0, G=9/12
chr9:26934858	G/A	G	A	SNV	PLAA	PLAA:intronic:NM 001031689.2	1	56		intronic	NM 00103	c.343+153	p.?	0.51					PFU (PLAA far	rs10738762		YES			Phospholip	cellular response to lipopolysaccharide,cytoplasm,extra	G=11, A=14	G=0.44, A=0.56	0.00001	134.35	25	G=4/7, A=8/6
chr9:26934895	CC/TG	CC	TG	MNV	PLAA	PLAA:intronic:NM 001031689.2	2	56		intronic	NM 00103	c.343+116	p.?	-0.01, -0.34					PFU (PLAA far	rs62544451,rs78658575		YES			Phospholip	cellular response to lipopolysaccharide,cytoplasm,extra	CC=11, TG=	CC=0.44, TG=0.56	0.00001	134.35	25	CC=4/7, TG=8/
chr9:27047246	C/C	T	C	SNV	IFT74	IFT74:intronic:NM 025103.3	1	100		intronic	NM 02510	c.1109-26	p.?	-0.27						rs10812520	0.2401	YES	8.765E-1 (pos.27047246)		Intraflagell	Notch signaling pathway,beta-tubulin binding,centrosor	T=0, C=28	T=0.0, C=1.0	0.00001	408.718	28	T=0/0, C=19/9
chr9:27173180	T/T	C	T	SNV	TEK	TEK:intronic:NM 000459.4	1	100		intronic	NM 00045	c.761-40C	p.?	-0.86					Fibronectin t	rs666478	0.4726	YES	2.956E-1 (pos.27173180)		TEK tyrosine	ATP bindin Arteriovenous Malf Ampicillin,N-(4-METHYL-3	C=0, T=44	C=0.0, T=1.0	0.00001	726.548	44	C=0/0, T=15/2
chr20:44596545	A/A	G	A	SNV	ZNF335	ZNF335:exonic:NM 022095.3	1	100	5	synonymous	NM 02209	c.642C>T	p.(=)	1.13				0.72(COSM44	C2H2-type zir	rs3848719	0.4298	YES	1.169E-3 (pos.44596545)	adenocarcinoi	Zinc finger p	RNA polymerase II core promoter sequenc Benign/Likely	G=0, A=58	G=0.0, A=1.0	0.00001	867.368	58	G=0/0, A=31/2
chr19:52468203	A/A	T	A	SNV	ZNF350,ZNF350-AS1	ZNF350:exonic:NM 021632.3, ZNF350-AS1:intronic nc:NR 1	1	100	5	missense	NR 10384	c.1503A>T	p.Arg501Ser	-0.26	0	110	0.064	0.18(COSM63	C2H2-type zir	rs2278415	0.1274	YES	2.2E-1 (pos.52468203)	carcinoma	Zinc finger p	DNA binding,RNA polymerase II intronic transcription rep	T=0, A=47	T=0.0, A=1.0	0.00001	789.312	47	T=0/0, A=28/1
chr5:178507069	C/C	T	C	SNV	ZNF354C	ZNF354C:exonic:NM 014594.2	1	100	5	missense	NM 01459	c.1636T>C	p.Phe546Leu	1.01	0.66	22	0		C2H2-type zir	rs1445846	0.3255	YES	8.764E-1 (pos.178507069)			DNA binding,DNA binding transcription factor activity,cyt	T=0, C=33	T=0.0, C=1.0	0.00001	504.598	33	T=0/0, C=13/2
chr5:178507090	A/A	G	A	SNV	ZNF354C	ZNF354C:exonic:NM 014594.2	1	100	5	missense	NM 01459	c.1657G>A	p.Glu553Lys	0.47	0.46	56	0.001		C2H2-type zir	rs1445845,rs775119(	0.3232	YES	8.76E-1;1.16E-4 (pos.178507090)			DNA binding,DNA binding transcription factor activity,cyt	G=0, A=32	G=0.0, A=1.0	0.00001	485.141	32	G=0/0, A=12/2
chr9:27525022	T/T	A	T	SNV	IFNK,MOB3B	IFNK:intronic:NM 020124.2, MOB3B:intronic:NM 024761.4	1	100		intronic	NM 02476	c.-198+45:	p.?, p.?	-1.17					Interferon al	rs700784					Interferon,	B cell differentiation,B cell proliferation,T cell activation	A=0, T=29	A=0.0, T=1.0	0.00001	427.603	29	A=0/0, T=12/1
chr19:37117302	A/G	A	G	SNV	ZNF382	ZNF382:exonic:NM 001256838.1	1	50	5	missense	NM 00125	c.500A>G	p.Glu167Gly	1.85	1	98	0.103	0.71(COSM37	C2H2-type zir	rs3108171	0.3844	YES	8.173E-1 (pos.37117302)	adenocarcinoi	Zinc finger p	DNA binding transcription factor activity,intracellular,m	A=16, G=16	A=0.5, G=0.5	0.00001	158.203	32	A=13/3, G=7/9
chr3:21462754	G/G	A	G	SNV	ZNF385D	ZNF385D:exonic:NM 024697.2	1	100	8	synonymous	NM 02469	c.1140T>C	p.(=)	-4.47					Zinc-finger dc	rs451242	0.2445	YES	9.078E-1;0.0 (pos.21462754)			RNA bindir Amphetamine-Related Disorders:Mental Dis	A=0, G=55	A=0.0, G=1.0	0.00001	814.861	55	A=0/0, G=18/3
chr9:33301098	C/T	C	T	SNV	NFX1	NFX1:intronic:NM 002504.5	1	58.33		intronic	NM 00250	c.1034-16:	p.?	0.46					NF-X1 type zi	rs7866990		YES			Nuclear tra	DNA binding transcription factor activity,RNA binding,RN	C=10, T=14	C=0.4167, T=0.5833	0.00001	135.266	24	C=9/1, T=8/6
chr9:33395196	G/G	A	G	SNV	AQP7	AQP7:intronic:NM 001170.2	1	100		intronic	NM 00117	c.27-3T>C	p.?	1.28				0.01(COSM41	Major intrins	rs642636	0		1.0 (pos.33395196)	neoplasm	Aquaporin-7	cell-cell ju Digestive System Diseases;Liver Cirrhosis;Liv	A=0, G=69	A=0.0, G=1.0	0.00001	1072.78	69	A=0/0, G=49/2
chr9:33447306	G/G	C	G	SNV	AQP3	AQP3:intronic:NM 004925.4	1	100		intronic	NM 00492	c.108+115	p.?	1.07					Major intrins	rs517210		YES			Aquaporin-3	basolatera Adnexal Diseases;Carcinoma;Carcinoma, Squ	C=0, G=24	C=0.0, G=1.0	0.00001	334.79	24	C=0/0, G=18/6
chr18:72347482	C/C	T	C	SNV	ZNF407	ZNF407:exonic:NM 017757.2	1	100	1	synonymous	NM 01775	c.4507T>C	p.(=)	1.2				0.51(COSM11	Zinc finger, C2	rs12327359	0.4302	YES	1.864E-1 (pos.72347482)	acute_myeloic	Zinc finger p	DNA binding,nucleic acid binding,nucleus, Benign/Likely	T=0, C=47	T=0.0, C=1.0	0.00001	789.328	47	T=0/0, C=25/2
chr9:33467983	A/A	G	A	SNV	MIR6851,NOL6	MIR6851:upstream:NR 106910.1, NOL6:intronic:NM 02291	1	100		intronic	NM 02291	c.1424+45	p.?	0.77					Nrap protein	rs679644	0		1.0 (pos.33467983)		Nucleolar p	CURI complex,RNA binding,UTP-C complex,condensed nu	G=0, A=119	G=0.0, A=1.0	0.00001	1917.69	119	G=0/0, A=63/5
chr9:33529314	G/A	G	A	SNV	ANKRD18B	ANKRD18B:intronic:NM 001353432.1	1	50		intronic	NM 00135	c.495+143	p.?	-0.32					Ankyrin repea	rs2985588		YES					G=28, A=28	G=0.5, A=0.5	0.00001	273.892	56	G=11/17, A=7/
chr18:72776121	A/A	G	A	SNV	ZNF407	ZNF407:exonic:NM 017757.2	1	100	8	synonymous	NM 01775	c.6444G>A	p.(=)	-5.43				0.04(COSM41	Zinc finger, C2	rs3744913	0.1456	YES	9.017E-1 (pos.72776121)	neoplasm	Zinc finger p	DNA binding,nucleic acid binding,nucleus, Likely benign	G=0, A=33	G=0.0, A=1.0	0.00001	504.506	33	G=0/0, A=13/2
chr19:53611802	T/G	T	G	SNV	ZNF415	ZNF415:exonic:NM 018355.3	1	50	4	missense	NM 01835	c.1496A>C	p.Asn499Thr	-2.27	0.13	65	0.426	0.01(COSM58	C2H2-type zir	rs61733666	0.0373	YES	0.0 (pos.53611802)	rhabdomyosarcoma		DNA binding,DNA binding transcription factor activity,cyt	T=23, G=23	T=0.5, G=0.5	0.00001	258.57	46	T=18/5, G=9/1
chr19:58420162	C/C	T	C	SNV	ZNF417	ZNF417:exonic:NM 152475.2	1	100	3	missense	NM 15247	c.1484A>G	p.Asn495Ser	-8.71	1	46	0		C2H2-type zir	rs10416584	0.2906	YES	2.875E-1 (pos.58420162)			DNA binding,intracellular,metal ion binding,nucleic acid	T=0, C=25	T=0.0, C=1.0	0.00001	353.018	25	T=0/0, C=3/22
chr19:58003488	G/G	A	G	SNV	ZNF419	ZNF419:exonic:NM 001098491.1	1	100	4	synonymous	NM 00109	c.210A>G	p.(=)	-0.35				0.01(COSM37	C2H2-type zir	rs2074070		YES	6.191E-1 (pos.58003488)	adenocarcinoi	Zinc finger p	DNA binding,intracellular,metal ion binding,nucleic acid	A=0, G=37	A=0.0, G=1.0	0.00001	583.704	37	A=0/0, G=11/2
chr19:58003580	G/G	A	G	SNV	ZNF419	ZNF419:splicesite 5:NM 001098491.1	1	100	4	splicesite	NM 00109	c.301+1A>	p.?	0.11				0(COSM3757:	C2H2-type zir	rs2074071	0.3056	YES	7.21E-1 (pos.58003580)	acute_myeloic	Zinc finger p	DNA binding,intracellular,metal ion binding,nucleic acid	A=0, G=38	A=0.0, G=1.0	0.00001	603.786	38	A=0/0, G=12/2
chr19:11943697	A/A	G	A	SNV	ZNF440	ZNF440:exonic:NM 152357.2	1	100	4	missense	NM 15235	c.1706G>A	p.Ser569Asn	-1.59	1	46	0		C2H2-type zir	rs400106	0.0714	YES	9.998E-1 (pos.11943697)			DNA binding,intracellular,metal ion binding,nucleic acid	G=0, A=24	G=0.0, A=1.0	0.00001	338.65	24	G=0/0, A=16/8
chr19:11891003	A/A	G	A	SNV	ZNF441	ZNF441:exonic:NM 152355.2	1	100	4	missense	NM 15235	c.364G>A	p.Val122Ile	-0.3	1	29	0	0(COSM3756:	C2H2-type zir	rs799193	0.2103	YES	9.472E-1:0.0 (pos.11891(	adenocarcinoma		DNA binding,intracellular,metal ion binding,nucleic acid	G=0, A=49	G=0.0, A=1.0	0.00001	831.633	49	G=0/0, A=27/2
chr19:44419030	G/G	A	G	SNV	ZNF45	ZNF45:exonic:NM 003425.3	1	100	10	synonymous	NM 00342	c.558T>C	p.(=)	-1.26					KRAB box,Zin	rs386809614,rs4202	1.00E-04		1.0;0.0 (pos.44419030)		Zinc finger p	DNA binding,DNA binding transcription factor activity,me	A=0, G=80	A=0.0, G=1.0	0.00001	1289.35	80	A=0/0, G=56/2
chr9:35062972	T/T	C	T	SNV	VCP	VCP:intronic:NM 007126.3	1	100		intronic	NM 00712	c.811+3G>	p.?	0.55				0.29(COSM15	AAA domain (	rs514492	0.2262	YES	4.643E-1 (pos.35062972)	acute_myeloic	Valosin-con	ADP bindir Amyotrophic Latera Phospha Benign	C=0, T=25	C=0.0, T=1.0	0.00001	353.017	25	C=0/0, T=12/1
chr9:35074917	C/C	T	C	SNV	FANCG	FANCG:intronic:NM 004629.1	1	100		intronic	NM 00462	c.1636+7A	p.?	0.23				0.13(COSM4408697)		rs587118	0.4993	YES	3.929E-3 (pos.35074917)	hepatocellula	X-ray repair	DNA repai Anemia;Anemia, Aplastic;Aner Benign	T=0, C=47	T=0.0, C=1.0	0.00001	789.314	47	T=0/0, C=25/2
chr9:35079361	T/T	G	T	SNV	FANCG	FANCG:intronic:NM 004629.1	1	100		intronic	NM 00462	c.84+77C>	p.?	-0.05						rs504082		YES			X-ray repair	DNA repai Anemia;Anemia, Aplastic;Anemia, Hypoplast	G=0, T=60	G=0.0, T=1.0	0.00001	903.53	60	G=0/0, T=43/1
chr9:35100569	G/G	A	G	SNV	STOML2	STOML2:intronic:NM 013442.2	1	100		intronic	NM 01344	c.933+26T	p.?	-0.21					C-terminal re	rs586017	0.2091	YES	4.78E-1 (pos.35100569)		Stomatin-lil	CD4-positi Dermatitis:Dermatitis, Contact:Skin Disease	A=0, G=29	A=0.0, G=1.0	0.00001	403.163	29	A=0/0, G=16/1
chr9:35101384	C/C	CAC	C	INDEL	STOML2	STOML2:intronic:NM 013442.2	2	100		intronic	NM 01344	c.579+39G	p.?	-1.52, -0.3, -0.61					C-terminal re	rs200078307,rs34240345,rs741		YES	0.0 (pos.35101386),4.725E-1 (pos.35101		Stomatin-lil	CD4-positi Dermatitis:Dermatitis, Contact:Skin Disease	CAC=0, C=2	CAC=0.0, C=1.0	0.00001	281.234	21	CAC=0/0, C=18
chr9:35556250	G/C	G	C	SNV	RUSC2	RUSC2:intronic:NM 014806.4	1	95.45		intronic	NM 01480	c.2843-55	p.?	0.56					RUN domain,	rs870978		YES			RUN and SH	Rab GTPase binding,biological_process,cytoplasmic vesi	G=1, C=21	G=0.0455, C=0.9545	0.00001	267.288	22	G=0/1, C=12/9
chr19:53344701	A/G	A	G	SNV	ZNF468	ZNF468:exonic:NM 001008801.1	1	50	4	synonymous	NM 00100	c.846T>C	p.(=)	-4.79				0.02(COSM14	C2H2-type zir	rs10420793,rs76603	0.295	YES	0.0 (pos.53344699),1.00:	acute_myeloic	Zinc finger p	DNA binding,DNA binding transcription factor activity,int	A=12, G=12	A=0.5, G=0.5	0.00001	106.709	24	A=7/5, G=6/6
chr16:88498221	T/T	C	T	SNV	ZNF469	ZNF469:exonic:NM 001127464.2	1	100	2	missense	NM 00112	c.4259C>T	p.Pro1420Leu	2.71	1	98	0	0.04(COSM37	Zinc finger, C2	rs4782300	0.0843	YES	8.795E-1 (pos.88498221)	adenocarcinoi	Zinc finger protein 469	Cardiovascular Diseases:Colla Benign	C=0, T=76	C=0.0, T=1.0	0.00001	1209.6	76	C=0/0, T=38/3
chr16:88498297	G/G	T	G	SNV	ZNF469	ZNF469:exonic:NM 001127464.2	1	100	2	synonymous	NM 00112	c.4335T>G	p.(=)	-3.14				0.03(COSM41	Zinc finger, C2	rs12445417	0.0292	YES	9.676E-1 (pos.88498297)	neoplasm	Zinc finger protein 469	Cardiovascular Diseases:Colla Benign	T=0, G=53	T=0.0, G=1.0	0.00001	782.03	53	T=0/0, G=22/3
chr9:35712823	A/A	G	A	SNV	TLN1	TLN1:intronic:NM 006289.3	1	100		intronic	NM 00628	c.3561+9C	p.?	-0.27					Clp amino ter	rs2249133	0.4086	YES	5.296E-1 (pos.35712823)		Talin 1	IRE1-medi Bone Diseases:Bone Diseases, Metabolic:Mu	G=0, A=24	G=0.0, A=1.0	0.00001	334.789	24	G=0/0, A=16/8
chr9:35739876	A/A	G	A	SNV	GBA2	GBA2:intronic:NM 020944.2	1	100		intronic	NM 02094	c.1410-79	p.?	0.09					Glycosyl-hydi	rs3750434		YES			Glucosidase	Golgi membrane,beta-glucosidase activity,bile acid met	G=0, A=35	G=0.0, A=1.0	0.00001	543.9	35	G=0/0, A=10/2
chr9:35806827	C/CA	C	CA	INDEL	NPR2,SPAG8	SPAG8:downstream:NM 172312.1, NPR2:intronic:NM 0039	1	95.65		intronic	NM 00399	c.2520-19:	p.?, p.?	-0.48					Adenylate an	rs35457827					Natiuretic p	ATP bindin Bone Diseases:Bon Erythrityl Tetranitrate,N	C=1, CA=22	C=0.0435, CA=0.956	0.00001	226.136	23	C=1/0, CA=18/
chr16:88502482	T/T	C	T	SNV	ZNF469	ZNF469:exonic:NM 001127464.2	1	100	2	synonymous	NM 00112	c.8520C>T	p.(=)	0.66				0.21(COSM43	Zinc finger, C2	rs3812953		YES	4.424E-1 (pos.88502482)	carcinoma,hei	Zinc finger protein 469	Cardiovascular Diseases:Colla Benign	C=0, T=20	C=0.0, T=1.0	0.00001	263.109	20	C=0/0, T=6/14
chr16:88504868	G/G	A	G	SNV	ZNF469	ZNF469:exonic:NM 001127464.2	1	100	2	missense	NM 00112	c.10906A>	p.Thr3636Ala	-1.4	0.36	58	0	0.01(COSM41	Zinc finger, C2	rs904783	0		1.0 (pos.88504868)	neoplasm	Zinc finger protein 469	Cardiovascular Diseases:Colla Benign	A=0, G=21	A=0.0, G=1.0	0.00001	281.567	21	A=0/0, G=15/6
chr16:88505635	G/G	A	G	SNV	ZNF469	ZNF469:exonic:NM 001127464.2	1	100	2	synonymous	NM 00112	c.11673A>	p.(=)	-1.3					Zinc finger, C2	rs4782301	0.2343	YES	7.313E-1 (pos.88505635)		Zinc finger protein 469	Cardiovascular Diseases:Colla Benign	A=0, G=22	A=0.0, G=1.0	0.00001	298.812	22	A=0/0, G=15/7
chr16:4815608	G/G	A	G	SNV	ZNF500	ZNF500:exonic:NM 021646.3	1	100	2	synonymous	NM 02164	c.372T>C	p.(=)	0.11					C2H2-type zir	rs4786549	0.0014	YES	9.999E-1 (pos.4815608)			DNA binding,DNA binding transcription factor activity,cyt	A=0, G=35	A=0.0, G=1.0	0.00001	538.735	35	A=0/0, G=12/2
chr9:37522672	A/A	G	A	SNV	FBXO10	FBXO10:intronic:NM 012166.2	1	100		intronic	NM 01216	c.1930+15	p.?	-0.14					F-box domair	rs10973394		YES			F-box only p	apoptotic process,cytoplasm,cytosol,post-translational	G=0, A=32	G=0.0, A=1.0	0.00001	485.141	32	G=0/0, A=16/1
chr2:27838058	C/A	C	A	SNV	ZNF512	ZNF512:exonic:NM 032434.3	1	95.45	11	synonymous	NM 03243	c.1155C>A	p.(=)	-0.33				0.1(COSM561	Zinc finger, C2	rs11127071	0.4944	YES	8.456E-1:0.0 (pos.27838(	ductal_carcinoma		DNA binding,metal ion binding,nucleic acid binding,nucle	C=1, A=21	C=0.0455, A=0.9545	0.00001	271.51	22	C=1/0, A=11/1
chr10:97920099	TC/TC	T	TC	INDEL	ZNF518A	ZNF518A:exonic:NM 014803.3	1	100	6	unknown	NM 014803.3			0.6						rs67530050	0	YES	1.0 (pos.97920099)		Zinc finger p	DNA binding,RNA polymerase II transcription factor activ	T=0, TC=32	T=0.0, TC=1.0	0.00001	465.387	32	T=0/0, TC=12/
chr4:10446385	T/T	C	T	SNV	ZNF518B	ZNF518B:exonic:NM 053042.2	1	100	3	missense	NM 05304	c.1568G>A	p.Ser523Asn	0.3		46				rs9291410	0		1.0;0.0 (pos.10446385)		Zinc finger p	DNA binding,RNA polymerase II transcription factor activ	C=0, T=44	C=0.0, T=1.0	0.00001	726.569	44	C=0/0, T=21/2
chr4:10446906	G/G	A	G	SNV	ZNF518B	ZNF518B:exonic:NM 053042.2	1	100	3	synonymous	NM 05304	c.1047T>C	p.(=)	-3.18						rs10016022	0.2559	YES	8.116E-1 (pos.10446906)		Zinc finger p	DNA binding,RNA polymerase II transcription factor activ	A=0, G=20	A=0.0, G=1.0	0.00001	263.785	20	A=0/0, G=10/1
chr18:14105923	C/C	T	C	SNV	ZNF519	ZNF519:exonic:NM 145287.3	1	98.99	3	missense	NM 14528	c.616A>G	p.Lys206Glu	0.31	1	56	0.063		C2H2-type zir	rs2159940	2.00E-04		1.0 (pos.14105923)			DNA binding,intracellular,metal ion binding,nucleic acid	T=2, C=196	T=0.0101, C=0.9899	0.00001	3269.99	198	T=2/0, C=79/1
chr19:52938459	G/G	A	G	SNV	ZNF534	ZNF534:exonic:NM 001143939.2	1	100	4	missense	NM 00114	c.307A>G	p.Thr103Ala	-1.29	0.48	58	0.012	0(COSM4000:	C2H2-type zir	rs11084163	0.0356	YES	6.699E-1:0.0 (pos.52938	acute_myeloid_leukaemia		DNA binding,DNA binding transcription factor activity,int	A=0, G=46	A=0.0, G=1.0	0.00001	768.304	46	A=0/0, G=36/1
chr19:52941171	C/C	T	C	SNV	ZNF534	ZNF534:exonic:NM 001143939.2	1	100	5	missense	NM 00114	c.497T>C	p.Phe166Ser	-0.48	0.6	155	0	0(COSM4000:	C2H2-type zir	rs1428180	0.0303	YES	6.714E-1 (pos.52941171)	acute_myeloid_leukaemia		DNA binding,DNA binding transcription factor activity,int	T=0, C=134	T=0.0, C=1.0	0.00001	2196.47	134	T=0/0, C=79/5
chr9:38414067	T/T	G	T	SNV	IGFBPL1	IGFBPL1:intronic:NM 001007563.2	1	100		intronic	NM 00100	c.570+24C	p.?	-0.85					Immunoglobi	rs12375529	0.302	YES	1.638E-1 (pos.38414067)		Insulin-like	cellular response to tumor cell,extracellular space,insul	G=0, T=24	G=0.0, T=1.0	0.00001	340.491	24	G=0/0, T=11/1
chr9:38423826	A/A	T	A	SNV	IGFBPL1	IGFBPL1:intronic:NM 001007563.2	1	100		intronic	NM 00100	c.460+136	p.?	-1.13					Immunoglobi	rs7039990		YES			Insulin-like	cellular response to tumor cell,extracellular space,insul	T=0, A=20	T=0.0, A=1.0	0.00001	263.786	20	T=0/0, A=7/13
chr19:52941847	T/T	A	T	SNV	ZNF534	ZNF534:exonic:NM 001143939.2	1	100	5	synonymous	NM 00114	c.1173A>T	p.(=)	-2.09				0.07(COSM40	C2H2-type zir	rs1366259	0.0324	YES	6.791E-1 (pos.52941847)	acute_myeloid_leukaemia		DNA binding,DNA binding transcription factor activity,int	A=0, T=41	A=0.0, T=1.0	0.00001	664.632	41	A=0/0, T=21/2
chr9:38600952	A/G	A	G	SNV	ANKRD18A	ANKRD18A:intronic:NM 147195.3	1	50		intronic	NM 14719	c.936+176	p.?	0.81					Ankyrin repea	rs9632891		YES				Lung Diseases:Lung Neoplasms:Neoplasms:N	A=16, G=16	A=0.5, G=0.5	0.00001	158.382	32	A=13/3, G=12/
chr19:38091932	T/T	A	T	SNV	ZNF540	ZNF540:exonic:NM 152606.4	1	100	4	missense	NM 15260	c.158A>T	p.Asp53Val	0.08	0.04	152	0.11	0.01(COSM43	C2H2-type zir	rs1975937	0.1773	YES	5.938E-1 (pos.38091932)	adenocarcinoi	Zinc finger p	DNA binding,DNA binding transcription factor activity,cyt	A=0, T=79	A=0.0, T=1.0	0.00001	1269.3	79	A=0/0, T=39/4
chr9:446352	C/C	G	C	SNV	DOCK8	DOCK8:intronic:NM 203447.3	1	100		intronic	NM 20344	c.5581-18	p.?	-1.56					C2 domain in	rs10814952	0.307	YES	9.884E-1;0.0 (pos.446352)		Dedicator o	blood coa Developmental Disabilities;He Benign	G=0, C=93	G=0.0, C=1.0	0.00001	1555.3	93	G=0/0, C=57/3
chr9:449700	G/T	G	T	SNV	DOCK8	DOCK8:intronic:NM 203447.3	1	45.83		intronic	NM 20344	c.5818-84	p.?	-0.27					C2 domain in	rs2031946		YES			Dedicator o	blood coa Developmental Disabilities:Hematologic Dis	G=13, T=11	G=0.5417, T=0.4583	0.00001	93.4392	24	G=11/2, T=8/3
chr9:4844507	T/T	G	T	SNV	RCL1	RCL1:intronic:NM 005772.4	1	100		intronic	NM 00577	c.711-18G	p.?	0.82					RNA 3'-termir	rs295259	0.1245	YES	9.995E-1 (pos.4844507)		RNA termin	RNA processing,RNA-3'-phosphate cyclase activity,biolog	G=0, T=148	G=0.0, T=1.0	0.00001	2513.67	148	G=0/0, T=87/6
chr19:38055612	T/T	A	T	SNV	ZNF540,ZNF571,ZNF571-AS1	ZNF540:intronic:NM 152606.4, ZNF571:exonic:NM 016536	1	100	4	missense	NR 03824	c.-72+130	p.?, p.Leu573His	4.74	0.01	99	1	0(COSM3756	C2H2-type zir	rs4802029	0.1856	YES	6.253E-1 (pos.38055612)	adenocarcinoi	Zinc finger p	DNA binding,DNA binding transcription factor activity,cyt	A=0, T=28	A=0.0, T=1.0	0.00001	408.715	28	A=0/0, T=15/1
chr9:5055015	T/T	C	T	SNV	JAK2	JAK2:intronic:NM 004972.3	1	100		intronic	NM 00497	c.936+131	p.?	-0.07					Protein kinas	rs1536799		YES			Janus kinas	ATP bindin Abortion, Spontane 2-(1,1-DIMETHYLETHYL)9-	C=0, T=86	C=0.0, T=1.0	0.00001	1410.9	86	C=0/0, T=27/5
chr9:5929167	C/C	T	C	SNV	KIAA2026	KIAA2026:intronic:NM 001017969.2	1	100		intronic	NM 00101	c.2383-8A	p.?	-1.09				0.26(COSM4418543)		rs7029054	0.0705	YES	6.823E-1 (pos.5929167)	acute_myeloid_leukaemia			T=0, C=152	T=0.0, C=1.0	0.00001	2435.17	152	T=0/0, C=87/6
chr19:38056574	A/A	T	A	SNV	ZNF540,ZNF571,ZNF571-AS1	ZNF540:intronic:NM 152606.4, ZNF571:exonic:NM 016536	1	100	4	missense	NR 03824	c.-72+140	p.?, p.Glu252Asp	-1.47	0.12	45	0.003	0(COSM3756	C2H2-type zir	rs28512414	0.1855	YES	6.236E-1 (pos.38056574)	adenocarcinoi	Zinc finger p	DNA binding,DNA binding transcription factor activity,cyt	T=0, A=39	T=0.0, A=1.0	0.00001	620.339	39	T=0/0, A=18/2
chr19:58774071	G/G	A	G	SNV	ZNF544	ZNF544:exonic:NM 014480.3	1	100	7	missense	NM 01448	c.2099A>G	p.Gln700Arg	-3.36	0.84	43	0.03	0(COSM4001(	C2H2-type zir	rs260462	0.4309	YES	5.637E-1 (pos.58774071)	acute_myeloid_leukaemia		DNA binding,DNA binding transcription factor activity,int	A=0, G=23	A=0.0, G=1.0	0.00001	316.747	23	A=0/0, G=15/8
chr19:57909872	A/A	G	A	SNV	ZNF548	ZNF548:exonic:NM 001172773.1	1	100	4	missense	NM 00117	c.253G>A	p.Ala85Thr	-0.56	1	58	0		C2H2-type zir	rs4801478	0.0643	YES	8.038E-1 (pos.57909872)			DNA binding,intracellular,metal ion binding,nucleic acid	G=0, A=35	G=0.0, A=1.0	0.00001	543.769	35	G=0/0, A=22/1
chr19:58038964	A/A	T	A	SNV	ZNF549	ZNF549:exonic:NM 001199295.1	1	100	1	missense	NM 00119	c.23T>A	p.Ile8Asn	0.44	0.73	149	0	0(COSM3757:	C2H2-type zir	rs12461014	0.0272	YES	1.0 (pos.58038964)	adenocarcinoma		DNA binding,intracellular,metal ion binding,nucleic acid	T=0, A=54	T=0.0, A=1.0	0.00001	798.091	54	T=0/0, A=32/2
chr9:71855122	G/A	G	A	SNV	TJP2	TJP2:intronic:NM 001170416.1	1	50		intronic	NM 00117	c.2659+59	p.?	-1.43					Guanylate kii	rs7035000		YES			Tight juncti	GDP meta Bile Duct Diseases;Biliary Tract Diseases;Chc	G=17, A=17	G=0.5, A=0.5	0.00001	172.009	34	G=10/7, A=3/1
chr9:72003036	T/C	T	C	SNV	FAM189A2	FAM189A2:intronic:NM 001127608.2	1	50		intronic	NM 00112	c.857-38T:	p.?	0.82					CD20-like fan	rs867366	0.3957	YES	3.906E-1 (pos.72003036)			biological_process,cellular_component,integral compor	T=39, C=39	T=0.5, C=0.5	0.00001	395.315	78	T=26/13, C=13
chr9:72064749	C/C	T	C	SNV	APBA1	APBA1:intronic:NM 001163.3	1	100		intronic	NM 00116	c.1969-37	p.?	-0.54					PDZ domain (	rs2781532	0.0815	YES	9.997E-1;0.0 (pos.72064749)		Amyloid bei	Golgi apparatus,amyloid-beta binding,axo-dendritic trar	T=0, C=42	T=0.0, C=1.0	0.00001	685.22	42	T=0/0, C=21/2
chr9:72338180	C/C	T	C	SNV	PTAR1	PTAR1:intronic:NM 001099666.1	1	100		intronic	NM 00109	c.947+62A	p.?	-0.26					Protein preny	rs11139996		YES				cytoplasm,protein prenylation,protein prenyltransferas	T=0, C=32	T=0.0, C=1.0	0.00001	485.062	32	T=0/0, C=17/1
chr9:72659677	T/C	T	C	SNV	MAMDC2	MAMDC2:intronic:NM 153267.4	1	50		intronic	NM 15326	c.148+64T	p.?	-1.47					MAM domain	rs545062040					MAM doma	endoplasmic reticulum,membrane,proteinaceous extra	T=9, C=14	T=0.3913, C=0.6087	0.00001	192.896	28	T=1/8, C=2/12
chr19:58058246	C/C	T	C	SNV	ZNF550	ZNF550:utr 3:NM 001277092.1	1	100	4	utr 3	NM 00127	c.1366A>G	p.?	-3.67					C2H2-type zir	rs1548475		YES				DNA binding,intracellular,metal ion binding,nucleic acid	T=0, C=21	T=0.0, C=1.0	0.00001	281.226	21	T=0/0, C=10/1
chr9:72783608	C/C	T	C	SNV	MAMDC2,MAMDC2-AS1	MAMDC2:intronic:NM 153267.4, MAMDC2-AS1:intronic nc:	1	100		intronic	NM 15326	c.1405-10	p.?	1.02					MAM domain	rs10780871	0.1236	YES	9.535E-1 (pos.72783608)		MAM doma	endoplasmic reticulum,membrane,proteinaceous extra	T=0, C=28	T=0.0, C=1.0	0.00001	408.718	28	T=0/0, C=22/6
chr19:58058536	G/G	A	G	SNV	ZNF550	ZNF550:exonic:NM 001277092.1	1	100	4	missense	NM 00127	c.1076T>C	p.Ile359Thr	-1.09	0.82	89	0		C2H2-type zir	rs1548476	0.4315	YES	7.503E-1 (pos.58058536)			DNA binding,intracellular,metal ion binding,nucleic acid	A=0, G=30	A=0.0, G=1.0	0.00001	446.629	30	A=0/0, G=18/1
chr9:732302	C/C	T	C	SNV	KANK1	KANK1:intronic:NM 015158.3	1	100		intronic	NM 01515	c.3006-76	p.?	0.47					Ankyrin repea	rs10815562		YES			KN motif-ar	beta-cate Brain Damage, Chronic;Brain Diseases;Centr	T=0, C=37	T=0.0, C=1.0	0.00001	583.487	37	T=0/0, C=22/1
chr9:73240312	G/G	C	G	SNV	TRPM3	TRPM3:intronic:NM 001007471.2	1	100		intronic	NM 00100	c.1633-65	p.?	-0.41					Ion transport	rs7035025,rs750745433		YES	0.0 (pos.73240294)		Transient re	G-protein Amphetamine-Related Disorders:Mental Dis	C=0, G=96	C=0.0, G=1.0	0.00001	1618.01	96	C=0/0, G=42/5
chr9:738238	A/A	G	A	SNV	KANK1	KANK1:intronic:NM 015158.3	1	100		intronic	NM 01515	c.3334-47	p.?	-1.09					Ankyrin repea	rs2296052	0.4155	YES	3.436E-1 (pos.738238)		KN motif-ar	beta-cate Brain Damage, Chronic:Brain Diseases:Centr	G=0, A=40	G=0.0, A=1.0	0.00001	644.286	40	G=0/0, A=18/2
chr19:36940760	T/T	G	T	SNV	ZNF566	ZNF566:exonic:NM 001145343.1	1	100	5	synonymous	NM 00114	c.379C>A	p.(=)	-0.4				0(COSM3756	C2H2-type zir	rs2967532	0.4088	YES	2.785E-1 (pos.36940760)	acute_myeloid_leukaemia		DNA binding,integral component of membrane,intracellu	G=0, T=57	G=0.0, T=1.0	0.00001	849.613	57	G=0/0, T=37/2
chr19:37487632	A/A	G	A	SNV	ZNF568	ZNF568:exonic:NM 001204838.1	1	100	10	missense	NM 00120	c.1039G>A	p.Ala347Thr	1.63	0	58	0.154	0.11(COSM3822847),0.16(C		rs935706		YES	3.353E-1 (pos.37487632)	acute_myeloic	Zinc finger p	DNA binding transcription factor activity,embryonic plac	G=0, A=55	G=0.0, A=1.0	0.00001	814.905	55	G=0/0, A=20/3
chr9:74485285	T/T	C	T	SNV	ABHD17B	ABHD17B:intronic:NM 016014.3	1	100		intronic	NM 01601	c.468-107	p.?	-0.55					Prolyl oligope	rs1524381		YES				extracellular region,membrane,negative regulation of p	C=0, T=36	C=0.0, T=1.0	0.00001	563.669	36	C=0/0, T=14/2
chr9:74675343	G/G	A	G	SNV	C9orf57	C9orf57:intronic:NM 001128618.1	1	100		intronic	NM 00112	c.13+70T>	p.?	0.07					Domain of un	rs10869090		YES				integral component of membrane	A=0, G=27	A=0.0, G=1.0	0.00001	389.988	27	A=0/0, G=8/19
chr9:74975711	C/C	T	C	SNV	ZFAND5	ZFAND5:intronic:NM 001102421.2	1	100		intronic	NM 00110	c.-8-8A>G	p.?	-1.17					A20-like zinc	rs1853276	1.00E-04		9.995E-1;0.0 (pos.74975711)		Zinc finger A	DNA bindi Genital Diseases, Male:Genital Neoplasms, I	T=0, C=25	T=0.0, C=1.0	0.00001	357.339	25	T=0/0, C=9/16
chr9:75541962	A/C	A	C	SNV	ALDH1A1	ALDH1A1:intronic:NM 000689.4	1	58.33		intronic	NM 00068	c.504+70T	p.?	0.36					Aldehyde del	rs8187925	0.0346	YES			Aldehyde de	GTPase ac Adenocarcinoma;B 1,4-Dithiothreitol,Glycer	A=10, C=14	A=0.4167, C=0.5833	0.00001	135.236	24	A=9/1, C=3/11
chr9:77245162	T/T	A	T	SNV	RORB	RORB:intronic:NM 006914.3	1	100		intronic	NM 00691	c.8-36A>T	p.?	-0.67					Ligand-bindir	rs2146777,rs780529:	0.0013	YES	9.997E-1;2.321E-4;0.0 (pos.77245162)		RAR-related	orphan rec Endometriosis;Eye I Melatonin	A=0, T=28	A=0.0, T=1.0	0.00001	408.704	28	A=0/0, T=25/3
chr9:77745424	T/C	T	C	SNV	OSTF1	OSTF1:intronic:NM 012383.4	1	52.38		intronic	NM 01238	c.133-70T:	p.?	-0.63					Ankyrin repea	rs2295861		YES			Osteoclast-	SH3 domain binding,extracellular exosome,extracellula	T=10, C=11	T=0.4762, C=0.5238	0.00001	95.3002	21	T=10/0, C=8/3
chr9:78799566	T/T	C	T	SNV	PCSK5	PCSK5:intronic:NM 001190482.1	1	100		intronic	NM 00119	c.2198-23	p.?	0.82					Growth facto	rs1537183	0.0354	YES	9.995E-1 (pos.78799566)		Proprotein	Golgi appa Abnormalities, Multiple:Anus, Imperforate:B	C=0, T=26	C=0.0, T=1.0	0.00001	371.42	26	C=0/0, T=22/4
chr9:79003440	A/A	G	A	SNV	RFK	RFK:intronic:NM 018339.5	1	100		intronic	NM 01833	c.337+30C	p.?	-0.32					Riboflavin kir	rs2501924	0.0549	YES	9.995E-1 (pos.79003440)		Riboflavin k	ATP bindin Genital Diseases, M Riboflavin,Riboflavin Mo	G=0, A=20	G=0.0, A=1.0	0.00001	263.782	20	G=0/0, A=7/13
chr19:37487723	A/A	G	A	SNV	ZNF568	ZNF568:exonic:NM 001204838.1	1	100	10	missense	NM 00120	c.1130G>A	p.Arg377His	-4.5	0.15	29	0	0(COSM439333)		rs935707		YES	4.528E-1 (pos.37487723)	acute_myeloic	Zinc finger p	DNA binding transcription factor activity,embryonic plac	G=0, A=48	G=0.0, A=1.0	0.00001	815.084	48	G=0/0, A=13/3
chr19:37487866	T/T	A	T	SNV	ZNF568	ZNF568:exonic:NM 001204838.1	1	100	10	missense	NM 00120	c.1273A>T	p.Ser425Cys	4.58	1	112	0	0.01(COSM4000699)		rs1667363		YES	0.0 (pos.37487863),4.23	acute_myeloic	Zinc finger p	DNA binding transcription factor activity,embryonic plac	A=0, T=30	A=0.0, T=1.0	0.00001	446.637	30	A=0/0, T=14/1
chr19:37487873	A/A	C	A	SNV	ZNF568	ZNF568:exonic:NM 001204838.1	1	100	10	missense	NM 00120	c.1280C>A	p.Ala427Glu	3.28	1	107	0	0.01(COSM4000700)		rs1667364		YES	0.0 (pos.37487863),4.21	acute_myeloic	Zinc finger p	DNA binding transcription factor activity,embryonic plac	C=0, A=30	C=0.0, A=1.0	0.00001	446.638	30	C=0/0, A=14/1
chr19:37488055	G/G	T	G	SNV	ZNF568	ZNF568:exonic:NM 001204838.1	1	100	10	missense	NM 00120	c.1462T>G	p.Tyr488Asp	7.11	0	160	1	0.97(COSM4417921)		rs10405238,rs746170450		YES	2.801E-1:0.0 (pos.37488(	acute_myeloic	Zinc finger p	DNA binding transcription factor activity,embryonic plac	T=0, G=37	T=0.0, G=1.0	0.00001	583.707	37	T=0/0, G=11/2
chr19:37488197	A/A	G	A	SNV	ZNF568	ZNF568:exonic:NM 001204838.1	1	100	10	missense	NM 00120	c.1604G>A	p.Cys535Tyr	0.02	1	194	0	0.02(COSM3822848)		rs1345748		YES	3.733E-1 (pos.37488197)	acute_myeloic	Zinc finger p	DNA binding transcription factor activity,embryonic plac	G=0, A=36	G=0.0, A=1.0	0.00001	563.732	36	G=0/0, A=17/1
chr19:37488481	G/C	G	C	SNV	ZNF568	ZNF568:exonic:NM 001204838.1	1	98.72	10	missense	NM 00120	c.1888G>C	p.Gly630Arg	-5.08	0.66	125	0	0(COSM4592589)		rs1363753		YES	5.392E-1;0.0 (pos.37488	squamous_cel	Zinc finger p	DNA binding transcription factor activity,embryonic plac	G=1, C=77	G=0.0128, C=0.9872	0.00001	1213.92	78	G=0/1, C=32/4
chr19:37488499	T/C	T	C	SNV	ZNF568	ZNF568:exonic:NM 001204838.1	1	98.72	10	stoploss	NM 00120	c.1906T>C	p.Ter636Arg	-1.96				0(COSM4592923)		rs1667366		YES	5.308E-1 (pos.37488499)	squamous_cel	Zinc finger p	DNA binding transcription factor activity,embryonic plac	T=1, C=77	T=0.0128, C=0.9872	0.00001	1213.93	78	T=0/1, C=32/4
chr19:2917186	C/C	T	C	SNV	ZNF57	ZNF57:exonic:NM 173480.2	1	100	4	synonymous	NM 17348	c.567T>C	p.(=)	-5.27					C2H2-type zir	rs2288959,rs776415(	0.103	YES	0.0 (pos.2917185),7.099E-1 (pos.2917186)			DNA binding,intracellular,metal ion binding,nucleic acid	T=0, C=31	T=0.0, C=1.0	0.00001	465.819	31	T=0/0, C=13/1
chr19:2917612	C/C	T	C	SNV	ZNF57	ZNF57:exonic:NM 173480.2	1	100	4	synonymous	NM 17348	c.993T>C	p.(=)	-4.59					C2H2-type zir	rs10410539	0.1284	YES	7.108E-1;0.0 (pos.2917612)			DNA binding,intracellular,metal ion binding,nucleic acid	T=0, C=25	T=0.0, C=1.0	0.00001	353.019	25	T=0/0, C=12/1
chr9:80863088	A/A	G	A	SNV	CEP78	CEP78:intronic:NM 001098802.2	1	100		intronic	NM 00109	c.893-119	p.?	-0.26					Leucine Rich	rs1929402		YES			Centrosoma	G2/M transition of mitotic cell cycle,centriole,centroson	G=0, A=69	G=0.0, A=1.0	0.00001	1072.78	69	G=0/0, A=29/4
chr9:82320887	T/T	A	T	SNV	TLE4	TLE4:intronic:NM 007005.4	1	100		intronic	NM 00700	c.783+30A	p.?	0.25					Groucho/TLE	rs4877147	0.1096	YES	8.063E-1;0.0 (pos.82320887)			Wnt signa Bipolar Disorder;Bipolar and Related Disorde	A=0, T=52	A=0.0, T=1.0	0.00001	767.013	52	A=0/0, T=26/2
chr9:84207857	A/G	A	G	SNV	TLE1	TLE1:intronic:NM 005077.4	1	50		intronic	NM 00507	c.1581+83	p.?	1.47					Groucho/TLE	rs2796471		YES			Transducin-	RNA polymerase II transcription corepressor activity,Wn	A=27, G=27	A=0.5, G=0.5	0.00001	262.859	54	A=12/15, G=1(
chr9:8436476	T/C	T	C	SNV	PTPRD	PTPRD:intronic:NM 002839.3	1	51.85		intronic	NM 00283	c.4086+11	p.?	-0.81					Fibronectin t	rs10977102		YES			Protein tyrc	cell adhes Attention Deficit Disorder with Hyperactivity	T=13, C=14	T=0.4815, C=0.5185	0.00001	132.61	27	T=6/7, C=11/3
chr9:847208	G/G	A	G	SNV	DMRT1	DMRT1:intronic:NM 021951.2	1	100		intronic	NM 02195	c.538+65A	p.?	-0.45					DM DNA bind	rs2273929		YES			Double sex	DNA bindi 46, XY Disorders of Sex Development:Congen	A=0, G=28	A=0.0, G=1.0	0.00001	408.681	28	A=0/0, G=20/8
chr19:52376952	A/A	T	A	SNV	ZNF577	ZNF577:exonic:NM 032679.2	1	100	7	synonymous	NM 03267	c.291A>T	p.(=)	-0.19					C2H2-type zir	rs8107969	0.3866	YES	3.013E-1 (pos.52376952)			DNA binding,DNA binding transcription factor activity,int	T=0, A=36	T=0.0, A=1.0	0.00001	563.741	36	T=0/0, A=20/1
chr9:85905666	G/G	A	G	SNV	FRMD3	FRMD3:intronic:NM 174938.5	1	100		intronic	NM 17493	c.1071-24	p.?	-1.38					FERM C-termi	rs942283	0.3787	YES	7.058E-1 (pos.85905666)		FERM doma	actomyosi Acneiform Eruptions;Chloracne;Skin Disease	A=0, G=46	A=0.0, G=1.0	0.00001	768.313	46	A=0/0, G=8/38
chr19:53014346	A/A	G	A	SNV	ZNF578	ZNF578:exonic:NM 001099694.1	1	100	6	missense	NM 00109	c.712G>A	p.Glu238Lys	0.24	1	56	0	0(COSM3733	C2H2-type zir	rs161931	0.3817	YES	6.165E-1 (pos.53014346)	acute_myeloid_leukaemia		DNA binding,DNA binding transcription factor activity,int	G=0, A=51	G=0.0, A=1.0	0.00001	753.641	51	G=0/0, A=12/3
chr9:86584903	G/G	A	G	SNV	HNRNPK,MIR7-1	HNRNPK:intronic:NM 031263.3, MIR7-1:upstream:NR 0296	1	100		intronic	NM 03126	c.1361+17	p.?	-0.81					KH domain,R	rs296885		YES			Heterogene	DNA bindi Breast Diseases;Breast Neoplasms;Endocrin	A=0, G=27	A=0.0, G=1.0	0.00001	389.985	27	A=0/0, G=11/1
chr9:88618462	G/G	C	G	SNV	NAA35	NAA35:intronic:NM 024635.3	1	100		intronic	NM 02463	c.1057-38	p.?	-0.81					Mak10 subur	rs1328923	0.4579	YES	2.269E-2 (pos.88618462)			N-terminal protein amino acid acetylation,NatC comple:	C=0, G=33	C=0.0, G=1.0	0.00001	504.598	33	C=0/0, G=18/1
chr9:90256827	G/G	A	G	SNV	DAPK1	DAPK1:intronic:NM 004938.3	1	100		intronic	NM 00493	c.829-57A	p.?	-0.31					Ankyrin repea	rs3118846		YES			Death-asso	ATP bindin Adenocarcinoma;A 5,6-Dihydro-BenzoHCinn	A=0, G=27	A=0.0, G=1.0	0.00001	389.92	27	A=0/0, G=20/7
chr9:90258537	A/A	G	A	SNV	DAPK1	DAPK1:intronic:NM 004938.3	1	100		intronic	NM 00493	c.1011+15	p.?	0.07					Ankyrin repea	rs3793656		YES			Death-asso	ATP bindin Adenocarcinoma;A 5,6-Dihydro-BenzoHCinn	G=0, A=69	G=0.0, A=1.0	0.00001	1072.75	69	G=0/0, A=56/1
chr19:53015384	A/G	A	G	SNV	ZNF578	ZNF578:exonic:NM 001099694.1	1	45.83	6	missense	NM 00109	c.1750A>G	p.Ile584Val	-9.3	0.59	29	0	0(COSM3757:	C2H2-type zir	rs4802965	0.2344	YES	4.845E-1:1.16E-4:0.0 (pos	acute_myeloid_leukaemia		DNA binding,DNA binding transcription factor activity,int	A=26, G=22	A=0.5417, G=0.4583	0.00001	240.971	48	A=12/14, G=1
chr9:90320954	A/A	G	A	SNV	DAPK1	DAPK1:intronic:NM 004938.3	1	100		intronic	NM 00493	c.3061-93	p.?	-1.81					Ankyrin repea	rs1927973		YES			Death-asso	ATP bindin Adenocarcinoma;A 5,6-Dihydro-BenzoHCinn	G=0, A=40	G=0.0, A=1.0	0.00001	644.286	40	G=0/0, A=30/1
chr19:58928786	A/G	A	G	SNV	ZNF584	ZNF584:exonic:NM 173548.2	1	57.45	4	missense	NM 17354	c.901A>G	p.Thr301Ala	-4.68	0.15	58	0.001		C2H2-type zir	rs7257872	0.272	YES	2.588E-1 (pos.58928786)			DNA binding,intracellular,metal ion binding,nucleic acid	A=20, G=27	A=0.4255, G=0.5745	0.00001	326.533	47	A=12/8, G=12/
chr9:93639803	G/G	A	G	SNV	SYK	SYK:intronic:NM 003177.6	1	100		intronic	NM 00317	c.1182-50	p.?	-1.45					Protein kinas	rs1760132		YES	1.0;0.0 (pos.93639803)		Spleen tyro	ATP bindin Adenoma;Arterial C 2-[(1R,2S)-2-aminocycloh	A=0, G=52	A=0.0, G=1.0	0.00001	766.883	52	A=0/0, G=30/2
chr19:58929052	G/A	G	A	SNV	ZNF584	ZNF584:exonic:NM 173548.2	1	45.45	4	synonymous	NM 17354	c.1167G>A	p.(=)	0.23					C2H2-type zir	rs3764535	0.4806	YES	3.199E-1 (pos.58929052)			DNA binding,intracellular,metal ion binding,nucleic acid	G=12, A=10	G=0.5455, A=0.4545	0.00001	81.8355	22	G=3/9, A=5/5
chr19:58352806	T/C	T	C	SNV	ZNF587B	ZNF587B:exonic:NM 001204818.1	1	57.14	3	missense	NM 00120	c.764T>C	p.Val255Ala	-1.77	0.51	64	0	0(COSM4597	C2H2-type zir	rs35313547		YES	2.223E-1 (pos.58352806)	squamous_cell_carcinoma		metal ion binding,negative regulation of transcription, D	T=18, C=24	T=0.4286, C=0.5714	0.00001	279.086	42	T=6/12, C=17/
chr19:58352522	G/A	G	A	SNV	ZNF587B	ZNF587B:exonic:NM 001204818.1	1	47.37	3	synonymous	NM 00120	c.480G>A	p.(=)	-2.67				0.01(COSM38	C2H2-type zir	rs12981244		YES	6.799E-1;0.0 (pos.58352	carcinoma		metal ion binding,negative regulation of transcription, D	G=20, A=18	G=0.5263, A=0.4737	0.00001	184.532	38	G=9/11, A=8/1
chr9:95397418	G/G	C	G	SNV	IPPK	IPPK:intronic:NM 022755.5	1	100		intronic	NM 02275	c.1067+22	p.?	-0.06					Inositol-pent	rs1111047	0.4264	YES	8.697E-1 (pos.95397418)			ATP binding,DNA-directed RNA polymerase I complex,cyt	C=0, G=63	C=0.0, G=1.0	0.00001	962.504	63	C=0/0, G=37/2
chr3:48309828	G/G	C	G	SNV	ZNF589	ZNF589:exonic:NM 016089.2	1	100	4	missense	NM 01608	c.647C>G	p.Thr216Arg	-1.96	0.73	71	0	0.01(COSM40	C2H2-type zir	rs11718329	0.3107	YES	1.196E-1:9.27E-4 (pos.48	acute_myeloic	Zinc finger p	RNA polymerase II regulatory region sequence-specific D	C=0, G=40	C=0.0, G=1.0	0.00001	644.281	40	C=0/0, G=20/2

chr4:86738	G/G	A	G	SNV	ZNF595,ZNF718	ZNF595:exonic:NM 001286054.1, ZNF718:intronic:NM 001	1	100	10	synonymous	NM 00128	c.792A>G,	p.(=), p.?	-1.9					C2H2-type zir	rs2353625			1.0 (pos.86738)			DNA binding,intracellular,metal ion binding,nucleic acid			A=0, G=27	A=0.0, G=1.0	0.00001	392.369	27	A=0/0, G=5/22
chr4:87277	C/C	G	C	SNV	ZNF595,ZNF718	ZNF595:exonic:NM 001286054.1, ZNF718:intronic:NM 001	1	100	10	missense	NM 00128	c.1331G>C	p.Gly444Ala, p.?	0.35		60			C2H2-type zir	rs2006764			1.0 (pos.87277)			DNA binding,intracellular,metal ion binding,nucleic acid			G=0, C=23	G=0.0, C=1.0	0.00001	316.747	23	G=0/0, C=8/15
chr9:95874070	C/C	G	C	SNV	CARD19	CARD19:intronic:NM 032310.4	1	100		intronic	NM 03231	c.305-91G	p.?	0.6						rs2027585		YES			Caspase re	CARD domain binding,cytosol,endoplasmic reticulum me			G=0, C=48	G=0.0, C=1.0	0.00001	814.248	48	G=0/0, C=19/2
chr9:96015377	G/G	A	G	SNV	WNK2	WNK2:intronic:NM 006648.3	1	100		intronic	NM 00664	c.2034+13	p.?	-1.3					Oxidative-str	rs386736361,rs4744	0.042	YES	9.142E-1 (pos.96015377)		WNK lysine deficient protein kinase 2				A=0, G=28	A=0.0, G=1.0	0.00001	408.711	28	A=0/0, G=16/1
chr9:96025771	A/A	G	A	SNV	WNK2	WNK2:intronic:NM 006648.3	1	100		intronic	NM 00664	c.3411-77	p.?	-1.09					Oxidative-str	rs6479467		YES			WNK lysine deficient protein kinase 2				G=0, A=54	G=0.0, A=1.0	0.00001	798.148	54	G=0/0, A=20/3
chr9:96030230	C/C	T	C	SNV	WNK2	WNK2:intronic:NM 006648.3	1	100		intronic	NM 00664	c.3867+32	p.?	-1.86					Oxidative-str	rs7856268	0.0539	YES	9.998E-1 (pos.96030230)		WNK lysine deficient protein kinase 2				T=0, C=47	T=0.0, C=1.0	0.00001	789.327	47	T=0/0, C=19/2
chr9:967981	C/C	G	C	SNV	DMRT1	DMRT1:intronic:NM 021951.2	1	100		intronic	NM 02195	c.968-4G>	p.?	0.98					DM DNA bind	rs279895	0.1664	YES	9.983E-1 (pos.967981)		Double sex	DNA bindi	46, XY Disorders of Sex Development;Congen		G=0, C=10(	G=0.0, C=1.0	0.00001	1702.11	100	G=0/0, C=53/4
chr9:97321127	C/C	T	C	SNV	FBP2,PCAT7	PCAT7:intronic nc:NR 121566.2, FBP2:utr 3:NM 003837.3	1	100		intronic	NR 12156	c.1113A>G	p.?	-3.85					Fructose-1-6-	rs1048510		YES			Fructose-1,	Z disc,cell junction,cytosol,dephosphorylation,extracell			T=0, C=38	T=0.0, C=1.0	0.00001	603.784	38	T=0/0, C=31/7
chr9:97822941	C/C	T	C	SNV	C9orf3	C9orf3:intronic:NM 001193329.1	1	100		intronic	NM 00119	c.2116-35	p.?	0.16					Leukotriene /	rs1888944	0.0281	YES	9.49E-1 (pos.97822941)			aminopep	Adnexal Diseases:Arrhythmias, Cardiac:Arth		T=0, C=61	T=0.0, C=1.0	0.00001	921.854	61	T=0/0, C=34/2
chr9:98678164	T/T	G	T	SNV	ERCC6L2	ERCC6L2:intronic:NM 001010895.2	1	98.72		intronic	NM 00101	c.983+53G	p.?	0.53					DEAD/DEAH b	rs683829		YES			ERCC6-like 2	ATP binding,DNA binding,cellular response to reactive o			G=2, T=154	G=0.0128, T=0.9872	0.00001	2432.76	156	G=0/2, T=64/9
chr4:87313	GA/GA	G	GA	INDEL	ZNF595,ZNF718	ZNF595:exonic:NM 001286054.1, ZNF718:intronic:NM 001	1	100	10	unknown	NM 00128	c.226+272	p.?	-1.1					C2H2-type zir	rs397952018,rs6015	5.00E-04	YES	1.0;0.0 (pos.87313)			DNA binding,intracellular,metal ion binding,nucleic acid			G=0, GA=24	G=0.0, GA=1.0	0.00001	332.882	24	G=0/0, GA=8/1
chr19:38202516	C/C	A	C	SNV	ZNF607	ZNF607:exonic:NM 032689.4	1	100	2	missense	NM 03268	c.4T>G	p.Ser2Ala	-0.3	1	99	0		C2H2-type zir	rs2909097,rs386809	0		9.685E-1 (pos.38202516)			DNA binding,DNA binding transcription factor activity,int			A=0, C=28	A=0.0, C=1.0	0.00001	406.161	28	A=0/0, C=10/1
chr15:64915106	T/T	C	T	SNV	ZNF609	ZNF609:exonic:NM 015042.1	1	100	2	synonymous	NM 01504	c.828C>T	p.(=)	-1.03						rs525878	5.00E-04		1.0 (pos.64915106)		Zinc finger p	integrator complex,metal ion binding,muscle organ deve			C=0, T=32	C=0.0, T=1.0	0.00001	484.937	32	C=0/0, T=22/1
chrX:10062079	A/A	T	A	SNV	WWC3	WWC3:intronic:NM 015691.3	1	100		intronic	NM 01569	c.508-93T	p.?	-0.84						rs5934746		YES				cytosol,kinase binding,negative regulation of hippo signa			T=0, A=20	T=0.0, A=1.0	0.00001	263.649	20	T=0/0, A=15/5
chrX:10062163	C/C	G	C	SNV	WWC3	WWC3:intronic:NM 015691.3	1	100		intronic	NM 01569	c.508-9G>	p.?	2.35						rs5934747	0.2757	YES	2.943E-2 (pos.10062163)			cytosol,kinase binding,negative regulation of hippo signa			G=0, C=21	G=0.0, C=1.0	0.00001	281.235	21	G=0/0, C=15/6
chr19:52519608	T/T	C	T	SNV	ZNF614	ZNF614:exonic:NM 025040.3	1	100	5	missense	NM 02504	c.1243G>A	p.Val415Ile	-1.52	1	29	0.004	0(COSM3757	C2H2-type zir	rs8104890	0.2664	YES	3.538E-1 (pos.52519608)	adenocarcinoma,diffuse_la		DNA binding,biological_process,cellular_component,int			C=0, T=34	C=0.0, T=1.0	0.00001	522.103	34	C=0/0, T=26/8
chr19:52521296	A/A	G	A	SNV	ZNF614	ZNF614:exonic:NM 025040.3	1	100	4	missense	NM 02504	c.203C>T	p.Thr68Ile	-0.22	0.71	89	0.001	0(COSM3757	C2H2-type zir	rs9636139	0.2707	YES	5.189E-1 (pos.52521296)	acute_myeloid_leukaemia		DNA binding,biological_process,cellular_component,int			G=0, A=42	G=0.0, A=1.0	0.00001	685.22	42	G=0/0, A=19/2
chr19:52496149	T/T	C	T	SNV	ZNF615	ZNF615:exonic:NM 001199324.1	1	100	7	missense	NM 00119	c.2213G>A	p.Arg738Lys	-0.21	0.15	26	0	0(COSM1487	C2H2-type zir	rs16983353	0.2774	YES	3.538E-1 (pos.52496149)	acute_myeloid_leukaemia		DNA binding,DNA binding transcription factor activity,int			C=0, T=66	C=0.0, T=1.0	0.00001	1015.4	66	C=0/0, T=33/3
chr19:52497250	A/A	G	A	SNV	ZNF615	ZNF615:exonic:NM 001199324.1	1	100	7	missense	NM 00119	c.1112C>T	p.Thr371Ile	-0.99	1	89	0	0(COSM1487	C2H2-type zir	rs1978717	0.2777	YES	3.532E-1 (pos.52497250)	acute_myeloid_leukaemia		DNA binding,DNA binding transcription factor activity,int			G=0, A=60	G=0.0, A=1.0	0.00001	903.53	60	G=0/0, A=40/2
chrX:115586325	A/A	G	A	SNV	SLC6A14	SLC6A14:intronic:NM 007231.4	1	100		intronic	NM 00723	c.1504+83	p.?	-0.43					Sodium:neur	rs5905287		YES			Solute carri	amino aci	Digestive System D	D-Serine,L-Proline,Valac	G=0, A=27	G=0.0, A=1.0	0.00001	389.987	27	G=0/0, A=13/1
chr19:52497943	A/A	G	A	SNV	ZNF615	ZNF615:exonic:NM 001199324.1	1	100	7	missense	NM 00119	c.419C>T	p.Thr140Met	0.56	0.13	81	0.005	0(COSM1487	C2H2-type zir	rs10500311	0.2781	YES	3.537E-1 (pos.52497943)	acute_myeloid_leukaemia		DNA binding,DNA binding transcription factor activity,int			G=0, A=45	G=0.0, A=1.0	0.00001	747.393	45	G=0/0, A=22/2
chrX:118974507	C/C	A	C	SNV	UPF3B	UPF3B:intronic:NM 080632.2	1	100		intronic	NM 08063	c.846+102	p.?	0.49					Smg-4/UPF3 f	rs2285552		YES			UPF3 regula	RNA bindir	Abnormalities, Multiple:Bone Diseases:Bone		A=0, C=21	A=0.0, C=1.0	0.00001	280.869	21	A=0/0, C=12/9
chr3:40519429	C/C	T	C	SNV	ZNF619	ZNF619:utr 5:NM 001145082.2	1	100	2	utr 5	NM 00114	c.-42T>C	p.?	-1.01					C2H2-type zir	rs4553952	0.0179	YES	1.0 (pos.40519429)			DNA binding,intracellular,metal ion binding,nucleic acid			T=0, C=27	T=0.0, C=1.0	0.00001	389.465	27	T=0/0, C=9/18
chrX:119065893	A/A	G	A	SNV	NKAP	NKAP:intronic:NM 024528.3	1	100		intronic	NM 02452	c.923+6C>	p.?	2.19				0.08(COSM54	NF-kappa-B-a	rs194302	0.4569	YES	5.958E-1 (pos.11906589	acute_myeloic	NFKB-activa	Notch signaling pathway,RNA binding,T cell differentiatic			G=0, A=65	G=0.0, A=1.0	0.00001	996.464	65	G=0/0, A=23/4
chr8:144732363	C/C	T	C	SNV	ZNF623	ZNF623:exonic:NM 014789.3	1	100	1	synonymous	NM 01478	c.321T>C	p.(=)	-1.59					C2H2-type zir	rs4875017	0		1.0 (pos.144732363)			DNA binding,DNA binding transcription factor activity,me			T=0, C=24	T=0.0, C=1.0	0.00001	334.788	24	T=0/0, C=11/1
chrX:123034227	G/G	A	G	SNV	XIAP	XIAP:intronic:NM 001204401.1	1	100		intronic	NM 00120	c.1100-11	p.?	-0.44					Inhibitor of A	rs5958338		YES			Inhibitor of	Wnt signa	Adnexal Diseases:A	1-3.3-Dimethyl-2-(2-Metl	A=0, G=35	A=0.0, G=1.0	0.00001	543.875	35	A=0/0, G=18/1
chr8:144732418	G/G	A	G	SNV	ZNF623	ZNF623:exonic:NM 014789.3	1	100	1	missense	NM 01478	c.376A>G	p.Asn126Asp	-1.16		23			C2H2-type zir	rs4874084	0		9.994E-1 (pos.144732418)			DNA binding,DNA binding transcription factor activity,me			A=0, G=27	A=0.0, G=1.0	0.00001	389.988	27	A=0/0, G=10/1
chr19:12256358	G/G	T	G	SNV	ZNF625,ZNF625-ZNF20	ZNF625-ZNF20:intronic nc:NR 037802.1, ZNF625:exonic:NM	1	100	4	synonymous	NR 03780	c.873A>C	p.(=)	-2.84				0.06(COSM41	C2H2-type zir	rs12972621	0.466	YES	6.103E-1 (pos.12256358)	neoplasm		DNA binding,intracellular,metal ion binding,nucleic acid			T=0, G=63	T=0.0, G=1.0	0.00001	963.137	63	T=0/0, G=19/4
chr19:20807438	G/G	A	G	SNV	ZNF626	ZNF626:exonic:NM 001076675.2	1	100	4	synonymous	NM 00107	c.1245T>C	p.(=)	-0.26				0.01(COSM13	C2H2-type zir	rs62107362,rs78263	0.1294	YES	0.0 (pos.20807433),0.0 (	adenocarcinoma,neoplasn		DNA binding,intracellular,metal ion binding,nucleic acid			A=0, G=31	A=0.0, G=1.0	0.00001	465.752	31	A=0/0, G=16/1
chr3:42947776	C/C	T	C	SNV	ZNF662	ZNF662:utr 5:NM 001134656.1	1	100	1	utr 5	NM 00113	c.-68T>C	p.?	0.99					C2H2-type zir	rs6789928		YES				DNA binding,intracellular,metal ion binding,nucleic acid			T=0, C=63	T=0.0, C=1.0	0.00001	958.913	63	T=0/0, C=46/1
chrX:125953932	G/G	A	G	SNV	PRR32	PRR32:intronic:NM 001122716.1	1	100		intronic	NM 00112	c.20+86A>	p.?	0.47					Domain of un	rs5931715		YES							A=0, G=32	A=0.0, G=1.0	0.00001	485.14	32	A=0/0, G=22/1
chrX:12693123	G/G	A	G	SNV	FRMPD4	FRMPD4:intronic:NM 014728.3	1	100		intronic	NM 01472	c.468+96A	p.?	0.53					FERM central	rs113303439		YES			FERM and P	cytoskeleton,dendritic spine,phosphatidylinositol-4,5-b			A=0, G=64	A=0.0, G=1.0	0.00001	977.576	64	A=0/0, G=19/4
chrX:12838934	G/G	A	G	SNV	PRPS2	PRPS2:intronic:NM 001039091.2	1	100		intronic	NM 00103	c.873+12A	p.?	0.68					N-terminal dc	rs917580	0.365	YES	4.622E-1;0.0 (pos.12838934)		Phosphorib	5-phosphoribose 1-diphosphate biosynthetic process,A(			A=0, G=59	A=0.0, G=1.0	0.00001	885.363	59	A=0/0, G=23/3
chr19:53669273	C/C	T	C	SNV	ZNF665	ZNF665:exonic:NM 024733.4	1	100	4	missense	NM 02473	c.470A>G	p.His157Arg	-4.81	0.44	29	0	0(COSM3701	C2H2-type zir	rs4801959	0.0335	YES	9.076E-1;0.0;0.0 (pos.53(	carcinoma		DNA binding,metal ion binding,nucleus,regulation of tra			T=0, C=40	T=0.0, C=1.0	0.00001	644.286	40	T=0/0, C=23/1
chrX:131231257	A/A	G	A	SNV	FRMD7	FRMD7:intronic:NM 194277.2	1	100		intronic	NM 19427	c.284+37C	p.?	0.13					FERM C-termi	rs2064650	0.1952	YES	9.508E-2 (pos.131231257)		FERM doma	cytoskelet	Congenital, Hereditary, and Neonatal Diseas		G=0, A=33	G=0.0, A=1.0	0.00001	504.586	33	G=0/0, A=17/1
chrX:131348510	G/G	A	G	SNV	RAP2C	RAP2C:intronic:NM 021183.4	1	100		intronic	NM 02118	c.274-36T	p.?	1.07					ADP-ribosyla	rs41310444	0.0758	YES	0.0 (pos.131348510)			GDP binding,GTP binding,GTPase activity,Rap protein sig			A=0, G=49	A=0.0, G=1.0	0.00001	831.628	49	A=0/0, G=21/2
chr19:56953963	A/A	G	A	SNV	ZNF667	ZNF667:exonic:NM 022103.3	1	100	5	missense	NM 02210	c.401C>T	p.Pro134Leu	-0.55	0.32	98	0.001	0.08(COSM41	C2H2-type zir	rs35914474	0.4666	YES	1.188E-1 (pos.56953963)	NS	Zinc finger p	DNA binding,intracellular,metal ion binding,nucleic acid			G=0, A=33	G=0.0, A=1.0	0.00001	504.59	33	G=0/0, A=15/1
chr19:56954103	A/A	C	A	SNV	ZNF667	ZNF667:exonic:NM 022103.3	1	100	5	synonymous	NM 02210	c.261G>T	p.(=)	-0.15					C2H2-type zir	rs7253217	0.1891	YES	6.7E-2 (pos.56954103)		Zinc finger p	DNA binding,intracellular,metal ion binding,nucleic acid			C=0, A=36	C=0.0, A=1.0	0.00001	563.686	36	C=0/0, A=31/5
chr19:56953863	T/C	T	C	SNV	ZNF667	ZNF667:exonic:NM 022103.3	1	95.45	5	synonymous	NM 02210	c.501A>G	p.(=)	-1.86					C2H2-type zir	rs8107758	0.041	YES	9.999E-1 (pos.56953863)		Zinc finger p	DNA binding,intracellular,metal ion binding,nucleic acid			T=3, C=63	T=0.0455, C=0.9545	0.00001	917.766	66	T=0/3, C=28/3
chr19:23869941	G/G	A	G	SNV	ZNF675	ZNF675:utr 5:NM 138330.2	1	100	1	utr 5	NM 13833	c.-106T>C	p.?	-1.21					C2H2-type zir	rs2364625		YES				DNA binding,I-kappaB kinase/NF-kappaB signaling,bone			A=0, G=39	A=0.0, G=1.0	0.00001	623.894	39	A=0/0, G=28/1
chrX:138714381	CAA/CAA	C	CAA	INDEL	MCF2	MCF2:intronic:NM 001171876.1	2	100		intronic	NM 00117	c.351+113	p.?	-0.66					Divergent CR	rs398122278,rs4007712,rs8669		YES			Oncogene M	G-protein coupled receptor signaling pathway,Rho guan			C=0, CAA=4	C=0.0, CAA=1.0	0.00001	719.059	44	C=0/0, CAA=25
chrX:138733848	G/G	A	G	SNV	MCF2	MCF2:intronic:NM 001171876.1	1	100		intronic	NM 00117	c.25+31T>	p.?	-0.03					Divergent CR	rs6635898	0.1732	YES	2.696E-1 (pos.138733848)		Oncogene M	G-protein coupled receptor signaling pathway,Rho guan			A=0, G=56	A=0.0, G=1.0	0.00001	832.073	56	A=0/0, G=28/2
chr19:23837365	A/C	A	C	SNV	ZNF675	ZNF675:exonic:NM 138330.2	1	98.85	4	missense	NM 13833	c.370T>G	p.Leu124Val	-0.85	1	32	0		C2H2-type zir	rs4380159	2.00E-04		1.0 (pos.23837365)			DNA binding,I-kappaB kinase/NF-kappaB signaling,bone			A=1, C=86	A=0.0115, C=0.9885	0.00001	1398.34	87	A=1/0, C=45/4
chrX:140983031	G/G	A	G	SNV	MAGEC3	MAGEC3:intronic:NM 138702.1	1	100		intronic	NM 13870	c.910-24A	p.?	-1.93						rs176023	0.0155	YES	5.298E-1 (pos.140983031)		Melanoma antigen, family C, 3				A=0, G=53	A=0.0, G=1.0	0.00001	789.051	53	A=0/0, G=29/2
chr19:23926708	G/G	A	G	SNV	ZNF681	ZNF681:exonic:NM 138286.2	1	100	4	synonymous	NM 13828	c.1644T>C	p.(=)	-1.23					C2H2-type zir	rs1852433	1.00E-04		1.0 (pos.23926708)			DNA binding,DNA binding transcription factor activity,me			A=0, G=45	A=0.0, G=1.0	0.00001	747.331	45	A=0/0, G=19/2
chrX:142121729	C/C	T	C	SNV	SPANXN4	SPANXN4:intronic:NM 001009613.2	1	100		intronic	NM 00100	c.79-82T>C	p.?	0.49					Sperm protei	rs4825148		YES			SPANX family, member N4				T=0, C=35	T=0.0, C=1.0	0.00001	543.903	35	T=0/0, C=25/1
chr19:23927252	T/T	C	T	SNV	ZNF681	ZNF681:exonic:NM 138286.2	1	100	4	missense	NM 13828	c.1100G>A	p.Arg367Lys	-1.47	1	26	0.001		C2H2-type zir	rs1818990	1.00E-04		1.0 (pos.23927252)			DNA binding,DNA binding transcription factor activity,me			C=0, T=99	C=0.0, T=1.0	0.00001	1681.13	99	C=0/0, T=43/5
chr19:23927468	G/G	A	G	SNV	ZNF681	ZNF681:exonic:NM 138286.2	1	100	4	missense	NM 13828	c.884T>C	p.Leu295Ser	-0.99	1	145	0		C2H2-type zir	rs1818989	1.00E-04		1.0 (pos.23927468)			DNA binding,DNA binding transcription factor activity,me			A=0, G=49	A=0.0, G=1.0	0.00001	831.563	49	A=0/0, G=25/2
chr8:144378868	G/G	A	G	SNV	ZNF696	ZNF696:exonic:NM 030895.2	1	100	3	synonymous	NM 03089	c.1023A>G	p.(=)	-0.26				0(COSM1755	Zinc finger, C	rs7386259	0.1577	YES	8.206E-1 (pos.14437886	NS,adenocarcinoma,neopl		DNA binding,DNA binding transcription factor activity,me			A=0, G=72	A=0.0, G=1.0	0.00001	1134	72	A=0/0, G=32/4
chrX:1460617	C/C	G	C	SNV	IL3RA	IL3RA:intronic:NM 002183.3	1	100		intronic	NM 00218	c.-37-4G>C	p.?	-0.28					Interleukin-6	rs17886180	0.0043	YES	8.253E-1 (pos.1460617)		Interleukin-	MAPK cascade,Ras guanyl-nucl		Sargramostim	G=0, C=52	G=0.0, C=1.0	0.00001	767.015	52	G=0/0, C=21/3
chrX:1460740	G/G	A	G	SNV	IL3RA	IL3RA:intronic:NM 002183.3	1	100		intronic	NM 00218	c.64+18A>	p.?	0.78					Interleukin-6	rs6647005	0.0679	YES	7.52E-1 (pos.1460740)		Interleukin-	MAPK cascade,Ras guanyl-nucl		Sargramostim	A=0, G=50	A=0.0, G=1.0	0.00001	852.748	50	A=0/0, G=19/3
chrX:1464184	T/T	G	T	SNV	IL3RA	IL3RA:intronic:NM 002183.3	1	100		intronic	NM 00218	c.65-25G>	p.?	-0.34					Interleukin-6	rs17884006	0.4483	YES	4.962E-1;0.0;0.0 (pos.1464184)		Interleukin-	MAPK cascade,Ras guanyl-nucl		Sargramostim	G=0, T=24	G=0.0, T=1.0	0.00001	334.792	24	G=0/0, T=12/1
chrX:1475086	ATCCTAAA/A	ATCCTAAA	A	INDEL	IL3RA	IL3RA:intronic:NM 002183.3	7	57.14		intronic	NM 00218	c.617-28A	p.?	0.34, 0.05, 0.81, 0.04, 0.84, 0.06, -0.25, 0.14					Interleukin-6	rs11280138,rs17881442,rs1833		YES	0.0 (pos.1475089),0.0 (pos.1475090),0.0		Interleukin-	MAPK cascade,Ras guanyl-nucl		Sargramostim	ATCCTAAA=	ATCCTAAA=0.4286, A	0.00001	161.77	28	ATCCTAAA=2/1
chr8:146068594	T/C	T	C	SNV	ZNF7	ZNF7:utr 3:NM 003416.3	1	50	5	utr 3	NM 00341	c.2102T>C	p.?	-1.02					C2H2-type zir	rs1735408	0.1892	YES	9.999E-1 (pos.146068594)		Zinc finger p	DNA binding,DNA binding transcription factor activity,int			T=11, C=11	T=0.5, C=0.5	0.00001	94.6063	22	T=0/11, C=4/7
chr8:144776109	G/G	A	G	SNV	ZNF707	ZNF707:exonic:NM 001100599.1	1	100	5	synonymous	NM 00110	c.525A>G	p.(=)	-2.92				0(COSM1455	KRAB box,Zin	rs11786757	1.00E-04		1.0 (pos.144776109)	NS,adenocarcinoma		DNA binding,intracellular,metal ion binding,nucleic acid			A=0, G=31	A=0.0, G=1.0	0.00001	465.818	31	A=0/0, G=24/7
chrX:149826503	A/A	G	A	SNV	MTM1	MTM1:intronic:NM 000252.2	1	100		intronic	NM 00025	c.1260+3G	p.?	2.44					GRAM domai	rs222410	0.4987	YES	3.229E-1 (pos.149826503)		Myotubular	I band,cyt	Muscular Diseases;Musculosk	Benign/Likely	G=0, A=38	G=0.0, A=1.0	0.00001	599.942	38	G=0/0, A=15/2
chr19:21477431	C/C	T	C	SNV	ZNF708	ZNF708:exonic:NM 021269.2	1	100	4	missense	NM 02126	c.337A>G	p.Lys113Glu	-0.33	0.17	56	0.004	0(COSM4000	C2H2-type zir	rs1781873	0.4784	YES	6.229E-1 (pos.21477431)	acute_myeloid_leukaemia					T=0, C=78	T=0.0, C=1.0	0.00001	1218.56	78	T=0/0, C=55/2
chrX:150789950	T/T	C	T	SNV	PASD1	PASD1:intronic:NM 173493.2	1	100		intronic	NM 17349	c.308-4C>	p.?	0.99					PAS domain	rs945815	0.0763	YES	9.723E-1 (pos.150789950)		PAS domain	Cry-Per complex,negative regulation of circadian rhythm			C=0, T=34	C=0.0, T=1.0	0.00001	524.183	34	C=0/0, T=16/1
chr3:75790444	A/A	G	A	SNV	ZNF717	ZNF717:exonic:NM 001128223.2	1	100	4	missense	NM 00112	c.260C>T	p.Pro87Leu	2.01	0.69	98	0.604	0.01(COSM41	C2H2-type zir	rs1971517		YES		adenocarcinoma,neoplasm					G=0, A=35	G=0.0, A=1.0	0.00001	543.893	35	G=0/0, A=28/7
chr3:75790513	C/C	T	C	SNV	ZNF717	ZNF717:exonic:NM 001128223.2	1	100	4	missense	NM 00112	c.191A>G	p.Tyr64Cys	-3.65	1	194	0	0(COSM4594	C2H2-type zir	rs2918519			0.0 (pos.75790513)	squamous_cell_carcinoma					T=0, C=32	T=0.0, C=1.0	0.00001	485.14	32	T=0/0, C=25/7
chr3:75790822	G/G	A	G	SNV	ZNF717	ZNF717:exonic:NM 001128223.2	1	100	3	synonymous	NM 00112	c.123T>C	p.(=)	-2.71				0(COSM4158	C2H2-type zir	rs2918520		YES		adenocarcinoma,neoplasm					A=0, G=137	A=0.0, G=1.0	0.00001	2277.17	137	A=0/0, G=55/8
chrX:152083089	G/G	T	G	SNV	ZNF185	ZNF185:intronic:NM 001178106.1	1	100		intronic	NM 00117	c.34+22T>	p.?	-0.22						rs6526143	0.2904	YES	1.426E-1 (pos.152083089)		Zinc finger p	actin cytoskeleton,actin filament binding,cytoplasm,foc			T=0, G=46	T=0.0, G=1.0	0.00001	768.313	46	T=0/0, G=24/2
chrX:152720979	G/A	G	A	SNV	HAUS7	HAUS7:intronic:NM 017518.7	1	95.45		intronic	NM 01751	c.960+21C	p.?	-0.15						rs6571304	0.0064	YES	0.0 (pos.152720979)		HAUS augm	G2/M transition of mitotic cell cycle,HAUS complex,cell c			G=1, A=21	G=0.0455, A=0.9545	0.00001	271.541	22	G=1/0, A=10/1
chrX:152728003	C/C	T	C	SNV	HAUS7	HAUS7:intronic:NM 017518.7	1	100		intronic	NM 01751	c.384+65A	p.?	-2.92						rs3020967	0.3117	YES			HAUS augm	G2/M transition of mitotic cell cycle,HAUS complex,cell c			T=0, C=23	T=0.0, C=1.0	0.00001	316.74	23	T=0/0, C=11/1
chrX:152807665	G/G	A	G	SNV	ATP2B3	ATP2B3:intronic:NM 001001344.2	1	100		intronic	NM 00100	c.665-116	p.?	-0.29					Cation transr	rs2980012		YES			ATPase, Ca	ATP bindin	Adenoma:Adrenal Gland Diseases:Adrenocor		A=0, G=20	A=0.0, G=1.0	0.00001	263.782	20	A=0/0, G=11/9
chrX:153073752	C/C	T	C	SNV	PDZD4	PDZD4:intronic:NM 032512.3	1	100		intronic	NM 03251	c.296+63A	p.?	0.03					PDZ domain,I	rs3761532		YES	8.293E-1 (pos.153073752)		PDZ domair	NAD binding,cell cortex,isocitrate dehydrogenase (NAD+			C=0, T=34	C=0.0, T=1.0	0.00001	523.932	34	T=0/0, C=21/1
chrX:153174819	TG/TG	T	TG	INDEL	ARHGAP4	ARHGAP4:intronic:NM 001666.4	1	100		intronic	NM 00166	c.2507+78	p.?	-1.45					Fes/CIP4, anc	rs11394527,rs375243502,rs869		YES				GTPase ac	HIV Infections;Immune System Diseases;Imm		T=0, TG=23	T=0.0, TG=1.0	0.00001	269.056	23	T=0/0, TG=8/1
chrX:153174867	TG/TG	T	TG	INDEL	ARHGAP4	ARHGAP4:intronic:NM 001666.4	1	100		intronic	NM 00166	c.2507+30	p.?	0.07					Fes/CIP4, anc	rs11405331,rs340460	2.00E-04		1.0;0.0 (pos.153174867)			GTPase ac	HIV Infections;Immune System Diseases;Imm		T=0, TG=24	T=0.0, TG=1.0	0.00001	298.078	24	T=0/0, TG=8/1
chr19:24116402	C/T	C	T	SNV	ZNF726	ZNF726:exonic:NM 001244038.1	1	57.14	4	missense	NM 00124	c.1484C>T	p.Thr495Ile	-0.89	0.51	89	0.003	0.04(COSM45	C2H2-type zir	rs145688653		YES	0.0 (pos.24116402)	squamous_cell_carcinoma		DNA binding,DNA binding transcription factor activity,int			C=15, T=20	C=0.4286, T=0.5714	0.00001	216.887	35	C=6/9, T=13/7
chr19:22498943	C/C	T	C	SNV	ZNF729	ZNF729:exonic:NM 001242680.1	1	100	4	synonymous	NM 00124	c.2724T>C	p.(=)	-2.27					C2H2-type zir	rs8104705		YES	1.232E-4;0.0;0.0 (pos.22498942),6.839E-1 (pos.2249			DNA binding,metal ion binding,nucleus,regulation of tra			T=0, C=43	T=0.0, C=1.0	0.00001	710.205	43	T=0/0, C=34/9
chrX:153696913	T/T	C	T	SNV	PLXNA3	PLXNA3:intronic:NM 017514.4	1	100		intronic	NM 01751	c.4220+11	p.?	-0.68					IPT/TIG doma	rs7886727	0.1313	YES	1.373E-1 (pos.153696913)			branchion	Endometrial Neoplasms;Female Urogenital D		C=0, T=36	C=0.0, T=1.0	0.00001	563.727	36	C=0/0, T=20/1
chrX:154774663	A/A	G	A	SNV	TMLHE	TMLHE:intronic:NM 018196.3	1	100		intronic	NM 01819	c.181+94C	p.?	-0.25					Protein of unl	rs2305517		YES			Epsilon-trin	carnitine biosynthetic process		Succinic acid,Vitamin C	G=0, A=43	G=0.0, A=1.0	0.00001	705.701	43	G=0/0, A=20/2
chrX:154774707	T/T	C	T	SNV	TMLHE	TMLHE:intronic:NM 018196.3	1	100		intronic	NM 01819	c.181+50G	p.?	-0.36					Protein of unl	rs2305518	0.235	YES	7.486E-1 (pos.154774707)		Epsilon-trin	carnitine biosynthetic process		Succinic acid,Vitamin C	C=0, T=50	C=0.0, T=1.0	0.00001	852.68	50	C=0/0, T=19/3
chr19:20748522	A/A	C	A	SNV	ZNF737	ZNF737:utr 5:NM 001159293.1	1	100	1	utr 5	NM 00115	c.-76G>T	p.?	0.19					C2H2-type zir	rs12979592	0.4403	YES			Zinc finger p	DNA binding,intracellular,metal ion binding,nucleic acid			C=0, A=41	C=0.0, A=1.0	0.00001	673.695	41	C=0/0, A=12/2
chrX:1554655	C/C	T	C	SNV	ASMTL	ASMTL:intronic:NM 004192.3	1	100		intronic	NM 00419	c.274-4A>	p.?	0.06					Dimerisation	rs28592995	0.3239	YES	3.879E-1 (pos.1554655)	acute_myeloid_leukaemia		O-methylt	Leukemia;Leukemia, Myeloid;Leukemia, Mye		T=0, C=50	T=0.0, C=1.0	0.00001	852.731	50	T=0/0, C=26/2
chrX:15800600	G/G	A	G	SNV	CA5B	CA5B:intronic:NM 007220.3	1	100		intronic	NM 00722	c.775-8A>	p.?	0.2					Eukaryotic-ty	rs4830548,rs755516	0.3384	YES	4.307E-1 (pos.15800600),5.62E-4 (pos.15		Carbonic ar	bicarbonate transport,carbon		Ellagic Acid,Zonisamide	A=0, G=38	A=0.0, G=1.0	0.00001	603.78	38	A=0/0, G=22/1
chr22:20759755	CG/C	CG	C	INDEL	ZNF74	ZNF74:exonic:NM 001256524.1	1	50	6	frameshiftDeletion	NM 00125	c.436delG	p.Ala146fs	-1.05, -1.77					C2H2-type zir	rs573923137,rs772198434			0.0 (pos.20759755),0.0 (pos.20759756)		Zinc finger p	DNA binding,DNA binding transcription factor activity,RN			CG=20, C=2	CG=0.5, C=0.5	0.00001	85.2418	40	CG=6/14, C=8/
chrX:16876980	A/A	G	A	SNV	RBBP7	RBBP7:intronic:NM 001198719.1	1	100		intronic	NM 00119	c.440-8C>	p.?	0.71					Histone-bind	rs4828536	0.4092	YES	8.701E-1 (pos.16876980)		Retinoblast	CENP-A containing nucleosome assembly,DNA replicatio			G=0, A=35	G=0.0, A=1.0	0.00001	543.903	35	G=0/0, A=15/2
chrX:1713168	C/T	C	T	SNV	AKAP17A	AKAP17A:intronic:NM 005088.2	1	57.14		intronic	NM 00508	c.762+51C	p.?	0.76						rs5989832	0.4688	YES	5.466E-1 (pos.1713168)		A kinase (PF	B cell activation,RNA binding,RNA splicing,cytosol,mRNA			C=18, T=24	C=0.4286, T=0.5714	0.00001	279.06	42	C=15/3, T=10/
chr19:57956828	G/G	T	G	SNV	ZNF749	ZNF749:exonic:NM 001023561.3	1	100	3	missense	NM 00102	c.2312T>G	p.Ile771Arg	-6.58	1	97	0		C2H2-type zir	rs7246856	0.3986	YES	7.577E-1 (pos.57956828)			DNA binding,metal ion binding,nucleus,regulation of tra			T=0, G=36	T=0.0, G=1.0	0.00001	563.743	36	T=0/0, G=15/2
chr6:35260530	T/T	C	T	SNV	ZNF76	ZNF76:exonic:NM 003427.4	1	100	10	synonymous	NM 00342	c.1131C>T	p.(=)	-0.32				0.28(COSM41	C2H2-type zir	rs1557568	0.1916	YES	6.635E-1 (pos.35260530)	carcinoma,ne	Zinc finger p	metal ion binding,nucleic acid binding,nucleus,positive			C=0, T=47	C=0.0, T=1.0	0.00001	789.327	47	C=0/0, T=6/41
chr19:53958126	G/G	T	G	SNV	ZNF761,ZNF765-ZNF761	ZNF765-ZNF761:exonic:NM 001350496.1, ZNF761:exonic:N	1	100	7	missense	NM 00135	c.365T>G,	p.Ile122Ser, p.Ile122Ser	-0.32	1.0, 1.0	142.0, 142	0.0, 0.0	0(COSM1564	C2H2-type zir	rs2708743	0.3181	YES	5.482E-1 (pos.53958126)	acute_myeloid_leukaemia		DNA binding,DNA binding transcription factor activity,int			T=0, G=84	T=0.0, G=1.0	0.00001	1370.02	84	T=0/0, G=32/5
chr19:2934282	C/C	T	C	SNV	ZNF77	ZNF77:exonic:NM 021217.2	1	100	4	synonymous	NM 02121	c.843A>G	p.(=)	-4.55					C2H2-type zir	rs3746073	0.0481	YES	5.384E-1;0.0 (pos.2934282)		Zinc finger p	DNA binding,biological_process,metal ion binding,molec			T=0, C=42	T=0.0, C=1.0	0.00001	685.219	42	T=0/0, C=22/2
chrX:21985464	T/T	C	T	SNV	SMS	SMS:intronic:NM 004595.4	1	100		intronic	NM 00459	c.170+30C	p.?	0.22					Spermidine s	rs3747276	0.3405	YES	3.25E-1 (pos.21985464)		Retinoic aci	cytosol,ex	Arthritis;Arthritis, F	Spermine	C=0, T=44	C=0.0, T=1.0	0.00001	726.564	44	C=0/0, T=23/2
chr7:150093701	A/C	A	C	SNV	ZNF775	ZNF775:exonic:NM 173680.3	1	46.51	3	synonymous	NM 17368	c.132A>C	p.(=)	-1.64				0.01(COSM32	C2H2-type zir	rs940453	0.0793	YES	7.965E-1 (pos.15009370:	hepatocellular_carcinoma		DNA binding,metal ion binding,nucleic acid binding,nucl			A=46, C=40	A=0.5349, C=0.4651	0.00001	418.34	86	A=24/22, C=26
chr16:89293271	C/C	A	C	SNV	ZNF778	ZNF778:exonic:NM 001201407.1	1	100	7	missense	NM 00120	c.575A>C	p.Lys192Thr	-0.09	0.39	78	0.51	0.17(COSM39	C2H2-type zir	rs28417933	0.1154	YES	5.572E-1;0.0 (pos.89293	acute_myeloid_leukaemia					A=0, C=74	A=0.0, C=1.0	0.00001	1170.13	74	A=0/0, C=47/2
chrX:23723261	C/C	T	C	SNV	ACOT9	ACOT9:intronic:NM 001037171.1	1	100		intronic	NM 00103	c.1020-64	p.?	0.29						rs2270142		YES			Acyl-CoA thi	acetyl-CoA hydrolase activity,acyl-CoA hydrolase activity			T=0, C=48	T=0.0, C=1.0	0.00001	810.435	48	T=0/0, C=20/2
chr16:89293614	G/G	A	G	SNV	ZNF778	ZNF778:exonic:NM 001201407.1	1	100	7	synonymous	NM 00120	c.918A>G	p.(=)	0.06				0(COSM6246	C2H2-type zir	rs4785626	0.0757	YES	8.848E-1 (pos.89293614)	neoplasm					A=0, G=26	A=0.0, G=1.0	0.00001	371.42	26	A=0/0, G=14/1
chrX:23897003	T/T	C	T	SNV	APOO	APOO:intronic:NM 024122.4	1	100		intronic	NM 02412	c.237+29G	p.?	-0.78					Apolipoprote	rs5970803	0.1766	YES	1.042E-1;0.0 (pos.23897003)		Apolipoprot	Golgi membrane,MICOS complex,cristae formation,cytos			C=0, T=71	C=0.0, T=1.0	0.00001	1111.48	71	C=0/0, T=61/1
chrX:2726396	G/G	A	G	SNV	XG	XG:intronic:NM 001141919.1	1	100		intronic	NM 00114	c.454+135	p.?	-1.26					CD99 antiger	rs6567645		YES			XG glycopro	biological_process,integral component of membrane,int			A=0, G=21	A=0.0, G=1.0	0.00001	281.23	21	A=0/0, G=6/15
chr16:89294184	A/A	G	A	SNV	ZNF778	ZNF778:exonic:NM 001201407.1	1	100	7	synonymous	NM 00120	c.1488G>A	p.(=)	-0.02					C2H2-type zir	rs4785627	0.0768	YES	8.848E-1 (pos.89294184)						G=0, A=36	G=0.0, A=1.0	0.00001	563.744	36	G=0/0, A=14/2
chr16:89294381	C/C	T	C	SNV	ZNF778	ZNF778:exonic:NM 001201407.1	1	100	7	missense	NM 00120	c.1685T>C	p.Ile562Thr	-1.66	0.03	89	0.481		C2H2-type zir	rs28415940	0.1156	YES	7.06E-1 (pos.89294381)						T=0, C=83	T=0.0, C=1.0	0.00001	1354.11	83	T=0/0, C=49/3
chr16:89294439	T/T	G	T	SNV	ZNF778	ZNF778:exonic:NM 001201407.1	1	100	7	missense	NM 00120	c.1743G>T	p.Gln581His	-1.06	0	24	0.016	0.01(COSM13	C2H2-type zir	rs9921361	0.1159	YES	7.057E-1 (pos.89294439)	adenocarcinoma					G=0, T=86	G=0.0, T=1.0	0.00001	1410.92	86	G=0/0, T=50/3
chr16:89294511	T/T	C	T	SNV	ZNF778	ZNF778:exonic:NM 001201407.1	1	100	7	synonymous	NM 00120	c.1815C>T	p.(=)	-5.43					C2H2-type zir	rs28638280	0.0766	YES	8.849E-1;0.0 (pos.89294511)						C=0, T=80	C=0.0, T=1.0	0.00001	1289.35	80	C=0/0, T=43/3
chr19:37310344	C/C	T	C	SNV	ZNF790,ZNF790-AS1	ZNF790:exonic:NM 001242800.1, ZNF790-AS1:intronic nc:N	1	100	5	missense	NR 04002	c.902A>G	p.Gln301Arg	0.64	0.07	43	0.081	0.01(COSM37	C2H2-type zir	rs3745775	0.2717	YES	2.276E-1 (pos.37310344)	acute_myeloid_leukaemia		DNA binding,DNA binding transcription factor activity,int			T=0, C=48	T=0.0, C=1.0	0.00001	810.428	48	T=0/0, C=20/2
chr3:113955164	T/G	T	G	SNV	ZNF80	ZNF80:exonic:NM 007136.3	1	58.33	1	missense	NM 00713	c.758A>C	p.Asp253Ala	-0.96		126			C2H2-type zir	rs3732782	0.3557	YES	5.37E-1 (pos.113955164)		Zinc finger p	DNA binding,metal ion binding,nucleus,regulation of tra			T=10, G=14	T=0.4167, G=0.5833	0.00001	135.339	24	T=2/8, G=4/10
chr3:113955320	C/T	C	T	SNV	ZNF80	ZNF80:exonic:NM 007136.3	1	50	1	missense	NM 00713	c.602G>A	p.Arg201His	-2.14		29			C2H2-type zir	rs6438191,rs754892	0.3555	YES	0.0;1.156E-4 (pos.113955306),5.367E-1 (		Zinc finger p	DNA binding,metal ion binding,nucleus,regulation of tra			C=12, T=12	C=0.5, T=0.5	0.00001	106.709	24	C=10/2, T=8/4
chr2:185802363	G/G	A	G	SNV	ZNF804A	ZNF804A:exonic:NM 194250.1	1	100	4	missense	NM 19425	c.2240A>G	p.His747Arg	0.08	0.29	29	0		Zinc-finger dc	rs12477430	0.291	YES	8.709E-1 (pos.185802363)		Zinc finger p	axon,cyto	Adenocarcinoma:Carcinoma:Carcinoma, Ren		A=0, G=62	A=0.0, G=1.0	0.00001	940.319	62	A=0/0, G=45/1
chr7:88964000	C/T	C	T	SNV	ZNF804B	ZNF804B:exonic:NM 181646.3	1	46.88	4	synonymous	NM 18164	c.1704C>T	p.(=)	-1.04				0.01(COSM37	Zinc-finger dc	rs801839	0.2341	YES	6.855E-1 (pos.88964000)	adenocarcinoma		metal ion binding,nucleic acid binding			C=34, T=30	C=0.5313, T=0.4688	0.00001	288.11	64	C=20/14, T=12
chr19:57760018	A/A	G	A	SNV	ZNF805	ZNF805:exonic:NM 001023563.3	1	100	3	missense	NM 00102	c.203G>A	p.Gly68Glu	0.26	0.16	98	0.682	0.04(COSM50	C2H2-type zir	rs2014572	0.4821	YES	2.354E-1 (pos.57760018)	adenocarcinoma,ductal_ca		DNA binding,metal ion binding,nucleus,regulation of tra			G=0, A=63	G=0.0, A=1.0	0.00001	958.917	63	G=0/0, A=36/2
chrX:308247	G/C	G	C	SNV	PPP2R3B	PPP2R3B:intronic:NM 013239.4	1	58.33		intronic	NM 01323	c.614+78C	p.?	-0.69					EF hand,EF-ha	rs1008697603					Protein pho	calcium ion binding,cell cycle arrest,nucleoplasm,nucle			G=10, C=14	G=0.4167, C=0.5833	0.00001	114.494	24	G=6/4, C=8/6
chr19:57764770	A/A	G	A	SNV	ZNF805	ZNF805:exonic:NM 001023563.3	1	100	4	missense	NM 00102	c.583G>A	p.Val195Ile	-2.46	0.66	29	0.026	0.02(COSM37	C2H2-type zir	rs10421632	0.4824	YES	2.14E-1 (pos.57764770)	adenocarcinoma,diffuse_la		DNA binding,metal ion binding,nucleus,regulation of tra			G=0, A=28	G=0.0, A=1.0	0.00001	408.718	28	G=0/0, A=10/1
chr19:57765432	G/G	A	G	SNV	ZNF805	ZNF805:exonic:NM 001023563.3	1	100	4	synonymous	NM 00102	c.1245A>G	p.(=)	-0.99				0.03(COSM37	C2H2-type zir	rs8100154		YES	1.54E-1 (pos.57765432)	acute_myeloid_leukaemia		DNA binding,metal ion binding,nucleus,regulation of tra			A=0, G=22	A=0.0, G=1.0	0.00001	298.892	22	A=0/0, G=9/13
chr19:57176198	G/G	T	G	SNV	ZNF835	ZNF835:exonic:NM 001005850.2	1	100	2	synonymous	NM 00100	c.369A>C	p.(=)	-4.54				0(COSM3757	Zinc finger, C	rs8108756	0.3034	YES	4.851E-1 (pos.57176198)	acute_myeloid_leukaemia		DNA binding,DNA binding transcription factor activity,me			T=0, G=28	T=0.0, G=1.0	0.00001	408.718	28	T=0/0, G=12/1
chr19:57176304	G/G	T	G	SNV	ZNF835	ZNF835:exonic:NM 001005850.2	1	100	2	missense	NM 00100	c.263A>C	p.Glu88Ala	-0.15		107		0.01(COSM37	Zinc finger, C	rs12460400,rs781000	0.2983	YES	0.0 (pos.57176303),4.83	acute_myeloid_leukaemia		DNA binding,DNA binding transcription factor activity,me			T=0, G=26	T=0.0, G=1.0	0.00001	371.353	26	T=0/0, G=8/18
chrX:3533962	C/C	T	C	SNV	PRKX	PRKX:intronic:NM 005044.4	1	100		intronic	NM 00504	c.874-29A	p.?	-0.21					Kinase-like,P	rs2302750	0.3958	YES	3.125E-1 (pos.3533962)		Protein kina	ATP binding,angiogenesis,cAMP-dependent protein kina			T=0, C=66	T=0.0, C=1.0	0.00001	1015.37	66	T=0/0, C=22/4
chr19:57176482	T/T	C	T	SNV	ZNF835	ZNF835:exonic:NM 001005850.2	1	100	2	missense	NM 00100	c.85G>A	p.Glu29Lys	0.14		56		0.04(COSM37	Zinc finger, C	rs12462469	0.2998	YES	4.861E-1 (pos.57176482)	acute_myeloid_leukaemia		DNA binding,DNA binding transcription factor activity,me			C=0, T=25	C=0.0, T=1.0	0.00001	351.885	25	C=0/0, T=7/18
chr19:58879965	G/G	A	G	SNV	ZNF837	ZNF837:exonic:NM 138466.1	1	100	3	synonymous	NM 13846	c.735T>C	p.(=)	-0.09				0(COSM3749	C2H2-type zir	rs7255489		YES	9.242E-1 (pos.58879965)	adenocarcinoma		DNA binding,metal ion binding,nucleus,regulation of tra			A=0, G=22	A=0.0, G=1.0	0.00001	298.762	22	A=0/0, G=14/8
chr19:58879976	T/T	C	T	SNV	ZNF837	ZNF837:exonic:NM 138466.1	1	100	3	missense	NM 13846	c.724G>A	p.Ala242Thr	-2.12		58		0(COSM3749	C2H2-type zir	rs7255596	0.0485	YES	9.221E-1 (pos.58879976)	adenocarcinoma		DNA binding,metal ion binding,nucleus,regulation of tra			C=0, T=21	C=0.0, T=1.0	0.00001	281.225	21	C=0/0, T=13/8
chr12:133634450	C/C	A	C	SNV	ZNF84	ZNF84:exonic:NM 001127372.2	1	100	5	synonymous	NM 00112	c.1149A>C	p.(=)	-1.79					C2H2-type zir	rs623100		YES				DNA binding,face morphogenesis,hemopoiesis,intracell			A=0, C=118	A=0.0, C=1.0	0.00001	1893.97	118	A=0/0, C=85/3
chrX:38280206	T/T	C	T	SNV	OTC	OTC:intronic:NM 000531.5	1	100		intronic	NM 00053	c.1006-70	p.?	1.05					Aspartate/or	rs1041718888,rs125	0.2196	YES			Ornithine tr	amino aci	ACMG Recommend:	L-Citrulline,N-(Phosphon	C=0, T=25	C=0.0, T=1.0	0.00001	353.015	25	C=0/0, T=21/4
chrX:44833841	A/A	C	A	SNV	KDM6A	KDM6A:intronic:NM 021140.3	1	100		intronic	NM 02114	c.335-70C	p.?	0.79					JmjC domain	rs6611055	0.4768	YES			Lysine (K)-sp	MLL3/4 co	Abnormalities, Multiple:Adenocarcinoma:Ca		C=0, A=44	C=0.0, A=1.0	0.00001	726.563	44	C=0/0, A=18/2
chrX:46949397	A/AT	A	AT	INDEL	RGN	RGN:intronic:NM 004683.5	1	100		intronic	NM 00468	c.562+7A>	p.?	1.78					SMP-30/Gluc	rs781897443			0.0 (pos.46949397)		Regucalcin	L-ascorbic	Digestive System D	Calcium Citrate,Calcium	A=0, AT=38	A=0.0, AT=1.0	0.00001	388.897	38	A=0/0, AT=31/
chrX:46949403	T/T	C	T	SNV	RGN	RGN:intronic:NM 004683.5	1	100		intronic	NM 00468	c.562+13C	p.?	-0.55					SMP-30/Gluc	rs6611330	0.3937	YES	7.424E-1;0.0 (pos.46949403)		Regucalcin	L-ascorbic	Digestive System D	Calcium Citrate,Calcium	C=0, T=42	C=0.0, T=1.0	0.00001	684.511	42	C=0/0, T=31/1
chrX:46952219	G/G	A	G	SNV	RGN	RGN:intronic:NM 004683.5	1	100		intronic	NM 00468	c.850-77A	p.?	0.39					SMP-30/Gluc	rs4480242		YES			Regucalcin	L-ascorbic	Digestive System D	Calcium Citrate,Calcium	A=0, G=44	A=0.0, G=1.0	0.00001	726.566	44	A=0/0, G=26/1
chr12:133617991	G/A	G	A	SNV	ZNF84	ZNF84:utr 5:NM 001127372.2	1	57.14	2	utr 5	NM 00112	c.-63G>A	p.?	-0.29					C2H2-type zir	rs7961667		YES				DNA binding,face morphogenesis,hemopoiesis,intracell			G=12, A=16	G=0.4286, A=0.5714	0.00001	159.244	28	G=9/3, A=6/10
chr19:37241141	G/G	A	G	SNV	ZNF850	ZNF850:exonic:NM 001193552.1	1	100	5	synonymous	NM 00119	c.801T>C	p.(=)	-2.73				0(COSM6353	C2H2-type zir	rs1673082		YES	5.397E-1 (pos.37241141)	carcinoma		DNA binding,intracellular,metal ion binding,nucleic acid			A=0, G=46	A=0.0, G=1.0	0.00001	767.957	46	A=0/0, G=29/1
chrX:48456962	T/T	C	T	SNV	WDR13	WDR13:intronic:NM 017883.5	1	100		intronic	NM 01788	c.42-143C	p.?	0.32					WD domain,	rs73487689		YES			WD repeat-	microtubule organizing center,negative regulation of typ			C=0, T=29	C=0.0, T=1.0	0.00001	427.603	29	C=0/0, T=17/1
chr19:20230076	C/A	C	A	SNV	ZNF90	ZNF90:exonic:NM 007138.1	1	53.13	4	synonymous	NM 00713	c.1713C>A	p.(=)	-1.32					C2H2-type zir	rs534439133			0.0 (pos.20230076)		Zinc finger p	DNA binding,DNA binding transcription factor activity,int			C=15, A=17	C=0.4688, A=0.5313	0.00001	173.579	32	C=8/7, A=13/4
chr19:23545004	G/A	G	A	SNV	ZNF91	ZNF91:exonic:NM 003430.3	1	50	4	synonymous	NM 00343	c.777C>T	p.(=)	-1.54				0.01(COSM37	C2H2-type zir	rs296092	0.2665	YES	3.83E-1;1.157E-4 (pos.23	adenocarcinoi	Zinc finger p	DNA binding,DNA binding transcription factor activity,int			G=23, A=23	G=0.5, A=0.5	0.00001	248.79	46	G=13/10, A=1
chrX:49069366	T/T	C	T	SNV	CACNA1F	CACNA1F:intronic:NM 001256789.2	1	100		intronic	NM 00125	c.3813+68	p.?	-0.38					Ion transport	rs2071316	0.2865	YES			Calcium cha	T cell hom	Albinism;Albinism,	Calcium Citrate,Calcium	C=0, T=37	C=0.0, T=1.0	0.00001	583.706	37	C=0/0, T=10/2
chr19:23545447	A/G	A	G	SNV	ZNF91	ZNF91:exonic:NM 003430.3	1	50	4	missense	NM 00343	c.334T>C	p.Tyr112His	-0.64	0	83	0.459	0.02(COSM40	C2H2-type zir	rs296091	0.0528	YES	3.742E-1 (pos.23545447)	acute_myeloic	Zinc finger p	DNA binding,DNA binding transcription factor activity,int			A=124, G=1	A=0.5, G=0.5	0.00001	1379.27	248	A=79/45, G=8
chrX:49459225	A/A	G	A	SNV	PAGE1	PAGE1:intronic:NM 003785.3	1	100		intronic	NM 00378	c.63+86C>	p.?	0.25					GAGE protein	rs553110220					P antigen fa	cellular de	Nervous System Diseases:Neuromuscular Di		G=0, A=172	G=0.0, A=1.0	0.00001	2819.92	172	G=0/0, A=79/9
chr20:47865372	A/A	G	A	SNV	ZNFX1	ZNFX1:exonic:NM 021035.2	1	100	14	synonymous	NM 02103	c.4189C>T	p.(=)	-1.82				0.17(COSM44	AAA domain	rs238208	0.1643	YES	6.309E-1 (pos.47865372)	acute_myeloid_leukaemia		DNA binding transcription factor activity,RNA binding,nu			G=0, A=56	G=0.0, A=1.0	0.00001	832.1	56	G=0/0, A=26/3
chr20:47886684	A/A	G	A	SNV	ZNFX1	ZNFX1:exonic:NM 021035.2	1	100	3	synonymous	NM 02103	c.1665C>T	p.(=)	-0.34				0.2(COSM441	AAA domain	rs57950	0.1642	YES	6.308E-1 (pos.47886684)	acute_myeloid_leukaemia		DNA binding transcription factor activity,RNA binding,nu			G=0, A=40	G=0.0, A=1.0	0.00001	644.285	40	G=0/0, A=25/1
chrX:53622078	A/A	T	A	SNV	HUWE1	HUWE1:intronic:NM 031407.6	1	100		intronic	NM 03140	c.3380+69	p.?	-0.01					Domain of Ur	rs1244413		YES			HECT, UBA, a	DNA bindi	Central Nervous System Diseases:Congenital		T=0, A=28	T=0.0, A=1.0	0.00001	408.677	28	T=0/0, A=18/1
chr17:34842521	G/G	A	G	SNV	ZNHIT3	ZNHIT3:utr 5:NM 004773.3	1	100	1	utr 5	NM 00477	c.-23A>G	p.?	-0.39					HIT zinc finge	rs4796224	0.4634	YES	4.842E-1 (pos.34842521)		Zinc finger H	cytoplasm	Autoimmune Diseases:Female Urogenital Di		A=0, G=105	A=0.0, G=1.0	0.00001	1613.46	105	A=0/0, G=34/7

chrX:55246965	T/T	C	T	SNV	PAGE5	PAGE5:intronic:NM 001013435.2	1	100		intronic	NM 00101	c.-8+80C>	p.?	-2.76					GAGE protein	rs5914272	0.0264	YES				protein binding			C=0, T=21	C=0.0, T=1.0	0.00001	281.205	21	C=0/0, T=9/12
chrX:55247030	C/G	C	G	SNV	PAGE5	PAGE5:intronic:NM 001013435.2	1	95.45		intronic	NM 00101	c.-8+145C	p.?	-0.47					GAGE protein	rs5914273	0.032	YES	9.998E-1;0.0 (pos.55247030)			protein binding			C=1, G=21	C=0.0455, G=0.9545	0.00001	271.337	22	C=1/0, G=5/16
chrX:55757744	G/G	T	G	SNV	RRAGB	RRAGB:intronic:NM 016656.3	1	100		intronic	NM 01665	c.378-53T	p.?	-0.46					ADP-ribosyla	rs5960480		YES			Ras-related	EGO complex,GTP binding,GTPase activity,GTPase bindin			T=0, G=26	T=0.0, G=1.0	0.00001	371.42	26	T=0/0, G=16/1
chrX:57474943	T/T	A	T	SNV	FAAH2	FAAH2:intronic:NM 174912.3	1	100		intronic	NM 17491	c.1229-14	p.?	0.04					Amidase	rs1821118	9.00E-04	YES	1.0 (pos.57474943)		Fatty acid a	anandami	Adenocarcinoma:Carcinoma:C	Benign	A=0, T=31	A=0.0, T=1.0	0.00001	465.819	31	A=0/0, T=17/1
chrX:63569979	T/T	A	T	SNV	MTMR8	MTMR8:intronic:NM 017677.3	1	100		intronic	NM 01767	c.469-29T	p.?	-1.22					Myotubulari	rs5964769,rs752391	0.0025,7.	YES	0.0 (pos.63569971),0.0 (pos.63569975),		Myotubular	cytosol,nuclear envelope,peptidyl-tyrosine dephosphor			A=0, T=21	A=0.0, T=1.0	0.00001	281.235	21	A=0/0, T=8/13
chrX:64959577	G/G	A	G	SNV	MSN	MSN:intronic:NM 002444.2	1	100		intronic	NM 00244	c.1570-14	p.?	-0.49					Ezrin/radixin,	rs5965007	0.0049	YES	0.0 (pos.64959577)		Moesin	T cell aggr	Arthritis;Arthritis, Juvenile;Autoimmune Dise		A=0, G=35	A=0.0, G=1.0	0.00001	543.865	35	A=0/0, G=17/1
chr11:60638470	G/G	A	G	SNV	ZP1	ZP1:exonic:NM 207341.3	1	100	5	synonymous	NM 20734	c.867A>G	p.(=)	-0.51				0.03(COSM45	Zona pelluci	rs566411		YES	1.0 (pos.60638470)	squamous cel	Zona pelluc	binding of sperm to zona pellucida,extracellular region,i			A=0, G=36	A=0.0, G=1.0	0.00001	563.725	36	A=0/0, G=14/2
chrX:69250308	T/T	C	T	SNV	EDA	EDA:intronic:NM 001399.4	1	100		intronic	NM 00139	c.742-11C	p.?	-0.37					TNF(Tumour N	rs2296765	0.3733	YES	1.082E-1 (pos.69250308)		Ectodysplas	apical par	Abnormalities, Multiple:Conge	Benign	C=0, T=23	C=0.0, T=1.0	0.00001	316.533	23	C=0/0, T=11/1
chrX:69639832	T/T	C	T	SNV	KIF4A	KIF4A:intronic:NM 012310.4	1	100		intronic	NM 01231	c.3496-80	p.?	-0.29					Kinesin moto	rs5980700		YES			Kinesin fam	ATP bindin	Autoimmune Diseases;Female Urogenital Di		C=0, T=23	C=0.0, T=1.0	0.00001	316.741	23	C=0/0, T=7/16
chr11:60637164	C/T	C	T	SNV	ZP1	ZP1:exonic:NM 207341.3	1	95.83	3	missense	NM 20734	c.473C>T	p.Thr158Ile	-0.26	0.11	89	0.061	0.05(COSM44	Zona pelluci	rs489172	0.1795	YES	3.946E-1 (pos.60637164)	acute myeloic	Zona pelluc	binding of sperm to zona pellucida,extracellular region,i			C=2, T=46	C=0.0417, T=0.9583	0.00001	746.233	48	C=1/1, T=21/2
chrX:74494483	G/G	A	G	SNV	UPRT	UPRT:intronic:NM 145052.3	1	100		intronic	NM 14505	c.386+8A>	p.?	-3.04					Uracil phosp	rs5981831	0.0278	YES	9.761E-1 (pos.74494483)		Uracil phos	GTP binding,UMP biosynthetic process,cytosol,female pr			A=0, G=38	A=0.0, G=1.0	0.00001	603.785	38	A=0/0, G=16/2
chrX:76709544	A/A	G	A	SNV	FGF16	FGF16:upstream:NM 003868.2	1	100		intronic	NM 00386	c.-103G>A	p.?	0.03						rs73224157					Fibroblast g	1-phospha	Adenocarcinoma:Carcinoma:Carcinoma, Ade		G=0, A=26	G=0.0, A=1.0	0.00001	371.178	26	G=0/0, A=10/1
chr1:33957152	G/G	T	G	SNV	ZSCAN20	ZSCAN20:exonic:NM 145238.3	1	100	6	missense	NM 14523	c.1294T>G	p.Tyr432Asp	2.47	1	160	0		C2H2-type zi	rs4403594	0.003		1.0 (pos.33957152)		Zinc finger-	DNA binding,DNA binding transcription factor activity,me			T=0, G=34	T=0.0, G=1.0	0.00001	523.856	34	T=0/0, G=12/2
chr1:33961147	T/G	T	G	SNV	ZSCAN20	ZSCAN20:utr 3:NM 145238.3	1	50	8	utr 3	NM 14523	c.3203T>G	p.?	0.43					C2H2-type zi	rs3795420		YES			Zinc finger-	DNA binding,DNA binding transcription factor activity,me			T=59, G=59	T=0.5, G=0.5	0.00001	615.601	118	T=31/28, G=37
chrX:83124802	T/T	A	T	SNV	CYLC1	CYLC1:intronic:NM 021118.2	1	100		intronic	NM 02111	c.18-71A>	p.?	-0.8					Cylicin N-ter	rs5968237		YES			Cylicin 1	acrosomal matrix,cell differentiation,cytoskeletal calyx,			A=0, T=61	A=0.0, T=1.0	0.00001	925.404	61	A=0/0, T=29/3
chrX:84358864	T/T	C	T	SNV	SATL1	SATL1:intronic:NM 001012980.2	1	100		intronic	NM 00101	c.1693+6G	p.?	-0.07						rs5922146	0.3184	YES	6.801E-1 (pos.84358864)			cytosol,diamine N-acetyltransferase activity,spermidine			C=0, T=81	C=0.0, T=1.0	0.00001	1309.48	81	C=0/0, T=51/3
chr7:99227172	A/G	A	G	SNV	ZSCAN25	ZSCAN25:exonic:NM 145115.2	1	57.14	8	synonymous	NM 14511	c.1164A>G	p.(=)	-2.02				0.06(COSM44	C2H2-type zi	rs1859690	0.0694	YES	2.951E-1 (pos.99227172)	acute myeloid leukaemia		DNA binding,DNA binding transcription factor activity,me			A=9, G=12	A=0.4286, G=0.5714	0.00001	109.101	21	A=4/5, G=5/7
chr1:45504720	C/C	G	C	SNV	ZSWIM5	ZSWIM5:exonic:NM 020883.1	1	100	8	synonymous	NM 02088	c.1782C>G	p.(=)	1.04				0.92(COSM37	SWIM zinc fin	rs7551698	0.4039	YES	5.086E-1 (pos.45504720)	acute myeloid leukaemia		extracellular space,zinc ion binding			G=0, C=38	G=0.0, C=1.0	0.00001	582.799	38	G=0/0, C=6/32
chrX:8501120	A/A	C	A	SNV	ANOS1	ANOS1:intronic:NM 000216.3	1	100		intronic	NM 00021	c.1985-26	p.?	-0.39					Fibronectin t	rs41303179	0.2977	YES	5.99E-1;0.0 (pos.8501120)			axon guidance,cell adhesion,chemotaxis,extracellular r			C=0, A=53	C=0.0, A=1.0	0.00001	782.026	53	C=0/0, A=24/2
chrX:86070935	C/C	T	C	SNV	DACH2	DACH2:intronic:NM 053281.3	1	100		intronic	NM 05328	c.1685-10	p.?	0.5					SKI/SNO/DAC	rs3761542		YES			Dachshund	DNA binding,RNA polymerase II transcriptional preinitiat			T=0, C=21	T=0.0, C=1.0	0.00001	281.235	21	T=0/0, C=13/8
chrX:9659766	A/A	G	A	SNV	TBL1X	TBL1X:intronic:NM 005647.3	1	100		intronic	NM 00564	c.749+15G	p.?	-1.02					Anaphase-pr	rs2301675,rs386823861		YES	2.126E-1;0.0 (pos.9659766)		Transducin-	beta-cate	Child Development Disorders, Pervasive:Mer		G=0, A=31	G=0.0, A=1.0	0.00001	465.819	31	G=0/0, A=18/1
chrX:9661110	G/G	C	G	SNV	TBL1X	TBL1X:intronic:NM 005647.3	1	100		intronic	NM 00564	c.892-79C	p.?	-0.04					Anaphase-pr	rs3747431		YES			Transducin-	beta-cate	Child Development Disorders, Pervasive:Mer		C=0, G=43	C=0.0, G=1.0	0.00001	693.396	43	C=0/0, G=31/1
chrX:9661165	T/T	G	T	SNV	TBL1X	TBL1X:intronic:NM 005647.3	1	100		intronic	NM 00564	c.892-24G	p.?	-0.02					Anaphase-pr	rs3747430	0.4318	YES	2.111E-1 (pos.9661165)		Transducin-	beta-cate	Child Development Disorders, Pervasive;Mer		G=0, T=53	G=0.0, T=1.0	0.00001	769.812	53	G=0/0, T=30/2

Supplementary Table 7. Functional enrichment analyses of the CNV-associated gene sets in ALS and SCA1-MN family members

Items	GO biological process name	Gene List	Reference (Human Genome)	P-value (Fisher's Exact)	FDR B&H
GO:0050896	Response to stimulus	89	543	5.15E-19	1.48E-15
GO:0007165	Signal transduction	137	1176	3.82E-15	1.76E-13
GO:0006508	Proteolysis	80	543	1.47E-14	1.41E-11
GO:0007155	Cell adhesion	79	556	1.53E-13	3.51E-12
GO:0055085	Transmembrane transport	81	630	1.47E-11	2.26E-10
GO:0007411	Axon guidance	48	307	3.52E-10	1.69E-07
GO:0006355	Regulation of transcription, DNA-depend	148	1609	1.41E-08	4.50E-06
GO:0006936	Muscle contraction	22	96	2.20E-08	6.33E-06
GO:0007049	Cell cycle	53	435	2.73E-07	3.13E-06
GO:0007229	Integrin-mediated signaling pathway	17	70	3.53E-07	7.82E-05
GO:0008152	Metabolic process	41	316	1.19E-06	2.45E-04
GO:0006810	Transport	64	604	2.44E-06	2.24E-05
GO:0006915	Apoptotic process	62	594	5.63E-06	8.54E-04
GO:0030154	Cell differentiation	55	519	1.21E-05	9.24E-05
GO:0006281	DNA repair	35	285	2.30E-05	2.76E-03
GO:0008219	Cell death	23	156	3.39E-05	2.23E-04
GO:0070371	ERK1 and ERK2 cascade	7	19	5.81E-05	5.07E-03
GO:0006468	Protein phosphorylation	43	398	6.60E-05	5.59E-03
GO:0006955	Immune response	41	382	1.11E-04	8.43E-03
GO:0006629	Lipid metabolic process	29	241	1.58E-04	9.10E-04
GO:0006898	receptor-mediated endocytosis (BP)	10	49	4.22E-04	2.33E-02
GO:0006511	ubiquitin-dependent protein catabolic pr	20	150	4.22E-04	2.29E-02
GO:0008283	Cell proliferation	33	312	6.38E-04	3.26E-03
GO:0048011	nerve growth factor receptor signaling pa	25	215	7.20E-04	3.35E-02
GO:0005975	Carbohydrate metabolic process	31	291	8.12E-04	3.74E-03
GO:0007268	synaptic transmission (BP)	38	383	8.91E-04	3.83E-02
GO:0002755	MyD88-dependent toll-like receptor sign	12	73	9.28E-04	3.87E-02
GO:0001525	angiogenesis (BP)	20	160	9.66E-04	3.86E-02
GO:0002224	toll-like receptor signaling pathway (BP)	12	74	1.05E-03	4.14E-02
GO:0007010	Cytoskeleton organization	15	106	1.15E-03	4.80E-03
GO:0007010	cytoskeleton organization (BP)	15	106	1.15E-03	4.35E-02
GO:0006974	response to DNA damage stimulus (BP)	17	129	1.26E-03	4.46E-02
GO:0050885	neuromuscular process controlling balan	8	38	1.27E-03	4.45E-02
GO:0007267	Cell-cell signaling	25	242	3.70E-03	1.42E-02
GO:0006259	DNA metabolic process	6	27	3.84E-03	1.36E-02
GO:0006461	Protein complex assembly	14	110	4.46E-03	1.47E-02
GO:0051301	Cell division	27	286	8.72E-03	2.51E-02
GO:0006464	Protein modification process	16	150	1.34E-02	3.42E-02

KEGG Pathway name	#Entities in gene list	#Entities total	pValue	FDR
Olfactory transduction	64	1975	4.21E-15	9.18E-13
ECM-receptor interaction	19	1975	2.40E-07	2.62E-05
PPAR signaling pathway	16	1975	1.82E-06	0.000132043
Axon guidance	22	1975	4.05E-06	0.000147009
Focal adhesion	29	1975	3.44E-06	1.50E-04
Dilated cardiomyopathy	18	1975	2.79E-06	1.52E-04
Pathways in cancer	38	1975	2.87E-05	8.94E-04
Glycosaminoglycan degradation	7	1975	5.76E-05	1.57E-03
Vascular smooth muscle contraction	18	1975	8.32E-05	2.01E-03
Lysosome	18	1975	2.03E-04	4.03E-03
Neurophilin interactions with VEGF and VEGFR	**4**	**4**		4.77E-03
Purine metabolism	21	1975	3.14E-04	5.26E-03
Apoptosis	14	1975	3.93E-04	6.13E-03
Regulation of actin cytoskeleton	25	1975	4.63E-04	6.73E-03
ABC transporters	9	1975	5.50E-04	7.50E-03
MAPK signaling pathway	29	1975	6.26E-04	7.58E-03
Toxoplasmosis	17	1975	7.19E-04	8.25E-03
Endocytosis	23	1975	8.06E-04	8.36E-03
Arrhythmogenic right ventricular cardiomyopathy (ARVC)	12	1975	9.15E-04	9.07E-03
Antigen processing and presentation	10	1975	1.25E-03	1.18E-02
GnRH signaling pathway	14	1975	1.48E-03	1.29E-02
Glutamatergic synapse	16	1975	2.11E-03	1.71E-02
Bile secretion	11	1975	2.41E-03	1.87E-02
Calcium signaling pathway	20	1975	2.80E-03	2.04E-02
Salivary secretion	12	1975	3.47E-03	2.29E-02
Gap junction	12	1975	4.62E-03	2.88E-02
Inositol phosphate metabolism	9	1975	5.11E-03	3.01E-02
NOD-like receptor signaling pathway	9	1975	5.11E-03	3.01E-02
Natural killer cell mediated cytotoxicity	15	1975	5.70E-03	3.27E-02
Bacterial invasion of epithelial cells	10	1975	6.72E-03	3.66E-02
Degradation of the extracellular matrix	34	148		4.00E-02
Gastric acid secretion	10	1975	8.19E-03	4.35E-02
Fatty acid metabolism	7	1975	8.44E-03	4.38E-02
Viral myocarditis	9	1975	9.87E-03	4.48E-02
VEGF signaling pathway	10	1975	1.08E-02	4.54E-02
**Terminal pathway of complement**	**4**	**8**		4.58E-02
Ubiquitin mediated proteolysis	15	1975	1.14E-02	4.67E-02
Cell cycle	14	1975	1.16E-02	4.68E-02
Peroxisome	10	1975	1.19E-02	4.70E-02

Supplementary Table 8. Protein-protein interaction network properties

Gene/Node name	Degree	BetweennessCentrality	Closeness Centrality	Eccentricity	Neighborhood Connectivity	Number Of Undirected Edges
**RPS27A**	122	0.07514753	0.46514161	6	54.68852459	122
**UBA52**	121	0.0791199	0.46413043	6	55.08264463	121
**UBC**	116	0.04700245	0.45717345	6	57.32758621	116
**UBB**	115	0.04498536	0.45668449	6	57.66956522	115
**CDC20**	94	0.0312473	0.43174924	7	68.5212766	94
**UBE2D1**	85	0.00629903	0.40018744	7	71.69411765	85
**CUL1**	85	0.00753495	0.3983209	7	72.81176471	85
**SKP1**	84	0.00419117	0.38159071	7	72.88095238	84
**CDC27**	81	0.00644129	0.38959854	7	74.83950617	81
**BTRC**	81	0.00122882	0.37821081	7	75.11111111	81
**RBX1**	81	0.00164246	0.38023152	7	74.16049383	81
**UBE2N**	81	0.01035728	0.40207156	7	73.39506173	81
**UBE2C**	80	0.00203727	0.37821081	7	74.7375	80
**UBE2D2**	80	0.00175558	0.37325175	7	73.25	80
**CBLB**	80	0.02713088	0.40978887	7	74.7	80
**CDC16**	79	0.00588572	0.38747731	7	75.87341772	79
**UBE2D3**	79	0.00182118	0.37489025	7	74.12658228	79
**ANAPC1**	78	0.00377074	0.38712602	7	76.83333333	78
**CDC23**	78	0.00377074	0.38712602	7	76.83333333	78
**ANAPC7**	78	9.74E-04	0.37720848	7	76.35897436	78
**ANAPC4**	78	9.74E-04	0.37720848	7	76.35897436	78
**VHL**	78	0.00622533	0.39906542	7	76.07692308	78
**SKP2**	77	5.87E-04	0.377542	7	77.05194805	77
**ANAPC10**	77	4.98E-04	0.37654321	7	77.24675325	77
**CDC26**	77	4.98E-04	0.37654321	7	77.24675325	77
**ANAPC5**	77	4.98E-04	0.37654321	7	77.24675325	77
**ANAPC2**	77	4.98E-04	0.37654321	7	77.24675325	77
**ANAPC11**	77	4.98E-04	0.37654321	7	77.24675325	77
**UBE3A**	77	0.00673732	0.37390543	7	75.7012987	77
**UBE2V2**	77	0.00173244	0.3735783	7	75.83116883	77
**TCEB1**	77	0.00130119	0.37390543	7	76.24675325	77
**TCEB2**	77	0.00130119	0.37390543	7	76.24675325	77
**FBXW11**	77	0.00301145	0.3836478	7	76.53246753	77
**UBR4**	77	0.01020987	0.38782925	7	76.35064935	77

**ITCH**	77	9.44E-04	0.37195122	7	76.05194805	77
**WWP1**	77	0.00309249	0.37195122	7	75.90909091	77
**SMURF2**	77	9.57E-04	0.37423313	7	76.27272727	77
**NEDD4L**	77	8.96E-04	0.37325175	7	75.76623377	77
**ATG7**	77	0.00726149	0.40900383	7	76.81818182	77
**UBA3**	76	0.00486115	0.37065972	7	76.57894737	76
**UBE2E1**	76	3.04E-04	0.37489025	7	78.06578947	76
**NEDD4**	76	9.06E-04	0.37325175	7	76.69736842	76
**KEAP1**	76	5.07E-04	0.37260035	7	76.84210526	76
**RBCK1**	76	3.93E-04	0.37195122	7	76.71052632	76
**PARK2**	76	0.00403024	0.3735783	7	76.39473684	76
**TRIM21**	76	0.00521145	0.37521968	6	76.40789474	76
**RNF111**	76	9.82E-04	0.37423313	7	76.78947368	76
**SMURF1**	75	0.00330783	0.3983209	7	77.98666667	75
**FBXL7**	75	1.47E-04	0.37065972	7	77.89333333	75
**CUL2**	75	1.47E-04	0.37065972	7	77.89333333	75
**CUL3**	75	1.47E-04	0.37065972	7	77.89333333	75
**MIB2**	75	7.59E-04	0.37098175	7	77.37333333	75
**LTN1**	75	0.00295197	0.37033825	7	77.16	75
**UBE2V1**	75	0.00228943	0.38433843	7	77.49333333	75
**UBA6**	75	0.00709232	0.37033825	7	77.10666667	75
**STUB1**	75	0.00250483	0.3722755	7	77.45333333	75
**UBE2S**	75	2.89E-04	0.3722755	7	77.69333333	75
**LMO7**	75	0.00453515	0.38125	7	77.53333333	75
**CCNF**	75	2.72E-04	0.37423313	7	78.10666667	75
**TRAIP**	74	1.56E-04	0.37001733	7	78.27027027	74
**UBE2L6**	74	0.00404431	0.37325175	7	78.16216216	74
**UBE4A**	74	0.00125248	0.37621145	7	78.44594595	74
**CDC34**	74	6.23E-05	0.37001733	7	78.39189189	74
**ASB18**	74	1.12E-04	0.37001733	7	78.39189189	74
**KLHL13**	74	1.12E-04	0.37001733	7	78.39189189	74
**FBXO2**	74	1.12E-04	0.37001733	7	78.39189189	74
**FBXW5**	74	1.12E-04	0.37001733	7	78.39189189	74
**LRSAM1**	74	0.00198093	0.37098175	7	78.14864865	74
**HECTD3**	73	0	0.36969697	7	79.20547945	73
**HECTD1**	73	0	0.36969697	7	79.20547945	73

**UBR2**	73	0	0.36969697	7	79.20547945	73
**RNF126**	73	0	0.36969697	7	79.20547945	73
**UBA7**	73	0	0.36969697	7	79.20547945	73
**RNF123**	73	0	0.36969697	7	79.20547945	73
**CDC42**	67	0.10762971	0.45619658	7	27.50746269	67
**CDK1**	58	0.02783562	0.42445328	6	51.43103448	58
**PLK1**	54	0.01886743	0.42277228	6	53.64814815	54
**RAC1**	50	0.03782751	0.3961039	7	17.82	50
**DYNC1H1**	50	0.02942825	0.42445328	6	28.2	50
**GNB1**	49	0.07600619	0.37065972	8	20.48979592	49
**SRC**	48	0.06976556	0.44248705	7	30.45833333	48
**GNGT1**	47	0.05197724	0.36186441	8	19.29787234	47
**PPP2R1A**	46	0.02518318	0.42914573	7	41.84782609	46
**CDC5L**	46	0.11370826	0.40397351	7	22.5	46
**DYNLL1**	45	0.01889765	0.39537037	6	27.02222222	45
**RHOA**	42	0.04946099	0.43131313	7	31.47619048	42
**PSMB4**	42	0.00307135	0.361558	7	72.83333333	42
**MAPK1**	40	0.04896351	0.41862745	6	23.775	40
**PPP2CA**	39	0.01767865	0.41617934	7	47.79487179	39
**FYN**	38	0.0408867	0.41216216	7	23.52631579	38
**DYNLL2**	38	0.02106816	0.39031079	6	27.68421053	38
**DYNC1I2**	37	0.00563688	0.39210285	6	30.13513514	37
**TP53**	35	0.02311291	0.40512334	7	42.71428571	35
**AKT1**	35	0.04610648	0.4194499	7	31.48571429	35
**PIK3R1**	35	0.01680002	0.39354839	7	22.97142857	35
**DYNC1LI1**	34	0.00723877	0.38853503	6	30.73529412	34
**DYNC1LI2**	33	0.00784205	0.40283019	6	32.78787879	33
**DCTN2**	33	0.00528767	0.36278675	6	27.03030303	33
**PTK2**	32	0.01304736	0.38572719	7	23.03125	32
**PIK3CA**	32	0.01476121	0.39647168	7	25.375	32
**GNAL**	31	8.12E-04	0.28278146	8	17.61290323	31
**PPP2R5A**	31	0.00603601	0.40744275	7	56.83870968	31
**SPTAN1**	31	0.03234266	0.38503156	6	27.35483871	31
**ACTR1A**	30	0.00395944	0.36186441	6	28.63333333	30
**DCTN3**	30	0.00197715	0.34857143	6	27.33333333	30
**ACTR10**	30	0.00593651	0.34914146	6	24.76666667	30

**ACTR2**	29	0.03678992	0.41903827	6	34.13793103	29
**PIK3R2**	28	0.01130971	0.38503156	7	25.64285714	28
**SMAD3**	27	0.01344869	0.37130435	7	59.66666667	27
**MAPK14**	26	0.03968788	0.40321058	7	24.73076923	26
**ITGB1**	25	0.03933866	0.35672515	7	19.4	25
**APP**	25	0.03152912	0.35260116	7	16.24	25
**VCP**	24	0.02136748	0.37390543	7	38.375	24
**ATM**	23	0.02297726	0.37033825	7	43.73913043	23
**ANK1**	23	0.00685629	0.3264526	6	23.7826087	23
**GOLGA2**	22	0.01095529	0.34132694	6	29.40909091	22
**SPTA1**	22	0.00378808	0.34659091	6	27.54545455	22
**CAPZA2**	22	0.00663594	0.34771987	6	28.5	22
**ZW10**	22	0.00517098	0.37489025	7	35.18181818	22
**REEP1**	21	8.42E-05	0.27406932	9	18.76190476	21
**VEGFA**	21	0.01264511	0.37423313	8	30.19047619	21
**CISH**	20	0	0.30763689	8	77	20
**ANK3**	20	9.70E-04	0.32299546	6	26.9	20
**COG1**	20	0.01453276	0.3237301	6	26.75	20
**CHUK**	20	8.06E-04	0.35347682	7	71.85	20
**TRAF2**	20	0.0089406	0.37292576	7	45.65	20
**GNAI2**	20	0.01284402	0.37065972	7	28.9	20
**REEP2**	19	8.25E-05	0.27371795	9	18.84210526	19
**RTP2**	19	8.25E-05	0.27371795	9	18.84210526	19
**RTP1**	19	8.25E-05	0.27371795	9	18.84210526	19
**KIF5B**	19	0.00352593	0.35057471	6	32.10526316	19
**KIF5A**	19	0.00352593	0.35057471	6	32.10526316	19
**HLA-DQB2**	19	0.00891684	0.34885621	6	28.68421053	19
**NUP85**	19	0.00699046	0.37325175	7	36.57894737	19
**OPTN**	19	0.00389212	0.35524126	7	32.84210526	19
**NUP107**	19	0.00699046	0.37325175	7	36.57894737	19
**REEP5**	18	8.25E-05	0.2735426	9	18.72222222	18
**REEP4**	18	8.25E-05	0.2735426	9	18.72222222	18
**RTP3**	18	8.25E-05	0.2735426	9	18.72222222	18
**REEP3**	18	8.25E-05	0.2735426	9	18.72222222	18
**RTP4**	18	8.25E-05	0.2735426	9	18.72222222	18
**REEP6**	18	8.25E-05	0.2735426	9	18.72222222	18

**RTP5**	18	8.25E-05	0.2735426	9	18.72222222	18
**CENPM**	18	6.17E-04	0.37325175	7	42.83333333	18
**TMEM115**	18	1.03E-04	0.32250755	6	29.55555556	18
**ACTA1**	17	0.00476261	0.35115132	7	25.41176471	17
**CBX4**	16	0.00151046	0.3346395	8	67.375	16
**PARP1**	16	0.00330313	0.35553705	7	58.875	16
**AP2A1**	16	0.02346863	0.36433447	6	40.4375	16
**TUBA4A**	16	0.00513298	0.33969769	7	31.5	16
**ACTN4**	15	0.00842205	0.36937716	7	29.8	15
**DNM1**	15	0.00270718	0.38193202	6	45.8	15
**GNAZ**	15	0.0101413	0.3646456	7	31.86666667	15
**OR2A5**	14	3.17E-05	0.27284345	9	23.64285714	14
**SFI1**	14	3.76E-06	0.32670237	7	32.78571429	14
**ARSB**	14	0.01081106	0.33333333	7	17.78571429	14
**OR8A1**	14	3.17E-05	0.27284345	9	23.64285714	14
**OR13F1**	14	3.17E-05	0.27284345	9	23.64285714	14
**OR10A6**	14	3.17E-05	0.27284345	9	23.64285714	14
**LRP2**	14	0.00776865	0.37098175	6	45.78571429	14
**OR14I1**	14	3.17E-05	0.27284345	9	23.64285714	14
**OR2L13**	14	3.17E-05	0.27284345	9	23.64285714	14
**SQSTM1**	14	3.17E-04	0.34105431	7	58.42857143	14
**OR13C5**	14	3.17E-05	0.27284345	9	23.64285714	14
**OR2T8**	14	3.17E-05	0.27284345	9	23.64285714	14
**CEP131**	14	3.76E-06	0.32670237	7	32.78571429	14
**GRN**	14	0.01047598	0.33359375	7	18.5	14
**NCOR2**	14	0.01106587	0.3637138	7	55.07142857	14
**OR11L1**	14	3.17E-05	0.27284345	9	23.64285714	14
**OR6S1**	14	3.17E-05	0.27284345	9	23.64285714	14
**OR1I1**	14	3.17E-05	0.27284345	9	23.64285714	14
**OR11G2**	14	3.17E-05	0.27284345	9	23.64285714	14
**OR4P4**	14	3.17E-05	0.27284345	9	23.64285714	14
**OR2L8**	14	3.17E-05	0.27284345	9	23.64285714	14
**OR2M7**	14	3.17E-05	0.27284345	9	23.64285714	14
**ODF2**	14	3.76E-06	0.32670237	7	32.78571429	14
**GLB1**	13	0.00934569	0.33255452	7	19.15384615	13
**NOS2**	13	0.00593332	0.35882353	7	64.07692308	13

**TMEM216**	13	0	0.32496195	7	33.92307692	13
**STAM**	13	2.46E-04	0.34743694	7	51.15384615	13
**SYNJ2**	13	0.00566744	0.34914146	7	45.30769231	13
**IGLL5**	13	0.01645612	0.36905791	7	29.76923077	13
**NSF**	12	0.00556707	0.32471483	6	19.5	12
**UBE2L3**	12	0	0.32323997	7	91	12
**NOTCH4**	12	0.00581561	0.3637138	7	72.91666667	12
**MAP2K3**	12	0.00883735	0.3735783	8	39.83333333	12
**TBK1**	12	0.00965099	0.34078212	7	51.75	12
**ERBB4**	11	0.00179612	0.35613011	7	42.54545455	11
**ACOX3**	11	0.01398555	0.32471483	7	68.54545455	11
**STK11IP**	11	0	0.33203733	7	22.45454545	11
**LCP2**	11	0.00175845	0.33969769	6	31.36363636	11
**MCM2**	11	0.00550246	0.33942766	7	70.54545455	11
**AGA**	11	0	0.33203733	7	22.45454545	11
**CYLD**	11	7.80E-05	0.33542812	7	70.18181818	11
**RHOT1**	11	9.52E-04	0.34659091	8	26.27272727	11
**PTGES2**	11	0	0.33203733	7	22.45454545	11
**CYB5R3**	11	0	0.33203733	7	22.45454545	11
**NHP2L1**	11	0.01460677	0.34491115	7	19.81818182	11
**CHMP2B**	10	9.70E-04	0.33049536	7	61.5	10
**CD86**	10	2.74E-04	0.34914146	8	40.1	10
**DECR2**	10	0	0.32397572	7	75.2	10
**FNBP1**	10	0	0.34602917	7	57.4	10
**WRN**	10	0.0028317	0.33177933	7	56.7	10
**AGXT**	10	0	0.32397572	7	75.2	10
**XRCC6**	10	0.00136572	0.31723626	8	27	10
RPL26L1	9	0.00438109	0.34407736	7	39.66666667	9
DCC	9	6.45E-04	0.34078212	7	36.44444444	9
FGF16	9	0.00114313	0.36094675	7	68.55555556	9
DLG4	9	0.00832916	0.32820907	8	24.44444444	9
MAP3K1	9	7.50E-04	0.35882353	7	33.44444444	9
EPHB1	9	0.00136621	0.32998454	6	30	9
PIK3C2G	9	7.42E-04	0.3162963	8	29.11111111	9
MDM4	9	2.52E-05	0.33100775	7	86.22222222	9
FUS	9	0.00125116	0.30391459	8	12.66666667	9

MSH2	9	0.00216405	0.34105431	8	42	9
PIKFYVE	8	0.00315147	0.32057057	7	18.125	8
DOCK1	8	7.13E-05	0.32998454	8	37.375	8
NAT10	8	0.01162551	0.25910194	8	4.875	8
FLT1	8	8.53E-05	0.31865672	8	25.125	8
WDR18	8	0.02244321	0.34352373	7	26	8
ADCY5	8	8.03E-05	0.28600134	8	25.125	8
ADCY4	8	8.03E-05	0.28600134	8	25.125	8
UPF1	8	5.15E-04	0.35231023	7	49.25	8
CDK9	8	9.86E-04	0.3416	7	43.375	8
DAG1	7	0	0.32820907	8	35.14285714	7
LAMA5	7	0	0.32820907	8	35.14285714	7
DNAH3	7	6.45E-05	0.29347079	7	30.85714286	7
DDX41	7	9.56E-04	0.34132694	7	86.85714286	7
CHMP4A	7	1.57E-06	0.32105263	7	82	7
TARDBP	7	9.08E-04	0.30305181	8	15.28571429	7
SACM1L	7	1.80E-04	0.30675287	7	14.85714286	7
TNC	7	0.00372152	0.31420162	8	17	7
GPC1	7	0.00143518	0.32348485	7	22.85714286	7
WDR34	7	1.81E-04	0.30155367	7	29.71428571	7
MUC6	6	0.00465086	0.22604553	9	4.16666667	6
VCAN	6	4.39E-05	0.26720901	8	9	6
KLRC2	6	0	0.31397059	7	32.33333333	6
MYH11	6	0.00571712	0.32008996	8	29	6
NOP9	6	0.00716208	0.25722892	8	4.33333333	6
CAPN2	6	3.33E-04	0.33307332	7	34.66666667	6
KIRREL	6	8.85E-05	0.32471483	7	29.83333333	6
PRPF40A	6	0.00468384	0.29550173	8	12.83333333	6
EDN2	6	0	0.3087491	8	36	6
MUC12	6	0.00465086	0.22604553	9	4.16666667	6
TAS2R46	6	0	0.28485657	8	26.16666667	6
INPP4B	6	1.60E-04	0.30653266	7	16.66666667	6
ITGA4	6	9.42E-05	0.3264526	8	36.5	6
SMG6	6	0	0.34105431	7	57.5	6
MUC7	6	0.00465086	0.22604553	9	4.16666667	6
C9orf72	6	2.06E-04	0.28561873	8	13.83333333	6

BCR	6	7.64E-06	0.3259542	8	39.5	6
MYH10	5	0.00468428	0.31985019	8	34	5
TIMELESS	5	3.45E-04	0.2781759	8	12.4	5
KRT76	5	0	1	1	5	5
KRT75	5	0	1	1	5	5
KRT6C	5	0	1	1	5	5
KRT71	5	0	1	1	5	5
HBS1L	5	3.83E-04	0.33888889	7	51	5
KRT1	5	0	1	1	5	5
KRT3	5	0	1	1	5	5
SEC24B	5	0.01909968	0.28638498	7	12.2	5
TTN	5	0.0055144	0.24554342	9	3.8	5
ITGB8	5	6.00E-04	0.31099782	8	22.6	5
MAPKAPK3	5	2.17E-04	0.33542812	8	31.8	5
TPM1	5	0.004571	0.27583979	8	9	5
SF3A2	5	0	0.29529737	8	15.4	5
NBAS	5	6.22E-05	0.29166667	7	15.2	5
CEP55	5	0	0.31841909	7	60.8	5
AMTN	5	0	0.26554726	8	9.6	5
ENAM	5	0	0.26554726	8	9.6	5
RBM5	5	0	0.29529737	8	15.4	5
PIP5KL1	5	1.17E-04	0.3047823	7	23	5
AMBN	5	0	0.26554726	8	9.6	5
CACNA2D2	4	2.10E-04	0.27945026	8	29.75	4
SCN1A	4	0	0.27963327	7	24	4
GNAT1	4	0	0.27853881	8	32.75	4
ABCE1	4	1.44E-05	0.32422172	7	64.25	4
TAF15	4	5.03E-04	0.31282051	8	23	4
COL6A2	4	0.00697628	0.26423267	8	9	4
ARHGEF19	4	0	0.31865672	8	42.5	4
IFT57	4	0	0.28485657	7	23.5	4
DCLRE1A	4	0	0.32008996	7	118.5	4
GALNT13	4	0.0277732	0.29027872	8	16	4
MYO9A	4	0	0.31865672	8	42.5	4
CD47	4	0.001264	0.33996815	7	39	4
MATR3	4	5.63E-06	0.29186603	8	16.5	4

WNK1	4	0	0.32008996	7	118.5	4
INTS1	4	7.73E-05	0.31841909	8	34.75	4
MYO5A	4	0.00486139	0.31443299	7	20.25	4
PFN1	4	0	0.31913303	8	44	4
CDON	4	9.15E-05	0.33075136	7	27.75	4
RPS6KA4	4	9.52E-04	0.33915806	6	32	4
SPAG9	4	0.00200776	0.3264526	8	24.75	4
MRPL1	4	0.00559104	0.32670237	7	34.25	4
TTC21B	4	0	0.28485657	7	23.5	4
COL17A1	4	0.00468384	0.20951914	9	3	4
CDC25B	4	6.90E-05	0.33516484	7	44.5	4
FAM13A	4	0	0.31865672	8	42.5	4
COL16A1	4	0.00697628	0.26423267	8	9	4
FARP2	4	0	0.32670237	8	50.75	4
TERF1	4	0.00468384	0.2716285	8	11	4
ARHGAP4	4	0	0.31865672	8	42.5	4
ARHGAP9	4	0	0.31865672	8	42.5	4
SCN7A	4	0	0.27963327	7	24	4
POLDIP3	4	3.66E-06	0.27249521	8	11	4
KIF1A	4	0	0.27963327	7	16.25	4
RRP7A	4	0.00385606	0.32795699	7	37.25	4
EPHA3	4	2.89E-06	0.32033008	7	34.25	4
GLE1	3	0	0.27232143	8	14	3
PPP2R3B	3	0	0.30176678	8	38.66666667	3
B4GALT5	3	0	0.18460873	10	6	3
MMRN1	3	0	0.29735376	8	20.33333333	3
DDX55	3	0	0.20618059	9	5.66666667	3
NVL	3	0	0.25676488	8	7.33333333	3
COL5A3	3	0	0.20941638	9	4	3
PCDHA10	3	0.00468384	0.18581375	10	2.33333333	3
RXRG	3	0	0.31747212	7	29.66666667	3
FYTTD1	3	0	0.27232143	8	14	3
ACADL	3	0.00935669	0.24582614	8	4.33333333	3
ESF1	3	0	0.20618059	9	5.66666667	3
TRAF3IP3	3	0	0.30964467	7	43.33333333	3
PTPN12	3	8.94E-05	0.31489676	8	35.66666667	3

E2F8	3	1.94E-06	0.32695253	7	62.33333333	3
MYO10	3	7.10E-05	0.30719424	7	18.33333333	3
MSH3	3	0	0.27745289	8	14	3
APEX1	3	1.24E-04	0.29008152	8	17.66666667	3
FLNB	3	0.00187551	0.30785869	8	33.66666667	3
MRPL36	3	2.02E-04	0.25785024	8	5	3
GALNTL5	3	0	0.18460873	10	6	3
PCDHA7	3	0.00466185	0.22773333	9	3	3
DNAH17	3	0	0.28390957	7	28.66666667	3
PCDHA4	3	0.01860342	0.29367263	8	14.66666667	3
PCDHA9	3	0.00466185	0.22773333	9	3	3
AGAP2	3	0	0.3047823	8	35	3
SCRIB	3	3.06E-04	0.33996815	8	51	3
IGLL1	3	0.00468384	0.27025316	8	5.33333333	3
ENG	3	2.10E-04	0.30587393	7	28.66666667	3
ST6GALNAC4	3	0	0.18460873	10	6	3
STXBP1	3	5.18E-04	0.28676964	7	12	3
MST1R	3	0.00131936	0.29694019	8	13	3
RAB36	3	0.00965253	0.25206612	7	8	3
RASSF1	2	0.00468384	0.27076728	8	12	2
SUGT1	2	2.22E-05	0.31259151	8	65	2
GBP3	2	0	0.28949153	7	47.5	2
MTIF2	2	0	0.24667822	8	3.5	2
CAMK2A	2	3.43E-05	0.26020719	9	6	2
PON1	2	0.00468384	0.26116208	8	13	2
MYH4	2	0	0.2166413	9	5	2
ALS2	2	8.27E-04	0.29367263	8	26	2
TRAPPC6A	2	0	0.25769463	7	13.5	2
HKDC1	2	0	0.24985372	8	7.5	2
USP48	2	4.57E-06	0.29286694	8	47.5	2
CHTF18	2	0	0.25630252	8	8	2
DOCK6	2	0	0.31723626	8	58.5	2
DIAPH3	2	0	0.3162963	8	54.5	2
NPC1L1	2	0.00934569	0.18302615	9	2	2
MSX2	2	0	0.27266922	8	76	2
ABCG8	2	0.00468384	0.15487849	10	1.5	2

MST1	2	0	0.2961165	8	19	2
SI	2	0	0.24985372	8	7.5	2
PCK1	2	0	0.2670419	8	8	2
AP5M1	2	0.00468384	0.26754386	7	8.5	2
NUAK1	2	0	0.29087193	8	29	2
SLC25A24	2	0	0.31077147	8	62.5	2
NEMF	2	1.67E-05	0.29166667	8	49.5	2
DNM1L	2	5.08E-05	0.3119065	8	61	2
ITGAE	2	0	0.26325524	8	15	2
STK10	2	0	0.29206566	8	40.5	2
RAB3D	2	0.00468384	0.20160529	8	2	2
PLXND1	2	1	1	1	1	2
FIG4	2	0	0.262124	7	15.5	2
HCLS1	2	0	0.31122449	7	31.5	2
CHL1	2	1.02E-04	0.27548387	7	24	2
MED17	2	0	0.2670419	8	8	2
SETX	2	7.29E-04	0.29448276	8	24	2
IQGAP3	2	0	0.31723626	8	58.5	2
SLC25A25	2	0	0.3114515	8	63.5	2
VPS54	2	0	0.25523013	7	16	2
SREBF2	2	0.01398555	0.22344322	8	3.5	2
HYAL3	2	6.49E-05	0.26309304	8	9	2
HYAL2	2	1.43E-04	0.25847458	8	8.5	2
TNFRSF13C	2	0	0.28773585	8	31	2
IGJ	2	0	0.27008223	8	8	2
LCN2	2	9.86E-05	0.31985019	7	52.5	2
RTDR1	2	0.00245127	0.27076728	8	9	2
NPRL2	2	0.00468384	0.25058685	8	7.5	2
SET	2	1.01E-04	0.2948895	8	21	2
ACSL4	1	0	0.197411	9	3	1
CYP2D6	1	0	1	1	1	1
TUSC2	1	0	0.28792987	8	46	1
VPREB1	1	0	0.21286142	9	3	1
TAF1B	1	0	1	1	1	1
DNAH9	1	0	0.28092105	7	38	1
SHANK2	1	0	0.24724957	9	9	1

LUC7L2	1	0	0.2282202	9	6	1
SHROOM3	1	0	0.28792987	8	46	1
TRDN	1	0	1	1	1	1
POLG	1	0	1	1	1	1
TTC27	1	0	0.20588235	9	8	1
BRWD1	1	0	1	1	1	1
TBCD	1	0	0.25371361	8	16	1
SPECC1	1	0	1	1	1	1
TBC1D13	1	0	1	1	1	1
FBN3	1	0	0.26309304	8	25	1
AZI2	1	0	0.25431805	8	12	1
TTF1	1	0	1	1	1	1
PARP4	1	0	0.28792987	8	46	1
SH3PXD2A	1	0	0.3069734	8	48	1
UBXN6	1	0	0.27232143	8	24	1
RYR3	1	0	1	1	1	1
C8B	1	0	1	1	1	1
SPAG17	1	0	1	1	1	1
TH	1	0	0.28754209	8	26	1
ACTR3B	1	0	0.29550173	7	29	1
PPP1R42	1	0	0.28792987	8	46	1
ACSL1	1	0	0.197411	9	3	1
DNHD1	1	0	0.28092105	7	38	1
MTRR	1	0	0.27042432	8	75	1
THSD7B	1	0	1	1	1	1
PTPN14	1	0	0.27093909	8	27	1
VAPB	1	0	1	1	1	1
POLR3C	1	0	0.20469799	9	6	1
ATP10A	1	0	0.27232143	8	77	1
NT5C3B	1	0	1	1	1	1
SYNE2	1	0	0.21371371	9	4	1
SPG11	1	0	0.21117705	8	2	1
SPIRE1	1	0	0.23934978	8	4	1
HPS4	1	0	1	1	1	1
ADAM9	1	0	0.17329545	10	4	1
PHIP	1	0	1	1	1	1

OSBP2	1	0	1	1	1	1
POLRMT	1	0	1	1	1	1
MAP3K6	1	0	0.27214786	9	12	1
UGT2B15	1	0	1	1	1	1
MTCH2	1	0	1	1	1	1
MYOF	1	0	0.27249521	9	21	1
ATIC	1	0	1	1	1	1
LFNG	1	0	0.266875	8	12	1
NEFH	1	0	0.28754209	8	26	1
C9orf114	1	0	1	1	1	1
MYOM2	1	0	0.19722864	10	5	1
FERMT2	1	0	0.29529737	7	40	1
DENND1B	1	0	0.29570637	8	35	1
SPEF2	1	0	1	1	1	1
FAT1	1	0	0.26423267	8	13	1
ABCA2	1	0	0.13415017	11	2	1
PCDHA6	1	0	0.15675477	11	3	1
BLOC1S3	1	0	1	1	1	1
C7	1	0	1	1	1	1
RNPC3	1	0	0.25661058	8	11	1
B3GALTL	1	0	1	1	1	1
ZFYVE16	1	0	0.27093909	8	27	1
SIGIRR	1	0	0.25431805	8	12	1
MAP1S	1	0	0.21318023	9	2	1
PREX2	1	0	0.28390957	8	50	1
NDUFA6	1	0	1	1	1	1
FAM129B	1	0	0.28792987	8	46	1
URM1	1	0	0.27059569	8	76	1
SEMA3B	1	0	0.66666667	2	2	1
SEMA3F	1	0	0.66666667	2	2	1
ZMYND10	1	0	0.20046948	9	2	1
NFAM1	1	0	0.16784591	9	2	1
SH2D3C	1	0	1	1	1	1
RIMBP3B	1	0	1	1	1	1
HYAL1	1	0	0.25014646	8	14	1
ZNF280B	1	0	1	1	1	1

ZNF280A	1	0	1	1	1	1
RIMBP3C	1	0	1	1	1	1
LPA	1	0	0.20718098	9	2	1
